# Causal relationship between gut microbiota and gastrointestinal diseases: a mendelian randomization study

**DOI:** 10.1186/s12967-024-04894-5

**Published:** 2024-01-23

**Authors:** Kaiwen Wu, Qiang Luo, Ye Liu, Aoshuang Li, Demeng Xia, Xiaobin Sun

**Affiliations:** 1 Department of Gastroenterology, The Third People’s Hospital of Chengdu, The Affiliated Hospital of Southwest Jiaotong University, Chengdu, Sichuan China; 2 Department of Rheumatology and Immunology, Ministry of Education Key Laboratory of Child Development and Disorders, National Clinical Research Center for Child Health and Disorders, International Science and Technology Cooperation base of Child Development and Critical Disorders, Chongqing Key Laboratory of Child Infection and Immunity, Children’s Hospital of Chongqing Medical University, Chongqing, China; 3 Department of Pharmacy, Seventh People’s Hospital of Shanghai University of Traditional Chinese Medicine, Shanghai, China

**Keywords:** Gut microbiota, Gastrointestinal disease, Mendelian randomization, SNPs

## Abstract

**Background:**

Recent research increasingly highlights a strong correlation between gut microbiota and the risk of gastrointestinal diseases. However, whether this relationship is causal or merely coincidental remains uncertain. To address this, a Mendelian randomization (MR) analysis was undertaken to explore the connections between gut microbiota and prevalent gastrointestinal diseases.

**Methods:**

Genome-wide association study (GWAS) summary statistics for gut microbiota, encompassing a diverse range of 211 taxa (131 genera, 35 families, 20 orders, 16 classes, and 9 phyla), were sourced from the comprehensive MiBioGen study. Genetic associations with 22 gastrointestinal diseases were gathered from the UK Biobank, FinnGen study, and various extensive GWAS studies. MR analysis was meticulously conducted to assess the causal relationship between genetically predicted gut microbiota and these gastrointestinal diseases. To validate the reliability of our findings, sensitivity analyses and tests for heterogeneity were systematically performed.

**Results:**

The MR analysis yielded significant evidence for 251 causal relationships between genetically predicted gut microbiota and the risk of gastrointestinal diseases. This included 98 associations with upper gastrointestinal diseases, 81 with lower gastrointestinal diseases, 54 with hepatobiliary diseases, and 18 with pancreatic diseases. Notably, these associations were particularly evident in taxa belonging to the genera *Ruminococcus* and *Eubacterium*. Further sensitivity analyses reinforced the robustness of these results.

**Conclusions:**

The findings of this study indicate a potential genetic predisposition linking gut microbiota to gastrointestinal diseases. These insights pave the way for designing future clinical trials focusing on microbiome-related interventions, including the use of microbiome-dependent metabolites, to potentially treat or manage gastrointestinal diseases and their associated risk factors.

**Supplementary Information:**

The online version contains supplementary material available at 10.1186/s12967-024-04894-5.

## Introduction

The human microbiome, a vast consortium of over 100 trillion microorganisms, coexists within the human body in a dynamic symbiotic or parasitic relationship. These microorganisms predominantly inhabit various niches such as the skin, respiratory, and gastrointestinal tracts [1, 2]. Extensive research has firmly established the crucial role of the gut microbiota in maintaining human health and modulating numerous physiological functions [3]. The gut microbiota is integral to processes like the breakdown and assimilation of nutrients, absorption of essential compounds, and synthesis of vital biological molecules like vitamins, providing the body with necessary energy and nutrients [4, 5]. Additionally, it plays a vital role in safeguarding the integrity of the intestinal barrier, protecting against pathogens, aiding immune system development, and regulating immune responses [6, 7]. Advancements in microbiota research have linked it to various diseases, notably gastrointestinal disorders like inflammatory bowel disease (IBD), irritable bowel syndrome (IBS), gastric ulcers, and gastroesophageal reflux disease (GERD) [8]. Changes in the microbiota’s abundance, diversity, and composition are thought to weaken the intestinal barrier, leading to increased inflammation, immune dysregulation, and metabolic issues, thus exacerbating these diseases [9–11].

However, research on the gut microbiota is often based on observational studies, which are susceptible to the influence of confounding factors and reverse causality [12]. Observational studies may be limited in their ability to establish causality due to the potential presence of unmeasured or unknown confounders that can distort the observed associations [13]. Additionally, the bidirectional relationship between the gut microbiota and host health further complicates the interpretation of observational findings [14]. While these studies provide valuable insights into the associations between the gut microbiota and various diseases, they cannot definitively establish causation. Randomized controlled trials (RCTs) play a crucial role in controlling potential confounding factors and providing robust evidence to support the relationship between the gut microbiota and diseases [15, 16]. However, RCTs need a substantial sample size and complex data analysis methods. The exorbitant costs, time constraints, and ethical considerations pose significant obstacles to microbiota research, thereby impeding causal inference in this field [17, 18]. Therefore, it is crucial to choose appropriate research methods to explore the causal relationship between gut microbiota and gastrointestinal diseases.

Mendelian Randomization (MR) analysis is a powerful analytical tool that leverages genetic variants as instrumental variables (IVs) to investigate the causal relationship between an exposure and an outcome. Because genetic variations are randomly allocated during conception, MR studies are less prone to the typical confounding factors and reverse causality issues that often affect conventional observational research [19]. Furthermore, MR methods are not influenced by subjective factors such as self-reporting and memory distortion, thereby reducing the potential for information bias [20, 21]. Recently, the MiBioGen consortium released numerous microbiome abundance-associated loci, offering an unprecedented chance to explore the causality between the gut microbiota and diseases. Using genetic variations (single nucleotide polymorphisms, SNPs) closely associated with the gut microbiota as instrumental variables, MR analysis is conducted to simulate the effects of random allocation. This approach allowed us to assess the impact of instrumental variables on specific diseases, thereby providing more robust evidence for the causal relationship between the gut microbiota and diseases by mitigating confounding factors through the process of randomization [22]. Currently, MR analysis has been increasingly applied to investigate the causal relationship between gut microbiota and various diseases, including cancer, psychiatric disorders, metabolic disorders, and autoimmune diseases, providing novel insights into the underlying mechanisms of microbiota-mediated diseases [23–26]. Previous MR studies have delved into the causal connections between the gut microbiota and several gastrointestinal diseases. However, there has been a dearth of comprehensive investigations into the potential impact of gut microbiota.

In the present study, based on large-scale genome-wide association studies (GWAS), we conducted a two-sample Mendelian randomization analysis to evaluate the potential causal relationships between gut microbiota and 22 gastrointestinal diseases, including upper gastrointestinal diseases, lower gastrointestinal diseases, hepatobiliary diseases, and pancreatic diseases. Our study contributes to a more comprehensive understanding of the role of gut microbiota in gastrointestinal health. It provides valuable insights and guidance for clinical practice and public health decision-making. These novel insights and strategies may contribute to enhancing individualized treatment approaches for gastrointestinal diseases, offering new directions and strategies to advance the prevention and management of gastrointestinal disorders.

## Methods

### Study design

**Fig. 1:**
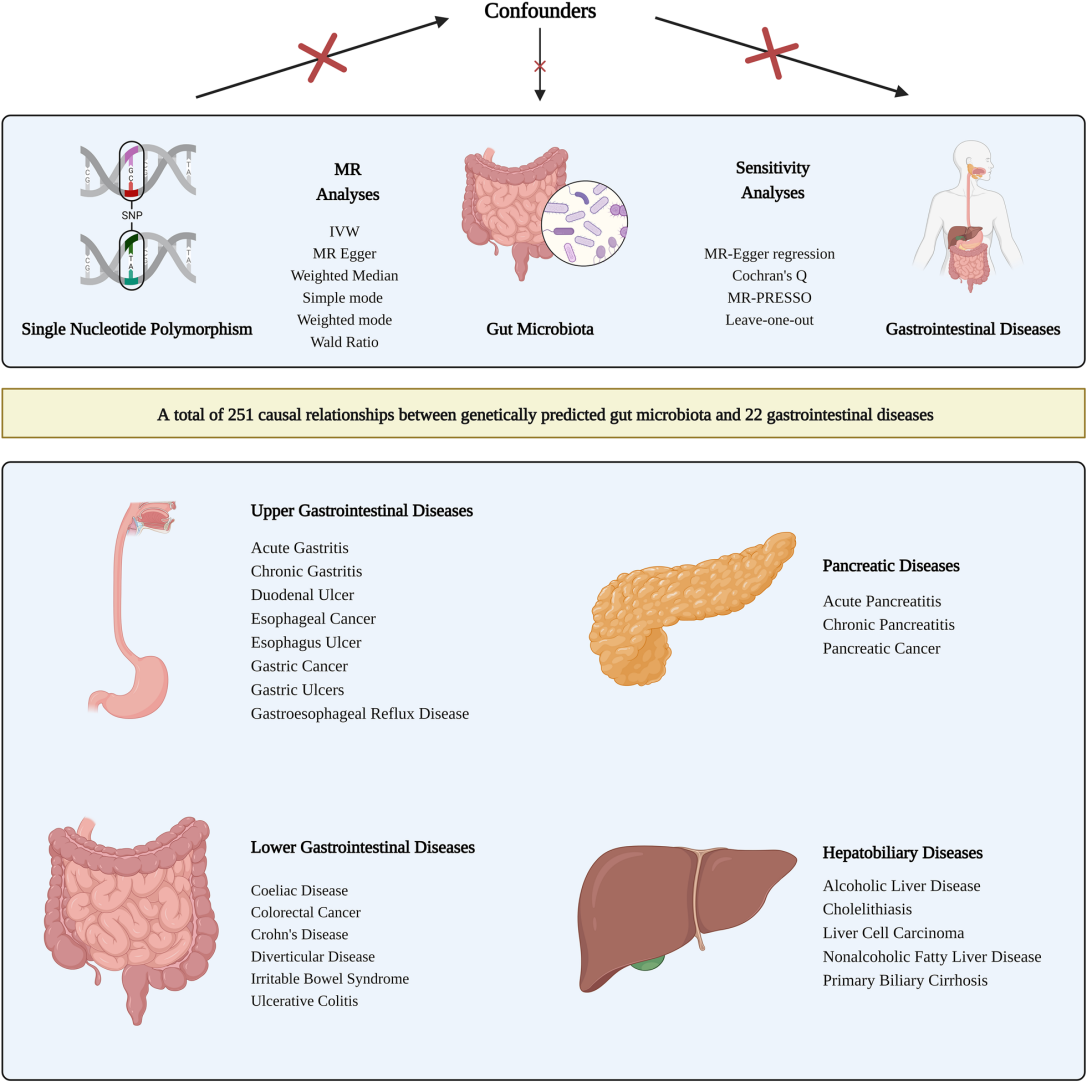
The design and flowchart of MR analysis in our study. This schematic representation emphasizes the research question that we attempted to answer, the analysis workflow and the data used. Based on large-scale publicly available genome-wide association study (GWAS) summary statistics data, we conducted Mendelian randomization (MR) analysis to explore the causal relationship between the gut microbiota and 22 gastrointestinal diseases, including upper gastrointestinal diseases, lower gastrointestinal diseases, liver and gallbladder diseases, and pancreatic diseases. Sensitivity analysis was used to verify the robustness of the MR results. SNP, single nucleotide polymorphism; MR-PRESSO, Mendelian randomization pleiotropy residual sum and outlier; MR, Mendelian randomization; IVW, inverse variance weighted

This study conducted a two sample MR analysis to investigate the potential causal relationship between gut microbiota and various gastrointestinal diseases. To ensure the validity of our findings, MR analysis needs to be grounded on three key assumptions: (1) genetic variants must exhibit a significant association with the exposure factor (gut microbiota); (2) genetic variants should not directly impact the outcome (gastrointestinal diseases); (3) genetic variants should have no causal relationship to any potential confounding factors [27]. The research process is depicted in Fig. 1.

### Data collection for gut microbiota

The SNPs associated with the composition of the human gut microbiome were selected from the GWAS dataset of the MiBioGen Consortium. Researchers performed a large-scale and genome-wide meta-analysis of the associations between autosomal human genetic variants and the gut microbiome [22]. This study stands as the most comprehensive large-scale association analysis of human gut microbiota composition to date, shedding light on the intricate relationships between genetic variants and the human gut microbiome. Employing a standardized pipeline, microbiome trait loci (mbTL) mapping was conducted to pinpoint genetic loci influencing the relative abundance or presence (microbiome Binary Trait loci) of microbial taxa. In the initial investigation, the gut microbiota summary statistics were classified into 257 taxa across five taxonomic levels: phylum, class, order, family, and genus. Among these, a subset of 211 taxa (comprising 9 phyla, 16 classes, 20 orders, 35 families, and 131 genera) meeting the criteria for microbial quantitative trait locus (mbQTL) mapping analysis was incorporated into the present study. To ensure consistent genetic backgrounds, minimize confounding variables related to lifestyle and environment, and enhance the reliability and interpretability of the results, we exclusively utilized data from the European ancestry within the MiBioGen Consortium. We obtained 14,306 participants of European ancestry GWAS summary data that collected profiles of sequencing for the 16 S ribosomal RNA gene and genotyping information (Additional file 1).

### Gastrointestinal disease data sources

The large-scale GWAS summary datasets for 22 gastrointestinal diseases were mainly collected from UK Biobank, FinnGen. Some studies of some large consortia were also included, such as the International Inflammatory Bowel Disease Genetics Consortium (IIBDGC), PanScan consortium and UK primary biliary cirrhosis consortium. Gastrointestinal diseases can be classified into four categories based on the site of occurrence: (1) Upper gastrointestinal diseases: gastroesophageal reflux disease (GERD), esophageal cancer, esophagus ulcer, gastric ulcers, duodenal ulcer, chronic gastritis, acute gastritis, and gastric cancer. (2) Lower gastrointestinal diseases: irritable bowel syndrome, diverticular disease, Crohn’s disease, ulcerative colitis, coeliac disease and colorectal cancer. (3) Hepatobiliary diseases: alcoholic liver disease, nonalcoholic fatty liver disease (NAFLD), primary biliary cirrhosis, liver cell carcinoma and cholelithiasis. (4) Pancreatic diseases: acute pancreatitis, chronic pancreatitis and pancreatic cancer. To mitigate the impact of population structure and diminish the confounding effects of ethnic and genetic diversity in Mendelian randomization, we restricted the use of GWAS summary statistics to those derived from European participants. All datasets were freely accessed from the IEU Open GWAS project (https://gwas.mrcieu.ac.uk/). Since detailed information about participants was not collected, ethical approval was not requisite for this study. Detailed dataset information is provided in Additional file 2.

### Instrumental variables selection

Bacterial taxa were analyzed at five levels (phylum, class, order, family, and genus). We selected SNPs that are closely associated with the gut microbiota as IVs. Initially, we screened the SNPs using a threshold of *P* < 5 × 10^−8^, resulting in only a small number of SNPs being included. Based on high-quality Mendelian randomization studies, we further selected SNPs using a threshold of *P* < 5 × 10^−5^. Furthermore, to reduce potential bias caused by allelic association, we also removed linkage disequilibrium (R^2^ > 0.001, kb = 10,000) to ensure the enhanced credibility of our results. To minimize the impact of weak instrument bias on causal inference, we used the following formula to calculate the F-statistic for each SNP: F_exposure_ = Beta^2^_exposure_ / SE^2^_exposure_ [28].

### MR analysis

A Two sample MR analysis was used to estimate the potential casual relationships between the gut microbiota and gastrointestinal diseases. The inverse-variance weighted (IVW) method was selected as the main approach for data analysis. IVW combines effect estimates from individual genetic variants by weighting them inversely to their variances. By assigning higher weight to more precise estimates, IVW enhances the reliability of the overall causal effect estimate [29, 30]. In cases where pleiotropy is lacking and instrumental validity is assumed, the IVW method provides unbiased estimates of a causal effect if horizontal pleiotropy is balanced. In conjunction with the primary IVW method, supplementary analytical approaches such as MR Egger, Weighted Median, Simple Mode, and Weighted Mode were employed to provide a comprehensive assessment. IF only one SNP could be used as IVs, the causal relationship between exposure and outcome would be estimated by the Wald ratio [28, 31]. Furthermore, we also performed genetic risk scores (GRS) to obtain the combined estimate of the relationship between gut microbiota and gastrointestinal diseases, aiming to incorporate the genetic influences of chosen SNPs on the focal exposure using the genotyping data accessible at the individual level. GRS can integrate information from multiple genetic loci rather than focusing on individual genes. This facilitates a more comprehensive assessment of the complexity of genetic risk, enhancing accuracy and sensitivity in estimating individual genetic predisposition [32].

### Pleiotropy and sensitivity analysis

MR Egger analysis was implemented for heterogeneity assessment and calculation of the Cochran’s Q value, crucial for evaluating the diversity among the genetic instruments used. Furthermore, MR-Egger regression was performed to assess potential horizontal pleiotropy effects in Mendelian randomization analysis. We also conducted Mendelian randomization pleiotropy residual sum and outlier (MR-PRESSO) to rigorously test for horizontal pleiotropy. Additionally, leave-one-out analysis and funnel plots were used to examine the robustness and accuracy of the MR results. All analyses were conducted using the TwoSampleMR package (version 0.5.6) and MR-PRESSO package (version 1.0) in R software (version 4.0.5). The gtx package was used for GRS analysis. Heatmaps were generated using the R package ComplexHeatmap (version 2.6.2).

## Results

**Fig. 2:**
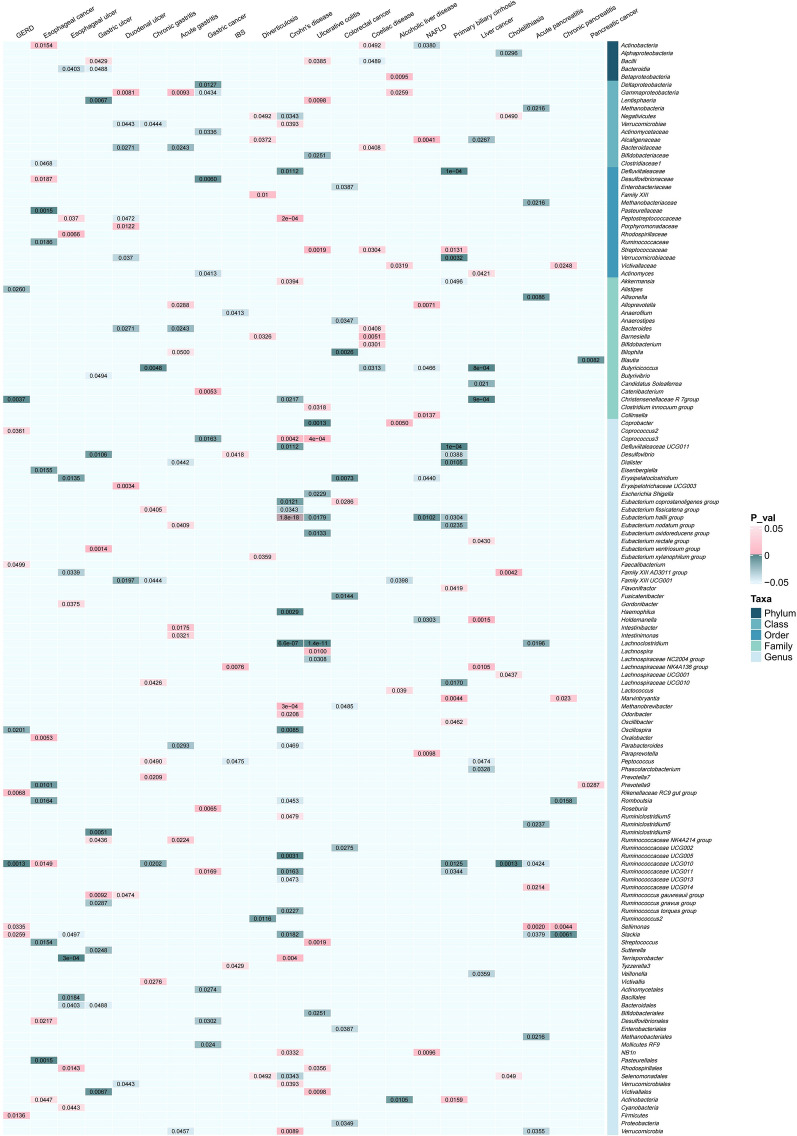
Heatmap of correlation coefficients between gut microbiota abundance and gastrointestinal diseases. Pink blocks represent an increase in the abundance of gut microbiota, which may be associated with an increased risk of developing outcome diseases. Blue blocks demonstrate gut microbiota abundance was negatively correlated with outcome diseases (P value < 0.05). Other blocks represent that there is no causal relationship between gut microbiota and gastrointestinal diseases (P value > 0.05)

**Fig. 3:**
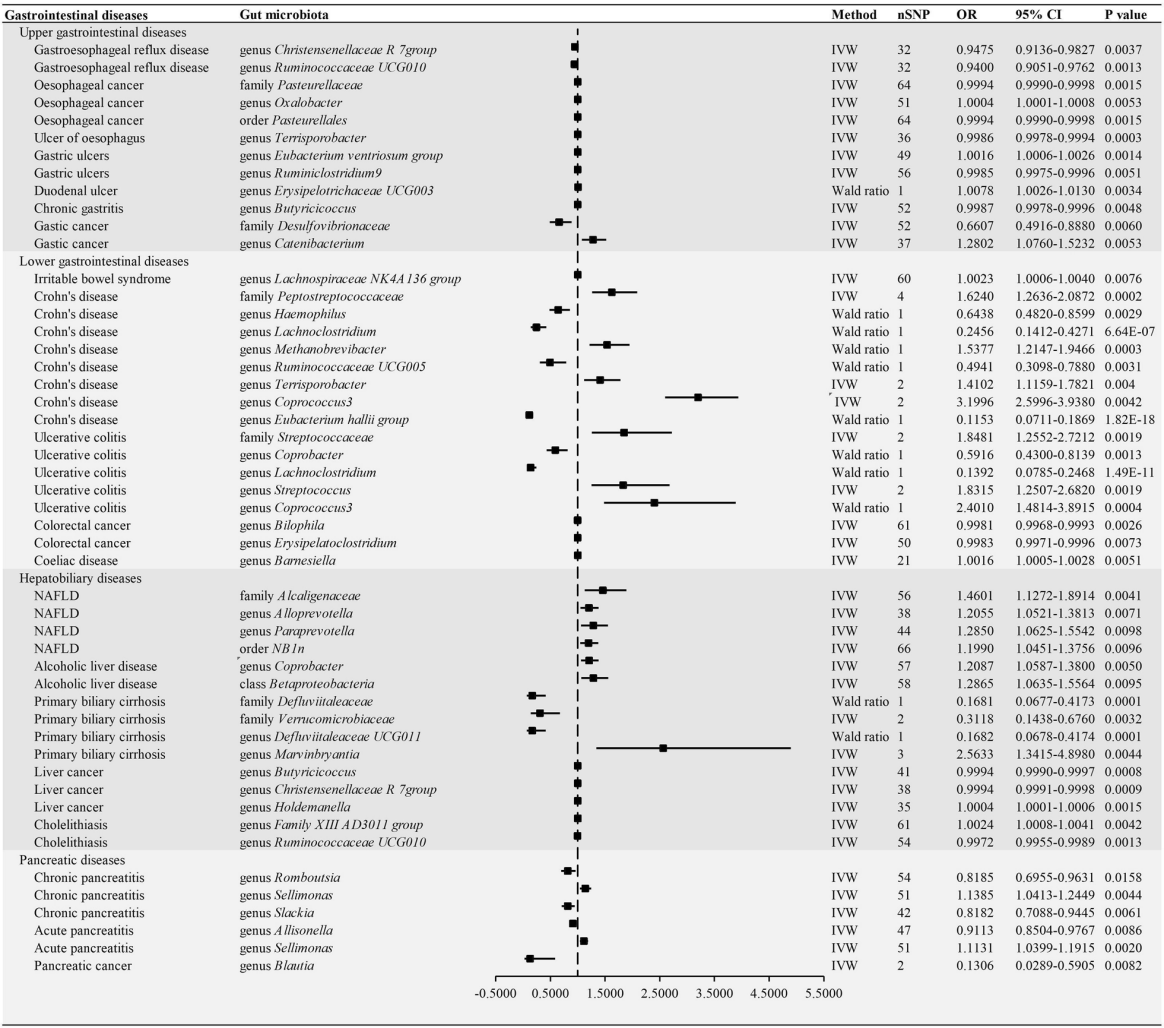
Forest plots of Mendelian randomization (MR) estimates between gut microbiota and gastrointestinal diseases according to P value corrected. MR results suggested 12 causal relationships in upper gastrointestinal diseases, 17 in lower gastrointestinal diseases, 15 in hepatobiliary diseases and 6 in pancreatic diseases according to P value corrected. NAFLD, nonalcoholic fatty liver disease; IVW, inverse variance weighted; CI, Confidence interval; OR, Odds ratios

The F-statistic of all IVs is greater than 10, indicating that there is no weak instrumental bias in our analysis (Additional file 3). MR analysis suggested 251 causal relationships between genetically predicted gut microbiota and 22 gastrointestinal diseases (Fig. 2 and Additional file 4). In upper gastrointestinal diseases, there were 10, 14, 14, 13, 11, 9, 12 and 15 causal relationships in GERD, esophageal cancer, esophagus ulcer, gastric ulcer, duodenal ulcer, chronic gastritis, acute gastritis and gastric cancer, respectively. In lower gastrointestinal diseases, we found 5, 9, 32, 17, 10 and 8 causal relationships, including IBS, diverticulosis, Crohn’s disease, ulcerative colitis, colorectal cancer and coeliac disease, respectively. In hepatobiliary diseases, we identified 7, 13, 17, 11 and 6 causal relationships in alcoholic liver disease, NAFLD, primary biliary cirrhosis, liver cancer and cholelithiasis, respectively. Within the pancreatic diseases, the results suggested 11, 5 and 2 causal relationships in acute pancreatitis, chronic pancreatitis and pancreatic cancer, respectively. Furthermore, we observed 12 causal relationships in upper gastrointestinal diseases, 17 in lower gastrointestinal diseases, 15 in hepatobiliary diseases and 6 in pancreatic diseases according to P value corrected (Fig. 3). In addition, the results of the GRS analysis still obtained similar results to IVW method (Additional files 5 and 6).

### Upper gastrointestinal diseases

Our results eindicated that genus *Christensenellaceae R 7group* (odds ratio [OR] = 0.9475, 95% confidence interval [CI] 0.9136–0.9827; *P* = 0.0037) and genus *Ruminococcaceae UCG010* (OR = 0.9400, 95%CI 0.9051–0.9762; *P* = 0.0013) may be protective factors for GRED. Genus *Pasteurellaceae* (OR = 0.9994, 95%CI 0.9990–0.9998; *P* = 0.0015) and order *Pasteurellales* (OR = 0.9994, 95%CI 0.9990–0.9998; *P* = 0.0015) were associated with a reduced risk of esophageal cancer, while genus *Oxalobacter* (OR = 1.0004, 95%CI 1.0001–1.0008; *P* = 0.0053) is related with the risk of esophageal cancer. The MR results demonstrated that genetically predicted increases in genus *Terrisporobacter* (OR = 1.0016, 95%CI 1.0006–1.0026; *P* = 0.0014) were associated with an increased risk of esophagus ulcers and that genus *Ruminiclostridium9* (OR = 0.9985, 95%CI 0.9975–0.9996; *P* = 0.0051) have causal relationships with gastric ulcers. Additionally, genus *Erysipelotrichaceae UCG003* (OR = 1.0078, 95%CI 1.0026–1.0130; *P* = 0.0034) was associated with a higher risk of duodenal ulcers. The results suggest that genus *Butyricicoccus* (OR = 0.9987. 95%CI 0.9978–0.9996; *P* = 0.0048) may be related to chronic gastritis. Moreover, genetic predictions showed that high abundance of family *Desulfovibrionaceae* (OR = 0.6607, 95%CI 0.4916–0.8880; *P* = 0.0060) was associated with a reduced risk of gastric cancer, while genus *Catenibacterium* (OR = 1.2802, 95%CI 1.0760–1.5232; *P* = 0.0053) may be associated with an increased risk of gastric cancer.

### Low gastrointestinal diseases

This Study have found that a high abundance of the genus *Lachnospiraceae NK4A136 group* (OR = 1.0023, 95%CI 1.0006–1.0040; *P* = 0.0076) may lead to an increased incidence of IBS. Family *Peptostreptococcaceae*, genus *Haemophilus*, genus *Lachnoclostridium*, genus *Methanobrevibacter*, genus *Ruminococcaceae*, genus *Terrisporobacter*, genus *Coprococcus3*, and genus *Eubacterium hallii group* with Crohn’s disease and genus *Coprobacter*, genus *Lachnoclostridium*, genus *Streptococcus*, and genus *Coprococcus3* were causally associated with ulcerative colitis. Genus *Lachnoclostridium* (OR = 0.2456, 95%CI 0.1412–0.4271;, *P* = 6.64E-07 for Crohn’s disease and OR = 0.1392, 95%CI 0.0785–0.2468; *P* = 1.49E-11 for ulcerative colitis) was negatively correlated IBD, whereas a higher genetically predicted abundance of genus *Coprococcus3* (OR = 3.1996, 95%CI 2.5996–3.9380; *P* = 0.0042 for Crohn’s disease and OR = 2.4010, 95%CI 1.4814–3.8915; *P* = 0.0004 for ulcerative colitis) was associated with an increased risk of Crohn’s disease and ulcerative colitis. In addition, genus *Bilophila* and genus *Erysipelatoclostridium* were associated with a reduced risk of colorectal cancer. Our study also demonstrated an inverse causal relationship between genetically predicted genus *Barnesiella* (OR = 1.0016, 95%CI 1.0005–1.0028; *P* = 0.0051) and the risk of coeliac disease.

### Hepatobiliary diseases

We found that the higher genetically predicted abundance of family *Alcaligenaceae* (OR = 1.4601, 95%CI 1.1272–1.8914; *P* = 0.0041), genus *Alloprevotella* (OR = 1.2055, 95%CI 1.0521–1.3813; *P* = 0.0071), genus *Paraprevotella* (OR = 1.2850, 95%CI 1.0625–1.5542; *P* = 0.0098) and order *NB1n* (OR = 1.1990, 95%CI 1.0451–1.3756; *P* = 0.0096) were linked to an increased risk of NAFLD. The results showed some potential causal associations between the gut microbiota and cancers. Higher genetically predicted abundances of genus *Coprobacter* (OR = 1.2087, 95%CI 1.0587-1.3800; *P* = 0.0050) and class *Betaproteobacteria* (OR = 1.2865, 95%CI 1.0635–1.5564; *P* = 0.0095) were associated with an increased risk of alcoholic liver disease. In addition, family *Defluviitaleaceae* (OR = 0.1681, 95%CI 0.0677–0.4173; *P* = 0.0001), genus *Defluviitaleaceae UCG011* (OR = 0.1682, 95%CI 0.0678–0.4174; *P* = 0.0001), family *Verrucomicrobiaceae* (OR = 0.3118, 95%CI 0.1438–0.6760; *P* = 0.0032), and genus *Marvinbryantia* (OR = 2.5633, 95%CI 1.3415–4.8980; *P* = 0.0001) had causal relationships with primary biliary cirrhosis. Furthermore, a high abundance of genus *Butyricicoccus* (OR = 0.9994, 95%CI 0.9990–0.9997; *P* = 0.0008) and genus *Christensenellaceae R 7group* (OR = 0.9994, 95%CI 0.9991–0.9998; *P* = 0.0009) are associated with a reduced risk of liver cancer. Additionally, the genus *Family XIII AD3011 group* (OR = 1.0024, 95%CI 1.0008–1.0041; *P* = 0.0042) and genus *Ruminococcaceae UCG010* (OR = 0.9972, 95%CI 0.9955–0.9989; *P* = 0.0013) were significantly associated with cholelithiasis at the genetic prediction level.

### Pancreatic diseases

In our MR analysis, genetically predicted genus *Romboutsia* (OR = 0.8185, 95% CI 0.6955–0.9631; *P* = 0.0158), genus *Selemonas* (OR = 1.1385, 95% CI 1.0413–1.2449; *P* = 0.0044), and genus *Slackia* (OR = 0.8182, 95% CI 0.7088–0.9445; *P* = 0.0061) were significantly correlated with chronic pancreatis. Genus *Allisonella* (OR = 0.9113, 95% CI 0.8504–0.9767; *P* = 0.0086) and genus *Selemonas* (OR = 1.1131, 95% CI 1.0399–1.1915; *P* = 0.0020) have a causal relationship with Acute pancreatis. Genus *Selemonas* may potentially serve as a risk factor for both acute and chronic pancreatitis. The genetic prediction level shows that genus *Blautia* (OR = 0.1306, 95% CI 0.0289–0.5905; *P* = 0.0082) may be a protective factor for Pancreative cancer.

### Sensitivity analyses

Sensitivity analyses confirmed the robustness of the findings. MR Egger regression intercepts deviated from zero, indicating no evidence of heterogeneity was observed (all intercepts *P* > 0.05). The MR-PRESSO test also confirmed that the results did not identify horizontal pleiotropy and revealed that there are no obvious outliers for the instrumental variables in this study. Furthermore, most of the Cochrane Q statistic outcomes showed no significant heterogeneity (*P* > 0.05). Additionally, the vast majority of results from Cochrane Q statistics showed no significant heterogeneity (*P* > 0.05). Funnel plots exhibited a symmetrical distribution of effect points corresponding to causal associations, suggesting that the causal association is less likely to be impacted by potential bias. The leave–one–out sensitivity also confirmed the above conclusion (Additional files 7 and 8).

## Discussion

In this study, based on the summary statistics of gut microbiota from the largest meta-analysis of GWAS conducted by the MiBioGen consortium, a two-sample MR analysis was conducted to evaluate the causal links between gut microbiota and 22 gastrointestinal diseases. The analysis revealed 251 genetically predicted causal relationships, highlighting the role of specific gut microbiota in influencing susceptibility to various gastrointestinal diseases. Besides, this finding emphasizes the complex relationship between gut microbiota and gastrointestinal health, potentially offering novel perspectives for public health interventions to mitigate the prevalence of these gastrointestinal disease risk factors.

Numerous studies have found a possible connection between the gut microbiota selected in our study and gastrointestinal diseases. Our research results suggested that some members of genus *Ruminiclostridium* and genus *Ruminococcaceae* (phylum *Firmicutes*) may act as protective agents against Crohn’ disease, gastric ulcers and GERD, which is consistent with previous reports [33–36]. Notably, *Ruminococcaceae* is one of the most abundant genera in the phylum Firmicutes, and it is also the most abundant bacterial group in the human genetically modified microbiome, encompassing numerous bacteria that produce short chain fatty acids (SCFAs), especially butyrate. In light of the evidence, SCFAs, produced by *Ruminococcaceae*, are widely believed to play a variety of important roles in maintaining gastrointestinal homeostasis, such as acting as special nutritional and energy components of the intestinal epithelium and enhancing gastrointestinal motor function [37–39]. SCFAs can significantly inhibit the production of pro-inflammatory cytokines, chemokines, and calprotectin in the intestinal tract, while downregulating myeloperoxidase, reactive oxygen species, and neutrophil extracellular traps formation. Furthermore, SCFAs help maintain the integrity of the intestinal epithelial barrier function by enhancing mucin secretion and other pathways. This explains the mechanism by which Ruminococcaceae may contribute to maintaining the integrity of the epithelial mucosa in disease of digestive tract such as GERD and Crohn’ disease [40–42]. Moreover, our research results suggested a negative causal relationship between the abundance of the genus *Ruminococcaceae UCG010* and the risk of cholelithiasis. This relationship may be attributed to the butyrate produced by *Ruminococcaceae*, which is known to enhance bile salt hydrolase (BSH) activity. Enhanced BSH activity promotes the excretion of bile acids through feces. To replenish the bile acids lost, hepatocytes synthesize new bile acids from blood cholesterol, subsequently lowering blood cholesterol levels and potentially reducing the incidence of cholelithiasis [43, 44]. Beyond their role in producing SCFAs, *Ruminococcaceae* are among the limited groups within the intestinal microbiota capable of generating secondary bile acids (SBAs). These specific bacterial species can transform primary bile acids (PBAs) into SBAs when bile acids reach the colon [45]. The SBAs produced by The SBAs produced by *Ruminococcaceae* are believed to play a pivotal role in mitigating intestinal inflammation by modulating bile acid homeostasis. There is increasing evidence that SBAs can interact with the intestinal farnesoid X receptor (FXR) in various intestinal immune cells, including dendritic cells (DCs), natural killer cells (NKCs), and macrophages. This interaction leads to the suppression of pro-inflammatory cytokines such as IL-1 and TNF-α, thereby alleviating inflammatory responses in the intestinal mucosa [46, 47]. In addition to FXR, SBAs can activate Takeda G protein-coupled receptor 5 (TGR5) to promote the polarization of NKT cells towards NKT10 cells, which secrete the anti-inflammatory cytokine IL-10. Additionally, numerous studies have demonstrated that SBAs can regulate the proliferation and differentiation of intestinal stem cells and the self-renewal of intestinal epithelial cells, they can maintain homeostasis of the mechanical barrier of the intestinal mucosa by stimulating the TGR5. This provides additional insight into how *Ruminococcaceae* protects against IBD by influencing the pathway of SBAs [48]. However, it is worth noting that though studies have shown that Rumen microbiomes are mostly beneficial, different strains may have different impacts on human health Notably, our study is currently the first report to revealed that the genus *Ruminococcus gauvreauii group* may be a risk factor for gastric and duodenal ulcers. Xu et al. highlighted a positive correlation between the *Ruminococcus gauvreauii group* and systemic immune responses mediated by pro-inflammatory cytokines such as TNF-α and IL-6. This implies that the *Ruminococcus gauvreauii group* has the potential to initiate an inflammatory signaling cascade [49, 50]. However, the exact mechanism is not yet clear. Therefore, more epidemiological and basic research is needed in the future to expound the associations and mechanisms among them.

The outcomes of our investigation also identified that the genus *Eubacterium hallii group* (*E. hallii*) may reduce the risk of IBD and these findings seem to be consistent with results of a double-blind trial, which reported a significantly lower abundance of *E. hallii* in the inflamed areas of the intestine in IBD patients compared to healthy individuals [51]. A large cohort study also suggested a negative correlation between the *E. hallii* and inflammatory markers such as IL-2 and C-reactive protein in IBD and *E. hallii* negatively correlated with the intestinal inflammatory response [52]. These lend further support to the evidence of a negative correlation between *E. hallii* and intestinal inflammatory response. Besides producing butyrate, *E. hallii* also contributes to intestinal mucosal integrity by producing propionate, which activates the NLRP3 inflammasome in intestinal epithelial cells and enhances colonic regulatory T cell expansion, thereby mitigating inflammatory symptoms in IBD [53]. Moreover, propionate boosts the expansion of colonic regulatory T cells by modulating cell surface G-protein coupled receptors [54]. These contribute to alleviating inflammatory manifestations in the intestinal epithelium in IBD. Our results also indicated a negative causal relationship between *E. hallii* and the risk of NAFLD, which is consistent with previous research findings. In vivo experiments show that propionate, produced by *E. hallii*, reduces triglyceride accumulation by modulating the expression of genes related to lipogenesis, fatty acid uptake, and oxidation [55–57]. This mechanism contributes to the amelioration of hepatic steatosis, alleviating the progression of non-alcoholic fatty liver disease (NAFLD).

Recognizing its potential, Caelus Health is currently collaborating with the Danish firm Korhansson in developing oral formulations incorporating *E. hallii* as biotherapeutic agents. The objective is to mitigate insulin resistance and prevent the onset of Type 2 Diabetes in individuals diagnosed with metabolic syndrome (ClinicalTrials.gov, 2020), underscoring the significant value of *E. hallii* in the realm of biotherapeutic development [58]. *E. hallii* holds substantial potential for future applications. In the forthcoming phases of research and development, harnessing *E. hallii* as a key component in the creation of biotherapeutics appears particularly protecting the gastrointestinal mucosa, alleviating gastrointestinal inflammation, and mitigating hepatic steatosis. The intrinsic properties of *E. hallii* that contribute to metabolic health improvement could extend to beneficial effects on the gastrointestinal system.

Furthermore, the MR results from our study further indicated that gut microbiota producing SCFAs, such as genus *Butyricicoccus*, genus *Christensenellaceae R 7group*, and genus *Lachnoclostridium*, play a protective role in diseases like chronic gastritis, liver cancer, and Crohn’s disease. This further emphasizes the significant role of gut microbiota producing SCFAs in maintaining the integrity of the gastrointestinal mucosa. These findings not only provide new insights into understanding the impact of gut microbiota on various diseases but also deepen our understanding of the crucial role of short-chain fatty acids in regulating the immune system and participating in anti-inflammatory processes. Further research holds the promise of revealing the specific mechanisms through which these microorganisms contribute to the protection observed in chronic gastritis, liver cancer, and Crohn’s disease.

Currently, research suggests that administering metabolites derived from the gut microbiota formulations, such as SCFA, via enemas or oral intake, help preserve the integrity of the intestinal epithelium and the mucosal barrier [59]. However, it is important to acknowledge that supplementing short-chain fatty acids directly faces challenges in achieving and maintaining adequate concentrations within the gastrointestinal tract. Furthermore, the diffusion of these acids on the intestinal surface can be hindered [60]. In light of these challenges, utilizing specific members from the gut microbiota as a biological formulation offers distinct advantages. Firstly, these members of beneficial bacteria like *Ruminococcaceae* naturally inhabit the gastrointestinal tract, demonstrating inherent adaptability to the gut environment, which facilitates their establishment and maintenance [51]. Secondly, the metabolites produced by beneficial gut microbiota during their fermentation processes exhibit a more stable release profile, ensuring a sustained concentration that aligns with physiological requirements [45].

However, it is worth noting that a number of limitations need to be noted regarding the present study. Firstly, though using exclusively European ancestry helps mitigate potential confounding effects related to population stratification, it may limit the generalizability of results to broader populations. Additionally, a relatively small sample size may limit the extent to which further conclusions may be drawn. Future investigations using larger-scale GWAS are necessary to enhance the robustness and applicability of these findings. Secondly, the GWAS database we used is publicly available, so detailed information on participants cannot be obtained for further subgroup analysis. Thirdly, the analysis of gut microbiota in this study was confined to broader taxonomic levels, namely phylum, class, order, family, and genus. Given the nascent stage of gut microbiota research, the limited number of SNPs available for certain microbiota could potentially introduce biases in the results. Lastly, despite employing various methodologies to assess horizontal pleiotropy, and achieving consistent results across multiple analytical approaches, it cannot be guaranteed that potential horizontal pleiotropy has been entirely eliminated.

In summary, we performed two sample MR analysis to evaluate the potential relationship between the gut microbiota and 22 gastrointestinal diseases for the first time. Our findings suggest a potential causal relationship between specific gut microbiota and gastrointestinal diseases. These novel findings offer promising directions for the development of preventive and therapeutic strategies for gastrointestinal disorders. In the future, more comprehensive epidemiological and foundational research is needed to unravel the intricate mechanisms by which gut microbiota may influence the onset and progression of gastrointestinal diseases.

### Supplementary information

**Additional file 1:** Information on GWAS samples of 22 gastrointestinal diseases used in this study.

**Additional file 2:** Detailed information on instrumental variables in the gut microbiota dataset.

**Additional file 3: **Detailed Information and F-statistics of Instrumental Variables Closely Associated with Gut Microbiota.

**Additional file 4:** The potential associations between gut microbiota and the risk of 22 gastrointestinal diseases.

**Additional file 5: **Genetic risk score (GRS) analysis for the associations of gut microbiota with gastrointestinal diseases.

**Additional file 6: **Genetic risk score GRS_gut microbiota_ for gastrointestinal diseasess.

**Additional file 7:** Heterogeneity, level pleiotropy test, and the power of Mendelian randomization analysis.

**Additional file 8: **The scatter plot effect size, leave-one-out analyses, and funnel plot for gut microbiota on gastrointestinal diseases in MR analysis.

## Data Availability

The datasets supporting the conclusions of this article are available in IEU open gwas project repository (https://gwas.mrcieu.ac.uk/).
