# Supplementary material for: Causal relationship between gut microbiota and gastrointestinal diseases: a mendelian randomization study
Source: J Transl Med. 2024 Jan 23;22:92. doi: 10.1186/s12967-024-04894-5 (PMC10804519; doi:10.1186/s12967-024-04894-5)
Supplement: Supplementary file 8 — Additional file 8: The scatter plot effect size, leave-one-out analyses, and funnel plot for gut microbiota on gastrointestinal diseases in MR analysis. [file 12967_2024_4894_MOESM8_ESM.pdf]

Figure 1 Leave-one-out analysis, funnel plot, MR effect size and scatter plot for gut microbiota abundance (genus Alistipes id.968) on gastroesophageal reflux disease

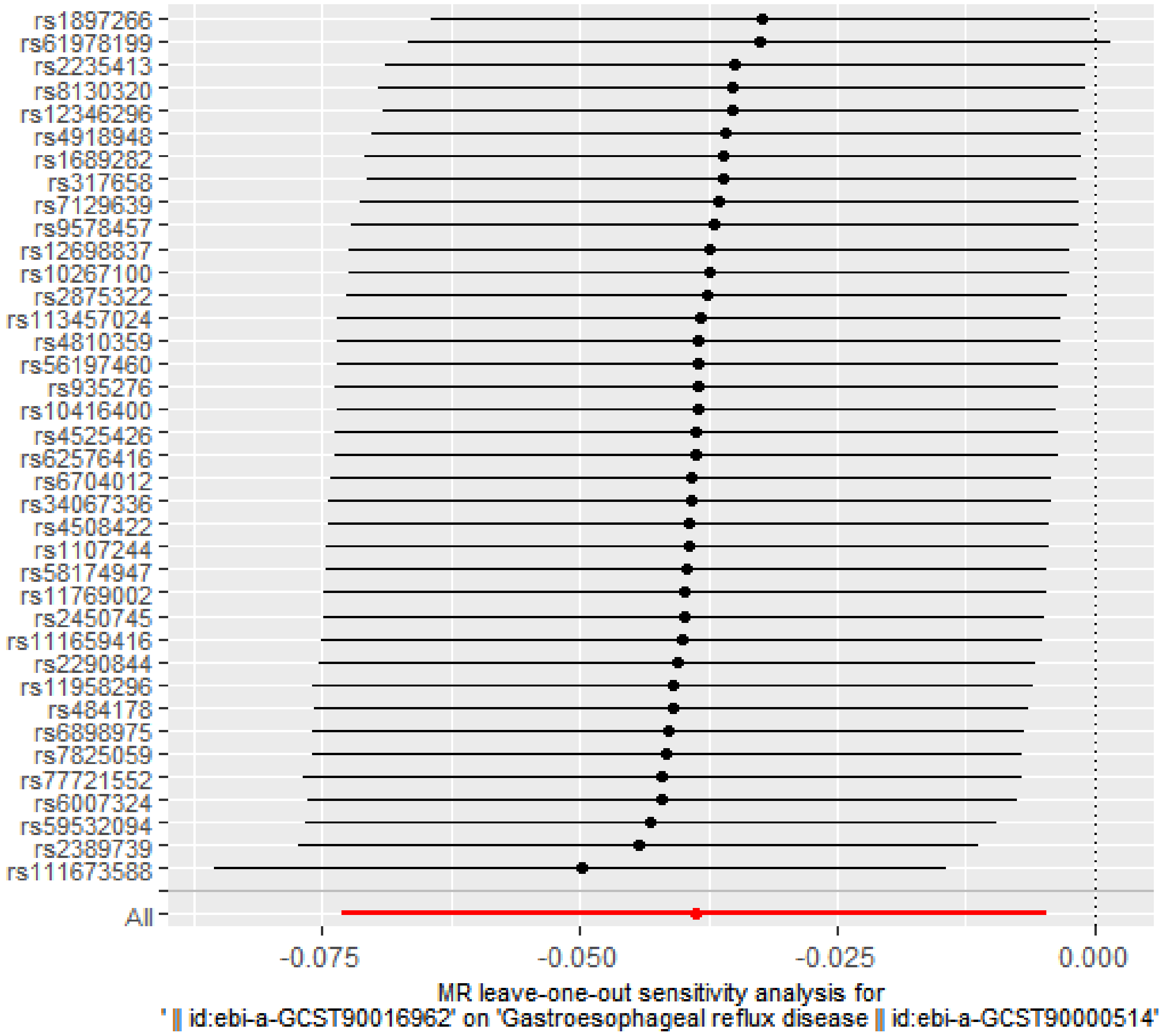

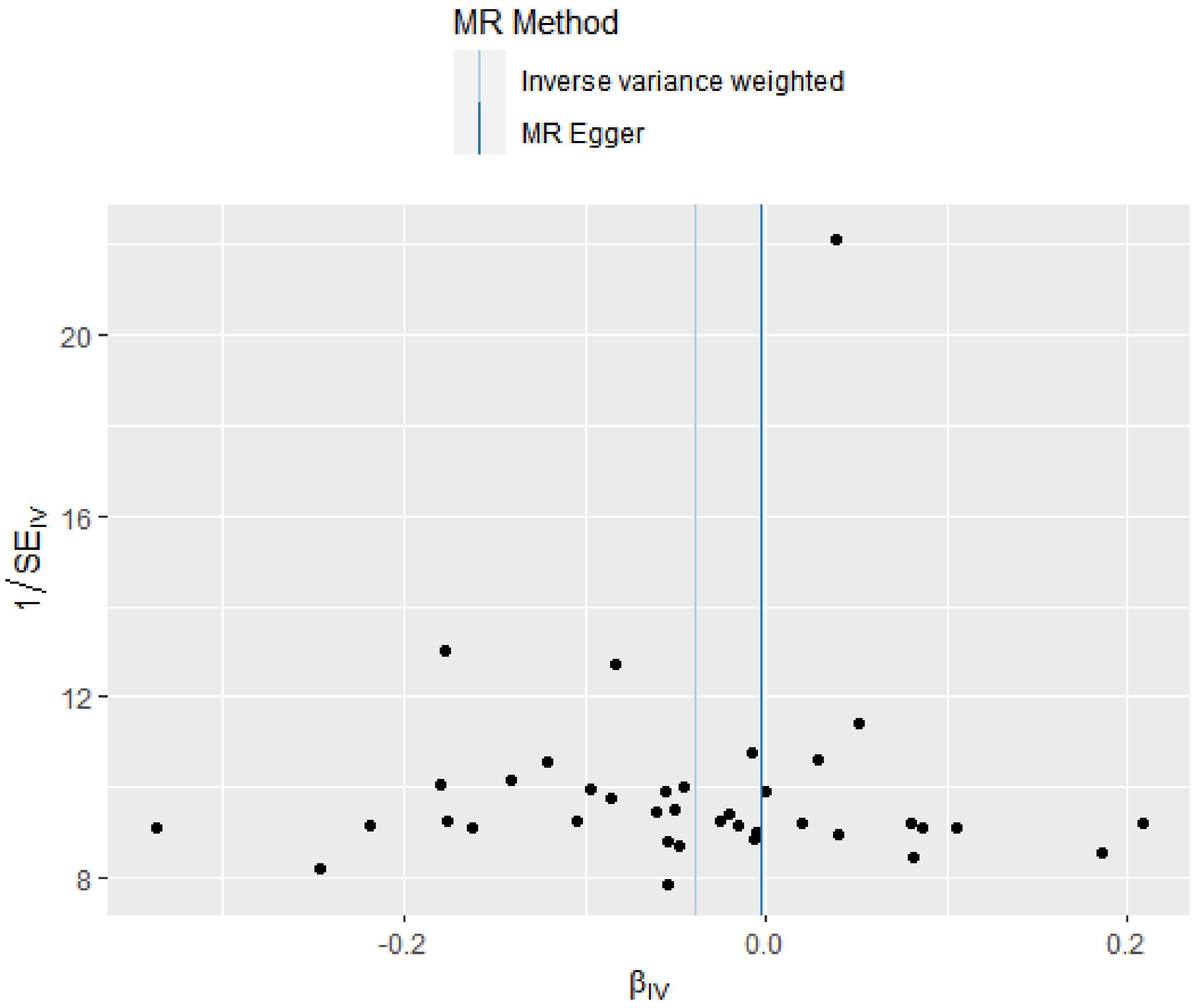
MR Method
Inverse variance weighted
MR Egger
20
16
12
8
-0.2
0.0
0.2
$1/SE_{IV}$
β_{IV}

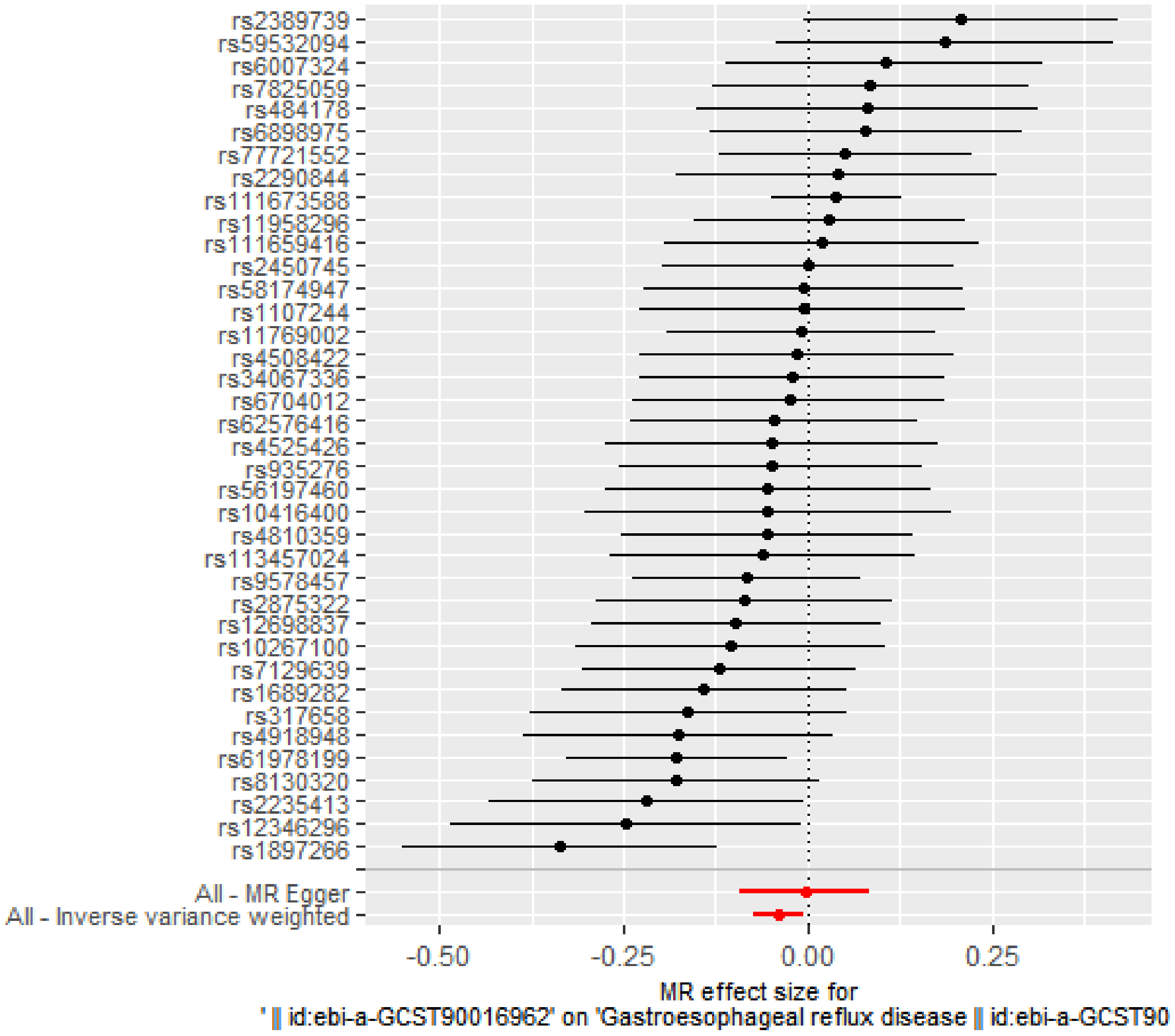

rs2389739
rs59532094
rs6007324
rs7825059
rs484178
rs6898975
rs77721552
rs2290844
rs111673588
rs11958296
rs111659416
rs2450745
rs58174947
rs1107244
rs11769002
rs4508422
rs34067336
rs6704012
rs62576416
rs4525426
rs935276
rs56197460
rs10416400
rs4810359
rs113457024
rs9578457
rs2875322
rs12698837
rs10267100
rs7129639
rs1689282
rs317658
rs4918948
rs61978199
rs8130320
rs2235413
rs12346296
rs1897266
All - MR Egger
All - Inverse variance weighted
-0.50
-0.25
0.00
0.25
MR effect size for
' || id:ebi-a-GCST90016962' on 'Gastroesophageal reflux disease || id:ebi-a-GCST90

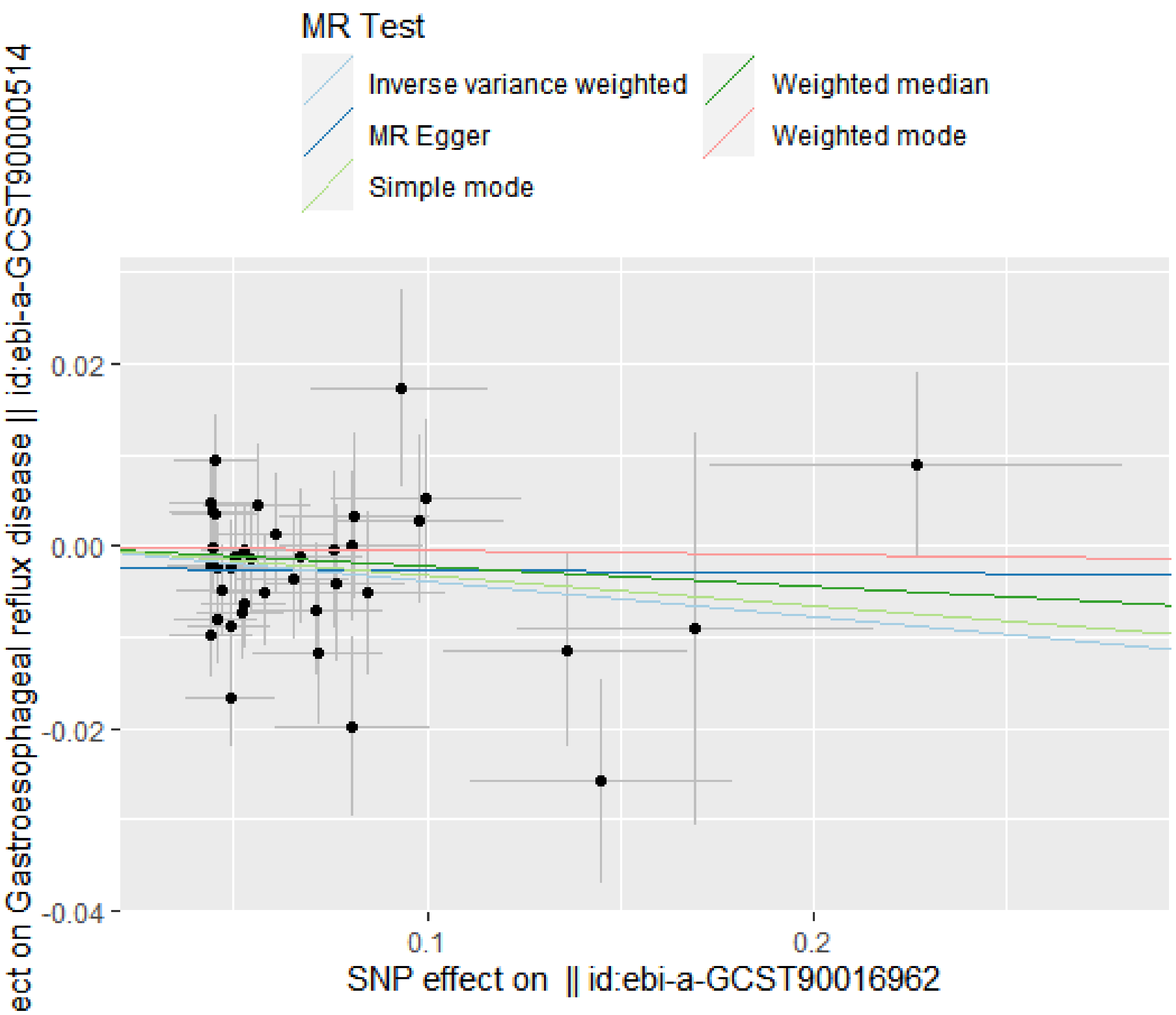

MR Test
Inverse variance weighted
MR Egger
Simple mode
Weighted median
Weighted mode
0.02
0.00
-0.02
-0.04
0.1
0.2
SNP effect on || id:ebi-a-GCST90016962
ect on Gastroesophageal reflux disease || id:ebi-a-GCST90000514

Figure 2 Leave-one-out analysis, funnel plot, MR effect size and scatter plot for gut microbiota abundance (genus Christensenellaceae R 7group id.11283) on gastroesophageal reflux disease

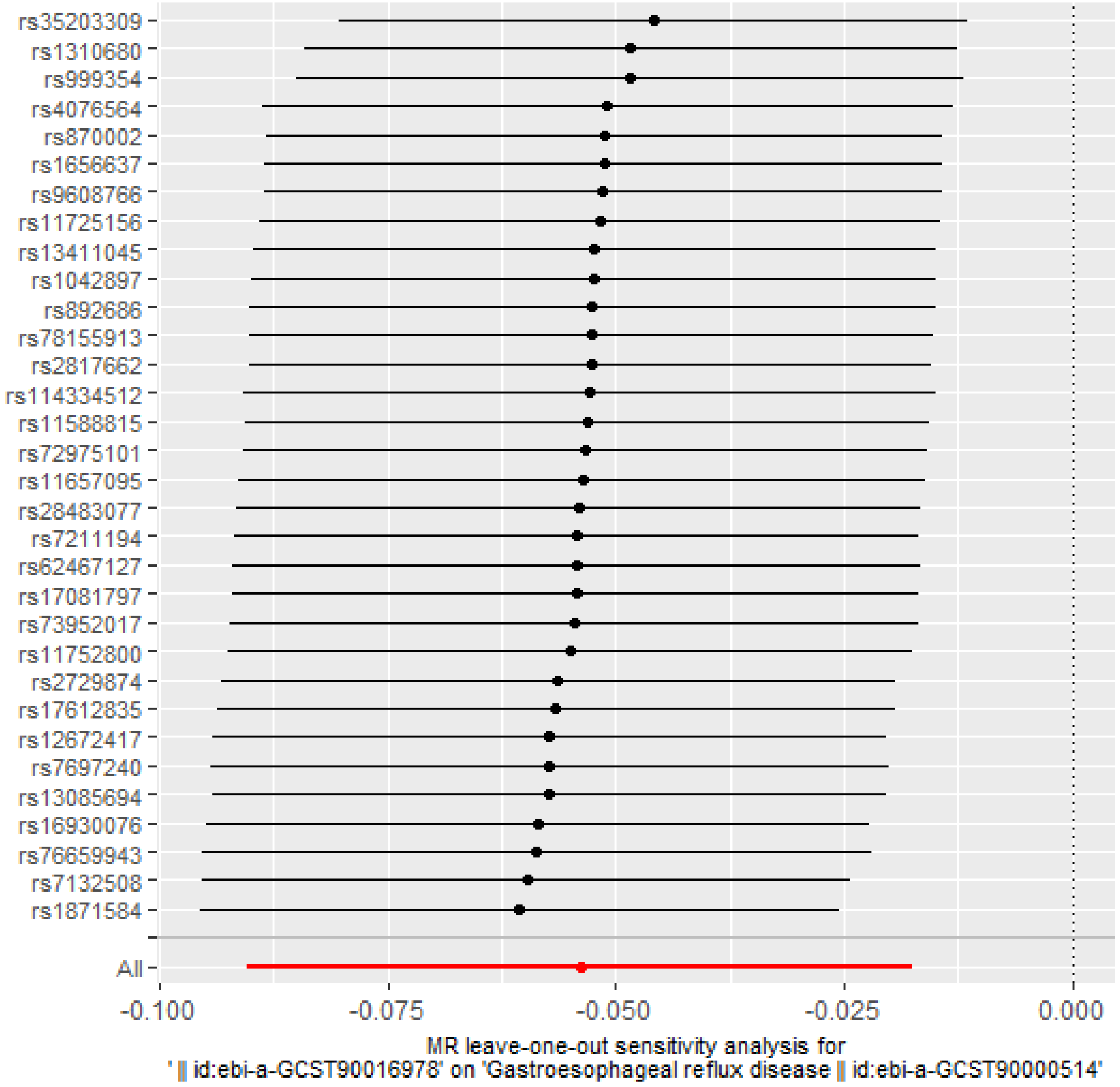

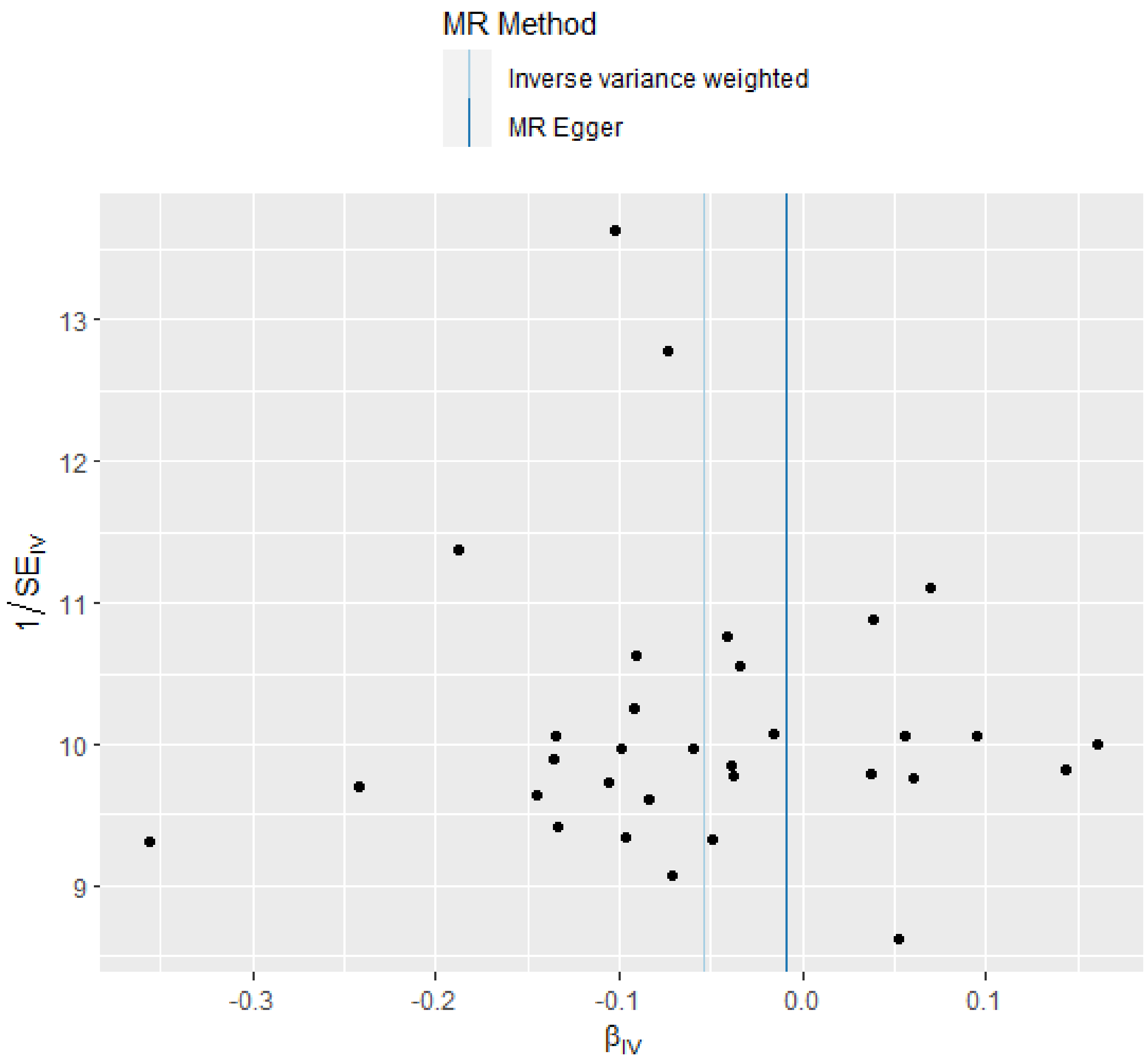

MR Method
Inverse variance weighted
MR Egger
13
12
11
10
9
$1/SE_{IV}$
-0.3
-0.2
-0.1
0.0
0.1
β_{IV}

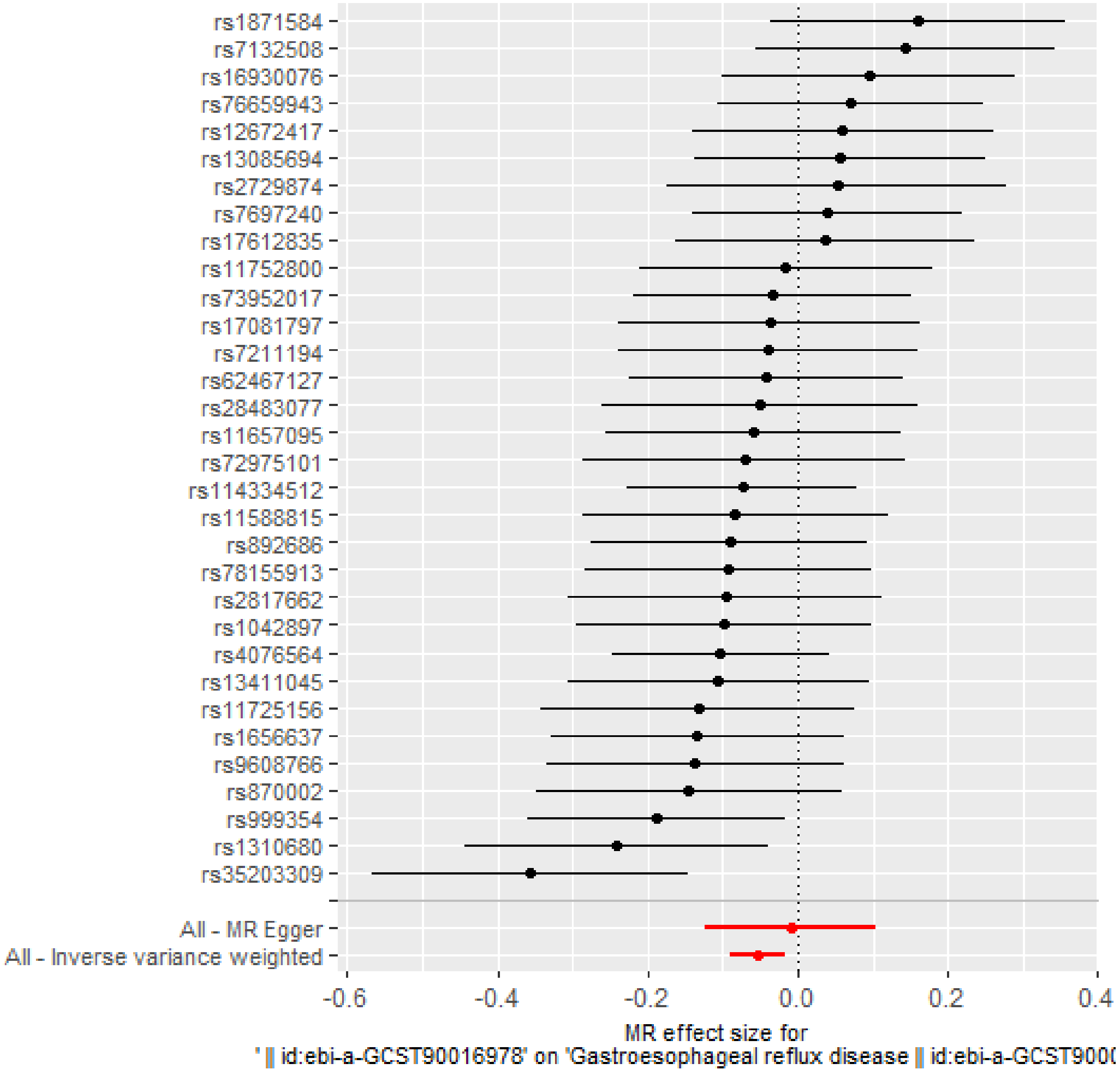

rs1871584
rs7132508
rs16930076
rs76659943
rs12672417
rs13085694
rs2729874
rs7697240
rs17612835
rs11752800
rs73952017
rs17081797
rs7211194
rs62467127
rs28483077
rs11657095
rs72975101
rs114334512
rs11588815
rs892686
rs78155913
rs2817662
rs1042897
rs4076564
rs13411045
rs11725156
rs1656637
rs9608766
rs870002
rs999354
rs1310680
rs35203309
All - MR Egger
All - Inverse variance weighted
-0.6
-0.4
-0.2
0.0
0.2
0.4
MR effect size for
' || id:ebi-a-GCST90016978' on 'Gastroesophageal reflux disease || id:ebi-a-GCST900(

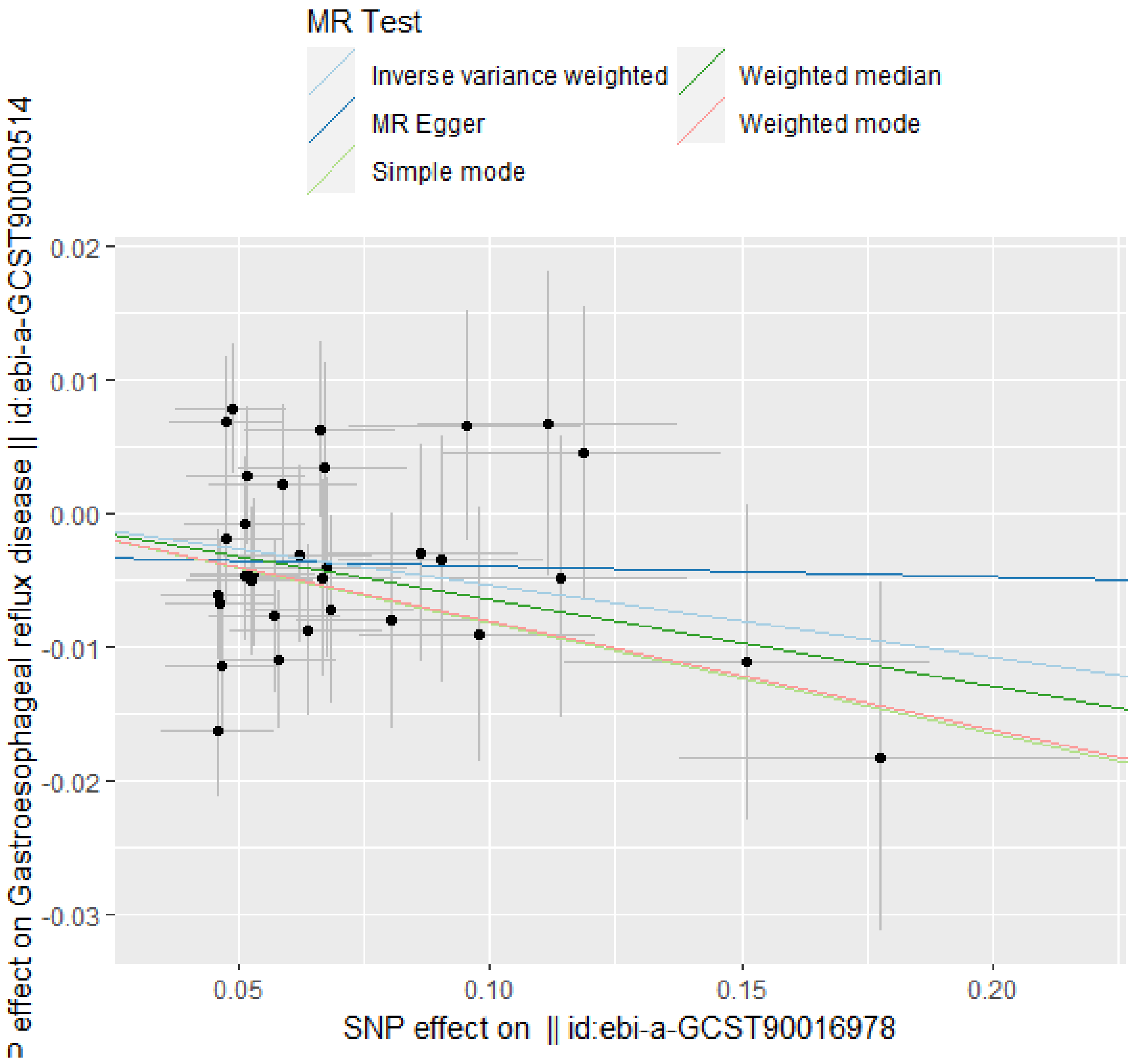
MR Test
Inverse variance weighted
MR Egger
Simple mode
Weighted median
Weighted mode
0.02
0.01
0.00
-0.01
-0.02
-0.03
0.05
0.10
0.15
0.20
SNP effect on || id:ebi-a-GCST90016978
effect on Gastroesophageal reflux disease || id:ebi-a-GCST90000514

Figure 3 Leave-one-out analysis, funnel plot, MR effect size and scatter plot for gut microbiota abundance (genus Coprococcus2 id.11302) on gastroesophageal reflux disease

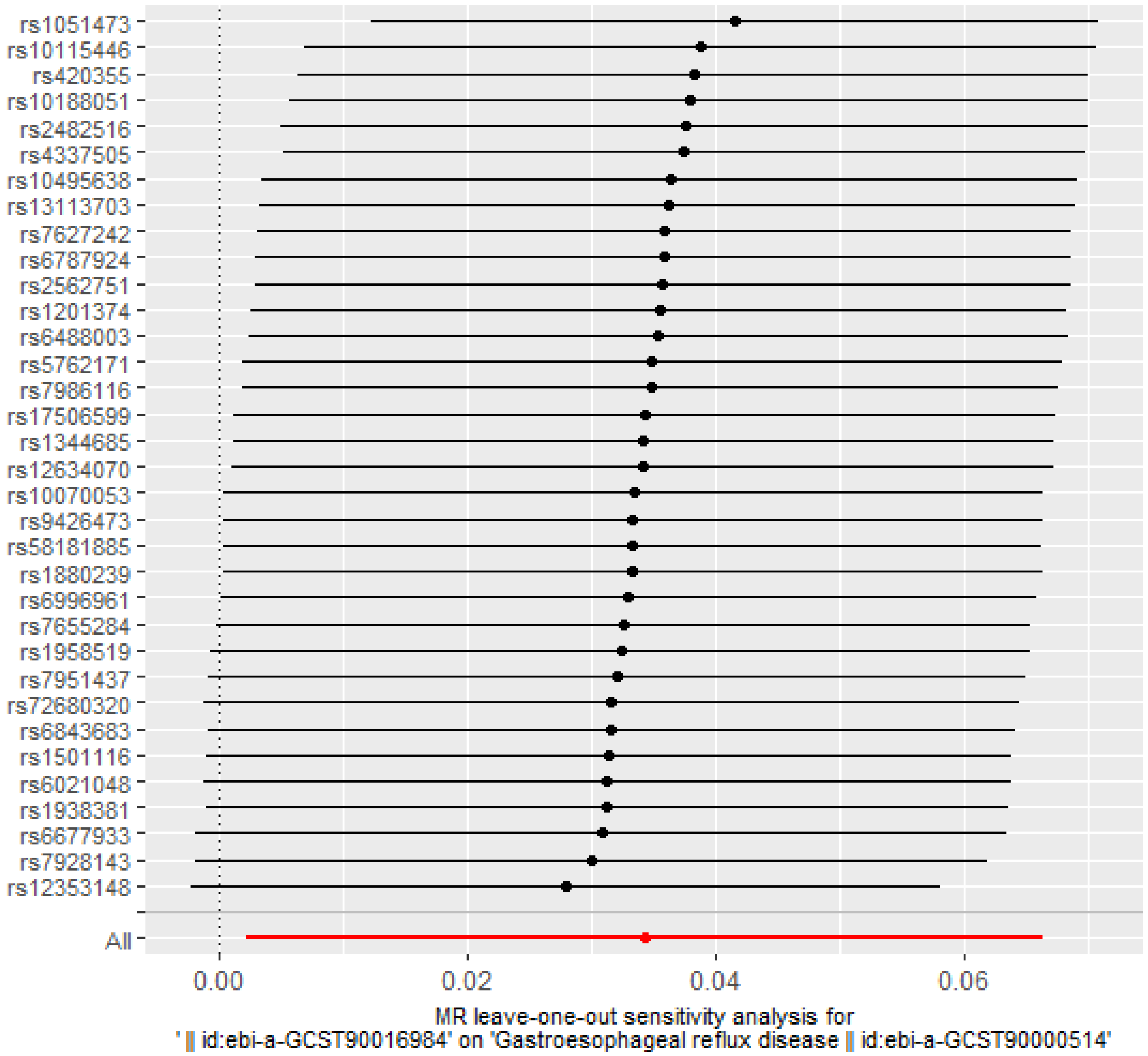

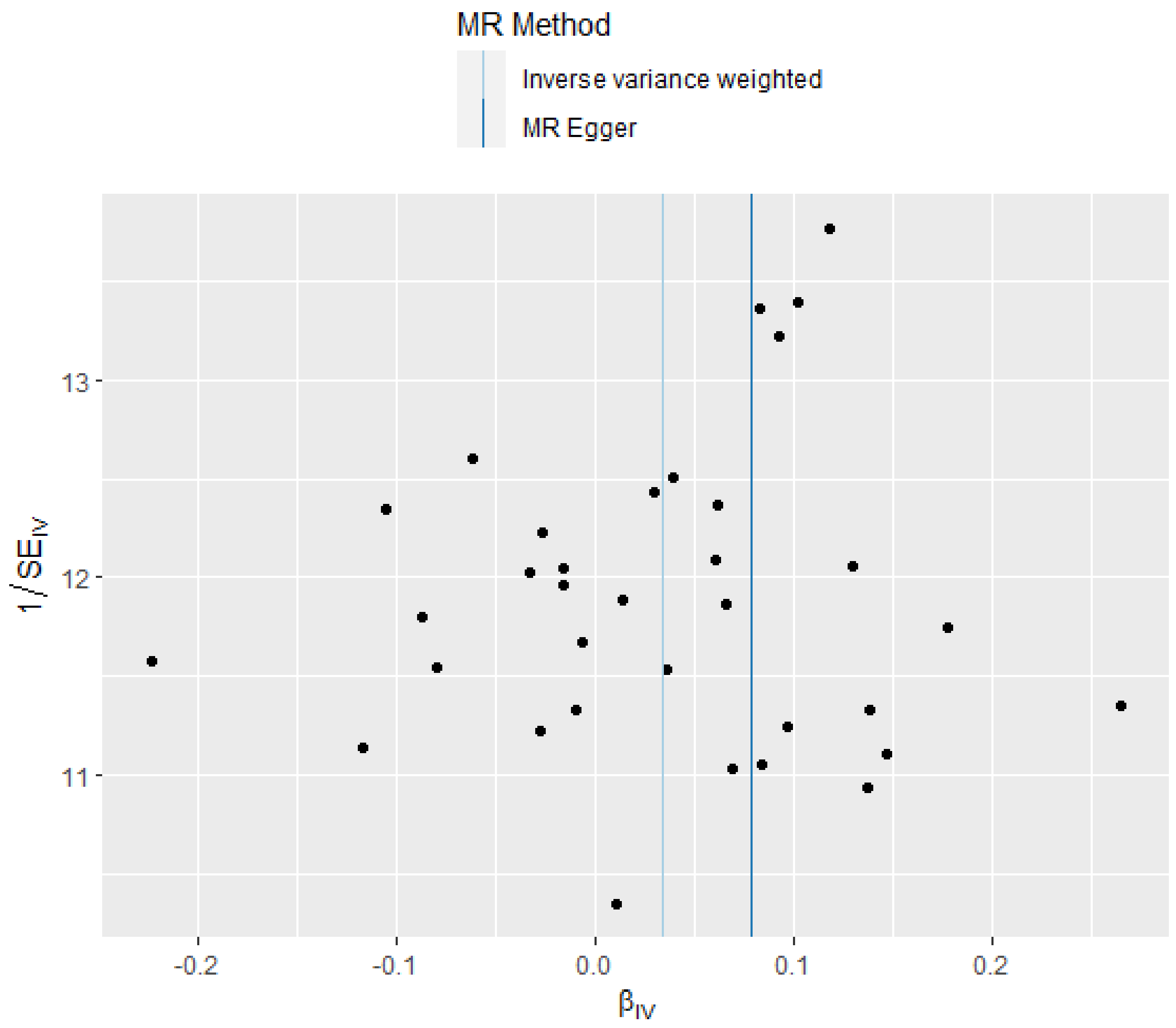

MR Method
Inverse variance weighted
MR Egger
13
12
11
1/SE_IV
-0.2
-0.1
0.0
0.1
0.2
β_IV

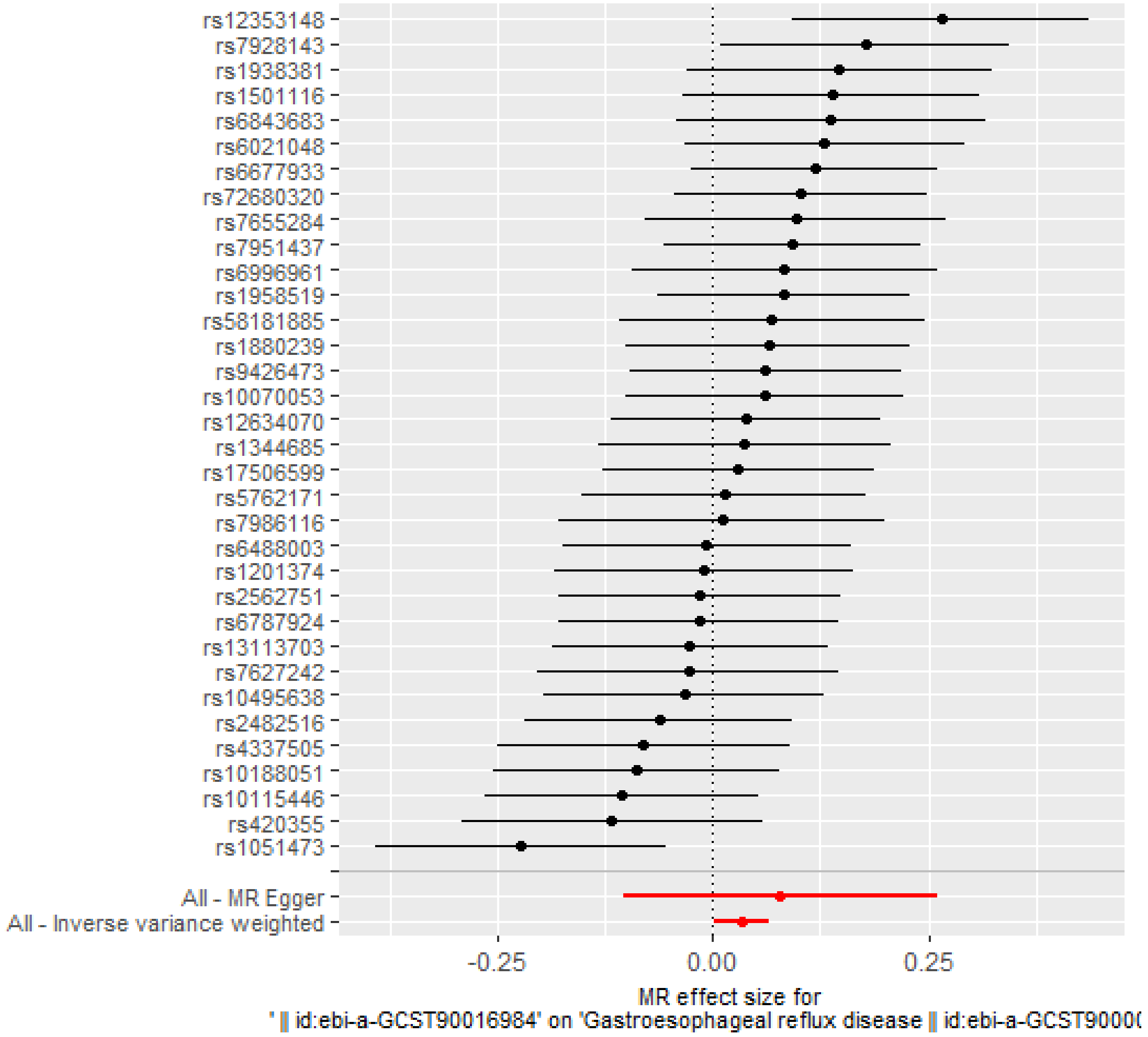

rs12353148
rs7928143
rs1938381
rs1501116
rs6843683
rs6021048
rs6677933
rs72680320
rs7655284
rs7951437
rs6996961
rs1958519
rs58181885
rs1880239
rs9426473
rs10070053
rs12634070
rs1344685
rs17506599
rs5762171
rs7986116
rs6488003
rs1201374
rs2562751
rs6787924
rs13113703
rs7627242
rs10495638
rs2482516
rs4337505
rs10188051
rs10115446
rs420355
rs1051473
All - MR Egger
All - Inverse variance weighted
-0.25
0.00
0.25
MR effect size for
' || id:ebi-a-GCST90016984' on 'Gastroesophageal reflux disease || id:ebi-a-GCST9000(

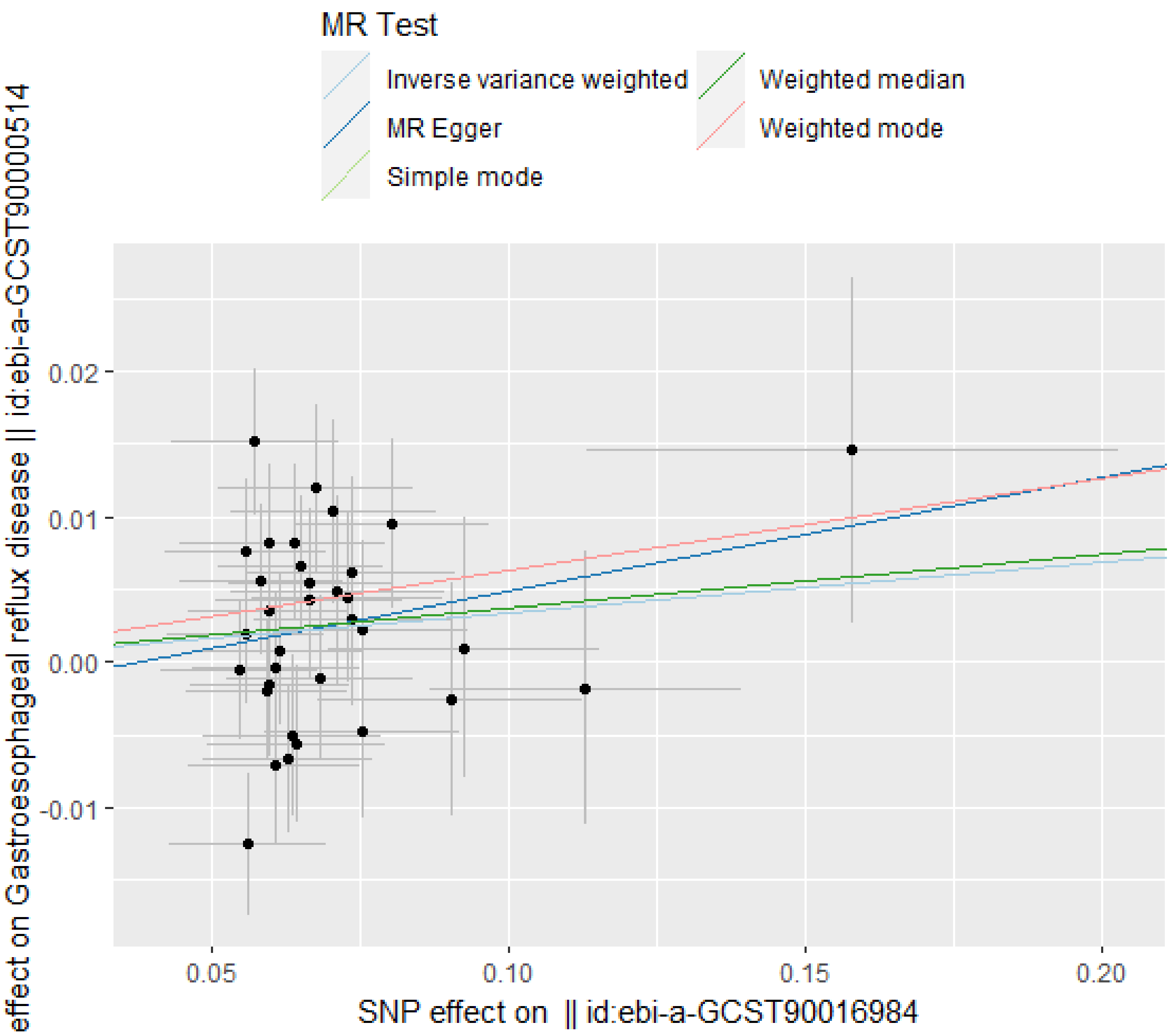

MR Test
Inverse variance weighted
MR Egger
Simple mode
Weighted median
Weighted mode
0.02
0.01
0.00
-0.01
0.05
0.10
0.15
0.20
SNP effect on || id:ebi-a-GCST90016984
effect on Gastroesophageal reflux disease || id:ebi-a-GCST90000514

Figure 4 Leave-one-out analysis, funnel plot, MR effect size and scatter plot for gut microbiota abundance (genus Faecalibacterium id.2057) on gastroesophageal reflux disease

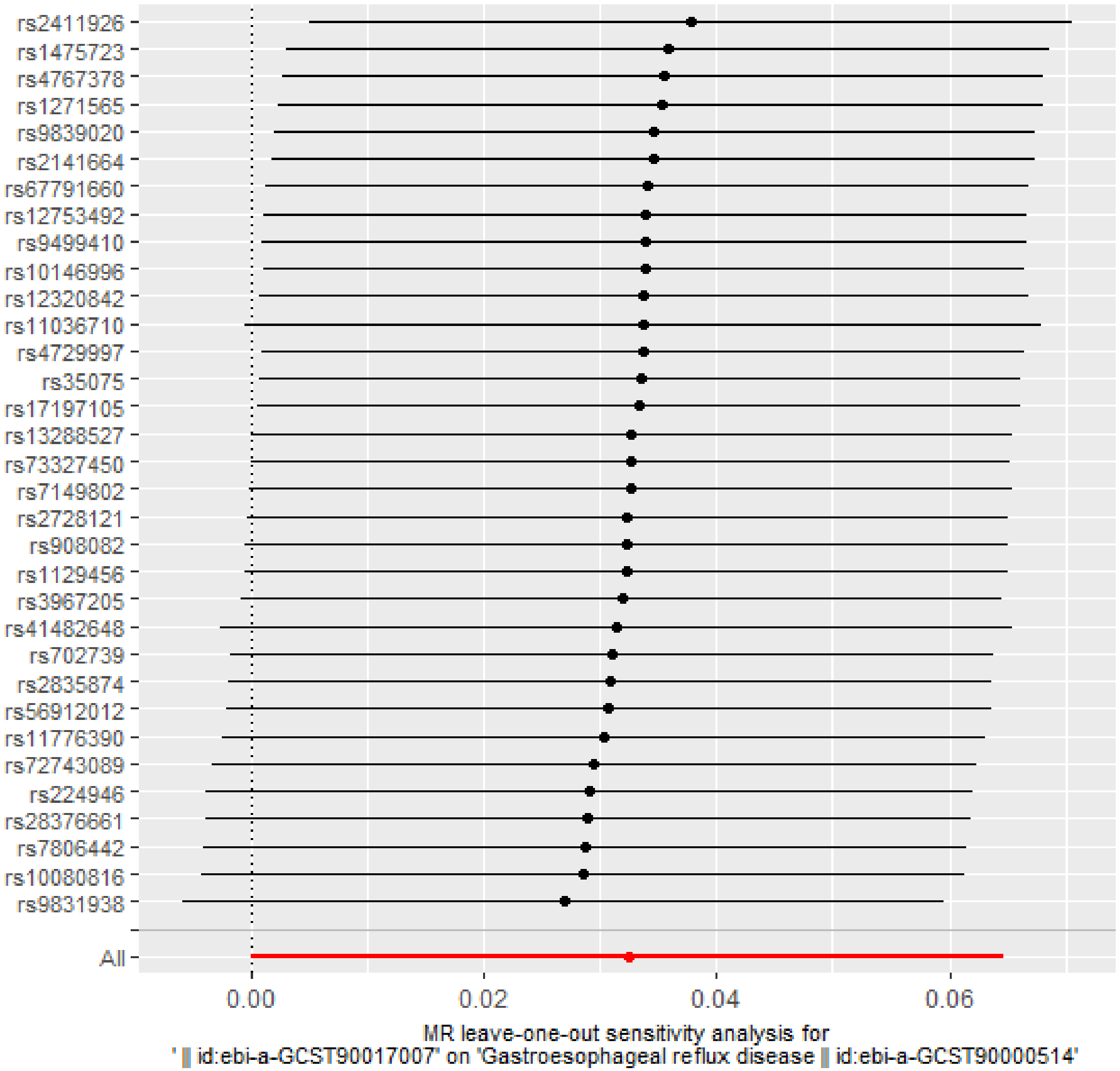

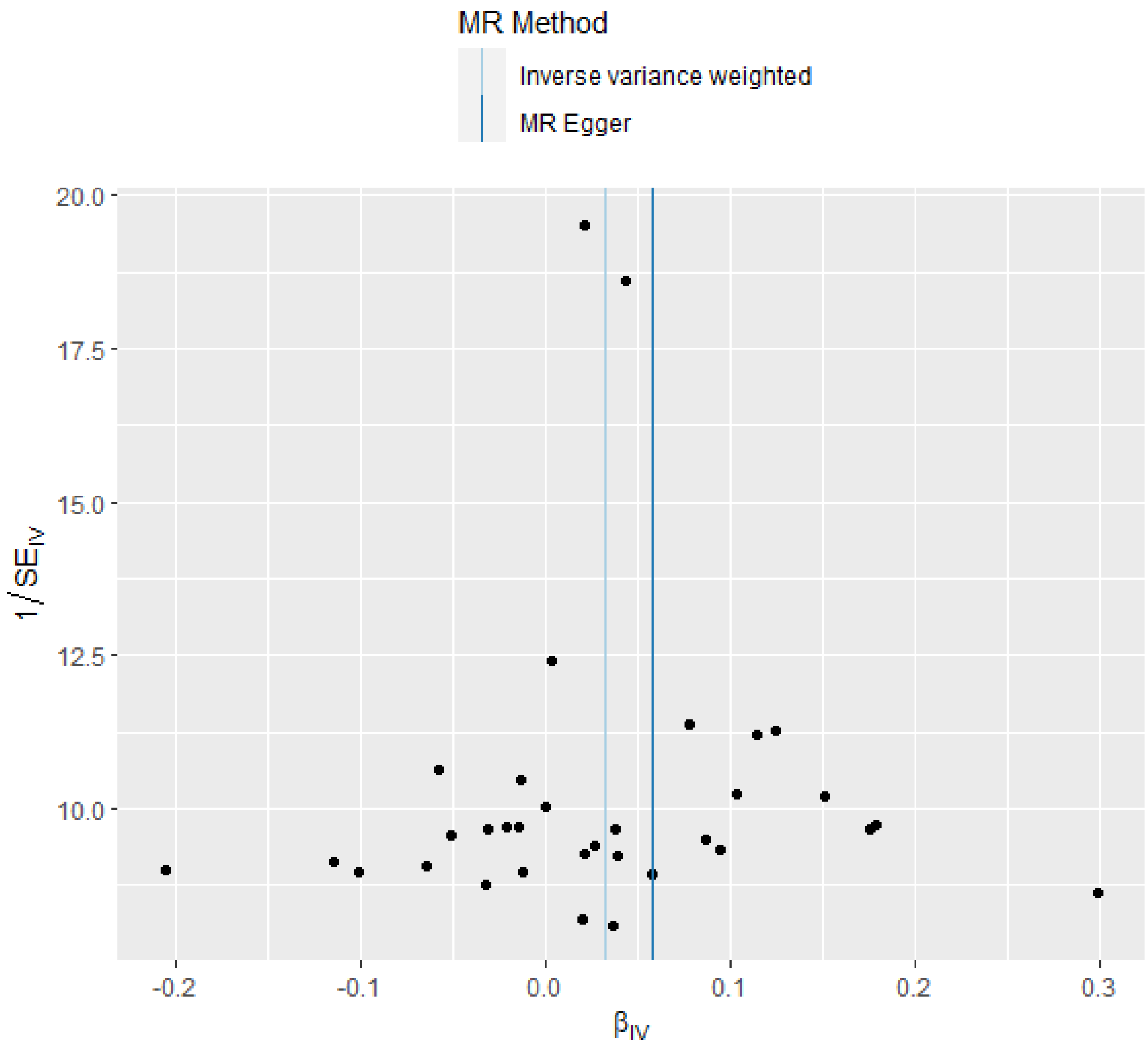

MR Method
Inverse variance weighted
MR Egger
20.0
17.5
15.0
12.5
10.0
1/SE_IV
-0.2
-0.1
0.0
0.1
0.2
0.3
β_IV

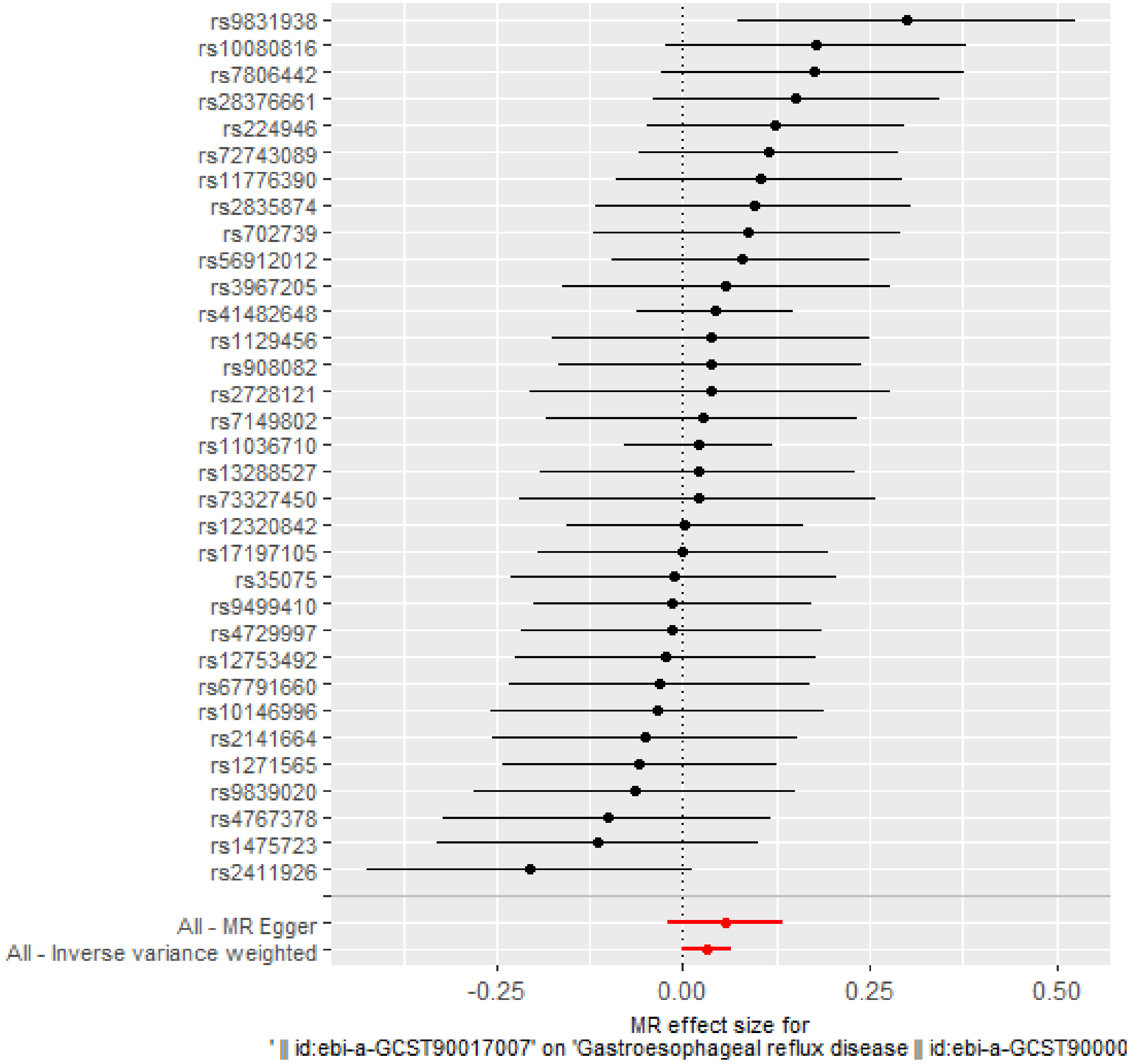

rs9831938
rs10080816
rs7806442
rs28376661
rs224946
rs72743089
rs11776390
rs2835874
rs702739
rs56912012
rs3967205
rs41482648
rs1129456
rs908082
rs2728121
rs7149802
rs11036710
rs13288527
rs73327450
rs12320842
rs17197105
rs35075
rs9499410
rs4729997
rs12753492
rs67791660
rs10146996
rs2141664
rs1271565
rs9839020
rs4767378
rs1475723
rs2411926
All - MR Egger
All - Inverse variance weighted
-0.25
0.00
0.25
0.50
MR effect size for
' || id:ebi-a-GCST90017007' on 'Gastroesophageal reflux disease || id:ebi-a-GCST90000

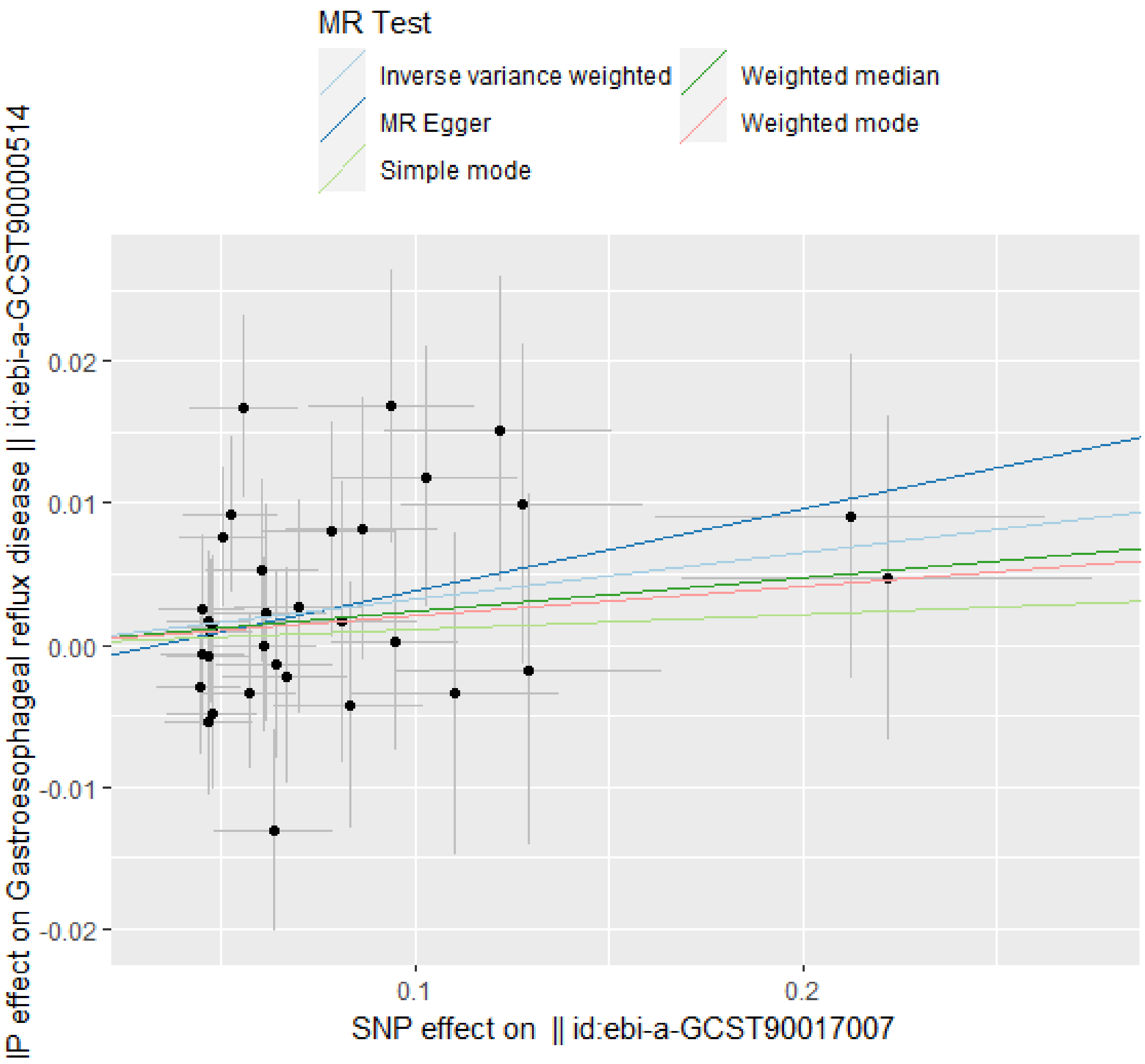
MR Test
Inverse variance weighted
MR Egger
Simple mode
Weighted median
Weighted mode
0.02
0.01
0.00
-0.01
-0.02
0.1
0.2
SNP effect on || id:ebi-a-GCST90017007
IP effect on Gastroesophageal reflux disease || id:ebi-a-GCST90000514

Figure 5 Leave-one-out analysis, funnel plot, MR effect size and scatter plot for gut microbiota abundance (genus Oscillospira id.2064) on gastroesophageal reflux disease

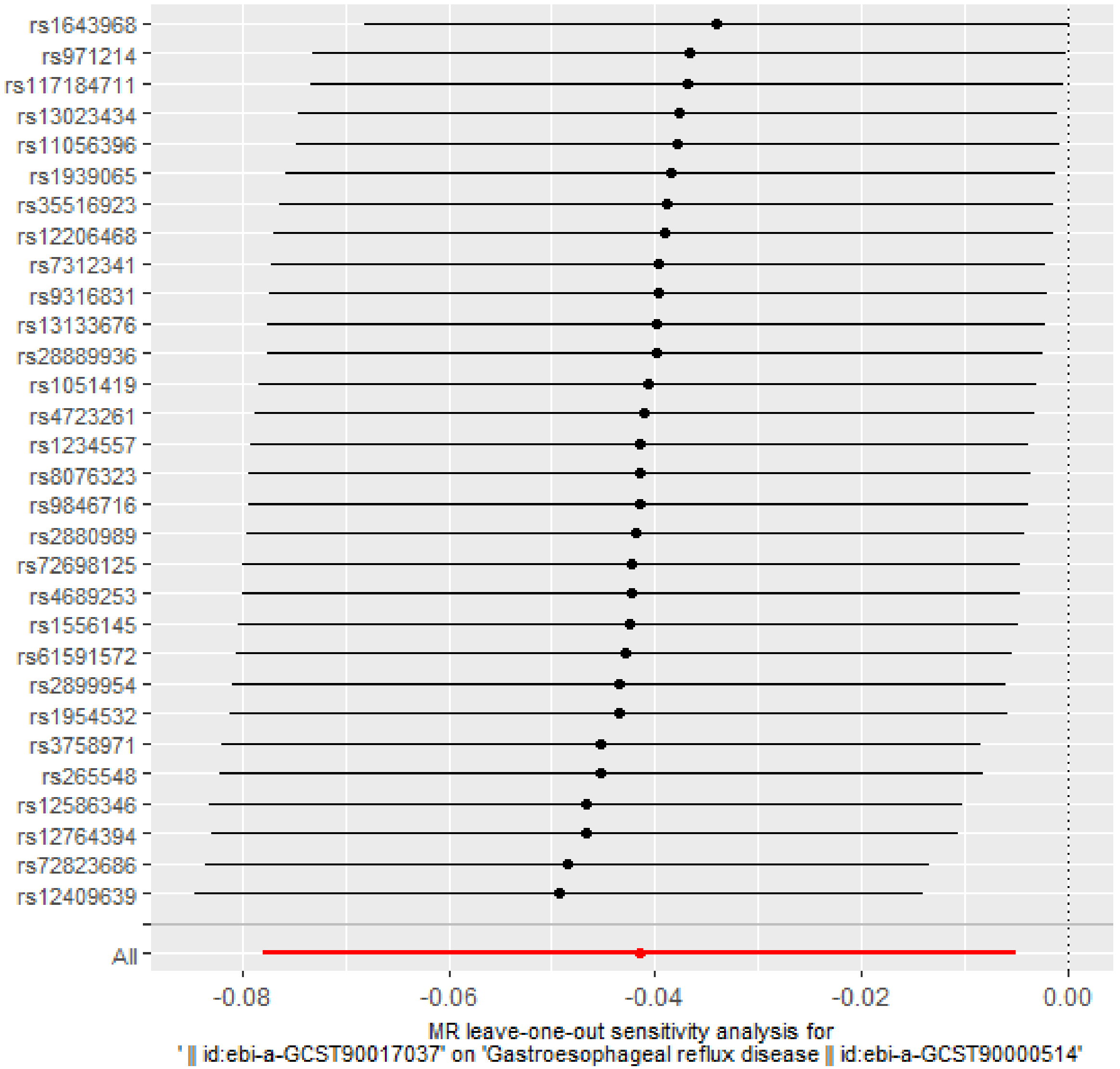

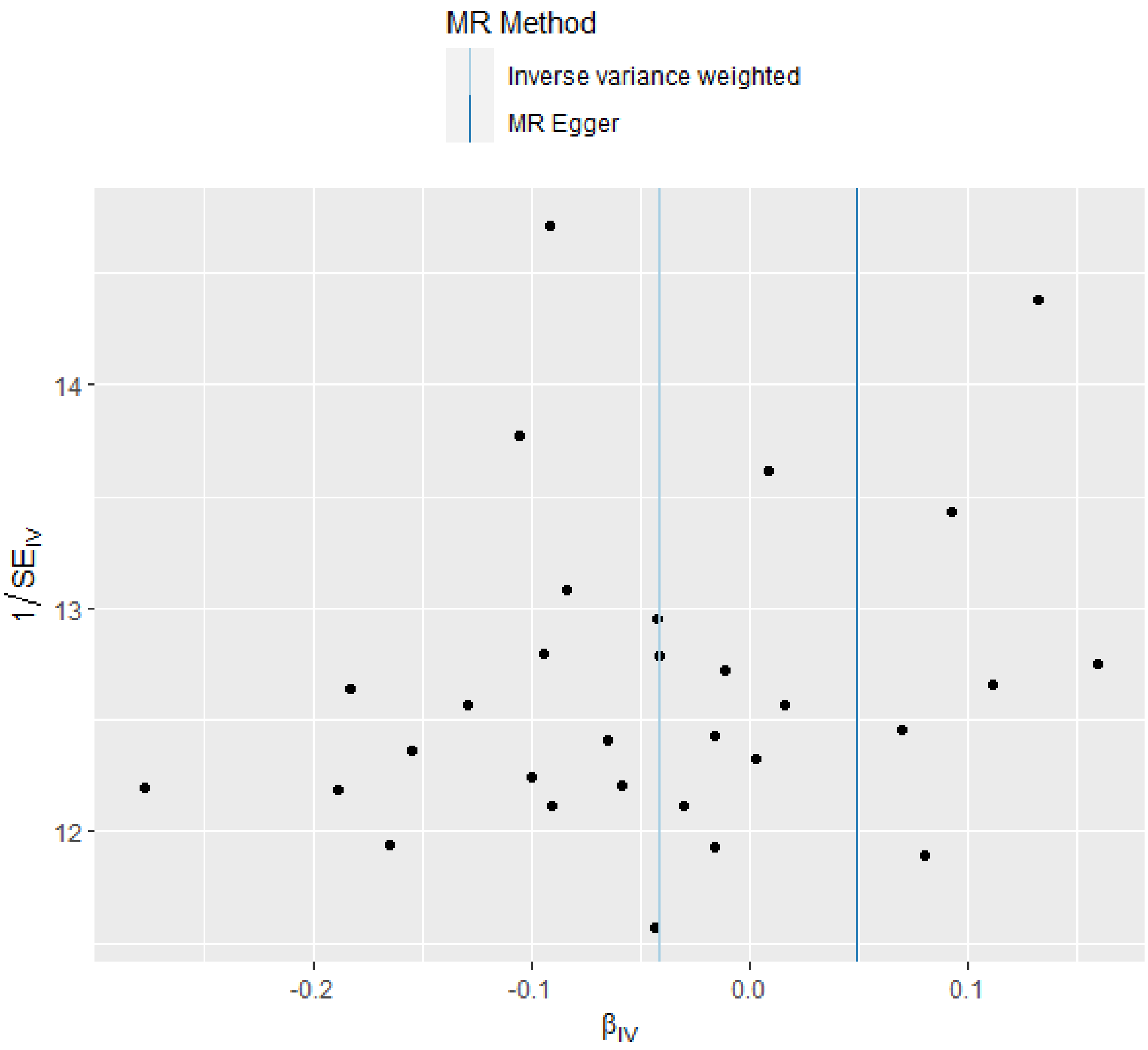

MR Method
Inverse variance weighted
MR Egger
14
13
12
-0.2
-0.1
0.0
0.1
$1/SE_{IV}$
β_{IV}

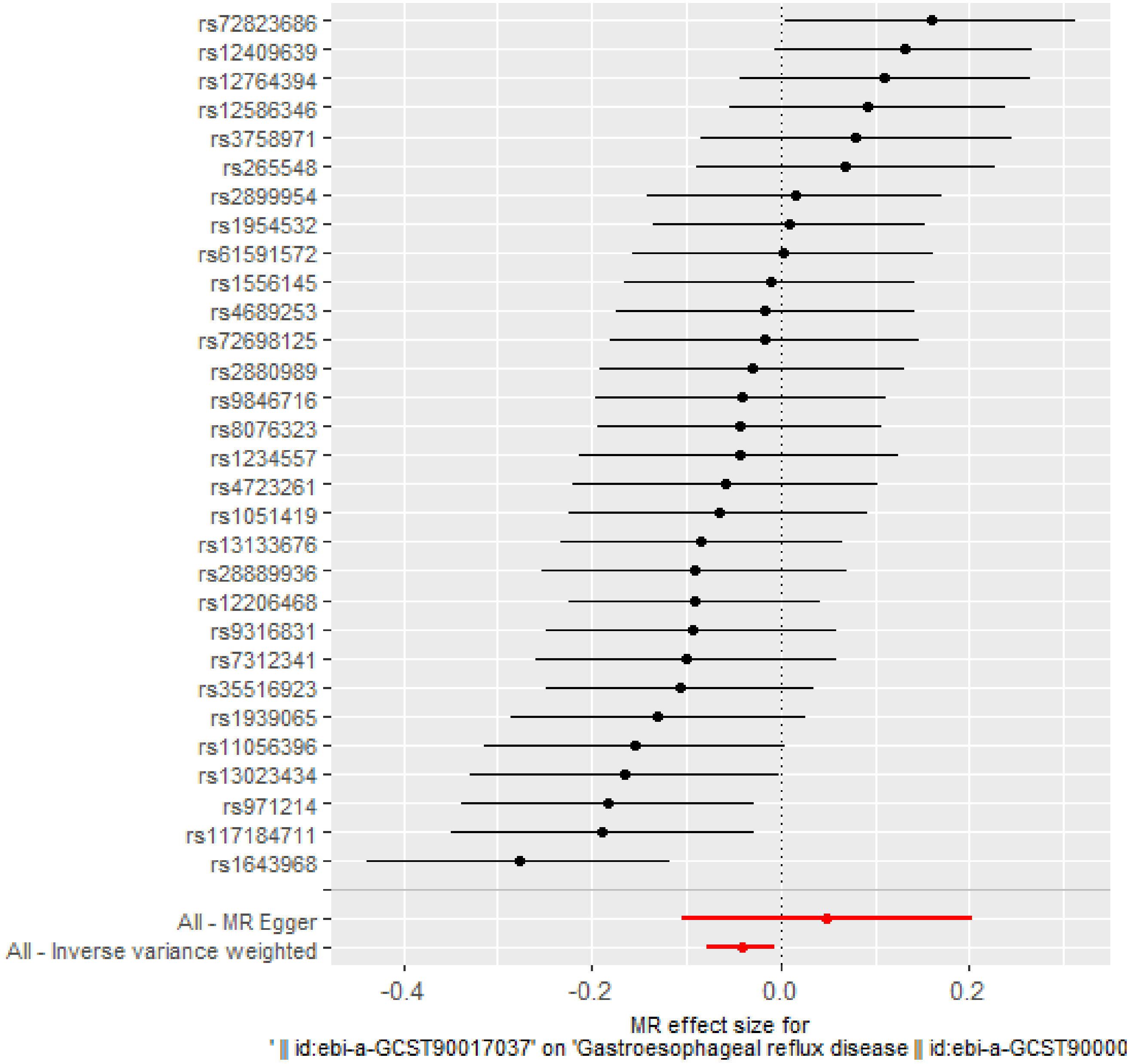
rs72823686
rs12409639
rs12764394
rs12586346
rs3758971
rs265548
rs2899954
rs1954532
rs61591572
rs1556145
rs4689253
rs72698125
rs2880989
rs9846716
rs8076323
rs1234557
rs4723261
rs1051419
rs13133676
rs28889936
rs12206468
rs9316831
rs7312341
rs35516923
rs1939065
rs11056396
rs13023434
rs971214
rs117184711
rs1643968
All - MR Egger
All - Inverse variance weighted
-0.4
-0.2
0.0
0.2
MR effect size for
' || id:ebi-a-GCST90017037' on 'Gastroesophageal reflux disease || id:ebi-a-GCST90000

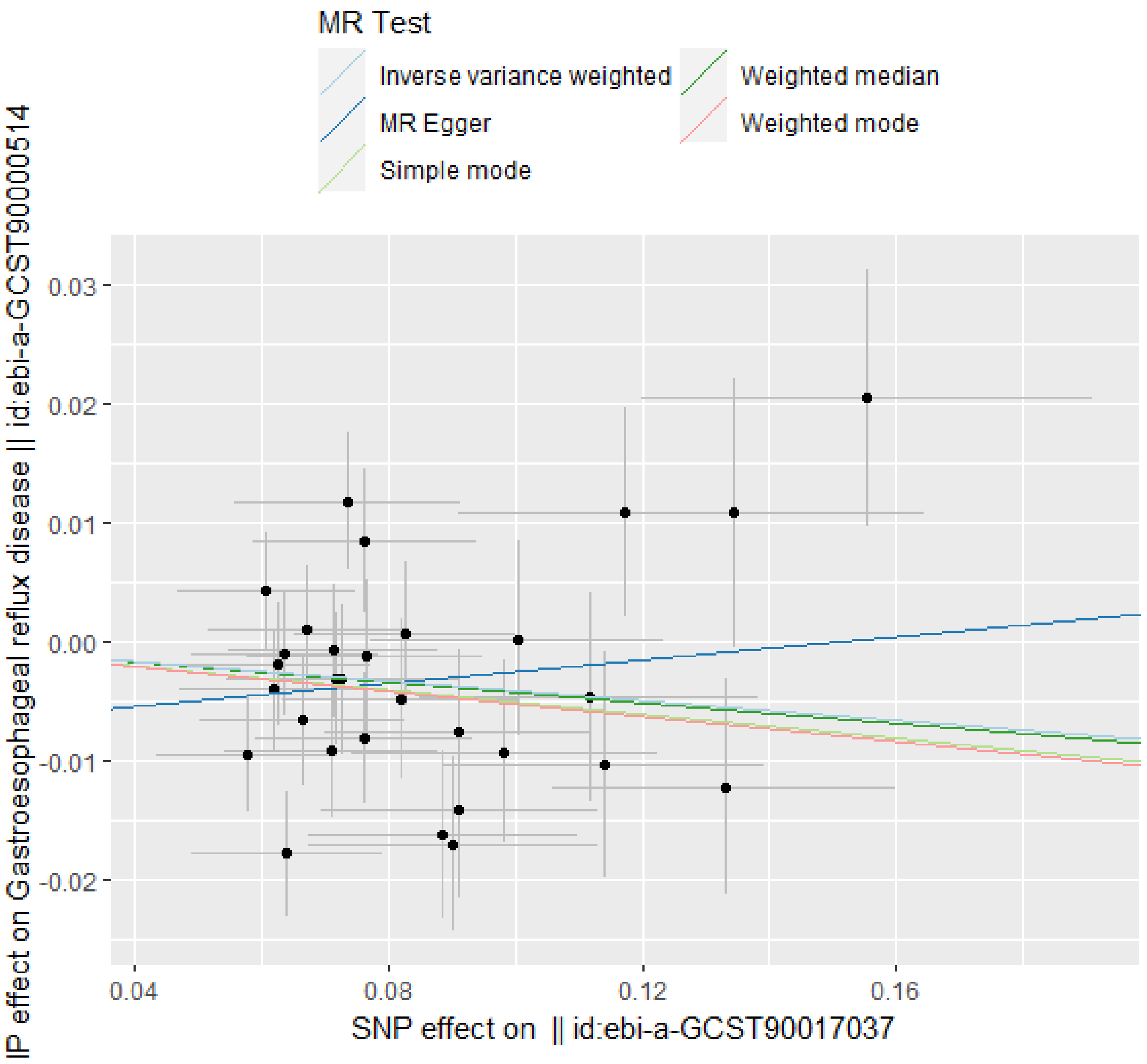

MR Test
Inverse variance weighted
MR Egger
Simple mode
Weighted median
Weighted mode
0.03
0.02
0.01
0.00
-0.01
-0.02
0.04
0.08
0.12
0.16
SNP effect on || id:ebi-a-GCST90017037
SNP effect on Gastroesophageal reflux disease || id:ebi-a-GCST90000514

Figure 6 Leave-one-out analysis, funnel plot, MR effect size and scatter plot for gut microbiota abundance (genus Rikenellaceae RC9 gut group id.11191) on gastroesophageal reflux disease

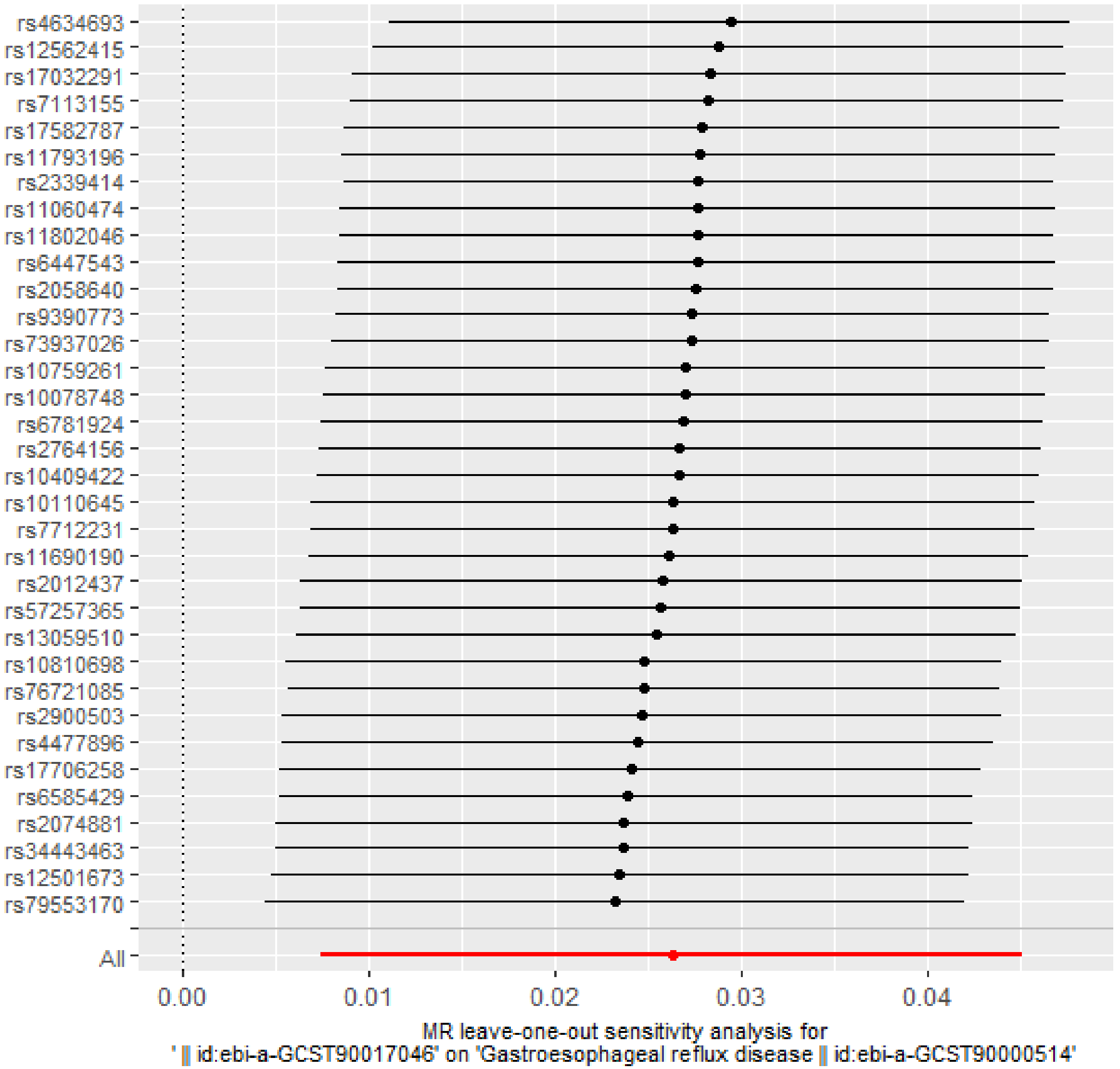

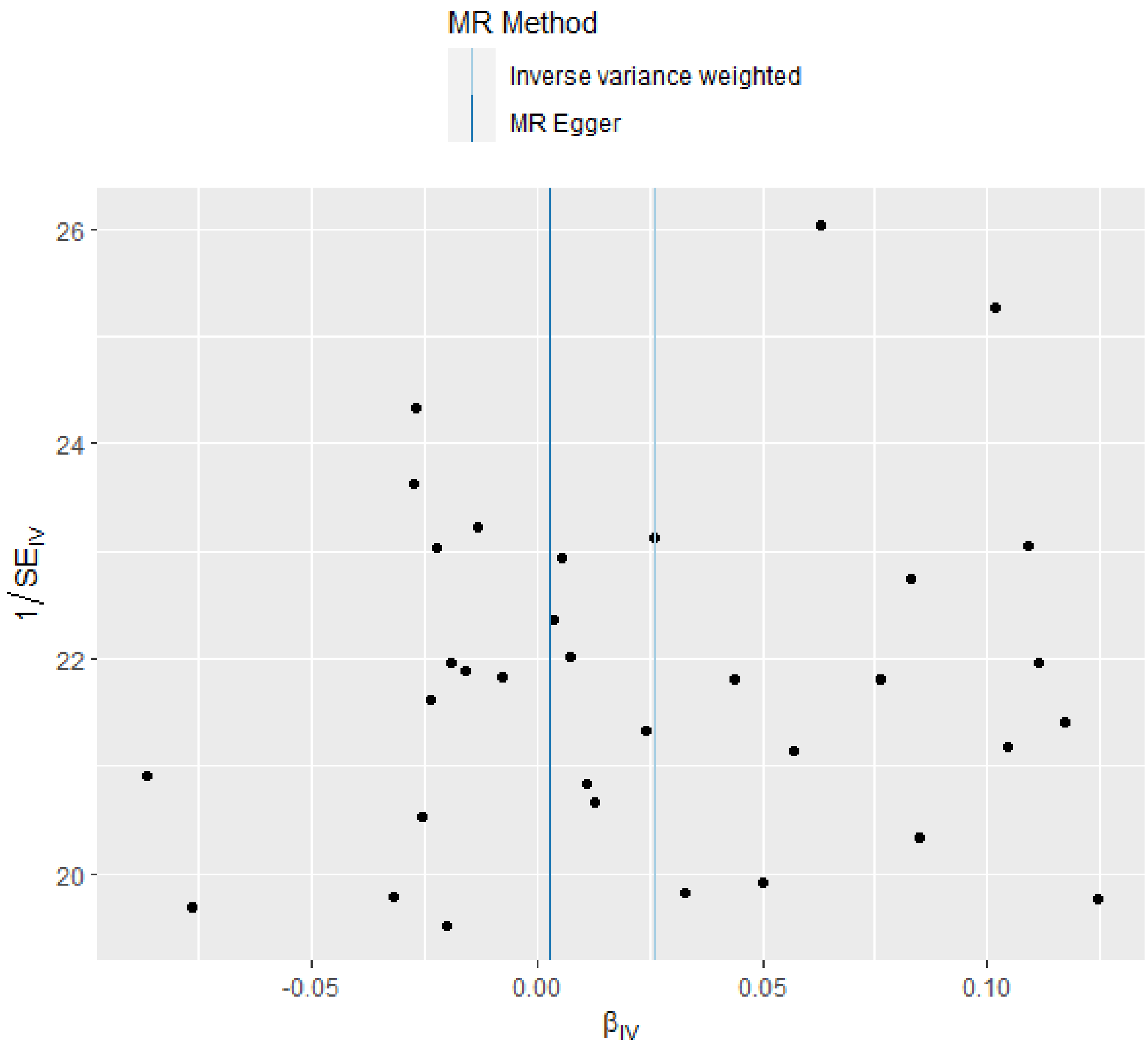

MR Method
Inverse variance weighted
MR Egger
26
24
22
20
1/SE_IV
-0.05
0.00
0.05
0.10
β_IV

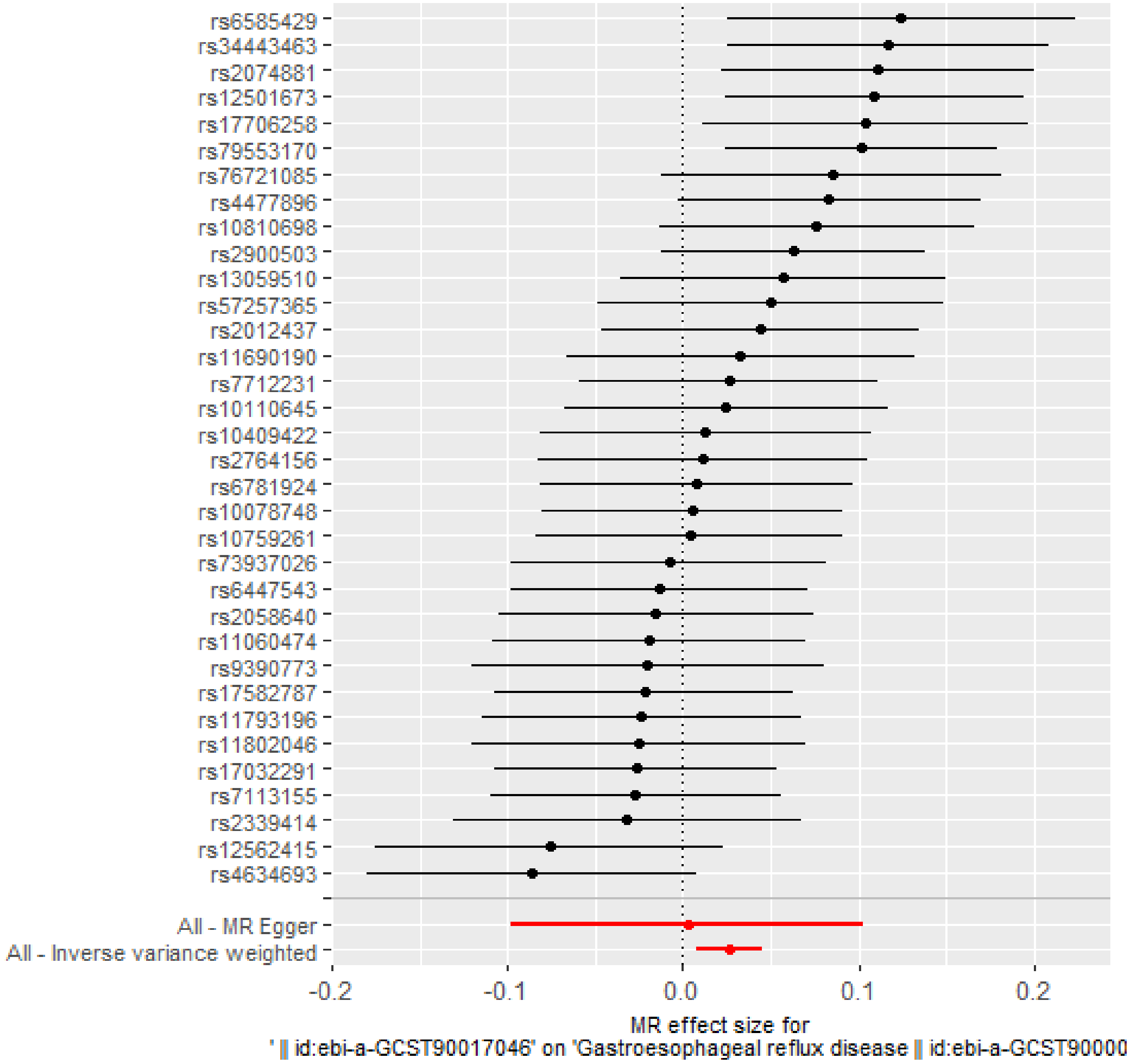

rs6585429
rs34443463
rs2074881
rs12501673
rs17706258
rs79553170
rs76721085
rs4477896
rs10810698
rs2900503
rs13059510
rs57257365
rs2012437
rs11690190
rs7712231
rs10110645
rs10409422
rs2764156
rs6781924
rs10078748
rs10759261
rs73937026
rs6447543
rs2058640
rs11060474
rs9390773
rs17582787
rs11793196
rs11802046
rs17032291
rs7113155
rs2339414
rs12562415
rs4634693
All - MR Egger
All - Inverse variance weighted
-0.2
-0.1
0.0
0.1
0.2
MR effect size for
' || id:ebi-a-GCST90017046' on 'Gastroesophageal reflux disease || id:ebi-a-GCST90000

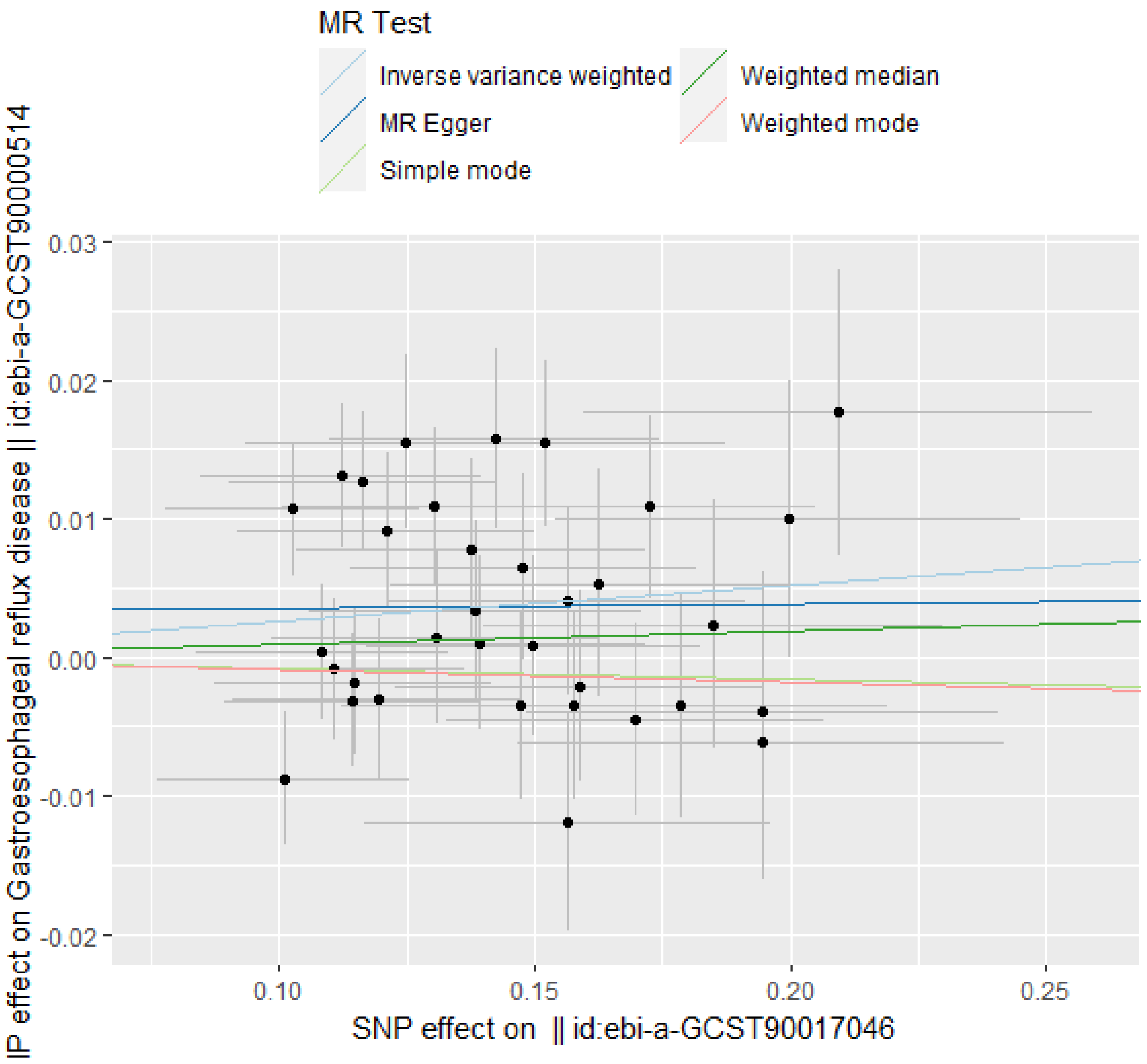

MR Test
Inverse variance weighted
MR Egger
Simple mode
Weighted median
Weighted mode
SNP effect on Gastroesophageal reflux disease || id:ebi-a-GCST90000514
0.03
0.02
0.01
0.00
-0.01
-0.02
0.10
0.15
0.20
0.25
SNP effect on || id:ebi-a-GCST90017046

Figure 7 Leave-one-out analysis, funnel plot, MR effect size and scatter plot for gut microbiota abundance (genus Ruminococcaceae UCG010 id.11367) on gastroesophageal reflux disease

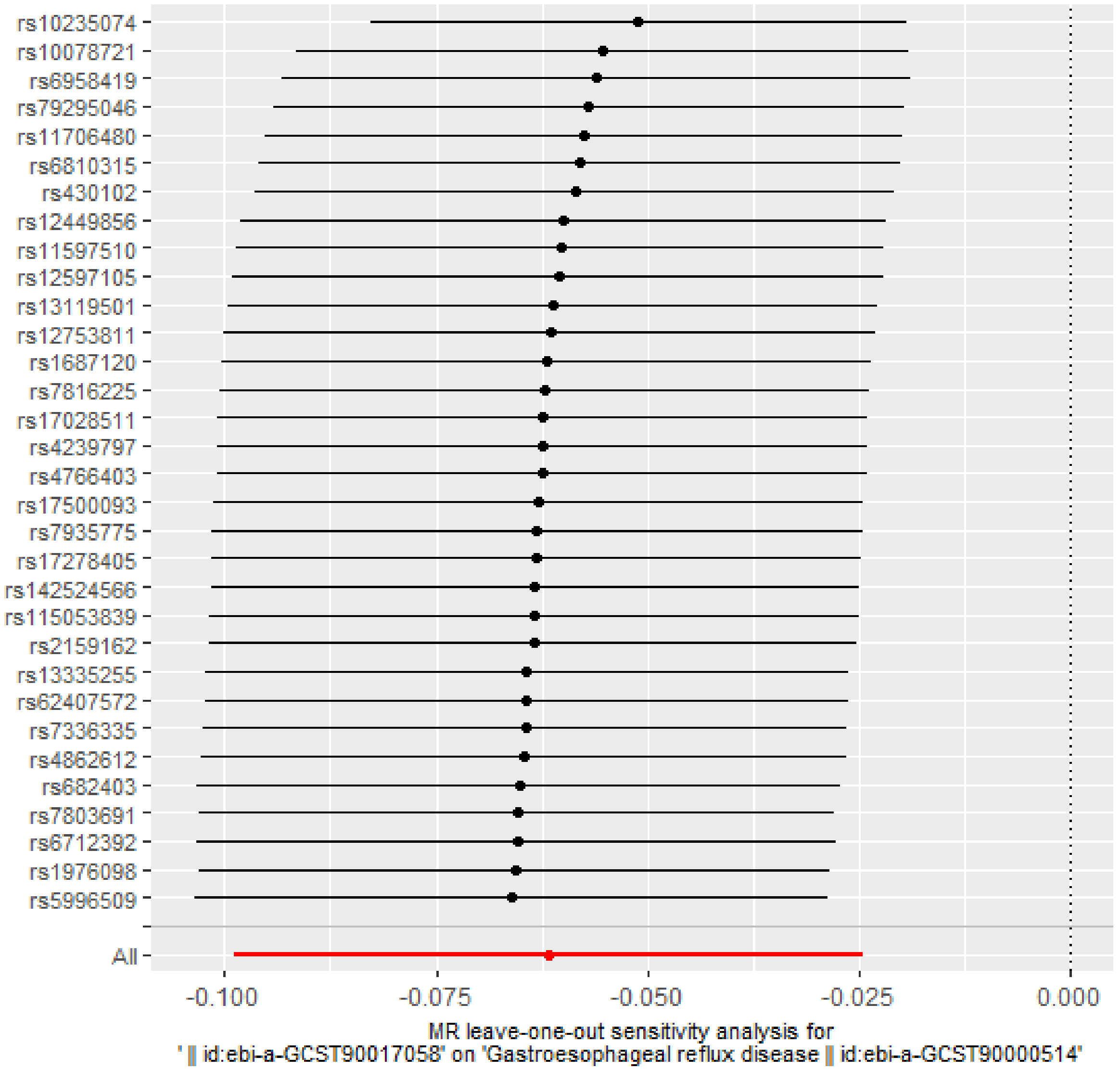

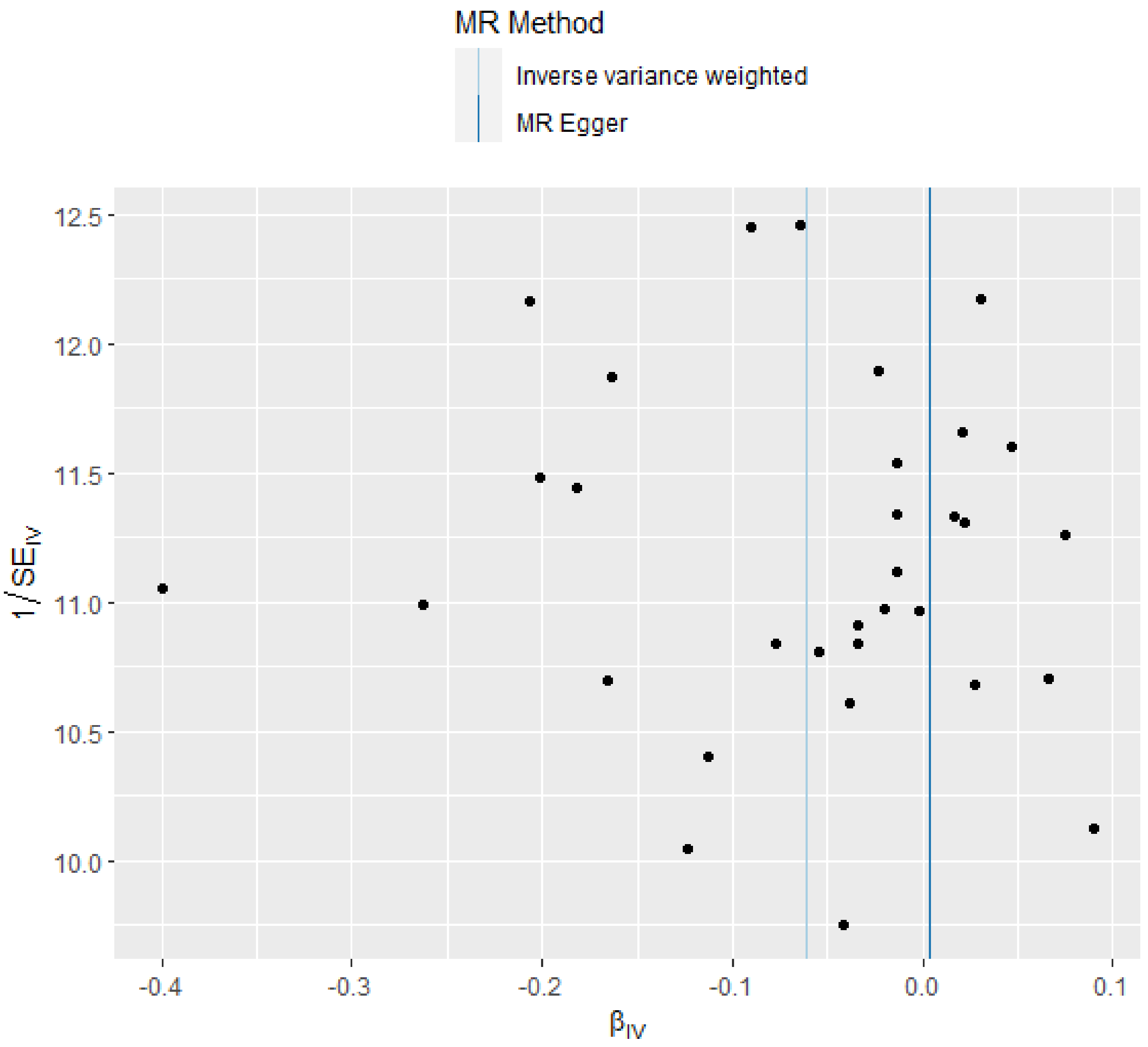

MR Method
Inverse variance weighted
MR Egger
12.5
12.0
11.5
11.0
10.5
10.0
-0.4
-0.3
-0.2
-0.1
0.0
0.1
$1/SE_{IV}$
β_{IV}

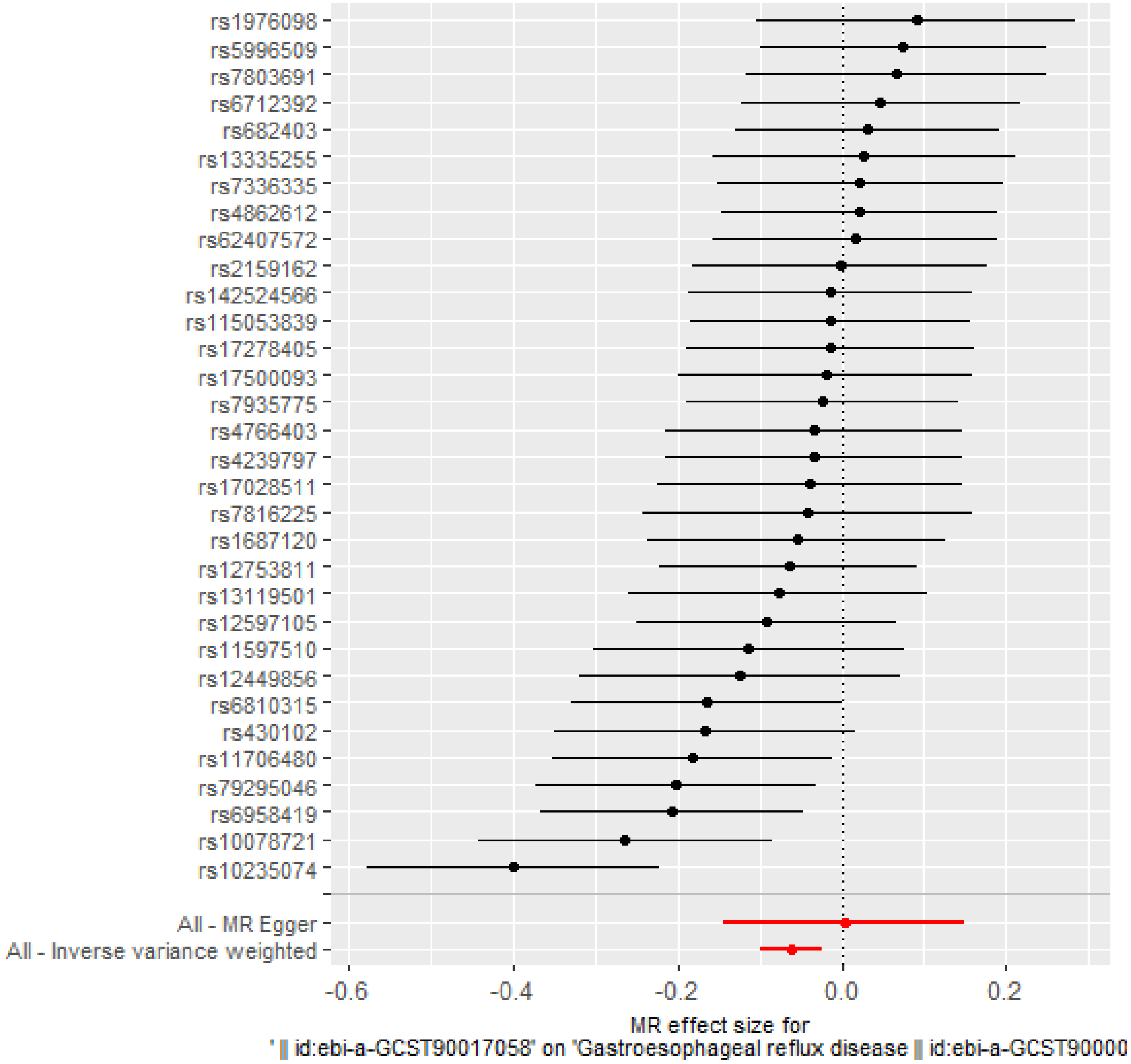
rs1976098
rs5996509
rs7803691
rs6712392
rs682403
rs13335255
rs7336335
rs4862612
rs62407572
rs2159162
rs142524566
rs115053839
rs17278405
rs17500093
rs7935775
rs4766403
rs4239797
rs17028511
rs7816225
rs1687120
rs12753811
rs13119501
rs12597105
rs11597510
rs12449856
rs6810315
rs430102
rs11706480
rs79295046
rs6958419
rs10078721
rs10235074
All - MR Egger
All - Inverse variance weighted
-0.6
-0.4
-0.2
0.0
0.2
MR effect size for
' || id:ebi-a-GCST90017058' on 'Gastroesophageal reflux disease || id:ebi-a-GCST90000

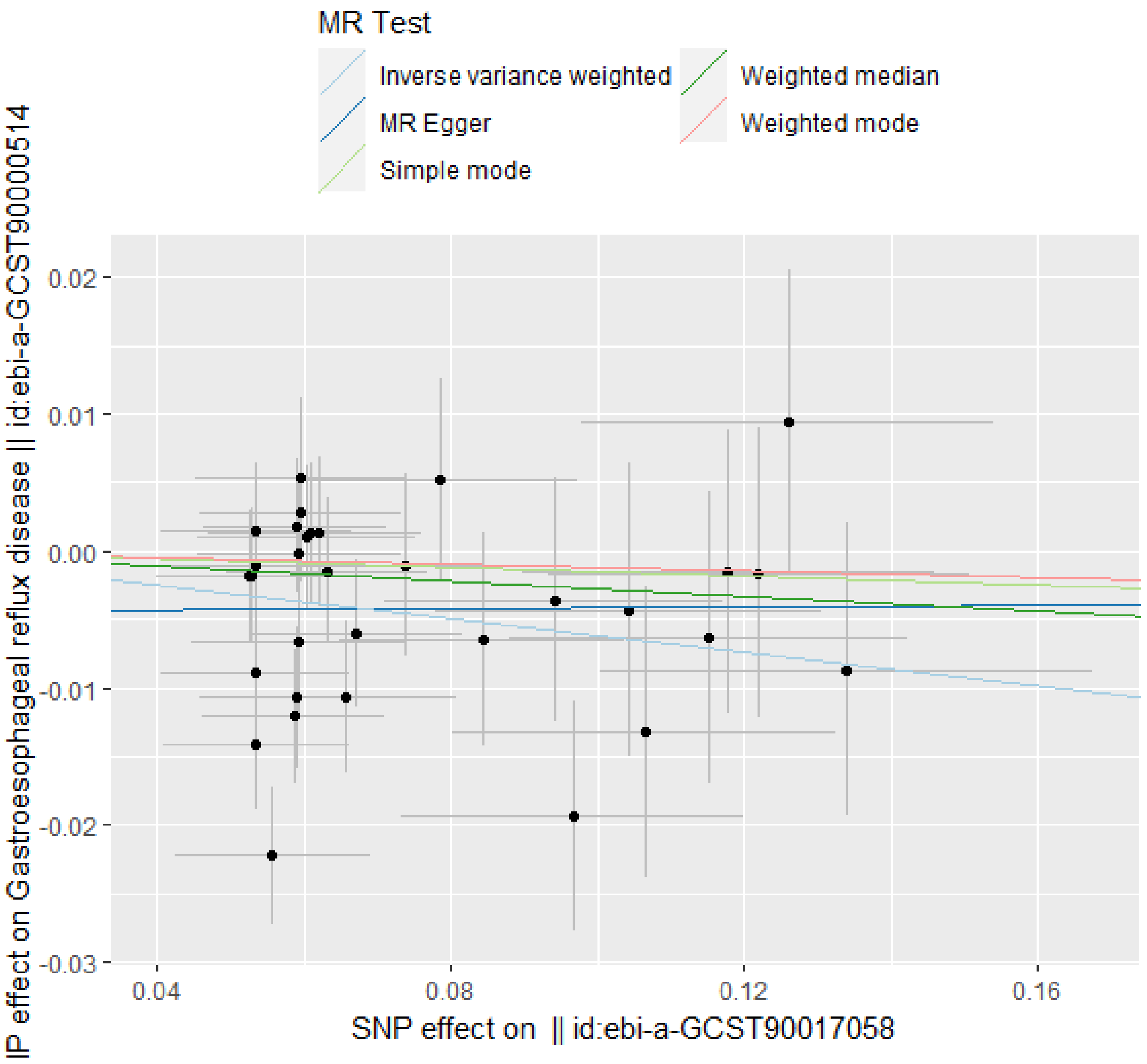

MR Test
Inverse variance weighted
MR Egger
Simple mode
Weighted median
Weighted mode
IP effect on Gastroesophageal reflux disease || id:ebi-a-GCST90000514
0.02
0.01
0.00
-0.01
-0.02
-0.03
0.04
0.08
0.12
0.16
SNP effect on || id:ebi-a-GCST90017058

Figure 8 Leave-one-out analysis, funnel plot, MR effect size and scatter plot for gut microbiota abundance (genus Sellimonas id.14369) on gastroesophageal reflux disease

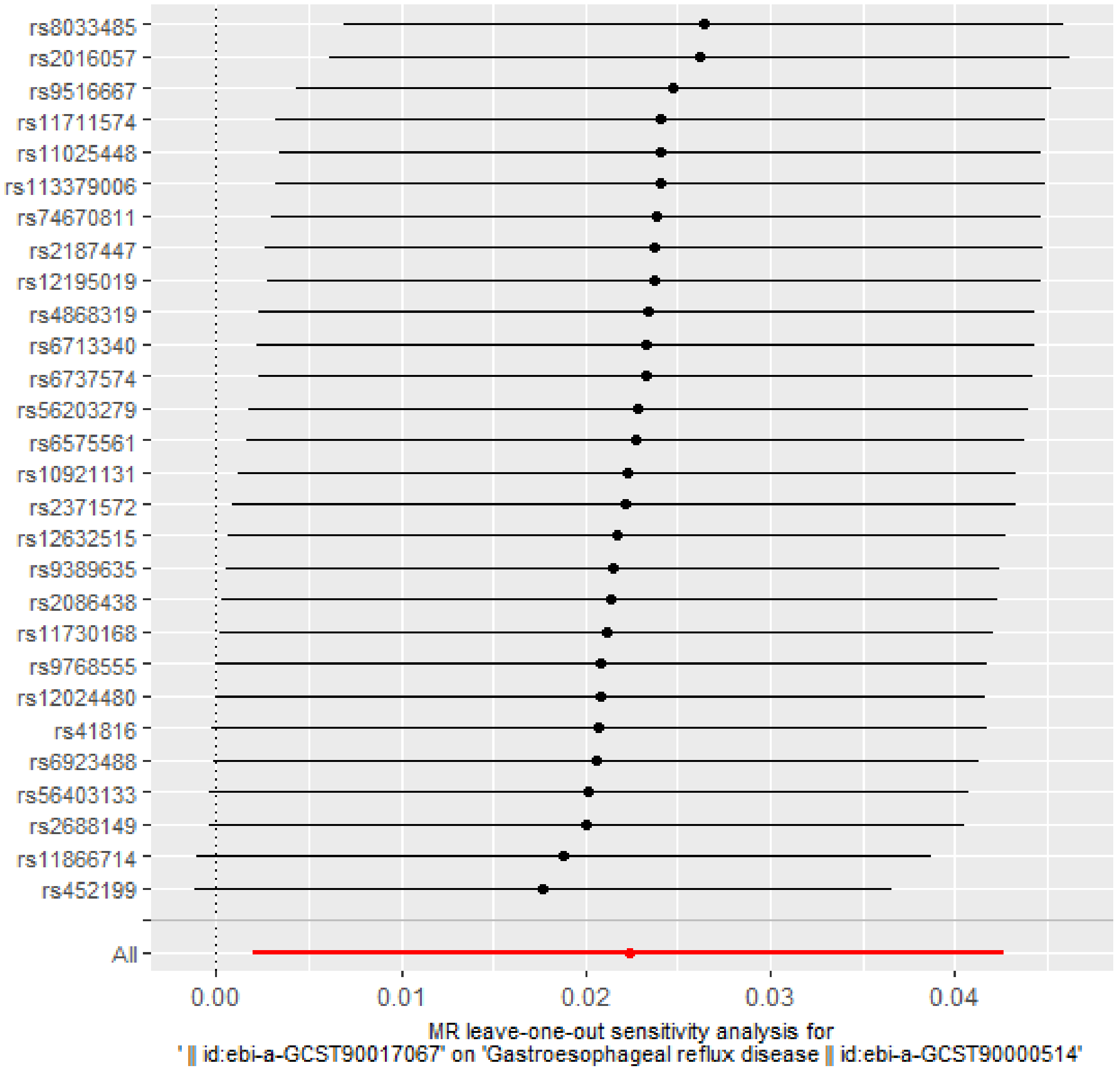

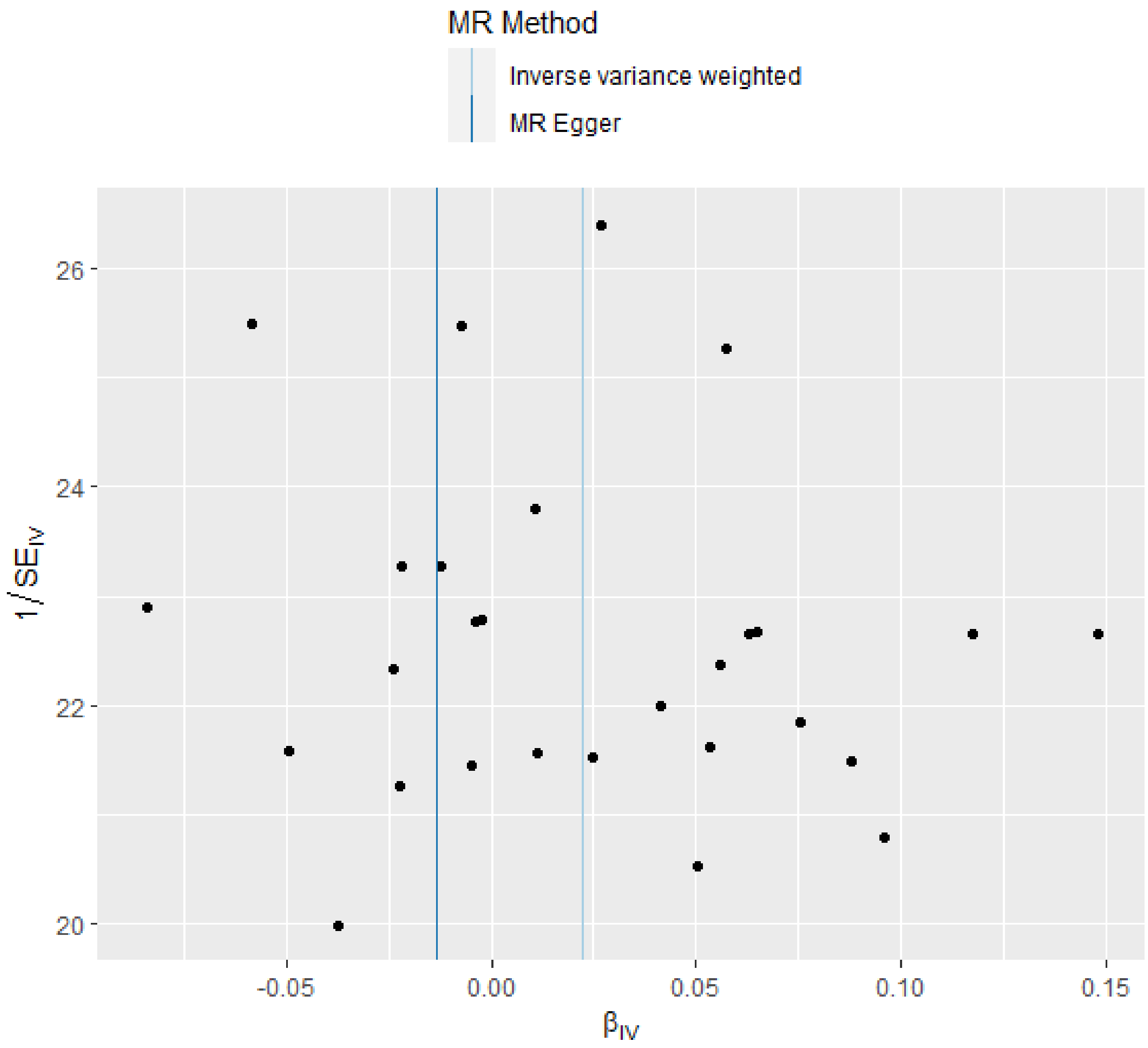

MR Method
Inverse variance weighted
MR Egger
26
24
22
20
1/SE$_{IV}$
-0.05
0.00
0.05
0.10
0.15
β$_{IV}$

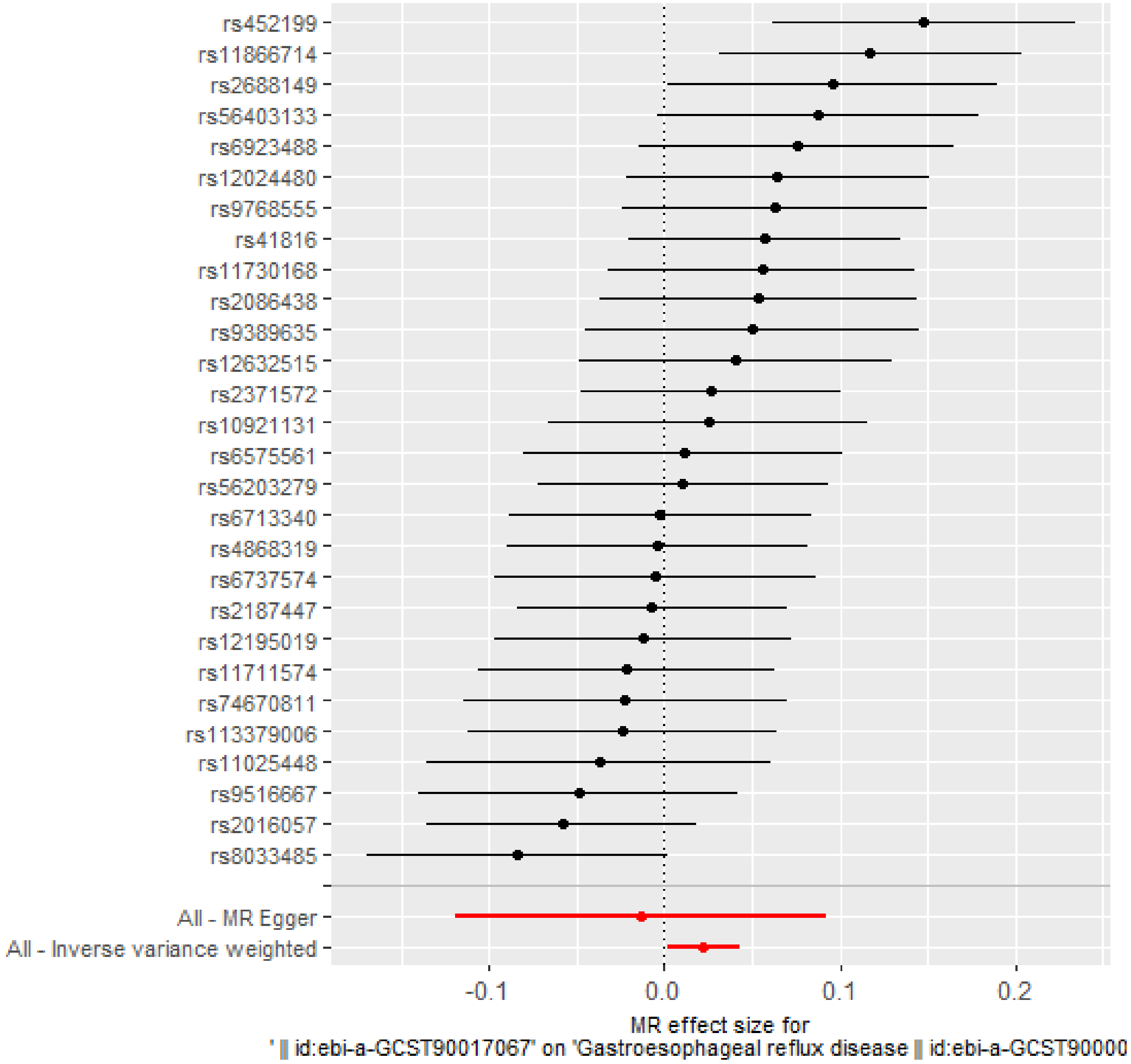
rs452199
rs11866714
rs2688149
rs56403133
rs6923488
rs12024480
rs9768555
rs41816
rs11730168
rs2086438
rs9389635
rs12632515
rs2371572
rs10921131
rs6575561
rs56203279
rs6713340
rs4868319
rs6737574
rs2187447
rs12195019
rs11711574
rs74670811
rs113379006
rs11025448
rs9516667
rs2016057
rs8033485
All - MR Egger
All - Inverse variance weighted
-0.1
0.0
0.1
0.2
MR effect size for
' || id:ebi-a-GCST90017067' on 'Gastroesophageal reflux disease || id:ebi-a-GCST90000

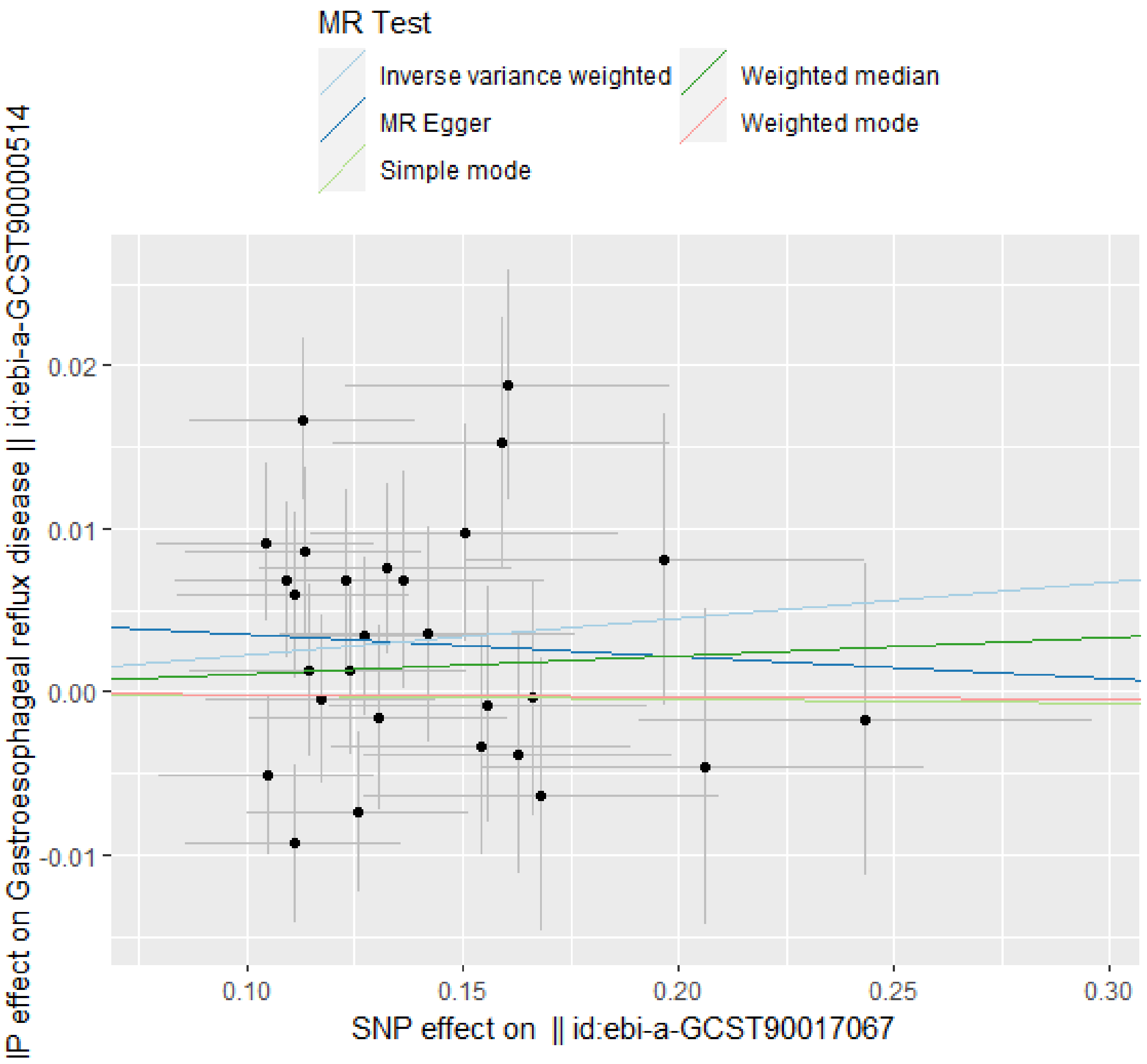

MR Test
Inverse variance weighted
MR Egger
Simple mode
Weighted median
Weighted mode
0.02
0.01
0.00
-0.01
0.10
0.15
0.20
0.25
0.30
SNP effect on || id:ebi-a-GCST90017067
IP effect on Gastroesophageal reflux disease || id:ebi-a-GCST90000514

Figure 9 Leave-one-out analysis, funnel plot, MR effect size and scatter plot for gut microbiota abundance (genus Slackia id.825) on gastroesophageal reflux disease

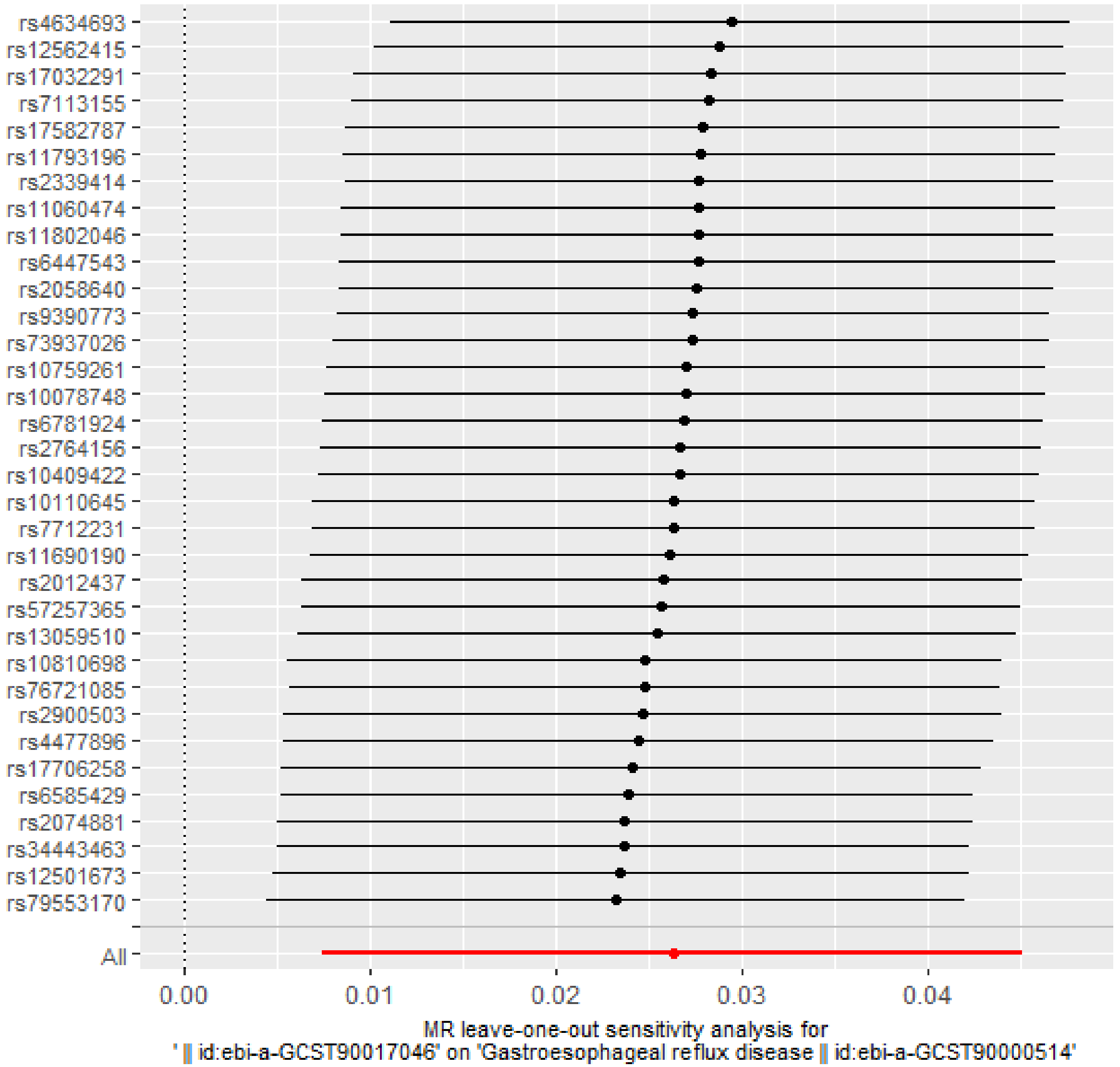

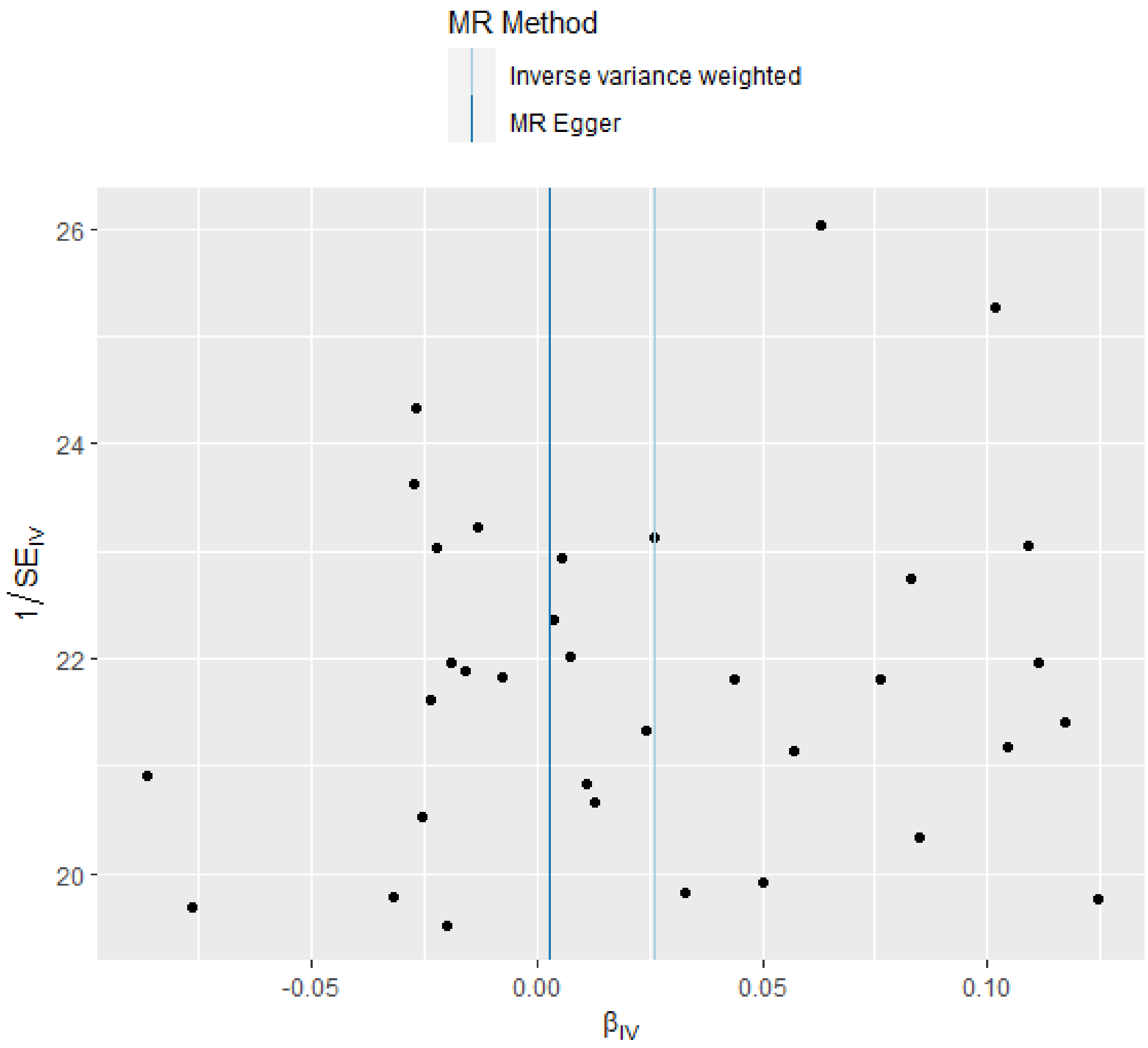
MR Method
Inverse variance weighted
MR Egger
26
24
22
20
1/SE_IV
-0.05
0.00
0.05
0.10
β_IV

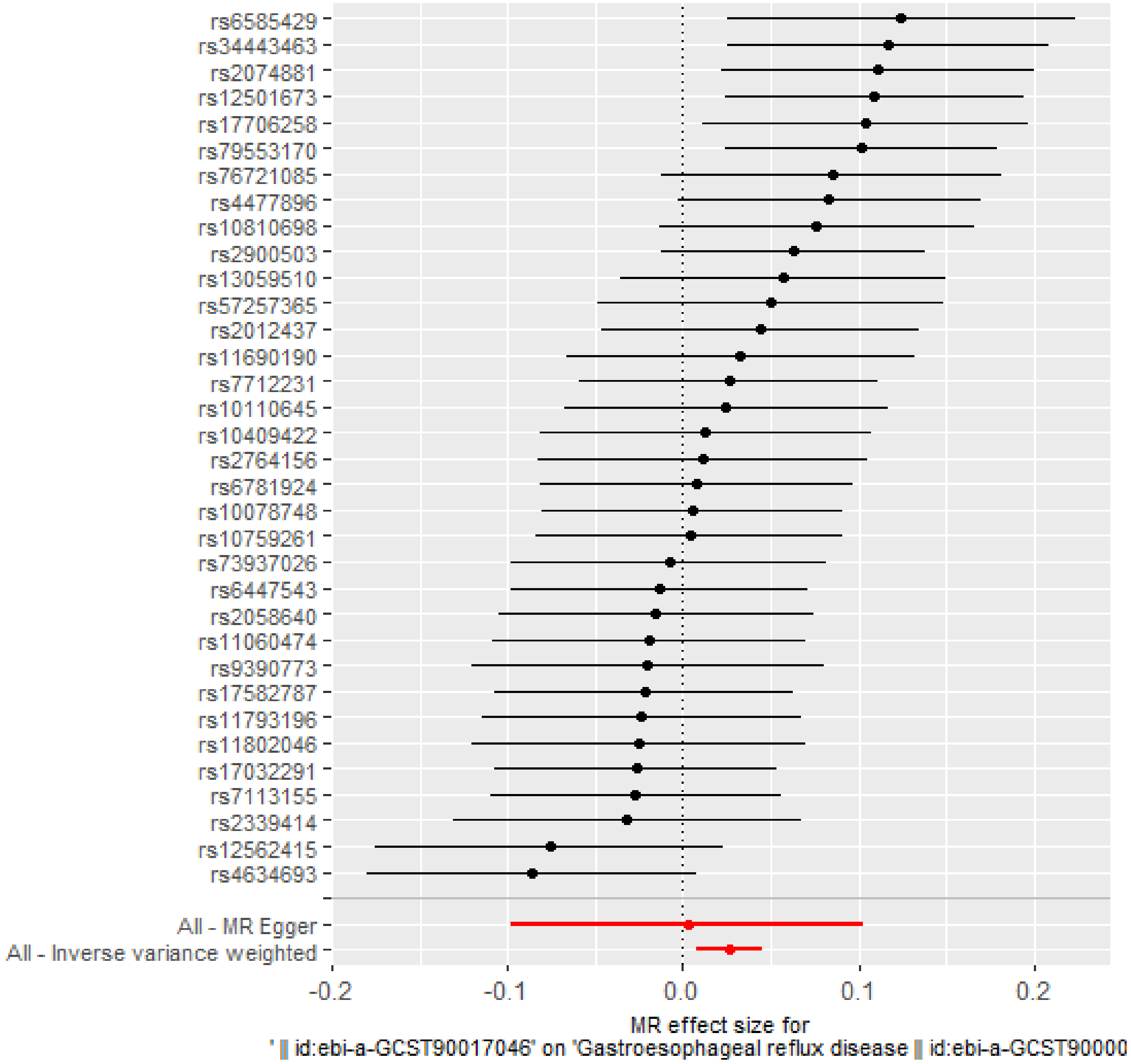

rs6585429
rs34443463
rs2074881
rs12501673
rs17706258
rs79553170
rs76721085
rs4477896
rs10810698
rs2900503
rs13059510
rs57257365
rs2012437
rs11690190
rs7712231
rs10110645
rs10409422
rs2764156
rs6781924
rs10078748
rs10759261
rs73937026
rs6447543
rs2058640
rs11060474
rs9390773
rs17582787
rs11793196
rs11802046
rs17032291
rs7113155
rs2339414
rs12562415
rs4634693
All - MR Egger
All - Inverse variance weighted
-0.2
-0.1
0.0
0.1
0.2
MR effect size for
' || id:ebi-a-GCST90017046' on 'Gastroesophageal reflux disease || id:ebi-a-GCST90000

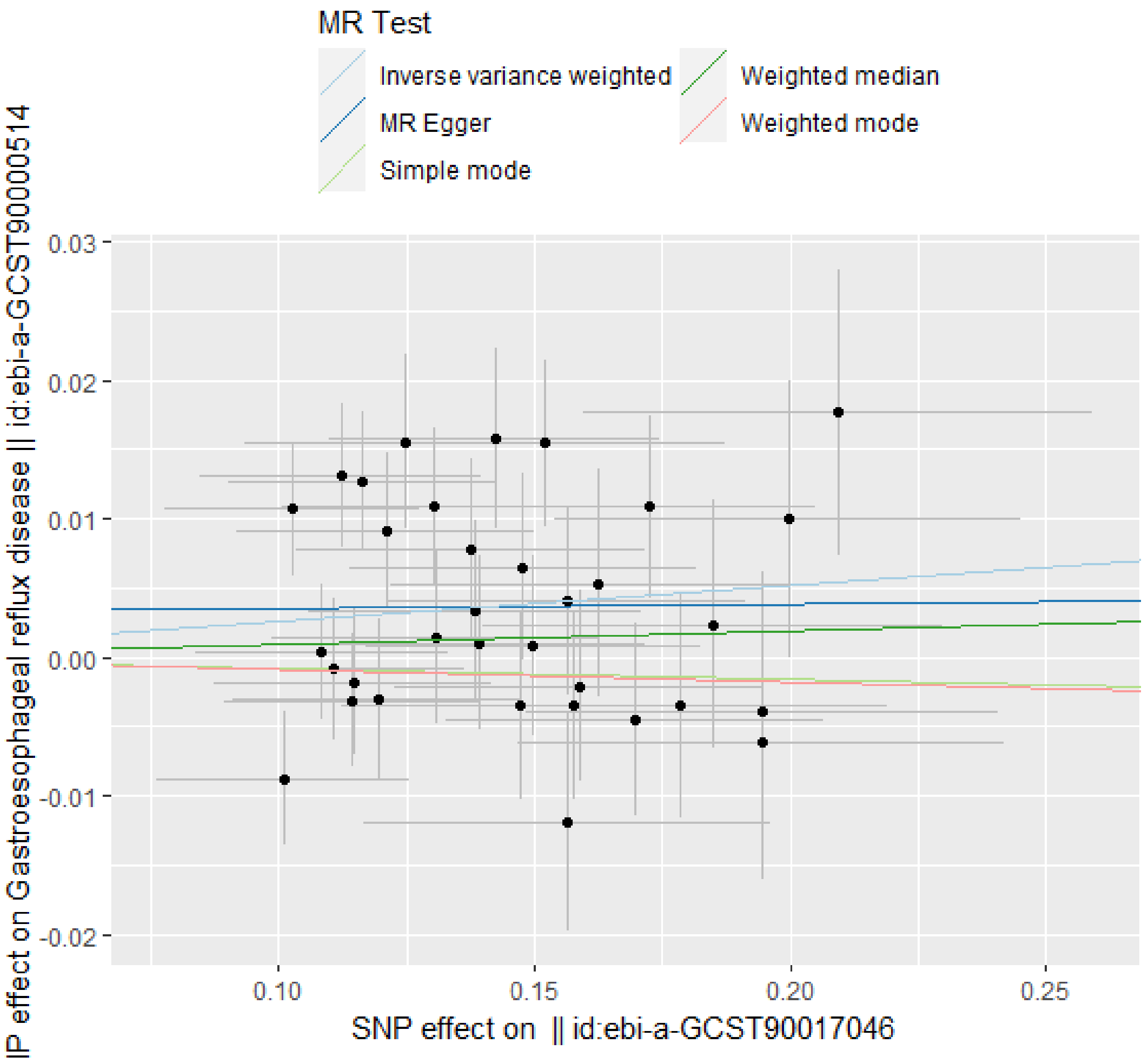
MR Test
Inverse variance weighted
MR Egger
Simple mode
Weighted median
Weighted mode
0.03
0.02
0.01
0.00
-0.01
-0.02
0.10
0.15
0.20
0.25
SNP effect on || id:ebi-a-GCST90017046
IP effect on Gastroesophageal reflux disease || id:ebi-a-GCST90000514

Figure 10 Leave-one-out analysis, funnel plot, MR effect size and scatter plot for gut microbiota abundance (phylum Firmicutes id.1672) on gastroesophageal reflux disease

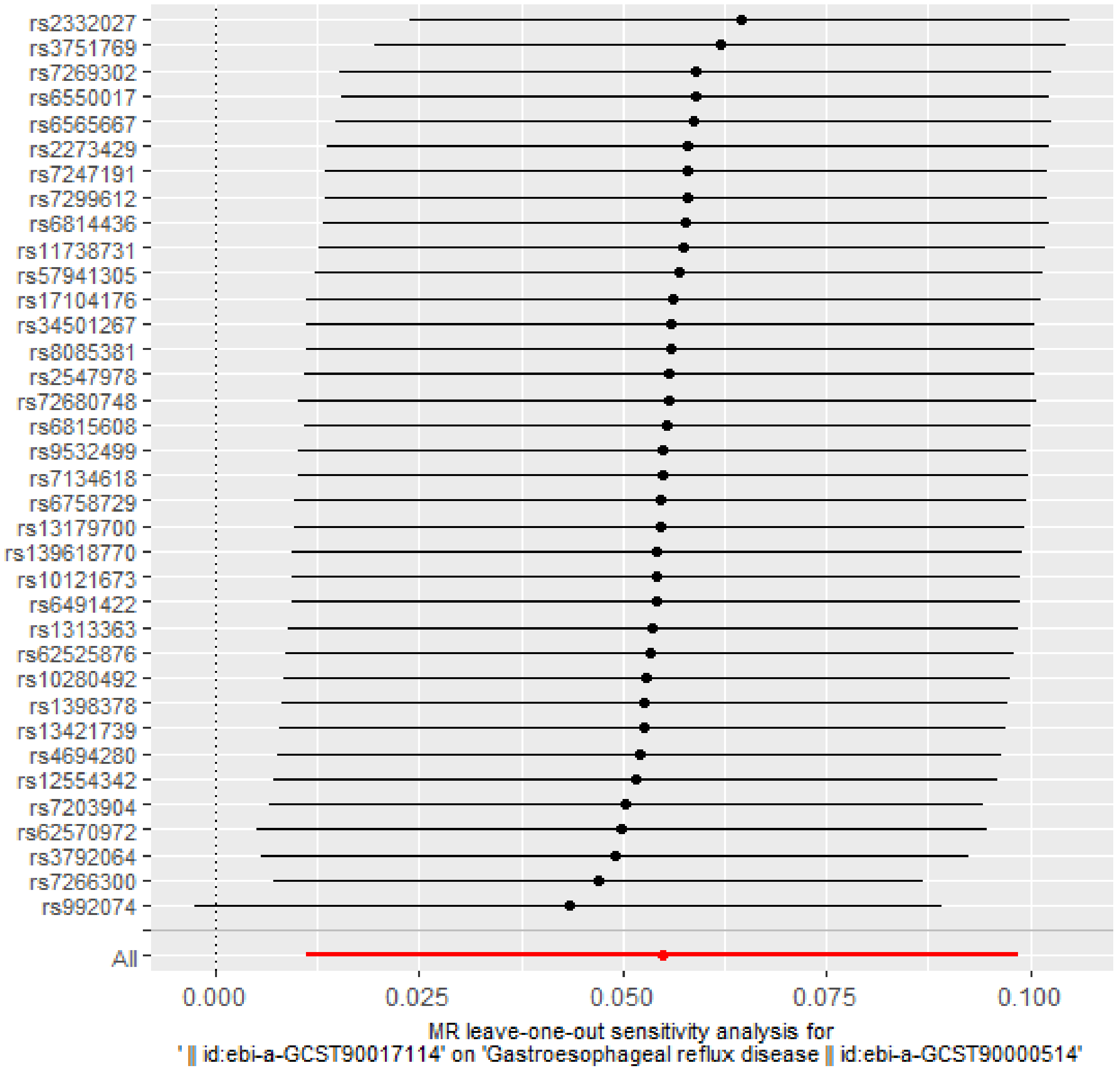

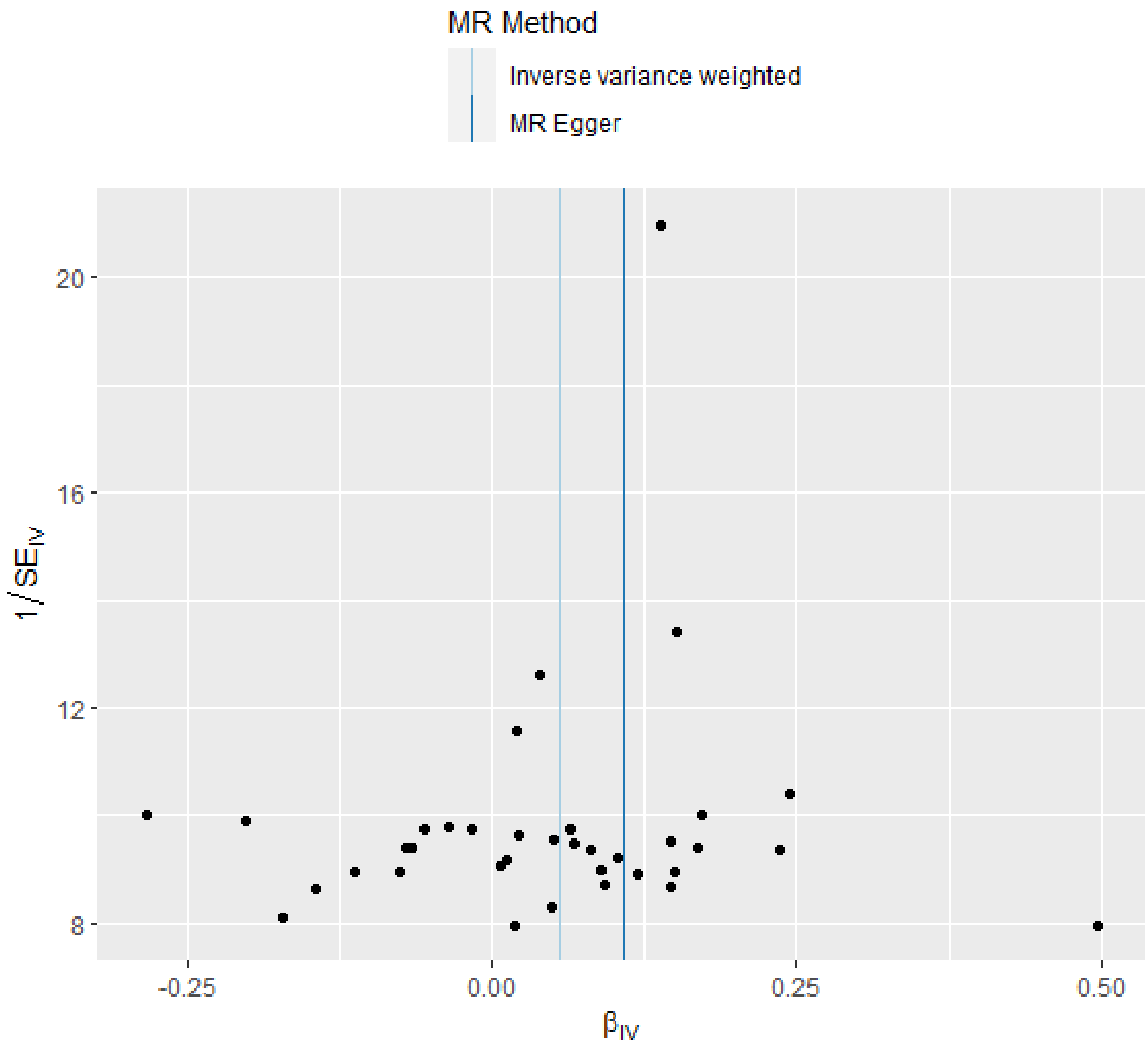
MR Method
Inverse variance weighted
MR Egger
20
16
12
8
-0.25
0.00
0.25
0.50
β_{IV}
$1/SE_{IV}$

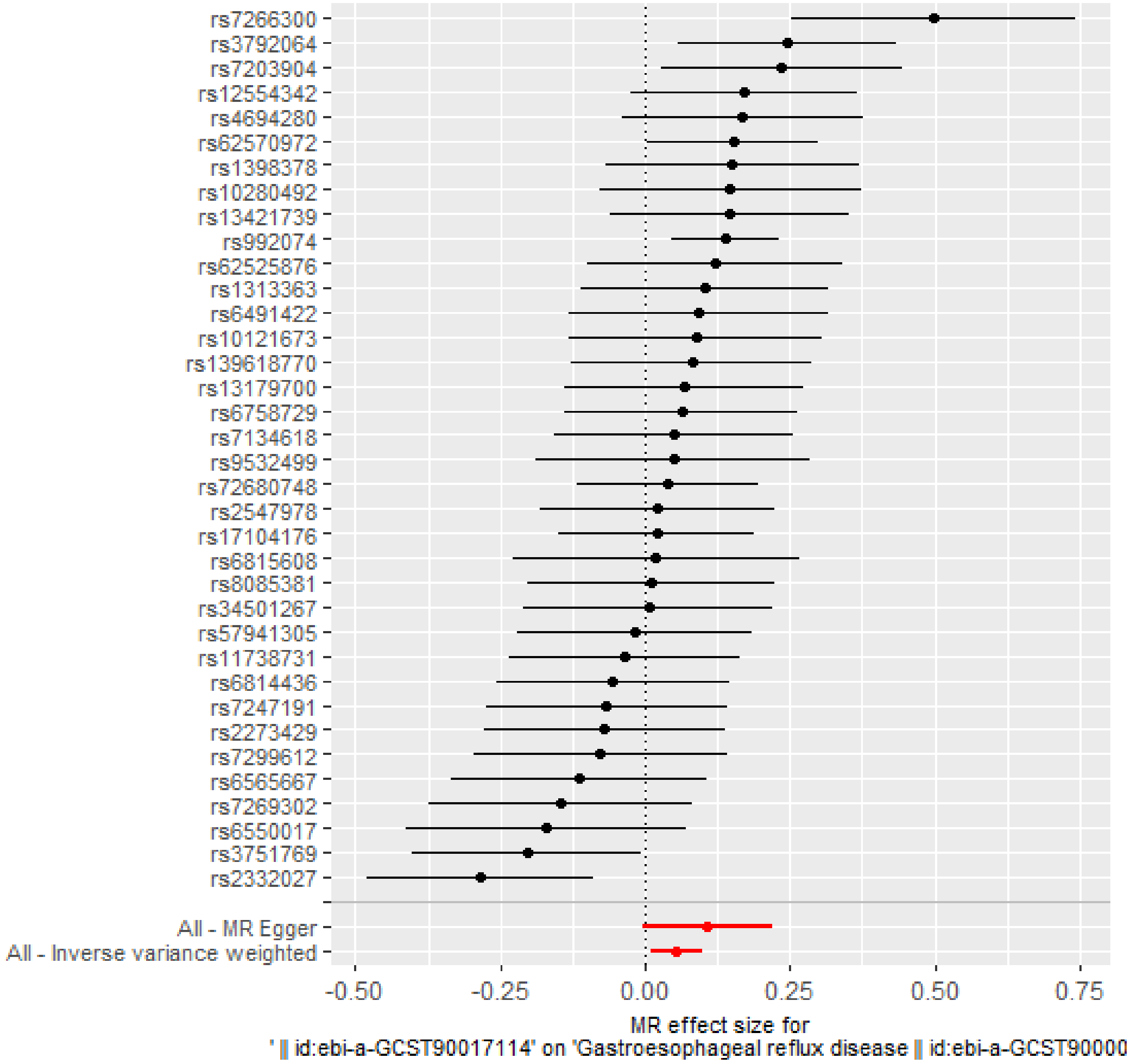
rs7266300
rs3792064
rs7203904
rs12554342
rs4694280
rs62570972
rs1398378
rs10280492
rs13421739
rs992074
rs62525876
rs1313363
rs6491422
rs10121673
rs139618770
rs13179700
rs6758729
rs7134618
rs9532499
rs72680748
rs2547978
rs17104176
rs6815608
rs8085381
rs34501267
rs57941305
rs11738731
rs6814436
rs7247191
rs2273429
rs7299612
rs6565667
rs7269302
rs6550017
rs3751769
rs2332027
All - MR Egger
All - Inverse variance weighted
-0.50
-0.25
0.00
0.25
0.50
0.75
MR effect size for
' || id:ebi-a-GCST90017114' on 'Gastroesophageal reflux disease || id:ebi-a-GCST90000

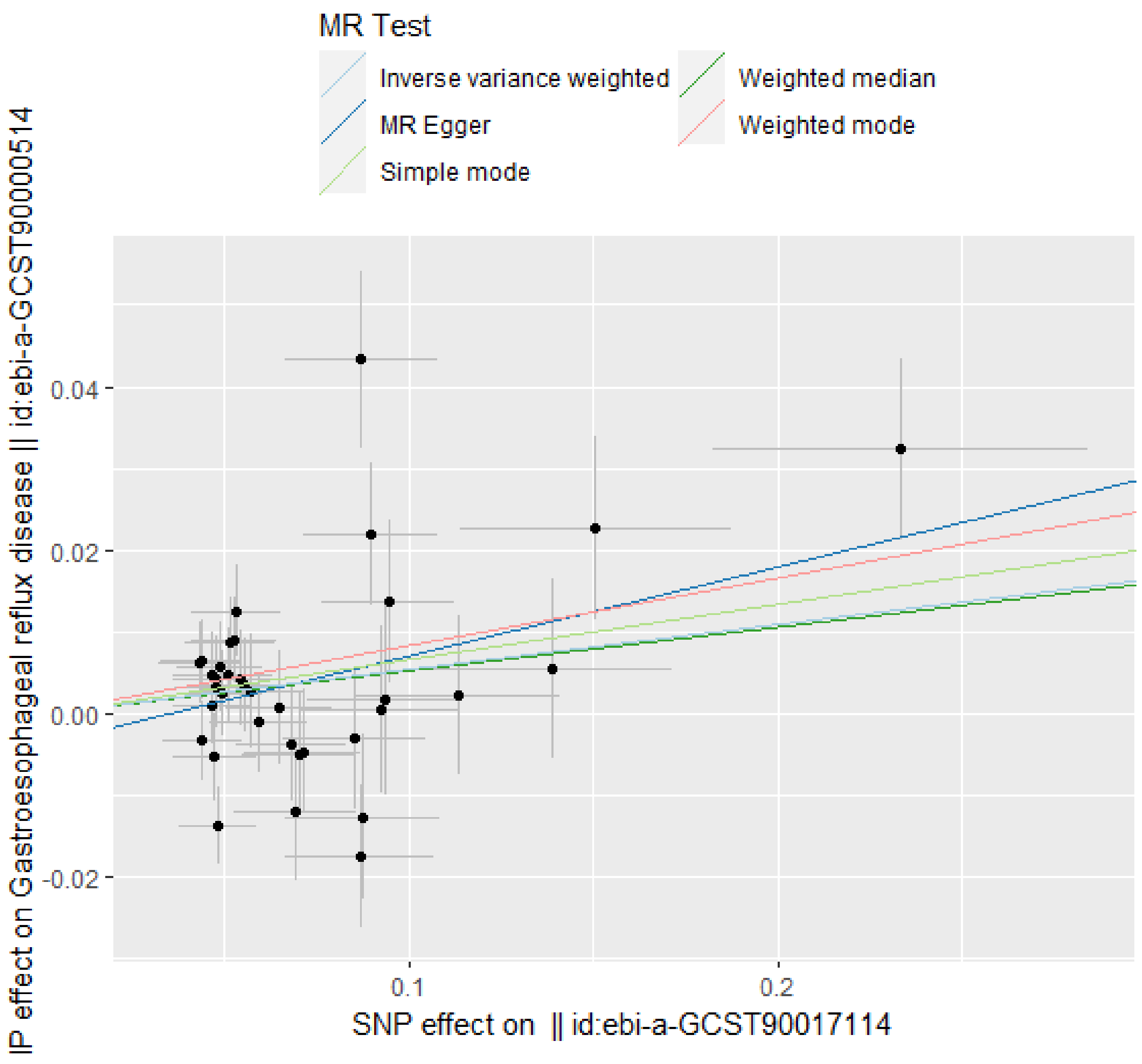

MR Test
Inverse variance weighted
MR Egger
Simple mode
Weighted median
Weighted mode
0.04
0.02
0.00
-0.02
0.1
0.2
SNP effect on || id:ebi-a-GCST90017114
IP effect on Gastroesophageal reflux disease || id:ebi-a-GCST90000514

Figure 11 Leave-one-out analysis, funnel plot, MR effect size and scatter plot for gut microbiota abundance (class Actinobacteria id.419) on oesophageal cancer

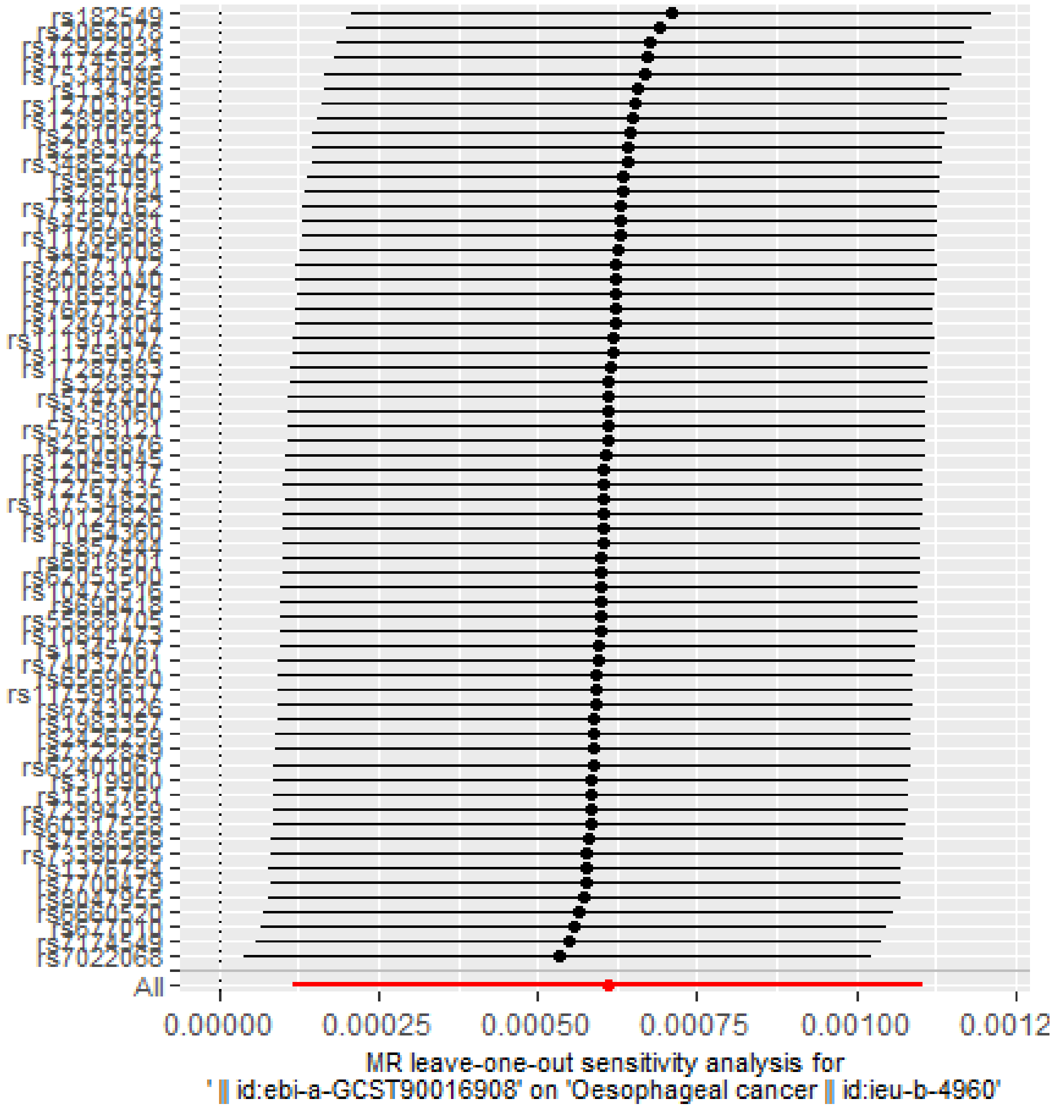

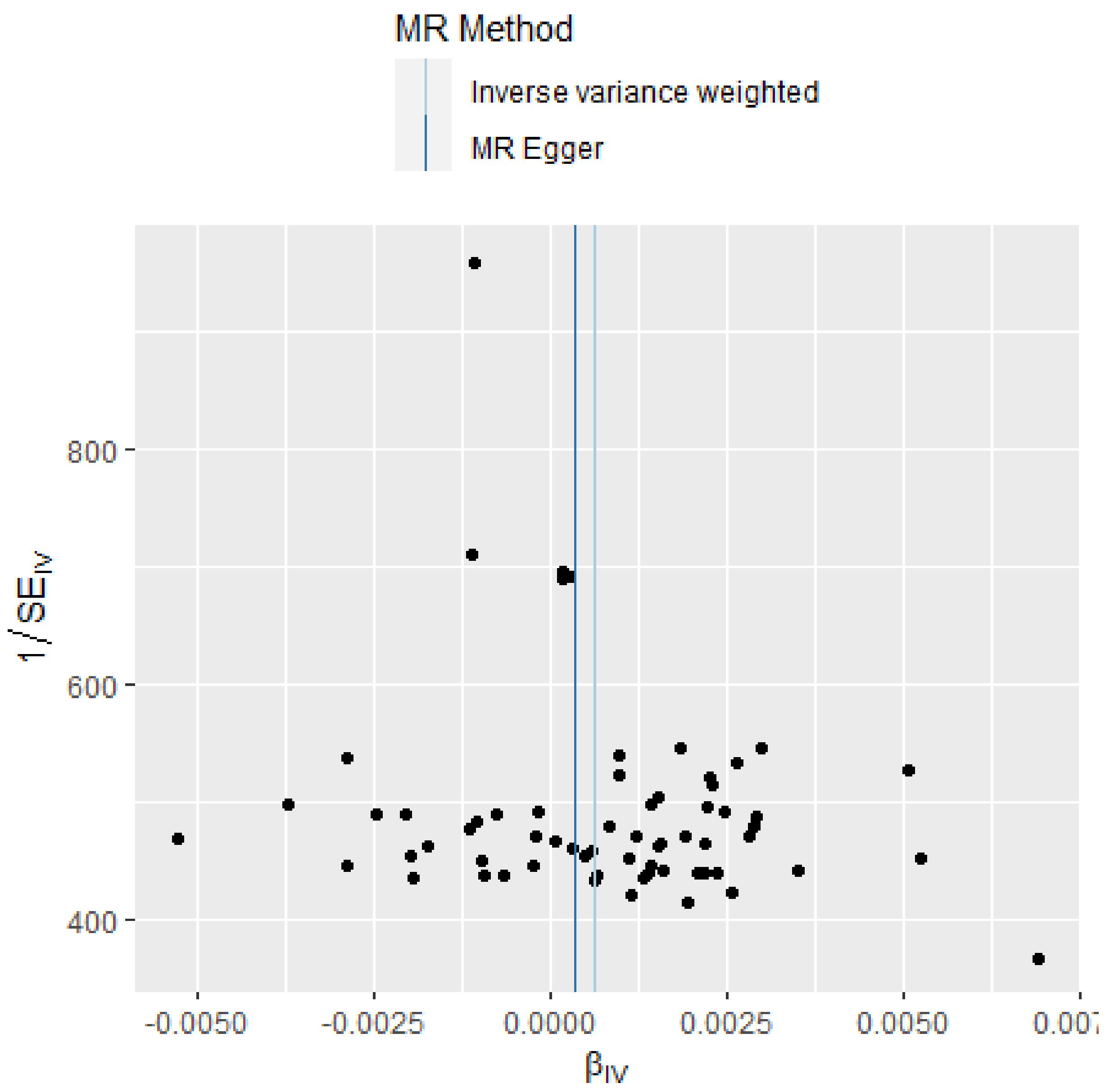

MR Method
Inverse variance weighted
MR Egger
800
600
400
1/SE_IV
-0.0050
-0.0025
0.0000
0.0025
0.0050
β_IV

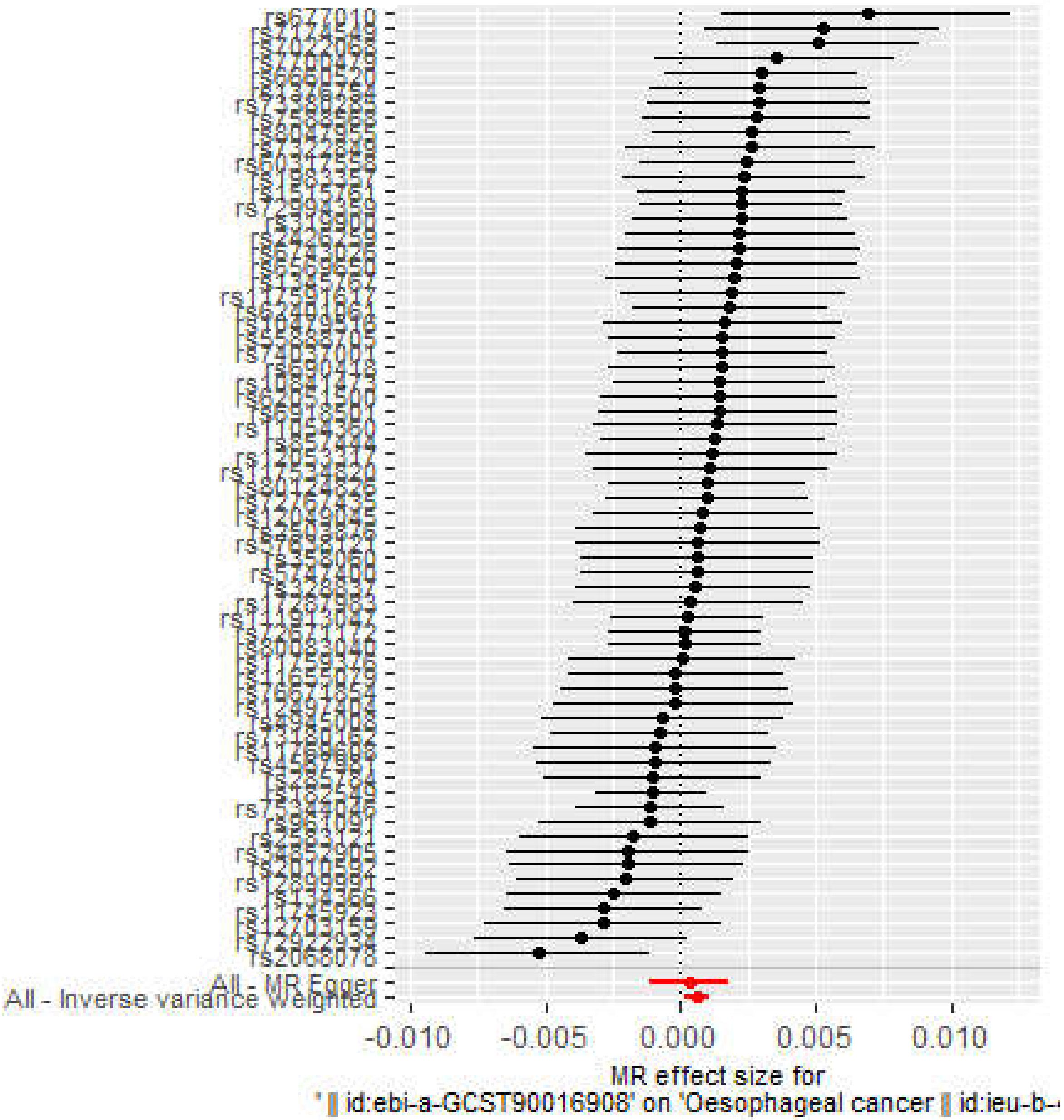
rs677010
rs2066078
All - MR Egger
All - Inverse variance weighted
-0.010
-0.005
0.000
0.005
0.010
MR effect size for
' || id:ebi-a-GCST90016908' on 'Oesophageal cancer || id:ieu-b-

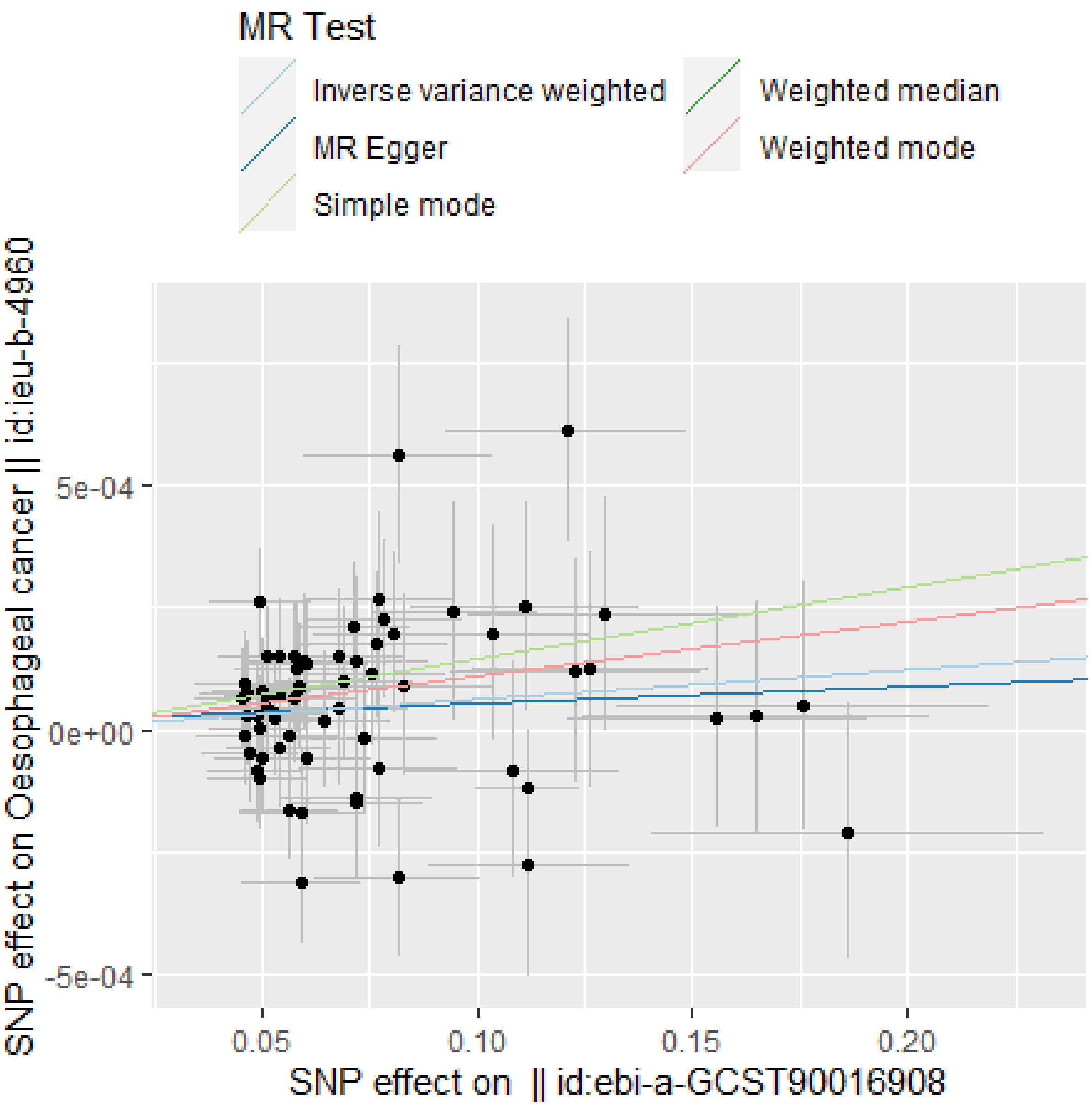
MR Test
Inverse variance weighted
MR Egger
Simple mode
Weighted median
Weighted mode
SNP effect on Oesophageal cancer || id:ieu-b-4960
5e-04
0e+00
-5e-04
0.05
0.10
0.15
0.20
SNP effect on || id:ebi-a-GCST90016908

Figure 12 Leave-one-out analysis, funnel plot, MR effect size and scatter plot for gut microbiota abundance (family Clostridiaceae1 id.1869) on oesophageal cancer

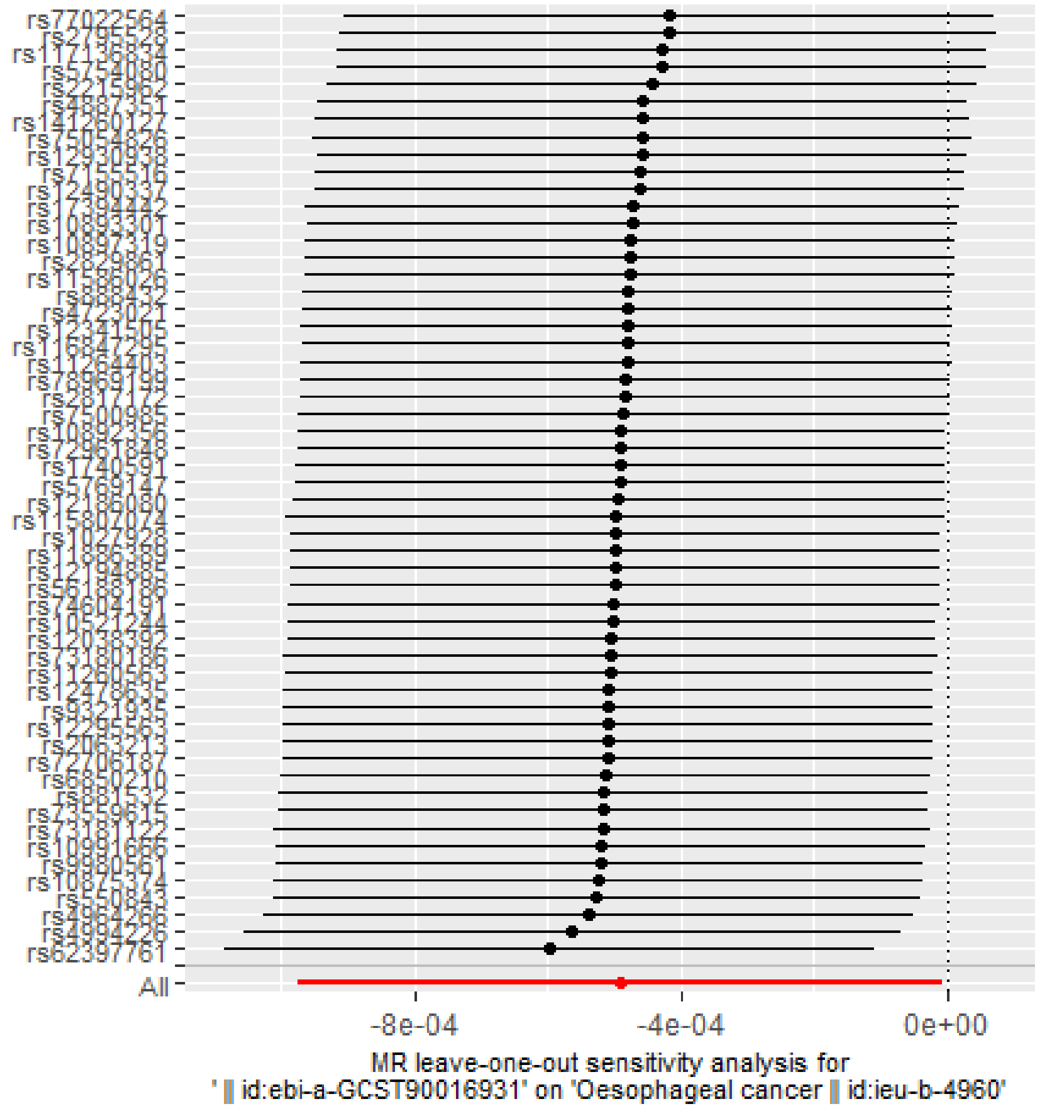

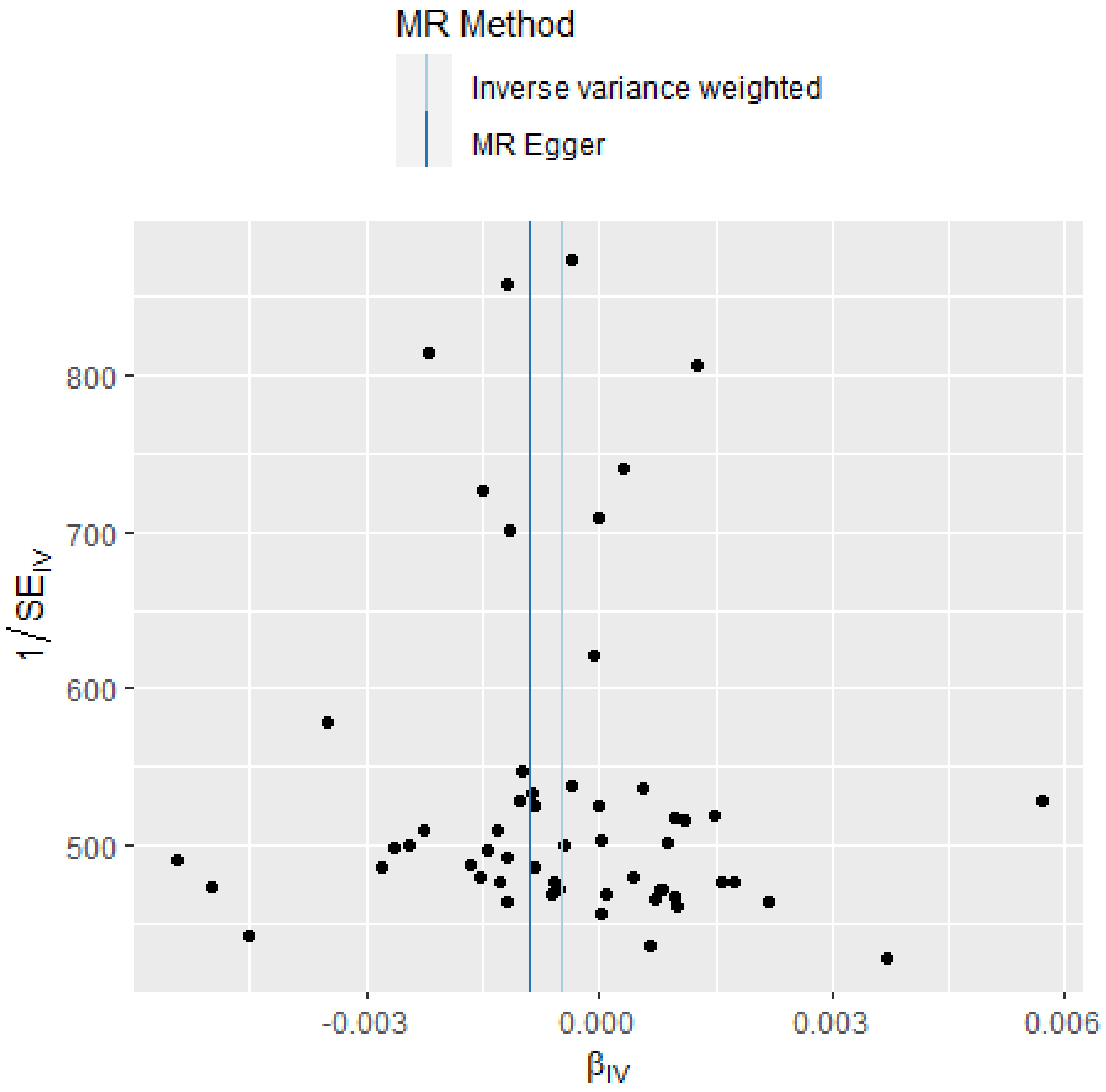

MR Method
Inverse variance weighted
MR Egger
800
700
600
500
1/SE$_{IV}$
-0.003
0.000
0.003
0.006
β$_{IV}$

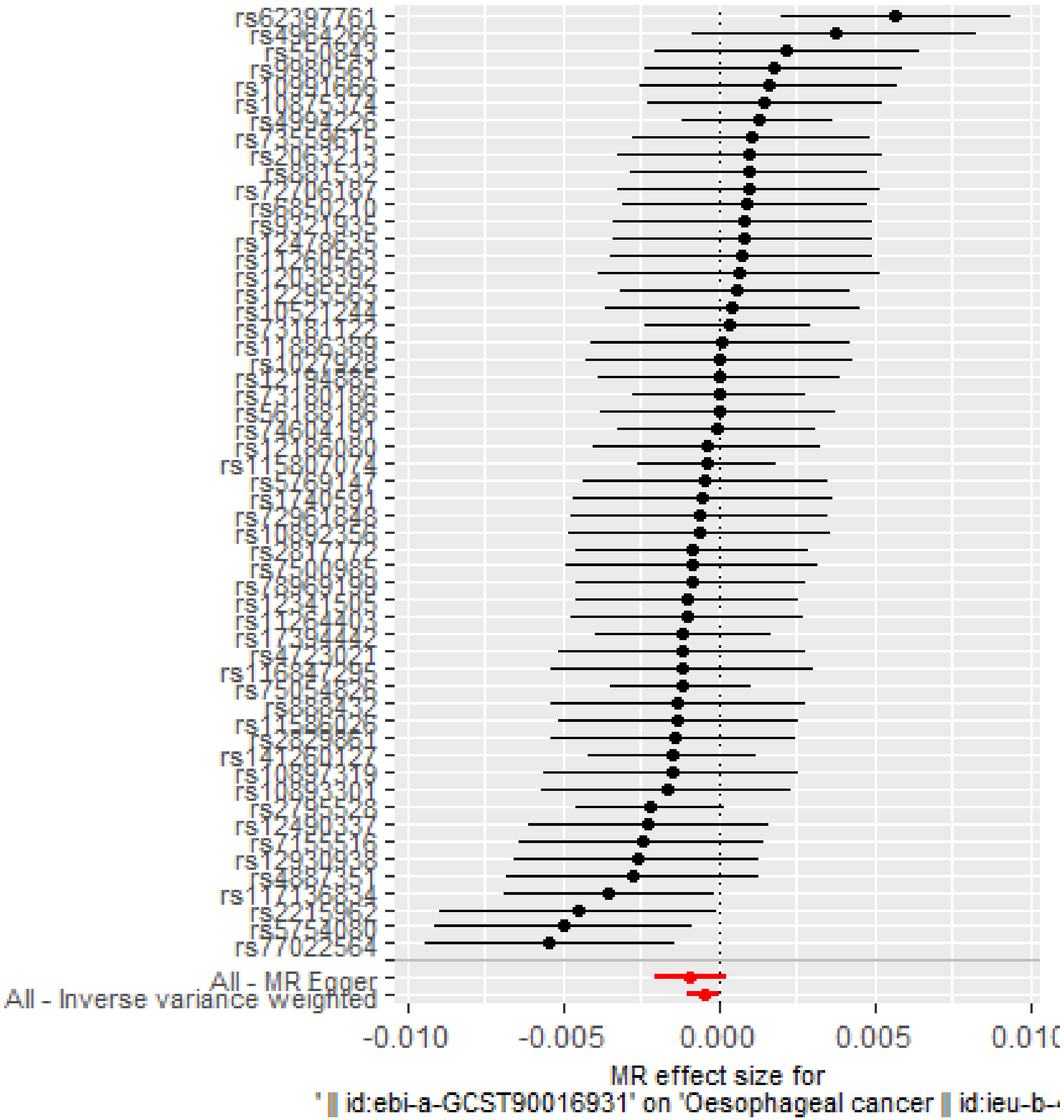

rs62397761
rs4964266
rs10875374
rs72706187
rs6850210
rs115807074
rs5769147
rs75054626
rs10897319
rs10893301
rs12490337
rs7155516
rs5754080
rs77022564
All - MR Egger
All - Inverse variance weighted
-0.010
-0.005
0.000
0.005
MR effect size for
' || id:ebi-a-GCST90016931' on 'Oesophageal cancer || id:ieu-b-

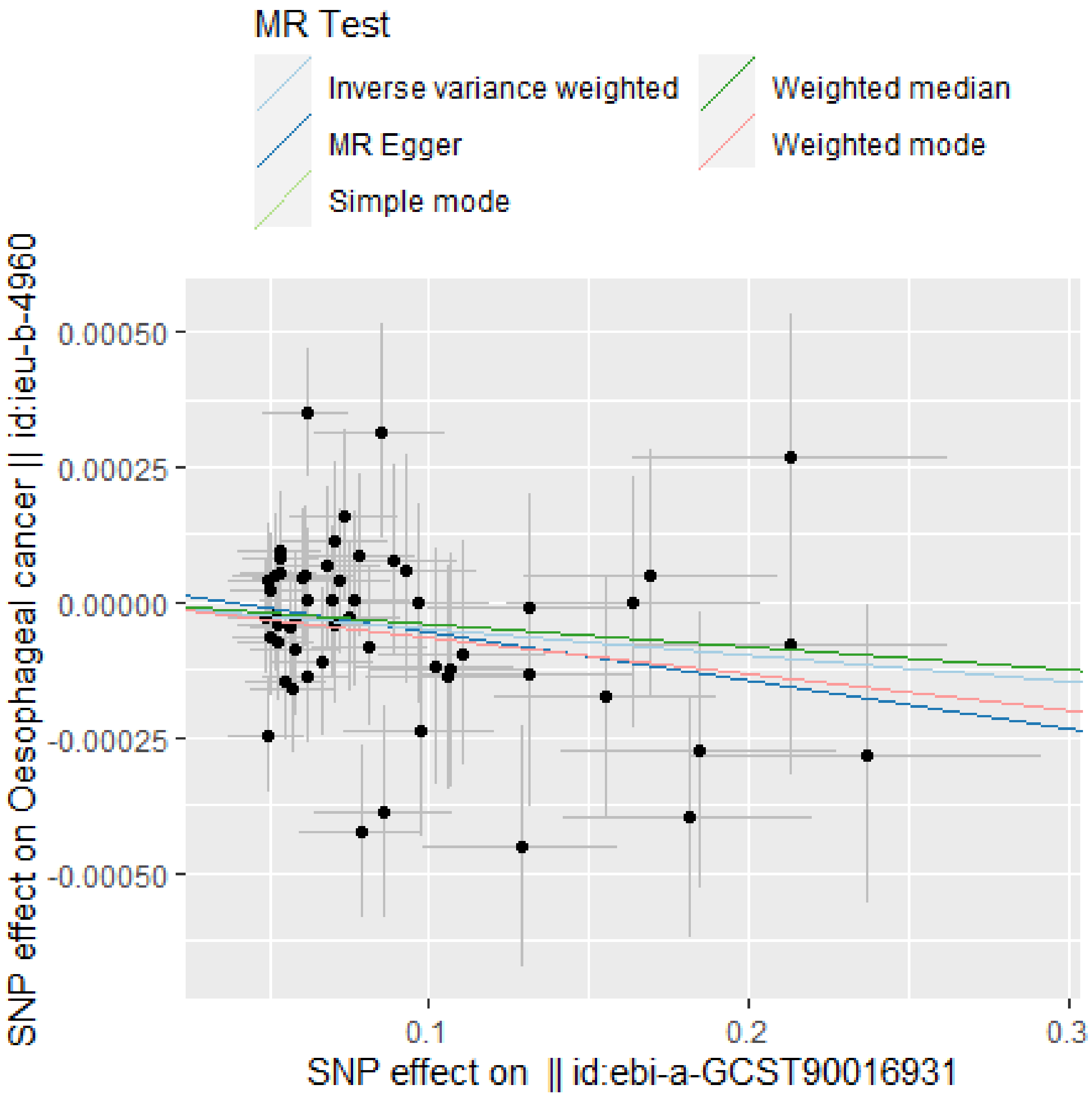

MR Test
Inverse variance weighted
MR Egger
Simple mode
Weighted median
Weighted mode
0.00050
0.00025
0.00000
-0.00025
-0.00050
SNP effect on Oesophageal cancer || id:ieu-b-4960
0.1
0.2
0.3
SNP effect on || id:ebi-a-GCST90016931

Figure 13 Leave-one-out analysis, funnel plot, MR effect size and scatter plot for gut microbiota abundance (family Desulfovibrionaceae id.3169) on oesophageal cancer

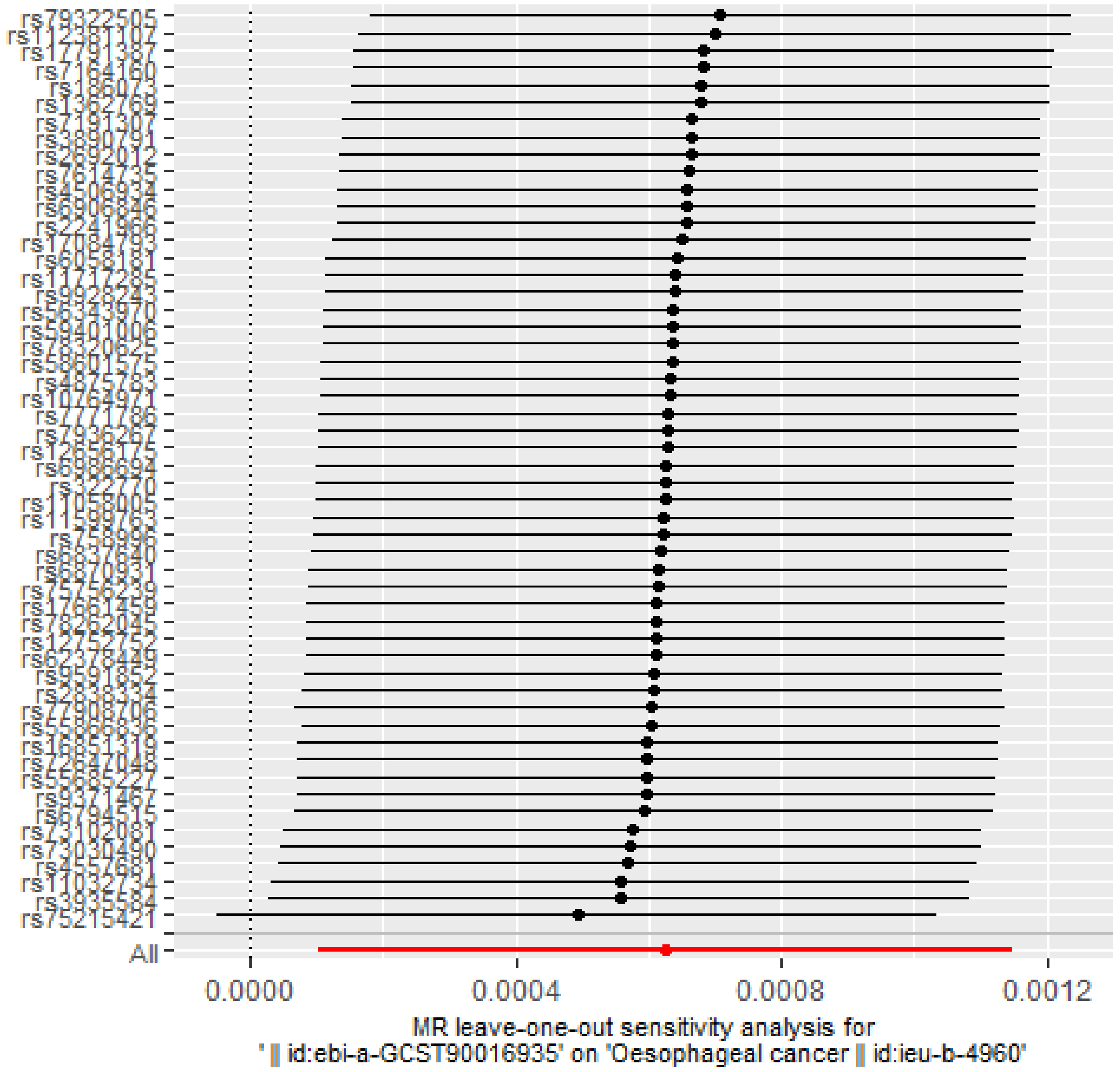

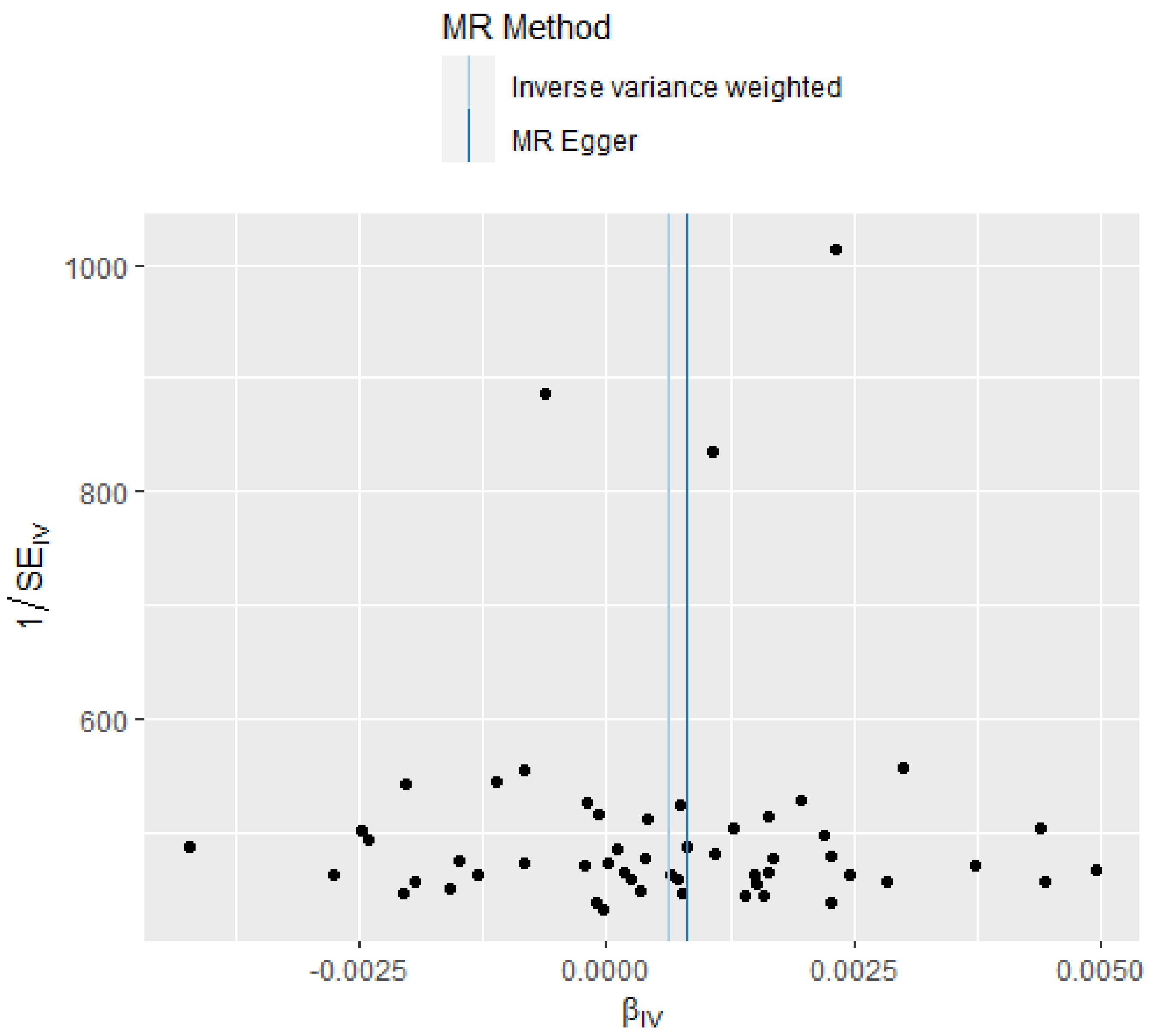

MR Method
Inverse variance weighted
MR Egger
1000
800
600
1/SE$_{IV}$
-0.0025
0.0000
0.0025
0.0050
β_{IV}

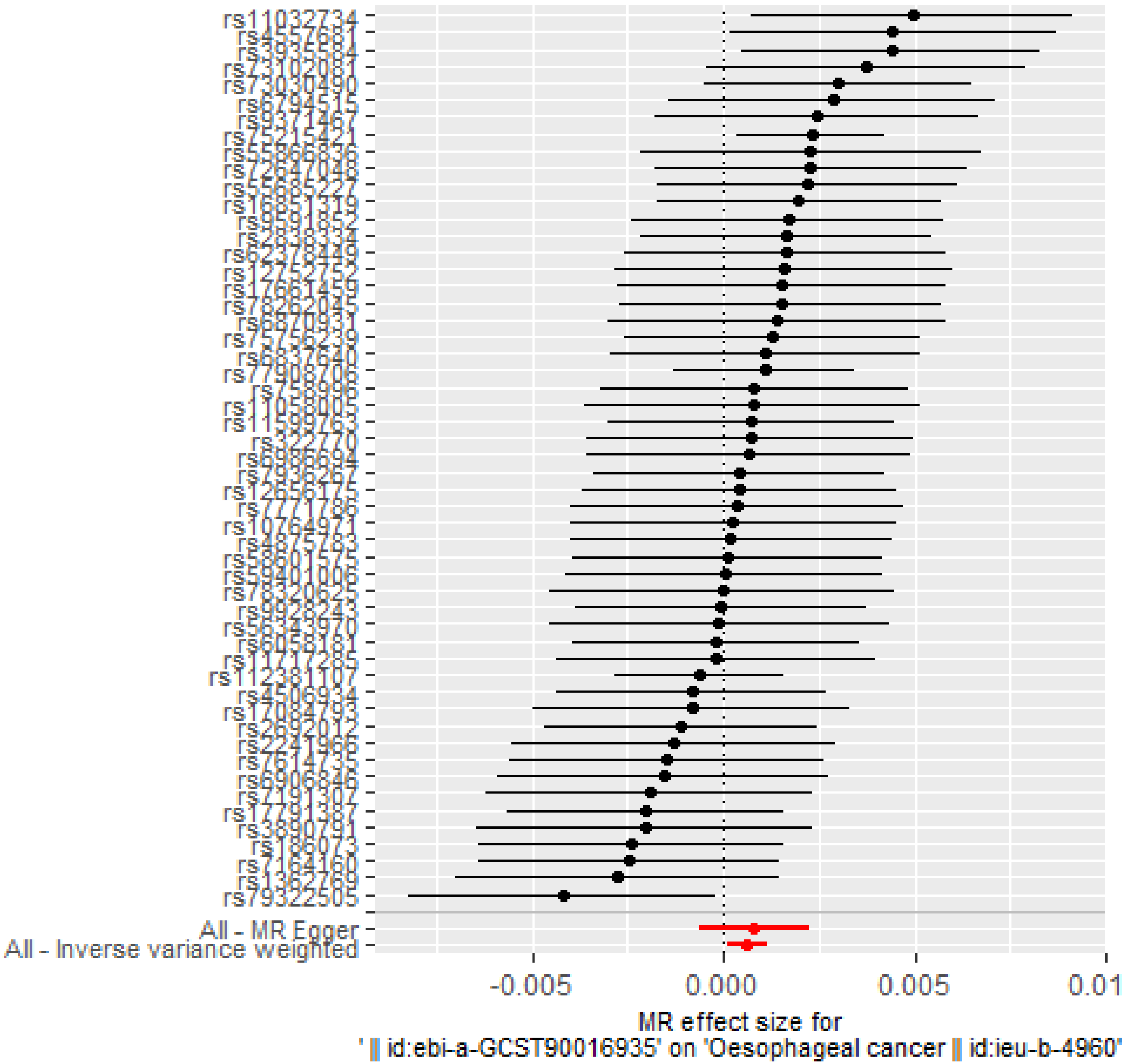
rs11032734
rs4557681
rs73102081
rs73030490
rs6794515
rs75215421
rs72647048
rs16851319
rs9591852
rs62378449
rs17661459
rs78262045
rs6870931
rs75756239
rs6837640
rs77908706
rs11058005
rs11599763
rs322770
rs6986694
rs12656175
rs10764971
rs59401006
rs9928243
rs56343970
rs6058181
rs112381107
rs4506934
rs2692012
rs2241966
rs7614735
rs7191307
rs17791387
rs3890791
rs186073
rs7164160
rs1362769
rs79322505
All - MR Egger
All - Inverse variance weighted
-0.005
0.000
0.005
0.01
MR effect size for
' || id:ebi-a-GCST90016935' on 'Oesophageal cancer || id:ieu-b-4960'

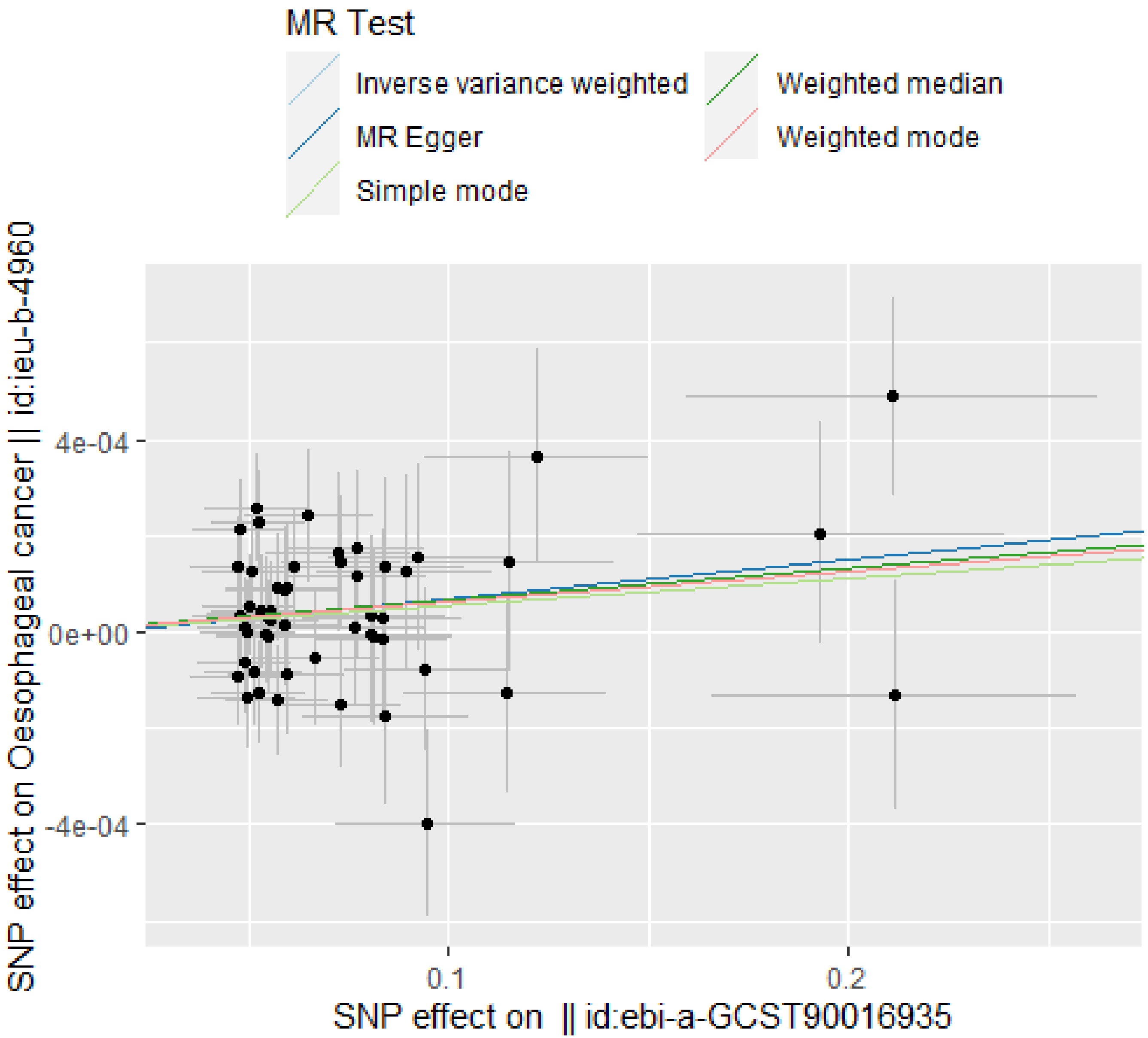

MR Test
Inverse variance weighted
MR Egger
Simple mode
Weighted median
Weighted mode
SNP effect on Oesophageal cancer || id:ieu-b-4960
4e-04
0e+00
-4e-04
0.1
0.2
SNP effect on || id:ebi-a-GCST90016935

Figure 14 Leave-one-out analysis, funnel plot, MR effect size and scatter plot for gut microbiota abundance (family Pasteurellaceae id.3689) on oesophageal cancer

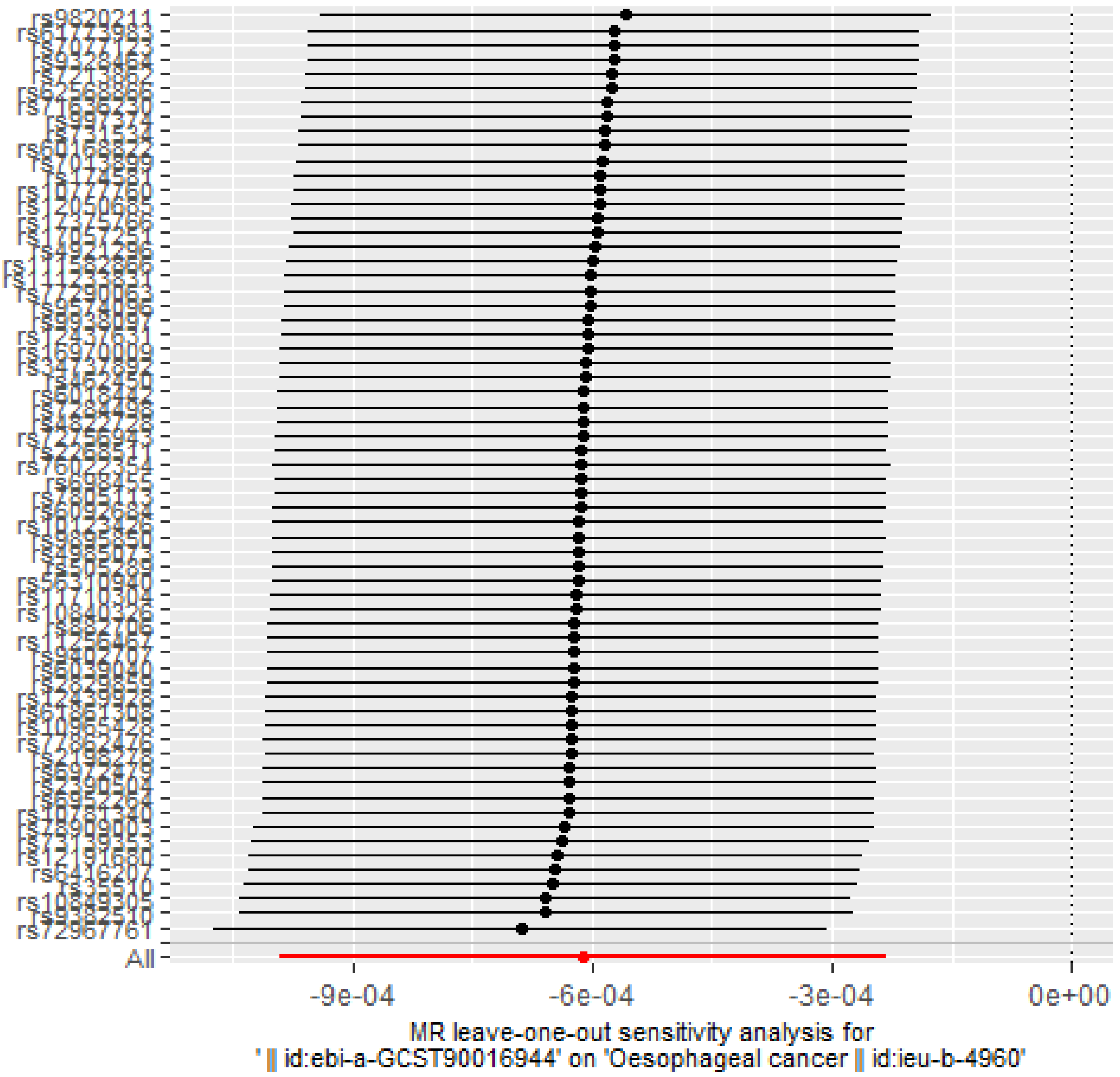

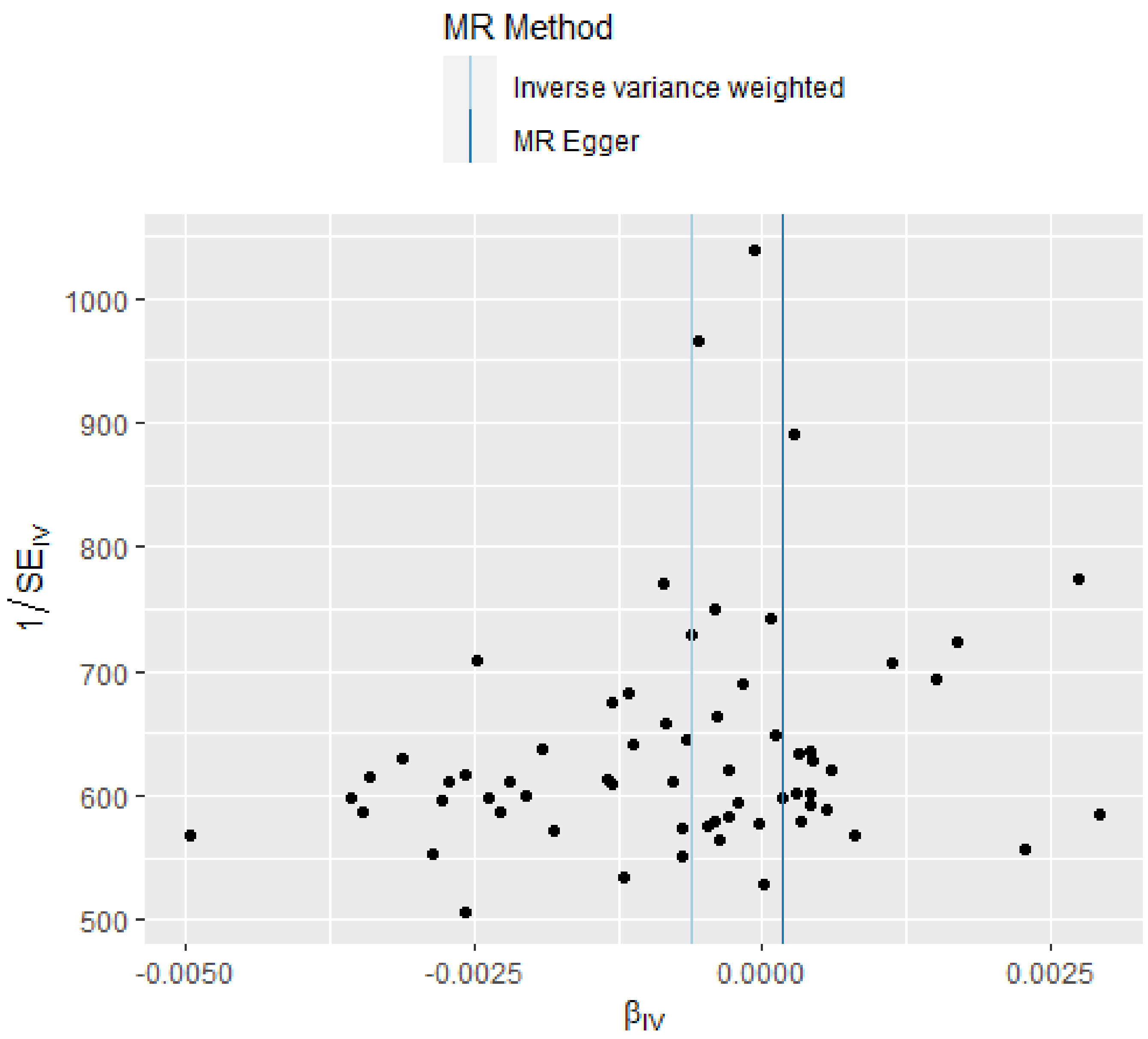
MR Method
Inverse variance weighted
MR Egger
1000
900
800
700
600
500
1/SE_IV
-0.0050
-0.0025
0.0000
0.0025
β_IV

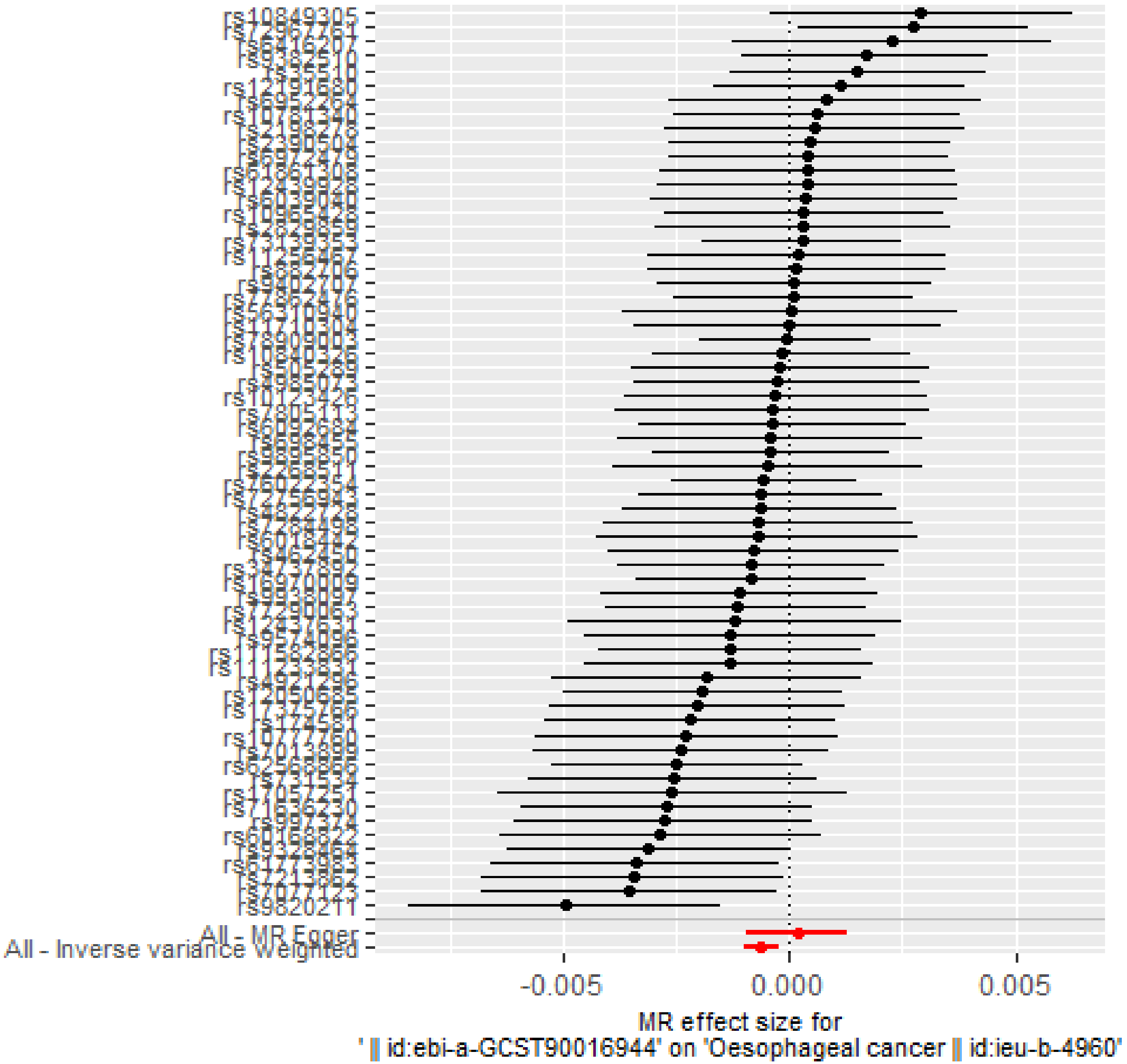
rs10849305
rs9820211
All - MR Egger
All - Inverse variance weighted
-0.005
0.000
0.005
MR effect size for
' || id:ebi-a-GCST90016944' on 'Oesophageal cancer || id:ieu-b-4960'

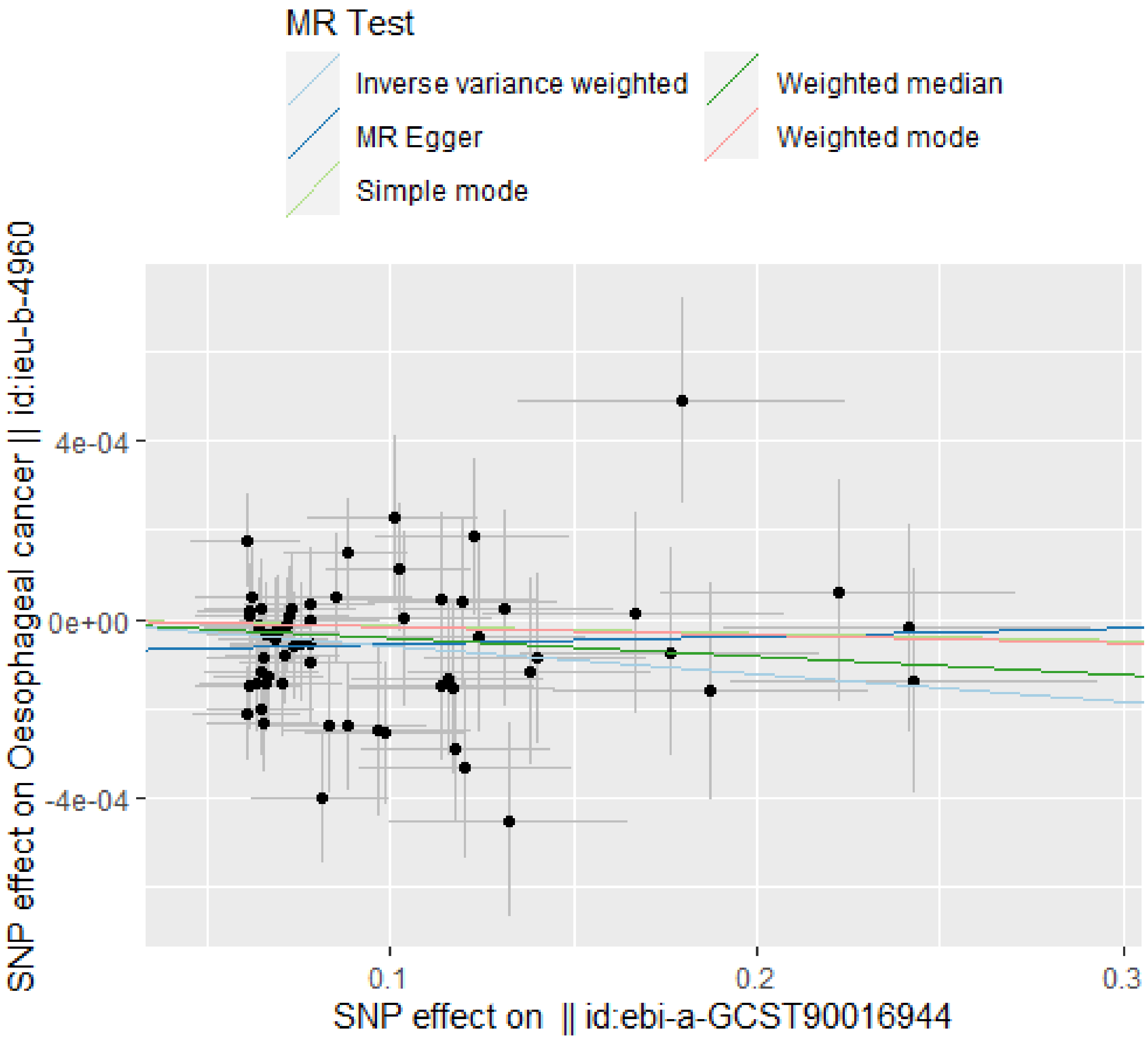

MR Test
Inverse variance weighted
MR Egger
Simple mode
Weighted median
Weighted mode
4e-04
0e+00
-4e-04
0.1
0.2
0.3
SNP effect on Oesophageal cancer || id:ieu-b-4960
SNP effect on || id:ebi-a-GCST90016944

Figure 15 Leave-one-out analysis, funnel plot, MR effect size and scatter plot for gut microbiota abundance (family Ruminococcaceae id.2050) on oesophageal cancer

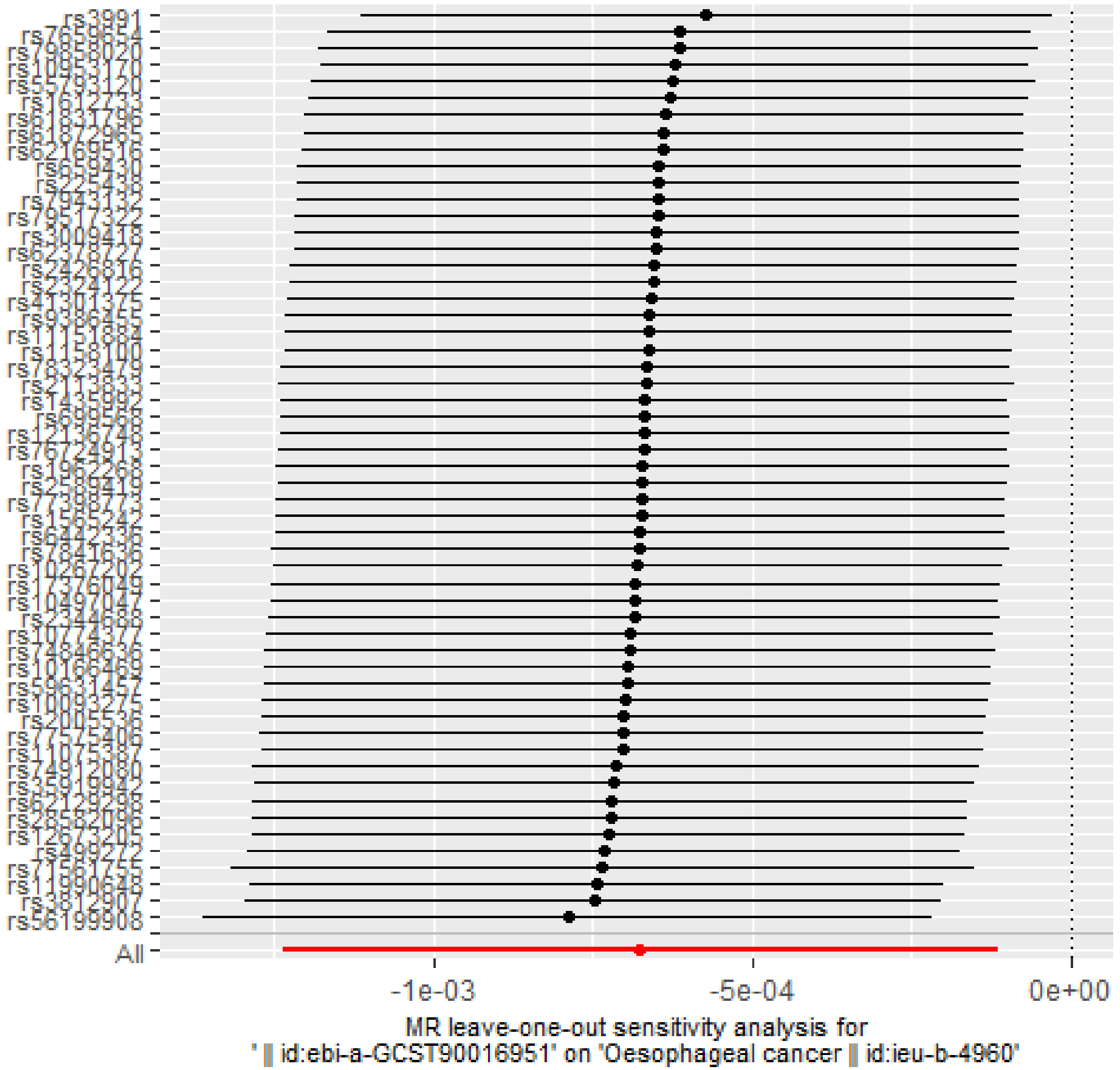

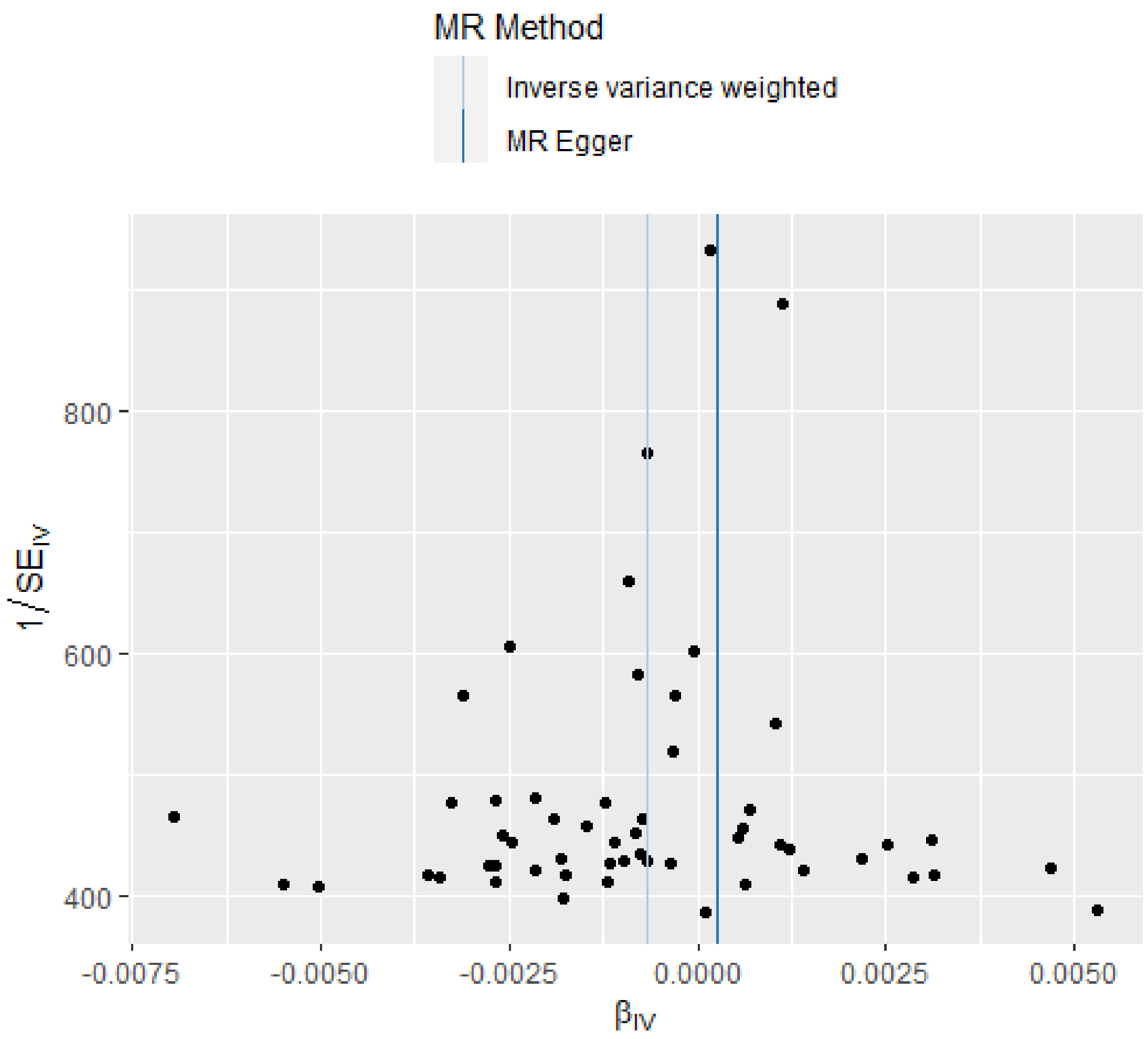

MR Method
Inverse variance weighted
MR Egger
800
600
400
1/SE_IV
-0.0075
-0.0050
-0.0025
0.0000
0.0025
0.0050
β_IV

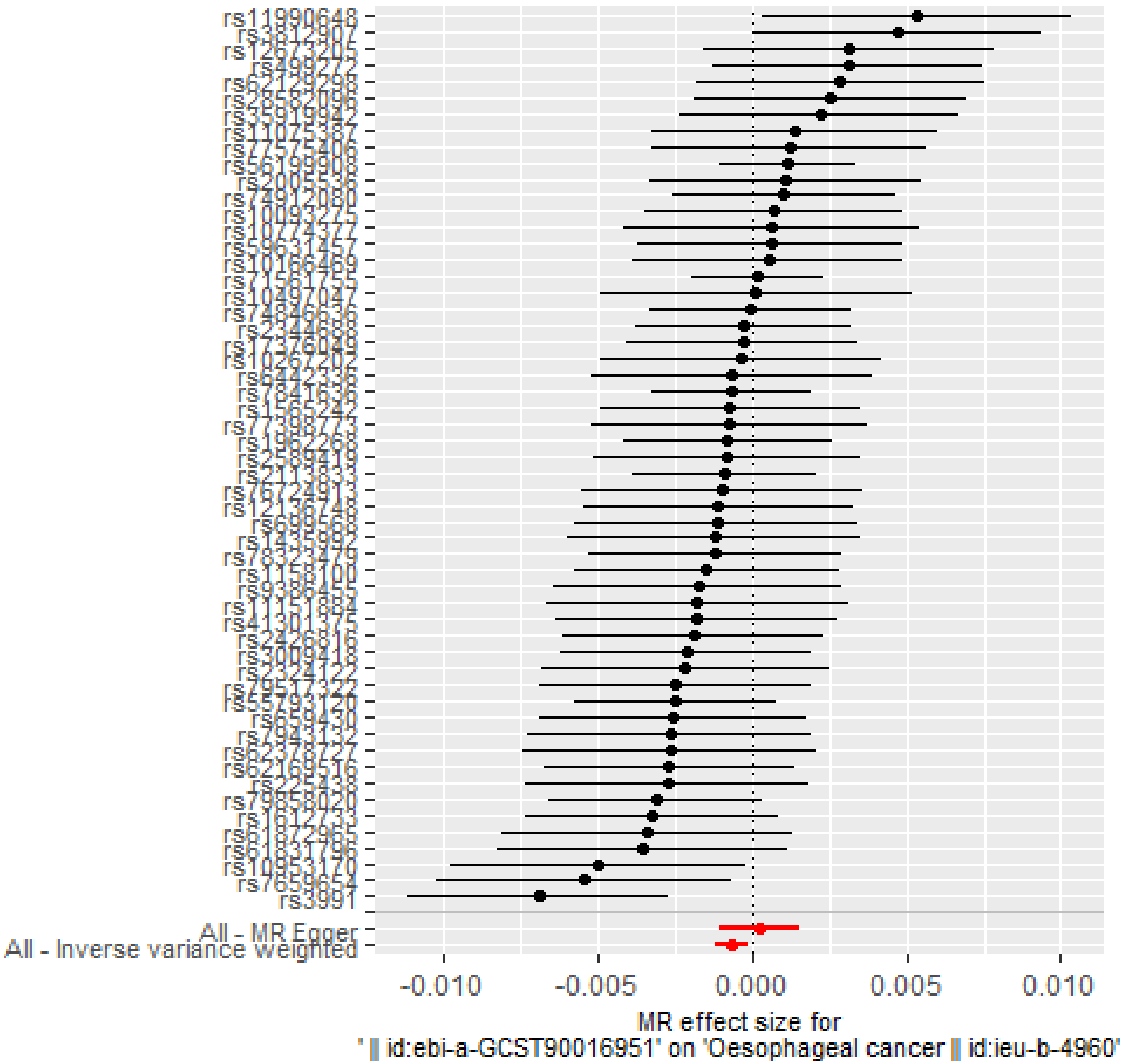
All - MR Egger
All - Inverse variance weighted
-0.010
-0.005
0.000
0.005
0.010
MR effect size for
' || id:ebi-a-GCST90016951' on 'Oesophageal cancer || id:ieu-b-4960'

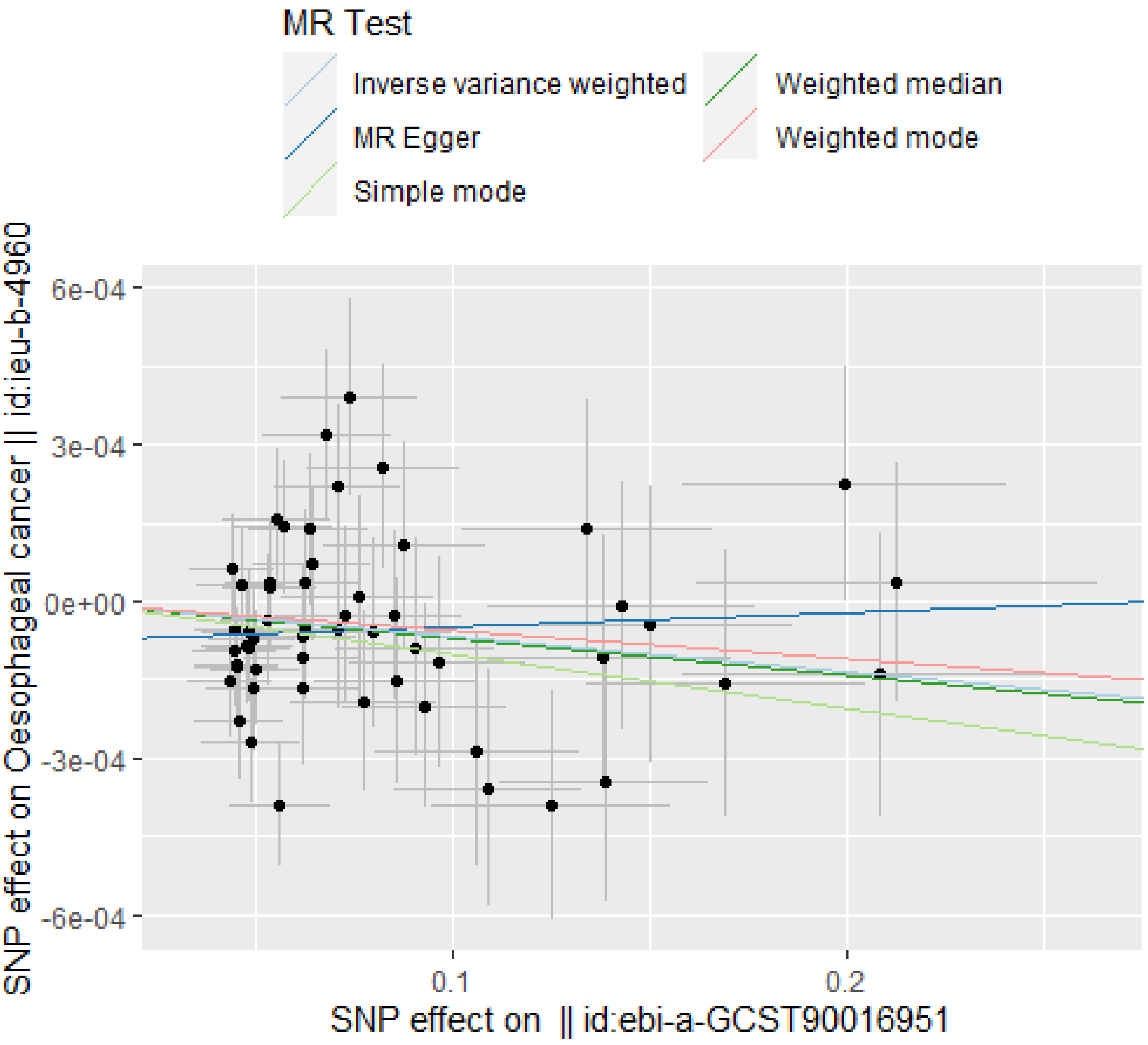
MR Test
Inverse variance weighted
MR Egger
Simple mode
Weighted median
Weighted mode
6e-04
3e-04
0e+00
-3e-04
-6e-04
0.1
0.2
SNP effect on Oesophageal cancer || id:ieu-b-4960
SNP effect on || id:ebi-a-GCST90016951

Figure 16 Leave-one-out analysis, funnel plot, MR effect size and scatter plot for gut microbiota abundance (genus Eisenbergiella id.11304) on oesophageal cancer

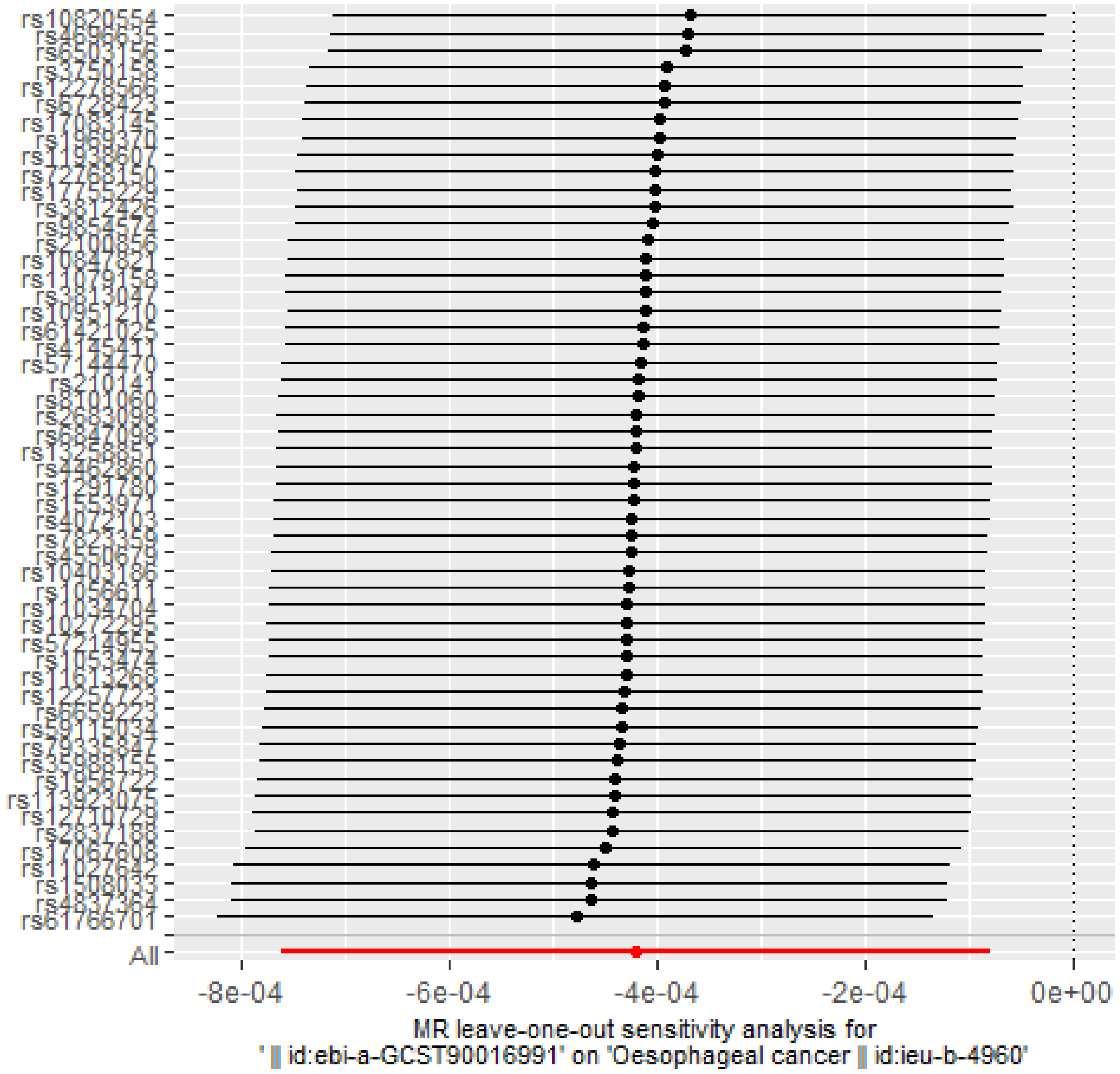

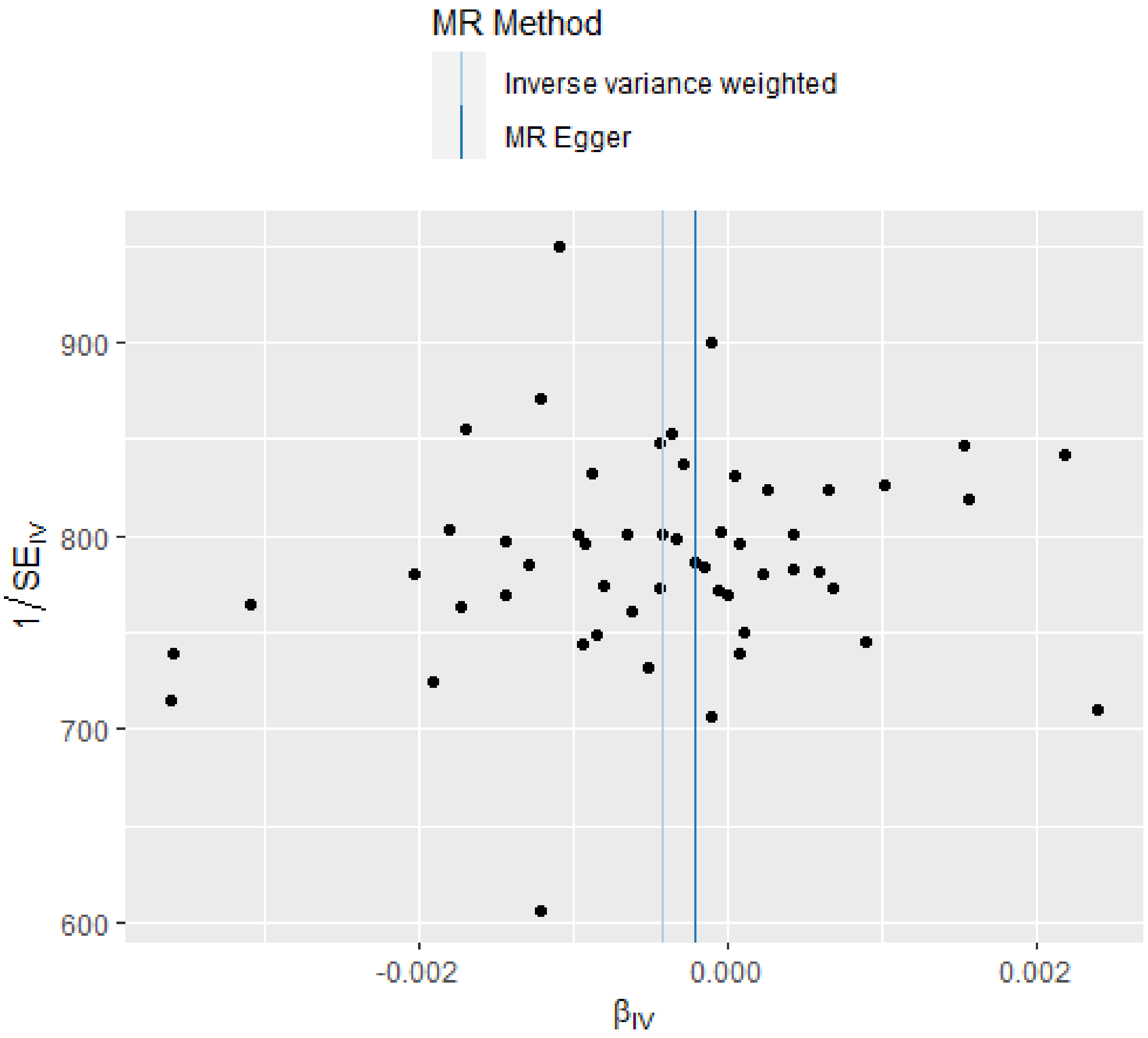
MR Method
Inverse variance weighted
MR Egger
900
800
700
600
-0.002
0.000
0.002
β_{IV}
$1/SE_{IV}$

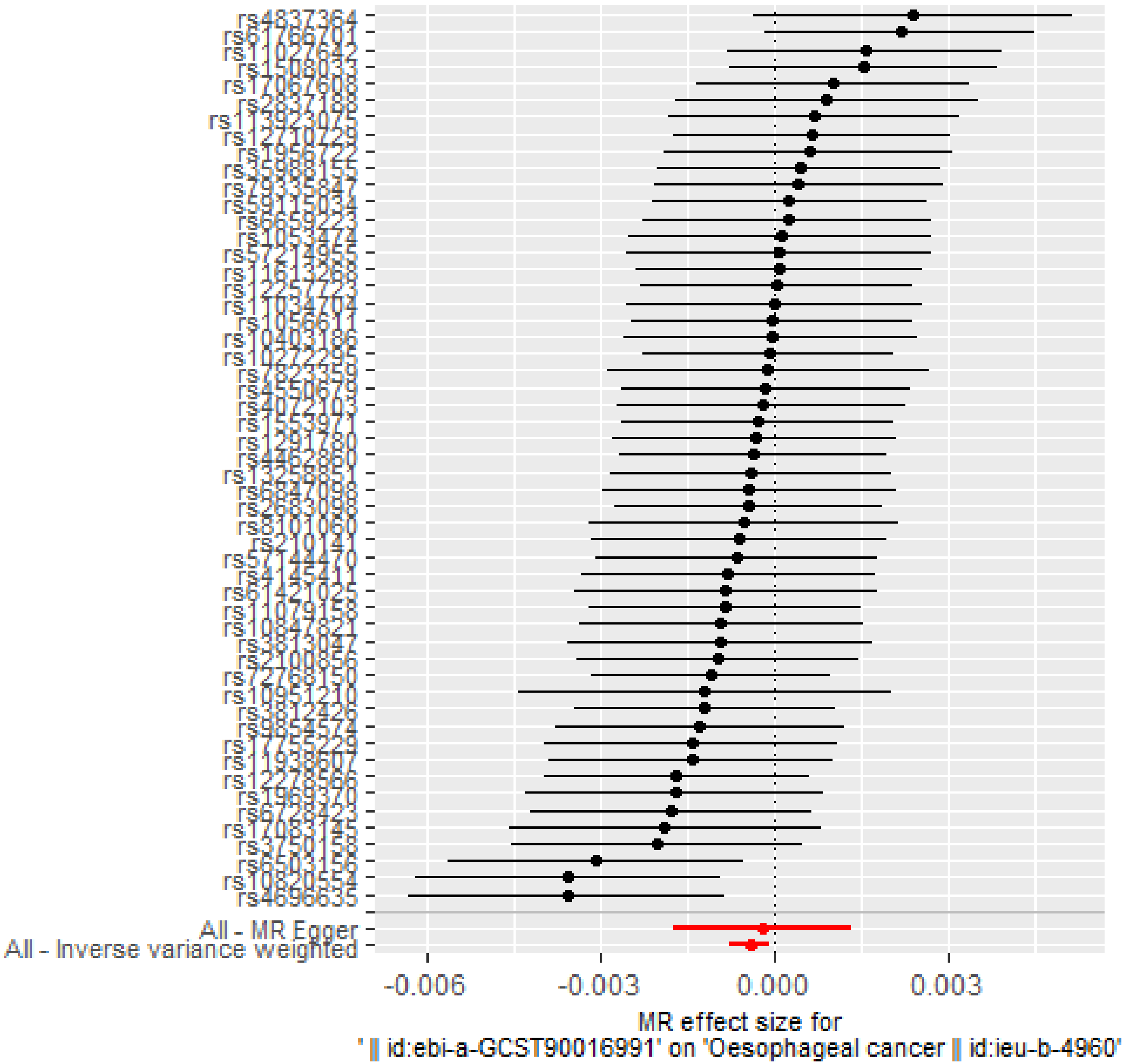
rs4837364
rs61766701
rs11027642
rs1508033
rs17067608
rs2837188
rs113923075
rs12710729
rs1956722
rs35988155
rs79335847
rs59115034
rs6659223
rs1053474
rs57214955
rs11613268
rs12257723
rs11034704
rs1056611
rs10403186
rs10272295
rs7823359
rs4550679
rs4072103
rs1553971
rs1291780
rs4462880
rs13256821
rs6847098
rs2683098
rs8101060
rs210141
rs57144470
rs4145411
rs61421025
rs11079158
rs10847821
rs3813047
rs2100856
rs72768150
rs10951210
rs3812428
rs9854574
rs17755229
rs11938607
rs12278566
rs1969370
rs6728423
rs17083145
rs3750158
rs6503156
rs10820554
rs4696635
All - MR Egger
All - Inverse variance weighted
-0.006
-0.003
0.000
0.003
MR effect size for
' || id:ebi-a-GCST90016991' on 'Oesophageal cancer || id:ieu-b-4960'

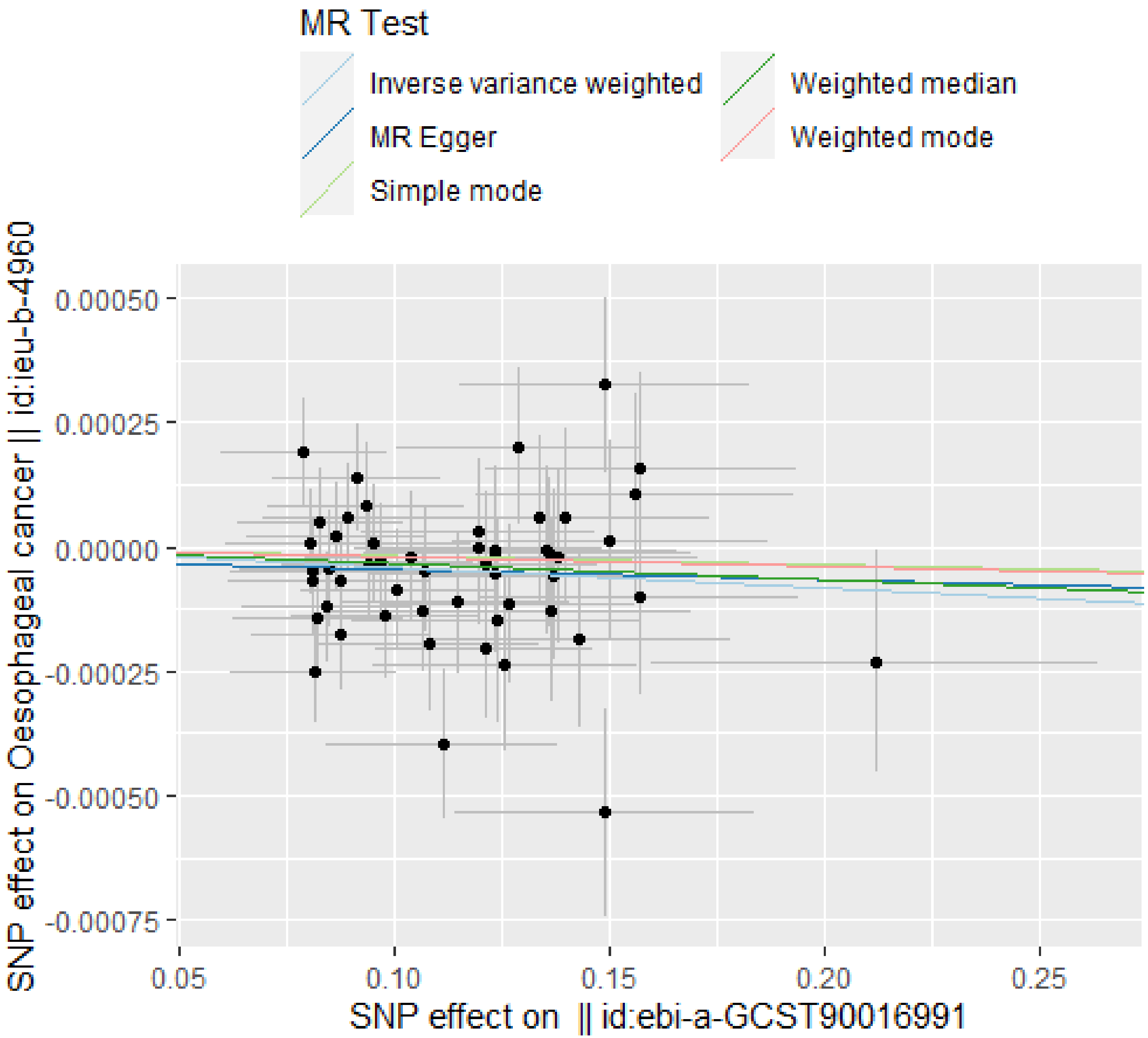
MR Test
Inverse variance weighted
MR Egger
Simple mode
Weighted median
Weighted mode
0.00050
0.00025
0.00000
-0.00025
-0.00050
-0.00075
SNP effect on Oesophageal cancer || id:ieu-b-4960
0.05
0.10
0.15
0.20
0.25
SNP effect on || id:ebi-a-GCST90016991

Figure 17 Leave-one-out analysis, funnel plot, MR effect size and scatter plot for gut microbiota abundance (genus Oxalobacter id.2978) on oesophageal cancer

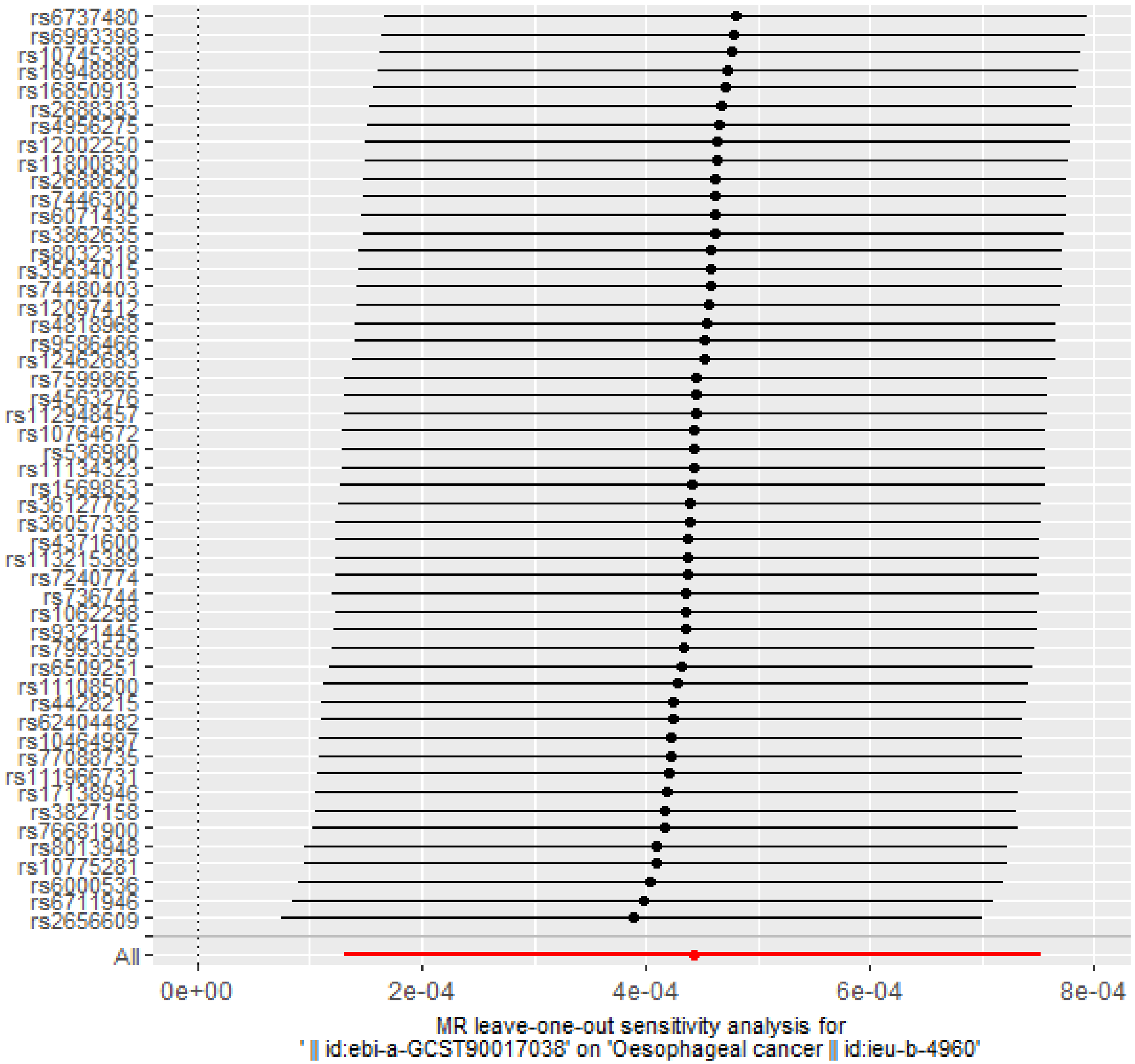

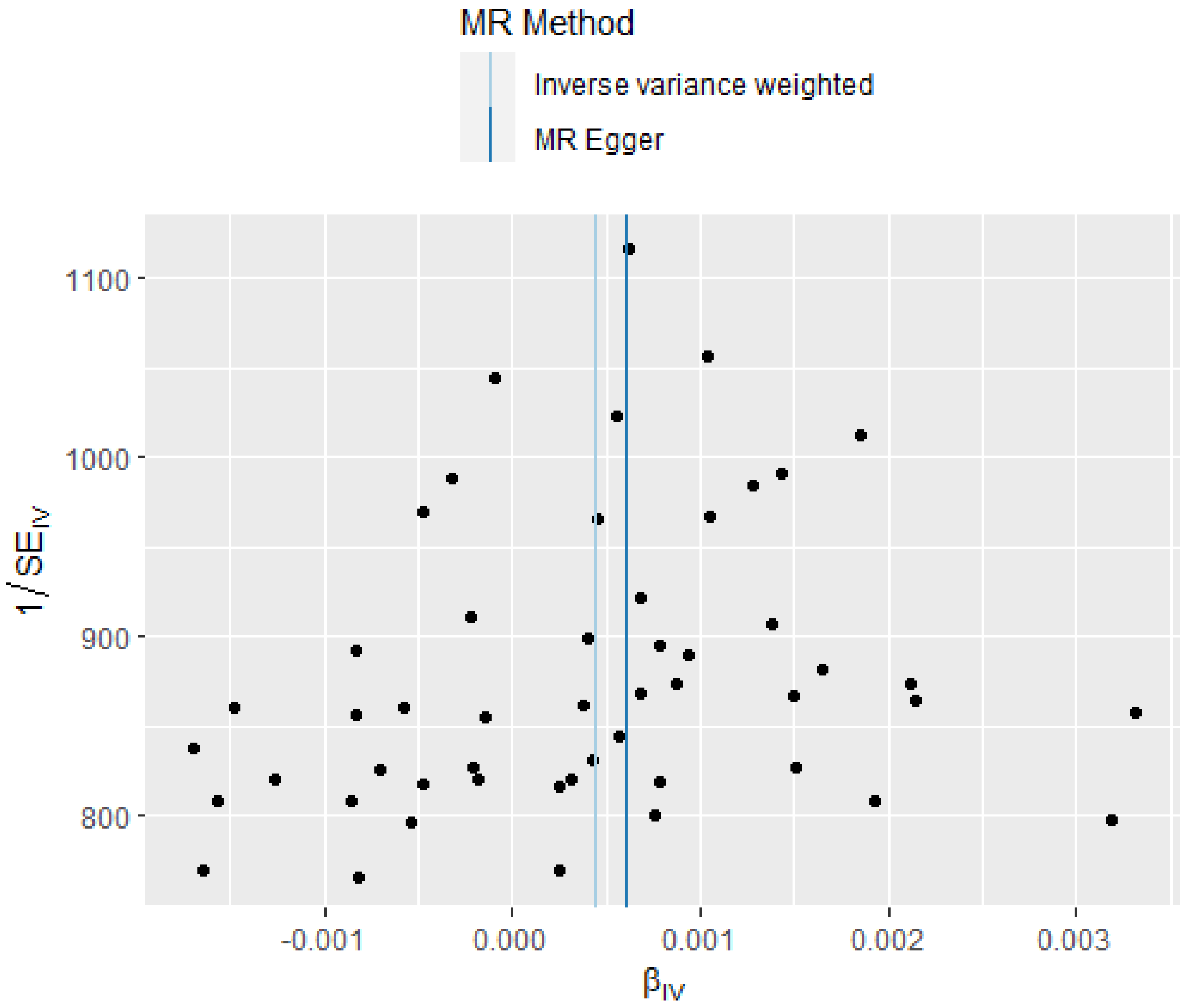

MR Method
Inverse variance weighted
MR Egger
1100
1000
900
800
$1/SE_{IV}$
-0.001
0.000
0.001
0.002
0.003
β_{IV}

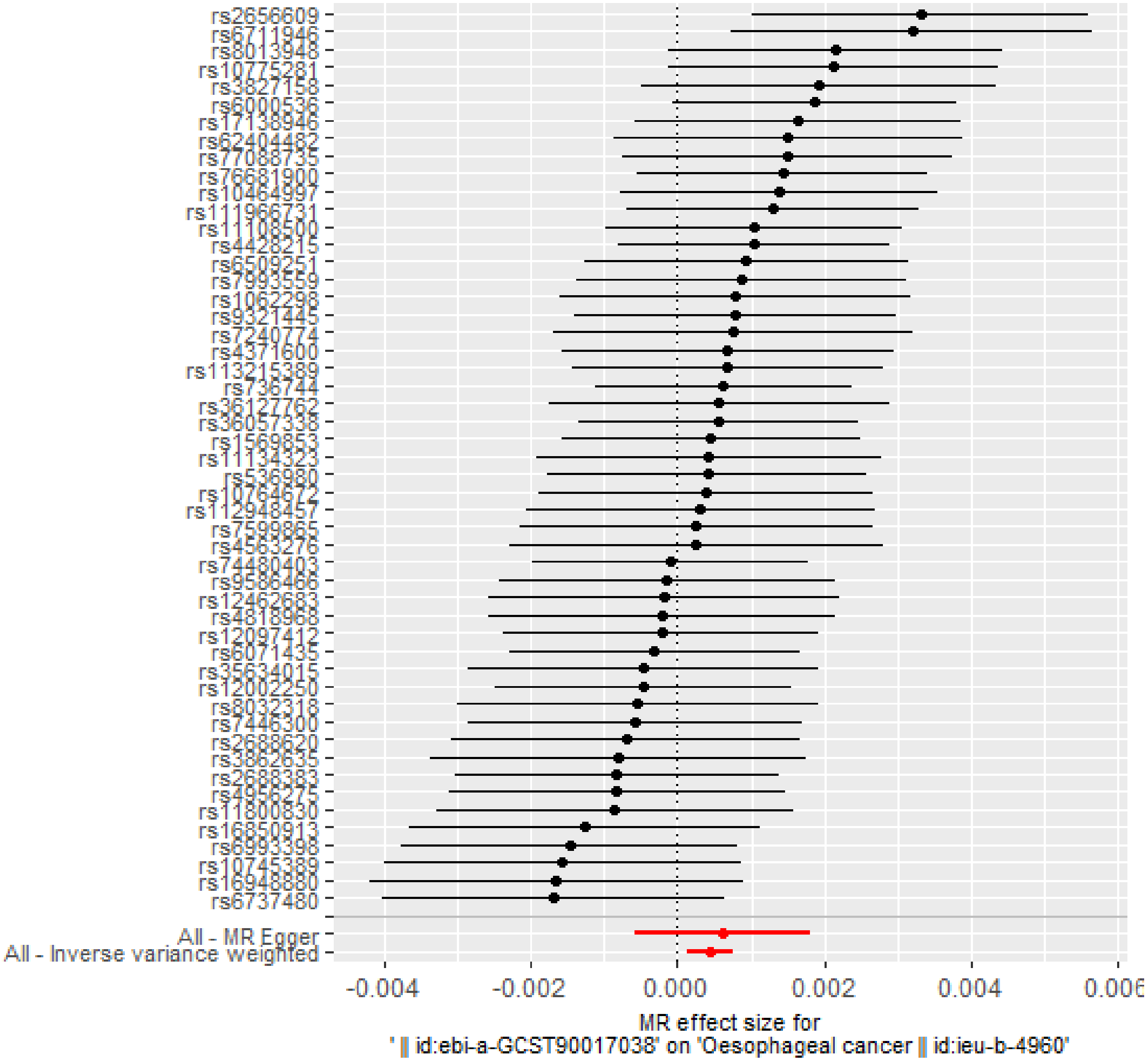
rs2656609
rs6711946
rs8013948
rs10775281
rs3827158
rs6000536
rs17138946
rs62404482
rs77088735
rs76681900
rs10464997
rs111966731
rs11108500
rs4428215
rs6509251
rs7993559
rs1062298
rs9321445
rs7240774
rs4371600
rs113215389
rs736744
rs36127762
rs36057338
rs1569853
rs11134323
rs536980
rs10764672
rs112948457
rs7599865
rs4563276
rs74480403
rs9586466
rs12462683
rs4818968
rs12097412
rs6071435
rs35634015
rs12002250
rs8032318
rs7446300
rs2688620
rs3862635
rs2688383
rs4956275
rs11800830
rs16850913
rs6993398
rs10745389
rs16948880
rs6737480
All - MR Egger
All - Inverse variance weighted
-0.004
-0.002
0.000
0.002
0.004
0.006
MR effect size for
' || id:ebi-a-GCST90017038' on 'Oesophageal cancer || id:ieu-b-4960'

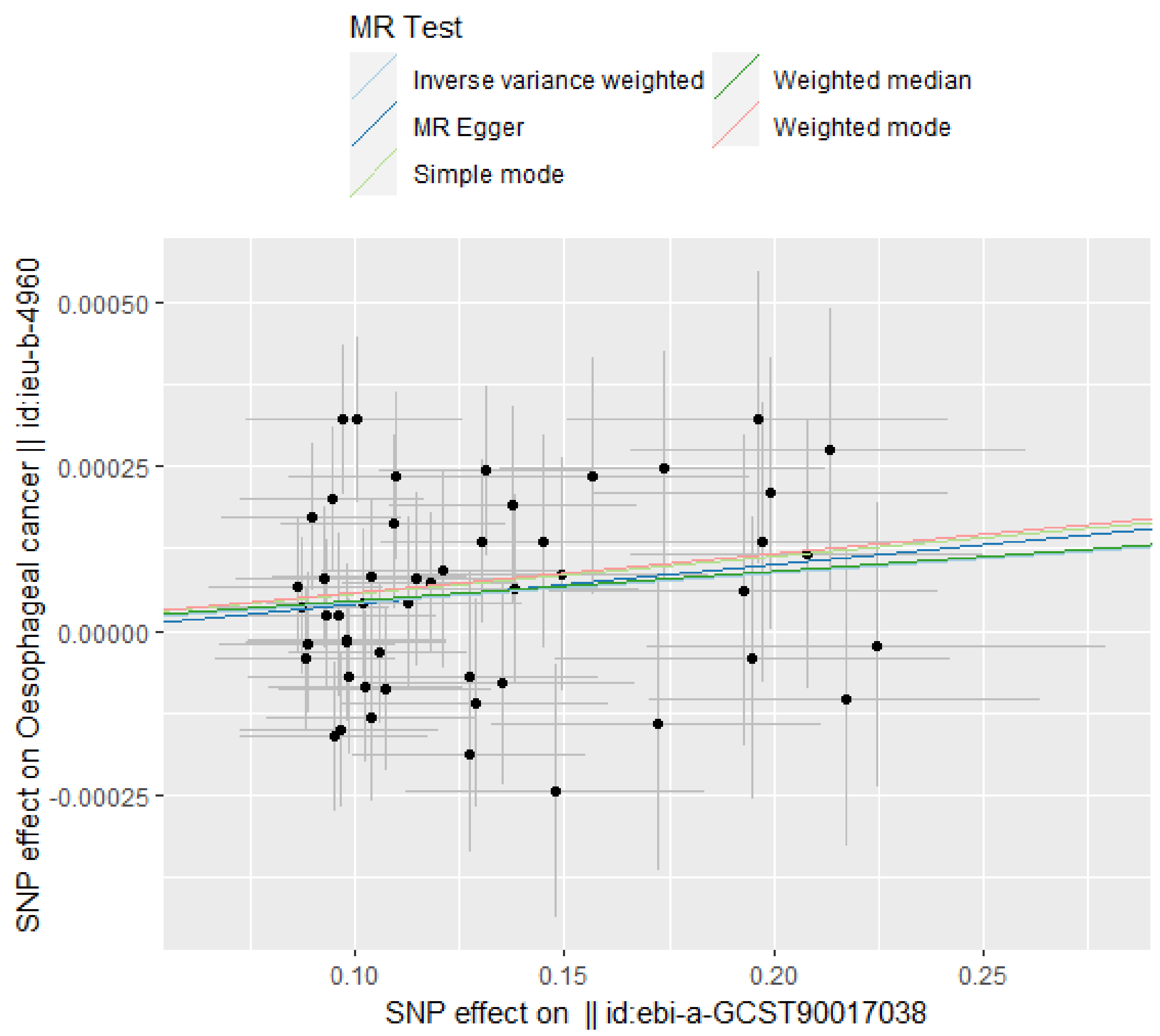

MR Test
Inverse variance weighted
MR Egger
Simple mode
Weighted median
Weighted mode
0.00050
0.00025
0.00000
-0.00025
SNP effect on Oesophageal cancer || id:ieu-b-4960
0.10
0.15
0.20
0.25
SNP effect on || id:ebi-a-GCST90017038

Figure 18 Leave-one-out analysis, funnel plot, MR effect size and scatter plot for gut microbiota abundance (genus Prevotella9 id.11183) on oesophageal cancer

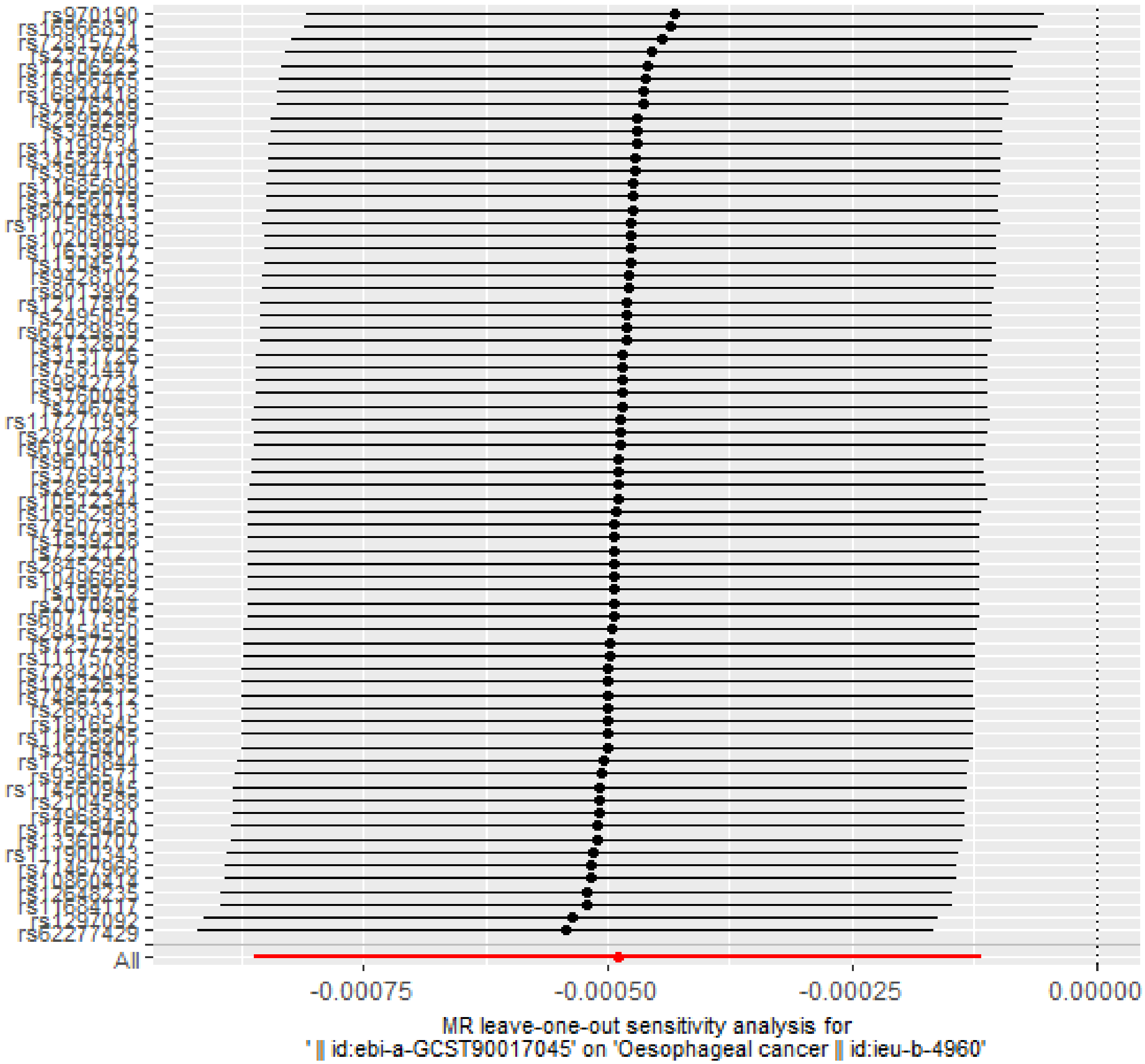

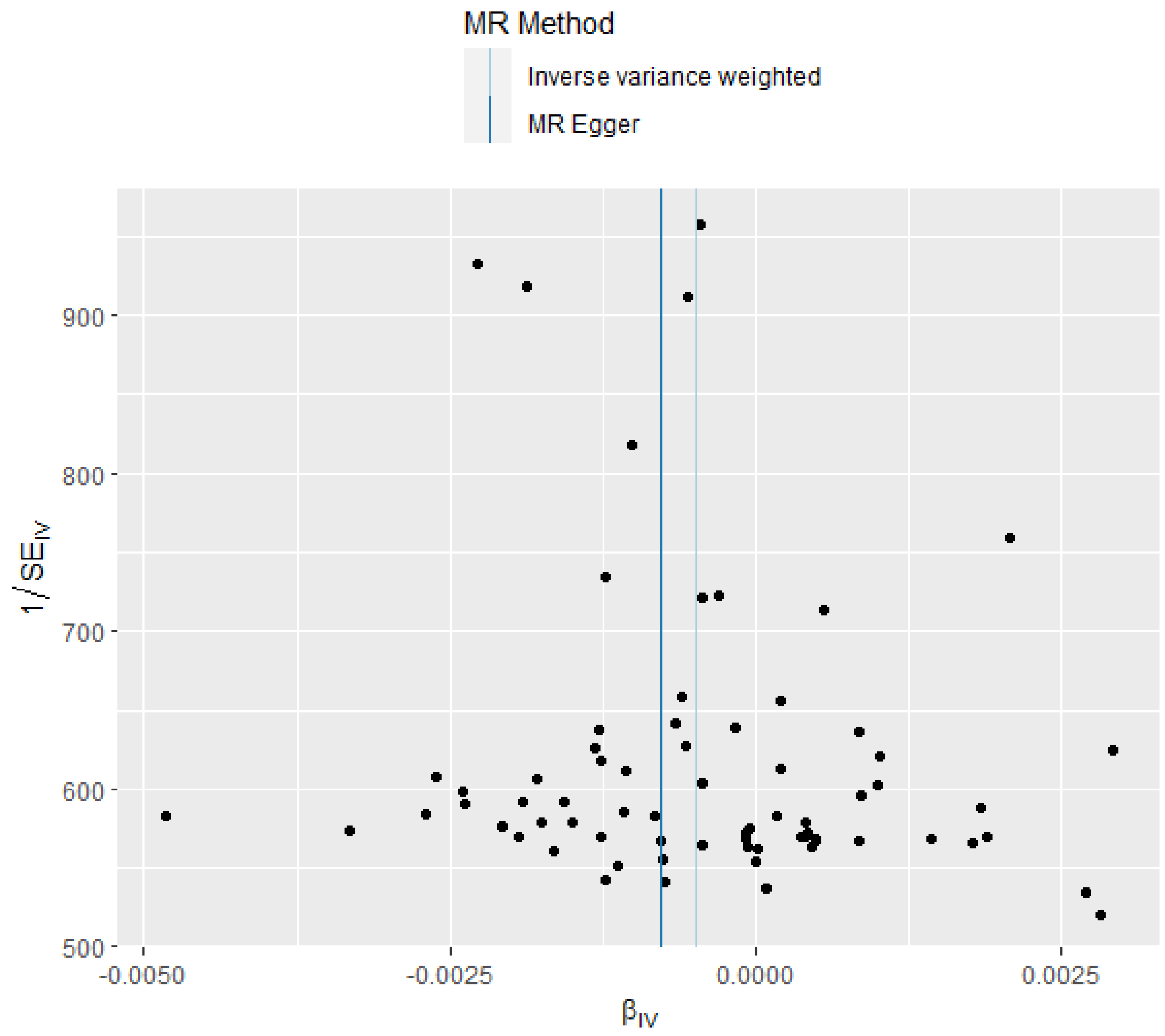
MR Method
Inverse variance weighted
MR Egger
900
800
700
600
500
-0.0050
-0.0025
0.0000
0.0025
$1/SE_{IV}$
β_{IV}

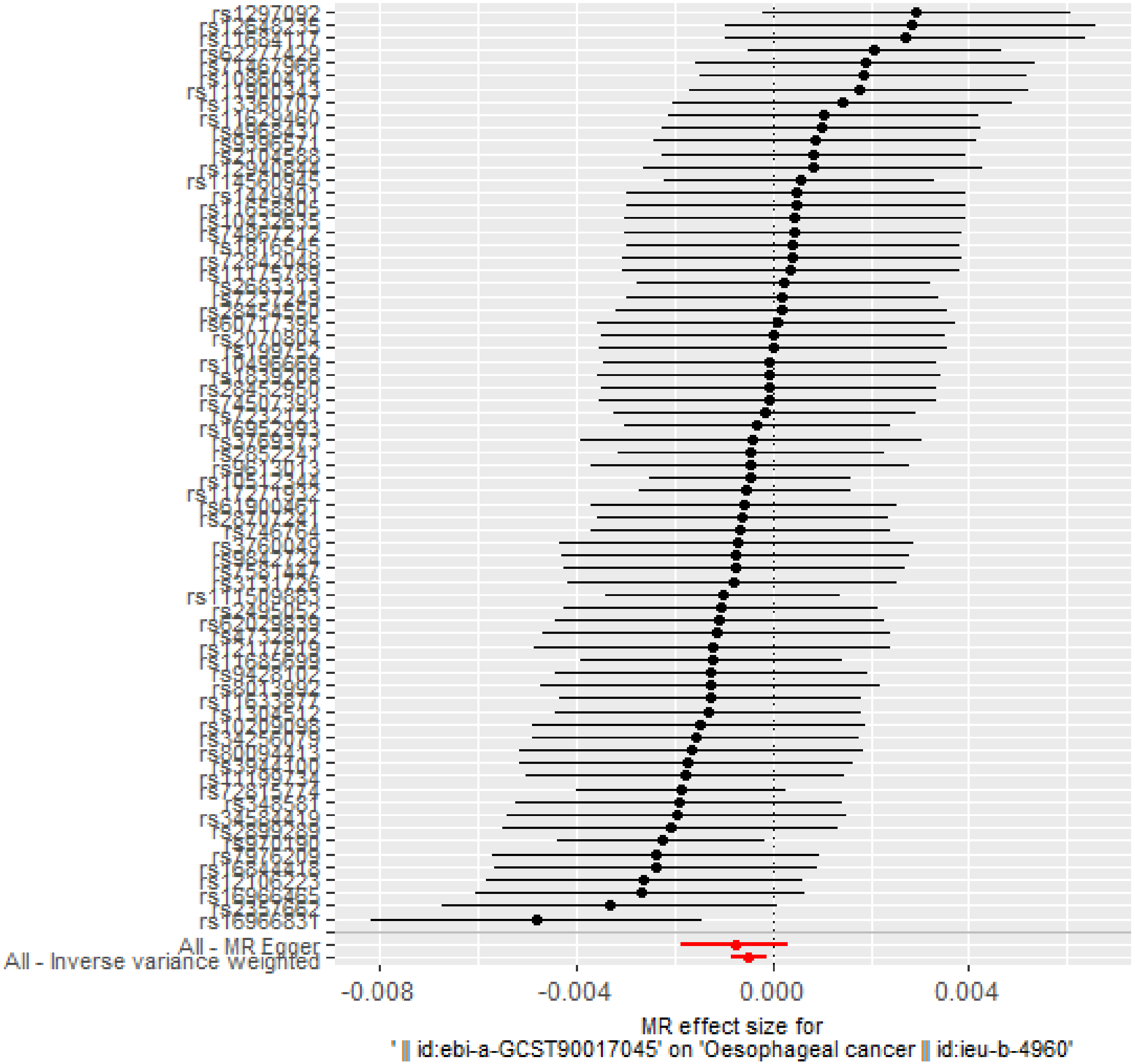

rs1297092
rs2070804
rs9613013
rs111509863
rs16966831
All - MR Egger
All - Inverse variance weighted
-0.008
-0.004
0.000
0.004
MR effect size for
' || id:ebi-a-GCST90017045' on 'Oesophageal cancer || id:ieu-b-4960'

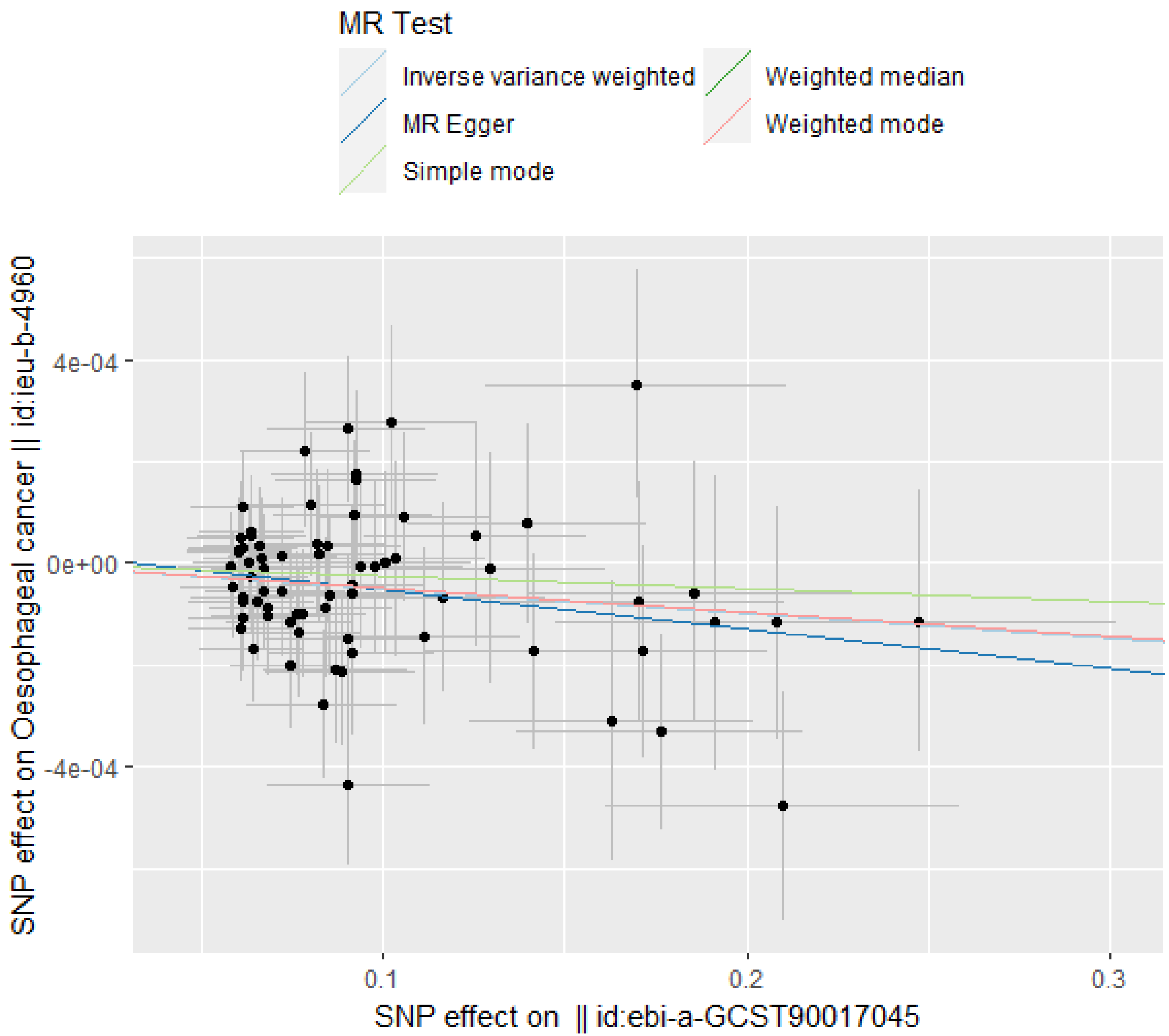

MR Test
Inverse variance weighted
MR Egger
Simple mode
Weighted median
Weighted mode
SNP effect on Oesophageal cancer || id:ieu-b-4960
4e-04
0e+00
-4e-04
0.1
0.2
0.3
SNP effect on || id:ebi-a-GCST90017045

Figure 19 Leave-one-out analysis, funnel plot, MR effect size and scatter plot for gut microbiota abundance (genus Romboutsia id.11347) on oesophageal cancer

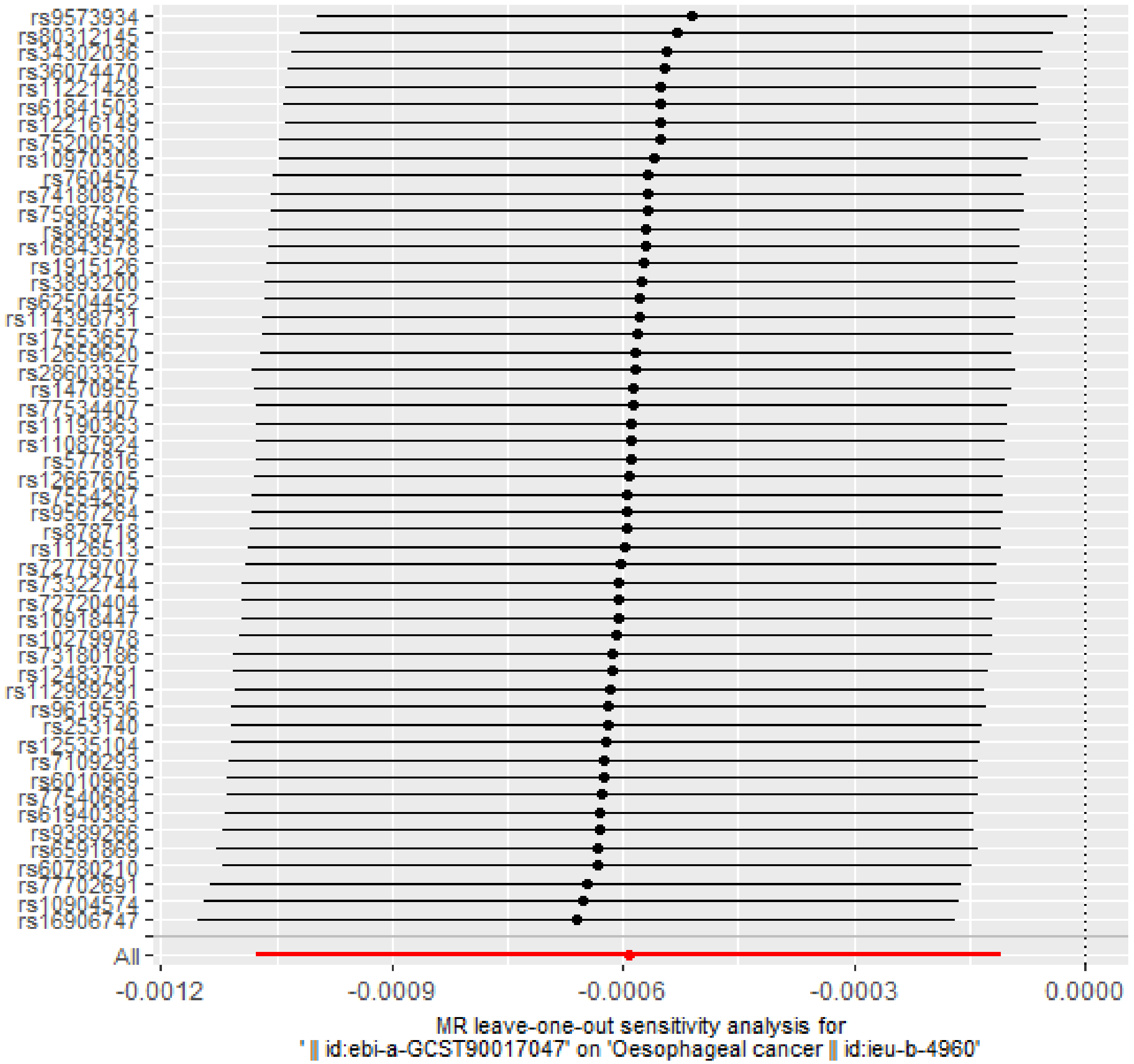

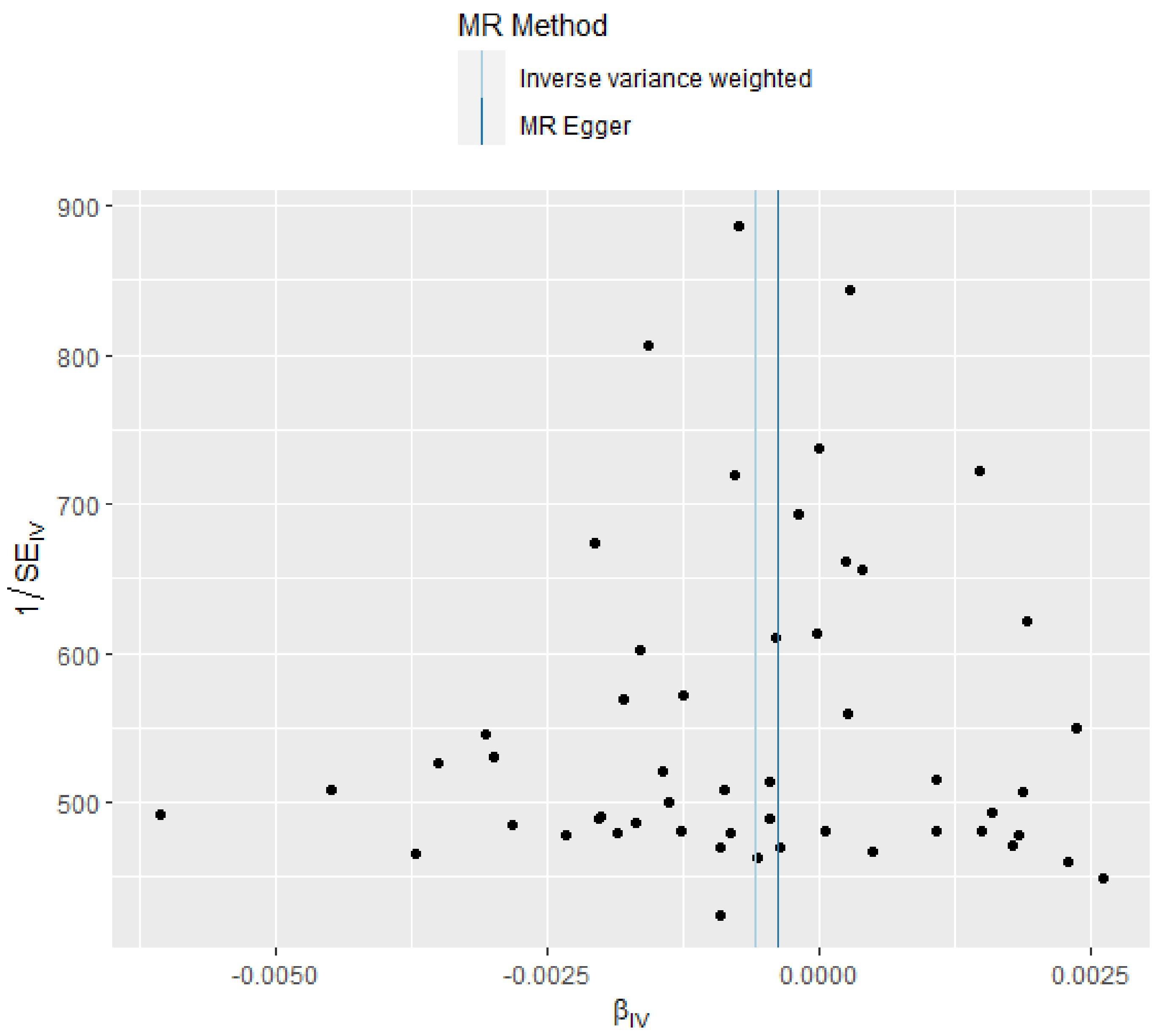

MR Method
Inverse variance weighted
MR Egger
900
800
700
600
500
-0.0050
-0.0025
0.0000
0.0025
β_{IV}
$1/SE_{IV}$

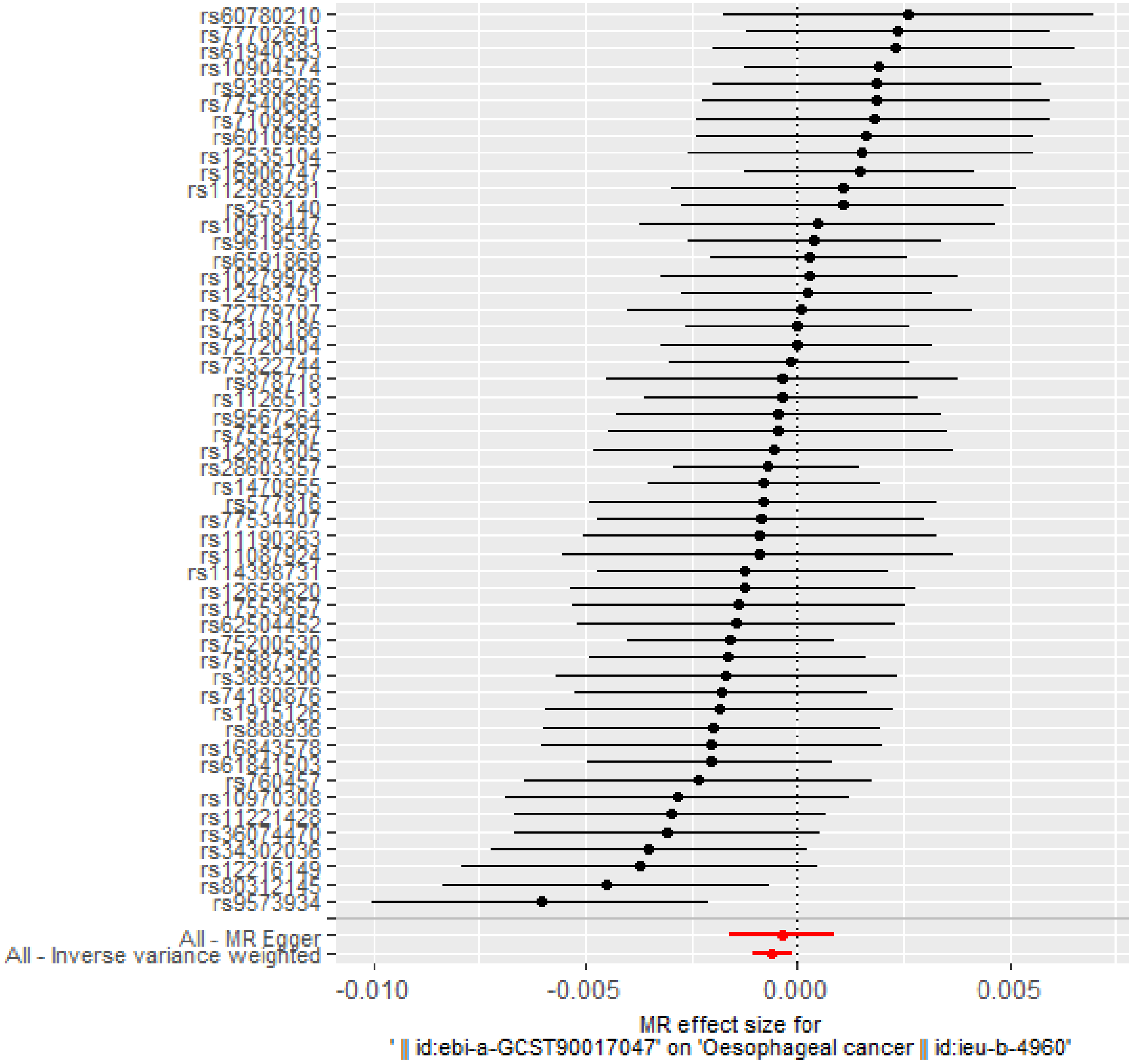

rs60780210
rs61940383
rs10904574
rs9389266
rs77540684
rs7109293
rs6010969
rs12535104
rs16906747
rs112989291
rs253140
rs9619536
rs6591869
rs10279978
rs12483791
rs72779707
rs73180186
rs72720404
rs73322744
rs878718
rs1126513
rs9567264
rs28603357
rs1470955
rs577816
rs77534407
rs11190363
rs11087924
rs114398731
rs12659620
rs62504452
rs75200530
rs3893200
rs74180876
rs1915126
rs888936
rs16843578
rs61841503
rs10970308
rs11221428
rs36074470
rs34302036
rs12216149
rs80312145
rs9573934
All - MR Egger
All - Inverse variance weighted
-0.010
-0.005
0.000
0.005
MR effect size for
' || id:ebi-a-GCST90017047' on 'Oesophageal cancer || id:ieu-b-4960'

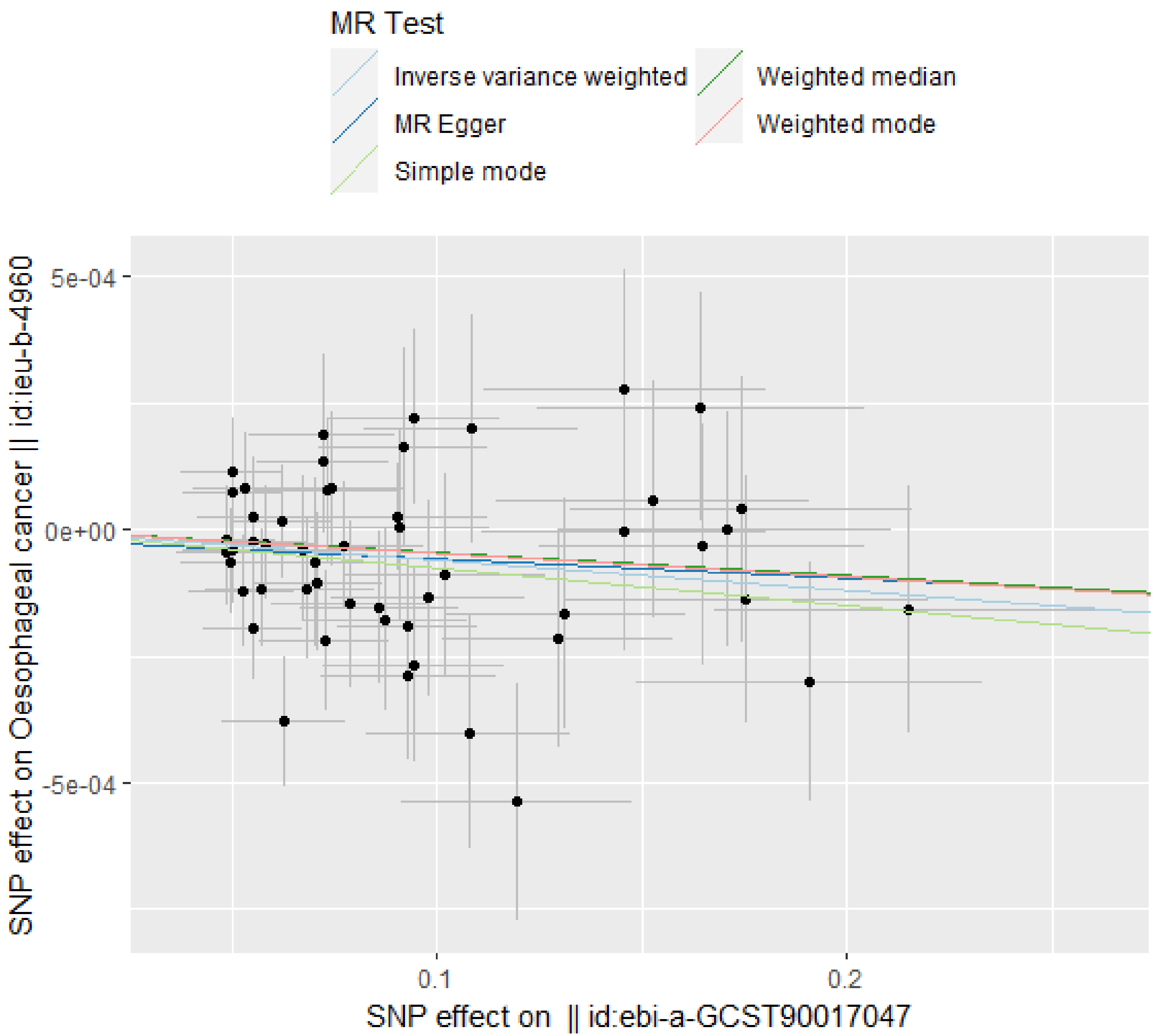
MR Test
Inverse variance weighted
MR Egger
Simple mode
Weighted median
Weighted mode
5e-04
0e+00
-5e-04
SNP effect on Oesophageal cancer || id:ieu-b-4960
0.1
0.2
SNP effect on || id:ebi-a-GCST90017047

Figure 20 Leave-one-out analysis, funnel plot, MR effect size and scatter plot for gut microbiota abundance (genus Ruminococcaceae UCG010 id.11367) on oesophageal cancer

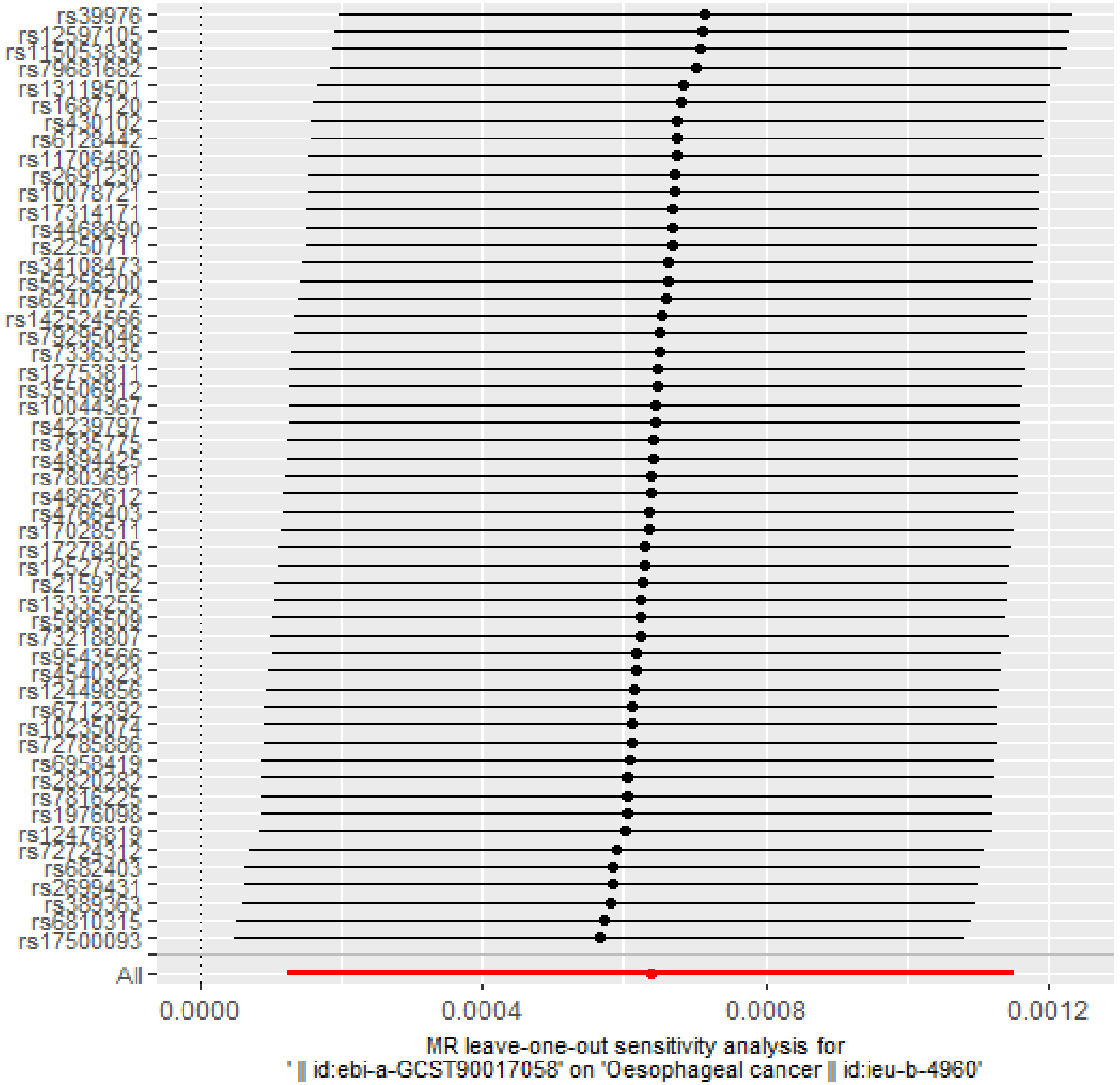

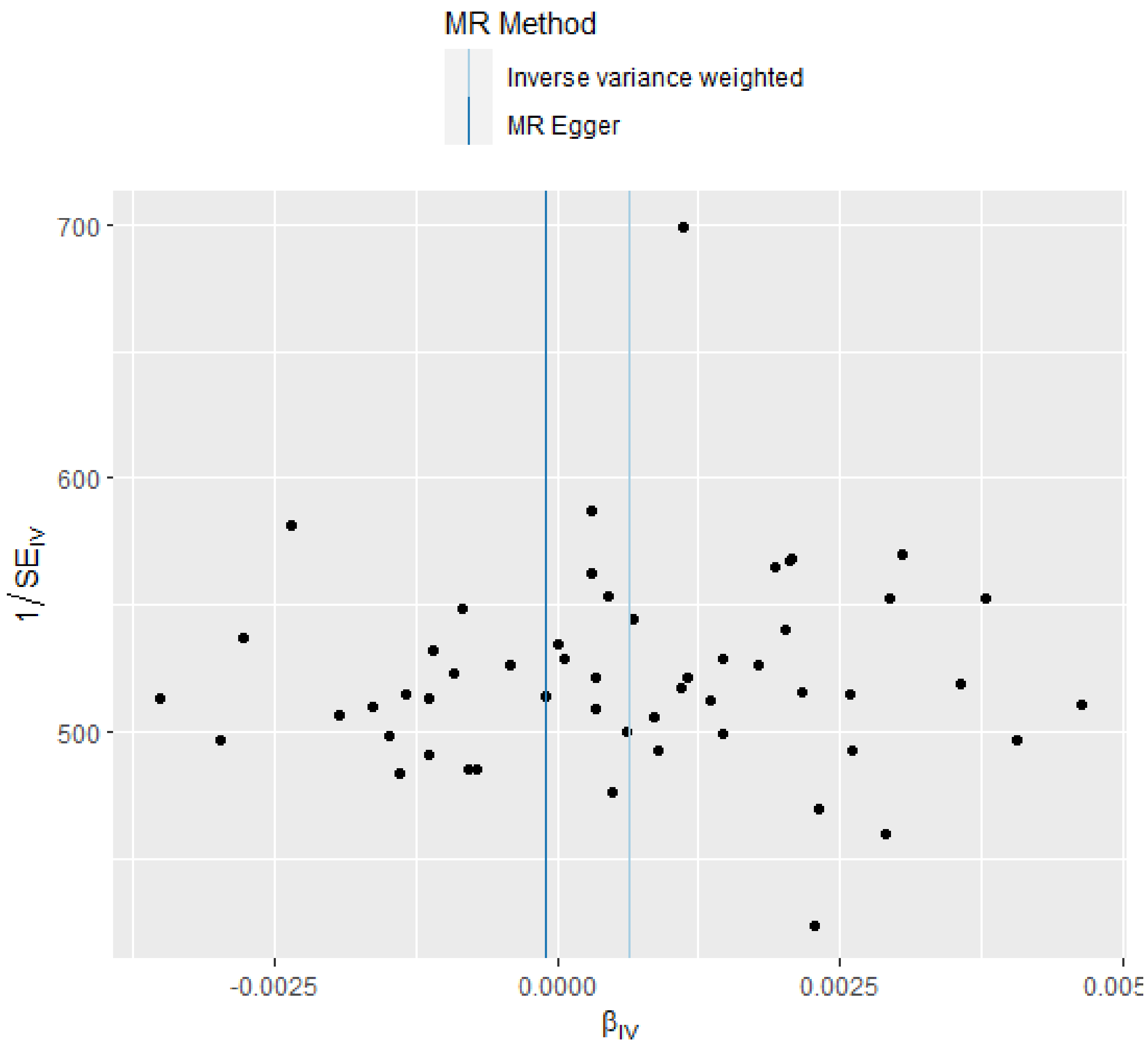
MR Method
Inverse variance weighted
MR Egger
700
600
500
1/SE_IV
-0.0025
0.0000
0.0025
0.005
β_IV

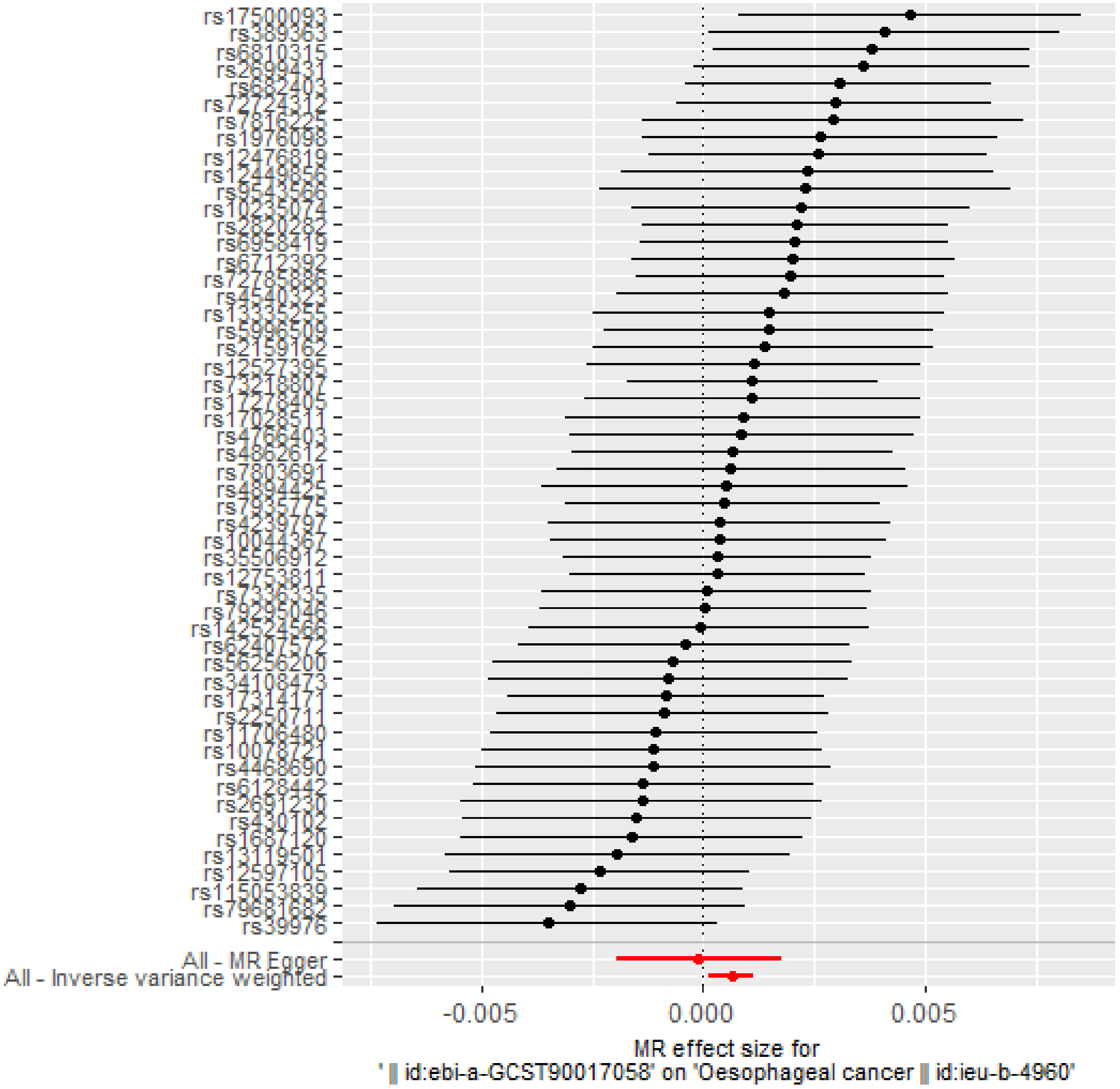
rs17500093
rs389363
rs6810315
rs2699431
rs582403
rs72724312
rs7816225
rs1976098
rs12476819
rs12449856
rs9543566
rs10235074
rs2820282
rs6958419
rs6712392
rs7278588
rs4540323
rs13335255
rs5996509
rs2159162
rs12527395
rs73218807
rs17278405
rs17028511
rs4766403
rs4862612
rs7803691
rs4894425
rs7935775
rs4239797
rs10044367
rs35506912
rs12753811
rs7336335
rs79295046
rs142524568
rs62407572
rs56256200
rs34108473
rs17314171
rs2250711
rs11706480
rs10078721
rs4468690
rs6128442
rs2691230
rs430102
rs1687120
rs13119501
rs12597105
rs115053839
rs79681682
rs39976
All - MR Egger
All - Inverse variance weighted
-0.005
0.000
0.005
MR effect size for
' || id:ebi-a-GCST90017058' on 'Oesophageal cancer || id:ieu-b-4960'

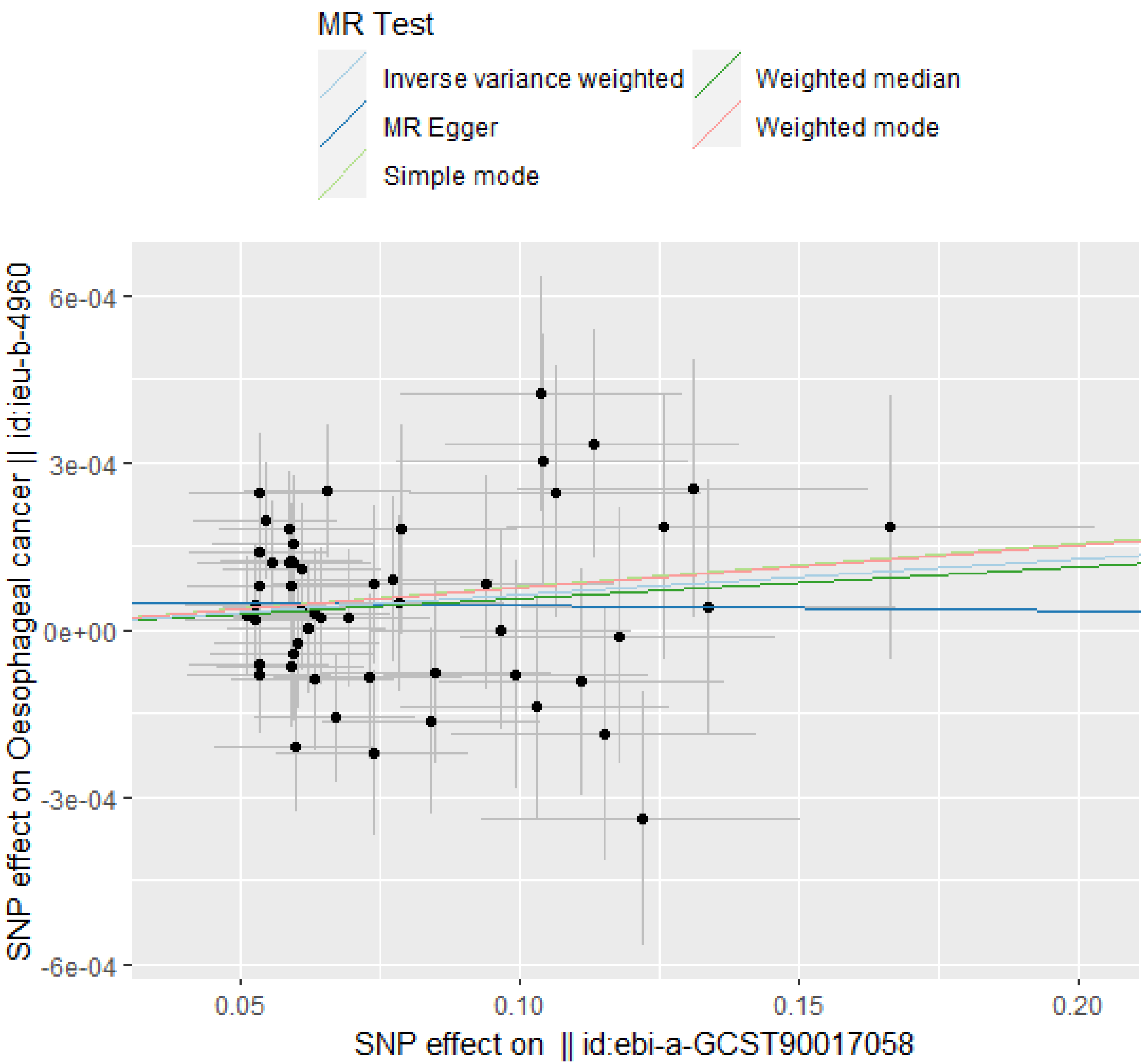

MR Test
Inverse variance weighted
MR Egger
Simple mode
Weighted median
Weighted mode
6e-04
3e-04
0e+00
-3e-04
-6e-04
SNP effect on Oesophageal cancer || id:ieu-b-4960
0.05
0.10
0.15
0.20
SNP effect on || id:ebi-a-GCST90017058

Figure 21 Leave-one-out analysis, funnel plot, MR effect size and scatter plot for gut microbiota abundance (genus Streptococcus id.1853) on oesophageal cancer

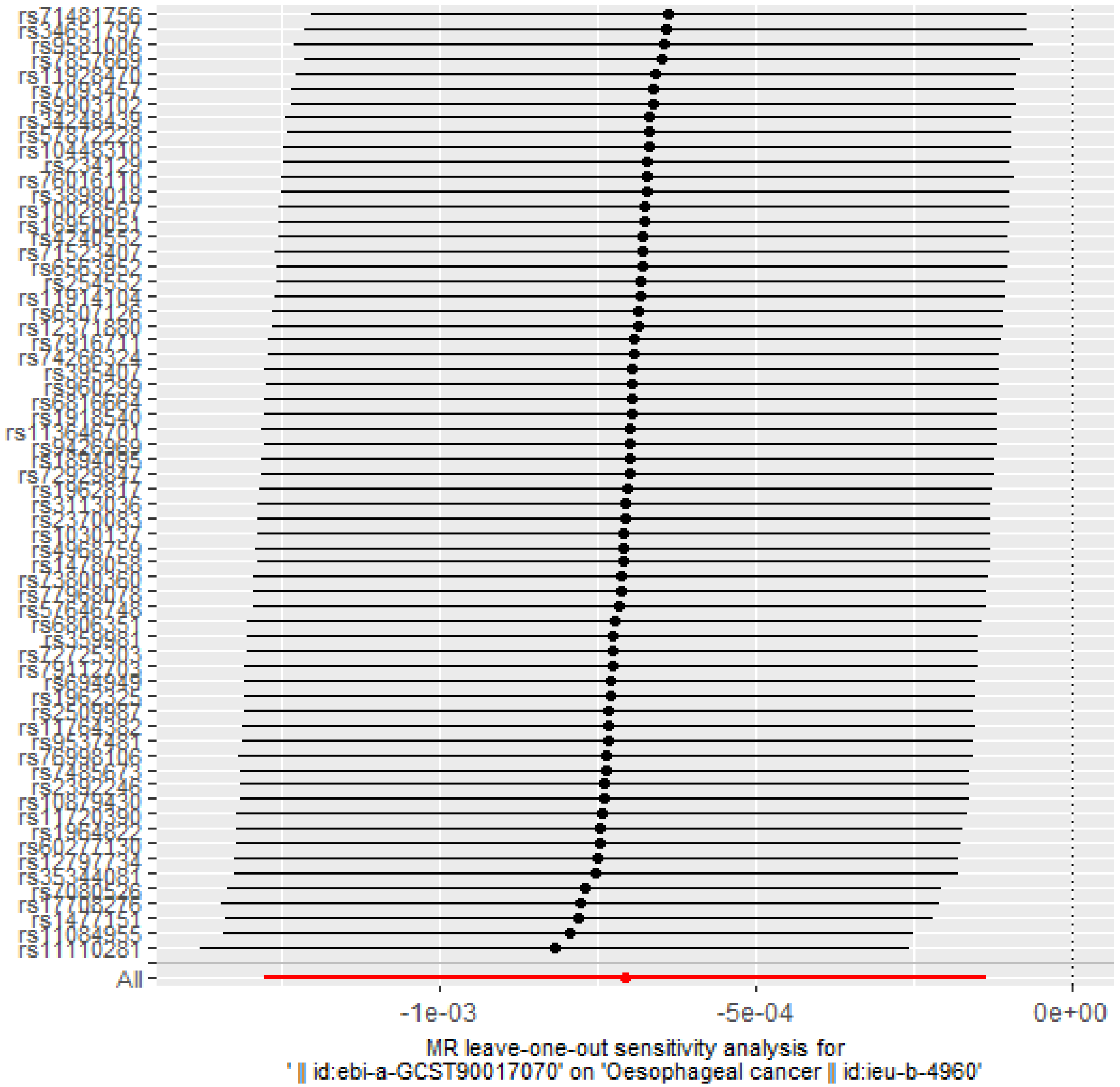

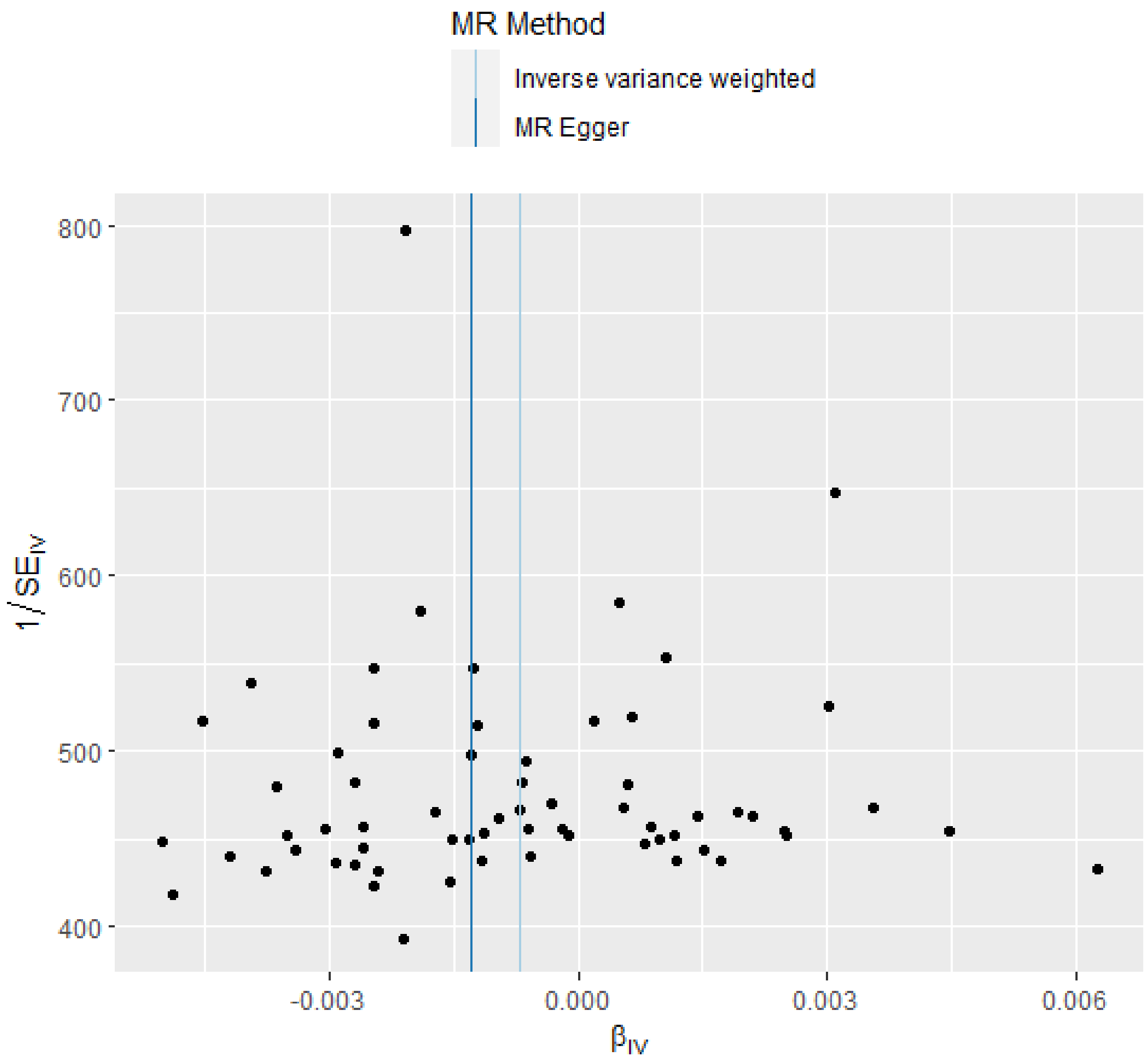

MR Method
Inverse variance weighted
MR Egger
800
700
600
500
400
-0.003
0.000
0.003
0.006
1/SE_IV
β_IV

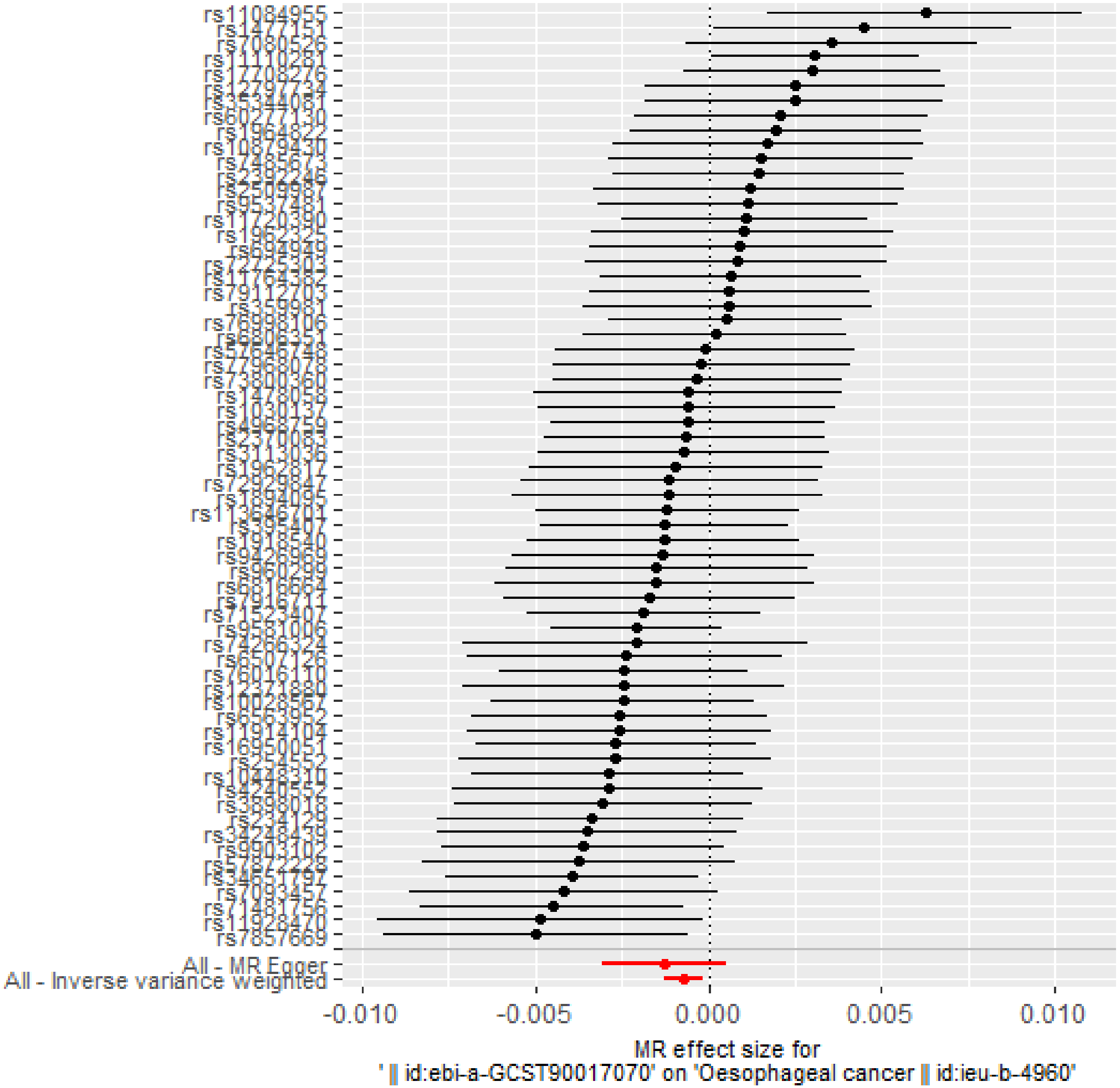
rs11084955
rs7857669
All - MR Egger
All - Inverse variance weighted
-0.010
-0.005
0.000
0.005
0.010
MR effect size for
' || id:ebi-a-GCST90017070' on 'Oesophageal cancer || id:ieu-b-4960'

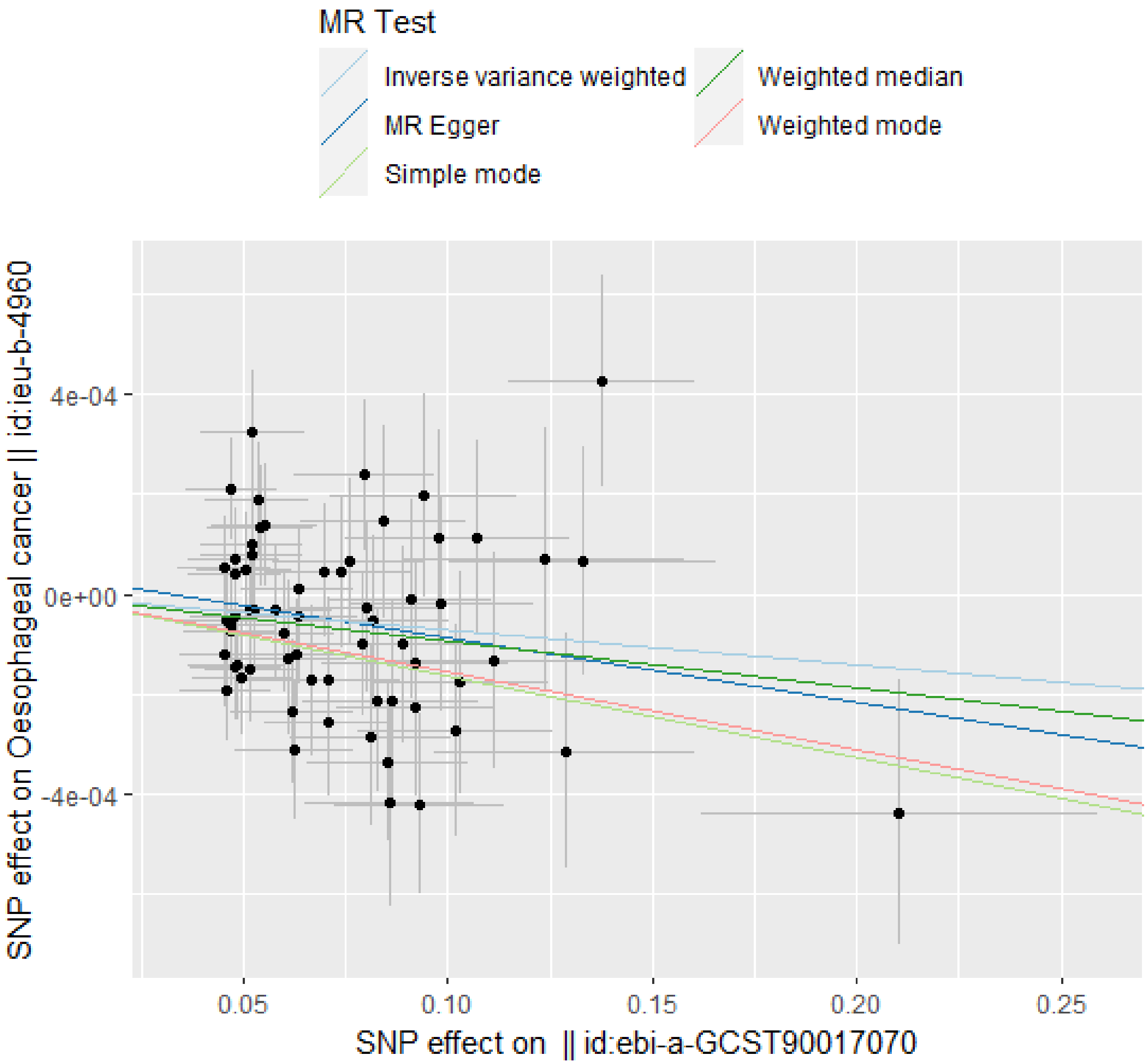
MR Test
Inverse variance weighted
MR Egger
Simple mode
Weighted median
Weighted mode
4e-04
0e+00
-4e-04
0.05
0.10
0.15
0.20
0.25
SNP effect on Oesophageal cancer || id:ieu-b-4960
SNP effect on || id:ebi-a-GCST90017070

Figure 22 Leave-one-out analysis, funnel plot, MR effect size and scatter plot for gut microbiota abundance (order Desulfovibrionales id.3156) on oesophageal cancer

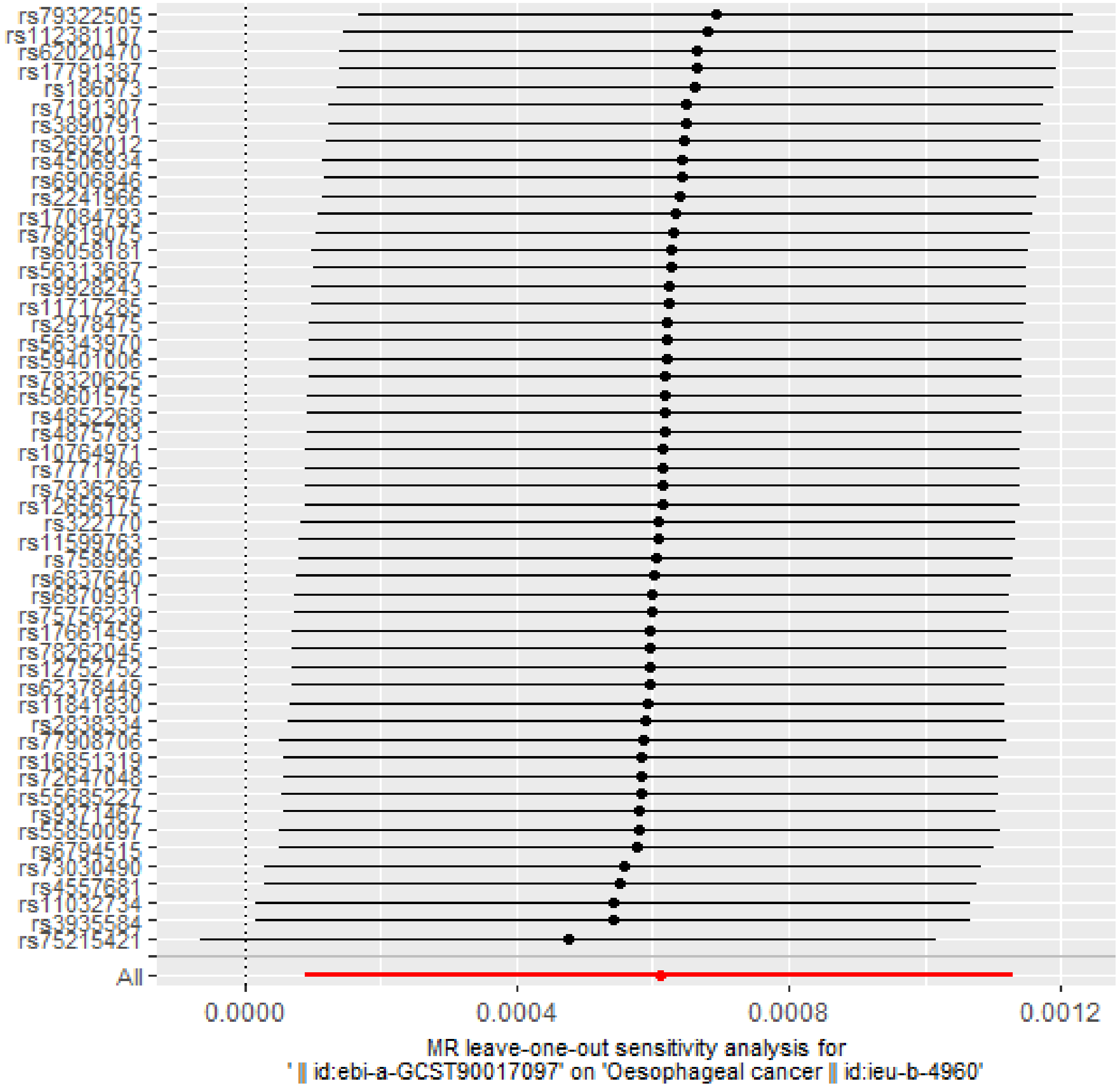

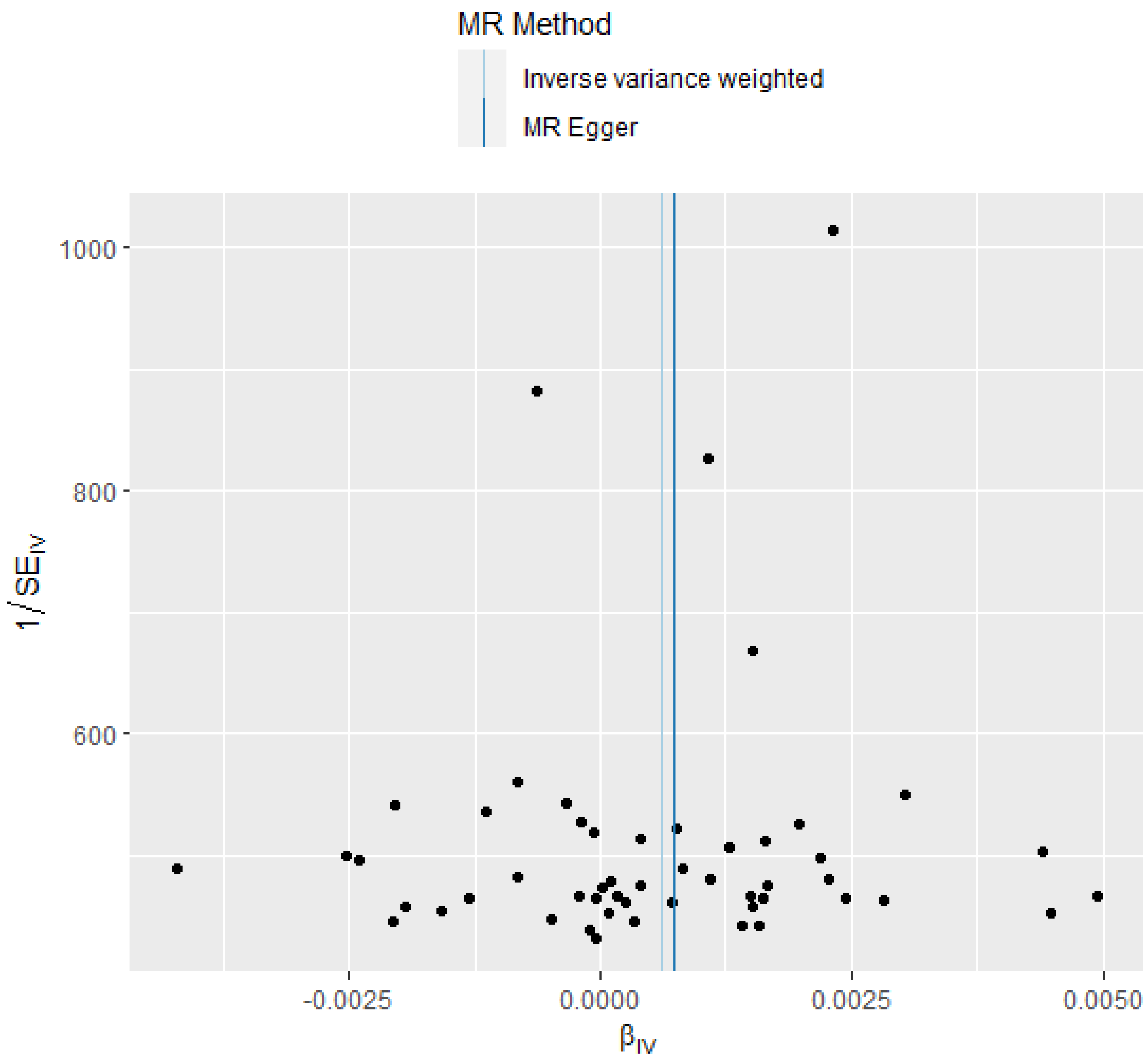

MR Method
Inverse variance weighted
MR Egger
1000
800
600
$1/SE_{IV}$
-0.0025
0.0000
0.0025
0.0050
β_{IV}

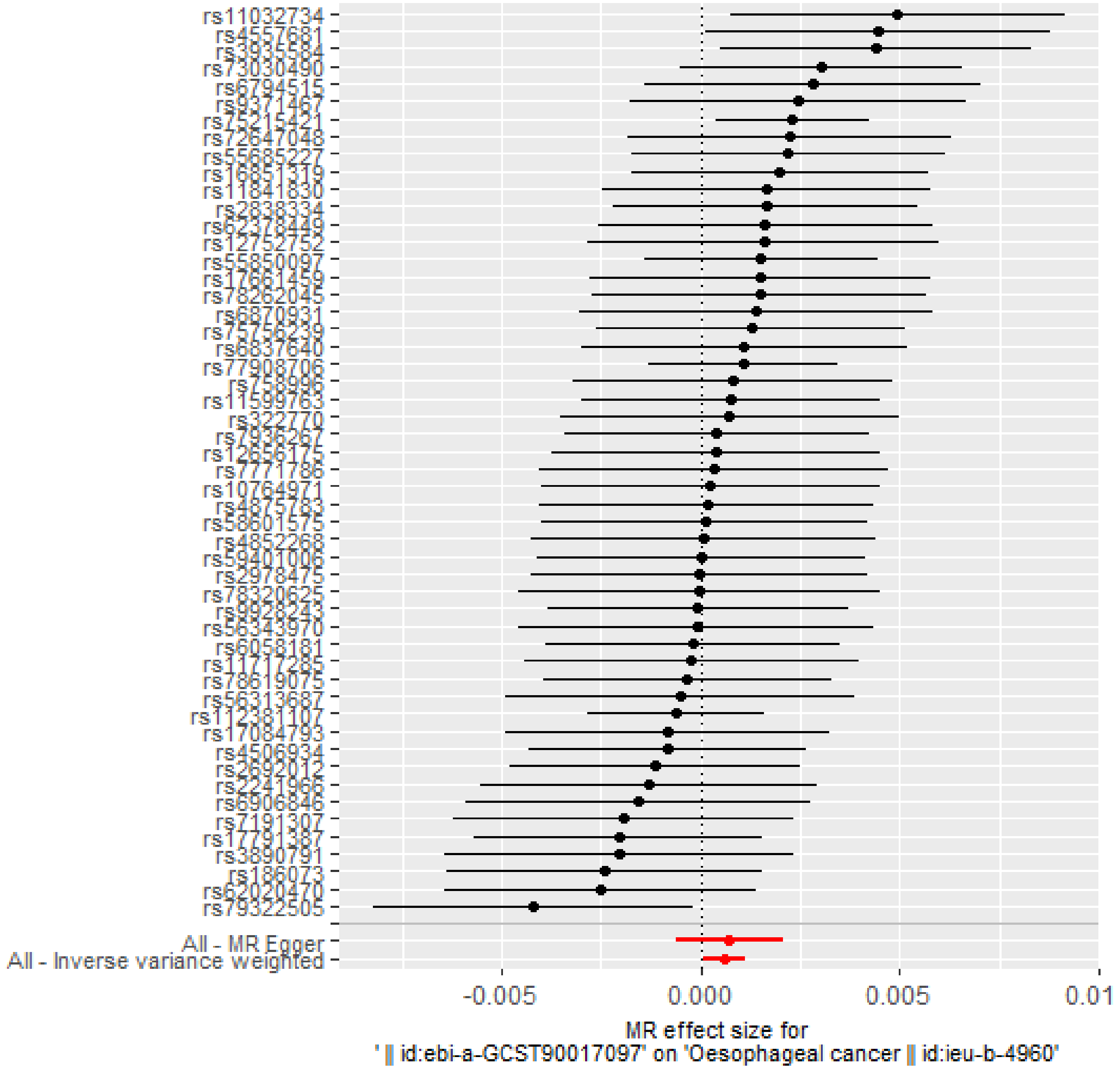
rs11032734
rs4557681
rs3935584
rs73030490
rs6794515
rs9371467
rs75215421
rs72647048
rs55685227
rs16851319
rs11841830
rs2838334
rs62378449
rs12752752
rs55850097
rs17661459
rs78262045
rs6870931
rs75756239
rs6837640
rs77908706
rs758996
rs11599763
rs322770
rs7935267
rs12656175
rs7771786
rs10764971
rs4875783
rs58601575
rs4852268
rs59401006
rs2978475
rs78320625
rs9928243
rs56343970
rs6058181
rs11717285
rs78619075
rs56313687
rs112381107
rs17084793
rs4506934
rs2692012
rs2241966
rs6906846
rs7191307
rs17791387
rs3890791
rs186073
rs62020470
rs79322505
All - MR Egger
All - Inverse variance weighted
-0.005
0.000
0.005
0.01
MR effect size for
' || id:ebi-a-GCST90017097' on 'Oesophageal cancer || id:ieu-b-4960'

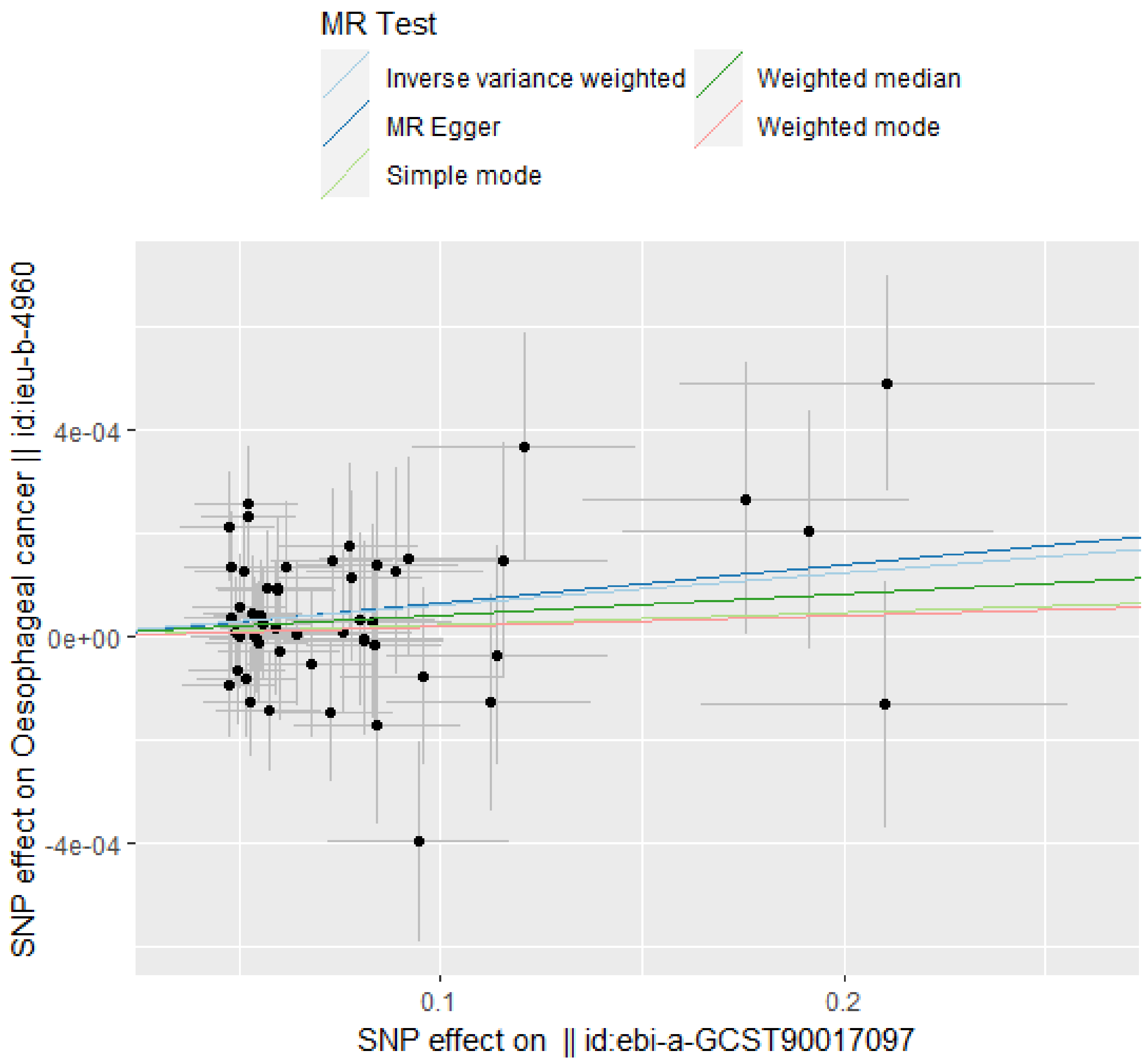
MR Test
Inverse variance weighted
MR Egger
Simple mode
Weighted median
Weighted mode
SNP effect on Oesophageal cancer || id:ieu-b-4960
4e-04
0e+00
-4e-04
0.1
0.2
SNP effect on || id:ebi-a-GCST90017097

Figure 23 Leave-one-out analysis, funnel plot, MR effect size and scatter plot for gut microbiota abundance (order Pasteurellales id.3688) on oesophageal cancer

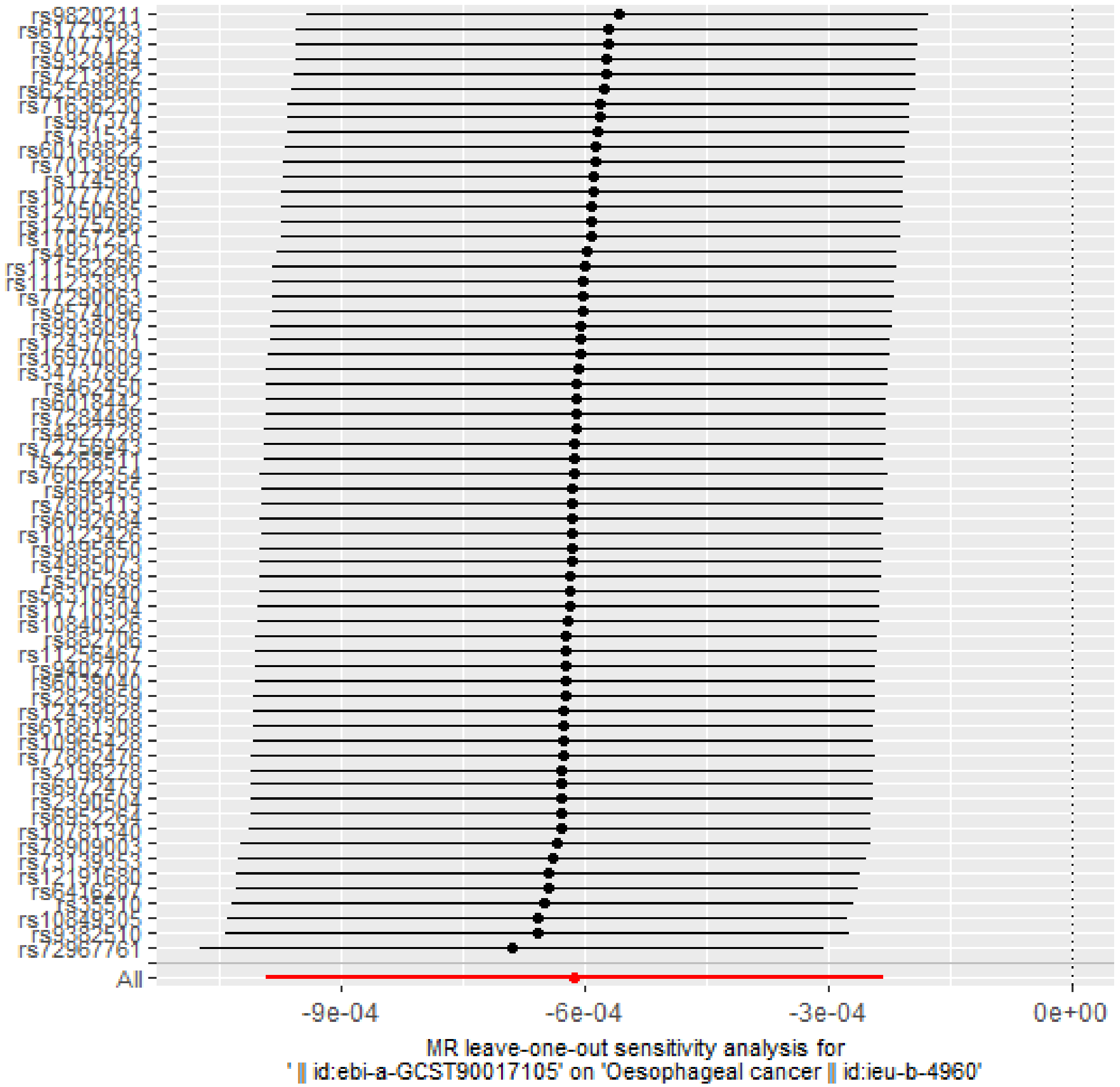

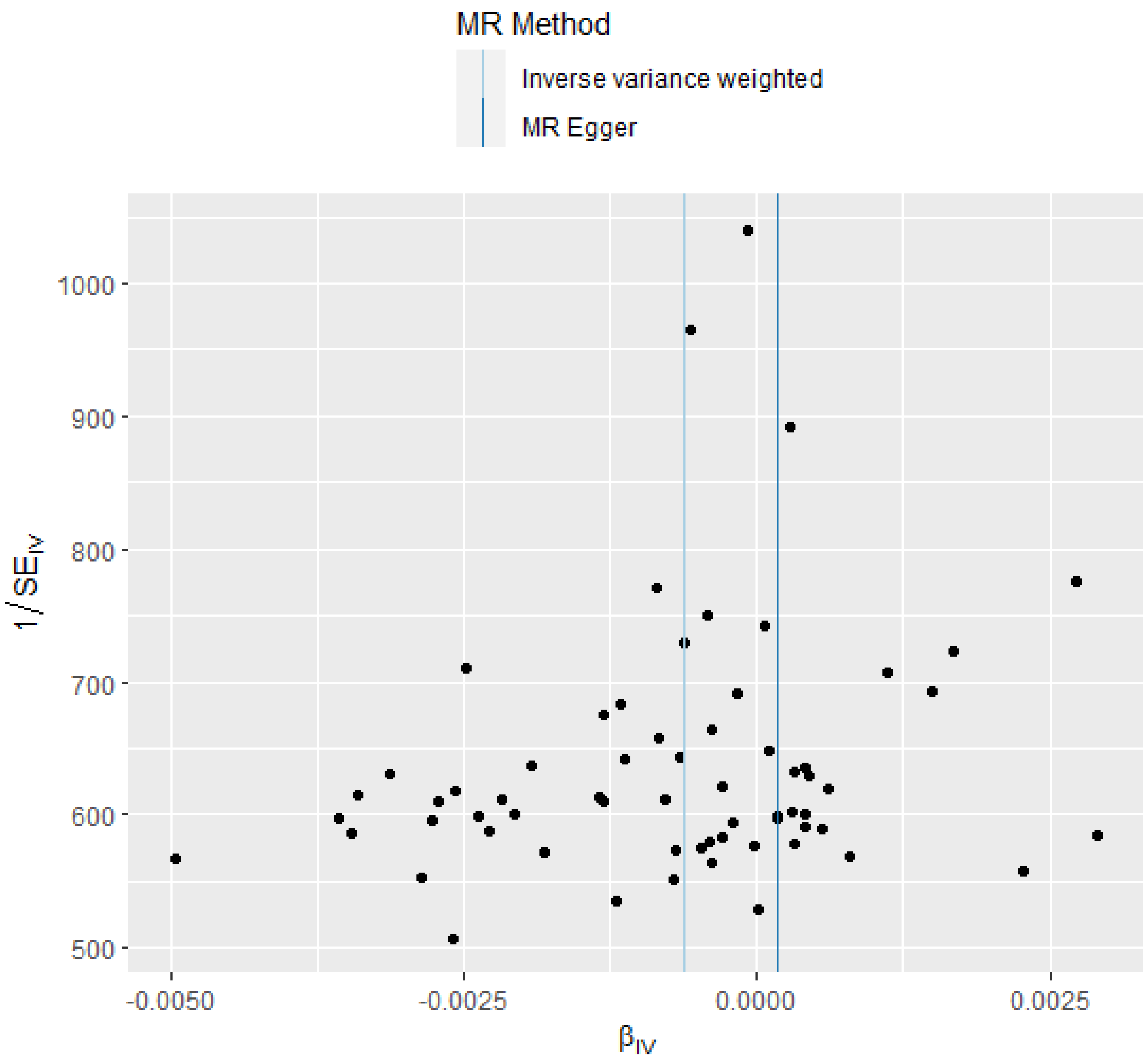

MR Method
Inverse variance weighted
MR Egger
1000
900
800
700
600
500
1/SE_IV
-0.0050
-0.0025
0.0000
0.0025
β_IV

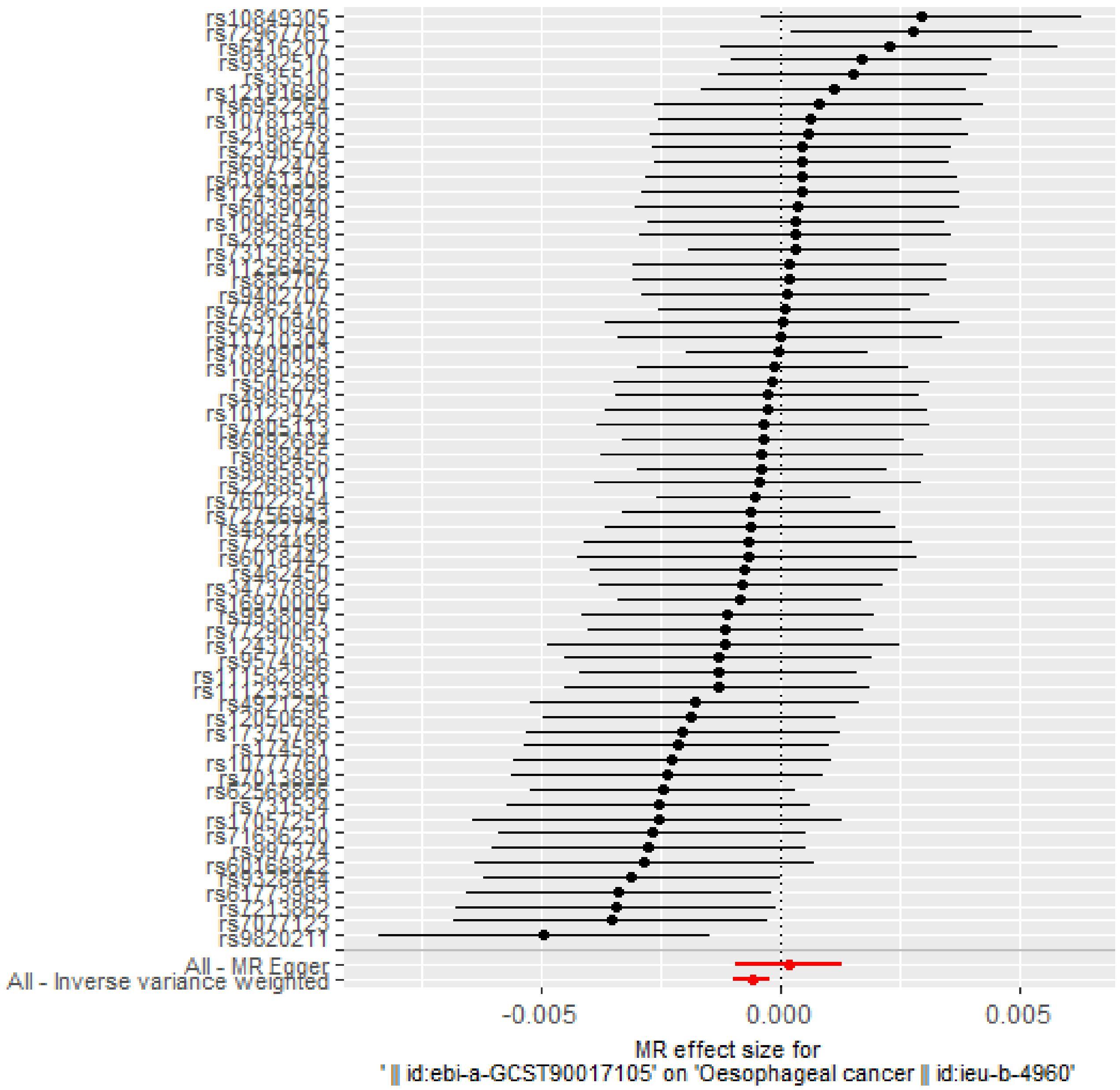
rs10849305
rs9820211
All - MR Egger
All - Inverse variance weighted
-0.005
0.000
0.005
MR effect size for
' || id:ebi-a-GCST90017105' on 'Oesophageal cancer || id:ieu-b-4960'

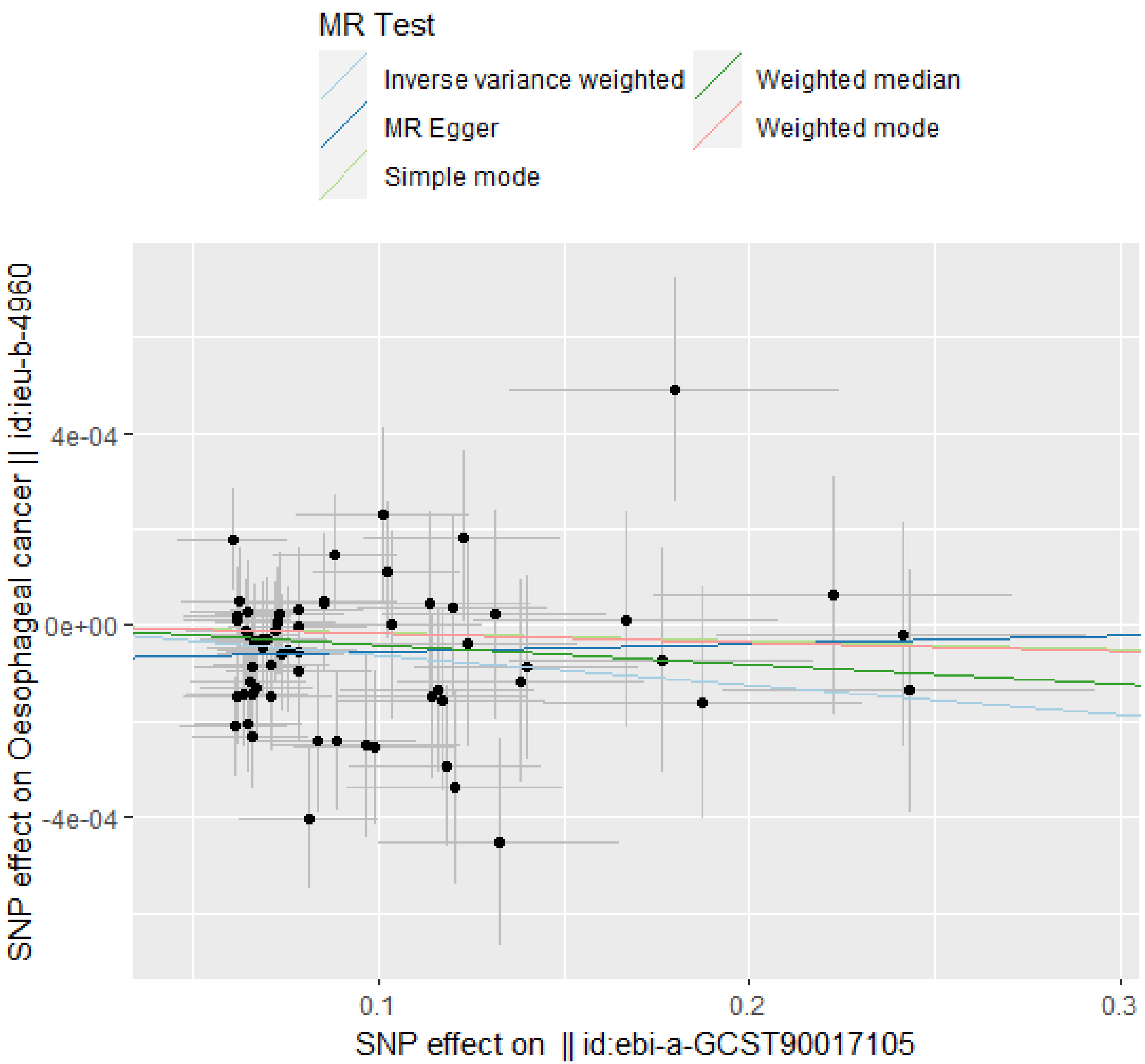
MR Test
Inverse variance weighted
MR Egger
Simple mode
Weighted median
Weighted mode
SNP effect on Oesophageal cancer || id:ieu-b-4960
4e-04
0e+00
-4e-04
0.1
0.2
0.3
SNP effect on || id:ebi-a-GCST90017105

Figure 24 Leave-one-out analysis, funnel plot, MR effect size and scatter plot for gut microbiota abundance (phylum Actinobacteria id.400) on oesophageal cancer

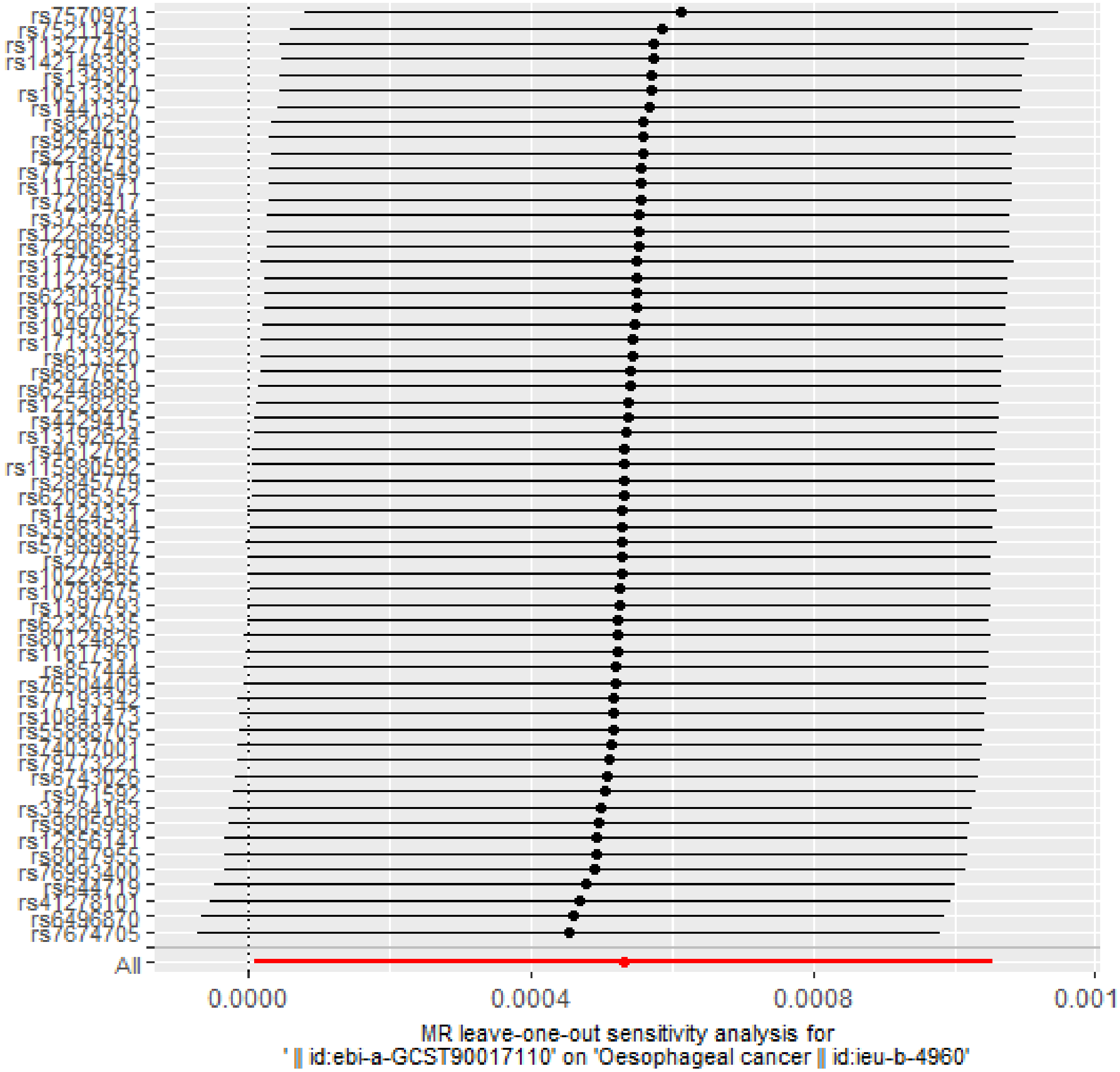

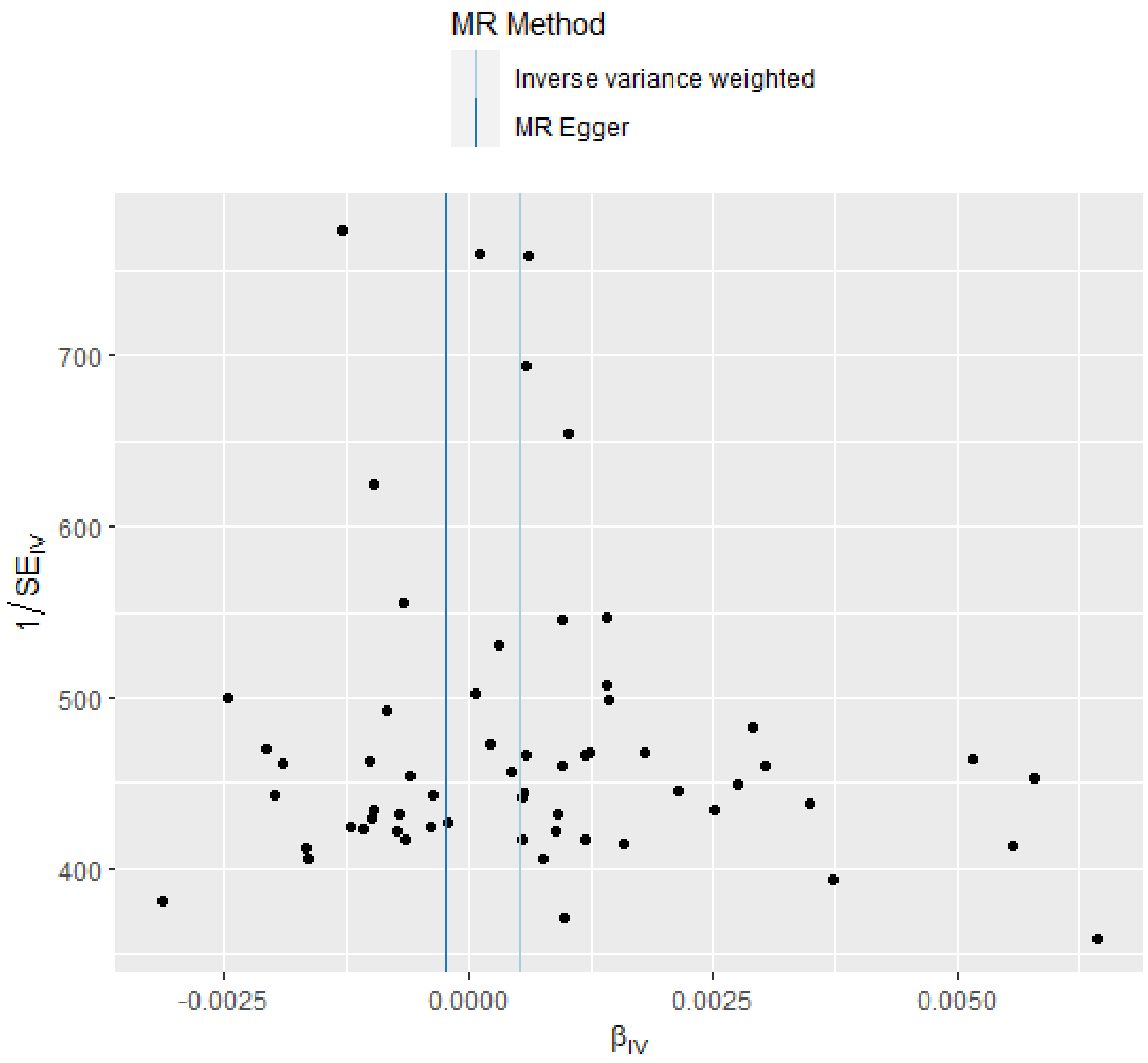

MR Method
Inverse variance weighted
MR Egger
700
600
500
400
1/SE_IV
-0.0025
0.0000
0.0025
0.0050
β_IV

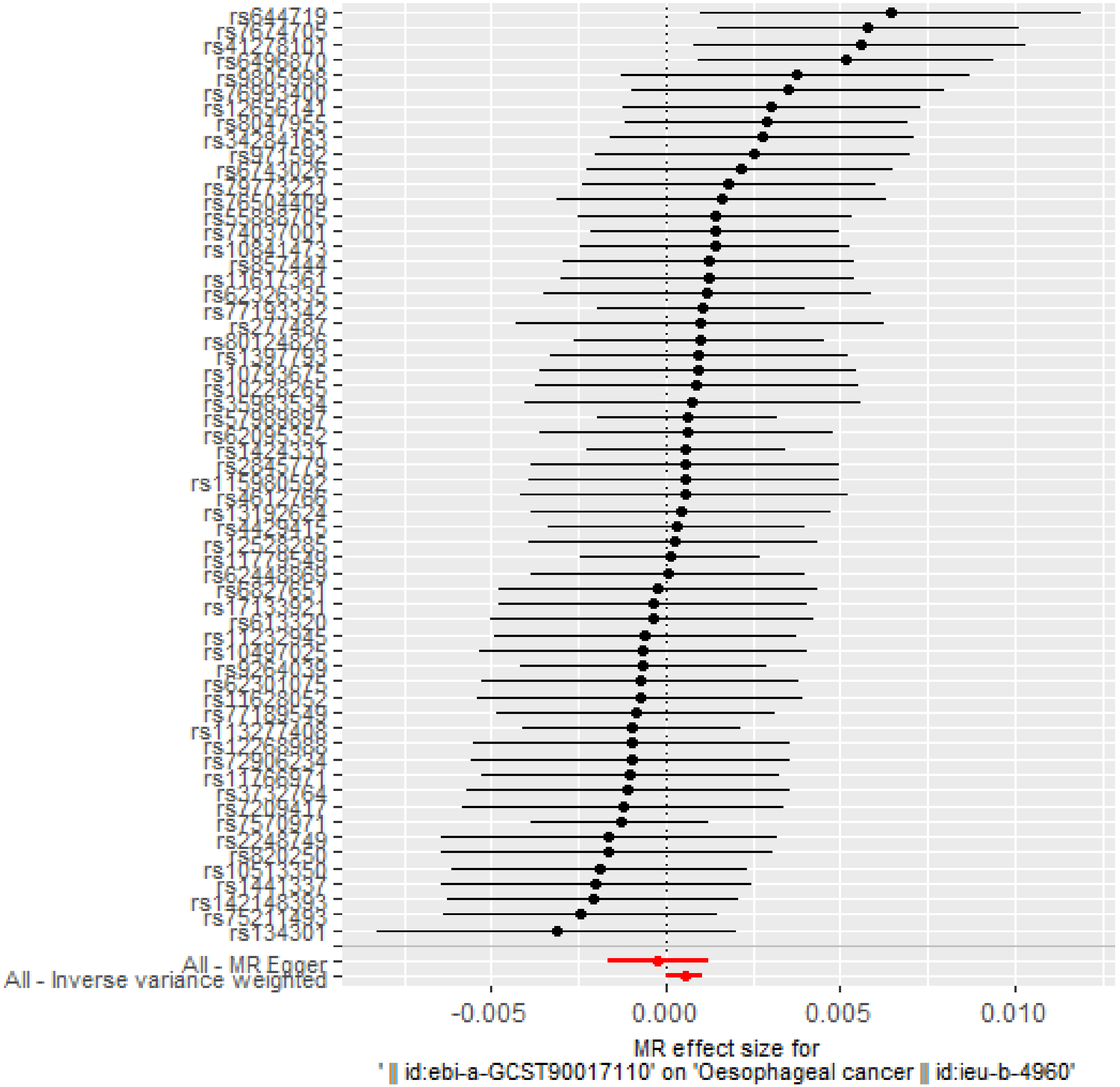
rs644719
rs134301
All - MR Egger
All - Inverse variance weighted
-0.005
0.000
0.005
0.010
MR effect size for
' || id:ebi-a-GCST90017110' on 'Oesophageal cancer || id:ieu-b-4960'

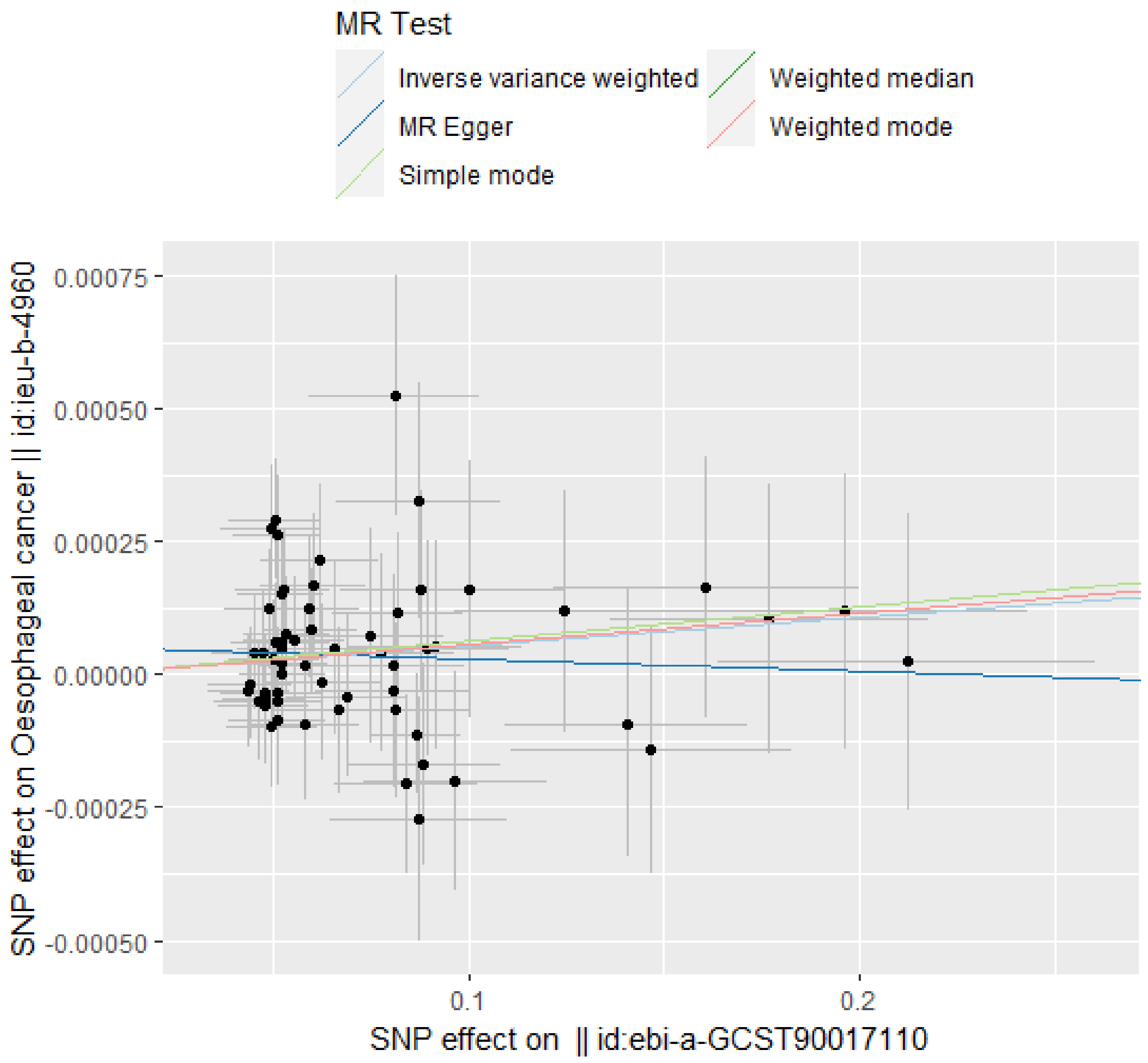

MR Test
Inverse variance weighted
MR Egger
Simple mode
Weighted median
Weighted mode
0.00075
0.00050
0.00025
0.00000
-0.00025
-0.00050
0.1
0.2
SNP effect on Oesophageal cancer || id:ieu-b-4960
SNP effect on || id:ebi-a-GCST90017110

Figure 25 Leave-one-out analysis, funnel plot, MR effect size and scatter plot for gut microbiota abundance (class Bacteroidia id.912) on ulcer of oesophagus

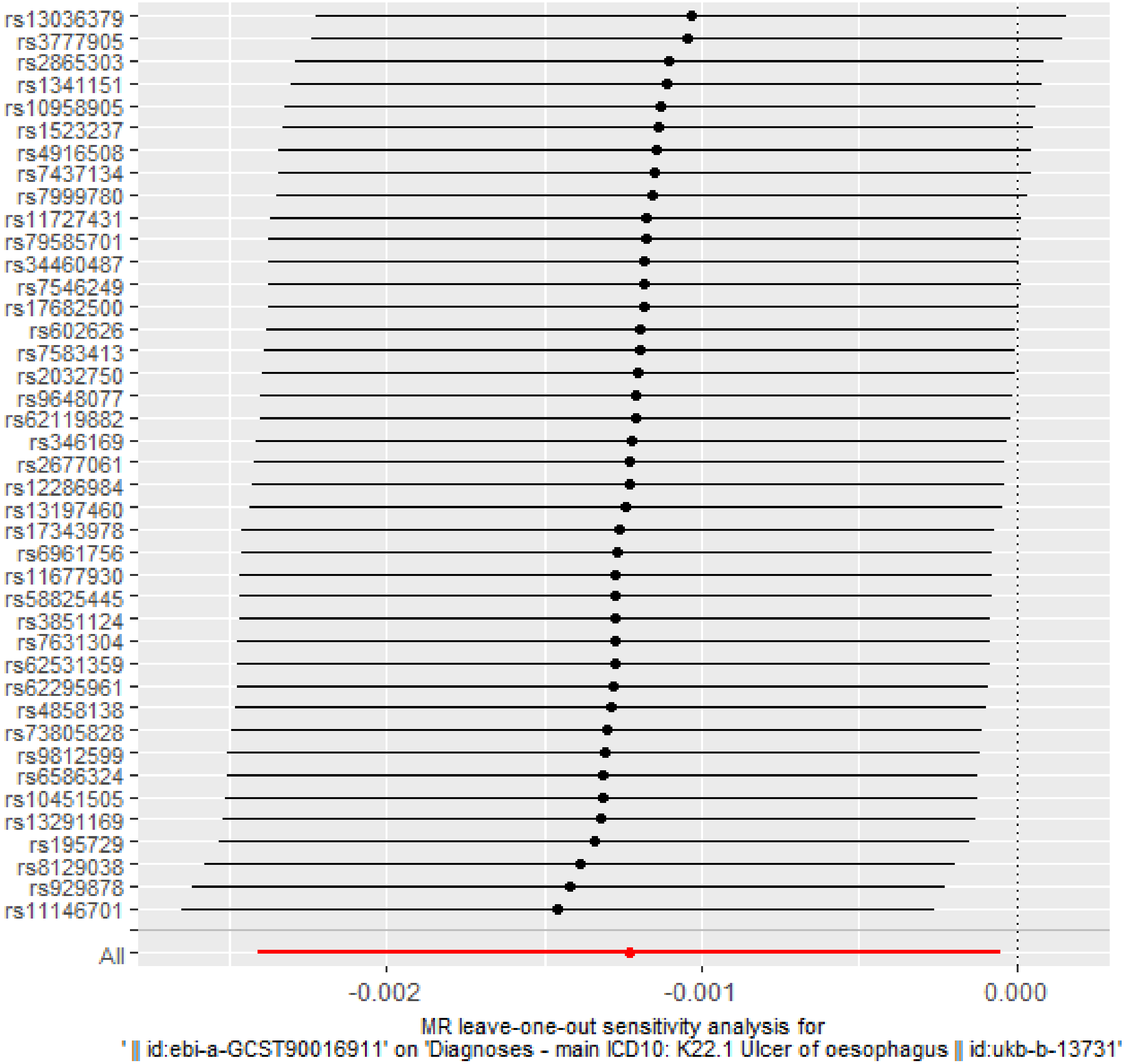

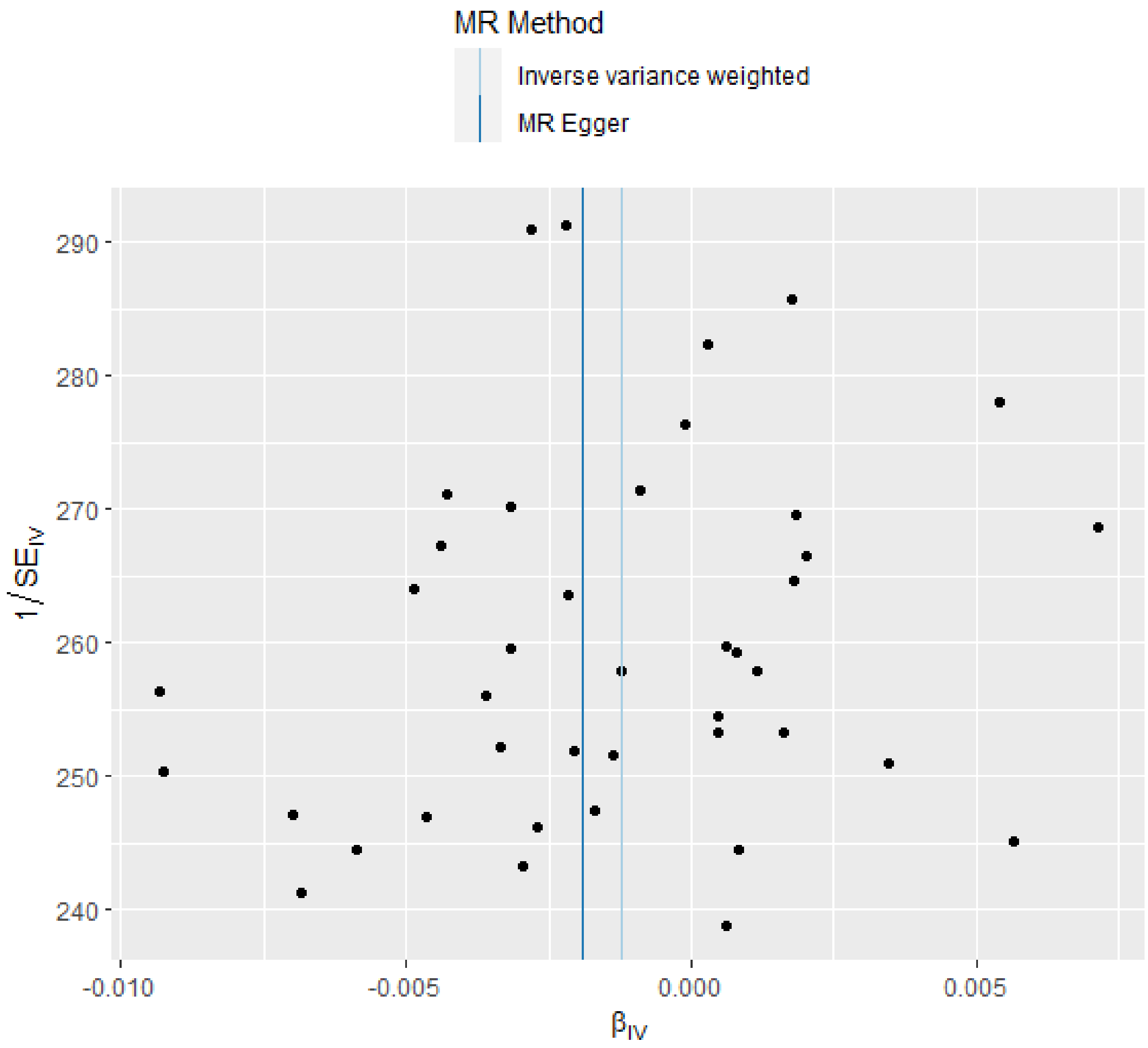

MR Method
Inverse variance weighted
MR Egger
290
280
270
260
250
240
1/SE$_{IV}$
-0.010
-0.005
0.000
0.005
β_{IV}

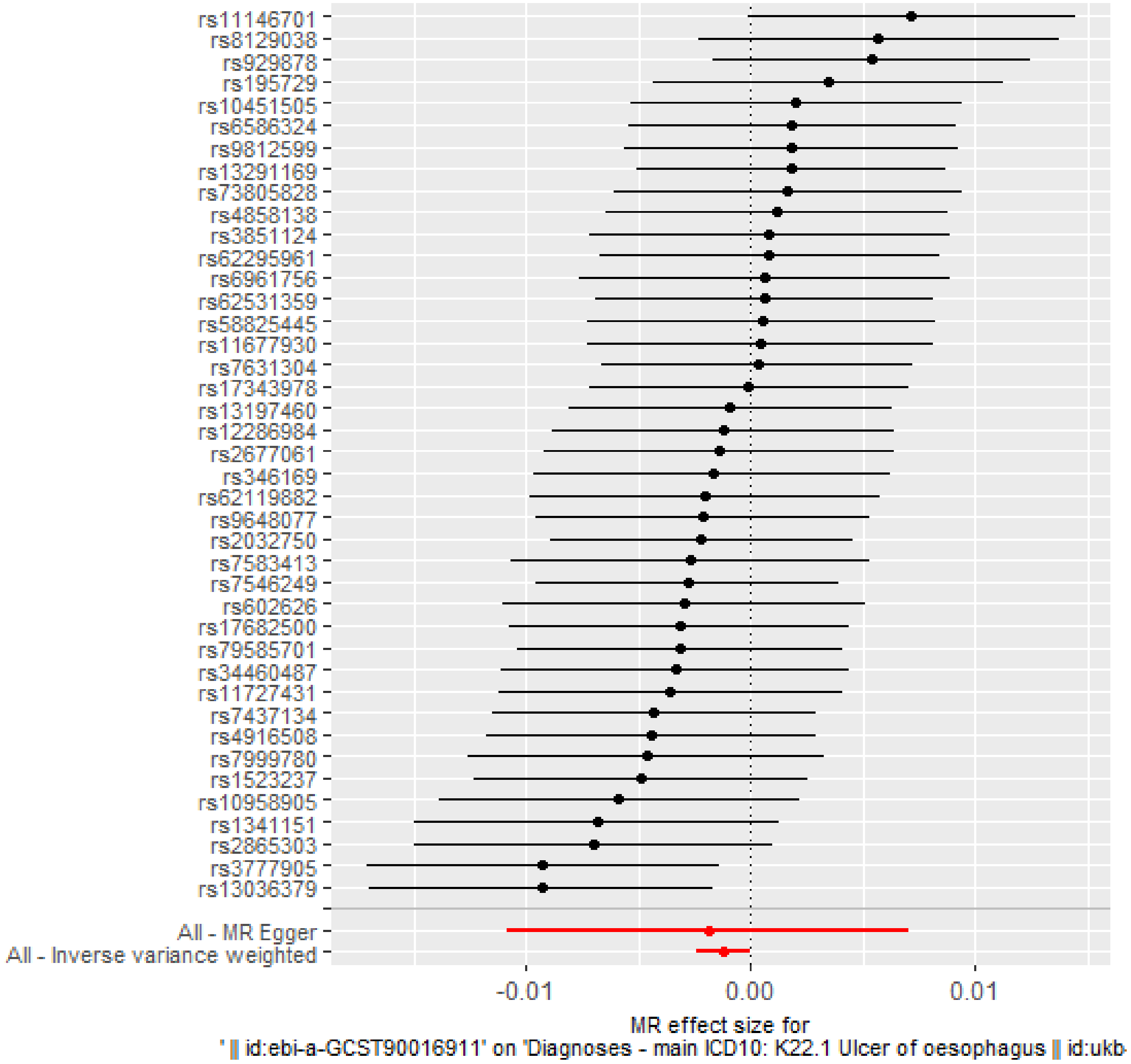
rs11146701
rs8129038
rs929878
rs195729
rs10451505
rs6586324
rs9812599
rs13291169
rs73805828
rs4858138
rs3851124
rs62295961
rs6961756
rs62531359
rs58825445
rs11677930
rs7631304
rs17343978
rs13197460
rs12286984
rs2677061
rs346169
rs62119882
rs9648077
rs2032750
rs7583413
rs7546249
rs602626
rs17682500
rs79585701
rs34460487
rs11727431
rs7437134
rs4916508
rs7999780
rs1523237
rs10958905
rs1341151
rs2865303
rs3777905
rs13036379
All - MR Egger
All - Inverse variance weighted
-0.01
0.00
0.01
MR effect size for
' || id:ebi-a-GCST90016911' on 'Diagnoses - main ICD10: K22.1 Ulcer of oesophagus || id:ukb-

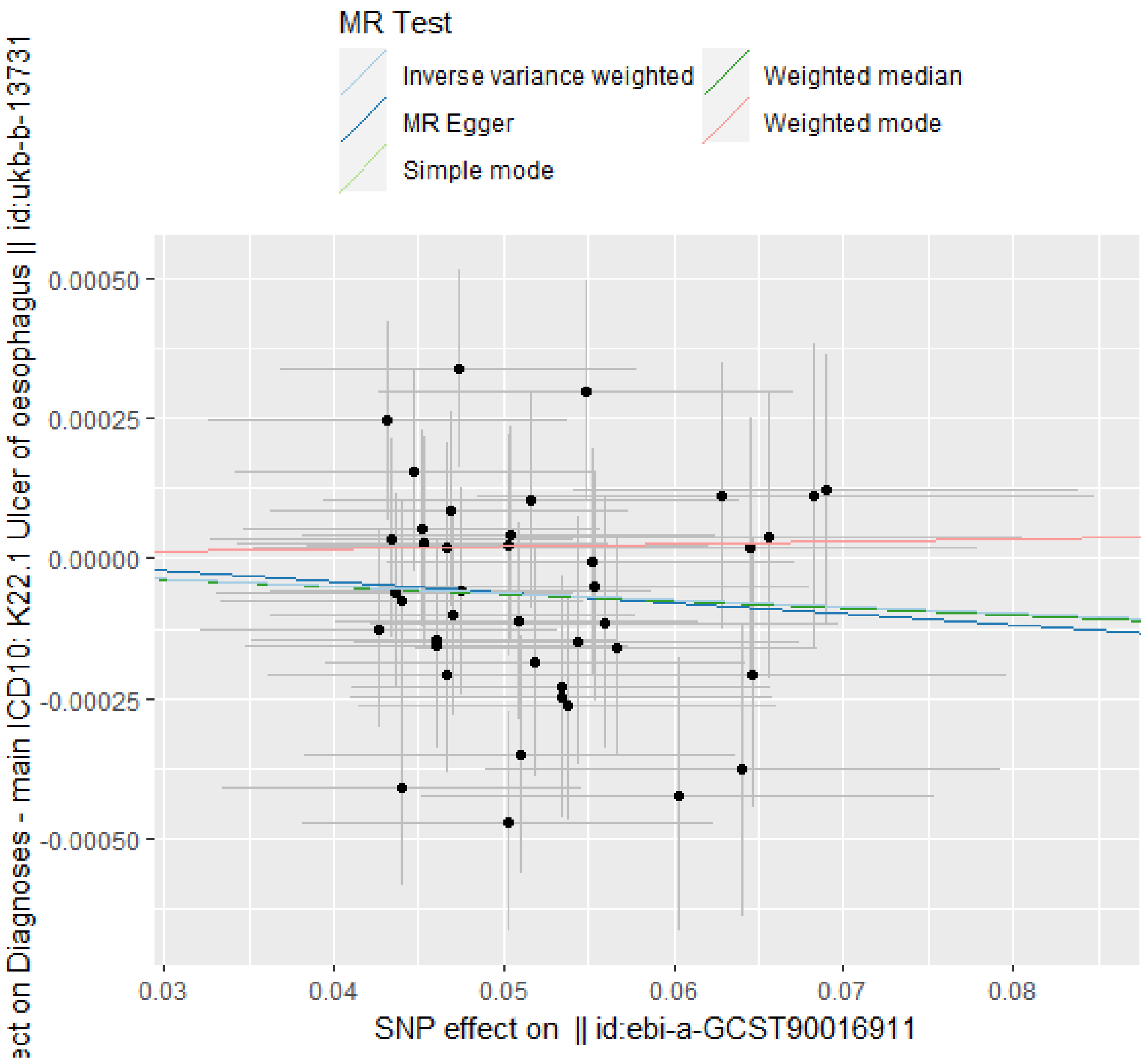

MR Test
Inverse variance weighted
MR Egger
Simple mode
Weighted median
Weighted mode
0.00050
0.00025
0.00000
-0.00025
-0.00050
0.03
0.04
0.05
0.06
0.07
0.08
SNP effect on || id:ebi-a-GCST90016911
ect on Diagnoses - main ICD10: K22.1 Ulcer of oesophagus || id:ukb-b-13731

Figure 26 Leave-one-out analysis, funnel plot, MR effect size and scatter plot for gut microbiota abundance (family Peptostreptococcaceae id.2042) on ulcer of oesophagus

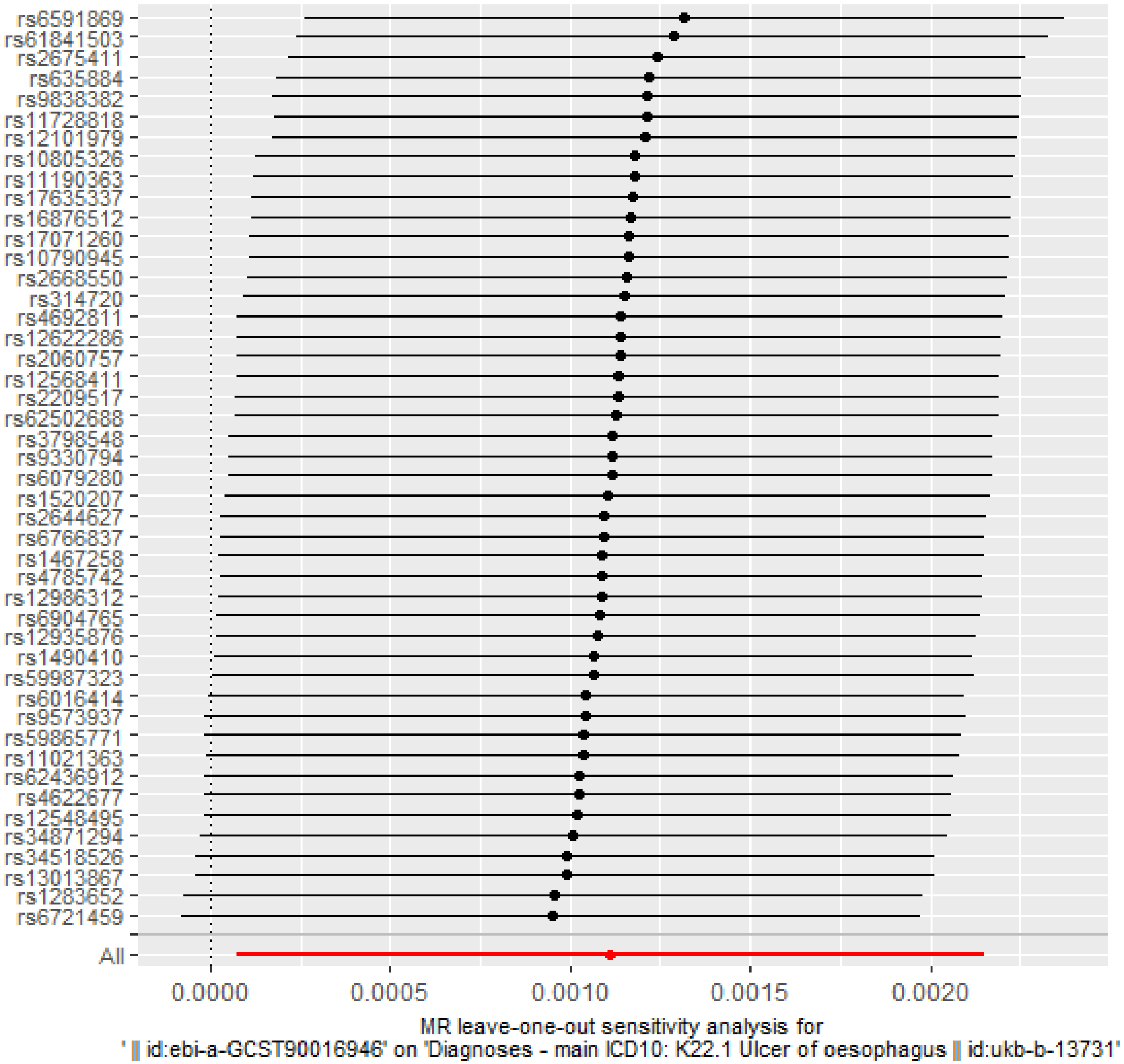

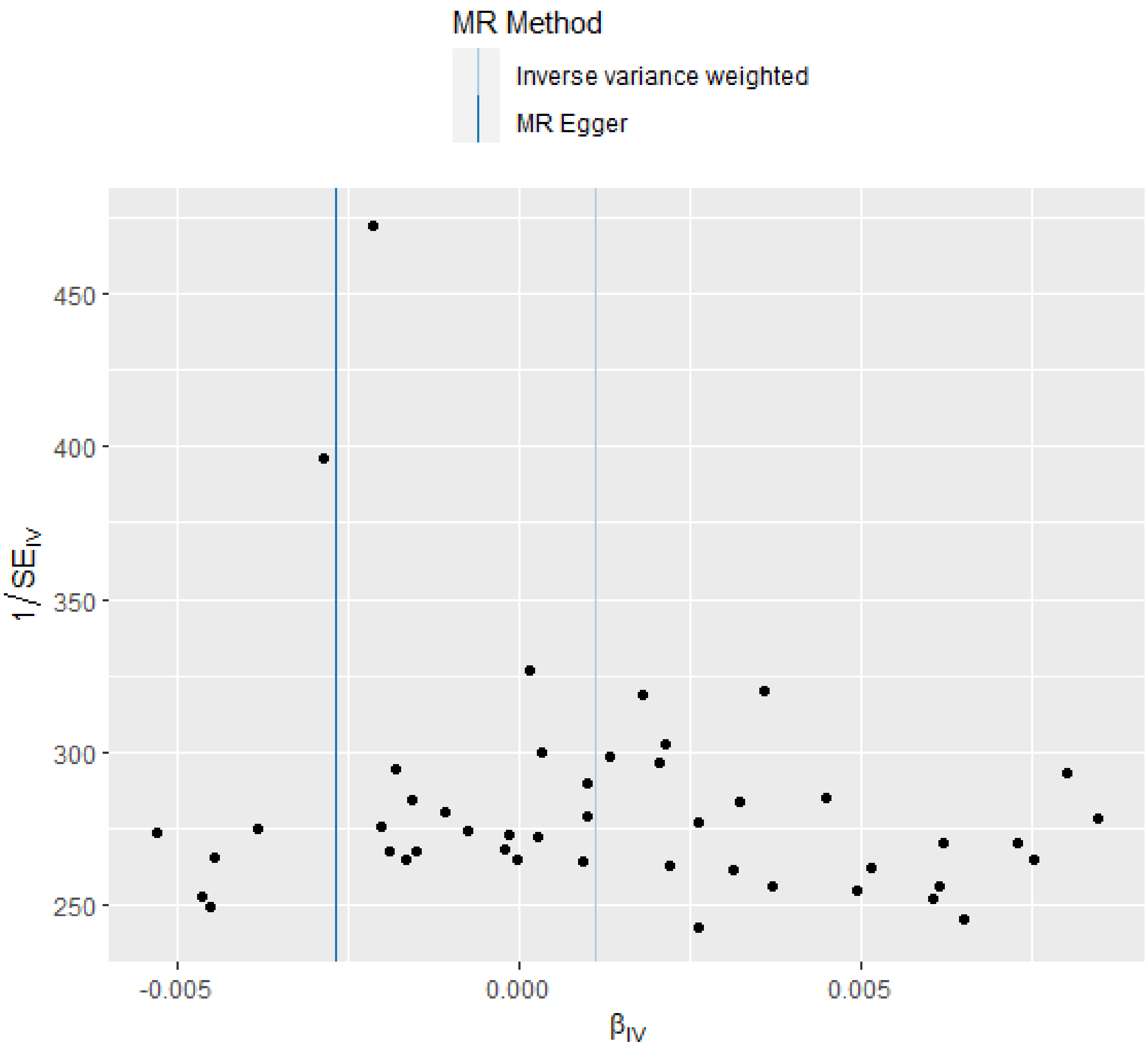

MR Method
Inverse variance weighted
MR Egger
450
400
350
300
250
-0.005
0.000
0.005
β_{IV}
$1/SE_{IV}$

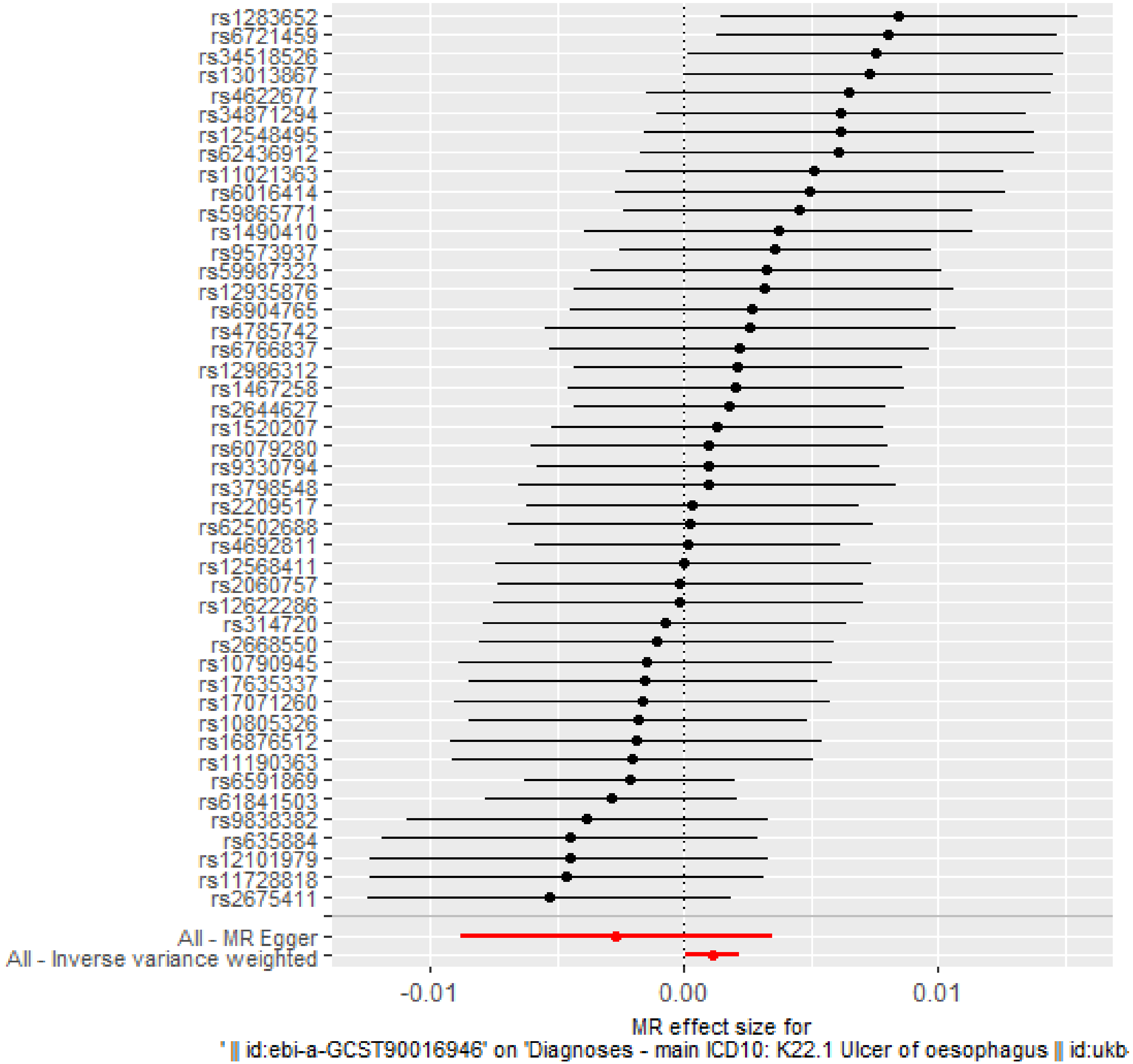

rs1283652
rs6721459
rs34518526
rs13013867
rs4622677
rs34871294
rs12548495
rs62436912
rs11021363
rs6016414
rs59865771
rs1490410
rs9573937
rs59987323
rs12935876
rs6904765
rs4785742
rs6766837
rs12986312
rs1467258
rs2644627
rs1520207
rs6079280
rs9330794
rs3798548
rs2209517
rs62502688
rs4692811
rs12568411
rs2060757
rs12622286
rs314720
rs2668550
rs10790945
rs17635337
rs17071260
rs10805326
rs16876512
rs11190363
rs6591869
rs61841503
rs9838382
rs635884
rs12101979
rs11728818
rs2675411
All - MR Egger
All - Inverse variance weighted
-0.01
0.00
0.01
MR effect size for
' || id:ebi-a-GCST90016946' on 'Diagnoses - main ICD10: K22.1 Ulcer of oesophagus || id:ukb-

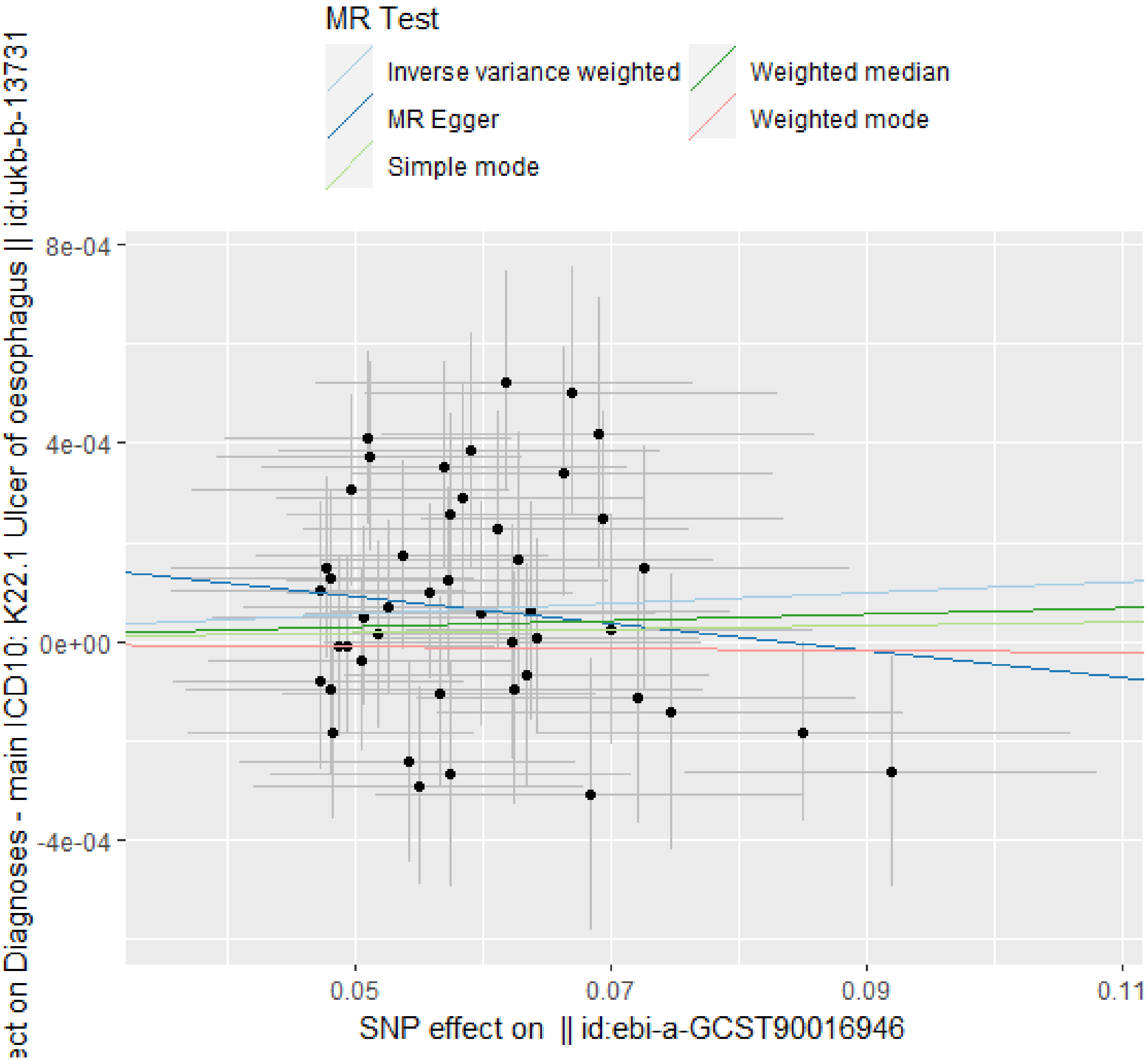

MR Test
Inverse variance weighted
MR Egger
Simple mode
Weighted median
Weighted mode
8e-04
4e-04
0e+00
-4e-04
0.05
0.07
0.09
0.11
SNP effect on || id:ebi-a-GCST90016946
ect on Diagnoses - main ICD10: K22.1 Ulcer of oesophagus || id:ukb-b-13731

Figure 27 Leave-one-out analysis, funnel plot, MR effect size and scatter plot for gut microbiota abundance (family Rhodospirillaceae id.2717) on ulcer of oesophagus

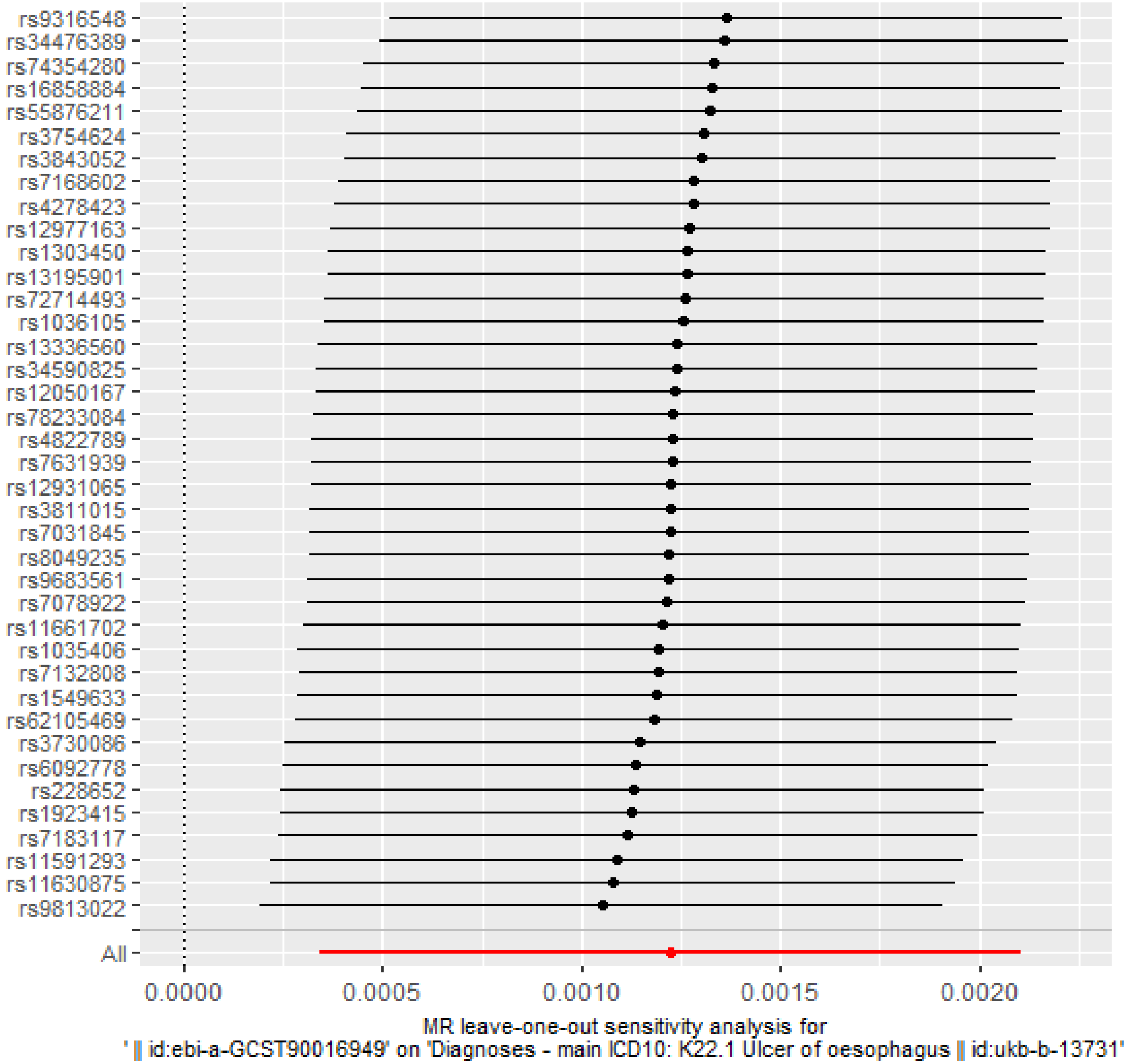

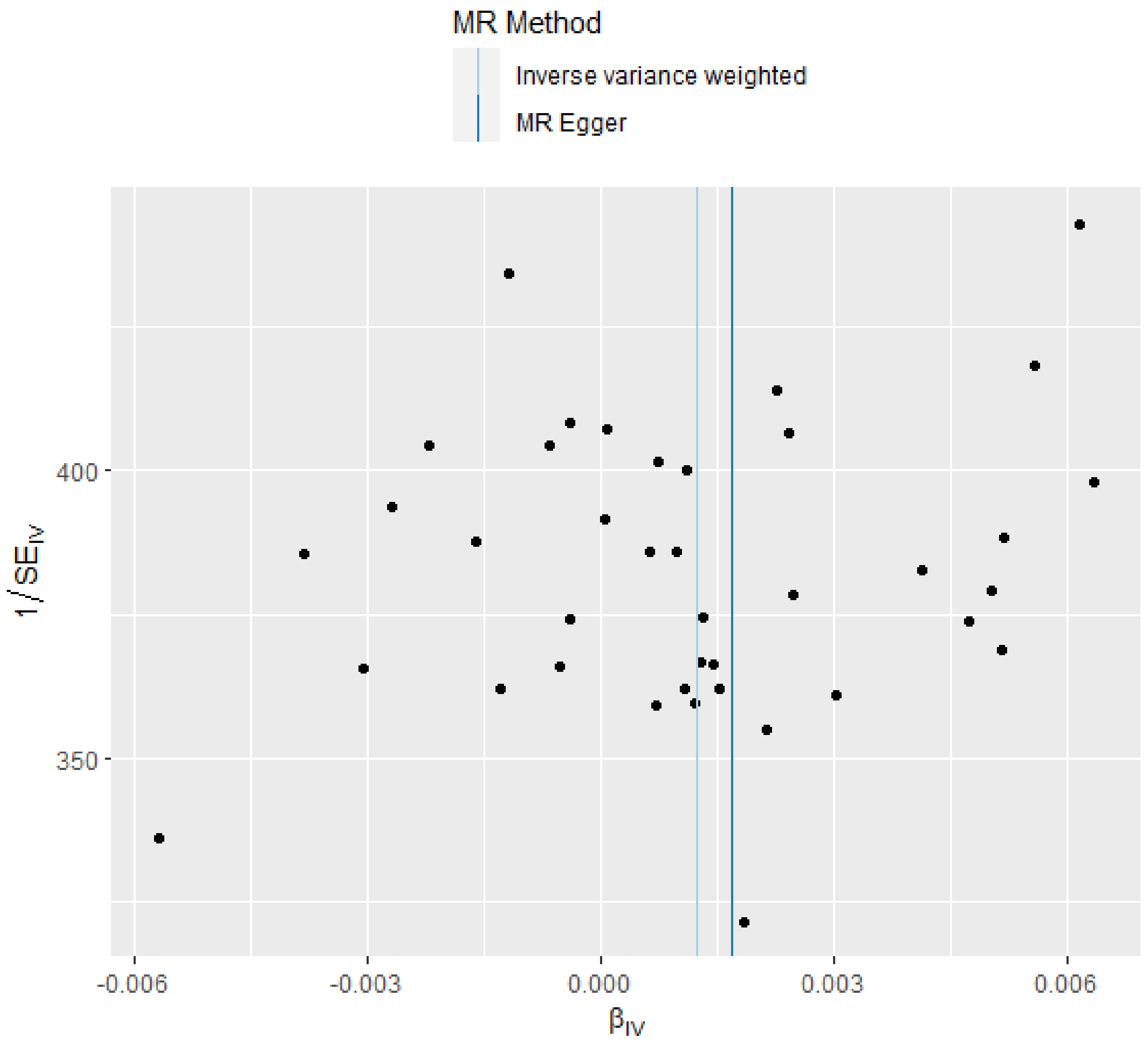

MR Method
Inverse variance weighted
MR Egger
400
350
$1/SE_{IV}$
-0.006
-0.003
0.000
0.003
0.006
β_{IV}

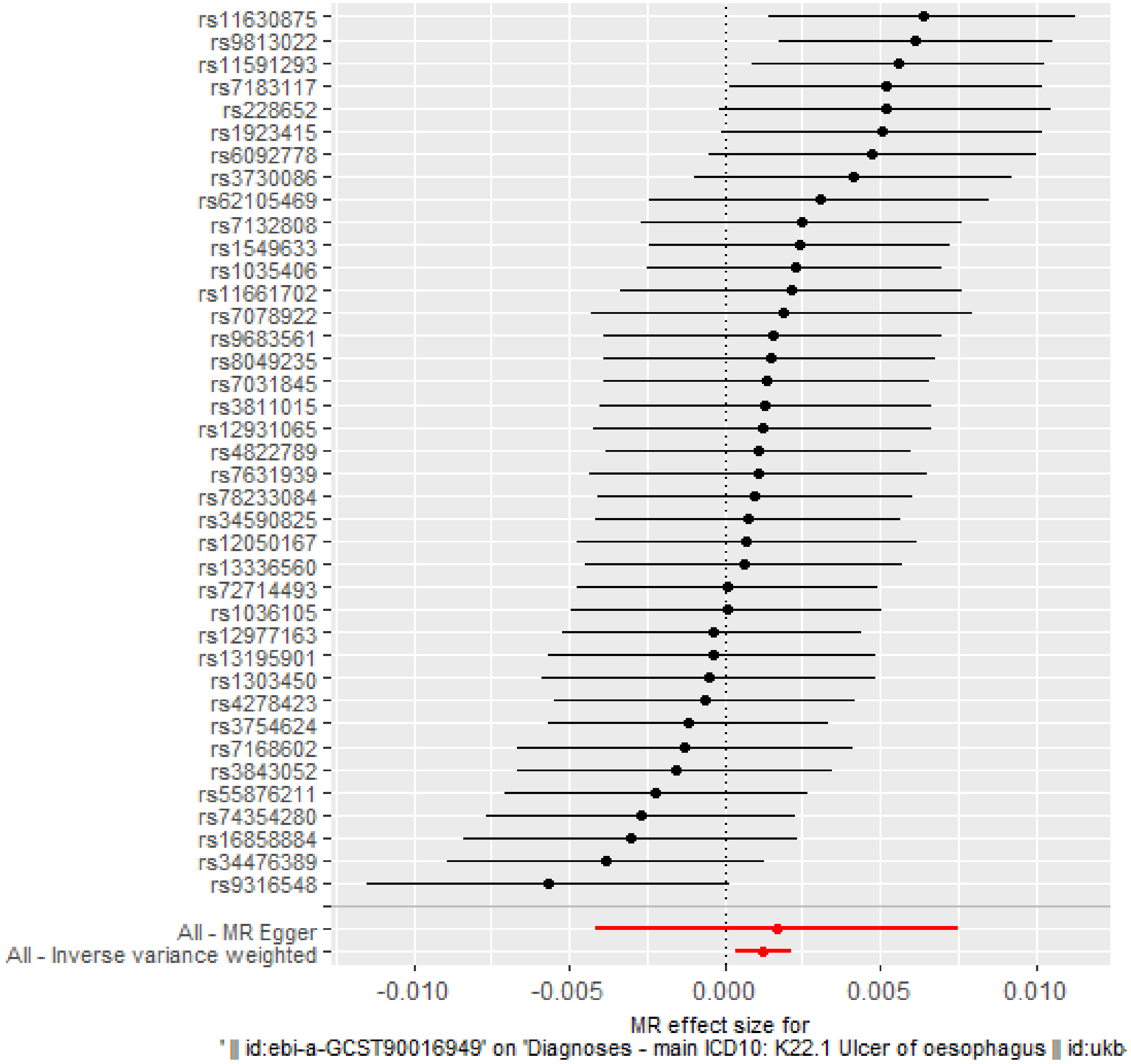
rs11630875
rs9813022
rs11591293
rs7183117
rs228652
rs1923415
rs6092778
rs3730086
rs62105469
rs7132808
rs1549633
rs1035406
rs11661702
rs7078922
rs9683561
rs8049235
rs7031845
rs3811015
rs12931065
rs4822789
rs7631939
rs78233084
rs34590825
rs12050167
rs13336560
rs72714493
rs1036105
rs12977163
rs13195901
rs1303450
rs4278423
rs3754624
rs7168602
rs3843052
rs55876211
rs74354280
rs16858884
rs34476389
rs9316548
All - MR Egger
All - Inverse variance weighted
-0.010
-0.005
0.000
0.005
0.010
MR effect size for
' || id:ebi-a-GCST90016949' on 'Diagnoses - main ICD10: K22.1 Ulcer of oesophagus || id:ukb-

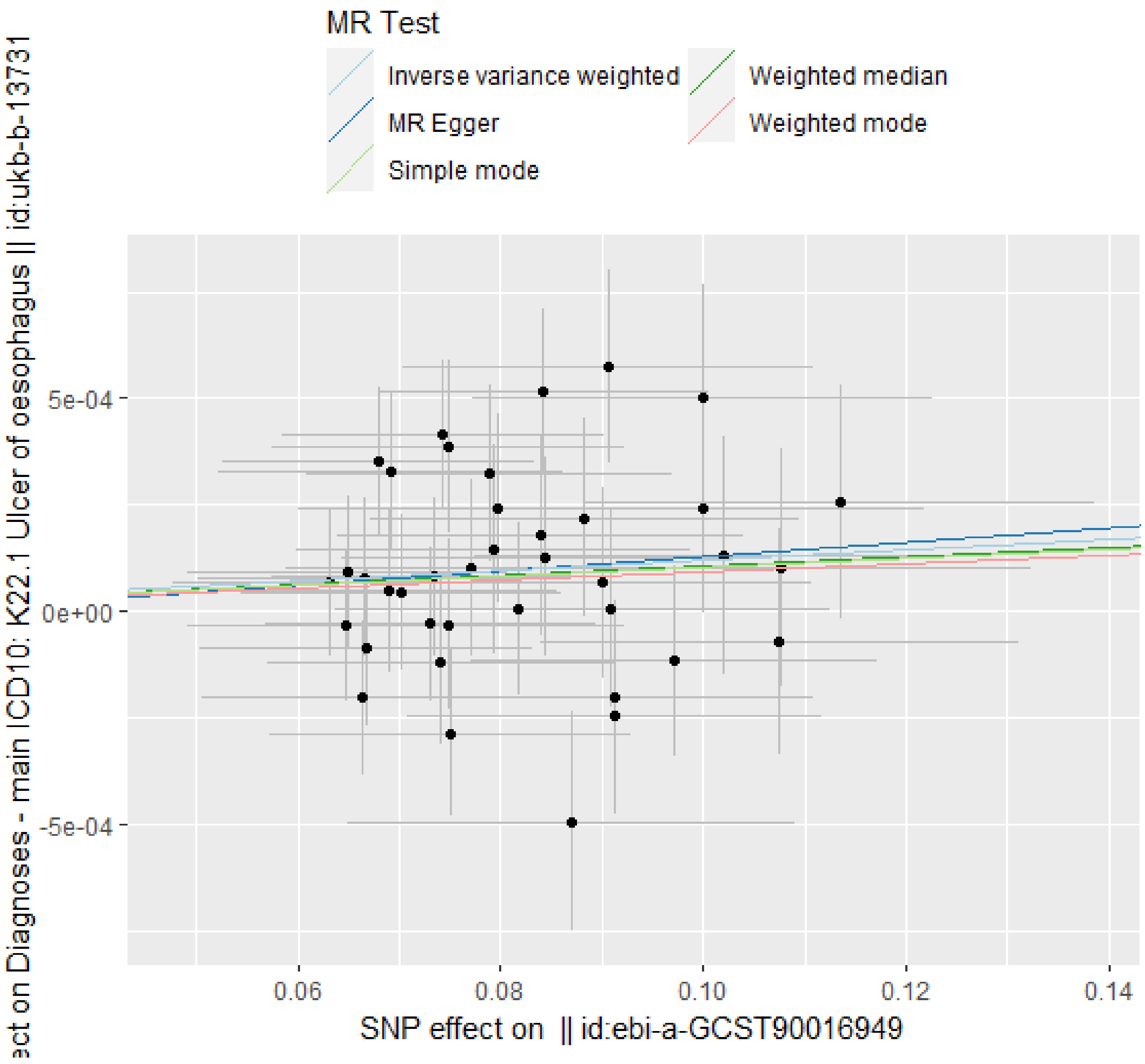

MR Test
Inverse variance weighted
MR Egger
Simple mode
Weighted median
Weighted mode
5e-04
0e+00
-5e-04
0.06
0.08
0.10
0.12
0.14
SNP effect on || id:ebi-a-GCST90016949
ect on Diagnoses - main ICD10: K22.1 Ulcer of oesophagus || id:ukb-b-13731

Figure 28 Leave-one-out analysis, funnel plot, MR effect size and scatter plot for gut microbiota abundance (genus Erysipelatoclostridium id.11381) on ulcer of oesophagus

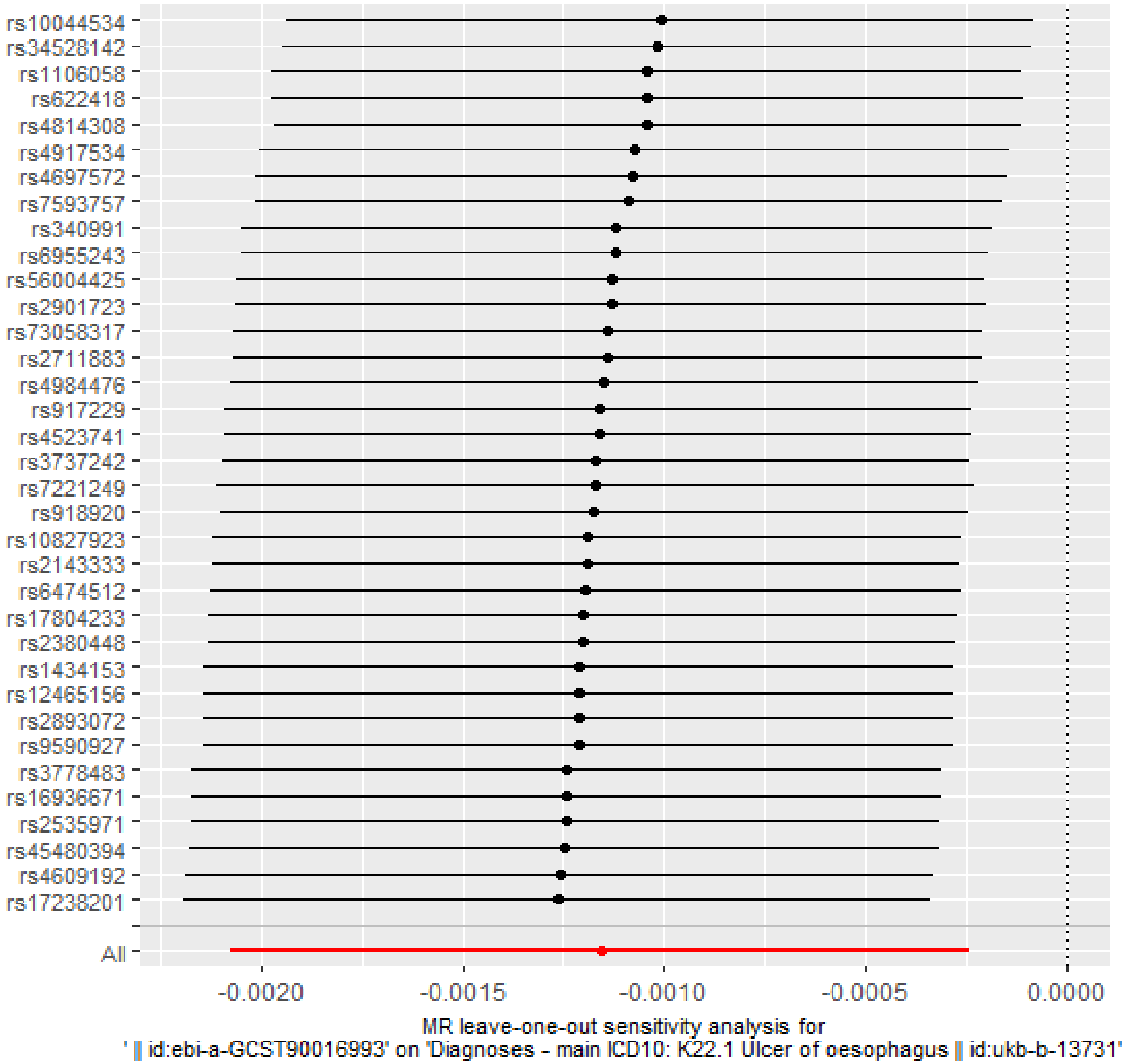

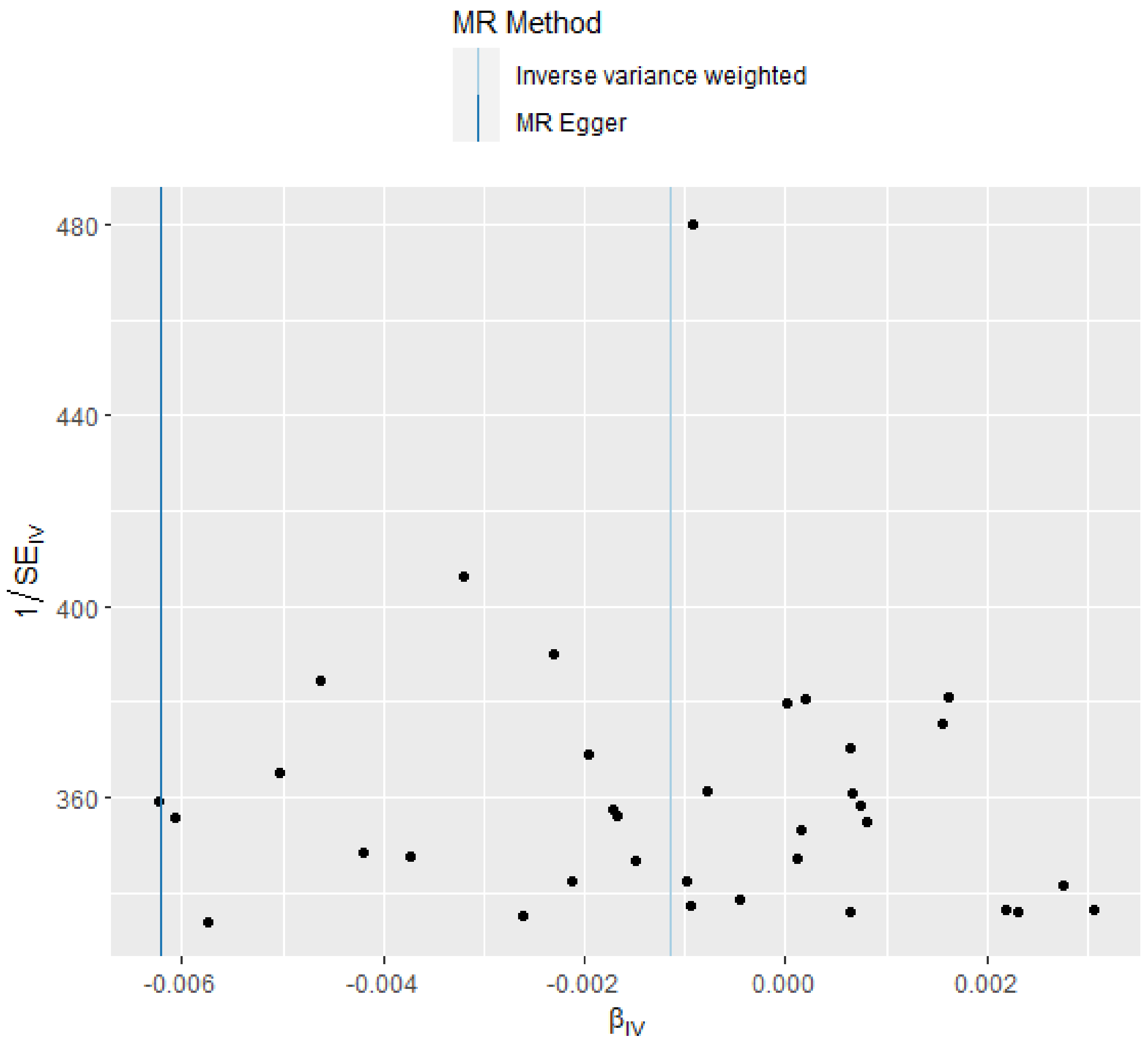
MR Method
Inverse variance weighted
MR Egger
480
440
400
360
1/SE_IV
-0.006
-0.004
-0.002
0.000
0.002
β_IV

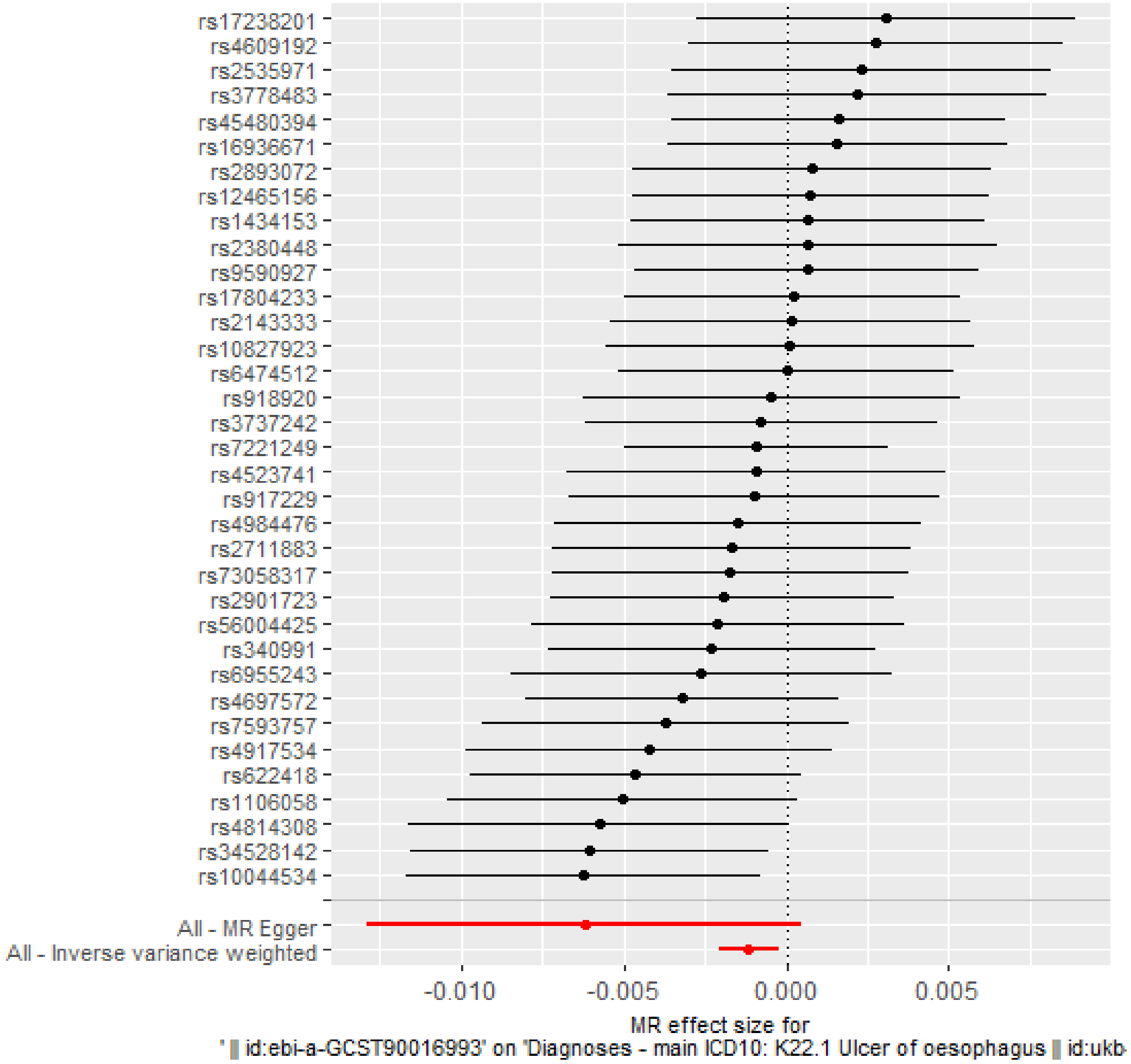
rs17238201
rs4609192
rs2535971
rs3778483
rs45480394
rs16936671
rs2893072
rs12465156
rs1434153
rs2380448
rs9590927
rs17804233
rs2143333
rs10827923
rs6474512
rs918920
rs3737242
rs7221249
rs4523741
rs917229
rs4984476
rs2711883
rs73058317
rs2901723
rs56004425
rs340991
rs6955243
rs4697572
rs7593757
rs4917534
rs622418
rs1106058
rs4814308
rs34528142
rs10044534
All - MR Egger
All - Inverse variance weighted
-0.010
-0.005
0.000
0.005
MR effect size for
' || id:ebi-a-GCST90016993' on 'Diagnoses - main ICD10: K22.1 Ulcer of oesophagus || id:ukb-

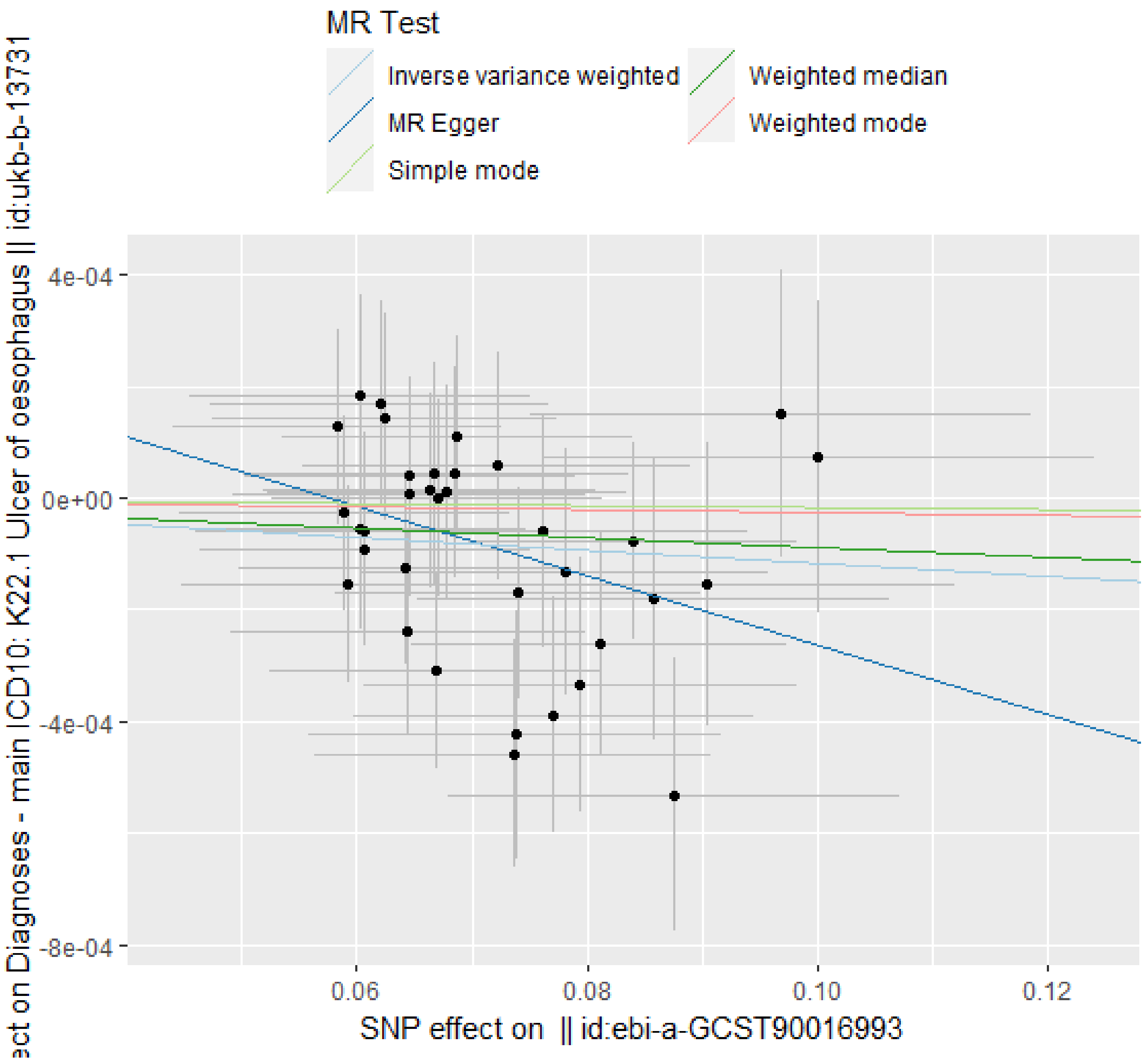

MR Test
Inverse variance weighted
MR Egger
Simple mode
Weighted median
Weighted mode
4e-04
0e+00
-4e-04
-8e-04
0.06
0.08
0.10
0.12
SNP effect on || id:ebi-a-GCST90016993
ect on Diagnoses - main ICD10: K22.1 Ulcer of oesophagus || id:ukb-b-13731

Figure 29 Leave-one-out analysis, funnel plot, MR effect size and scatter plot for gut microbiota abundance (genus Family XIII AD3011 group id.11293) on ulcer of oesophagus

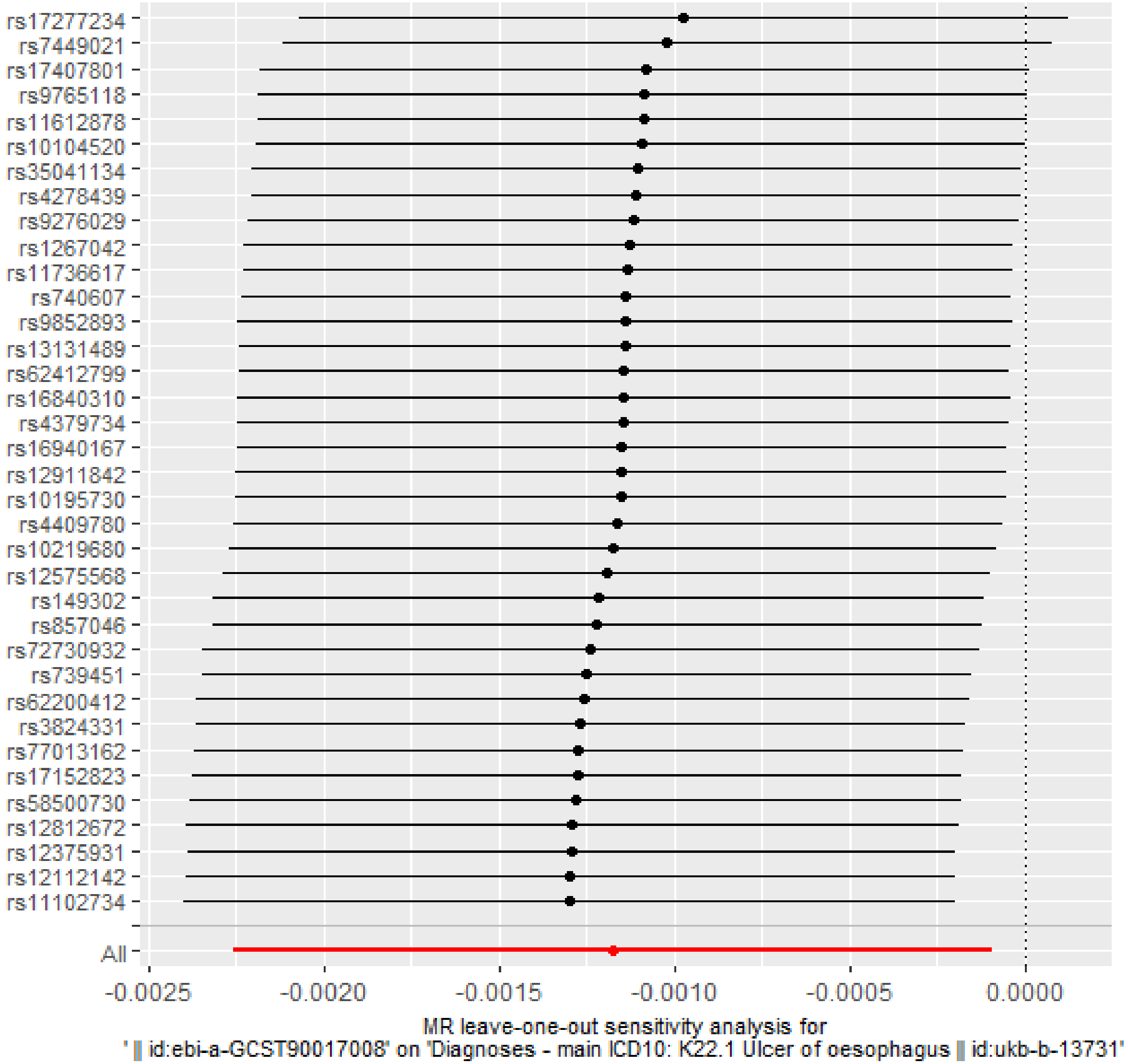

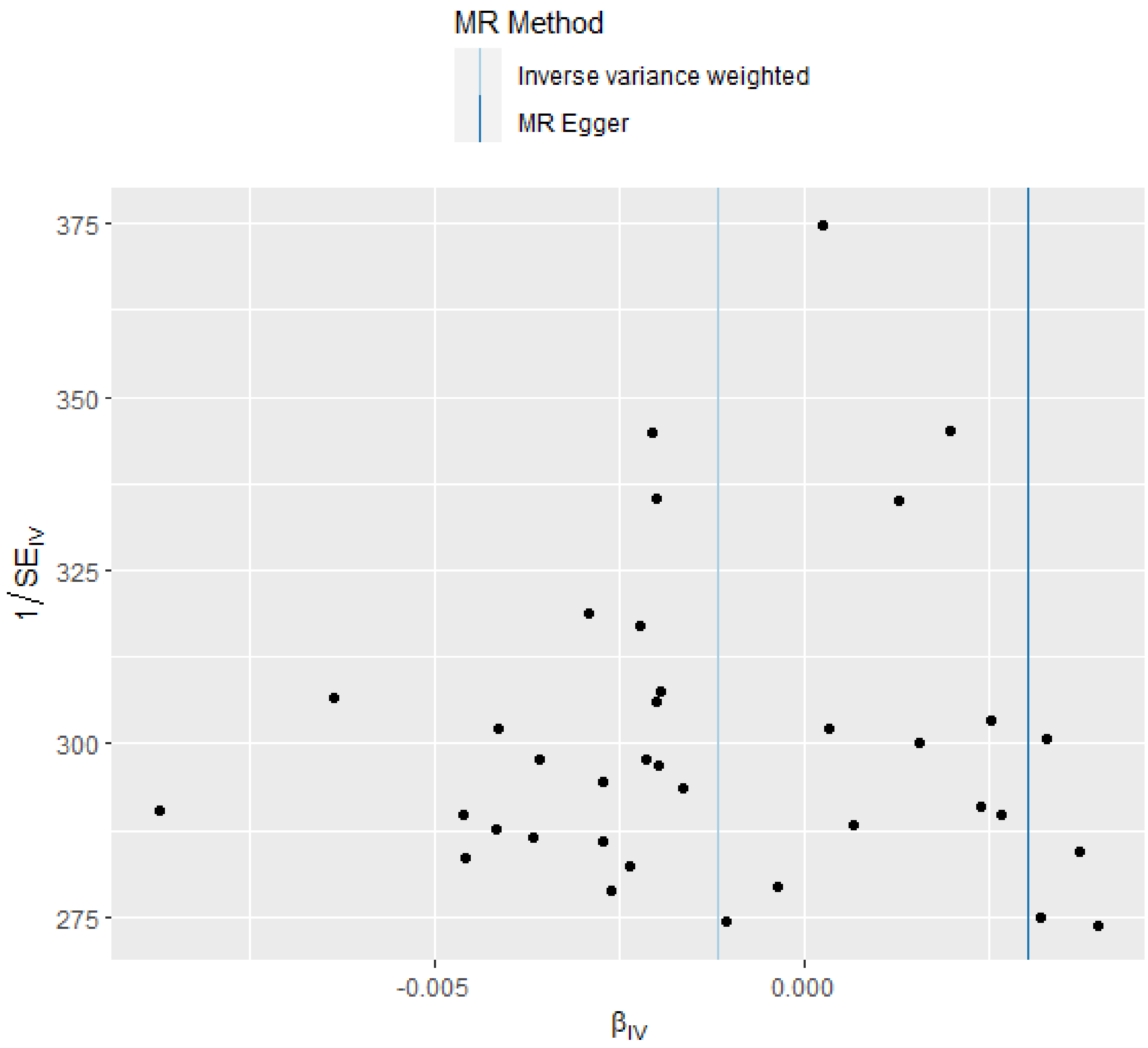

MR Method
Inverse variance weighted
MR Egger
375
350
325
300
275
1/SE_IV
-0.005
0.000
β_IV

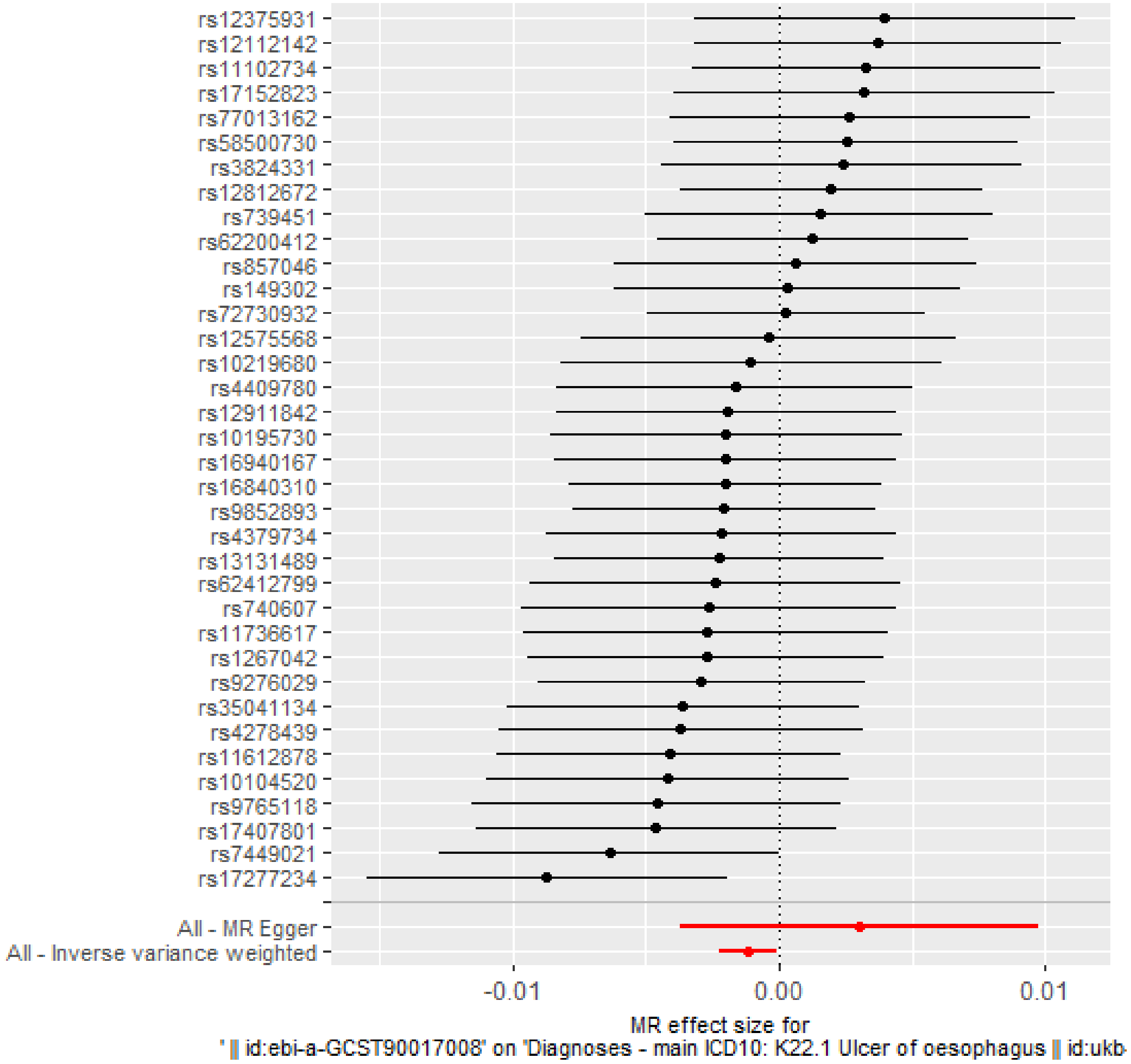
rs12375931
rs12112142
rs11102734
rs17152823
rs77013162
rs58500730
rs3824331
rs12812672
rs739451
rs62200412
rs857046
rs149302
rs72730932
rs12575568
rs10219680
rs4409780
rs12911842
rs10195730
rs16940167
rs16840310
rs9852893
rs4379734
rs13131489
rs62412799
rs740607
rs11736617
rs1267042
rs9276029
rs35041134
rs4278439
rs11612878
rs10104520
rs9765118
rs17407801
rs7449021
rs17277234
All - MR Egger
All - Inverse variance weighted
-0.01
0.00
0.01
MR effect size for
' || id:ebi-a-GCST90017008' on 'Diagnoses - main ICD10: K22.1 Ulcer of oesophagus || id:ukb-

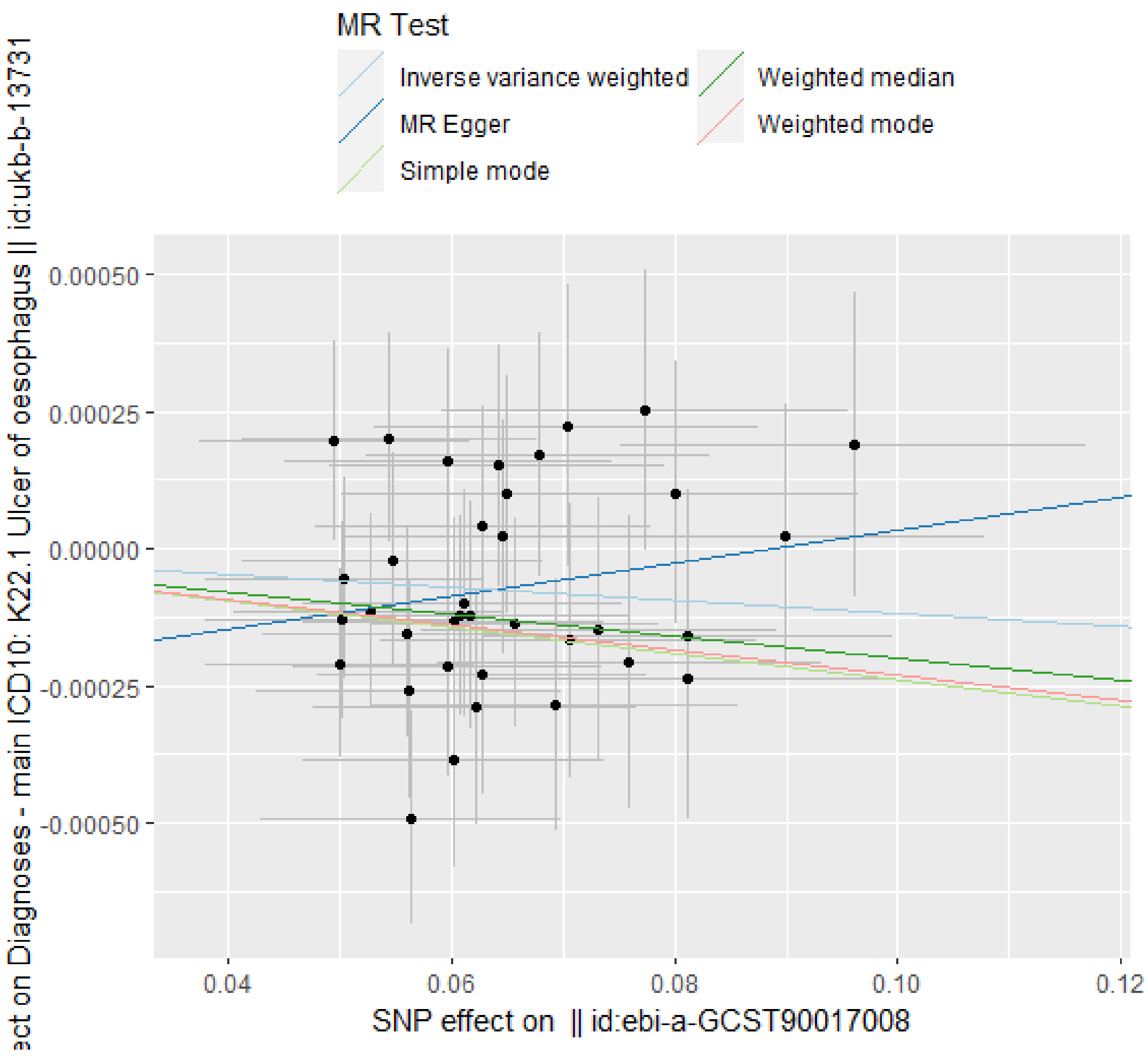

MR Test
Inverse variance weighted
MR Egger
Simple mode
Weighted median
Weighted mode
0.00050
0.00025
0.00000
-0.00025
-0.00050
0.04
0.06
0.08
0.10
0.12
SNP effect on || id:ebi-a-GCST90017008
ect on Diagnoses - main ICD10: K22.1 Ulcer of oesophagus || id:ukb-b-13731

Figure 30 Leave-one-out analysis, funnel plot, MR effect size and scatter plot for gut microbiota abundance (genus Gordonibacter id.821) on ulcer of oesophagus

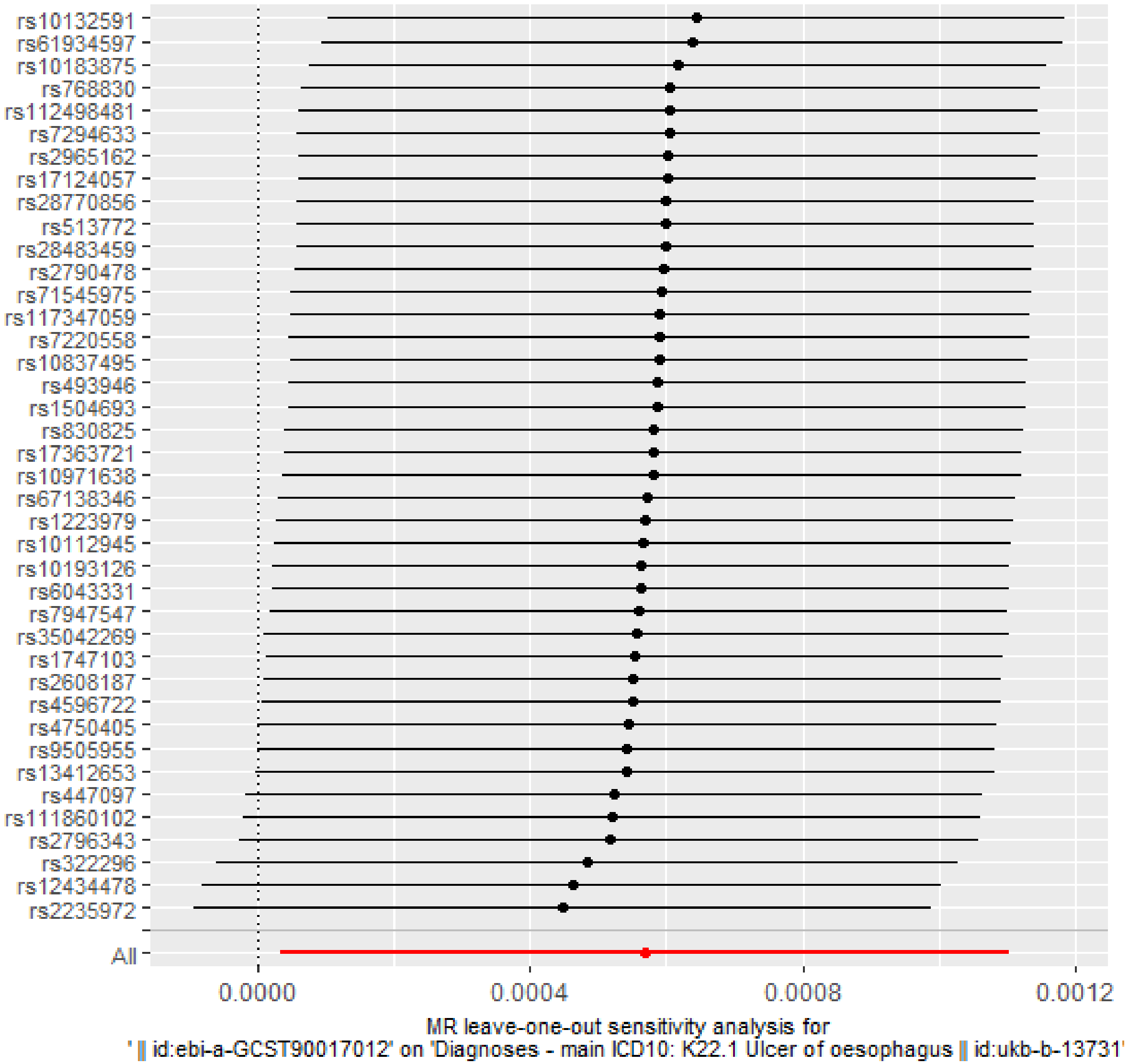

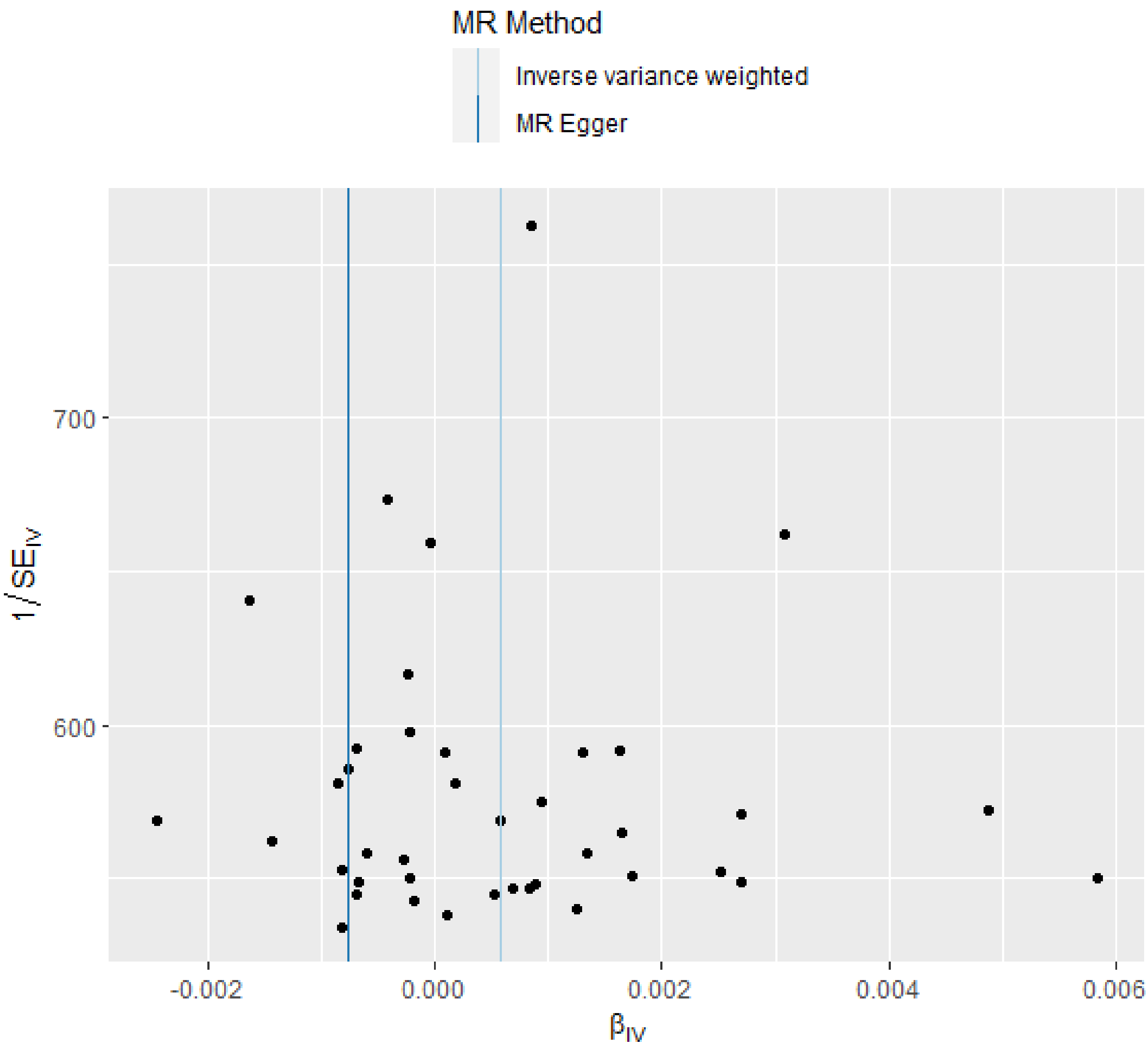

MR Method
Inverse variance weighted
MR Egger
700
600
-0.002
0.000
0.002
0.004
0.006
$1/SE_{IV}$
β_{IV}

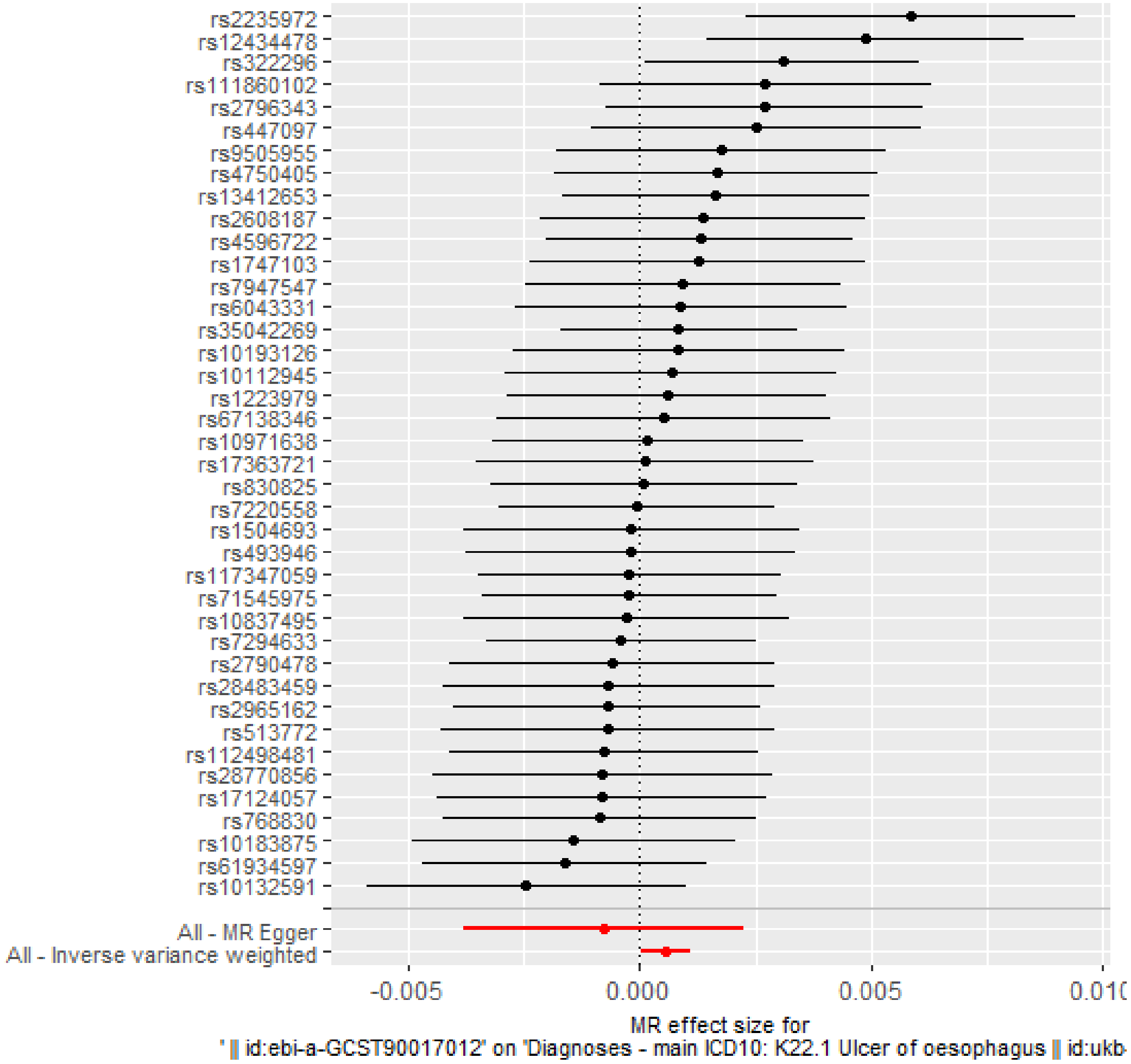
rs2235972
rs12434478
rs322296
rs111860102
rs2796343
rs447097
rs9505955
rs4750405
rs13412653
rs2608187
rs4596722
rs1747103
rs7947547
rs6043331
rs35042269
rs10193126
rs10112945
rs1223979
rs67138346
rs10971638
rs17363721
rs830825
rs7220558
rs1504693
rs493946
rs117347059
rs71545975
rs10837495
rs7294633
rs2790478
rs28483459
rs2965162
rs513772
rs112498481
rs28770856
rs17124057
rs768830
rs10183875
rs61934597
rs10132591
All - MR Egger
All - Inverse variance weighted
-0.005
0.000
0.005
MR effect size for
' || id:ebi-a-GCST90017012' on 'Diagnoses - main ICD10: K22.1 Ulcer of oesophagus || id:ukb

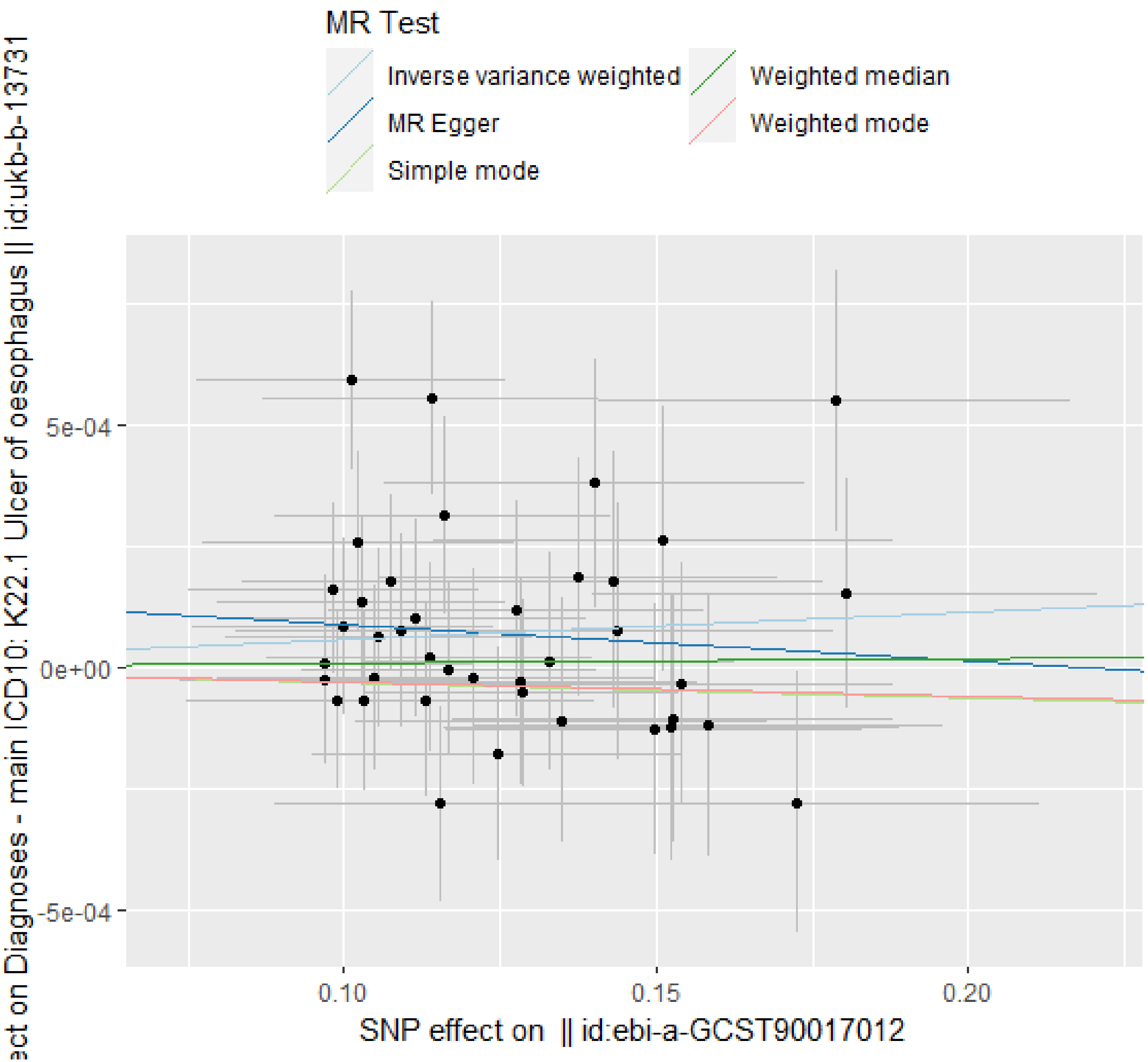

MR Test
Inverse variance weighted
MR Egger
Simple mode
Weighted median
Weighted mode
5e-04
0e+00
-5e-04
0.10
0.15
0.20
SNP effect on || id:ebi-a-GCST90017012
act on Diagnoses - main ICD10: K22.1 Ulcer of oesophagus || id:ukb-b-13731

Figure 31 Leave-one-out analysis, funnel plot, MR effect size and scatter plot for gut microbiota abundance (genus Slackia id.825) on ulcer of oesophagus

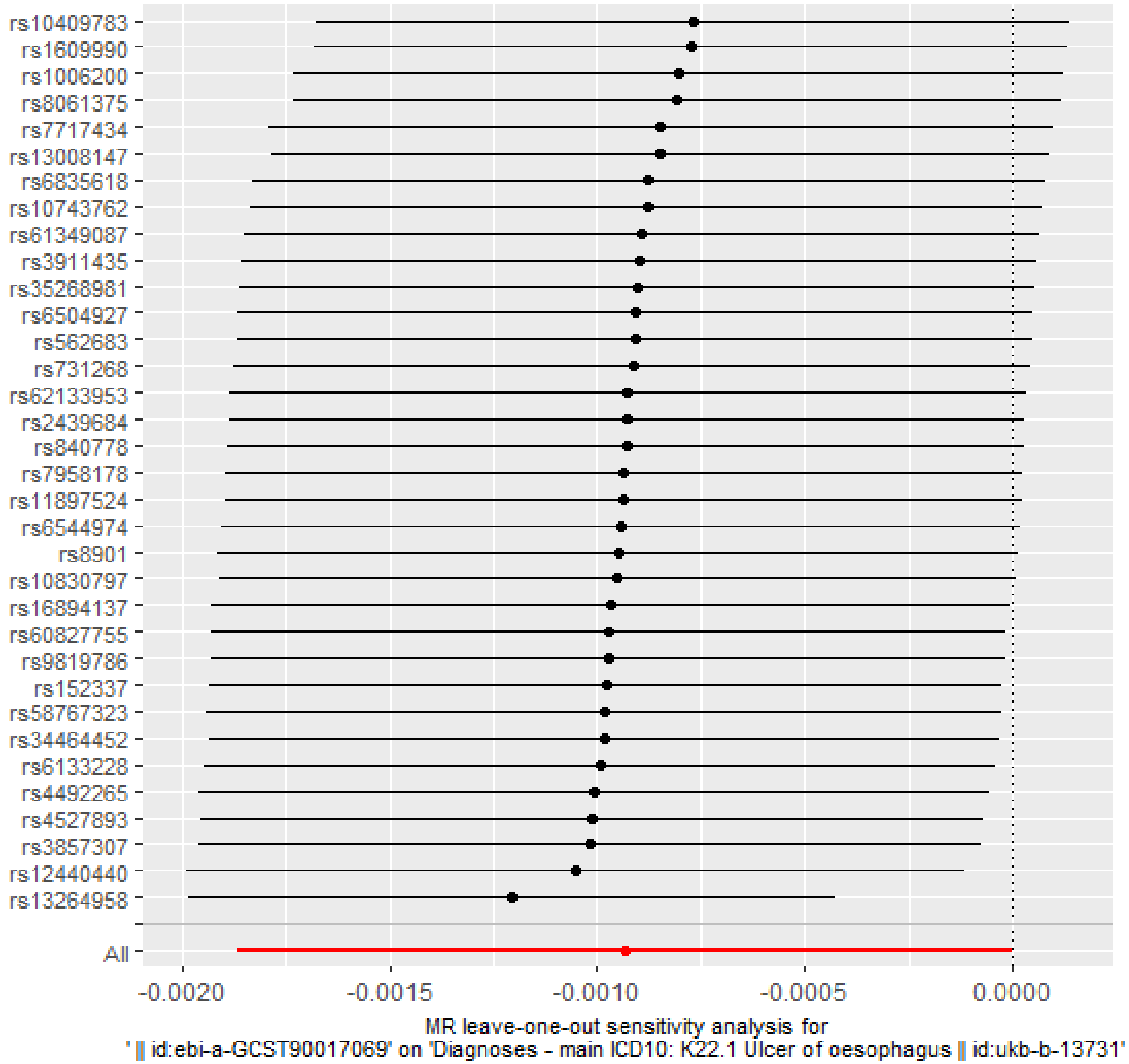

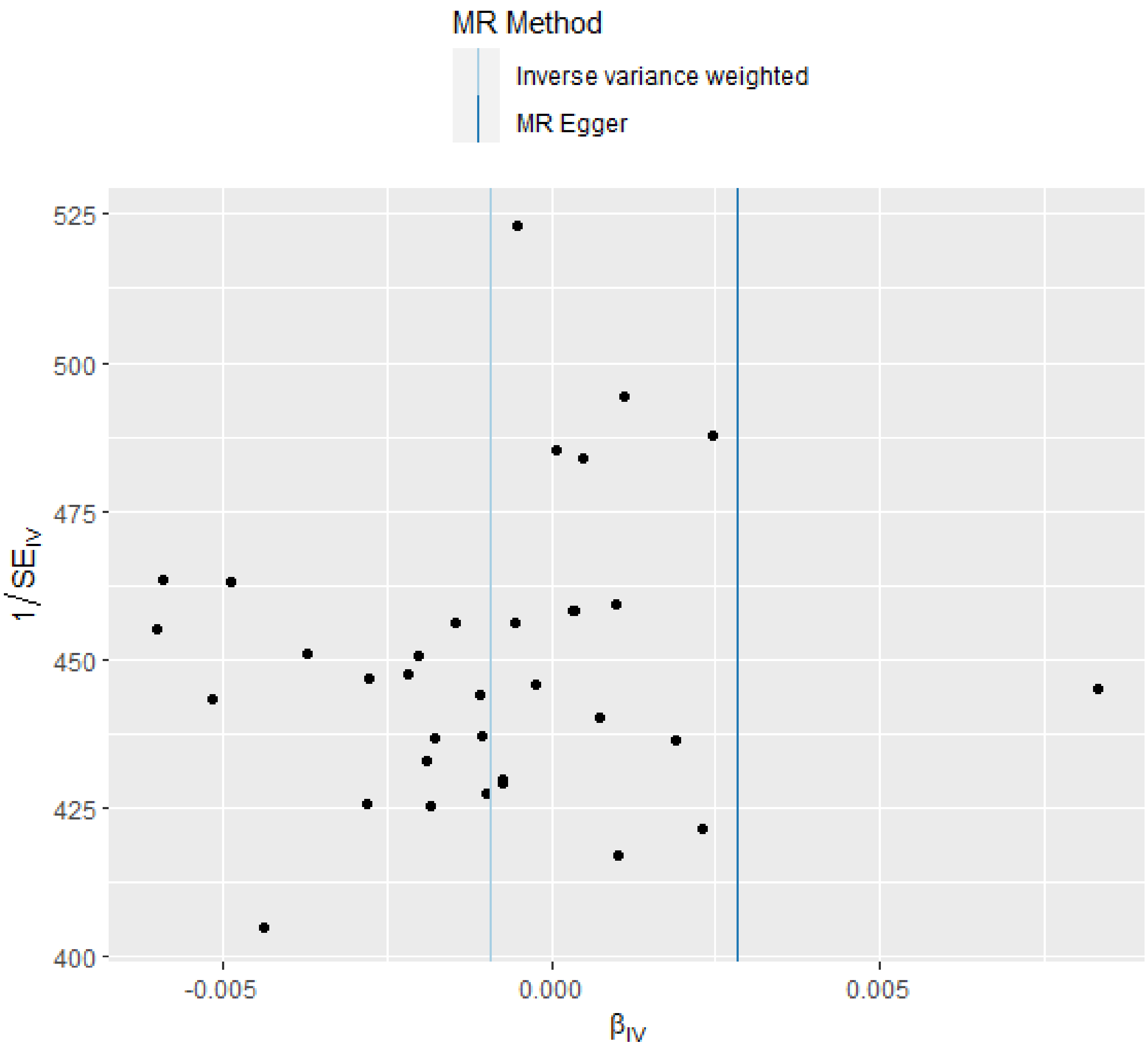
MR Method
Inverse variance weighted
MR Egger
525
500
475
450
425
400
1/SE_IV
-0.005
0.000
0.005
β_IV

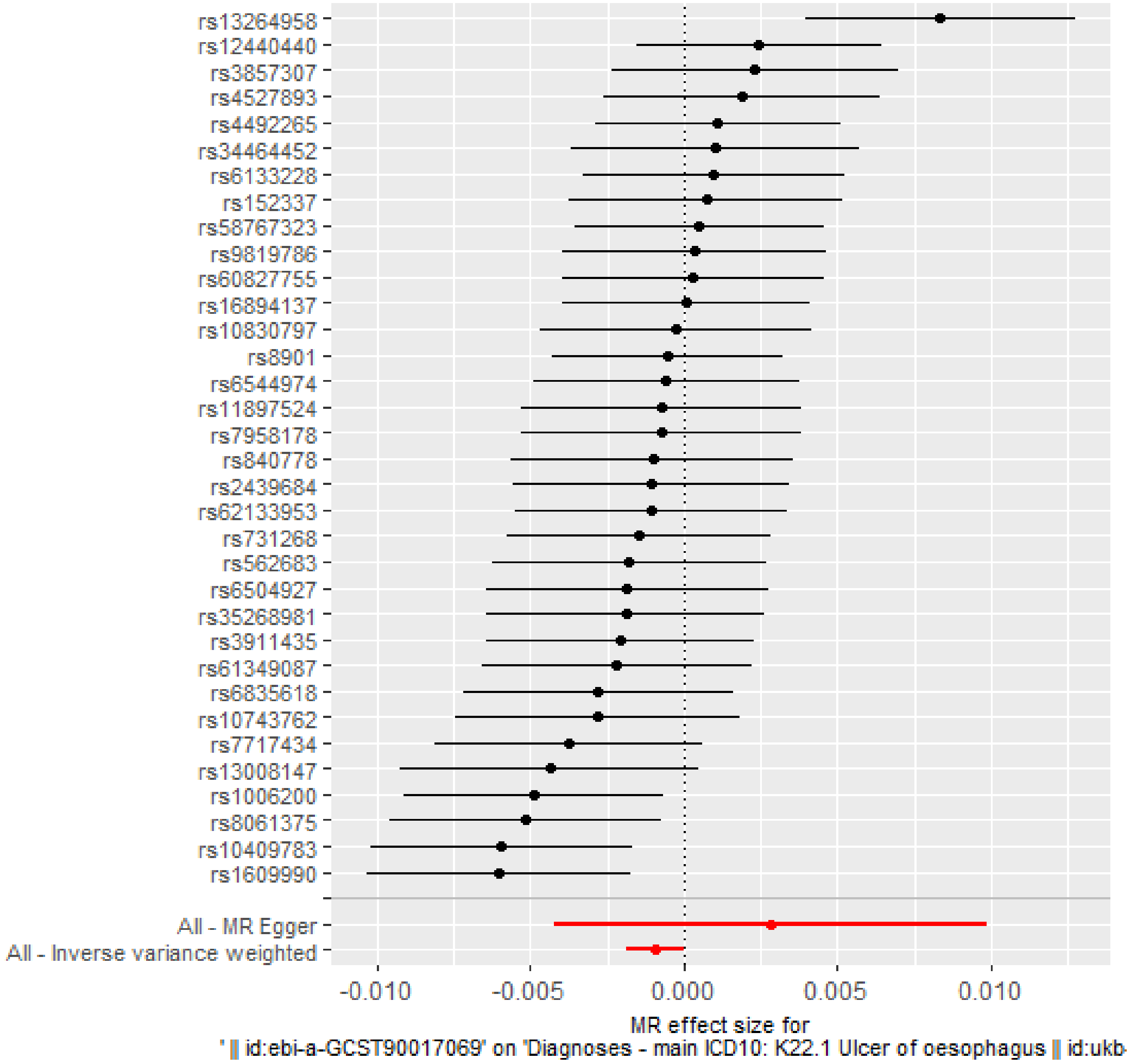

rs13264958
rs12440440
rs3857307
rs4527893
rs4492265
rs34464452
rs6133228
rs152337
rs58767323
rs9819786
rs60827755
rs16894137
rs10830797
rs8901
rs6544974
rs11897524
rs7958178
rs840778
rs2439684
rs62133953
rs731268
rs562683
rs6504927
rs35268981
rs3911435
rs61349087
rs6835618
rs10743762
rs7717434
rs13008147
rs1006200
rs8061375
rs10409783
rs1609990
All - MR Egger
All - Inverse variance weighted
-0.010
-0.005
0.000
0.005
0.010
MR effect size for
' || id:ebi-a-GCST90017069' on 'Diagnoses - main ICD10: K22.1 Ulcer of oesophagus || id:ukb-

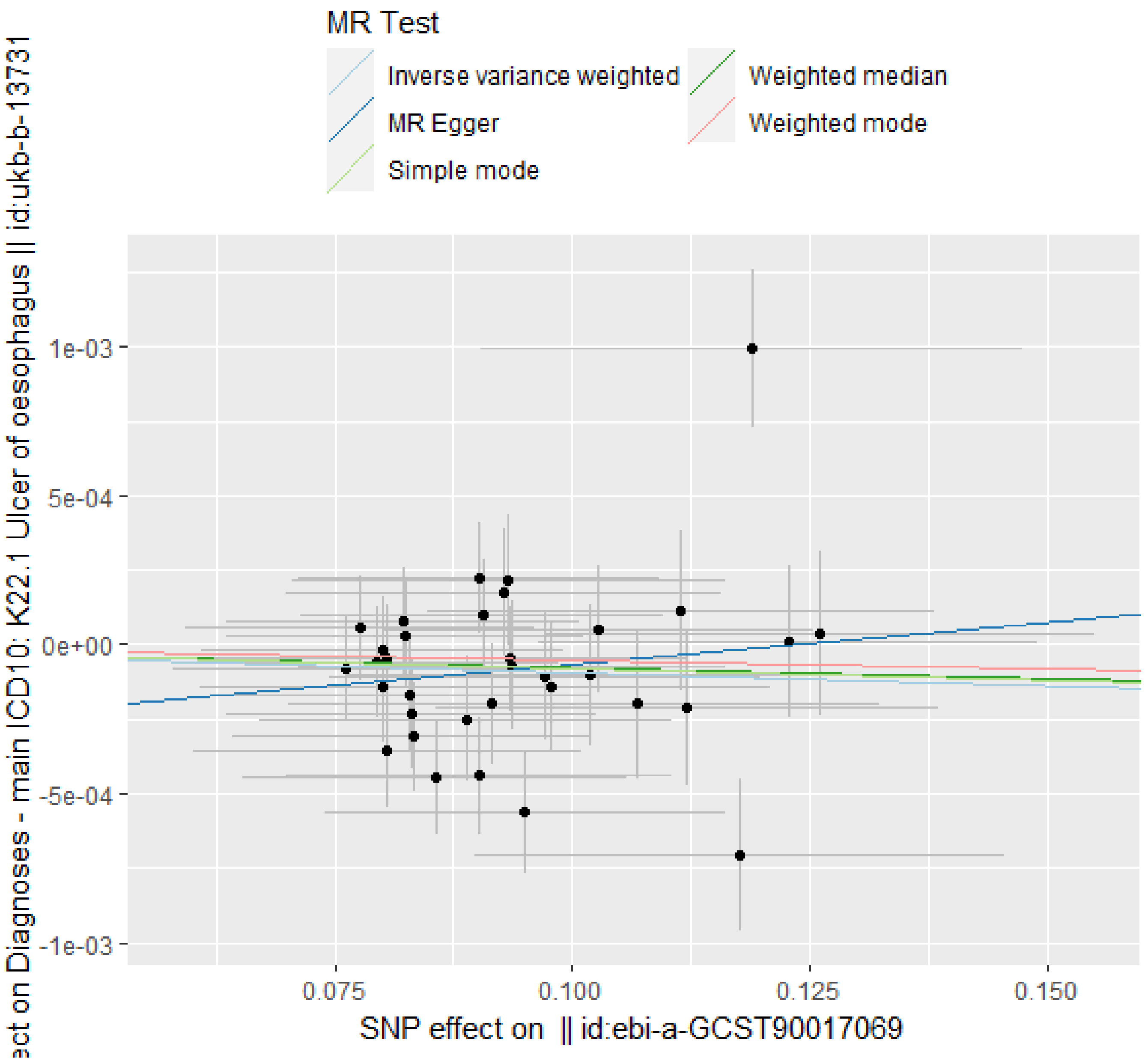
MR Test
Inverse variance weighted
MR Egger
Simple mode
Weighted median
Weighted mode
1e-03
5e-04
0e+00
-5e-04
-1e-03
0.075
0.100
0.125
0.150
SNP effect on || id:ebi-a-GCST90017069
ect on Diagnoses - main ICD10: K22.1 Ulcer of oesophagus || id:ukb-b-13731

Figure 32 Leave-one-out analysis, funnel plot, MR effect size and scatter plot for gut microbiota abundance (genus Terrisporobacter id.11348) on ulcer of oesophagus

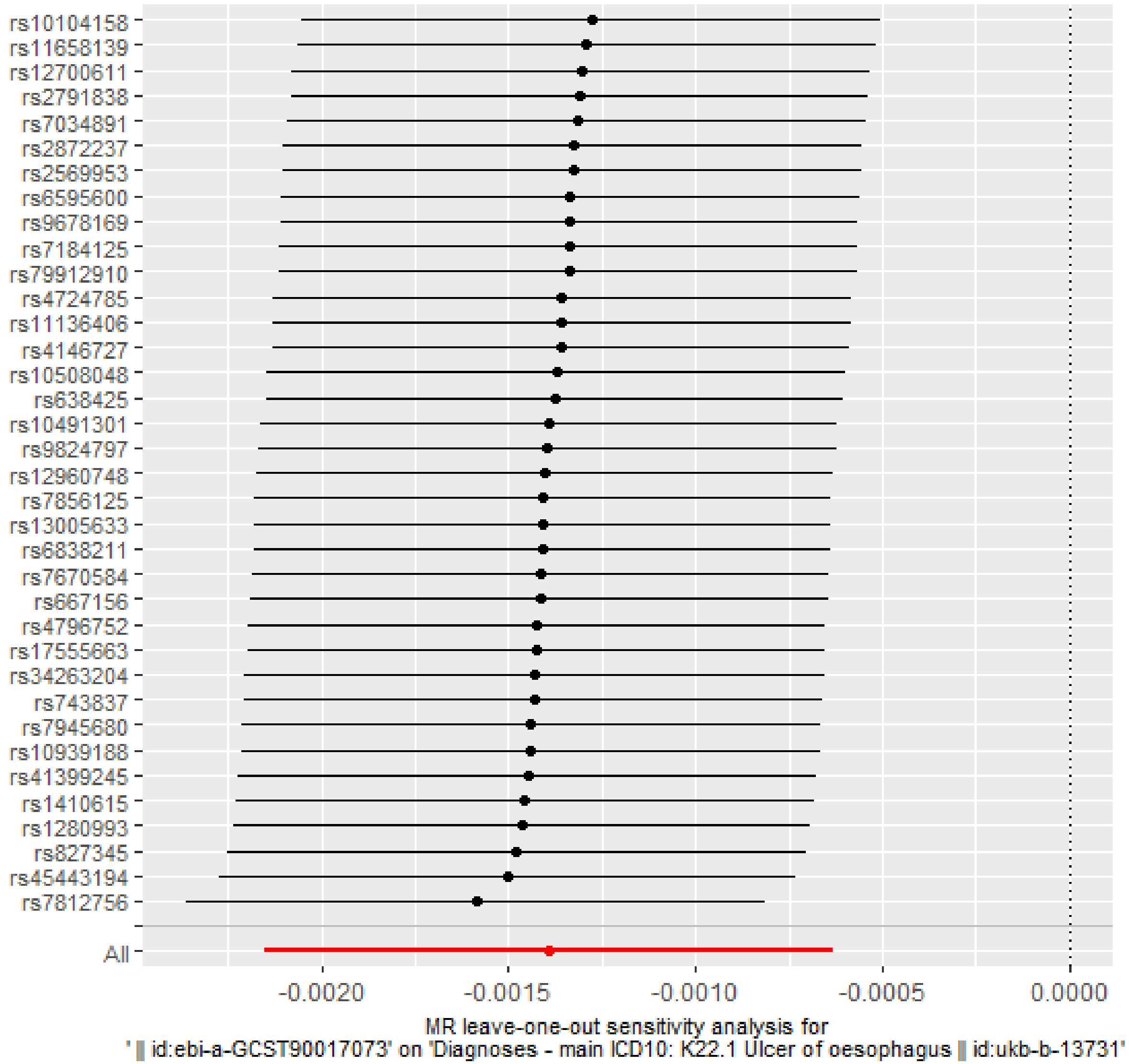

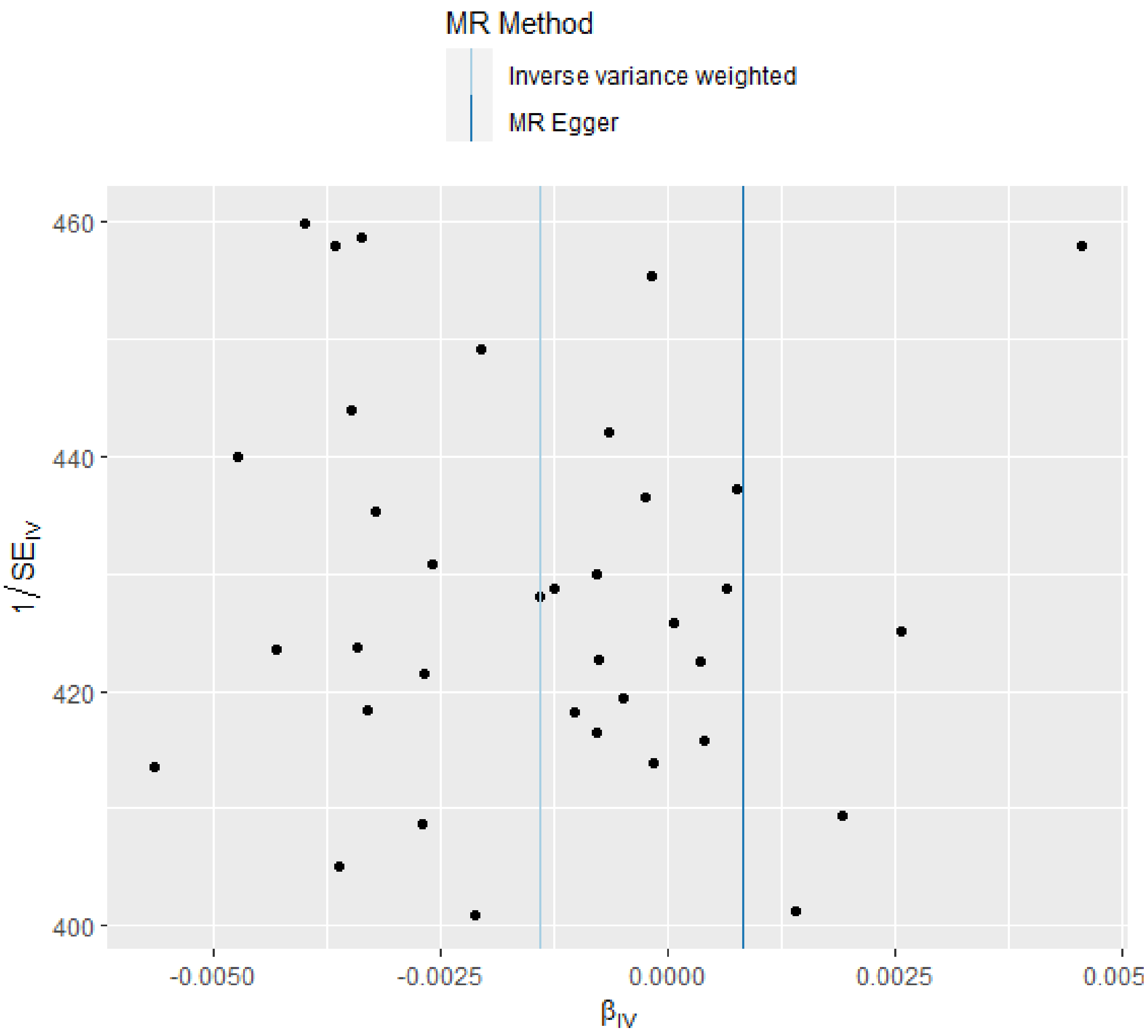

MR Method
Inverse variance weighted
MR Egger
460
440
420
400
1/SE_IV
-0.0050
-0.0025
0.0000
0.0025
0.005
β_IV

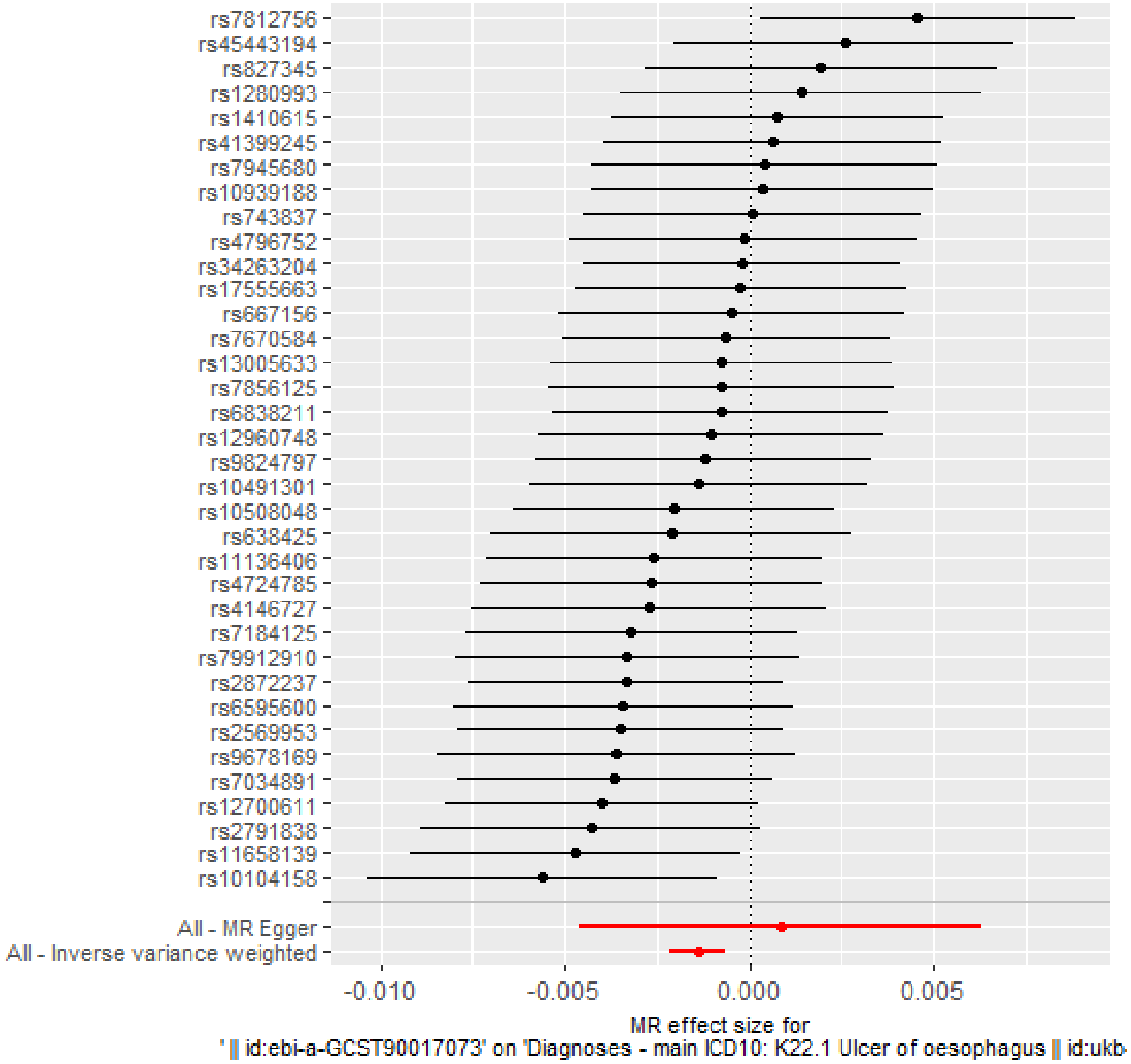
rs7812756
rs45443194
rs827345
rs1280993
rs1410615
rs41399245
rs7945680
rs10939188
rs743837
rs4796752
rs34263204
rs17555663
rs667156
rs7670584
rs13005633
rs7856125
rs6838211
rs12960748
rs9824797
rs10491301
rs10508048
rs638425
rs11136406
rs4724785
rs4146727
rs7184125
rs79912910
rs2872237
rs6595600
rs2569953
rs9678169
rs7034891
rs12700611
rs2791838
rs11658139
rs10104158
All - MR Egger
All - Inverse variance weighted
-0.010
-0.005
0.000
0.005
MR effect size for
' || id:ebi-a-GCST90017073' on 'Diagnoses - main ICD10: K22.1 Ulcer of oesophagus || id:ukb-

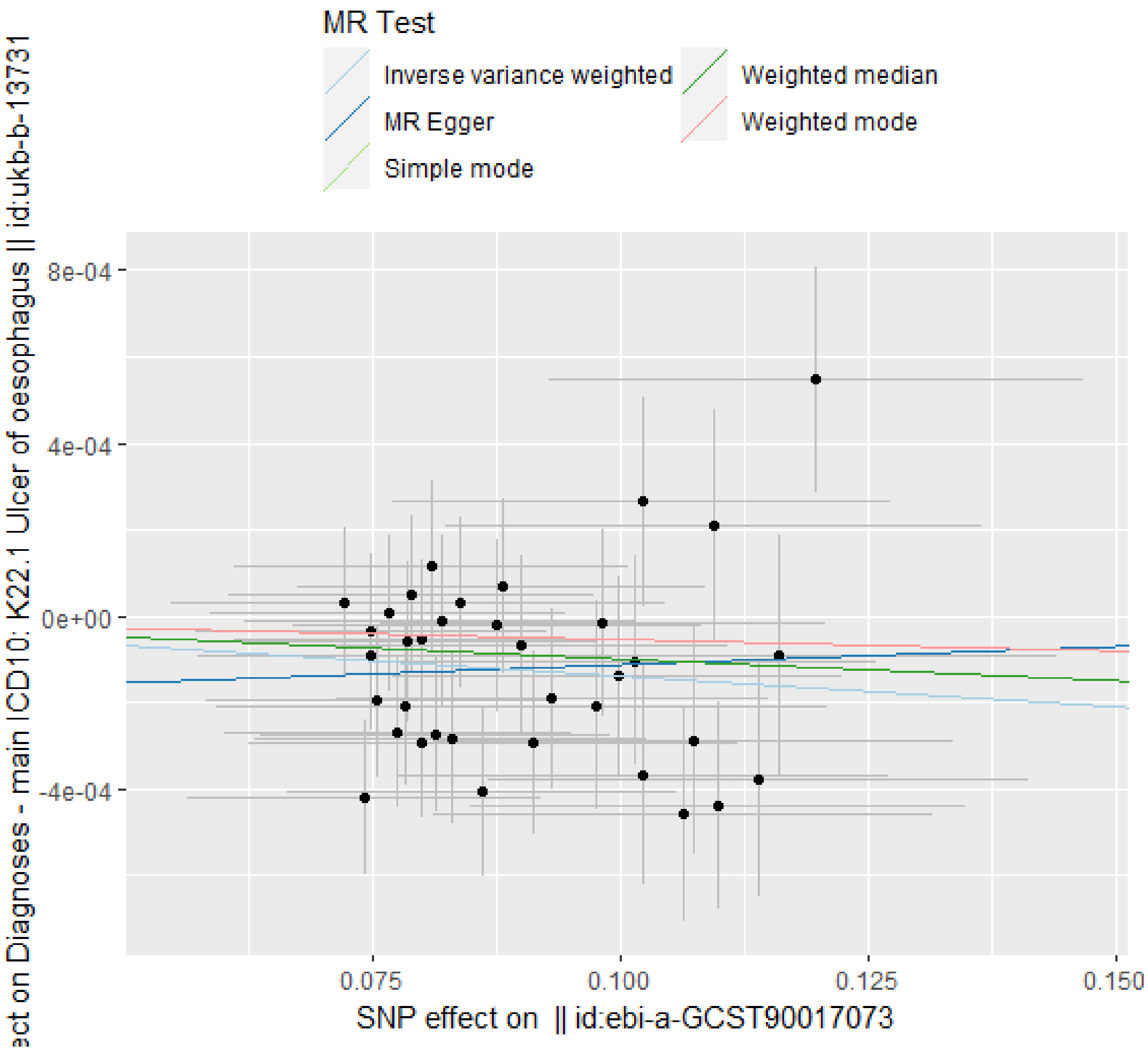

MR Test
Inverse variance weighted
MR Egger
Simple mode
Weighted median
Weighted mode
8e-04
4e-04
0e+00
-4e-04
0.075
0.100
0.125
0.150
SNP effect on || id:ebi-a-GCST90017073
ect on Diagnoses - main ICD10: K22.1 Ulcer of oesophagus || id:ukb-b-13731

Figure 33 Leave-one-out analysis, funnel plot, MR effect size and scatter plot for gut microbiota abundance (unknown genus id.2001) on ulcer of oesophagus

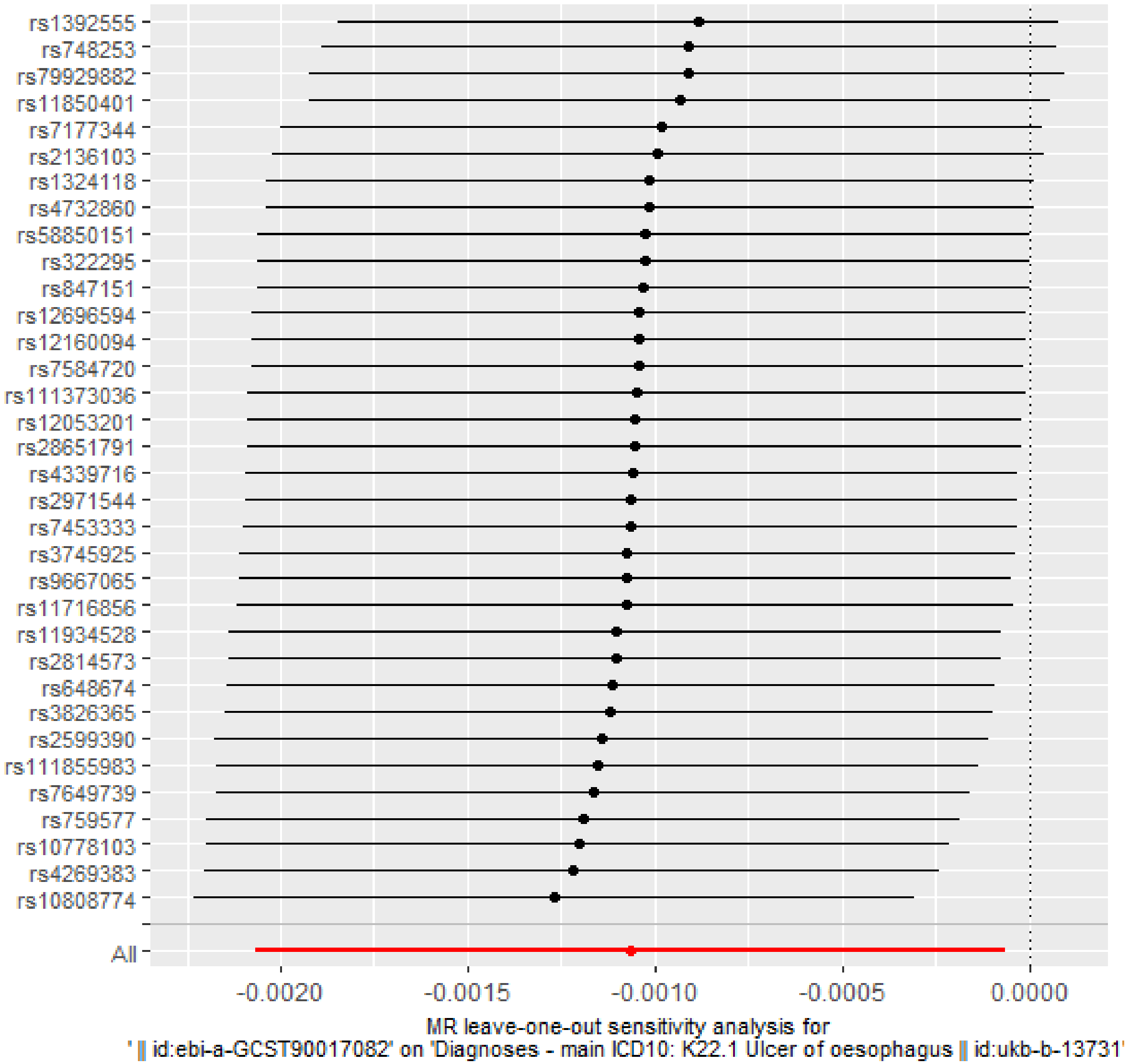

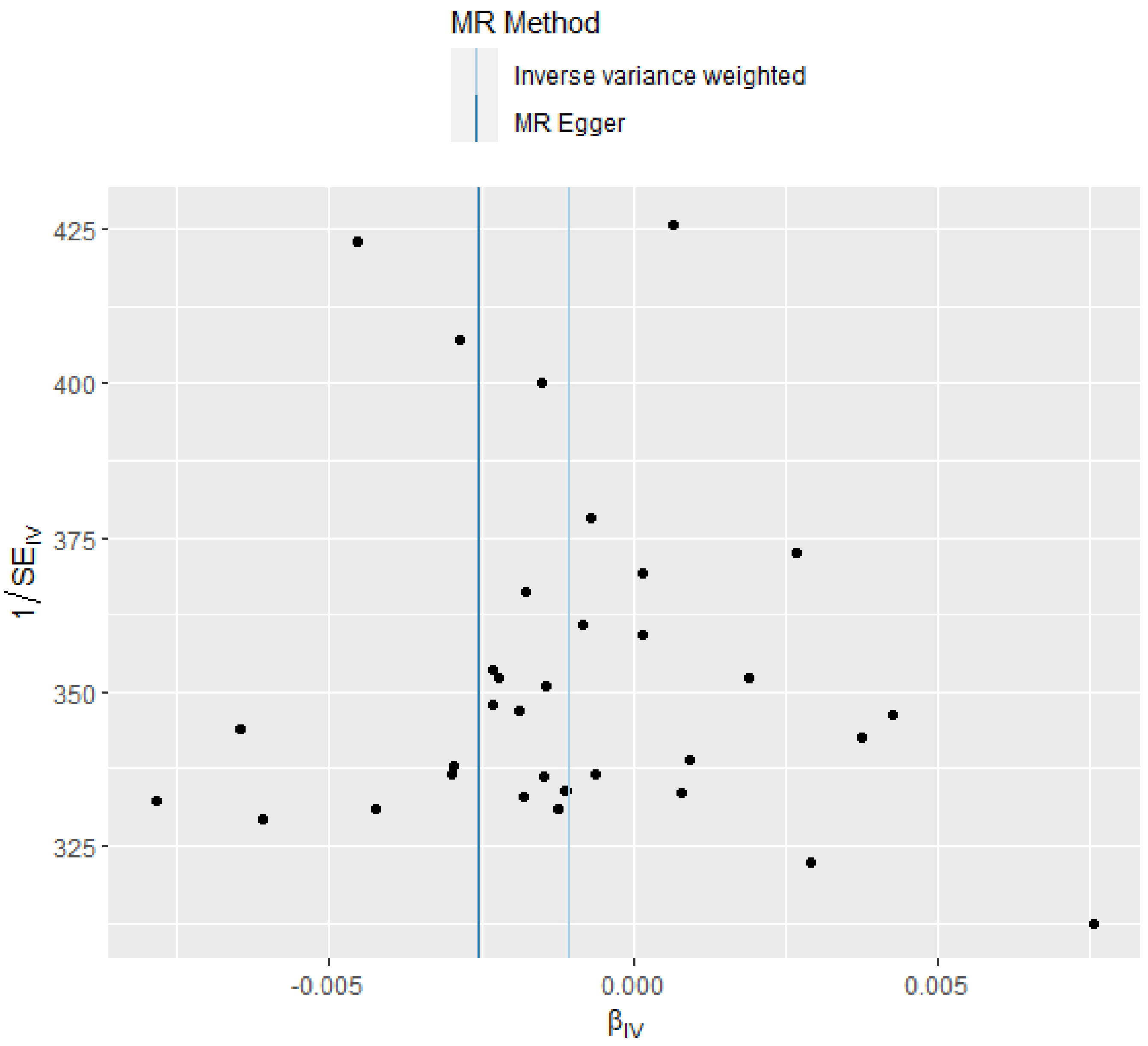

MR Method
Inverse variance weighted
MR Egger
425
400
375
350
325
1/SE_IV
-0.005
0.000
0.005
β_IV

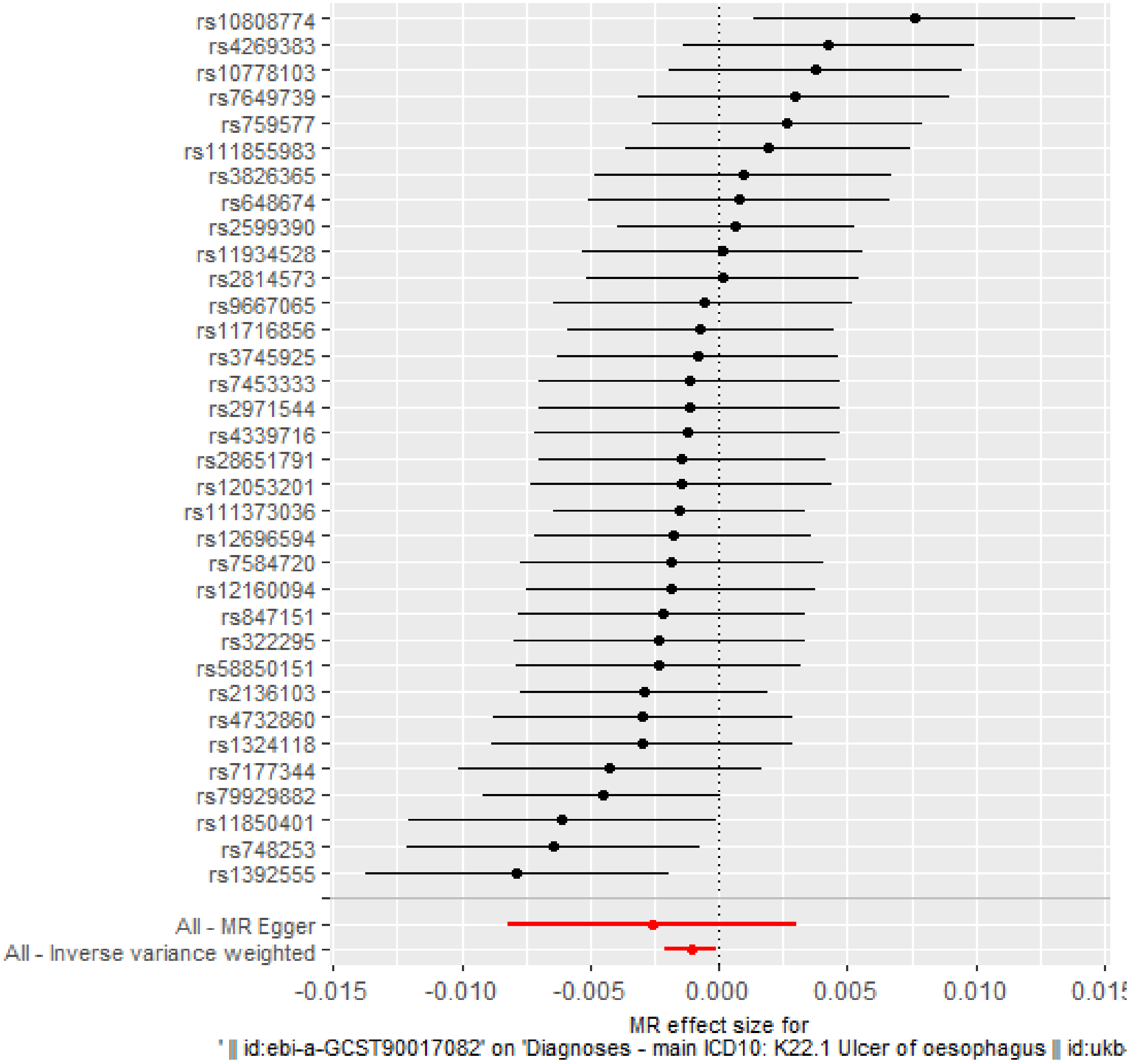

rs10808774
rs4269383
rs10778103
rs7649739
rs759577
rs111855983
rs3826365
rs648674
rs2599390
rs11934528
rs2814573
rs9667065
rs11716856
rs3745925
rs7453333
rs2971544
rs4339716
rs28651791
rs12053201
rs111373036
rs12696594
rs7584720
rs12160094
rs847151
rs322295
rs58850151
rs2136103
rs4732860
rs1324118
rs7177344
rs79929882
rs11850401
rs748253
rs1392555
All - MR Egger
All - Inverse variance weighted
-0.015
-0.010
-0.005
0.000
0.005
0.010
MR effect size for
' || id:ebi-a-GCST90017082' on 'Diagnoses - main ICD10: K22.1 Ulcer of oesophagus || id:ukb

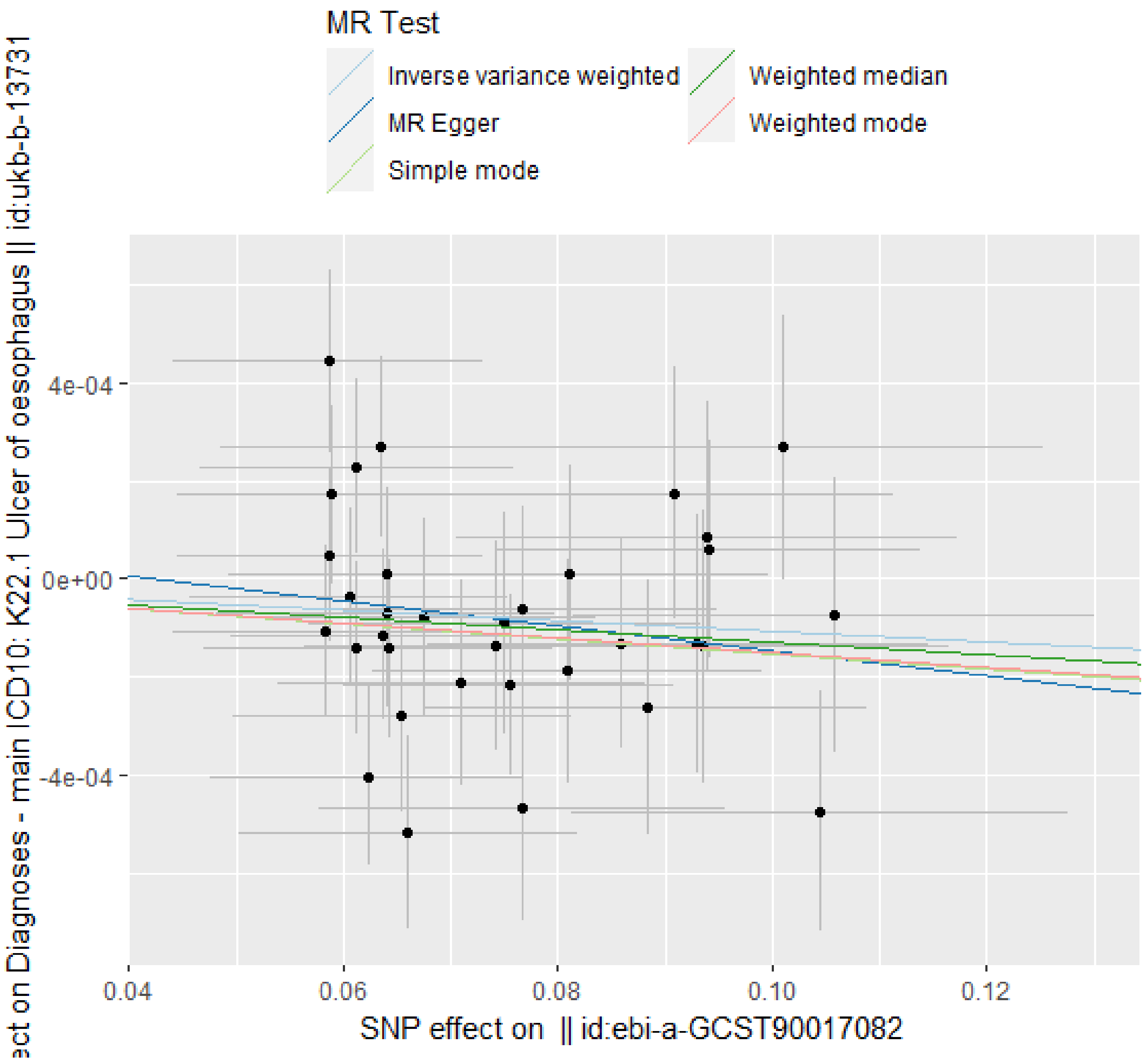
MR Test
Inverse variance weighted
MR Egger
Simple mode
Weighted median
Weighted mode
4e-04
0e+00
-4e-04
0.04
0.06
0.08
0.10
0.12
SNP effect on || id:ebi-a-GCST90017082
ect on Diagnoses - main ICD10: K22.1 Ulcer of oesophagus || id:ukb-b-13731

Figure 34 Leave-one-out analysis, funnel plot, MR effect size and scatter plot for gut microbiota abundance (unknown genus id.2755) on ulcer of oesophagus

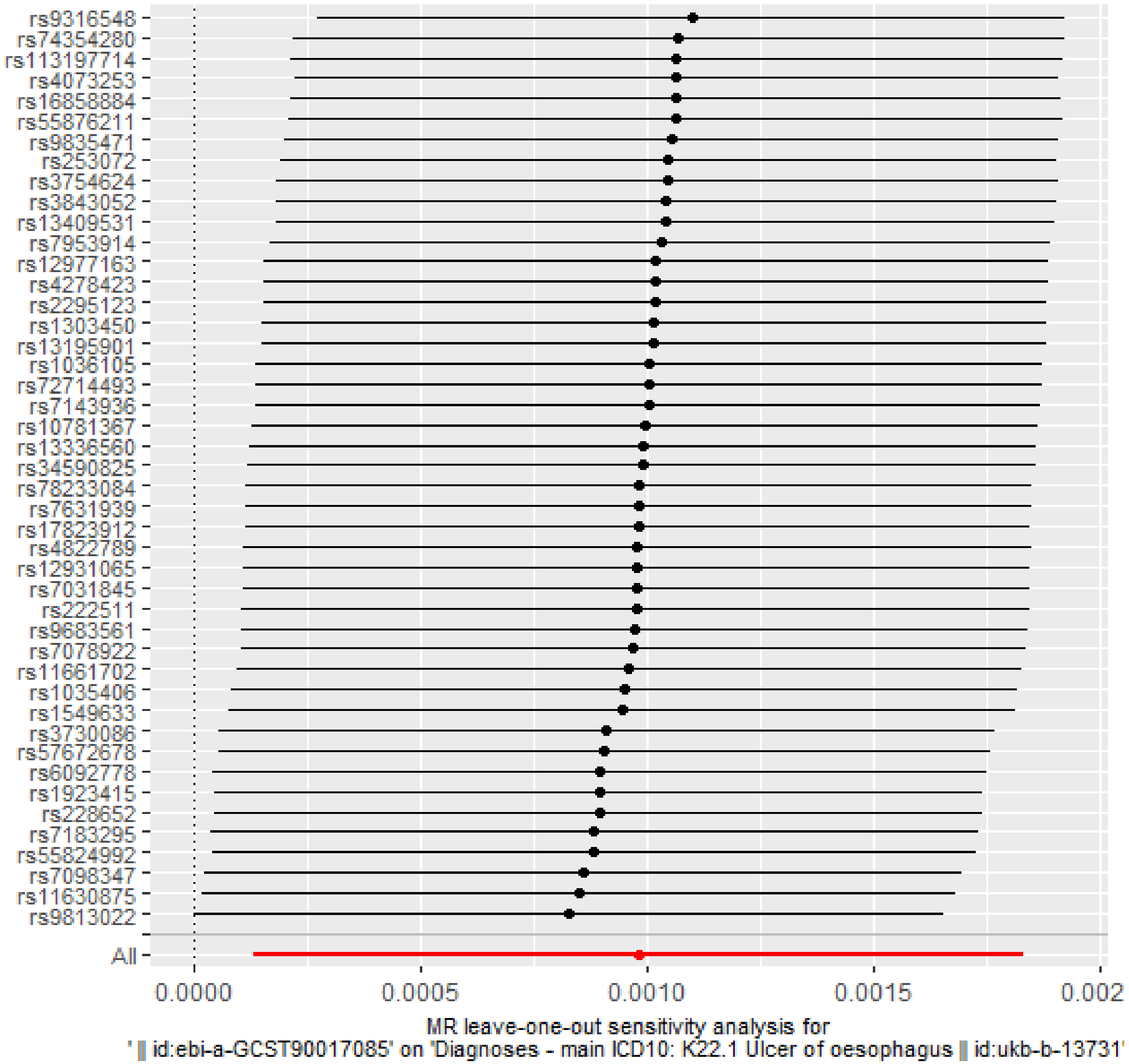

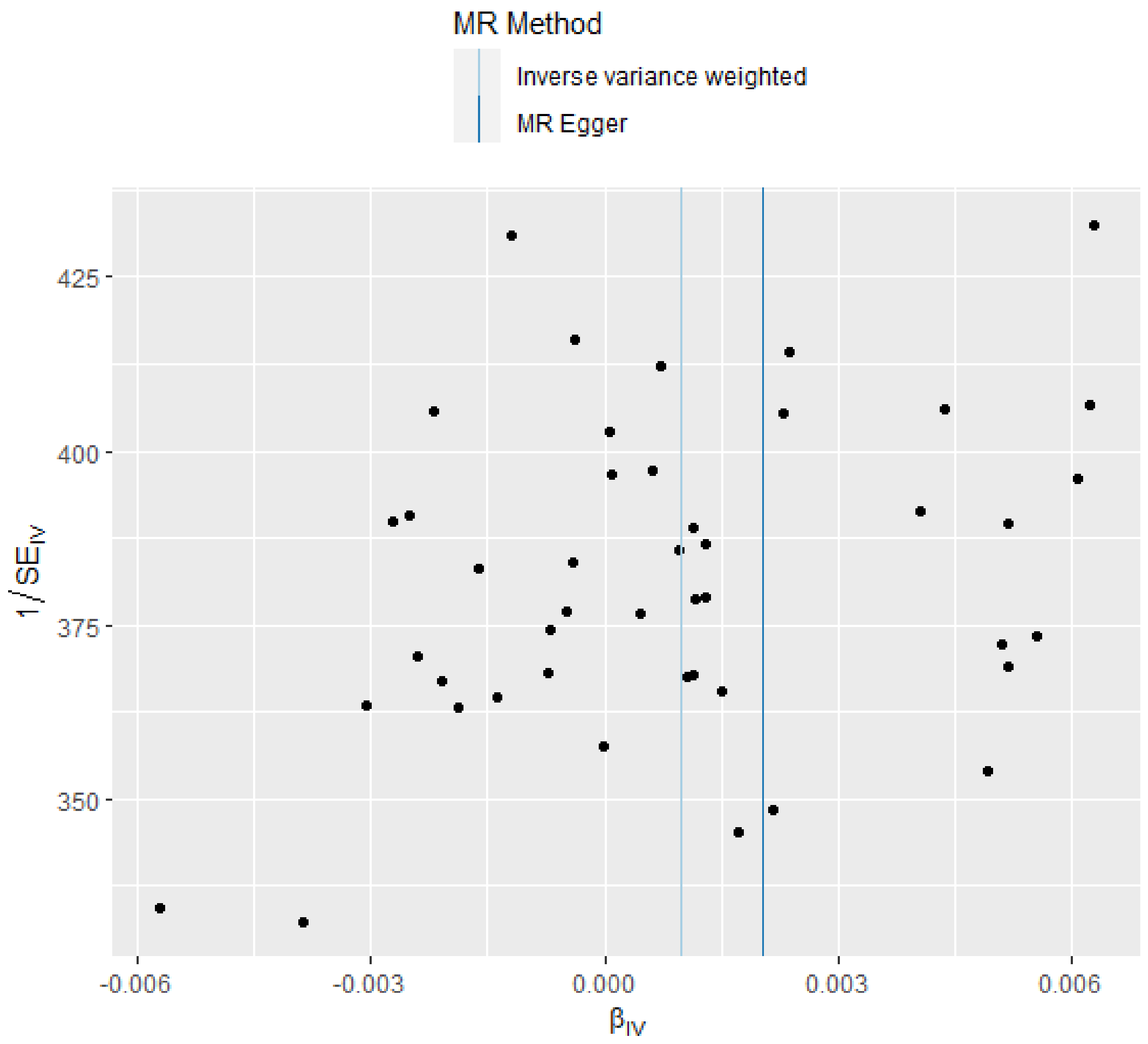
MR Method
Inverse variance weighted
MR Egger
425
400
375
350
$1/SE_{IV}$
-0.006
-0.003
0.000
0.003
0.006
β_{IV}

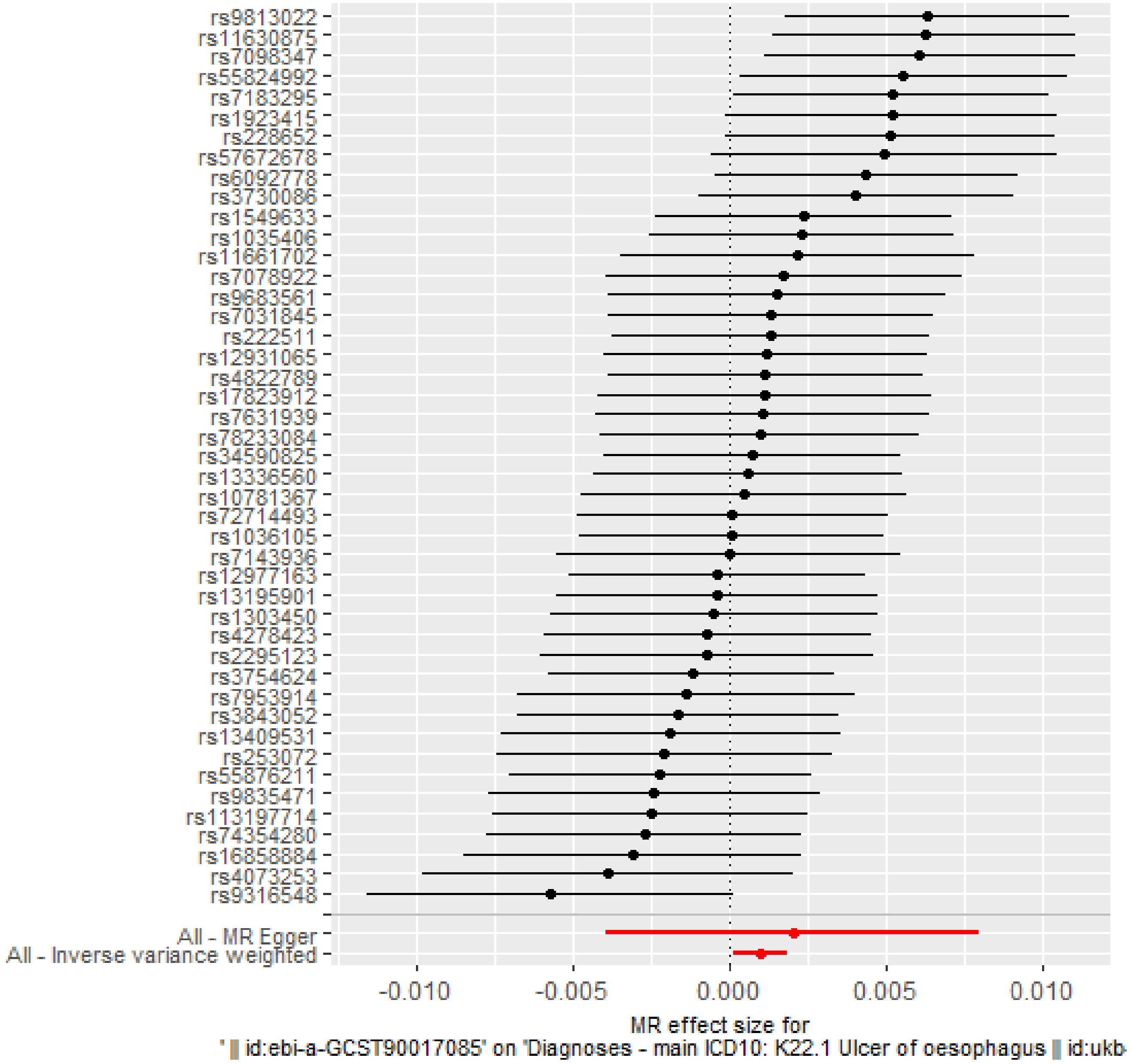
rs9813022
rs11630875
rs7098347
rs55824992
rs7183295
rs1923415
rs228652
rs57672678
rs6092778
rs3730086
rs1549633
rs1035406
rs11661702
rs7078922
rs9683561
rs7031845
rs222511
rs12931065
rs4822789
rs17823912
rs7631939
rs78233084
rs34590825
rs13336560
rs10781367
rs72714493
rs1036105
rs7143936
rs12977163
rs13195901
rs1303450
rs4278423
rs2295123
rs3754624
rs7953914
rs3843052
rs13409531
rs253072
rs55876211
rs9835471
rs113197714
rs74354280
rs16858884
rs4073253
rs9316548
All - MR Egger
All - Inverse variance weighted
-0.010
-0.005
0.000
0.005
0.010
MR effect size for
' || id:ebi-a-GCST90017085' on 'Diagnoses - main ICD10: K22.1 Ulcer of oesophagus || id:ukb-

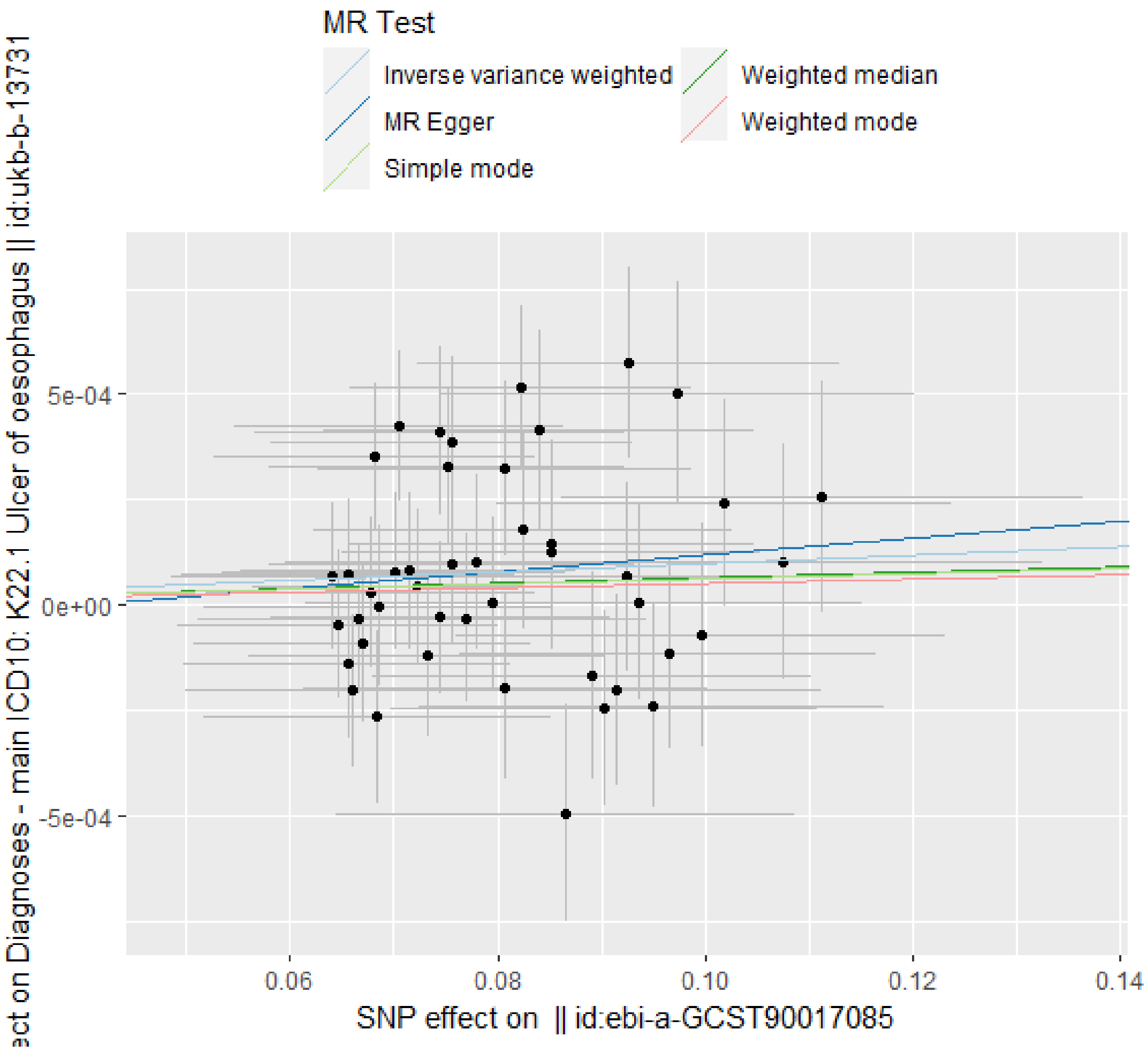
MR Test
Inverse variance weighted
MR Egger
Simple mode
Weighted median
Weighted mode
5e-04
0e+00
-5e-04
0.06
0.08
0.10
0.12
0.14
SNP effect on || id:ebi-a-GCST90017085
ect on Diagnoses - main ICD10: K22.1 Ulcer of oesophagus || id:ukb-b-13731

Figure 35 Leave-one-out analysis, funnel plot, MR effect size and scatter plot for gut microbiota abundance (order Bacillales id.1674) on ulcer of oesophagus

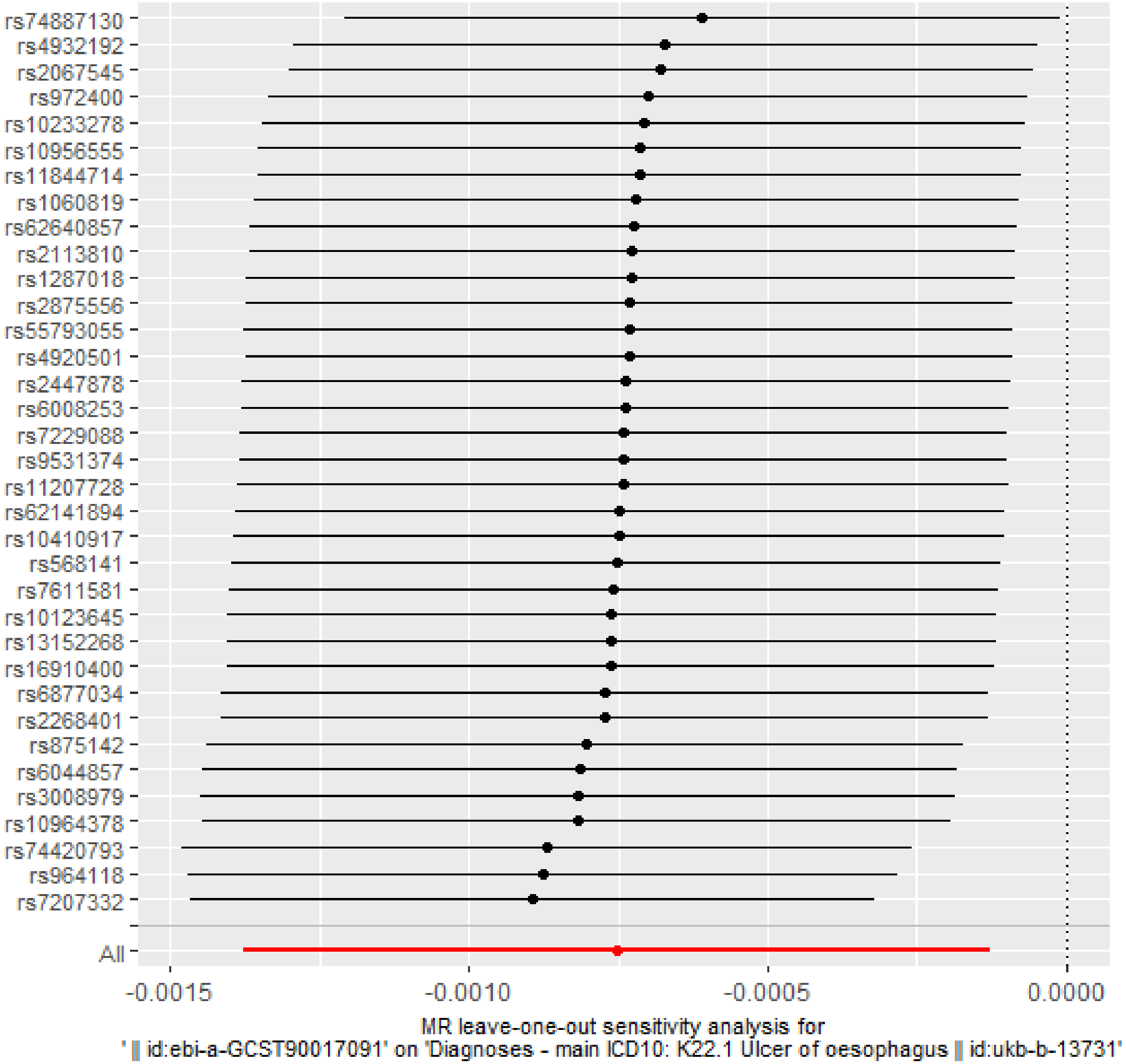

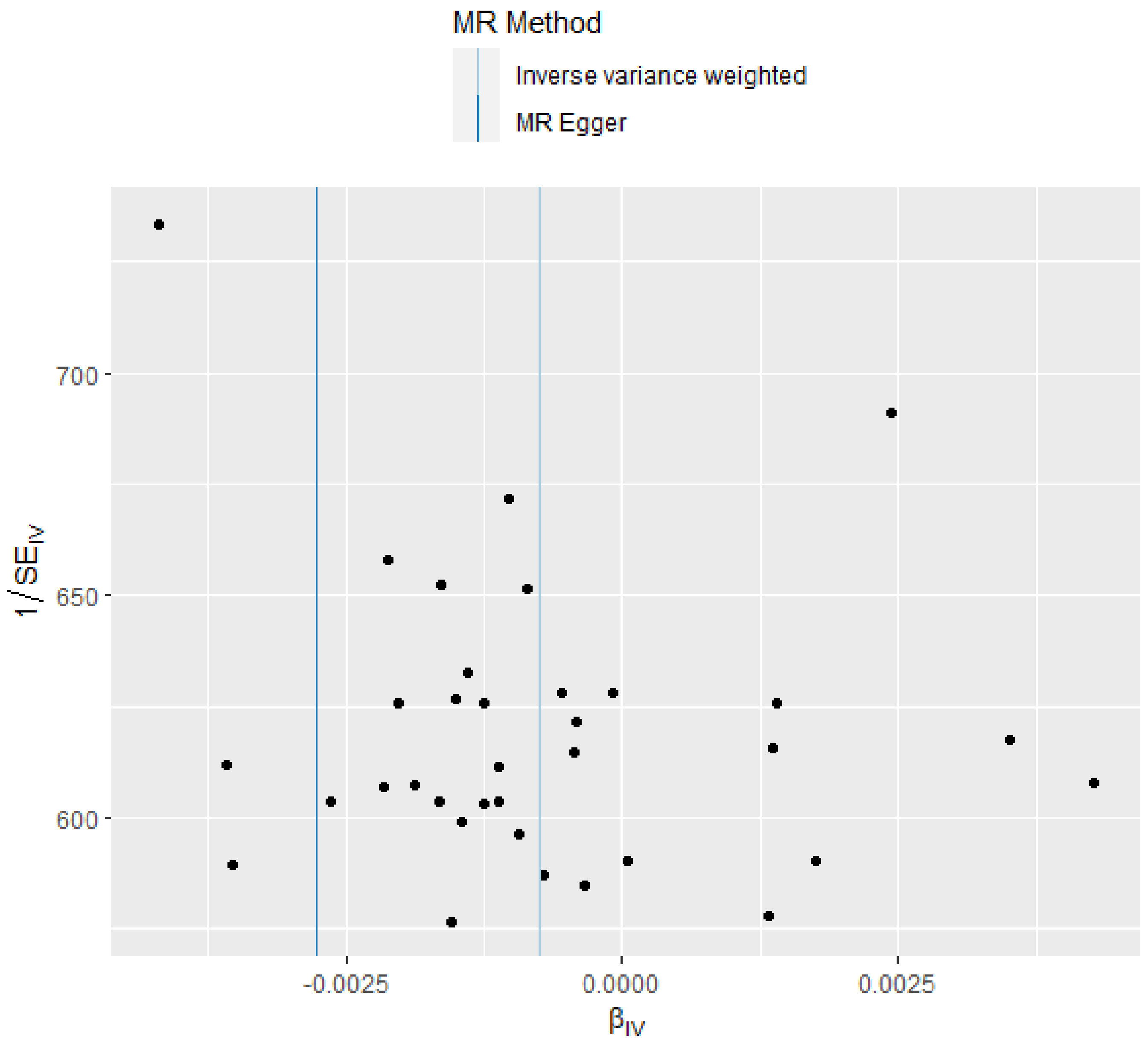

MR Method
Inverse variance weighted
MR Egger
700
650
600
$1/SE_{IV}$
-0.0025
0.0000
0.0025
β_{IV}

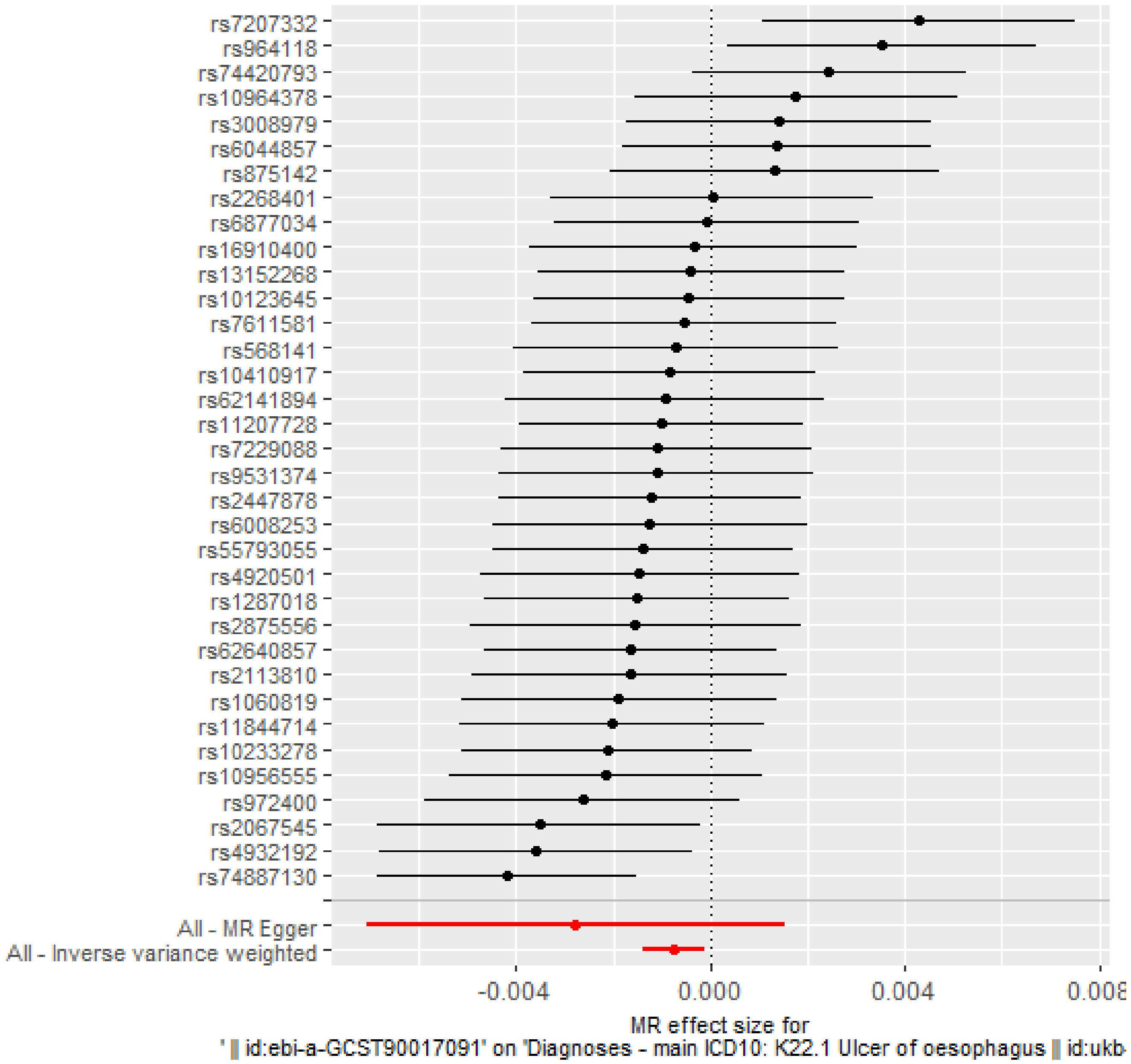

rs7207332
rs964118
rs74420793
rs10964378
rs3008979
rs6044857
rs875142
rs2268401
rs6877034
rs16910400
rs13152268
rs10123645
rs7611581
rs568141
rs10410917
rs62141894
rs11207728
rs7229088
rs9531374
rs2447878
rs6008253
rs55793055
rs4920501
rs1287018
rs2875556
rs62640857
rs2113810
rs1060819
rs11844714
rs10233278
rs10956555
rs972400
rs2067545
rs4932192
rs74887130
All - MR Egger
All - Inverse variance weighted
-0.004
0.000
0.004
0.008
MR effect size for
' || id:ebi-a-GCST90017091' on 'Diagnoses - main ICD10: K22.1 Ulcer of oesophagus || id:ukb-

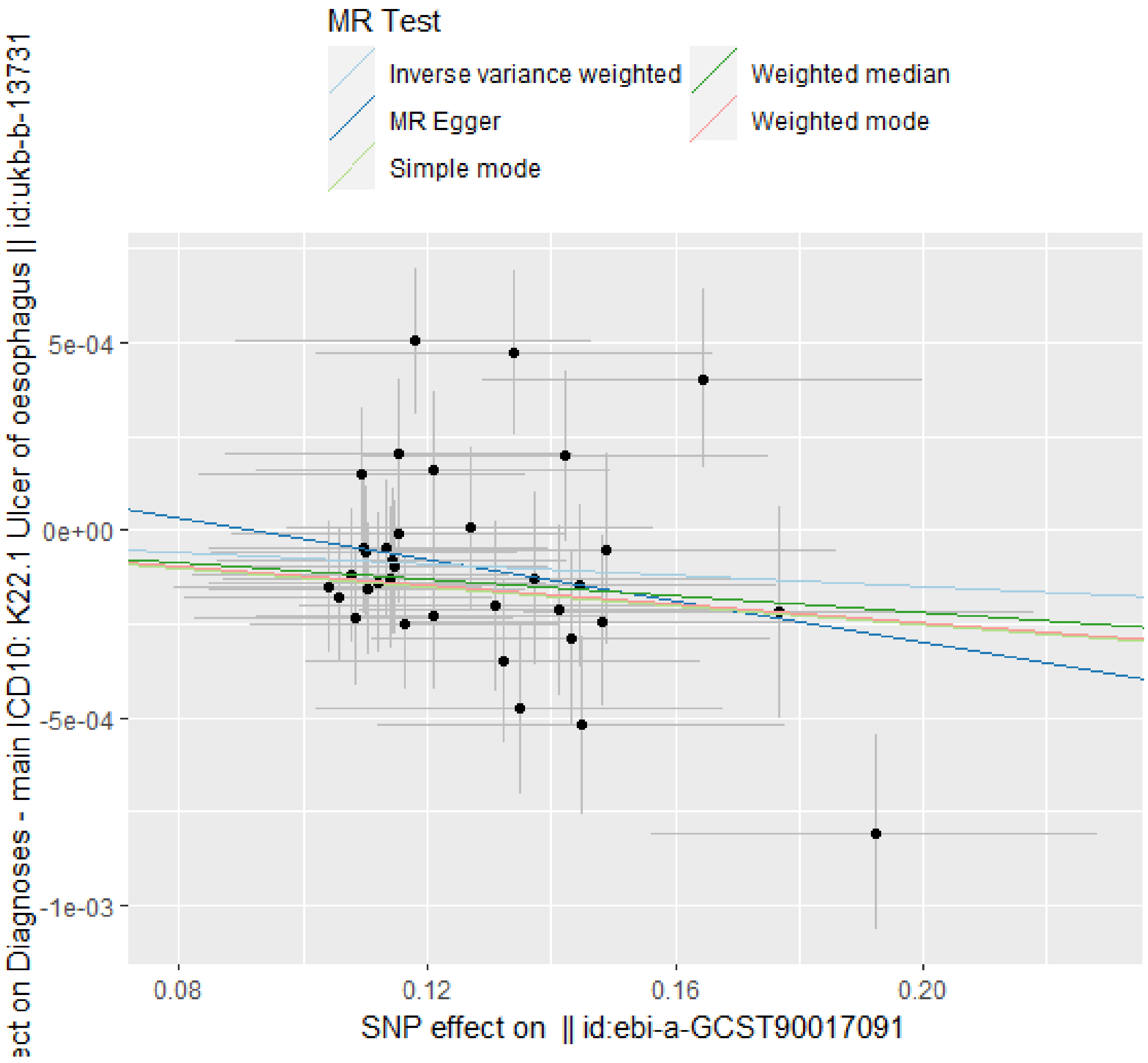

MR Test
Inverse variance weighted
MR Egger
Simple mode
Weighted median
Weighted mode
5e-04
0e+00
-5e-04
-1e-03
0.08
0.12
0.16
0.20
SNP effect on || id:ebi-a-GCST90017091
ect on Diagnoses - main ICD10: K22.1 Ulcer of oesophagus || id:ukb-b-13731

Figure 36 Leave-one-out analysis, funnel plot, MR effect size and scatter plot for gut microbiota abundance (order Bacteroidales id.913) on ulcer of oesophagus

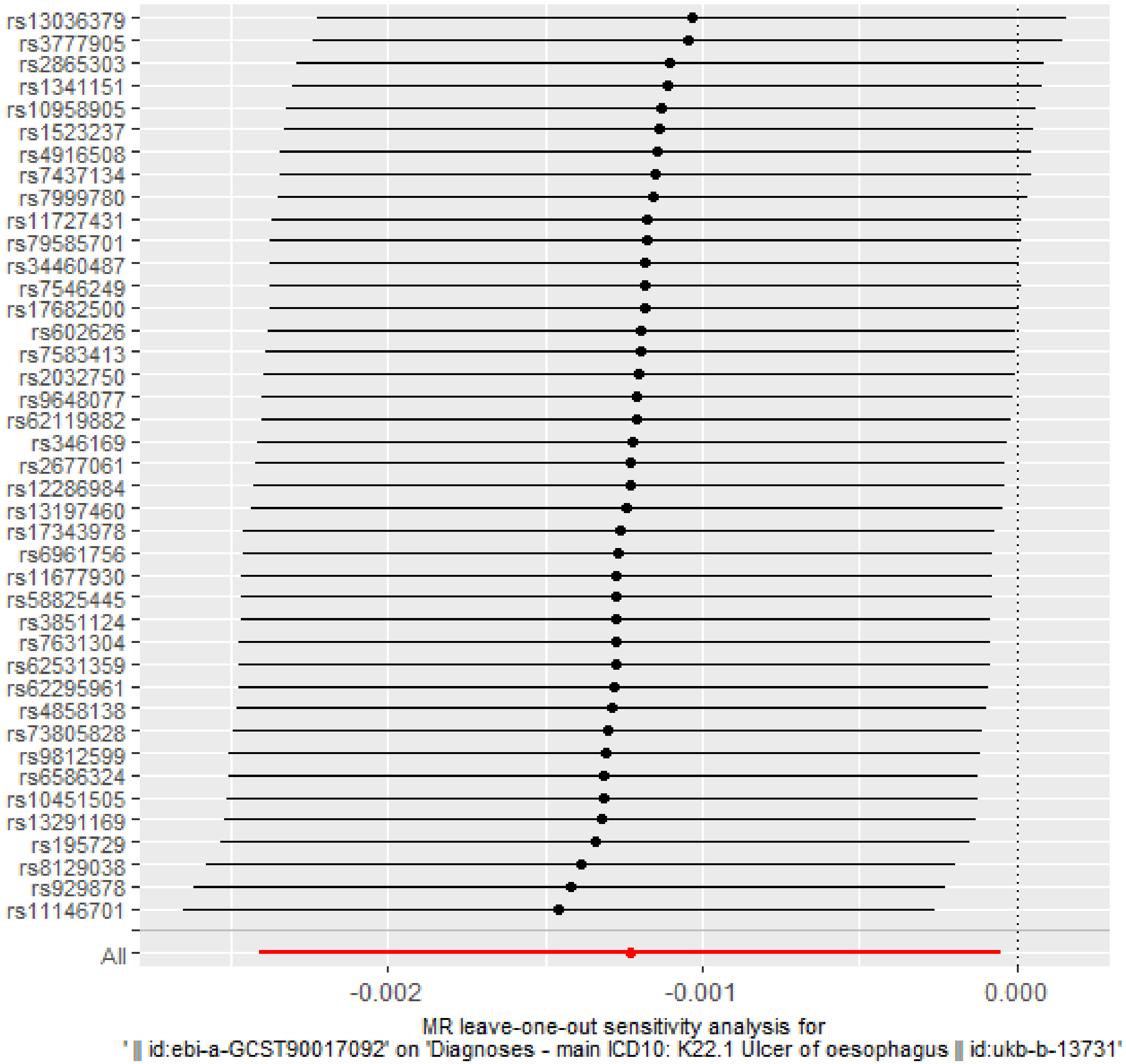

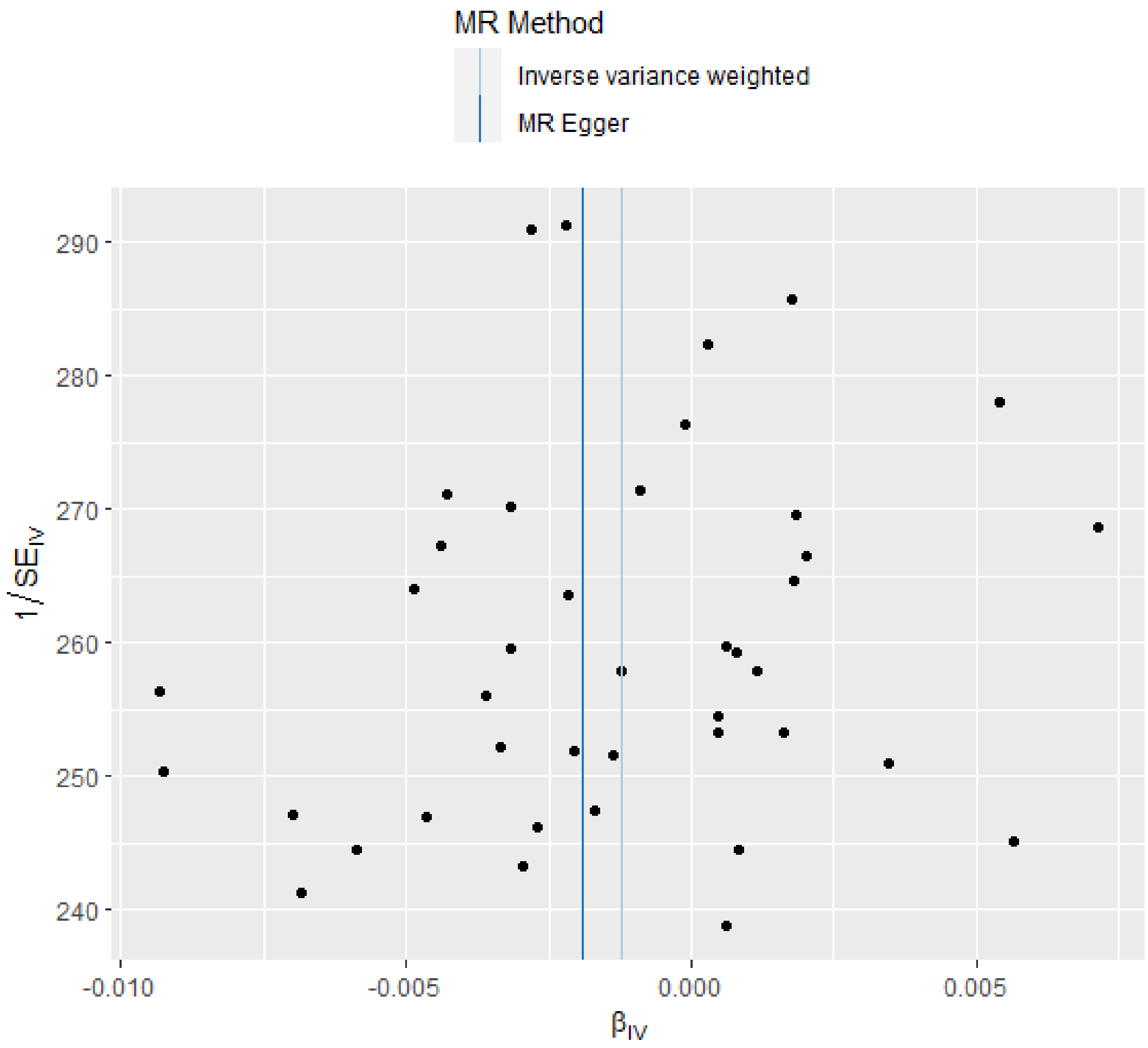
MR Method
Inverse variance weighted
MR Egger
290
280
270
260
250
240
1/SE$_{IV}$
-0.010
-0.005
0.000
0.005
β_{IV}

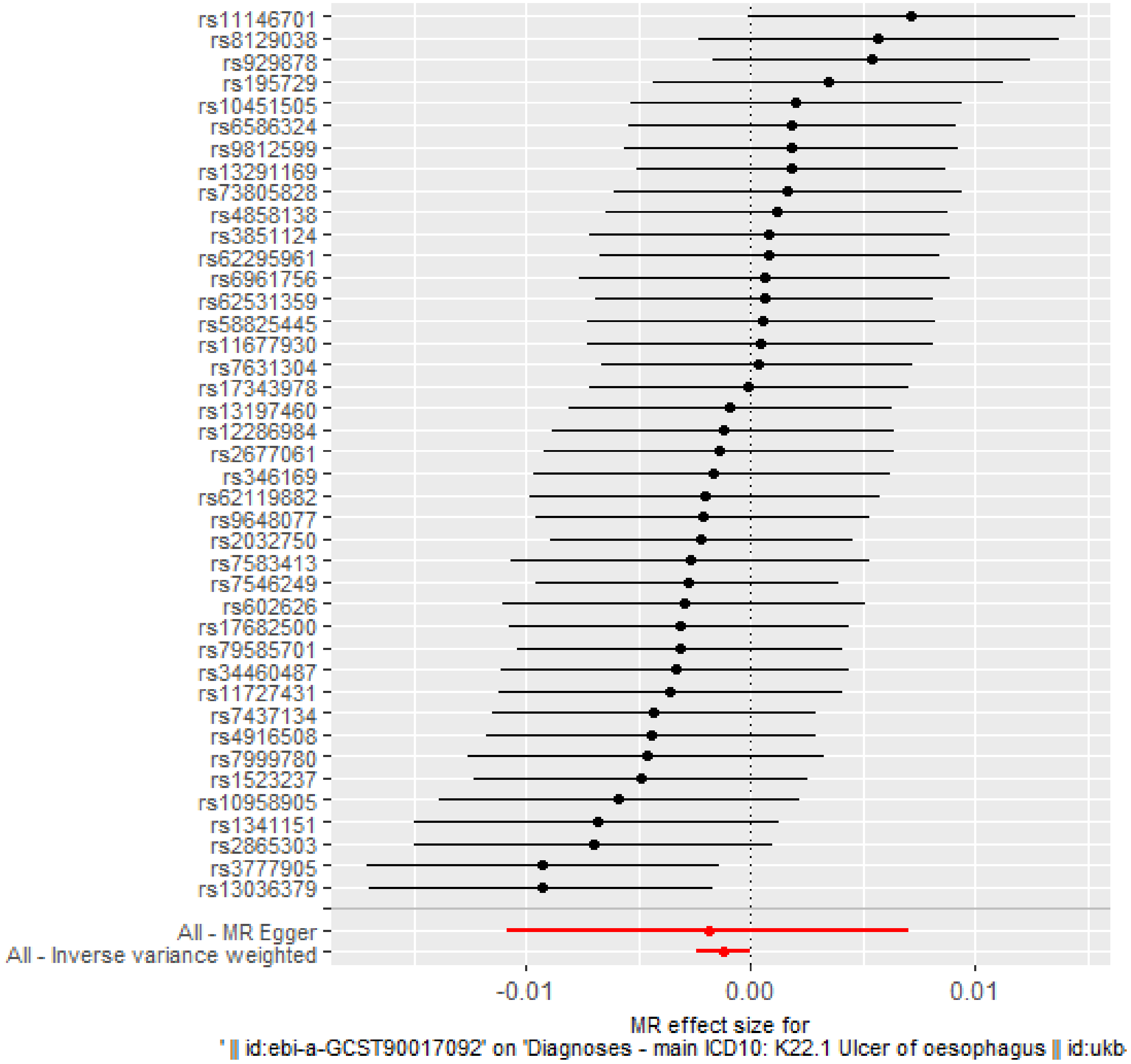
rs11146701
rs8129038
rs929878
rs195729
rs10451505
rs6586324
rs9812599
rs13291169
rs73805828
rs4858138
rs3851124
rs62295961
rs6961756
rs62531359
rs58825445
rs11677930
rs7631304
rs17343978
rs13197460
rs12286984
rs2677061
rs346169
rs62119882
rs9648077
rs2032750
rs7583413
rs7546249
rs602626
rs17682500
rs79585701
rs34460487
rs11727431
rs7437134
rs4916508
rs7999780
rs1523237
rs10958905
rs1341151
rs2865303
rs3777905
rs13036379
All - MR Egger
All - Inverse variance weighted
-0.01
0.00
0.01
MR effect size for
' || id:ebi-a-GCST90017092' on 'Diagnoses - main ICD10: K22.1 Ulcer of oesophagus || id:ukb-

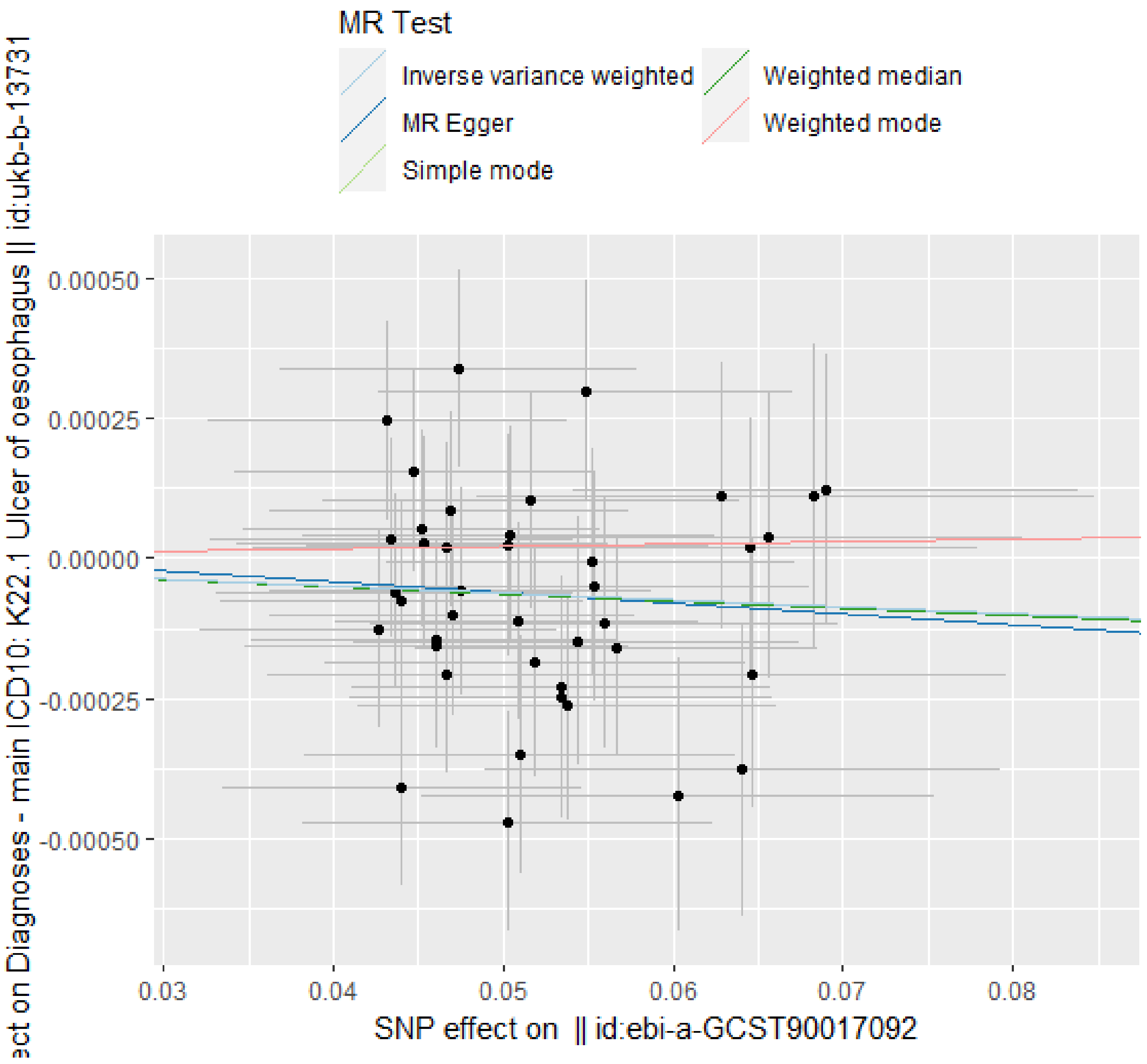

MR Test
Inverse variance weighted
MR Egger
Simple mode
Weighted median
Weighted mode
0.00050
0.00025
0.00000
-0.00025
-0.00050
0.03
0.04
0.05
0.06
0.07
0.08
SNP effect on || id:ebi-a-GCST90017092
ect on Diagnoses - main ICD10: K22.1 Ulcer of oesophagus || id:ukb-b-13731

Figure 37 Leave-one-out analysis, funnel plot, MR effect size and scatter plot for gut microbiota abundance (order Rhodospirillales id.2667) on ulcer of oesophagus

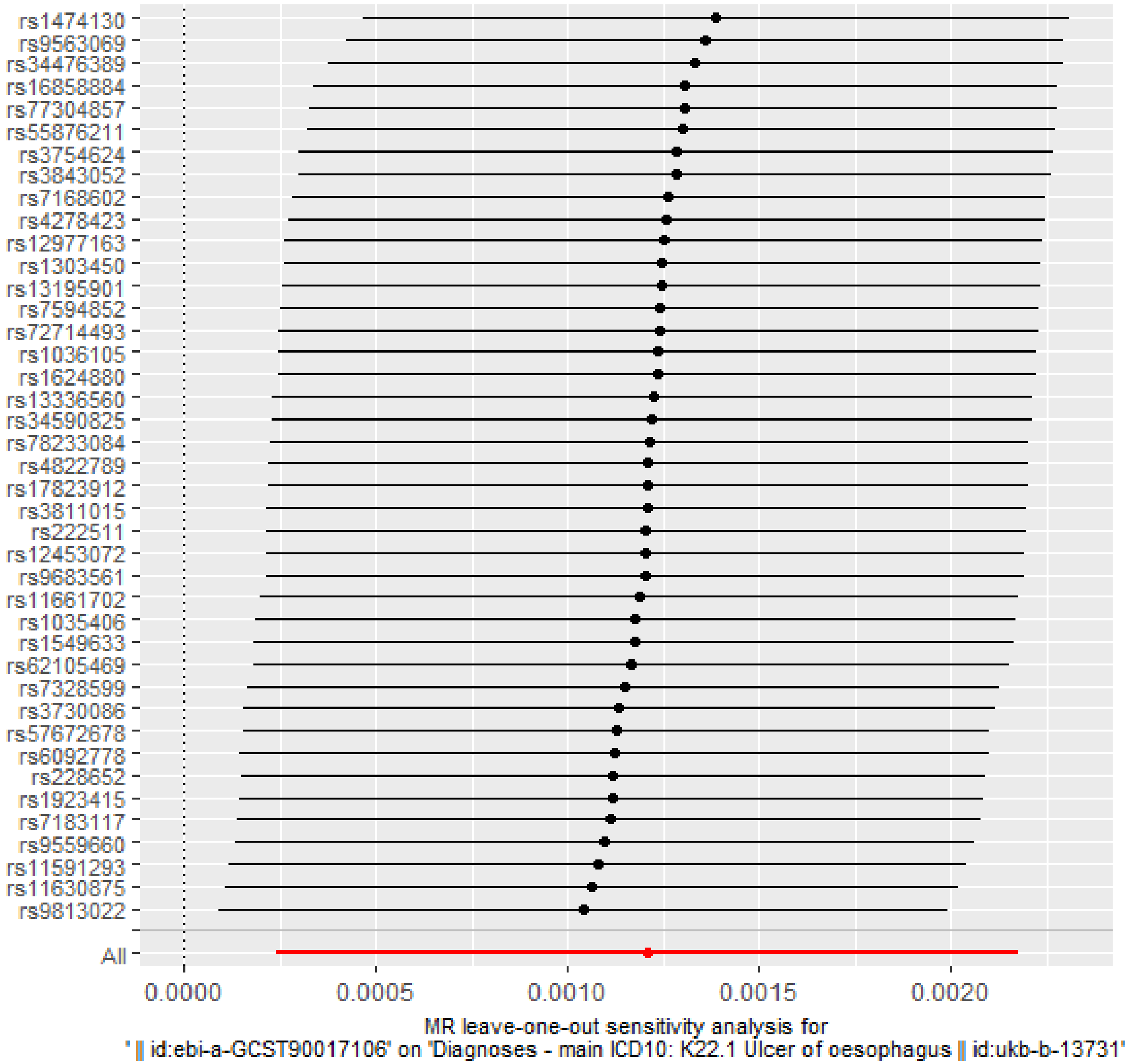

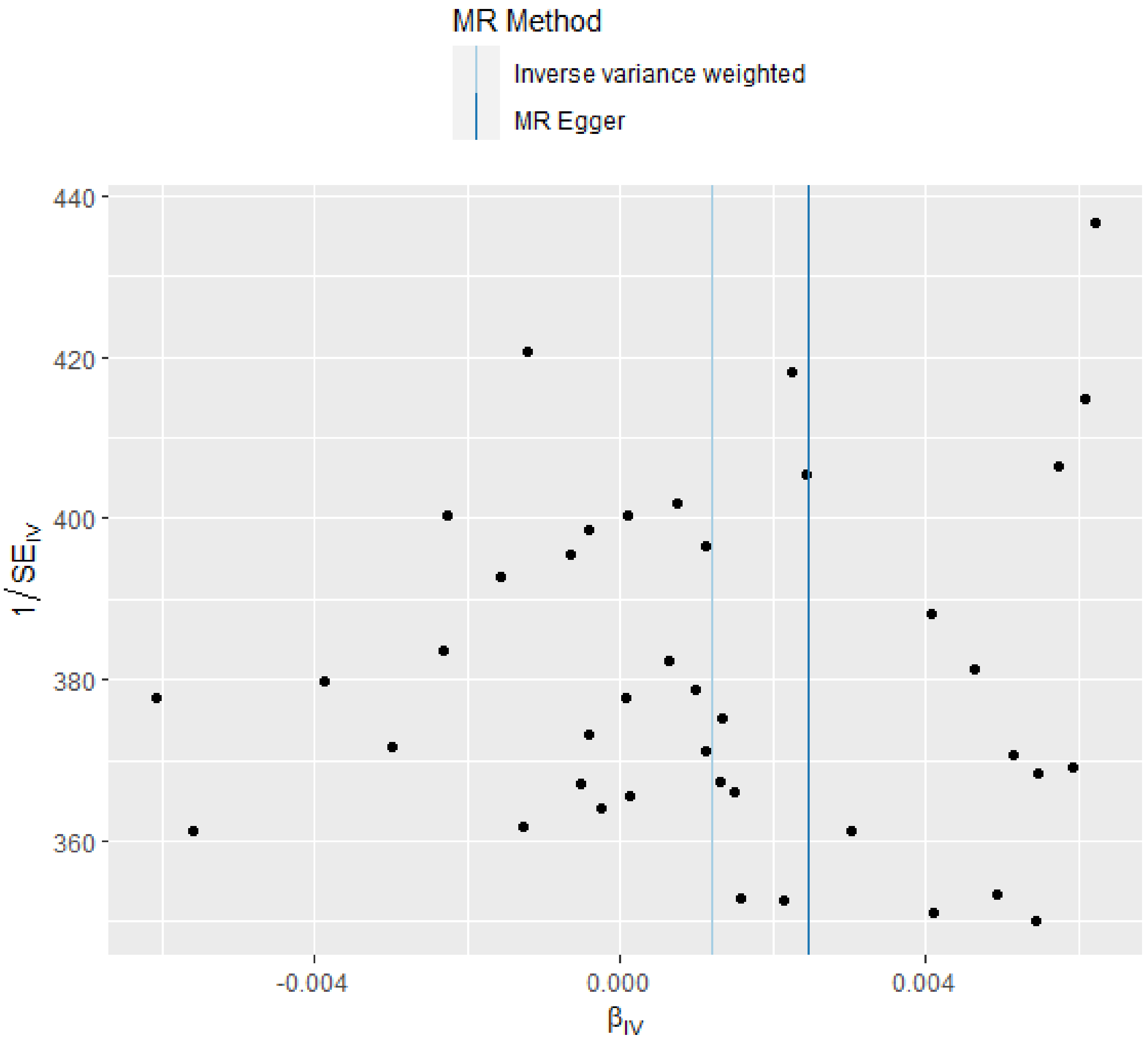
MR Method
Inverse variance weighted
MR Egger
440
420
400
380
360
1/SE_IV
-0.004
0.000
0.004
β_IV

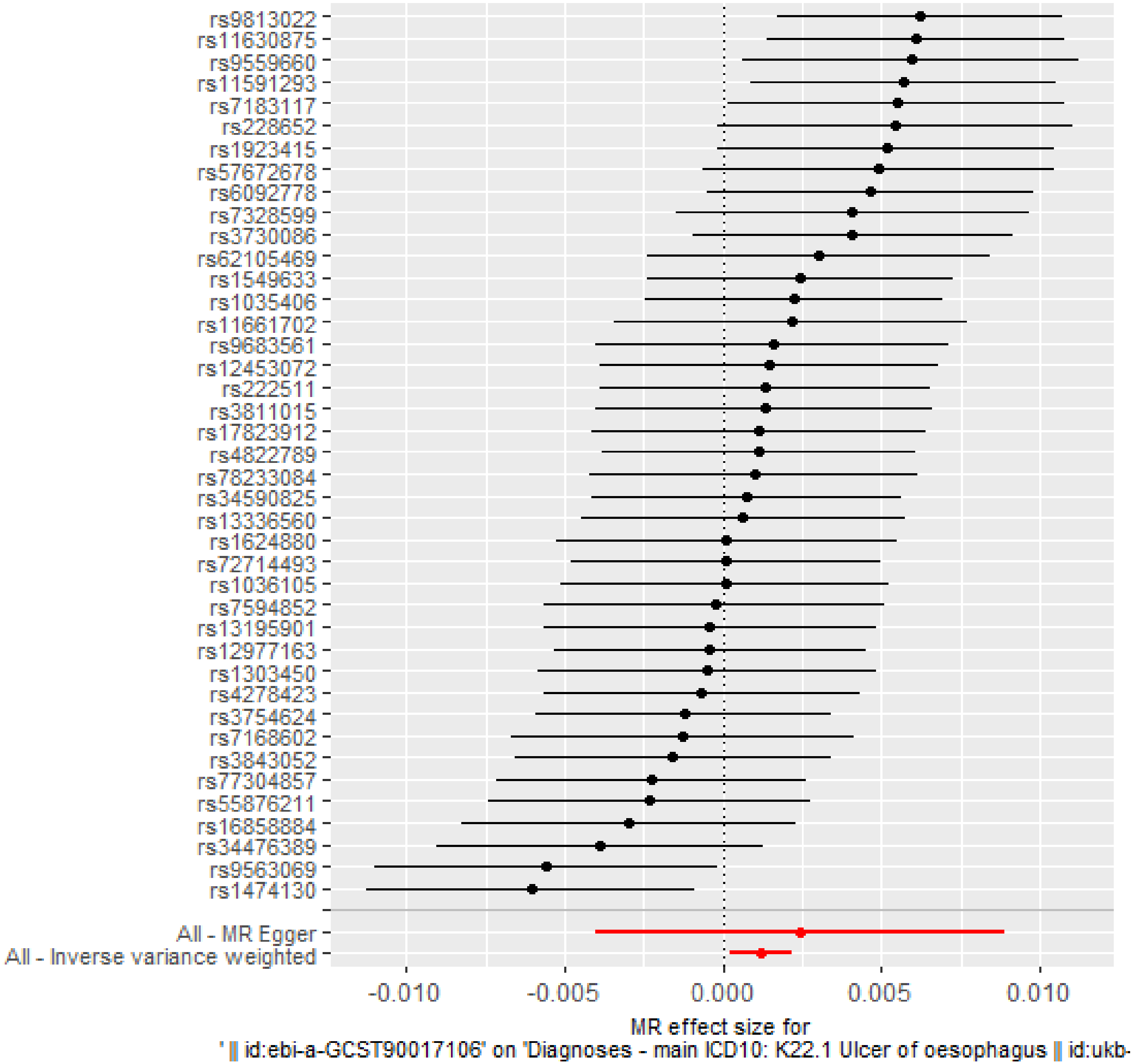
rs9813022
rs11630875
rs9559660
rs11591293
rs7183117
rs228652
rs1923415
rs57672678
rs6092778
rs7328599
rs3730086
rs62105469
rs1549633
rs1035406
rs11661702
rs9683561
rs12453072
rs222511
rs3811015
rs17823912
rs4822789
rs78233084
rs34590825
rs13336560
rs1624880
rs72714493
rs1036105
rs7594852
rs13195901
rs12977163
rs1303450
rs4278423
rs3754624
rs7168602
rs3843052
rs77304857
rs55876211
rs16858884
rs34476389
rs9563069
rs1474130
All - MR Egger
All - Inverse variance weighted
-0.010
-0.005
0.000
0.005
0.010
MR effect size for
' || id:ebi-a-GCST90017106' on 'Diagnoses - main ICD10: K22.1 Ulcer of oesophagus || id:ukb-

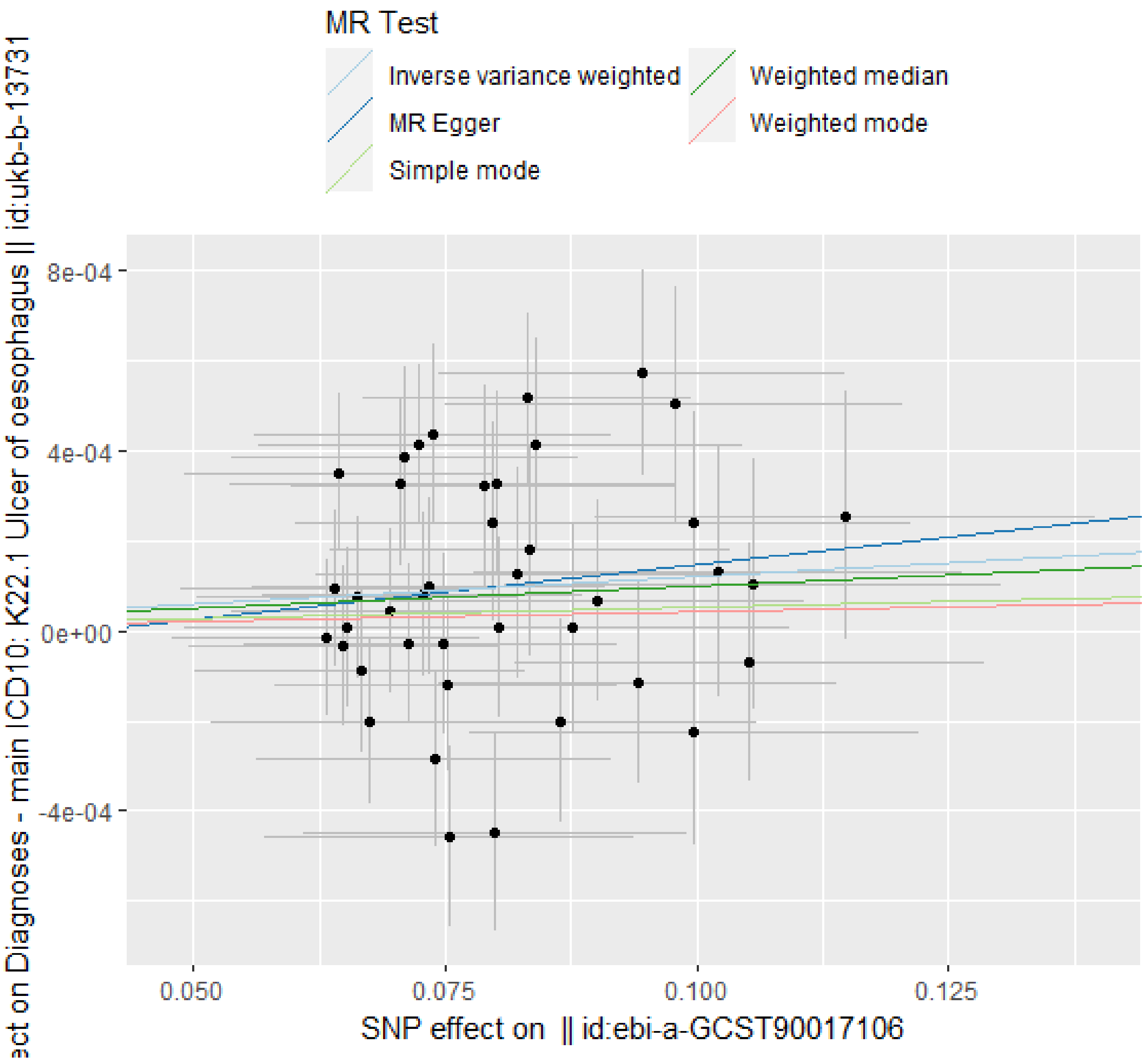

MR Test
Inverse variance weighted
MR Egger
Simple mode
Weighted median
Weighted mode
8e-04
4e-04
0e+00
-4e-04
0.050
0.075
0.100
0.125
SNP effect on || id:ebi-a-GCST90017106
ect on Diagnoses - main ICD10: K22.1 Ulcer of oesophagus || id:ukb-b-13731

Figure 38 Leave-one-out analysis, funnel plot, MR effect size and scatter plot for gut microbiota abundance (phylum Cyanobacteria id.1500) on ulcer of oesophagus

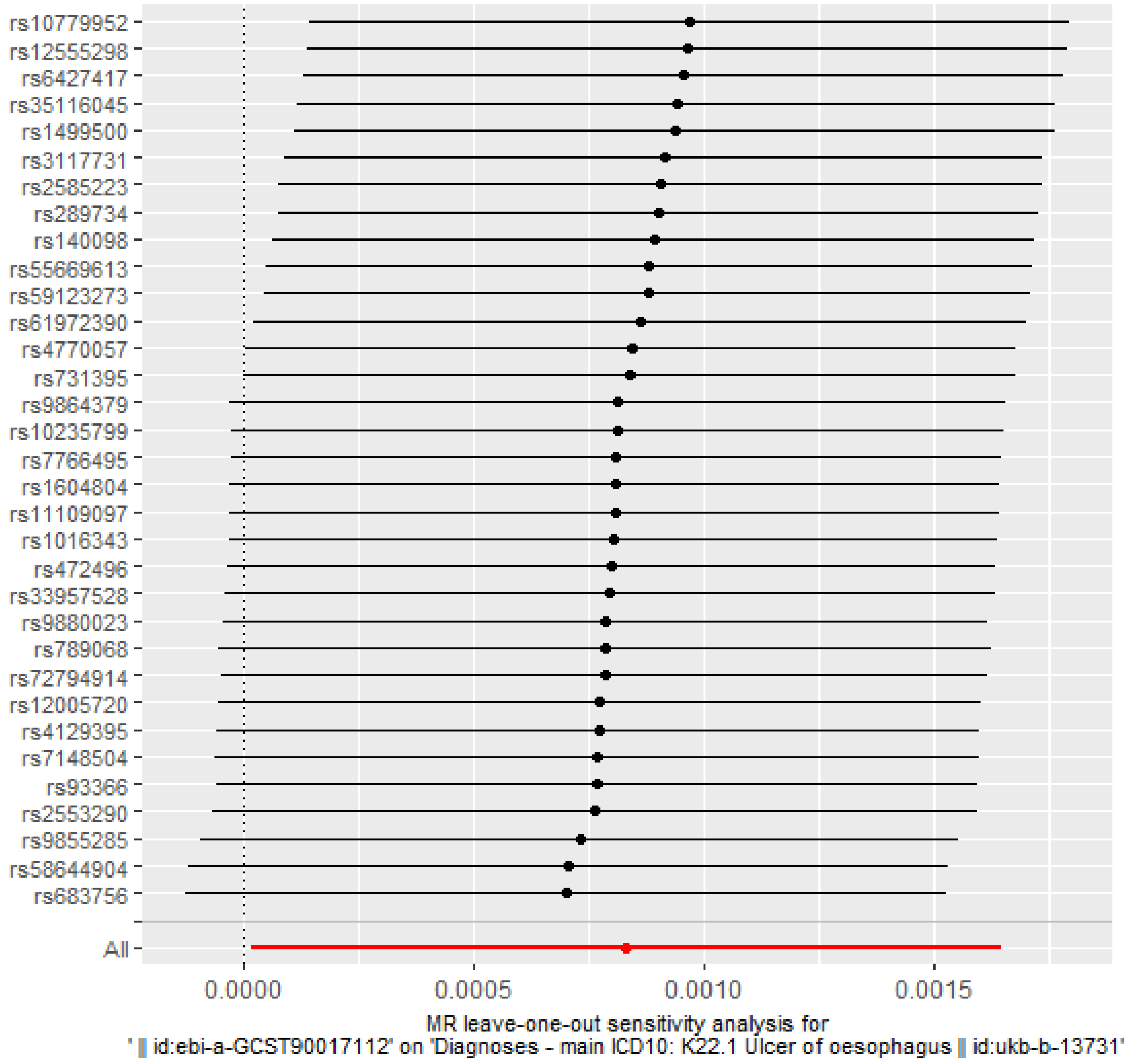

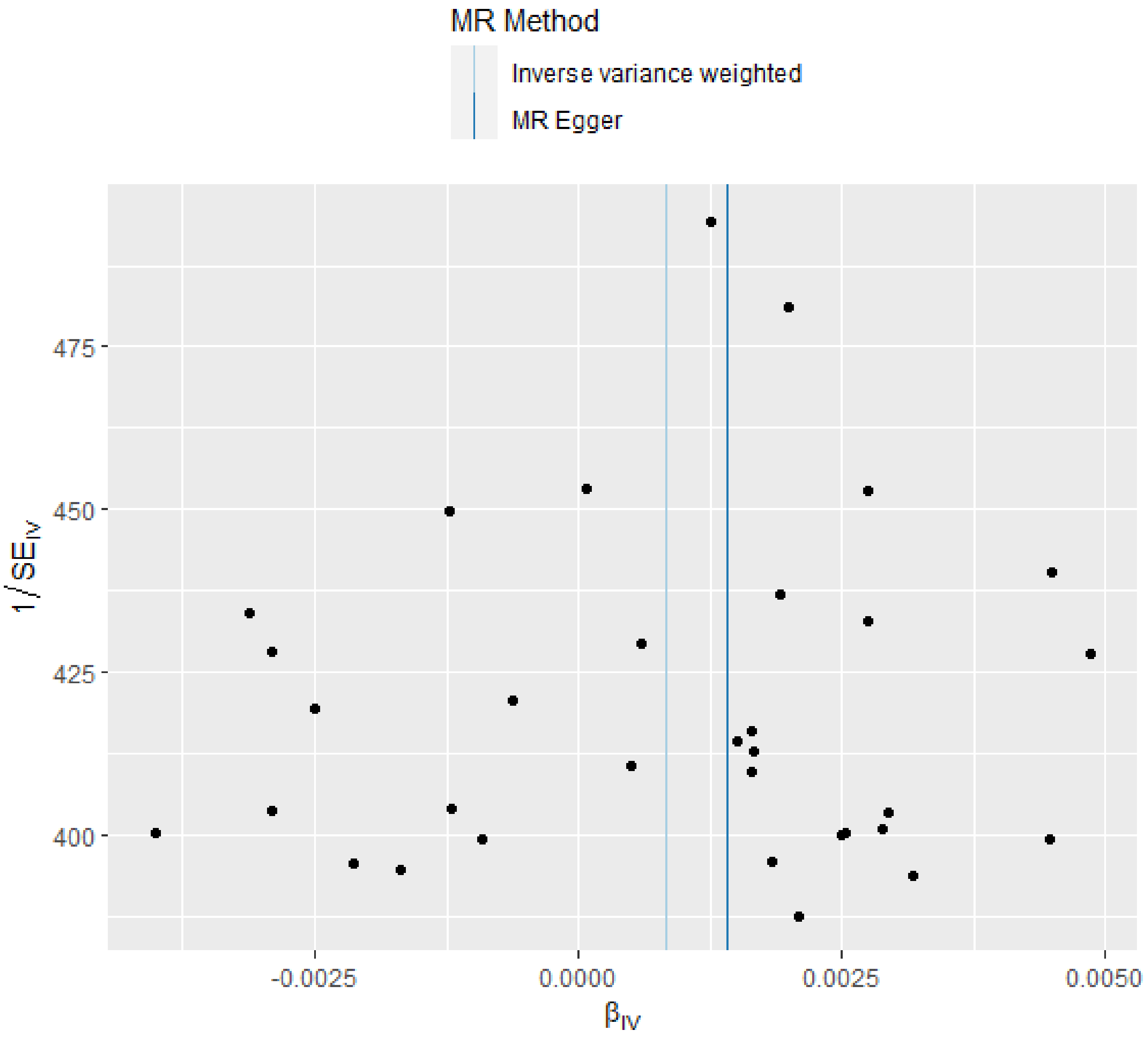
MR Method
Inverse variance weighted
MR Egger
475
450
425
400
1/SE_IV
-0.0025
0.0000
0.0025
0.0050
β_IV

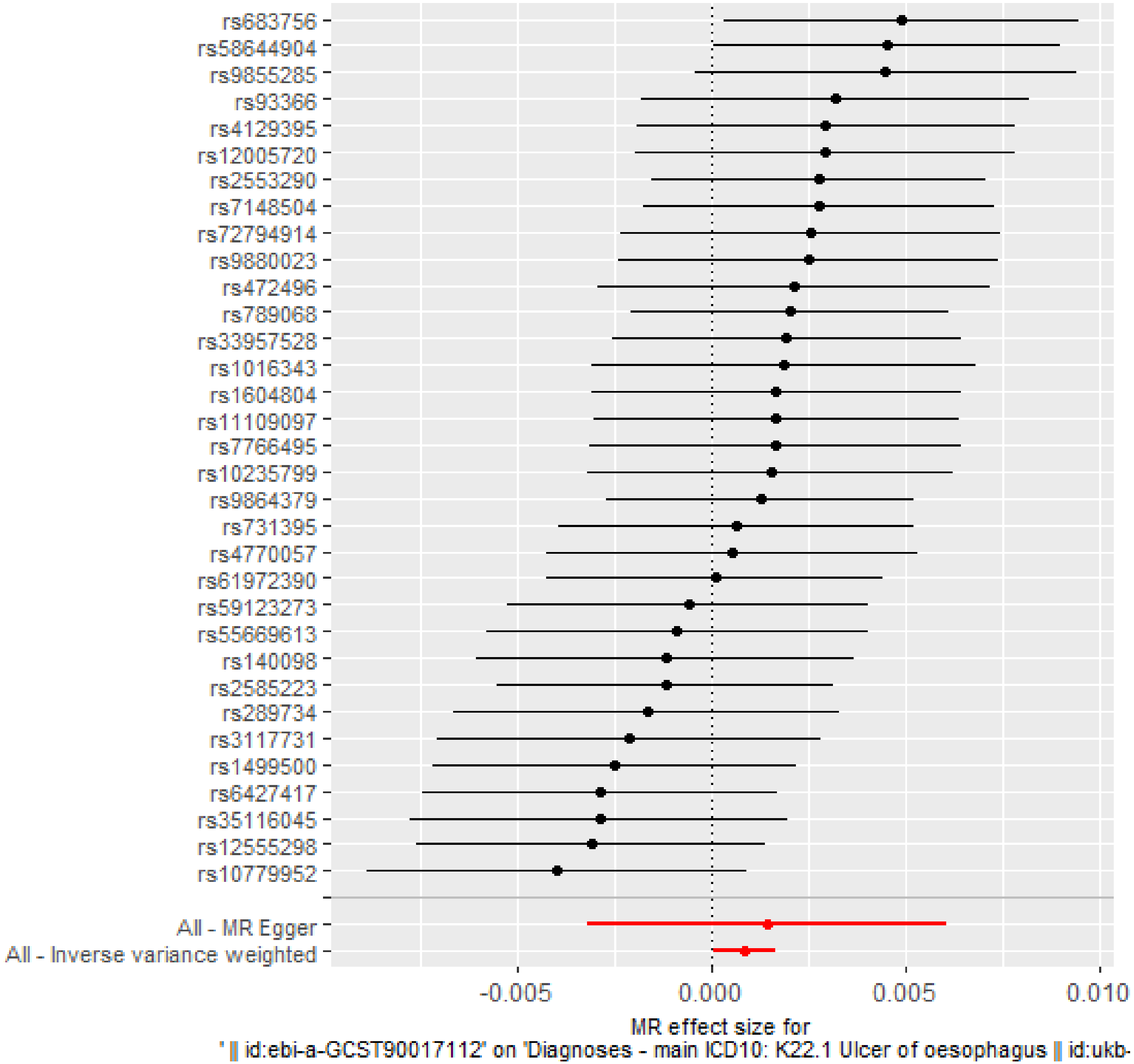

rs683756
rs58644904
rs9855285
rs93366
rs4129395
rs12005720
rs2553290
rs7148504
rs72794914
rs9880023
rs472496
rs789068
rs33957528
rs1016343
rs1604804
rs11109097
rs7766495
rs10235799
rs9864379
rs731395
rs4770057
rs61972390
rs59123273
rs55669613
rs140098
rs2585223
rs289734
rs3117731
rs1499500
rs6427417
rs35116045
rs12555298
rs10779952
All - MR Egger
All - Inverse variance weighted
-0.005
0.000
0.005
0.010
MR effect size for
' || id:ebi-a-GCST90017112' on 'Diagnoses - main ICD10: K22.1 Ulcer of oesophagus || id:ukb-

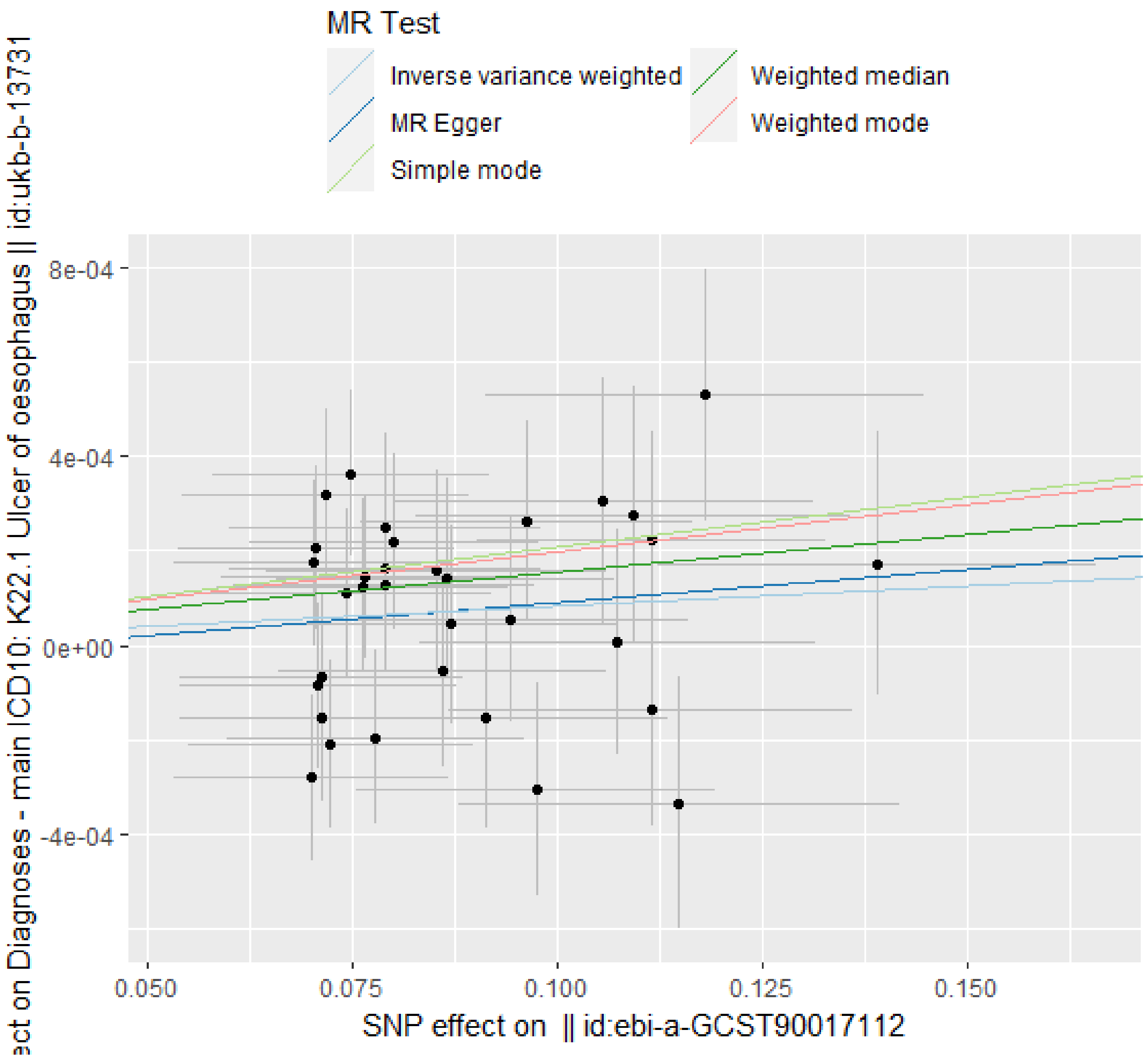

MR Test
Inverse variance weighted
MR Egger
Simple mode
Weighted median
Weighted mode
8e-04
4e-04
0e+00
-4e-04
0.050
0.075
0.100
0.125
0.150
SNP effect on || id:ebi-a-GCST90017112
ect on Diagnoses - main ICD10: K22.1 Ulcer of oesophagus || id:ukb-b-13731

Figure 39 Leave-one-out analysis, funnel plot, MR effect size and scatter plot for gut microbiota abundance (class Bacilli id.1673) on gastric ulcer

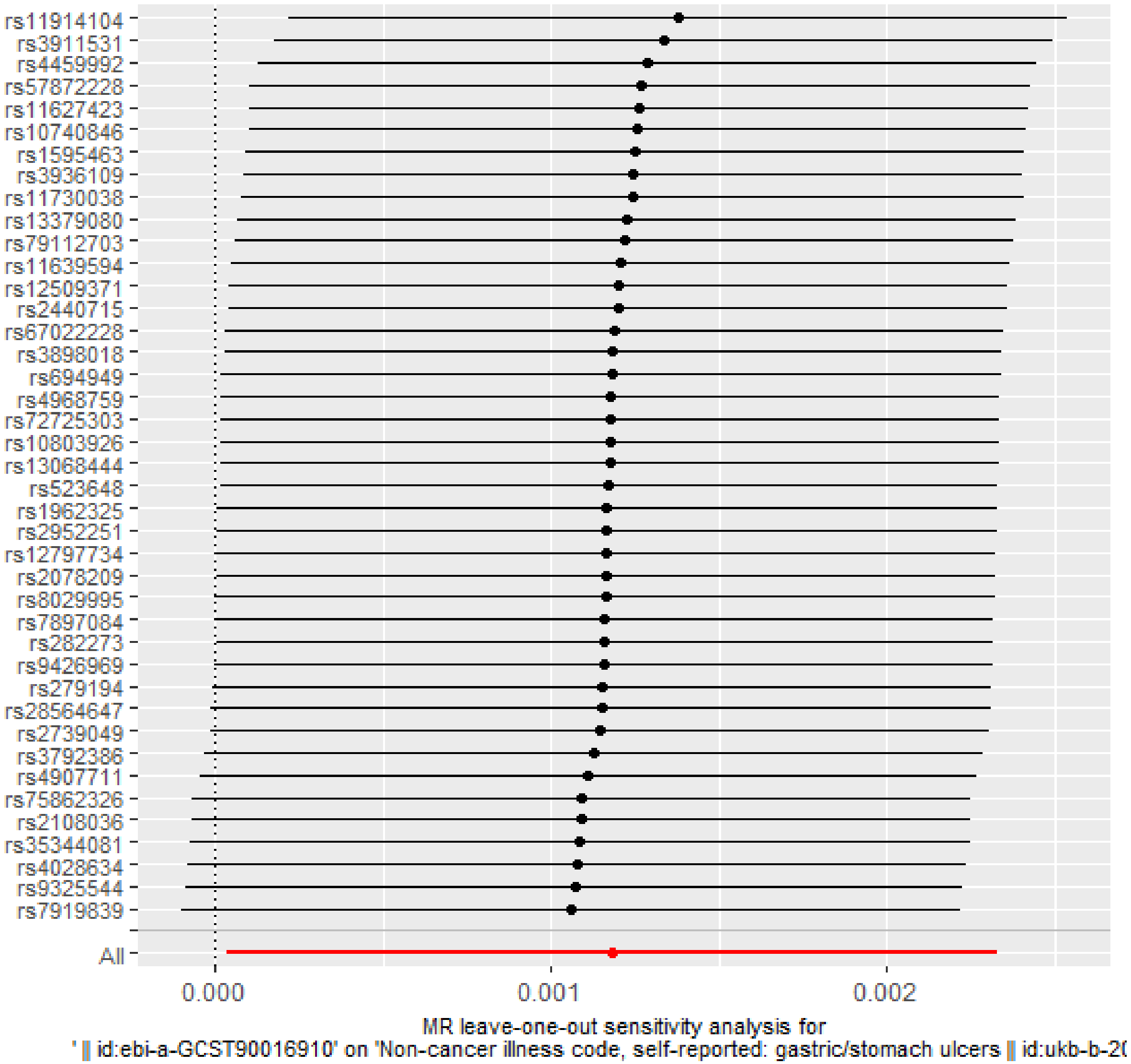

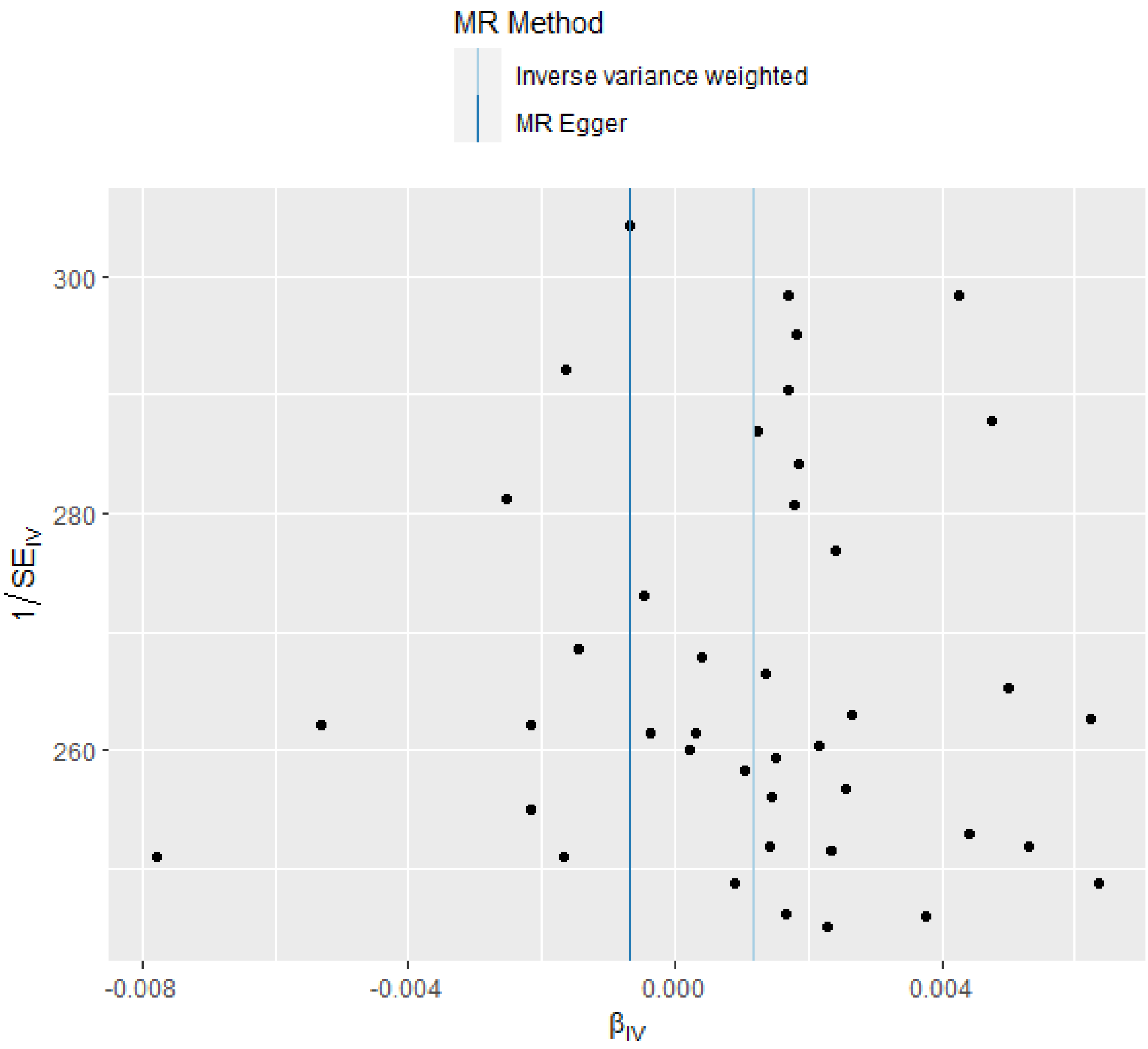

MR Method
Inverse variance weighted
MR Egger
300
280
260
1/SE$_{IV}$
-0.008
-0.004
0.000
0.004
β_{IV}

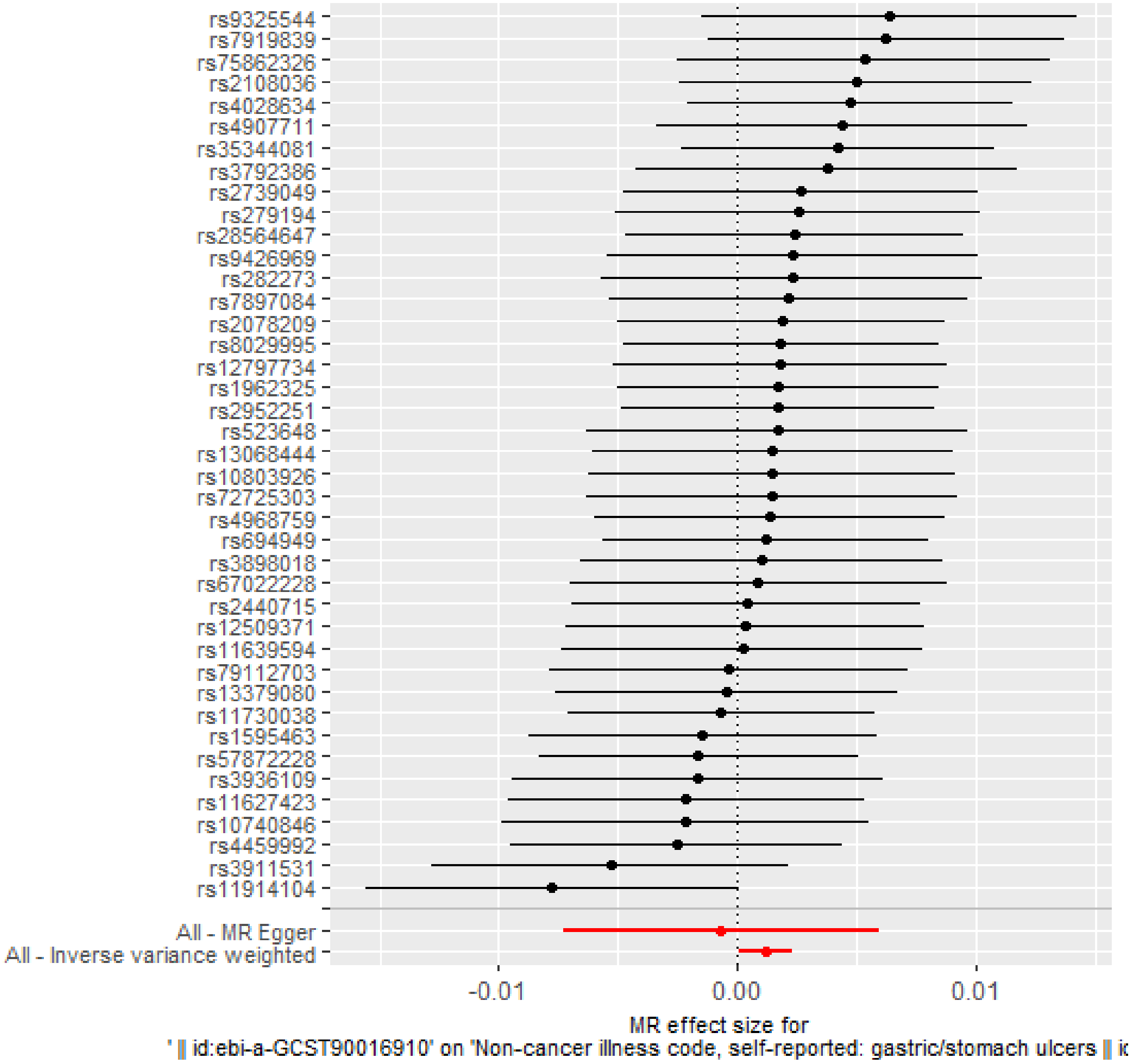

rs9325544
rs7919839
rs75862326
rs2108036
rs4028634
rs4907711
rs35344081
rs3792386
rs2739049
rs279194
rs28564647
rs9426969
rs282273
rs7897084
rs2078209
rs8029995
rs12797734
rs1962325
rs2952251
rs523648
rs13068444
rs10803926
rs72725303
rs4968759
rs694949
rs3898018
rs67022228
rs2440715
rs12509371
rs11639594
rs79112703
rs13379080
rs11730038
rs1595463
rs57872228
rs3936109
rs11627423
rs10740846
rs4459992
rs3911531
rs11914104
All - MR Egger
All - Inverse variance weighted
-0.01
0.00
0.01
MR effect size for
' || id:ebi-a-GCST90016910' on 'Non-cancer illness code, self-reported: gastric/stomach ulcers || ic

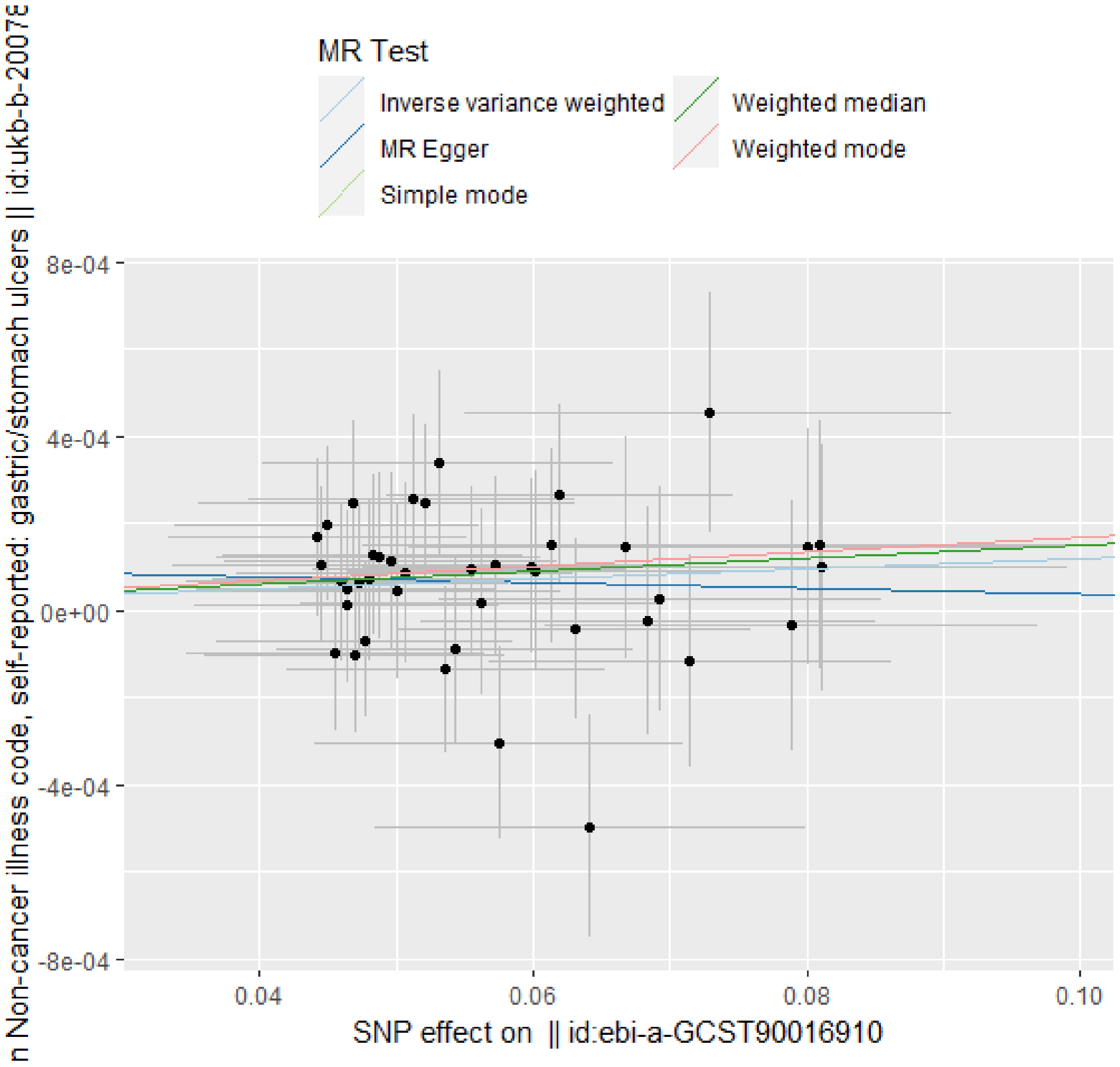

MR Test
Inverse variance weighted
MR Egger
Simple mode
Weighted median
Weighted mode
8e-04
4e-04
0e+00
-4e-04
-8e-04
0.04
0.06
0.08
0.10
SNP effect on || id:ebi-a-GCST90016910
n Non-cancer illness code, self-reported: gastric/stomach ulcers || id:ukb-b-2007

Figure 40 Leave-one-out analysis, funnel plot, MR effect size and scatter plot for gut microbiota abundance (class Bacteroidia id.912) on gastric ulcer

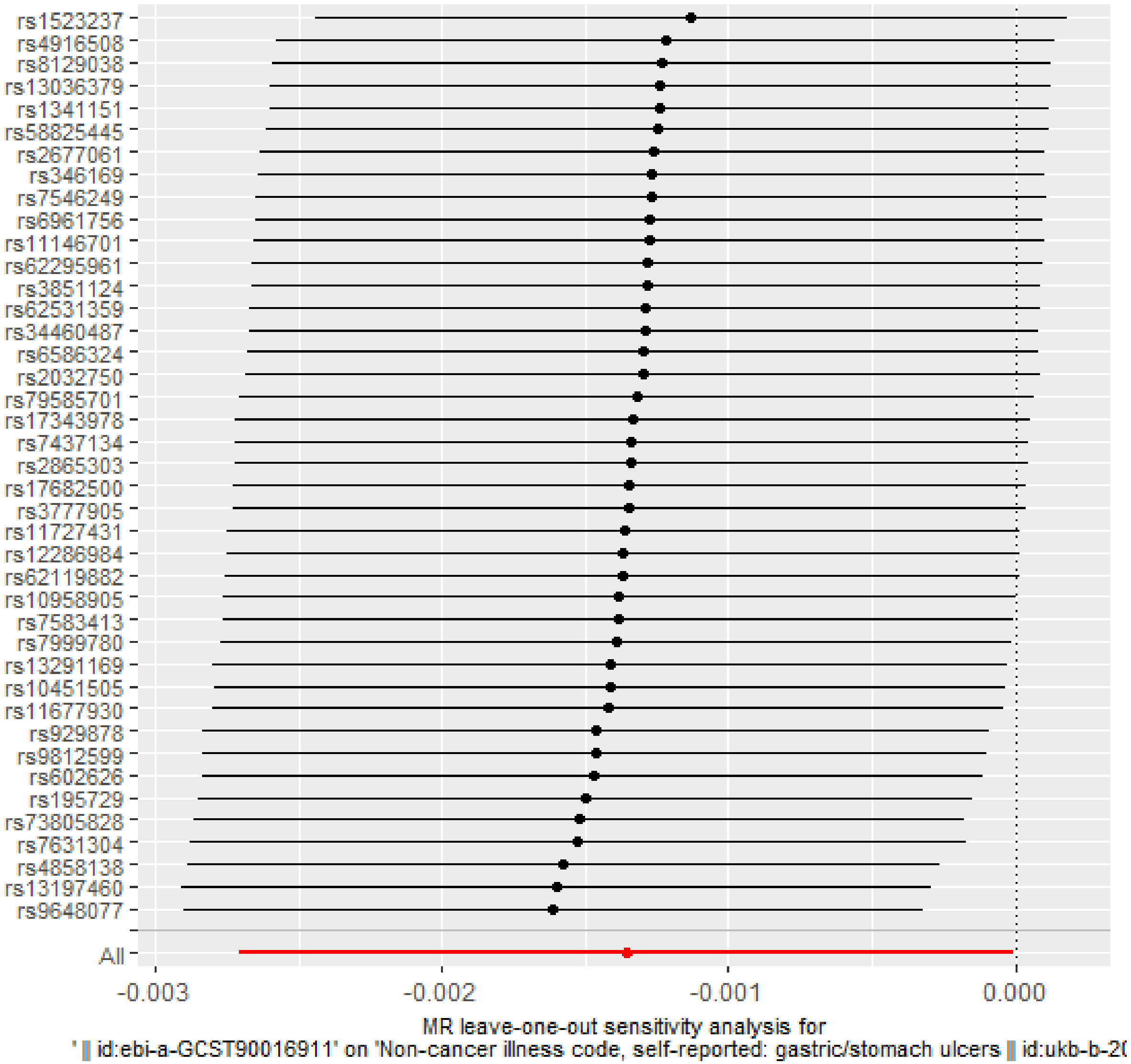

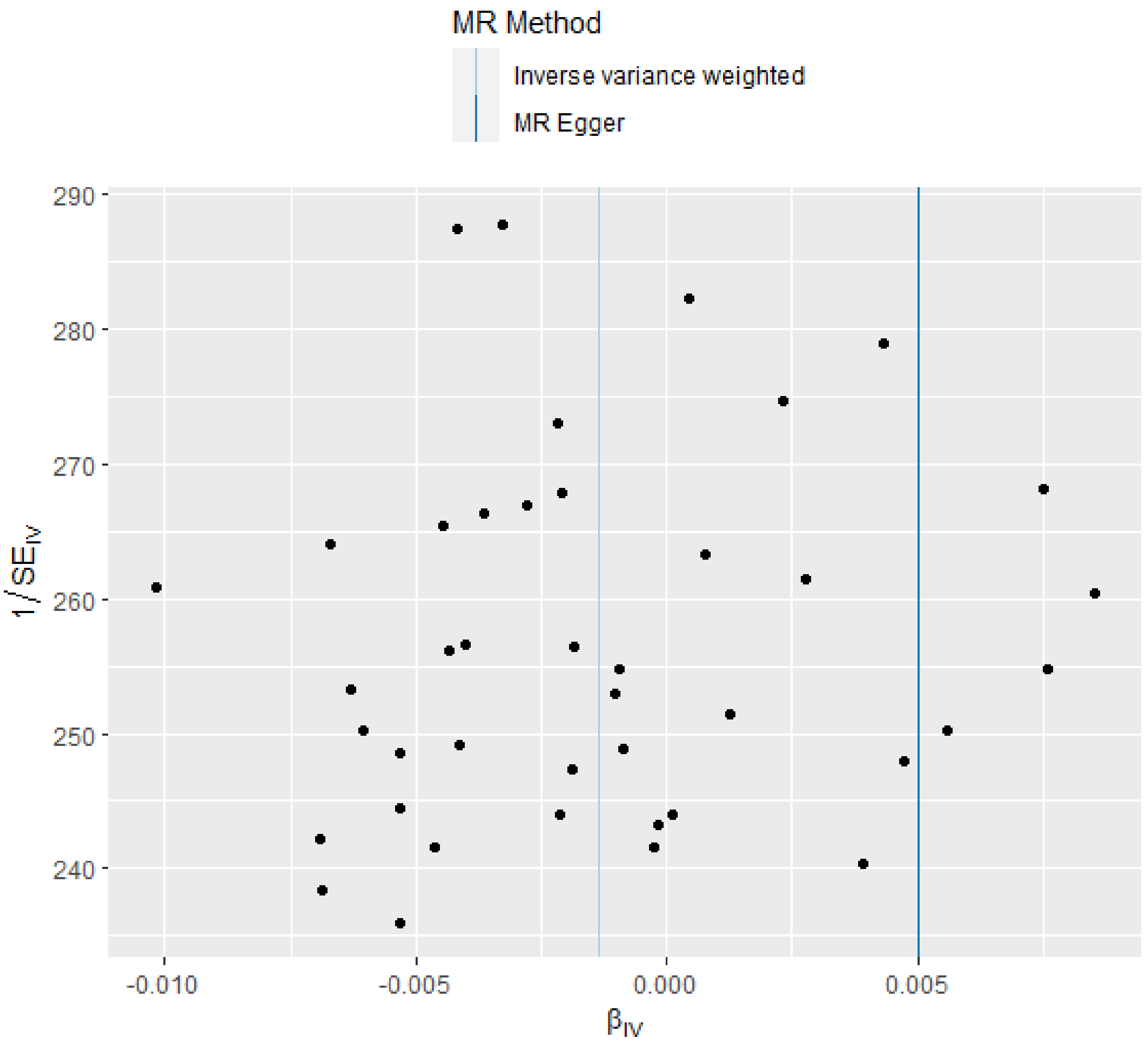

MR Method
Inverse variance weighted
MR Egger
290
280
270
260
250
240
1/SE_IV
-0.010
-0.005
0.000
0.005
β_IV

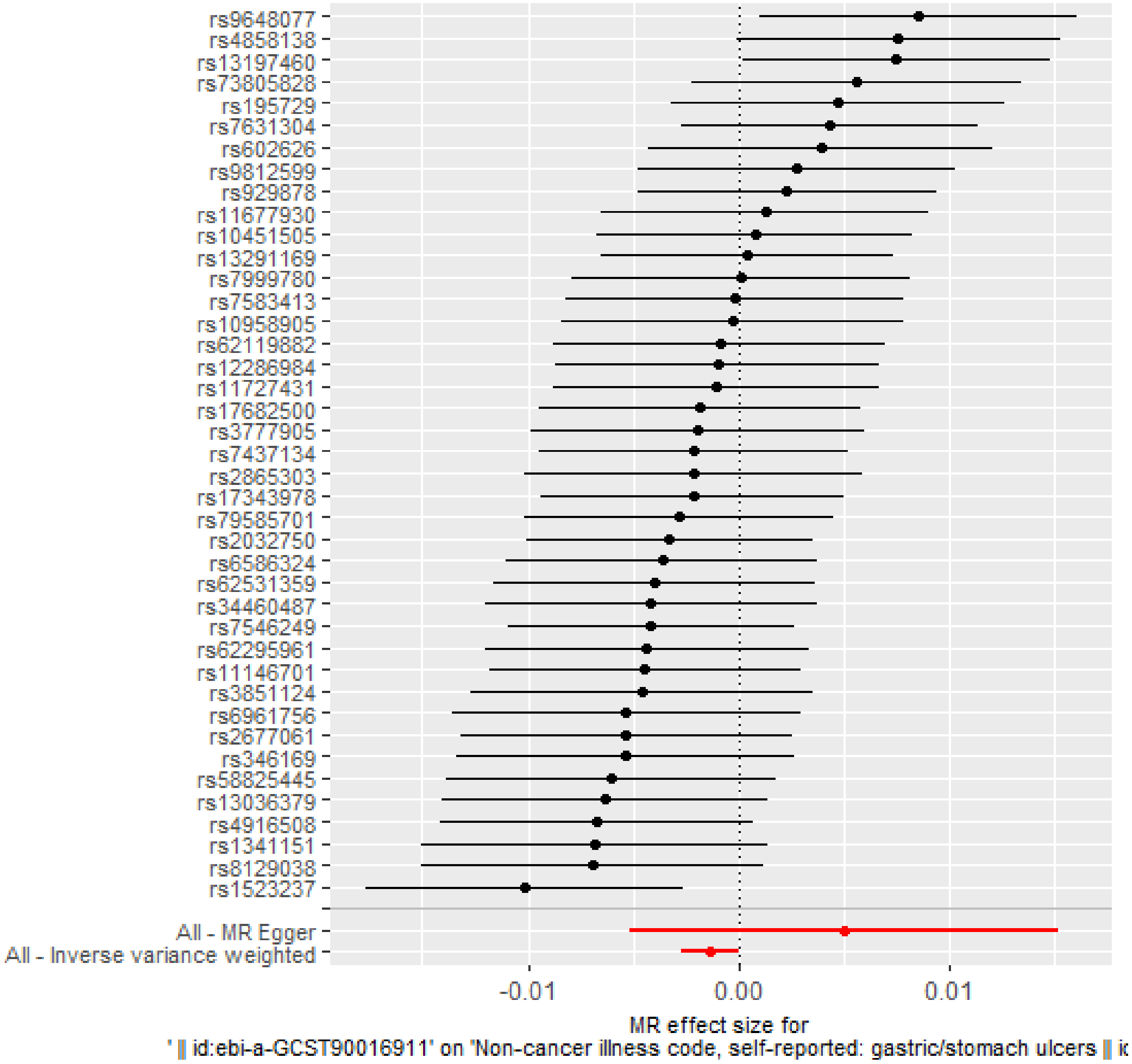
rs9648077
rs4858138
rs13197460
rs73805828
rs195729
rs7631304
rs602626
rs9812599
rs929878
rs11677930
rs10451505
rs13291169
rs7999780
rs7583413
rs10958905
rs62119882
rs12286984
rs11727431
rs17682500
rs3777905
rs7437134
rs2865303
rs17343978
rs79585701
rs2032750
rs6586324
rs62531359
rs34460487
rs7546249
rs62295961
rs11146701
rs3851124
rs6961756
rs2677061
rs346169
rs58825445
rs13036379
rs4916508
rs1341151
rs8129038
rs1523237
All - MR Egger
All - Inverse variance weighted
-0.01
0.00
0.01
MR effect size for
' || id:ebi-a-GCST90016911' on 'Non-cancer illness code, self-reported: gastric/stomach ulcers || id

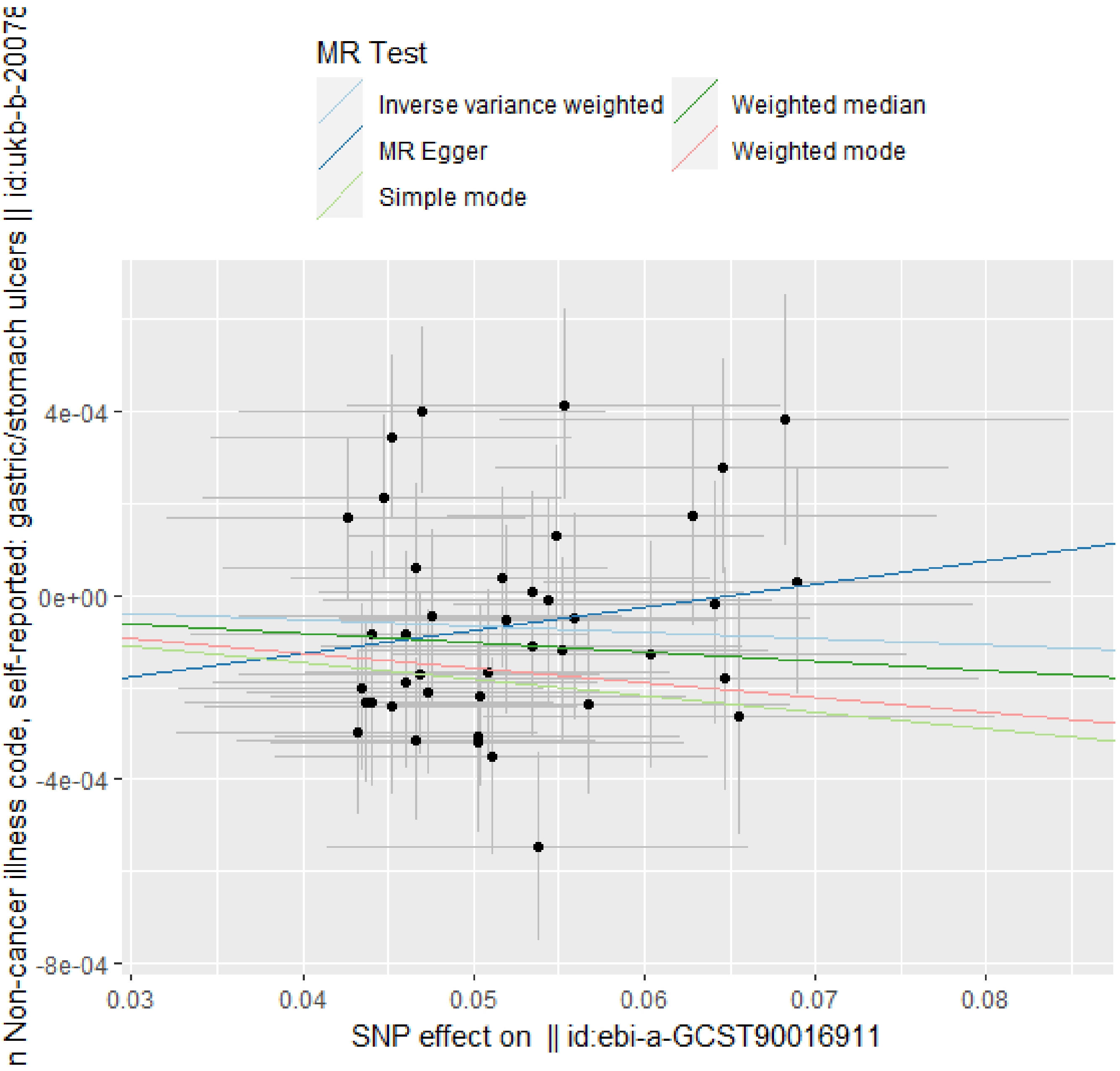

MR Test
Inverse variance weighted
MR Egger
Simple mode
Weighted median
Weighted mode
4e-04
0e+00
-4e-04
-8e-04
0.03
0.04
0.05
0.06
0.07
0.08
SNP effect on || id:ebi-a-GCST90016911
n Non-cancer illness code, self-reported: gastric/stomach ulcers || id:ukb-b-2007€

Figure 41 Leave-one-out analysis, funnel plot, MR effect size and scatter plot for gut microbiota abundance (class Lentisphaeria id.2250) on gastric ulcer

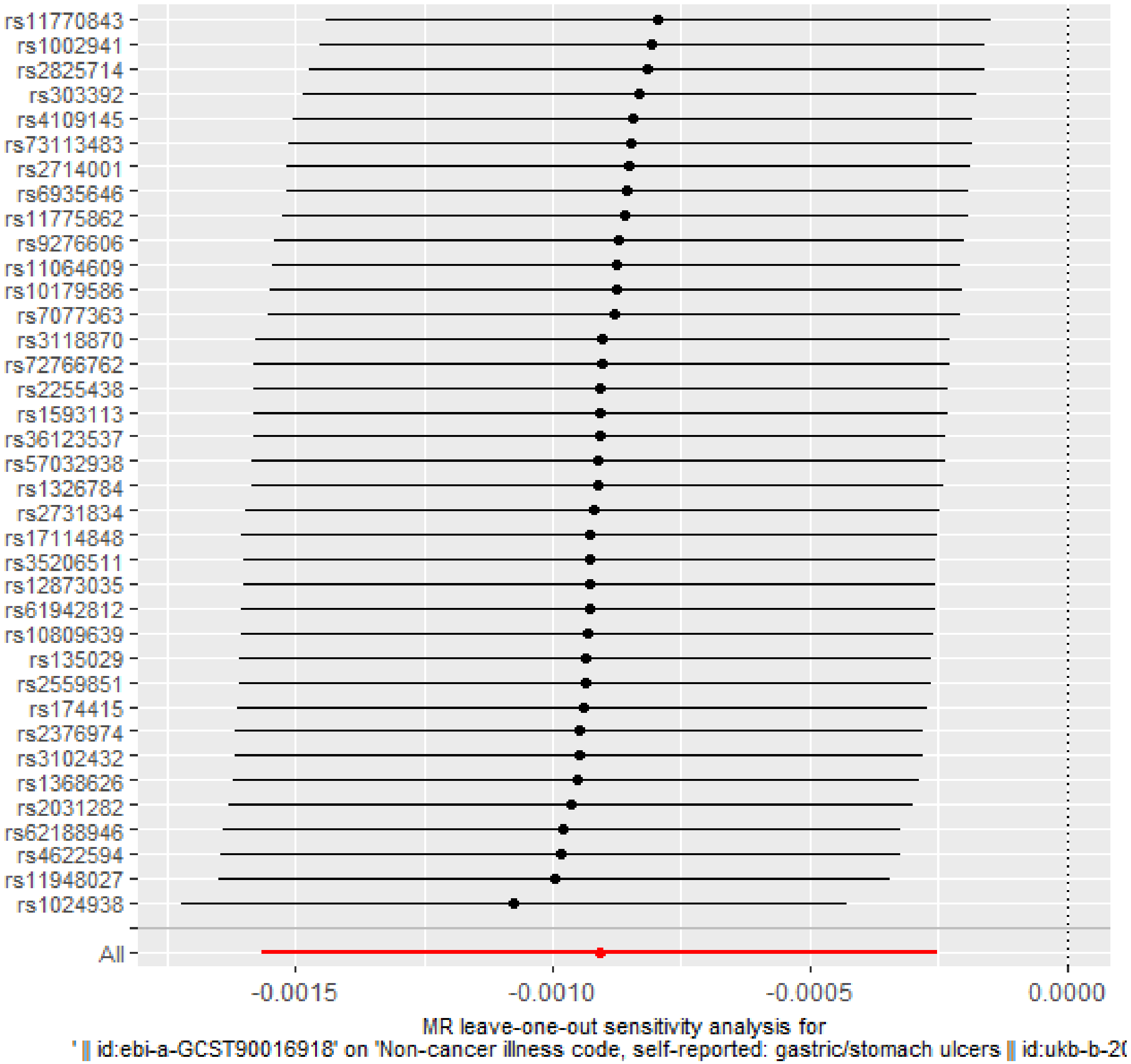

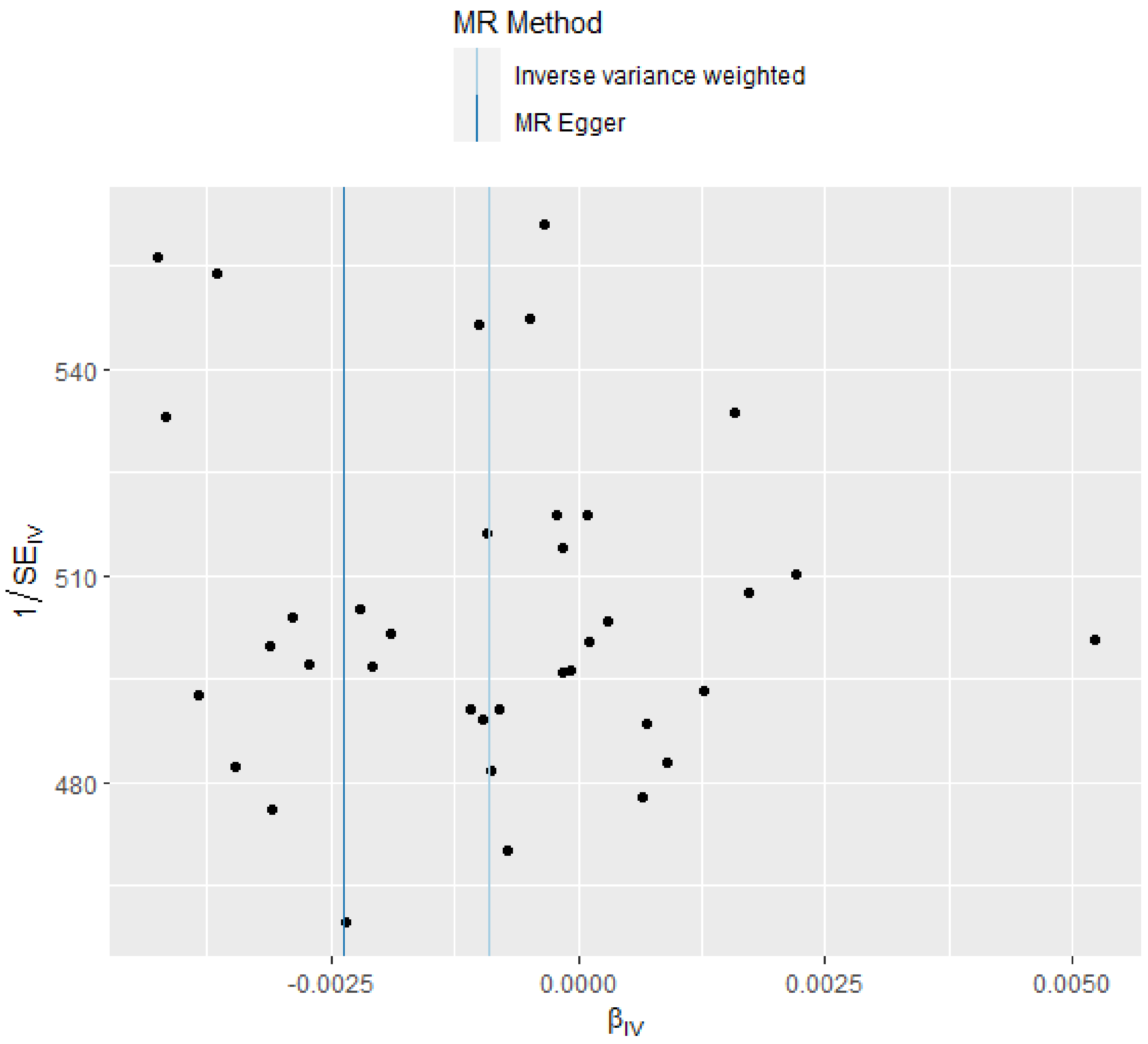

MR Method
Inverse variance weighted
MR Egger
540
510
480
$1/SE_{IV}$
-0.0025
0.0000
0.0025
0.0050
β_{IV}

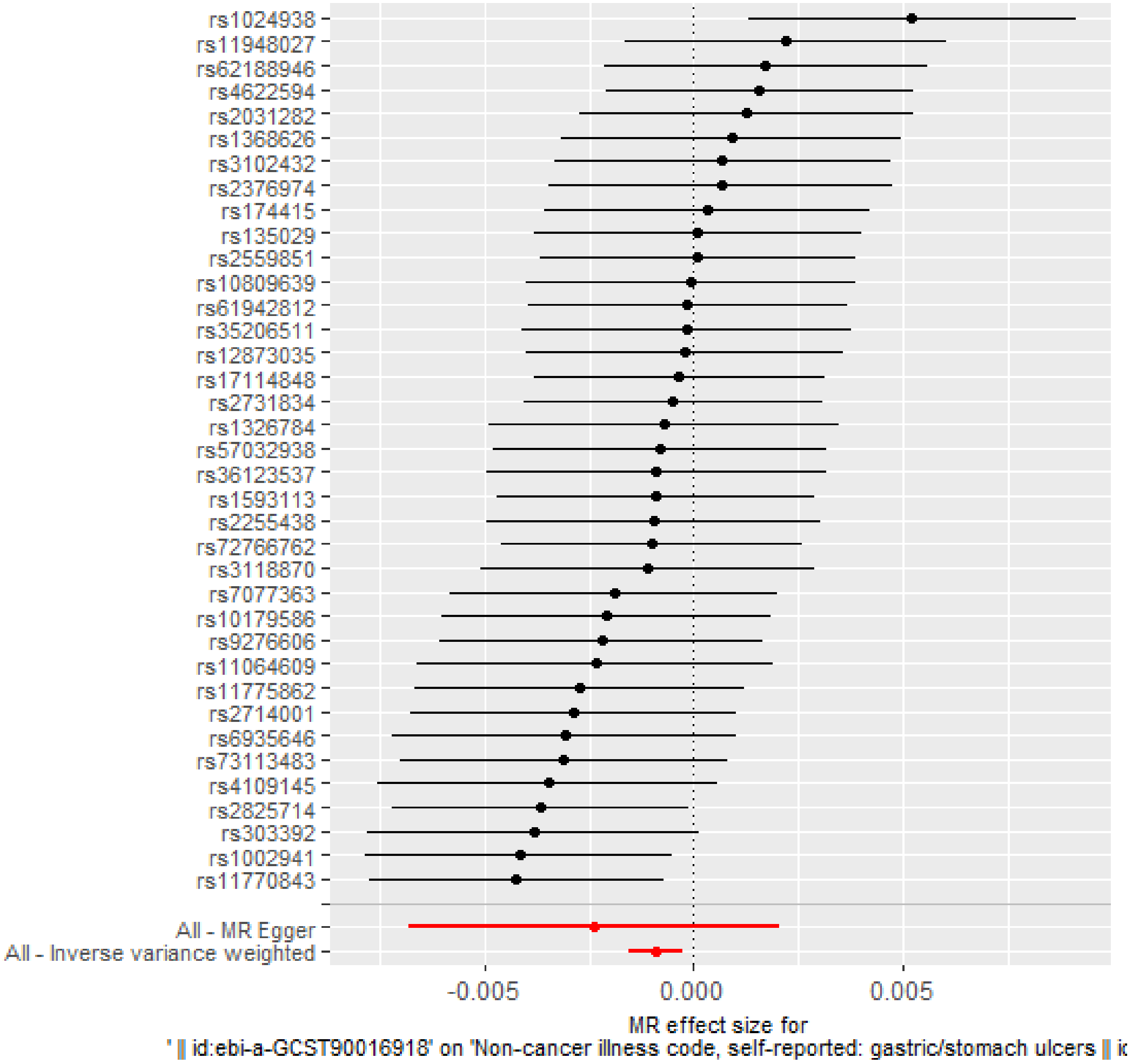
rs1024938
rs11948027
rs62188946
rs4622594
rs2031282
rs1368626
rs3102432
rs2376974
rs174415
rs135029
rs2559851
rs10809639
rs61942812
rs35206511
rs12873035
rs17114848
rs2731834
rs1326784
rs57032938
rs36123537
rs1593113
rs2255438
rs72766762
rs3118870
rs7077363
rs10179586
rs9276606
rs11064609
rs11775862
rs2714001
rs6935646
rs73113483
rs4109145
rs2825714
rs303392
rs1002941
rs11770843
All - MR Egger
All - Inverse variance weighted
-0.005
0.000
0.005
MR effect size for
' || id:ebi-a-GCST90016918' on 'Non-cancer illness code, self-reported: gastric/stomach ulcers || ic

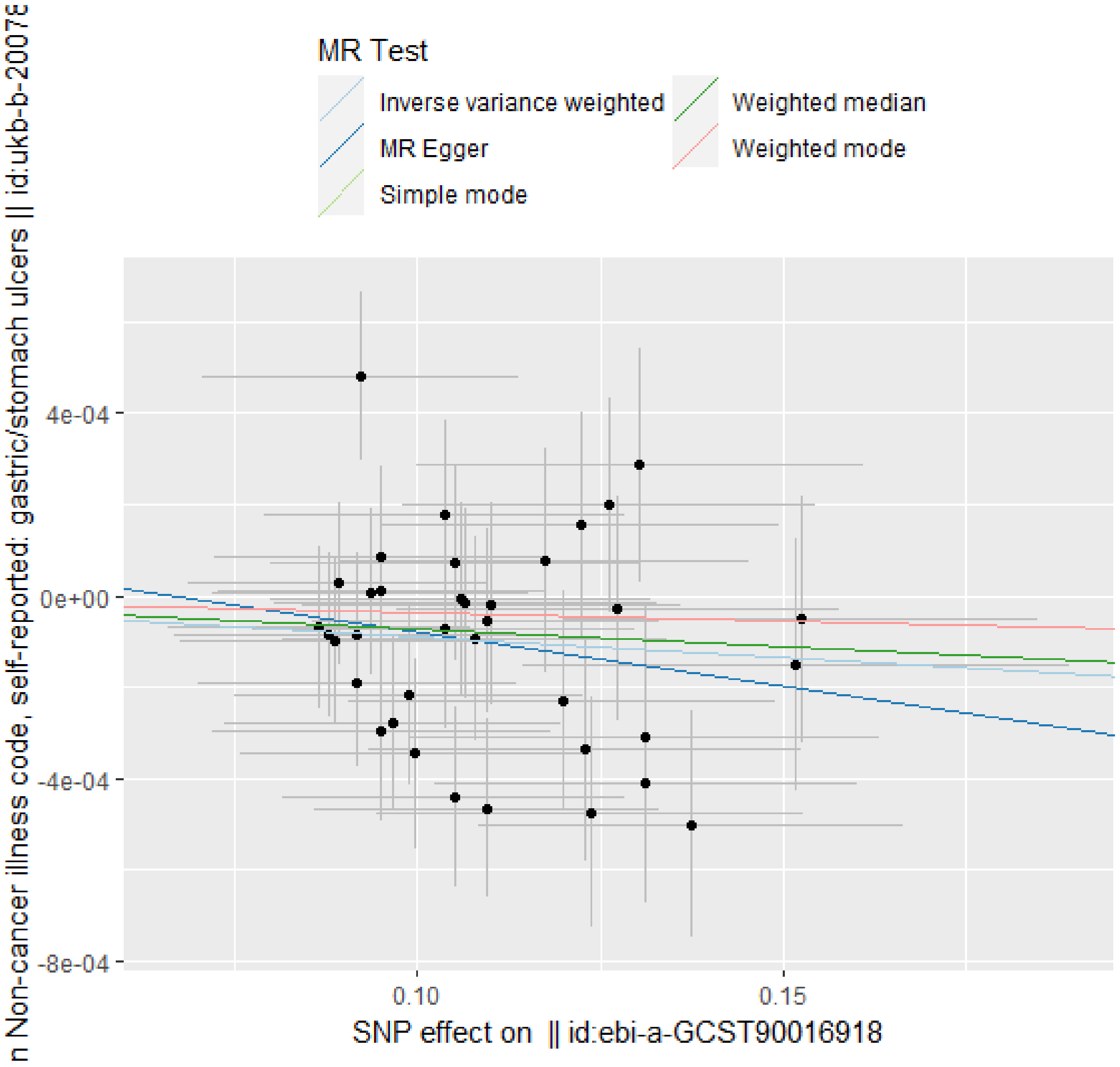

MR Test
Inverse variance weighted
MR Egger
Simple mode
Weighted median
Weighted mode
4e-04
0e+00
-4e-04
-8e-04
0.10
0.15
SNP effect on || id:ebi-a-GCST90016918
n Non-cancer illness code, self-reported: gastric/stomach ulcers || id:ukb-b-2007

Figure 42 Leave-one-out analysis, funnel plot, MR effect size and scatter plot for gut microbiota abundance (genus Butyrivibrio id.1993) on gastric ulcer

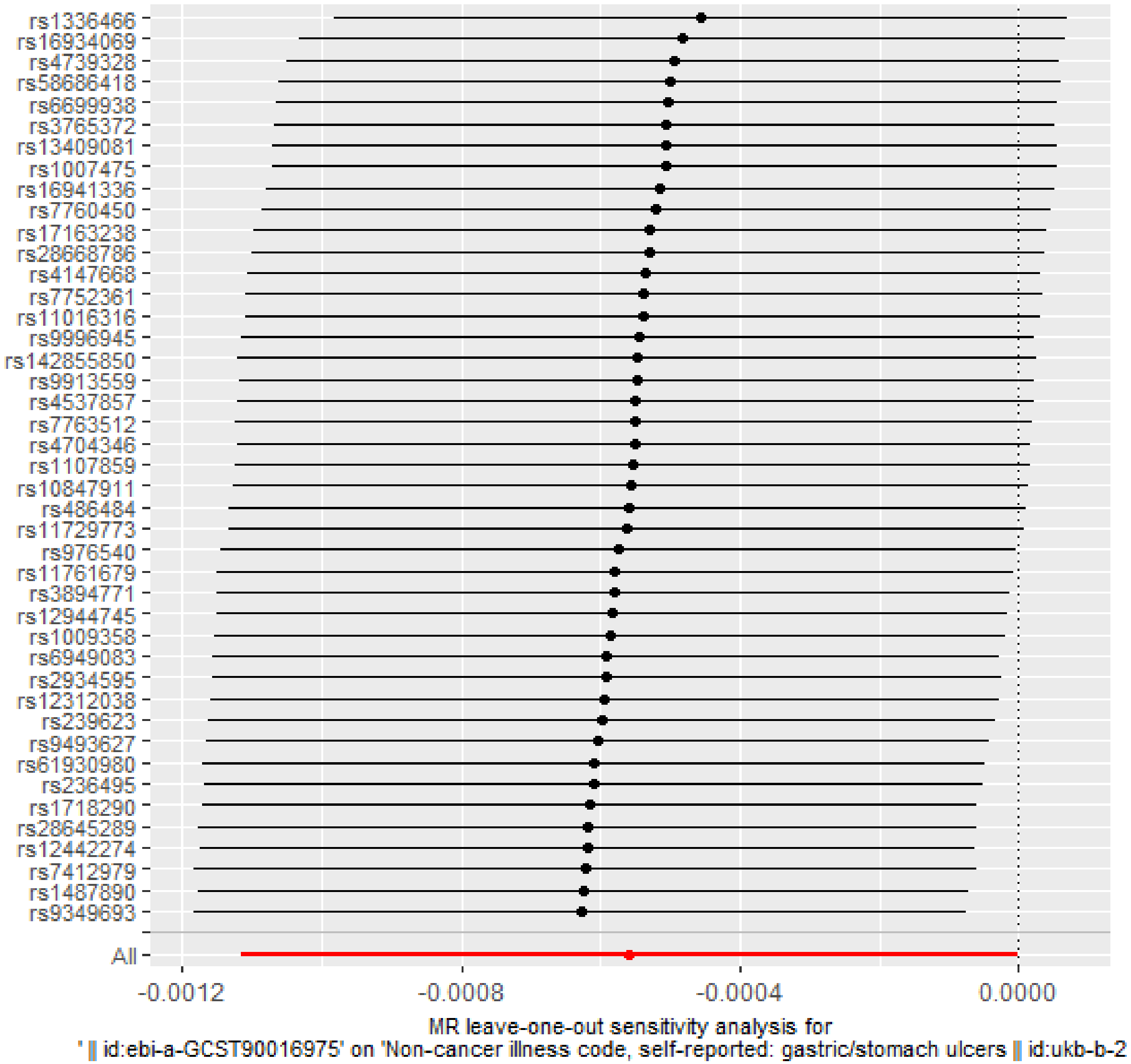

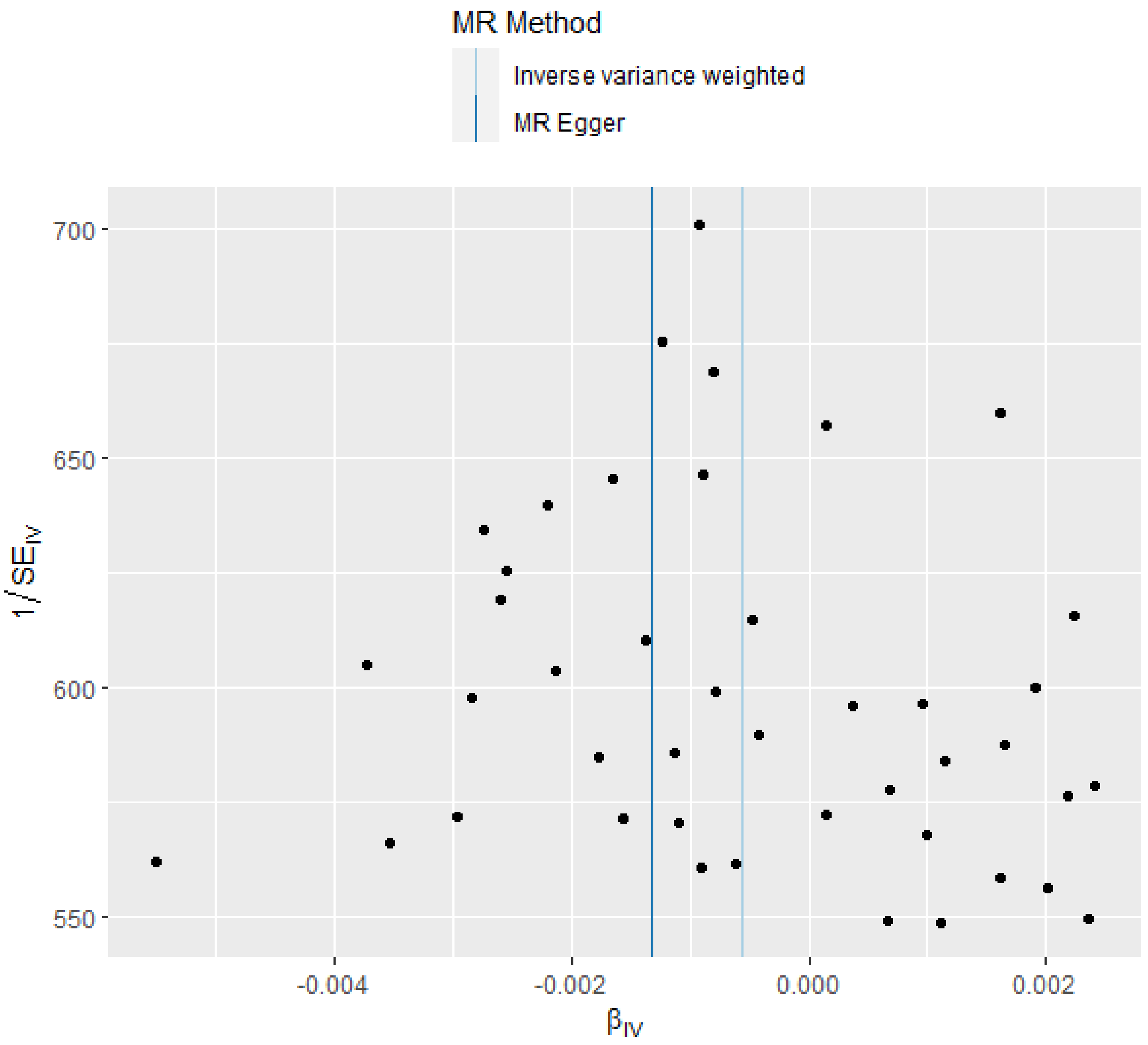

MR Method
Inverse variance weighted
MR Egger
700
650
600
550
1/SE_IV
-0.004
-0.002
0.000
0.002
β_IV

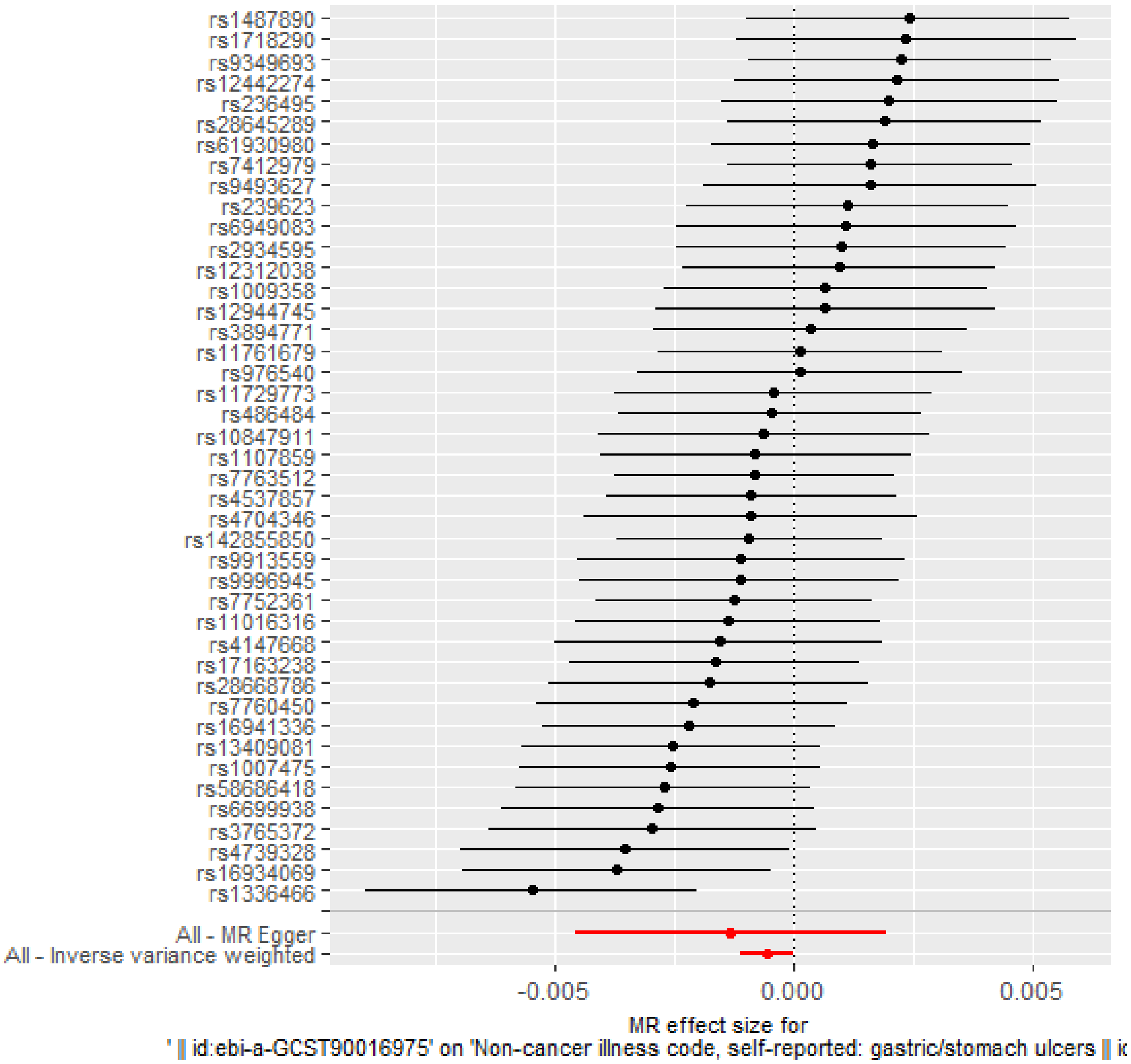
rs1487890
rs1718290
rs9349693
rs12442274
rs236495
rs28645289
rs61930980
rs7412979
rs9493627
rs239623
rs6949083
rs2934595
rs12312038
rs1009358
rs12944745
rs3894771
rs11761679
rs976540
rs11729773
rs486484
rs10847911
rs1107859
rs7763512
rs4537857
rs4704346
rs142855850
rs9913559
rs9996945
rs7752361
rs11016316
rs4147668
rs17163238
rs28668786
rs7760450
rs16941336
rs13409081
rs1007475
rs58686418
rs6699938
rs3765372
rs4739328
rs16934069
rs1336466
All - MR Egger
All - Inverse variance weighted
-0.005
0.000
0.005
MR effect size for
' || id:ebi-a-GCST90016975' on 'Non-cancer illness code, self-reported: gastric/stomach ulcers || ic

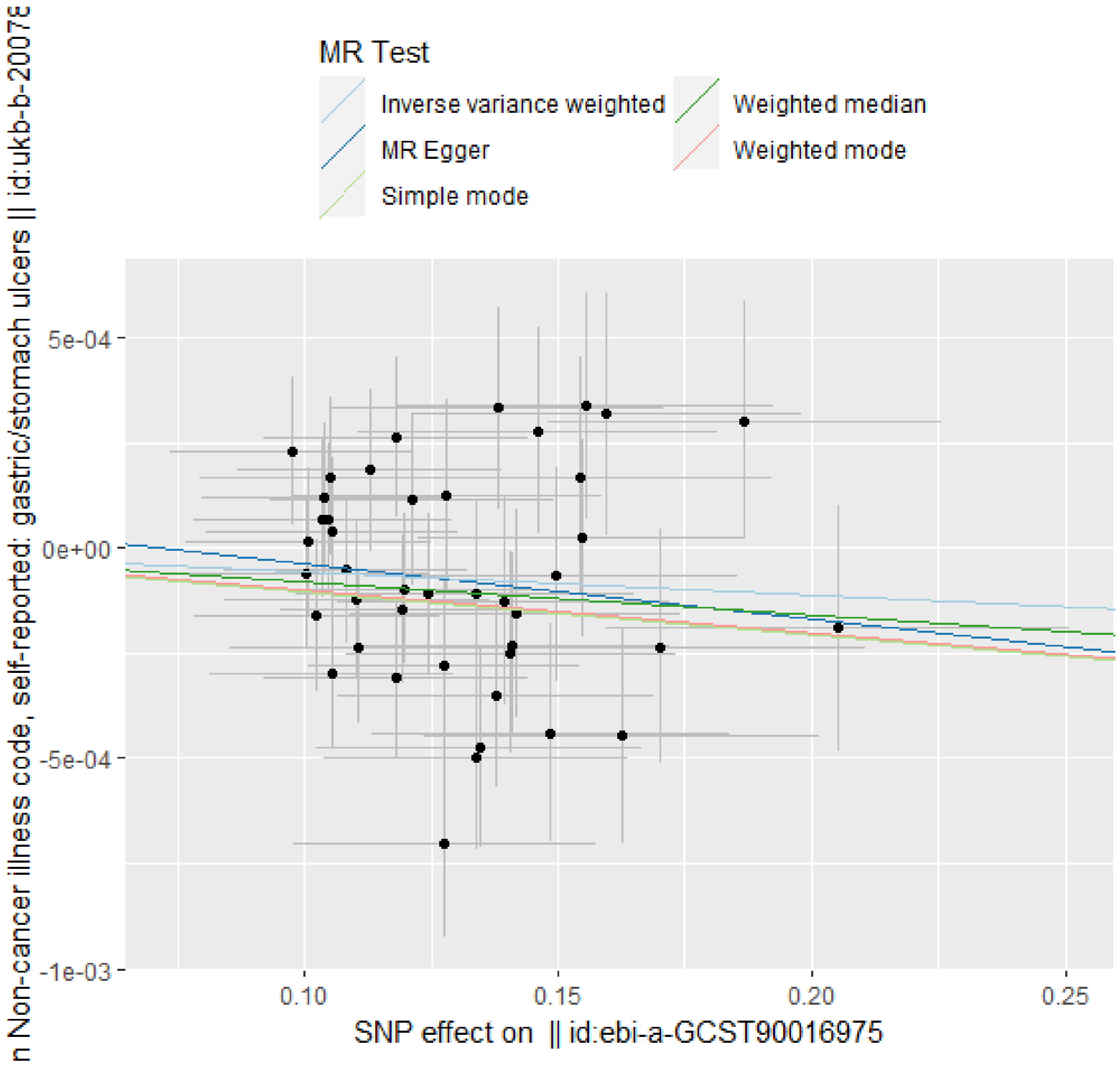
MR Test
Inverse variance weighted
MR Egger
Simple mode
Weighted median
Weighted mode
5e-04
0e+00
-5e-04
-1e-03
0.10
0.15
0.20
0.25
SNP effect on || id:ebi-a-GCST90016975
n Non-cancer illness code, self-reported: gastric/stomach ulcers || id:ukb-b-20078

Figure 43 Leave-one-out analysis, funnel plot, MR effect size and scatter plot for gut microbiota abundance (genus Desulfovibrio id.3173) on gastric ulcer

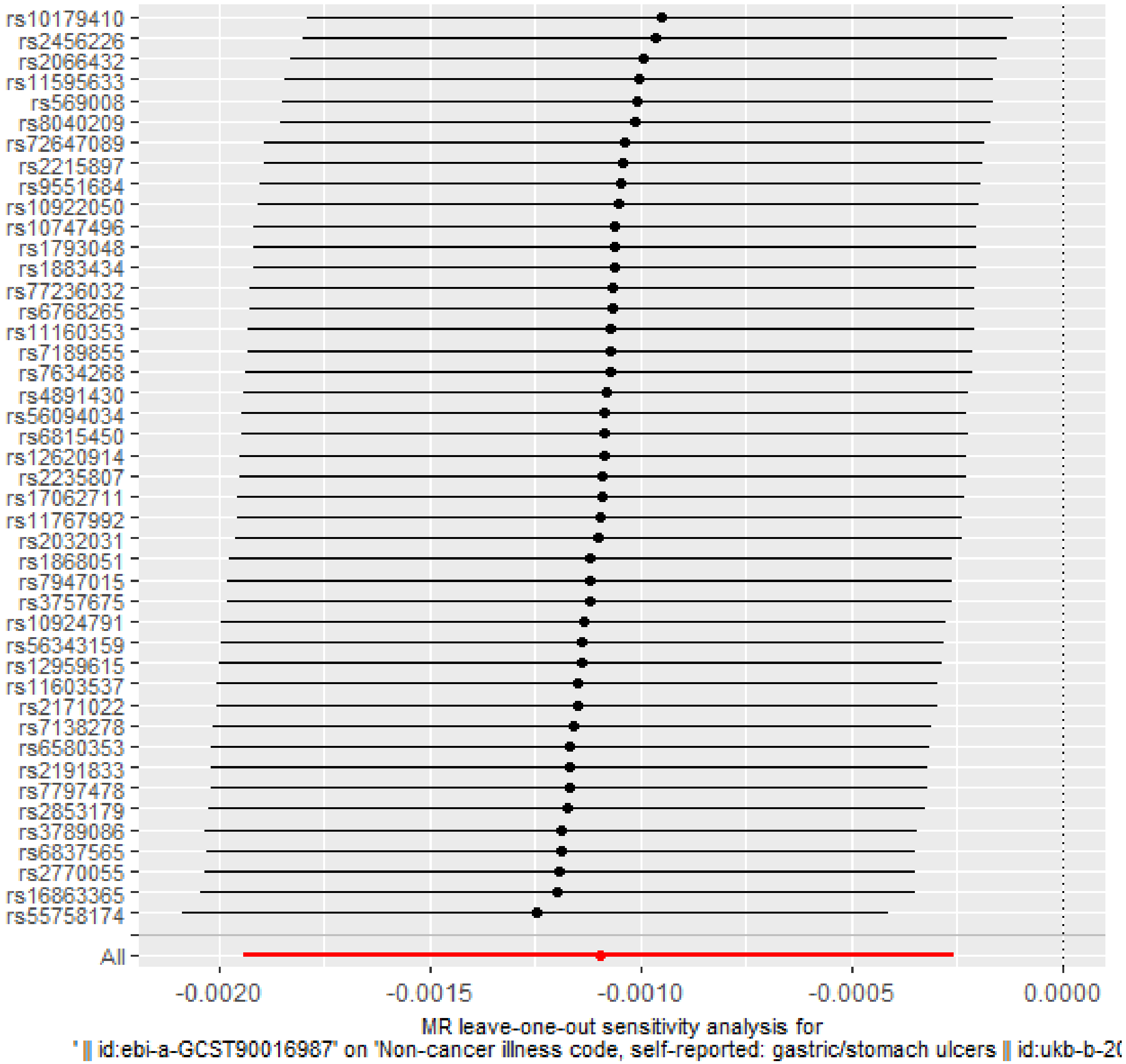

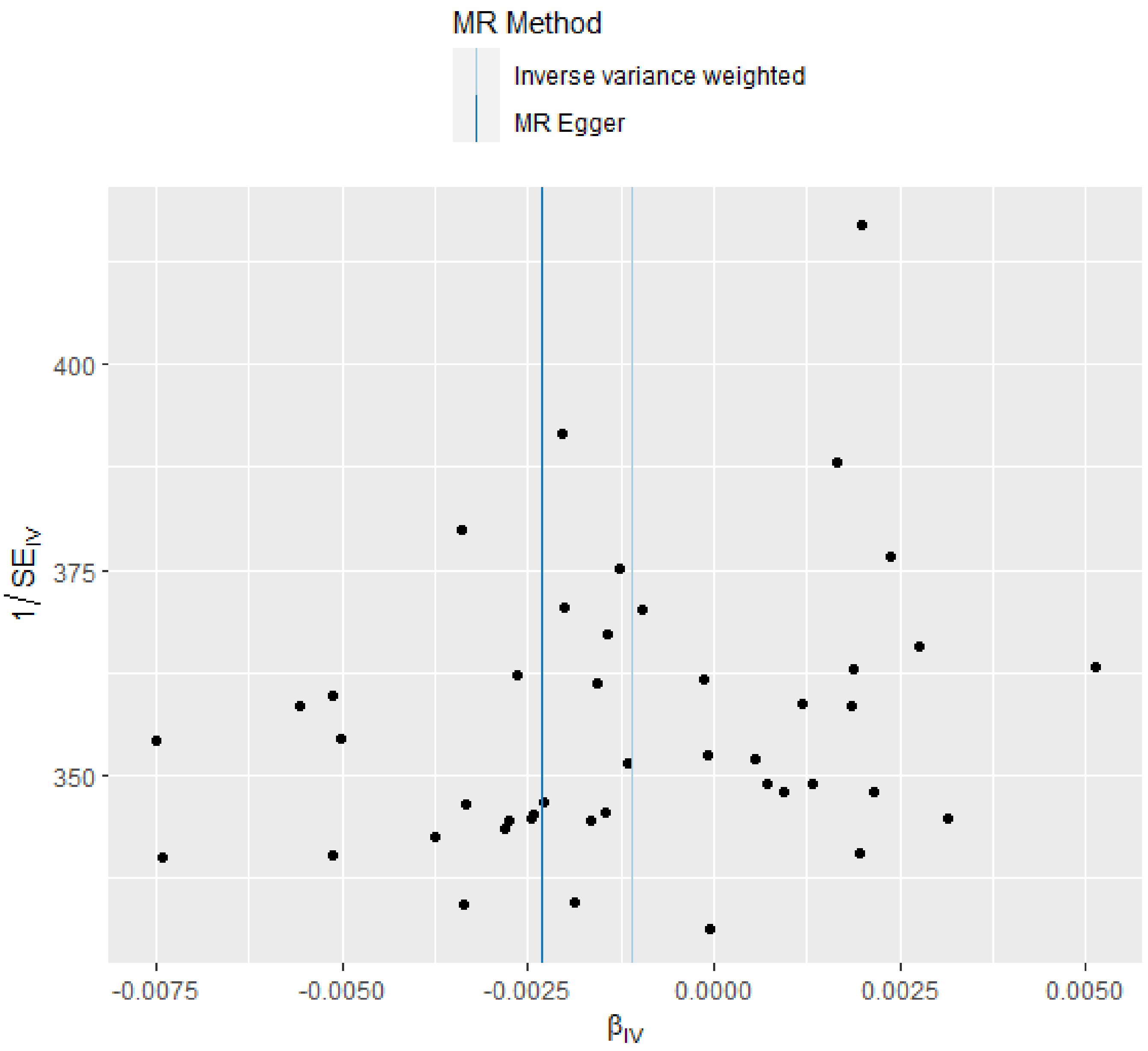

MR Method
Inverse variance weighted
MR Egger
400
375
350
$1/SE_{IV}$
-0.0075
-0.0050
-0.0025
0.0000
0.0025
0.0050
β_{IV}

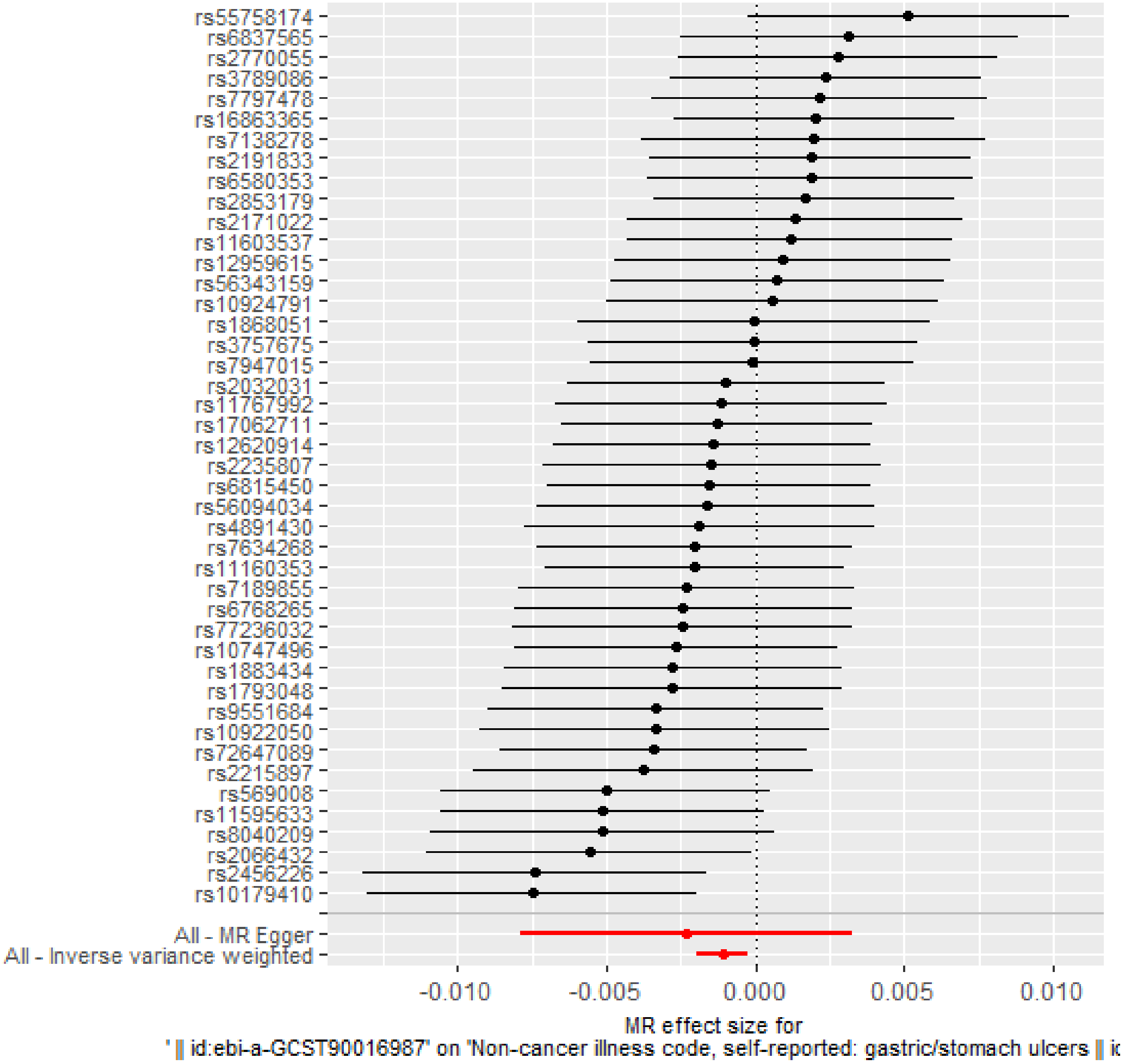
rs55758174
rs6837565
rs2770055
rs3789086
rs7797478
rs16863365
rs7138278
rs2191833
rs6580353
rs2853179
rs2171022
rs11603537
rs12959615
rs56343159
rs10924791
rs1868051
rs3757675
rs7947015
rs2032031
rs11767992
rs17062711
rs12620914
rs2235807
rs6815450
rs56094034
rs4891430
rs7634268
rs11160353
rs7189855
rs6768265
rs77236032
rs10747496
rs1883434
rs1793048
rs9551684
rs10922050
rs72647089
rs2215897
rs569008
rs11595633
rs8040209
rs2066432
rs2456226
rs10179410
All - MR Egger
All - Inverse variance weighted
-0.010
-0.005
0.000
0.005
0.010
MR effect size for
' || id:ebi-a-GCST90016987' on 'Non-cancer illness code, self-reported: gastric/stomach ulcers || id

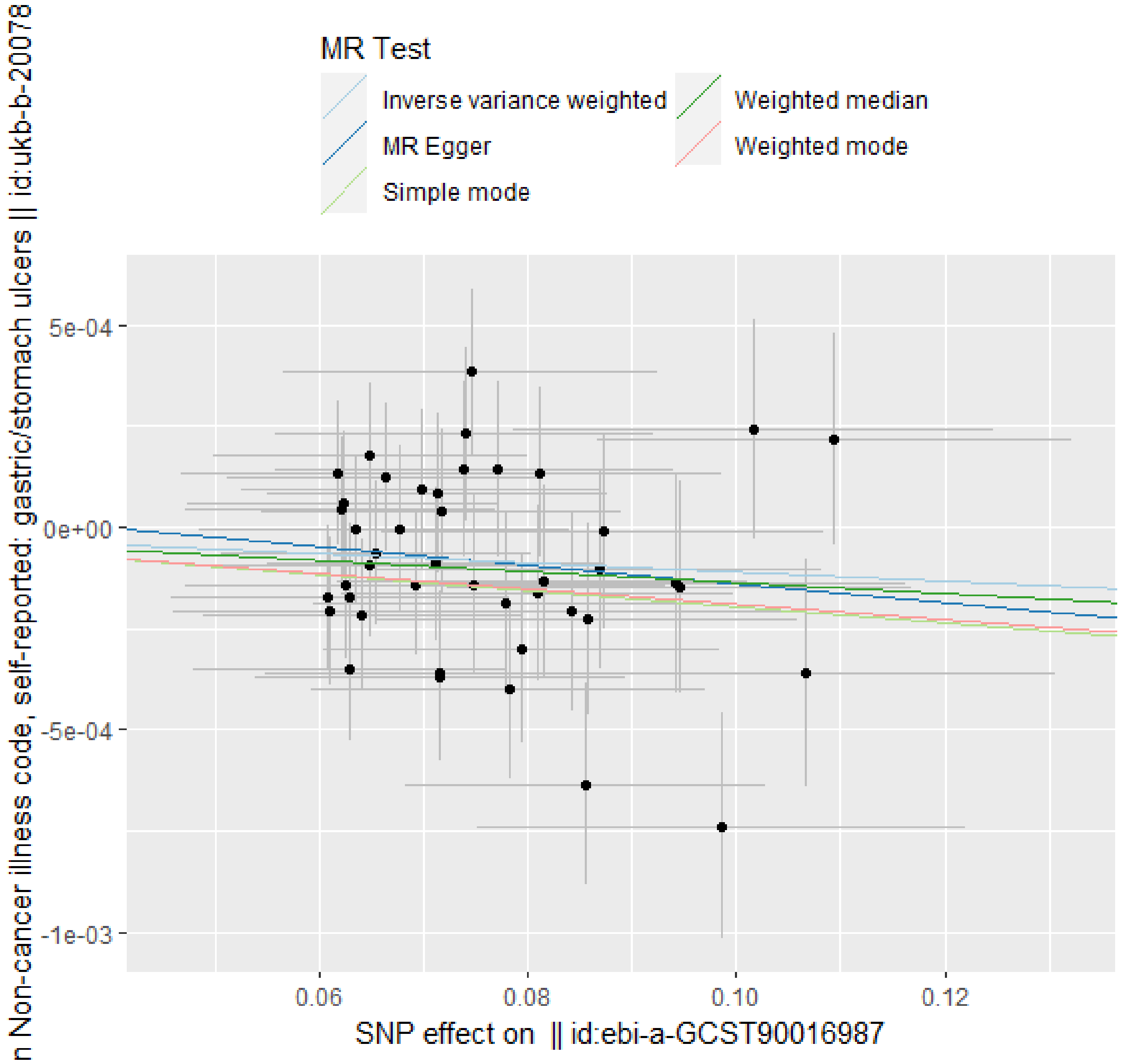

MR Test
Inverse variance weighted
MR Egger
Simple mode
Weighted median
Weighted mode
5e-04
0e+00
-5e-04
-1e-03
0.06
0.08
0.10
0.12
SNP effect on || id:ebi-a-GCST90016987
n Non-cancer illness code, self-reported: gastric/stomach ulcers || id:ukb-b-20078

Figure 44 Leave-one-out analysis, funnel plot, MR effect size and scatter plot for gut microbiota abundance (genus Eubacterium ventriosum group id.11341) on gastric ulcer

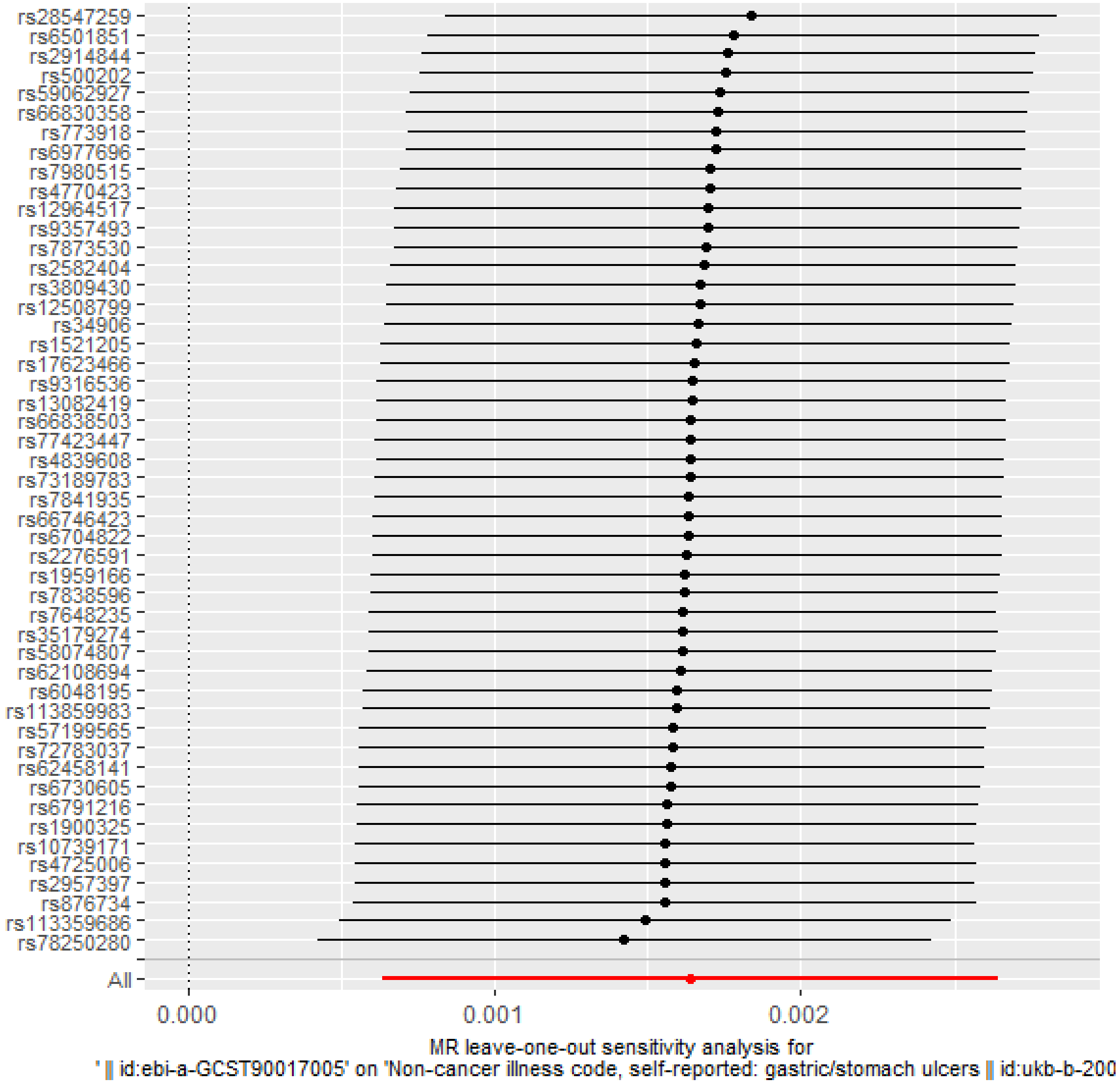

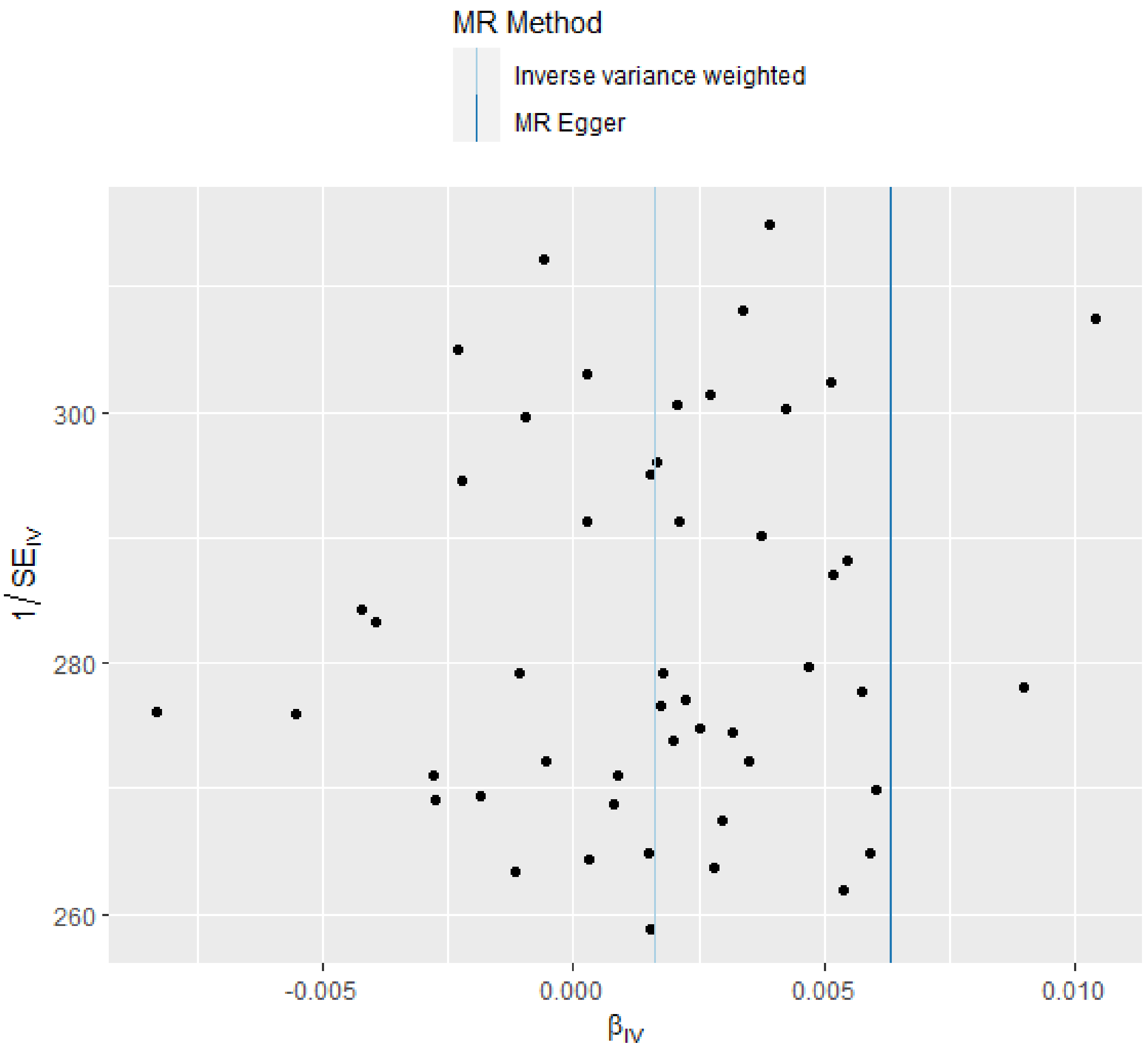
MR Method
Inverse variance weighted
MR Egger
300
280
260
$1/SE_{IV}$
-0.005
0.000
0.005
0.010
β_{IV}

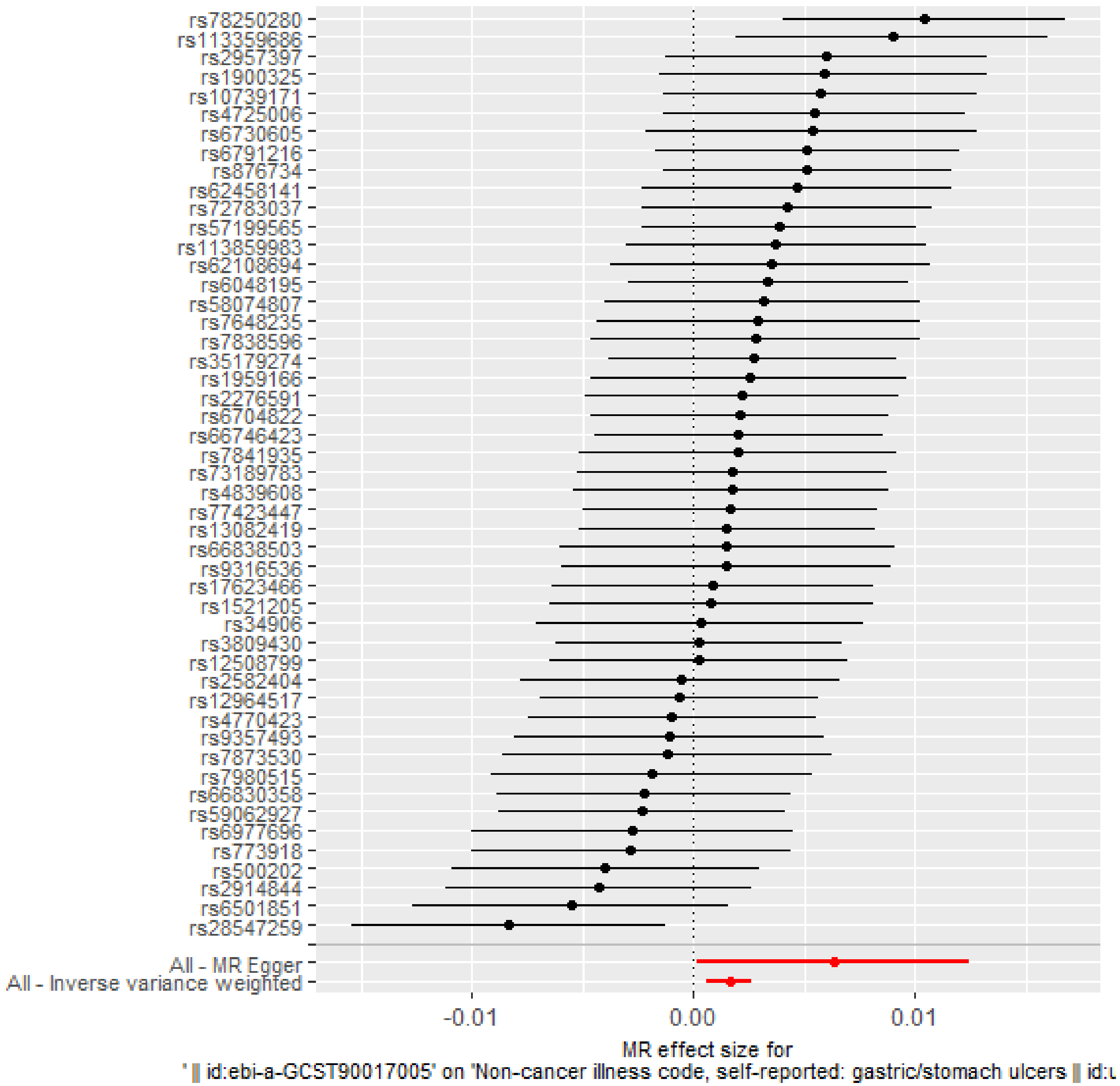

rs78250280
rs113359686
rs2957397
rs1900325
rs10739171
rs4725006
rs6730605
rs6791216
rs876734
rs62458141
rs72783037
rs57199565
rs113859983
rs62108694
rs6048195
rs58074807
rs7648235
rs7838596
rs35179274
rs1959166
rs2276591
rs6704822
rs66746423
rs7841935
rs73189783
rs4839608
rs77423447
rs13082419
rs66838503
rs9316536
rs17623466
rs1521205
rs34906
rs3809430
rs12508799
rs2582404
rs12964517
rs4770423
rs9357493
rs7873530
rs7980515
rs66830358
rs59062927
rs6977696
rs773918
rs500202
rs2914844
rs6501851
rs28547259
All - MR Egger
All - Inverse variance weighted
-0.01
0.00
0.01
MR effect size for
' || id:ebi-a-GCST90017005' on 'Non-cancer illness code, self-reported: gastric/stomach ulcers || id:u

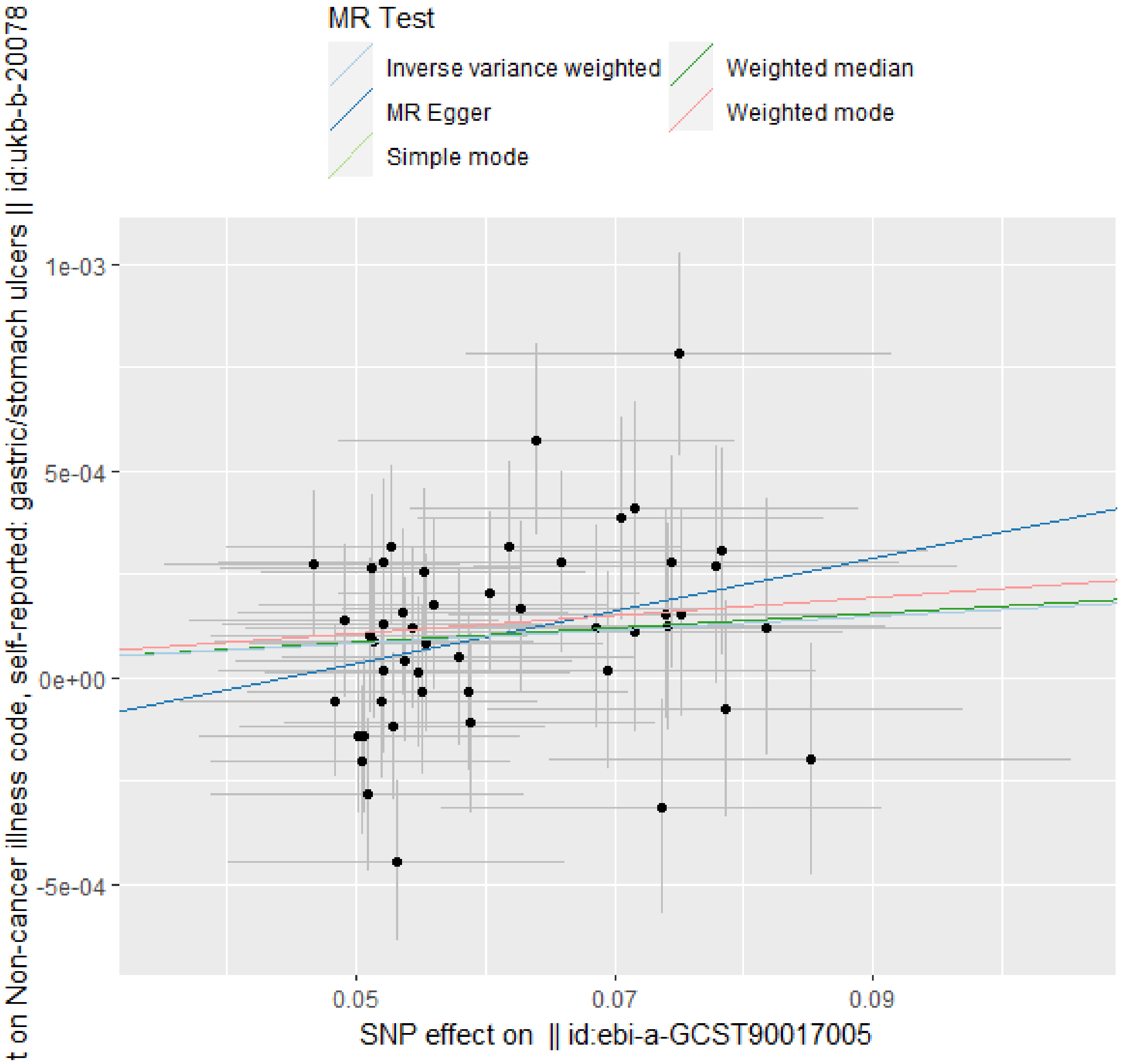

MR Test
Inverse variance weighted
MR Egger
Simple mode
Weighted median
Weighted mode
1e-03
5e-04
0e+00
-5e-04
0.05
0.07
0.09
SNP effect on || id:ebi-a-GCST90017005
t on Non-cancer illness code, self-reported: gastric/stomach ulcers || id:ukb-b-20078

Figure 45 Leave-one-out analysis, funnel plot, MR effect size and scatter plot for gut microbiota abundance (genus Ruminiclostridium9 id.11357) on gastric ulcer

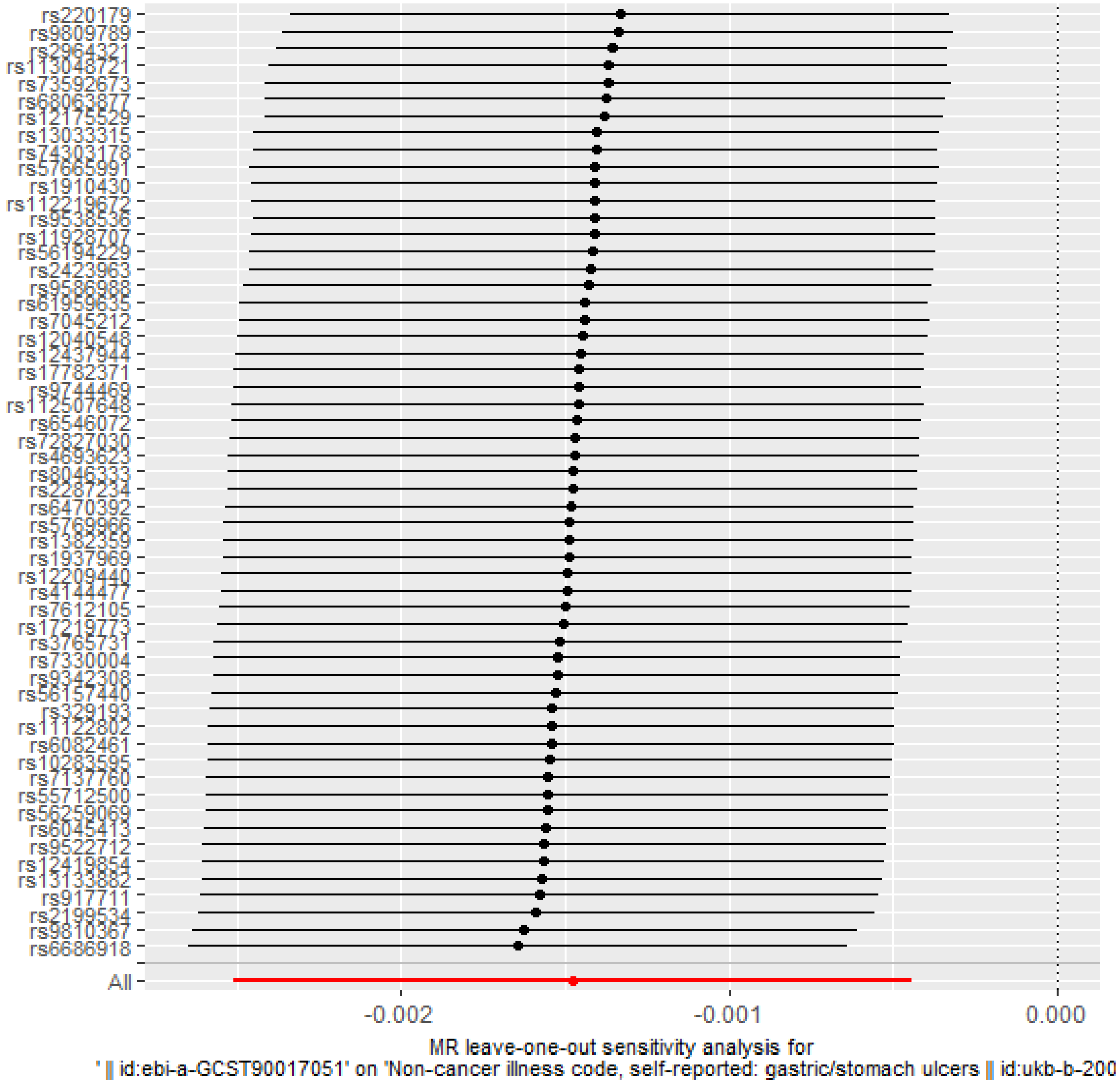

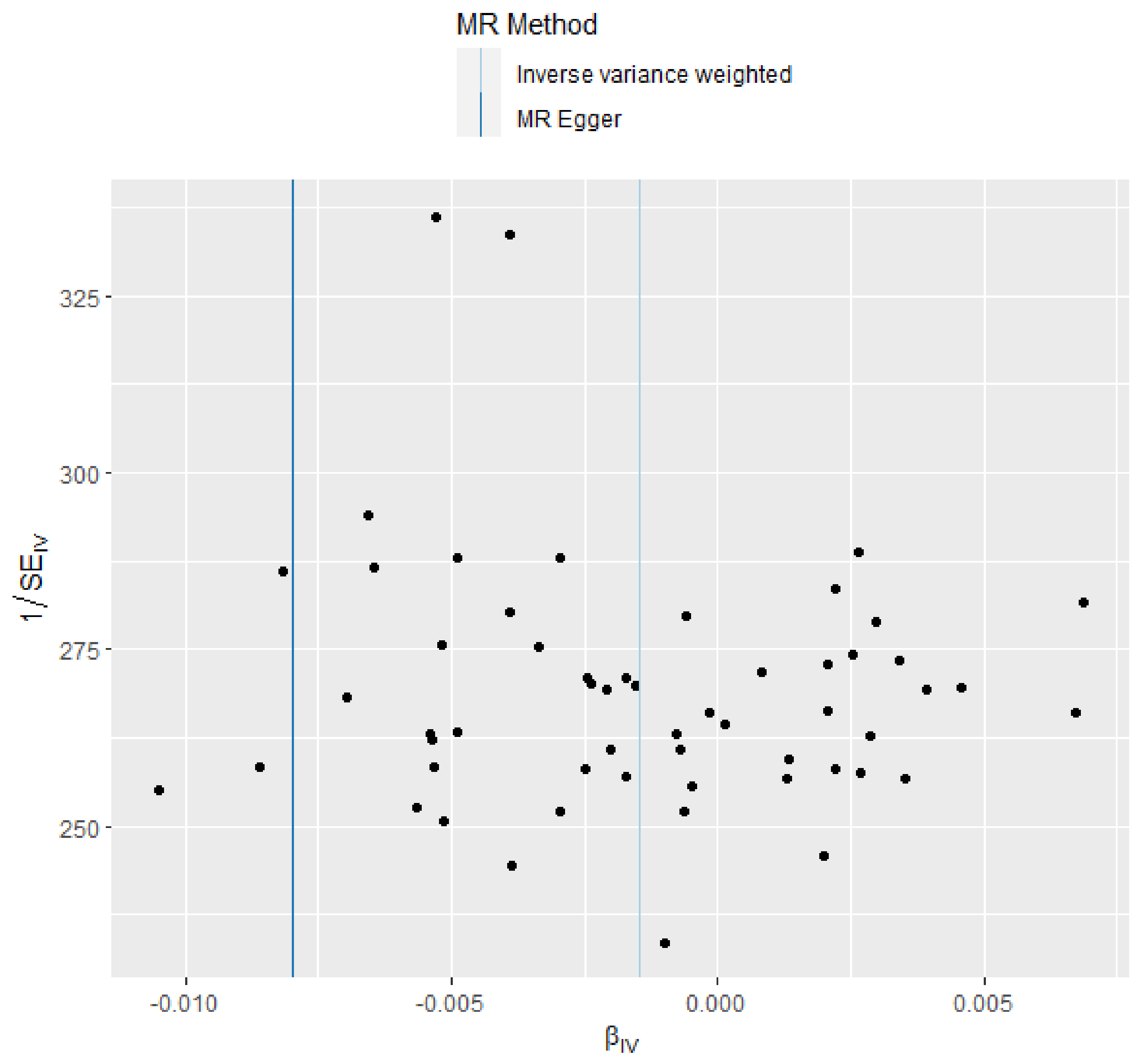

MR Method
Inverse variance weighted
MR Egger
325
300
275
250
1/SE_IV
-0.010
-0.005
0.000
0.005
β_IV

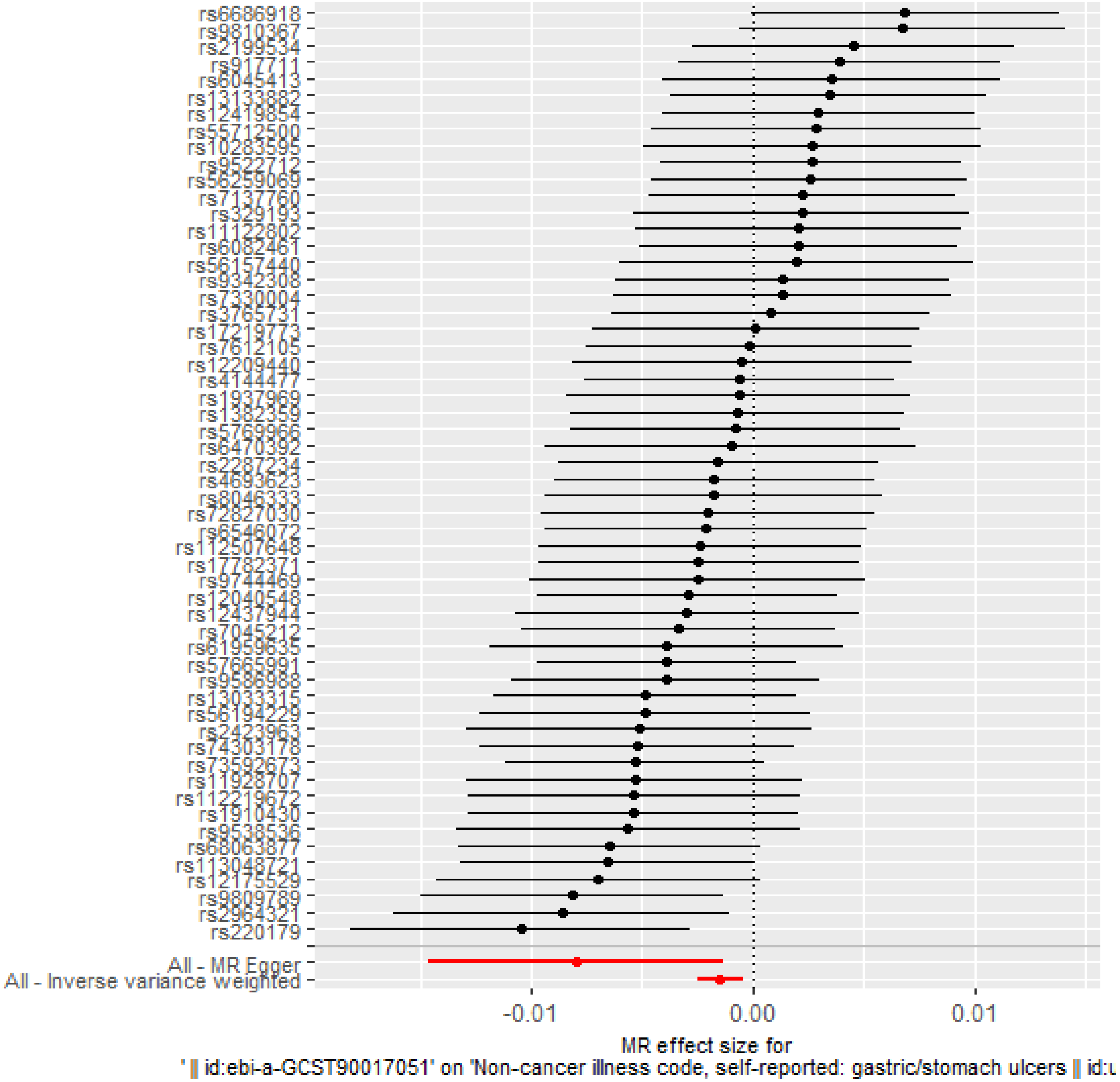
rs6686918
rs9810367
rs2199534
rs917711
rs6045413
rs13133882
rs12419854
rs55712500
rs10283595
rs9522712
rs56259069
rs7137760
rs329193
rs11122802
rs6082461
rs56157440
rs9342308
rs7330004
rs3765731
rs17219773
rs7612105
rs12209440
rs4144477
rs1937969
rs1382359
rs5769966
rs6470392
rs2287234
rs4693623
rs8046333
rs72827030
rs6546072
rs112507648
rs17782371
rs9744469
rs12040548
rs12437944
rs7045212
rs61959635
rs57665991
rs9586988
rs13033315
rs56194229
rs2423963
rs74303178
rs73592673
rs11928707
rs112219672
rs1910430
rs9538536
rs68063877
rs113048721
rs12175529
rs9809789
rs2964321
rs220179
All - MR Egger
All - Inverse variance weighted
-0.01
0.00
0.01
MR effect size for
' || id:ebi-a-GCST90017051' on 'Non-cancer illness code, self-reported: gastric/stomach ulcers || id:

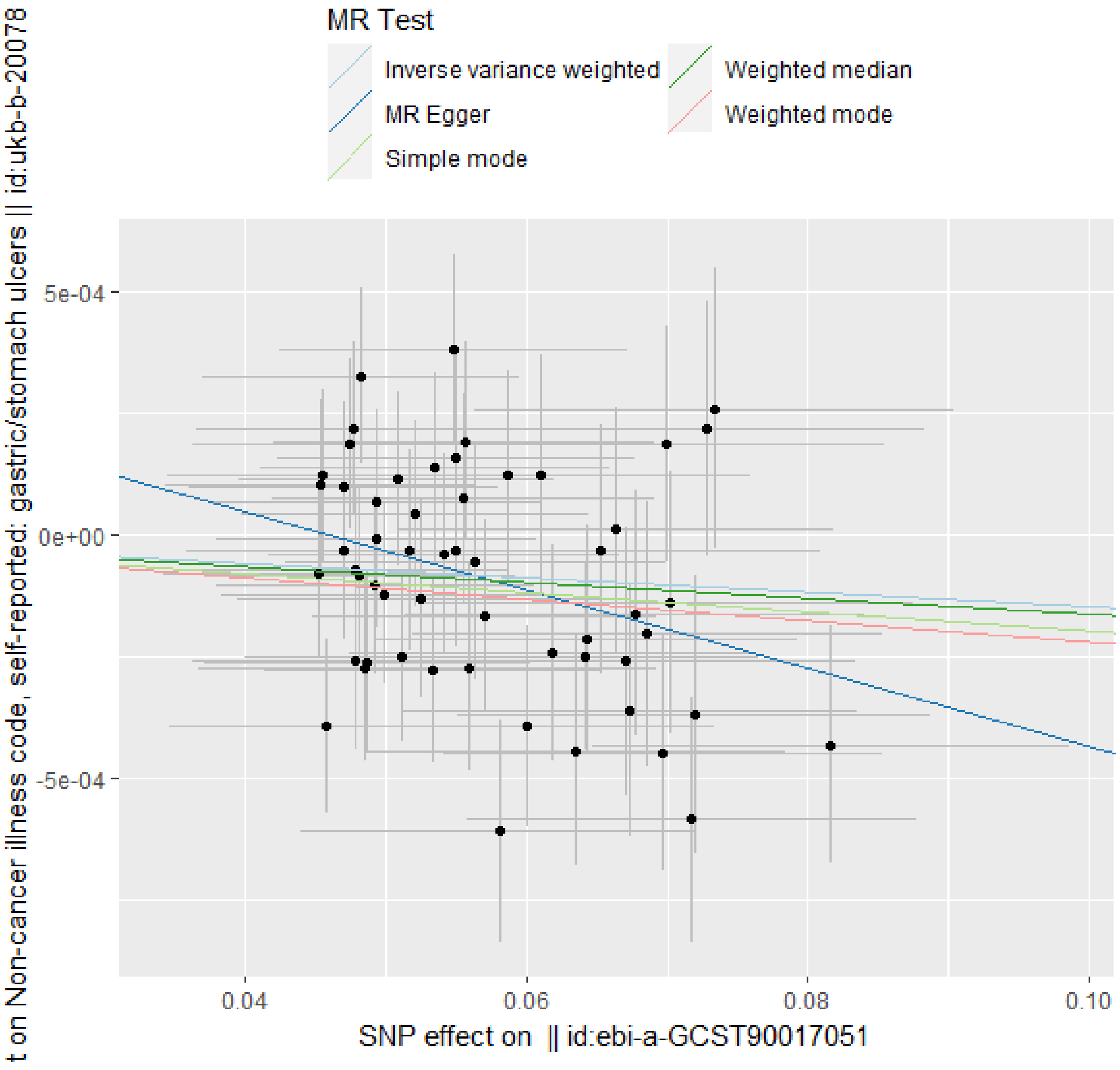

MR Test
Inverse variance weighted
MR Egger
Simple mode
Weighted median
Weighted mode
5e-04
0e+00
-5e-04
0.04
0.06
0.08
0.10
SNP effect on || id:ebi-a-GCST90017051
t on Non-cancer illness code, self-reported: gastric/stomach ulcers || id:ukb-b-20078

Figure 46 Leave-one-out analysis, funnel plot, MR effect size and scatter plot for gut microbiota abundance (genus Ruminococcaceae NK4A214 group id.11358) on gastric ulcer

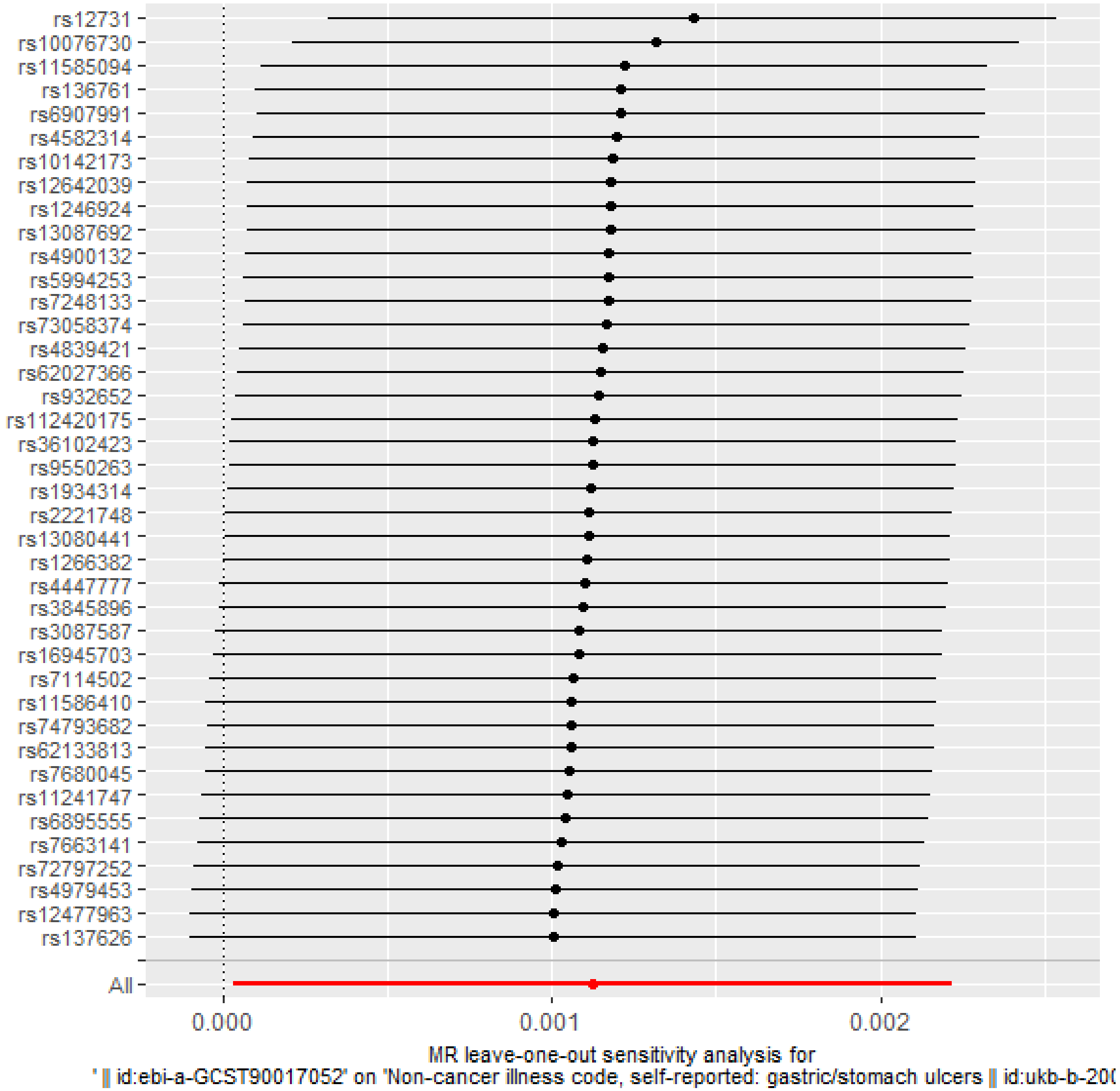

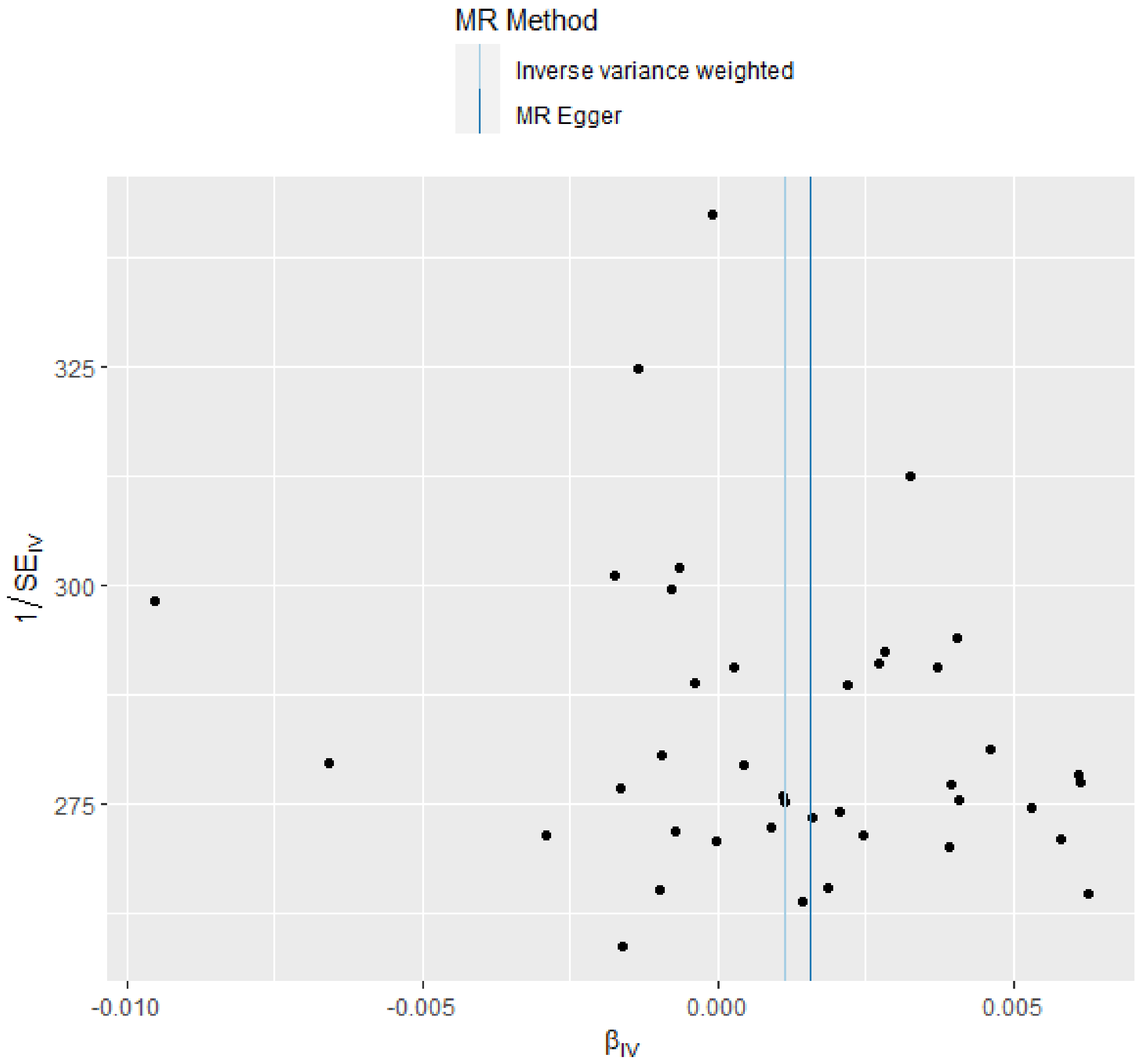
MR Method
Inverse variance weighted
MR Egger
325
300
275
1/SE$_{IV}$
-0.010
-0.005
0.000
0.005
β$_{IV}$

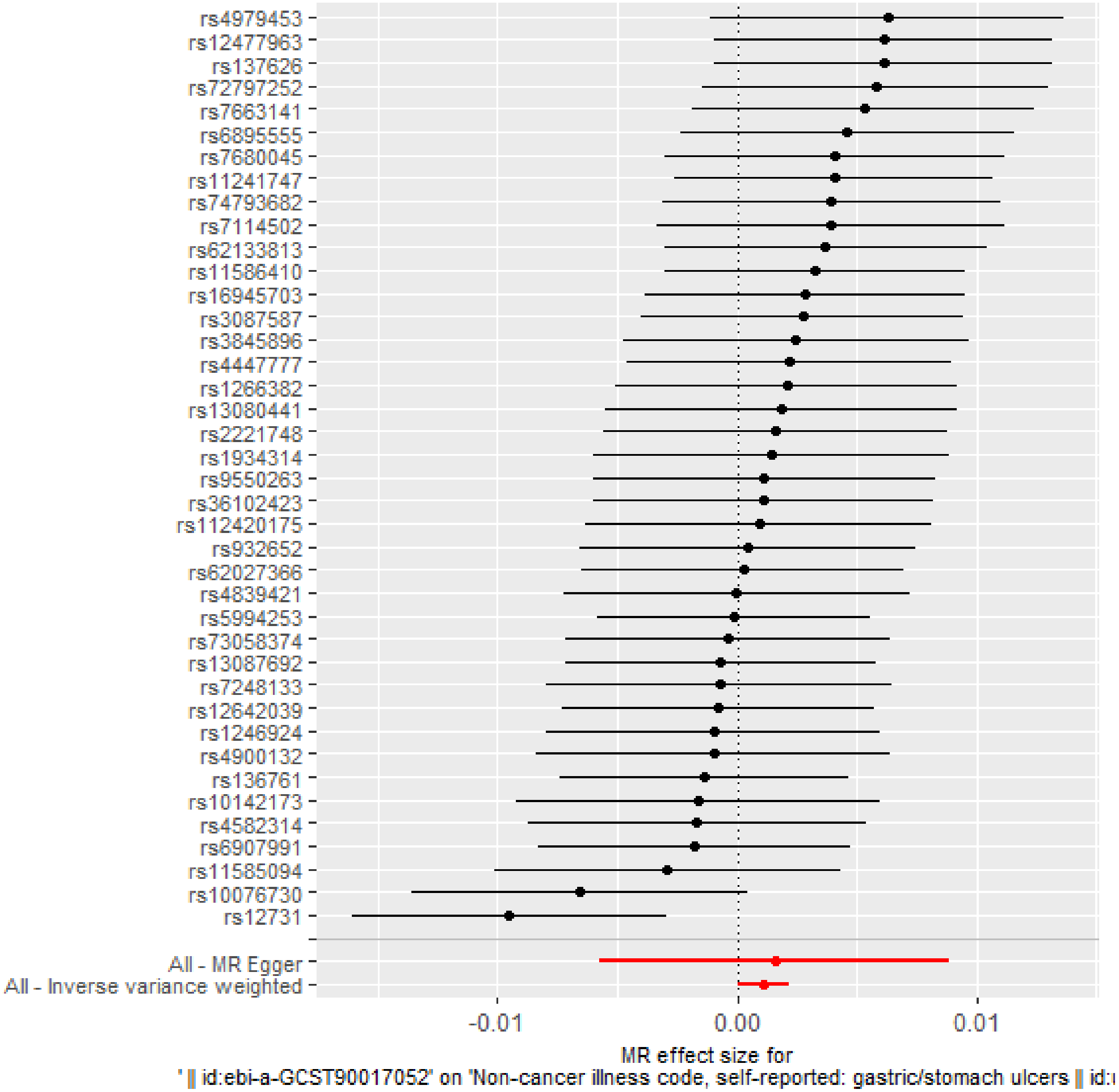

rs4979453
rs12477963
rs137626
rs72797252
rs7663141
rs6895555
rs7680045
rs11241747
rs74793682
rs7114502
rs62133813
rs11586410
rs16945703
rs3087587
rs3845896
rs4447777
rs1266382
rs13080441
rs2221748
rs1934314
rs9550263
rs36102423
rs112420175
rs932652
rs62027366
rs4839421
rs5994253
rs73058374
rs13087692
rs7248133
rs12642039
rs1246924
rs4900132
rs136761
rs10142173
rs4582314
rs6907991
rs11585094
rs10076730
rs12731
All - MR Egger
All - Inverse variance weighted
-0.01
0.00
0.01
MR effect size for
' || id:ebi-a-GCST90017052' on 'Non-cancer illness code, self-reported: gastric/stomach ulcers || id:

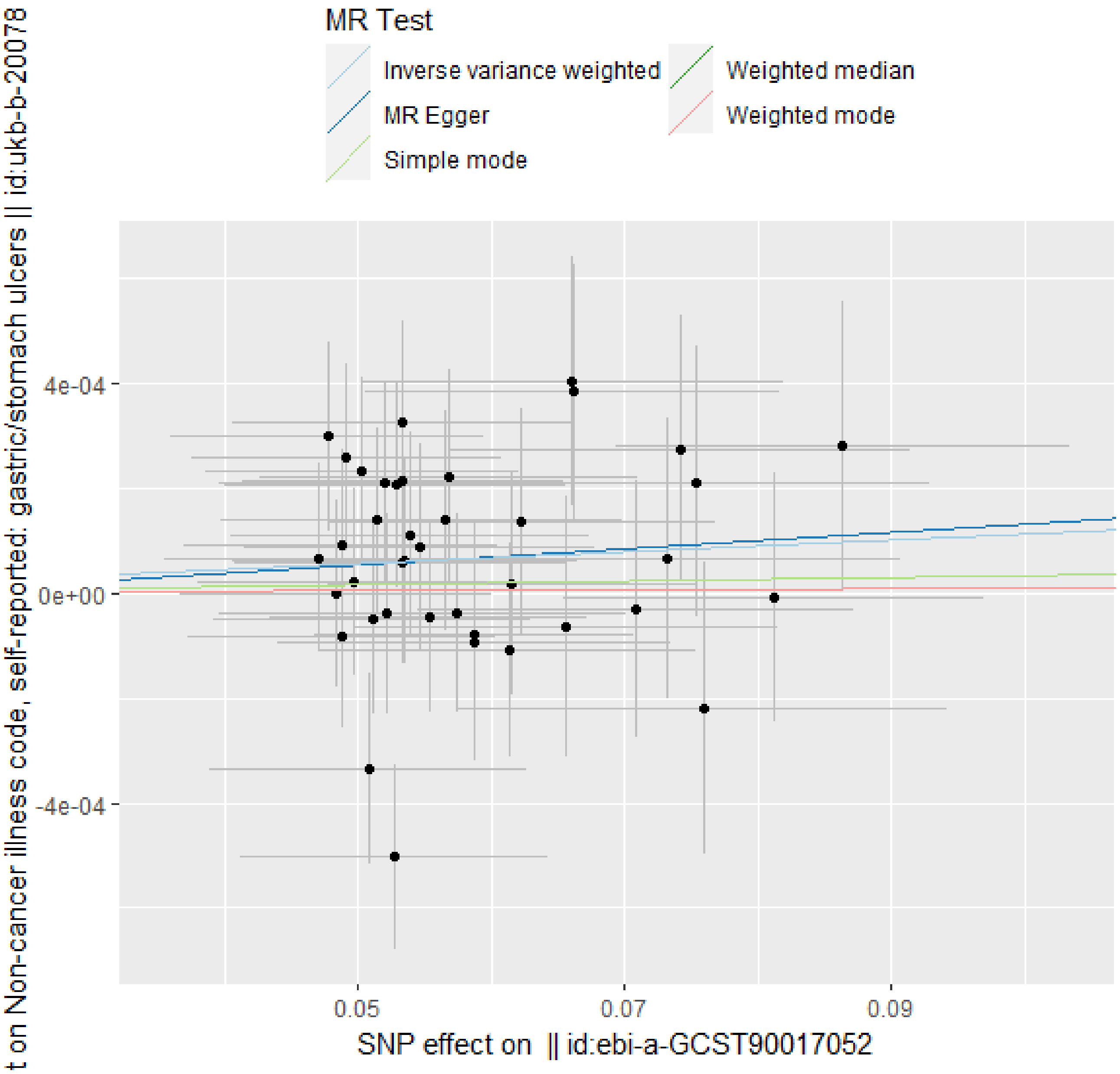

MR Test
Inverse variance weighted
MR Egger
Simple mode
Weighted median
Weighted mode
4e-04
0e+00
-4e-04
0.05
0.07
0.09
SNP effect on || id:ebi-a-GCST90017052
t on Non-cancer illness code, self-reported: gastric/stomach ulcers || id:ukb-b-20078

Figure 47 Leave-one-out analysis, funnel plot, MR effect size and scatter plot for gut microbiota abundance (genus Ruminococcus gauvreauii group id.11342) on gastric ulcer

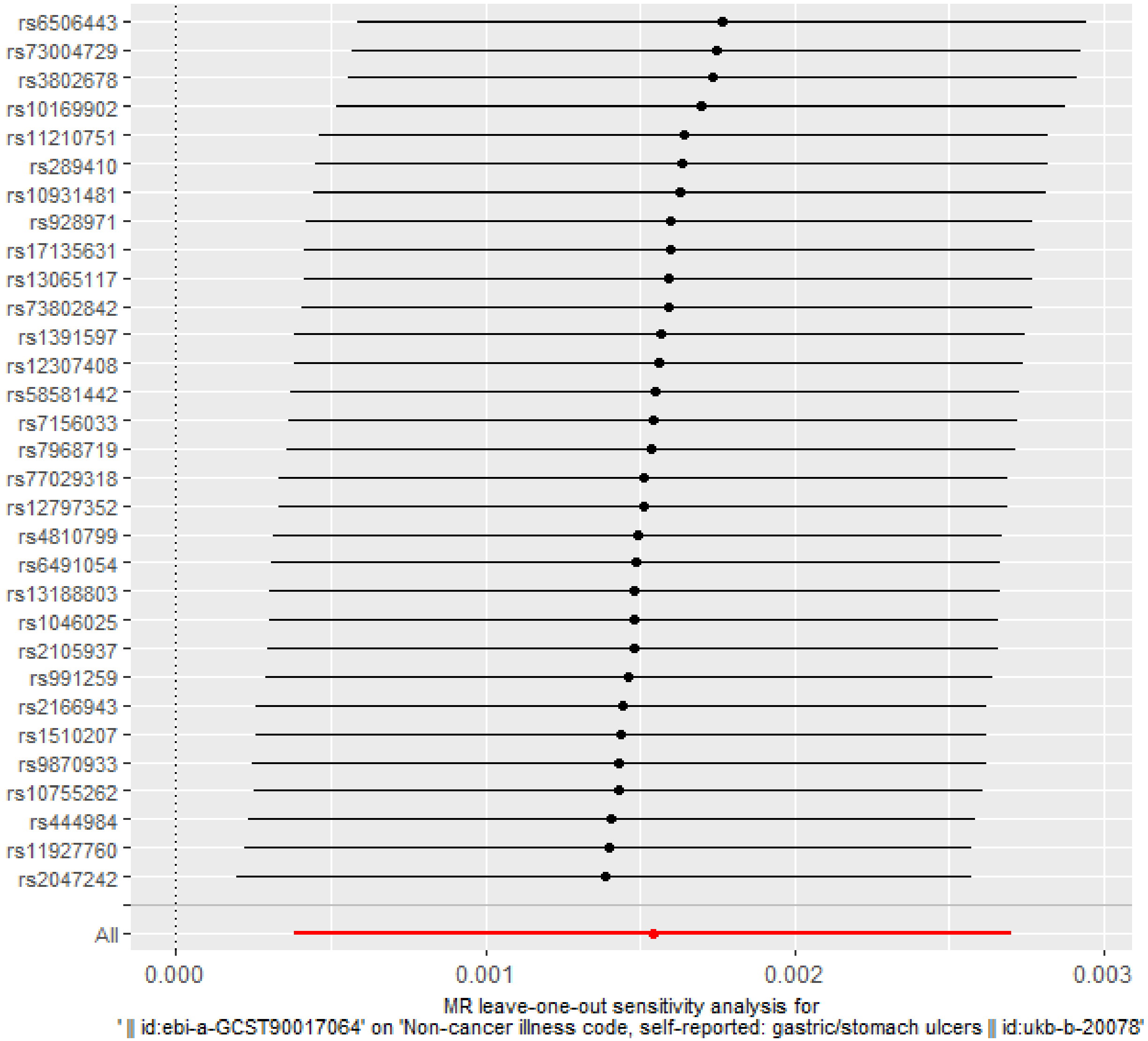

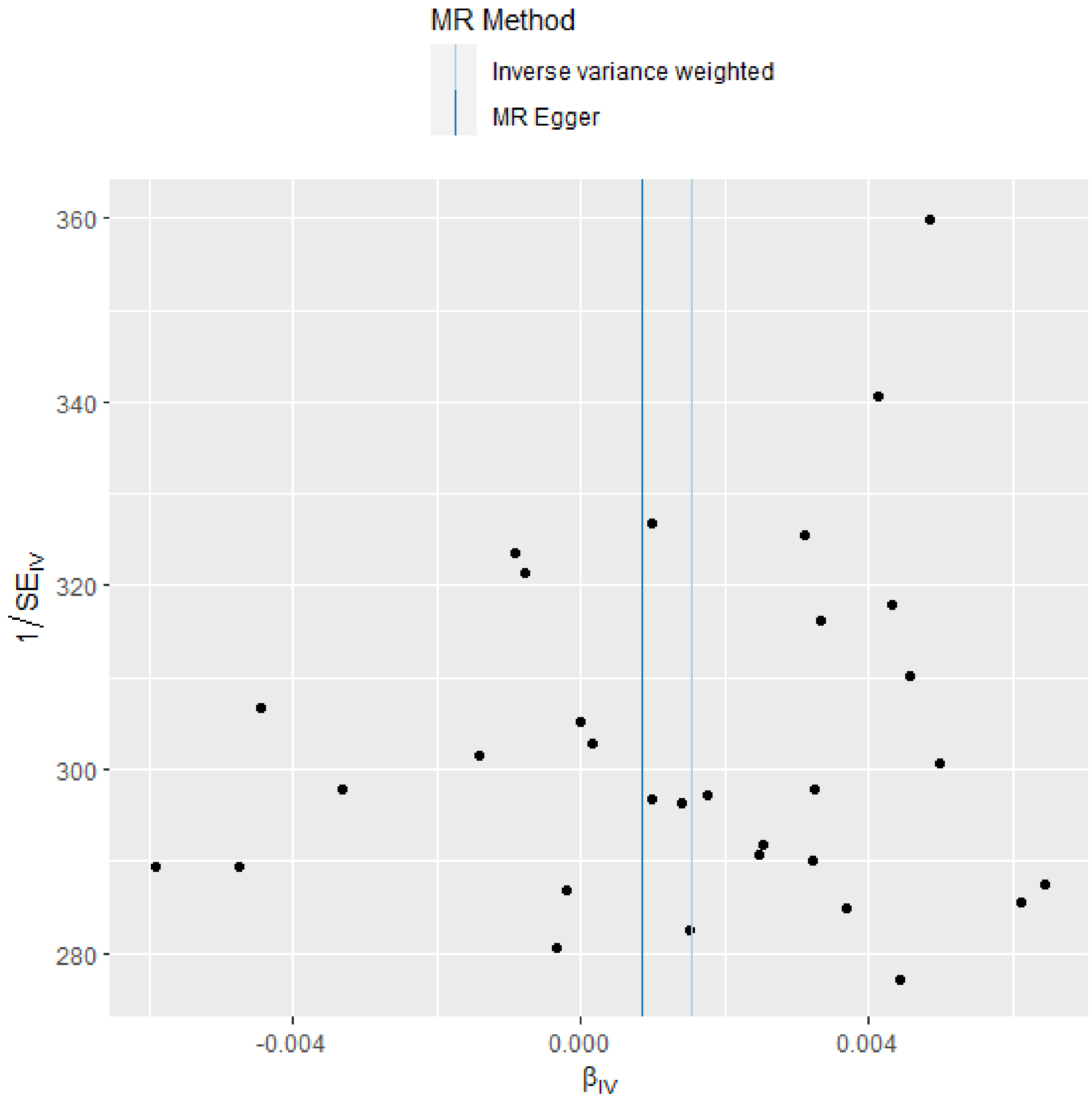

MR Method
Inverse variance weighted
MR Egger
360
340
320
300
280
$1/SE_{IV}$
-0.004
0.000
0.004
β_{IV}

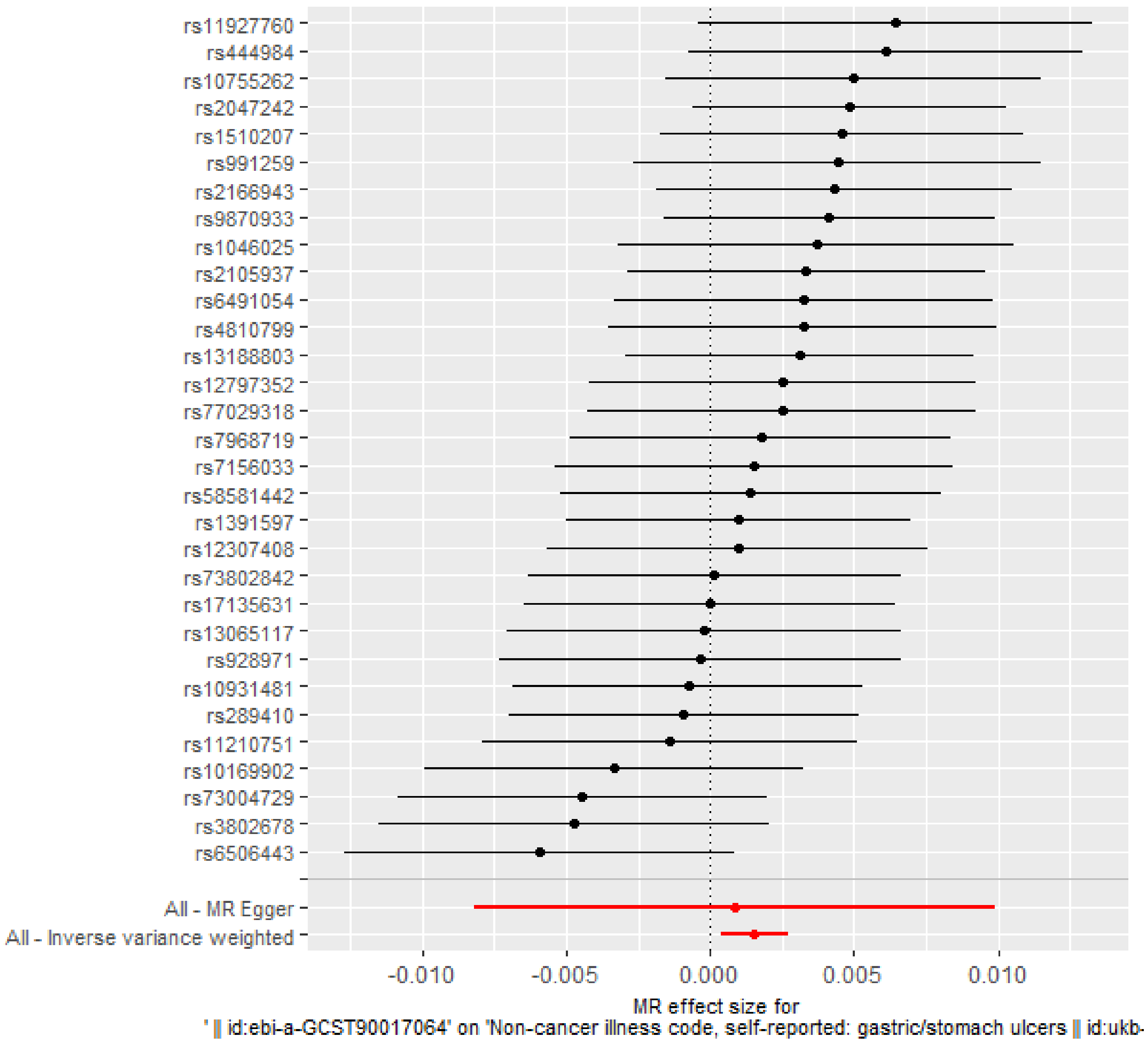

rs11927760
rs444984
rs10755262
rs2047242
rs1510207
rs991259
rs2166943
rs9870933
rs1046025
rs2105937
rs6491054
rs4810799
rs13188803
rs12797352
rs77029318
rs7968719
rs7156033
rs58581442
rs1391597
rs12307408
rs73802842
rs17135631
rs13065117
rs928971
rs10931481
rs289410
rs11210751
rs10169902
rs73004729
rs3802678
rs6506443
All - MR Egger
All - Inverse variance weighted
-0.010
-0.005
0.000
0.005
0.010
MR effect size for
' || id:ebi-a-GCST90017064' on 'Non-cancer illness code, self-reported: gastric/stomach ulcers || id:ukb-

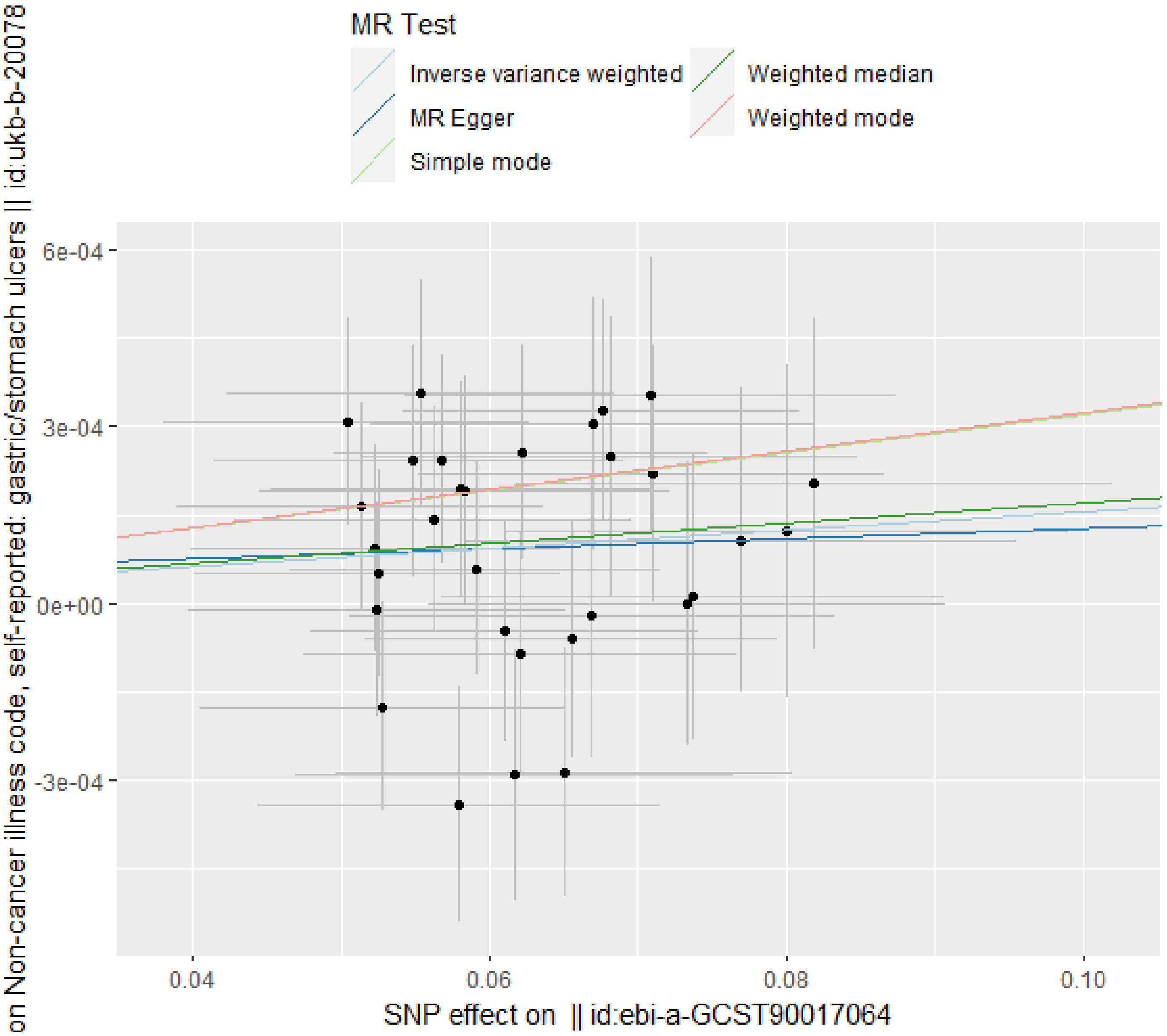

MR Test
Inverse variance weighted
MR Egger
Simple mode
Weighted median
Weighted mode
6e-04
3e-04
0e+00
-3e-04
0.04
0.06
0.08
0.10
SNP effect on || id:ebi-a-GCST90017064
on Non-cancer illness code, self-reported: gastric/stomach ulcers || id:ukb-b-20078

Figure 48 Leave-one-out analysis, funnel plot, MR effect size and scatter plot for gut microbiota abundance (genus Ruminococcus gnavus group id.14376) on gastric ulcer

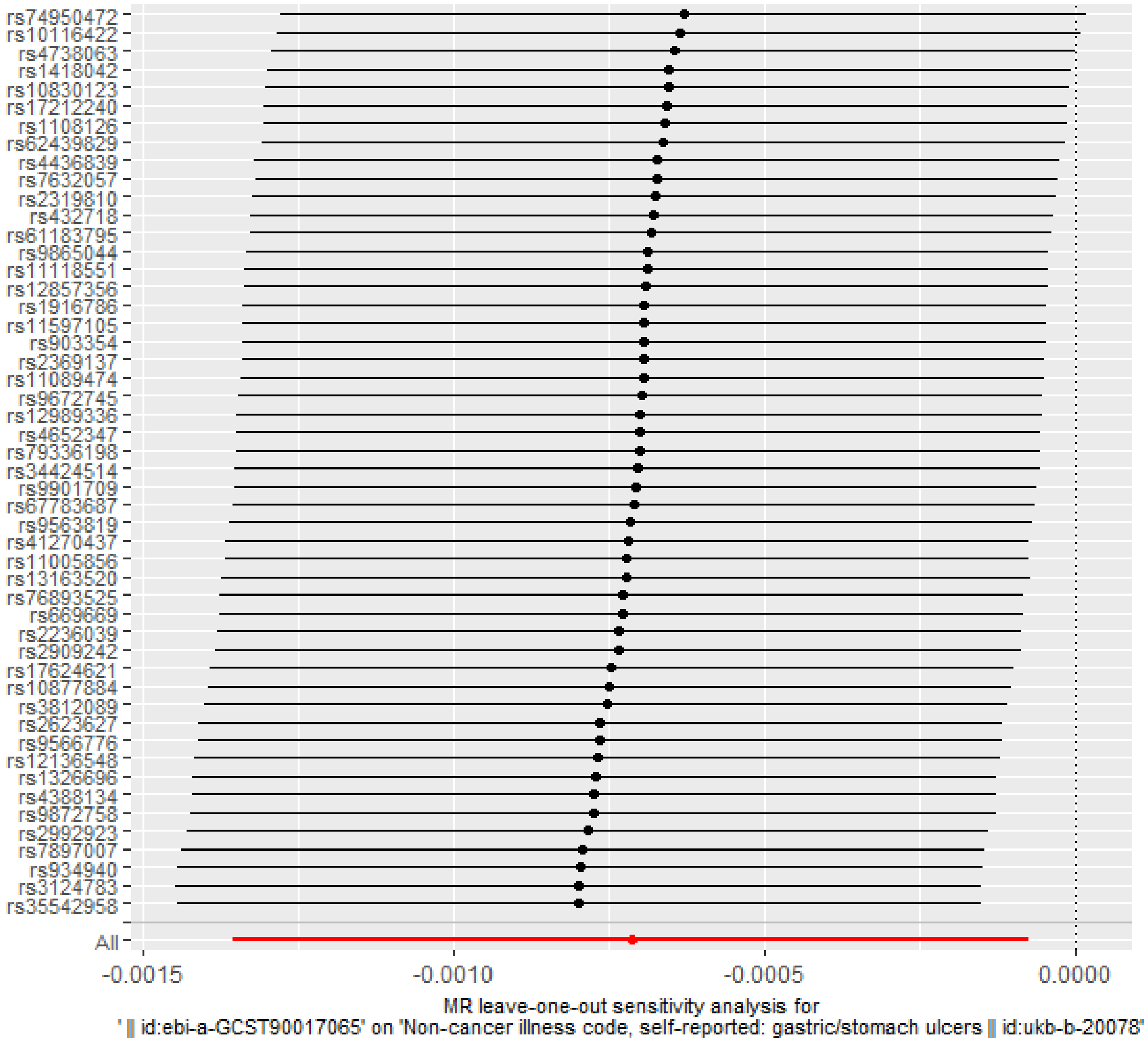

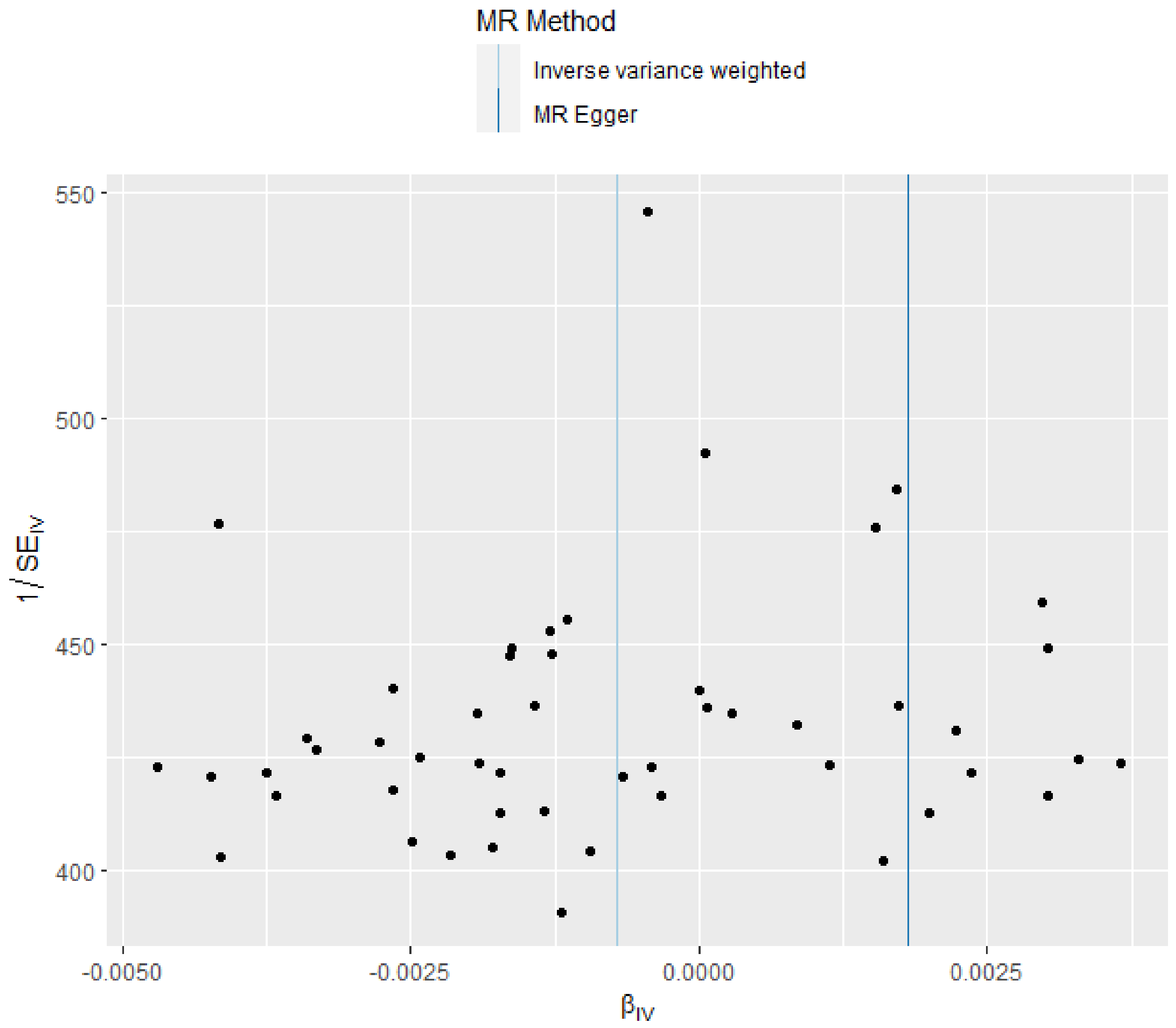

MR Method
Inverse variance weighted
MR Egger
550
500
450
400
1/SE$_{IV}$
-0.0050
-0.0025
0.0000
0.0025
β$_{IV}$

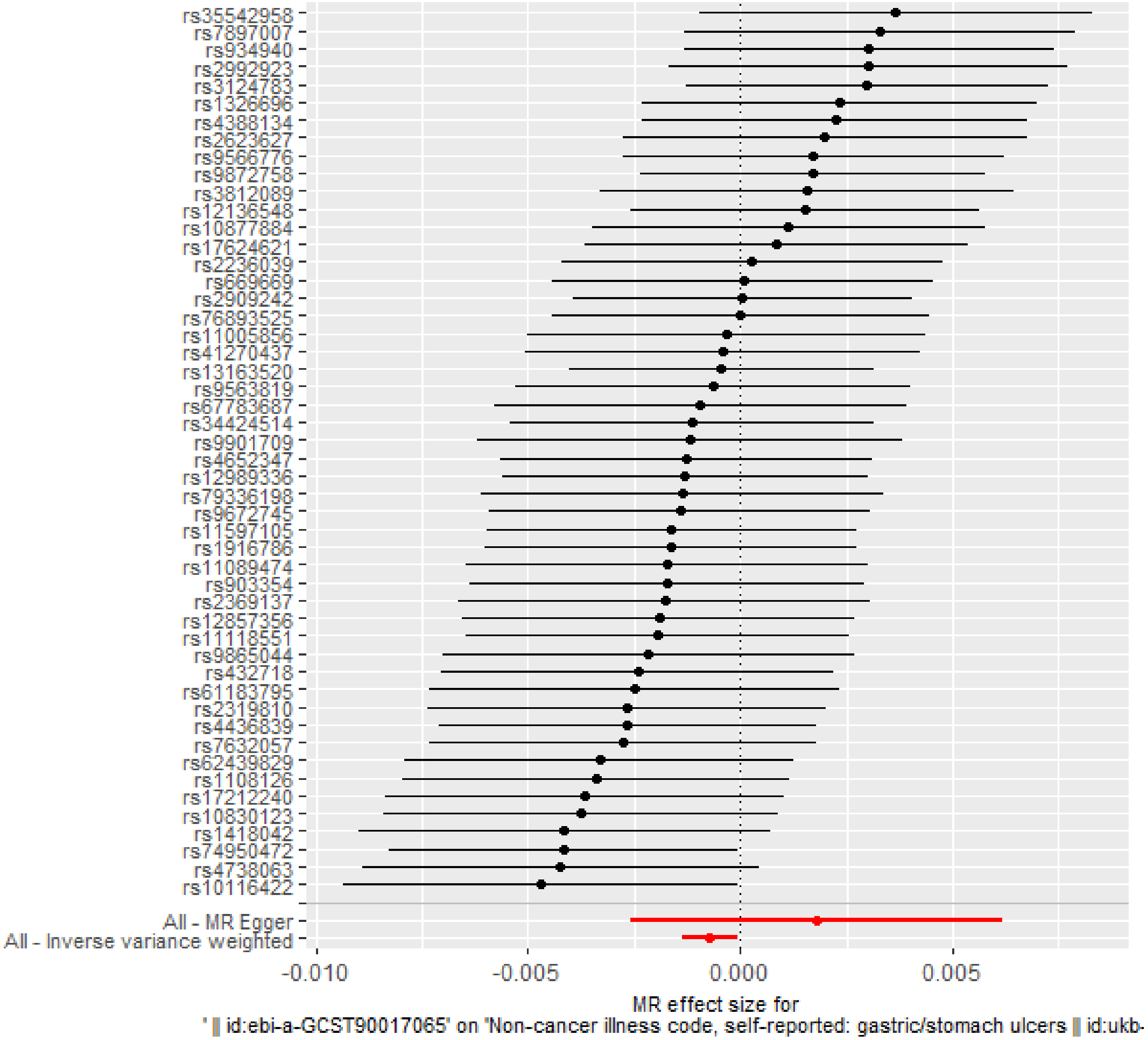

rs35542958
rs7897007
rs934940
rs2992923
rs3124783
rs1326696
rs4388134
rs2623627
rs9566776
rs9872758
rs3812089
rs12136548
rs10877884
rs17624621
rs2236039
rs669669
rs2909242
rs76893525
rs11005856
rs41270437
rs13163520
rs9563819
rs67783687
rs34424514
rs9901709
rs4652347
rs12989336
rs79336198
rs9672745
rs11597105
rs1916786
rs11089474
rs903354
rs2369137
rs12857356
rs11118551
rs9865044
rs432718
rs61183795
rs2319810
rs4436839
rs7632057
rs62439829
rs1108126
rs17212240
rs10830123
rs1418042
rs74950472
rs4738063
rs10116422
All - MR Egger
All - Inverse variance weighted
-0.010
-0.005
0.000
0.005
MR effect size for
' || id:ebi-a-GCST90017065' on 'Non-cancer illness code, self-reported: gastric/stomach ulcers || id:ukb-

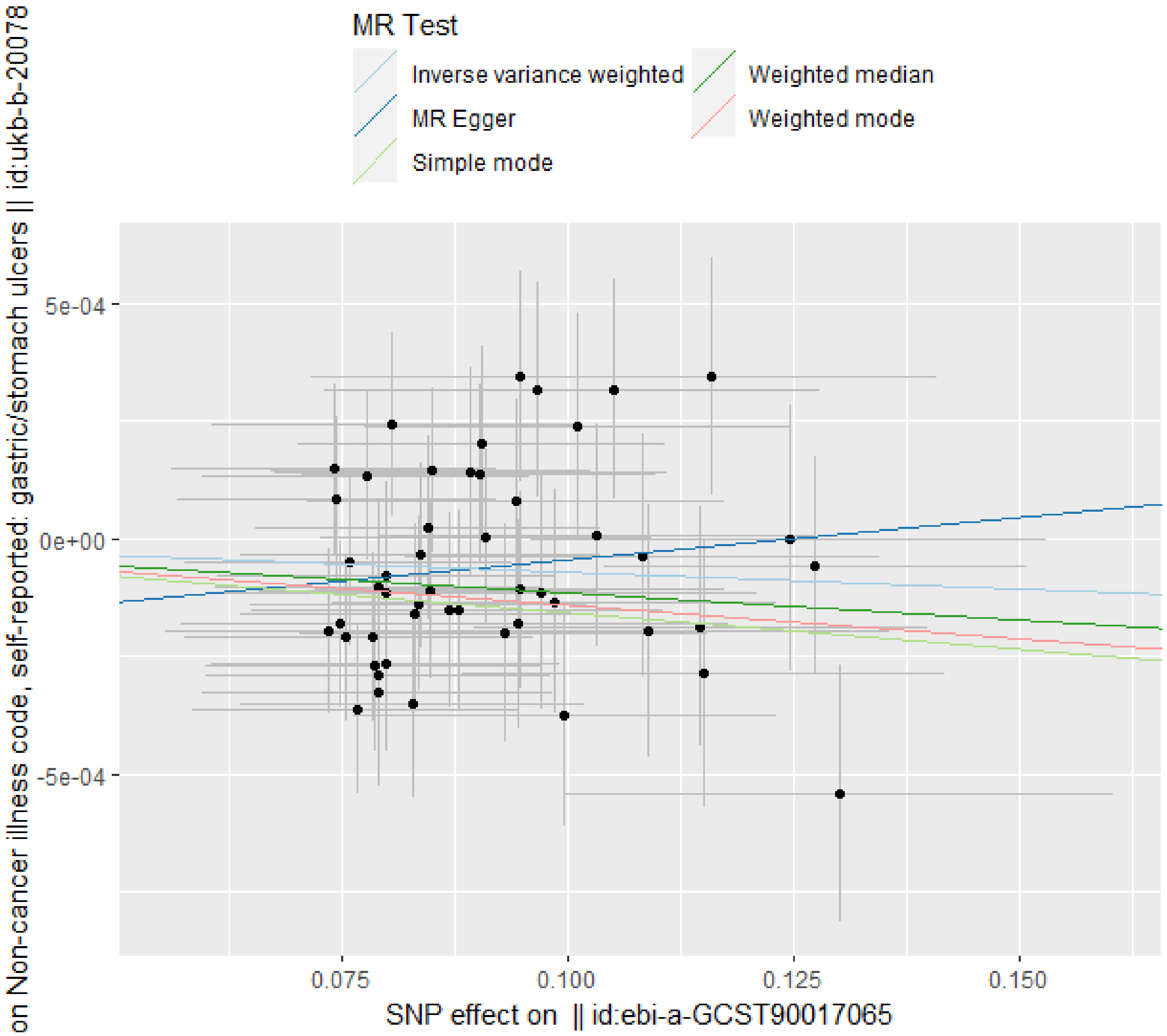

MR Test
Inverse variance weighted
MR Egger
Simple mode
Weighted median
Weighted mode
5e-04
0e+00
-5e-04
0.075
0.100
0.125
0.150
SNP effect on || id:ebi-a-GCST90017065
on Non-cancer illness code, self-reported: gastric/stomach ulcers || id:ukb-b-20078

Figure 49 Leave-one-out analysis, funnel plot, MR effect size and scatter plot for gut microbiota abundance (genus Sutterella id.2896) on gastric ulcer

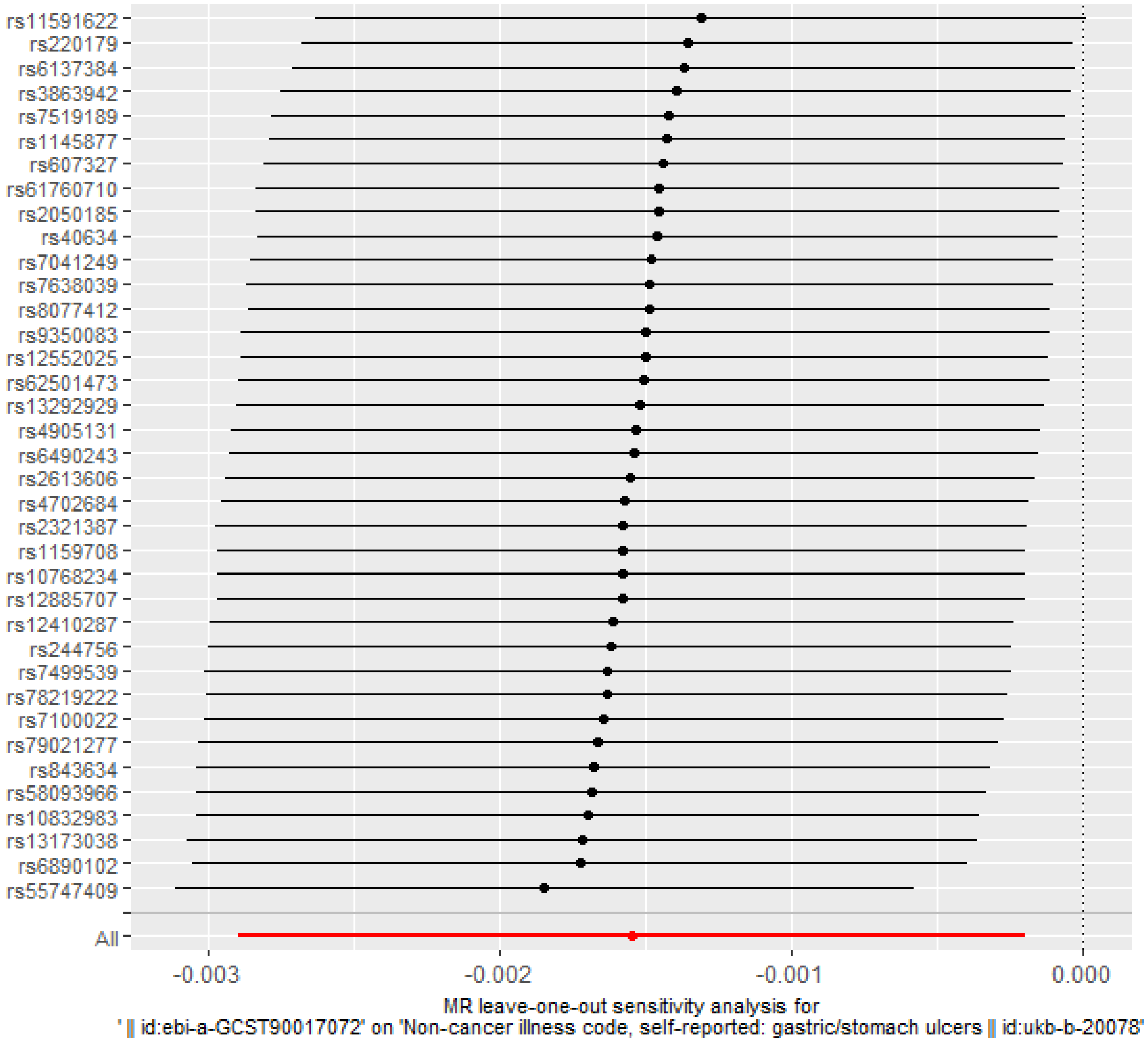

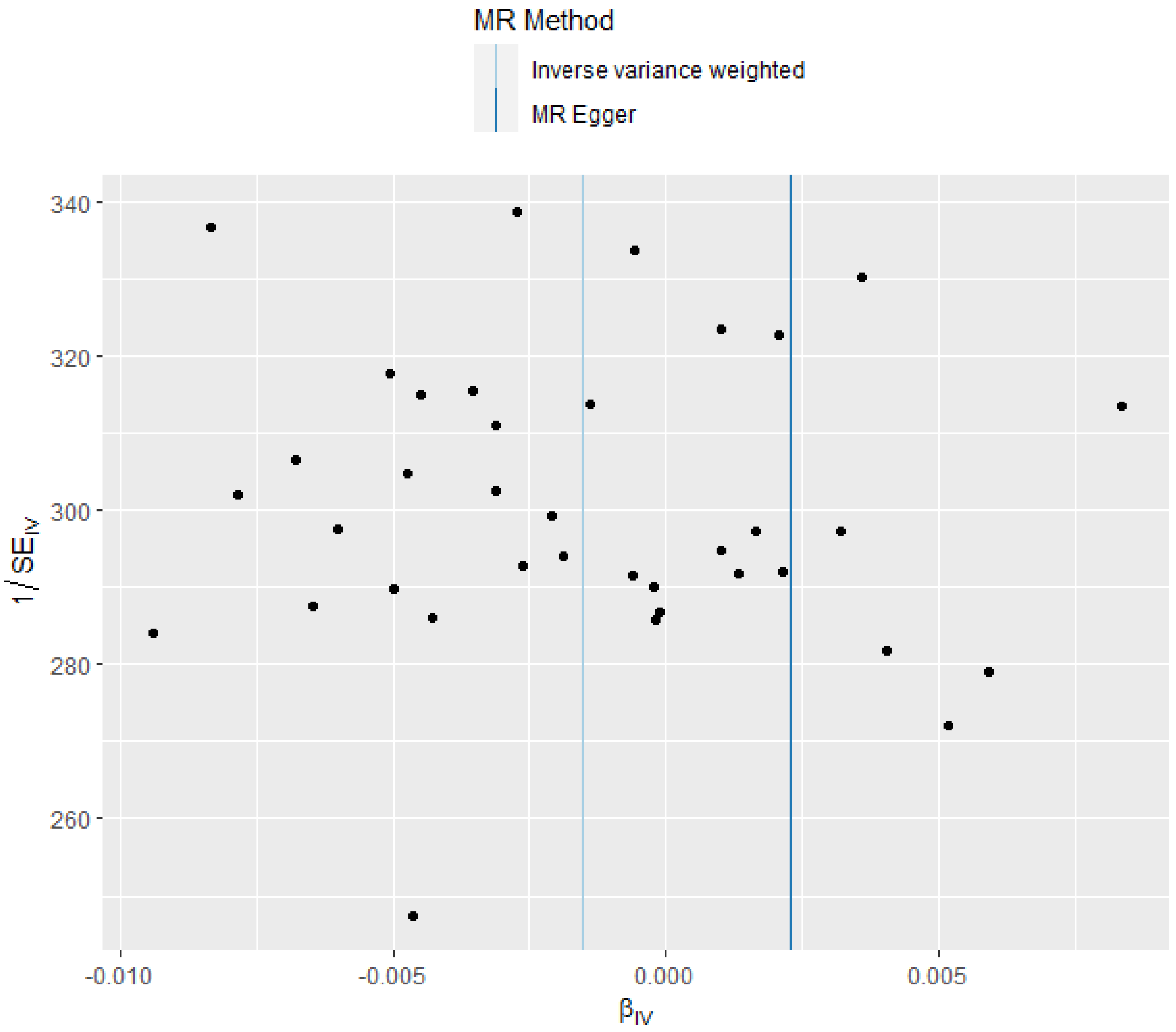
MR Method
Inverse variance weighted
MR Egger
340
320
300
280
260
1/SE_IV
-0.010
-0.005
0.000
0.005
β_IV

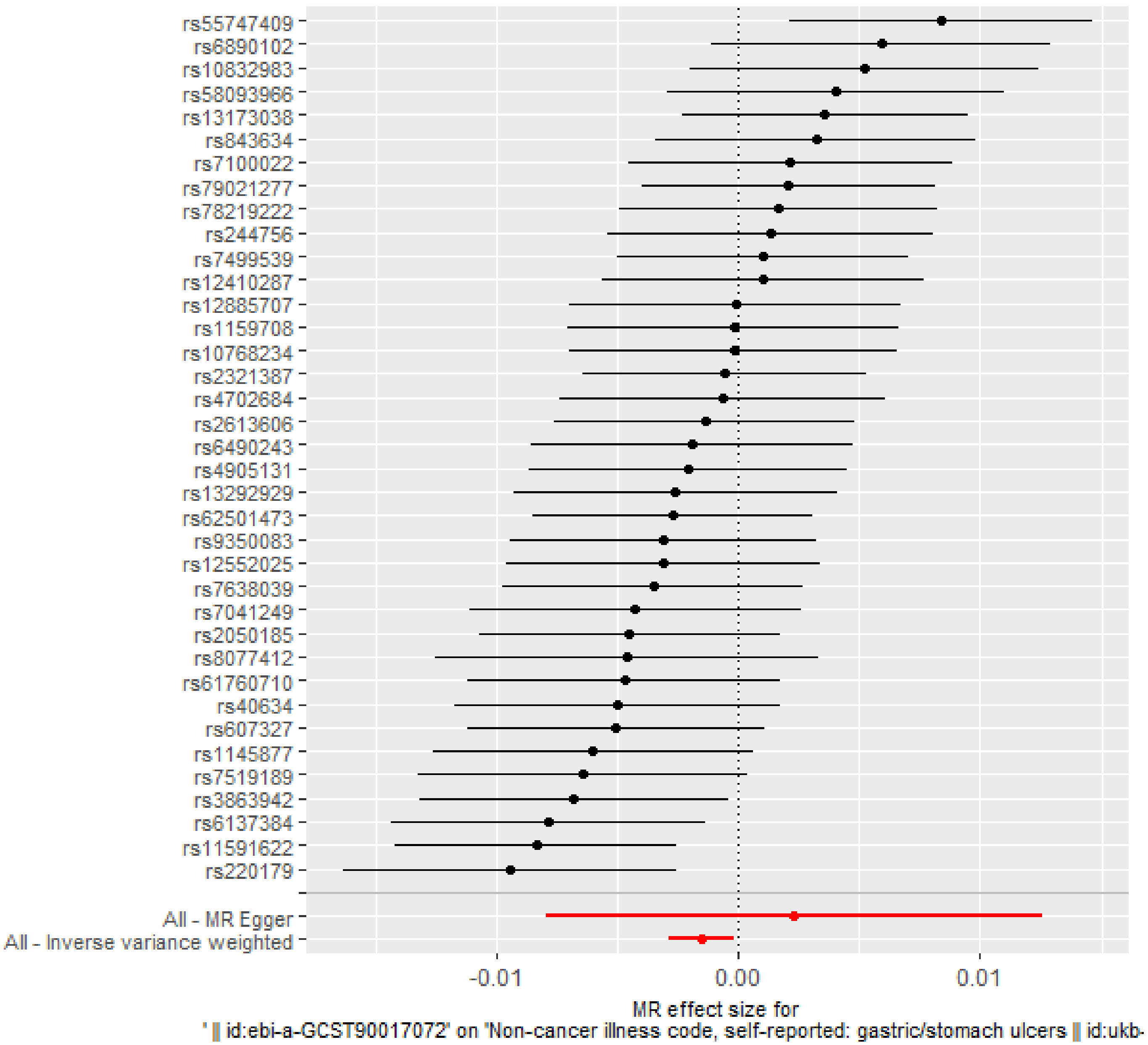
rs55747409
rs6890102
rs10832983
rs58093966
rs13173038
rs843634
rs7100022
rs79021277
rs78219222
rs244756
rs7499539
rs12410287
rs12885707
rs1159708
rs10768234
rs2321387
rs4702684
rs2613606
rs6490243
rs4905131
rs13292929
rs62501473
rs9350083
rs12552025
rs7638039
rs7041249
rs2050185
rs8077412
rs61760710
rs40634
rs607327
rs1145877
rs7519189
rs3863942
rs6137384
rs11591622
rs220179
All - MR Egger
All - Inverse variance weighted
-0.01
0.00
0.01
MR effect size for
' || id:ebi-a-GCST90017072' on 'Non-cancer illness code, self-reported: gastric/stomach ulcers || id:ukb-

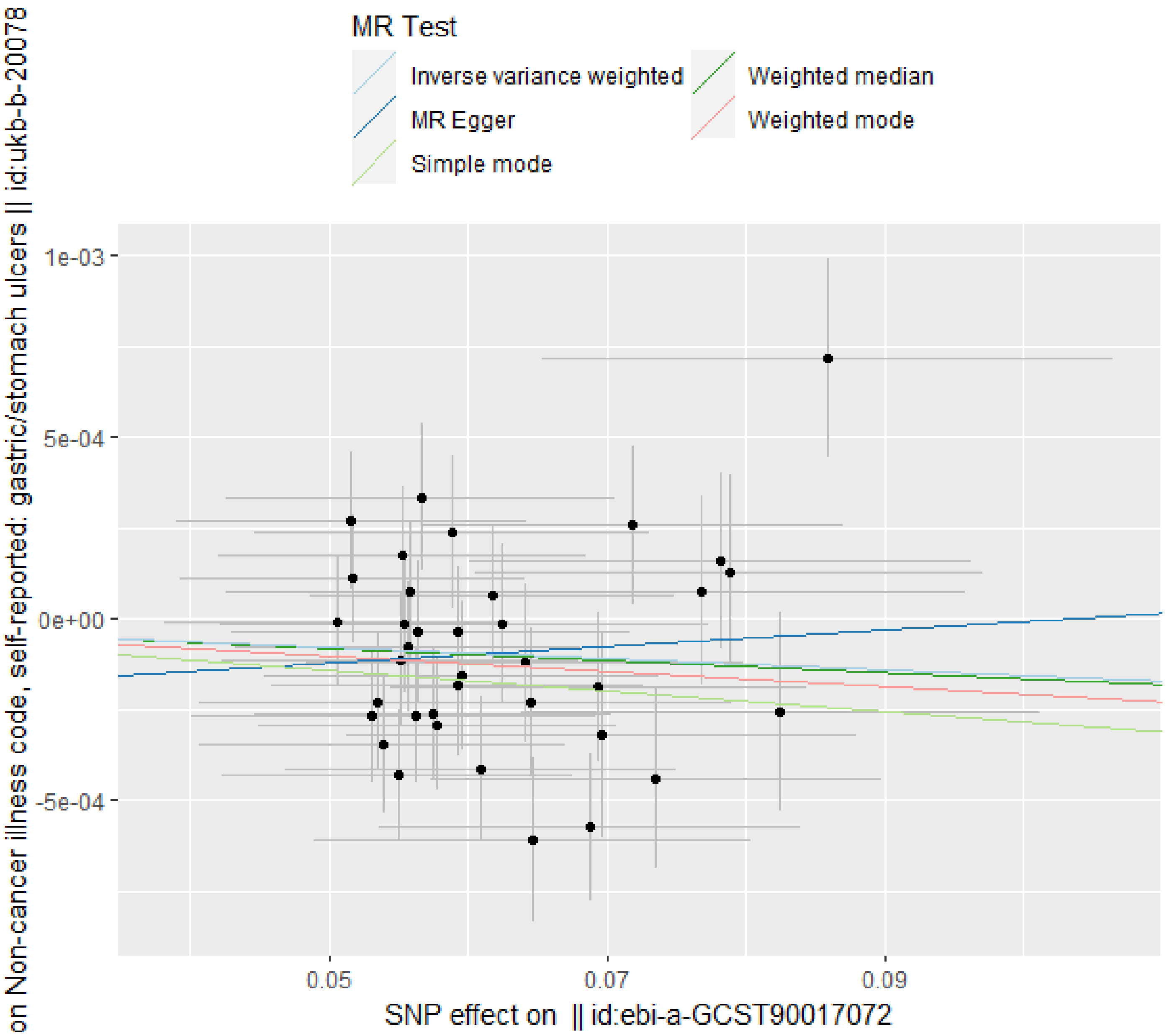

MR Test
Inverse variance weighted
MR Egger
Simple mode
Weighted median
Weighted mode
1e-03
5e-04
0e+00
-5e-04
0.05
0.07
0.09
SNP effect on || id:ebi-a-GCST90017072
on Non-cancer illness code, self-reported: gastric/stomach ulcers || id:ukb-b-20078

Figure 50 Leave-one-out analysis, funnel plot, MR effect size and scatter plot for gut microbiota abundance (order Bacteroidales id.913) on gastric ulcer

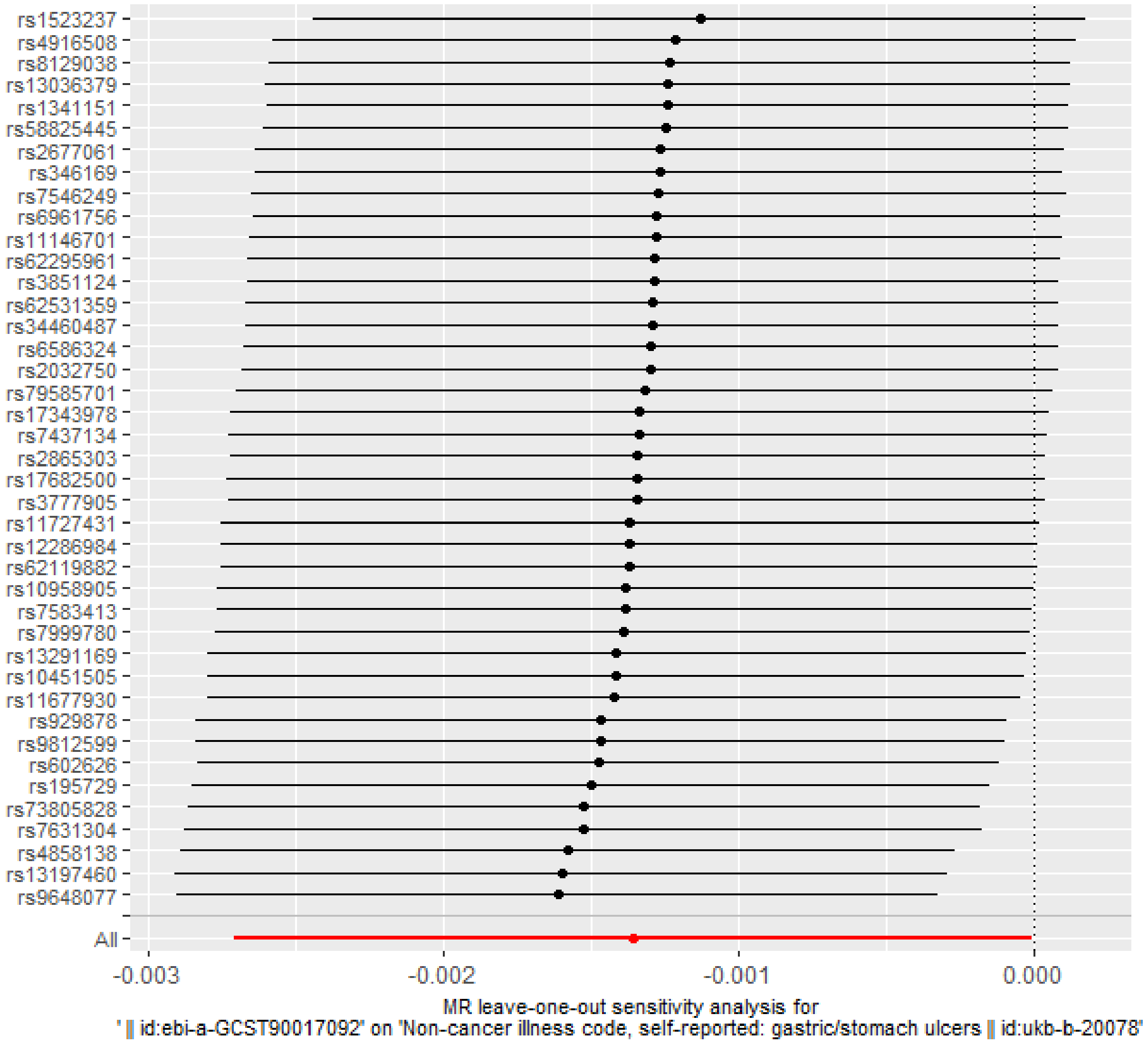

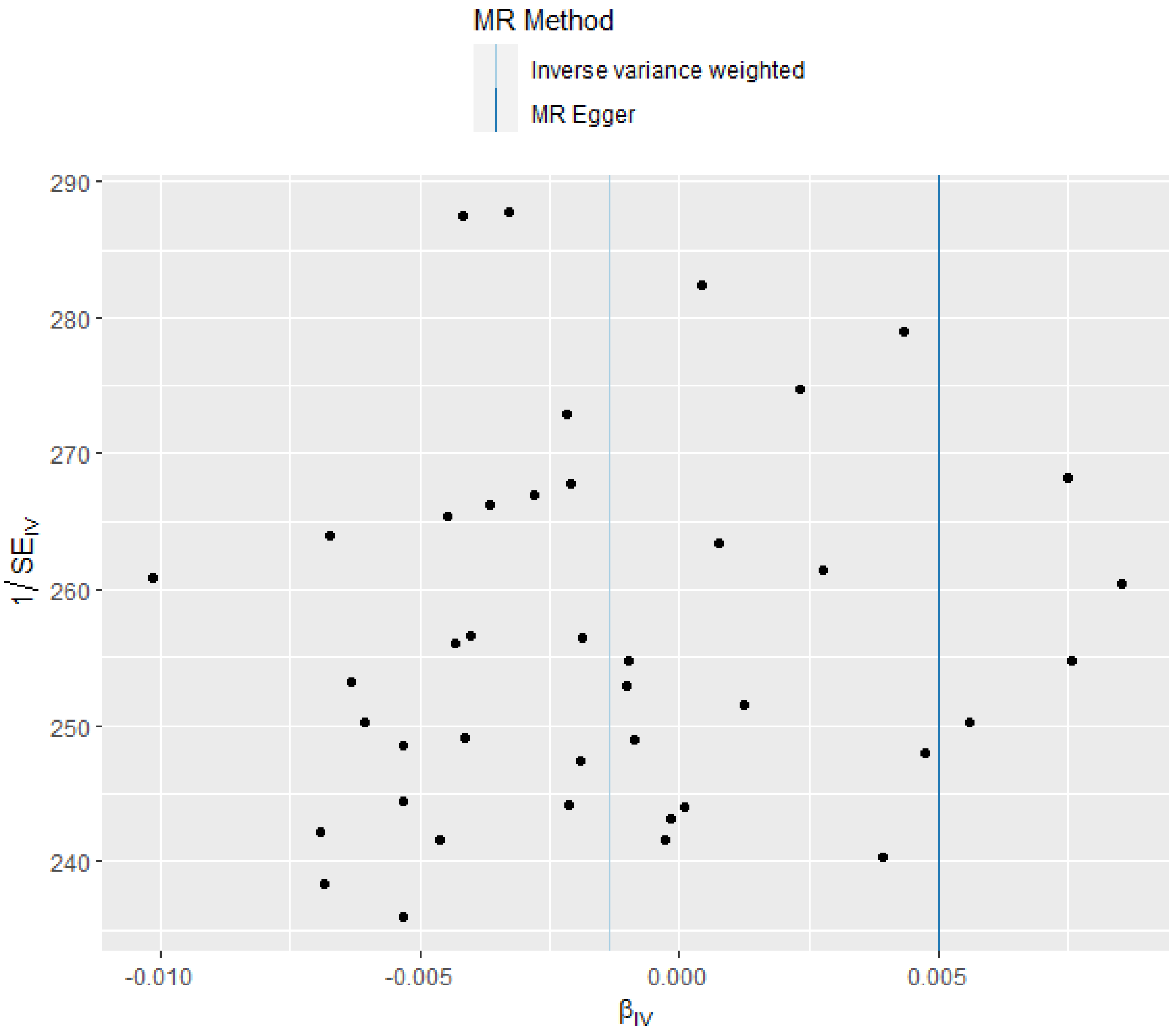

MR Method
Inverse variance weighted
MR Egger
290
280
270
260
250
240
1/SE_IV
-0.010
-0.005
0.000
0.005
β_IV

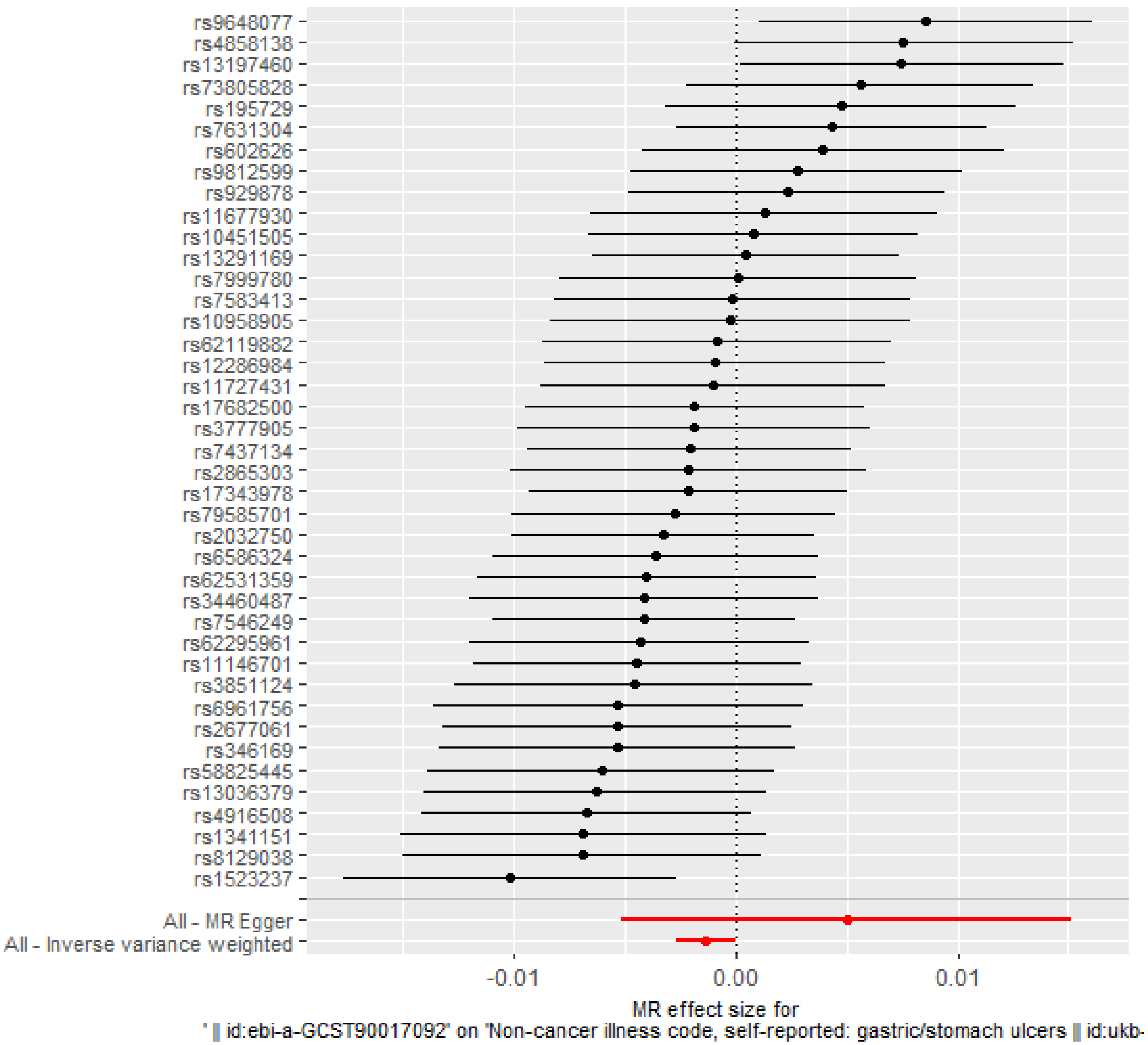

rs9648077
rs4858138
rs13197460
rs73805828
rs195729
rs7631304
rs602626
rs9812599
rs929878
rs11677930
rs10451505
rs13291169
rs7999780
rs7583413
rs10958905
rs62119882
rs12286984
rs11727431
rs17682500
rs3777905
rs7437134
rs2865303
rs17343978
rs79585701
rs2032750
rs6586324
rs62531359
rs34460487
rs7546249
rs62295961
rs11146701
rs3851124
rs6961756
rs2677061
rs346169
rs58825445
rs13036379
rs4916508
rs1341151
rs8129038
rs1523237
All - MR Egger
All - Inverse variance weighted
-0.01
0.00
0.01
MR effect size for
' || id:ebi-a-GCST90017092' on 'Non-cancer illness code, self-reported: gastric/stomach ulcers || id:ukb-

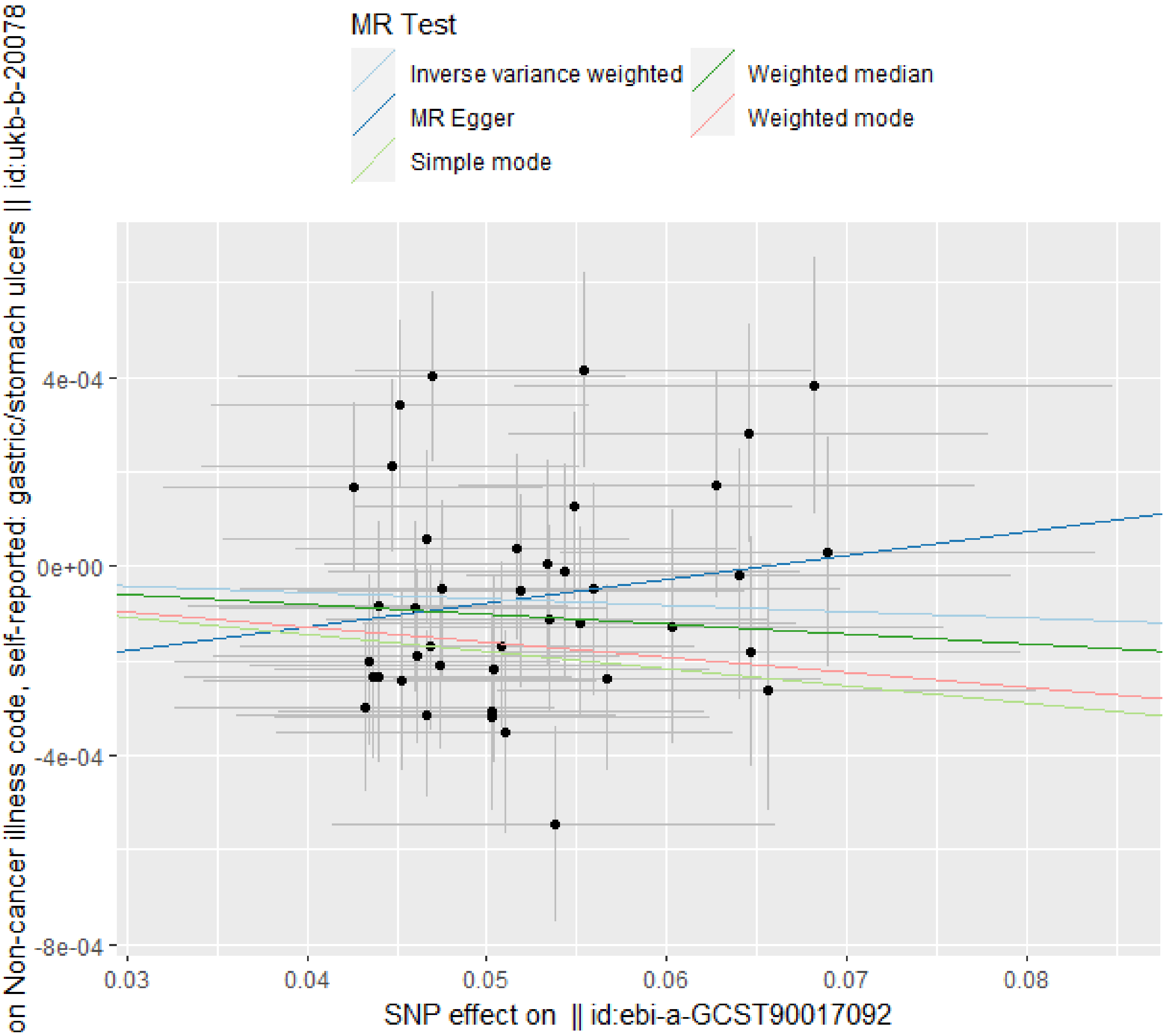

MR Test
Inverse variance weighted
MR Egger
Simple mode
Weighted median
Weighted mode
on Non-cancer illness code, self-reported: gastric/stomach ulcers || id:ukb-b-20078
4e-04
0e+00
-4e-04
-8e-04
0.03
0.04
0.05
0.06
0.07
0.08
SNP effect on || id:ebi-a-GCST90017092

Figure 51 Leave-one-out analysis, funnel plot, MR effect size and scatter plot for gut microbiota abundance (order Victivallales id.2254) on gastric ulcer

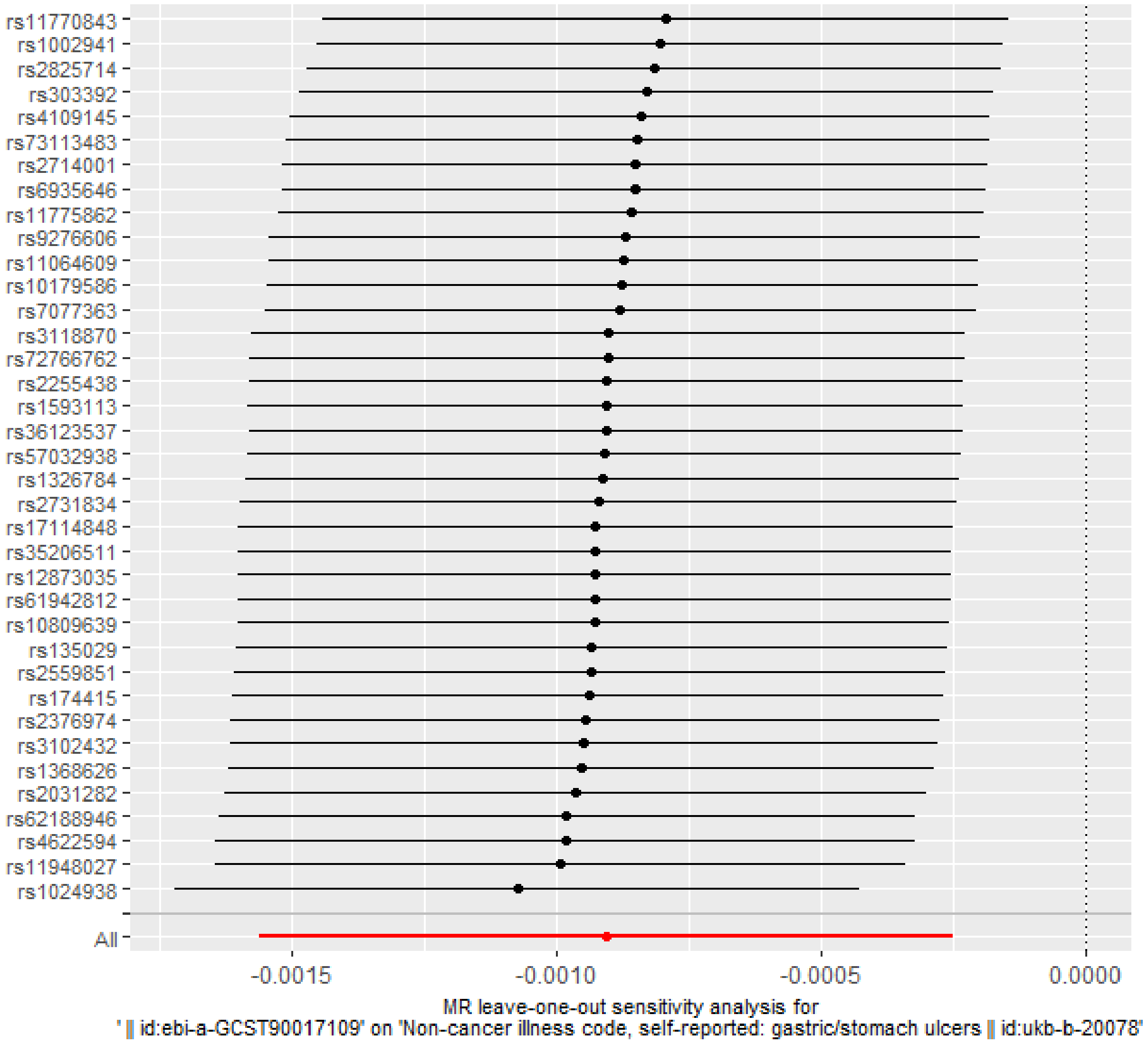

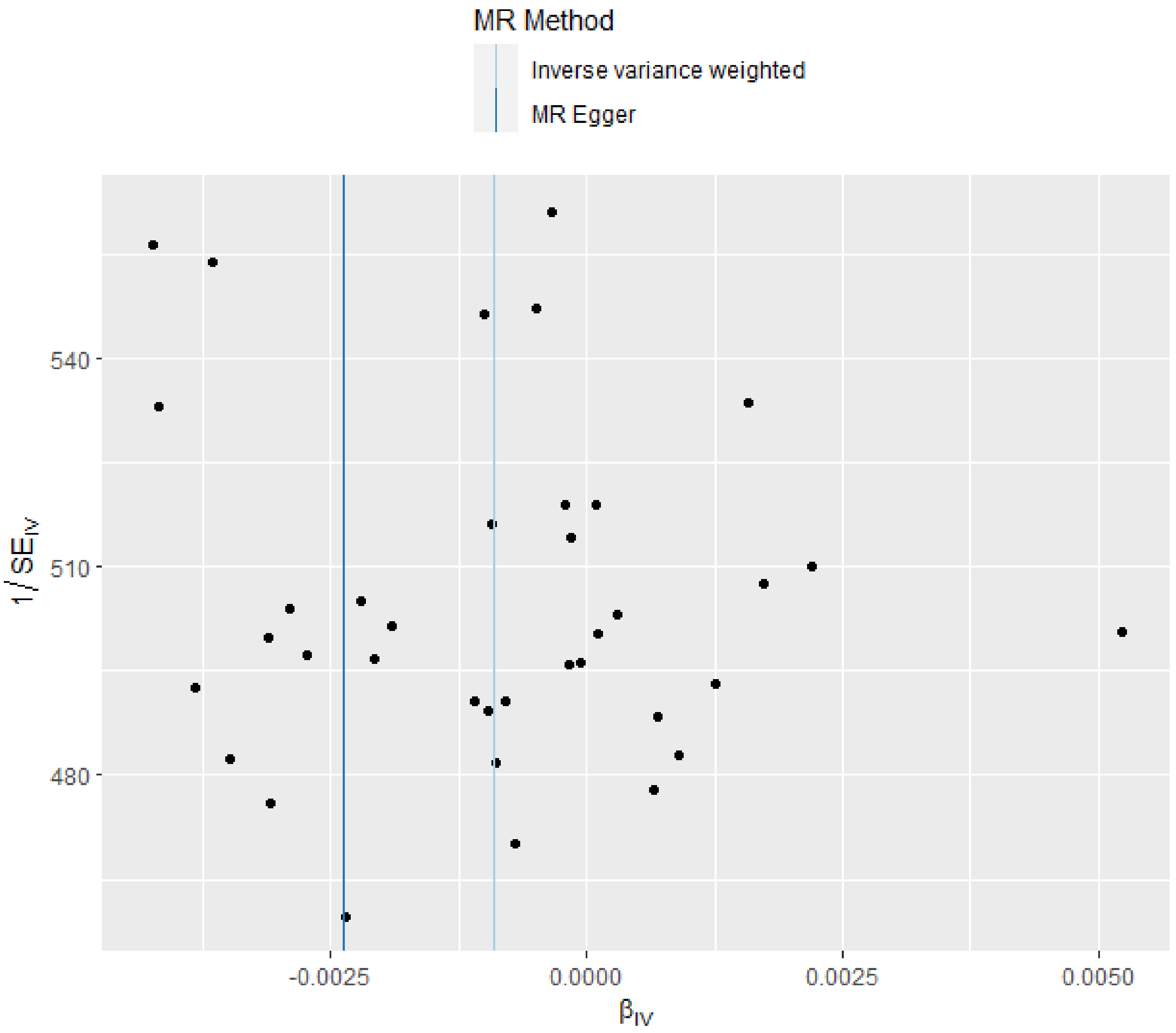

MR Method
Inverse variance weighted
MR Egger
540
510
480
1/SE_IV
-0.0025
0.0000
0.0025
0.0050
β_IV

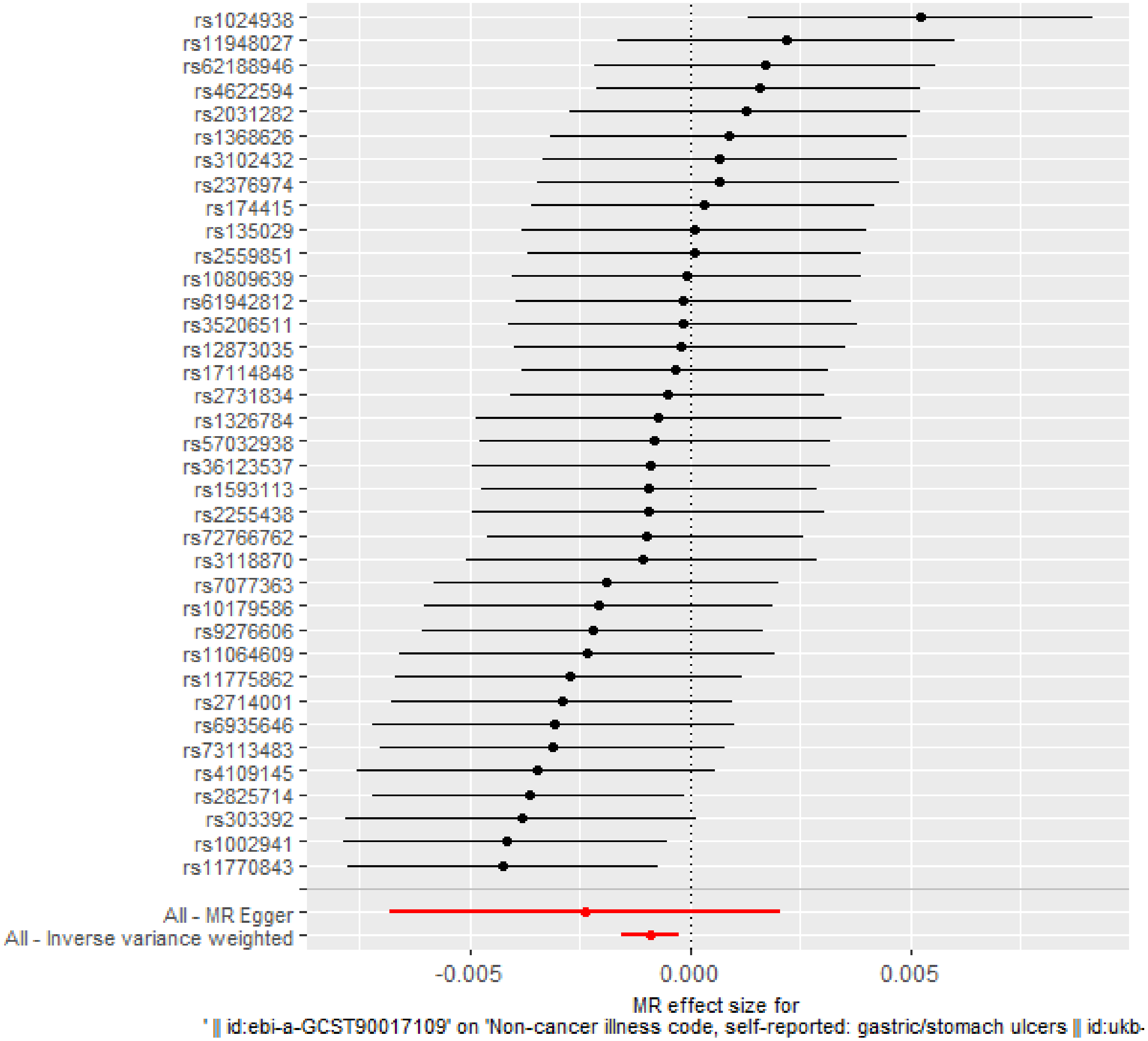

rs1024938
rs11948027
rs62188946
rs4622594
rs2031282
rs1368626
rs3102432
rs2376974
rs174415
rs135029
rs2559851
rs10809639
rs61942812
rs35206511
rs12873035
rs17114848
rs2731834
rs1326784
rs57032938
rs36123537
rs1593113
rs2255438
rs72766762
rs3118870
rs7077363
rs10179586
rs9276606
rs11064609
rs11775862
rs2714001
rs6935646
rs73113483
rs4109145
rs2825714
rs303392
rs1002941
rs11770843
All - MR Egger
All - Inverse variance weighted
-0.005
0.000
0.005
MR effect size for
' || id:ebi-a-GCST90017109' on 'Non-cancer illness code, self-reported: gastric/stomach ulcers || id:ukb-

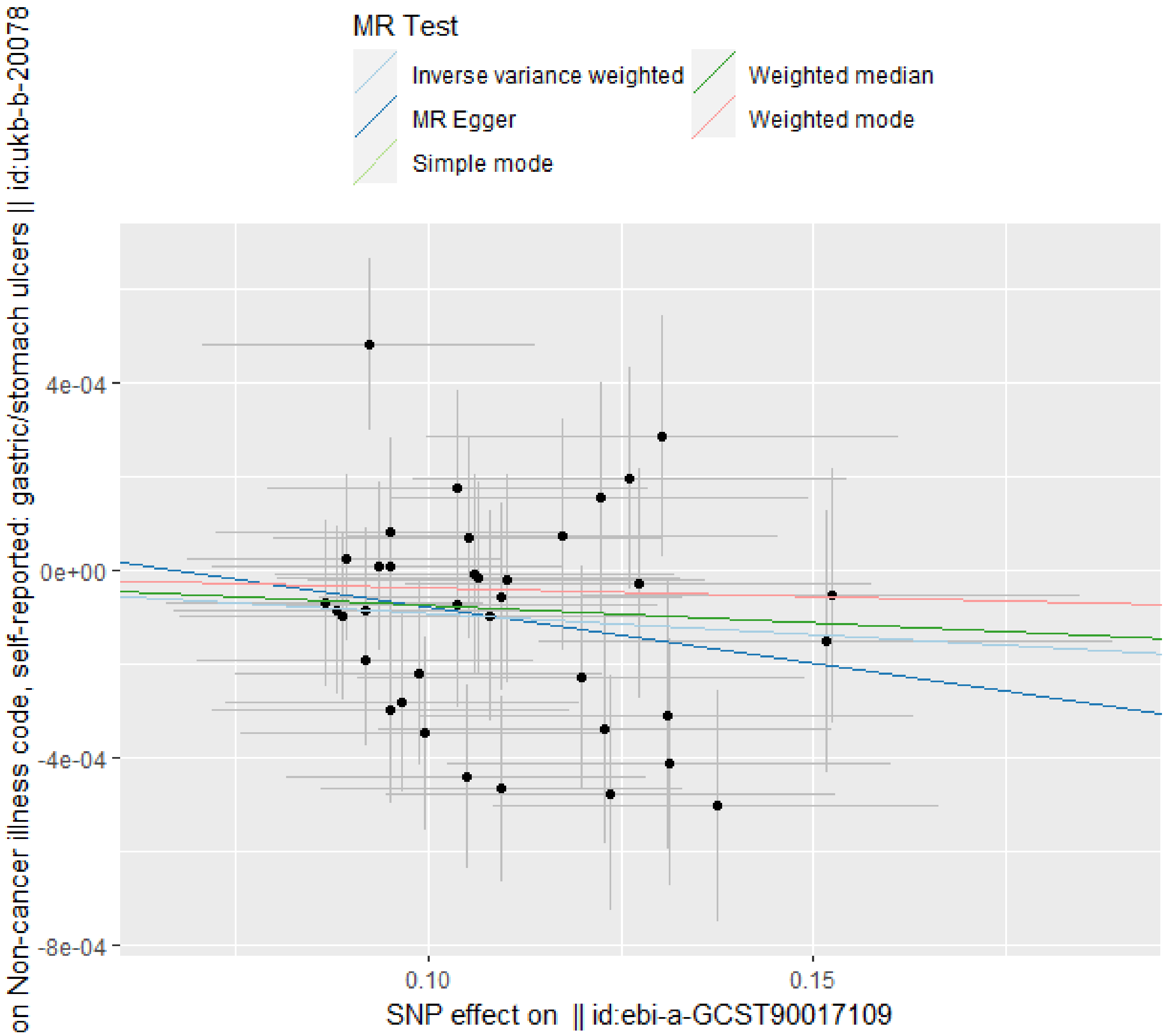

MR Test
Inverse variance weighted
MR Egger
Simple mode
Weighted median
Weighted mode
4e-04
0e+00
-4e-04
-8e-04
0.10
0.15
SNP effect on || id:ebi-a-GCST90017109
on Non-cancer illness code, self-reported: gastric/stomach ulcers || id:ukb-b-20078

Figure 52 Leave-one-out analysis, funnel plot, MR effect size and scatter plot for gut microbiota abundance (class Gammaproteobacteria id.3303) on duodenal ulcer

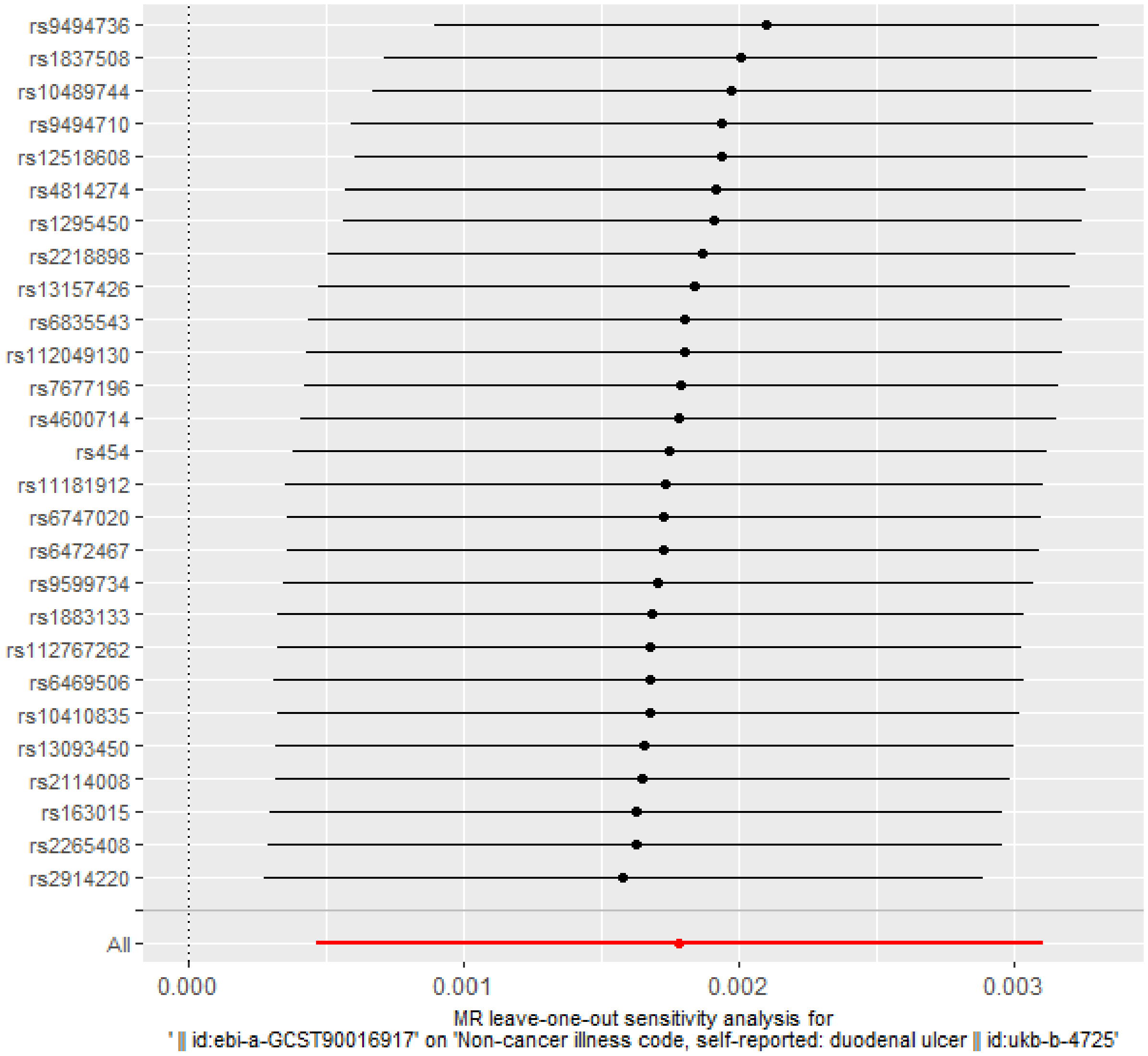

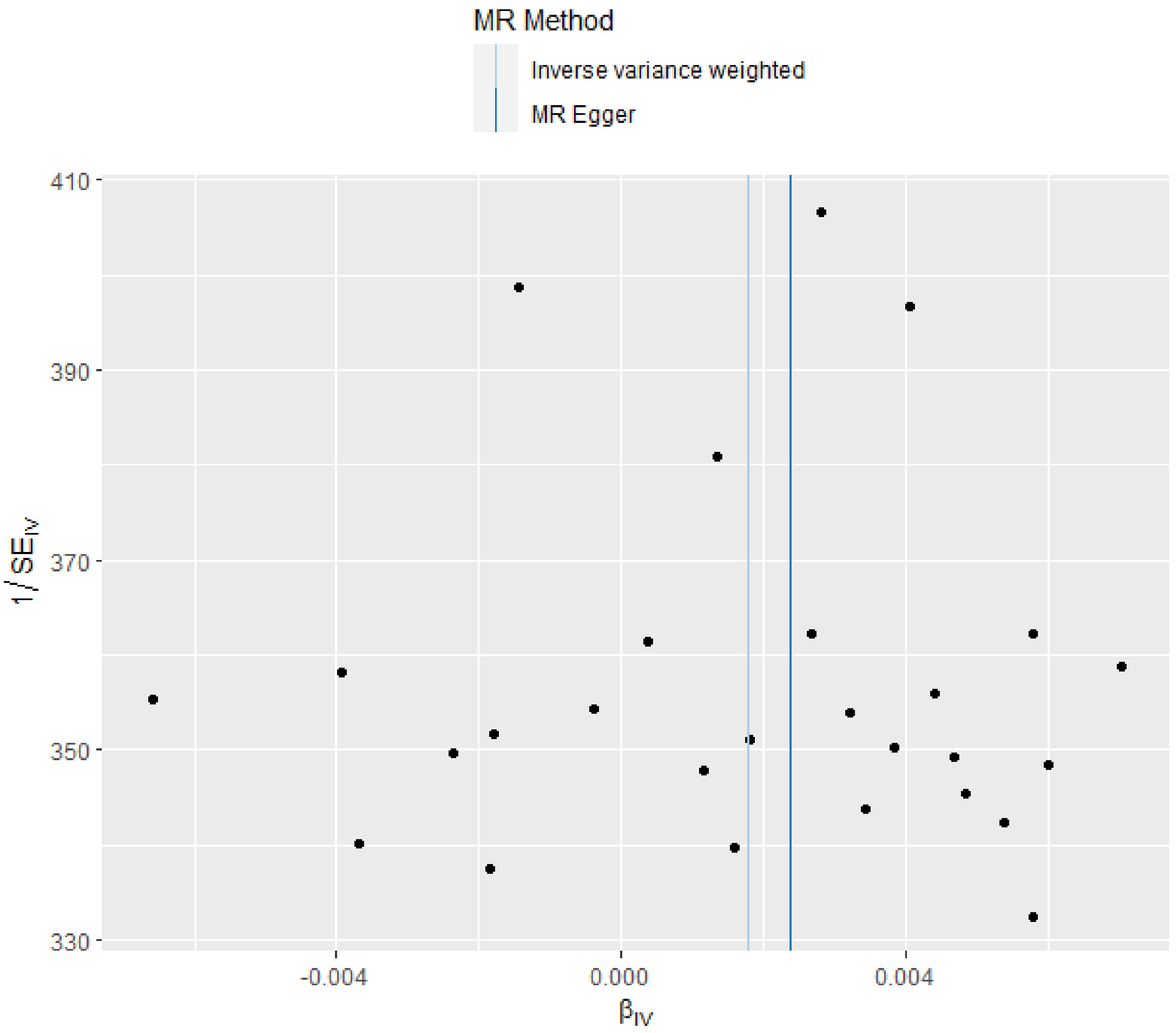

MR Method
Inverse variance weighted
MR Egger
410
390
370
350
330
1/SE_IV
-0.004
0.000
0.004
β_IV

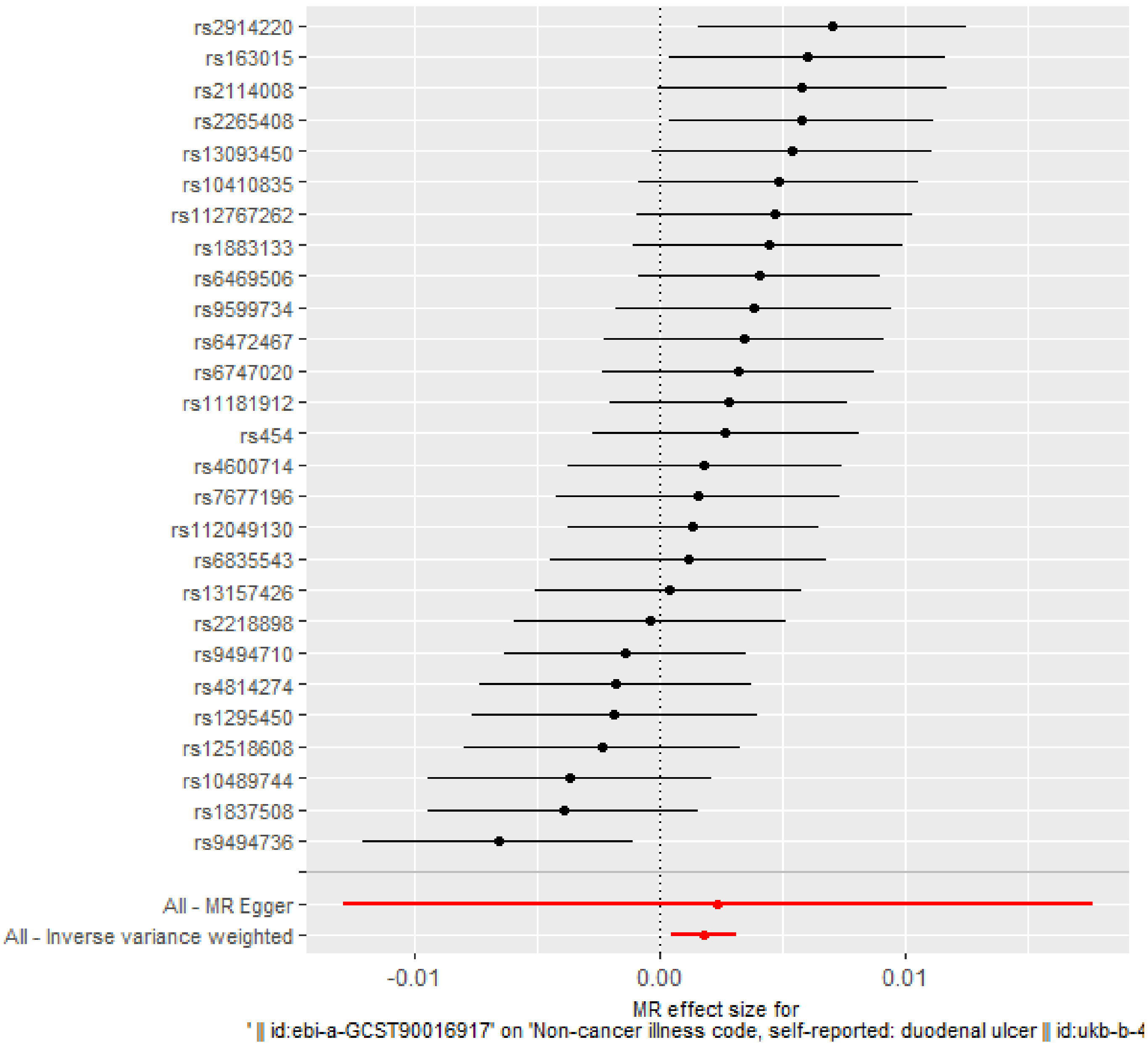

rs2914220
rs163015
rs2114008
rs2265408
rs13093450
rs10410835
rs112767262
rs1883133
rs6469506
rs9599734
rs6472467
rs6747020
rs11181912
rs454
rs4600714
rs7677196
rs112049130
rs6835543
rs13157426
rs2218898
rs9494710
rs4814274
rs1295450
rs12518608
rs10489744
rs1837508
rs9494736
All - MR Egger
All - Inverse variance weighted
-0.01
0.00
0.01
MR effect size for
' || id:ebi-a-GCST90016917' on 'Non-cancer illness code, self-reported: duodenal ulcer || id:ukb-b-4

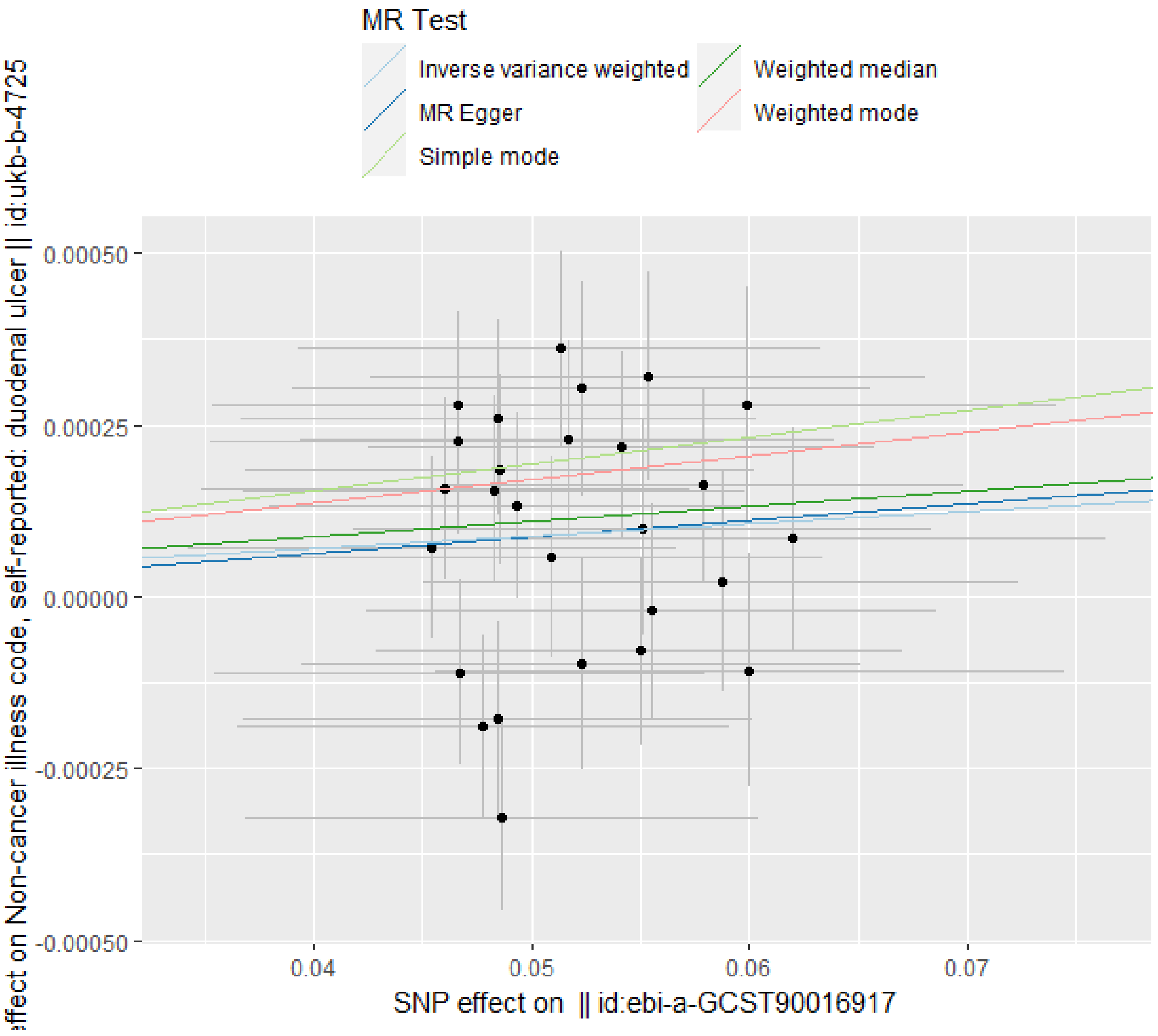

MR Test
Inverse variance weighted
MR Egger
Simple mode
Weighted median
Weighted mode
0.00050
0.00025
0.00000
-0.00025
-0.00050
0.04
0.05
0.06
0.07
SNP effect on || id:ebi-a-GCST90016917
ffect on Non-cancer illness code, self-reported: duodenal ulcer || id:ukb-b-4725

Figure 53 Leave-one-out analysis, funnel plot, MR effect size and scatter plot for gut microbiota abundance (class Verrucomicrobiae id.4029) on duodenal ulcer

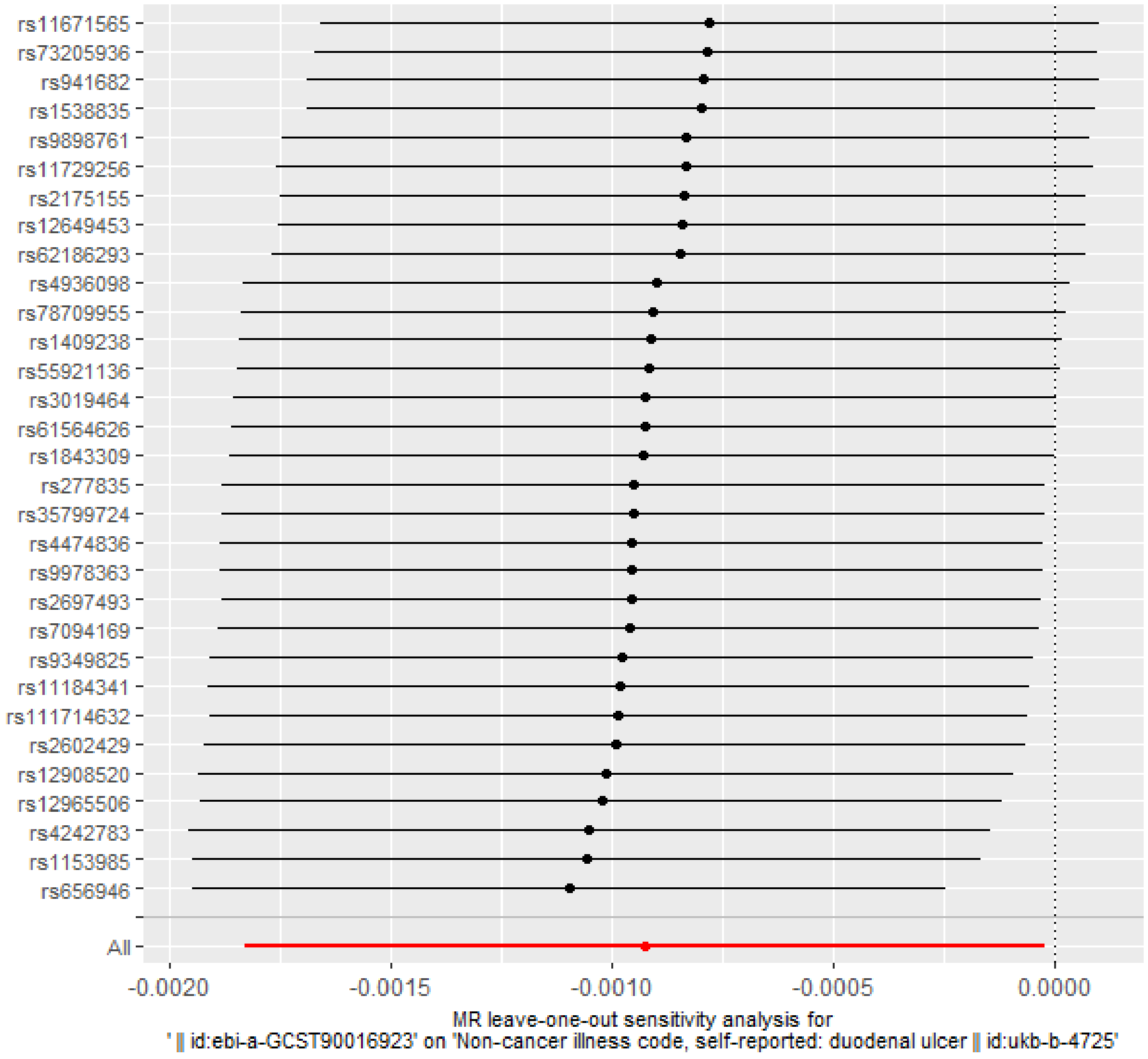

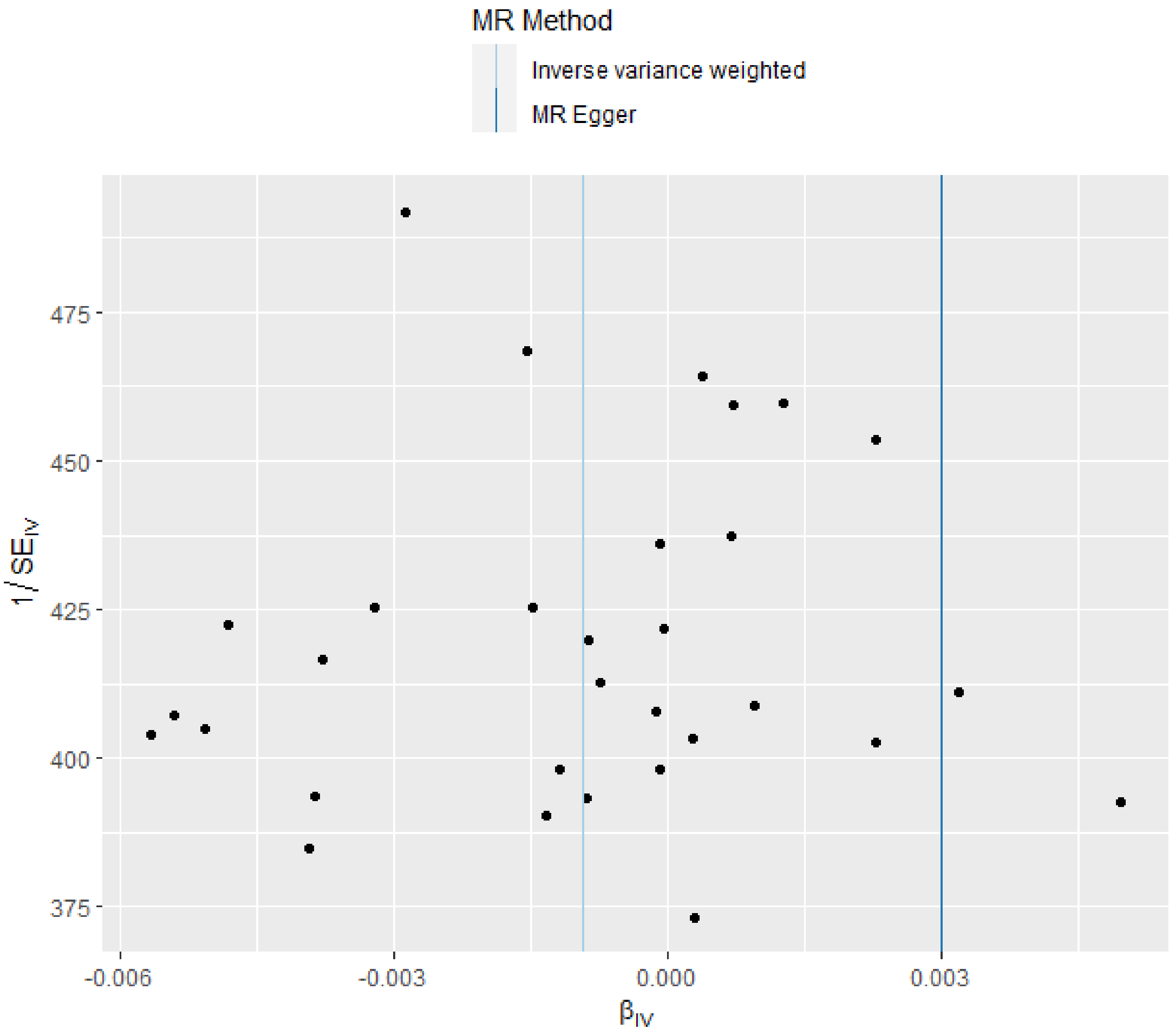
MR Method
Inverse variance weighted
MR Egger
475
450
425
400
375
1/SE_IV
-0.006
-0.003
0.000
0.003
β_IV

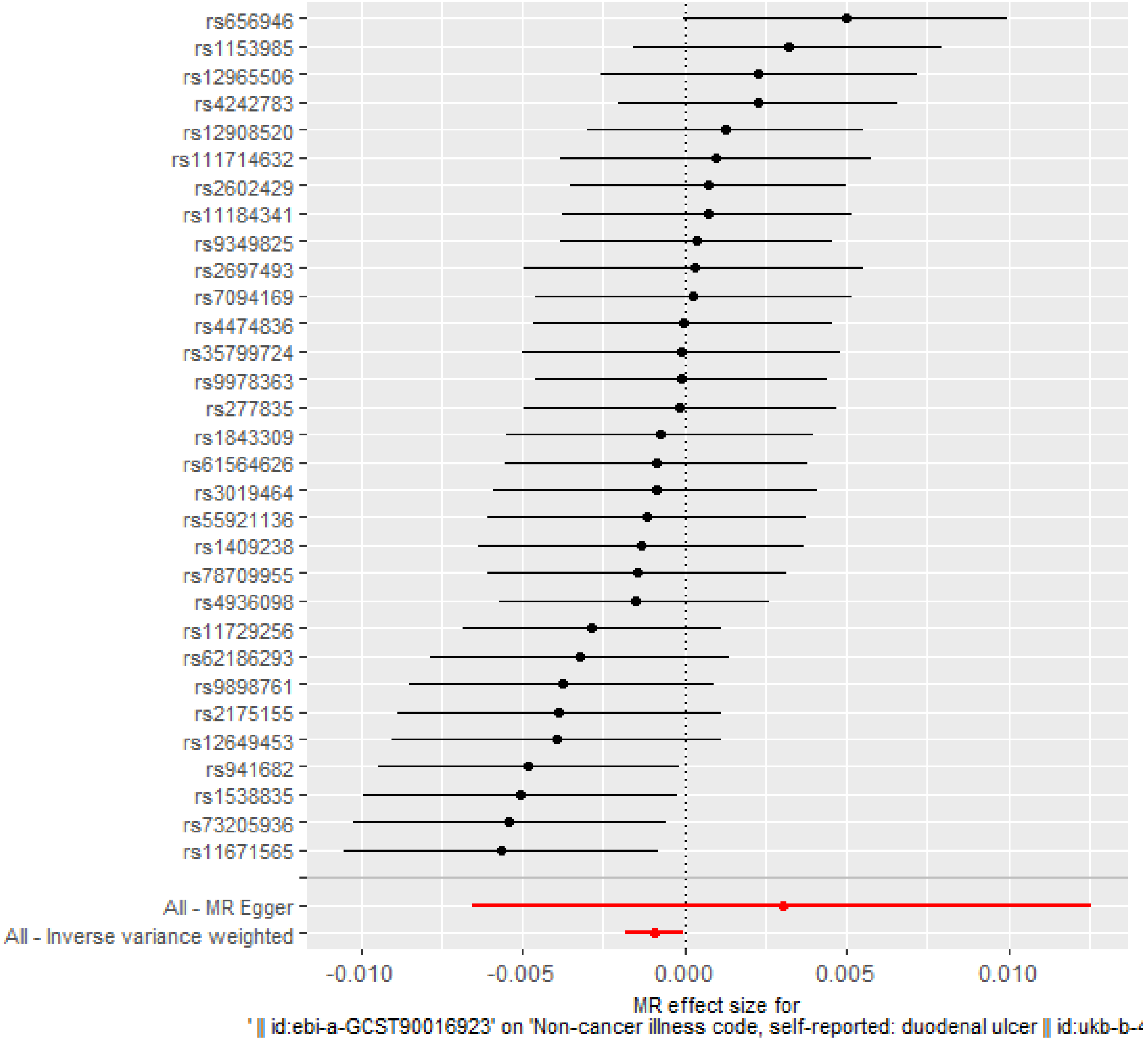

rs656946
rs1153985
rs12965506
rs4242783
rs12908520
rs111714632
rs2602429
rs11184341
rs9349825
rs2697493
rs7094169
rs4474836
rs35799724
rs9978363
rs277835
rs1843309
rs61564626
rs3019464
rs55921136
rs1409238
rs78709955
rs4936098
rs11729256
rs62186293
rs9898761
rs2175155
rs12649453
rs941682
rs1538835
rs73205936
rs11671565
All - MR Egger
All - Inverse variance weighted
-0.010
-0.005
0.000
0.005
0.010
MR effect size for
' || id:ebi-a-GCST90016923' on 'Non-cancer illness code, self-reported: duodenal ulcer || id:ukb-b-

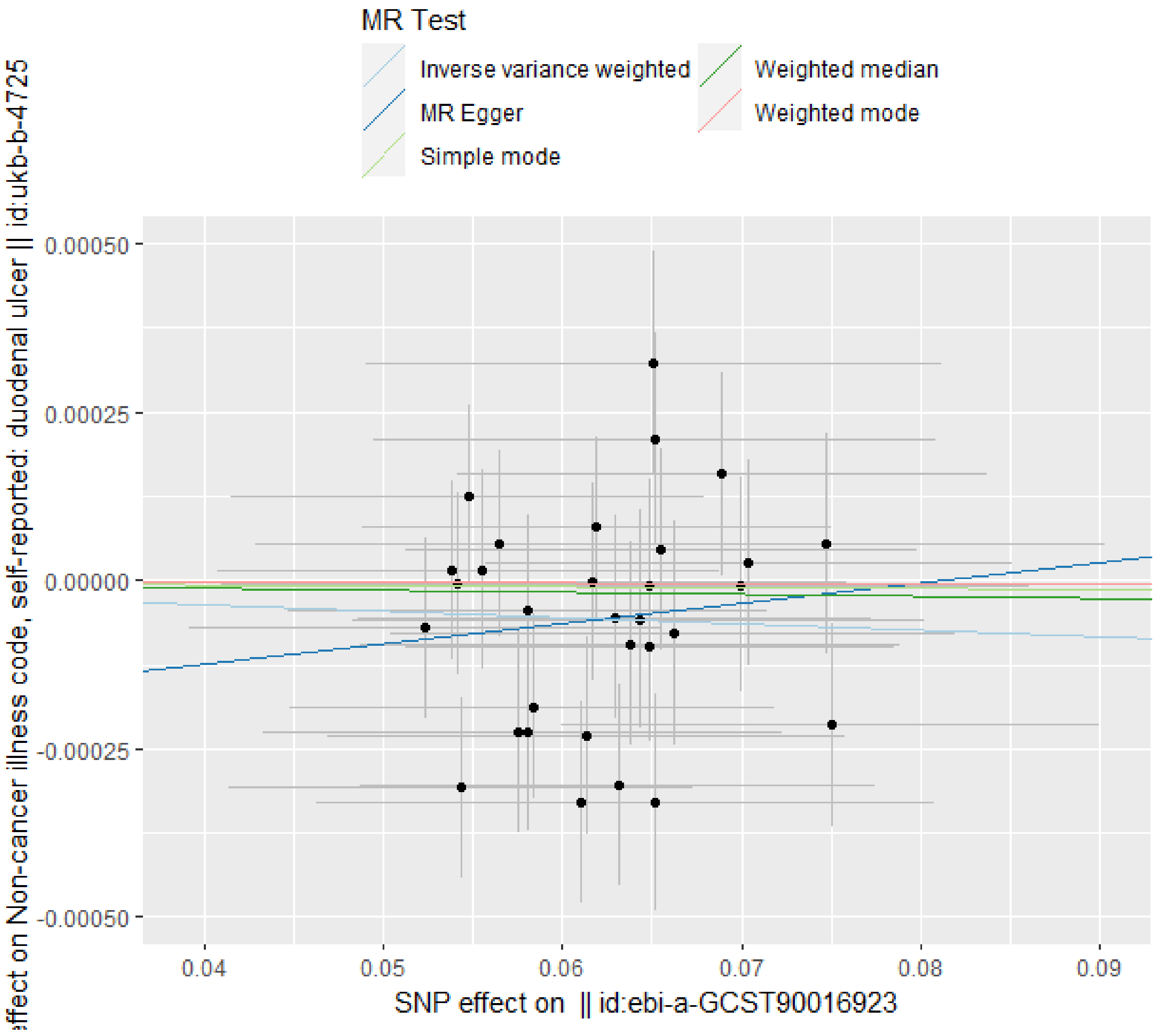

MR Test
Inverse variance weighted
MR Egger
Simple mode
Weighted median
Weighted mode
effect on Non-cancer illness code, self-reported: duodenal ulcer || id:ukb-b-4725
0.00050
0.00025
0.00000
-0.00025
-0.00050
0.04
0.05
0.06
0.07
0.08
0.09
SNP effect on || id:ebi-a-GCST90016923

Figure 54 Leave-one-out analysis, funnel plot, MR effect size and scatter plot for gut microbiota abundance (family Bacteroidaceae id.917) on duodenal ulcer

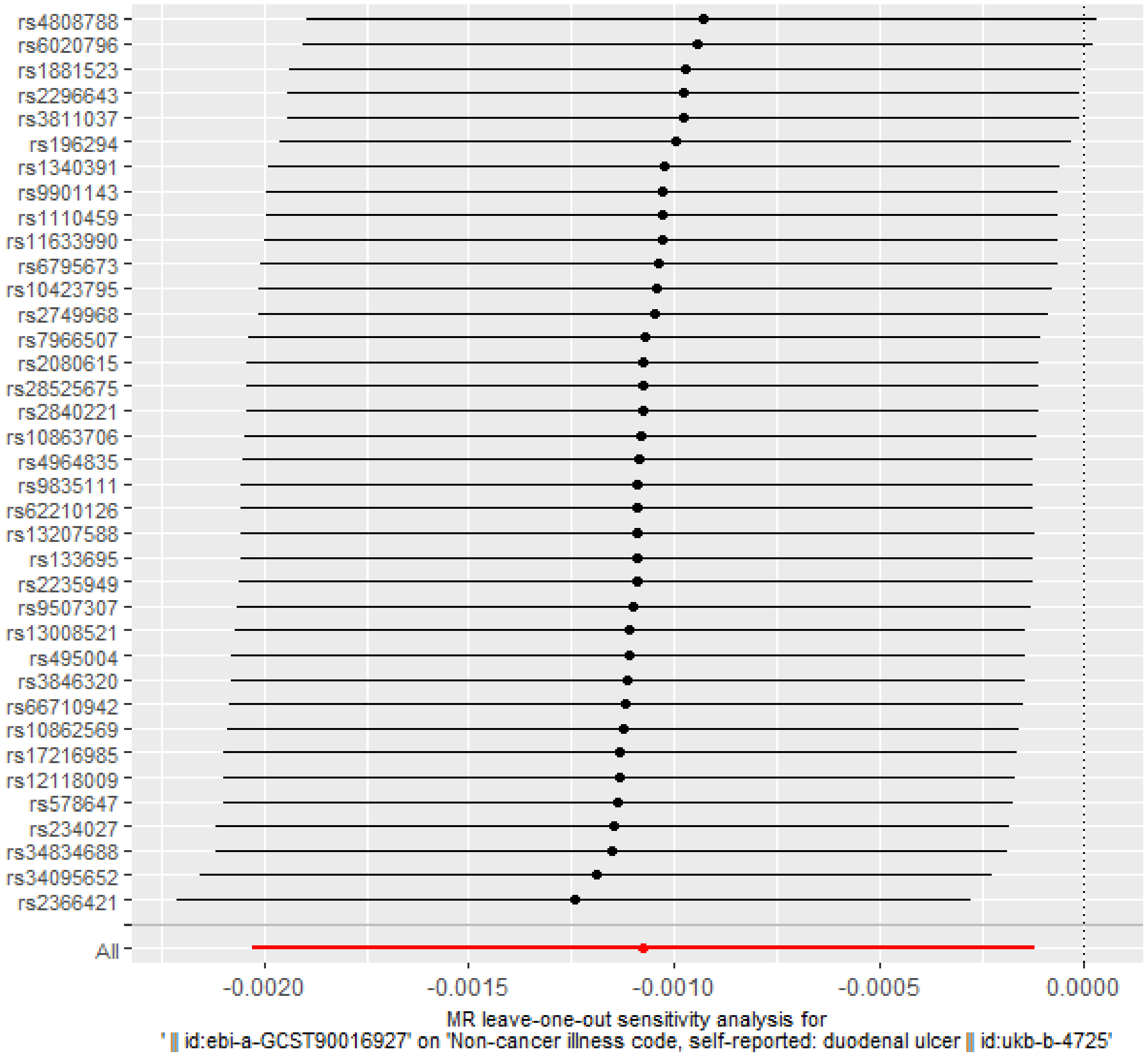

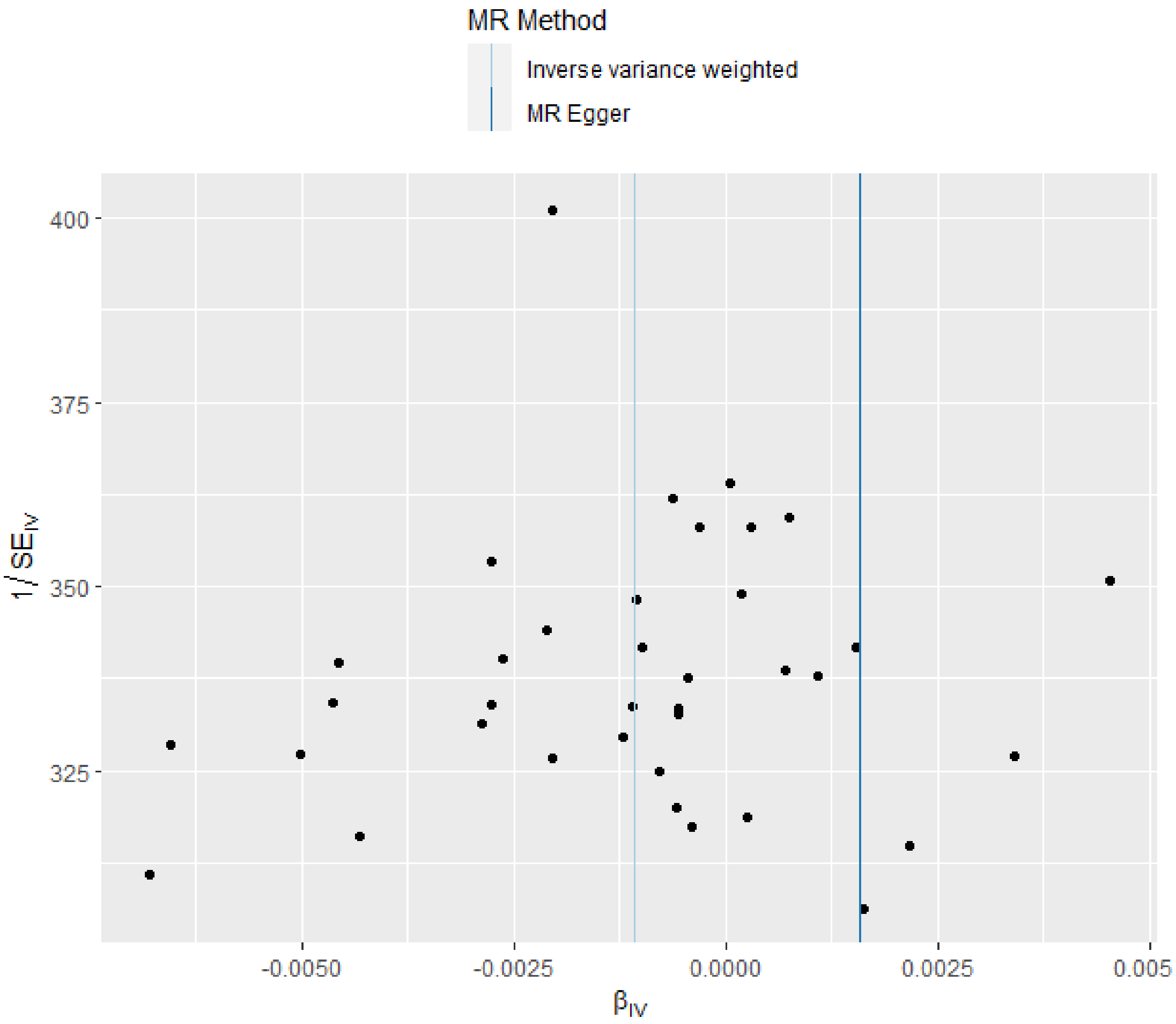

MR Method
Inverse variance weighted
MR Egger
400
375
350
325
1/SE_IV
-0.0050
-0.0025
0.0000
0.0025
0.005
β_IV

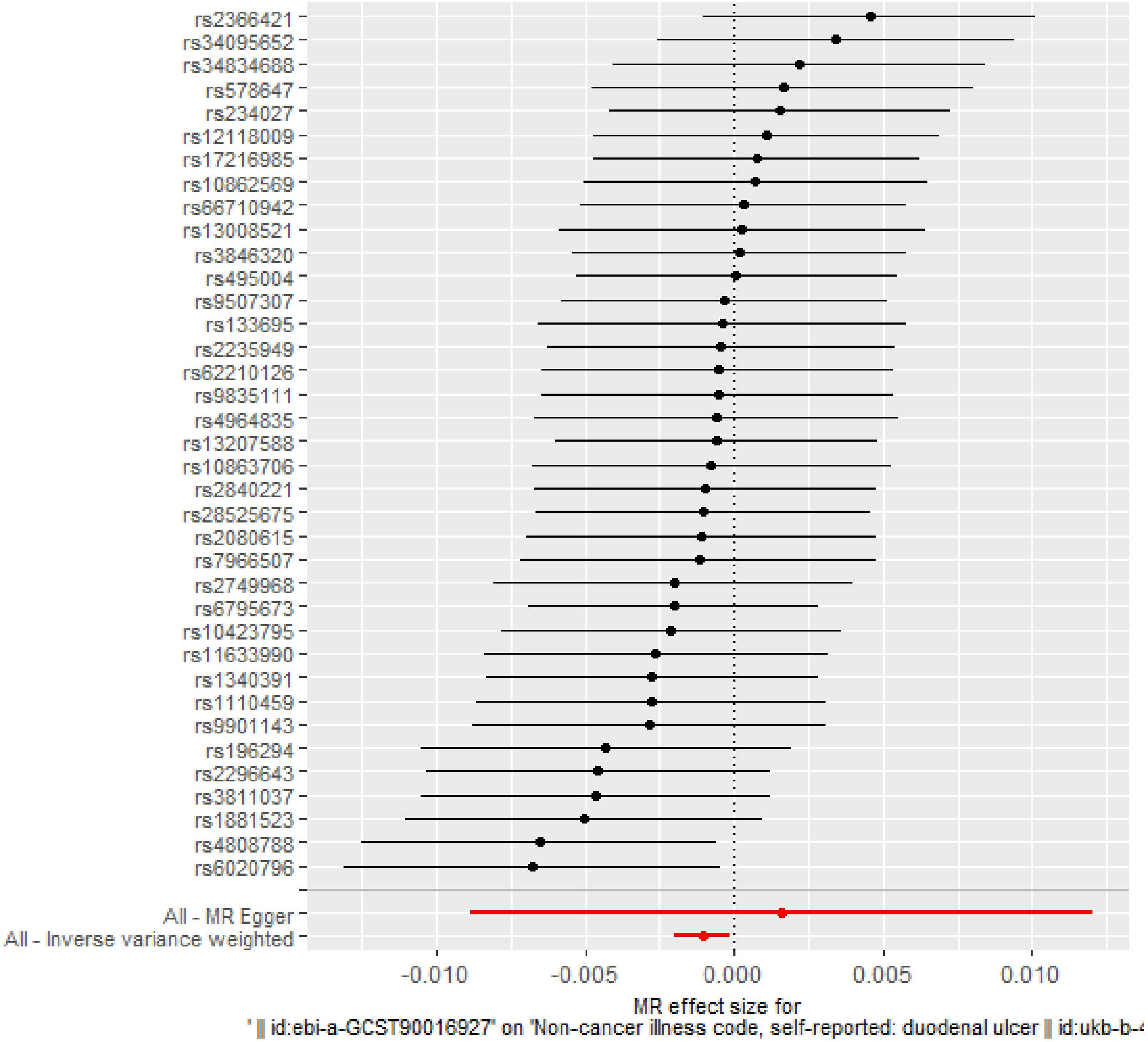

rs2366421
rs34095652
rs34834688
rs578647
rs234027
rs12118009
rs17216985
rs10862569
rs66710942
rs13008521
rs3846320
rs495004
rs9507307
rs133695
rs2235949
rs62210126
rs9835111
rs4964835
rs13207588
rs10863706
rs2840221
rs28525675
rs2080615
rs7966507
rs2749968
rs6795673
rs10423795
rs11633990
rs1340391
rs1110459
rs9901143
rs196294
rs2296643
rs3811037
rs1881523
rs4808788
rs6020796
All - MR Egger
All - Inverse variance weighted
-0.010
-0.005
0.000
0.005
0.010
MR effect size for
' || id:ebi-a-GCST90016927' on 'Non-cancer illness code, self-reported: duodenal ulcer || id:ukb-b-

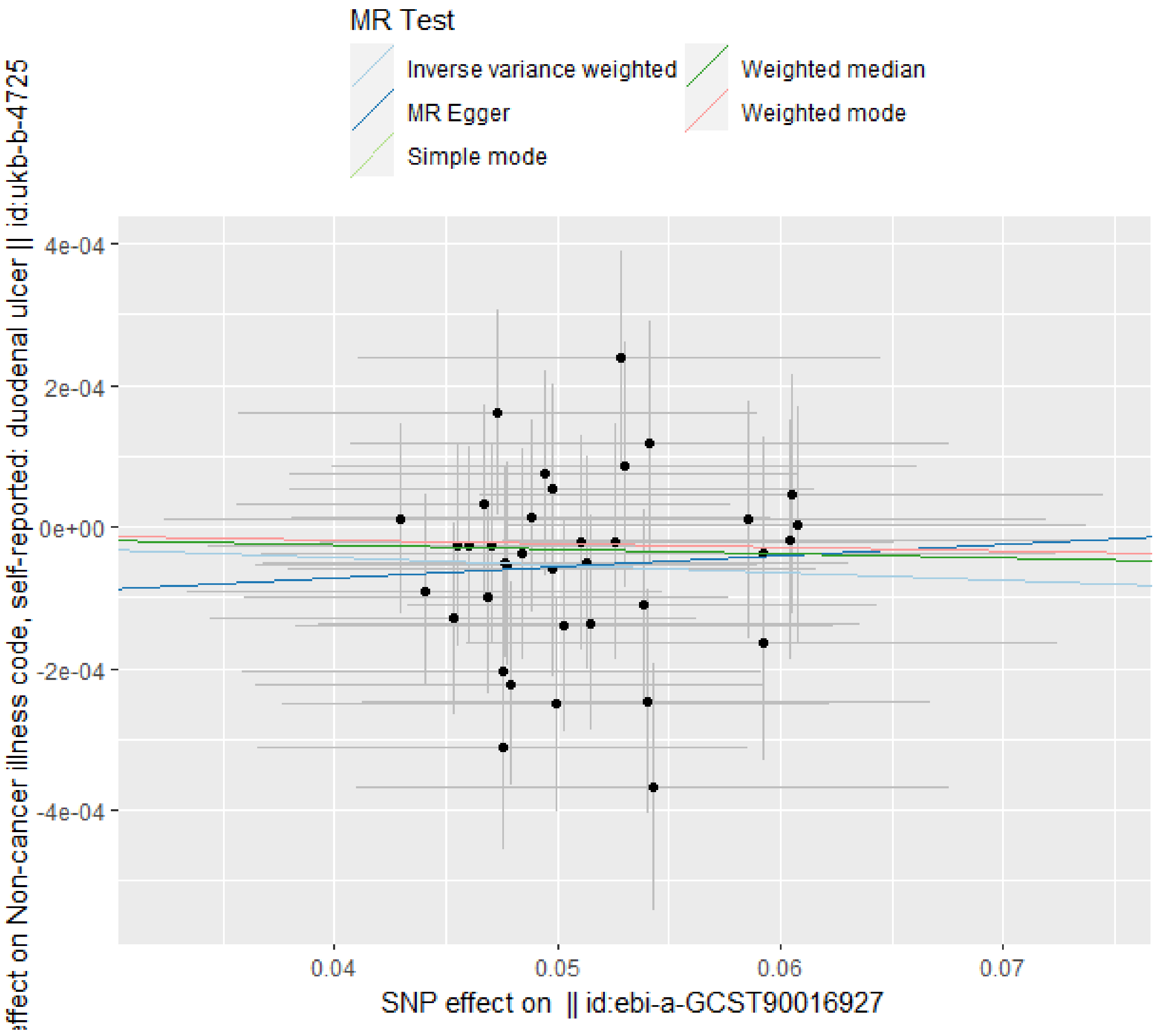

MR Test
Inverse variance weighted
MR Egger
Simple mode
Weighted median
Weighted mode
4e-04
2e-04
0e+00
-2e-04
-4e-04
0.04
0.05
0.06
0.07
SNP effect on || id:ebi-a-GCST90016927
ffect on Non-cancer illness code, self-reported: duodenal ulcer || id:ukb-b-4725

Figure 55 Leave-one-out analysis, funnel plot, MR effect size and scatter plot for gut microbiota abundance (family Peptostreptococcaceae id.2042) on duodenal ulcer

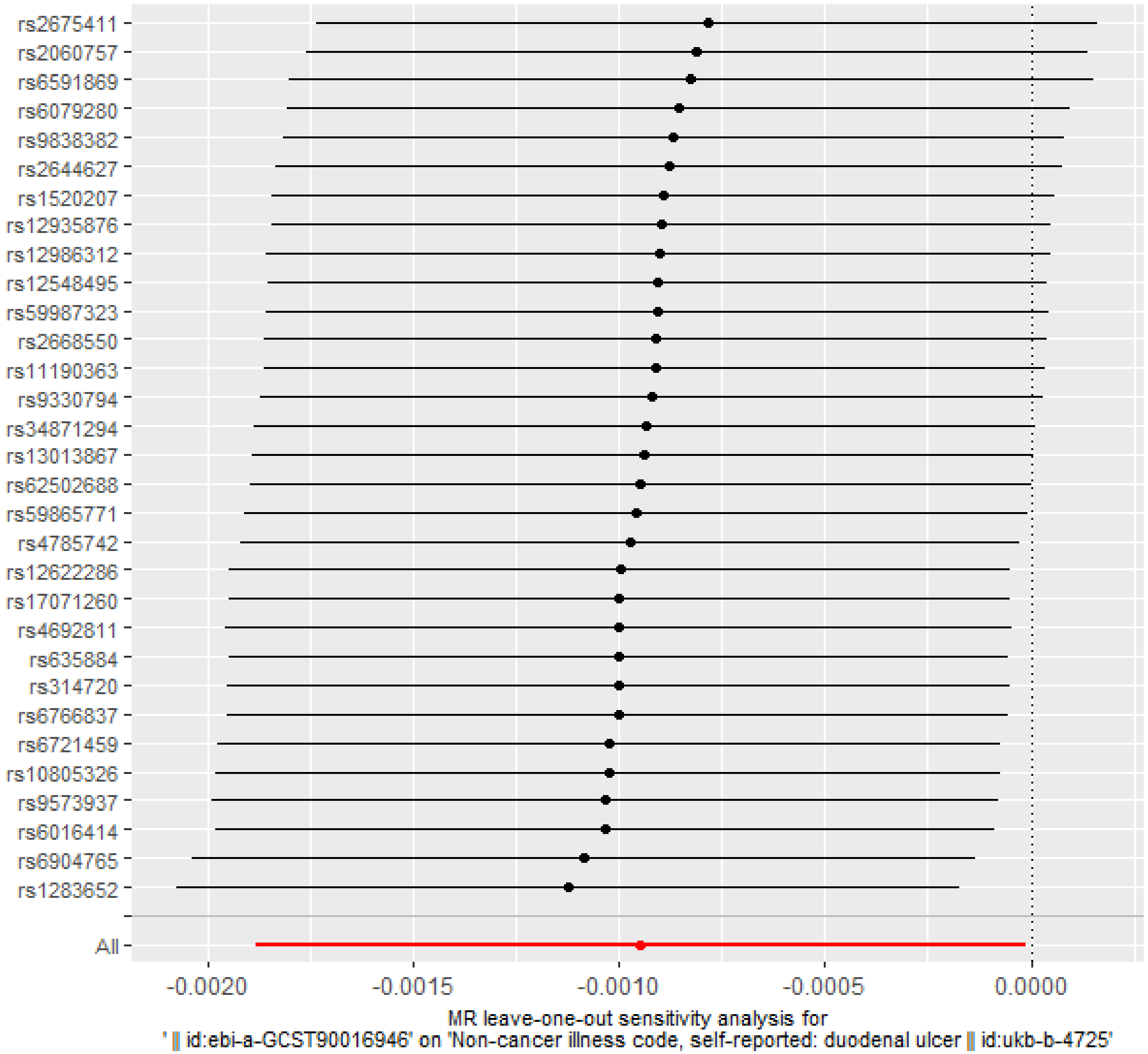

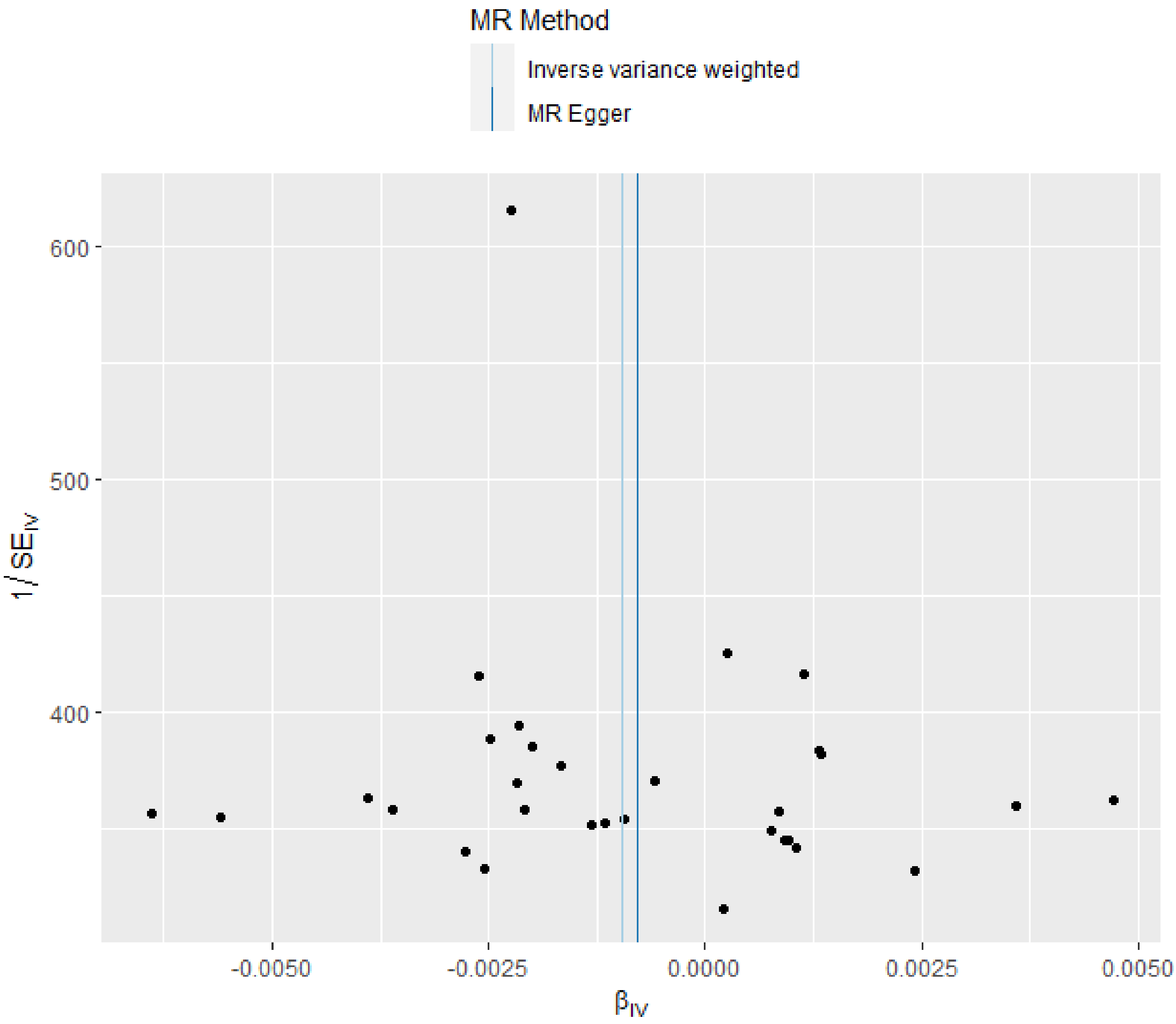
MR Method
Inverse variance weighted
MR Egger
600
500
400
$1/SE_{IV}$
-0.0050
-0.0025
0.0000
0.0025
0.0050
β_{IV}

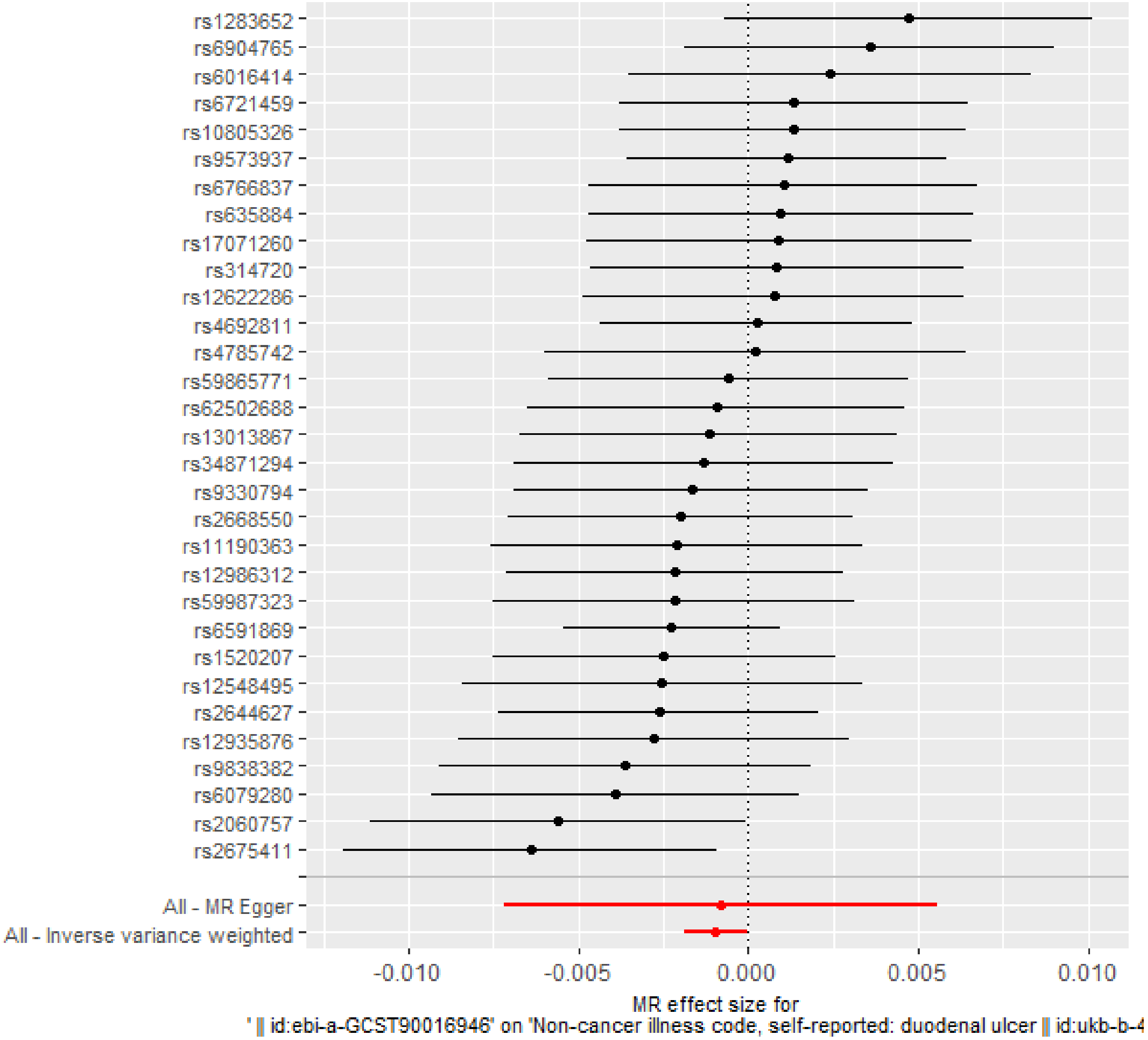
rs1283652
rs6904765
rs6016414
rs6721459
rs10805326
rs9573937
rs6766837
rs635884
rs17071260
rs314720
rs12622286
rs4692811
rs4785742
rs59865771
rs62502688
rs13013867
rs34871294
rs9330794
rs2668550
rs11190363
rs12986312
rs59987323
rs6591869
rs1520207
rs12548495
rs2644627
rs12935876
rs9838382
rs6079280
rs2060757
rs2675411
All - MR Egger
All - Inverse variance weighted
-0.010
-0.005
0.000
0.005
0.010
MR effect size for
' || id:ebi-a-GCST90016946' on 'Non-cancer illness code, self-reported: duodenal ulcer || id:ukb-b-4

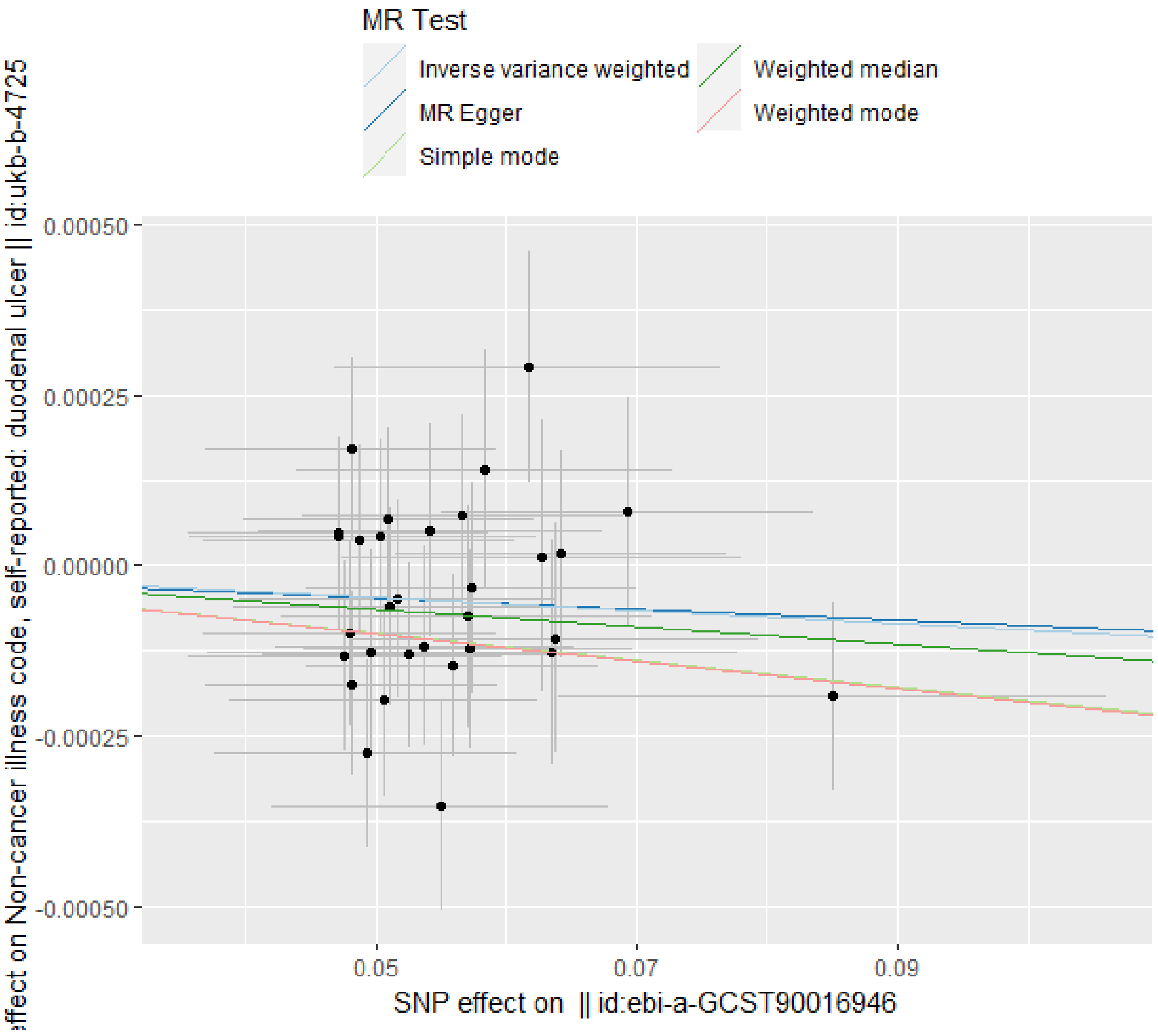
MR Test
Inverse variance weighted
MR Egger
Simple mode
Weighted median
Weighted mode
0.00050
0.00025
0.00000
-0.00025
-0.00050
0.05
0.07
0.09
SNP effect on || id:ebi-a-GCST90016946
ffect on Non-cancer illness code, self-reported: duodenal ulcer || id:ukb-b-4725

Figure 56 Leave-one-out analysis, funnel plot, MR effect size and scatter plot for gut microbiota abundance (family Porphyromonadaceae id.943) on duodenal ulcer

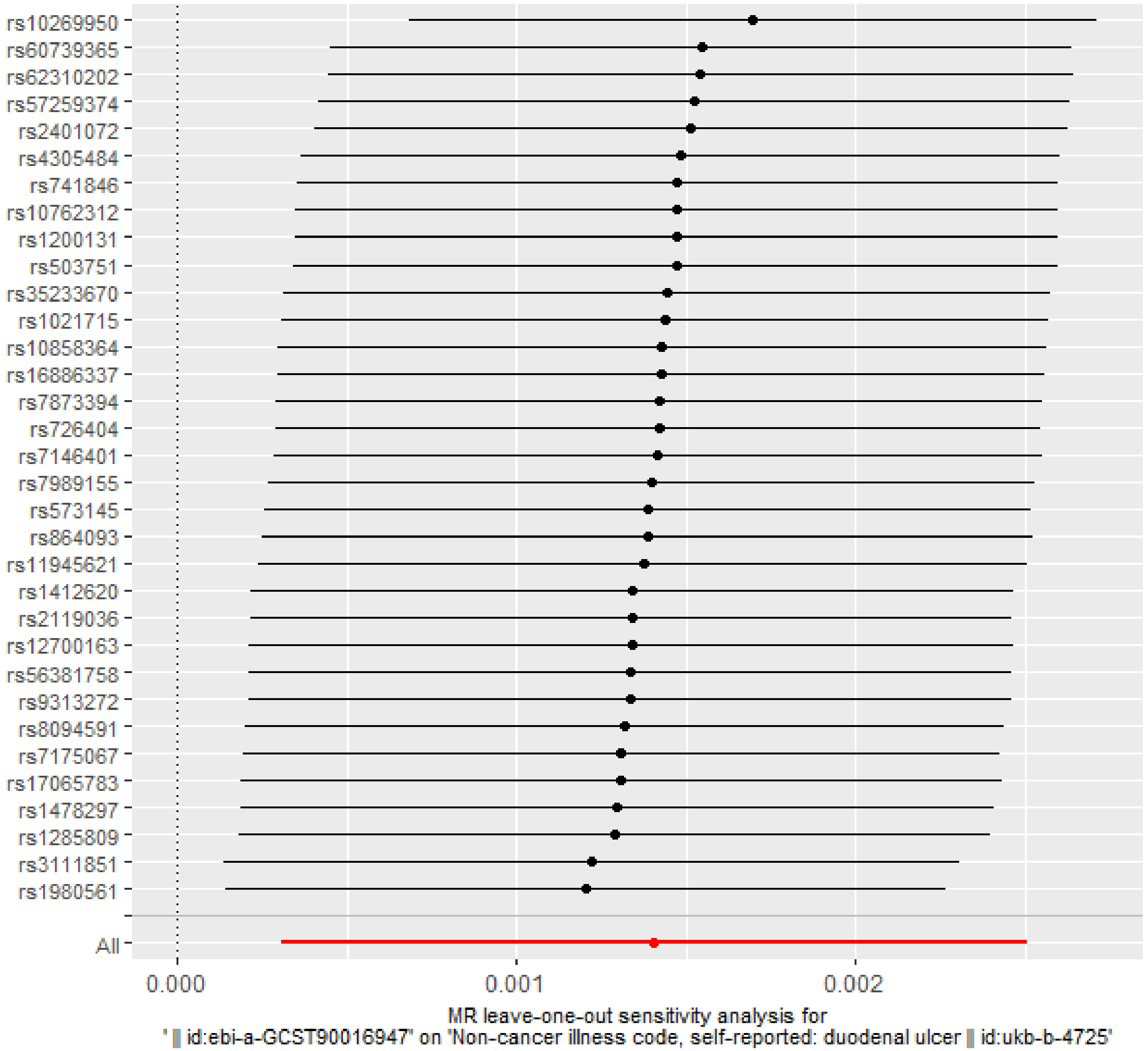

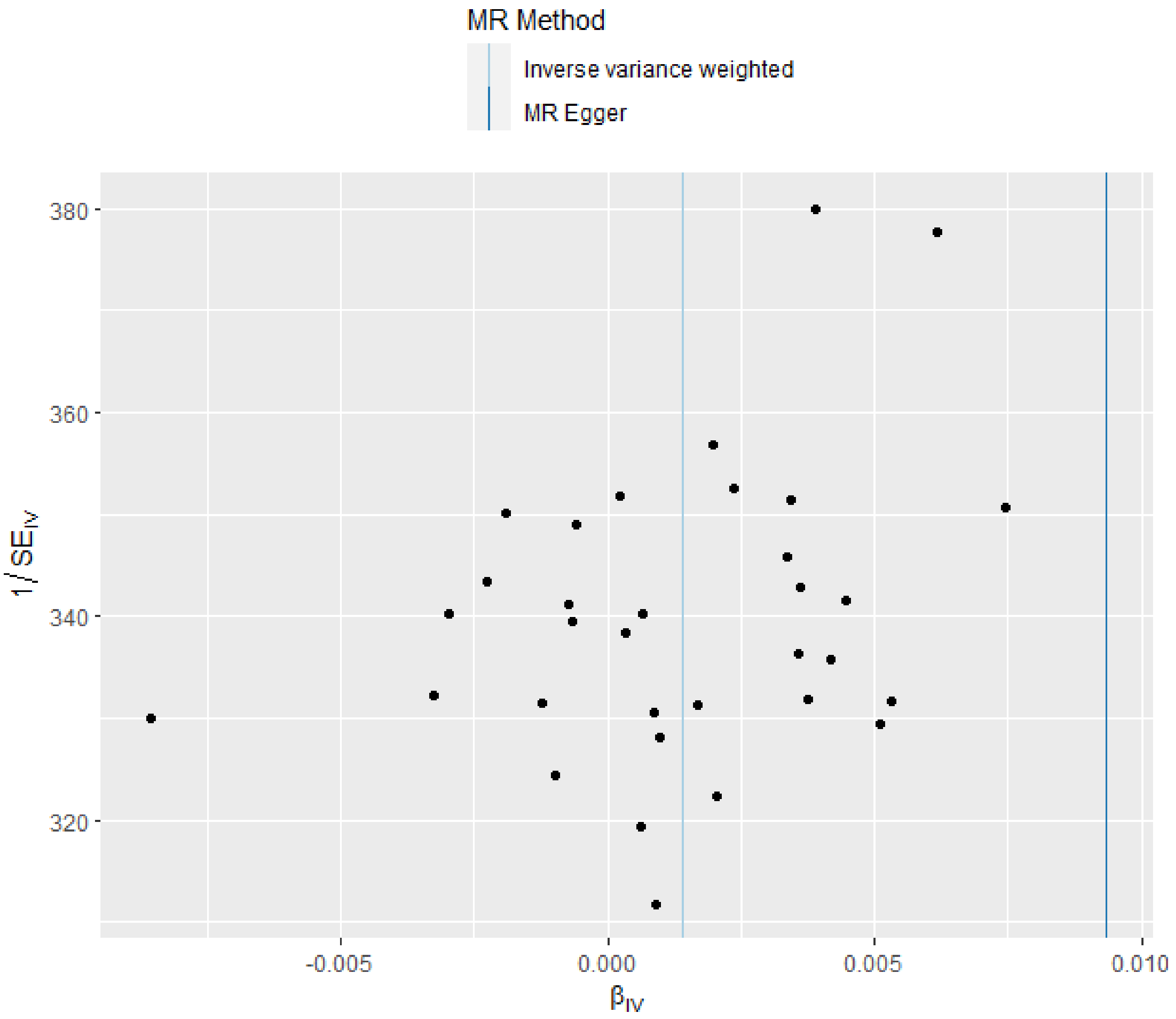
MR Method
Inverse variance weighted
MR Egger
380
360
340
320
-0.005
0.000
0.005
0.010
βIV
1/SEIV

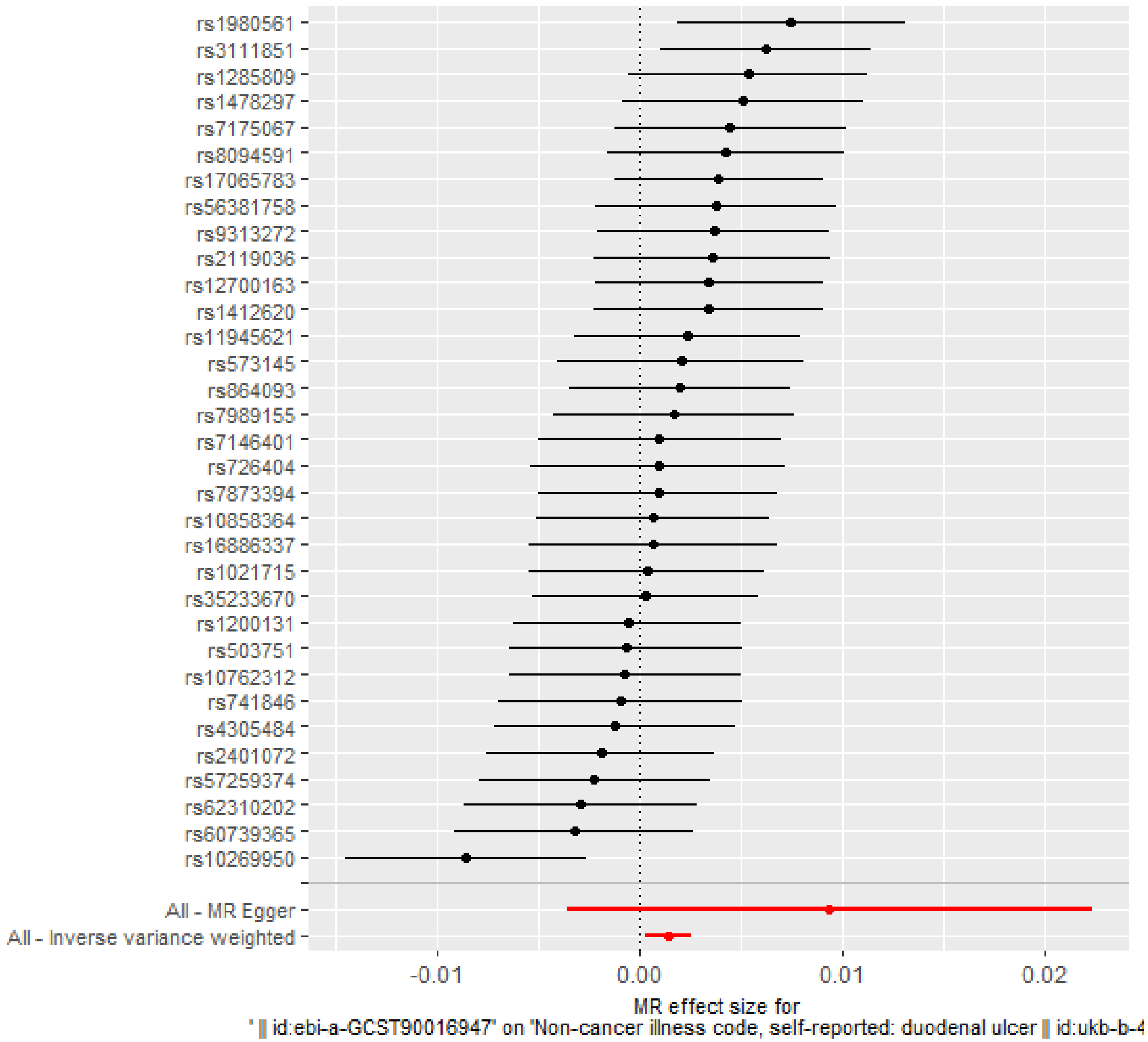
rs1980561
rs3111851
rs1285809
rs1478297
rs7175067
rs8094591
rs17065783
rs56381758
rs9313272
rs2119036
rs12700163
rs1412620
rs11945621
rs573145
rs864093
rs7989155
rs7146401
rs726404
rs7873394
rs10858364
rs16886337
rs1021715
rs35233670
rs1200131
rs503751
rs10762312
rs741846
rs4305484
rs2401072
rs57259374
rs62310202
rs60739365
rs10269950
All - MR Egger
All - Inverse variance weighted
-0.01
0.00
0.01
0.02
MR effect size for
' || id:ebi-a-GCST90016947' on 'Non-cancer illness code, self-reported: duodenal ulcer || id:ukb-b-4

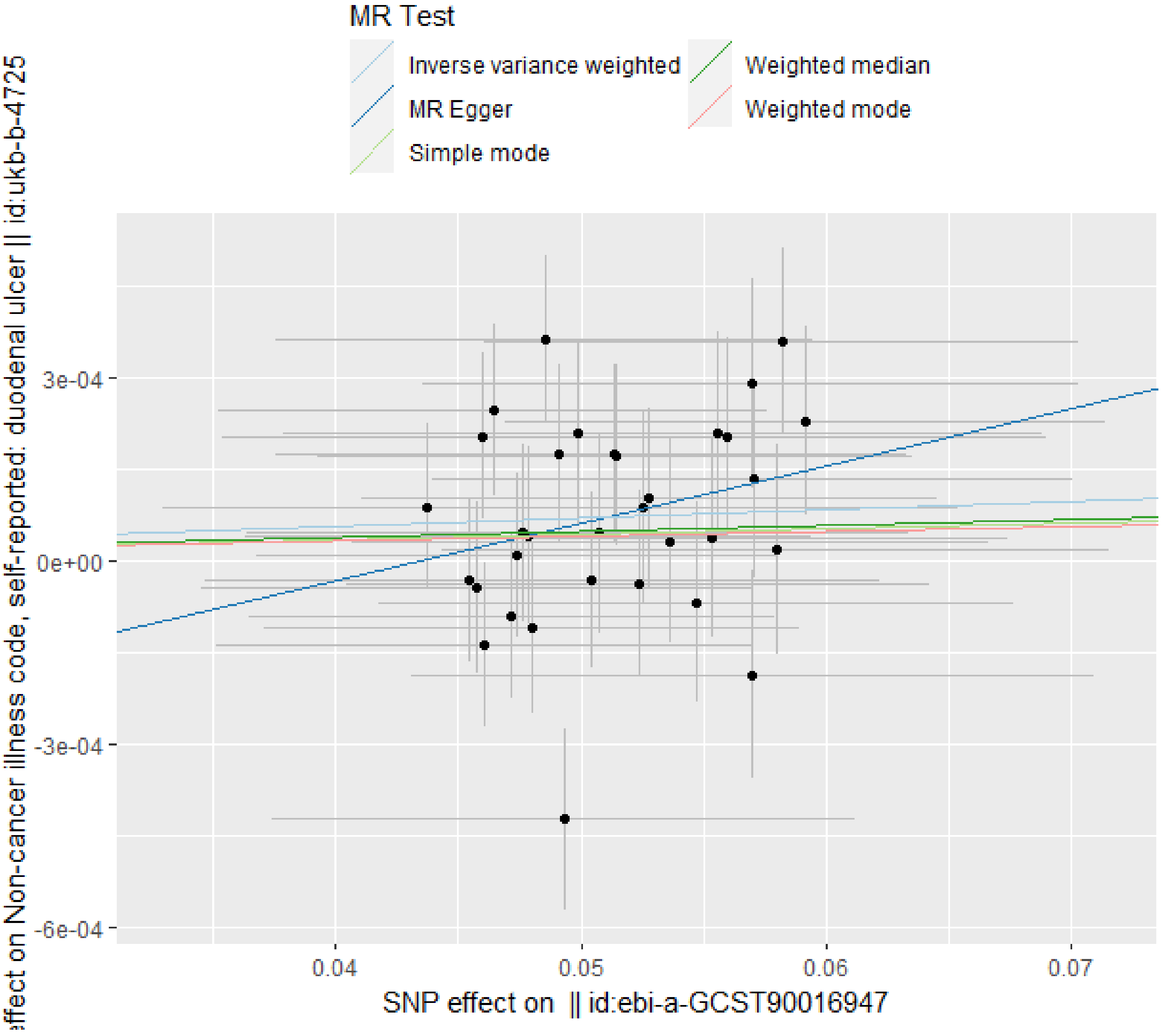

MR Test
Inverse variance weighted
MR Egger
Simple mode
Weighted median
Weighted mode
3e-04
0e+00
-3e-04
-6e-04
0.04
0.05
0.06
0.07
SNP effect on || id:ebi-a-GCST90016947
ffect on Non-cancer illness code, self-reported: duodenal ulcer || id:ukb-b-4725

Figure 57 Leave-one-out analysis, funnel plot, MR effect size and scatter plot for gut microbiota abundance (family Verrucomicrobiaceae id.4036) on duodenal ulcer

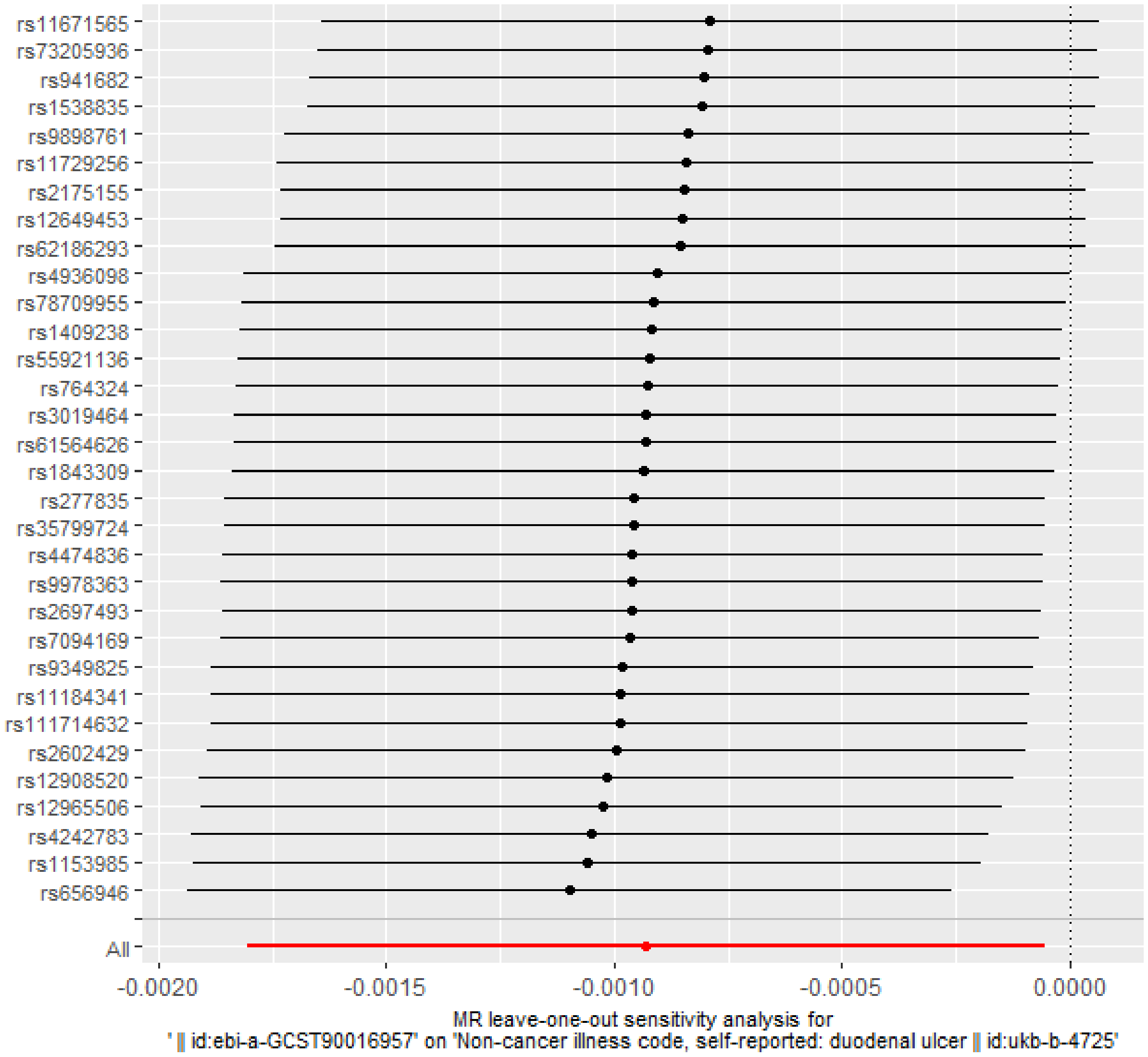

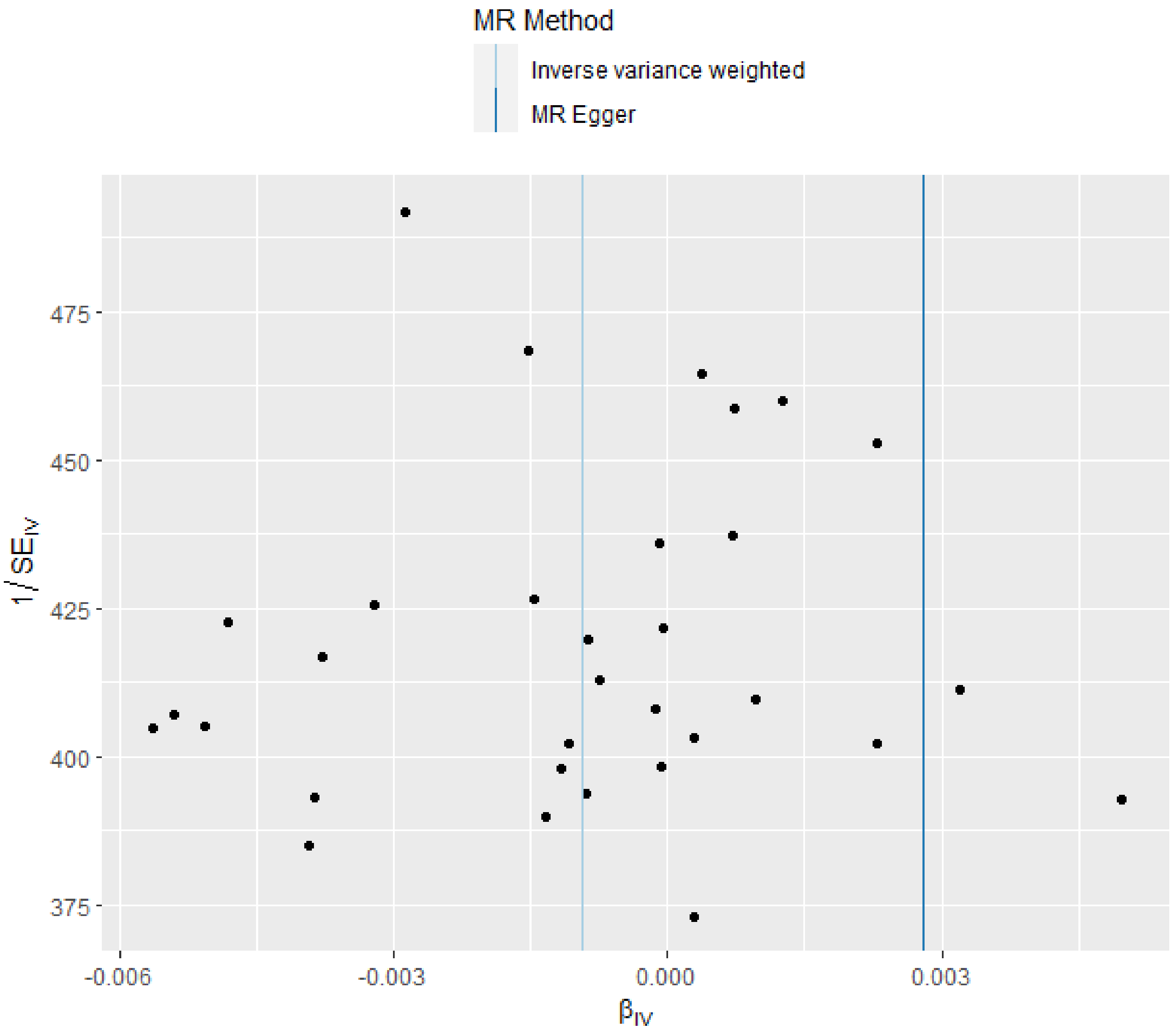
MR Method
Inverse variance weighted
MR Egger
475
450
425
400
375
$1/SE_{IV}$
-0.006
-0.003
0.000
0.003
β_{IV}

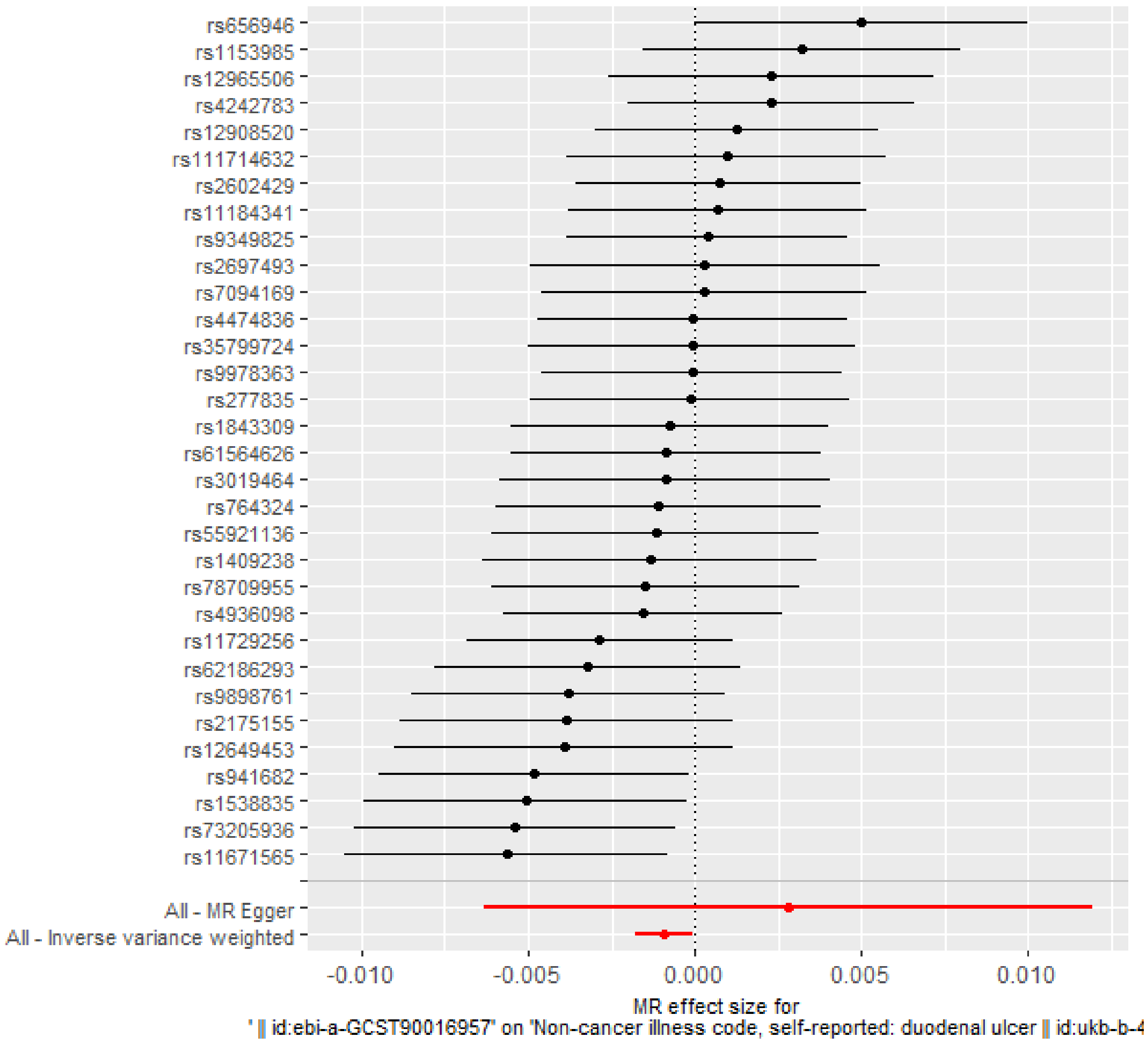
rs656946
rs1153985
rs12965506
rs4242783
rs12908520
rs111714632
rs2602429
rs11184341
rs9349825
rs2697493
rs7094169
rs4474836
rs35799724
rs9978363
rs277835
rs1843309
rs61564626
rs3019464
rs764324
rs55921136
rs1409238
rs78709955
rs4936098
rs11729256
rs62186293
rs9898761
rs2175155
rs12649453
rs941682
rs1538835
rs73205936
rs11671565
All - MR Egger
All - Inverse variance weighted
-0.010
-0.005
0.000
0.005
0.010
MR effect size for
' || id:ebi-a-GCST90016957' on 'Non-cancer illness code, self-reported: duodenal ulcer || id:ukb-b-4

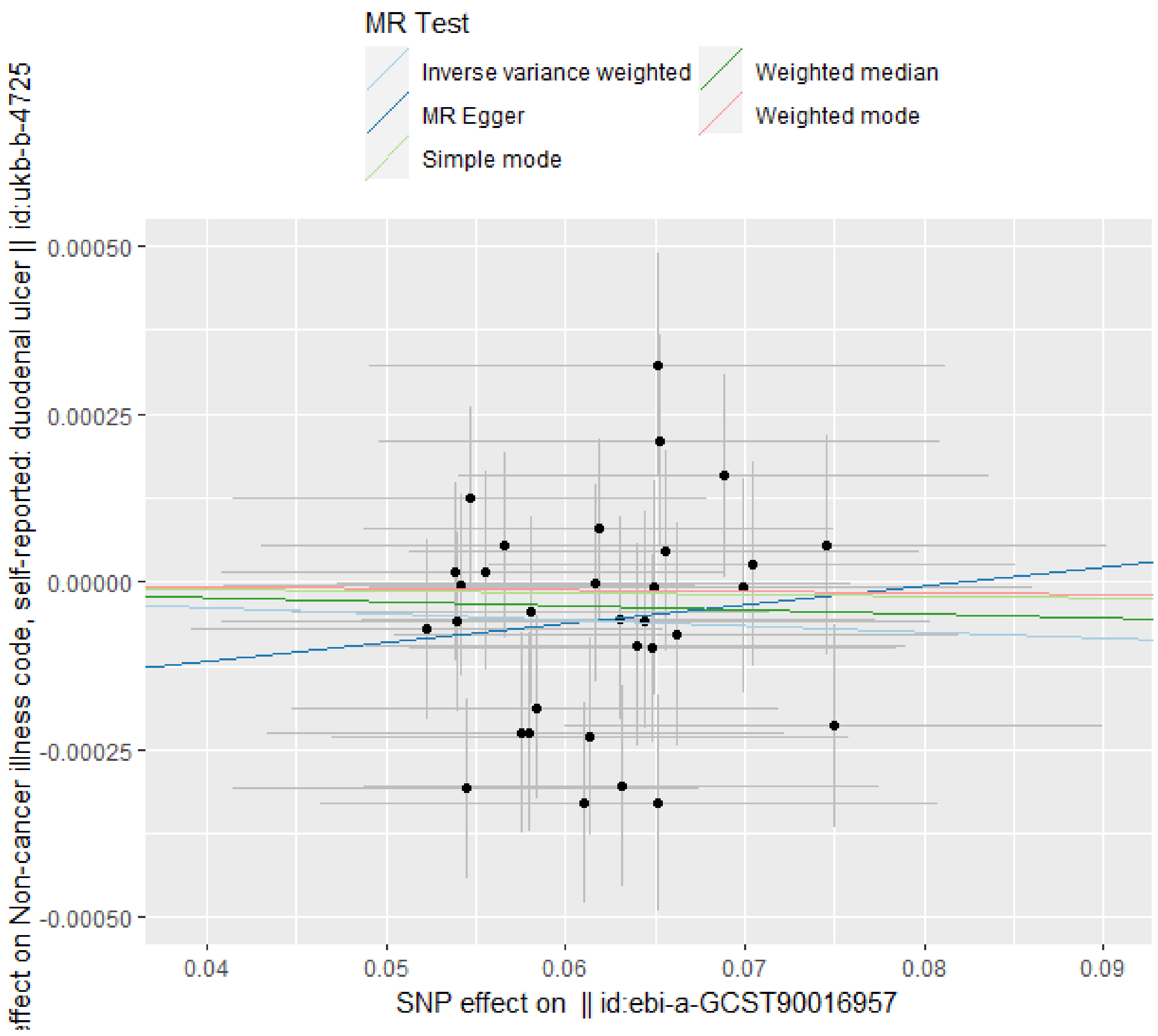

MR Test
Inverse variance weighted
MR Egger
Simple mode
Weighted median
Weighted mode
0.00050
0.00025
0.00000
-0.00025
-0.00050
0.04
0.05
0.06
0.07
0.08
0.09
SNP effect on || id:ebi-a-GCST90016957
ffect on Non-cancer illness code, self-reported: duodenal ulcer || id:ukb-b-4725

Figure 58 Leave-one-out analysis, funnel plot, MR effect size and scatter plot for gut microbiota abundance (genus Bacteroides id.918) on duodenal ulcer

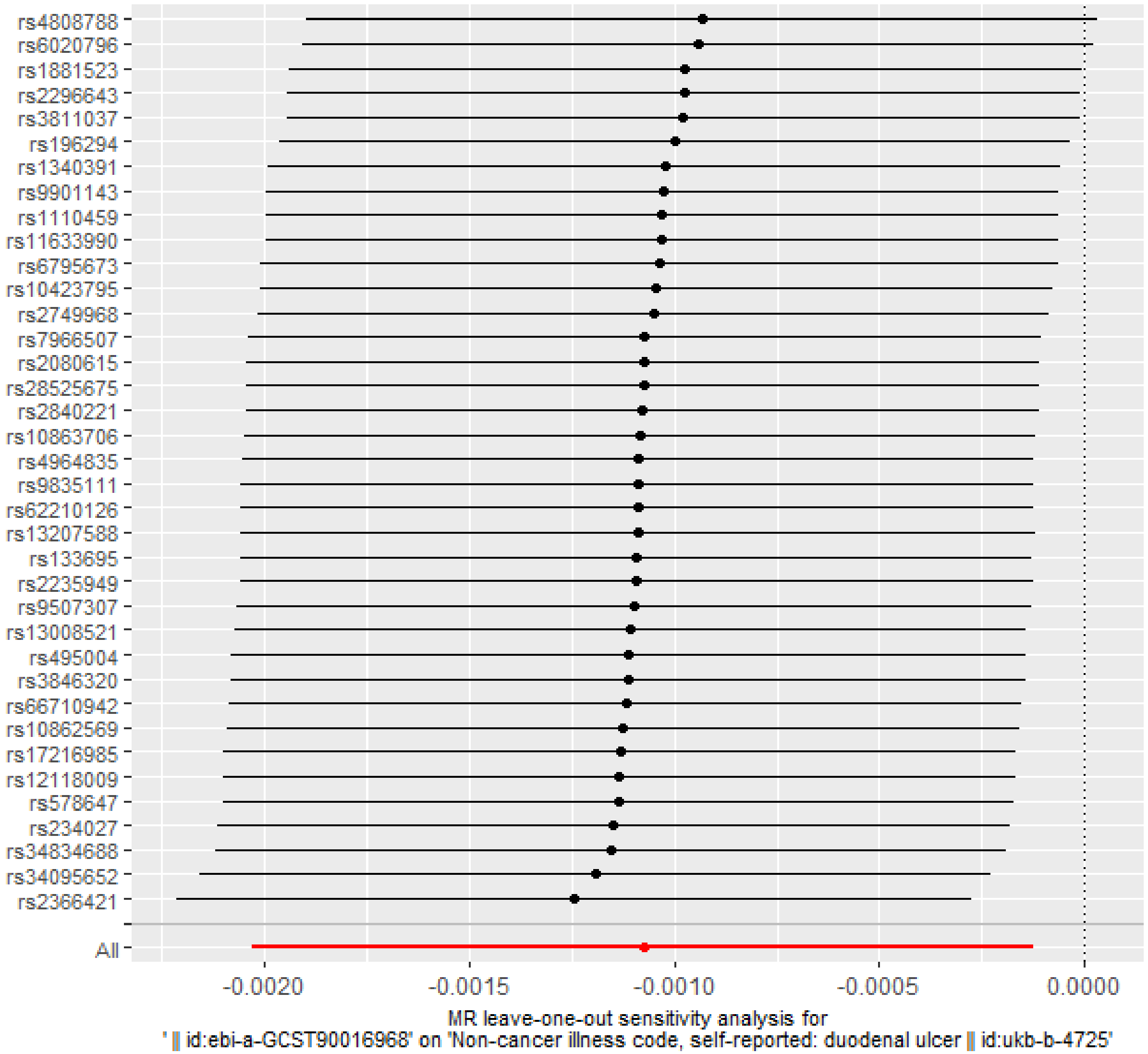

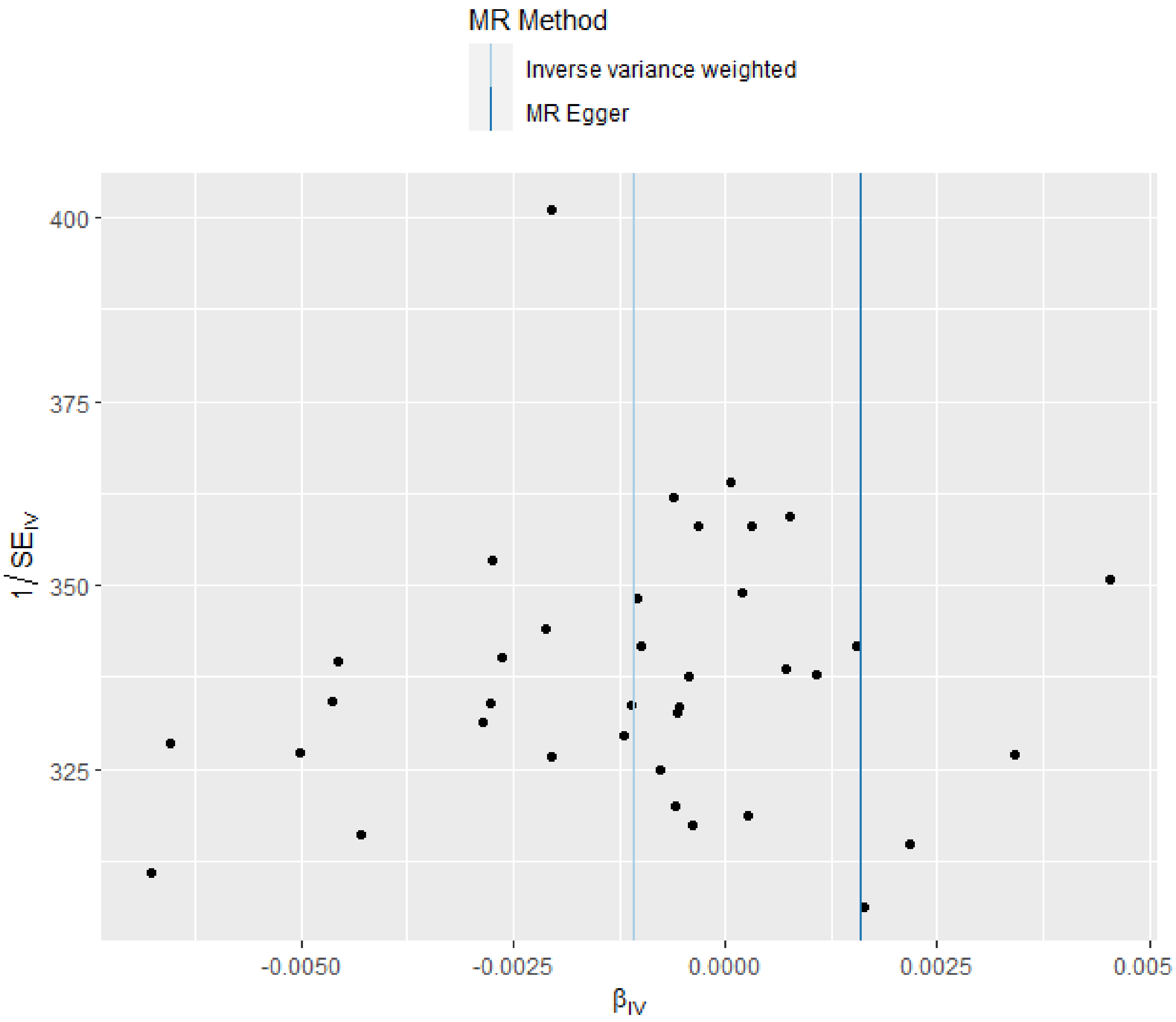

MR Method
Inverse variance weighted
MR Egger
400
375
350
325
-0.0050
-0.0025
0.0000
0.0025
0.005
β_{IV}
$1/SE_{IV}$

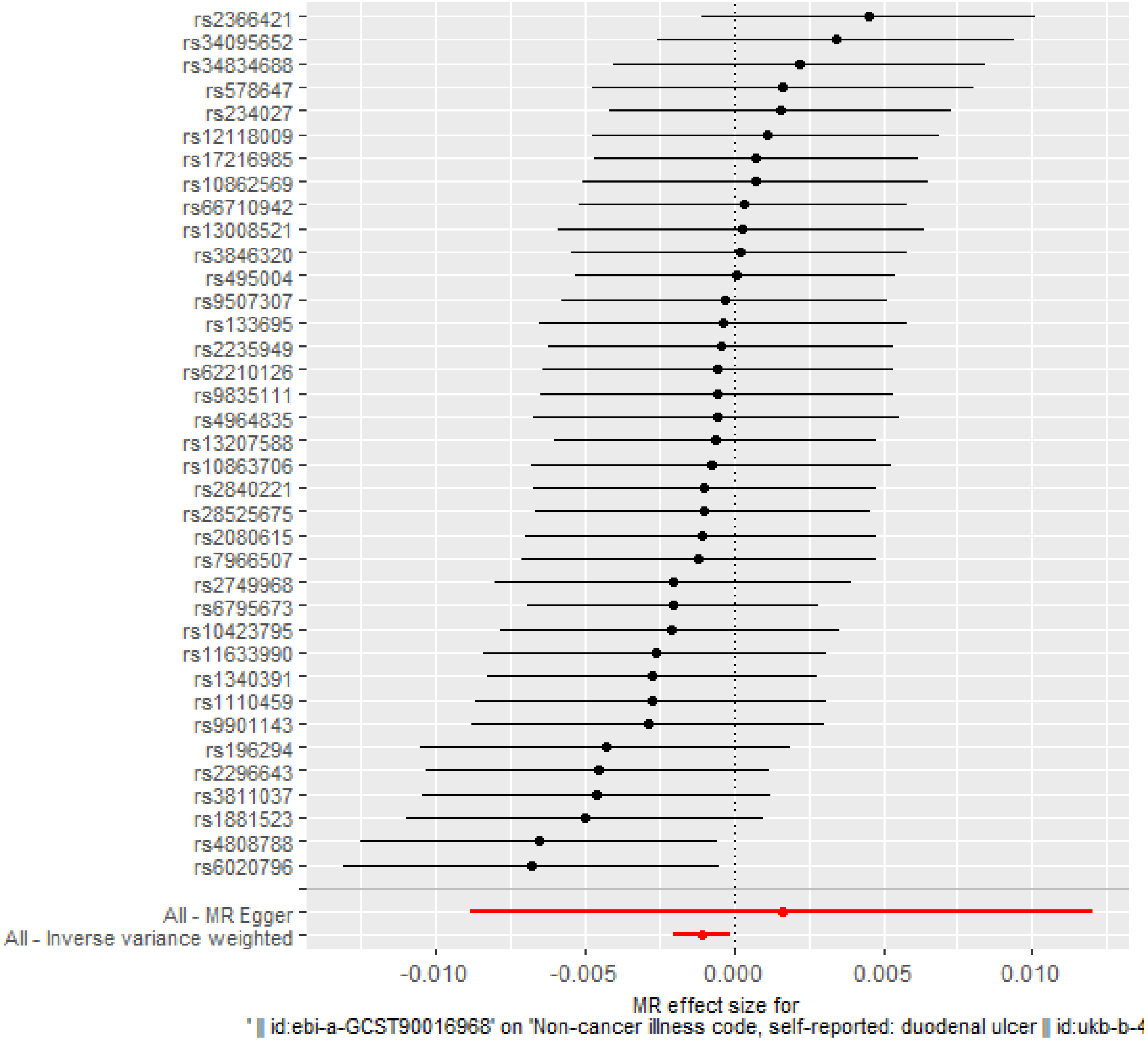
rs2366421
rs34095652
rs34834688
rs578647
rs234027
rs12118009
rs17216985
rs10862569
rs66710942
rs13008521
rs3846320
rs495004
rs9507307
rs133695
rs2235949
rs62210126
rs9835111
rs4964835
rs13207588
rs10863706
rs2840221
rs28525675
rs2080615
rs7966507
rs2749968
rs6795673
rs10423795
rs11633990
rs1340391
rs1110459
rs9901143
rs196294
rs2296643
rs3811037
rs1881523
rs4808788
rs6020796
All - MR Egger
All - Inverse variance weighted
-0.010
-0.005
0.000
0.005
0.010
MR effect size for
' || id:ebi-a-GCST90016968' on 'Non-cancer illness code, self-reported: duodenal ulcer || id:ukb-b-4

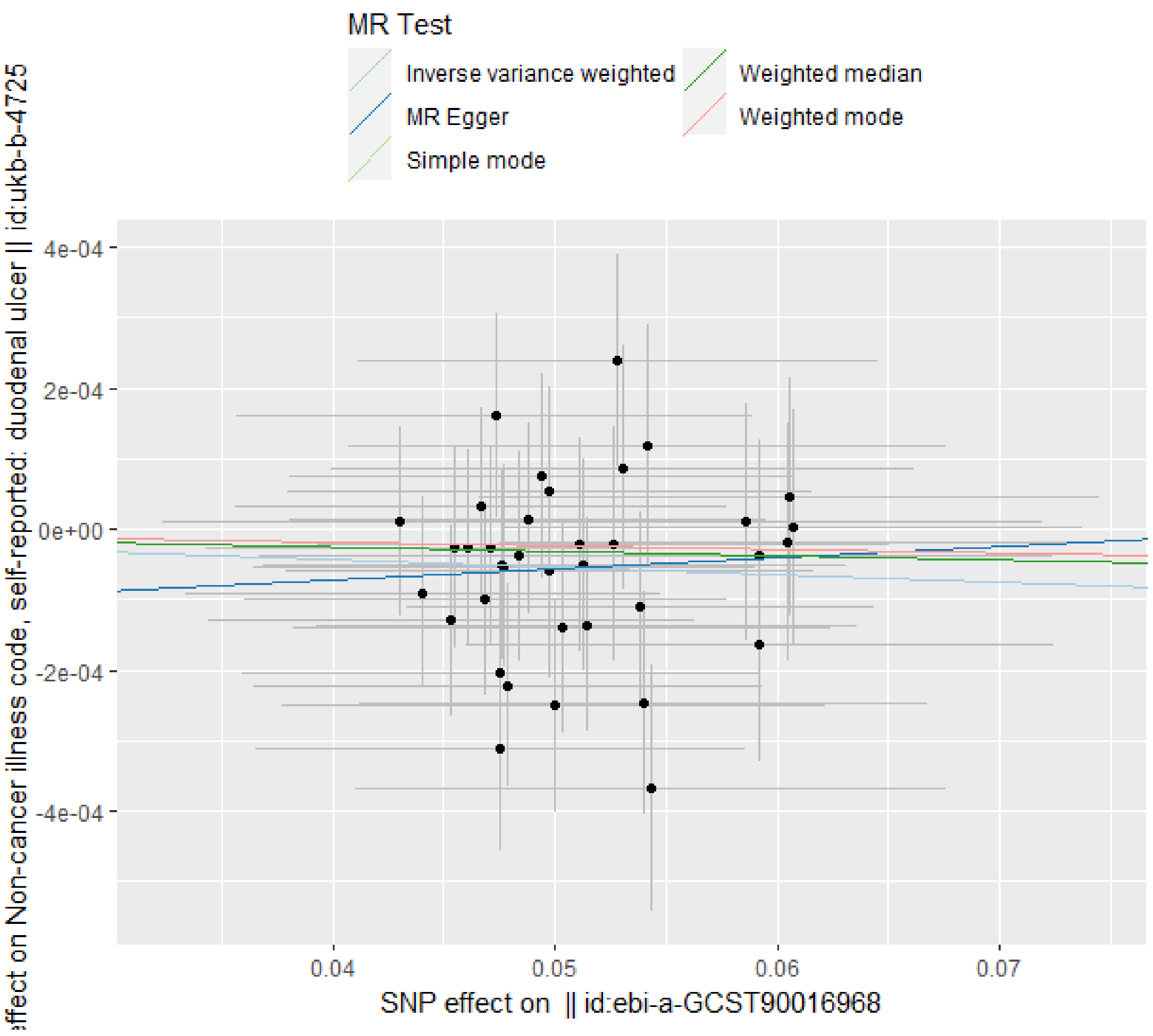
MR Test
Inverse variance weighted
MR Egger
Simple mode
Weighted median
Weighted mode
4e-04
2e-04
0e+00
-2e-04
-4e-04
0.04
0.05
0.06
0.07
SNP effect on || id:ebi-a-GCST90016968
ffect on Non-cancer illness code, self-reported: duodenal ulcer || id:ukb-b-4725

Figure 59 Leave-one-out analysis, funnel plot, MR effect size and scatter plot for gut microbiota abundance (genus Family XIII UCG001 id.11294) on duodenal ulcer

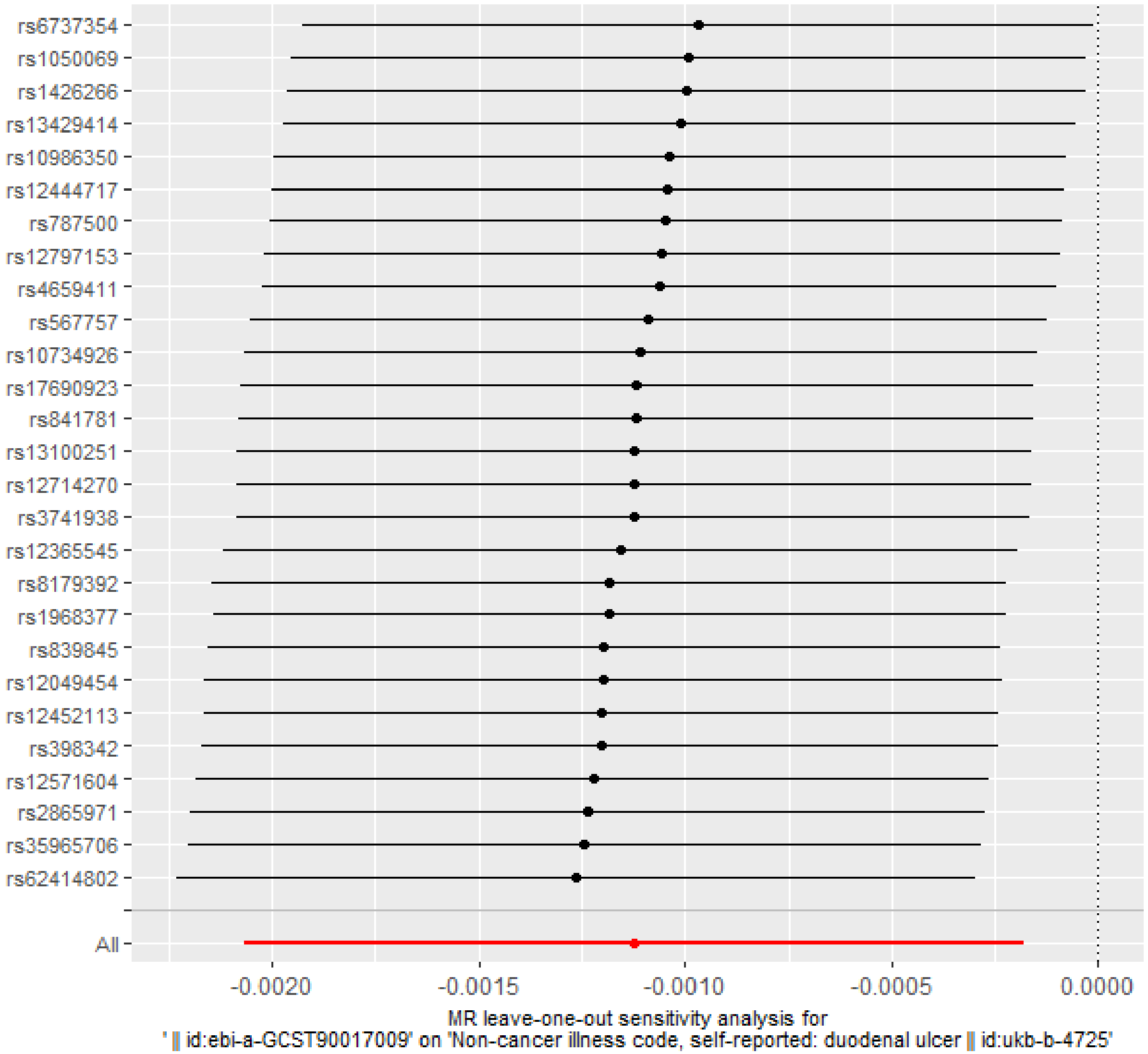

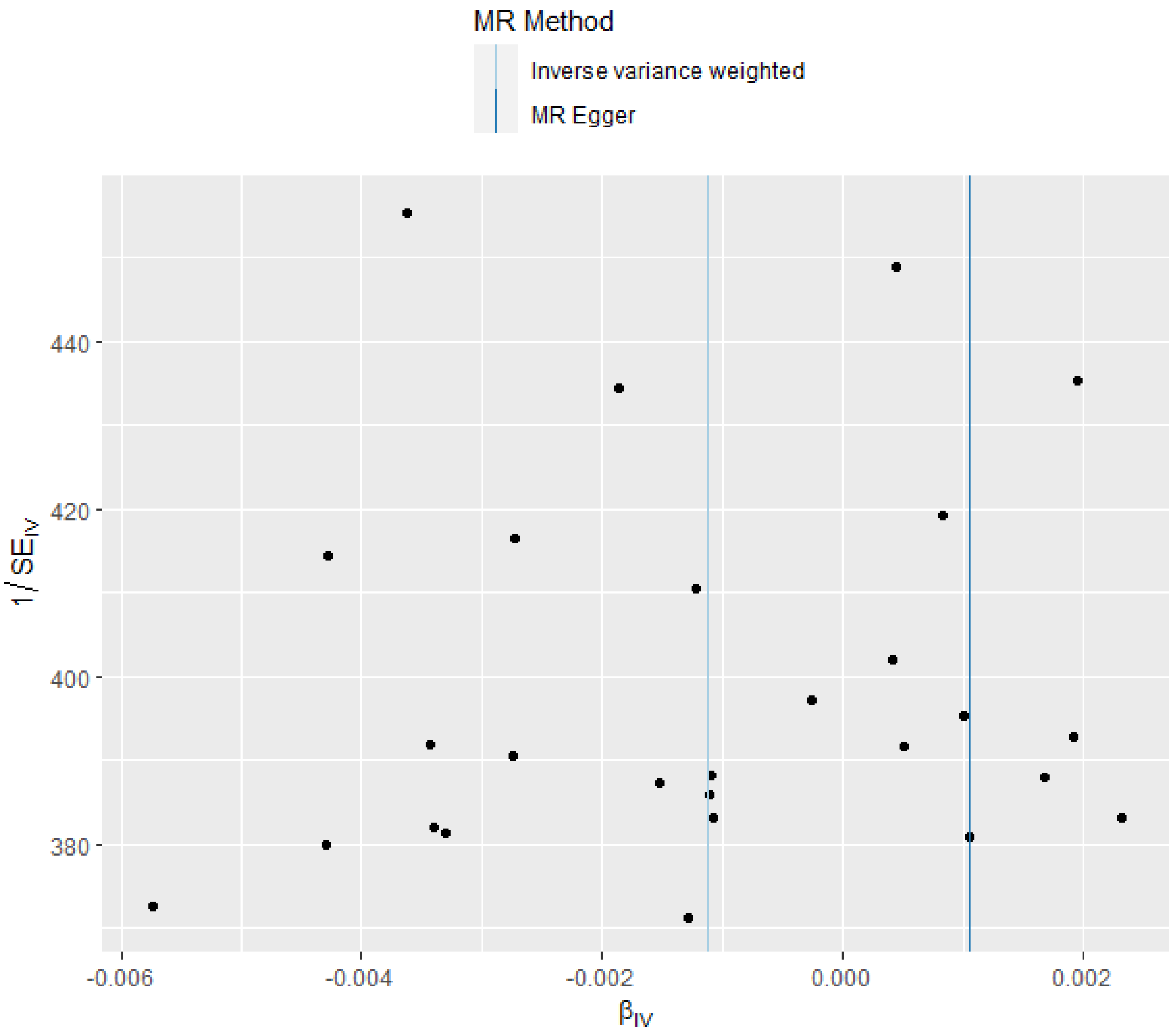

MR Method
Inverse variance weighted
MR Egger
440
420
400
380
-0.006
-0.004
-0.002
0.000
0.002
β_{IV}
$1/SE_{IV}$

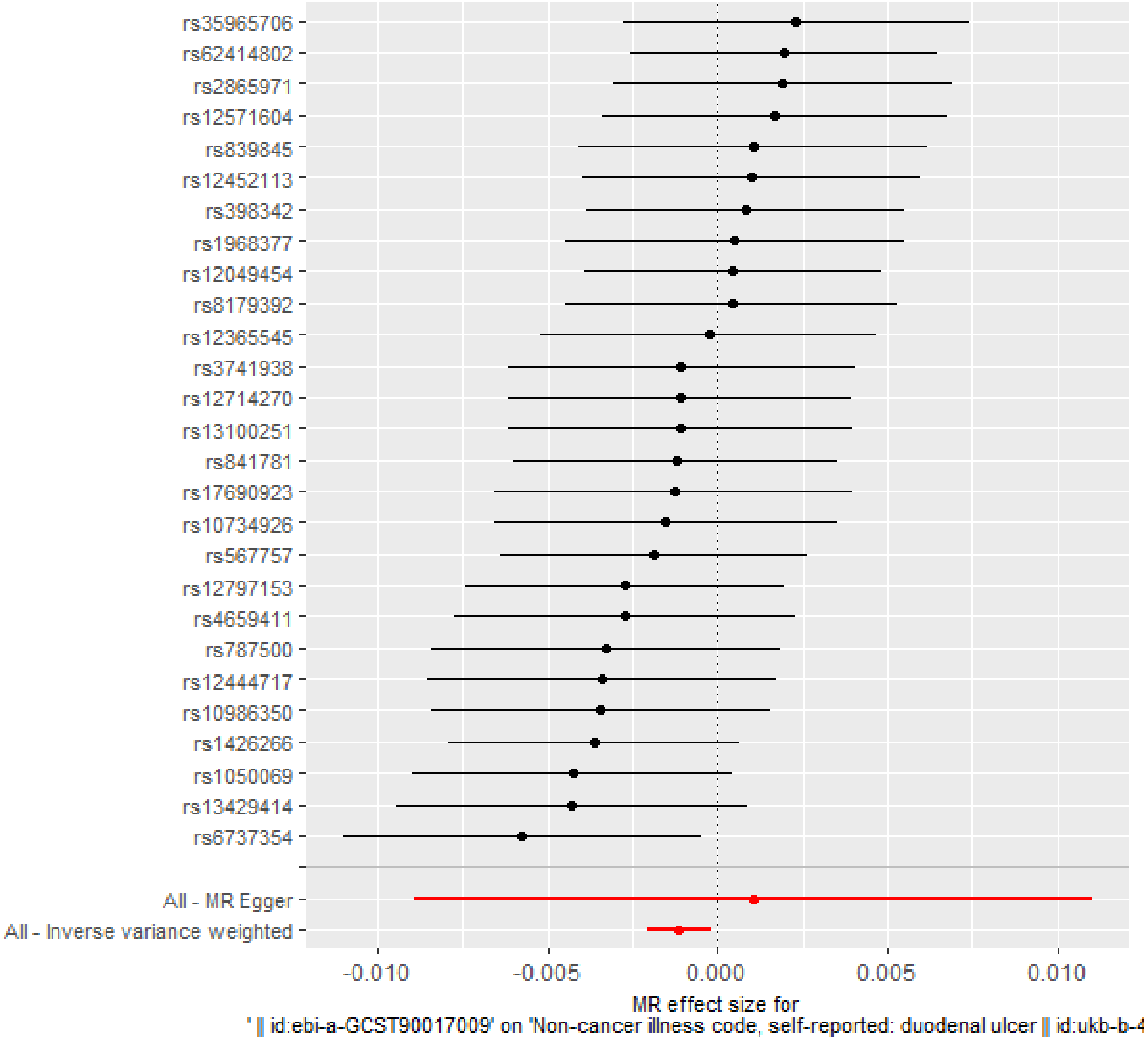

rs35965706
rs62414802
rs2865971
rs12571604
rs839845
rs12452113
rs398342
rs1968377
rs12049454
rs8179392
rs12365545
rs3741938
rs12714270
rs13100251
rs841781
rs17690923
rs10734926
rs567757
rs12797153
rs4659411
rs787500
rs12444717
rs10986350
rs1426266
rs1050069
rs13429414
rs6737354
All - MR Egger
All - Inverse variance weighted
-0.010
-0.005
0.000
0.005
0.010
MR effect size for
' || id:ebi-a-GCST90017009' on 'Non-cancer illness code, self-reported: duodenal ulcer || id:ukb-b-4

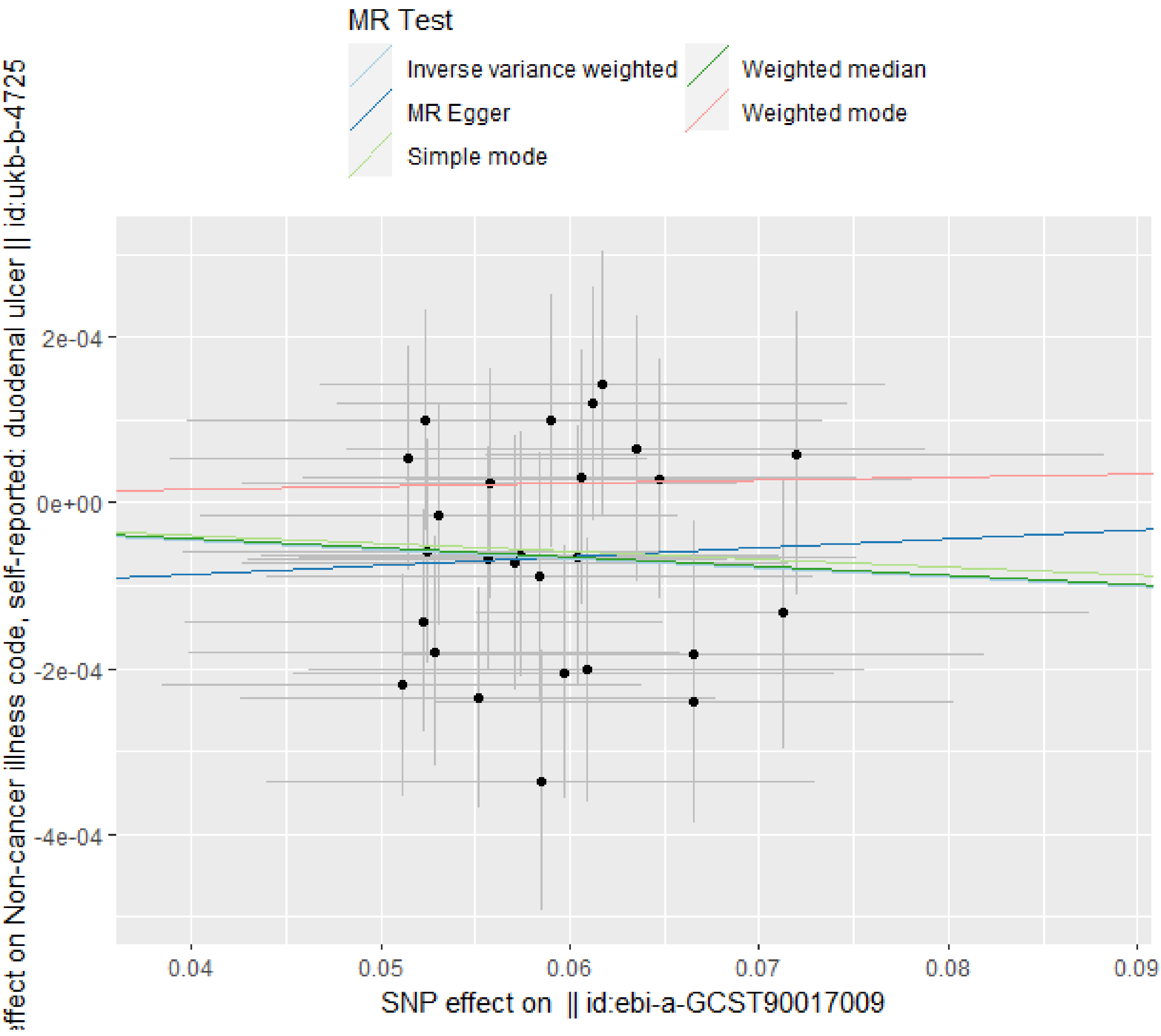

MR Test
Inverse variance weighted
MR Egger
Simple mode
Weighted median
Weighted mode
2e-04
0e+00
-2e-04
-4e-04
0.04
0.05
0.06
0.07
0.08
0.09
SNP effect on || id:ebi-a-GCST90017009
SNP effect on Non-cancer illness code, self-reported: duodenal ulcer || id:ukb-b-4725

Figure 60 Leave-one-out analysis, funnel plot, MR effect size and scatter plot for gut microbiota abundance (genus Ruminococcus gauvreauii group id.11342) on duodenal ulcer

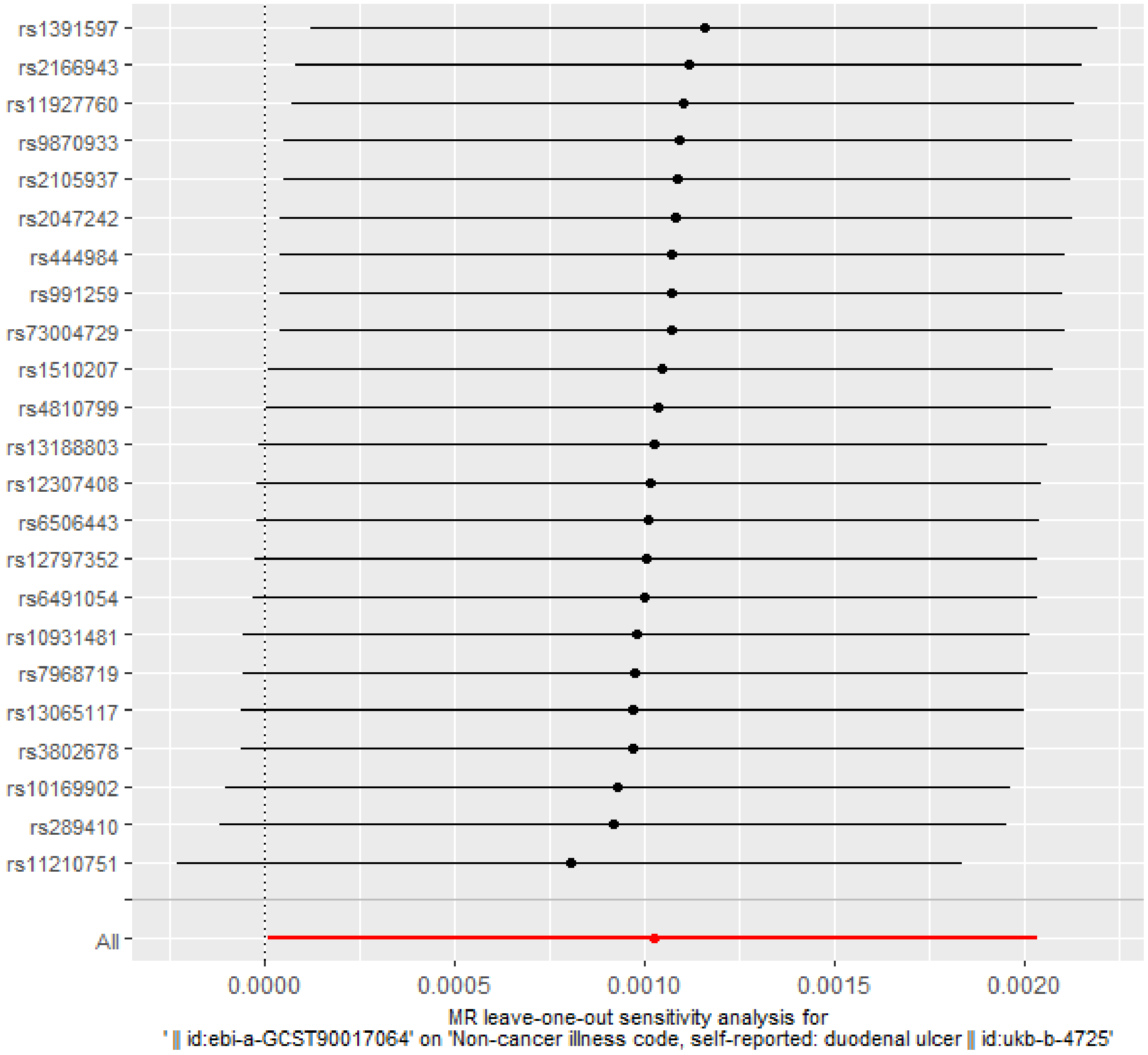

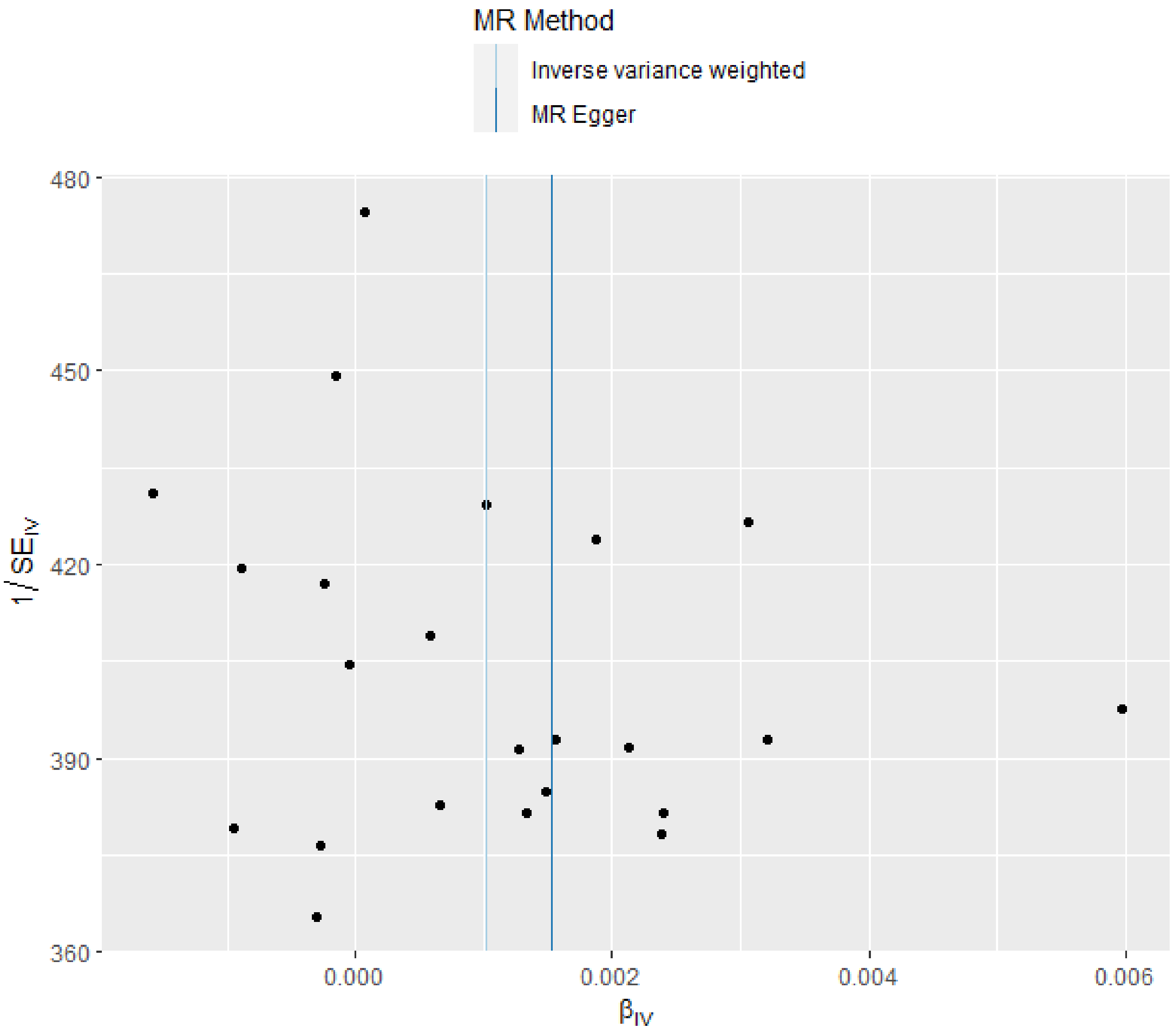
MR Method
Inverse variance weighted
MR Egger
480
450
420
390
360
1/SE_IV
0.000
0.002
0.004
0.006
β_IV

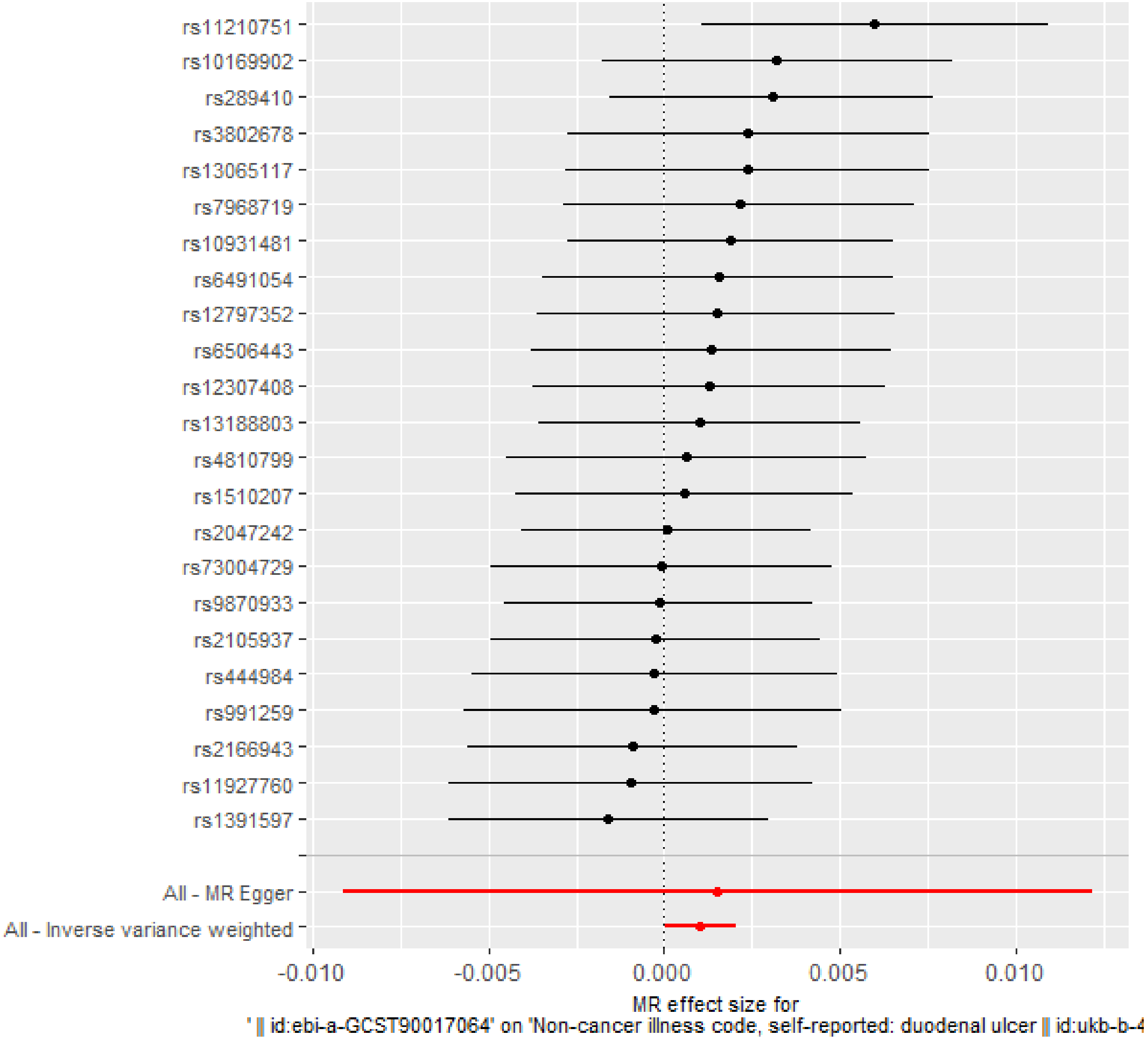

rs11210751
rs10169902
rs289410
rs3802678
rs13065117
rs7968719
rs10931481
rs6491054
rs12797352
rs6506443
rs12307408
rs13188803
rs4810799
rs1510207
rs2047242
rs73004729
rs9870933
rs2105937
rs444984
rs991259
rs2166943
rs11927760
rs1391597
All - MR Egger
All - Inverse variance weighted
-0.010
-0.005
0.000
0.005
0.010
MR effect size for
' || id:ebi-a-GCST90017064' on 'Non-cancer illness code, self-reported: duodenal ulcer || id:ukb-b-4

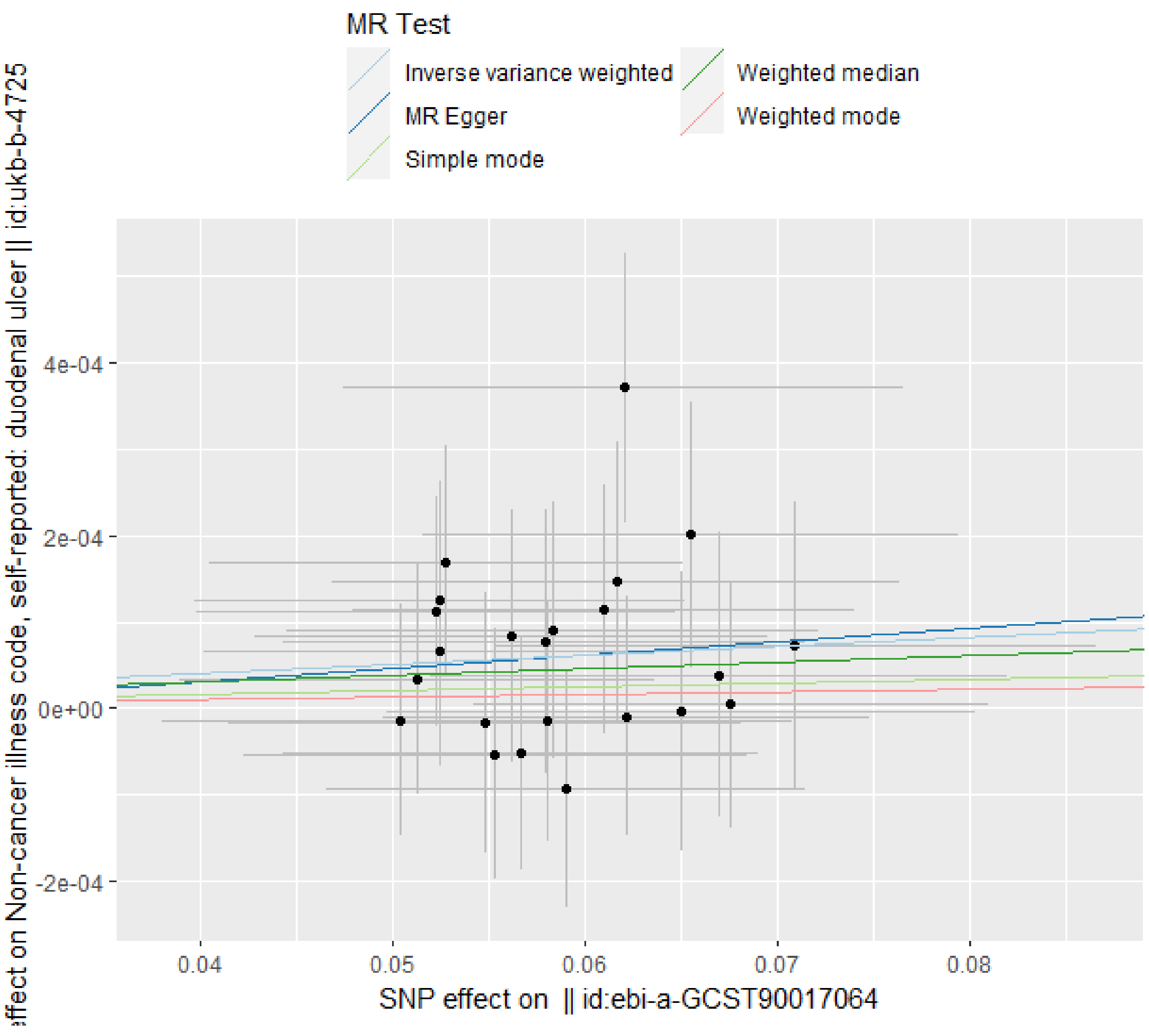

MR Test
Inverse variance weighted
MR Egger
Simple mode
Weighted median
Weighted mode
4e-04
2e-04
0e+00
-2e-04
0.04
0.05
0.06
0.07
0.08
SNP effect on || id:ebi-a-GCST90017064
effect on Non-cancer illness code, self-reported: duodenal ulcer || id:ukb-b-4725

Figure 61 Leave-one-out analysis, funnel plot, MR effect size and scatter plot for gut microbiota abundance (order Verrucomicrobiales id.4030) on duodenal ulcer

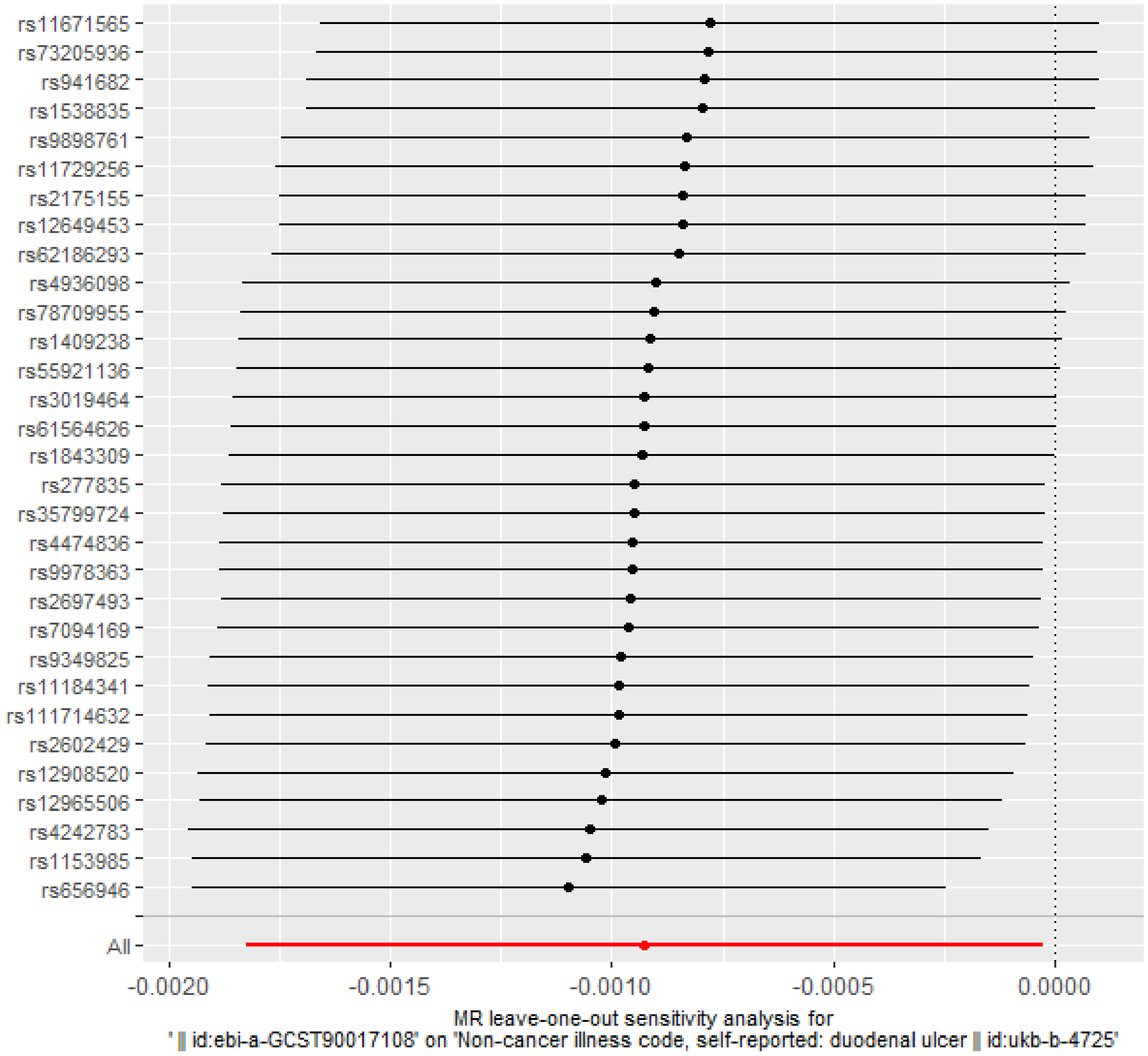

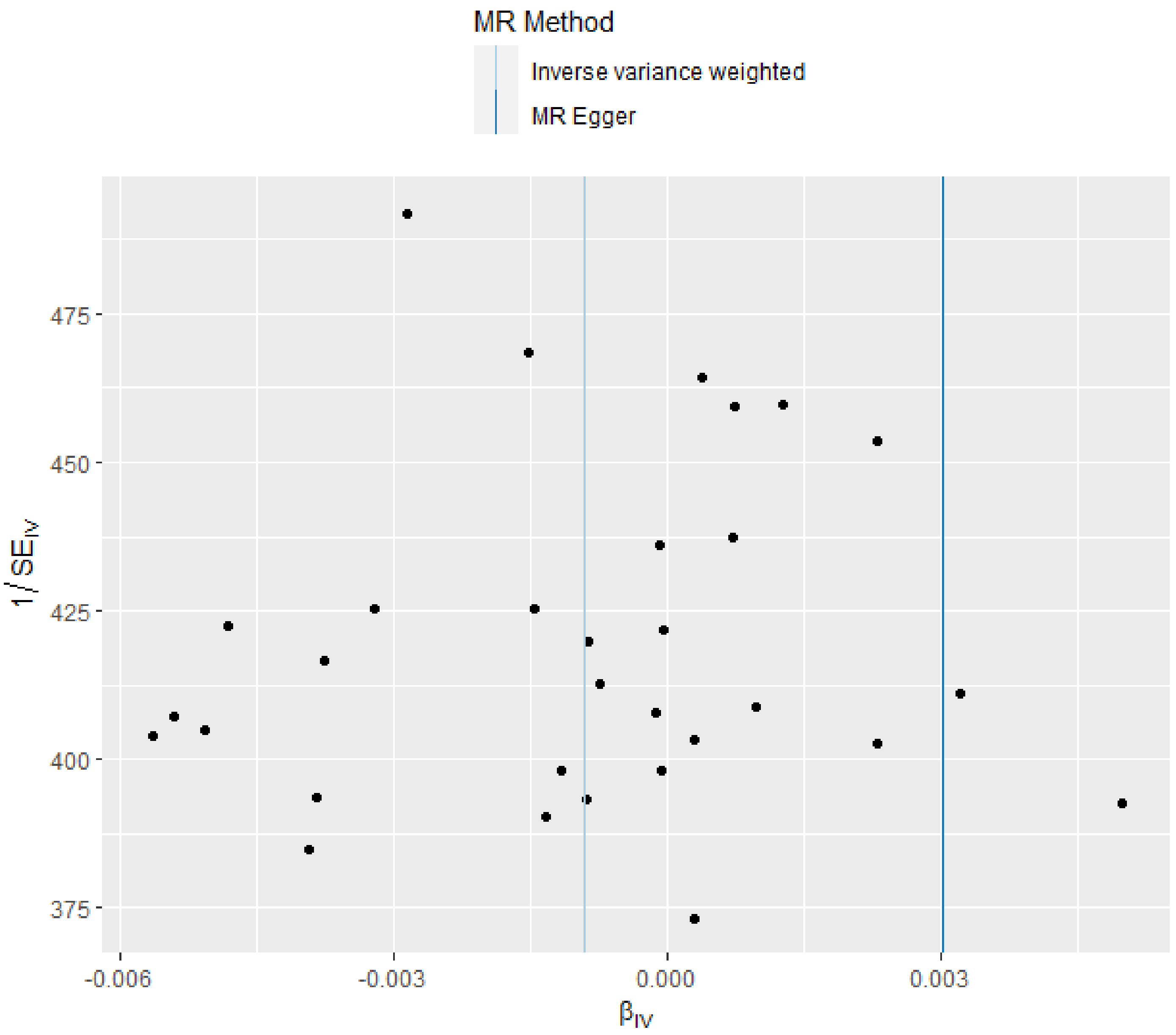

MR Method
Inverse variance weighted
MR Egger
475
450
425
400
375
1/SE_IV
-0.006
-0.003
0.000
0.003
β_IV

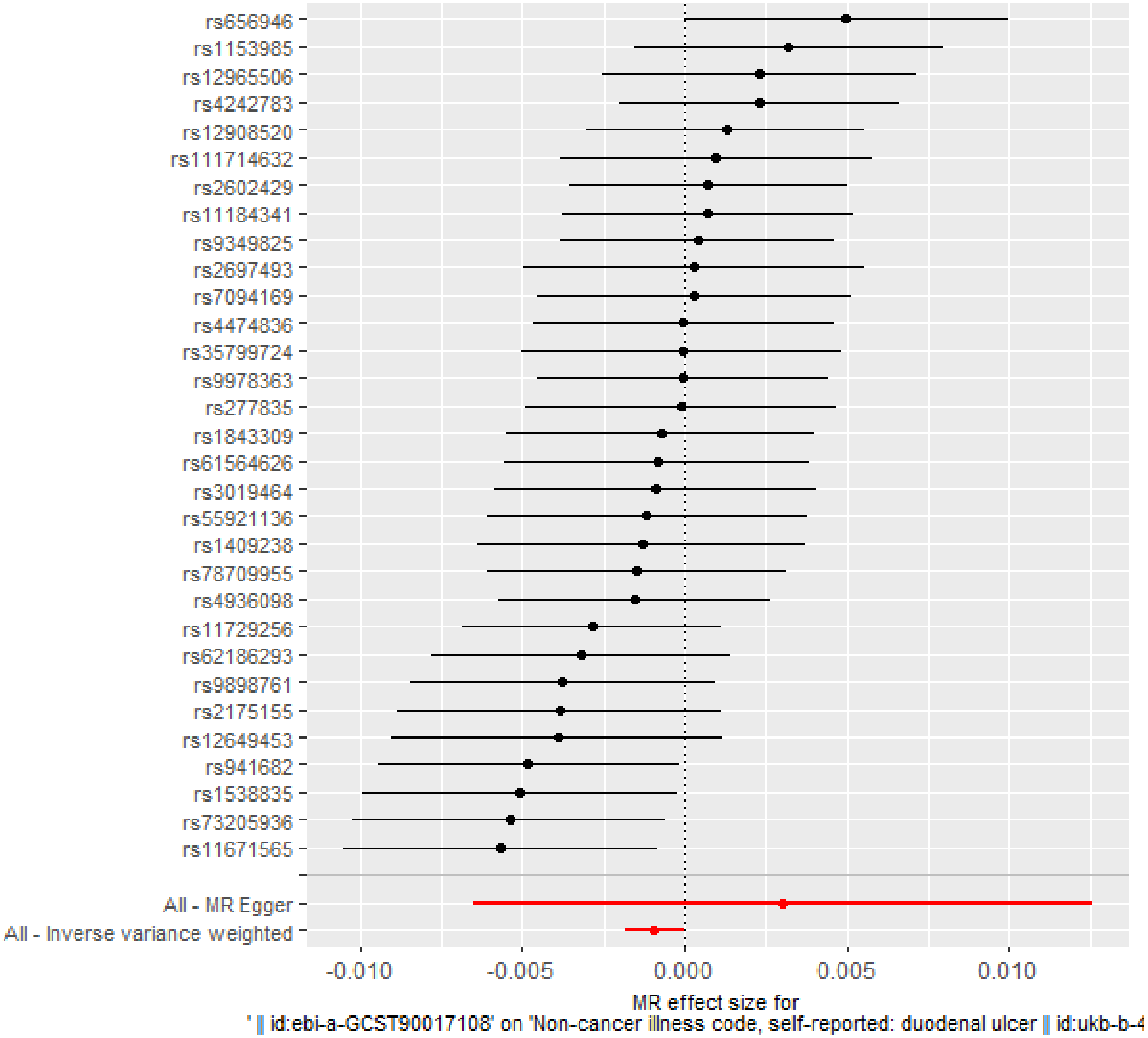
rs656946
rs1153985
rs12965506
rs4242783
rs12908520
rs111714632
rs2602429
rs11184341
rs9349825
rs2697493
rs7094169
rs4474836
rs35799724
rs9978363
rs277835
rs1843309
rs61564626
rs3019464
rs55921136
rs1409238
rs78709955
rs4936098
rs11729256
rs62186293
rs9898761
rs2175155
rs12649453
rs941682
rs1538835
rs73205936
rs11671565
All - MR Egger
All - Inverse variance weighted
-0.010
-0.005
0.000
0.005
0.010
MR effect size for
' || id:ebi-a-GCST90017108' on 'Non-cancer illness code, self-reported: duodenal ulcer || id:ukb-b-4

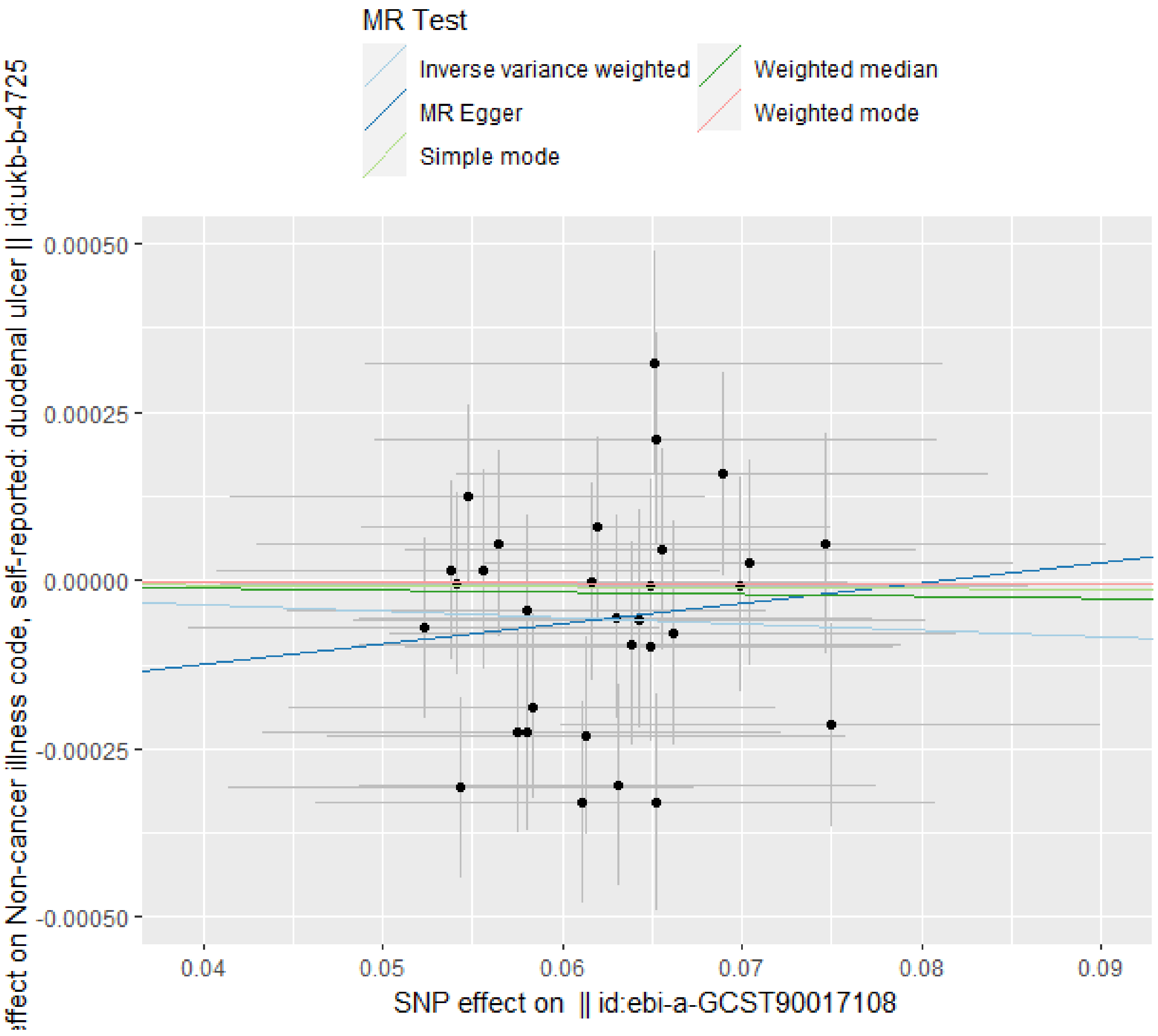

MR Test
Inverse variance weighted
MR Egger
Simple mode
Weighted median
Weighted mode
effect on Non-cancer illness code, self-reported: duodenal ulcer || id:ukb-b-4725
0.00050
0.00025
0.00000
-0.00025
-0.00050
0.04
0.05
0.06
0.07
0.08
0.09
SNP effect on || id:ebi-a-GCST90017108

Figure 62 Leave-one-out analysis, funnel plot, MR effect size and scatter plot for gut microbiota abundance (class Verrucomicrobiae id.4029) on chronic gastritis

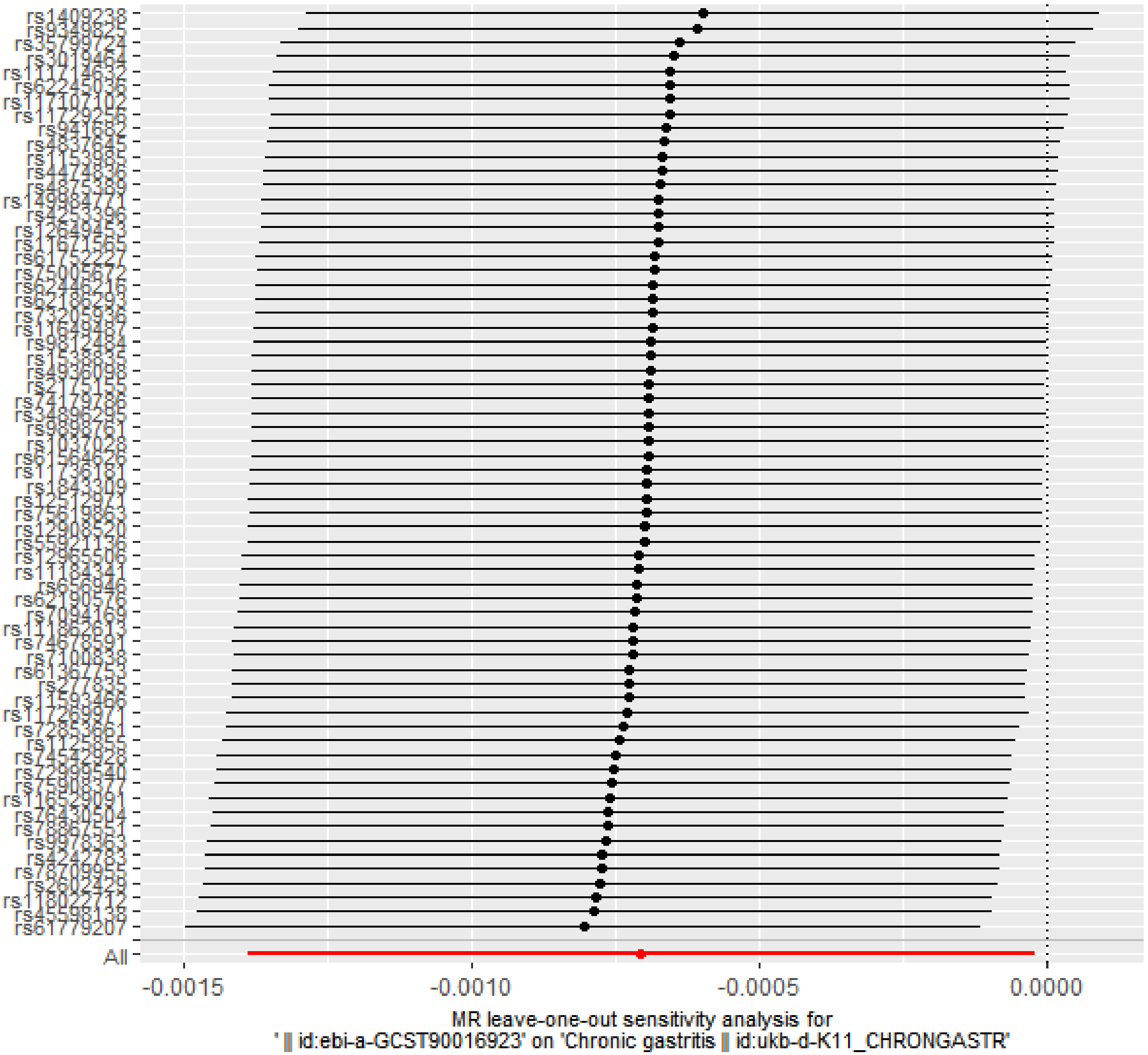

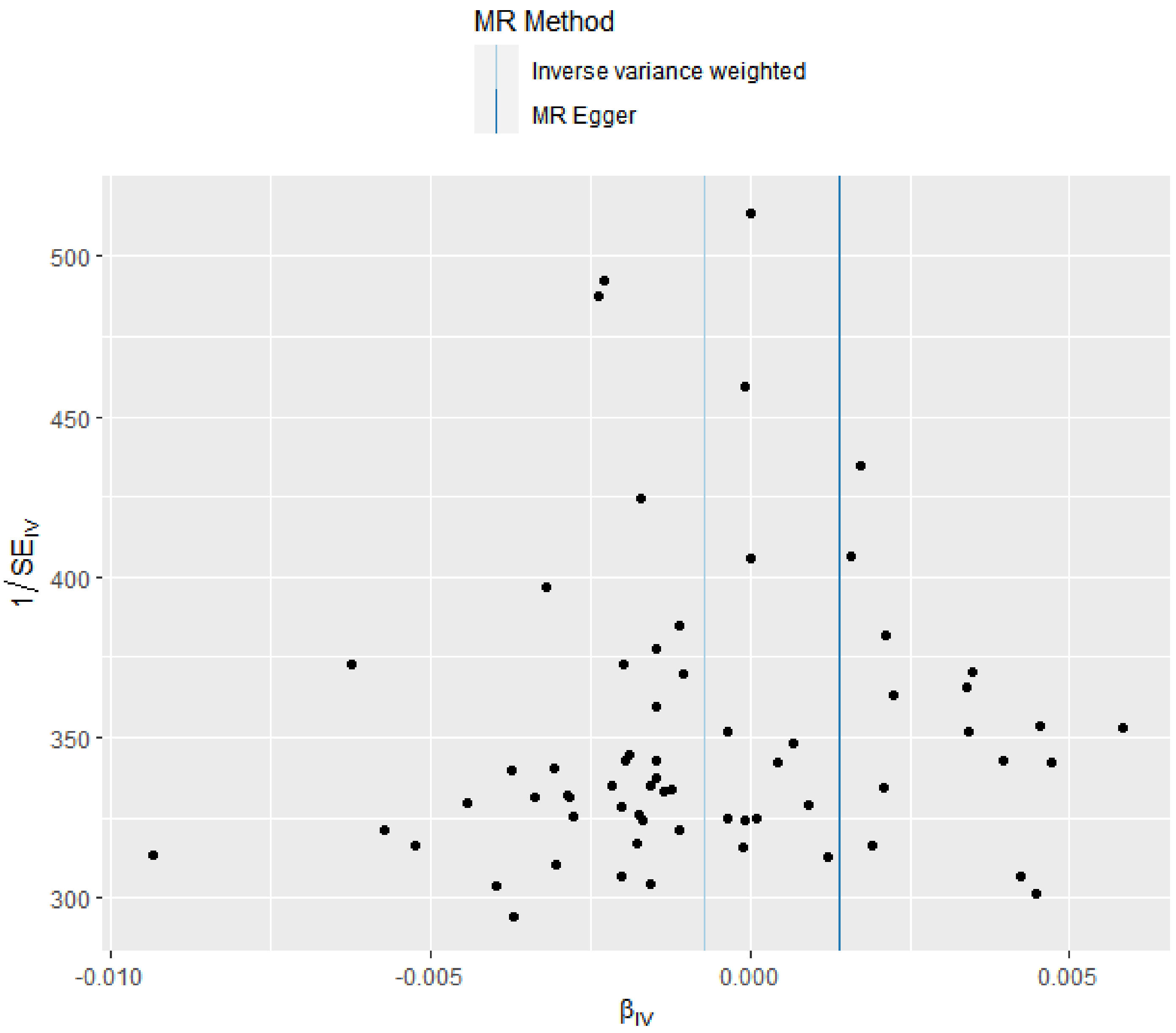

MR Method
Inverse variance weighted
MR Egger
500
450
400
350
300
1/SE_IV
-0.010
-0.005
0.000
0.005
β_IV

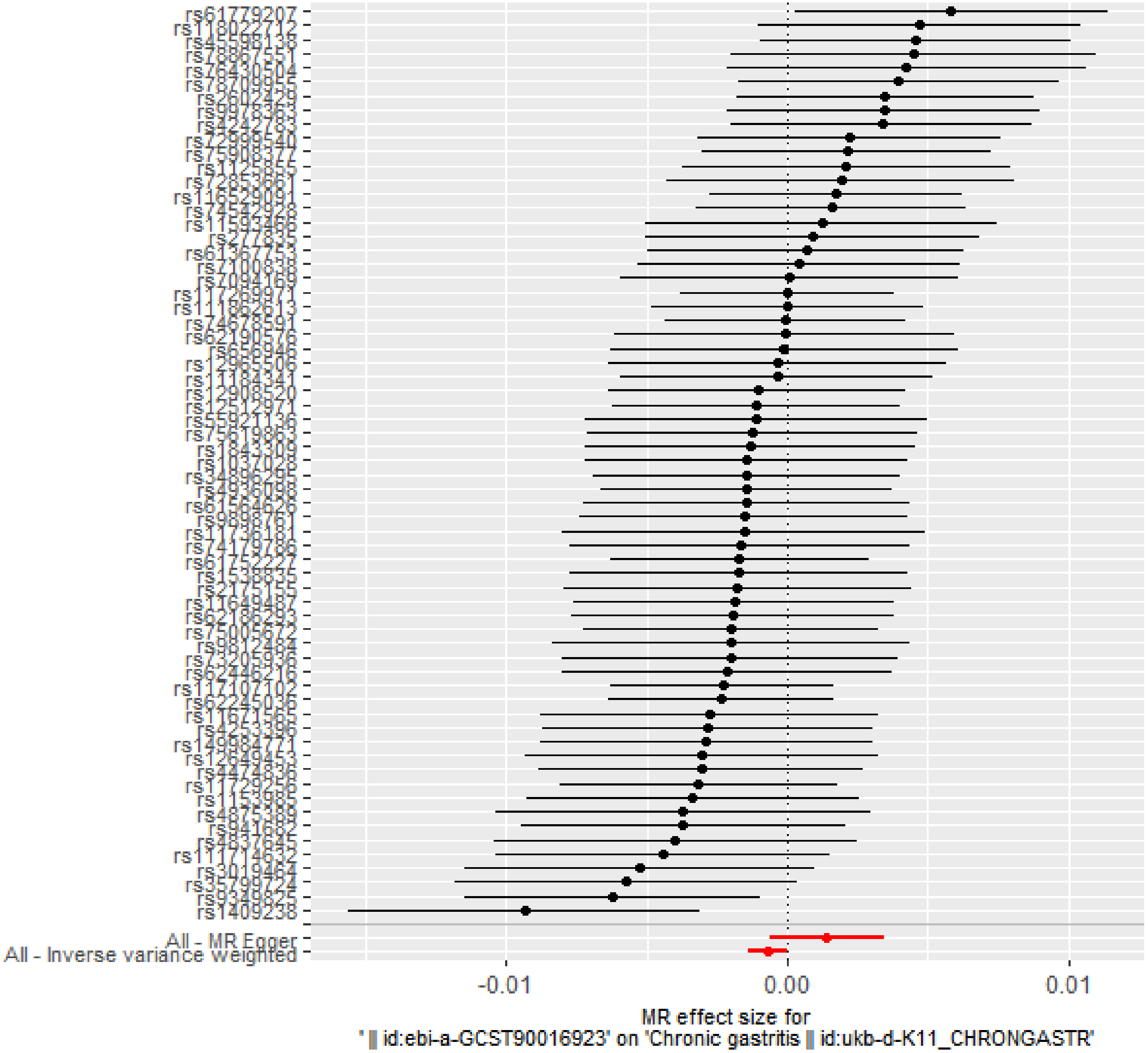
rs61779207
rs1409238
All - MR Egger
All - Inverse variance weighted
-0.01
0.00
0.01
MR effect size for
' || id:ebi-a-GCST90016923' on 'Chronic gastritis || id:ukb-d-K11_CHRONGASTR'

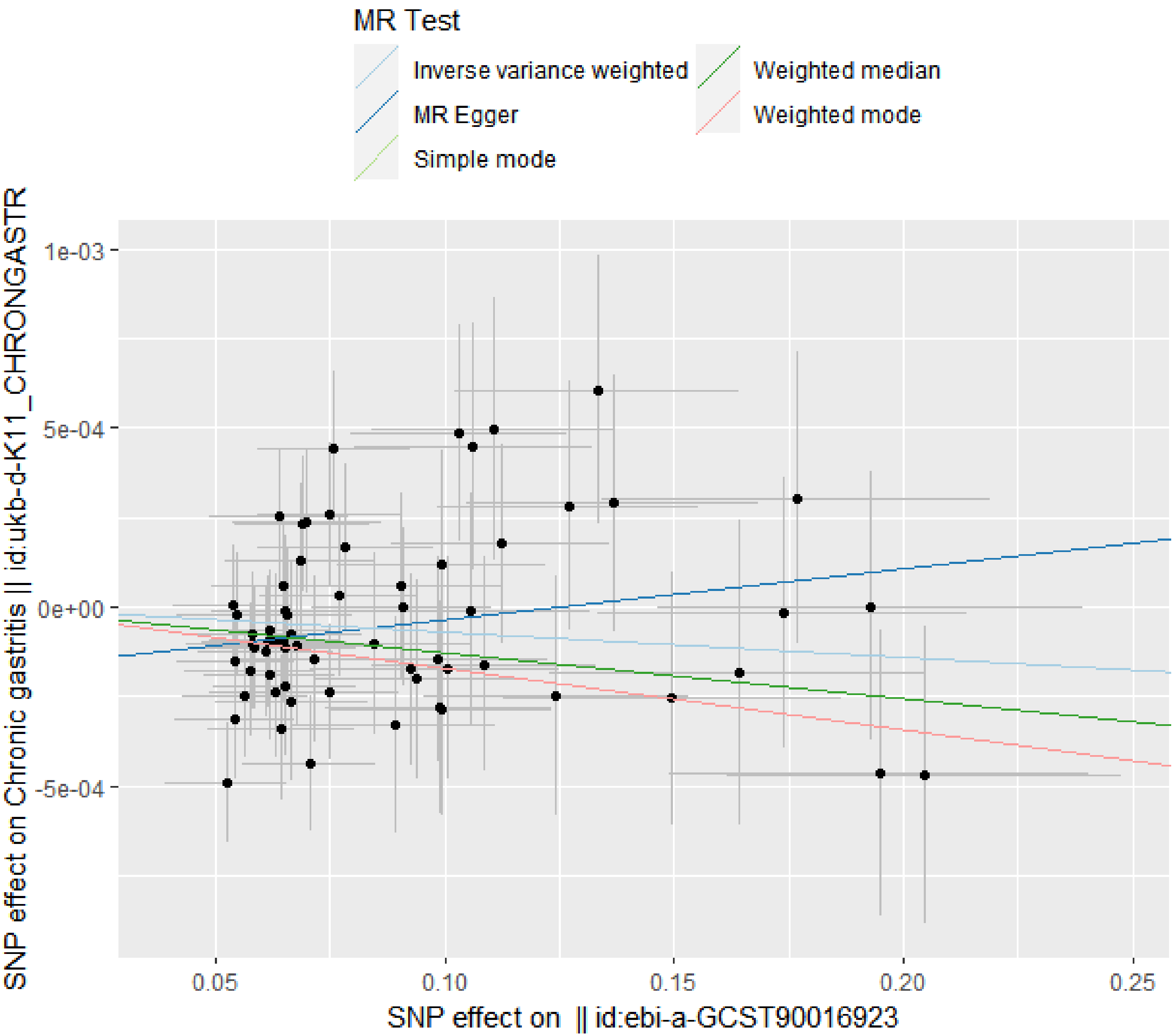

MR Test
Inverse variance weighted
MR Egger
Simple mode
Weighted median
Weighted mode
1e-03
5e-04
0e+00
-5e-04
0.05
0.10
0.15
0.20
0.25
SNP effect on Chronic gastritis || id:ukb-d-K11_CHRONGASTR
SNP effect on || id:ebi-a-GCST90016923

Figure 63 Leave-one-out analysis, funnel plot, MR effect size and scatter plot for gut microbiota abundance (genus Butyricicoccus id.2055) on chronic gastritis

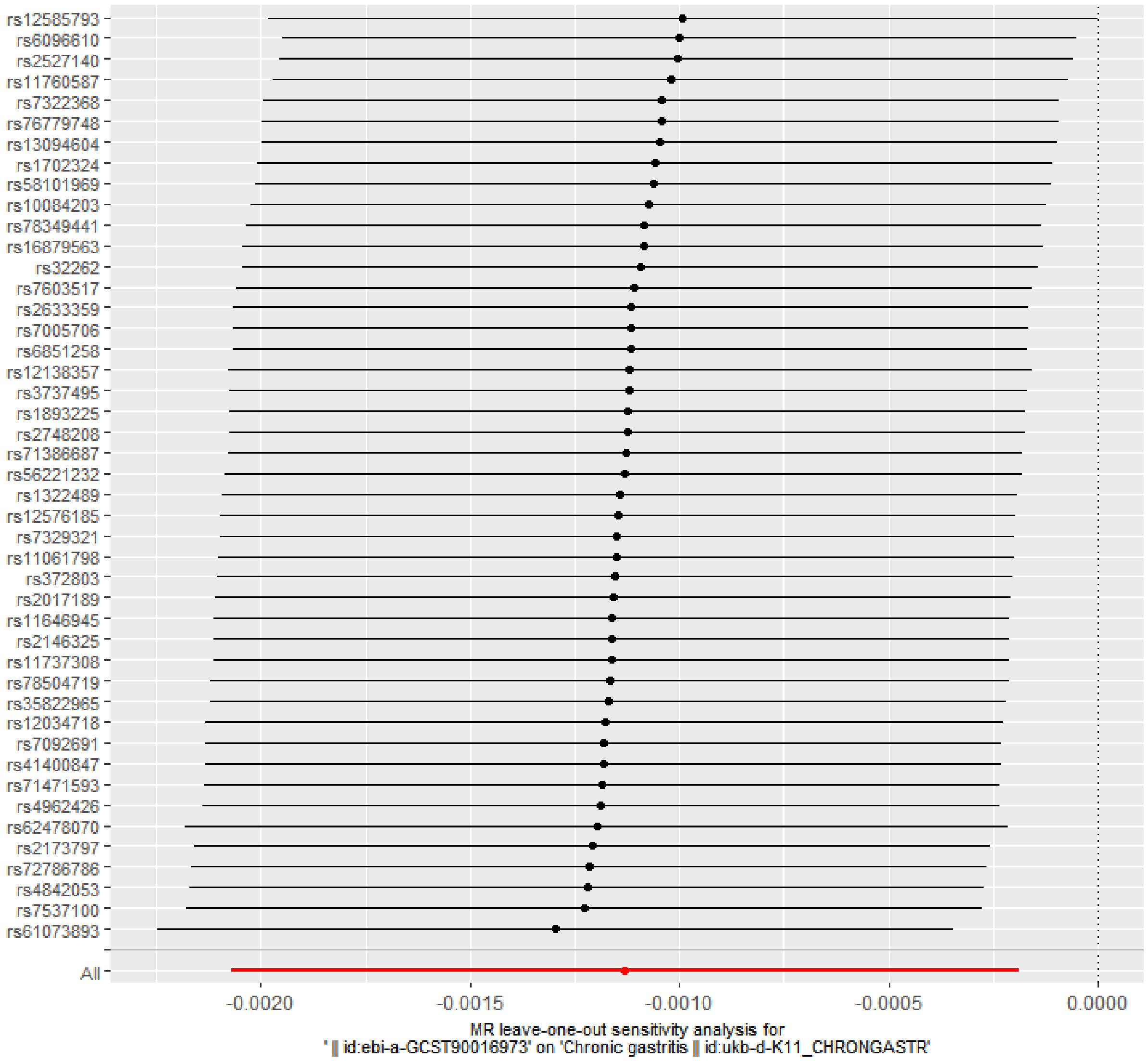

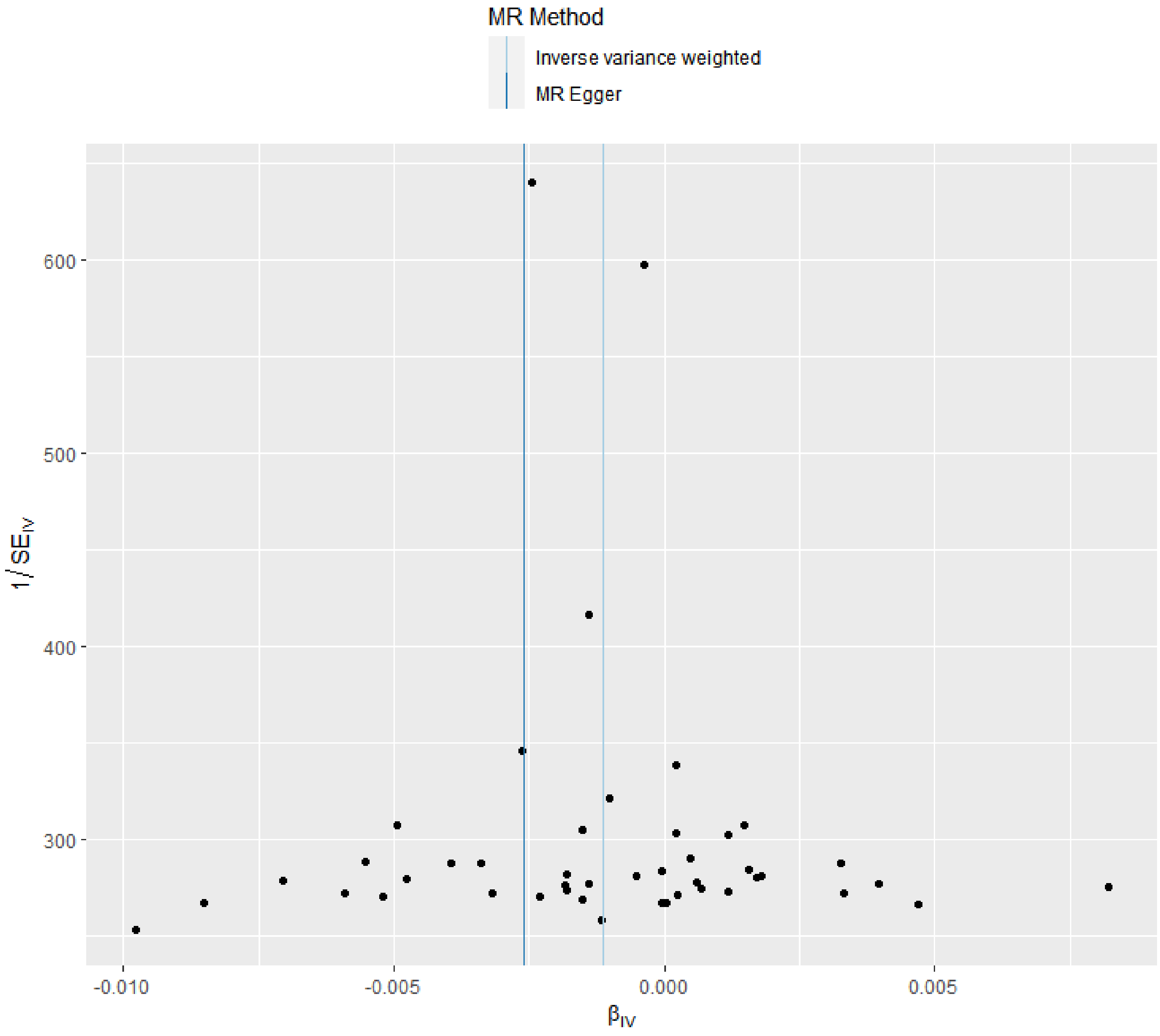

MR Method
Inverse variance weighted
MR Egger
600
500
400
300
1/SE_IV
-0.010
-0.005
0.000
0.005
β_IV

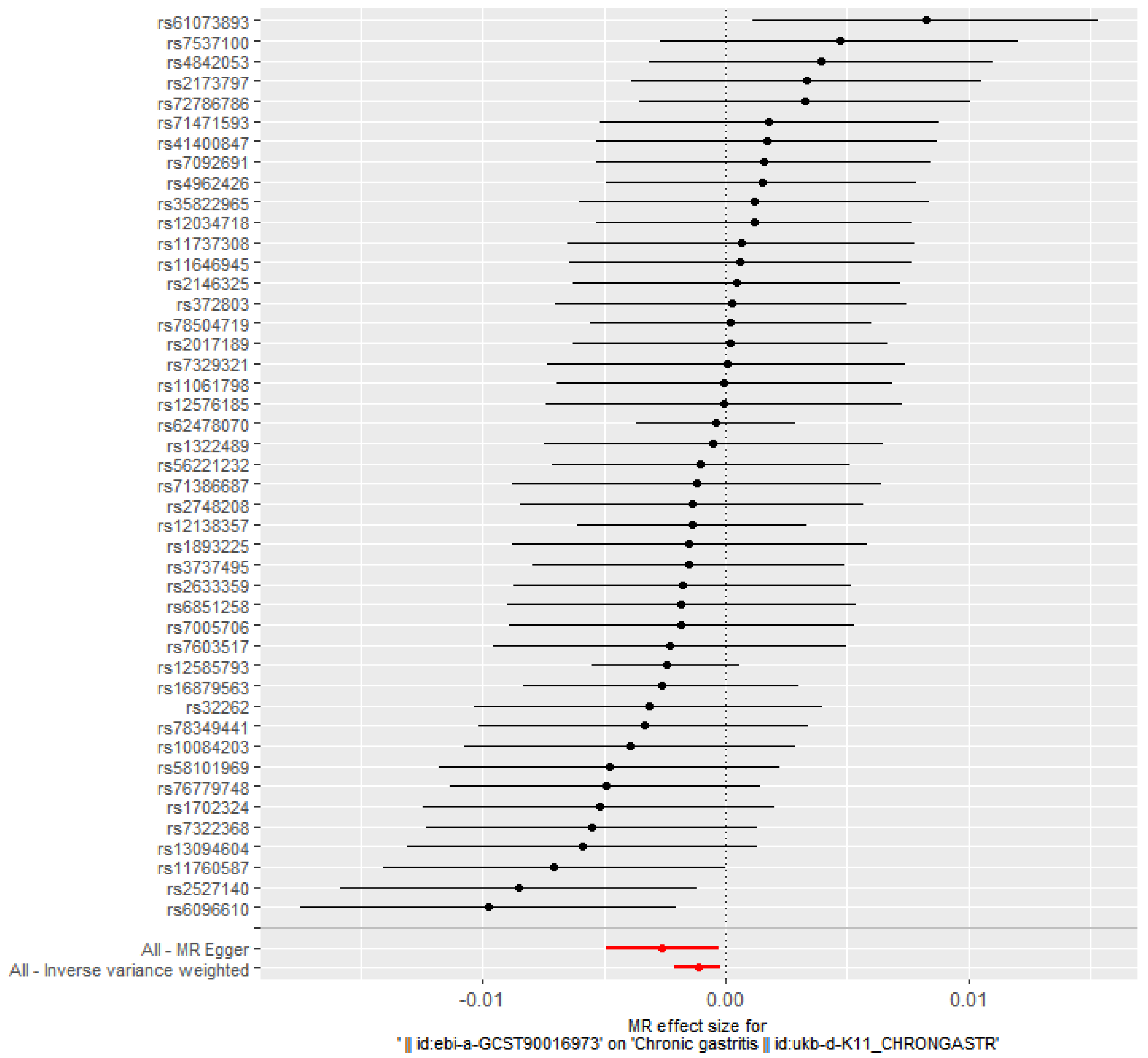
rs61073893
rs7537100
rs4842053
rs2173797
rs72786786
rs71471593
rs41400847
rs7092691
rs4962426
rs35822965
rs12034718
rs11737308
rs11646945
rs2146325
rs372803
rs78504719
rs2017189
rs7329321
rs11061798
rs12576185
rs62478070
rs1322489
rs56221232
rs71386687
rs2748208
rs12138357
rs1893225
rs3737495
rs2633359
rs6851258
rs7005706
rs7603517
rs12585793
rs16879563
rs32262
rs78349441
rs10084203
rs58101969
rs76779748
rs1702324
rs7322368
rs13094604
rs11760587
rs2527140
rs6096610
All - MR Egger
All - Inverse variance weighted
-0.01
0.00
0.01
MR effect size for
'|| id:ebi-a-GCST90016973' on 'Chronic gastritis || id:ukb-d-K11_CHRONGASTR'

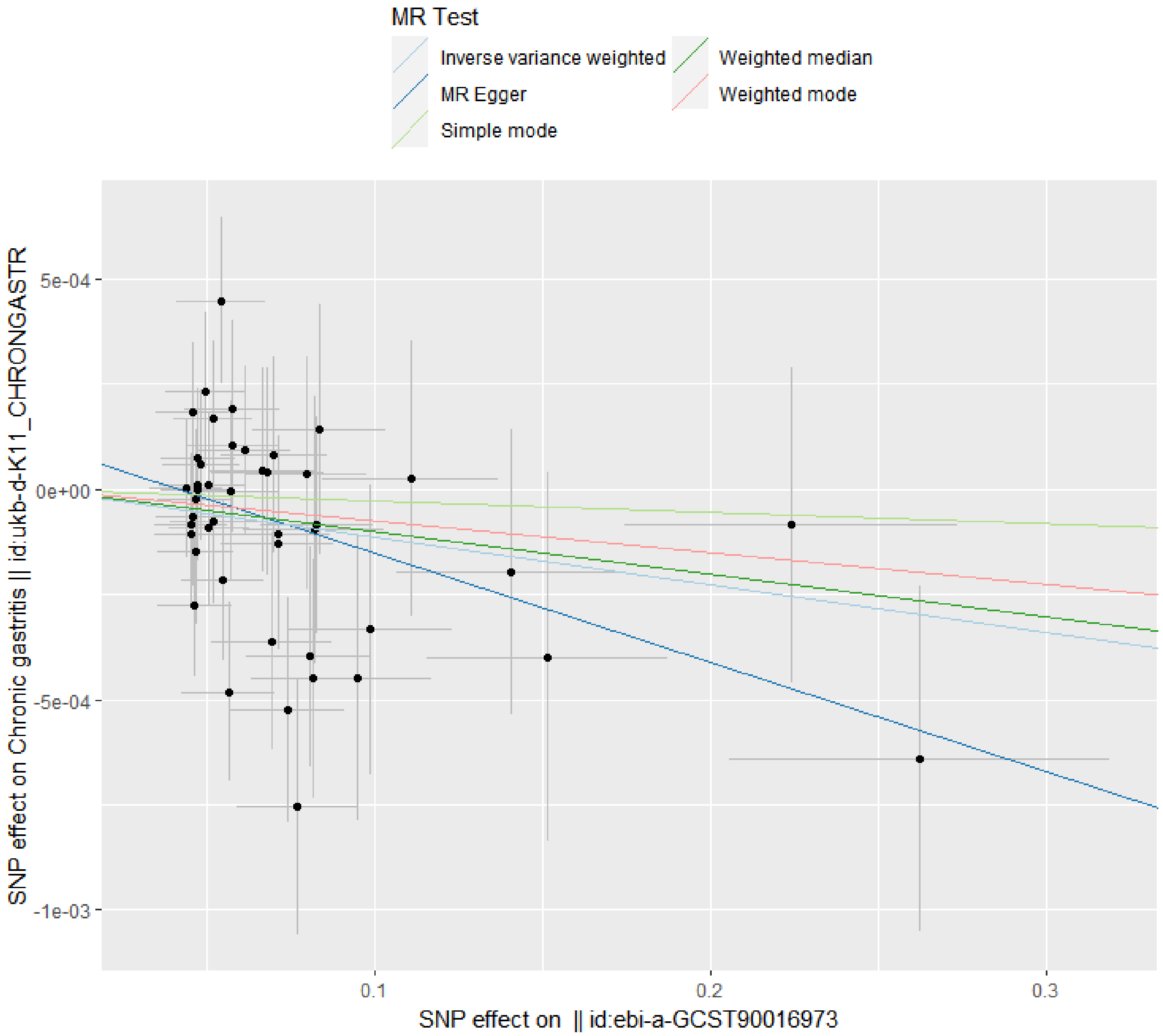

MR Test
Inverse variance weighted
MR Egger
Simple mode
Weighted median
Weighted mode
5e-04
0e+00
-5e-04
-1e-03
0.1
0.2
0.3
SNP effect on Chronic gastritis || id:ukb-d-K11_CHRONGASTR
SNP effect on || id:ebi-a-GCST90016973

Figure 64 Leave-one-out analysis, funnel plot, MR effect size and scatter plot for gut microbiota abundance (genus Eubacterium fissicatena group id.14373) on chronic gastritis

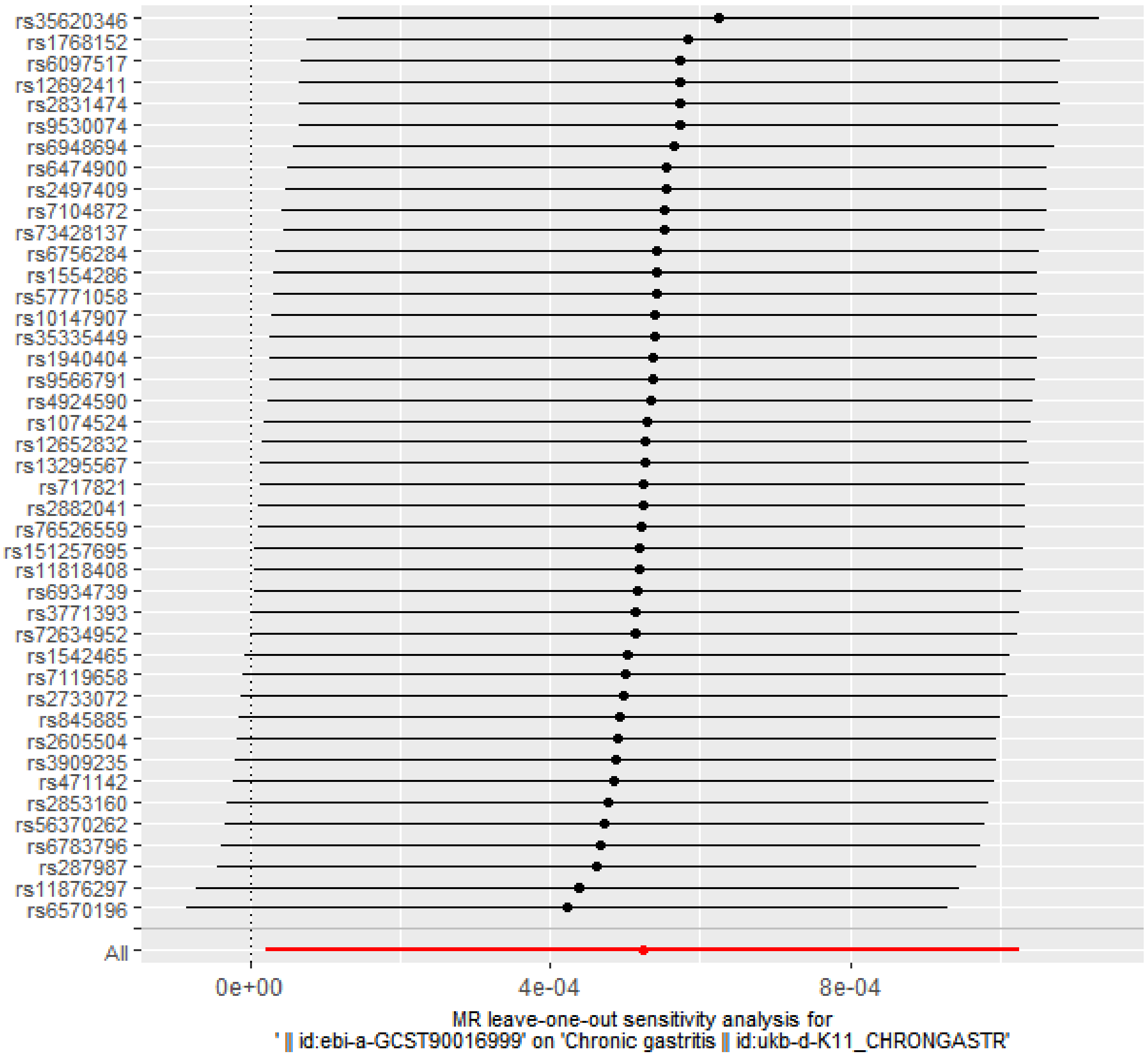

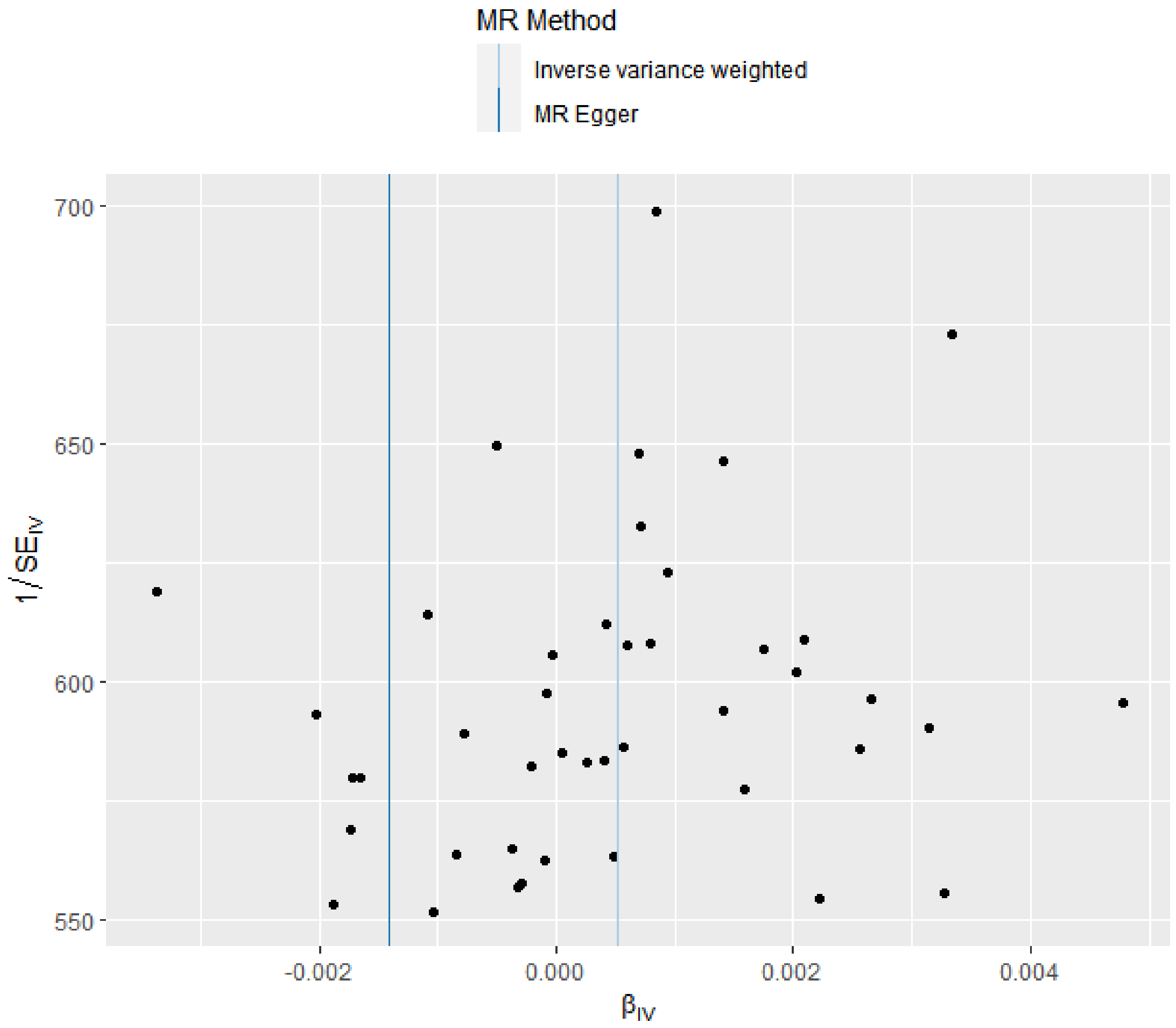
MR Method
Inverse variance weighted
MR Egger
700
650
600
550
1/SE$_{IV}$
-0.002
0.000
0.002
0.004
β_{IV}

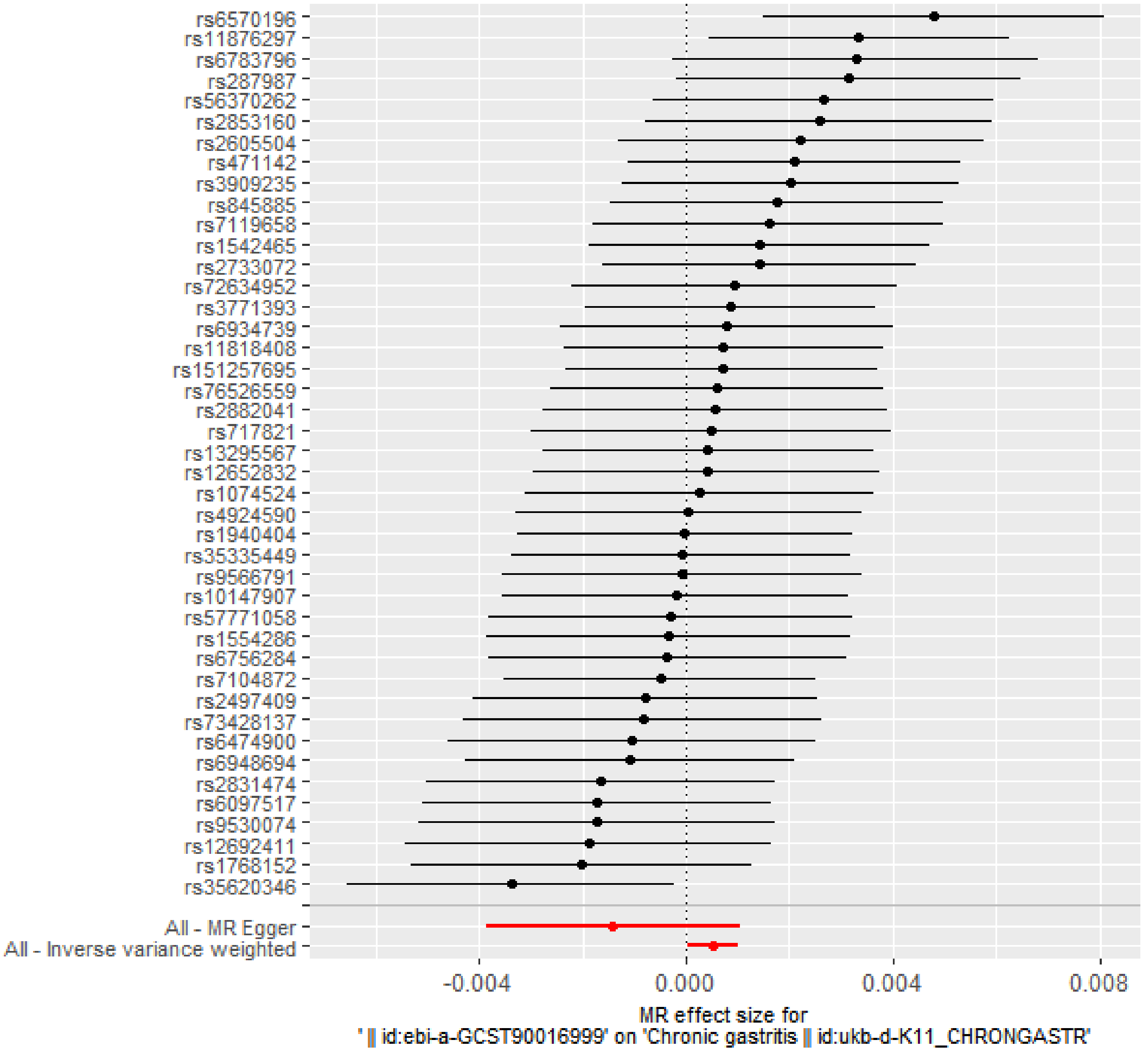
rs6570196
rs11876297
rs6783796
rs287987
rs56370262
rs2853160
rs2605504
rs471142
rs3909235
rs845885
rs7119658
rs1542465
rs2733072
rs72634952
rs3771393
rs6934739
rs11818408
rs151257695
rs76526559
rs2882041
rs717821
rs13295567
rs12652832
rs1074524
rs4924590
rs1940404
rs35335449
rs9566791
rs10147907
rs57771058
rs1554286
rs6756284
rs7104872
rs2497409
rs73428137
rs6474900
rs6948694
rs2831474
rs6097517
rs9530074
rs12692411
rs1768152
rs35620346
All - MR Egger
All - Inverse variance weighted
-0.004
0.000
0.004
0.008
MR effect size for
' || id:ebi-a-GCST90016999' on 'Chronic gastritis || id:ukb-d-K11_CHRONGASTR'

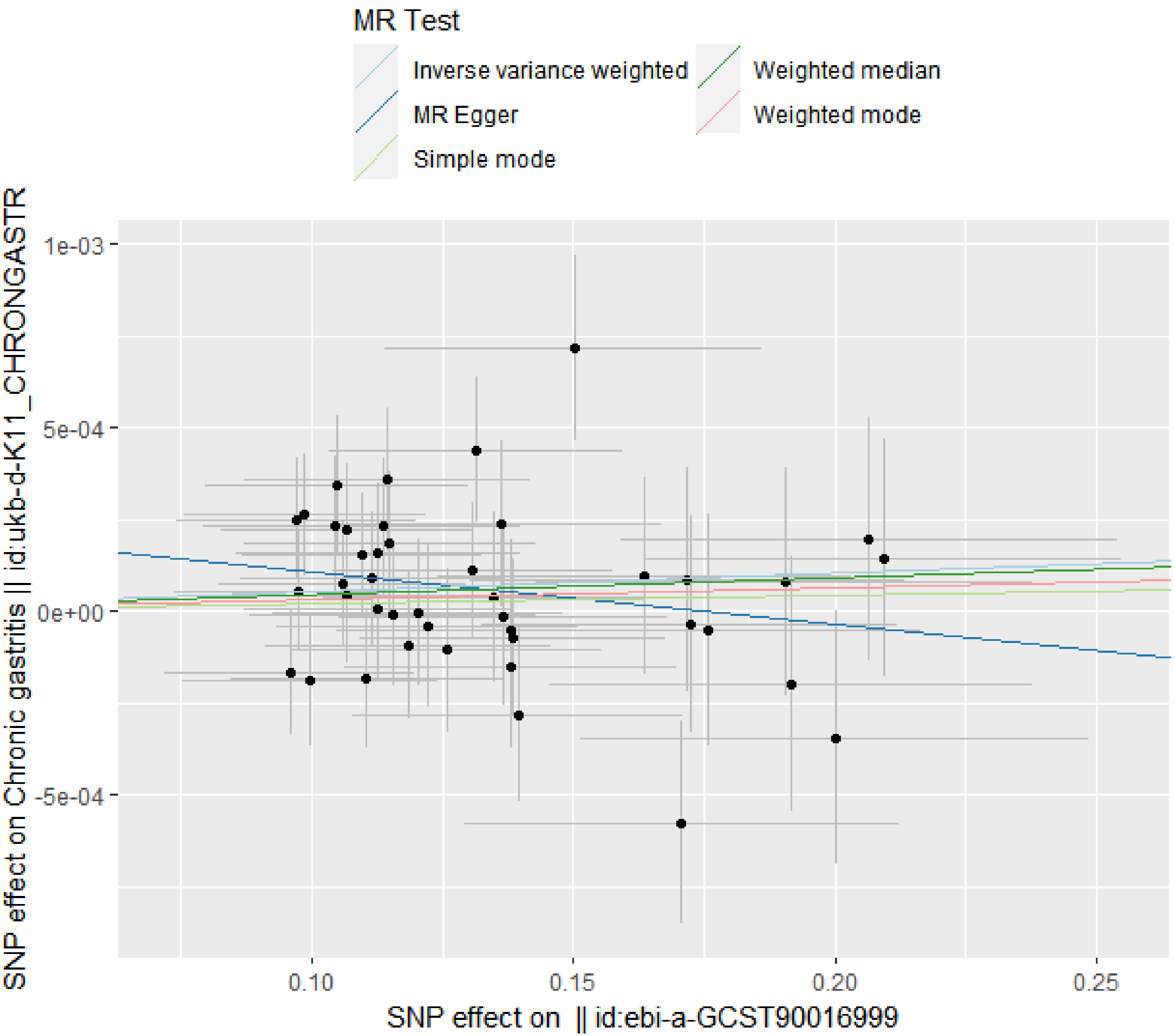
MR Test
Inverse variance weighted
MR Egger
Simple mode
Weighted median
Weighted mode
SNP effect on Chronic gastritis || id:ukb-d-K11_CHRONGASTR
1e-03
5e-04
0e+00
-5e-04
0.10
0.15
0.20
0.25
SNP effect on || id:ebi-a-GCST90016999

Figure 65 Leave-one-out analysis, funnel plot, MR effect size and scatter plot for gut microbiota abundance (genus Lachnospiraceae UCG010 id.11330) on chronic gastritis

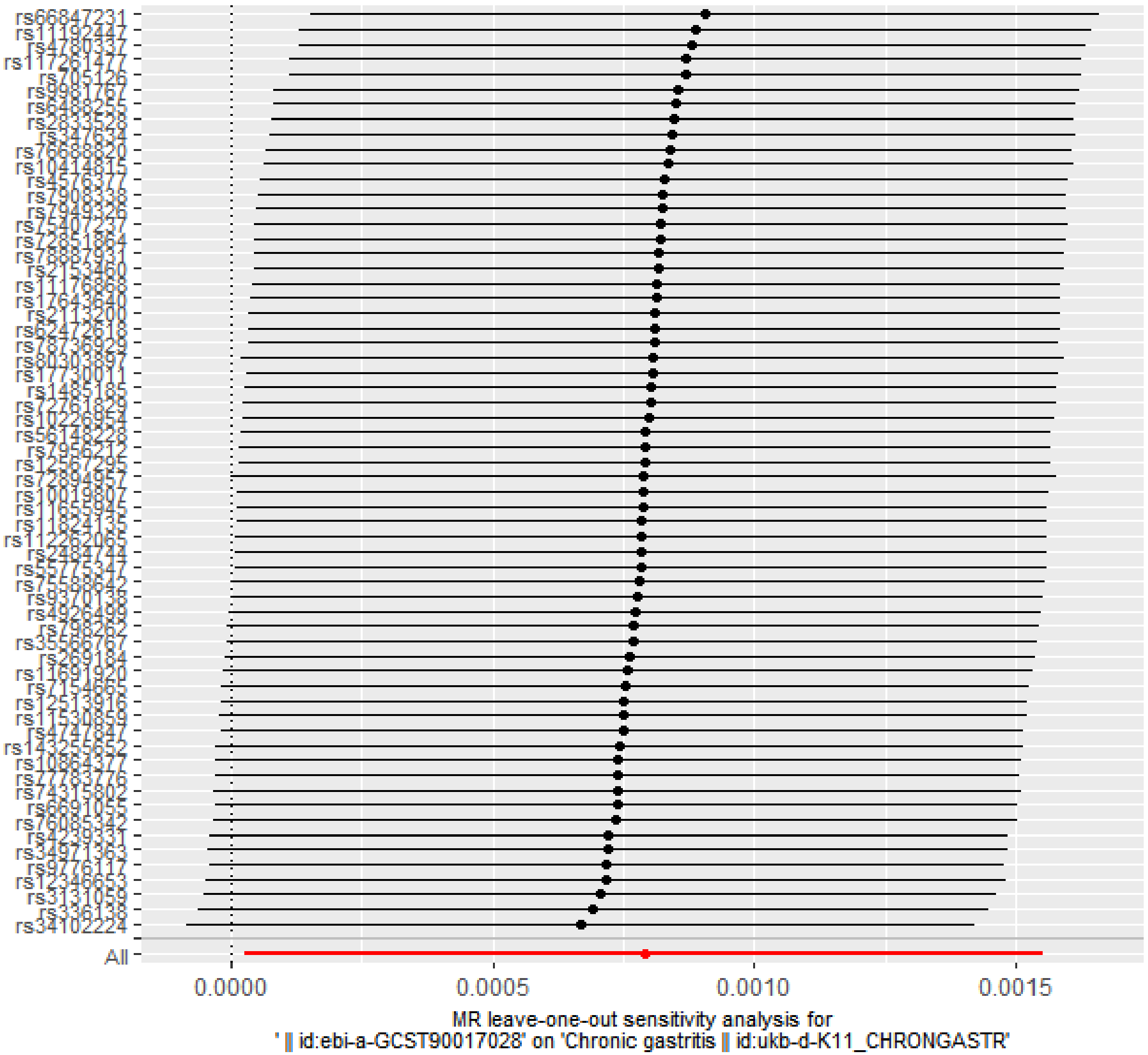

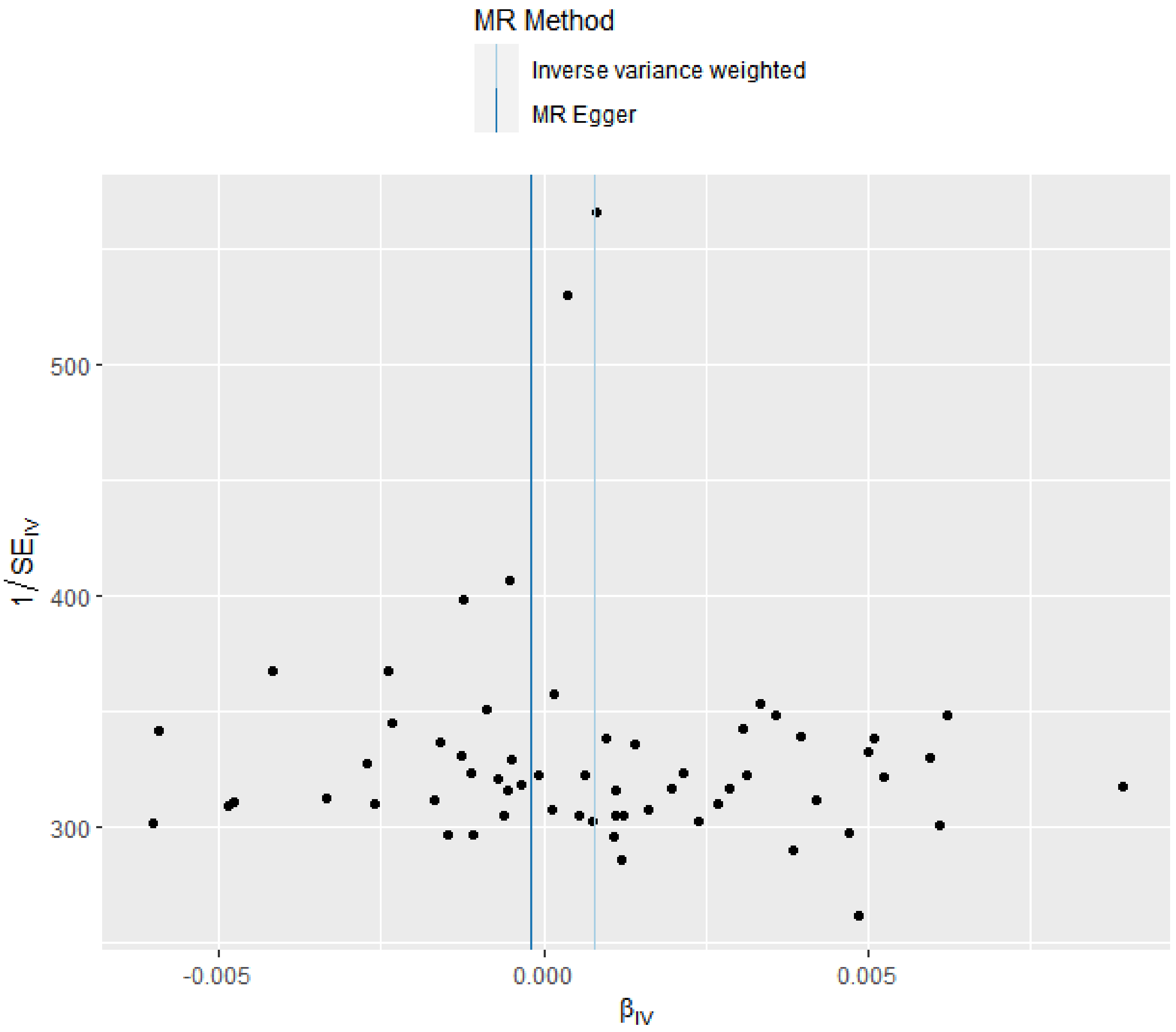

MR Method
Inverse variance weighted
MR Egger
500
400
300
-0.005
0.000
0.005
$1/SE_{IV}$
β_{IV}

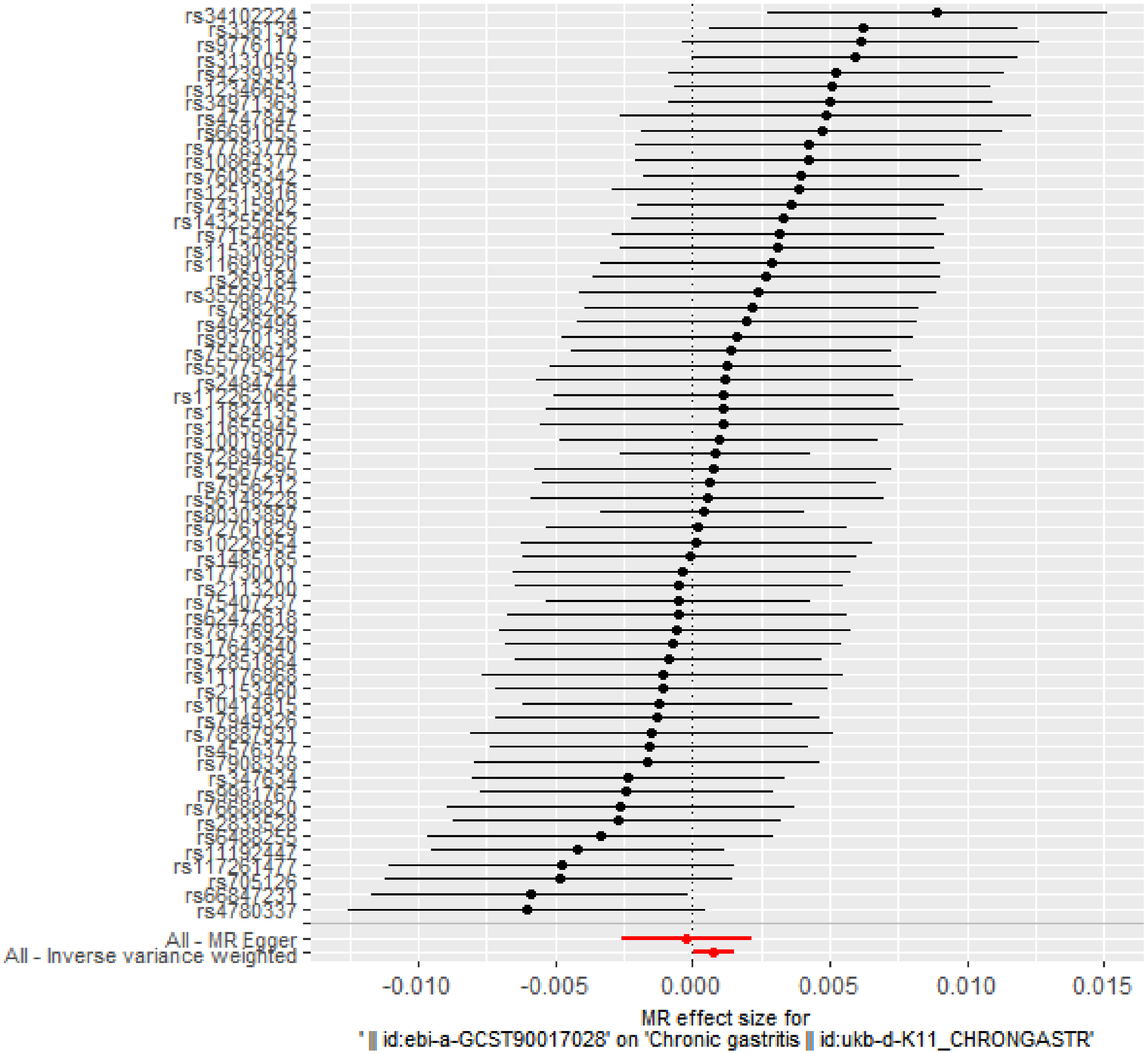

rs34102224
rs4780337
All - MR Egger
All - Inverse variance weighted
-0.010
-0.005
0.000
0.005
0.010
0.015
MR effect size for
' || id:ebi-a-GCST90017028' on 'Chronic gastritis || id:ukb-d-K11_CHRONGASTR'

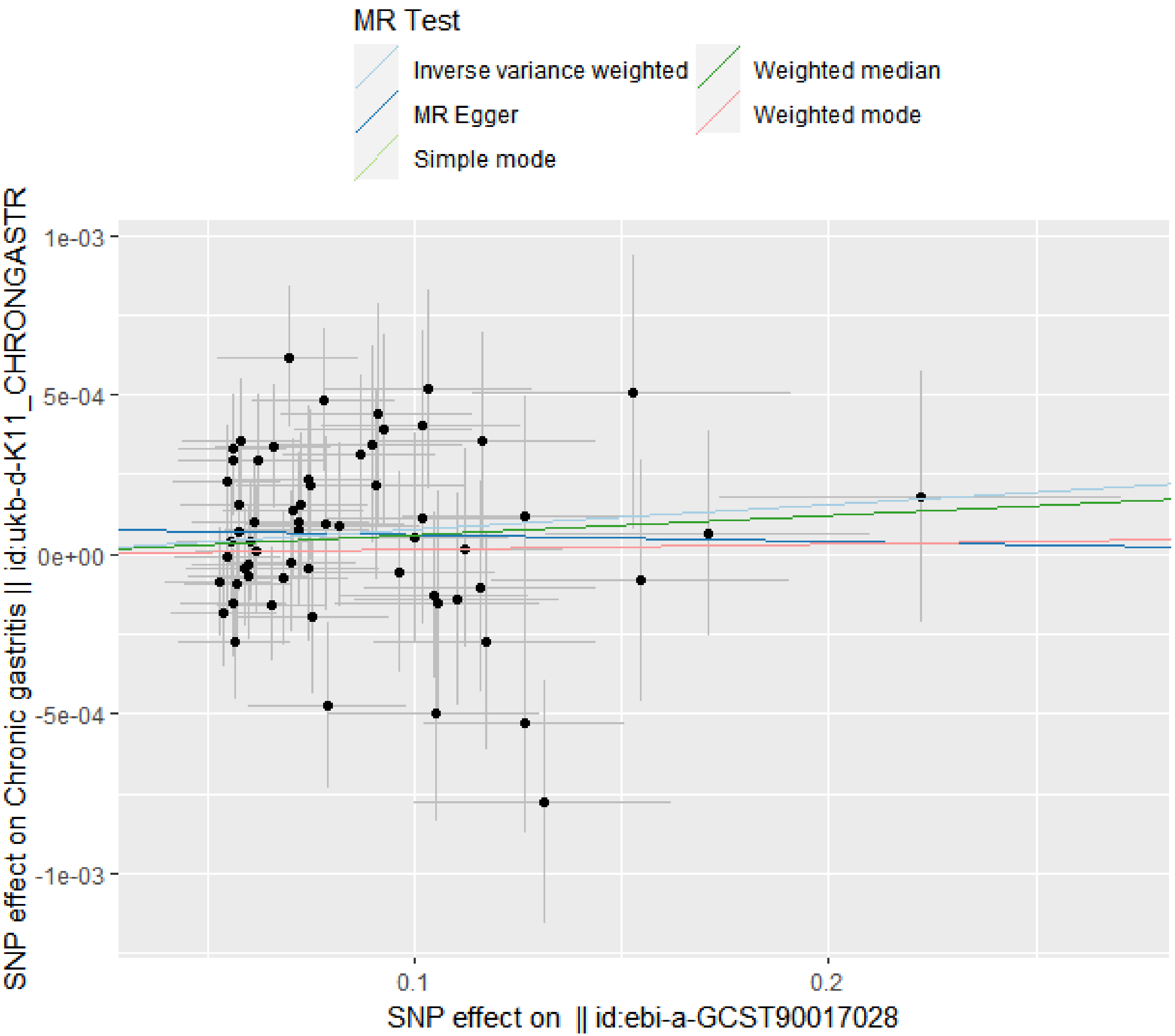

MR Test
Inverse variance weighted
MR Egger
Simple mode
Weighted median
Weighted mode
SNP effect on Chronic gastritis || id:ukb-d-K11_CHRONGASTR
1e-03
5e-04
0e+00
-5e-04
-1e-03
0.1
0.2
SNP effect on || id:ebi-a-GCST90017028

Figure 66 Leave-one-out analysis, funnel plot, MR effect size and scatter plot for gut microbiota abundance (genus Peptococcus id.2037) on chronic gastritis

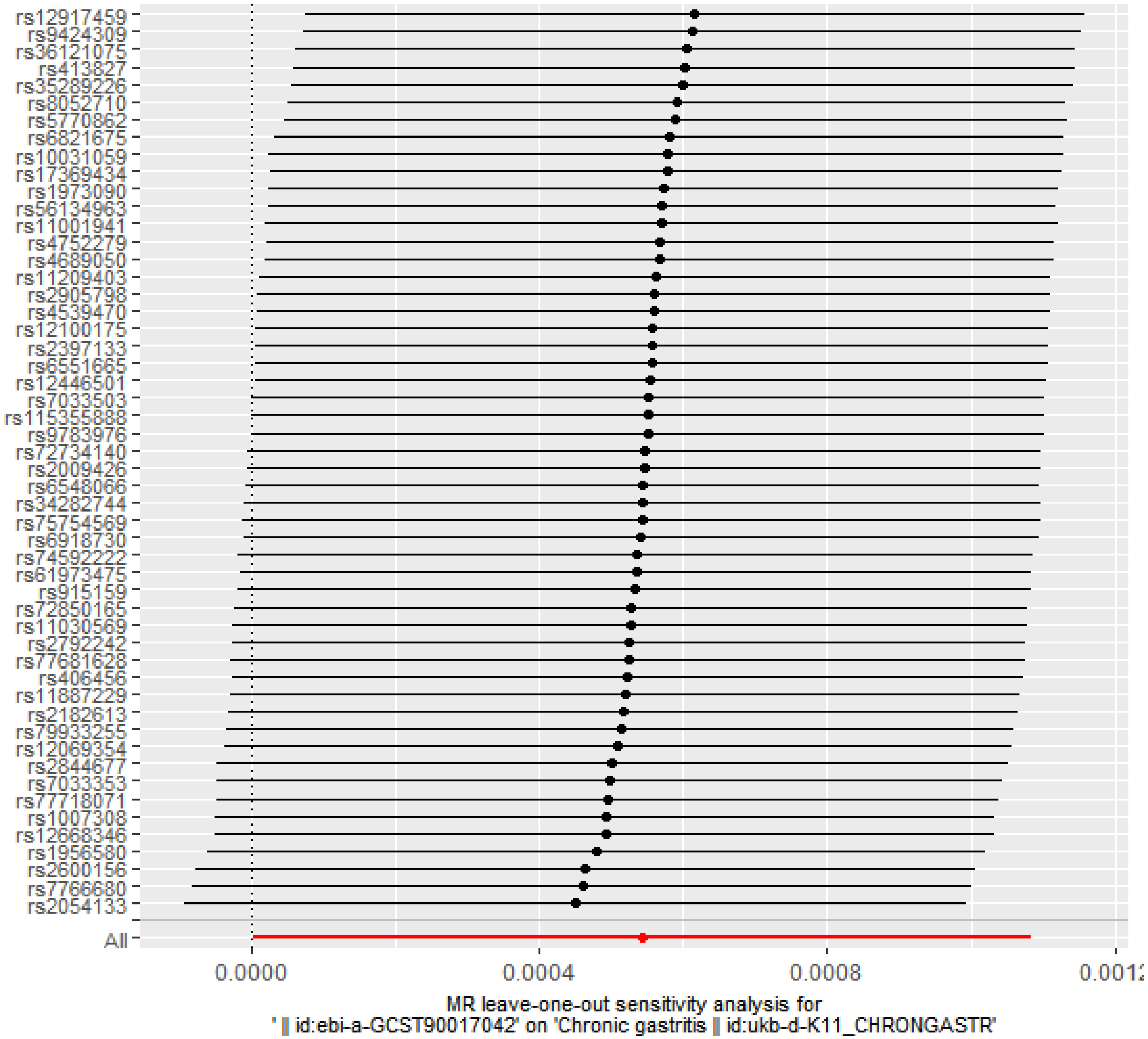

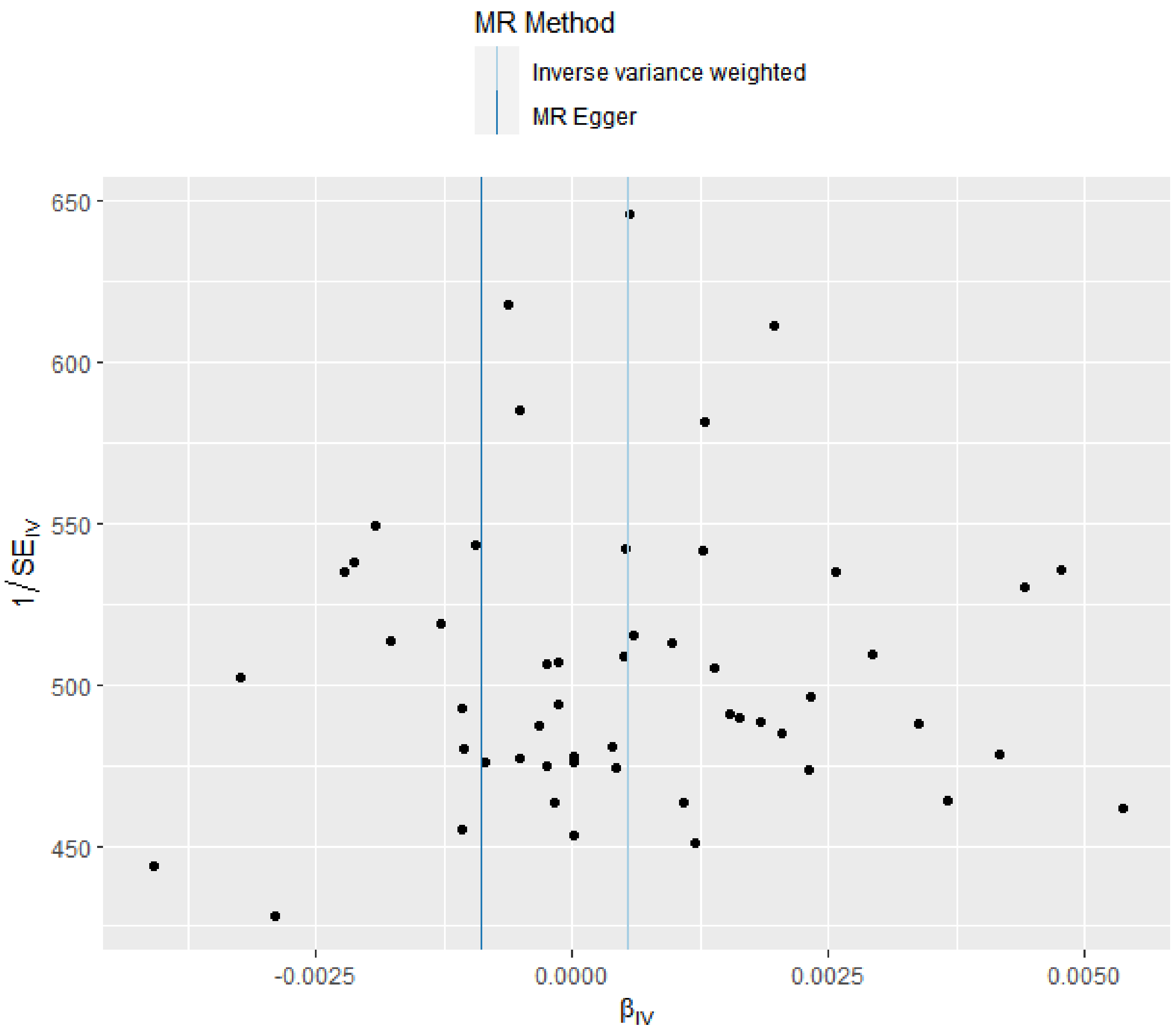
MR Method
Inverse variance weighted
MR Egger
650
600
550
500
450
1/SE_IV
-0.0025
0.0000
0.0025
0.0050
β_IV

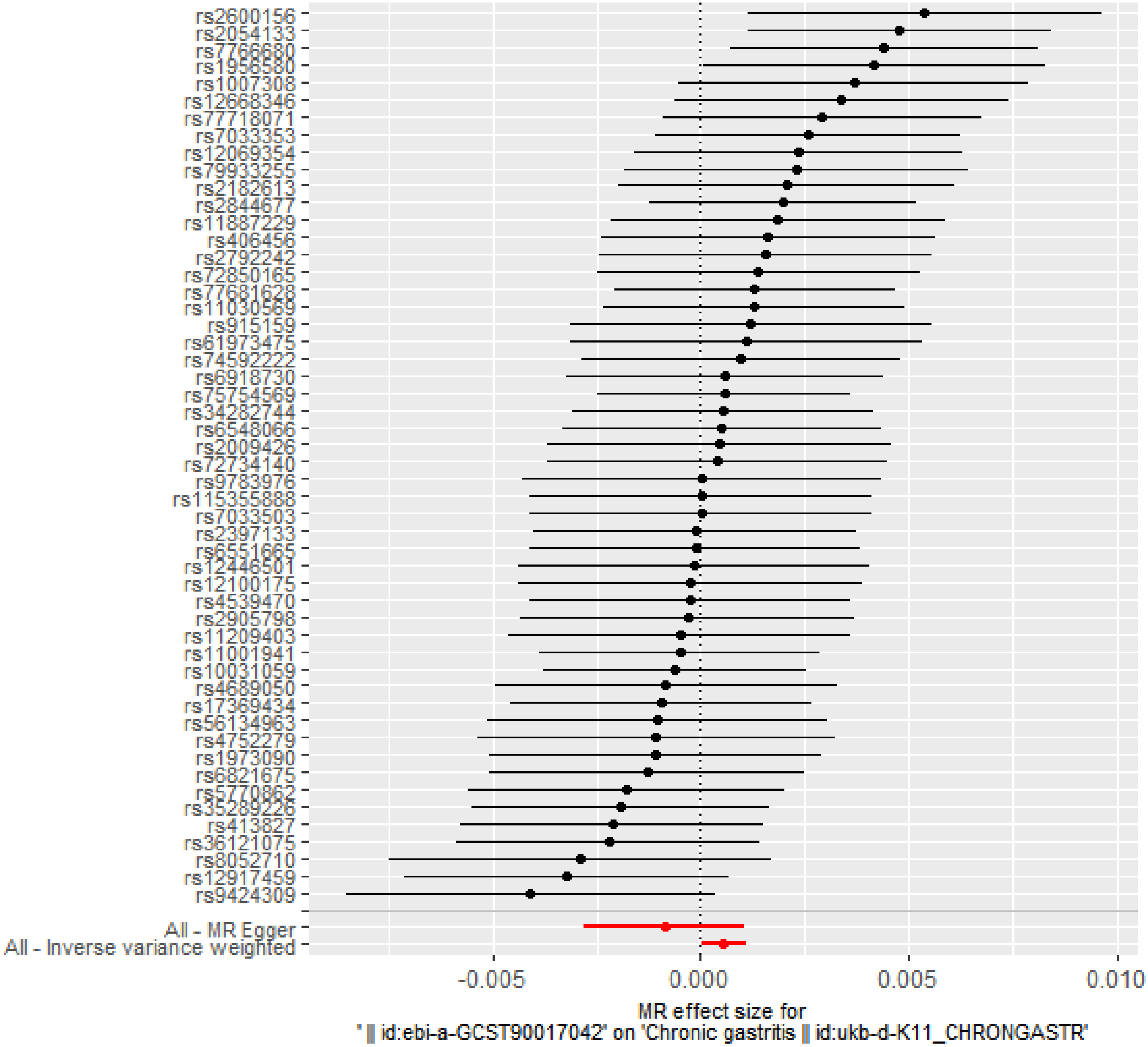
rs2600156
rs2054133
rs7766680
rs1956580
rs1007308
rs12668346
rs77718071
rs7033353
rs12069354
rs79933255
rs2182613
rs2844677
rs11887229
rs406456
rs2792242
rs72850165
rs77681628
rs11030569
rs915159
rs61973475
rs74592222
rs6918730
rs75754569
rs34282744
rs6548066
rs2009426
rs72734140
rs9783976
rs115355888
rs7033503
rs2397133
rs6551665
rs12446501
rs12100175
rs4539470
rs2905798
rs11209403
rs11001941
rs10031059
rs4689050
rs17369434
rs56134963
rs4752279
rs1973090
rs6821675
rs5770862
rs35289226
rs413827
rs36121075
rs8052710
rs12917459
rs9424309
All - MR Egger
All - Inverse variance weighted
-0.005
0.000
0.005
0.010
MR effect size for
' || id:ebi-a-GCST90017042' on 'Chronic gastritis || id:ukb-d-K11_CHRONGASTR'

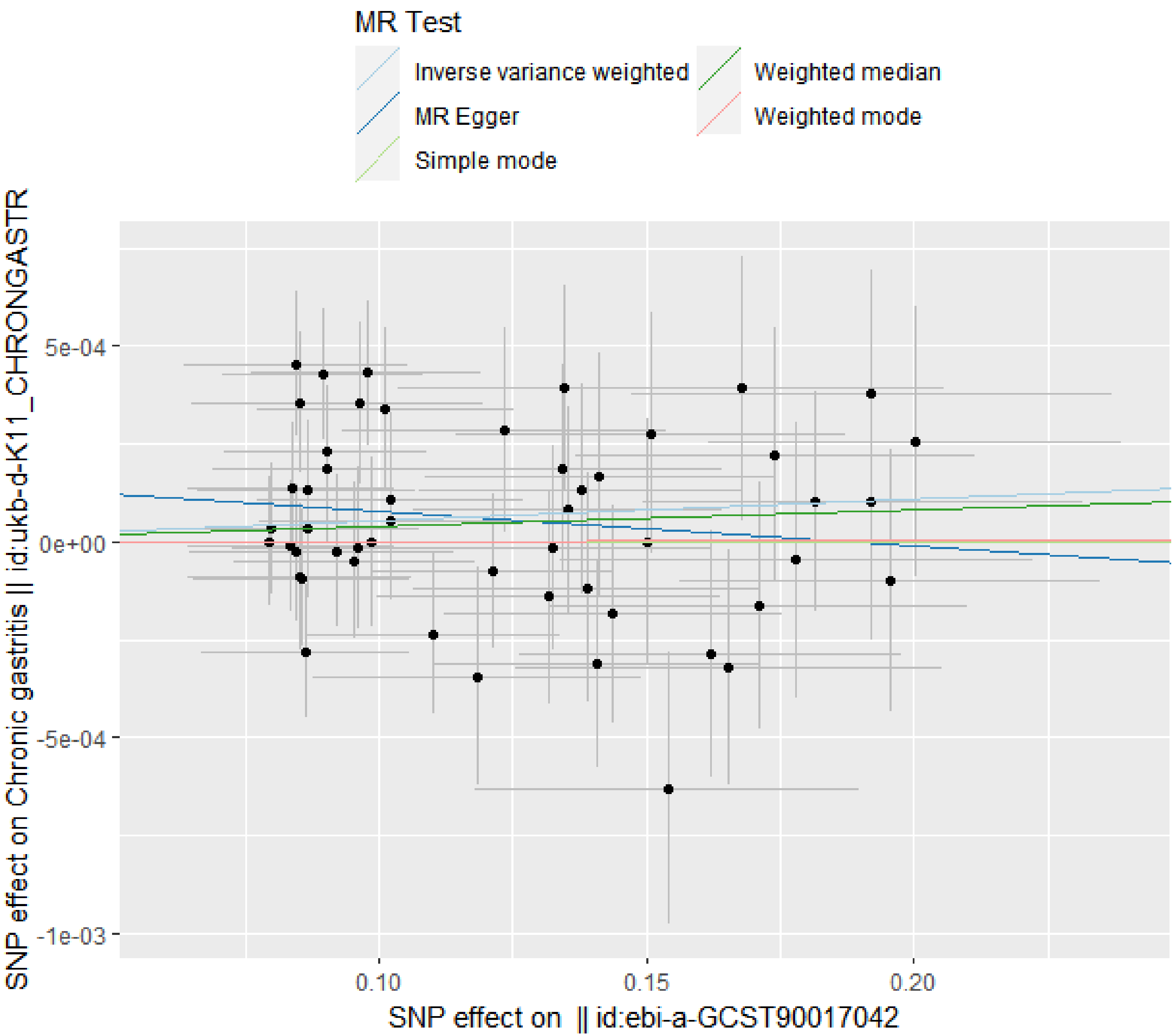

MR Test
Inverse variance weighted
MR Egger
Simple mode
Weighted median
Weighted mode
SNP effect on Chronic gastritis || id:ukb-d-K11_CHRONGASTR
5e-04
0e+00
-5e-04
-1e-03
0.10
0.15
0.20
SNP effect on || id:ebi-a-GCST90017042

Figure 67 Leave-one-out analysis, funnel plot, MR effect size and scatter plot for gut microbiota abundance (genus Prevotella7 id.11182) on chronic gastritis

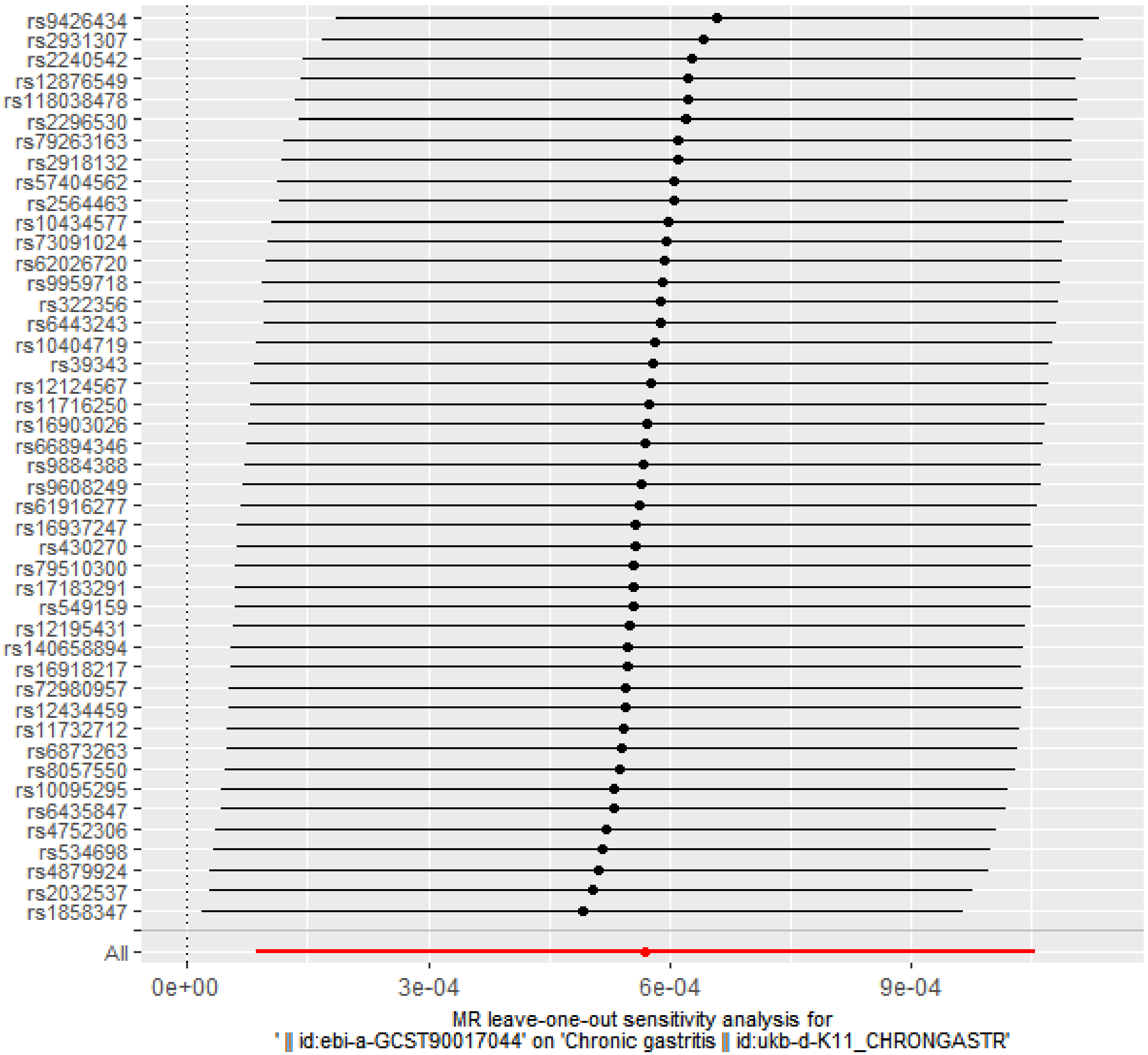

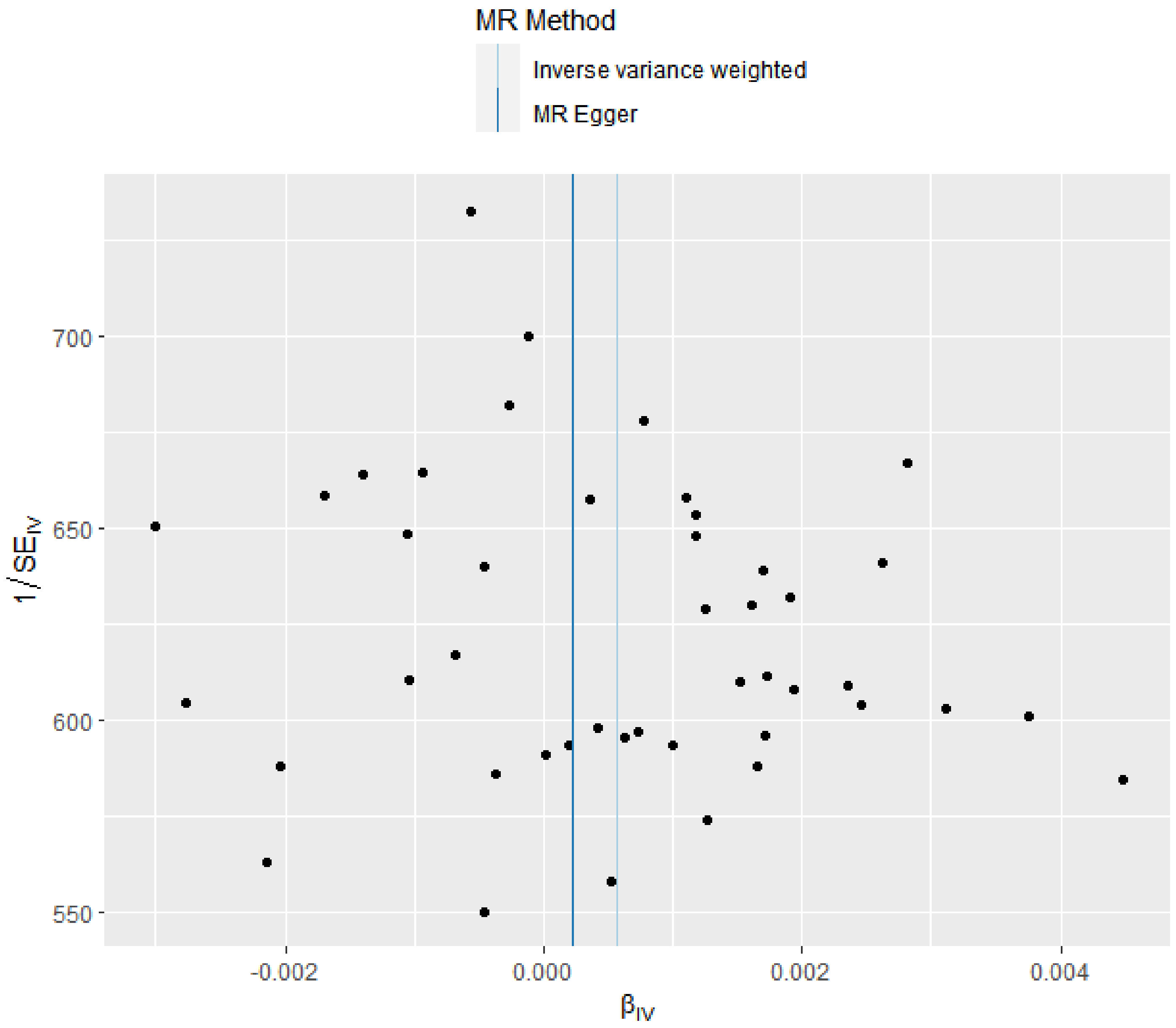

MR Method
Inverse variance weighted
MR Egger
700
650
600
550
1/SE_IV
-0.002
0.000
0.002
0.004
β_IV

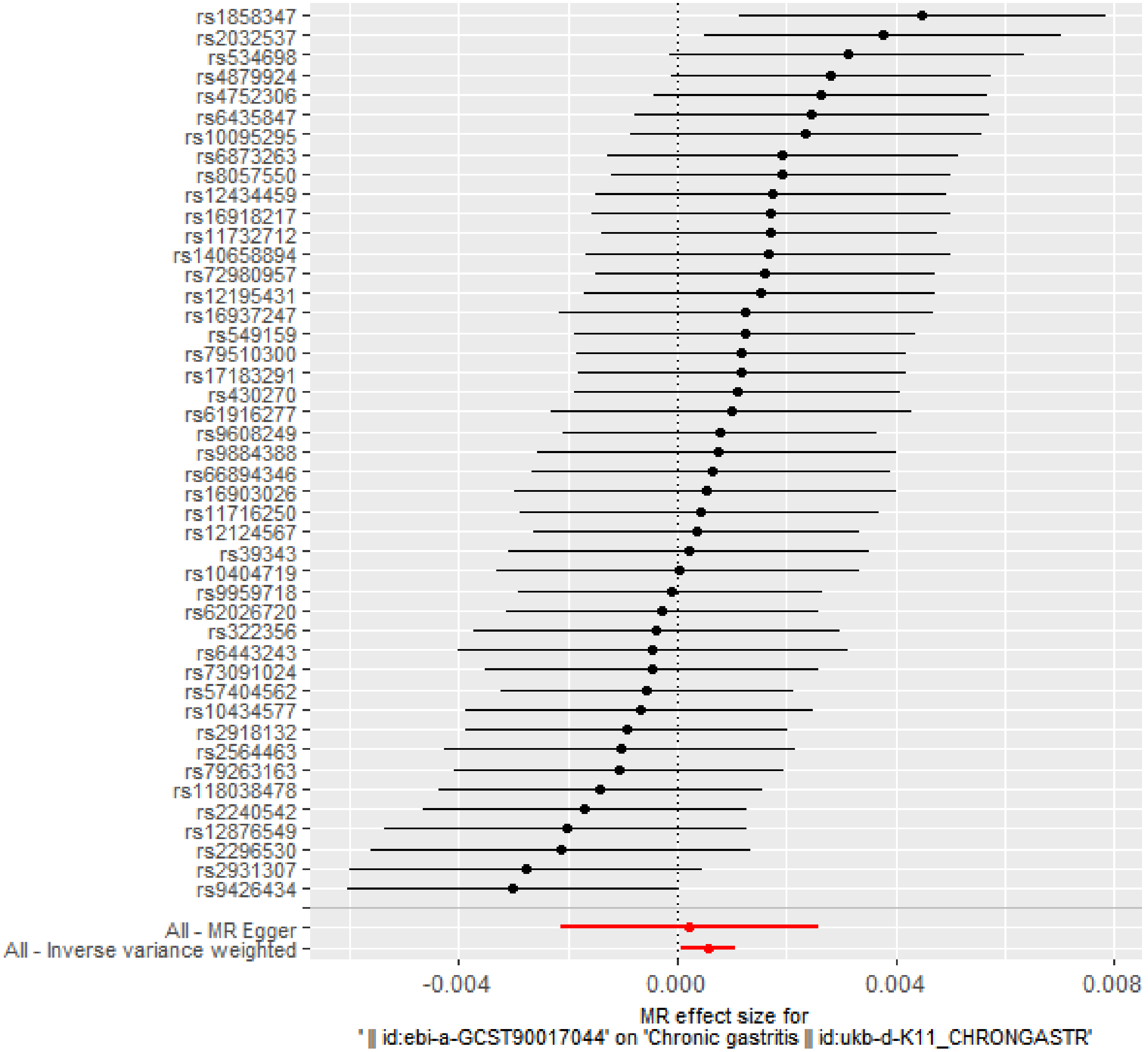
rs1858347
rs2032537
rs534698
rs4879924
rs4752306
rs6435847
rs10095295
rs6873263
rs8057550
rs12434459
rs16918217
rs11732712
rs140658894
rs72980957
rs12195431
rs16937247
rs549159
rs79510300
rs17183291
rs430270
rs61916277
rs9608249
rs9884388
rs66894346
rs16903026
rs11716250
rs12124567
rs39343
rs10404719
rs9959718
rs62026720
rs322356
rs6443243
rs73091024
rs57404562
rs10434577
rs2918132
rs2564463
rs79263163
rs118038478
rs2240542
rs12876549
rs2296530
rs2931307
rs9426434
All - MR Egger
All - Inverse variance weighted
-0.004
0.000
0.004
0.008
MR effect size for
' || id:ebi-a-GCST90017044' on 'Chronic gastritis || id:ukb-d-K11_CHRONGASTR'

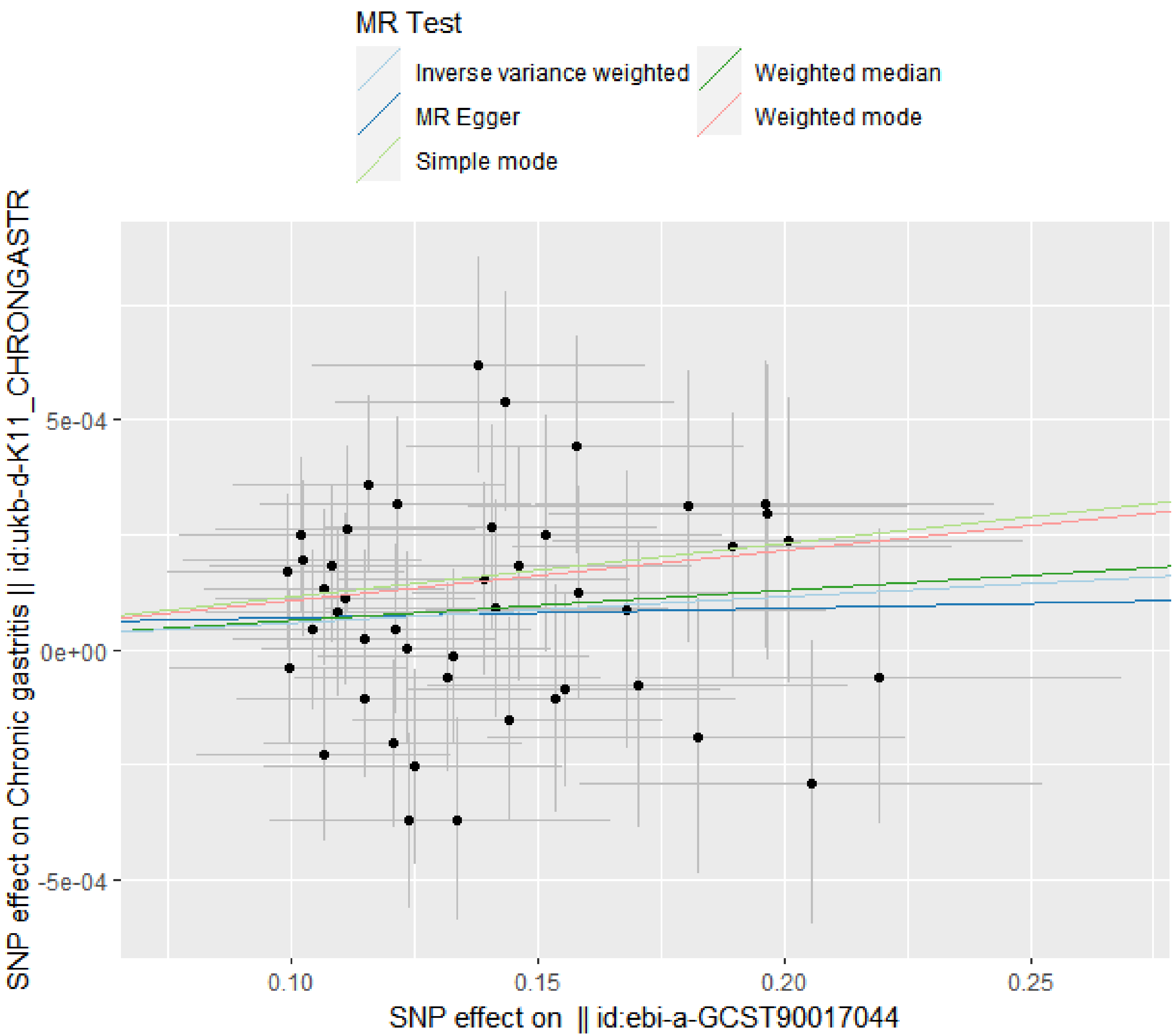
MR Test
Inverse variance weighted
MR Egger
Simple mode
Weighted median
Weighted mode
SNP effect on Chronic gastritis || id:ukb-d-K11_CHRONGASTR
5e-04
0e+00
-5e-04
0.10
0.15
0.20
0.25
SNP effect on || id:ebi-a-GCST90017044

Figure 68 Leave-one-out analysis, funnel plot, MR effect size and scatter plot for gut microbiota abundance (genus Ruminococcaceae UCG010 id.11367) on chronic gastritis

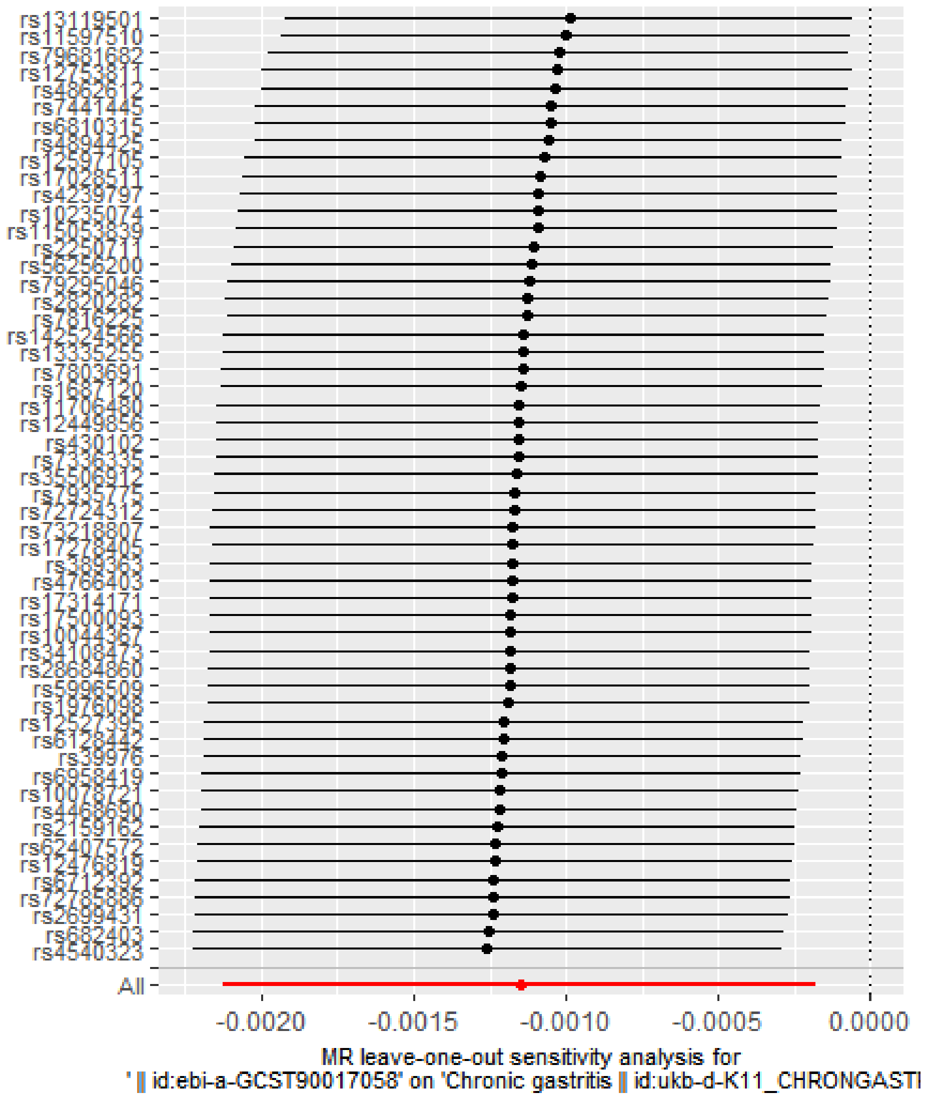

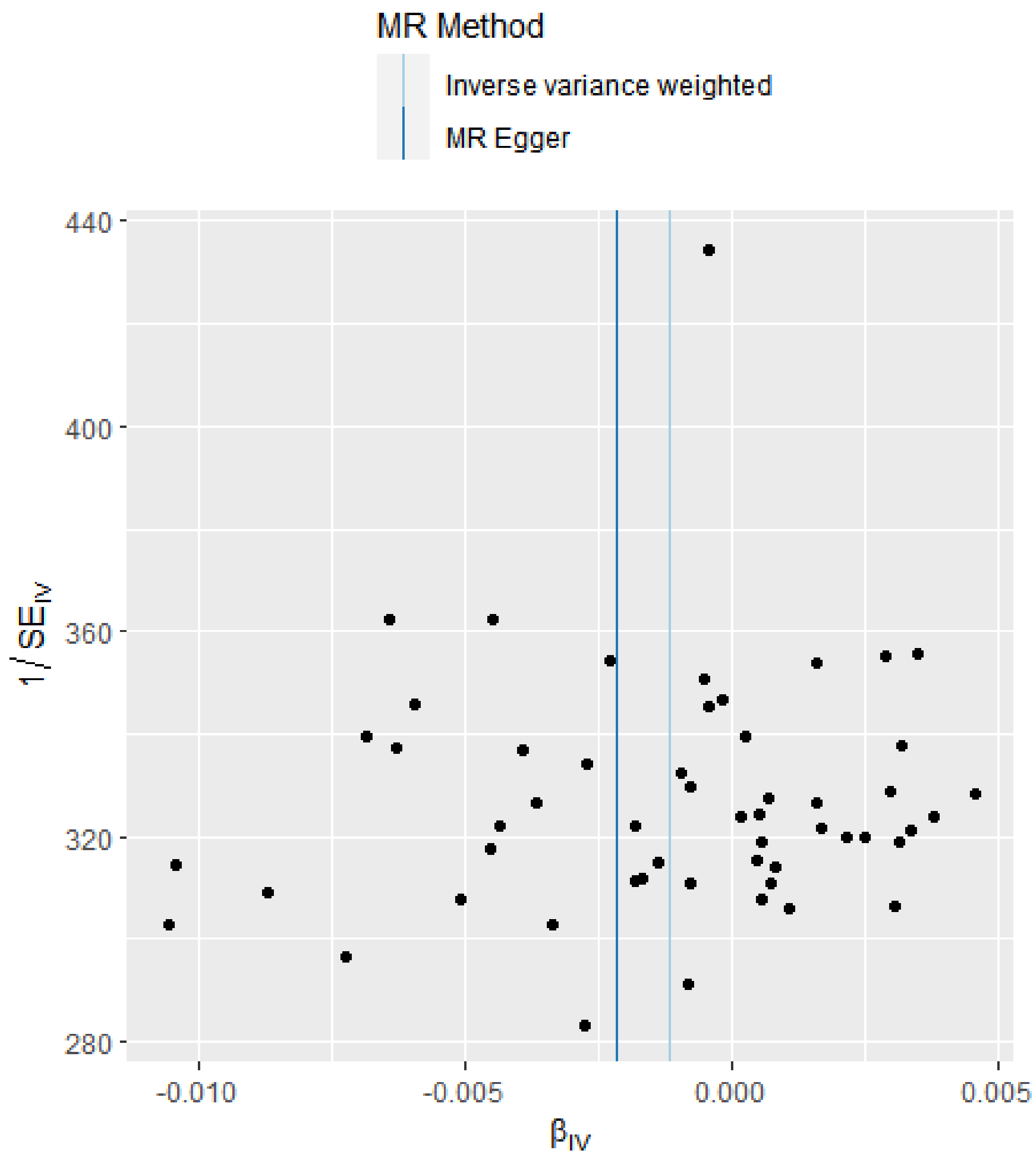

MR Method
Inverse variance weighted
MR Egger
440
400
360
320
280
1/SE_IV
-0.010
-0.005
0.000
0.005
β_IV

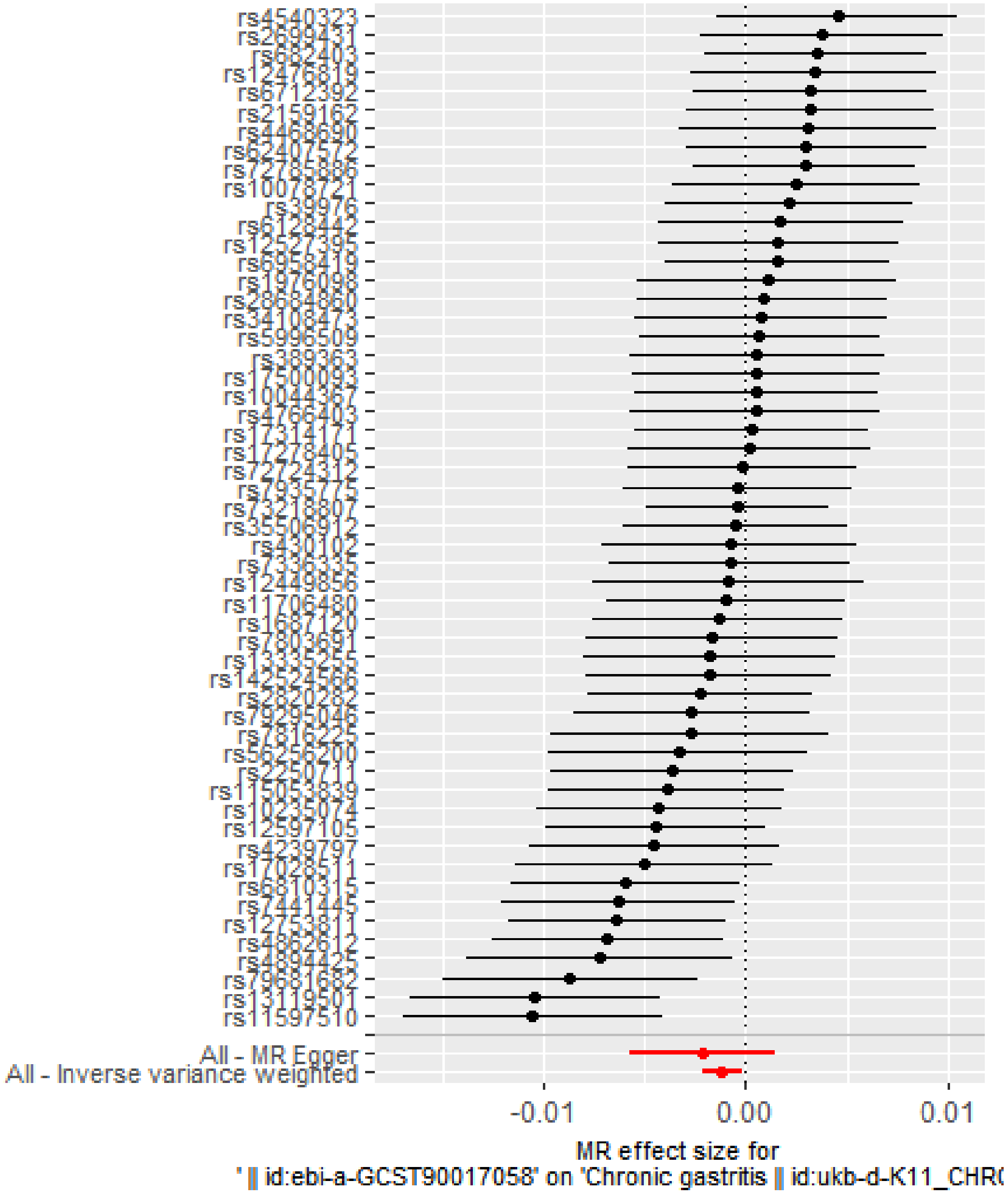

rs4540323
rs2699431
rs12476819
rs6712392
rs2159162
rs4468690
rs62407572
rs10078721
rs39976
rs6128442
rs12527395
rs6955419
rs1976098
rs28684860
rs34108473
rs5996509
rs389363
rs17500093
rs10044367
rs4766403
rs17314171
rs17278405
rs72724312
rs7935775
rs73218807
rs35506912
rs430102
rs7336335
rs12449856
rs11706480
rs1687120
rs7803691
rs142524566
rs2820282
rs79295046
rs7816225
rs56256200
rs2250711
rs115053839
rs10235074
rs12597105
rs4239797
rs17028511
rs6810315
rs7441445
rs12753811
rs4862612
rs4894425
rs79881682
rs13119501
rs11597510
All - MR Egger
All - Inverse variance weighted
-0.01
0.00
0.01
MR effect size for
' || id:ebi-a-GCST90017058' on 'Chronic gastritis || id:ukb-d-K11_CHR(

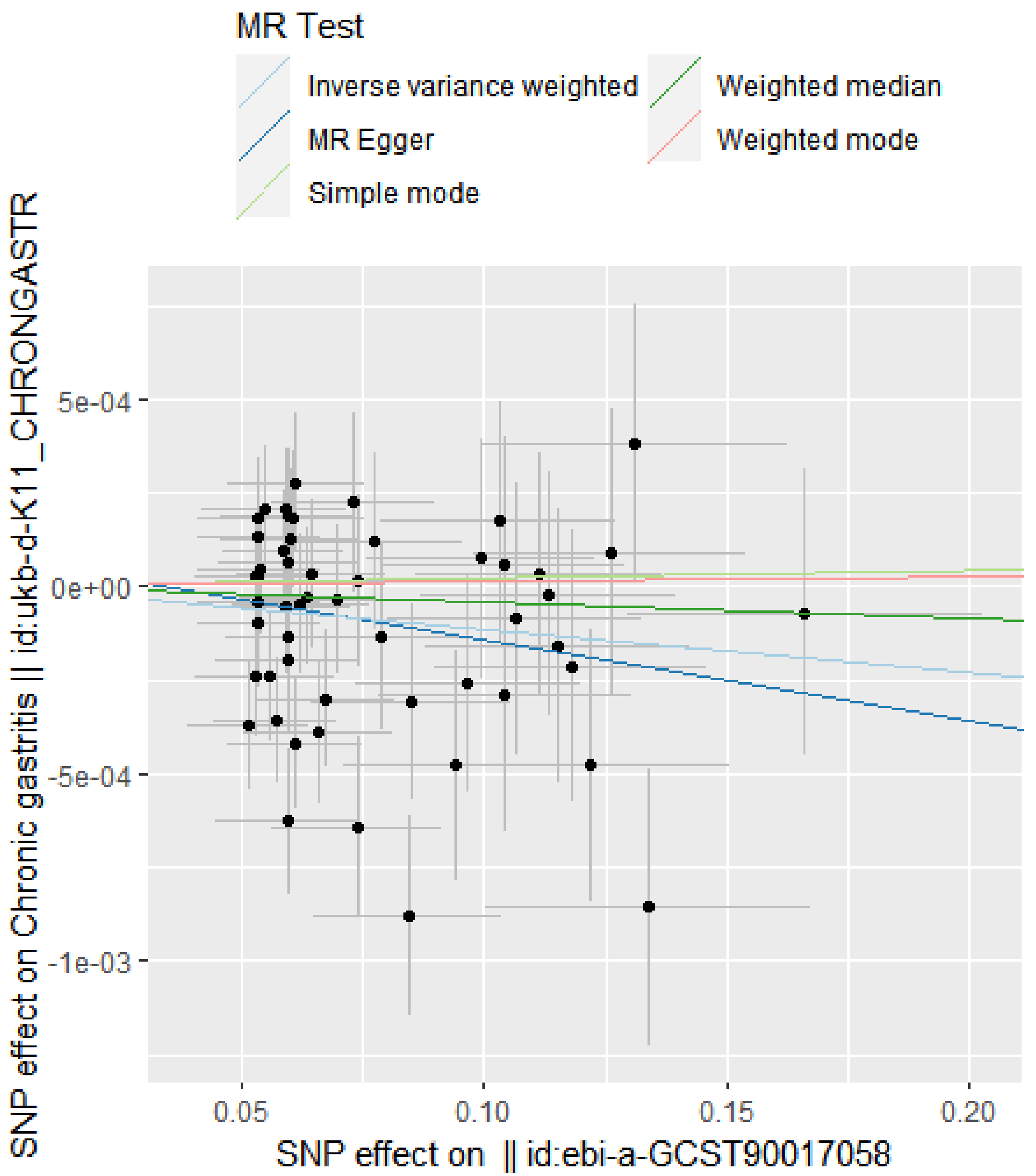
MR Test
Inverse variance weighted
MR Egger
Simple mode
Weighted median
Weighted mode
5e-04
0e+00
-5e-04
-1e-03
0.05
0.10
0.15
0.20
SNP effect on Chronic gastritis || id:ukb-d-K11_CHRONGASTR
SNP effect on || id:ebi-a-GCST90017058

Figure 69 Leave-one-out analysis, funnel plot, MR effect size and scatter plot for gut microbiota abundance (genus Victivallis id.2256) on chronic gastritis

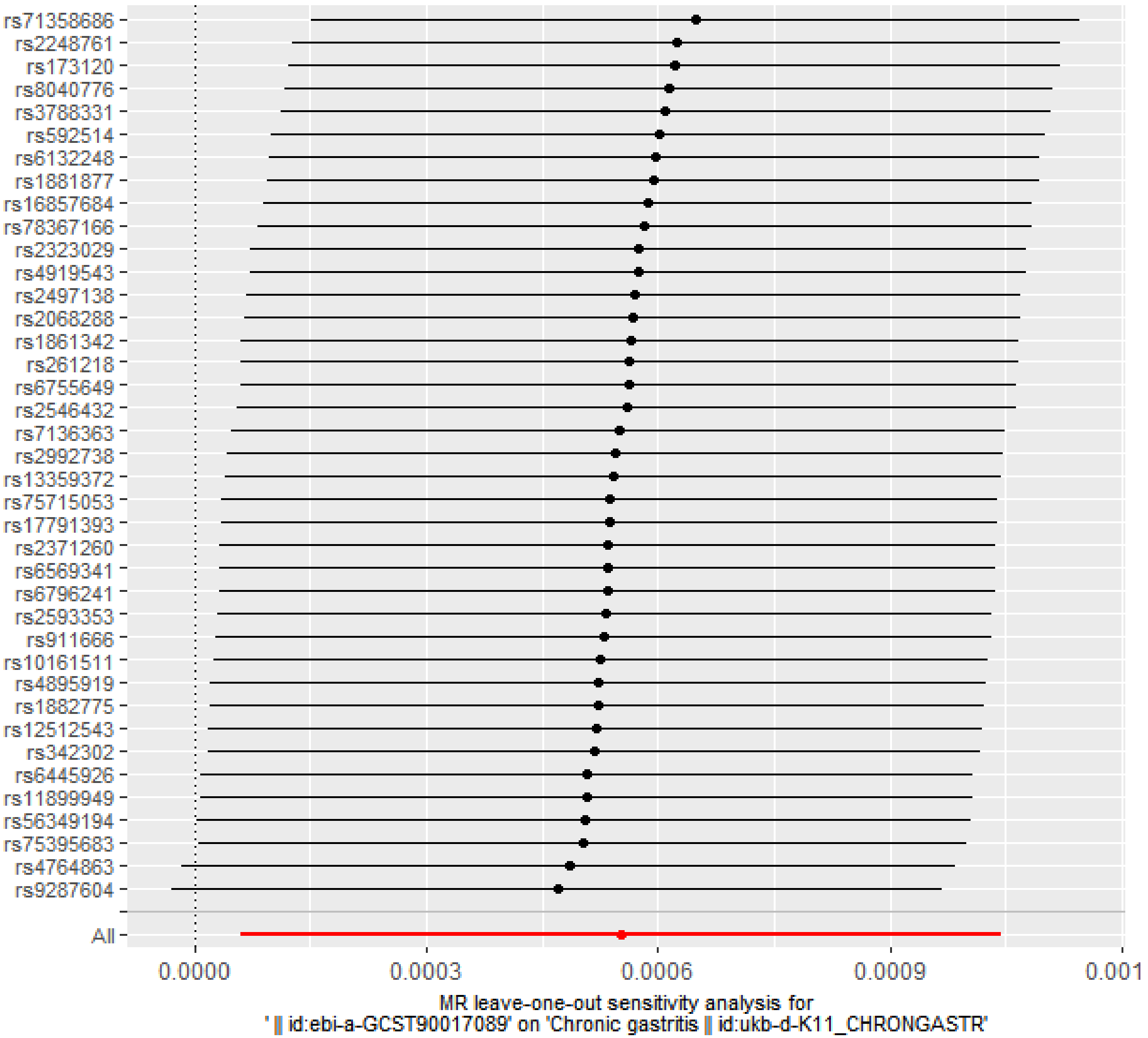

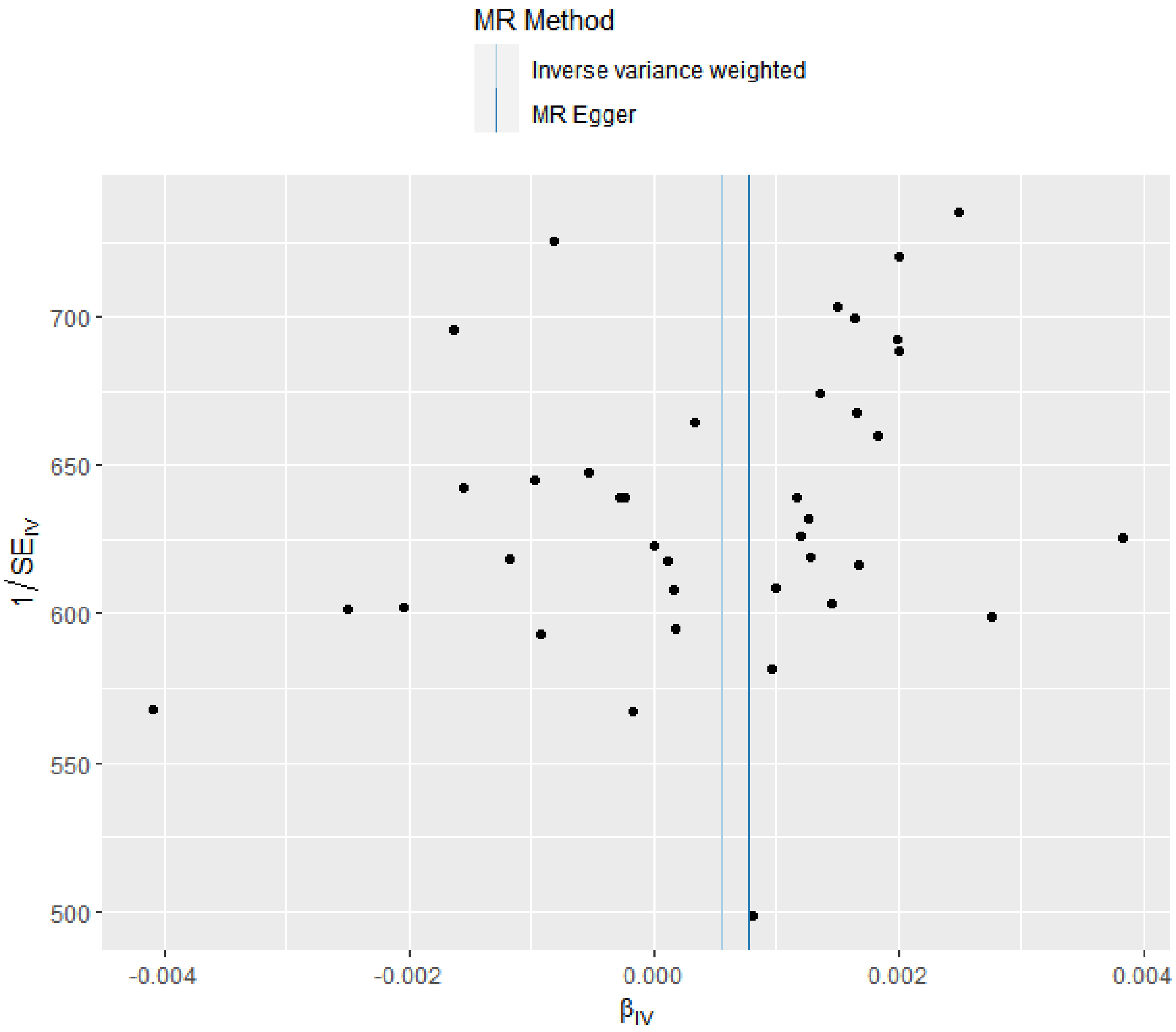

MR Method
Inverse variance weighted
MR Egger
700
650
600
550
500
-0.004
-0.002
0.000
0.002
0.004
$1/SE_{IV}$
β_{IV}

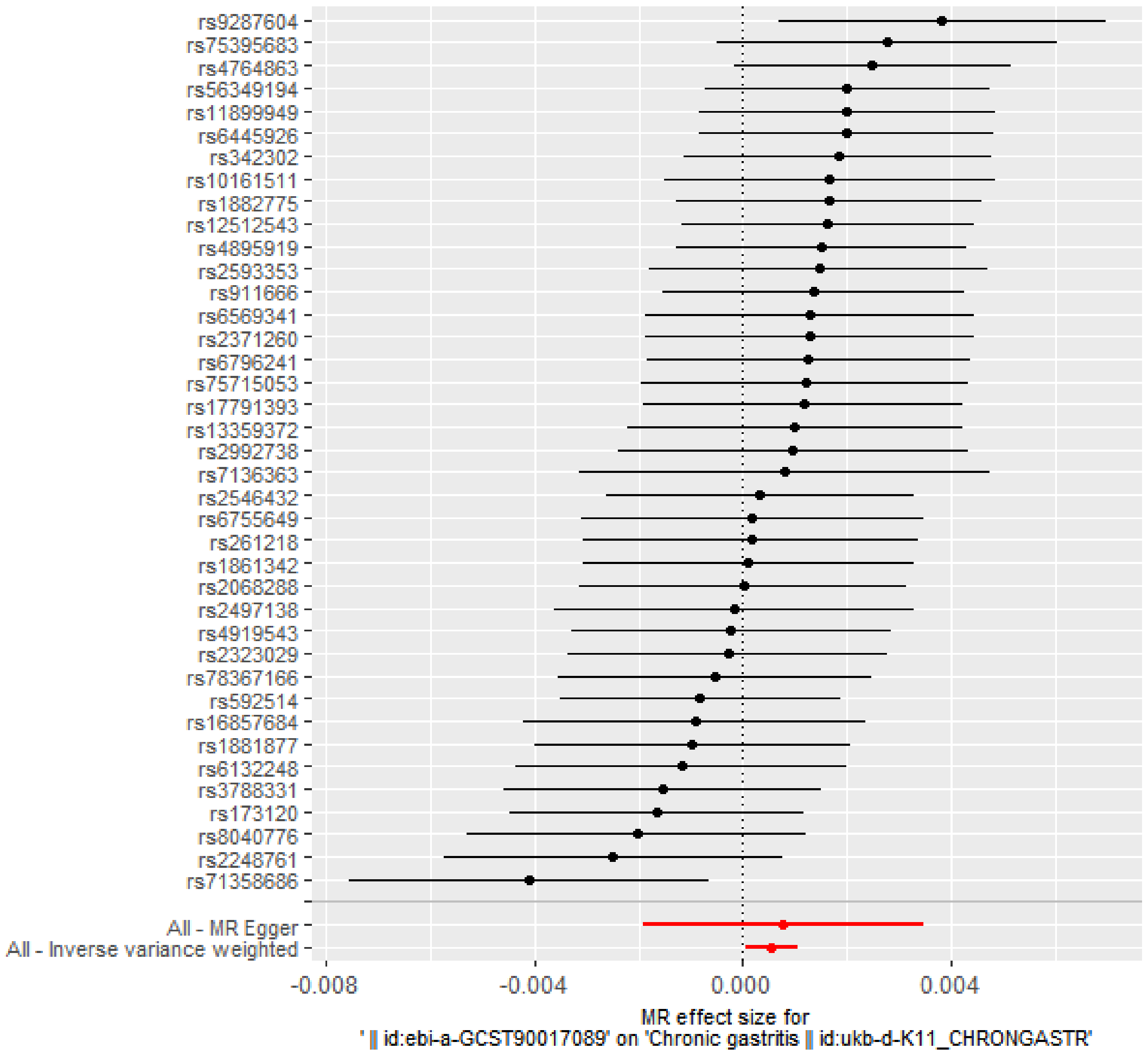
rs9287604
rs75395683
rs4764863
rs56349194
rs11899949
rs6445926
rs342302
rs10161511
rs1882775
rs12512543
rs4895919
rs2593353
rs911666
rs6569341
rs2371260
rs6796241
rs75715053
rs17791393
rs13359372
rs2992738
rs7136363
rs2546432
rs6755649
rs261218
rs1861342
rs2068288
rs2497138
rs4919543
rs2323029
rs78367166
rs592514
rs16857684
rs1881877
rs6132248
rs3788331
rs173120
rs8040776
rs2248761
rs71358686
All - MR Egger
All - Inverse variance weighted
-0.008
-0.004
0.000
0.004
MR effect size for
' || id:ebi-a-GCST90017089' on 'Chronic gastritis || id:ukb-d-K11_CHRONGASTR'

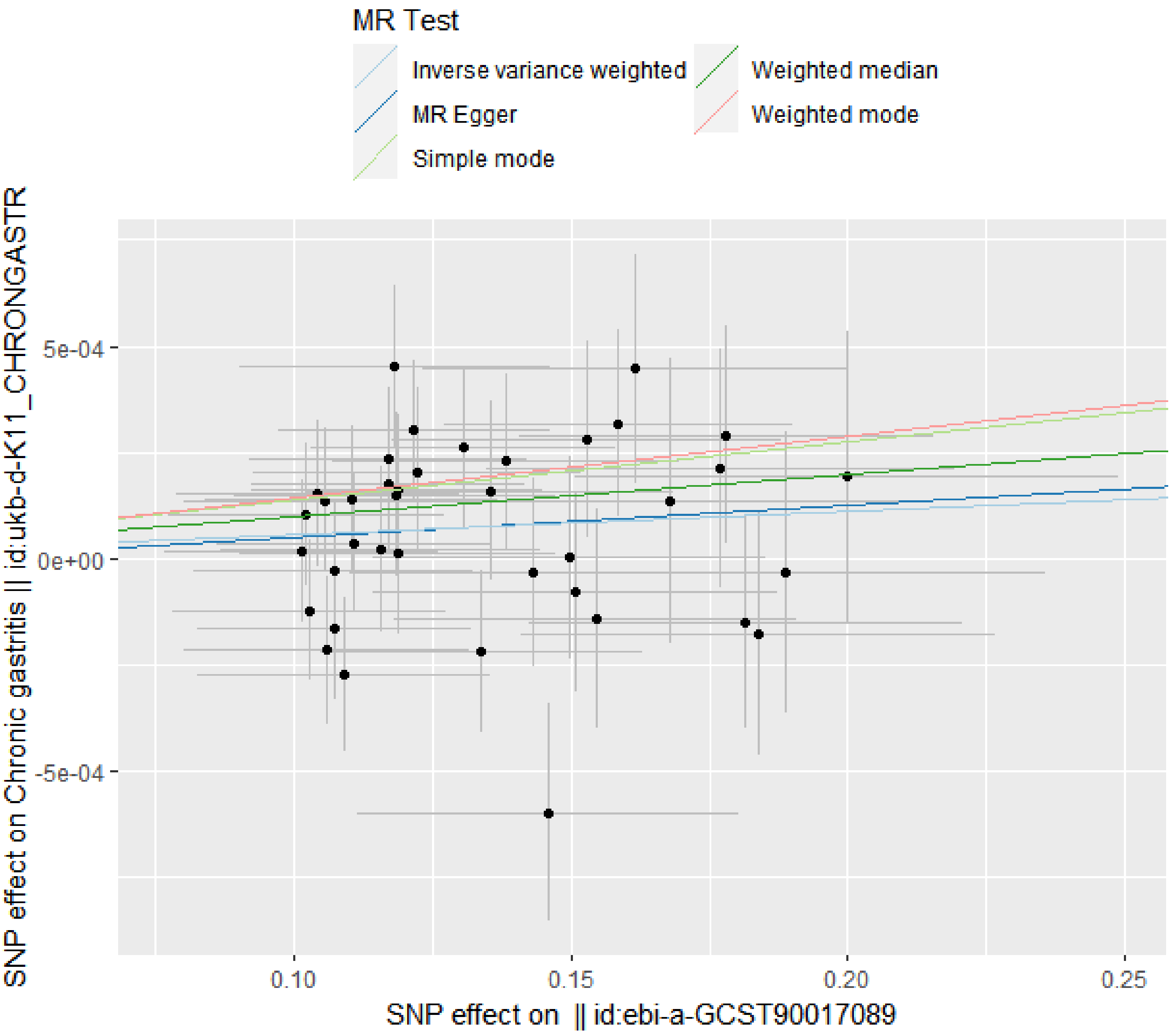
MR Test
Inverse variance weighted
MR Egger
Simple mode
Weighted median
Weighted mode
SNP effect on Chronic gastritis || id:ukb-d-K11_CHRONGASTR
5e-04
0e+00
-5e-04
0.10
0.15
0.20
0.25
SNP effect on || id:ebi-a-GCST90017089

Figure 70 Leave-one-out analysis, funnel plot, MR effect size and scatter plot for gut microbiota abundance (order Verrucomicrobiales id.4030) on chronic gastritis

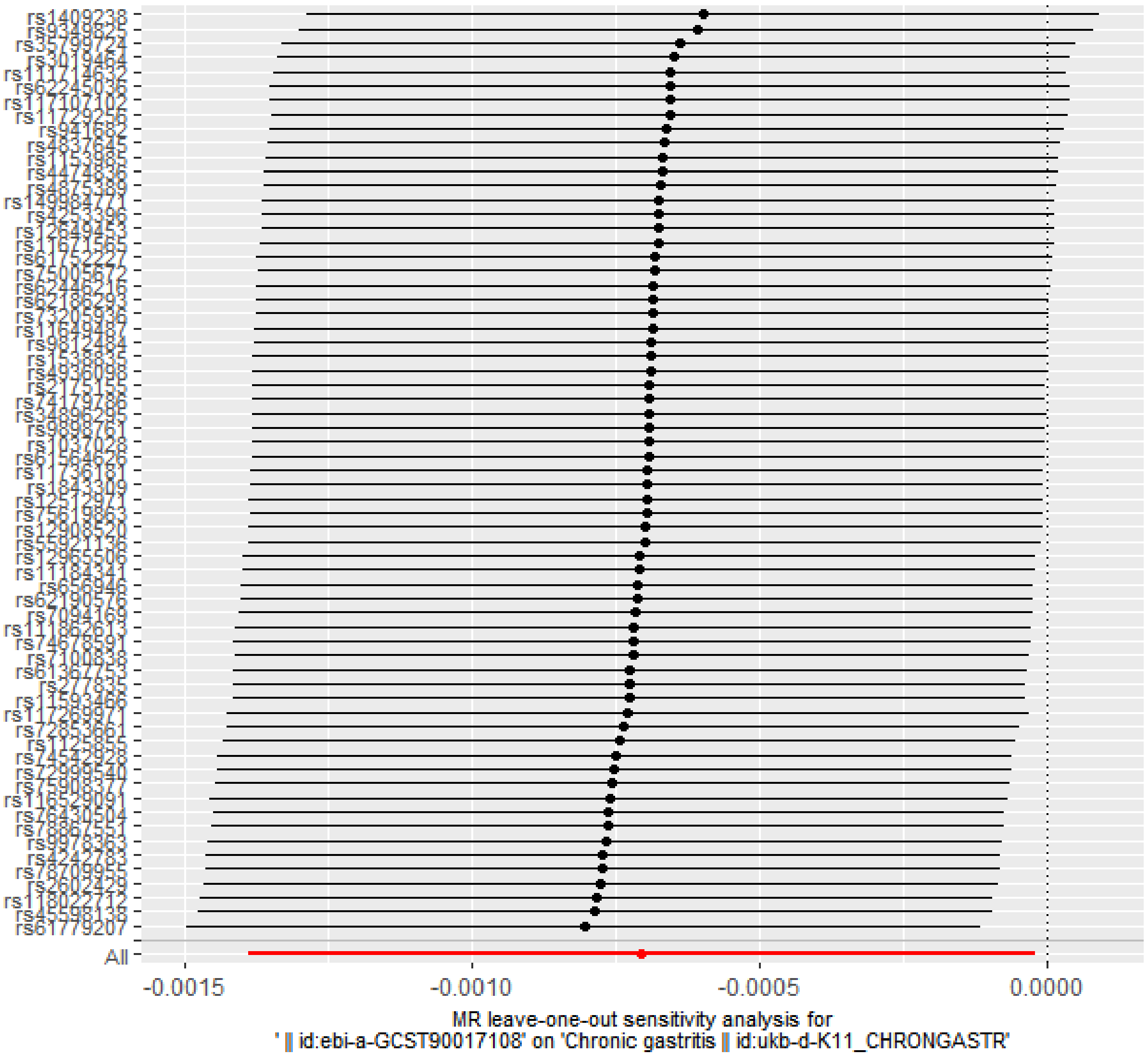

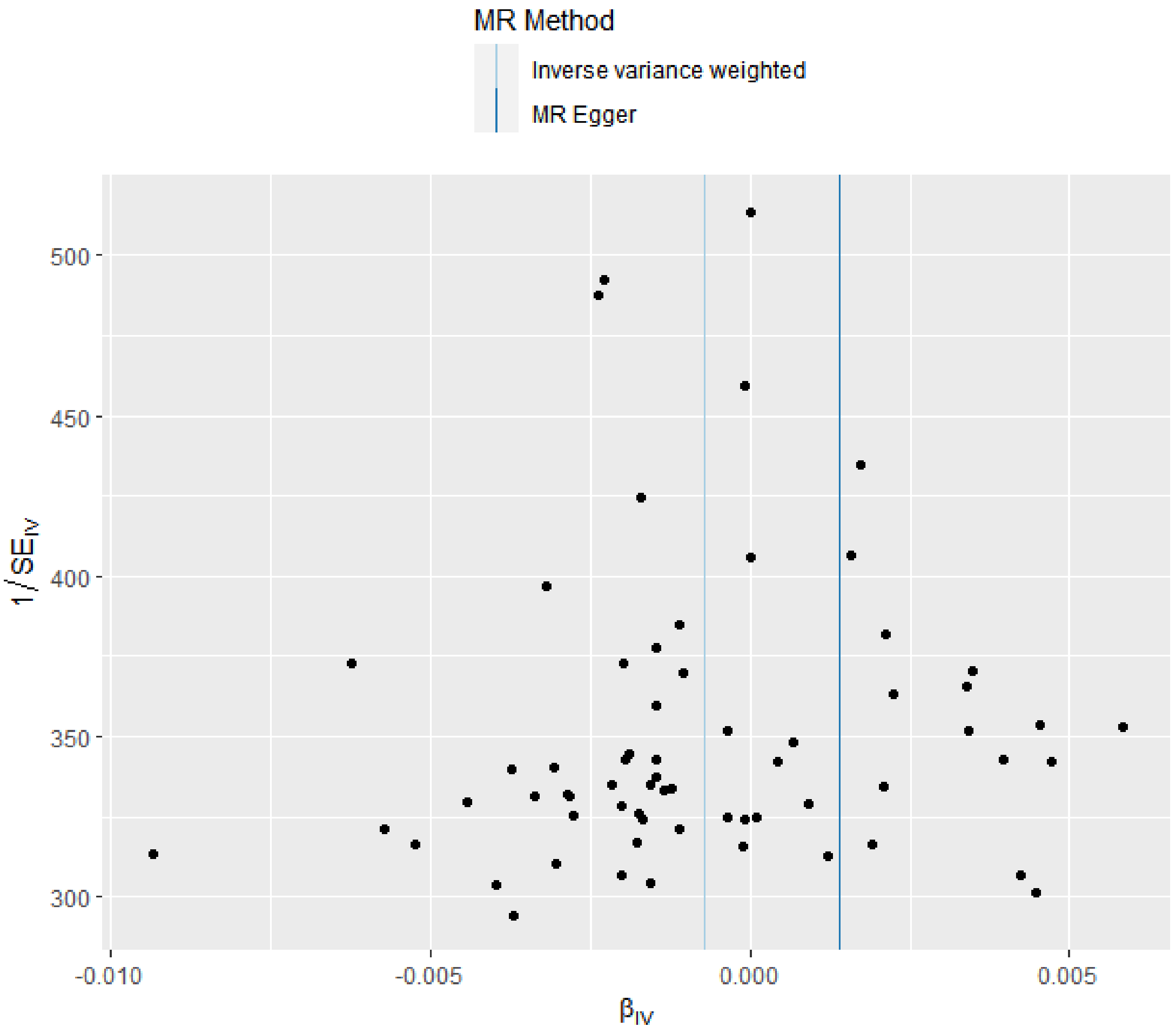

MR Method
Inverse variance weighted
MR Egger
500
450
400
350
300
$1/SE_{IV}$
-0.010
-0.005
0.000
0.005
β_{IV}

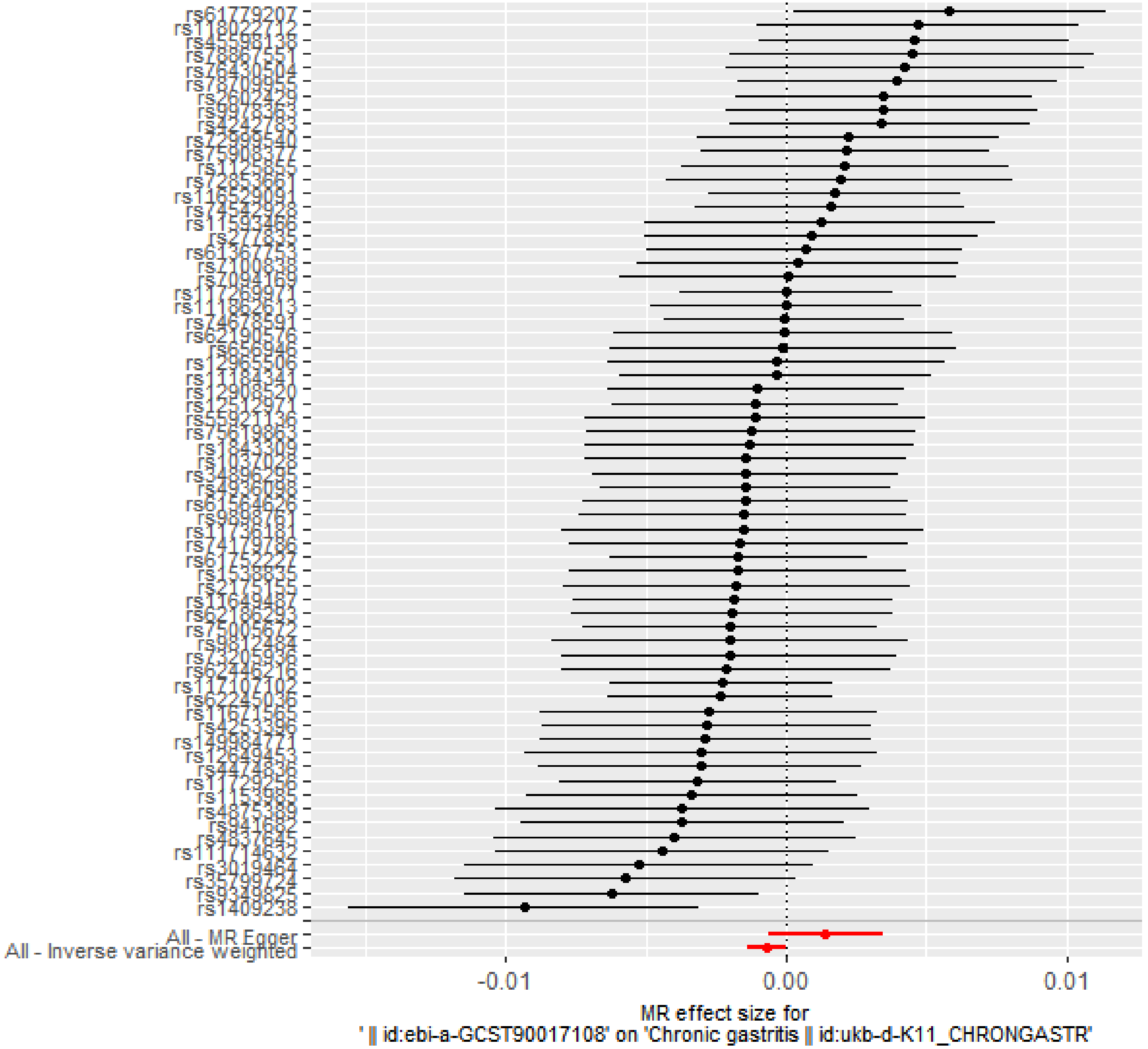

rs61779207
rs1409238
All - MR Egger
All - Inverse variance weighted
-0.01
0.00
0.01
MR effect size for
' || id:ebi-a-GCST90017108' on 'Chronic gastritis || id:ukb-d-K11_CHRONGASTR'

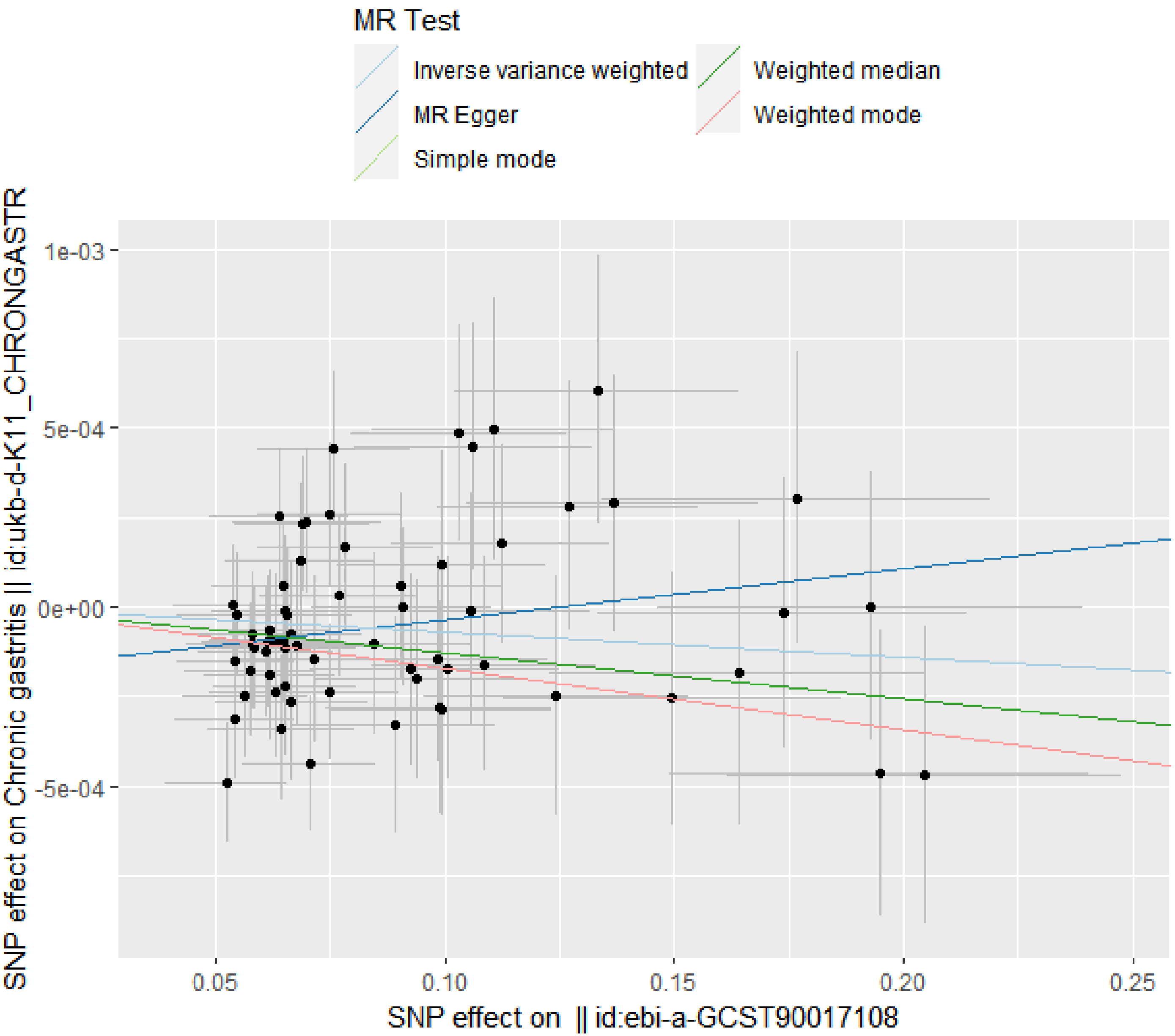

MR Test
Inverse variance weighted
MR Egger
Simple mode
Weighted median
Weighted mode
SNP effect on Chronic gastritis || id:ukb-d-K11_CHRONGASTR
1e-03
5e-04
0e+00
-5e-04
0.05
0.10
0.15
0.20
0.25
SNP effect on || id:ebi-a-GCST90017108

Figure 71 Leave-one-out analysis, funnel plot, MR effect size and scatter plot for gut microbiota abundance (class Gammaproteobacteria id.3303) on acute gastritis

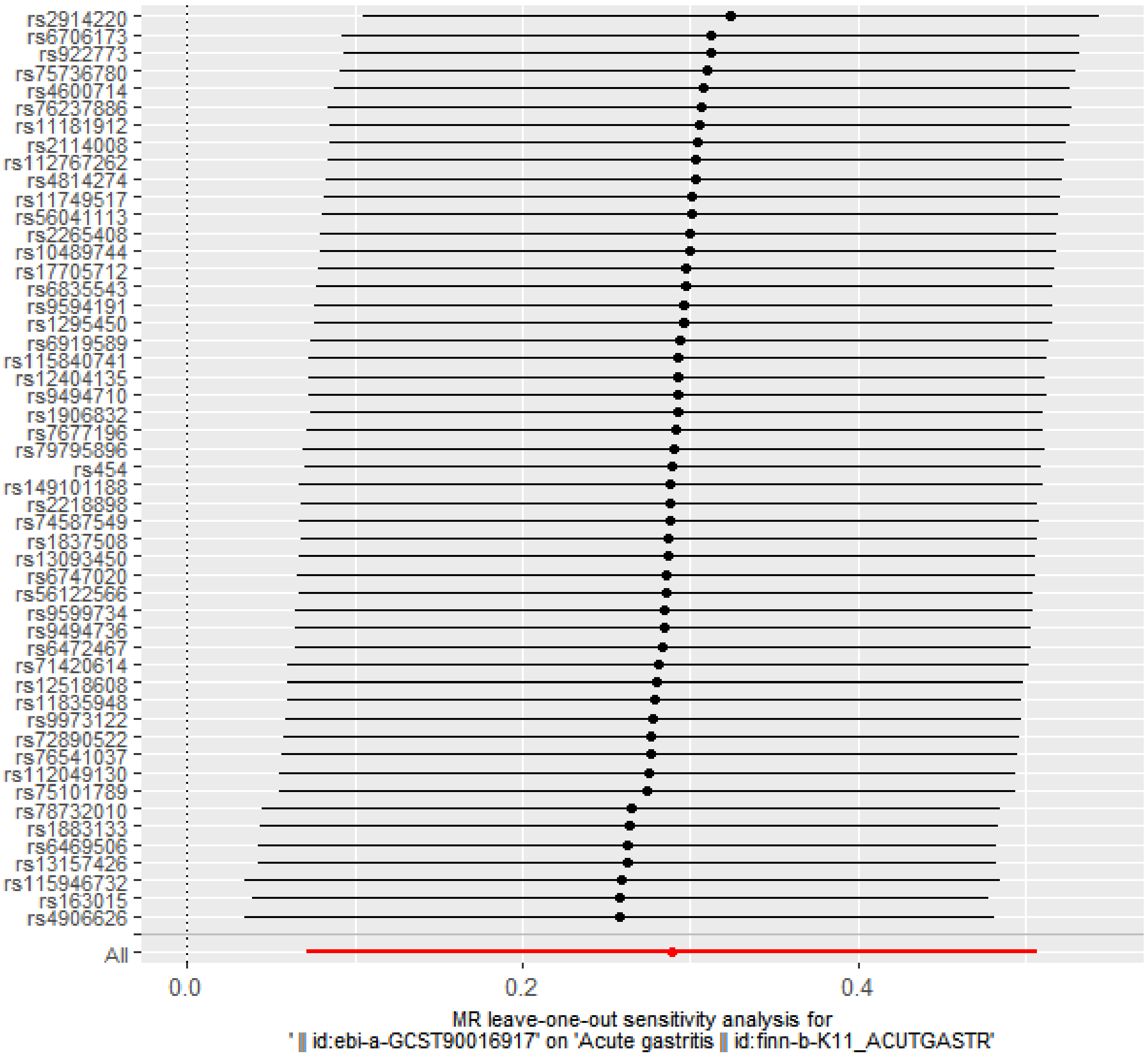

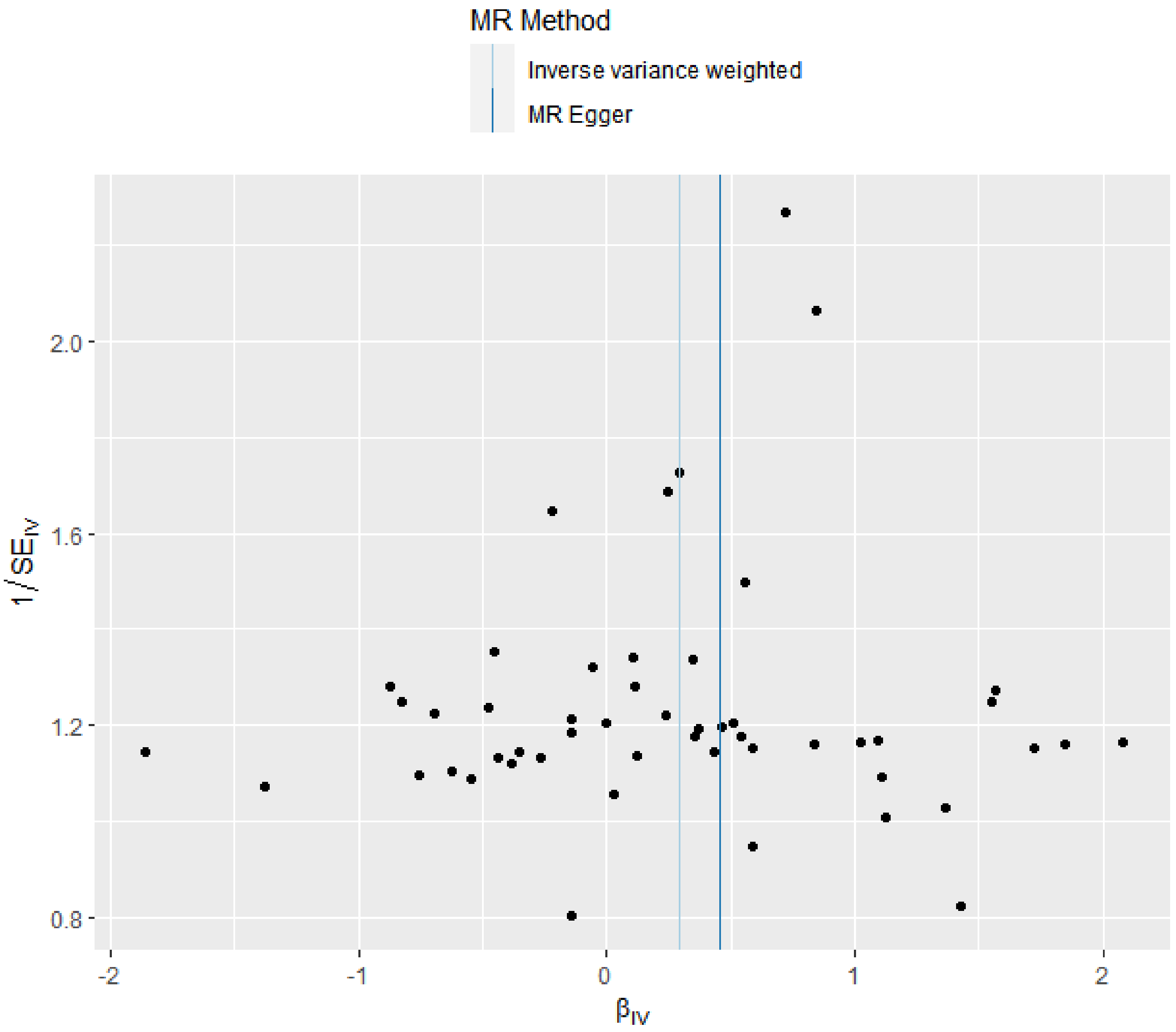
MR Method
Inverse variance weighted
MR Egger
2.0
1.6
1.2
0.8
$1/SE_{IV}$
-2
-1
0
1
2
β_{IV}

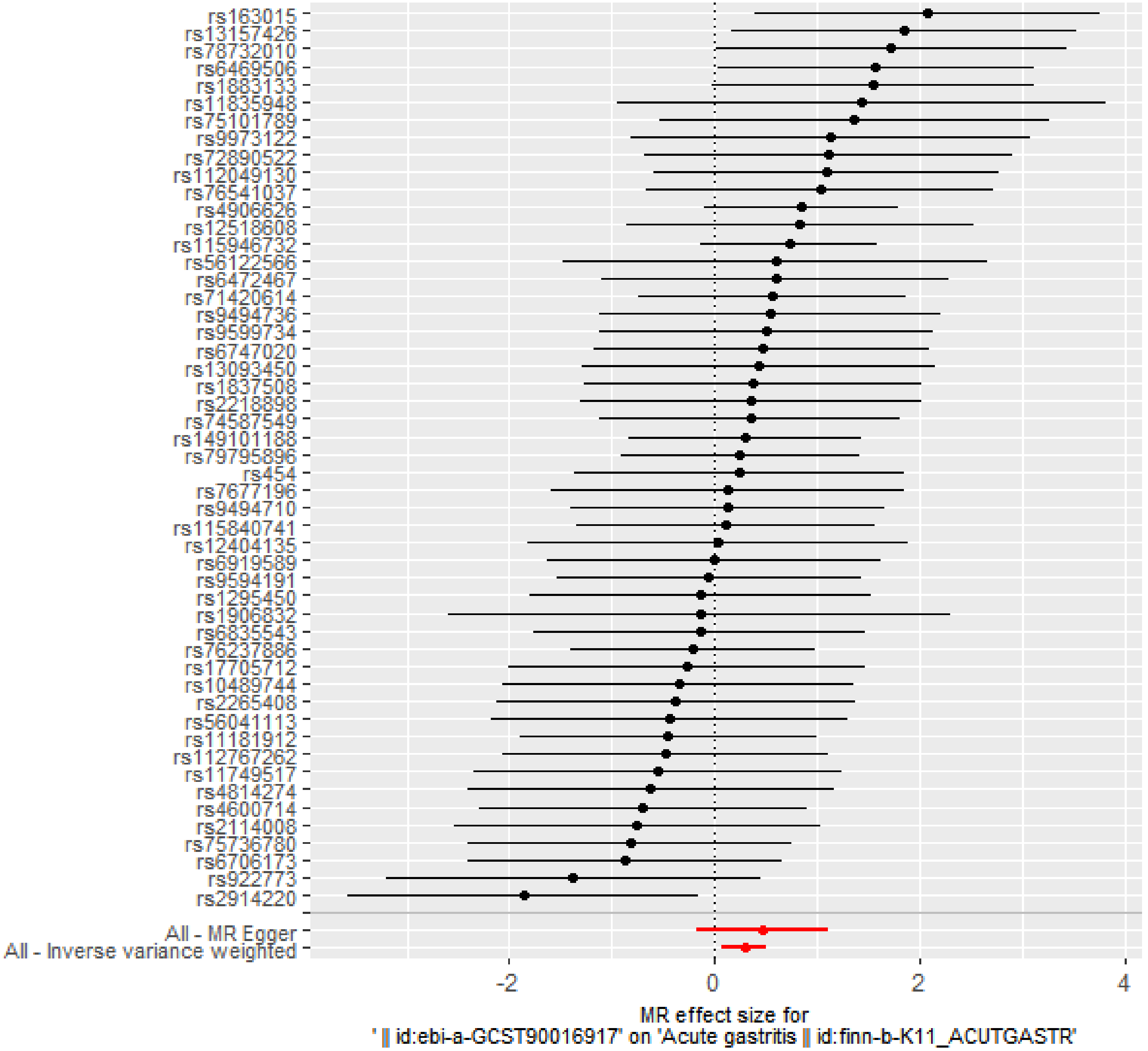

rs163015
rs13157426
rs78732010
rs6469506
rs1883133
rs11835948
rs75101789
rs9973122
rs72890522
rs112049130
rs76541037
rs4906626
rs12518608
rs115946732
rs56122566
rs6472467
rs71420614
rs9494736
rs9599734
rs6747020
rs13093450
rs1837508
rs2218898
rs74587549
rs149101188
rs79795896
rs454
rs7677196
rs9494710
rs115840741
rs12404135
rs6919589
rs9594191
rs1295450
rs1906832
rs6835543
rs76237886
rs17705712
rs10489744
rs2265408
rs56041113
rs11181912
rs112767262
rs11749517
rs4814274
rs4600714
rs2114008
rs75736780
rs6706173
rs922773
rs2914220
All - MR Egger
All - Inverse variance weighted
-2
0
2
4
MR effect size for
' || id:ebi-a-GCST90016917' on 'Acute gastritis || id:finn-b-K11_ACUTGASTR'

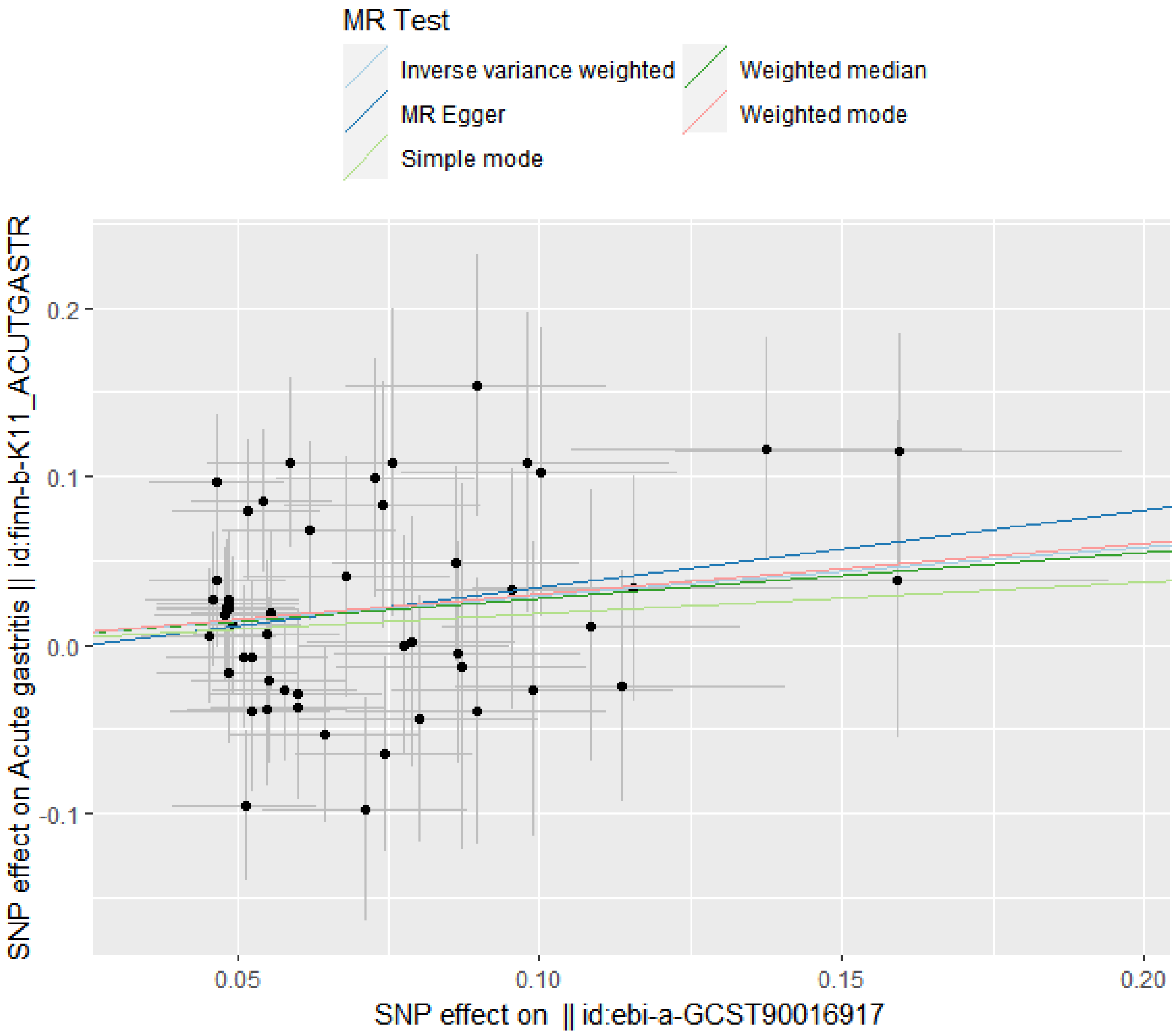

MR Test
Inverse variance weighted
MR Egger
Simple mode
Weighted median
Weighted mode
SNP effect on Acute gastritis || id:finn-b-K11_ACUTGASTR
0.2
0.1
0.0
-0.1
0.05
0.10
0.15
0.20
SNP effect on || id:ebi-a-GCST90016917

Figure 72 Leave-one-out analysis, funnel plot, MR effect size and scatter plot for gut microbiota abundance (family Bacteroidaceae id.917) on acute gastritis

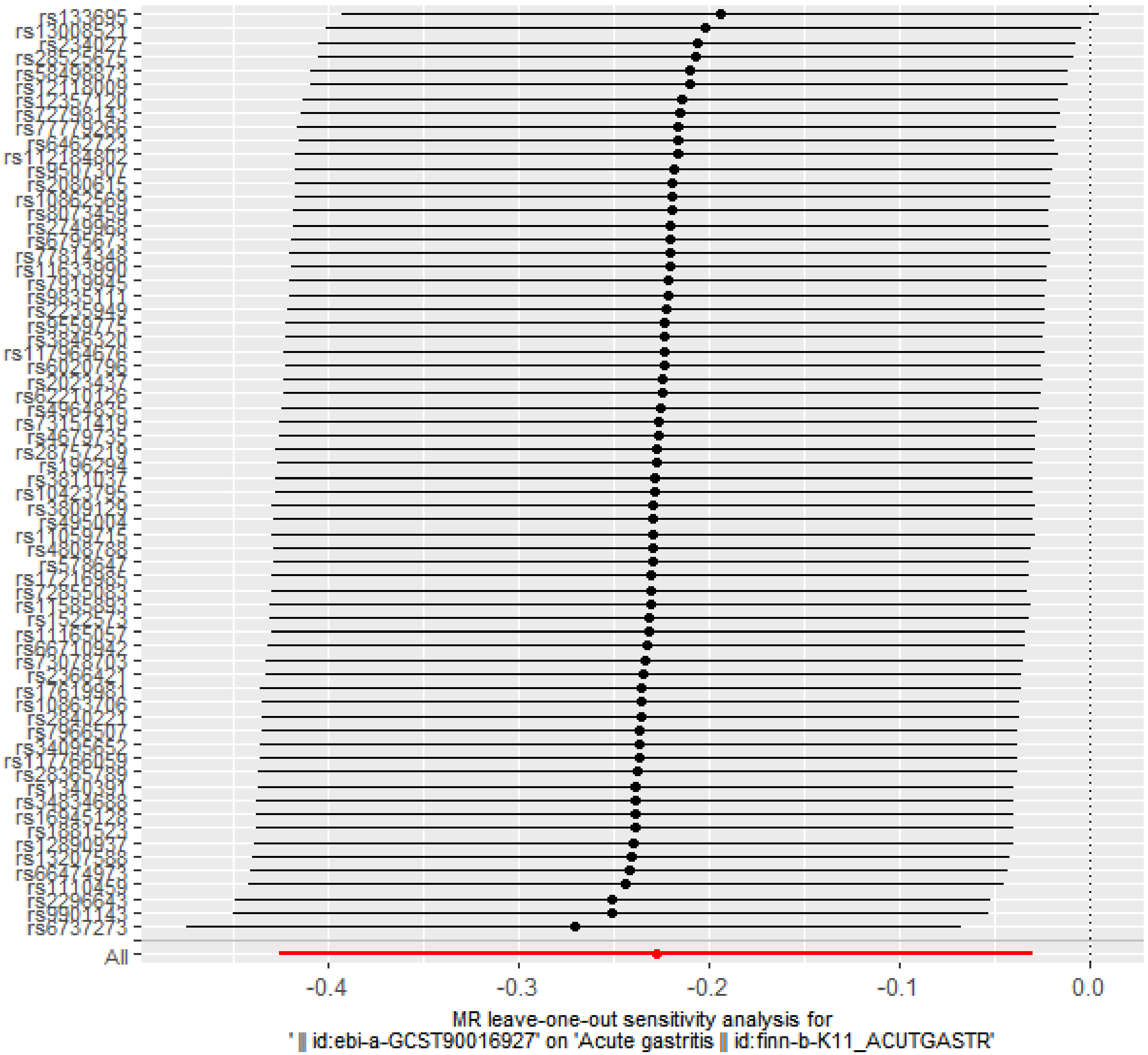

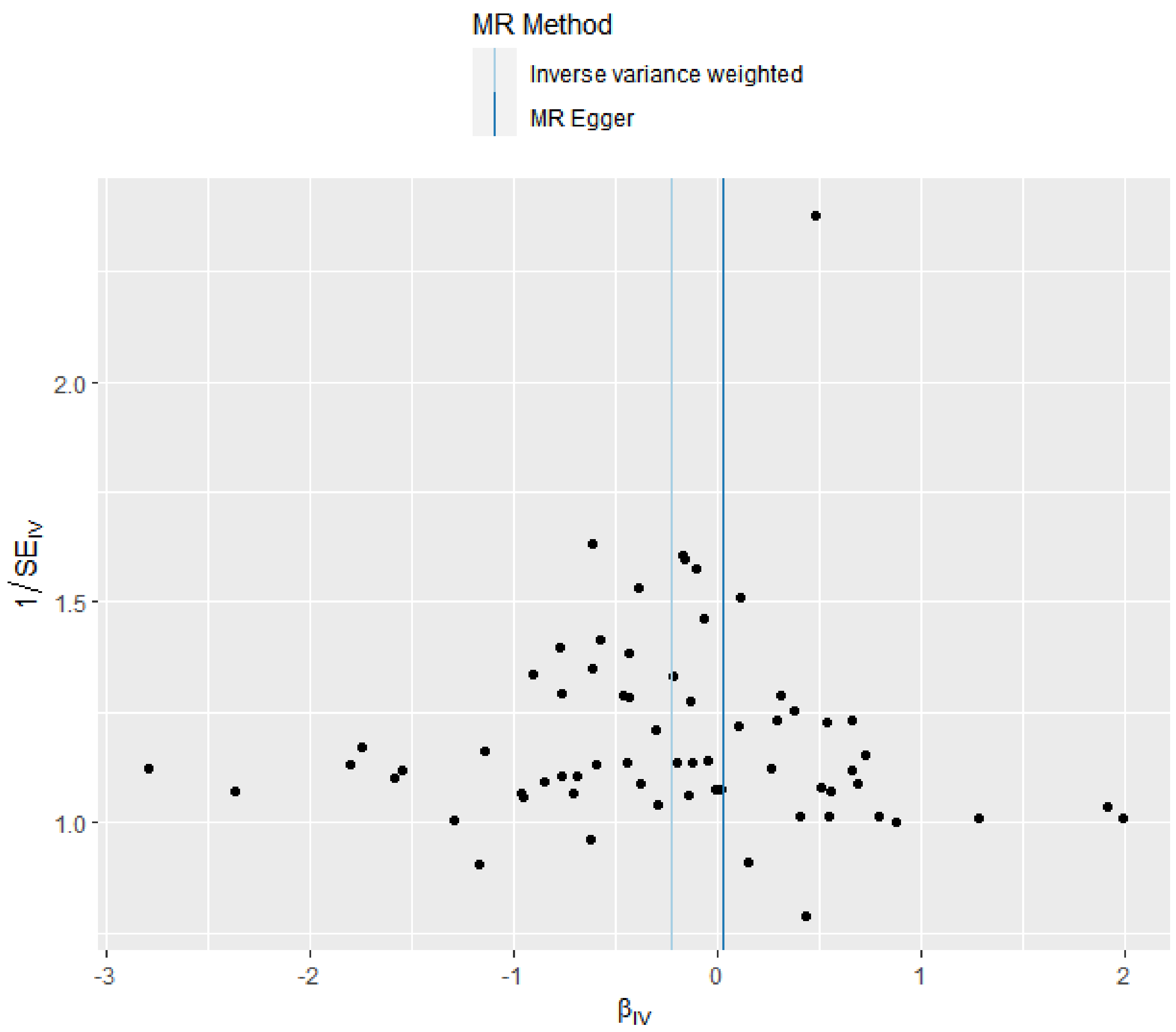
MR Method
Inverse variance weighted
MR Egger
2.0
1.5
1.0
$1/SE_{IV}$
-3
-2
-1
0
1
2
β_{IV}

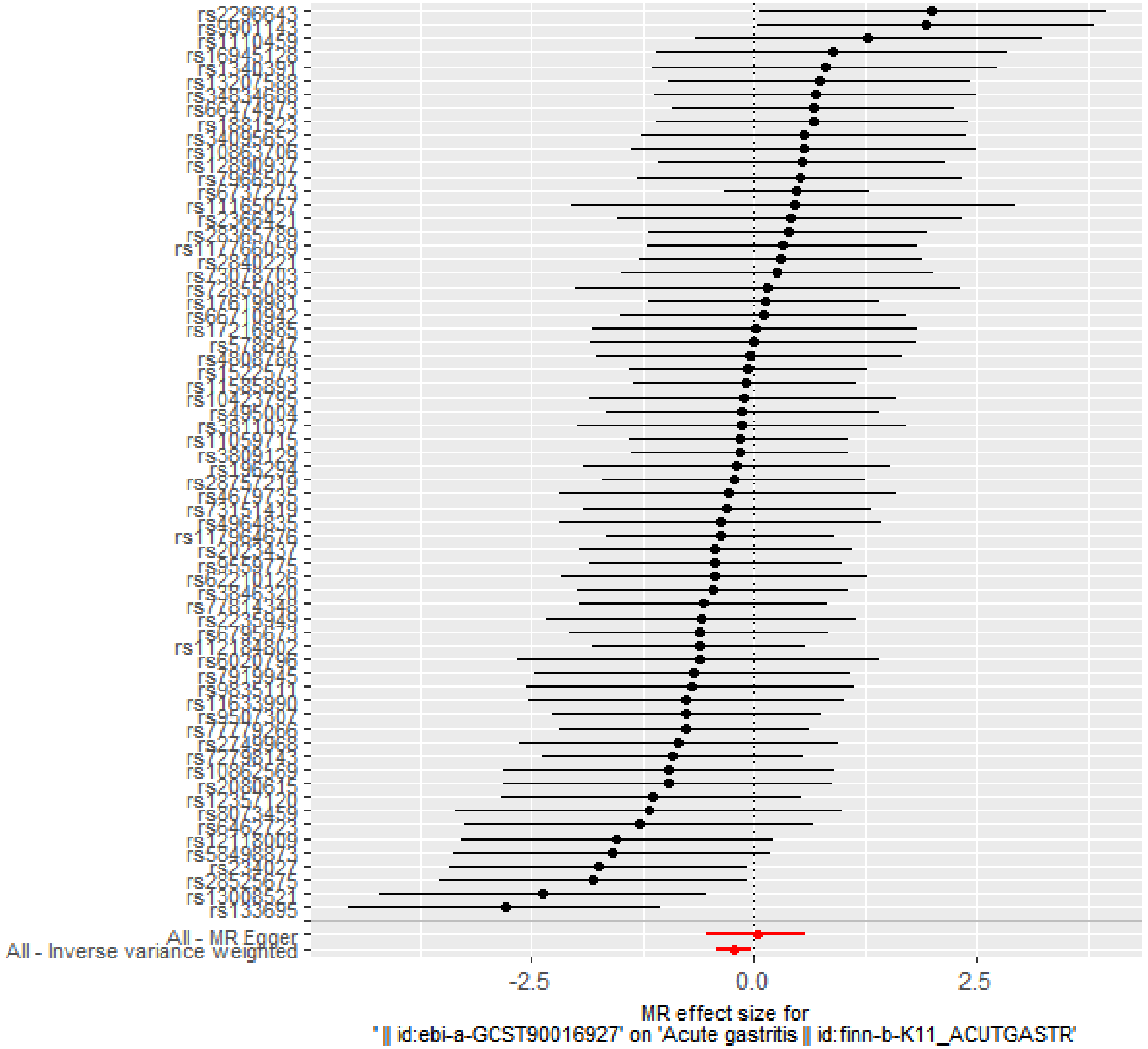
All - MR Egger
All - Inverse variance weighted
-2.5
0.0
2.5
MR effect size for
' || id:ebi-a-GCST90016927' on 'Acute gastritis || id:finn-b-K11_ACUTGASTR'

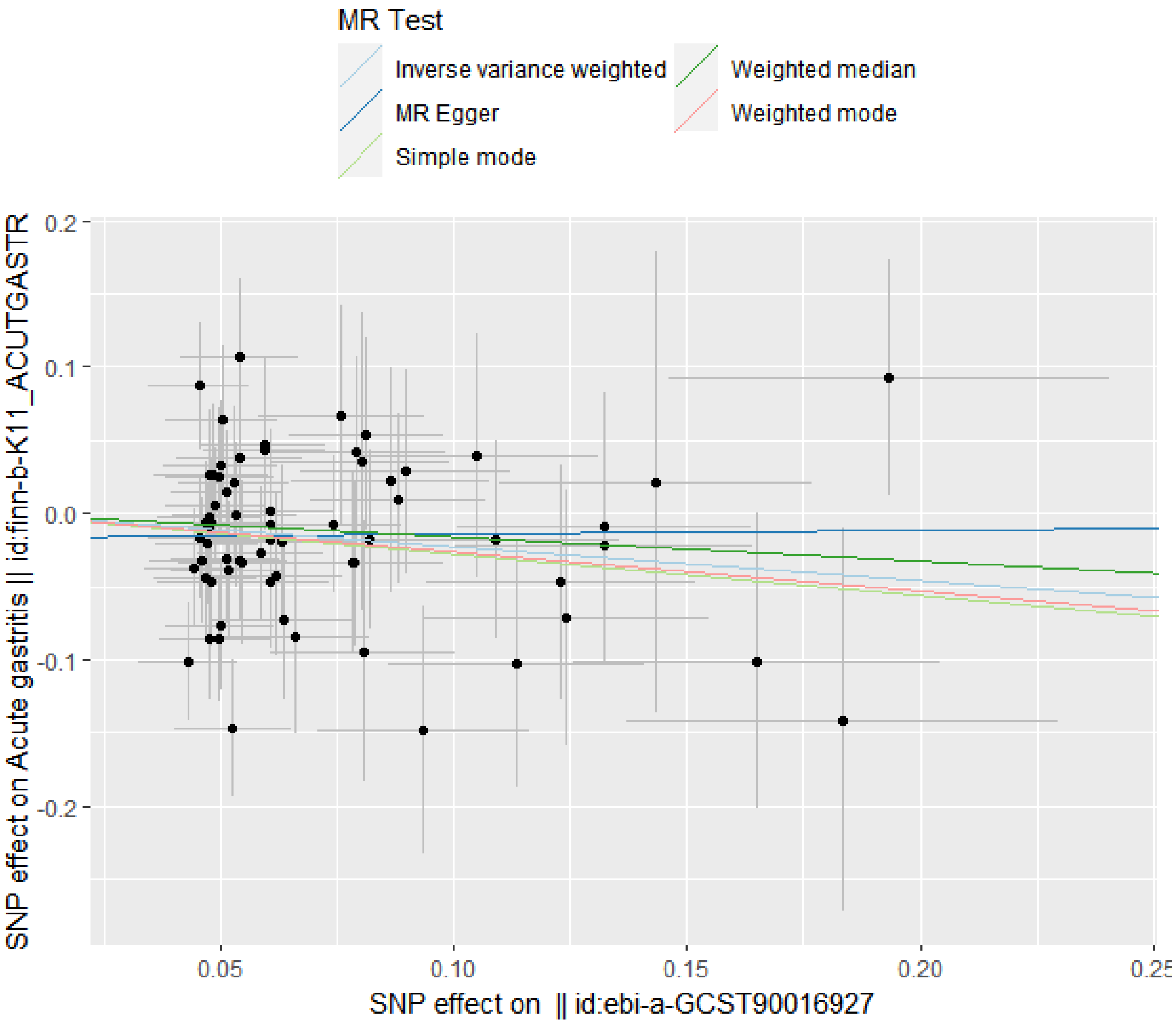

MR Test
Inverse variance weighted
MR Egger
Simple mode
Weighted median
Weighted mode
SNP effect on Acute gastritis || id:finn-b-K11_ACUTGASTR
0.2
0.1
0.0
-0.1
-0.2
0.05
0.10
0.15
0.20
0.25
SNP effect on || id:ebi-a-GCST90016927

Figure 73 Leave-one-out analysis, funnel plot, MR effect size and scatter plot for gut microbiota abundance (genus Alloprevotella id.961) on acute gastritis

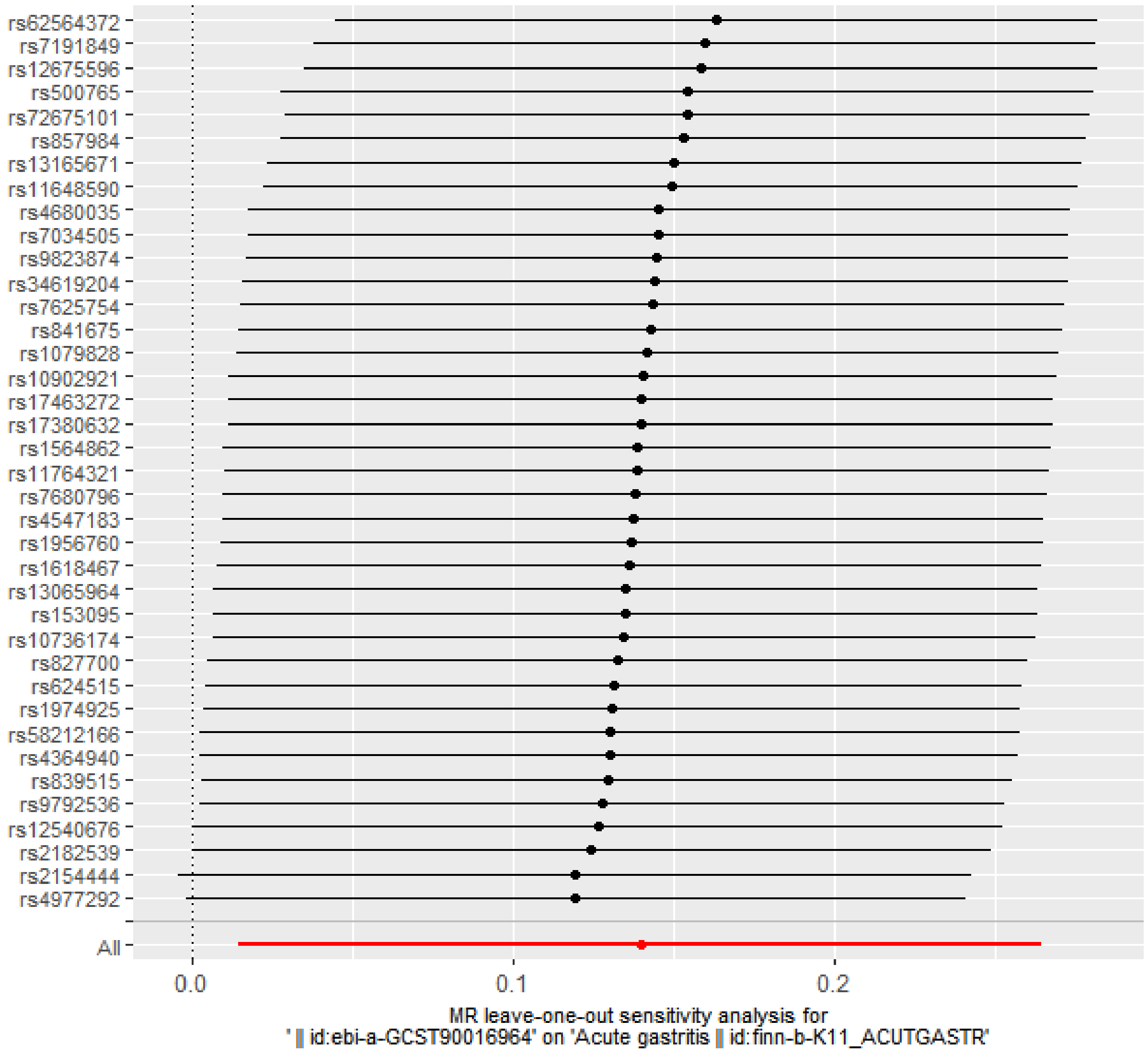

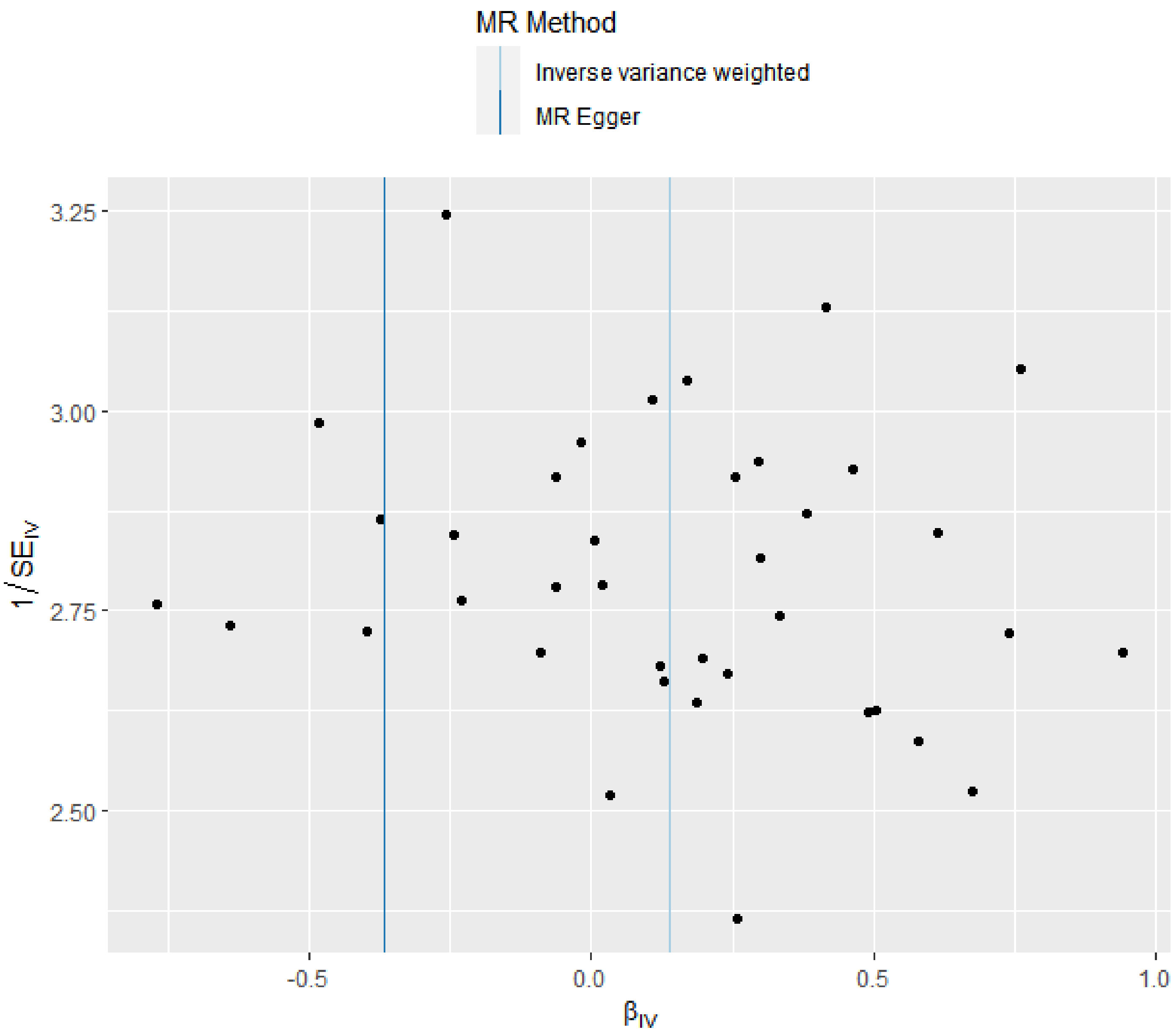
MR Method
Inverse variance weighted
MR Egger
3.25
3.00
2.75
2.50
1/SE$_{IV}$
-0.5
0.0
0.5
1.0
β$_{IV}$

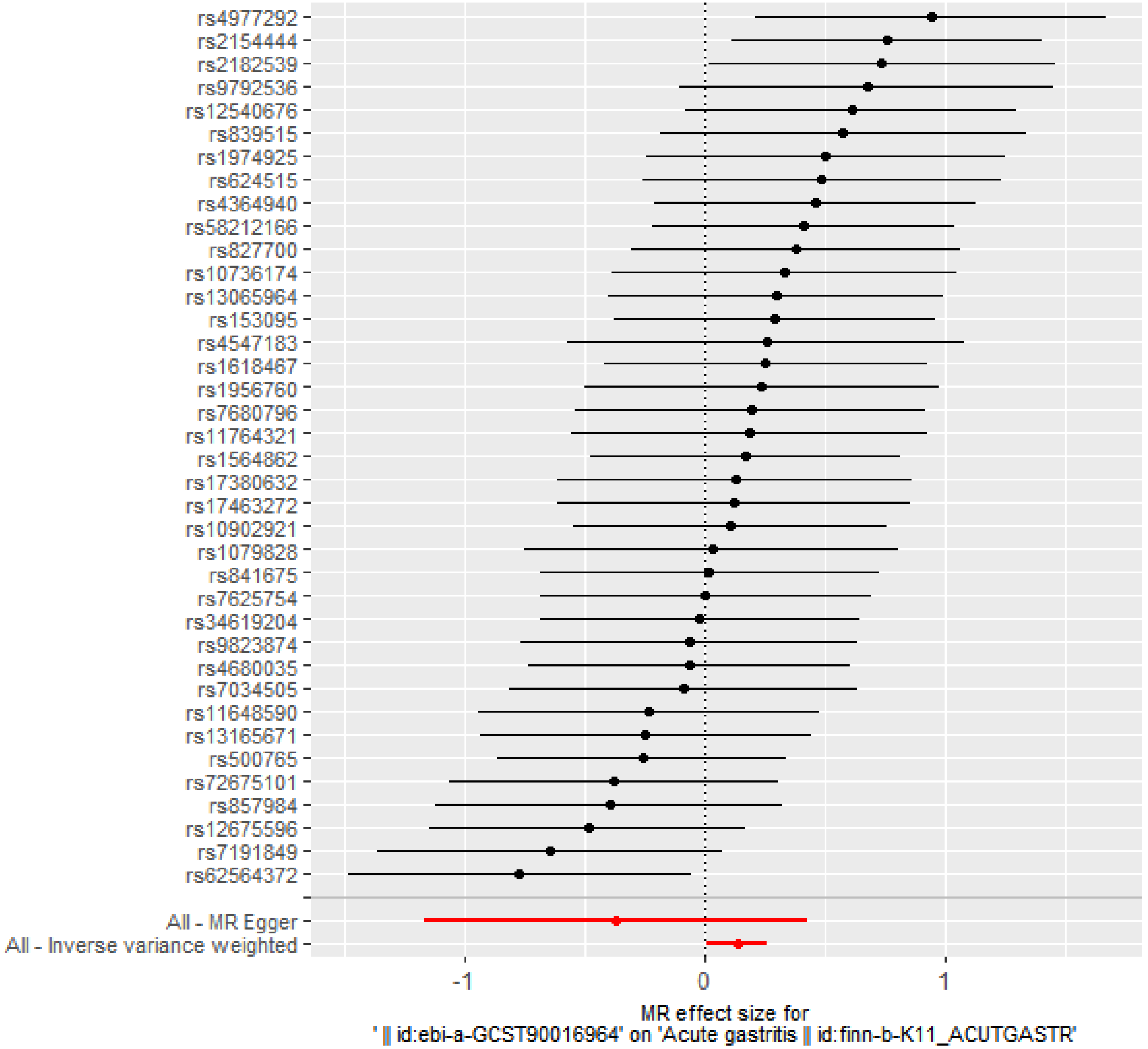

rs4977292
rs2154444
rs2182539
rs9792536
rs12540676
rs839515
rs1974925
rs624515
rs4364940
rs58212166
rs827700
rs10736174
rs13065964
rs153095
rs4547183
rs1618467
rs1956760
rs7680796
rs11764321
rs1564862
rs17380632
rs17463272
rs10902921
rs1079828
rs841675
rs7625754
rs34619204
rs9823874
rs4680035
rs7034505
rs11648590
rs13165671
rs500765
rs72675101
rs857984
rs12675596
rs7191849
rs62564372
All - MR Egger
All - Inverse variance weighted
-1
0
1
MR effect size for
' || id:ebi-a-GCST90016964' on 'Acute gastritis || id:finn-b-K11_ACUTGASTR'

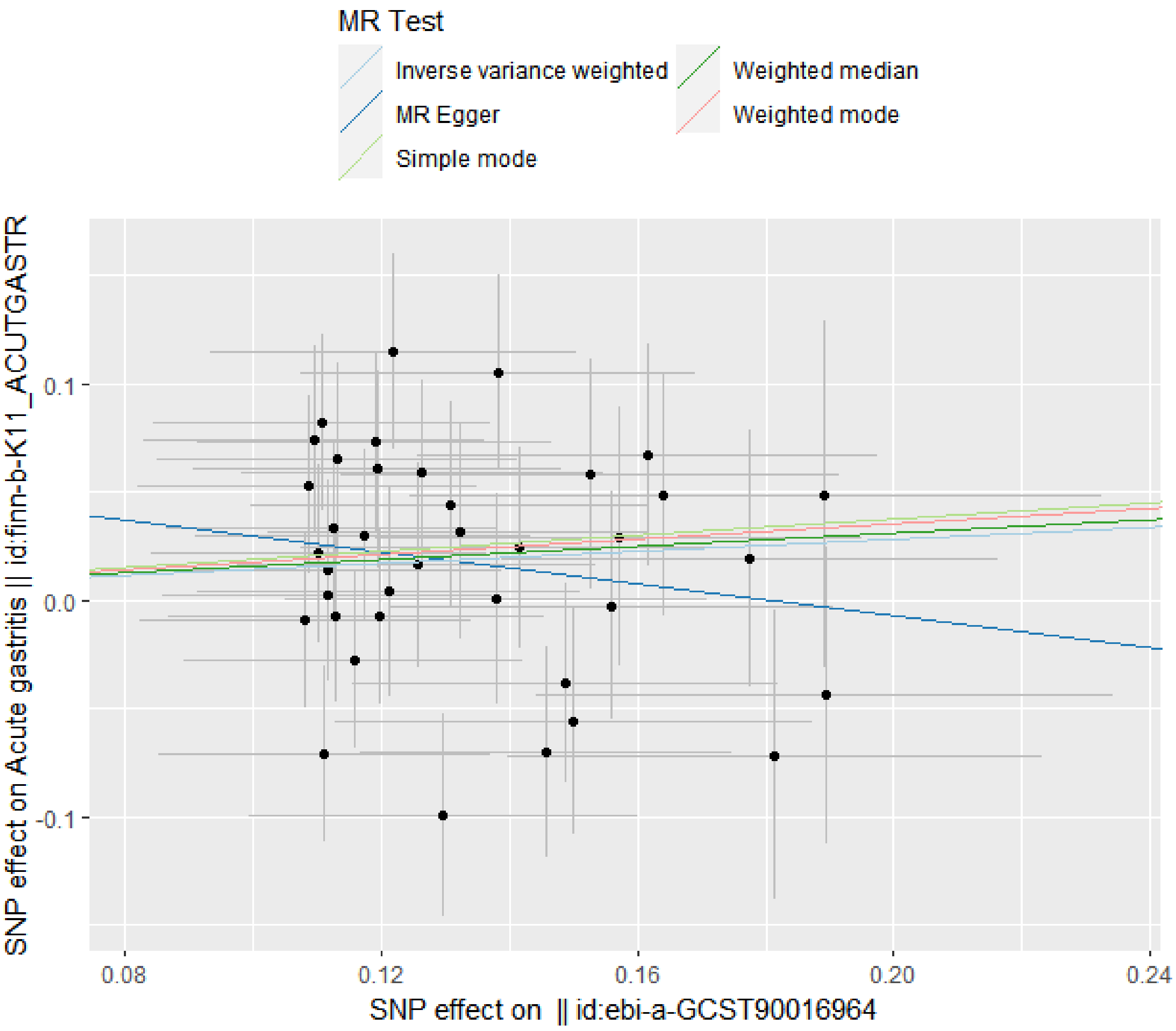

MR Test
Inverse variance weighted
MR Egger
Simple mode
Weighted median
Weighted mode
0.1
0.0
-0.1
0.08
0.12
0.16
0.20
0.24
SNP effect on Acute gastritis || id:finn-b-K11_ACUTGASTR
SNP effect on || id:ebi-a-GCST90016964

Figure 74 Leave-one-out analysis, funnel plot, MR effect size and scatter plot for gut microbiota abundance (genus Bacteroides id.918) on acute gastritis

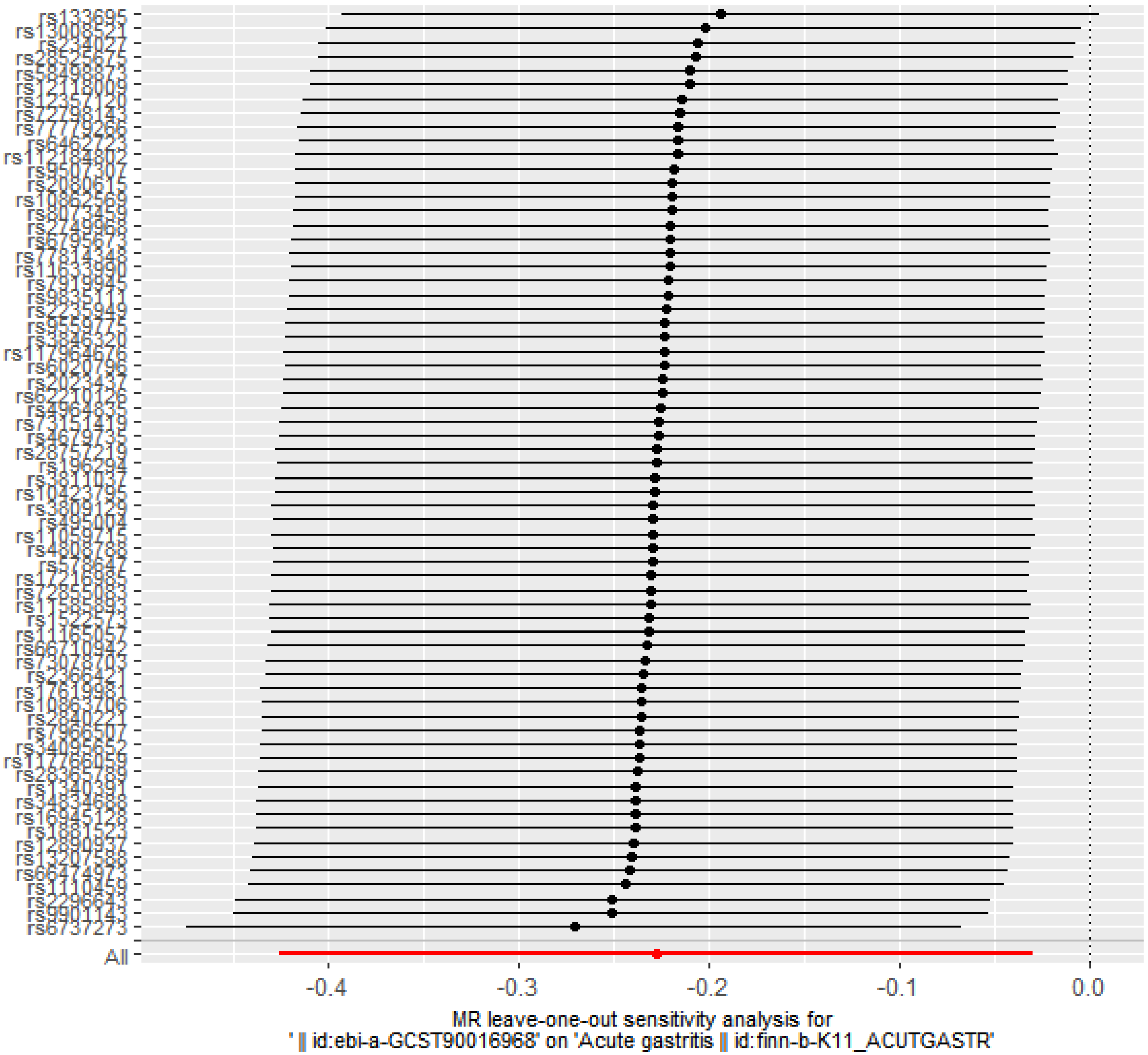

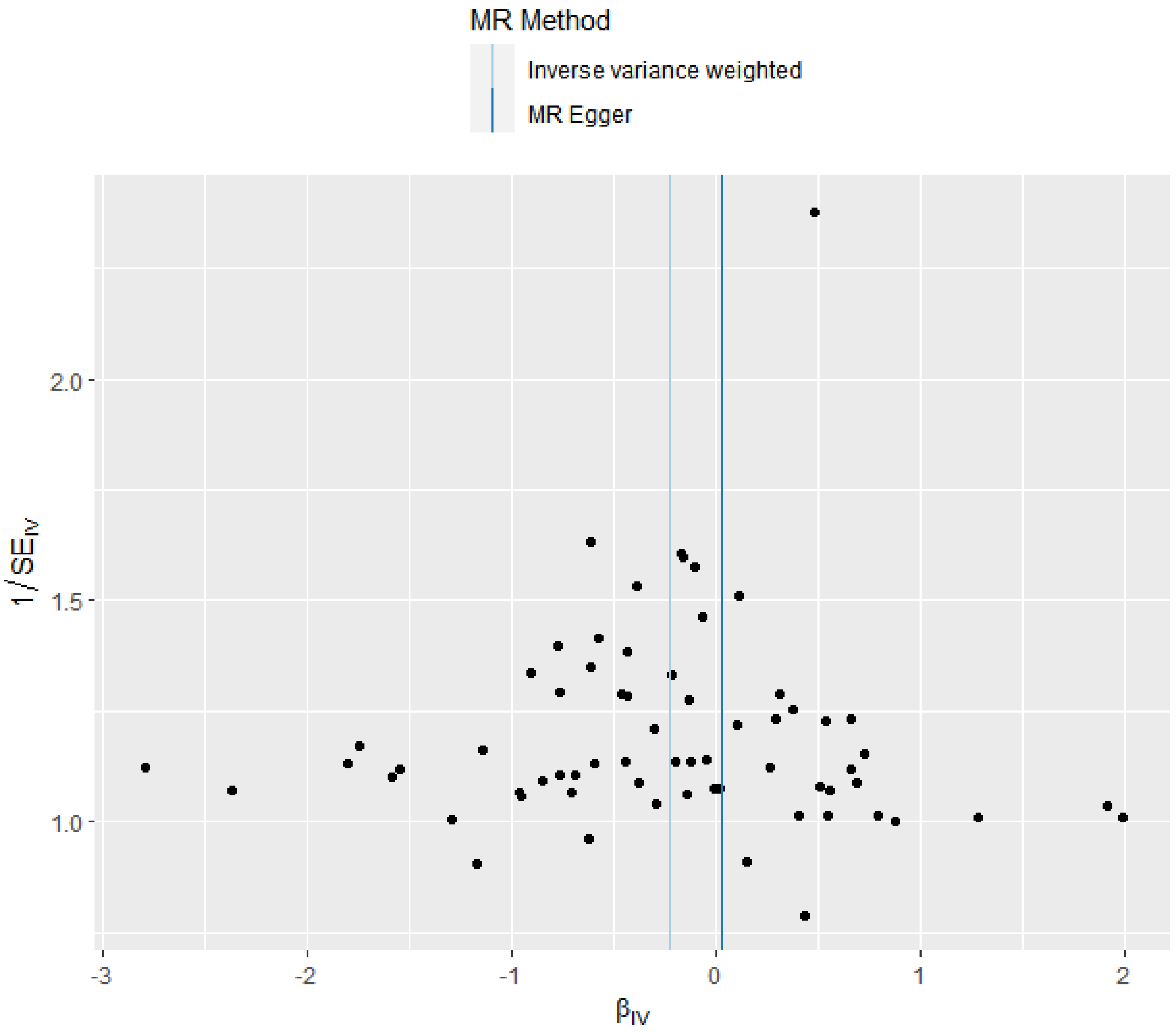
MR Method
Inverse variance weighted
MR Egger
2.0
1.5
1.0
1/SE_IV
-3
-2
-1
0
1
2
β_IV

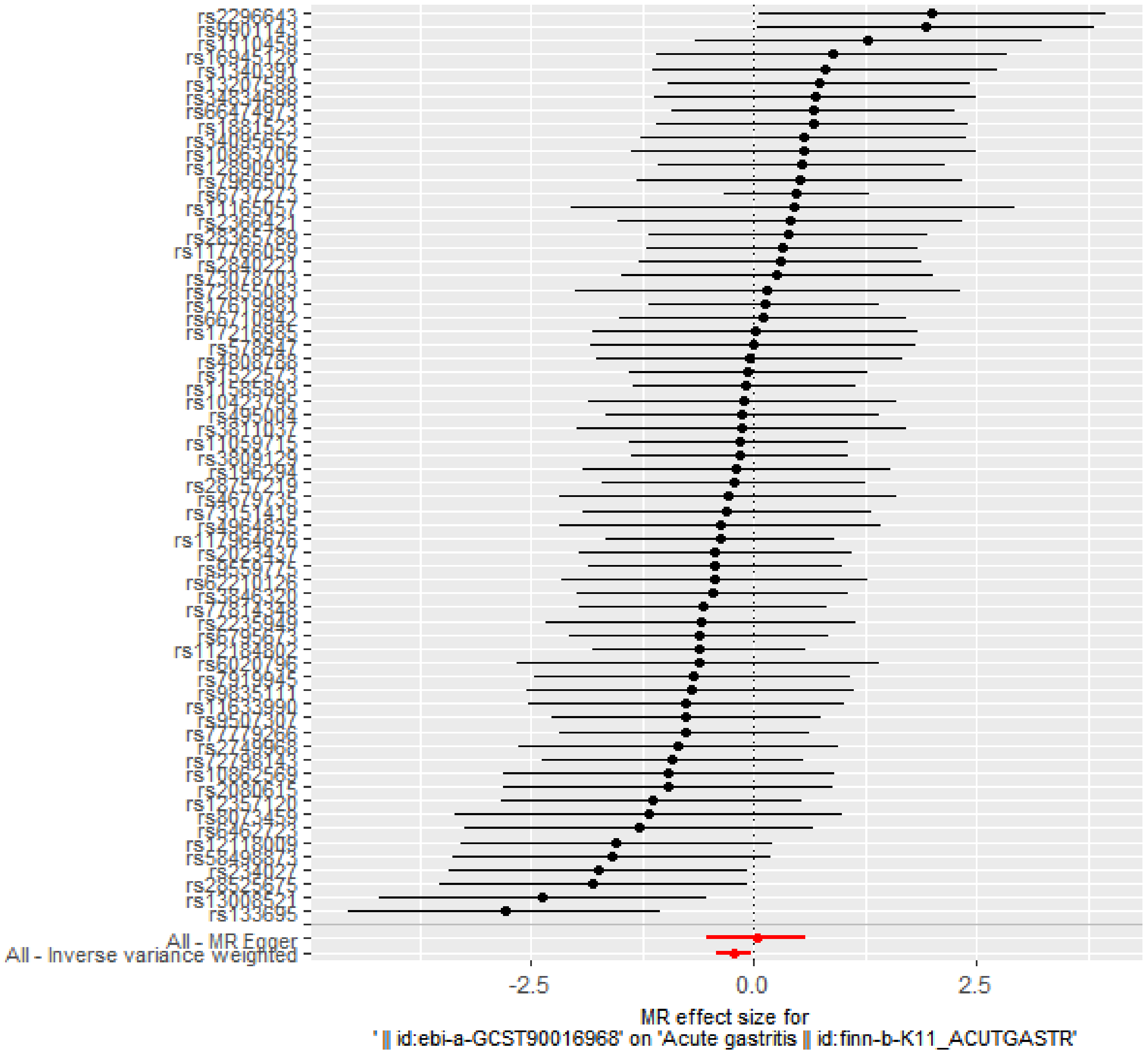

rs2296643
rs9901143
rs1110459
rs16945128
rs133695
All - MR Egger
All - Inverse variance weighted
-2.5
0.0
2.5
MR effect size for
' || id:ebi-a-GCST90016968' on 'Acute gastritis || id:finn-b-K11_ACUTGASTR'

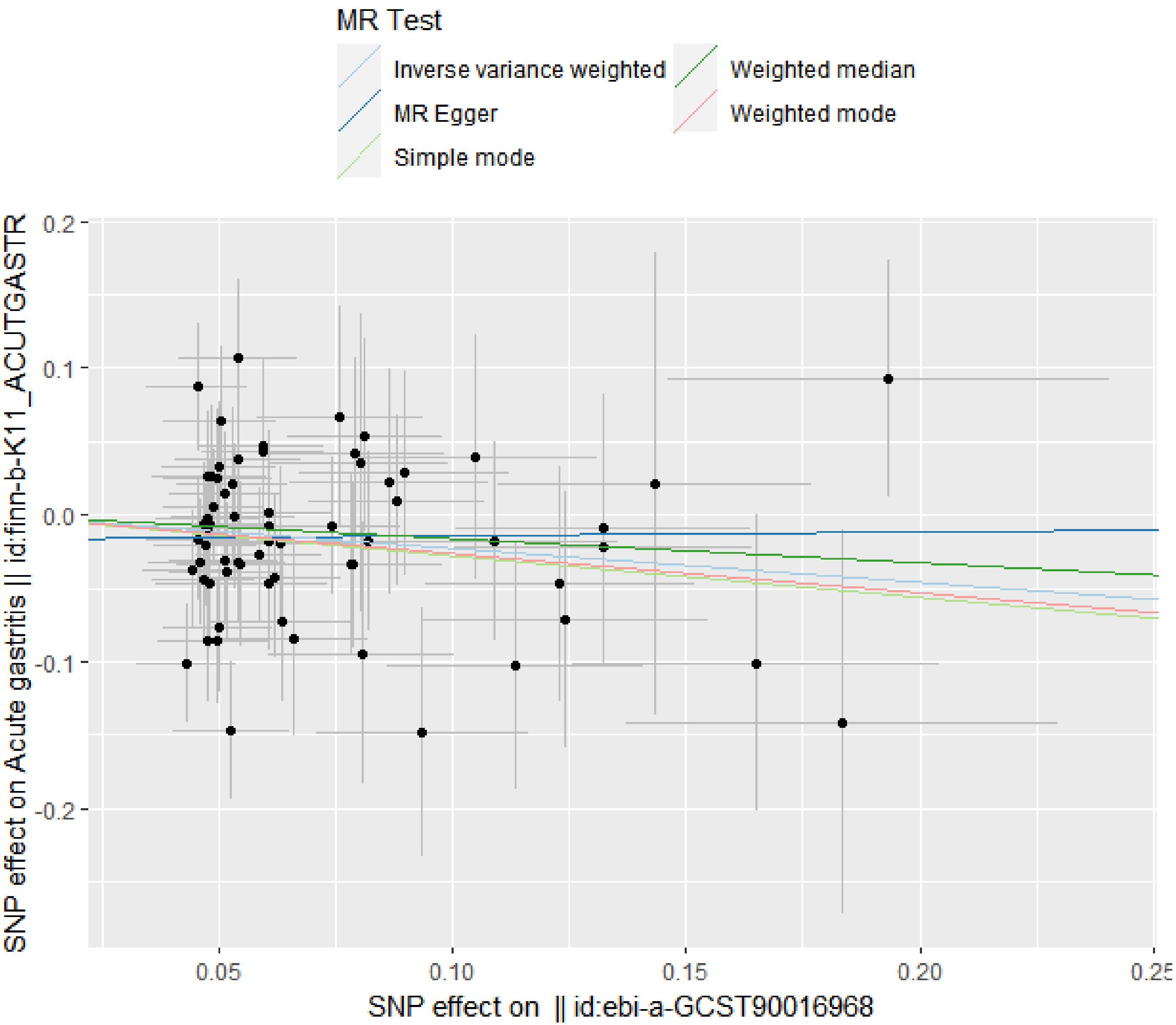
MR Test
Inverse variance weighted
MR Egger
Simple mode
Weighted median
Weighted mode
0.2
0.1
0.0
-0.1
-0.2
SNP effect on Acute gastritis || id:finn-b-K11_ACUTGASTR
0.05
0.10
0.15
0.20
0.25
SNP effect on || id:ebi-a-GCST90016968

Figure 75 Leave-one-out analysis, funnel plot, MR effect size and scatter plot for gut microbiota abundance (genus Bilophila id.3170) on acute gastritis

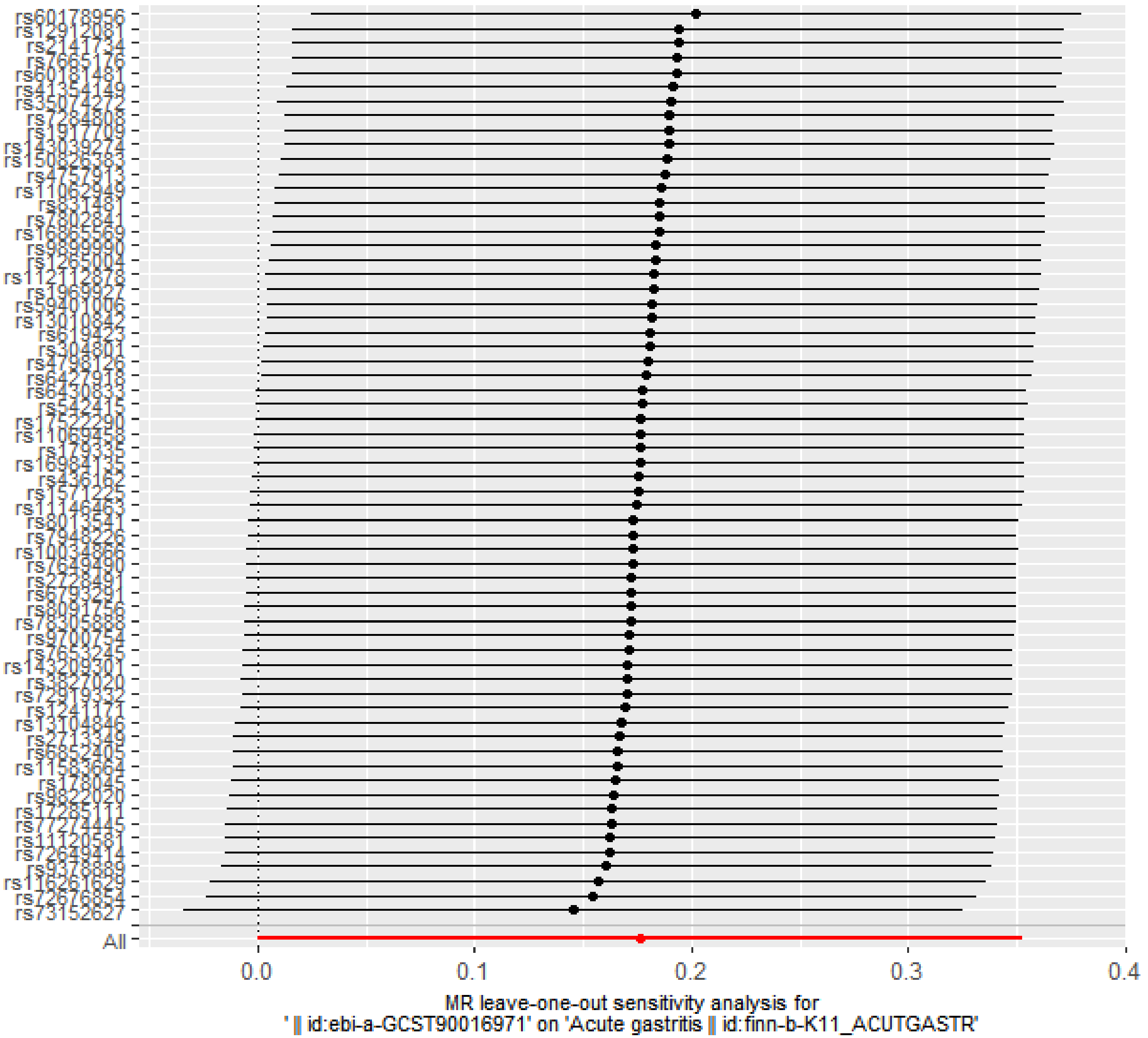

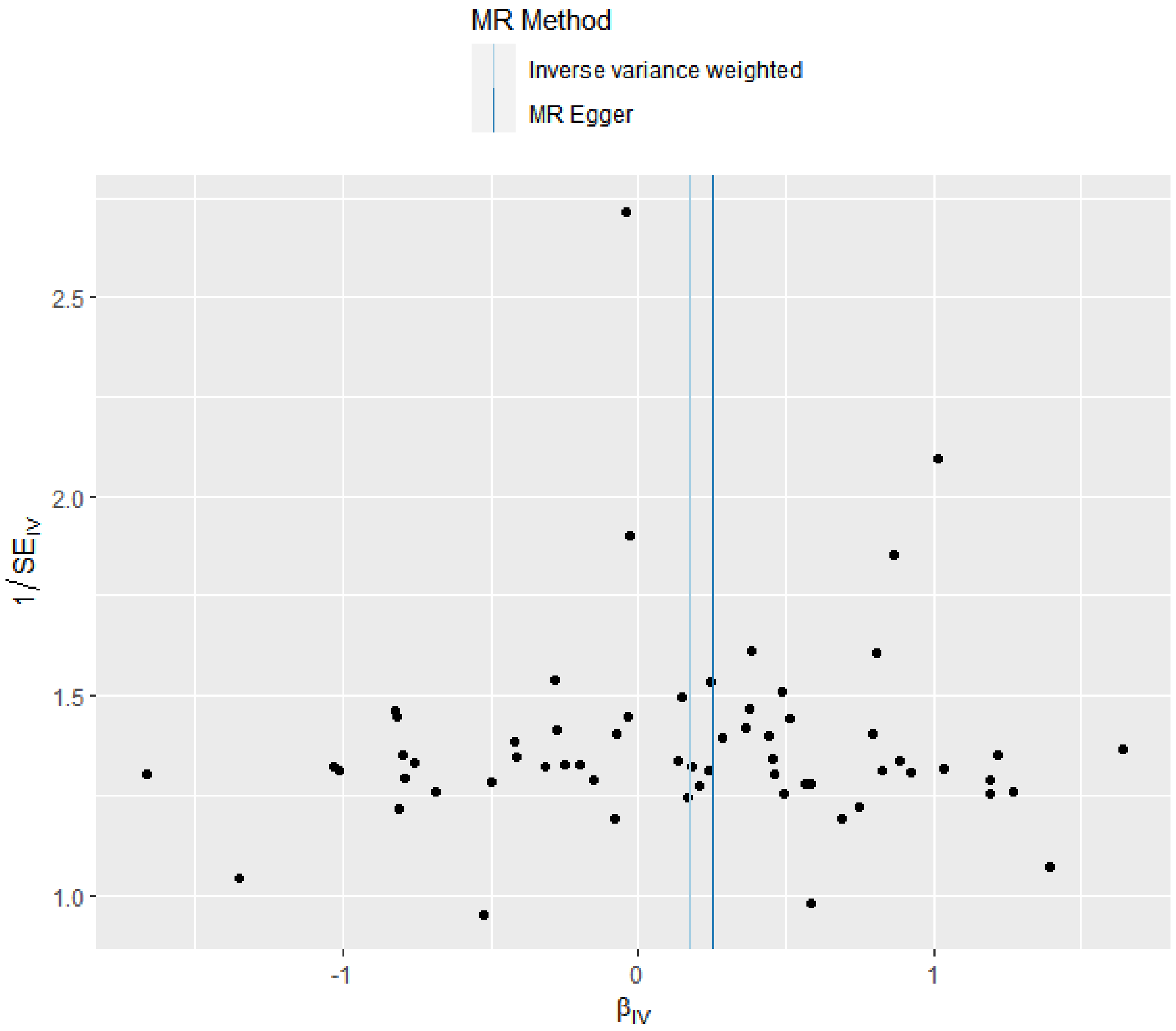
MR Method
Inverse variance weighted
MR Egger
2.5
2.0
1.5
1.0
-1
0
1
$1/SE_{IV}$
β_{IV}

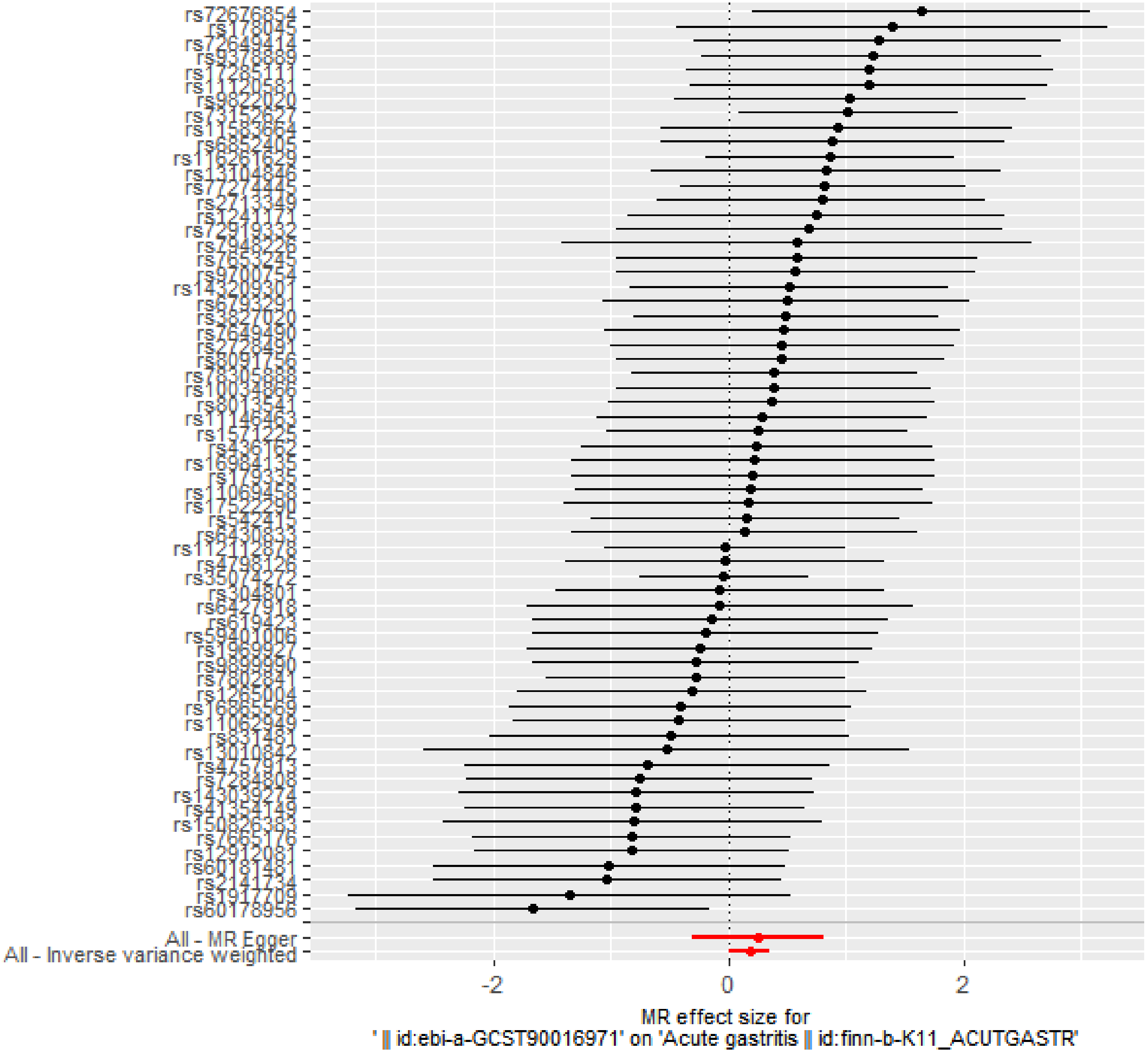

All - MR Egger
All - Inverse variance weighted
-2
0
2
MR effect size for
' || id:ebi-a-GCST90016971' on 'Acute gastritis || id:finn-b-K11_ACUTGASTR'

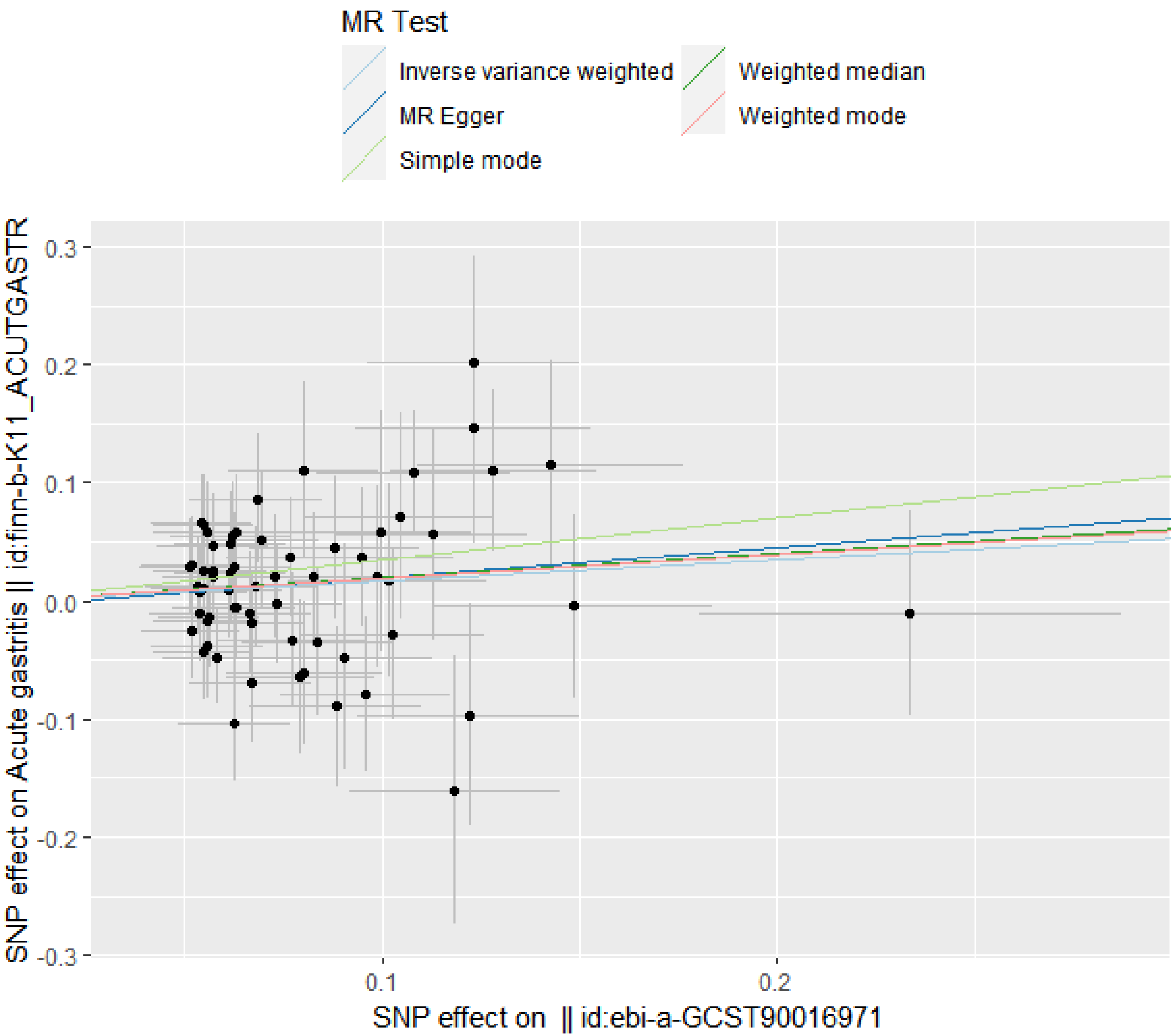

MR Test
Inverse variance weighted
MR Egger
Simple mode
Weighted median
Weighted mode
SNP effect on Acute gastritis || id:finn-b-K11_ACUTGASTR
0.3
0.2
0.1
0.0
-0.1
-0.2
-0.3
0.1
0.2
SNP effect on || id:ebi-a-GCST90016971

Figure 76 Leave-one-out analysis, funnel plot, MR effect size and scatter plot for gut microbiota abundance (genus Dialister id.2183) on acute gastritis

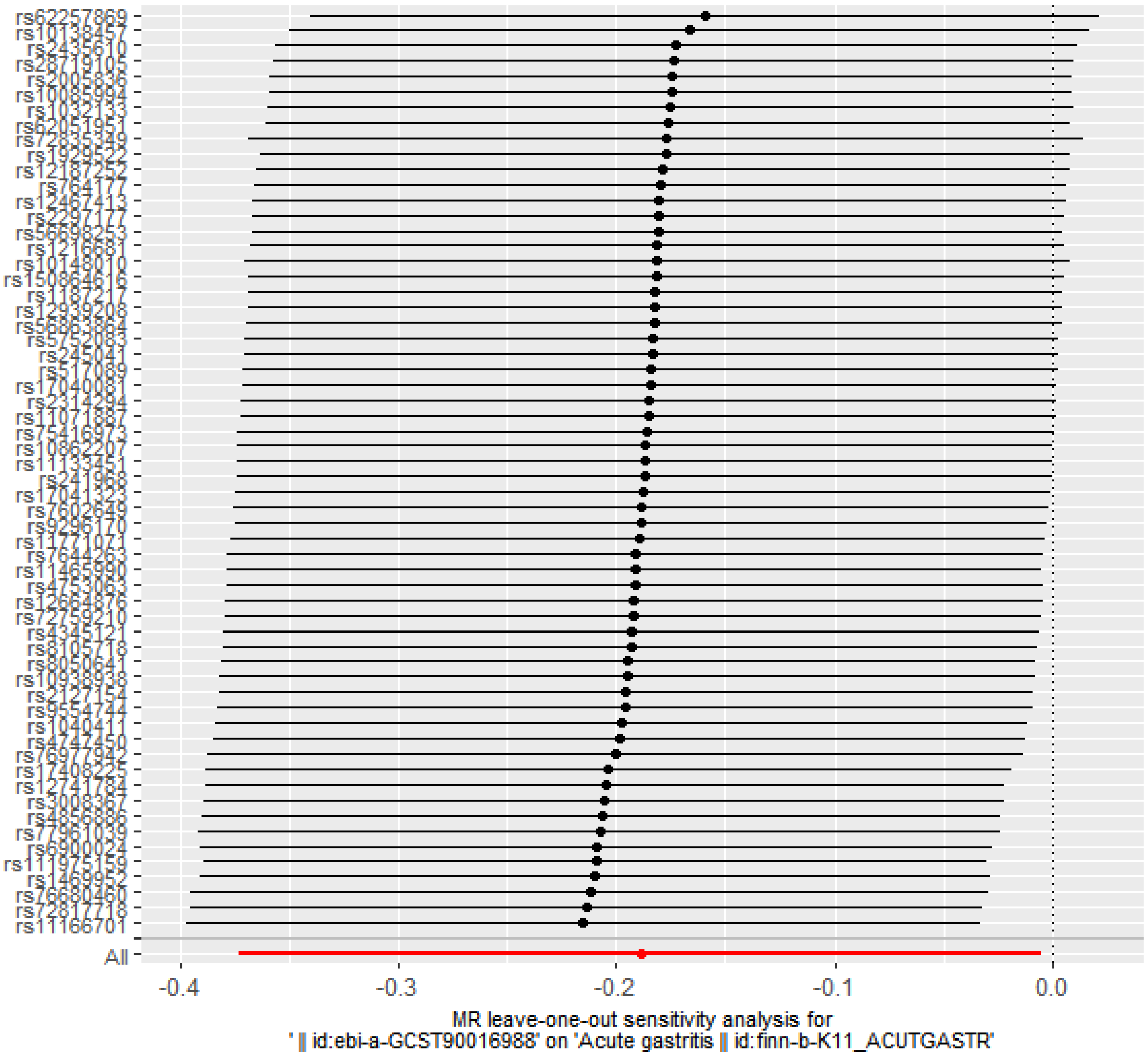

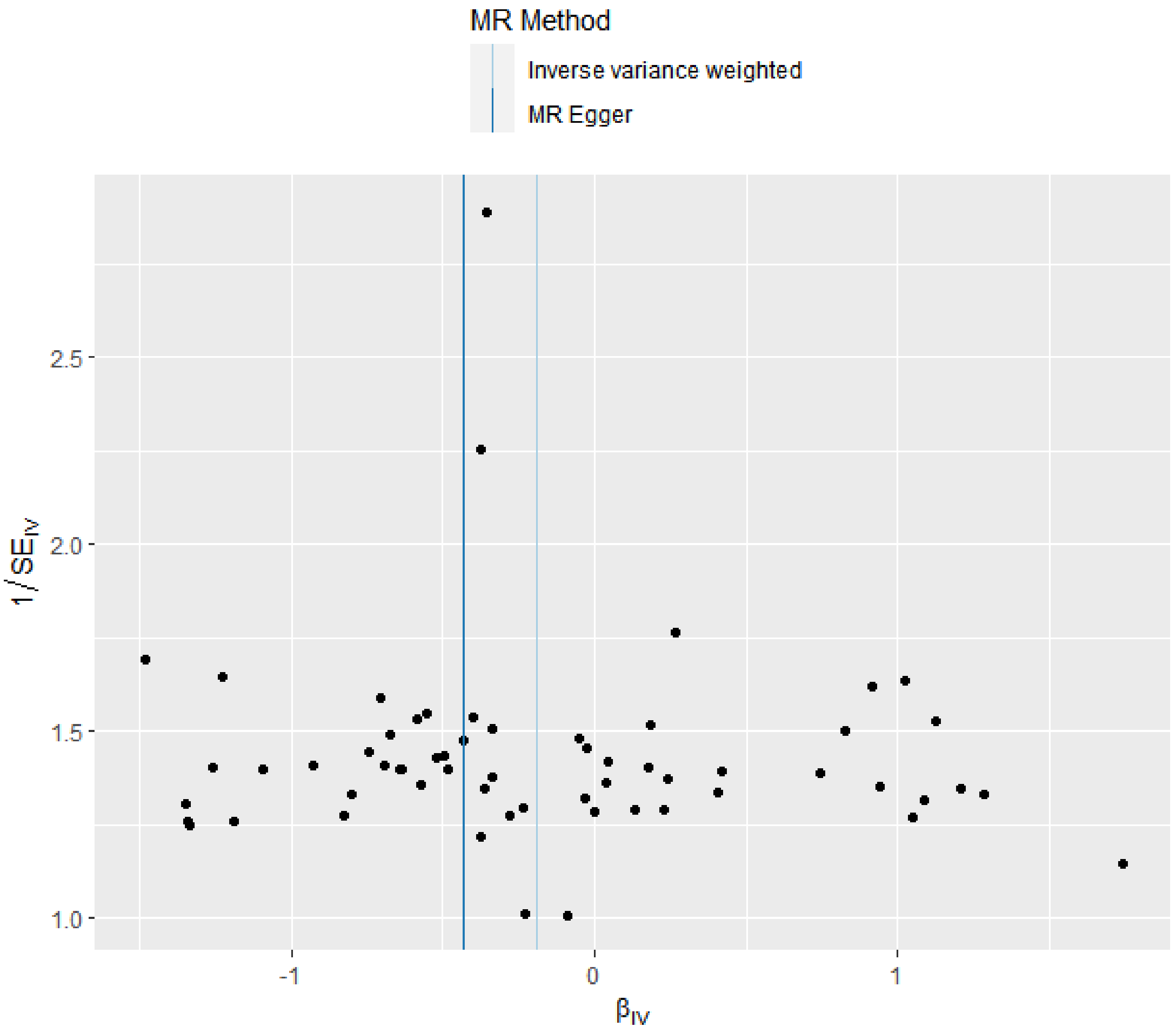

MR Method
Inverse variance weighted
MR Egger
2.5
2.0
1.5
1.0
1/SE_IV
-1
0
1
β_IV

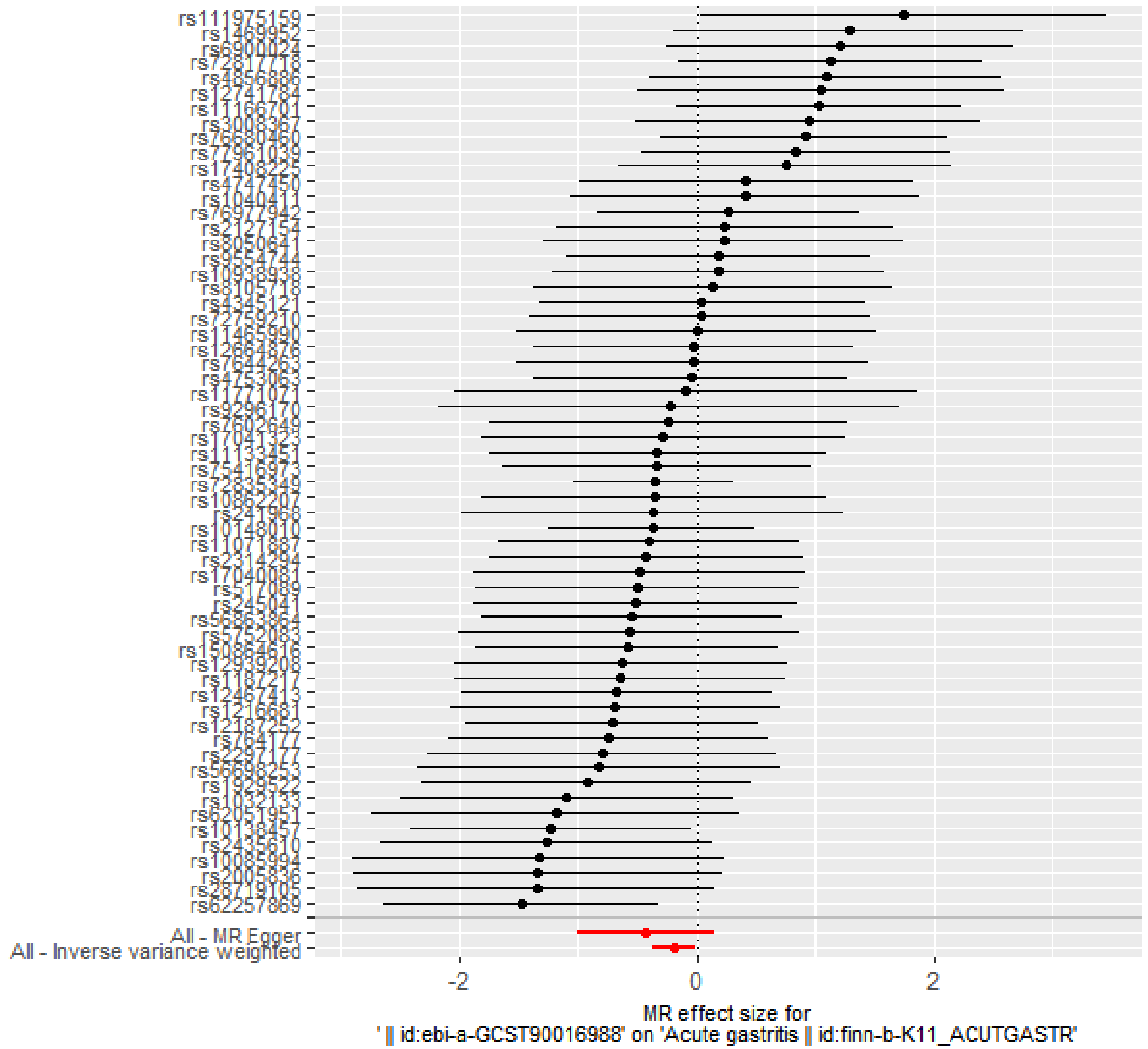

rs111975159
rs1469952
rs6900024
rs72817718
rs12741784
rs11166701
rs3008367
rs76680460
rs77961039
rs4747450
rs1040411
rs76977942
rs2127154
rs8050641
rs8105718
rs4345121
rs72759210
rs11465990
rs12664876
rs4753063
rs11771071
rs9296170
rs7602649
rs17041323
rs11133451
rs75416973
rs72835349
rs10862207
rs10148010
rs11071887
rs2314294
rs17040081
rs517089
rs245041
rs5752083
rs150864616
rs1187217
rs12467413
rs1216681
rs764177
rs2297177
rs1929522
rs1052133
rs62051951
rs10138457
rs2435610
rs10085994
rs2005836
rs28719105
rs62257869
All - MR Egger
All - Inverse variance weighted
-2
0
2
MR effect size for
' || id:ebi-a-GCST90016988' on 'Acute gastritis || id:finn-b-K11_ACUTGASTR'

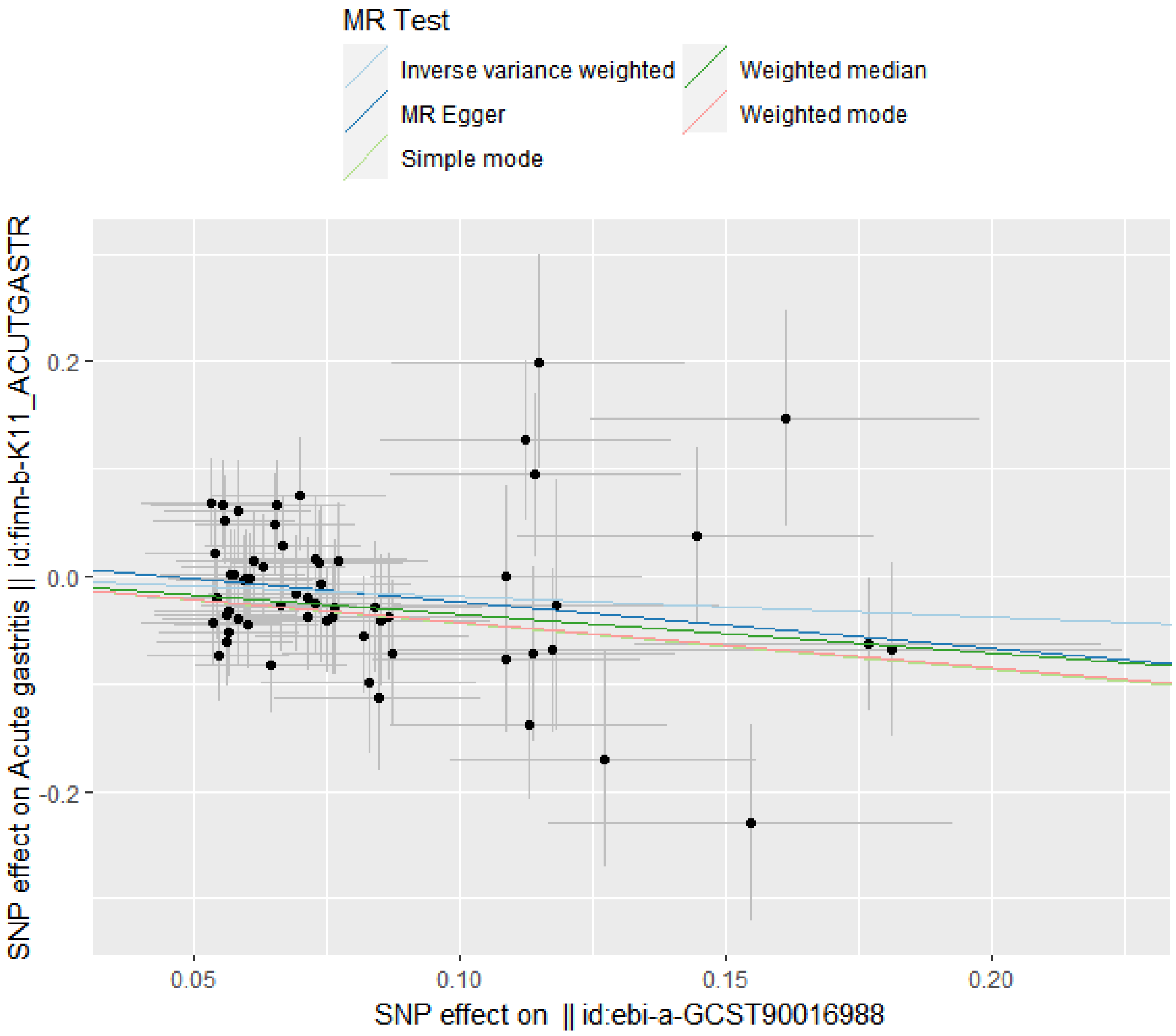

MR Test
Inverse variance weighted
MR Egger
Simple mode
Weighted median
Weighted mode
0.2
0.0
-0.2
0.05
0.10
0.15
0.20
SNP effect on Acute gastritis || id:finn-b-K11_ACUTGASTR
SNP effect on || id:ebi-a-GCST90016988

Figure 77 Leave-one-out analysis, funnel plot, MR effect size and scatter plot for gut microbiota abundance (genus Eubacterium nodatum group id.11297) on acute gastritis

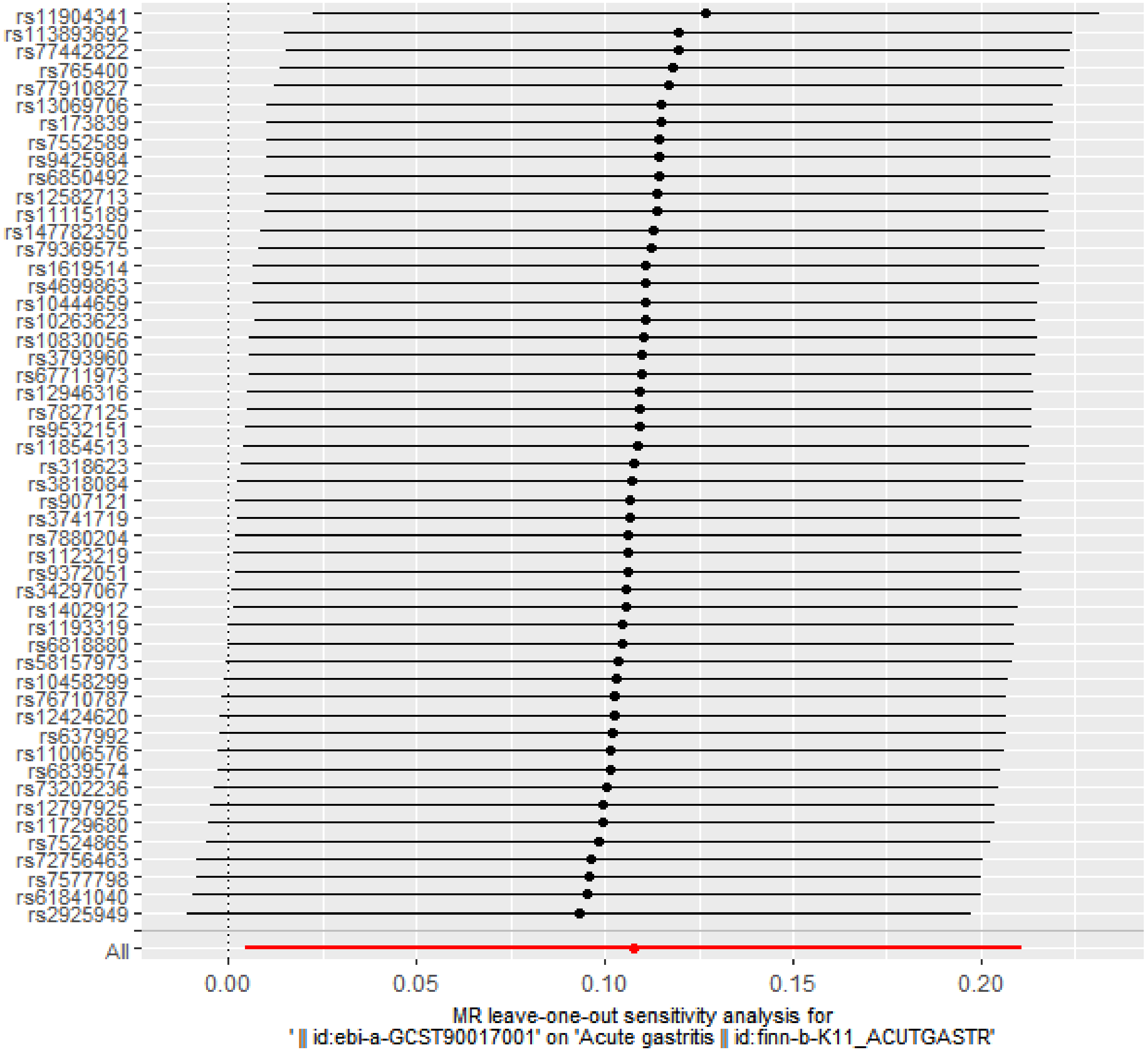

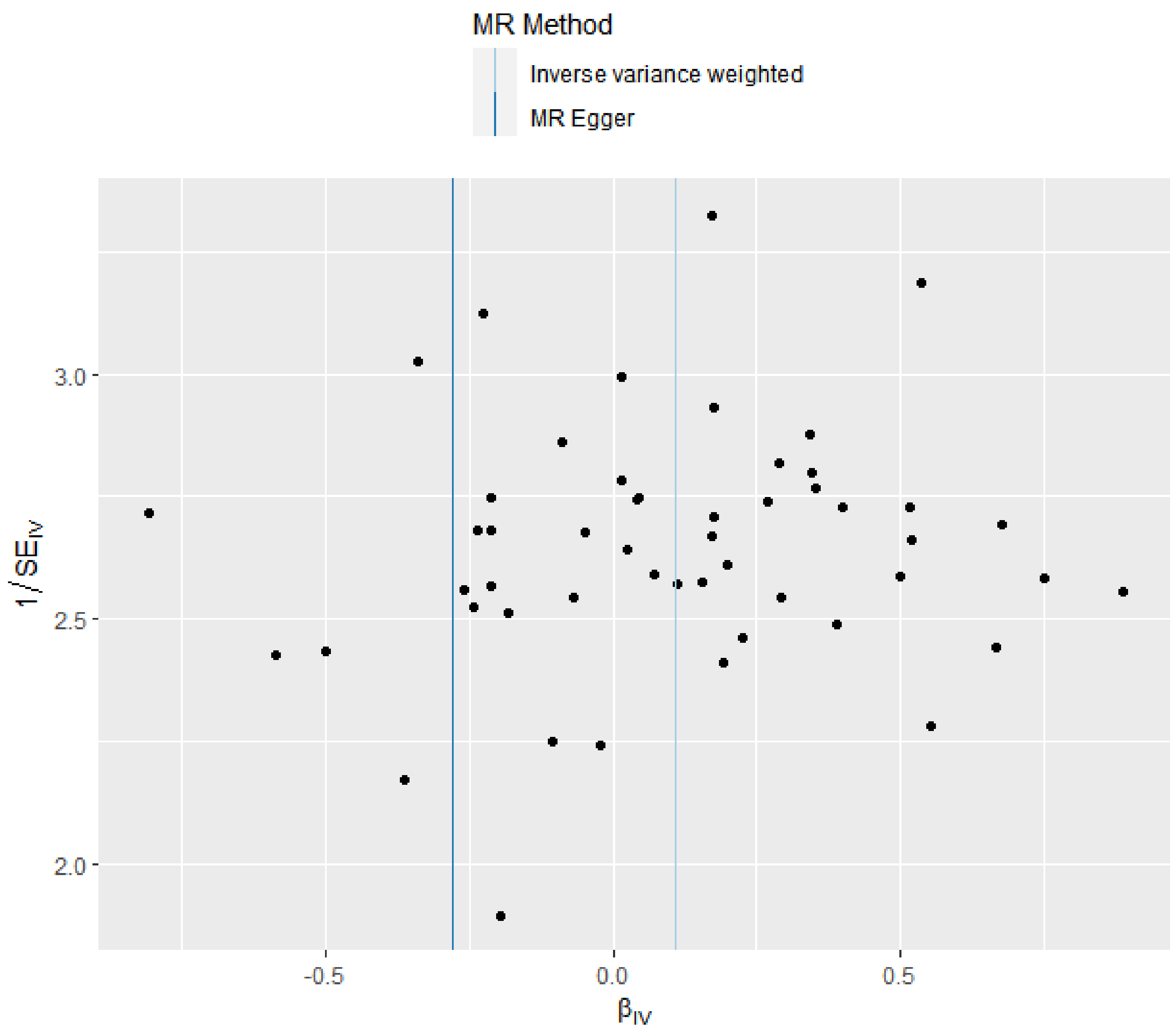
MR Method
Inverse variance weighted
MR Egger
3.0
2.5
2.0
-0.5
0.0
0.5
$1/SE_{IV}$
β_{IV}

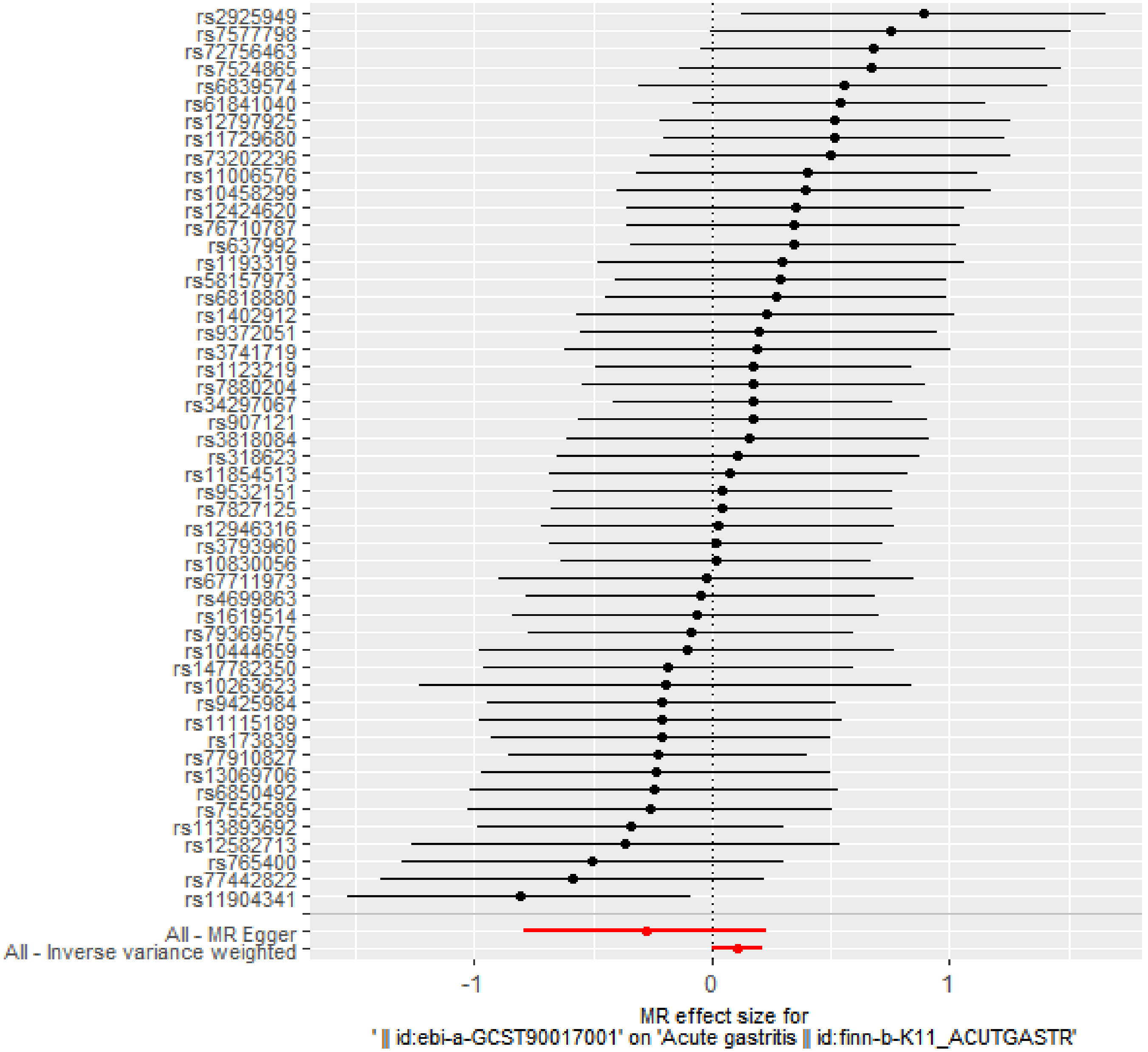
rs2925949
rs7577798
rs72756463
rs7524865
rs6839574
rs61841040
rs12797925
rs11729680
rs73202236
rs11006576
rs10458299
rs12424620
rs76710787
rs637992
rs1193319
rs58157973
rs6818880
rs1402912
rs9372051
rs3741719
rs1123219
rs7880204
rs34297067
rs907121
rs3818084
rs318623
rs11854513
rs9532151
rs7827125
rs12946316
rs3793960
rs10830056
rs67711973
rs4699863
rs1619514
rs79369575
rs10444659
rs147782350
rs10263623
rs9425984
rs11115189
rs173839
rs77910827
rs13069706
rs6850492
rs7552589
rs113893692
rs12582713
rs765400
rs77442822
rs11904341
All - MR Egger
All - Inverse variance weighted
-1
0
1
MR effect size for
' || id:ebi-a-GCST90017001' on 'Acute gastritis || id:finn-b-K11_ACUTGASTR'

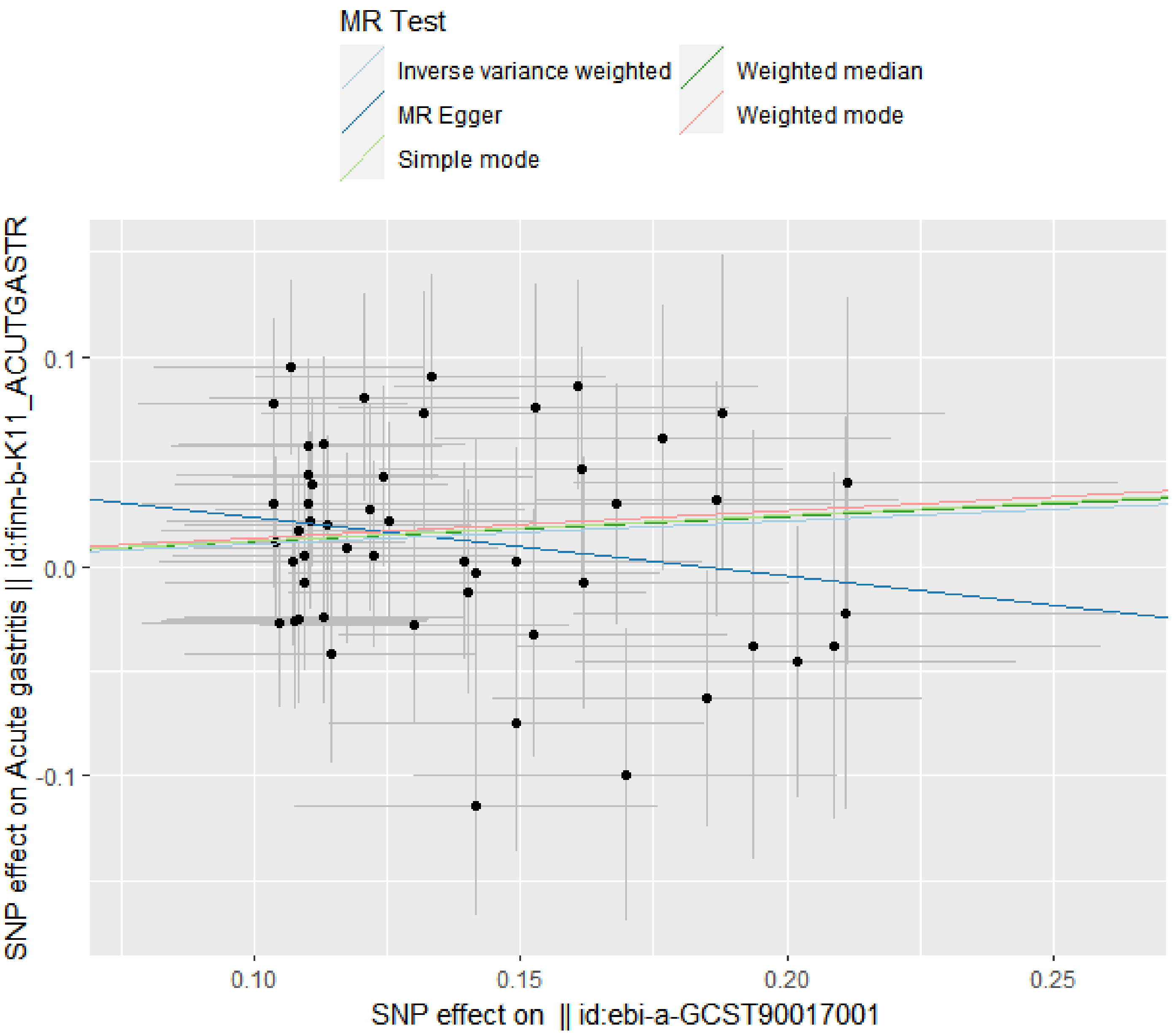
MR Test
Inverse variance weighted
MR Egger
Simple mode
Weighted median
Weighted mode
SNP effect on Acute gastritis || id:finn-b-K11_ACUTGASTR
0.1
0.0
-0.1
0.10
0.15
0.20
0.25
SNP effect on || id:ebi-a-GCST90017001

Figure 78 Leave-one-out analysis, funnel plot, MR effect size and scatter plot for gut microbiota abundance (genus Intestinibacter id.11345) on acute gastritis

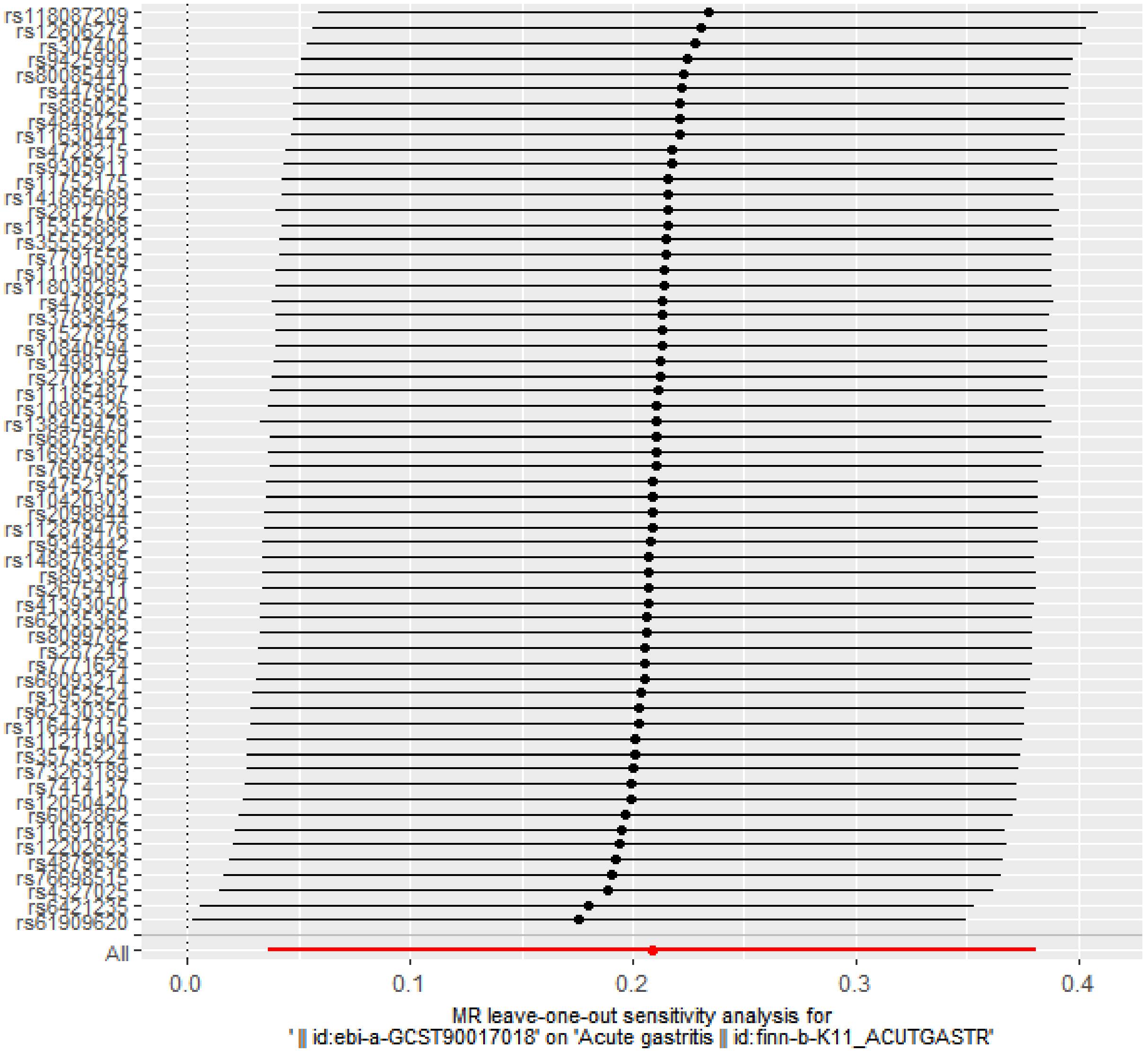

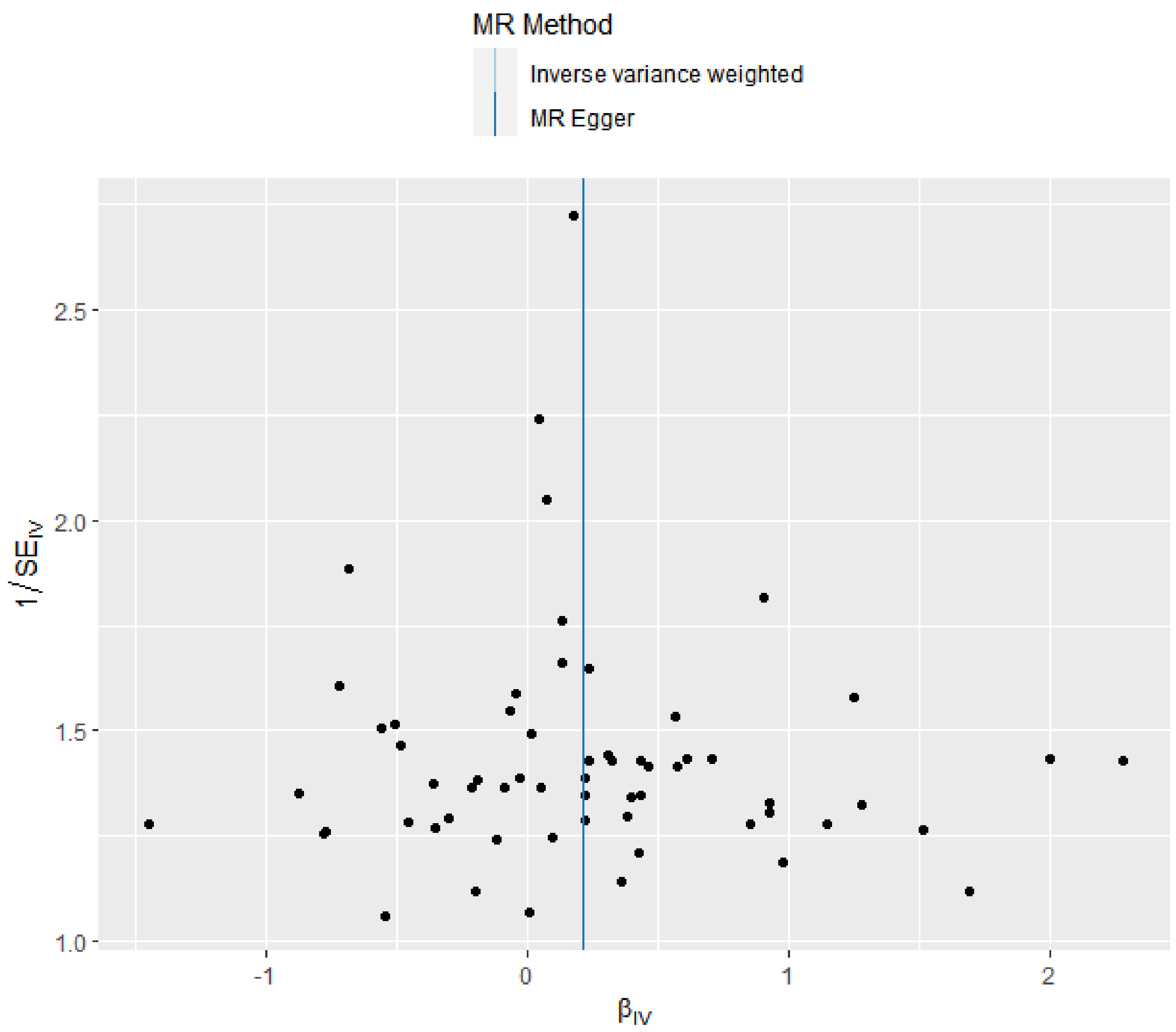

MR Method
Inverse variance weighted
MR Egger
2.5
2.0
1.5
1.0
1/SE_IV
-1
0
1
2
β_IV

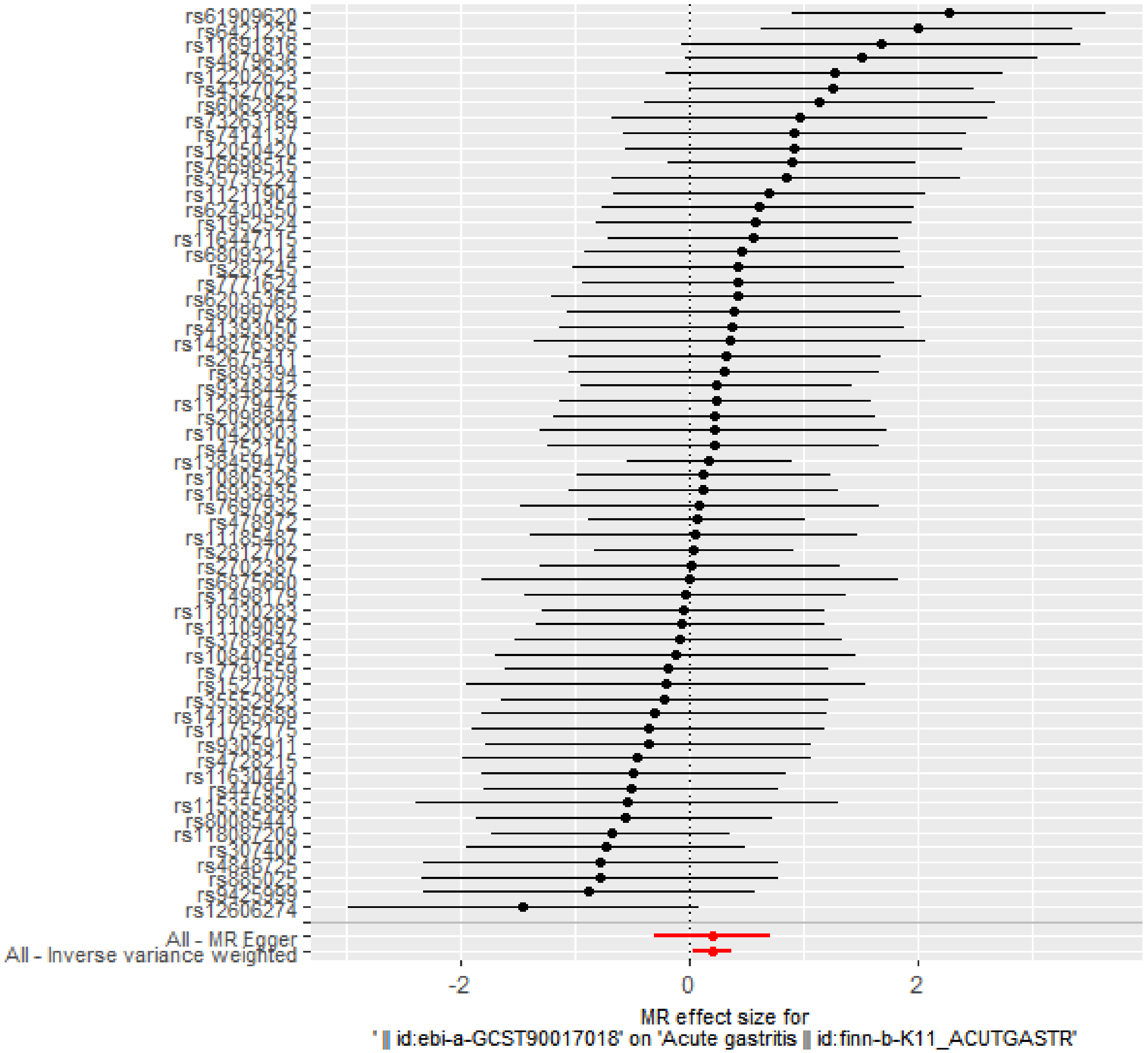
rs61909620
rs12050420
rs62430350
rs116447115
rs68093214
rs112879476
rs10420303
rs4752150
rs138459479
rs118030283
rs11109097
rs10840594
rs141865689
rs9305911
rs11630441
rs447950
rs80085441
rs118087209
rs307400
rs12606274
All - MR Egger
All - Inverse variance weighted
-2
0
2
MR effect size for
' || id:ebi-a-GCST90017018' on 'Acute gastritis || id:finn-b-K11_ACUTGASTR'

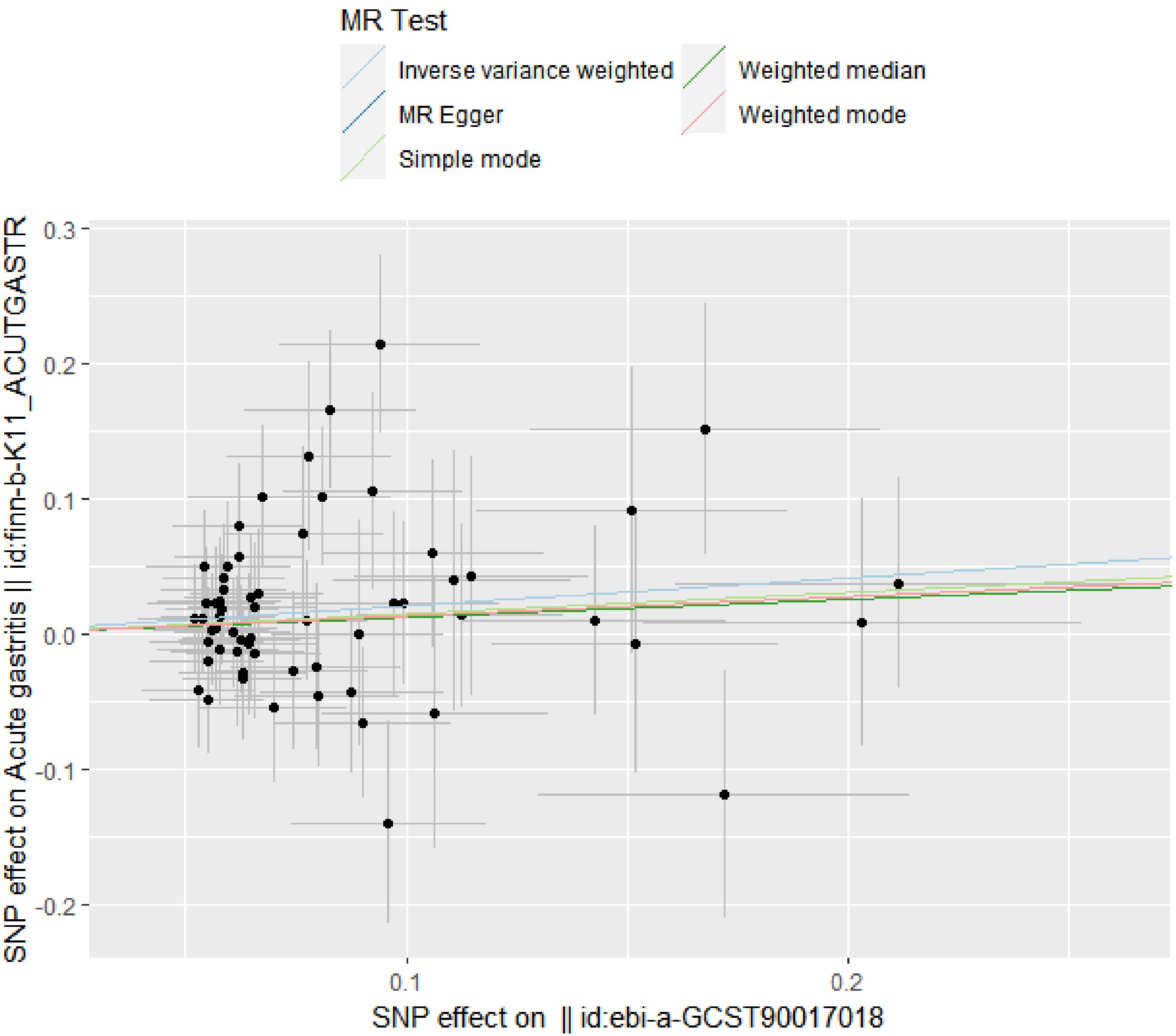

MR Test
Inverse variance weighted
MR Egger
Simple mode
Weighted median
Weighted mode
0.3
0.2
0.1
0.0
-0.1
-0.2
SNP effect on Acute gastritis || id:finn-b-K11_ACUTGASTR
0.1
0.2
SNP effect on || id:ebi-a-GCST90017018

Figure 79 Leave-one-out analysis, funnel plot, MR effect size and scatter plot for gut microbiota abundance (genus Intestinimonas id.2062) on acute gastritis

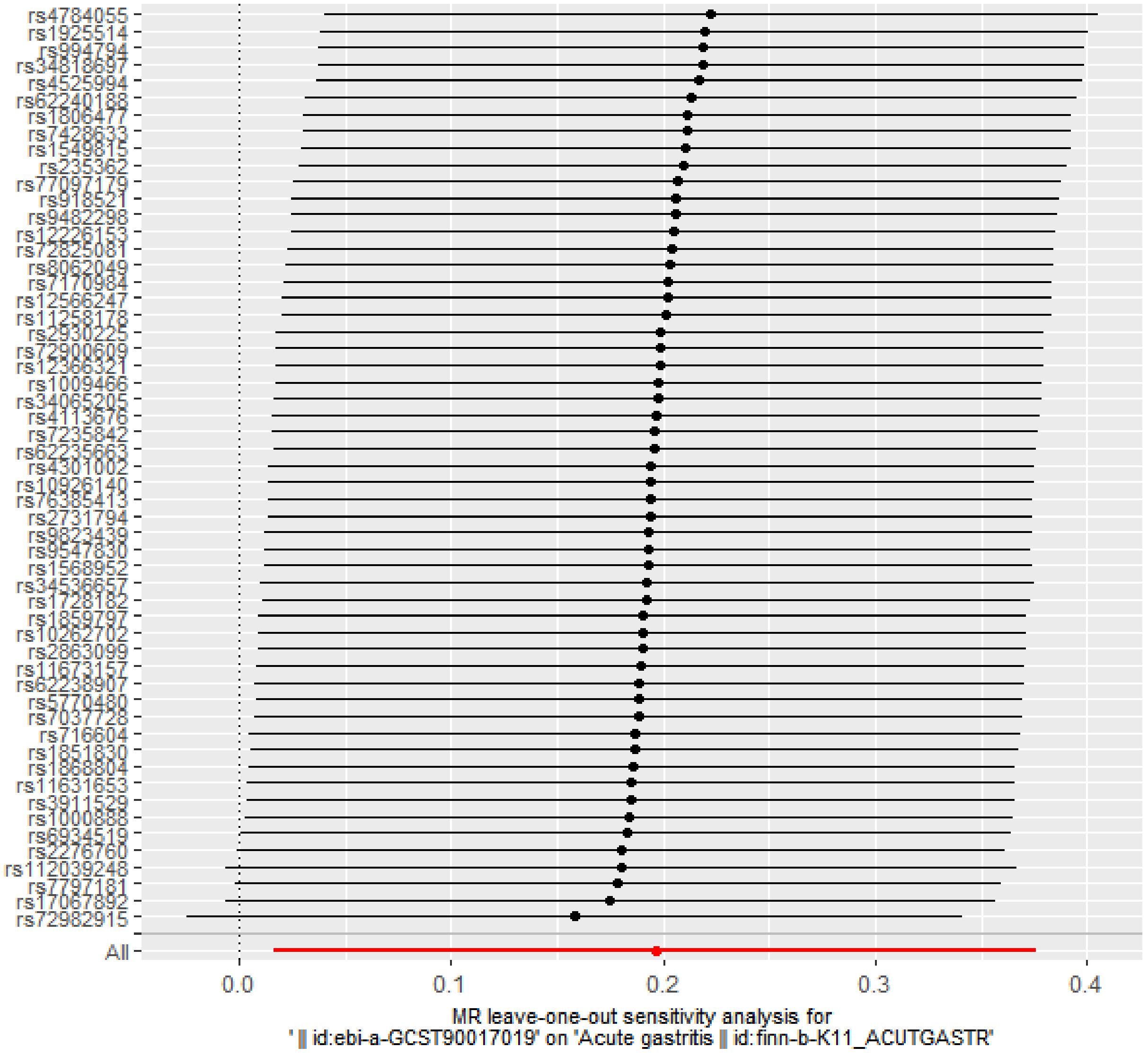

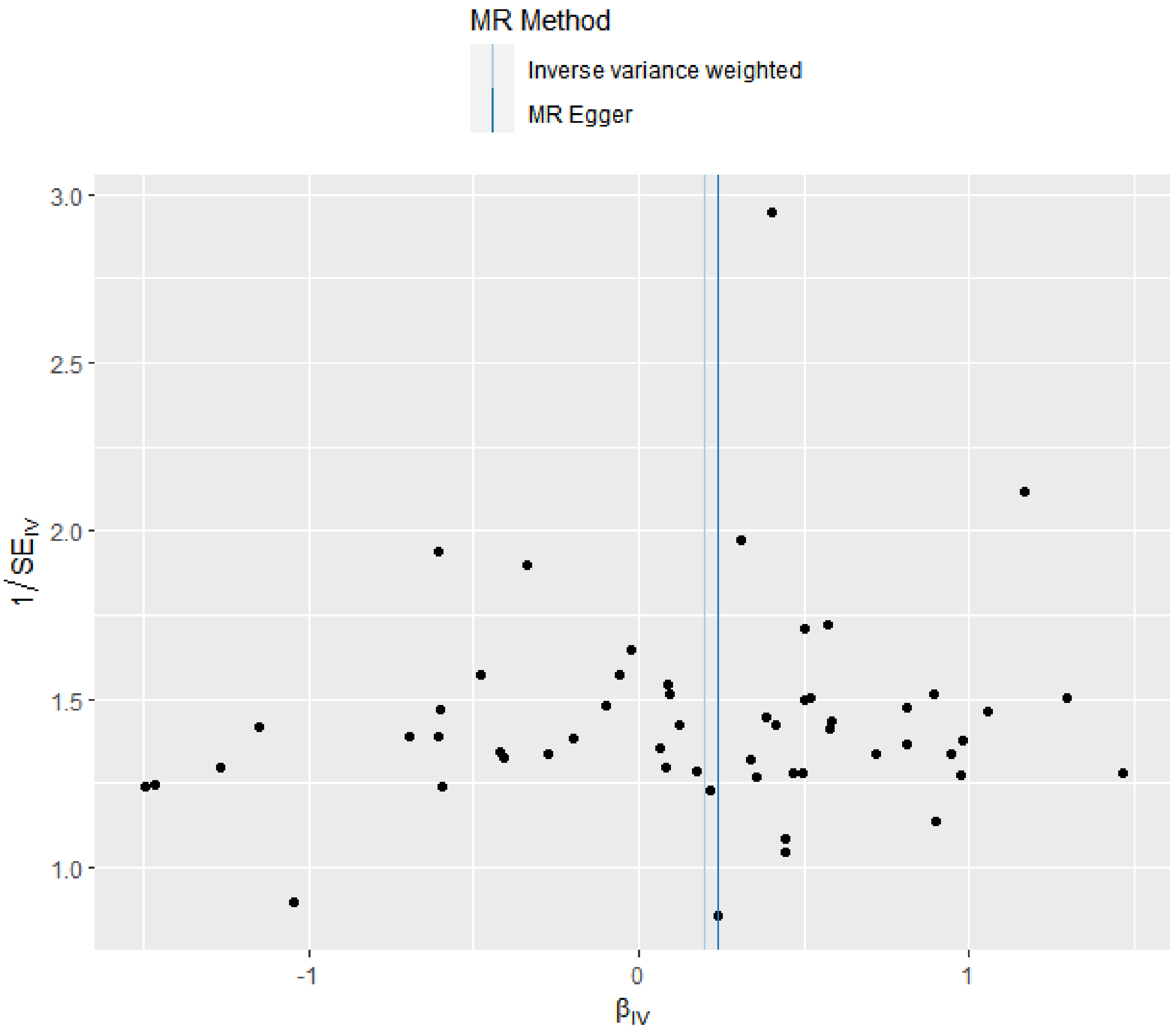

MR Method
Inverse variance weighted
MR Egger
3.0
2.5
2.0
1.5
1.0
$1/SE_{IV}$
-1
0
1
β_{IV}

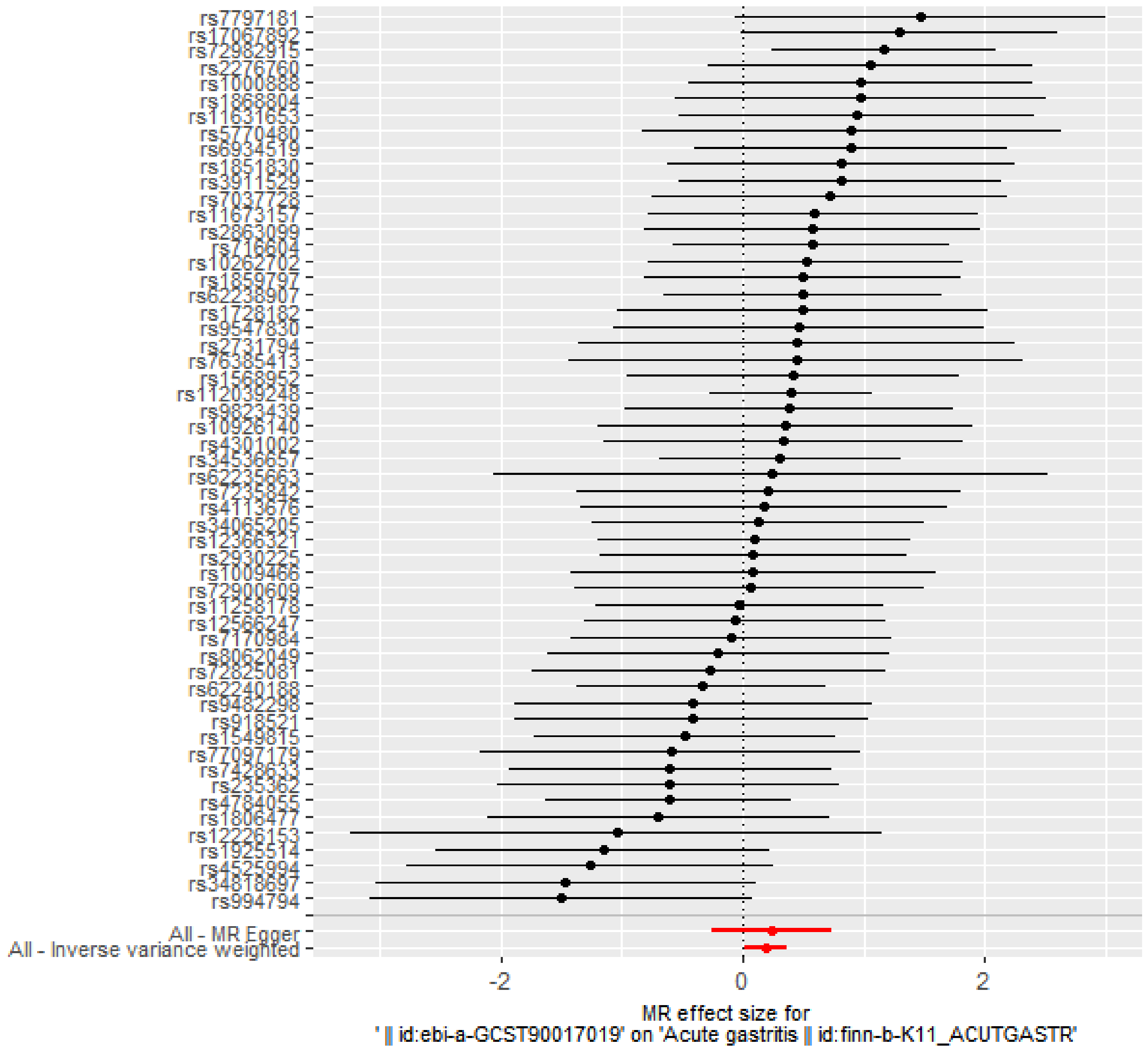
rs7797181
rs17067892
rs72982915
rs2276760
rs1000888
rs1868804
rs11631653
rs5770480
rs6934519
rs1851830
rs3911529
rs7037728
rs11673157
rs2863099
rs716604
rs10262702
rs1859797
rs62238907
rs1728182
rs9547830
rs2731794
rs76385413
rs1568952
rs112039248
rs9823439
rs10926140
rs4301002
rs34536657
rs62235663
rs4113676
rs34065205
rs12366321
rs2930225
rs1009466
rs72900609
rs11258178
rs12566247
rs7170984
rs8062049
rs72825081
rs62240188
rs9482298
rs918521
rs1549815
rs77097179
rs235362
rs4784055
rs1806477
rs12226153
rs1925514
rs4525994
rs34818697
rs994794
All - MR Egger
All - Inverse variance weighted
-2
0
2
MR effect size for
' || id:ebi-a-GCST90017019' on 'Acute gastritis || id:finn-b-K11_ACUTGASTR'

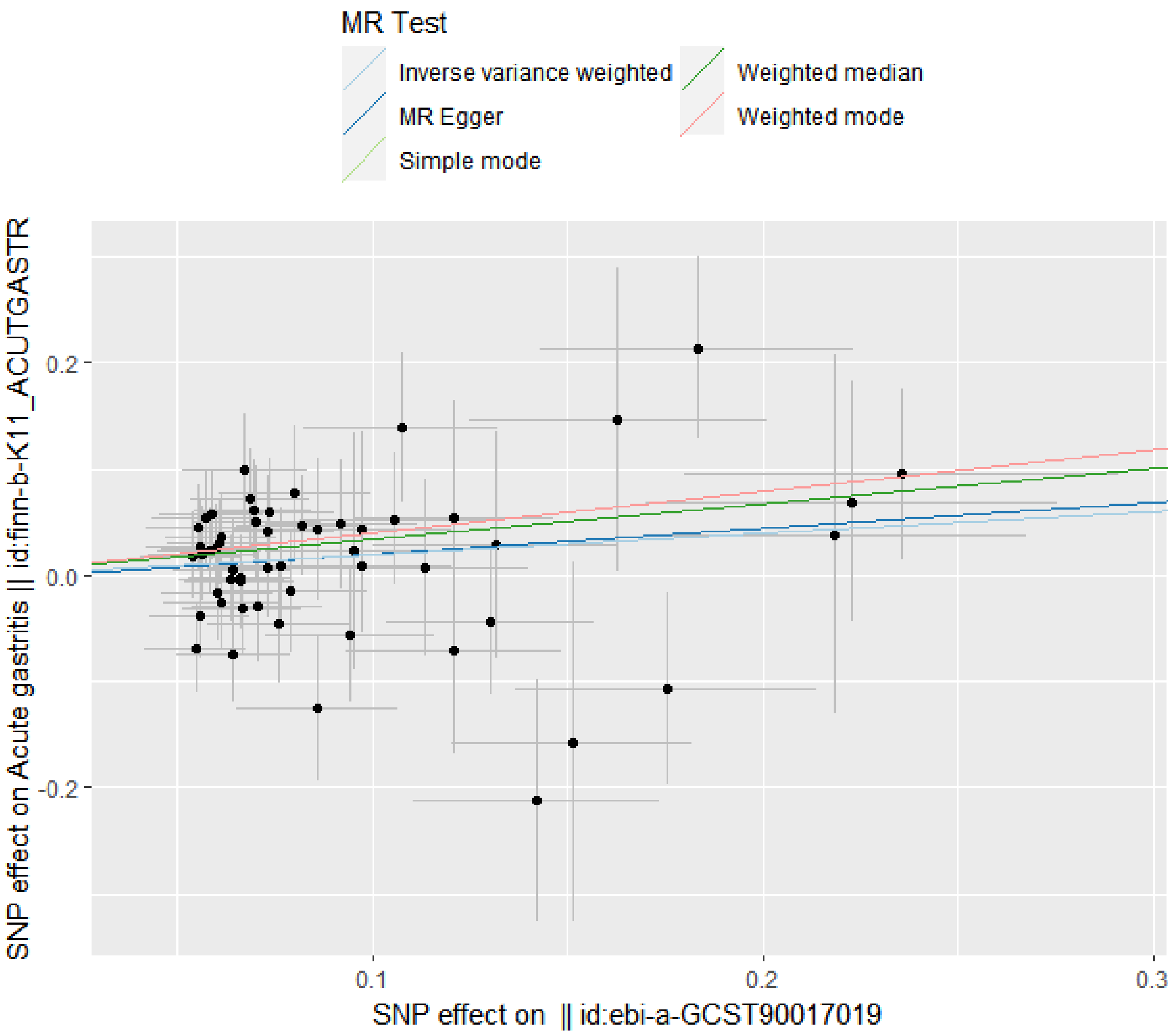
MR Test
Inverse variance weighted
MR Egger
Simple mode
Weighted median
Weighted mode
SNP effect on Acute gastritis || id:finn-b-K11_ACUTGASTR
0.2
0.0
-0.2
0.1
0.2
0.3
SNP effect on || id:ebi-a-GCST90017019

Figure 80 Leave-one-out analysis, funnel plot, MR effect size and scatter plot for gut microbiota abundance (genus Parabacteroides id.954) on acute gastritis

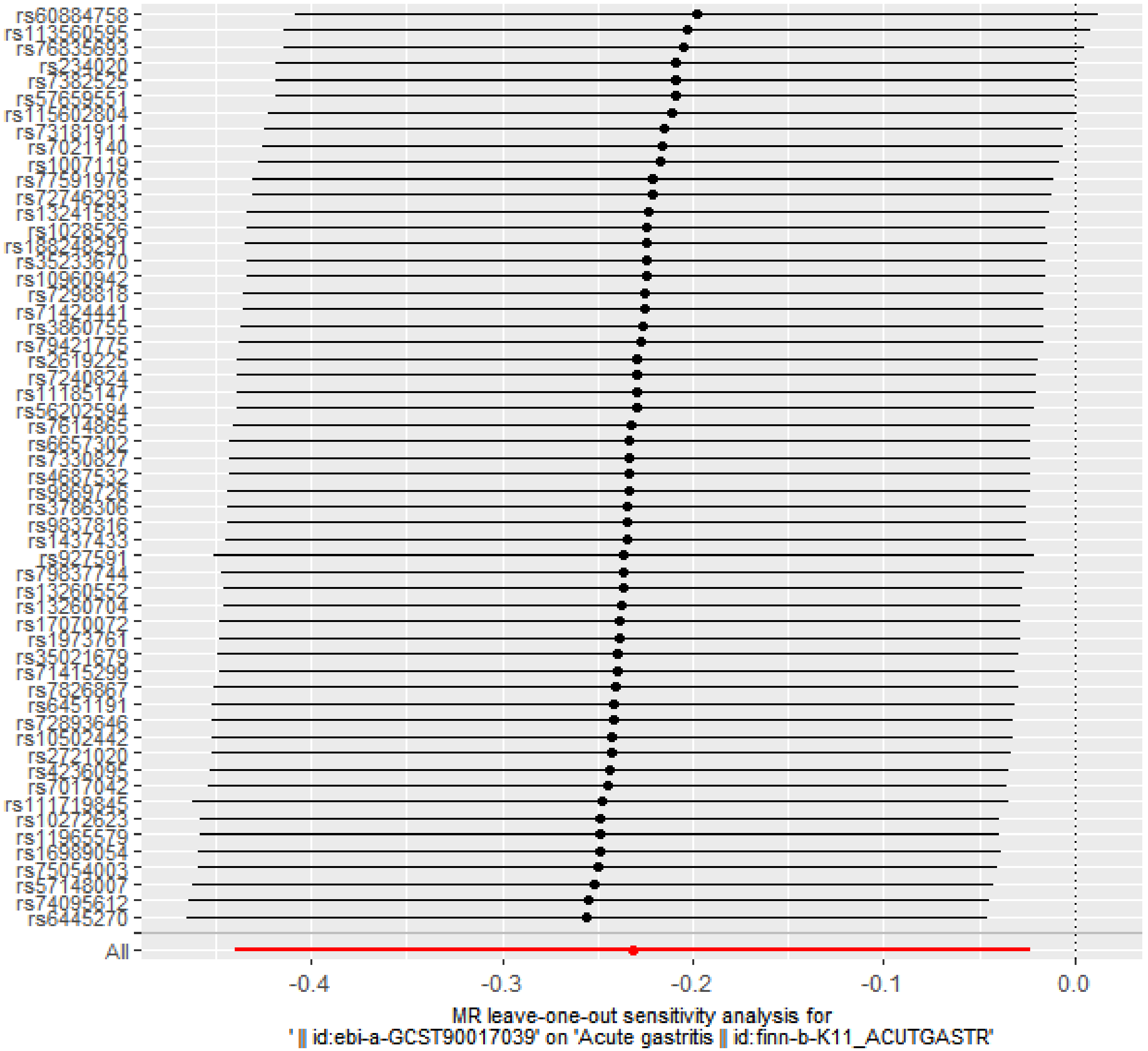

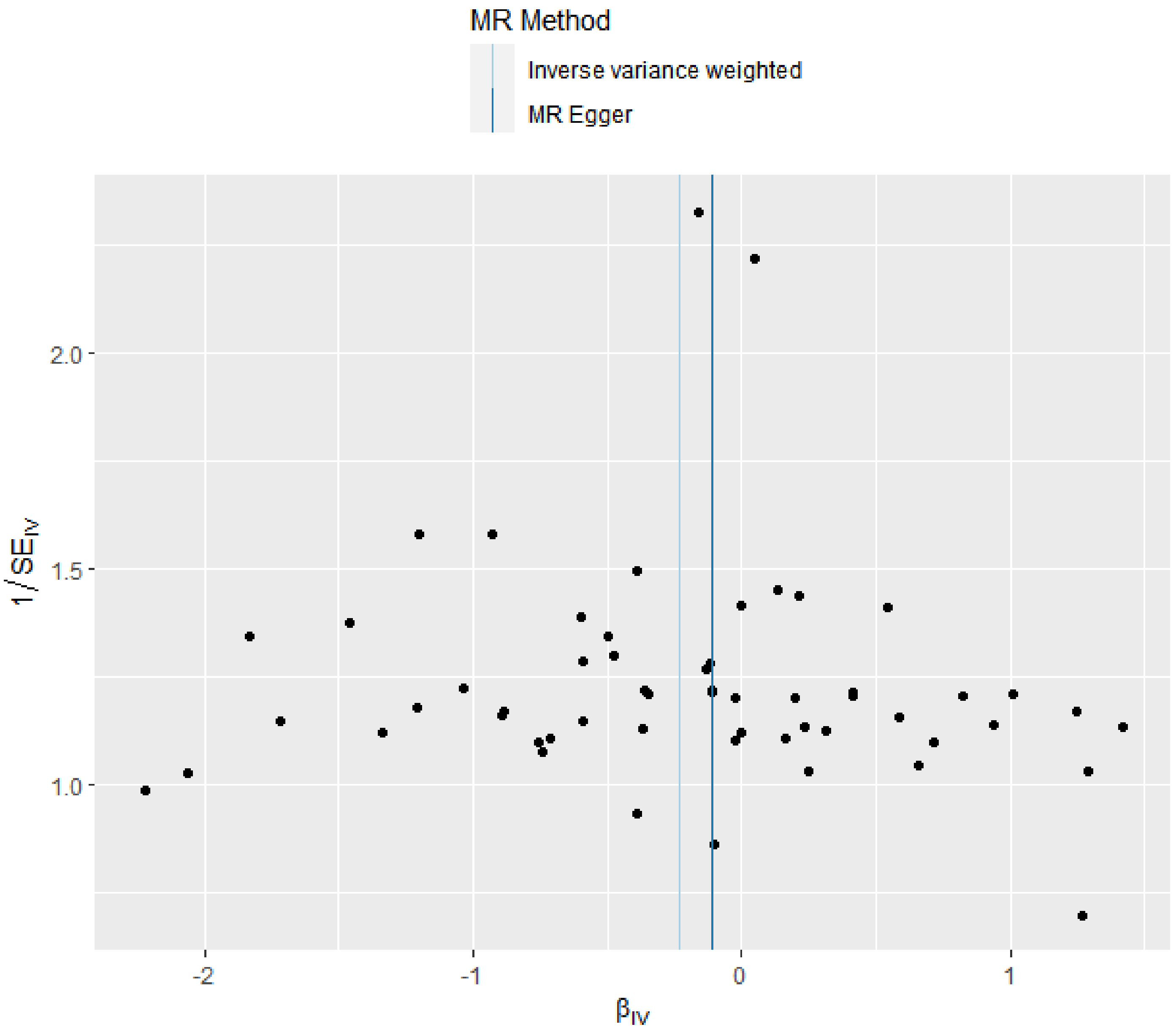
MR Method
Inverse variance weighted
MR Egger
2.0
1.5
1.0
1/SE_IV
-2
-1
0
1
β_IV

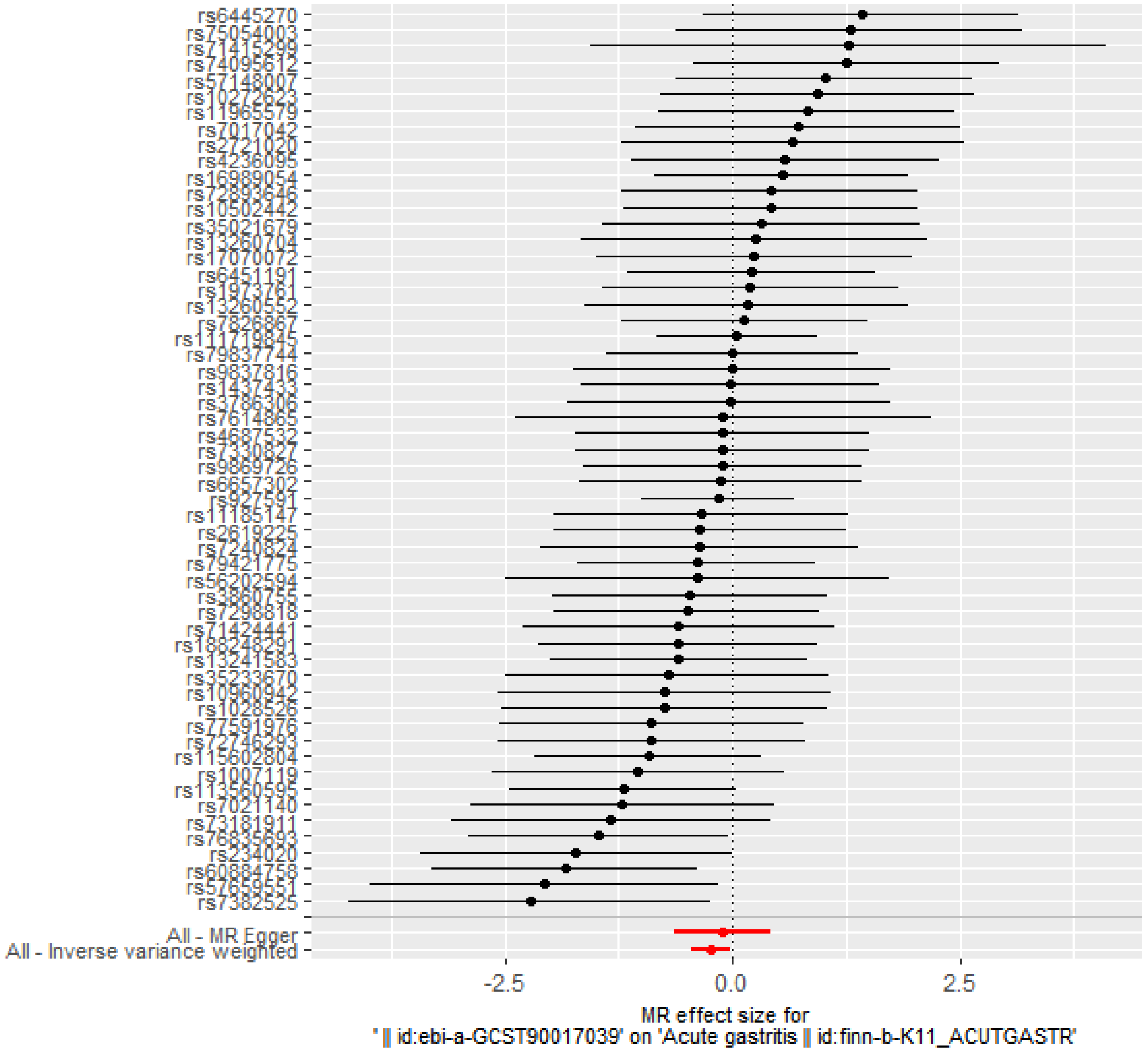
rs6445270
rs75054003
rs11965579
rs7017042
rs4236095
rs16989054
rs72893646
rs10502442
rs35021679
rs13260704
rs17070072
rs6451191
rs1973761
rs13260552
rs7826867
rs79837744
rs9837816
rs3786306
rs7614865
rs4687532
rs7330827
rs9869726
rs6657302
rs927591
rs11185147
rs2619225
rs79421775
rs56202594
rs7298818
rs71424441
rs188248291
rs13241583
rs35233670
rs10960942
rs1028526
rs77591976
rs72746293
rs115602804
rs1007119
rs7021140
rs73181911
rs234020
rs60884758
rs7382525
All - MR Egger
All - Inverse variance weighted
-2.5
0.0
2.5
MR effect size for
' || id:ebi-a-GCST90017039' on 'Acute gastritis || id:finn-b-K11_ACUTGASTR'

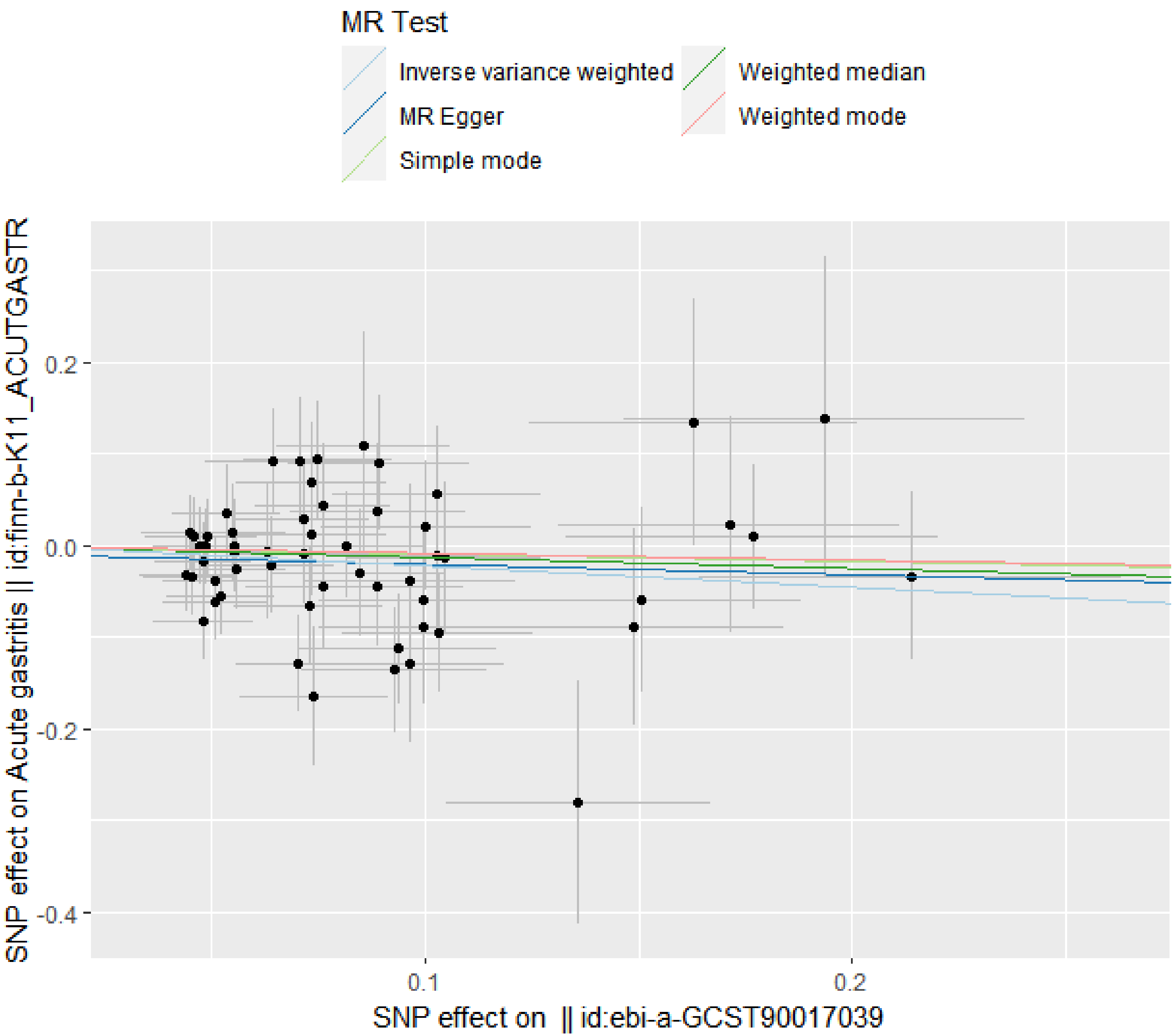

MR Test
Inverse variance weighted
MR Egger
Simple mode
Weighted median
Weighted mode
SNP effect on Acute gastritis || id:finn-b-K11_ACUTGASTR
0.2
0.0
-0.2
-0.4
0.1
0.2
SNP effect on || id:ebi-a-GCST90017039

Figure 81 Leave-one-out analysis, funnel plot, MR effect size and scatter plot for gut microbiota abundance (genus Ruminococcaceae NK4A214 group id.11358) on acute gastritis

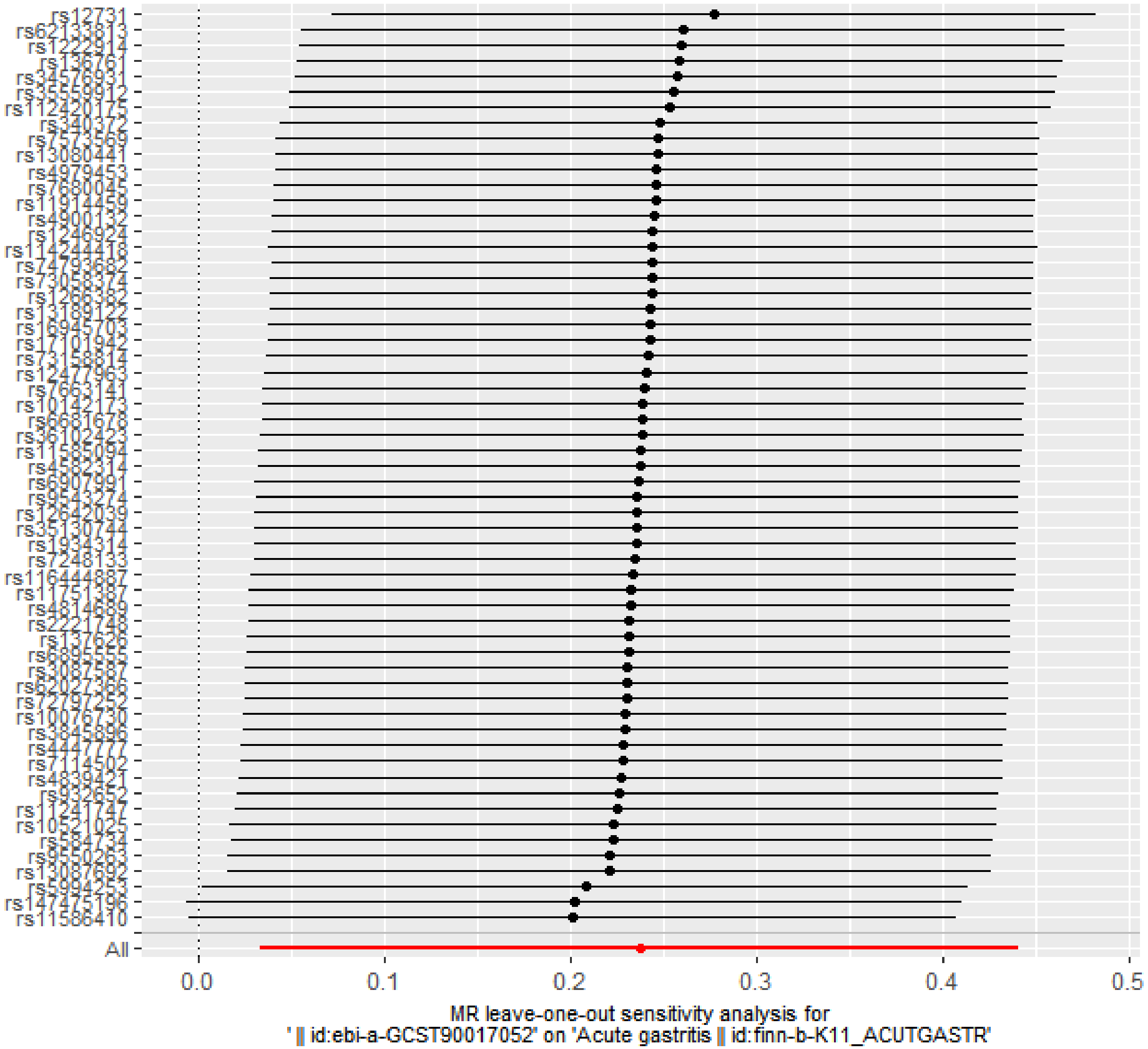

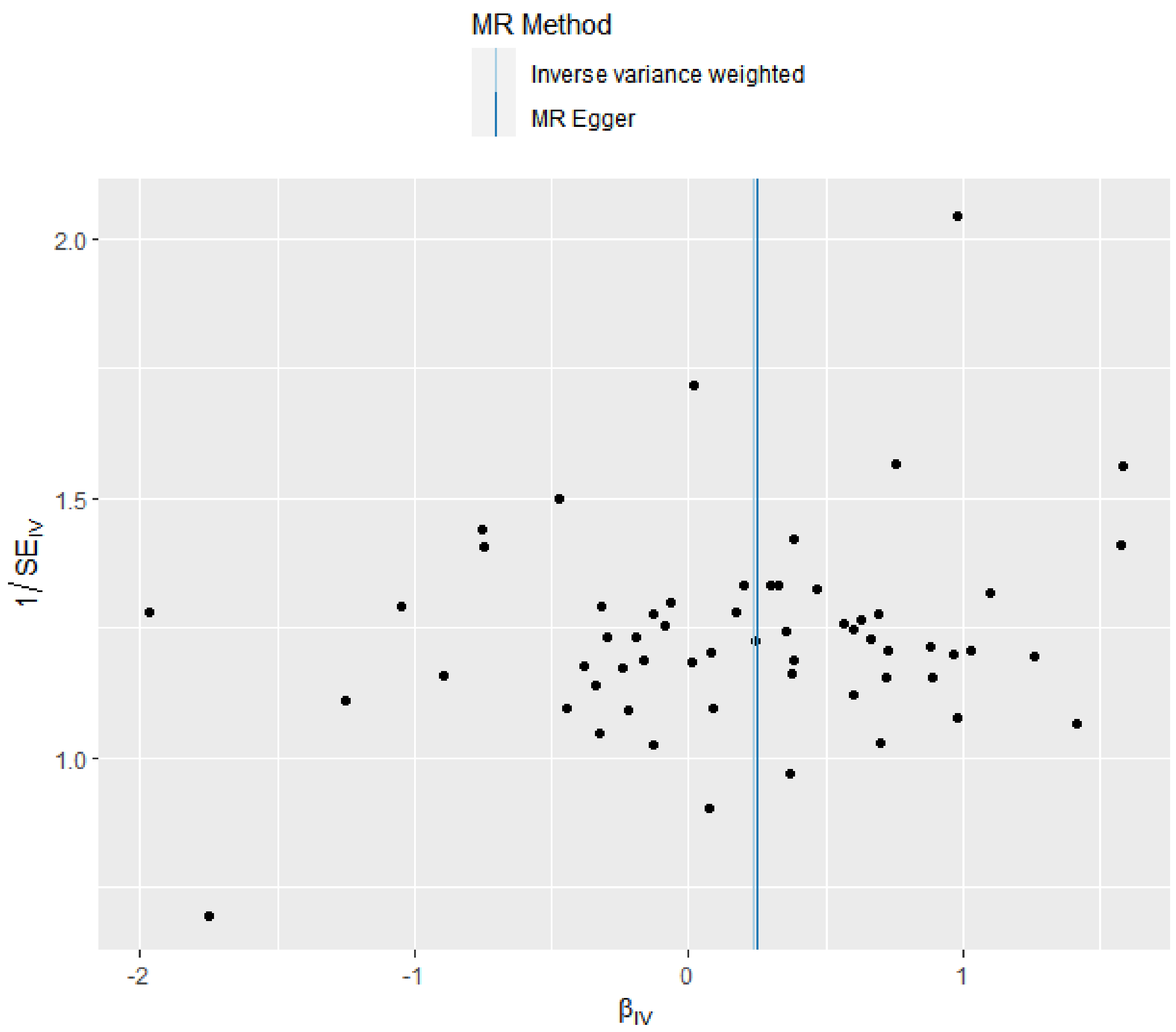

MR Method
Inverse variance weighted
MR Egger
2.0
1.5
1.0
1/SE_IV
-2
-1
0
1
β_IV

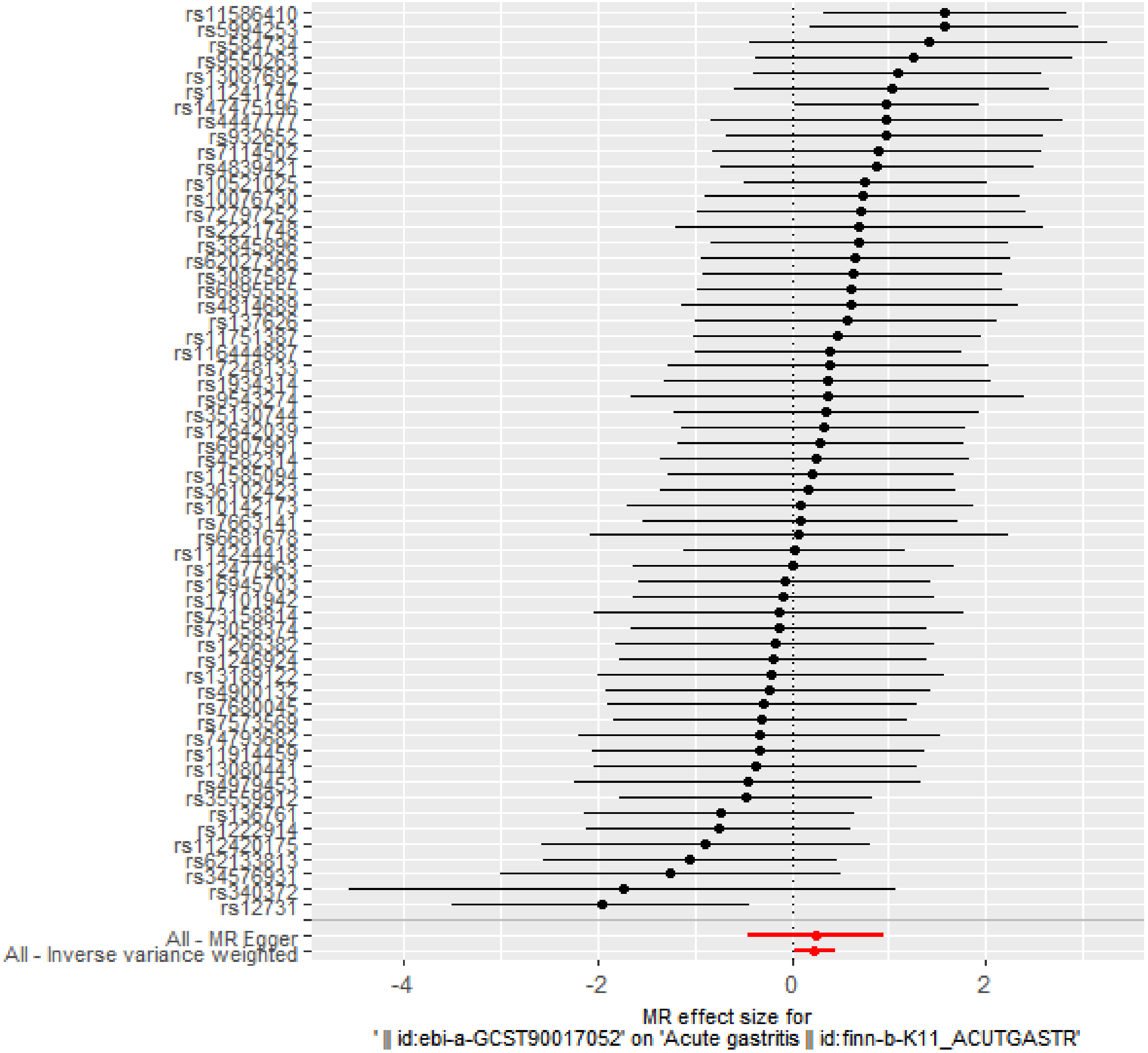
All - MR Egger
All - Inverse variance weighted
-4
-2
0
2
MR effect size for
' || id:ebi-a-GCST90017052' on 'Acute gastritis || id:finn-b-K11_ACUTGASTR'

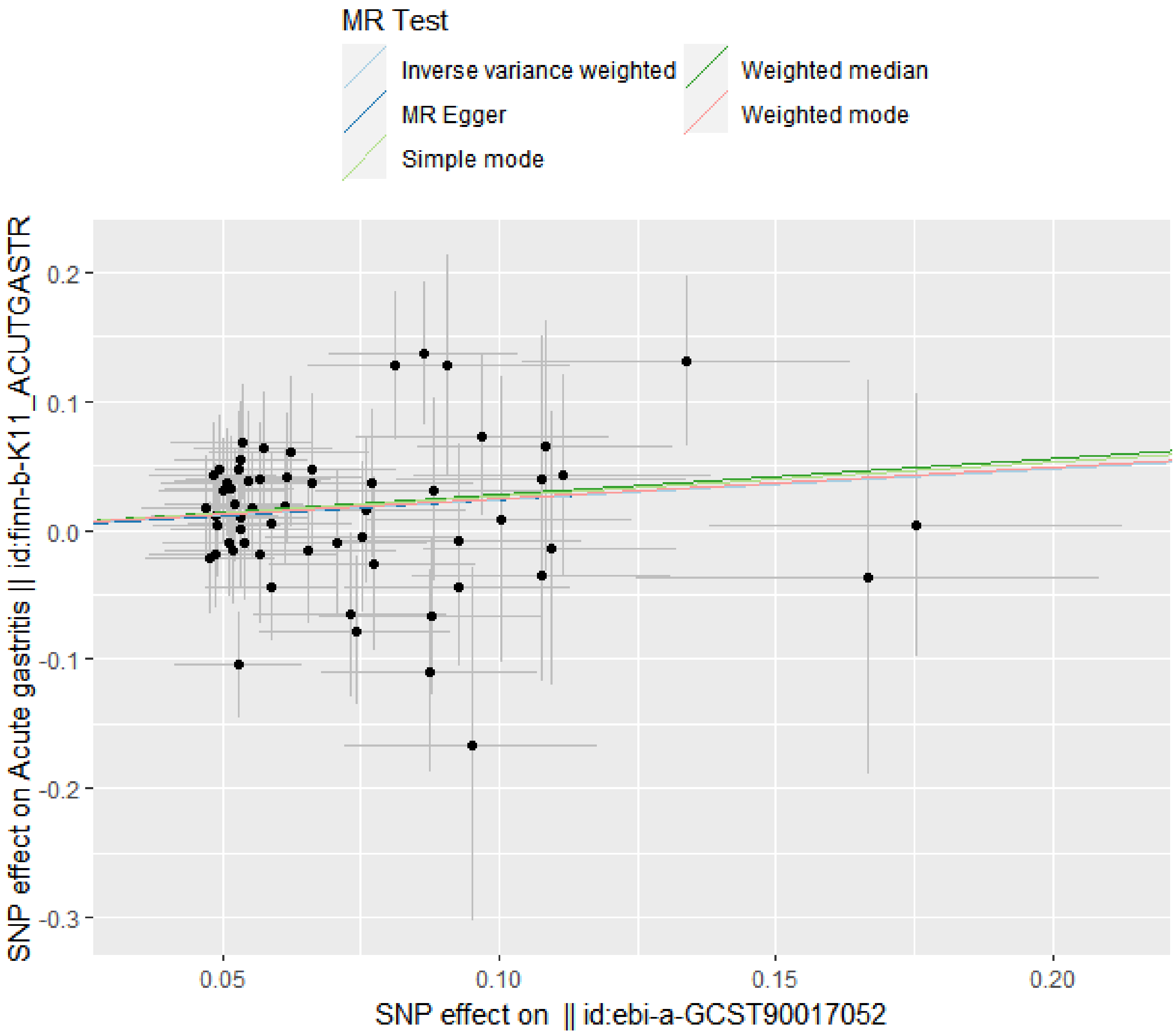

MR Test
Inverse variance weighted
MR Egger
Simple mode
Weighted median
Weighted mode
SNP effect on Acute gastritis || id:finn-b-K11_ACUTGASTR
0.2
0.1
0.0
-0.1
-0.2
-0.3
0.05
0.10
0.15
0.20
SNP effect on || id:ebi-a-GCST90017052

Figure 82 Leave-one-out analysis, funnel plot, MR effect size and scatter plot for gut microbiota abundance (phylum Verrucomicrobia id.3982) on acute gastritis

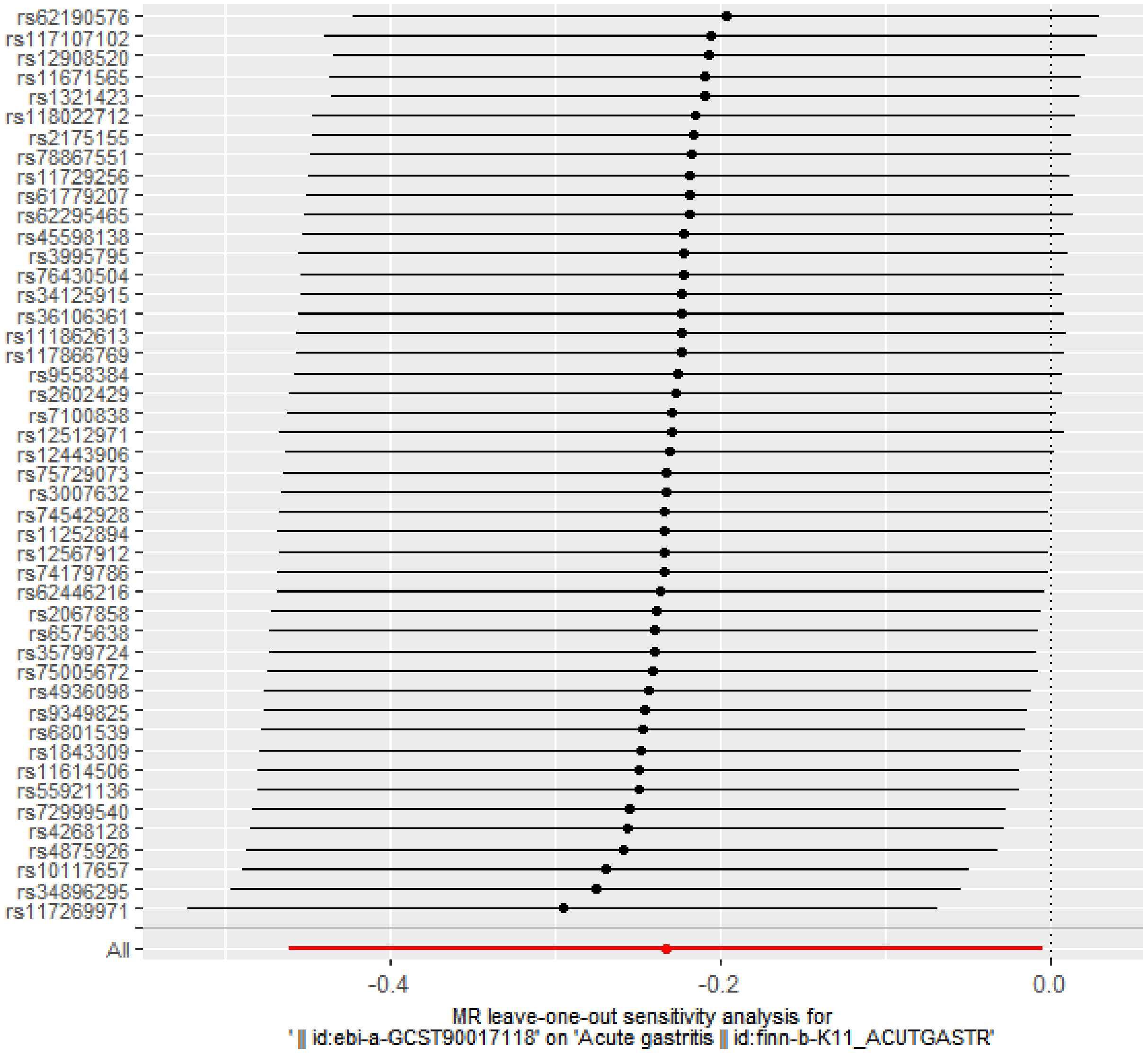

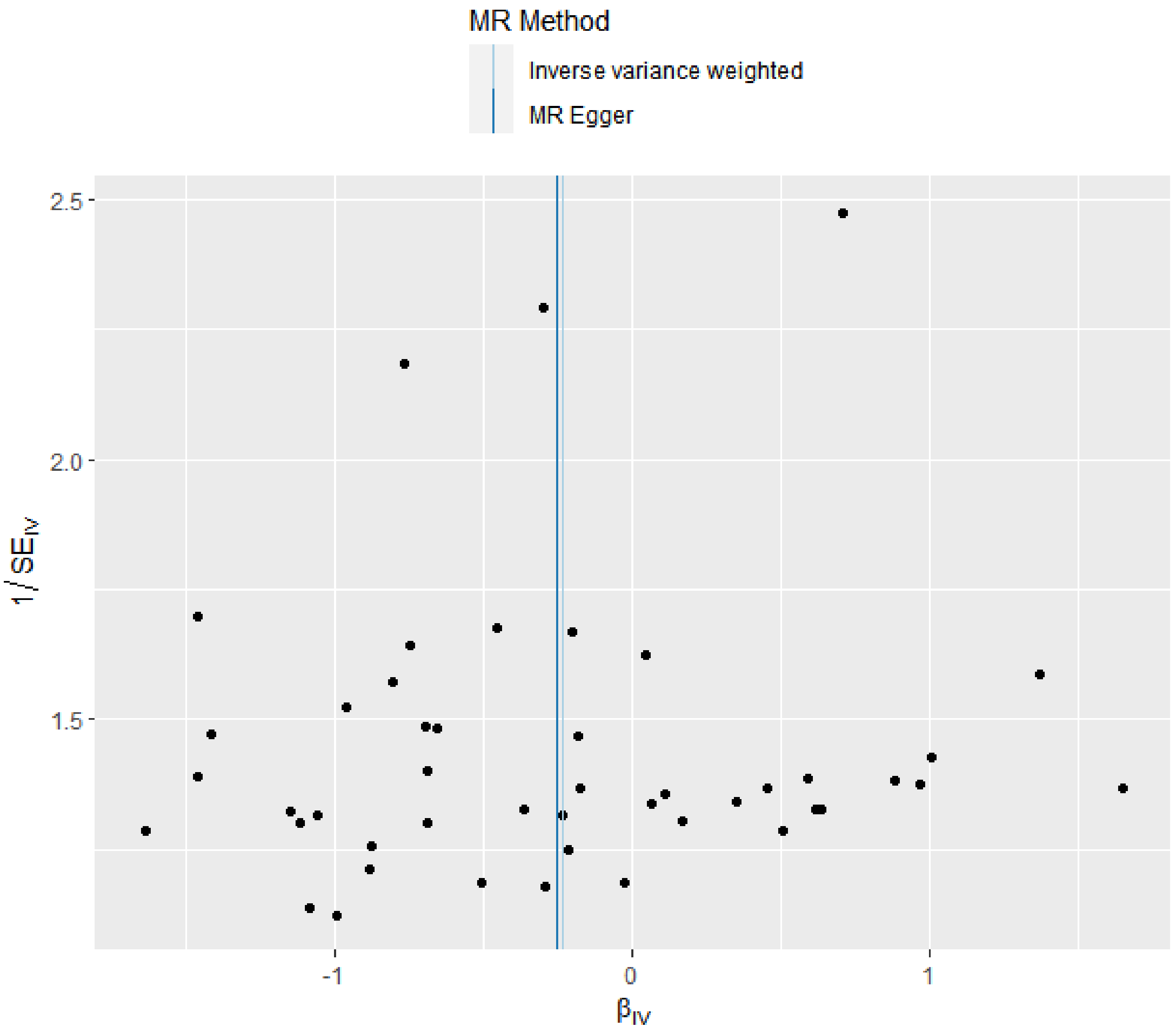

MR Method
Inverse variance weighted
MR Egger
2.5
2.0
1.5
1/SE_IV
-1
0
1
β_IV

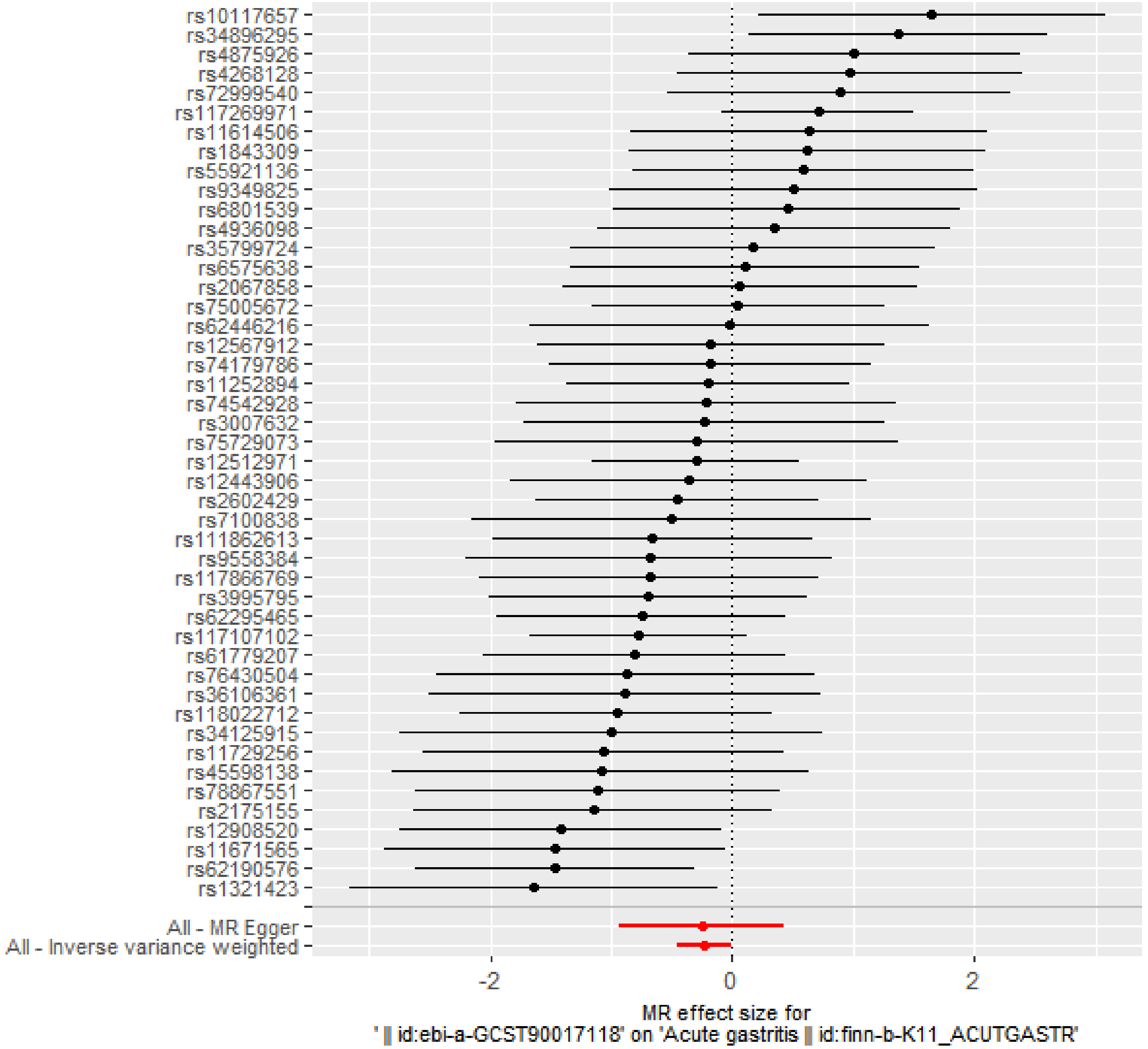
rs10117657
rs34896295
rs4875926
rs4268128
rs72999540
rs117269971
rs11614506
rs1843309
rs55921136
rs9349825
rs6801539
rs4936098
rs35799724
rs6575638
rs2067858
rs75005672
rs62446216
rs12567912
rs74179786
rs11252894
rs74542928
rs3007632
rs75729073
rs12512971
rs12443906
rs2602429
rs7100838
rs111862613
rs9558384
rs117866769
rs3995795
rs62295465
rs117107102
rs61779207
rs76430504
rs36106361
rs118022712
rs34125915
rs11729256
rs45598138
rs78867551
rs2175155
rs12908520
rs11671565
rs62190576
rs1321423
All - MR Egger
All - Inverse variance weighted
-2
0
2
MR effect size for
' || id:ebi-a-GCST90017118' on 'Acute gastritis || id:finn-b-K11_ACUTGASTR'

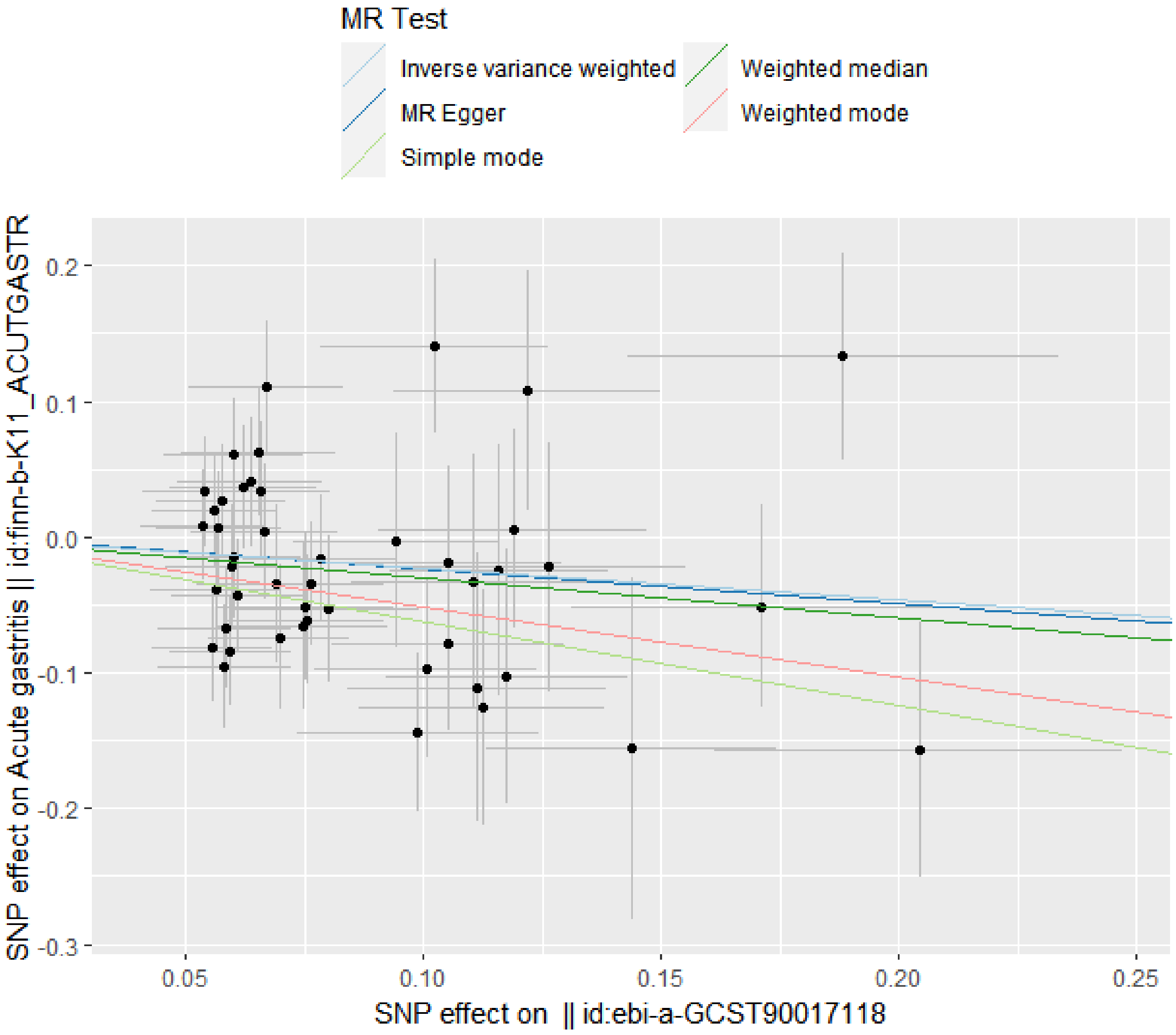
MR Test
Inverse variance weighted
MR Egger
Simple mode
Weighted median
Weighted mode
0.2
0.1
0.0
-0.1
-0.2
-0.3
0.05
0.10
0.15
0.20
0.25
SNP effect on Acute gastritis || id:finn-b-K11_ACUTGASTR
SNP effect on || id:ebi-a-GCST90017118

Figure 83 Leave-one-out analysis, funnel plot, MR effect size and scatter plot for gut microbiota abundance (class Deltaproteobacteria id.3087) on gastric cancer

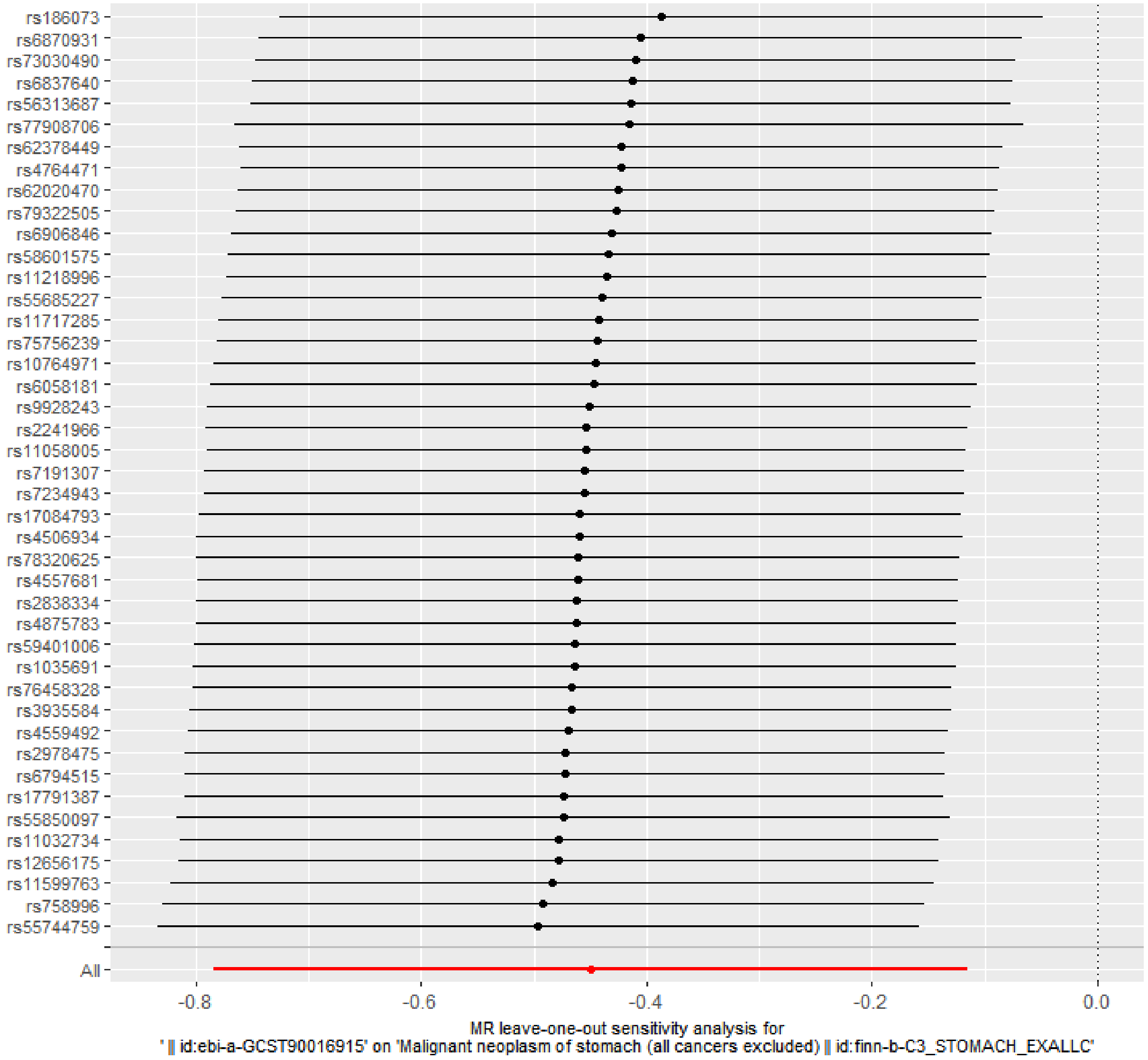

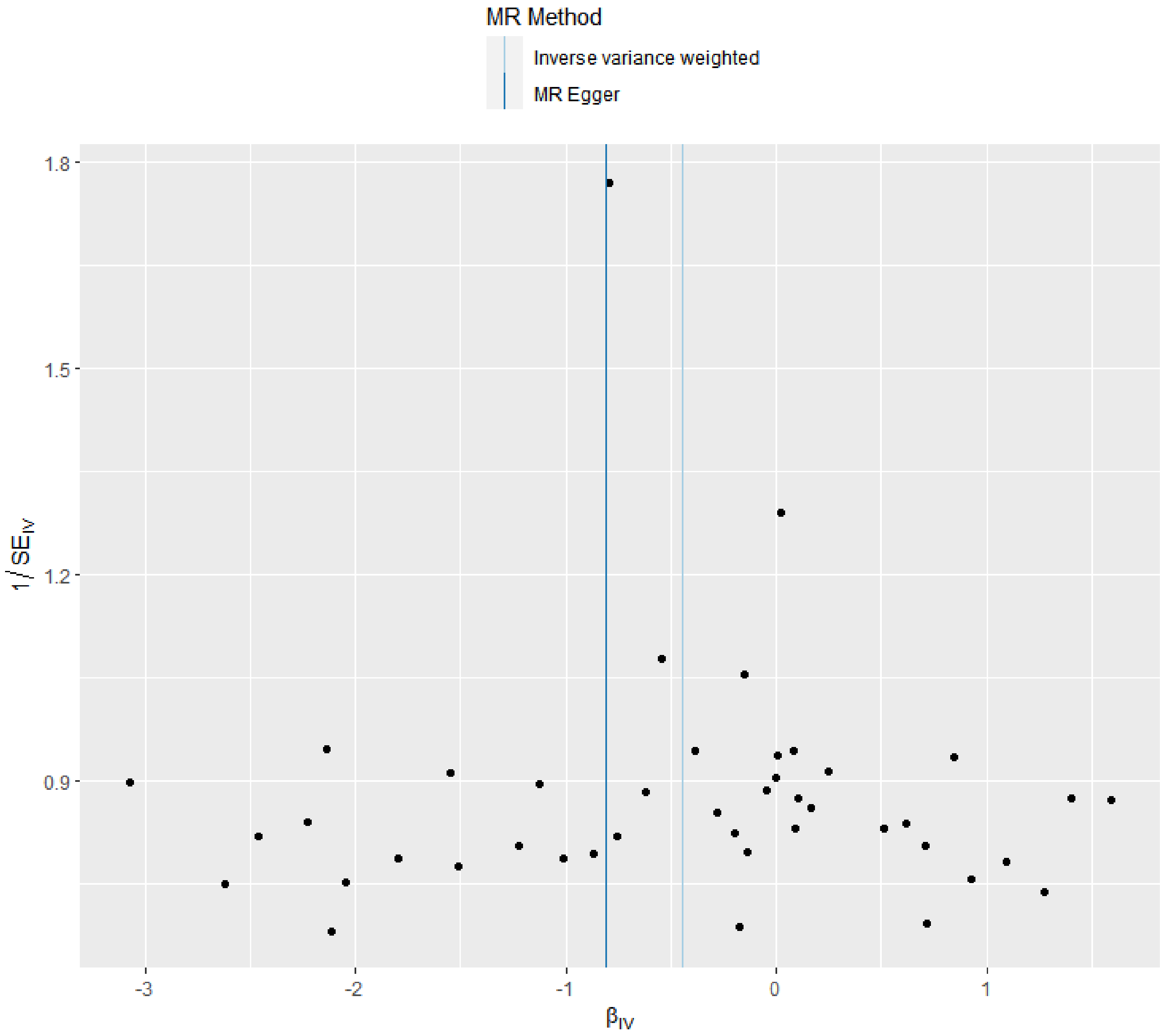

MR Method
Inverse variance weighted
MR Egger
1.8
1.5
1.2
0.9
1/SE_IV
-3
-2
-1
0
1
β_IV

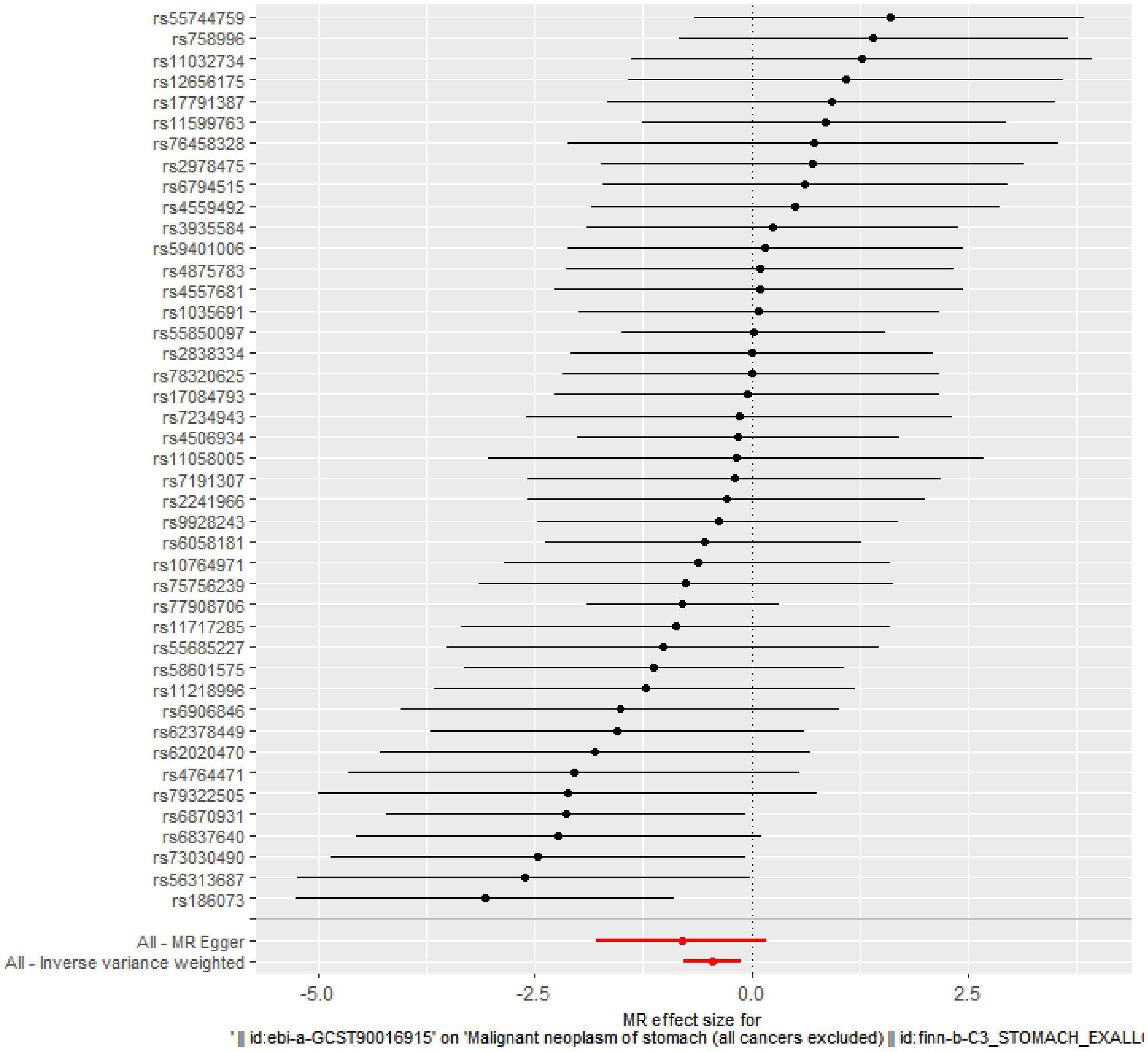

rs55744759
rs758996
rs11032734
rs12656175
rs17791387
rs11599763
rs76458328
rs2978475
rs6794515
rs4559492
rs3935584
rs59401006
rs4875783
rs4557681
rs1035691
rs55850097
rs2838334
rs78320625
rs17084793
rs7234943
rs4506934
rs11058005
rs7191307
rs2241966
rs9928243
rs6058181
rs10764971
rs75756239
rs77908706
rs11717285
rs55685227
rs58601575
rs11218996
rs6906846
rs62378449
rs62020470
rs4764471
rs79322505
rs6870931
rs6837640
rs73030490
rs56313687
rs186073
All - MR Egger
All - Inverse variance weighted
-5.0
-2.5
0.0
2.5
MR effect size for
' || id:ebi-a-GCST90016915' on 'Malignant neoplasm of stomach (all cancers excluded) || id:finn-b-C3_STOMACH_EXALL

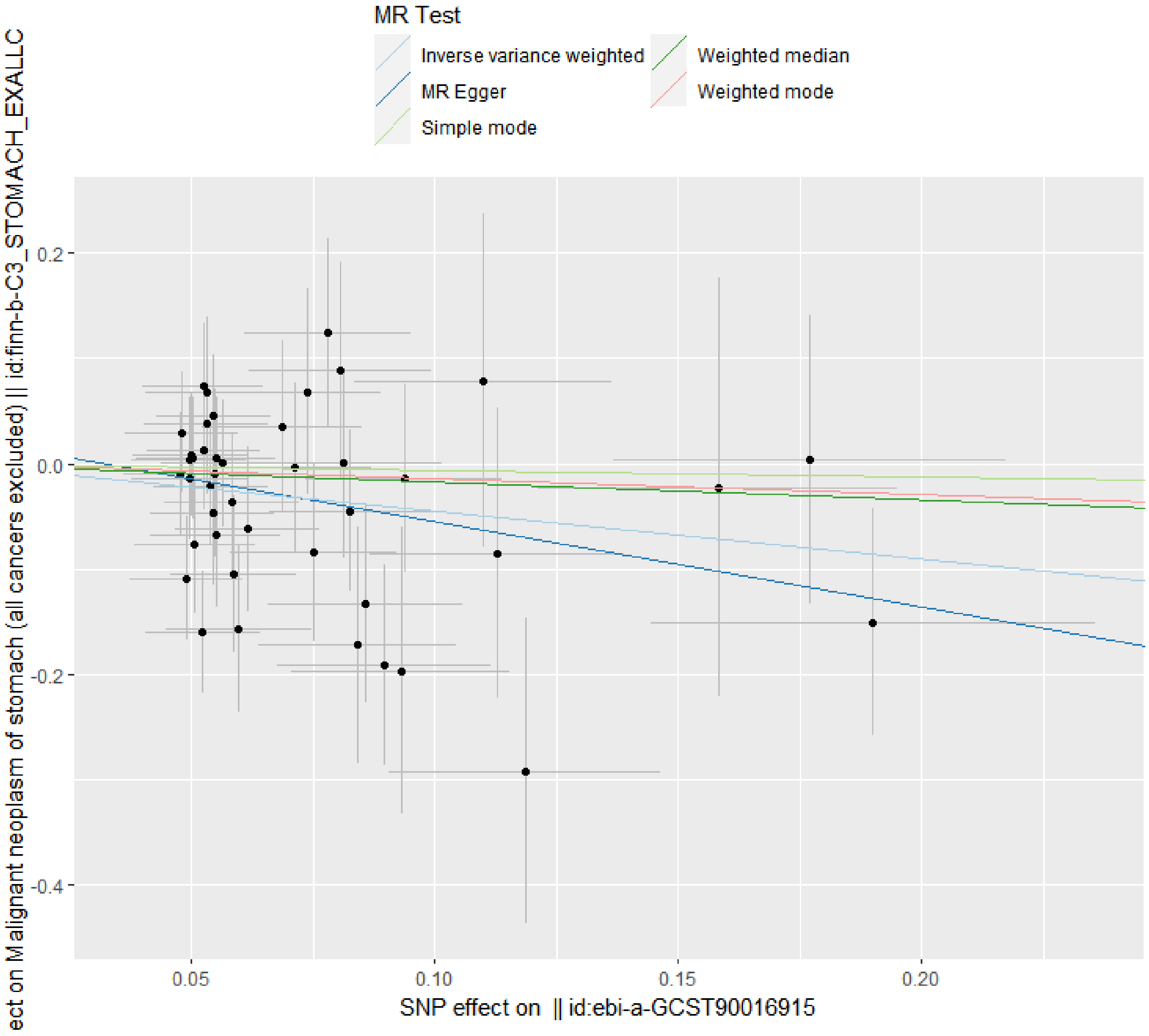

MR Test
Inverse variance weighted
MR Egger
Simple mode
Weighted median
Weighted mode
ect on Malignant neoplasm of stomach (all cancers excluded) || id:finn-b-C3_STOMACH_EXALLC
0.2
0.0
-0.2
-0.4
0.05
0.10
0.15
0.20
SNP effect on || id:ebi-a-GCST90016915

Figure 84 Leave-one-out analysis, funnel plot, MR effect size and scatter plot for gut microbiota abundance (class Gammaproteobacteria id.3303) on gastric cancer

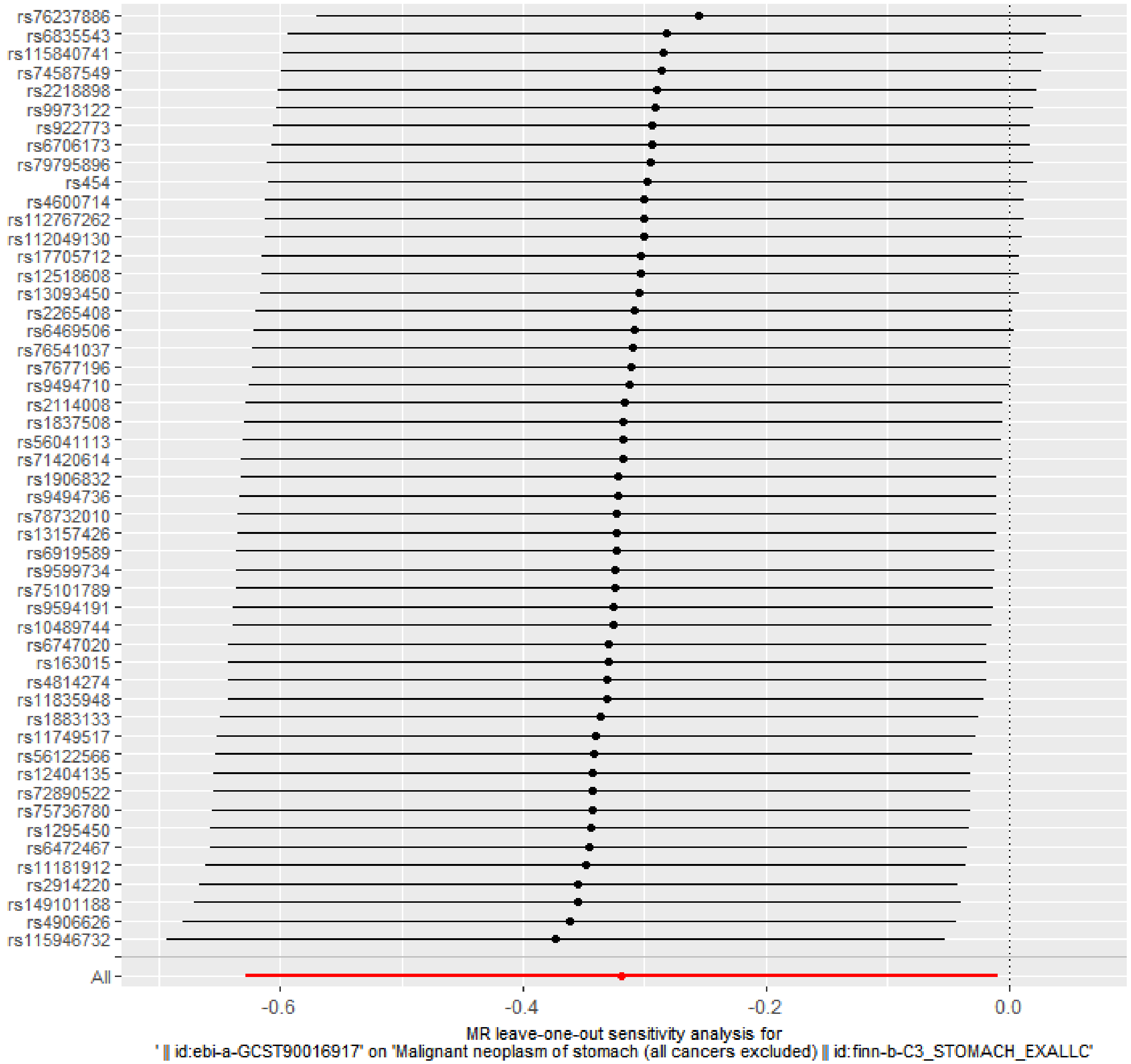

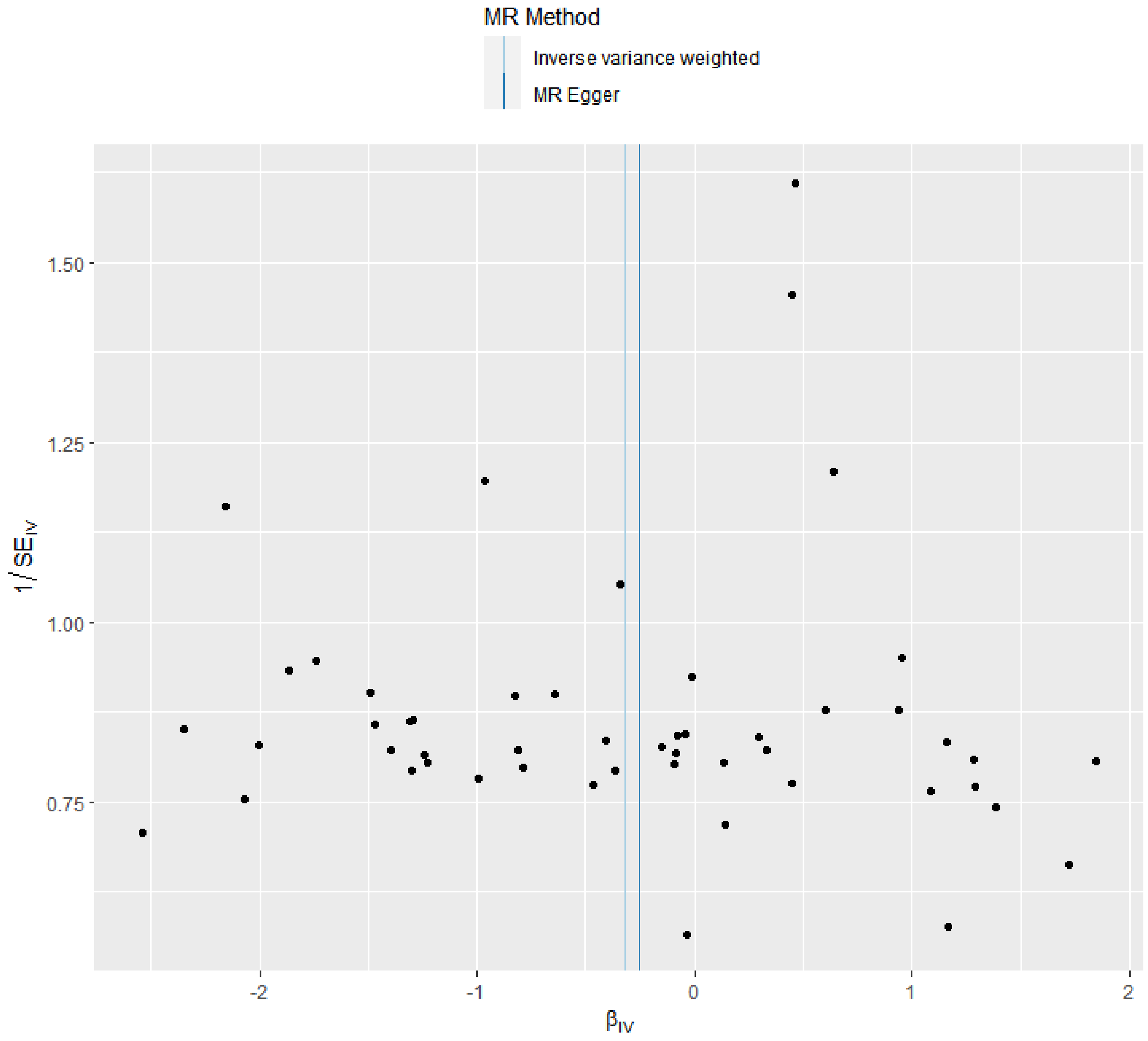
MR Method
Inverse variance weighted
MR Egger
1.50
1.25
1.00
0.75
-2
-1
0
1
2
β_{IV}
$1/SE_{IV}$

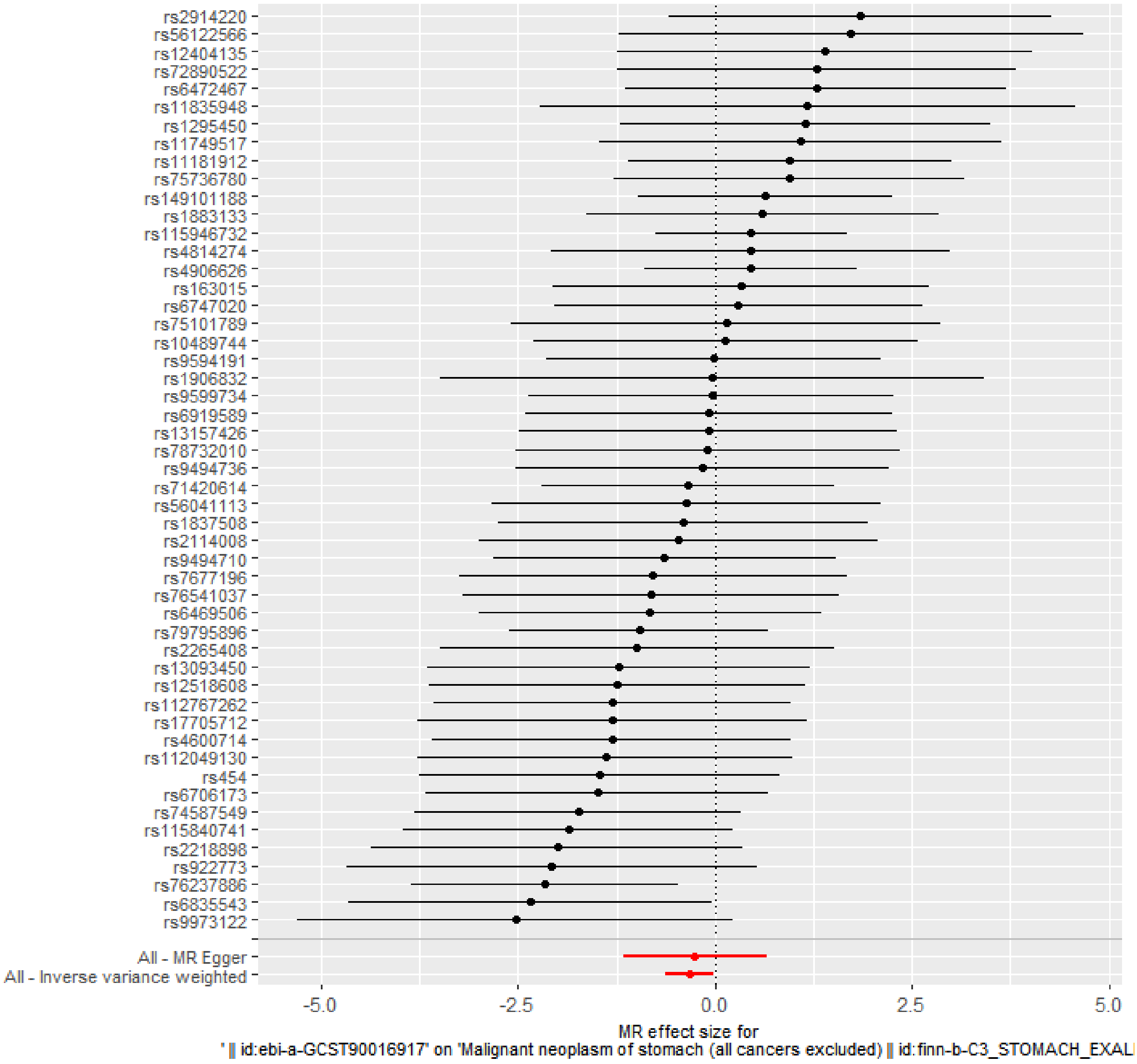
rs2914220
rs56122566
rs12404135
rs72890522
rs6472467
rs11835948
rs1295450
rs11749517
rs11181912
rs75736780
rs149101188
rs1883133
rs115946732
rs4814274
rs4906626
rs163015
rs6747020
rs75101789
rs10489744
rs9594191
rs1906832
rs9599734
rs6919589
rs13157426
rs78732010
rs9494736
rs71420614
rs56041113
rs1837508
rs2114008
rs9494710
rs7677196
rs76541037
rs6469506
rs79795896
rs2265408
rs13093450
rs12518608
rs112767262
rs17705712
rs4600714
rs112049130
rs454
rs6706173
rs74587549
rs115840741
rs2218898
rs922773
rs76237886
rs6835543
rs9973122
All - MR Egger
All - Inverse variance weighted
-5.0
-2.5
0.0
2.5
5.0
MR effect size for
' || id:ebi-a-GCST90016917' on 'Malignant neoplasm of stomach (all cancers excluded) || id:finn-b-C3_STOMACH_EXALL

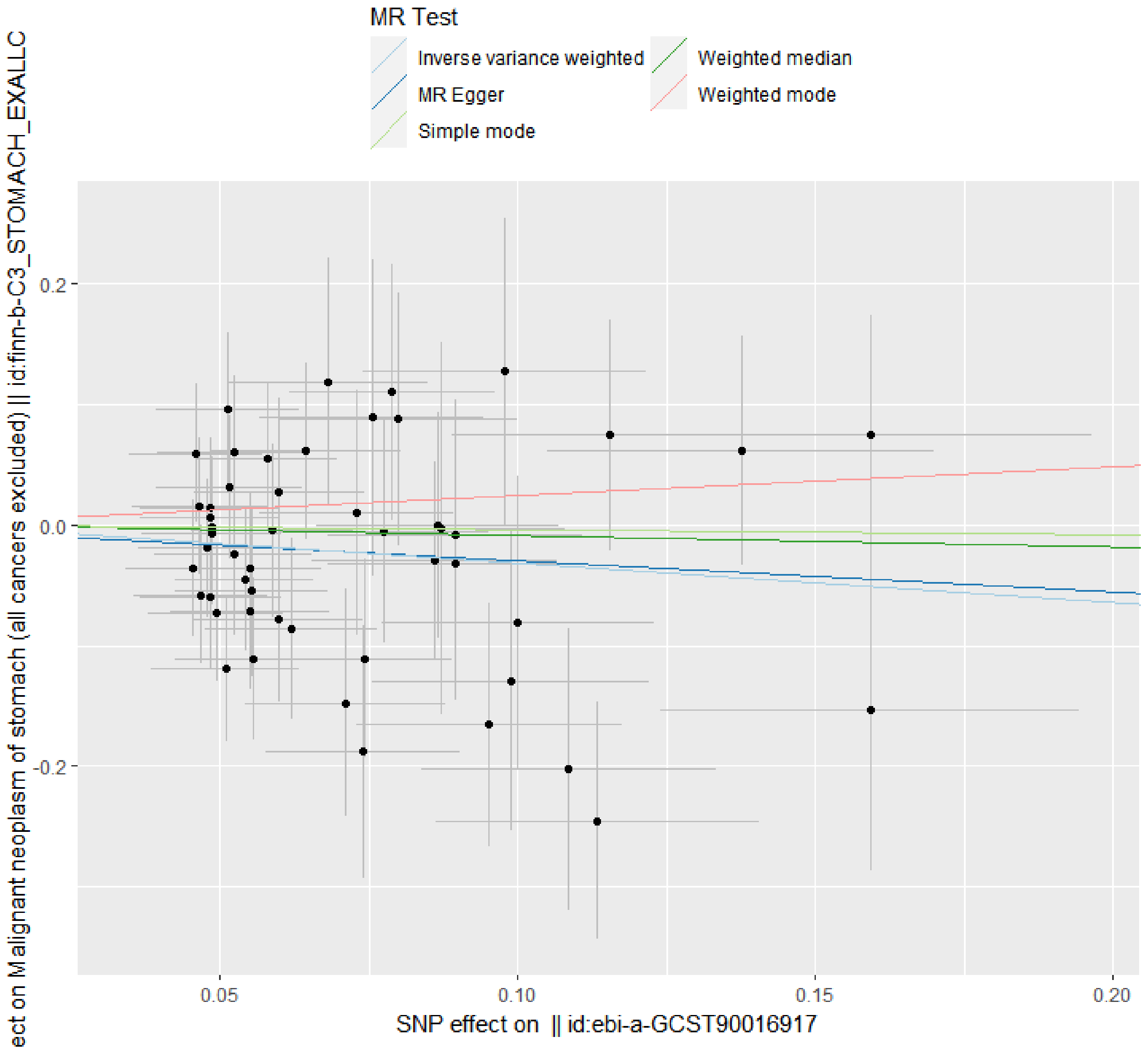
MR Test
Inverse variance weighted
MR Egger
Simple mode
Weighted median
Weighted mode
0.2
0.0
-0.2
0.05
0.10
0.15
0.20
SNP effect on || id:ebi-a-GCST90016917
ect on Malignant neoplasm of stomach (all cancers excluded) || id:finn-b-C3_STOMACH_EXALLC

Figure 85 Leave-one-out analysis, funnel plot, MR effect size and scatter plot for gut microbiota abundance (family Actinomycetaceae id.421) on gastric cancer

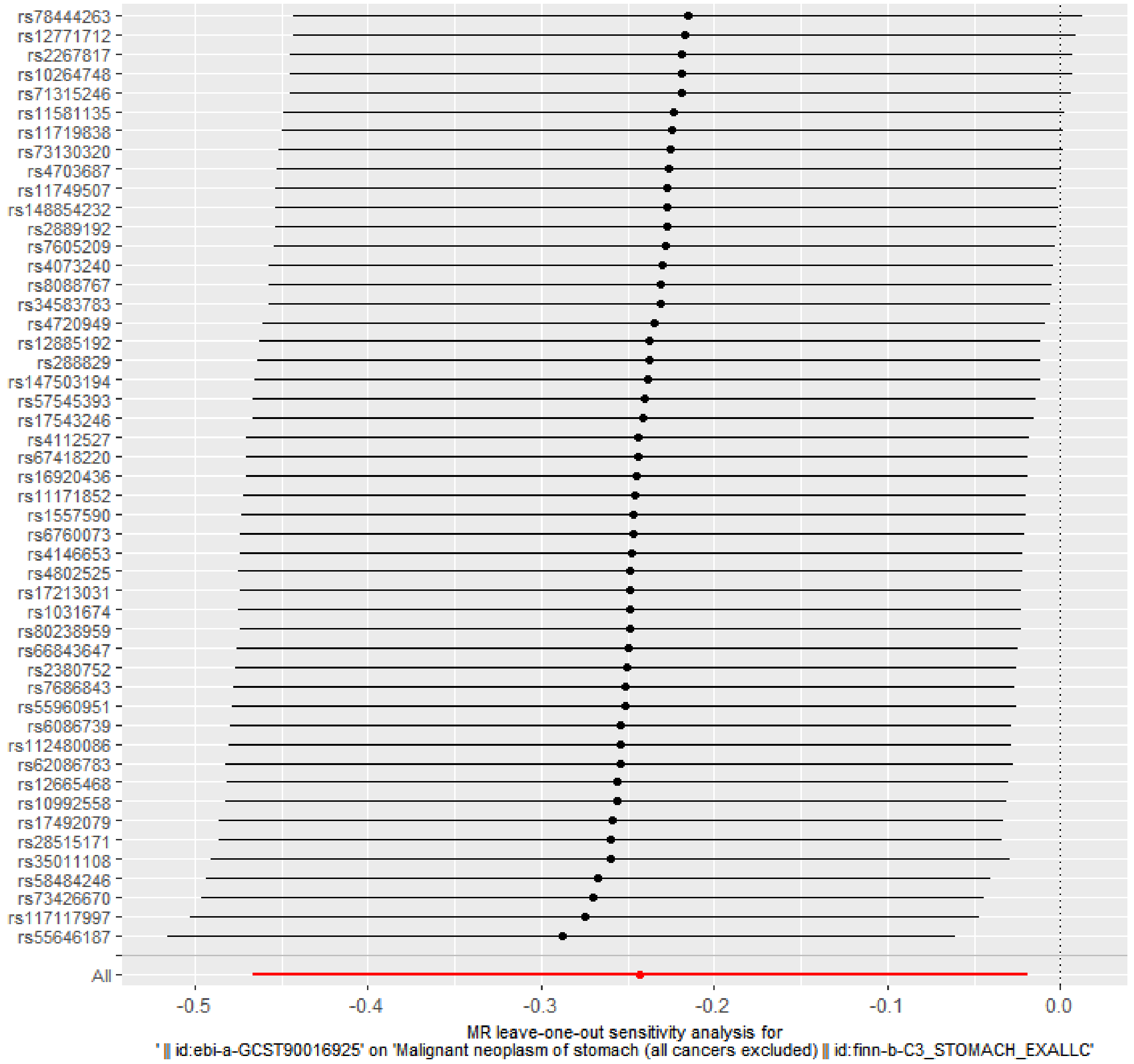

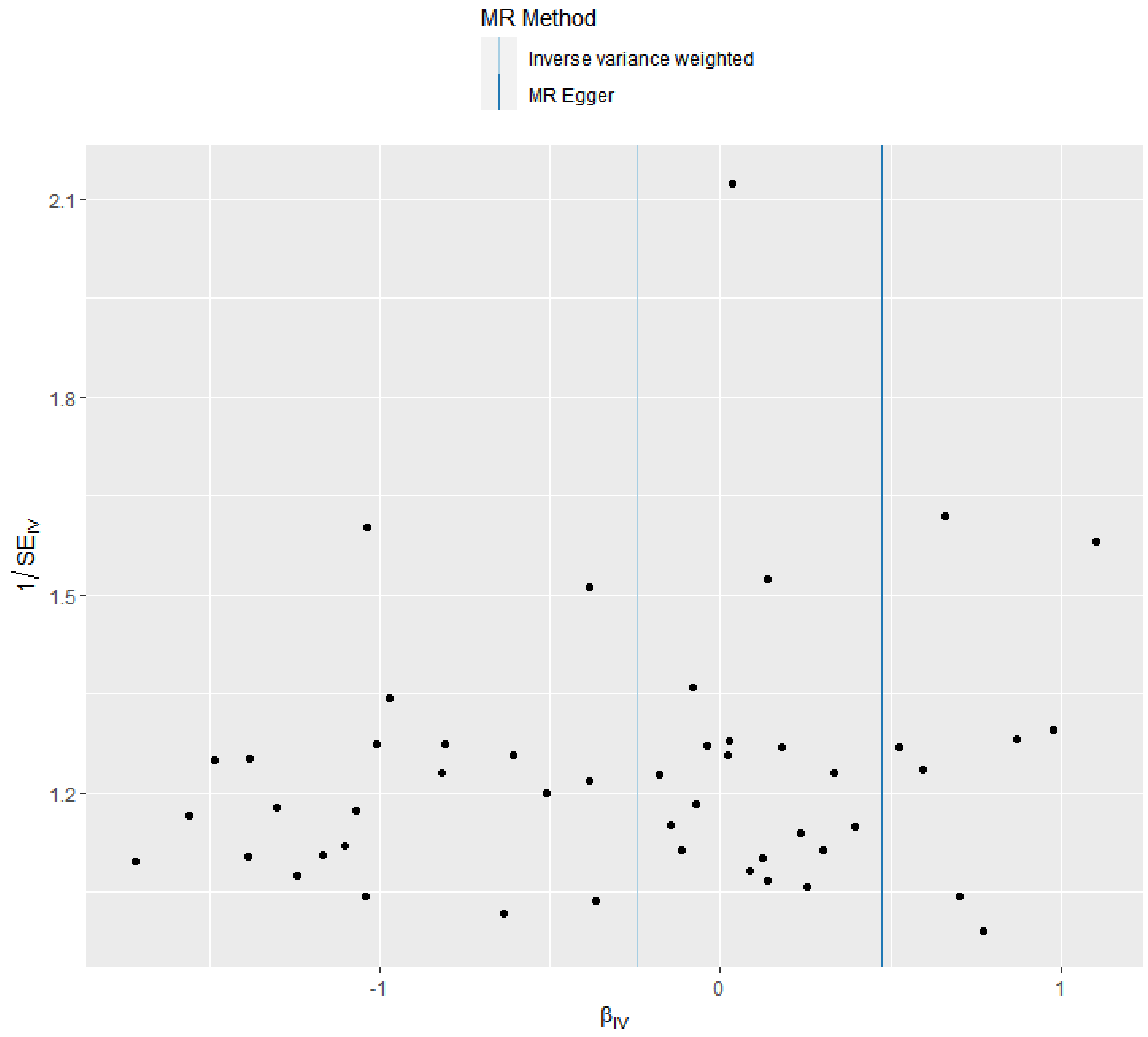

MR Method
Inverse variance weighted
MR Egger
2.1
1.8
1.5
1.2
$1/SE_{IV}$
-1
0
1
β_{IV}

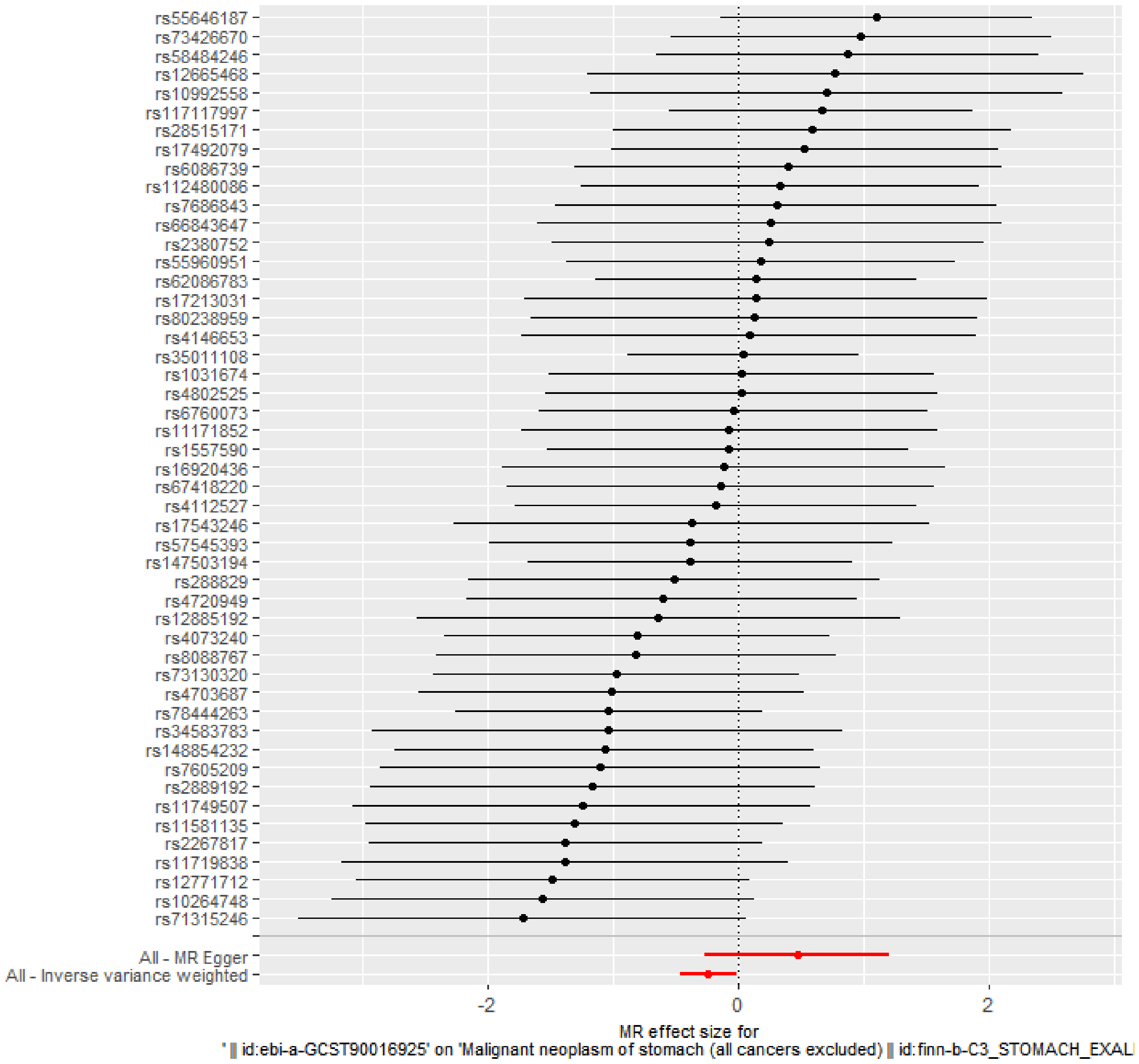
rs55646187
rs73426670
rs58484246
rs12665468
rs10992558
rs117117997
rs28515171
rs17492079
rs6086739
rs112480086
rs7686843
rs66843647
rs2380752
rs55960951
rs62086783
rs17213031
rs80238959
rs4146653
rs35011108
rs1031674
rs4802525
rs6760073
rs11171852
rs1557590
rs16920436
rs67418220
rs4112527
rs17543246
rs57545393
rs147503194
rs288829
rs4720949
rs12885192
rs4073240
rs8088767
rs73130320
rs4703687
rs78444263
rs34583783
rs148854232
rs7605209
rs2889192
rs11749507
rs11581135
rs2267817
rs11719838
rs12771712
rs10264748
rs71315246
All - MR Egger
All - Inverse variance weighted
-2
0
2
MR effect size for
' || id:ebi-a-GCST90016925' on 'Malignant neoplasm of stomach (all cancers excluded) || id:finn-b-C3_STOMACH_EXALL

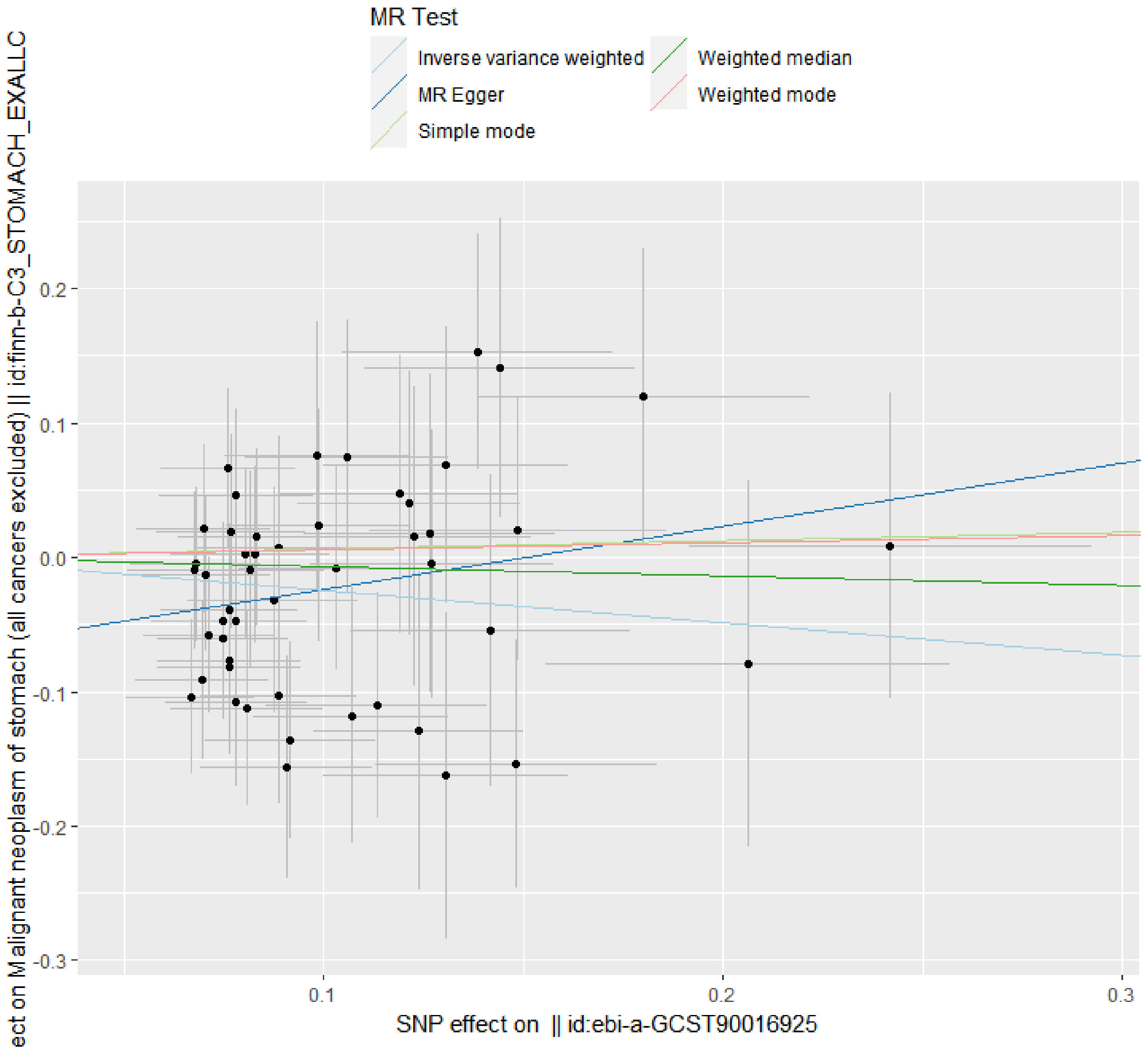

MR Test
Inverse variance weighted
MR Egger
Simple mode
Weighted median
Weighted mode
0.2
0.1
0.0
-0.1
-0.2
-0.3
0.1
0.2
0.3
SNP effect on || id:ebi-a-GCST90016925
ect on Malignant neoplasm of stomach (all cancers excluded) || id:finn-b-C3_STOMACH_EXALLC

Figure 86 Leave-one-out analysis, funnel plot, MR effect size and scatter plot for gut microbiota abundance (family Desulfovibrionaceae id.3169) on gastric cancer

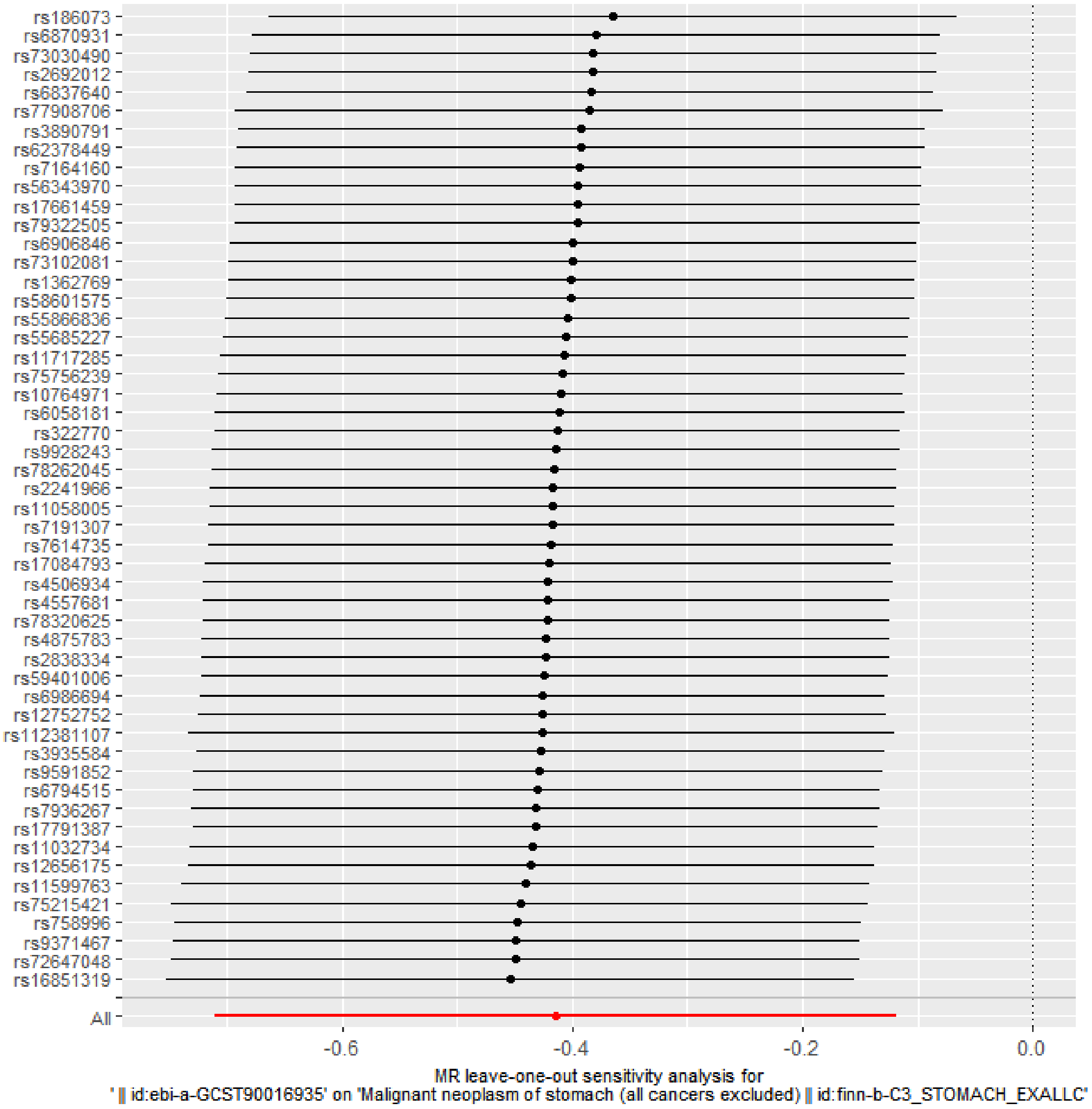

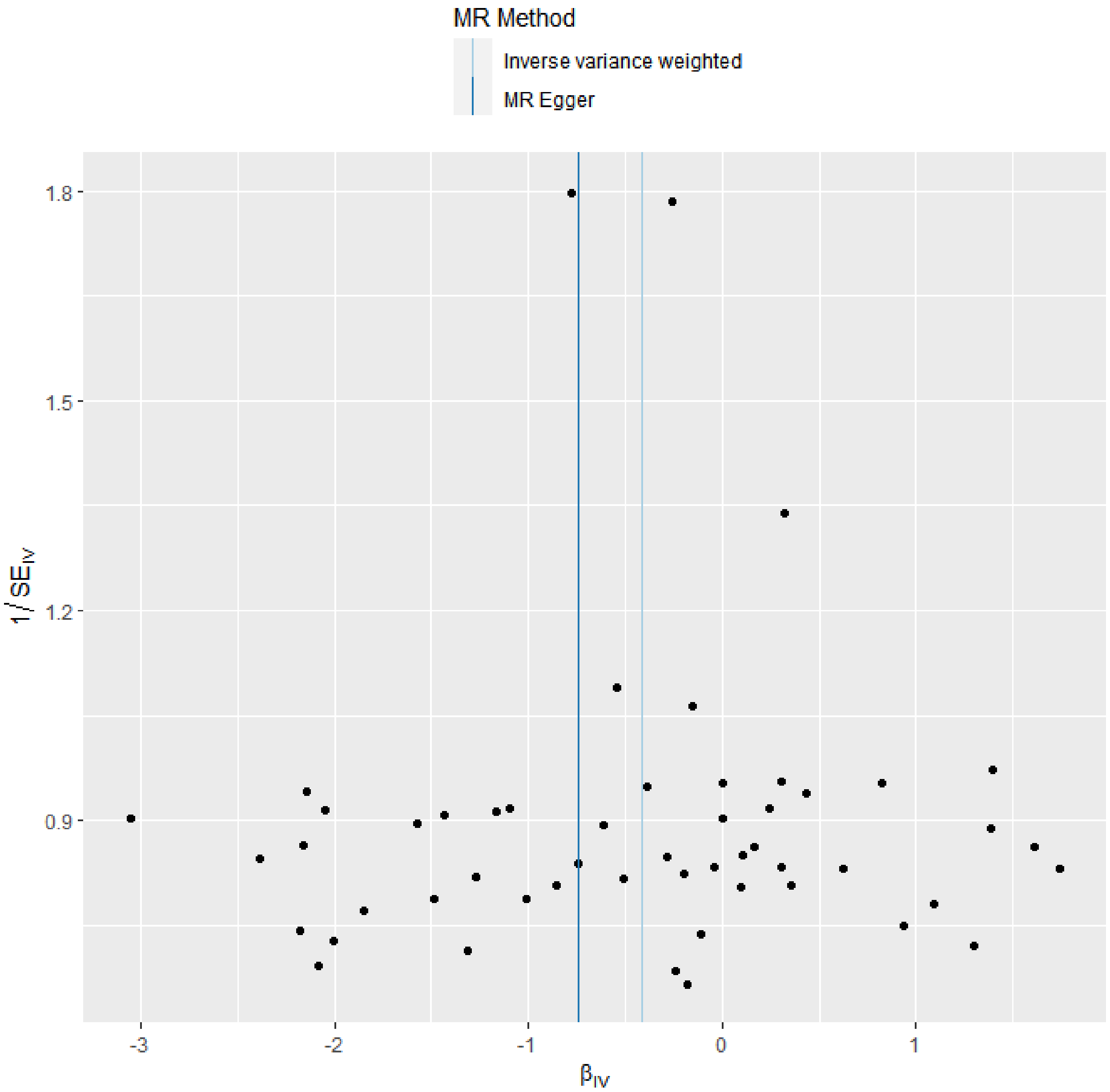
MR Method
Inverse variance weighted
MR Egger
1.8
1.5
1.2
0.9
1/SE_IV
-3
-2
-1
0
1
β_IV

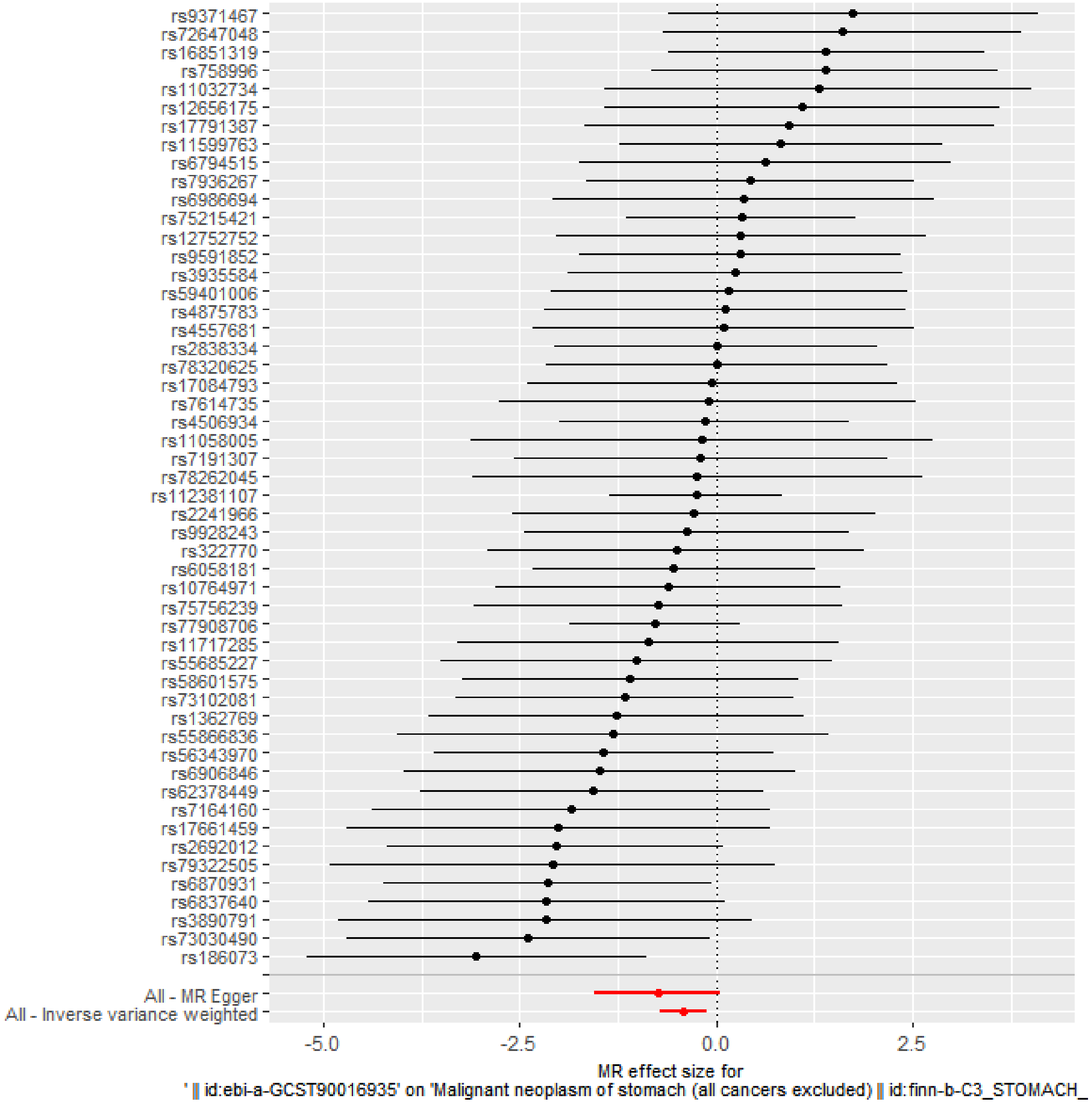
rs9371467
rs72647048
rs16851319
rs758996
rs11032734
rs12656175
rs17791387
rs11599763
rs6794515
rs7936267
rs6986694
rs75215421
rs12752752
rs9591852
rs3935584
rs59401006
rs4875783
rs4557681
rs2838334
rs78320625
rs17084793
rs7614735
rs4506934
rs11058005
rs7191307
rs78262045
rs112381107
rs2241966
rs9928243
rs322770
rs6058181
rs10764971
rs75756239
rs77908706
rs11717285
rs55685227
rs58601575
rs73102081
rs1362769
rs55866836
rs56343970
rs6906846
rs62378449
rs7164160
rs17661459
rs2692012
rs79322505
rs6870931
rs6837640
rs3890791
rs73030490
rs186073
All - MR Egger
All - Inverse variance weighted
-5.0
-2.5
0.0
2.5
MR effect size for
' ‖ id:ebi-a-GCST90016935' on 'Malignant neoplasm of stomach (all cancers excluded) ‖ id:finn-b-C3_STOMACH_

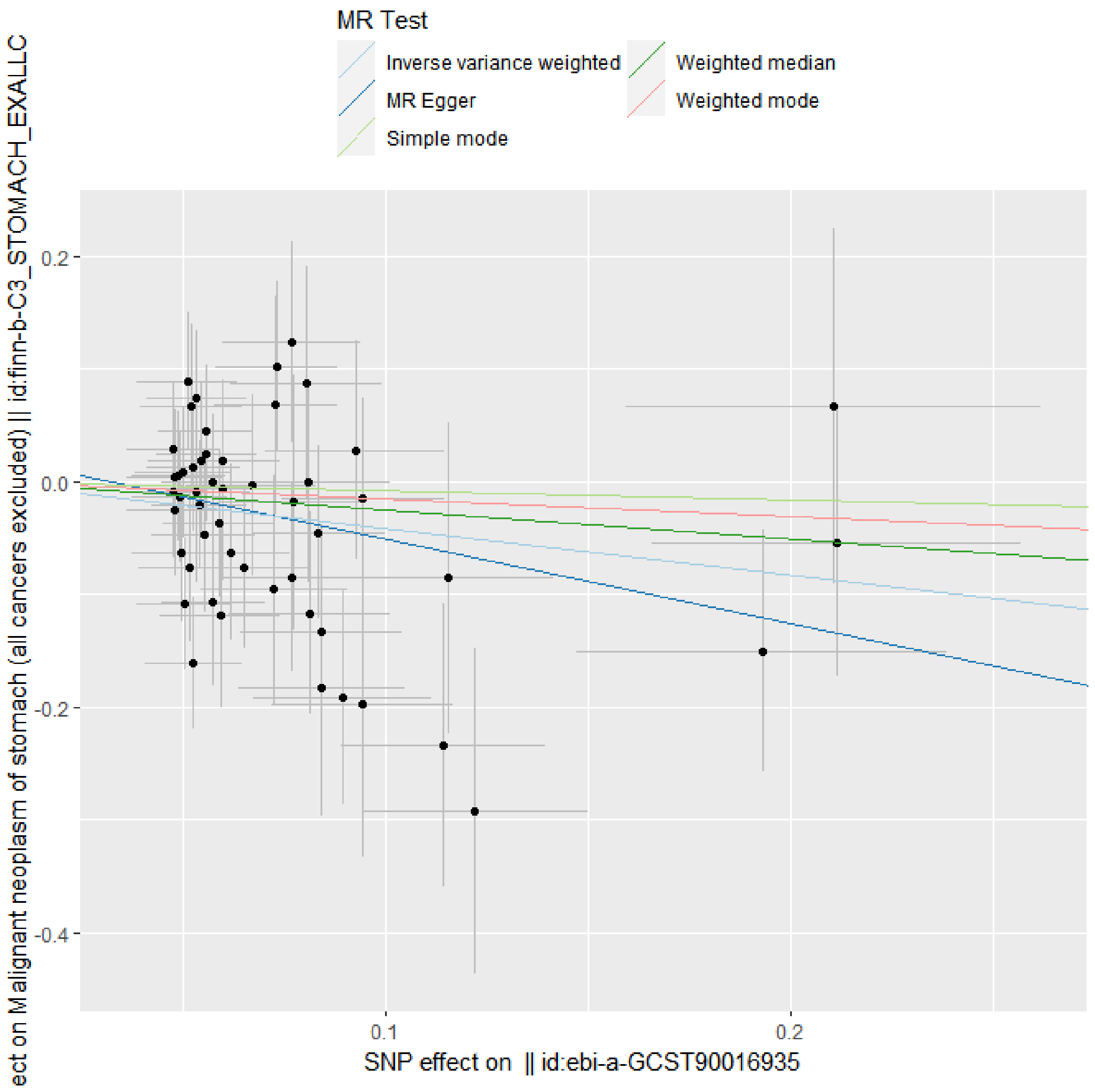

MR Test
Inverse variance weighted
MR Egger
Simple mode
Weighted median
Weighted mode
0.2
0.0
-0.2
-0.4
0.1
0.2
SNP effect on || id:ebi-a-GCST90016935
ect on Malignant neoplasm of stomach (all cancers excluded) || id:finn-b-C3_STOMACH_EXALLC

Figure 87 Leave-one-out analysis, funnel plot, MR effect size and scatter plot for gut microbiota abundance (unknown family id.1000005471) on gastric cancer

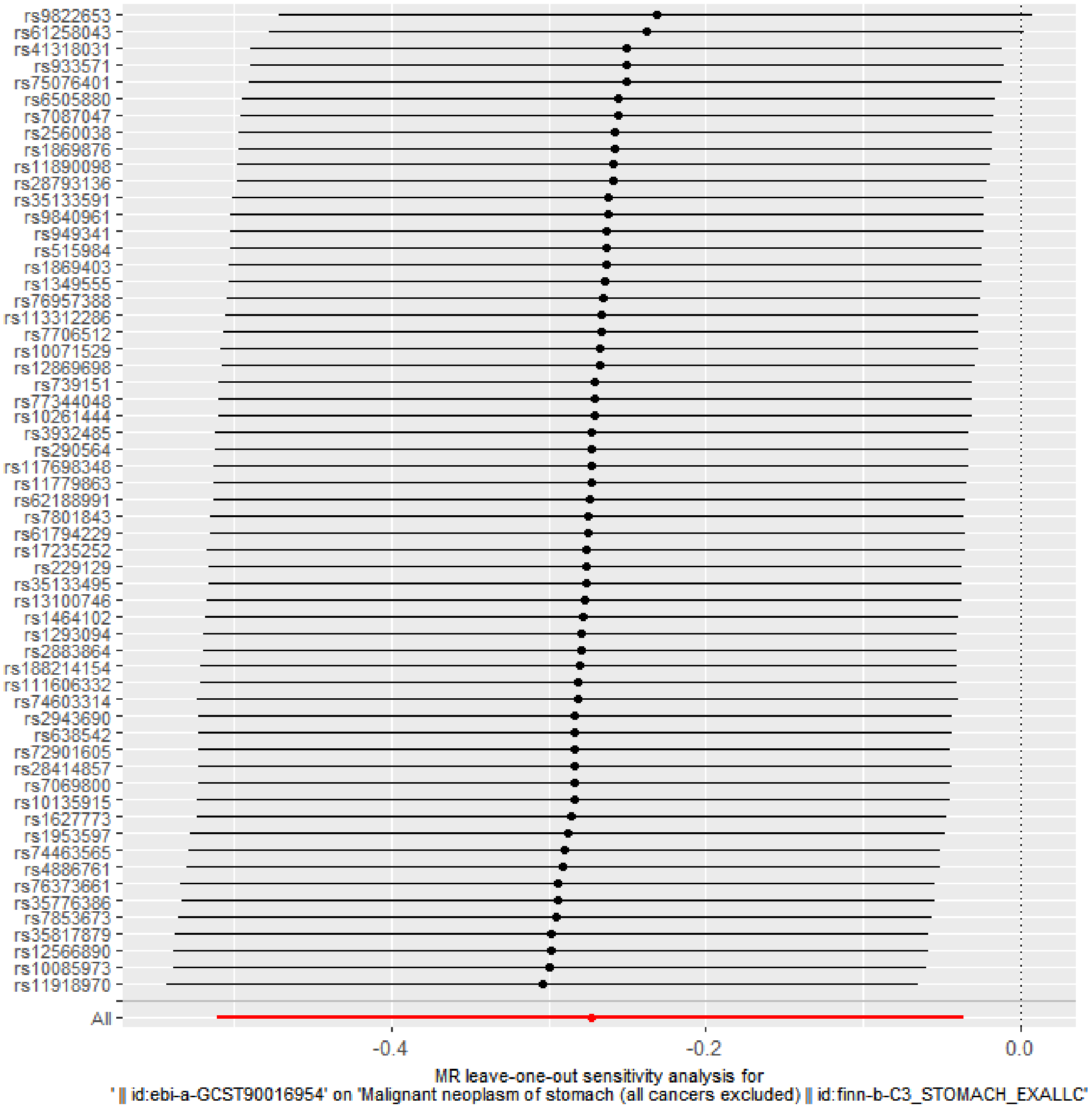

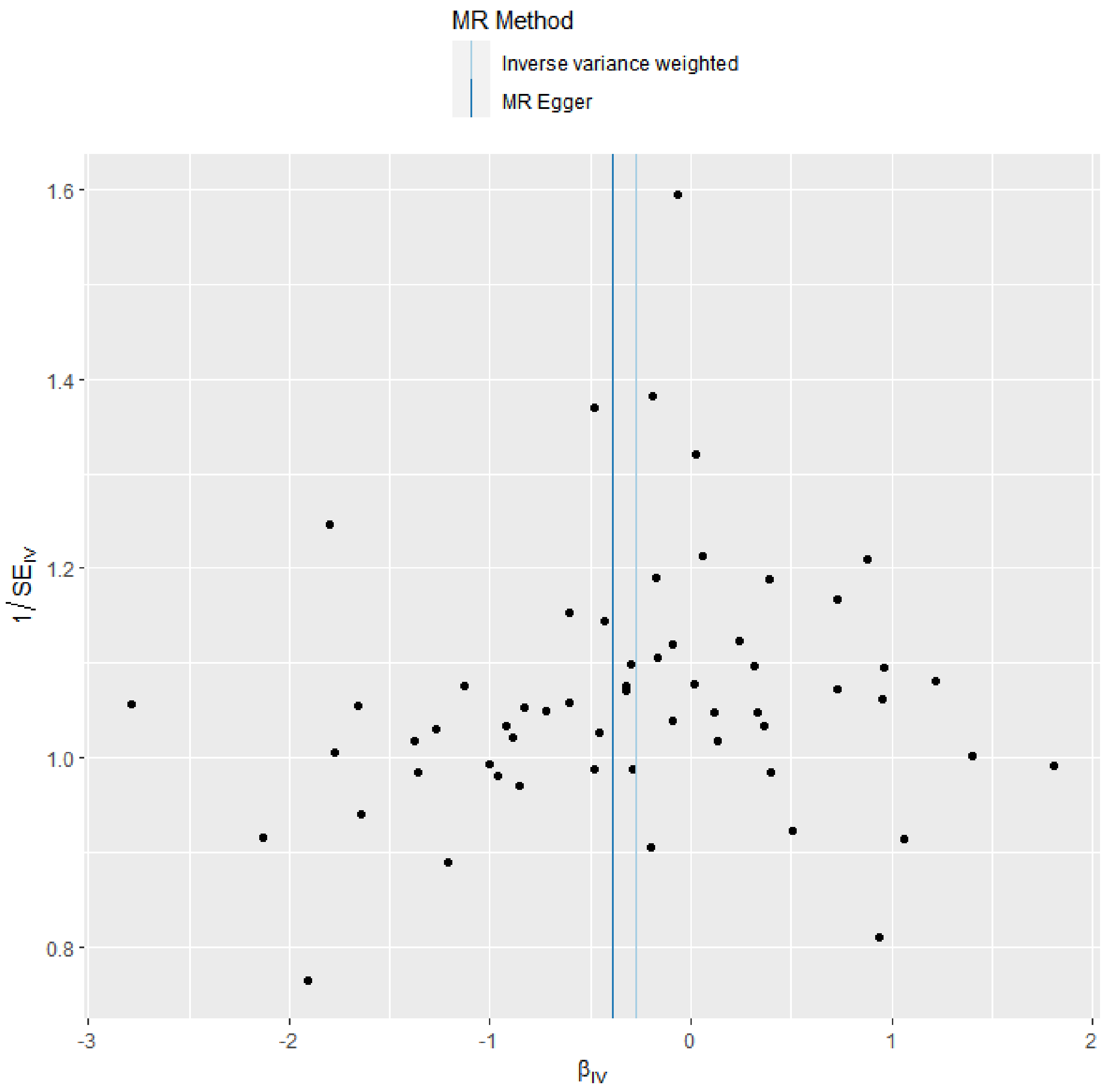

MR Method
Inverse variance weighted
MR Egger
1.6
1.4
1.2
1.0
0.8
1/SE$_{IV}$
-3
-2
-1
0
1
2
β$_{IV}$

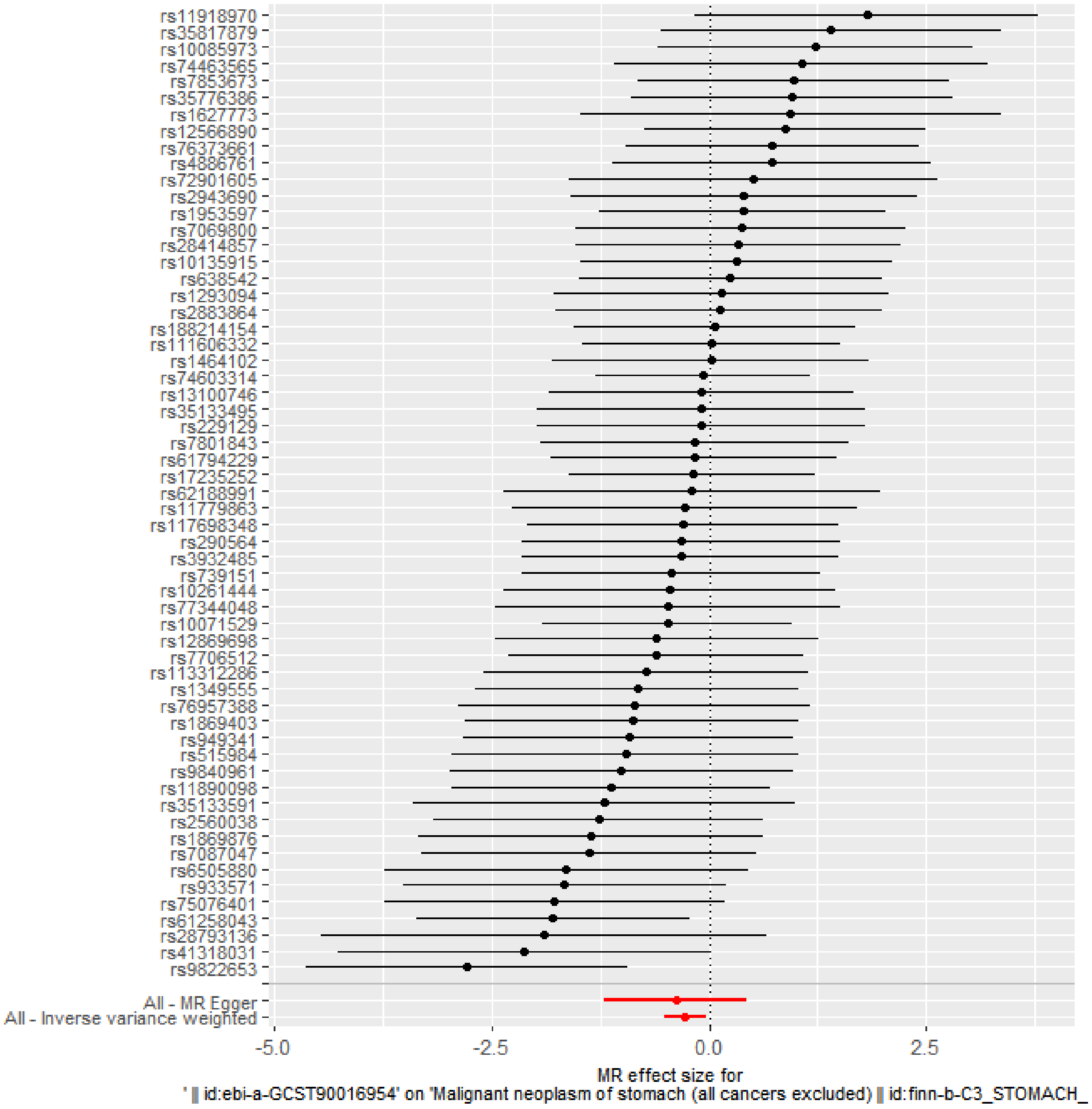

rs11918970
rs35817879
rs10085973
rs74463565
rs7853673
rs35776386
rs1627773
rs12566890
rs76373661
rs4886761
rs72901605
rs2943690
rs1953597
rs7069800
rs28414857
rs10135915
rs638542
rs1293094
rs2883864
rs188214154
rs111606332
rs1464102
rs74603314
rs13100746
rs35133495
rs229129
rs7801843
rs61794229
rs17235252
rs62188991
rs11779863
rs117698348
rs290564
rs3932485
rs739151
rs10261444
rs77344048
rs10071529
rs12869698
rs7706512
rs113312286
rs1349555
rs76957388
rs1869403
rs949341
rs515984
rs9840961
rs11890098
rs35133591
rs2560038
rs1869876
rs7087047
rs6505880
rs933571
rs75076401
rs61258043
rs28793136
rs41318031
rs9822653
All - MR Egger
All - Inverse variance weighted
-5.0
-2.5
0.0
2.5
MR effect size for
' || id:ebi-a-GCST90016954' on 'Malignant neoplasm of stomach (all cancers excluded) || id:finn-b-C3_STOMACH_

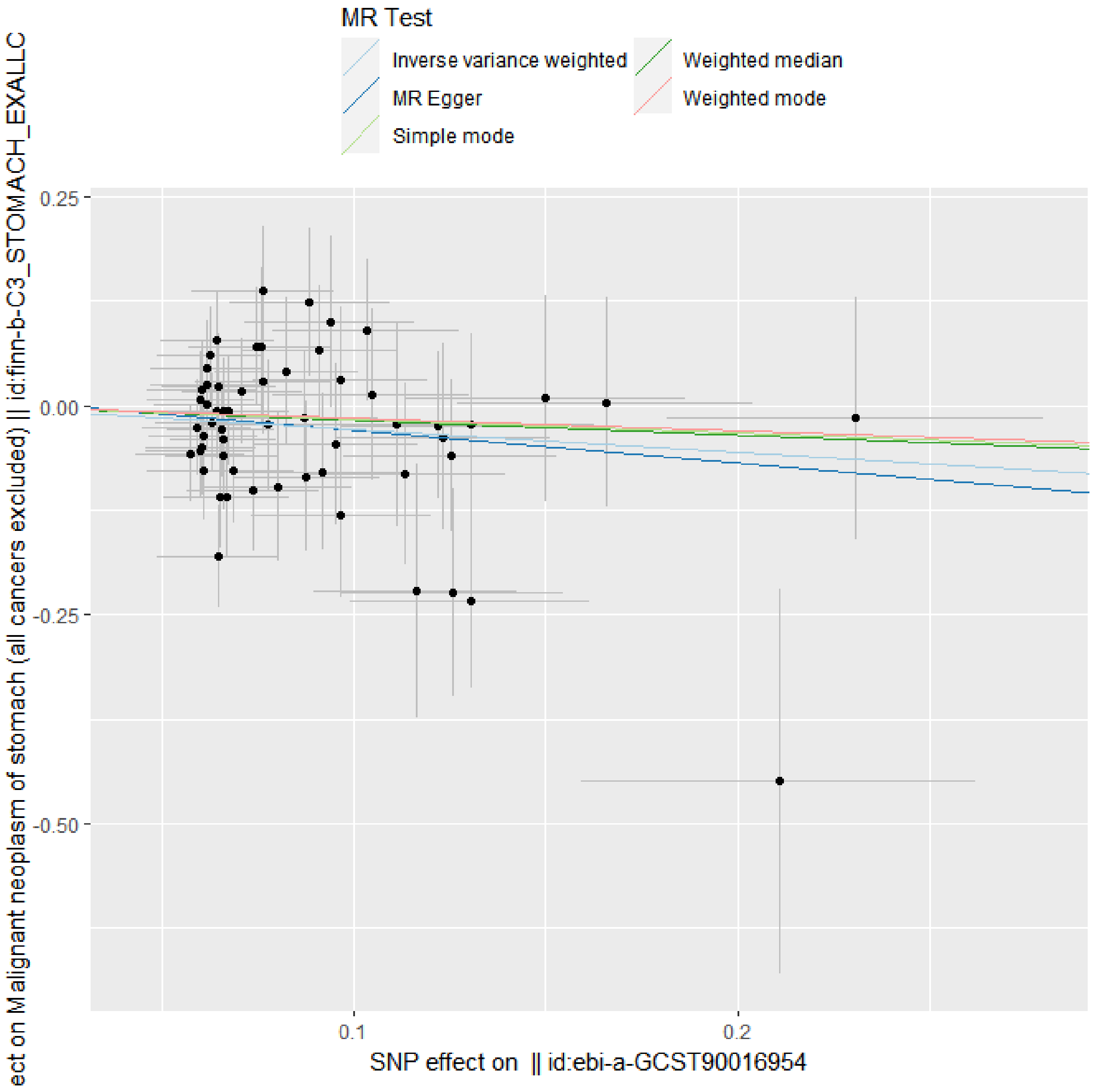

MR Test
Inverse variance weighted
MR Egger
Simple mode
Weighted median
Weighted mode
0.25
0.00
-0.25
-0.50
0.1
0.2
SNP effect on || id:ebi-a-GCST90016954
ect on Malignant neoplasm of stomach (all cancers excluded) || id:finn-b-C3_STOMACH_EXALLC

Figure 88 Leave-one-out analysis, funnel plot, MR effect size and scatter plot for gut microbiota abundance (genus Actinomyces id.423) on gastric cancer

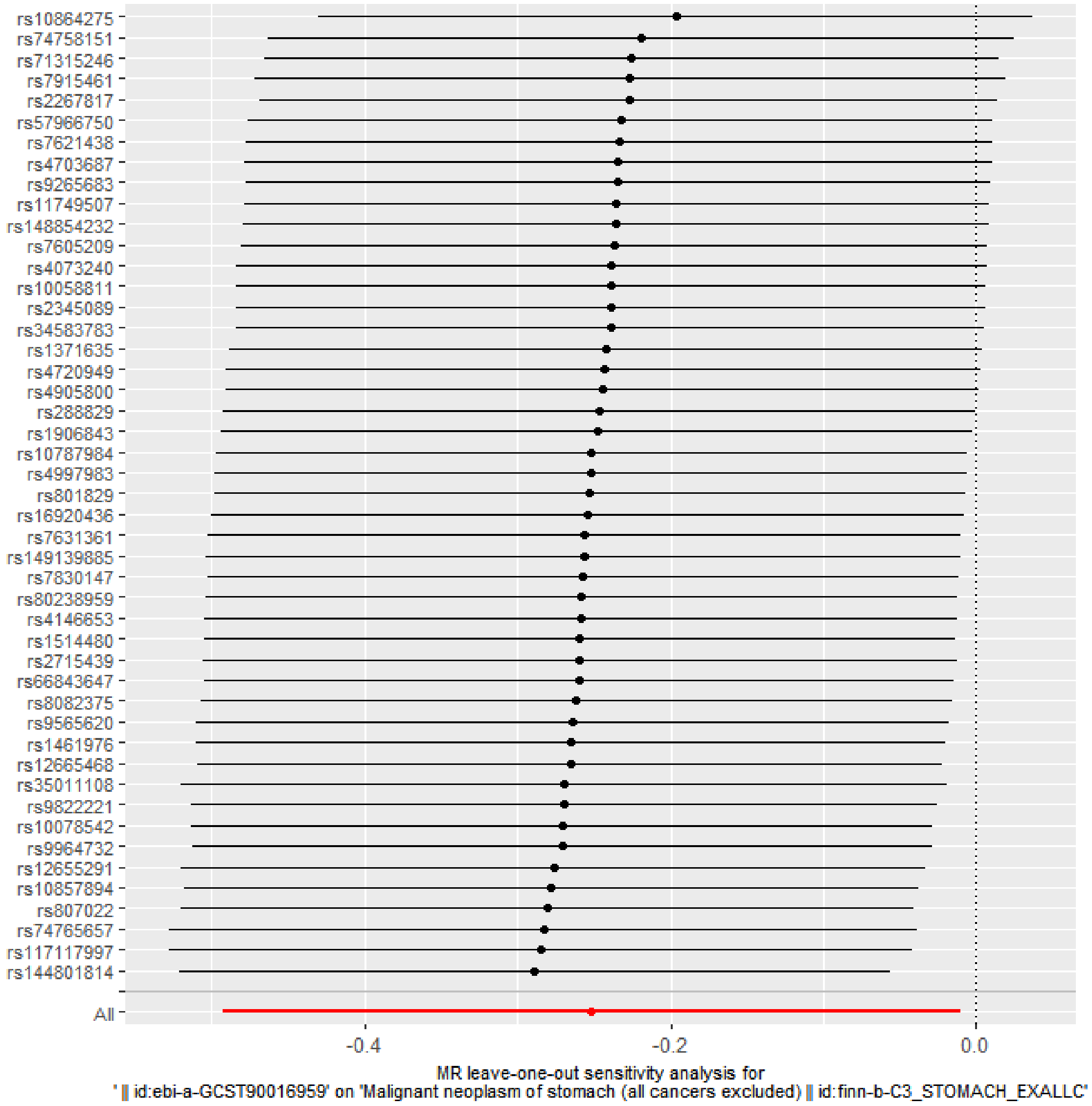

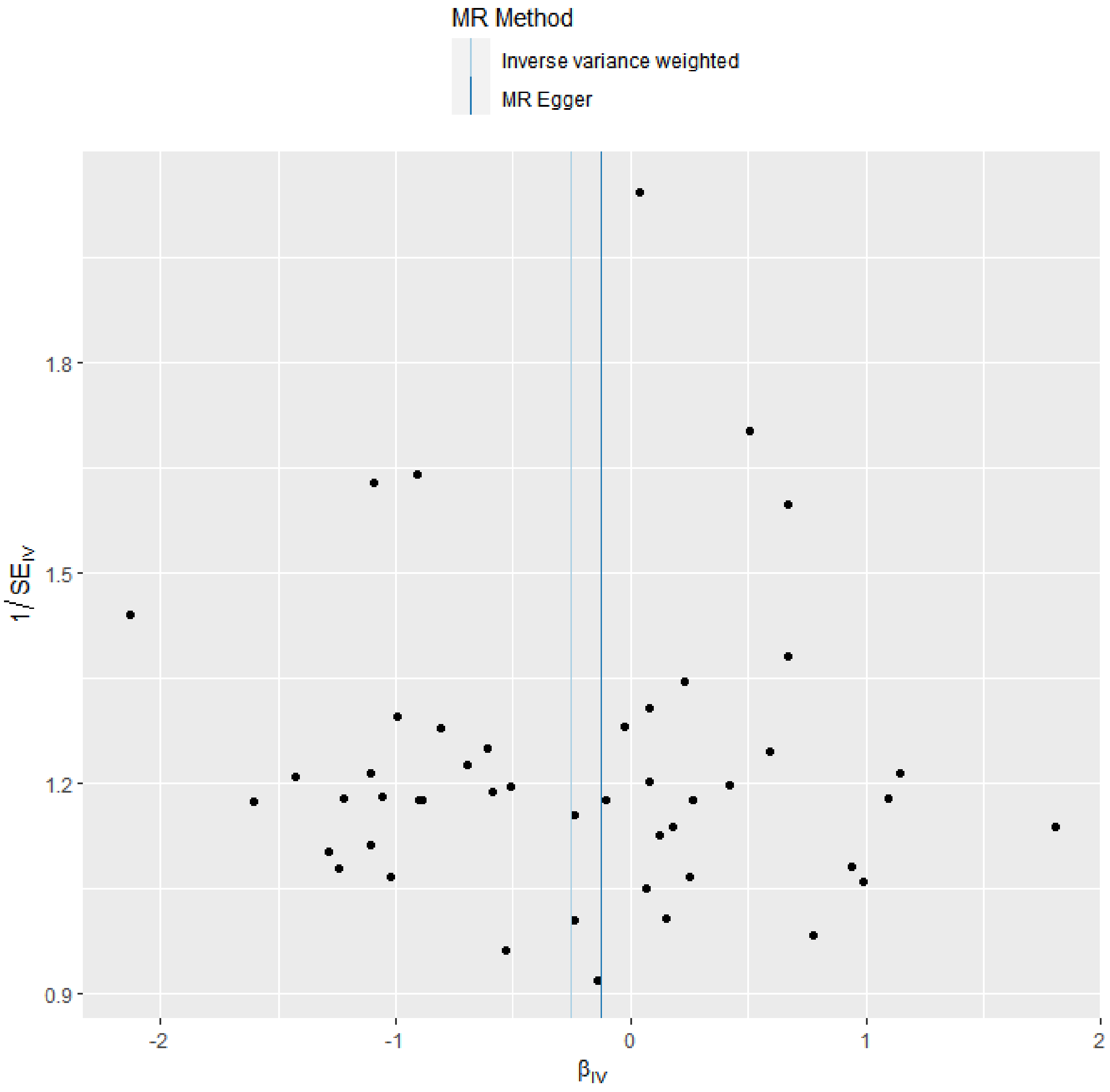

MR Method
Inverse variance weighted
MR Egger
1.8
1.5
1.2
0.9
1/SE_IV
-2
-1
0
1
2
β_IV

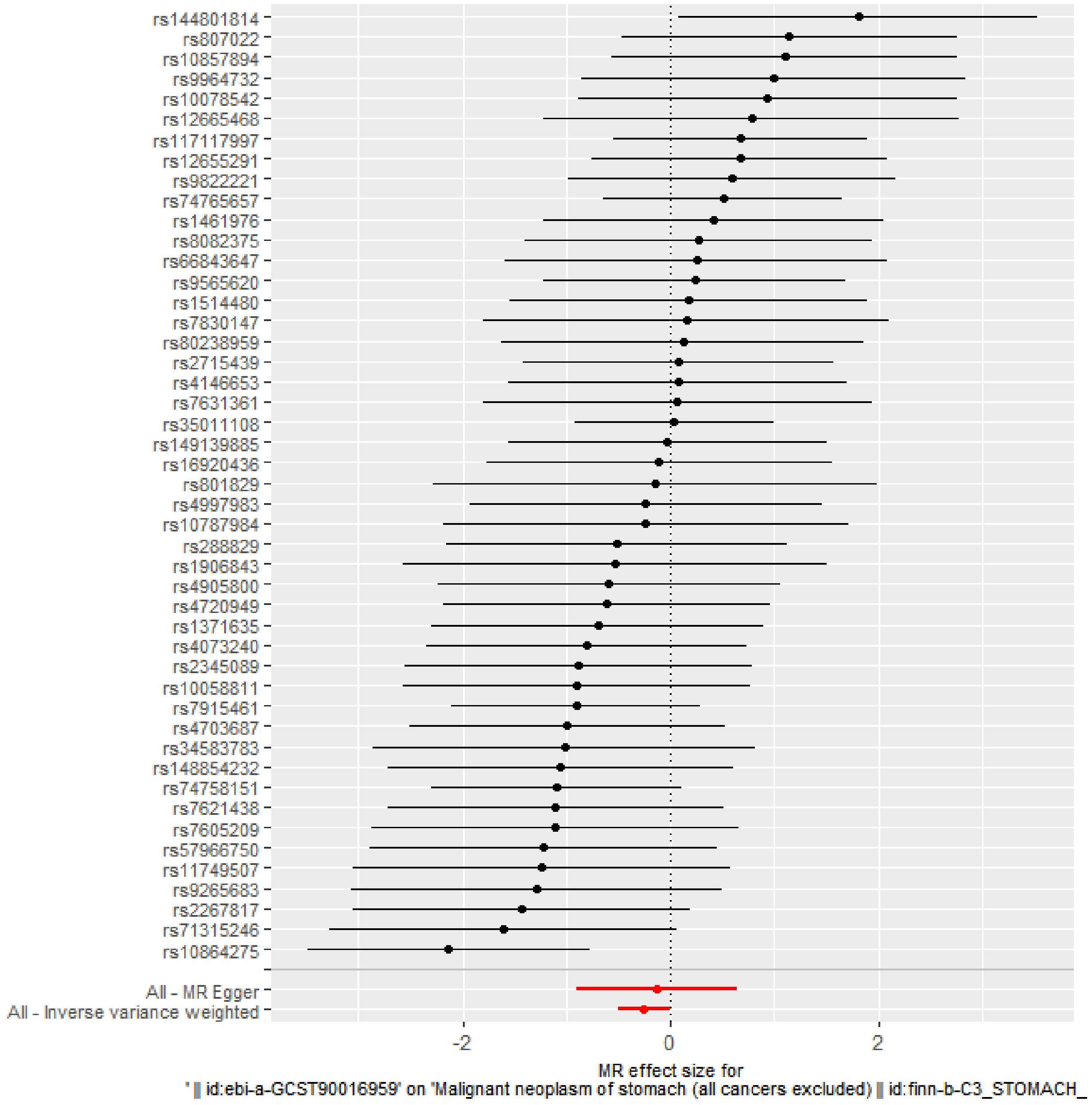

rs144801814
rs807022
rs10857894
rs9964732
rs10078542
rs12665468
rs117117997
rs12655291
rs9822221
rs74765657
rs1461976
rs8082375
rs66843647
rs9565620
rs1514480
rs7830147
rs80238959
rs2715439
rs4146653
rs7631361
rs35011108
rs149139885
rs16920436
rs801829
rs4997983
rs10787984
rs288829
rs1906843
rs4905800
rs4720949
rs1371635
rs4073240
rs2345089
rs10058811
rs7915461
rs4703687
rs34583783
rs148854232
rs74758151
rs7621438
rs7605209
rs57966750
rs11749507
rs9265683
rs2267817
rs71315246
rs10864275
All - MR Egger
All - Inverse variance weighted
-2
0
2
MR effect size for
' || id:ebi-a-GCST90016959' on 'Malignant neoplasm of stomach (all cancers excluded) || id:finn-b-C3_STOMACH_

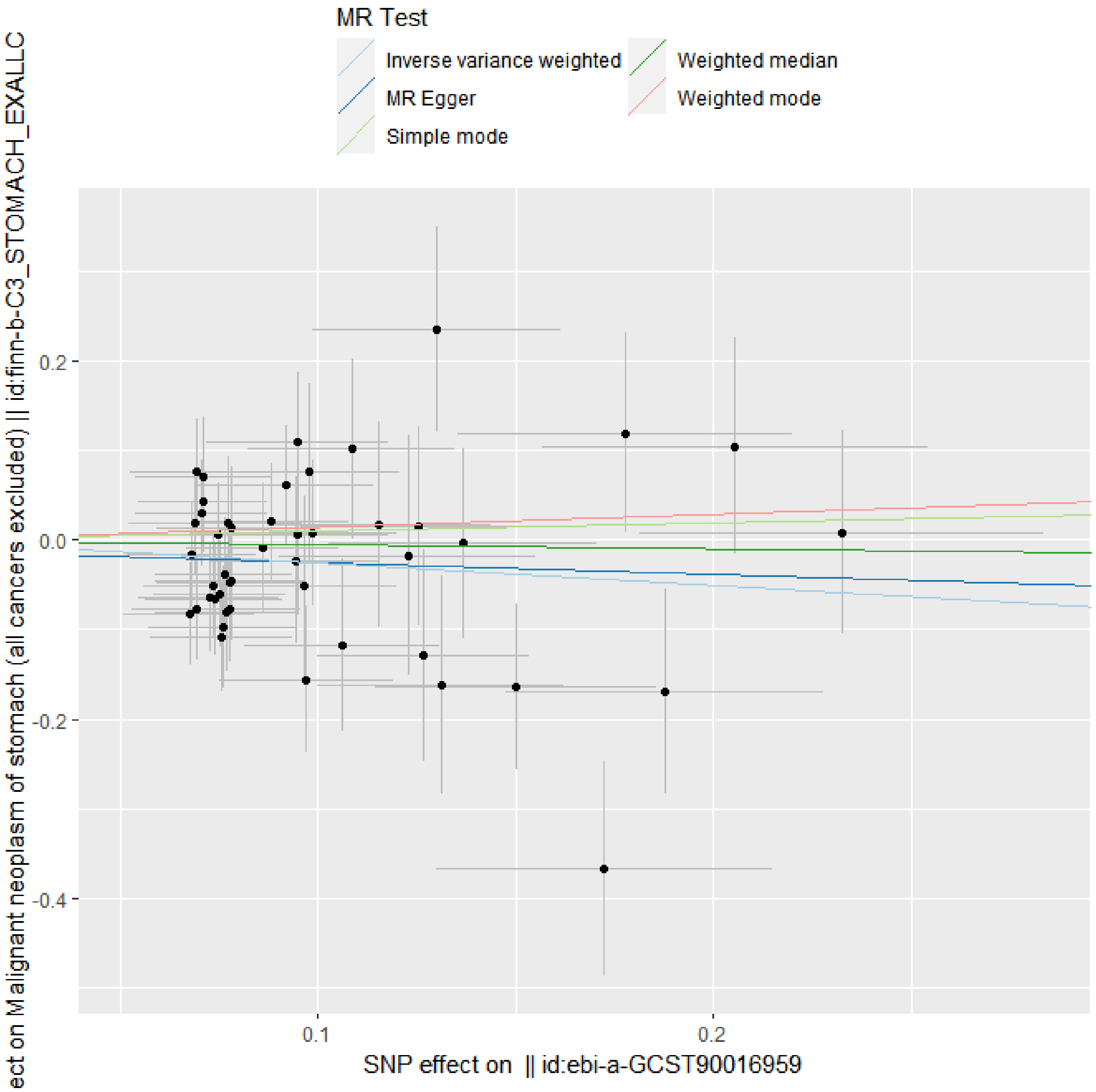
MR Test
Inverse variance weighted
MR Egger
Simple mode
Weighted median
Weighted mode
0.2
0.0
-0.2
-0.4
0.1
0.2
SNP effect on || id:ebi-a-GCST90016959
ect on Malignant neoplasm of stomach (all cancers excluded) || id:finn-b-C3_STOMACH_EXALLC

Figure 89 Leave-one-out analysis, funnel plot, MR effect size and scatter plot for gut microbiota abundance (genus Catenibacterium id.2153) on gastric cancer

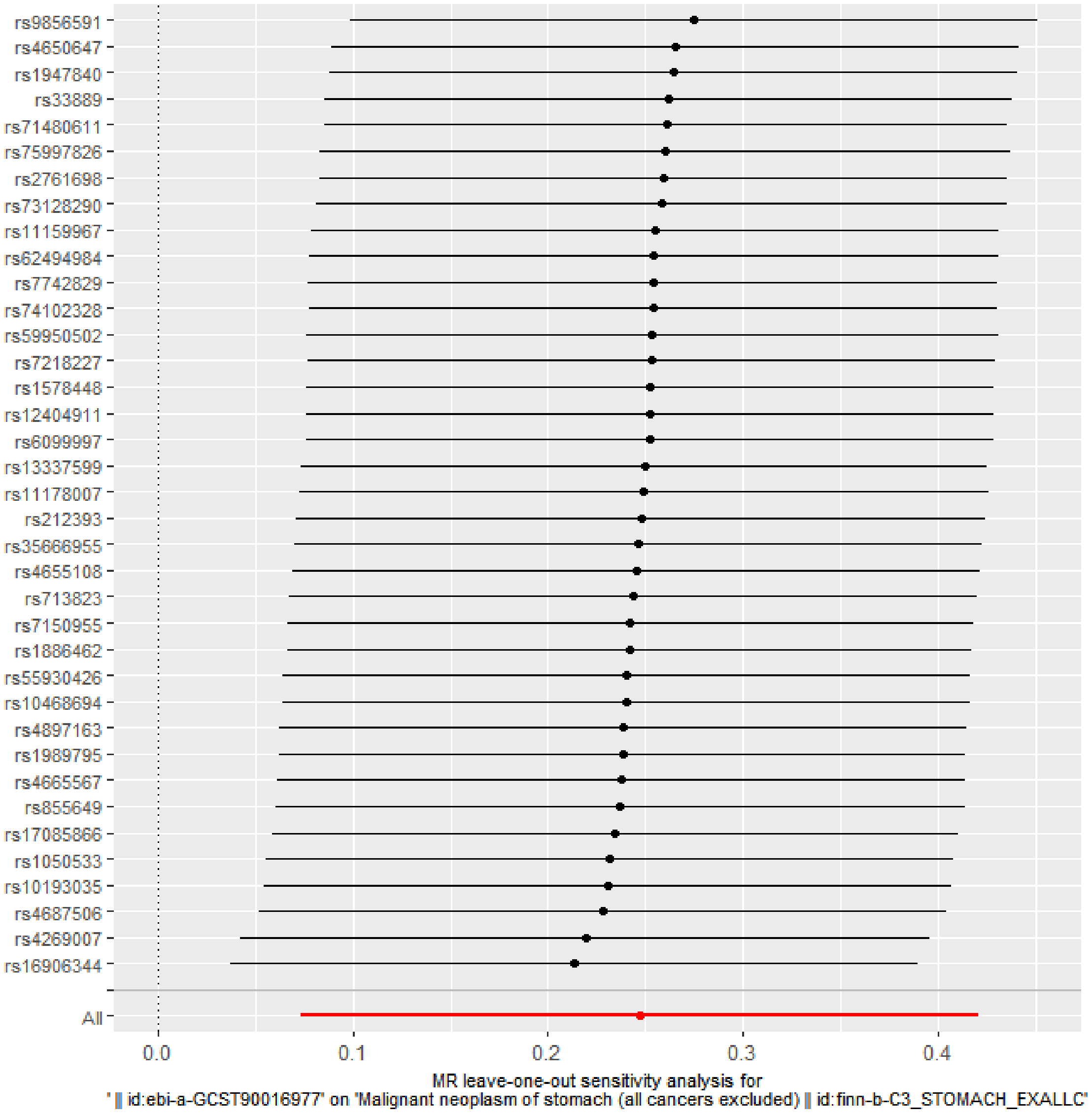

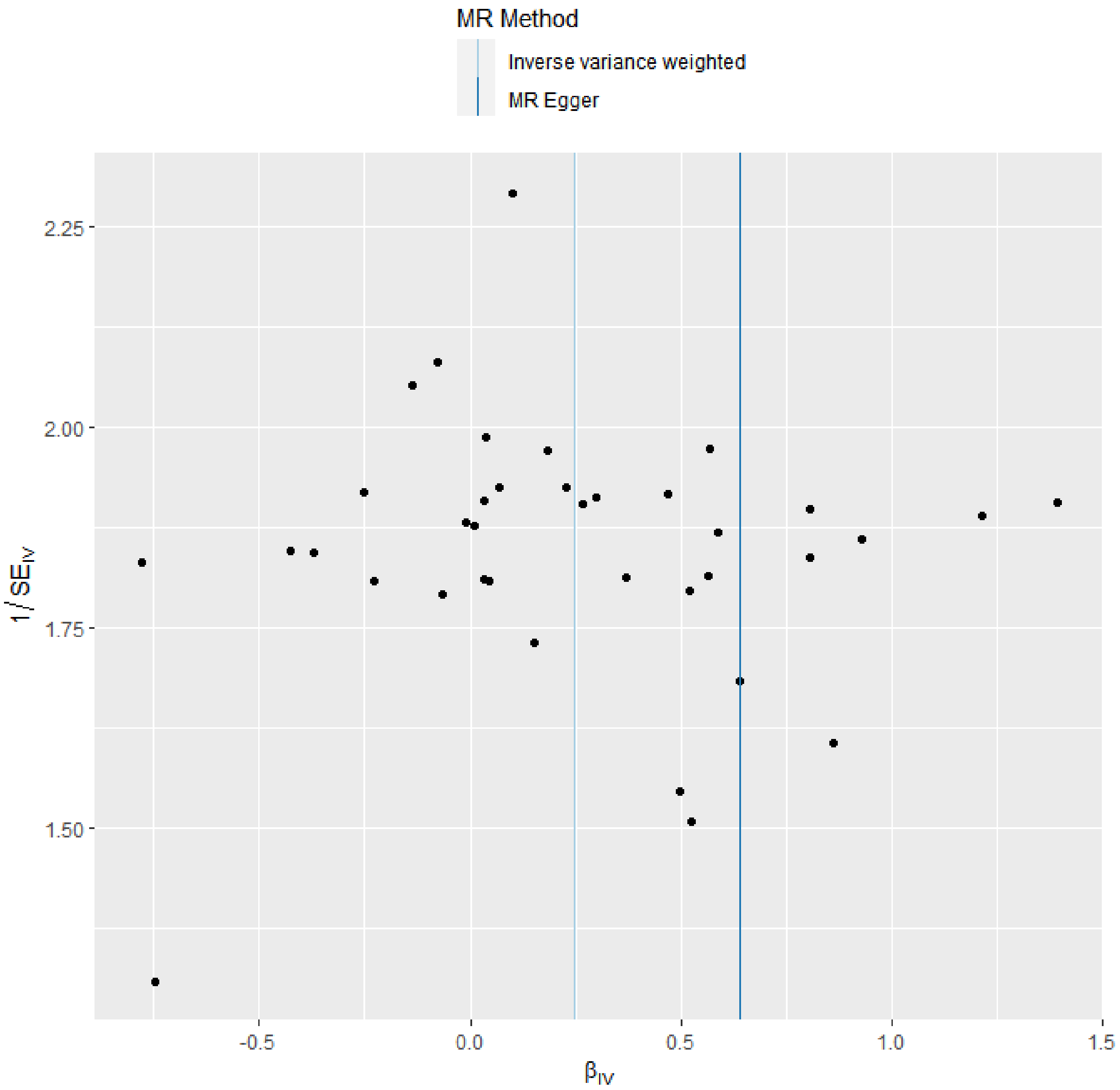

MR Method
Inverse variance weighted
MR Egger
2.25
2.00
1.75
1.50
1/SE_IV
-0.5
0.0
0.5
1.0
1.5
β_IV

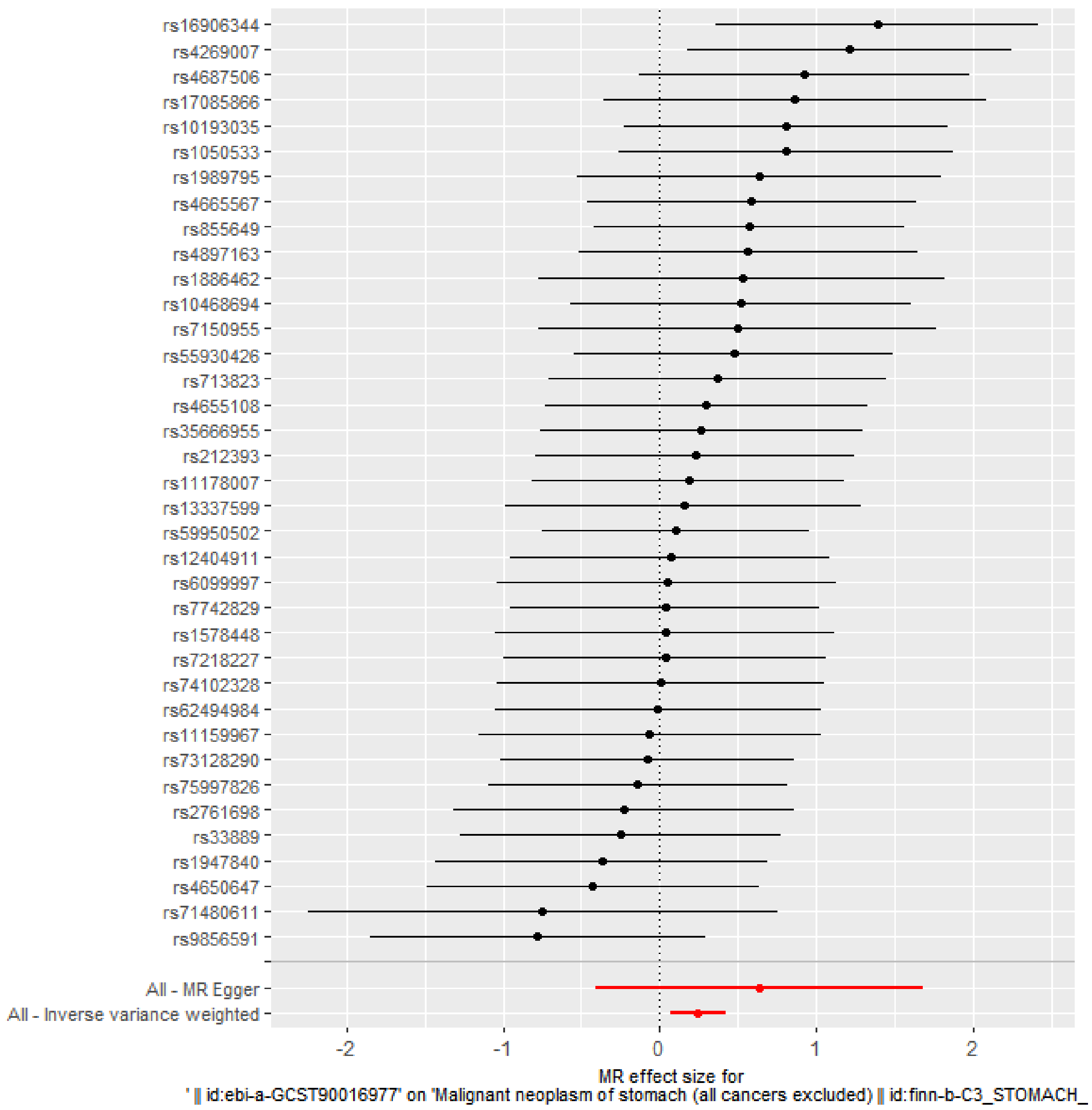
rs16906344
rs4269007
rs4687506
rs17085866
rs10193035
rs1050533
rs1989795
rs4665567
rs855649
rs4897163
rs1886462
rs10468694
rs7150955
rs55930426
rs713823
rs4655108
rs35666955
rs212393
rs11178007
rs13337599
rs59950502
rs12404911
rs6099997
rs7742829
rs1578448
rs7218227
rs74102328
rs62494984
rs11159967
rs73128290
rs75997826
rs2761698
rs33889
rs1947840
rs4650647
rs71480611
rs9856591
All - MR Egger
All - Inverse variance weighted
-2
-1
0
1
2
MR effect size for
' || id:ebi-a-GCST90016977' on 'Malignant neoplasm of stomach (all cancers excluded) || id:finn-b-C3_STOMACH_

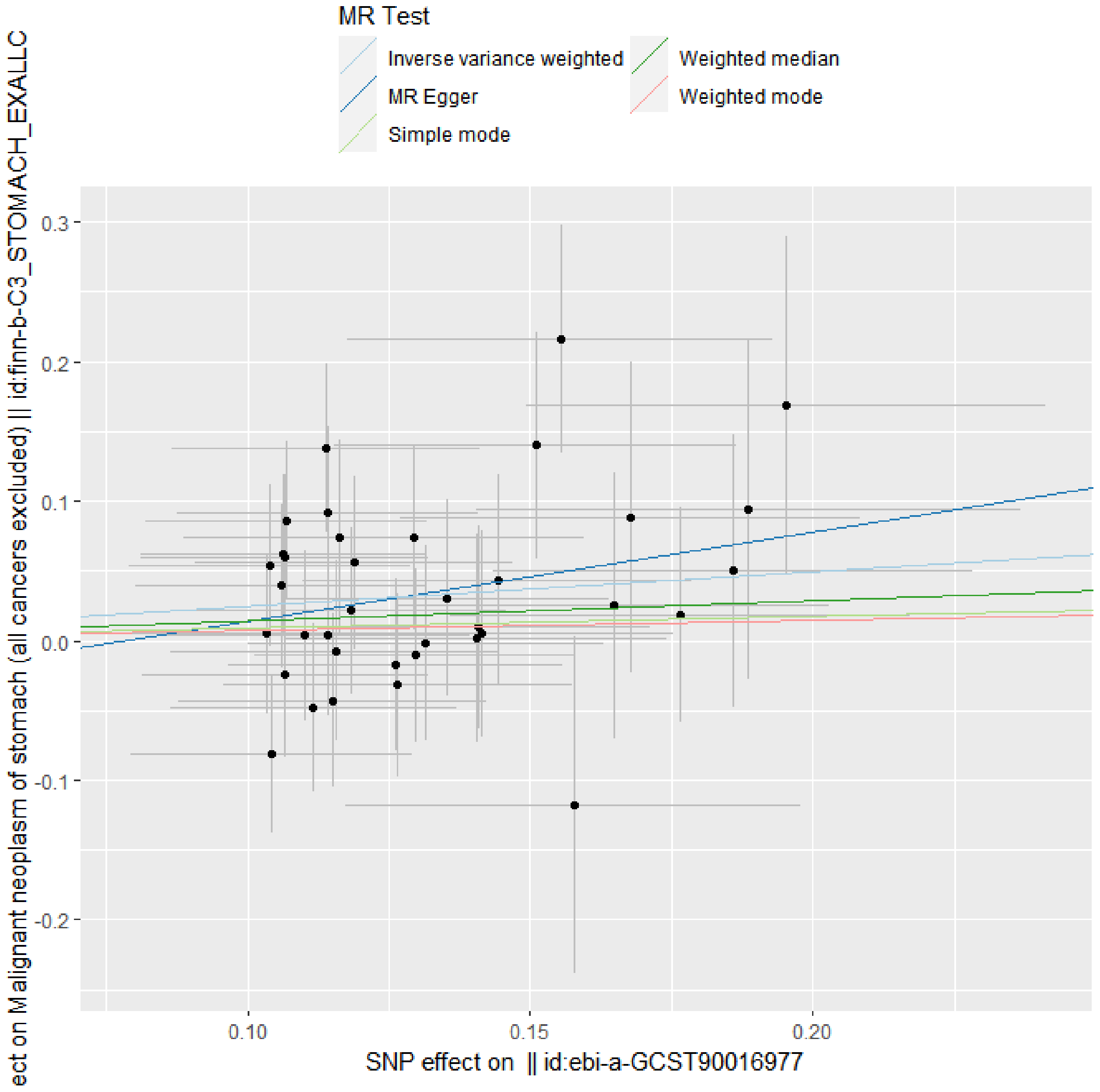
MR Test
Inverse variance weighted
MR Egger
Simple mode
Weighted median
Weighted mode
ect on Malignant neoplasm of stomach (all cancers excluded) || id:finn-b-C3_STOMACH_EXALLC
0.3
0.2
0.1
0.0
-0.1
-0.2
0.10
0.15
0.20
SNP effect on || id:ebi-a-GCST90016977

Figure 90 Leave-one-out analysis, funnel plot, MR effect size and scatter plot for gut microbiota abundance (genus Coprococcus3 id.11303) on gastric cancer

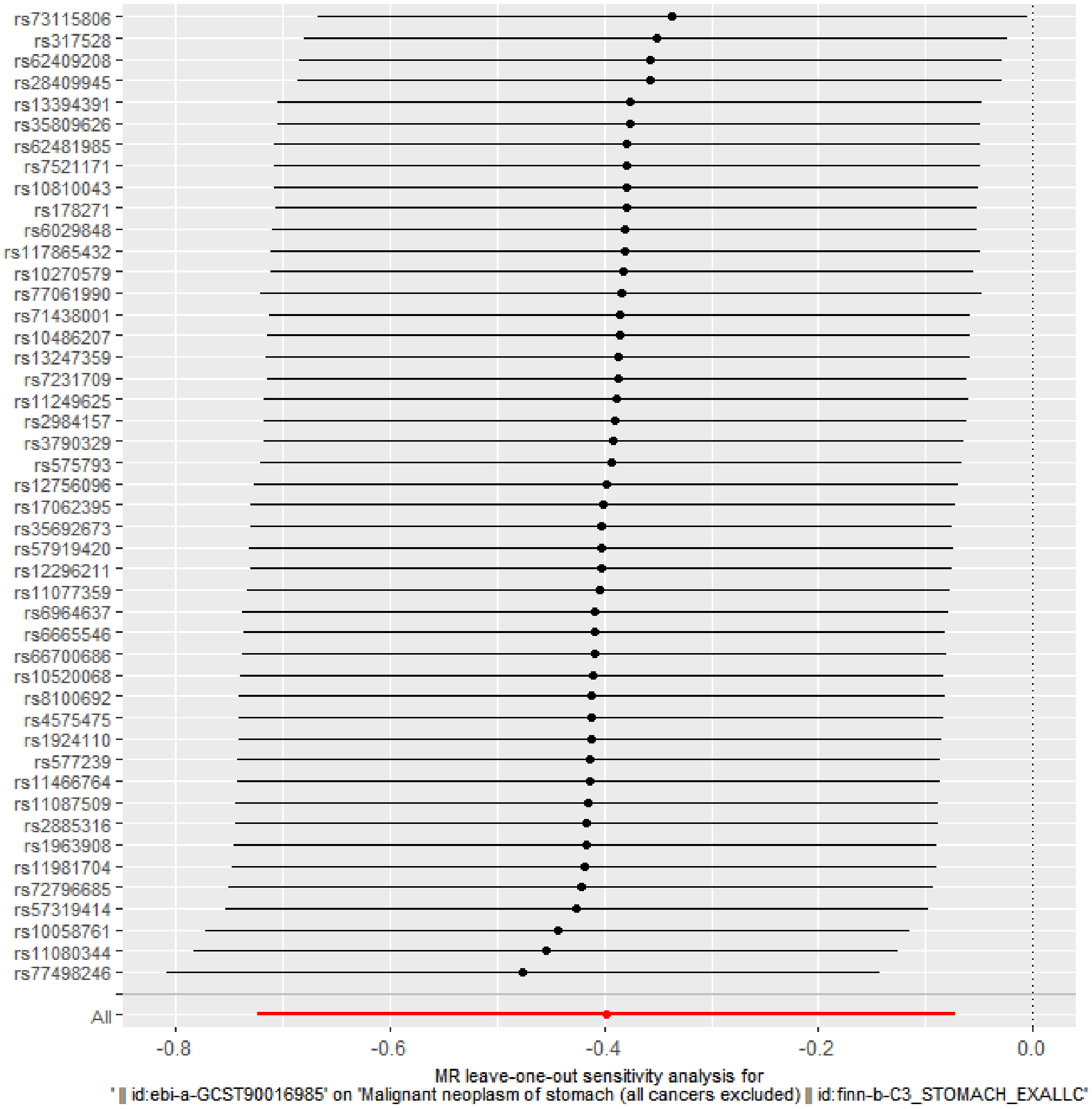

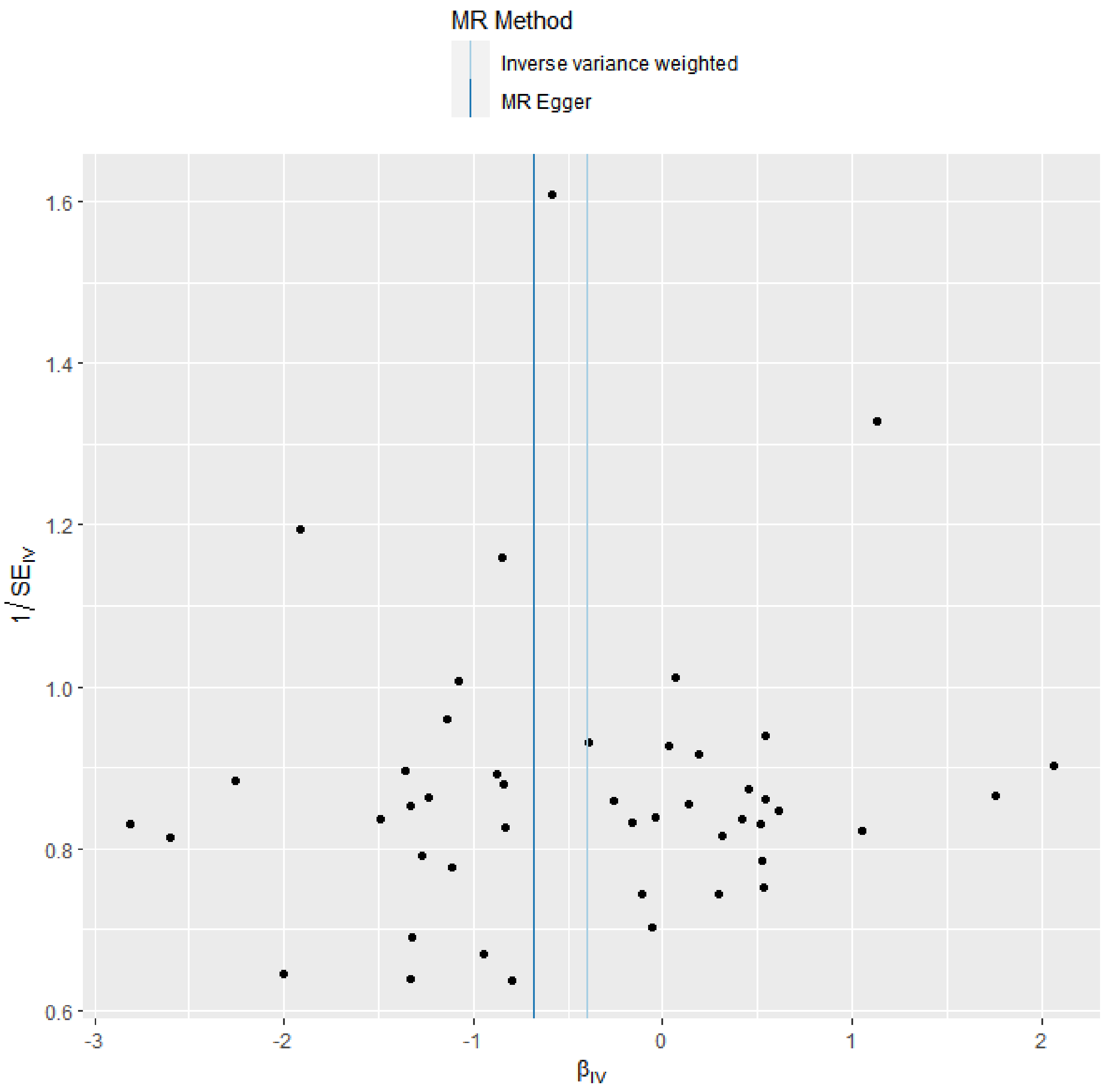
MR Method
Inverse variance weighted
MR Egger
1.6
1.4
1.2
1.0
0.8
0.6
-3
-2
-1
0
1
2
$1/SE_{IV}$
β_{IV}

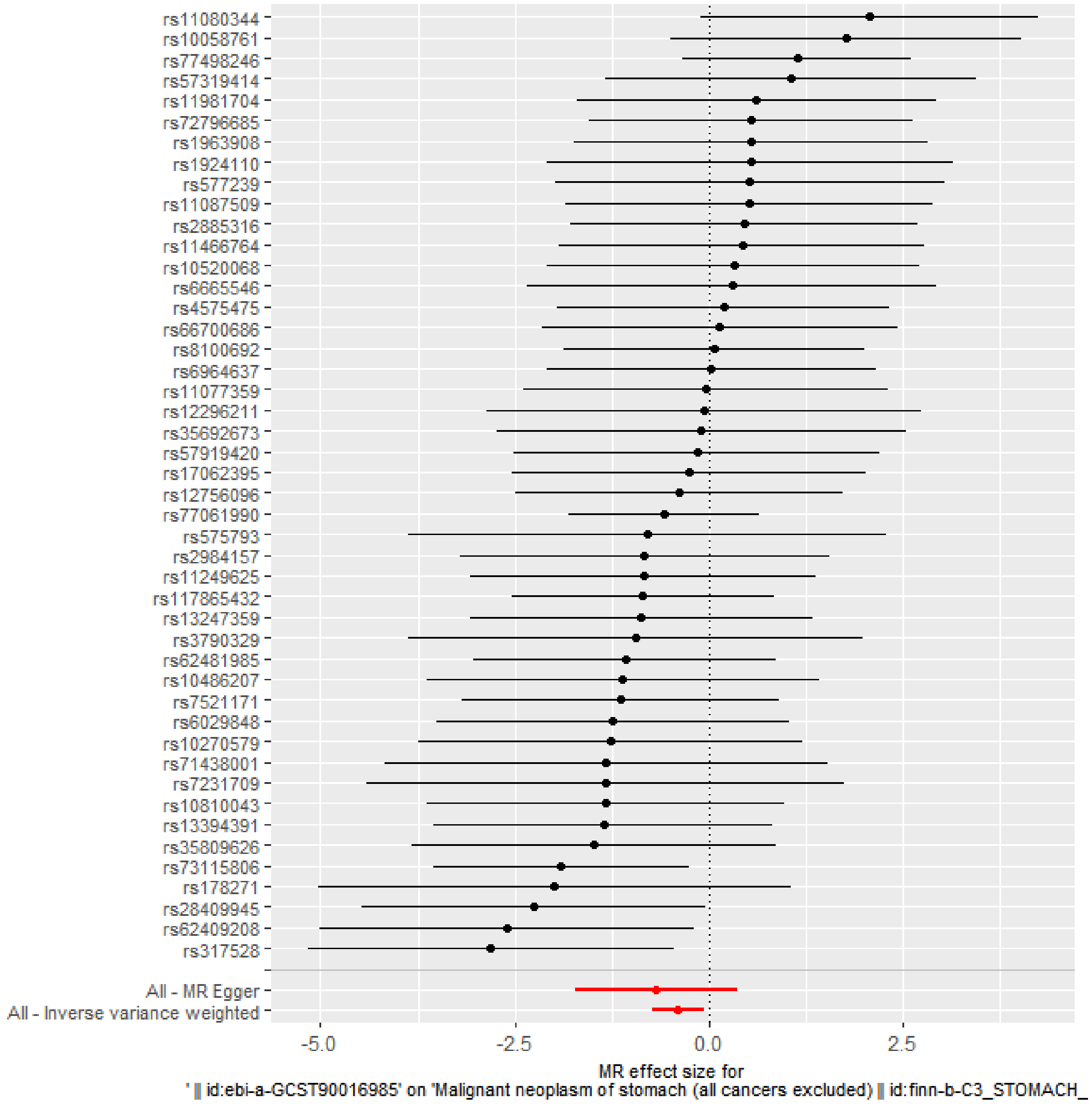
rs11080344
rs10058761
rs77498246
rs57319414
rs11981704
rs72796685
rs1963908
rs1924110
rs577239
rs11087509
rs2885316
rs11466764
rs10520068
rs6665546
rs4575475
rs66700686
rs8100692
rs6964637
rs11077359
rs12296211
rs35692673
rs57919420
rs17062395
rs12756096
rs77061990
rs575793
rs2984157
rs11249625
rs117865432
rs13247359
rs3790329
rs62481985
rs10486207
rs7521171
rs6029848
rs10270579
rs71438001
rs7231709
rs10810043
rs13394391
rs35809626
rs73115806
rs178271
rs28409945
rs62409208
rs317528
All - MR Egger
All - Inverse variance weighted
-5.0
-2.5
0.0
2.5
MR effect size for
' || id:ebi-a-GCST90016985' on 'Malignant neoplasm of stomach (all cancers excluded) || id:finn-b-C3_STOMACH_

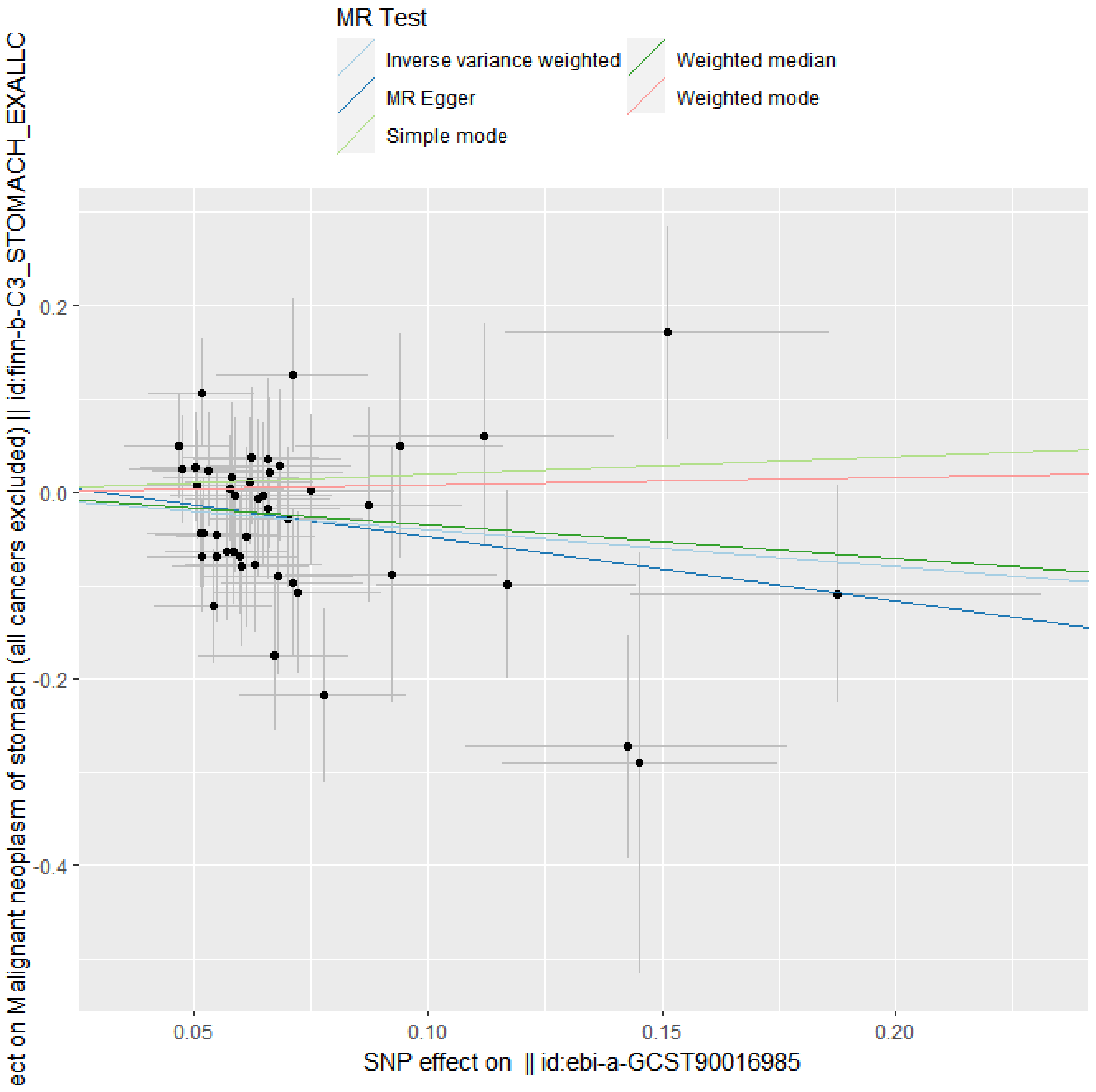
MR Test
Inverse variance weighted
MR Egger
Simple mode
Weighted median
Weighted mode
0.2
0.0
-0.2
-0.4
0.05
0.10
0.15
0.20
SNP effect on || id:ebi-a-GCST90016985
ect on Malignant neoplasm of stomach (all cancers excluded) || id:finn-b-C3_STOMACH_EXALLC

Figure 91 Leave-one-out analysis, funnel plot, MR effect size and scatter plot for gut microbiota abundance (genus Roseburia id.2012) on gastric cancer

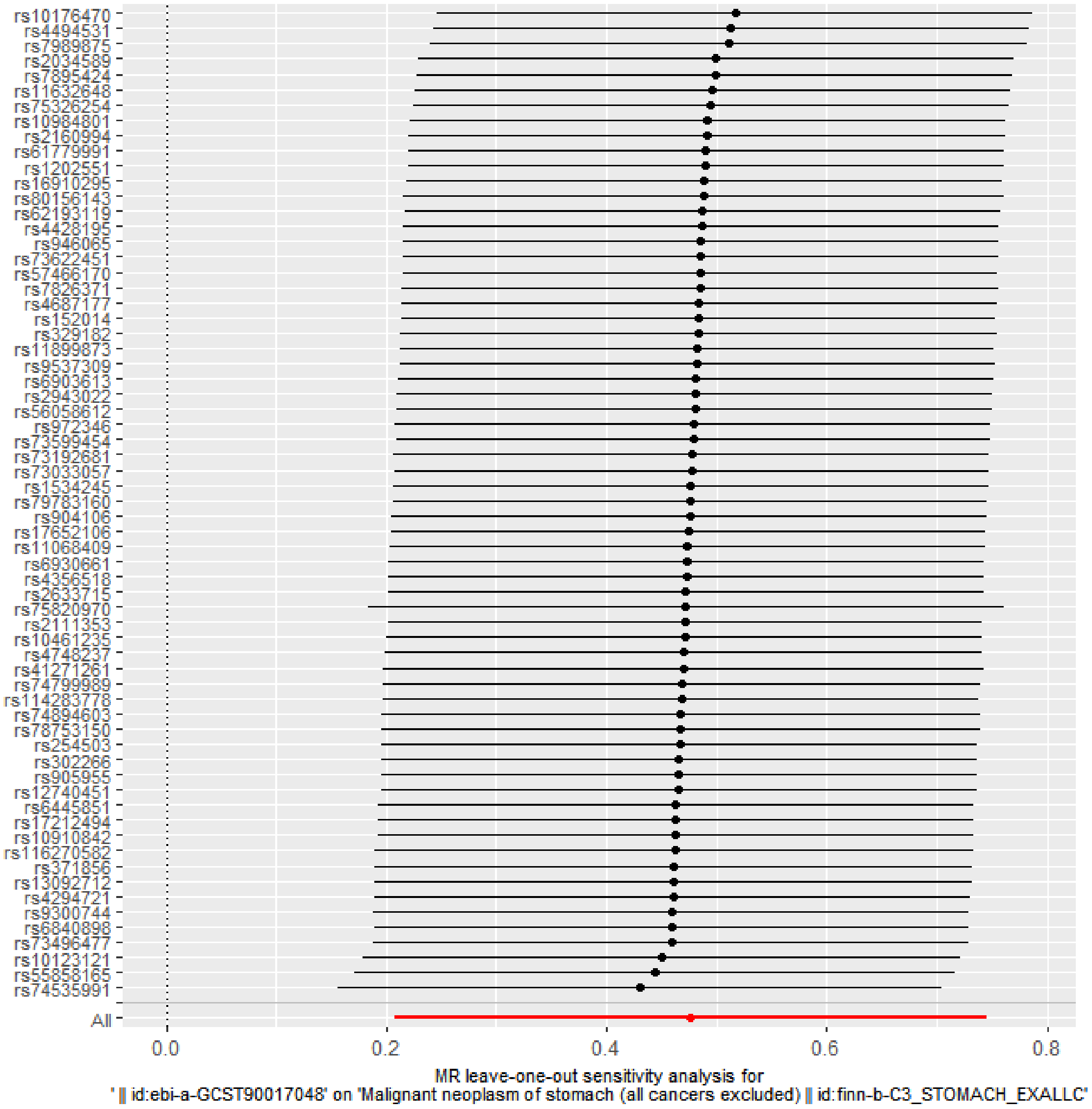

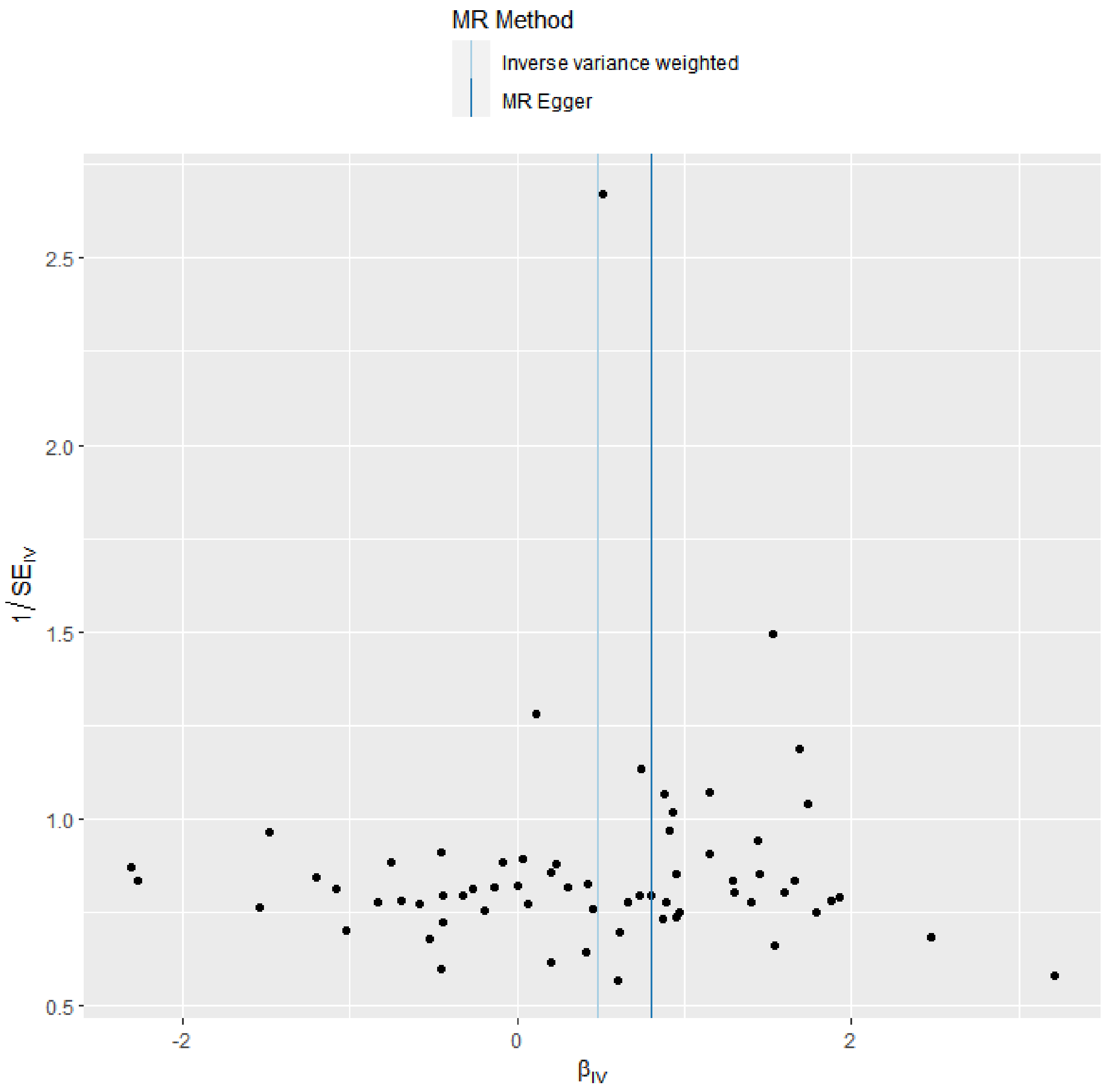
MR Method
Inverse variance weighted
MR Egger
2.5
2.0
1.5
1.0
0.5
-2
0
2
$1/SE_{IV}$
β_{IV}

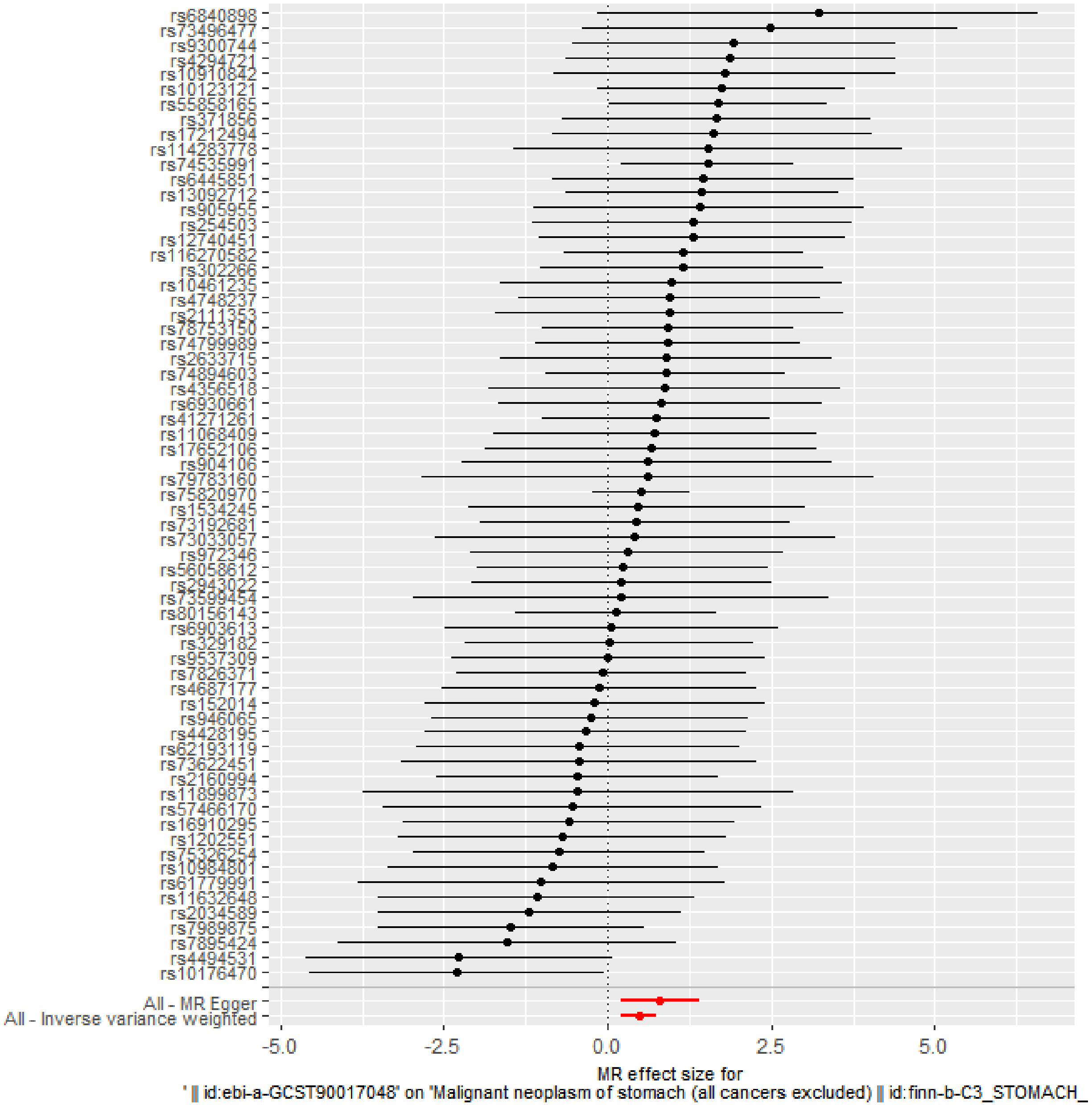
rs6840898
rs73496477
rs9300744
rs4294721
rs10910842
rs10123121
rs55858165
rs371856
rs17212494
rs114283778
rs74535991
rs6445851
rs13092712
rs905955
rs254503
rs12740451
rs116270582
rs302266
rs10461235
rs4748237
rs2111353
rs78753150
rs74799989
rs2633715
rs74894603
rs4356518
rs6930661
rs41271261
rs11068409
rs17652106
rs904106
rs79783160
rs75820970
rs1534245
rs73192681
rs73033057
rs972346
rs56058612
rs2943022
rs73599454
rs80156143
rs6903613
rs329182
rs9537309
rs7826371
rs4687177
rs152014
rs946065
rs4428195
rs62193119
rs73622451
rs2160994
rs11899873
rs57466170
rs16910295
rs1202551
rs75326254
rs10984801
rs61779991
rs11632648
rs2034589
rs7989875
rs7895424
rs4494531
rs10176470
All - MR Egger
All - Inverse variance weighted
-5.0
-2.5
0.0
2.5
5.0
MR effect size for
' || id:ebi-a-GCST90017048' on 'Malignant neoplasm of stomach (all cancers excluded) || id:finn-b-C3_STOMACH_

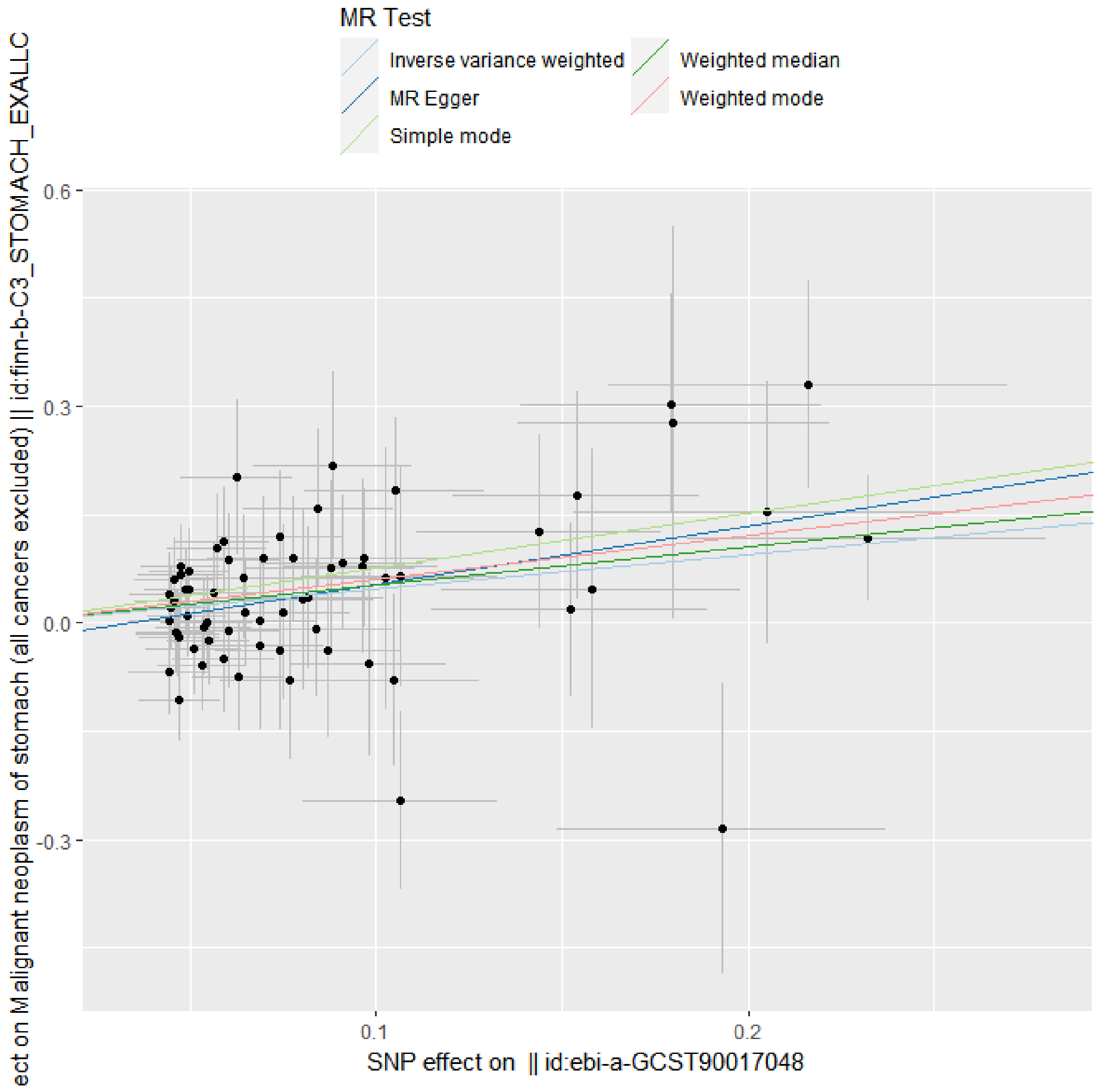

MR Test
Inverse variance weighted
MR Egger
Simple mode
Weighted median
Weighted mode
0.6
0.3
0.0
-0.3
0.1
0.2
SNP effect on || id:ebi-a-GCST90017048
ect on Malignant neoplasm of stomach (all cancers excluded) || id:finn-b-C3_STOMACH_EXALLC

Figure 92 Leave-one-out analysis, funnel plot, MR effect size and scatter plot for gut microbiota abundance (genus Ruminococcaceae UCG011 id.11368) on gastric cancer

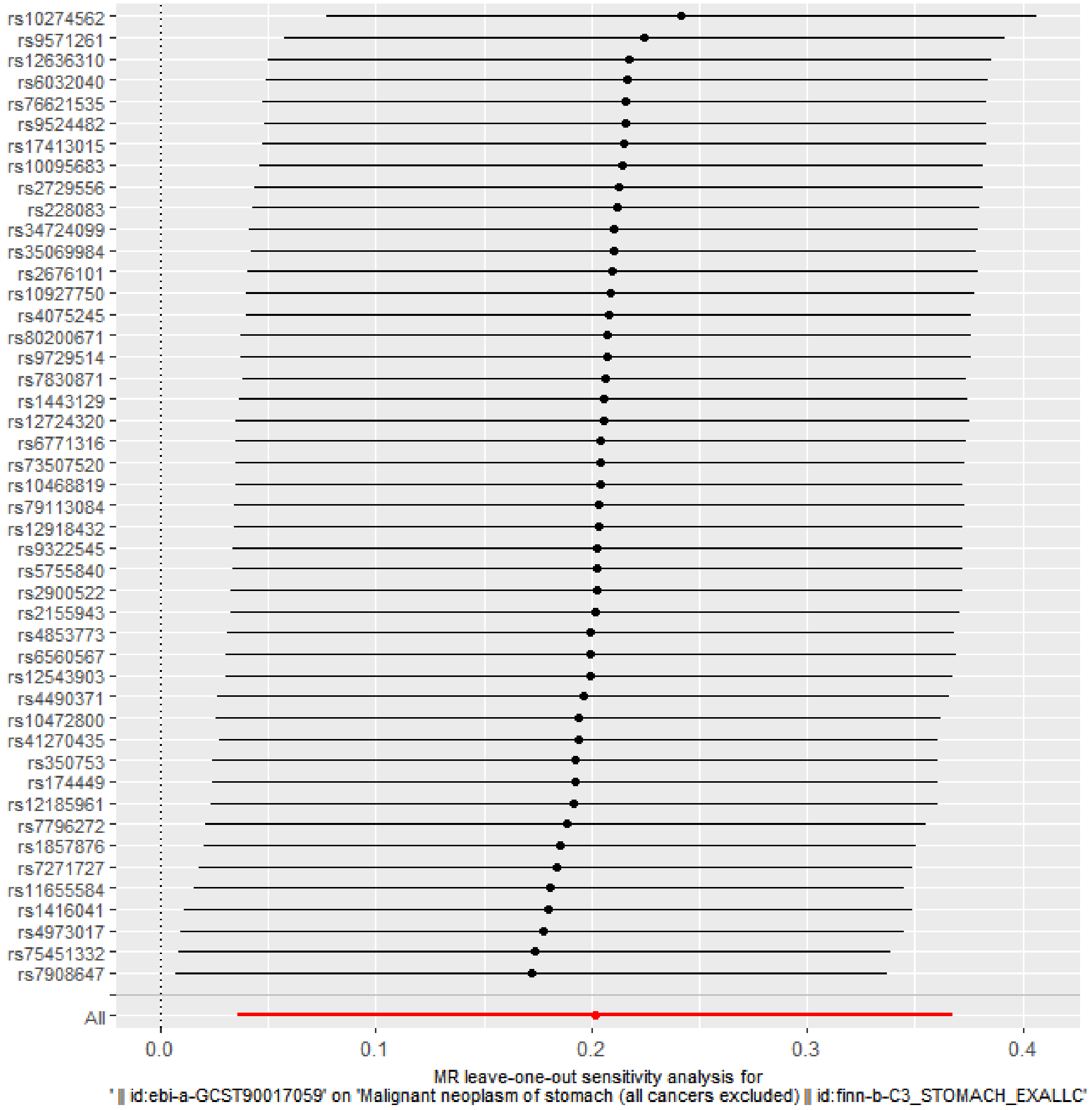

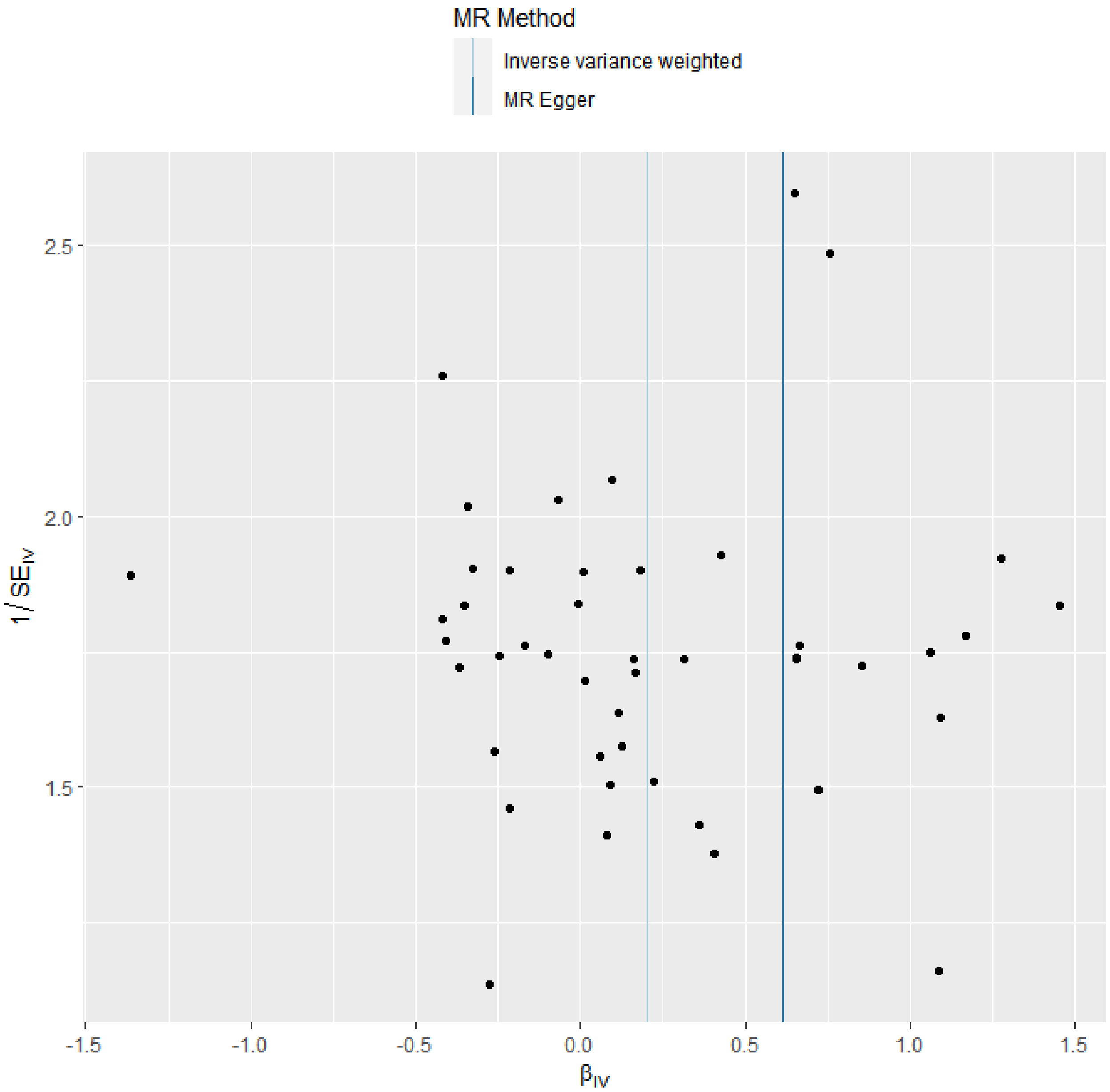

MR Method
Inverse variance weighted
MR Egger
2.5
2.0
1.5
1/SE_IV
-1.5
-1.0
-0.5
0.0
0.5
1.0
1.5
β_IV

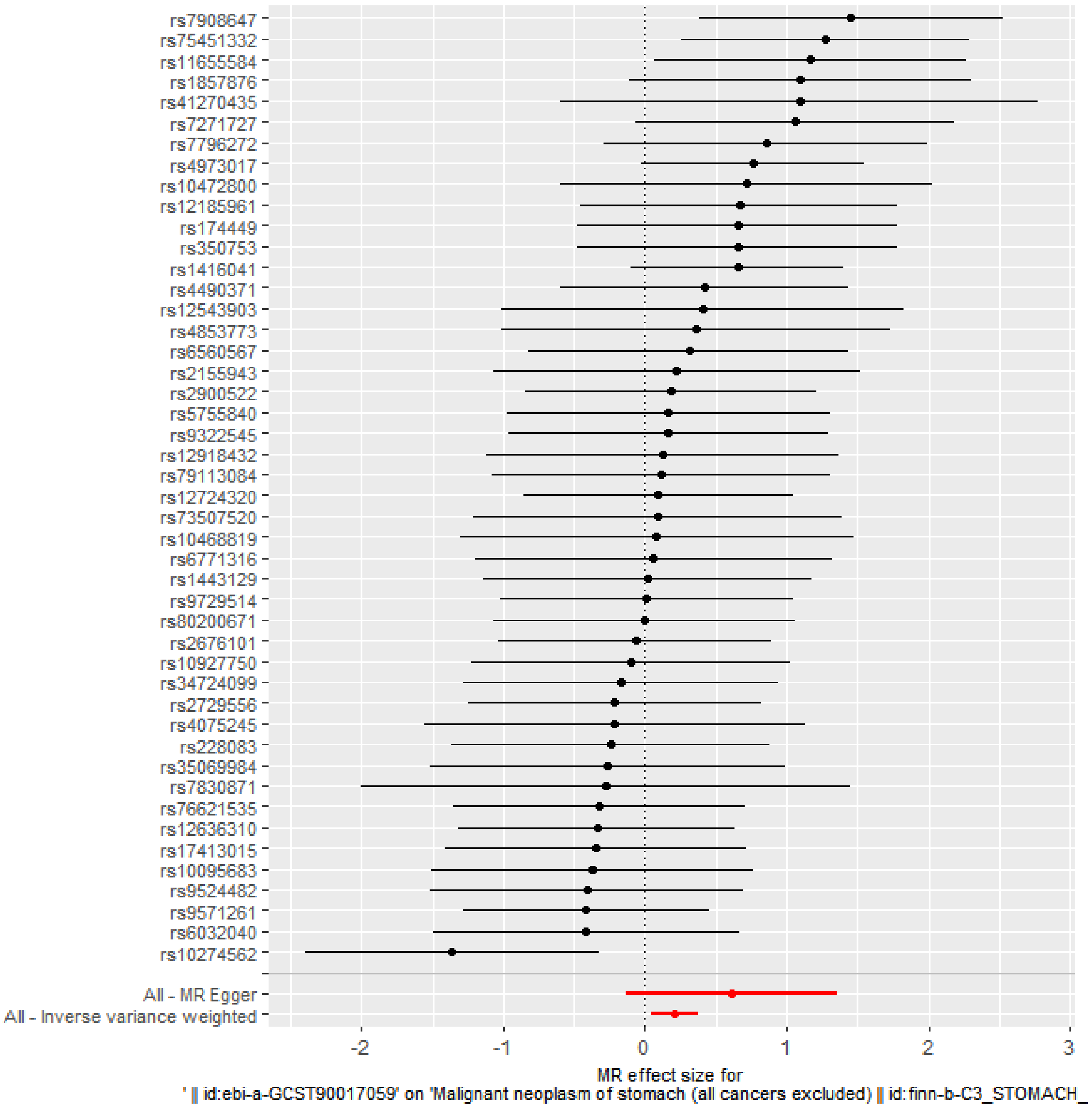

rs7908647
rs75451332
rs11655584
rs1857876
rs41270435
rs7271727
rs7796272
rs4973017
rs10472800
rs12185961
rs174449
rs350753
rs1416041
rs4490371
rs12543903
rs4853773
rs6560567
rs2155943
rs2900522
rs5755840
rs9322545
rs12918432
rs79113084
rs12724320
rs73507520
rs10468819
rs6771316
rs1443129
rs9729514
rs80200671
rs2676101
rs10927750
rs34724099
rs2729556
rs4075245
rs228083
rs35069984
rs7830871
rs76621535
rs12636310
rs17413015
rs10095683
rs9524482
rs9571261
rs6032040
rs10274562
All - MR Egger
All - Inverse variance weighted
-2
-1
0
1
2
3
MR effect size for
' || id:ebi-a-GCST90017059' on 'Malignant neoplasm of stomach (all cancers excluded) || id:finn-b-C3_STOMACH_

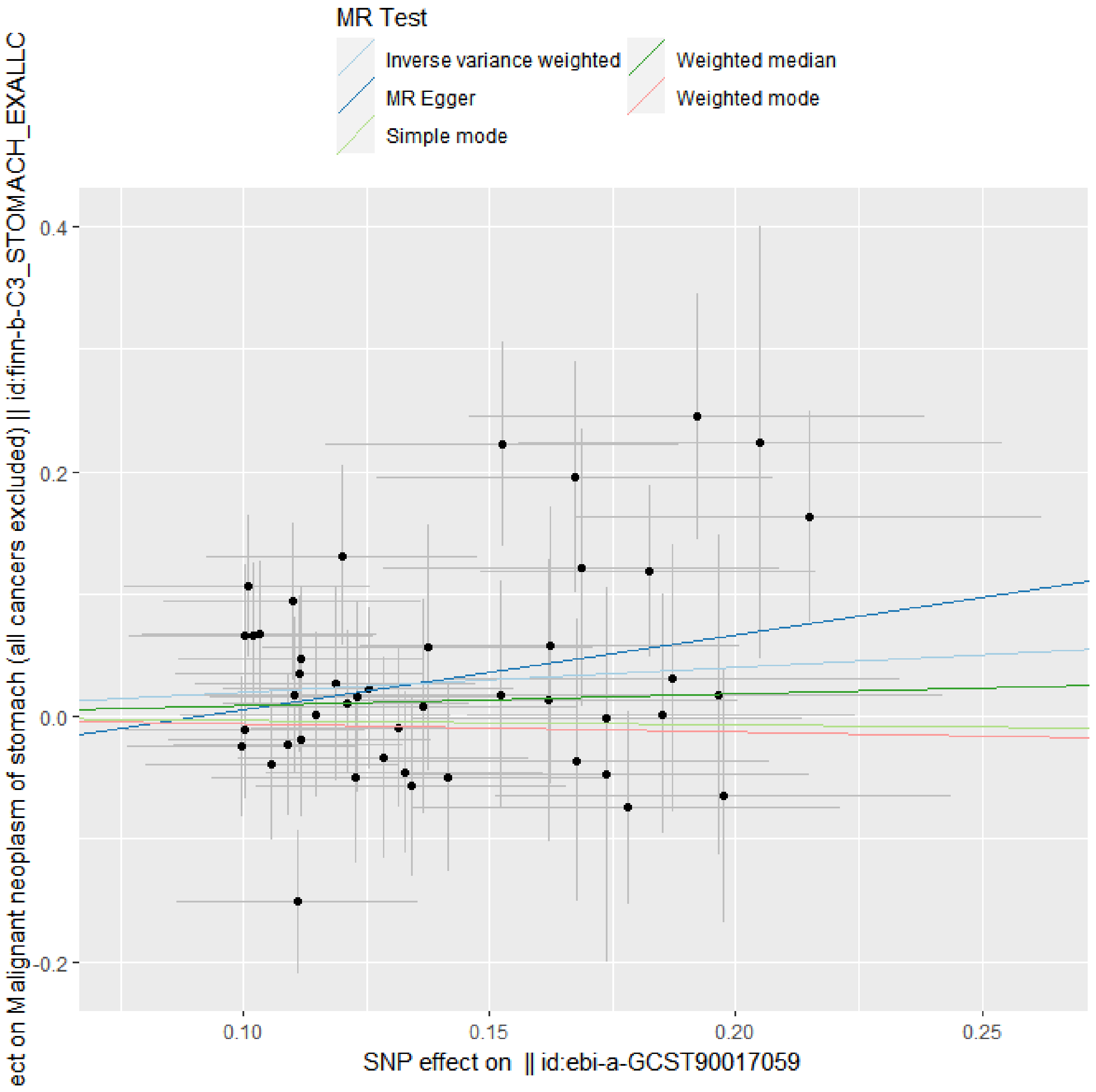

MR Test
Inverse variance weighted
MR Egger
Simple mode
Weighted median
Weighted mode
0.4
0.2
0.0
-0.2
0.10
0.15
0.20
0.25
SNP effect on || id:ebi-a-GCST90017059
ect on Malignant neoplasm of stomach (all cancers excluded) || id:finn-b-C3_STOMACH_EXALLC

Figure 93 Leave-one-out analysis, funnel plot, MR effect size and scatter plot for gut microbiota abundance (unknown genus id.1000005472) on gastric cancer

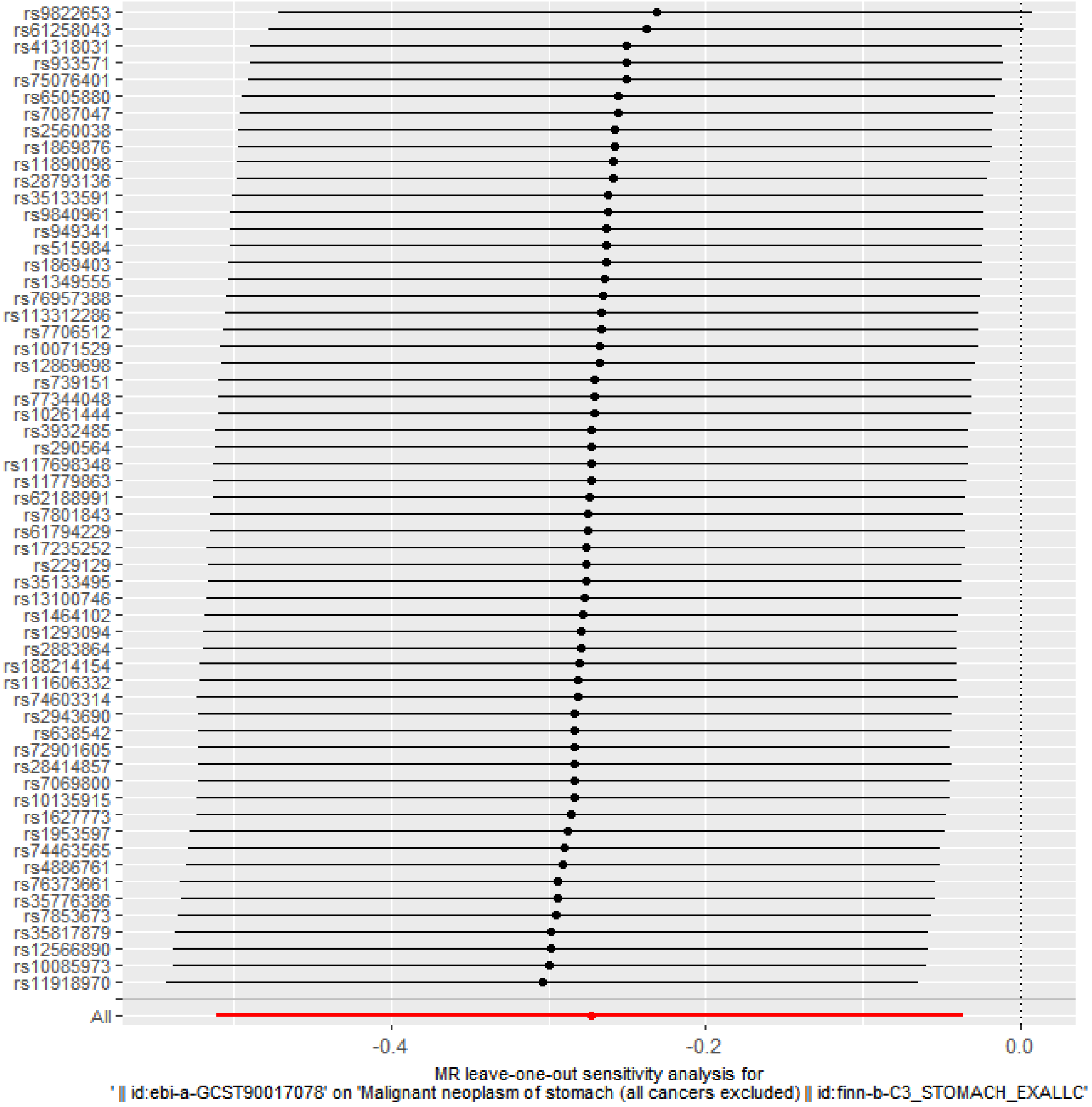

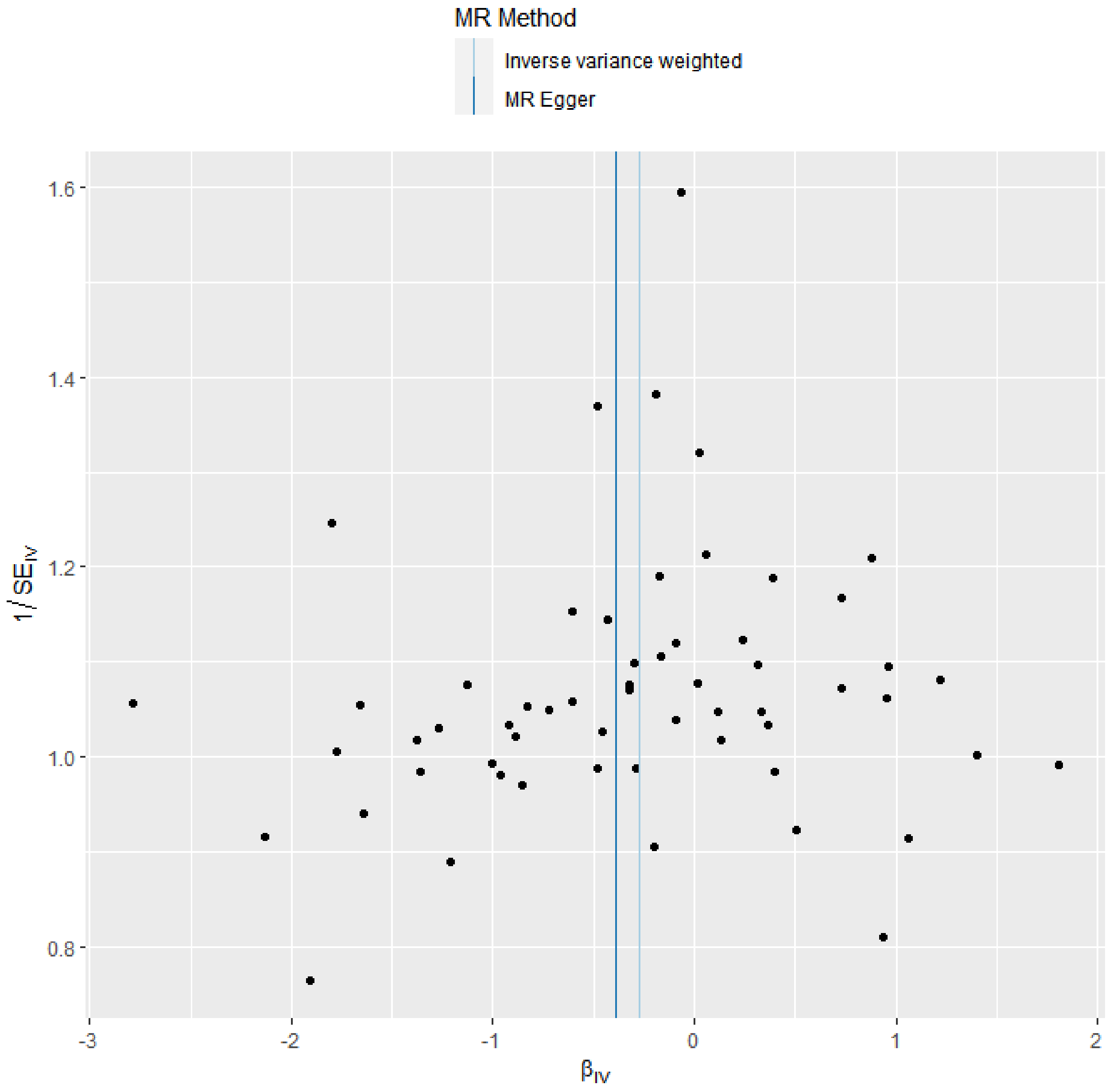
MR Method
Inverse variance weighted
MR Egger
1.6
1.4
1.2
1.0
0.8
-3
-2
-1
0
1
2
β_{IV}
$1/SE_{IV}$

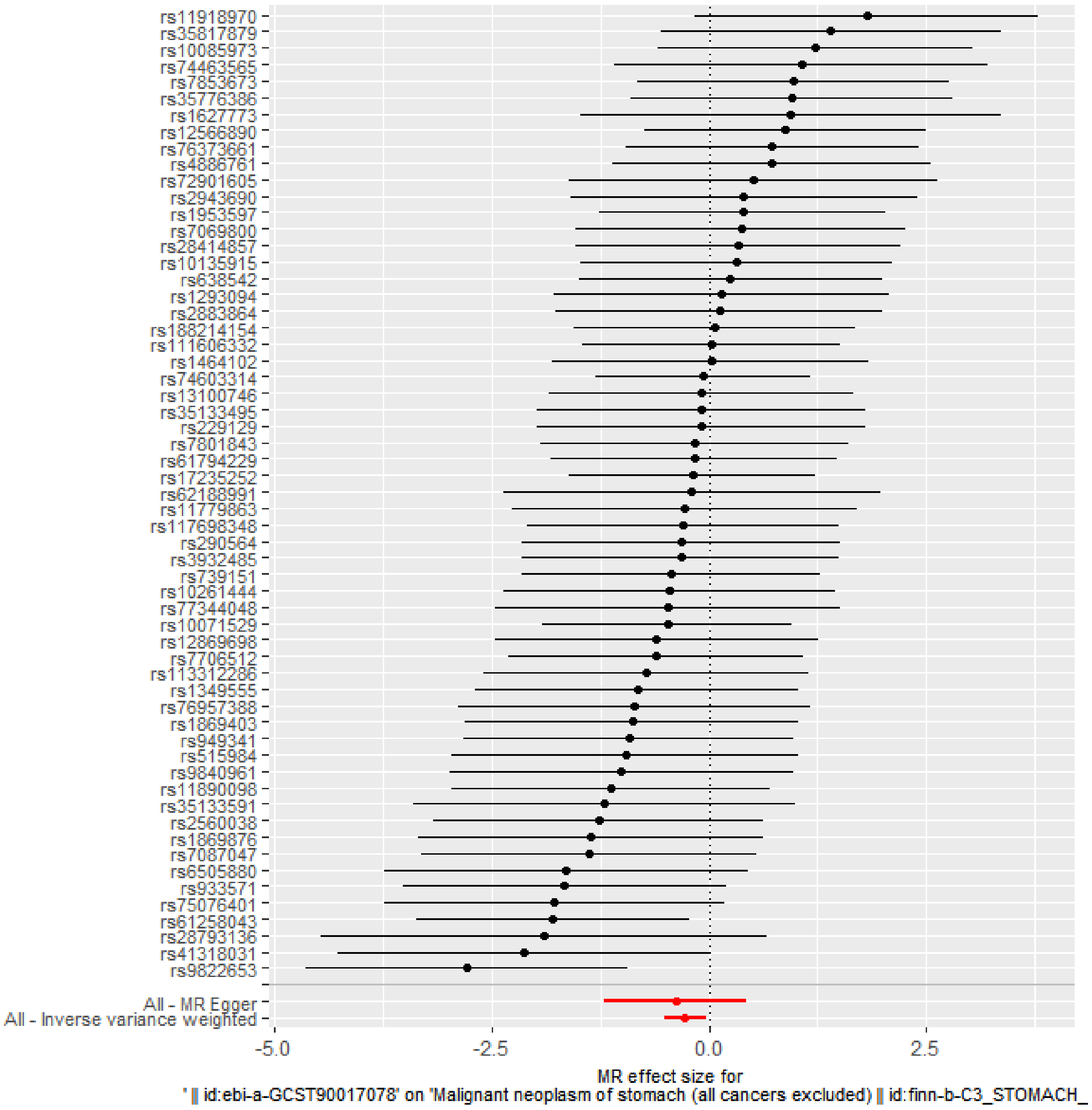

rs11918970
rs35817879
rs10085973
rs74463565
rs7853673
rs35776386
rs1627773
rs12566890
rs76373661
rs4886761
rs72901605
rs2943690
rs1953597
rs7069800
rs28414857
rs10135915
rs638542
rs1293094
rs2883864
rs188214154
rs111606332
rs1464102
rs74603314
rs13100746
rs35133495
rs229129
rs7801843
rs61794229
rs17235252
rs62188991
rs11779863
rs117698348
rs290564
rs3932485
rs739151
rs10261444
rs77344048
rs10071529
rs12869698
rs7706512
rs113312286
rs1349555
rs76957388
rs1869403
rs949341
rs515984
rs9840961
rs11890098
rs35133591
rs2560038
rs1869876
rs7087047
rs6505880
rs933571
rs75076401
rs61258043
rs28793136
rs41318031
rs9822653
All - MR Egger
All - Inverse variance weighted
-5.0
-2.5
0.0
2.5
MR effect size for
' || id:ebi-a-GCST90017078' on 'Malignant neoplasm of stomach (all cancers excluded) || id:finn-b-C3_STOMACH_

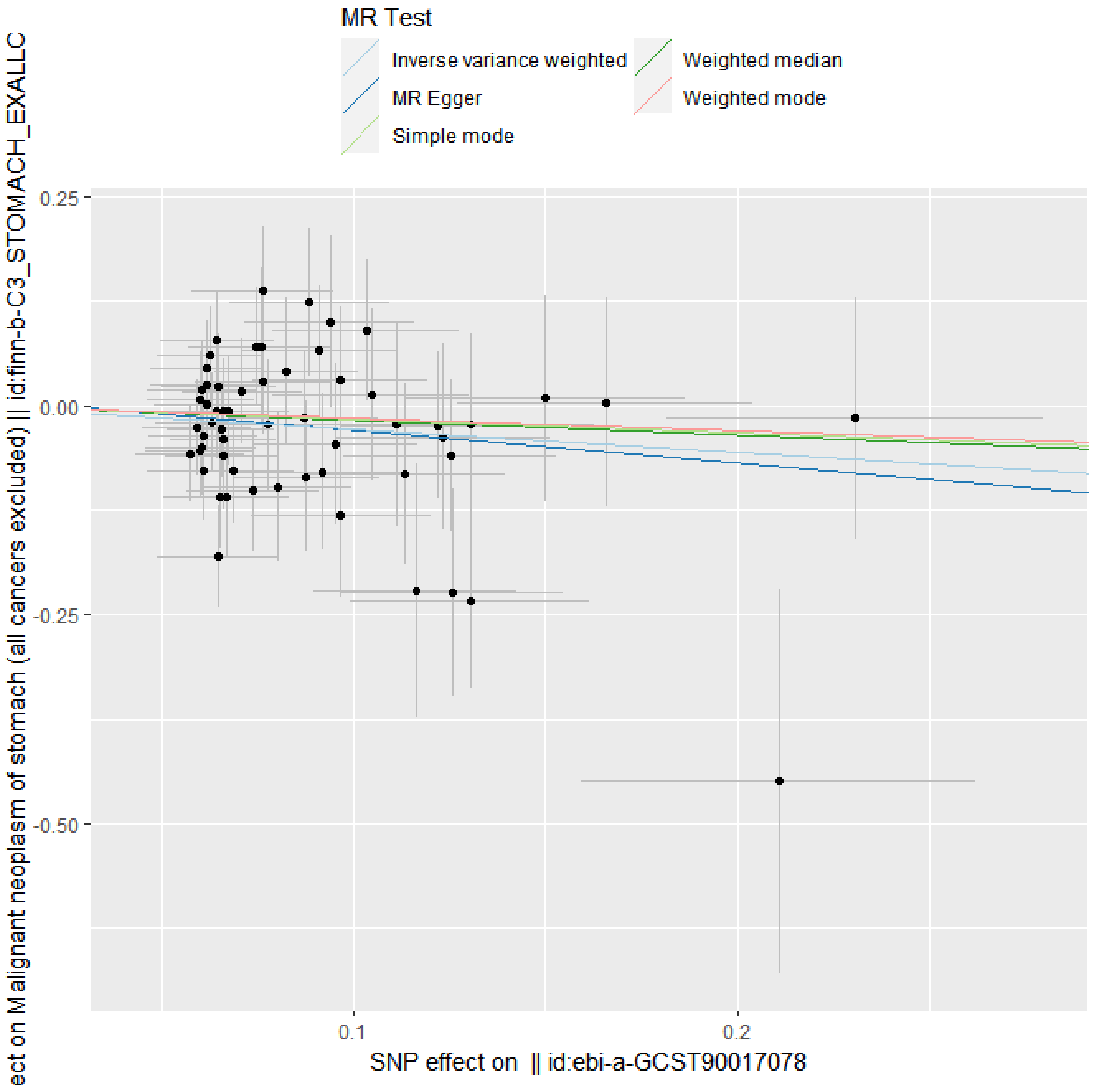

MR Test
Inverse variance weighted
MR Egger
Simple mode
Weighted median
Weighted mode
0.25
0.00
-0.25
-0.50
0.1
0.2
SNP effect on || id:ebi-a-GCST90017078
ect on Malignant neoplasm of stomach (all cancers excluded) || id:finn-b-C3_STOMACH_EXALLC

Figure 94 Leave-one-out analysis, funnel plot, MR effect size and scatter plot for gut microbiota abundance (unknown genus id.2755) on gastric cancer

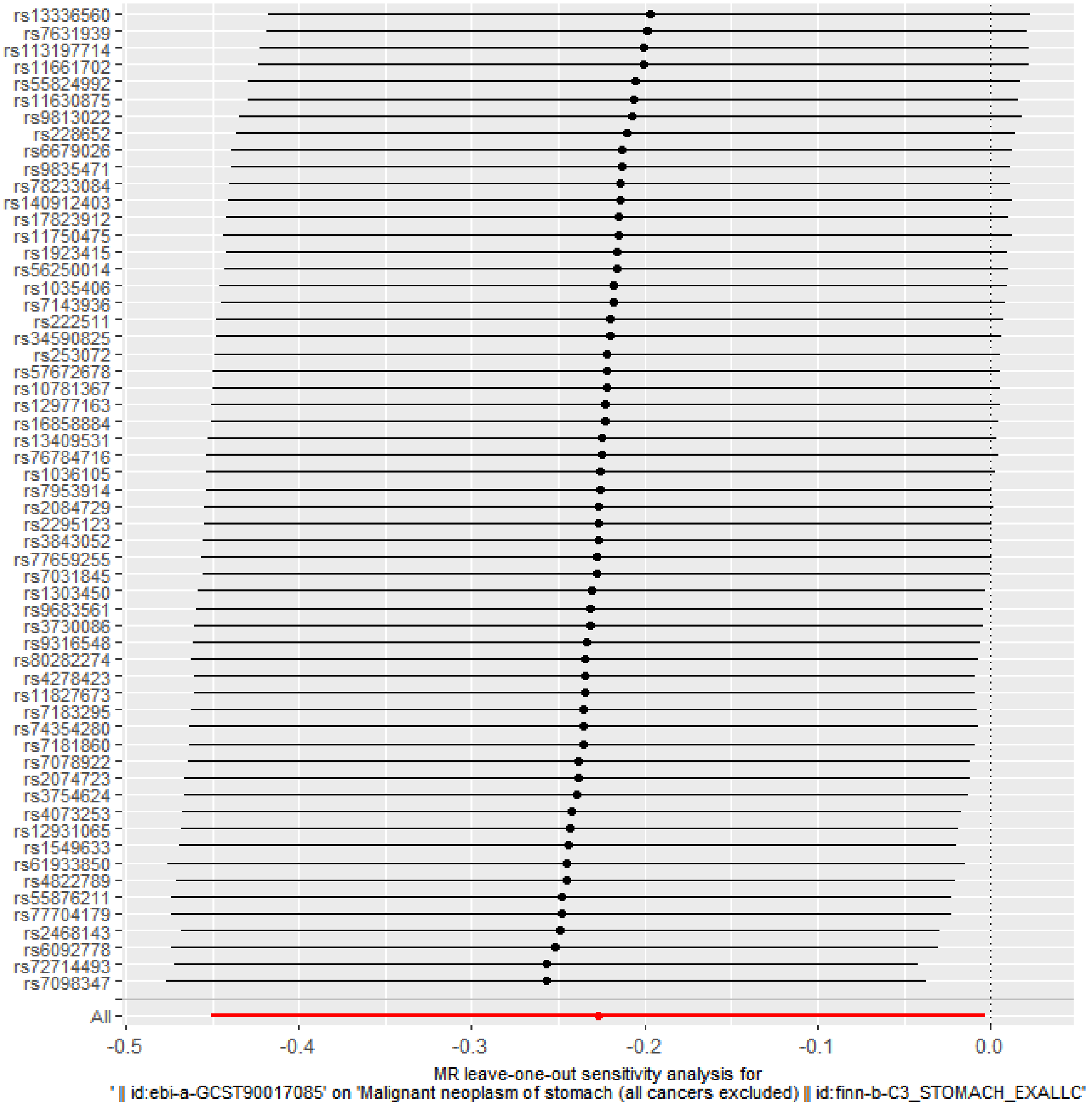

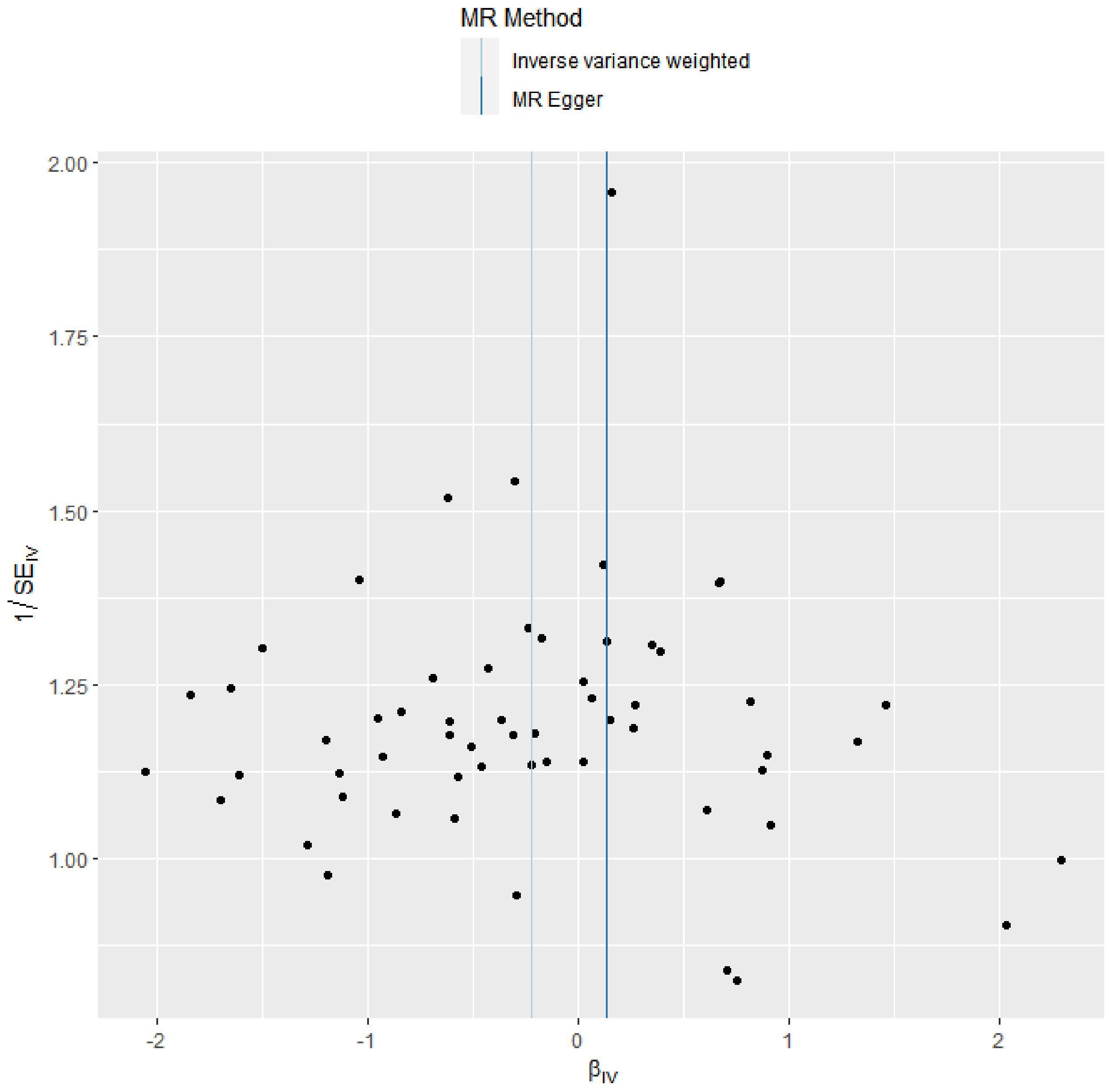
MR Method
Inverse variance weighted
MR Egger
2.00
1.75
1.50
1.25
1.00
1/SE_IV
-2
-1
0
1
2
β_IV

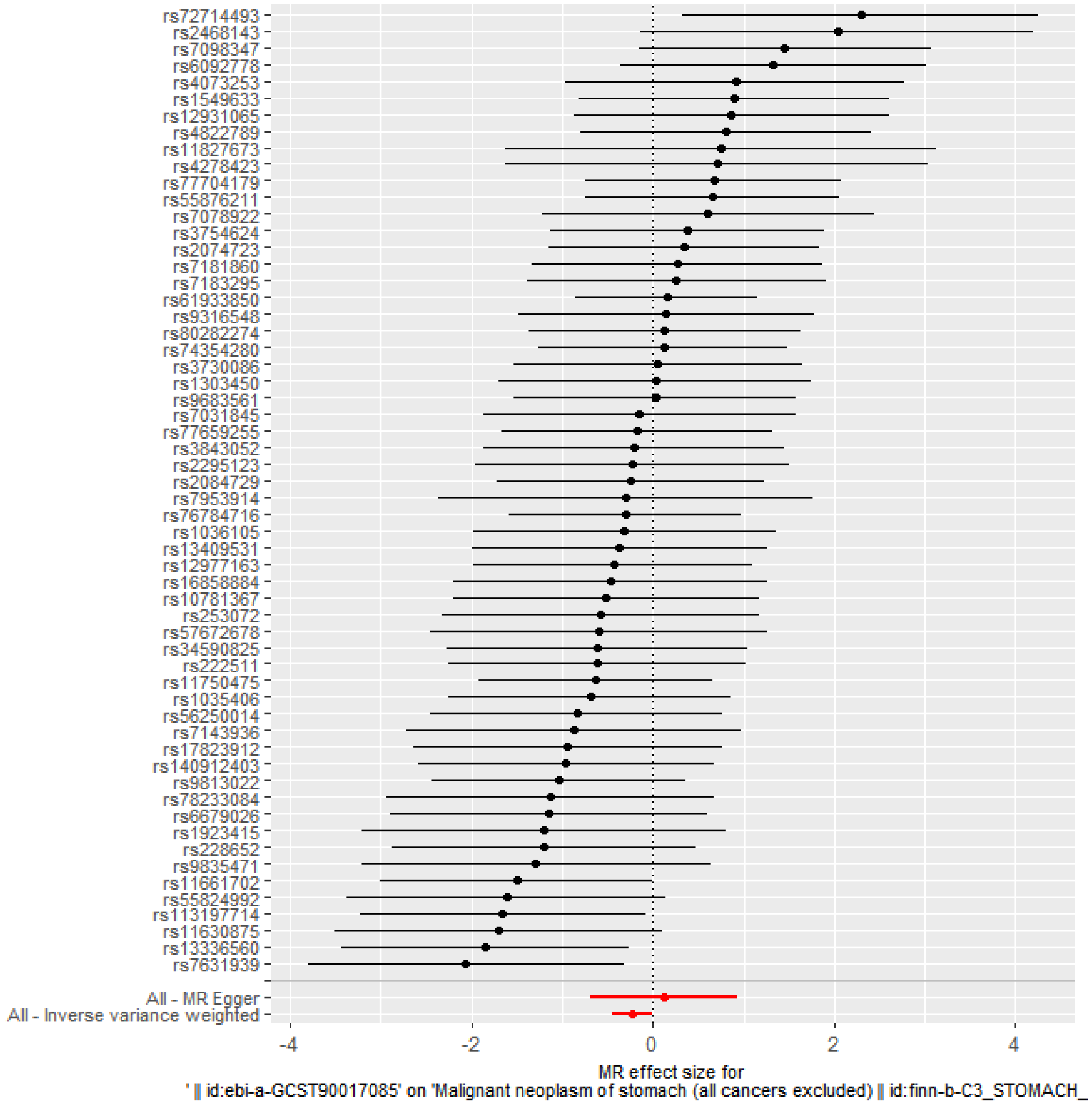
rs72714493
rs2468143
rs7098347
rs6092778
rs4073253
rs1549633
rs12931065
rs4822789
rs11827673
rs4278423
rs77704179
rs55876211
rs7078922
rs3754624
rs2074723
rs7181860
rs7183295
rs61933850
rs9316548
rs80282274
rs74354280
rs3730086
rs1303450
rs9683561
rs7031845
rs77659255
rs3843052
rs2295123
rs2084729
rs7953914
rs76784716
rs1036105
rs13409531
rs12977163
rs16858884
rs10781367
rs253072
rs57672678
rs34590825
rs222511
rs11750475
rs1035406
rs56250014
rs7143936
rs17823912
rs140912403
rs9813022
rs78233084
rs6679026
rs1923415
rs228652
rs9835471
rs11661702
rs55824992
rs113197714
rs11630875
rs13336560
rs7631939
All - MR Egger
All - Inverse variance weighted
-4
-2
0
2
4
MR effect size for
' || id:ebi-a-GCST90017085' on 'Malignant neoplasm of stomach (all cancers excluded) || id:finn-b-C3_STOMACH_

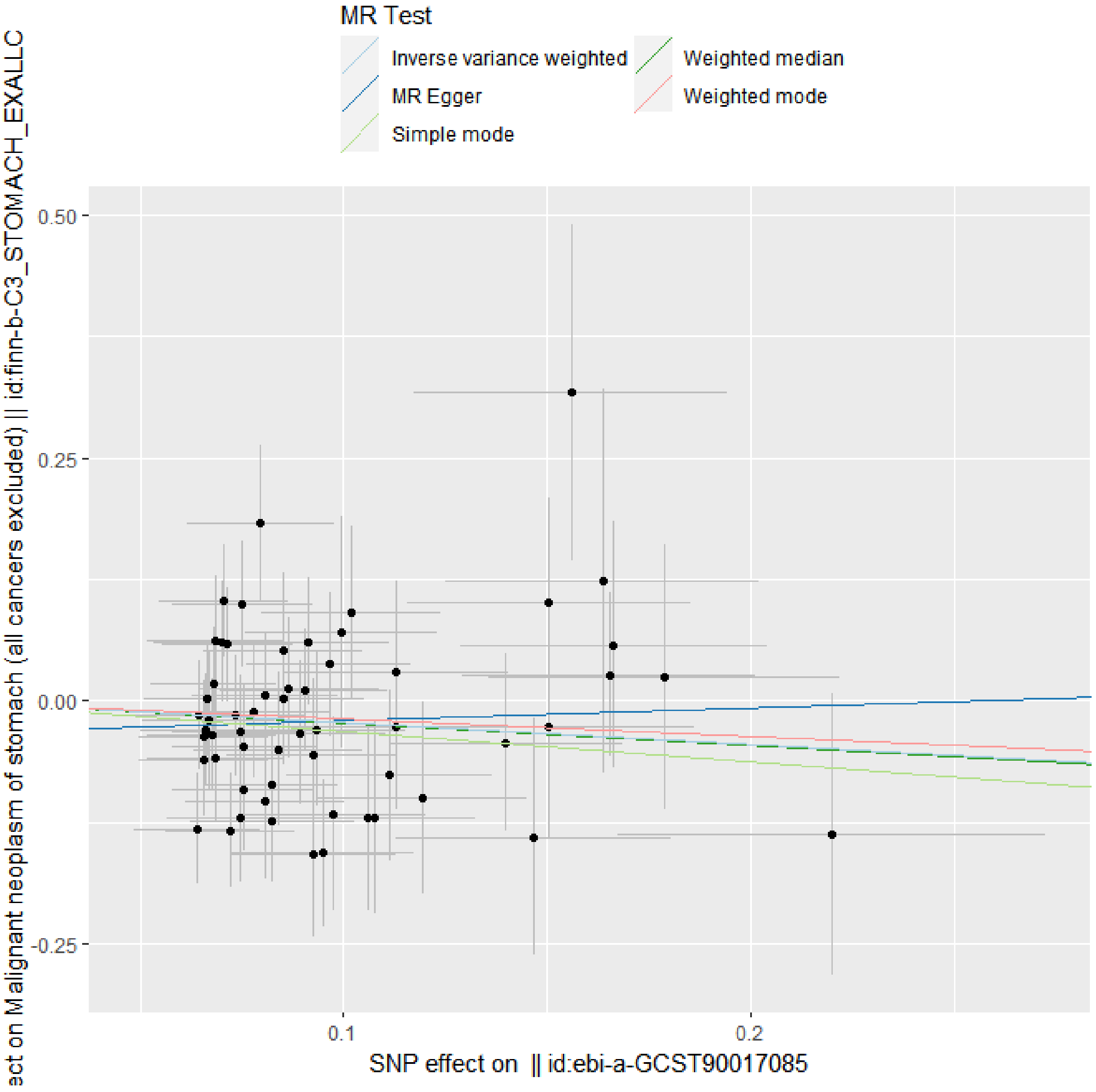

MR Test
Inverse variance weighted
MR Egger
Simple mode
Weighted median
Weighted mode
0.50
0.25
0.00
-0.25
0.1
0.2
SNP effect on || id:ebi-a-GCST90017085
act on Malignant neoplasm of stomach (all cancers excluded) || id:finn-b-C3_STOMACH_EXALLC

Figure 95 Leave-one-out analysis, funnel plot, MR effect size and scatter plot for gut microbiota abundance (order Actinomycetales id.420) on gastric cancer

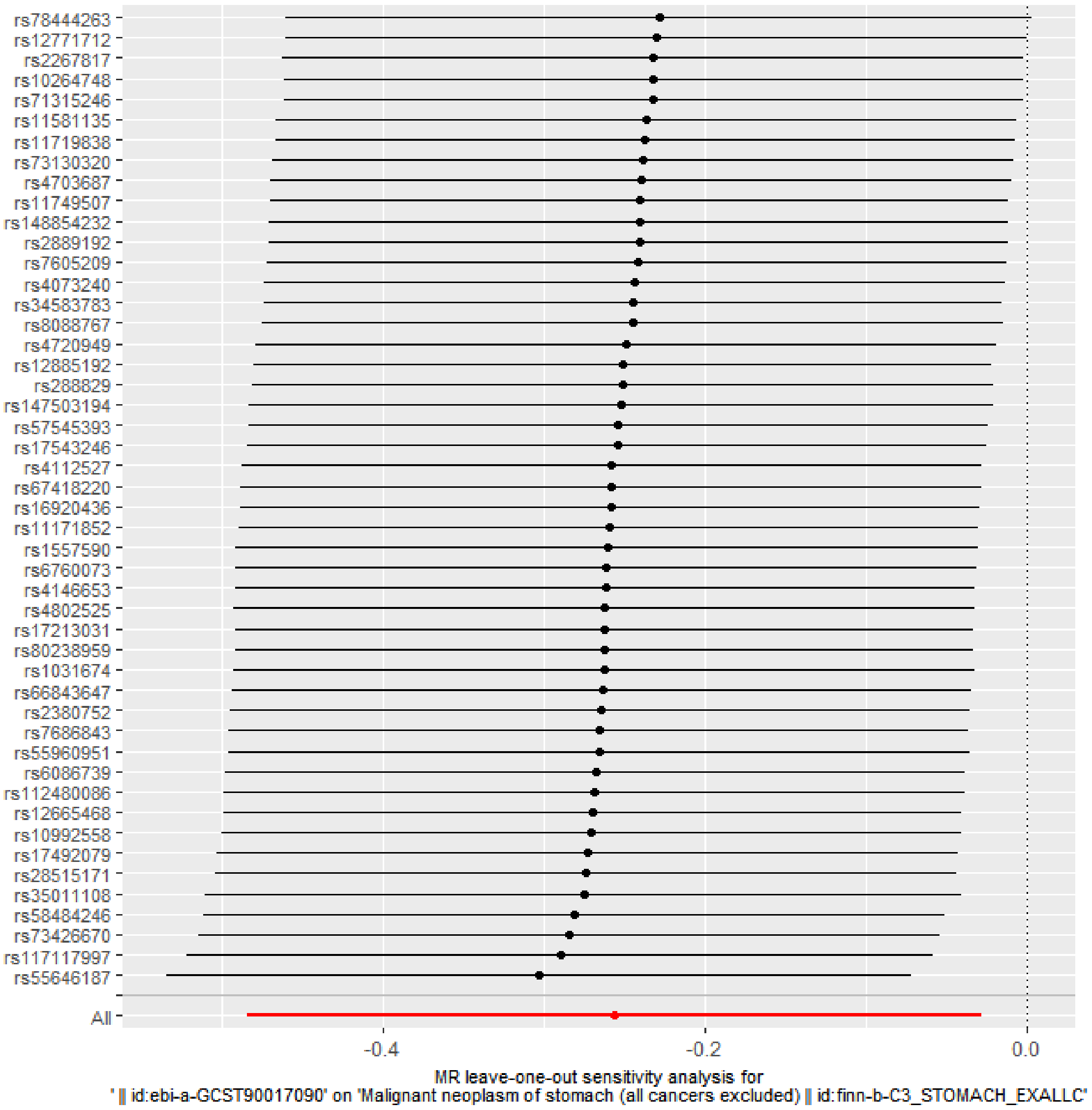

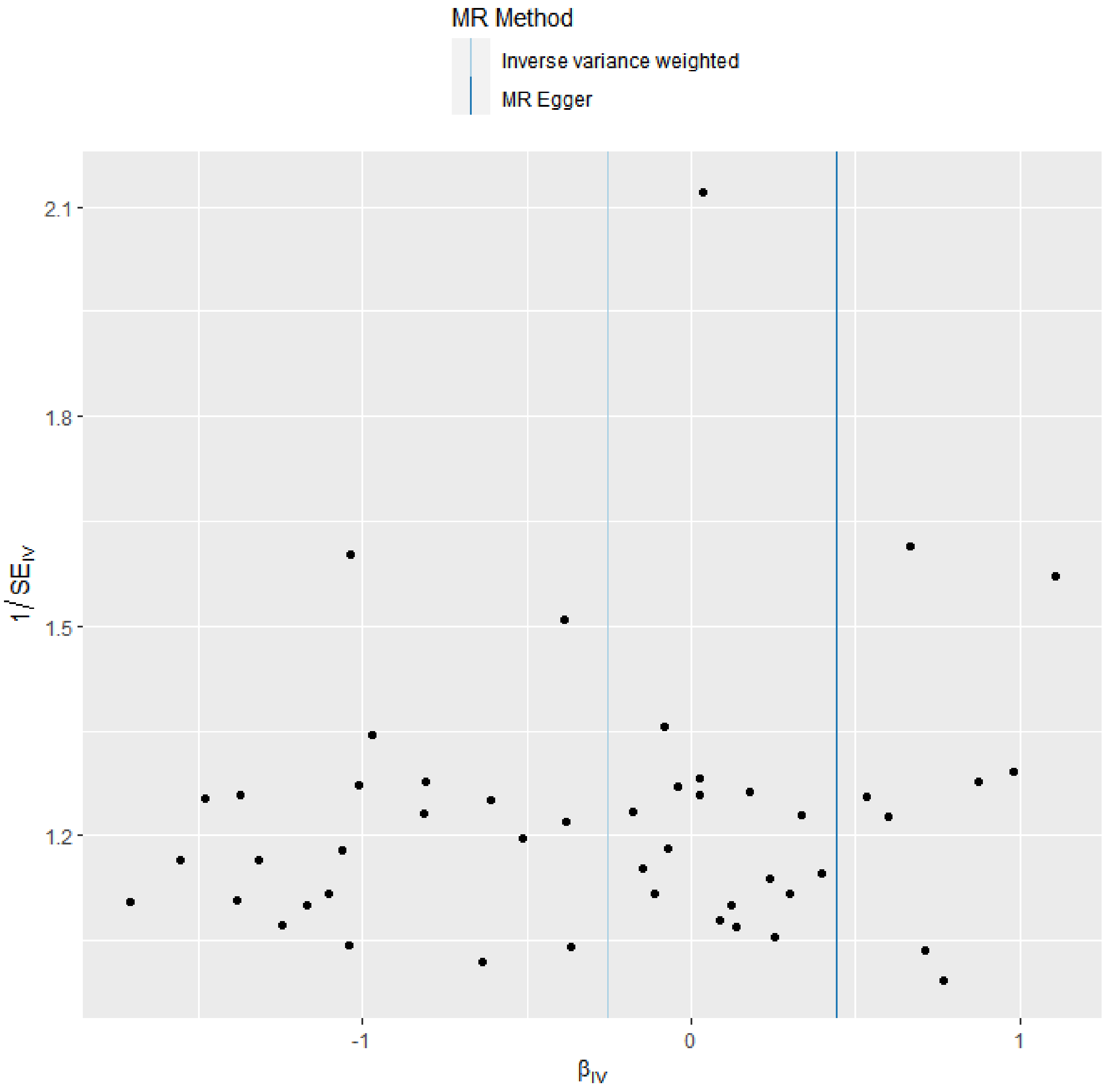
MR Method
Inverse variance weighted
MR Egger
2.1
1.8
1.5
1.2
1/SE_IV
-1
0
1
β_IV

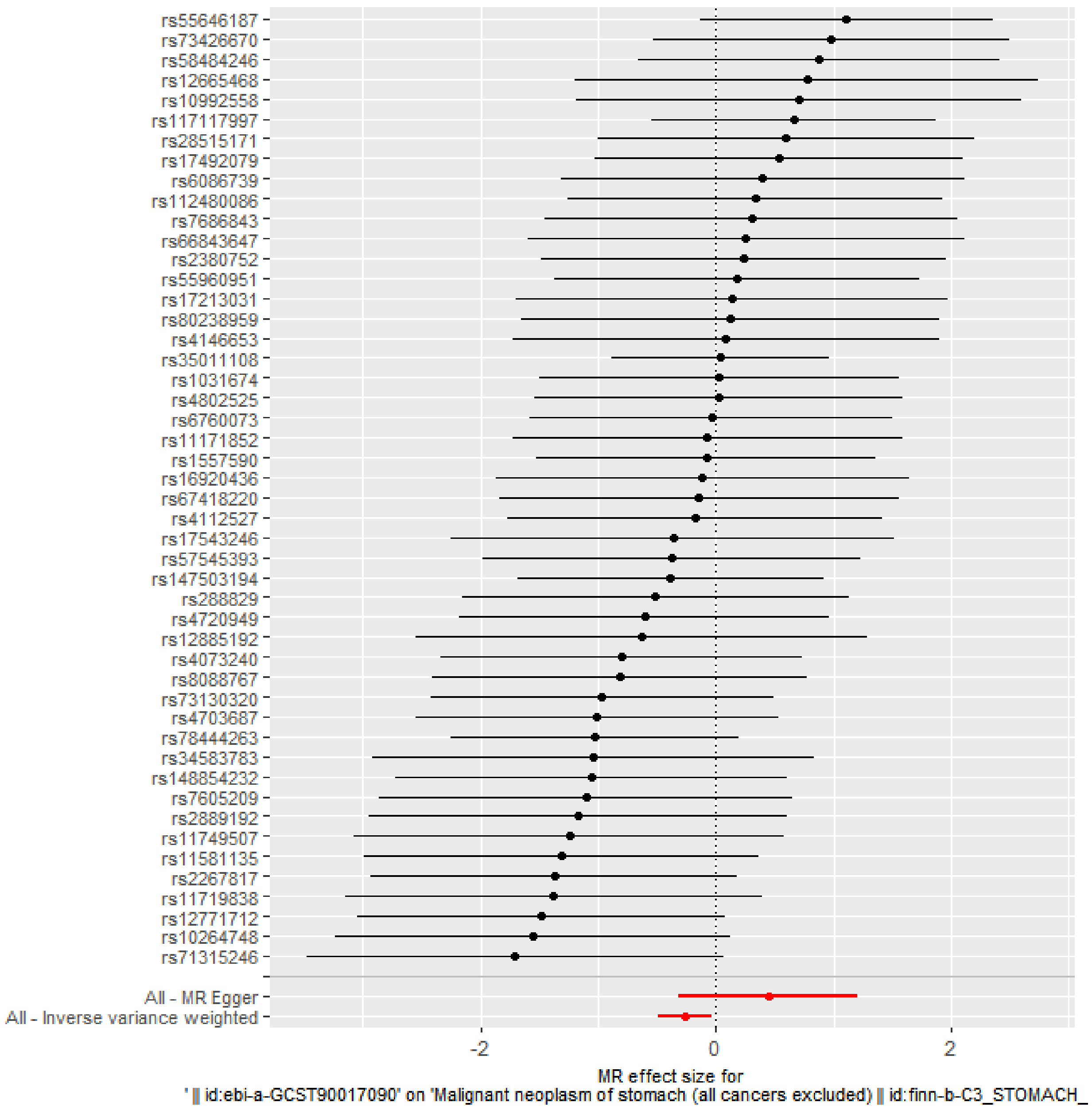
rs55646187
rs73426670
rs58484246
rs12665468
rs10992558
rs117117997
rs28515171
rs17492079
rs6086739
rs112480086
rs7686843
rs66843647
rs2380752
rs55960951
rs17213031
rs80238959
rs4146653
rs35011108
rs1031674
rs4802525
rs6760073
rs11171852
rs1557590
rs16920436
rs67418220
rs4112527
rs17543246
rs57545393
rs147503194
rs288829
rs4720949
rs12885192
rs4073240
rs8088767
rs73130320
rs4703687
rs78444263
rs34583783
rs148854232
rs7605209
rs2889192
rs11749507
rs11581135
rs2267817
rs11719838
rs12771712
rs10264748
rs71315246
All - MR Egger
All - Inverse variance weighted
-2
0
2
MR effect size for
' || id:ebi-a-GCST90017090' on 'Malignant neoplasm of stomach (all cancers excluded) || id:finn-b-C3_STOMACH_

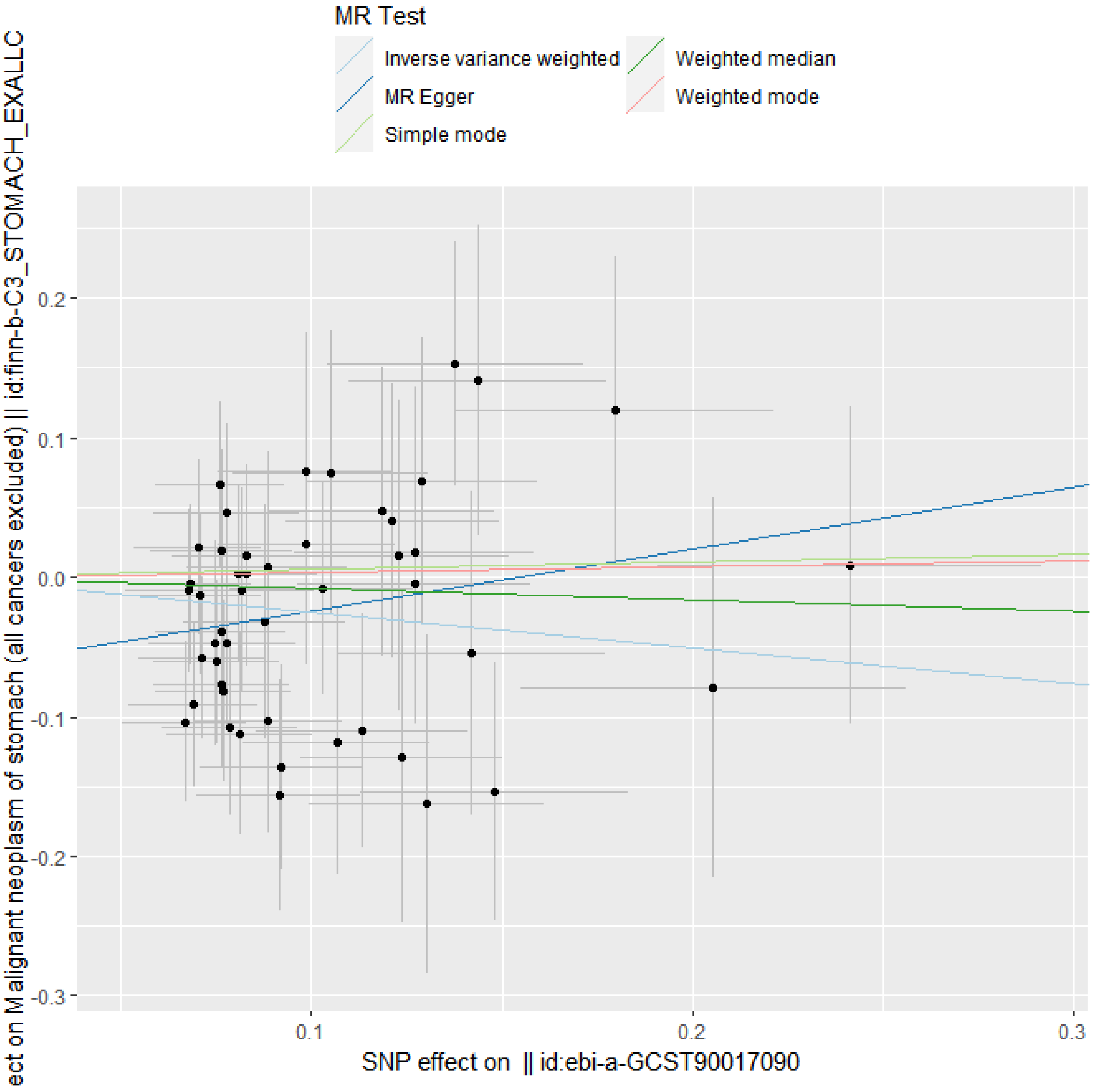
MR Test
Inverse variance weighted
MR Egger
Simple mode
Weighted median
Weighted mode
ect on Malignant neoplasm of stomach (all cancers excluded) || id:finn-b-C3_STOMACH_EXALLC
0.2
0.1
0.0
-0.1
-0.2
-0.3
0.1
0.2
0.3
SNP effect on || id:ebi-a-GCST90017090

Figure 96 Leave-one-out analysis, funnel plot, MR effect size and scatter plot for gut microbiota abundance (order Desulfovibrionales id.3156) on gastric cancer

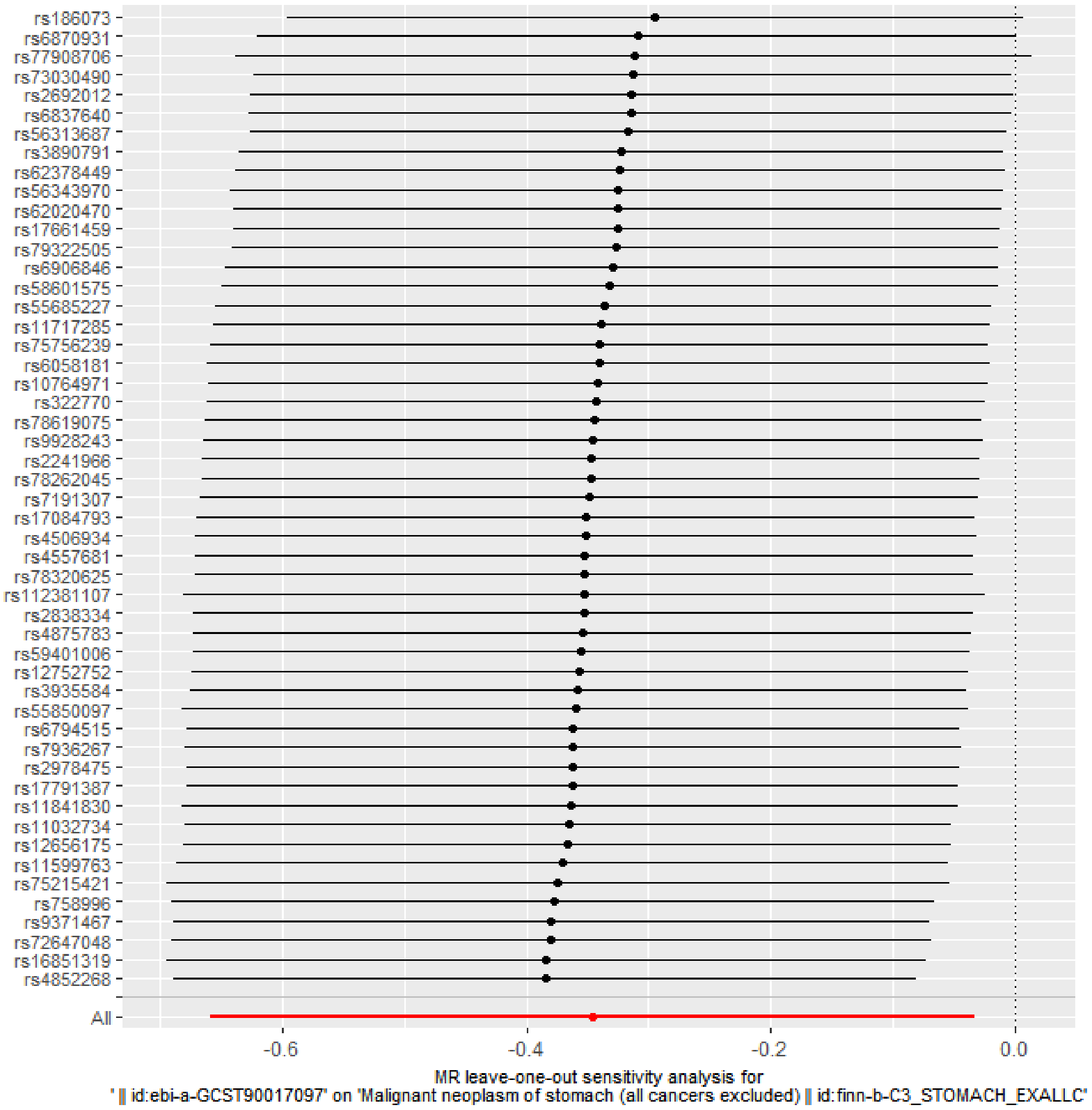

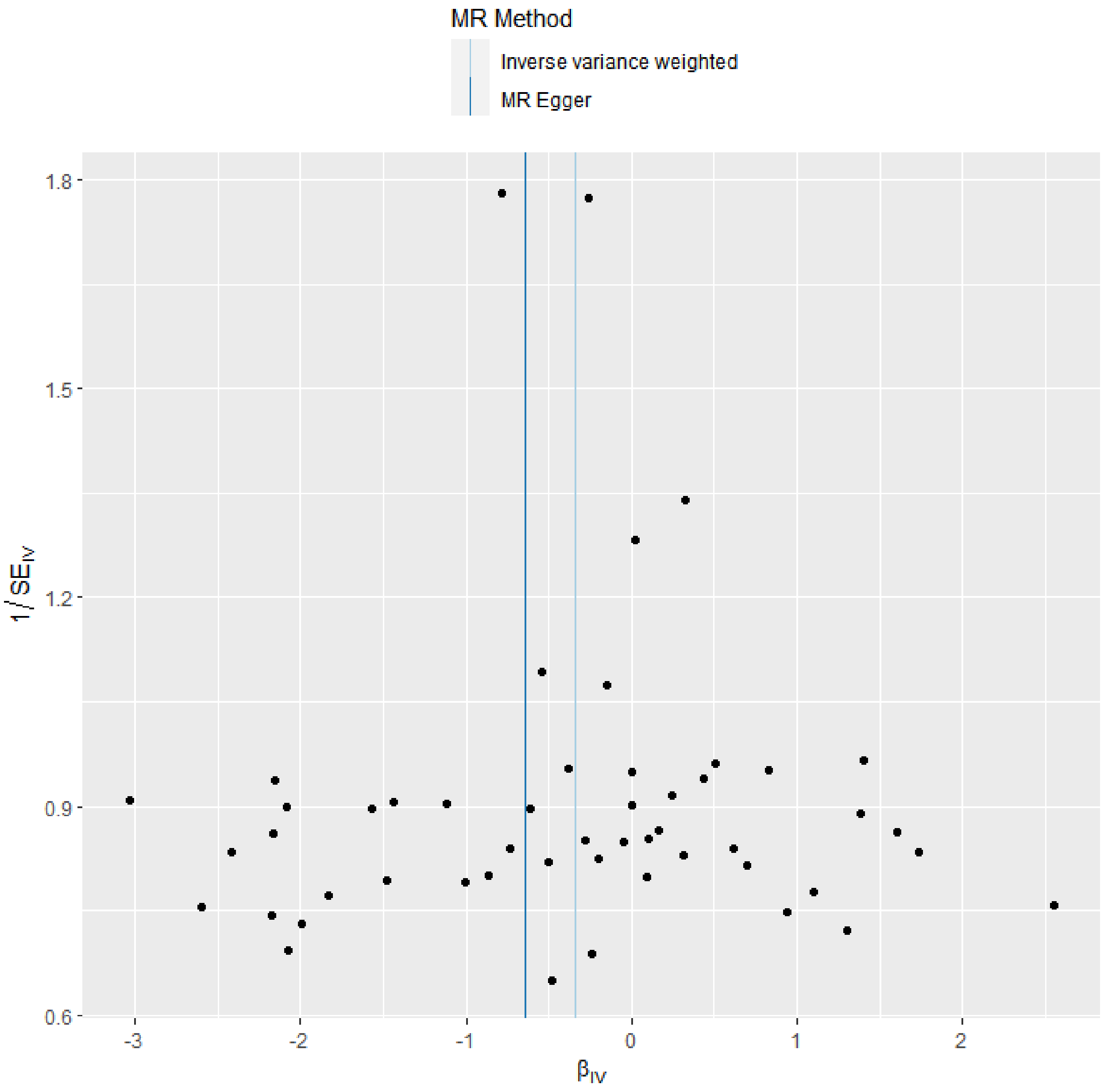

MR Method
Inverse variance weighted
MR Egger
1.8
1.5
1.2
0.9
0.6
$1/SE_{IV}$
-3
-2
-1
0
1
2
β_{IV}

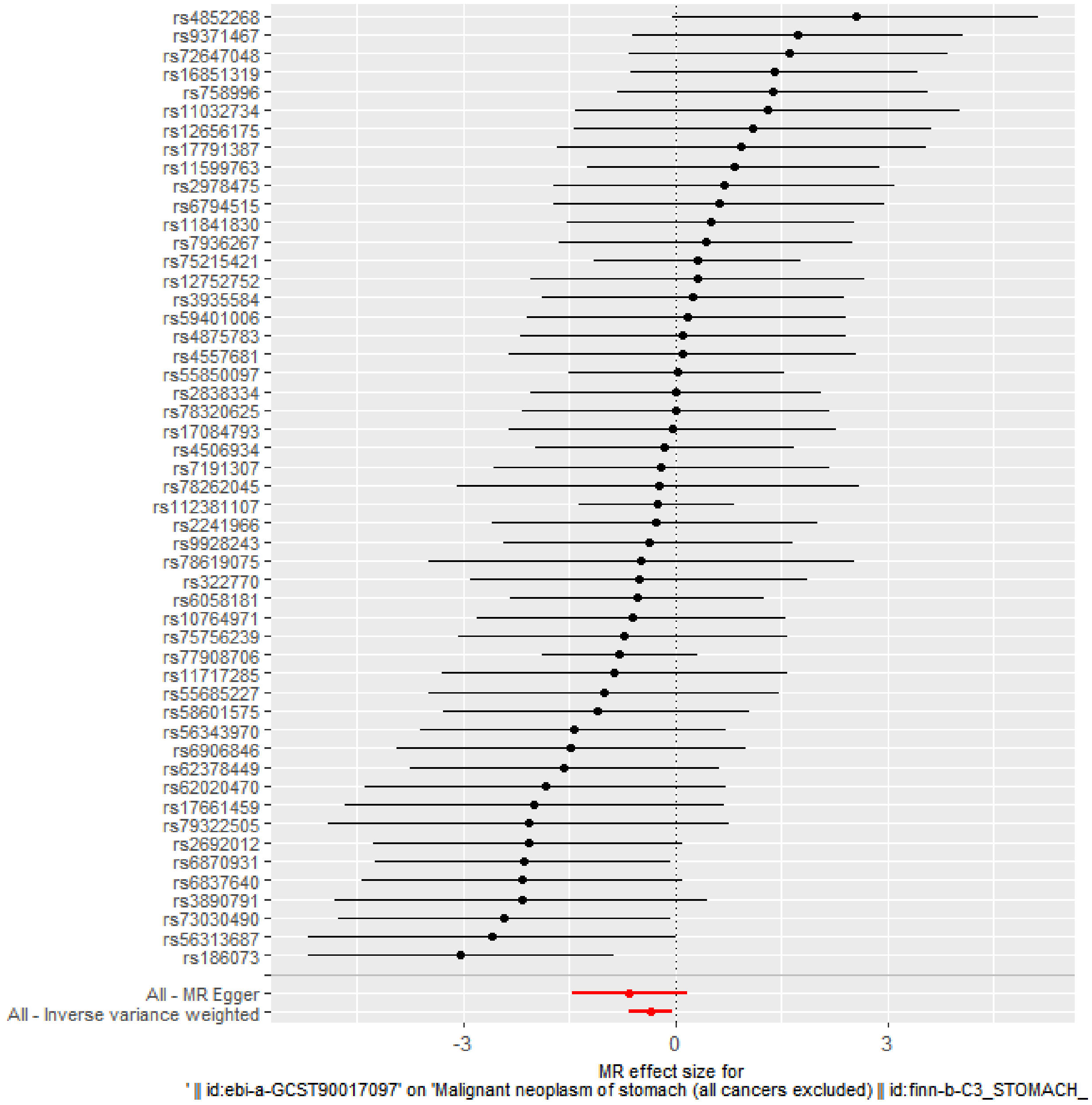

rs4852268
rs9371467
rs72647048
rs16851319
rs758996
rs11032734
rs12656175
rs17791387
rs11599763
rs2978475
rs6794515
rs11841830
rs7936267
rs75215421
rs12752752
rs3935584
rs59401006
rs4875783
rs4557681
rs55850097
rs2838334
rs78320625
rs17084793
rs4506934
rs7191307
rs78262045
rs112381107
rs2241966
rs9928243
rs78619075
rs322770
rs6058181
rs10764971
rs75756239
rs77908706
rs11717285
rs55685227
rs58601575
rs56343970
rs6906846
rs62378449
rs62020470
rs17661459
rs79322505
rs2692012
rs6870931
rs6837640
rs3890791
rs73030490
rs56313687
rs186073
All - MR Egger
All - Inverse variance weighted
-3
0
3
MR effect size for
' || id:ebi-a-GCST90017097' on 'Malignant neoplasm of stomach (all cancers excluded) || id:finn-b-C3_STOMACH_

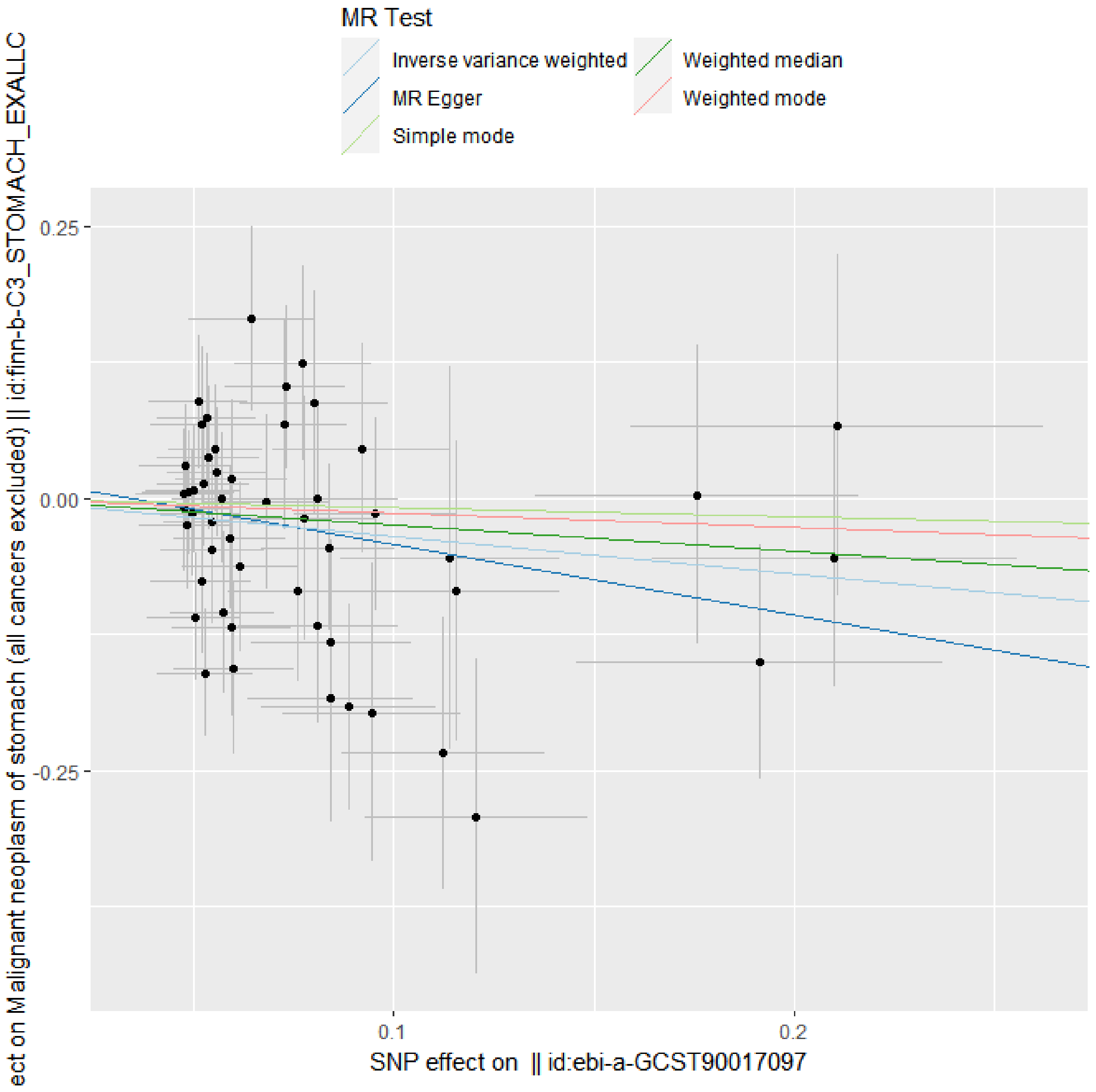
MR Test
Inverse variance weighted
MR Egger
Simple mode
Weighted median
Weighted mode
0.25
0.00
-0.25
0.1
0.2
SNP effect on || id:ebi-a-GCST90017097
ect on Malignant neoplasm of stomach (all cancers excluded) || id:finn-b-C3_STOMACH_EXALLC

Figure 97 Leave-one-out analysis, funnel plot, MR effect size and scatter plot for gut microbiota abundance (order Mollicutes RF9 id.11579) on gastric cancer

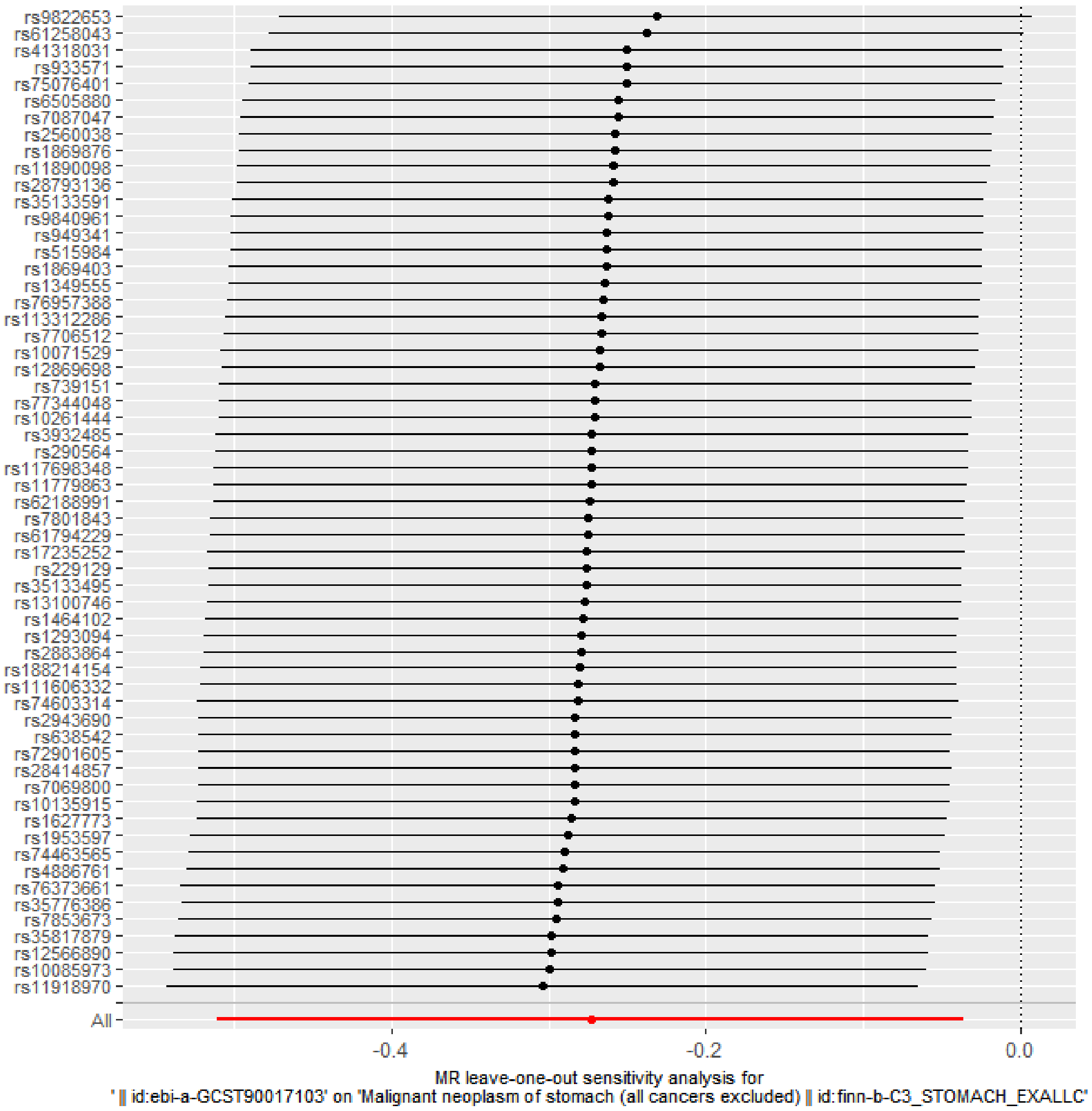

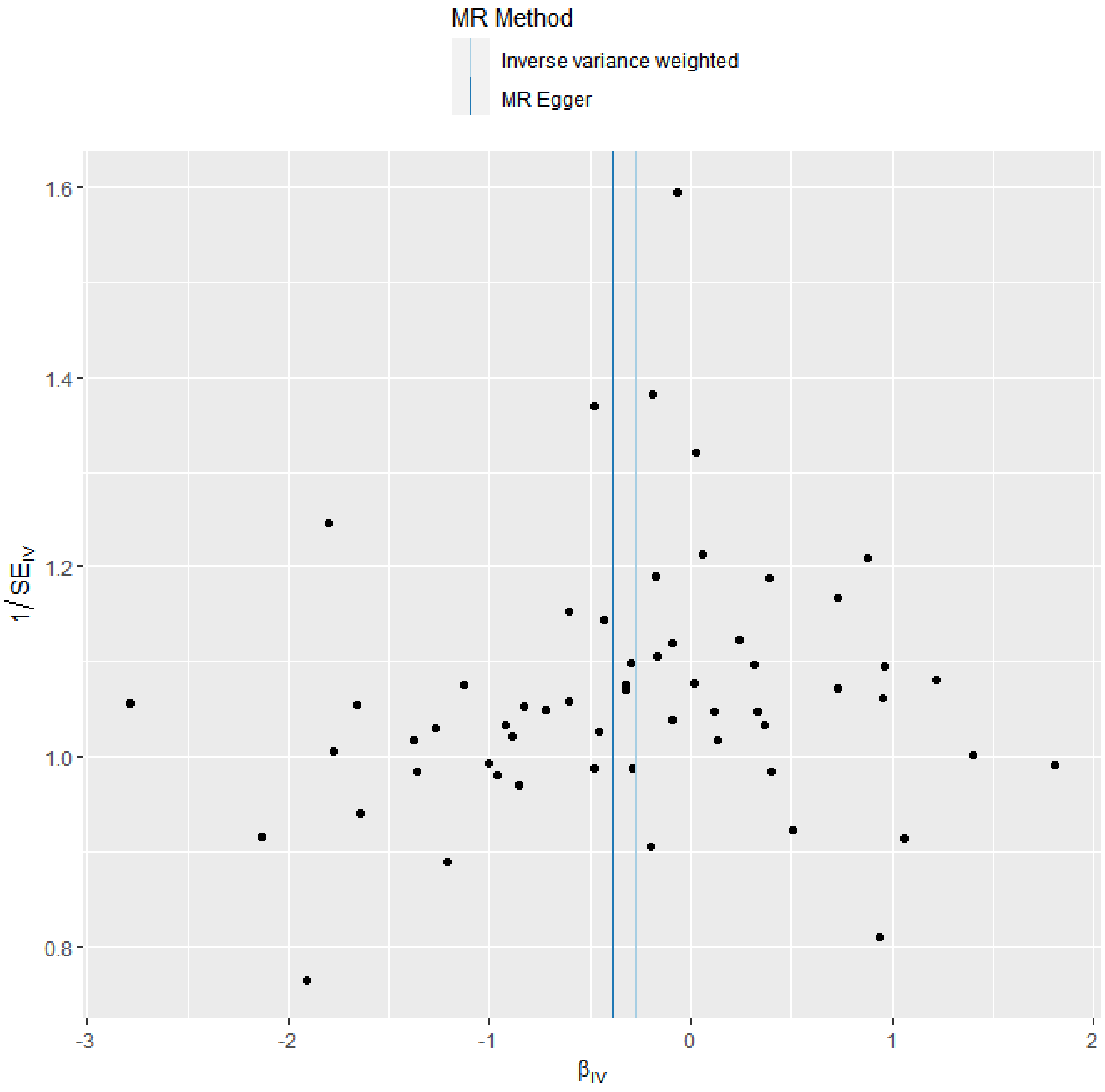
MR Method
Inverse variance weighted
MR Egger
1.6
1.4
1.2
1.0
0.8
-3
-2
-1
0
1
2
$1/SE_{IV}$
β_{IV}

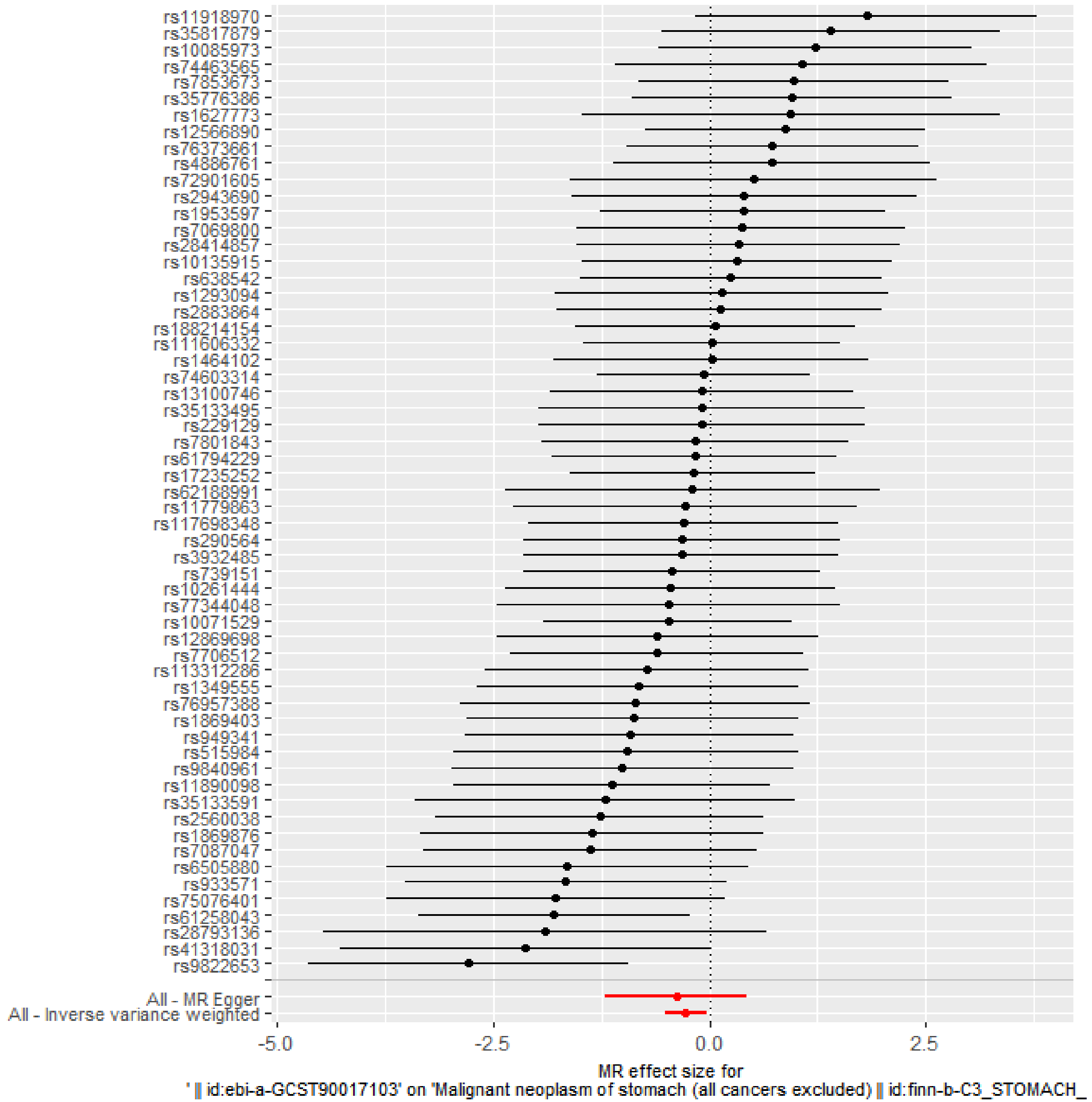

rs11918970
rs35817879
rs10085973
rs74463565
rs7853673
rs35776386
rs1627773
rs12566890
rs76373661
rs4886761
rs72901605
rs2943690
rs1953597
rs7069800
rs28414857
rs10135915
rs638542
rs1293094
rs2883864
rs188214154
rs111606332
rs1464102
rs74603314
rs13100746
rs35133495
rs229129
rs7801843
rs61794229
rs17235252
rs62188991
rs11779863
rs117698348
rs290564
rs3932485
rs739151
rs10261444
rs77344048
rs10071529
rs12869698
rs7706512
rs113312286
rs1349555
rs76957388
rs1869403
rs949341
rs515984
rs9840961
rs11890098
rs35133591
rs2560038
rs1869876
rs7087047
rs6505880
rs933571
rs75076401
rs61258043
rs28793136
rs41318031
rs9822653
All - MR Egger
All - Inverse variance weighted
-5.0
-2.5
0.0
2.5
MR effect size for
' || id:ebi-a-GCST90017103' on 'Malignant neoplasm of stomach (all cancers excluded) || id:finn-b-C3_STOMACH_

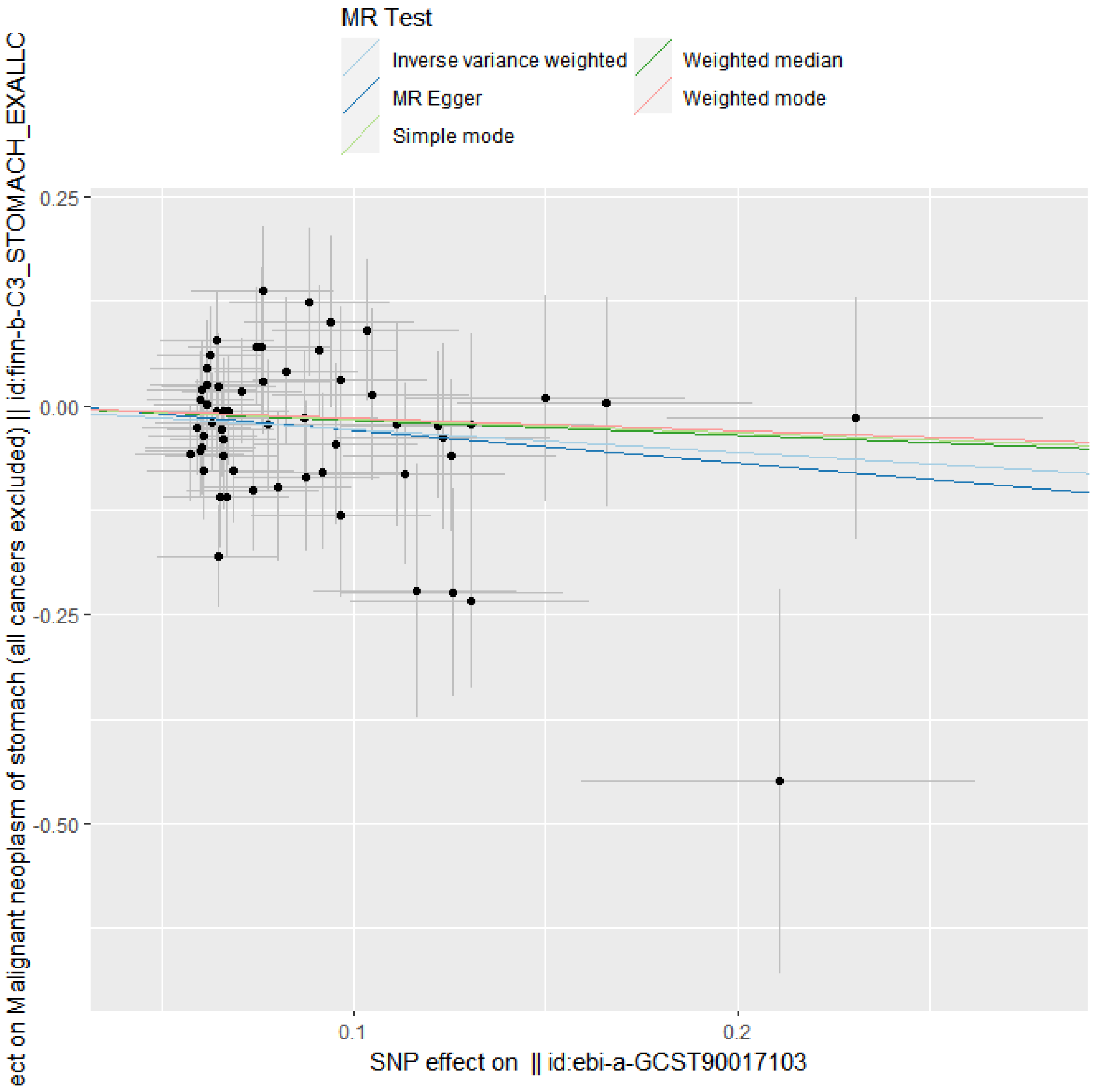

MR Test
Inverse variance weighted
MR Egger
Simple mode
Weighted median
Weighted mode
ect on Malignant neoplasm of stomach (all cancers excluded) || id:finn-b-C3_STOMACH_EXALLC
0.25
0.00
-0.25
-0.50
0.1
0.2
SNP effect on || id:ebi-a-GCST90017103

Figure 98 Leave-one-out analysis, funnel plot, MR effect size and scatter plot for gut microbiota abundance (genus Anaerofilum id.2053) on irritable bowel syndrome

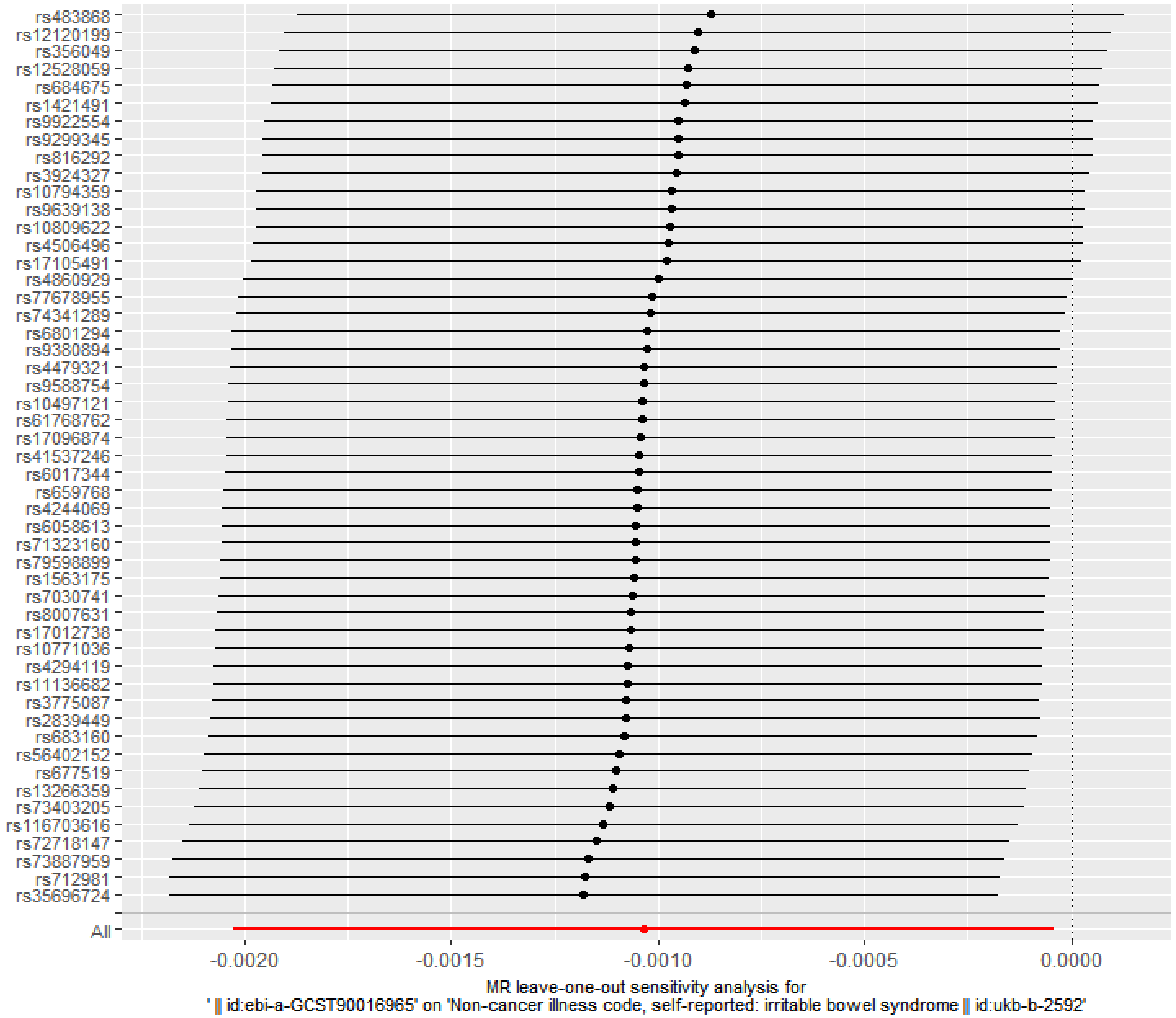

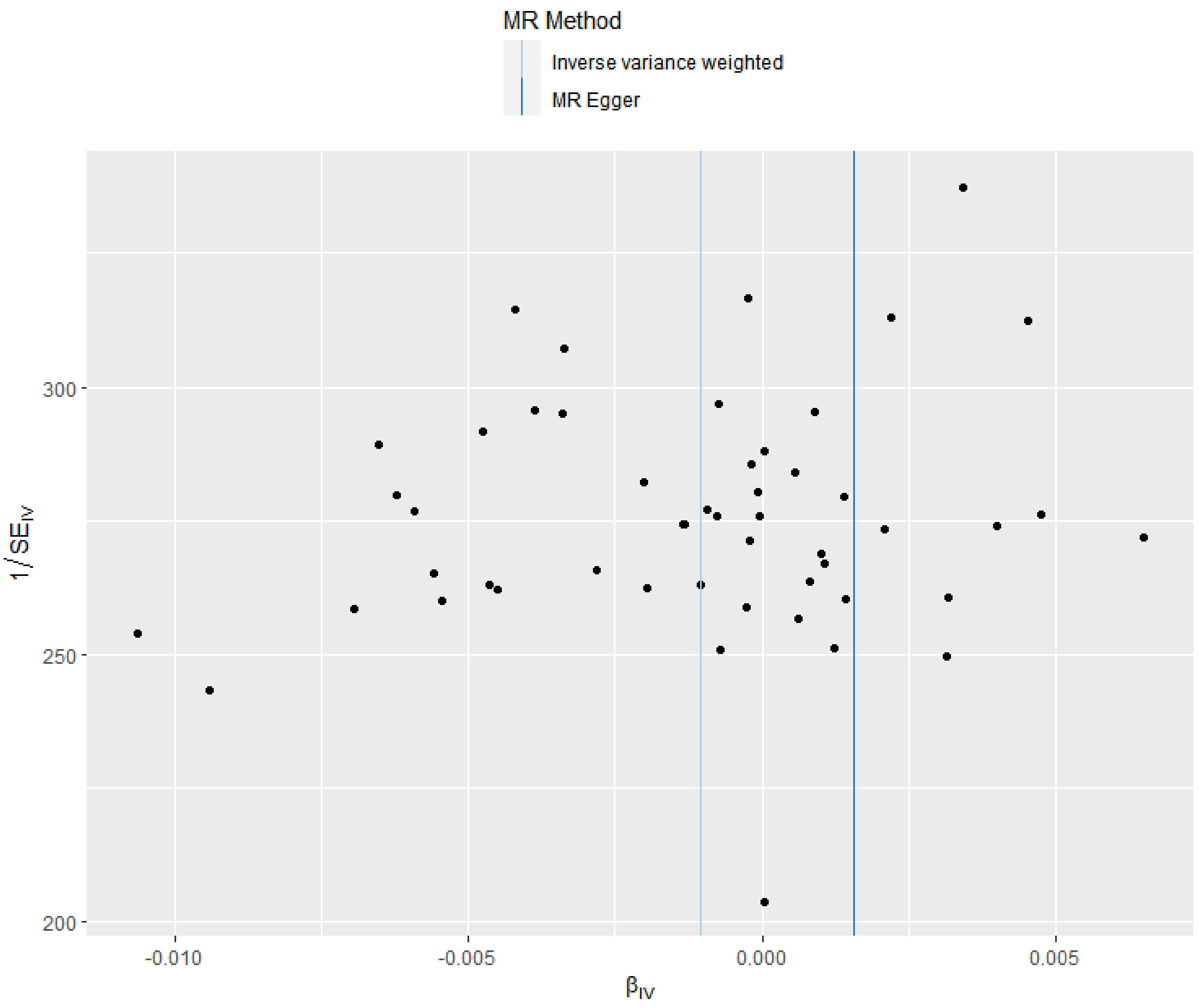
MR Method
Inverse variance weighted
MR Egger
300
250
200
-0.010
-0.005
0.000
0.005
$1/SE_{IV}$
β_{IV}

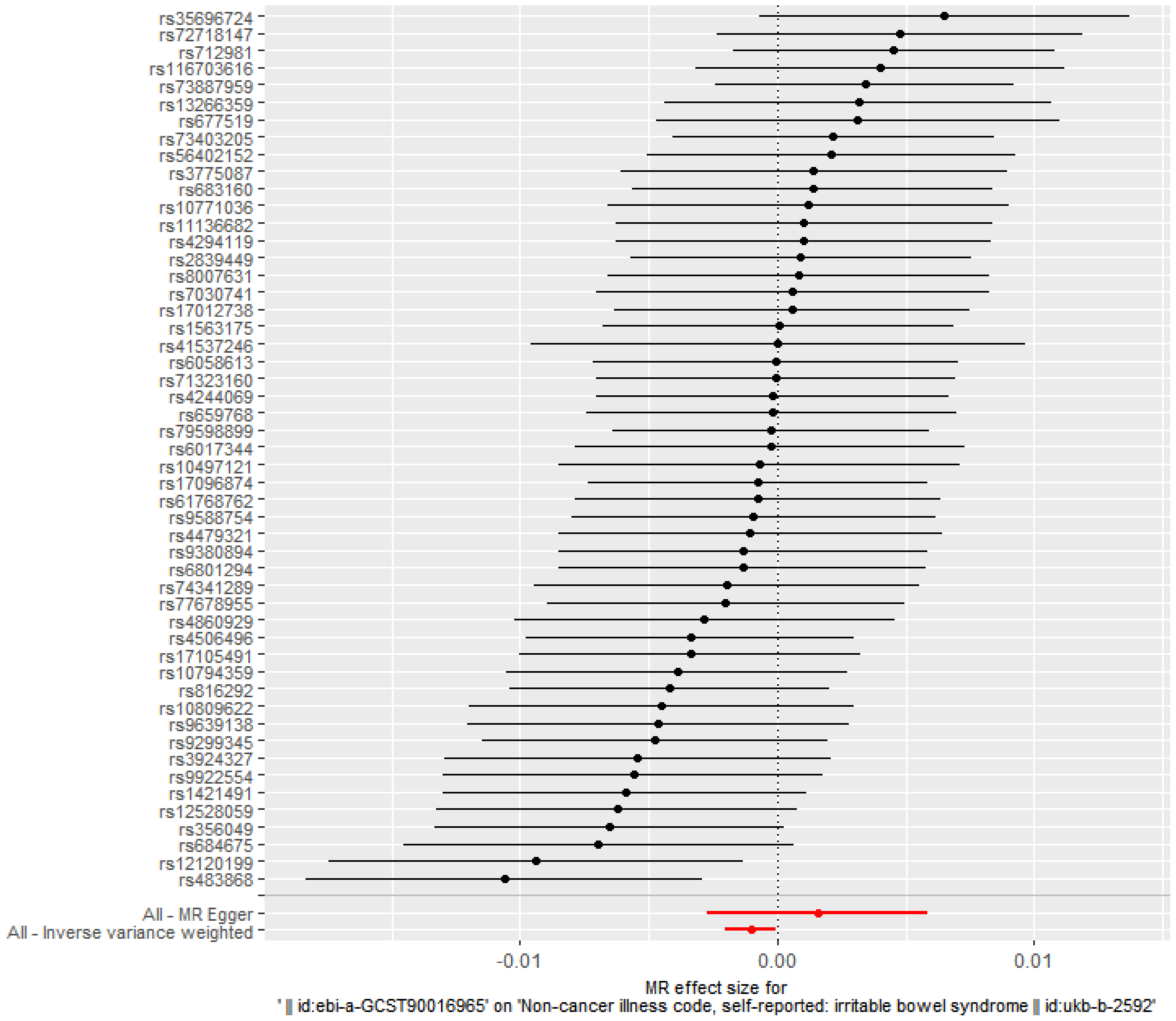
rs35696724
rs72718147
rs712981
rs116703616
rs73887959
rs13266359
rs677519
rs73403205
rs56402152
rs3775087
rs683160
rs10771036
rs11136682
rs4294119
rs2839449
rs8007631
rs7030741
rs17012738
rs1563175
rs41537246
rs6058613
rs71323160
rs4244069
rs659768
rs79598899
rs6017344
rs10497121
rs17096874
rs61768762
rs9588754
rs4479321
rs9380894
rs6801294
rs74341289
rs77678955
rs4860929
rs4506496
rs17105491
rs10794359
rs816292
rs10809622
rs9639138
rs9299345
rs3924327
rs9922554
rs1421491
rs12528059
rs356049
rs684675
rs12120199
rs483868
All - MR Egger
All - Inverse variance weighted
-0.01
0.00
0.01
MR effect size for
' || id:ebi-a-GCST90016965' on 'Non-cancer illness code, self-reported: irritable bowel syndrome || id:ukb-b-2592'

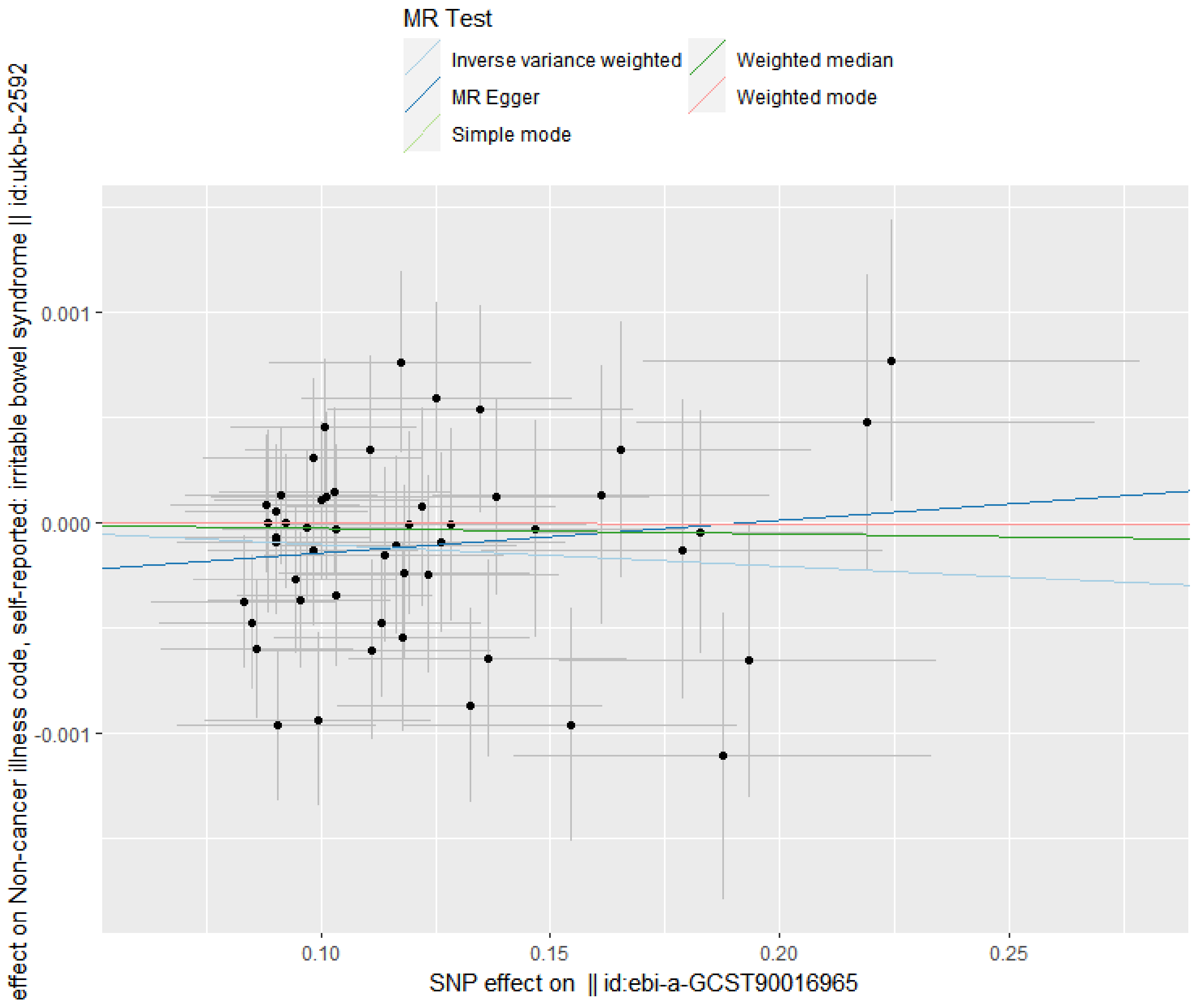

MR Test
Inverse variance weighted
MR Egger
Simple mode
Weighted median
Weighted mode
0.001
0.000
-0.001
0.10
0.15
0.20
0.25
SNP effect on || id:ebi-a-GCST90016965
effect on Non-cancer illness code, self-reported: irritable bowel syndrome || id:ukb-b-2592

Figure 99 Leave-one-out analysis, funnel plot, MR effect size and scatter plot for gut microbiota abundance (genus Desulfovibrio id.3173) on irritable bowel syndrome

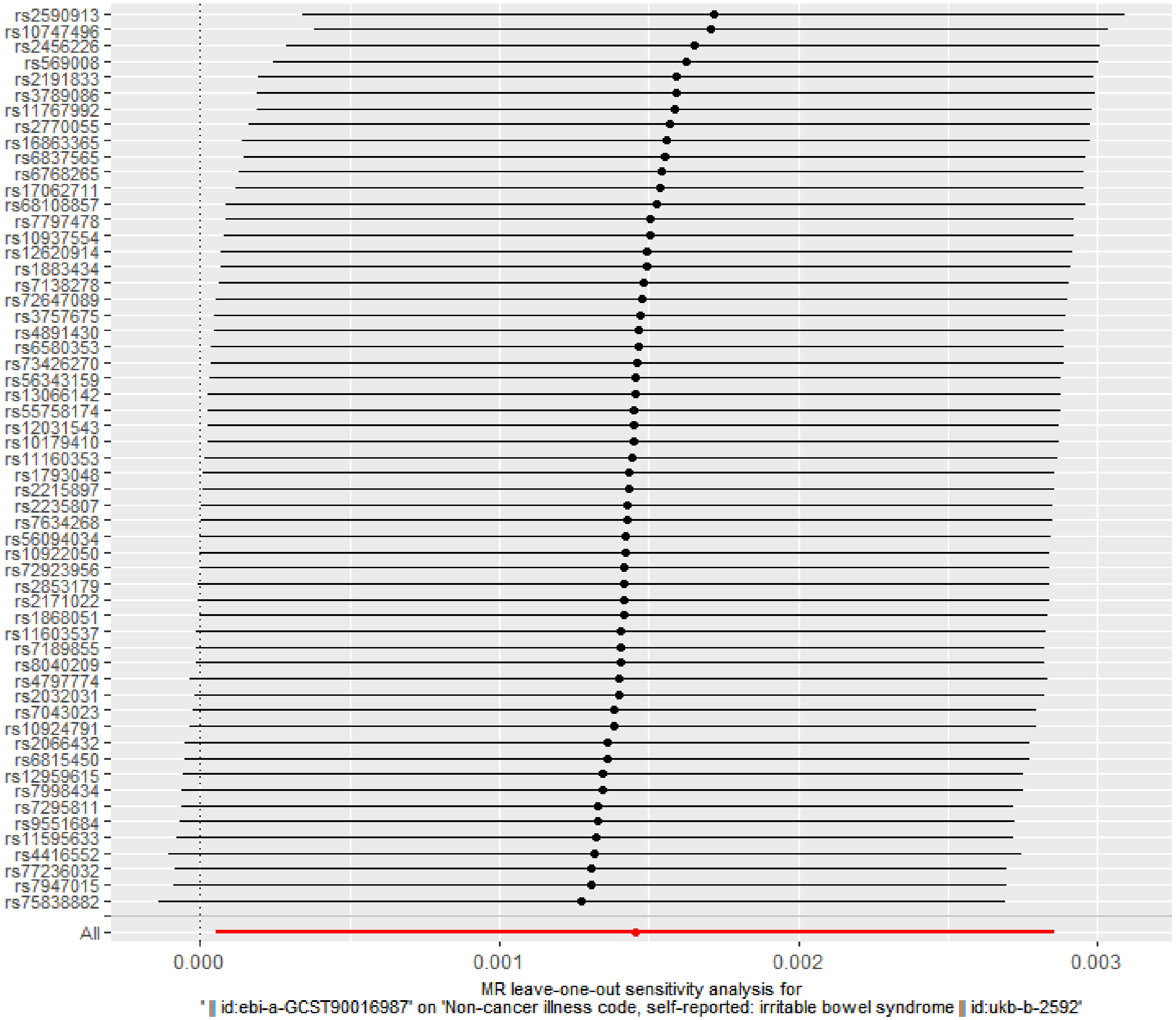

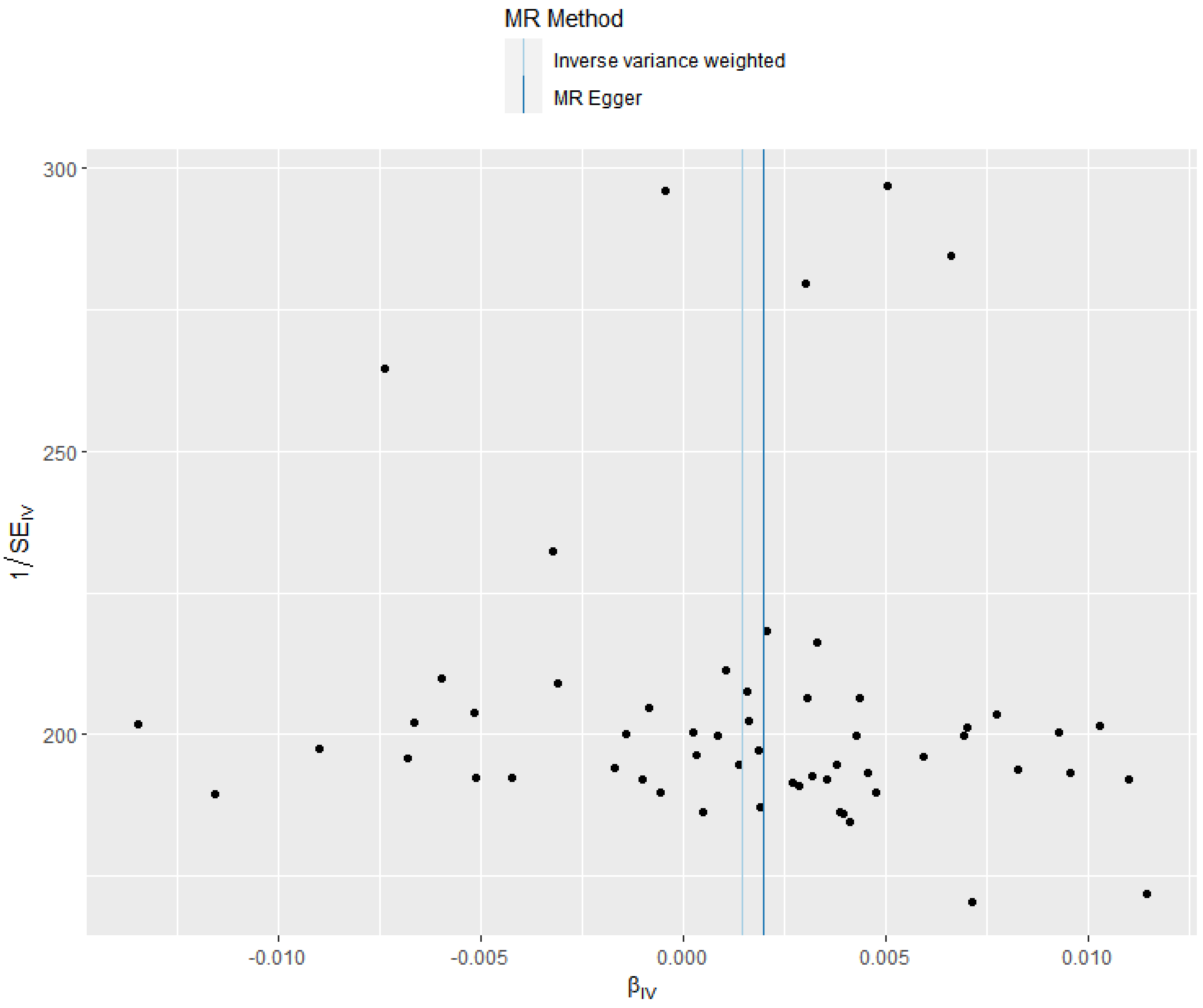

MR Method
Inverse variance weighted
MR Egger
300
250
200
1/SE$_{IV}$
-0.010
-0.005
0.000
0.005
0.010
β$_{IV}$

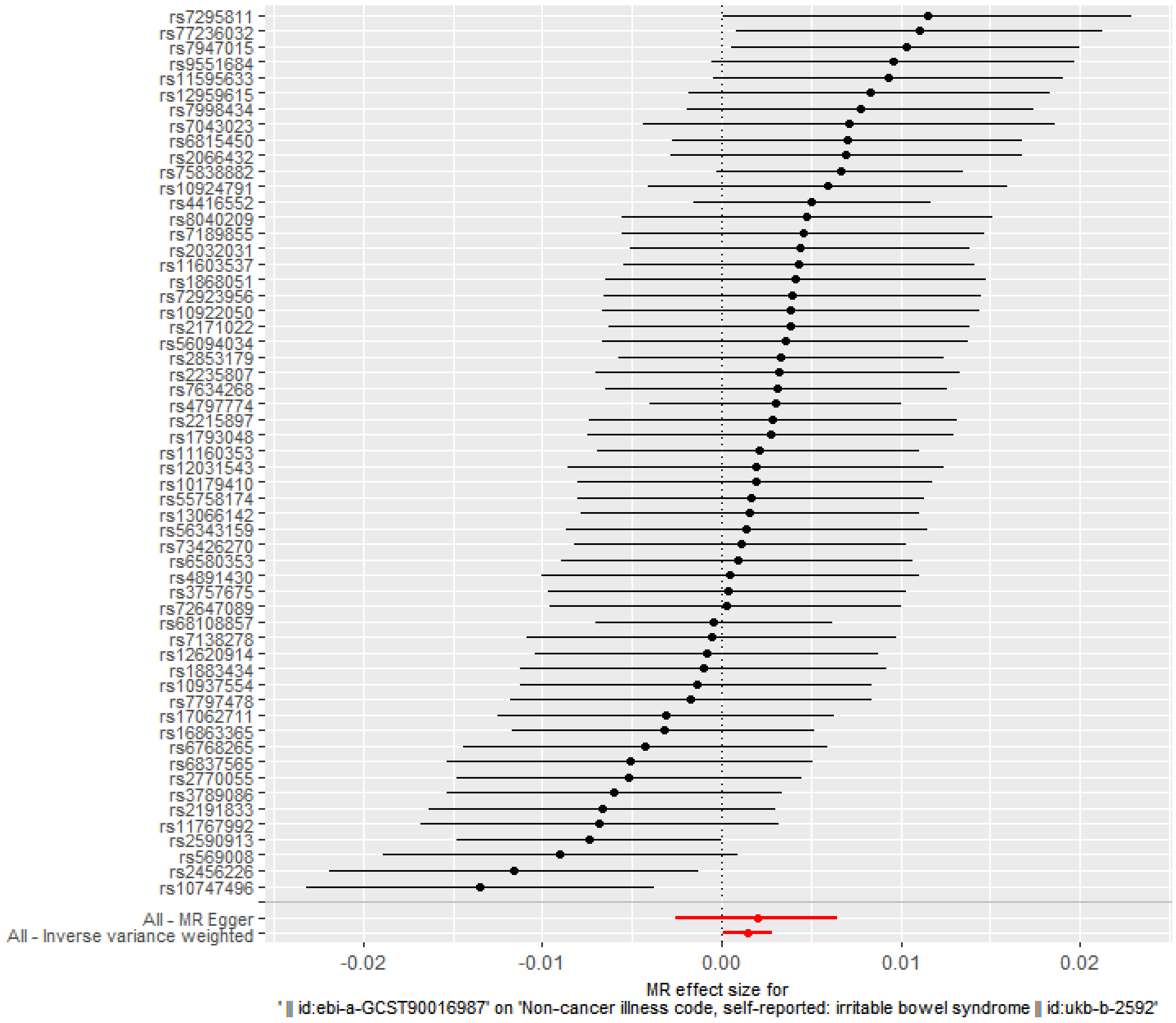
rs7295811
rs77236032
rs7947015
rs9551684
rs11595633
rs12959615
rs7998434
rs7043023
rs6815450
rs2066432
rs75838882
rs10924791
rs4416552
rs8040209
rs7189855
rs2032031
rs11603537
rs1868051
rs72923956
rs10922050
rs2171022
rs56094034
rs2853179
rs2235807
rs7634268
rs4797774
rs2215897
rs1793048
rs11160353
rs12031543
rs10179410
rs55758174
rs13066142
rs56343159
rs73426270
rs6580353
rs4891430
rs3757675
rs72647089
rs68108857
rs7138278
rs12620914
rs1883434
rs10937554
rs7797478
rs17062711
rs16863365
rs6768265
rs6837565
rs2770055
rs3789086
rs2191833
rs11767992
rs2590913
rs569008
rs2456226
rs10747496
All - MR Egger
All - Inverse variance weighted
-0.02
-0.01
0.00
0.01
0.02
MR effect size for
' || id:ebi-a-GCST90016987' on 'Non-cancer illness code, self-reported: irritable bowel syndrome || id:ukb-b-2592'

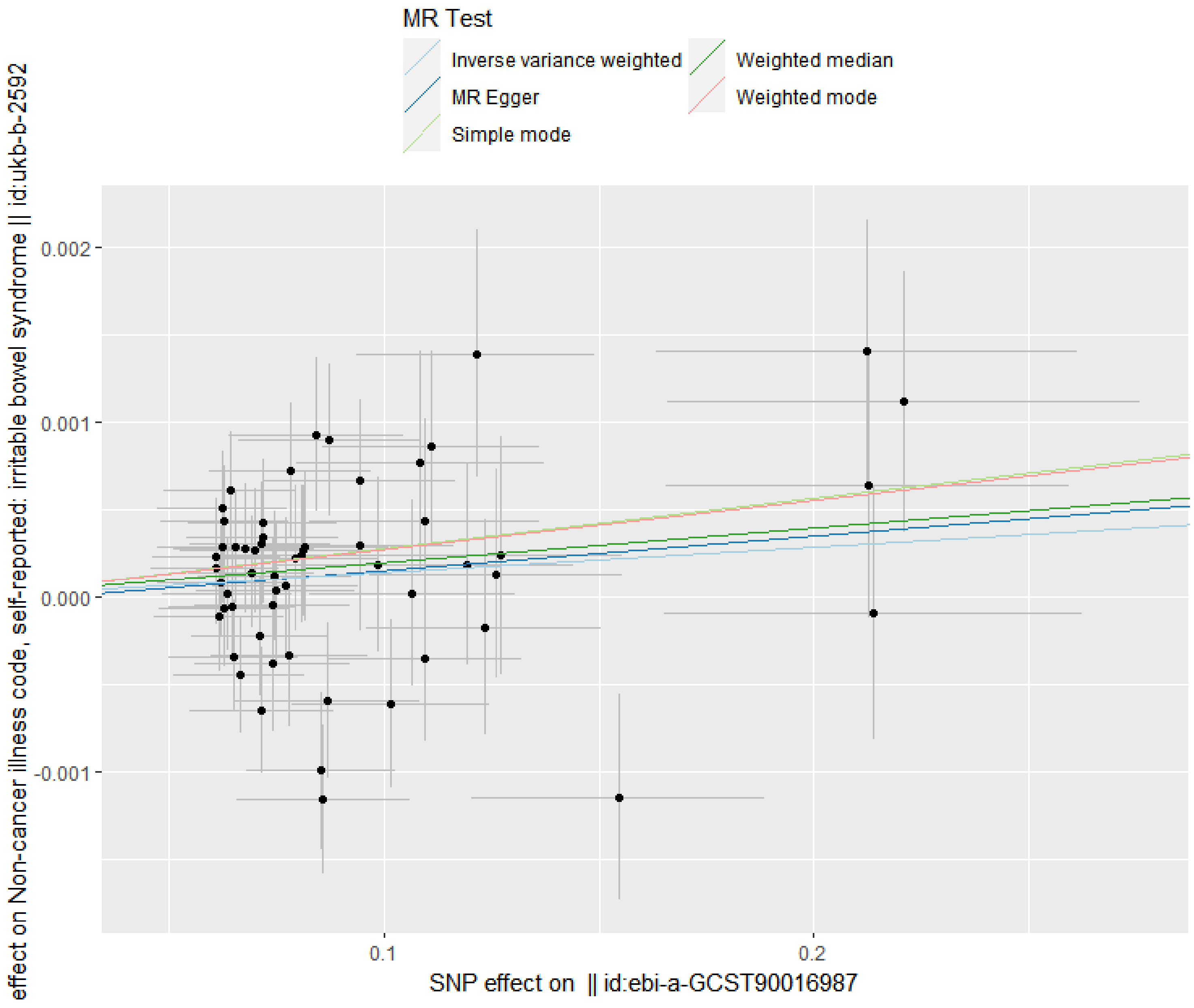

MR Test
Inverse variance weighted
MR Egger
Simple mode
Weighted median
Weighted mode
0.002
0.001
0.000
-0.001
0.1
0.2
SNP effect on || id:ebi-a-GCST90016987
effect on Non-cancer illness code, self-reported: irritable bowel syndrome || id:ukb-b-2592

Figure 100 Leave-one-out analysis, funnel plot, MR effect size and scatter plot for gut microbiota abundance (genus Lachnospiraceae NK4A136 group id.11319) on irritable bowel syndrome

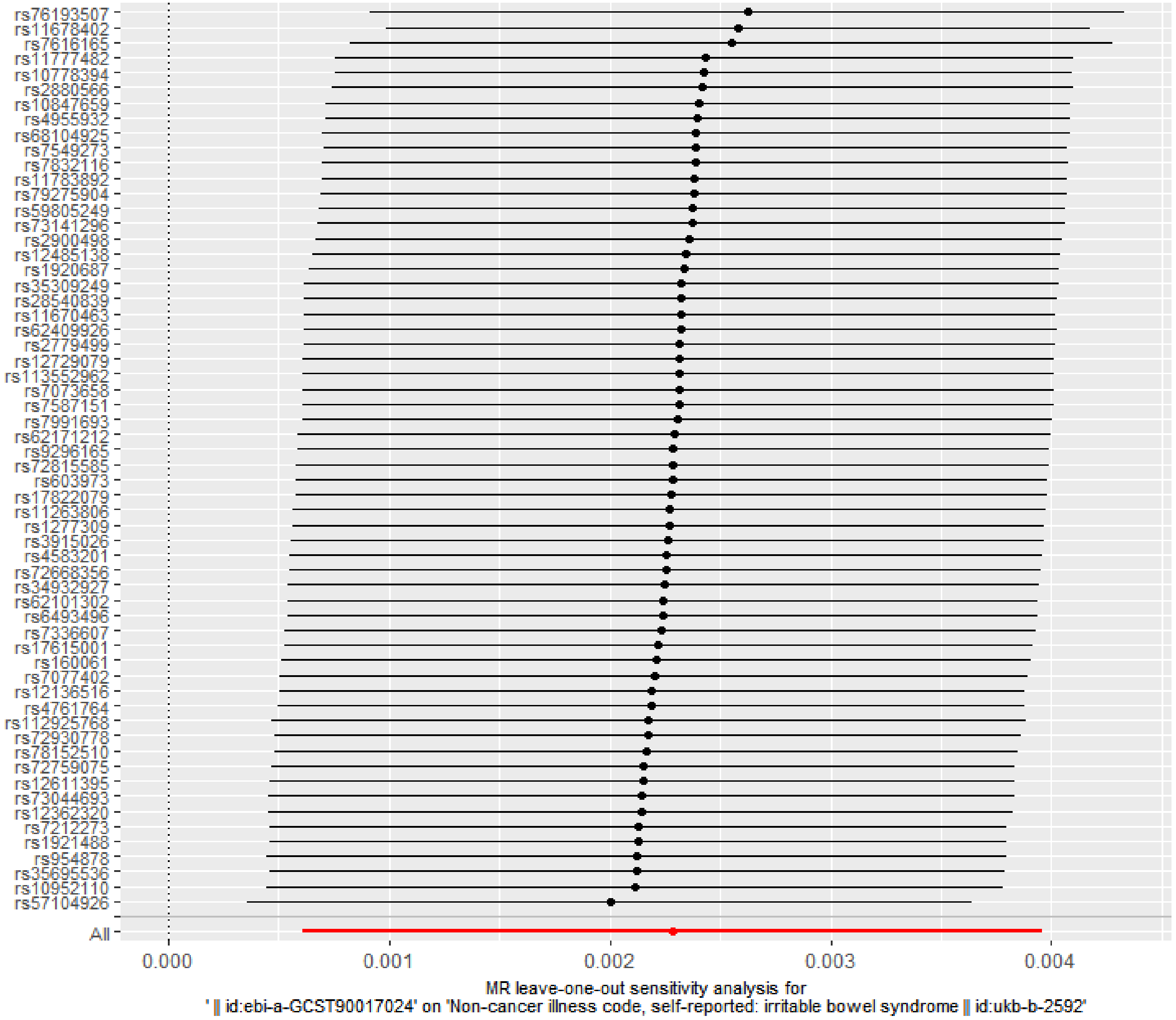

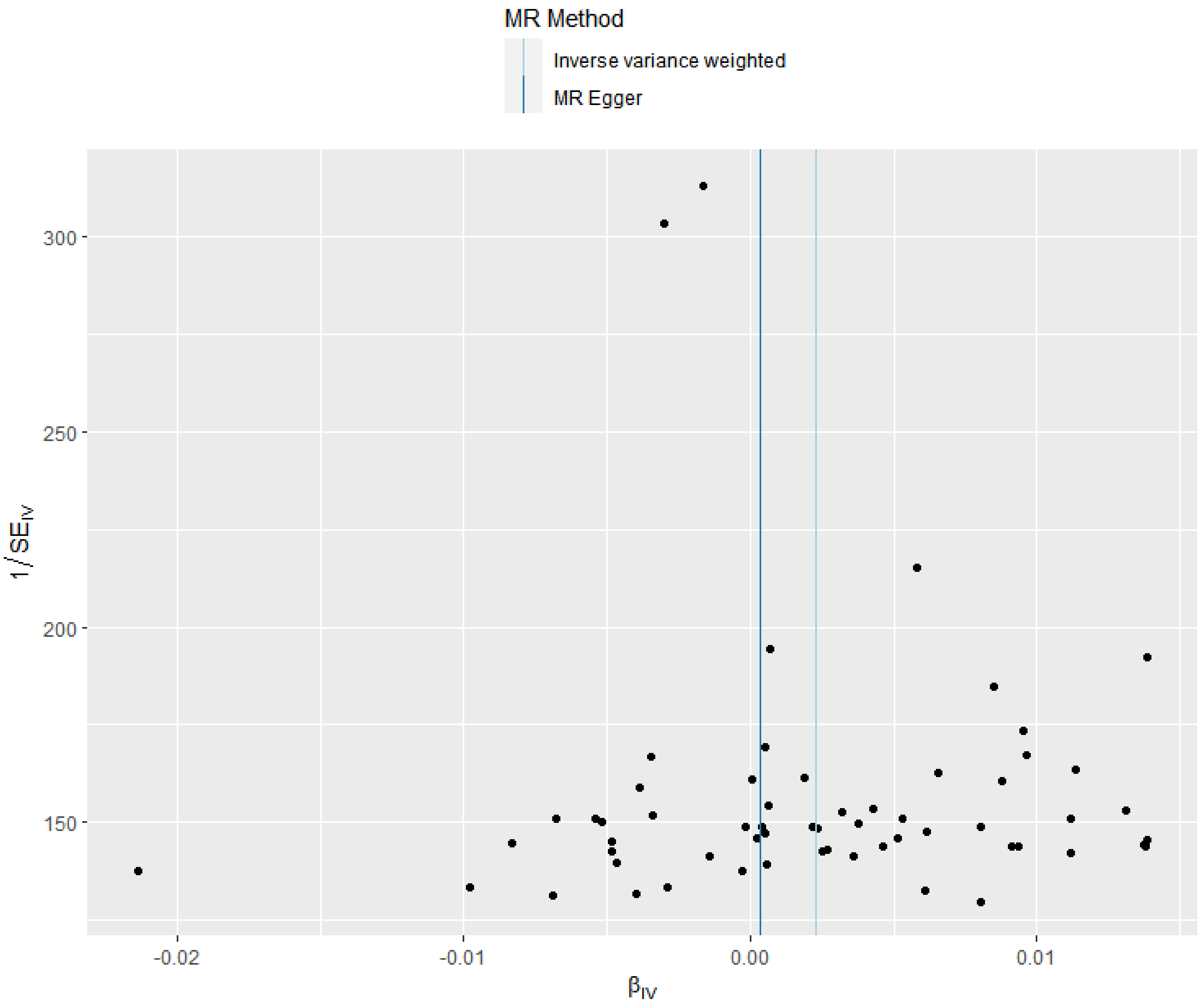

MR Method
Inverse variance weighted
MR Egger
300
250
200
150
1/SE_IV
-0.02
-0.01
0.00
0.01
β_IV

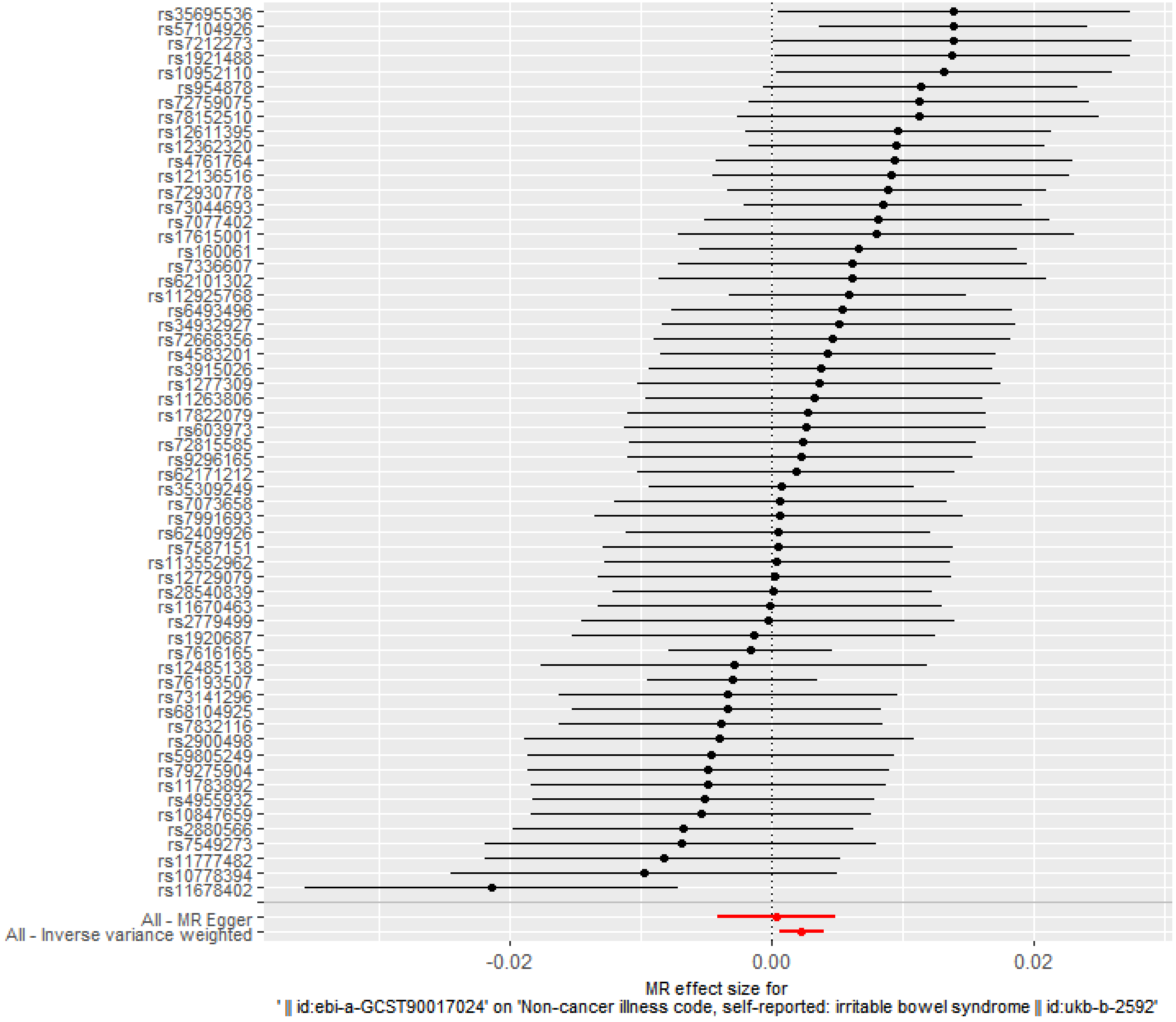
rs35695536
rs57104926
rs7212273
rs1921488
rs10952110
rs954878
rs72759075
rs78152510
rs12611395
rs12362320
rs4761764
rs12136516
rs72930778
rs73044693
rs7077402
rs17615001
rs160061
rs7336607
rs62101302
rs112925768
rs6493496
rs34932927
rs72668356
rs4583201
rs3915026
rs1277309
rs11263806
rs17822079
rs603973
rs72815585
rs9296165
rs62171212
rs35309249
rs7073658
rs7991693
rs62409926
rs7587151
rs113552962
rs12729079
rs28540839
rs11670463
rs2779499
rs1920687
rs7616165
rs12485138
rs76193507
rs73141296
rs68104925
rs7832116
rs2900498
rs59805249
rs79275904
rs11783892
rs4955932
rs10847659
rs2880566
rs7549273
rs11777482
rs10778394
rs11678402
All - MR Egger
All - Inverse variance weighted
-0.02
0.00
0.02
MR effect size for
' || id:ebi-a-GCST90017024' on 'Non-cancer illness code, self-reported: irritable bowel syndrome || id:ukb-b-2592'

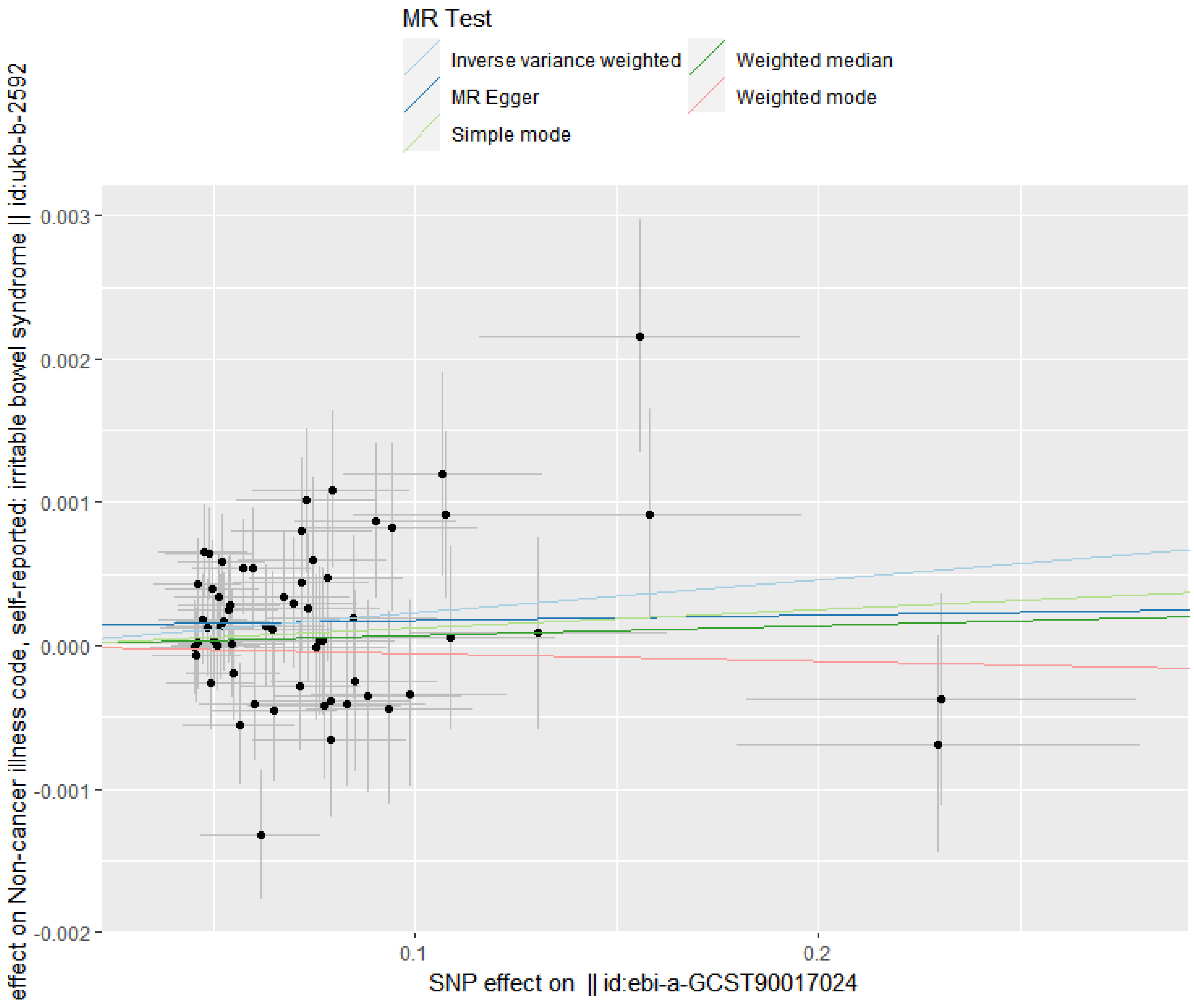

MR Test
Inverse variance weighted
MR Egger
Simple mode
Weighted median
Weighted mode
0.003
0.002
0.001
0.000
-0.001
-0.002
0.1
0.2
SNP effect on || id:ebi-a-GCST90017024
effect on Non-cancer illness code, self-reported: irritable bowel syndrome || id:ukb-b-2592

Figure 101 Leave-one-out analysis, funnel plot, MR effect size and scatter plot for gut microbiota abundance (genus Peptococcus id.2037) on irritable bowel syndrome

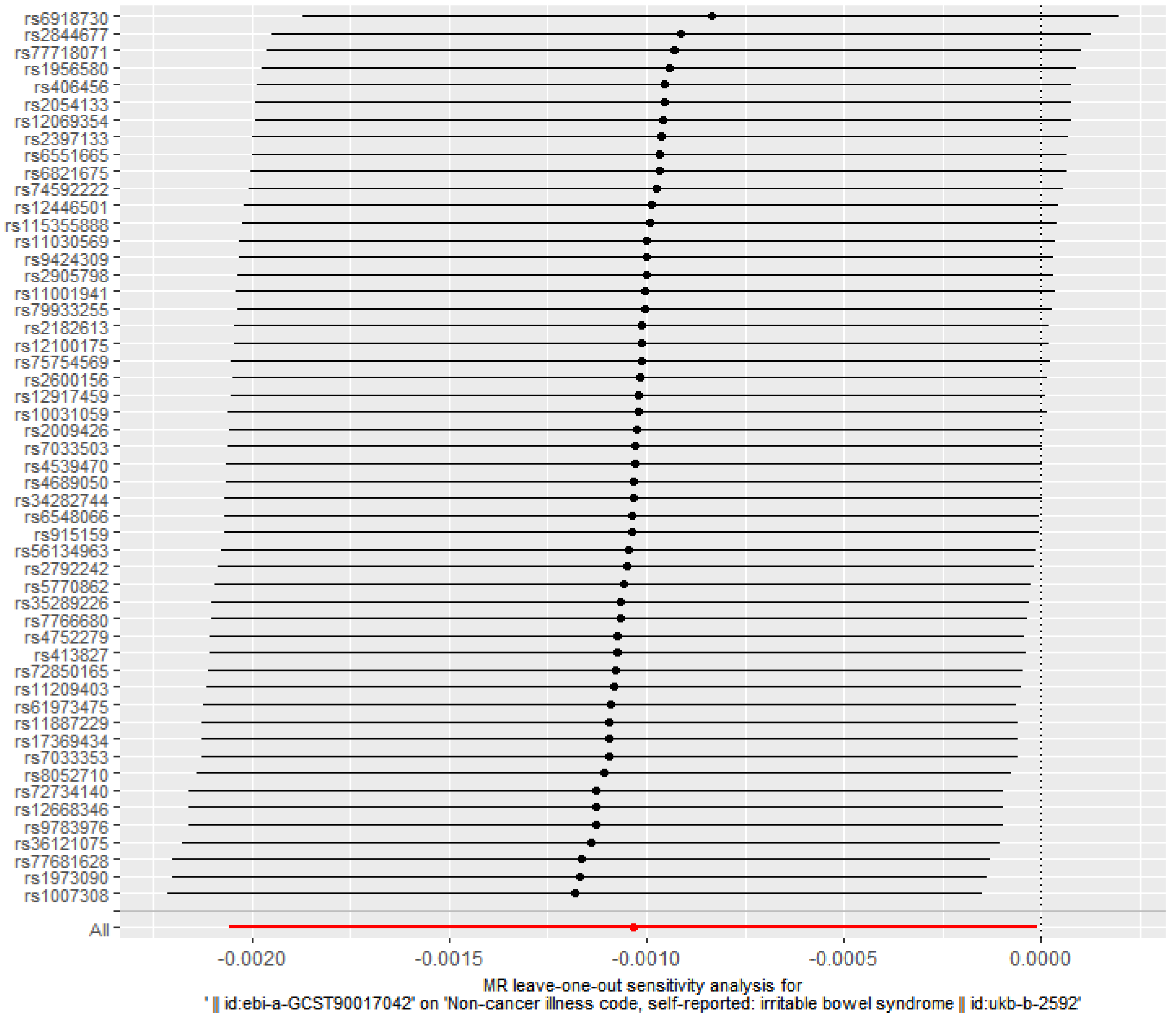

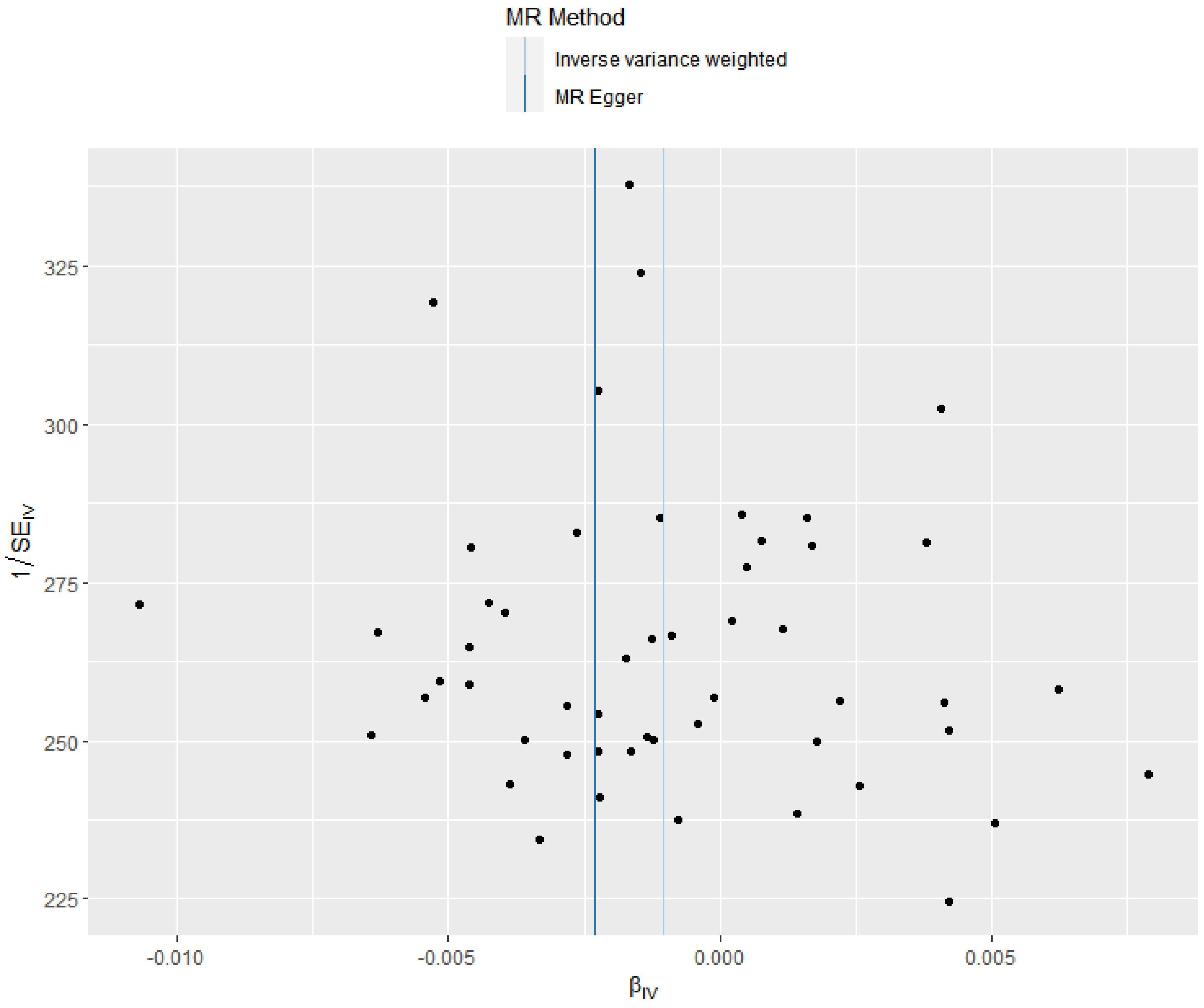

MR Method
Inverse variance weighted
MR Egger
325
300
275
250
225
1/SE_IV
-0.010
-0.005
0.000
0.005
β_IV

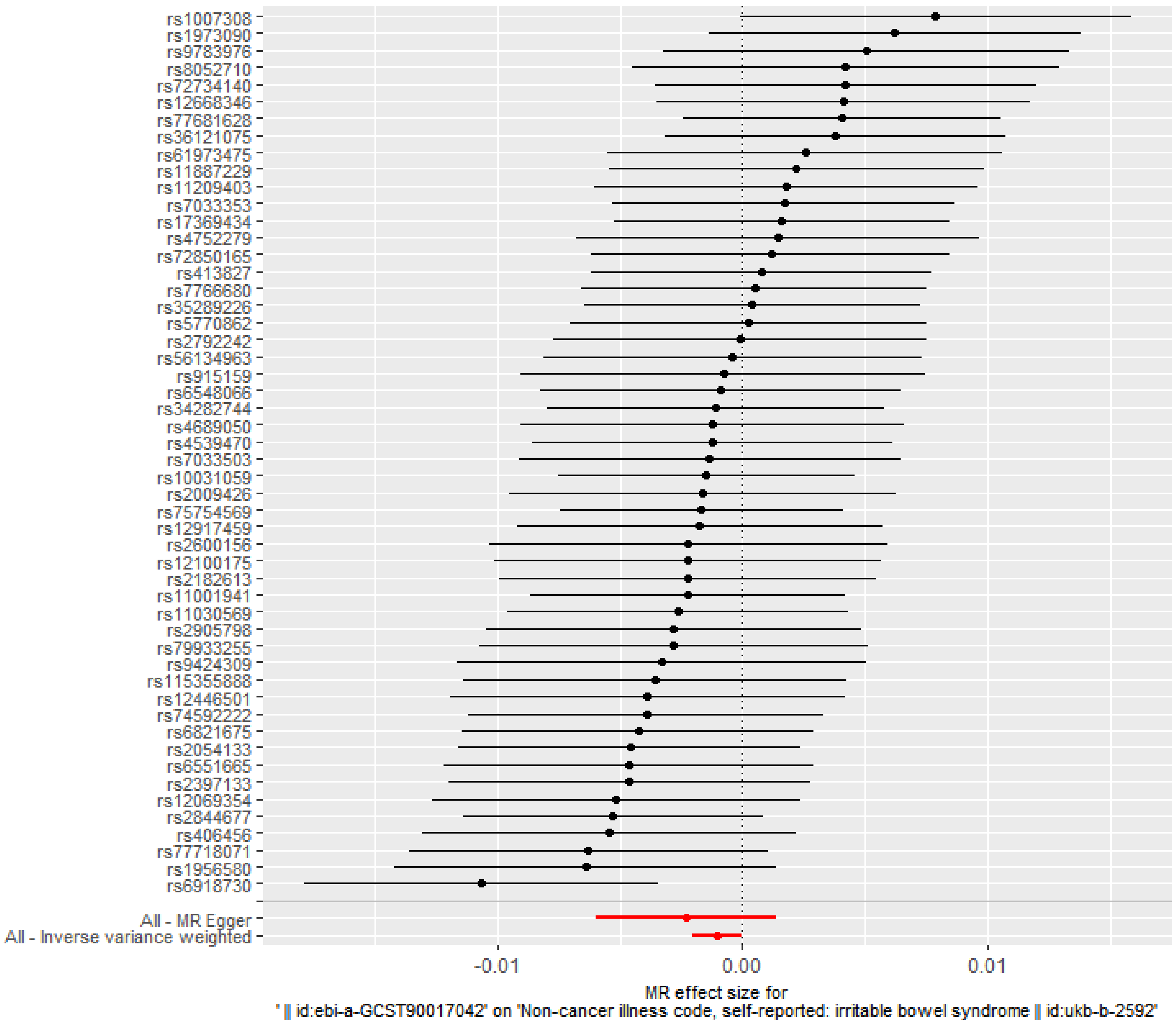

rs1007308
rs1973090
rs9783976
rs8052710
rs72734140
rs12668346
rs77681628
rs36121075
rs61973475
rs11887229
rs11209403
rs7033353
rs17369434
rs4752279
rs72850165
rs413827
rs7766680
rs35289226
rs5770862
rs2792242
rs56134963
rs915159
rs6548066
rs34282744
rs4689050
rs4539470
rs7033503
rs10031059
rs2009426
rs75754569
rs12917459
rs2600156
rs12100175
rs2182613
rs11001941
rs11030569
rs2905798
rs79933255
rs9424309
rs115355888
rs12446501
rs74592222
rs6821675
rs2054133
rs6551665
rs2397133
rs12069354
rs2844677
rs406456
rs77718071
rs1956580
rs6918730
All - MR Egger
All - Inverse variance weighted
-0.01
0.00
0.01
MR effect size for
' || id:ebi-a-GCST90017042' on 'Non-cancer illness code, self-reported: irritable bowel syndrome || id:ukb-b-2592'

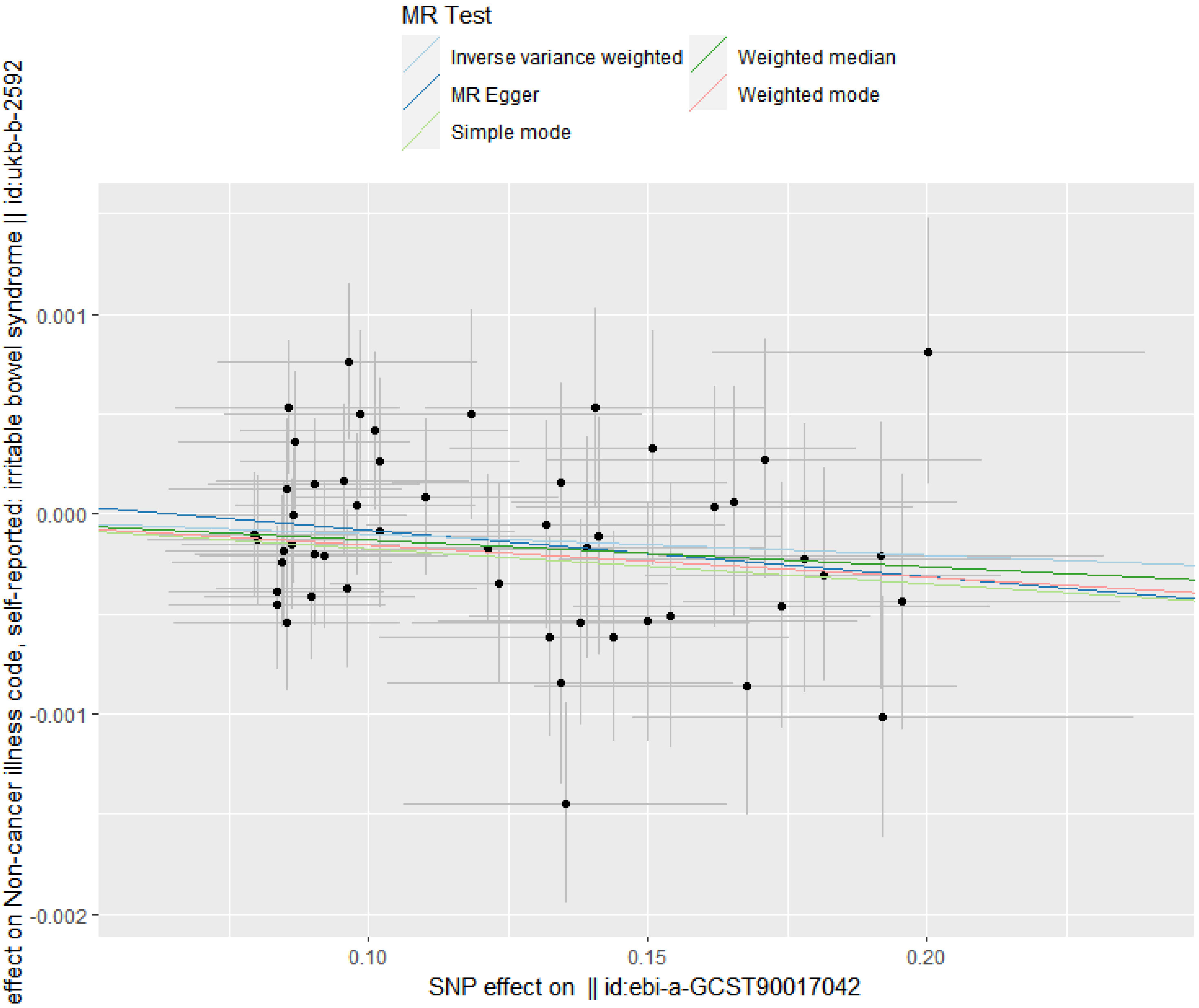

MR Test
Inverse variance weighted
MR Egger
Simple mode
Weighted median
Weighted mode
0.001
0.000
-0.001
-0.002
0.10
0.15
0.20
SNP effect on || id:ebi-a-GCST90017042
effect on Non-cancer illness code, self-reported: irritable bowel syndrome || id:ukb-b-2592

Figure 102 Leave-one-out analysis, funnel plot, MR effect size and scatter plot for gut microbiota abundance (genus Tyzzerella3 id.11335) on irritable bowel syndrome

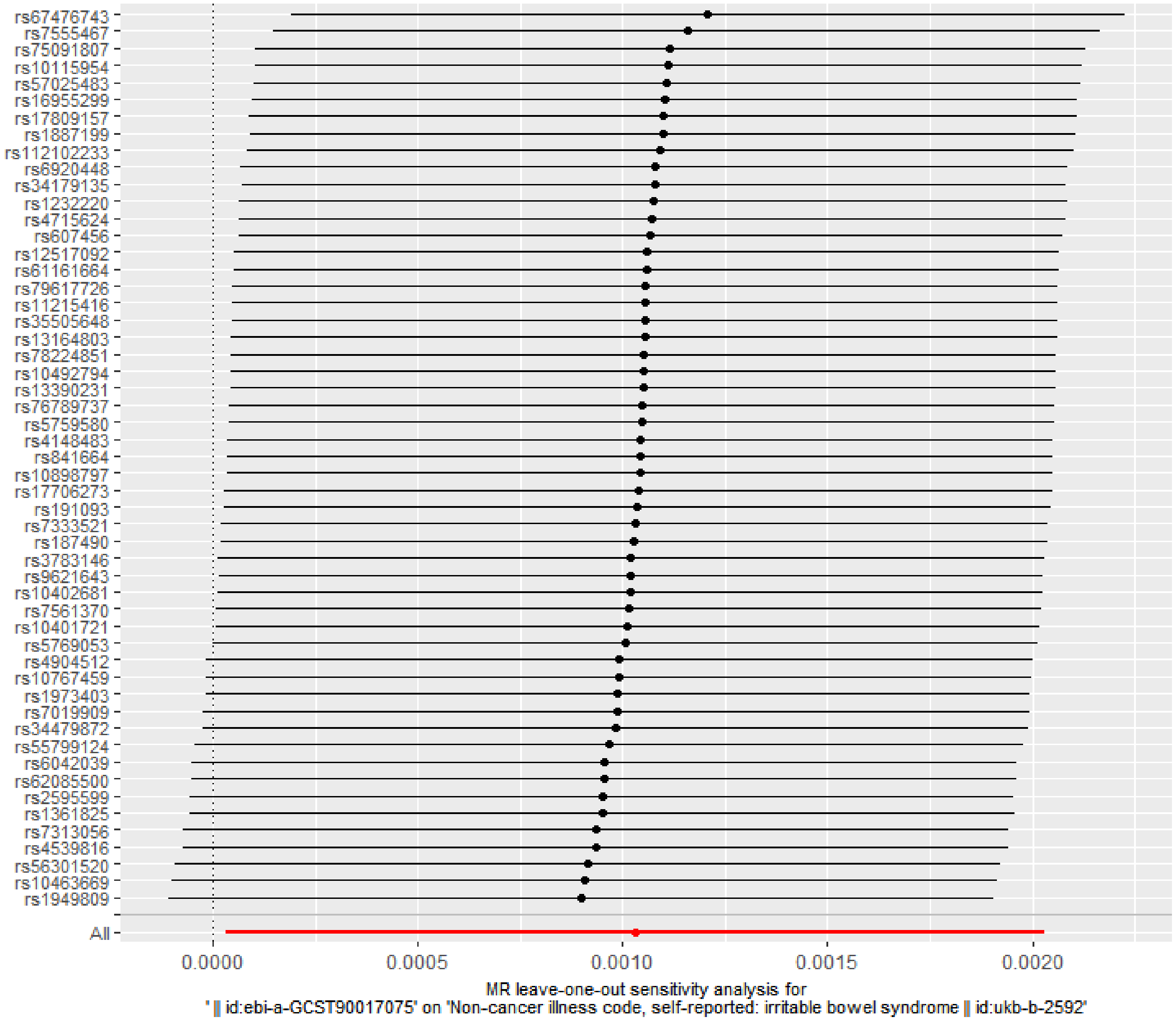

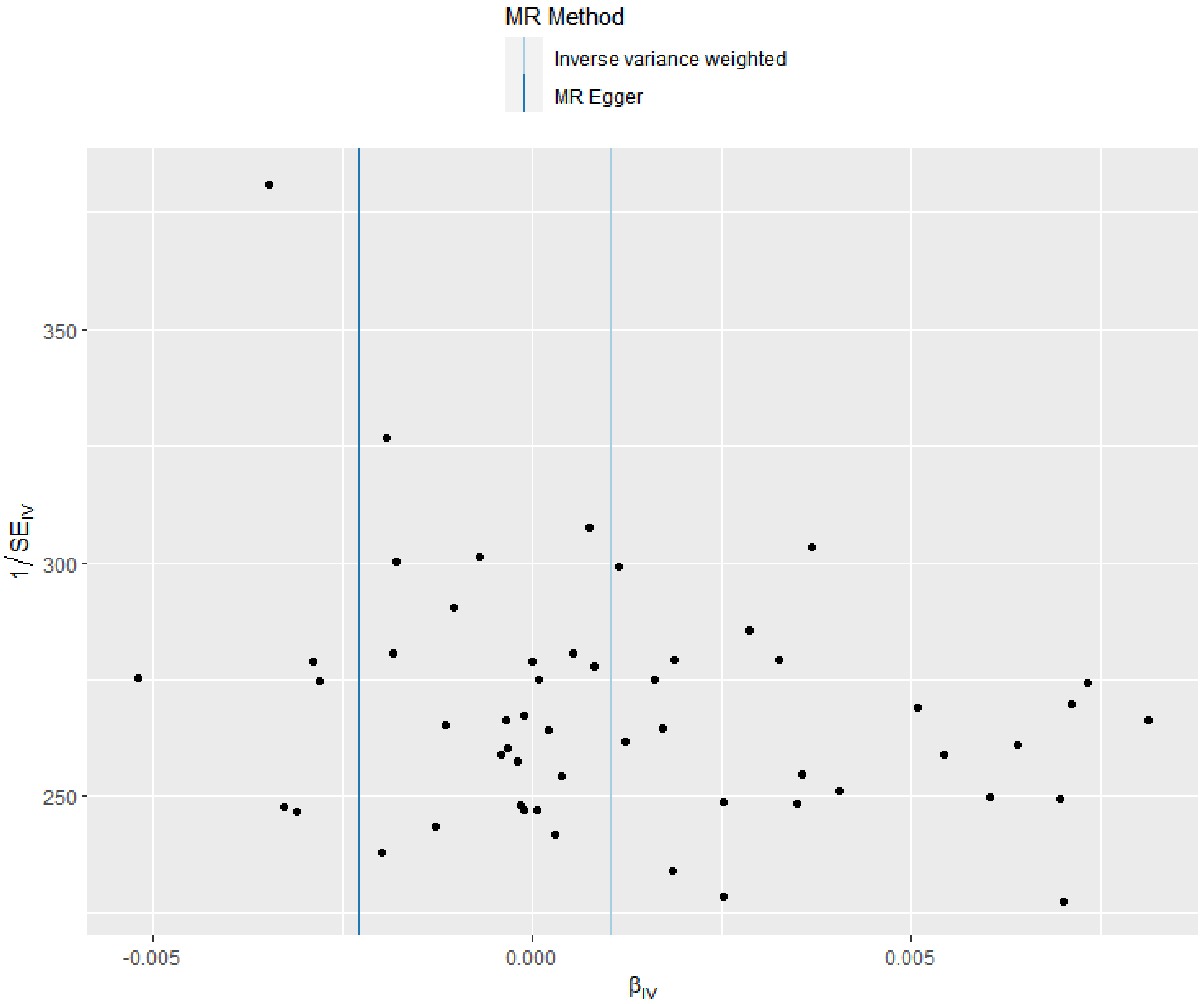

MR Method
Inverse variance weighted
MR Egger
350
300
250
-0.005
0.000
0.005
$1/SE_{IV}$
β_{IV}

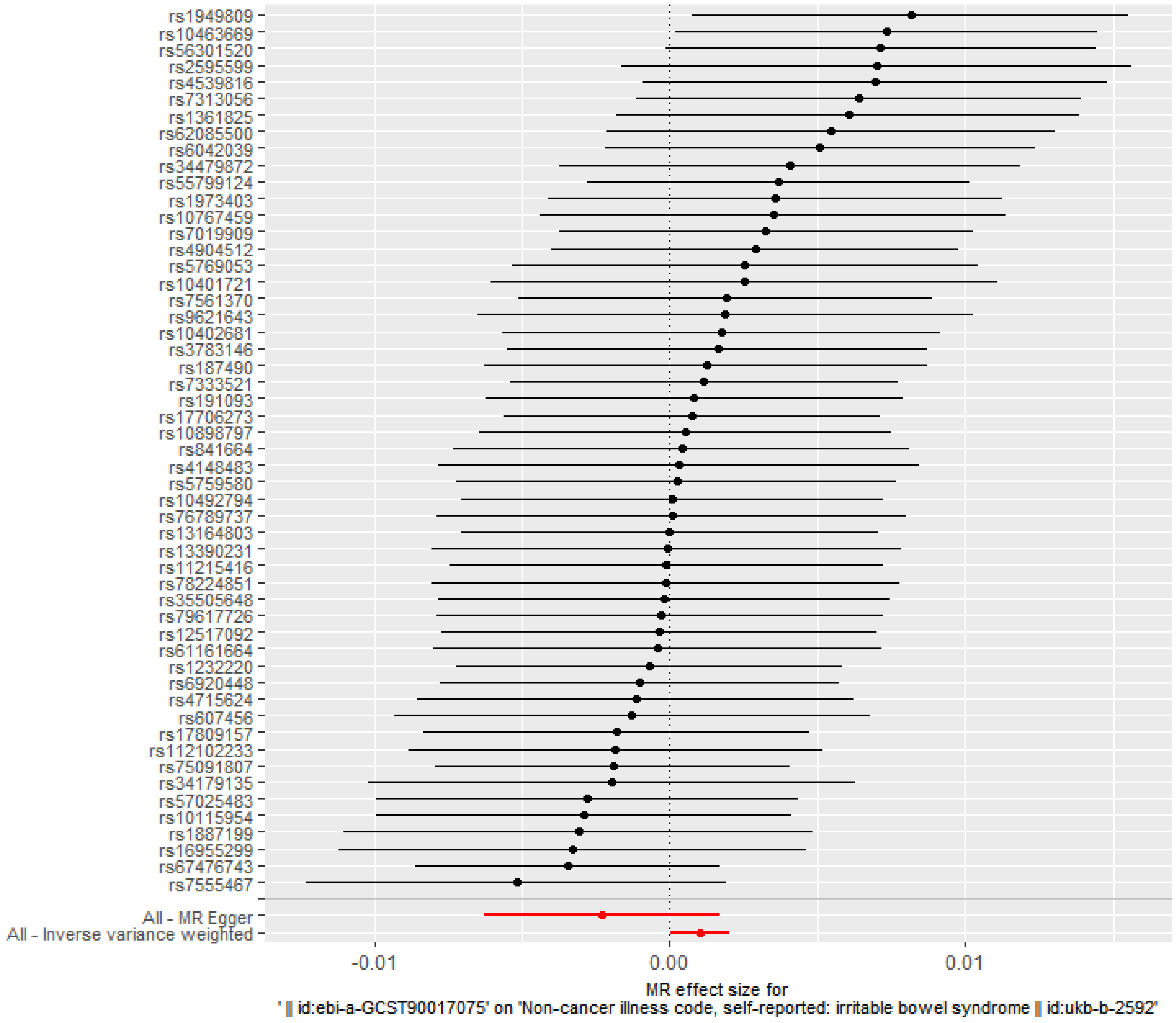

rs1949809
rs10463669
rs56301520
rs2595599
rs4539816
rs7313056
rs1361825
rs62085500
rs6042039
rs34479872
rs55799124
rs1973403
rs10767459
rs7019909
rs4904512
rs5769053
rs10401721
rs7561370
rs9621643
rs10402681
rs3783146
rs187490
rs7333521
rs191093
rs17706273
rs10898797
rs841664
rs4148483
rs5759580
rs10492794
rs76789737
rs13164803
rs13390231
rs11215416
rs78224851
rs35505648
rs79617726
rs12517092
rs61161664
rs1232220
rs6920448
rs4715624
rs607456
rs17809157
rs112102233
rs75091807
rs34179135
rs57025483
rs10115954
rs1887199
rs16955299
rs67476743
rs7555467
All - MR Egger
All - Inverse variance weighted
-0.01
0.00
0.01
MR effect size for
' || id:ebi-a-GCST90017075' on 'Non-cancer illness code, self-reported: irritable bowel syndrome || id:ukb-b-2592'

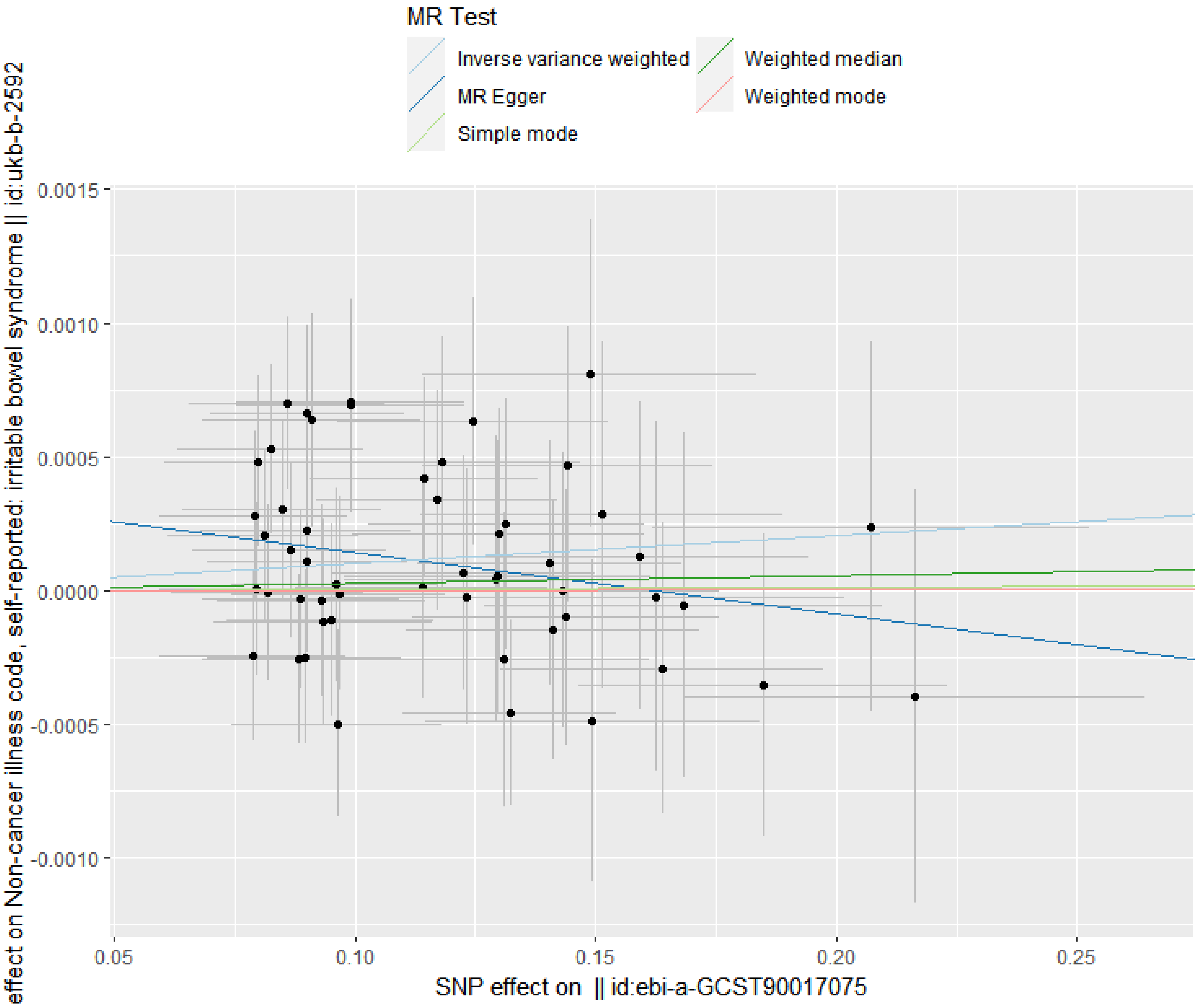
MR Test
Inverse variance weighted
MR Egger
Simple mode
Weighted median
Weighted mode
effect on Non-cancer illness code, self-reported: irritable bowel syndrome || id:ukb-b-2592
0.0015
0.0010
0.0005
0.0000
-0.0005
-0.0010
0.05
0.10
0.15
0.20
0.25
SNP effect on || id:ebi-a-GCST90017075

Figure 103 Leave-one-out analysis, funnel plot, MR effect size and scatter plot for gut microbiota abundance (class Negativicutes id.2164) on diverticular disease

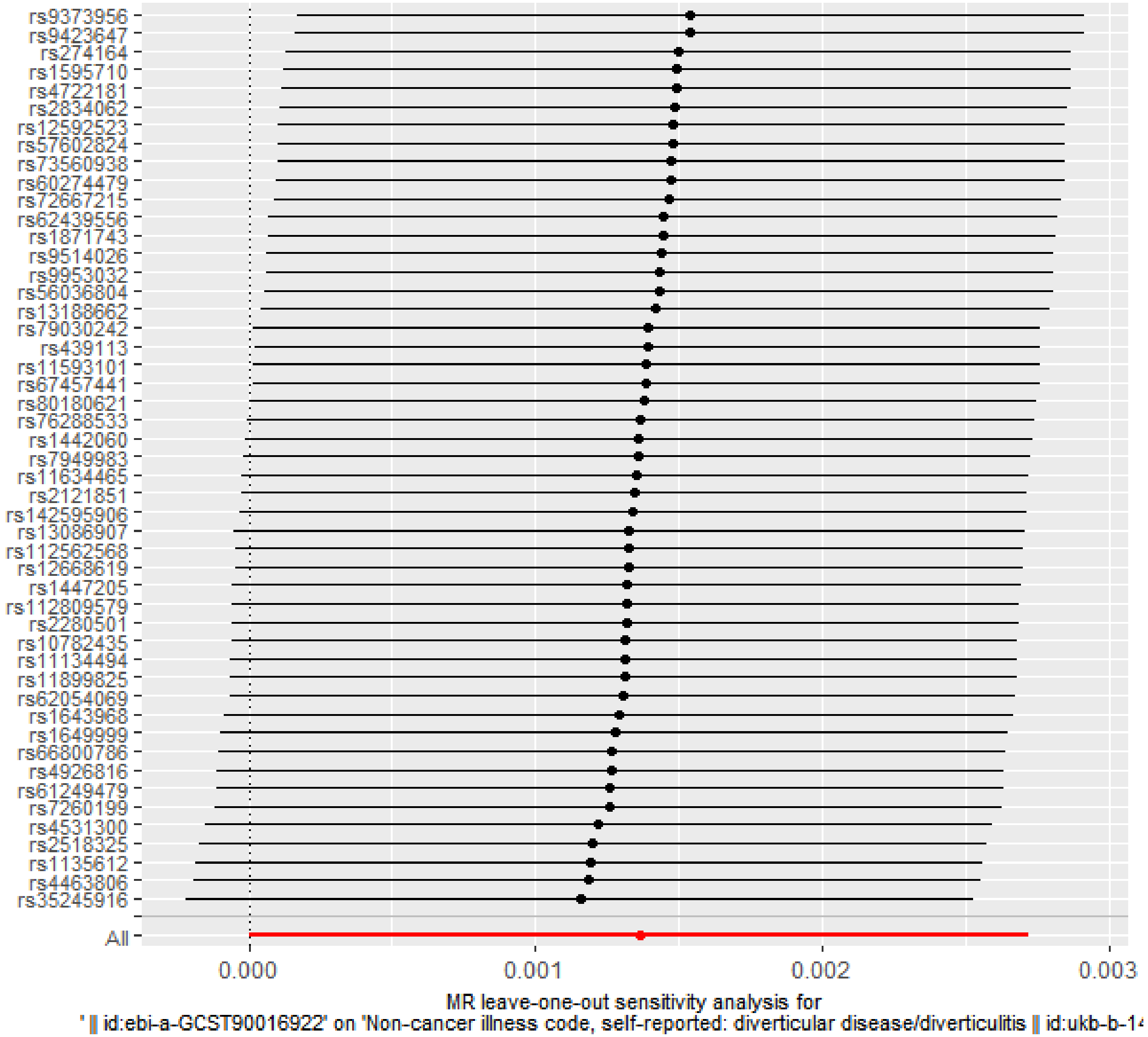

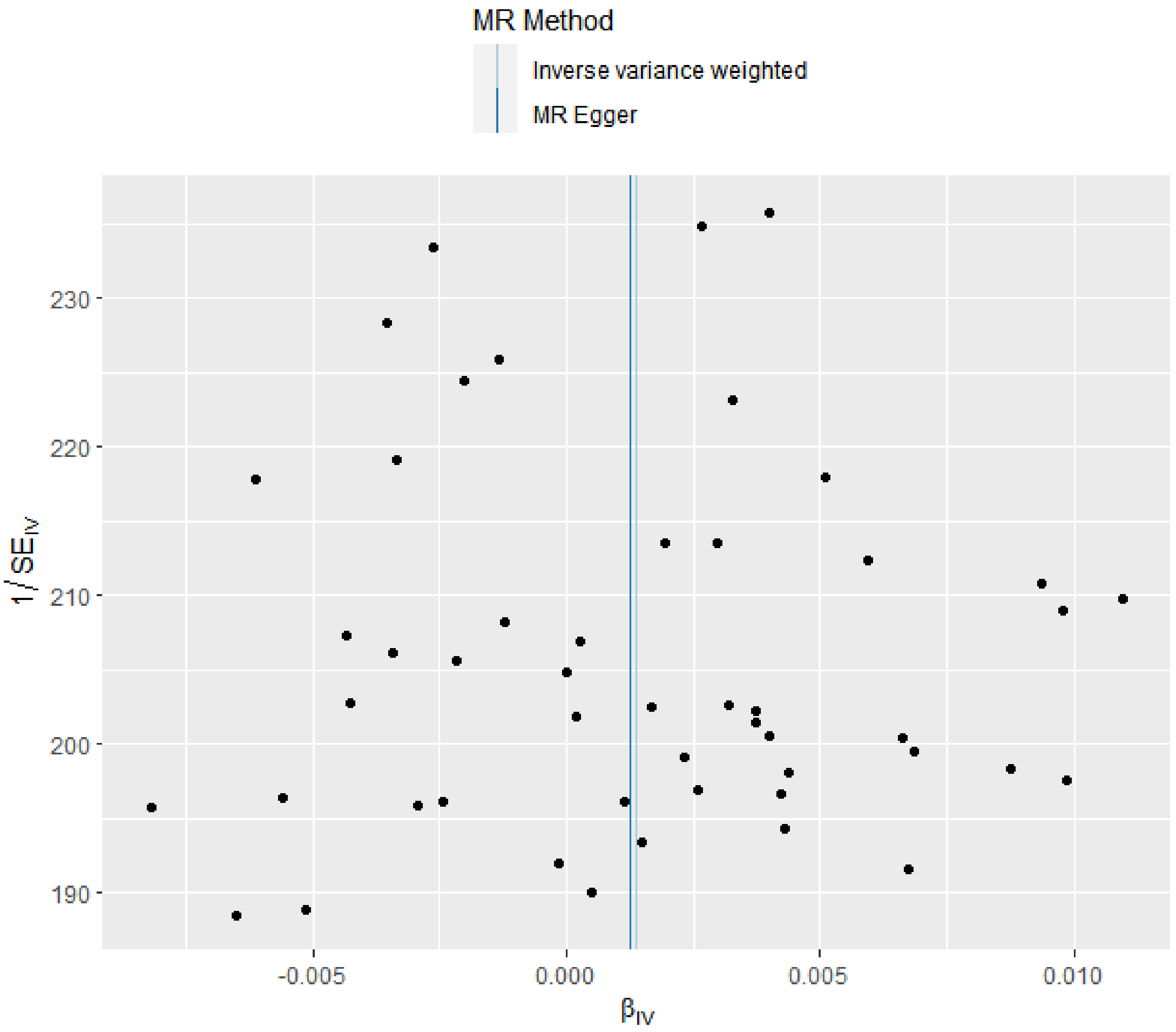

MR Method
Inverse variance weighted
MR Egger
230
220
210
200
190
1/SE_IV
-0.005
0.000
0.005
0.010
β_IV

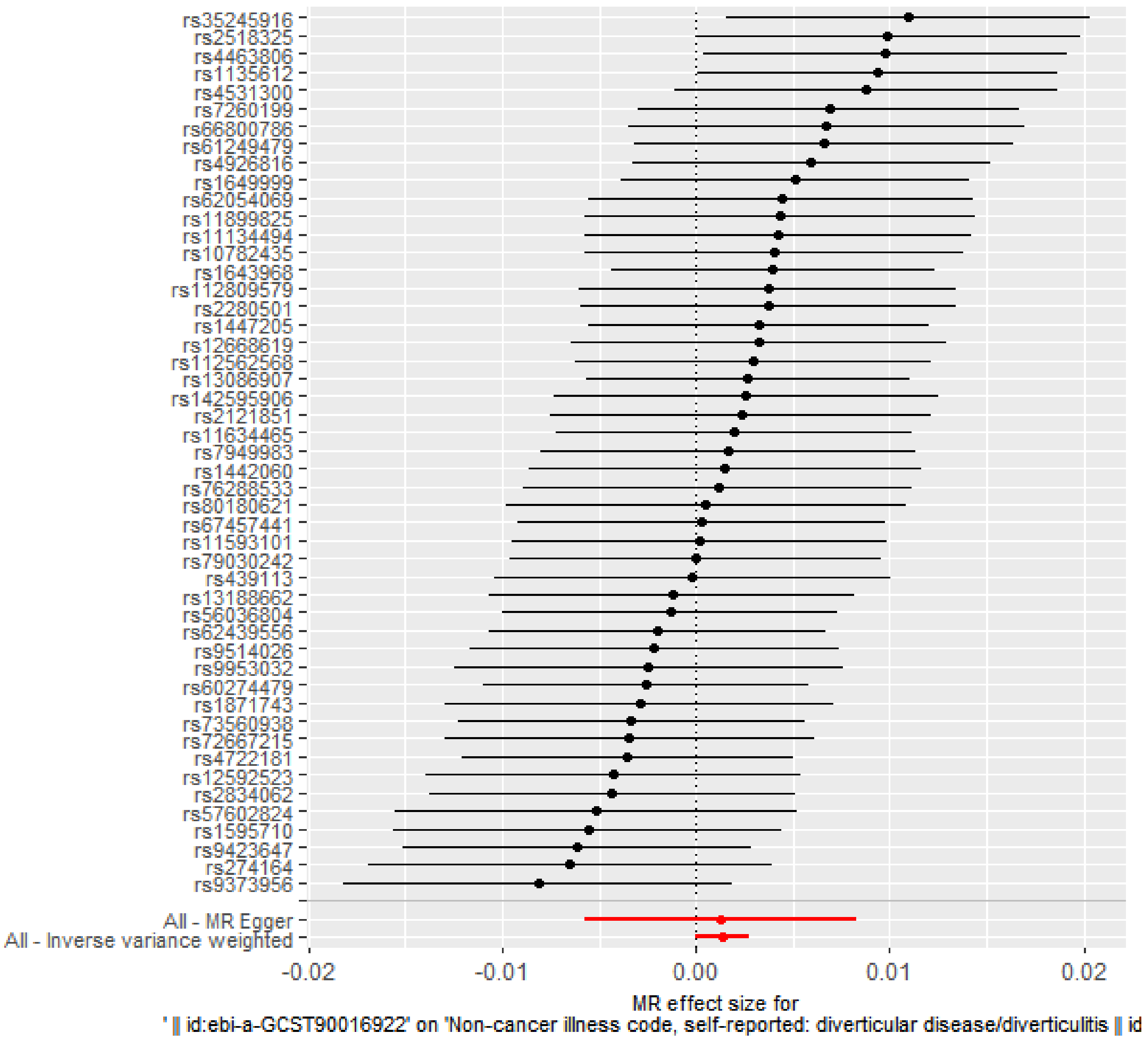

rs35245916
rs2518325
rs4463806
rs1135612
rs4531300
rs7260199
rs66800786
rs61249479
rs4926816
rs1649999
rs62054069
rs11899825
rs11134494
rs10782435
rs1643968
rs112809579
rs2280501
rs1447205
rs12668619
rs112562568
rs13086907
rs142595906
rs2121851
rs11634465
rs7949983
rs1442060
rs76288533
rs80180621
rs67457441
rs11593101
rs79030242
rs439113
rs13188662
rs56036804
rs62439556
rs9514026
rs9953032
rs60274479
rs1871743
rs73560938
rs72667215
rs4722181
rs12592523
rs2834062
rs57602824
rs1595710
rs9423647
rs274164
rs9373956
All - MR Egger
All - Inverse variance weighted
-0.02
-0.01
0.00
0.01
0.02
MR effect size for
' || id:ebi-a-GCST90016922' on 'Non-cancer illness code, self-reported: diverticular disease/diverticulitis || id

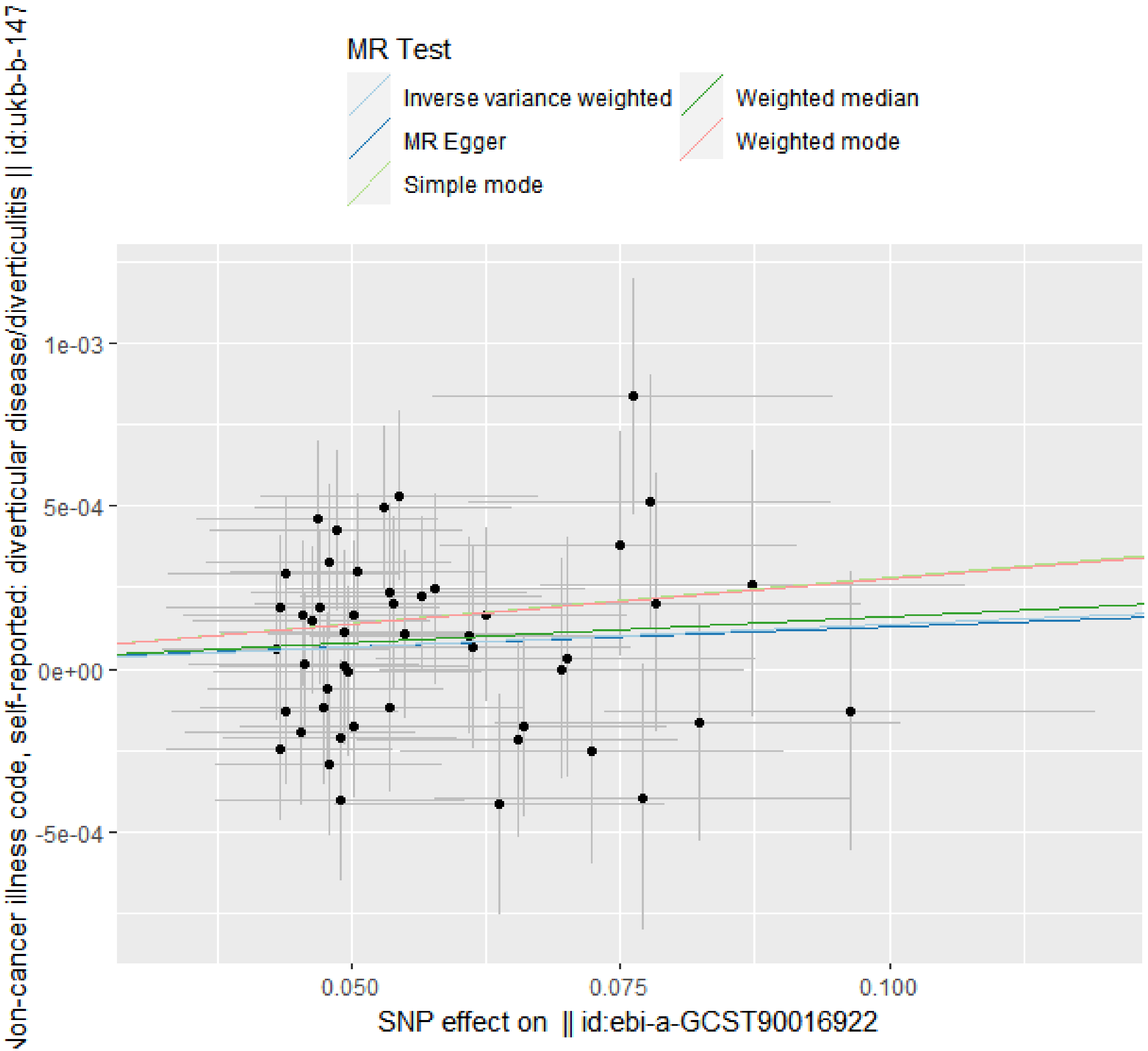
MR Test
Inverse variance weighted
MR Egger
Simple mode
Weighted median
Weighted mode
1e-03
5e-04
0e+00
-5e-04
0.050
0.075
0.100
SNP effect on || id:ebi-a-GCST90016922
Non-cancer illness code, self-reported: diverticular disease/diverticulitis || id:ukb-b-147

Figure 104 Leave-one-out analysis, funnel plot, MR effect size and scatter plot for gut microbiota abundance (family Alcaligenaceae id.2875) on diverticular disease

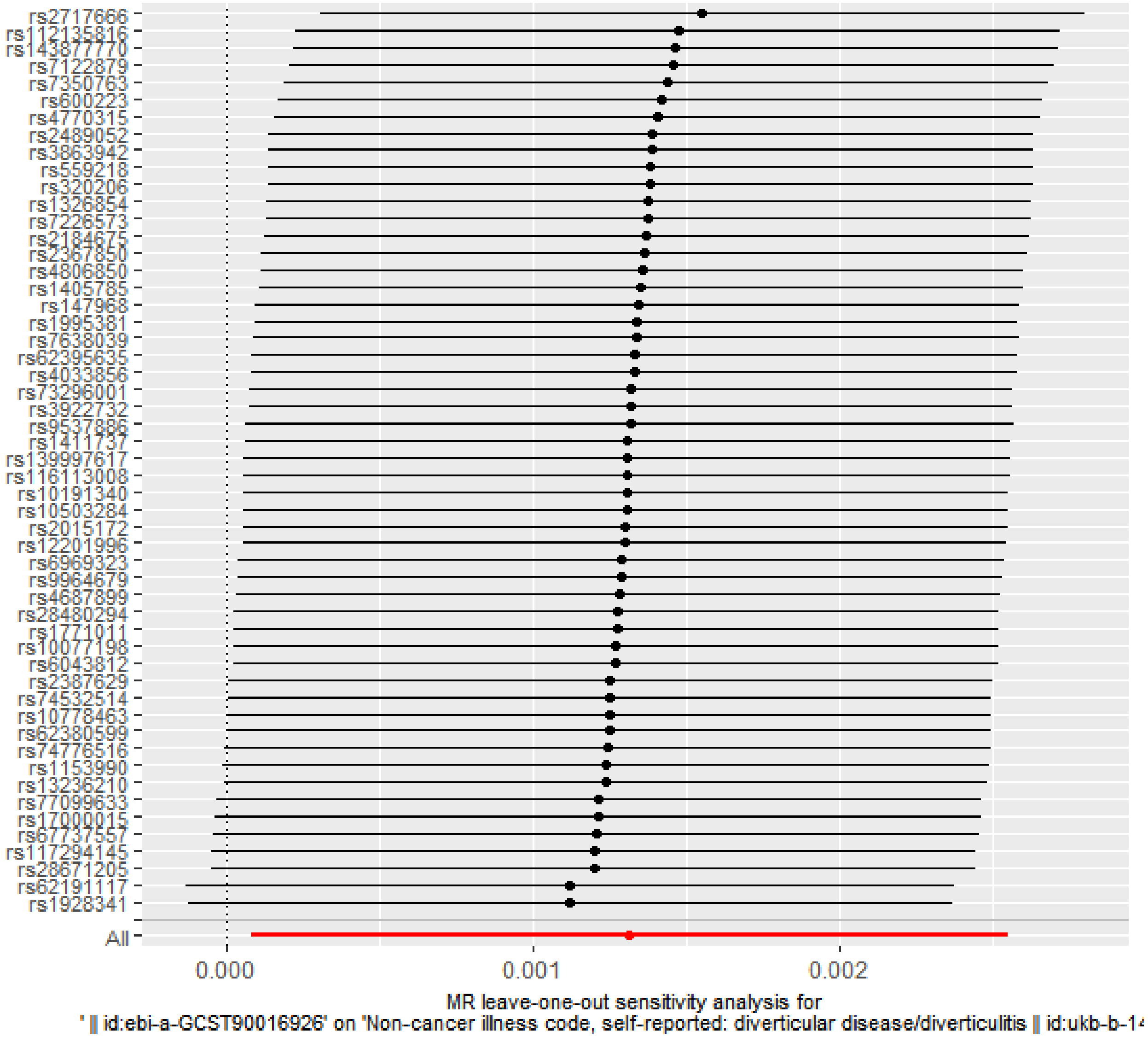

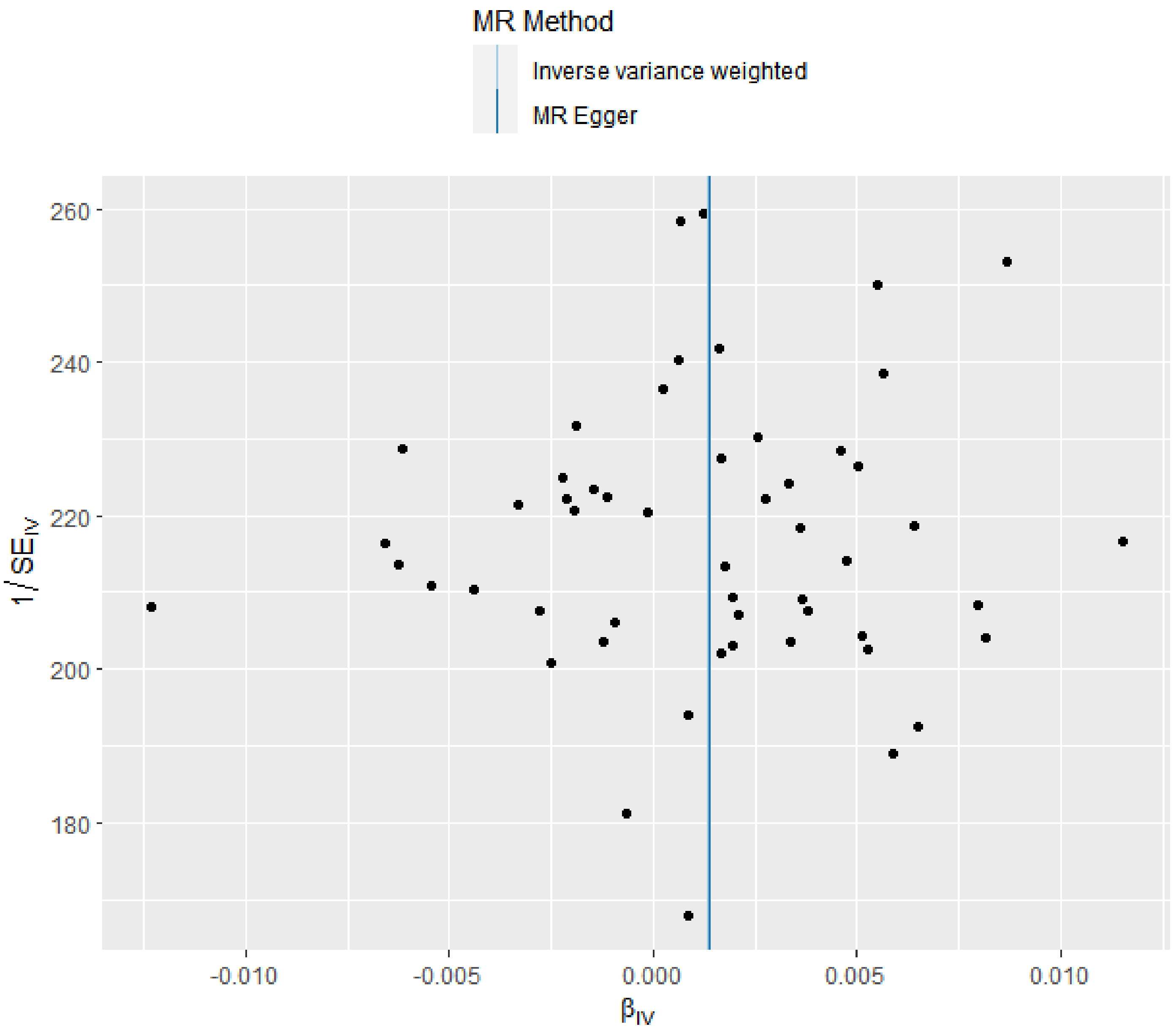

MR Method
Inverse variance weighted
MR Egger
260
240
220
200
180
$1/SE_{IV}$
-0.010
-0.005
0.000
0.005
0.010
β_{IV}

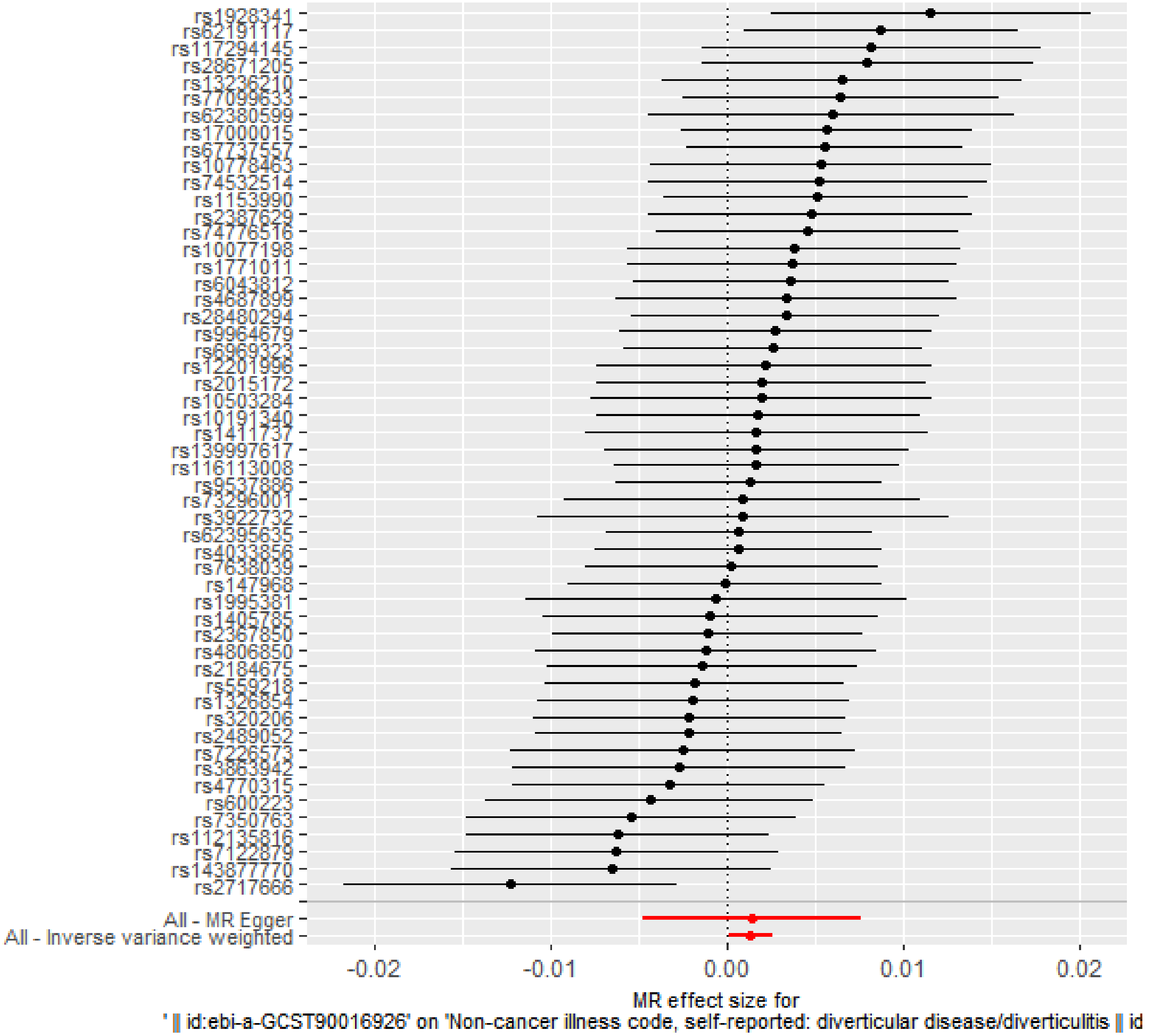

rs1928341
rs62191117
rs117294145
rs28671205
rs13236210
rs77099633
rs62380599
rs17000015
rs67737557
rs10778463
rs74532514
rs1153990
rs2387629
rs74776516
rs10077198
rs1771011
rs6043812
rs4687899
rs28480294
rs9964679
rs6969323
rs12201996
rs2015172
rs10503284
rs10191340
rs1411737
rs139997617
rs116113008
rs9537886
rs73296001
rs3922732
rs62395635
rs4033856
rs7638039
rs147968
rs1995381
rs1405785
rs2367850
rs4806850
rs2184675
rs559218
rs1326854
rs320206
rs2489052
rs7226573
rs3863942
rs4770315
rs600223
rs7350763
rs112135816
rs7122879
rs143877770
rs2717666
All - MR Egger
All - Inverse variance weighted
-0.02
-0.01
0.00
0.01
0.02
MR effect size for
' || id:ebi-a-GCST90016926' on 'Non-cancer illness code, self-reported: diverticular disease/diverticulitis || id

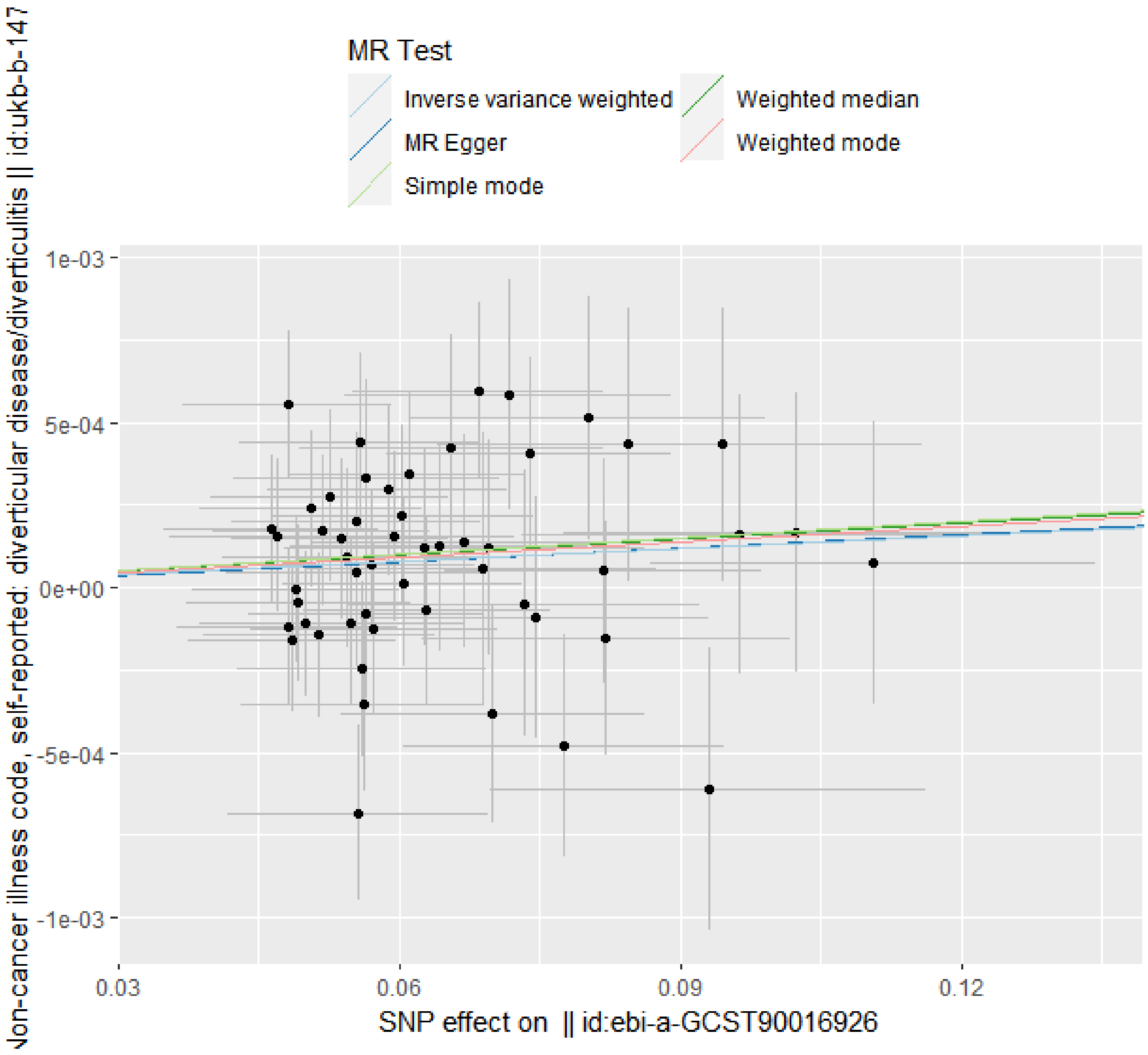

MR Test
Inverse variance weighted
MR Egger
Simple mode
Weighted median
Weighted mode
1e-03
5e-04
0e+00
-5e-04
-1e-03
0.03
0.06
0.09
0.12
SNP effect on || id:ebi-a-GCST90016926
Non-cancer illness code, self-reported: diverticular disease/diverticulitis || id:ukb-b-147

Figure 105 Leave-one-out analysis, funnel plot, MR effect size and scatter plot for gut microbiota abundance (family Family XIII id.1957) on diverticular disease

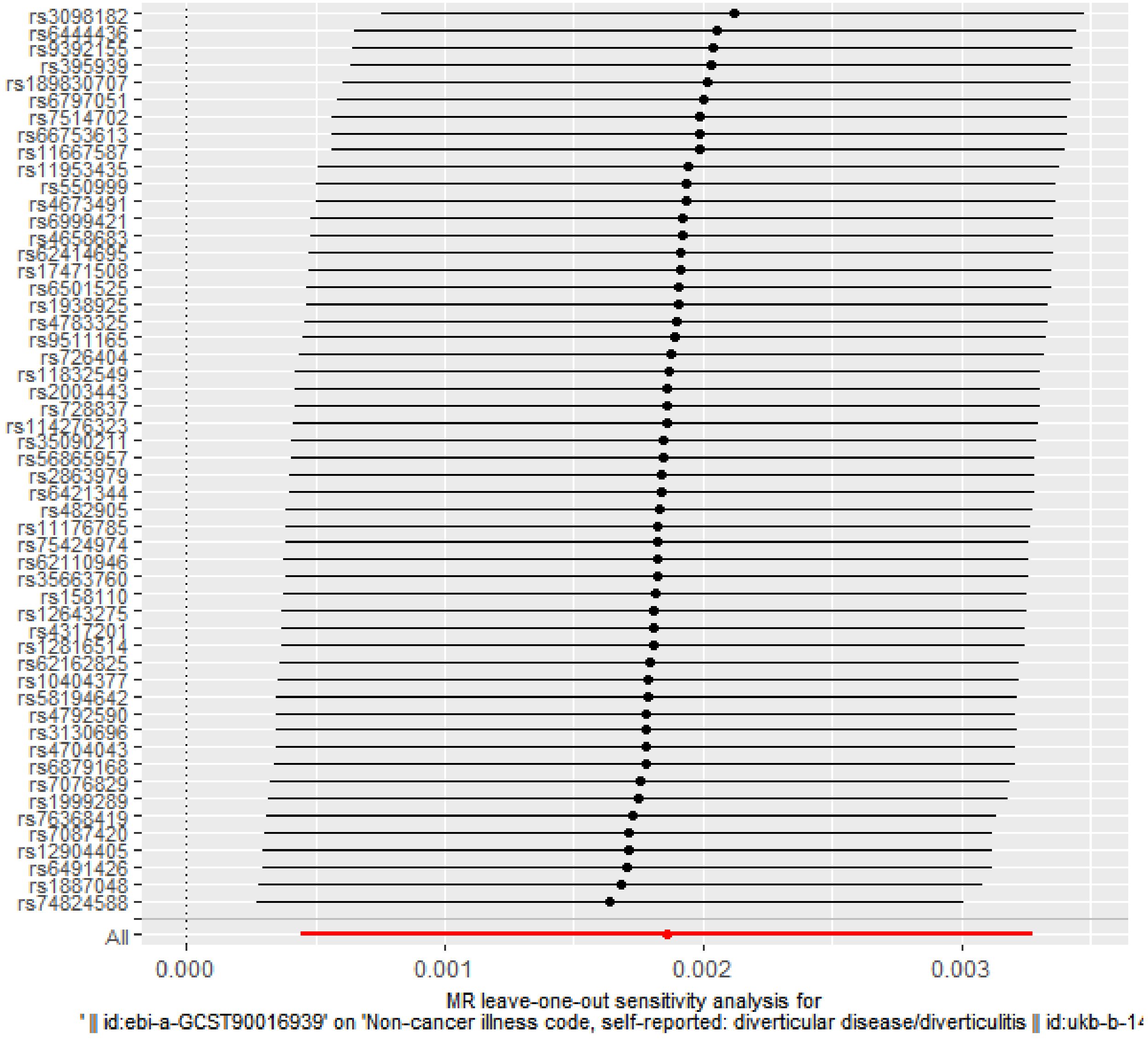

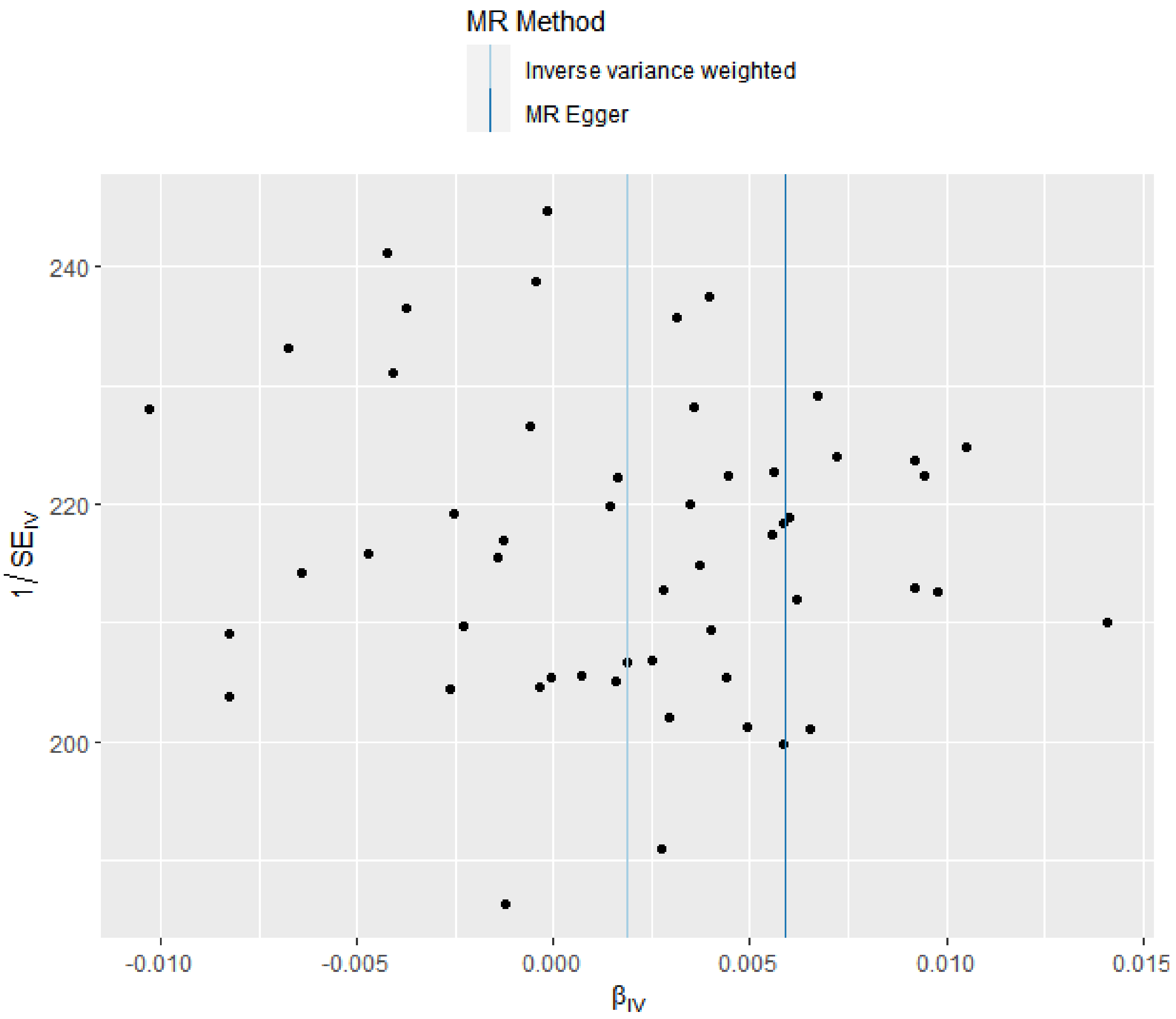
MR Method
Inverse variance weighted
MR Egger
240
220
200
-0.010
-0.005
0.000
0.005
0.010
0.015
β_{IV}
$1/SE_{IV}$

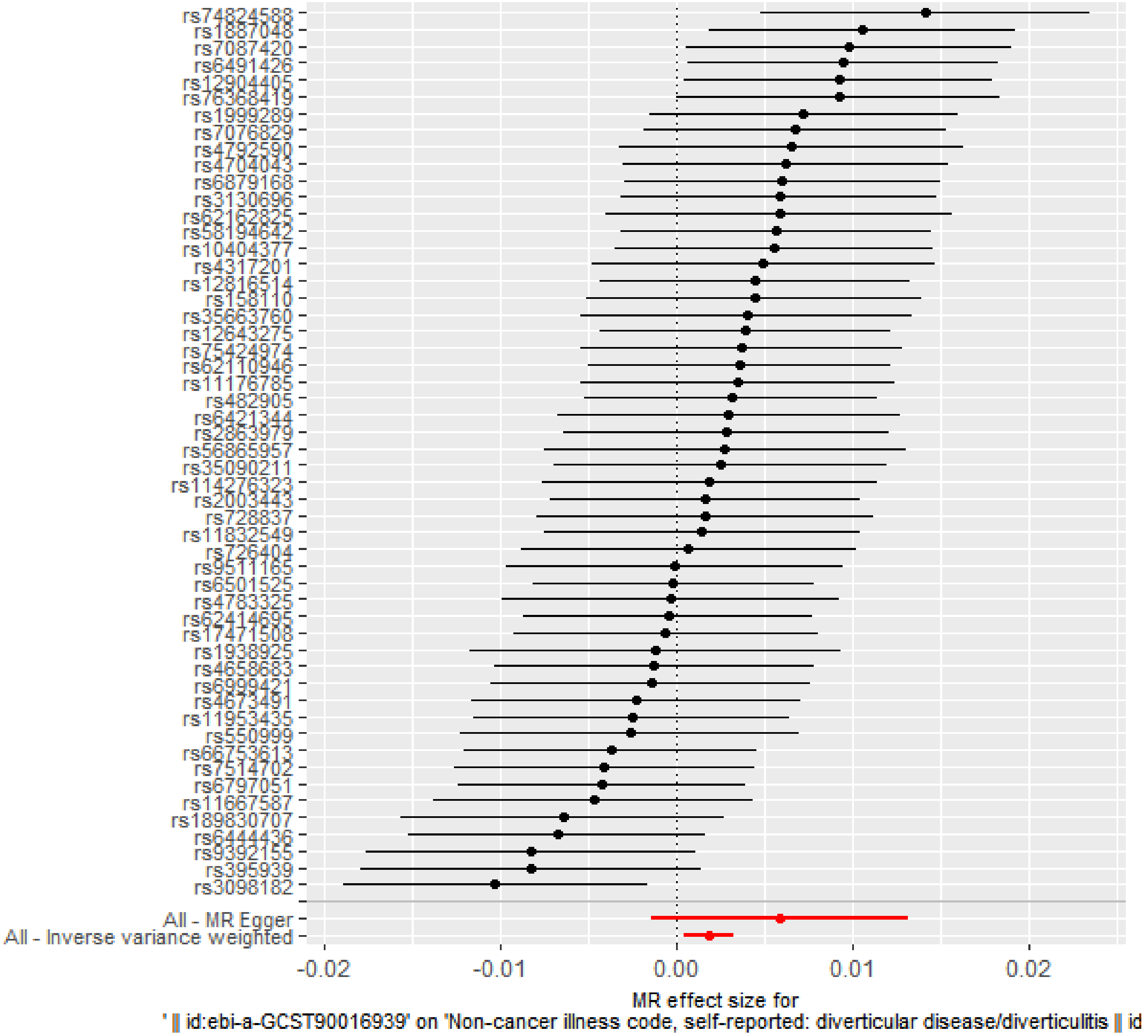
rs74824588
rs1887048
rs7087420
rs6491426
rs12904405
rs76368419
rs1999289
rs7076829
rs4792590
rs4704043
rs6879168
rs3130696
rs62162825
rs58194642
rs10404377
rs4317201
rs12816514
rs158110
rs35663760
rs12643275
rs75424974
rs62110946
rs11176785
rs482905
rs6421344
rs2863979
rs56865957
rs35090211
rs114276323
rs2003443
rs728837
rs11832549
rs726404
rs9511165
rs6501525
rs4783325
rs62414695
rs17471508
rs1938925
rs4658683
rs6999421
rs4673491
rs11953435
rs550999
rs66753613
rs7514702
rs6797051
rs11667587
rs189830707
rs6444436
rs9392155
rs395939
rs3098182
All - MR Egger
All - Inverse variance weighted
-0.02
-0.01
0.00
0.01
0.02
MR effect size for
' || id:ebi-a-GCST90016939' on 'Non-cancer illness code, self-reported: diverticular disease/diverticulitis || id

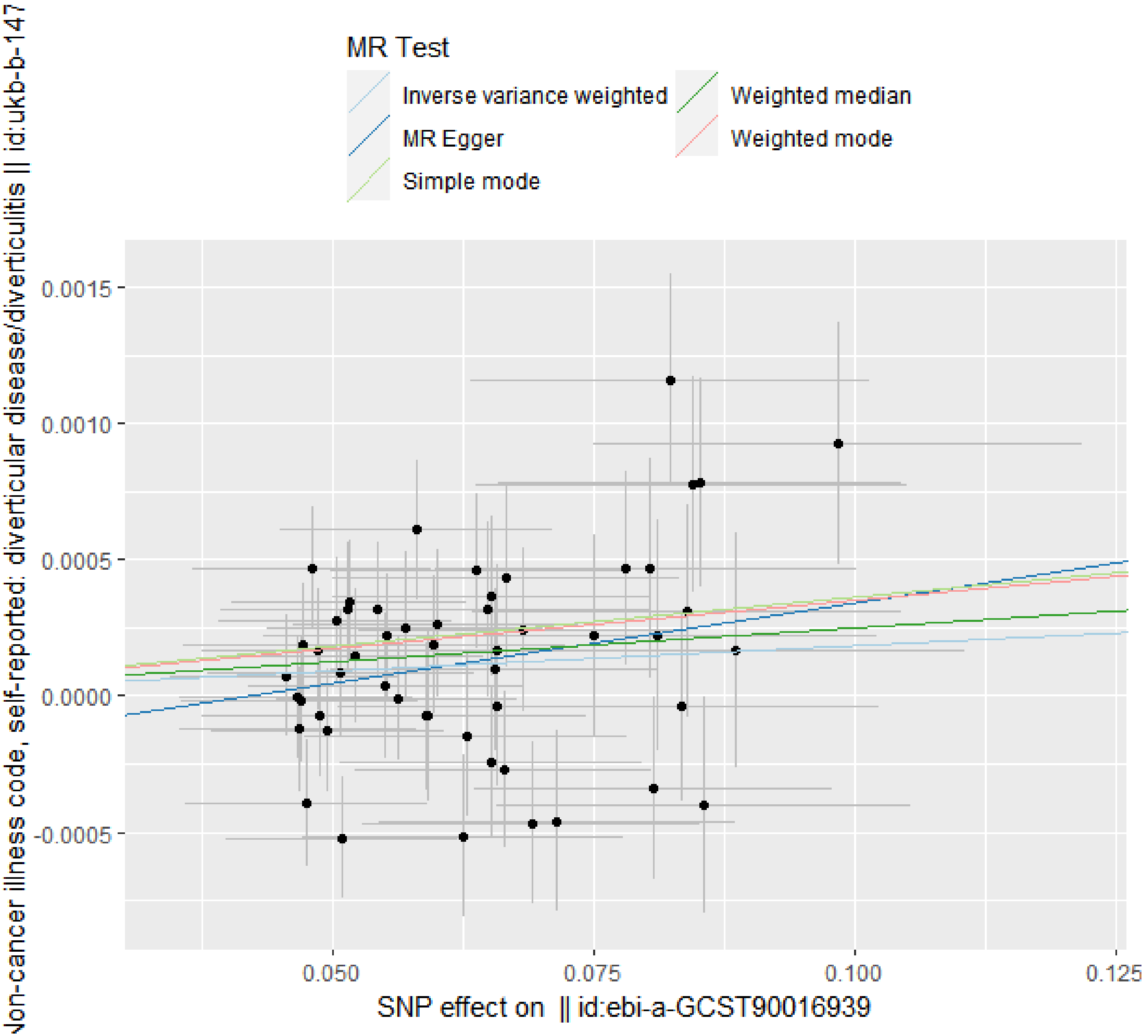
MR Test
Inverse variance weighted
MR Egger
Simple mode
Weighted median
Weighted mode
0.0015
0.0010
0.0005
0.0000
-0.0005
0.050
0.075
0.100
0.125
SNP effect on || id:ebi-a-GCST90016939
Non-cancer illness code, self-reported: diverticular disease/diverticulitis || id:ukb-b-147

Figure 106 Leave-one-out analysis, funnel plot, MR effect size and scatter plot for gut microbiota abundance (genus Barnesiella id.944) on diverticular disease

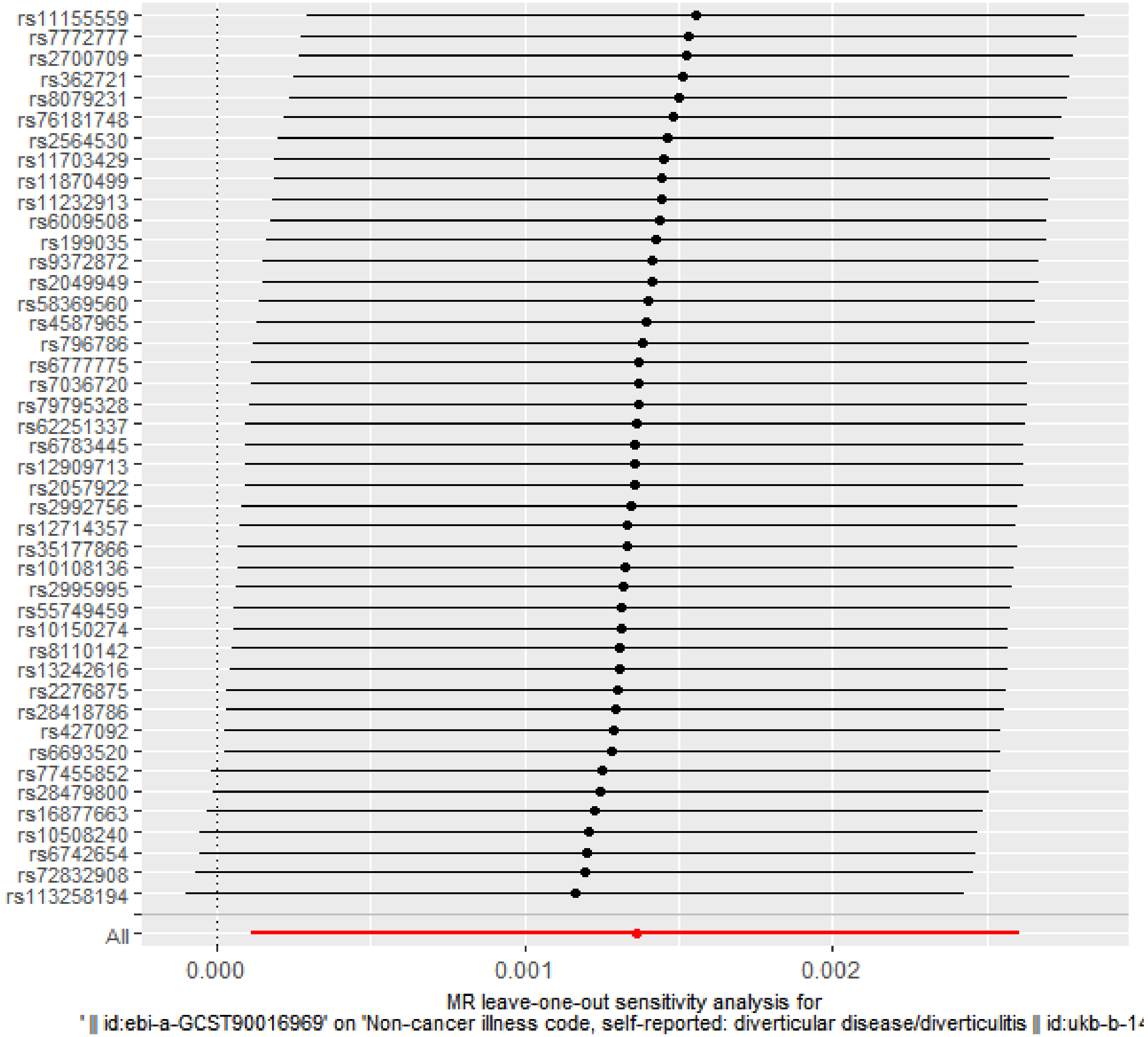

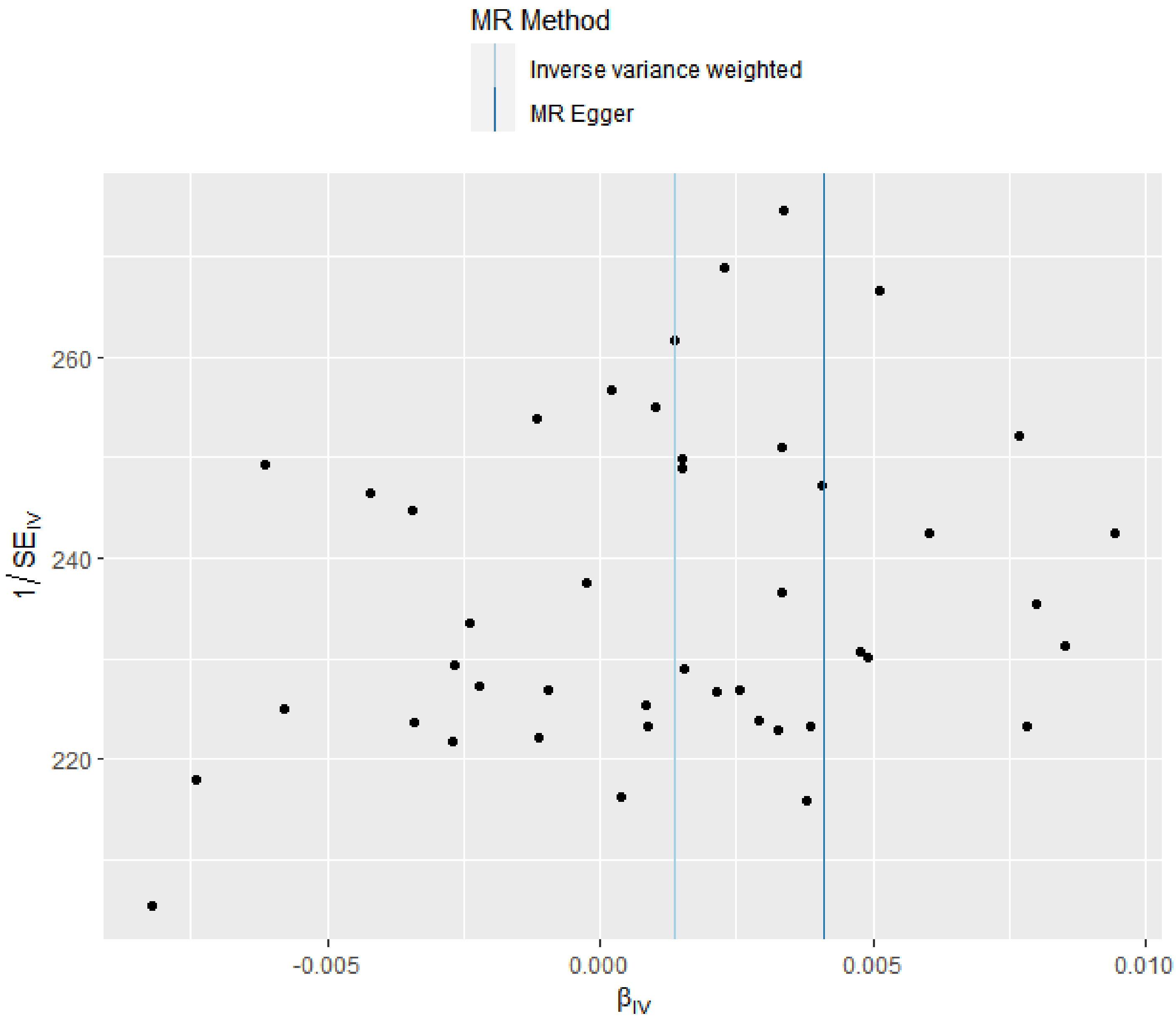

MR Method
Inverse variance weighted
MR Egger
260
240
220
1/SE_IV
-0.005
0.000
0.005
0.010
β_IV

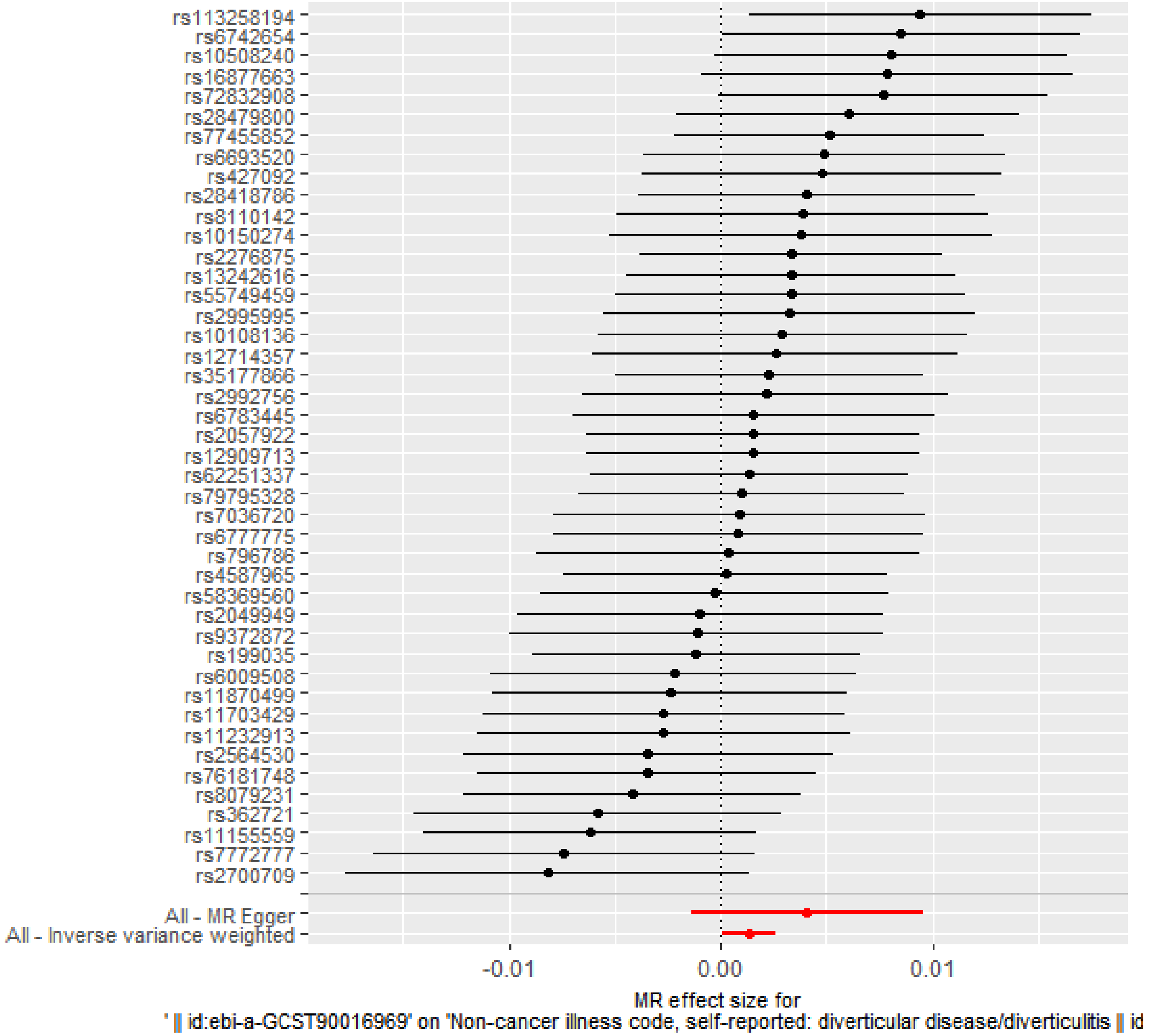

rs113258194
rs6742654
rs10508240
rs16877663
rs72832908
rs28479800
rs77455852
rs6693520
rs427092
rs28418786
rs8110142
rs10150274
rs2276875
rs13242616
rs55749459
rs2995995
rs10108136
rs12714357
rs35177866
rs2992756
rs6783445
rs2057922
rs12909713
rs62251337
rs79795328
rs7036720
rs6777775
rs796786
rs4587965
rs58369560
rs2049949
rs9372872
rs199035
rs6009508
rs11870499
rs11703429
rs11232913
rs2564530
rs76181748
rs8079231
rs362721
rs11155559
rs7772777
rs2700709
All - MR Egger
All - Inverse variance weighted
-0.01
0.00
0.01
MR effect size for
' || id:ebi-a-GCST90016969' on 'Non-cancer illness code, self-reported: diverticular disease/diverticulitis || id

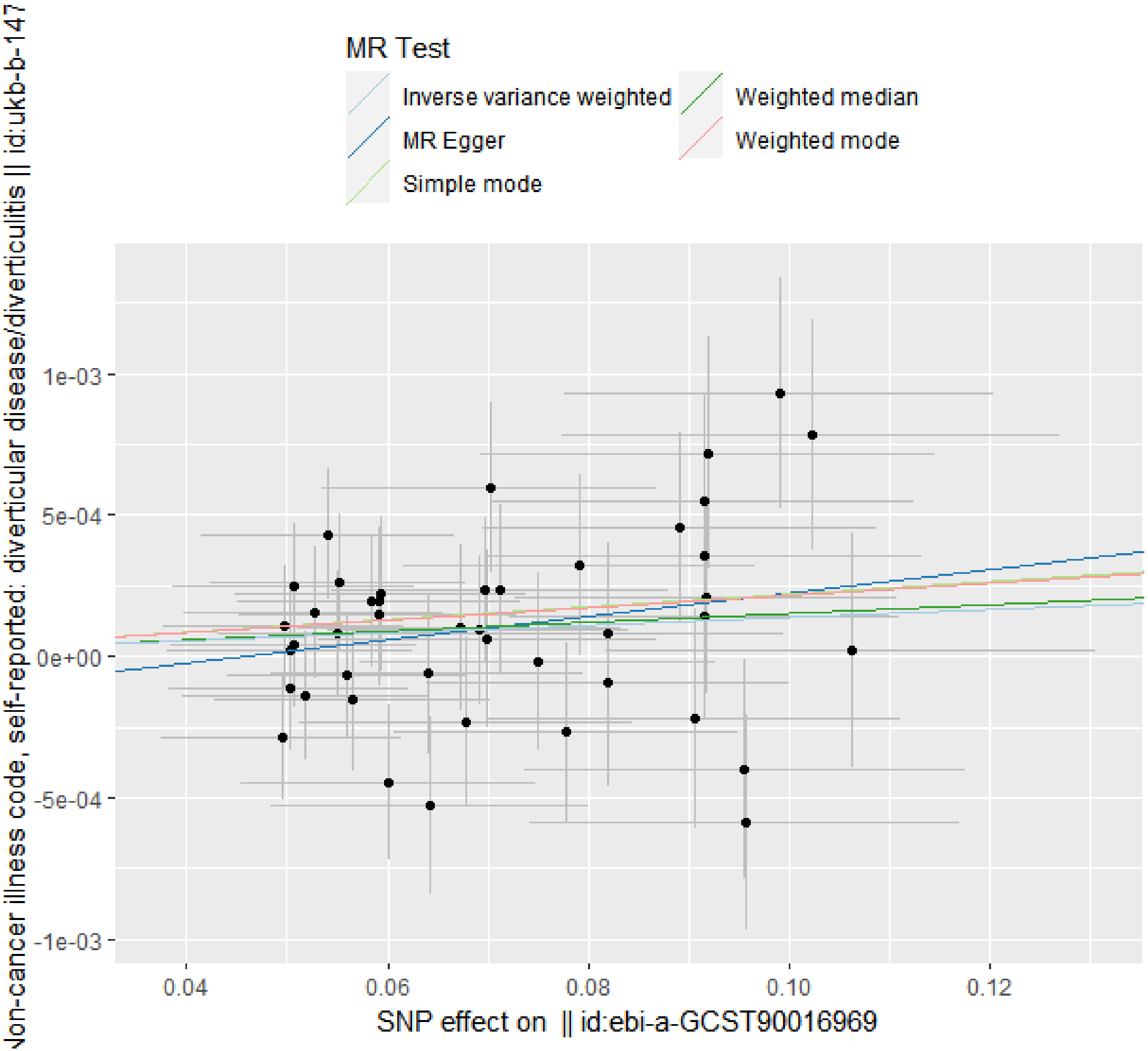
MR Test
Inverse variance weighted
MR Egger
Simple mode
Weighted median
Weighted mode
1e-03
5e-04
0e+00
-5e-04
-1e-03
0.04
0.06
0.08
0.10
0.12
SNP effect on || id:ebi-a-GCST90016969
Non-cancer illness code, self-reported: diverticular disease/diverticulitis || id:ukb-b-147

Figure 107 Leave-one-out analysis, funnel plot, MR effect size and scatter plot for gut microbiota abundance (genus Eubacterium xylanophilum group id.14375) on diverticular disease

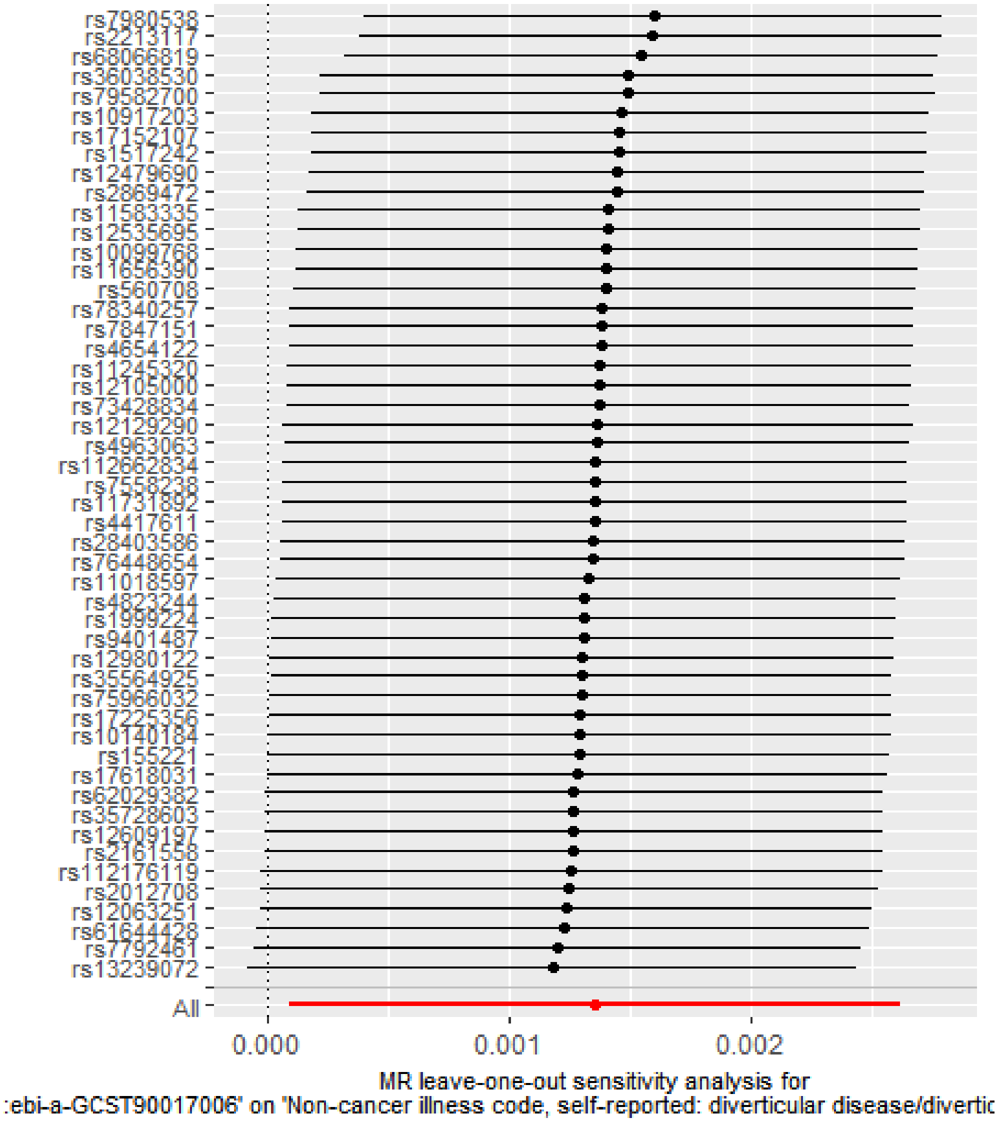

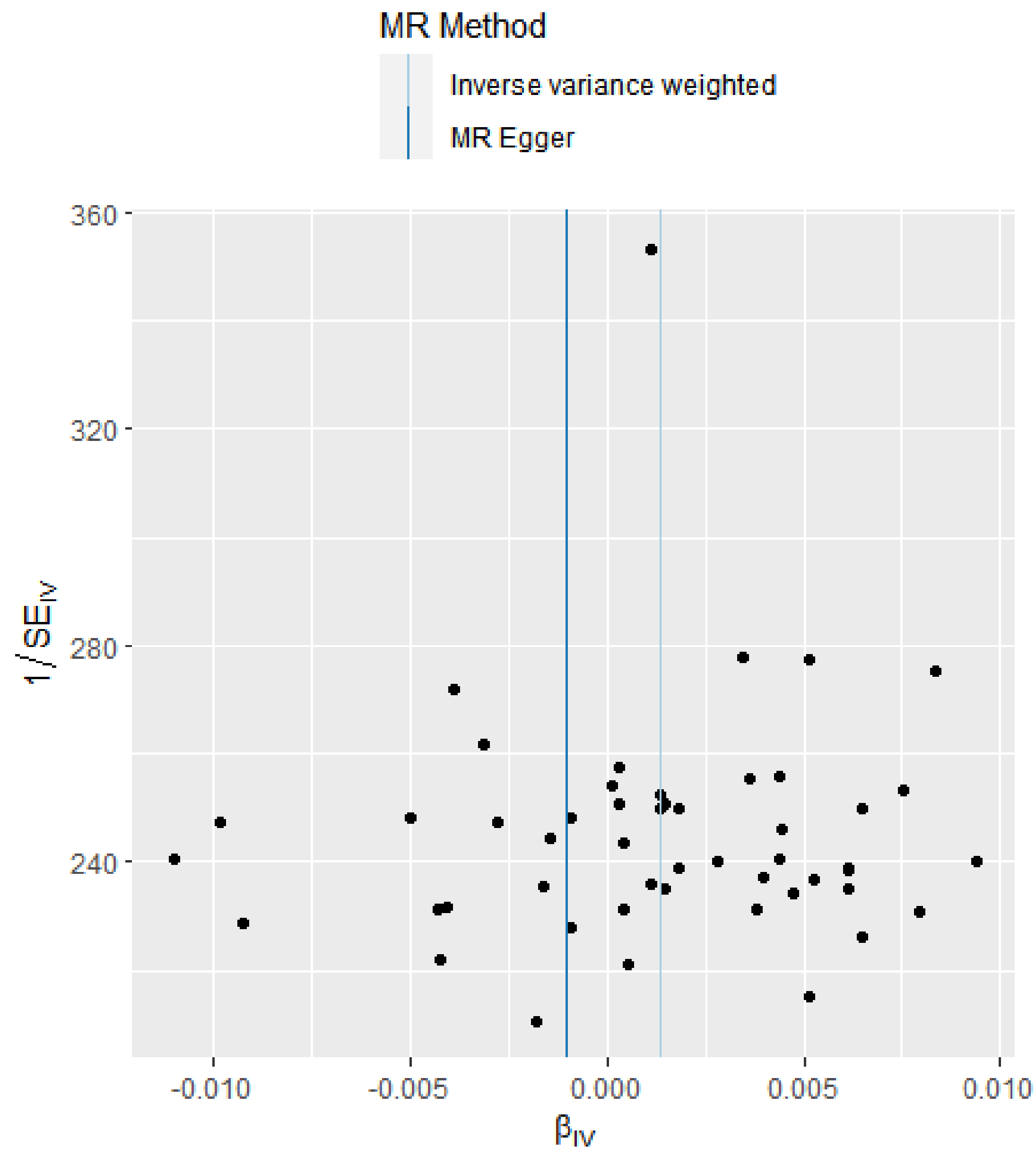

MR Method
Inverse variance weighted
MR Egger
360
320
280
240
-0.010
-0.005
0.000
0.005
0.010
$1/SE_{IV}$
β_{IV}

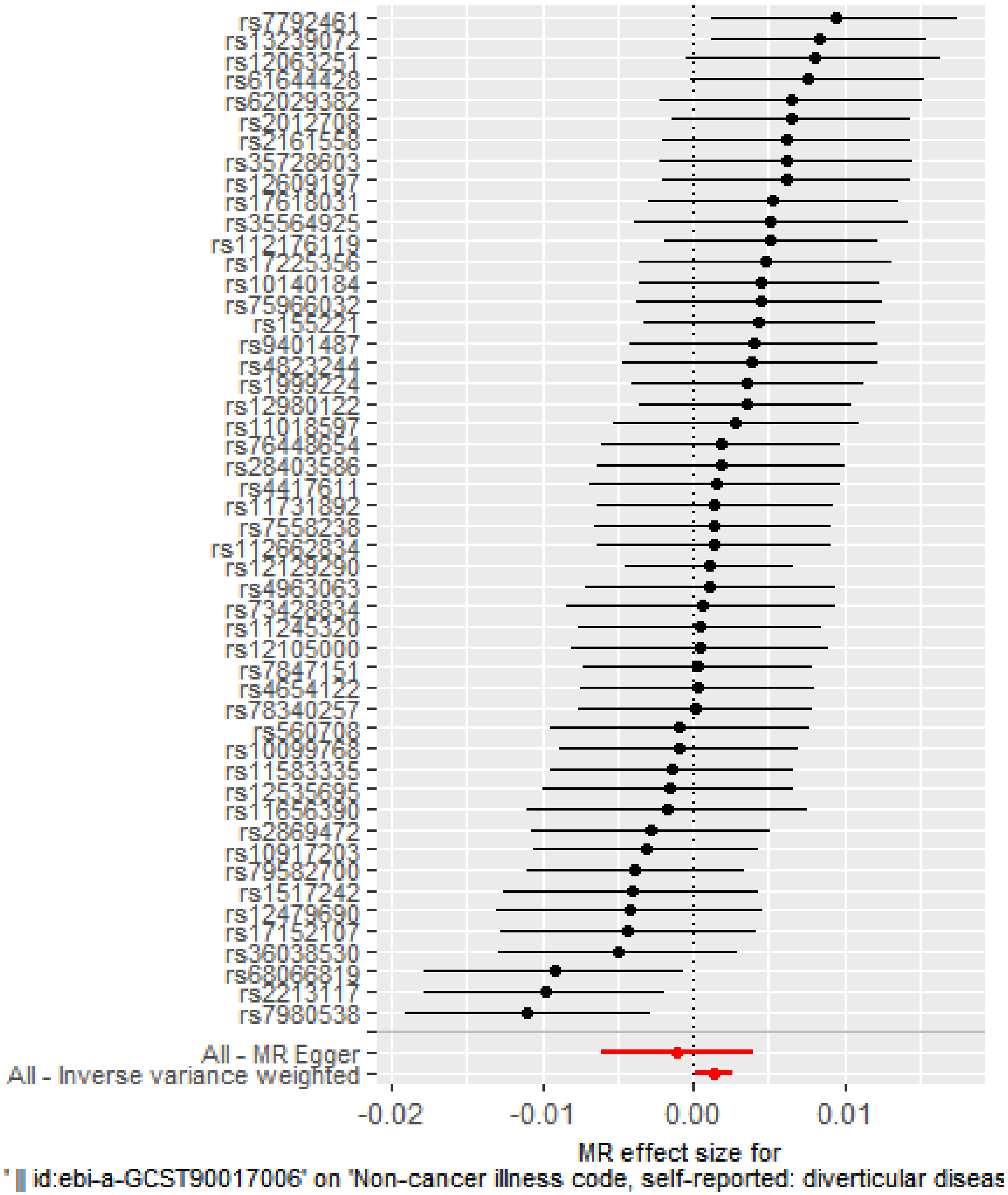

rs7792461
rs13239072
rs12063251
rs61644428
rs62029382
rs2012708
rs2161558
rs35728603
rs12609197
rs17618031
rs35564925
rs112176119
rs17225356
rs10140184
rs75966032
rs155221
rs9401487
rs4823244
rs1999224
rs12980122
rs11018597
rs76448654
rs28403586
rs4417611
rs11731892
rs7558238
rs112662834
rs12129290
rs4963063
rs73428834
rs11245320
rs12105000
rs7847151
rs4654122
rs78340257
rs560708
rs10099768
rs11583335
rs12535695
rs11656390
rs2869472
rs10917203
rs79582700
rs1517242
rs12479690
rs17152107
rs36038530
rs68066819
rs2213117
rs7980538
All - MR Egger
All - Inverse variance weighted
-0.02
-0.01
0.00
0.01
MR effect size for
' || id:ebi-a-GCST90017006' on 'Non-cancer illness code, self-reported: diverticular diseas

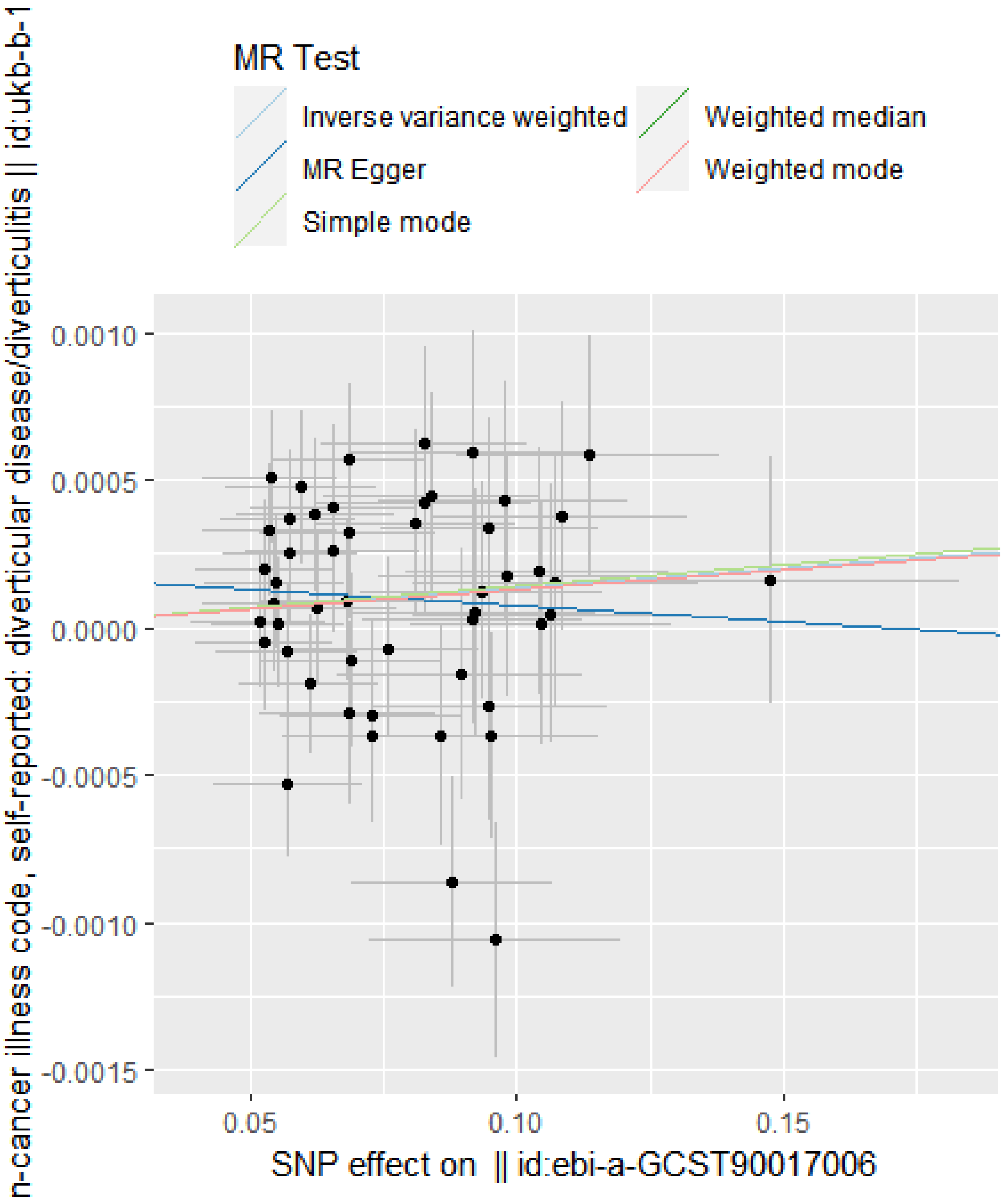

MR Test
Inverse variance weighted
MR Egger
Simple mode
Weighted median
Weighted mode
0.0010
0.0005
0.0000
-0.0005
-0.0010
-0.0015
0.05
0.10
0.15
SNP effect on || id:ebi-a-GCST90017006
n-cancer illness code, self-reported: diverticular disease/diverticulitis || id:ukb-b-1

Figure 108 Leave-one-out analysis, funnel plot, MR effect size and scatter plot for gut microbiota abundance (genus Ruminococcus2 id.11374) on diverticular disease

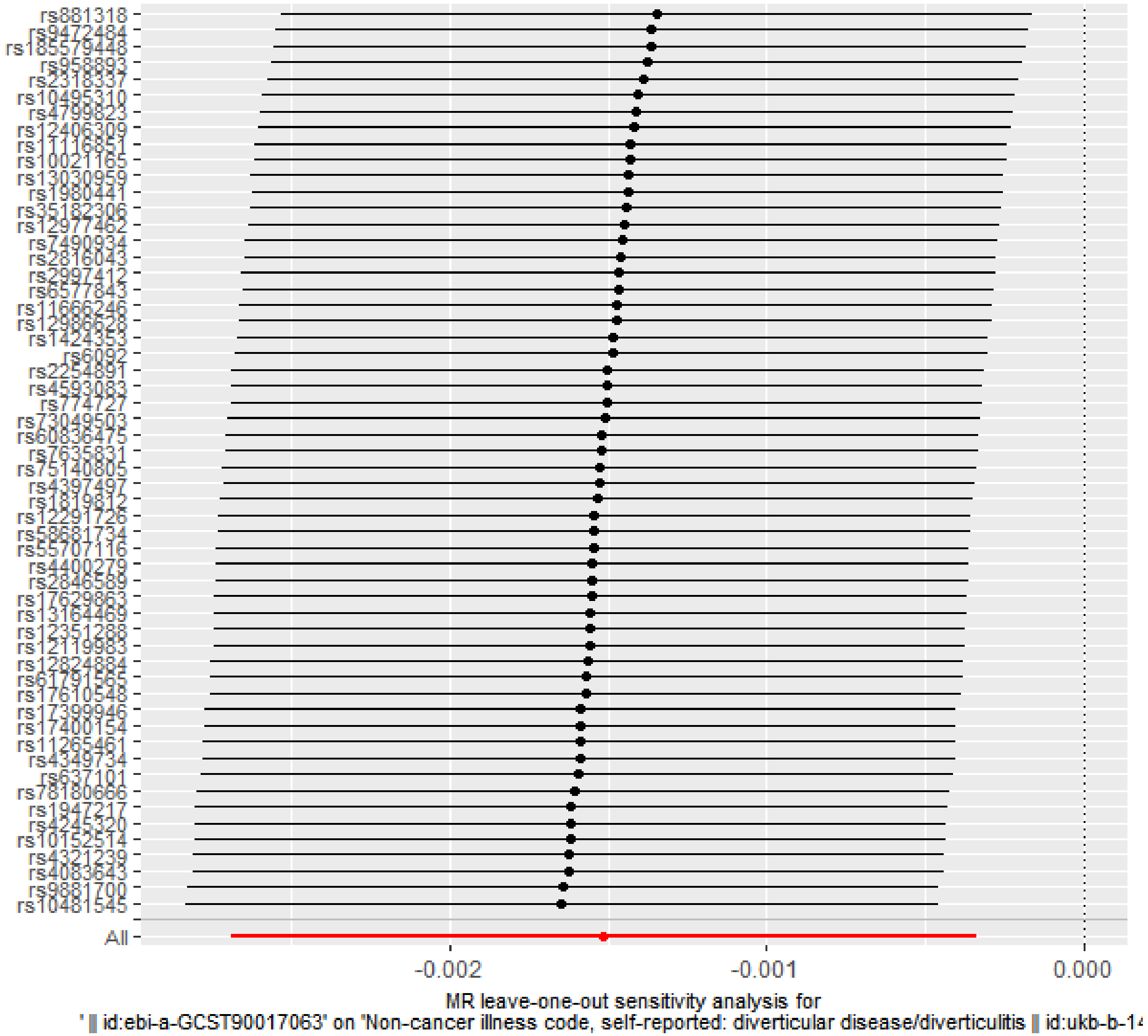

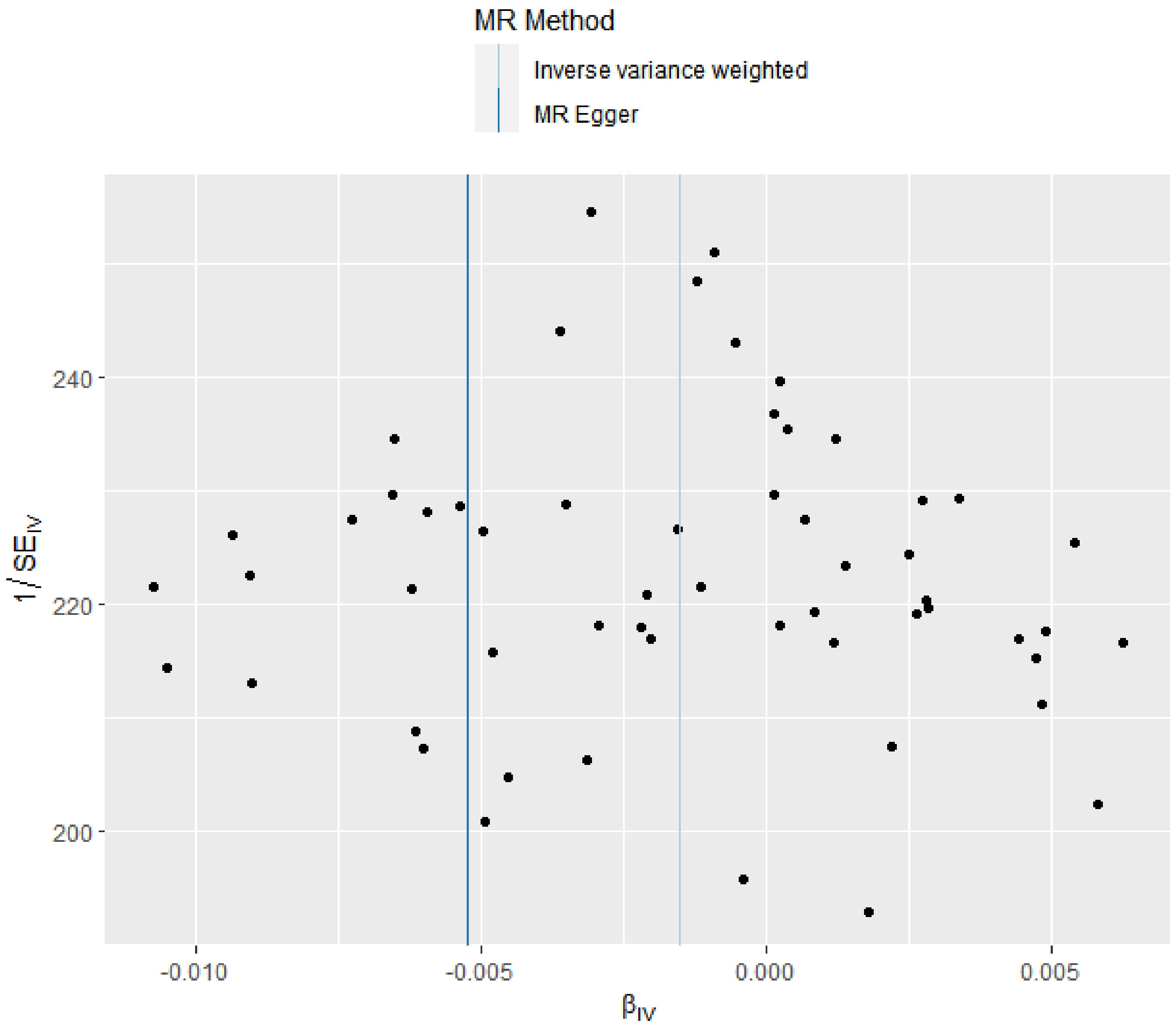

MR Method
Inverse variance weighted
MR Egger
240
220
200
1/SE_IV
-0.010
-0.005
0.000
0.005
β_IV

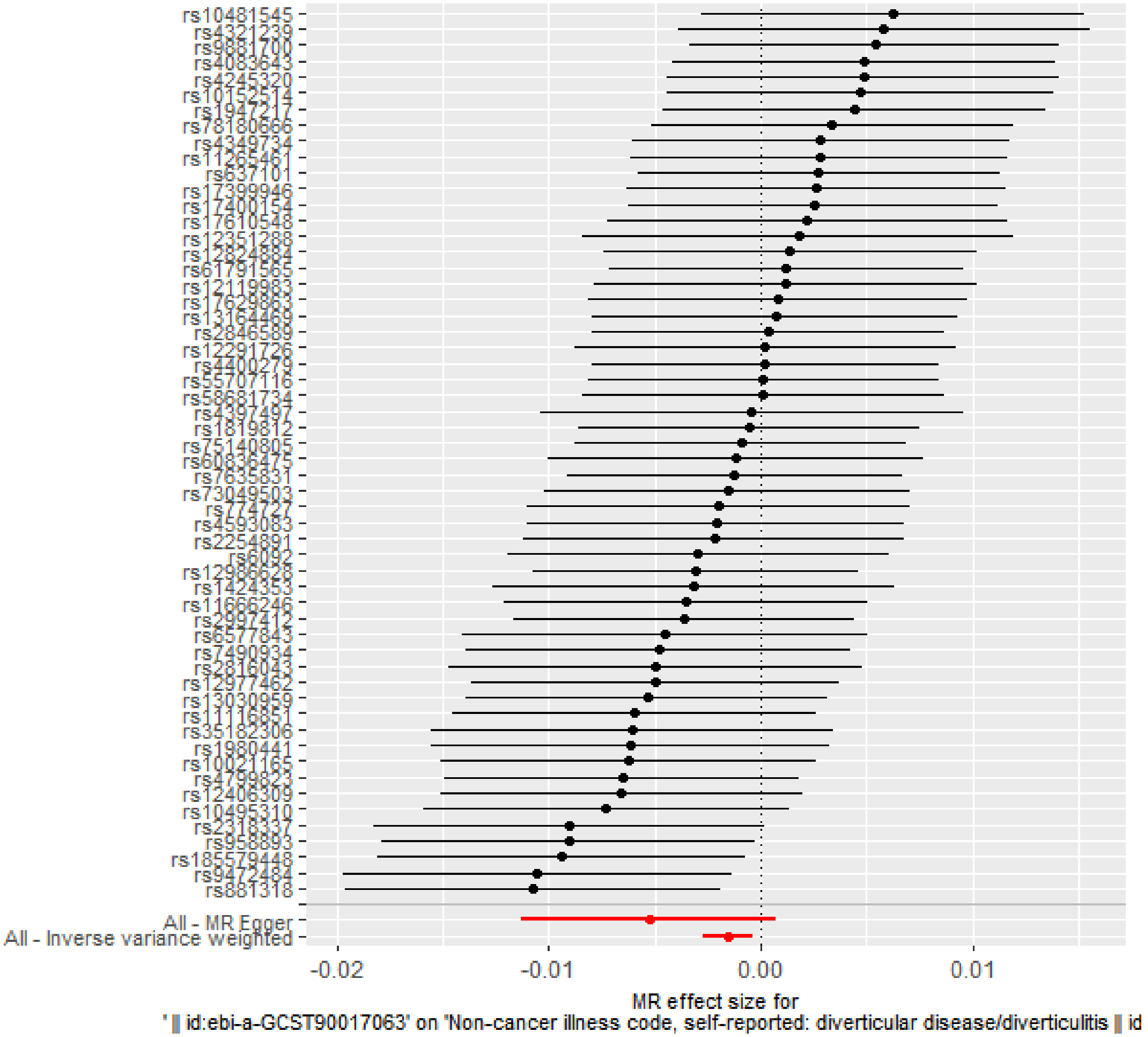
rs10481545
rs4321239
rs9881700
rs4083643
rs4245320
rs10152514
rs1947217
rs78180666
rs4349734
rs11265461
rs637101
rs17399946
rs17400154
rs17610548
rs12351288
rs61791565
rs12119983
rs13164469
rs2846589
rs12291726
rs4400279
rs55707116
rs58681734
rs4397497
rs1819812
rs75140805
rs60836475
rs7635831
rs73049503
rs774727
rs4593083
rs2254891
rs6092
rs12986628
rs1424353
rs11666246
rs2997412
rs6577843
rs7490934
rs2816043
rs12977462
rs13030959
rs11116851
rs35182306
rs1980441
rs10021165
rs4799823
rs12406309
rs10495310
rs2318337
rs958893
rs185579448
rs9472484
rs881318
All - MR Egger
All - Inverse variance weighted
-0.02
-0.01
0.00
0.01
MR effect size for
' || id:ebi-a-GCST90017063' on 'Non-cancer illness code, self-reported: diverticular disease/diverticulitis || id

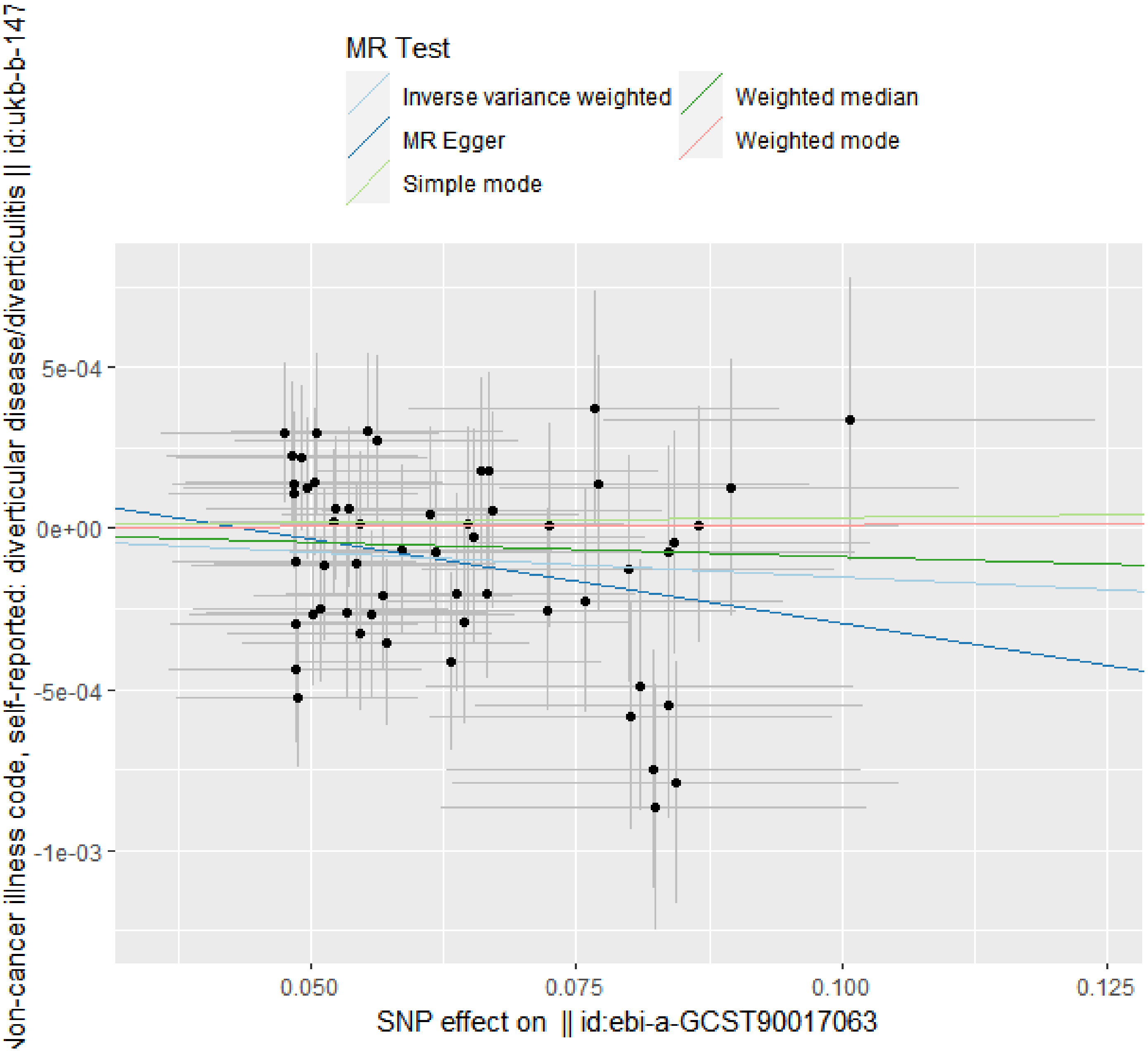

MR Test
Inverse variance weighted
MR Egger
Simple mode
Weighted median
Weighted mode
5e-04
0e+00
-5e-04
-1e-03
0.050
0.075
0.100
0.125
SNP effect on || id:ebi-a-GCST90017063
Non-cancer illness code, self-reported: diverticular disease/diverticulitis || id:ukb-b-147

Figure 109 Leave-one-out analysis, funnel plot, MR effect size and scatter plot for gut microbiota abundance (unknown genus id.1868) on diverticular disease

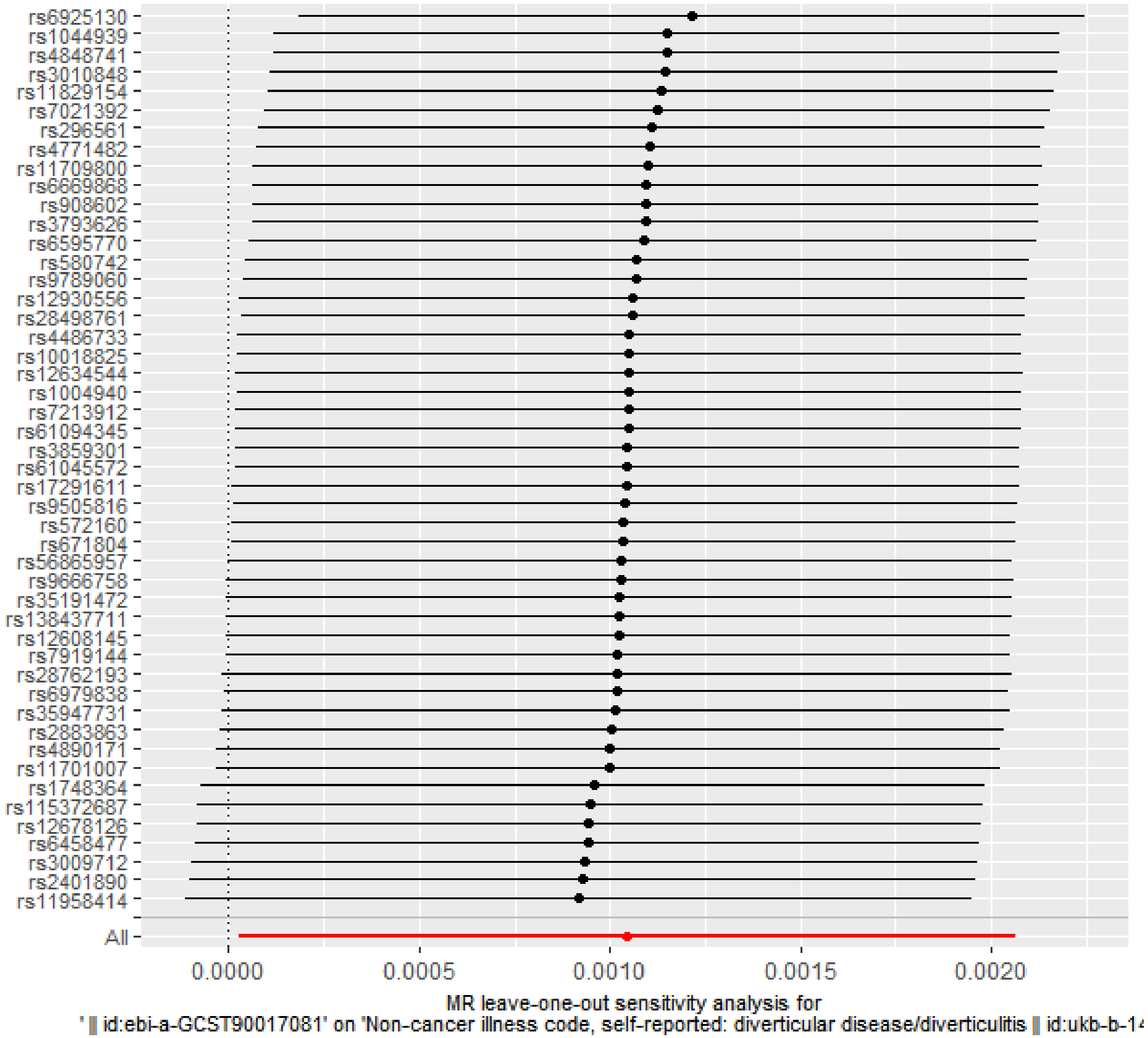

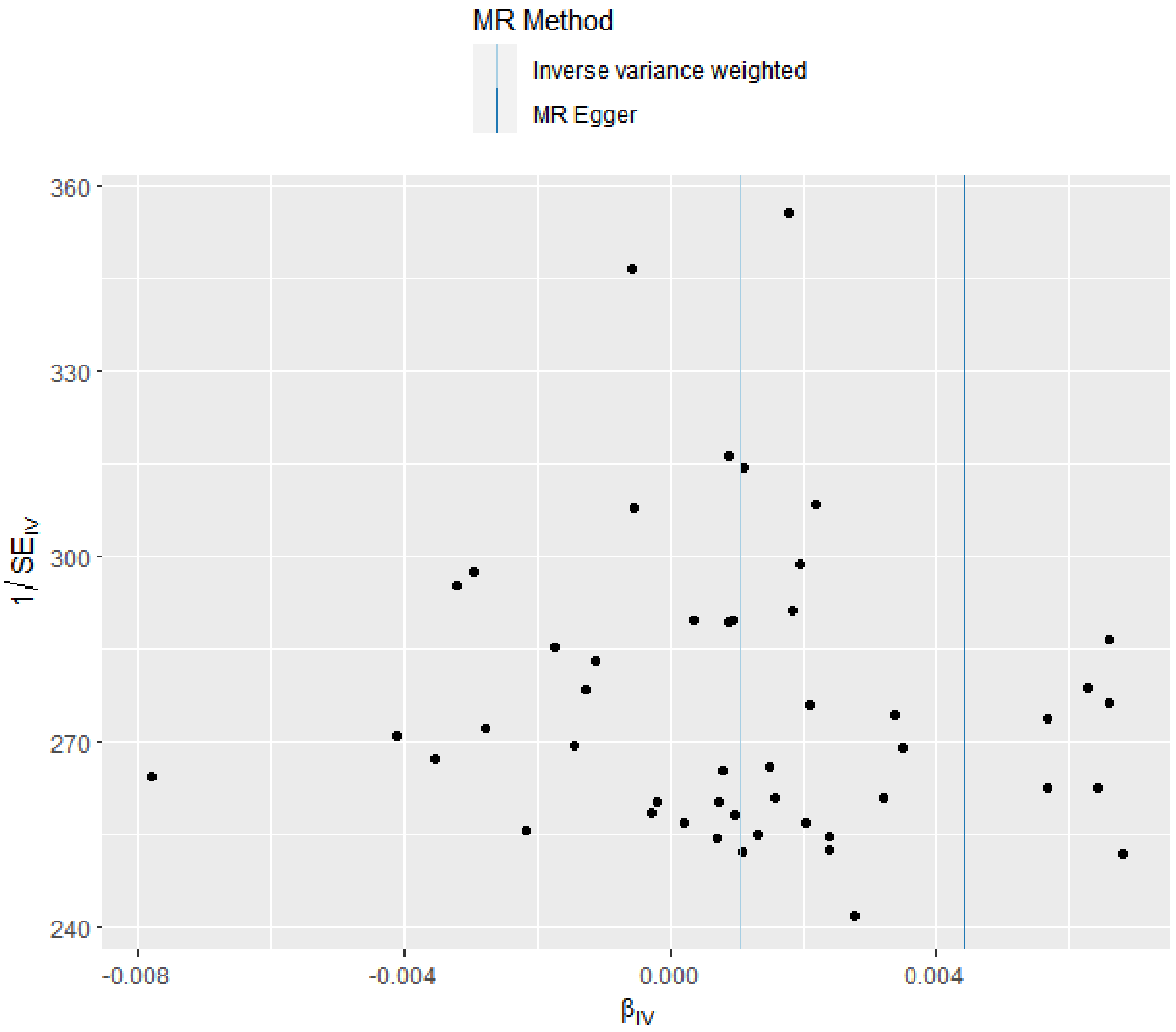

MR Method
Inverse variance weighted
MR Egger
360
330
300
270
240
1/SE_IV
-0.008
-0.004
0.000
0.004
β_IV

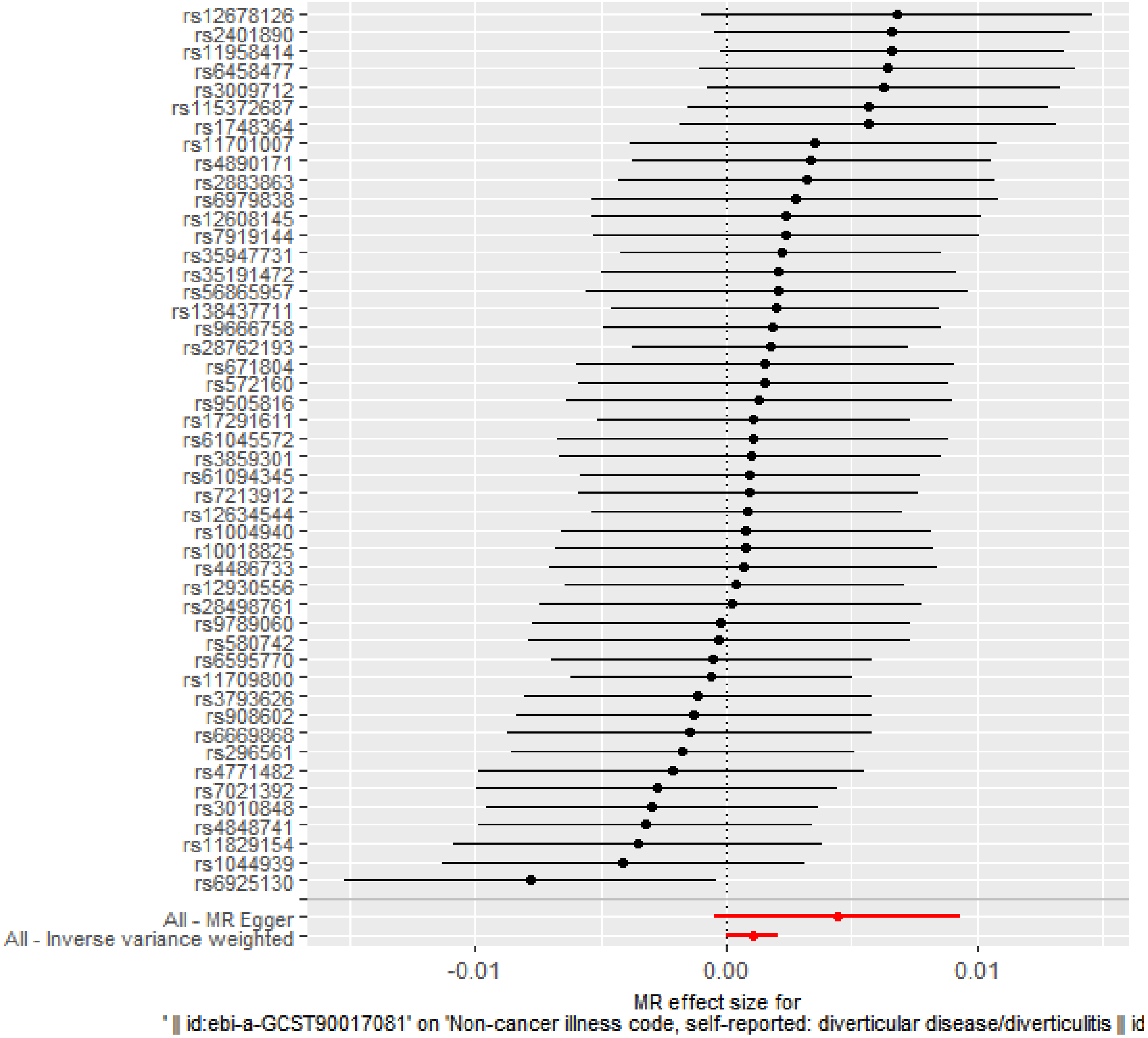

rs12678126
rs2401890
rs11958414
rs6458477
rs3009712
rs115372687
rs1748364
rs11701007
rs4890171
rs2883863
rs6979838
rs12608145
rs7919144
rs35947731
rs35191472
rs56865957
rs138437711
rs9666758
rs28762193
rs671804
rs572160
rs9505816
rs17291611
rs61045572
rs3859301
rs61094345
rs7213912
rs12634544
rs1004940
rs10018825
rs4486733
rs12930556
rs28498761
rs9789060
rs580742
rs6595770
rs11709800
rs3793626
rs908602
rs6669868
rs296561
rs4771482
rs7021392
rs3010848
rs4848741
rs11829154
rs1044939
rs6925130
All - MR Egger
All - Inverse variance weighted
-0.01
0.00
0.01
MR effect size for
' || id:ebi-a-GCST90017081' on 'Non-cancer illness code, self-reported: diverticular disease/diverticulitis || id

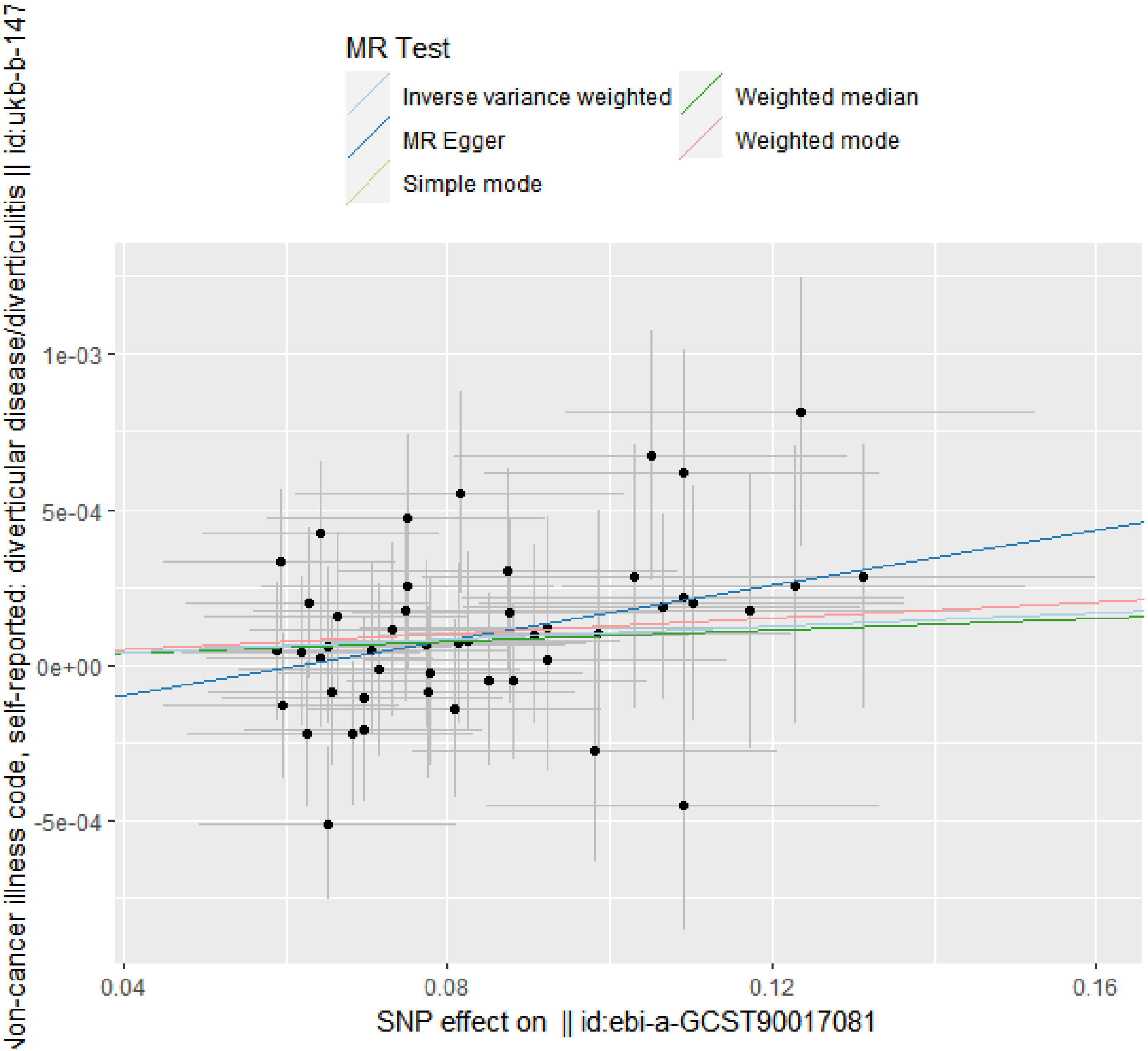

MR Test
Inverse variance weighted
MR Egger
Simple mode
Weighted median
Weighted mode
1e-03
5e-04
0e+00
-5e-04
0.04
0.08
0.12
0.16
SNP effect on || id:ebi-a-GCST90017081
Non-cancer illness code, self-reported: diverticular disease/diverticulitis || id:ukb-b-147

Figure 110 Leave-one-out analysis, funnel plot, MR effect size and scatter plot for gut microbiota abundance (unknown genus id.959) on diverticular disease

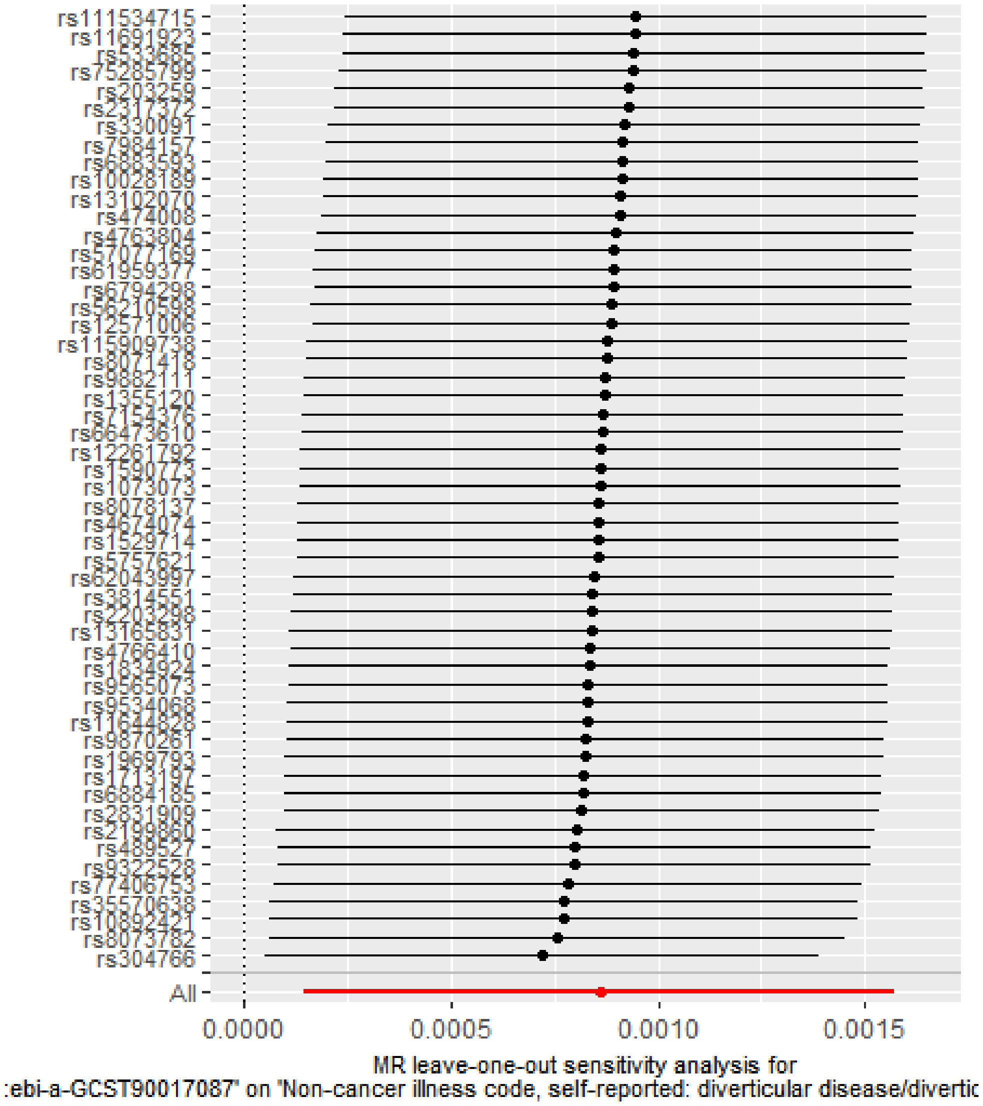

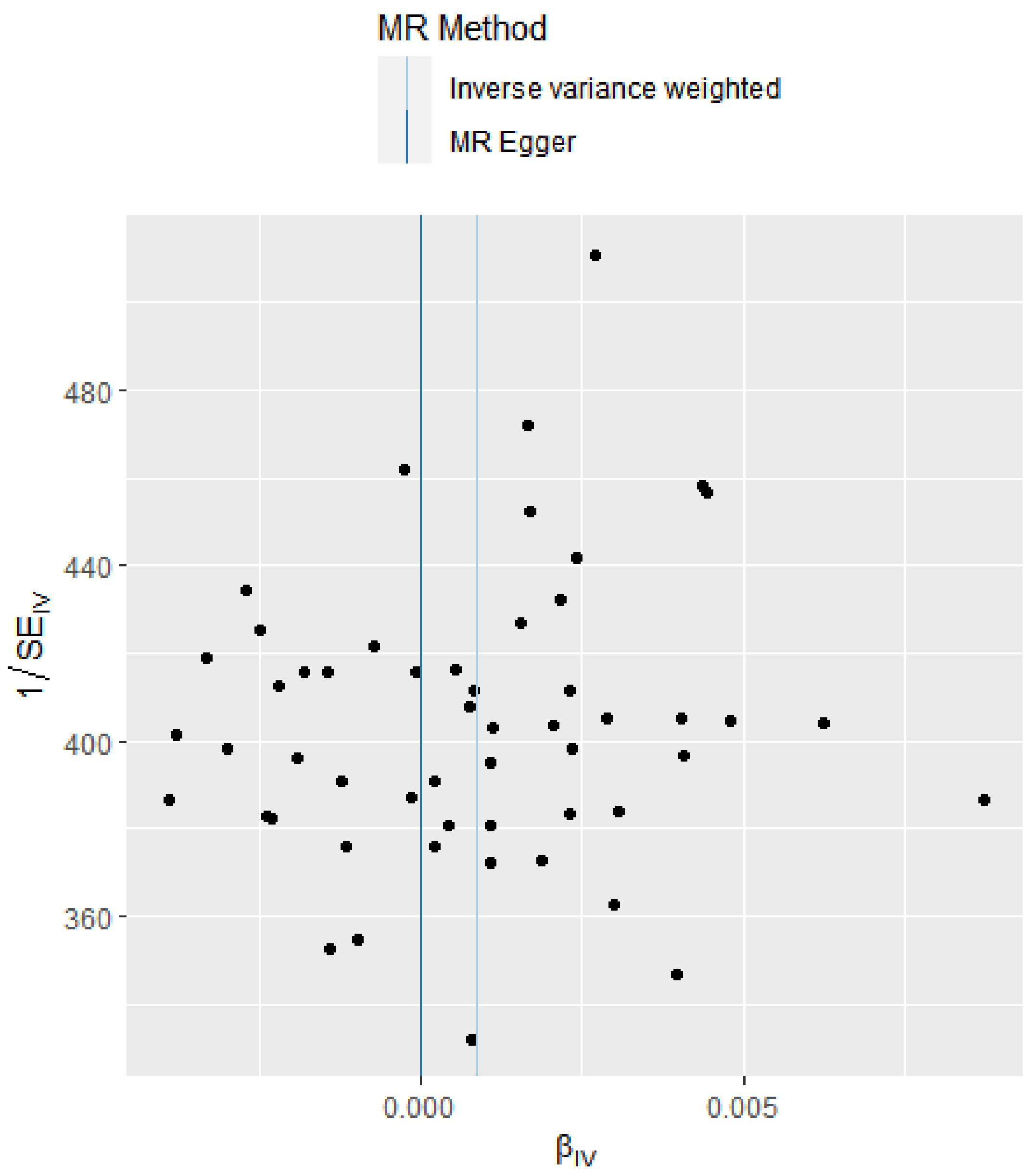
MR Method
Inverse variance weighted
MR Egger
480
440
400
360
1/SE_IV
0.000
0.005
β_IV

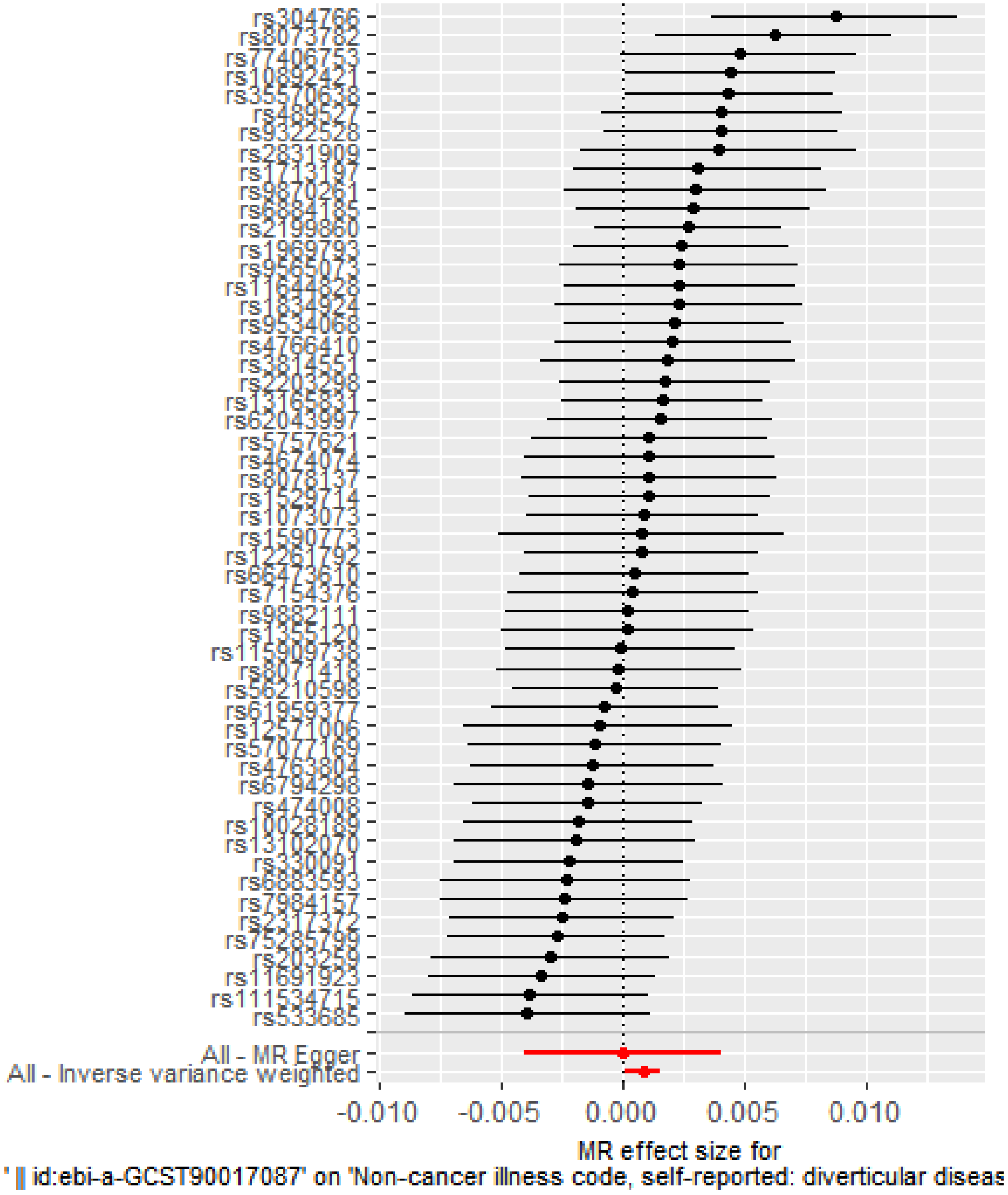

All - MR Egger
All - Inverse variance weighted
-0.010
-0.005
0.000
0.005
0.010
MR effect size for
' || id:ebi-a-GCST90017087' on 'Non-cancer illness code, self-reported: diverticular diseas

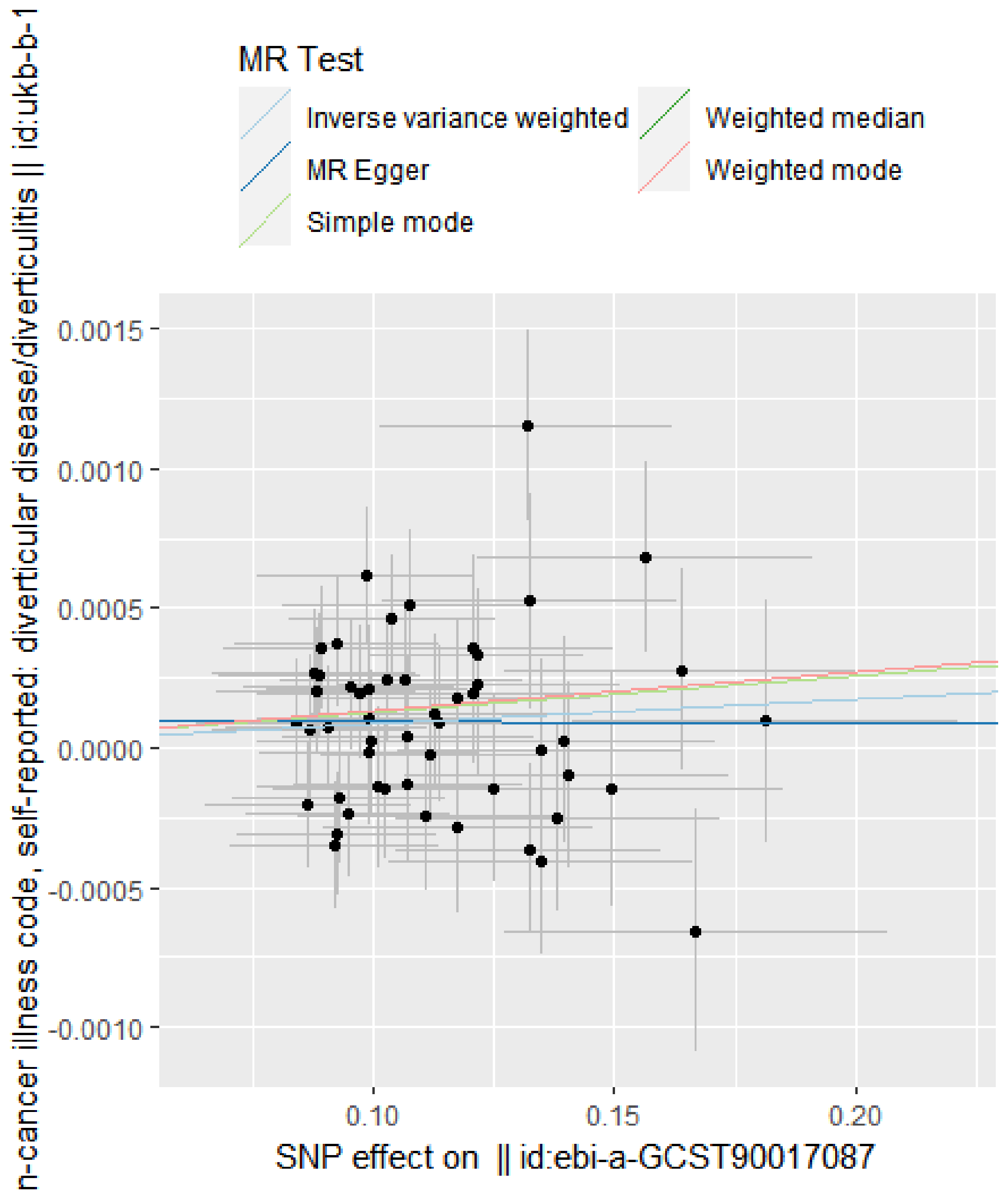
MR Test
Inverse variance weighted
MR Egger
Simple mode
Weighted median
Weighted mode
0.0015
0.0010
0.0005
0.0000
-0.0005
-0.0010
0.10
0.15
0.20
SNP effect on || id:ebi-a-GCST90017087
n-cancer illness code, self-reported: diverticular disease/diverticulitis || id:ukb-b-1

Figure 111 Leave-one-out analysis, funnel plot, MR effect size and scatter plot for gut microbiota abundance (order Selenomonadales id.2165) on diverticular disease

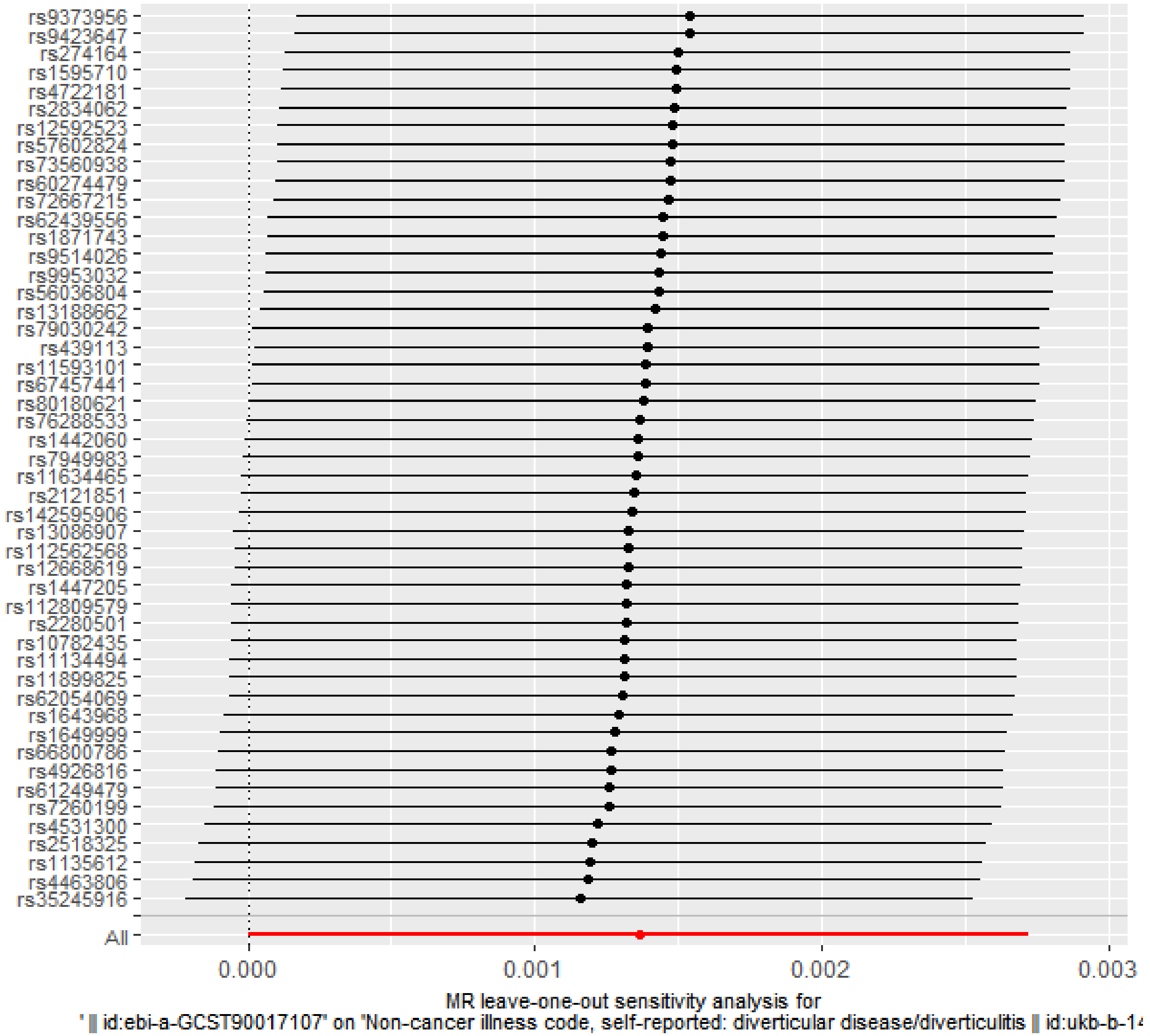

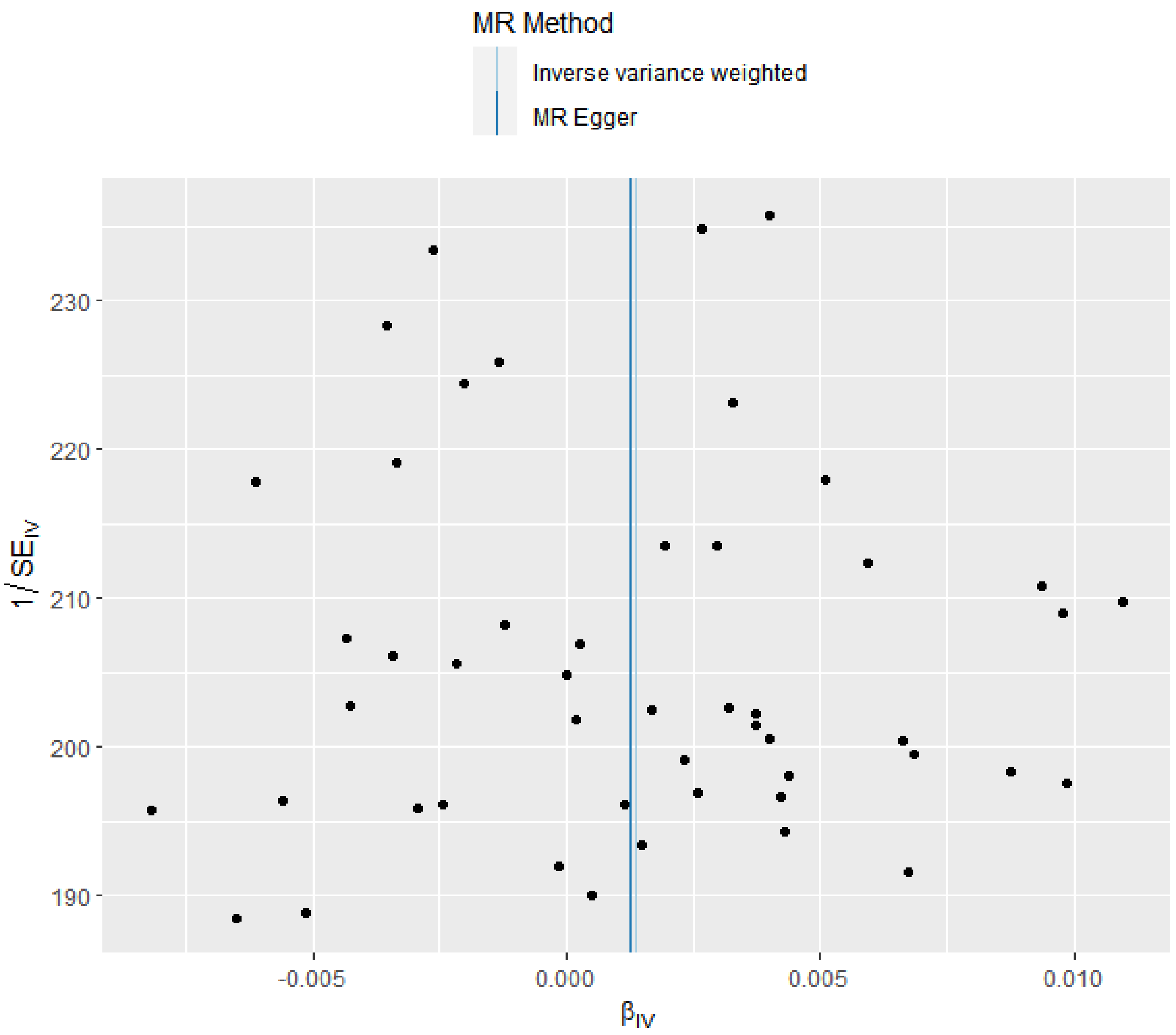

MR Method
Inverse variance weighted
MR Egger
230
220
210
200
190
1/SEIV
-0.005
0.000
0.005
0.010
βIV

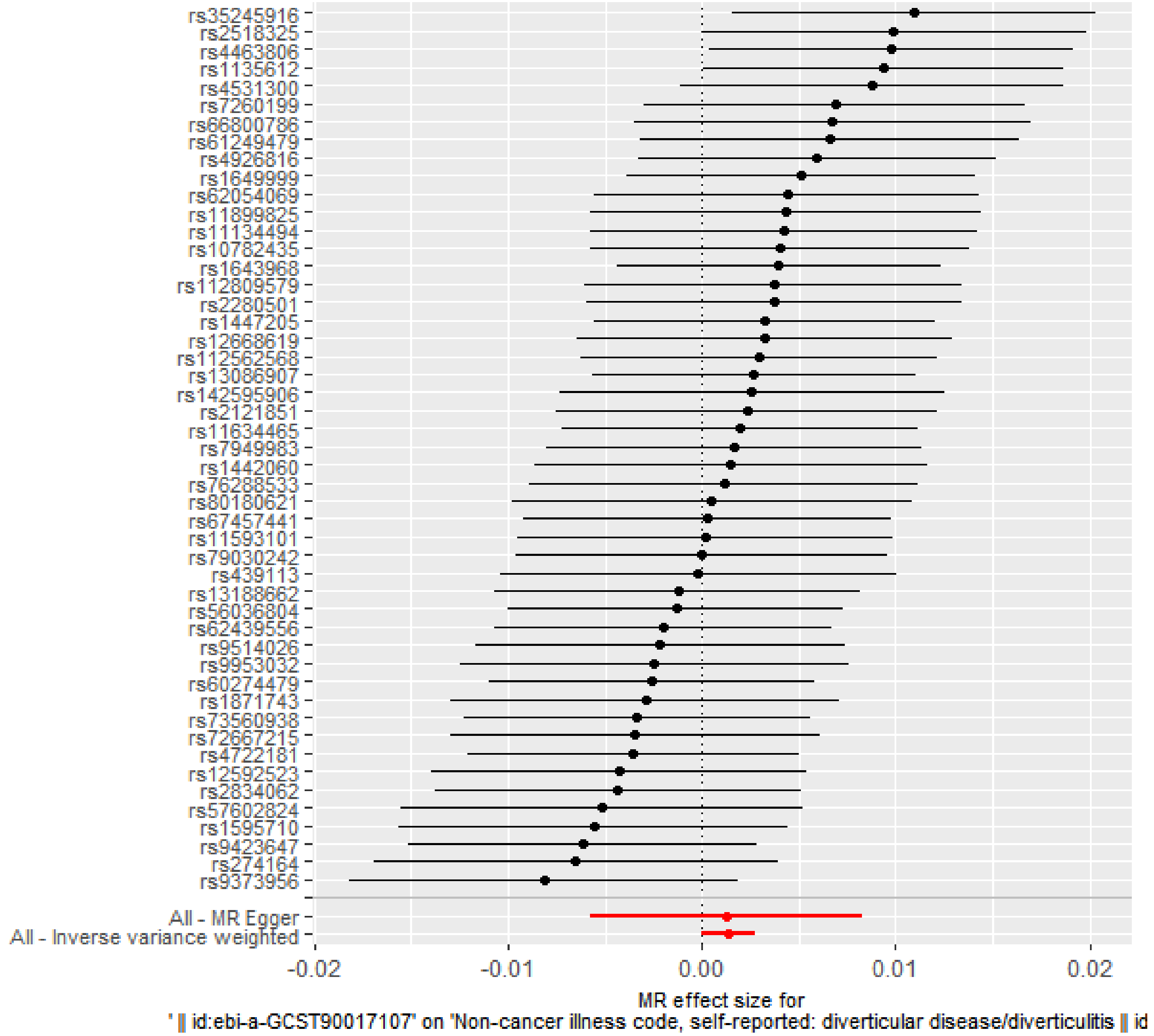

rs35245916
rs2518325
rs4463806
rs1135612
rs4531300
rs7260199
rs66800786
rs61249479
rs4926816
rs1649999
rs62054069
rs11899825
rs11134494
rs10782435
rs1643968
rs112809579
rs2280501
rs1447205
rs12668619
rs112562568
rs13086907
rs142595906
rs2121851
rs11634465
rs7949983
rs1442060
rs76288533
rs80180621
rs67457441
rs11593101
rs79030242
rs439113
rs13188662
rs56036804
rs62439556
rs9514026
rs9953032
rs60274479
rs1871743
rs73560938
rs72667215
rs4722181
rs12592523
rs2834062
rs57602824
rs1595710
rs9423647
rs274164
rs9373956
All - MR Egger
All - Inverse variance weighted
-0.02
-0.01
0.00
0.01
0.02
MR effect size for
' || id:ebi-a-GCST90017107' on 'Non-cancer illness code, self-reported: diverticular disease/diverticulitis || id

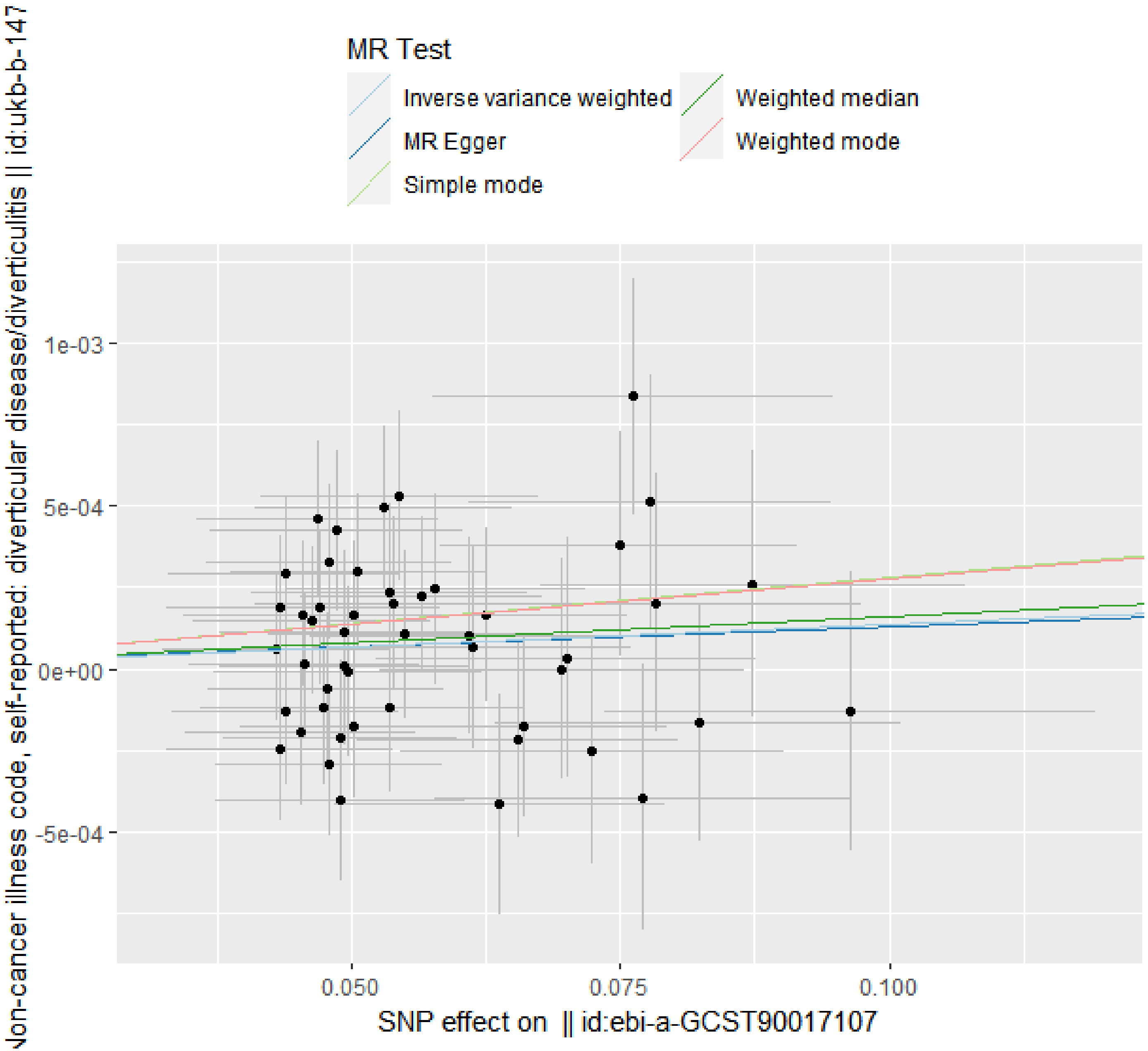
MR Test
Inverse variance weighted
MR Egger
Simple mode
Weighted median
Weighted mode
1e-03
5e-04
0e+00
-5e-04
0.050
0.075
0.100
SNP effect on || id:ebi-a-GCST90017107
Non-cancer illness code, self-reported: diverticular disease/diverticulitis || id:ukb-b-147

Figure 112 Leave-one-out analysis, funnel plot, MR effect size and scatter plot for gut microbiota abundance (class Verrucomicrobiae id.4029) on crohn's disease

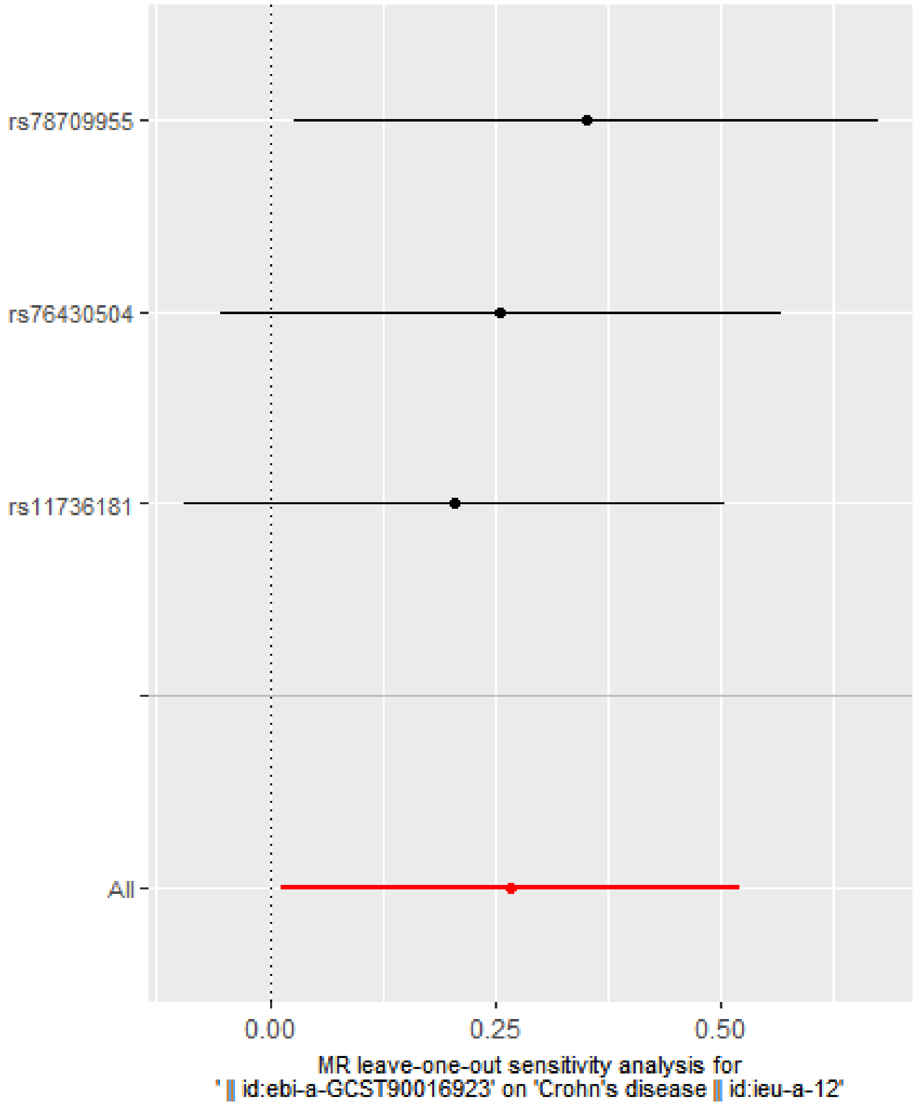

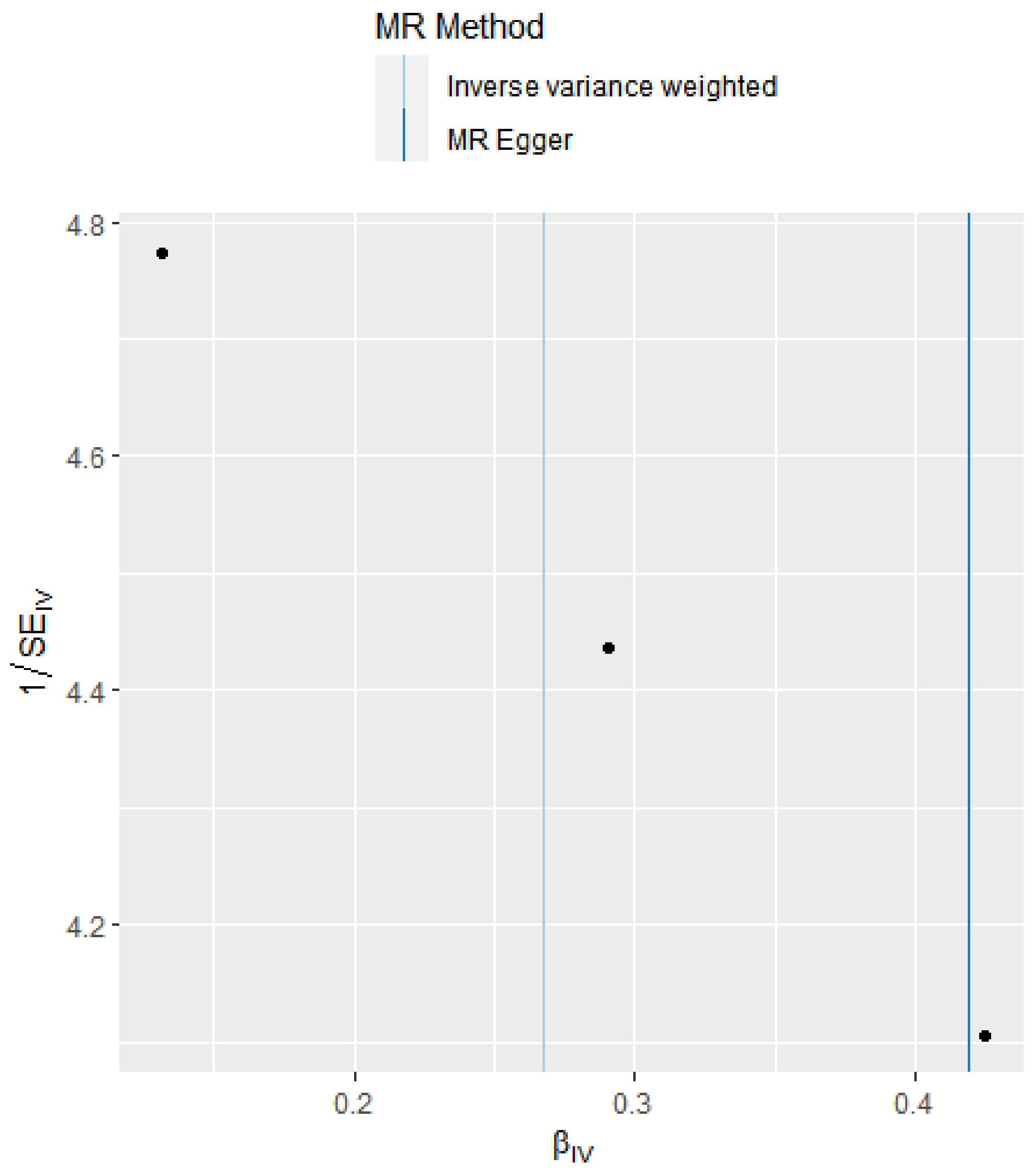

MR Method
Inverse variance weighted
MR Egger
4.8
4.6
4.4
4.2
0.2
0.3
0.4
1/SE_IV
β_IV

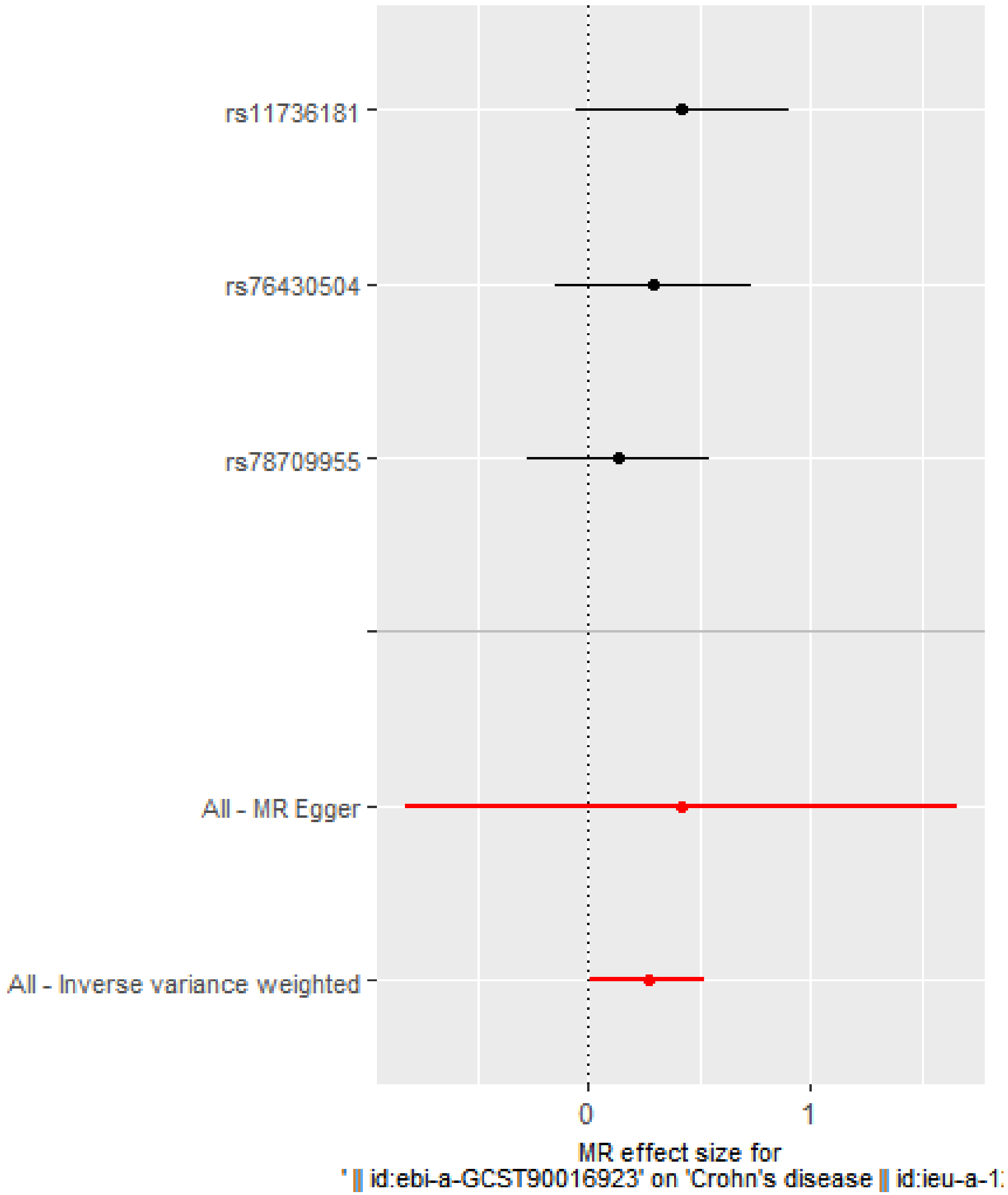

rs11736181
rs76430504
rs78709955
All - MR Egger
All - Inverse variance weighted
0
1
MR effect size for
' || id:ebi-a-GCST90016923' on 'Crohn's disease || id:ieu-a-1

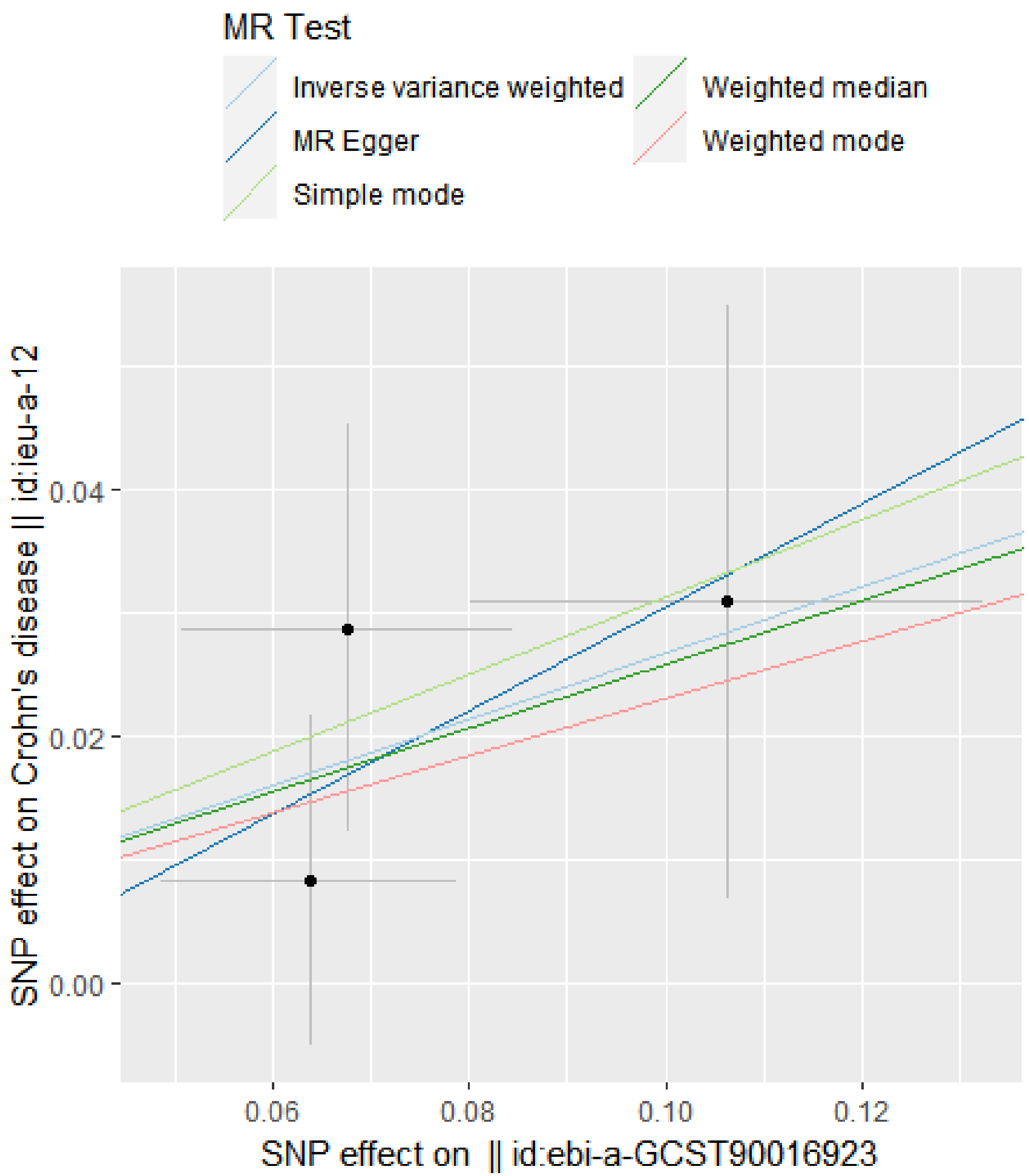
MR Test
Inverse variance weighted
MR Egger
Simple mode
Weighted median
Weighted mode
SNP effect on Crohn's disease || id:ieu-a-12
0.04
0.02
0.00
0.06
0.08
0.10
0.12
SNP effect on || id:ebi-a-GCST90016923

Figure 113 Leave-one-out analysis, funnel plot, MR effect size and scatter plot for gut microbiota abundance (family Peptostreptococcaceae id.2042) on crohn's disease

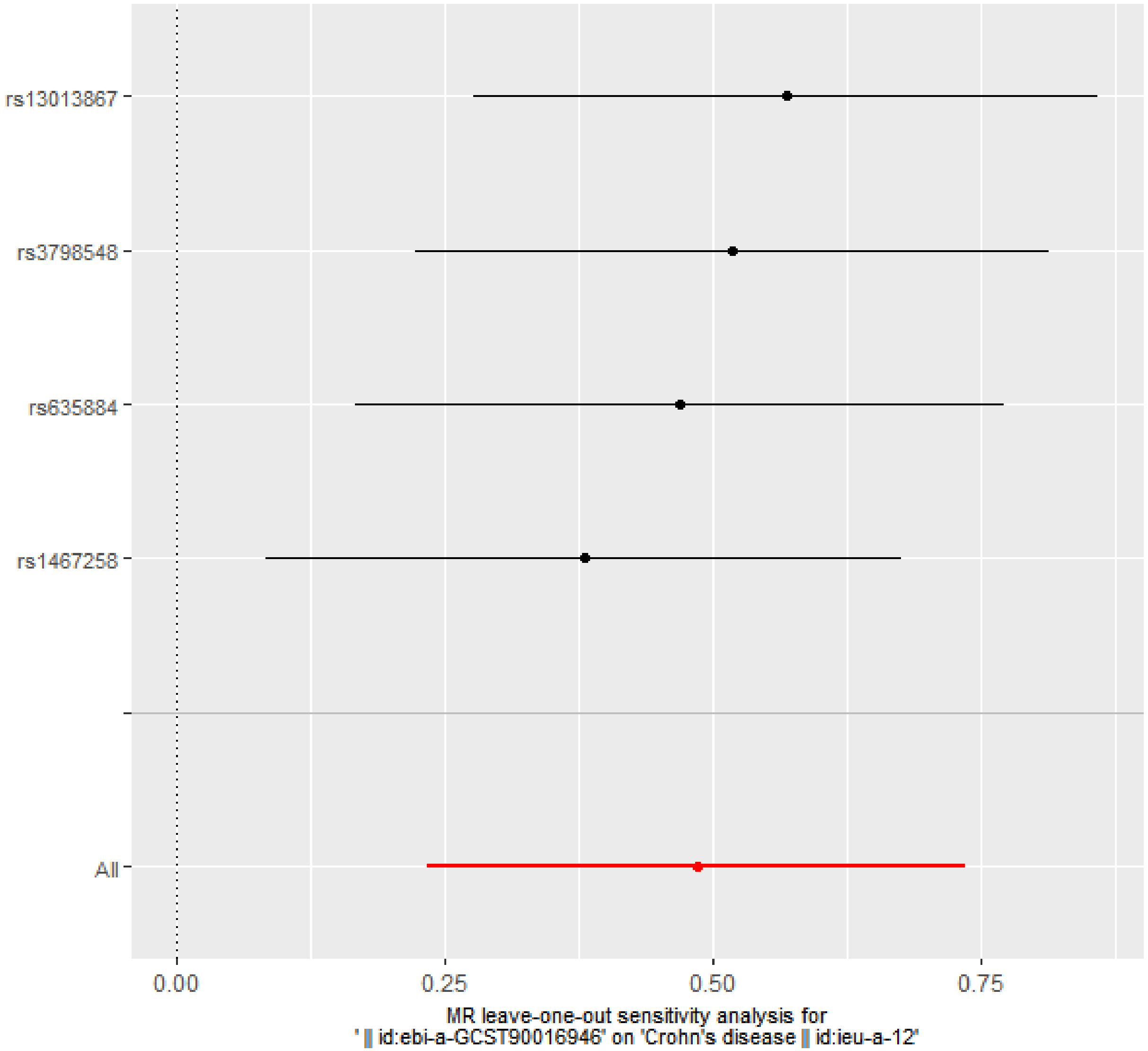

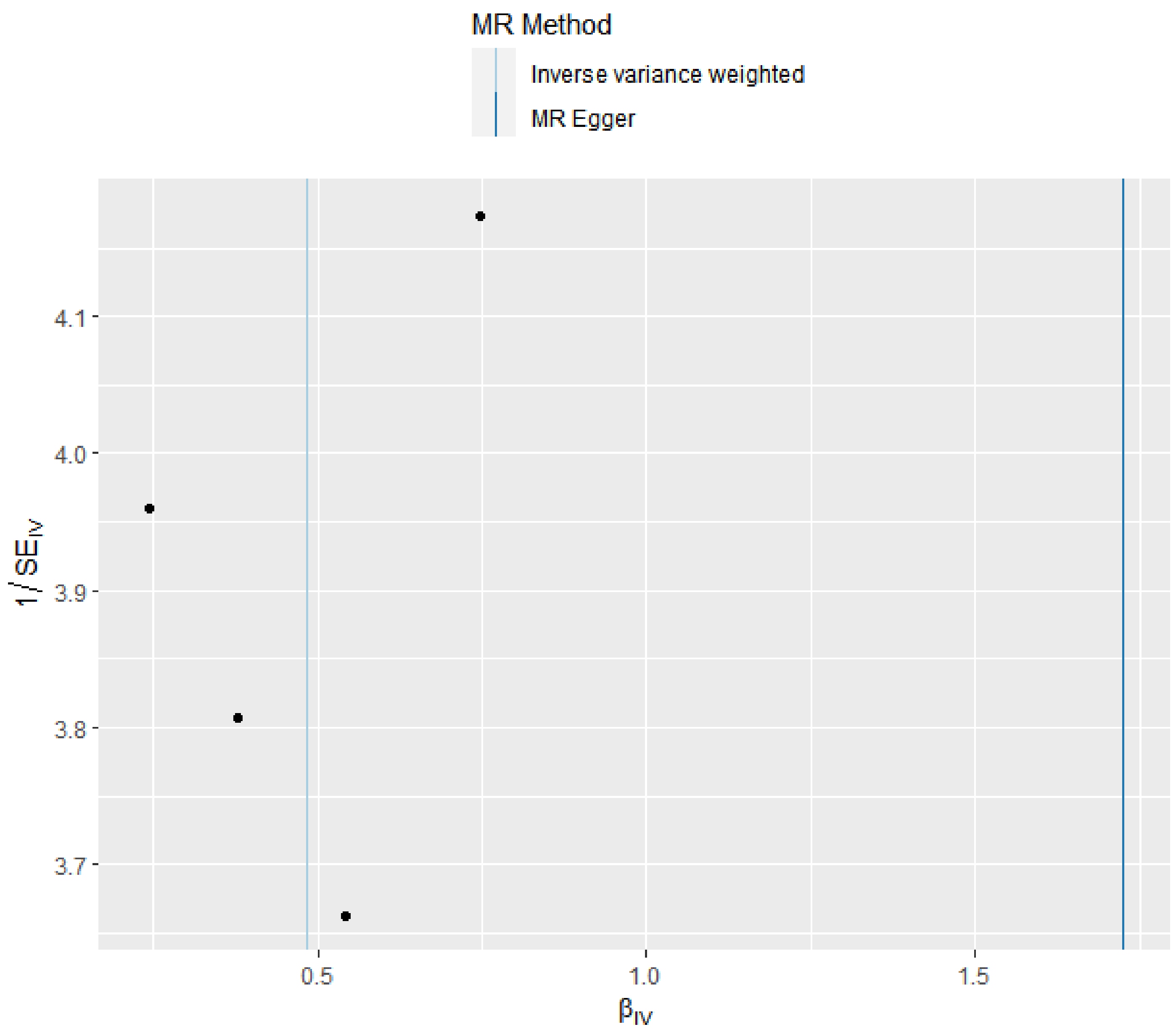

MR Method
Inverse variance weighted
MR Egger
4.1
4.0
3.9
3.8
3.7
1/SE_IV
0.5
1.0
1.5
β_IV

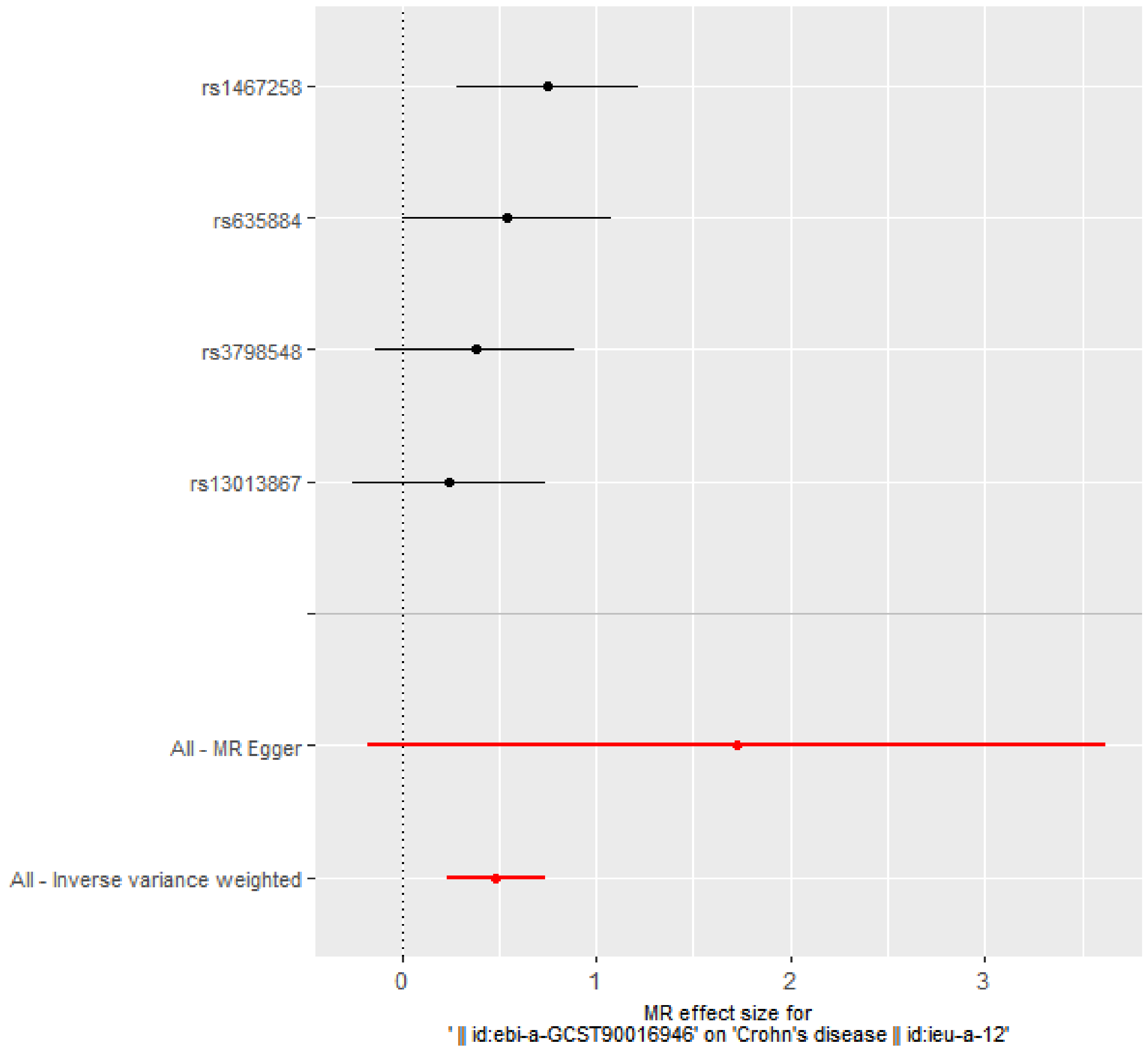
rs1467258
rs635884
rs3798548
rs13013867
All - MR Egger
All - Inverse variance weighted
0
1
2
3
MR effect size for
' || id:ebi-a-GCST90016946' on 'Crohn's disease || id:ieu-a-12'

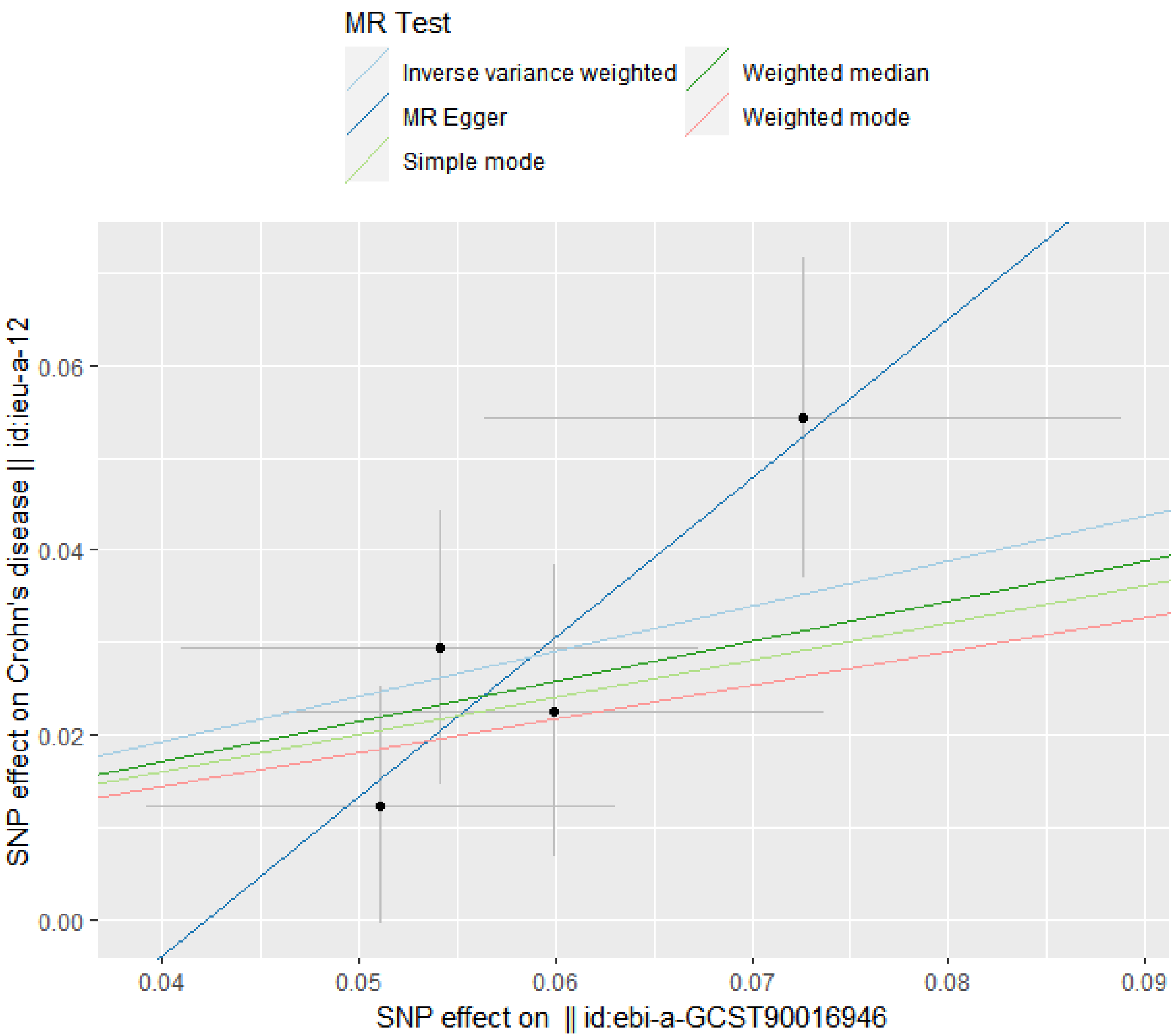
MR Test
Inverse variance weighted
MR Egger
Simple mode
Weighted median
Weighted mode
0.06
0.04
0.02
0.00
SNP effect on Crohn's disease || id:ieu-a-12
0.04
0.05
0.06
0.07
0.08
0.09
SNP effect on || id:ebi-a-GCST90016946

Figure 114 Leave-one-out analysis, funnel plot, MR effect size and scatter plot for gut microbiota abundance (unknown family id.1000006161) on crohn's disease

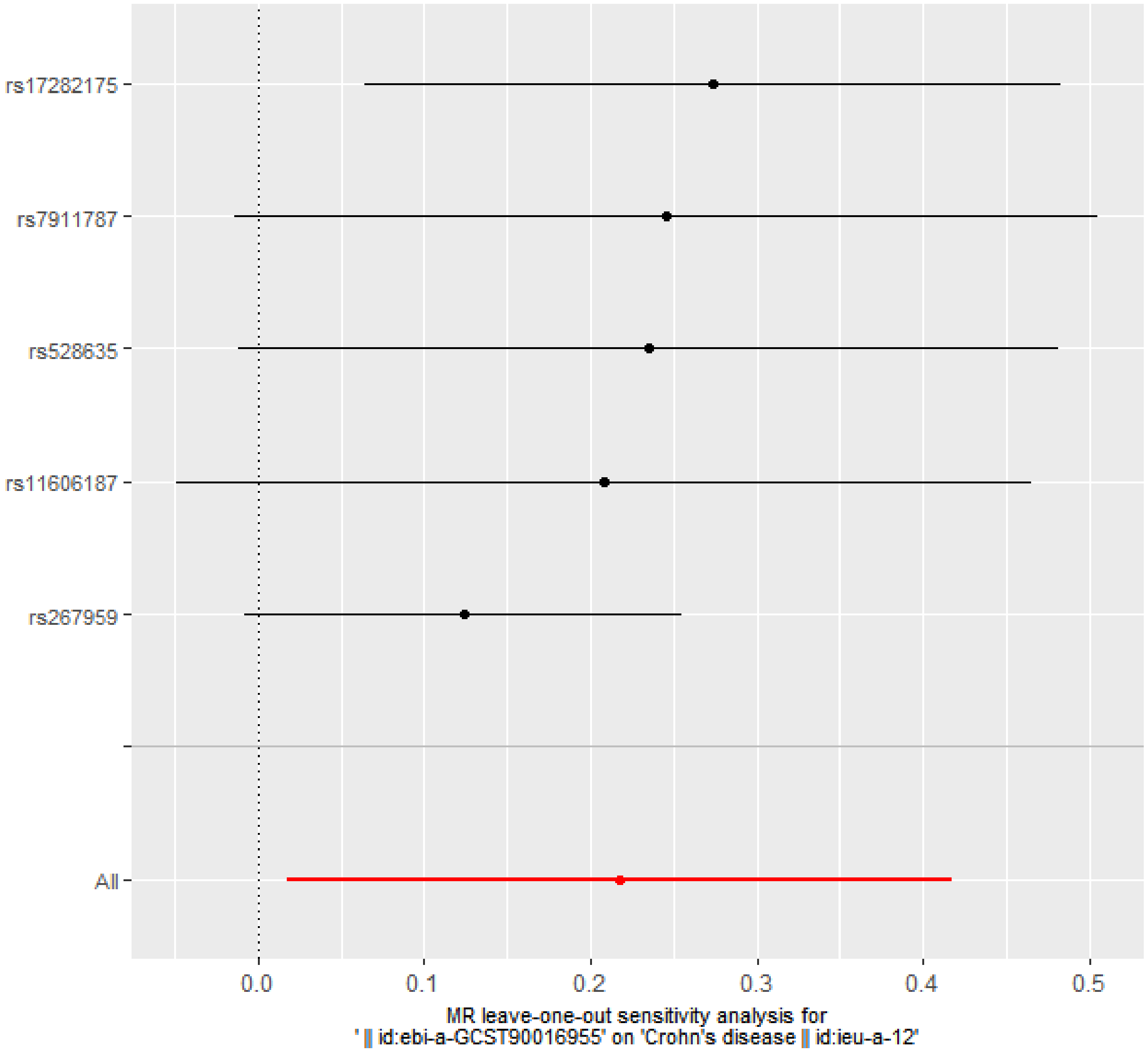

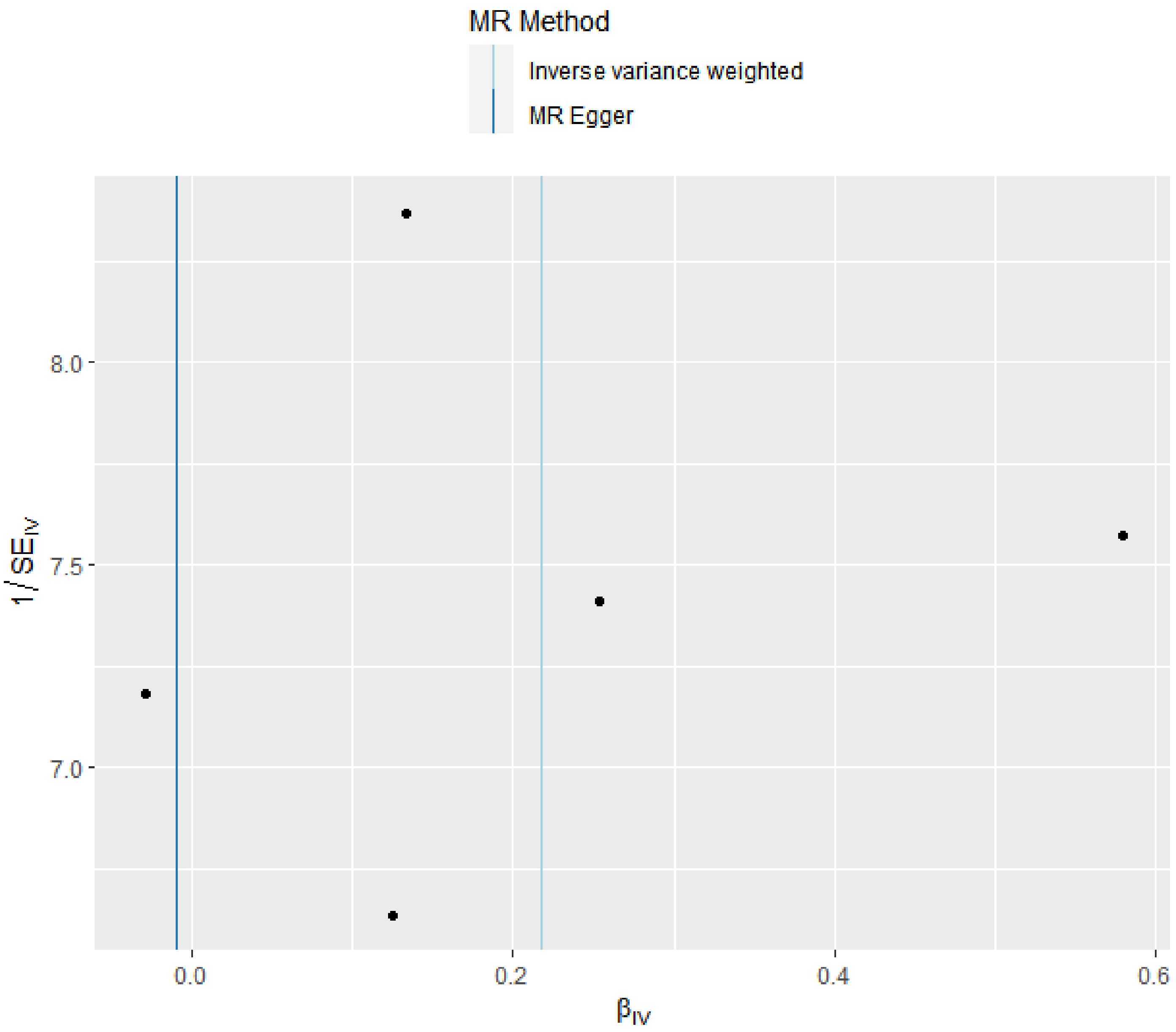
MR Method
Inverse variance weighted
MR Egger
8.0
7.5
7.0
1/SE_IV
0.0
0.2
0.4
0.6
β_IV

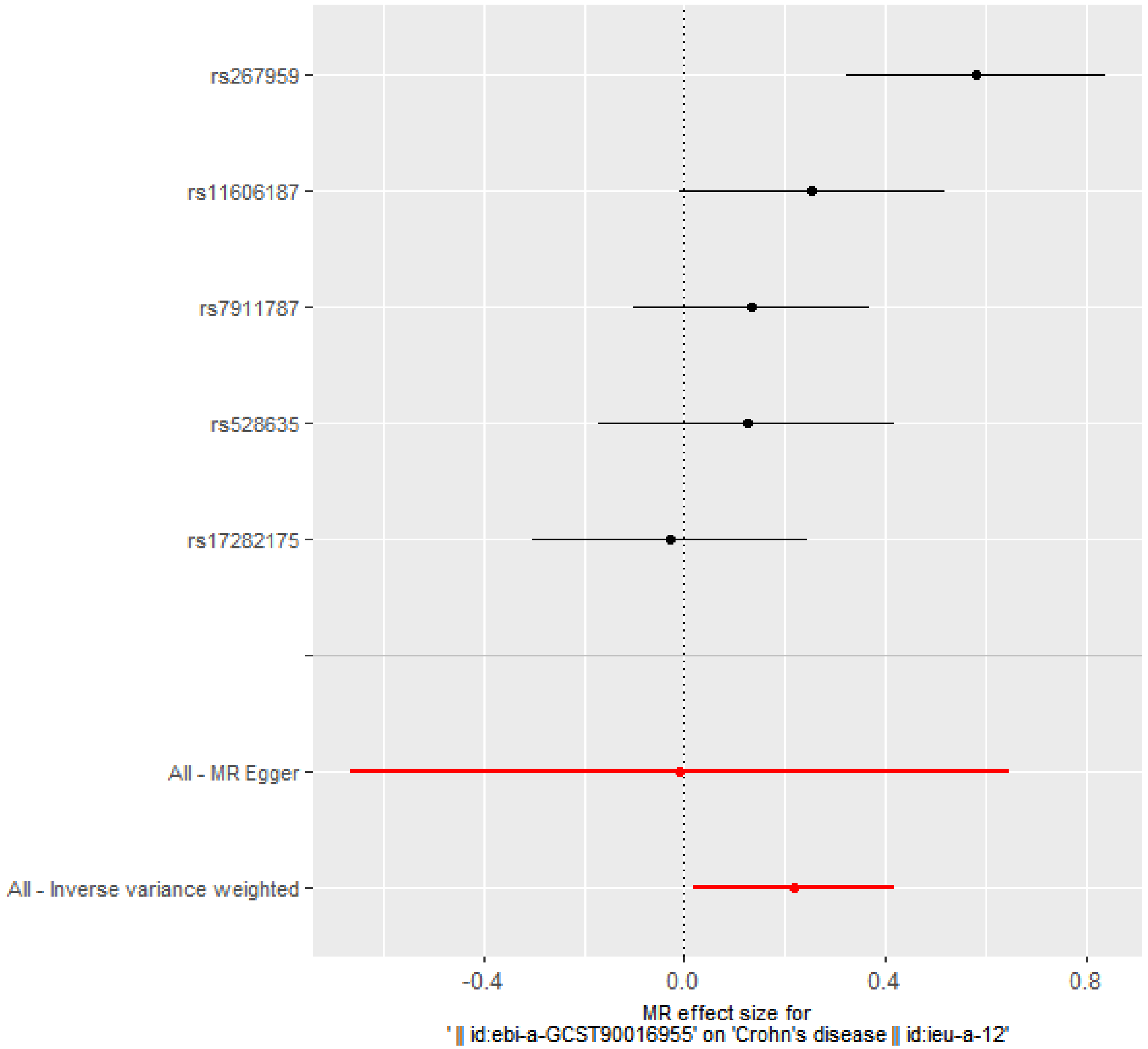
rs267959
rs11606187
rs7911787
rs528635
rs17282175
All - MR Egger
All - Inverse variance weighted
-0.4
0.0
0.4
0.8
MR effect size for
' || id:ebi-a-GCST90016955' on 'Crohn's disease || id:ieu-a-12'

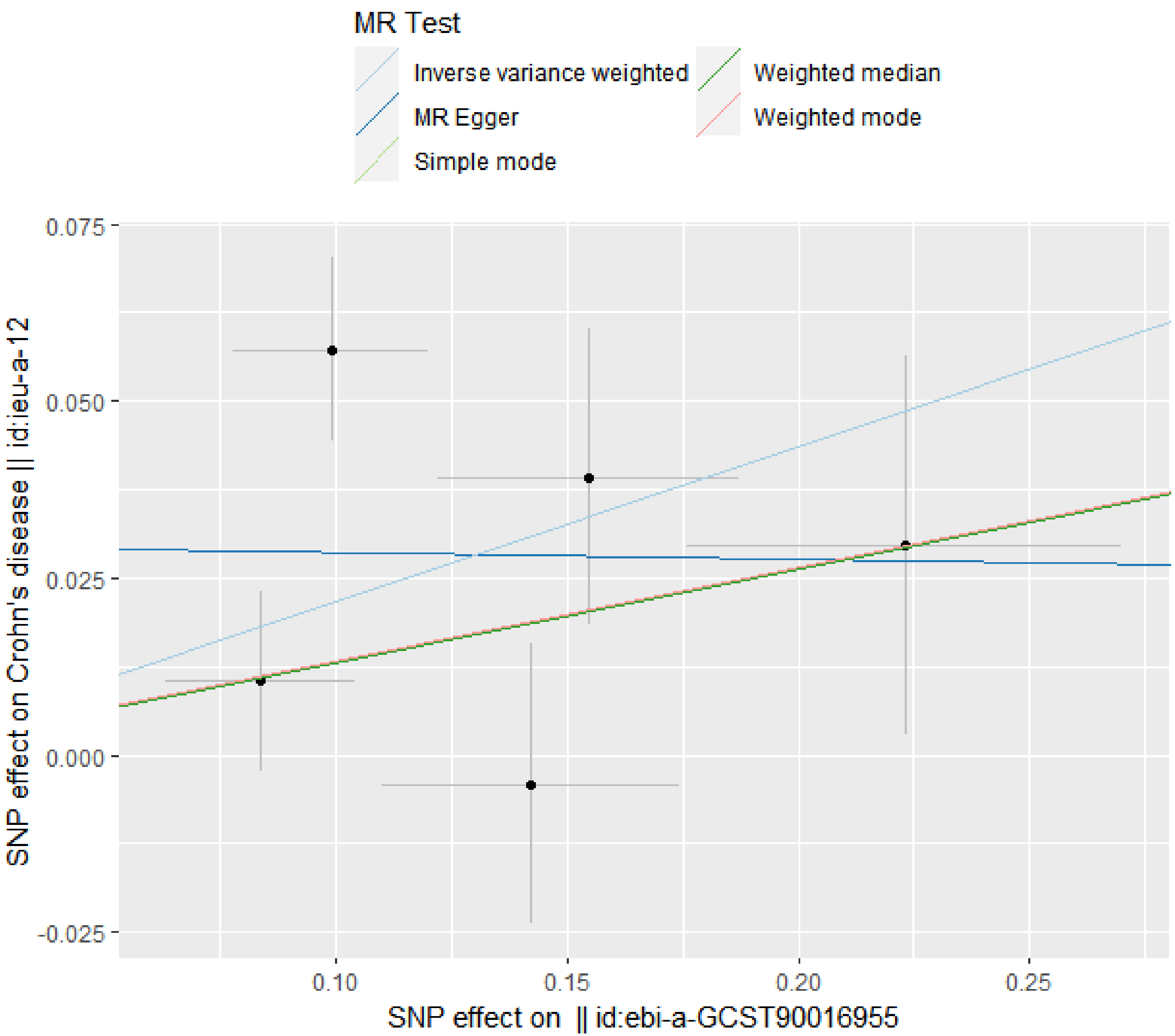
MR Test
Inverse variance weighted
MR Egger
Simple mode
Weighted median
Weighted mode
0.075
0.050
0.025
0.000
-0.025
SNP effect on Crohn's disease || id:ieu-a-12
0.10
0.15
0.20
0.25
SNP effect on || id:ebi-a-GCST90016955

Figure 115 Leave-one-out analysis, funnel plot, MR effect size and scatter plot for gut microbiota abundance (genus Akkermansia id.4037) on crohn's disease

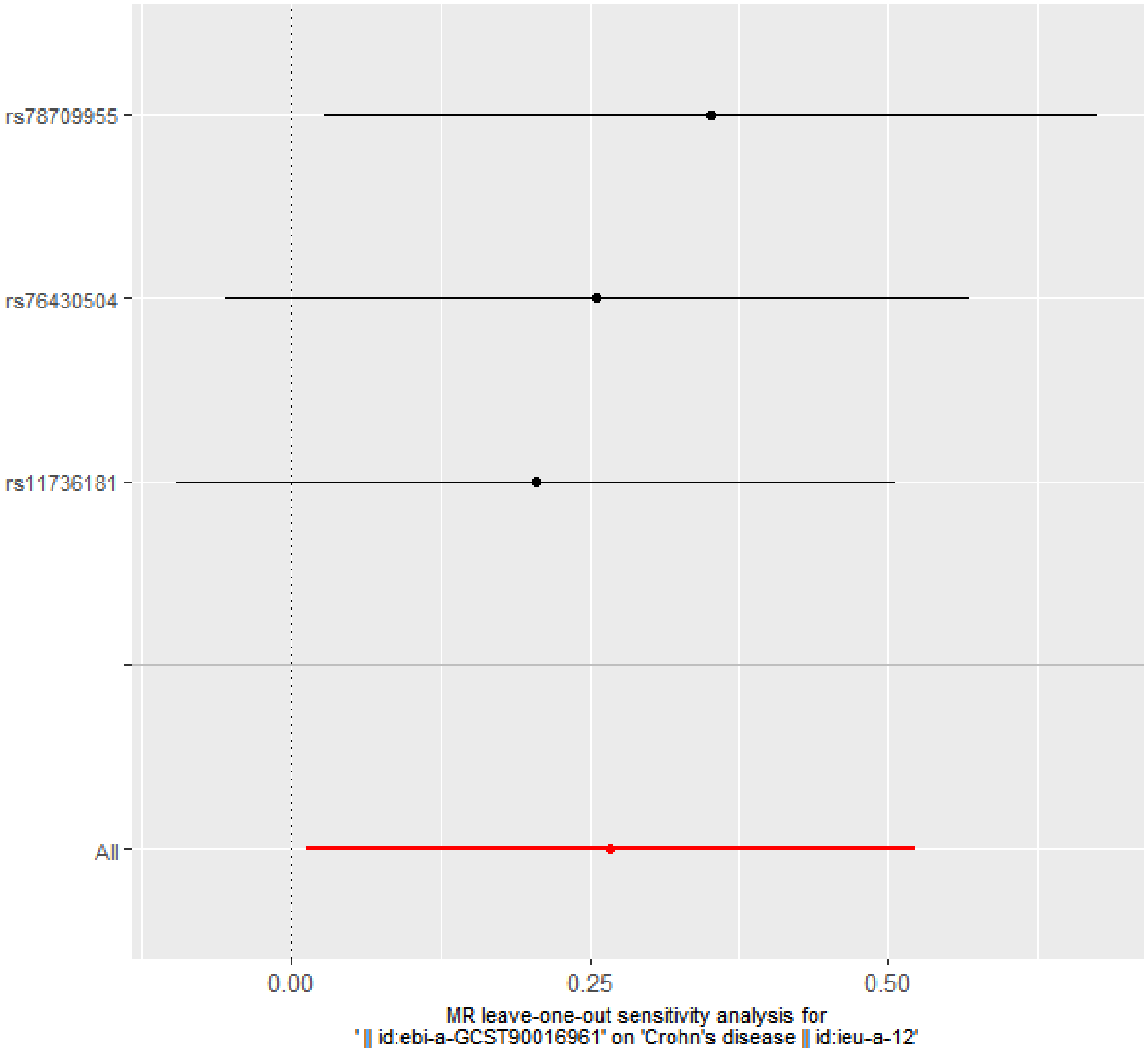

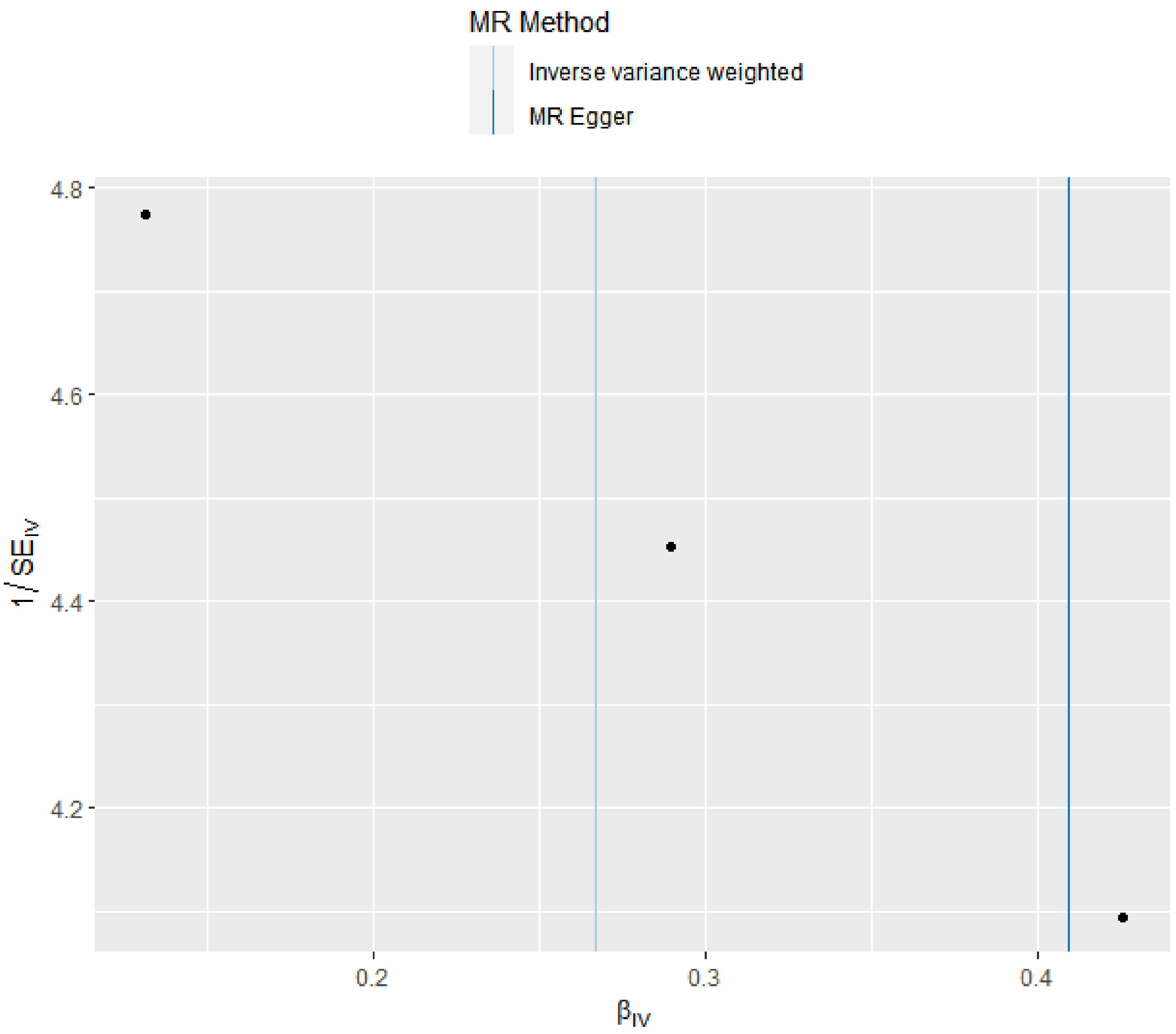

MR Method
Inverse variance weighted
MR Egger
4.8
4.6
4.4
4.2
1/SE_IV
0.2
0.3
0.4
β_IV

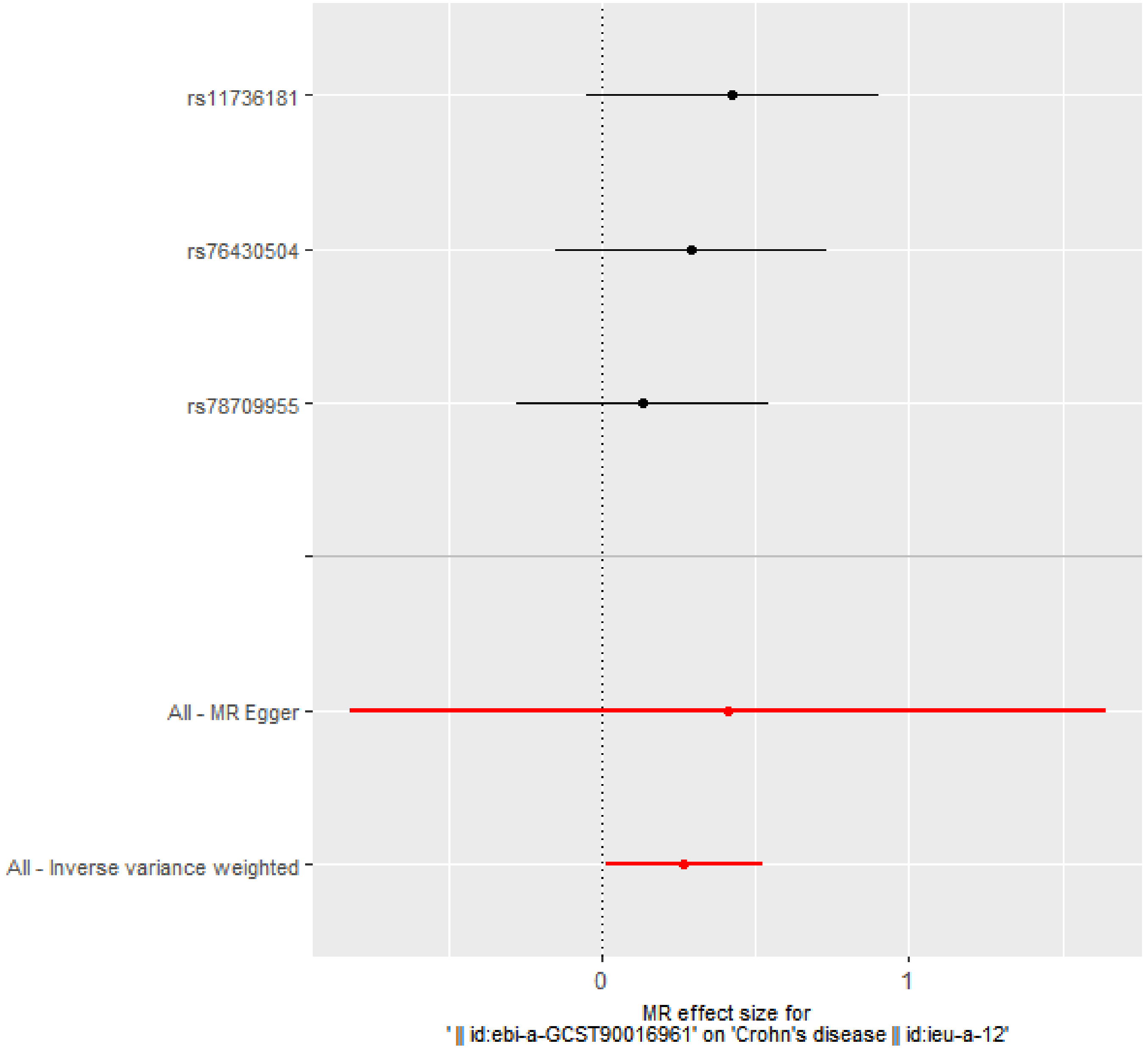
rs11736181
rs76430504
rs78709955
All - MR Egger
All - Inverse variance weighted
0
1
MR effect size for
' || id:ebi-a-GCST90016961' on 'Crohn's disease || id:ieu-a-12'

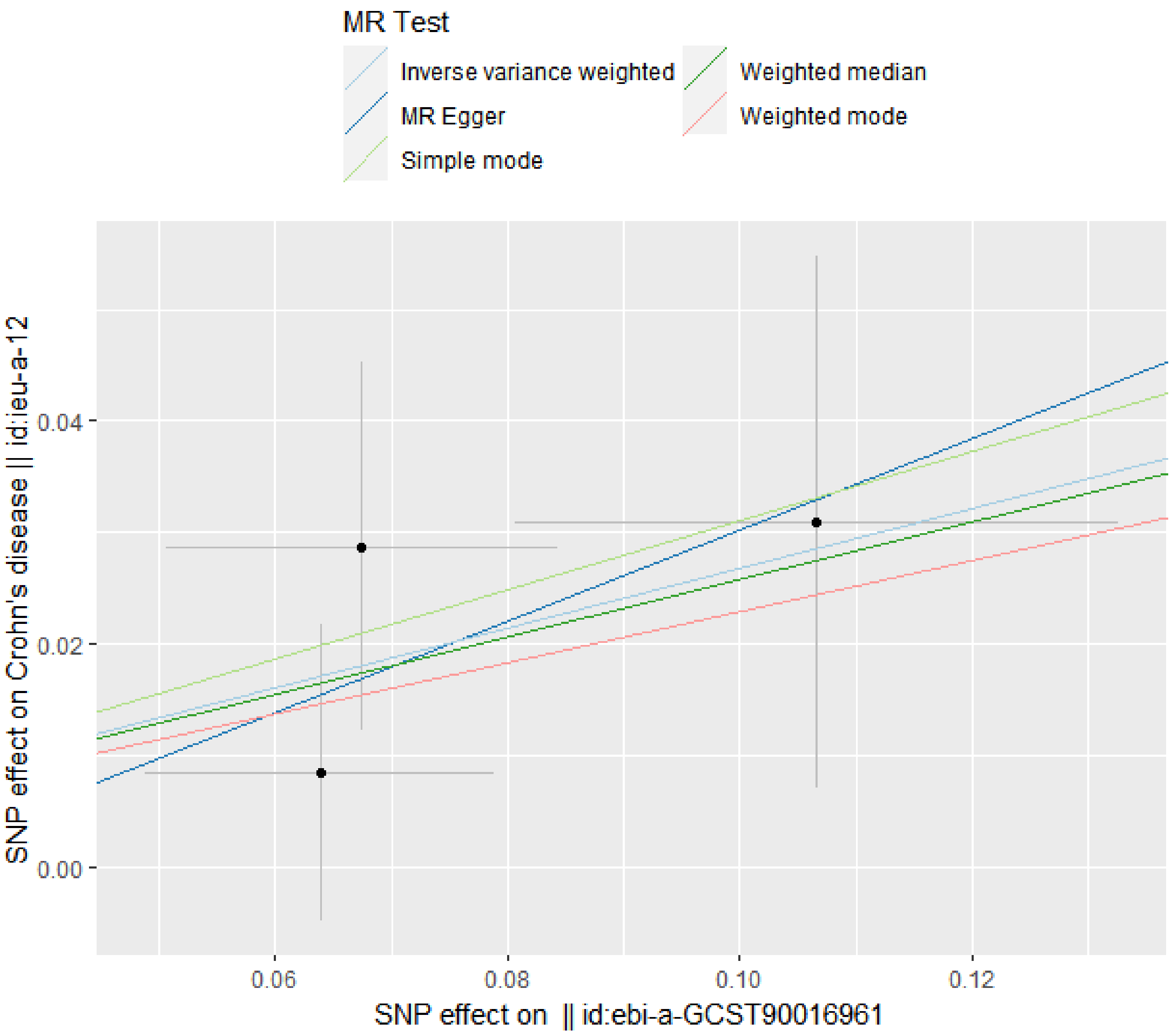

MR Test
Inverse variance weighted
MR Egger
Simple mode
Weighted median
Weighted mode
SNP effect on Crohn's disease || id:ieu-a-12
0.04
0.02
0.00
0.06
0.08
0.10
0.12
SNP effect on || id:ebi-a-GCST90016961

Figure 116 Leave-one-out analysis, funnel plot, MR effect size and scatter plot for gut microbiota abundance (genus Christensenellaceae R 7group id.11283) on crohn's disease

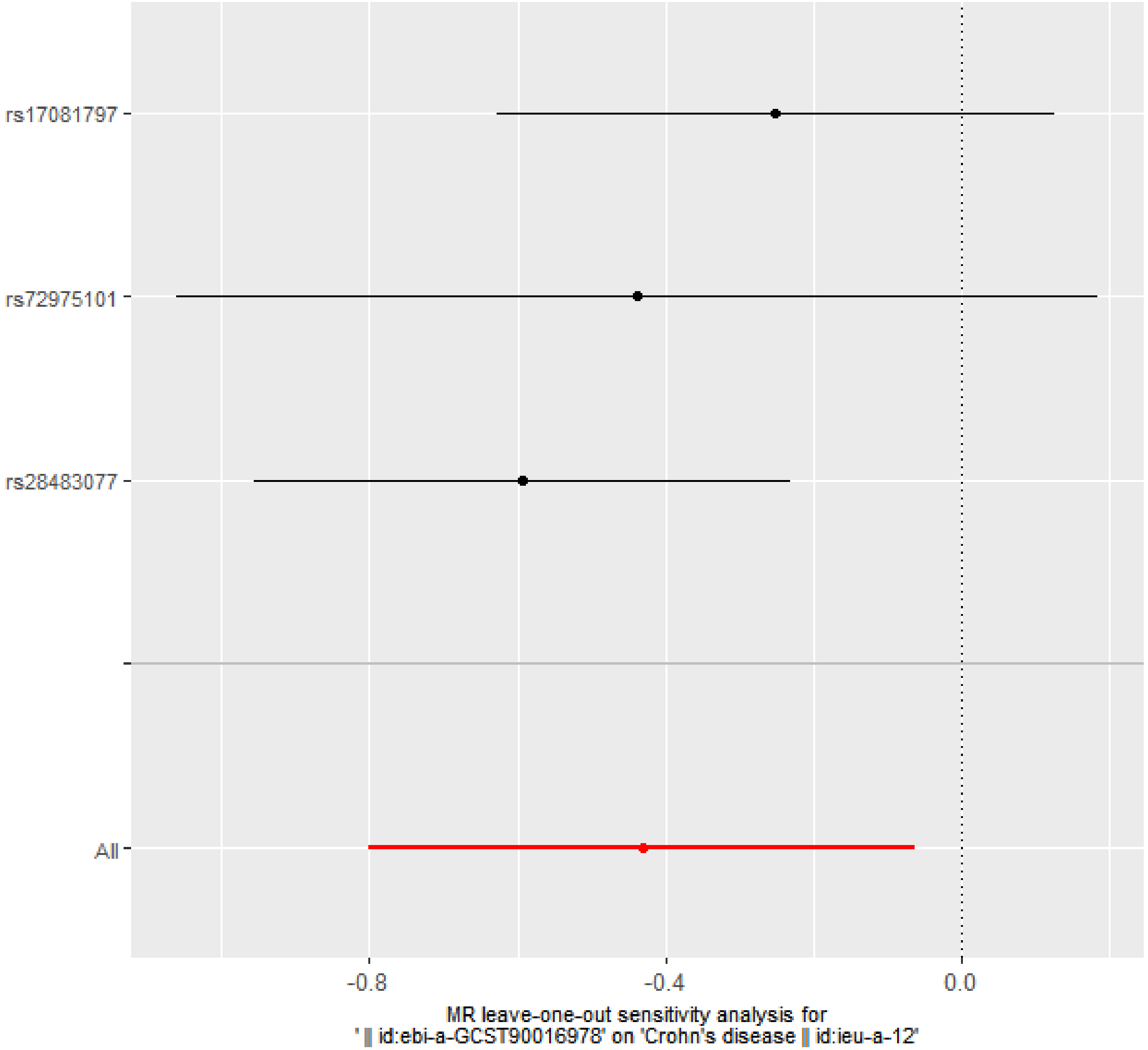

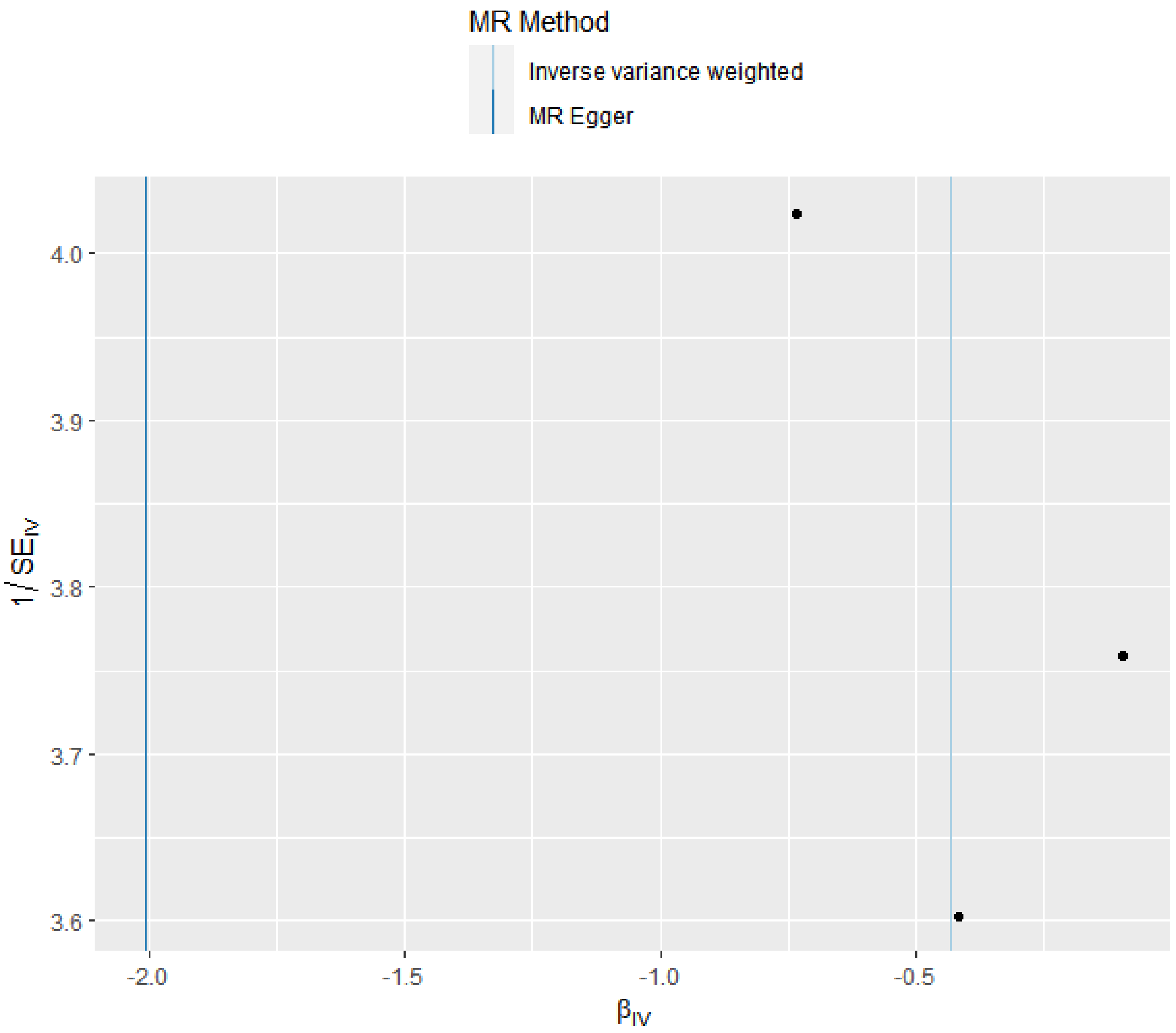

MR Method
Inverse variance weighted
MR Egger
4.0
3.9
3.8
3.7
3.6
1/SE_IV
-2.0
-1.5
-1.0
-0.5
β_IV

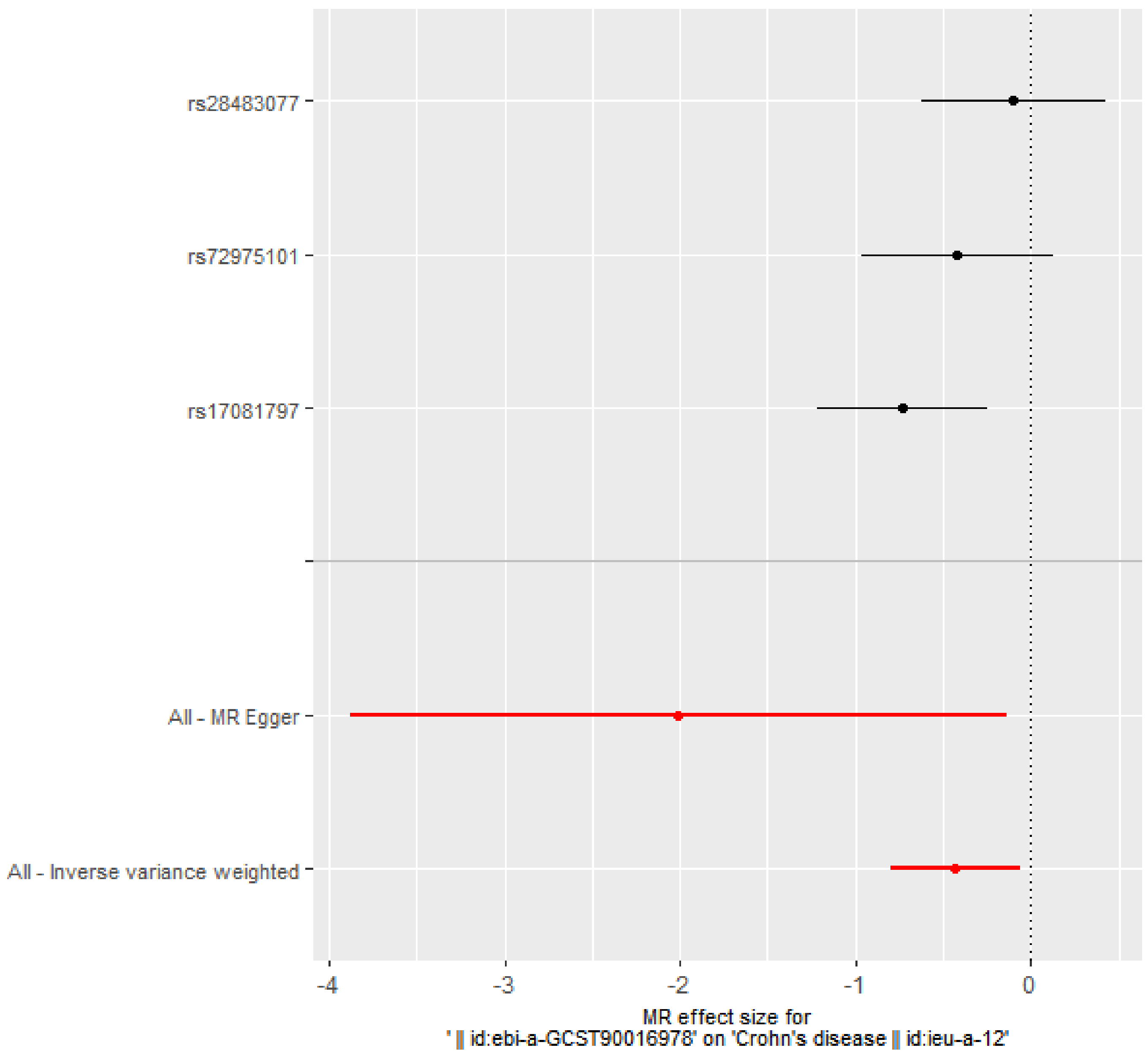
rs28483077
rs72975101
rs17081797
All - MR Egger
All - Inverse variance weighted
-4
-3
-2
-1
0
MR effect size for
' || id:ebi-a-GCST90016978' on 'Crohn's disease || id:ieu-a-12'

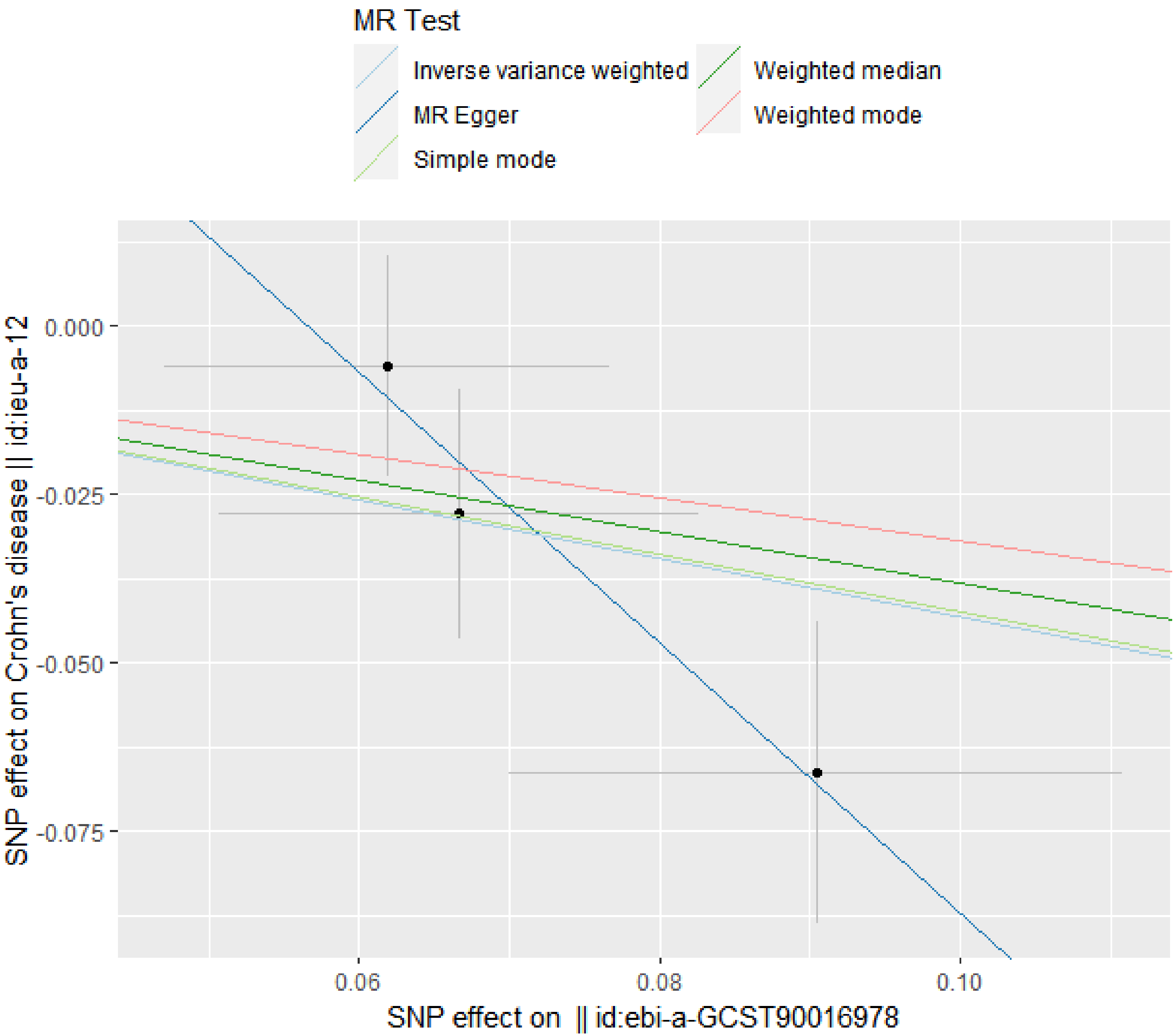

MR Test
Inverse variance weighted
MR Egger
Simple mode
Weighted median
Weighted mode
0.000
-0.025
-0.050
-0.075
SNP effect on Crohn's disease || id:ieu-a-12
0.06
0.08
0.10
SNP effect on || id:ebi-a-GCST90016978

Figure 117 Leave-one-out analysis, funnel plot, MR effect size and scatter plot for gut microbiota abundance (genus Eubacterium coprostanoligenes group id.11375) on crohn's disease

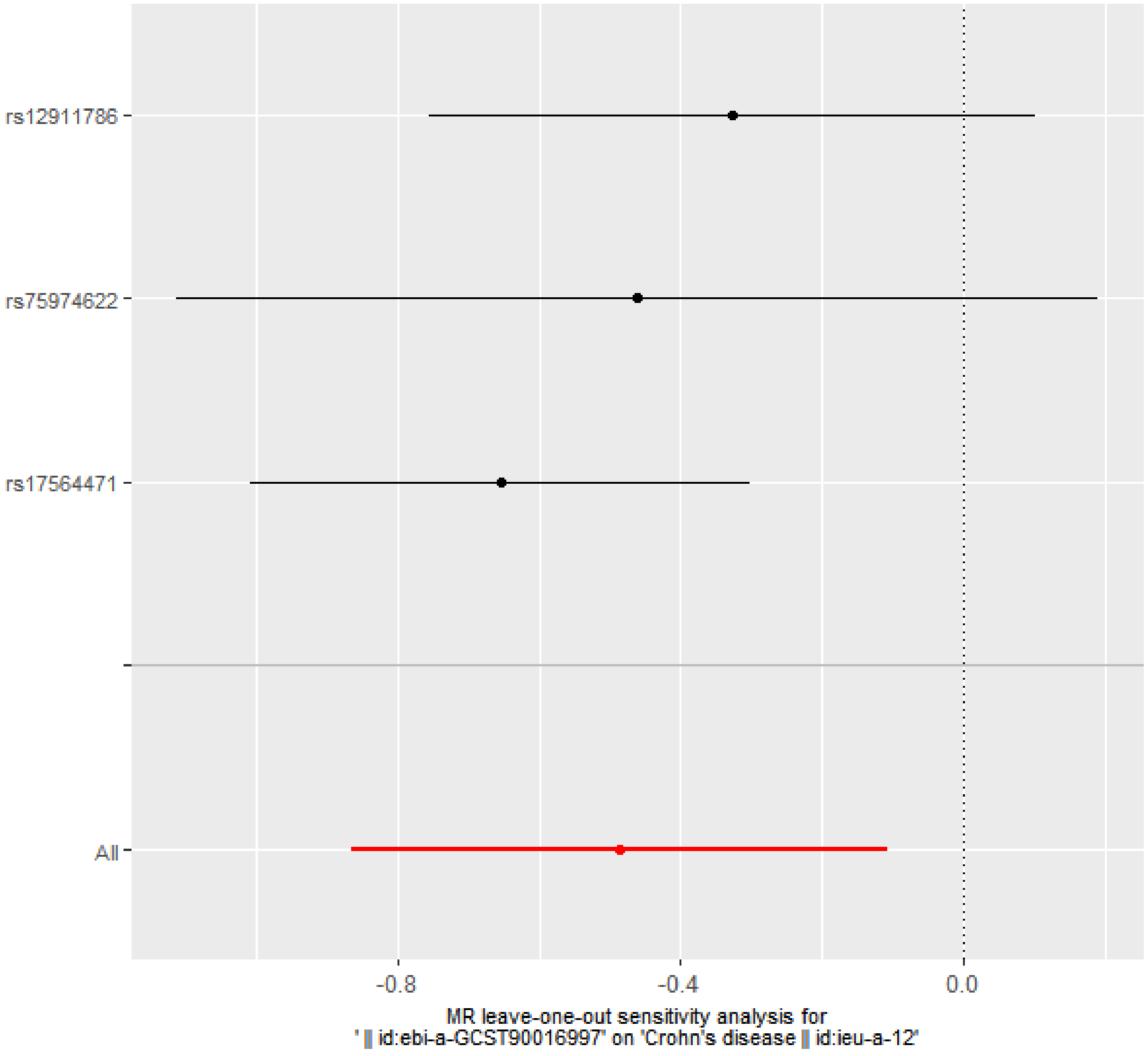

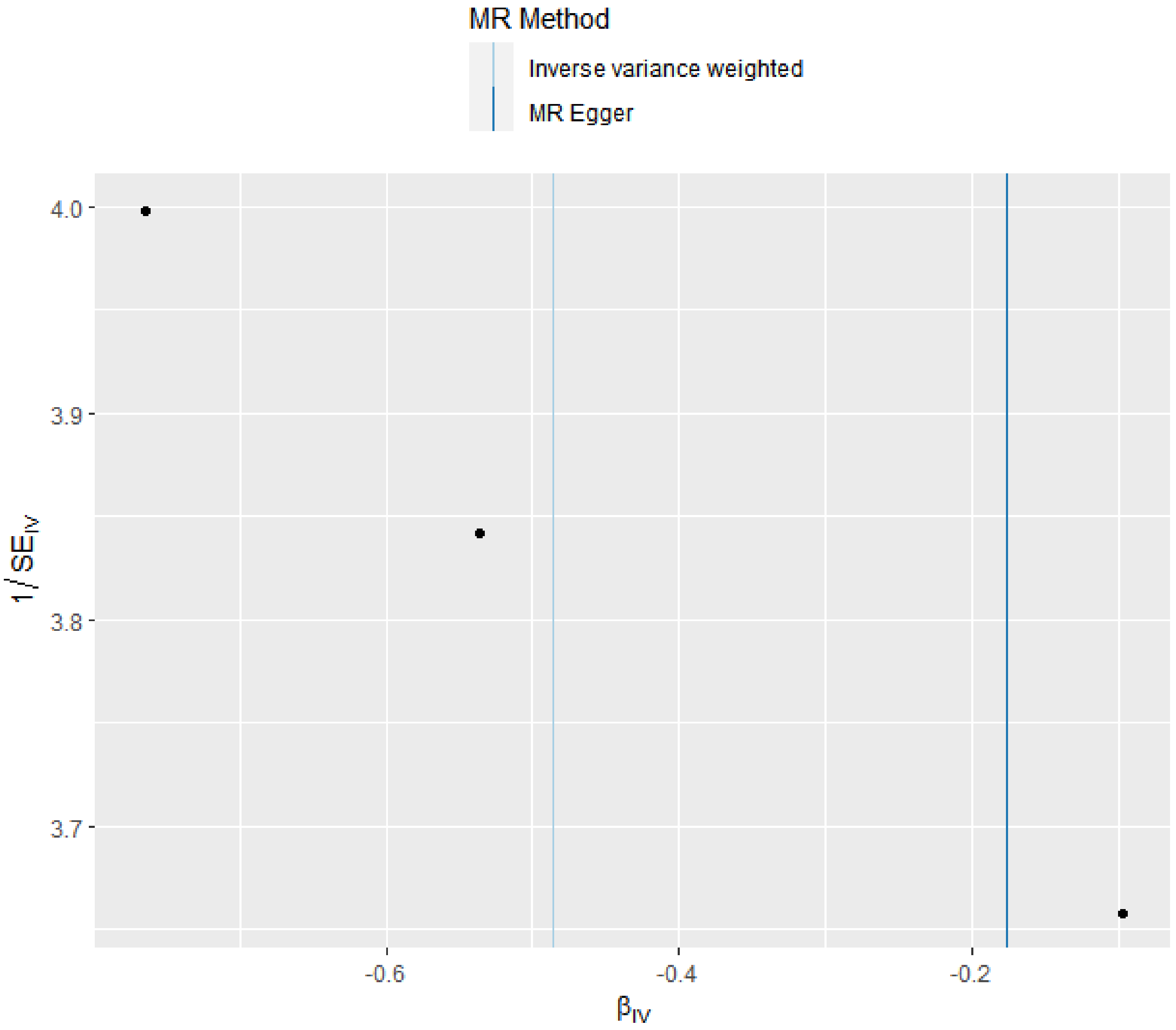

MR Method
Inverse variance weighted
MR Egger
4.0
3.9
3.8
3.7
1/SE_IV
-0.6
-0.4
-0.2
β_IV

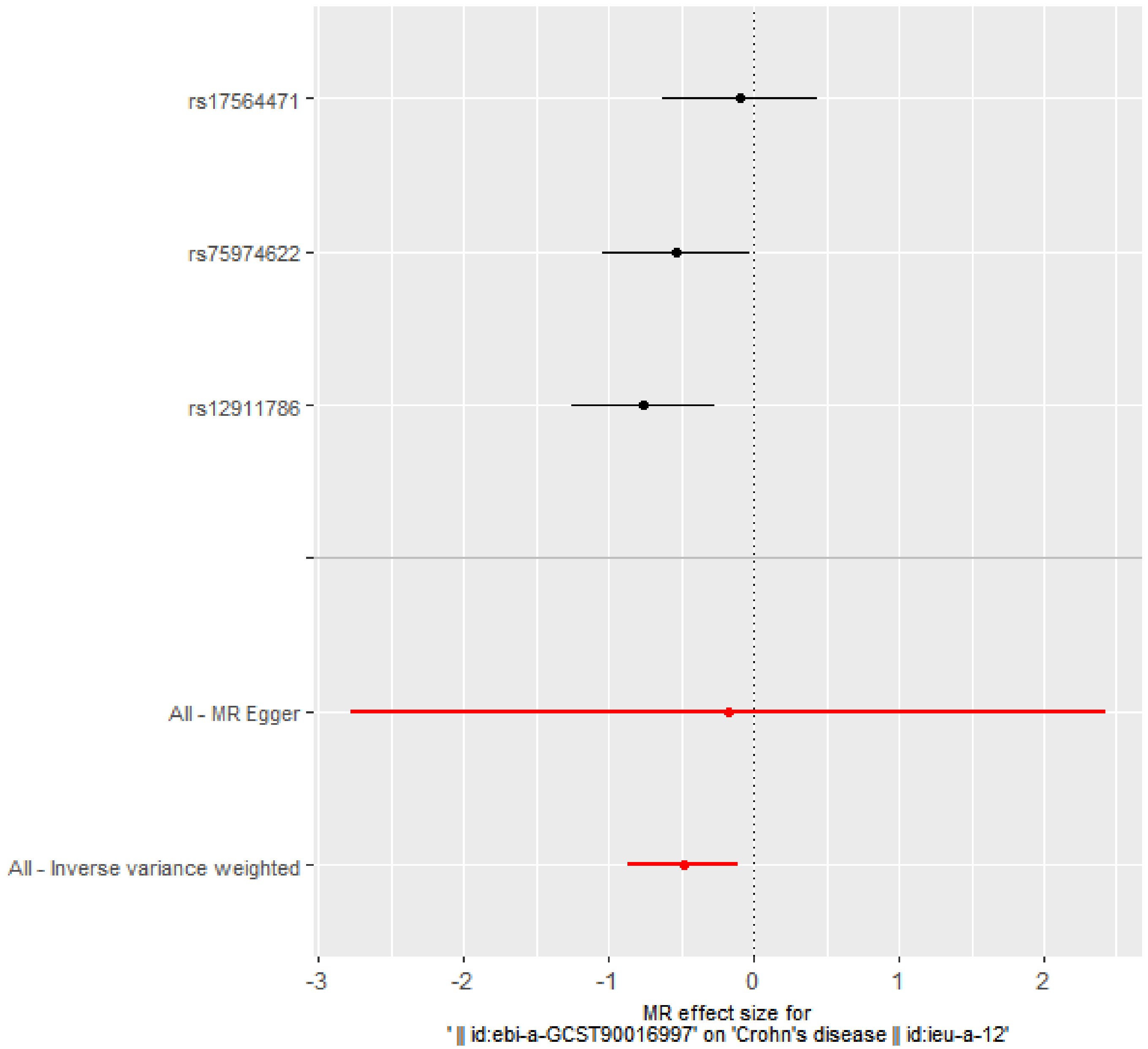
rs17564471
rs75974622
rs12911786
All - MR Egger
All - Inverse variance weighted
-3
-2
-1
0
1
2
MR effect size for
' || id:ebi-a-GCST90016997' on 'Crohn's disease || id:ieu-a-12'

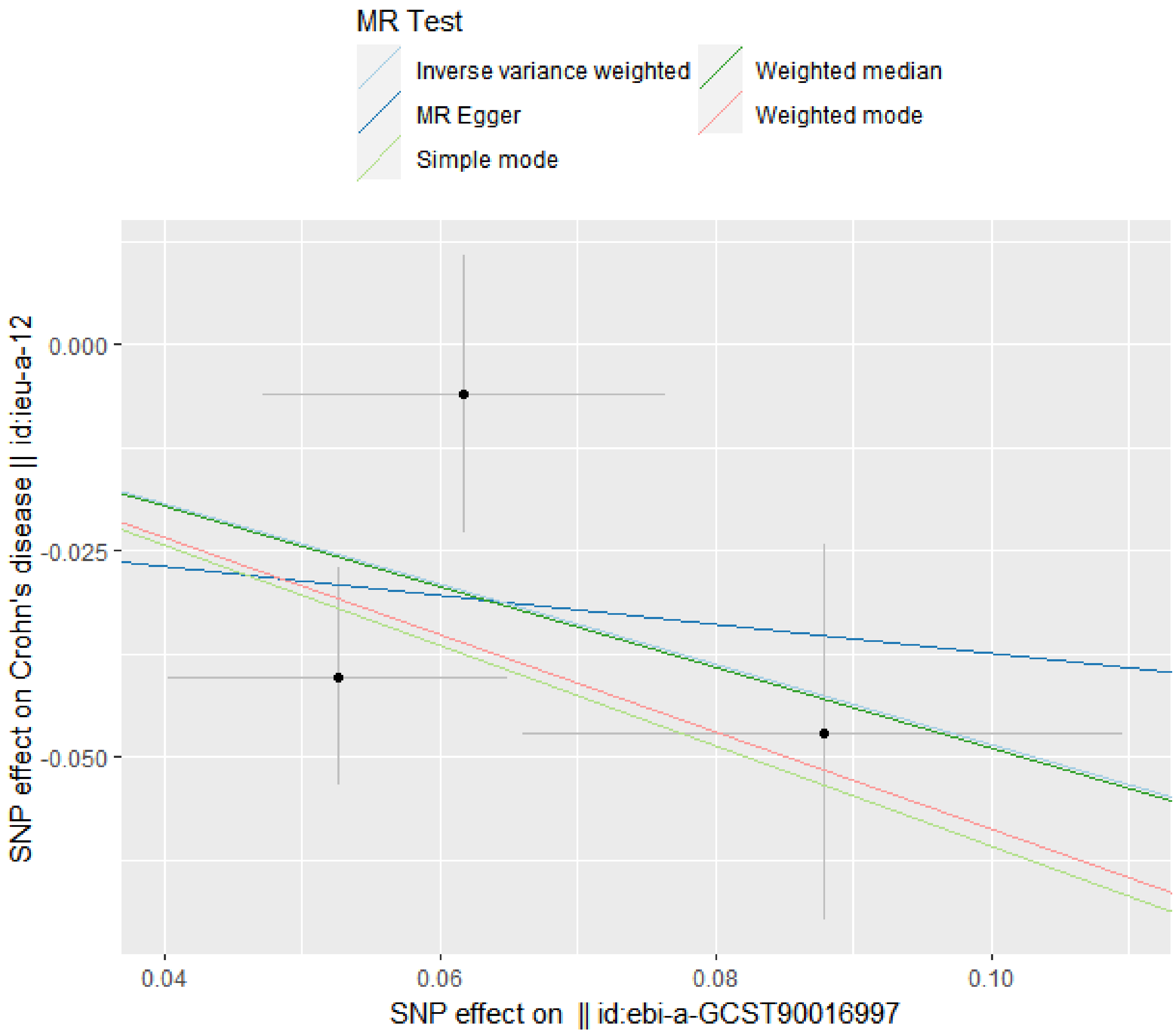

MR Test
Inverse variance weighted
MR Egger
Simple mode
Weighted median
Weighted mode
0.000
-0.025
-0.050
0.04
0.06
0.08
0.10
SNP effect on Crohn's disease || id:ieu-a-12
SNP effect on || id:ebi-a-GCST90016997

Figure 118 Leave-one-out analysis, funnel plot, MR effect size and scatter plot for gut microbiota abundance (genus Eubacterium fissicatena group id.14373) on crohn's disease

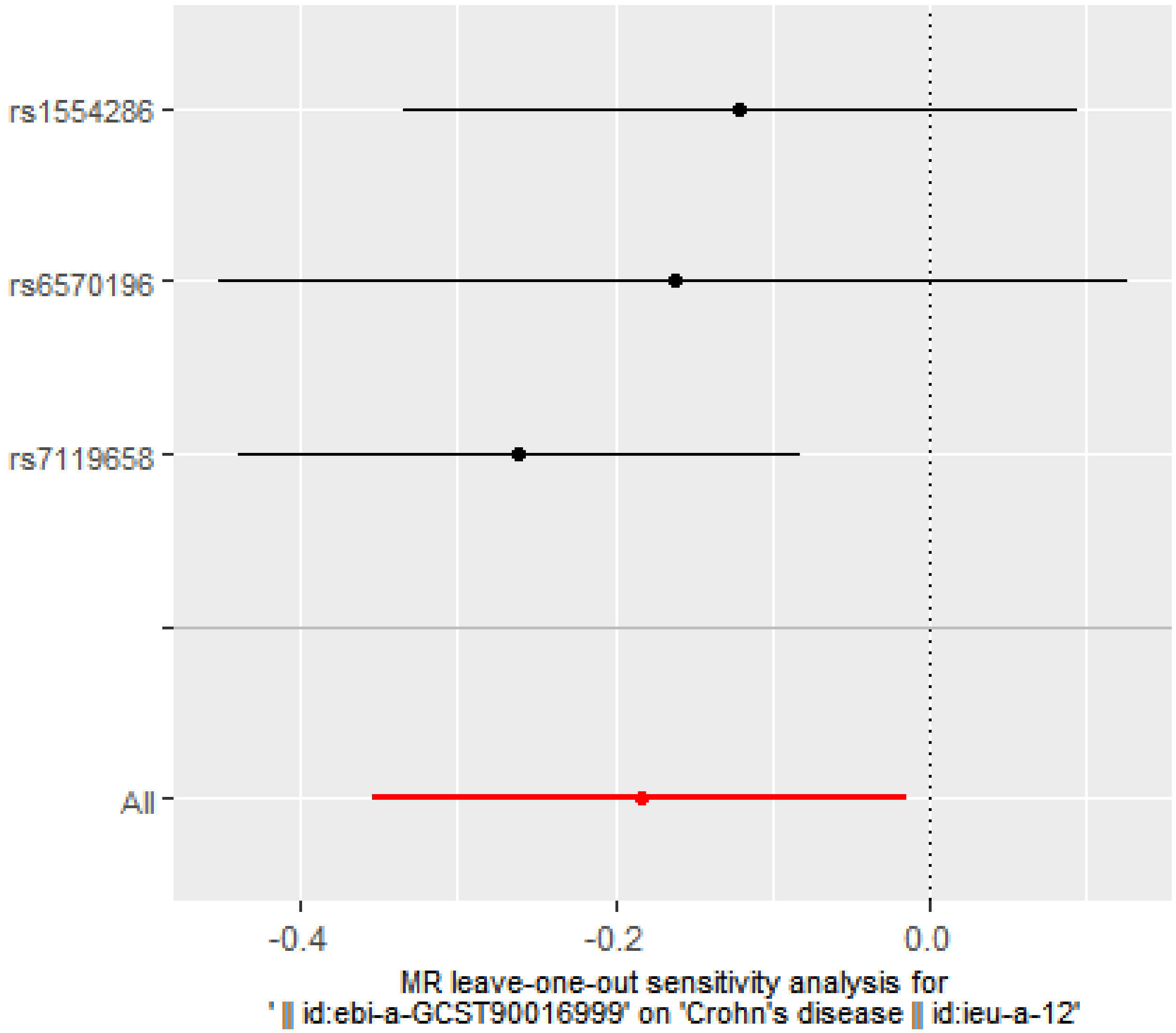

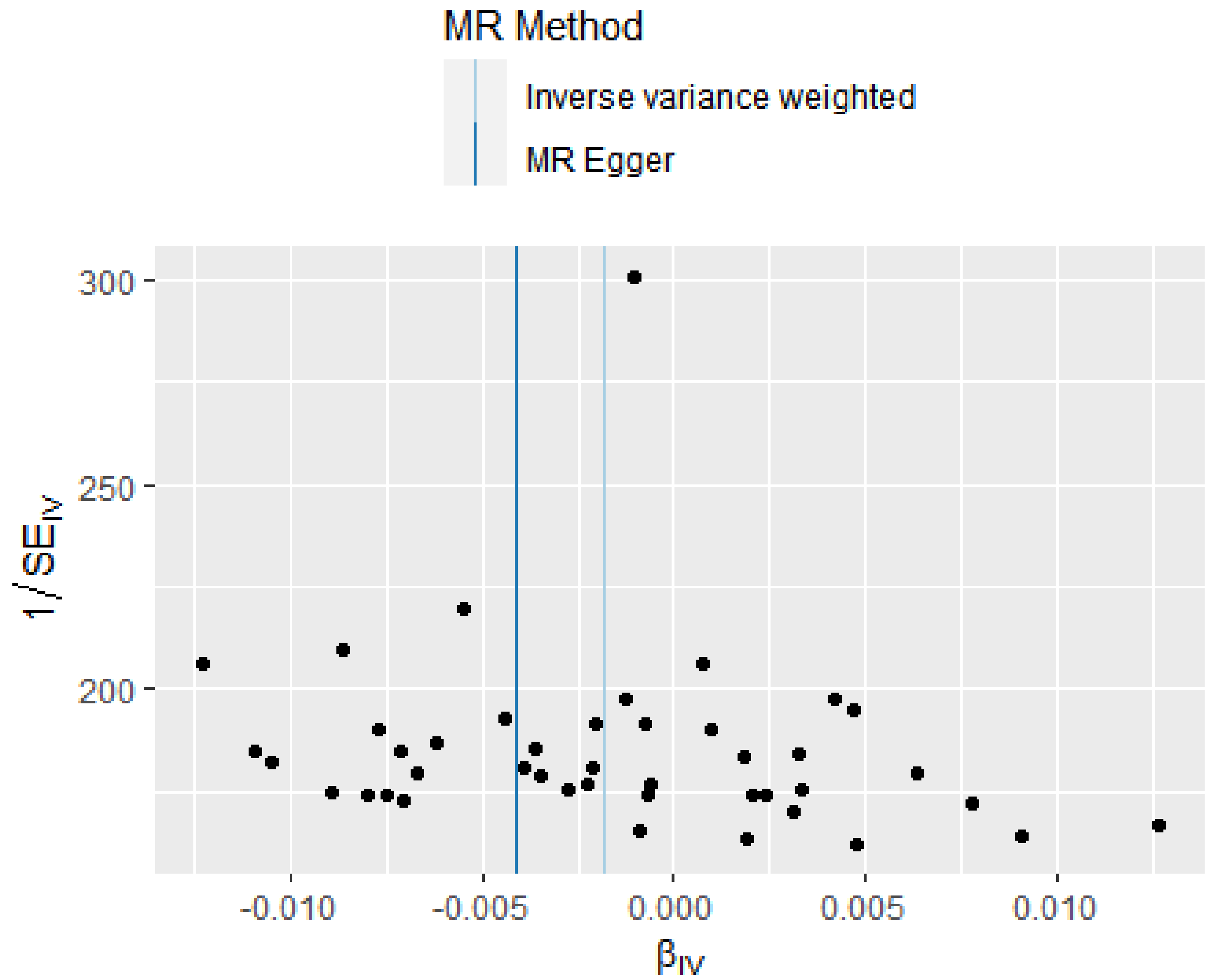

MR Method
Inverse variance weighted
MR Egger
300
250
200
1/SE_IV
-0.010
-0.005
0.000
0.005
0.010
β_IV

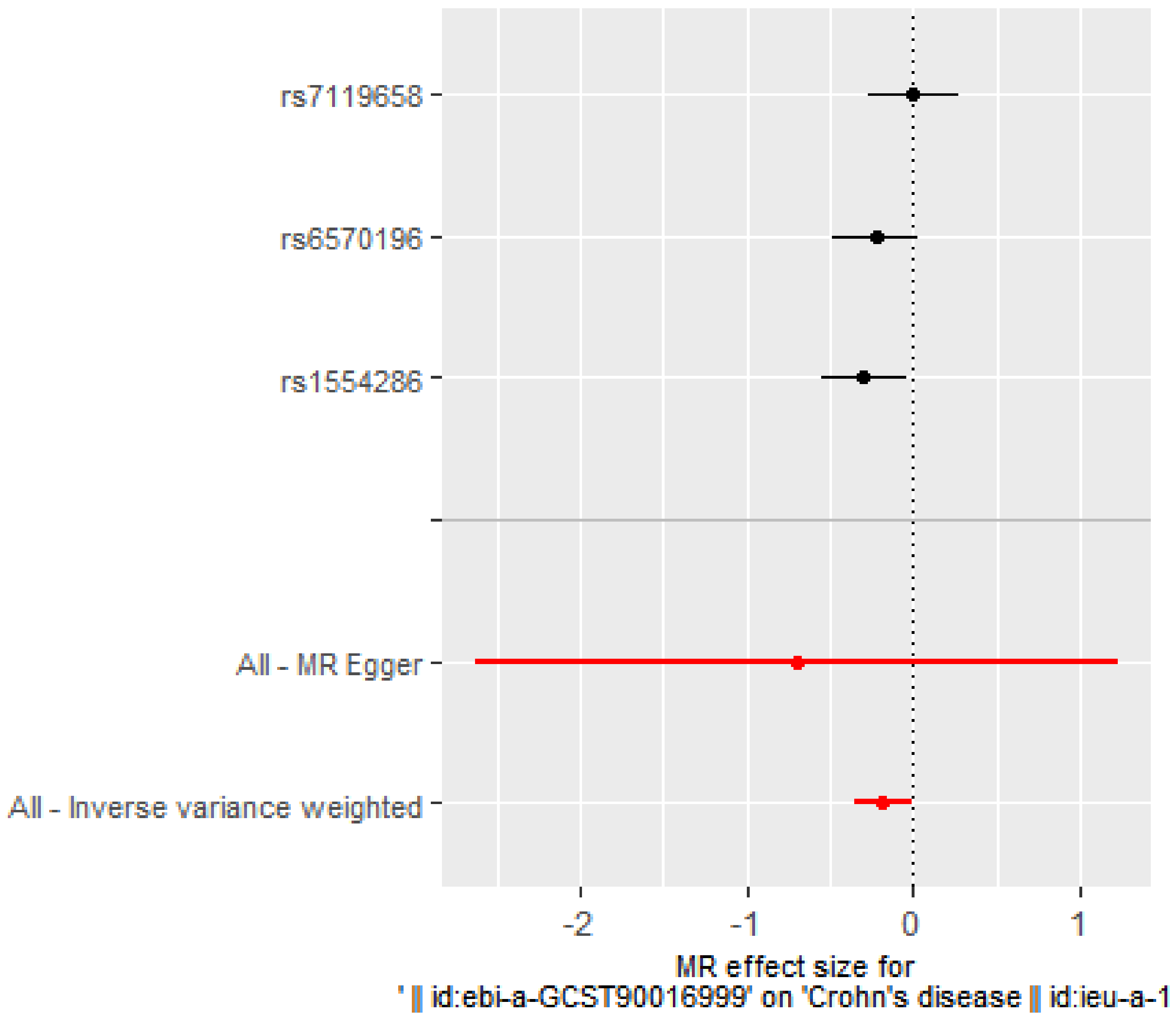
rs7119658
rs6570196
rs1554286
All - MR Egger
All - Inverse variance weighted
-2
-1
0
1
MR effect size for
' || id:ebi-a-GCST90016999' on 'Crohn's disease || id:ieu-a-1

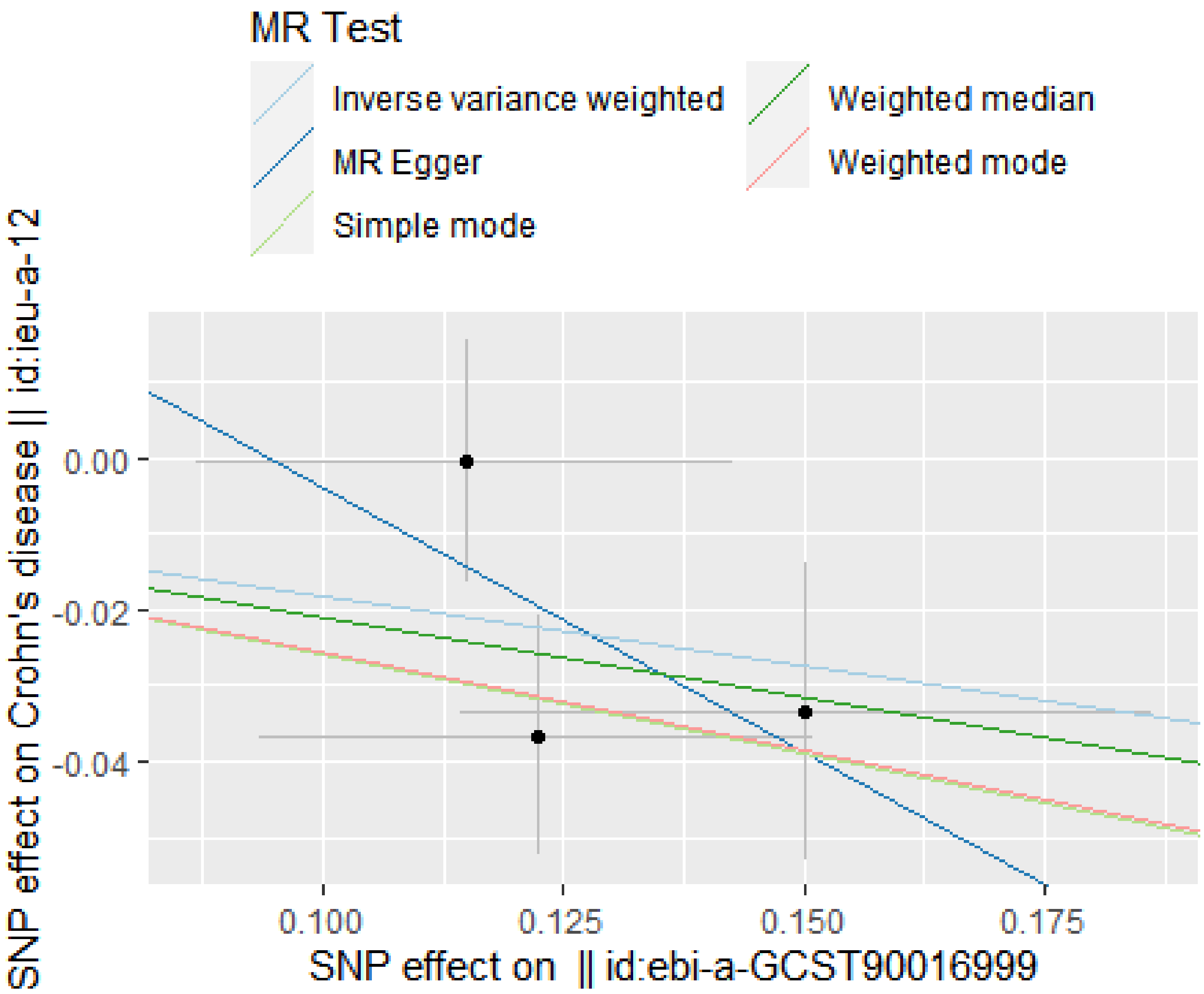
MR Test
Inverse variance weighted
MR Egger
Simple mode
Weighted median
Weighted mode
0.00
-0.02
-0.04
0.100
0.125
0.150
0.175
SNP effect on || id:ebi-a-GCST90016999
SNP effect on Crohn's disease || id:ieu-a-12

Figure 119 Leave-one-out analysis, funnel plot, MR effect size and scatter plot for gut microbiota abundance (genus Oscillospira id.2064) on crohn's disease

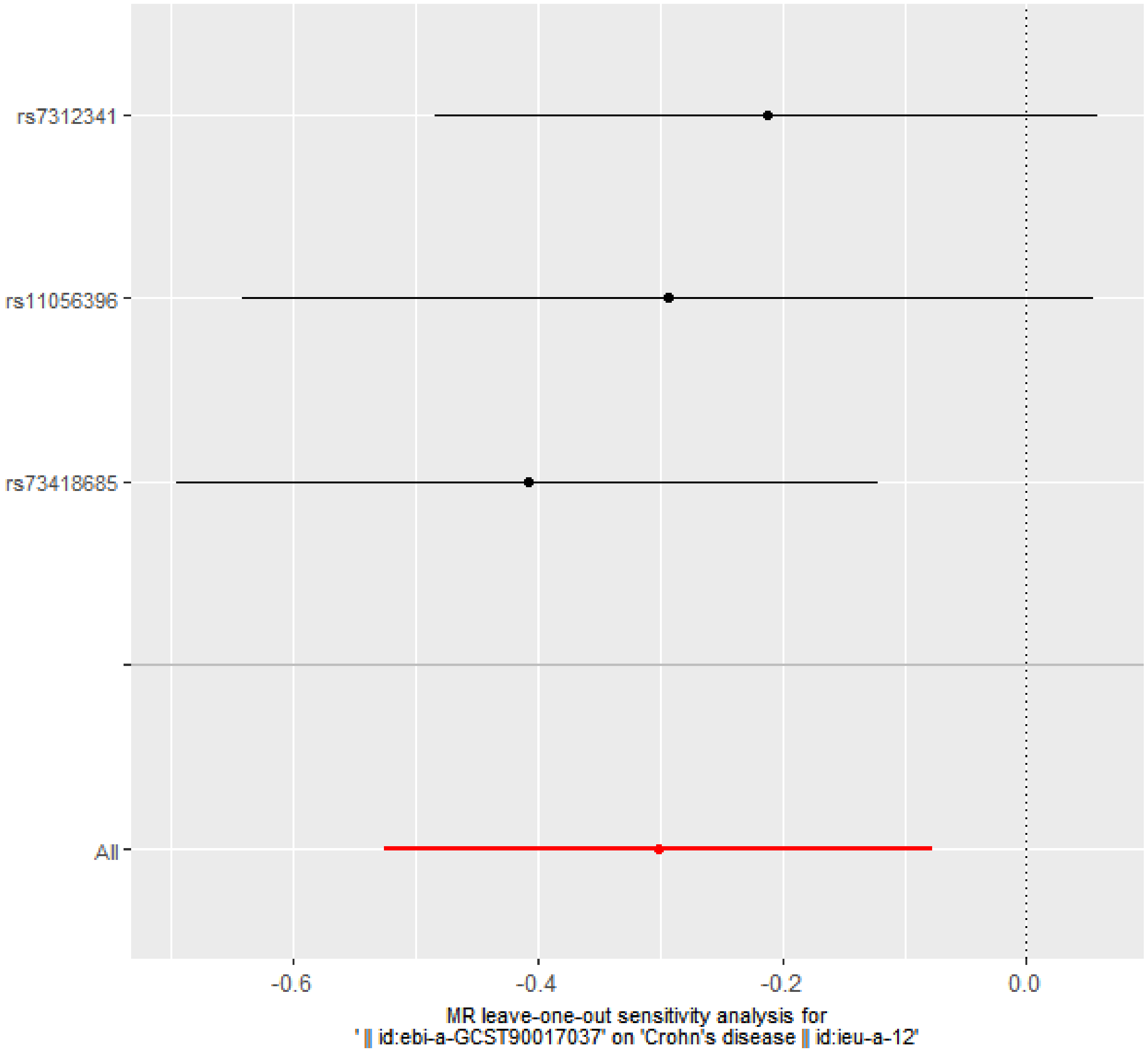

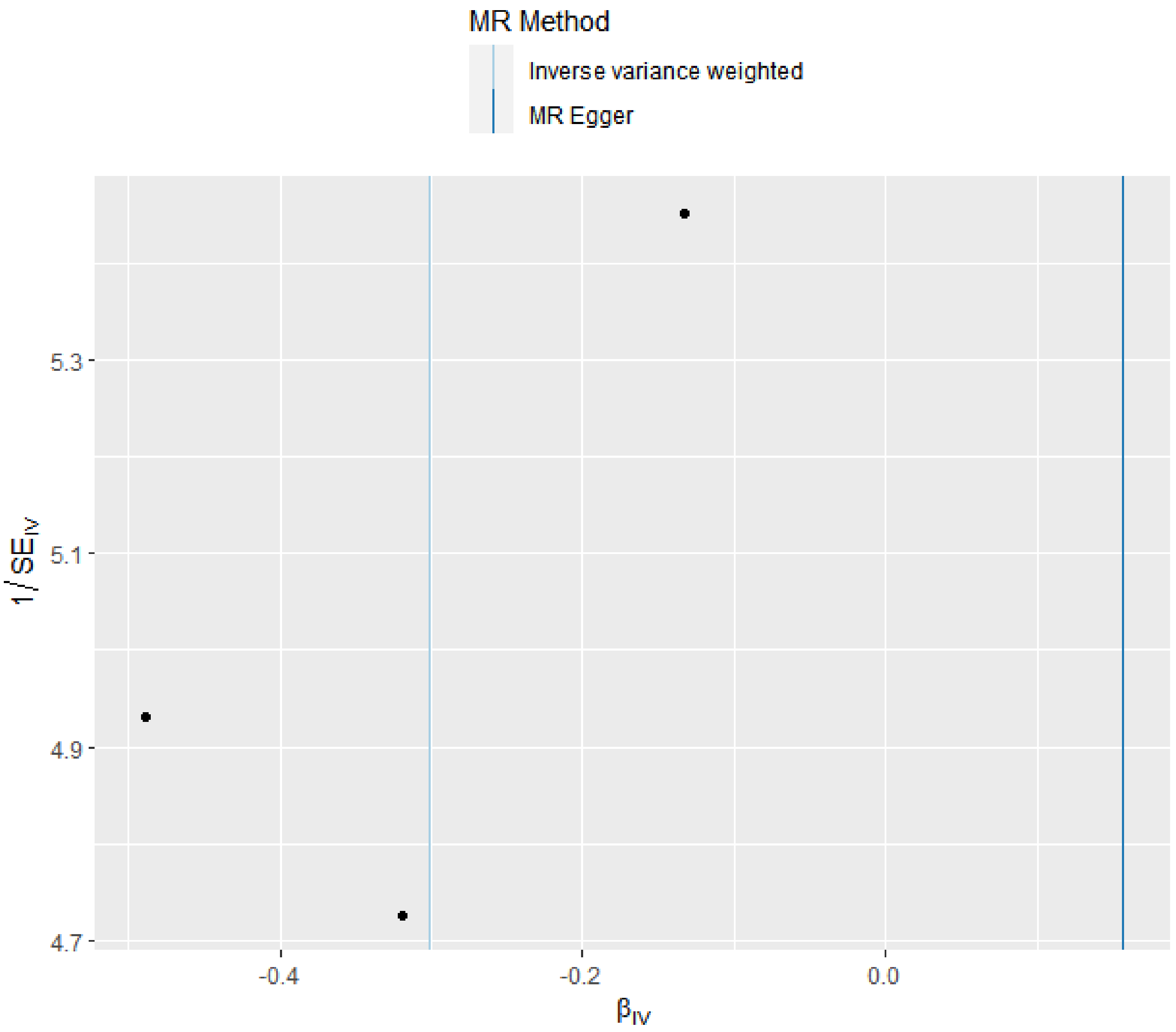

MR Method
Inverse variance weighted
MR Egger
5.3
5.1
4.9
4.7
1/SE_IV
-0.4
-0.2
0.0
β_IV

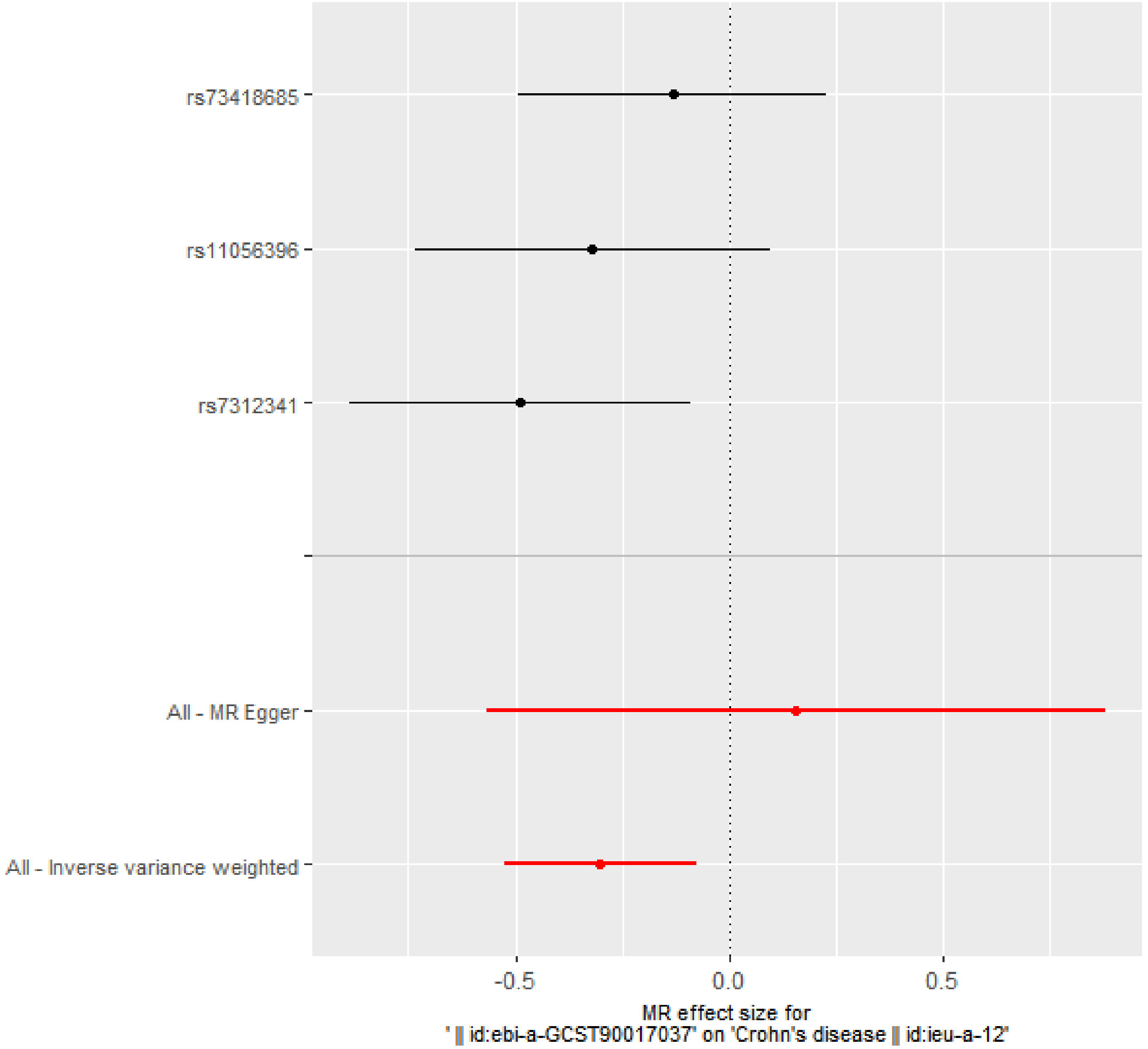

rs73418685
rs11056396
rs7312341
All - MR Egger
All - Inverse variance weighted
-0.5
0.0
0.5
MR effect size for
' || id:ebi-a-GCST90017037' on 'Crohn's disease || id:ieu-a-12'

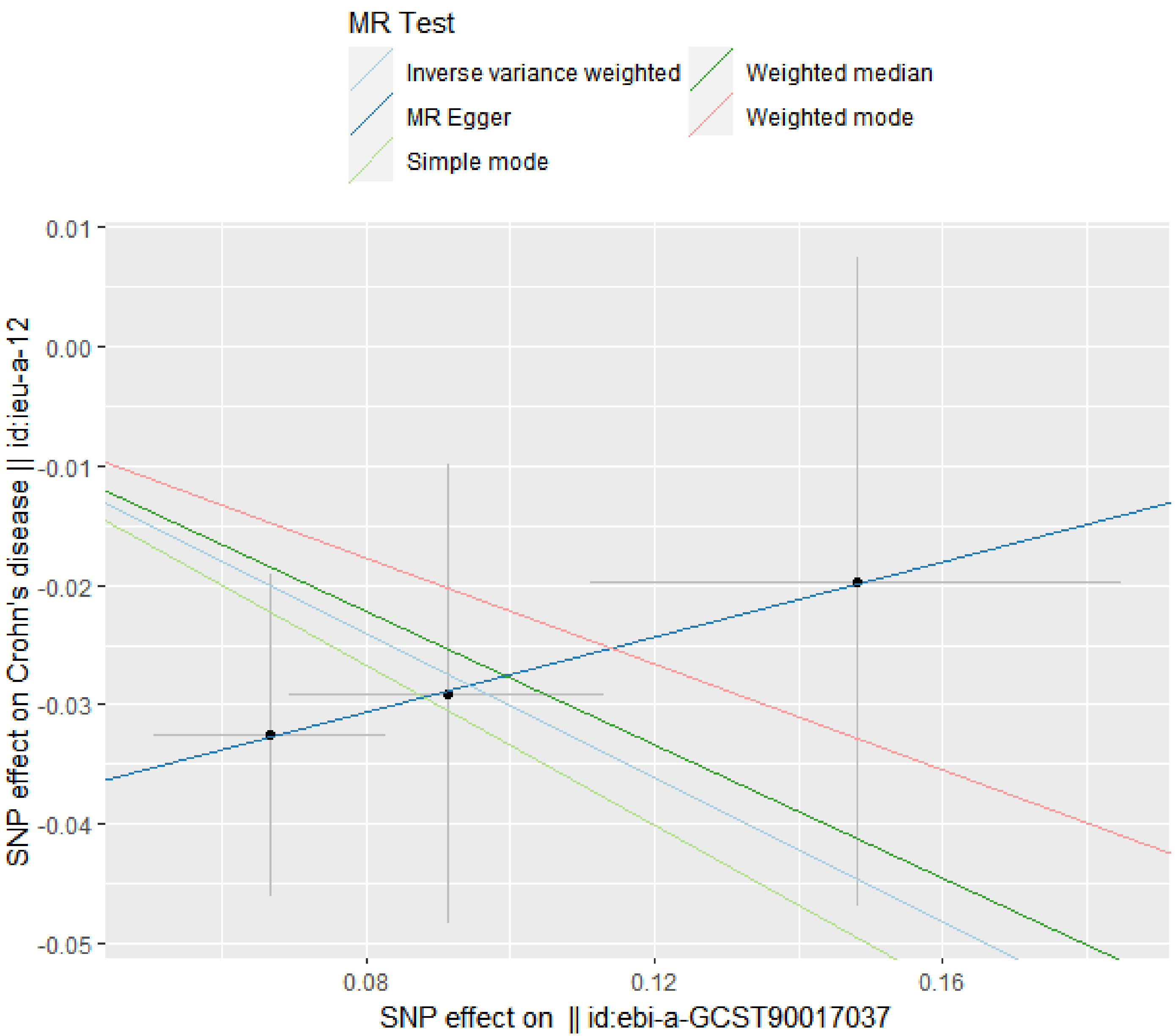

MR Test
Inverse variance weighted
MR Egger
Simple mode
Weighted median
Weighted mode
0.01
0.00
-0.01
-0.02
-0.03
-0.04
-0.05
SNP effect on Crohn's disease || id:ieu-a-12
0.08
0.12
0.16
SNP effect on || id:ebi-a-GCST90017037

Figure 120 Leave-one-out analysis, funnel plot, MR effect size and scatter plot for gut microbiota abundance (genus Ruminococcaceae UCG013 id.11370) on crohn's disease

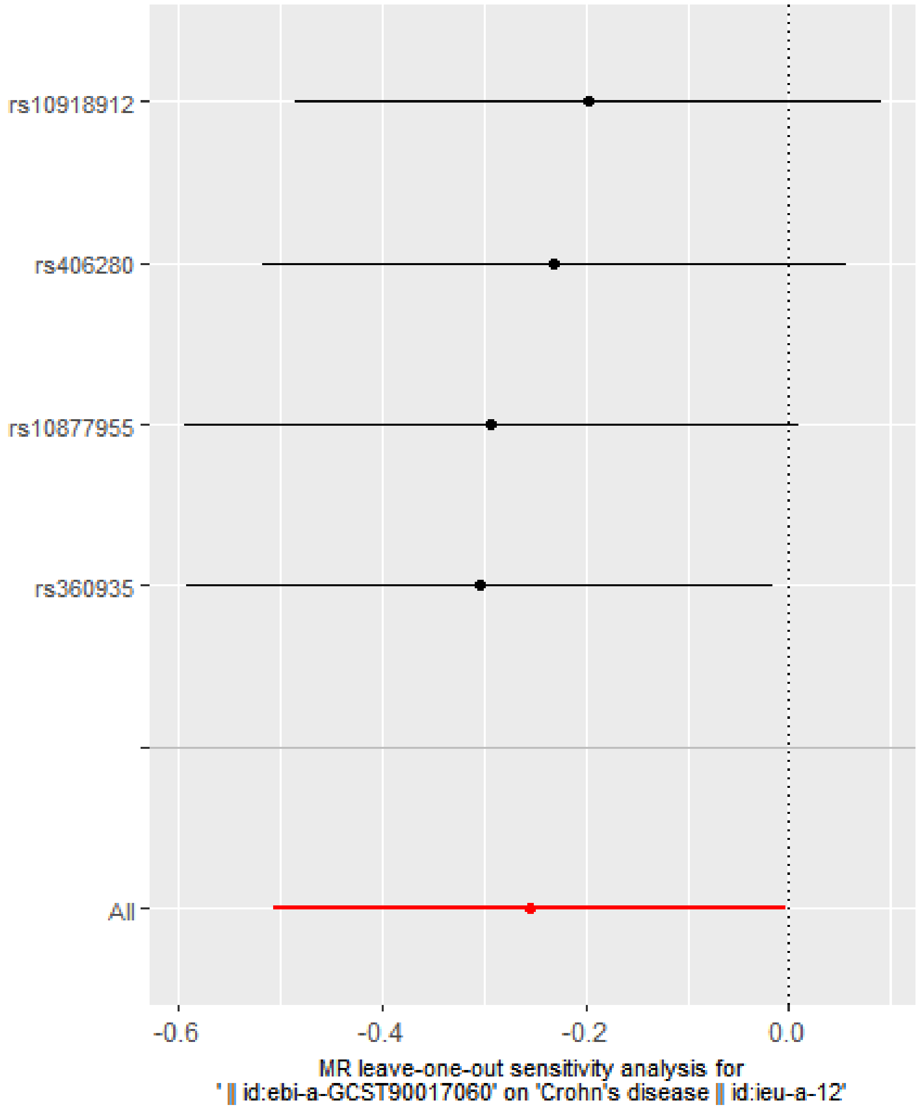

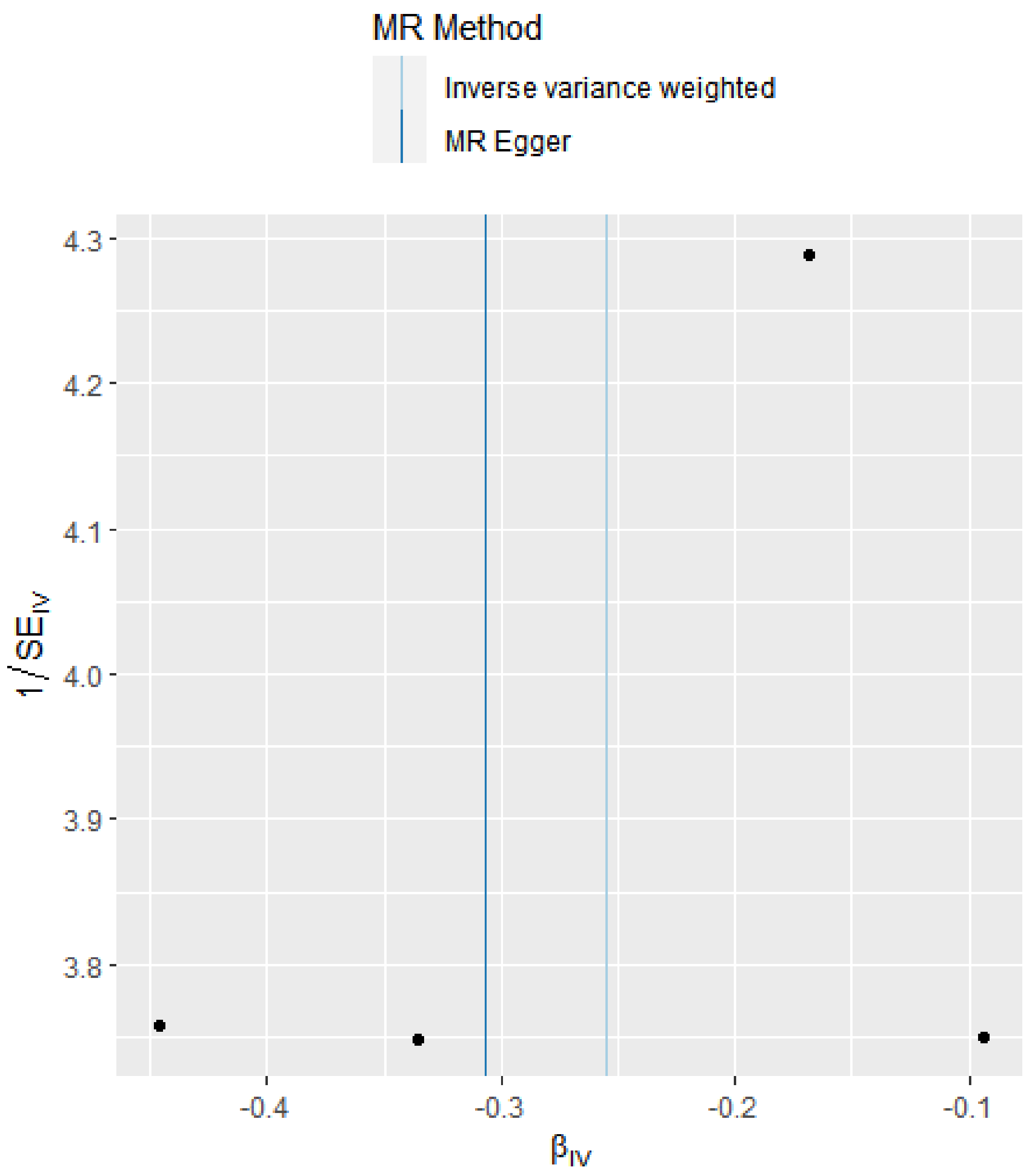

MR Method
Inverse variance weighted
MR Egger
4.3
4.2
4.1
4.0
3.9
3.8
1/SE_IV
-0.4
-0.3
-0.2
-0.1
β_IV

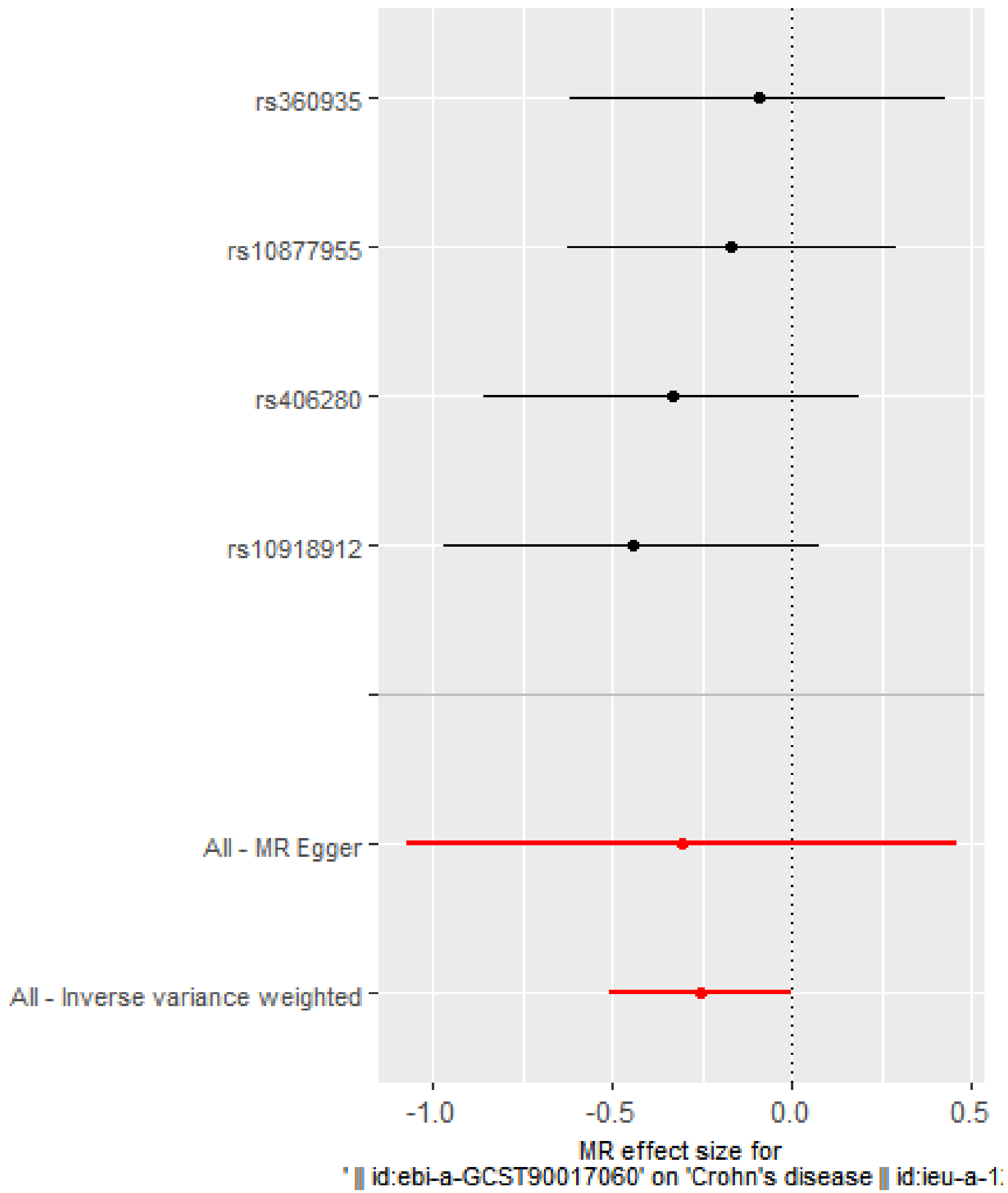
rs360935
rs10877955
rs406280
rs10918912
All - MR Egger
All - Inverse variance weighted
-1.0
-0.5
0.0
0.5
MR effect size for
' || id:ebi-a-GCST90017060' on 'Crohn's disease || id:ieu-a-1

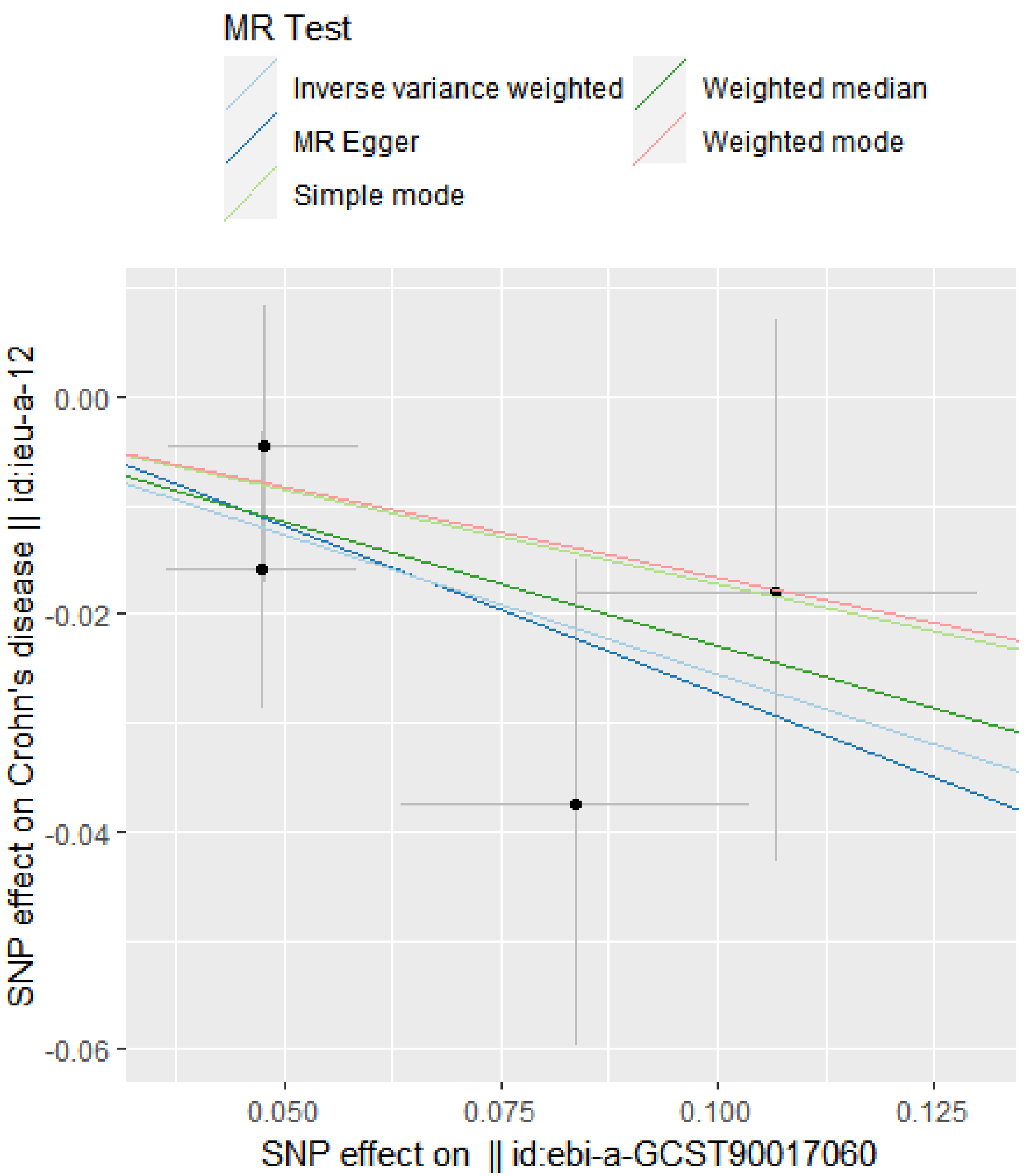

MR Test
Inverse variance weighted
MR Egger
Simple mode
Weighted median
Weighted mode
0.00
-0.02
-0.04
-0.06
0.050
0.075
0.100
0.125
SNP effect on Crohn's disease || id:ieu-a-12
SNP effect on || id:ebi-a-GCST90017060

Figure 121 Leave-one-out analysis, funnel plot, MR effect size and scatter plot for gut microbiota abundance (genus Ruminococcus torques group id.14377) on crohn's disease

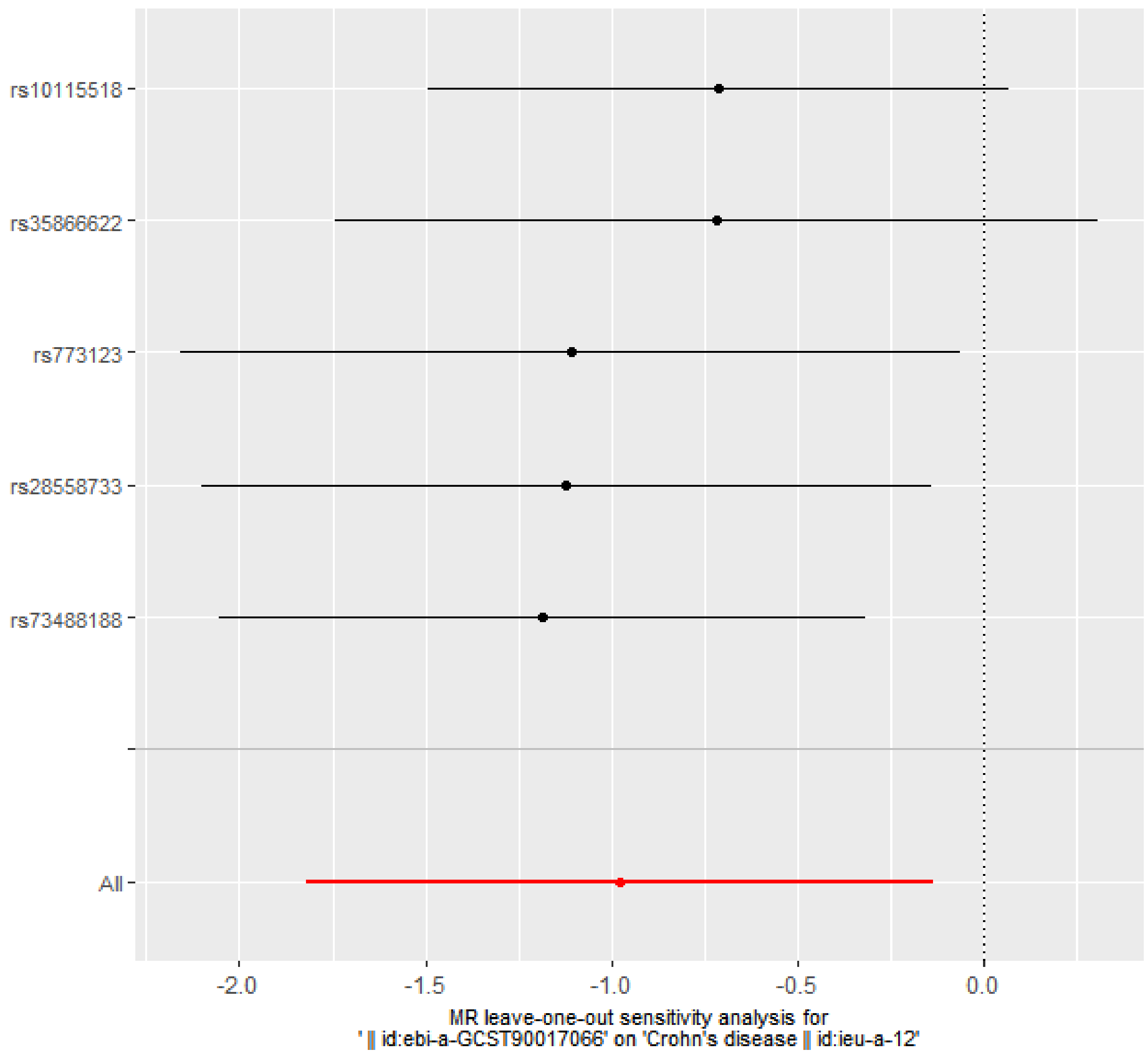

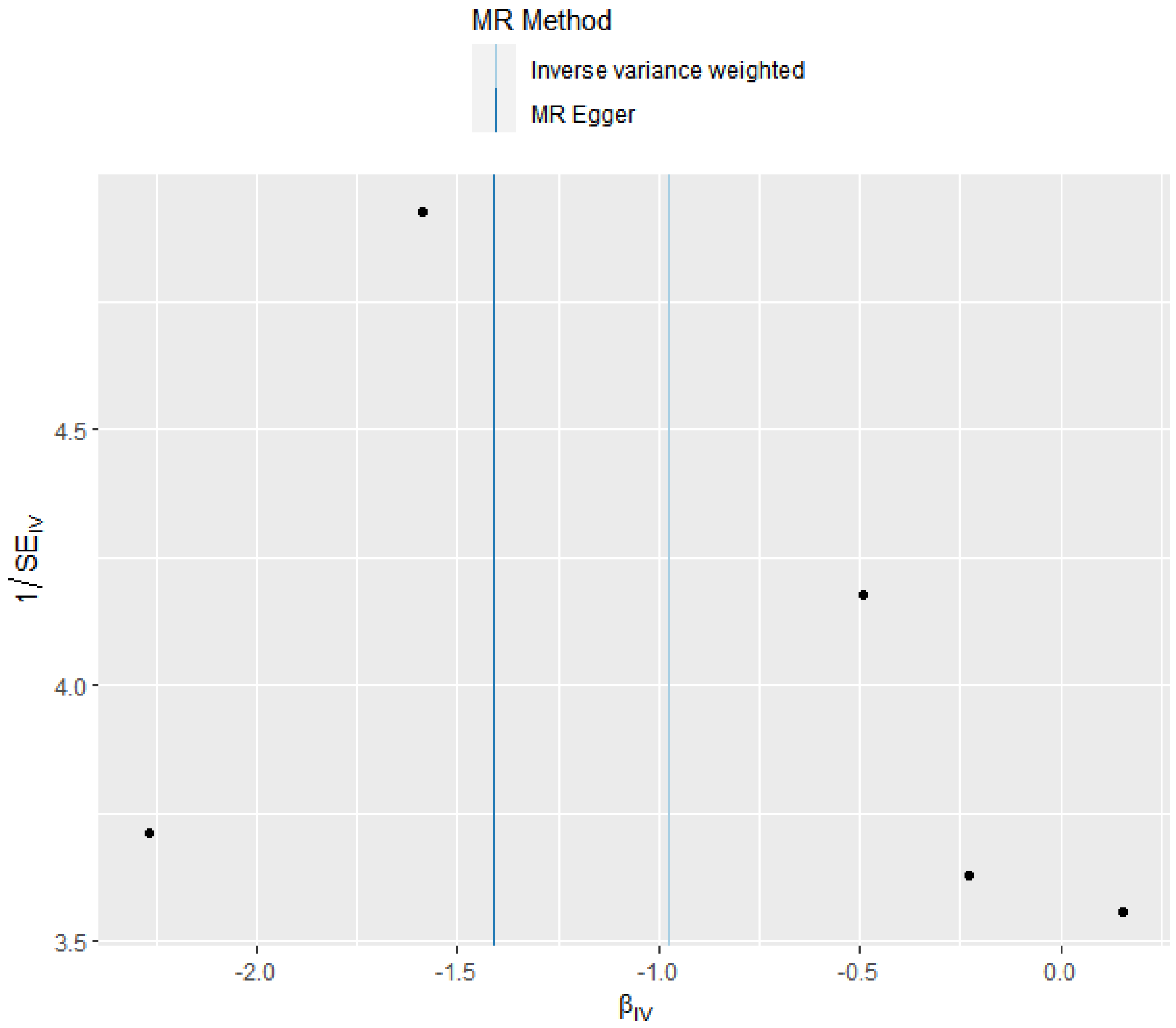

MR Method
Inverse variance weighted
MR Egger
4.5
4.0
3.5
1/SE_IV
-2.0
-1.5
-1.0
-0.5
0.0
β_IV

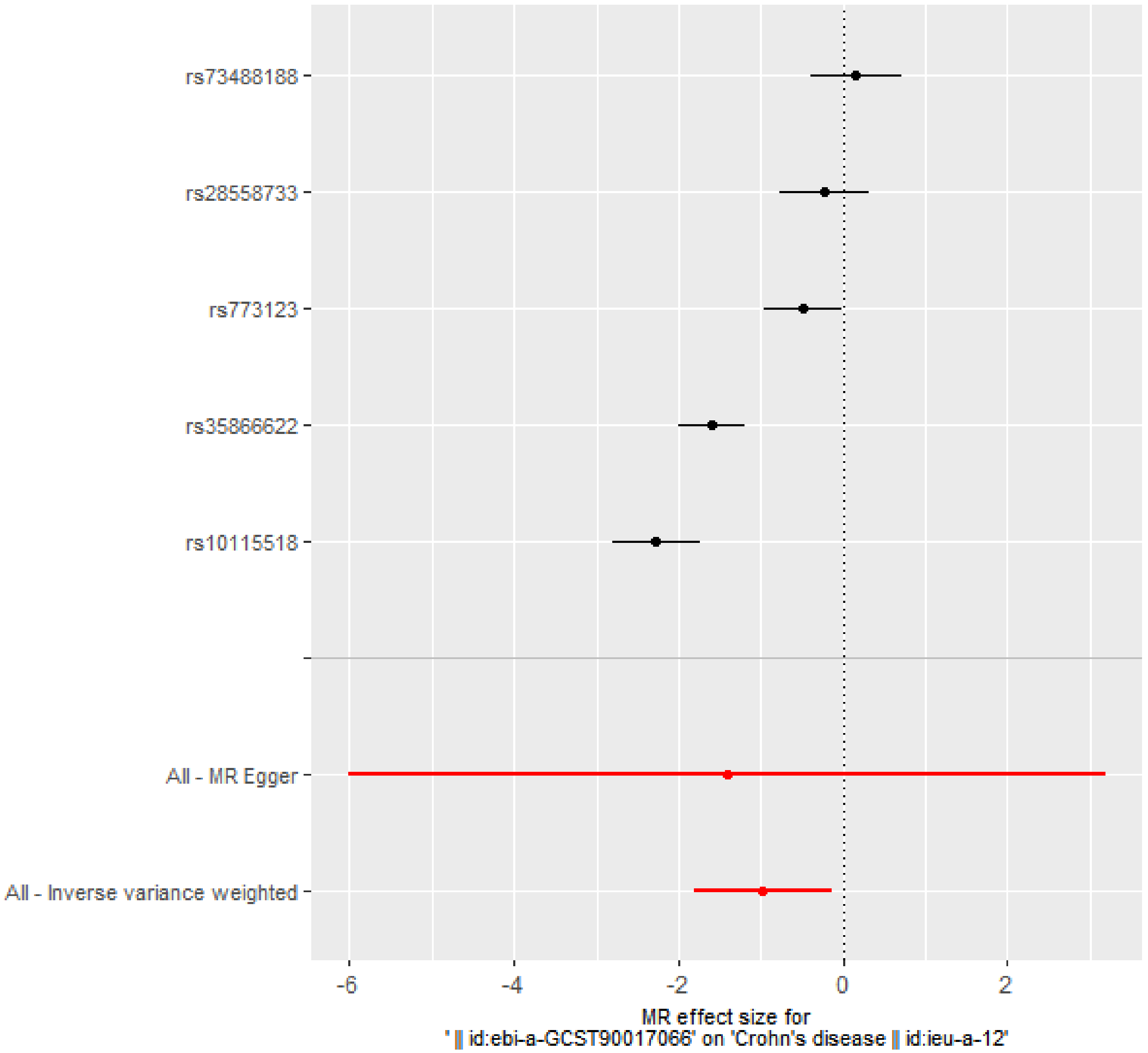

rs73488188
rs28558733
rs773123
rs35866622
rs10115518
All - MR Egger
All - Inverse variance weighted
-6
-4
-2
0
2
MR effect size for
' || id:ebi-a-GCST90017066' on 'Crohn's disease || id:ieu-a-12'

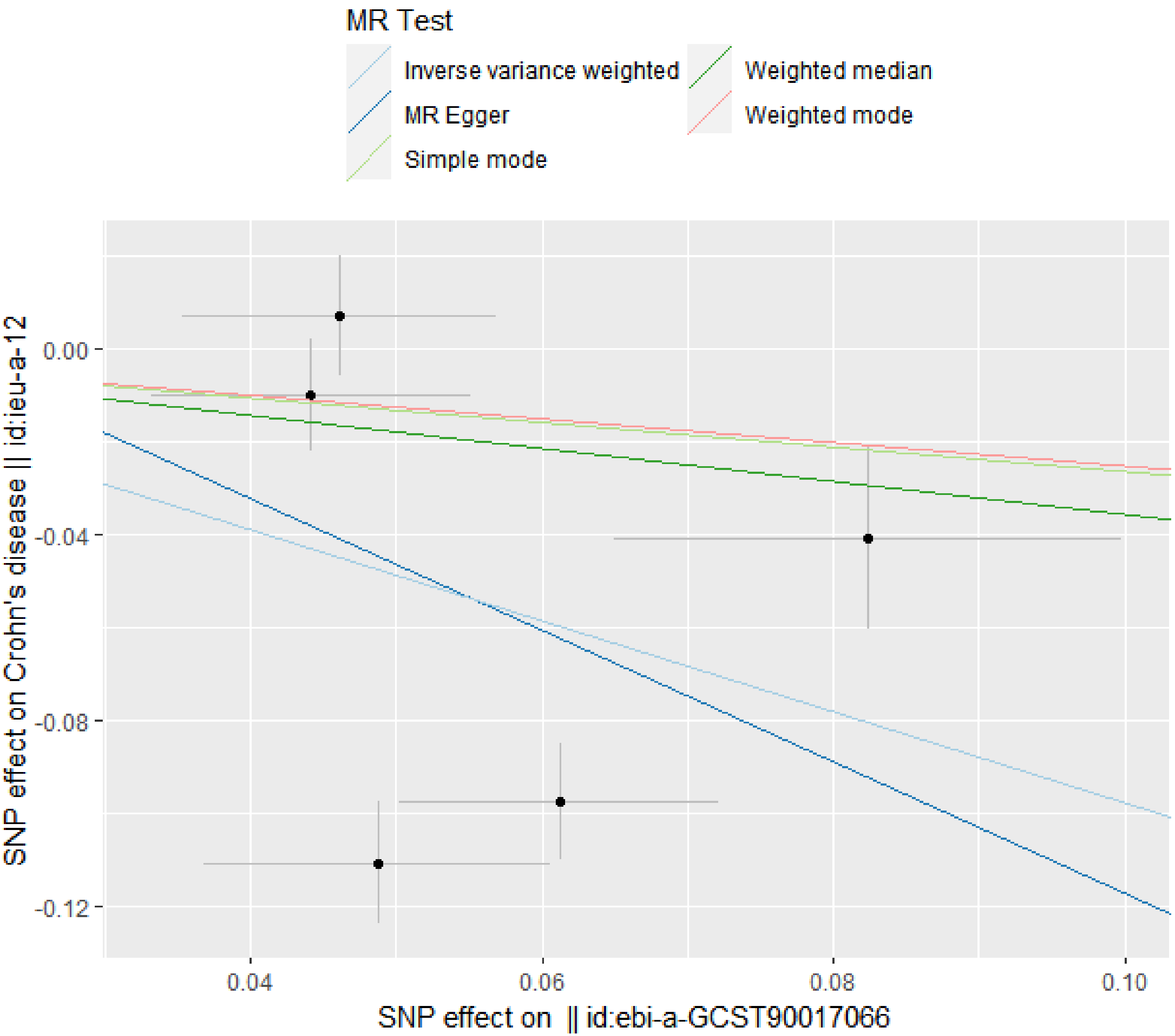
MR Test
Inverse variance weighted
MR Egger
Simple mode
Weighted median
Weighted mode
0.00
-0.04
-0.08
-0.12
0.04
0.06
0.08
0.10
SNP effect on Crohn's disease || id:ieu-a-12
SNP effect on || id:ebi-a-GCST90017066

Figure 122 Leave-one-out analysis, funnel plot, MR effect size and scatter plot for gut microbiota abundance (unknown genus id.1000006162) on crohn's disease

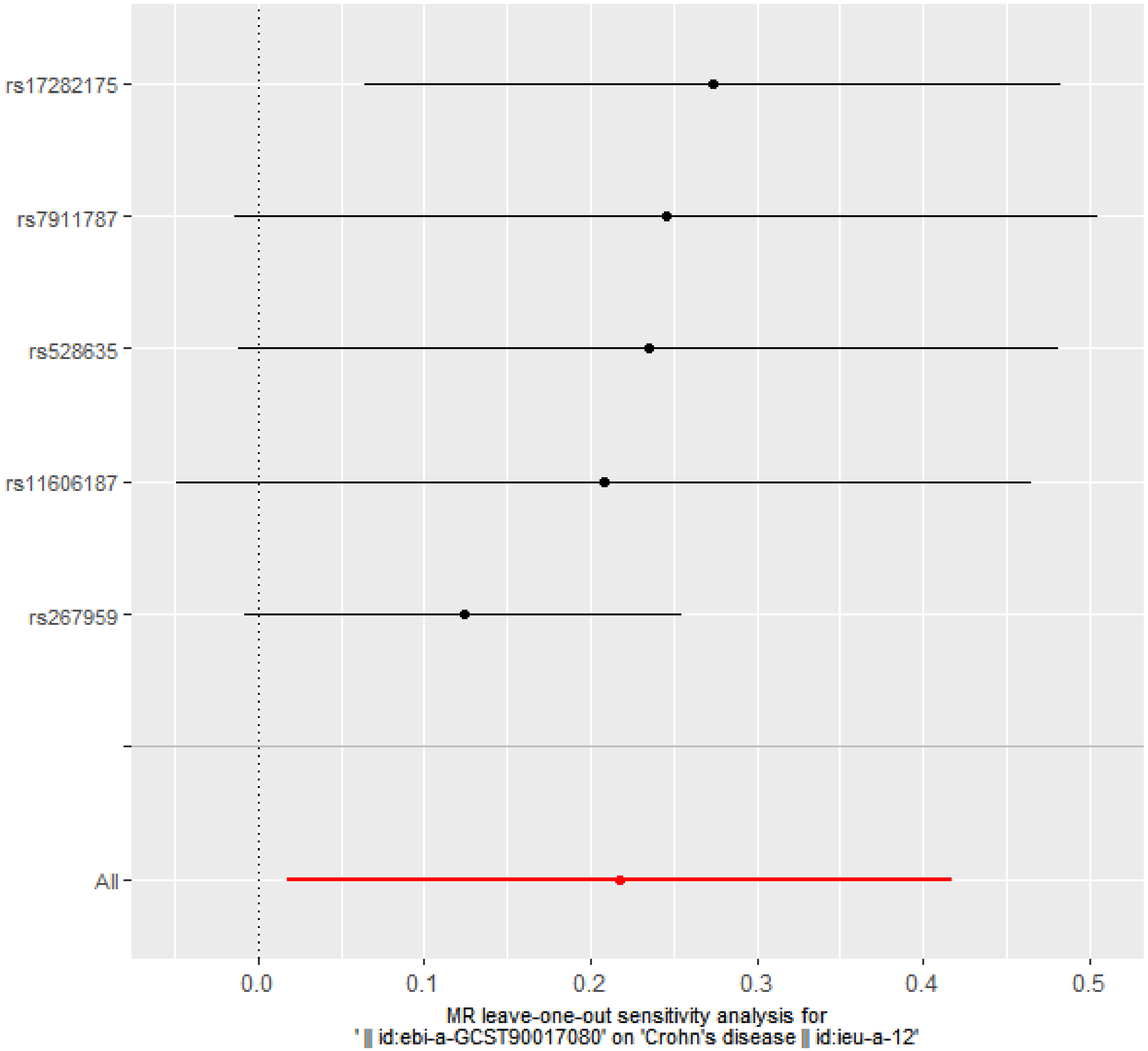

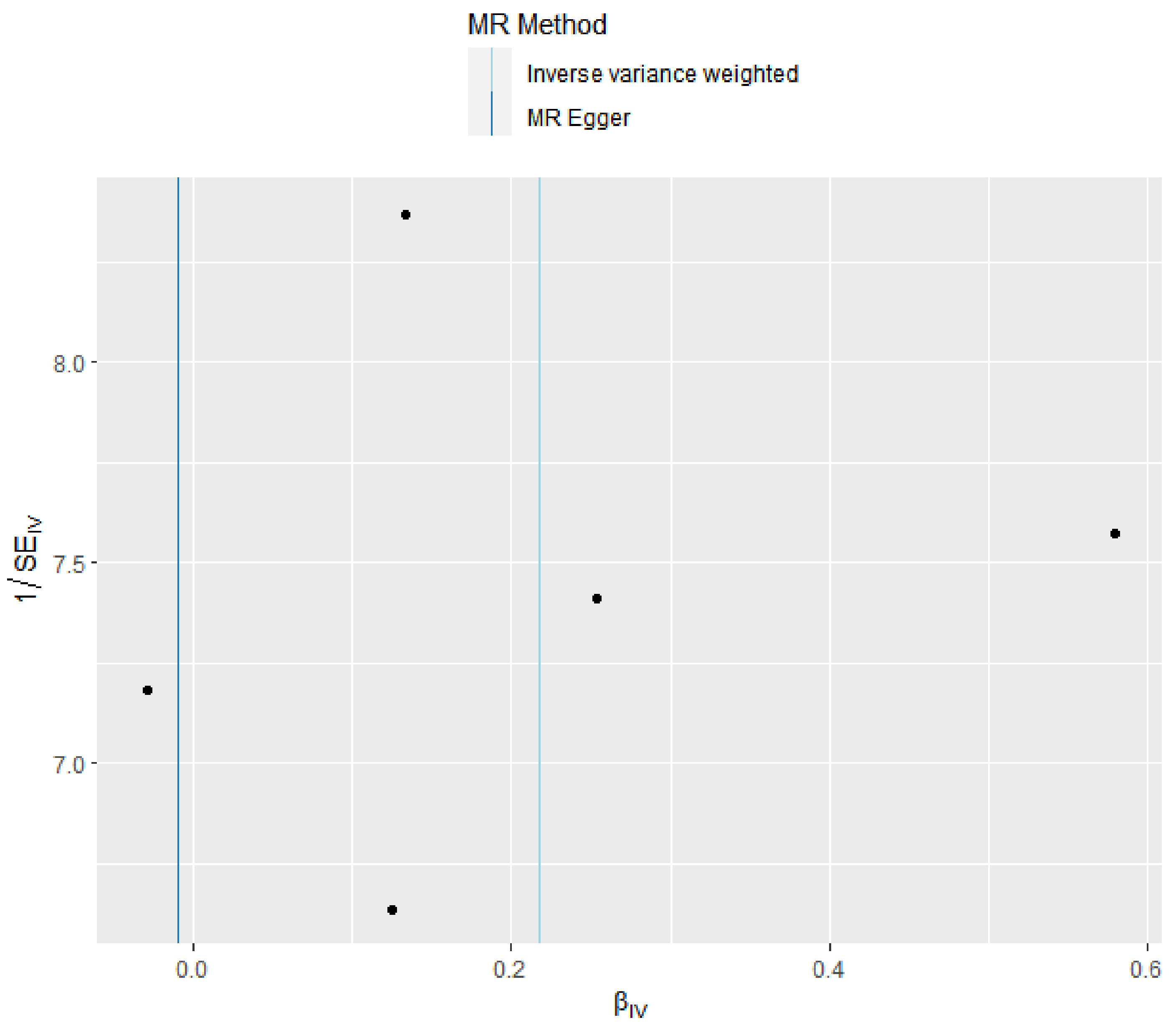

MR Method
Inverse variance weighted
MR Egger
8.0
7.5
7.0
0.0
0.2
0.4
0.6
1/SE_IV
β_IV

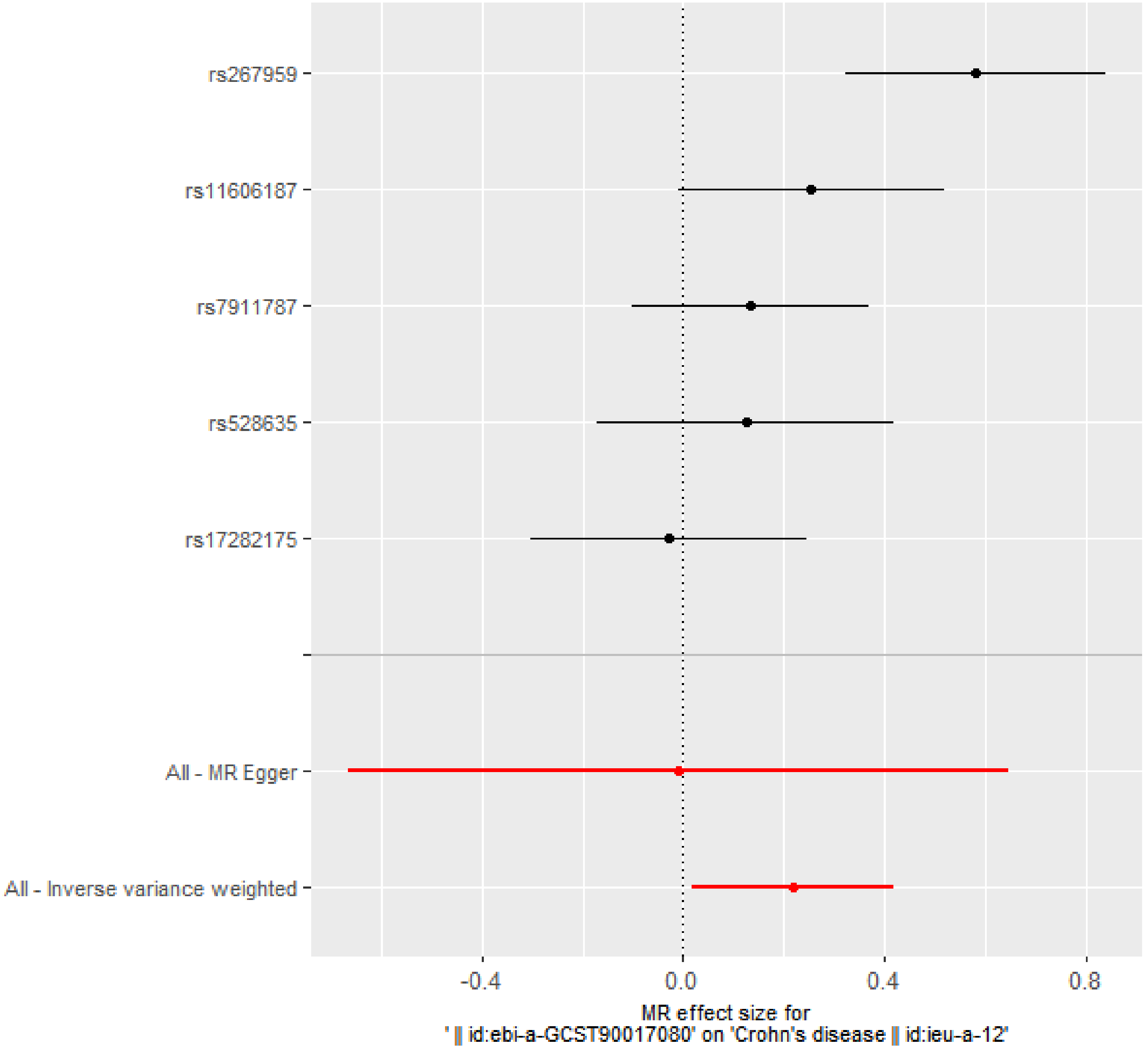

rs267959
rs11606187
rs7911787
rs528635
rs17282175
All - MR Egger
All - Inverse variance weighted
-0.4
0.0
0.4
0.8
MR effect size for
' || id:ebi-a-GCST90017080' on 'Crohn's disease || id:ieu-a-12'

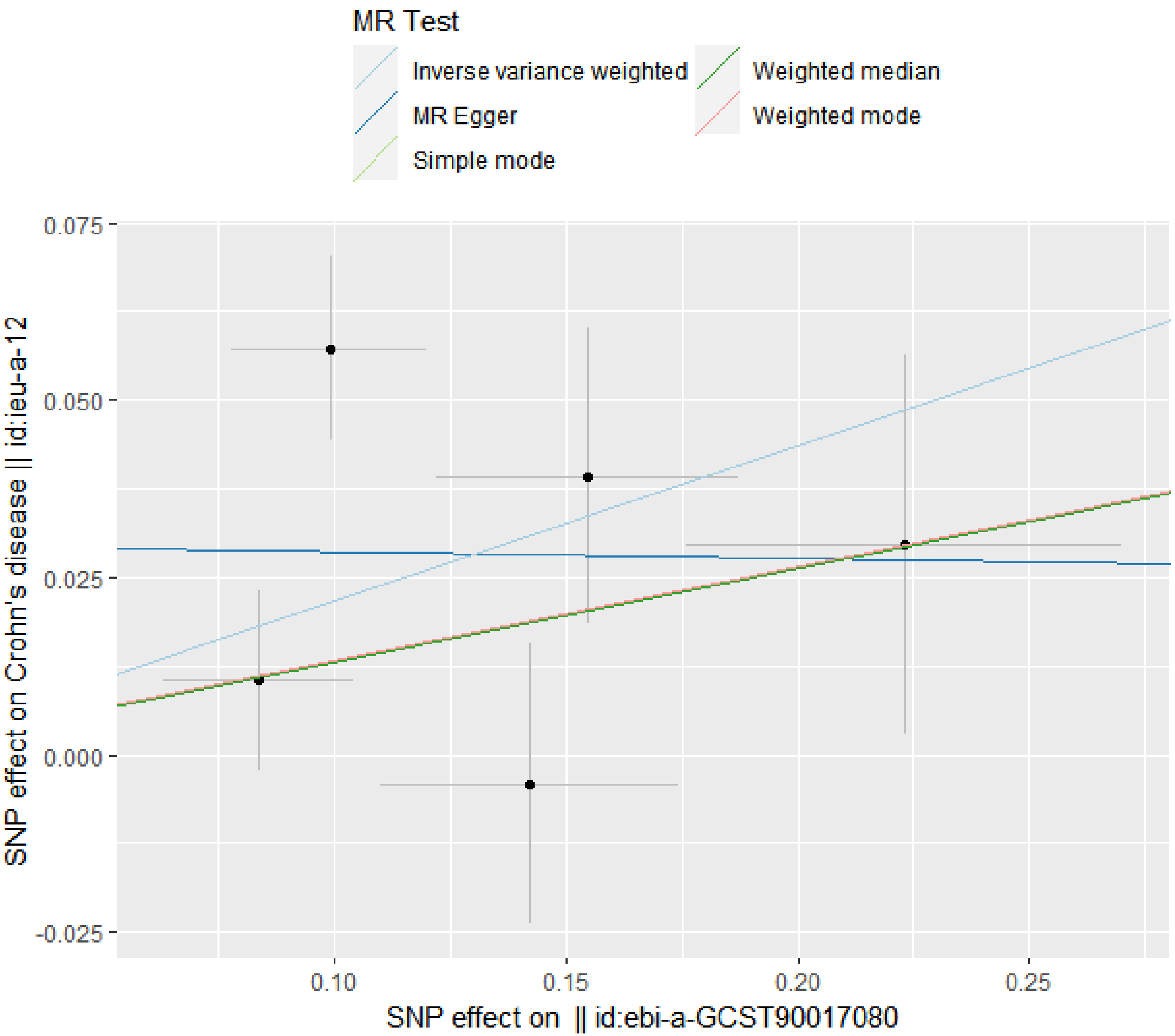

MR Test
Inverse variance weighted
MR Egger
Simple mode
Weighted median
Weighted mode
0.075
0.050
0.025
0.000
-0.025
SNP effect on Crohn's disease || id:ieu-a-12
0.10
0.15
0.20
0.25
SNP effect on || id:ebi-a-GCST90017080

Figure 123 Leave-one-out analysis, funnel plot, MR effect size and scatter plot for gut microbiota abundance (unknown genus id.2001) on crohn's disease

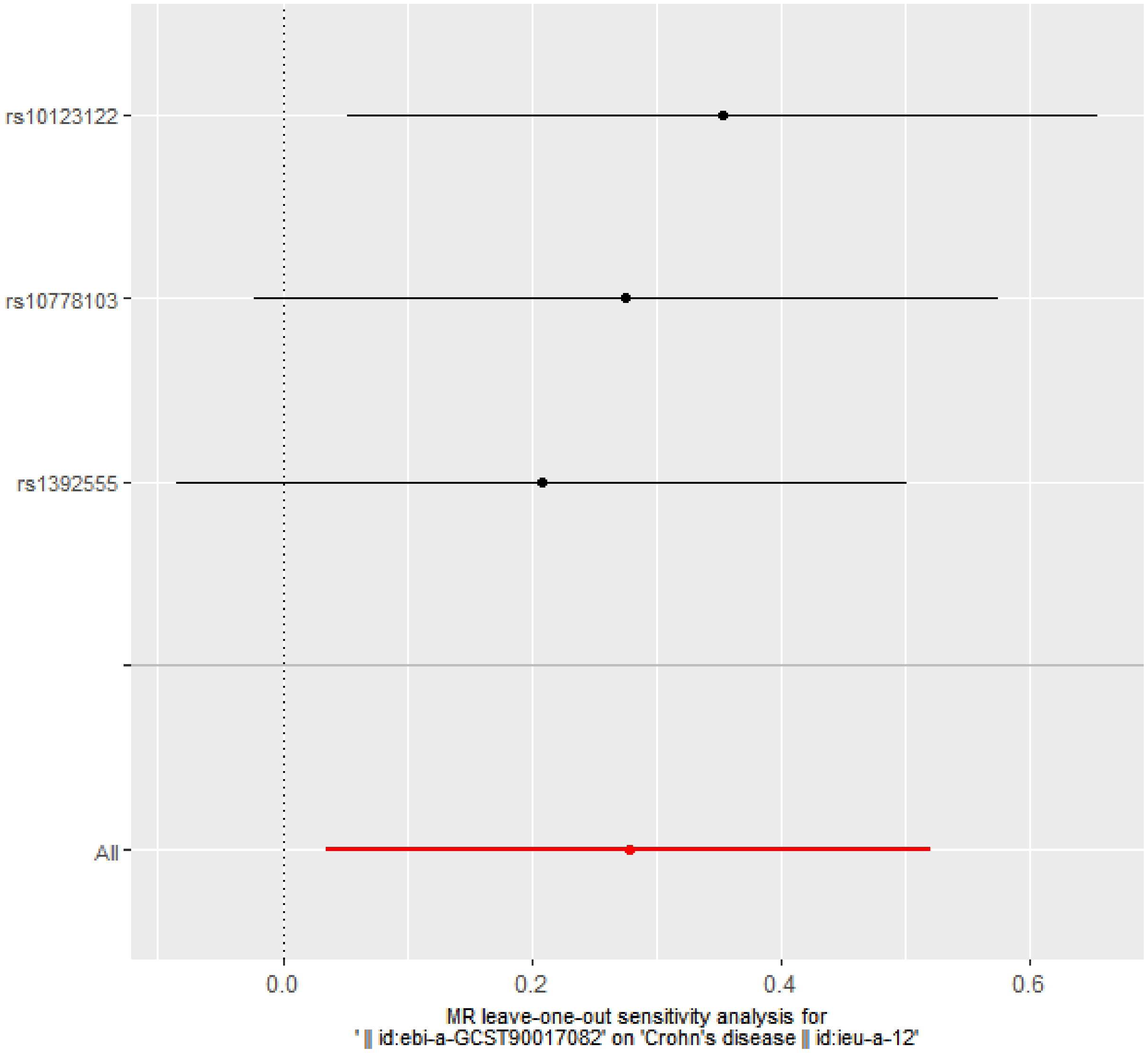

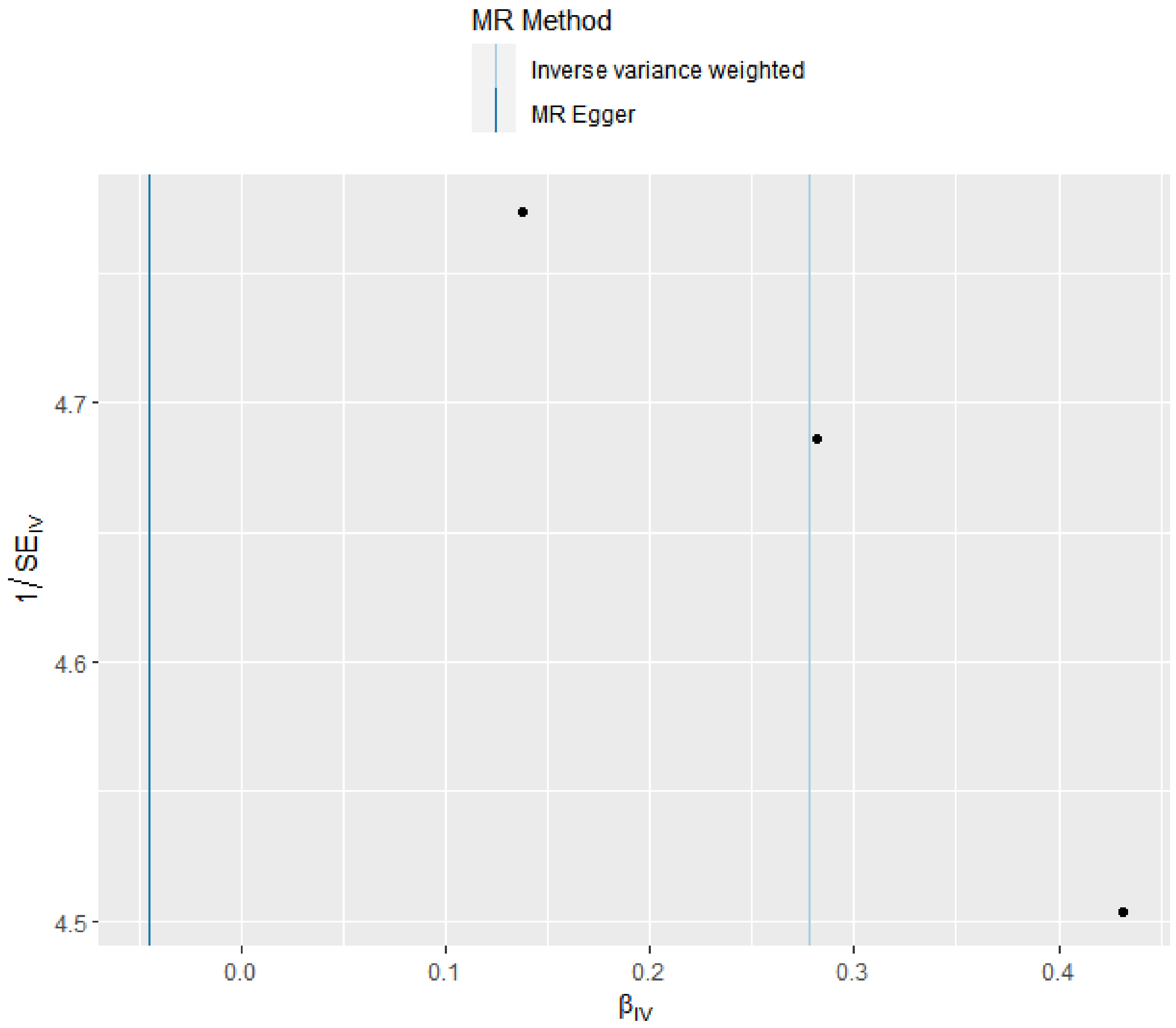

MR Method
Inverse variance weighted
MR Egger
4.7
4.6
4.5
1/SE_IV
0.0
0.1
0.2
0.3
0.4
β_IV

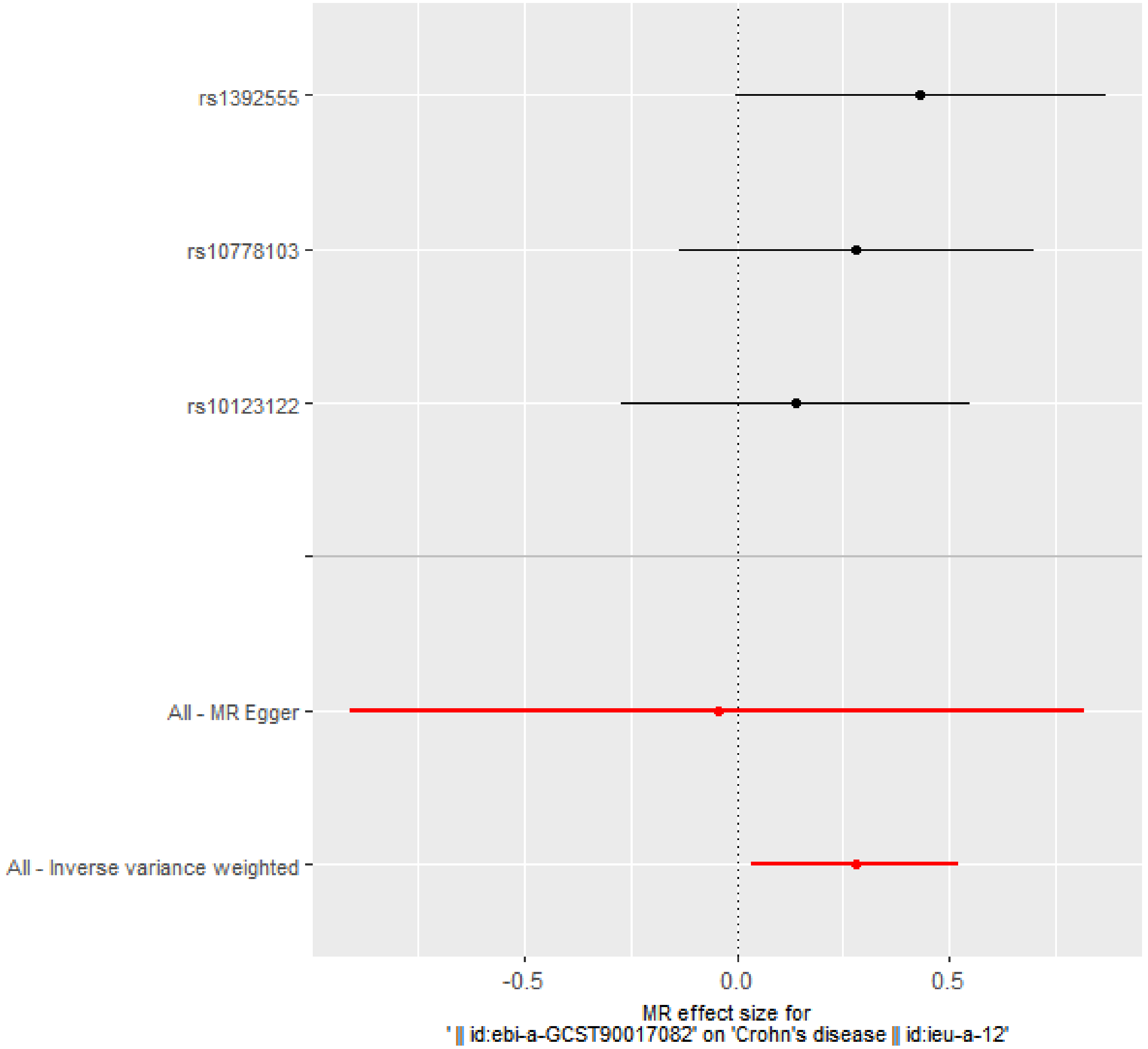
rs1392555
rs10778103
rs10123122
All - MR Egger
All - Inverse variance weighted
-0.5
0.0
0.5
MR effect size for
' || id:ebi-a-GCST90017082' on 'Crohn's disease || id:ieu-a-12'

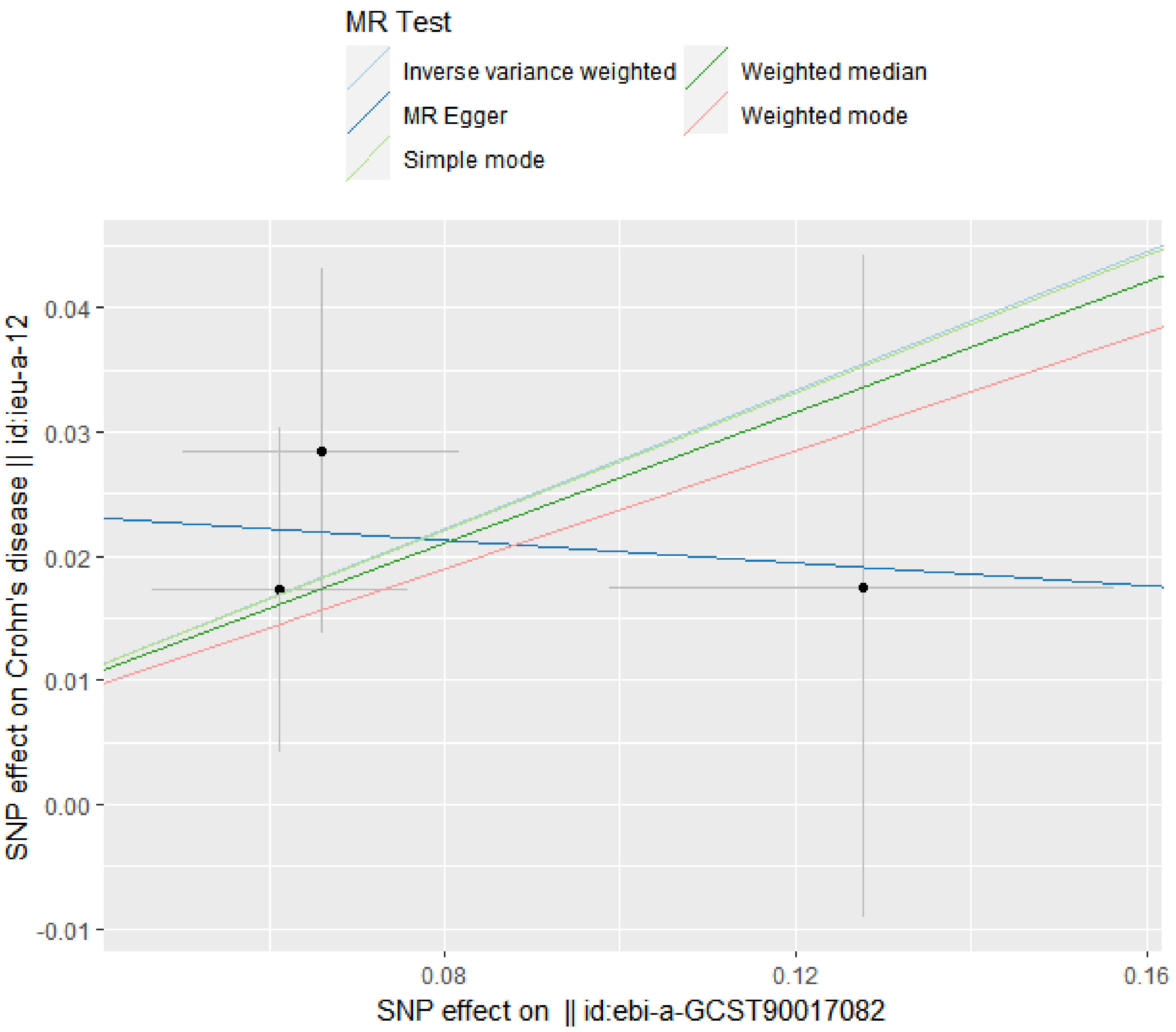
MR Test
Inverse variance weighted
MR Egger
Simple mode
Weighted median
Weighted mode
0.04
0.03
0.02
0.01
0.00
-0.01
0.08
0.12
0.16
SNP effect on Crohn's disease || id:ieu-a-12
SNP effect on || id:ebi-a-GCST90017082

Figure 124 Leave-one-out analysis, funnel plot, MR effect size and scatter plot for gut microbiota abundance (order NB1n id.3953) on crohn's disease

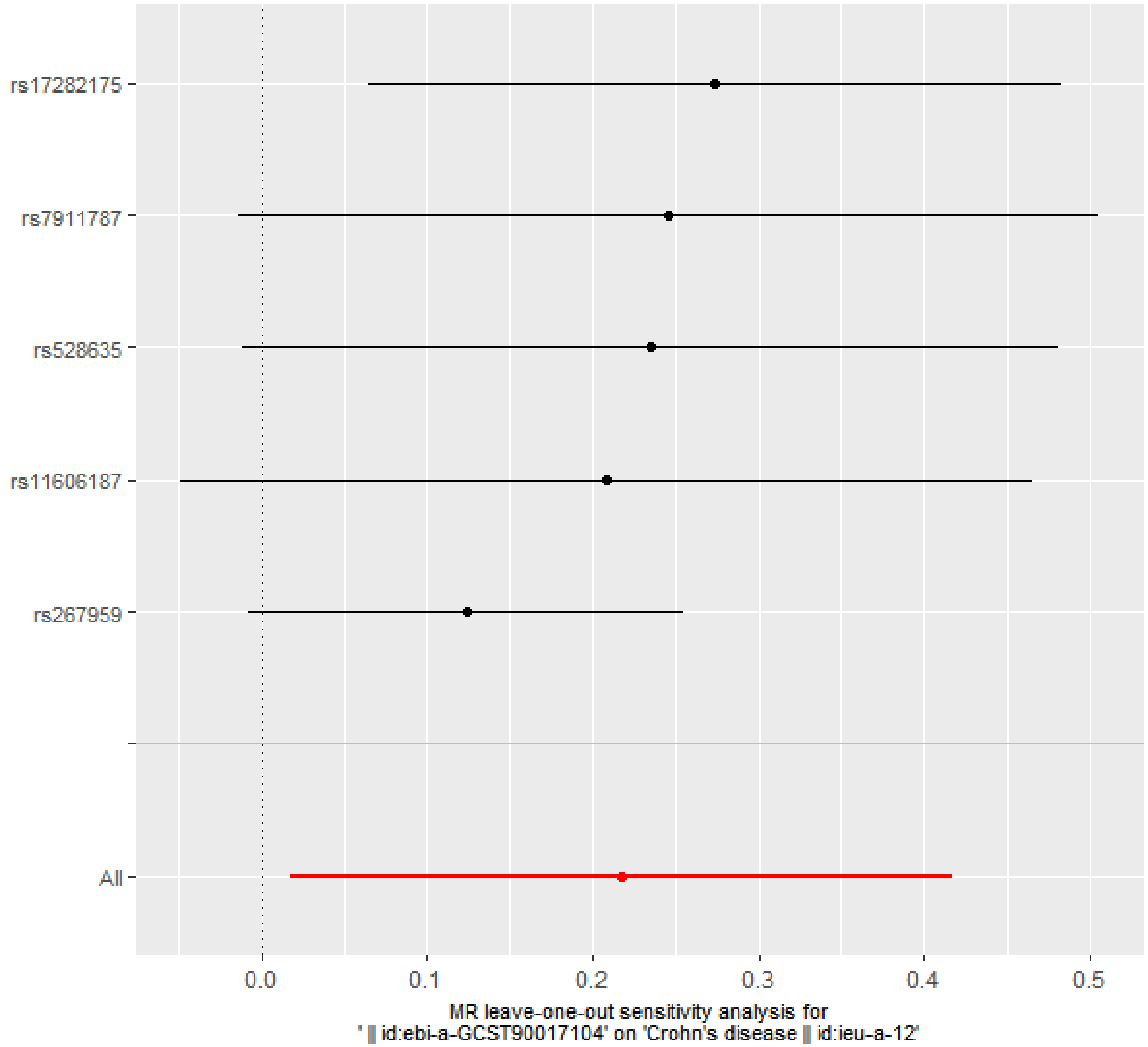

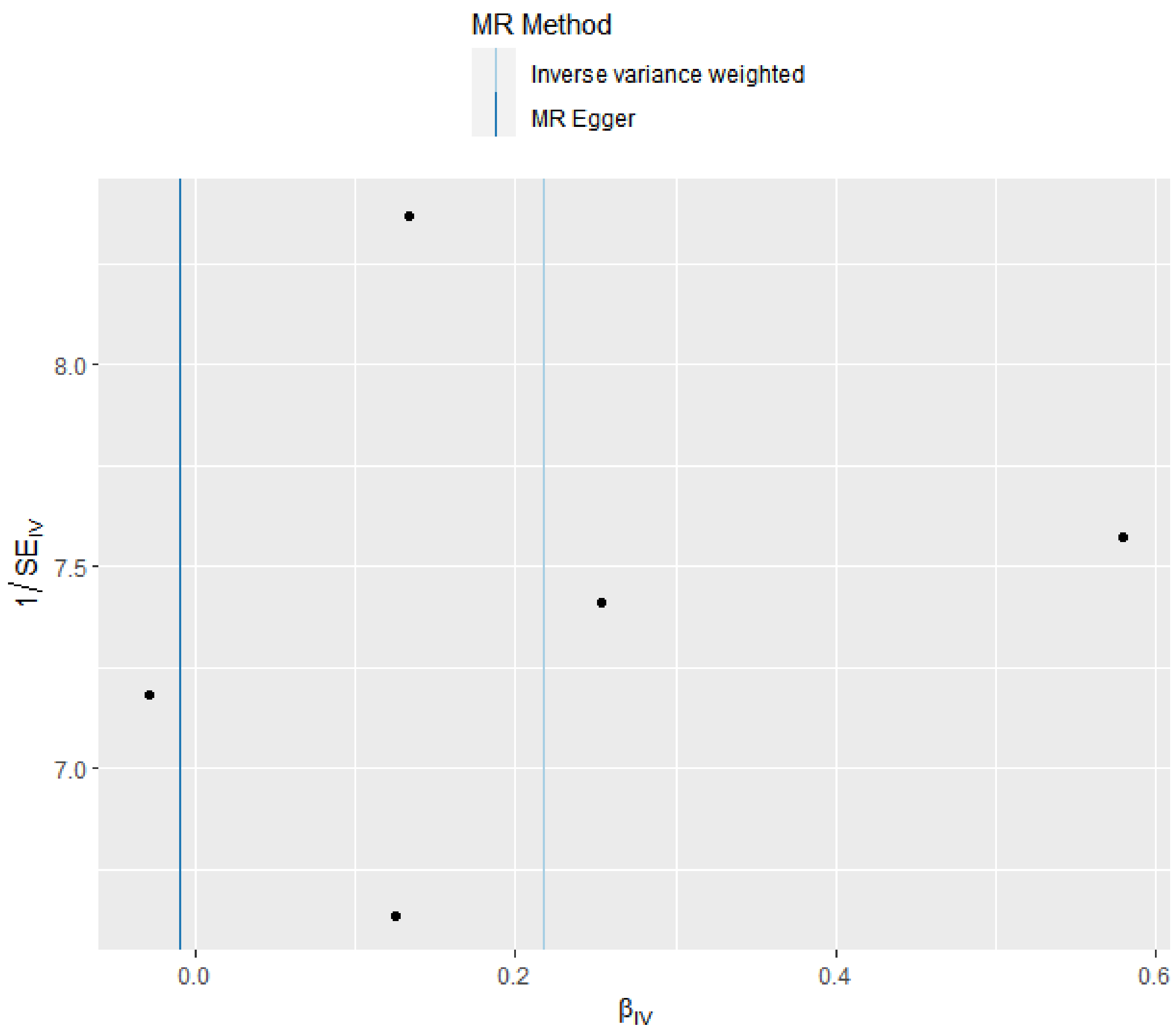
MR Method
Inverse variance weighted
MR Egger
8.0
7.5
7.0
0.0
0.2
0.4
0.6
$1/SE_{IV}$
β_{IV}

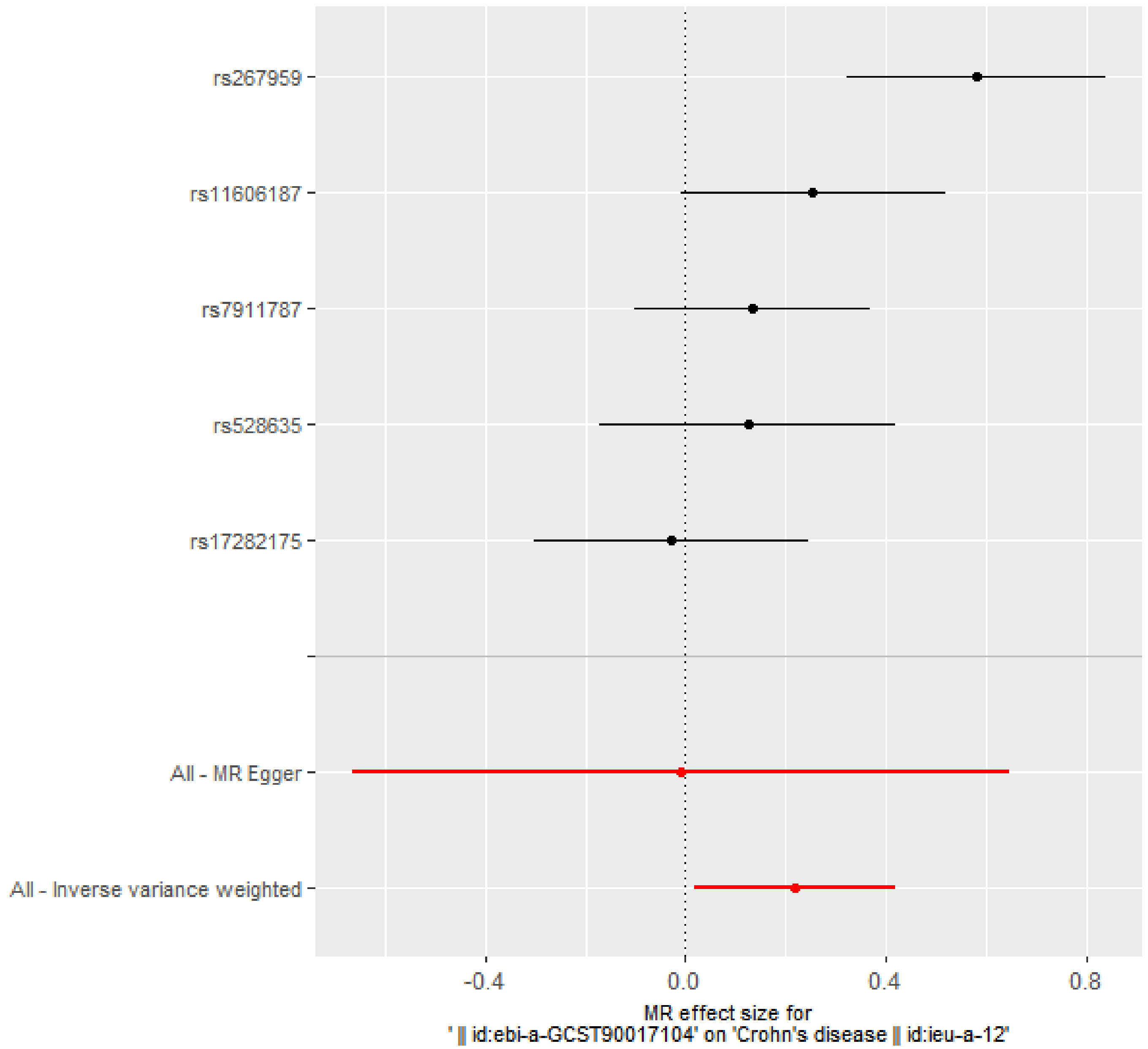
rs267959
rs11606187
rs7911787
rs528635
rs17282175
All - MR Egger
All - Inverse variance weighted
-0.4
0.0
0.4
0.8
MR effect size for
' || id:ebi-a-GCST90017104' on 'Crohn's disease || id:ieu-a-12'

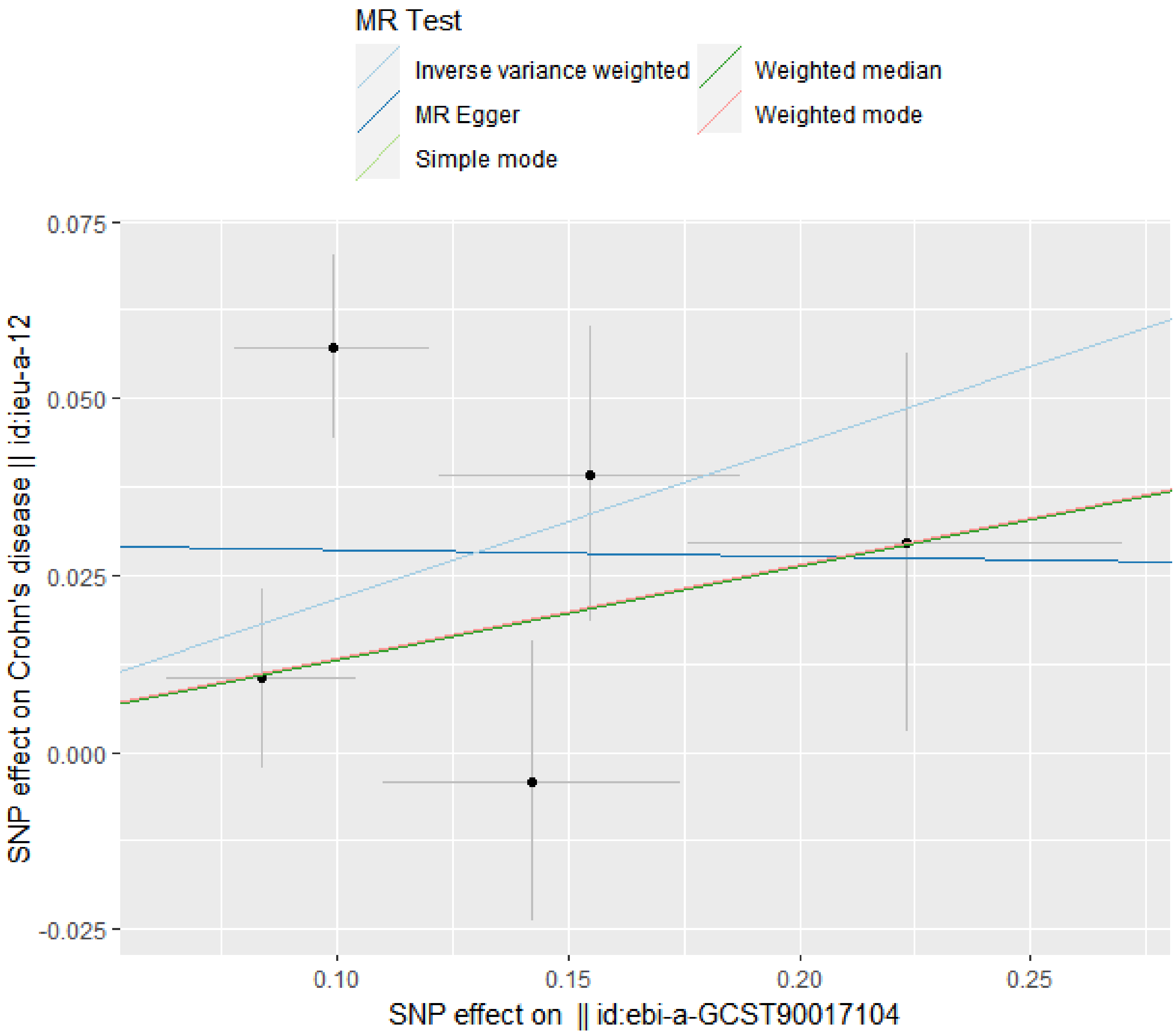
MR Test
Inverse variance weighted
MR Egger
Simple mode
Weighted median
Weighted mode
0.075
0.050
0.025
0.000
-0.025
SNP effect on Crohn's disease || id:ieu-a-12
0.10
0.15
0.20
0.25
SNP effect on || id:ebi-a-GCST90017104

Figure 125 Leave-one-out analysis, funnel plot, MR effect size and scatter plot for gut microbiota abundance (order Verrucomicrobiales id.4030) on crohn's disease

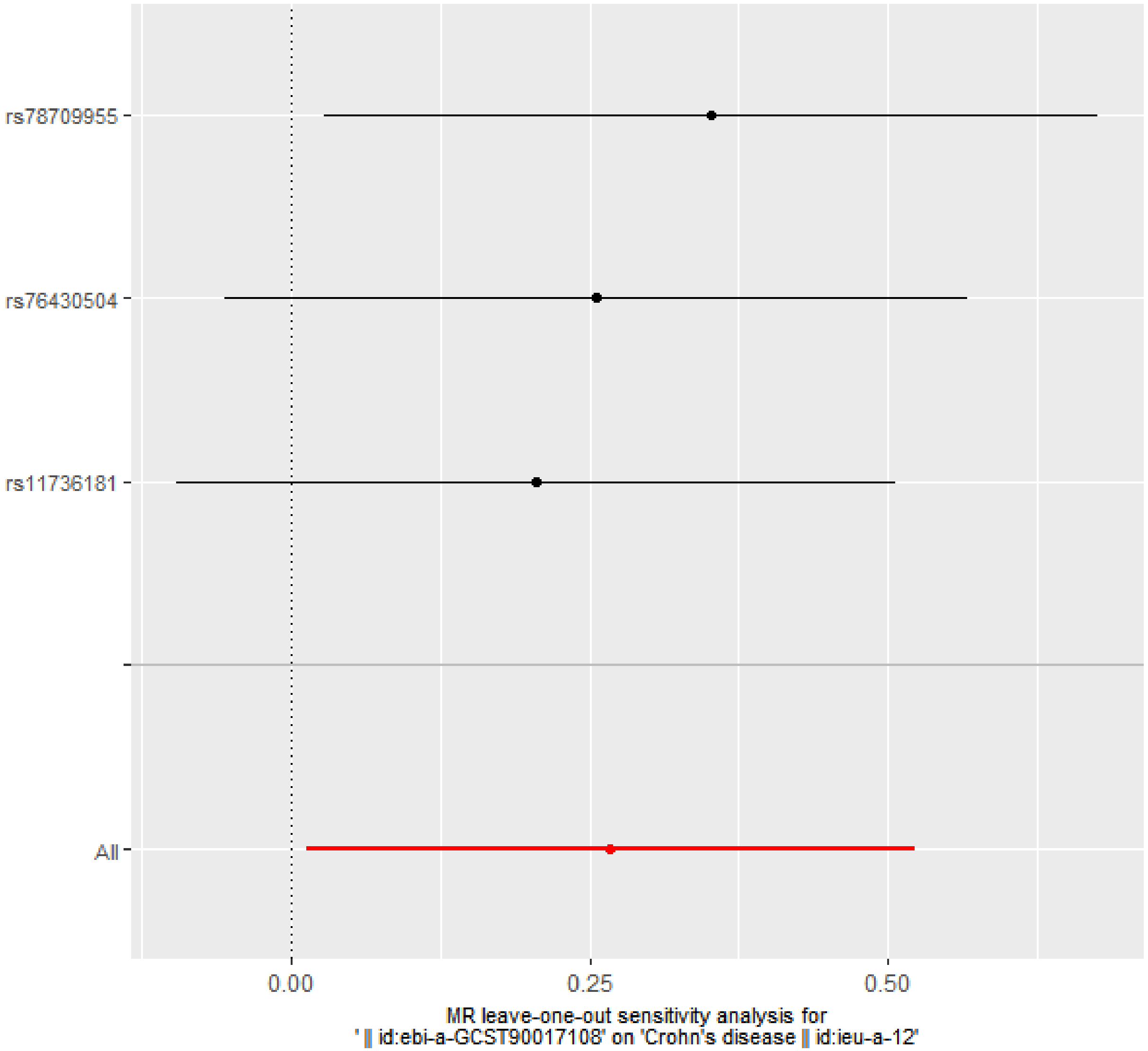

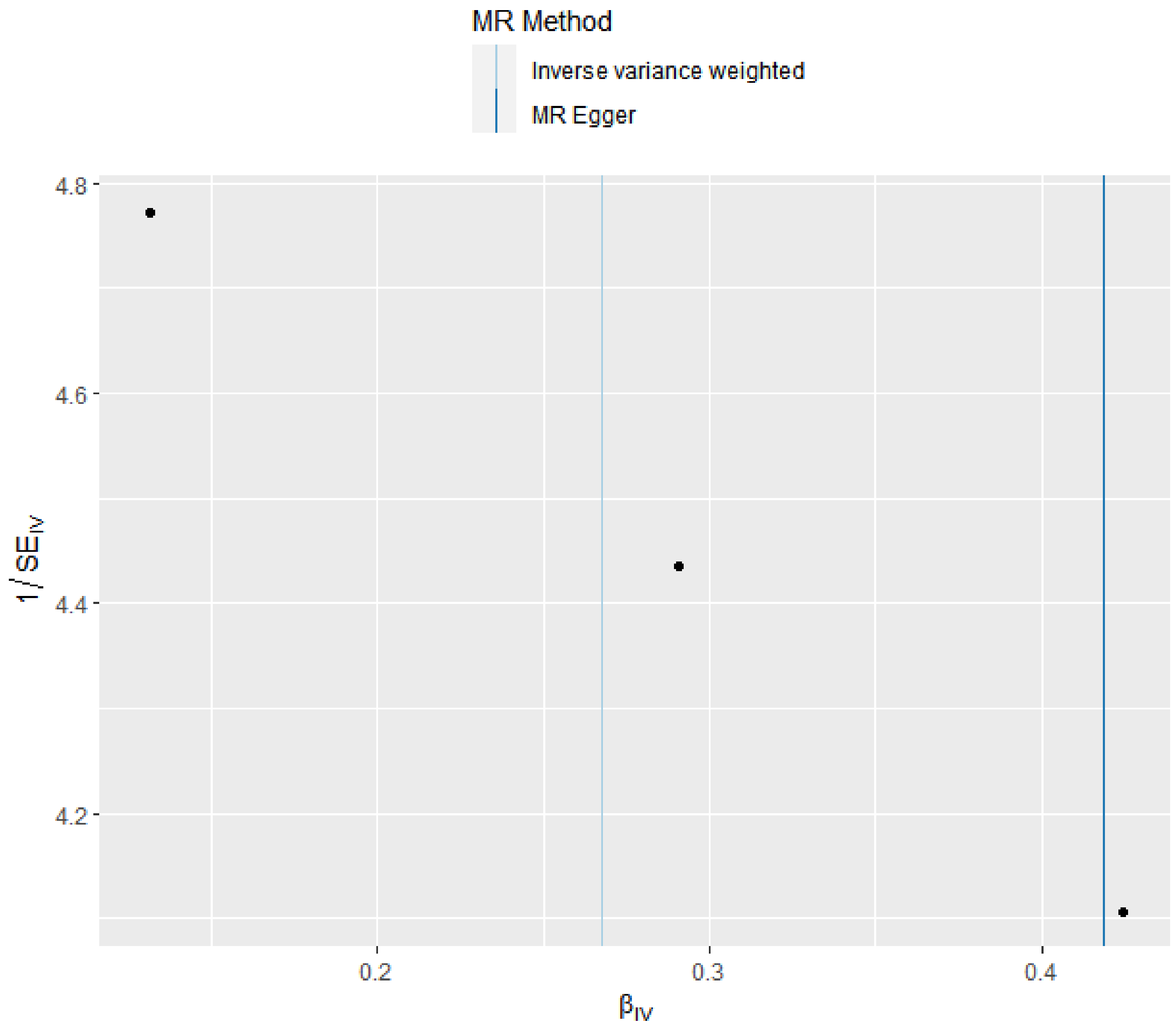

MR Method
Inverse variance weighted
MR Egger
4.8
4.6
4.4
4.2
0.2
0.3
0.4
β_{IV}
$1/SE_{IV}$

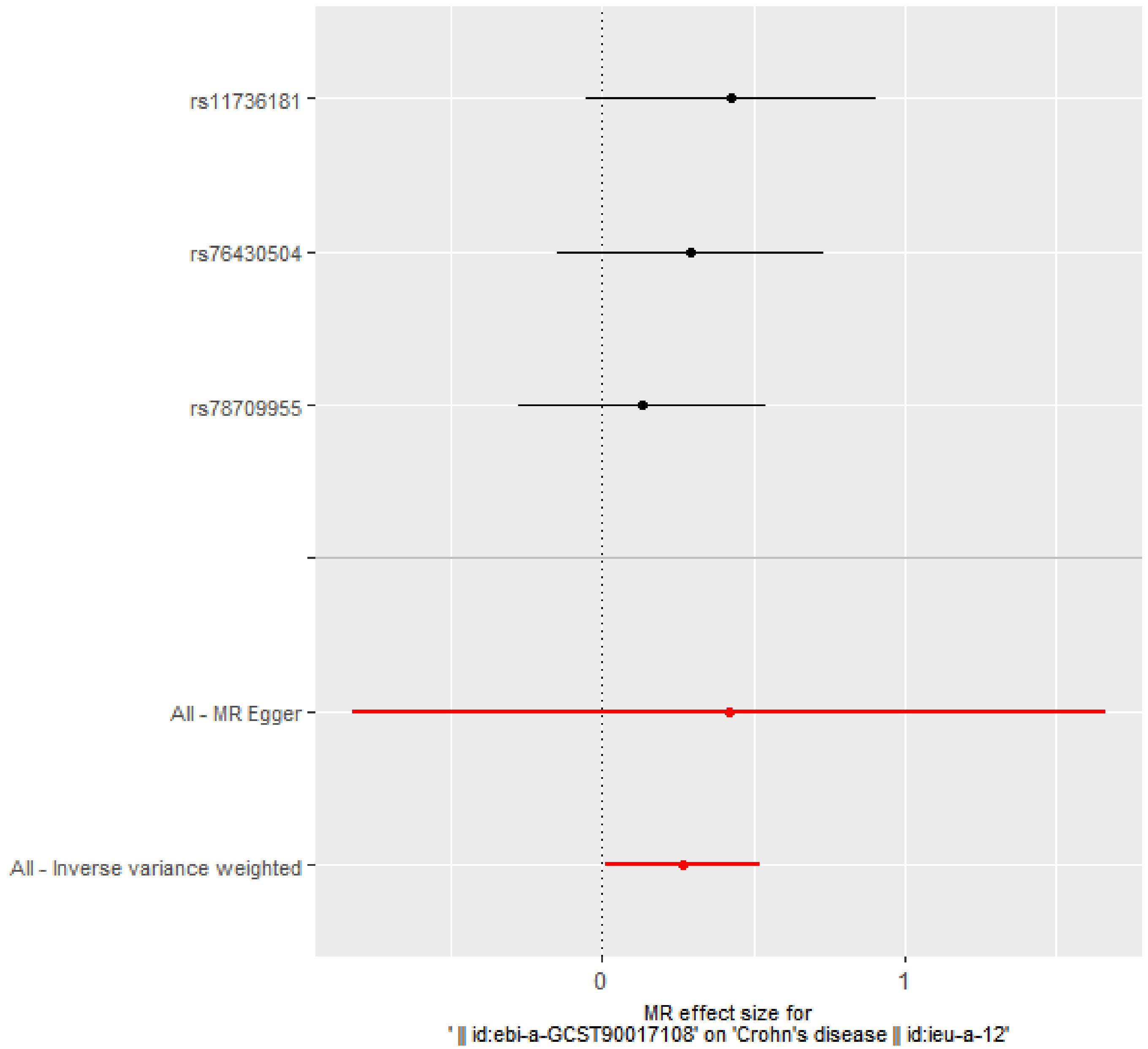
rs11736181
rs76430504
rs78709955
All - MR Egger
All - Inverse variance weighted
0
1
MR effect size for
' || id:ebi-a-GCST90017108' on 'Crohn's disease || id:ieu-a-12'

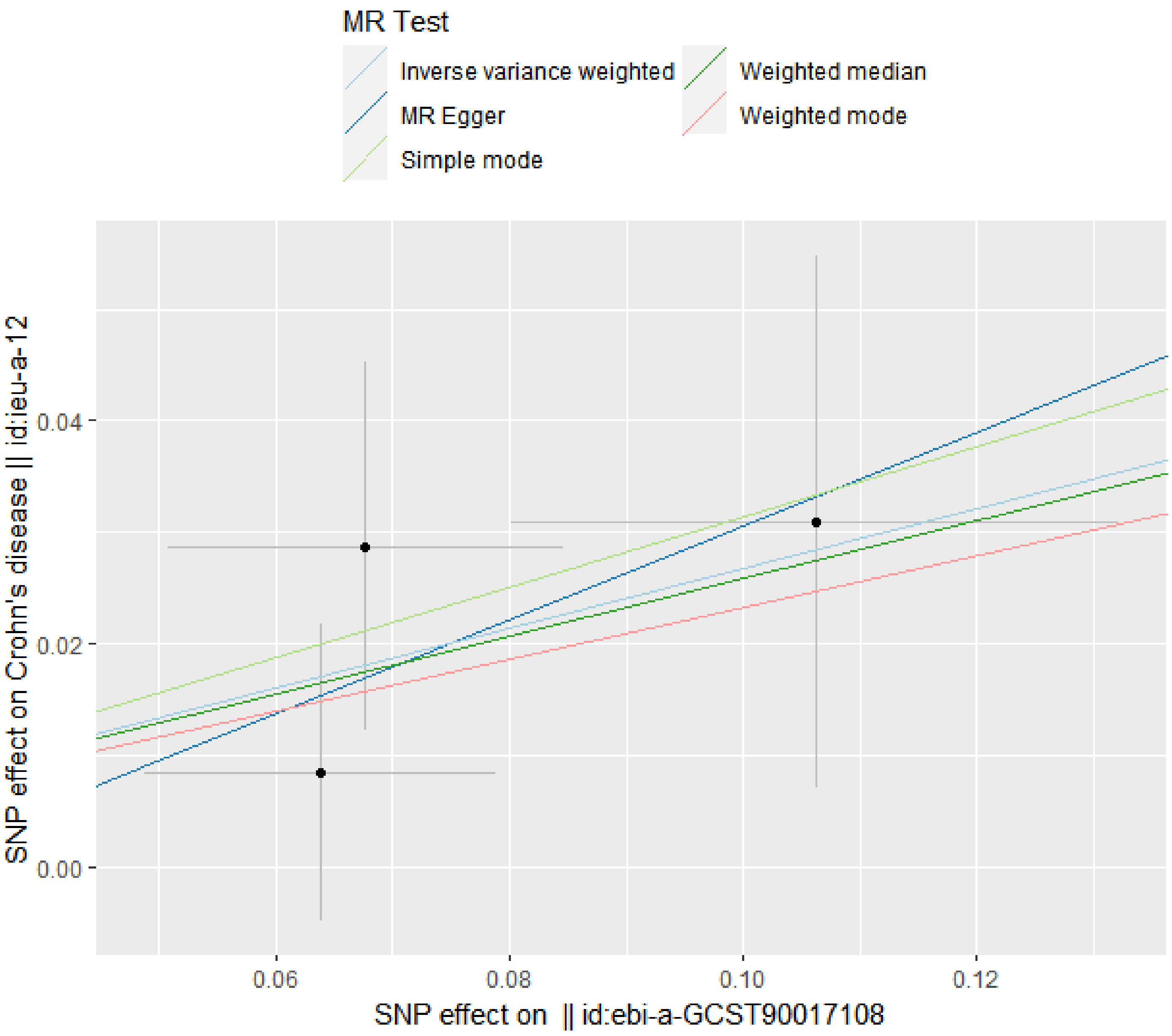

MR Test
Inverse variance weighted
MR Egger
Simple mode
Weighted median
Weighted mode
SNP effect on Crohn's disease || id:ieu-a-12
0.04
0.02
0.00
0.06
0.08
0.10
0.12
SNP effect on || id:ebi-a-GCST90017108

Figure 126 Leave-one-out analysis, funnel plot, MR effect size and scatter plot for gut microbiota abundance (phylum Verrucomicrobia id.3982) on crohn's disease

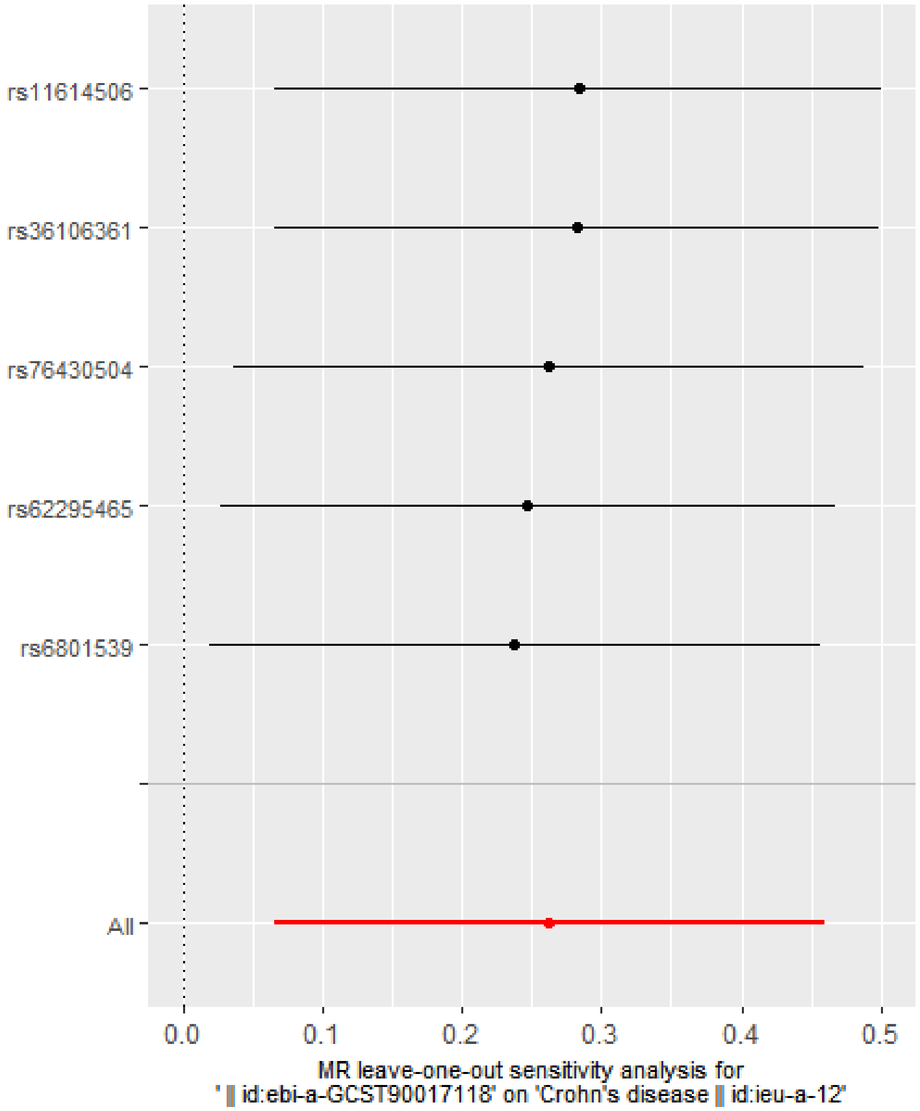

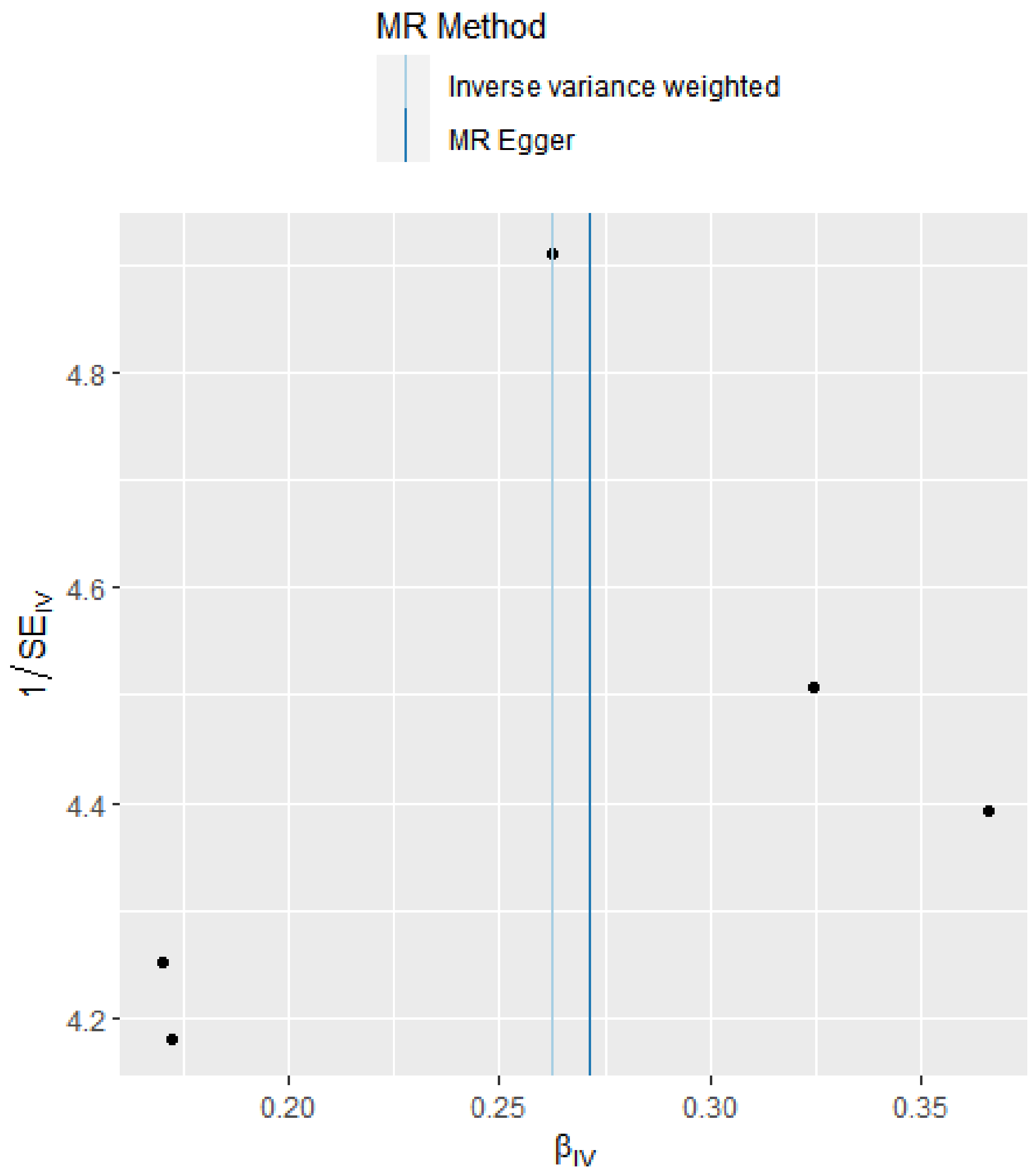

MR Method
Inverse variance weighted
MR Egger
4.8
4.6
4.4
4.2
$1/SE_{IV}$
0.20
0.25
0.30
0.35
β_{IV}

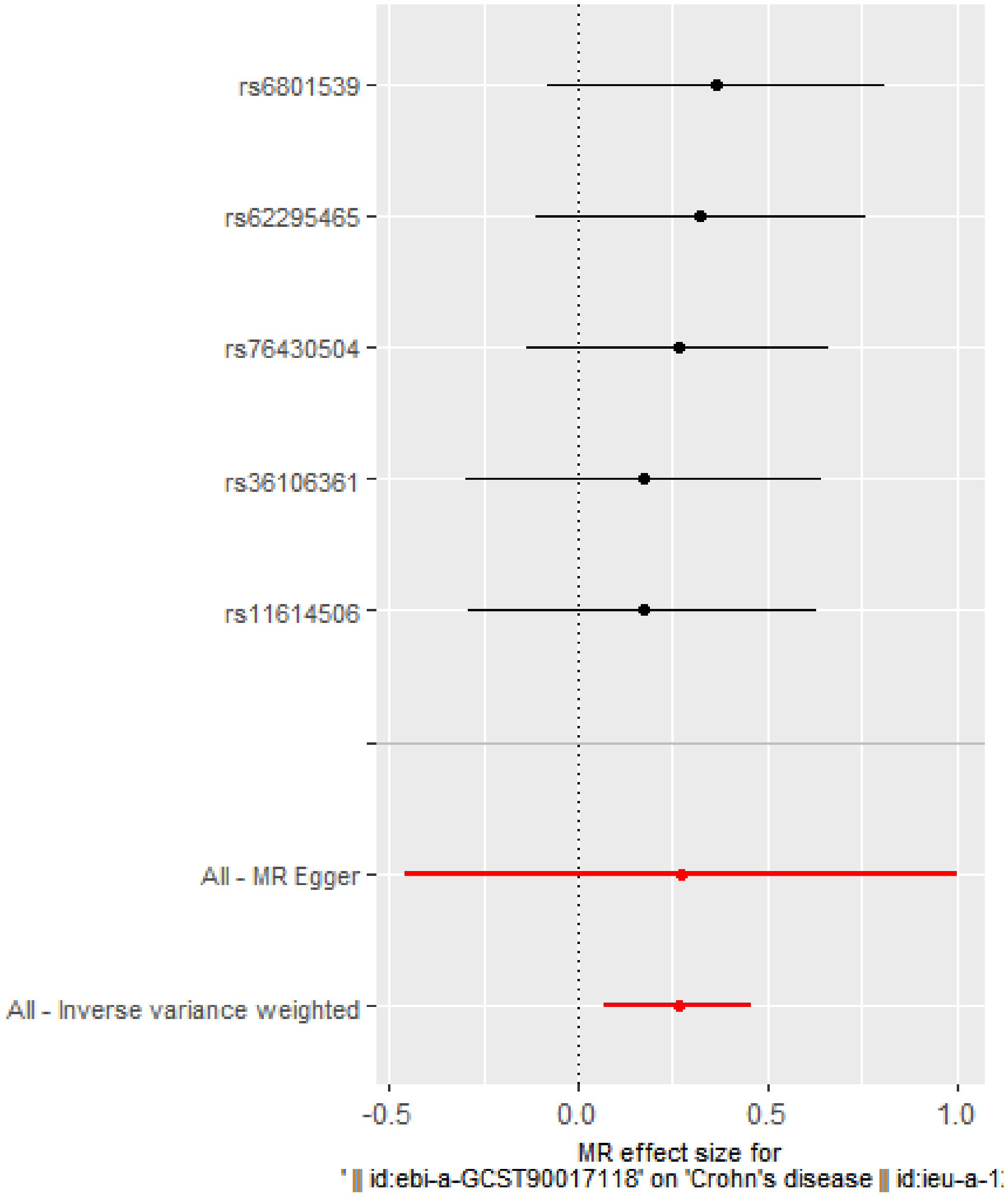
rs6801539
rs62295465
rs76430504
rs36106361
rs11614506
All - MR Egger
All - Inverse variance weighted
-0.5
0.0
0.5
1.0
MR effect size for
' || id:ebi-a-GCST90017118' on 'Crohn's disease || id:ieu-a-1:

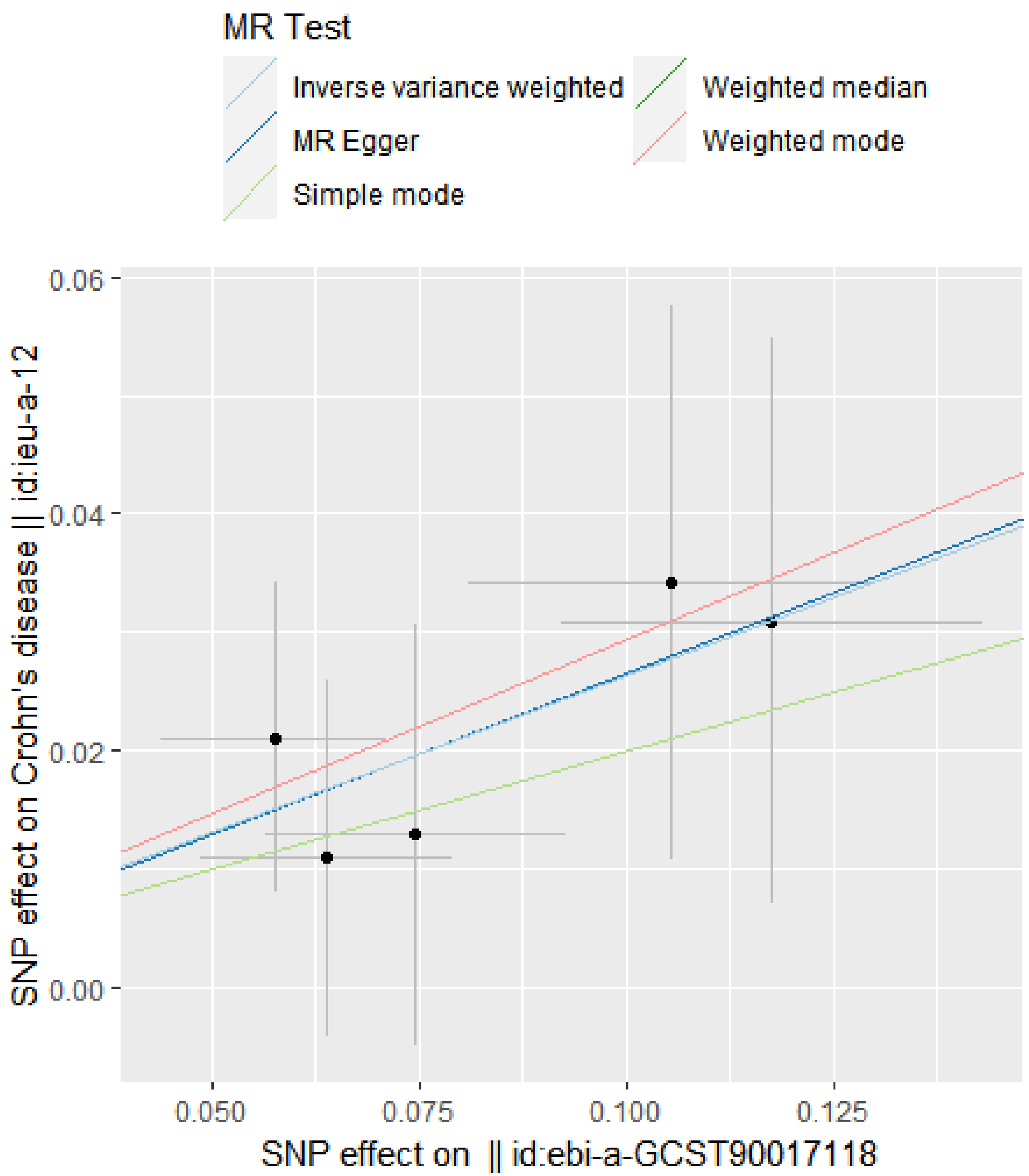

MR Test
Inverse variance weighted
MR Egger
Simple mode
Weighted median
Weighted mode
0.06
0.04
0.02
0.00
SNP effect on Crohn's disease || id:ieu-a-12
0.050
0.075
0.100
0.125
SNP effect on || id:ebi-a-GCST90017118

Figure 127 Leave-one-out analysis, funnel plot, MR effect size and scatter plot for gut microbiota abundance (class Bacilli id.1673) on ulcerative colitis

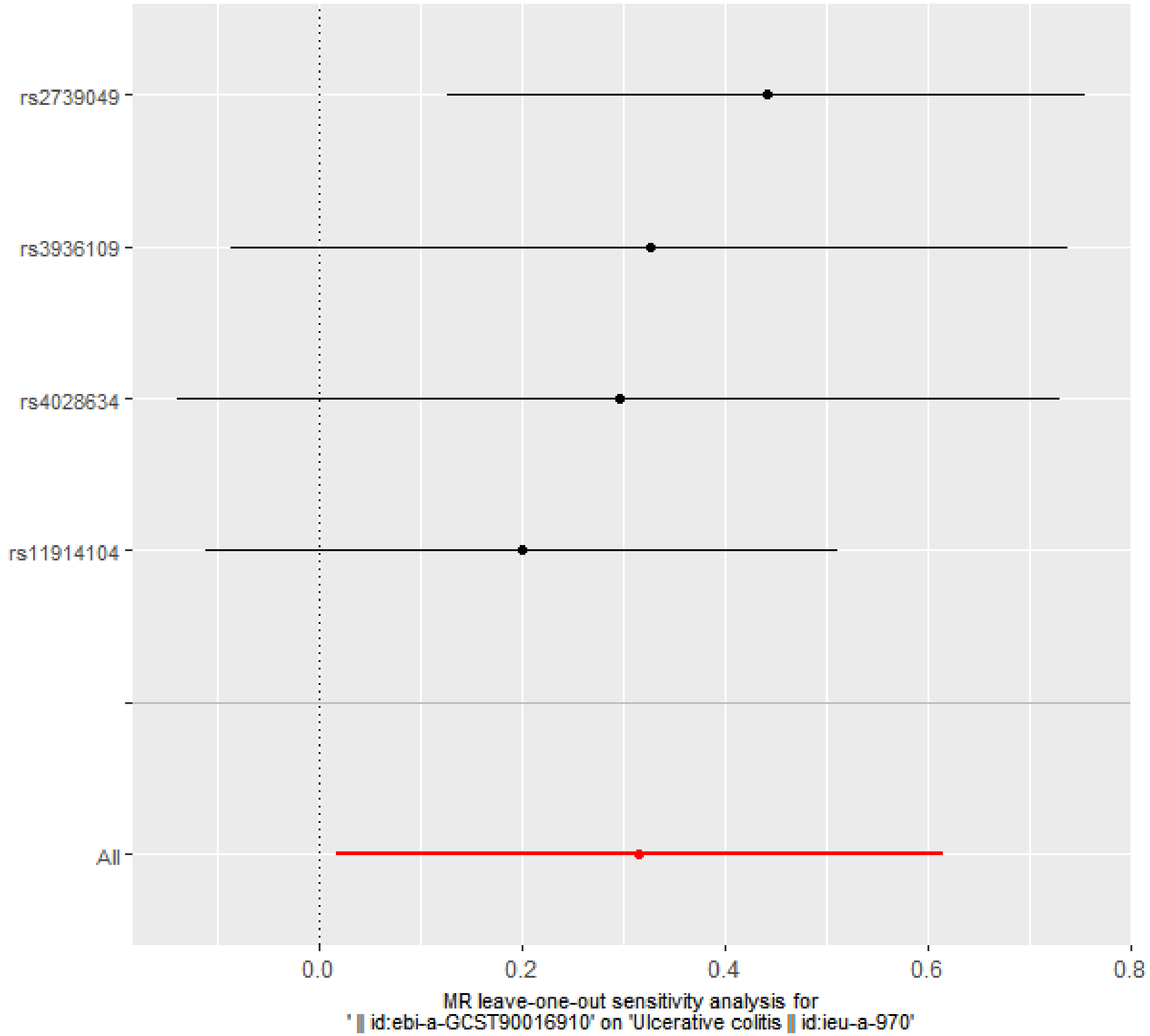

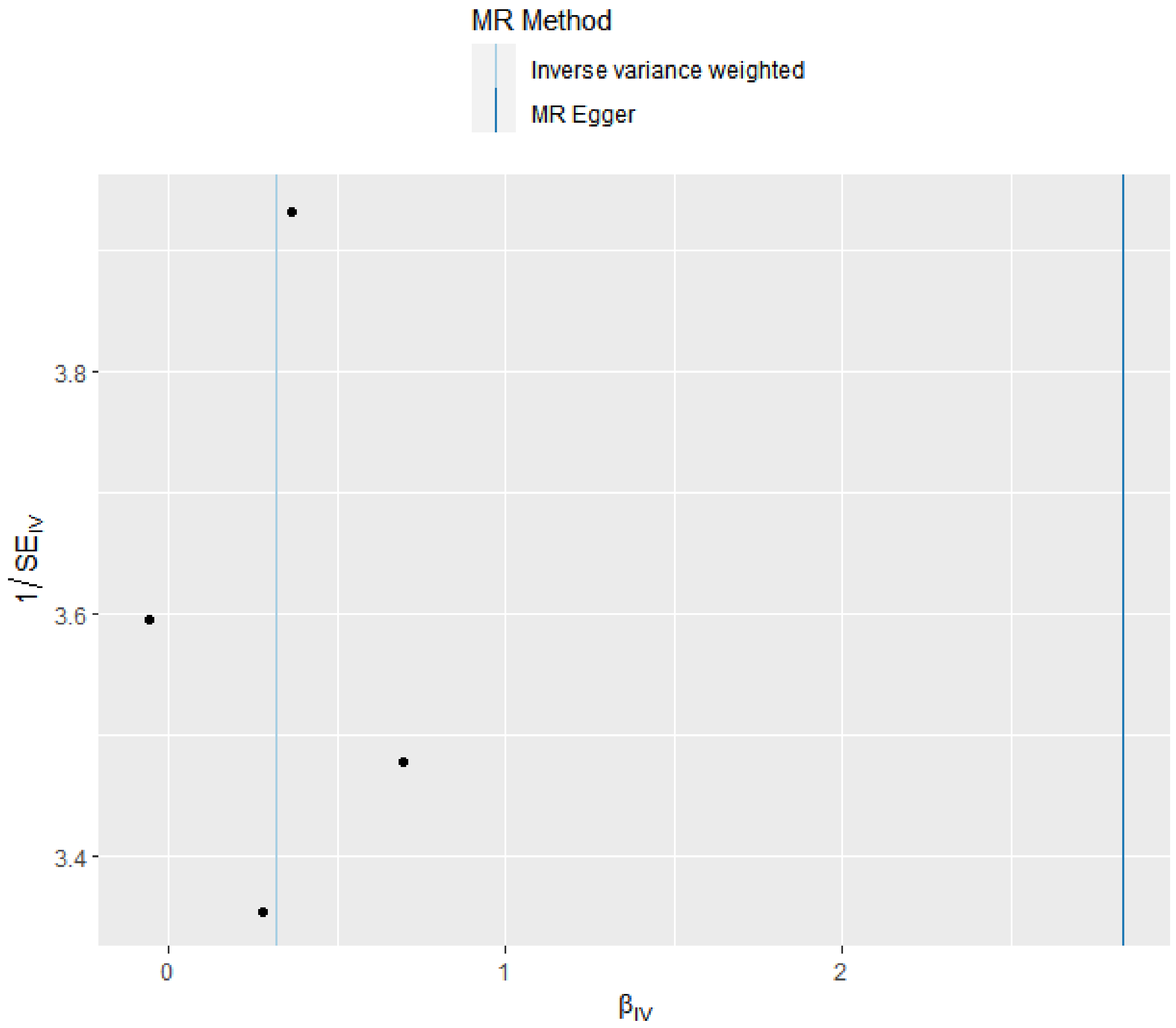
MR Method
Inverse variance weighted
MR Egger
3.8
3.6
3.4
0
1
2
$1/SE_{IV}$
$β_{IV}$

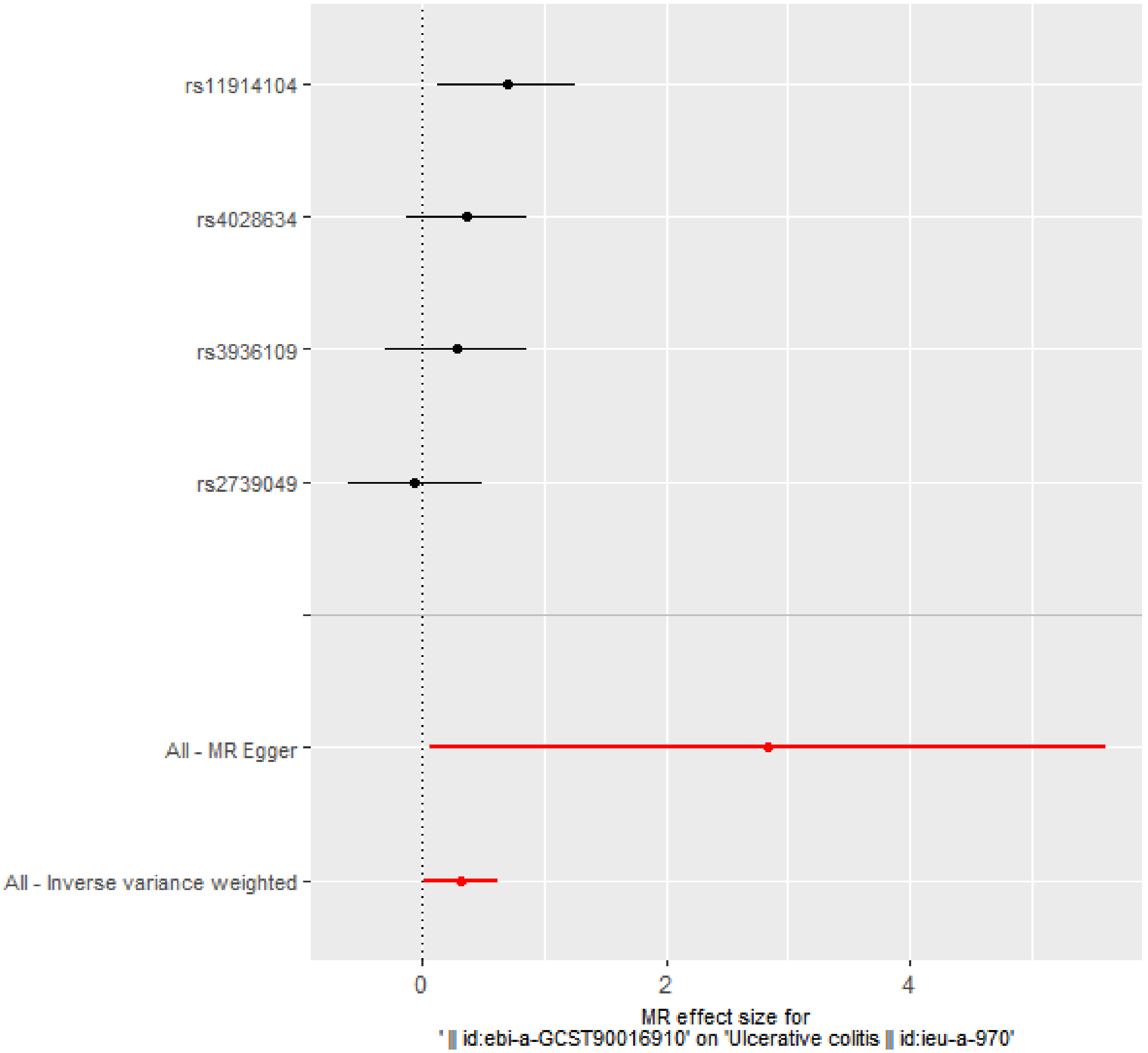

rs11914104
rs4028634
rs3936109
rs2739049
All - MR Egger
All - Inverse variance weighted
0
2
4
MR effect size for
' || id:ebi-a-GCST90016910' on 'Ulcerative colitis || id:ieu-a-970'

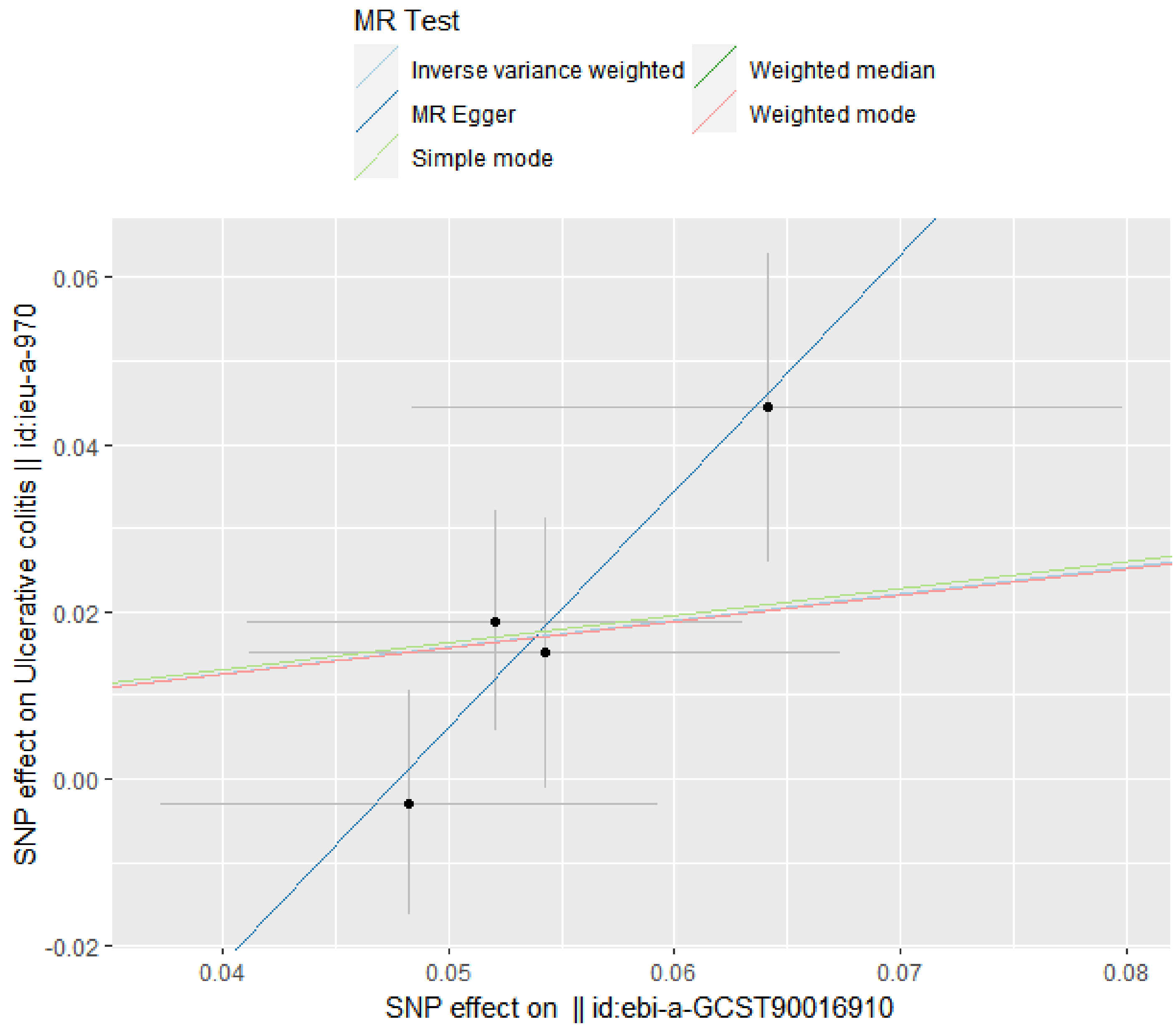

MR Test
Inverse variance weighted
MR Egger
Simple mode
Weighted median
Weighted mode
0.06
0.04
0.02
0.00
-0.02
SNP effect on Ulcerative colitis || id:ieu-a-970
0.04
0.05
0.06
0.07
0.08
SNP effect on || id:ebi-a-GCST90016910

Figure 128 Leave-one-out analysis, funnel plot, MR effect size and scatter plot for gut microbiota abundance (genus Eubacterium oxidoreducens group id.11339) on ulcerative colitis

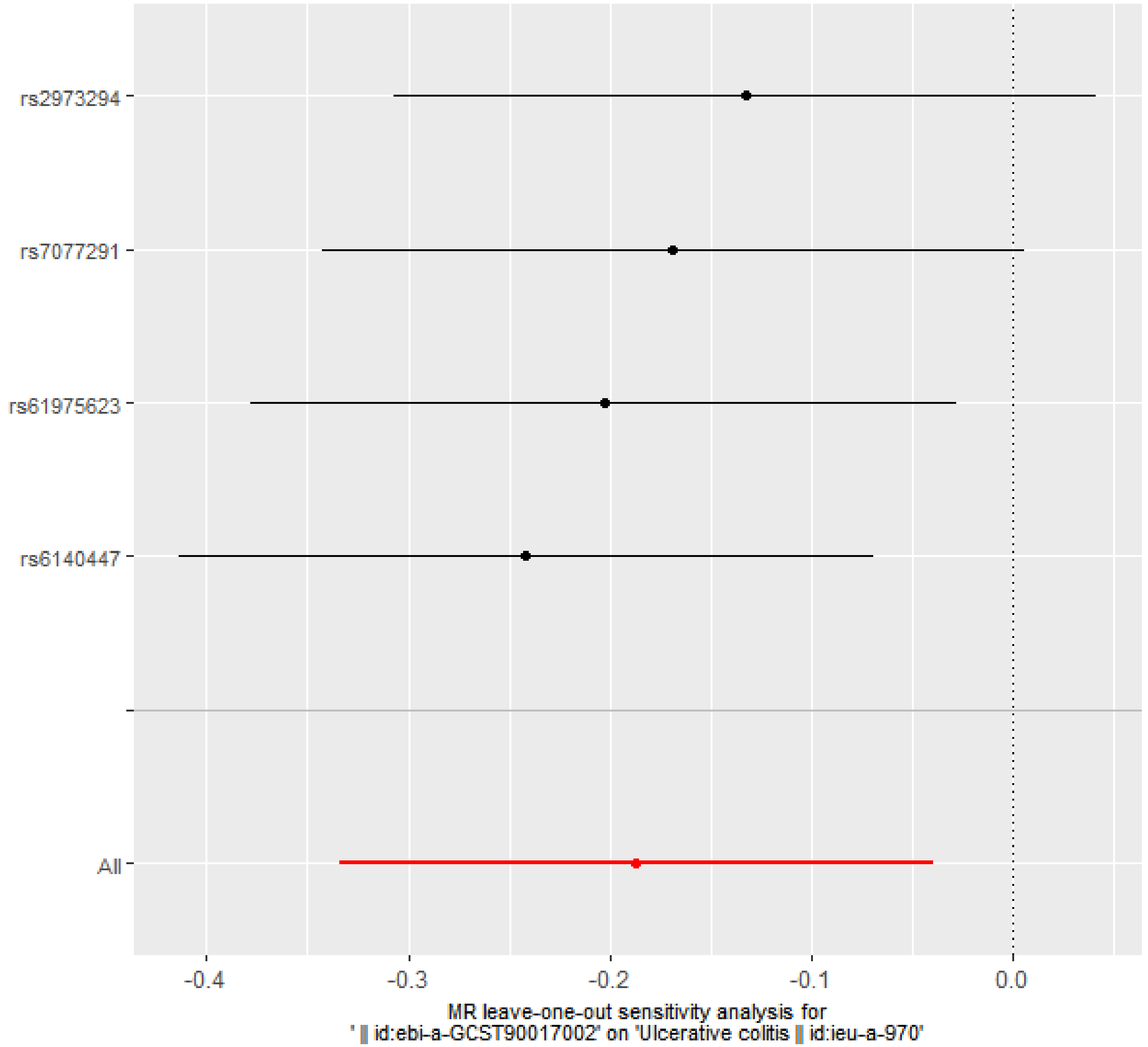

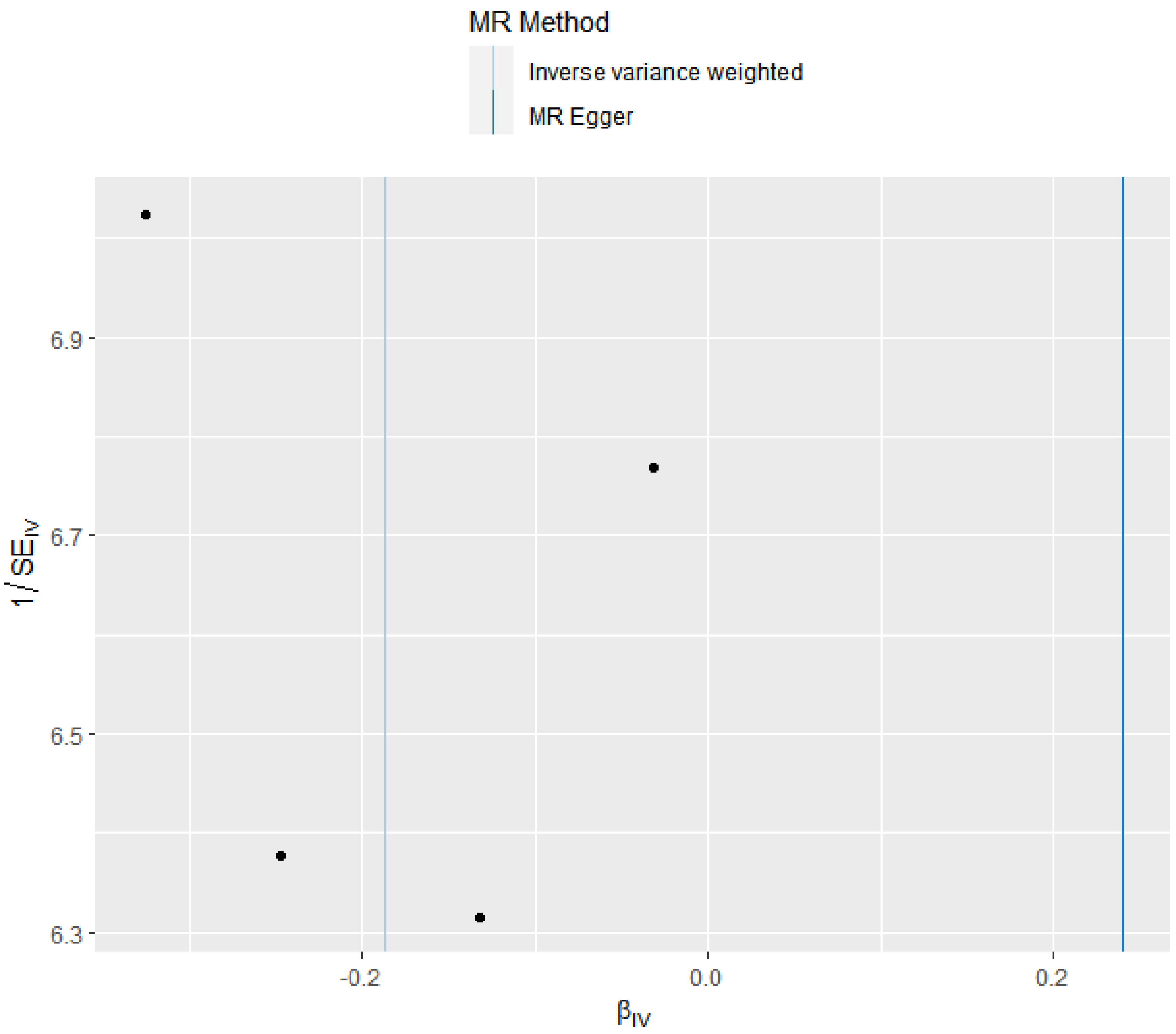

MR Method
Inverse variance weighted
MR Egger
6.9
6.7
6.5
6.3
1/SE_IV
-0.2
0.0
0.2
β_IV

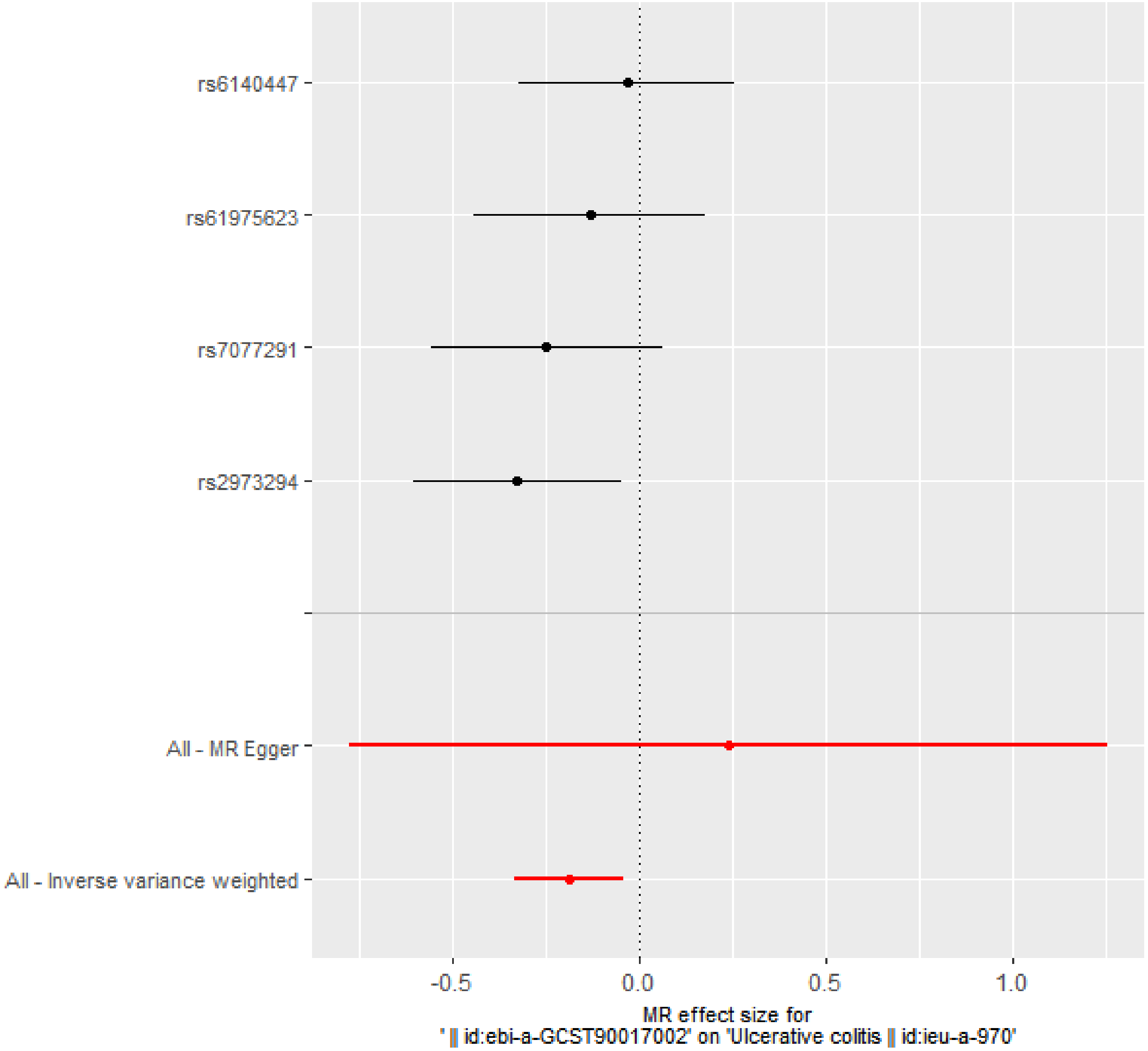
rs6140447
rs61975623
rs7077291
rs2973294
All - MR Egger
All - Inverse variance weighted
-0.5
0.0
0.5
1.0
MR effect size for
' || id:ebi-a-GCST90017002' on 'Ulcerative colitis || id:ieu-a-970'

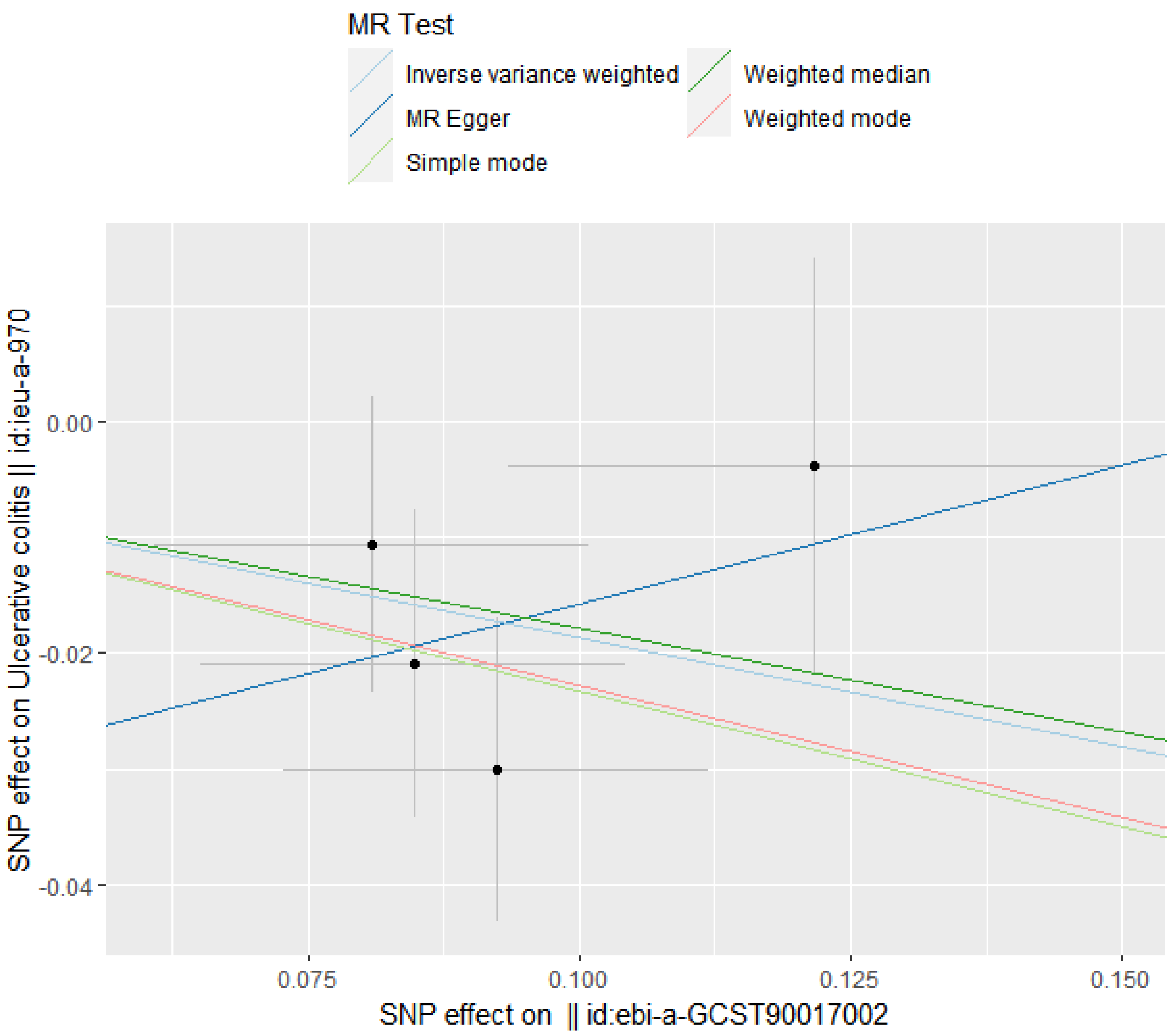
MR Test
Inverse variance weighted
MR Egger
Simple mode
Weighted median
Weighted mode
0.00
-0.02
-0.04
SNP effect on Ulcerative colitis || id:ieu-a-970
0.075
0.100
0.125
0.150
SNP effect on || id:ebi-a-GCST90017002

Figure 129 Leave-one-out analysis, funnel plot, MR effect size and scatter plot for gut microbiota abundance (genus Lachnospiraceae NC2004 group id.11316) on ulcerative colitis

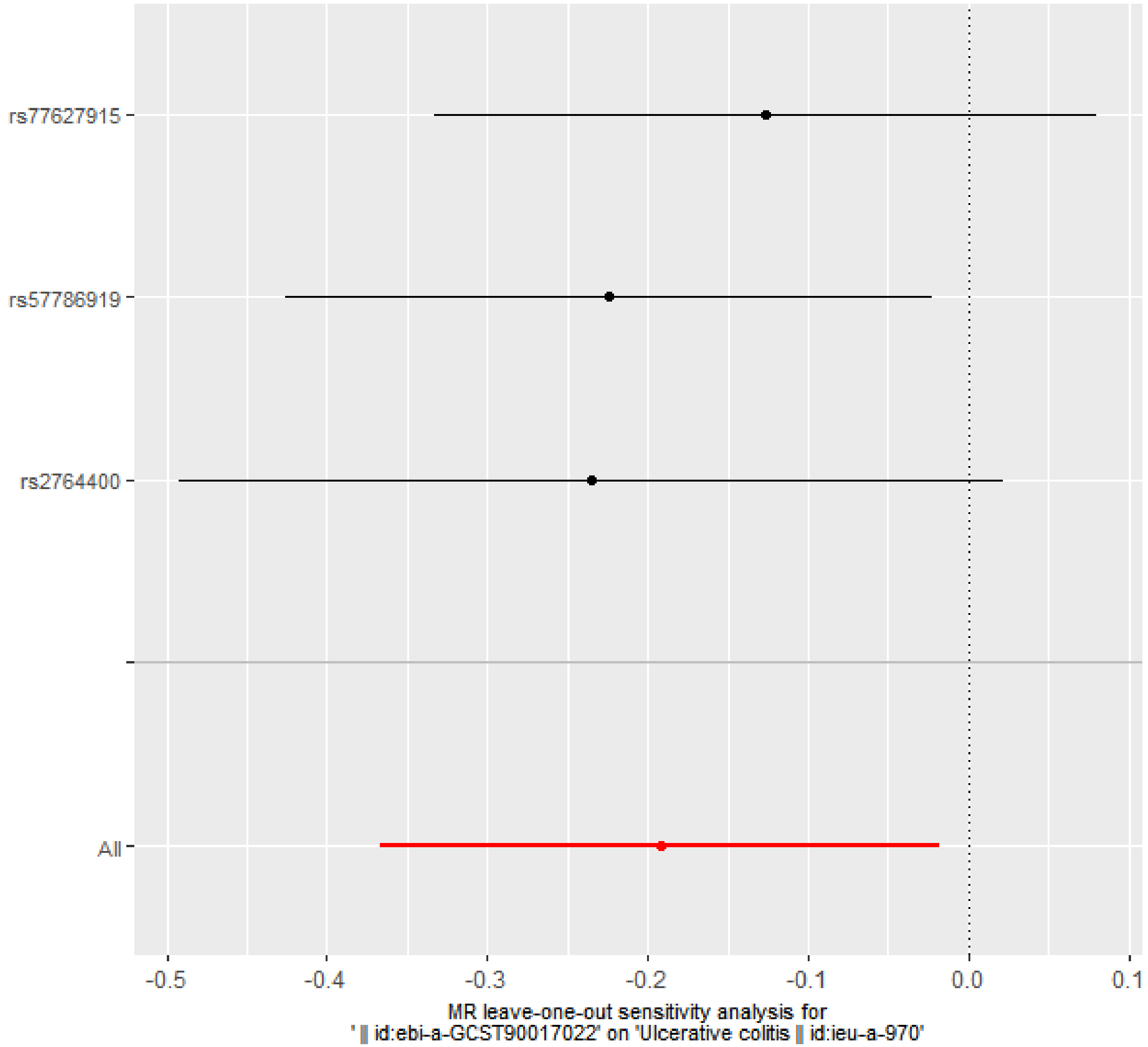

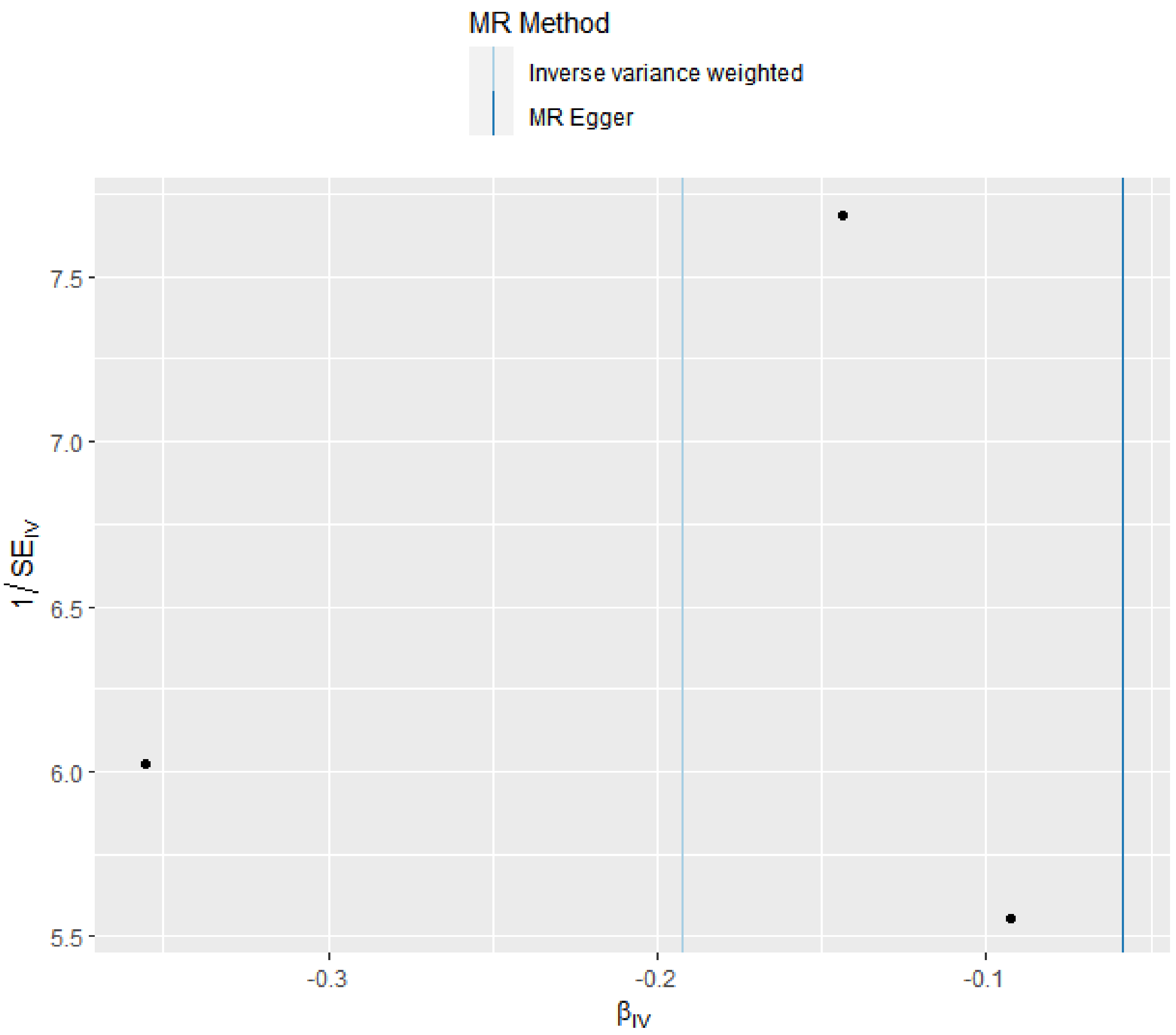

MR Method
Inverse variance weighted
MR Egger
7.5
7.0
6.5
6.0
5.5
1/SE_IV
-0.3
-0.2
-0.1
β_IV

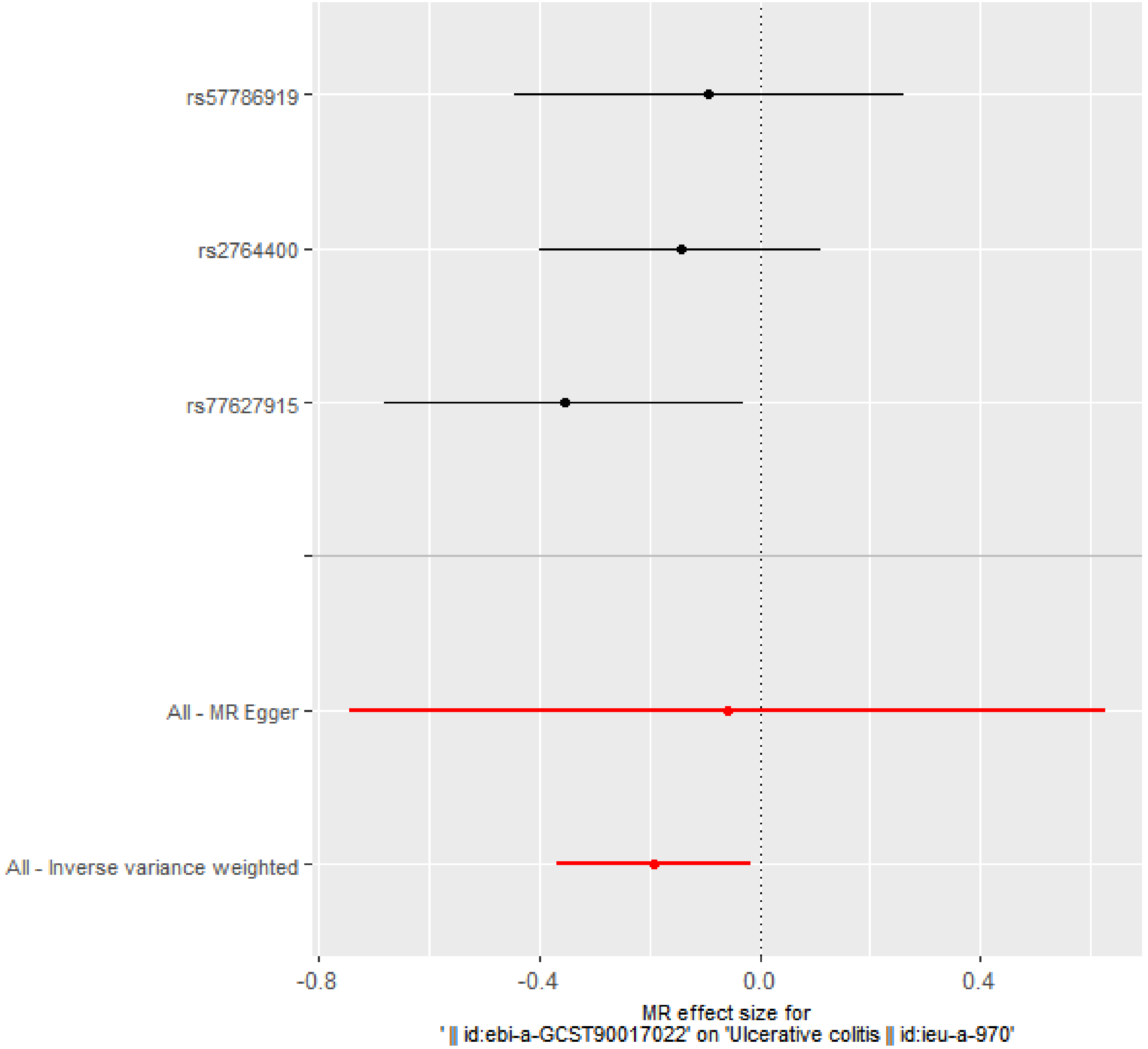
rs57786919
rs2764400
rs77627915
All - MR Egger
All - Inverse variance weighted
-0.8
-0.4
0.0
0.4
MR effect size for
' || id:ebi-a-GCST90017022' on 'Ulcerative colitis || id:ieu-a-970'

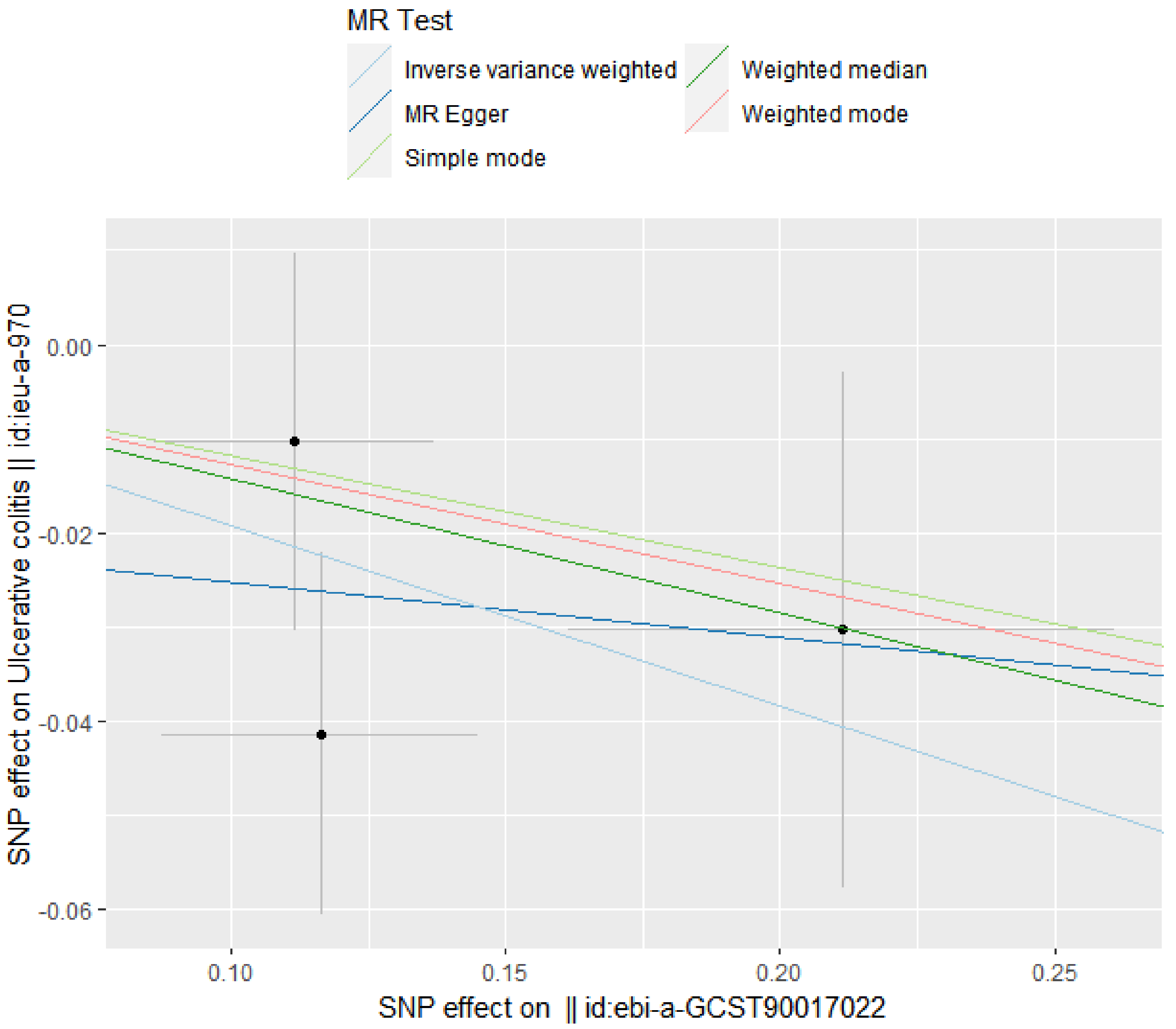
MR Test
Inverse variance weighted
MR Egger
Simple mode
Weighted median
Weighted mode
0.00
-0.02
-0.04
-0.06
0.10
0.15
0.20
0.25
SNP effect on Ulcerative colitis || id:ieu-a-970
SNP effect on || id:ebi-a-GCST90017022

Figure 130 Leave-one-out analysis, funnel plot, MR effect size and scatter plot for gut microbiota abundance (order Rhodospirillales id.2667) on ulcerative colitis

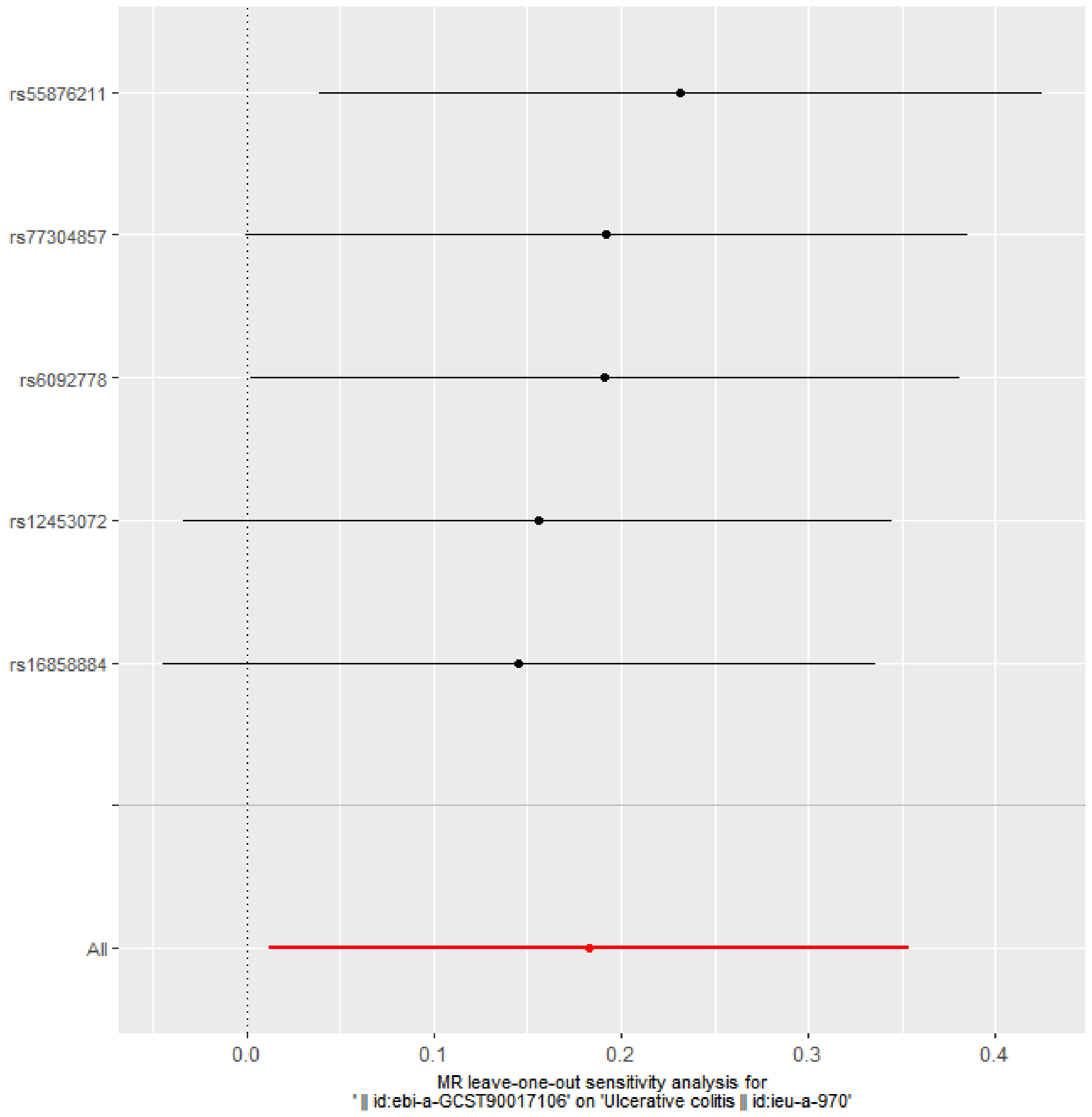

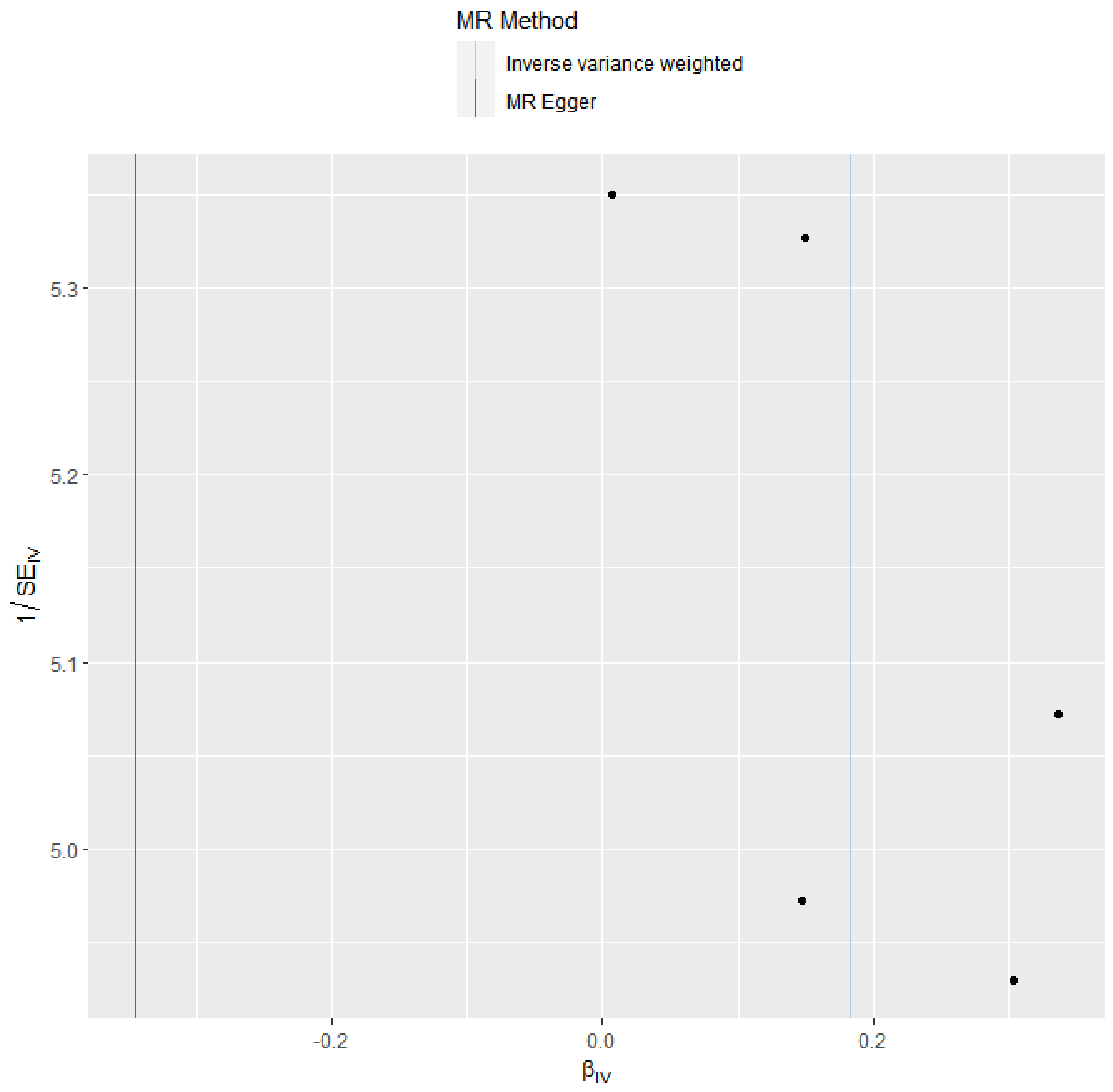
MR Method
Inverse variance weighted
MR Egger
5.3
5.2
5.1
5.0
$1/SE_{IV}$
-0.2
0.0
0.2
β_{IV}

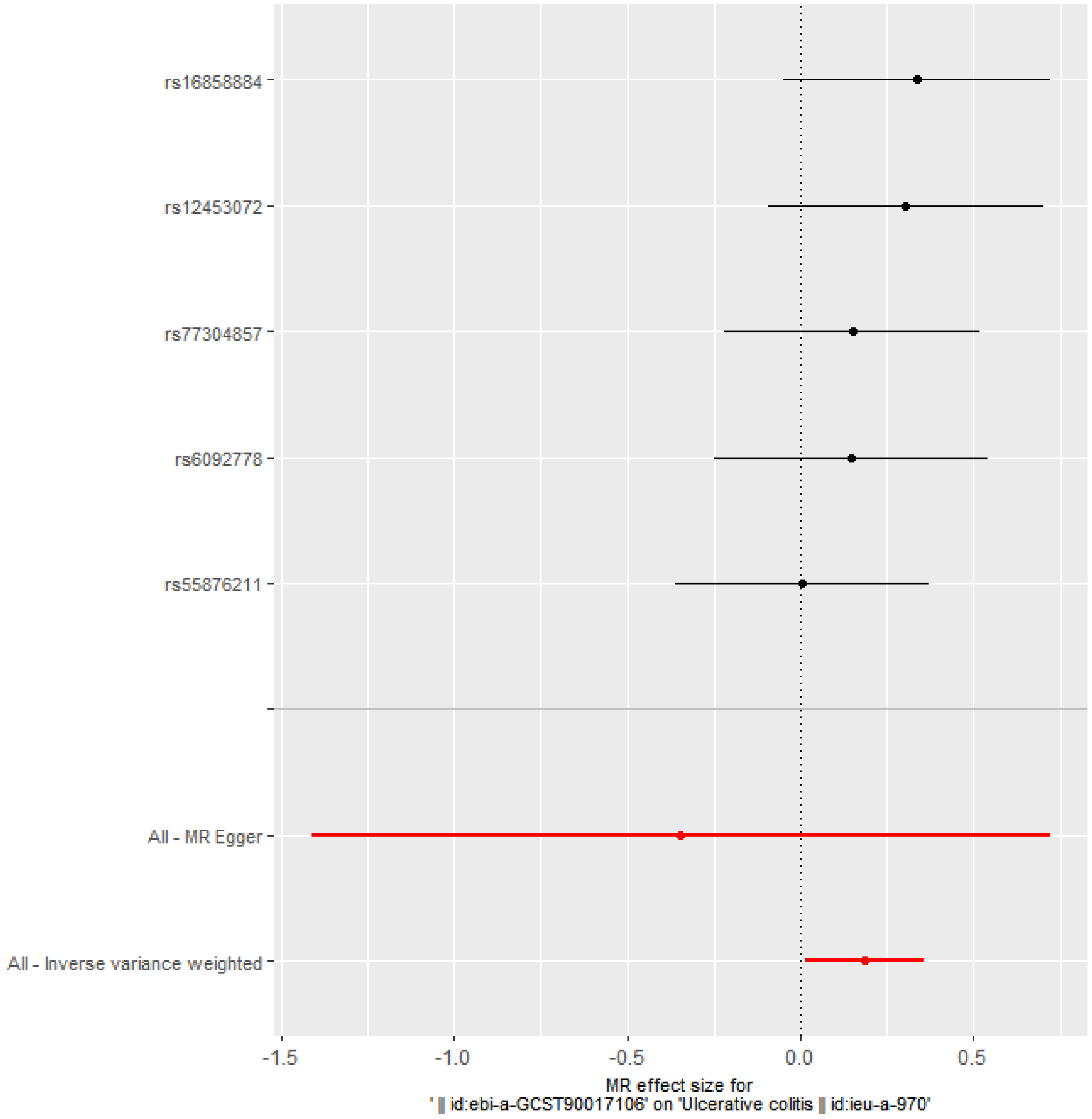

rs16858884
rs12453072
rs77304857
rs6092778
rs55876211
All - MR Egger
All - Inverse variance weighted
-1.5
-1.0
-0.5
0.0
0.5
MR effect size for
' || id:ebi-a-GCST90017106' on 'Ulcerative colitis || id:ieu-a-970'

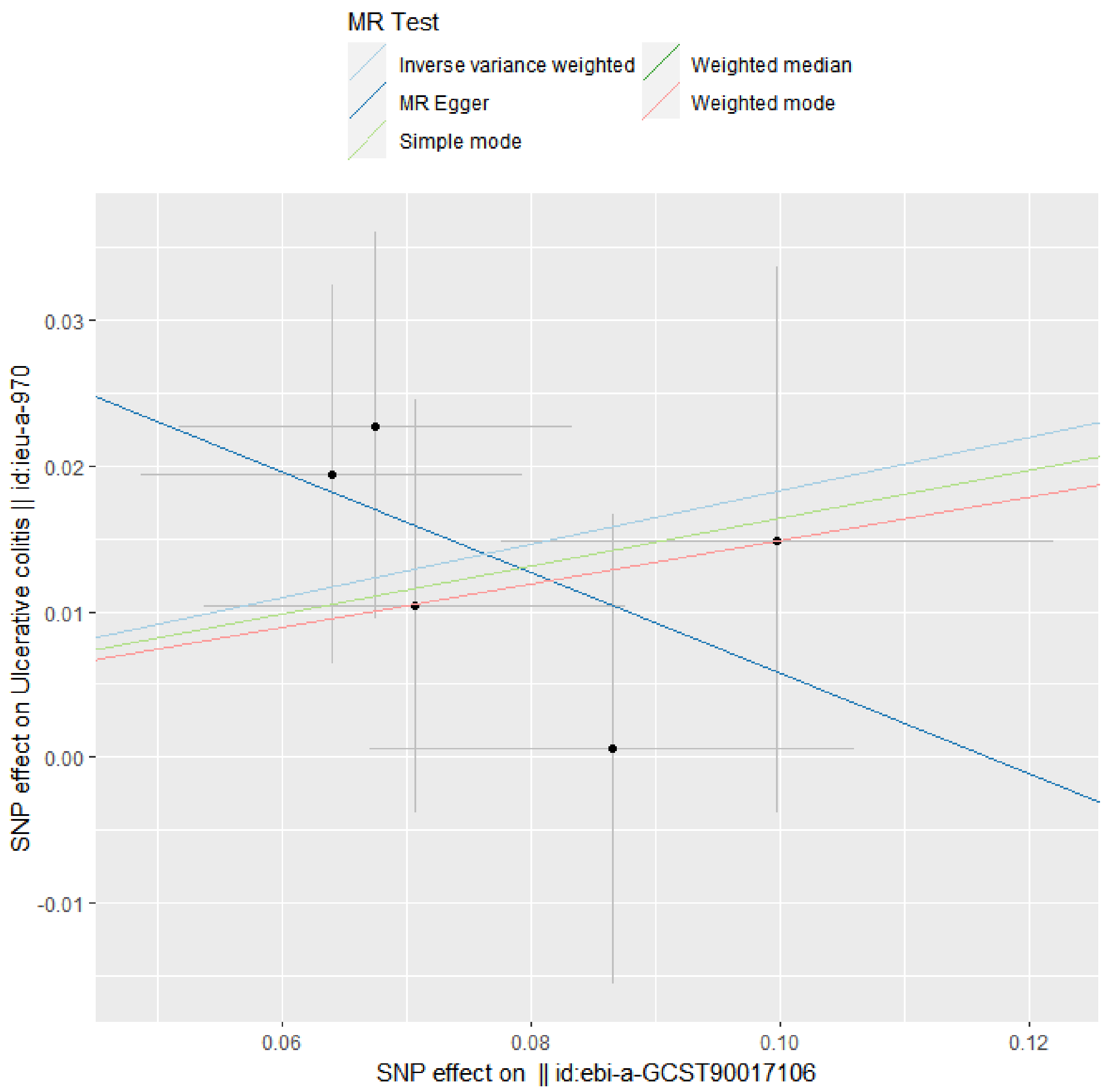

MR Test
Inverse variance weighted
MR Egger
Simple mode
Weighted median
Weighted mode
0.03
0.02
0.01
0.00
-0.01
0.06
0.08
0.10
0.12
SNP effect on Ulcerative colitis || id:ieu-a-970
SNP effect on || id:ebi-a-GCST90017106

Figure 131 Leave-one-out analysis, funnel plot, MR effect size and scatter plot for gut microbiota abundance (family Enterobacteriaceae id.3469) on colorectal cancer

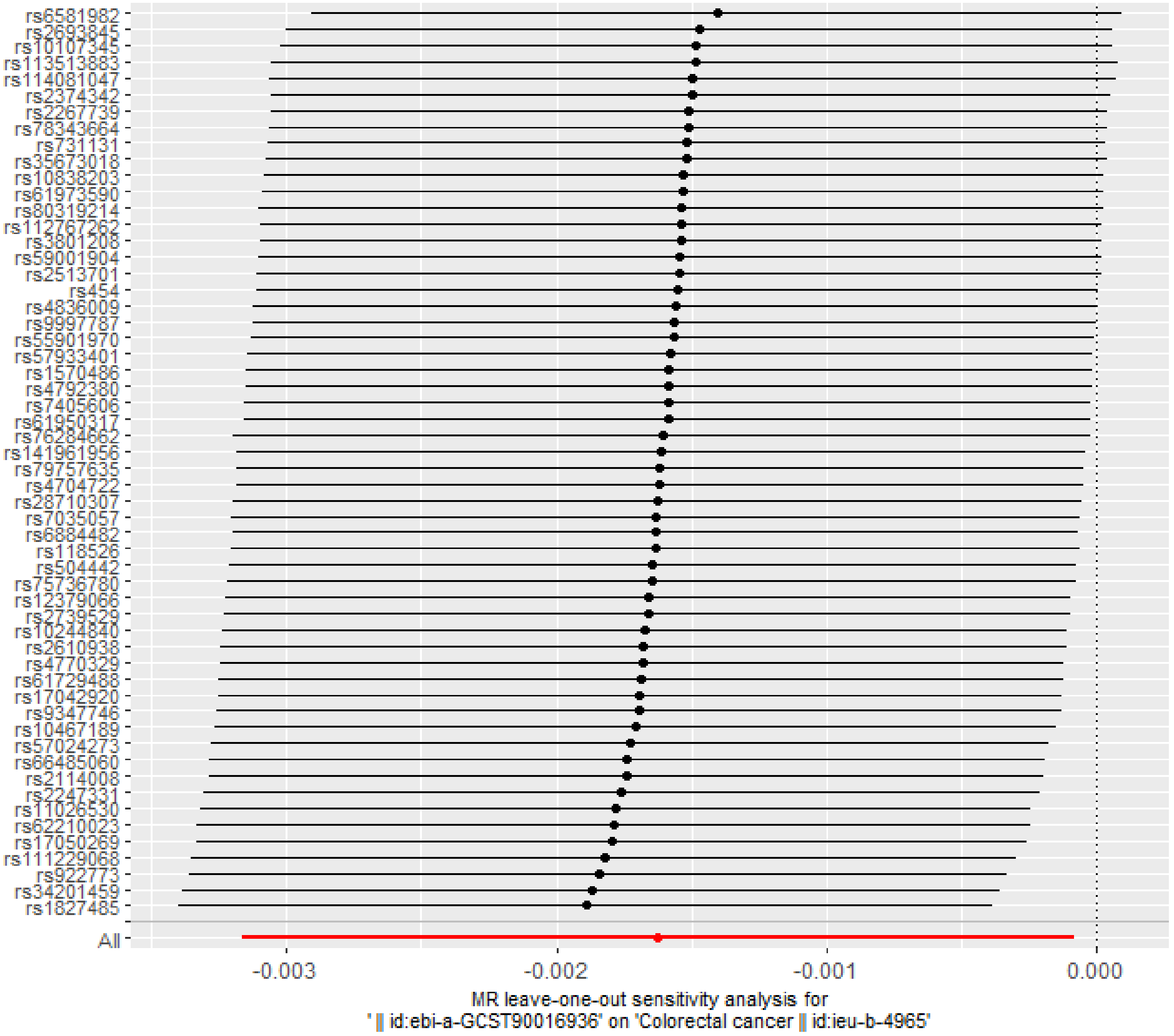

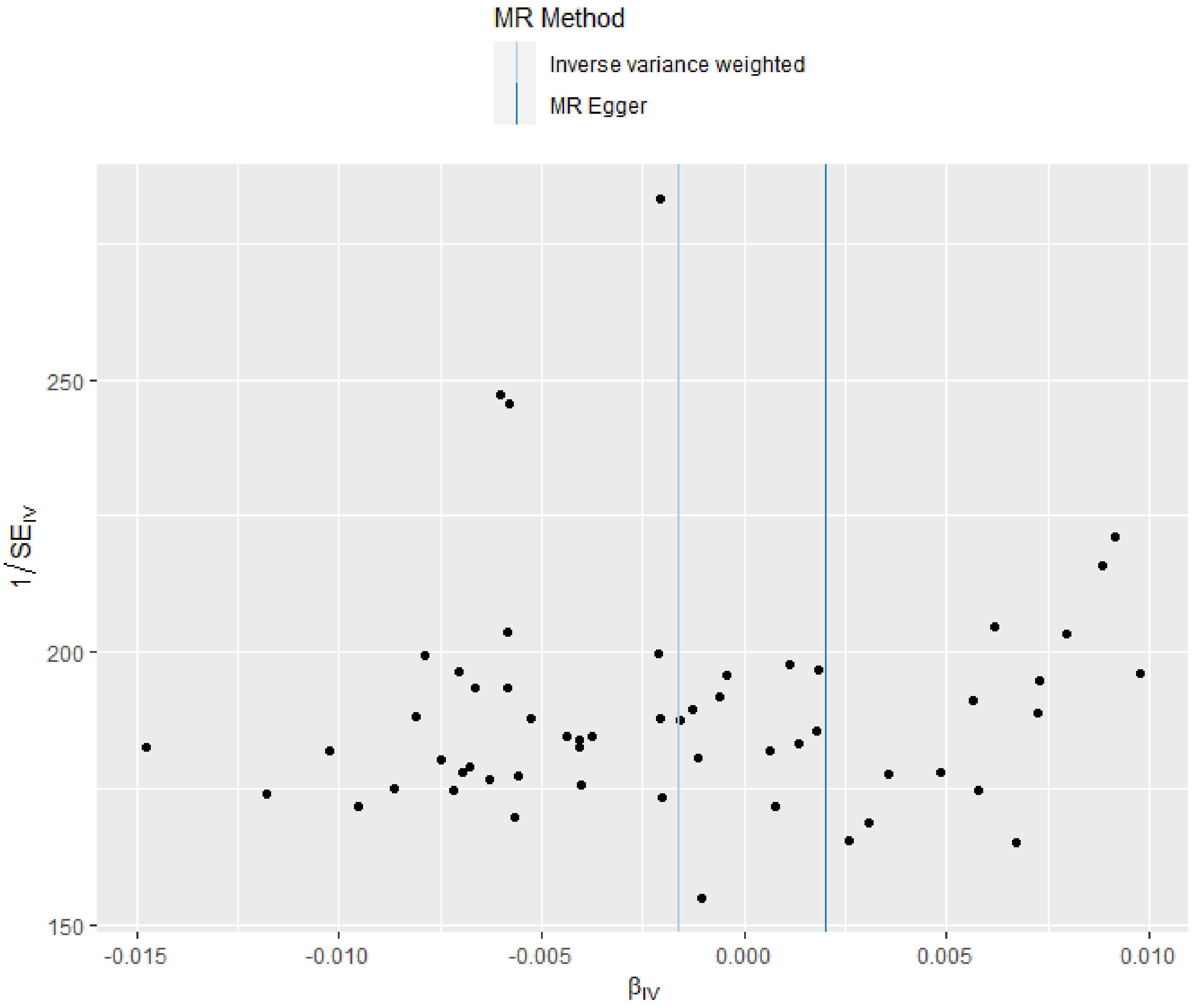
MR Method
Inverse variance weighted
MR Egger
250
200
150
1/SE_IV
-0.015
-0.010
-0.005
0.000
0.005
0.010
β_IV

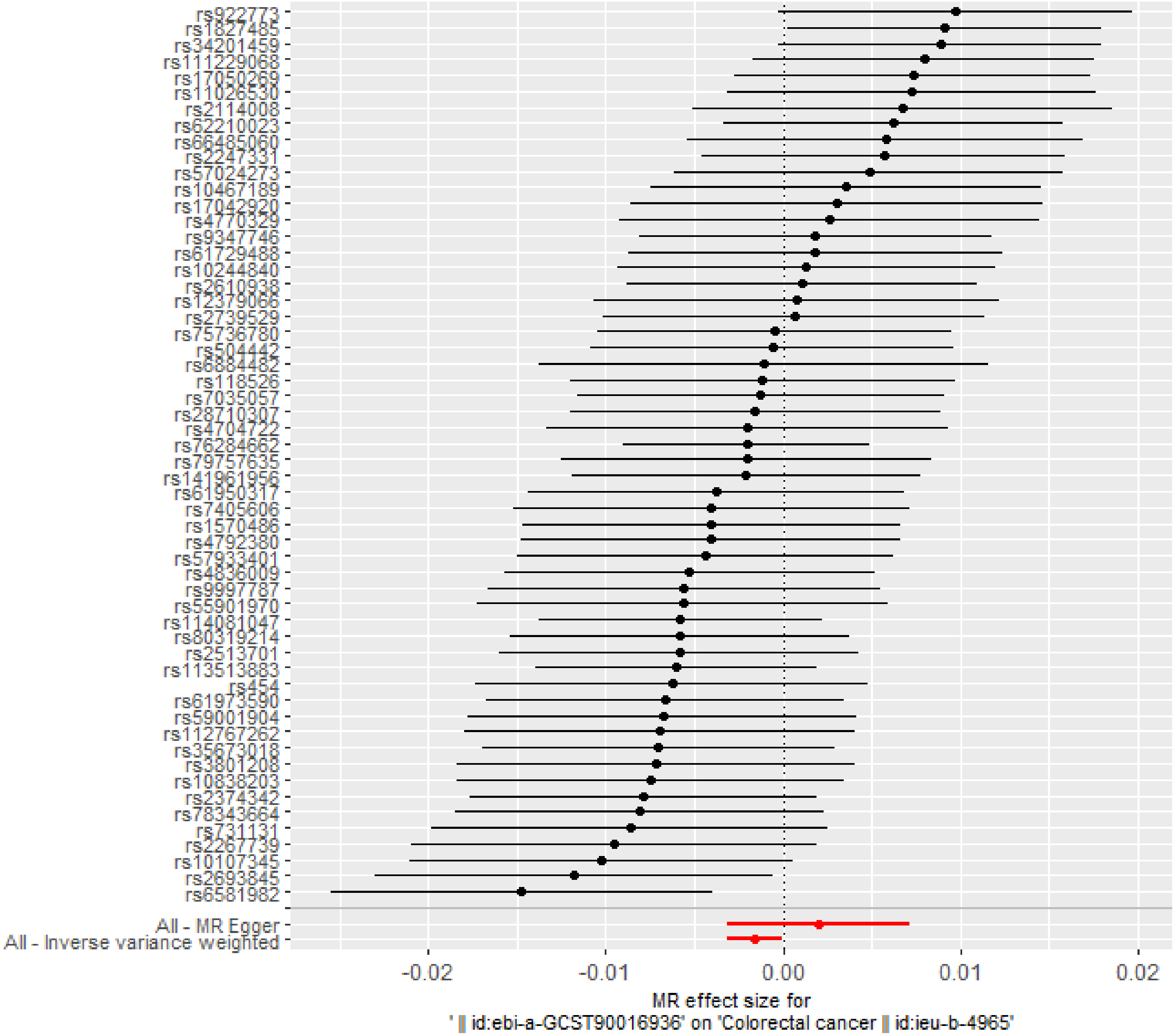

rs922773
rs1827485
rs34201459
rs111229068
rs17050269
rs11026530
rs2114008
rs62210023
rs66485060
rs2247331
rs57024273
rs10467189
rs17042920
rs4770329
rs9347746
rs61729488
rs10244840
rs2610938
rs12379066
rs2739529
rs75736780
rs504442
rs6884482
rs118526
rs7035057
rs28710307
rs4704722
rs76284662
rs79757635
rs141961956
rs61950317
rs7405606
rs1570486
rs4792380
rs57933401
rs4836009
rs9997787
rs55901970
rs114081047
rs80319214
rs2513701
rs113513883
rs454
rs61973590
rs59001904
rs112767262
rs35673018
rs3801208
rs10838203
rs2374342
rs78343664
rs731131
rs2267739
rs10107345
rs2693845
rs6581982
All - MR Egger
All - Inverse variance weighted
-0.02
-0.01
0.00
0.01
0.02
MR effect size for
' || id:ebi-a-GCST90016936' on 'Colorectal cancer || id:ieu-b-4965'

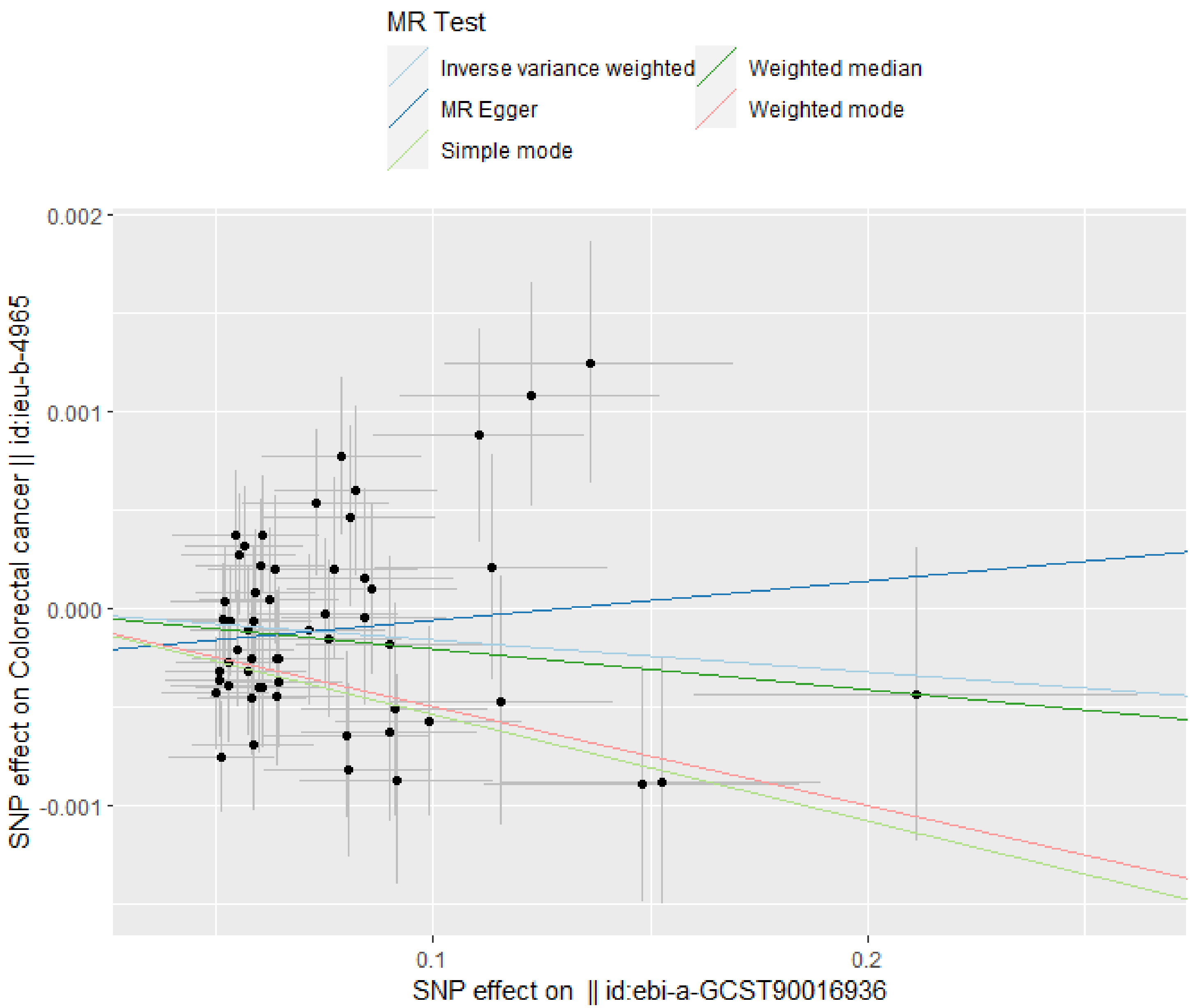

MR Test
Inverse variance weighted
MR Egger
Simple mode
Weighted median
Weighted mode
0.002
0.001
0.000
-0.001
SNP effect on Colorectal cancer || id:ieu-b-4965
0.1
0.2
SNP effect on || id:ebi-a-GCST90016936

Figure 132 Leave-one-out analysis, funnel plot, MR effect size and scatter plot for gut microbiota abundance (genus Anaerostipes id.1991) on colorectal cancer

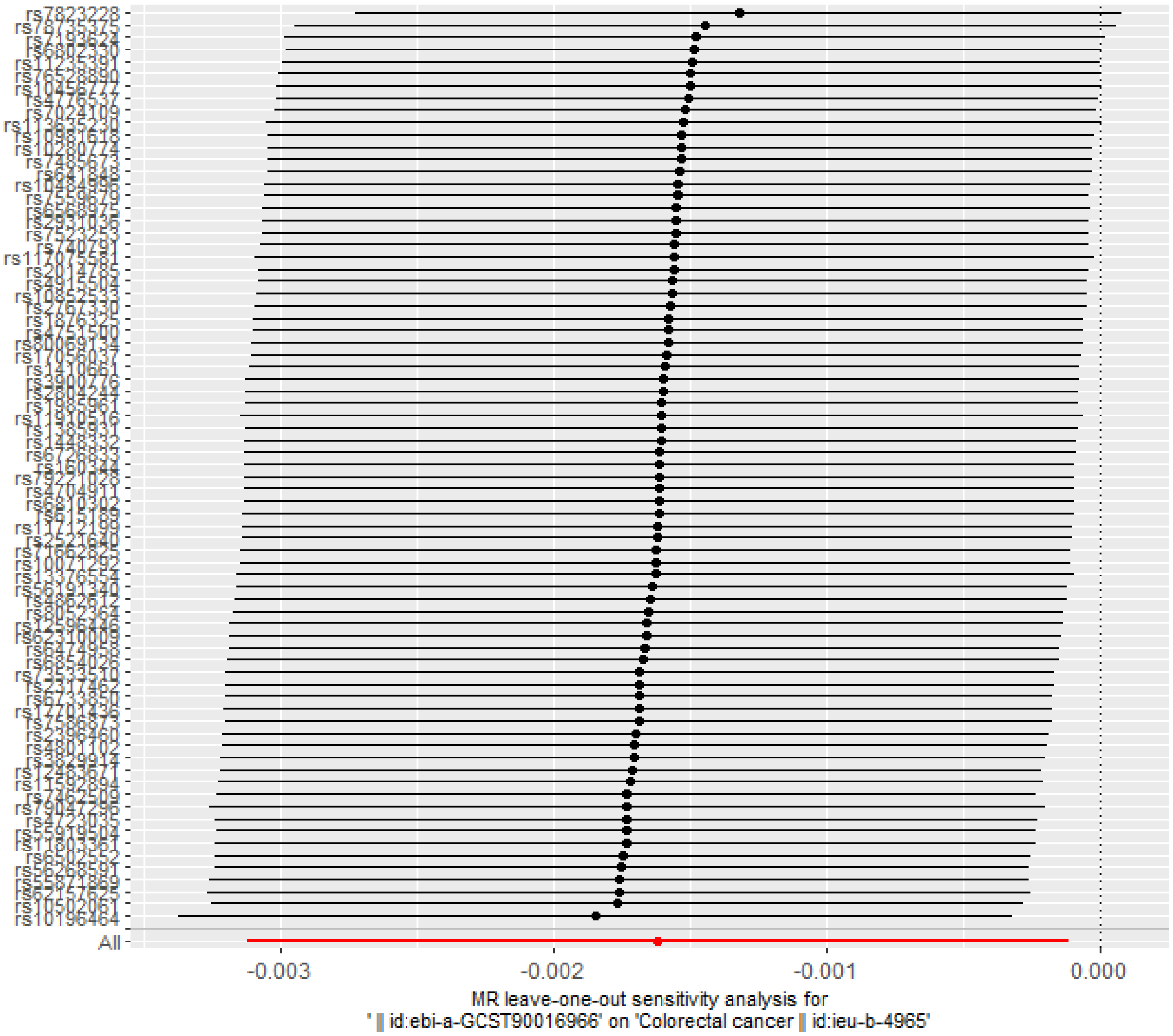

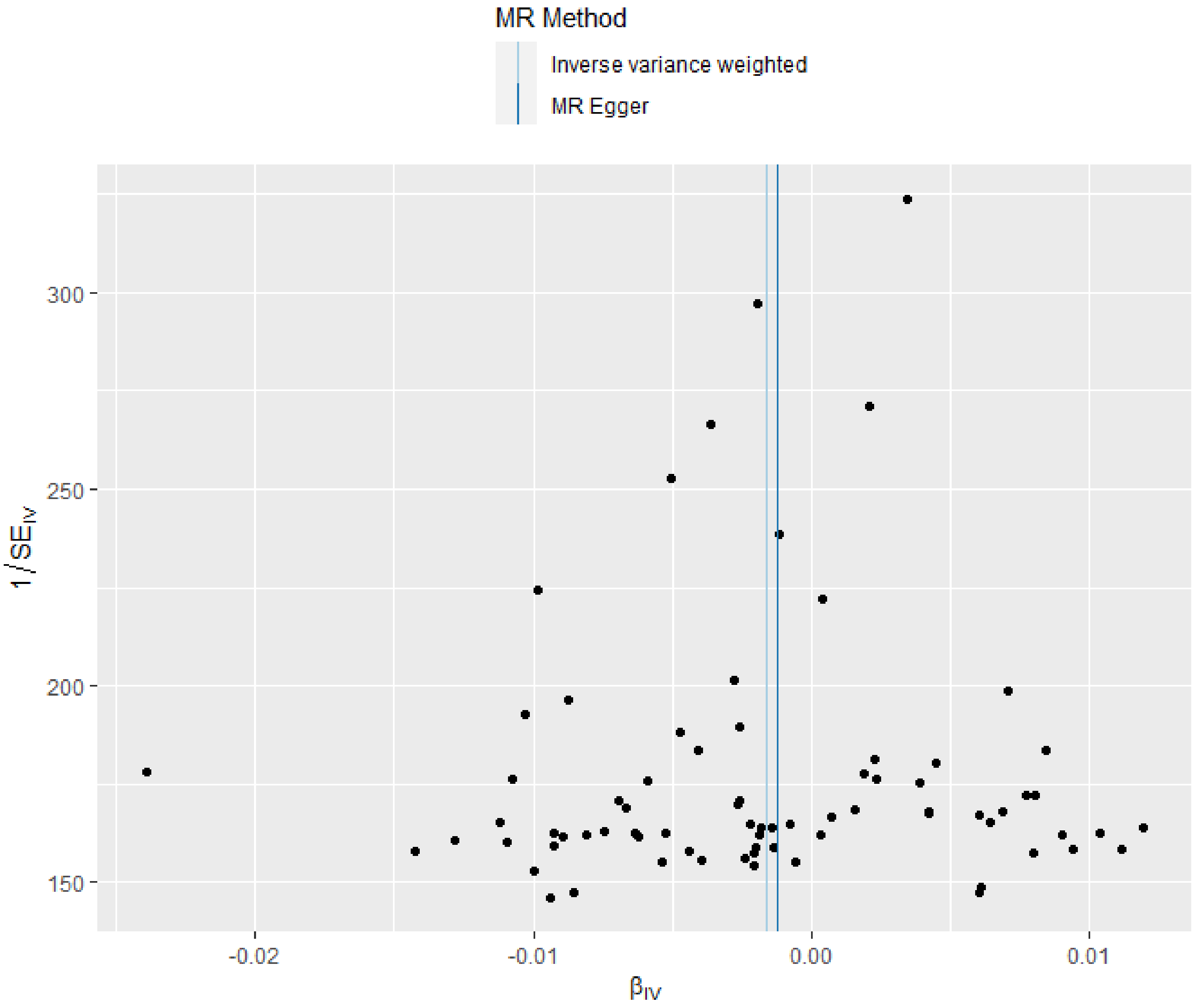

MR Method
Inverse variance weighted
MR Egger
300
250
200
150
-0.02
-0.01
0.00
0.01
1/SE_IV
β_IV

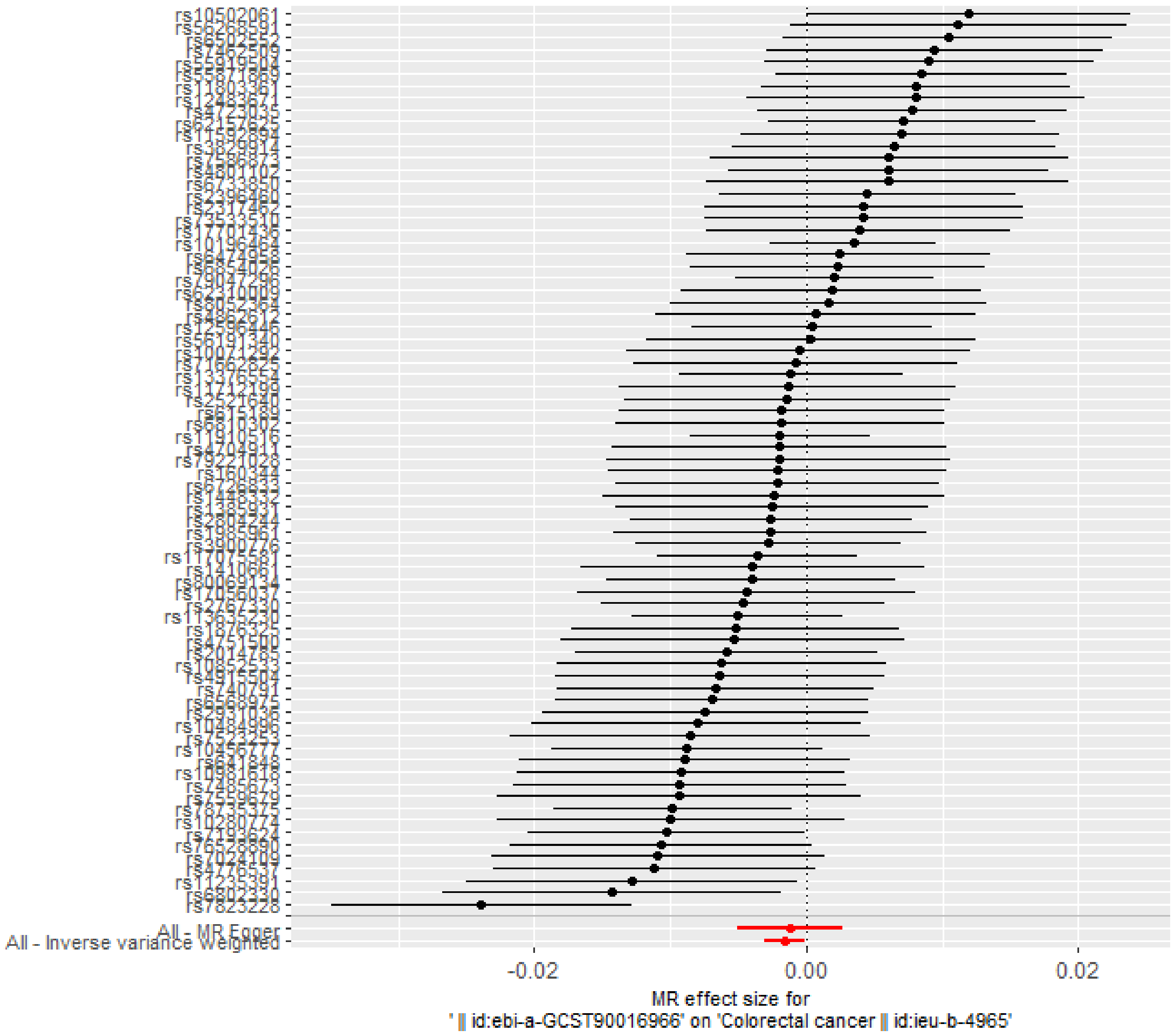

rs10502061
All - MR Egger
All - Inverse variance weighted
-0.02
0.00
0.02
MR effect size for
' || id:ebi-a-GCST90016966' on 'Colorectal cancer || id:ieu-b-4965'

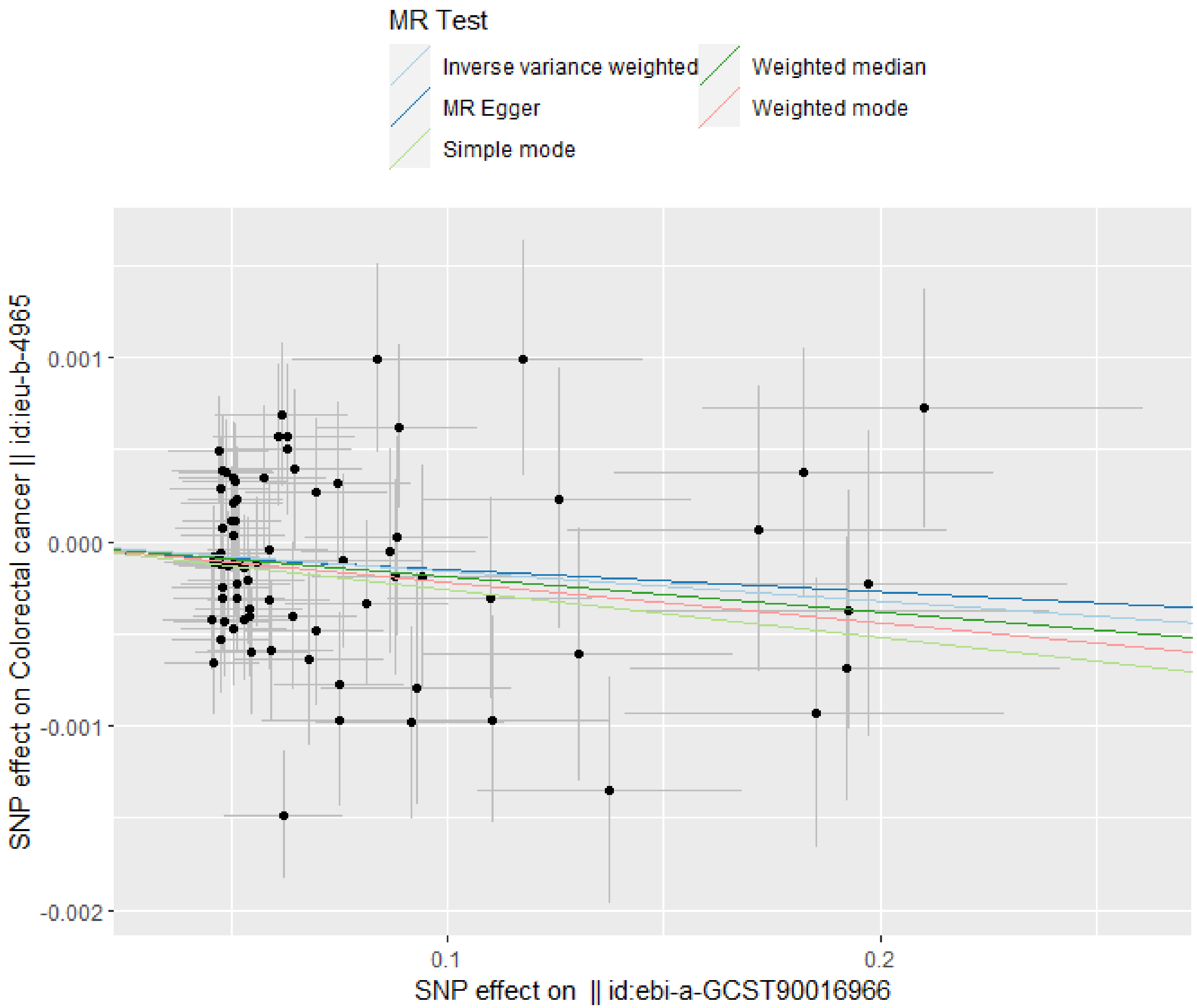
MR Test
Inverse variance weighted
MR Egger
Simple mode
Weighted median
Weighted mode
0.001
0.000
-0.001
-0.002
0.1
0.2
SNP effect on Colorectal cancer || id:ieu-b-4965
SNP effect on || id:ebi-a-GCST90016966

Figure 133 Leave-one-out analysis, funnel plot, MR effect size and scatter plot for gut microbiota abundance (genus Bilophila id.3170) on colorectal cancer

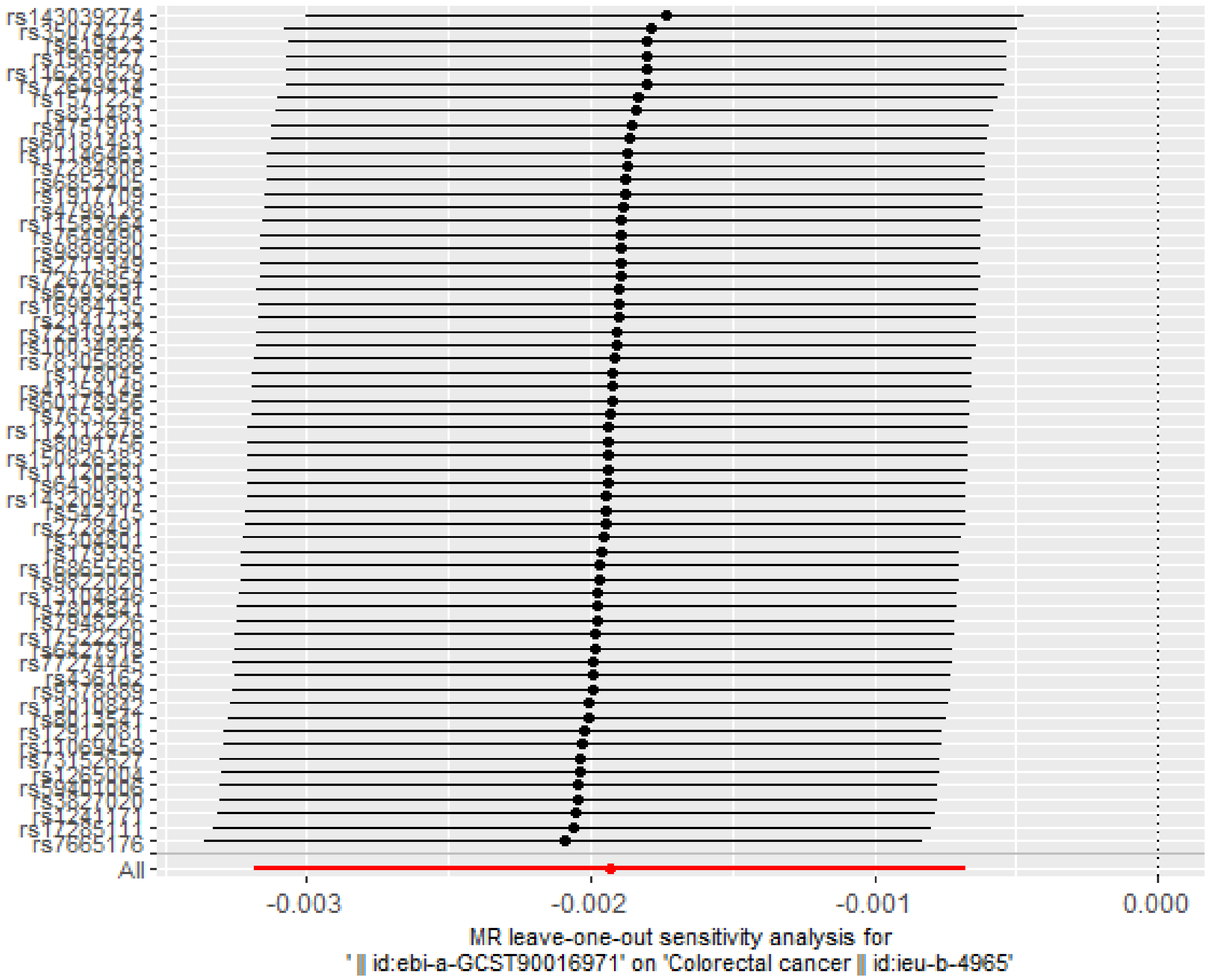

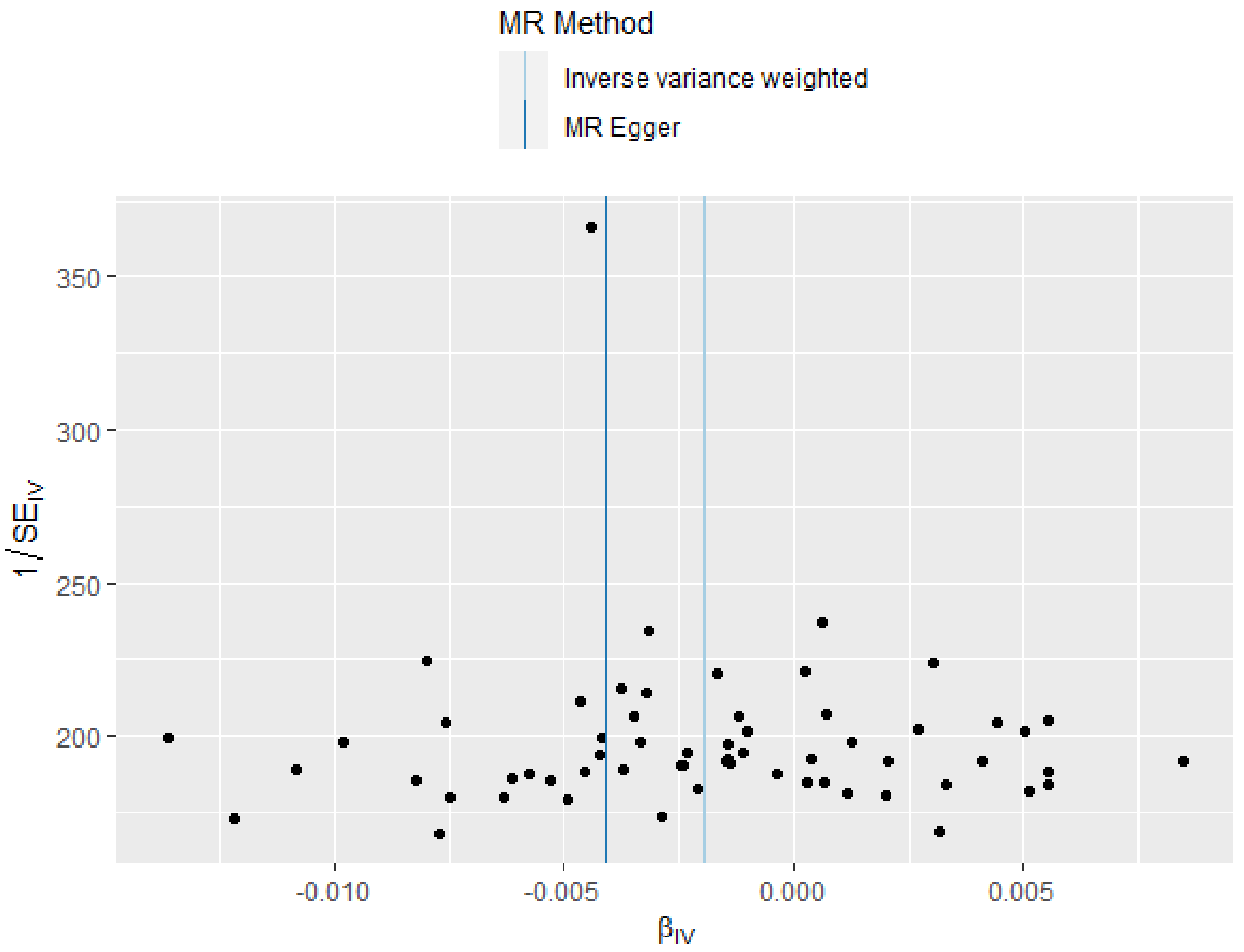

MR Method
Inverse variance weighted
MR Egger
350
300
250
200
1/SE_IV
-0.010
-0.005
0.000
0.005
β_IV

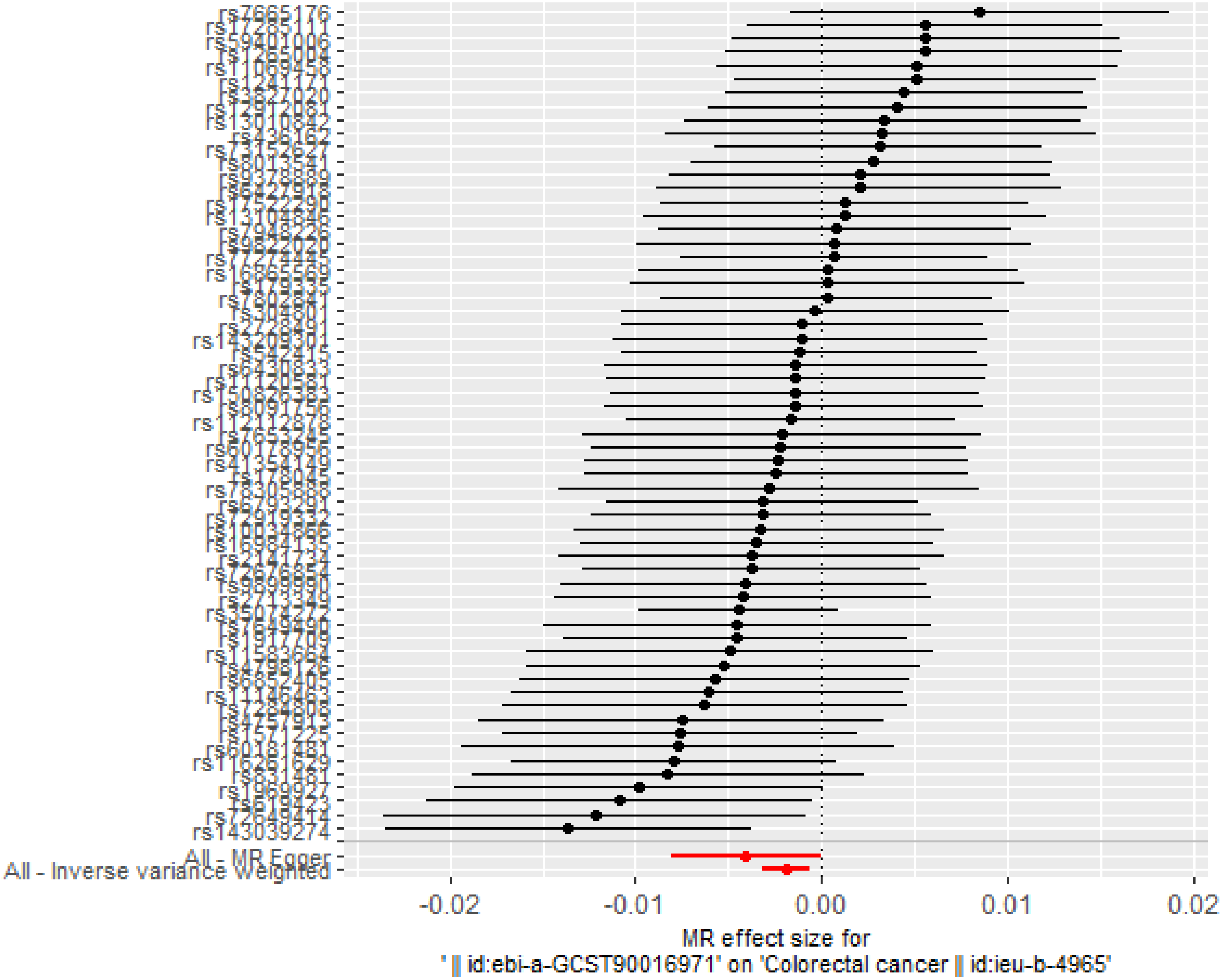
rs7665176
rs143039274
All - MR Egger
All - Inverse variance weighted
-0.02
-0.01
0.00
0.01
0.02
MR effect size for
' || id:ebi-a-GCST90016971' on 'Colorectal cancer || id:ieu-b-4965'

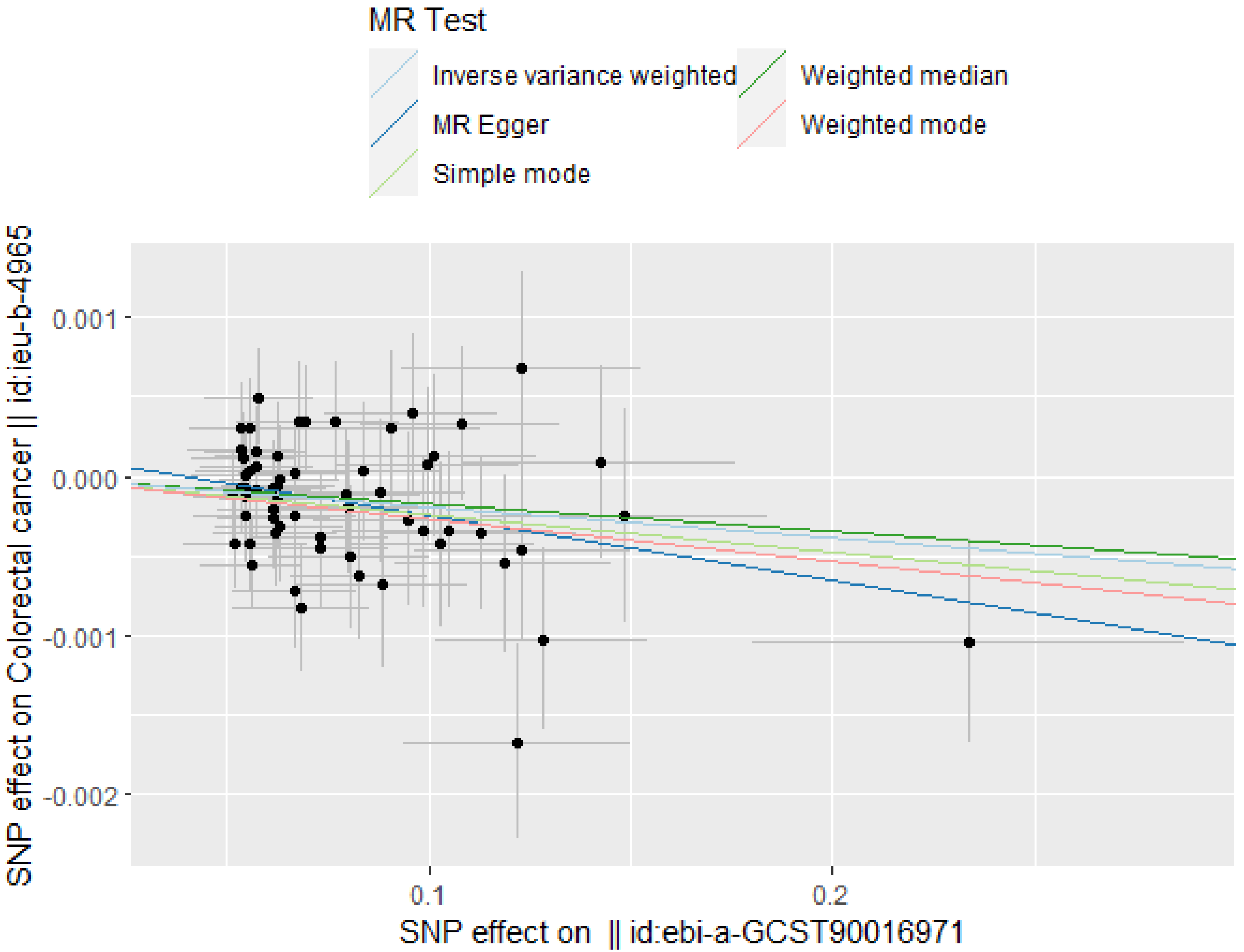
MR Test
Inverse variance weighted
MR Egger
Simple mode
Weighted median
Weighted mode
SNP effect on Colorectal cancer || id:ieu-b-4965
0.001
0.000
-0.001
-0.002
0.1
0.2
SNP effect on || id:ebi-a-GCST90016971

Figure 134 Leave-one-out analysis, funnel plot, MR effect size and scatter plot for gut microbiota abundance (genus Erysipelatoclostridium id.11381) on colorectal cancer

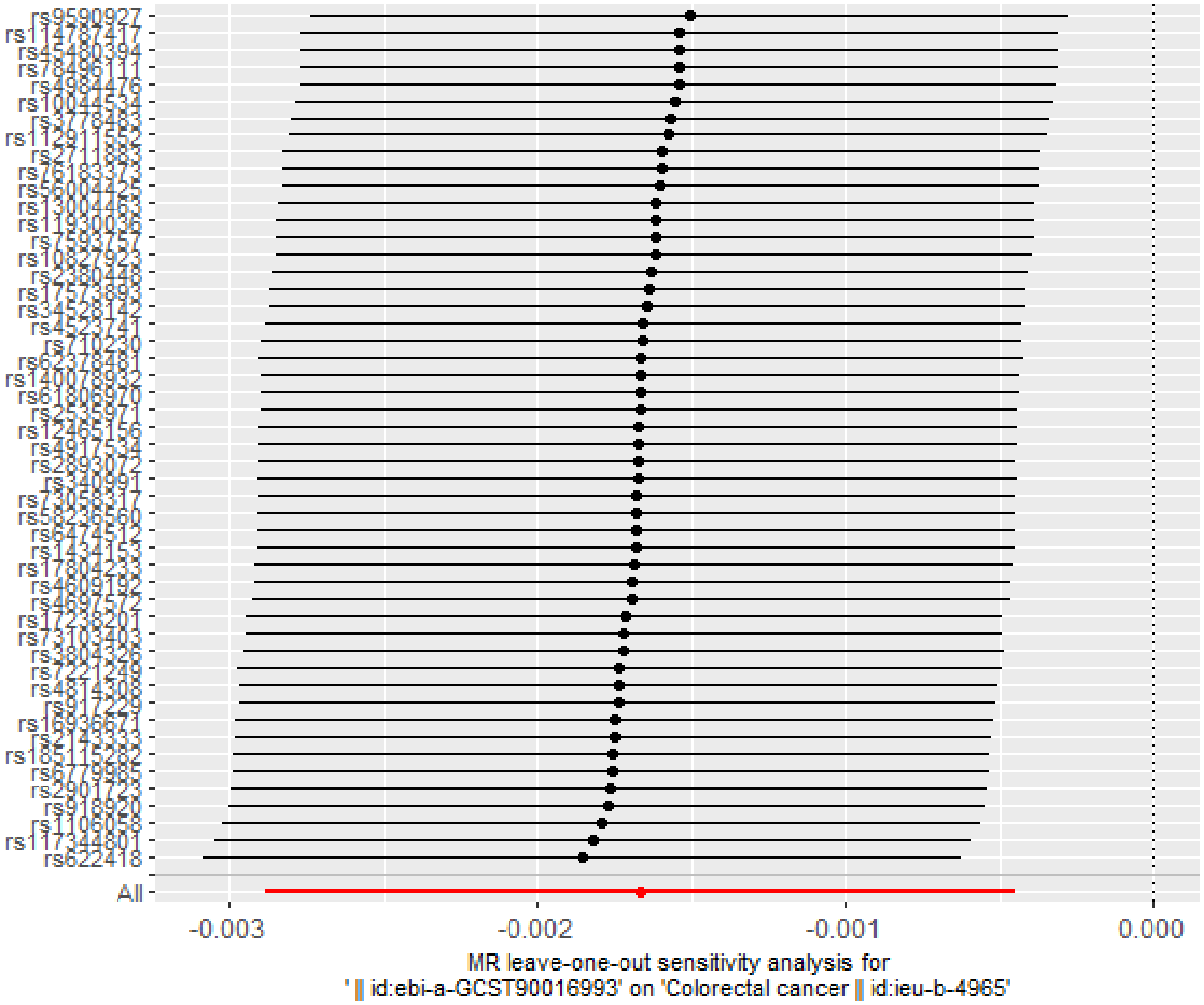

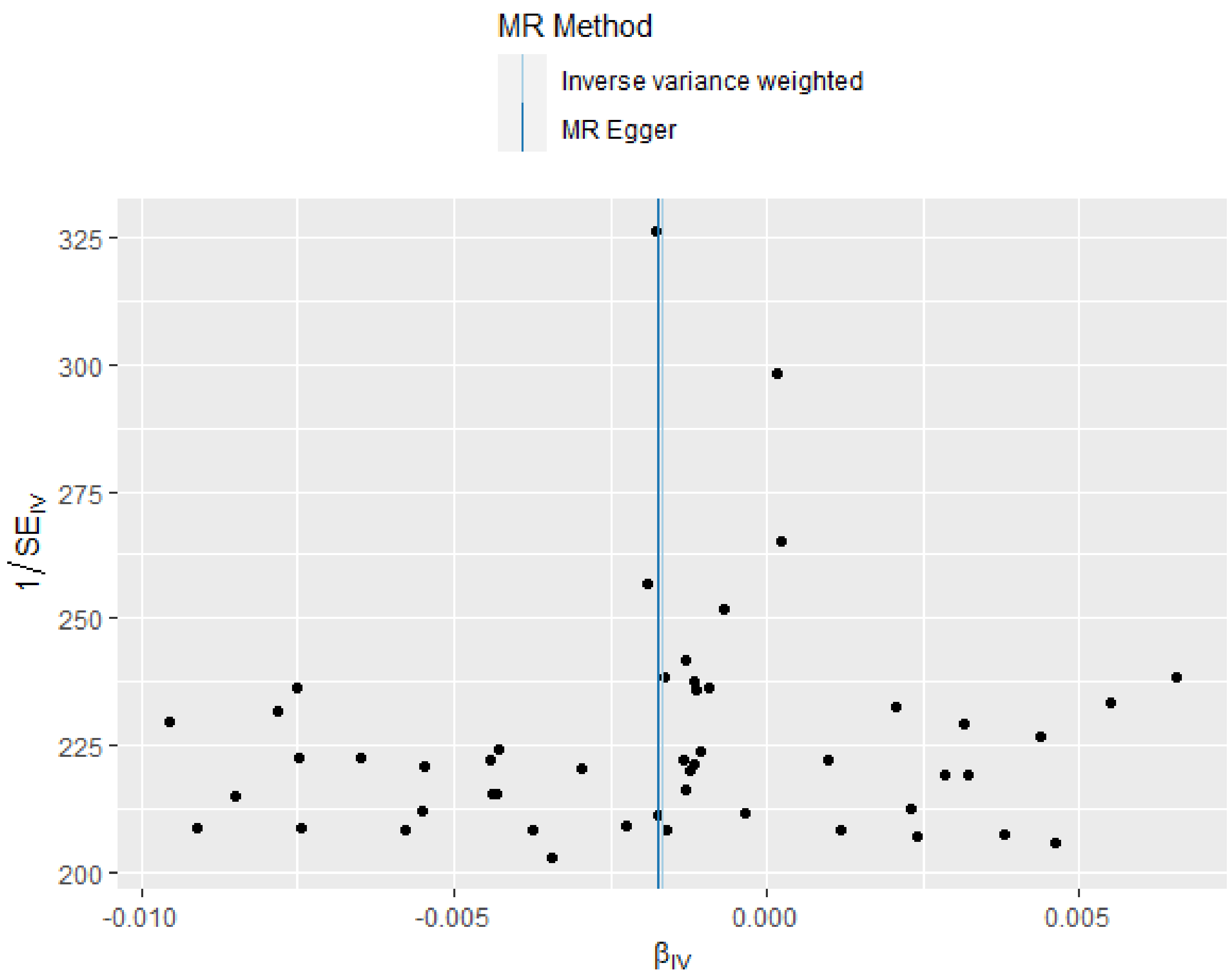

MR Method
Inverse variance weighted
MR Egger
325
300
275
250
225
200
$1/SE_{IV}$
-0.010
-0.005
0.000
0.005
β_{IV}

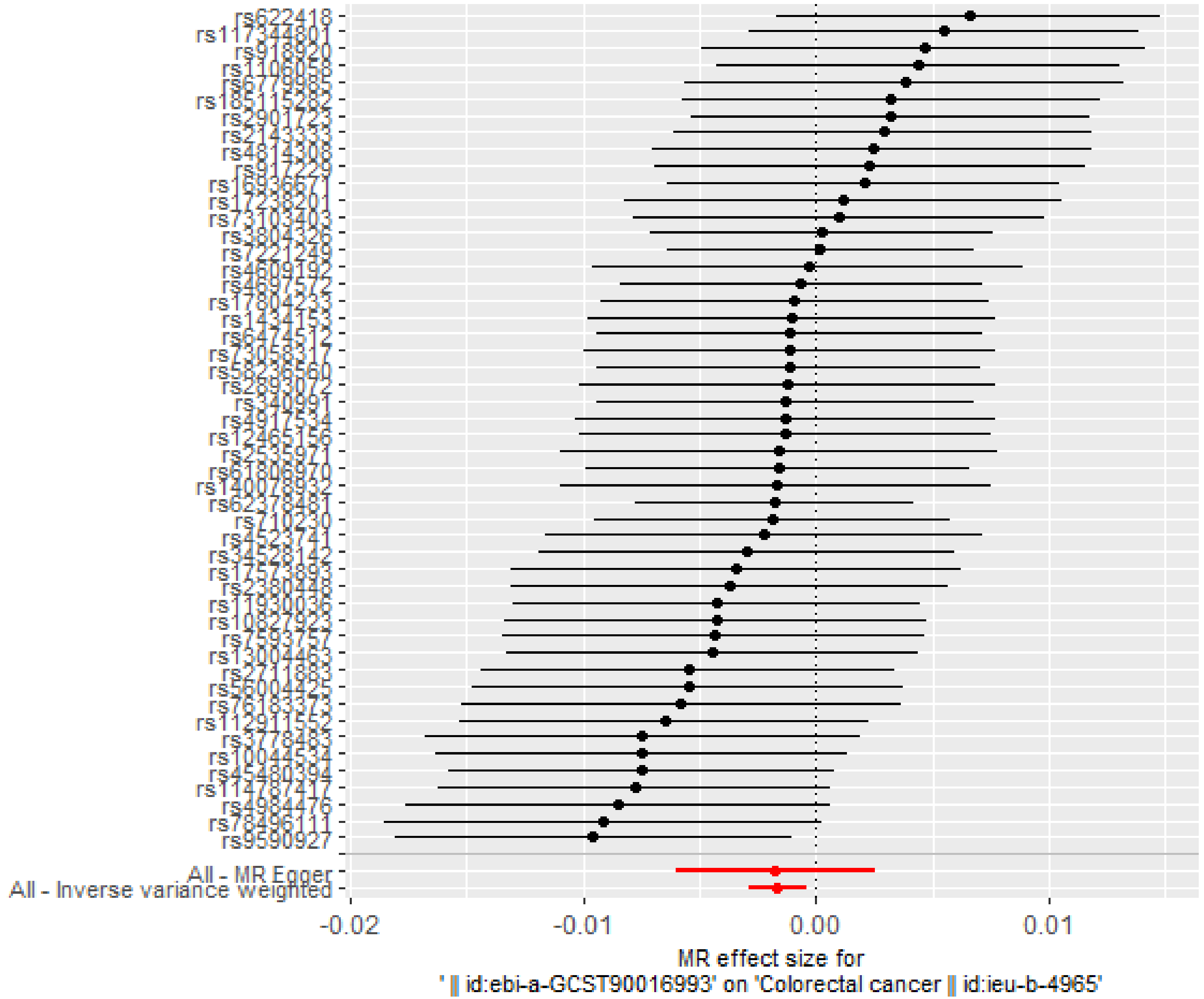

rs622418
rs117344801
rs918920
rs1106058
rs6779985
rs185115282
rs2901723
rs2143333
rs4814308
rs917229
rs16936671
rs17238201
rs73103403
rs3804326
rs7221249
rs4609192
rs4697572
rs17804253
rs1434153
rs6474512
rs73058317
rs58236560
rs2693072
rs340991
rs4917534
rs12465156
rs2535971
rs61806970
rs140078932
rs62378481
rs710230
rs4523741
rs34528142
rs17573893
rs2380448
rs11930036
rs10827923
rs7593757
rs13004463
rs2711883
rs56004425
rs76183373
rs112911552
rs3778483
rs10044534
rs45480394
rs114787417
rs4984476
rs78496111
rs9590927
All - MR Egger
All - Inverse variance weighted
-0.02
-0.01
0.00
0.01
MR effect size for
' || id:ebi-a-GCST90016993' on 'Colorectal cancer || id:ieu-b-4965'

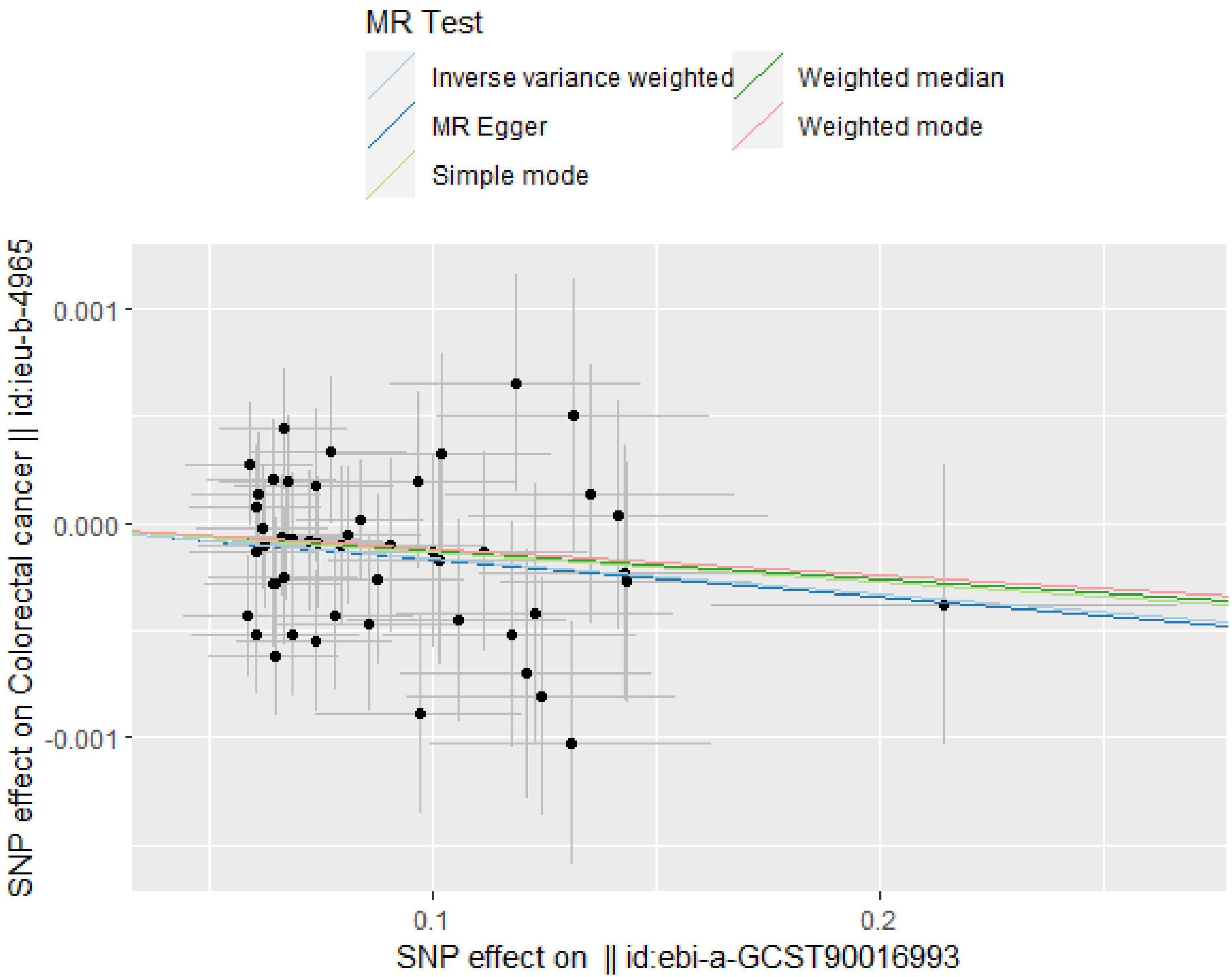

MR Test
Inverse variance weighted
MR Egger
Simple mode
Weighted median
Weighted mode
0.001
0.000
-0.001
0.1
0.2
SNP effect on Colorectal cancer || id:ieu-b-4965
SNP effect on || id:ebi-a-GCST90016993

Figure 135 Leave-one-out analysis, funnel plot, MR effect size and scatter plot for gut microbiota abundance (genus Eubacterium coprostanoligenes group id.11375) on colorectal cancer

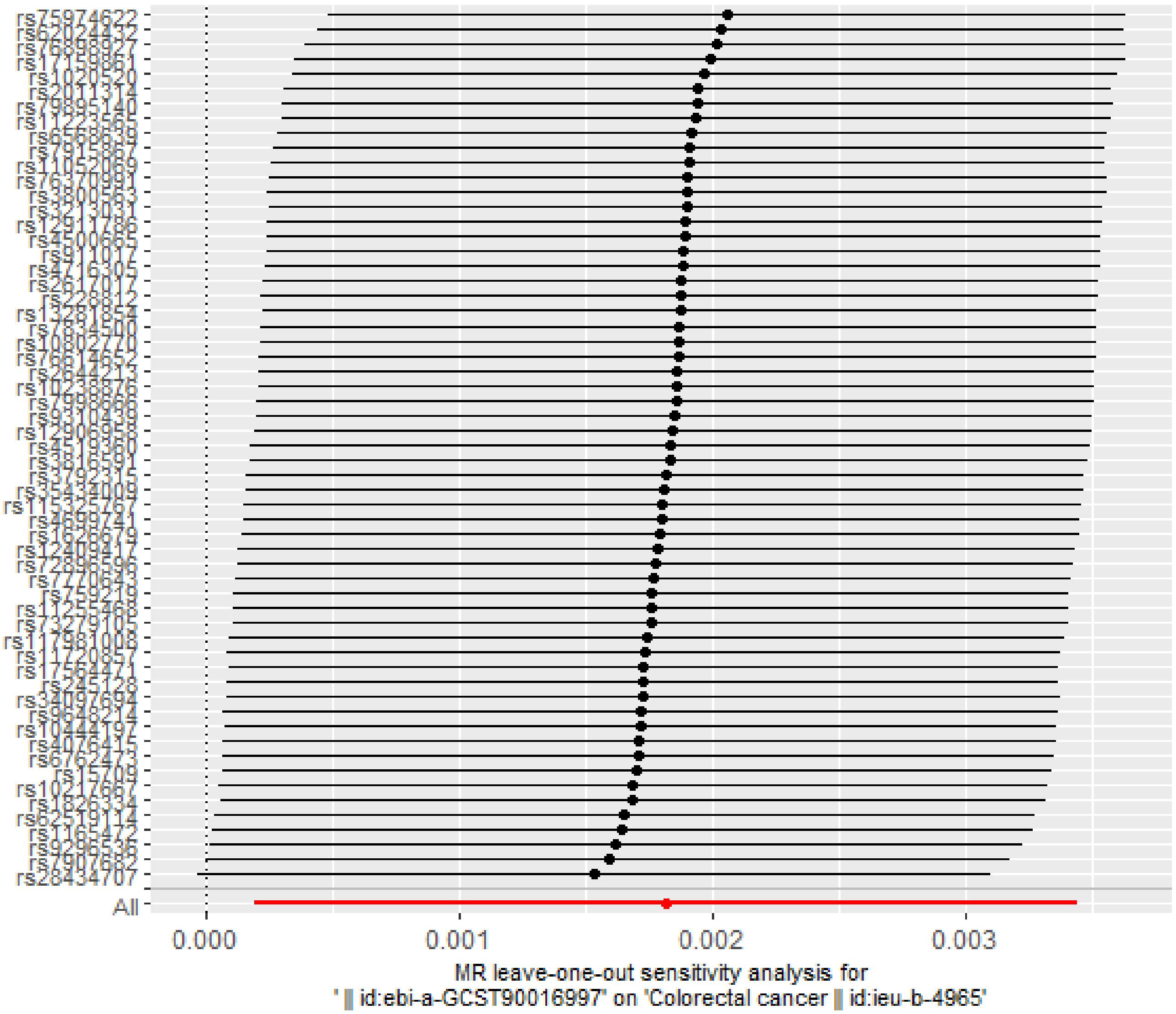

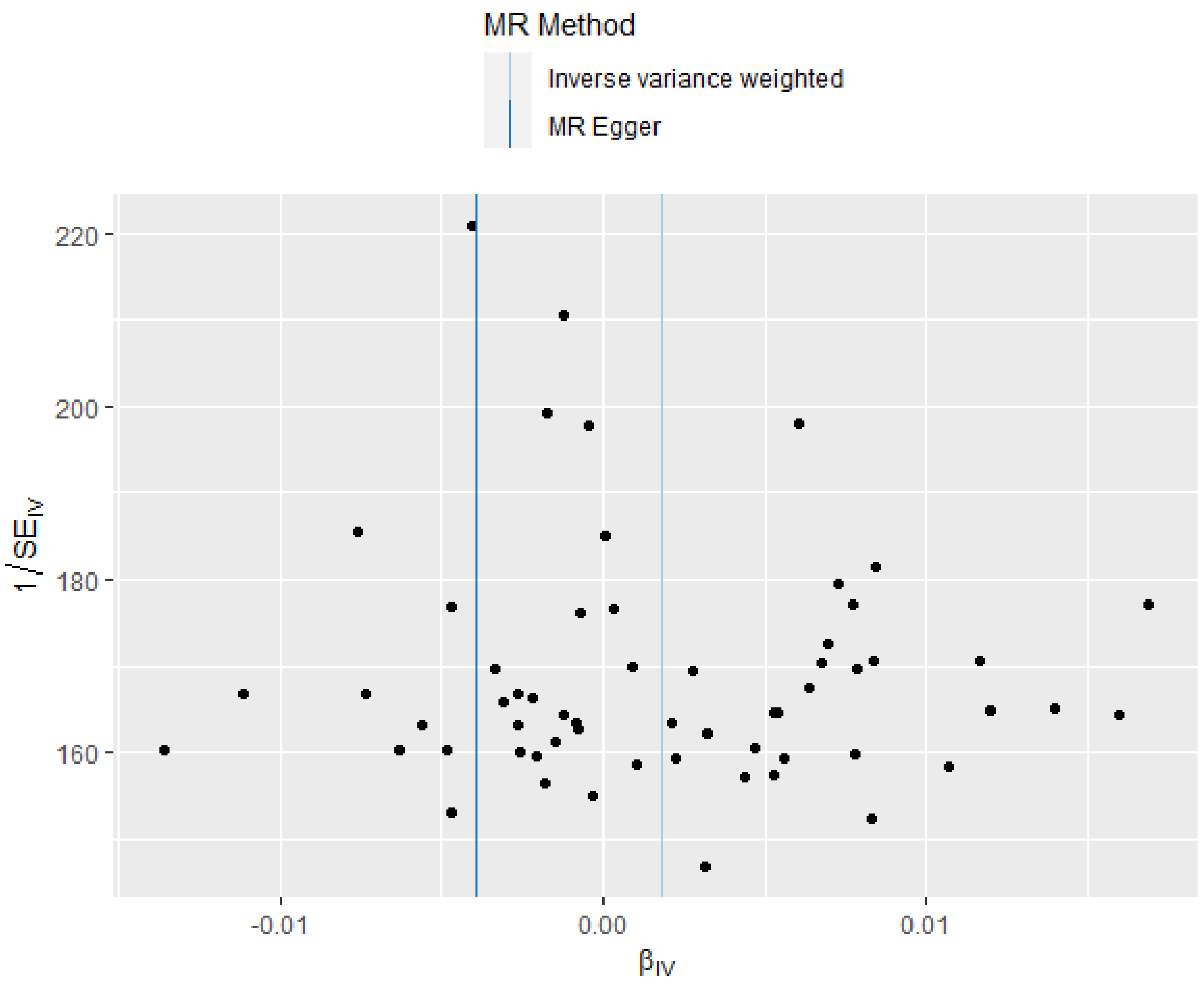

MR Method
Inverse variance weighted
MR Egger
220
200
180
160
1/SE_IV
-0.01
0.00
0.01
β_IV

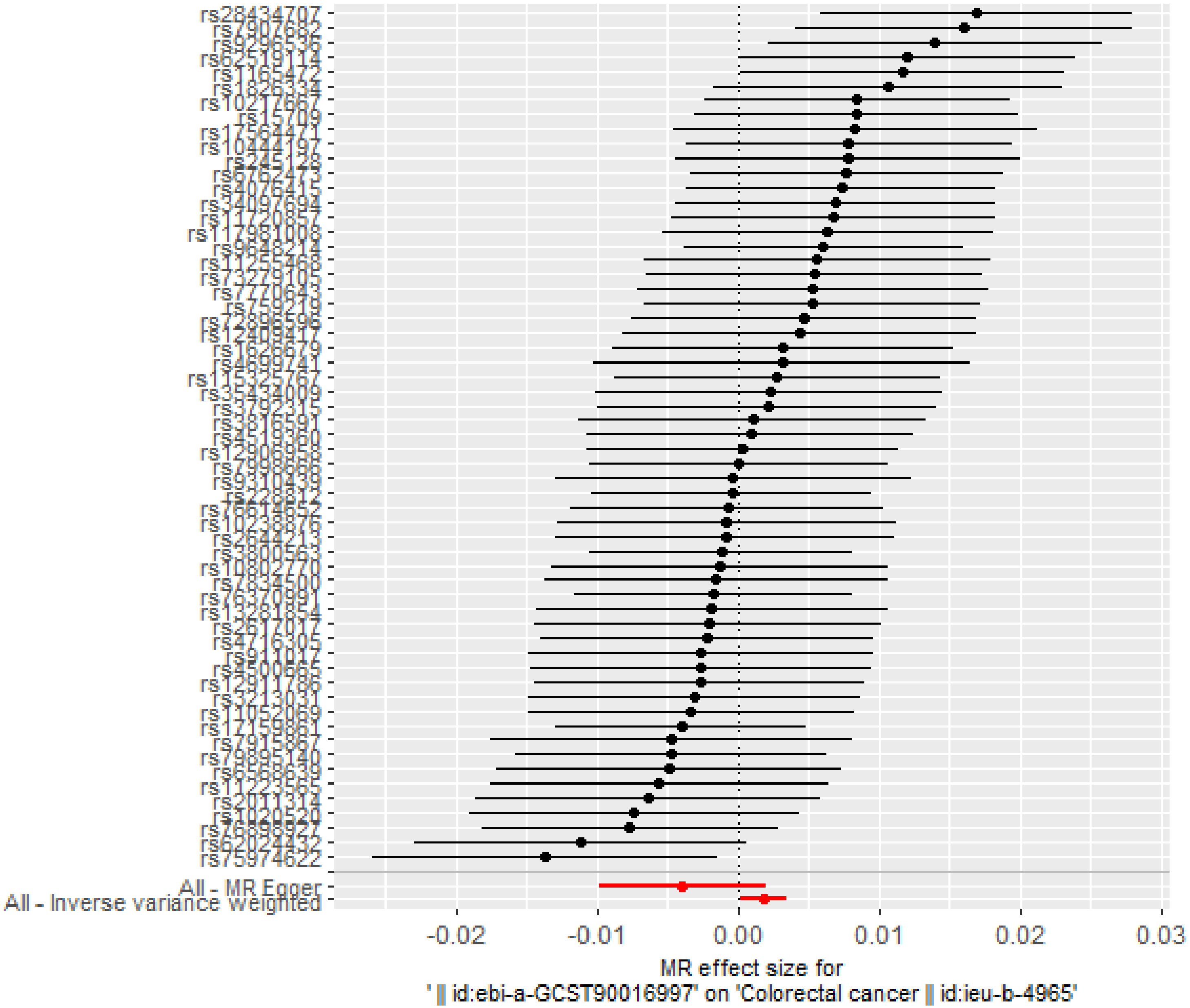
rs28434707
rs75974622
All - MR Egger
All - Inverse variance weighted
-0.02
-0.01
0.00
0.01
0.02
0.03
MR effect size for
' || id:ebi-a-GCST90016997' on 'Colorectal cancer || id:ieu-b-4965'

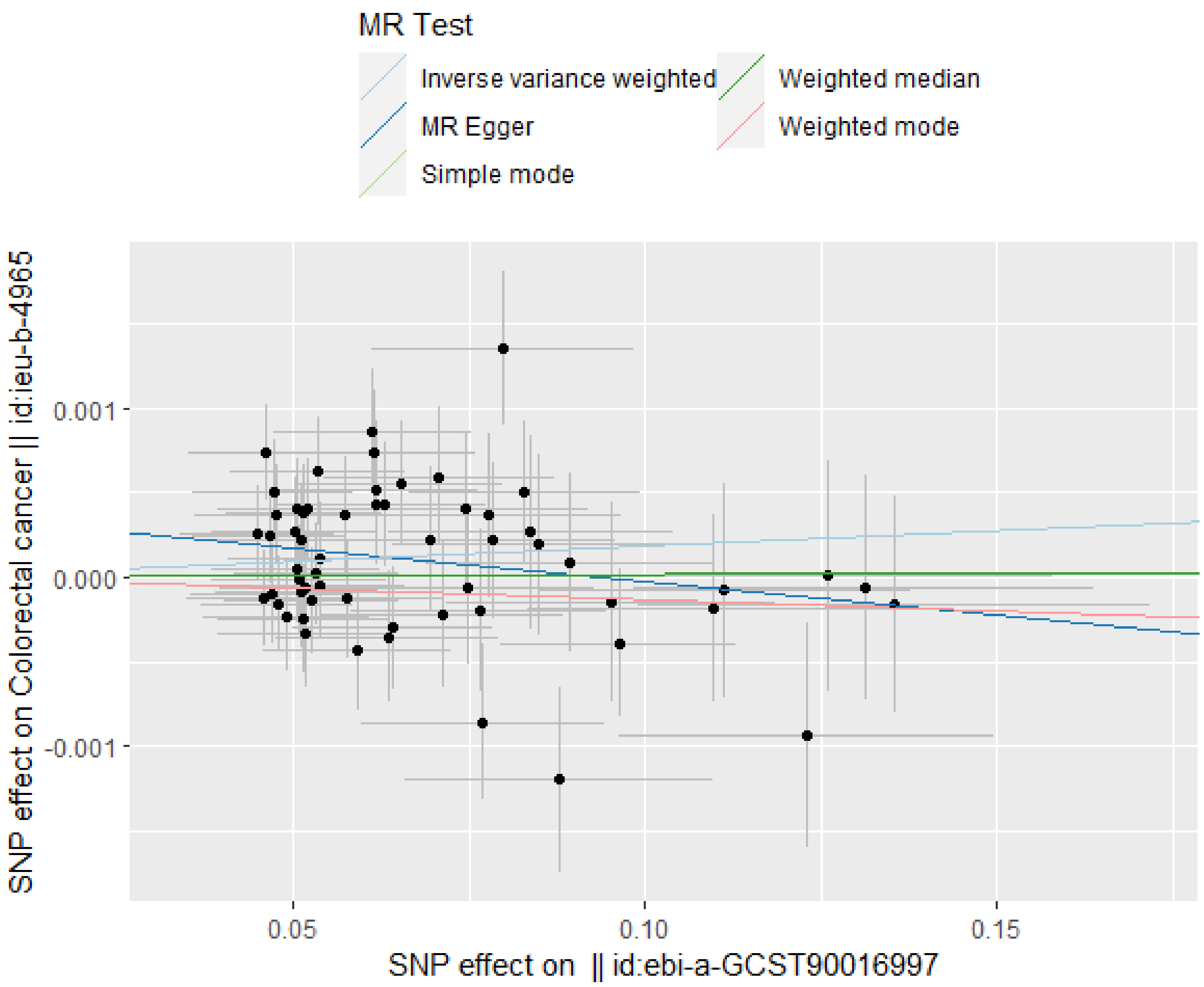

MR Test
Inverse variance weighted
MR Egger
Simple mode
Weighted median
Weighted mode
SNP effect on Colorectal cancer || id:ieu-b-4965
0.001
0.000
-0.001
0.05
0.10
0.15
SNP effect on || id:ebi-a-GCST90016997

Figure 136 Leave-one-out analysis, funnel plot, MR effect size and scatter plot for gut microbiota abundance (genus Fusicatenibacter id.11305) on colorectal cancer

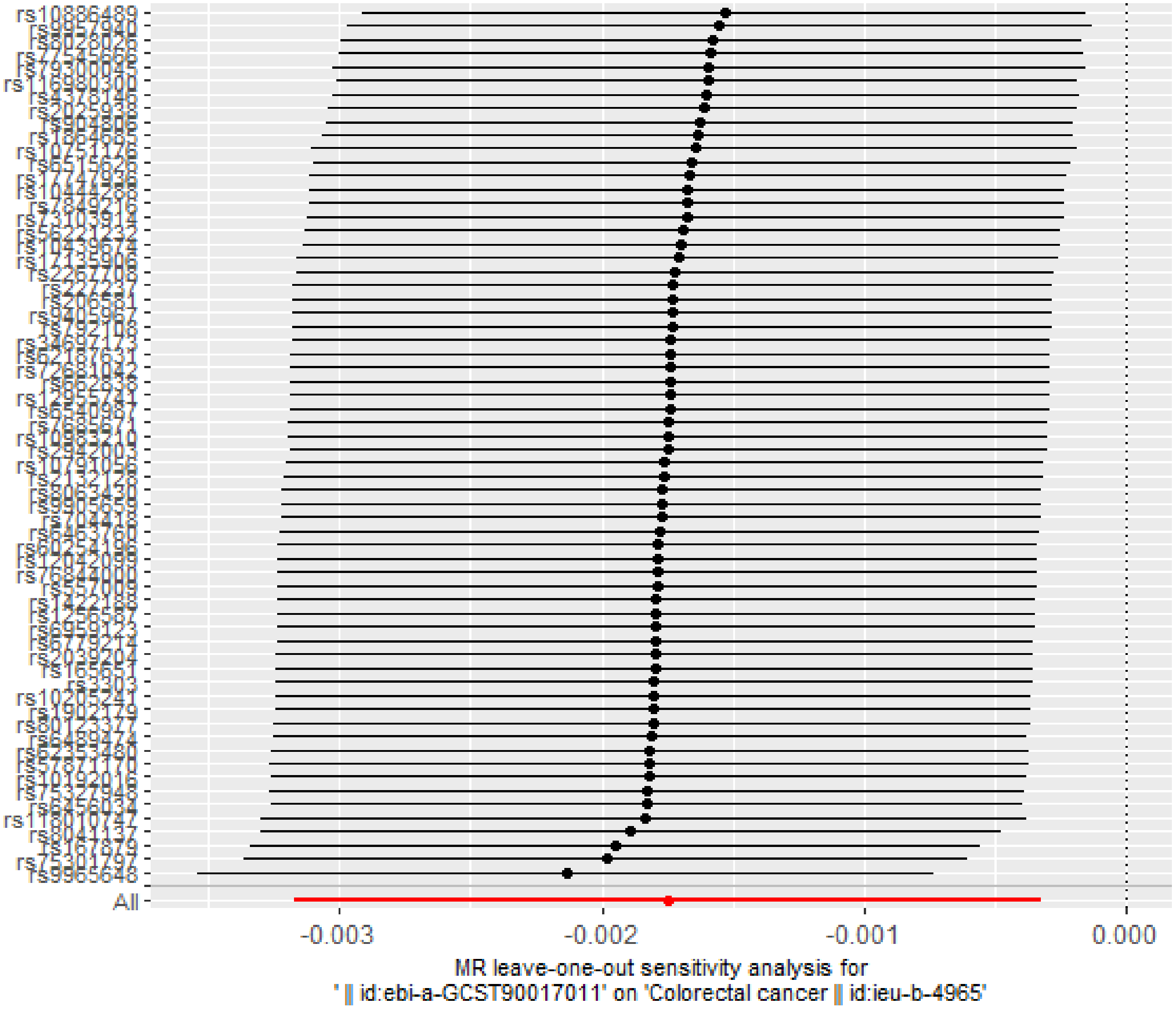

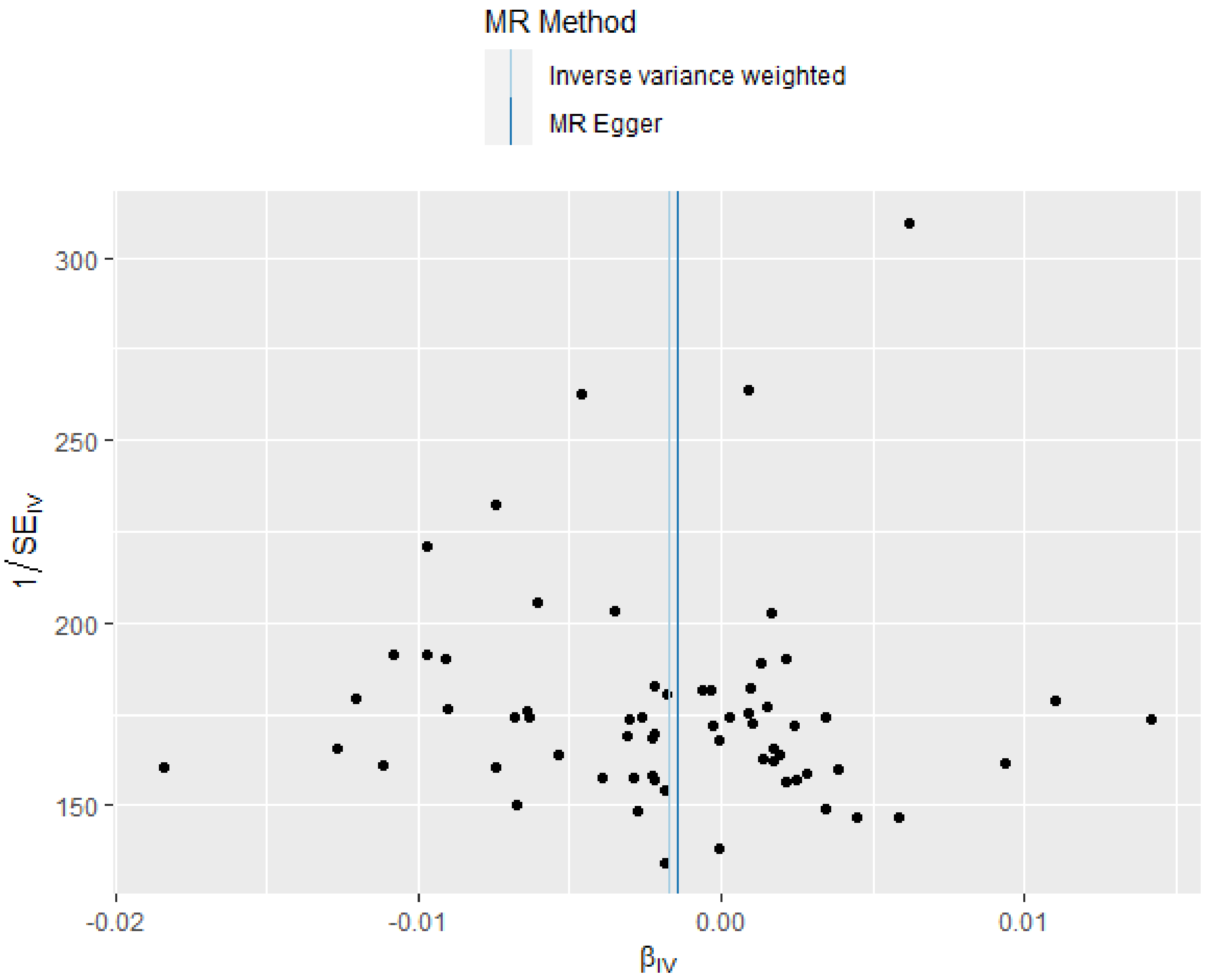

MR Method
Inverse variance weighted
MR Egger
300
250
200
150
1/SE_IV
-0.02
-0.01
0.00
0.01
β_IV

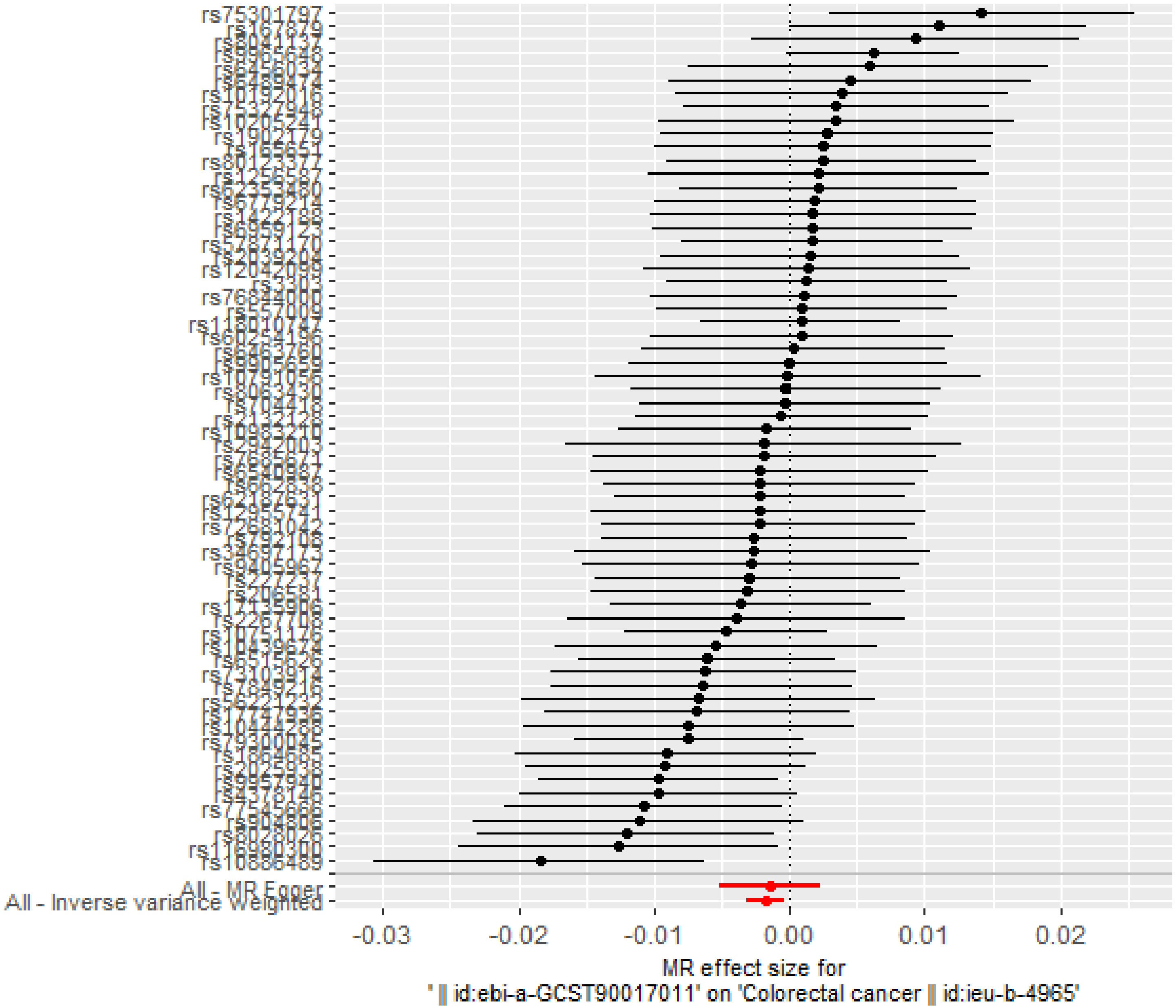
rs75301797
All - MR Egger
All - Inverse variance weighted
-0.03
-0.02
-0.01
0.00
0.01
0.02
MR effect size for
' || id:ebi-a-GCST90017011' on 'Colorectal cancer || id:ieu-b-4965'

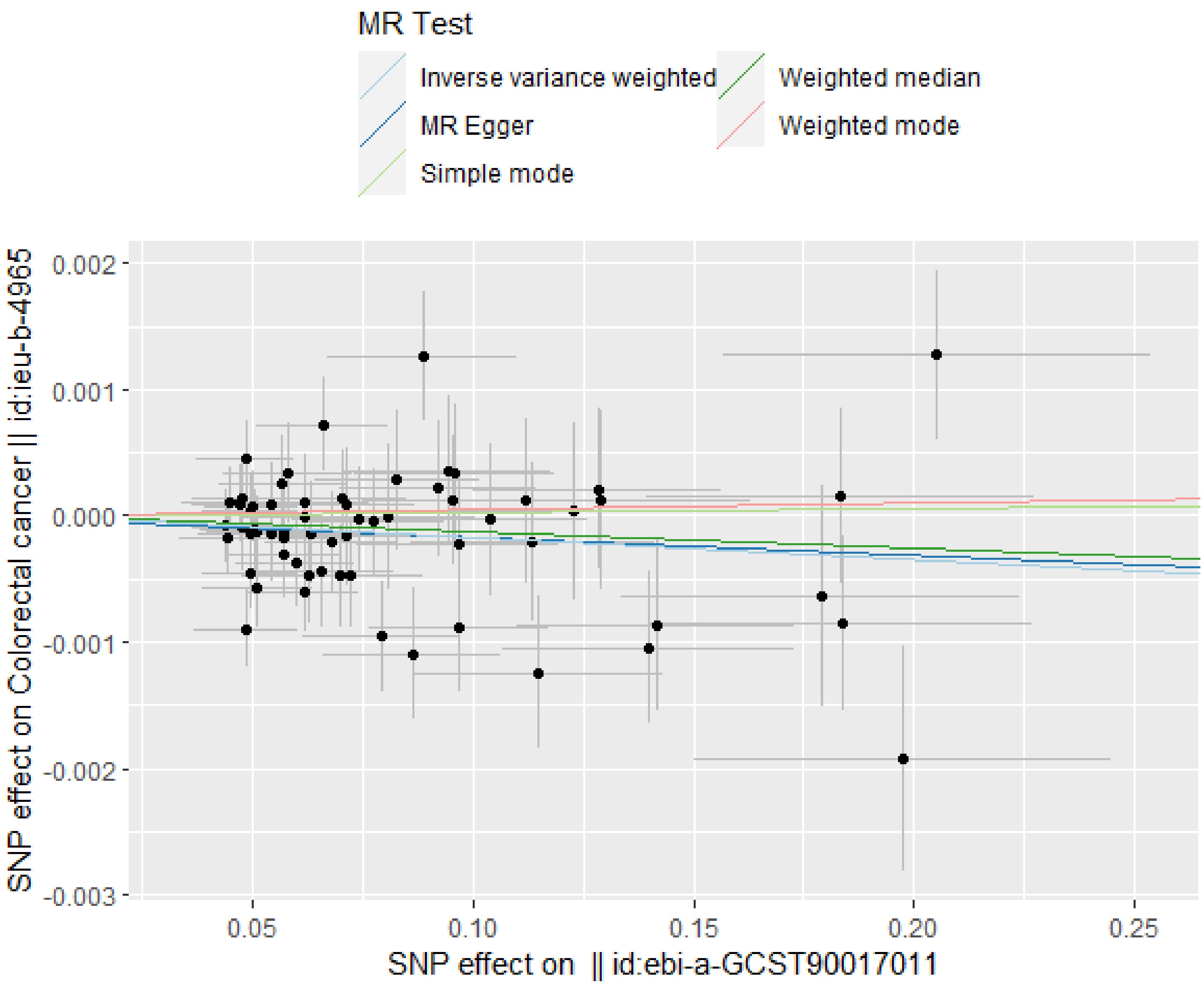
MR Test
Inverse variance weighted
MR Egger
Simple mode
Weighted median
Weighted mode
0.002
0.001
0.000
-0.001
-0.002
-0.003
SNP effect on Colorectal cancer || id:ieu-b-4965
0.05
0.10
0.15
0.20
0.25
SNP effect on || id:ebi-a-GCST90017011

Figure 137 Leave-one-out analysis, funnel plot, MR effect size and scatter plot for gut microbiota abundance (genus Methanobrevibacter id.123) on colorectal cancer

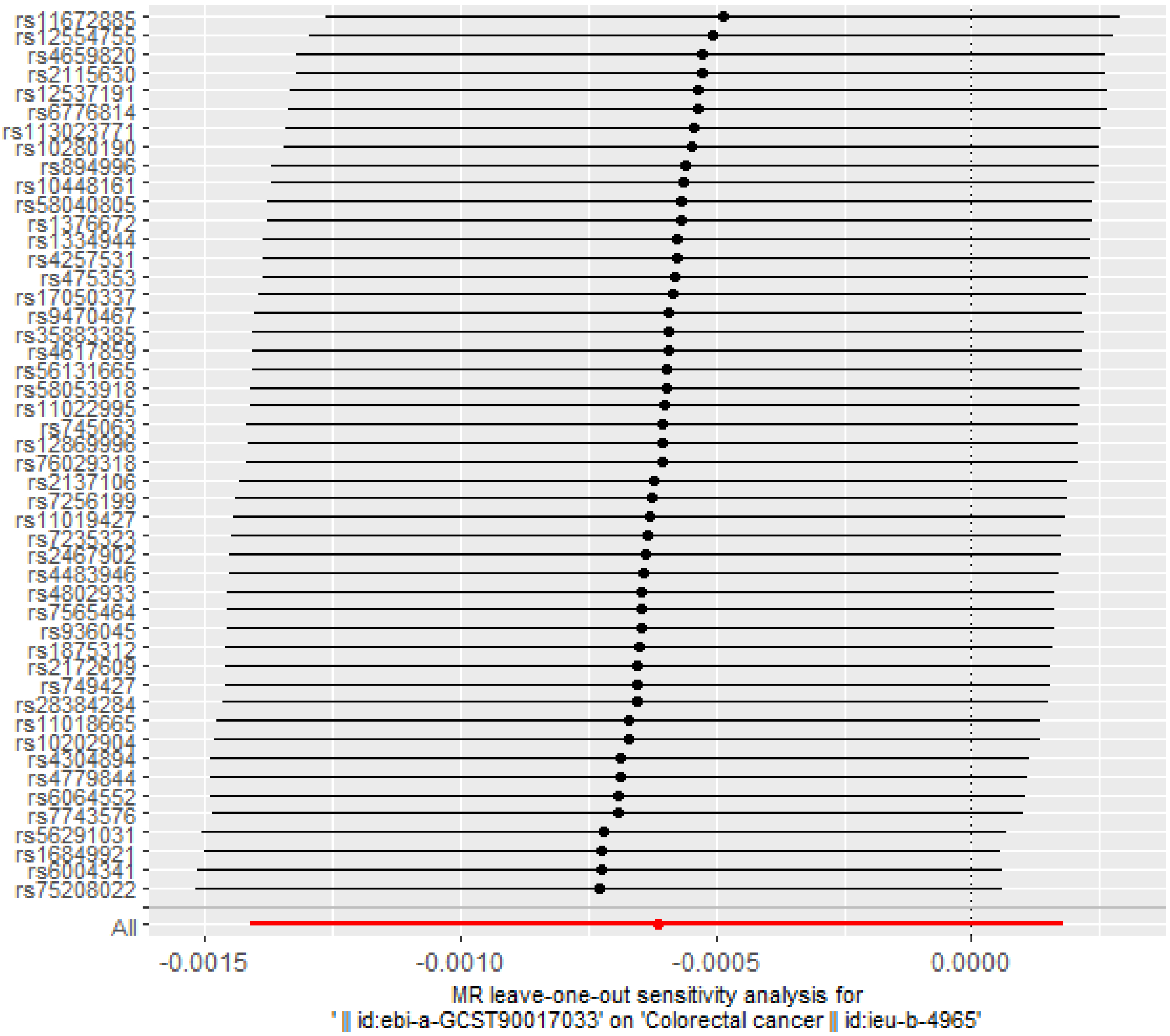

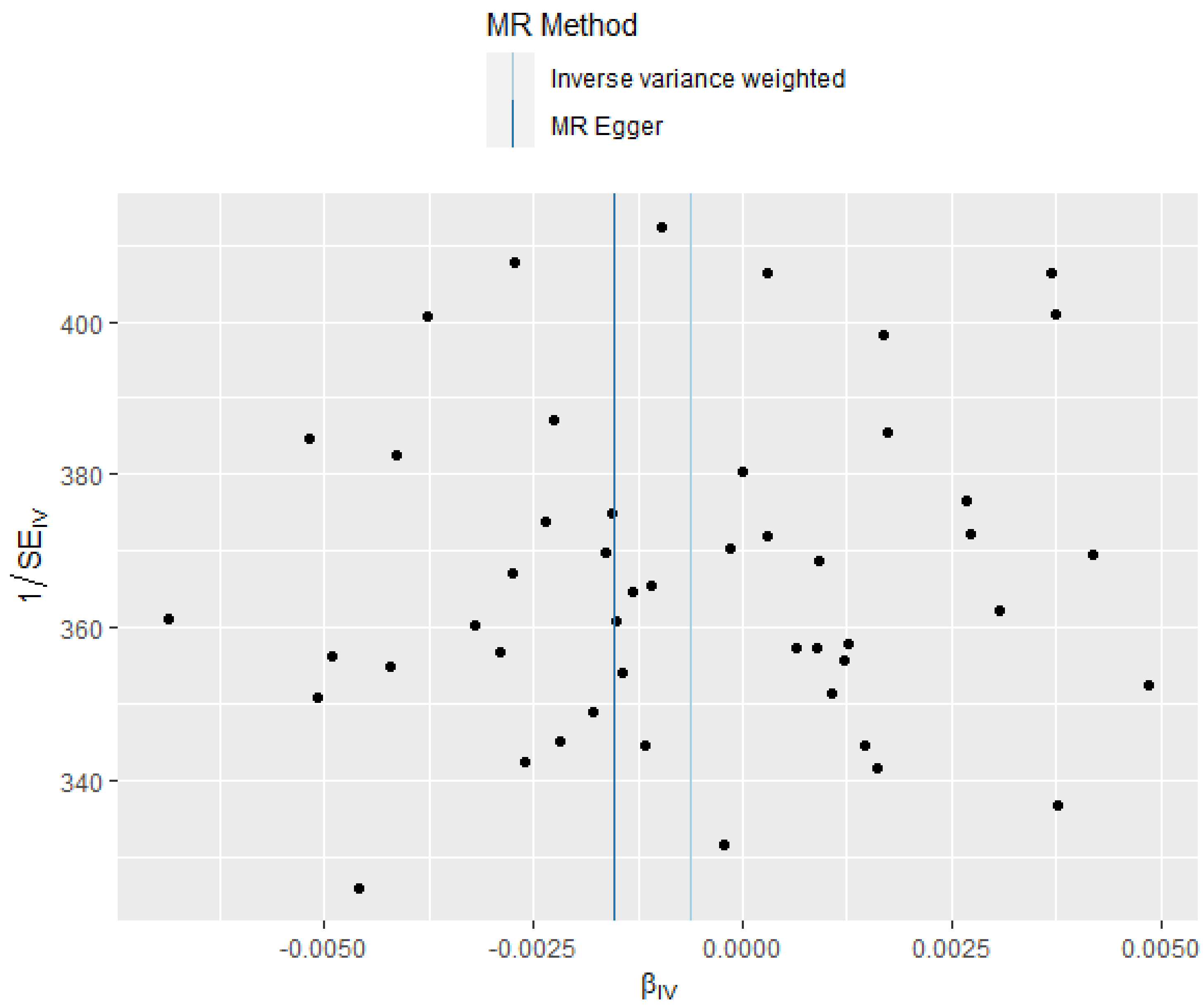
MR Method
Inverse variance weighted
MR Egger
400
380
360
340
1/SE_IV
-0.0050
-0.0025
0.0000
0.0025
0.0050
β_IV

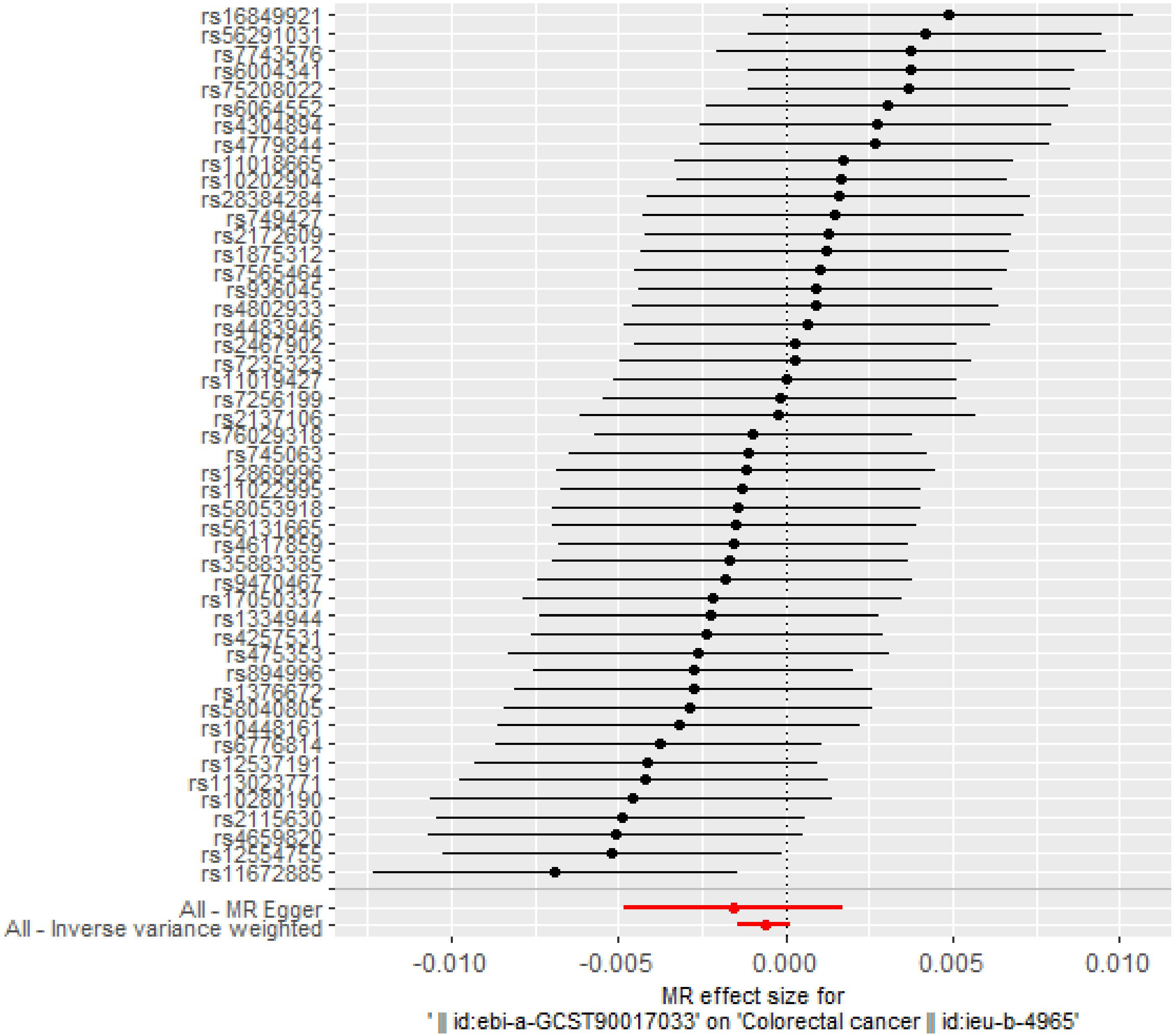
rs16849921
rs56291031
rs7743576
rs6004341
rs75208022
rs6064552
rs4304894
rs4779844
rs11018665
rs10202904
rs28384284
rs749427
rs2172609
rs1875312
rs7565464
rs936045
rs4802933
rs4483946
rs2467902
rs7235323
rs11019427
rs7256199
rs2137106
rs76029318
rs745063
rs12869996
rs11022995
rs58053918
rs56131665
rs4617859
rs35883385
rs9470467
rs17050337
rs1334944
rs4257531
rs475353
rs894996
rs1376672
rs58040805
rs10448161
rs6776814
rs12537191
rs113023771
rs10280190
rs2115630
rs4659820
rs12554755
rs11672885
All - MR Egger
All - Inverse variance weighted
-0.010
-0.005
0.000
0.005
0.010
MR effect size for
' || id:ebi-a-GCST90017033' on 'Colorectal cancer || id:ieu-b-4965'

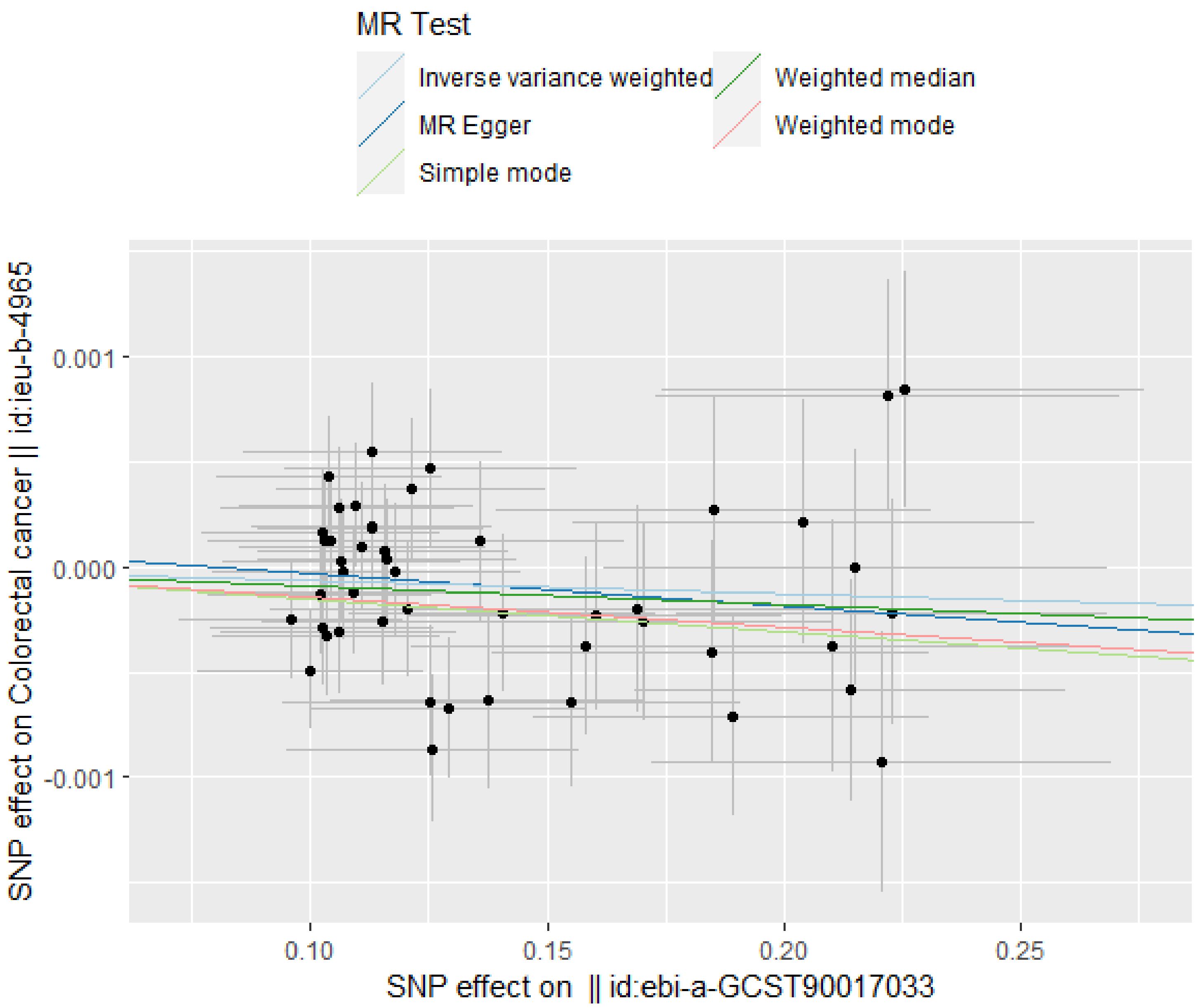

MR Test
Inverse variance weighted
MR Egger
Simple mode
Weighted median
Weighted mode
0.001
0.000
-0.001
SNP effect on Colorectal cancer || id:ieu-b-4965
0.10
0.15
0.20
0.25
SNP effect on || id:ebi-a-GCST90017033

Figure 138 Leave-one-out analysis, funnel plot, MR effect size and scatter plot for gut microbiota abundance (genus Ruminococcaceae UCG002 id.11360) on colorectal cancer

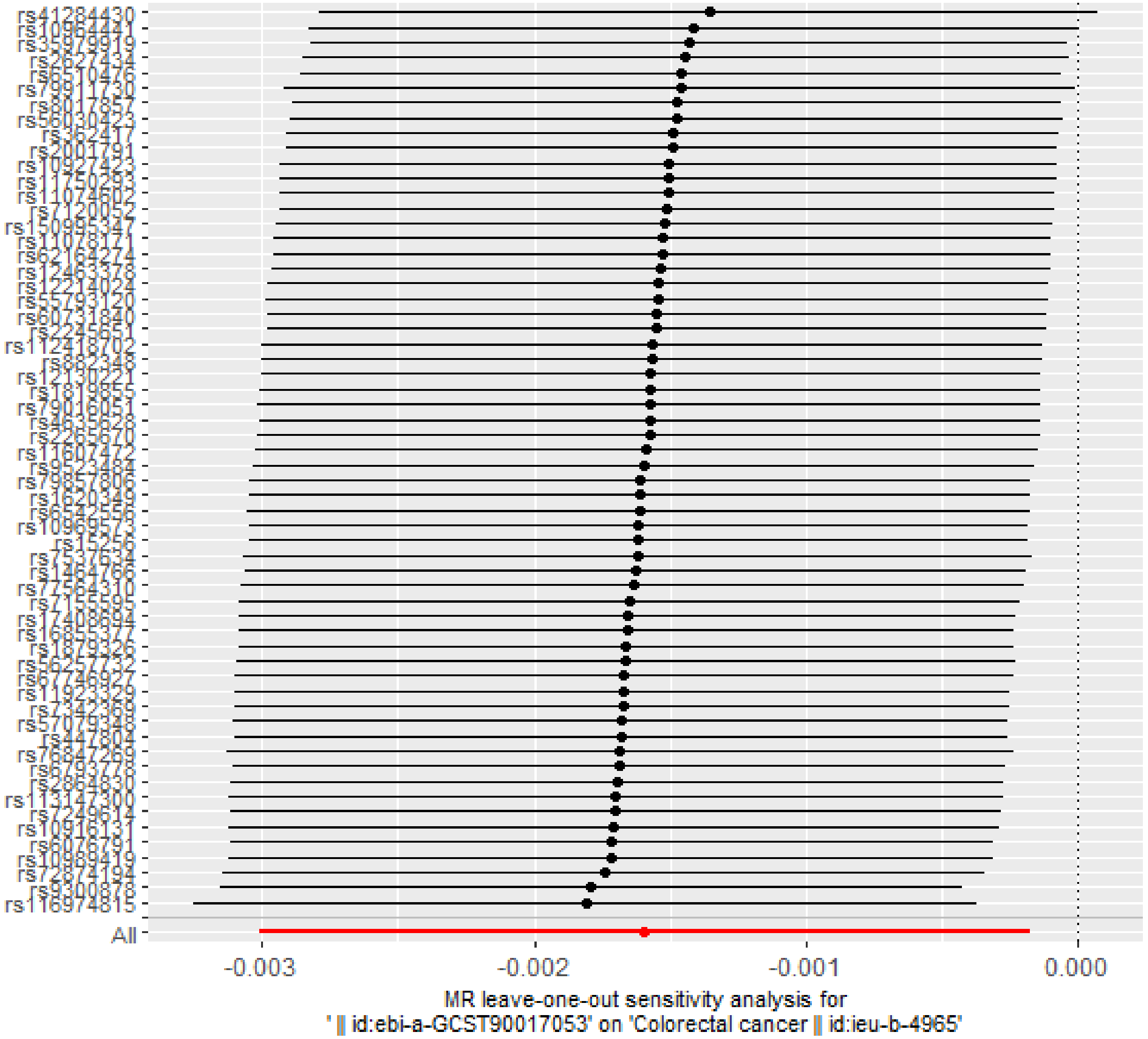

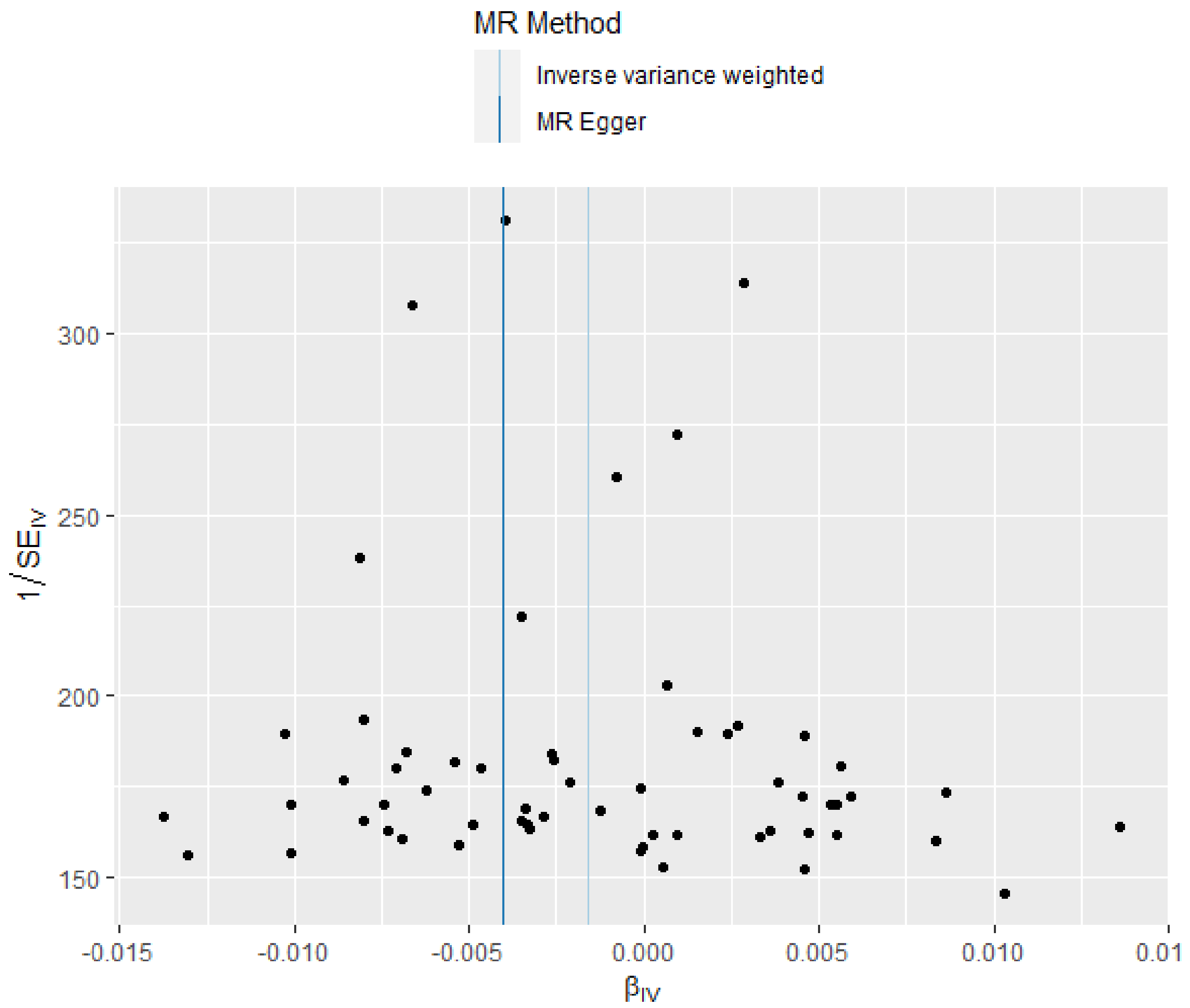

MR Method
Inverse variance weighted
MR Egger
300
250
200
150
1/SE$_{IV}$
-0.015
-0.010
-0.005
0.000
0.005
0.010
0.01
β$_{IV}$

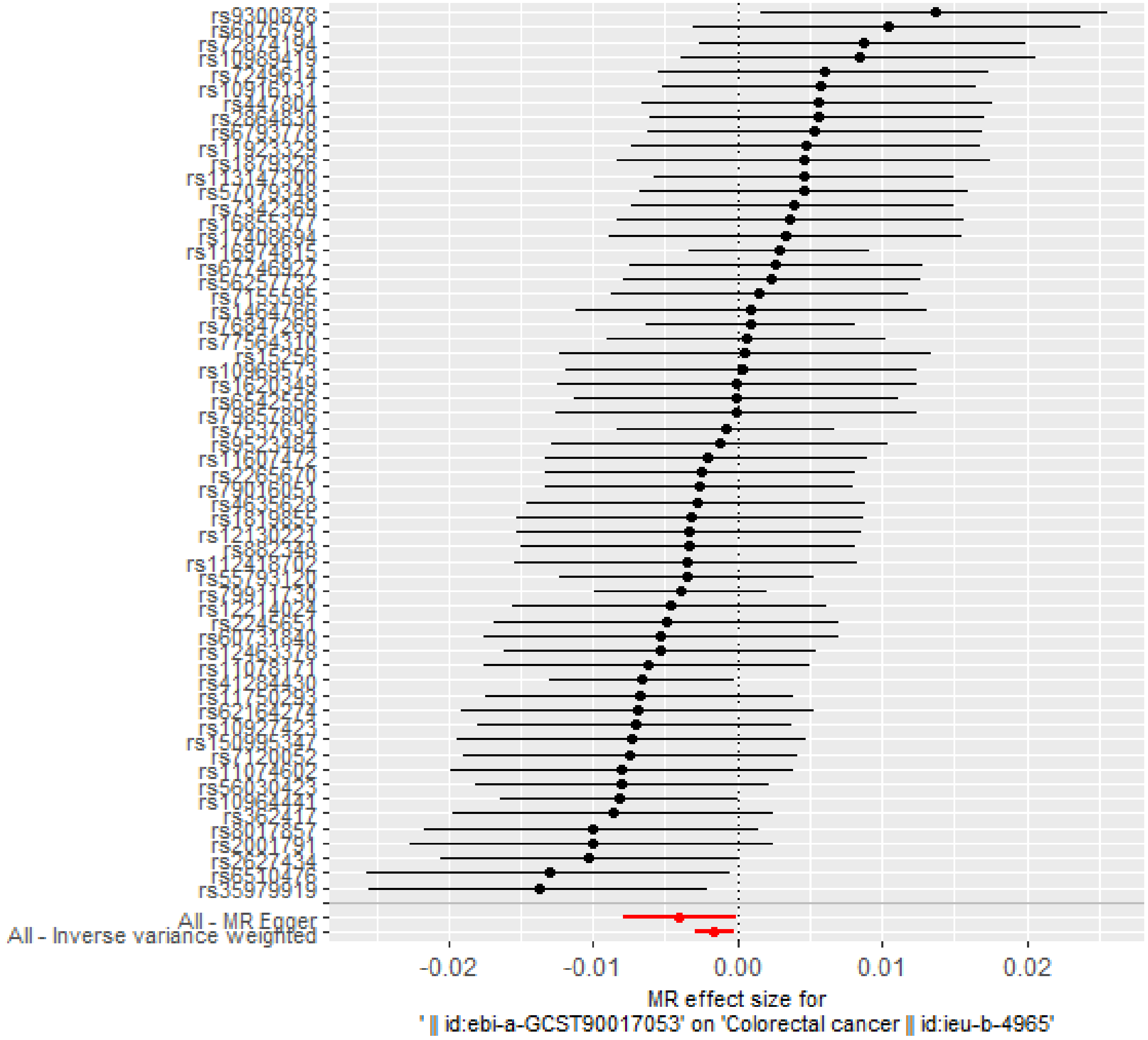
All - MR Egger
All - Inverse variance weighted
-0.02
-0.01
0.00
0.01
0.02
MR effect size for
' || id:ebi-a-GCST90017053' on 'Colorectal cancer || id:ieu-b-4965'

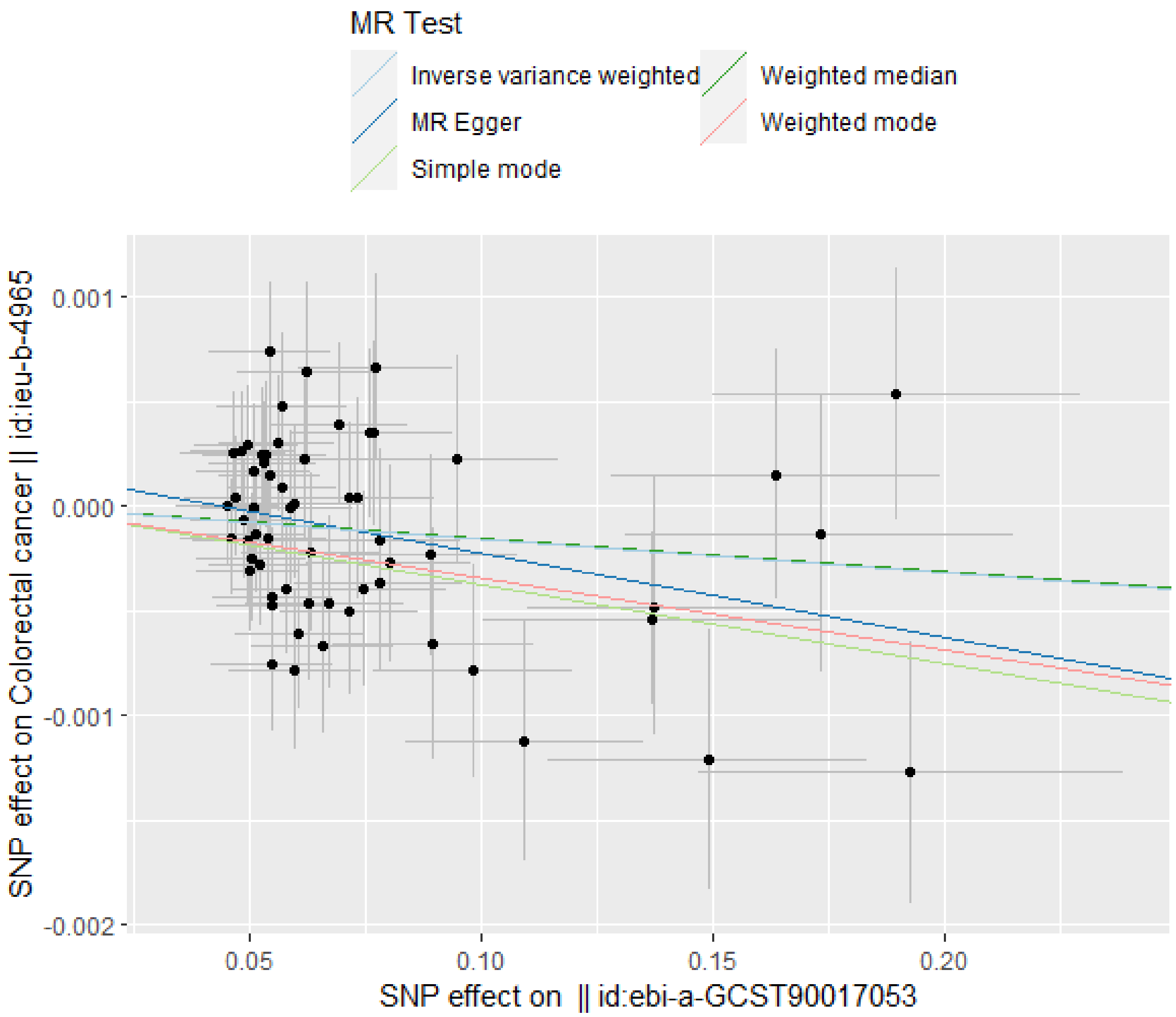

MR Test
Inverse variance weighted
MR Egger
Simple mode
Weighted median
Weighted mode
0.001
0.000
-0.001
-0.002
0.05
0.10
0.15
0.20
SNP effect on Colorectal cancer || id:ieu-b-4965
SNP effect on || id:ebi-a-GCST90017053

Figure 139 Leave-one-out analysis, funnel plot, MR effect size and scatter plot for gut microbiota abundance (order Enterobacteriales id.3468) on colorectal cancer

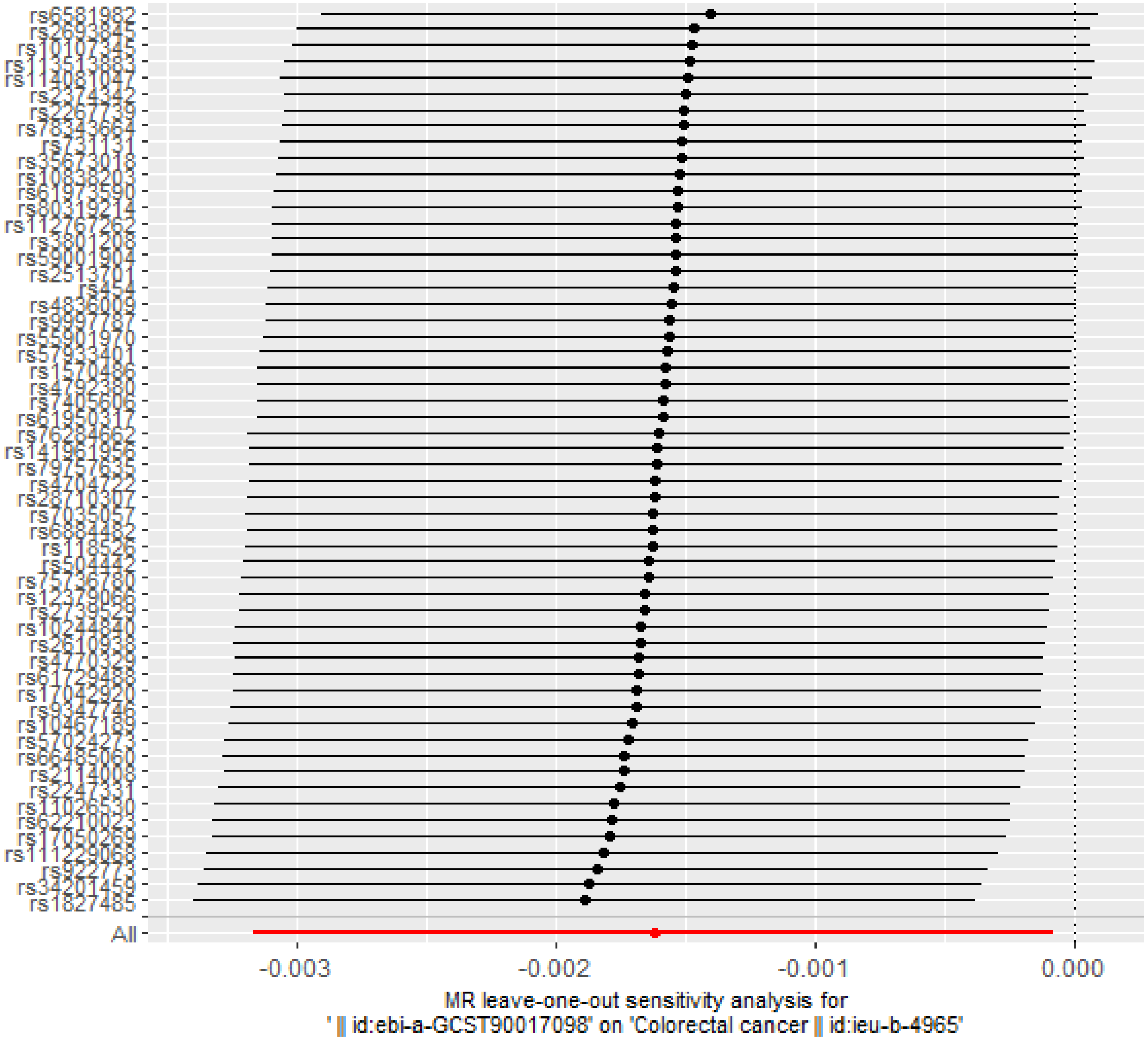

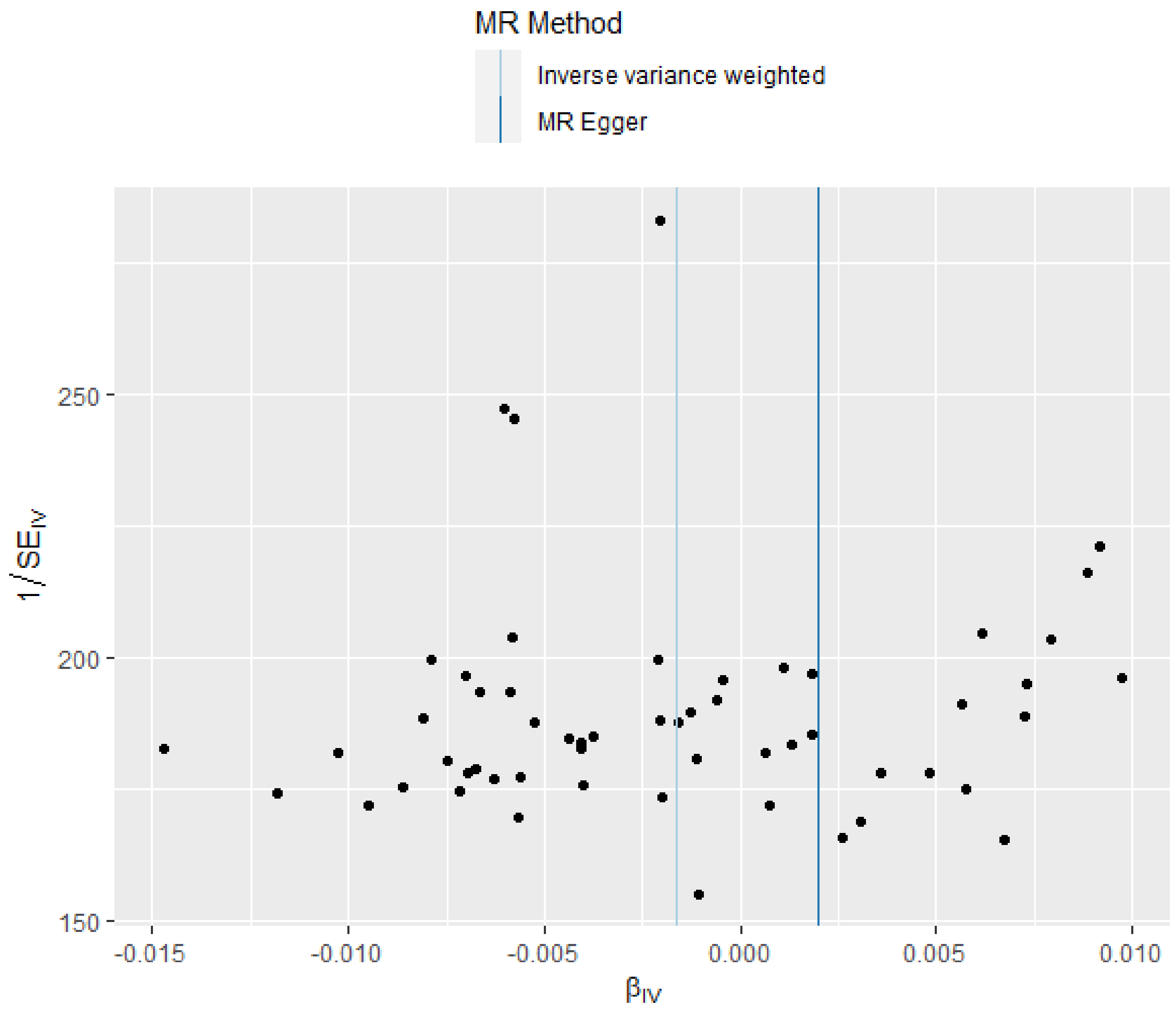

MR Method
Inverse variance weighted
MR Egger
250
200
150
$1/SE_{IV}$
-0.015
-0.010
-0.005
0.000
0.005
0.010
β_{IV}

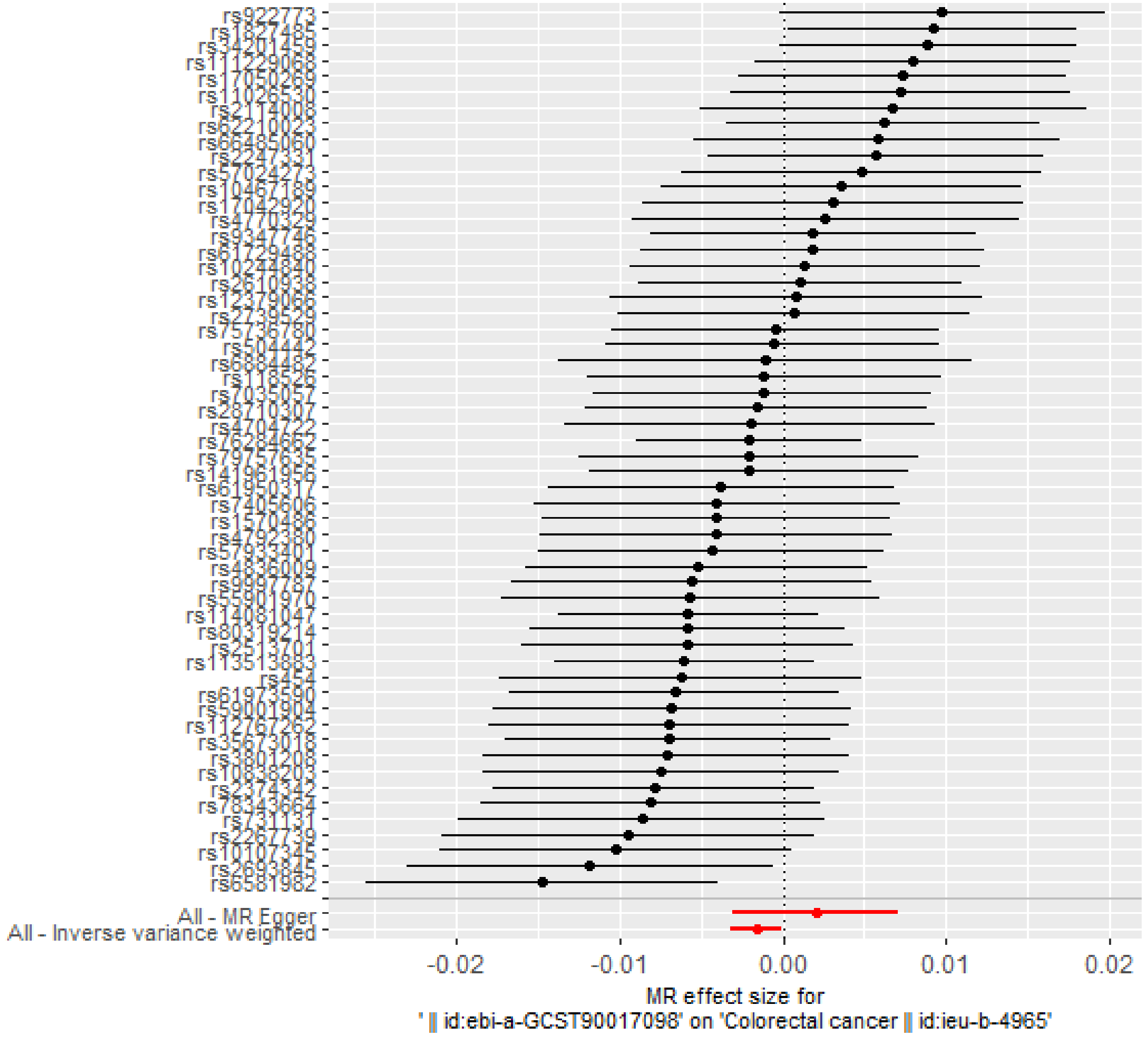

rs922773
rs1827485
rs34201459
rs111229068
rs17050269
rs11026530
rs2114008
rs62210023
rs66485060
rs2247331
rs57024273
rs10467189
rs17042920
rs4770329
rs9347746
rs61729438
rs10244840
rs2610938
rs12279058
rs2739529
rs75736780
rs504442
rs6884482
rs118526
rs7035057
rs28710307
rs4704722
rs76284662
rs79757635
rs141961956
rs61950317
rs7405606
rs1570486
rs4792380
rs57932401
rs4836009
rs9997787
rs55901970
rs114081047
rs80319214
rs2513701
rs113513883
rs454
rs61973590
rs59001904
rs112767262
rs35673018
rs3801209
rs10838209
rs2374342
rs78343664
rs731131
rs2267739
rs10107345
rs2693845
rs6581982
All - MR Egger
All - Inverse variance weighted
-0.02
-0.01
0.00
0.01
0.02
MR effect size for
' || id:ebi-a-GCST90017098' on 'Colorectal cancer || id:ieu-b-4965'

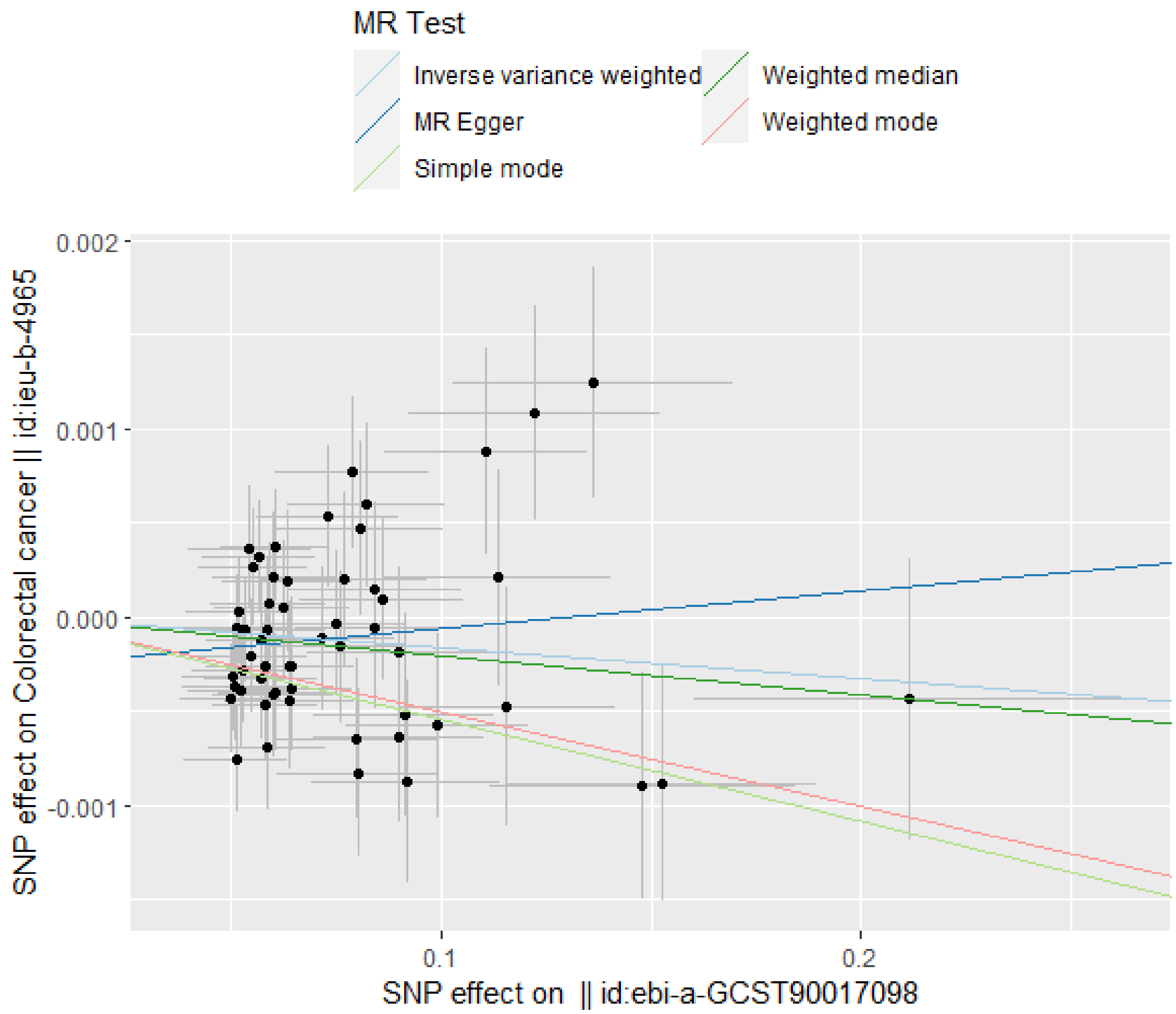

MR Test
Inverse variance weighted
MR Egger
Simple mode
Weighted median
Weighted mode
0.002
0.001
0.000
-0.001
SNP effect on Colorectal cancer || id:ieu-b-4965
0.1
0.2
SNP effect on || id:ebi-a-GCST90017098

Figure 140 Leave-one-out analysis, funnel plot, MR effect size and scatter plot for gut microbiota abundance (phylum Proteobacteria id.2375) on colorectal cancer

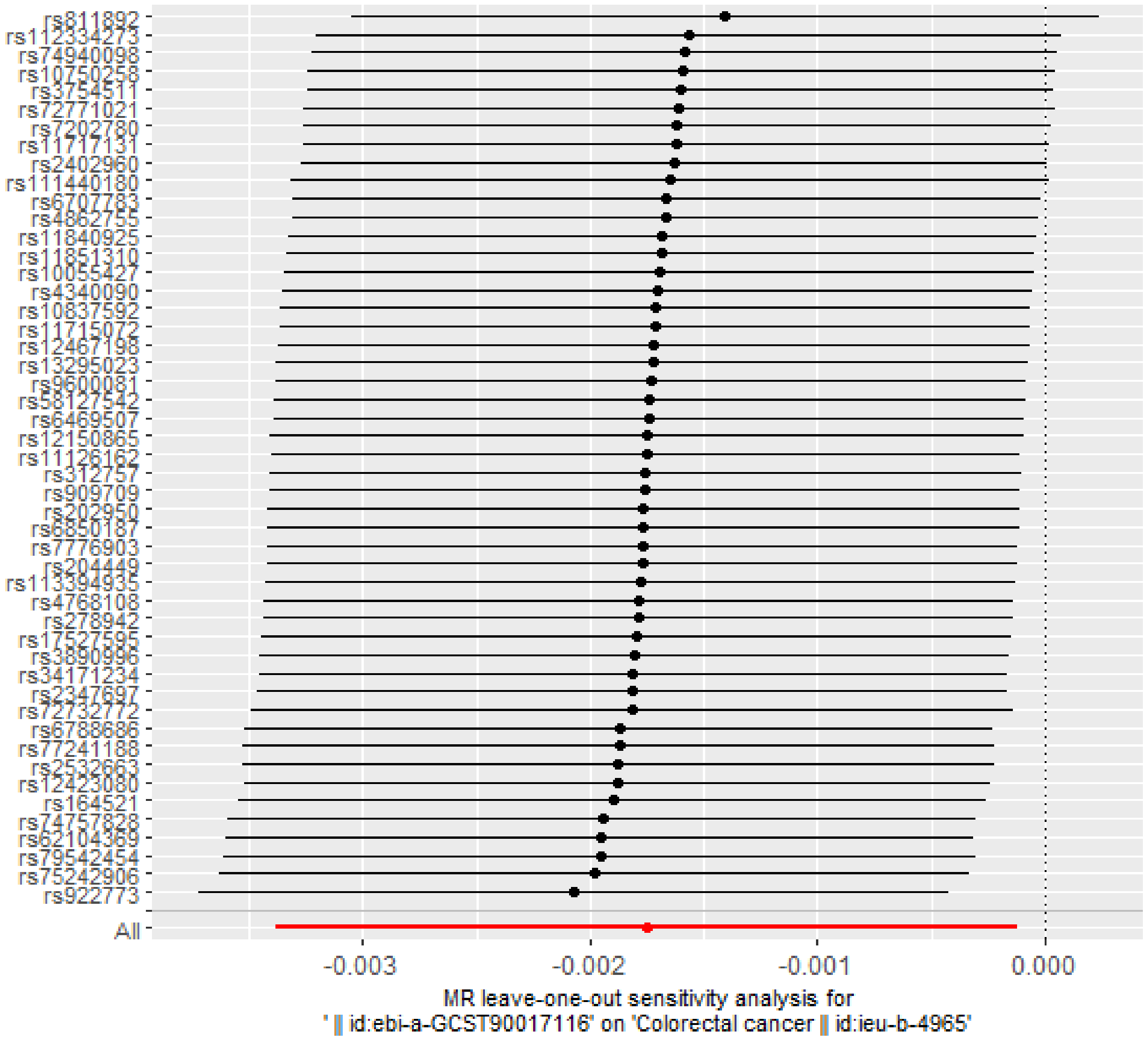

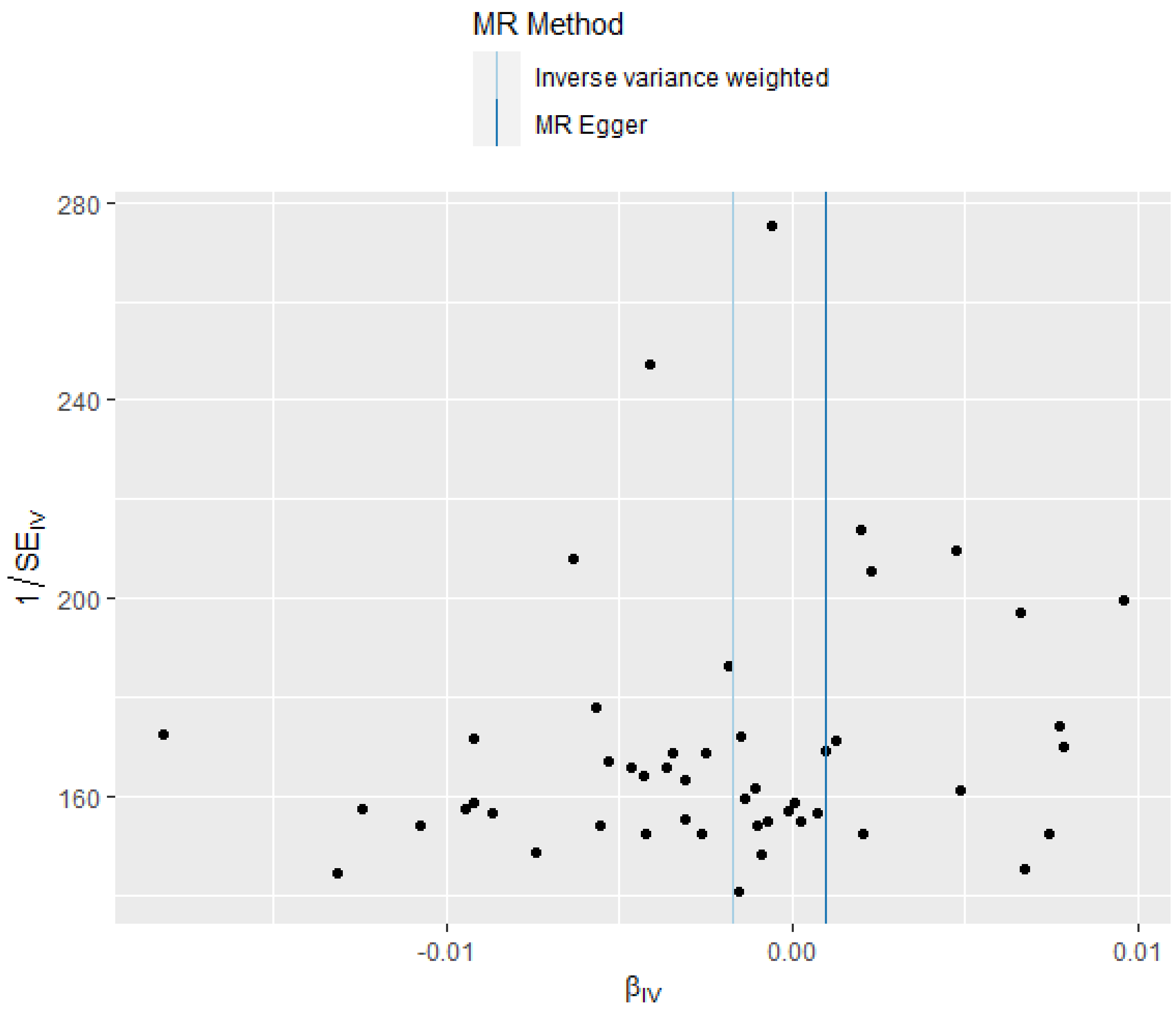

MR Method
Inverse variance weighted
MR Egger
280
240
200
160
1/SE_IV
-0.01
0.00
0.01
β_IV

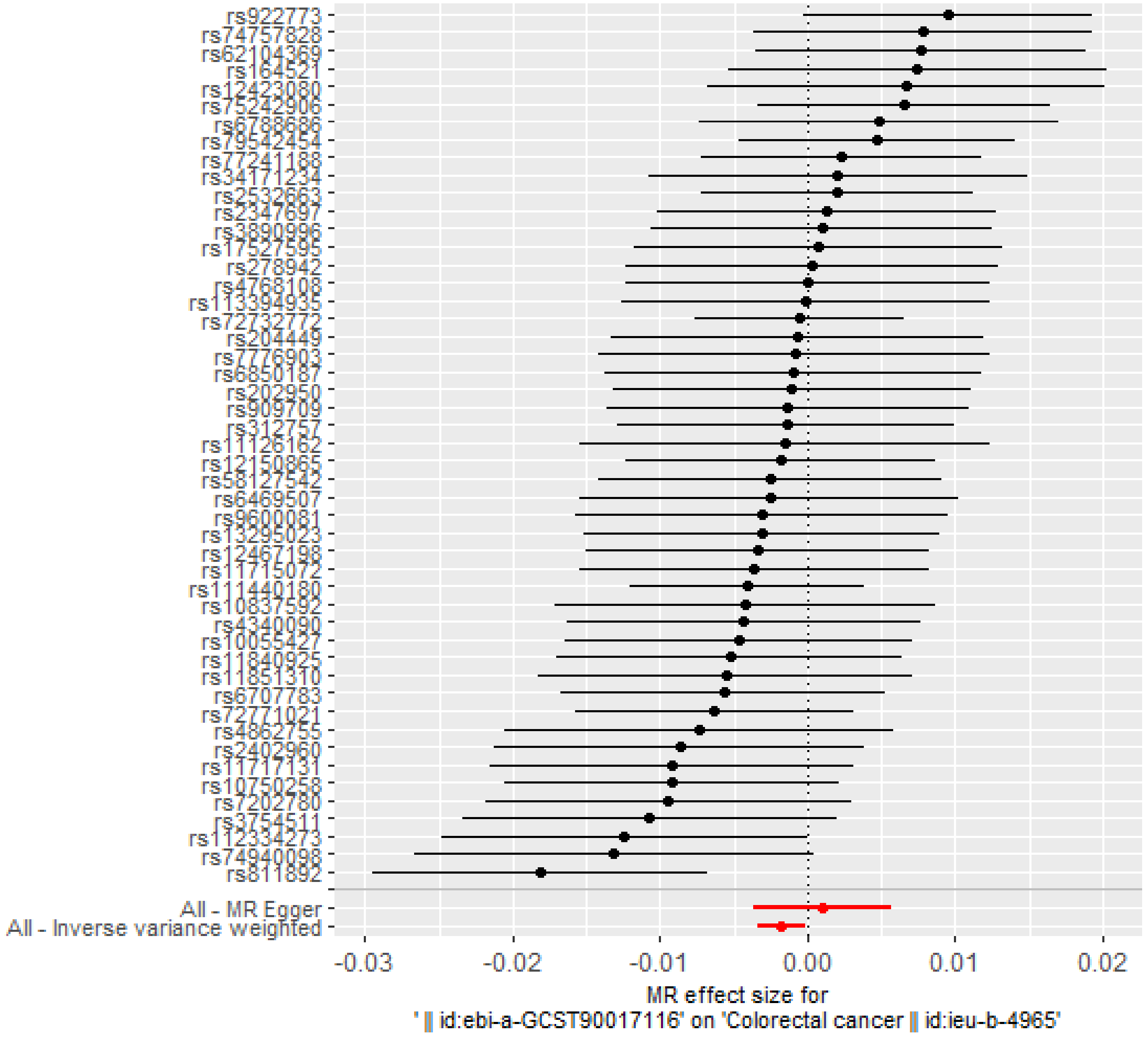
rs922773
rs74757828
rs62104369
rs164521
rs12423080
rs75242906
rs6788686
rs79542454
rs77241188
rs34171234
rs2532663
rs2347697
rs3890996
rs17527595
rs278942
rs4768108
rs113394935
rs72732772
rs204449
rs7776903
rs6850187
rs202950
rs909709
rs312757
rs11126162
rs12150865
rs58127542
rs6469507
rs9600081
rs13295023
rs12467198
rs11715072
rs111440180
rs10837592
rs4340090
rs10055427
rs11840925
rs11851310
rs6707783
rs72771021
rs4862755
rs2402960
rs11717131
rs10750258
rs7202780
rs3754511
rs112334273
rs74940098
rs811892
All - MR Egger
All - Inverse variance weighted
-0.03
-0.02
-0.01
0.00
0.01
0.02
MR effect size for
' || id:ebi-a-GCST90017116' on 'Colorectal cancer || id:ieu-b-4965'

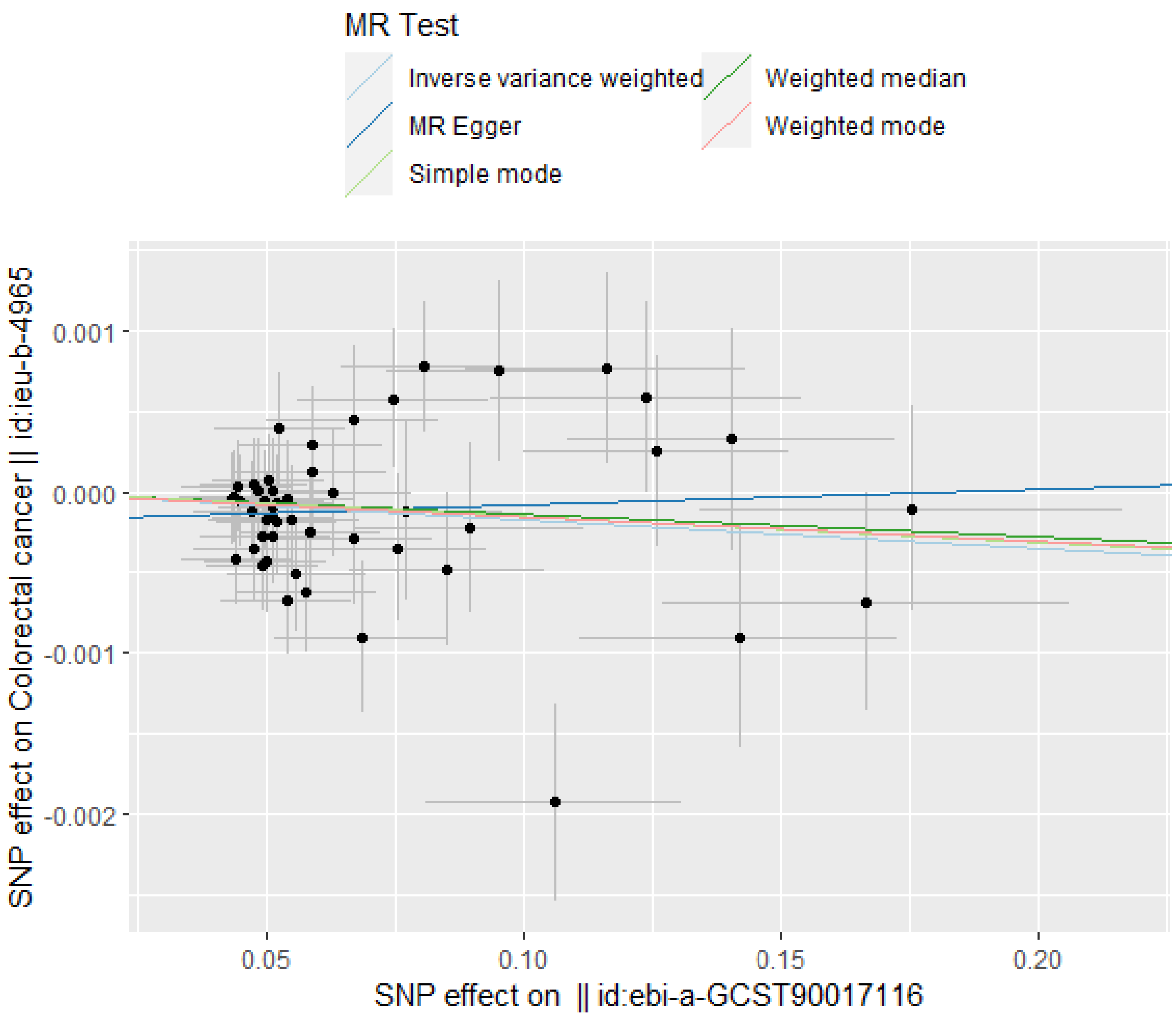
MR Test
Inverse variance weighted
MR Egger
Simple mode
Weighted median
Weighted mode
SNP effect on Colorectal cancer || id:ieu-b-4965
0.001
0.000
-0.001
-0.002
0.05
0.10
0.15
0.20
SNP effect on || id:ebi-a-GCST90017116

Figure 141 Leave-one-out analysis, funnel plot, MR effect size and scatter plot for gut microbiota abundance (class Actinobacteria id.419) on coeliac disease

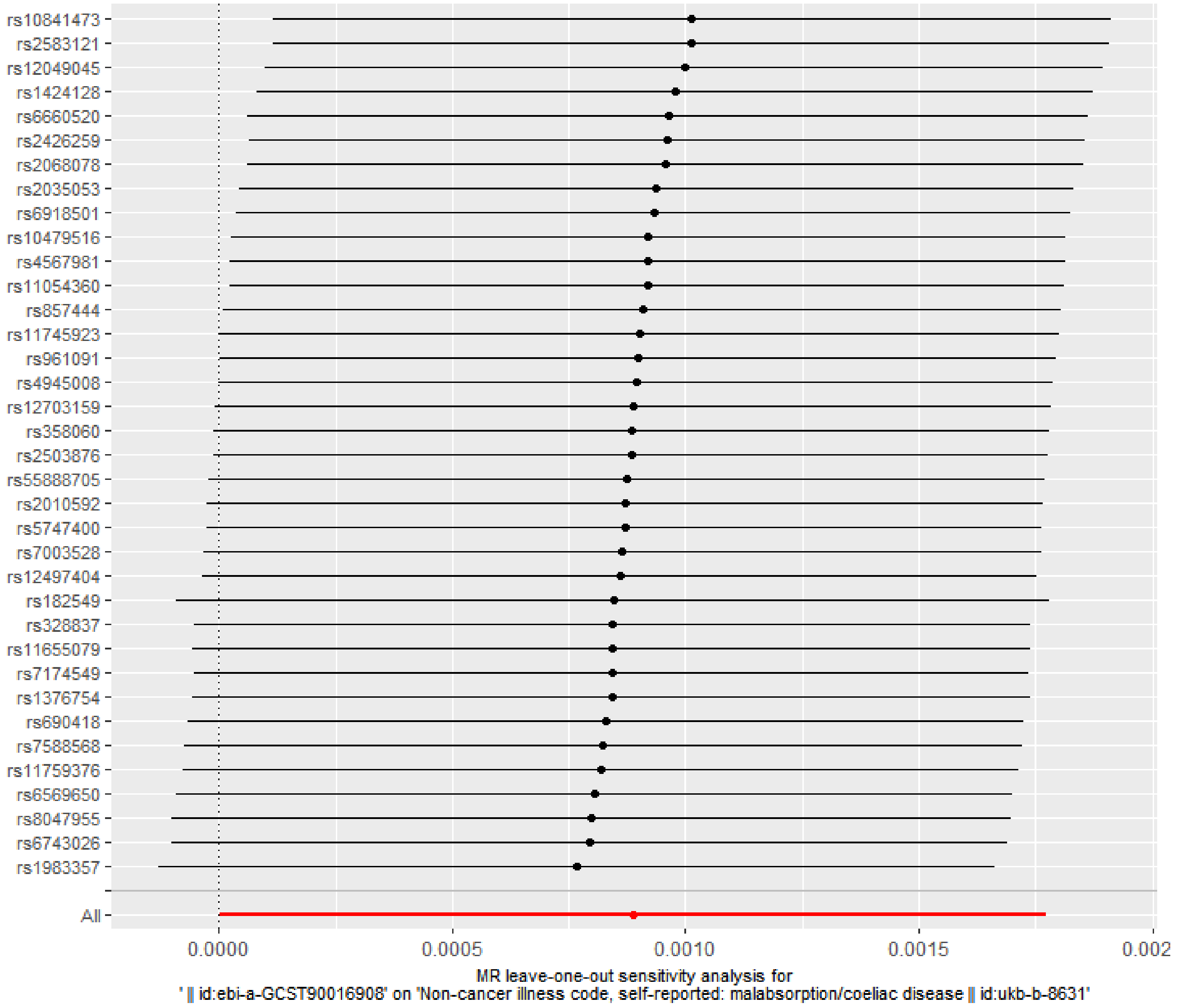

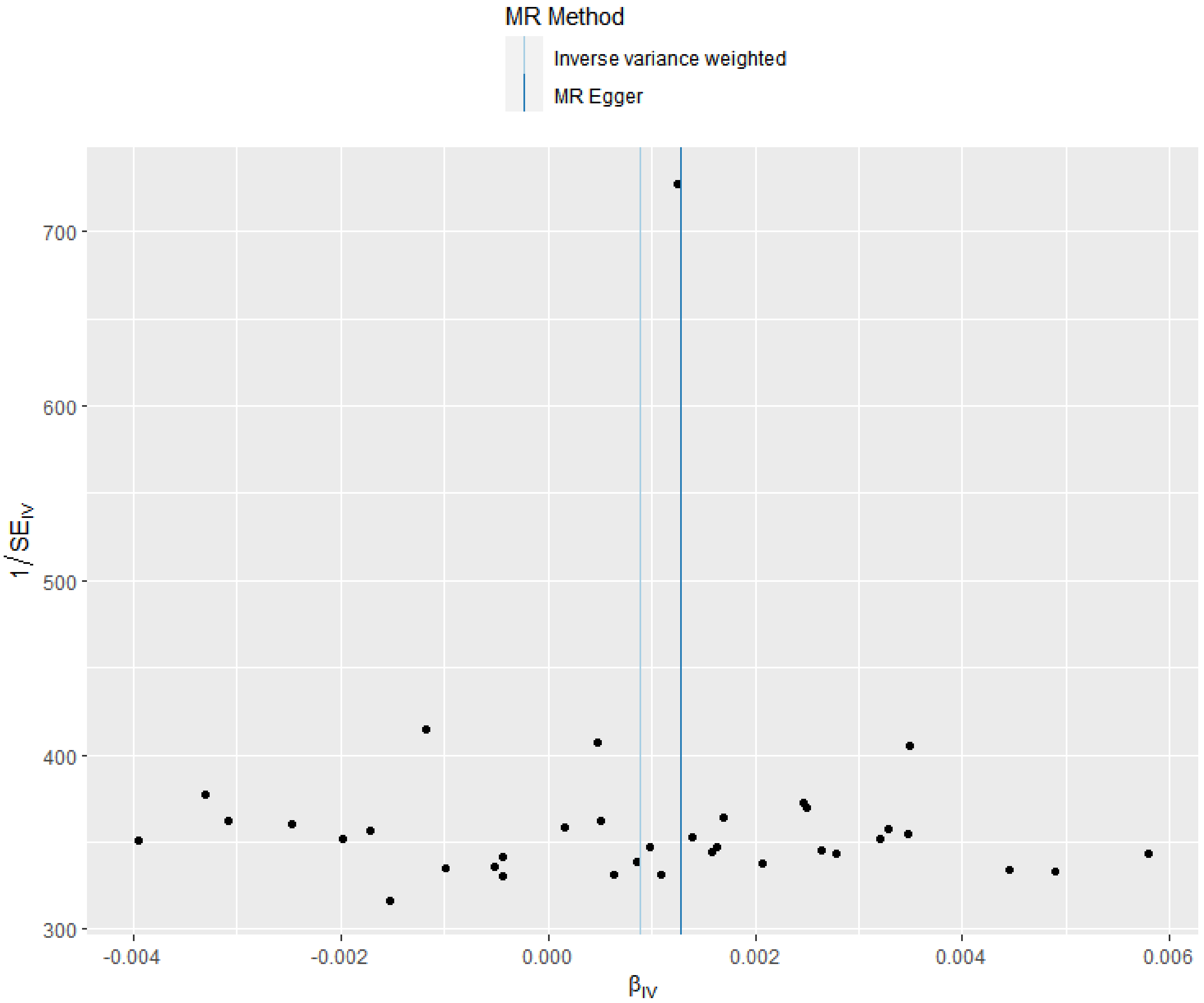
MR Method
Inverse variance weighted
MR Egger
700
600
500
400
300
1/SE_IV
-0.004
-0.002
0.000
0.002
0.004
0.006
β_IV

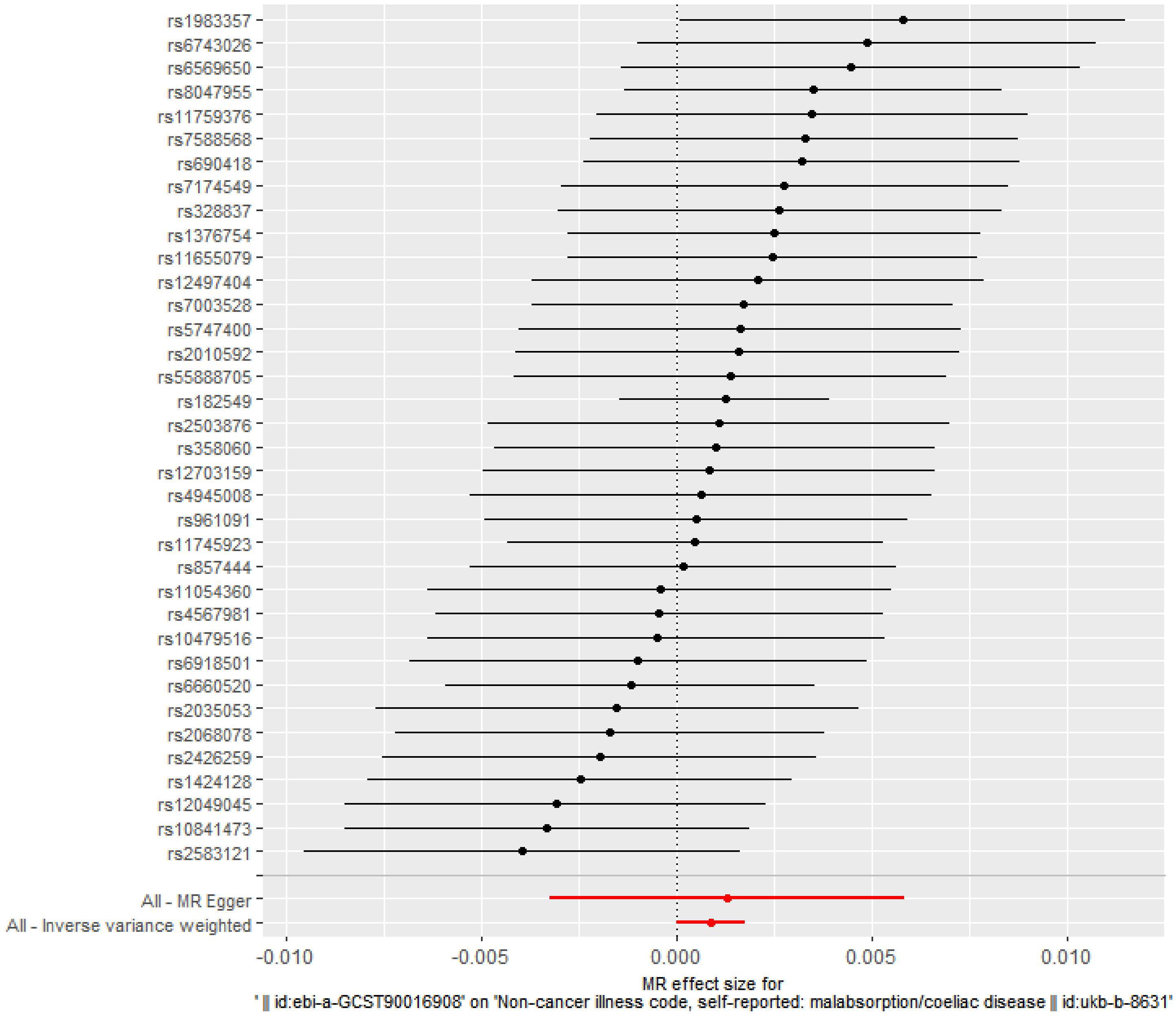
rs1983357
rs6743026
rs6569650
rs8047955
rs11759376
rs7588568
rs690418
rs7174549
rs328837
rs1376754
rs11655079
rs12497404
rs7003528
rs5747400
rs2010592
rs55888705
rs182549
rs2503876
rs358060
rs12703159
rs4945008
rs961091
rs11745923
rs857444
rs11054360
rs4567981
rs10479516
rs6918501
rs6660520
rs2035053
rs2068078
rs2426259
rs1424128
rs12049045
rs10841473
rs2583121
All - MR Egger
All - Inverse variance weighted
-0.010
-0.005
0.000
0.005
0.010
MR effect size for
' || id:ebi-a-GCST90016908' on 'Non-cancer illness code, self-reported: malabsorption/coeliac disease || id:ukb-b-8631'

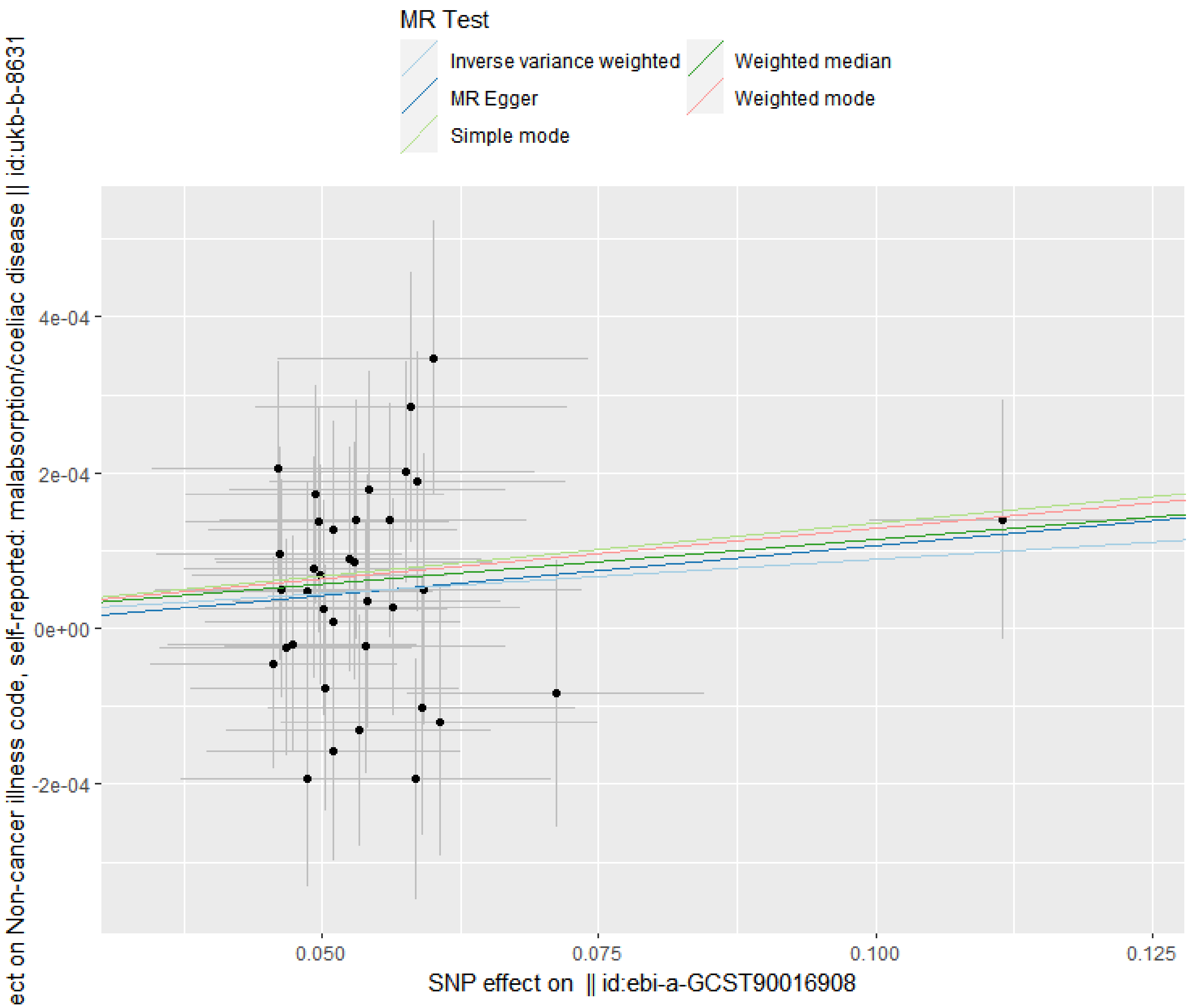

MR Test
Inverse variance weighted
MR Egger
Simple mode
Weighted median
Weighted mode
4e-04
2e-04
0e+00
-2e-04
0.050
0.075
0.100
0.125
SNP effect on || id:ebi-a-GCST90016908
ect on Non-cancer illness code, self-reported: malabsorption/coeliac disease || id:ukb-b-8631

Figure 142 Leave-one-out analysis, funnel plot, MR effect size and scatter plot for gut microbiota abundance (class Bacilli id.1673) on coeliac disease

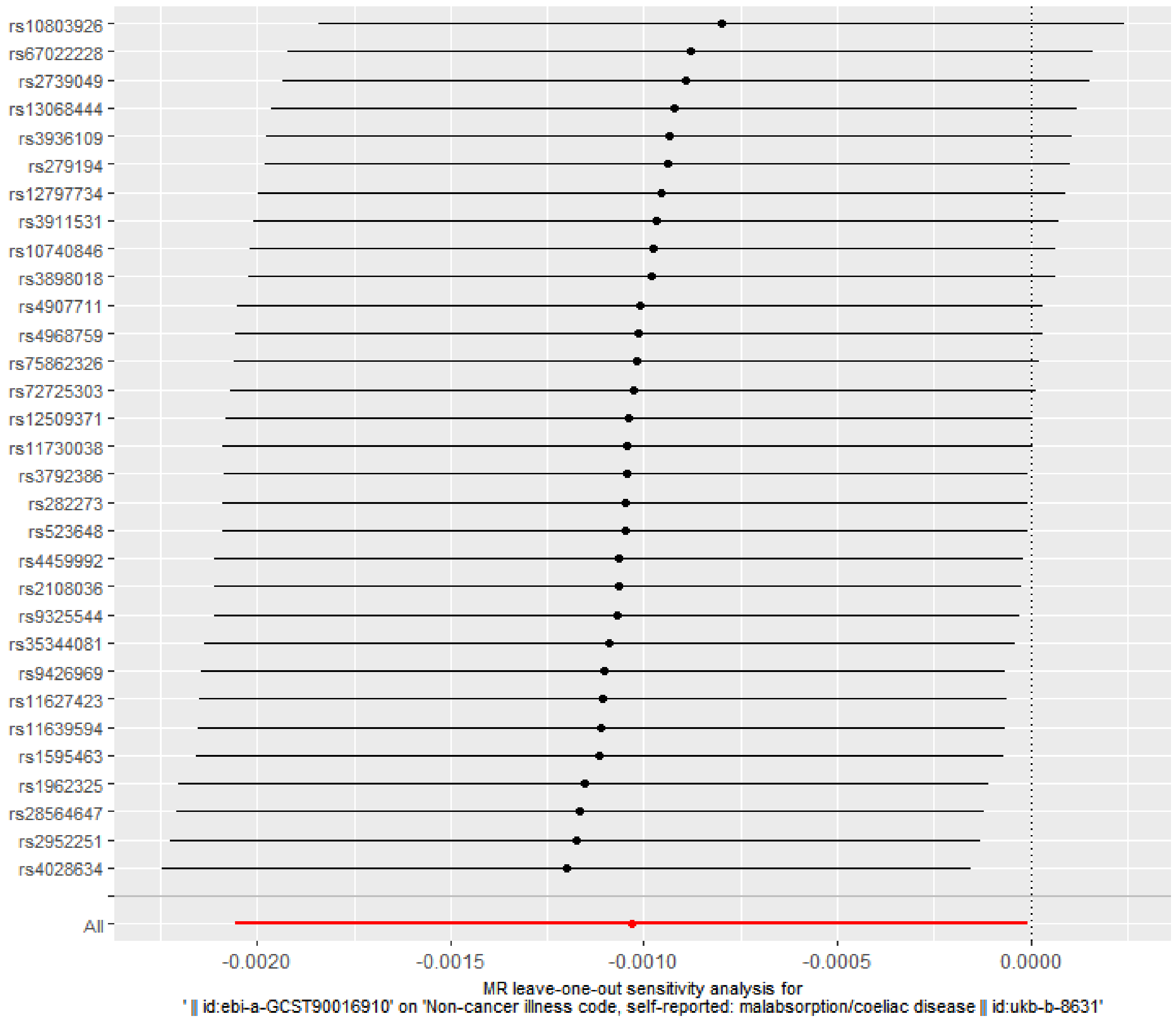

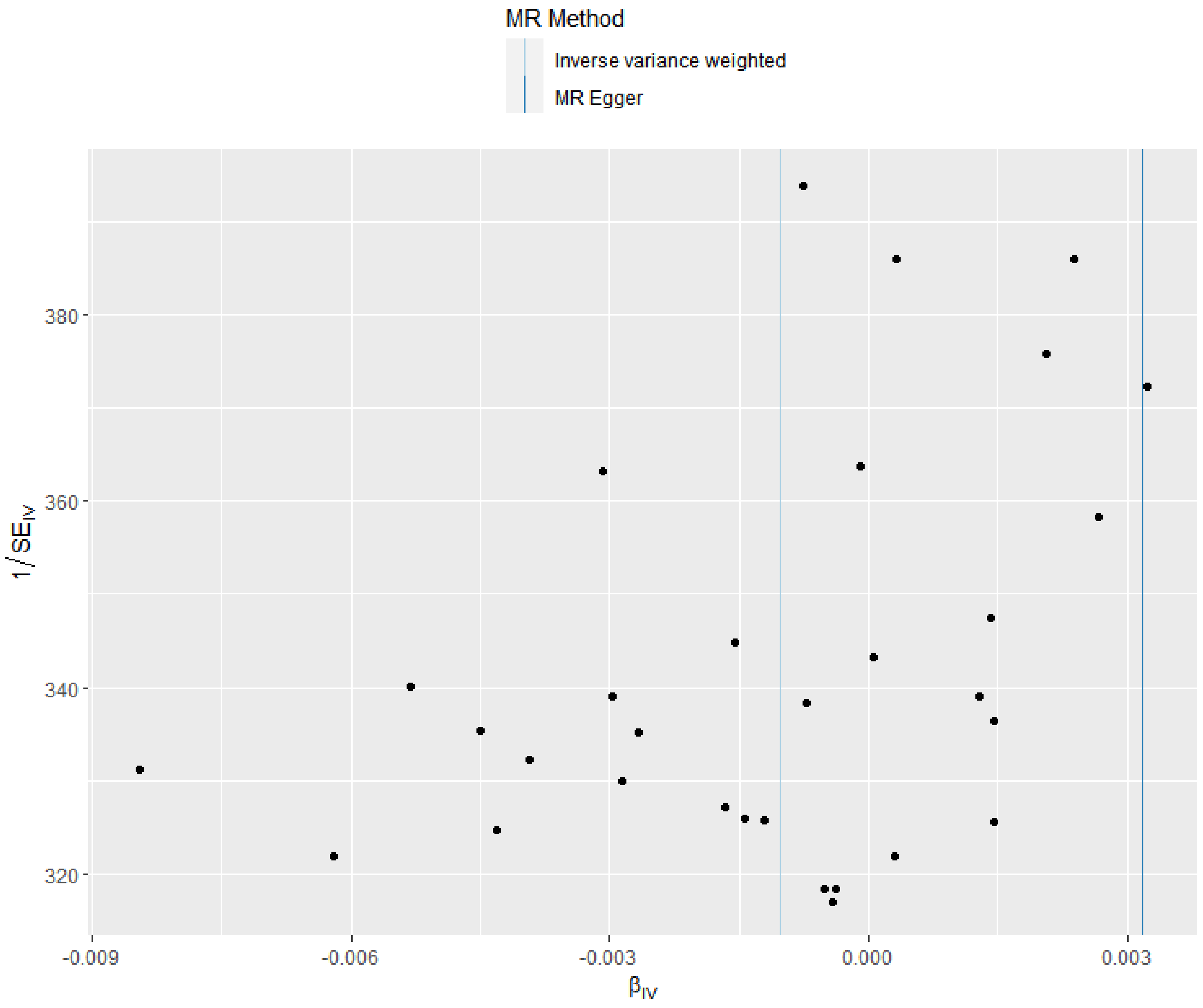

MR Method
Inverse variance weighted
MR Egger
380
360
340
320
1/SE$_{IV}$
-0.009
-0.006
-0.003
0.000
0.003
β$_{IV}$

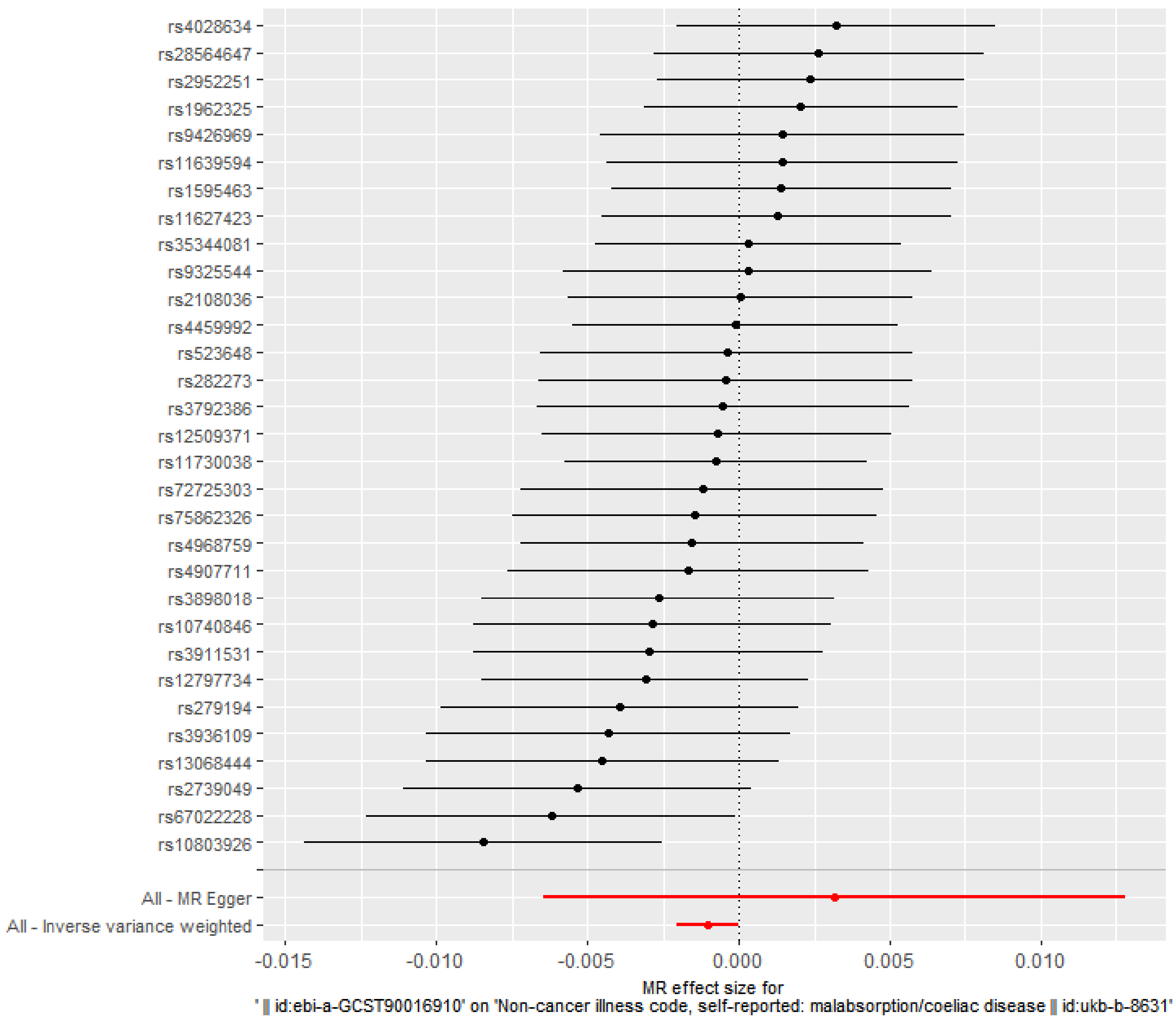

rs4028634
rs28564647
rs2952251
rs1962325
rs9426969
rs11639594
rs1595463
rs11627423
rs35344081
rs9325544
rs2108036
rs4459992
rs523648
rs282273
rs3792386
rs12509371
rs11730038
rs72725303
rs75862326
rs4968759
rs4907711
rs3898018
rs10740846
rs3911531
rs12797734
rs279194
rs3936109
rs13068444
rs2739049
rs67022228
rs10803926
All - MR Egger
All - Inverse variance weighted
-0.015
-0.010
-0.005
0.000
0.005
0.010
MR effect size for
' || id:ebi-a-GCST90016910' on 'Non-cancer illness code, self-reported: malabsorption/coeliac disease || id:ukb-b-8631'

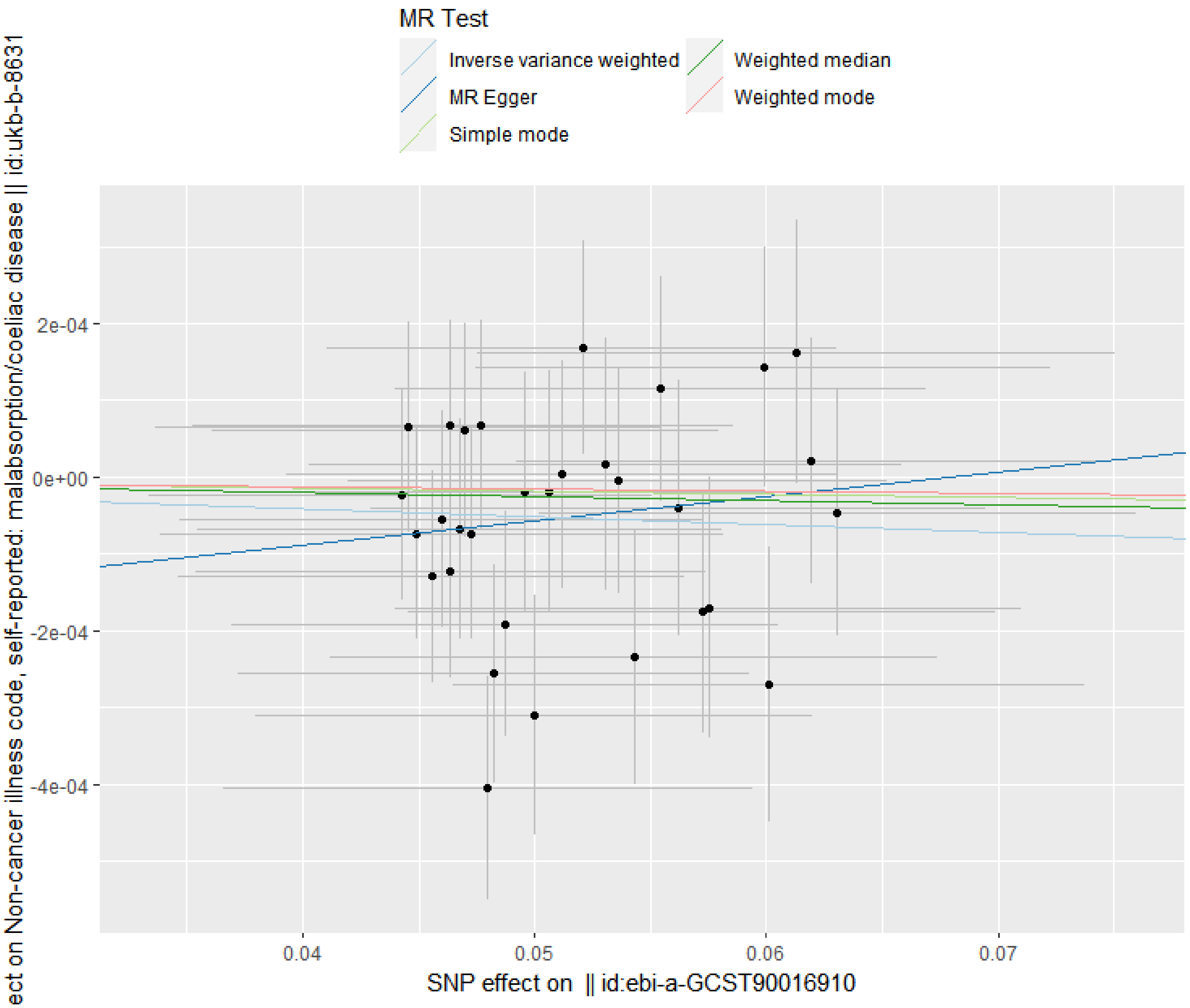

MR Test
Inverse variance weighted
MR Egger
Simple mode
Weighted median
Weighted mode
2e-04
0e+00
-2e-04
-4e-04
0.04
0.05
0.06
0.07
SNP effect on || id:ebi-a-GCST90016910
ect on Non-cancer illness code, self-reported: malabsorption/coeliac disease || id:ukb-b-8631

Figure 143 Leave-one-out analysis, funnel plot, MR effect size and scatter plot for gut microbiota abundance (family Bacteroidaceae id.917) on coeliac disease

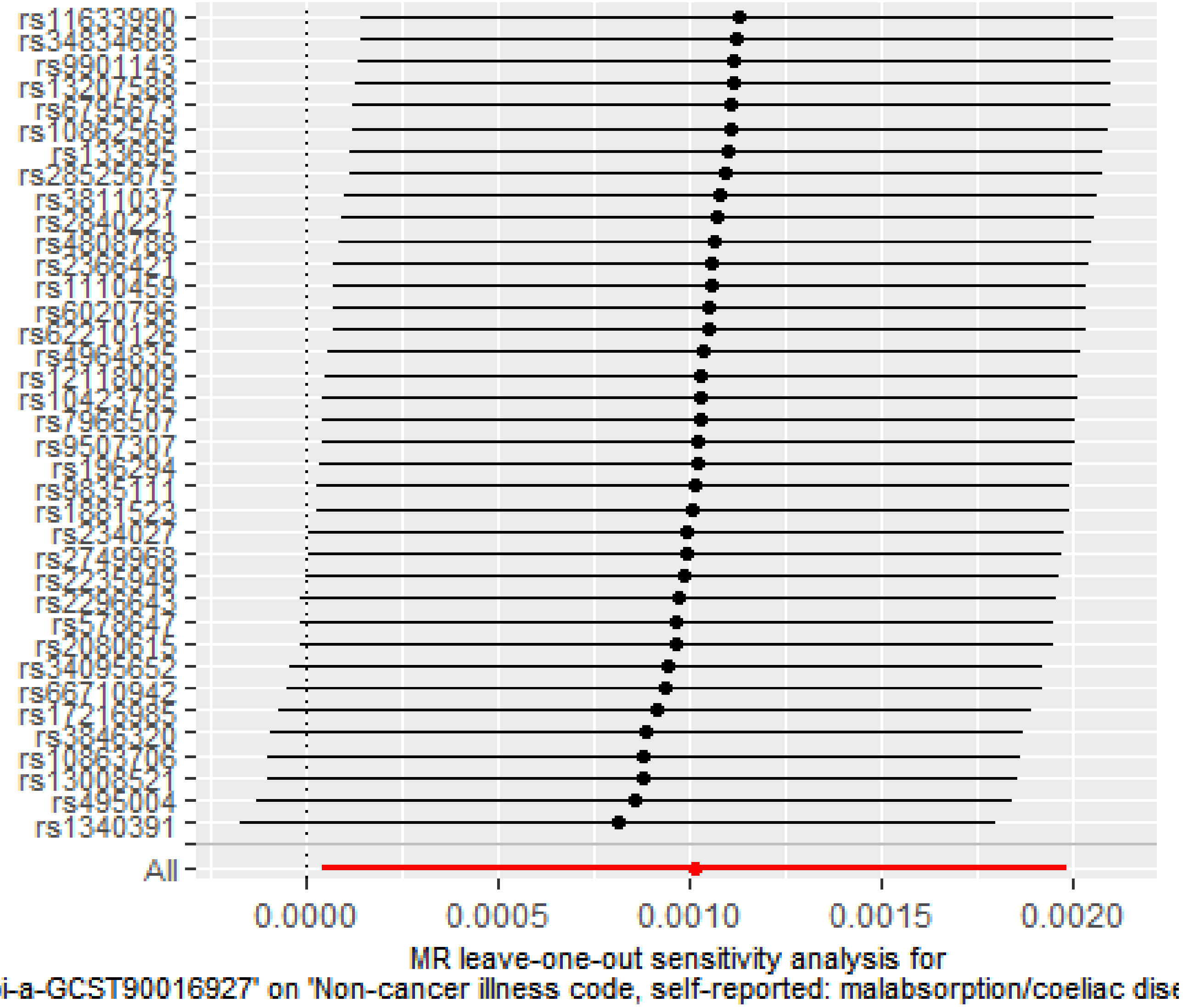

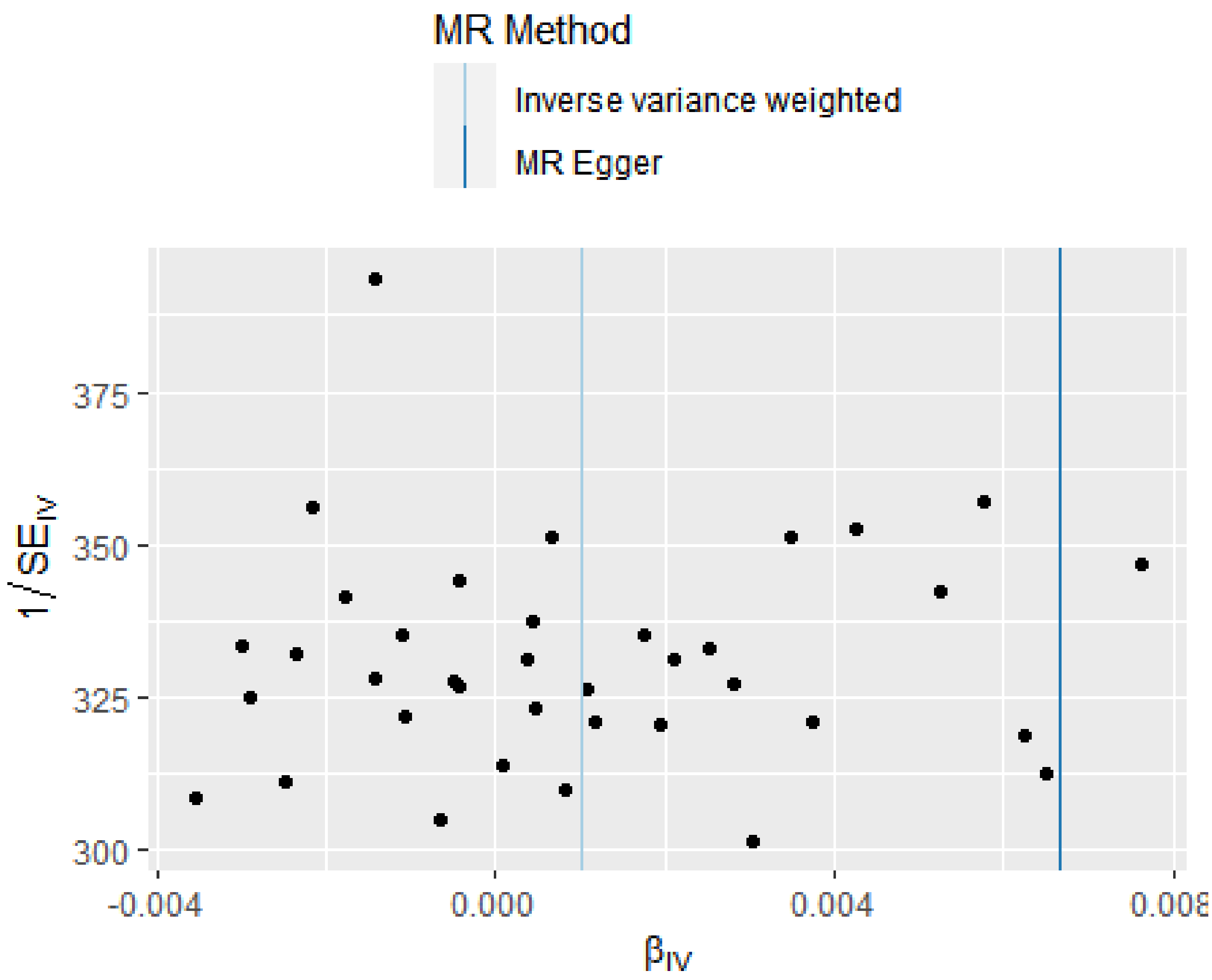
MR Method
Inverse variance weighted
MR Egger
375
350
325
300
-0.004
0.000
0.004
0.008
$1/SE_{IV}$
β_{IV}

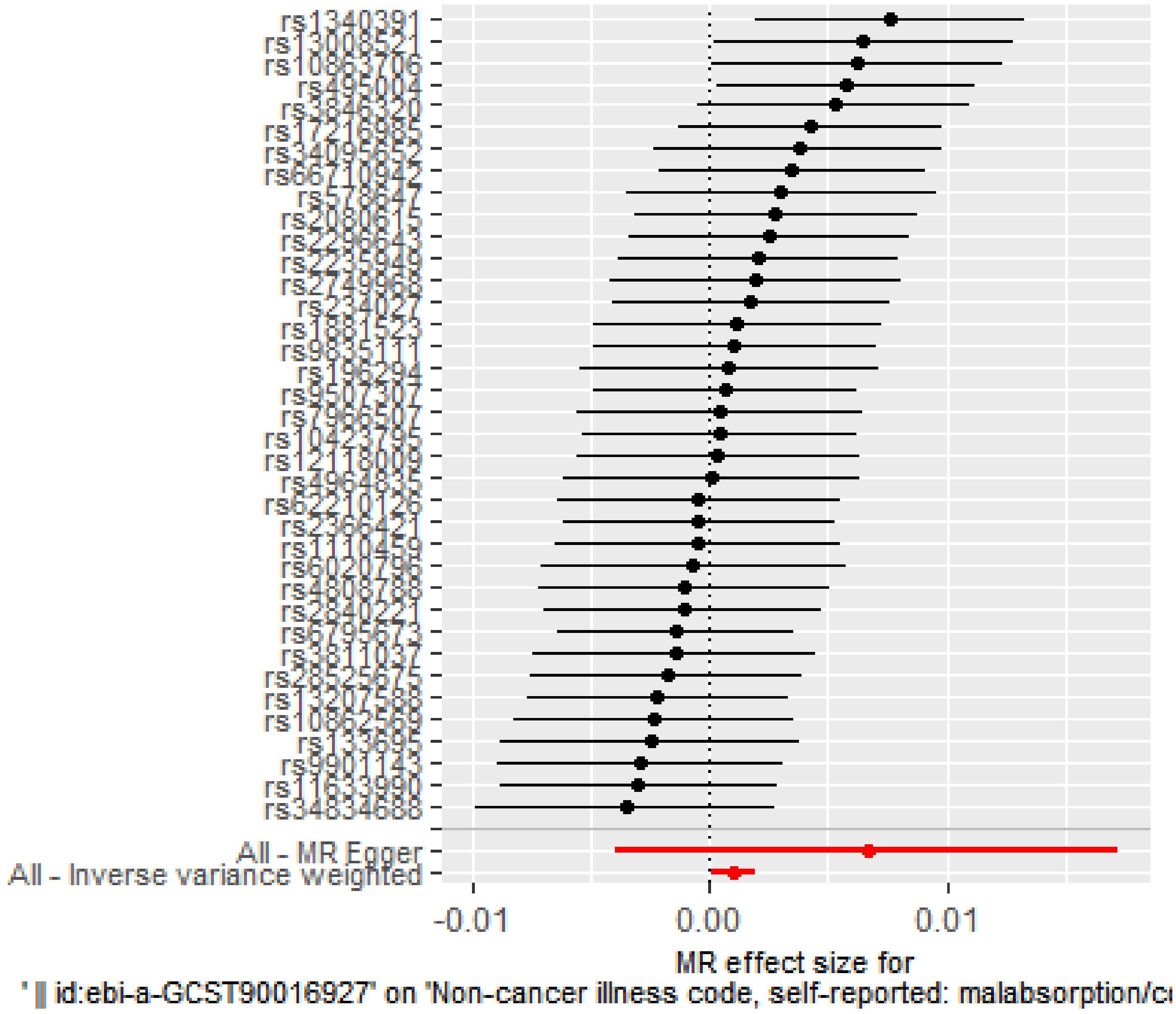
rs1340391
rs13008521
rs10863706
rs495004
rs3846320
rs17216985
rs34095552
rs66710942
rs578647
rs2080615
rs2296643
rs2235949
rs2749968
rs234027
rs1881523
rs9835111
rs196294
rs9507307
rs7966507
rs10423795
rs12118009
rs4964835
rs62210126
rs2366421
rs1110459
rs6020796
rs4808788
rs2840221
rs6795673
rs3811037
rs28525675
rs13207588
rs10862569
rs133695
rs9901143
rs11633990
rs34834688
All - MR Egger
All - Inverse variance weighted
-0.01
0.00
0.01
MR effect size for
' || id:ebi-a-GCST90016927' on 'Non-cancer illness code, self-reported: malabsorption/c

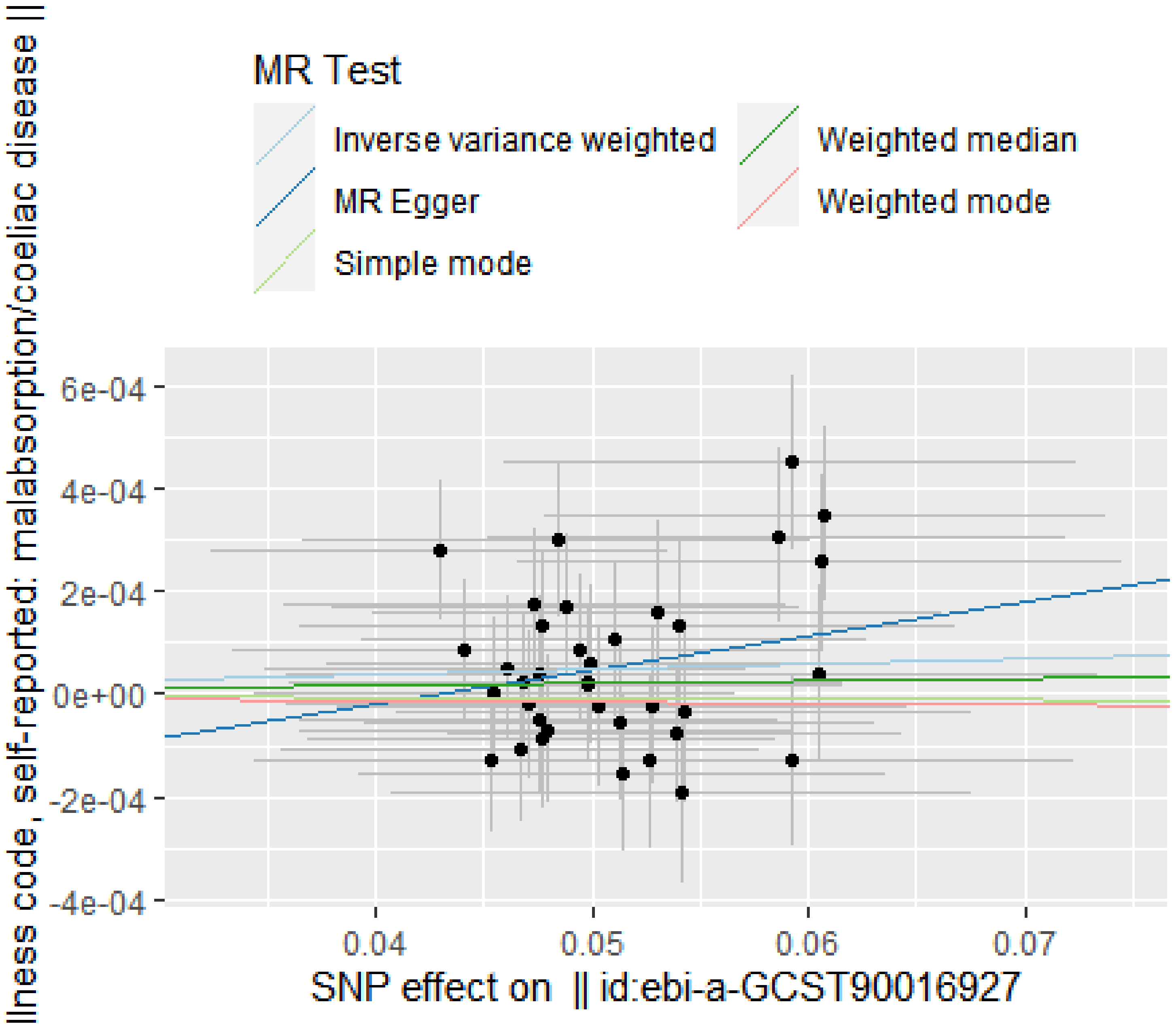

MR Test
Inverse variance weighted
MR Egger
Simple mode
Weighted median
Weighted mode
6e-04
4e-04
2e-04
0e+00
-2e-04
-4e-04
0.04
0.05
0.06
0.07
SNP effect on || id:ebi-a-GCST90016927
Illness code, self-reported: malabsorption/coeliac disease ||

Figure 144 Leave-one-out analysis, funnel plot, MR effect size and scatter plot for gut microbiota abundance (family Streptococcaceae id.1850) on coeliac disease

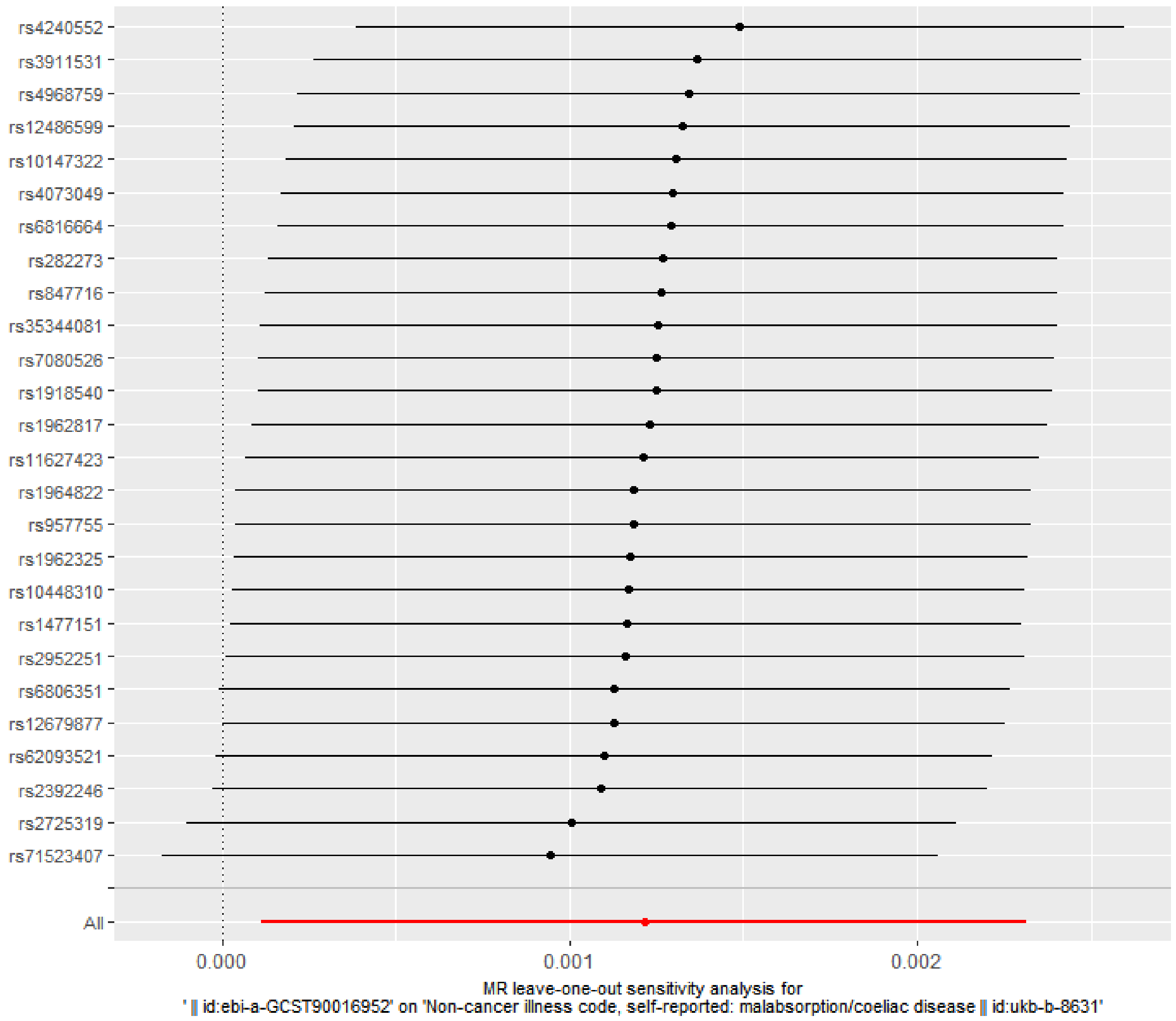

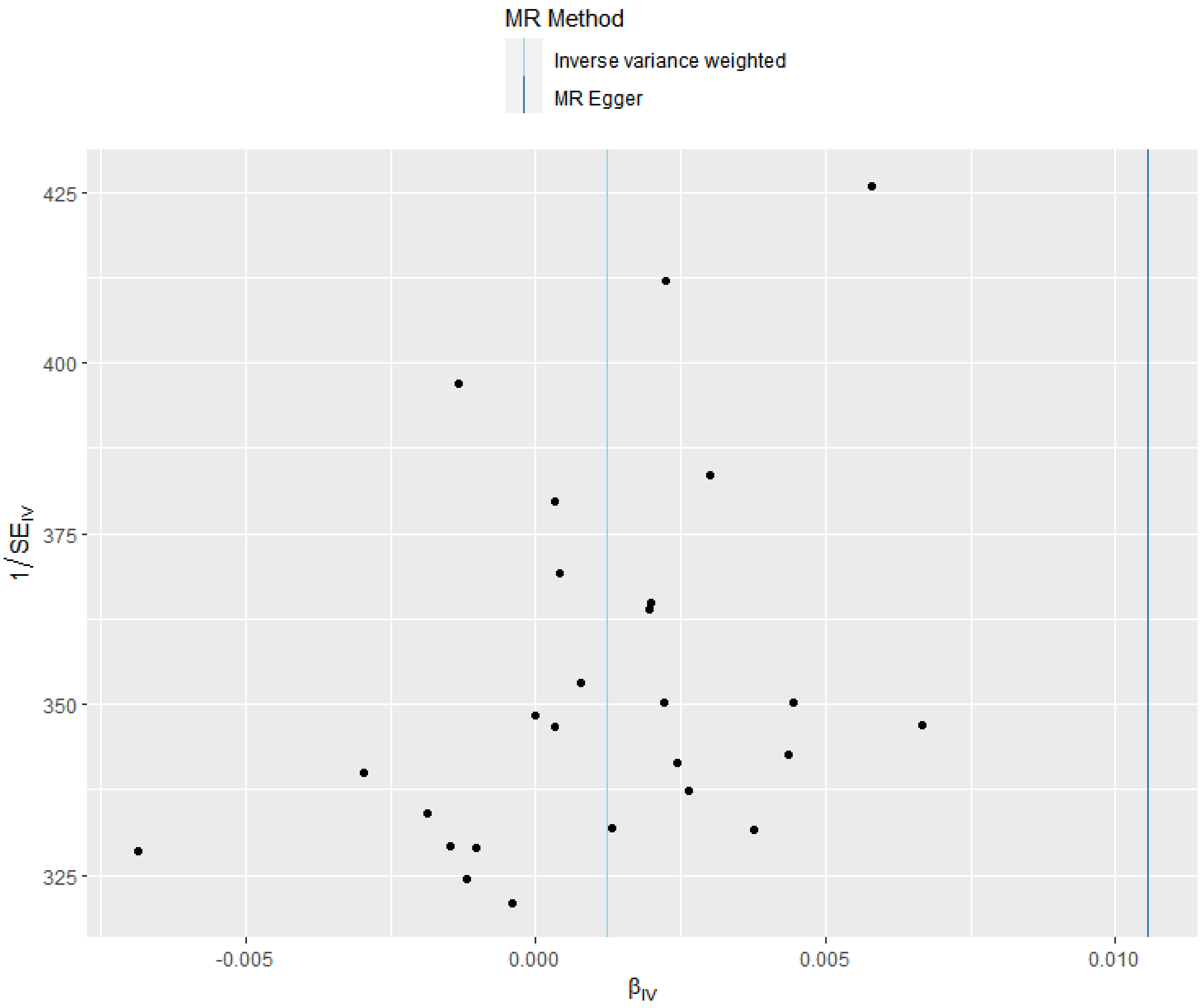
MR Method
Inverse variance weighted
MR Egger
425
400
375
350
325
1/SE_IV
-0.005
0.000
0.005
0.010
β_IV

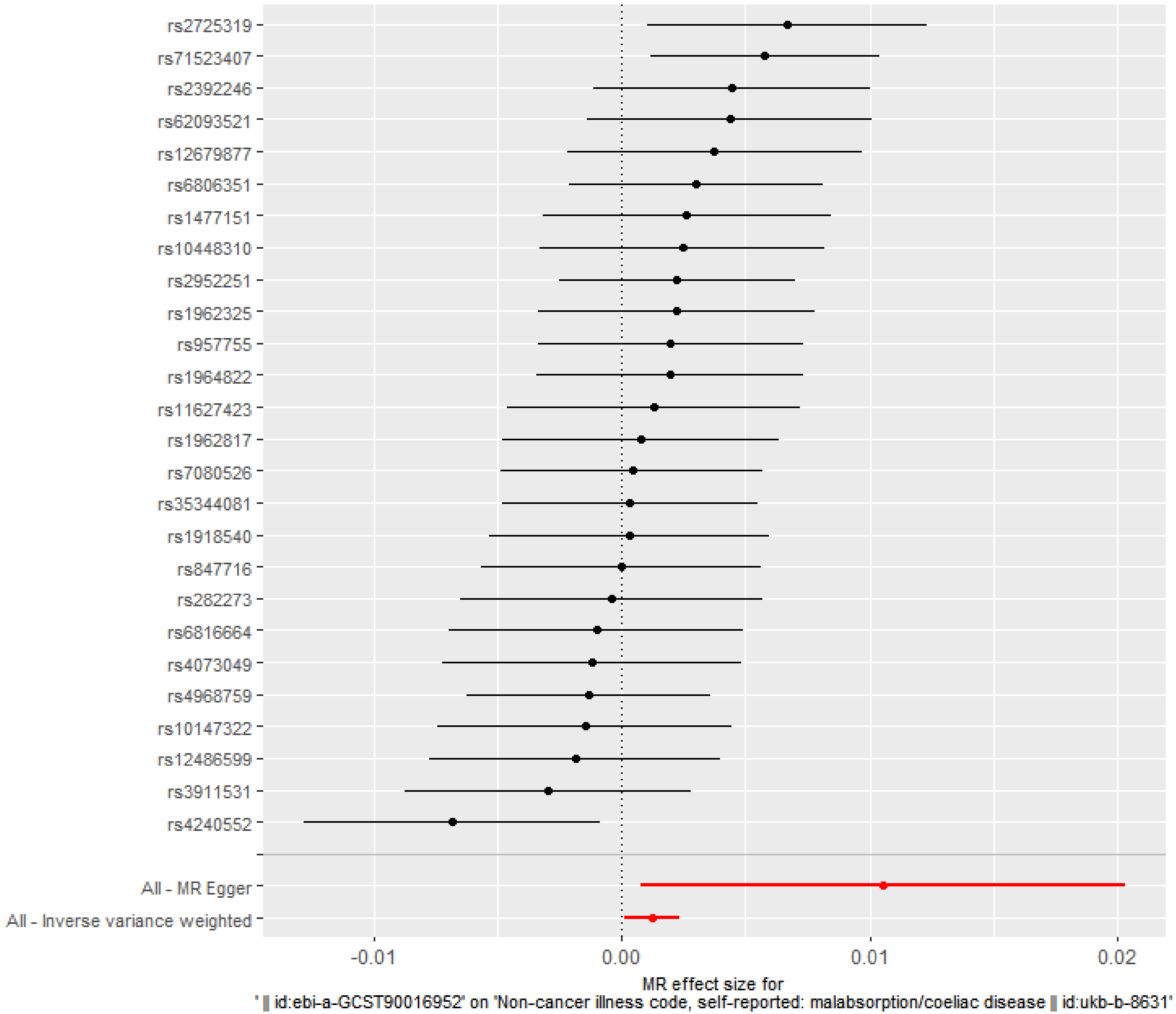

rs2725319
rs71523407
rs2392246
rs62093521
rs12679877
rs6806351
rs1477151
rs10448310
rs2952251
rs1962325
rs957755
rs1964822
rs11627423
rs1962817
rs7080526
rs35344081
rs1918540
rs847716
rs282273
rs6816664
rs4073049
rs4968759
rs10147322
rs12486599
rs3911531
rs4240552
All - MR Egger
All - Inverse variance weighted
-0.01
0.00
0.01
0.02
MR effect size for
' || id:ebi-a-GCST90016952' on 'Non-cancer illness code, self-reported: malabsorption/coeliac disease || id:ukb-b-8631'

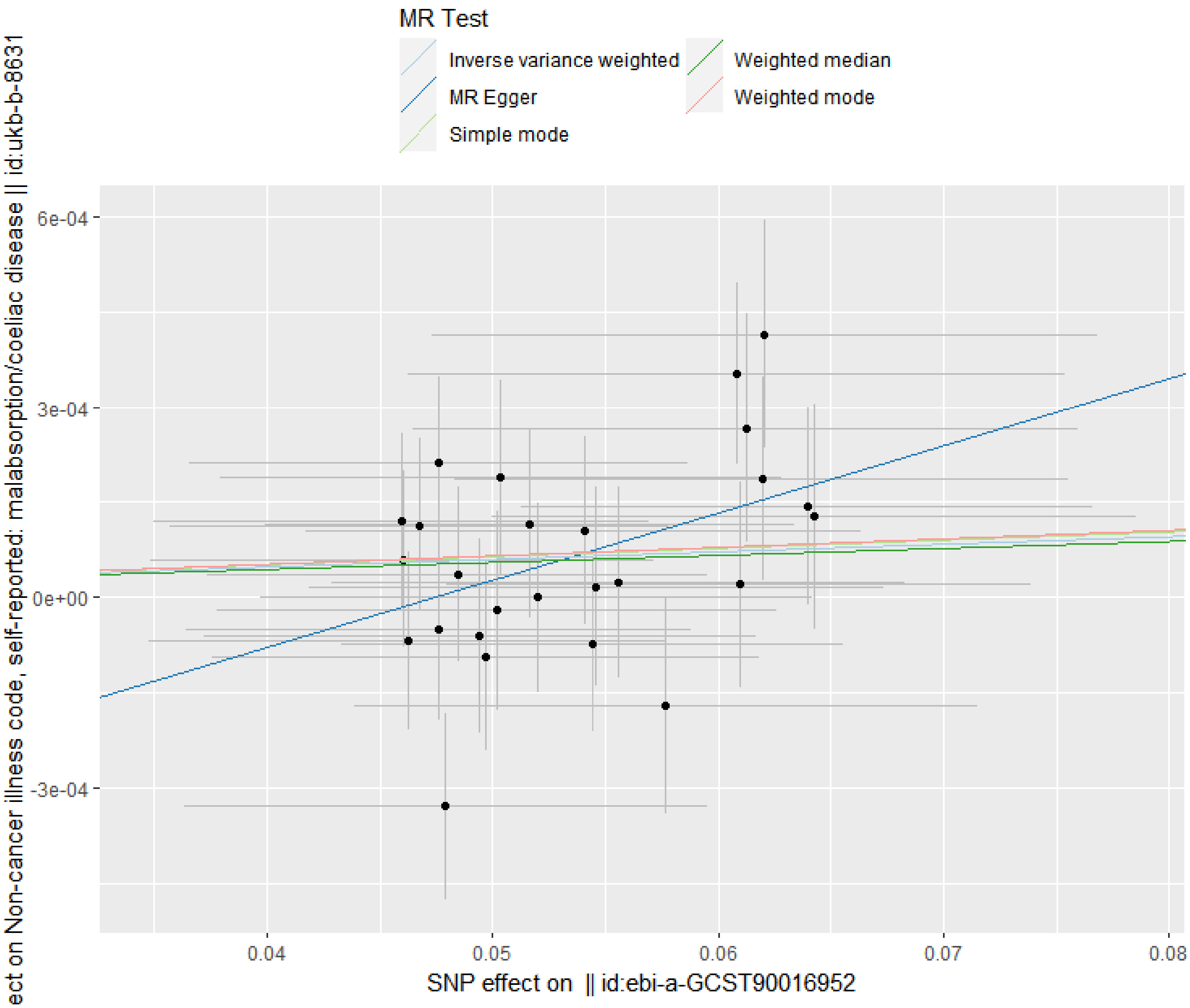

MR Test
Inverse variance weighted
MR Egger
Simple mode
Weighted median
Weighted mode
6e-04
3e-04
0e+00
-3e-04
0.04
0.05
0.06
0.07
0.08
SNP effect on || id:ebi-a-GCST90016952
ect on Non-cancer illness code, self-reported: malabsorption/coeliac disease || id:ukb-b-8631

Figure 145 Leave-one-out analysis, funnel plot, MR effect size and scatter plot for gut microbiota abundance (genus Bacteroides id.918) on coeliac disease

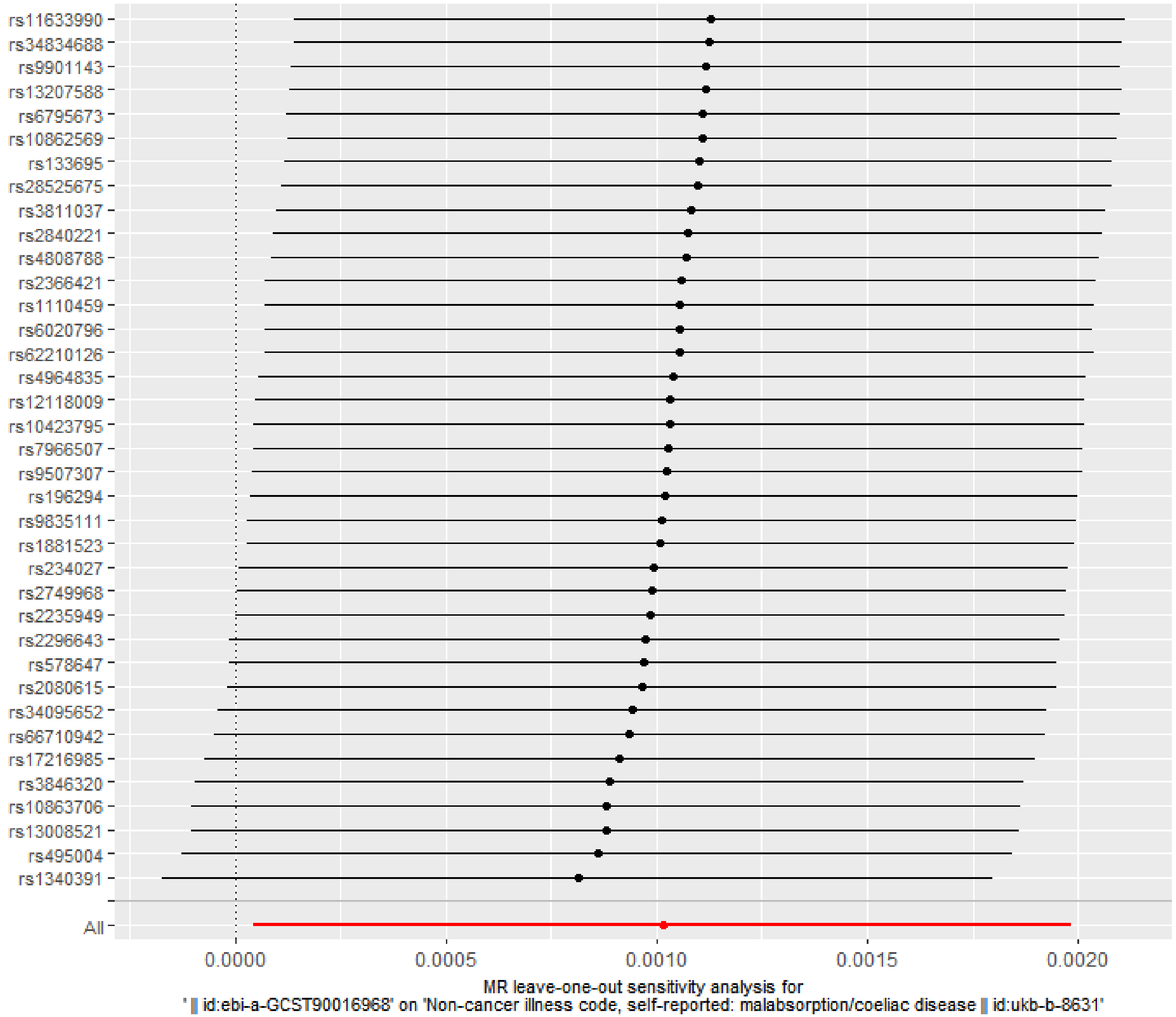

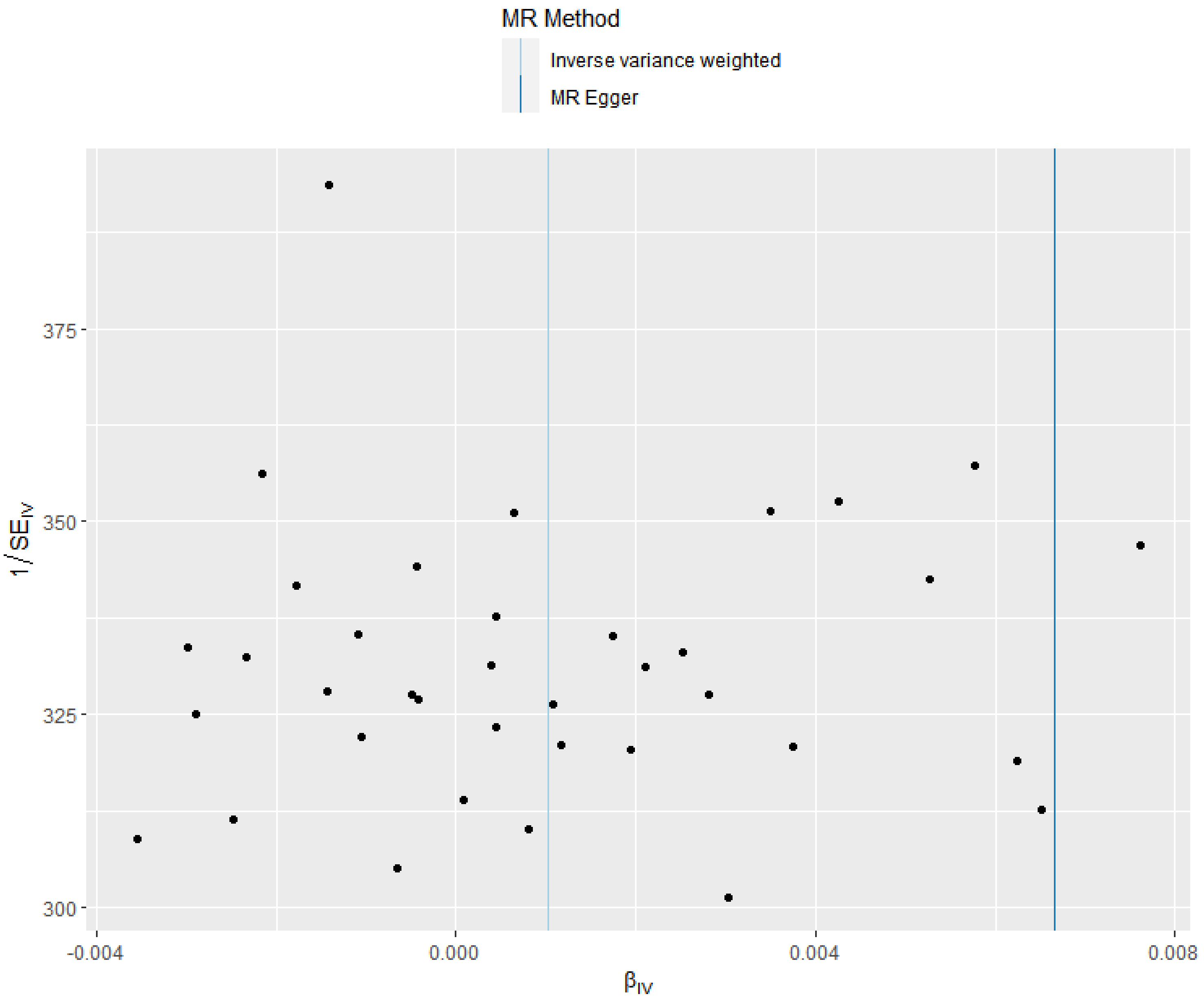

MR Method
Inverse variance weighted
MR Egger
375
350
325
300
1/SE_IV
-0.004
0.000
0.004
0.008
β_IV

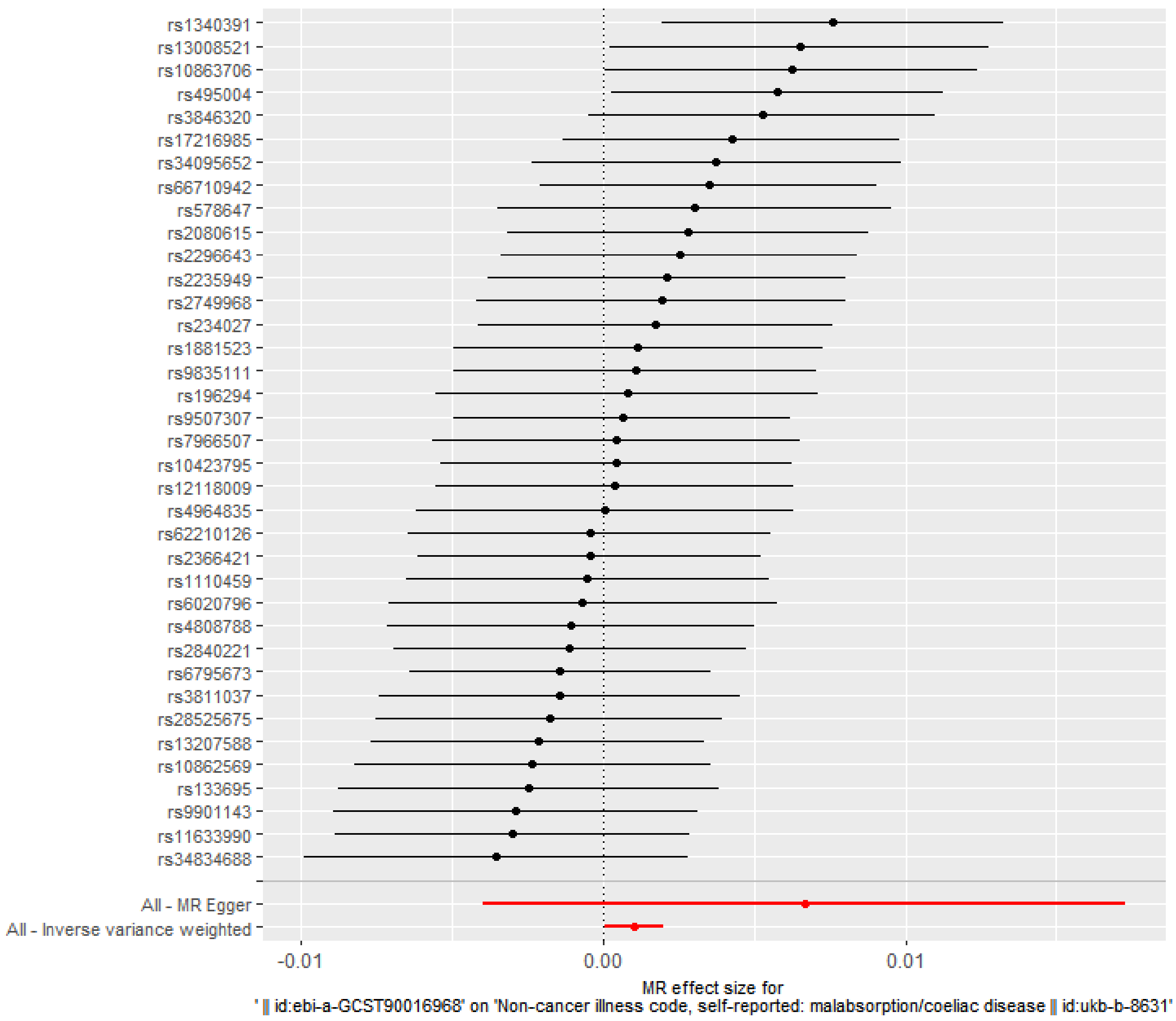

rs1340391
rs13008521
rs10863706
rs495004
rs3846320
rs17216985
rs34095652
rs66710942
rs578647
rs2080615
rs2296643
rs2235949
rs2749968
rs234027
rs1881523
rs9835111
rs196294
rs9507307
rs7966507
rs10423795
rs12118009
rs4964835
rs62210126
rs2366421
rs1110459
rs6020796
rs4808788
rs2840221
rs6795673
rs3811037
rs28525675
rs13207588
rs10862569
rs133695
rs9901143
rs11633990
rs34834688
All - MR Egger
All - Inverse variance weighted
-0.01
0.00
0.01
MR effect size for
' || id:ebi-a-GCST90016968' on 'Non-cancer illness code, self-reported: malabsorption/coeliac disease || id:ukb-b-8631'

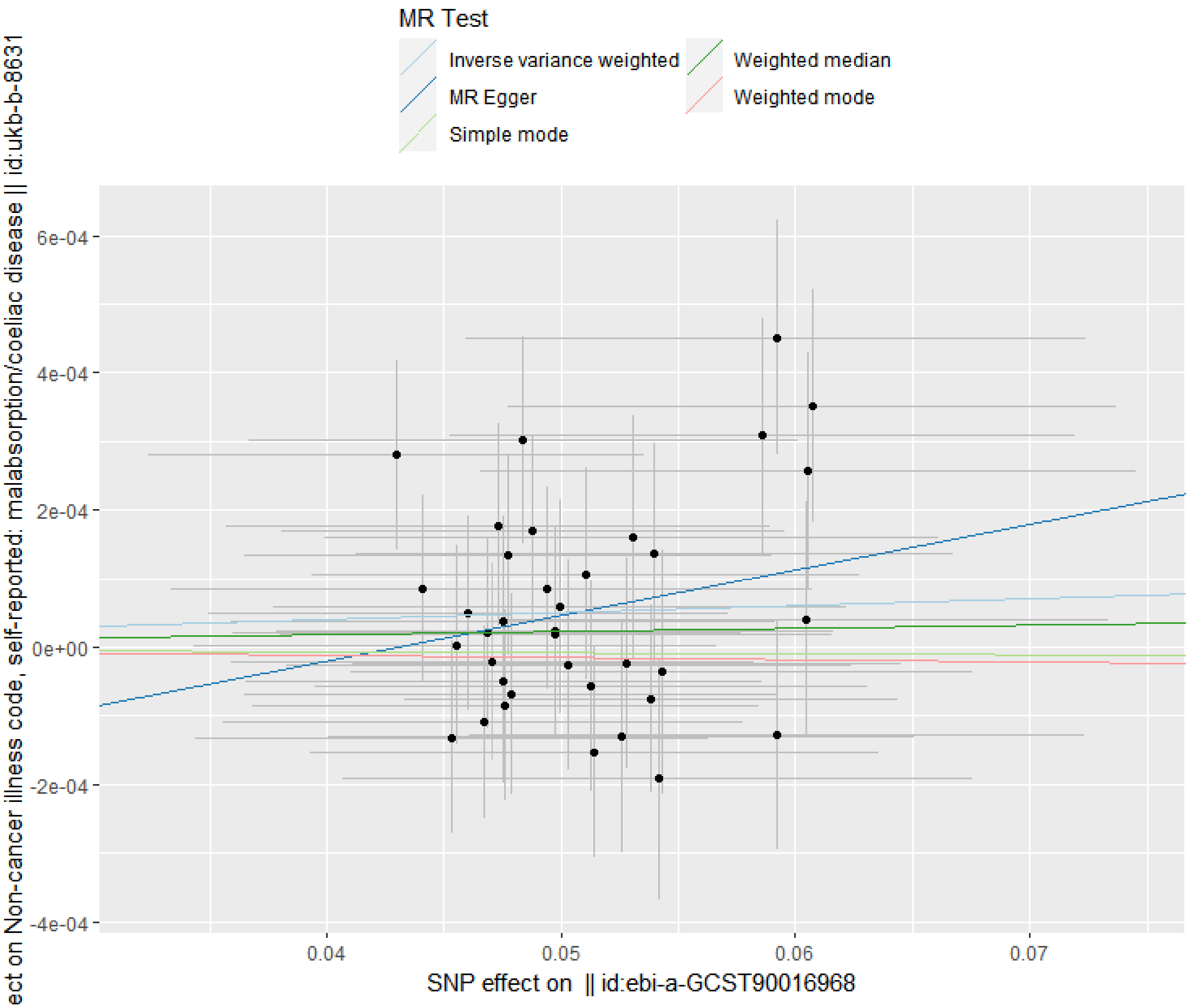

MR Test
Inverse variance weighted
MR Egger
Simple mode
Weighted median
Weighted mode
6e-04
4e-04
2e-04
0e+00
-2e-04
-4e-04
0.04
0.05
0.06
0.07
SNP effect on || id:ebi-a-GCST90016968
ect on Non-cancer illness code, self-reported: malabsorption/coeliac disease || id:ukb-b-8631

Figure 146 Leave-one-out analysis, funnel plot, MR effect size and scatter plot for gut microbiota abundance (genus Barnesiella id.944) on coeliac disease

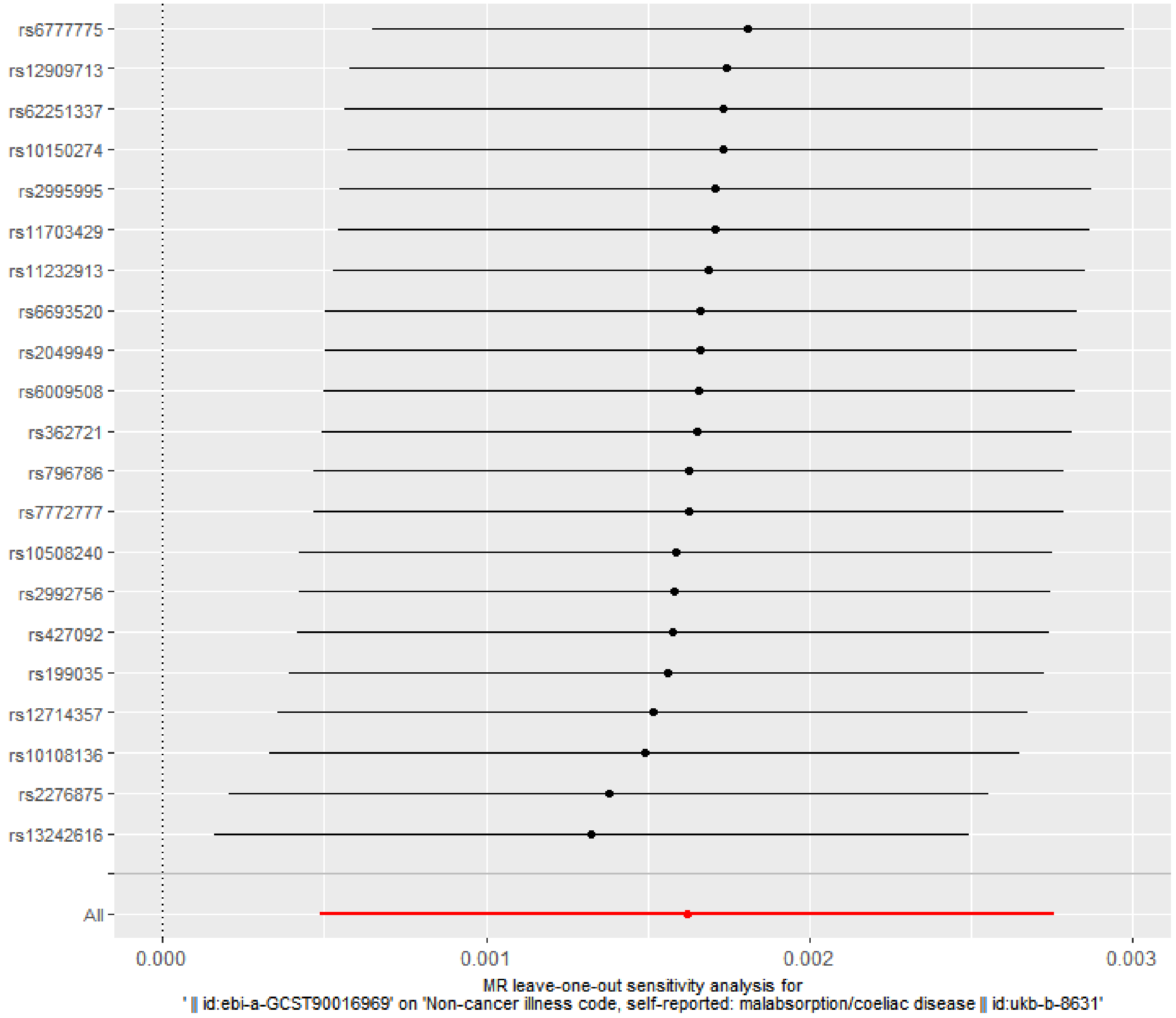

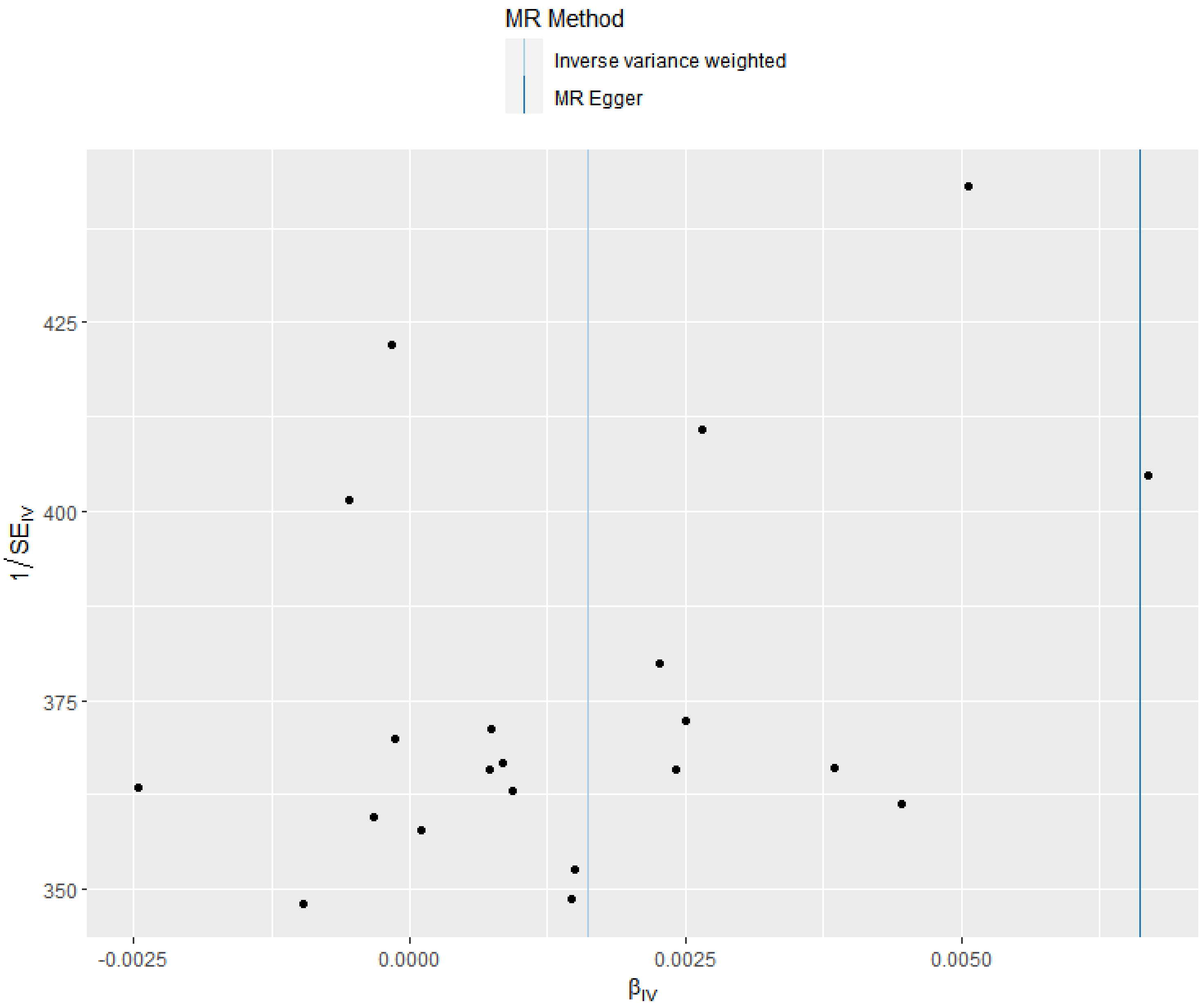
MR Method
Inverse variance weighted
MR Egger
425
400
375
350
1/SE$_{IV}$
-0.0025
0.0000
0.0025
0.0050
β$_{IV}$

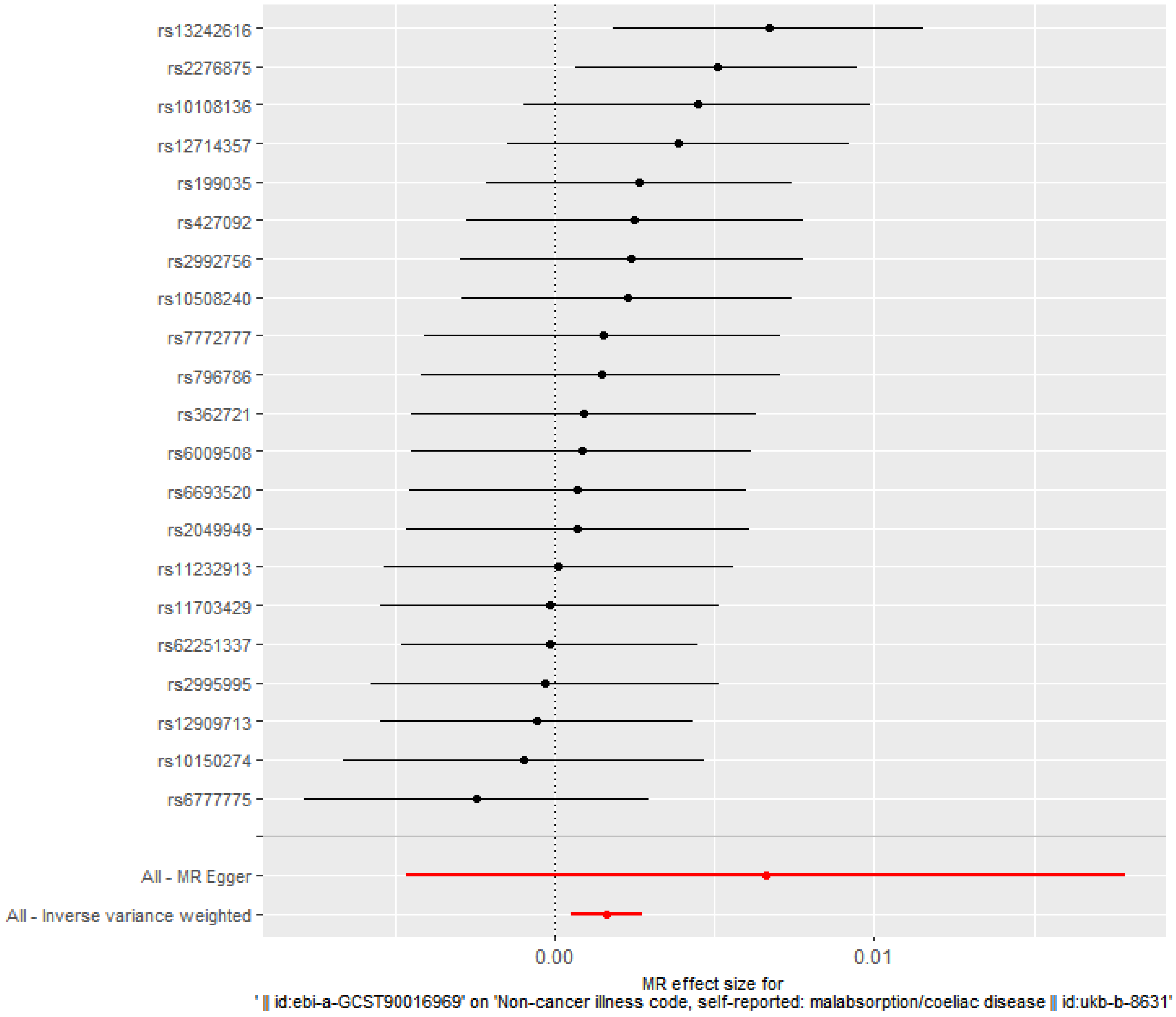

rs13242616
rs2276875
rs10108136
rs12714357
rs199035
rs427092
rs2992756
rs10508240
rs7772777
rs796786
rs362721
rs6009508
rs6693520
rs2049949
rs11232913
rs11703429
rs62251337
rs2995995
rs12909713
rs10150274
rs6777775
All - MR Egger
All - Inverse variance weighted
0.00
0.01
MR effect size for
' || id:ebi-a-GCST90016969' on 'Non-cancer illness code, self-reported: malabsorption/coeliac disease || id:ukb-b-8631'

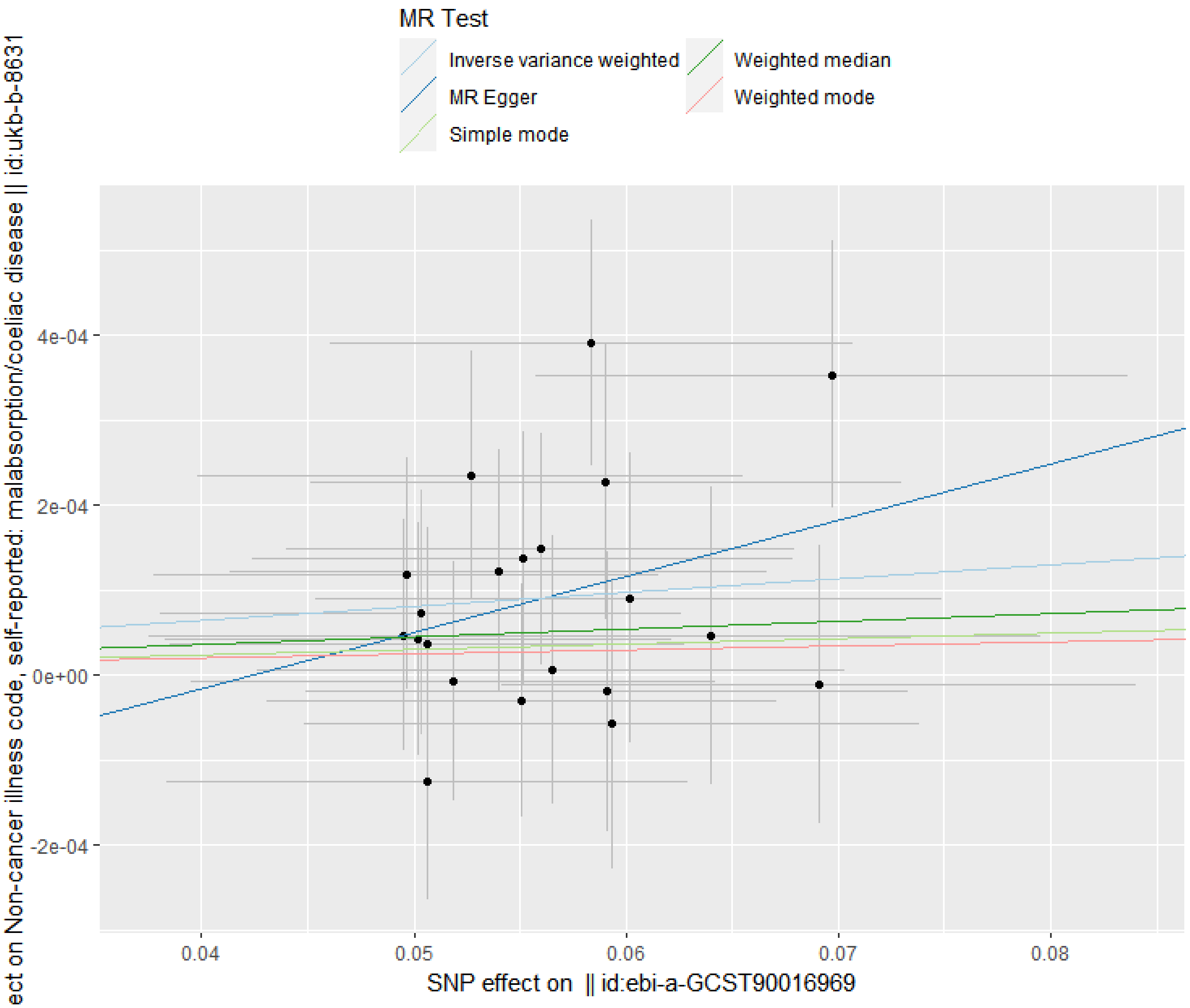
MR Test
Inverse variance weighted
MR Egger
Simple mode
Weighted median
Weighted mode
4e-04
2e-04
0e+00
-2e-04
0.04
0.05
0.06
0.07
0.08
SNP effect on || id:ebi-a-GCST90016969
ect on Non-cancer illness code, self-reported: malabsorption/coeliac disease || id:ukb-b-8631

Figure 147 Leave-one-out analysis, funnel plot, MR effect size and scatter plot for gut microbiota abundance (genus Bifidobacterium id.436) on coeliac disease

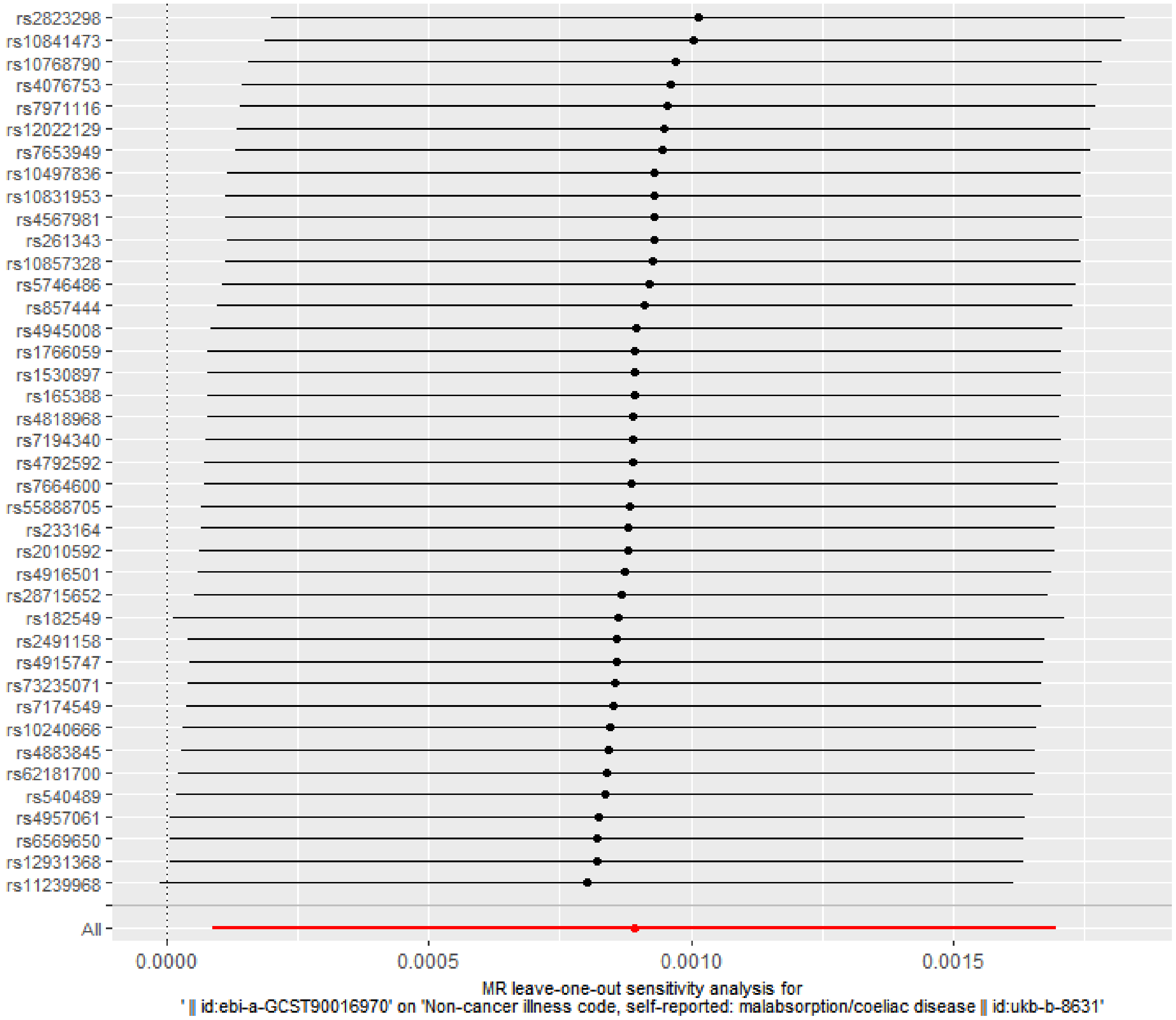

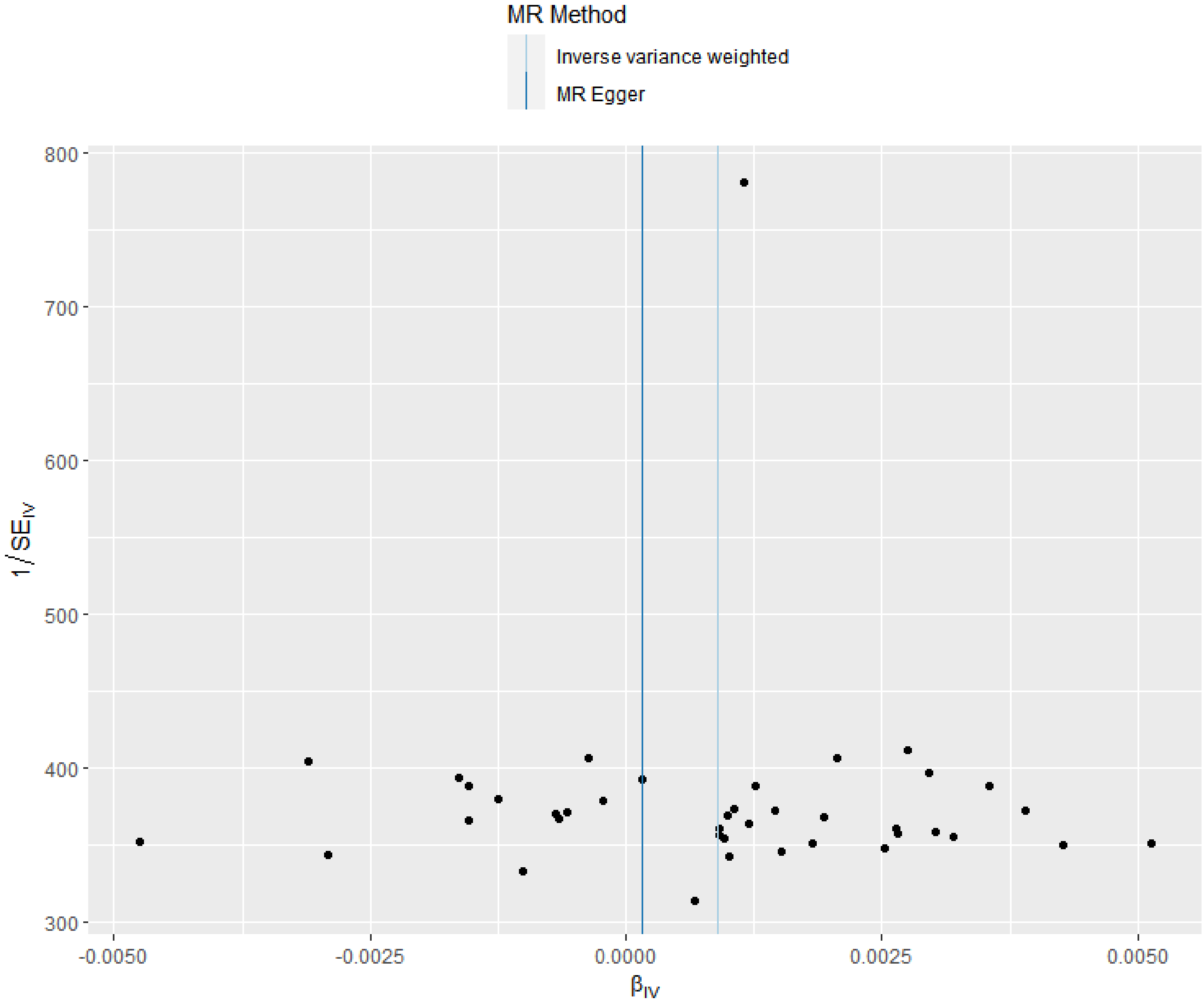
MR Method
Inverse variance weighted
MR Egger
800
700
600
500
400
300
1/SE_IV
-0.0050
-0.0025
0.0000
0.0025
0.0050
β_IV

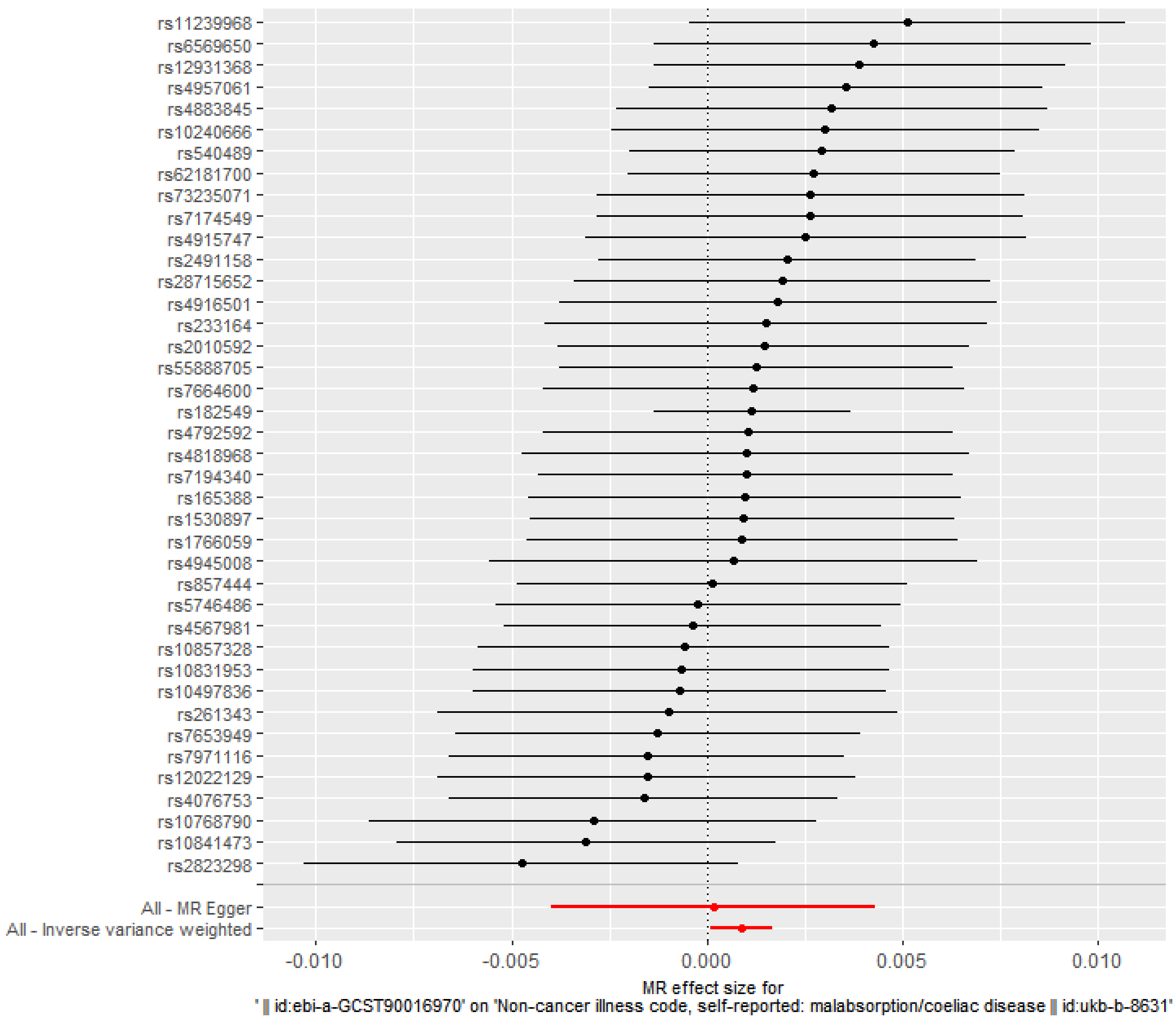

rs11239968
rs6569650
rs12931368
rs4957061
rs4883845
rs10240666
rs540489
rs62181700
rs73235071
rs7174549
rs4915747
rs2491158
rs28715652
rs4916501
rs233164
rs2010592
rs55888705
rs7664600
rs182549
rs4792592
rs4818968
rs7194340
rs165388
rs1530897
rs1766059
rs4945008
rs857444
rs5746486
rs4567981
rs10857328
rs10831953
rs10497836
rs261343
rs7653949
rs7971116
rs12022129
rs4076753
rs10768790
rs10841473
rs2823298
All - MR Egger
All - Inverse variance weighted
-0.010
-0.005
0.000
0.005
0.010
MR effect size for
' ‖ id:ebi-a-GCST90016970' on 'Non-cancer illness code, self-reported: malabsorption/coeliac disease ‖ id:ukb-b-8631'

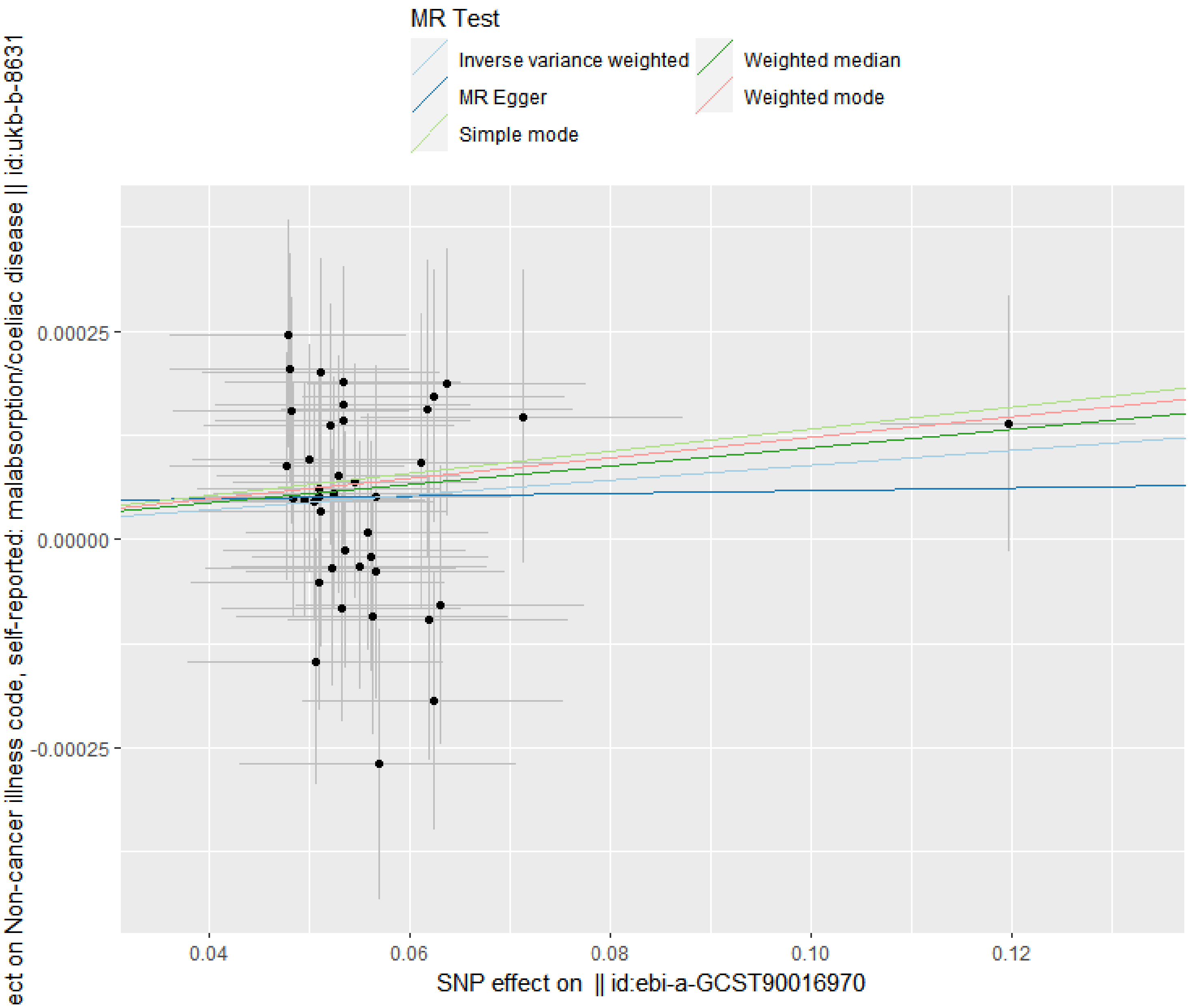

MR Test
Inverse variance weighted
MR Egger
Simple mode
Weighted median
Weighted mode
0.00025
0.00000
-0.00025
0.04
0.06
0.08
0.10
0.12
SNP effect on || id:ebi-a-GCST90016970
ect on Non-cancer illness code, self-reported: malabsorption/coeliac disease || id:ukb-b-8631

Figure 148 Leave-one-out analysis, funnel plot, MR effect size and scatter plot for gut microbiota abundance (genus Butyricicoccus id.2055) on coeliac disease

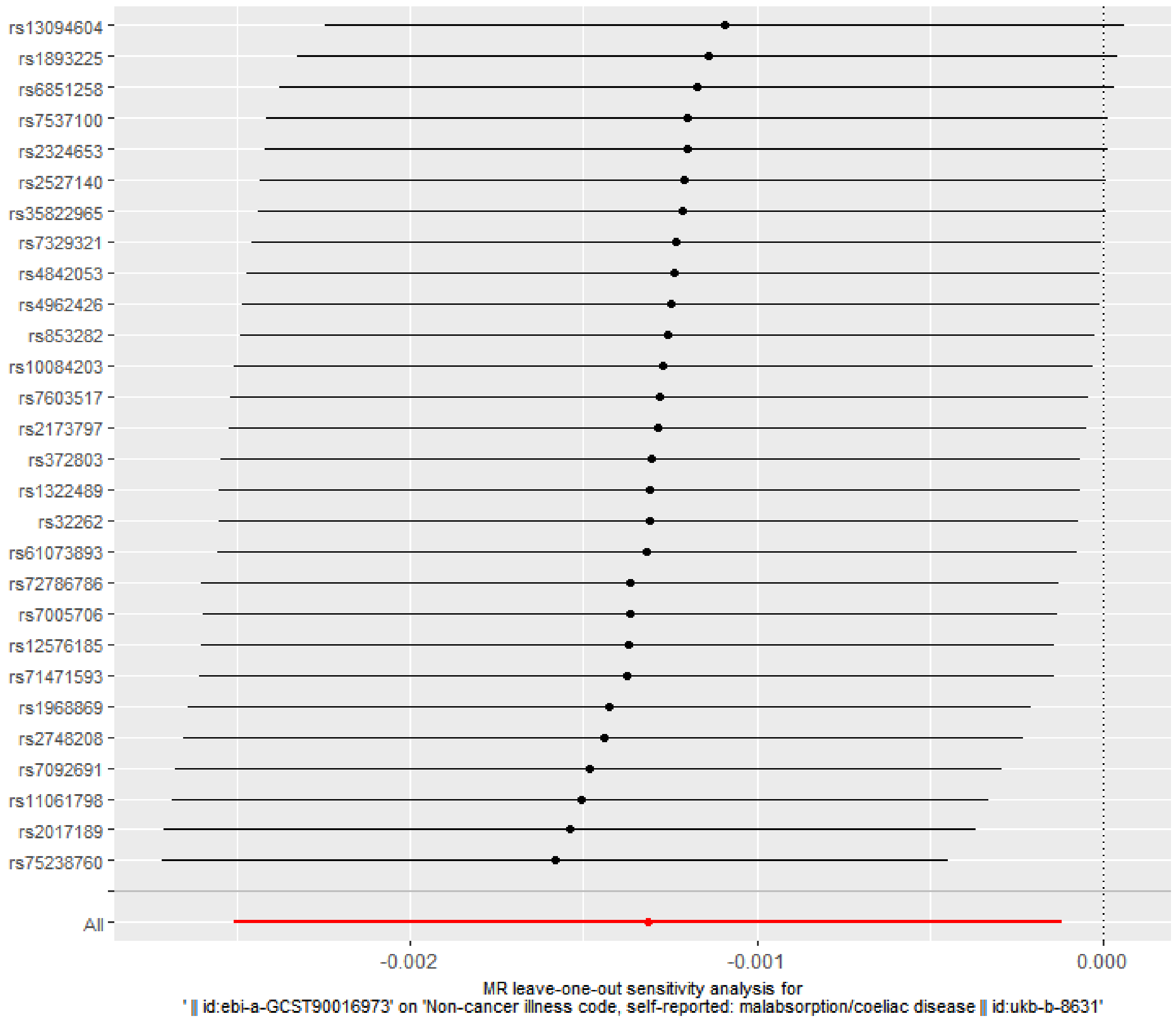

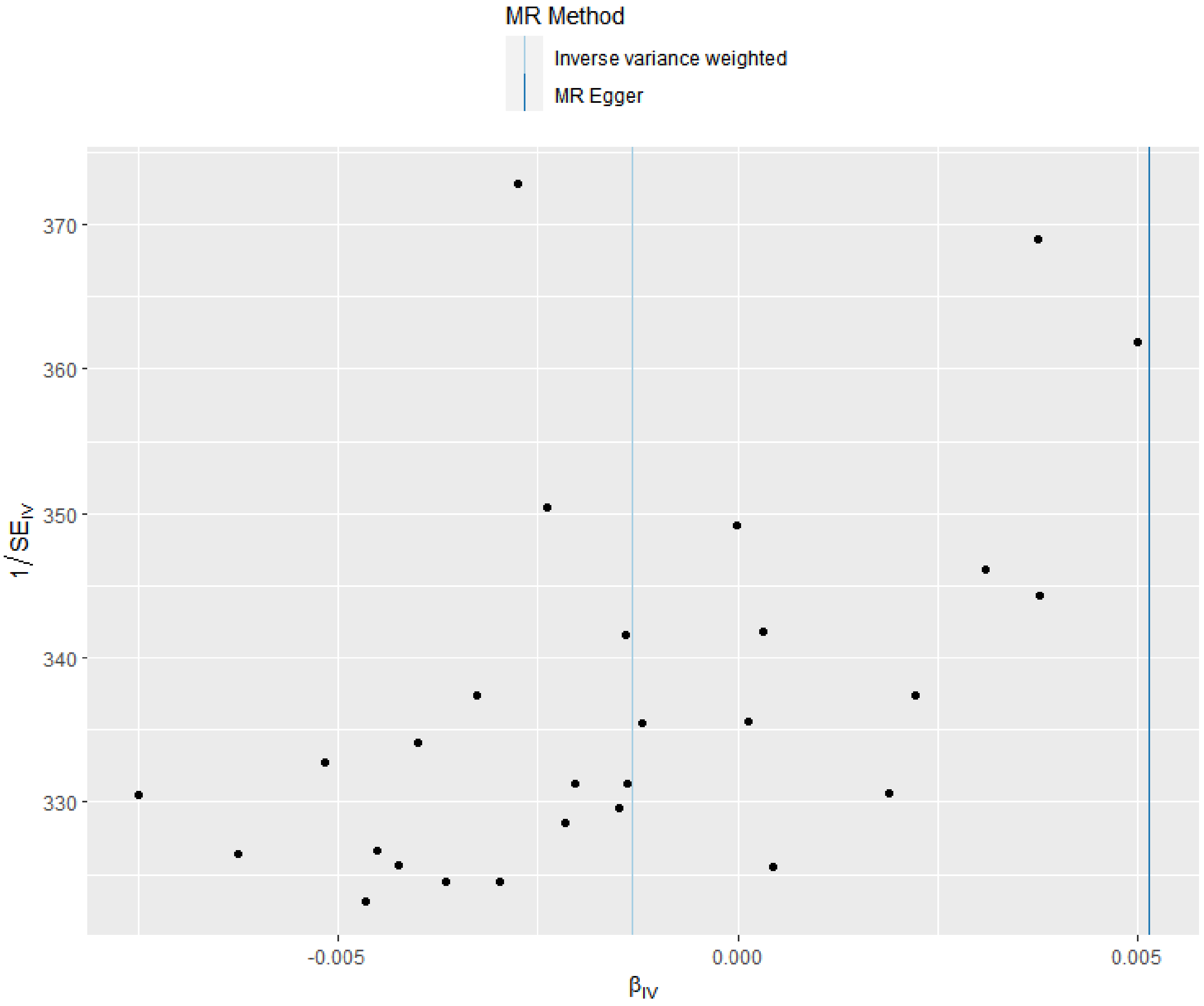

MR Method
Inverse variance weighted
MR Egger
370
360
350
340
330
1/SE_IV
-0.005
0.000
0.005
β_IV

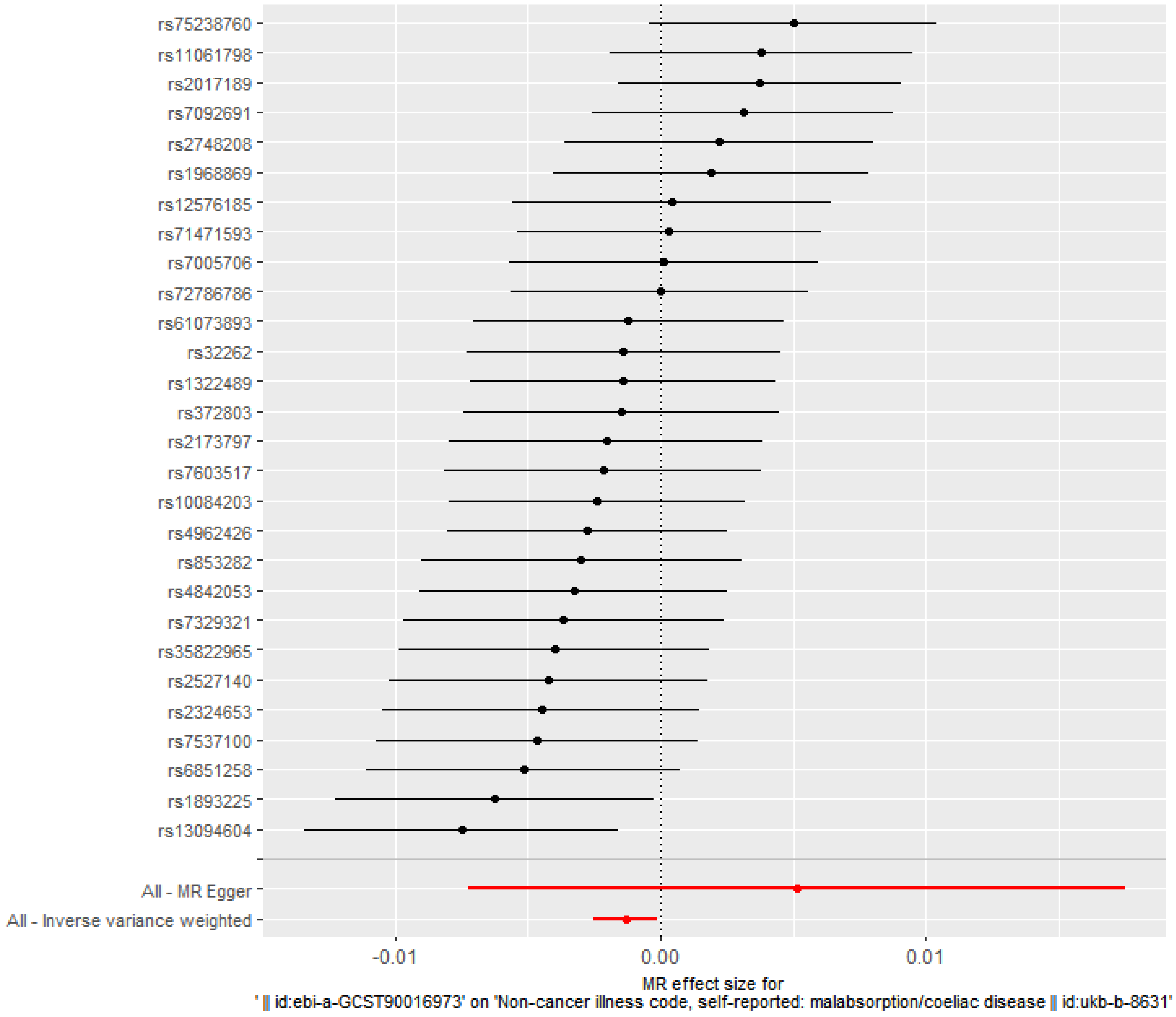
rs75238760
rs11061798
rs2017189
rs7092691
rs2748208
rs1968869
rs12576185
rs71471593
rs7005706
rs72786786
rs61073893
rs32262
rs1322489
rs372803
rs2173797
rs7603517
rs10084203
rs4962426
rs853282
rs4842053
rs7329321
rs35822965
rs2527140
rs2324653
rs7537100
rs6851258
rs1893225
rs13094604
All - MR Egger
All - Inverse variance weighted
-0.01
0.00
0.01
MR effect size for
' || id:ebi-a-GCST90016973' on 'Non-cancer illness code, self-reported: malabsorption/coeliac disease || id:ukb-b-8631'

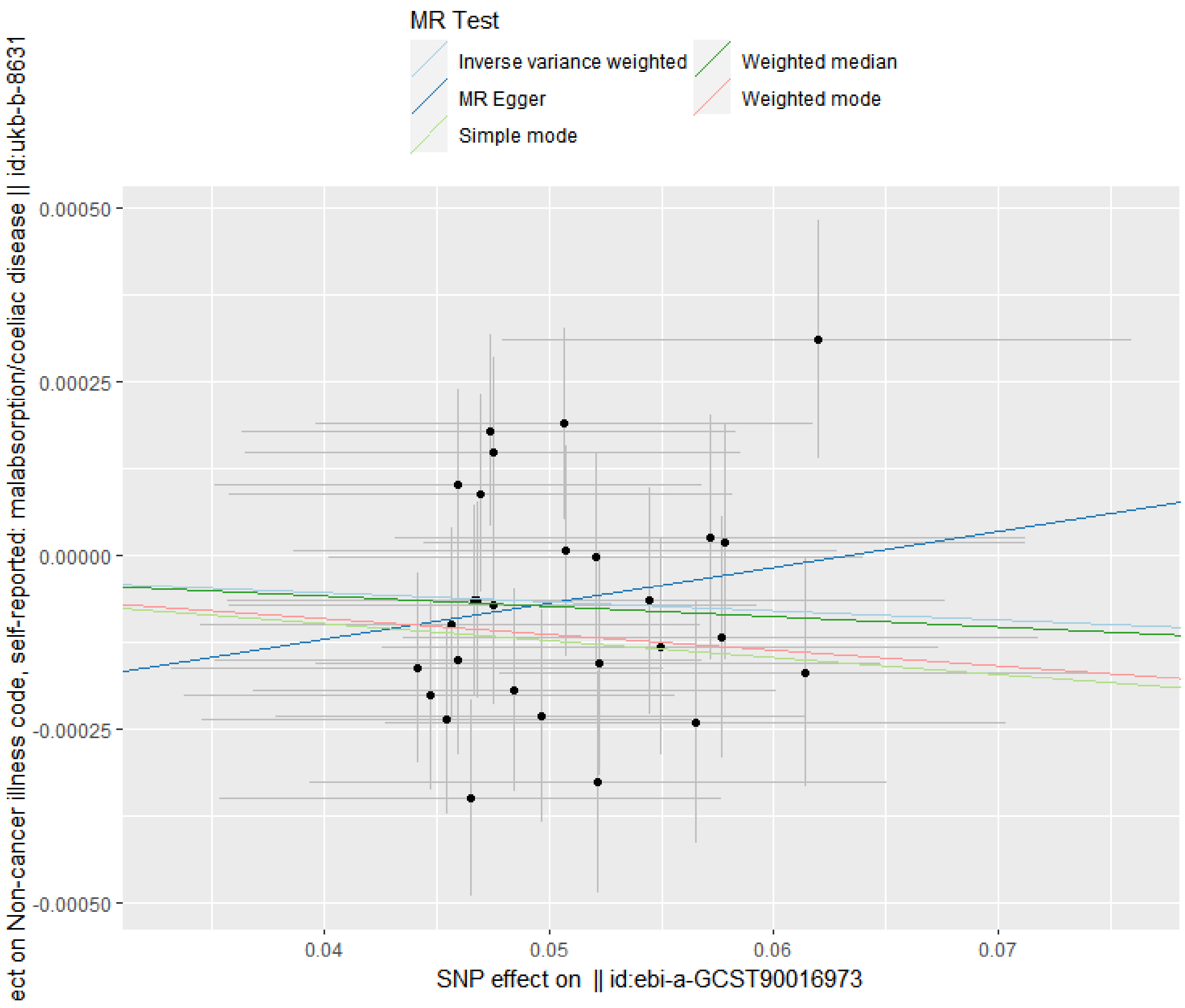

MR Test
Inverse variance weighted
MR Egger
Simple mode
Weighted median
Weighted mode
0.00050
0.00025
0.00000
-0.00025
-0.00050
0.04
0.05
0.06
0.07
SNP effect on || id:ebi-a-GCST90016973
ect on Non-cancer illness code, self-reported: malabsorption/coeliac disease || id:ukb-b-8631

Figure 149 Leave-one-out analysis, funnel plot, MR effect size and scatter plot for gut microbiota abundance (class Actinobacteria id.419) on nonalcoholic fatty liver disease

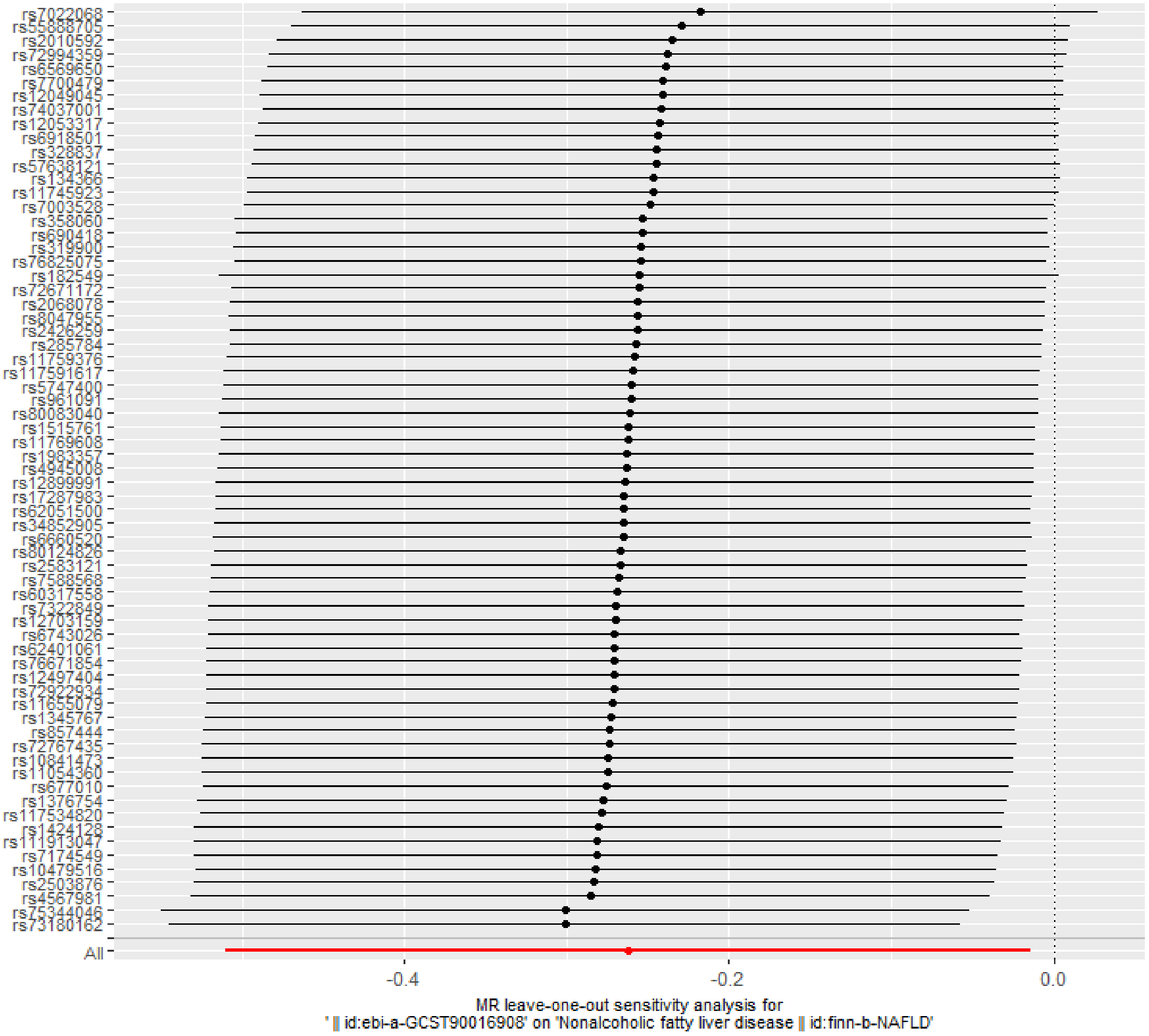

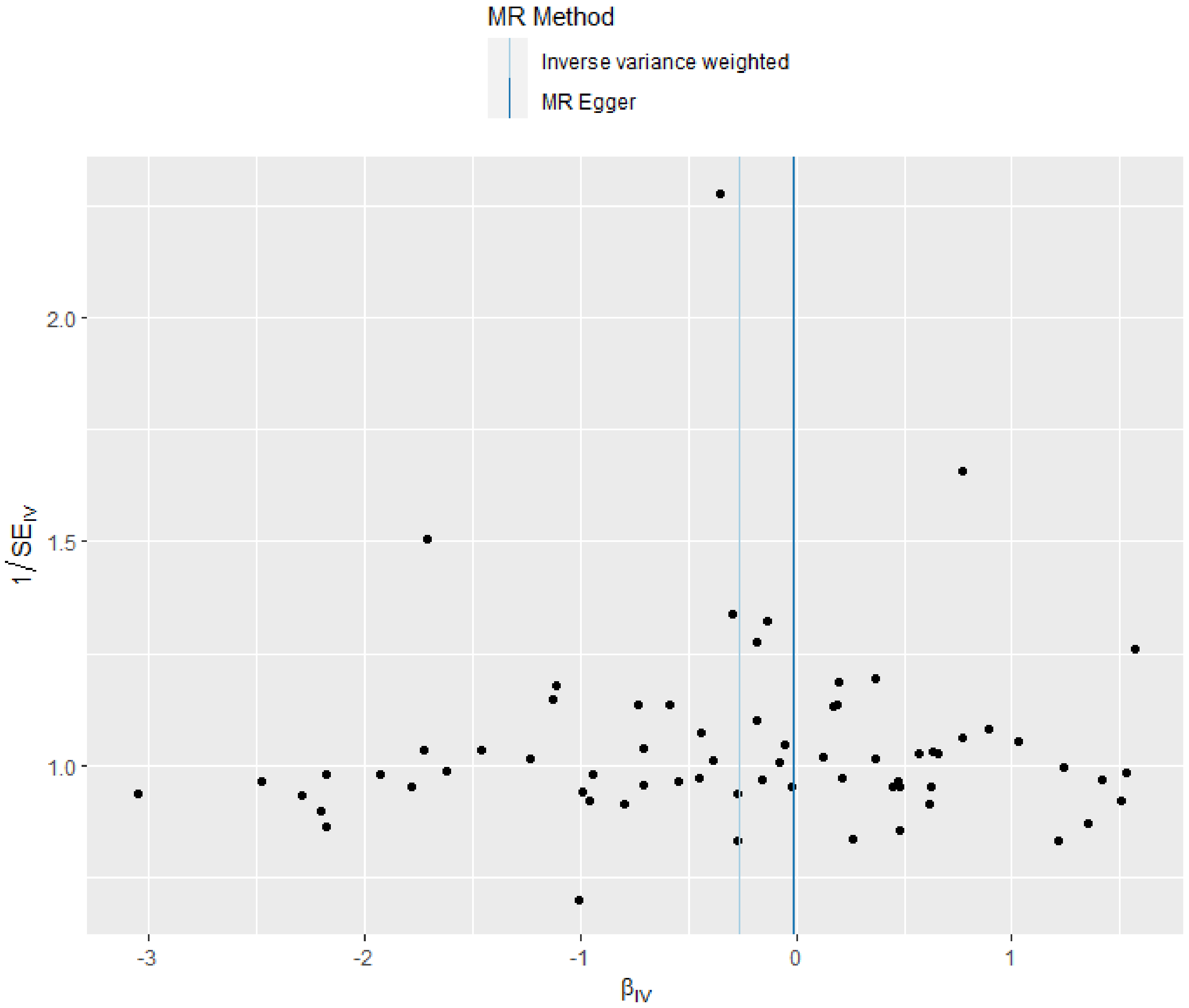
MR Method
Inverse variance weighted
MR Egger
2.0
1.5
1.0
-3
-2
-1
0
1
$1/SE_{IV}$
β_{IV}

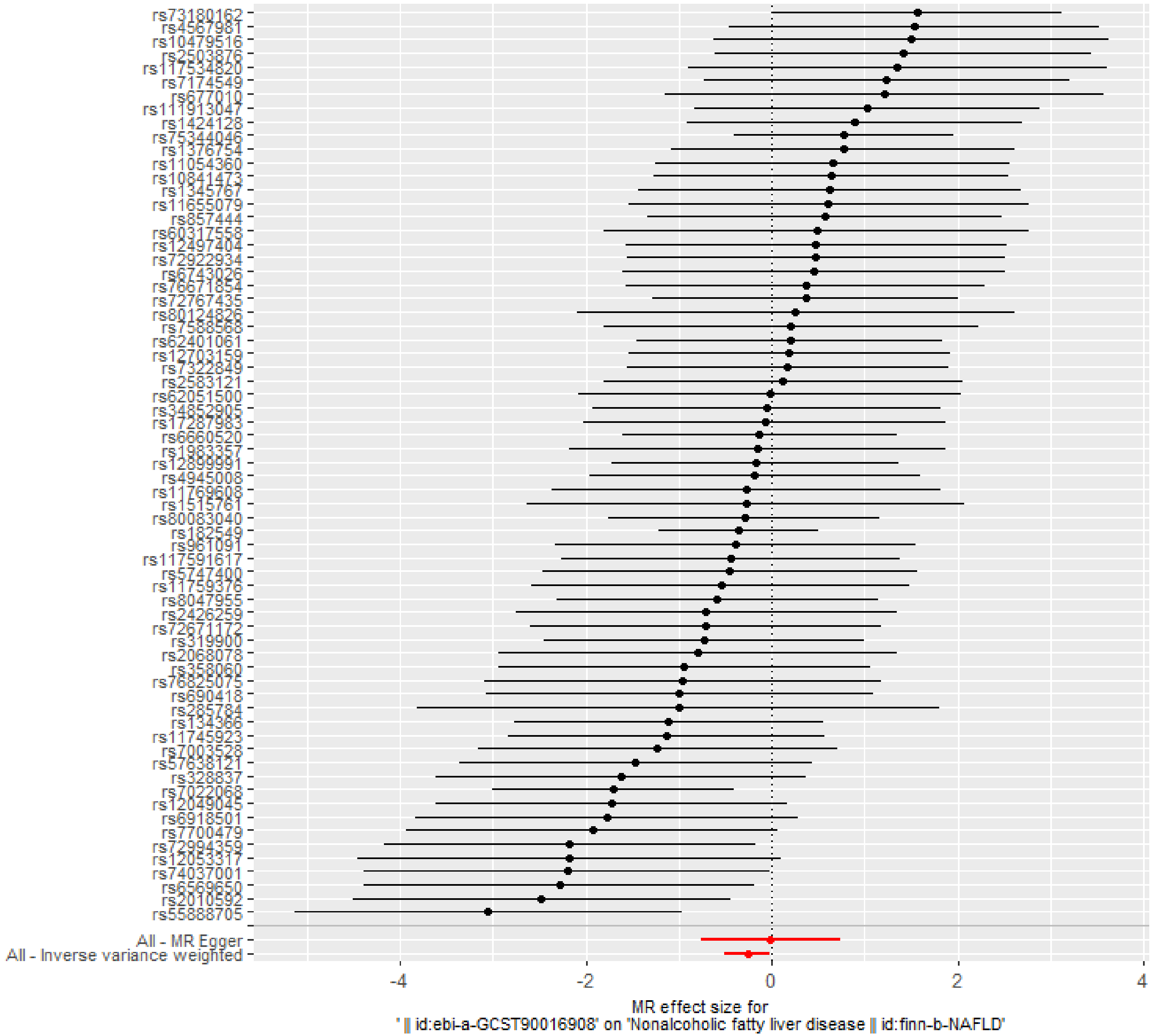
rs73180162
rs4567981
rs10479516
rs2503876
rs117534820
rs7174549
rs677010
rs111913047
rs1424128
rs75344046
rs1376754
rs11054360
rs10841473
rs1345767
rs11655079
rs857444
rs60317558
rs12497404
rs72922934
rs6743026
rs76671854
rs72767435
rs80124826
rs7588568
rs62401061
rs12703159
rs7322849
rs2583121
rs62051500
rs34852905
rs17287983
rs6660520
rs1983357
rs12899991
rs4945008
rs11769608
rs1515761
rs80083040
rs182549
rs961091
rs117591617
rs5747400
rs11759376
rs8047955
rs2426259
rs72671172
rs319900
rs2068078
rs358060
rs76825075
rs690418
rs285784
rs134366
rs11745923
rs7003528
rs57638121
rs328837
rs7022068
rs12049045
rs6918501
rs7700479
rs72994359
rs12053317
rs74037001
rs6569650
rs2010592
rs55888705
All - MR Egger
All - Inverse variance weighted
-4
-2
0
2
4
MR effect size for
' || id:ebi-a-GCST90016908' on 'Nonalcoholic fatty liver disease || id:finn-b-NAFLD'

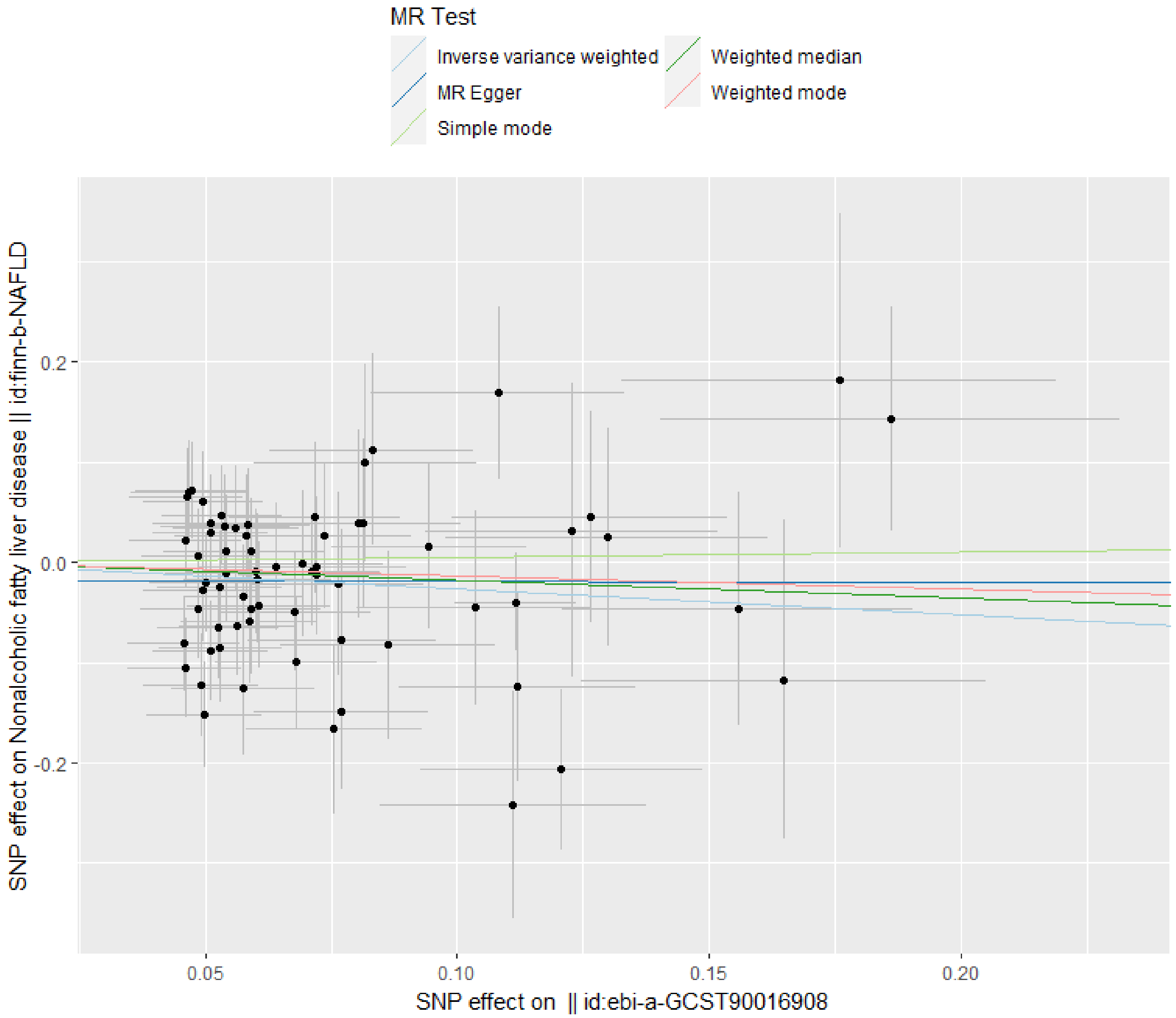

MR Test
Inverse variance weighted
MR Egger
Simple mode
Weighted median
Weighted mode
0.2
0.0
-0.2
0.05
0.10
0.15
0.20
SNP effect on Nonalcoholic fatty liver disease || id:finn-b-NAFLD
SNP effect on || id:ebi-a-GCST90016908

Figure 150 Leave-one-out analysis, funnel plot, MR effect size and scatter plot for gut microbiota abundance (family Alcaligenaceae id.2875) on nonalcoholic fatty liver disease

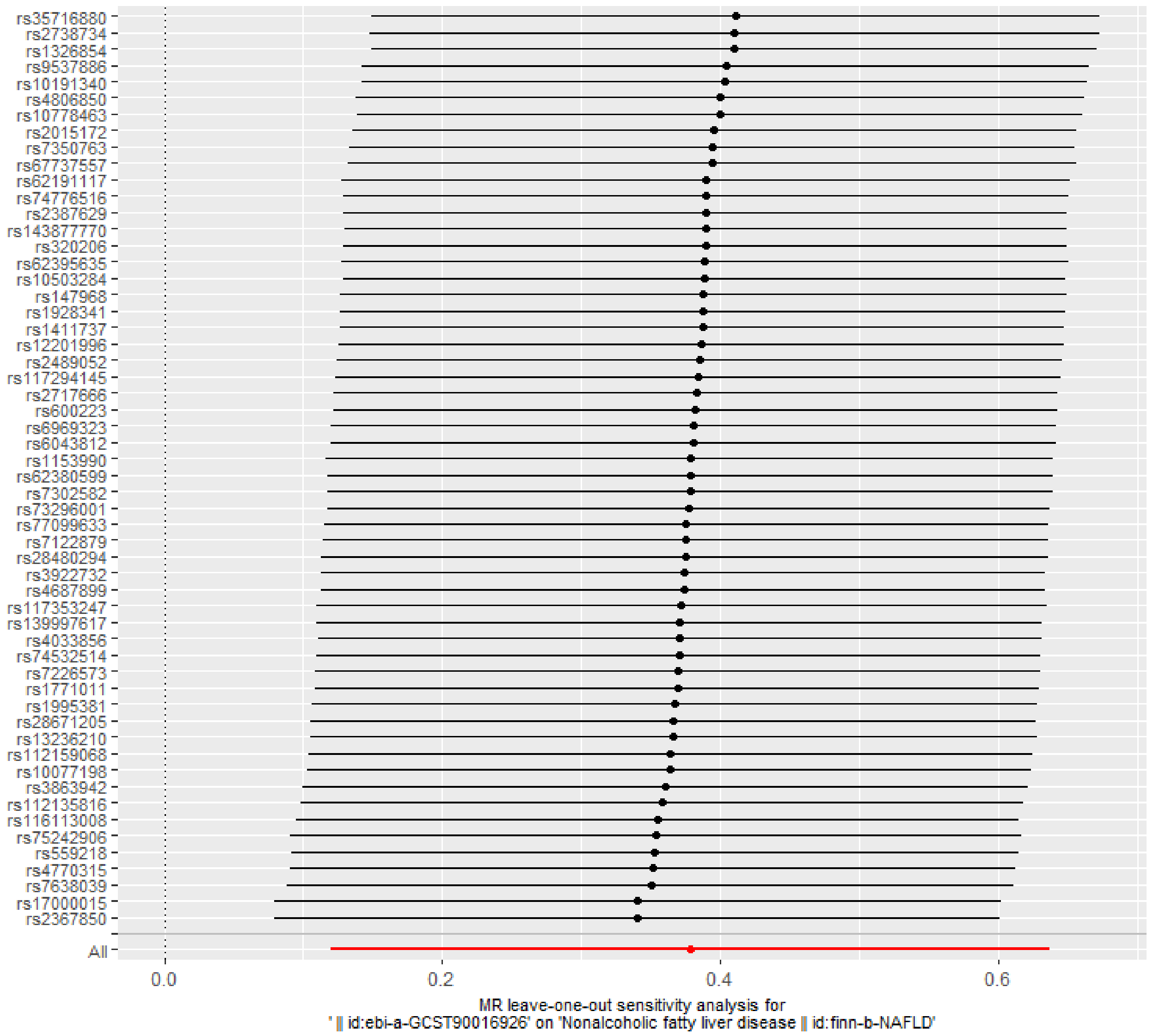

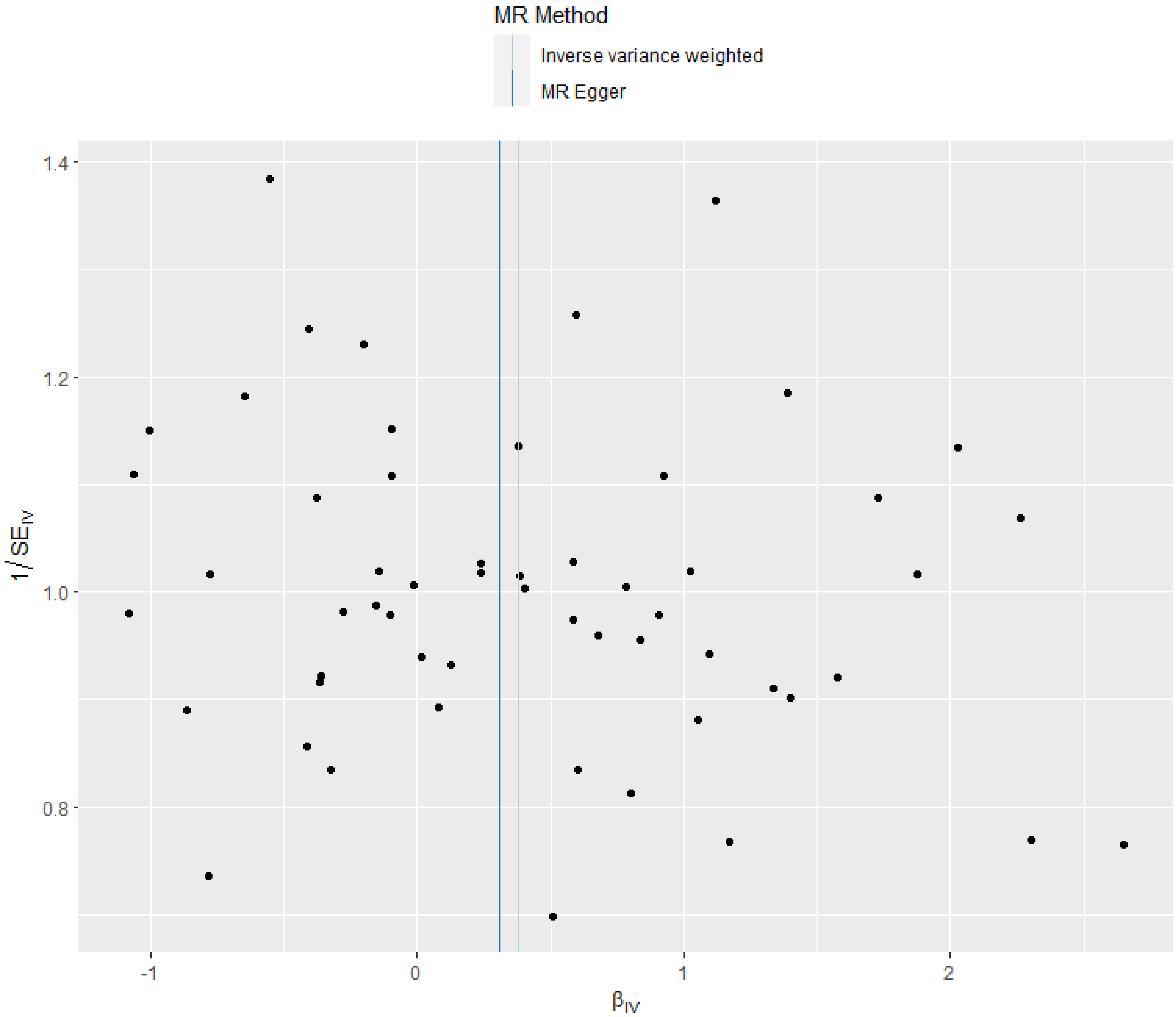

MR Method
Inverse variance weighted
MR Egger
1.4
1.2
1.0
0.8
1/SE_IV
-1
0
1
2
β_IV

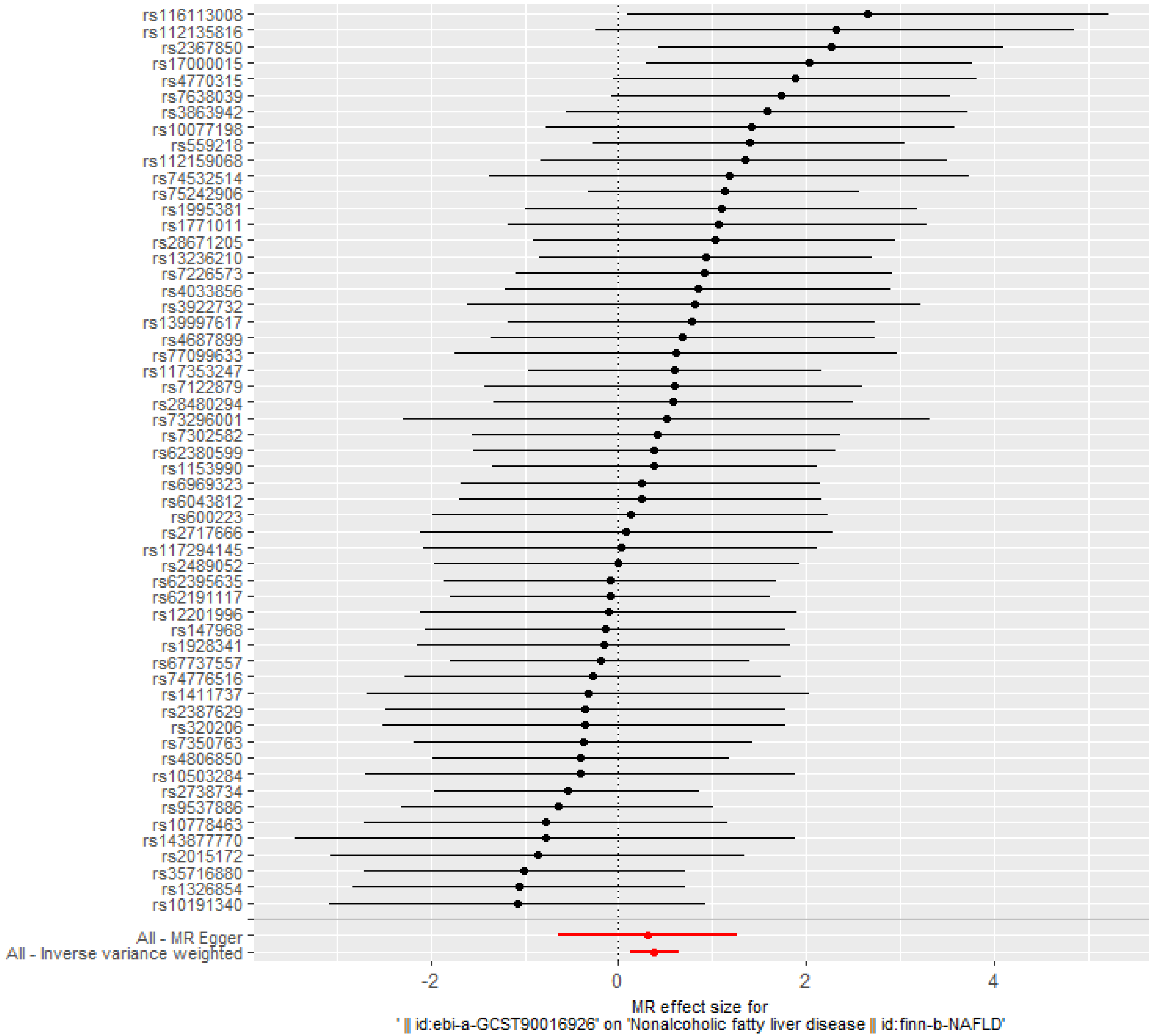
rs116113008
rs112135816
rs2367850
rs17000015
rs4770315
rs7638039
rs3863942
rs10077198
rs559218
rs112159068
rs74532514
rs75242906
rs1995381
rs1771011
rs28671205
rs13236210
rs7226573
rs4033856
rs3922732
rs139997617
rs4687899
rs77099633
rs117353247
rs7122879
rs28480294
rs73296001
rs7302582
rs62380599
rs1153990
rs6969323
rs6043812
rs600223
rs2717666
rs117294145
rs2489052
rs62395635
rs62191117
rs12201996
rs147968
rs1928341
rs67737557
rs74776516
rs1411737
rs2387629
rs320206
rs7350763
rs4806850
rs10503284
rs2738734
rs9537886
rs10778463
rs143877770
rs2015172
rs35716880
rs1326854
rs10191340
All - MR Egger
All - Inverse variance weighted
-2
0
2
4
MR effect size for
' || id:ebi-a-GCST90016926' on 'Nonalcoholic fatty liver disease || id:finn-b-NAFLD'

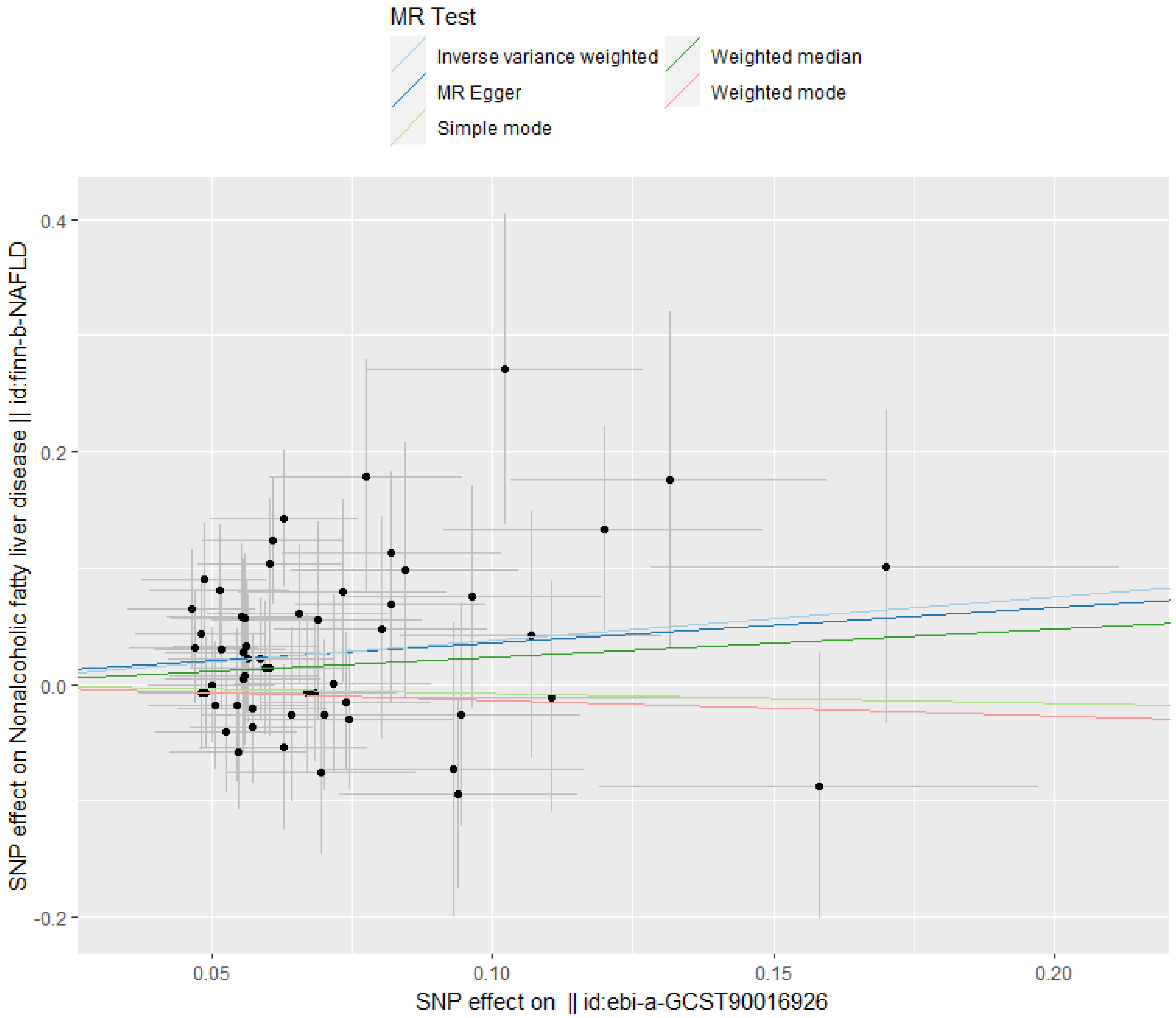
MR Test
Inverse variance weighted
MR Egger
Simple mode
Weighted median
Weighted mode
0.4
0.2
0.0
-0.2
0.05
0.10
0.15
0.20
SNP effect on Nonalcoholic fatty liver disease || id:finn-b-NAFLD
SNP effect on || id:ebi-a-GCST90016926

Figure 151 Leave-one-out analysis, funnel plot, MR effect size and scatter plot for gut microbiota abundance (unknown family id.1000006161) on nonalcoholic fatty liver disease

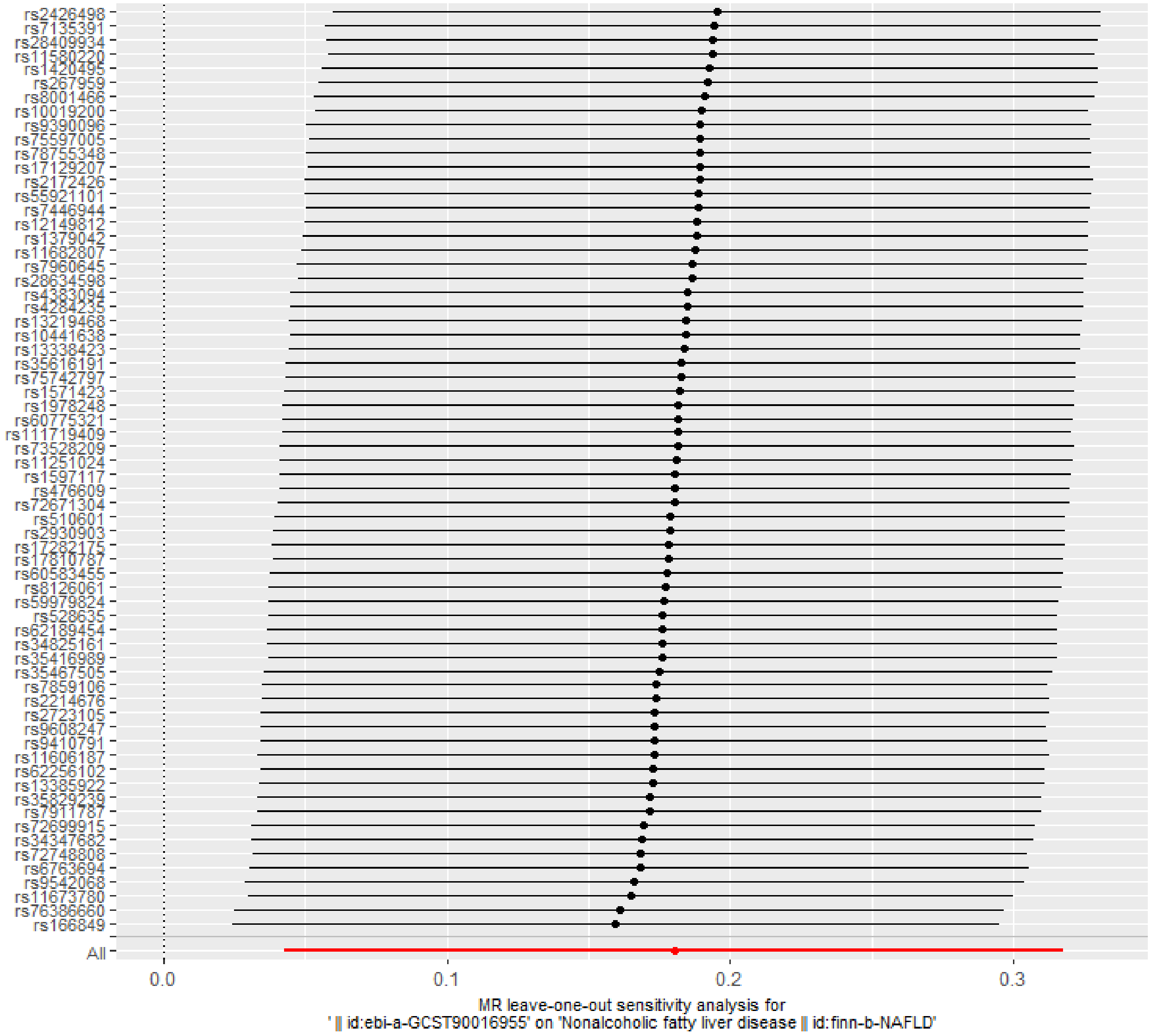

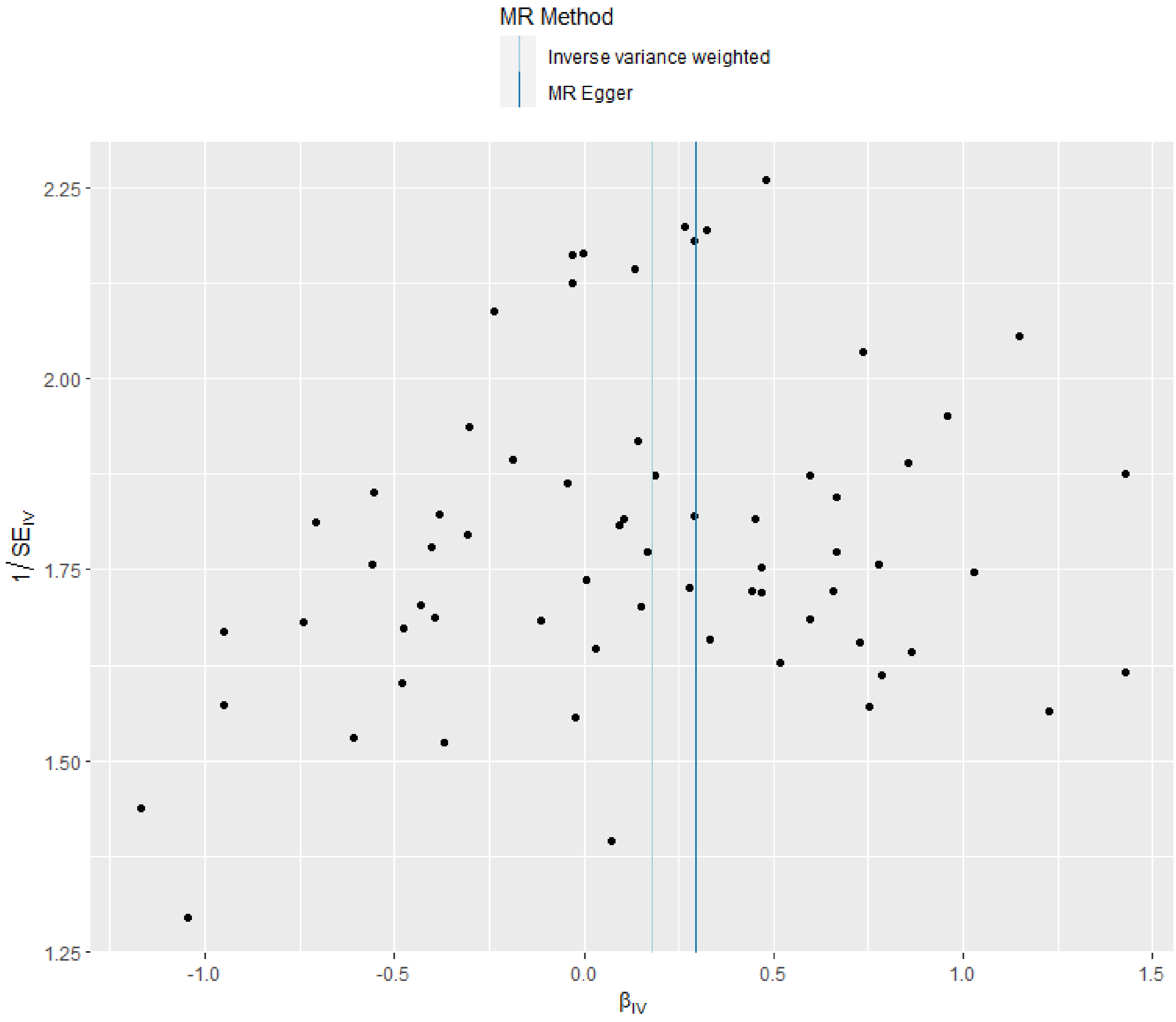

MR Method
Inverse variance weighted
MR Egger
2.25
2.00
1.75
1.50
1.25
1/SE_IV
-1.0
-0.5
0.0
0.5
1.0
1.5
β_IV

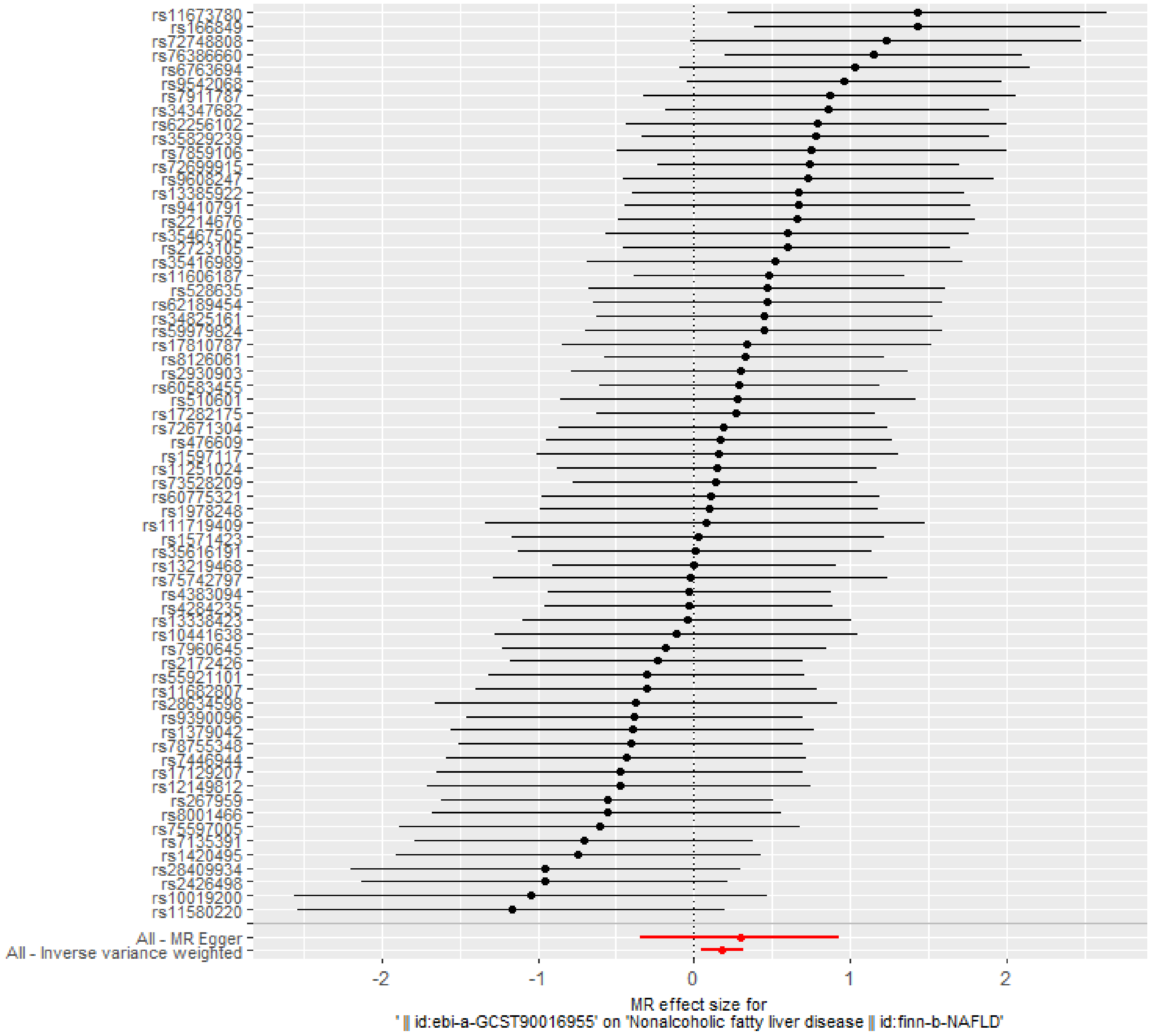

rs11673780
rs166849
rs72748808
rs76386660
rs6763694
rs9542068
rs7911787
rs34347682
rs62256102
rs35829239
rs7859106
rs72699915
rs9608247
rs13385922
rs9410791
rs2214676
rs35467505
rs2723105
rs35416989
rs11606187
rs528635
rs62189454
rs34825161
rs59979824
rs17810787
rs8126061
rs2930903
rs60583455
rs510601
rs17282175
rs72671304
rs476609
rs1597117
rs11251024
rs73528209
rs60775321
rs1978248
rs111719409
rs1571423
rs35616191
rs13219468
rs75742797
rs4383094
rs4284235
rs13338423
rs10441638
rs7960645
rs2172426
rs55921101
rs11682807
rs28634598
rs9390096
rs1379042
rs78755348
rs7446944
rs17129207
rs12149812
rs267959
rs8001466
rs75597005
rs7135391
rs1420495
rs28409934
rs2426498
rs10019200
rs11580220
All - MR Egger
All - Inverse variance weighted
-2
-1
0
1
2
MR effect size for
' || id:ebi-a-GCST90016955' on 'Nonalcoholic fatty liver disease || id:finn-b-NAFLD'

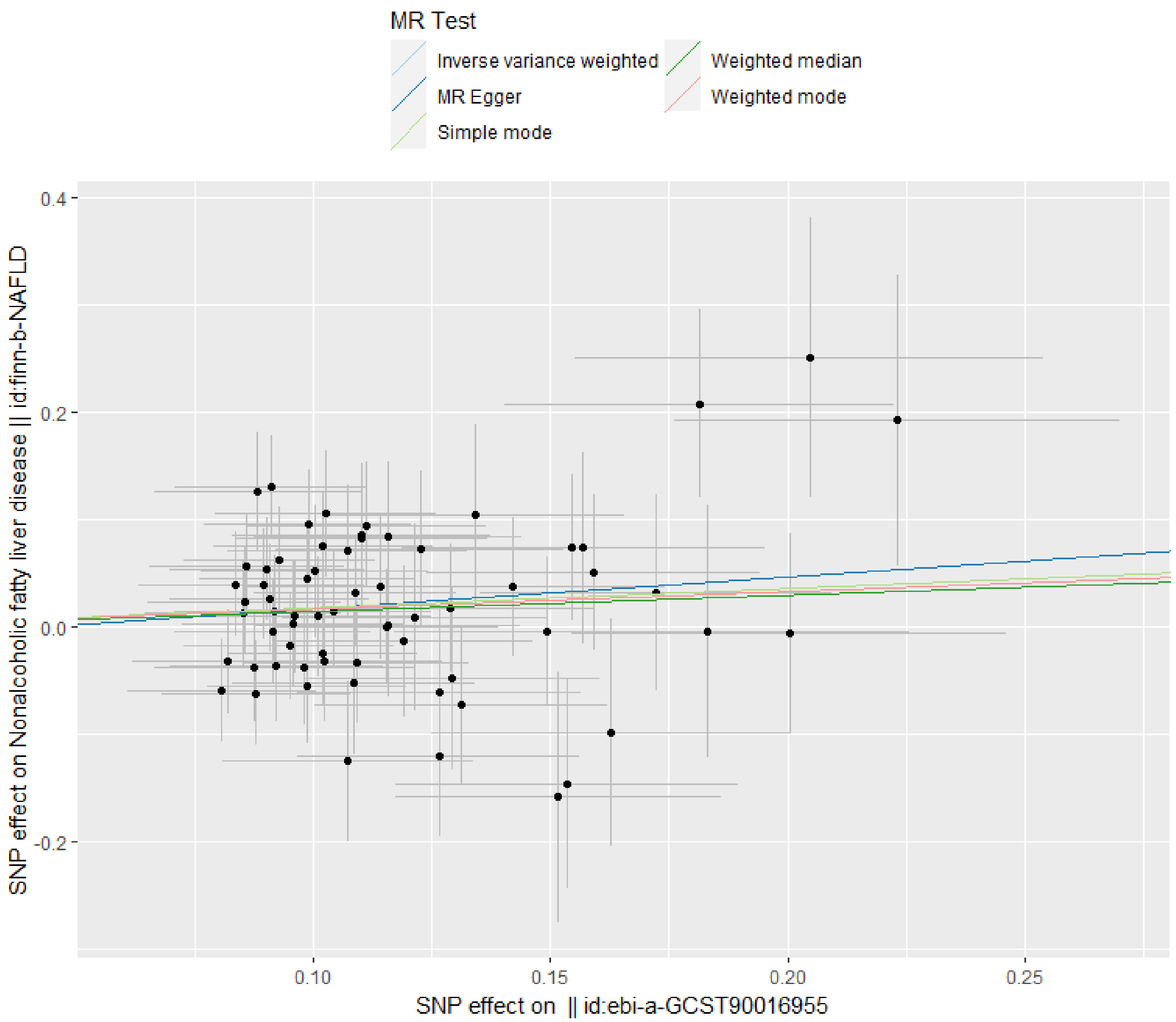

MR Test
Inverse variance weighted
MR Egger
Simple mode
Weighted median
Weighted mode
0.4
0.2
0.0
-0.2
SNP effect on Nonalcoholic fatty liver disease || id:finn-b-NAFLD
0.10
0.15
0.20
0.25
SNP effect on || id:ebi-a-GCST90016955

Figure 152 Leave-one-out analysis, funnel plot, MR effect size and scatter plot for gut microbiota abundance (genus Alloprevotella id.961) on nonalcoholic fatty liver disease

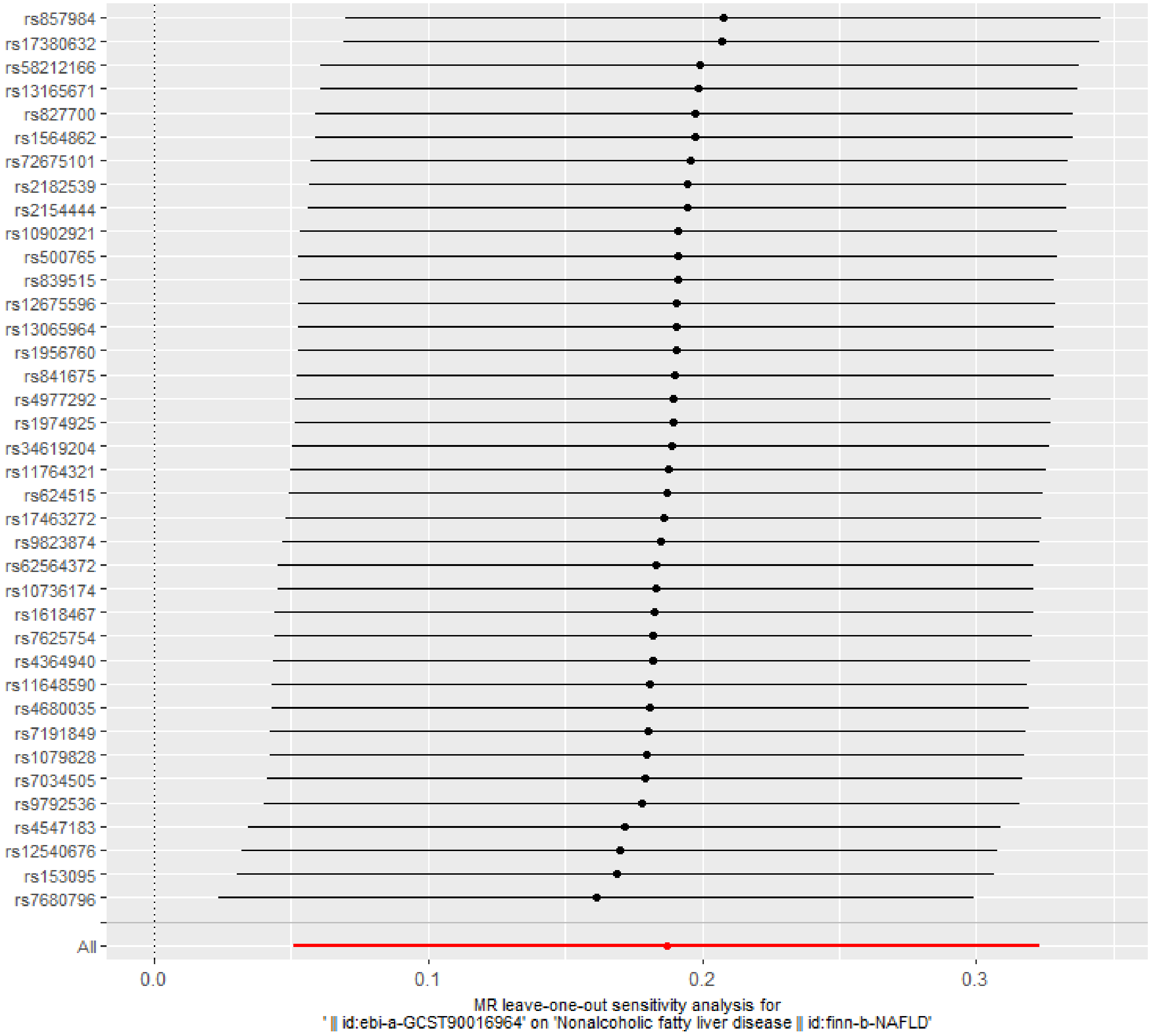

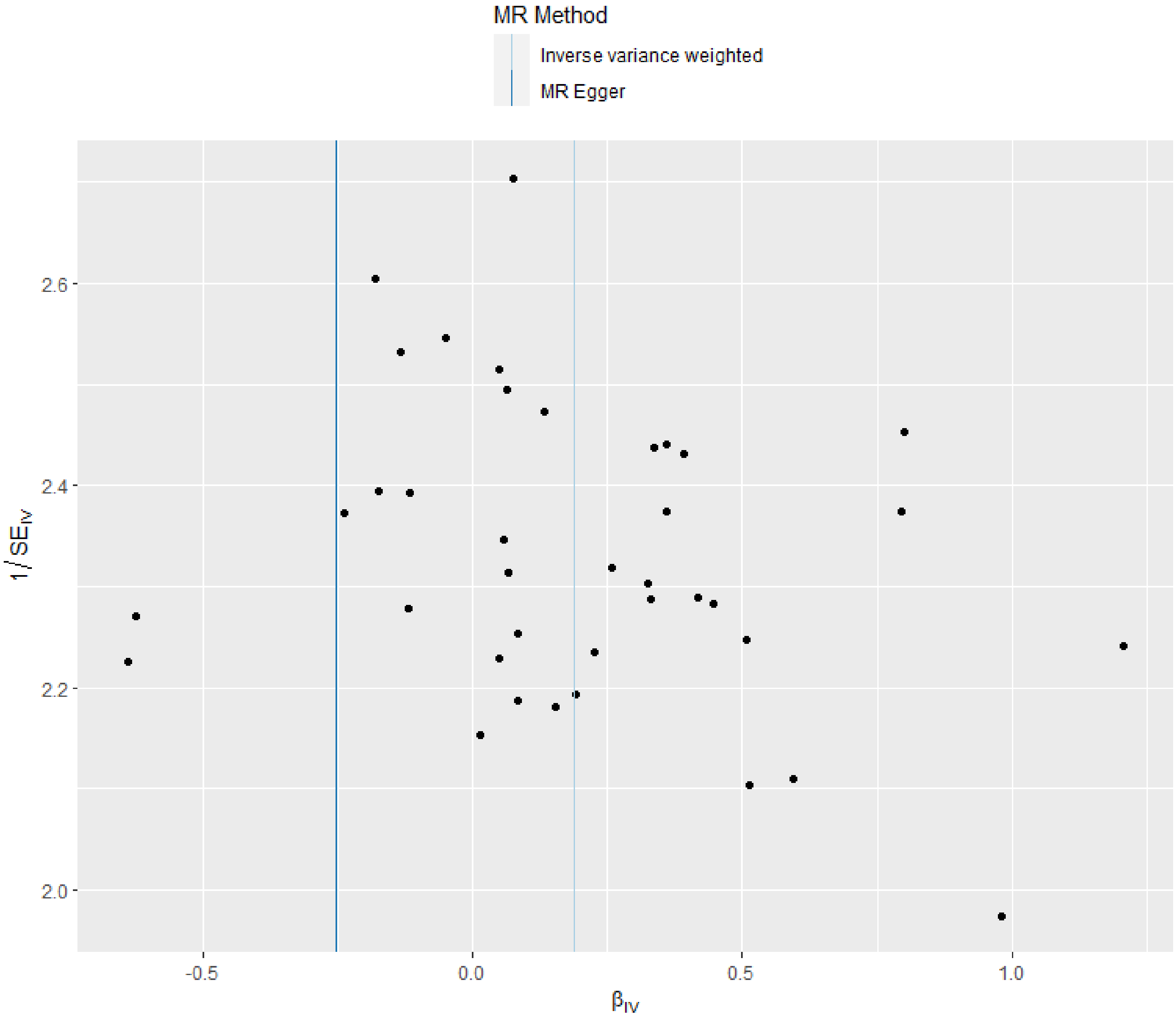
MR Method
Inverse variance weighted
MR Egger
2.6
2.4
2.2
2.0
1/SE_IV
-0.5
0.0
0.5
1.0
β_IV

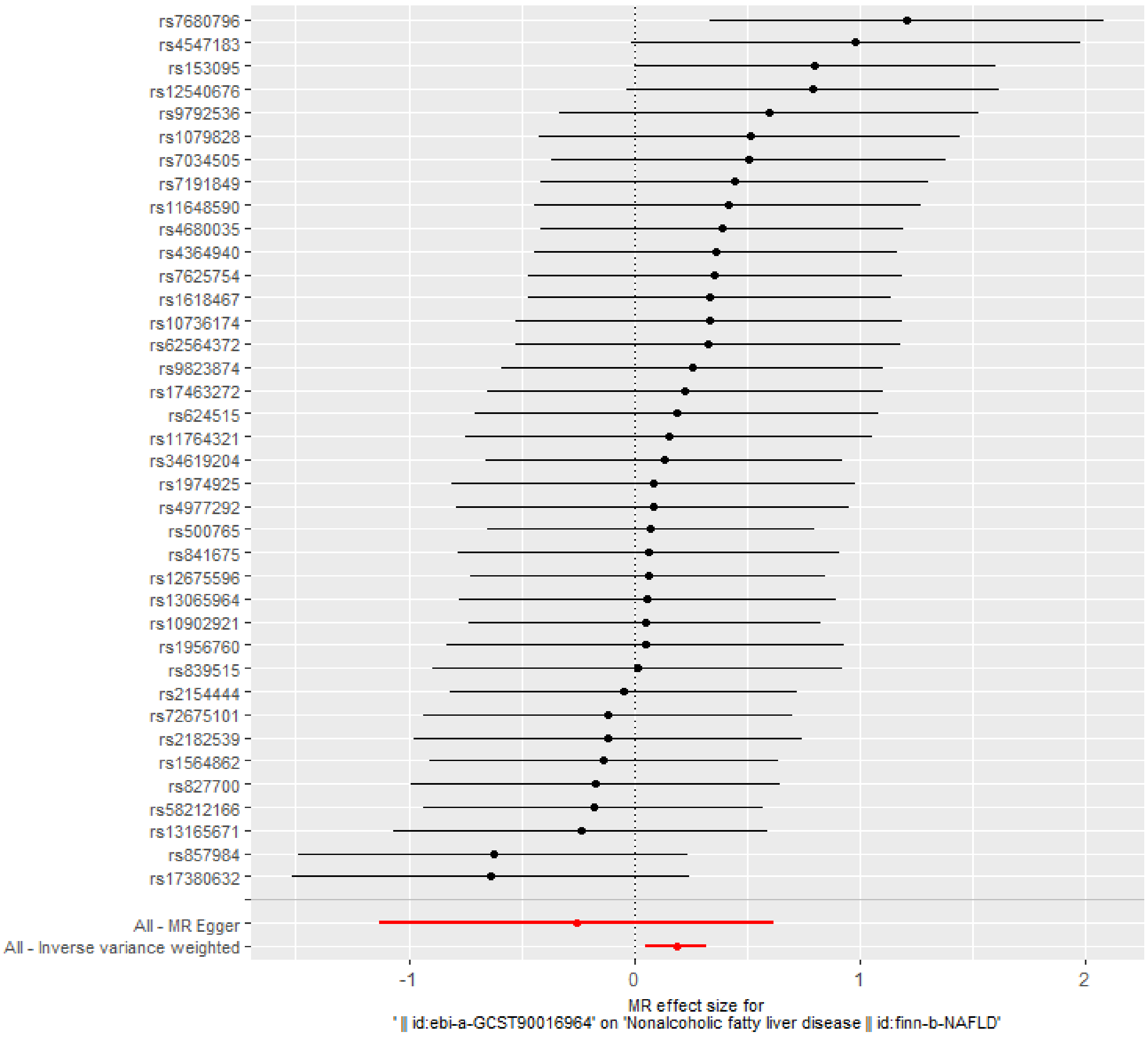

rs7680796
rs4547183
rs153095
rs12540676
rs9792536
rs1079828
rs7034505
rs7191849
rs11648590
rs4680035
rs4364940
rs7625754
rs1618467
rs10736174
rs62564372
rs9823874
rs17463272
rs624515
rs11764321
rs34619204
rs1974925
rs4977292
rs500765
rs841675
rs12675596
rs13065964
rs10902921
rs1956760
rs839515
rs2154444
rs72675101
rs2182539
rs1564862
rs827700
rs58212166
rs13165671
rs857984
rs17380632
All - MR Egger
All - Inverse variance weighted
-1
0
1
2
MR effect size for
' || id:ebi-a-GCST90016964' on 'Nonalcoholic fatty liver disease || id:finn-b-NAFLD'

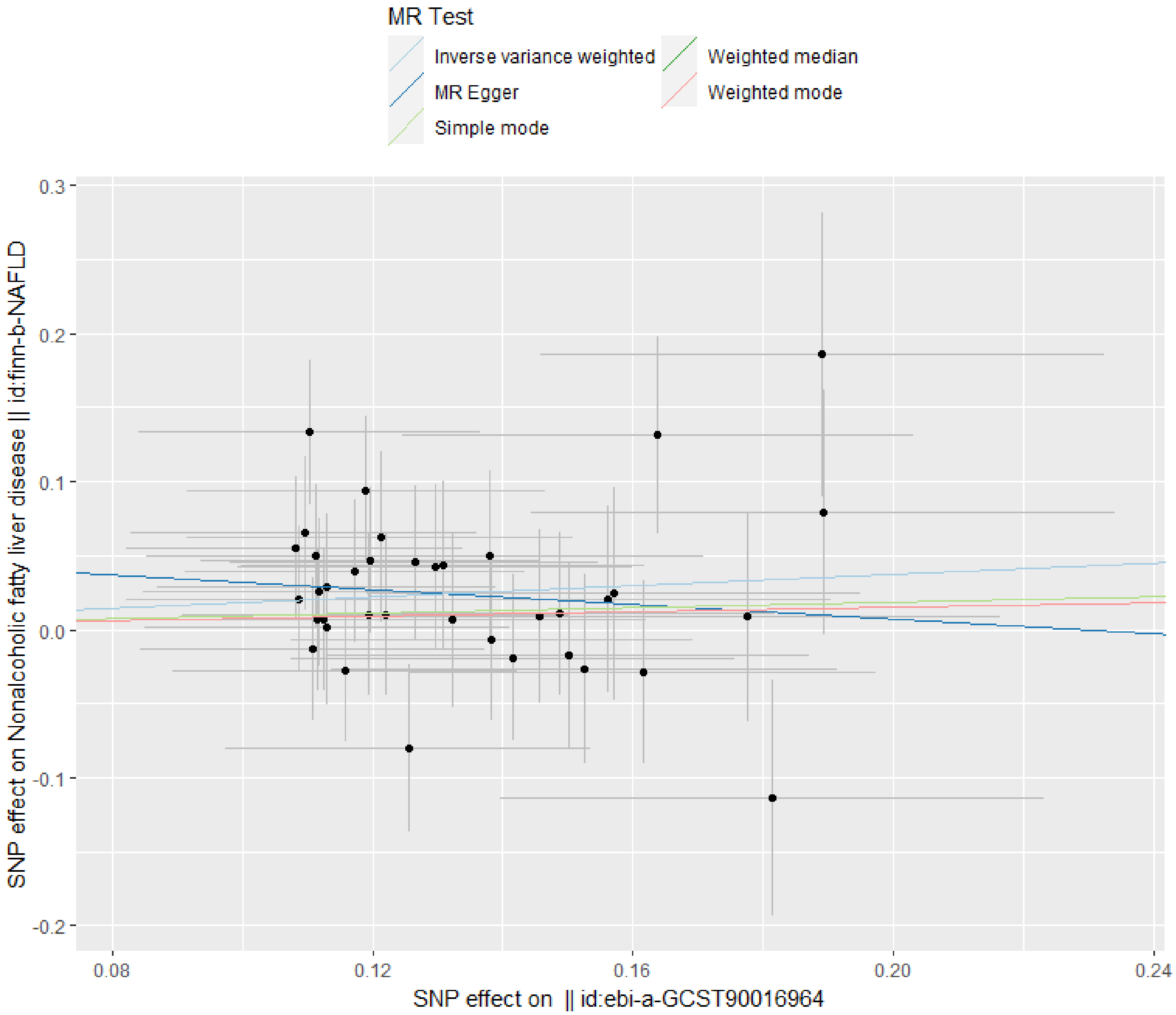
MR Test
Inverse variance weighted
MR Egger
Simple mode
Weighted median
Weighted mode
SNP effect on Nonalcoholic fatty liver disease || id:finn-b-NAFLD
0.3
0.2
0.1
0.0
-0.1
-0.2
0.08
0.12
0.16
0.20
0.24
SNP effect on || id:ebi-a-GCST90016964

Figure 153 Leave-one-out analysis, funnel plot, MR effect size and scatter plot for gut microbiota abundance (genus Butyricicoccus id.2055) on nonalcoholic fatty liver disease

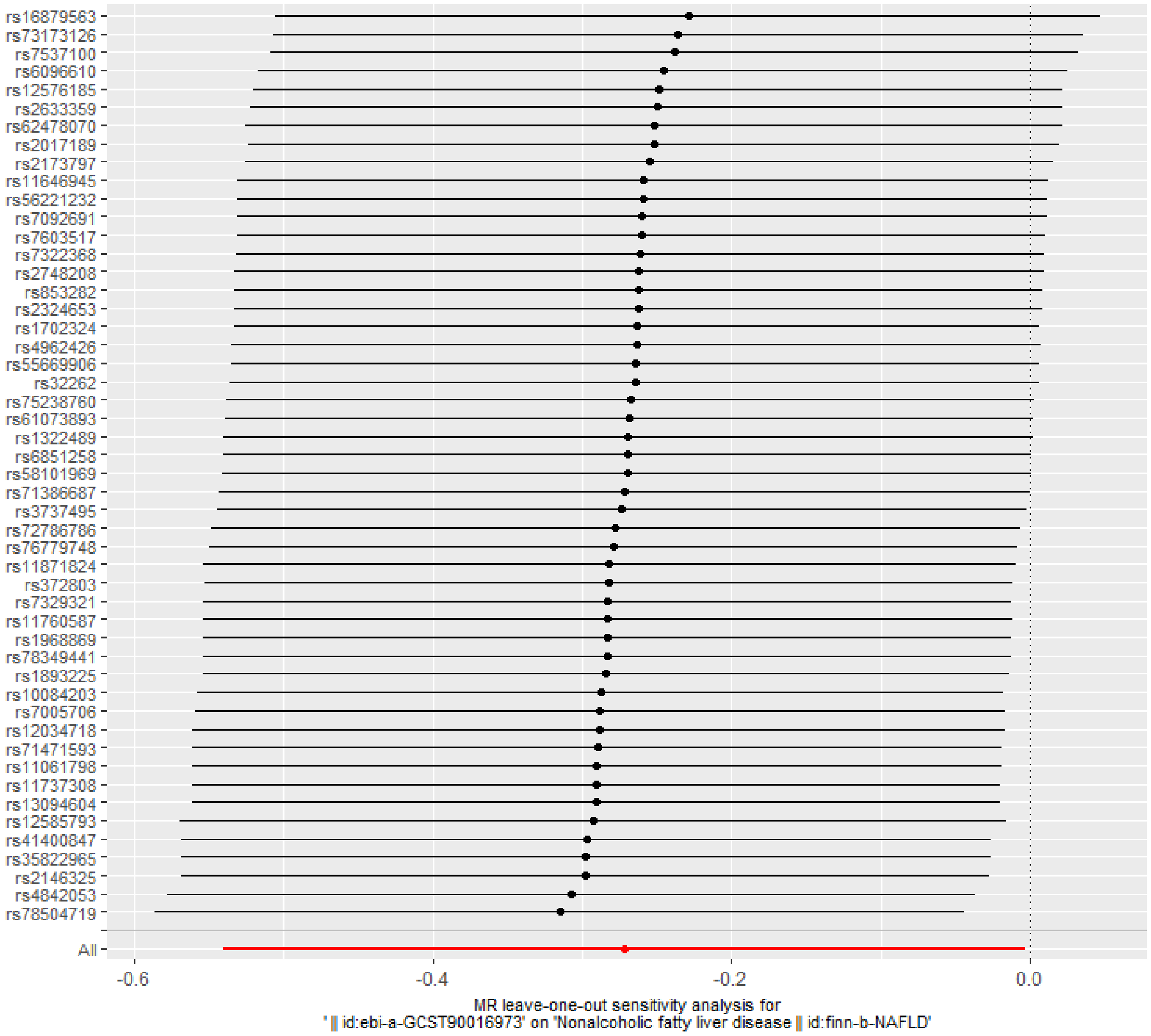

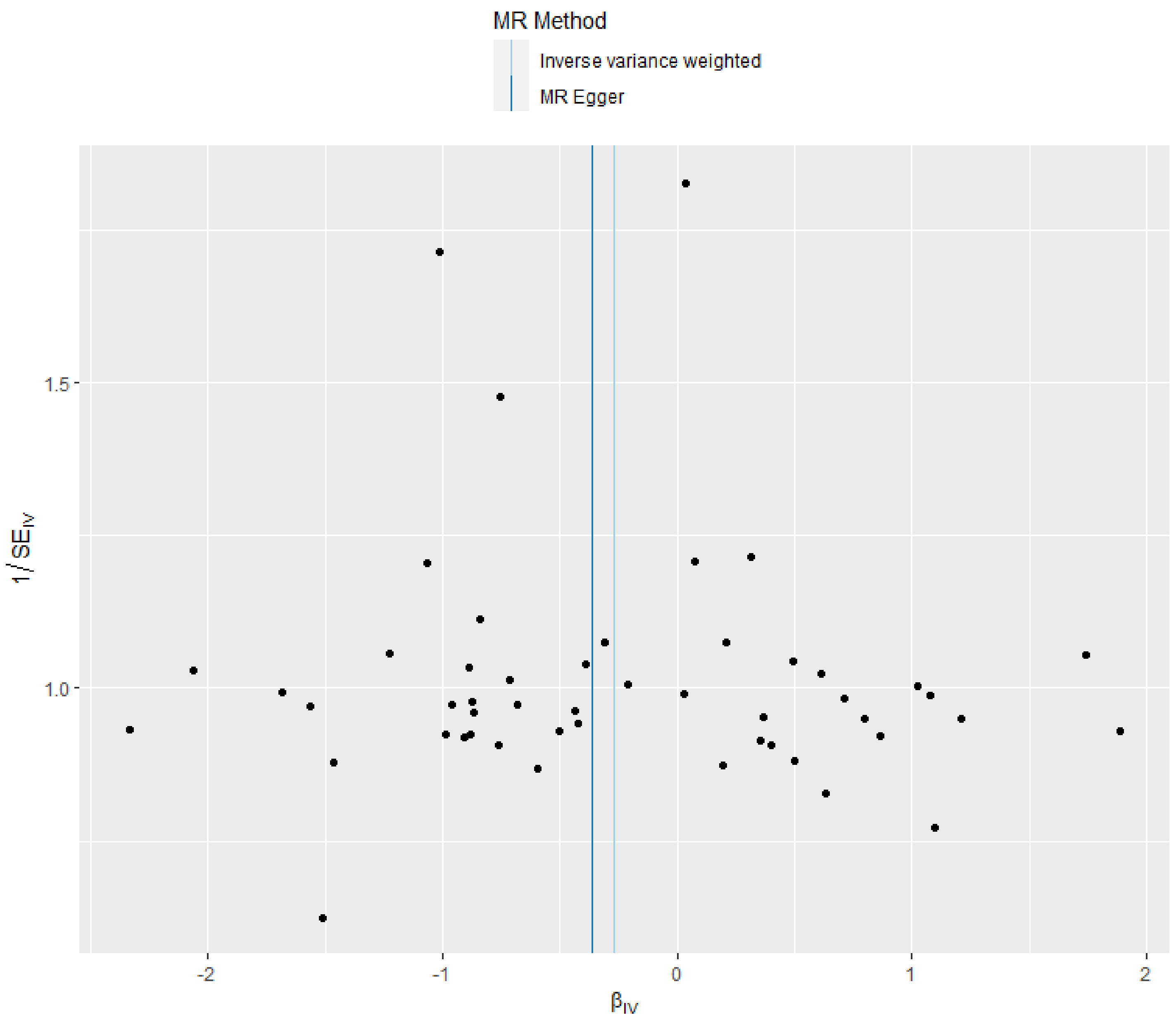

MR Method
Inverse variance weighted
MR Egger
1.5
1.0
-2
-1
0
1
2
$1/SE_{IV}$
β_{IV}

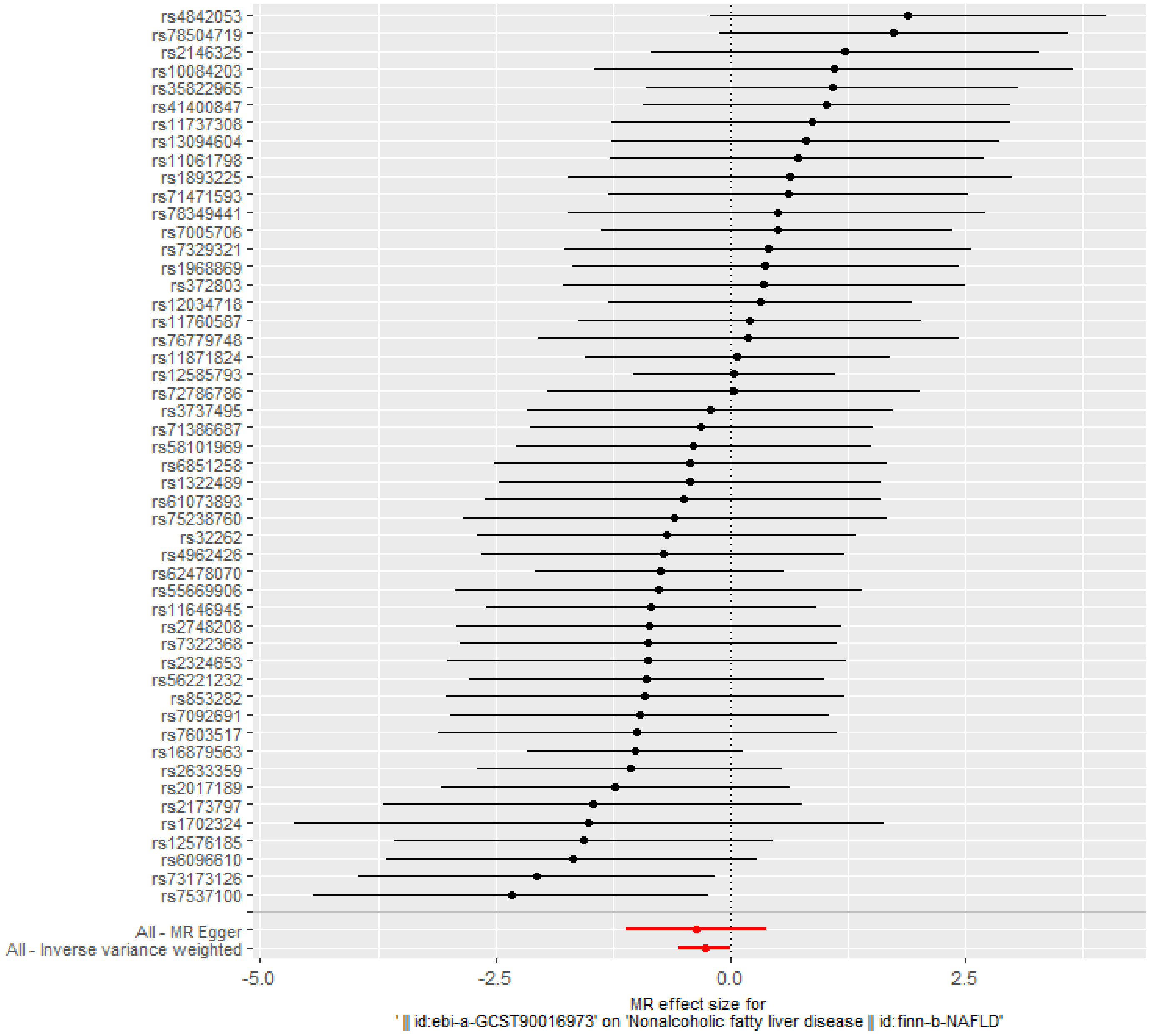
rs4842053
rs78504719
rs2146325
rs10084203
rs35822965
rs41400847
rs11737308
rs13094604
rs11061798
rs1893225
rs71471593
rs78349441
rs7005706
rs7329321
rs1968869
rs372803
rs12034718
rs11760587
rs76779748
rs11871824
rs12585793
rs72786786
rs3737495
rs71386687
rs58101969
rs6851258
rs1322489
rs61073893
rs75238760
rs32262
rs4962426
rs62478070
rs55669906
rs11646945
rs2748208
rs7322368
rs2324653
rs56221232
rs853282
rs7092691
rs7603517
rs16879563
rs2633359
rs2017189
rs2173797
rs1702324
rs12576185
rs6096610
rs73173126
rs7537100
All - MR Egger
All - Inverse variance weighted
-5.0
-2.5
0.0
2.5
MR effect size for
' || id:ebi-a-GCST90016973' on 'Nonalcoholic fatty liver disease || id:finn-b-NAFLD'

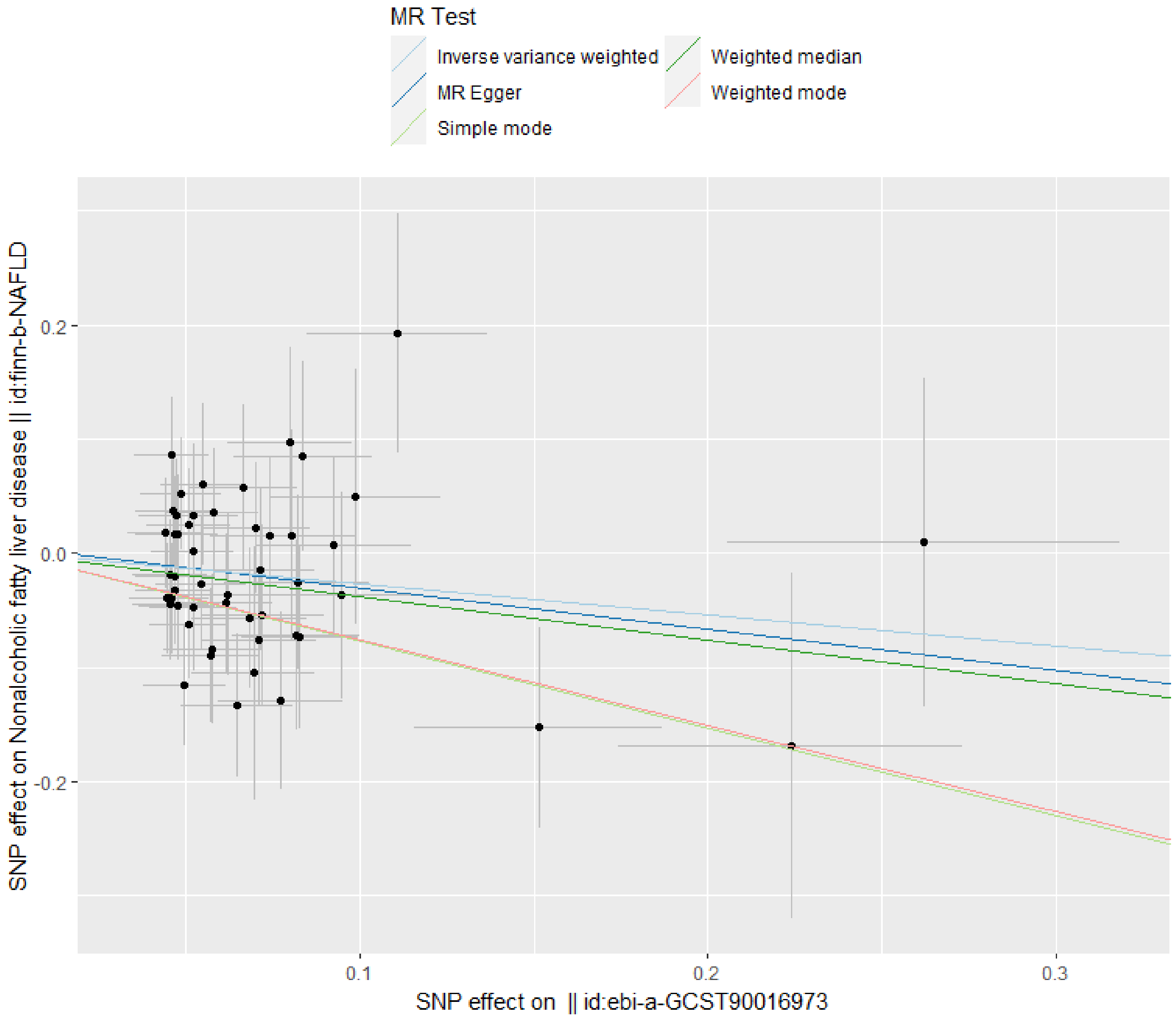

MR Test
Inverse variance weighted
MR Egger
Simple mode
Weighted median
Weighted mode
SNP effect on Nonalcoholic fatty liver disease || id:finn-b-NAFLD
SNP effect on || id:ebi-a-GCST90016973
0.2
0.0
-0.2
0.1
0.2
0.3

Figure 154 Leave-one-out analysis, funnel plot, MR effect size and scatter plot for gut microbiota abundance (genus Collinsella id.815) on nonalcoholic fatty liver disease

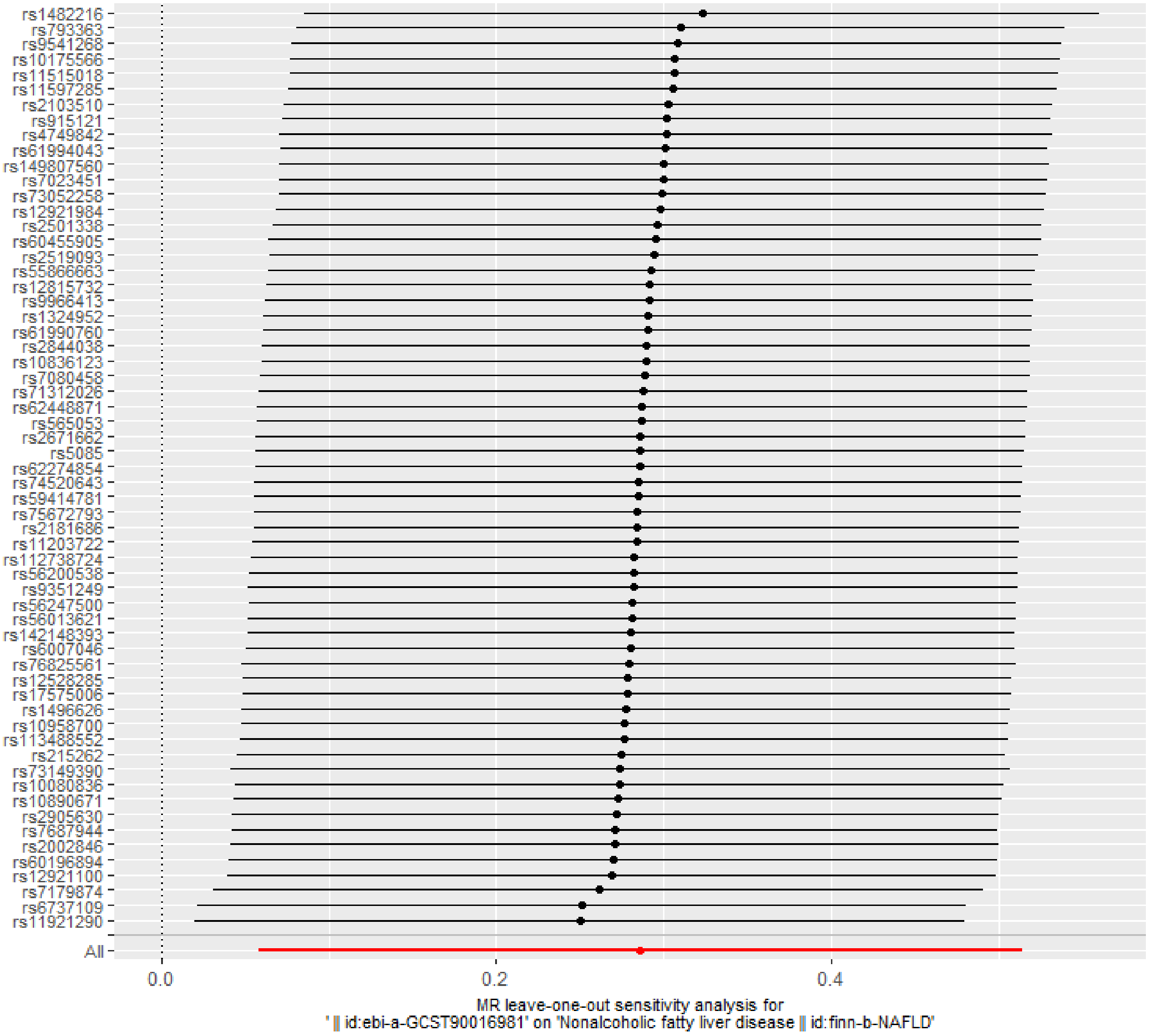

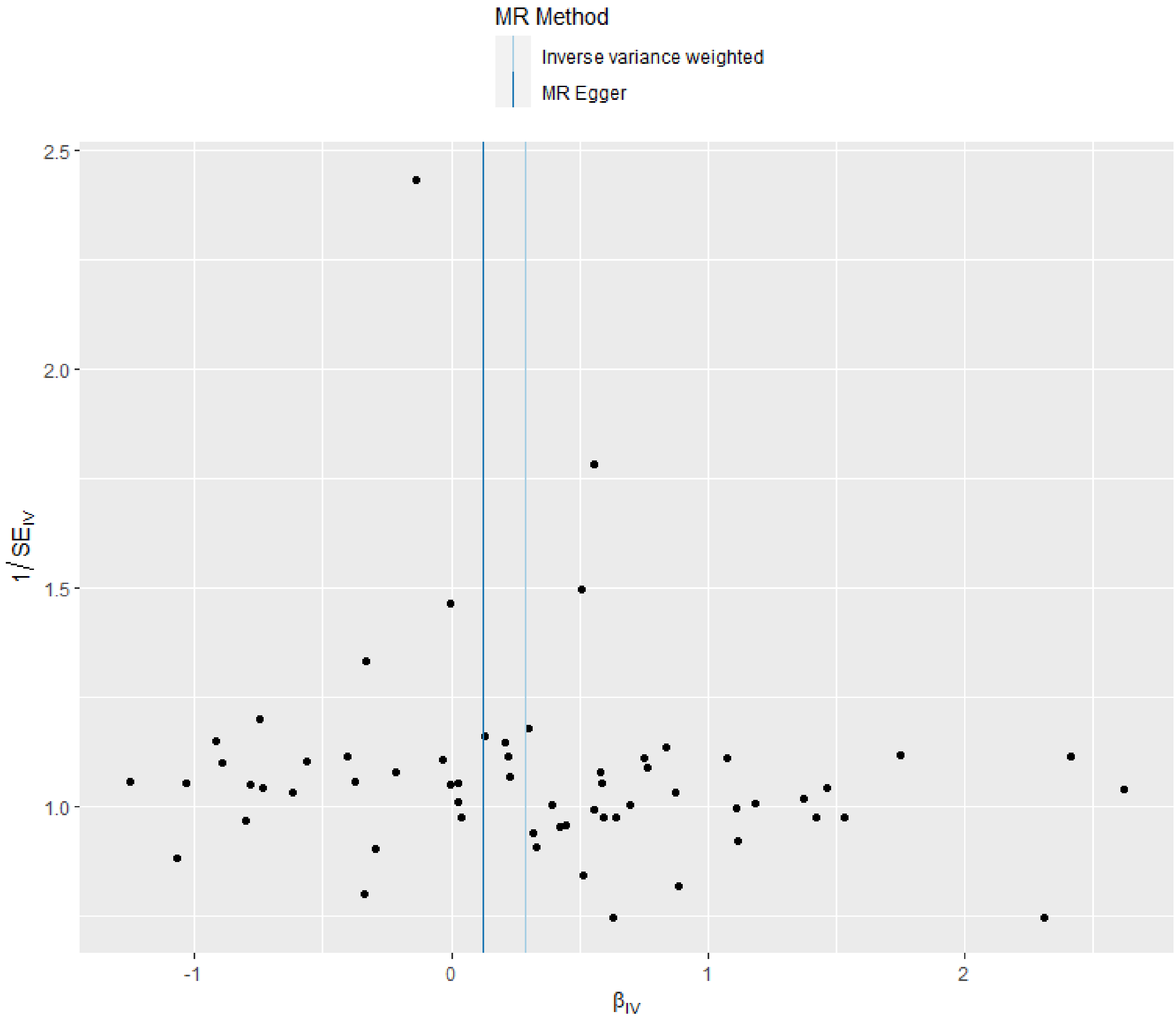

MR Method
Inverse variance weighted
MR Egger
2.5
2.0
1.5
1.0
-1
0
1
2
$1/SE_{IV}$
β_{IV}

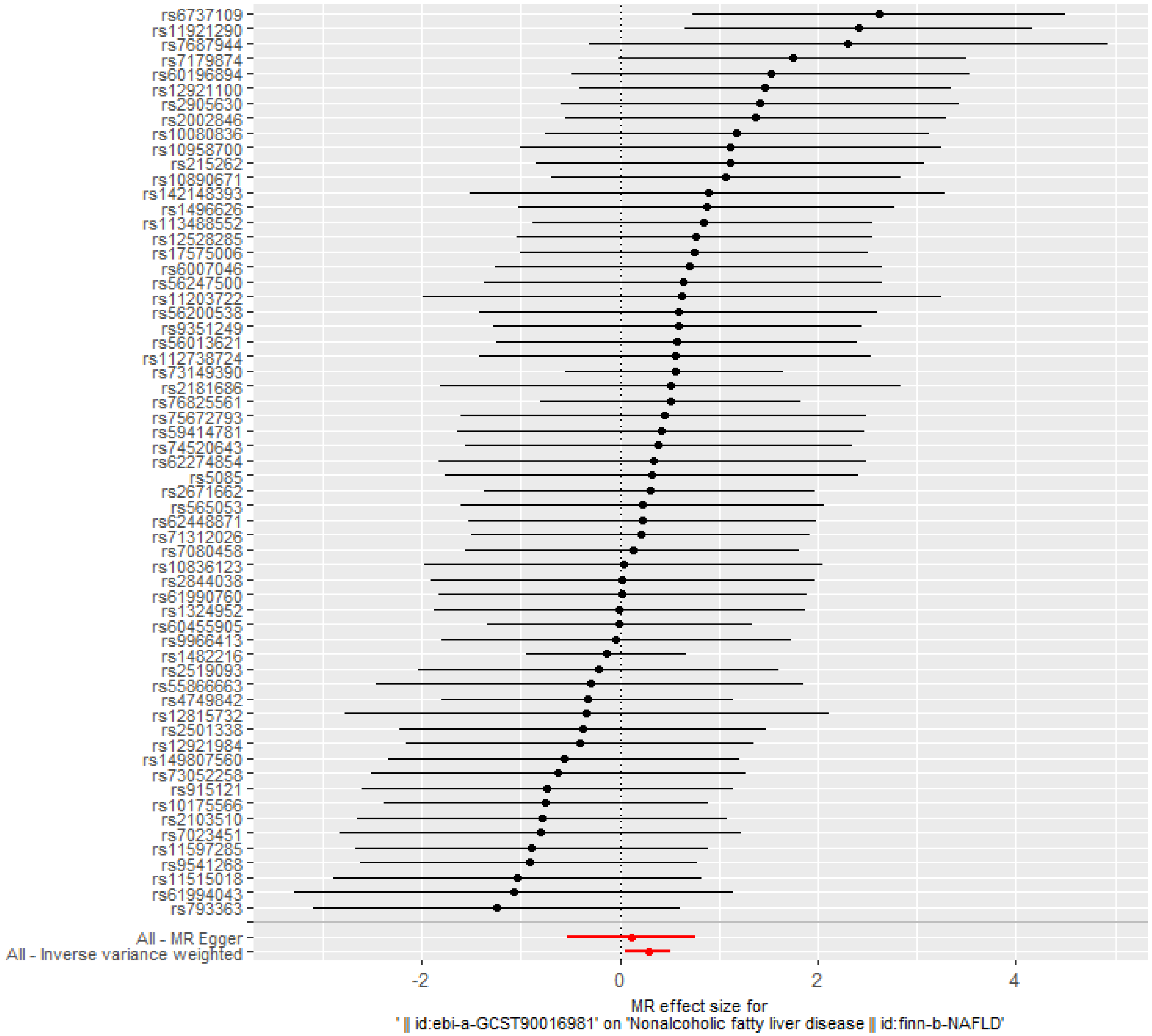
rs6737109
rs11921290
rs7687944
rs7179874
rs60196894
rs12921100
rs2905630
rs2002846
rs10080836
rs10958700
rs215262
rs10890671
rs142148393
rs1496626
rs113488552
rs12528285
rs17575006
rs6007046
rs56247500
rs11203722
rs56200538
rs9351249
rs56013621
rs112738724
rs73149390
rs2181686
rs76825561
rs75672793
rs59414781
rs74520643
rs62274854
rs5085
rs2671662
rs565053
rs62448871
rs71312026
rs7080458
rs10836123
rs2844038
rs61990760
rs1324952
rs60455905
rs9966413
rs1482216
rs2519093
rs55866663
rs4749842
rs12815732
rs2501338
rs12921984
rs149807560
rs73052258
rs915121
rs10175566
rs2103510
rs7023451
rs11597285
rs9541268
rs11515018
rs61994043
rs793363
All - MR Egger
All - Inverse variance weighted
-2
0
2
4
MR effect size for
' || id:ebi-a-GCST90016981' on 'Nonalcoholic fatty liver disease || id:finn-b-NAFLD'

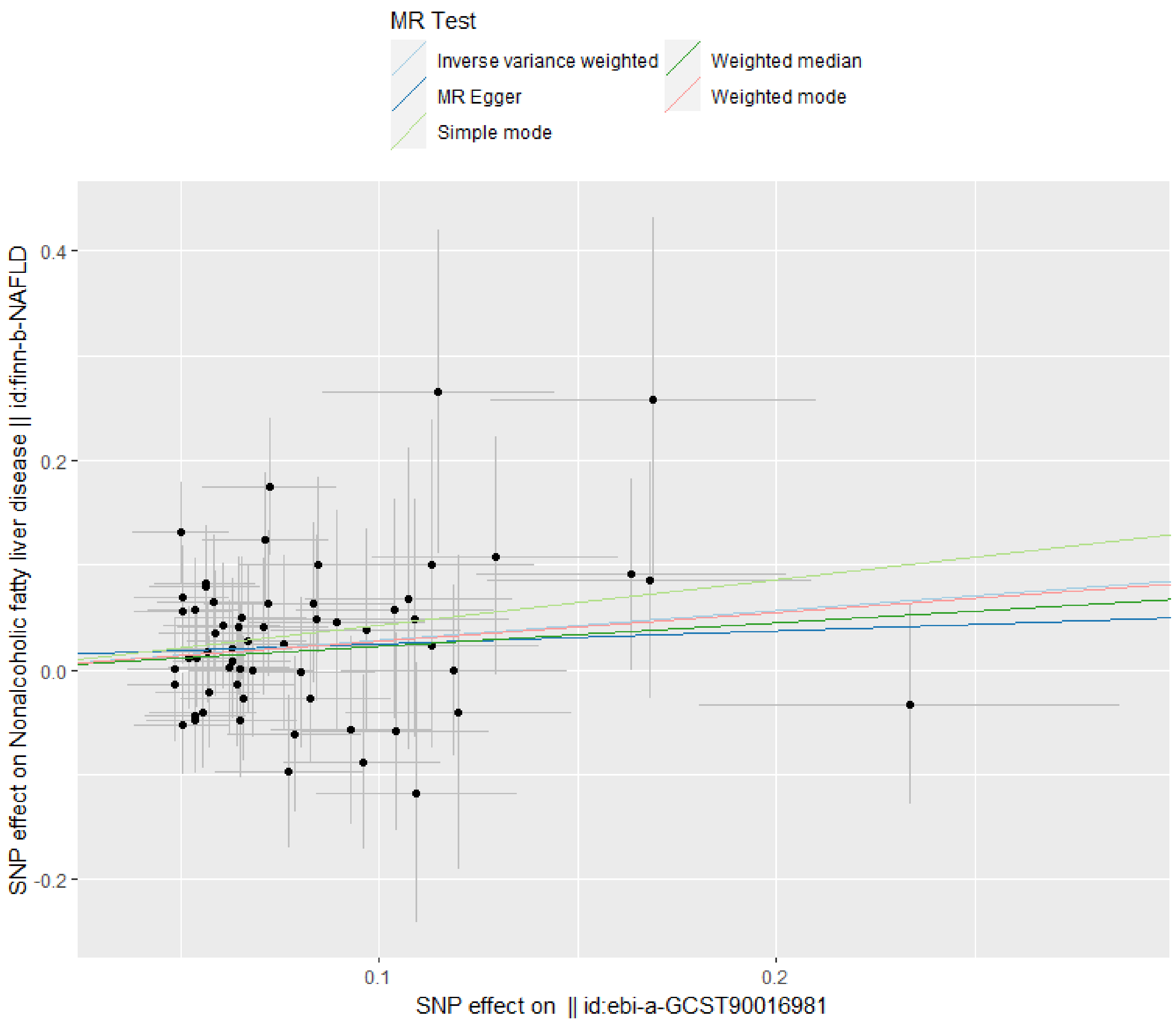

MR Test
Inverse variance weighted
MR Egger
Simple mode
Weighted median
Weighted mode
SNP effect on Nonalcoholic fatty liver disease || id:finn-b-NAFLD
0.4
0.2
0.0
-0.2
0.1
0.2
SNP effect on || id:ebi-a-GCST90016981

Figure 155 Leave-one-out analysis, funnel plot, MR effect size and scatter plot for gut microbiota abundance (genus Erysipelatoclostridium id.11381) on nonalcoholic fatty liver disease

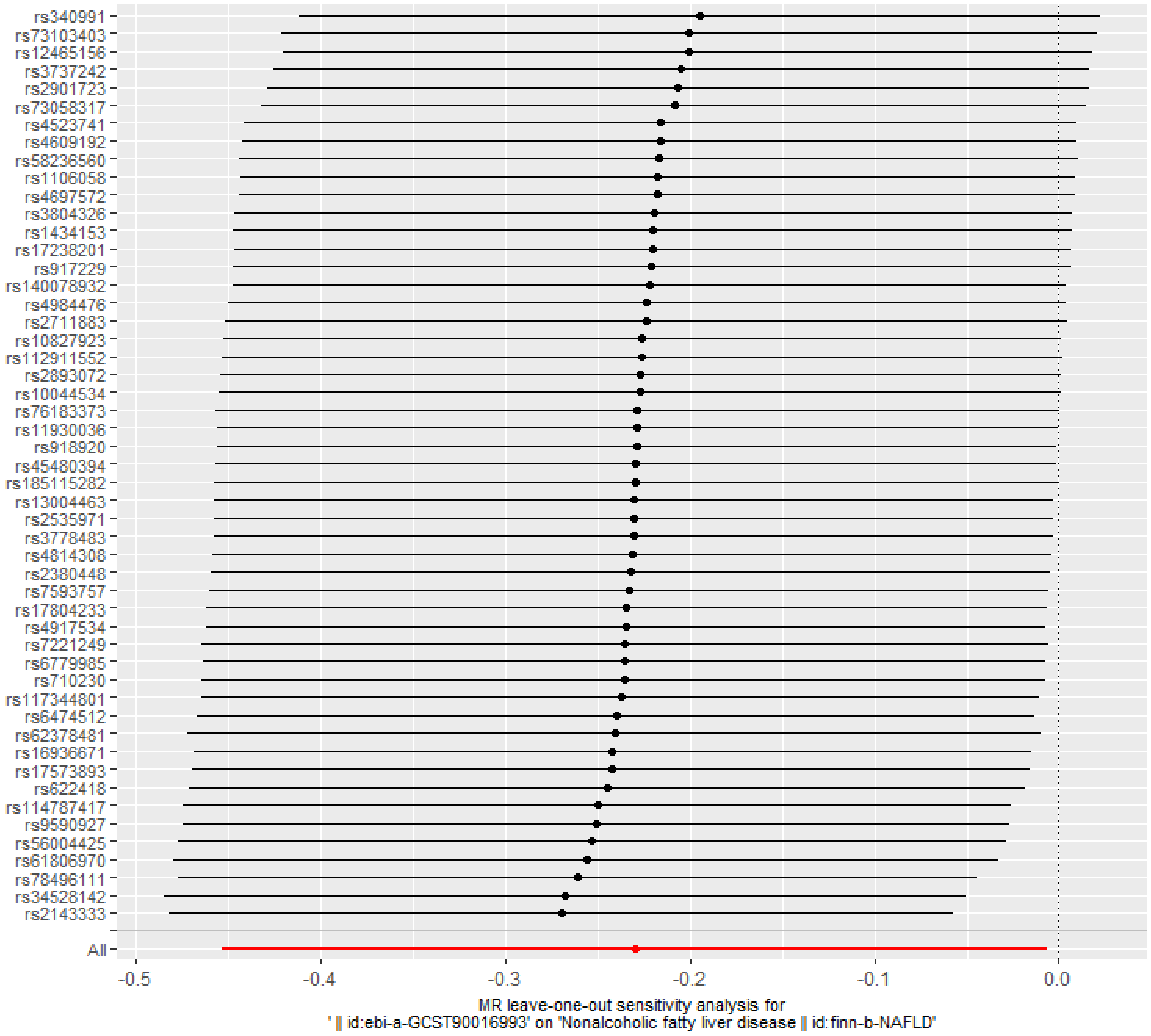

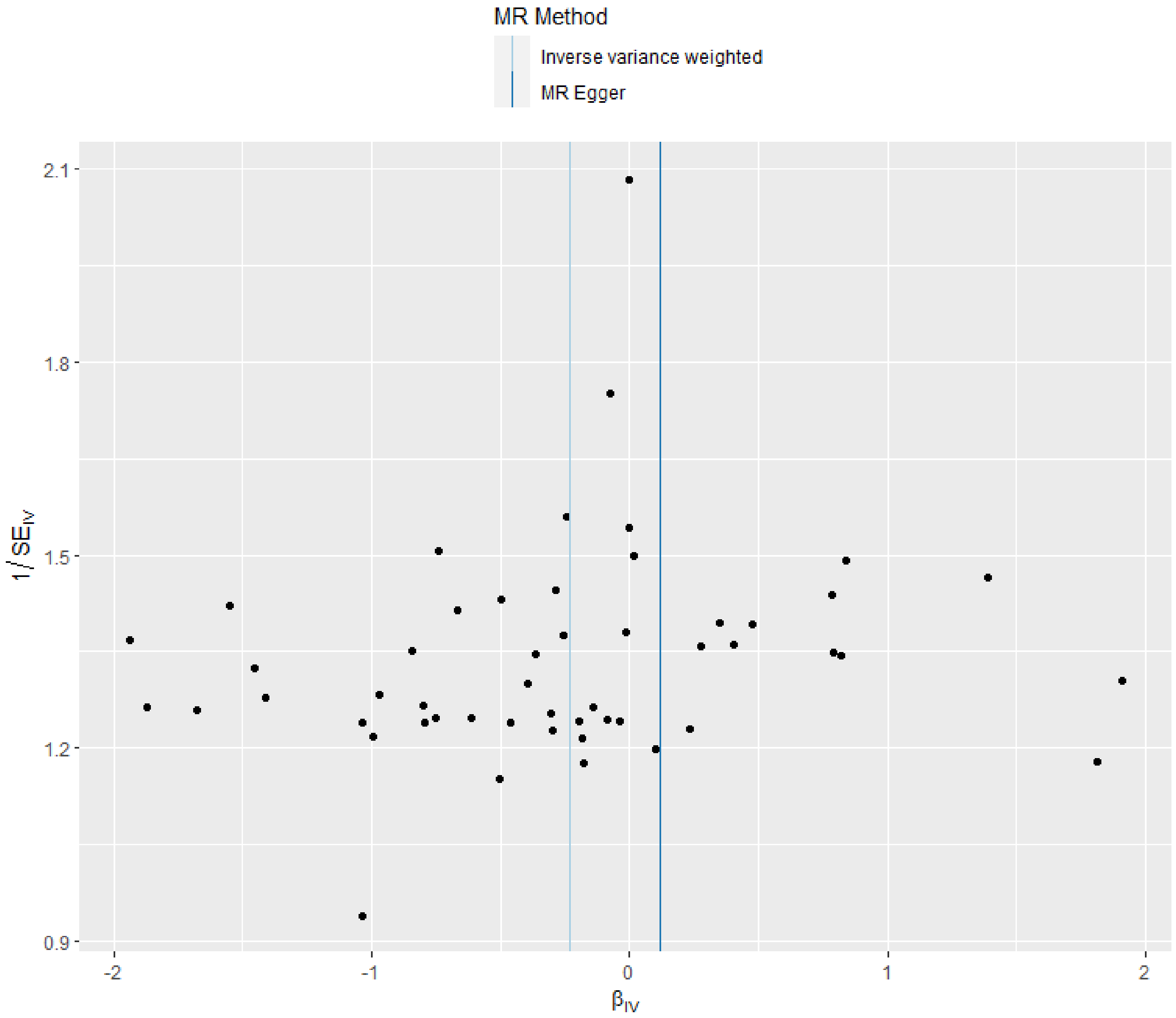
MR Method
Inverse variance weighted
MR Egger
2.1
1.8
1.5
1.2
0.9
1/SE_IV
-2
-1
0
1
2
β_IV

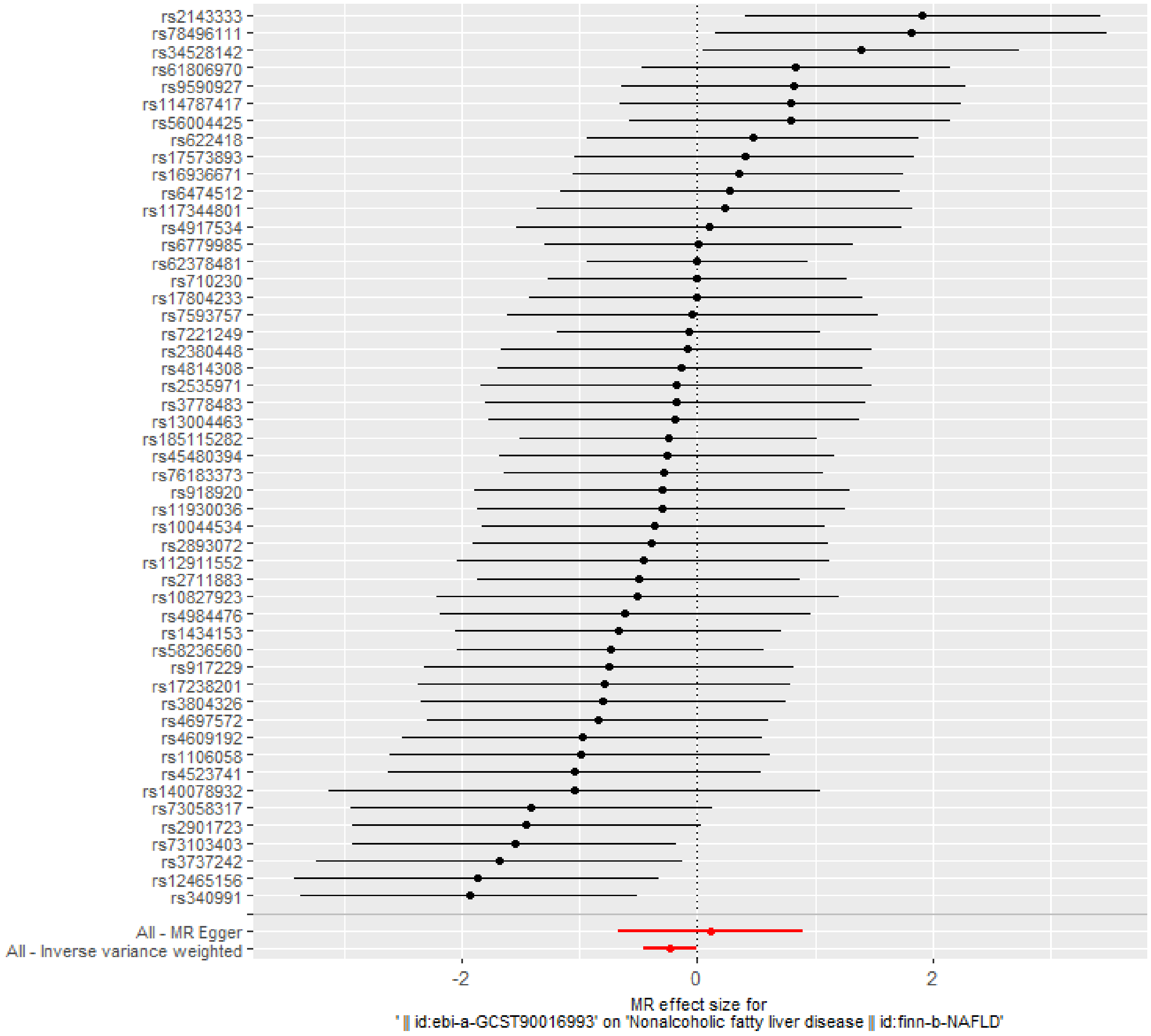
rs2143333
rs78496111
rs34528142
rs61806970
rs9590927
rs114787417
rs56004425
rs622418
rs17573893
rs16936671
rs6474512
rs117344801
rs4917534
rs6779985
rs62378481
rs710230
rs17804233
rs7593757
rs7221249
rs2380448
rs4814308
rs2535971
rs3778483
rs13004463
rs185115282
rs45480394
rs76183373
rs918920
rs11930036
rs10044534
rs2893072
rs112911552
rs2711883
rs10827923
rs4984476
rs1434153
rs58236560
rs917229
rs17238201
rs3804326
rs4697572
rs4609192
rs1106058
rs4523741
rs140078932
rs73058317
rs2901723
rs73103403
rs3737242
rs12465156
rs340991
All - MR Egger
All - Inverse variance weighted
-2
0
2
MR effect size for
' || id:ebi-a-GCST90016993' on 'Nonalcoholic fatty liver disease || id:finn-b-NAFLD'

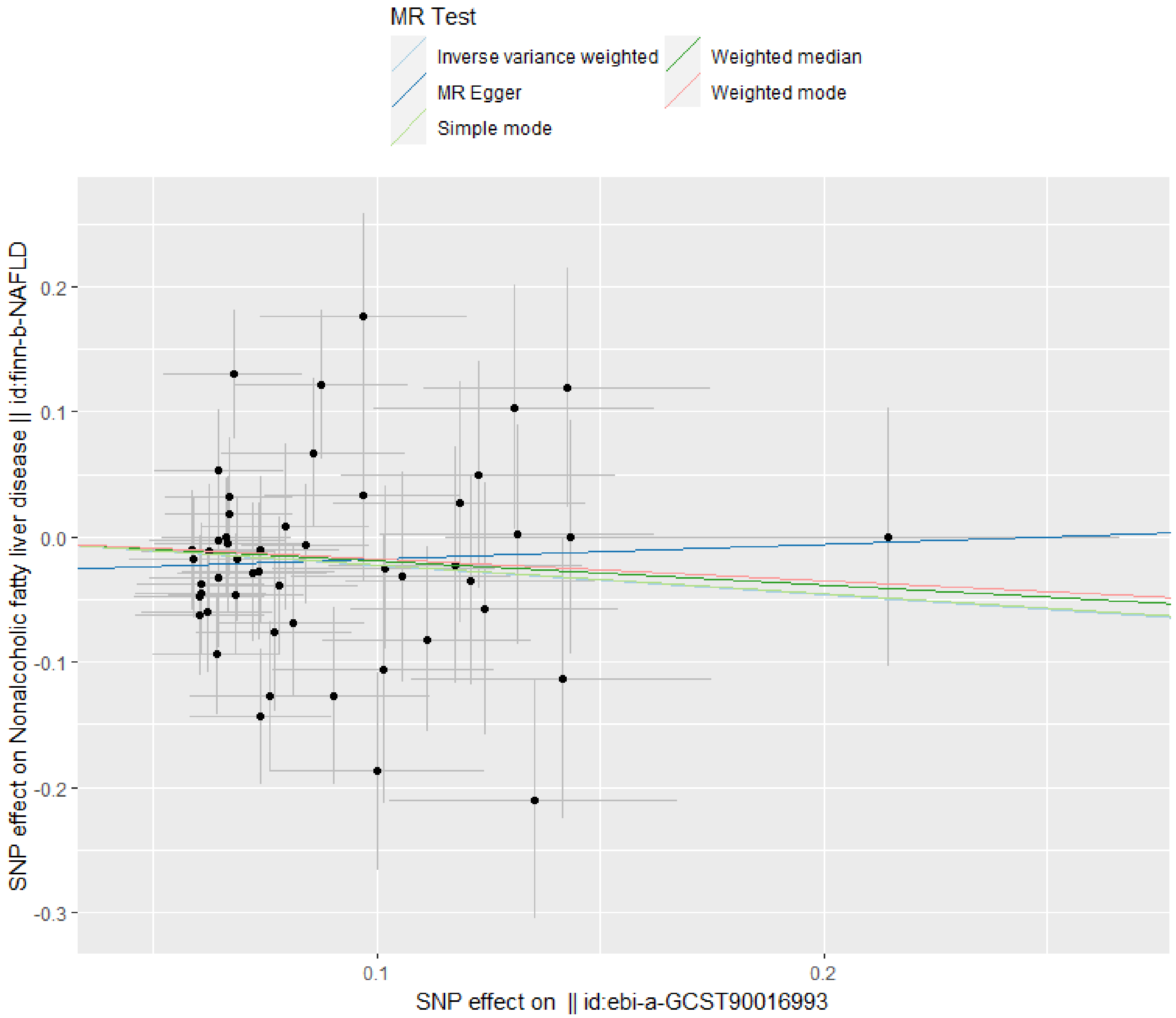

MR Test
Inverse variance weighted
MR Egger
Simple mode
Weighted median
Weighted mode
SNP effect on Nonalcoholic fatty liver disease || id:finn-b-NAFLD
0.2
0.1
0.0
-0.1
-0.2
-0.3
0.1
0.2
SNP effect on || id:ebi-a-GCST90016993

Figure 156 Leave-one-out analysis, funnel plot, MR effect size and scatter plot for gut microbiota abundance (genus Eubacterium hallii group id.11338) on nonalcoholic fatty liver disease

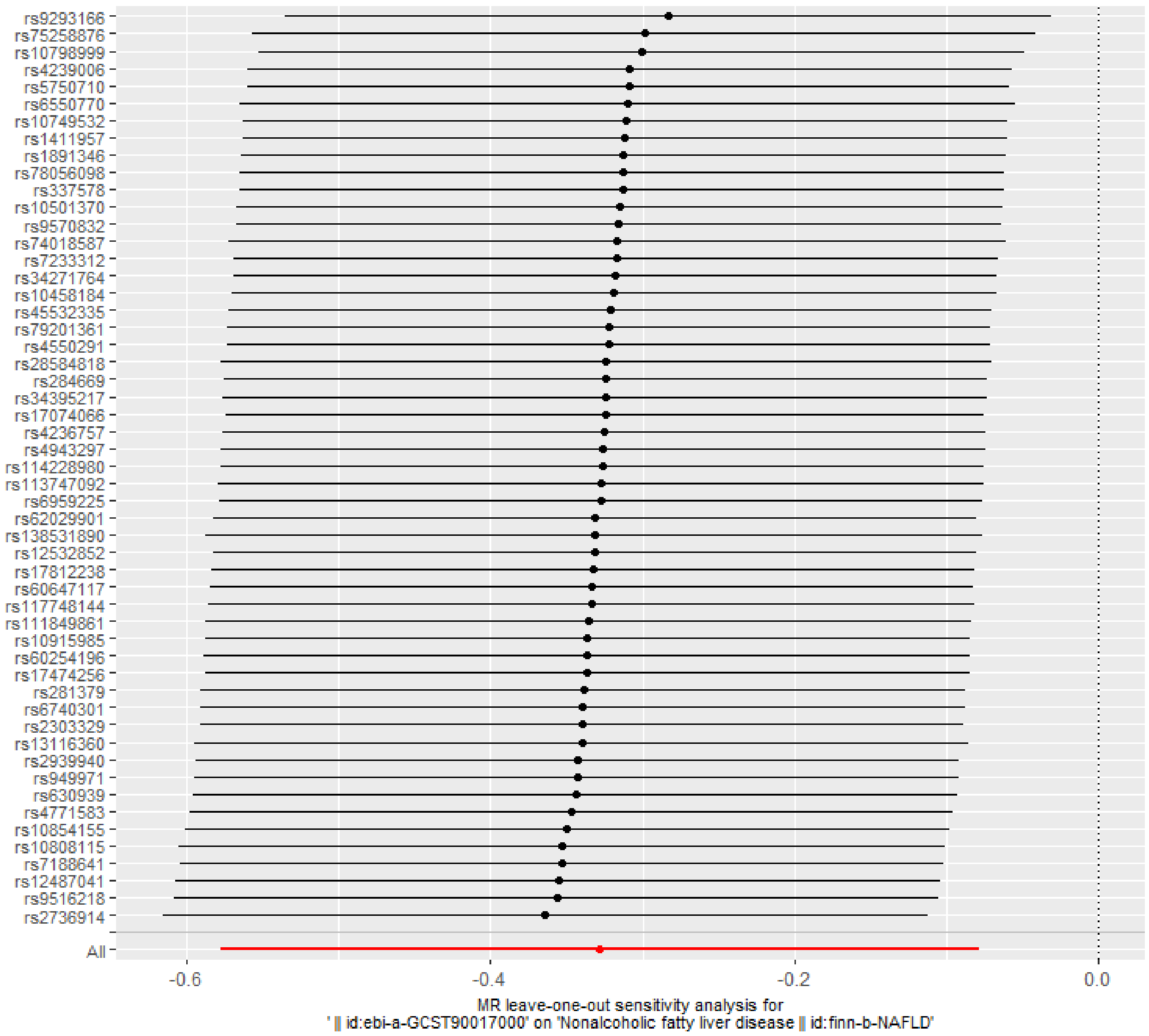

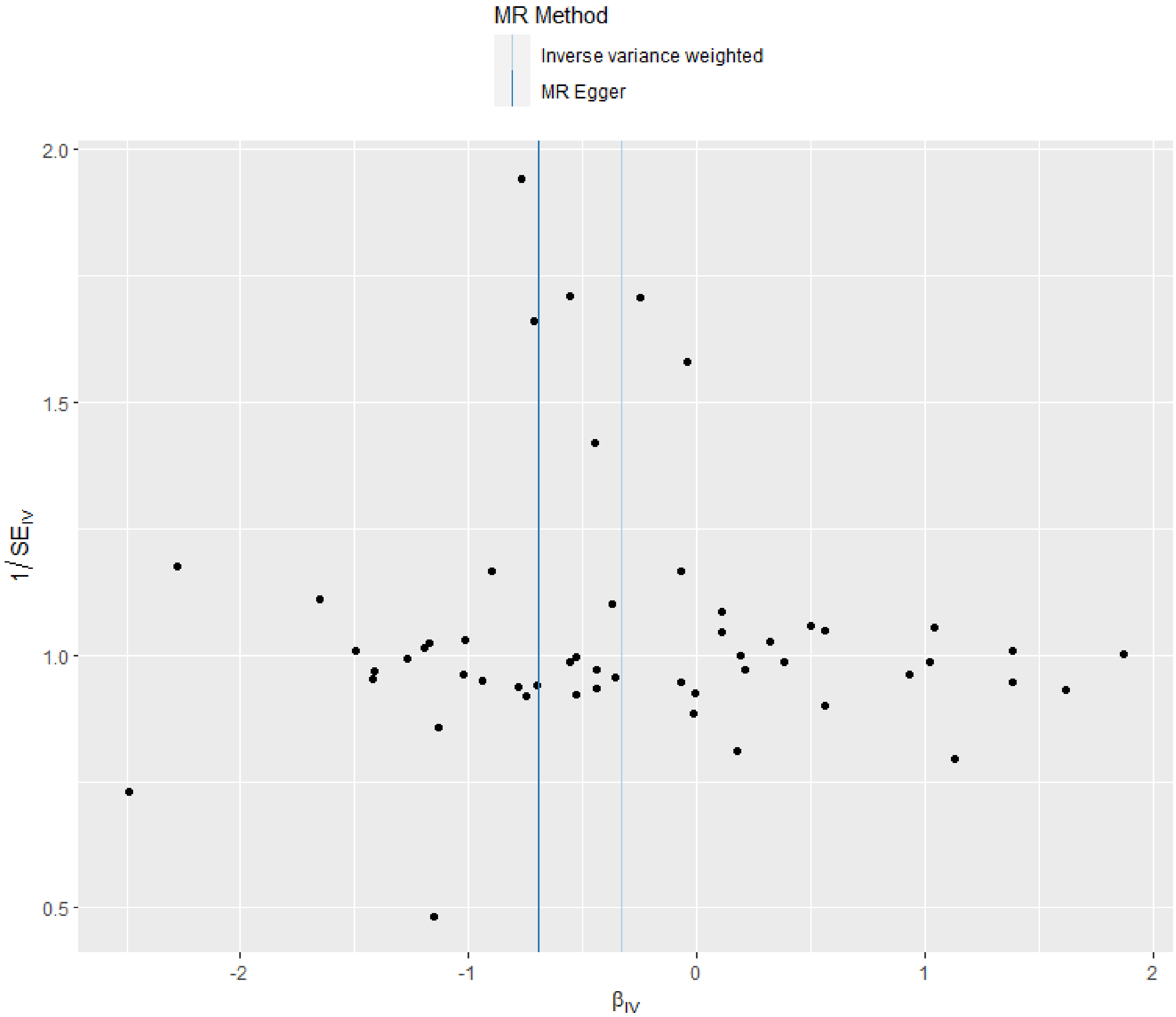
MR Method
Inverse variance weighted
MR Egger
2.0
1.5
1.0
0.5
$1/SE_{IV}$
-2
-1
0
1
2
β_{IV}

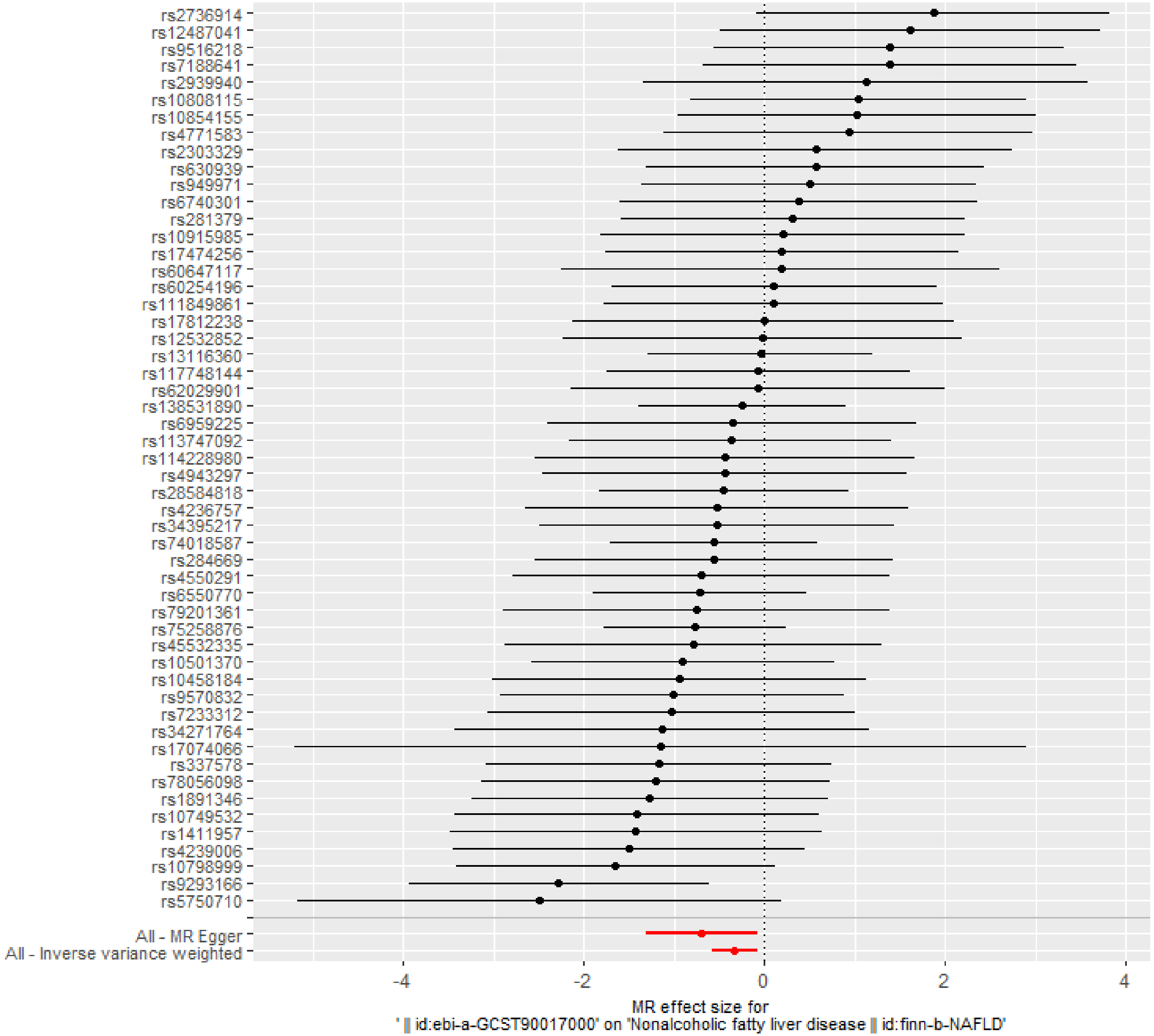

rs2736914
rs12487041
rs9516218
rs7188641
rs2939940
rs10808115
rs10854155
rs4771583
rs2303329
rs630939
rs949971
rs6740301
rs281379
rs10915985
rs17474256
rs60647117
rs60254196
rs111849861
rs17812238
rs12532852
rs13116360
rs117748144
rs62029901
rs138531890
rs6959225
rs113747092
rs114228980
rs4943297
rs28584818
rs4236757
rs34395217
rs74018587
rs284669
rs4550291
rs6550770
rs79201361
rs75258876
rs45532335
rs10501370
rs10458184
rs9570832
rs7233312
rs34271764
rs17074066
rs337578
rs78056098
rs1891346
rs10749532
rs1411957
rs4239006
rs10798999
rs9293166
rs5750710
All - MR Egger
All - Inverse variance weighted
-4
-2
0
2
4
MR effect size for
' || id:ebi-a-GCST90017000' on 'Nonalcoholic fatty liver disease || id:finn-b-NAFLD'

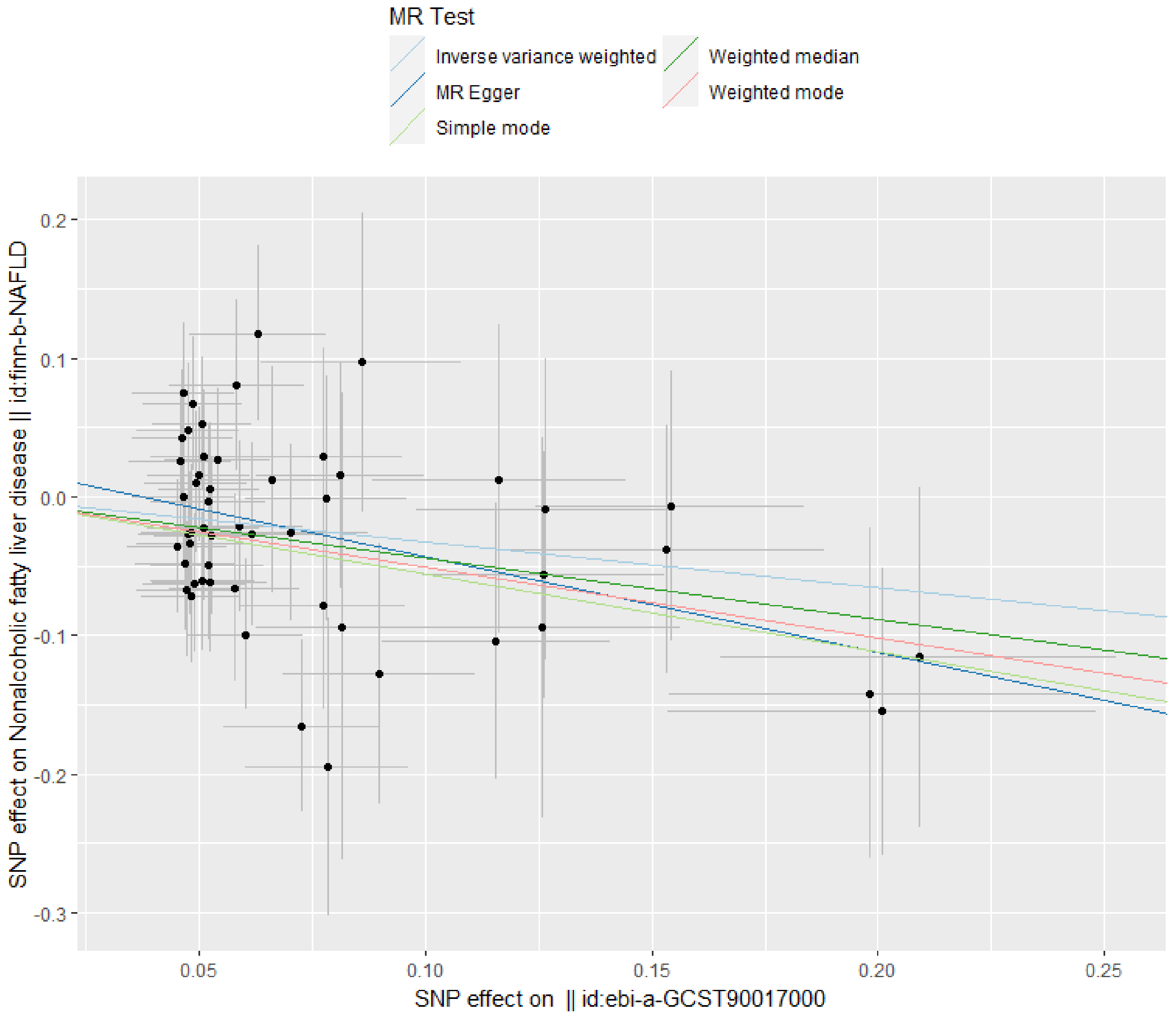

MR Test
Inverse variance weighted
MR Egger
Simple mode
Weighted median
Weighted mode
0.2
0.1
0.0
-0.1
-0.2
-0.3
0.05
0.10
0.15
0.20
0.25
SNP effect on Nonalcoholic fatty liver disease || id:finn-b-NAFLD
SNP effect on || id:ebi-a-GCST90017000

Figure 157 Leave-one-out analysis, funnel plot, MR effect size and scatter plot for gut microbiota abundance (genus Holdemanella id.11393) on nonalcoholic fatty liver disease

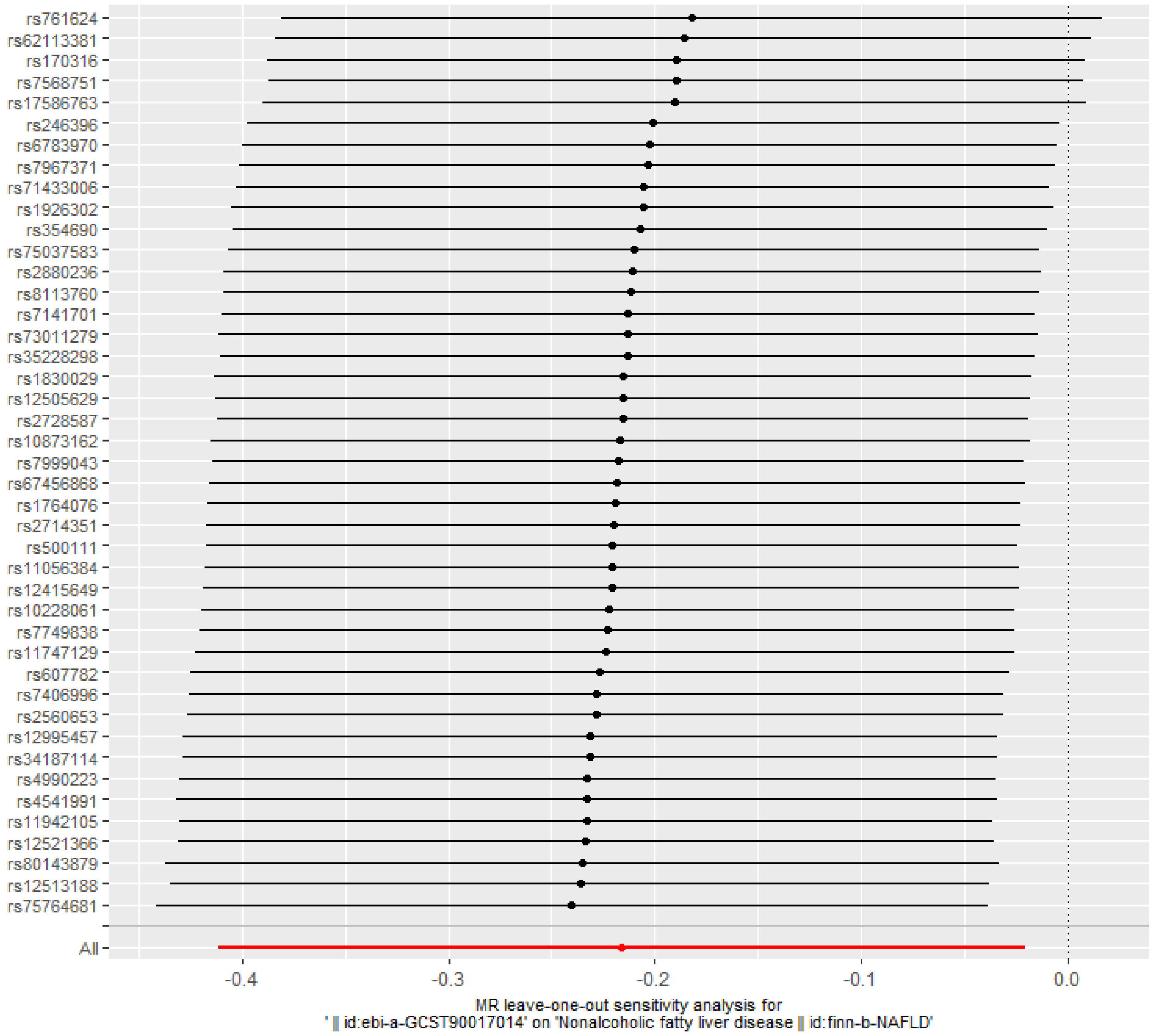

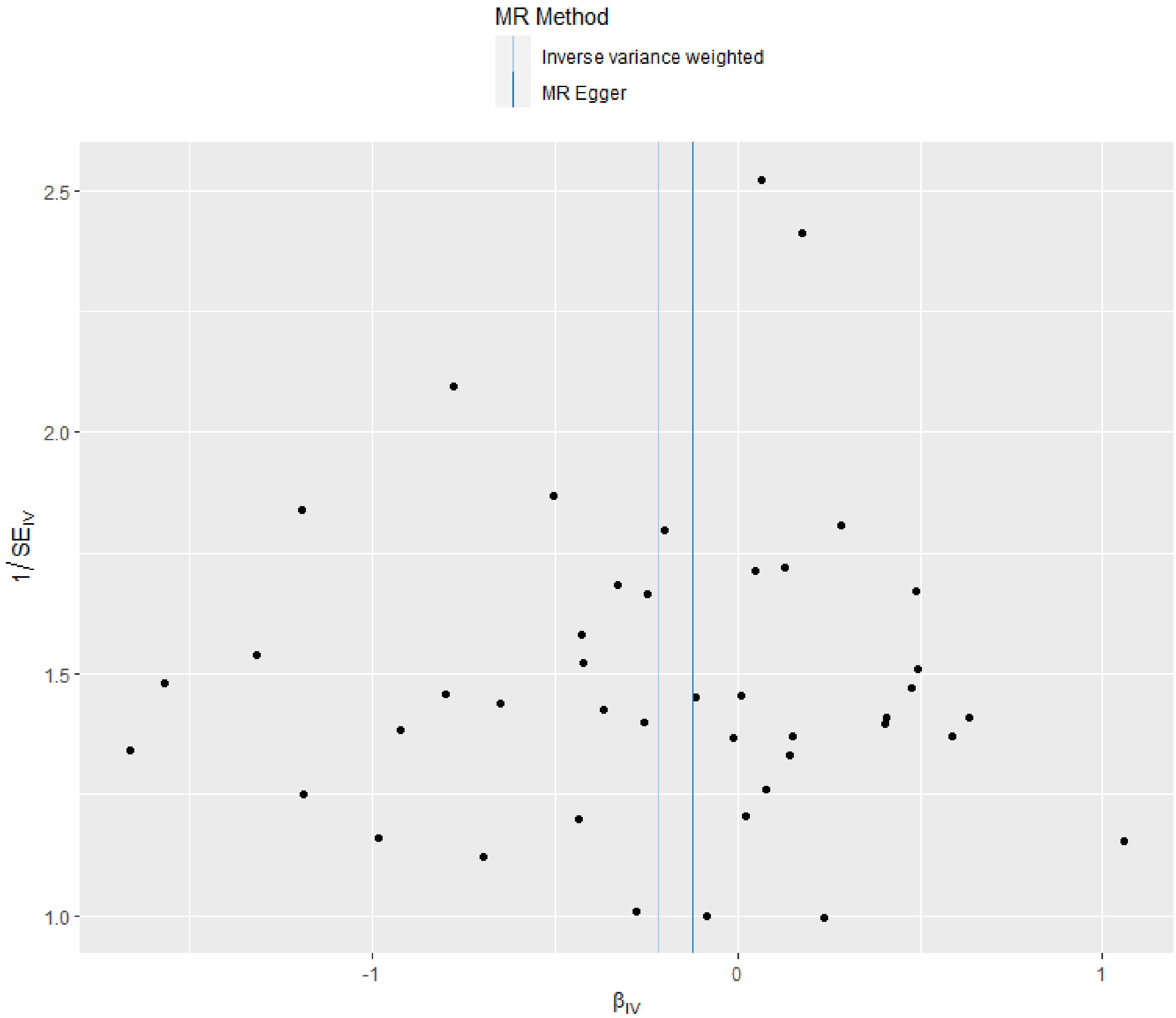

MR Method
Inverse variance weighted
MR Egger
2.5
2.0
1.5
1.0
1/SE_IV
-1
0
1
β_IV

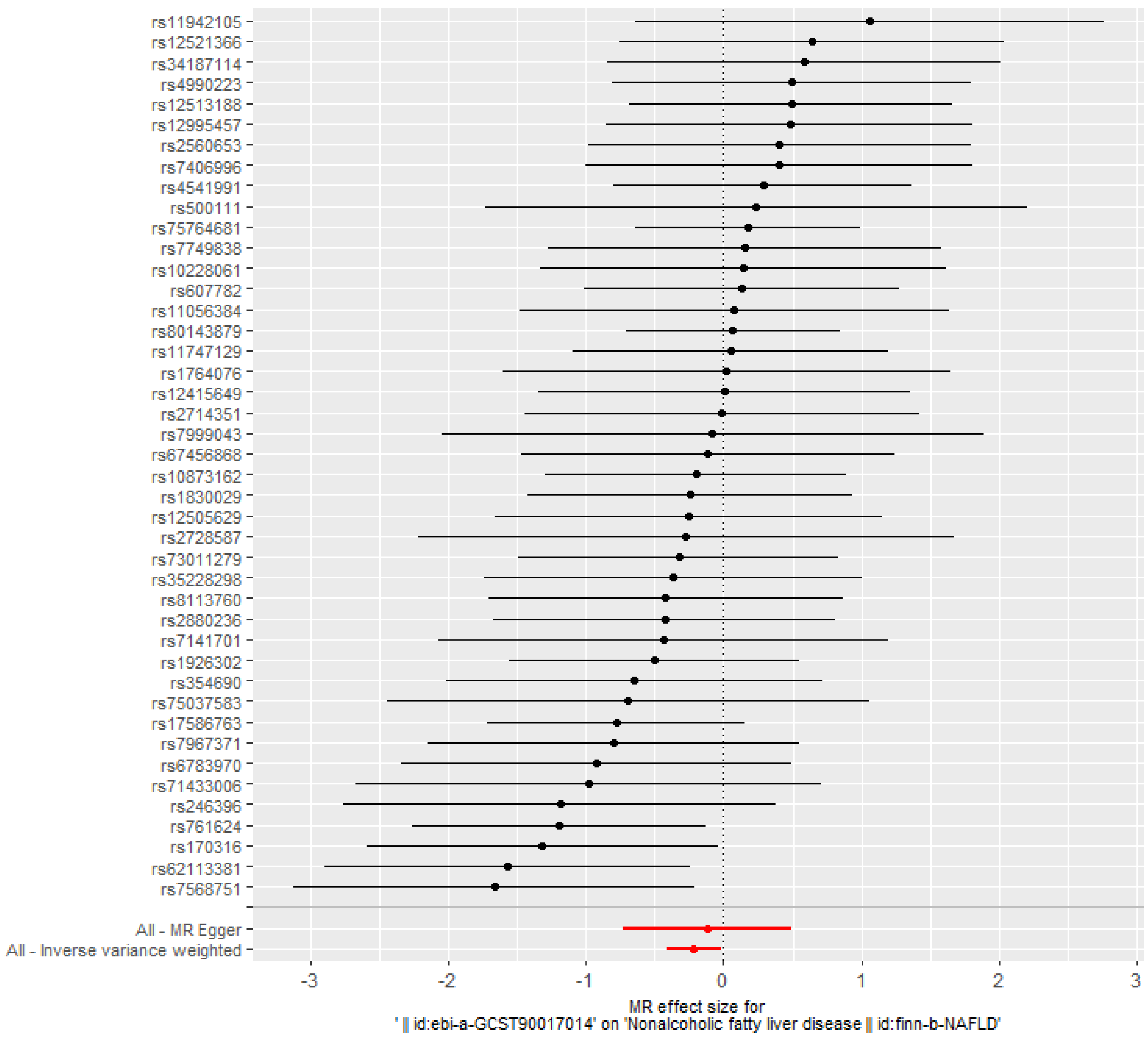
rs11942105
rs12521366
rs34187114
rs4990223
rs12513188
rs12995457
rs2560653
rs7406996
rs4541991
rs500111
rs75764681
rs7749838
rs10228061
rs607782
rs11056384
rs80143879
rs11747129
rs1764076
rs12415649
rs2714351
rs7999043
rs67456868
rs10873162
rs1830029
rs12505629
rs2728587
rs73011279
rs35228298
rs8113760
rs2880236
rs7141701
rs1926302
rs354690
rs75037583
rs17586763
rs7967371
rs6783970
rs71433006
rs246396
rs761624
rs170316
rs62113381
rs7568751
All - MR Egger
All - Inverse variance weighted
-3
-2
-1
0
1
2
3
MR effect size for
' || id:ebi-a-GCST90017014' on 'Nonalcoholic fatty liver disease || id:finn-b-NAFLD'

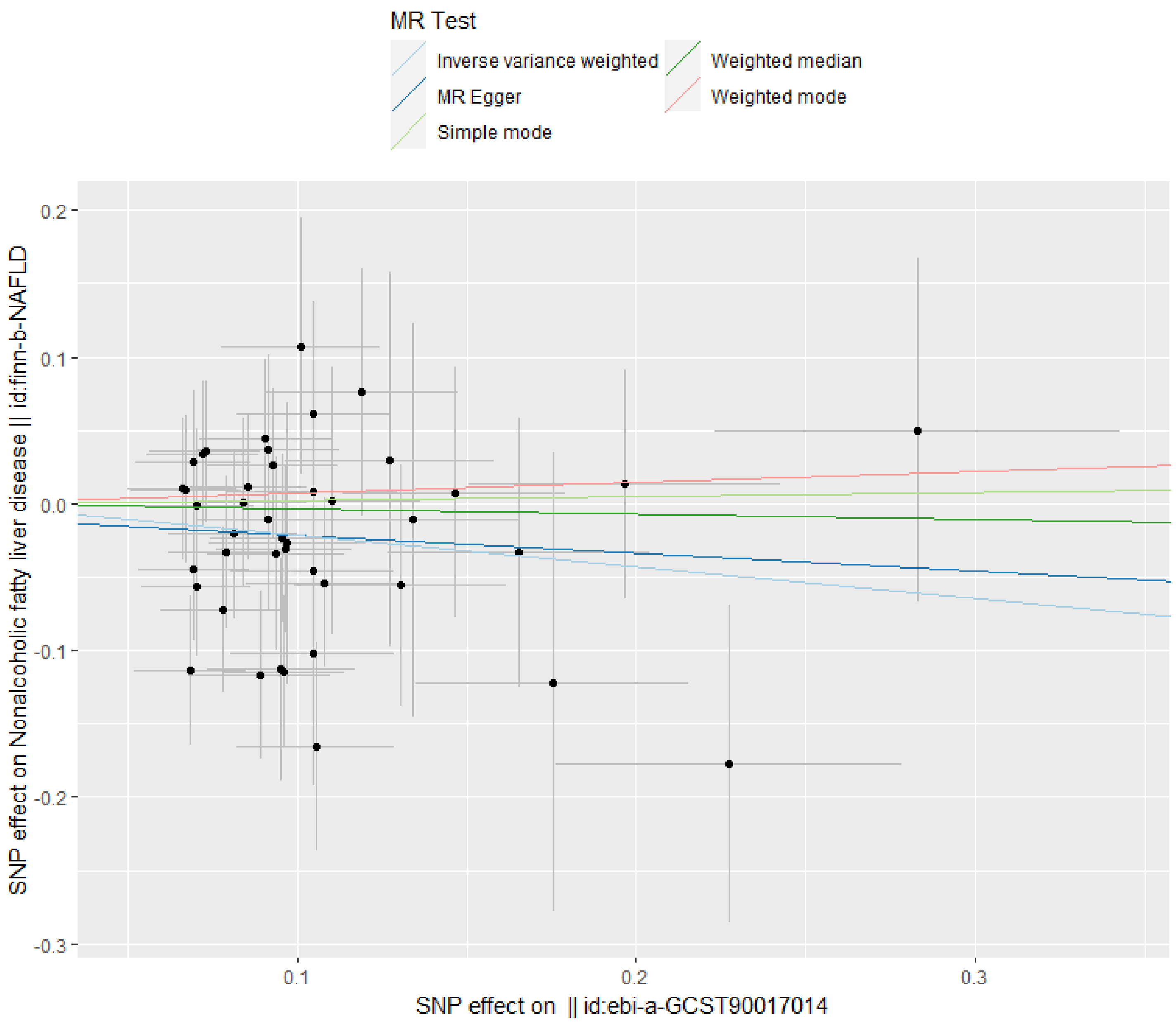
MR Test
Inverse variance weighted
MR Egger
Simple mode
Weighted median
Weighted mode
0.2
0.1
0.0
-0.1
-0.2
-0.3
SNP effect on Nonalcoholic fatty liver disease || id:finn-b-NAFLD
0.1
0.2
0.3
SNP effect on || id:ebi-a-GCST90017014

Figure 158 Leave-one-out analysis, funnel plot, MR effect size and scatter plot for gut microbiota abundance (genus Paraprevotella id.962) on nonalcoholic fatty liver disease

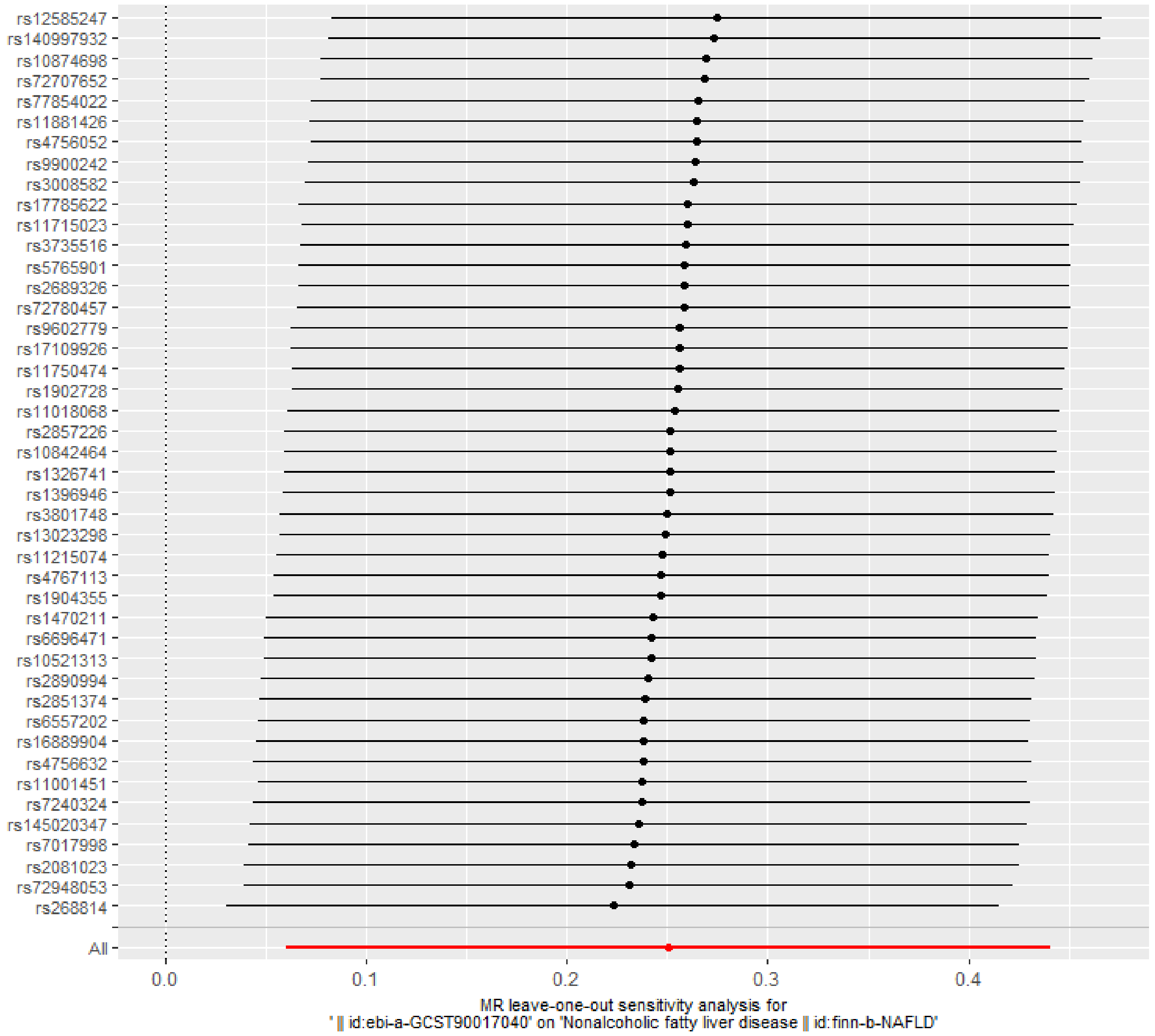

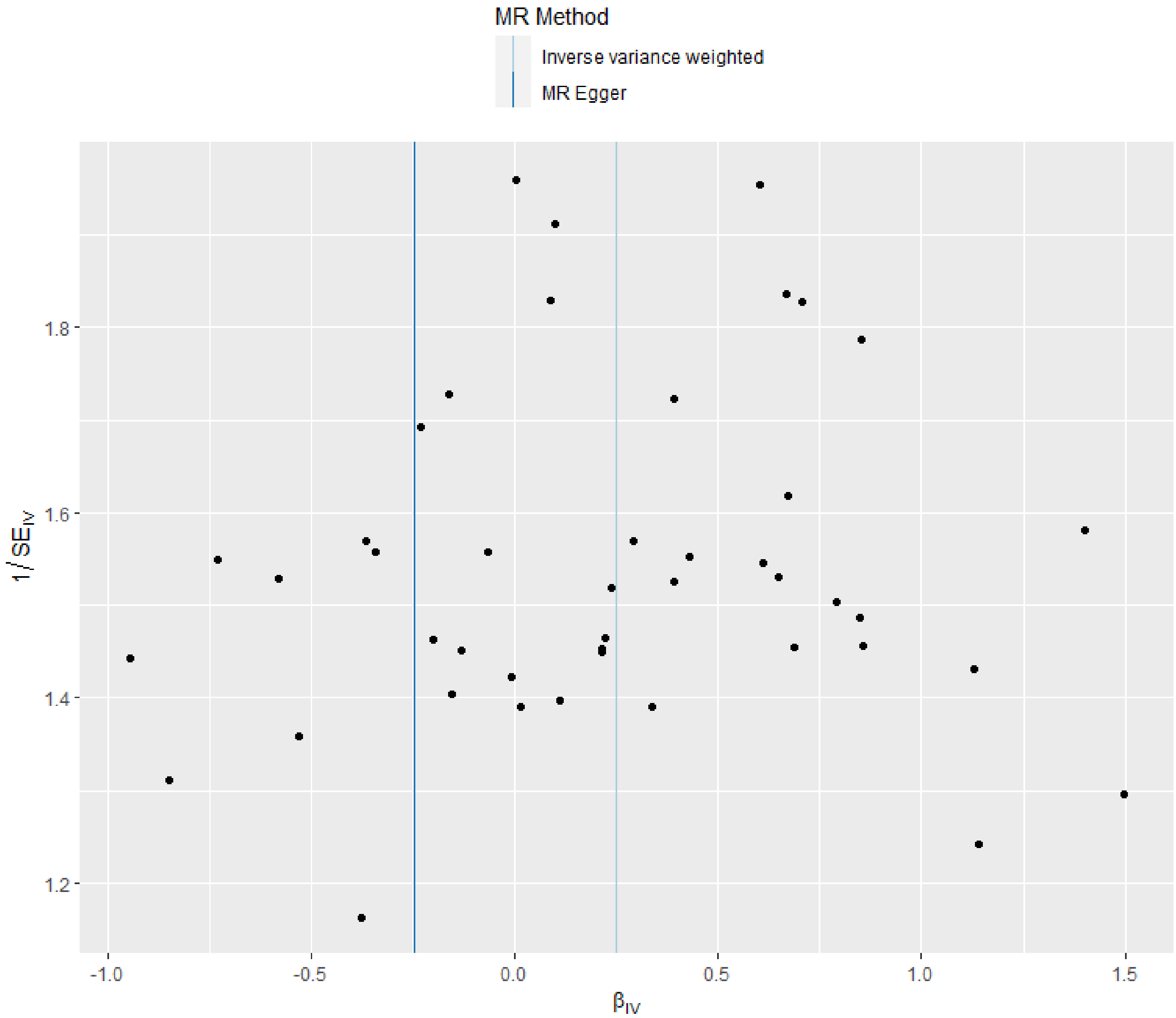

MR Method
Inverse variance weighted
MR Egger
1.8
1.6
1.4
1.2
1/SE_IV
-1.0
-0.5
0.0
0.5
1.0
1.5
β_IV

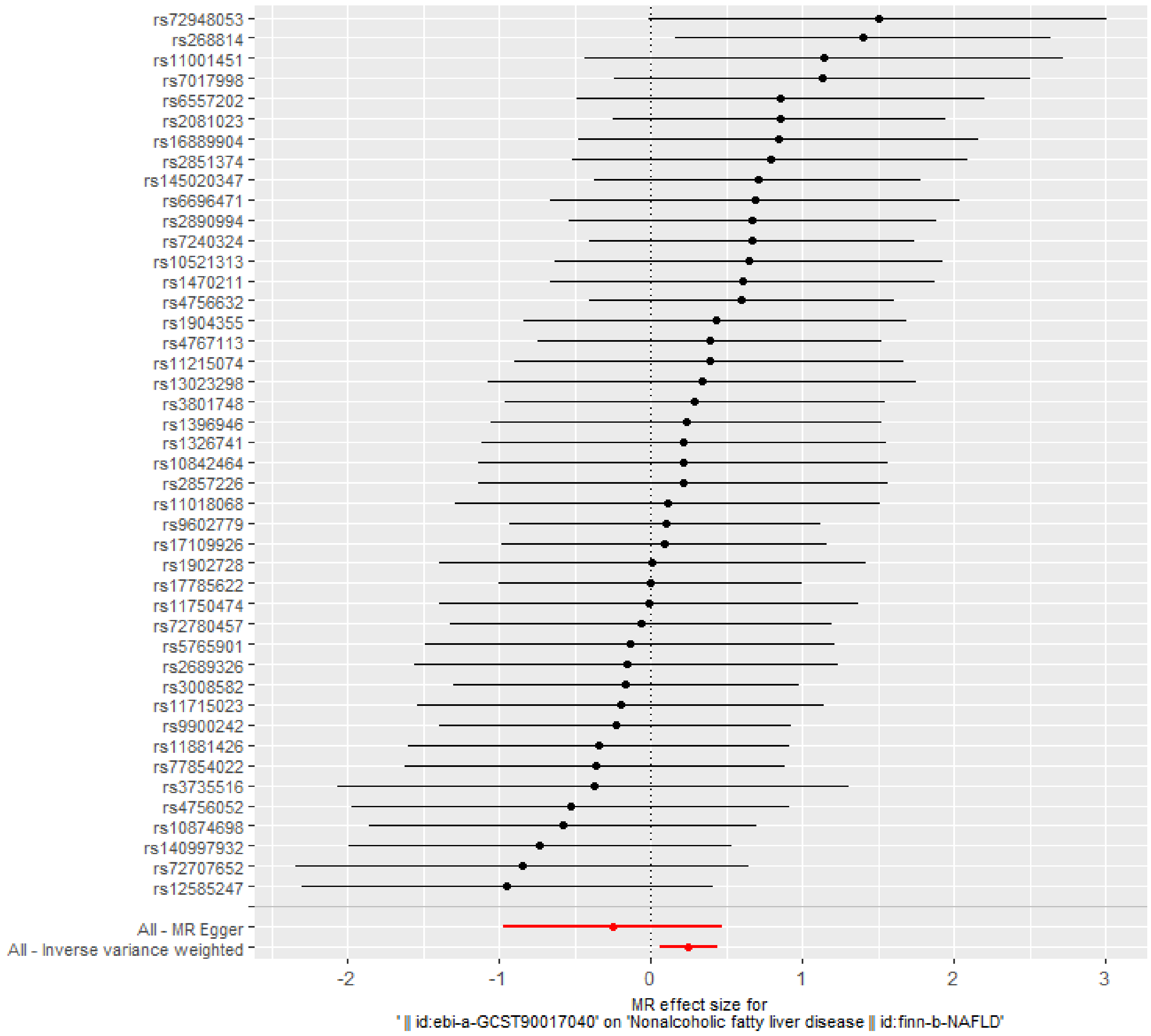
rs72948053
rs268814
rs11001451
rs7017998
rs6557202
rs2081023
rs16889904
rs2851374
rs145020347
rs6696471
rs2890994
rs7240324
rs10521313
rs1470211
rs4756632
rs1904355
rs4767113
rs11215074
rs13023298
rs3801748
rs1396946
rs1326741
rs10842464
rs2857226
rs11018068
rs9602779
rs17109926
rs1902728
rs17785622
rs11750474
rs72780457
rs5765901
rs2689326
rs3008582
rs11715023
rs9900242
rs11881426
rs77854022
rs3735516
rs4756052
rs10874698
rs140997932
rs72707652
rs12585247
All - MR Egger
All - Inverse variance weighted
-2
-1
0
1
2
3
MR effect size for
' || id:ebi-a-GCST90017040' on 'Nonalcoholic fatty liver disease || id:finn-b-NAFLD'

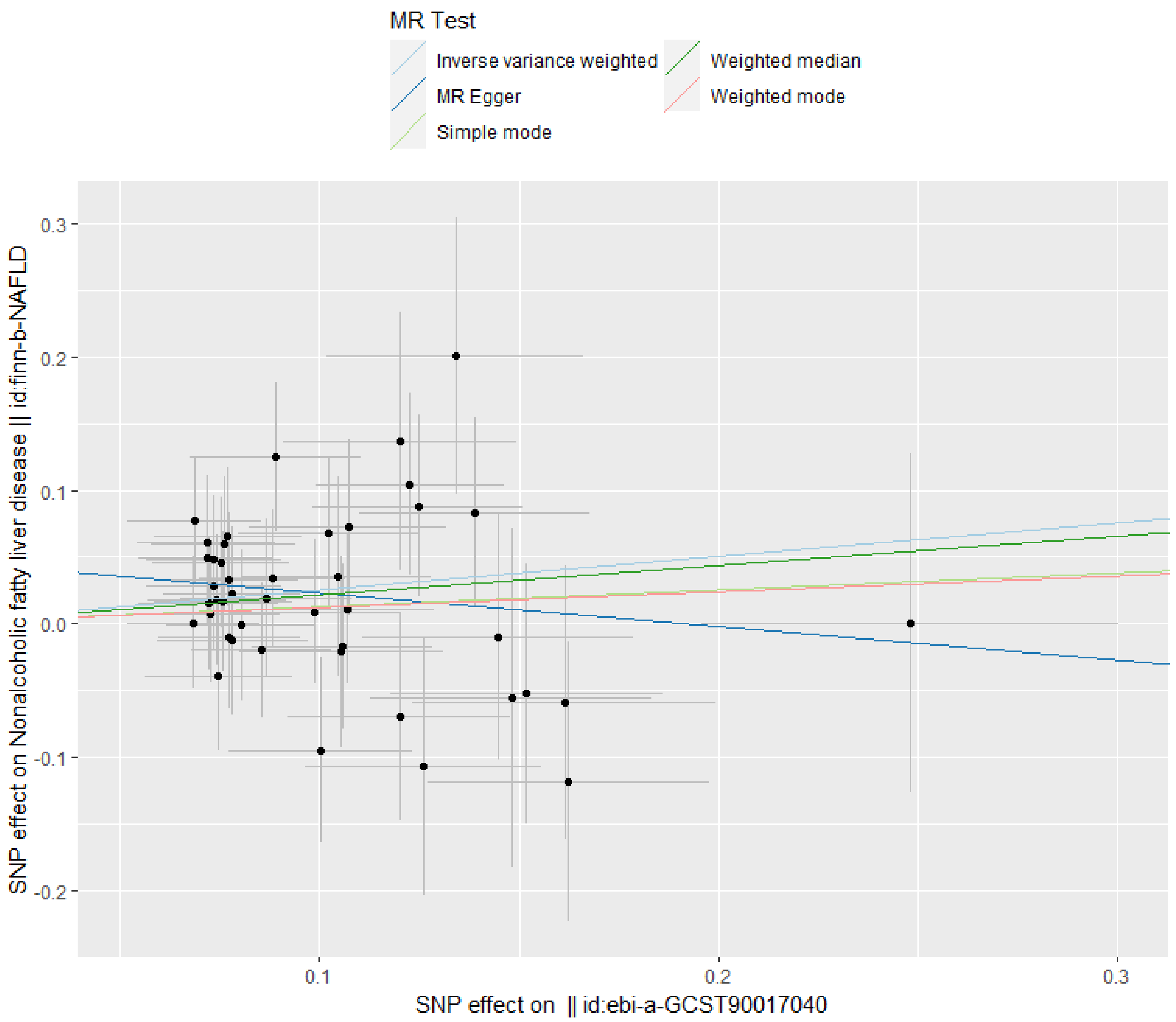

MR Test
Inverse variance weighted
MR Egger
Simple mode
Weighted median
Weighted mode
0.3
0.2
0.1
0.0
-0.1
-0.2
SNP effect on Nonalcoholic fatty liver disease || id:finn-b-NAFLD
0.1
0.2
0.3
SNP effect on || id:ebi-a-GCST90017040

Figure 159 Leave-one-out analysis, funnel plot, MR effect size and scatter plot for gut microbiota abundance (unknown genus id.1000006162) on nonalcoholic fatty liver disease

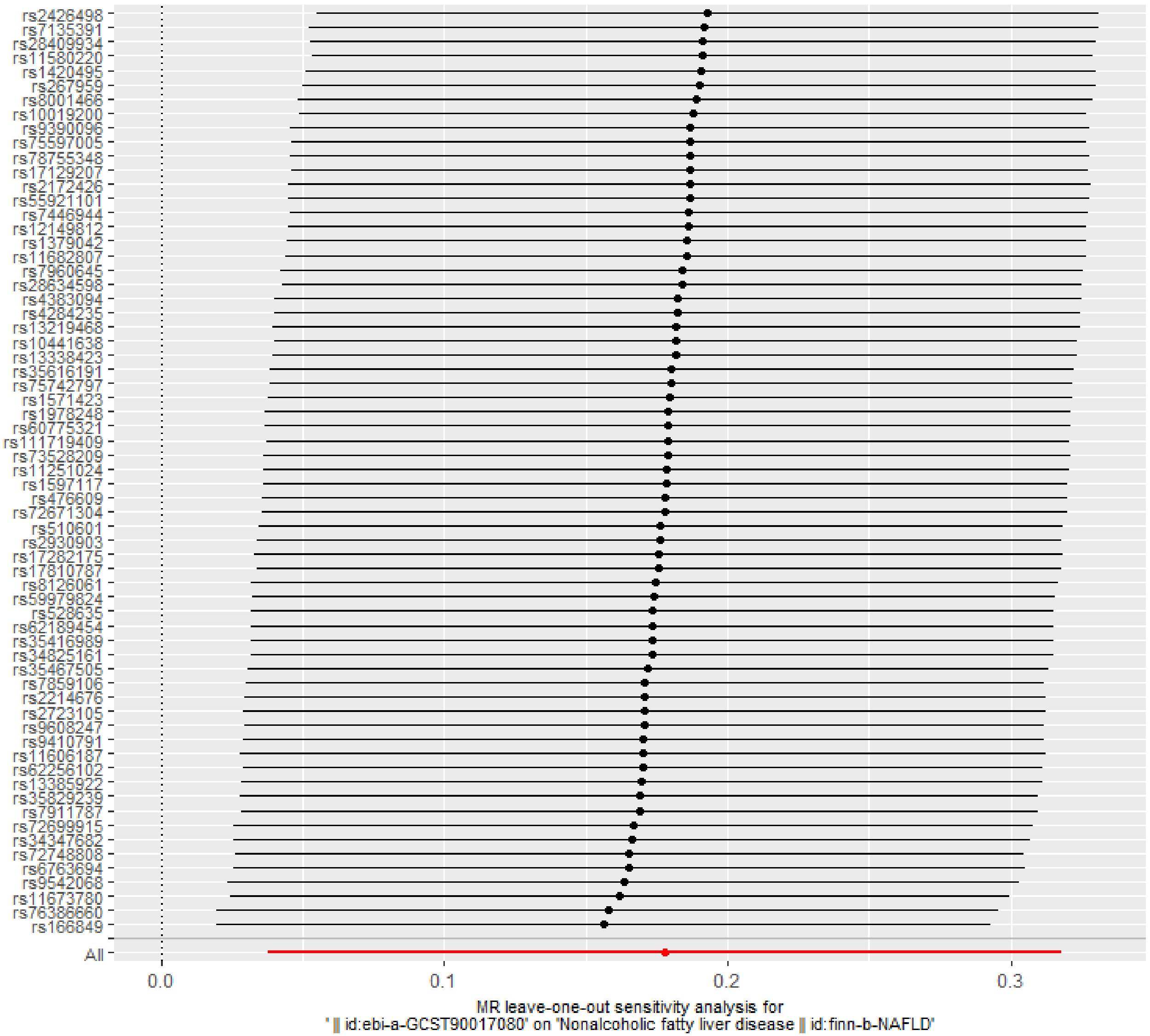

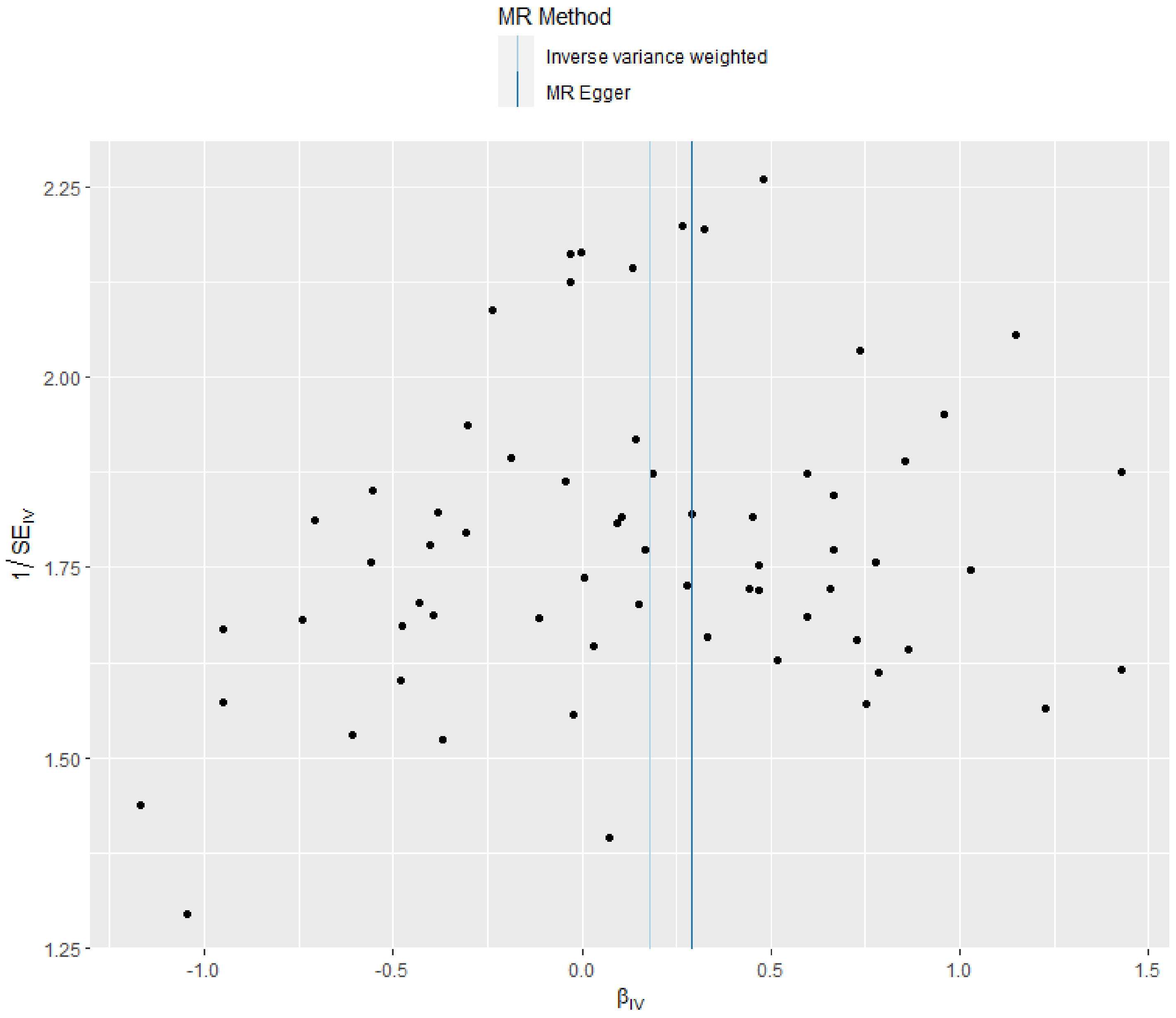

MR Method
Inverse variance weighted
MR Egger
2.25
2.00
1.75
1.50
1.25
1/SE_IV
-1.0
-0.5
0.0
0.5
1.0
1.5
β_IV

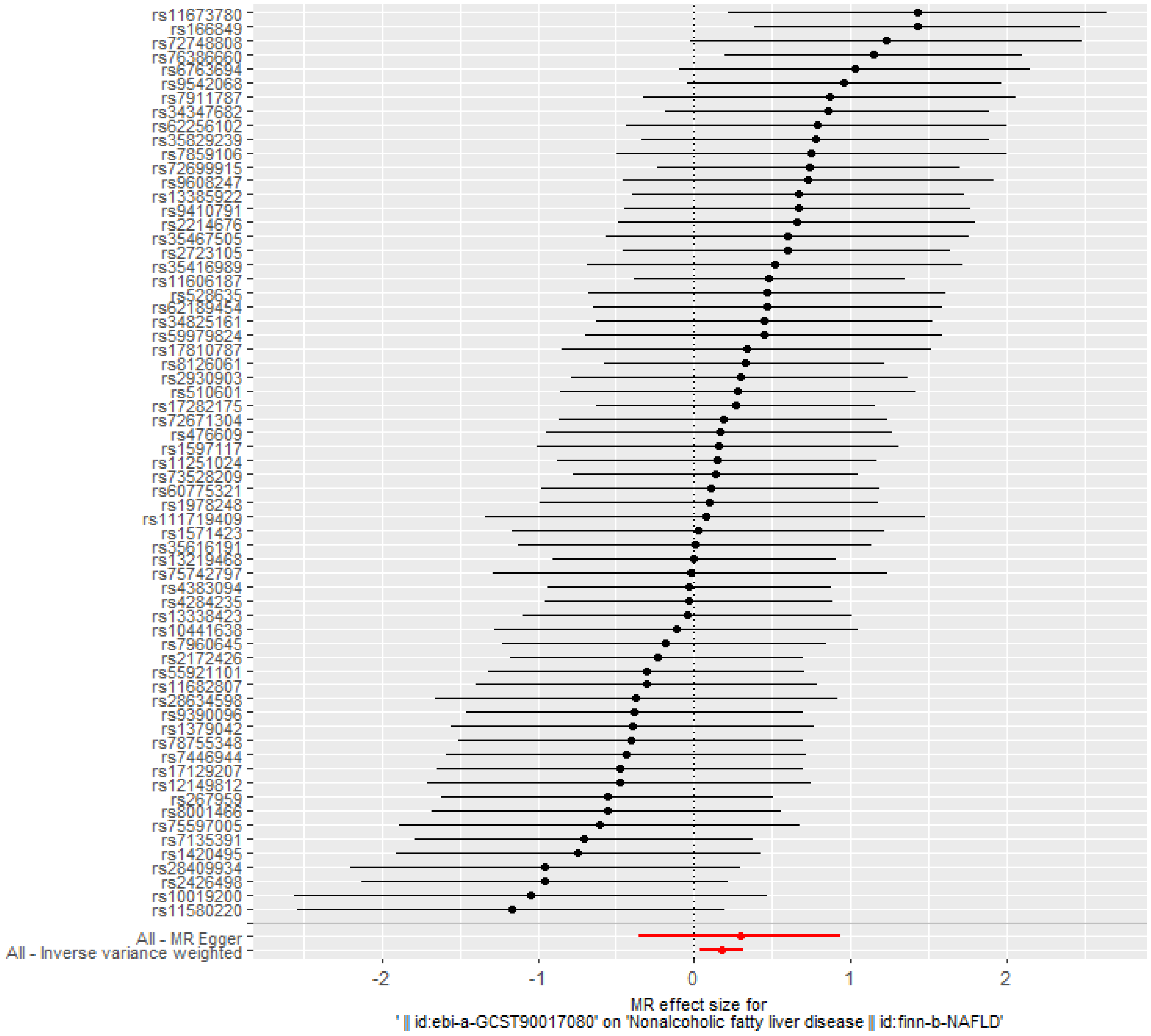
rs11673780
rs166849
rs72748808
rs76386660
rs6763694
rs9542068
rs7911787
rs34347682
rs62256102
rs35829239
rs7859106
rs72699915
rs9608247
rs13385922
rs9410791
rs2214676
rs35467505
rs2723105
rs35416989
rs11606187
rs528635
rs62189454
rs34825161
rs59979824
rs17810787
rs8126061
rs2930903
rs510601
rs17282175
rs72671304
rs476609
rs1597117
rs11251024
rs73528209
rs60775321
rs1978248
rs111719409
rs1571423
rs35616191
rs13219468
rs75742797
rs4383094
rs4284235
rs13338423
rs10441638
rs7960645
rs2172426
rs55921101
rs11682807
rs28634598
rs9390096
rs1379042
rs78755348
rs7446944
rs17129207
rs12149812
rs267959
rs8001466
rs75597005
rs7135391
rs1420495
rs28409934
rs2426498
rs10019200
rs11580220
All - MR Egger
All - Inverse variance weighted
-2
-1
0
1
2
MR effect size for
' || id:ebi-a-GCST90017080' on 'Nonalcoholic fatty liver disease || id:finn-b-NAFLD'

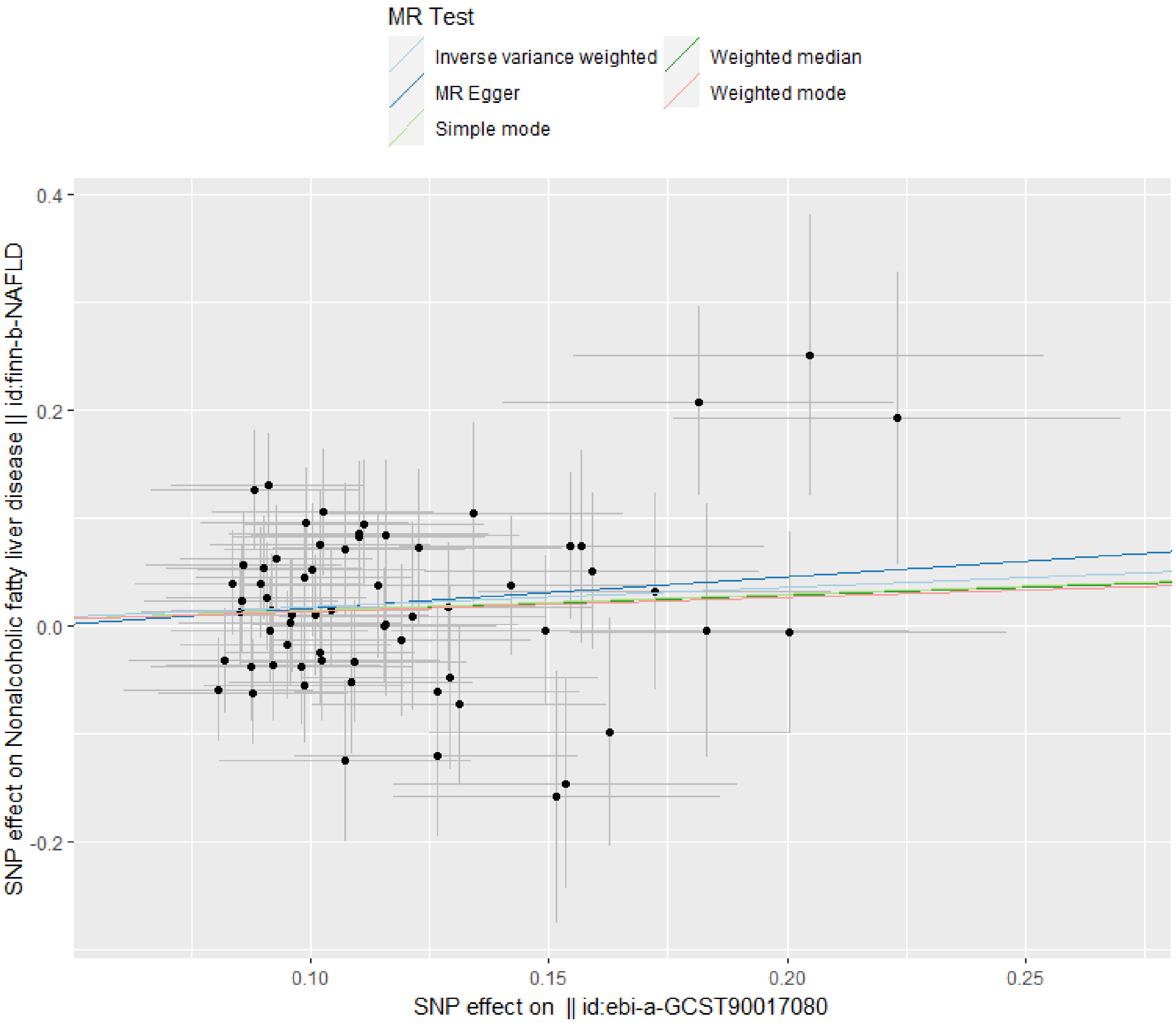

MR Test
Inverse variance weighted
MR Egger
Simple mode
Weighted median
Weighted mode
SNP effect on Nonalcoholic fatty liver disease || id:finn-b-NAFLD
0.4
0.2
0.0
-0.2
0.10
0.15
0.20
0.25
SNP effect on || id:ebi-a-GCST90017080

Figure 160 Leave-one-out analysis, funnel plot, MR effect size and scatter plot for gut microbiota abundance (unknown genus id.959) on nonalcoholic fatty liver disease

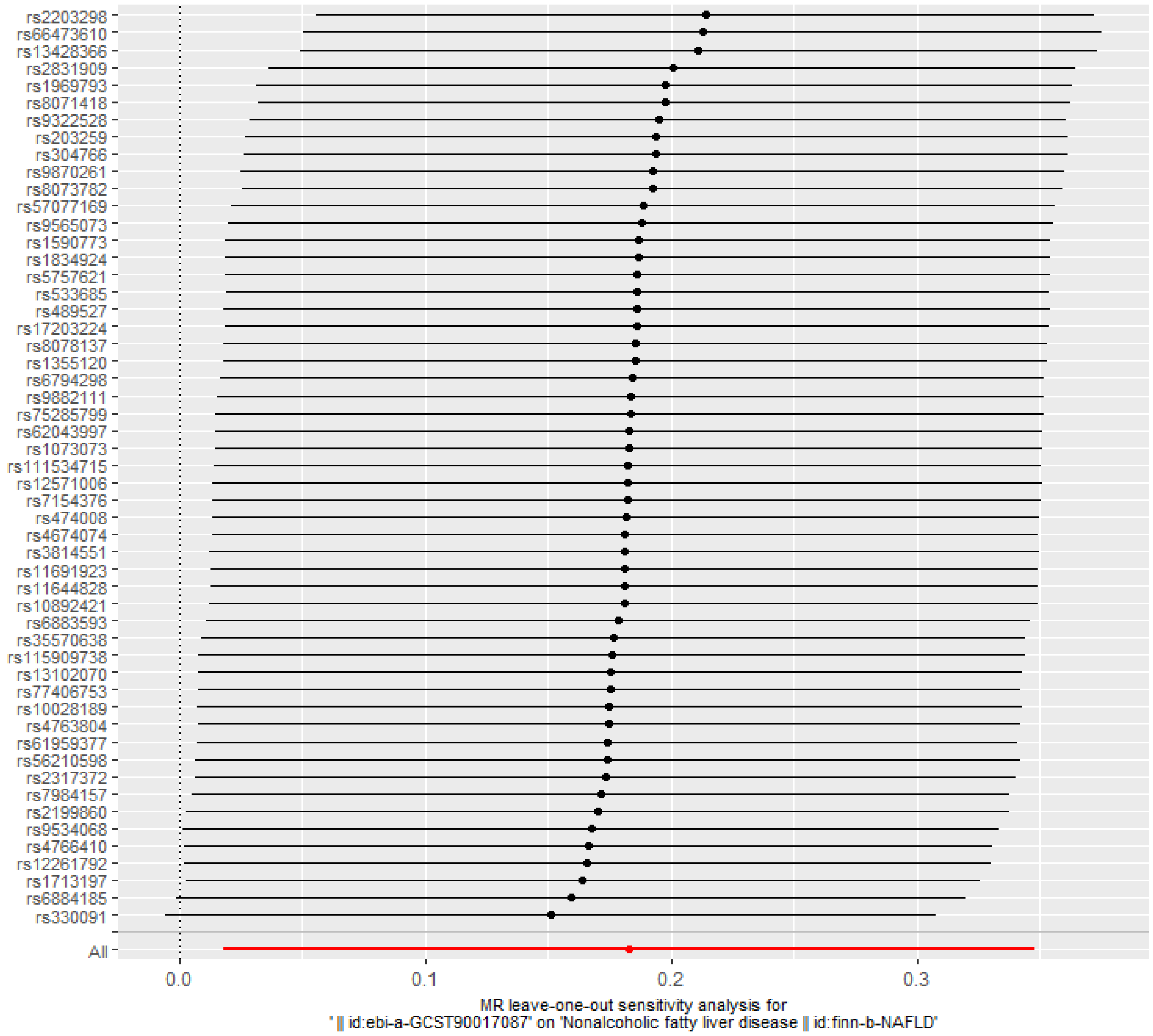

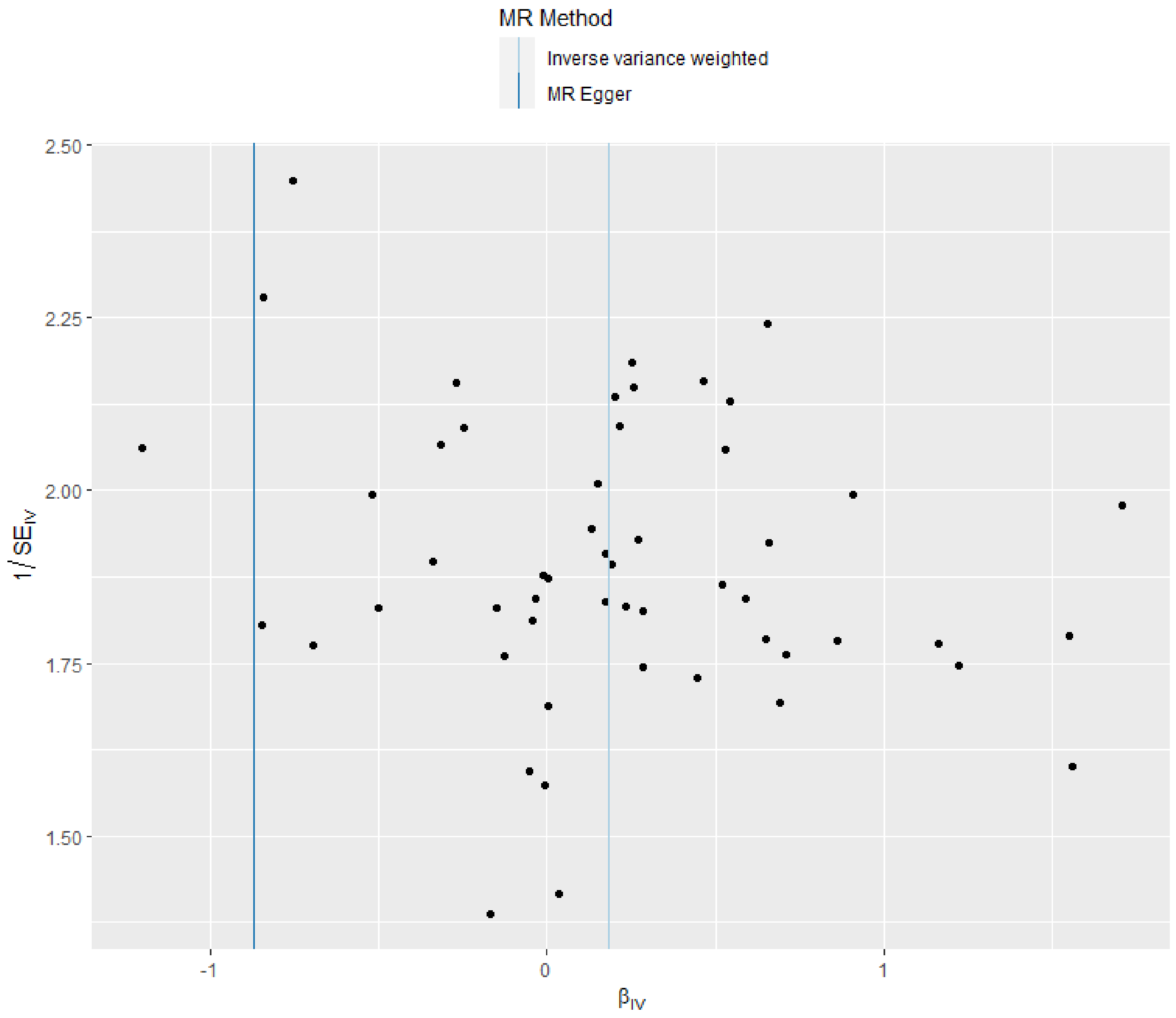

MR Method
Inverse variance weighted
MR Egger
2.50
2.25
2.00
1.75
1.50
-1
0
1
1/SE_IV
β_IV

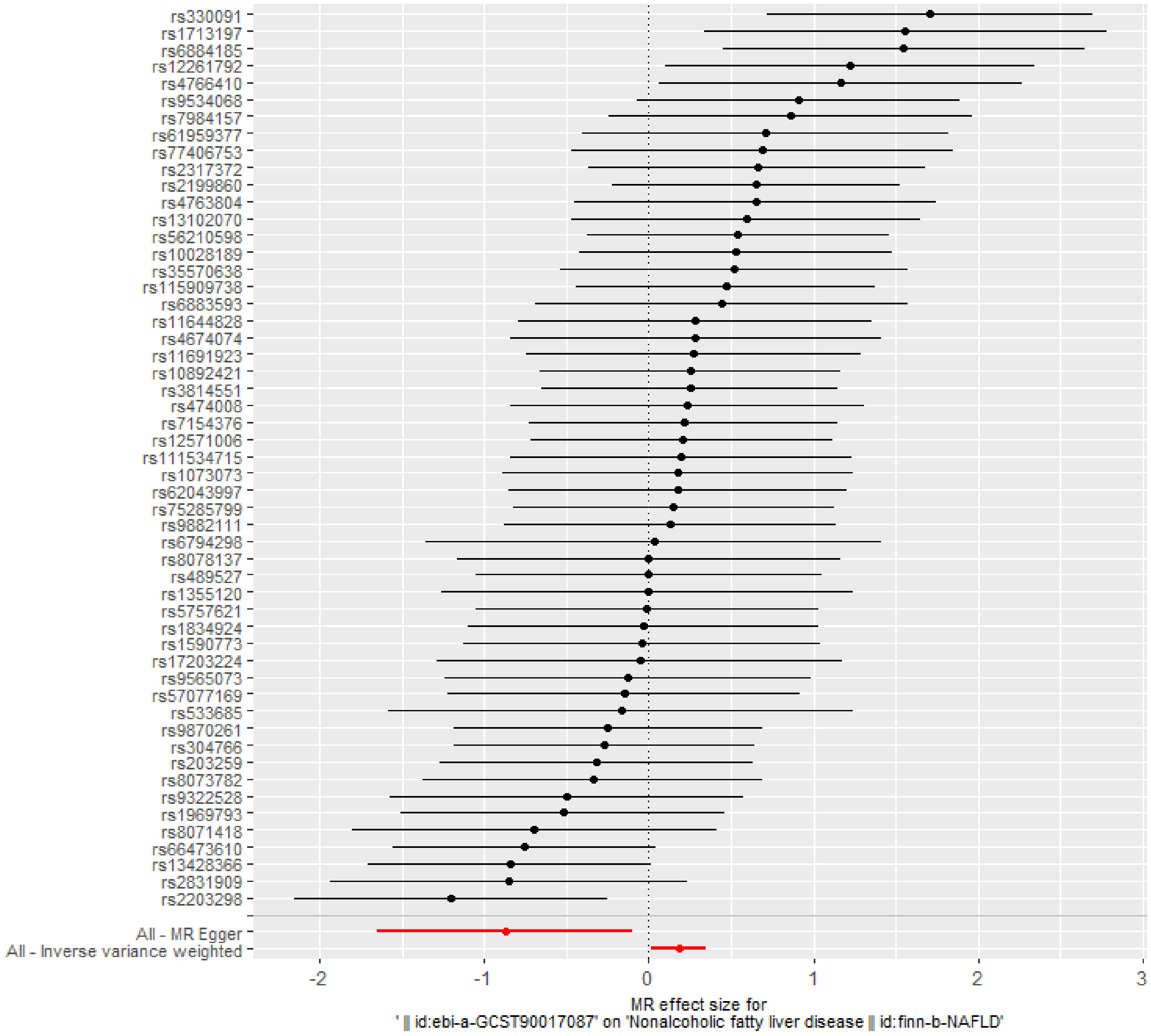

rs330091
rs1713197
rs6884185
rs12261792
rs4766410
rs9534068
rs7984157
rs61959377
rs77406753
rs2317372
rs2199860
rs4763804
rs13102070
rs56210598
rs10028189
rs35570638
rs115909738
rs6883593
rs11644828
rs4674074
rs11691923
rs10892421
rs3814551
rs474008
rs7154376
rs12571006
rs111534715
rs1073073
rs62043997
rs75285799
rs9882111
rs6794298
rs8078137
rs489527
rs1355120
rs5757621
rs1834924
rs1590773
rs17203224
rs9565073
rs57077169
rs533685
rs9870261
rs304766
rs203259
rs8073782
rs9322528
rs1969793
rs8071418
rs66473610
rs13428366
rs2831909
rs2203298
All - MR Egger
All - Inverse variance weighted
-2
-1
0
1
2
3
MR effect size for
' || id:ebi-a-GCST90017087' on 'Nonalcoholic fatty liver disease || id:finn-b-NAFLD'

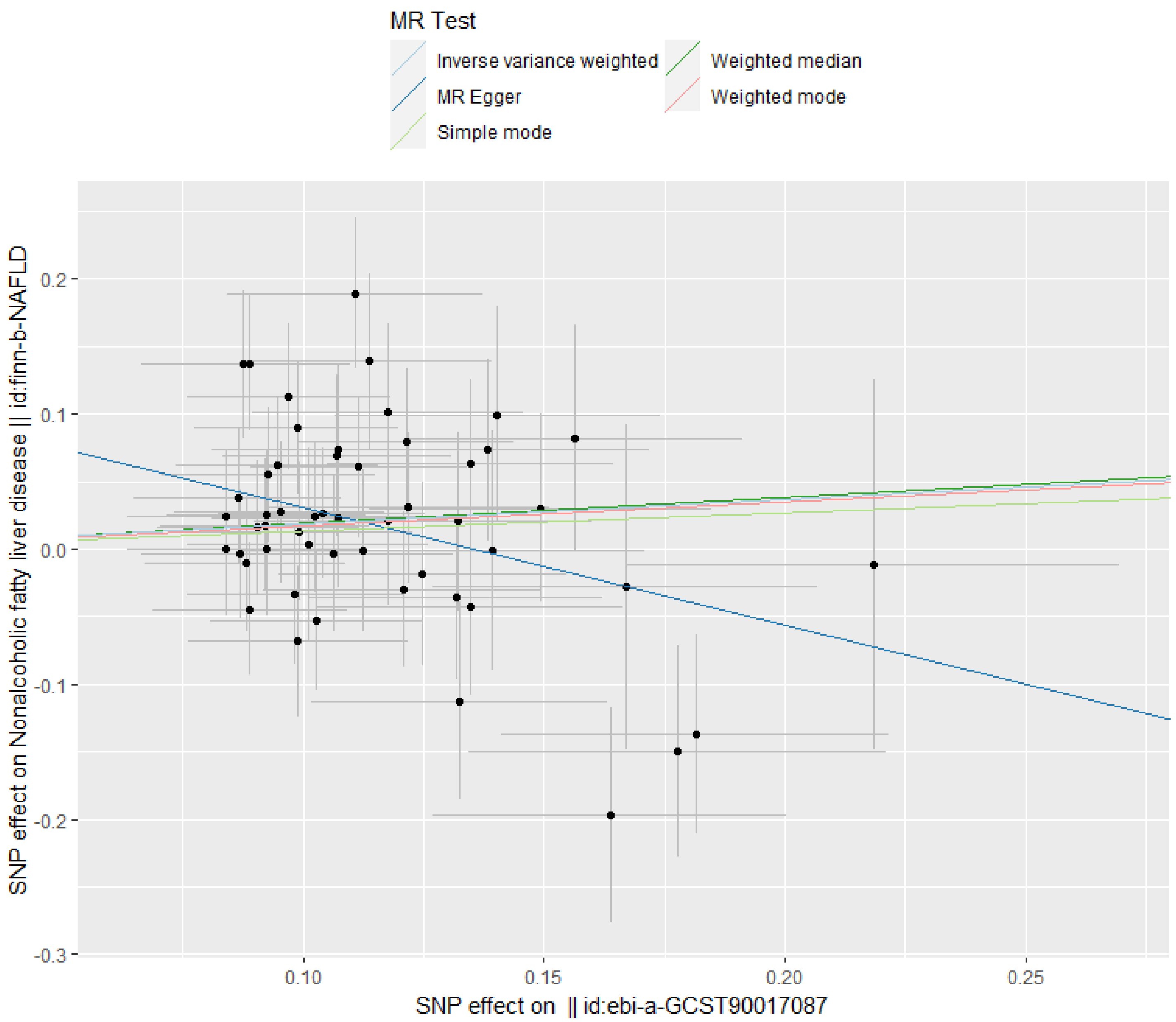

MR Test
Inverse variance weighted
MR Egger
Simple mode
Weighted median
Weighted mode
SNP effect on Nonalcoholic fatty liver disease || id:finn-b-NAFLD
0.2
0.1
0.0
-0.1
-0.2
-0.3
0.10
0.15
0.20
0.25
SNP effect on || id:ebi-a-GCST90017087

Figure 161 Leave-one-out analysis, funnel plot, MR effect size and scatter plot for gut microbiota abundance (order NB1n id.3953) on nonalcoholic fatty liver disease

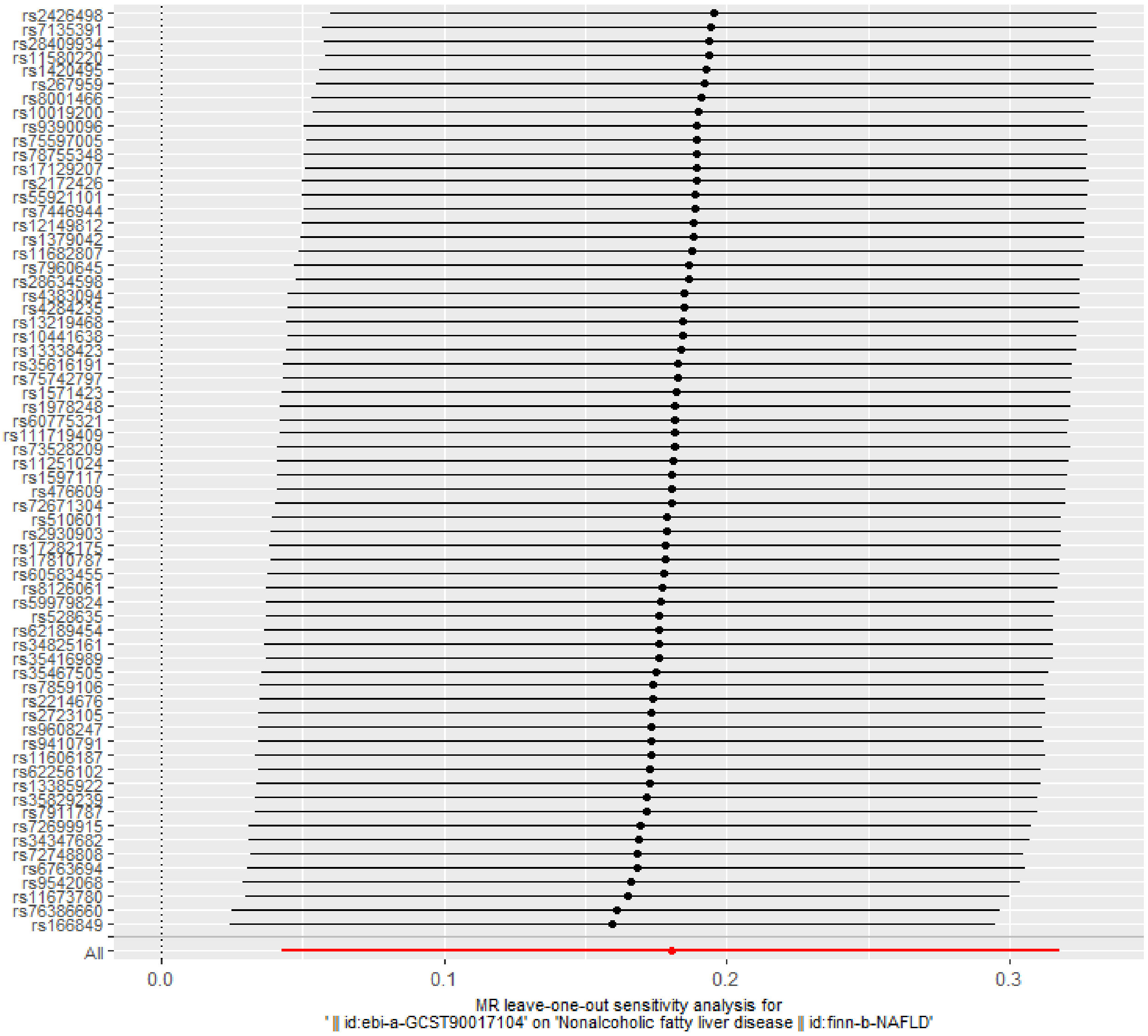

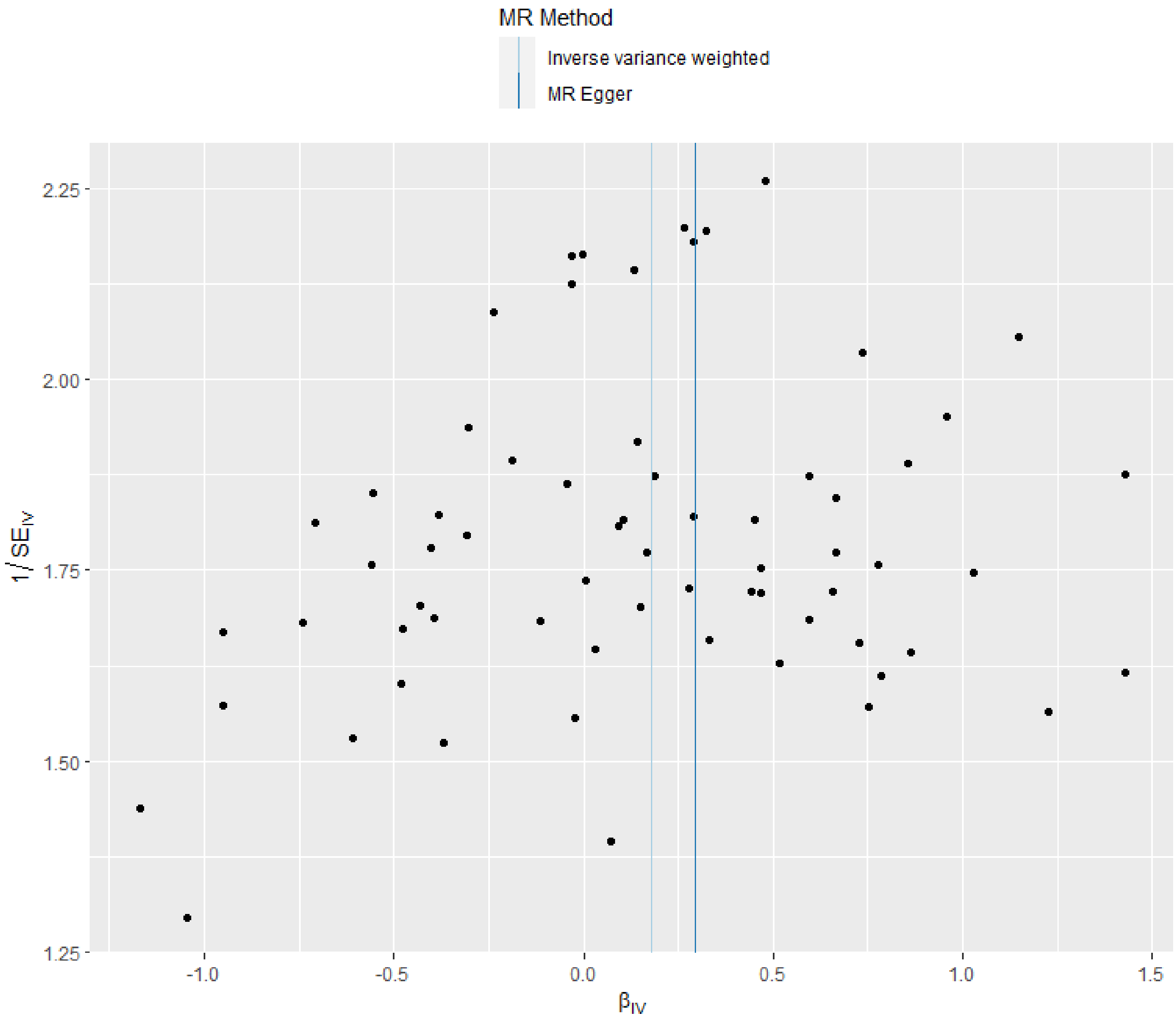

MR Method
Inverse variance weighted
MR Egger
2.25
2.00
1.75
1.50
1.25
1/SE_IV
-1.0
-0.5
0.0
0.5
1.0
1.5
β_IV

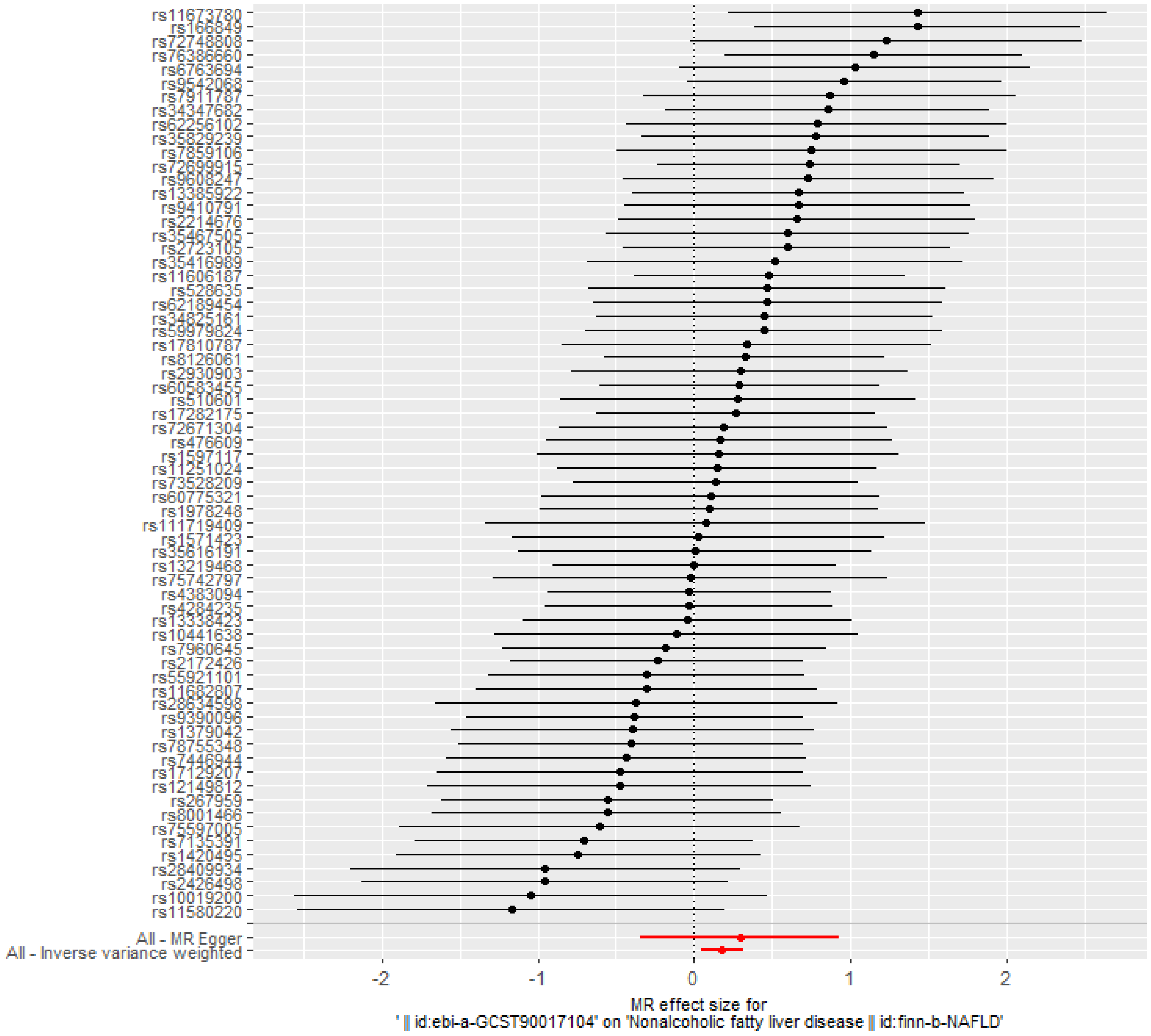

rs11673780
rs166849
rs72748808
rs76386660
rs6763694
rs9542068
rs7911787
rs34347682
rs62256102
rs35829239
rs7859106
rs72699915
rs9608247
rs13385922
rs9410791
rs2214676
rs35467505
rs2723105
rs35416989
rs11606187
rs528635
rs62189454
rs34825161
rs59979824
rs17810787
rs8126061
rs2930903
rs60583455
rs510601
rs17282175
rs72671304
rs476609
rs1597117
rs11251024
rs73528209
rs60775321
rs1978248
rs111719409
rs1571423
rs35616191
rs13219468
rs75742797
rs4383094
rs4284235
rs13338423
rs10441638
rs7960645
rs2172426
rs55921101
rs11682807
rs28634598
rs9390096
rs1379042
rs78755348
rs7446944
rs17129207
rs12149812
rs267959
rs8001466
rs75597005
rs7135391
rs1420495
rs28409934
rs2426498
rs10019200
rs11580220
All - MR Egger
All - Inverse variance weighted
-2
-1
0
1
2
MR effect size for
' || id:ebi-a-GCST90017104' on 'Nonalcoholic fatty liver disease || id:finn-b-NAFLD'

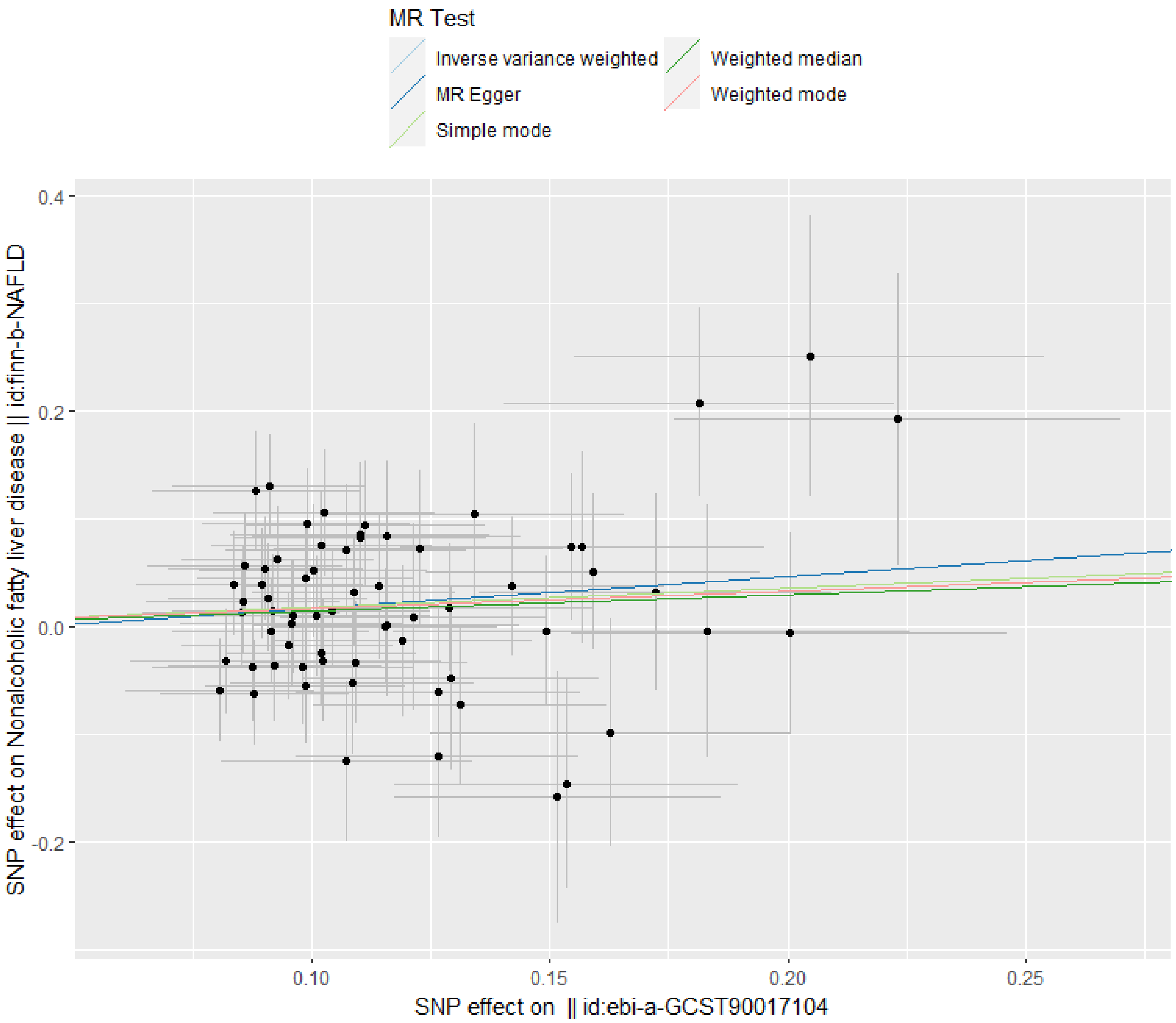

MR Test
Inverse variance weighted
MR Egger
Simple mode
Weighted median
Weighted mode
0.4
0.2
0.0
-0.2
SNP effect on Nonalcoholic fatty liver disease || id:finn-b-NAFLD
0.10
0.15
0.20
0.25
SNP effect on || id:ebi-a-GCST90017104

Figure 162 Leave-one-out analysis, funnel plot, MR effect size and scatter plot for gut microbiota abundance (class Gammaproteobacteria id.3303) on alcoholic liver disease

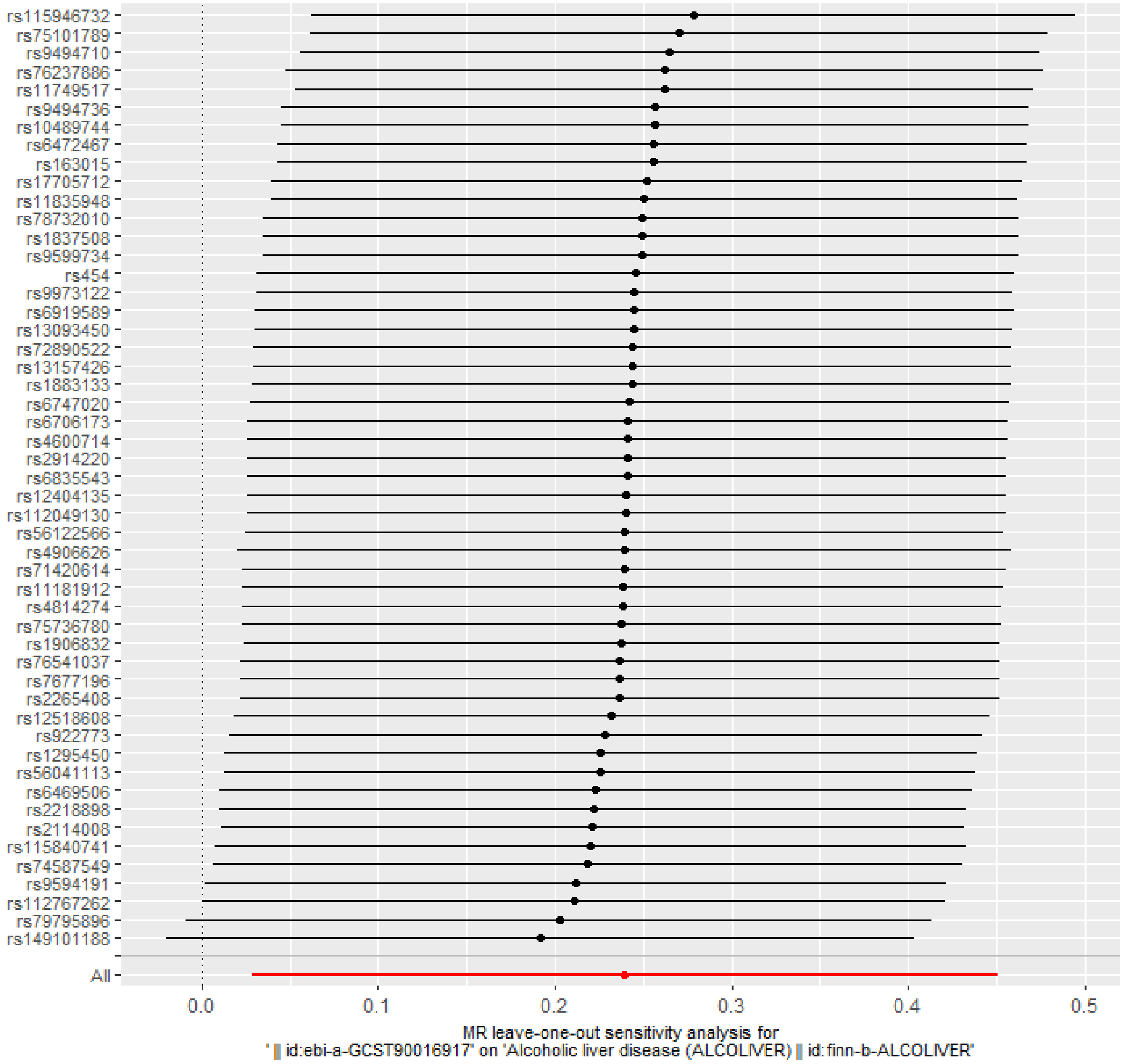

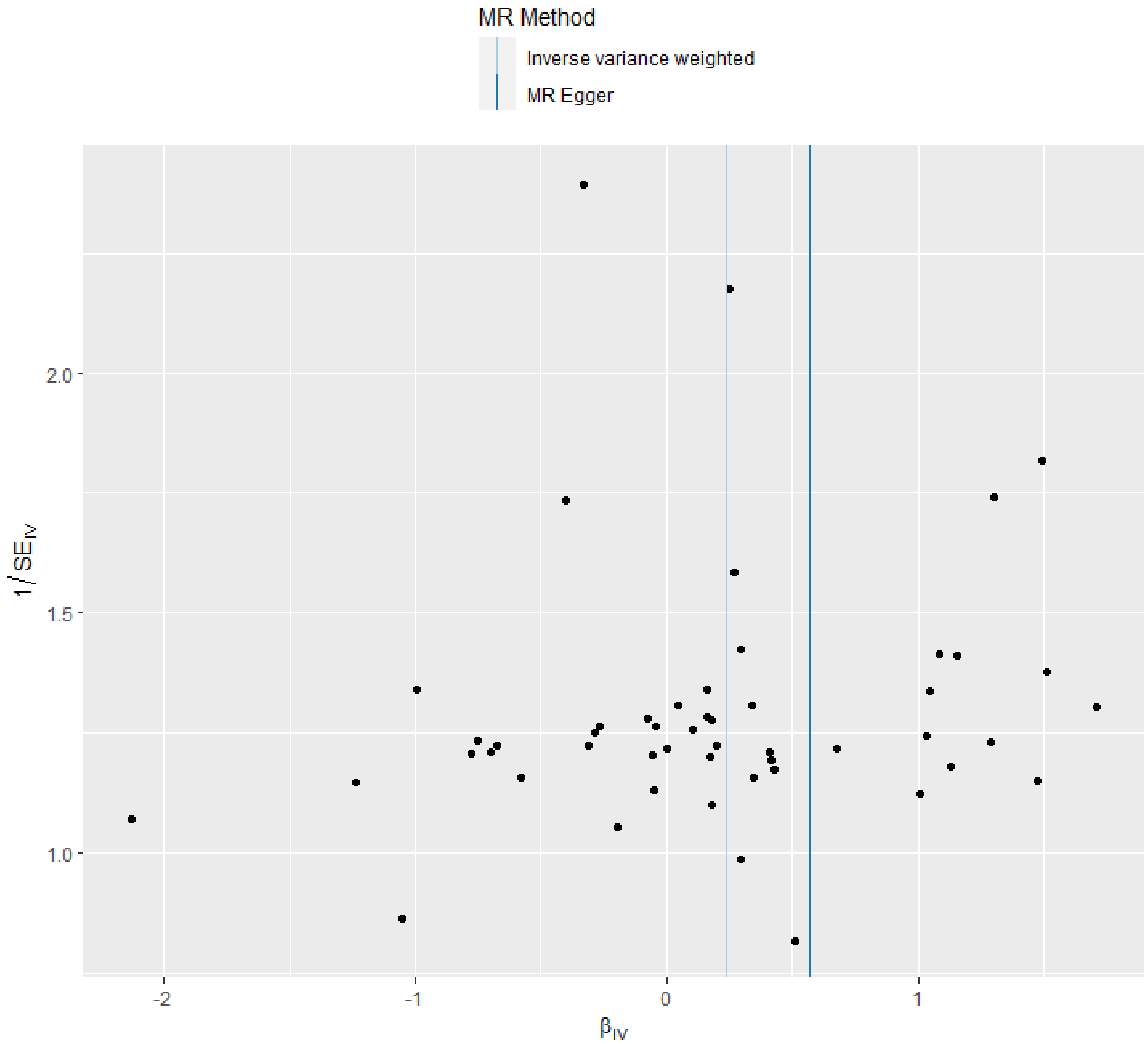

MR Method
Inverse variance weighted
MR Egger
2.0
1.5
1.0
-2
-1
0
1
$1/SE_{IV}$
β_{IV}

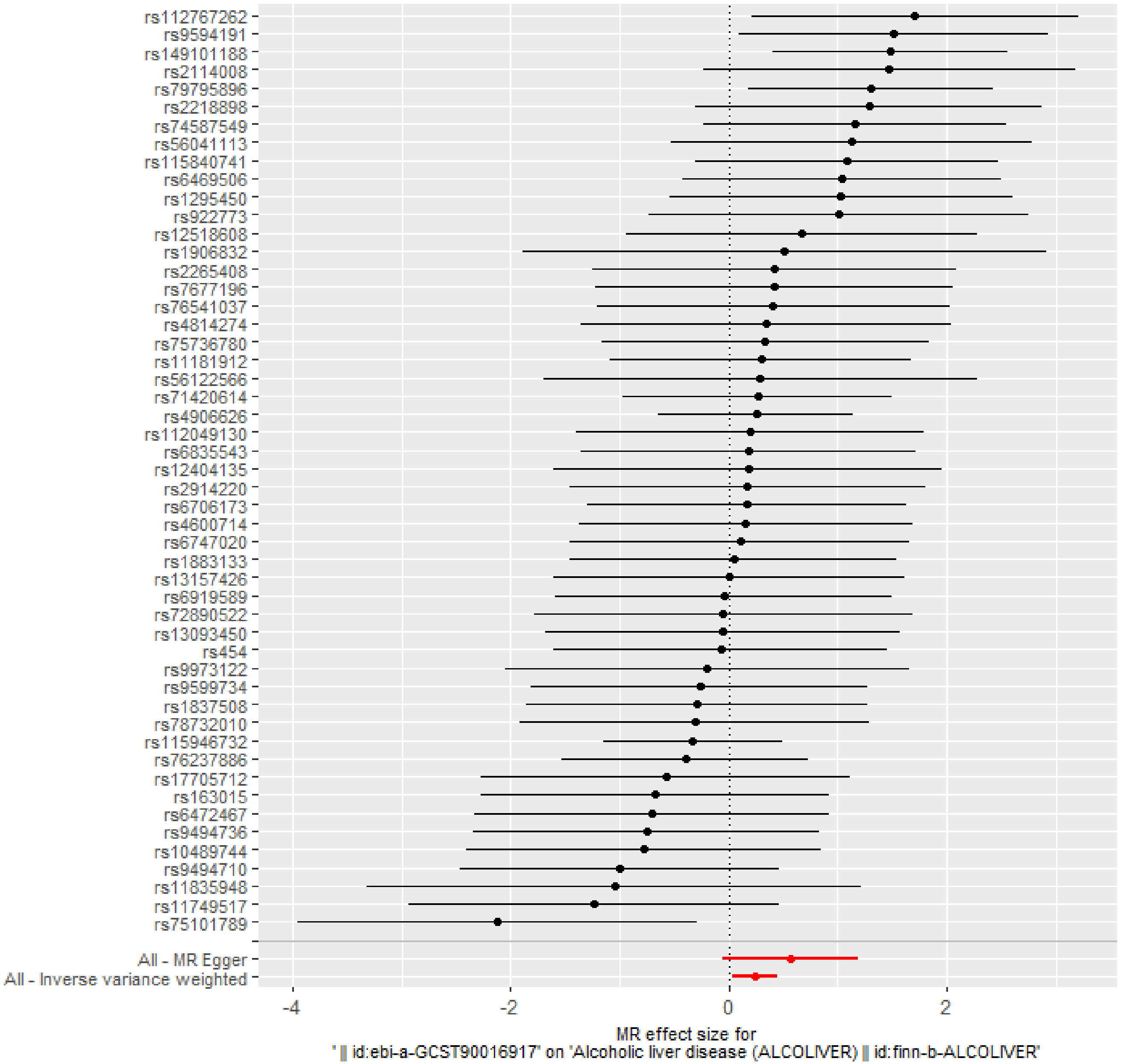
rs112767262
rs9594191
rs149101188
rs2114008
rs79795896
rs2218898
rs74587549
rs56041113
rs115840741
rs6469506
rs1295450
rs922773
rs12518608
rs1906832
rs2265408
rs7677196
rs76541037
rs4814274
rs75736780
rs11181912
rs56122566
rs71420614
rs4906626
rs112049130
rs6835543
rs12404135
rs2914220
rs6706173
rs4600714
rs6747020
rs1883133
rs13157426
rs6919589
rs72890522
rs13093450
rs454
rs9973122
rs9599734
rs1837508
rs78732010
rs115946732
rs76237886
rs17705712
rs163015
rs6472467
rs9494736
rs10489744
rs9494710
rs11835948
rs11749517
rs75101789
All - MR Egger
All - Inverse variance weighted
-4
-2
0
2
MR effect size for
' || id:ebi-a-GCST90016917' on 'Alcoholic liver disease (ALCOLIVER) || id:finn-b-ALCOLIVER'

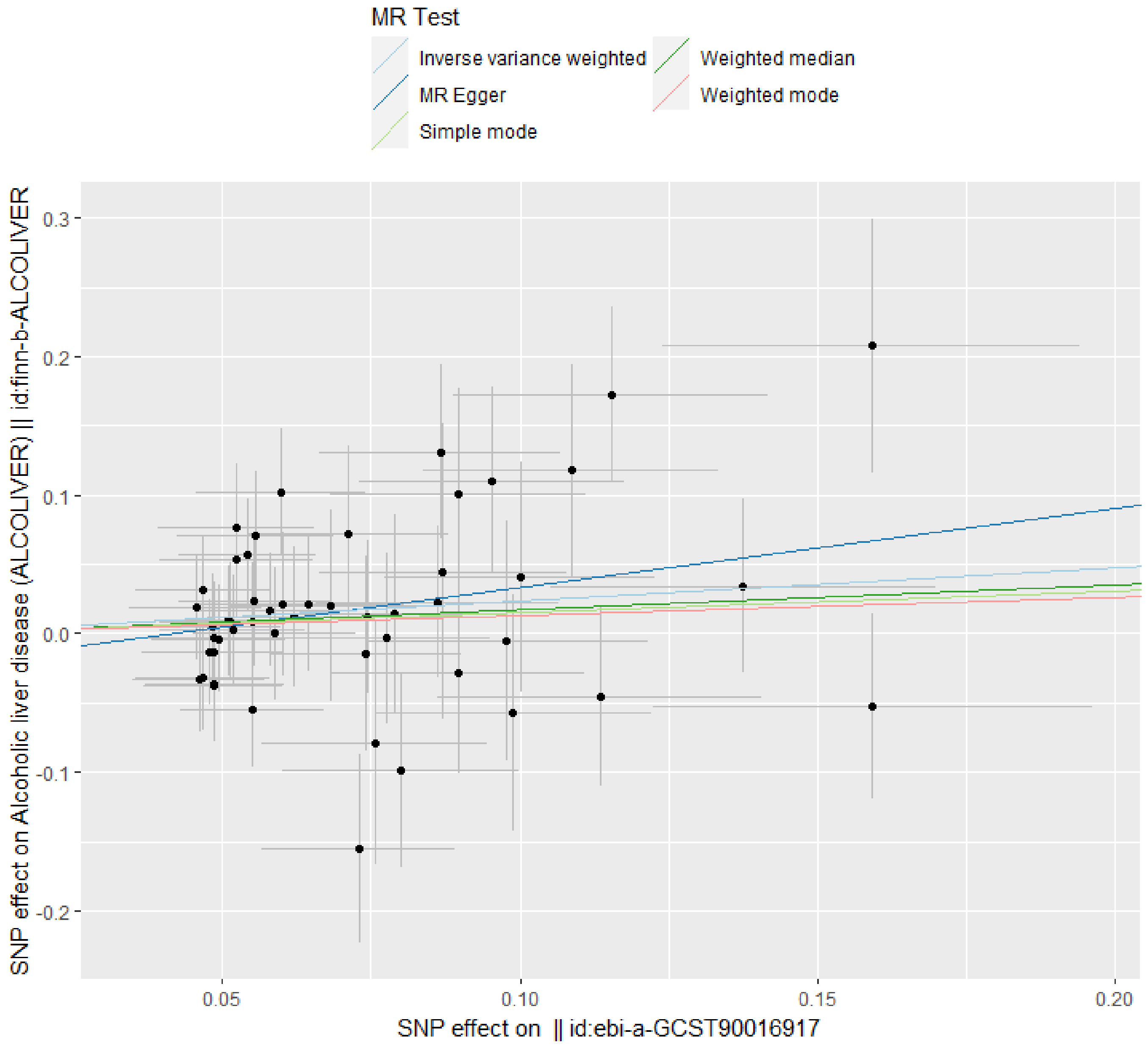

MR Test
Inverse variance weighted
MR Egger
Simple mode
Weighted median
Weighted mode
SNP effect on Alcoholic liver disease (ALCOLIVER) || id:finn-b-ALCOLIVER
0.3
0.2
0.1
0.0
-0.1
-0.2
0.05
0.10
0.15
0.20
SNP effect on || id:ebi-a-GCST90016917

Figure 163 Leave-one-out analysis, funnel plot, MR effect size and scatter plot for gut microbiota abundance (family Victivallaceae id.2255) on alcoholic liver disease

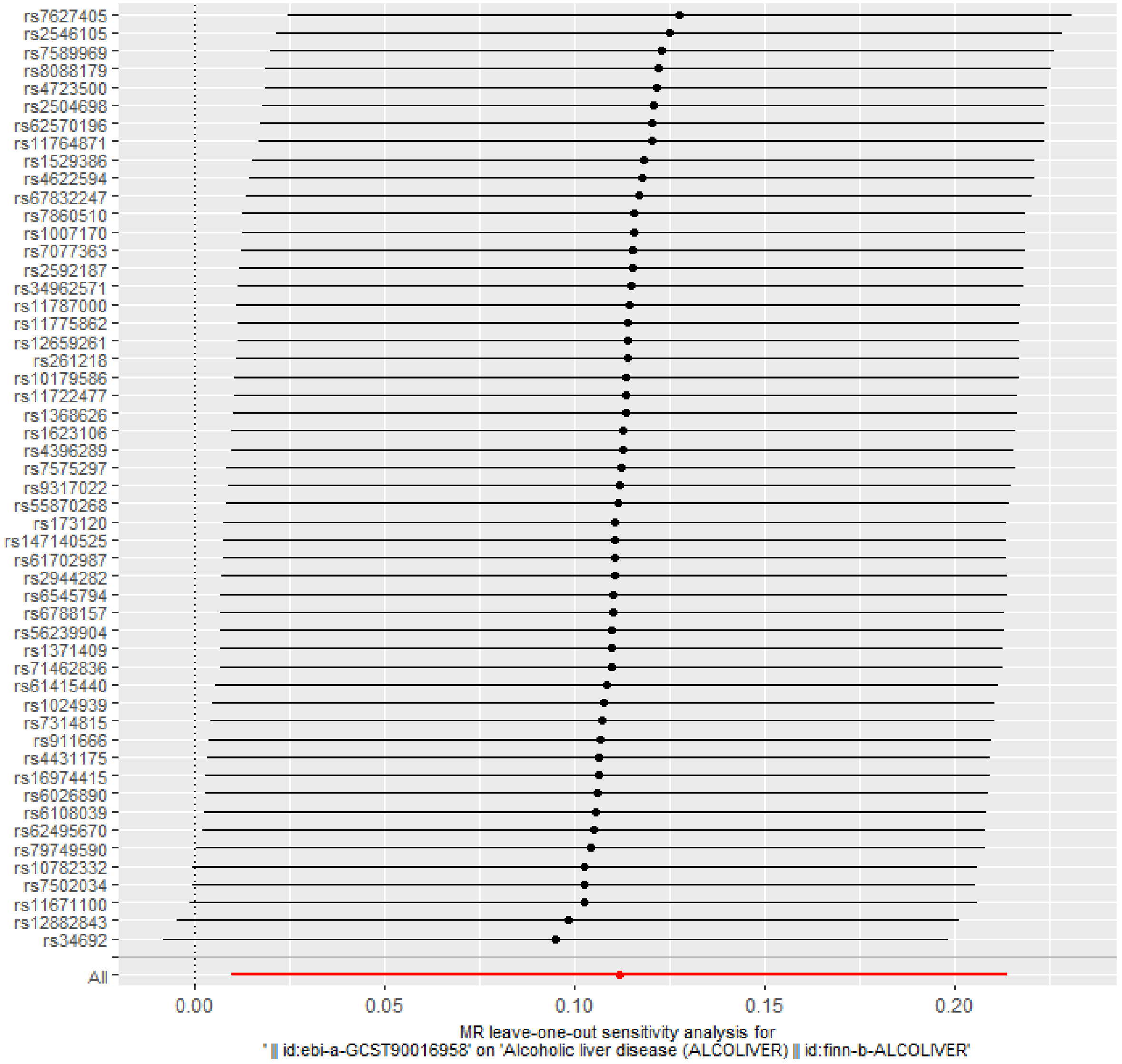

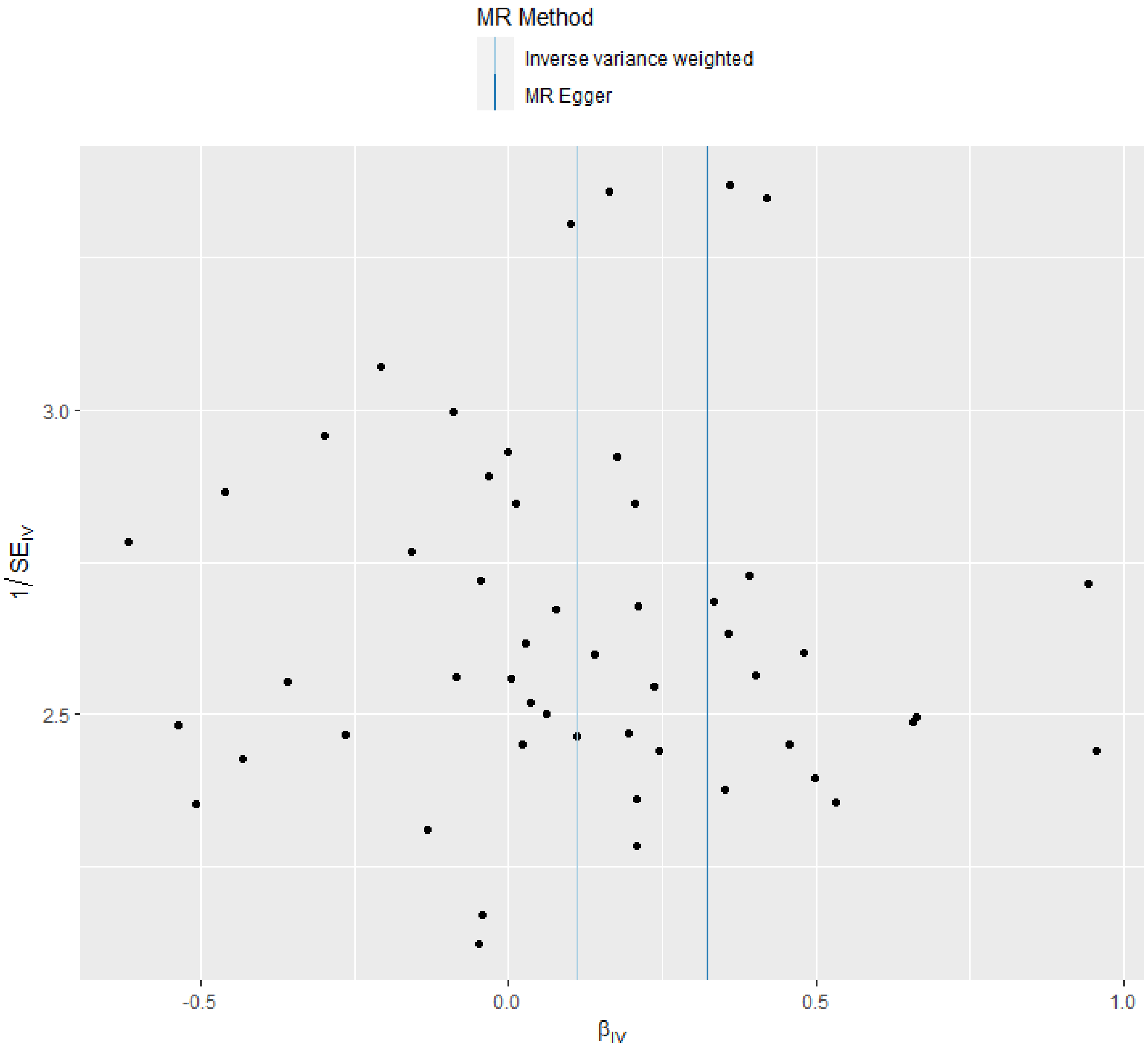

MR Method
Inverse variance weighted
MR Egger
3.0
2.5
1/SE$_{IV}$
-0.5
0.0
0.5
1.0
β$_{IV}$

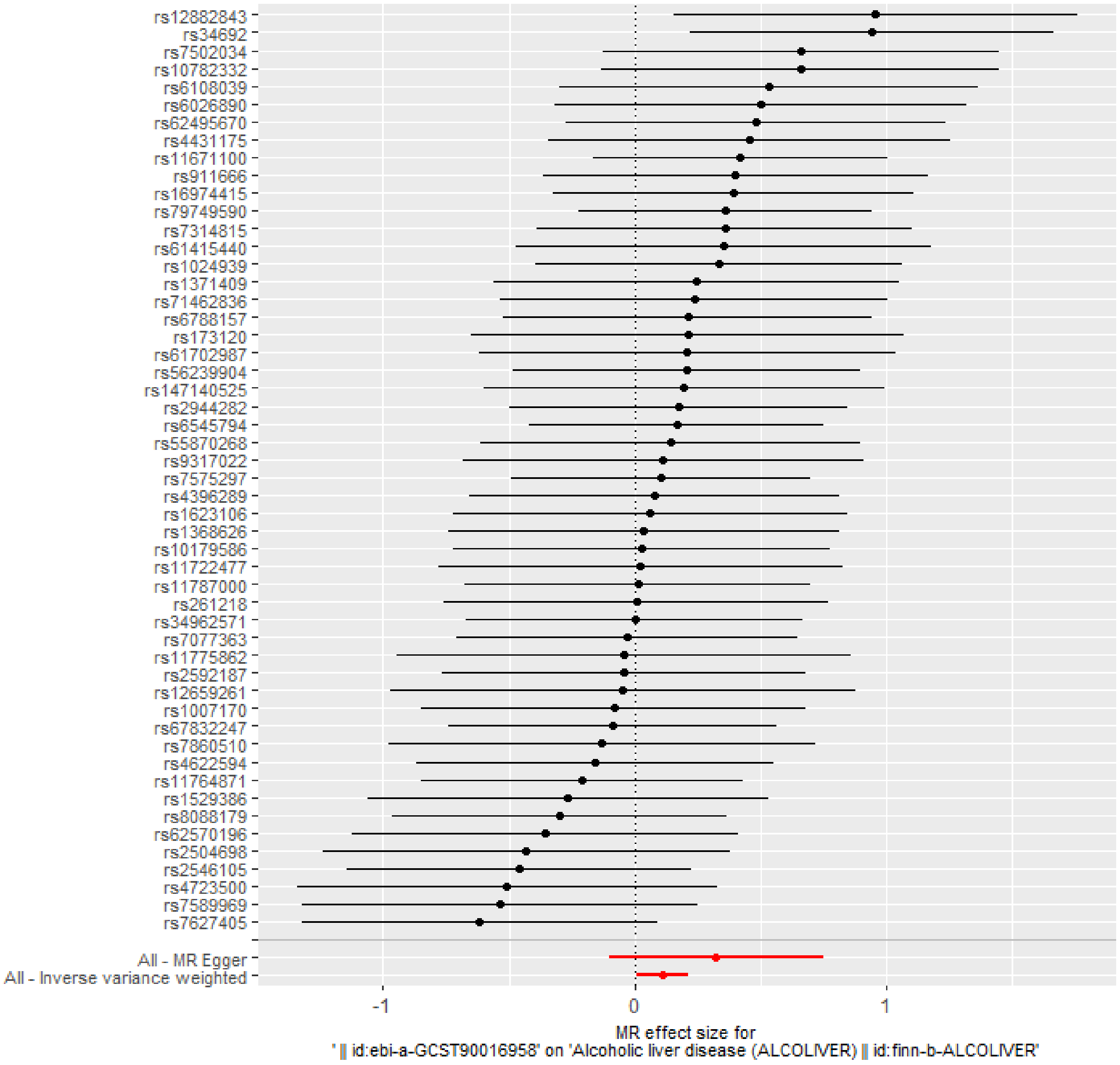

rs12882843
rs34692
rs7502034
rs10782332
rs6108039
rs6026890
rs62495670
rs4431175
rs11671100
rs911666
rs16974415
rs79749590
rs7314815
rs61415440
rs1024939
rs1371409
rs71462836
rs6788157
rs173120
rs61702987
rs56239904
rs147140525
rs2944282
rs6545794
rs55870268
rs9317022
rs7575297
rs4396289
rs1623106
rs1368626
rs10179586
rs11722477
rs11787000
rs261218
rs34962571
rs7077363
rs11775862
rs2592187
rs12659261
rs1007170
rs67832247
rs7860510
rs4622594
rs11764871
rs1529386
rs8088179
rs62570196
rs2504698
rs2546105
rs4723500
rs7589969
rs7627405
All - MR Egger
All - Inverse variance weighted
-1
0
1
MR effect size for
' || id:ebi-a-GCST90016958' on 'Alcoholic liver disease (ALCOLIVER) || id:finn-b-ALCOLIVER'

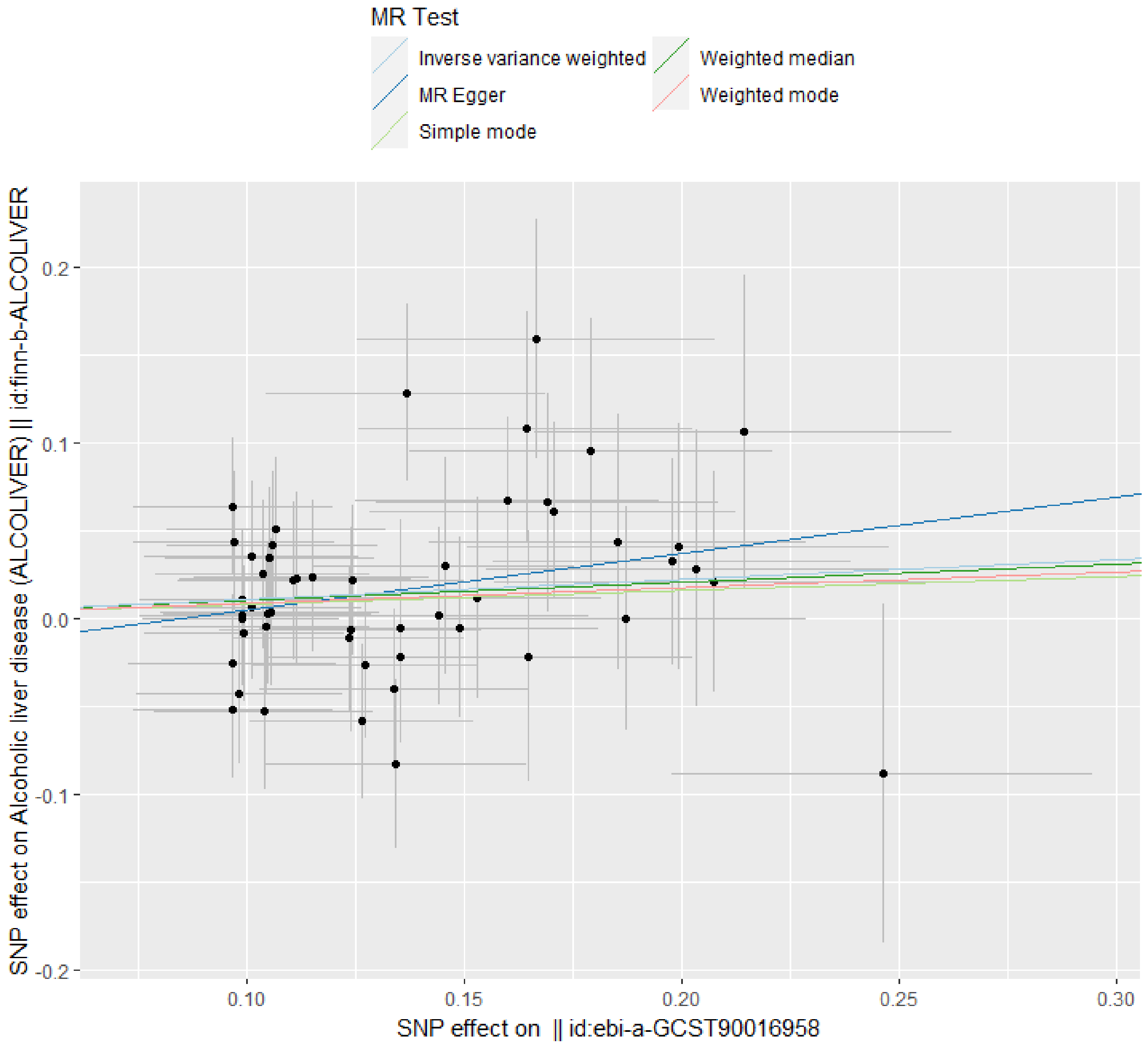

MR Test
Inverse variance weighted
MR Egger
Simple mode
Weighted median
Weighted mode
SNP effect on Alcoholic liver disease (ALCOLIVER) || id:finn-b-ALCOLIVER
0.2
0.1
0.0
-0.1
-0.2
0.10
0.15
0.20
0.25
0.30
SNP effect on || id:ebi-a-GCST90016958

Figure 164 Leave-one-out analysis, funnel plot, MR effect size and scatter plot for gut microbiota abundance (genus Coprobacter id.949) on alcoholic liver disease

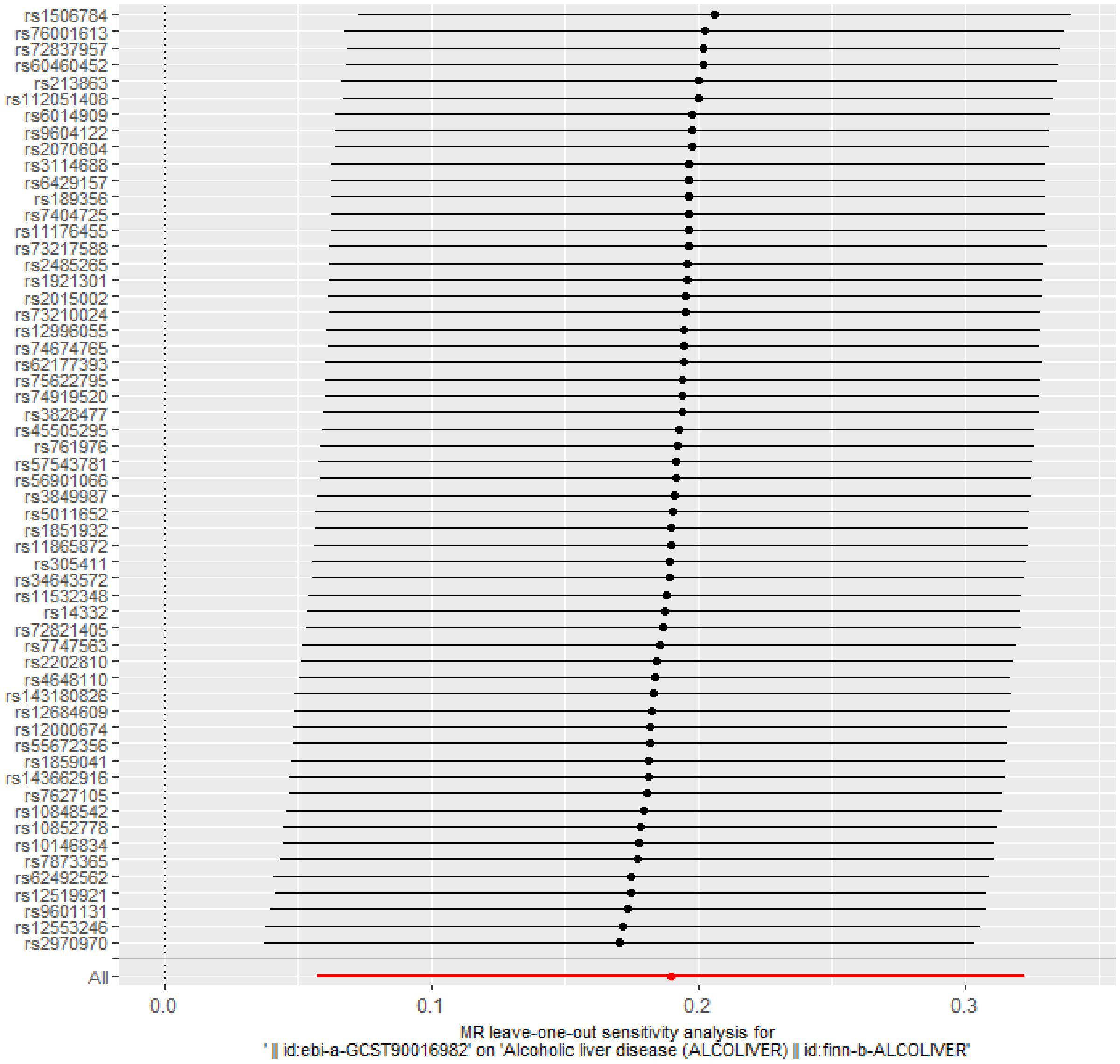

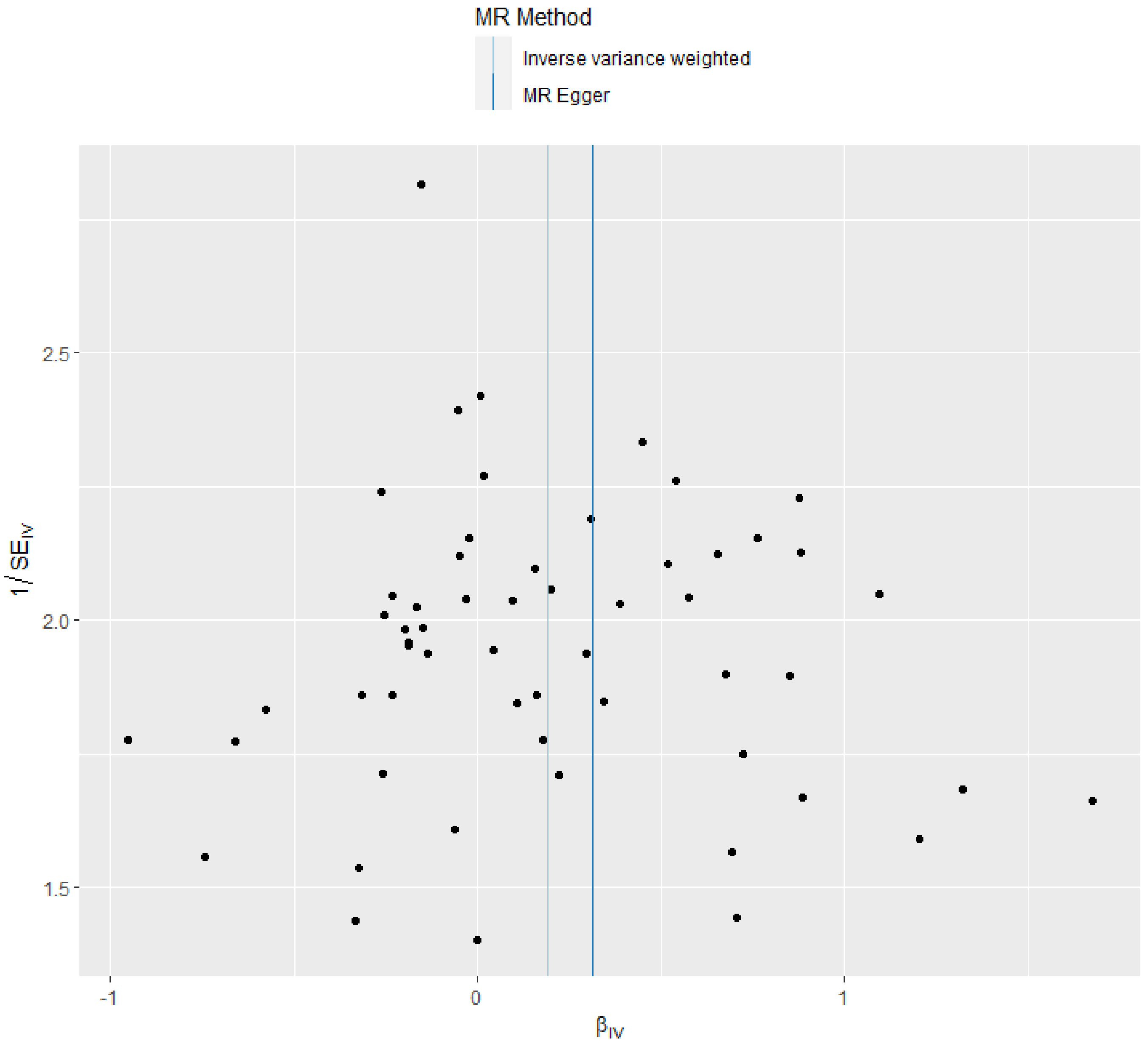

MR Method
Inverse variance weighted
MR Egger
2.5
2.0
1.5
-1
0
1
$1/SE_{IV}$
β_{IV}

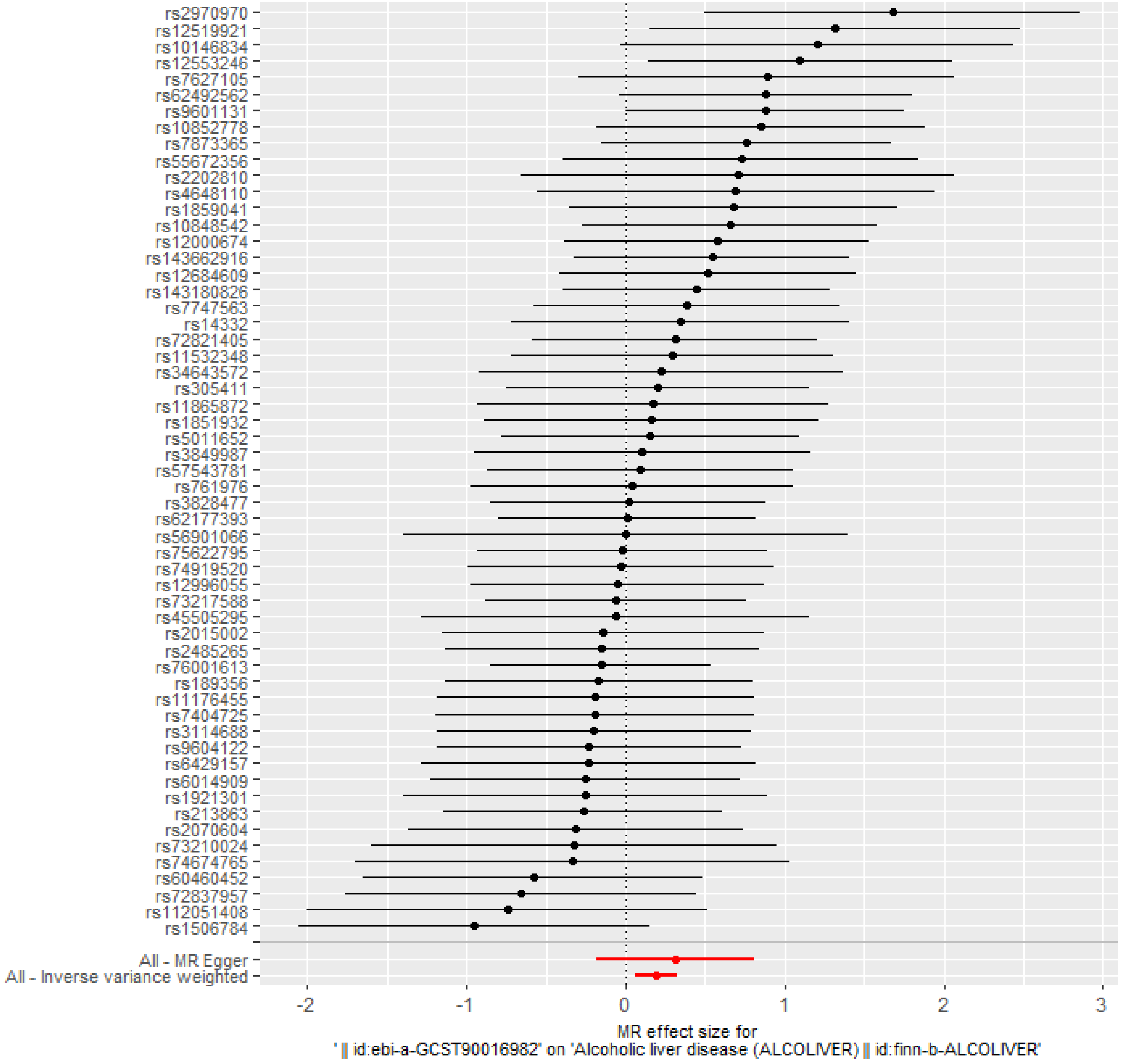

rs2970970
rs12519921
rs10146834
rs12553246
rs7627105
rs62492562
rs9601131
rs10852778
rs7873365
rs55672356
rs2202810
rs4648110
rs1859041
rs10848542
rs12000674
rs143662916
rs12684609
rs143180826
rs7747563
rs14332
rs72821405
rs11532348
rs34643572
rs305411
rs11865872
rs1851932
rs5011652
rs3849987
rs57543781
rs761976
rs3828477
rs62177393
rs56901066
rs75622795
rs74919520
rs12996055
rs73217588
rs45505295
rs2015002
rs2485265
rs76001613
rs189356
rs11176455
rs7404725
rs3114688
rs9604122
rs6429157
rs6014909
rs1921301
rs213863
rs2070604
rs73210024
rs74674765
rs60460452
rs72837957
rs112051408
rs1506784
All - MR Egger
All - Inverse variance weighted
-2
-1
0
1
2
3
MR effect size for
' || id:ebi-a-GCST90016982' on 'Alcoholic liver disease (ALCOLIVER) || id:finn-b-ALCOLIVER'

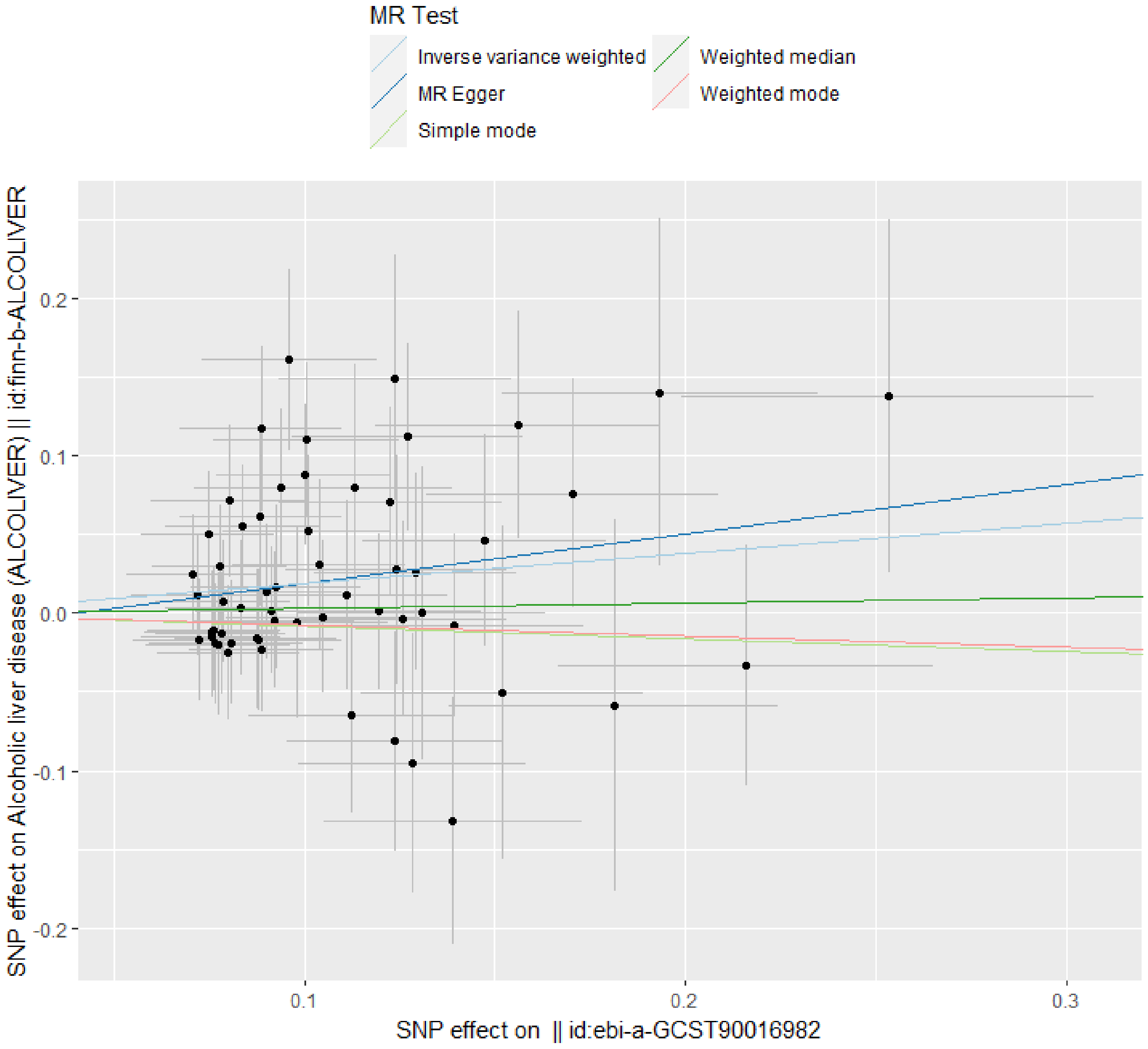

MR Test
Inverse variance weighted
MR Egger
Simple mode
Weighted median
Weighted mode
SNP effect on Alcoholic liver disease (ALCOLIVER) || id:finn-b-ALCOLIVER
0.2
0.1
0.0
-0.1
-0.2
0.1
0.2
0.3
SNP effect on || id:ebi-a-GCST90016982

Figure 165 Leave-one-out analysis, funnel plot, MR effect size and scatter plot for gut microbiota abundance (genus Family XIII UCG001 id.11294) on alcoholic liver disease

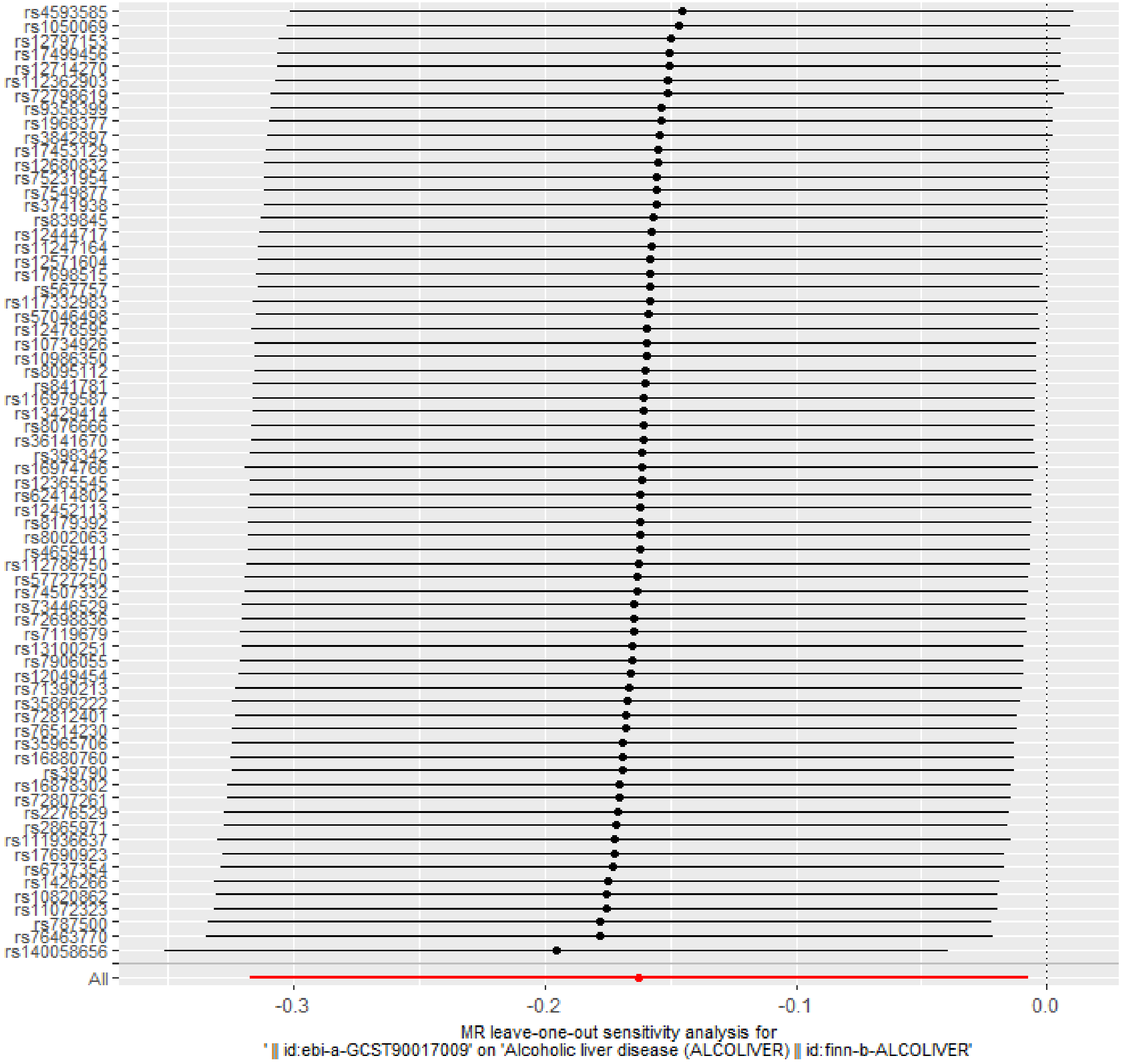

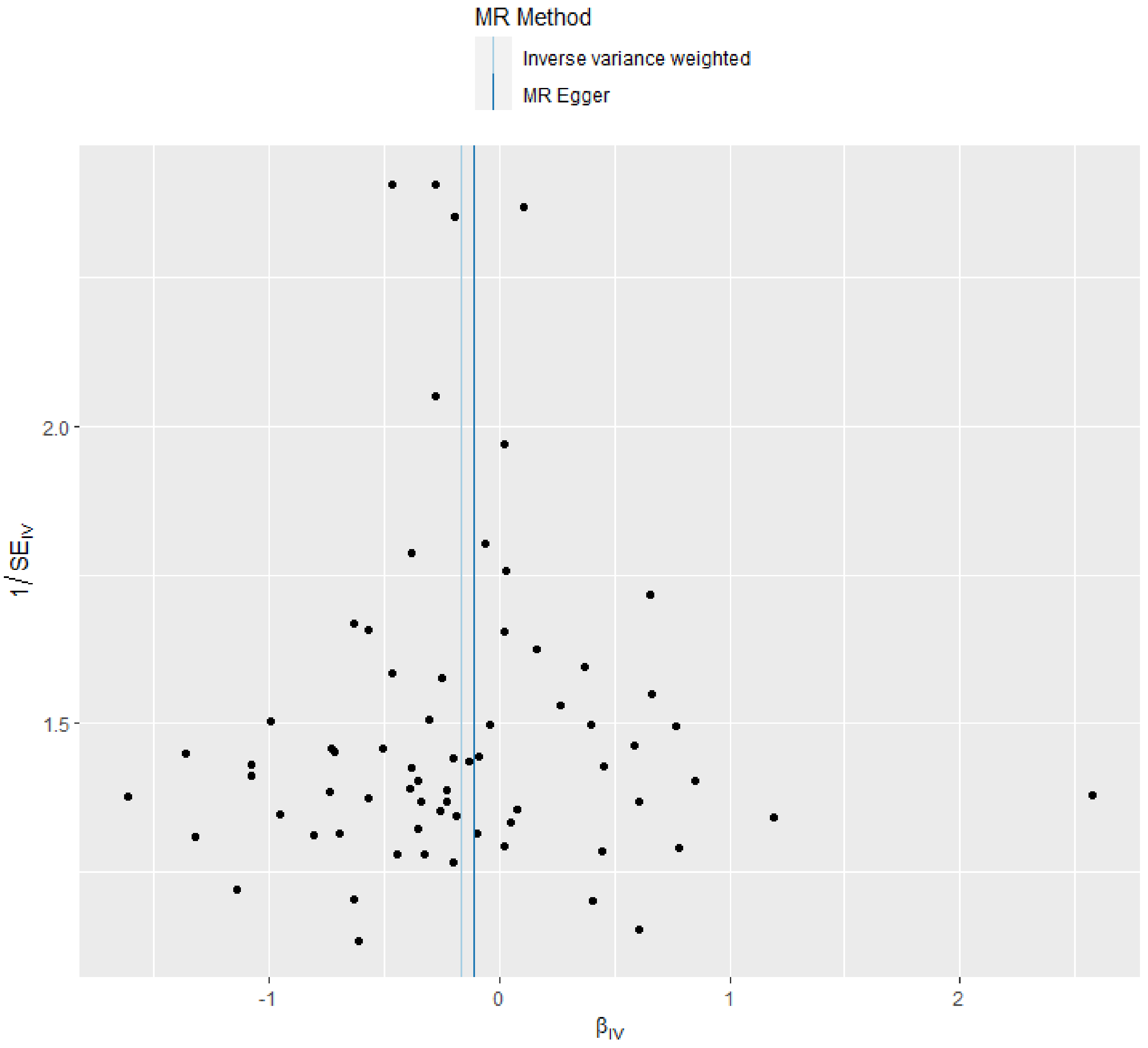

MR Method
Inverse variance weighted
MR Egger
2.0
1.5
-1
0
1
2
β_{IV}
$1/SE_{IV}$

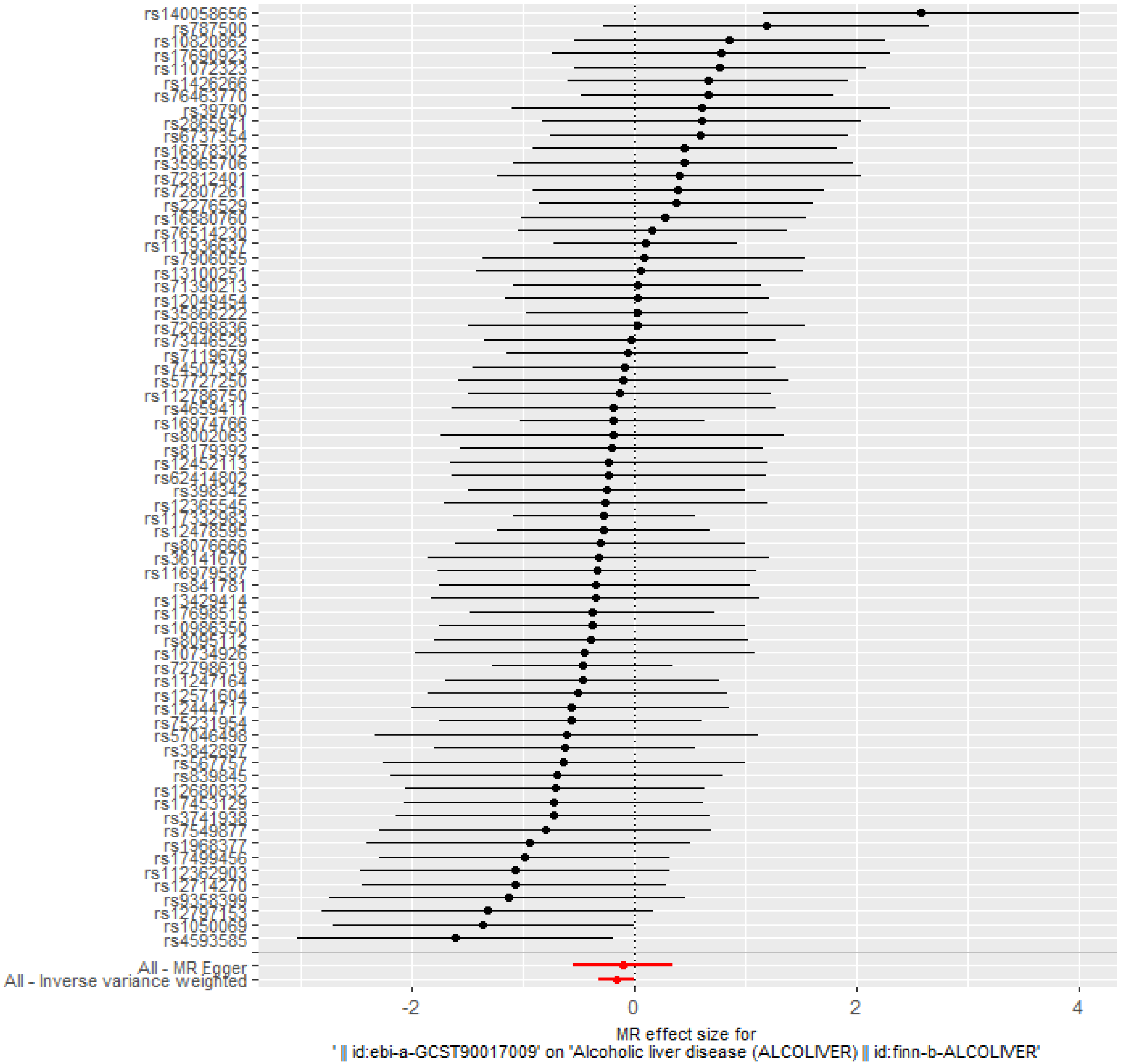
All - MR Egger
All - Inverse variance weighted
-2
0
2
4
MR effect size for
' || id:ebi-a-GCST90017009' on 'Alcoholic liver disease (ALCOLIVER) || id:finn-b-ALCOLIVER'

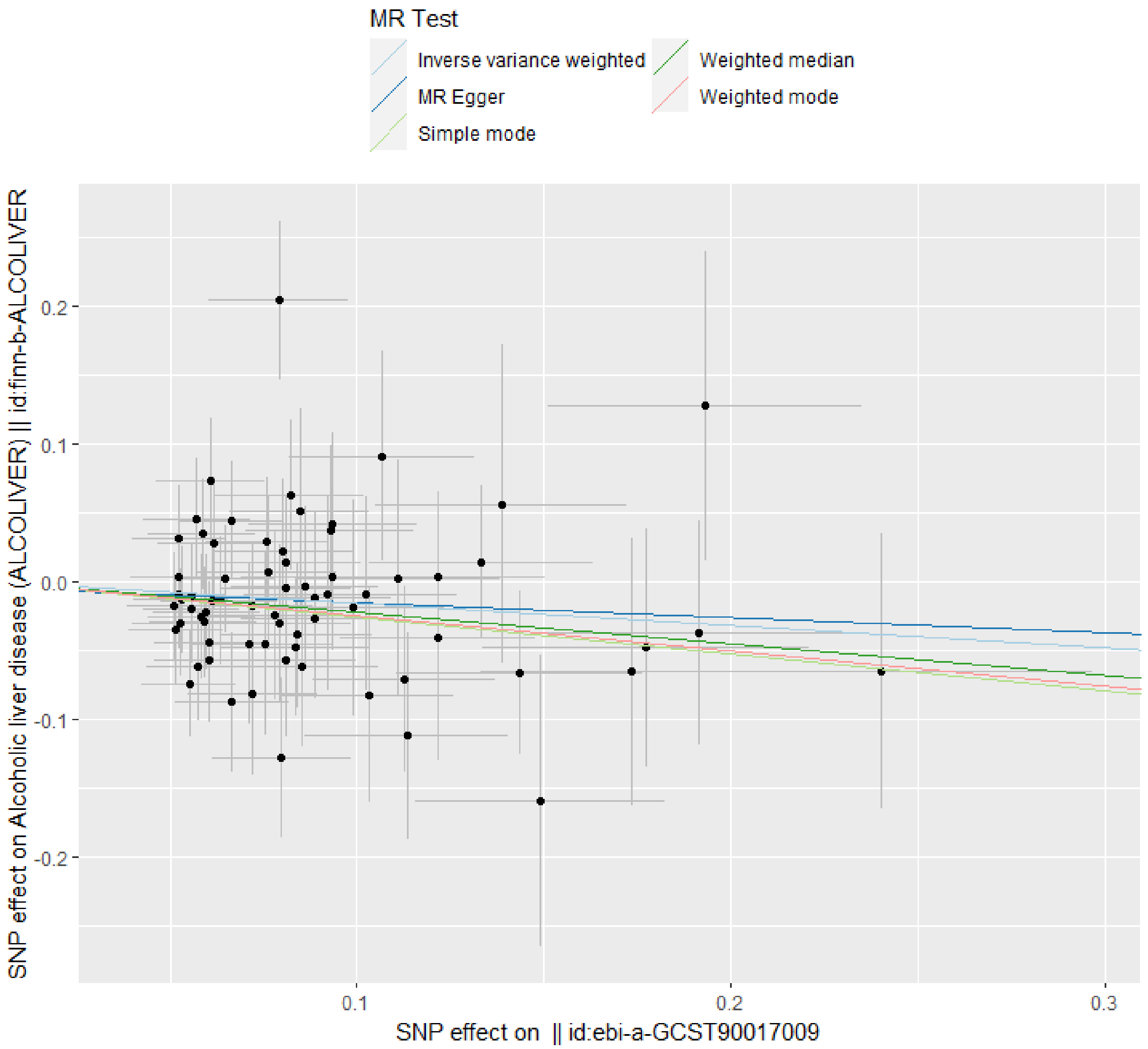
MR Test
Inverse variance weighted
MR Egger
Simple mode
Weighted median
Weighted mode
SNP effect on Alcoholic liver disease (ALCOLIVER) || id:finn-b-ALCOLIVER
0.2
0.1
0.0
-0.1
-0.2
0.1
0.2
0.3
SNP effect on || id:ebi-a-GCST90017009

Figure 166 Leave-one-out analysis, funnel plot, MR effect size and scatter plot for gut microbiota abundance (genus Lactococcus id.1851) on alcoholic liver disease

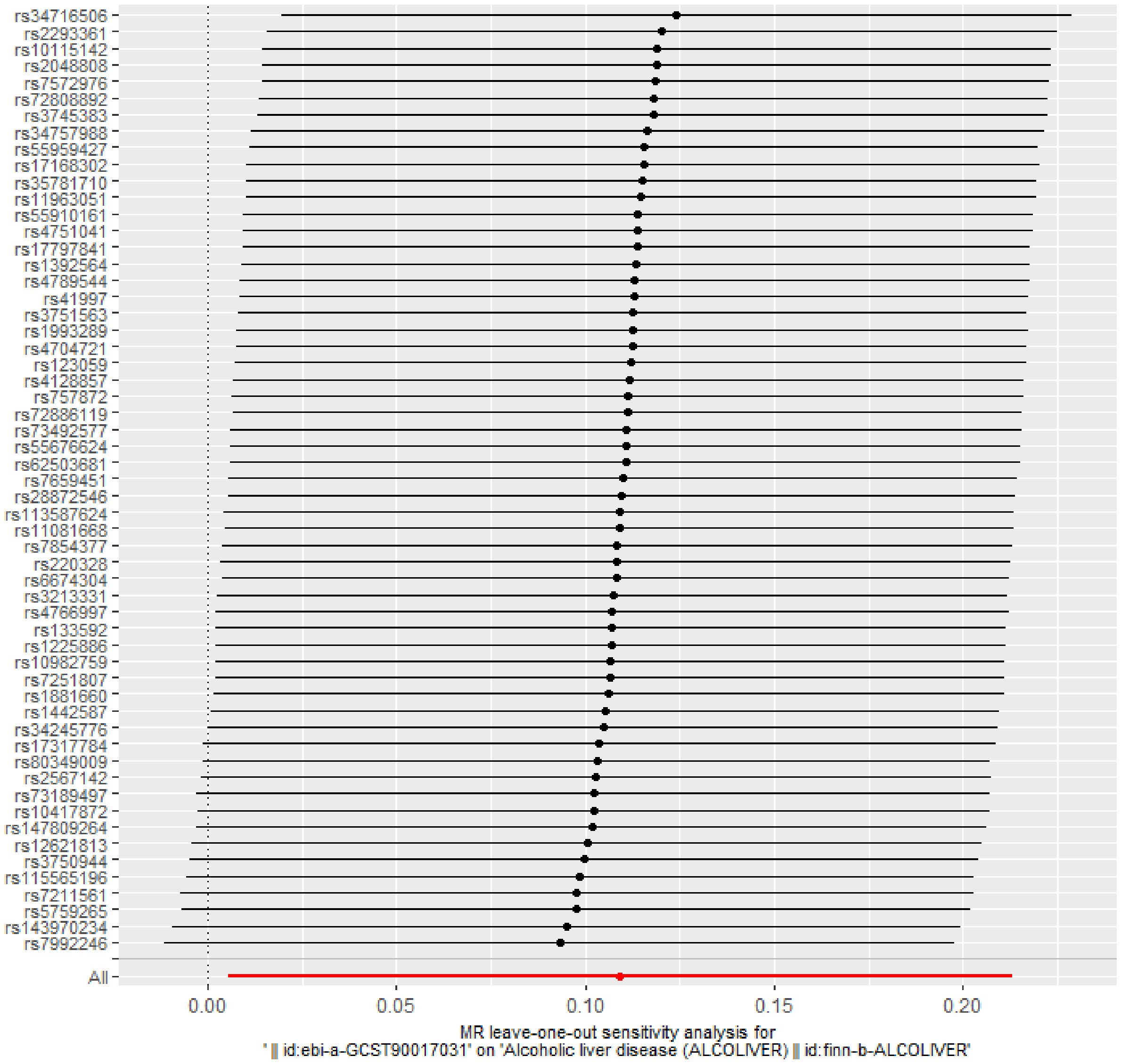

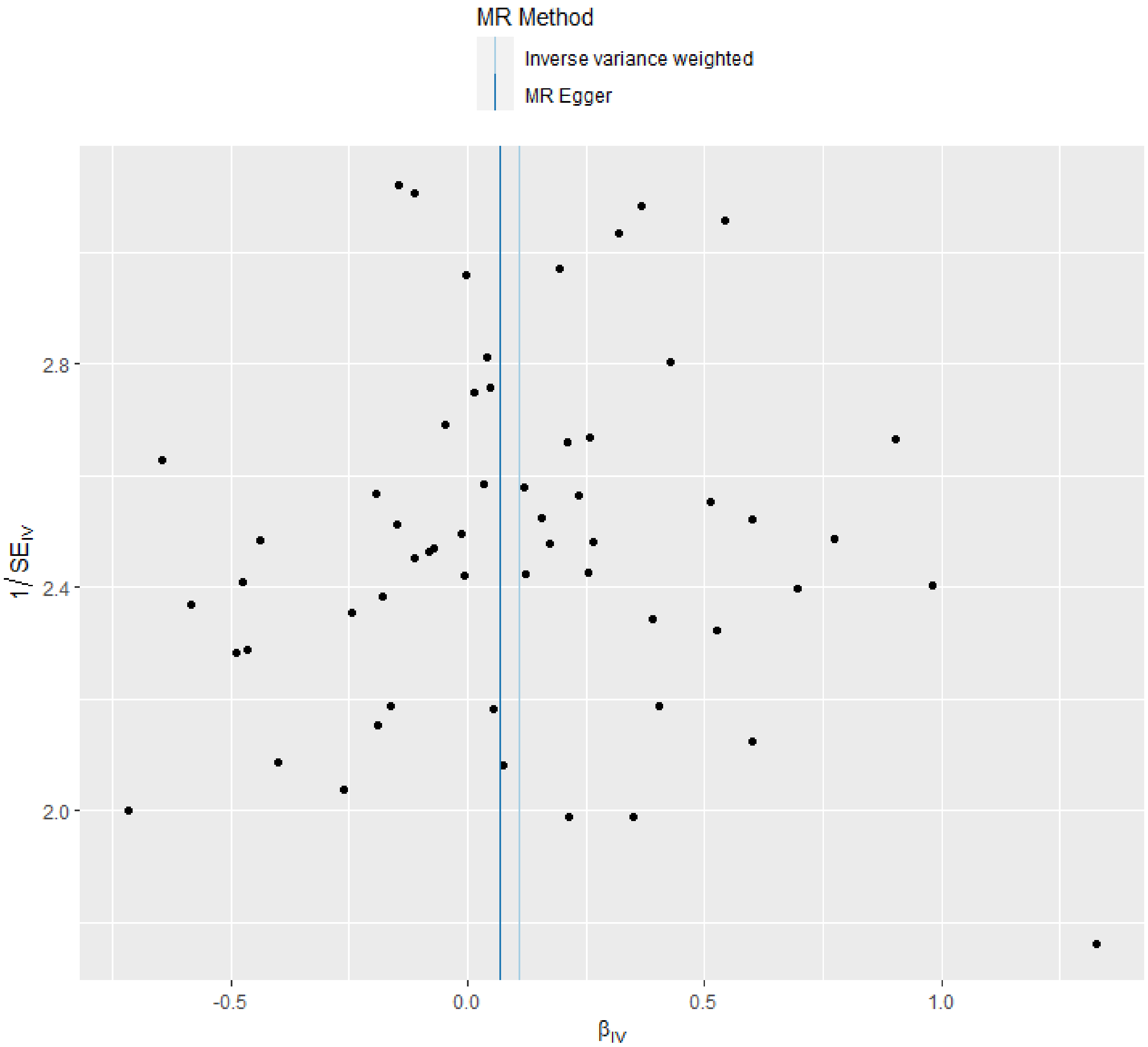

MR Method
Inverse variance weighted
MR Egger
2.8
2.4
2.0
1/SE_IV
-0.5
0.0
0.5
1.0
β_IV

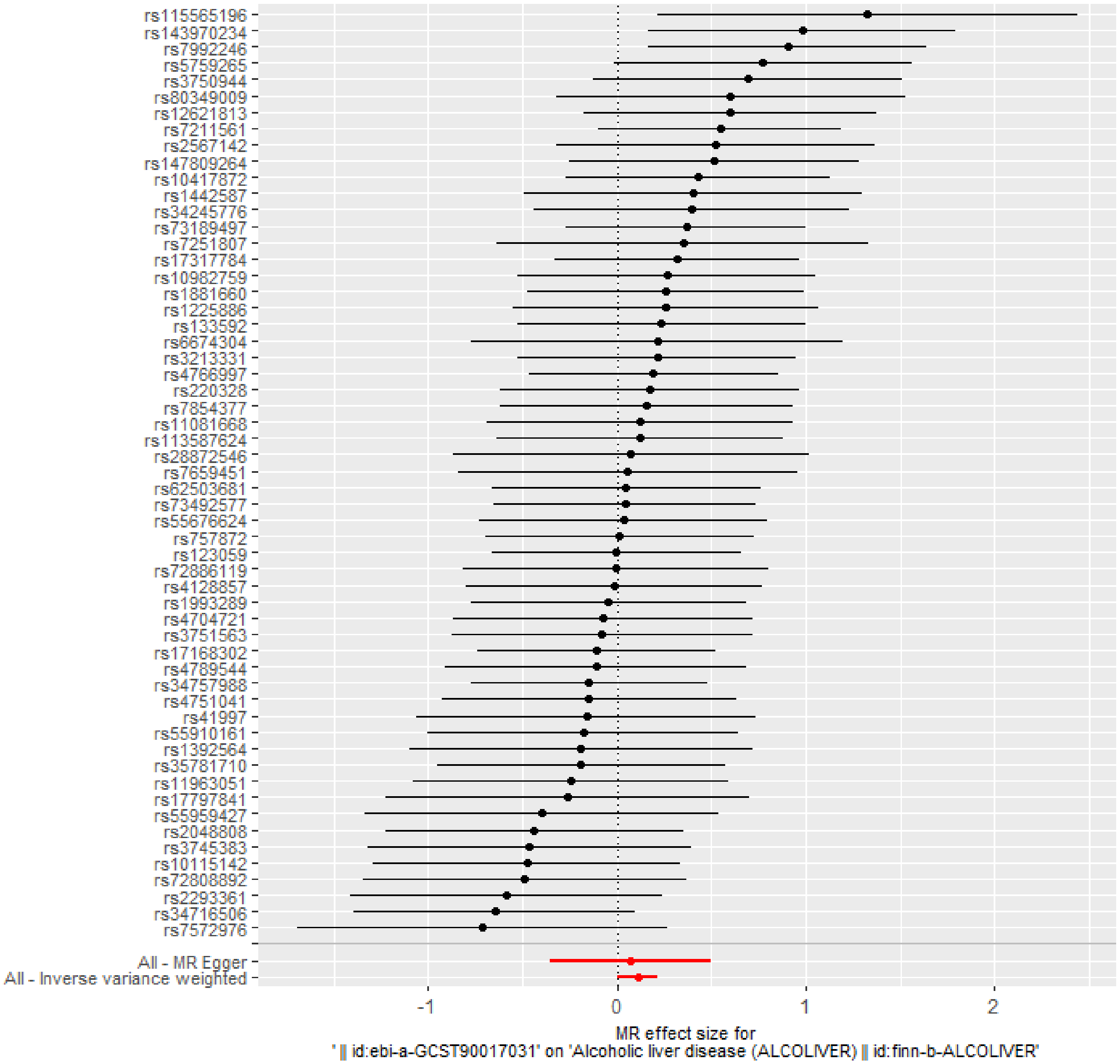
rs115565196
rs143970234
rs7992246
rs5759265
rs3750944
rs80349009
rs12621813
rs7211561
rs2567142
rs147809264
rs10417872
rs1442587
rs34245776
rs73189497
rs7251807
rs17317784
rs10982759
rs1881660
rs1225886
rs133592
rs6674304
rs3213331
rs4766997
rs220328
rs7854377
rs11081668
rs113587624
rs28872546
rs7659451
rs62503681
rs73492577
rs55676624
rs757872
rs123059
rs72886119
rs4128857
rs1993289
rs4704721
rs3751563
rs17168302
rs4789544
rs34757988
rs4751041
rs41997
rs55910161
rs1392564
rs35781710
rs11963051
rs17797841
rs55959427
rs2048808
rs3745383
rs10115142
rs72808892
rs2293361
rs34716506
rs7572976
All - MR Egger
All - Inverse variance weighted
-1
0
1
2
MR effect size for
' || id:ebi-a-GCST90017031' on 'Alcoholic liver disease (ALCOLIVER) || id:finn-b-ALCOLIVER'

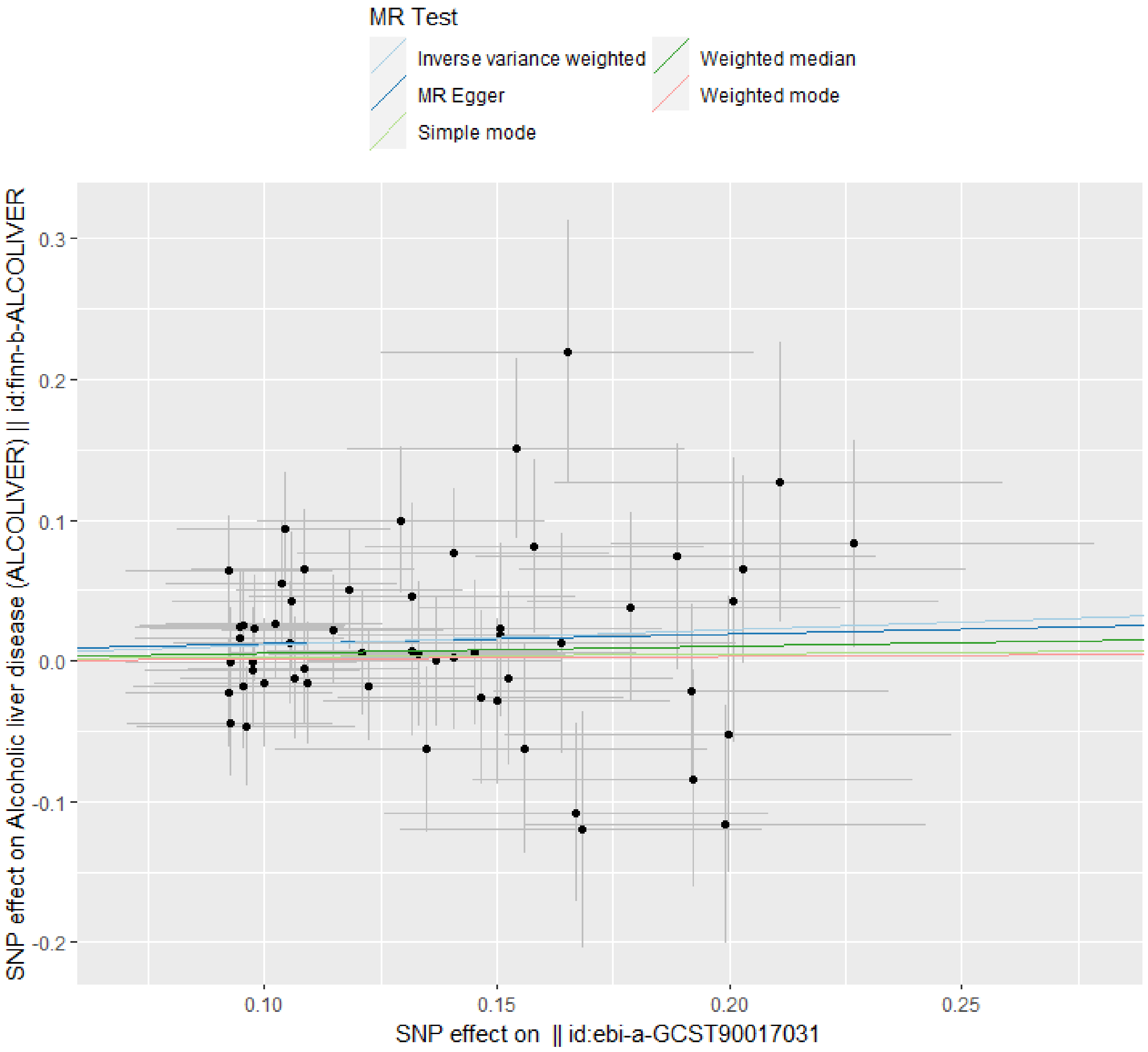

MR Test
Inverse variance weighted
MR Egger
Simple mode
Weighted median
Weighted mode
SNP effect on Alcoholic liver disease (ALCOLIVER) || id:finn-b-ALCOLIVER
0.3
0.2
0.1
0.0
-0.1
-0.2
0.10
0.15
0.20
0.25
SNP effect on || id:ebi-a-GCST90017031

Figure 167 Leave-one-out analysis, funnel plot, MR effect size and scatter plot for gut microbiota abundance (phylum Actinobacteria id.400) on alcoholic liver disease

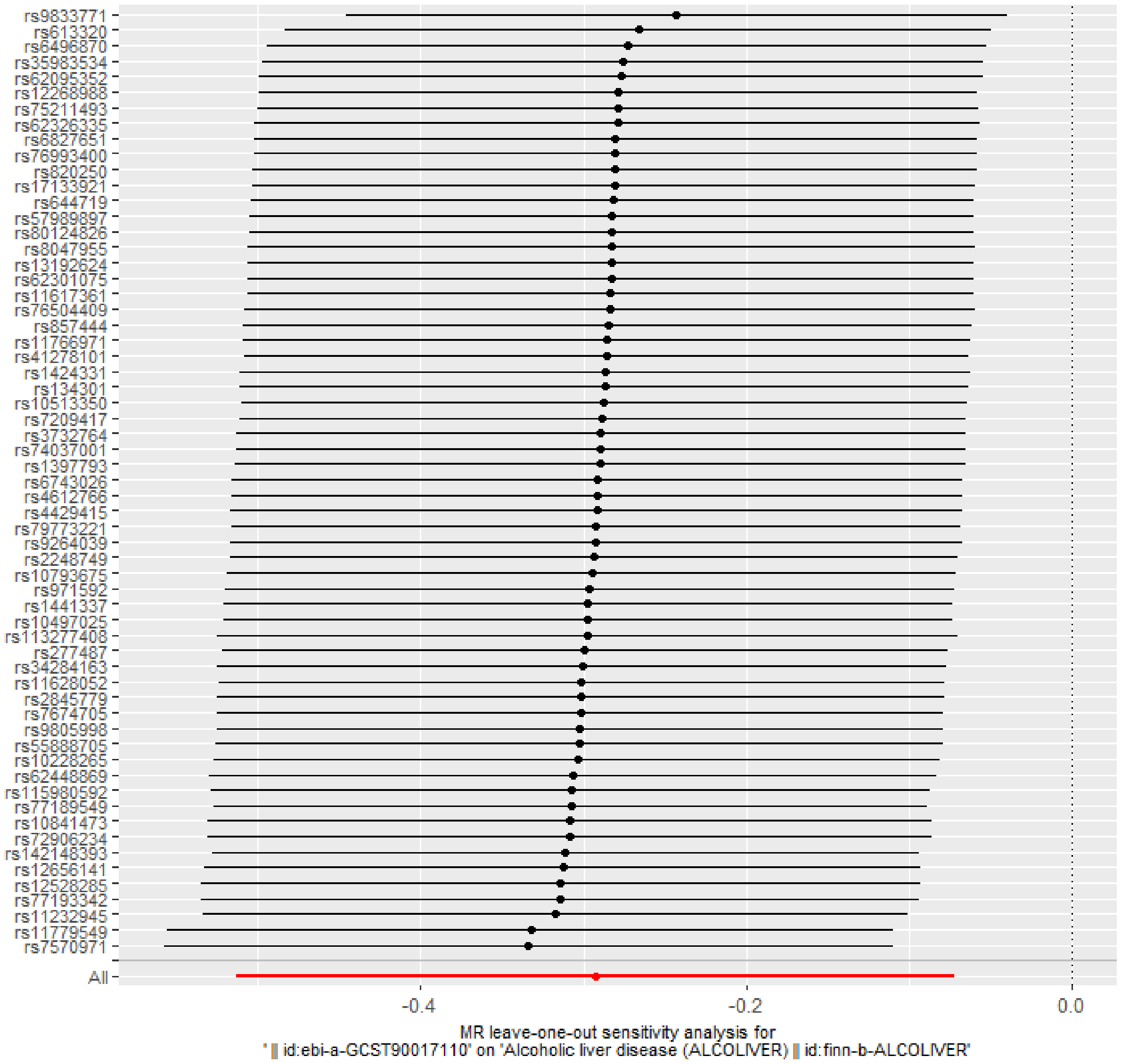

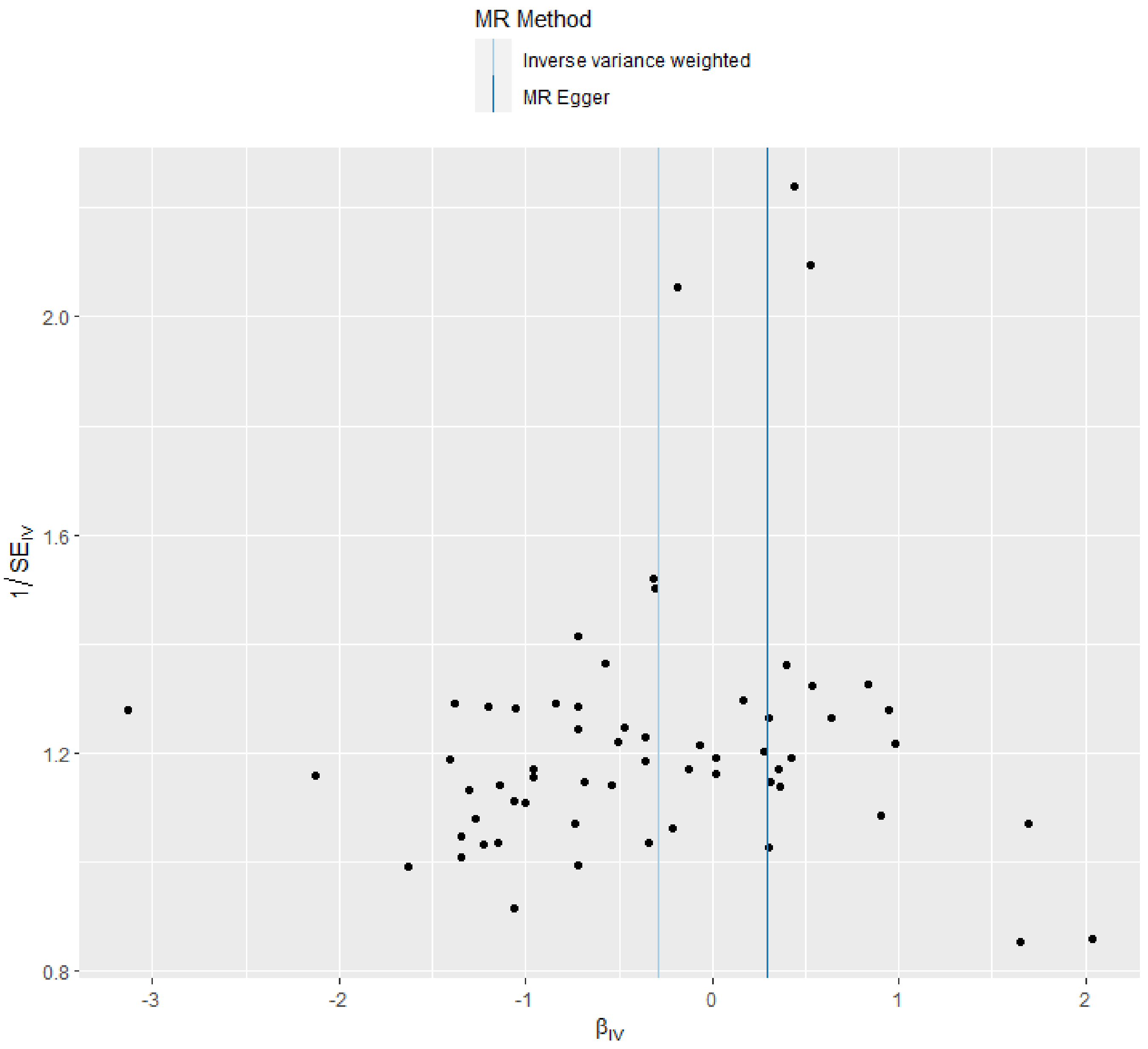
MR Method
Inverse variance weighted
MR Egger
2.0
1.6
1.2
0.8
-3
-2
-1
0
1
2
β_{IV}
$1/SE_{IV}$

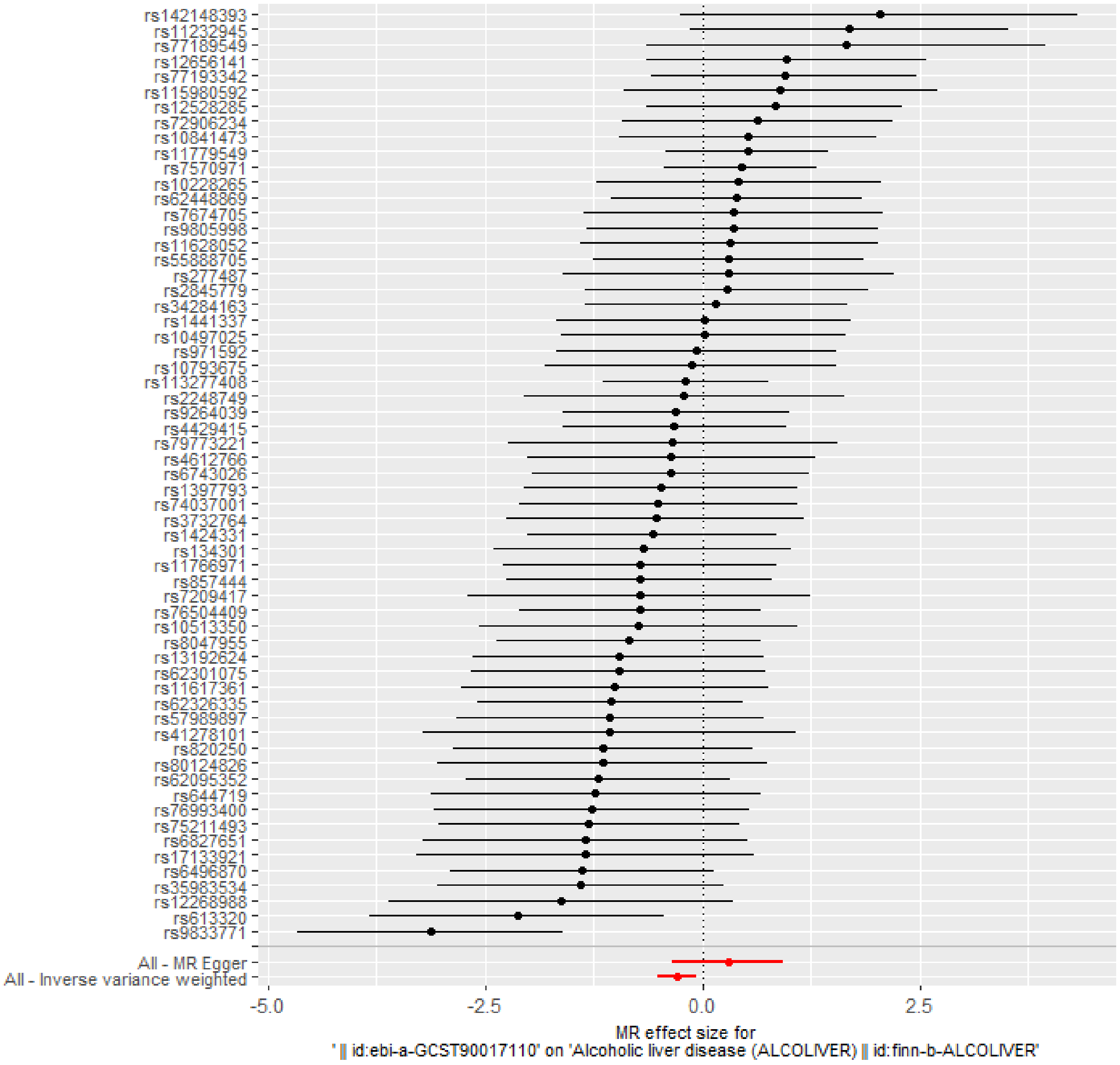

rs142148393
rs11232945
rs77189549
rs12656141
rs77193342
rs115980592
rs12528285
rs72906234
rs10841473
rs11779549
rs7570971
rs10228265
rs62448869
rs7674705
rs9805998
rs11628052
rs55888705
rs277487
rs2845779
rs34284163
rs1441337
rs10497025
rs971592
rs10793675
rs113277408
rs2248749
rs9264039
rs4429415
rs79773221
rs4612766
rs6743026
rs1397793
rs74037001
rs3732764
rs1424331
rs134301
rs11766971
rs857444
rs7209417
rs76504409
rs10513350
rs8047955
rs13192624
rs62301075
rs11617361
rs62326335
rs57989897
rs41278101
rs820250
rs80124826
rs62095352
rs644719
rs76993400
rs75211493
rs6827651
rs17133921
rs6496870
rs35983534
rs12268988
rs613320
rs9833771
All - MR Egger
All - Inverse variance weighted
-5.0
-2.5
0.0
2.5
MR effect size for
' || id:ebi-a-GCST90017110' on 'Alcoholic liver disease (ALCOLIVER) || id:finn-b-ALCOLIVER'

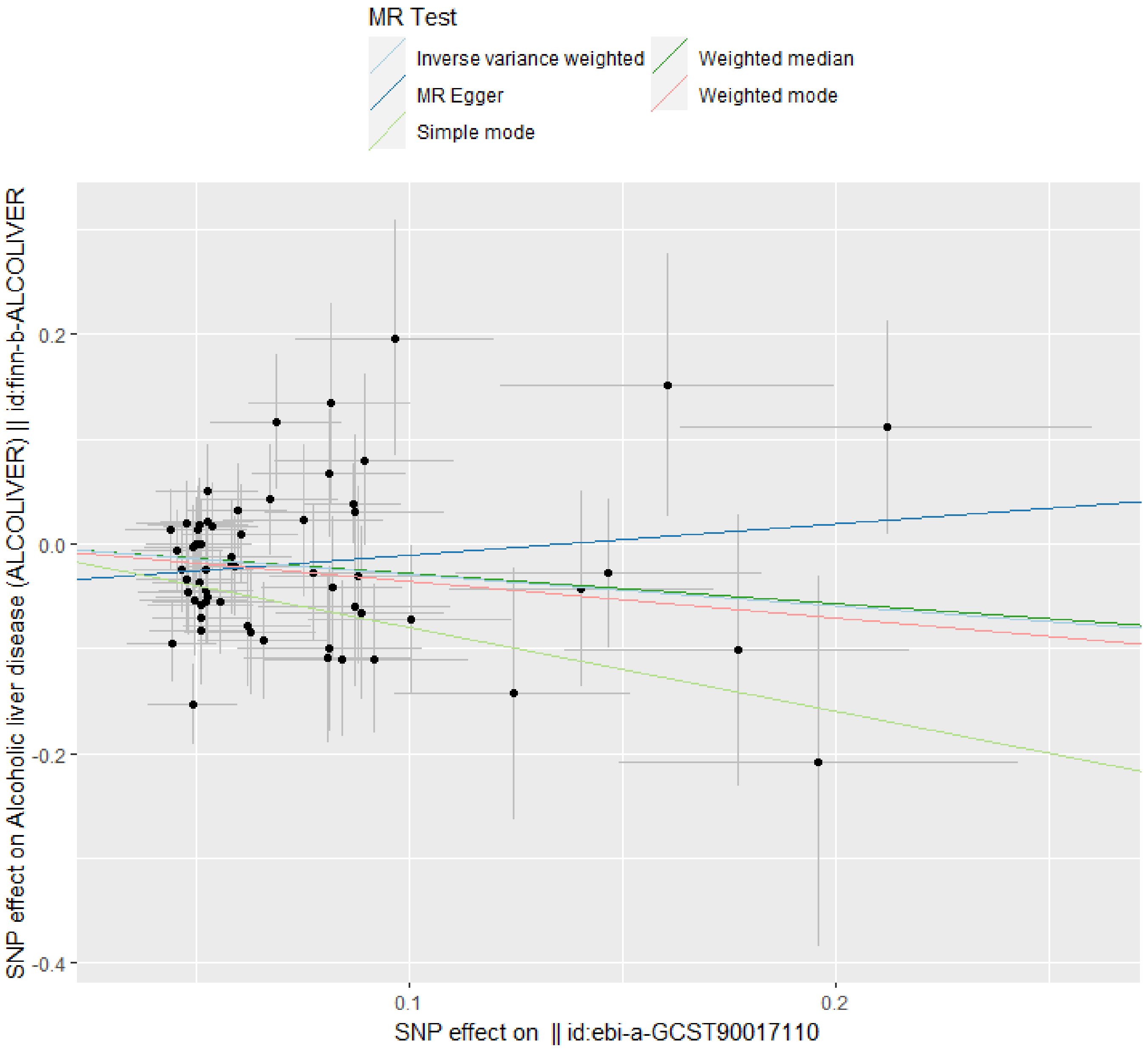

MR Test
Inverse variance weighted
MR Egger
Simple mode
Weighted median
Weighted mode
SNP effect on Alcoholic liver disease (ALCOLIVER) || id:finn-b-ALCOLIVER
0.2
0.0
-0.2
-0.4
0.1
0.2
SNP effect on || id:ebi-a-GCST90017110

Figure 168 Leave-one-out analysis, funnel plot, MR effect size and scatter plot for gut microbiota abundance (class Betaproteobacteria id.2867) on alcoholic liver disease

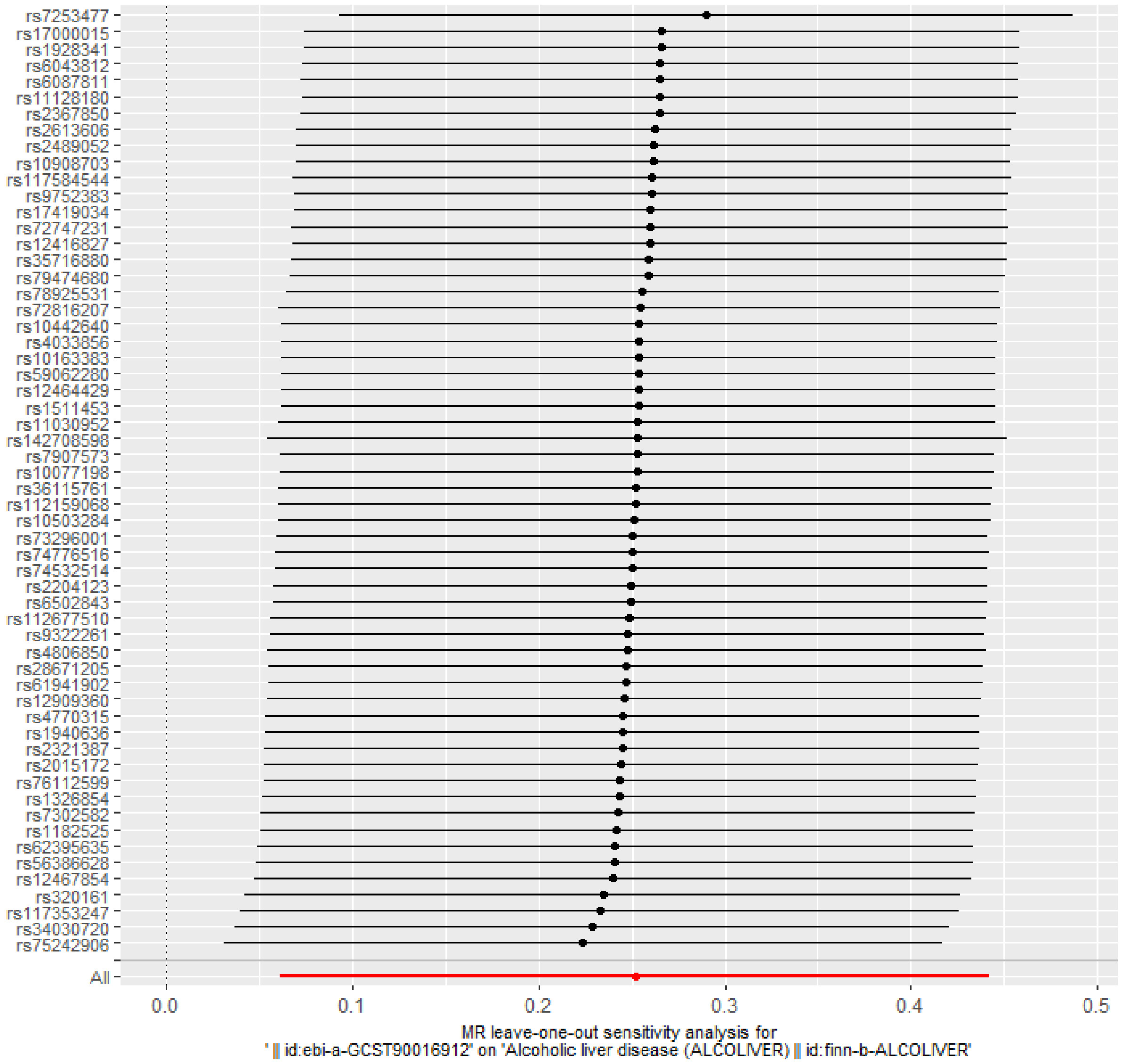

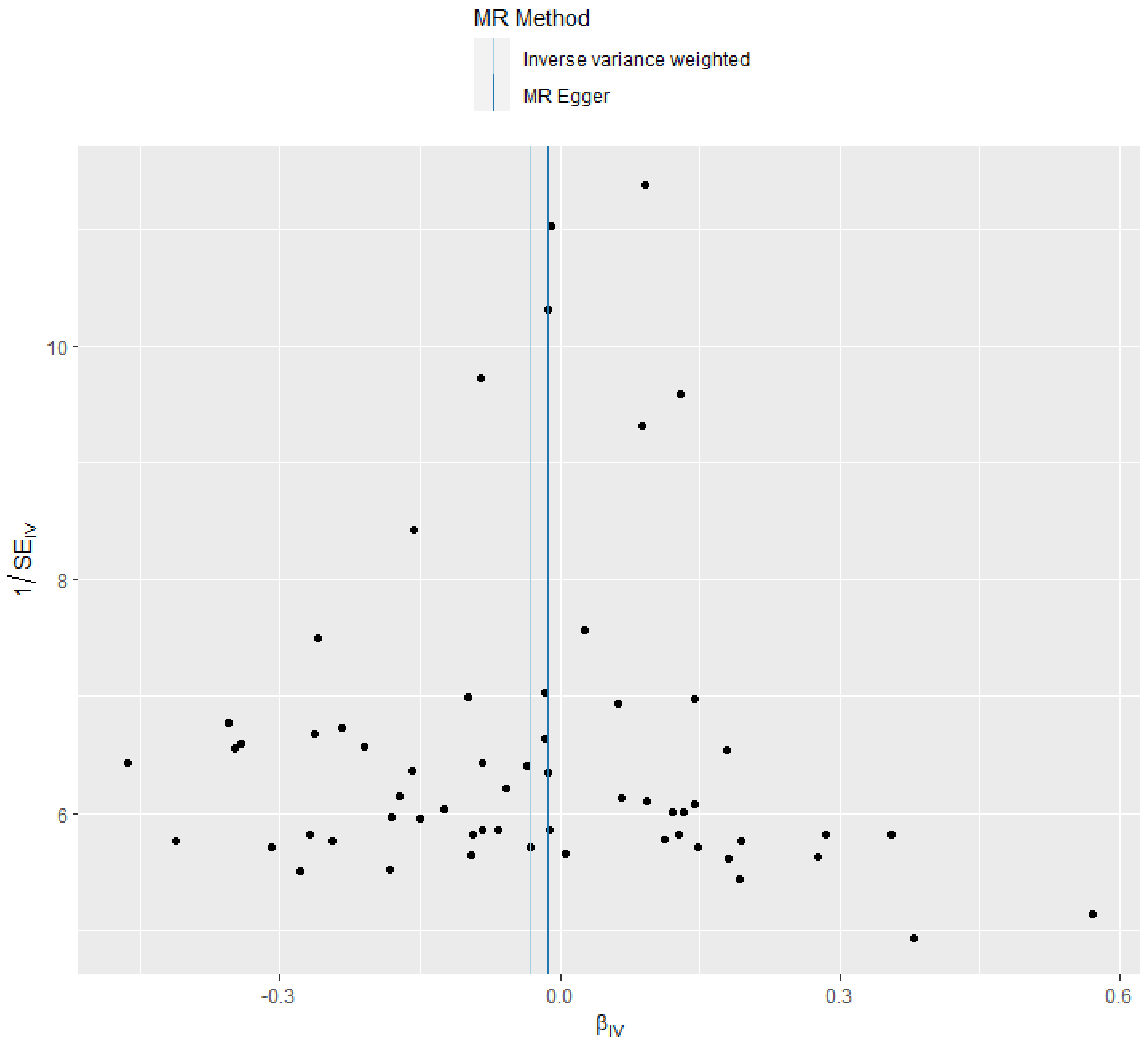

MR Method
Inverse variance weighted
MR Egger
10
8
6
-0.3
0.0
0.3
0.6
1/SE_IV
β_IV

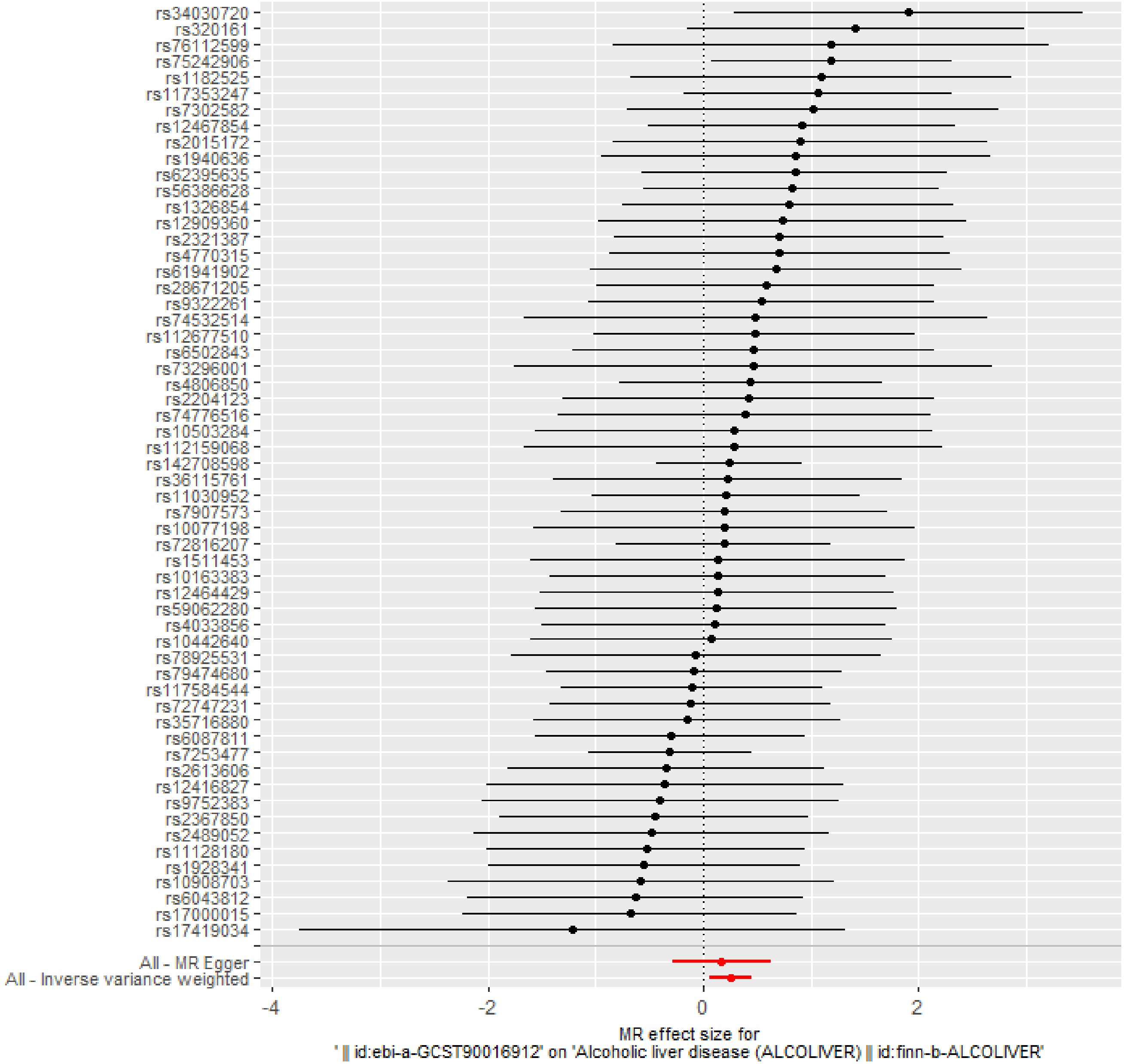

rs34030720
rs320161
rs76112599
rs75242906
rs1182525
rs117353247
rs7302582
rs12467854
rs2015172
rs1940636
rs62395635
rs56386628
rs1326854
rs12909360
rs2321387
rs4770315
rs61941902
rs28671205
rs9322261
rs74532514
rs112677510
rs6502843
rs73296001
rs4806850
rs2204123
rs74776516
rs10503284
rs112159068
rs142708598
rs36115761
rs11030952
rs7907573
rs10077198
rs72816207
rs1511453
rs10163383
rs12464429
rs59062280
rs4033856
rs10442640
rs78925531
rs79474680
rs117584544
rs72747231
rs35716880
rs6087811
rs7253477
rs2613606
rs12416827
rs9752383
rs2367850
rs2489052
rs11128180
rs1928341
rs10908703
rs6043812
rs17000015
rs17419034
All - MR Egger
All - Inverse variance weighted
-4
-2
0
2
MR effect size for
' || id:ebi-a-GCST90016912' on 'Alcoholic liver disease (ALCOLIVER) || id:finn-b-ALCOLIVER'

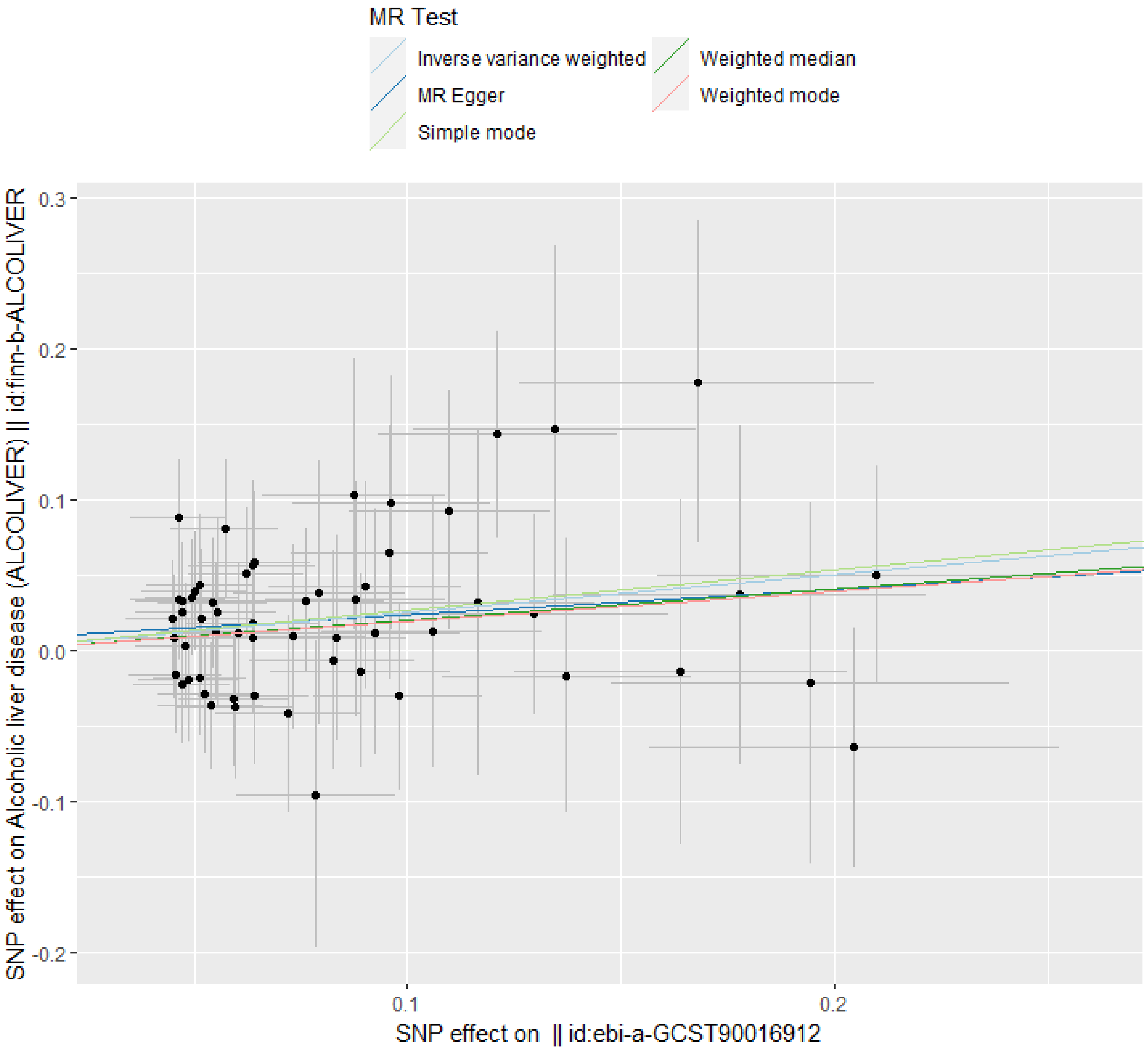

MR Test
Inverse variance weighted
MR Egger
Simple mode
Weighted median
Weighted mode
SNP effect on Alcoholic liver disease (ALCOLIVER) || id:finn-b-ALCOLIVER
0.3
0.2
0.1
0.0
-0.1
-0.2
0.1
0.2
SNP effect on || id:ebi-a-GCST90016912

Figure 169 Leave-one-out analysis, funnel plot, MR effect size and scatter plot for gut microbiota abundance (family Streptococcaceae id.1850) on primary biliary cirrhosis

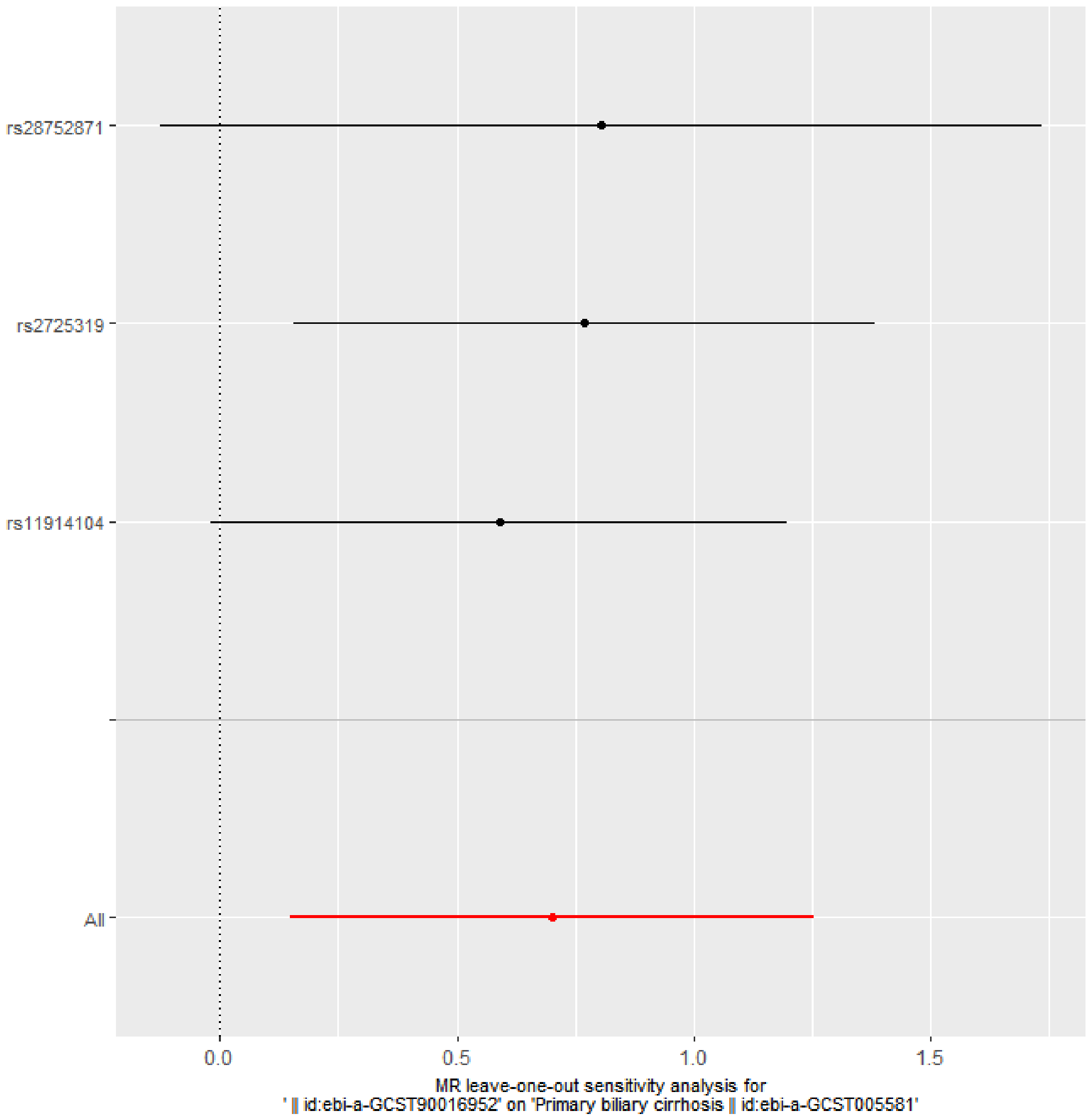

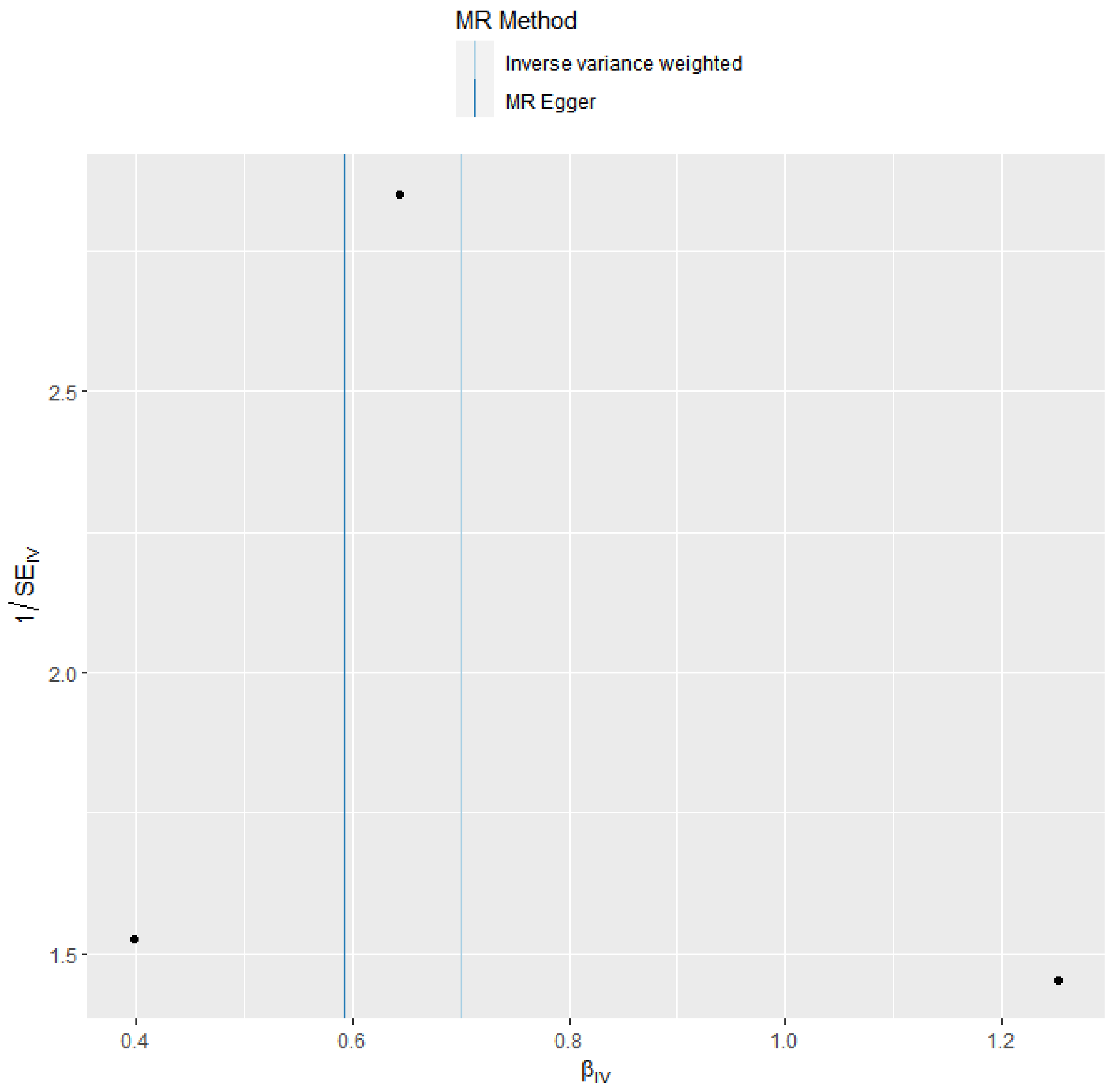

MR Method
Inverse variance weighted
MR Egger
2.5
2.0
1.5
0.4
0.6
0.8
1.0
1.2
$1/SE_{IV}$
β_{IV}

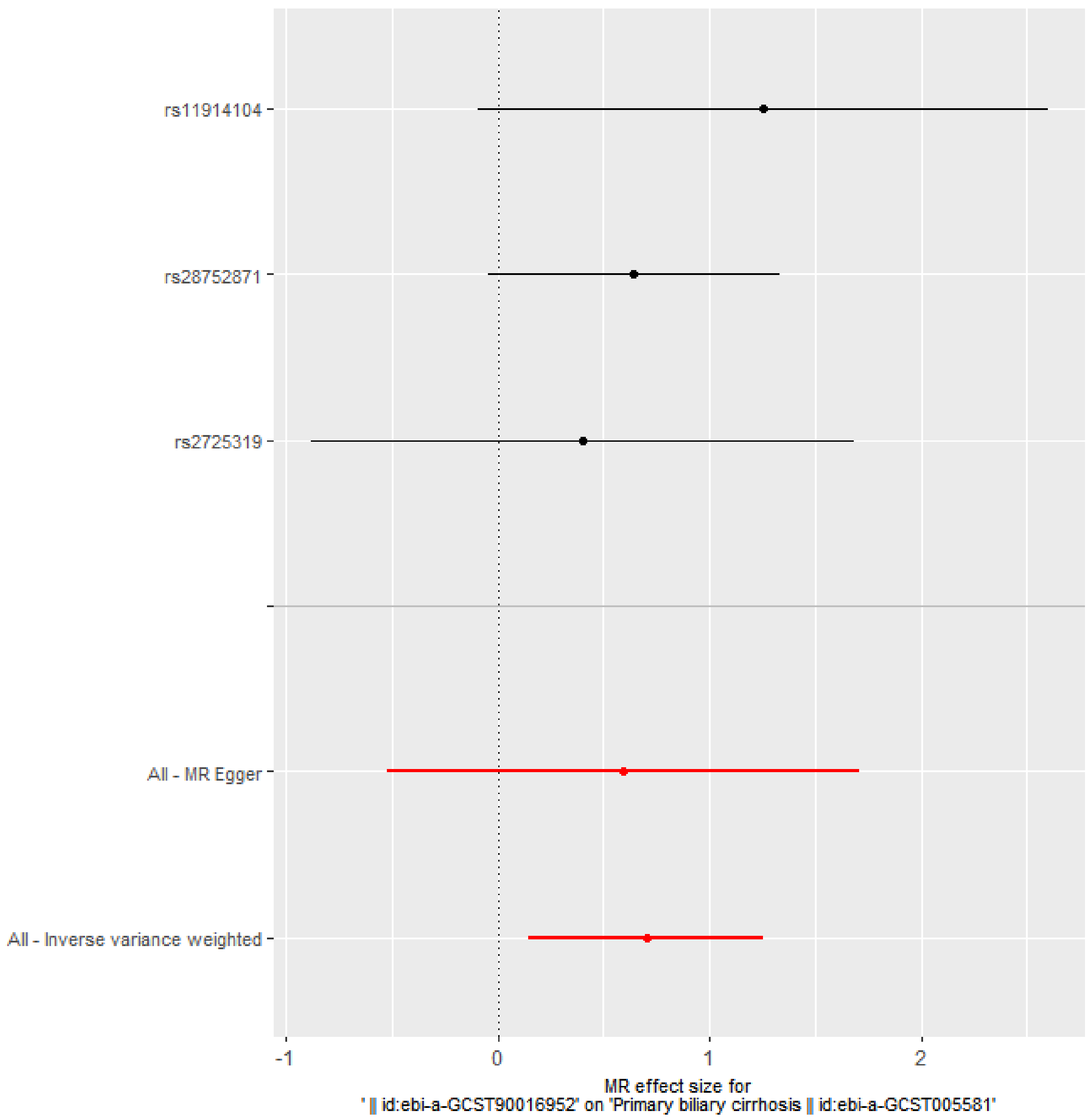
rs11914104
rs28752871
rs2725319
All - MR Egger
All - Inverse variance weighted
-1
0
1
2
MR effect size for
' || id:ebi-a-GCST90016952' on 'Primary biliary cirrhosis || id:ebi-a-GCST005581'

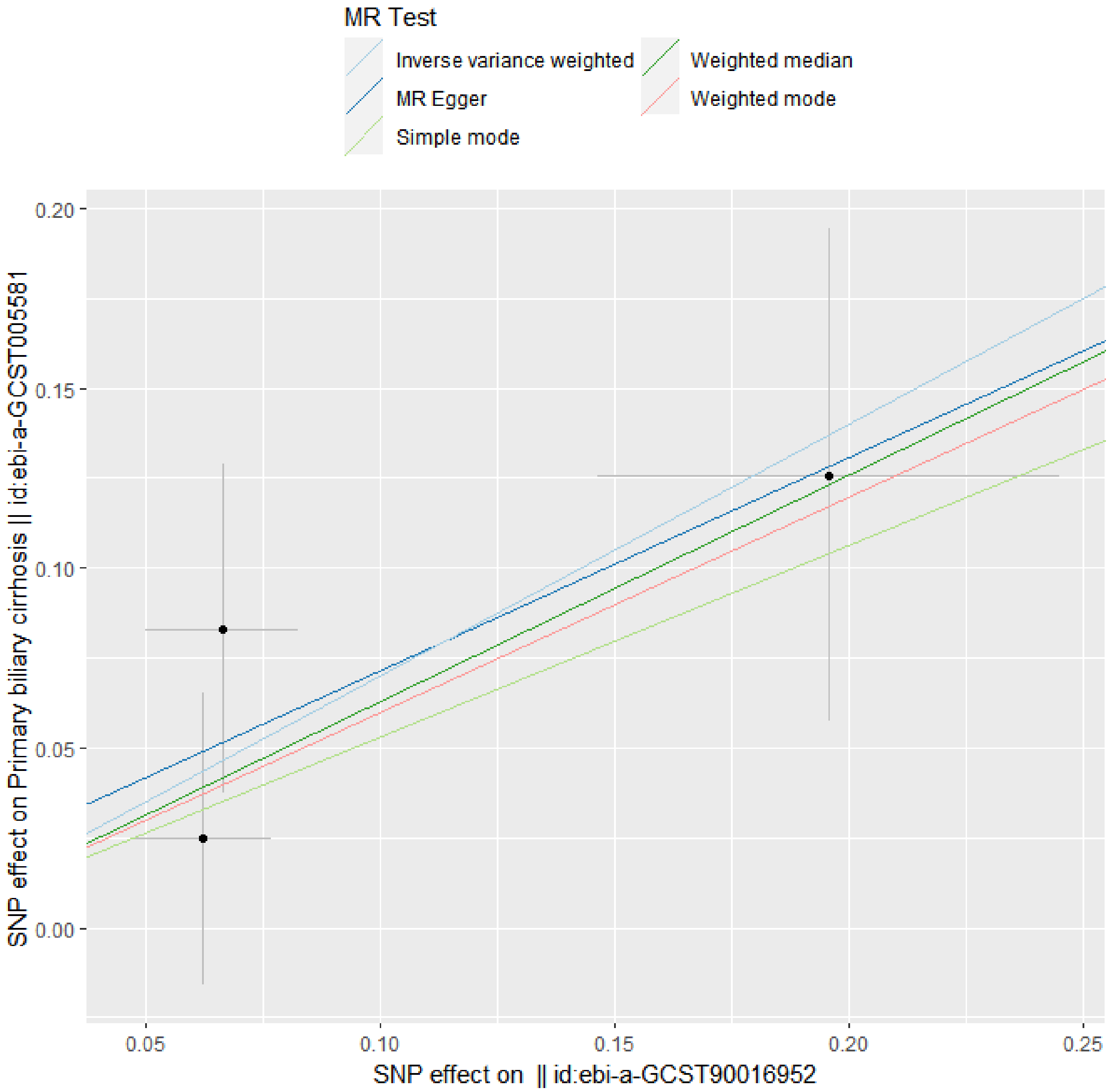

MR Test
Inverse variance weighted
MR Egger
Simple mode
Weighted median
Weighted mode
0.20
0.15
0.10
0.05
0.00
SNP effect on Primary biliary cirrhosis || id:ebi-a-GCST005581
0.05
0.10
0.15
0.20
0.25
SNP effect on || id:ebi-a-GCST90016952

Figure 170 Leave-one-out analysis, funnel plot, MR effect size and scatter plot for gut microbiota abundance (genus Akkermansia id.4037) on primary biliary cirrhosis

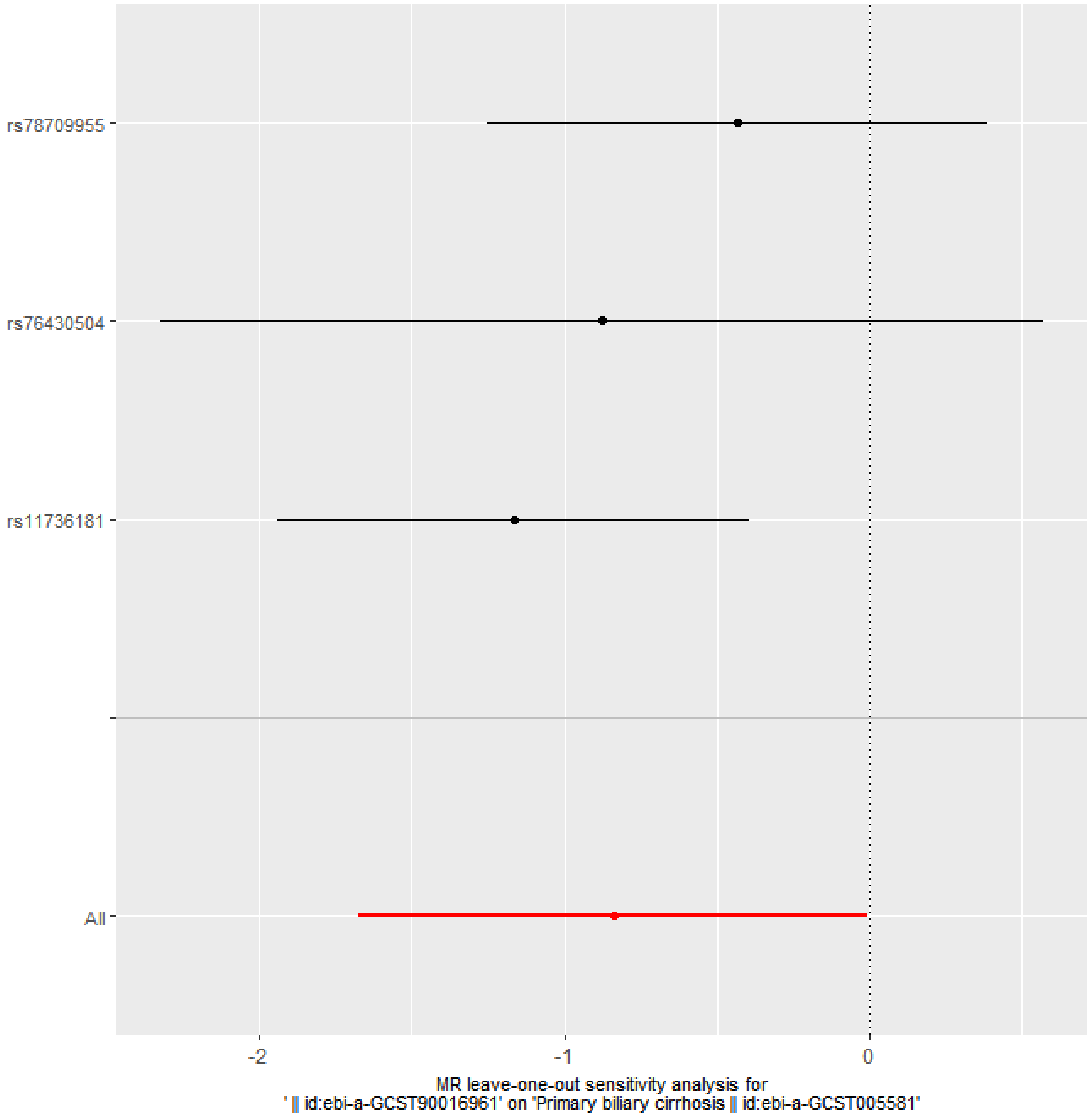

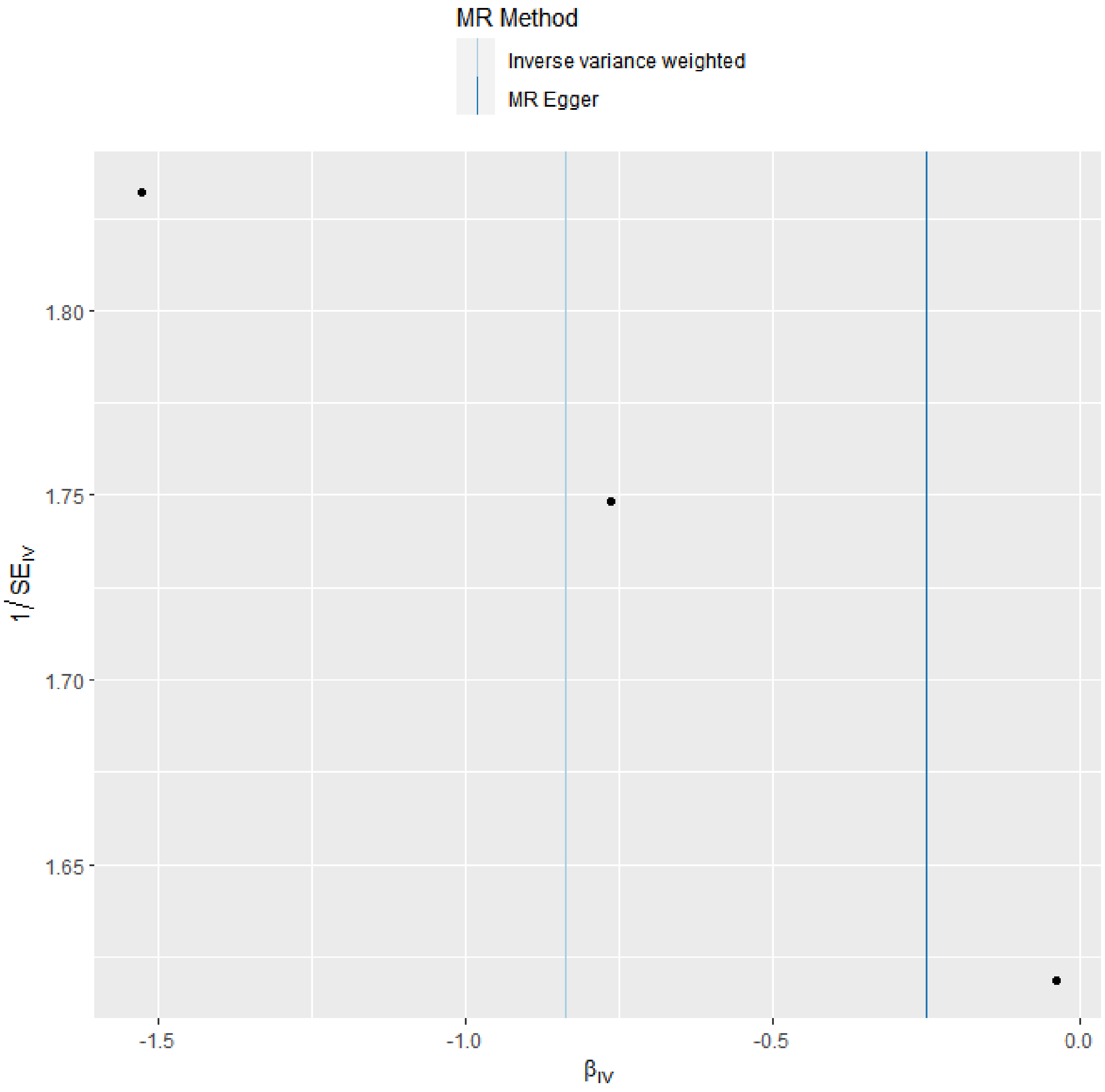

MR Method
Inverse variance weighted
MR Egger
1.80
1.75
1.70
1.65
$1/SE_{IV}$
-1.5
-1.0
-0.5
0.0
β_{IV}

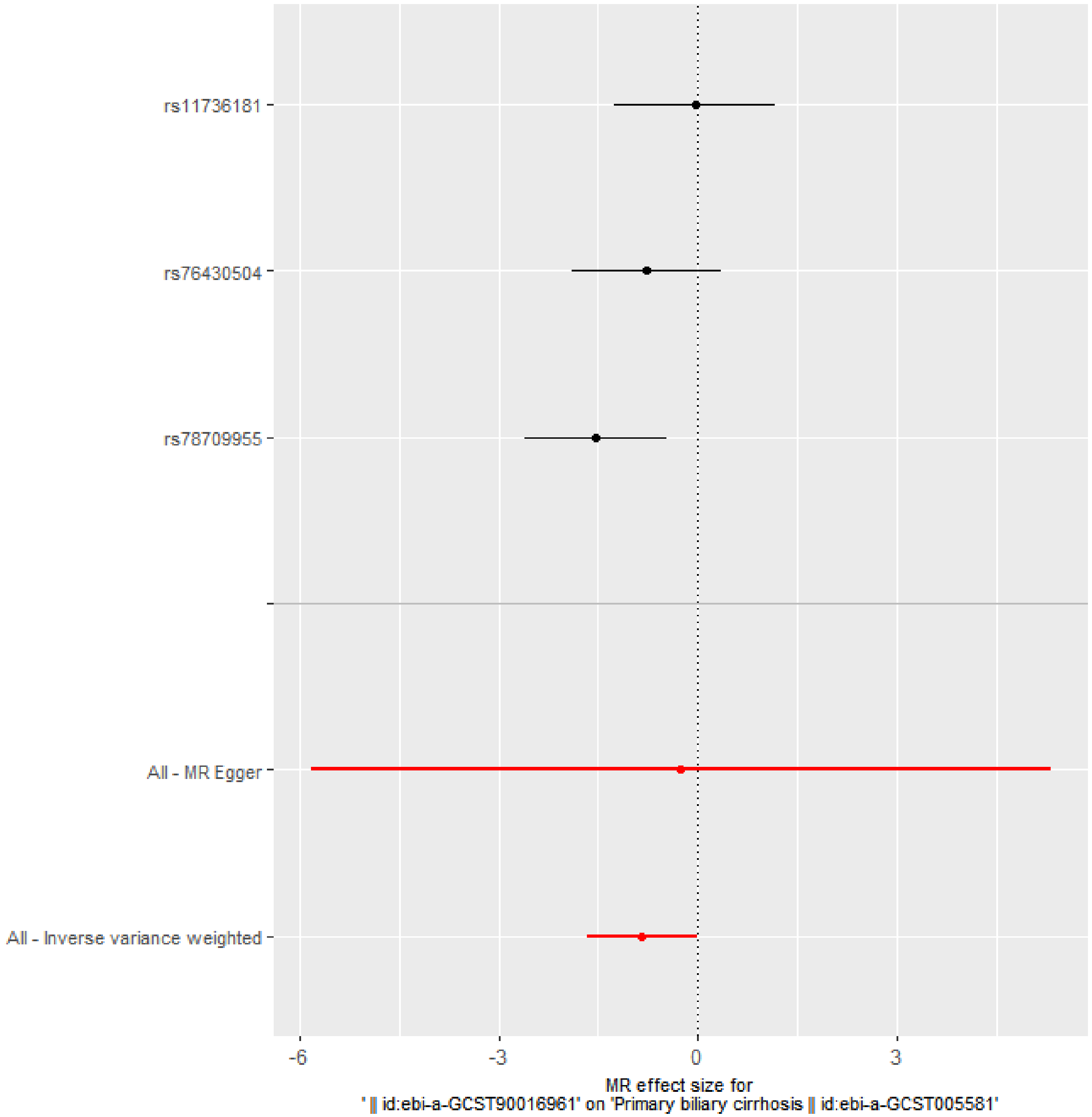
rs11736181
rs76430504
rs78709955
All - MR Egger
All - Inverse variance weighted
-6
-3
0
3
MR effect size for
' || id:ebi-a-GCST90016961' on 'Primary biliary cirrhosis || id:ebi-a-GCST005581'

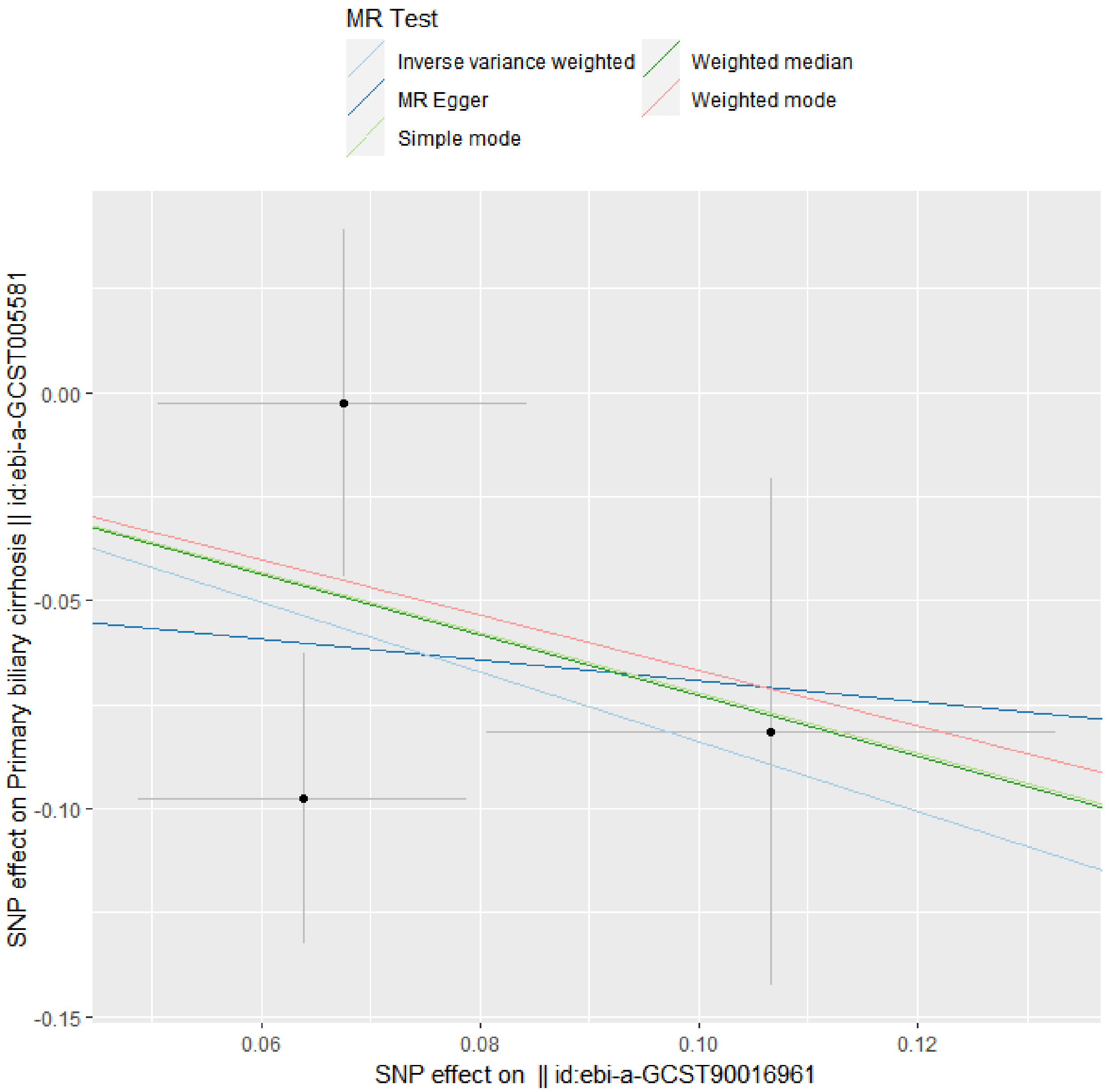

MR Test
Inverse variance weighted
MR Egger
Simple mode
Weighted median
Weighted mode
0.00
-0.05
-0.10
-0.15
0.06
0.08
0.10
0.12
SNP effect on Primary biliary cirrhosis || id:ebi-a-GCST005581
SNP effect on || id:ebi-a-GCST90016961

Figure 171 Leave-one-out analysis, funnel plot, MR effect size and scatter plot for gut microbiota abundance (genus Desulfovibrio id.3173) on primary biliary cirrhosis

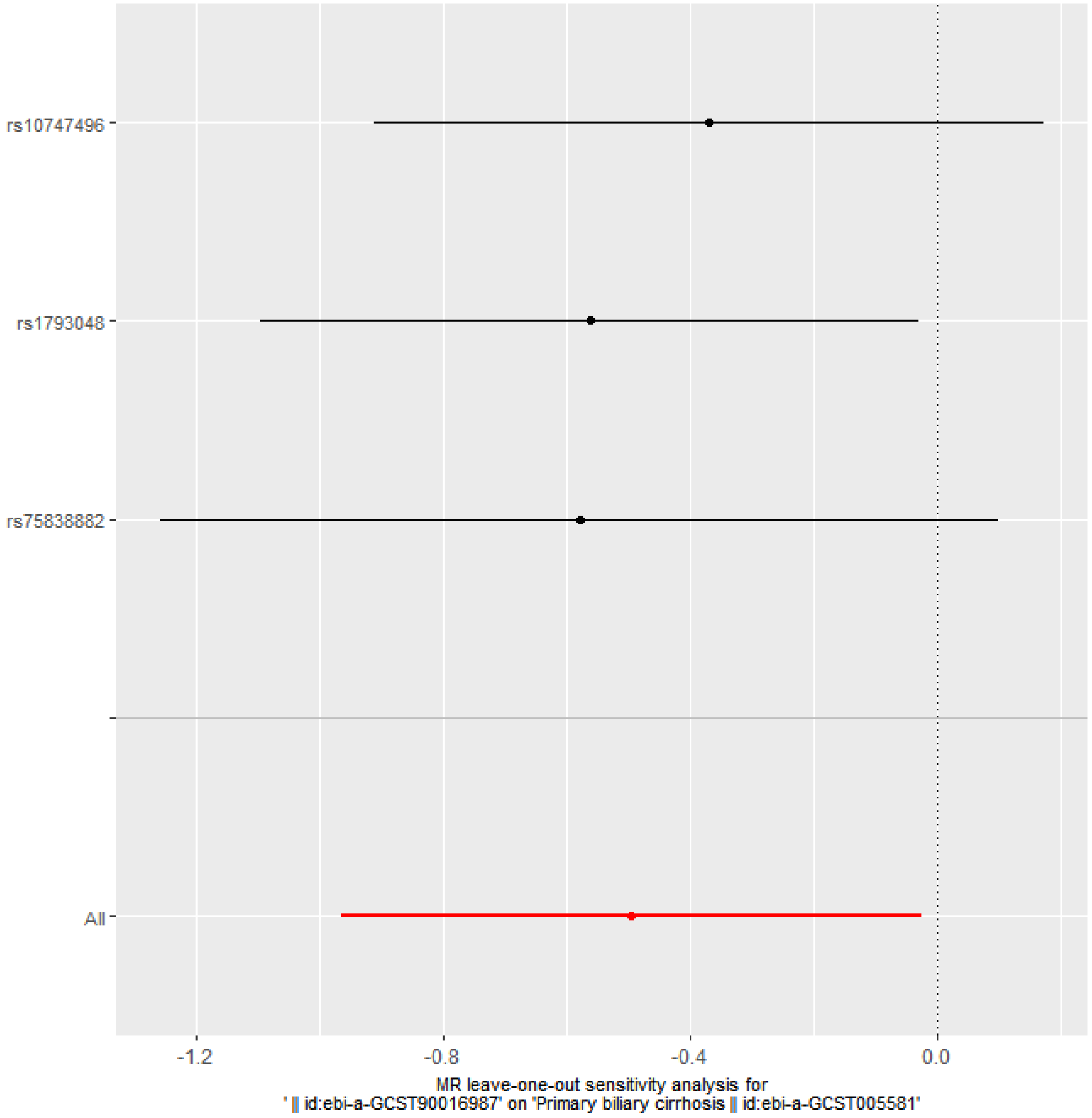

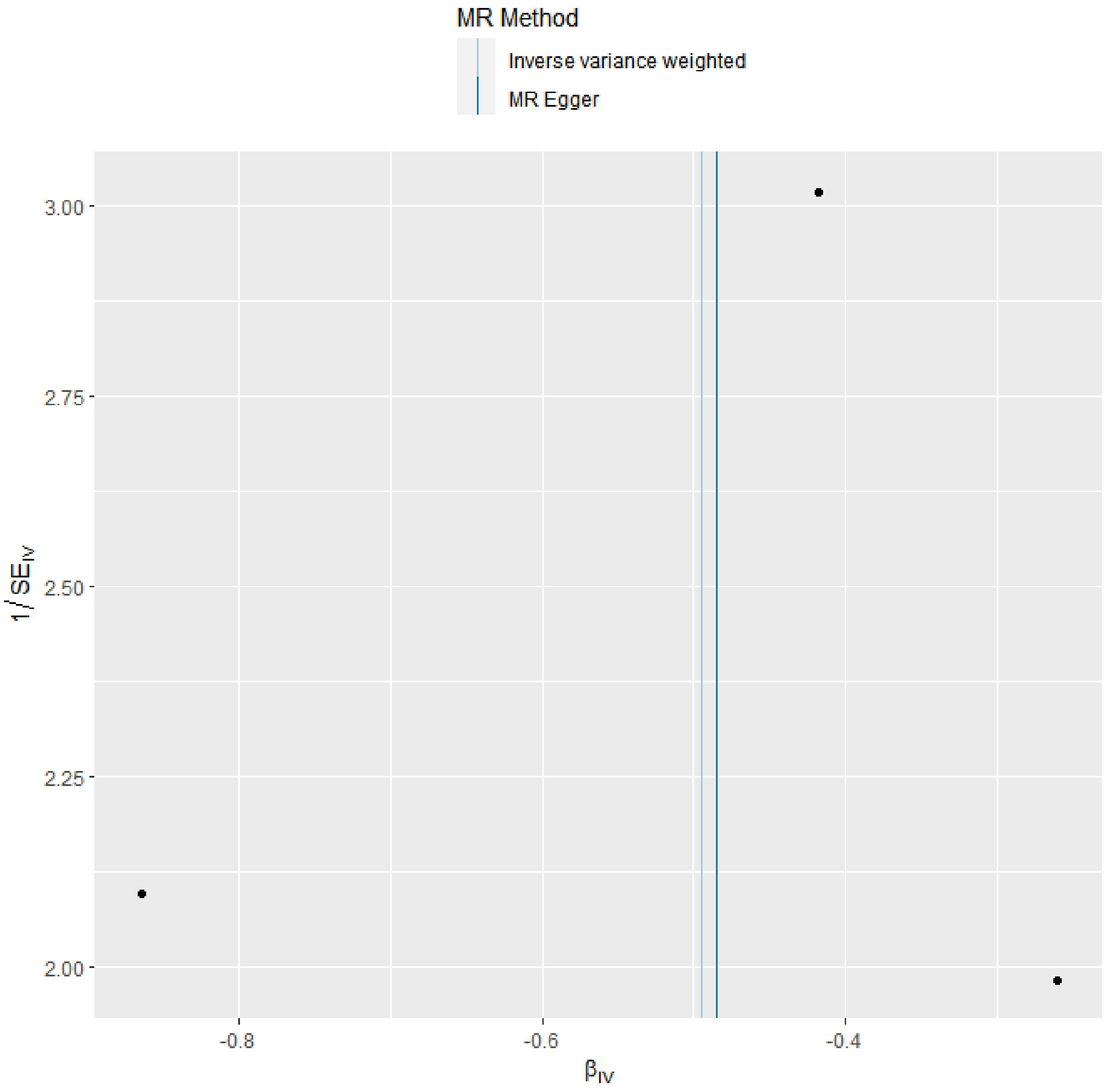

MR Method
Inverse variance weighted
MR Egger
3.00
2.75
2.50
2.25
2.00
1/SE_IV
-0.8
-0.6
-0.4
β_IV

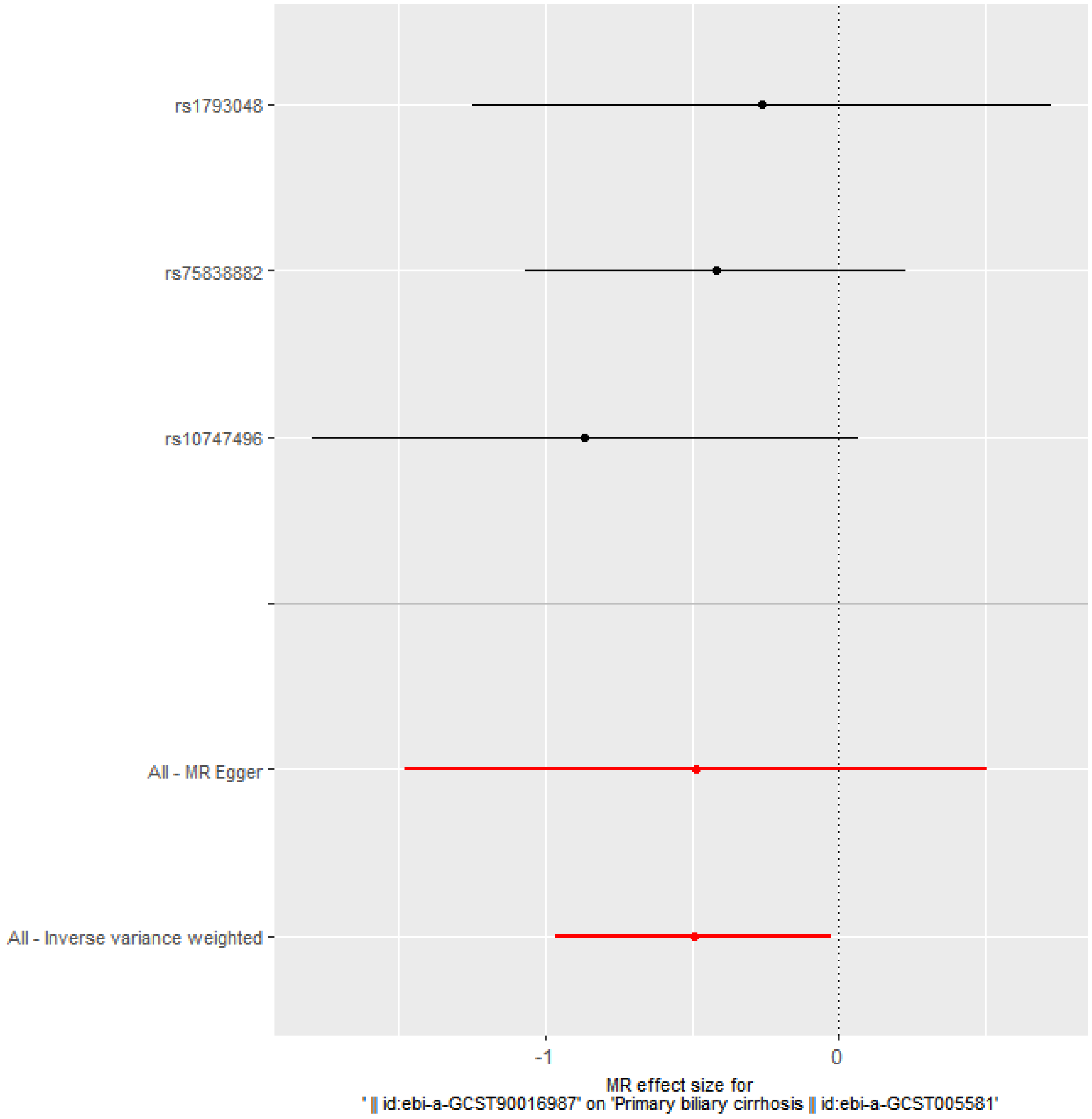

rs1793048
rs75838882
rs10747496
All - MR Egger
All - Inverse variance weighted
-1
0
MR effect size for
' || id:ebi-a-GCST90016987' on 'Primary biliary cirrhosis || id:ebi-a-GCST005581'

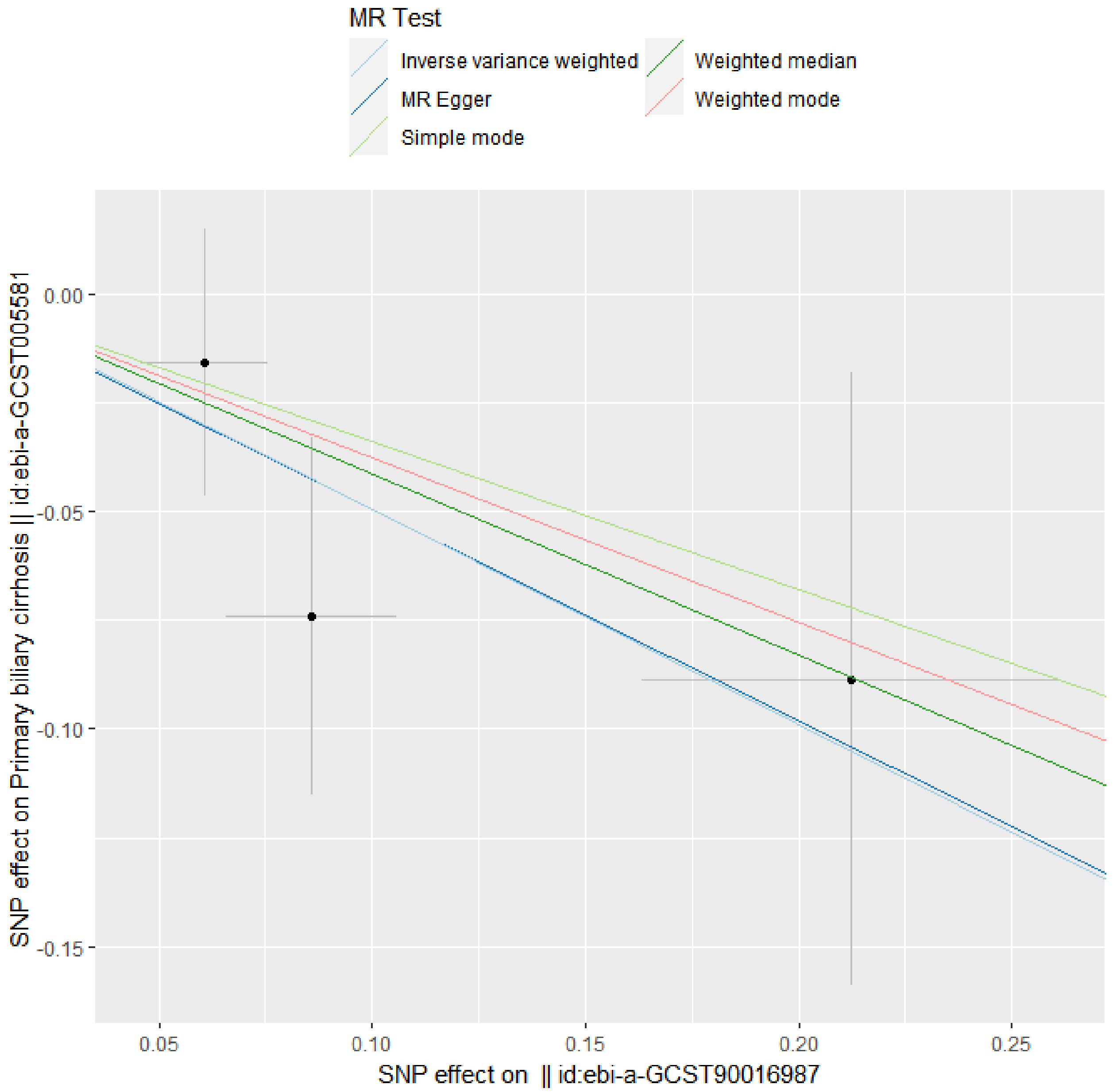
MR Test
Inverse variance weighted
MR Egger
Simple mode
Weighted median
Weighted mode
SNP effect on Primary biliary cirrhosis || id:ebi-a-GCST005581
0.00
-0.05
-0.10
-0.15
0.05
0.10
0.15
0.20
0.25
SNP effect on || id:ebi-a-GCST90016987

Figure 172 Leave-one-out analysis, funnel plot, MR effect size and scatter plot for gut microbiota abundance (genus Dialister id.2183) on primary biliary cirrhosis

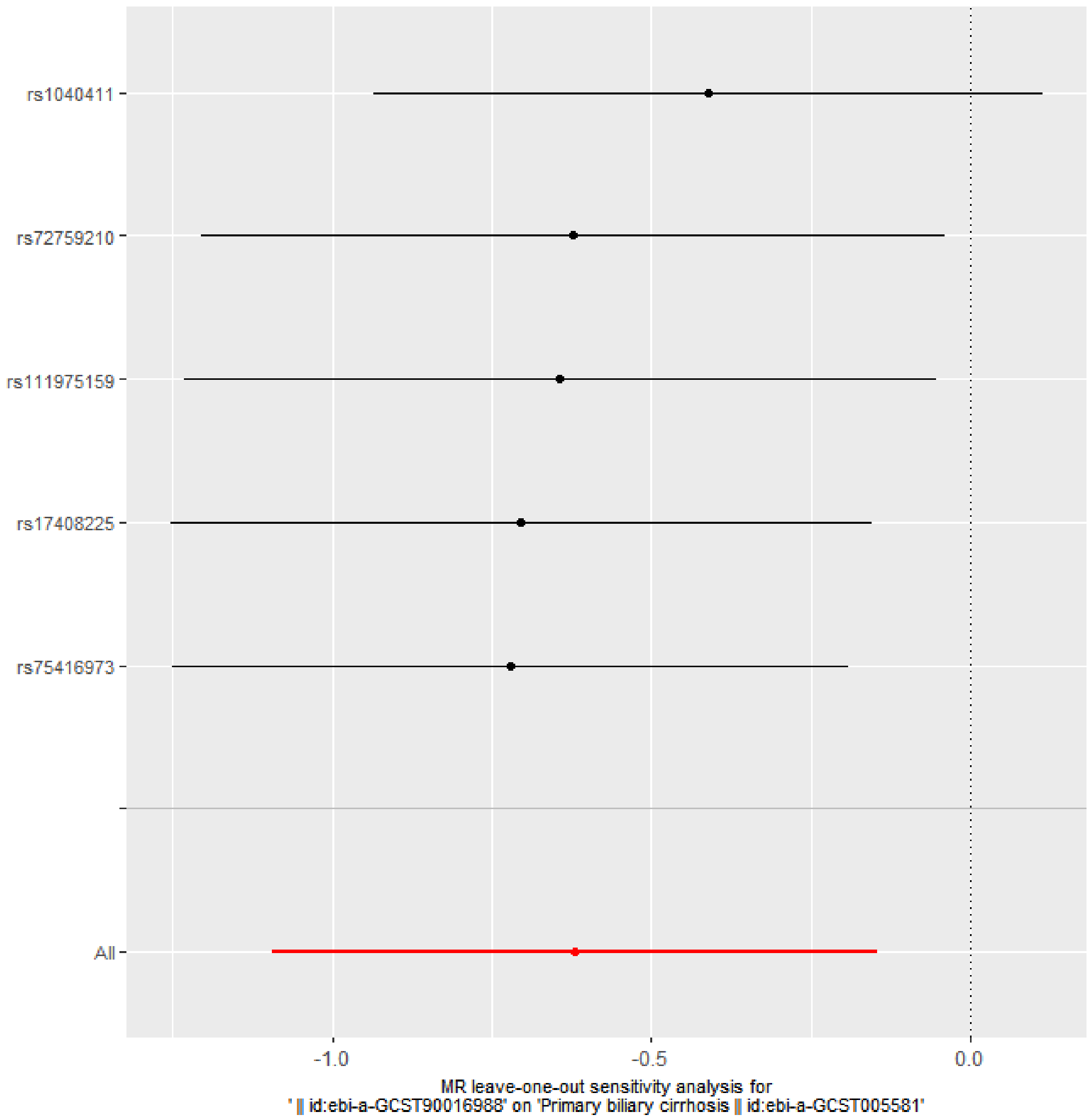

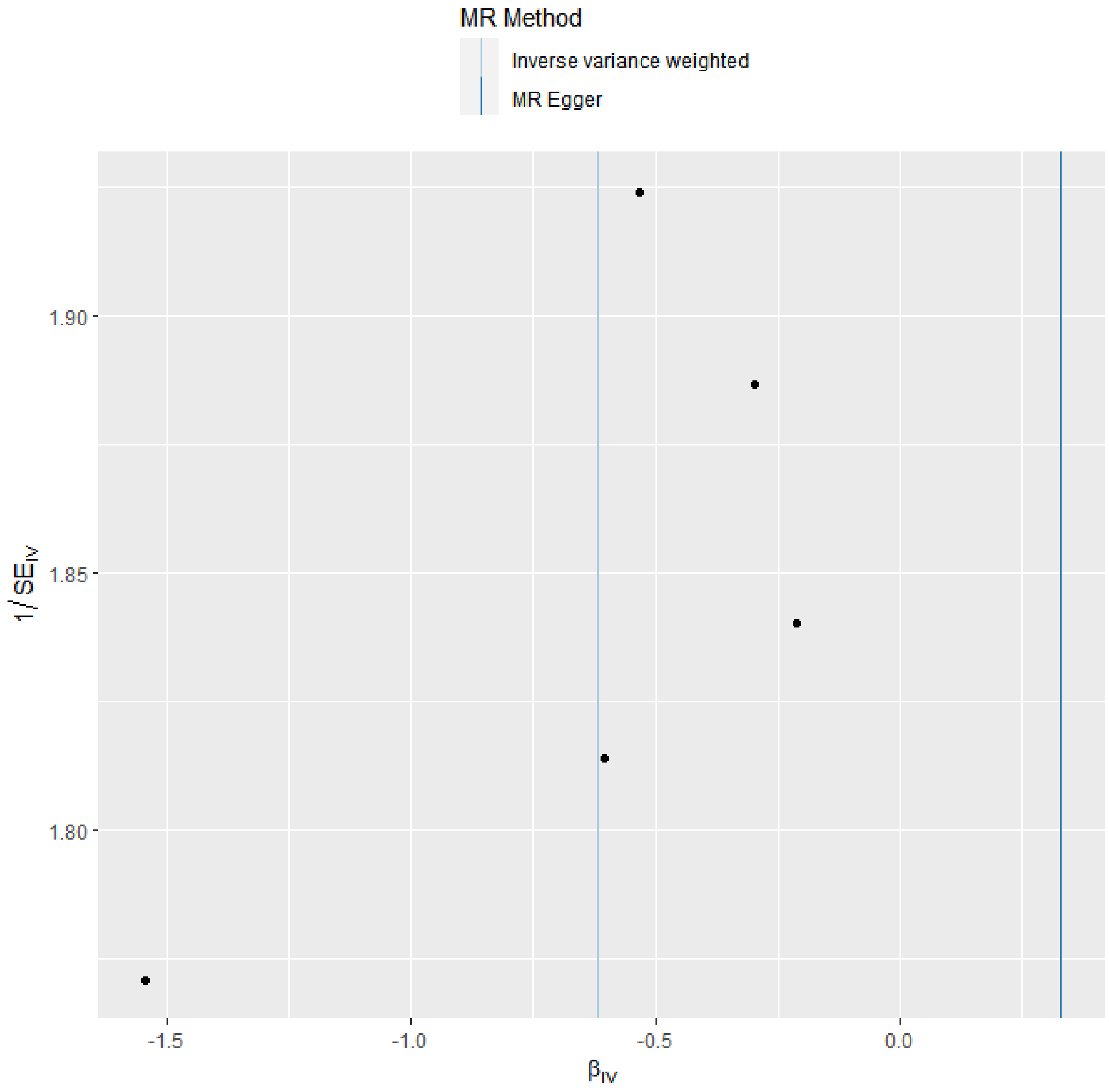

MR Method
Inverse variance weighted
MR Egger
1.90
1.85
1.80
-1.5
-1.0
-0.5
0.0
β IV
1/SE IV

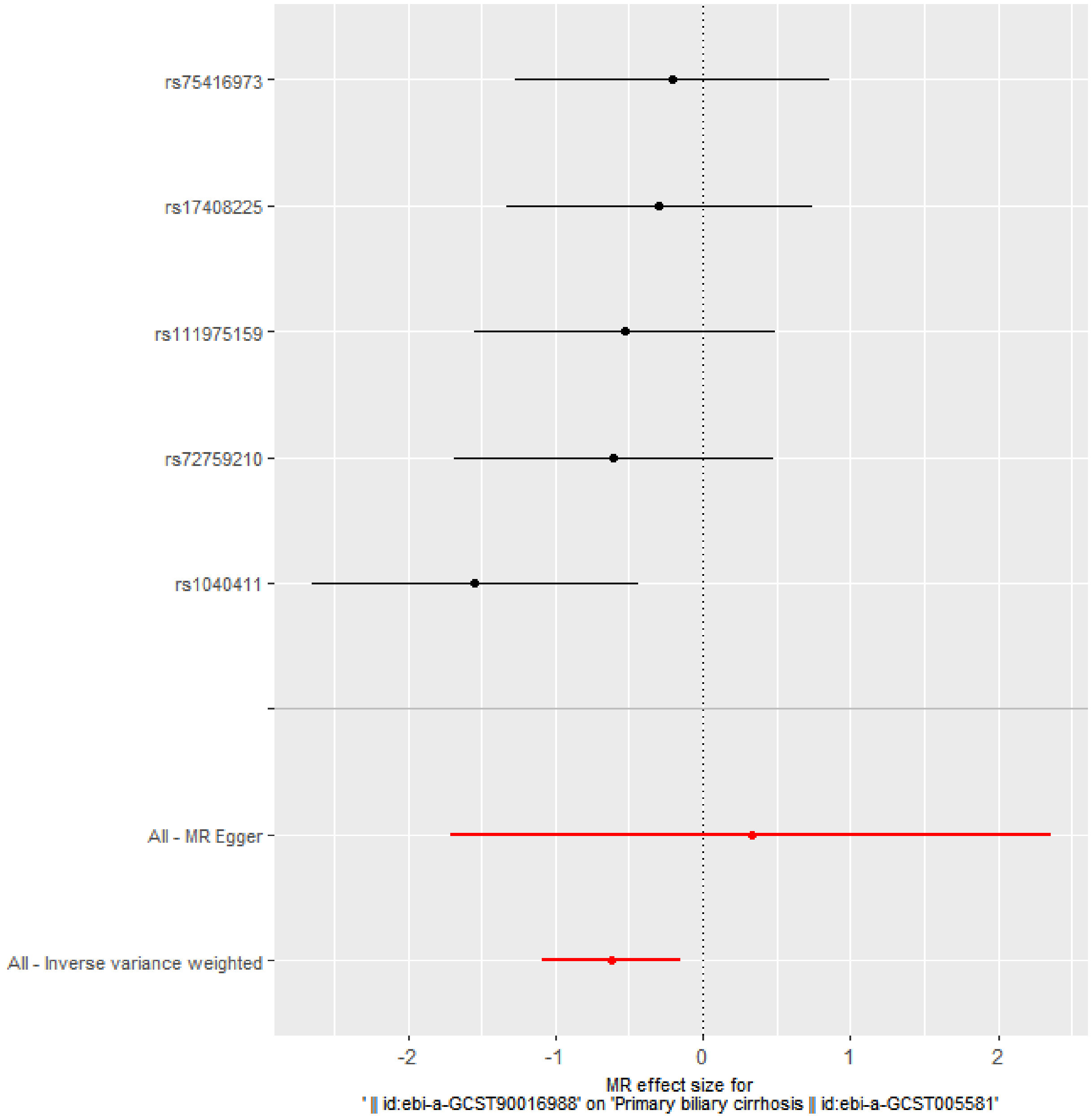

rs75416973
rs17408225
rs111975159
rs72759210
rs1040411
All - MR Egger
All - Inverse variance weighted
-2
-1
0
1
2
MR effect size for
' || id:ebi-a-GCST90016988' on 'Primary biliary cirrhosis || id:ebi-a-GCST005581'

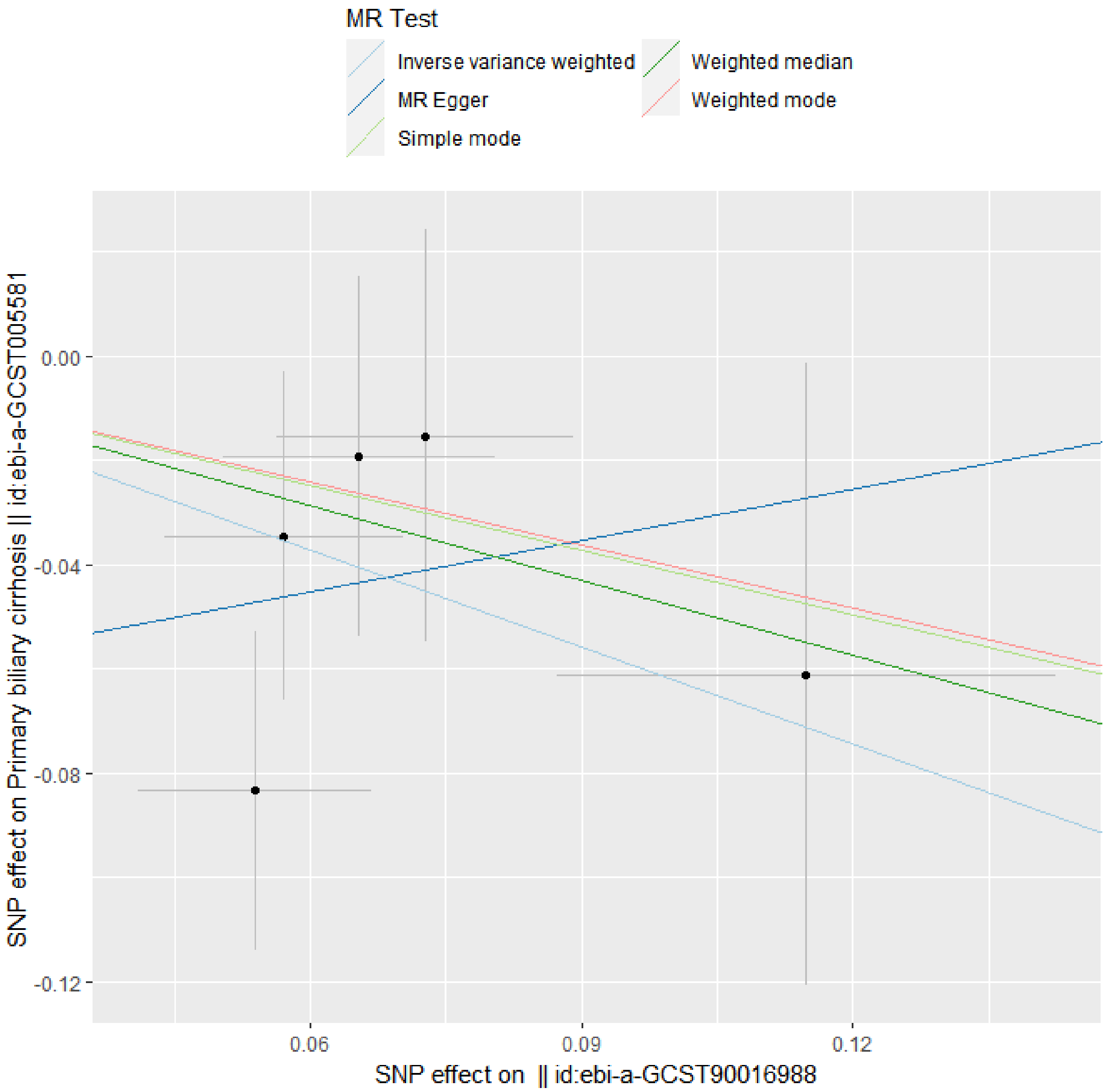
MR Test
Inverse variance weighted
MR Egger
Simple mode
Weighted median
Weighted mode
SNP effect on Primary biliary cirrhosis || id:ebi-a-GCST005581
0.00
-0.04
-0.08
-0.12
0.06
0.09
0.12
SNP effect on || id:ebi-a-GCST90016988

Figure 173 Leave-one-out analysis, funnel plot, MR effect size and scatter plot for gut microbiota abundance (genus Eubacterium nodatum group id.11297) on primary biliary cirrhosis

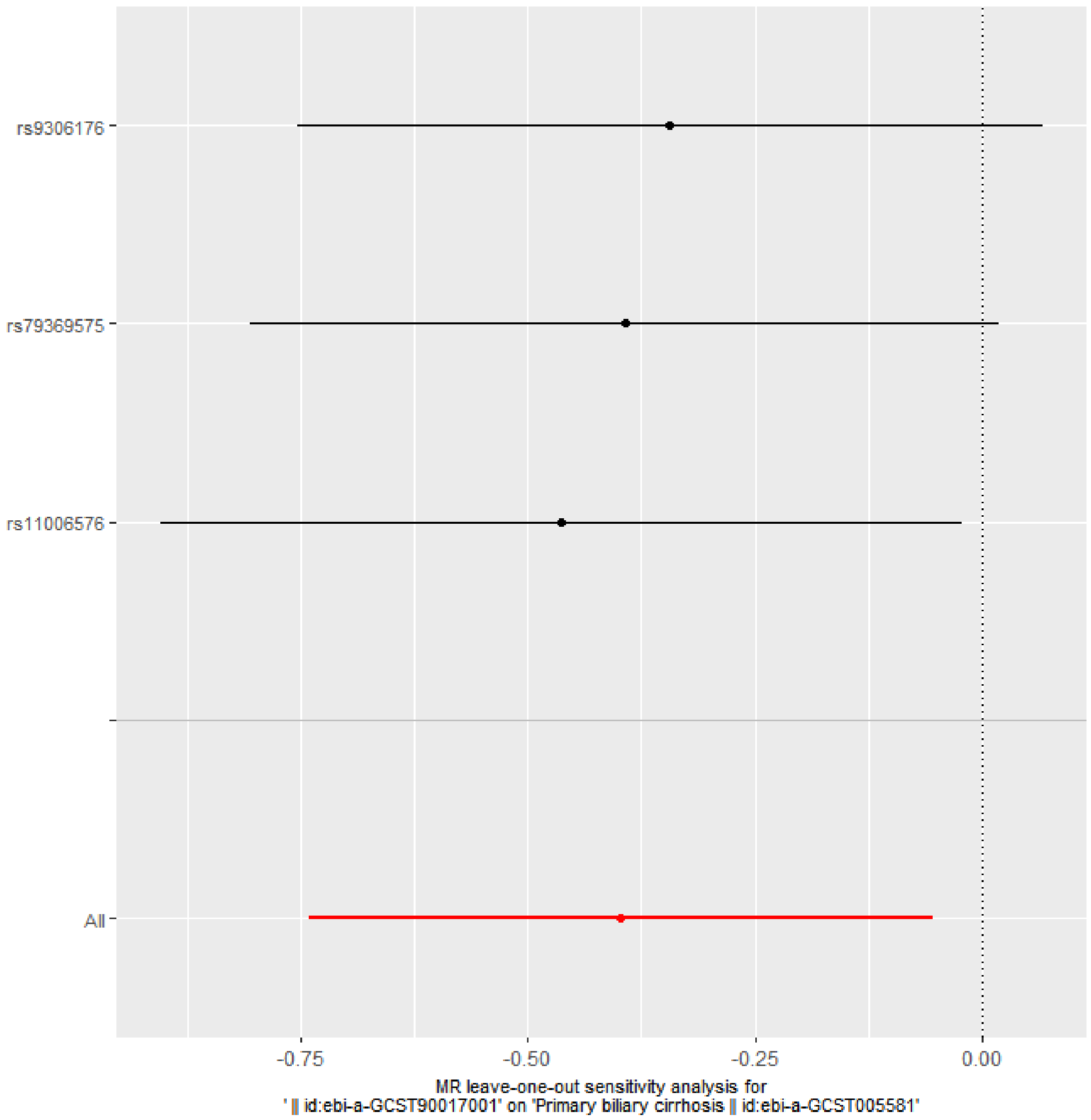

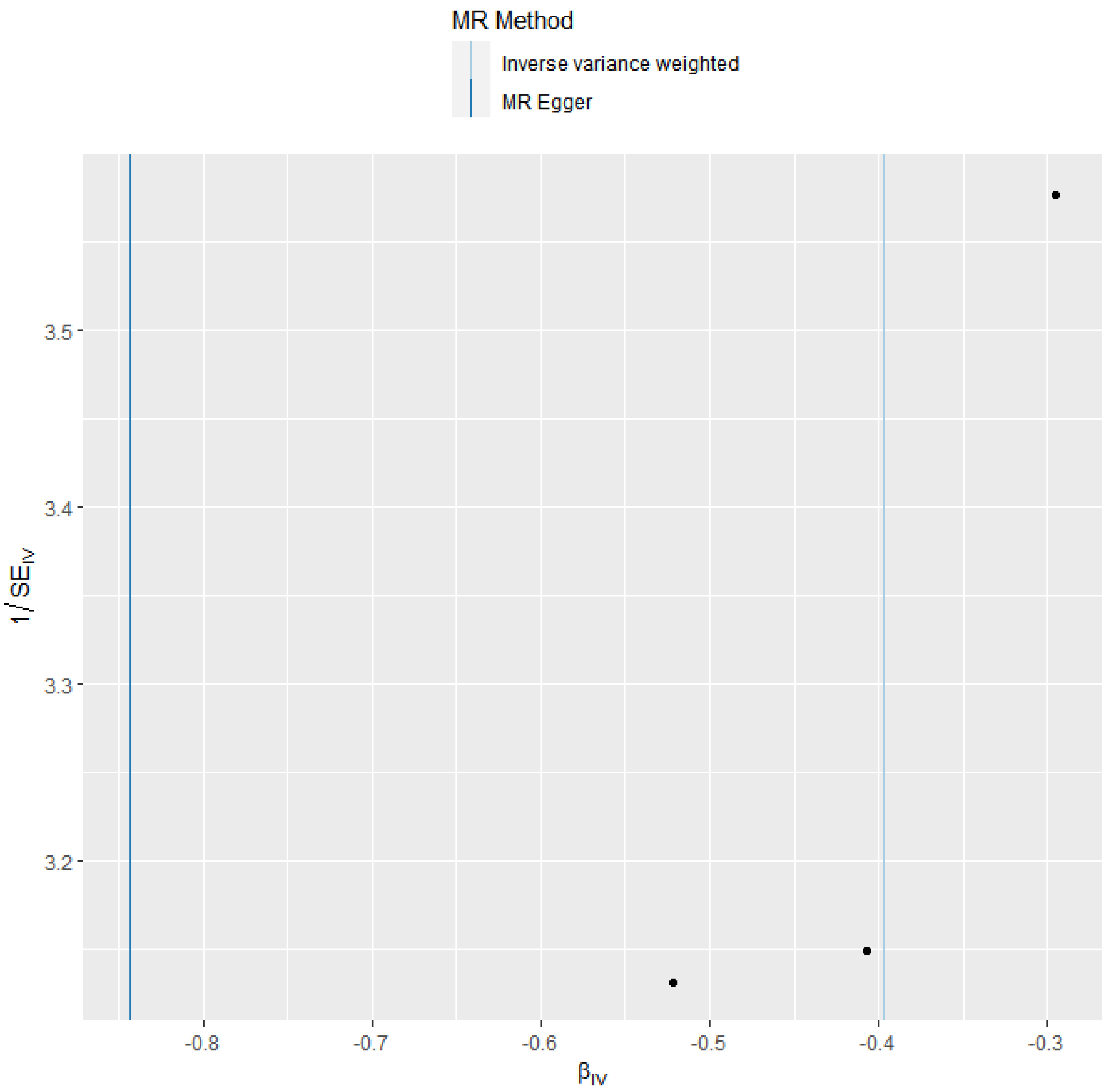
MR Method
Inverse variance weighted
MR Egger
3.5
3.4
3.3
3.2
1/SE_IV
-0.8
-0.7
-0.6
-0.5
-0.4
-0.3
β_IV

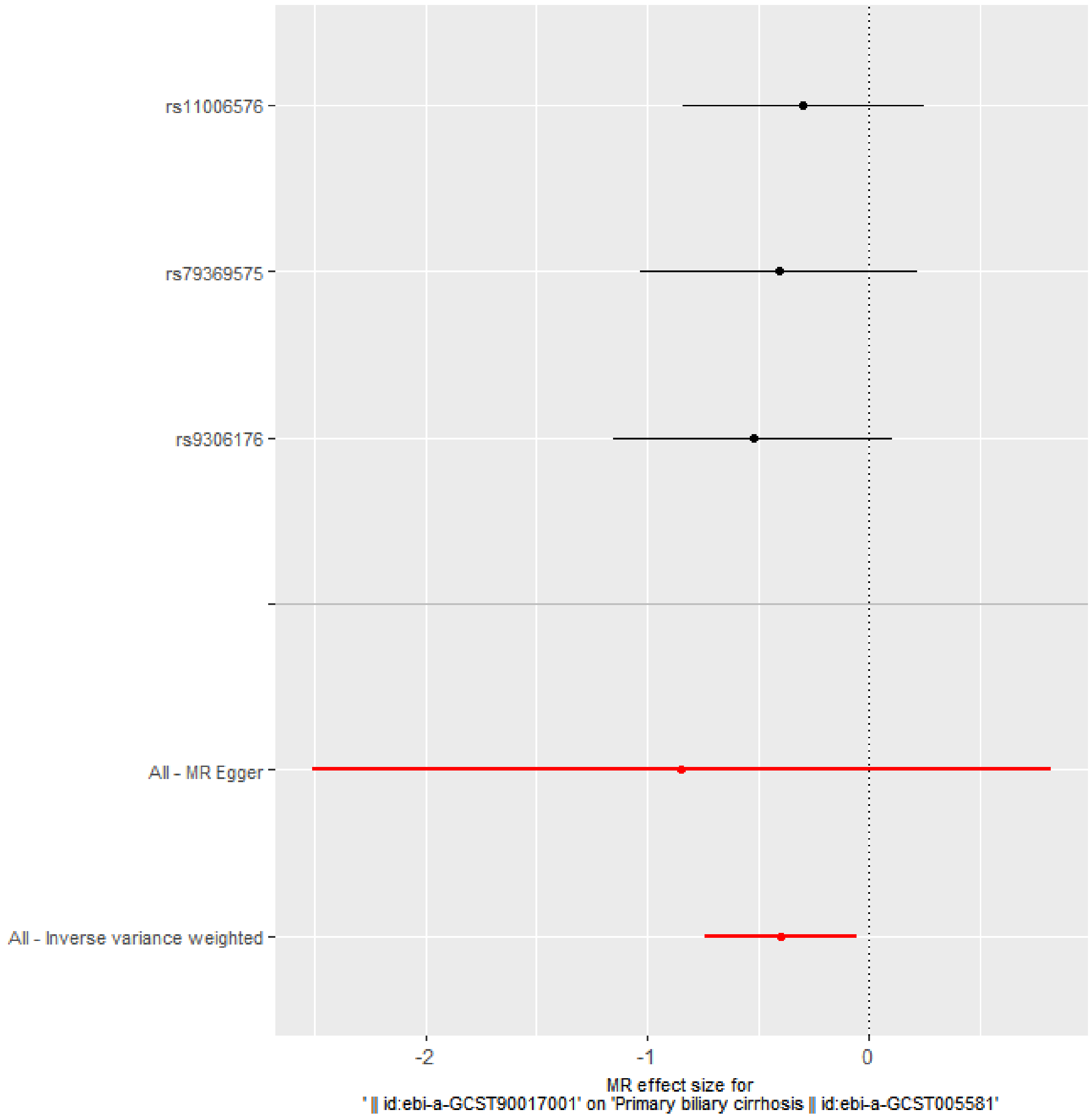
rs11006576
rs79369575
rs9306176
All - MR Egger
All - Inverse variance weighted
-2
-1
0
MR effect size for
' || id:ebi-a-GCST90017001' on 'Primary biliary cirrhosis || id:ebi-a-GCST005581'

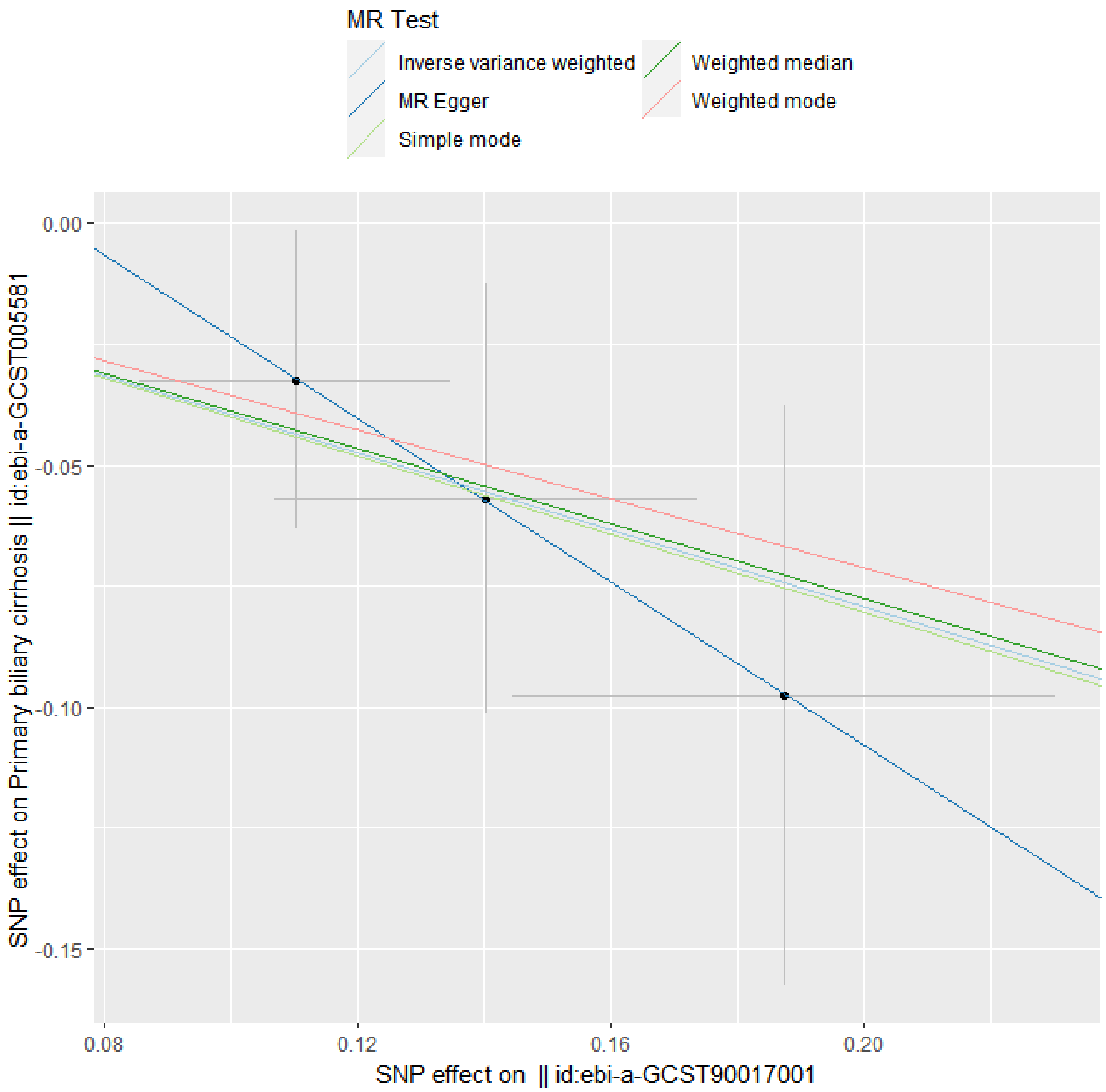

MR Test
Inverse variance weighted
MR Egger
Simple mode
Weighted median
Weighted mode
0.00
-0.05
-0.10
-0.15
SNP effect on Primary biliary cirrhosis || id:ebi-a-GCST005581
0.08
0.12
0.16
0.20
SNP effect on || id:ebi-a-GCST90017001

Figure 174 Leave-one-out analysis, funnel plot, MR effect size and scatter plot for gut microbiota abundance (genus Lachnospiraceae UCG010 id.11330) on primary biliary cirrhosis

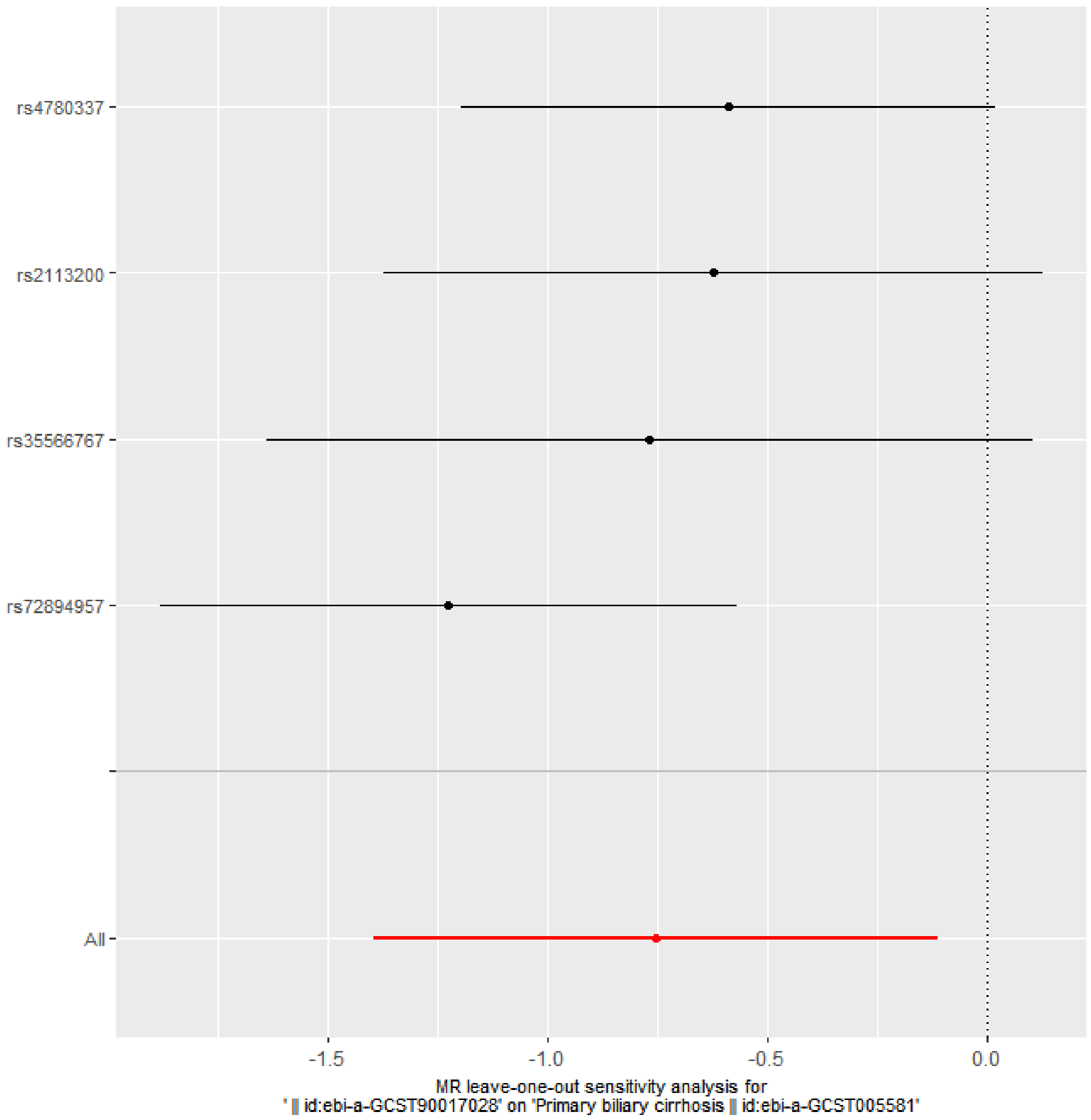

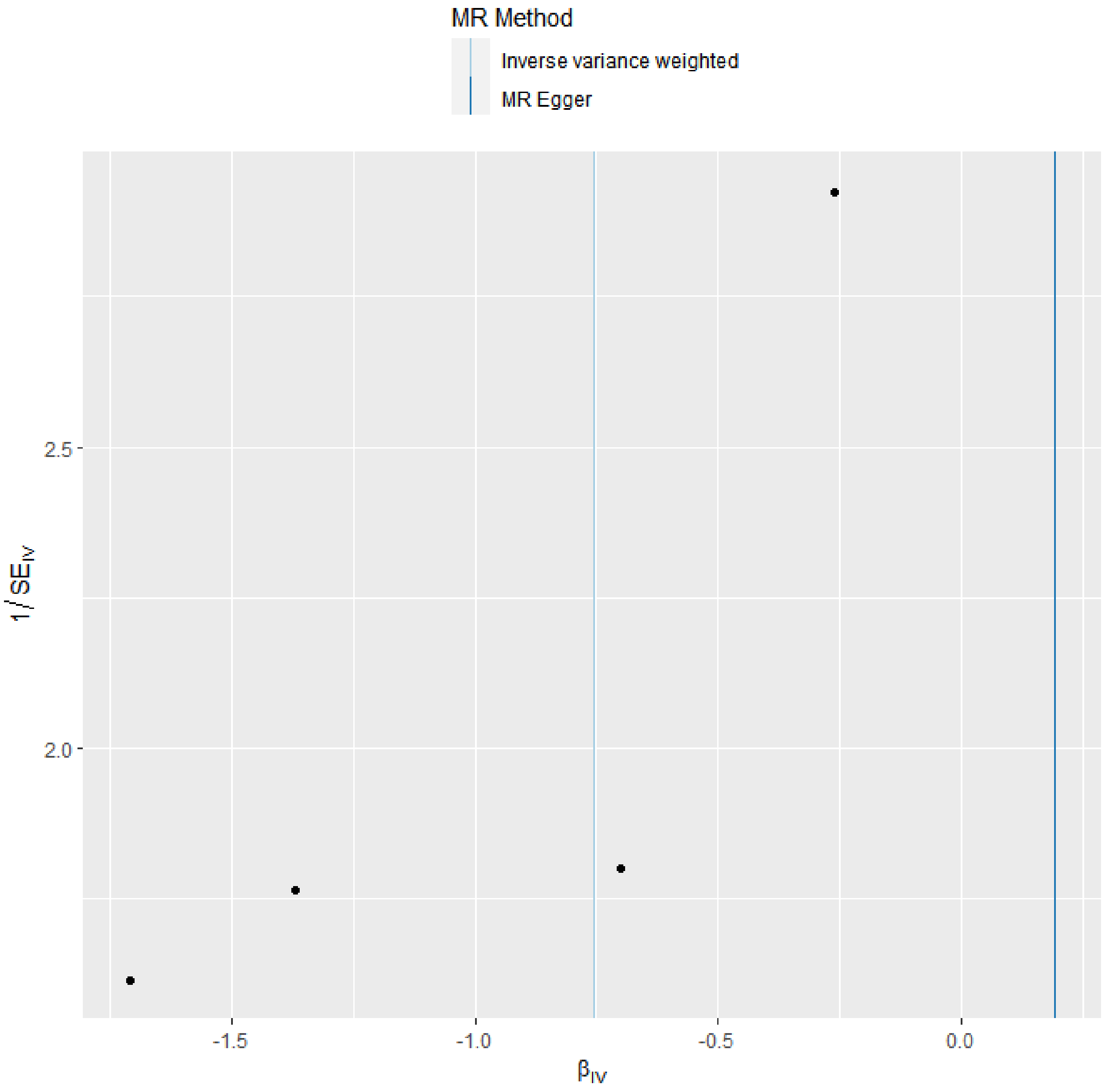

MR Method
Inverse variance weighted
MR Egger
2.5
2.0
1/SE_IV
-1.5
-1.0
-0.5
0.0
β_IV

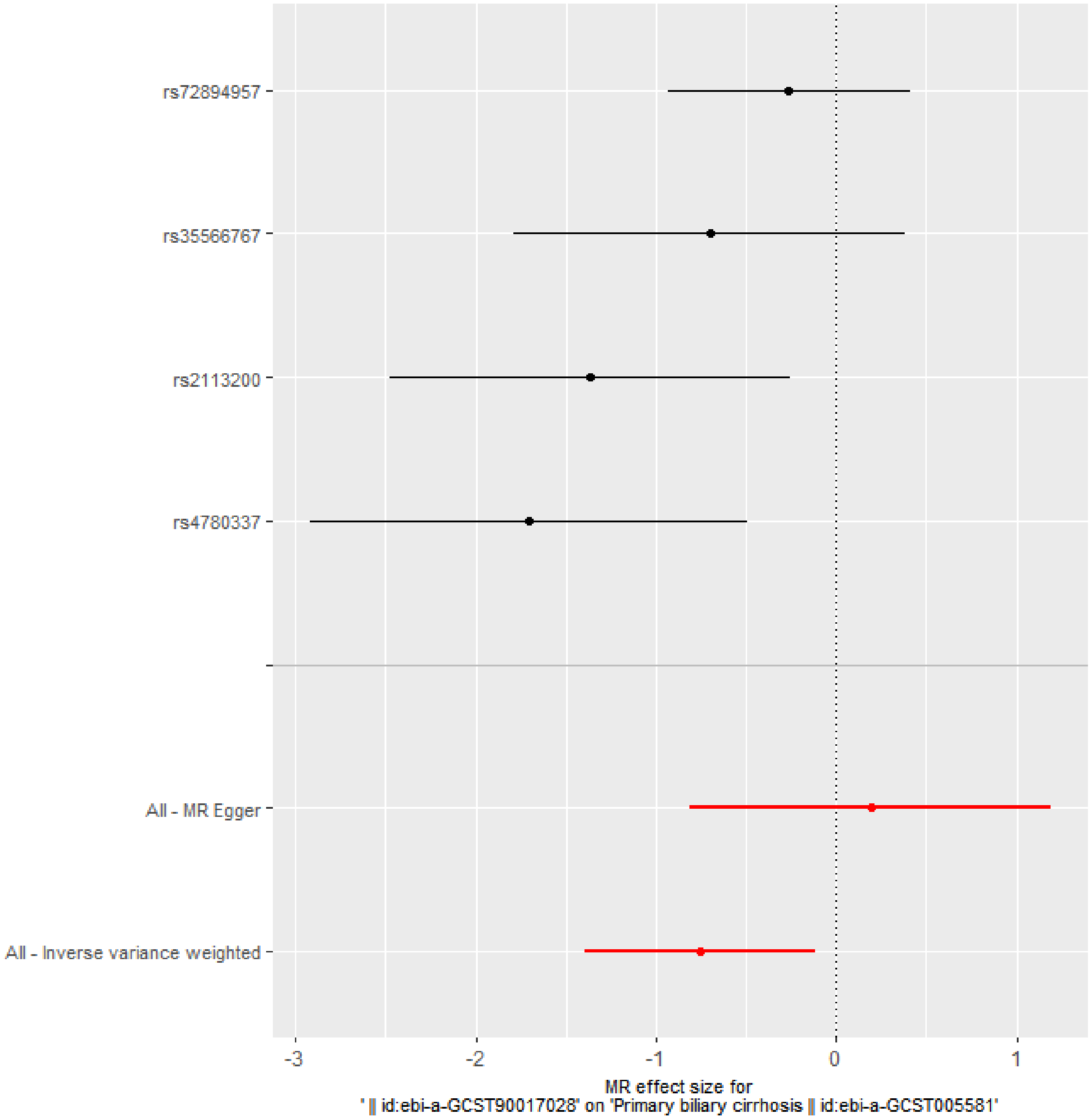

rs72894957
rs35566767
rs2113200
rs4780337
All - MR Egger
All - Inverse variance weighted
-3
-2
-1
0
1
MR effect size for
' || id:ebi-a-GCST90017028' on 'Primary biliary cirrhosis || id:ebi-a-GCST005581'

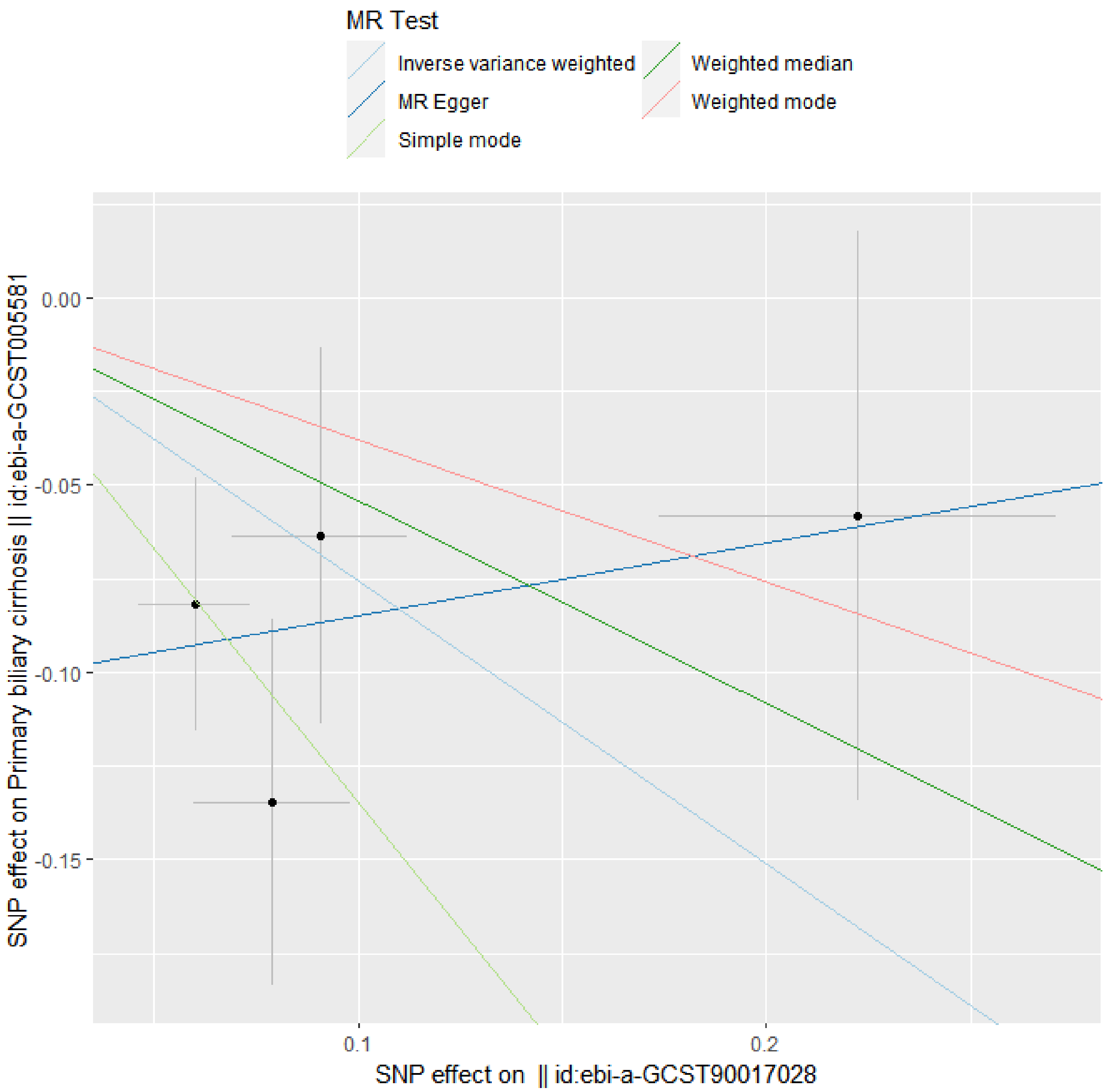

MR Test
Inverse variance weighted
MR Egger
Simple mode
Weighted median
Weighted mode
0.00
-0.05
-0.10
-0.15
0.1
0.2
SNP effect on Primary biliary cirrhosis || id:ebi-a-GCST005581
SNP effect on || id:ebi-a-GCST90017028

Figure 175 Leave-one-out analysis, funnel plot, MR effect size and scatter plot for gut microbiota abundance (genus Marvinbryantia id.2005) on primary biliary cirrhosis

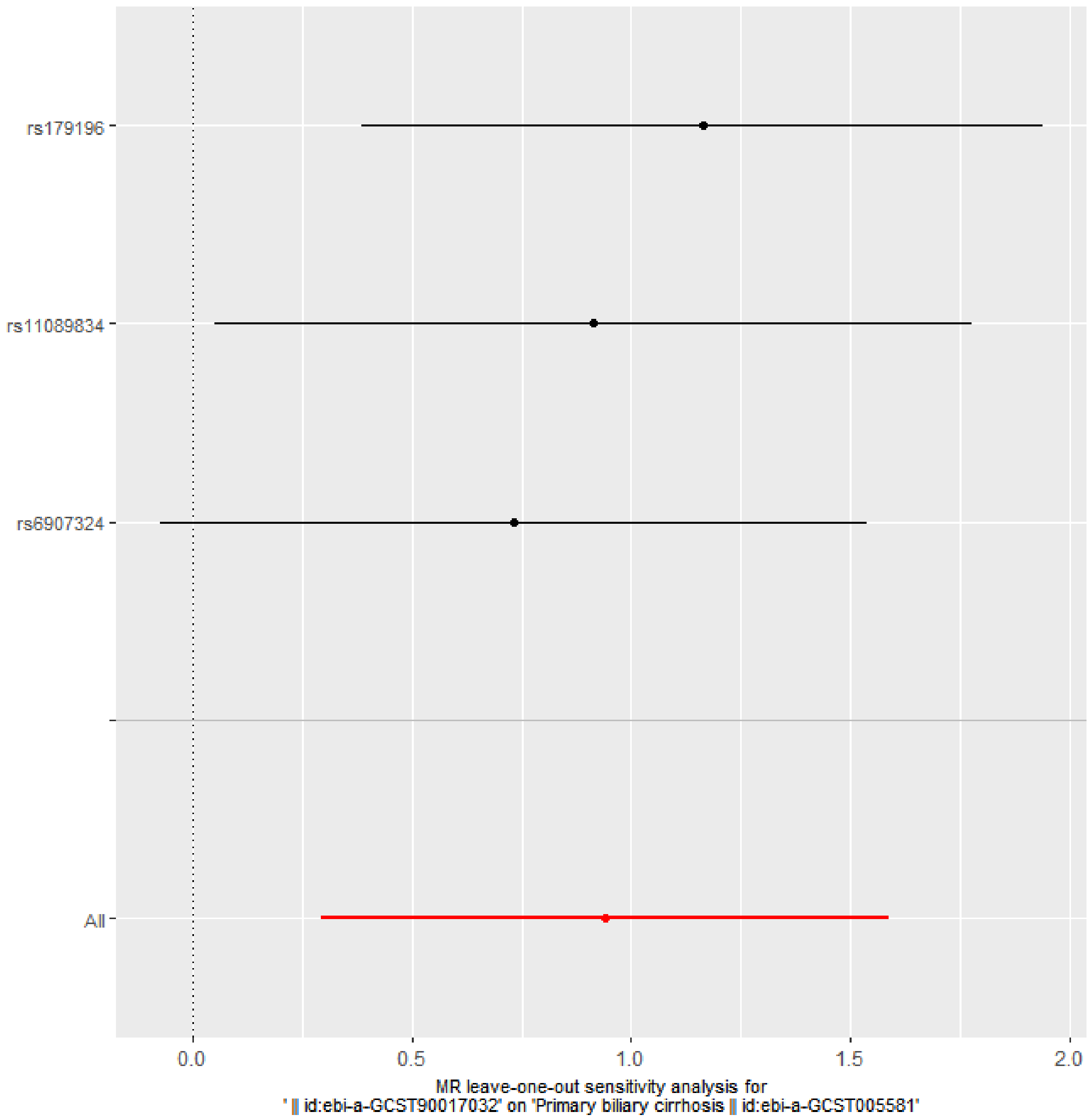

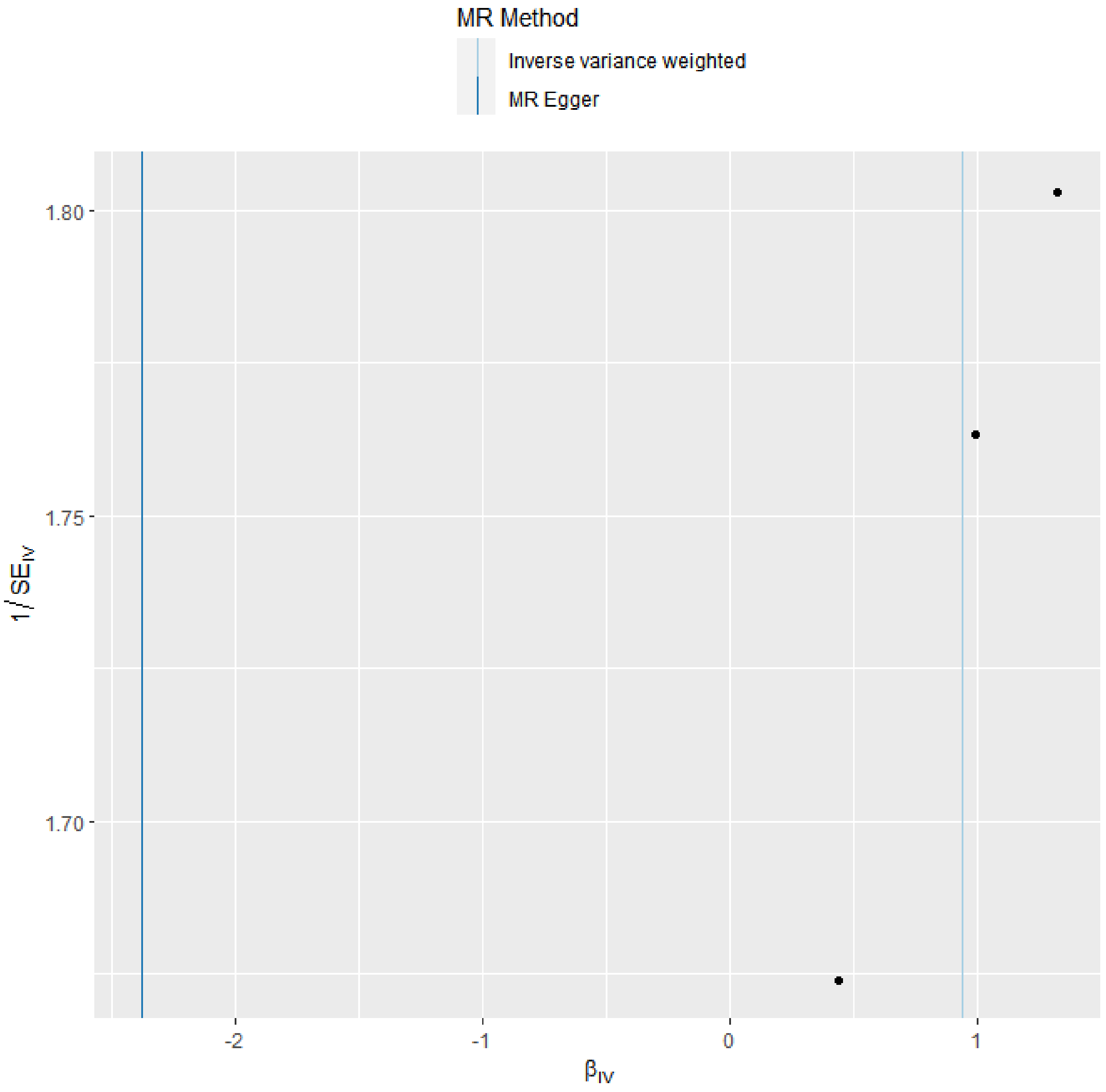

MR Method
Inverse variance weighted
MR Egger
1.80
1.75
1.70
1/SE$_{IV}$
-2
-1
0
1
β$_{IV}$

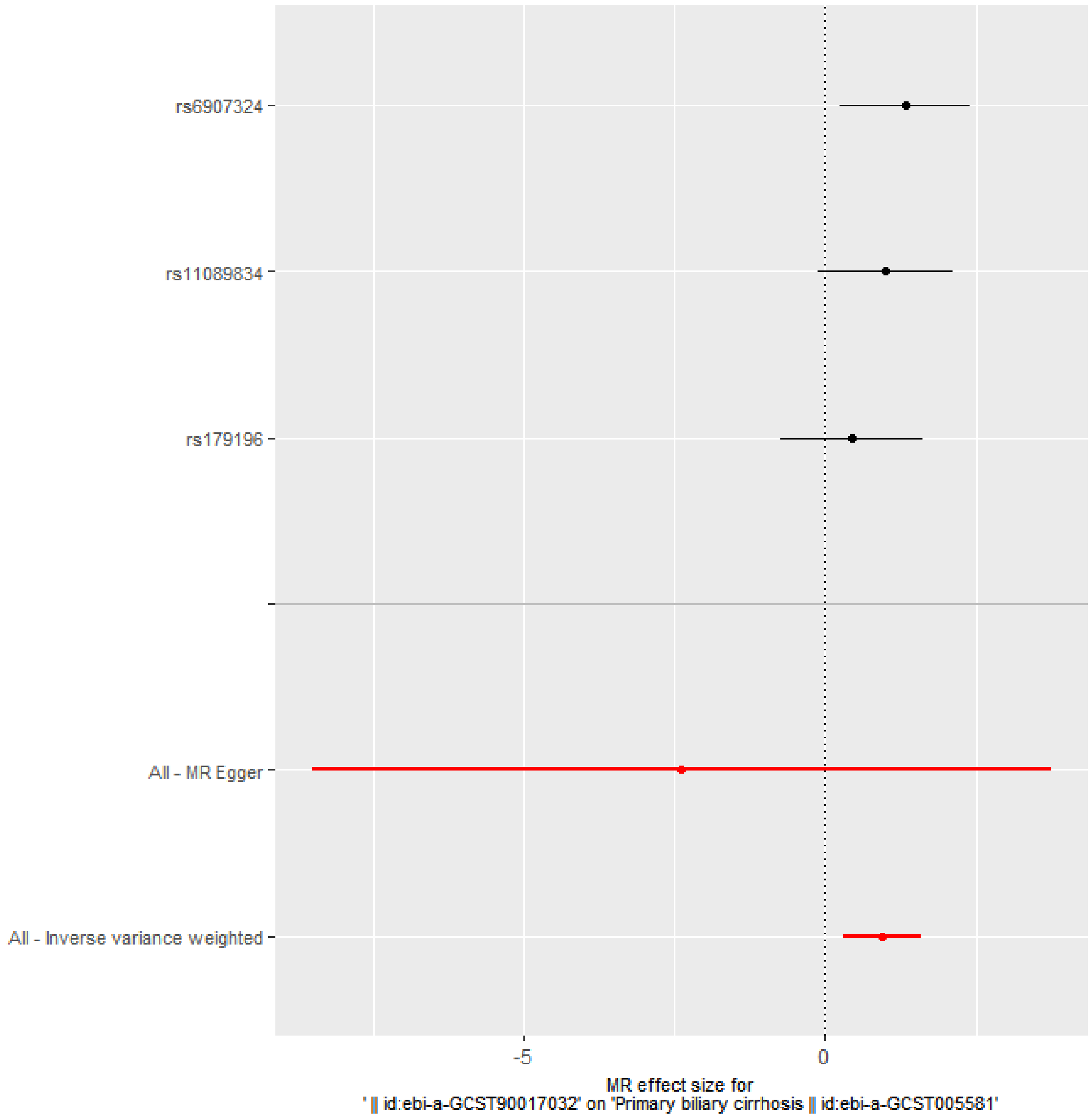
rs6907324
rs11089834
rs179196
All - MR Egger
All - Inverse variance weighted
-5
0
MR effect size for
' || id:ebi-a-GCST90017032' on 'Primary biliary cirrhosis || id:ebi-a-GCST005581'

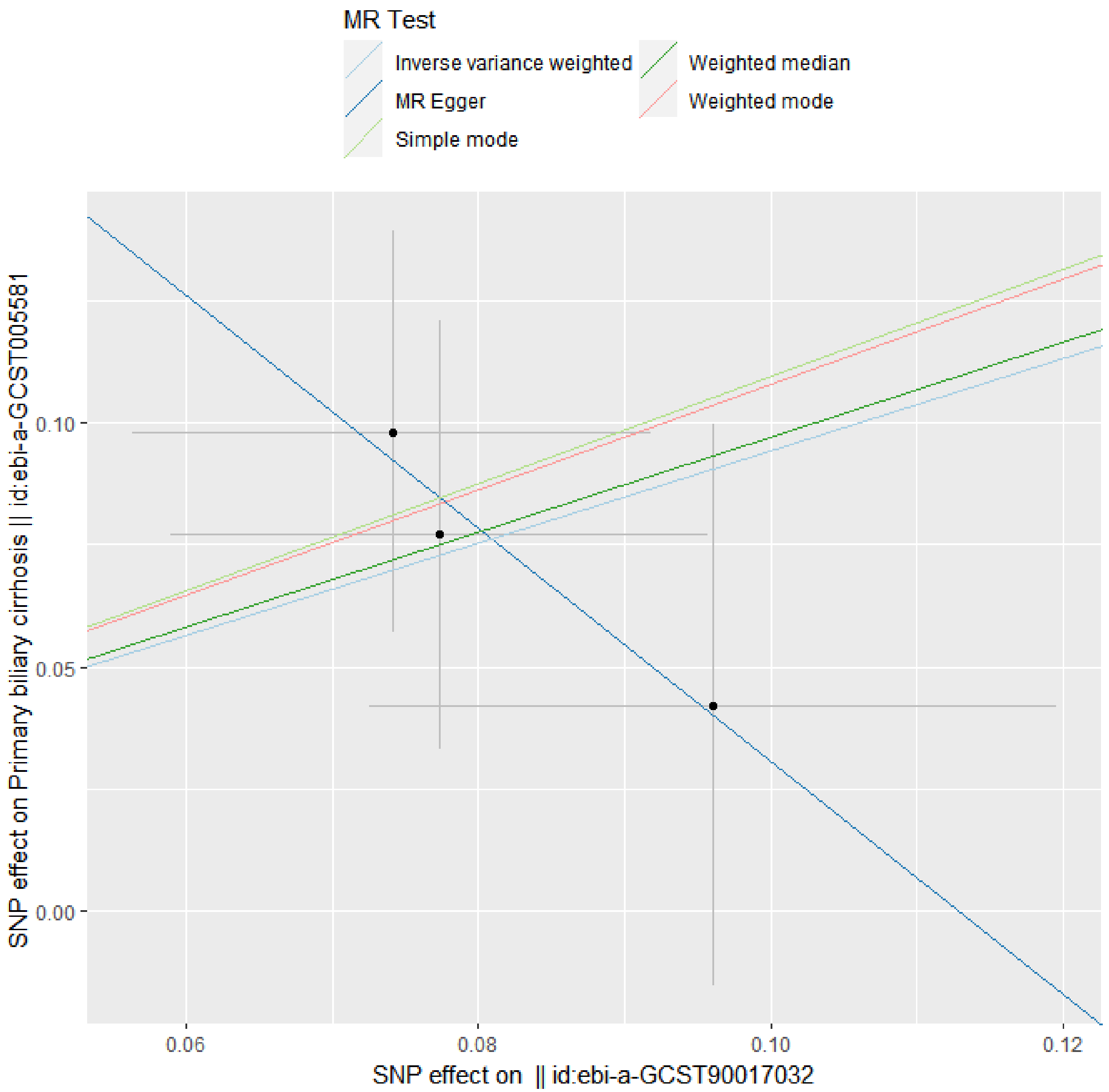

MR Test
Inverse variance weighted
MR Egger
Simple mode
Weighted median
Weighted mode
SNP effect on Primary biliary cirrhosis || id:ebi-a-GCST005581
0.10
0.05
0.00
0.06
0.08
0.10
0.12
SNP effect on || id:ebi-a-GCST90017032

Figure 176 Leave-one-out analysis, funnel plot, MR effect size and scatter plot for gut microbiota abundance (genus Oscillibacter id.2063) on primary biliary cirrhosis

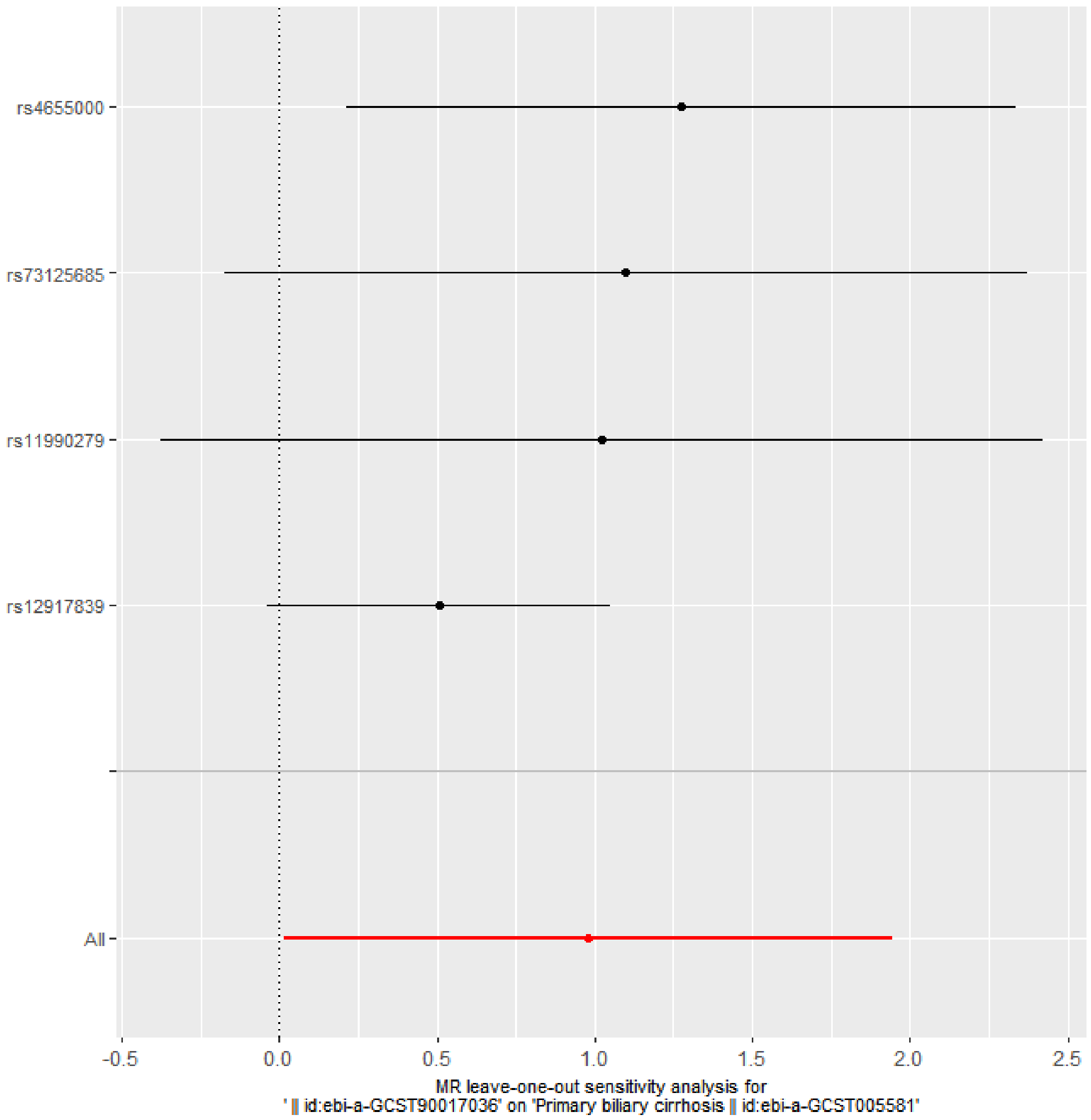

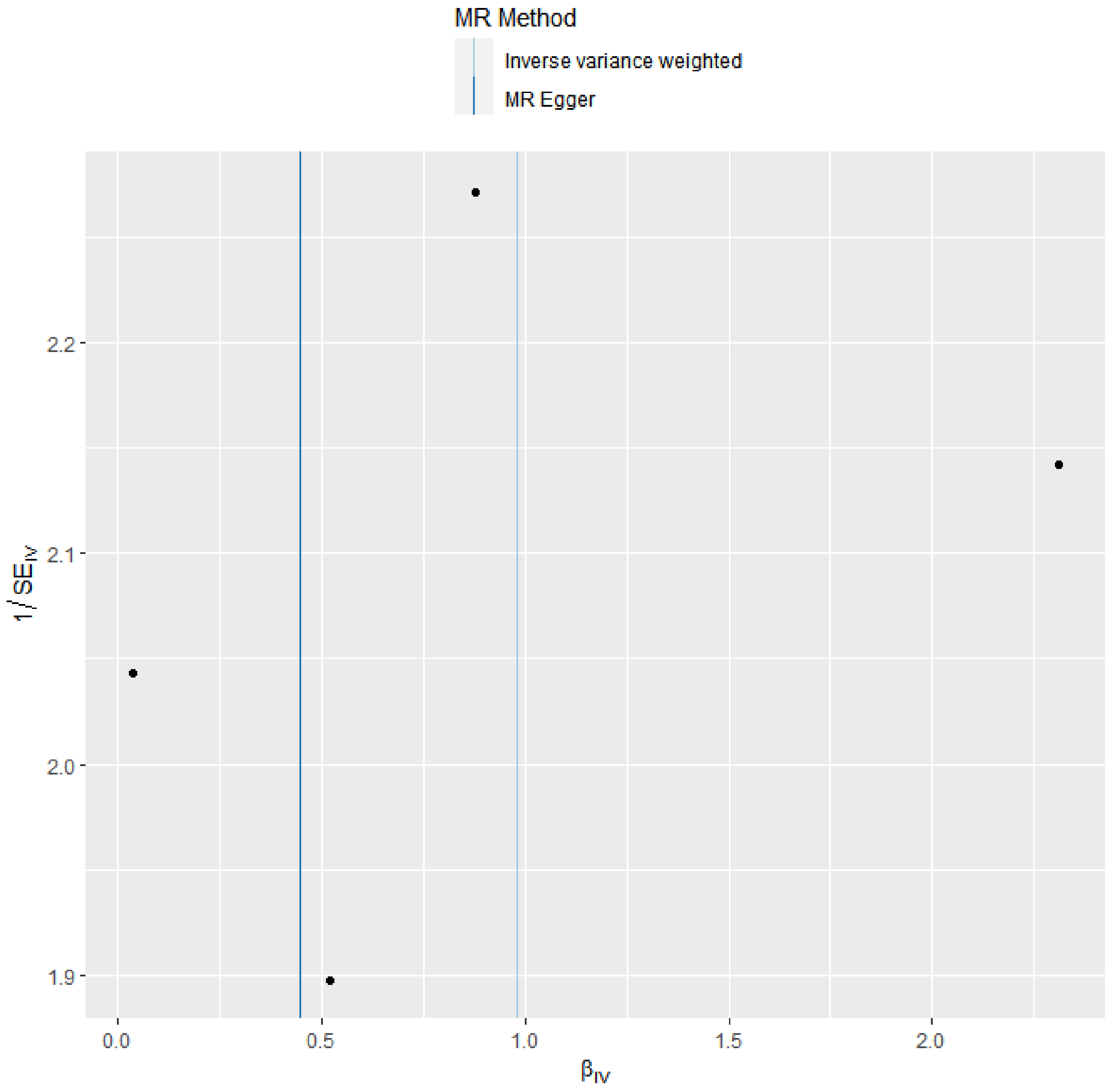

MR Method
Inverse variance weighted
MR Egger
2.2
2.1
2.0
1.9
1/SE_IV
0.0
0.5
1.0
1.5
2.0
β_IV

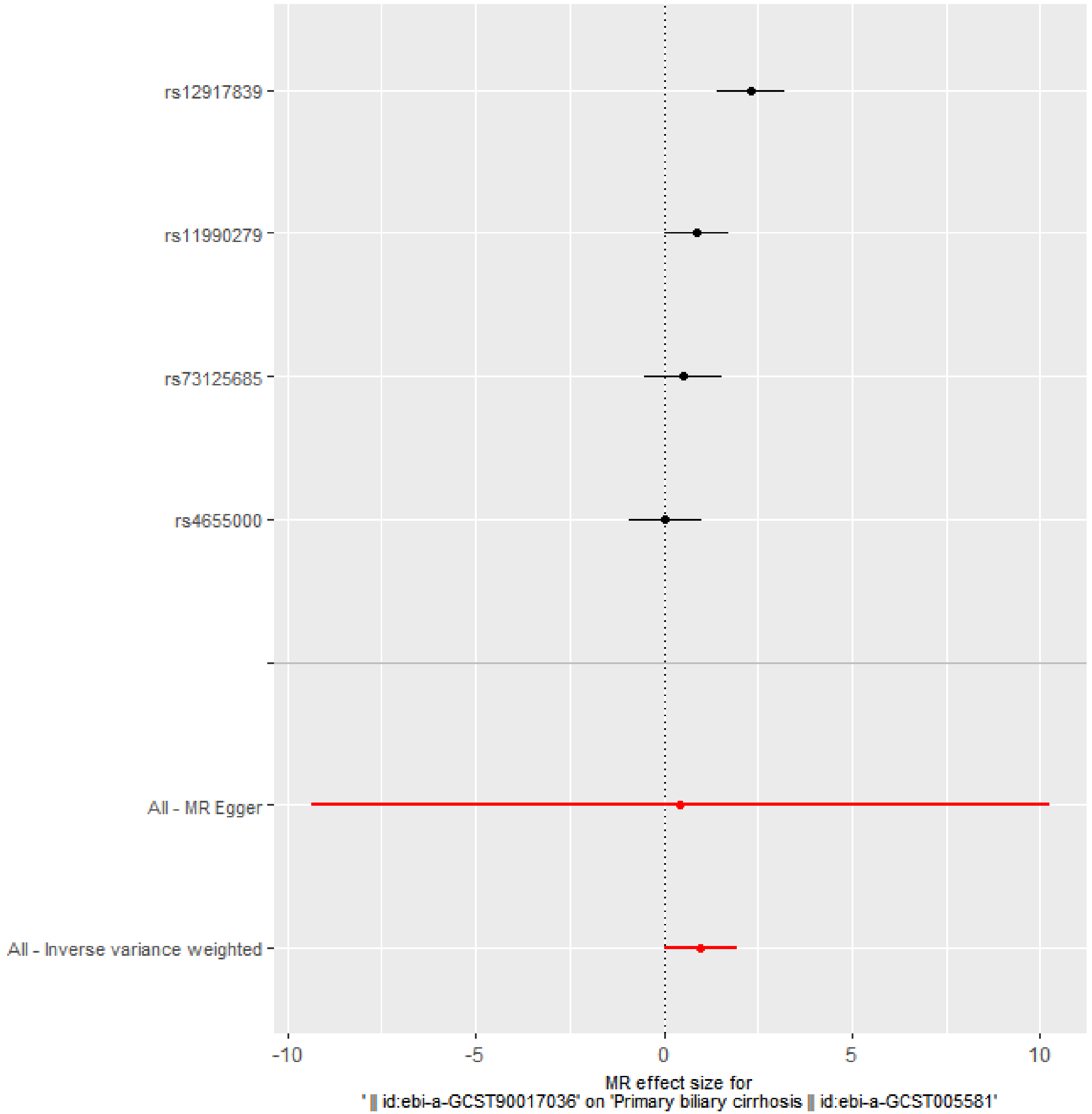
rs12917839
rs11990279
rs73125685
rs4655000
All - MR Egger
All - Inverse variance weighted
-10
-5
0
5
10
MR effect size for
' || id:ebi-a-GCST90017036' on 'Primary biliary cirrhosis || id:ebi-a-GCST005581'

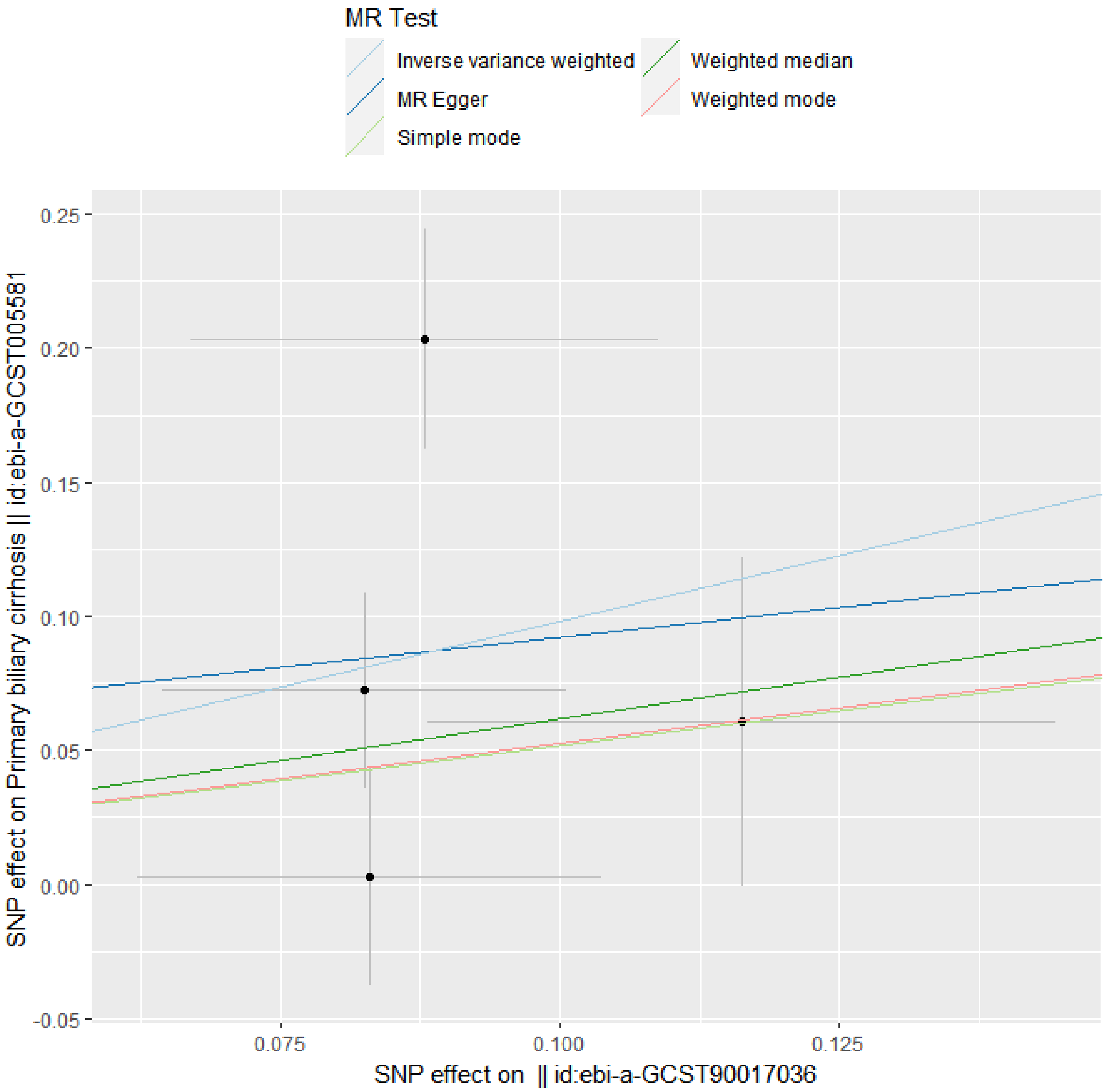

MR Test
Inverse variance weighted
MR Egger
Simple mode
Weighted median
Weighted mode
0.25
0.20
0.15
0.10
0.05
0.00
-0.05
0.075
0.100
0.125
SNP effect on Primary biliary cirrhosis || id:ebi-a-GCST005581
SNP effect on || id:ebi-a-GCST90017036

Figure 177 Leave-one-out analysis, funnel plot, MR effect size and scatter plot for gut microbiota abundance (genus Ruminococcaceae UCG010 id.11367) on primary biliary cirrhosis

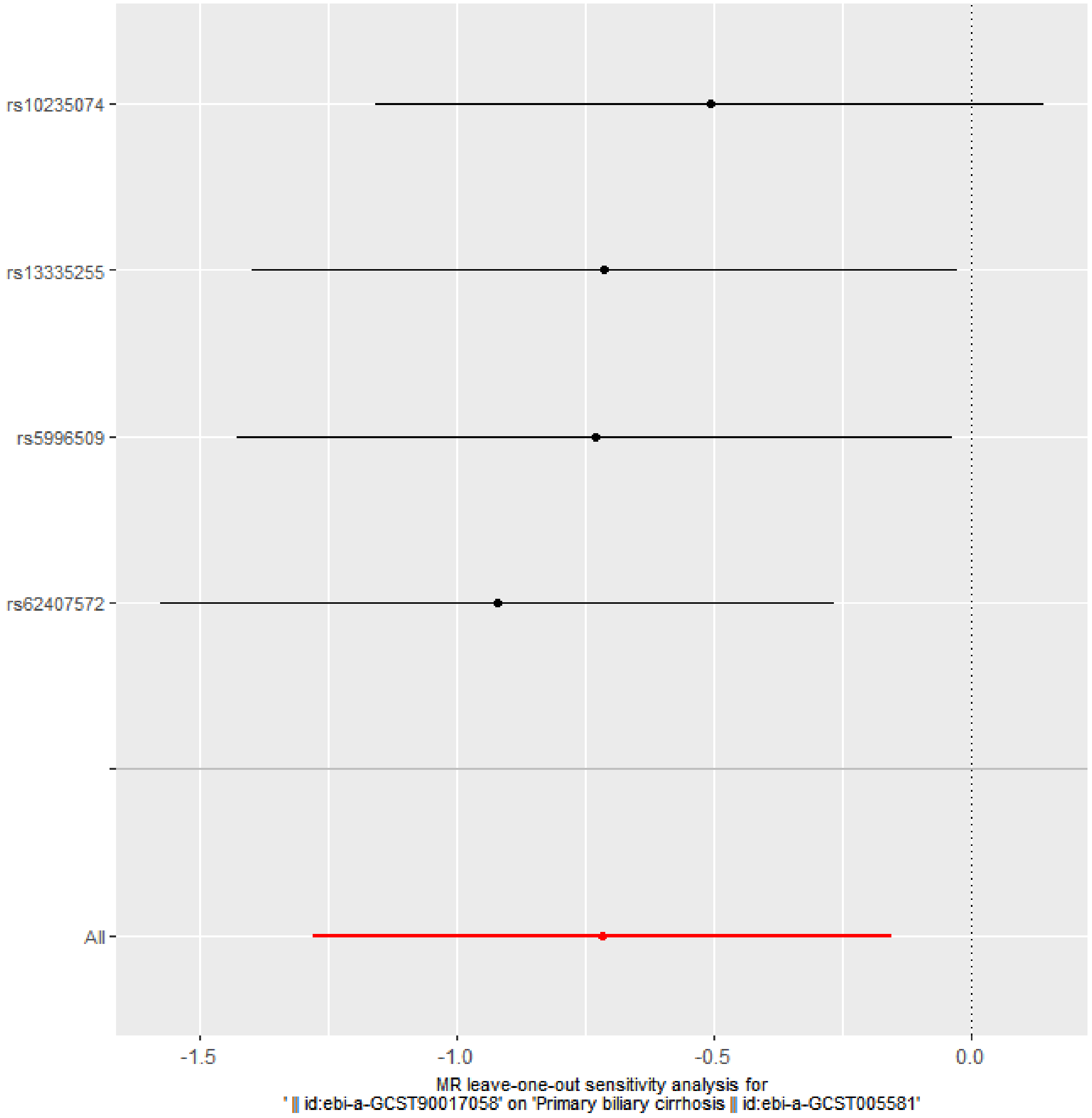

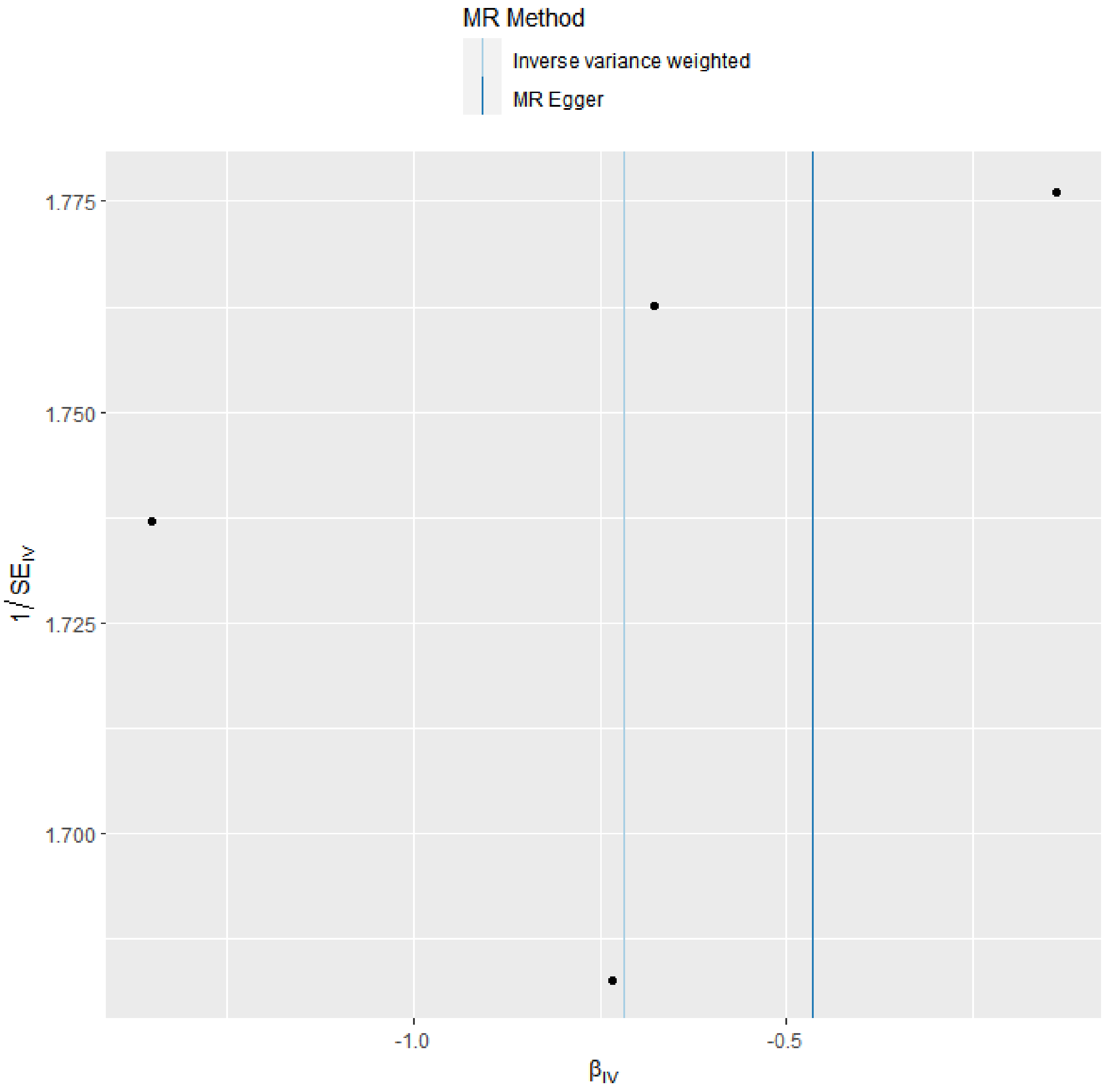

MR Method
Inverse variance weighted
MR Egger
1.775
1.750
1.725
1.700
-1.0
-0.5
β_{IV}
$1/SE_{IV}$

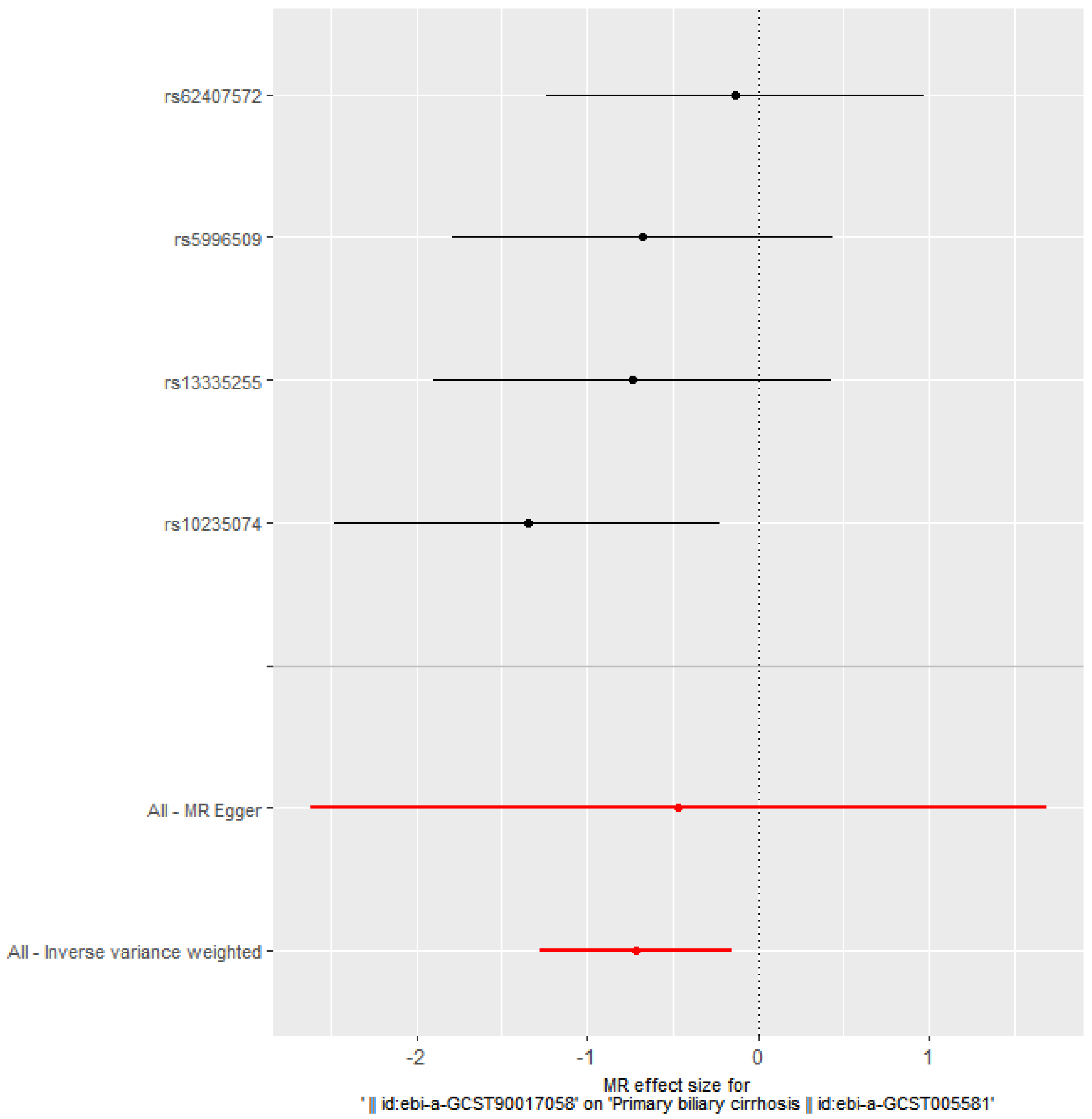
rs62407572
rs5996509
rs13335255
rs10235074
All - MR Egger
All - Inverse variance weighted
-2
-1
0
1
MR effect size for
' || id:ebi-a-GCST90017058' on 'Primary biliary cirrhosis || id:ebi-a-GCST005581'

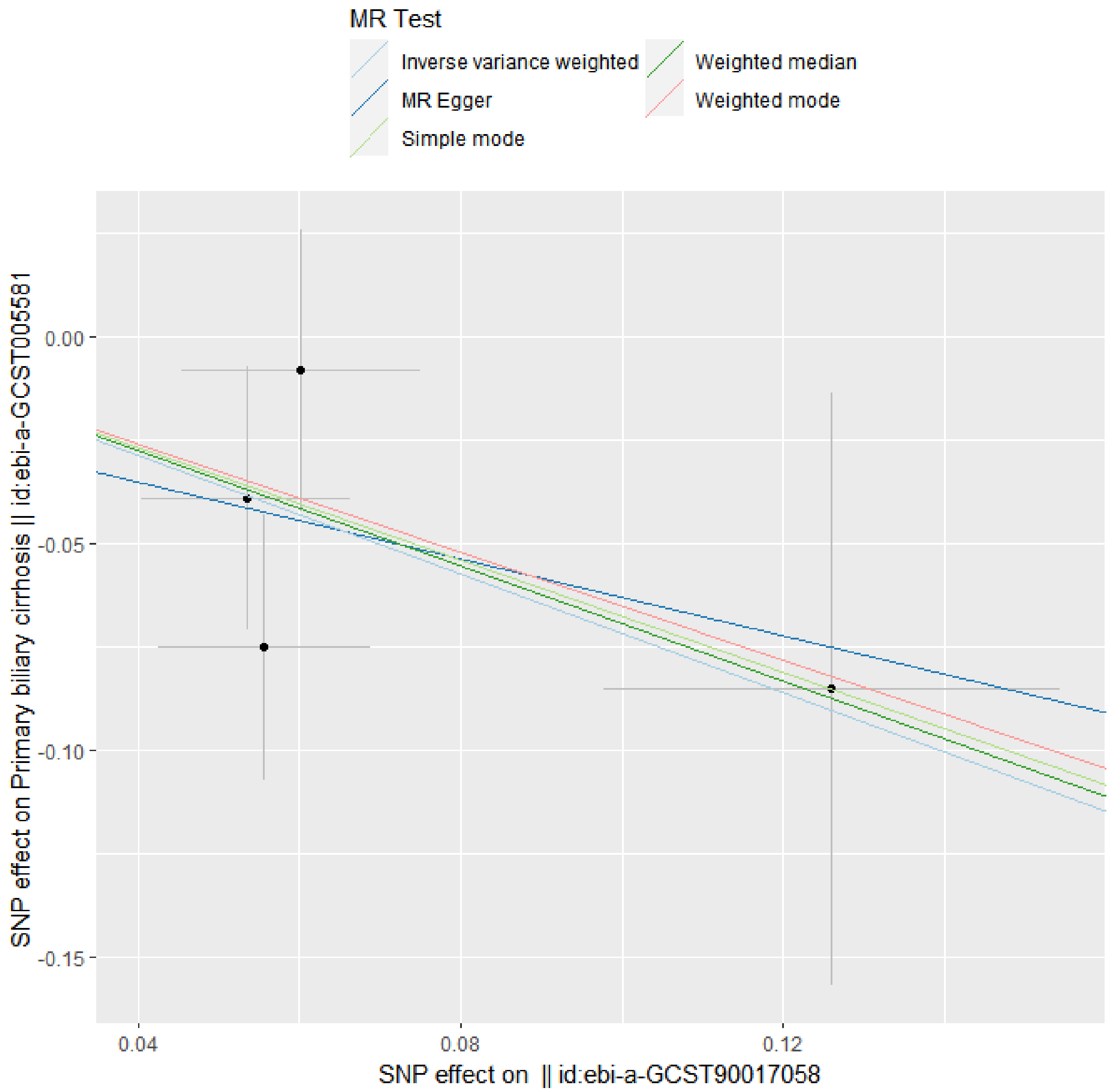

MR Test
Inverse variance weighted
MR Egger
Simple mode
Weighted median
Weighted mode
SNP effect on Primary biliary cirrhosis || id:ebi-a-GCST005581
0.00
-0.05
-0.10
-0.15
0.04
0.08
0.12
SNP effect on || id:ebi-a-GCST90017058

Figure 178 Leave-one-out analysis, funnel plot, MR effect size and scatter plot for gut microbiota abundance (unknown genus id.826) on primary biliary cirrhosis

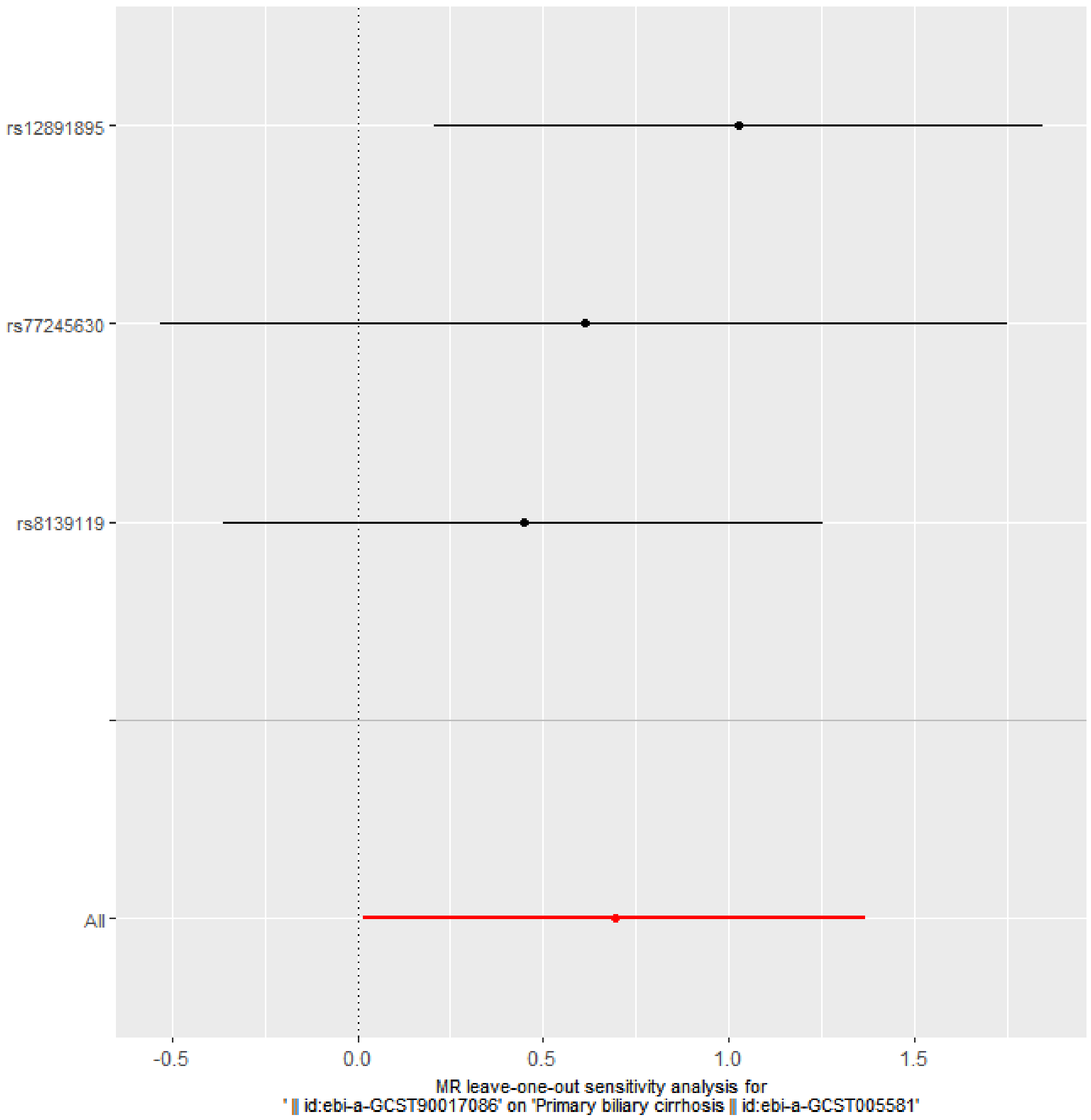

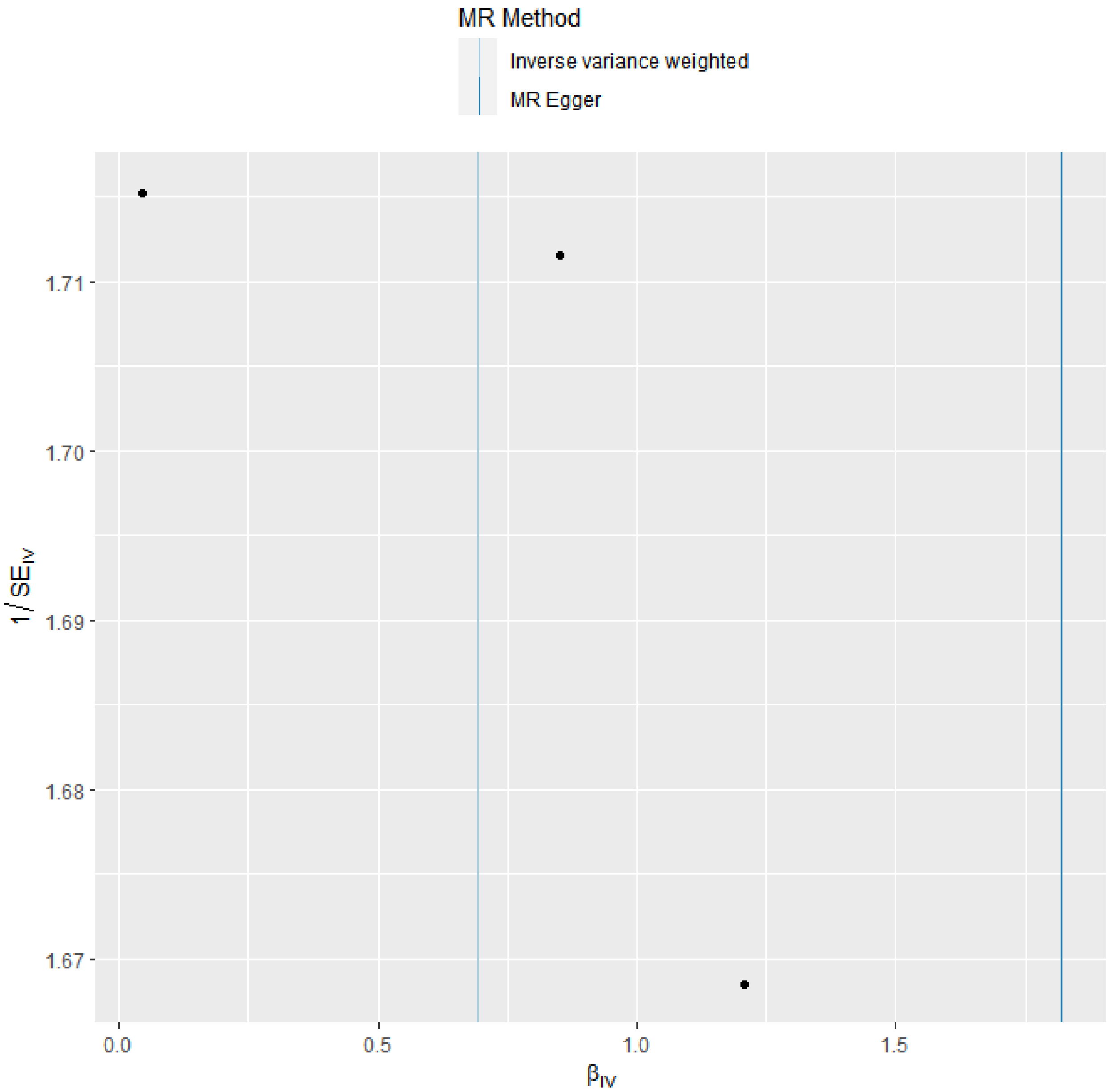

MR Method
Inverse variance weighted
MR Egger
1.71
1.70
1.69
1.68
1.67
0.0
0.5
1.0
1.5
β_{IV}
$1/SE_{IV}$

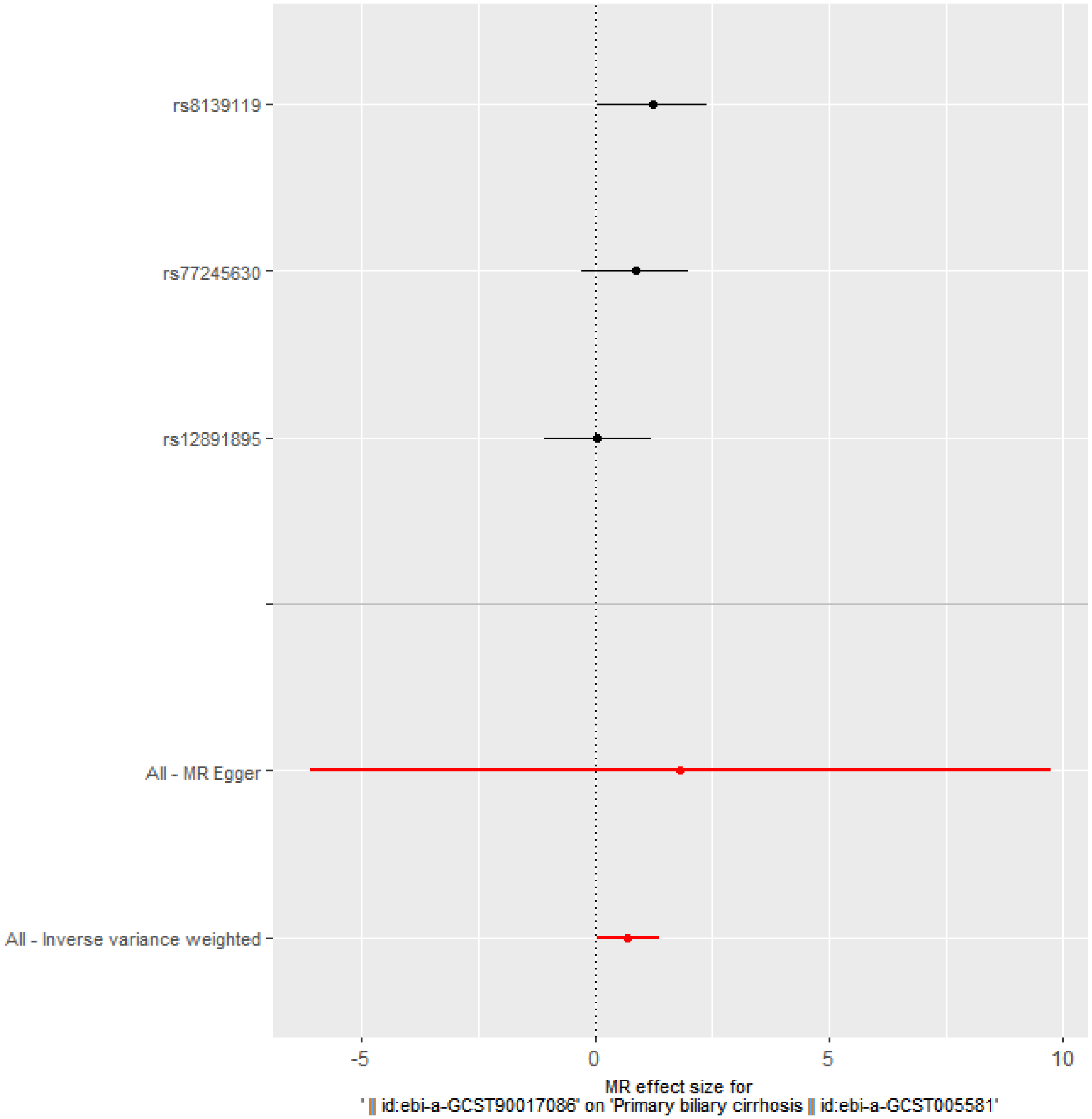

rs8139119
rs77245630
rs12891895
All - MR Egger
All - Inverse variance weighted
-5
0
5
10
MR effect size for
' || id:ebi-a-GCST90017086' on 'Primary biliary cirrhosis || id:ebi-a-GCST005581'

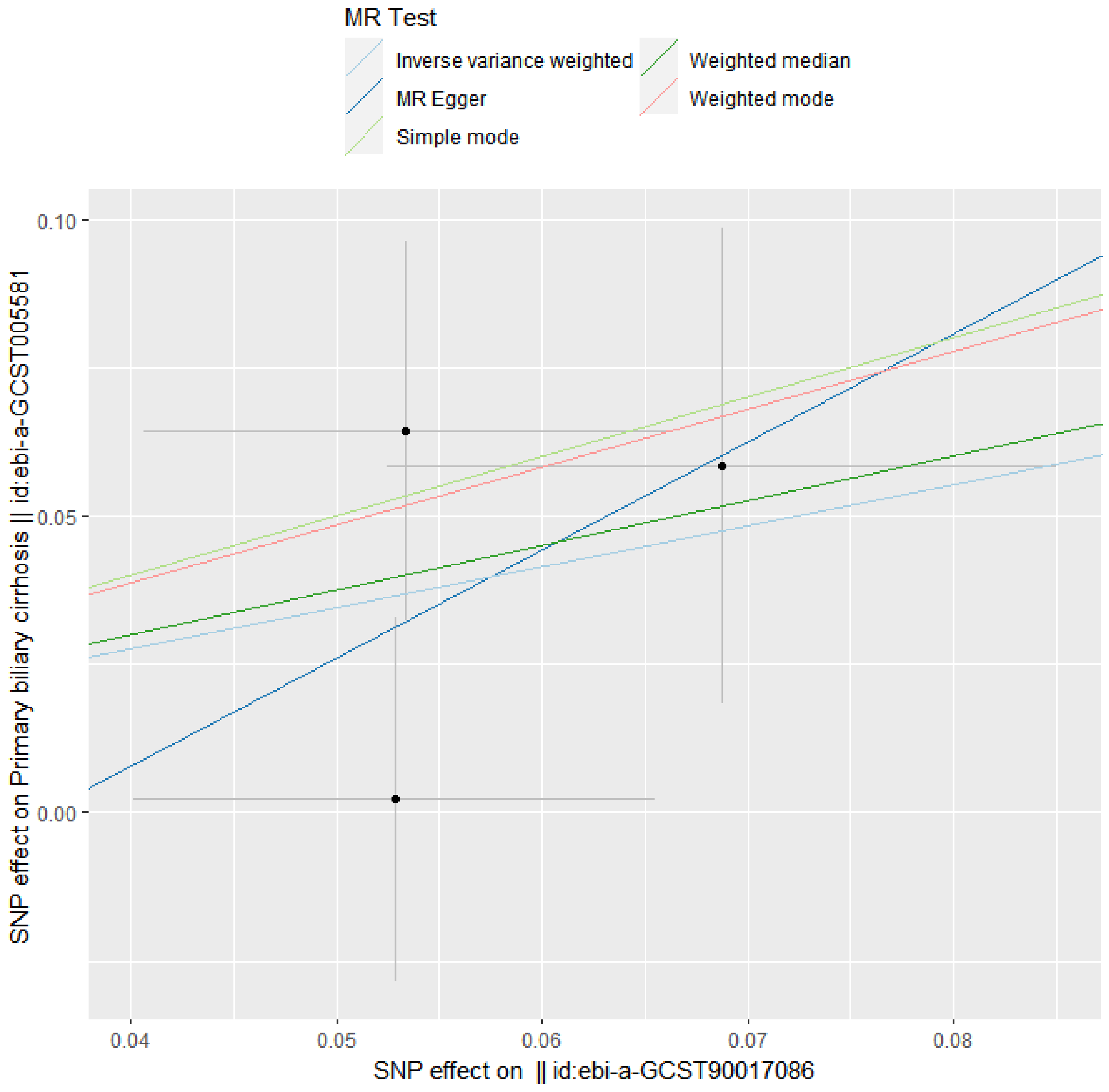

MR Test
Inverse variance weighted
MR Egger
Simple mode
Weighted median
Weighted mode
0.10
0.05
0.00
SNP effect on Primary biliary cirrhosis || id:ebi-a-GCST005581
0.04
0.05
0.06
0.07
0.08
SNP effect on || id:ebi-a-GCST90017086

Figure 179 Leave-one-out analysis, funnel plot, MR effect size and scatter plot for gut microbiota abundance (family Alcaligenaceae id.2875) on liver cell carcinoma

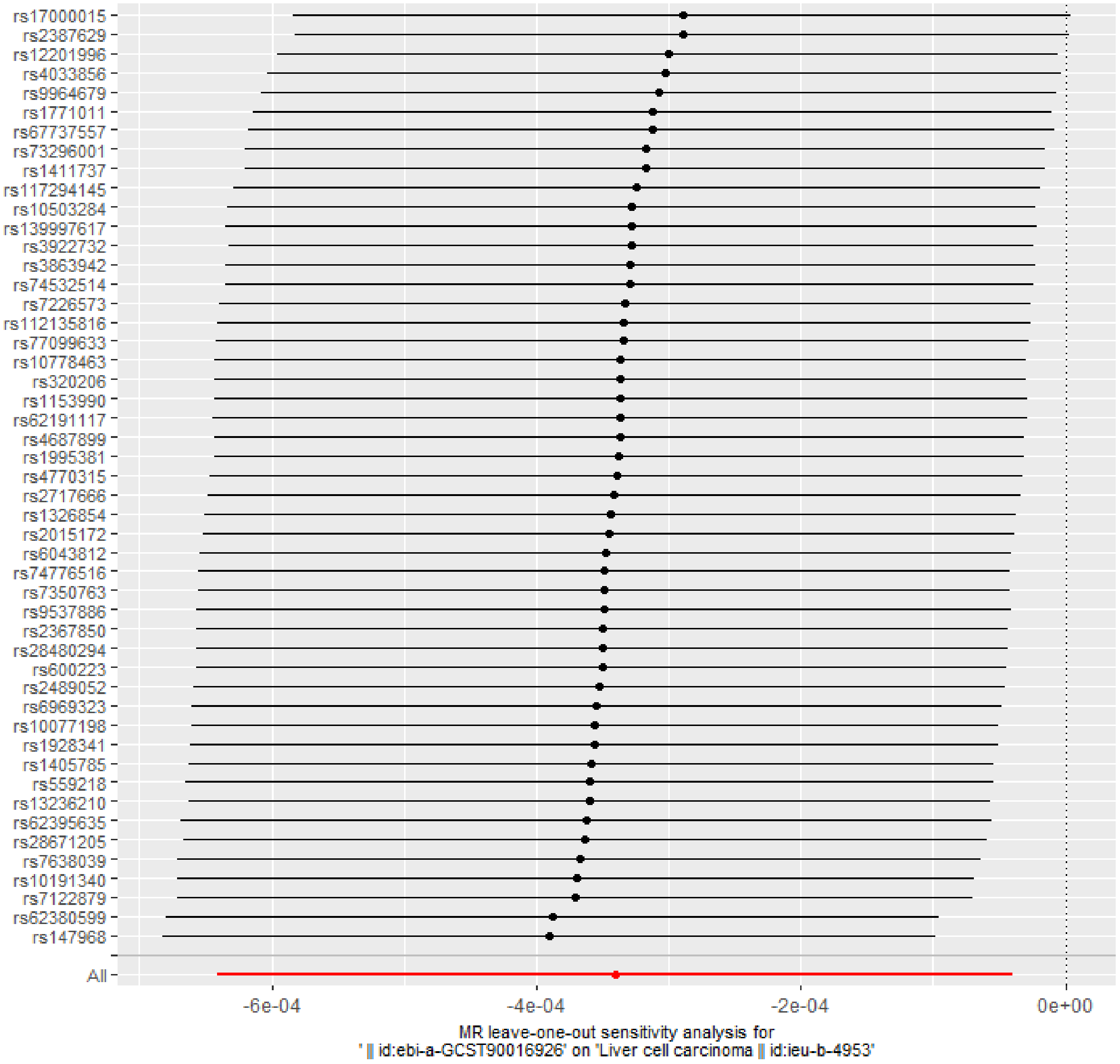

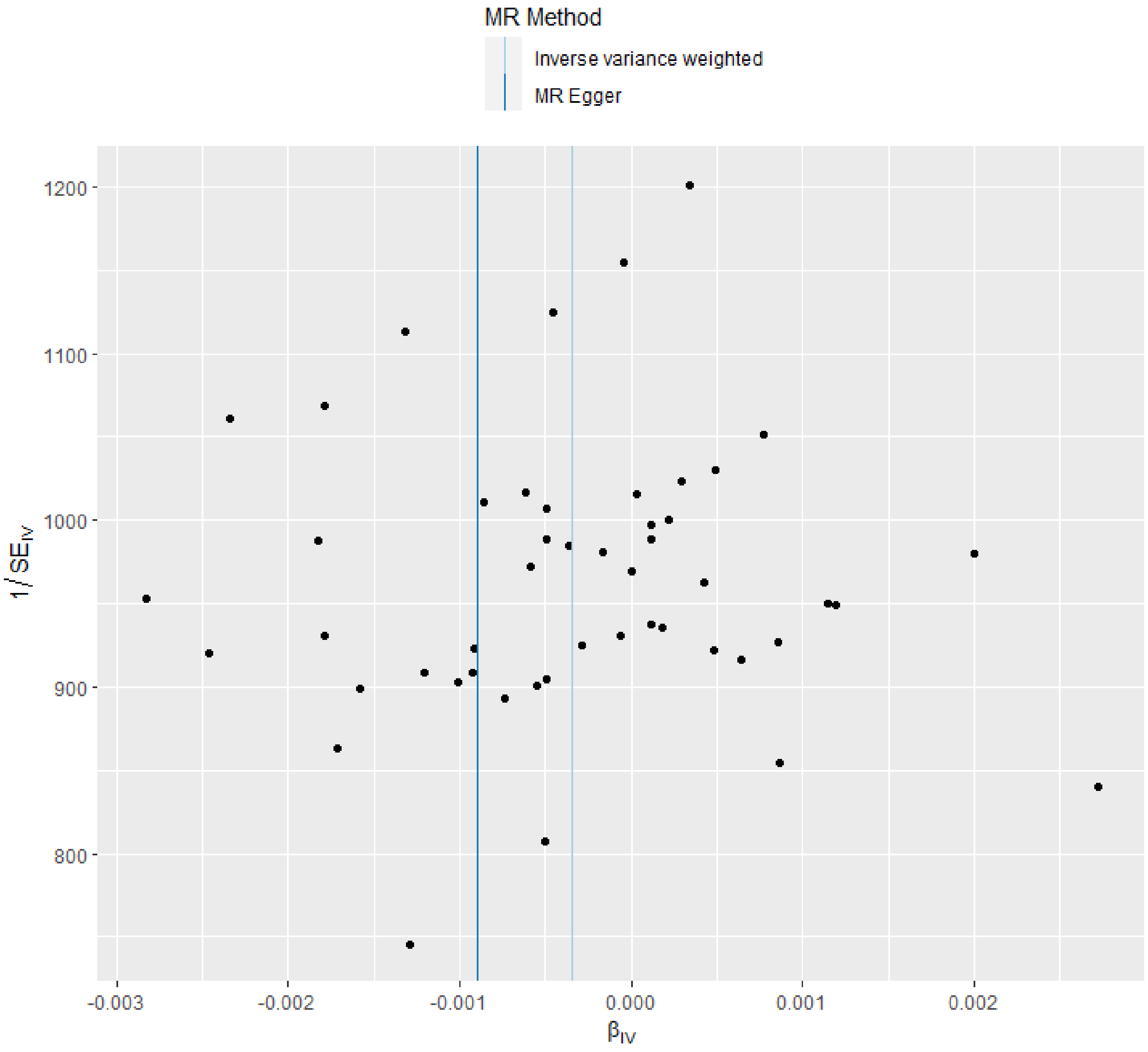
MR Method
Inverse variance weighted
MR Egger
1200
1100
1000
900
800
-0.003
-0.002
-0.001
0.000
0.001
0.002
$1/SE_{IV}$
β_{IV}

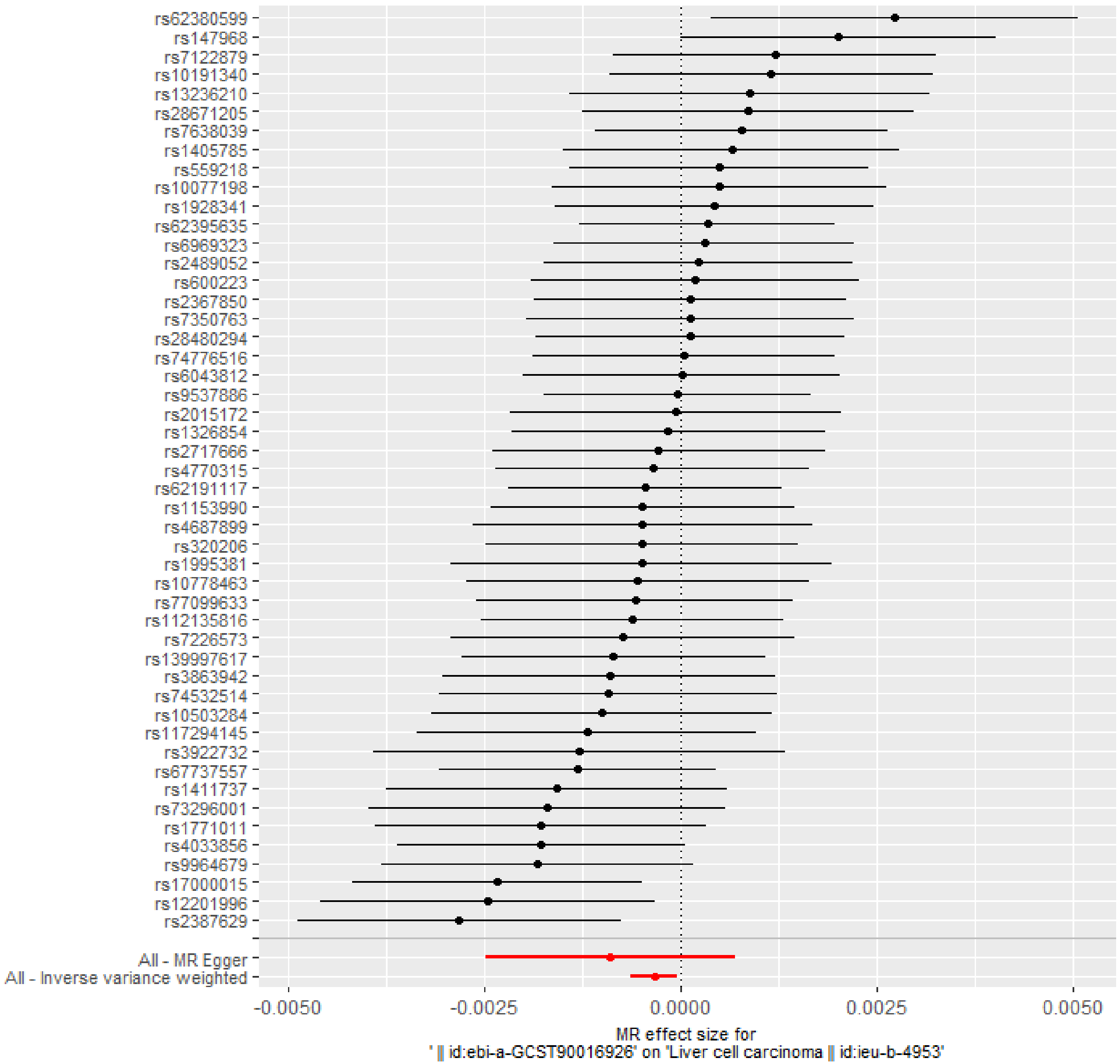
rs62380599
rs147968
rs7122879
rs10191340
rs13236210
rs28671205
rs7638039
rs1405785
rs559218
rs10077198
rs1928341
rs62395635
rs6969323
rs2489052
rs600223
rs2367850
rs7350763
rs28480294
rs74776516
rs6043812
rs9537886
rs2015172
rs1326854
rs2717666
rs4770315
rs62191117
rs1153990
rs4687899
rs320206
rs1995381
rs10778463
rs77099633
rs112135816
rs7226573
rs139997617
rs3863942
rs74532514
rs10503284
rs117294145
rs3922732
rs67737557
rs1411737
rs73296001
rs1771011
rs4033856
rs9964679
rs17000015
rs12201996
rs2387629
All - MR Egger
All - Inverse variance weighted
-0.0050
-0.0025
0.0000
0.0025
0.0050
MR effect size for
' || id:ebi-a-GCST90016926' on 'Liver cell carcinoma || id:ieu-b-4953'

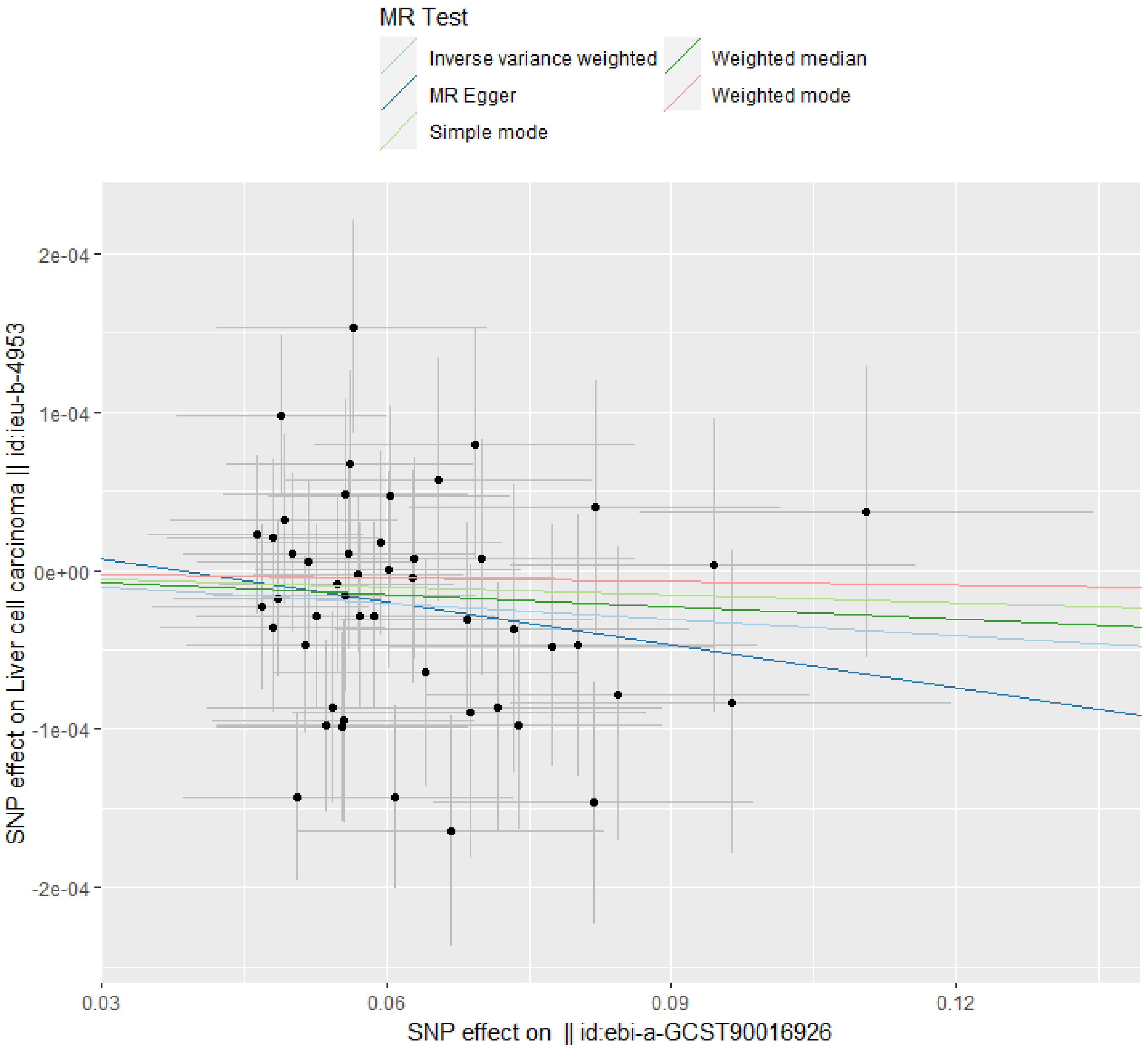

MR Test
Inverse variance weighted
MR Egger
Simple mode
Weighted median
Weighted mode
2e-04
1e-04
0e+00
-1e-04
-2e-04
SNP effect on Liver cell carcinoma || id:ieu-b-4953
0.03
0.06
0.09
0.12
SNP effect on || id:ebi-a-GCST90016926

Figure 180 Leave-one-out analysis, funnel plot, MR effect size and scatter plot for gut microbiota abundance (genus Actinomyces id.423) on liver cell carcinoma

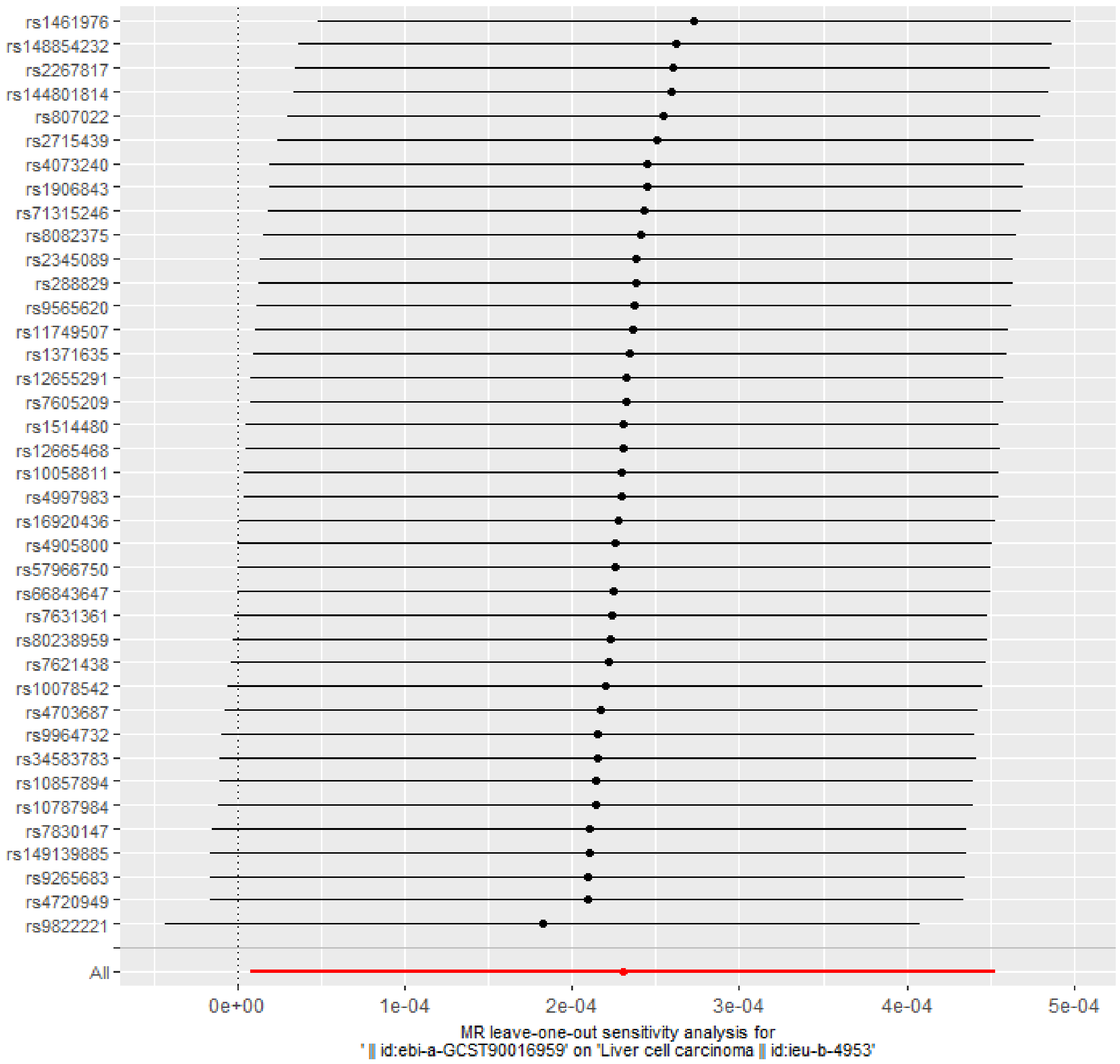

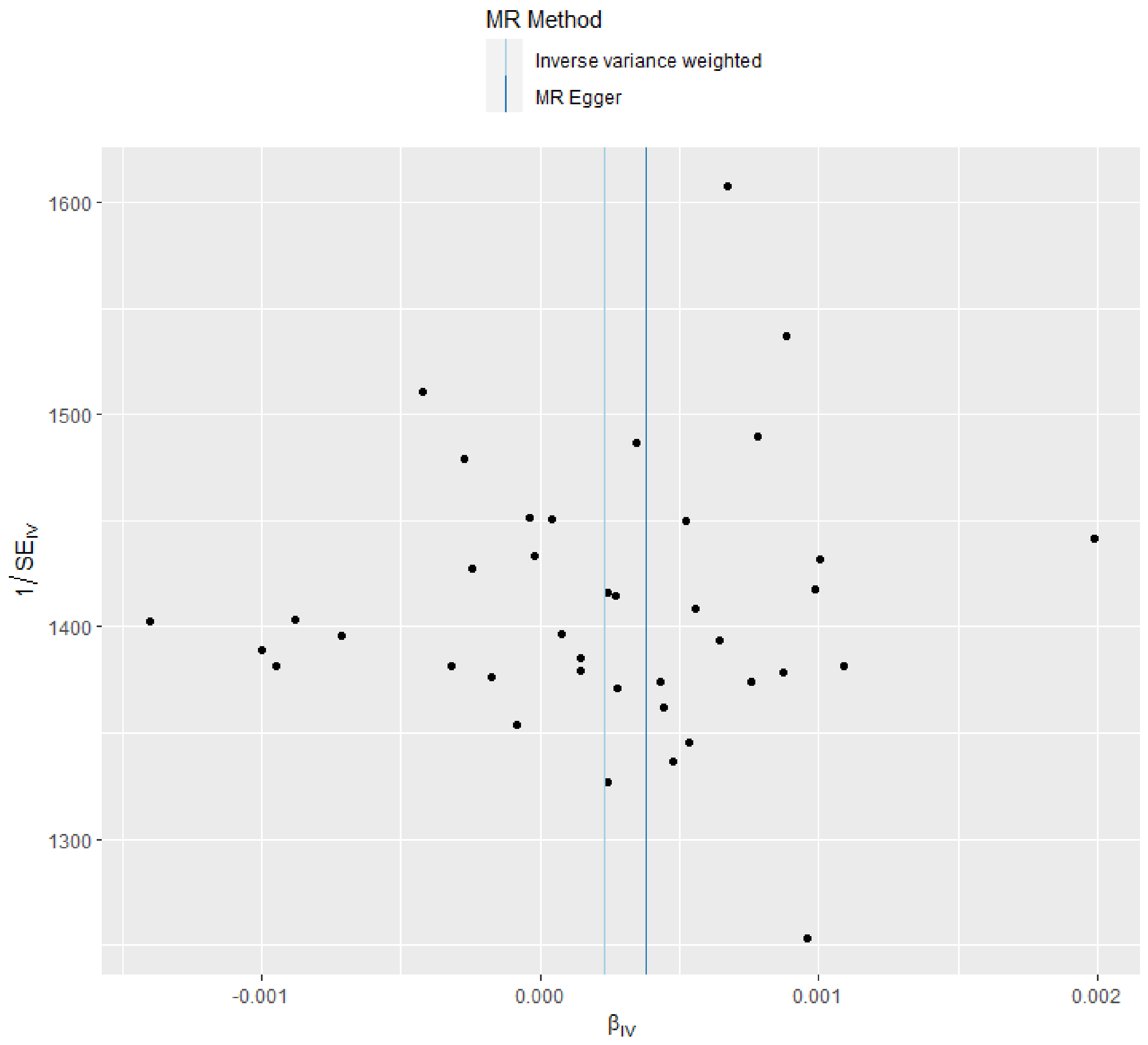

MR Method
Inverse variance weighted
MR Egger
1600
1500
1400
1300
-0.001
0.000
0.001
0.002
β_{IV}
$1/SE_{IV}$

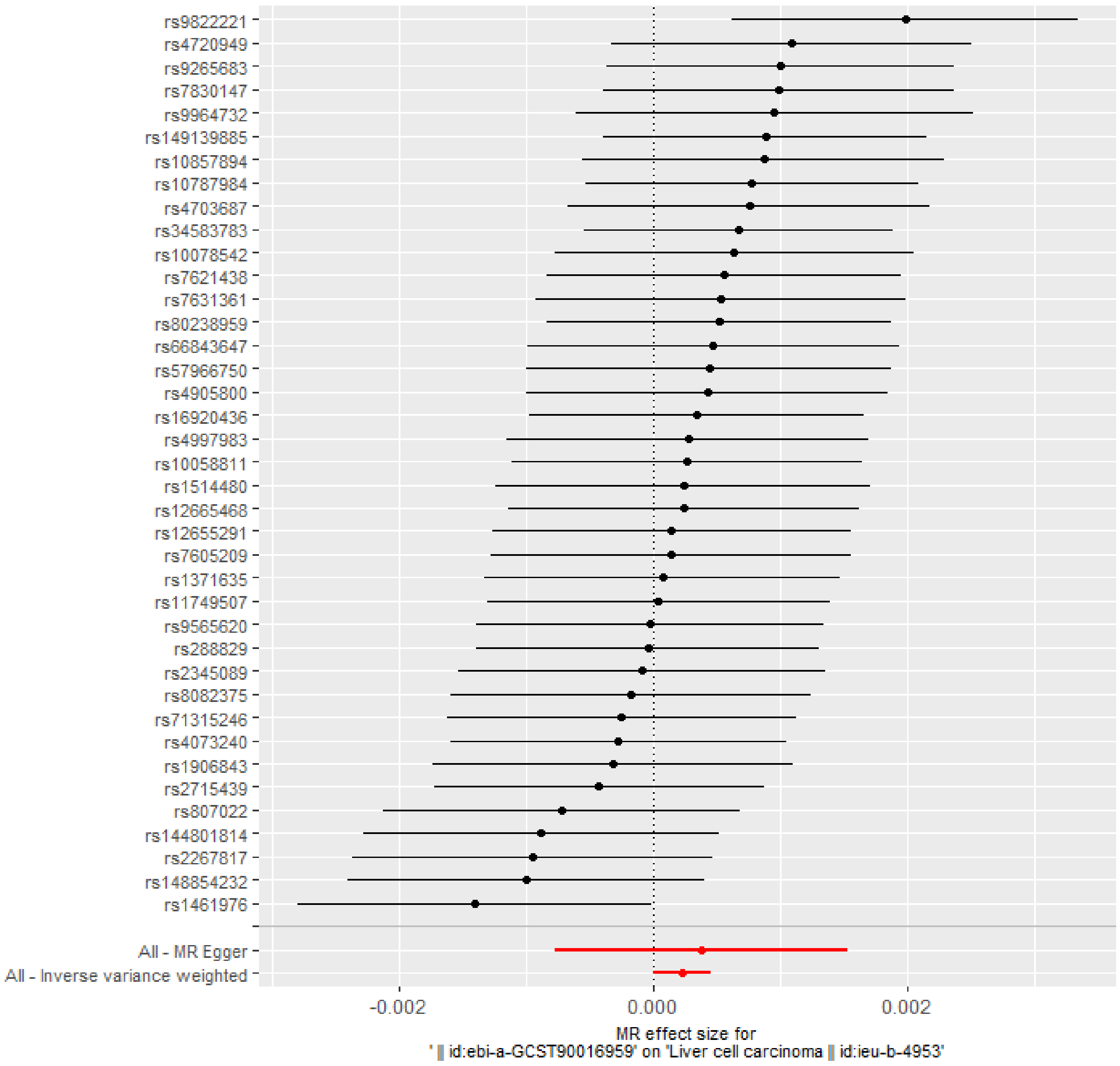

rs9822221
rs4720949
rs9265683
rs7830147
rs9964732
rs149139885
rs10857894
rs10787984
rs4703687
rs34583783
rs10078542
rs7621438
rs7631361
rs80238959
rs66843647
rs57966750
rs4905800
rs16920436
rs4997983
rs10058811
rs1514480
rs12665468
rs12655291
rs7605209
rs1371635
rs11749507
rs9565620
rs288829
rs2345089
rs8082375
rs71315246
rs4073240
rs1906843
rs2715439
rs807022
rs144801814
rs2267817
rs148854232
rs1461976
All - MR Egger
All - Inverse variance weighted
-0.002
0.000
0.002
MR effect size for
' || id:ebi-a-GCST90016959' on 'Liver cell carcinoma || id:ieu-b-4953'

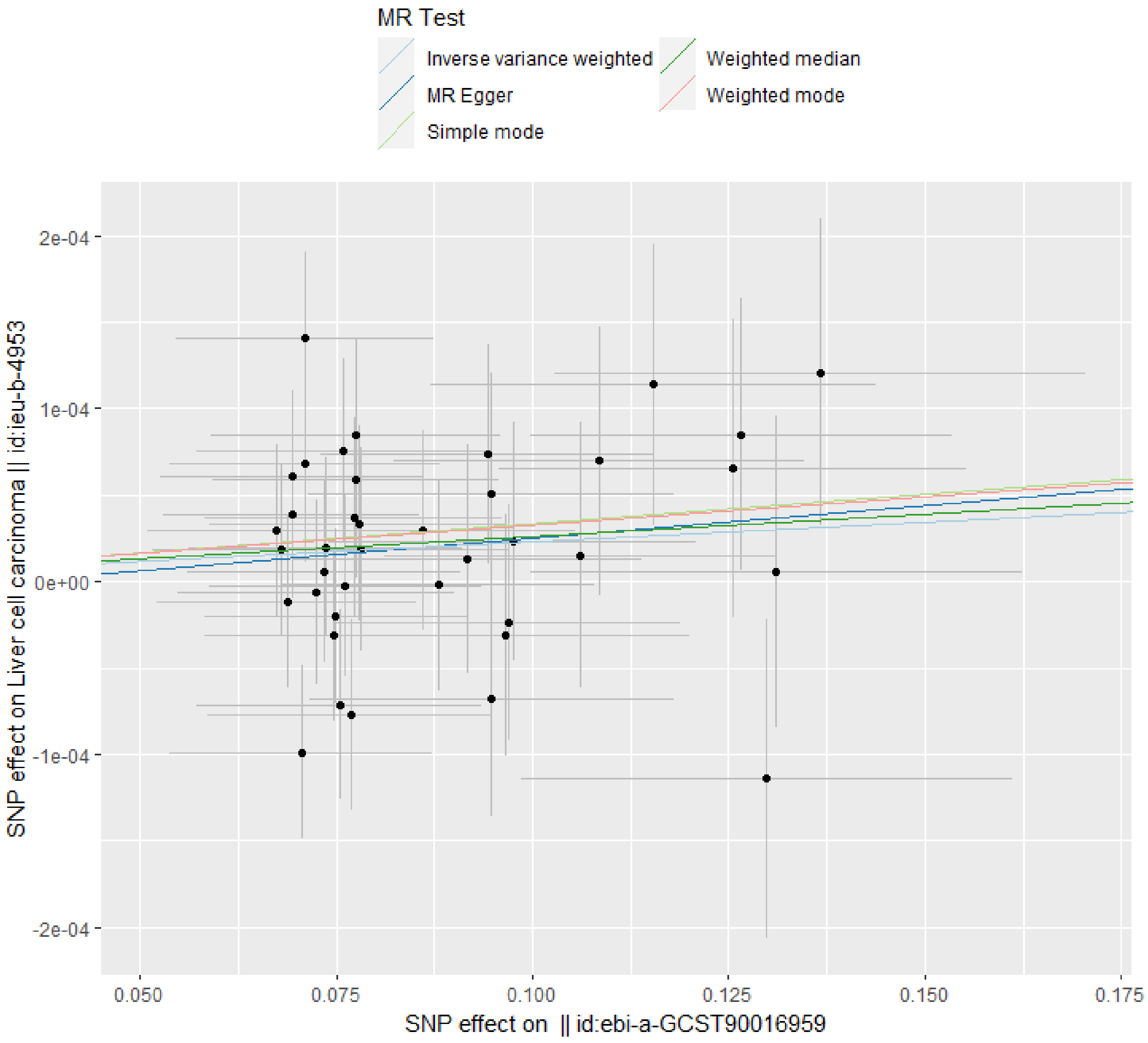
MR Test
Inverse variance weighted
MR Egger
Simple mode
Weighted median
Weighted mode
2e-04
1e-04
0e+00
-1e-04
-2e-04
0.050
0.075
0.100
0.125
0.150
0.175
SNP effect on Liver cell carcinoma || id:ieu-b-4953
SNP effect on || id:ebi-a-GCST90016959

Figure 181 Leave-one-out analysis, funnel plot, MR effect size and scatter plot for gut microbiota abundance (genus Butyricicoccus id.2055) on liver cell carcinoma

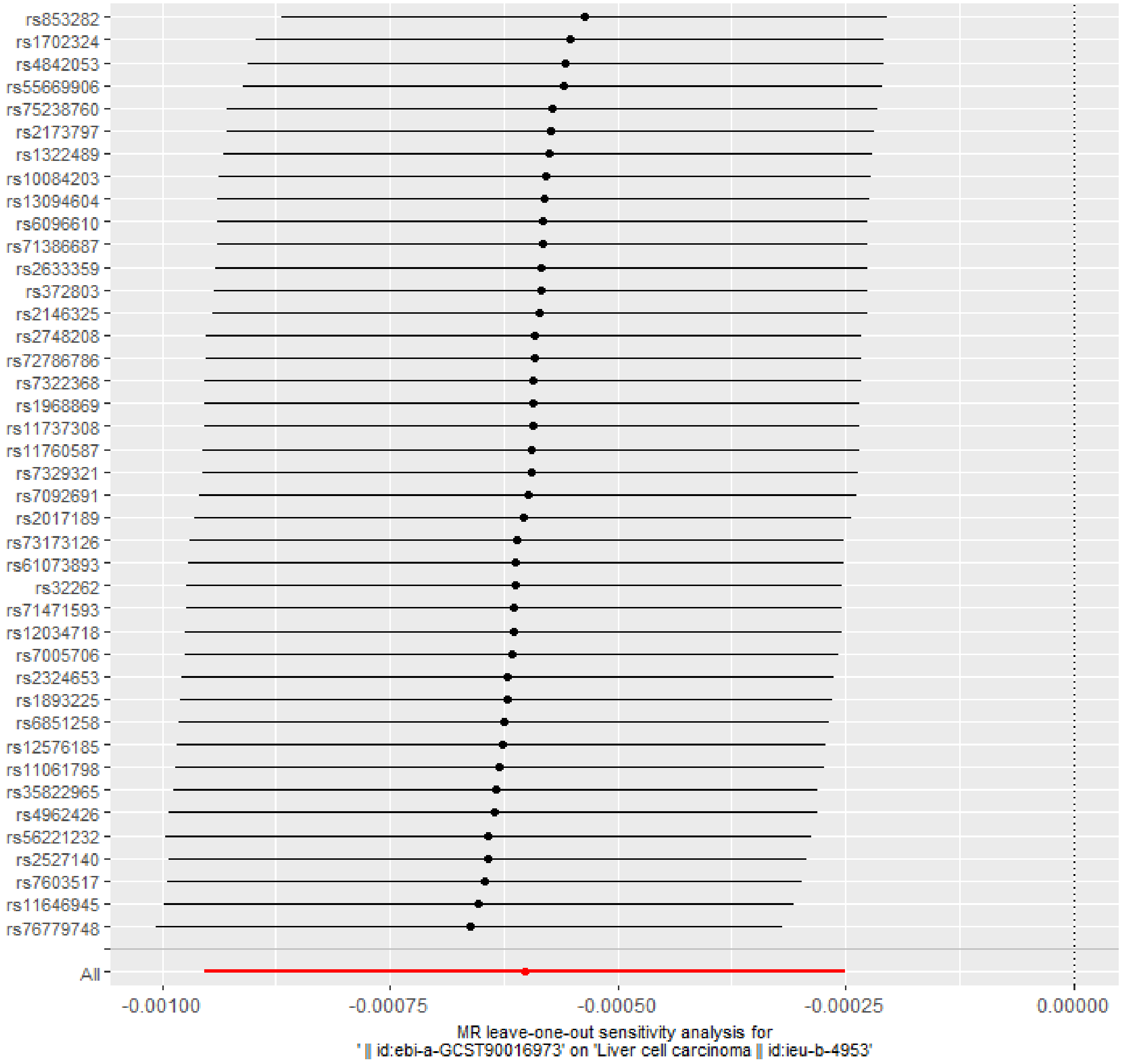

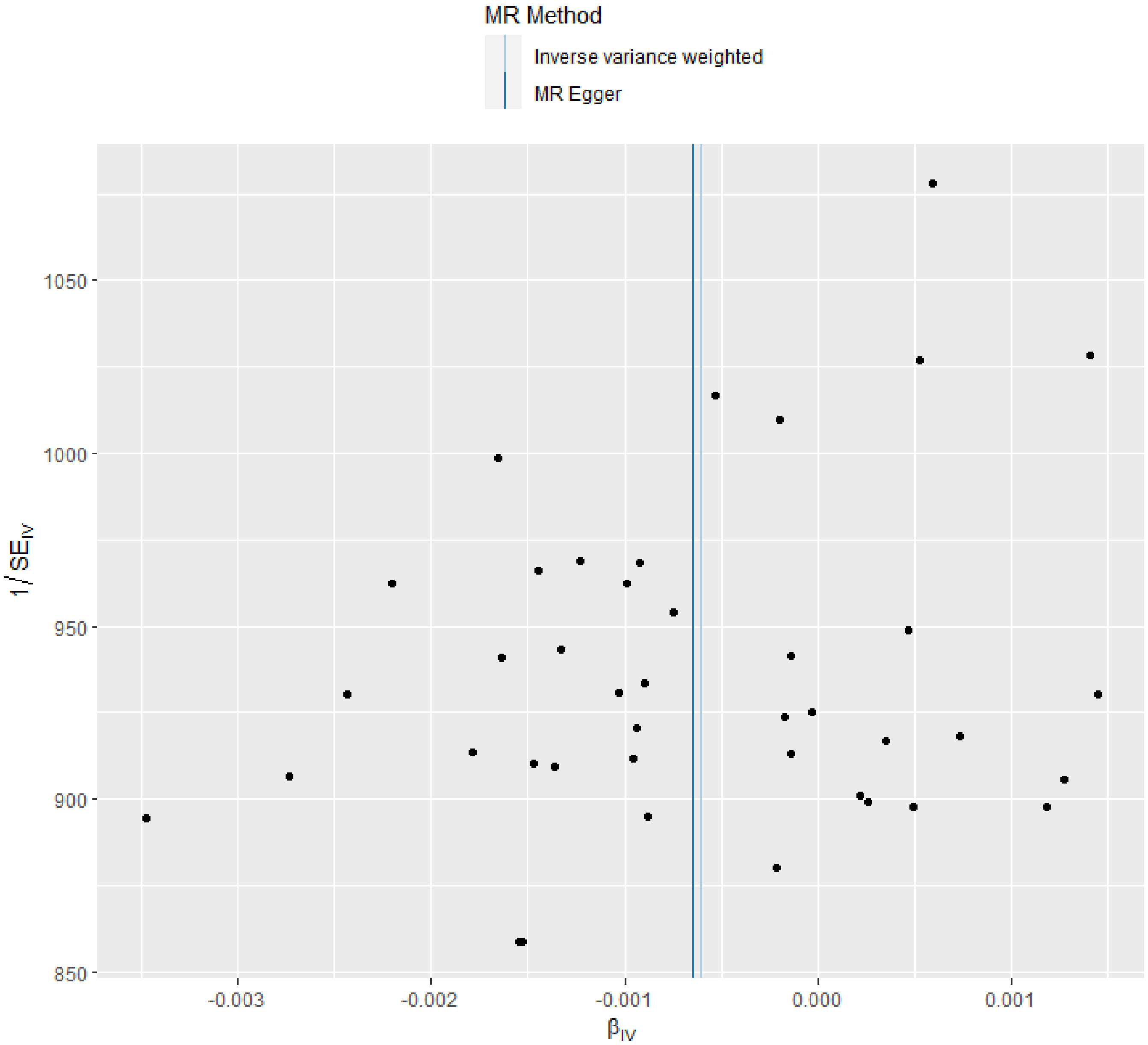
MR Method
Inverse variance weighted
MR Egger
1050
1000
950
900
850
-0.003
-0.002
-0.001
0.000
0.001
β_{IV}
$1/SE_{IV}$

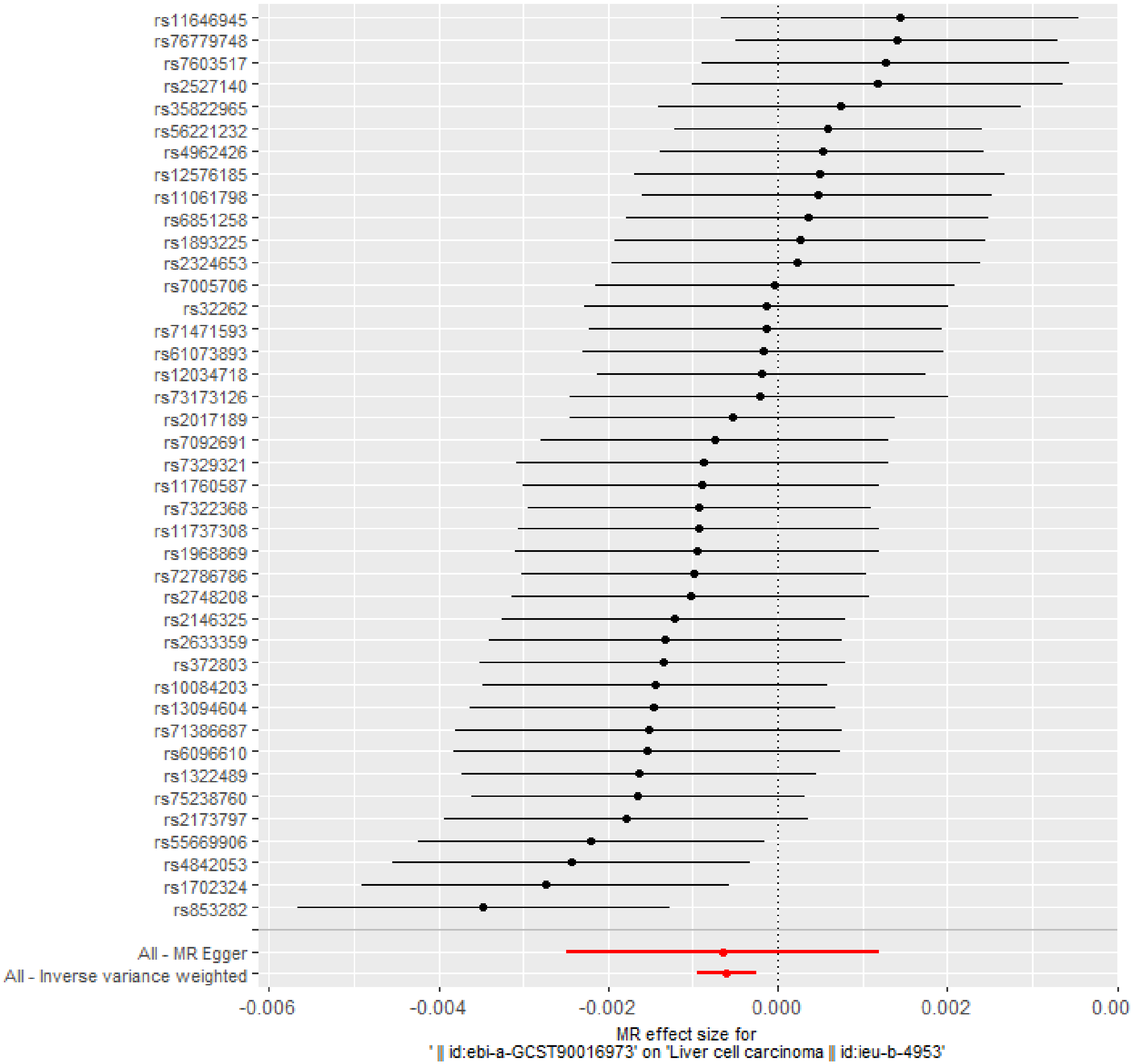
rs11646945
rs76779748
rs7603517
rs2527140
rs35822965
rs56221232
rs4962426
rs12576185
rs11061798
rs6851258
rs1893225
rs2324653
rs7005706
rs32262
rs71471593
rs61073893
rs12034718
rs73173126
rs2017189
rs7092691
rs7329321
rs11760587
rs7322368
rs11737308
rs1968869
rs72786786
rs2748208
rs2146325
rs2633359
rs372803
rs10084203
rs13094604
rs71386687
rs6096610
rs1322489
rs75238760
rs2173797
rs55669906
rs4842053
rs1702324
rs853282
All - MR Egger
All - Inverse variance weighted
-0.006
-0.004
-0.002
0.000
0.002
0.00
MR effect size for
' || id:ebi-a-GCST90016973' on 'Liver cell carcinoma || id:ieu-b-4953'

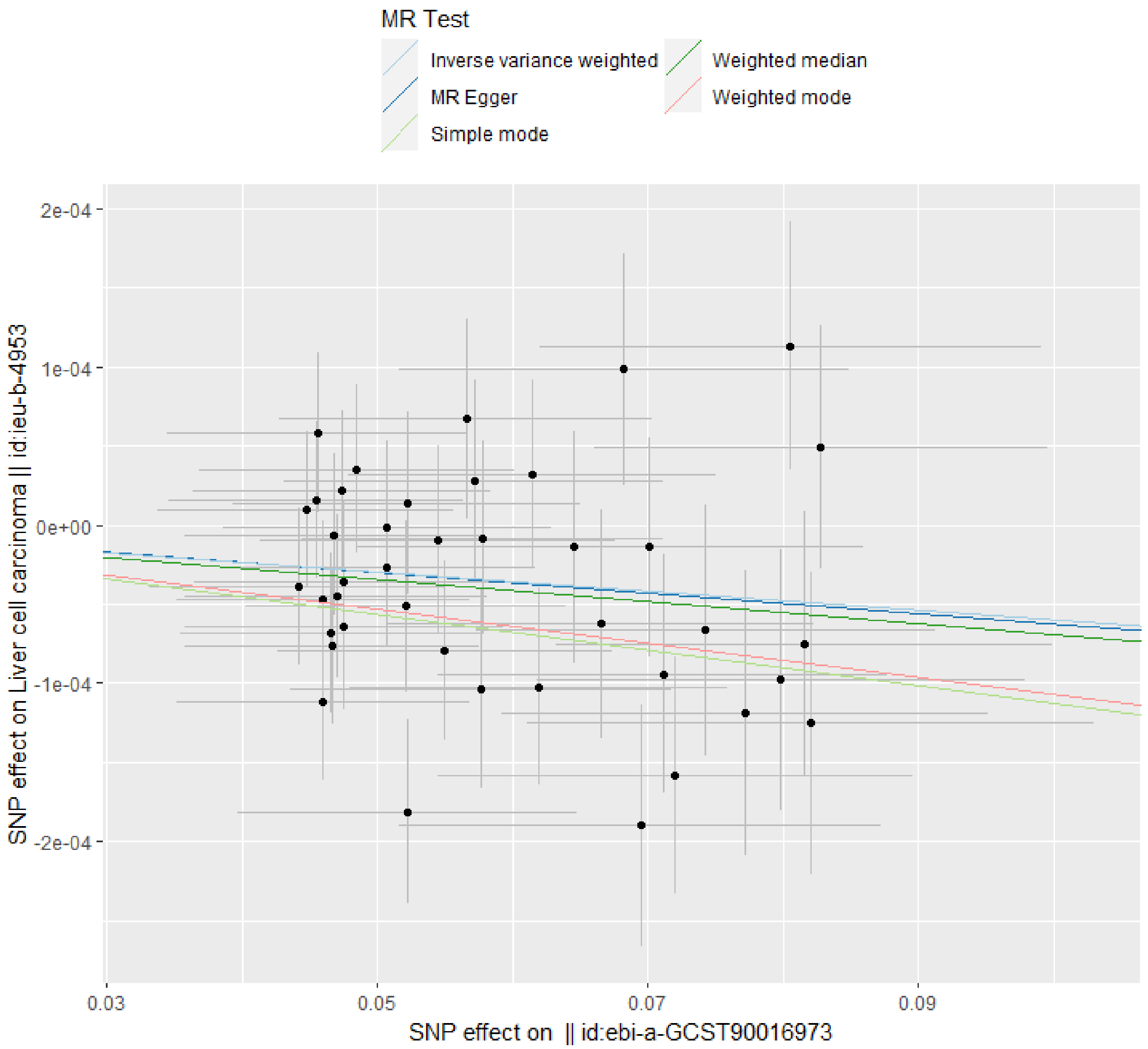
MR Test
Inverse variance weighted
MR Egger
Simple mode
Weighted median
Weighted mode
2e-04
1e-04
0e+00
-1e-04
-2e-04
SNP effect on Liver cell carcinoma || id:ieu-b-4953
0.03
0.05
0.07
0.09
SNP effect on || id:ebi-a-GCST90016973

Figure 182 Leave-one-out analysis, funnel plot, MR effect size and scatter plot for gut microbiota abundance (genus Candidatus Soleaferrea id.11350) on liver cell carcinoma

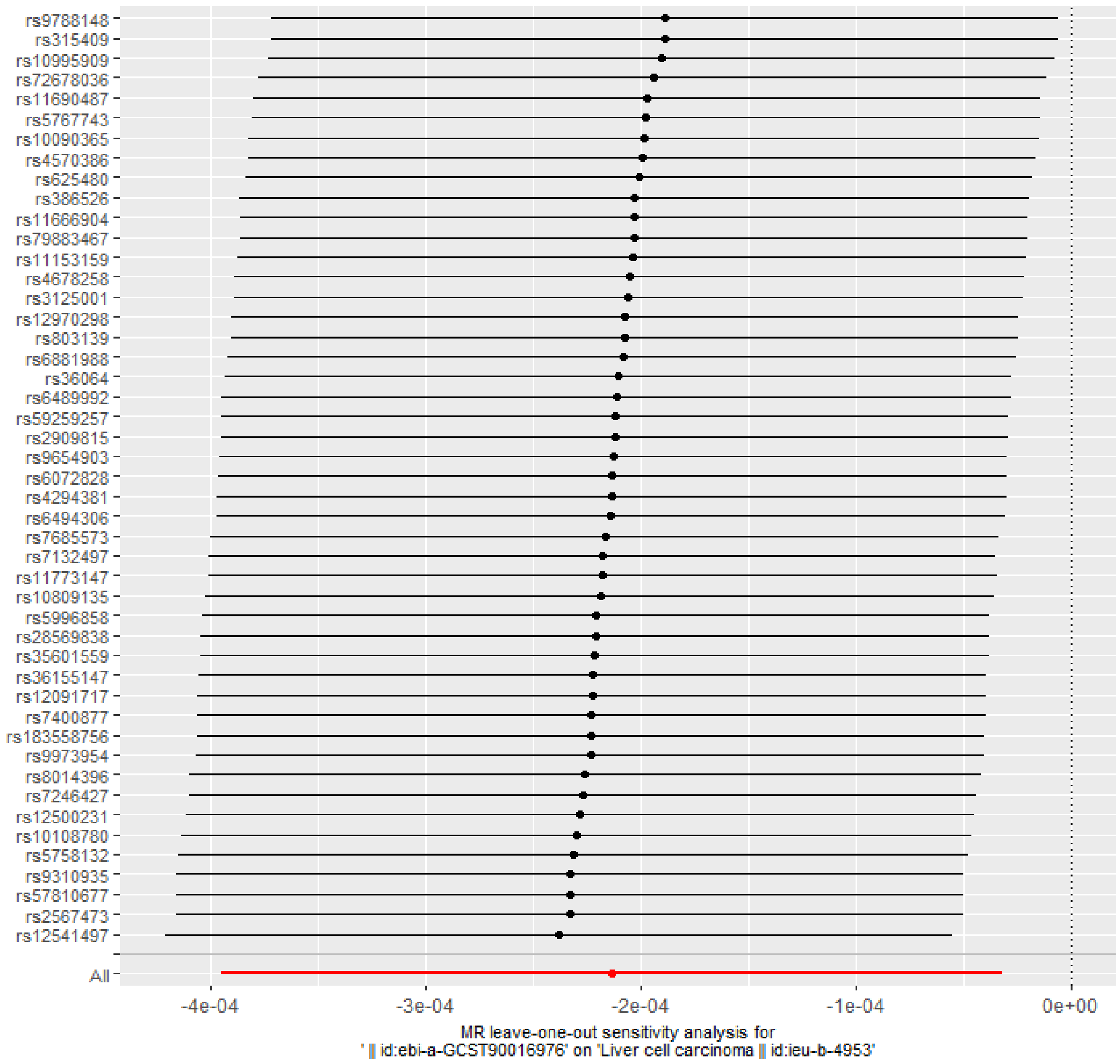

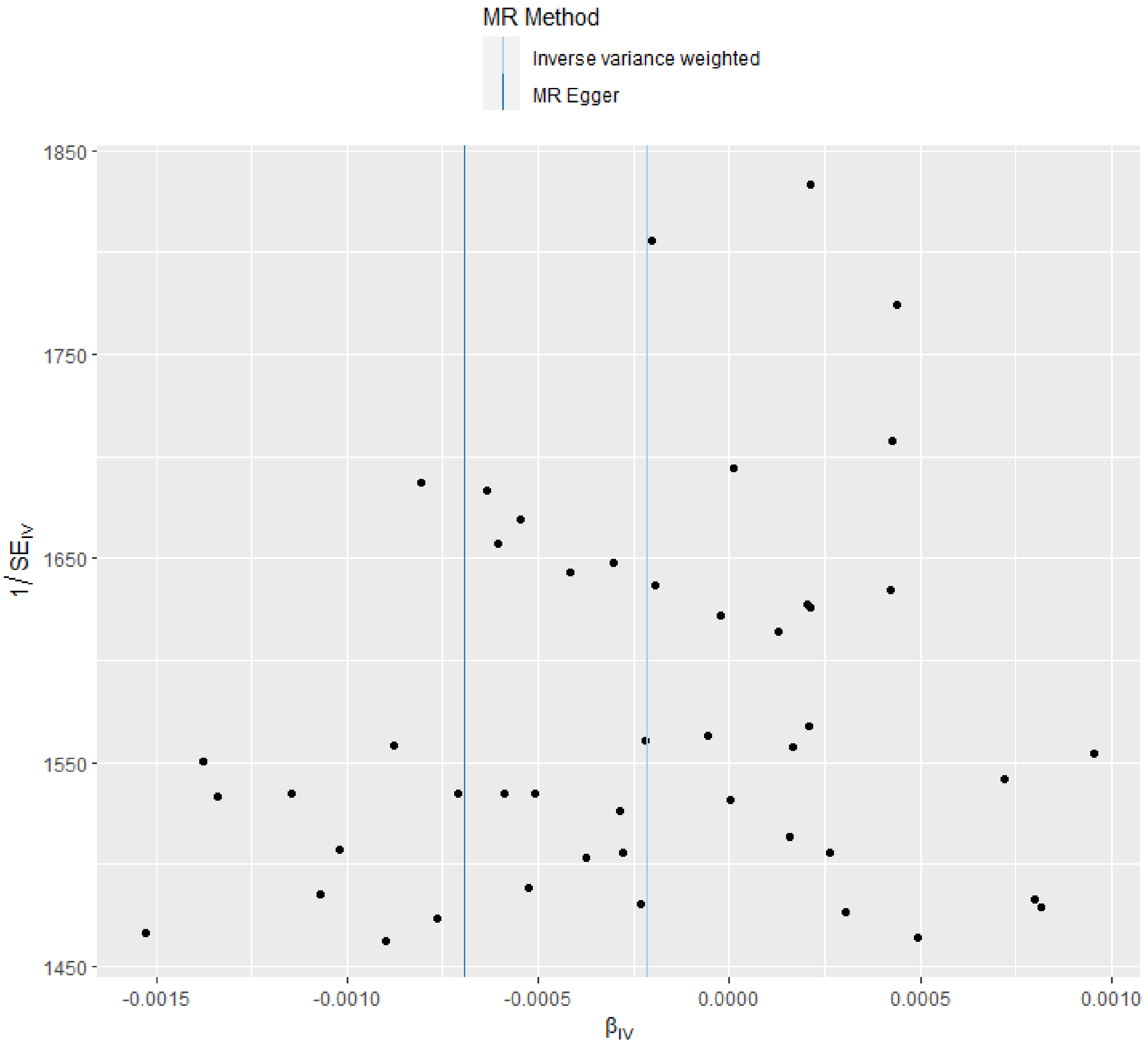

MR Method
Inverse variance weighted
MR Egger
1850
1750
1650
1550
1450
1/SE_IV
-0.0015
-0.0010
-0.0005
0.0000
0.0005
0.0010
β_IV

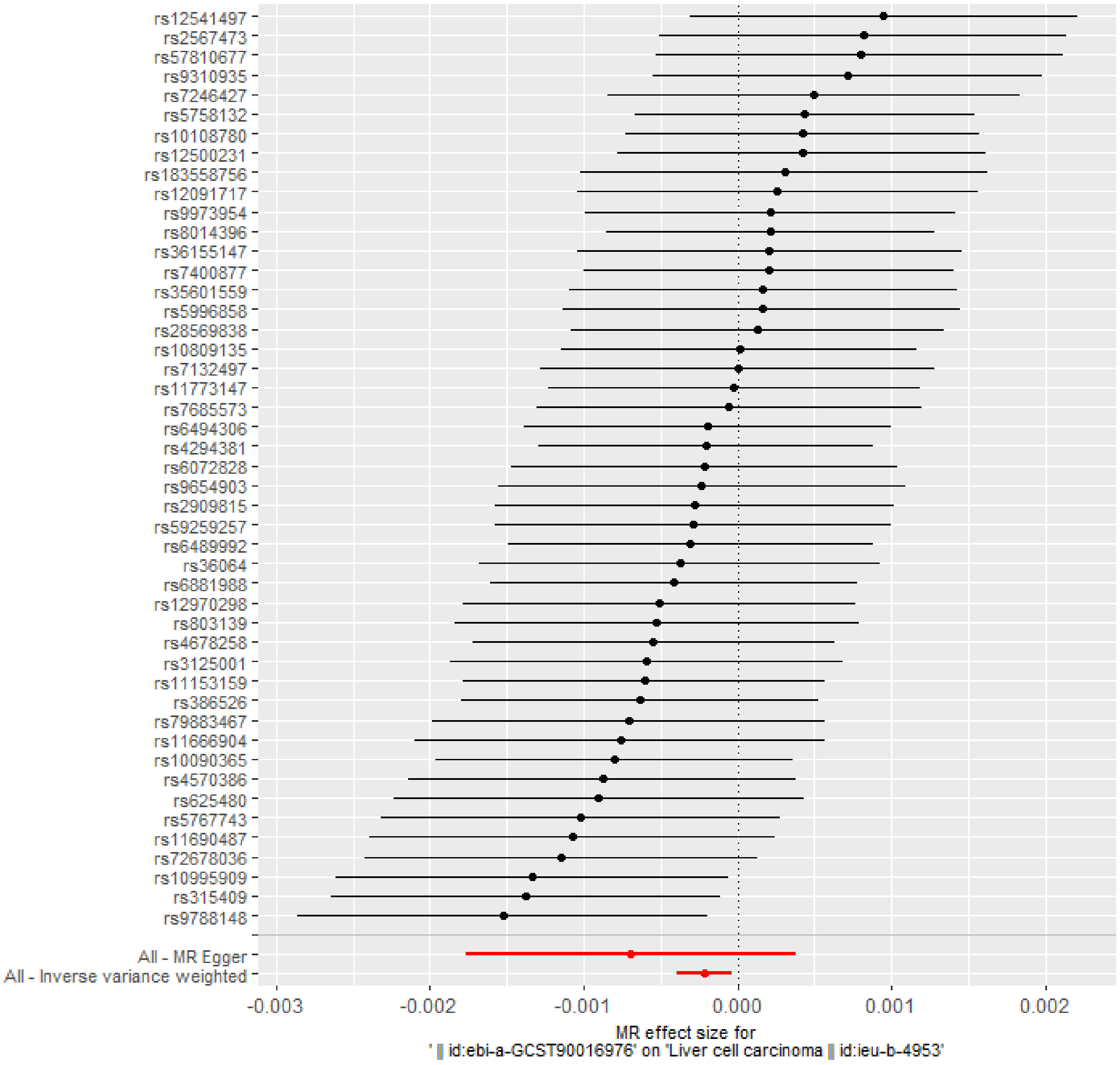
rs12541497
rs2567473
rs57810677
rs9310935
rs7246427
rs5758132
rs10108780
rs12500231
rs183558756
rs12091717
rs9973954
rs8014396
rs36155147
rs7400877
rs35601559
rs5996858
rs28569838
rs10809135
rs7132497
rs11773147
rs7685573
rs6494306
rs4294381
rs6072828
rs9654903
rs2909815
rs59259257
rs6489992
rs36064
rs6881988
rs12970298
rs803139
rs4678258
rs3125001
rs11153159
rs386526
rs79883467
rs11666904
rs10090365
rs4570386
rs625480
rs5767743
rs11690487
rs72678036
rs10995909
rs315409
rs9788148
All - MR Egger
All - Inverse variance weighted
-0.003
-0.002
-0.001
0.000
0.001
0.002
MR effect size for
' || id:ebi-a-GCST90016976' on 'Liver cell carcinoma || id:ieu-b-4953'

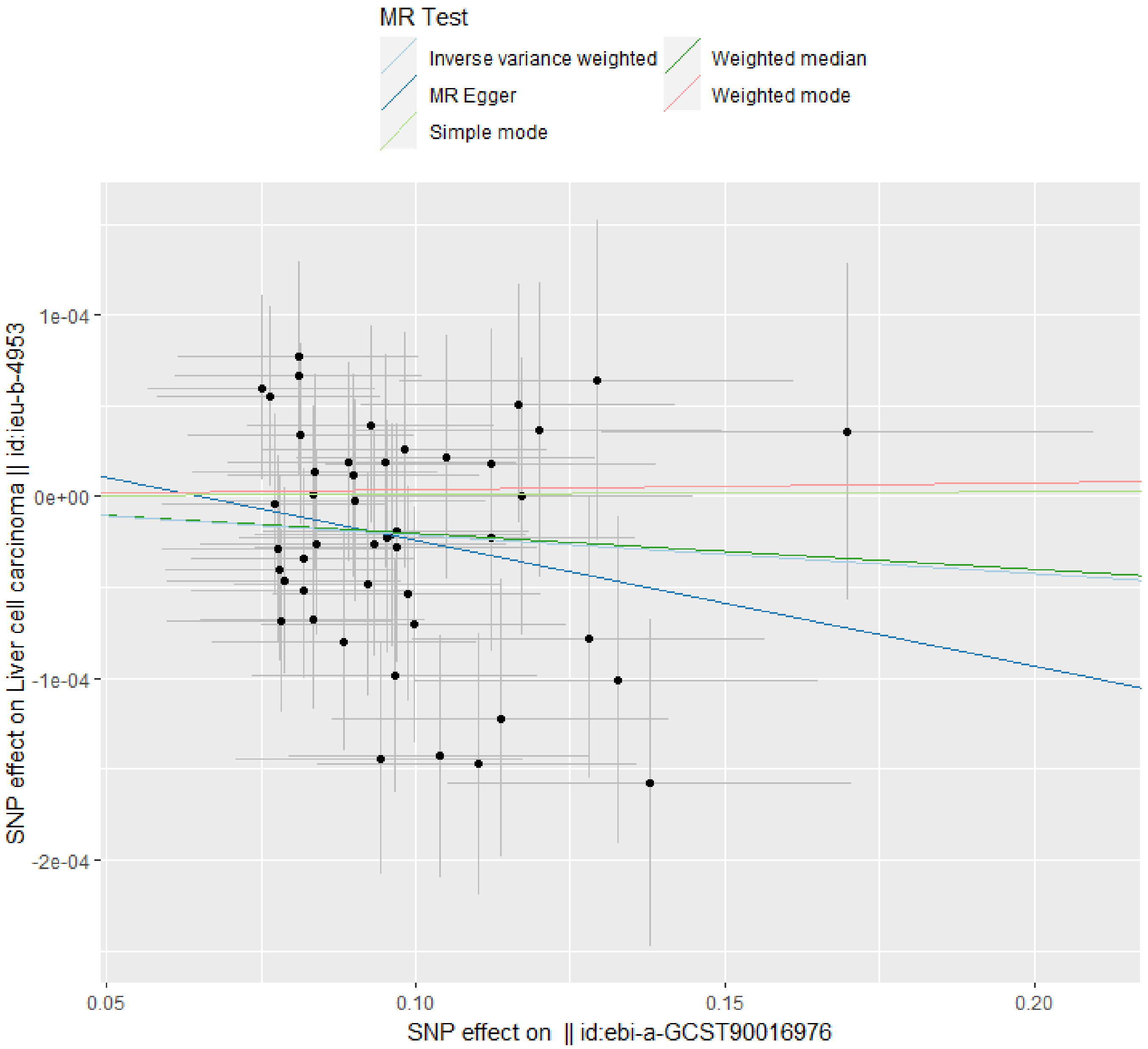
MR Test
Inverse variance weighted
MR Egger
Simple mode
Weighted median
Weighted mode
1e-04
0e+00
-1e-04
-2e-04
SNP effect on Liver cell carcinoma || id:ieu-b-4953
0.05
0.10
0.15
0.20
SNP effect on || id:ebi-a-GCST90016976

Figure 183 Leave-one-out analysis, funnel plot, MR effect size and scatter plot for gut microbiota abundance (genus Christensenellaceae R 7group id.11283) on liver cell carcinoma

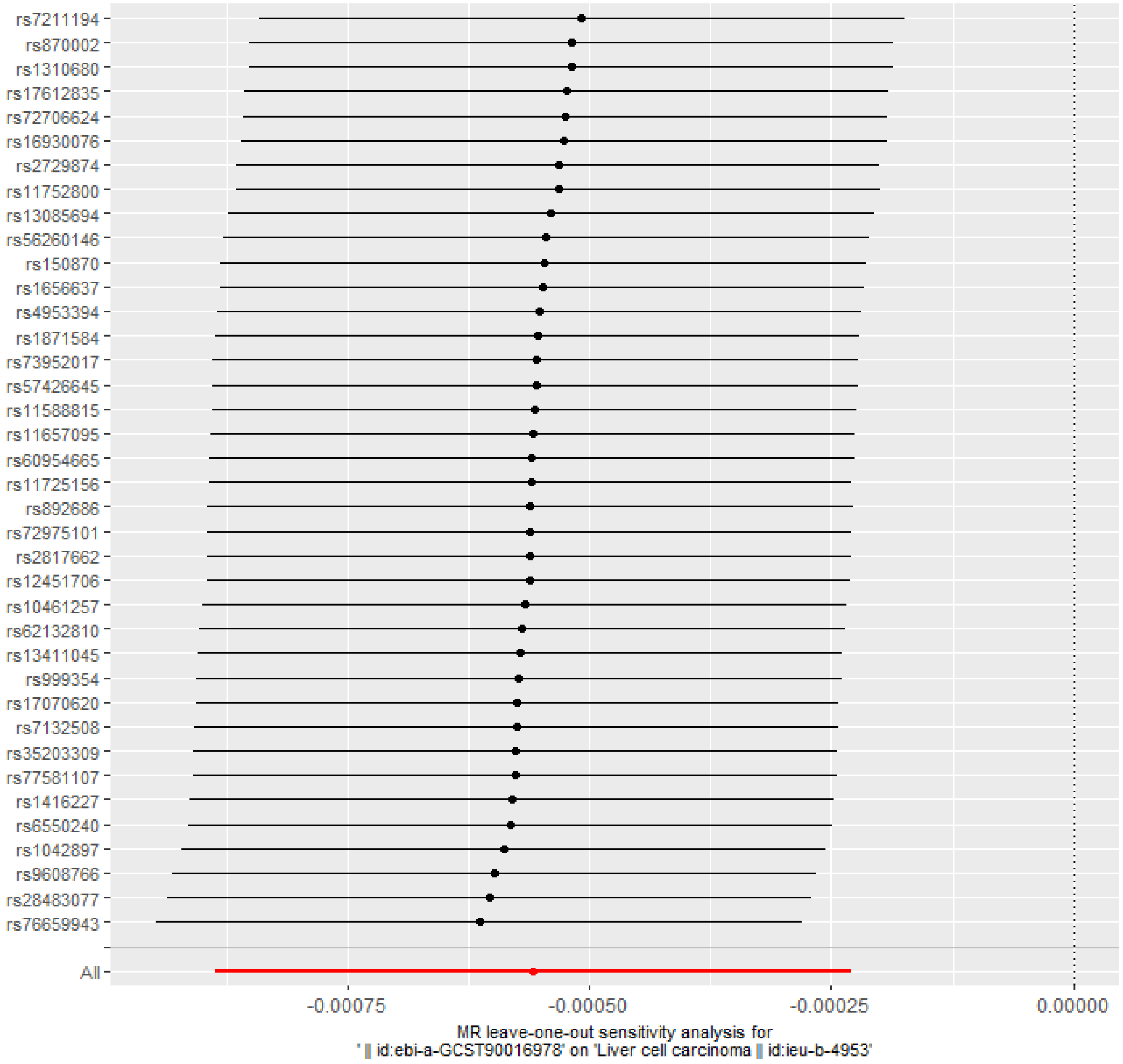

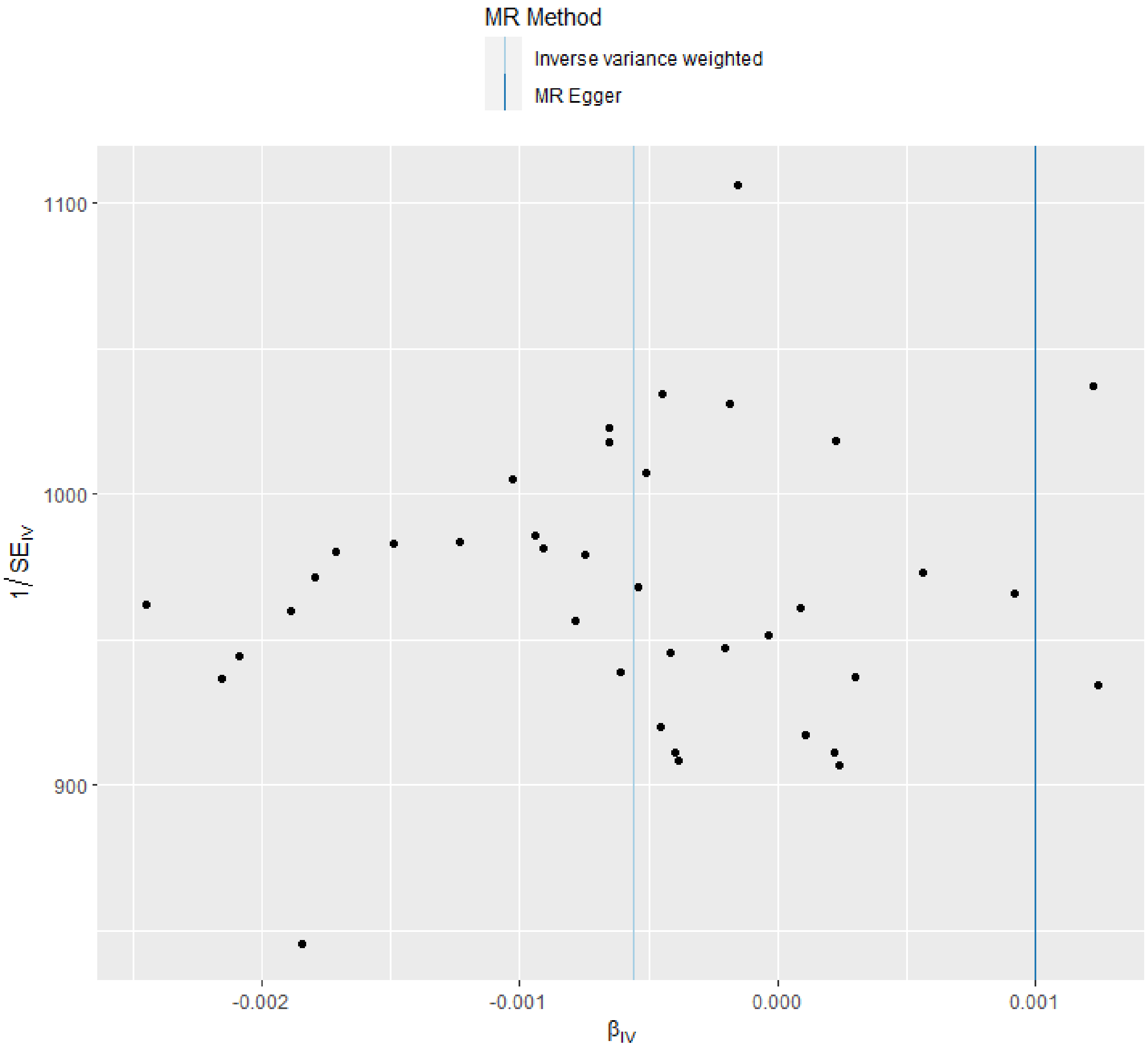
MR Method
Inverse variance weighted
MR Egger
1100
1000
900
-0.002
-0.001
0.000
0.001
β_{IV}
$1/SE_{IV}$

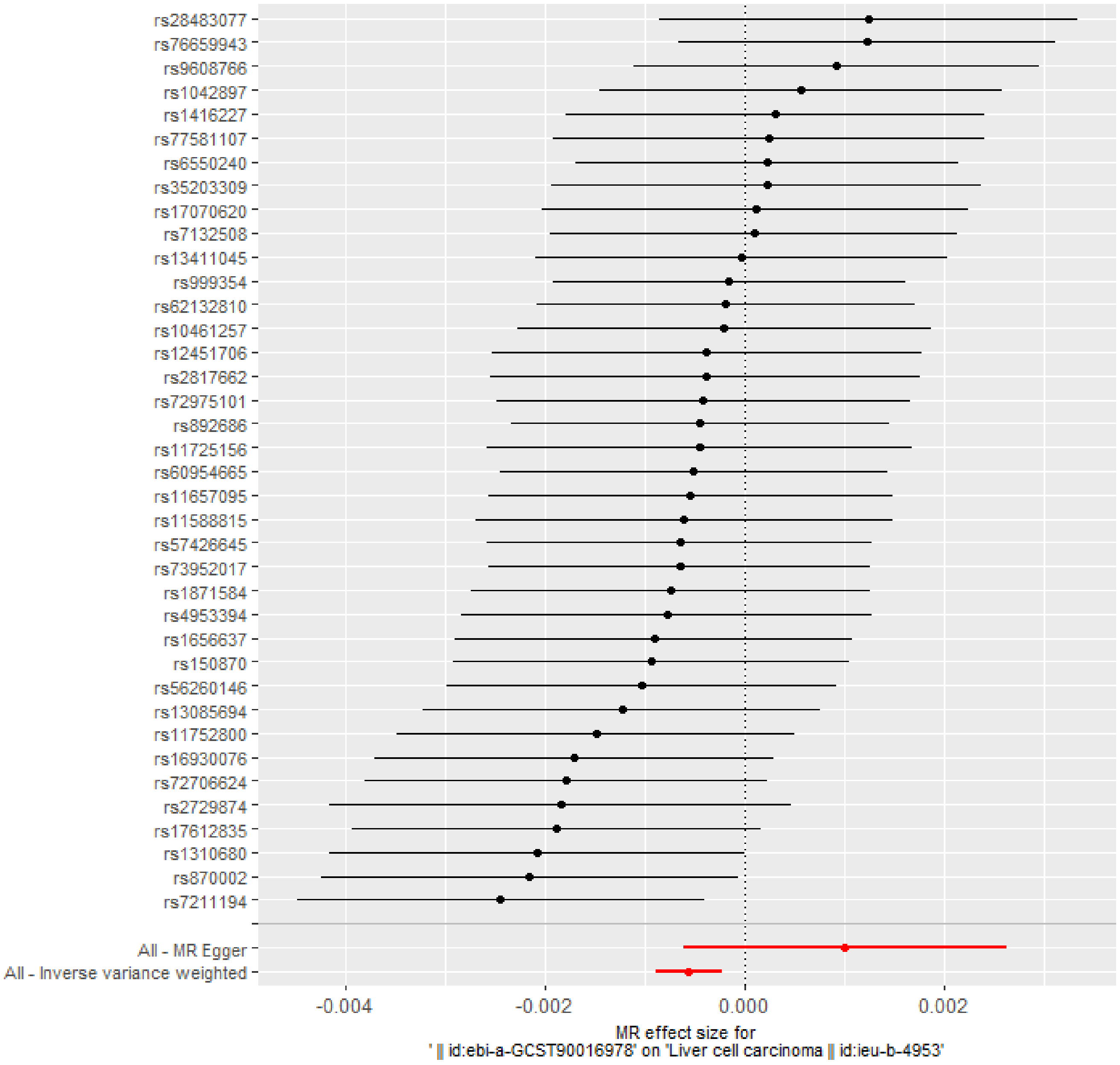

rs28483077
rs76659943
rs9608766
rs1042897
rs1416227
rs77581107
rs6550240
rs35203309
rs17070620
rs7132508
rs13411045
rs999354
rs62132810
rs10461257
rs12451706
rs2817662
rs72975101
rs892686
rs11725156
rs60954665
rs11657095
rs11588815
rs57426645
rs73952017
rs1871584
rs4953394
rs1656637
rs150870
rs56260146
rs13085694
rs11752800
rs16930076
rs72706624
rs2729874
rs17612835
rs1310680
rs870002
rs7211194
All - MR Egger
All - Inverse variance weighted
-0.004
-0.002
0.000
0.002
MR effect size for
' || id:ebi-a-GCST90016978' on 'Liver cell carcinoma || id:ieu-b-4953'

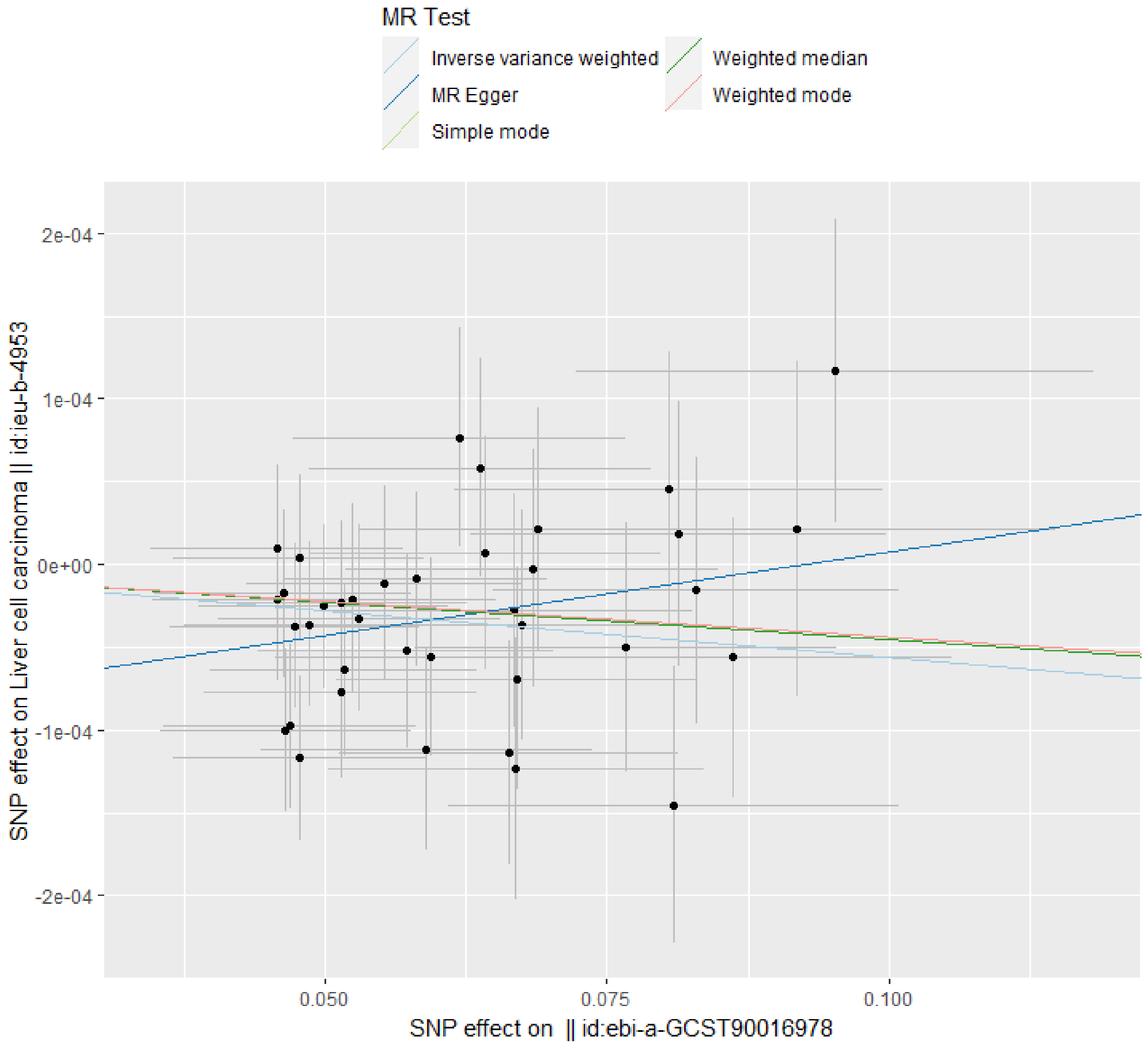
MR Test
Inverse variance weighted
MR Egger
Simple mode
Weighted median
Weighted mode
2e-04
1e-04
0e+00
-1e-04
-2e-04
SNP effect on Liver cell carcinoma || id:ieu-b-4953
0.050
0.075
0.100
SNP effect on || id:ebi-a-GCST90016978

Figure 184 Leave-one-out analysis, funnel plot, MR effect size and scatter plot for gut microbiota abundance (genus Eubacterium rectale group id.14374) on liver cell carcinoma

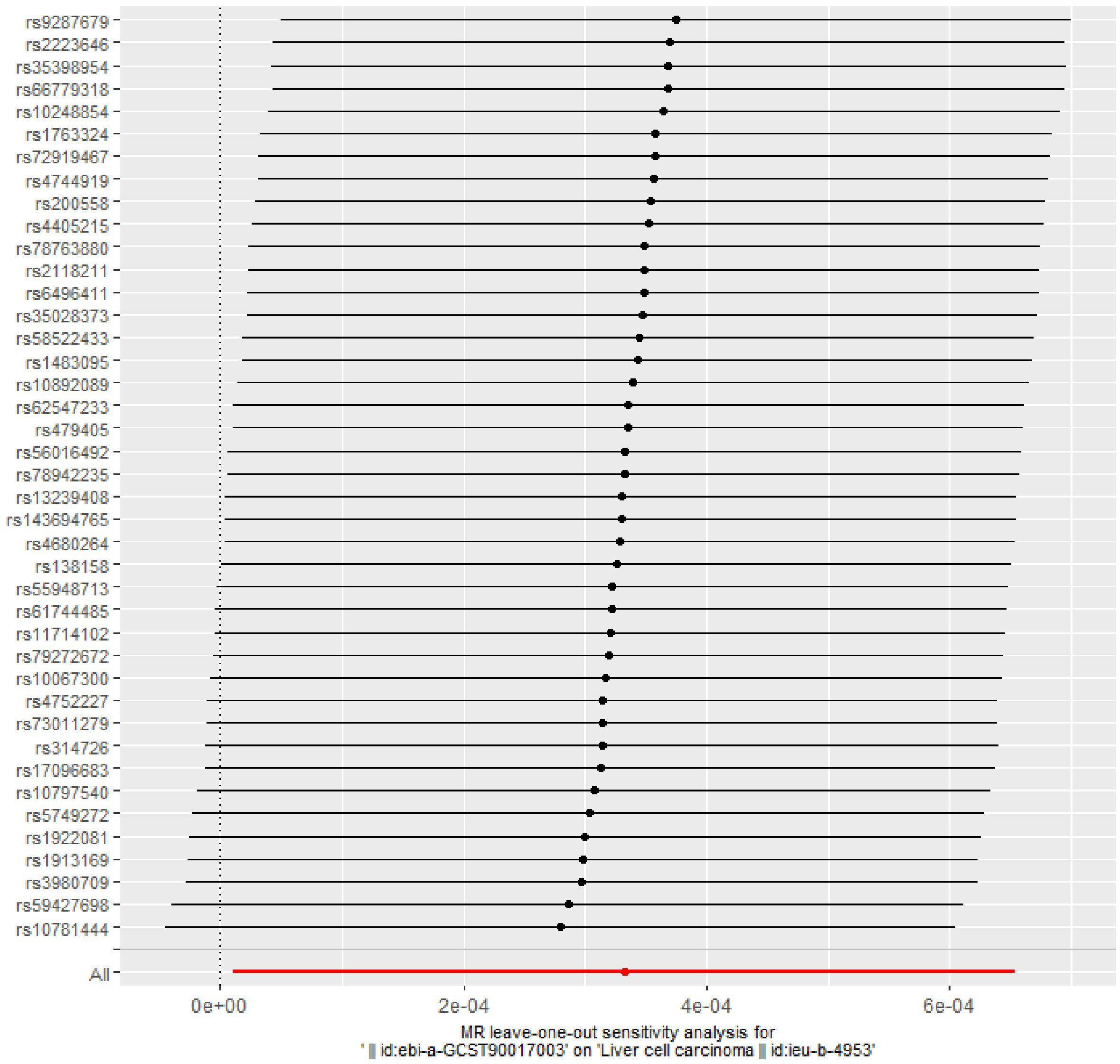

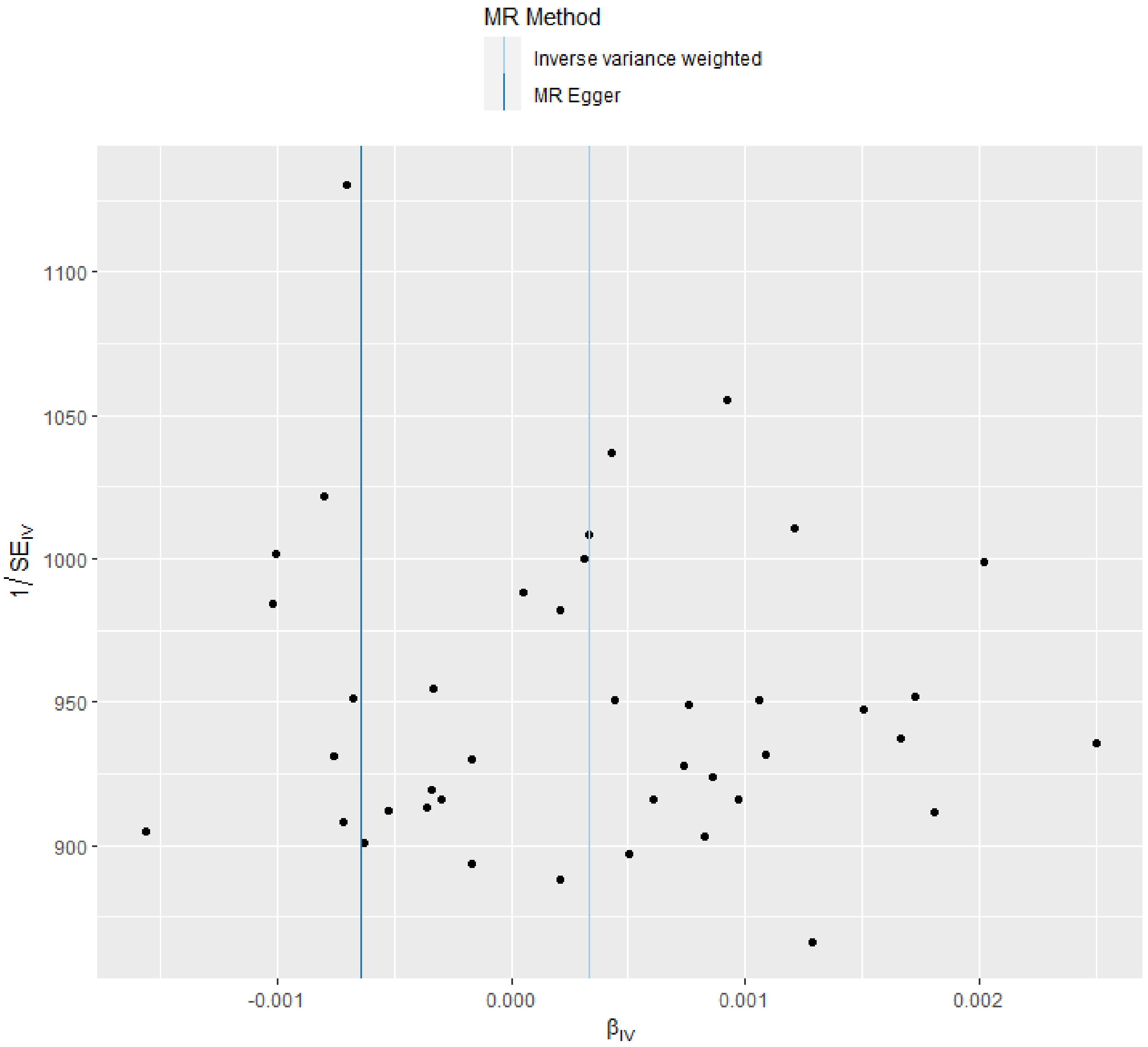
MR Method
Inverse variance weighted
MR Egger
1100
1050
1000
950
900
-0.001
0.000
0.001
0.002
$1/SE_{IV}$
β_{IV}

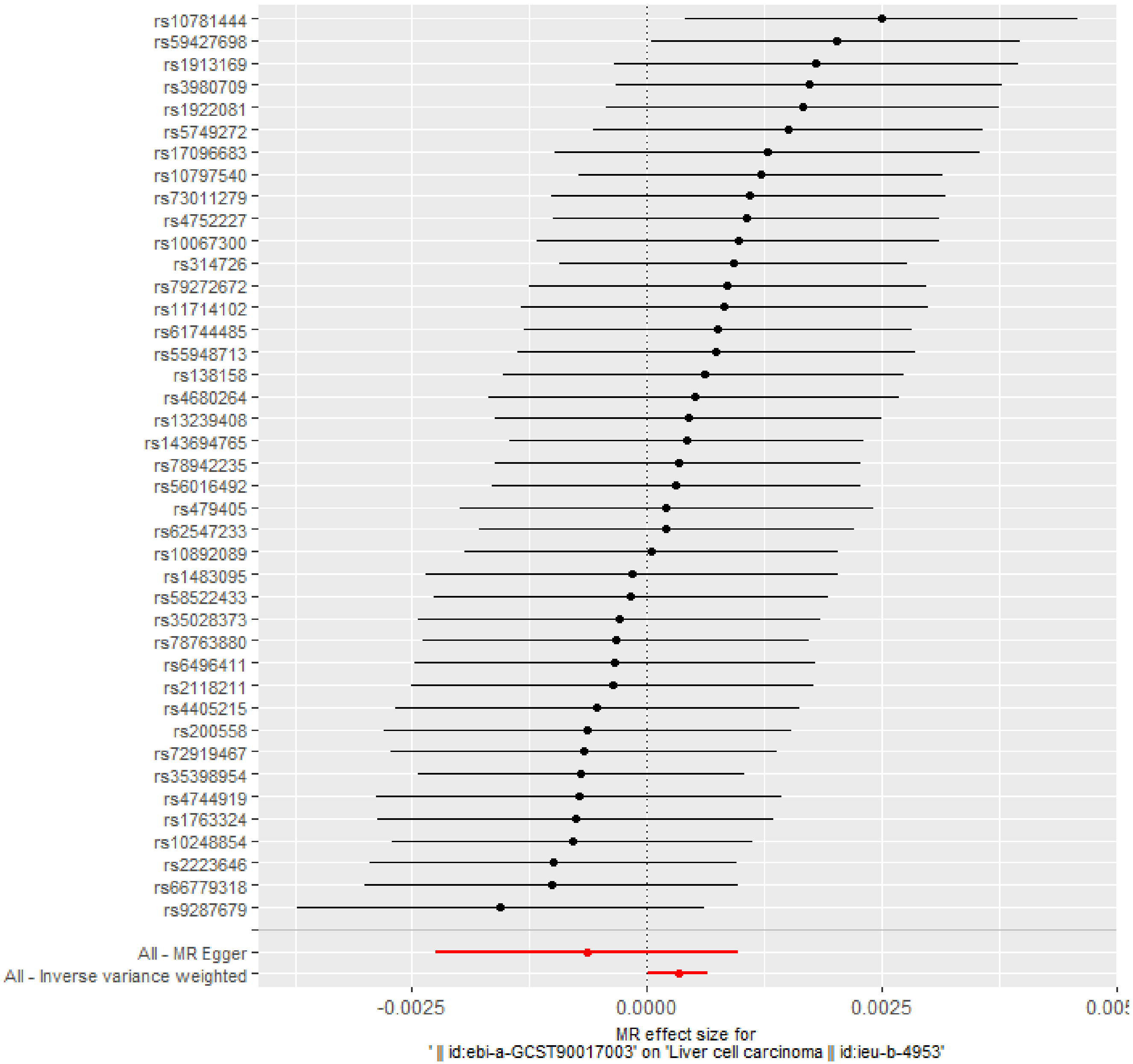
rs10781444
rs59427698
rs1913169
rs3980709
rs1922081
rs5749272
rs17096683
rs10797540
rs73011279
rs4752227
rs10067300
rs314726
rs79272672
rs11714102
rs61744485
rs55948713
rs138158
rs4680264
rs13239408
rs143694765
rs78942235
rs56016492
rs479405
rs62547233
rs10892089
rs1483095
rs58522433
rs35028373
rs78763880
rs6496411
rs2118211
rs4405215
rs200558
rs72919467
rs35398954
rs4744919
rs1763324
rs10248854
rs2223646
rs66779318
rs9287679
All - MR Egger
All - Inverse variance weighted
-0.0025
0.0000
0.0025
MR effect size for
' || id:ebi-a-GCST90017003' on 'Liver cell carcinoma || id:ieu-b-4953'

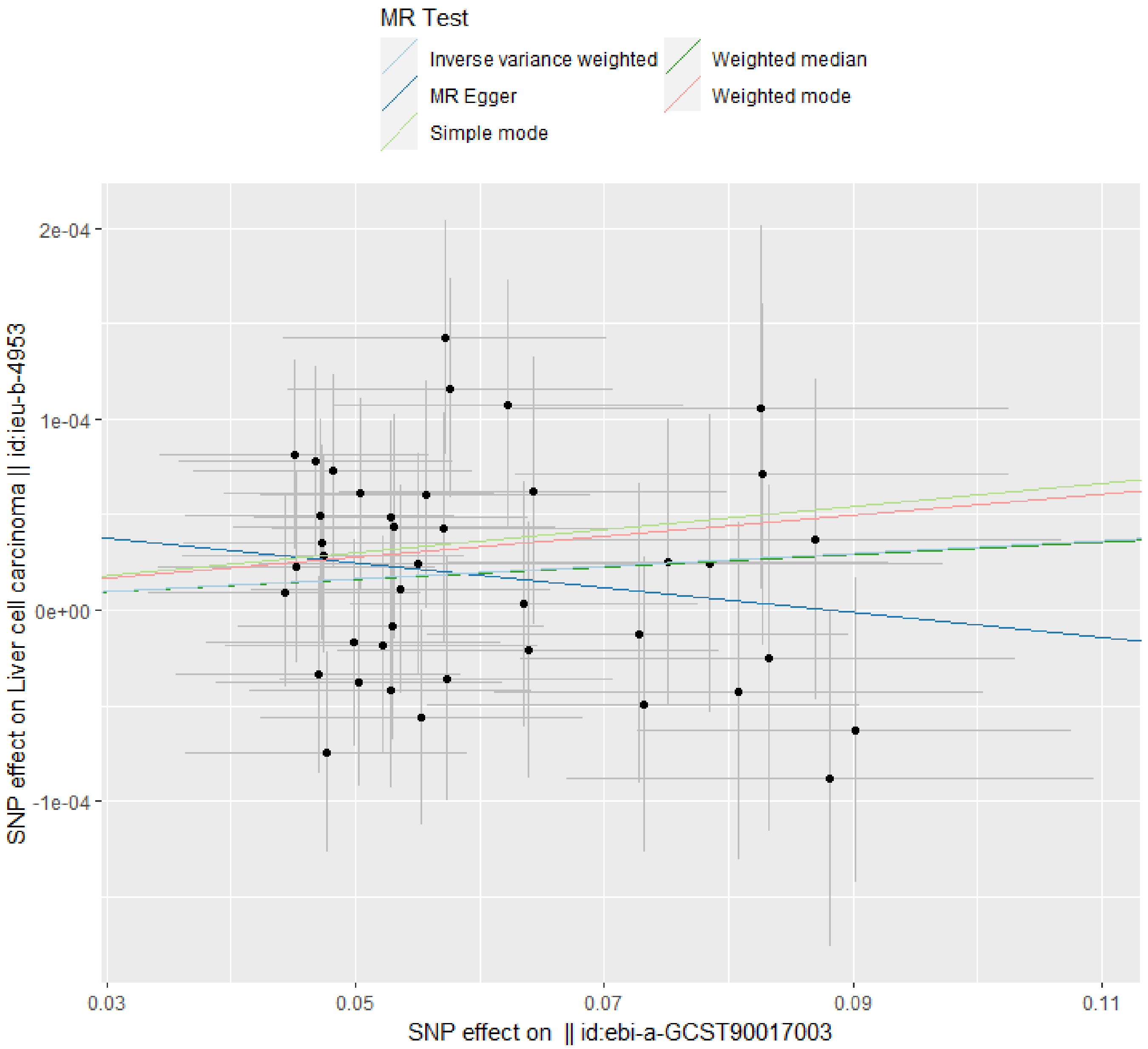
MR Test
Inverse variance weighted
MR Egger
Simple mode
Weighted median
Weighted mode
2e-04
1e-04
0e+00
-1e-04
0.03
0.05
0.07
0.09
0.11
SNP effect on Liver cell carcinoma || id:ieu-b-4953
SNP effect on || id:ebi-a-GCST90017003

Figure 185 Leave-one-out analysis, funnel plot, MR effect size and scatter plot for gut microbiota abundance (genus Holdemanella id.11393) on liver cell carcinoma

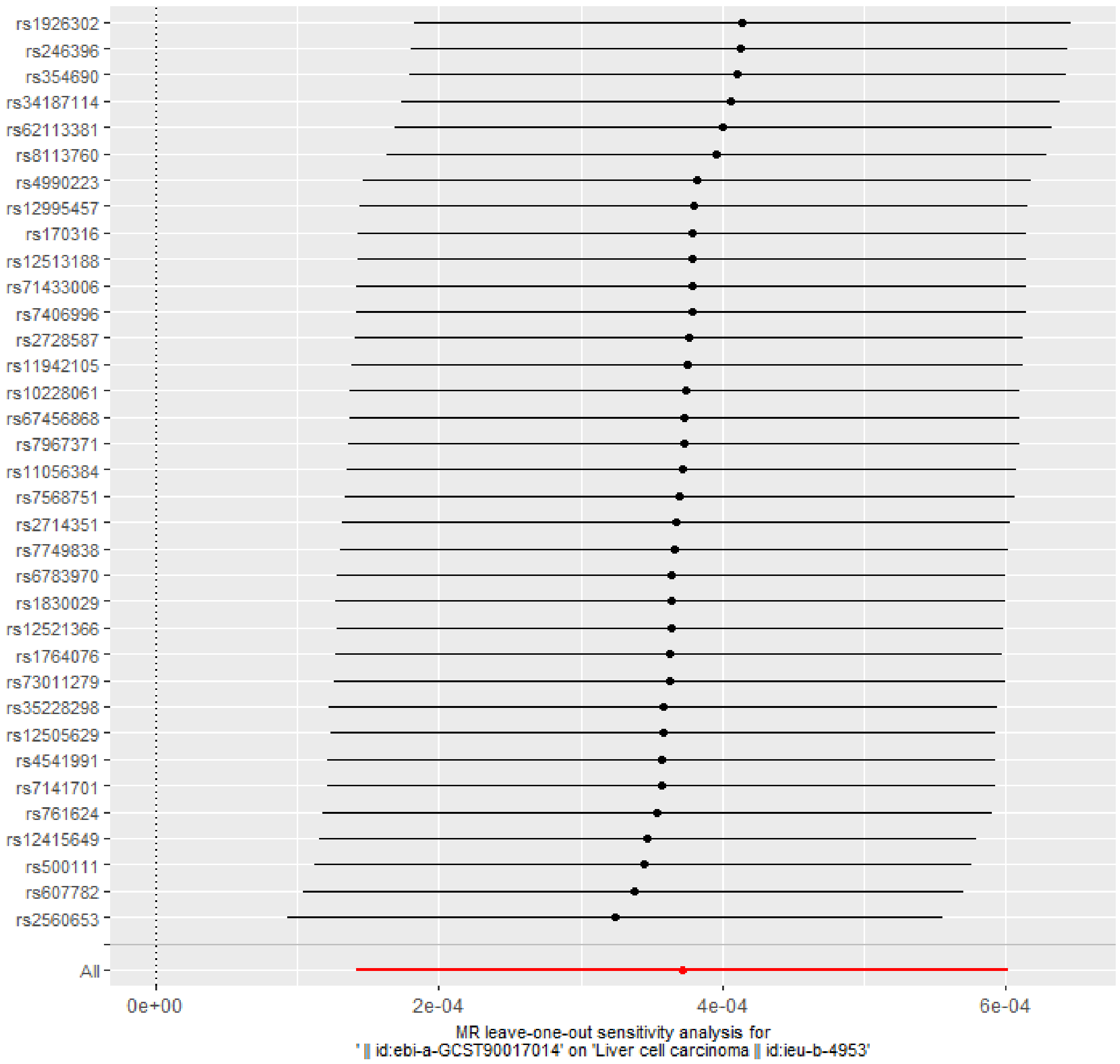

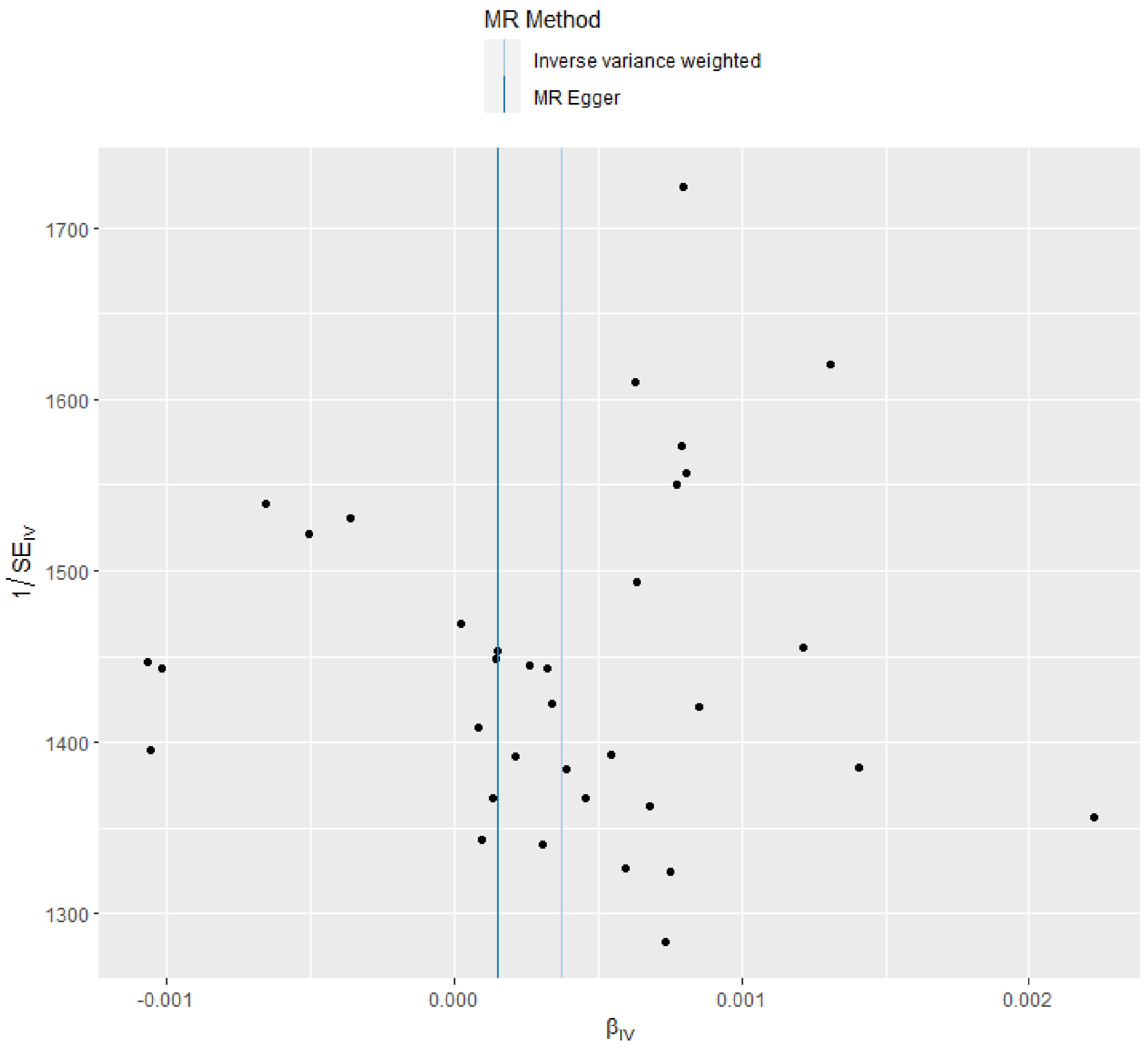

MR Method
Inverse variance weighted
MR Egger
1700
1600
1500
1400
1300
1/SE$_{IV}$
-0.001
0.000
0.001
0.002
β_{IV}

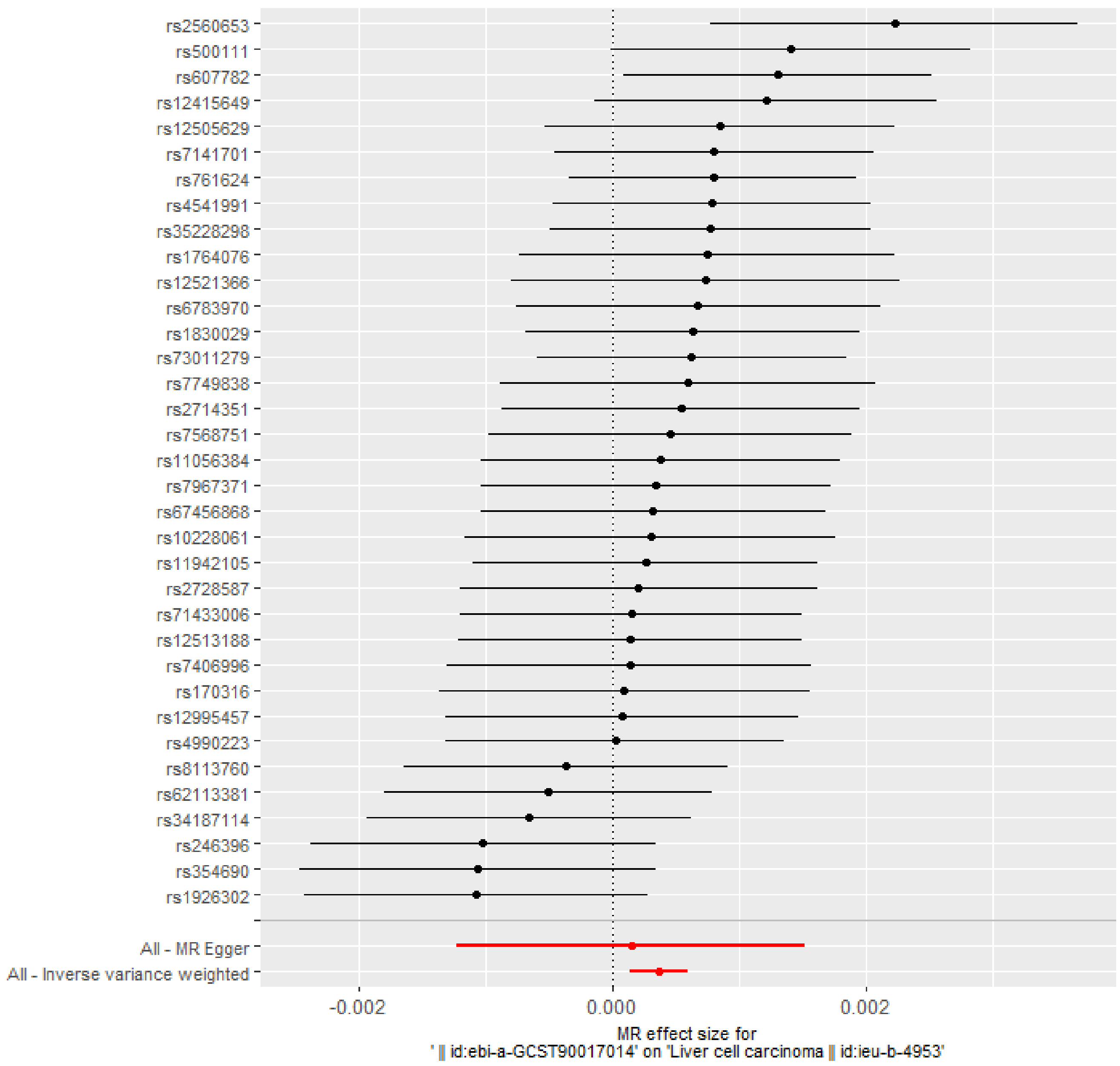
rs2560653
rs500111
rs607782
rs12415649
rs12505629
rs7141701
rs761624
rs4541991
rs35228298
rs1764076
rs12521366
rs6783970
rs1830029
rs73011279
rs7749838
rs2714351
rs7568751
rs11056384
rs7967371
rs67456868
rs10228061
rs11942105
rs2728587
rs71433006
rs12513188
rs7406996
rs170316
rs12995457
rs4990223
rs8113760
rs62113381
rs34187114
rs246396
rs354690
rs1926302
All - MR Egger
All - Inverse variance weighted
-0.002
0.000
0.002
MR effect size for
' || id:ebi-a-GCST90017014' on 'Liver cell carcinoma || id:ieu-b-4953'

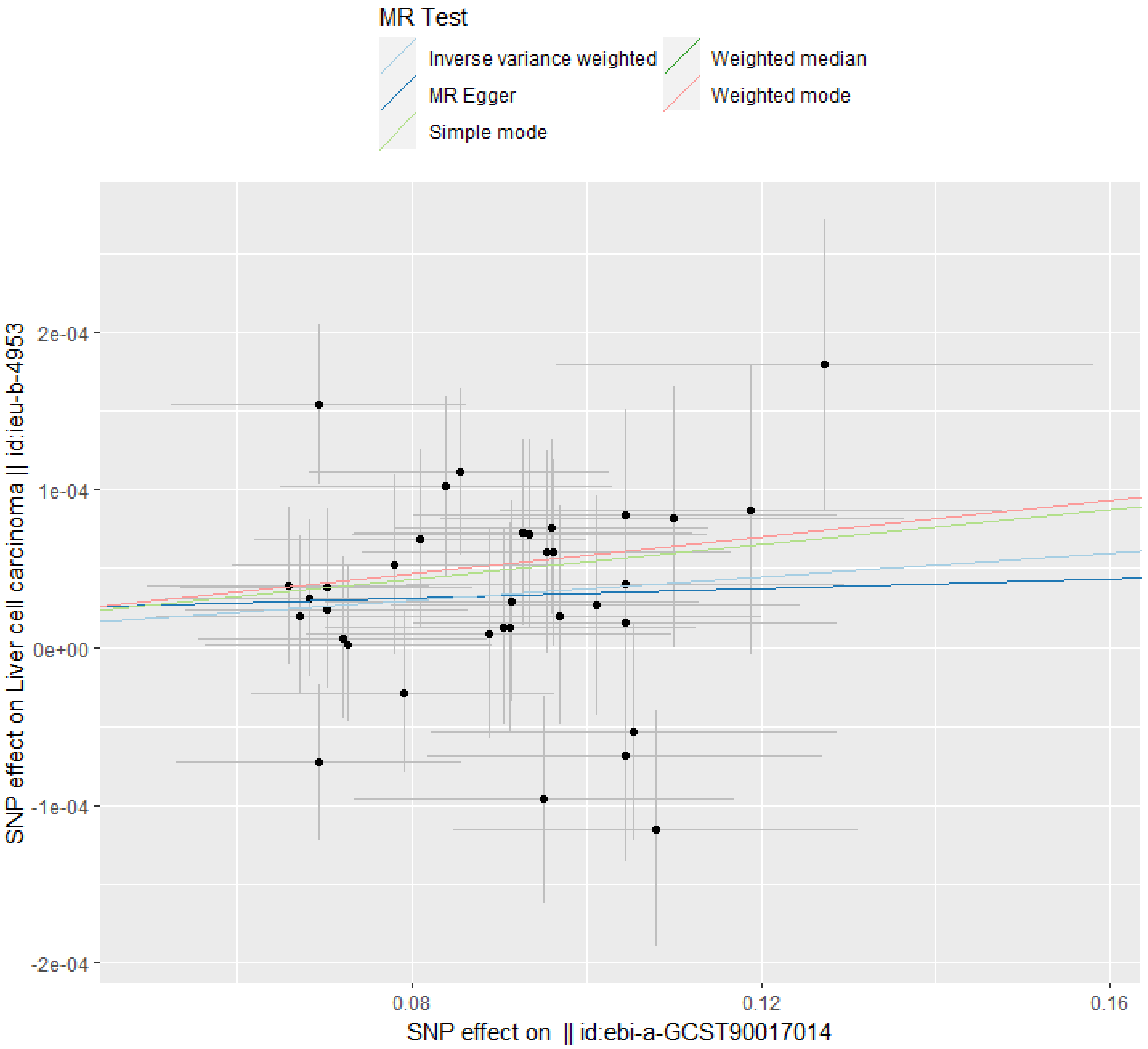

MR Test
Inverse variance weighted
MR Egger
Simple mode
Weighted median
Weighted mode
2e-04
1e-04
0e+00
-1e-04
-2e-04
SNP effect on Liver cell carcinoma || id:ieu-b-4953
0.08
0.12
0.16
SNP effect on || id:ebi-a-GCST90017014

Figure 186 Leave-one-out analysis, funnel plot, MR effect size and scatter plot for gut microbiota abundance (genus Lachnospiraceae NK4A136 group id.11319) on liver cell carcinoma

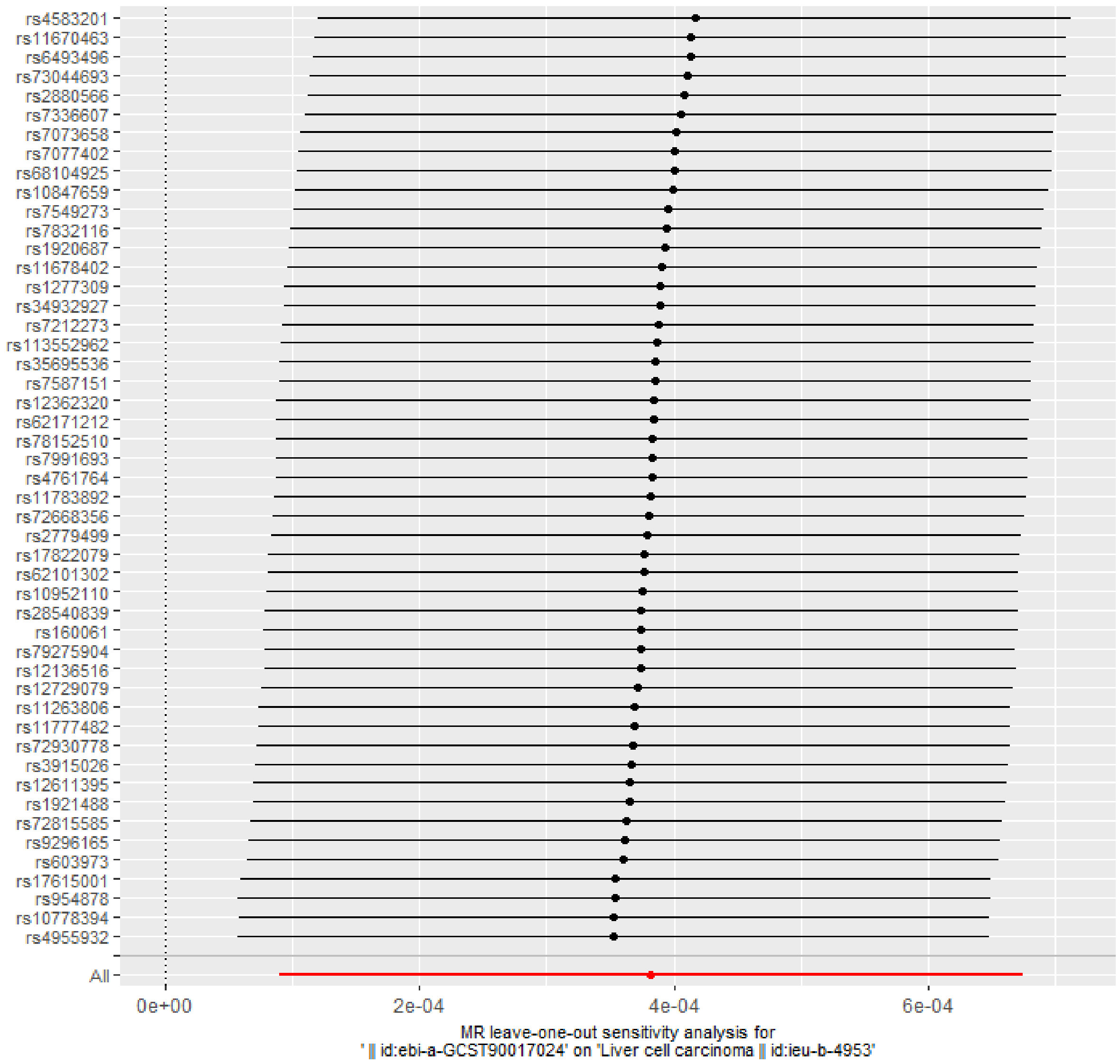

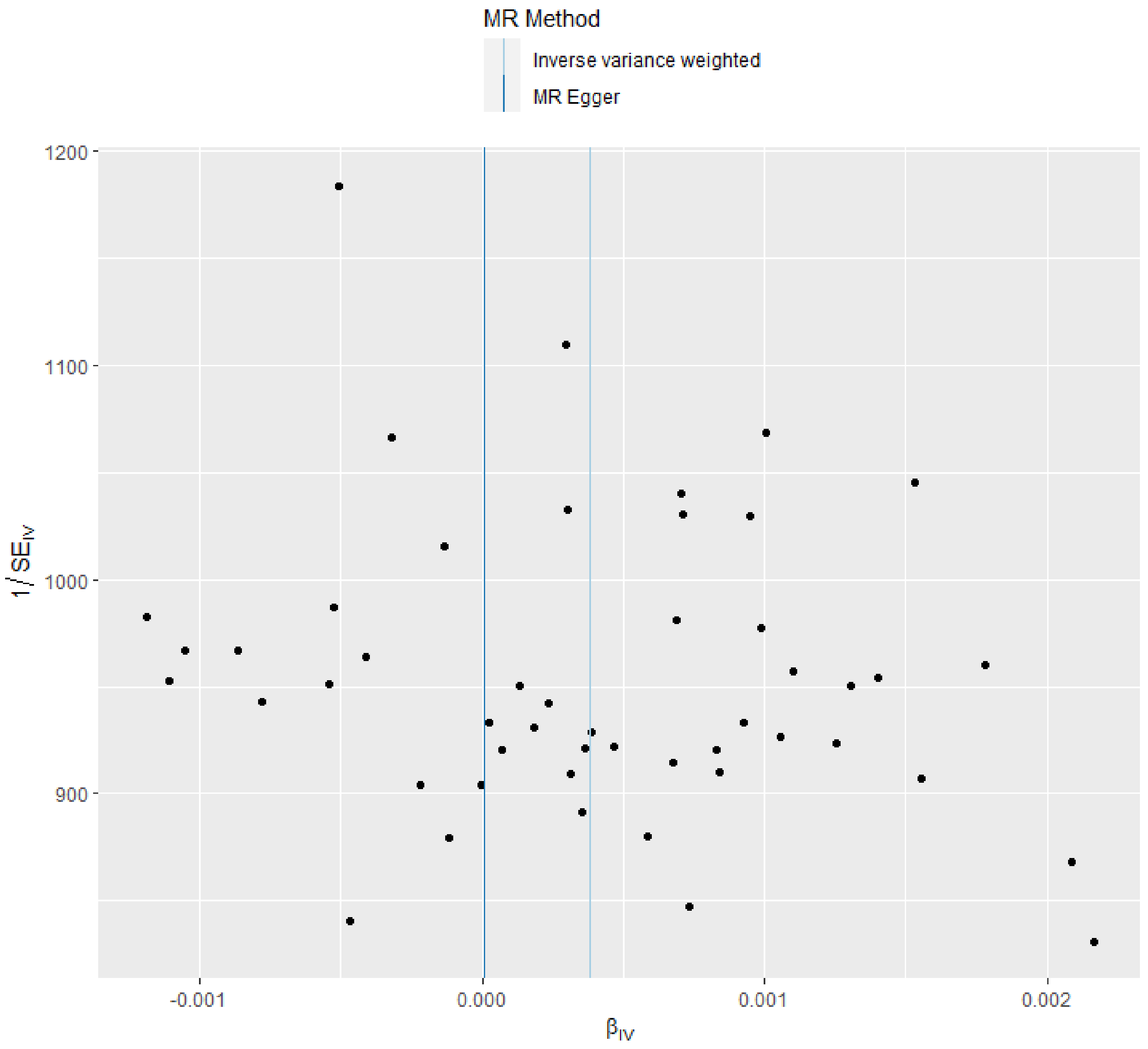

MR Method
Inverse variance weighted
MR Egger
1200
1100
1000
900
1/SE_IV
-0.001
0.000
0.001
0.002
β_IV

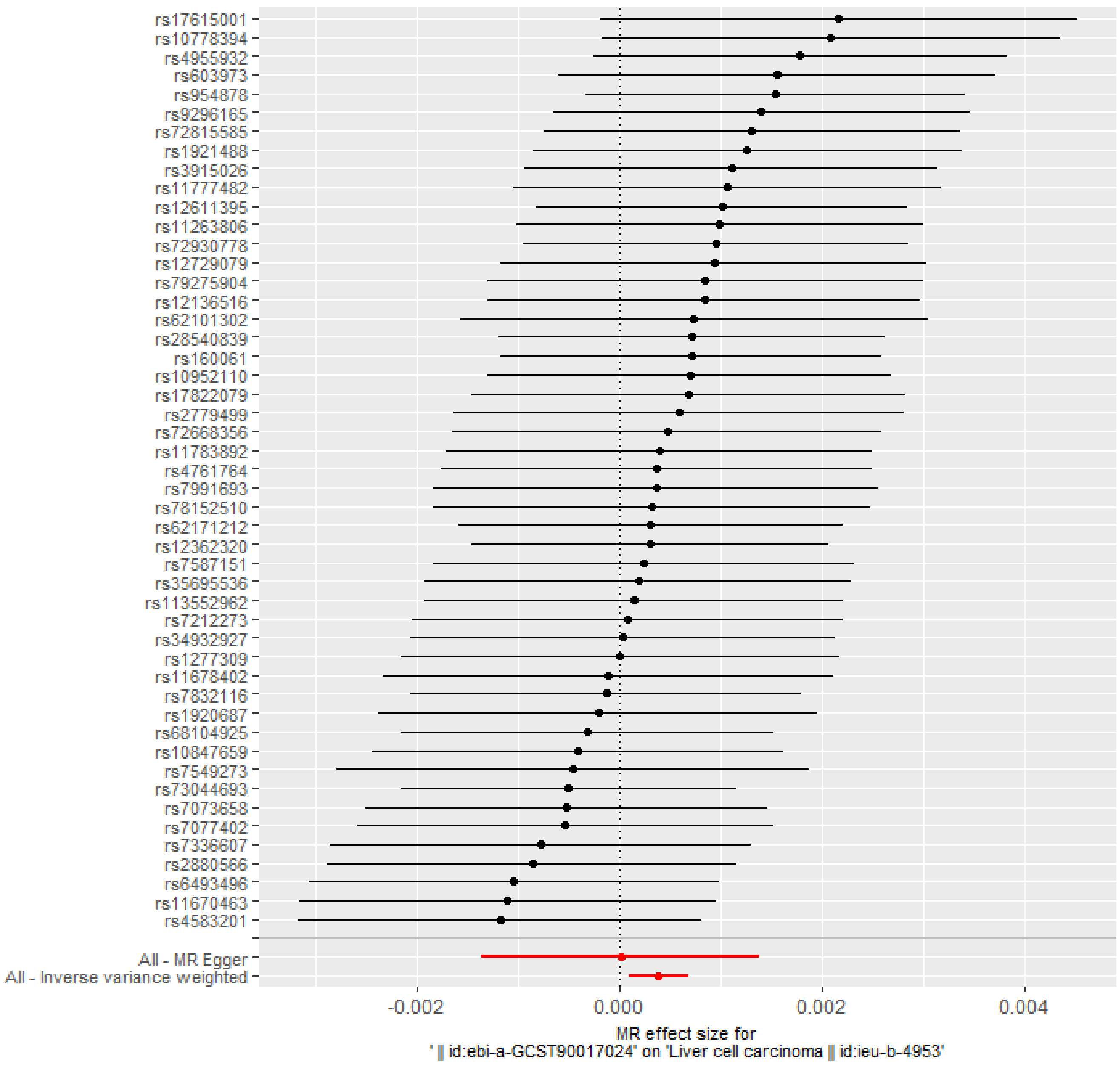

rs17615001
rs10778394
rs4955932
rs603973
rs954878
rs9296165
rs72815585
rs1921488
rs3915026
rs11777482
rs12611395
rs11263806
rs72930778
rs12729079
rs79275904
rs12136516
rs62101302
rs28540839
rs160061
rs10952110
rs17822079
rs2779499
rs72668356
rs11783892
rs4761764
rs7991693
rs78152510
rs62171212
rs12362320
rs7587151
rs35695536
rs113552962
rs7212273
rs34932927
rs1277309
rs11678402
rs7832116
rs1920687
rs68104925
rs10847659
rs7549273
rs73044693
rs7073658
rs7077402
rs7336607
rs2880566
rs6493496
rs11670463
rs4583201
All - MR Egger
All - Inverse variance weighted
-0.002
0.000
0.002
0.004
MR effect size for
' || id:ebi-a-GCST90017024' on 'Liver cell carcinoma || id:ieu-b-4953'

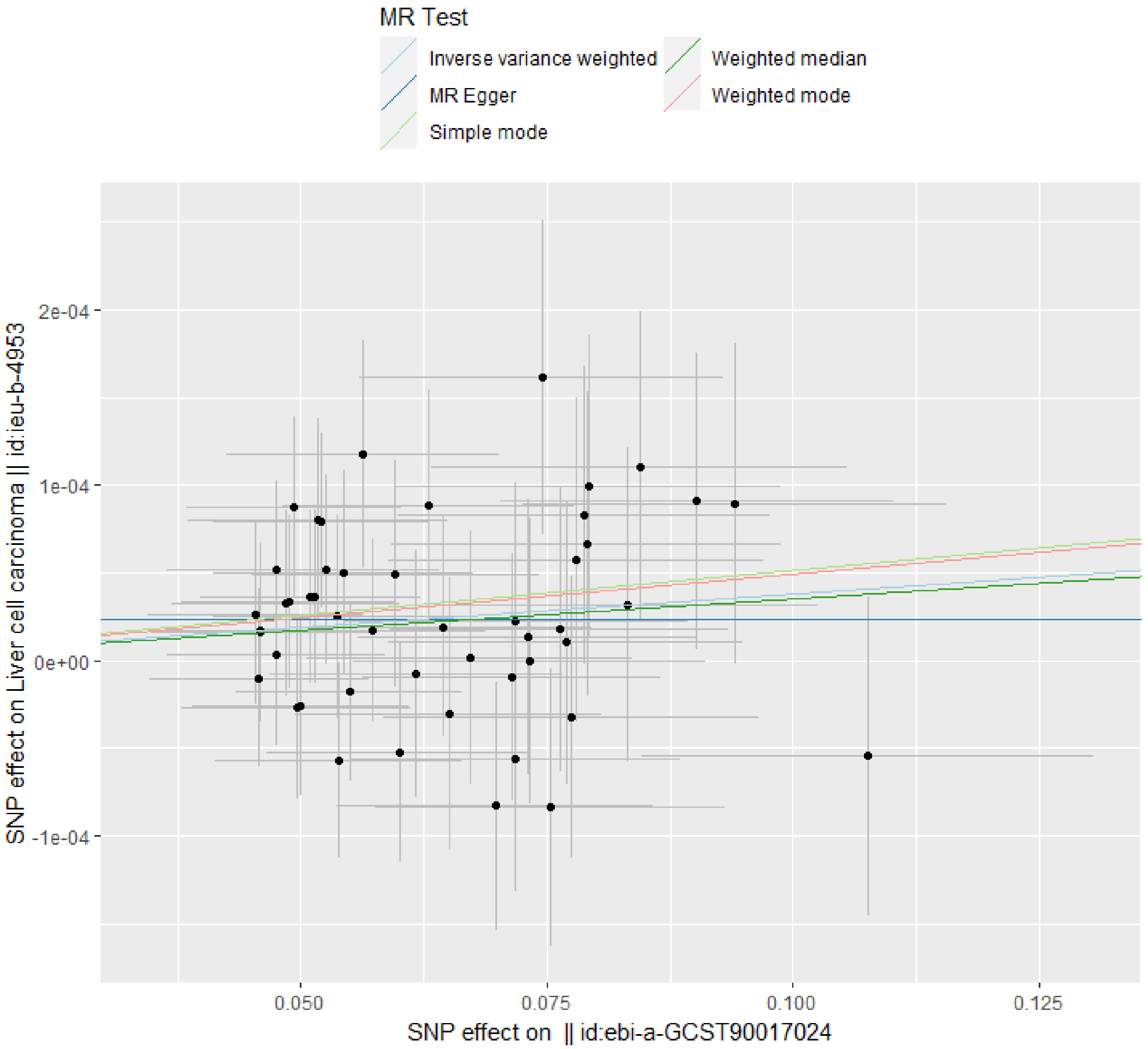
MR Test
Inverse variance weighted
MR Egger
Simple mode
Weighted median
Weighted mode
2e-04
1e-04
0e+00
-1e-04
0.050
0.075
0.100
0.125
SNP effect on Liver cell carcinoma || id:ieu-b-4953
SNP effect on || id:ebi-a-GCST90017024

Figure 187 Leave-one-out analysis, funnel plot, MR effect size and scatter plot for gut microbiota abundance (genus Peptococcus id.2037) on liver cell carcinoma

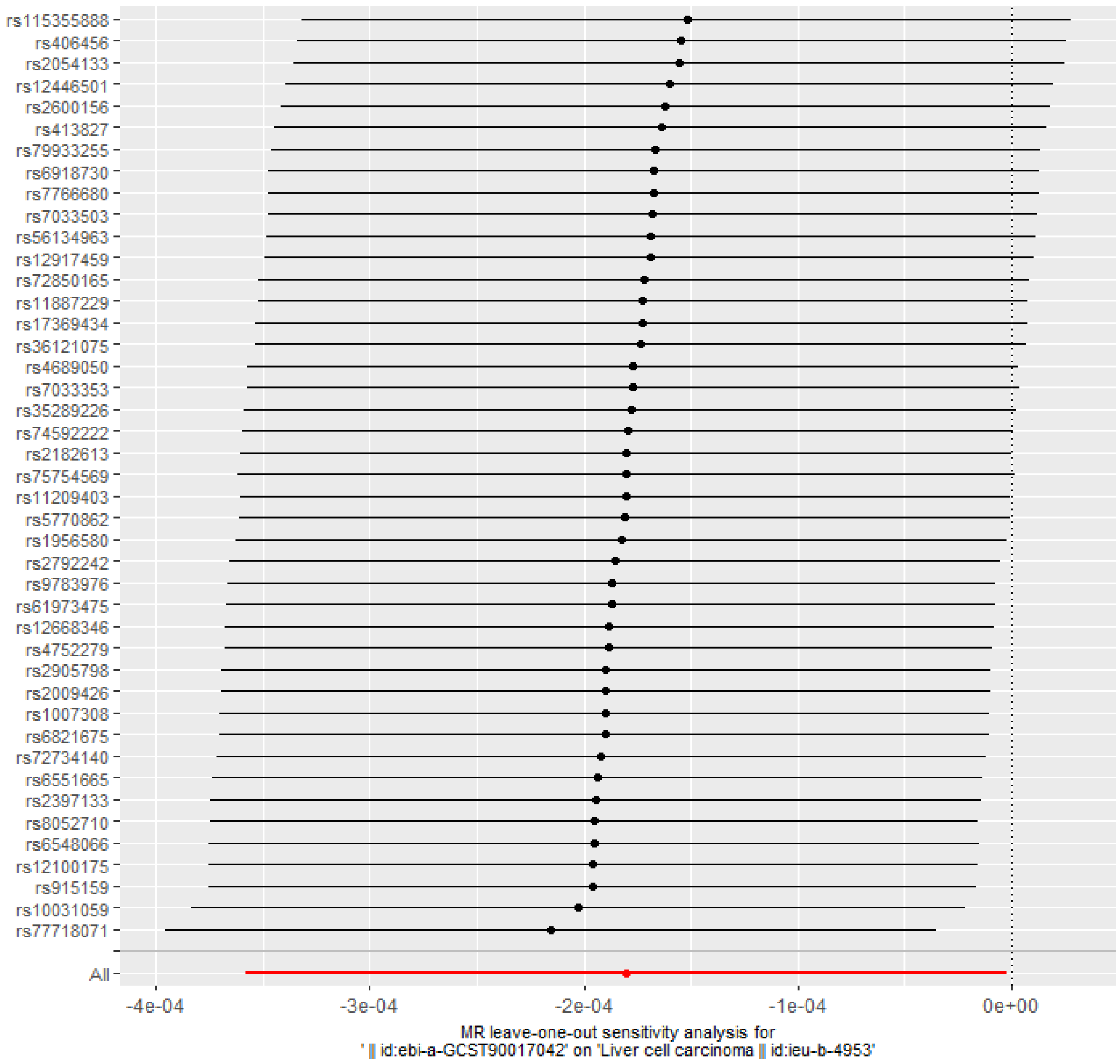

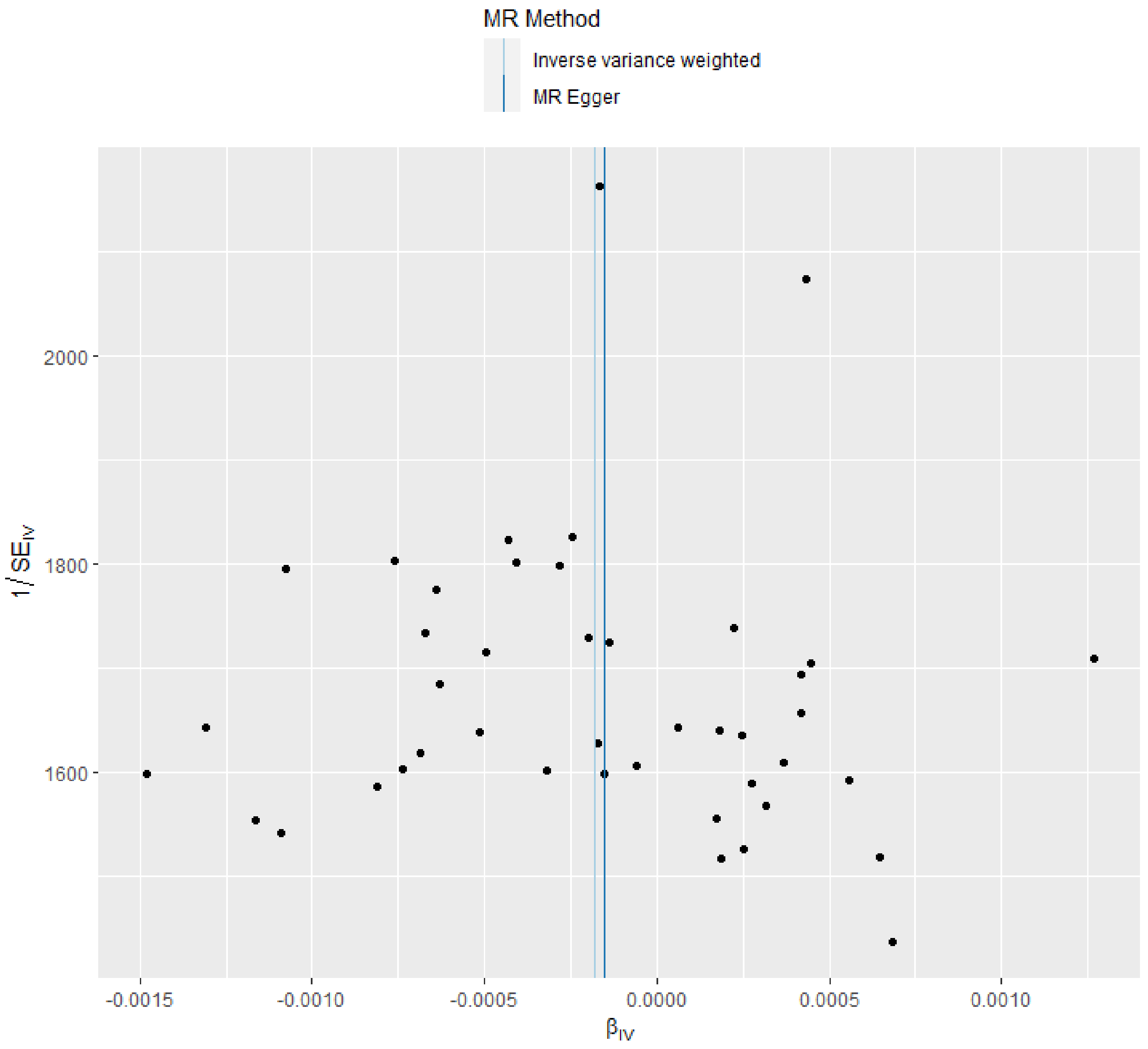

MR Method
Inverse variance weighted
MR Egger
2000
1800
1600
1/SE_IV
-0.0015
-0.0010
-0.0005
0.0000
0.0005
0.0010
β_IV

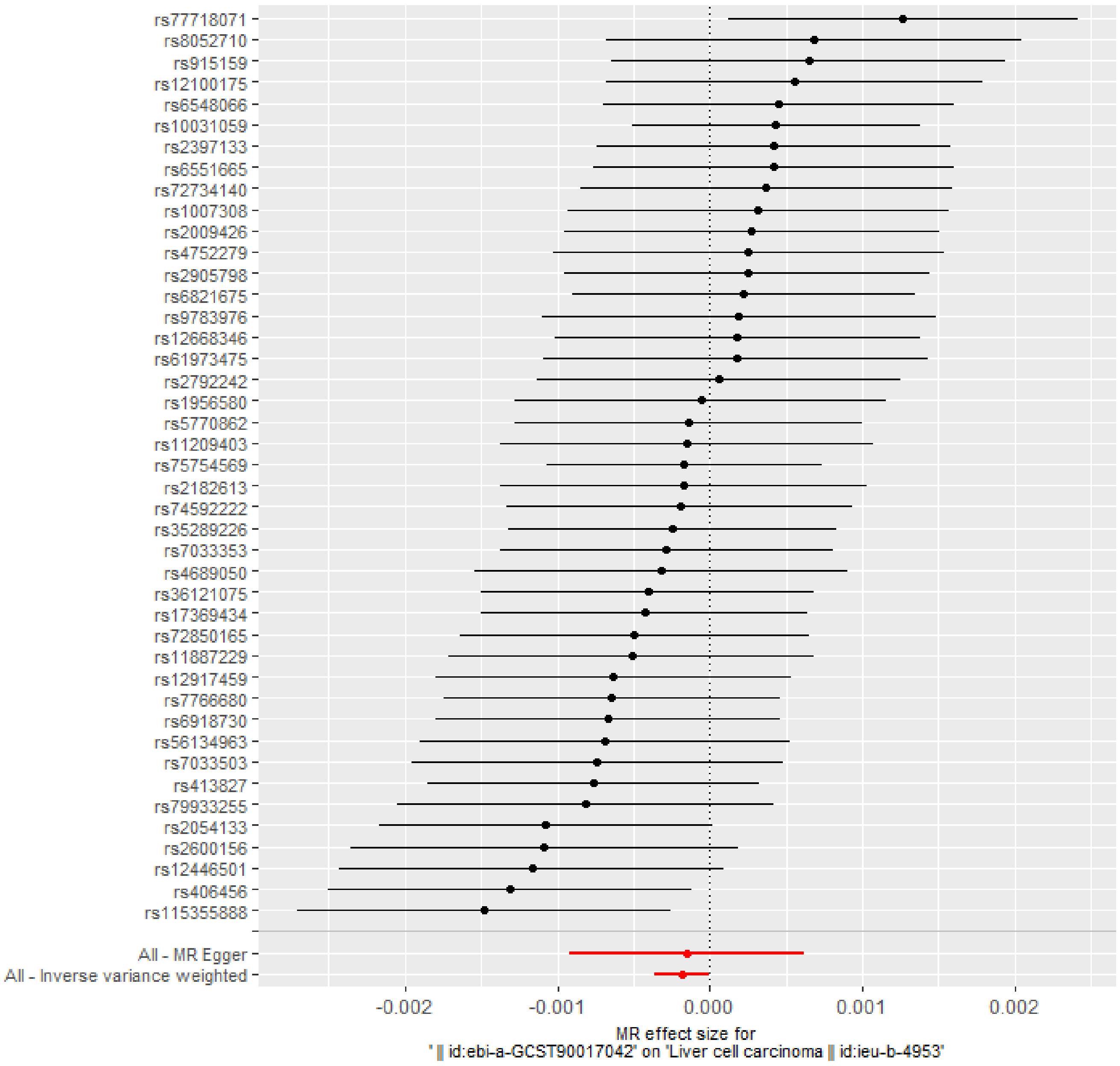

rs77718071
rs8052710
rs915159
rs12100175
rs6548066
rs10031059
rs2397133
rs6551665
rs72734140
rs1007308
rs2009426
rs4752279
rs2905798
rs6821675
rs9783976
rs12668346
rs61973475
rs2792242
rs1956580
rs5770862
rs11209403
rs75754569
rs2182613
rs74592222
rs35289226
rs7033353
rs4689050
rs36121075
rs17369434
rs72850165
rs11887229
rs12917459
rs7766680
rs6918730
rs56134963
rs7033503
rs413827
rs79933255
rs2054133
rs2600156
rs12446501
rs406456
rs115355888
All - MR Egger
All - Inverse variance weighted
-0.002
-0.001
0.000
0.001
0.002
MR effect size for
' || id:ebi-a-GCST90017042' on 'Liver cell carcinoma || id:ieu-b-4953'

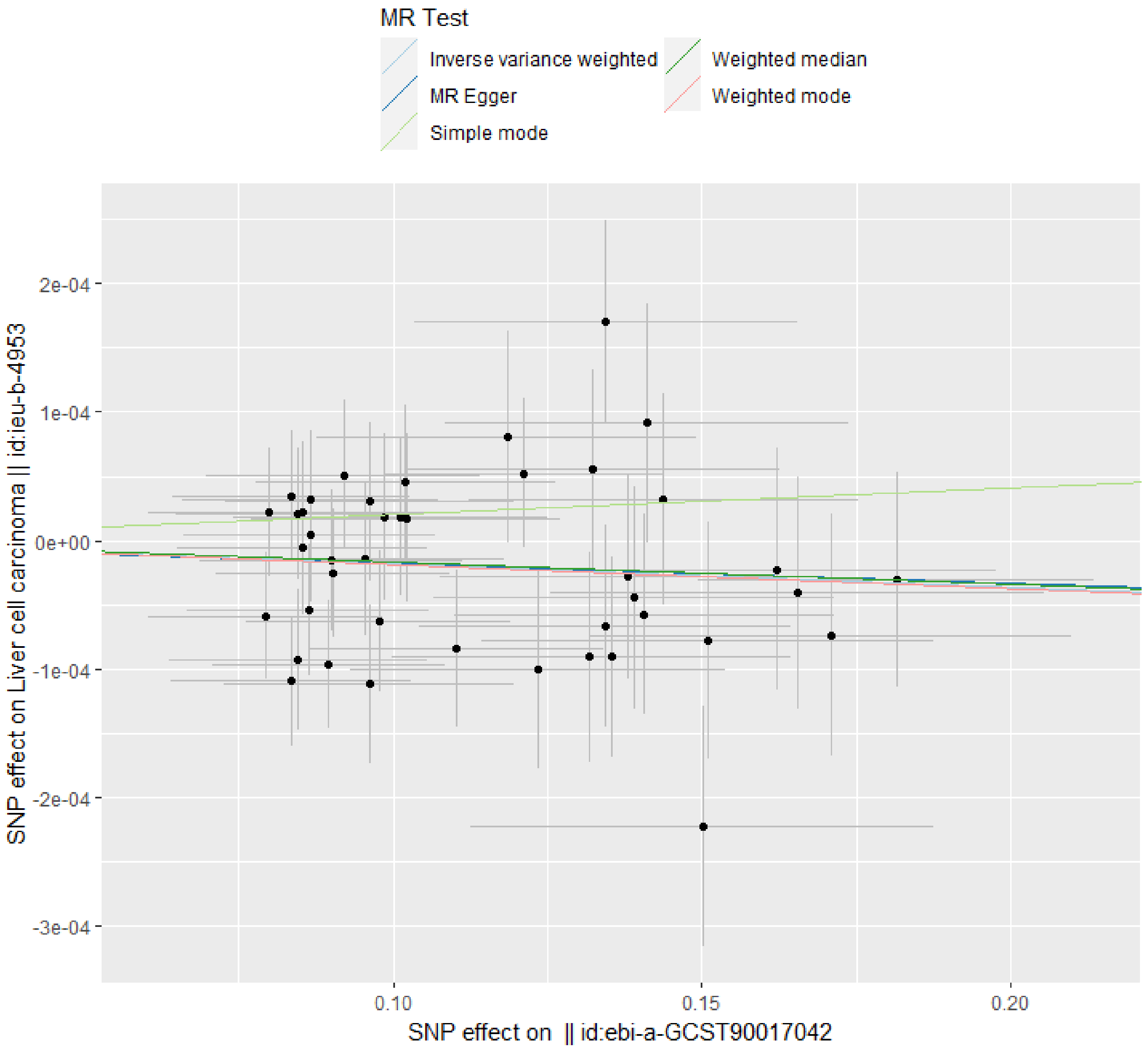
MR Test
Inverse variance weighted
MR Egger
Simple mode
Weighted median
Weighted mode
2e-04
1e-04
0e+00
-1e-04
-2e-04
-3e-04
SNP effect on Liver cell carcinoma || id:ieu-b-4953
0.10
0.15
0.20
SNP effect on || id:ebi-a-GCST90017042

Figure 188 Leave-one-out analysis, funnel plot, MR effect size and scatter plot for gut microbiota abundance (genus Phascolarctobacterium id.2168) on liver cell carcinoma

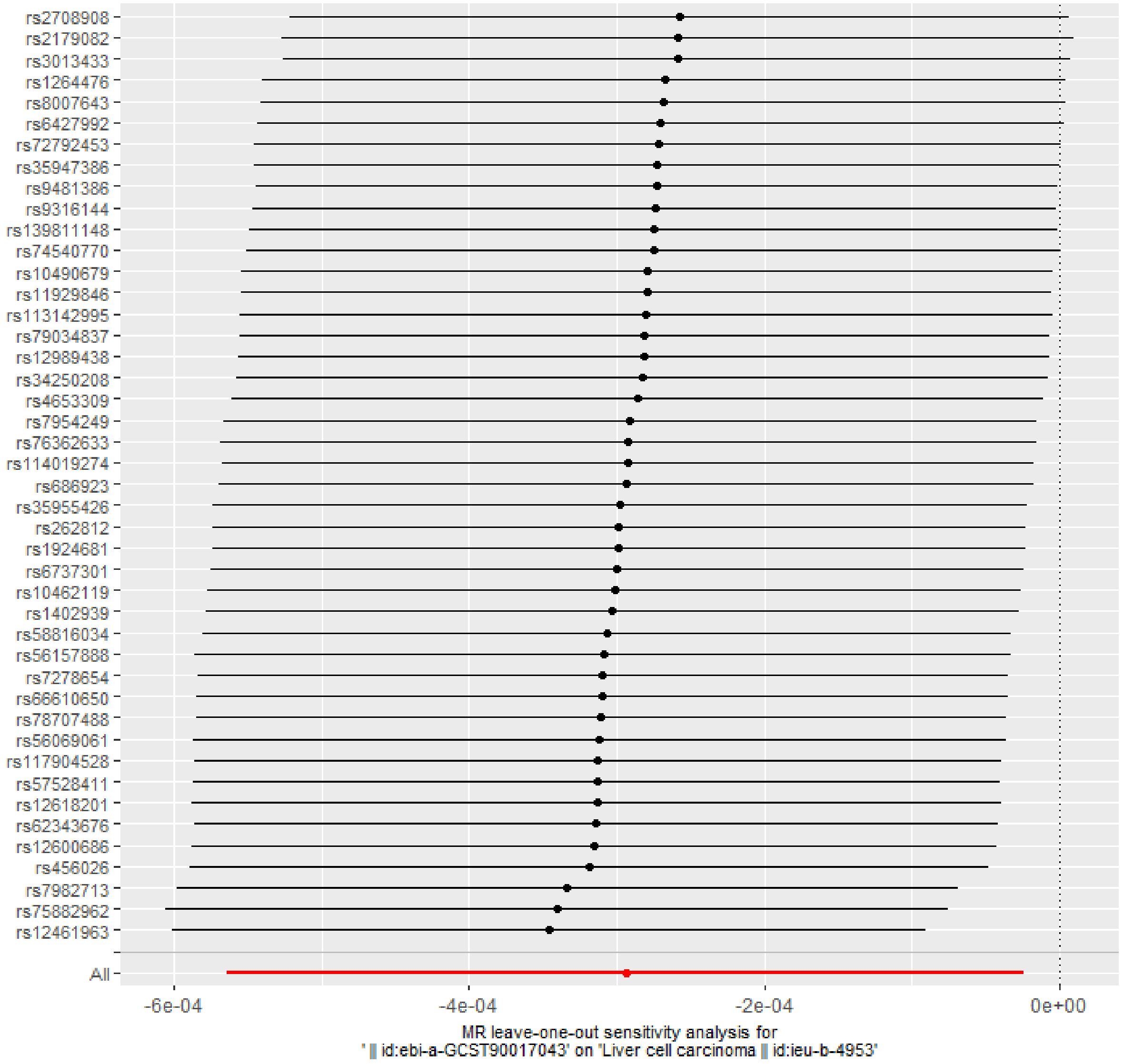

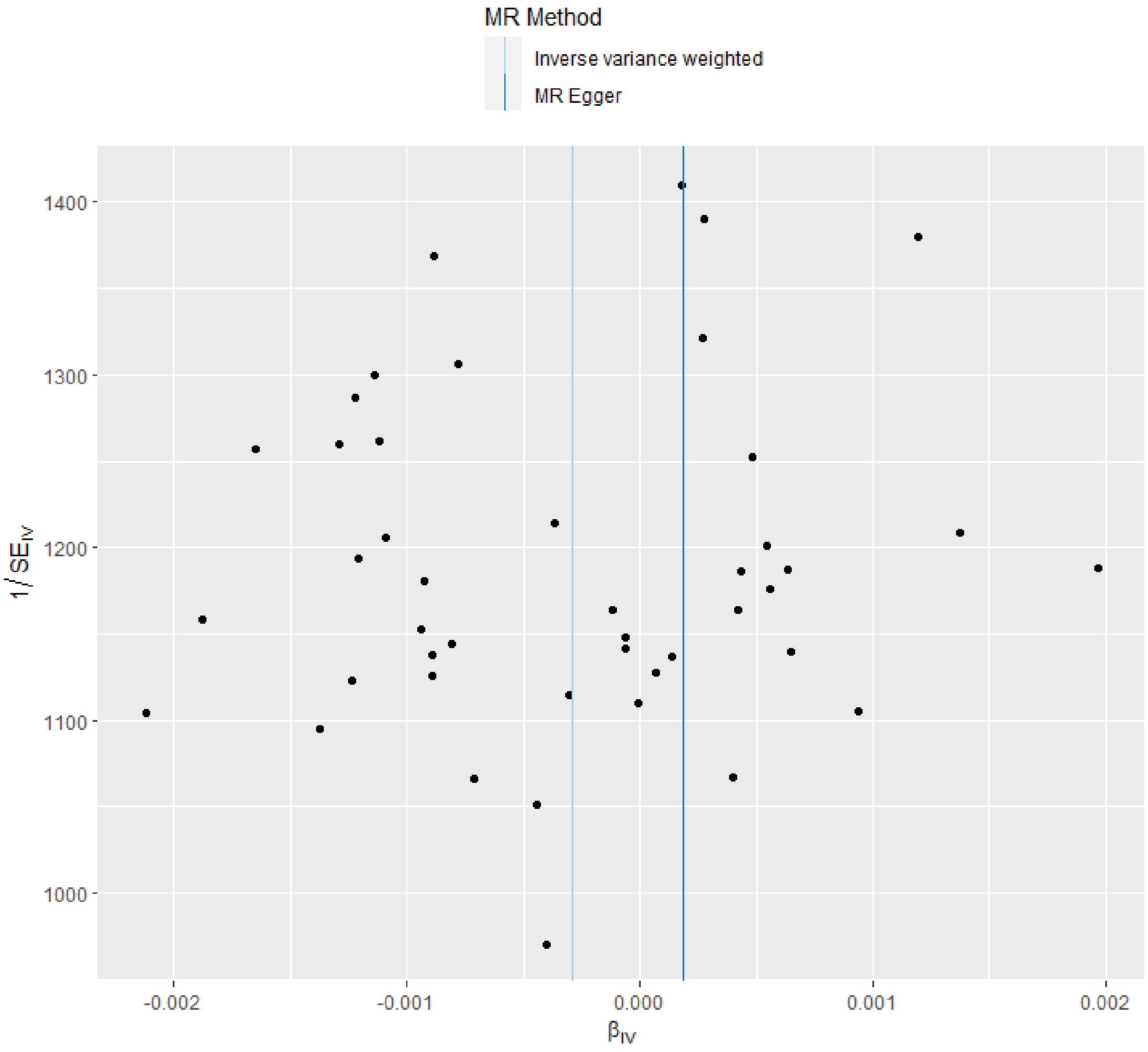
MR Method
Inverse variance weighted
MR Egger
1400
1300
1200
1100
1000
1/SE_IV
-0.002
-0.001
0.000
0.001
0.002
β_IV

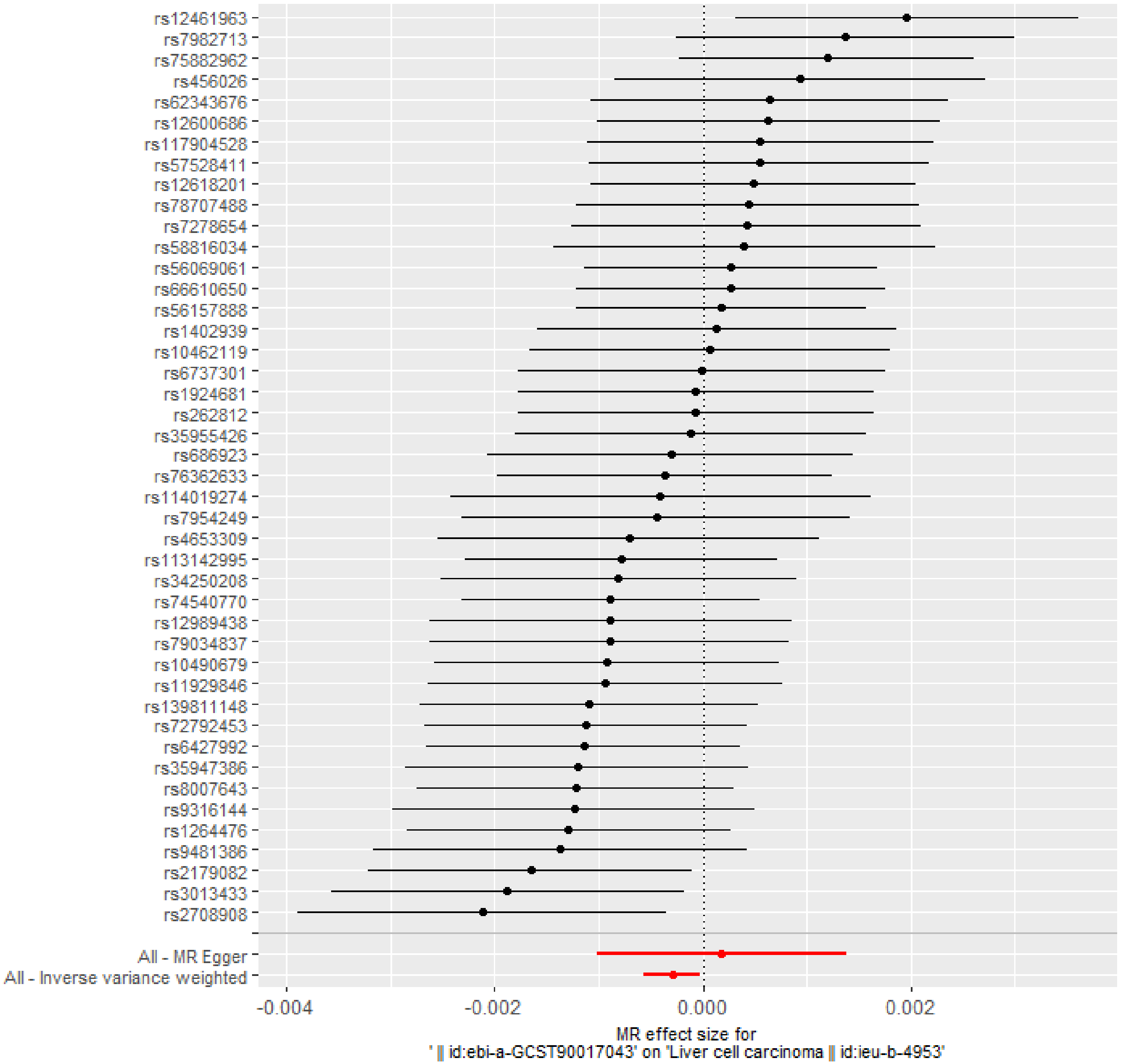

rs12461963
rs7982713
rs75882962
rs456026
rs62343676
rs12600686
rs117904528
rs57528411
rs12618201
rs78707488
rs7278654
rs58816034
rs56069061
rs66610650
rs56157888
rs1402939
rs10462119
rs6737301
rs1924681
rs262812
rs35955426
rs686923
rs76362633
rs114019274
rs7954249
rs4653309
rs113142995
rs34250208
rs74540770
rs12989438
rs79034837
rs10490679
rs11929846
rs139811148
rs72792453
rs6427992
rs35947386
rs8007643
rs9316144
rs1264476
rs9481386
rs2179082
rs3013433
rs2708908
All - MR Egger
All - Inverse variance weighted
-0.004
-0.002
0.000
0.002
MR effect size for
' || id:ebi-a-GCST90017043' on 'Liver cell carcinoma || id:ieu-b-4953'

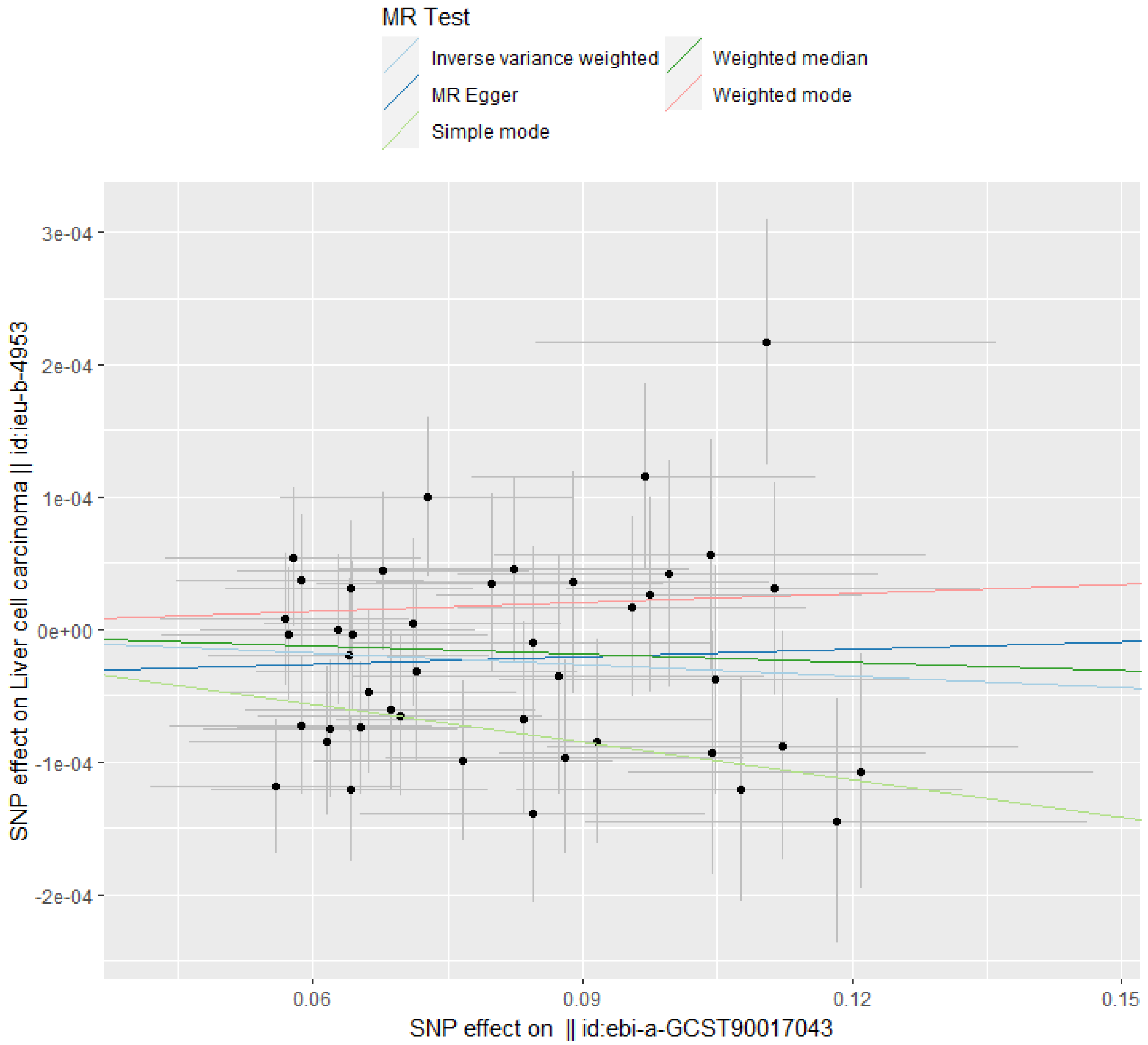

MR Test
Inverse variance weighted
MR Egger
Simple mode
Weighted median
Weighted mode
3e-04
2e-04
1e-04
0e+00
-1e-04
-2e-04
SNP effect on Liver cell carcinoma || id:ieu-b-4953
0.06
0.09
0.12
0.15
SNP effect on || id:ebi-a-GCST90017043

Figure 189 Leave-one-out analysis, funnel plot, MR effect size and scatter plot for gut microbiota abundance (genus Veillonella id.2198) on liver cell carcinoma

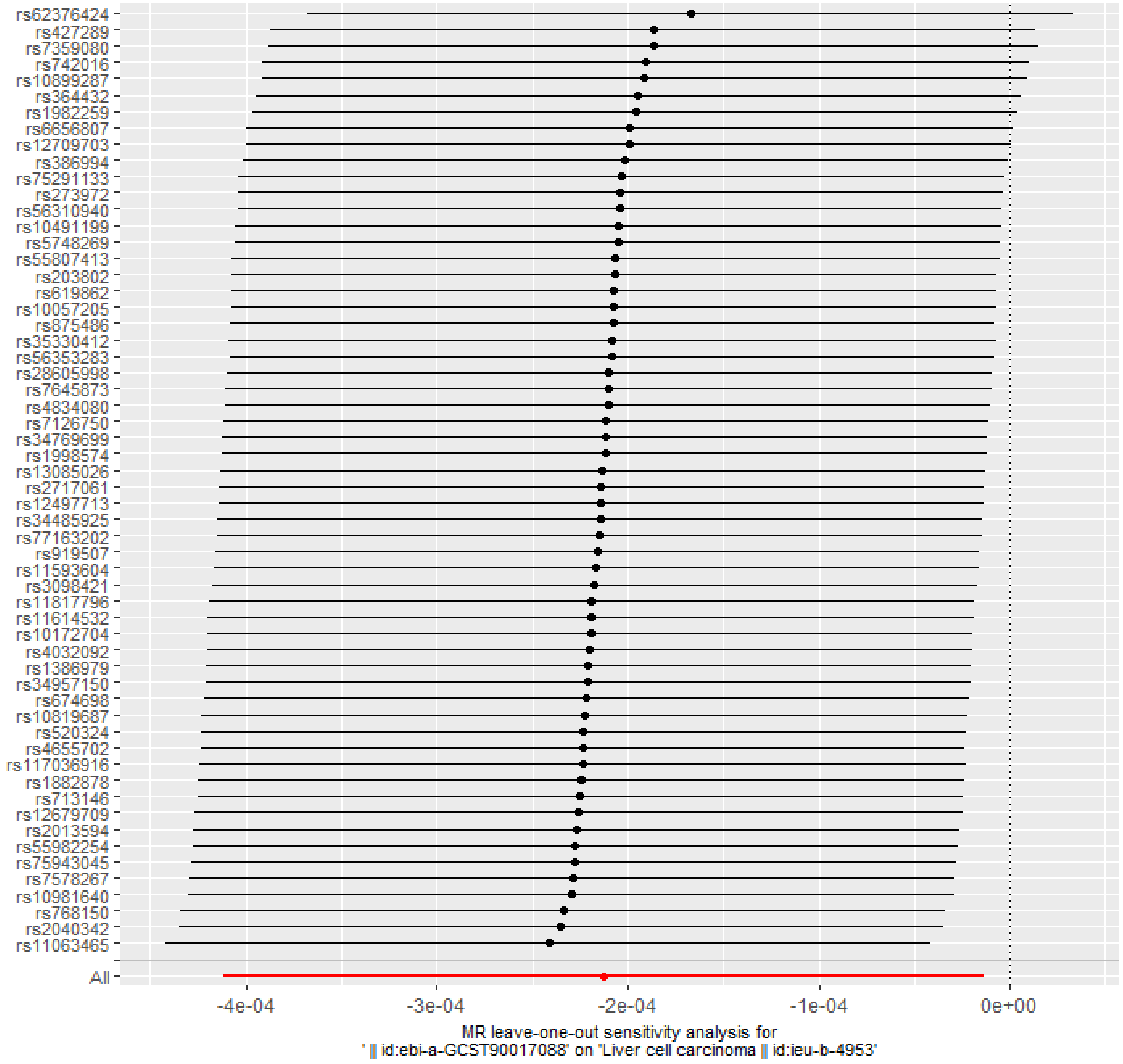

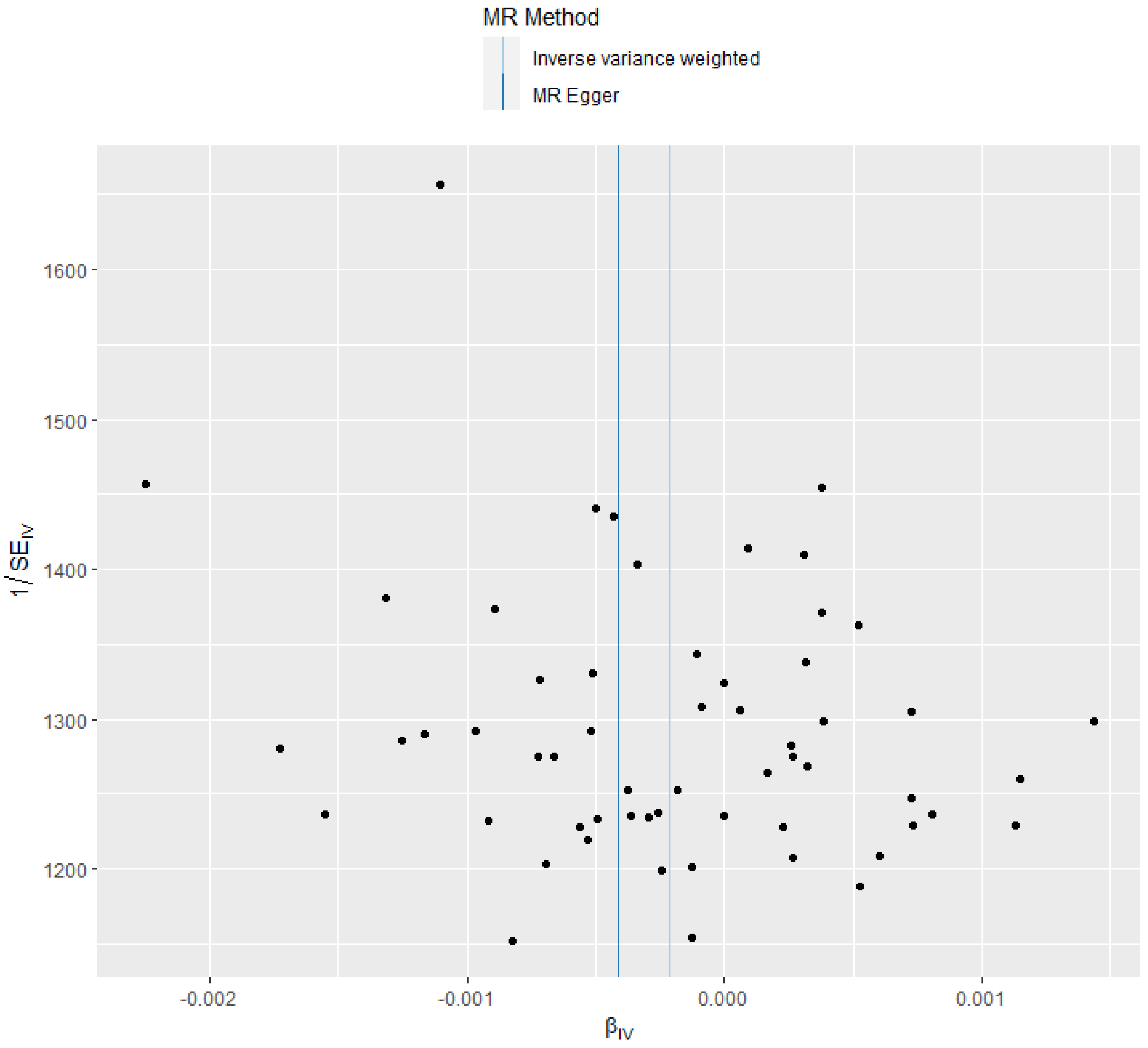
MR Method
Inverse variance weighted
MR Egger
1600
1500
1400
1300
1200
$1/SE_{IV}$
-0.002
-0.001
0.000
0.001
β_{IV}

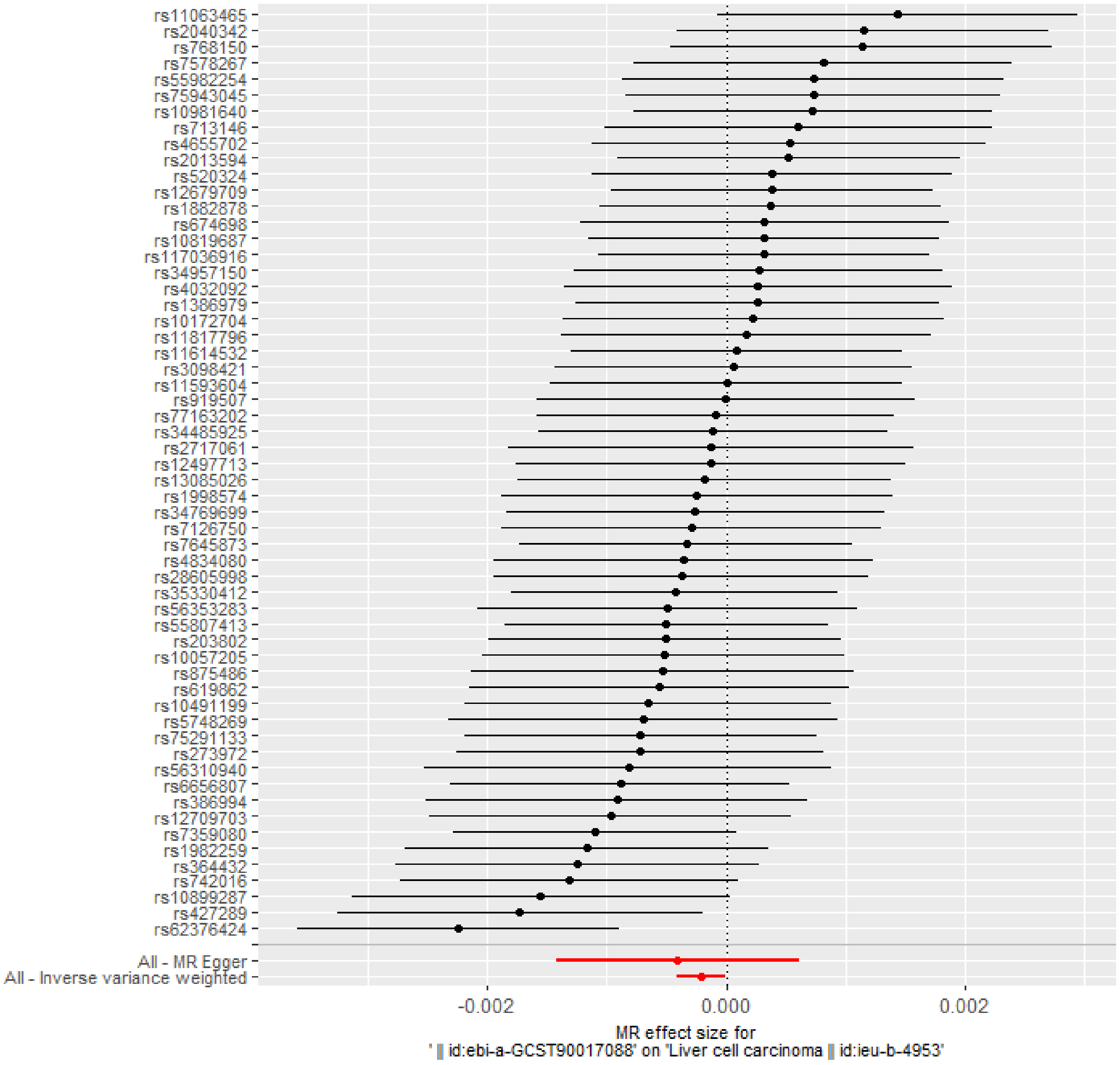
rs11063465
rs2040342
rs768150
rs7578267
rs55982254
rs75943045
rs10981640
rs713146
rs4655702
rs2013594
rs520324
rs12679709
rs1882878
rs674698
rs10819687
rs117036916
rs34957150
rs4032092
rs1386979
rs10172704
rs11817796
rs11614532
rs3098421
rs11593604
rs919507
rs77163202
rs34485925
rs2717061
rs12497713
rs13085026
rs1998574
rs34769699
rs7126750
rs7645873
rs4834080
rs28605998
rs35330412
rs56353283
rs55807413
rs203802
rs10057205
rs875486
rs619862
rs10491199
rs5748269
rs75291133
rs273972
rs56310940
rs6656807
rs386994
rs12709703
rs7359080
rs1982259
rs364432
rs742016
rs10899287
rs427289
rs62376424
All - MR Egger
All - Inverse variance weighted
-0.002
0.000
0.002
MR effect size for
' ‖ id:ebi-a-GCST90017088' on 'Liver cell carcinoma ‖ id:ieu-b-4953'

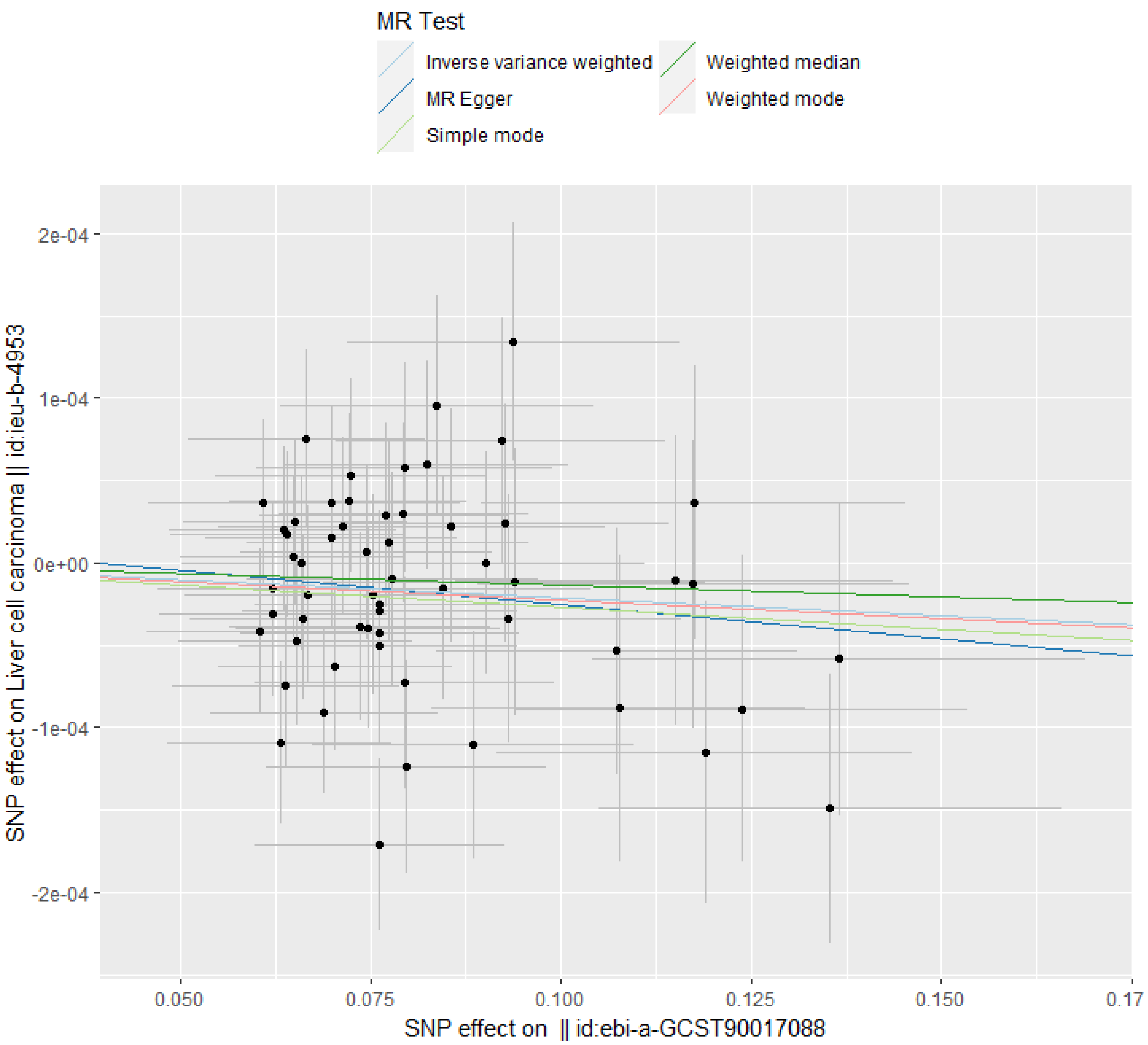

MR Test
Inverse variance weighted
MR Egger
Simple mode
Weighted median
Weighted mode
2e-04
1e-04
0e+00
-1e-04
-2e-04
0.050
0.075
0.100
0.125
0.150
0.17
SNP effect on Liver cell carcinoma || id:ieu-b-4953
SNP effect on || id:ebi-a-GCST90017088

Figure 190 Leave-one-out analysis, funnel plot, MR effect size and scatter plot for gut microbiota abundance (class Alphaproteobacteria id.2379) on cholelithiasis

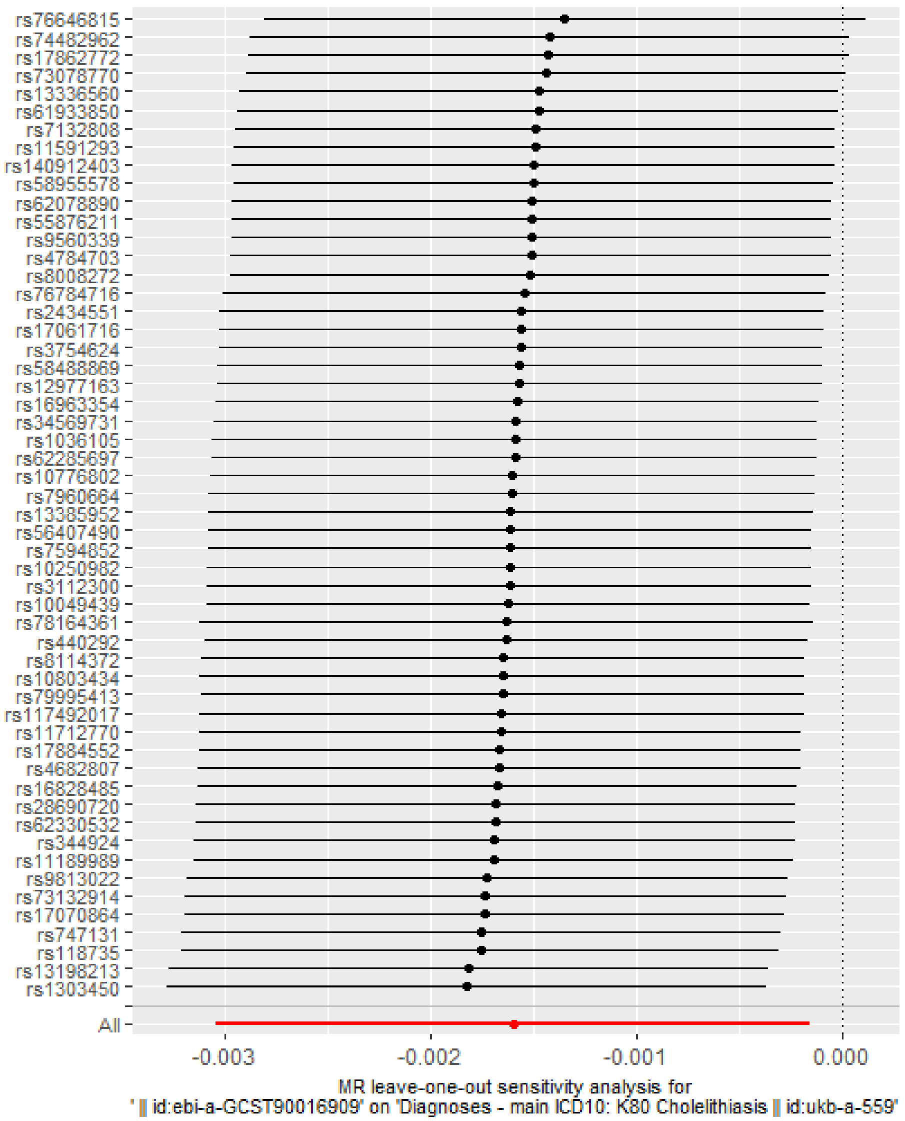

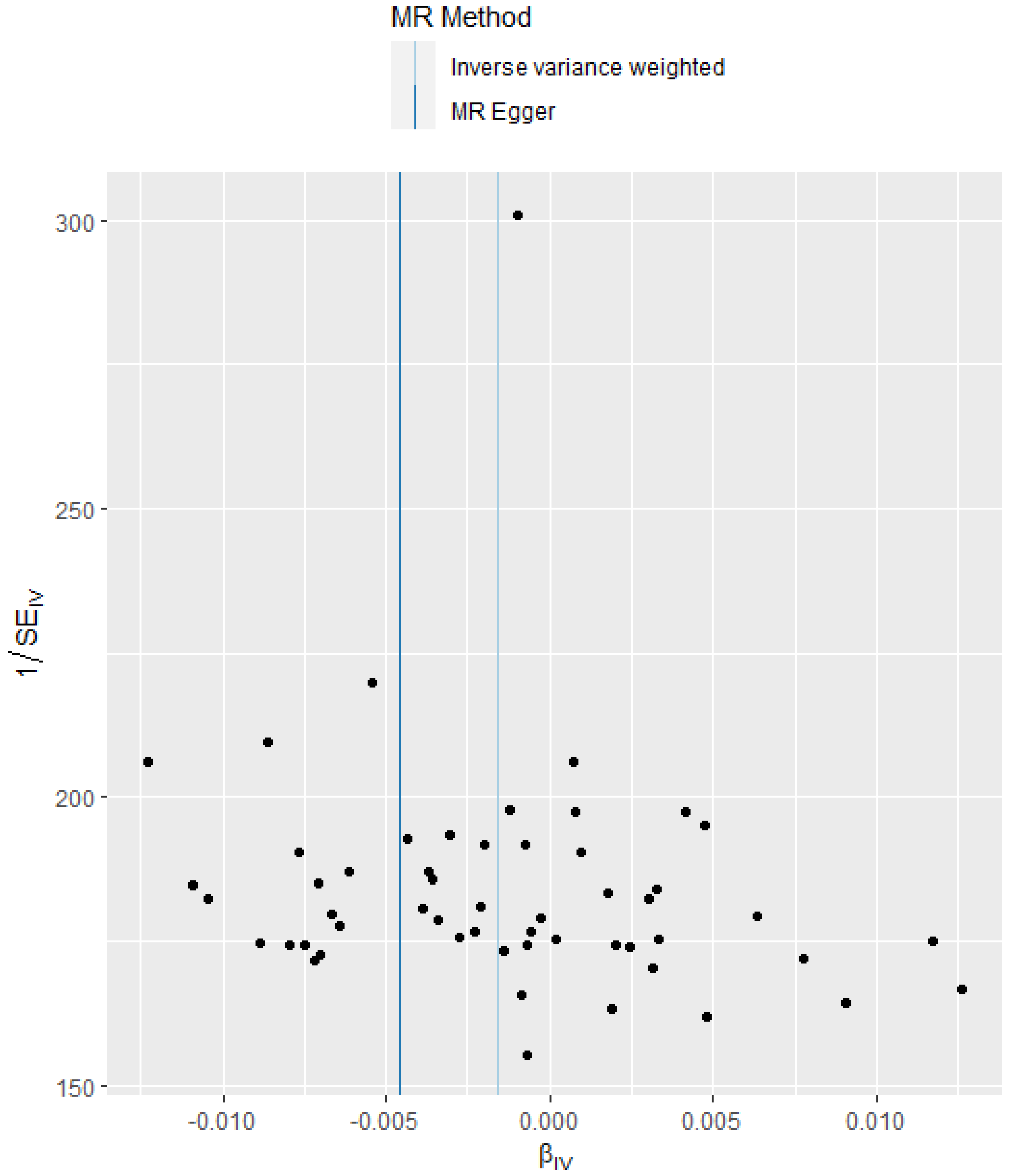

MR Method
Inverse variance weighted
MR Egger
300
250
200
150
1/SE_IV
-0.010
-0.005
0.000
0.005
0.010
β_IV

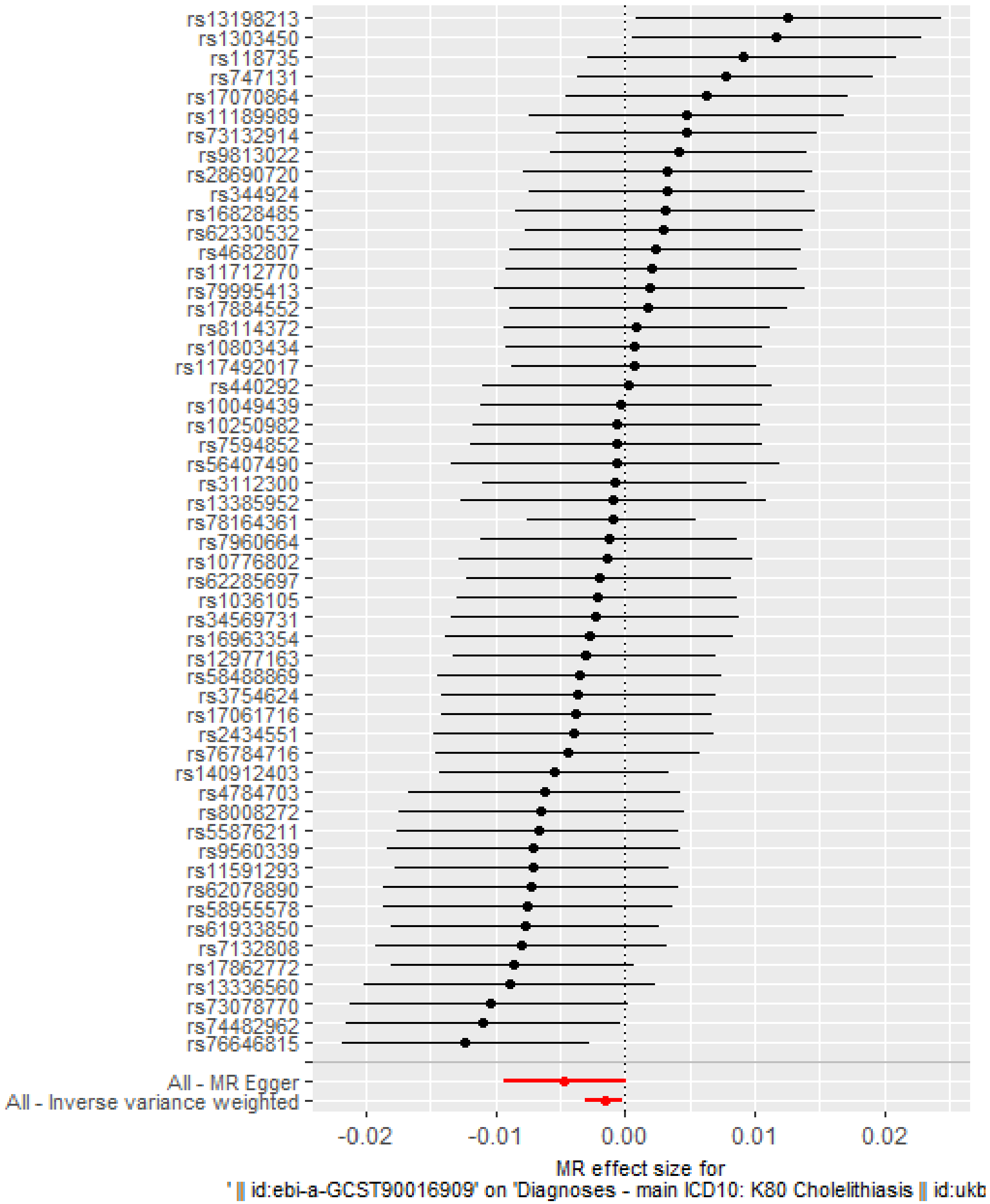

rs13198213
rs1303450
rs118735
rs747131
rs17070864
rs11189989
rs73132914
rs9813022
rs28690720
rs344924
rs16828485
rs62330532
rs4682807
rs11712770
rs79995413
rs17884552
rs8114372
rs10803434
rs117492017
rs440292
rs10049439
rs10250982
rs7594852
rs56407490
rs3112300
rs13385952
rs78164361
rs7960664
rs10776802
rs62285697
rs1036105
rs34569731
rs16963354
rs12977163
rs58488869
rs3754624
rs17061716
rs2434551
rs76784716
rs140912403
rs4784703
rs8008272
rs55876211
rs9560339
rs11591293
rs62078890
rs58955578
rs61933850
rs7132808
rs17862772
rs13336560
rs73078770
rs74482962
rs76646815
All - MR Egger
All - Inverse variance weighted
-0.02
-0.01
0.00
0.01
0.02
MR effect size for
' || id:ebi-a-GCST90016909' on 'Diagnoses - main ICD10: K80 Cholelithiasis || id:ukb

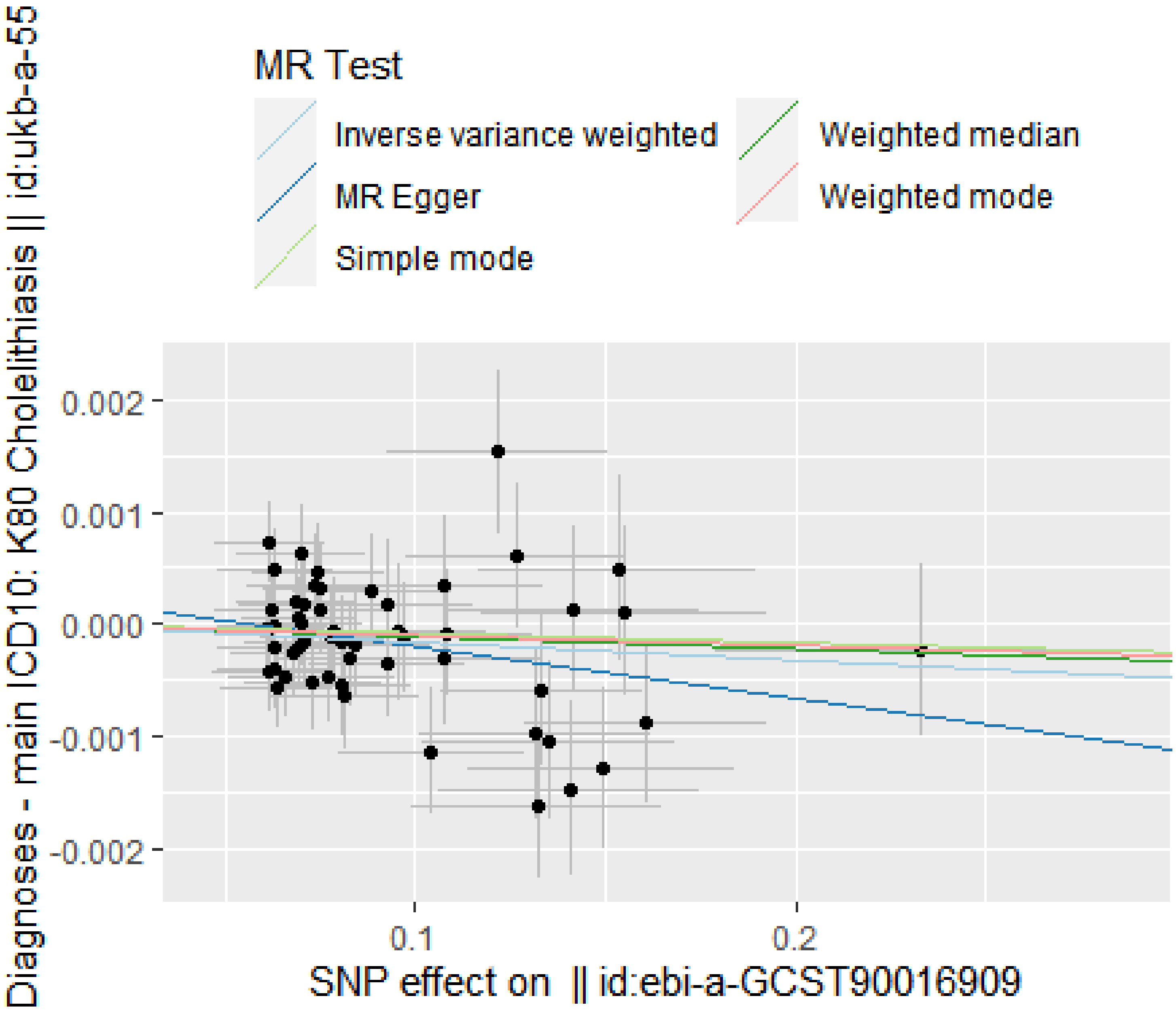
MR Test
Inverse variance weighted
MR Egger
Simple mode
Weighted median
Weighted mode
0.002
0.001
0.000
-0.001
-0.002
0.1
0.2
SNP effect on || id:ebi-a-GCST90016909
Diagnoses - main ICD10: K80 Cholelithiasis || id:ukb-a-55

Figure 191 Leave-one-out analysis, funnel plot, MR effect size and scatter plot for gut microbiota abundance (class Negativicutes id.2164) on cholelithiasis

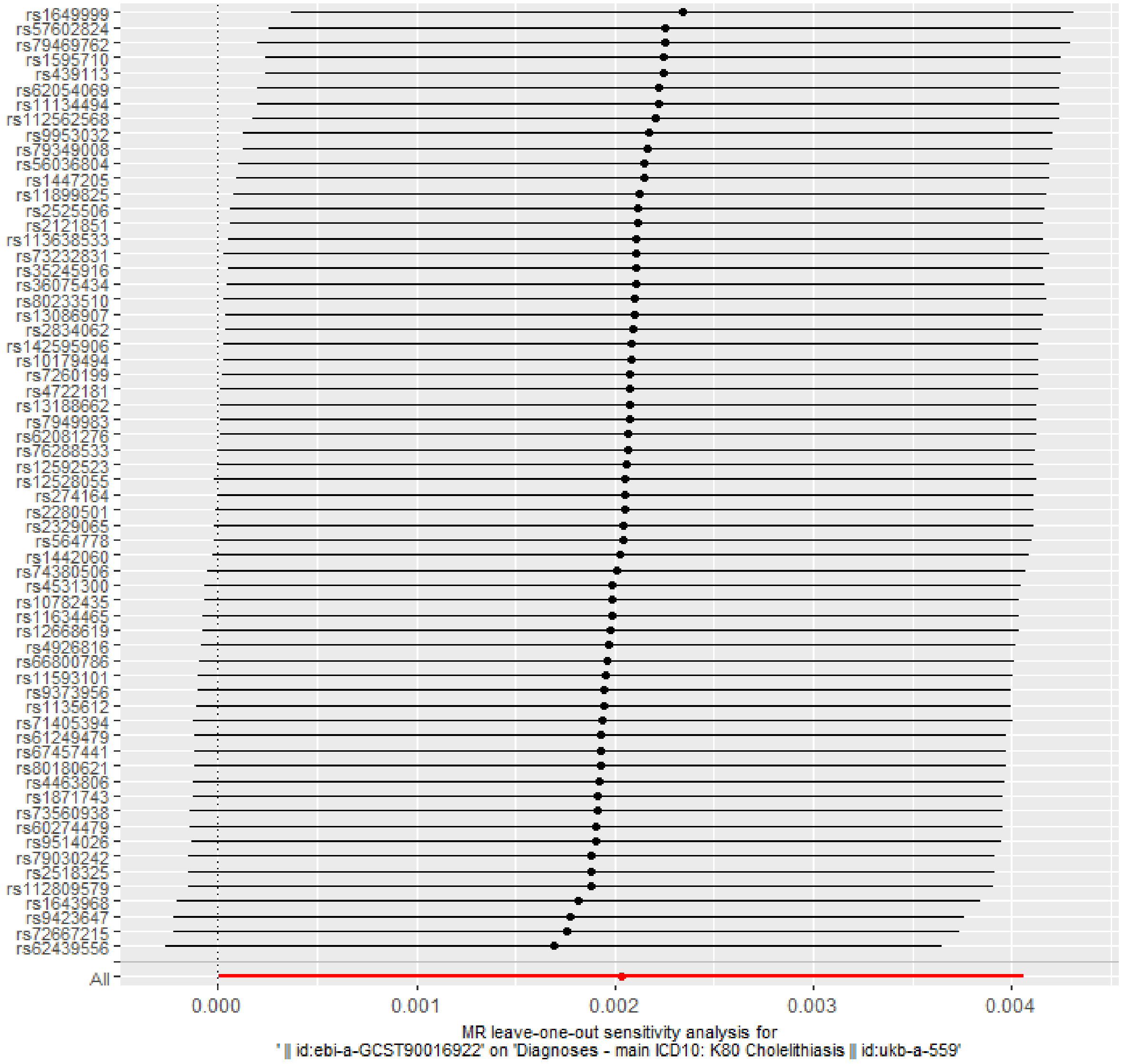

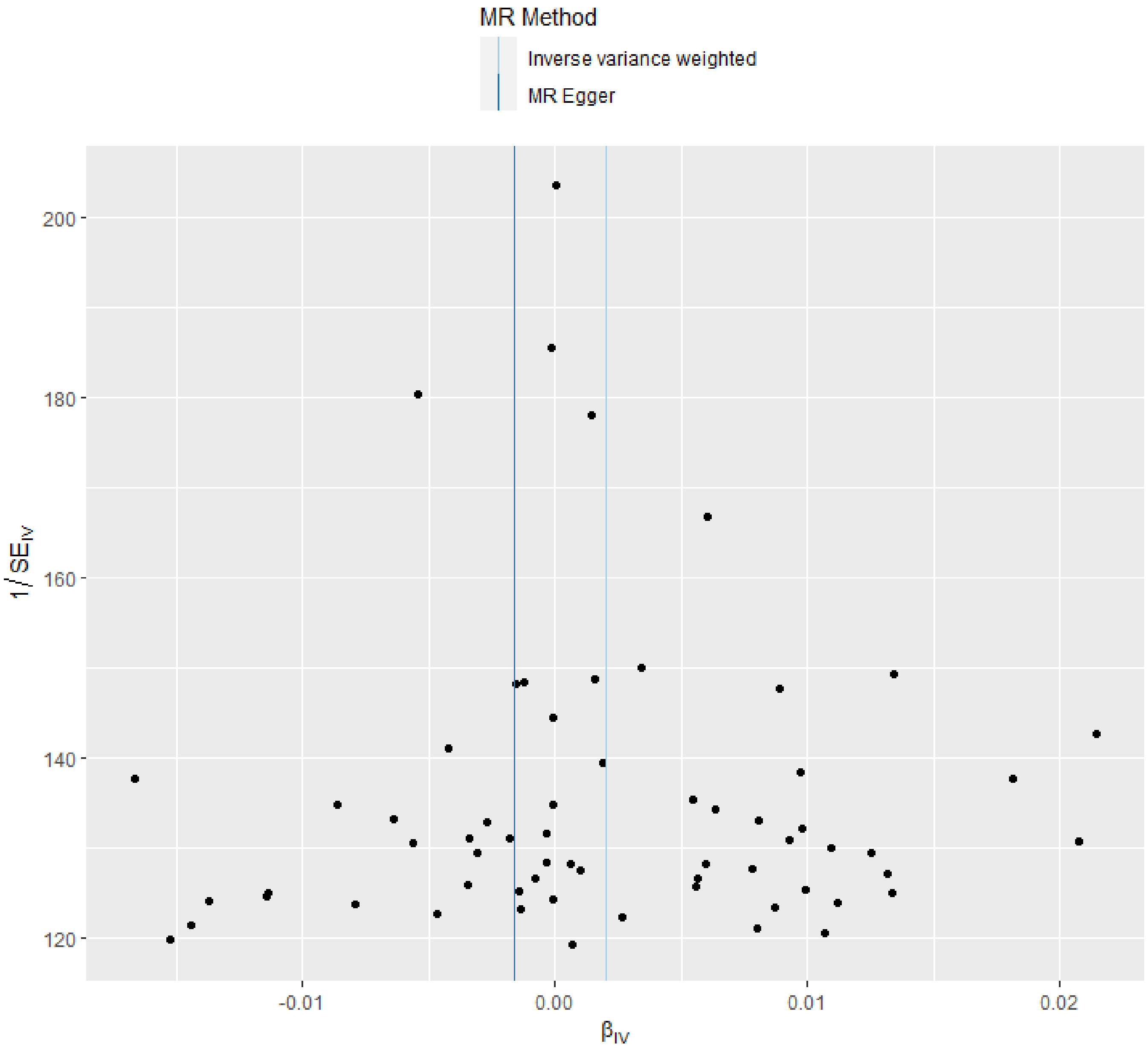
MR Method
Inverse variance weighted
MR Egger
200
180
160
140
120
1/SE_IV
-0.01
0.00
0.01
0.02
β_IV

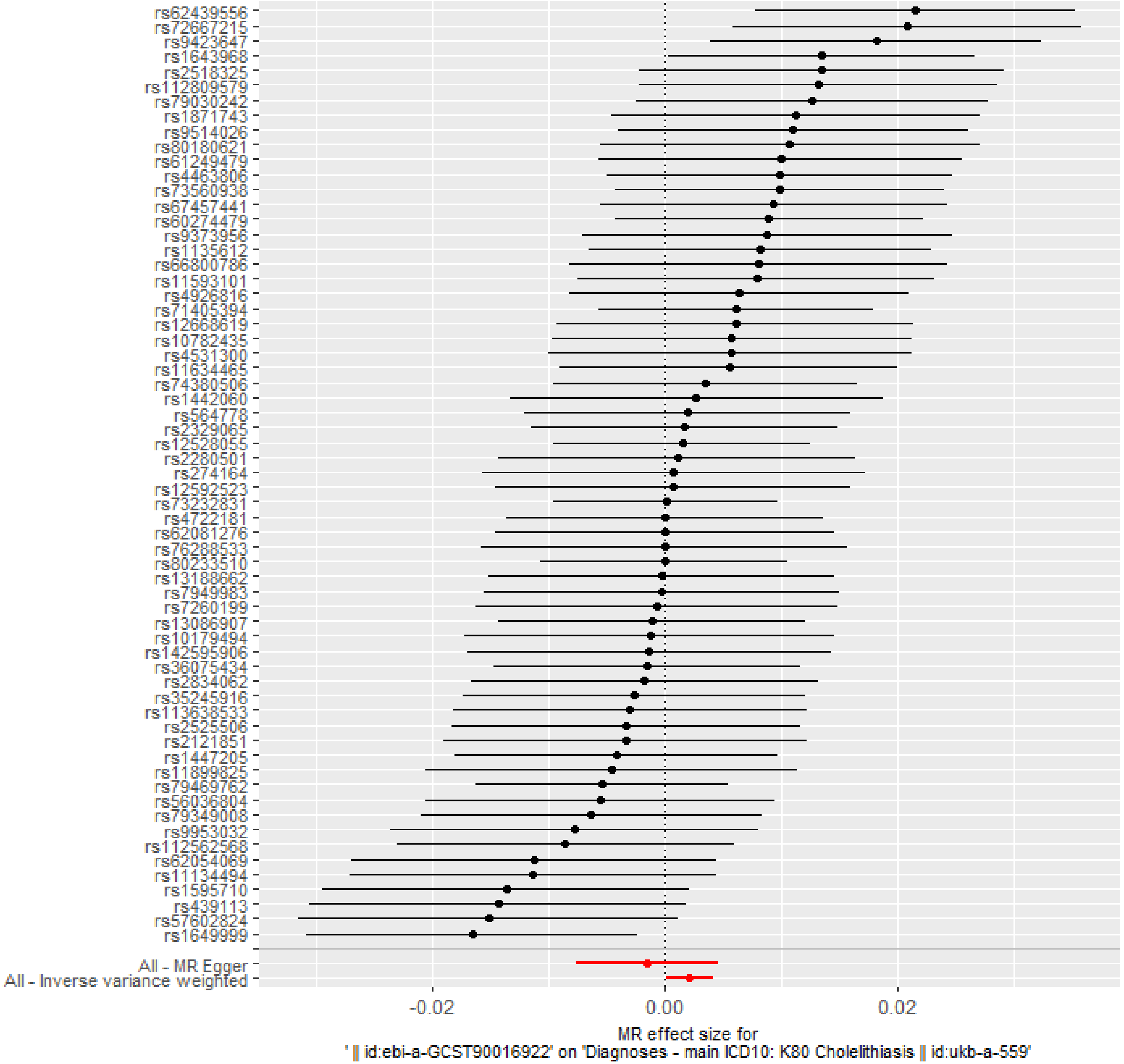
rs62439556
rs72667215
rs9423647
rs1643968
rs2518325
rs112809579
rs79030242
rs1871743
rs9514026
rs80180621
rs61249479
rs4463806
rs73560938
rs67457441
rs60274479
rs9373956
rs1135612
rs66800786
rs11593101
rs4926816
rs71405394
rs12668619
rs10782435
rs4531300
rs11634465
rs74380506
rs1442060
rs564778
rs2329065
rs12528055
rs2280501
rs274164
rs12592523
rs73232831
rs4722181
rs62081276
rs76288533
rs80233510
rs13188662
rs7949983
rs7260199
rs13086907
rs10179494
rs142595906
rs36075434
rs2834062
rs35245916
rs113638533
rs2525506
rs2121851
rs1447205
rs11899825
rs79469762
rs56036804
rs79349008
rs9953032
rs112562568
rs62054069
rs11134494
rs1595710
rs439113
rs57602824
rs1649999
All - MR Egger
All - Inverse variance weighted
-0.02
0.00
0.02
MR effect size for
' || id:ebi-a-GCST90016922' on 'Diagnoses - main ICD10: K80 Cholelithiasis || id:ukb-a-559'

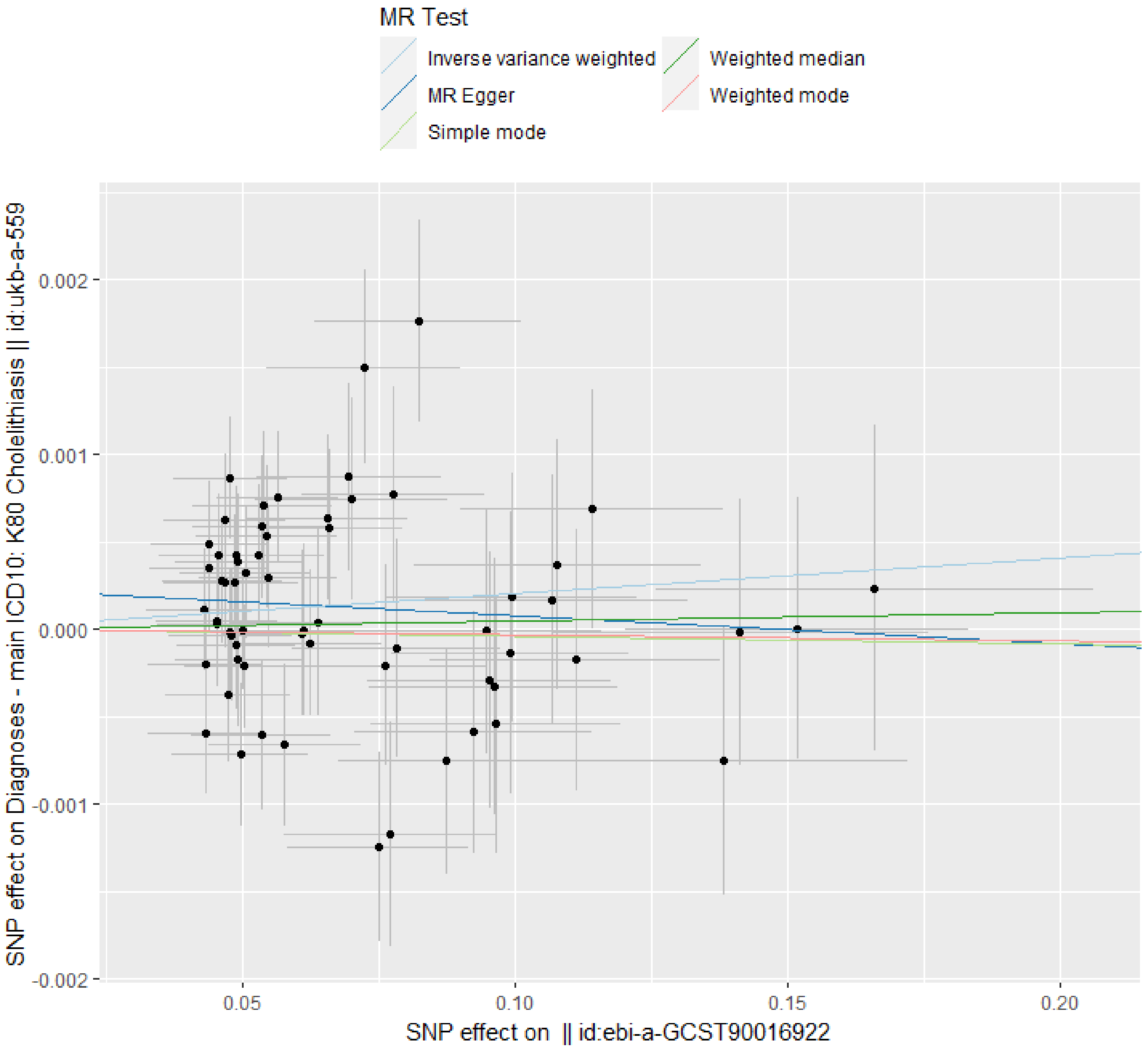

MR Test
Inverse variance weighted
MR Egger
Simple mode
Weighted median
Weighted mode
0.002
0.001
0.000
-0.001
-0.002
0.05
0.10
0.15
0.20
SNP effect on Diagnoses - main ICD10: K80 Cholelithiasis || id:ukb-a-559
SNP effect on || id:ebi-a-GCST90016922

Figure 192 Leave-one-out analysis, funnel plot, MR effect size and scatter plot for gut microbiota abundance (genus Family XIII AD3011 group id.11293) on cholelithiasis

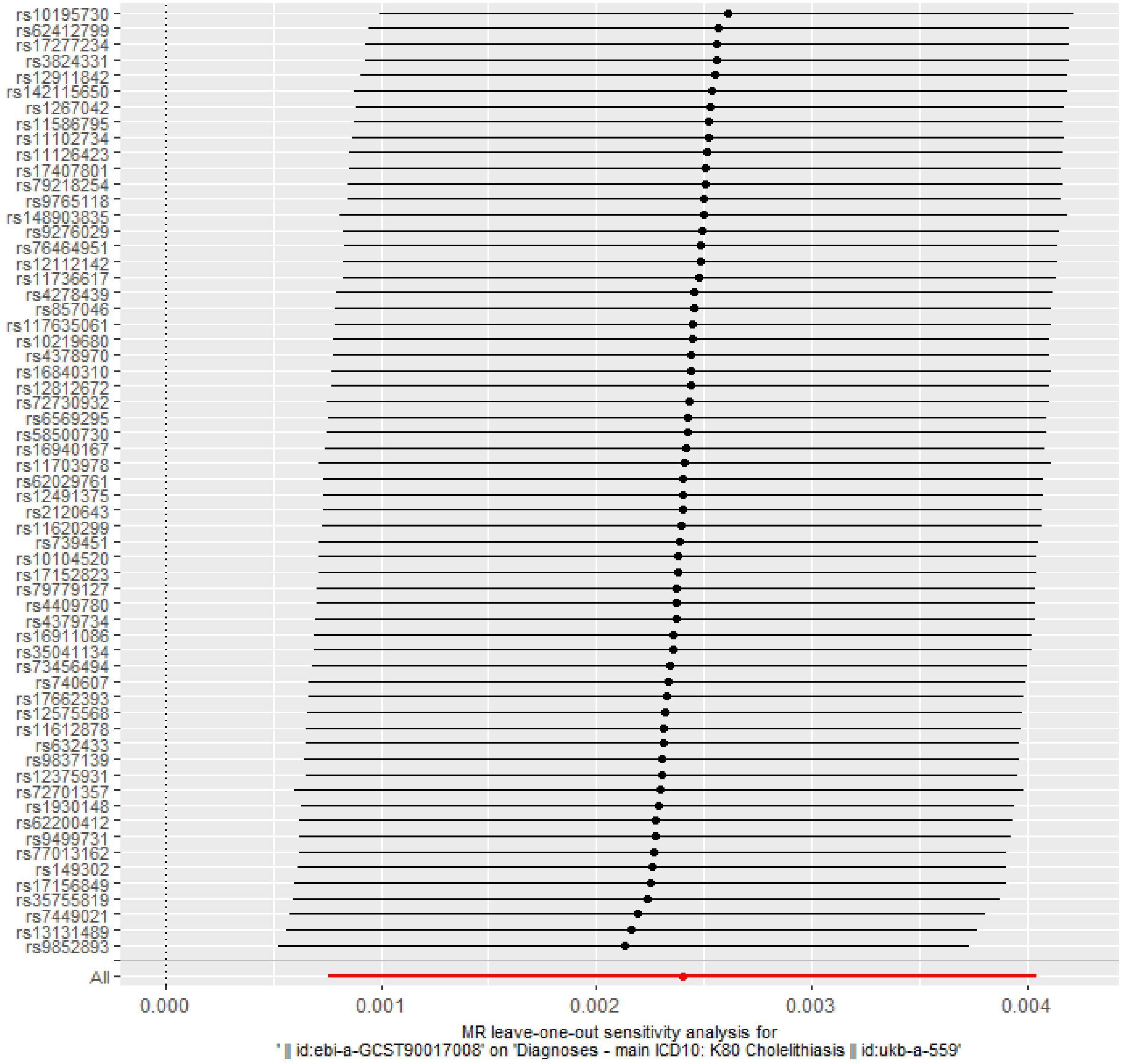

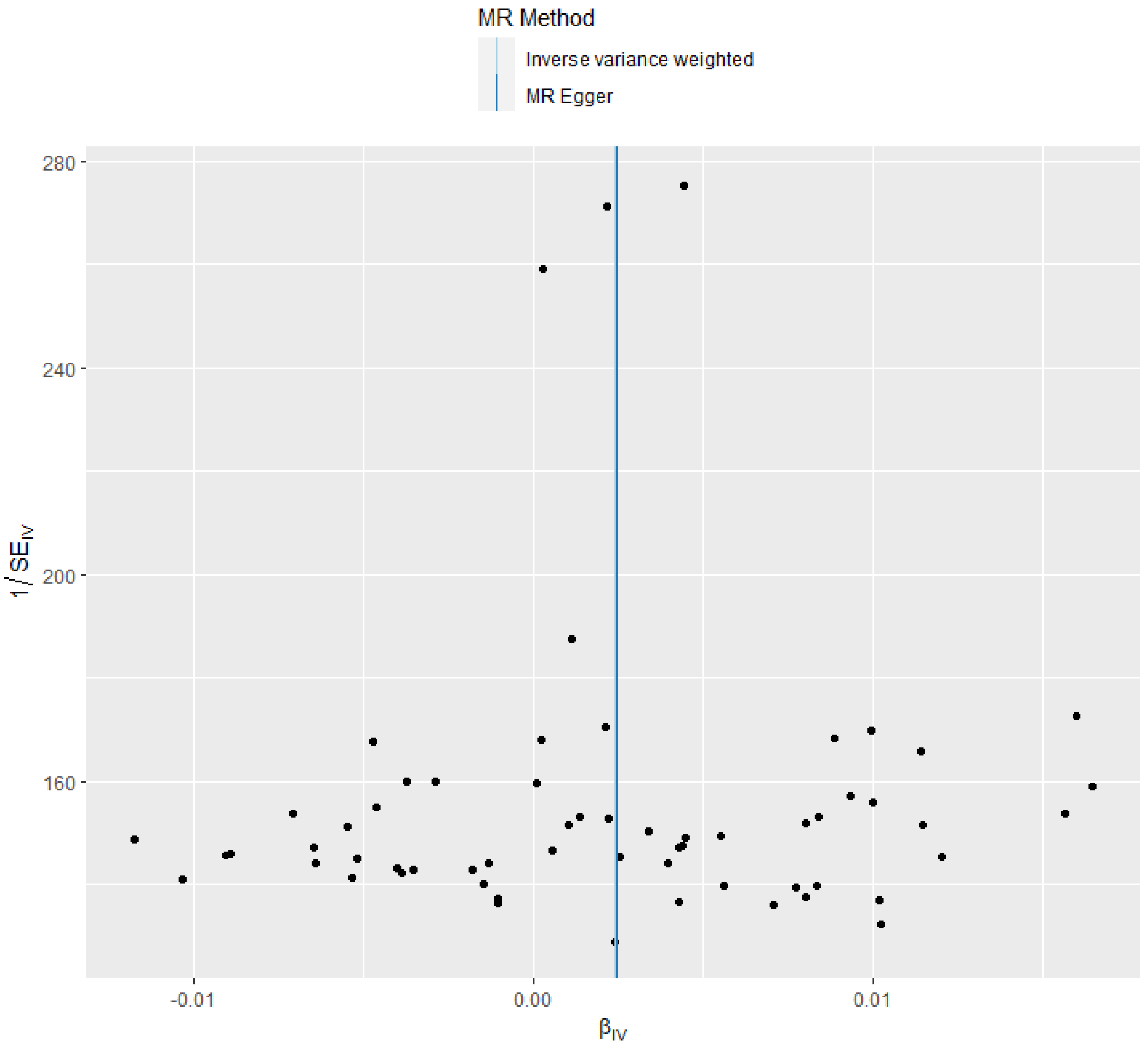
MR Method
Inverse variance weighted
MR Egger
280
240
200
160
1/SE_IV
-0.01
0.00
0.01
β_IV

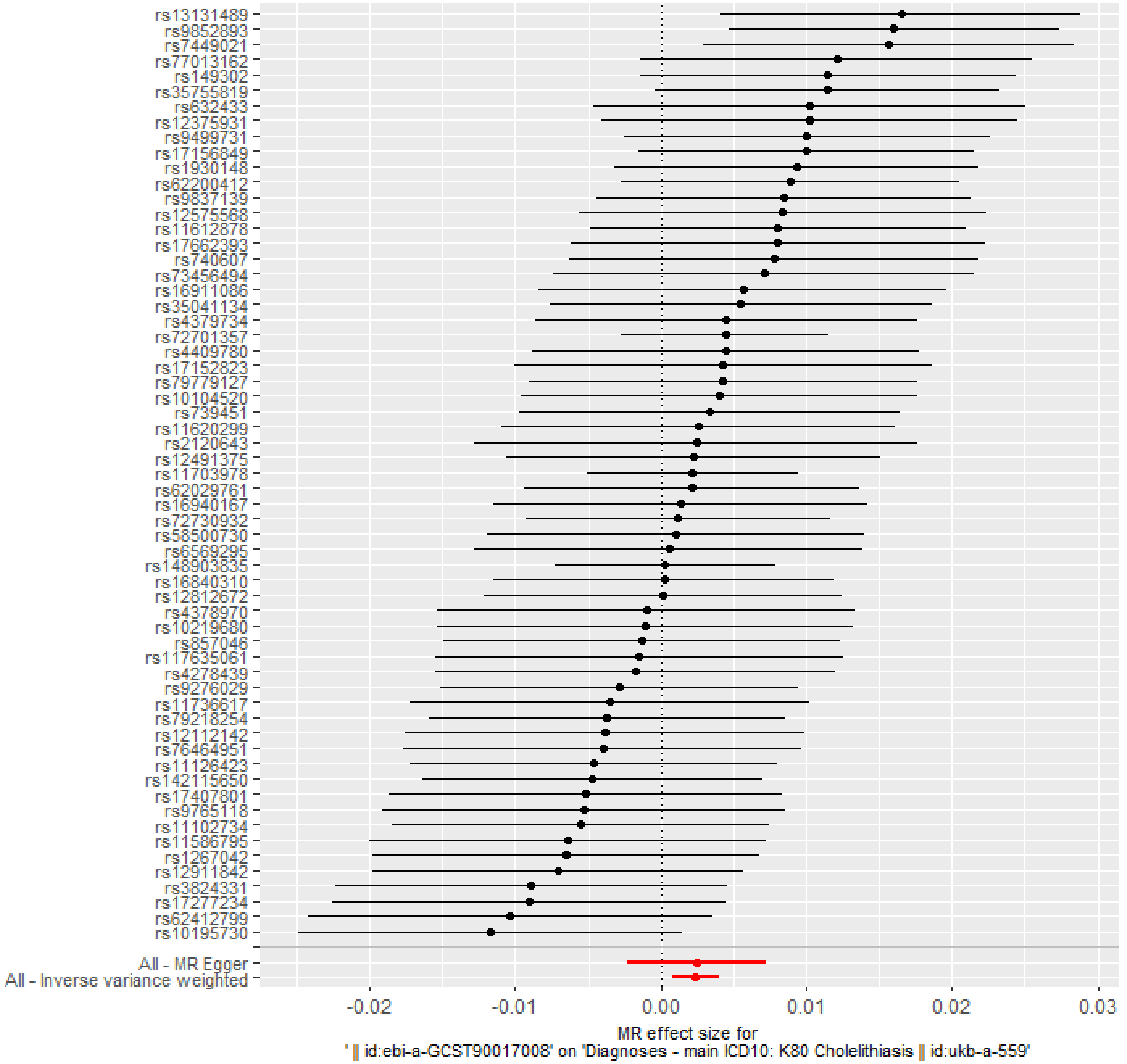

rs13131489
rs9852893
rs7449021
rs77013162
rs149302
rs35755819
rs632433
rs12375931
rs9499731
rs17156849
rs1930148
rs62200412
rs9837139
rs12575568
rs11612878
rs17662393
rs740607
rs73456494
rs16911086
rs35041134
rs4379734
rs72701357
rs4409780
rs17152823
rs79779127
rs10104520
rs739451
rs11620299
rs2120643
rs12491375
rs11703978
rs62029761
rs16940167
rs72730932
rs58500730
rs6569295
rs148903835
rs16840310
rs12812672
rs4378970
rs10219680
rs857046
rs117635061
rs4278439
rs9276029
rs11736617
rs79218254
rs12112142
rs76464951
rs11126423
rs142115650
rs17407801
rs9765118
rs11102734
rs11586795
rs1267042
rs12911842
rs3824331
rs17277234
rs62412799
rs10195730
All - MR Egger
All - Inverse variance weighted
-0.02
-0.01
0.00
0.01
0.02
0.03
MR effect size for
' || id:ebi-a-GCST90017008' on 'Diagnoses - main ICD10: K80 Cholelithiasis || id:ukb-a-559'

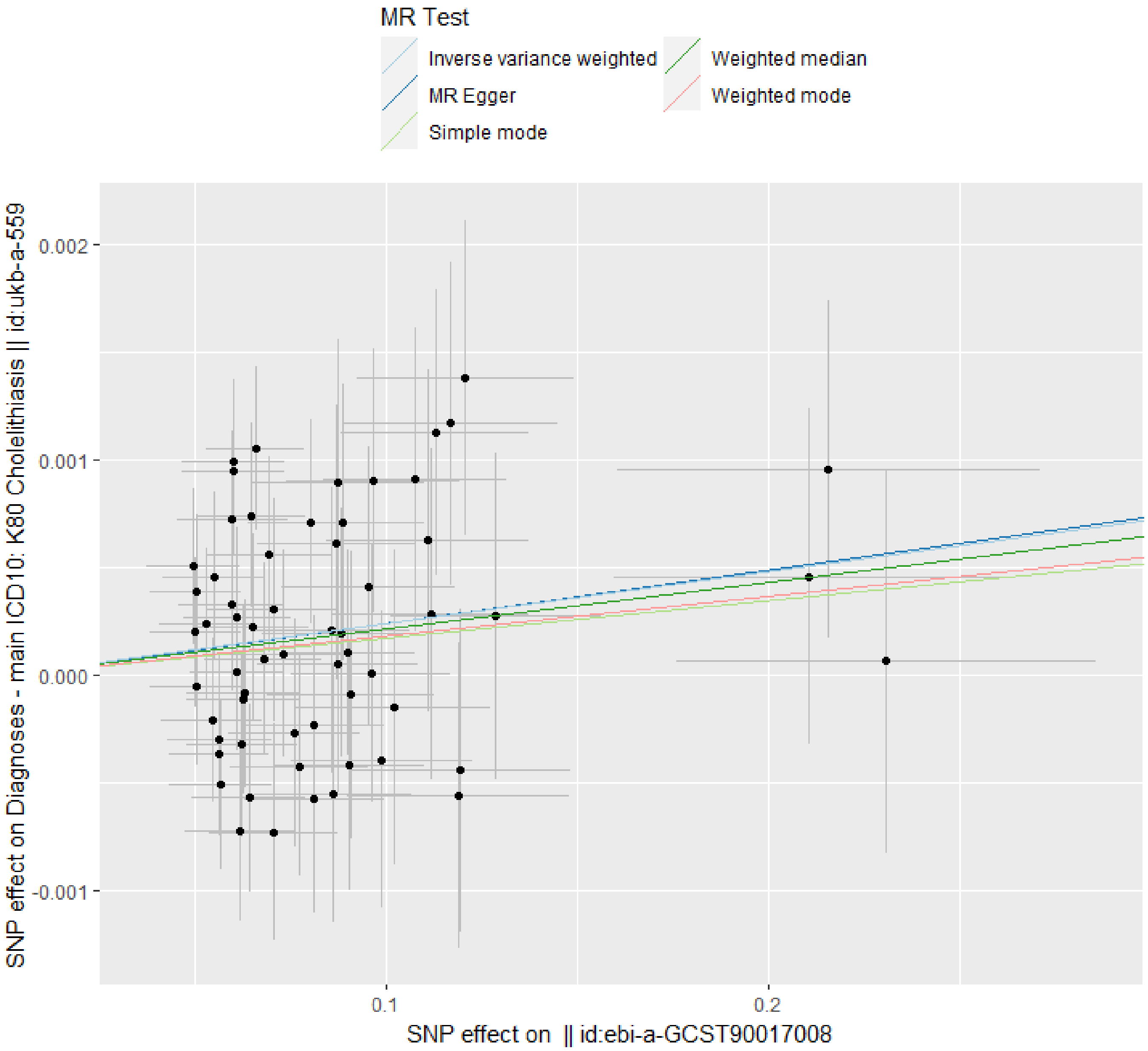

MR Test
Inverse variance weighted
MR Egger
Simple mode
Weighted median
Weighted mode
0.002
0.001
0.000
-0.001
0.1
0.2
SNP effect on Diagnoses - main ICD10: K80 Cholelithiasis || id:ukb-a-559
SNP effect on || id:ebi-a-GCST90017008

Figure 193 Leave-one-out analysis, funnel plot, MR effect size and scatter plot for gut microbiota abundance (genus Lachnospiraceae UCG001 id.11321) on cholelithiasis

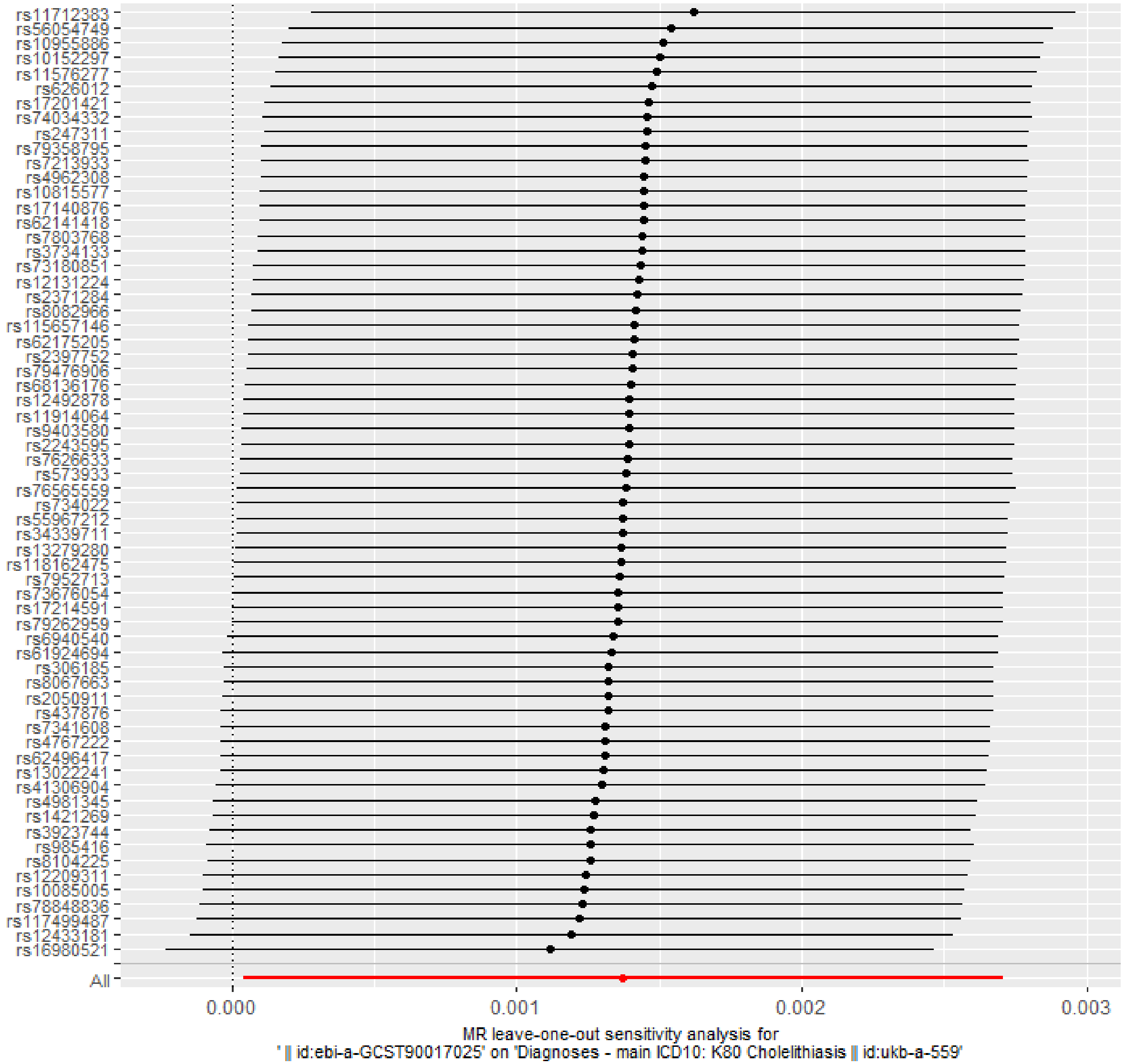

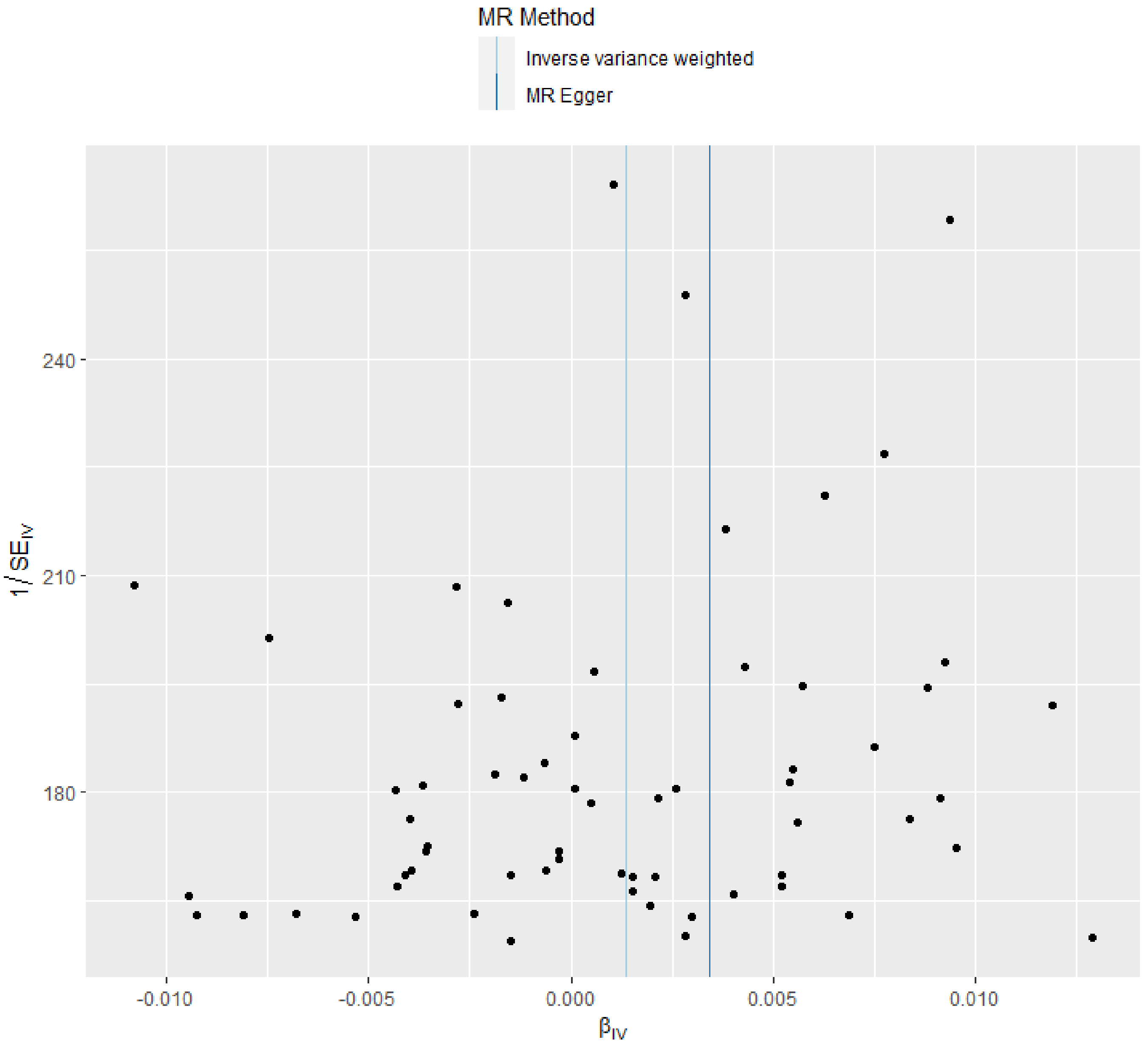
MR Method
Inverse variance weighted
MR Egger
240
210
180
-0.010
-0.005
0.000
0.005
0.010
$1/SE_{IV}$
β_{IV}

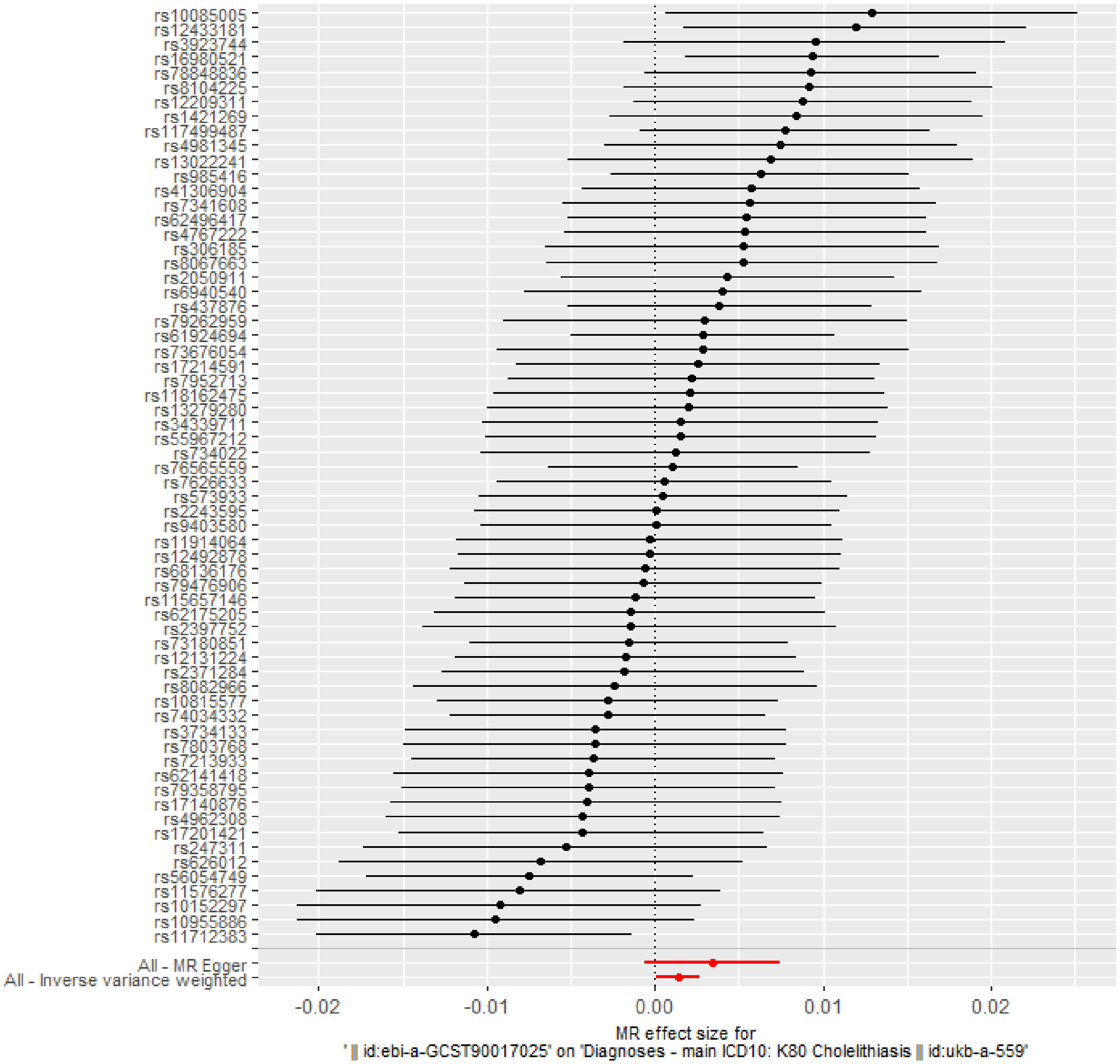
rs10085005
rs12433181
rs3923744
rs16980521
rs78848836
rs8104225
rs12209311
rs1421269
rs117499487
rs4981345
rs13022241
rs985416
rs41306904
rs7341608
rs62496417
rs4767222
rs306185
rs8067663
rs2050911
rs6940540
rs437876
rs79262959
rs61924694
rs73676054
rs17214591
rs7952713
rs118162475
rs13279280
rs34339711
rs55967212
rs734022
rs76565559
rs7626633
rs573933
rs2243595
rs9403580
rs11914064
rs12492878
rs68136176
rs79476906
rs115657146
rs62175205
rs2397752
rs73180851
rs12131224
rs2371284
rs8082966
rs10815577
rs74034332
rs3734133
rs7803768
rs7213933
rs62141418
rs79358795
rs17140876
rs4962308
rs17201421
rs247311
rs626012
rs56054749
rs11576277
rs10152297
rs10955886
rs11712383
All - MR Egger
All - Inverse variance weighted
-0.02
-0.01
0.00
0.01
0.02
MR effect size for
' || id:ebi-a-GCST90017025' on 'Diagnoses - main ICD10: K80 Cholelithiasis || id:ukb-a-559'

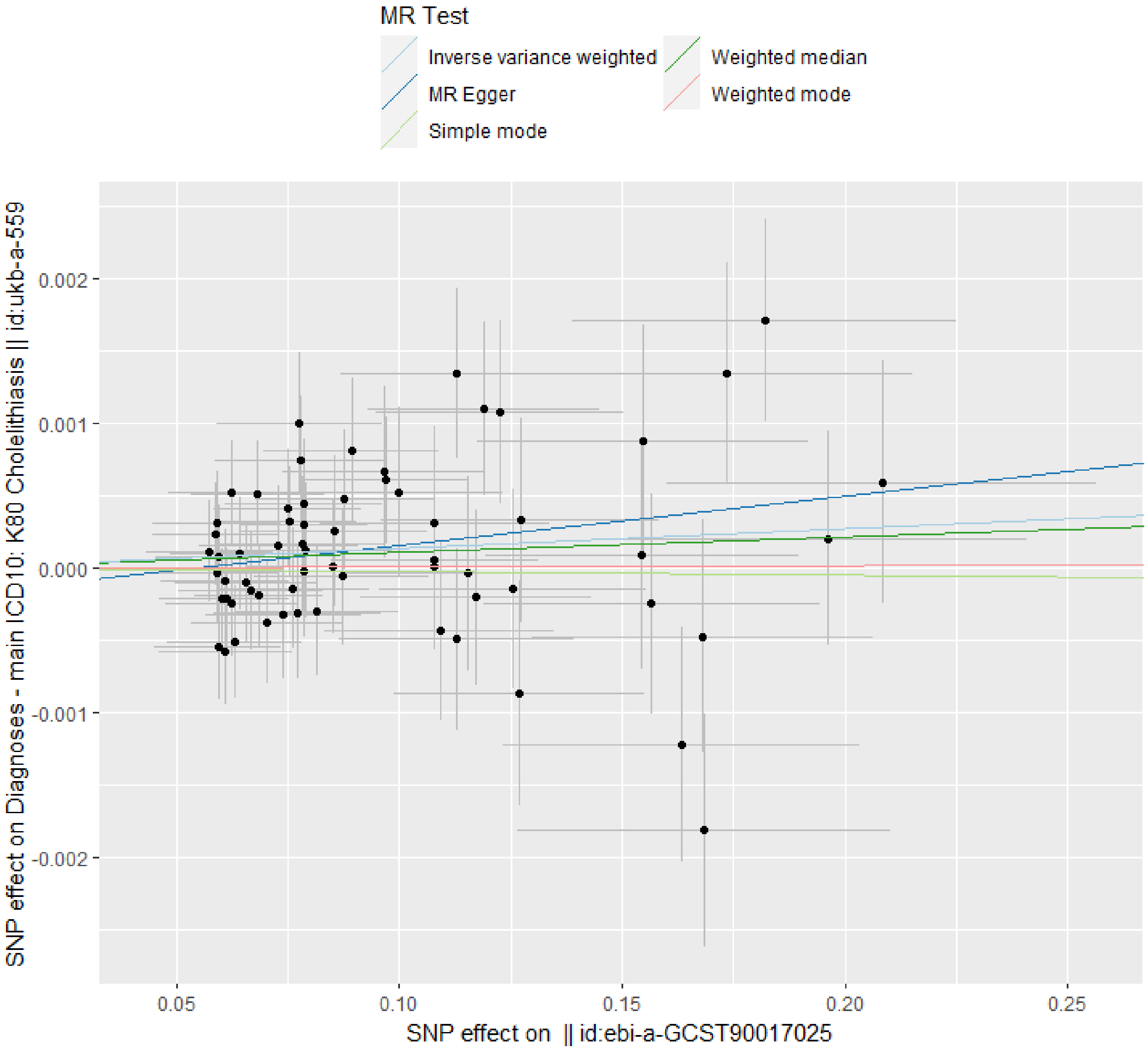

MR Test
Inverse variance weighted
MR Egger
Simple mode
Weighted median
Weighted mode
SNP effect on Diagnoses - main ICD10: K80 Cholelithiasis || id:ukb-a-559
0.002
0.001
0.000
-0.001
-0.002
0.05
0.10
0.15
0.20
0.25
SNP effect on || id:ebi-a-GCST90017025

Figure 194 Leave-one-out analysis, funnel plot, MR effect size and scatter plot for gut microbiota abundance (genus Ruminococcaceae UCG010 id.11367) on cholelithiasis

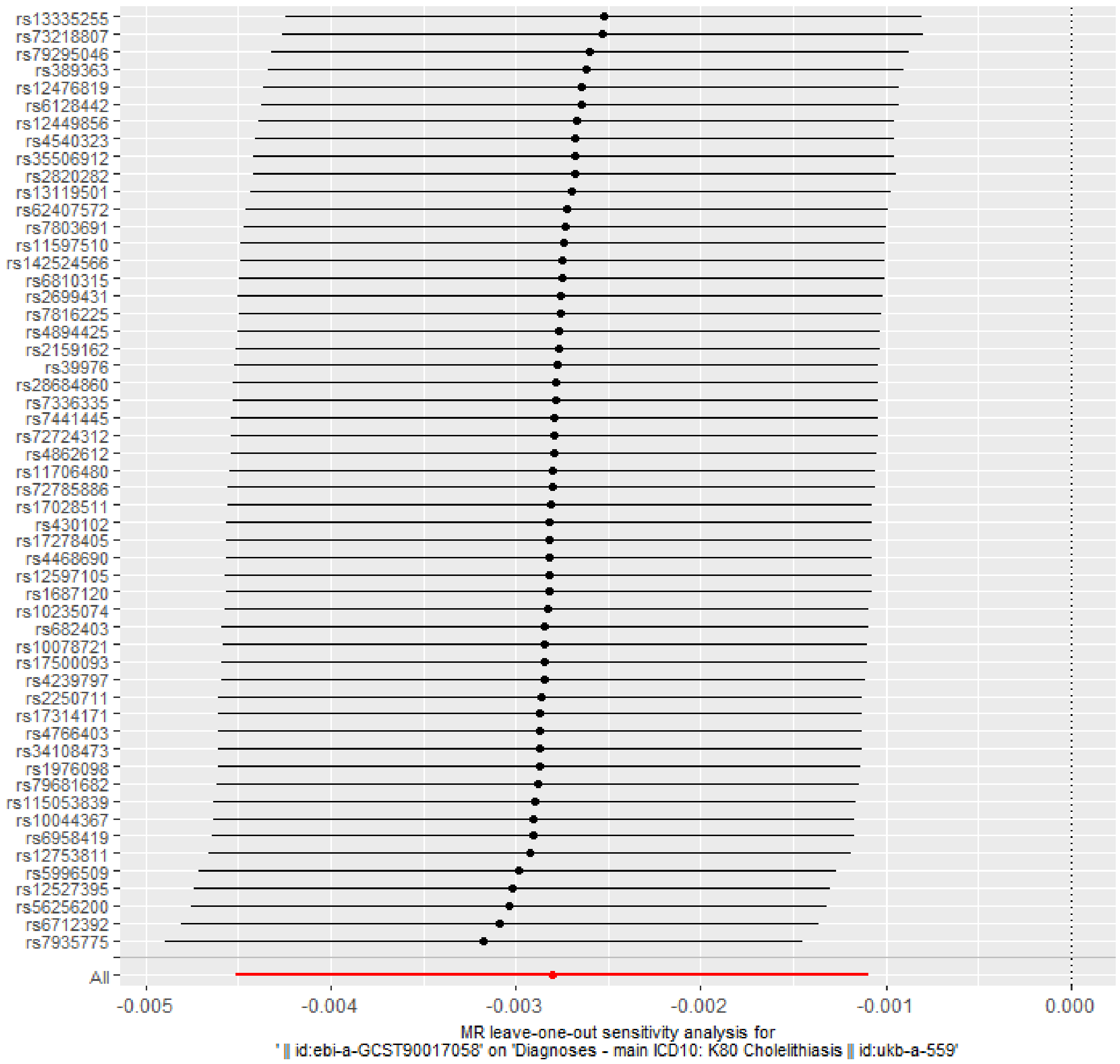

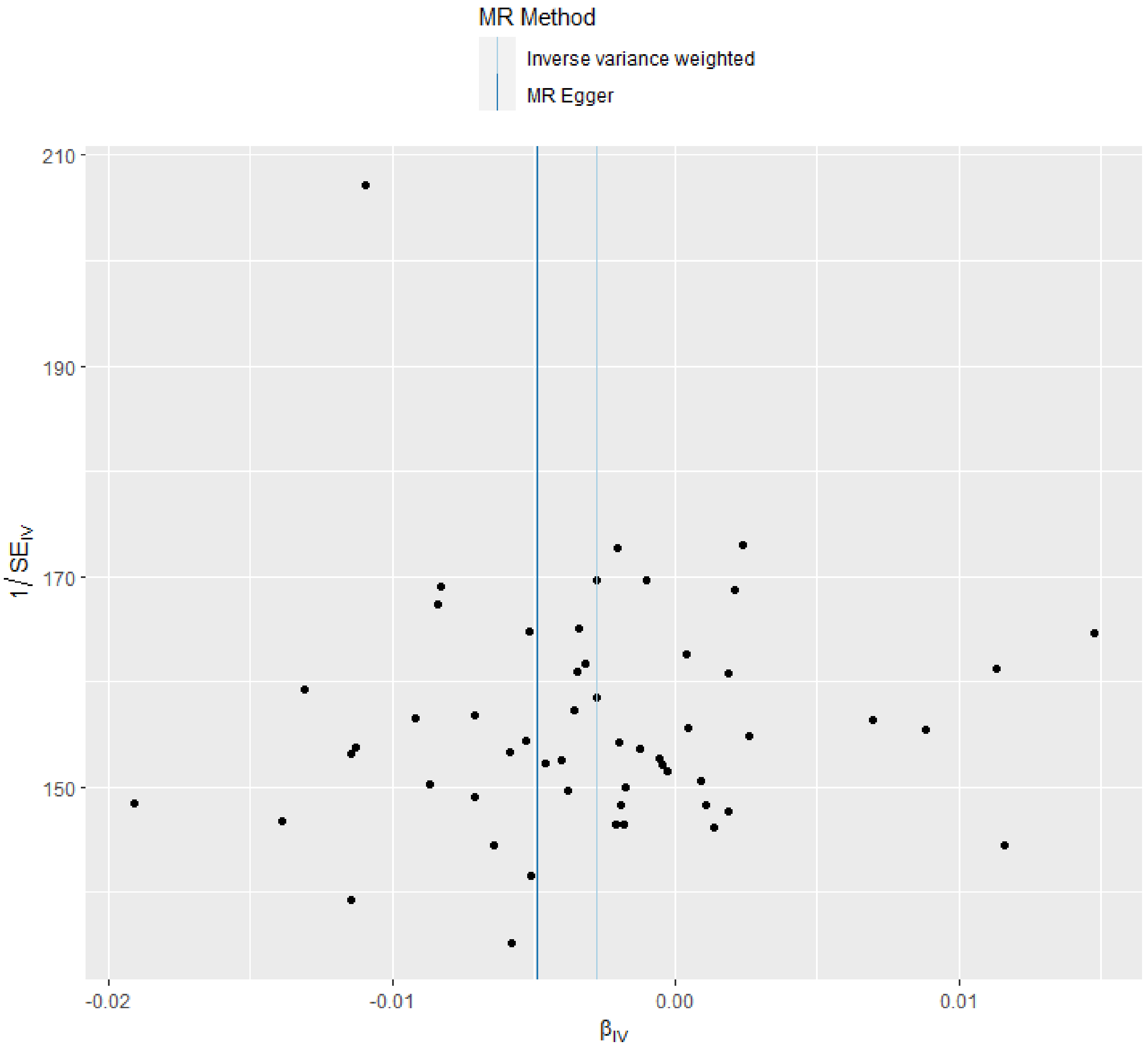
MR Method
Inverse variance weighted
MR Egger
210
190
170
150
1/SE_IV
-0.02
-0.01
0.00
0.01
β_IV

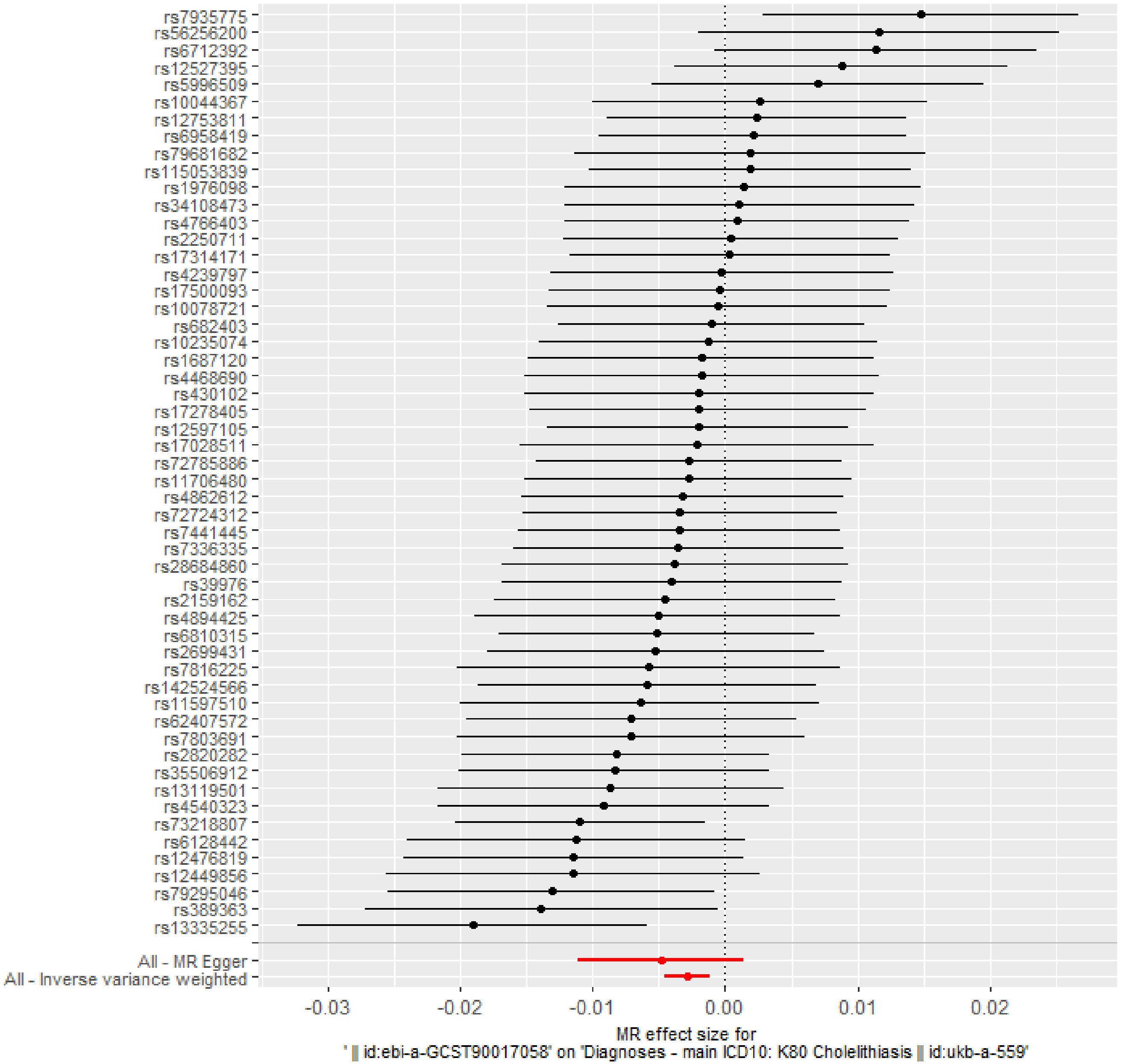

rs7935775
rs56256200
rs6712392
rs12527395
rs5996509
rs10044367
rs12753811
rs6958419
rs79681682
rs115053839
rs1976098
rs34108473
rs4766403
rs2250711
rs17314171
rs4239797
rs17500093
rs10078721
rs682403
rs10235074
rs1687120
rs4468690
rs430102
rs17278405
rs12597105
rs17028511
rs72785886
rs11706480
rs4862612
rs72724312
rs7441445
rs7336335
rs28684860
rs39976
rs2159162
rs4894425
rs6810315
rs2699431
rs7816225
rs142524566
rs11597510
rs62407572
rs7803691
rs2820282
rs35506912
rs13119501
rs4540323
rs73218807
rs6128442
rs12476819
rs12449856
rs79295046
rs389363
rs13335255
All - MR Egger
All - Inverse variance weighted
-0.03
-0.02
-0.01
0.00
0.01
0.02
MR effect size for
' || id:ebi-a-GCST90017058' on 'Diagnoses - main ICD10: K80 Cholelithiasis || id:ukb-a-559'

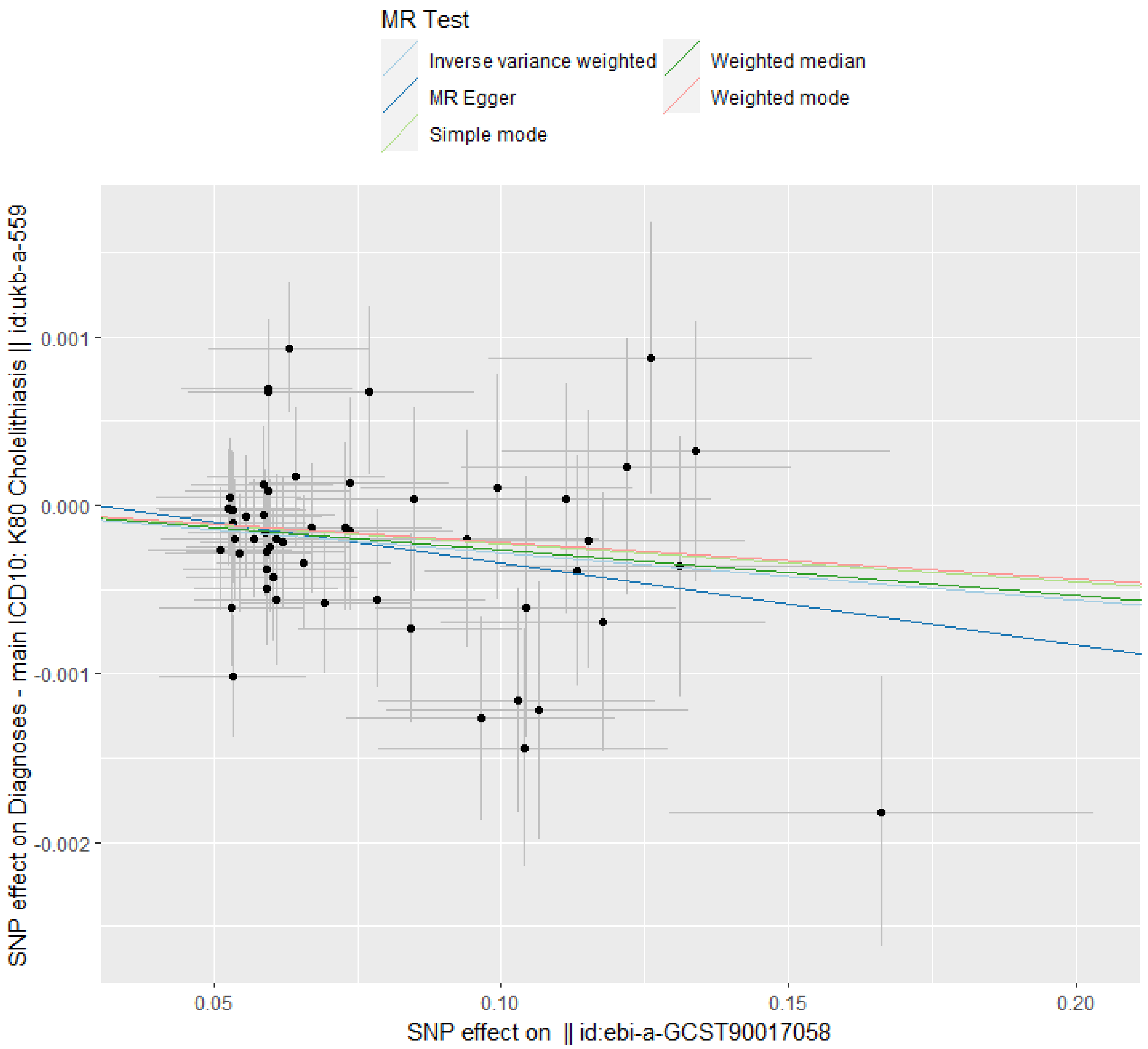
MR Test
Inverse variance weighted
MR Egger
Simple mode
Weighted median
Weighted mode
0.001
0.000
-0.001
-0.002
SNP effect on Diagnoses - main ICD10: K80 Cholelithiasis || id:ukb-a-559
0.05
0.10
0.15
0.20
SNP effect on || id:ebi-a-GCST90017058

Figure 195 Leave-one-out analysis, funnel plot, MR effect size and scatter plot for gut microbiota abundance (order Selenomonadales id.2165) on cholelithiasis

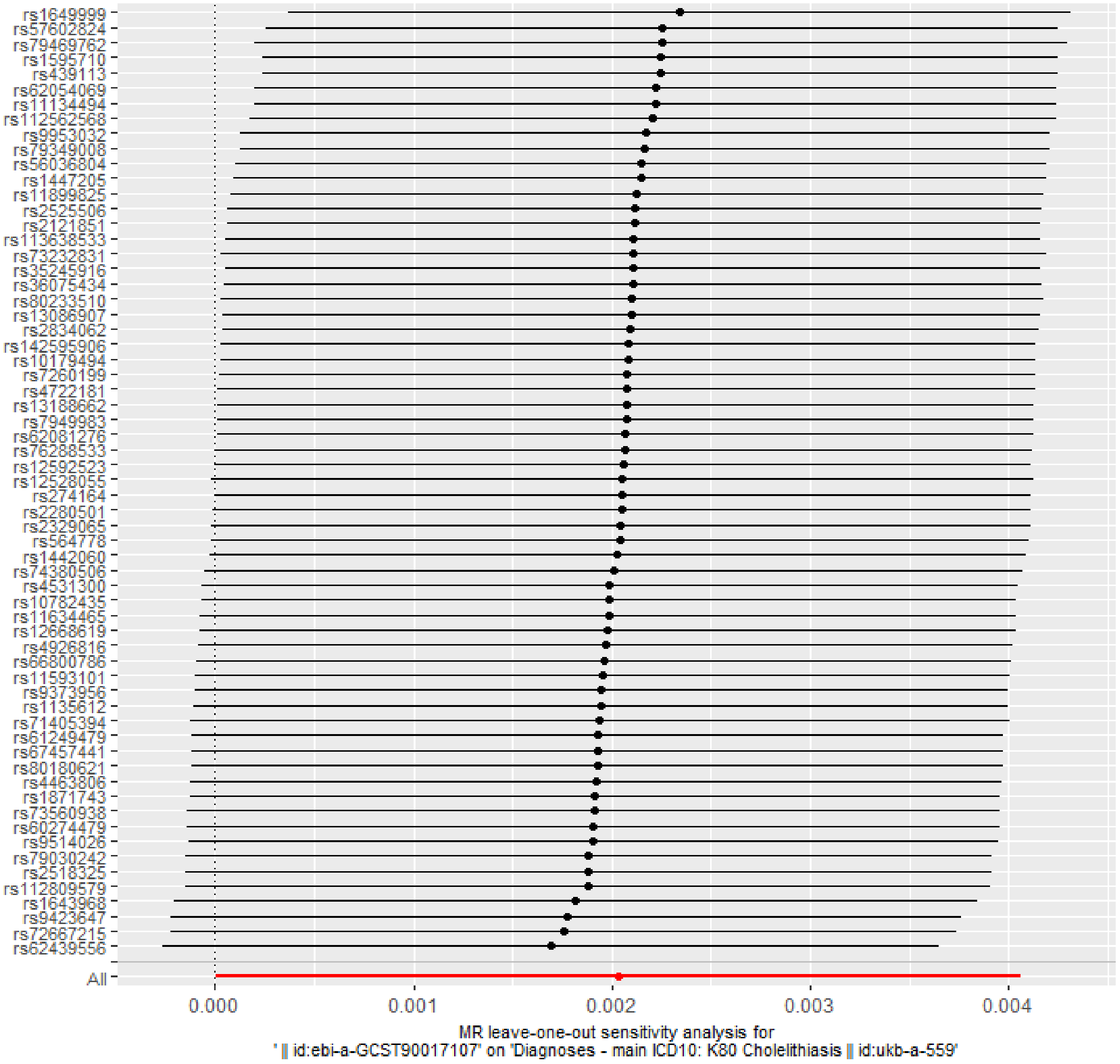

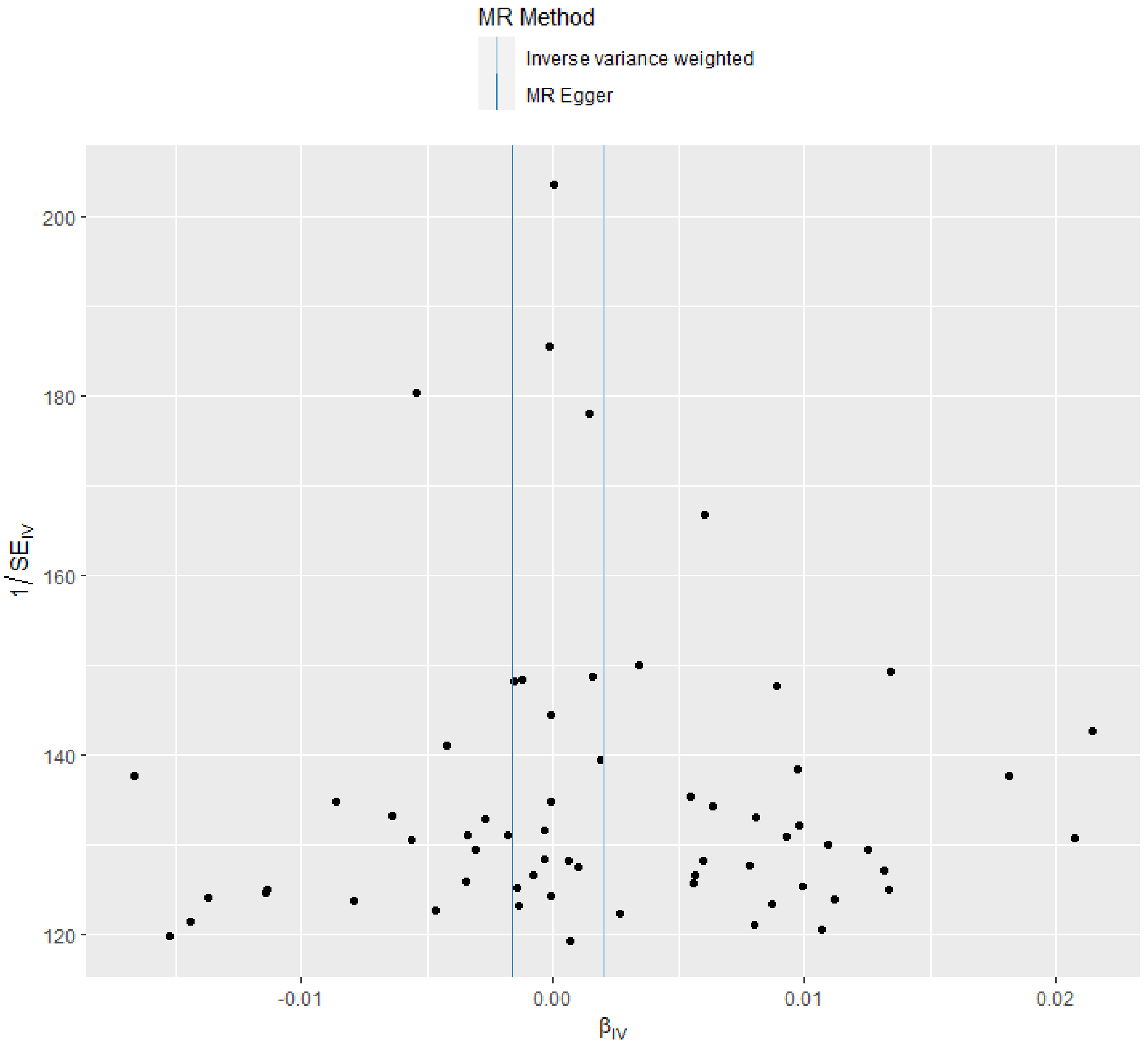
MR Method
Inverse variance weighted
MR Egger
200
180
160
140
120
$1/SE_{IV}$
-0.01
0.00
0.01
0.02
β_{IV}

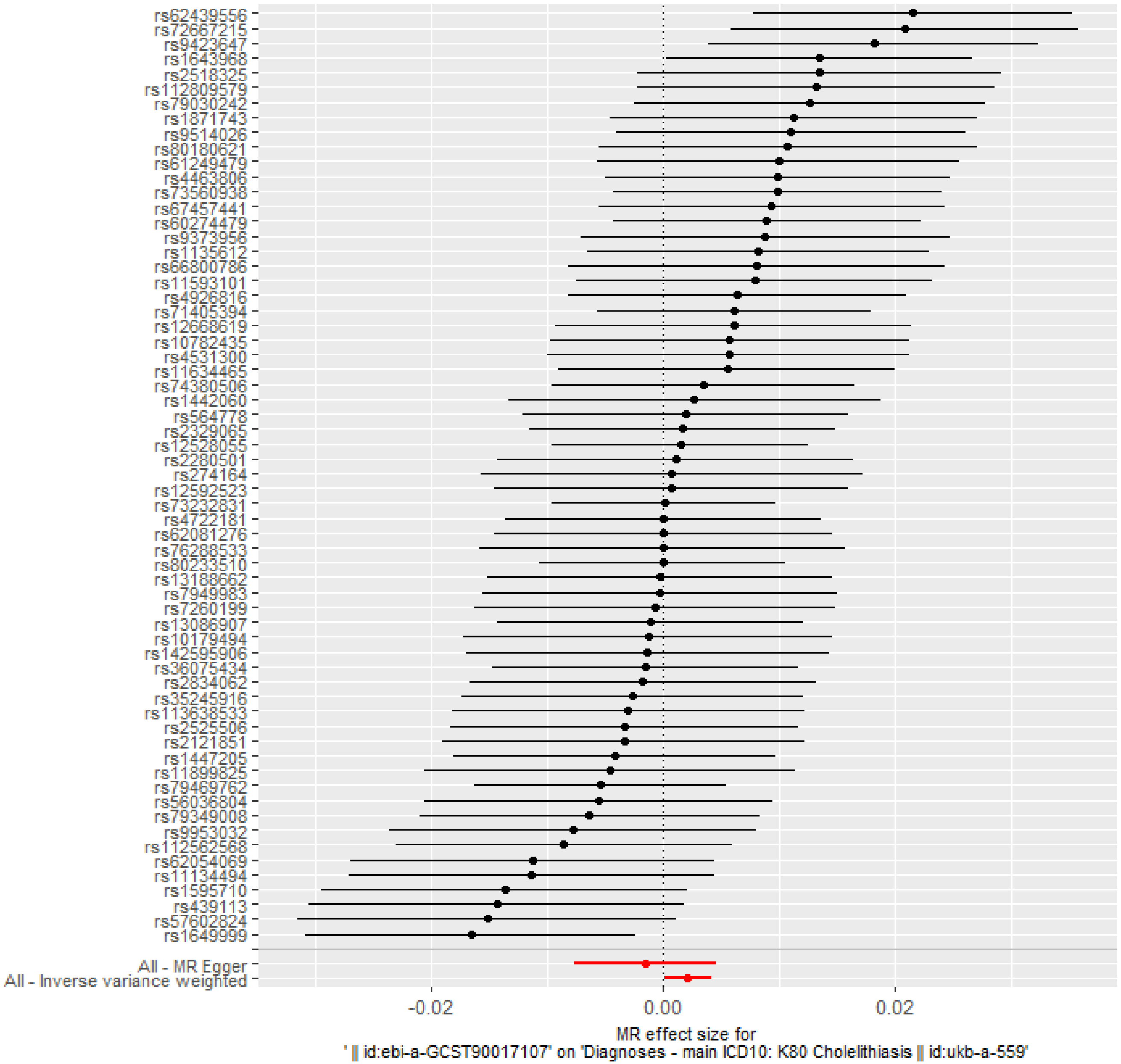

rs62439556
rs72667215
rs9423647
rs1643968
rs2518325
rs112809579
rs79030242
rs1871743
rs9514026
rs80180621
rs61249479
rs4463806
rs73560938
rs67457441
rs60274479
rs9373956
rs1135612
rs66800786
rs11593101
rs4926816
rs71405394
rs12668619
rs10782435
rs4531300
rs11634465
rs74380506
rs1442060
rs564778
rs2329065
rs12528055
rs2280501
rs274164
rs12592523
rs73232831
rs4722181
rs62081276
rs76288533
rs80233510
rs13188662
rs7949983
rs7260199
rs13086907
rs10179494
rs142595906
rs36075434
rs2834062
rs35245916
rs113638533
rs2525506
rs2121851
rs1447205
rs11899825
rs79469762
rs56036804
rs79349008
rs9953032
rs112562568
rs62054069
rs11134494
rs1595710
rs439113
rs57602824
rs1649999
All - MR Egger
All - Inverse variance weighted
-0.02
0.00
0.02
MR effect size for
'|| id:ebi-a-GCST90017107' on 'Diagnoses - main ICD10: K80 Cholelithiasis || id:ukb-a-559'

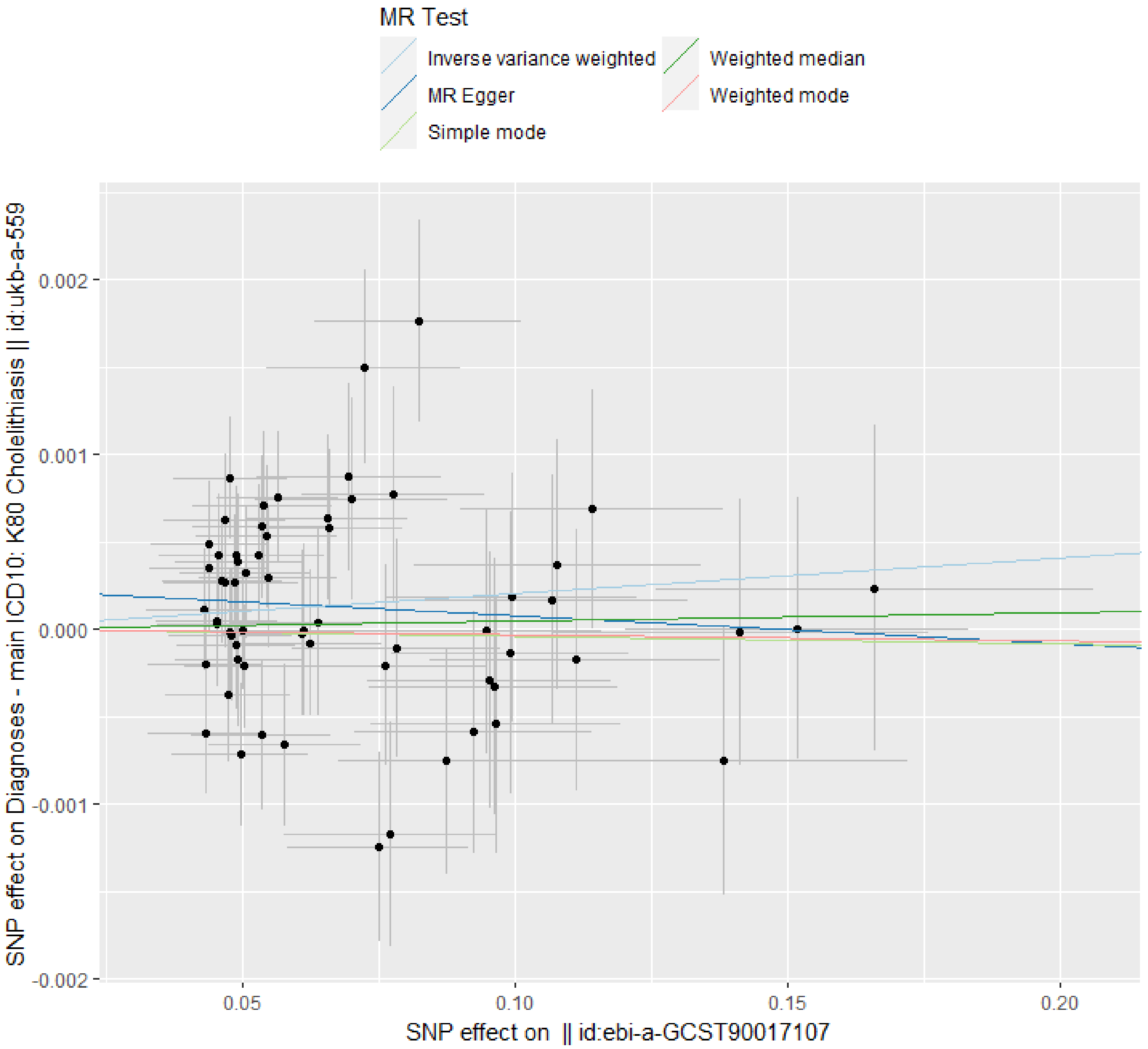
MR Test
Inverse variance weighted
MR Egger
Simple mode
Weighted median
Weighted mode
SNP effect on Diagnoses - main ICD10: K80 Cholelithiasis || id:ukb-a-559
0.002
0.001
0.000
-0.001
-0.002
0.05
0.10
0.15
0.20
SNP effect on || id:ebi-a-GCST90017107

Figure 196 Leave-one-out analysis, funnel plot, MR effect size and scatter plot for gut microbiota abundance (class Methanobacteria id.119) on acute pancreatitis

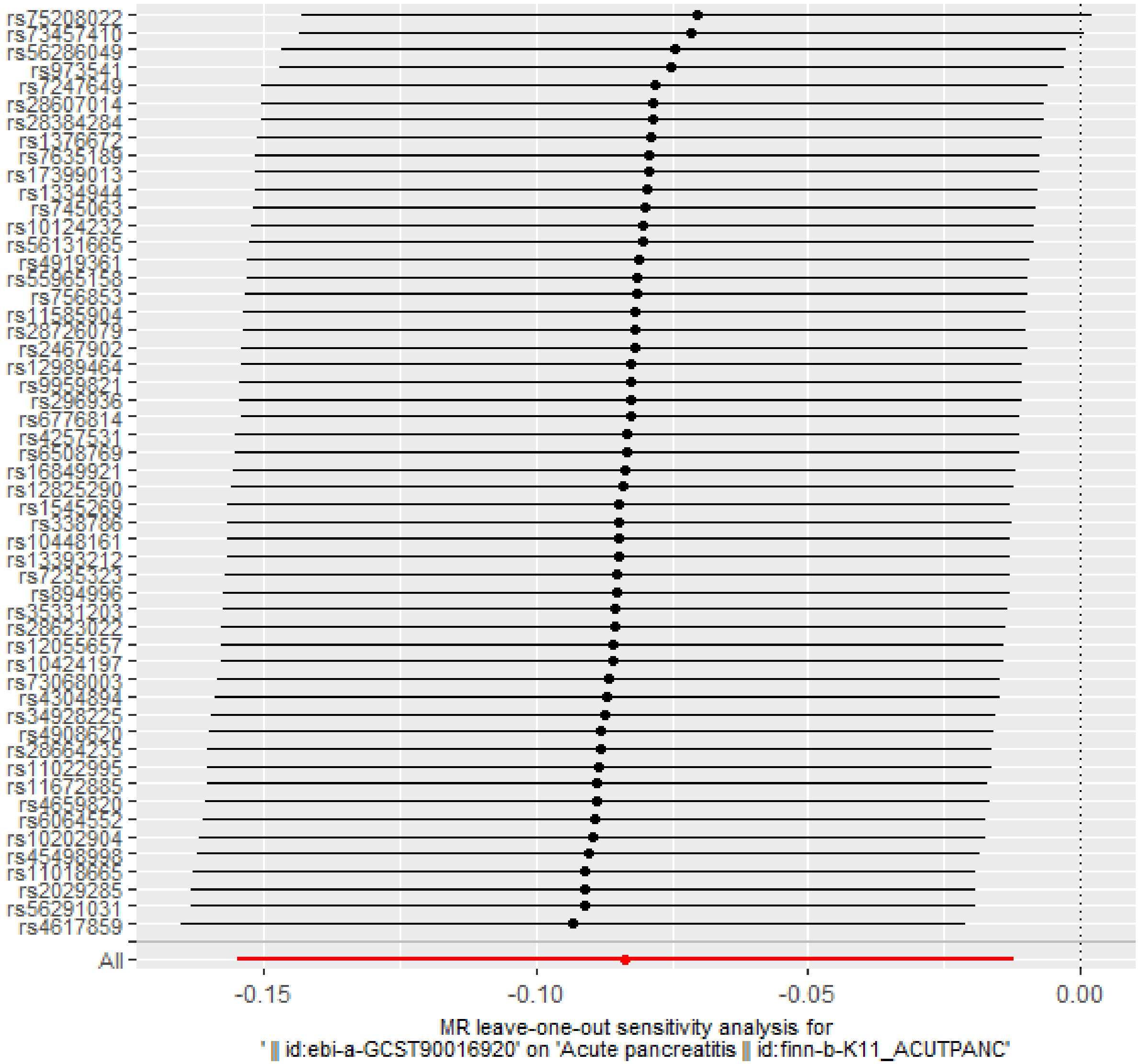

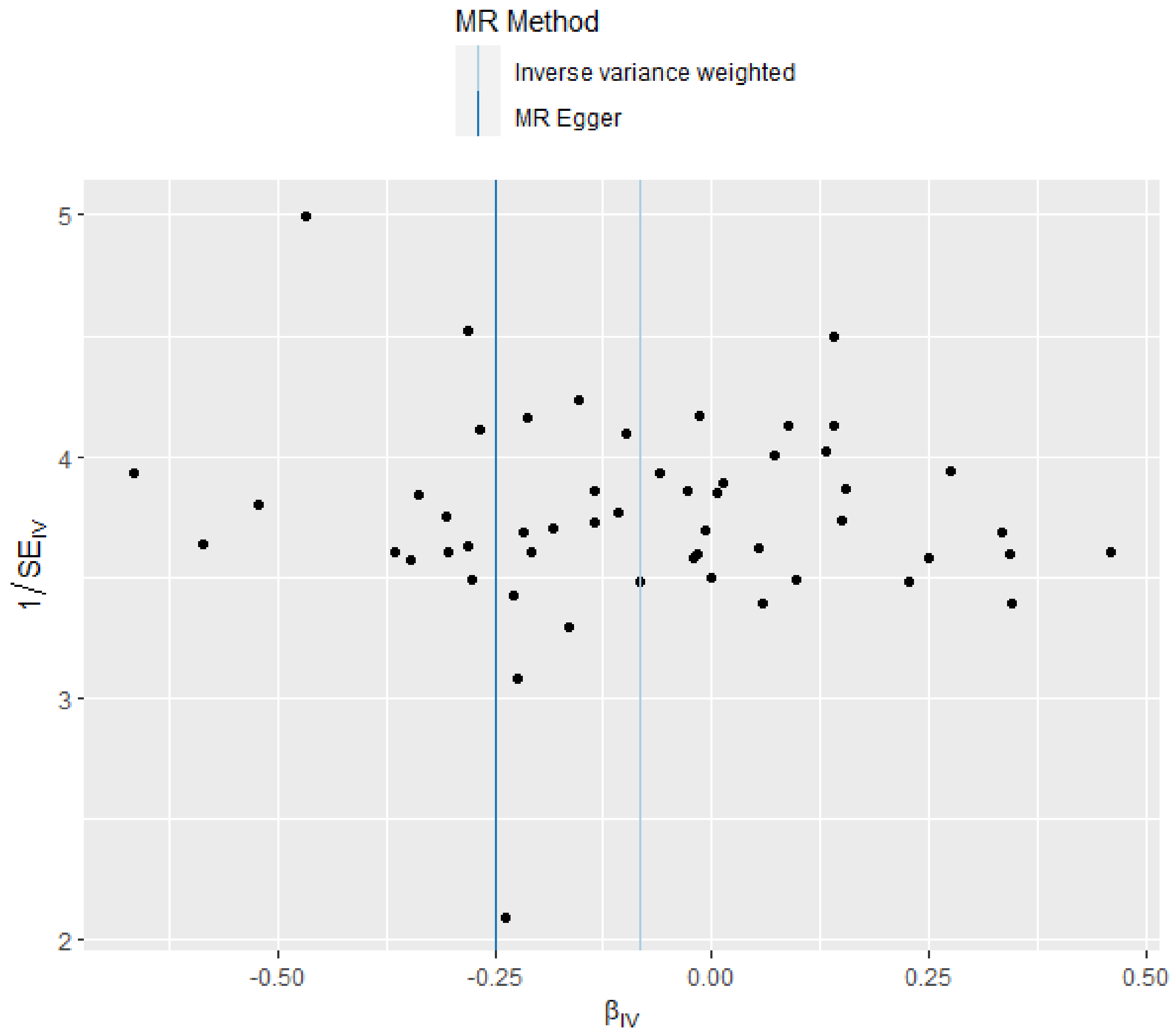

MR Method
Inverse variance weighted
MR Egger
5
4
3
2
1/SE_IV
-0.50
-0.25
0.00
0.25
0.50
β_IV

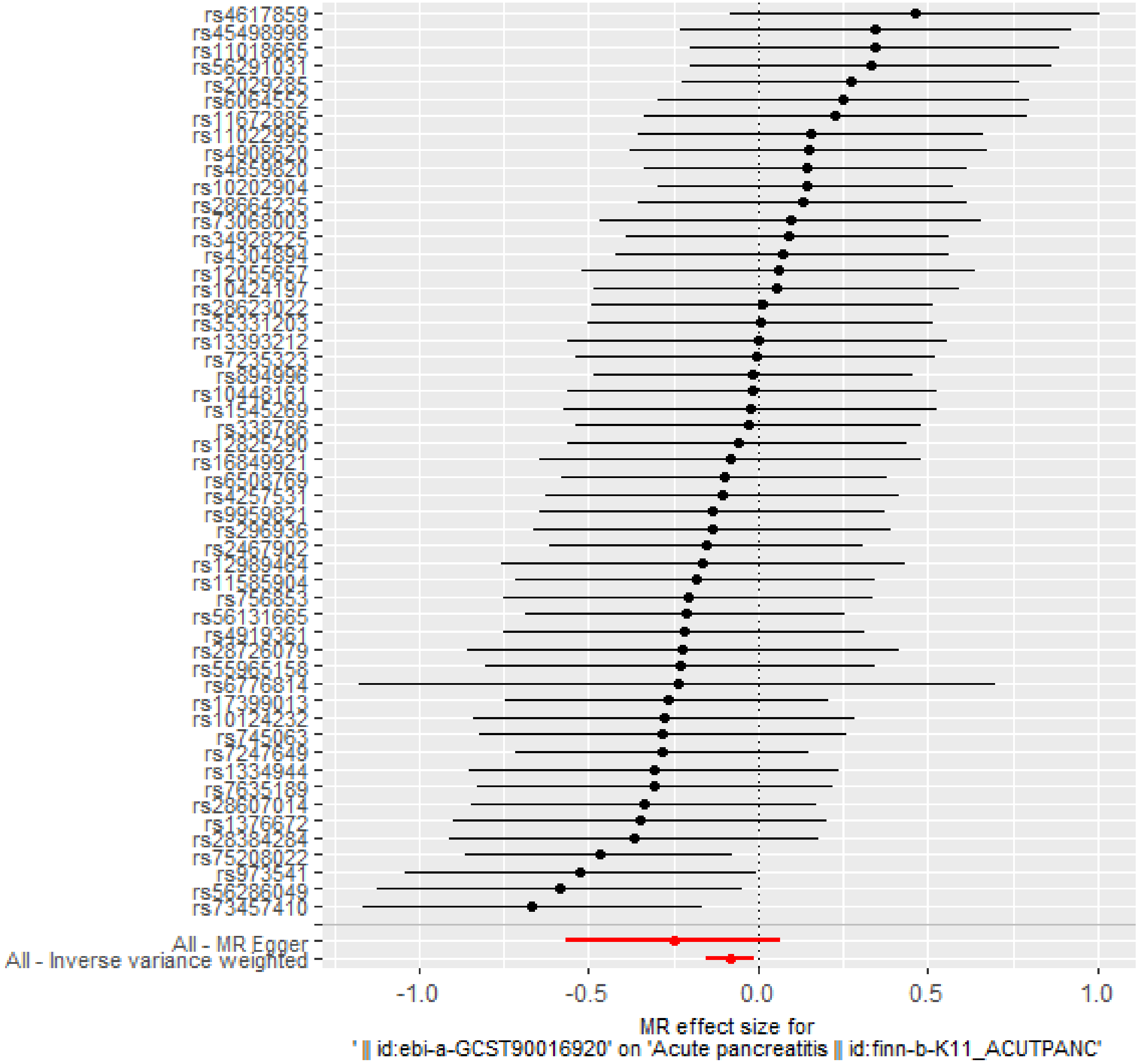
rs4617859
rs45498998
rs11018665
rs56291031
rs2029285
rs6064552
rs11672885
rs11022995
rs4908620
rs4659820
rs10202904
rs28664235
rs73068003
rs34928225
rs4304894
rs12055657
rs10424197
rs28623022
rs35331203
rs13393212
rs7235323
rs894996
rs10448161
rs1545269
rs338786
rs12825290
rs16849921
rs6508769
rs4257531
rs9959821
rs296936
rs2467902
rs12989464
rs11585904
rs756853
rs56131665
rs4919361
rs28726079
rs55965158
rs6776814
rs17399013
rs10124232
rs745063
rs7247649
rs1334944
rs7635189
rs28607014
rs1376672
rs28384284
rs75208022
rs973541
rs56286049
rs73457410
All - MR Egger
All - Inverse variance weighted
-1.0
-0.5
0.0
0.5
1.0
MR effect size for
' || id:ebi-a-GCST90016920' on 'Acute pancreatitis || id:finn-b-K11_ACUTPANC'

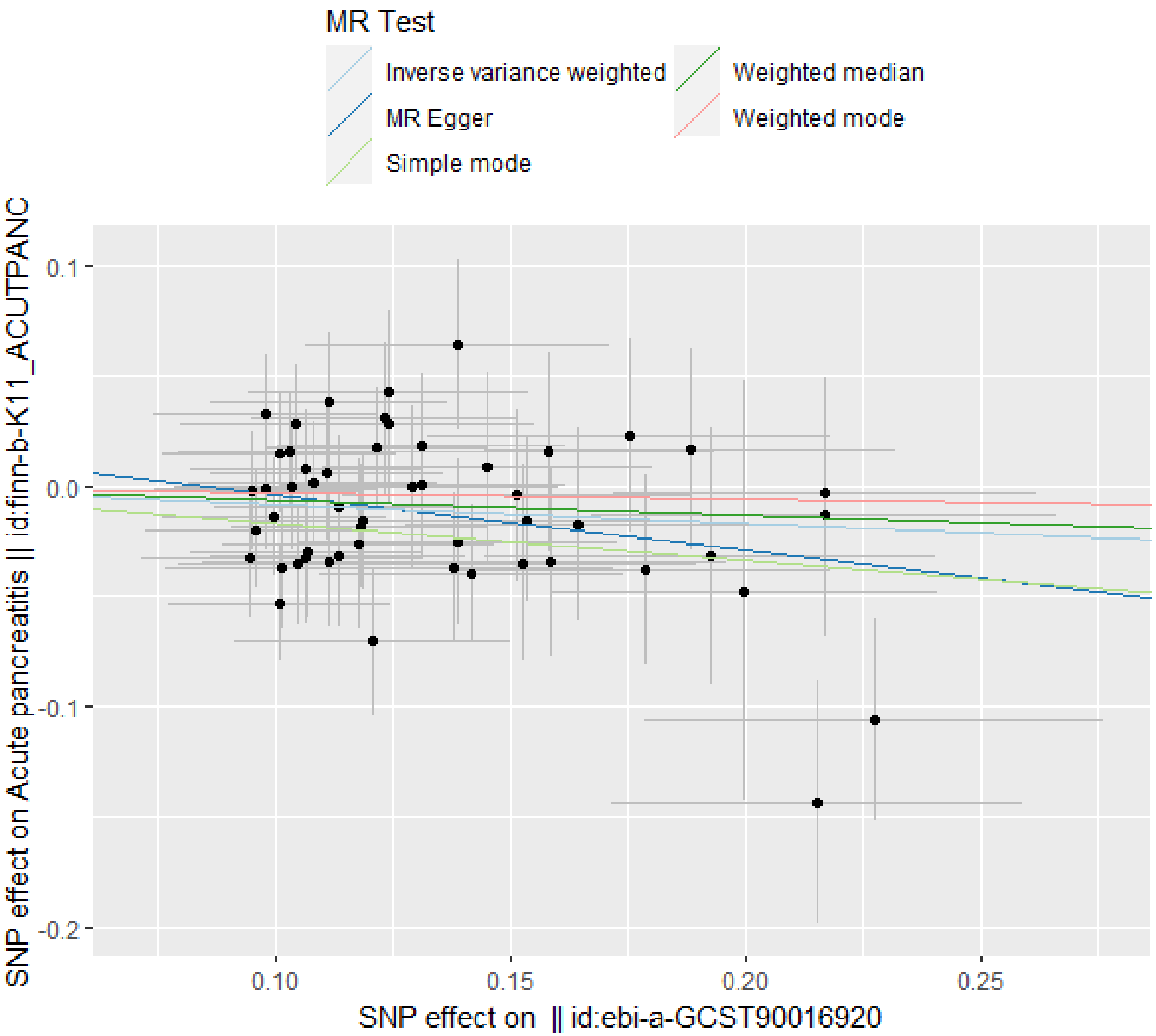

MR Test
Inverse variance weighted
MR Egger
Simple mode
Weighted median
Weighted mode
SNP effect on Acute pancreatitis || id:finn-b-K11_ACUTPANC
0.1
0.0
-0.1
-0.2
0.10
0.15
0.20
0.25
SNP effect on || id:ebi-a-GCST90016920

Figure 197 Leave-one-out analysis, funnel plot, MR effect size and scatter plot for gut microbiota abundance (family Methanobacteriaceae id.121) on acute pancreatitis

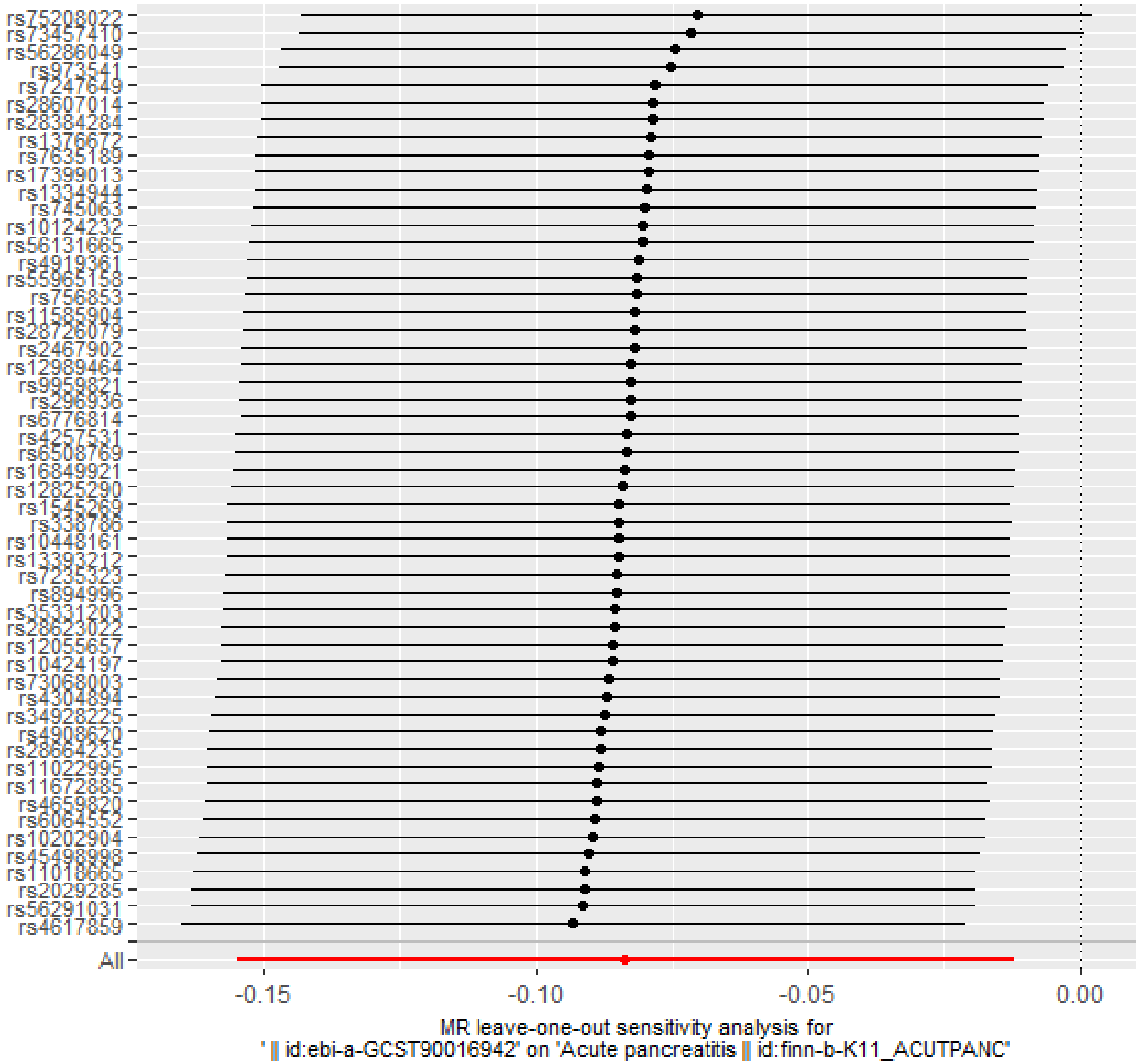

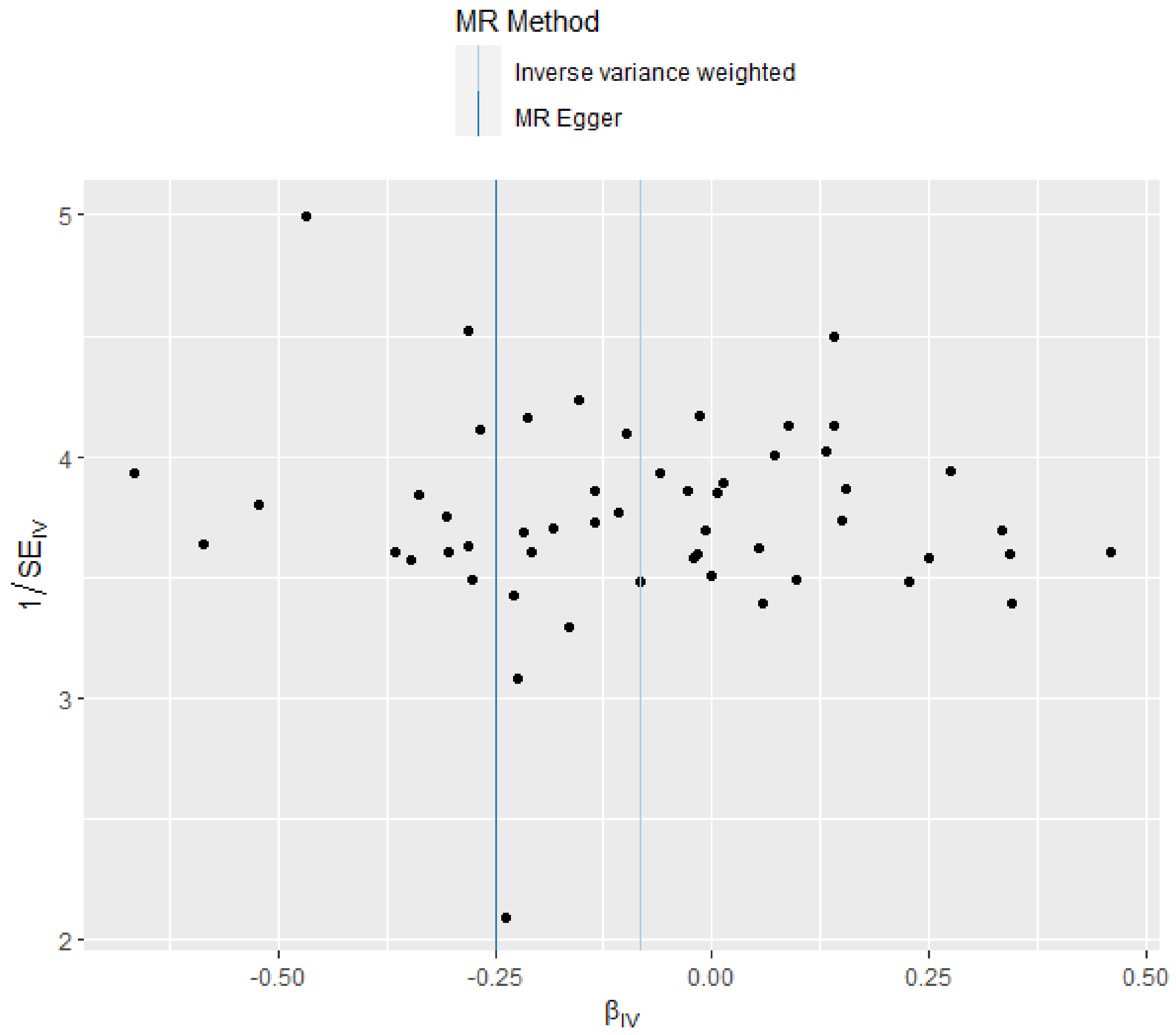

MR Method
Inverse variance weighted
MR Egger
5
4
3
2
1/SE_IV
-0.50
-0.25
0.00
0.25
0.50
β_IV

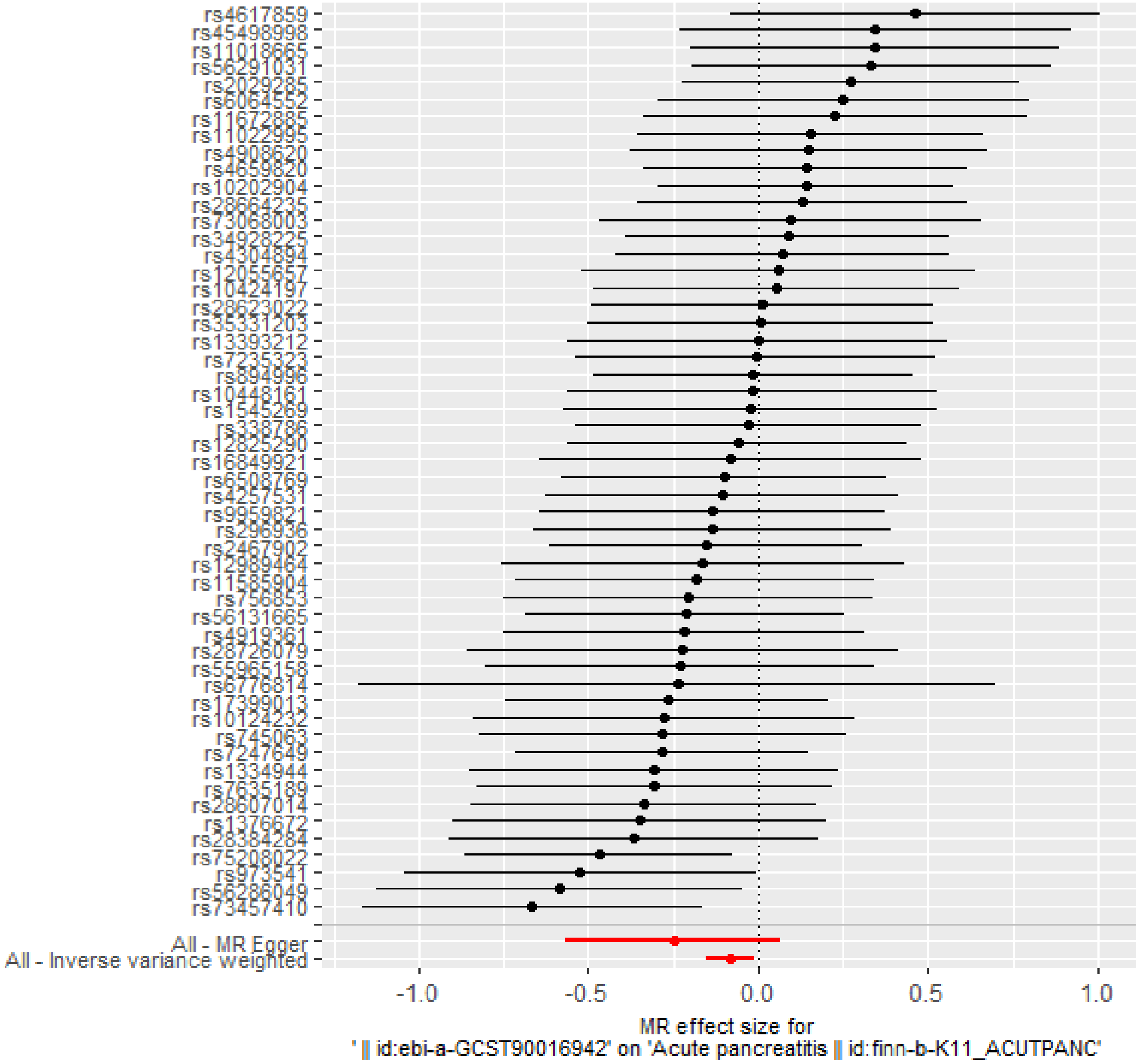

rs4617859
rs45498998
rs11018665
rs56291031
rs2029285
rs6064552
rs11672885
rs11022995
rs4908620
rs4659820
rs10202904
rs28664235
rs73068003
rs34928225
rs4304894
rs12055657
rs10424197
rs28623022
rs35331203
rs13393212
rs7235323
rs894996
rs10448161
rs1545269
rs338786
rs12825290
rs16849921
rs6508769
rs4257531
rs9959821
rs296936
rs2467902
rs12989464
rs11585904
rs756853
rs56131665
rs4919361
rs28726079
rs55965158
rs6776814
rs17399013
rs10124232
rs745063
rs7247649
rs1334944
rs7635189
rs28607014
rs1376672
rs28384284
rs75208022
rs973541
rs56286049
rs73457410
All - MR Egger
All - Inverse variance weighted
-1.0
-0.5
0.0
0.5
1.0
MR effect size for
' || id:ebi-a-GCST90016942' on 'Acute pancreatitis || id:finn-b-K11_ACUTPANC'

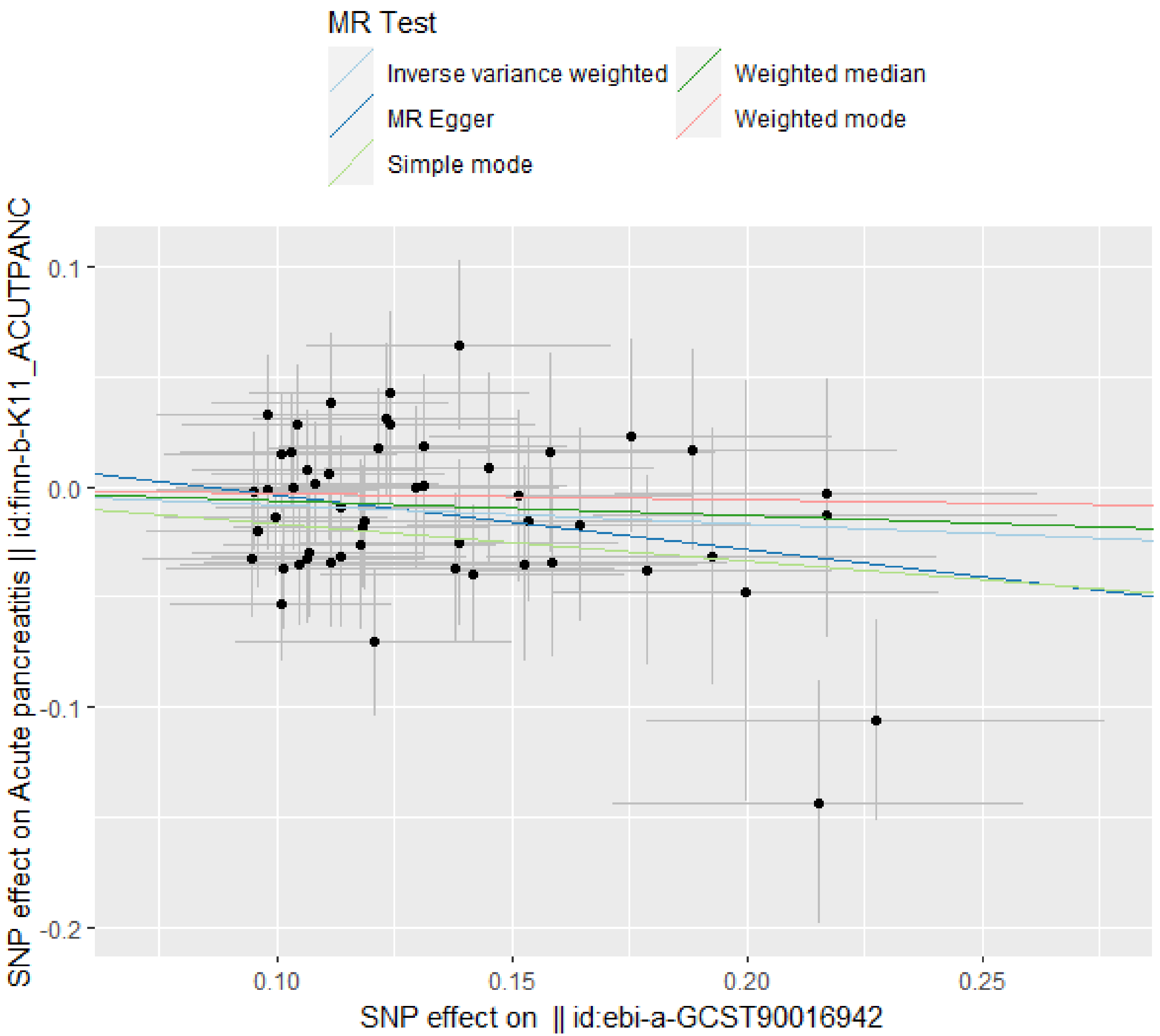

MR Test
Inverse variance weighted
MR Egger
Simple mode
Weighted median
Weighted mode
SNP effect on Acute pancreatitis || id:finn-b-K11_ACUTPANC
0.1
0.0
-0.1
-0.2
0.10
0.15
0.20
0.25
SNP effect on || id:ebi-a-GCST90016942

Figure 198 Leave-one-out analysis, funnel plot, MR effect size and scatter plot for gut microbiota abundance (genus Allisonella id.2174) on acute pancreatitis

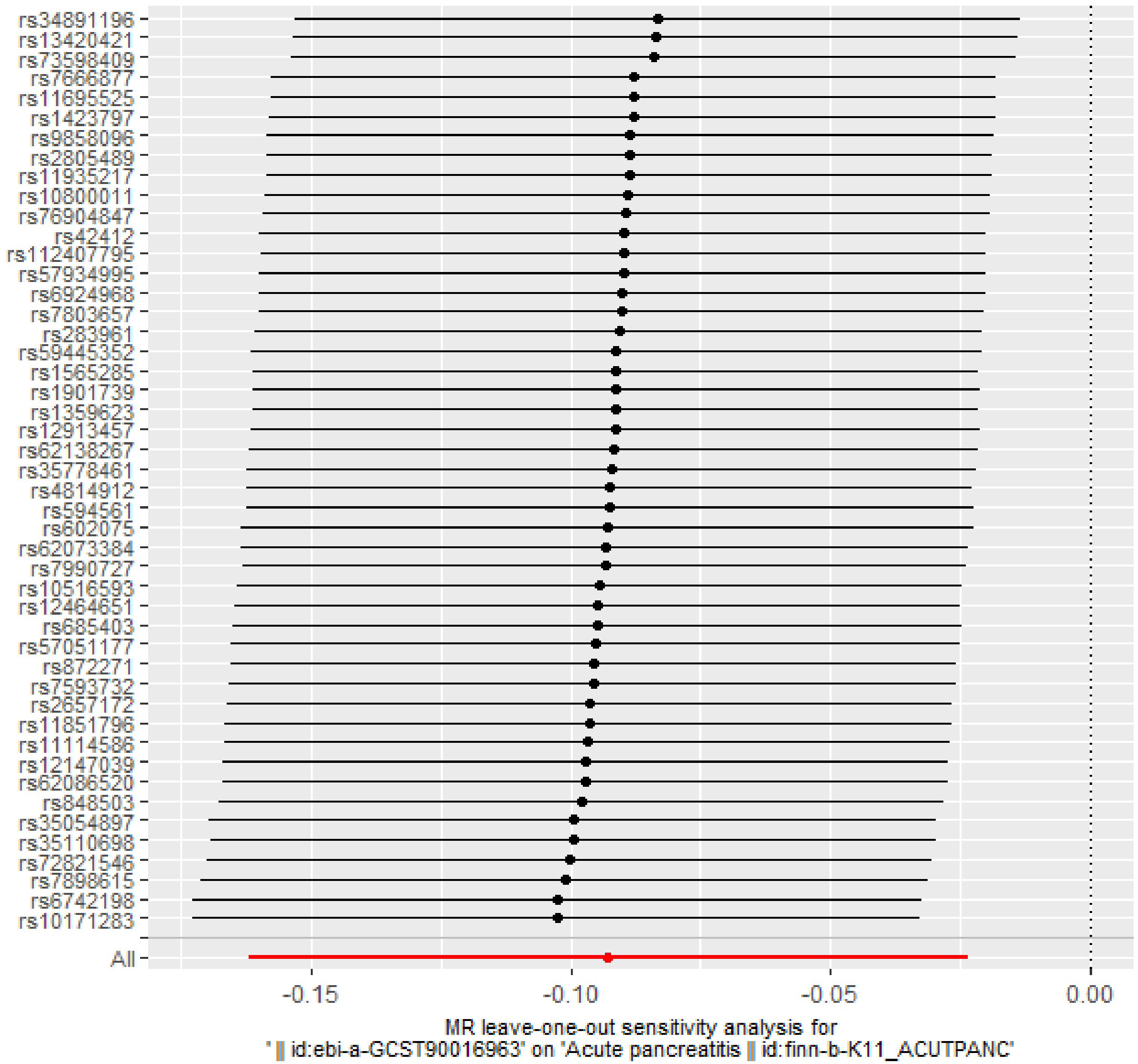

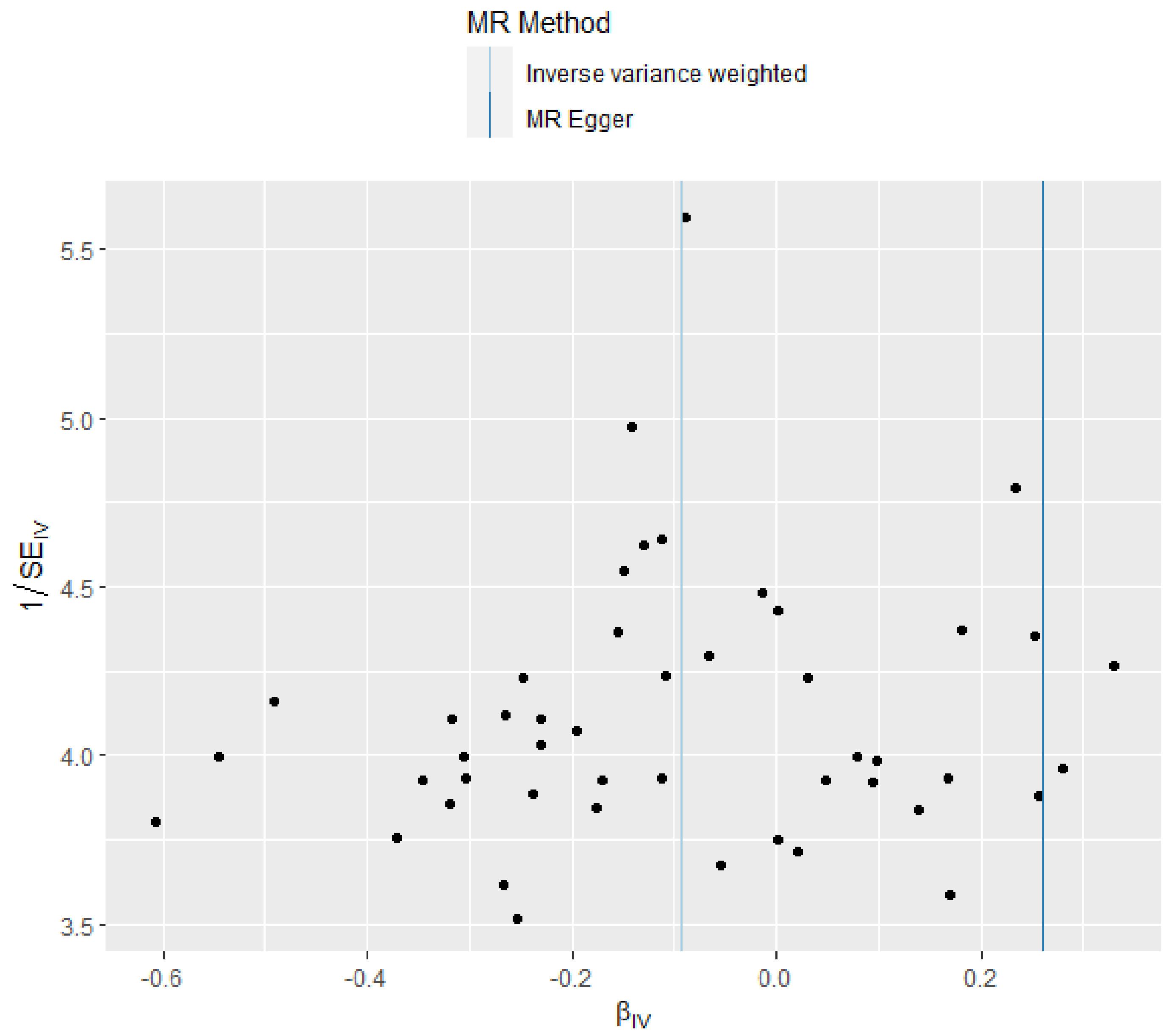

MR Method
Inverse variance weighted
MR Egger
5.5
5.0
4.5
4.0
3.5
1/SE_IV
-0.6
-0.4
-0.2
0.0
0.2
β_IV

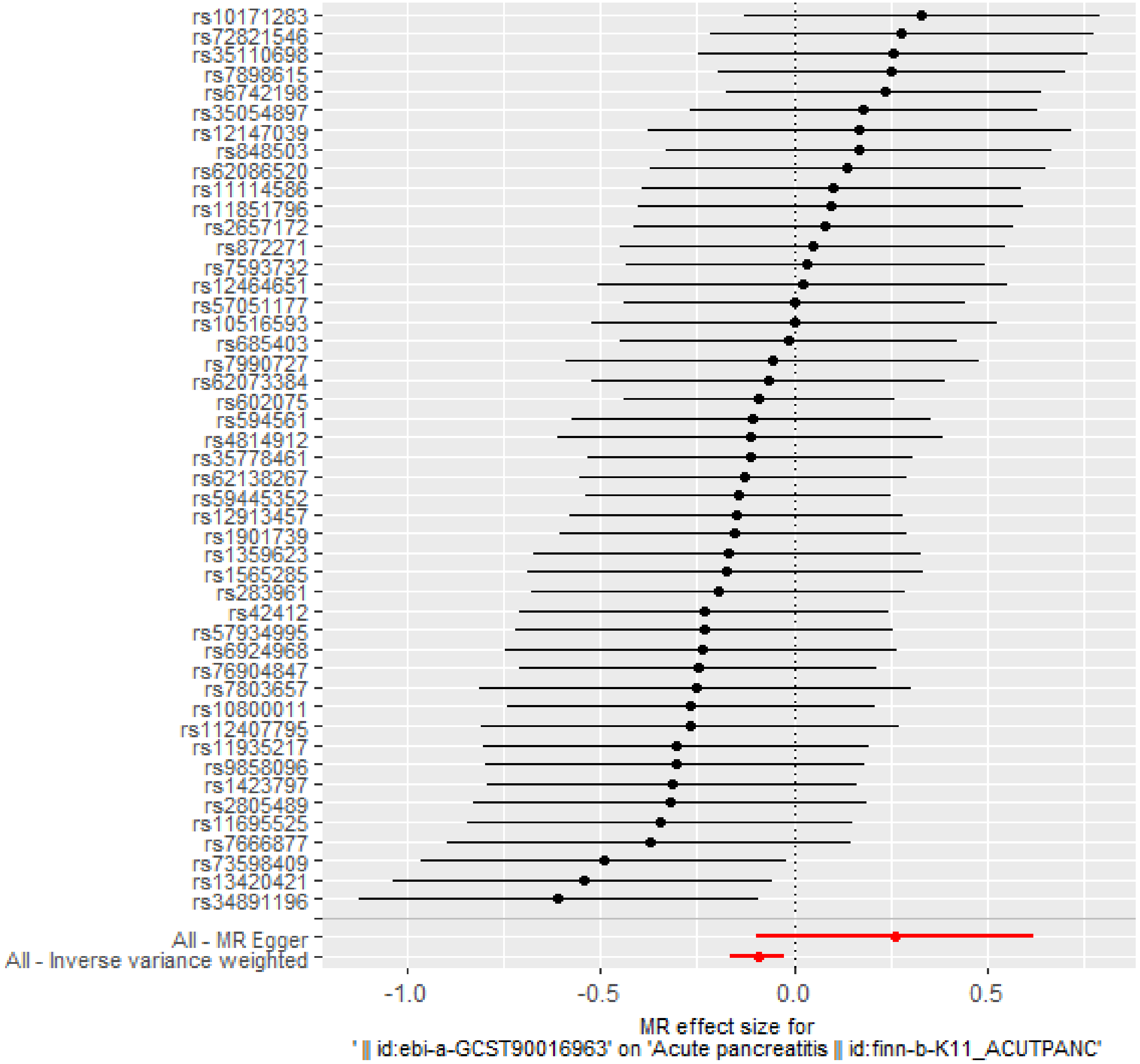

rs10171283
rs72821546
rs35110698
rs7898615
rs6742198
rs35054897
rs12147039
rs848503
rs62086520
rs11114586
rs11851796
rs2657172
rs872271
rs7593732
rs12464651
rs57051177
rs10516593
rs685403
rs7990727
rs62073384
rs602075
rs594561
rs4814912
rs35778461
rs62138267
rs59445352
rs12913457
rs1901739
rs1359623
rs1565285
rs283961
rs42412
rs57934995
rs6924968
rs76904847
rs7803657
rs10800011
rs112407795
rs11935217
rs9858096
rs1423797
rs2805489
rs11695525
rs7666877
rs73598409
rs13420421
rs34891196
All - MR Egger
All - Inverse variance weighted
-1.0
-0.5
0.0
0.5
MR effect size for
' || id:ebi-a-GCST90016963' on 'Acute pancreatitis || id:finn-b-K11_ACUTPANC'

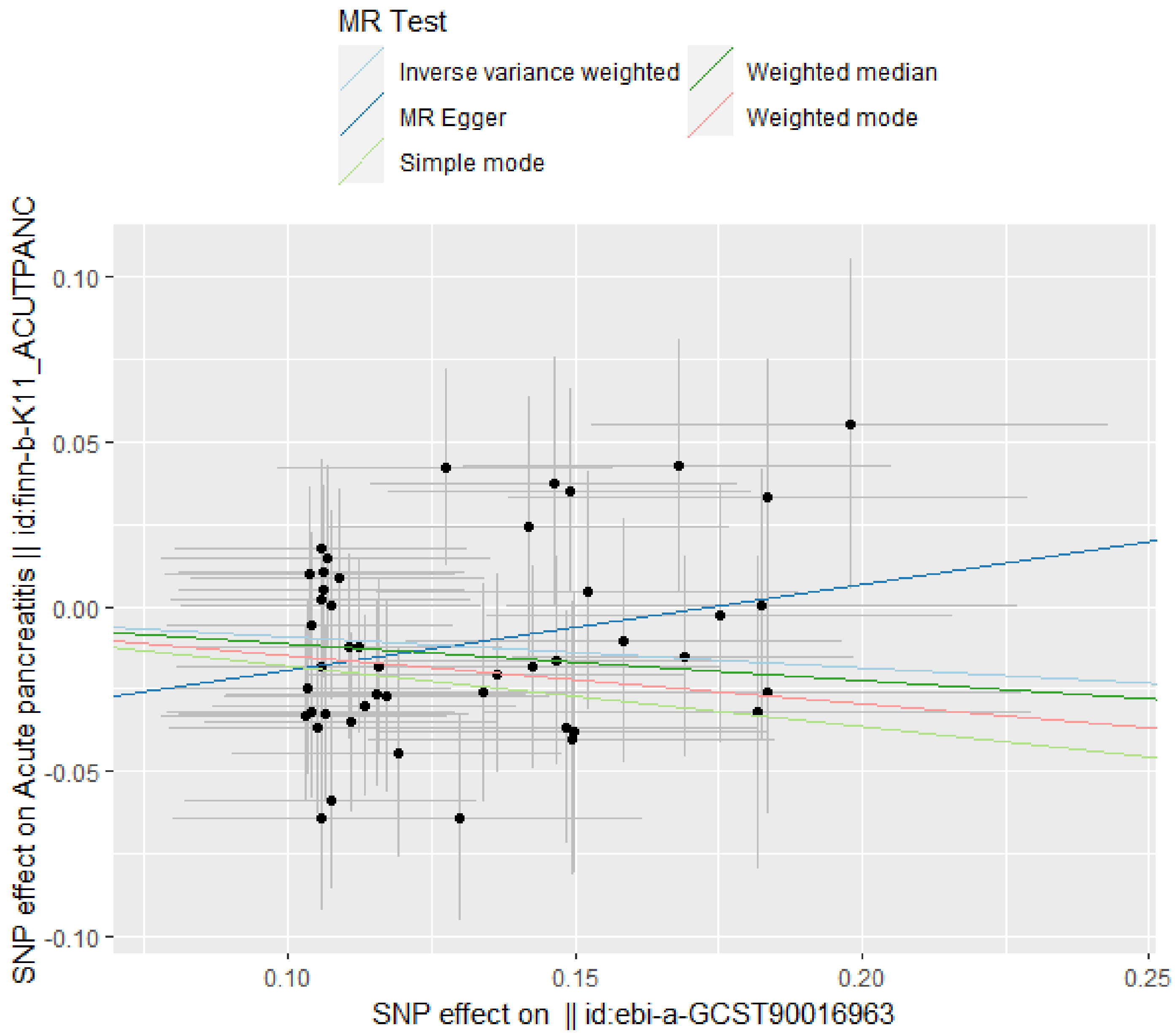

MR Test
Inverse variance weighted
MR Egger
Simple mode
Weighted median
Weighted mode
SNP effect on Acute pancreatitis || id:finn-b-K11_ACUTPANC
0.10
0.05
0.00
-0.05
-0.10
0.10
0.15
0.20
0.25
SNP effect on || id:ebi-a-GCST90016963

Figure 199 Leave-one-out analysis, funnel plot, MR effect size and scatter plot for gut microbiota abundance (genus Lachnoclostridium id.11308) on acute pancreatitis

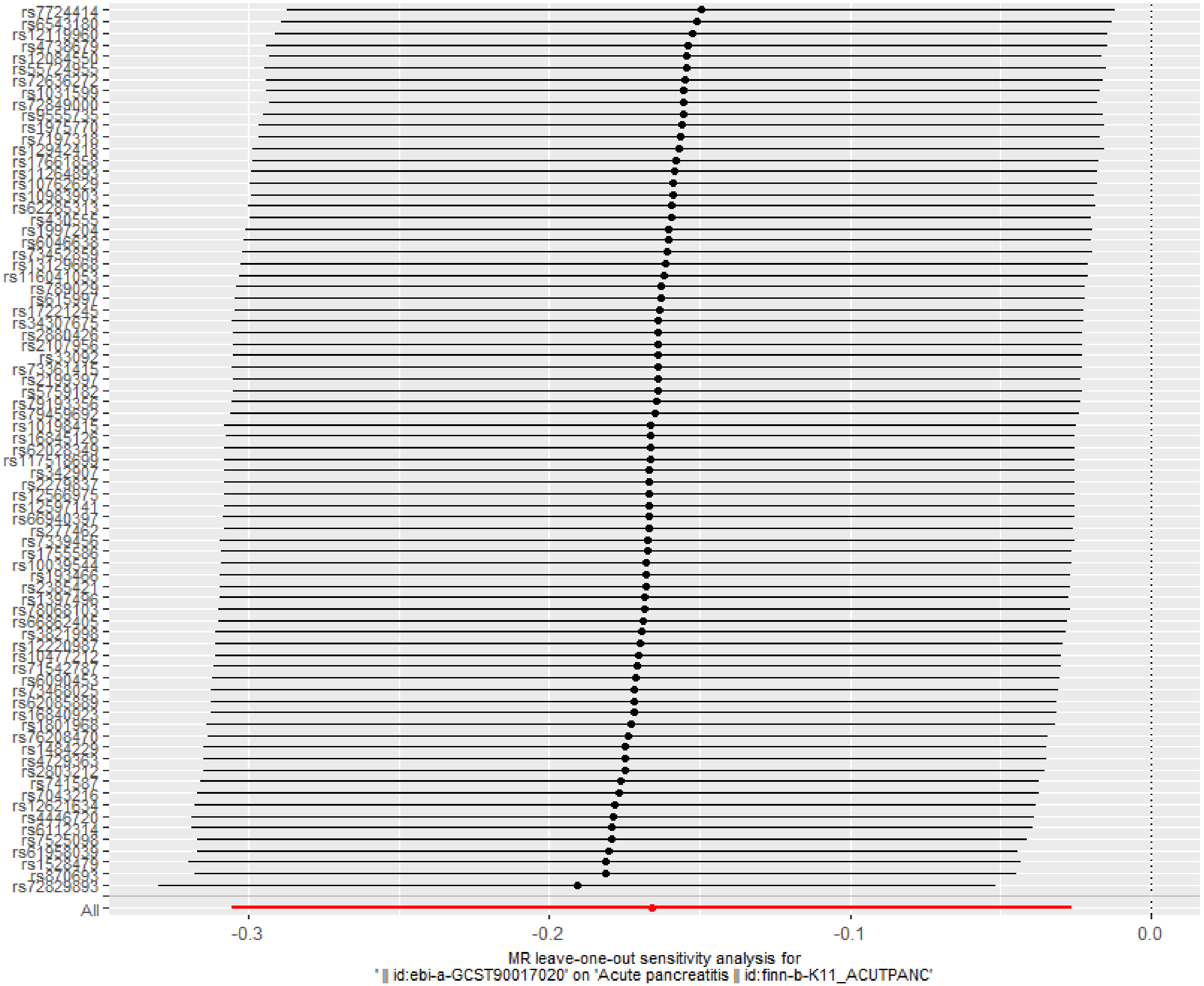

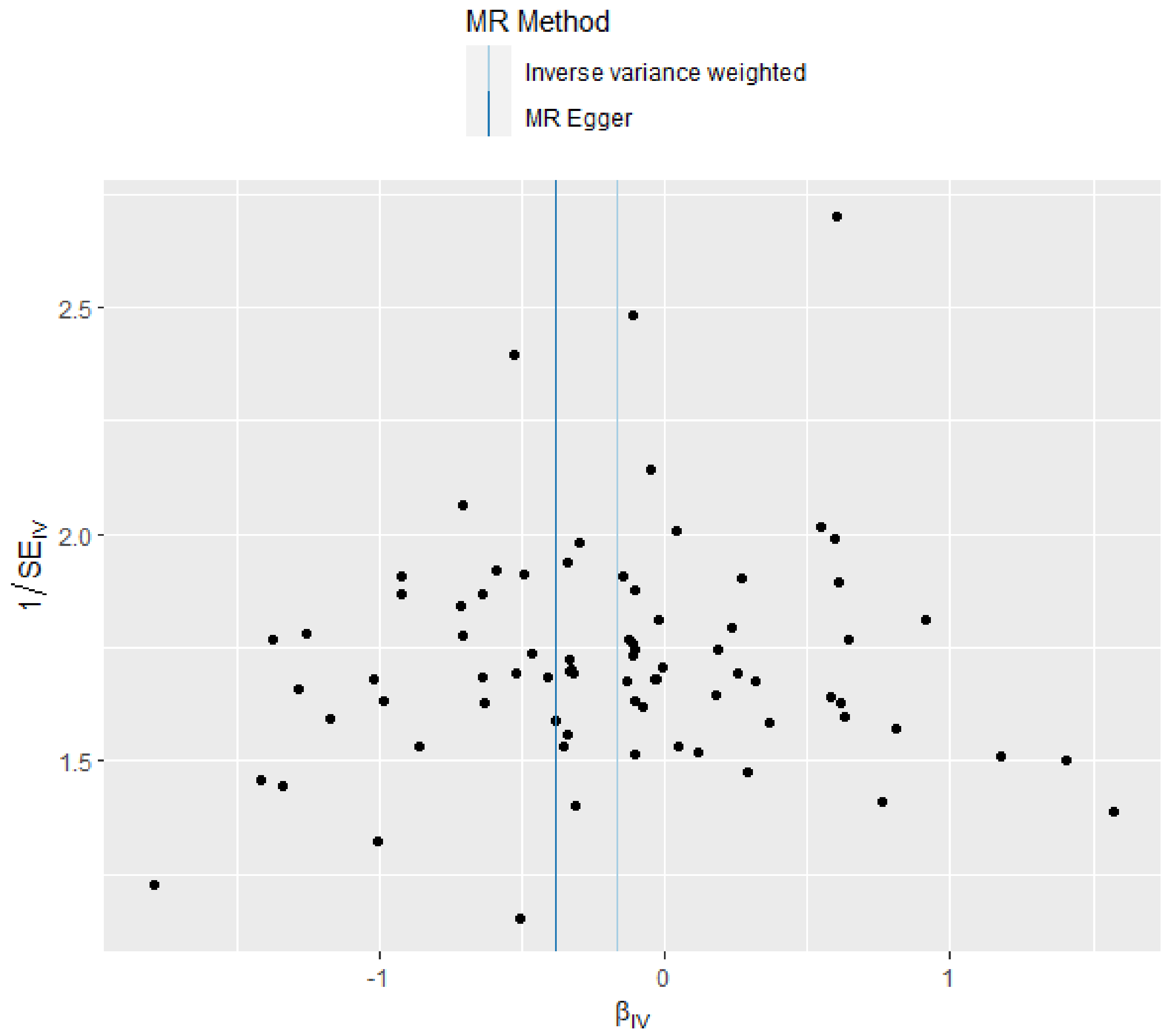
MR Method
Inverse variance weighted
MR Egger
2.5
2.0
1.5
-1
0
1
$1/SE_{IV}$
β_{IV}

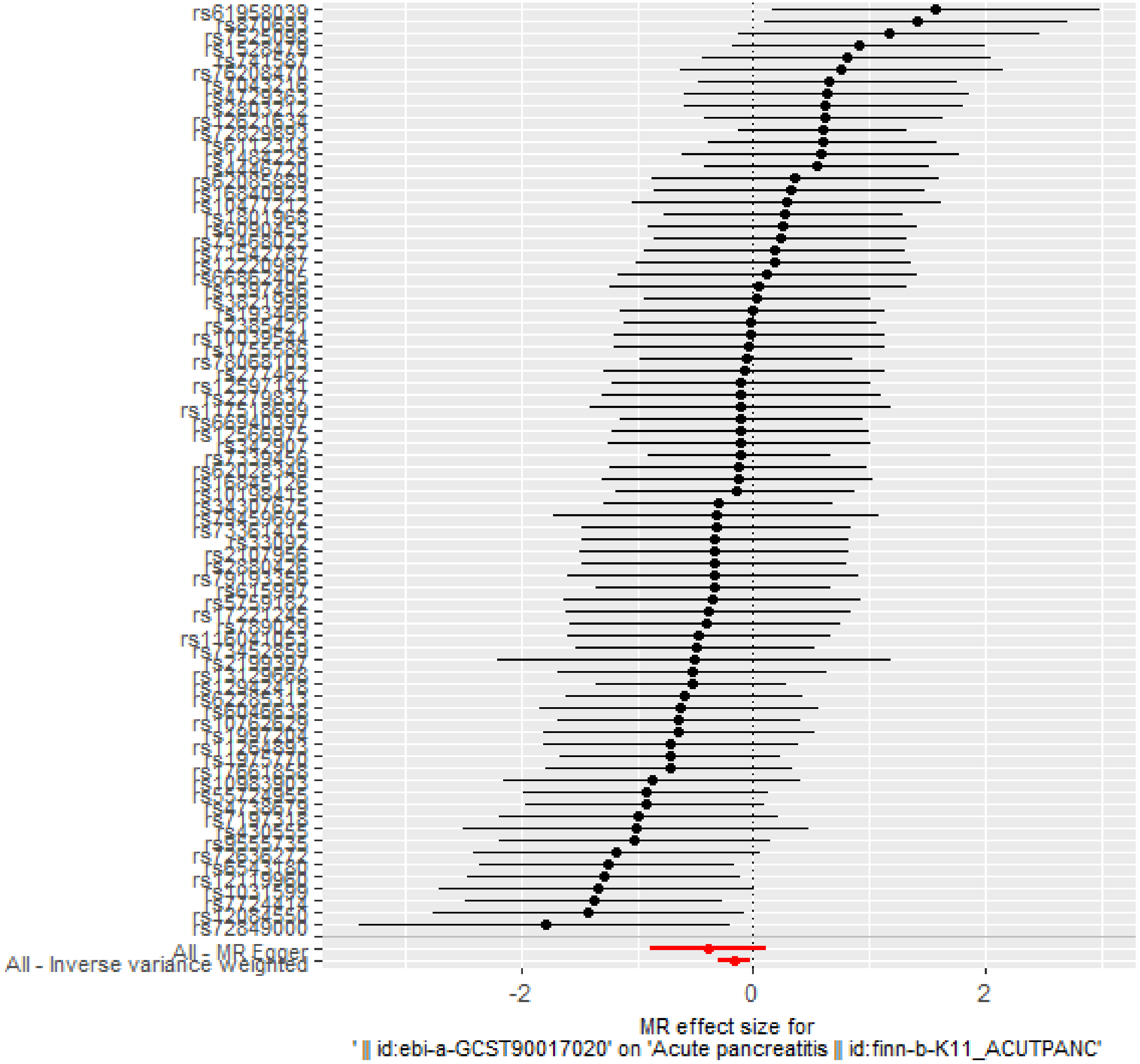
rs61958039
rs72849000
All - MR Egger
All - Inverse variance weighted
-2
0
2
MR effect size for
' || id:ebi-a-GCST90017020' on 'Acute pancreatitis || id:finn-b-K11_ACUTPANC'

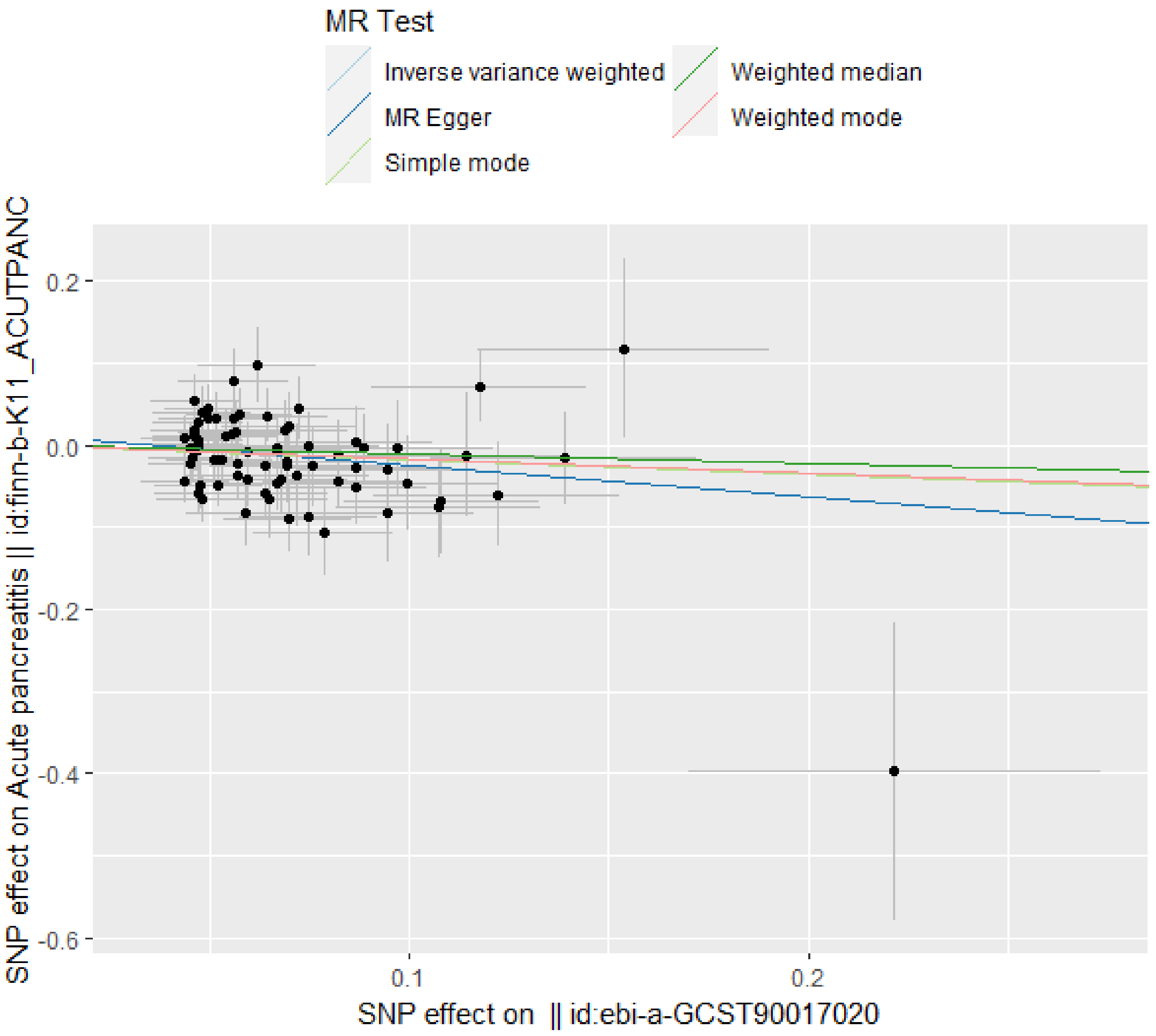
MR Test
Inverse variance weighted
MR Egger
Simple mode
Weighted median
Weighted mode
SNP effect on Acute pancreatitis || id:finn-b-K11_ACUTPANC
0.2
0.0
-0.2
-0.4
-0.6
0.1
0.2
SNP effect on || id:ebi-a-GCST90017020

Figure 200 Leave-one-out analysis, funnel plot, MR effect size and scatter plot for gut microbiota abundance (genus Ruminiclostridium6 id.11356) on acute pancreatitis

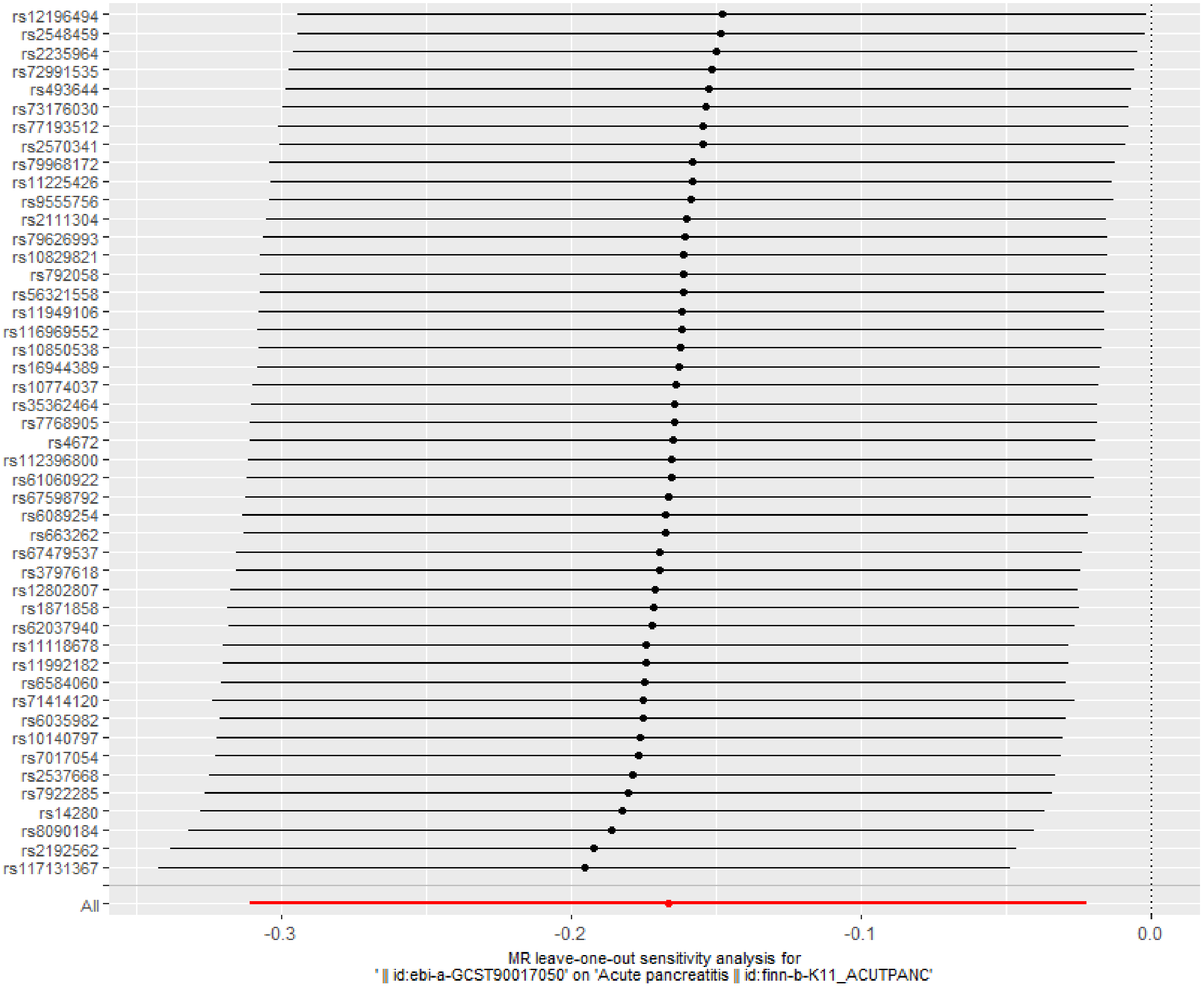

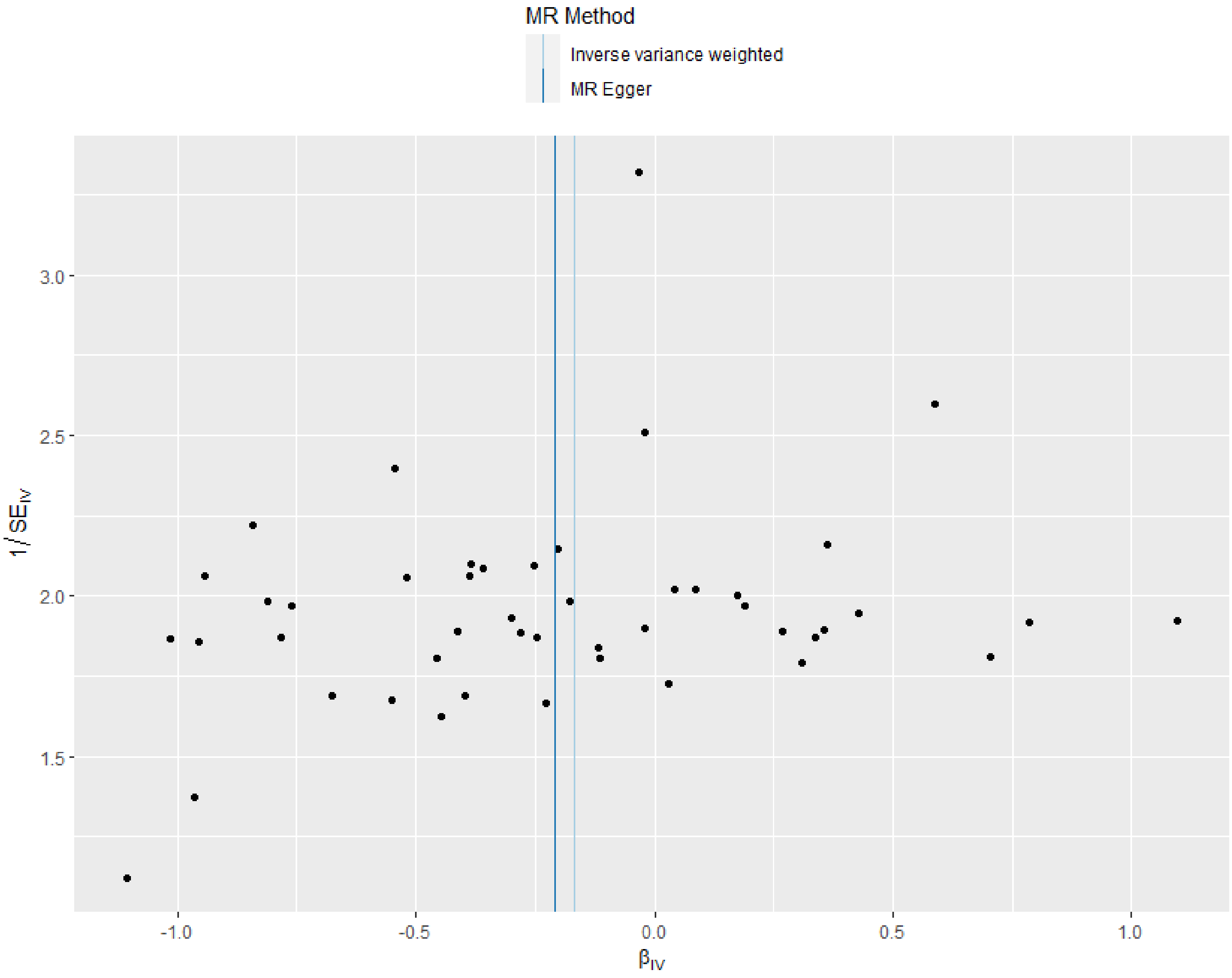

MR Method
Inverse variance weighted
MR Egger
3.0
2.5
2.0
1.5
$1/SE_{IV}$
-1.0
-0.5
0.0
0.5
1.0
β_{IV}

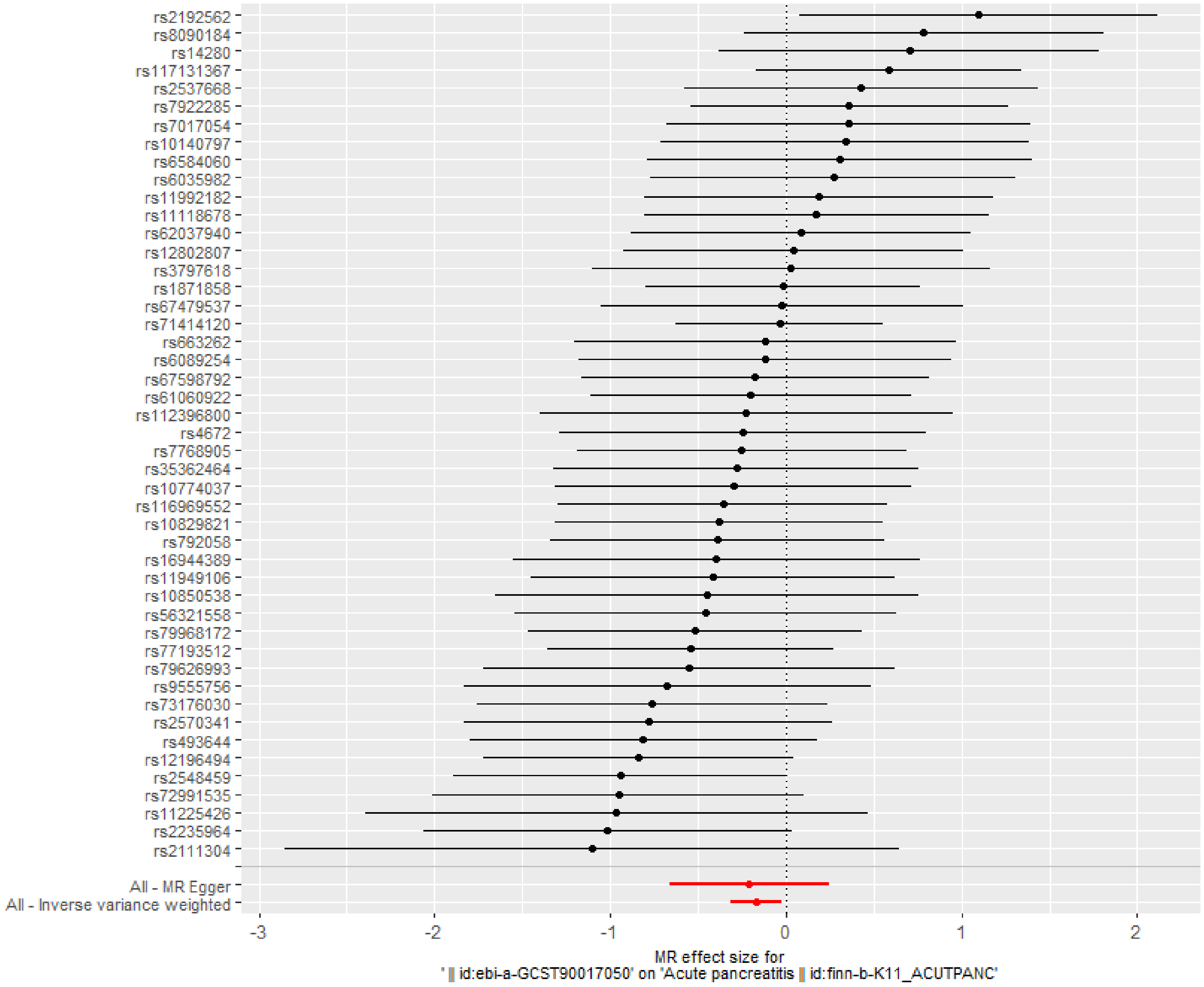

rs2192562
rs8090184
rs14280
rs117131367
rs2537668
rs7922285
rs7017054
rs10140797
rs6584060
rs6035982
rs11992182
rs11118678
rs62037940
rs12802807
rs3797618
rs1871858
rs67479537
rs71414120
rs663262
rs6089254
rs67598792
rs61060922
rs112396800
rs4672
rs7768905
rs35362464
rs10774037
rs116969552
rs10829821
rs792058
rs16944389
rs11949106
rs10850538
rs56321558
rs79968172
rs77193512
rs79626993
rs9555756
rs73176030
rs2570341
rs493644
rs12196494
rs2548459
rs72991535
rs11225426
rs2235964
rs2111304
All - MR Egger
All - Inverse variance weighted
-3
-2
-1
0
1
2
MR effect size for
' || id:ebi-a-GCST90017050' on 'Acute pancreatitis || id:finn-b-K11_ACUTPANC'

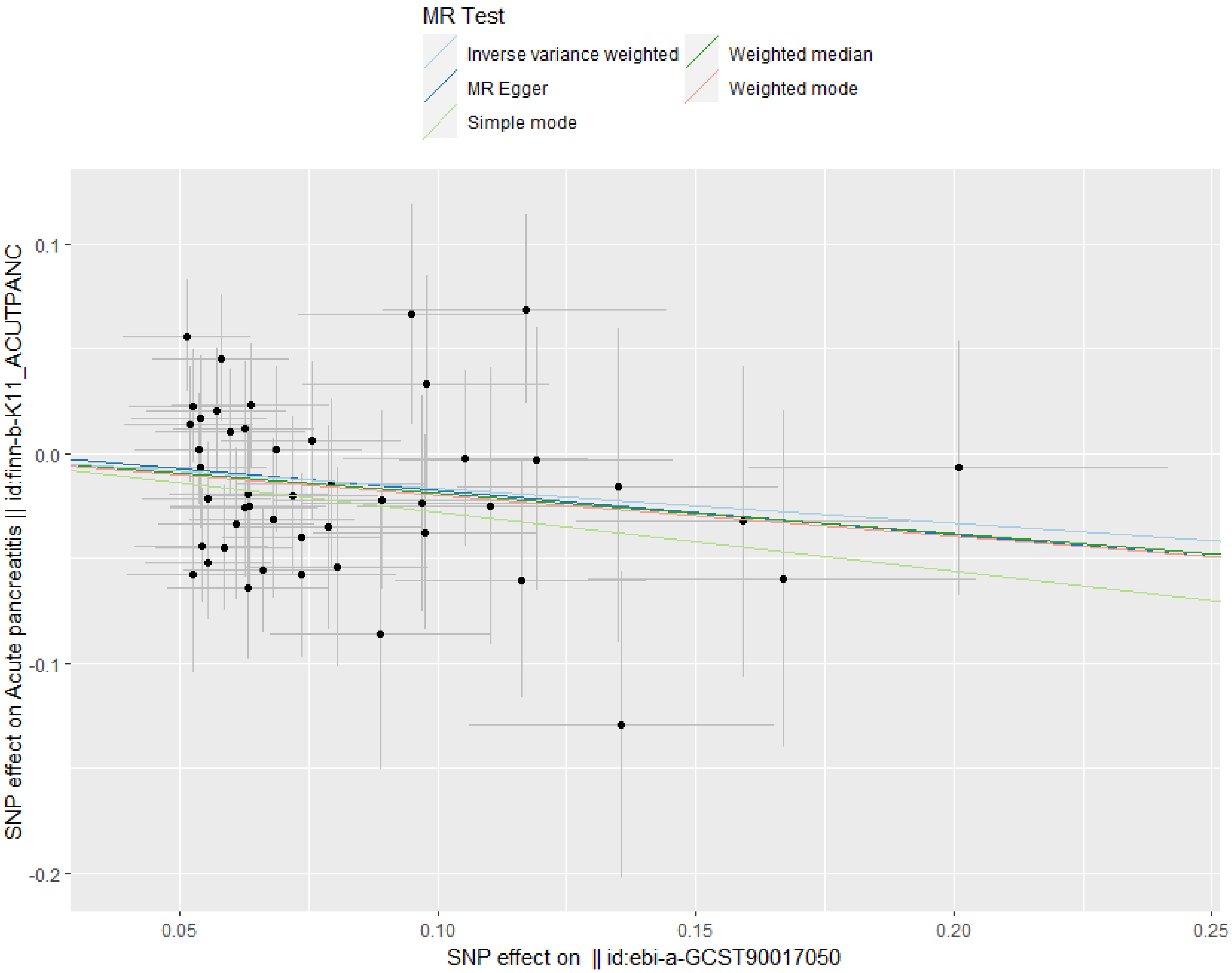
MR Test
Inverse variance weighted
MR Egger
Simple mode
Weighted median
Weighted mode
SNP effect on Acute pancreatitis || id:finn-b-K11_ACUTPANC
0.1
0.0
-0.1
-0.2
0.05
0.10
0.15
0.20
0.25
SNP effect on || id:ebi-a-GCST90017050

Figure 201 Leave-one-out analysis, funnel plot, MR effect size and scatter plot for gut microbiota abundance (genus Ruminococcaceae UCG010 id.11367) on acute pancreatitis

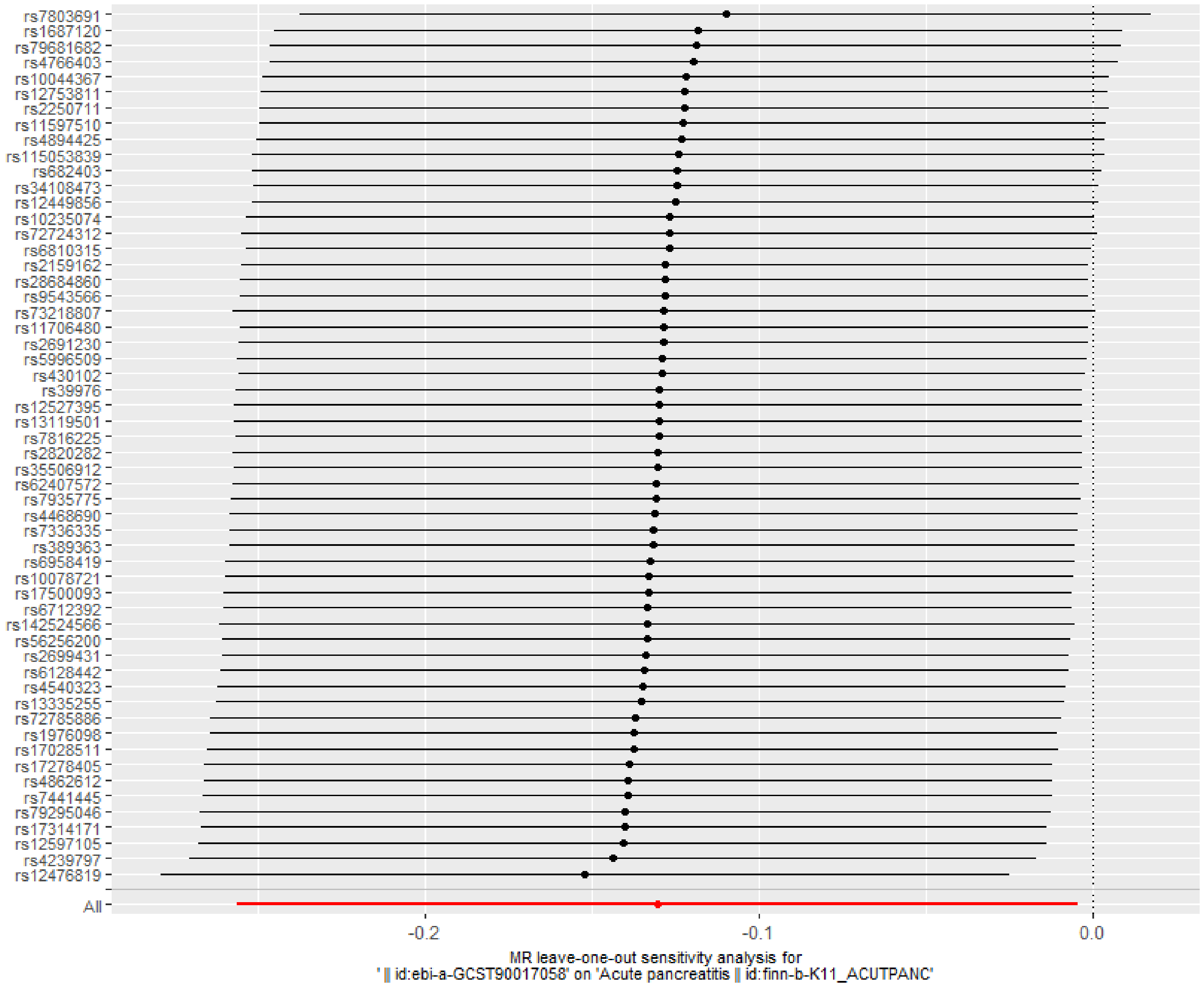

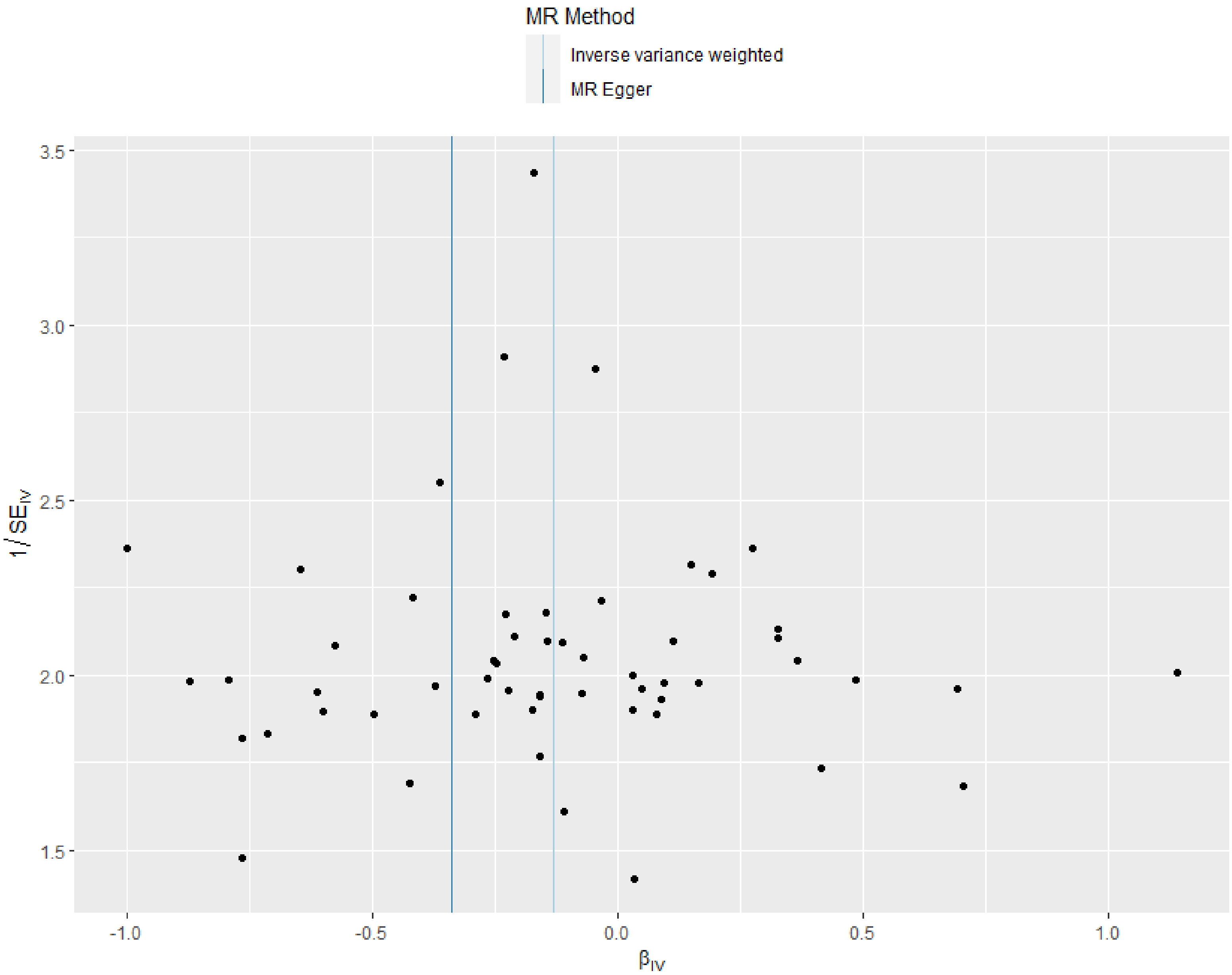

MR Method
Inverse variance weighted
MR Egger
3.5
3.0
2.5
2.0
1.5
1/SE_IV
-1.0
-0.5
0.0
0.5
1.0
β_IV

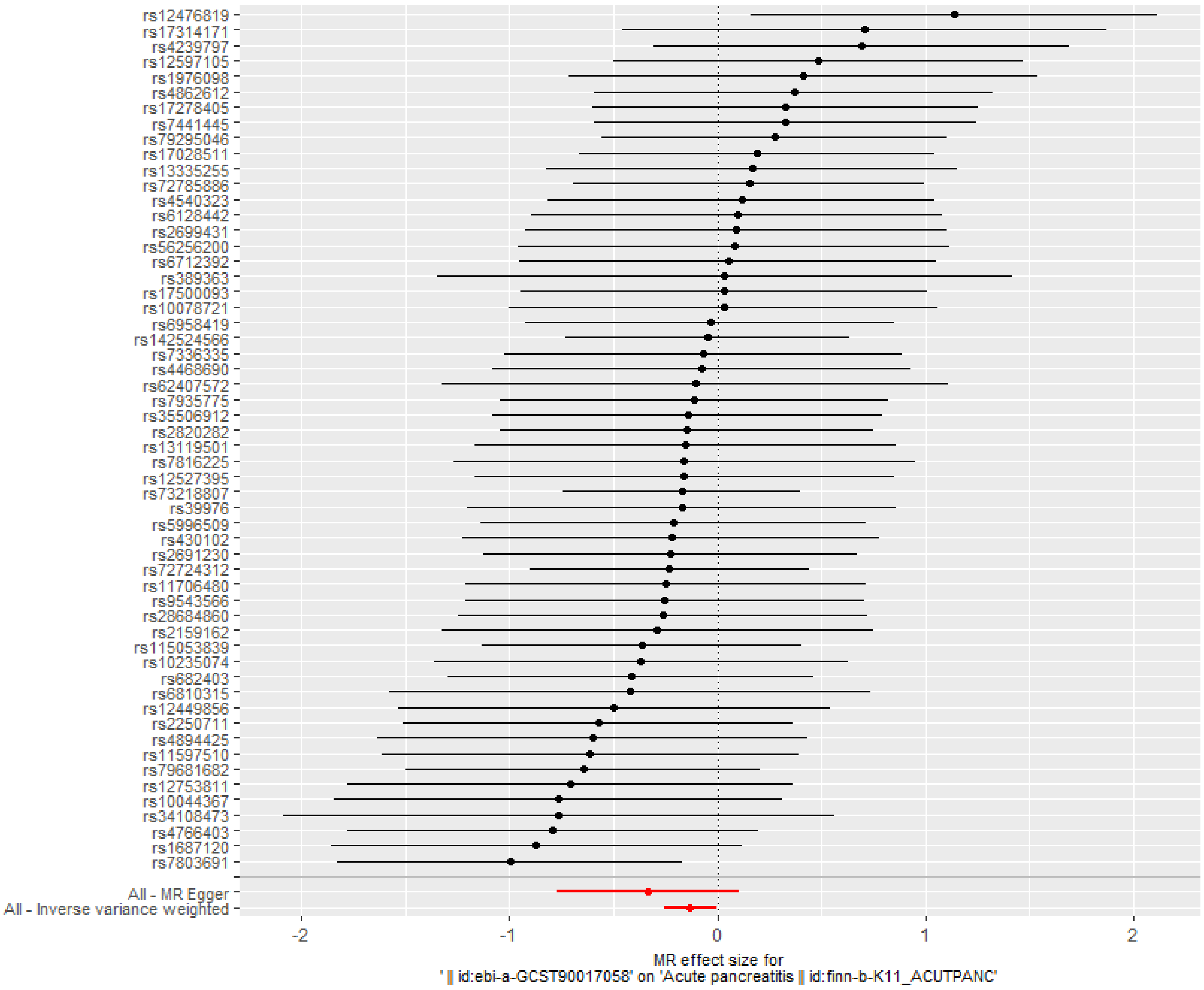
rs12476819
rs17314171
rs4239797
rs12597105
rs1976098
rs4862612
rs17278405
rs7441445
rs79295046
rs17028511
rs13335255
rs72785886
rs4540323
rs6128442
rs2699431
rs56256200
rs6712392
rs389363
rs17500093
rs10078721
rs6958419
rs142524566
rs7336335
rs4468690
rs62407572
rs7935775
rs35506912
rs2820282
rs13119501
rs7816225
rs12527395
rs73218807
rs39976
rs5996509
rs430102
rs2691230
rs72724312
rs11706480
rs9543566
rs28684860
rs2159162
rs115053839
rs10235074
rs682403
rs6810315
rs12449856
rs2250711
rs4894425
rs11597510
rs79681682
rs12753811
rs10044367
rs34108473
rs4766403
rs1687120
rs7803691
All - MR Egger
All - Inverse variance weighted
-2
-1
0
1
2
MR effect size for
' || id:ebi-a-GCST90017058' on 'Acute pancreatitis || id:finn-b-K11_ACUTPANC'

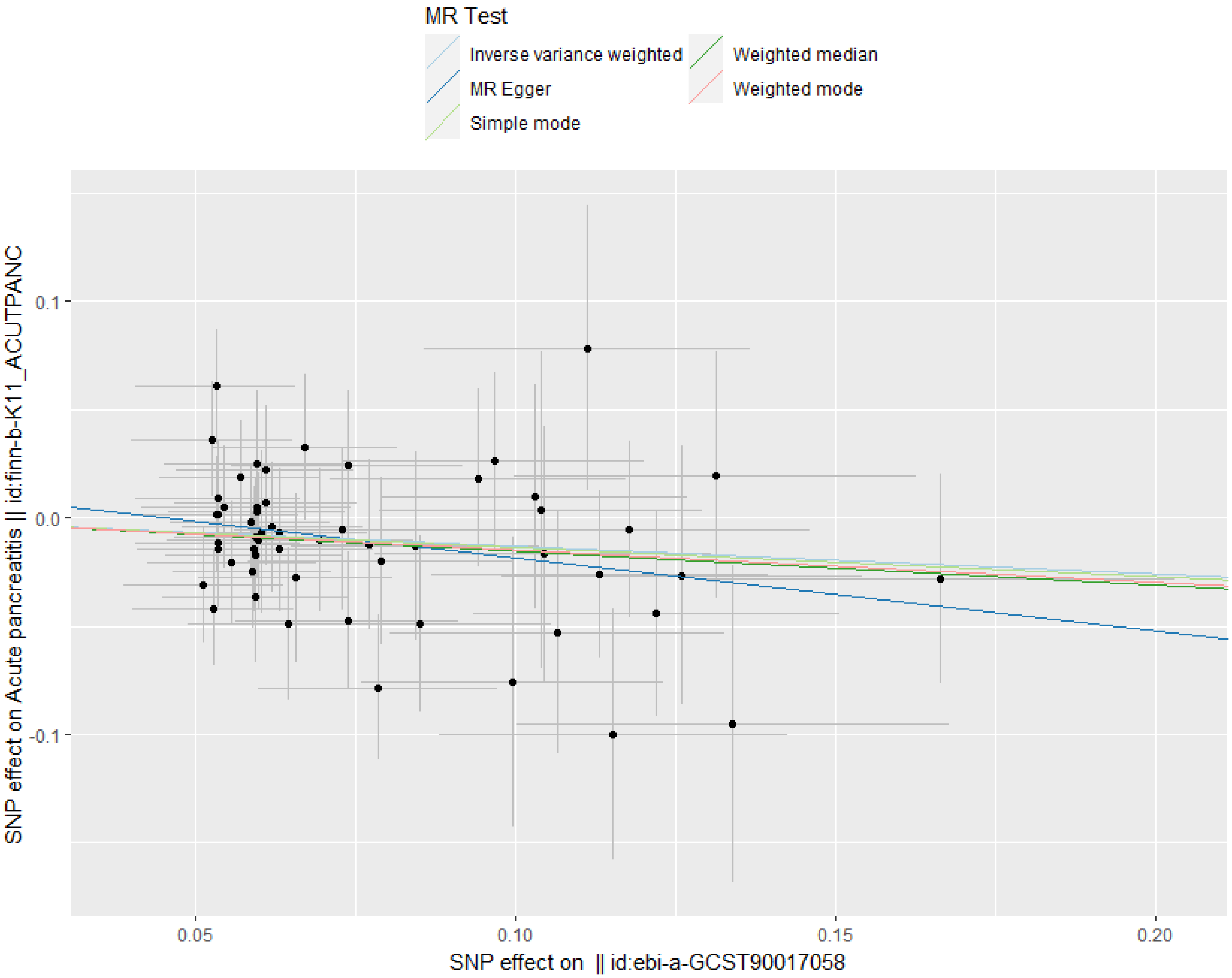

MR Test
Inverse variance weighted
MR Egger
Simple mode
Weighted median
Weighted mode
0.1
0.0
-0.1
SNP effect on Acute pancreatitis || id:finn-b-K11_ACUTPANC
0.05
0.10
0.15
0.20
SNP effect on || id:ebi-a-GCST90017058

Figure 202 Leave-one-out analysis, funnel plot, MR effect size and scatter plot for gut microbiota abundance (genus Ruminococcaceae UCG014 id.11371) on acute pancreatitis

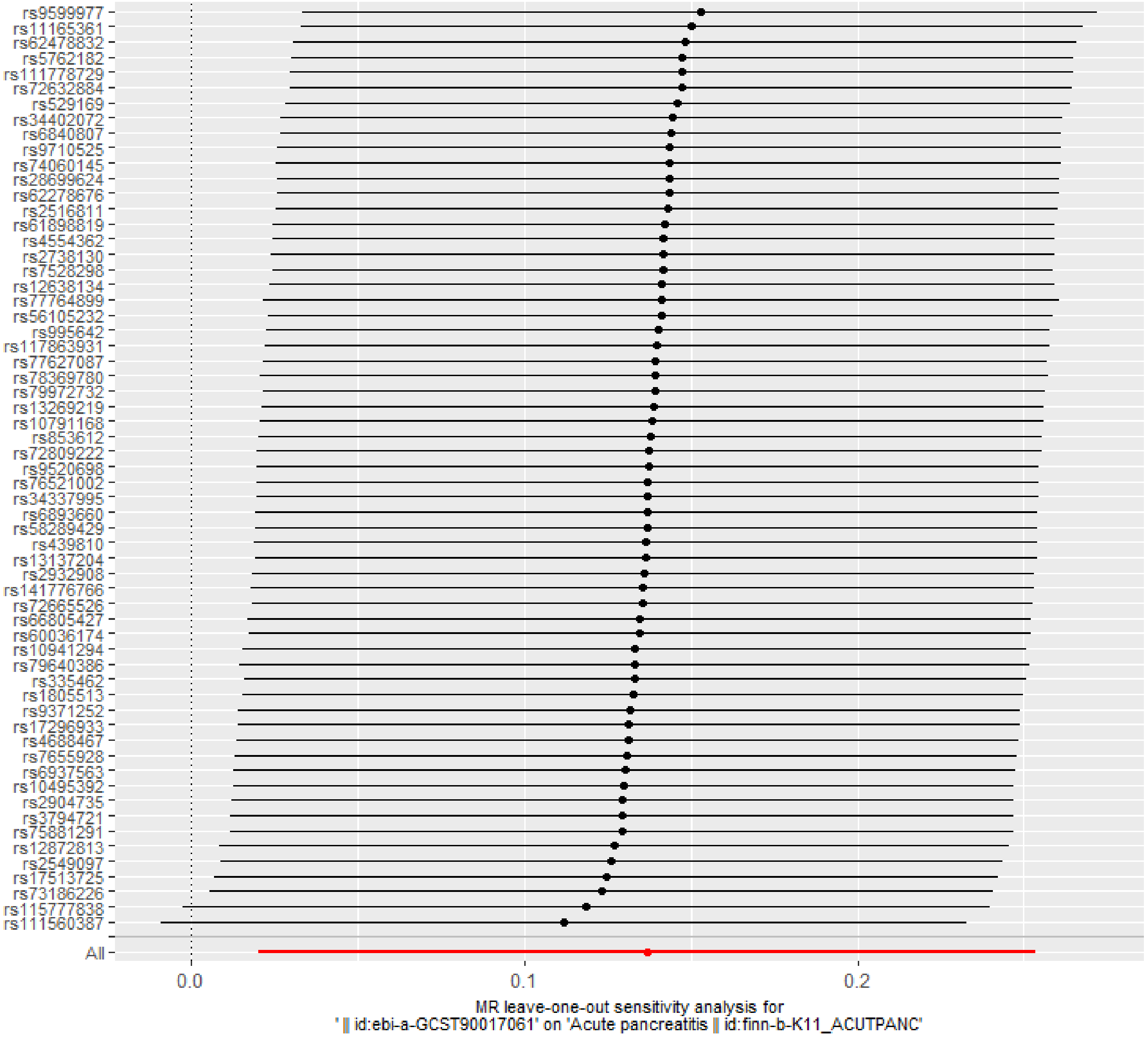

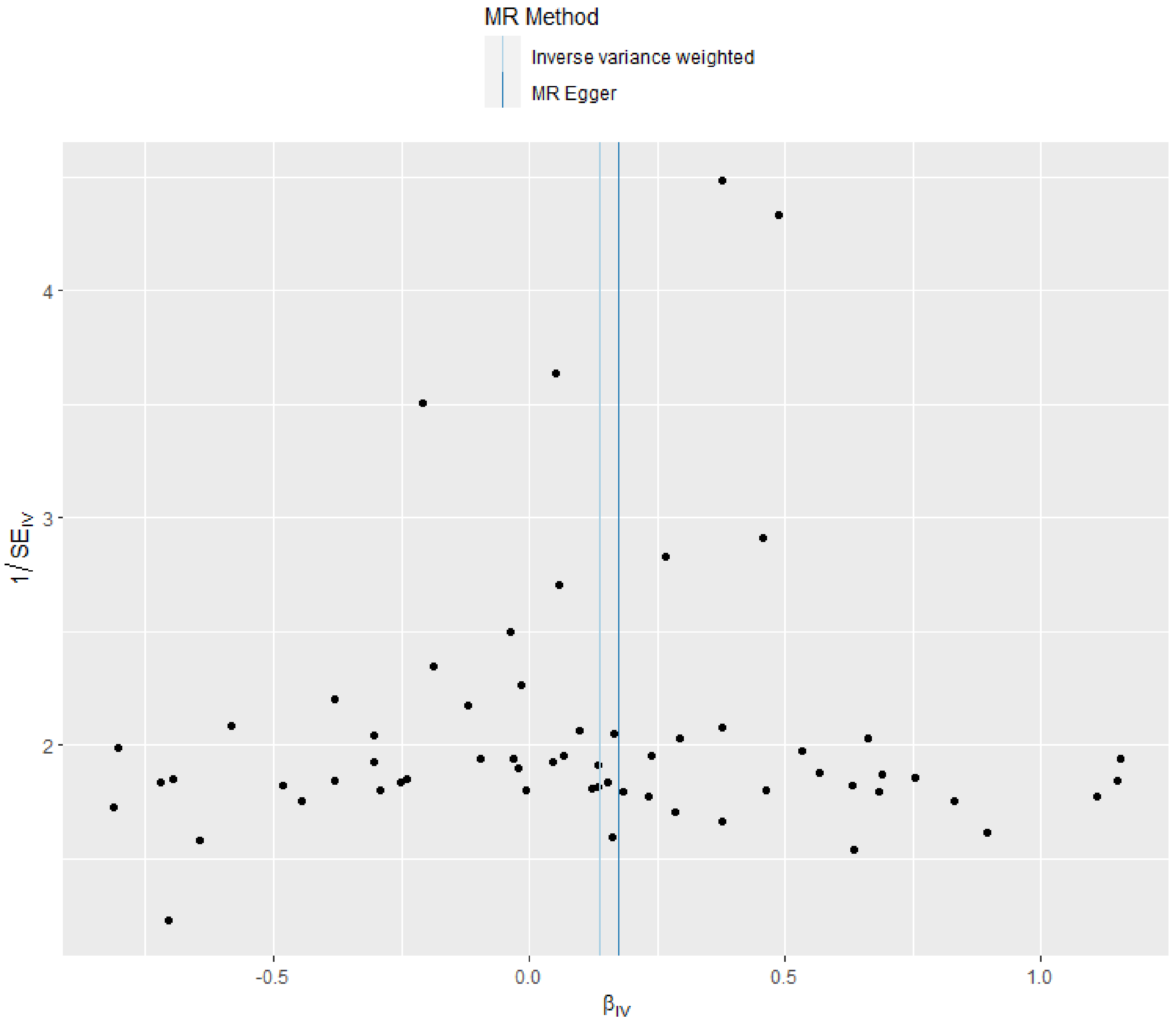
MR Method
Inverse variance weighted
MR Egger
4
3
2
$1/SE_{IV}$
-0.5
0.0
0.5
1.0
β_{IV}

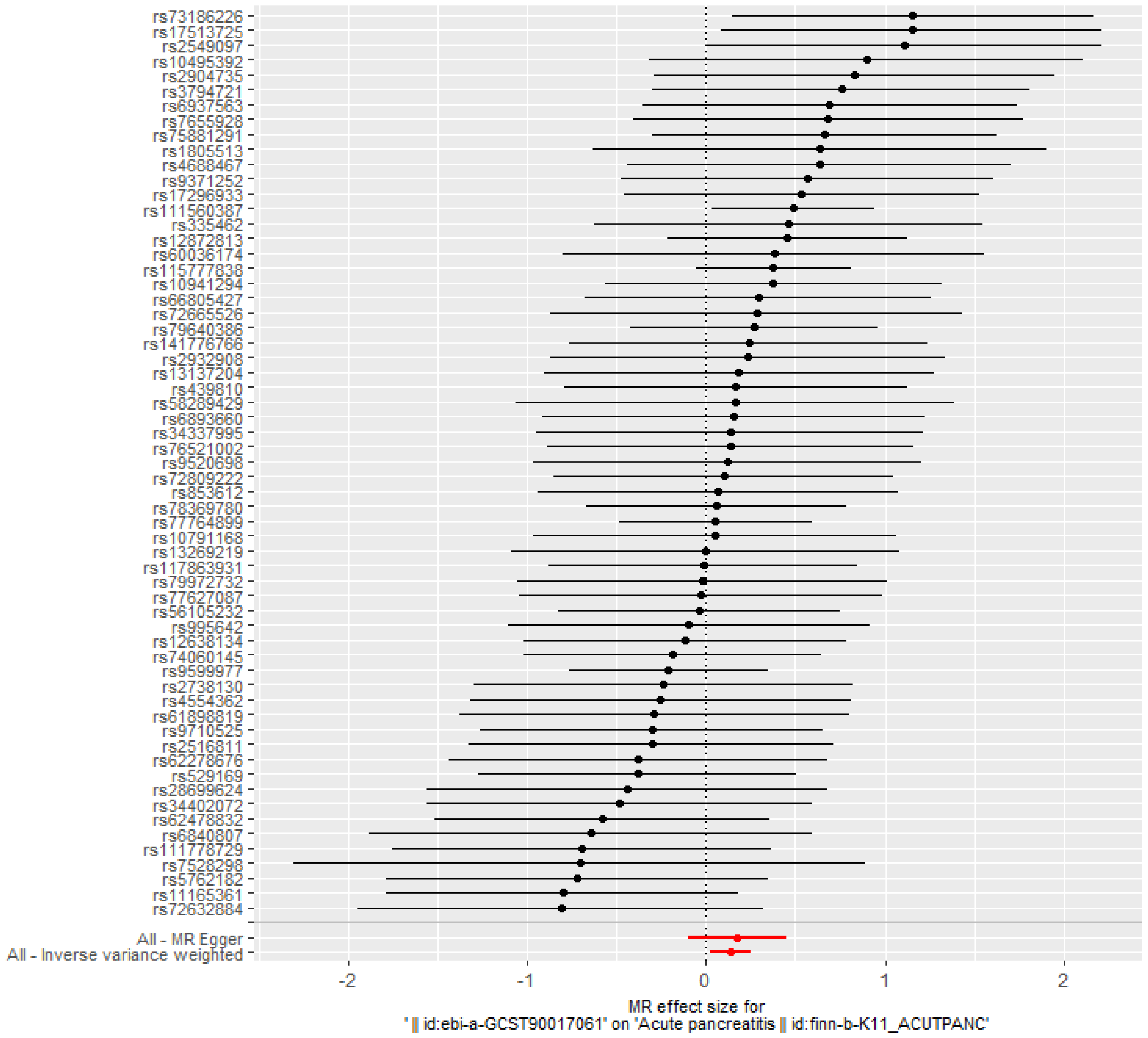

rs73186226
rs17513725
rs2549097
rs10495392
rs2904735
rs3794721
rs6937563
rs7655928
rs75881291
rs1805513
rs4688467
rs9371252
rs17296933
rs111560387
rs335462
rs12872813
rs60036174
rs115777838
rs10941294
rs66805427
rs72665526
rs79640386
rs141776766
rs2932908
rs13137204
rs439810
rs58289429
rs6893660
rs34337995
rs76521002
rs9520698
rs72809222
rs853612
rs78369780
rs77764899
rs10791168
rs13269219
rs117863931
rs79972732
rs77627087
rs56105232
rs995642
rs12638134
rs74060145
rs9599977
rs2738130
rs4554362
rs61898819
rs9710525
rs2516811
rs62278676
rs529169
rs28699624
rs34402072
rs62478832
rs6840807
rs111778729
rs7528298
rs5762182
rs11165361
rs72632884
All - MR Egger
All - Inverse variance weighted
-2
-1
0
1
2
MR effect size for
' || id:ebi-a-GCST90017061' on 'Acute pancreatitis || id:finn-b-K11_ACUTPANC'

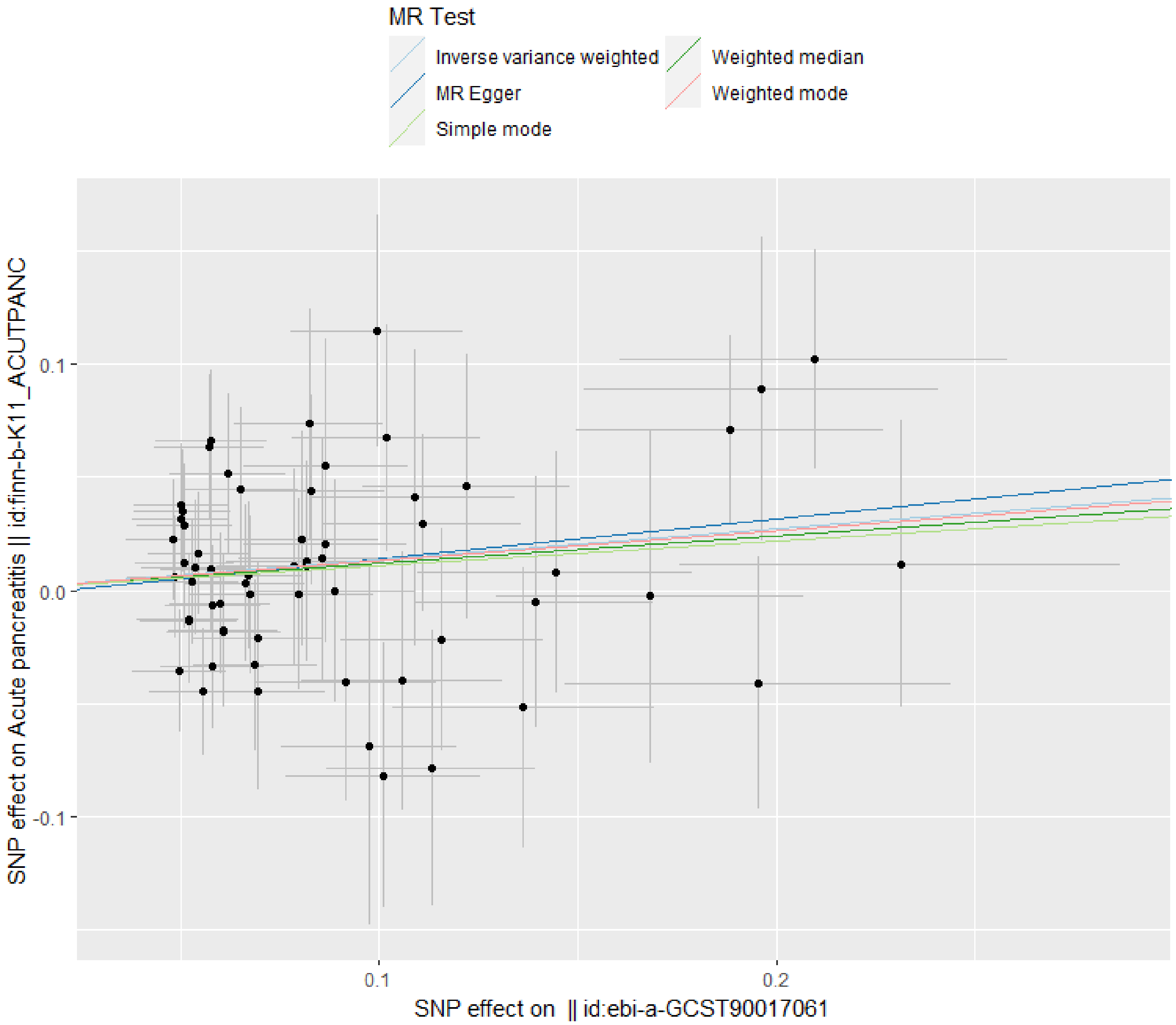
MR Test
Inverse variance weighted
MR Egger
Simple mode
Weighted median
Weighted mode
0.1
0.0
-0.1
0.1
0.2
SNP effect on Acute pancreatitis || id:finn-b-K11_ACUTPANC
SNP effect on || id:ebi-a-GCST90017061

Figure 203 Leave-one-out analysis, funnel plot, MR effect size and scatter plot for gut microbiota abundance (genus Sellimonas id.14369) on acute pancreatitis

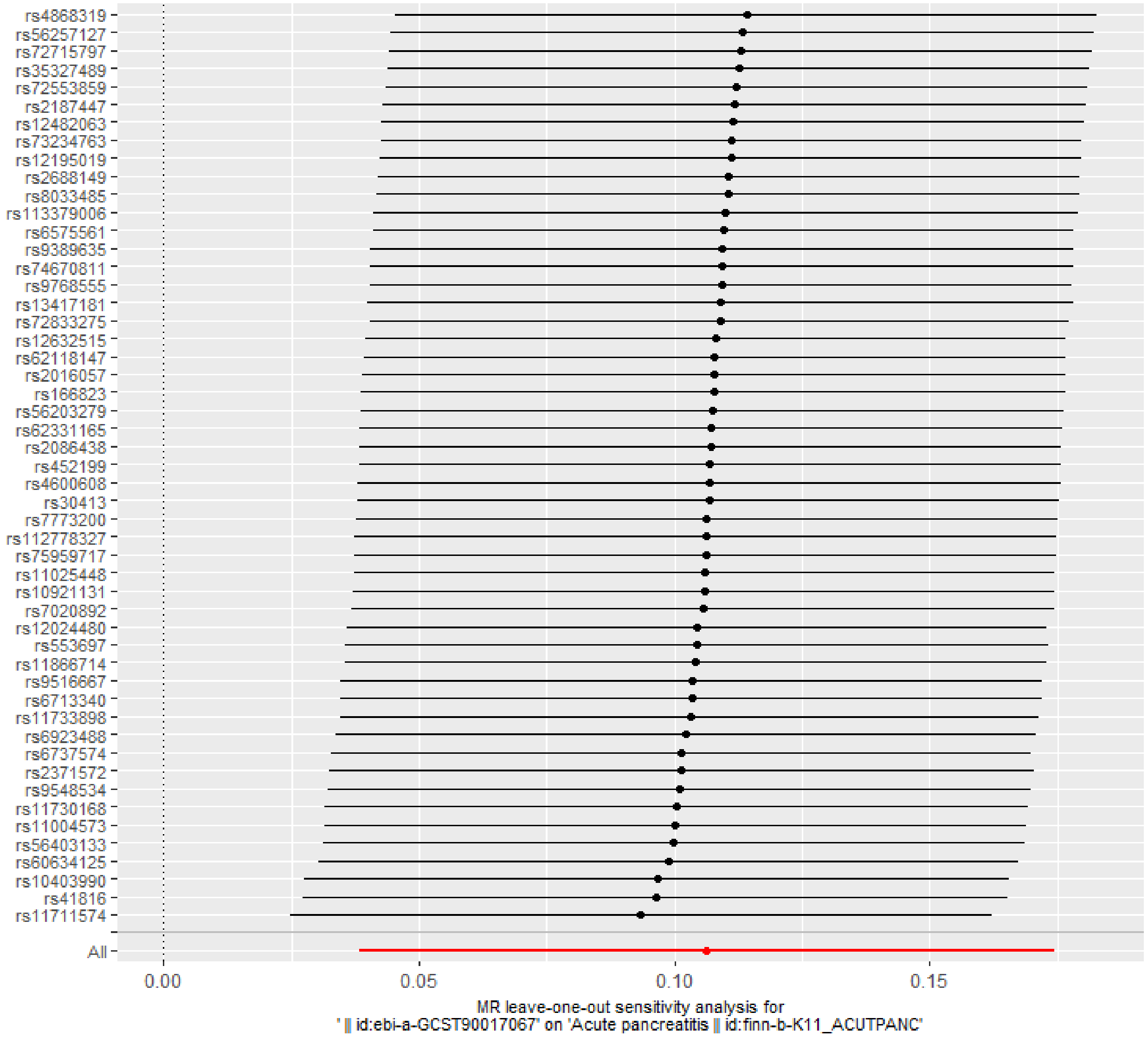

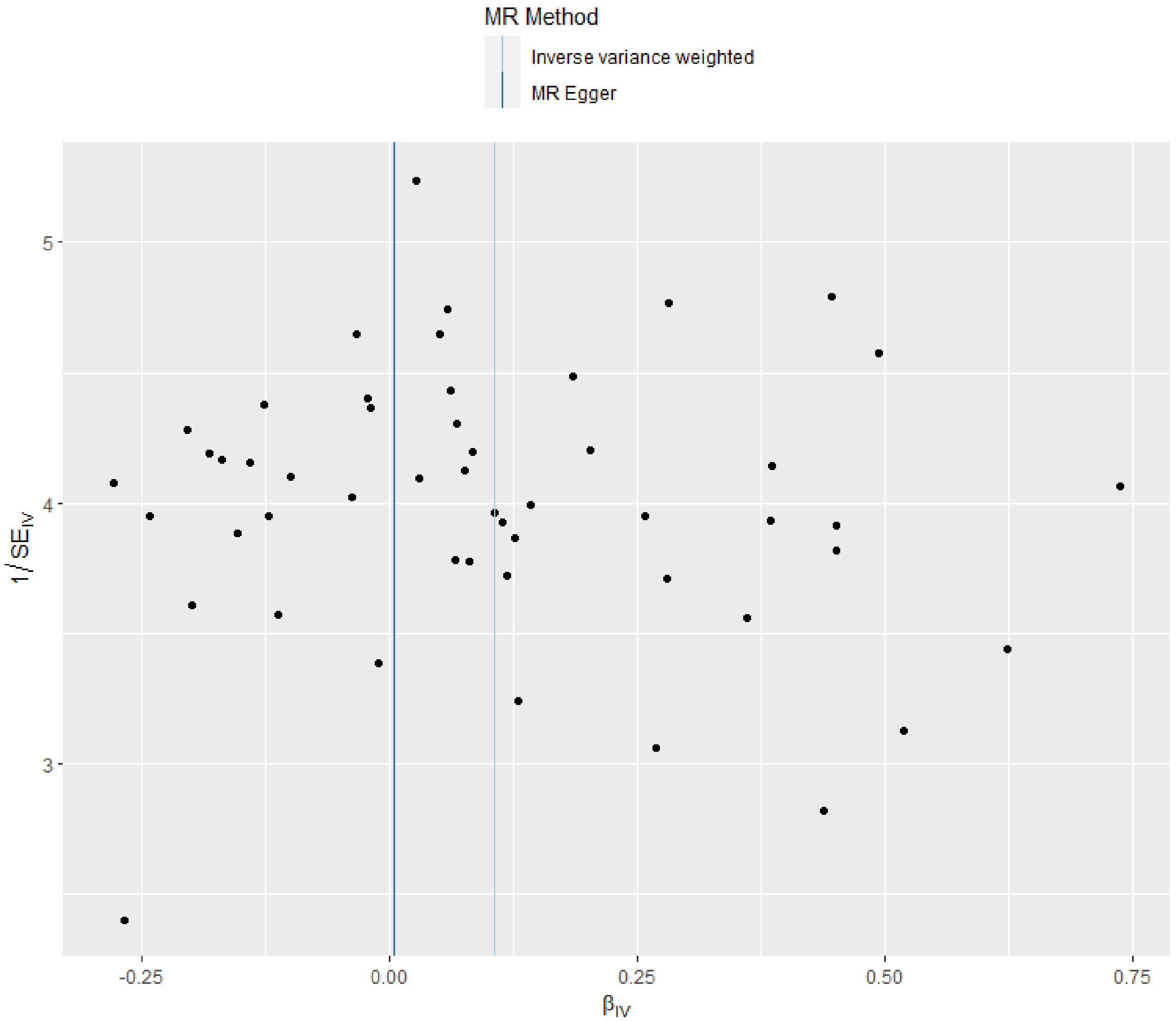

MR Method
Inverse variance weighted
MR Egger
5
4
3
1/SE_IV
-0.25
0.00
0.25
0.50
0.75
β_IV

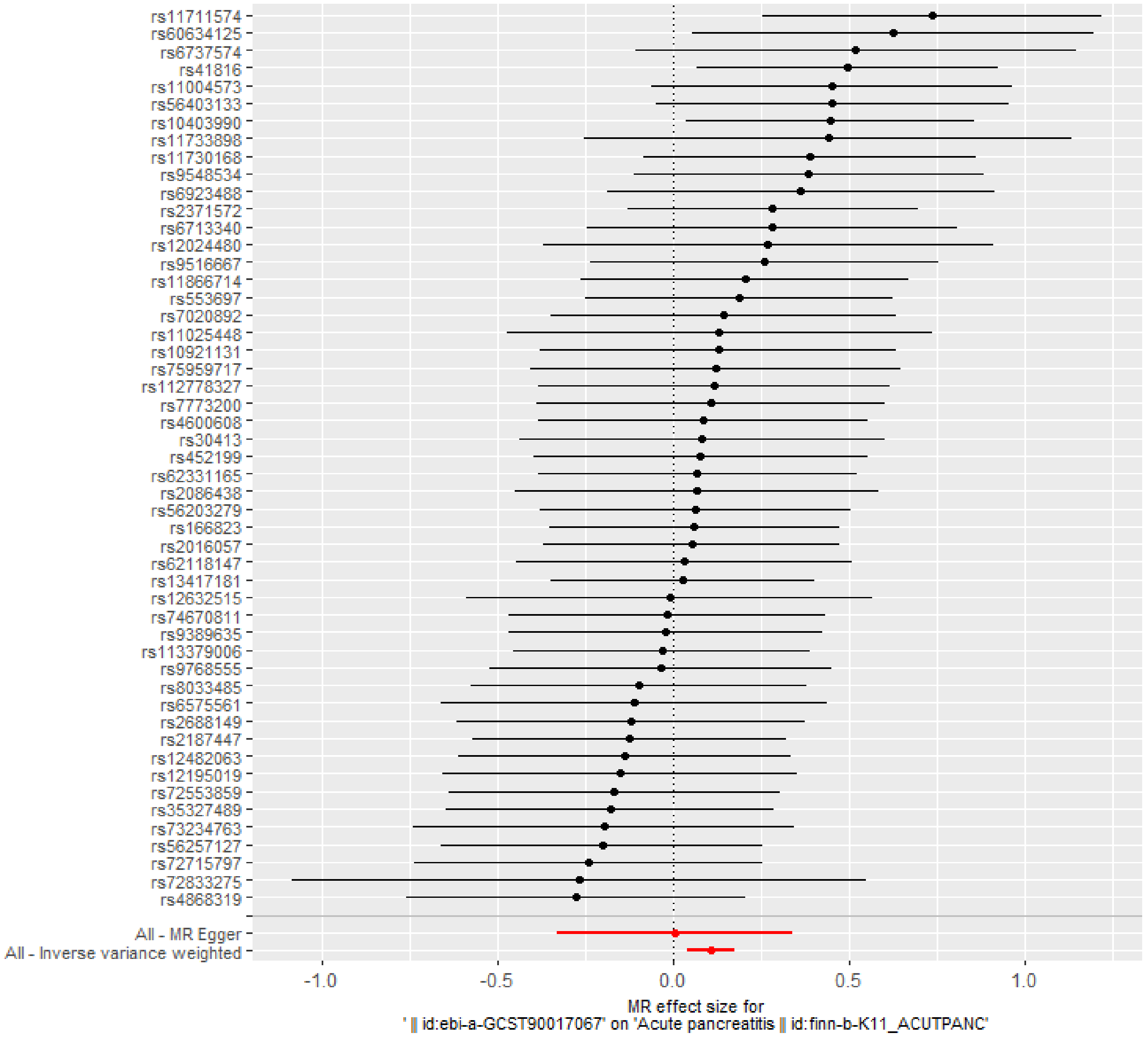

rs11711574
rs60634125
rs6737574
rs41816
rs11004573
rs56403133
rs10403990
rs11733898
rs11730168
rs9548534
rs6923488
rs2371572
rs6713340
rs12024480
rs9516667
rs11866714
rs553697
rs7020892
rs11025448
rs10921131
rs75959717
rs112778327
rs7773200
rs4600608
rs30413
rs452199
rs62331165
rs2086438
rs56203279
rs166823
rs2016057
rs62118147
rs13417181
rs12632515
rs74670811
rs9389635
rs113379006
rs9768555
rs8033485
rs6575561
rs2688149
rs2187447
rs12482063
rs12195019
rs72553859
rs35327489
rs73234763
rs56257127
rs72715797
rs72833275
rs4868319
All - MR Egger
All - Inverse variance weighted
-1.0
-0.5
0.0
0.5
1.0
MR effect size for
' || id:ebi-a-GCST90017067' on 'Acute pancreatitis || id:finn-b-K11_ACUTPANC'

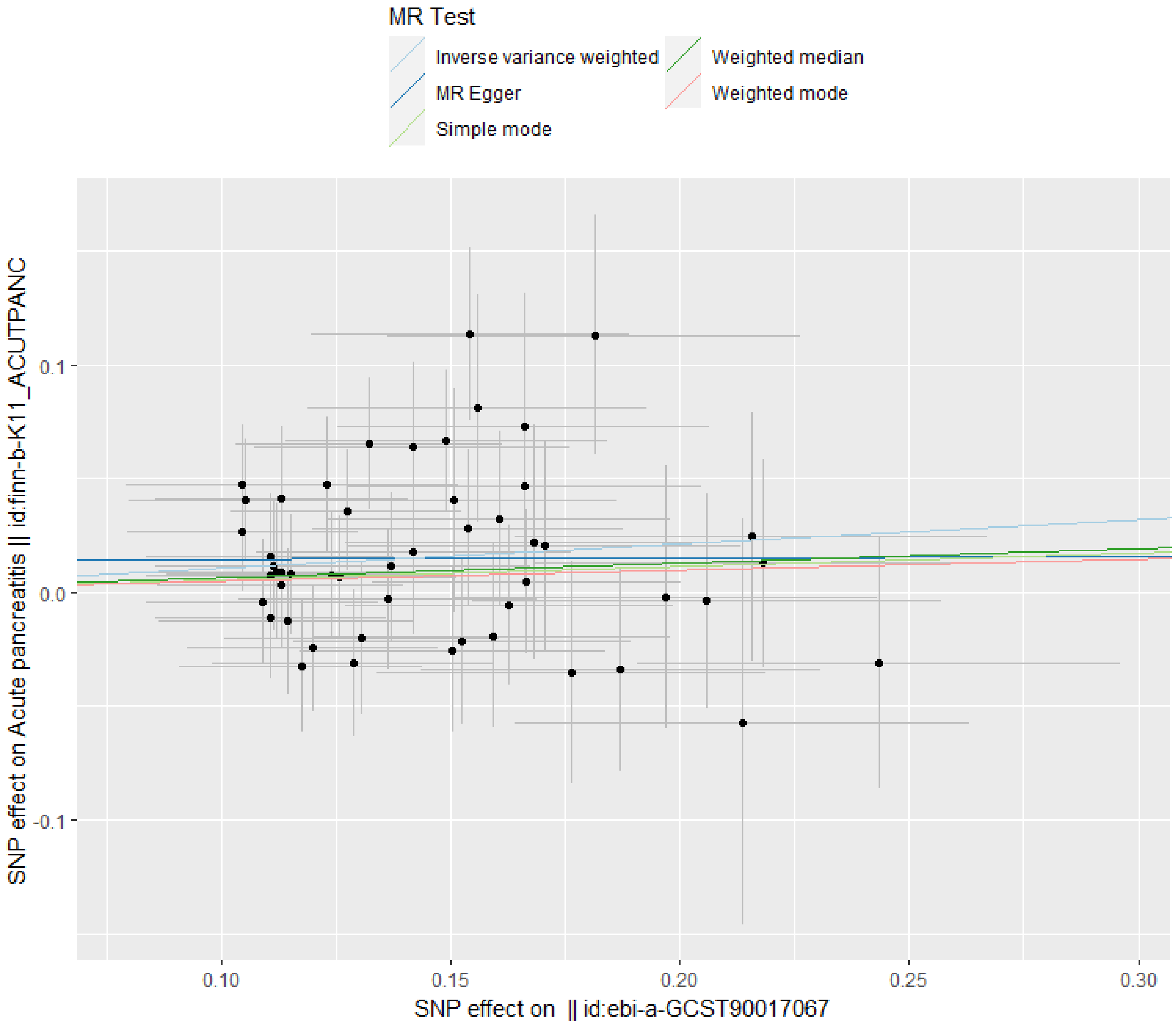
MR Test
Inverse variance weighted
MR Egger
Simple mode
Weighted median
Weighted mode
0.1
0.0
-0.1
SNP effect on Acute pancreatitis || id:finn-b-K11_ACUTPANC
0.10
0.15
0.20
0.25
0.30
SNP effect on || id:ebi-a-GCST90017067

Figure 204 Leave-one-out analysis, funnel plot, MR effect size and scatter plot for gut microbiota abundance (genus Slackia id.825) on acute pancreatitis

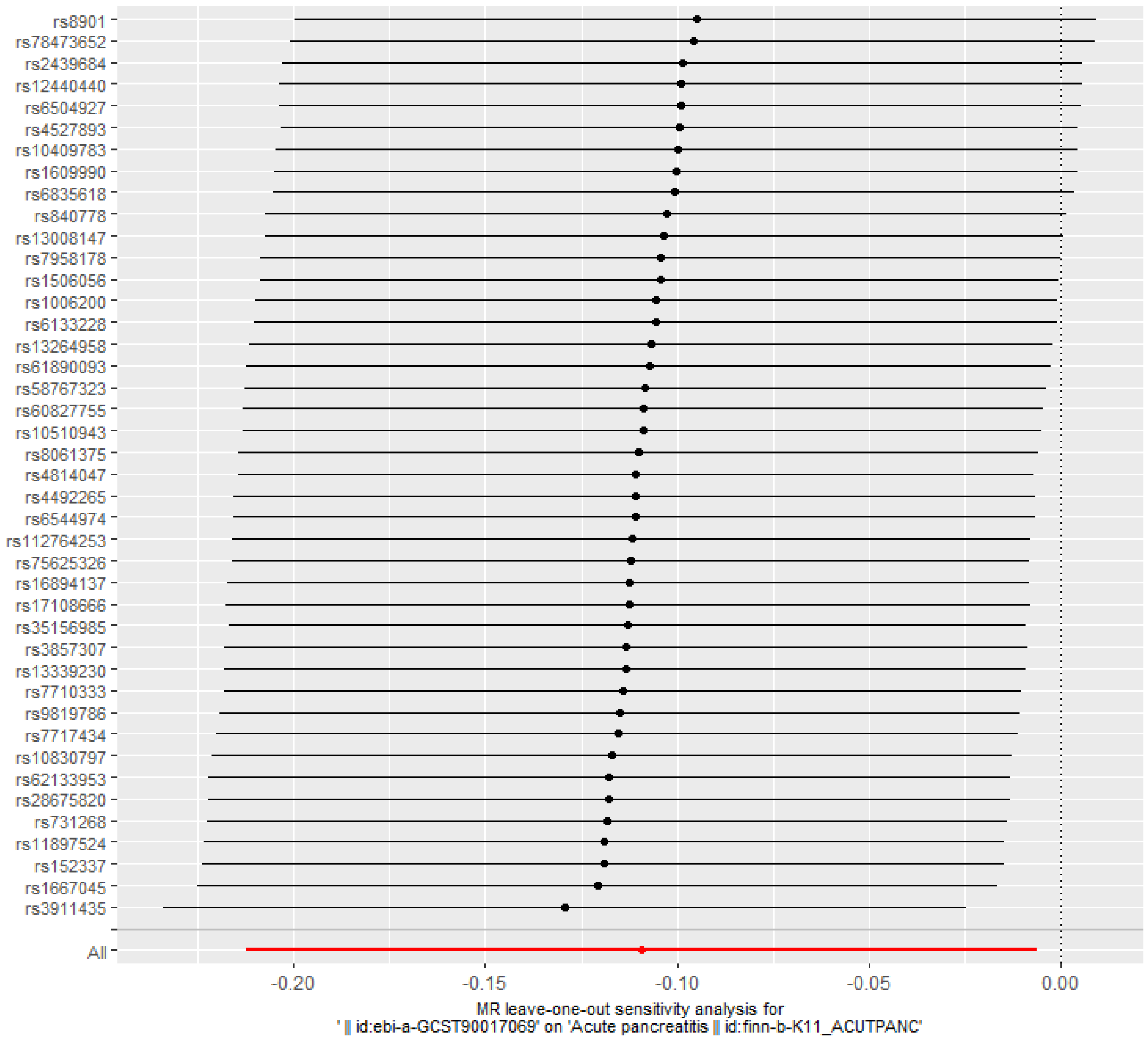

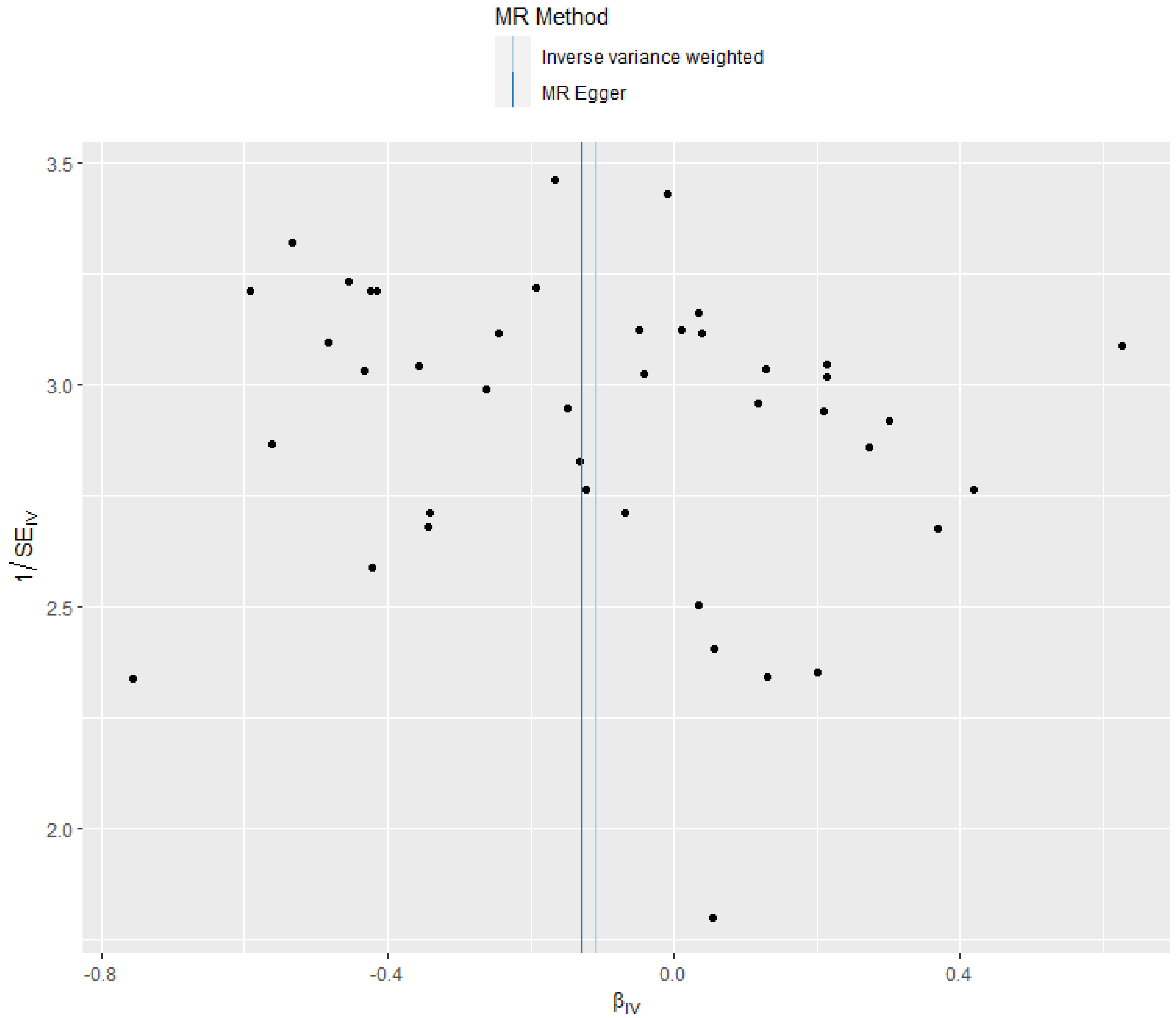
MR Method
Inverse variance weighted
MR Egger
3.5
3.0
2.5
2.0
1/SE_IV
-0.8
-0.4
0.0
0.4
β_IV

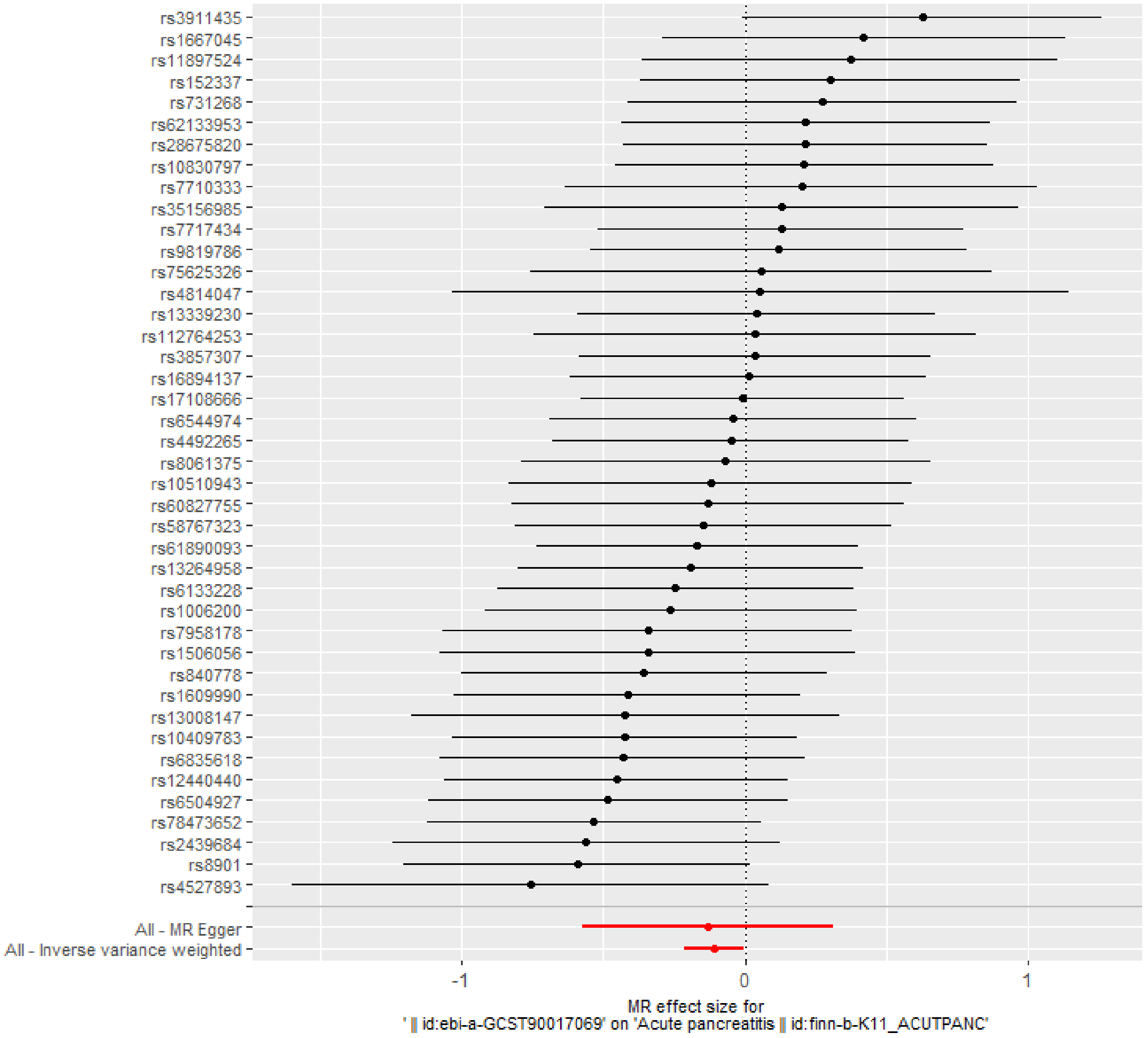

rs3911435
rs1667045
rs11897524
rs152337
rs731268
rs62133953
rs28675820
rs10830797
rs7710333
rs35156985
rs7717434
rs9819786
rs75625326
rs4814047
rs13339230
rs112764253
rs3857307
rs16894137
rs17108666
rs6544974
rs4492265
rs8061375
rs10510943
rs60827755
rs58767323
rs61890093
rs13264958
rs6133228
rs1006200
rs7958178
rs1506056
rs840778
rs1609990
rs13008147
rs10409783
rs6835618
rs12440440
rs6504927
rs78473652
rs2439684
rs8901
rs4527893
All - MR Egger
All - Inverse variance weighted
-1
0
1
MR effect size for
' || id:ebi-a-GCST90017069' on 'Acute pancreatitis || id:finn-b-K11_ACUTPANC'

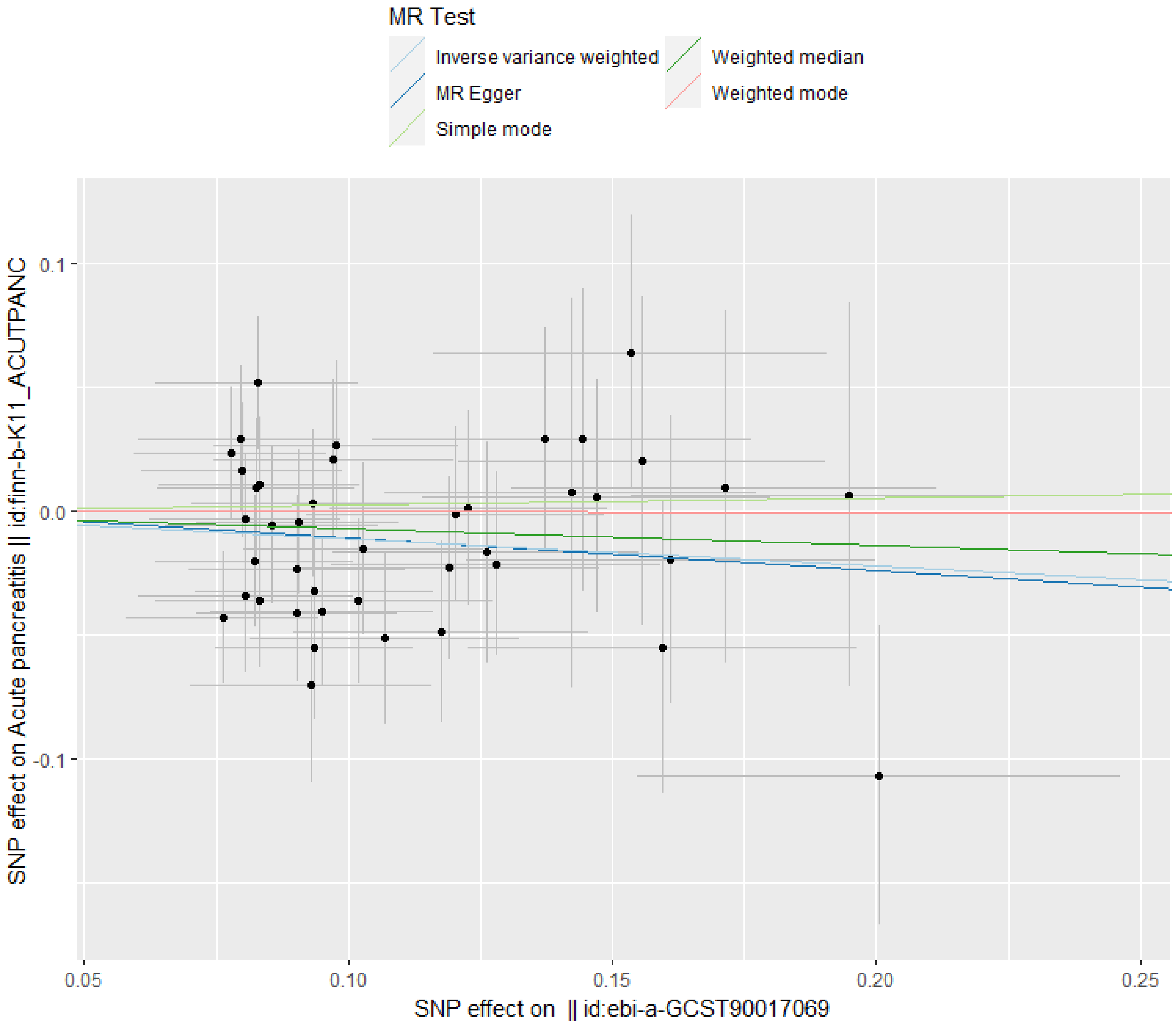

MR Test
Inverse variance weighted
MR Egger
Simple mode
Weighted median
Weighted mode
0.1
0.0
-0.1
SNP effect on Acute pancreatitis || id:finn-b-K11_ACUTPANC
0.05
0.10
0.15
0.20
0.25
SNP effect on || id:ebi-a-GCST90017069

Figure 205 Leave-one-out analysis, funnel plot, MR effect size and scatter plot for gut microbiota abundance (order Methanobacteriales id.120) on acute pancreatitis

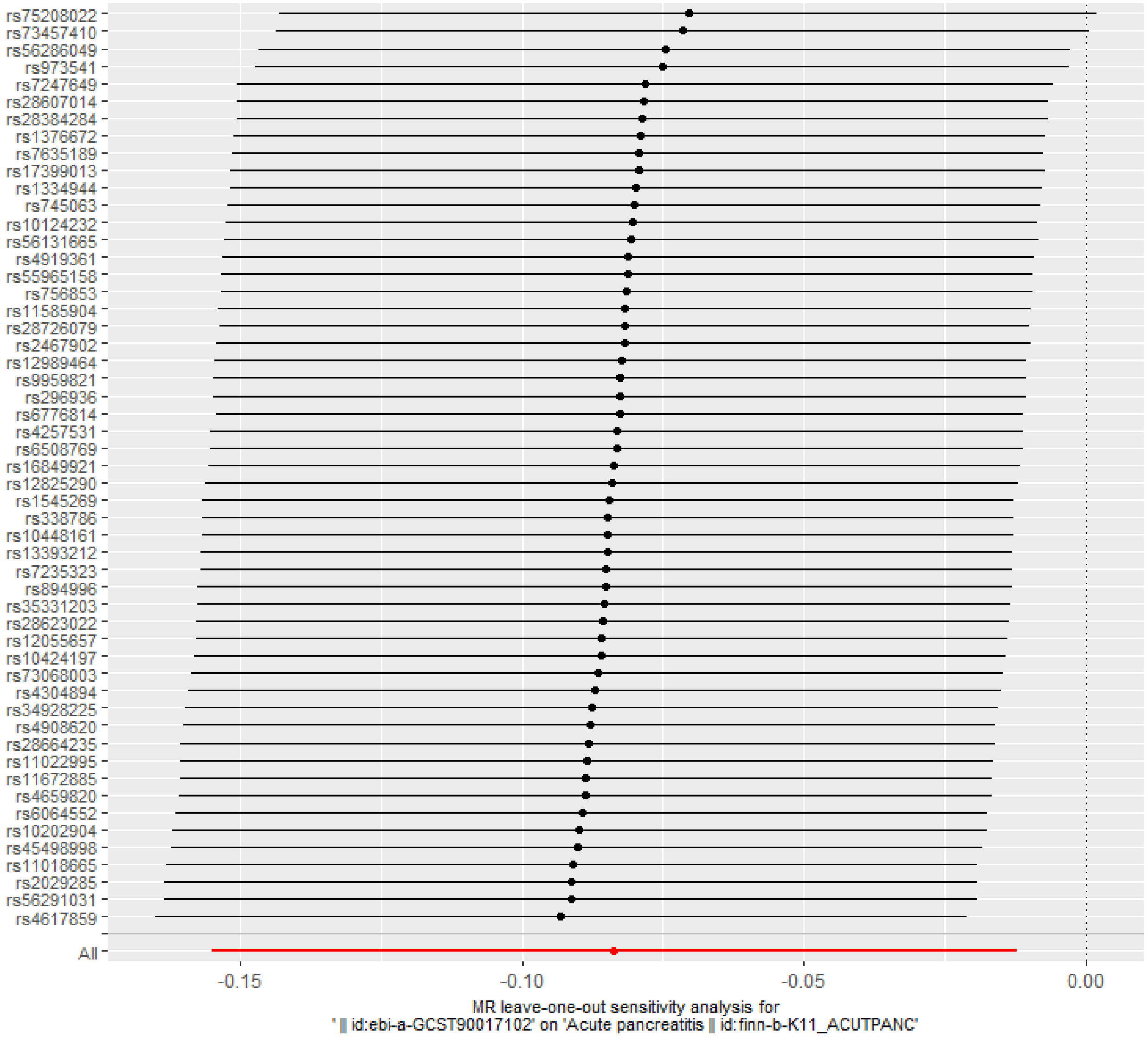

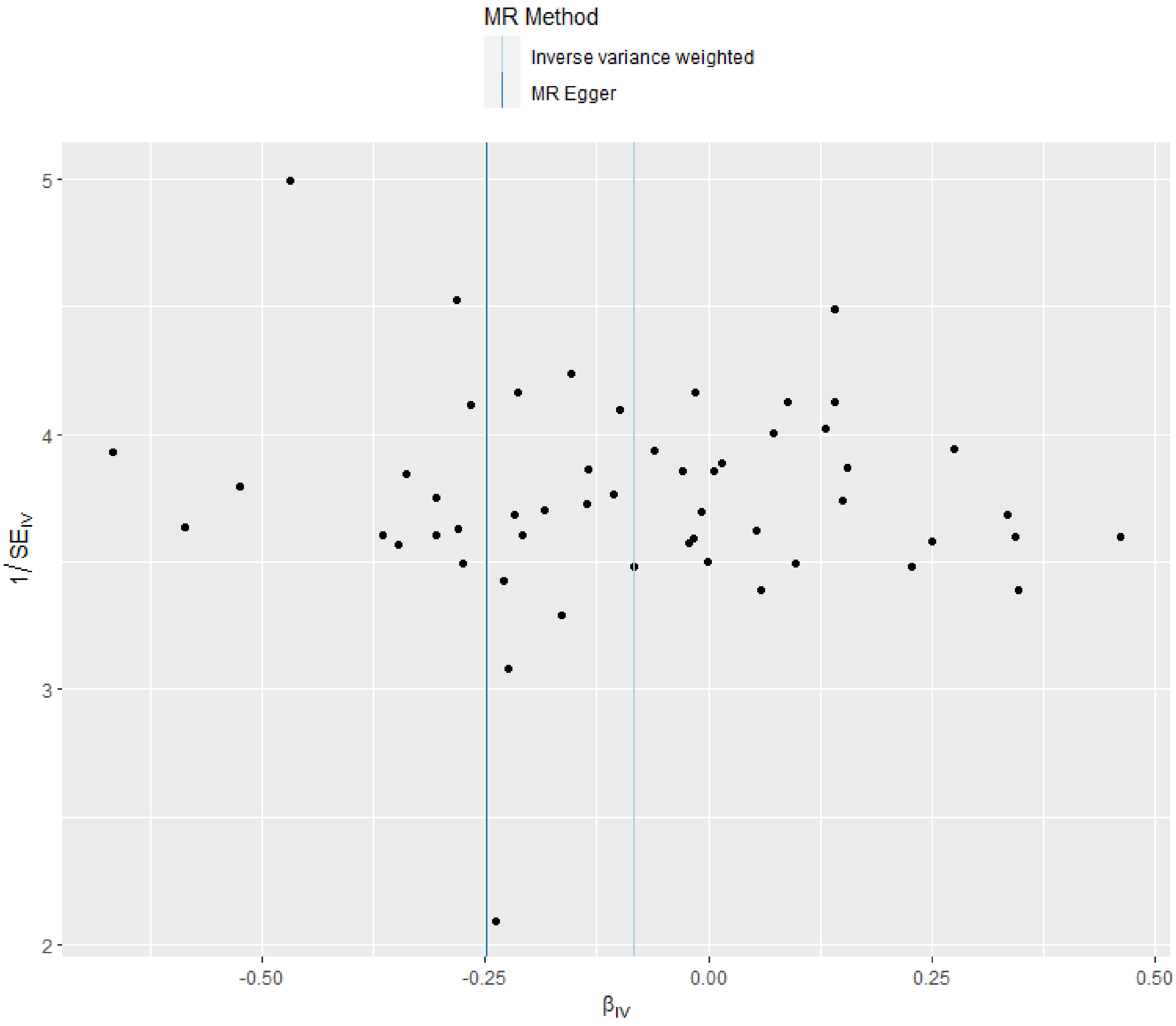

MR Method
Inverse variance weighted
MR Egger
5
4
3
2
-0.50
-0.25
0.00
0.25
0.50
1/SE_IV
β_IV

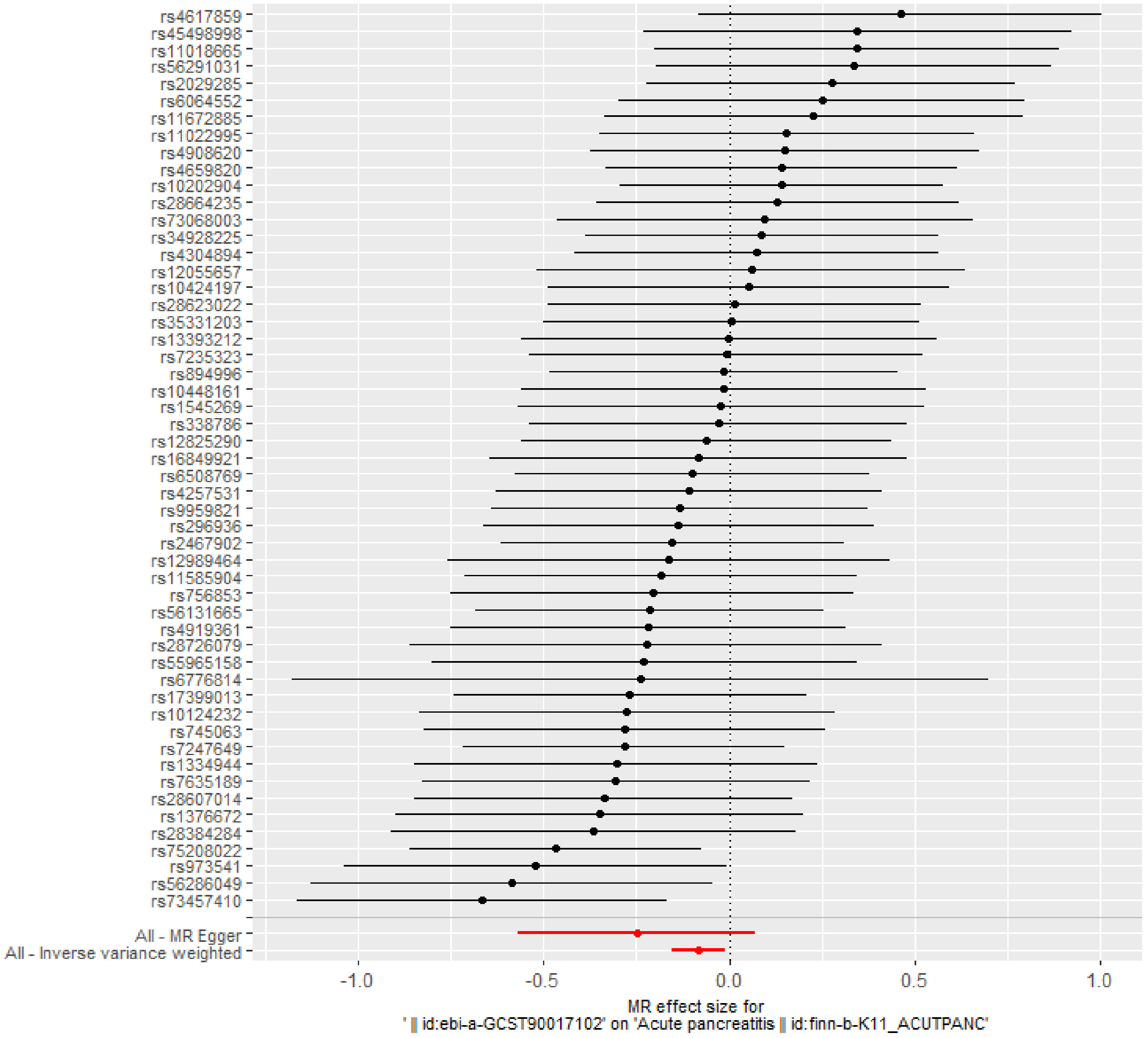
rs4617859
rs45498998
rs11018665
rs56291031
rs2029285
rs6064552
rs11672885
rs11022995
rs4908620
rs4659820
rs10202904
rs28664235
rs73068003
rs34928225
rs4304894
rs12055657
rs10424197
rs28623022
rs35331203
rs13393212
rs7235323
rs894996
rs10448161
rs1545269
rs338786
rs12825290
rs16849921
rs6508769
rs4257531
rs9959821
rs296936
rs2467902
rs12989464
rs11585904
rs756853
rs56131665
rs4919361
rs28726079
rs55965158
rs6776814
rs17399013
rs10124232
rs745063
rs7247649
rs1334944
rs7635189
rs28607014
rs1376672
rs28384284
rs75208022
rs973541
rs56286049
rs73457410
All - MR Egger
All - Inverse variance weighted
-1.0
-0.5
0.0
0.5
1.0
MR effect size for
' || id:ebi-a-GCST90017102' on 'Acute pancreatitis || id:finn-b-K11_ACUTPANC'

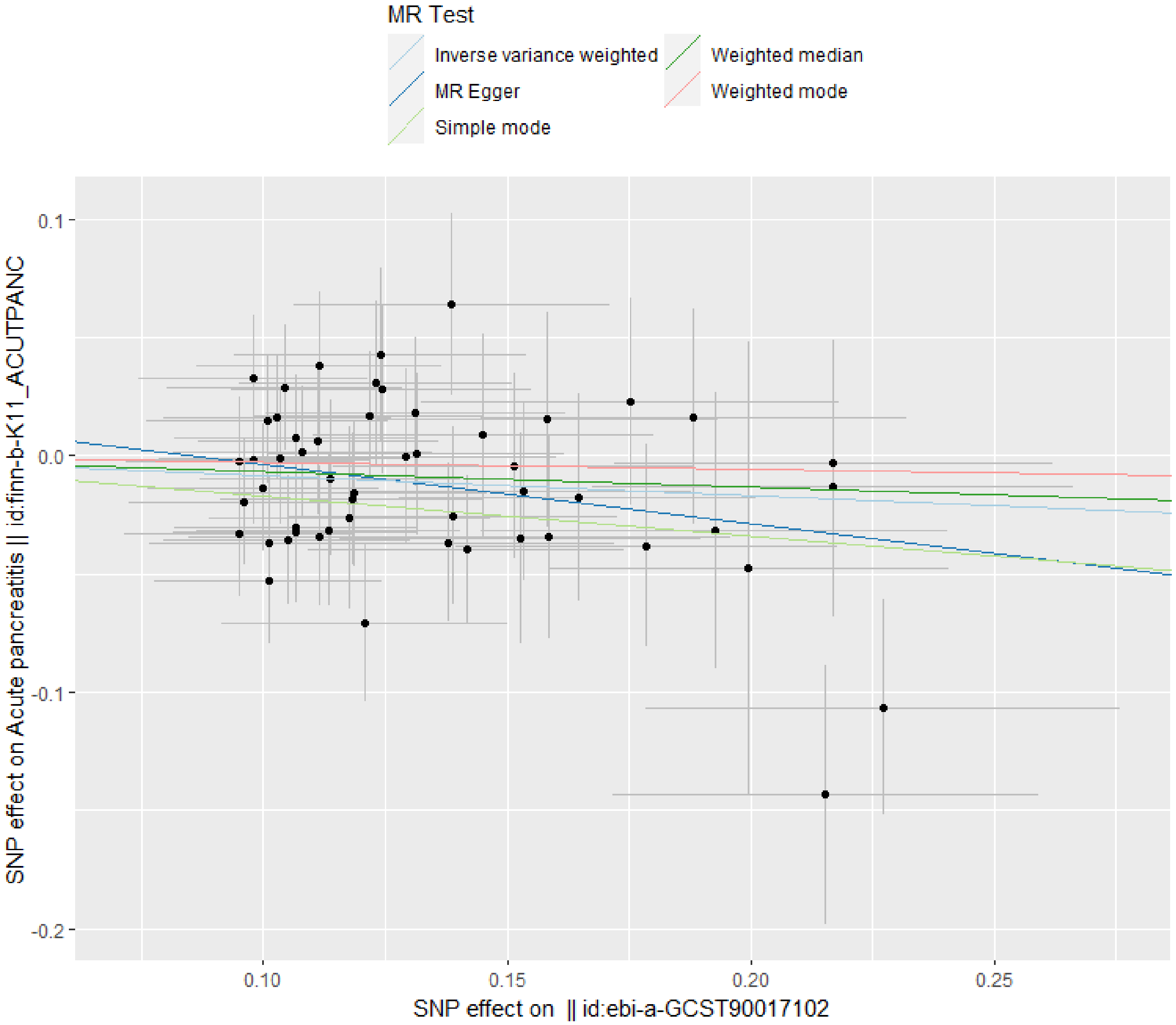

MR Test
Inverse variance weighted
MR Egger
Simple mode
Weighted median
Weighted mode
0.1
0.0
-0.1
-0.2
0.10
0.15
0.20
0.25
SNP effect on Acute pancreatitis || id:finn-b-K11_ACUTPANC
SNP effect on || id:ebi-a-GCST90017102

Figure 206 Leave-one-out analysis, funnel plot, MR effect size and scatter plot for gut microbiota abundance (phylum Verrucomicrobia id.3982) on acute pancreatitis

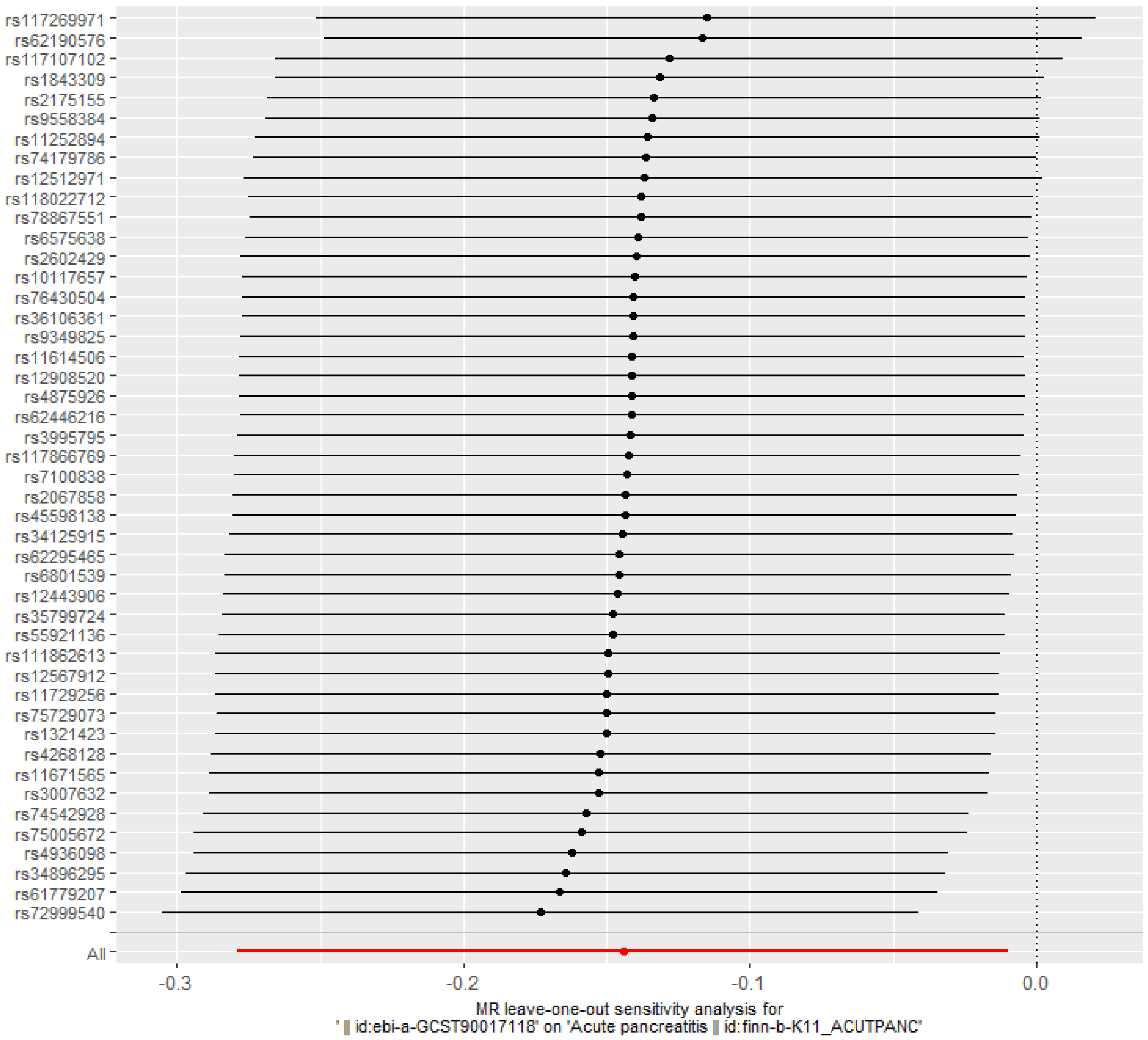

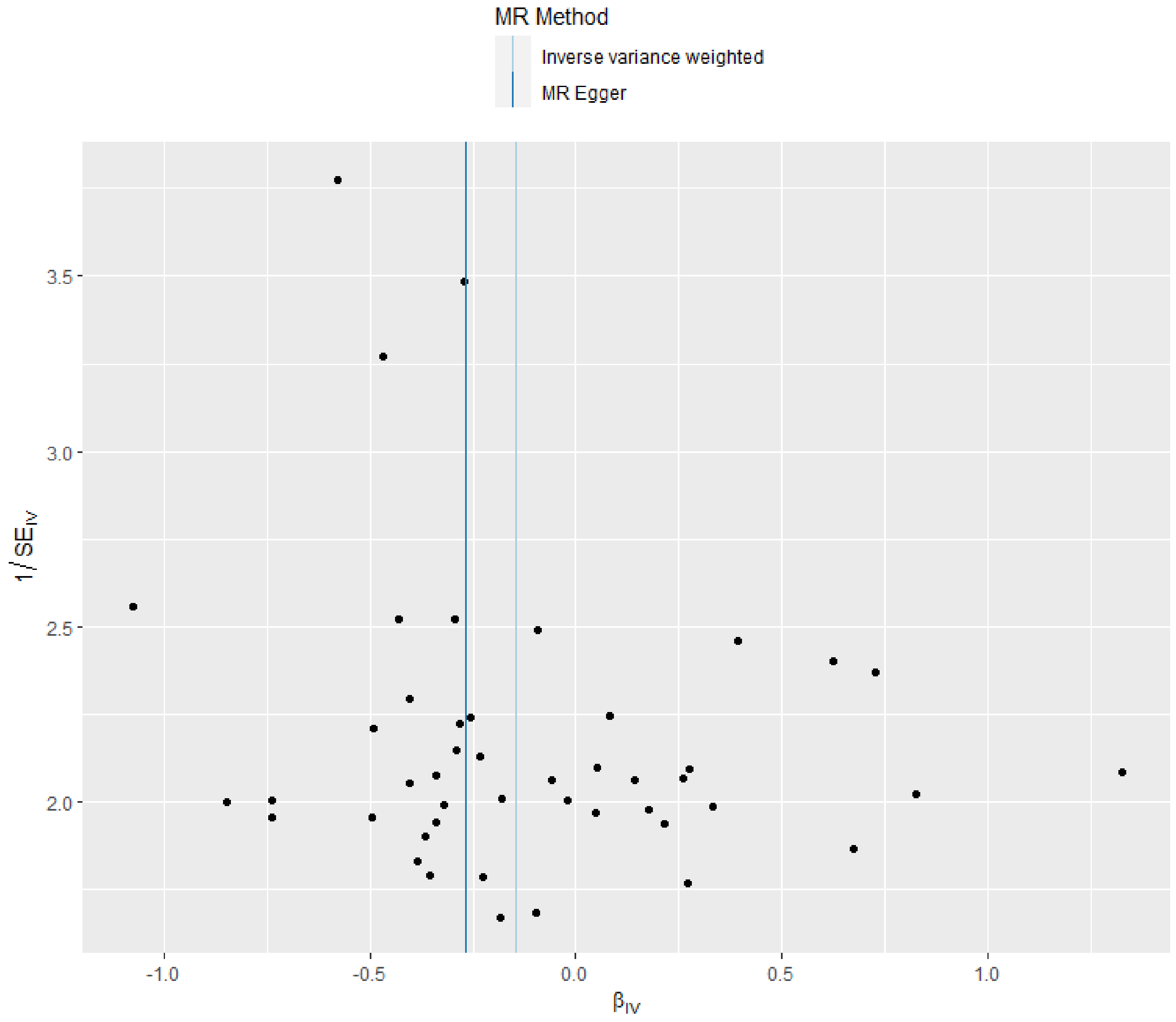

MR Method
Inverse variance weighted
MR Egger
3.5
3.0
2.5
2.0
1/SE_IV
-1.0
-0.5
0.0
0.5
1.0
β_IV

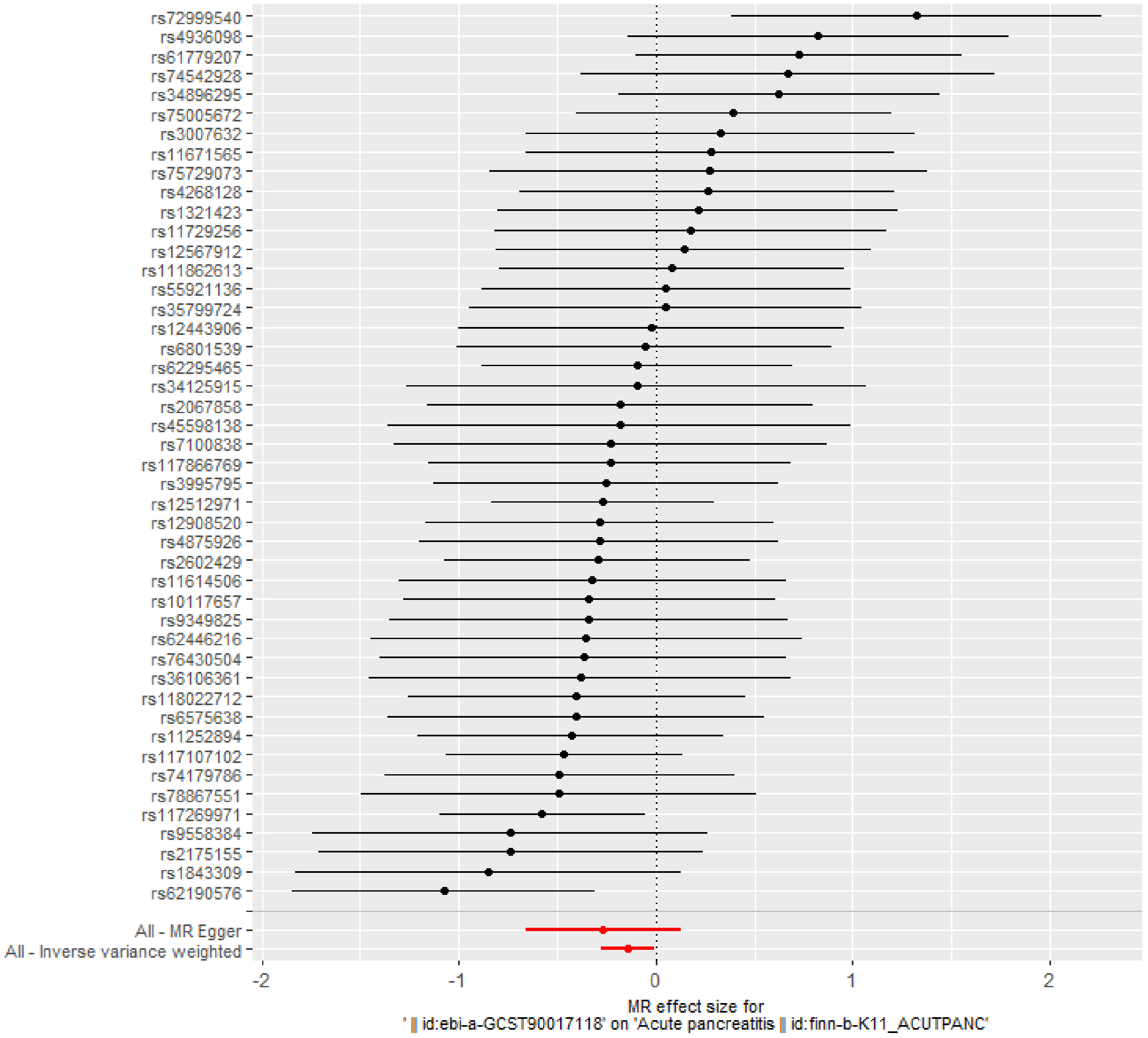
rs72999540
rs4936098
rs61779207
rs74542928
rs34896295
rs75005672
rs3007632
rs11671565
rs75729073
rs4268128
rs1321423
rs11729256
rs12567912
rs111862613
rs55921136
rs35799724
rs12443906
rs6801539
rs62295465
rs34125915
rs2067858
rs45598138
rs7100838
rs117866769
rs3995795
rs12512971
rs12908520
rs4875926
rs2602429
rs11614506
rs10117657
rs9349825
rs62446216
rs76430504
rs36106361
rs118022712
rs6575638
rs11252894
rs117107102
rs74179786
rs78867551
rs117269971
rs9558384
rs2175155
rs1843309
rs62190576
All - MR Egger
All - Inverse variance weighted
-2
-1
0
1
2
MR effect size for
' || id:ebi-a-GCST90017118' on 'Acute pancreatitis || id:finn-b-K11_ACUTPANC'

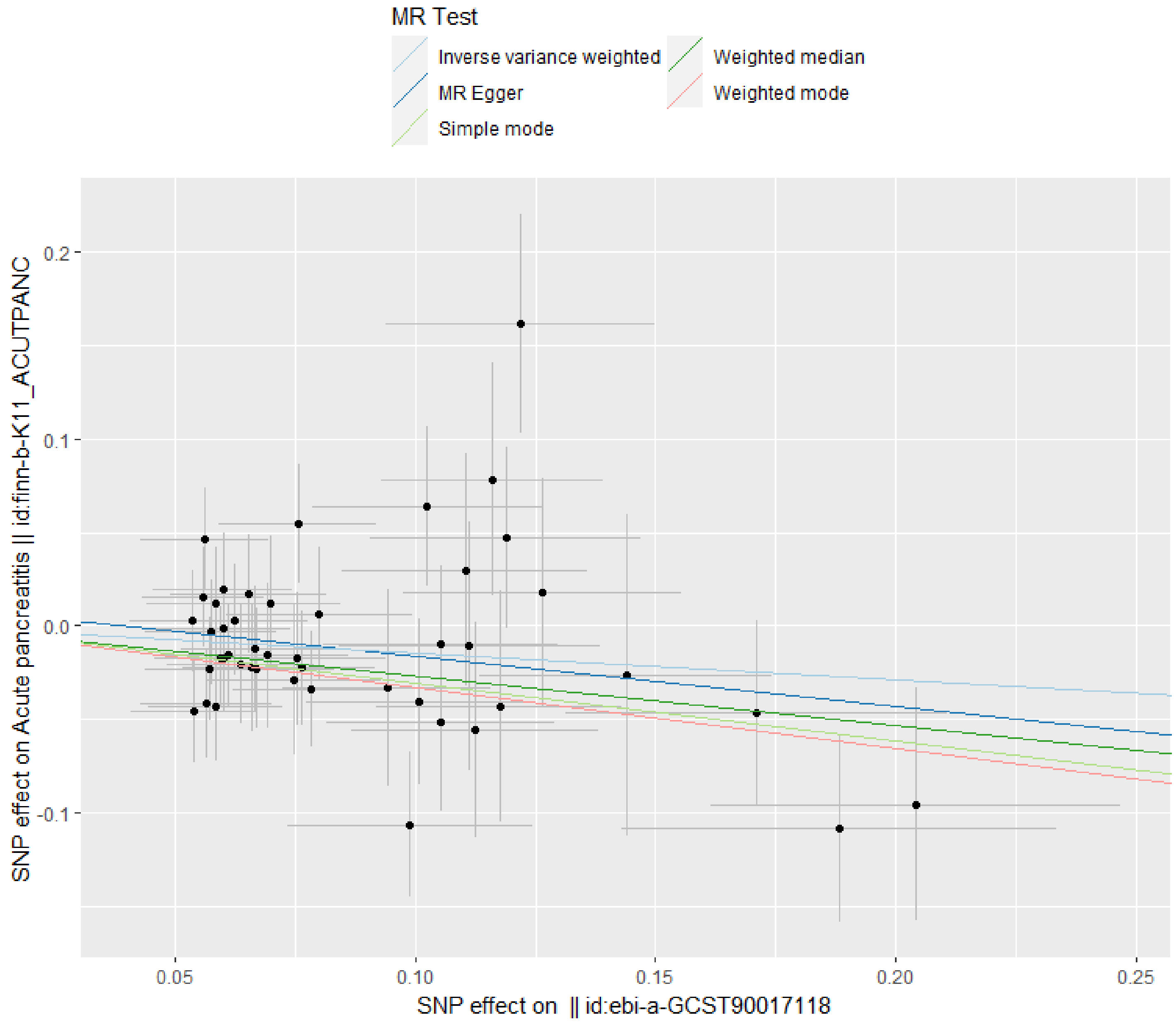
MR Test
Inverse variance weighted
MR Egger
Simple mode
Weighted median
Weighted mode
SNP effect on Acute pancreatitis || id:finn-b-K11_ACUTPANC
0.2
0.1
0.0
-0.1
0.05
0.10
0.15
0.20
0.25
SNP effect on || id:ebi-a-GCST90017118

Figure 207 Leave-one-out analysis, funnel plot, MR effect size and scatter plot for gut microbiota abundance (family Victivallaceae id.2255) on chronic pancreatitis

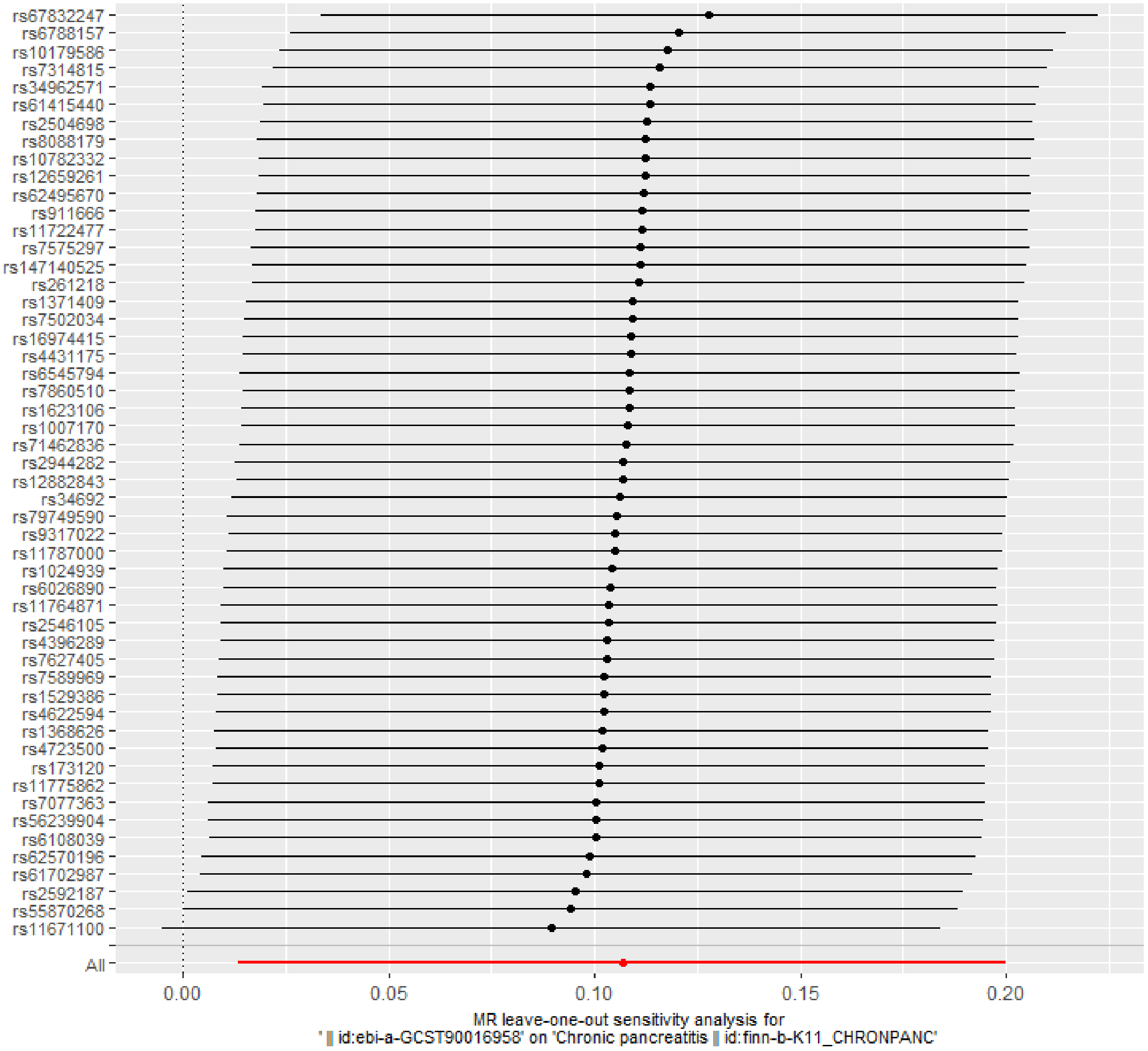

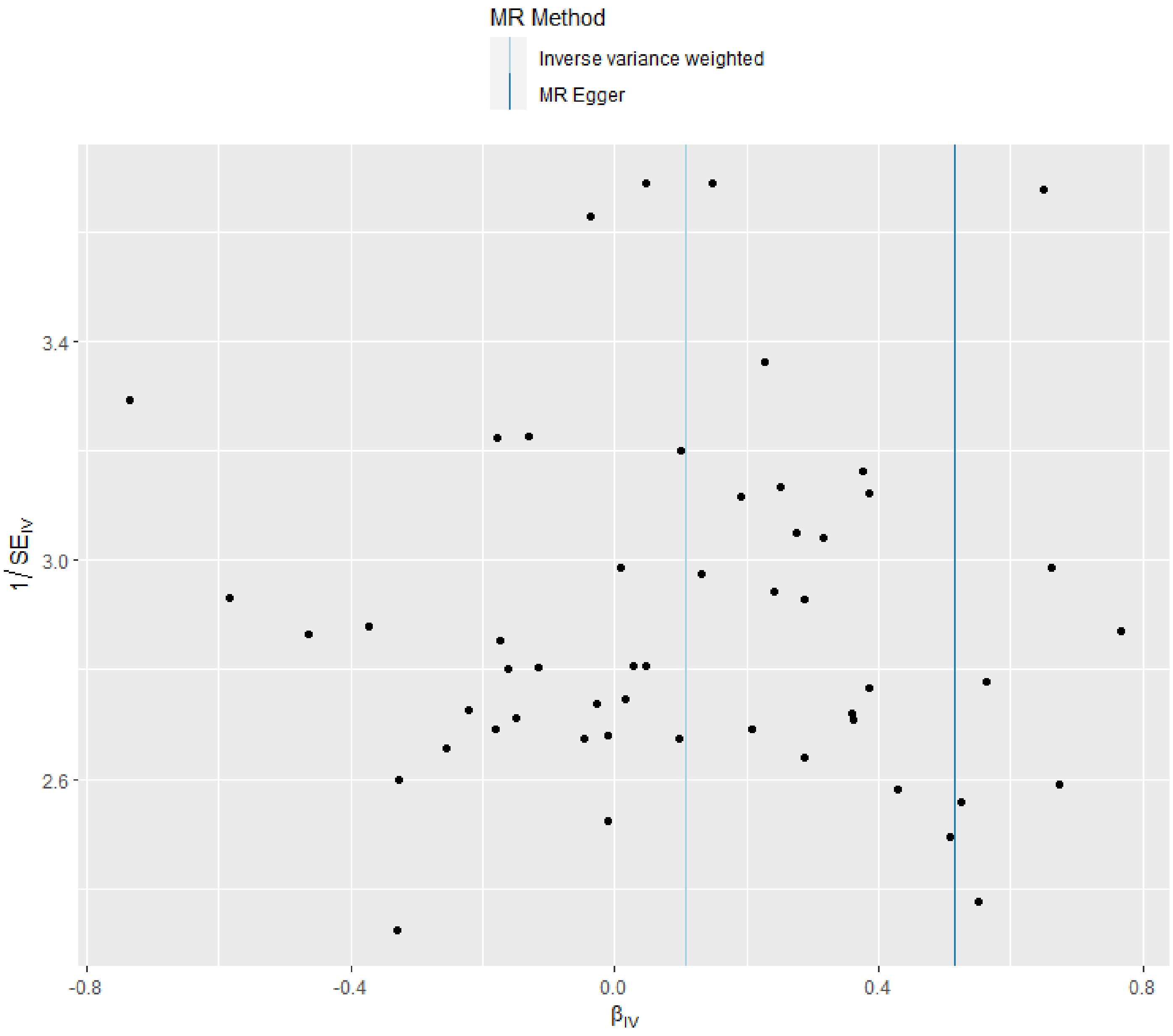

MR Method
Inverse variance weighted
MR Egger
3.4
3.0
2.6
1/SE_IV
-0.8
-0.4
0.0
0.4
0.8
β_IV

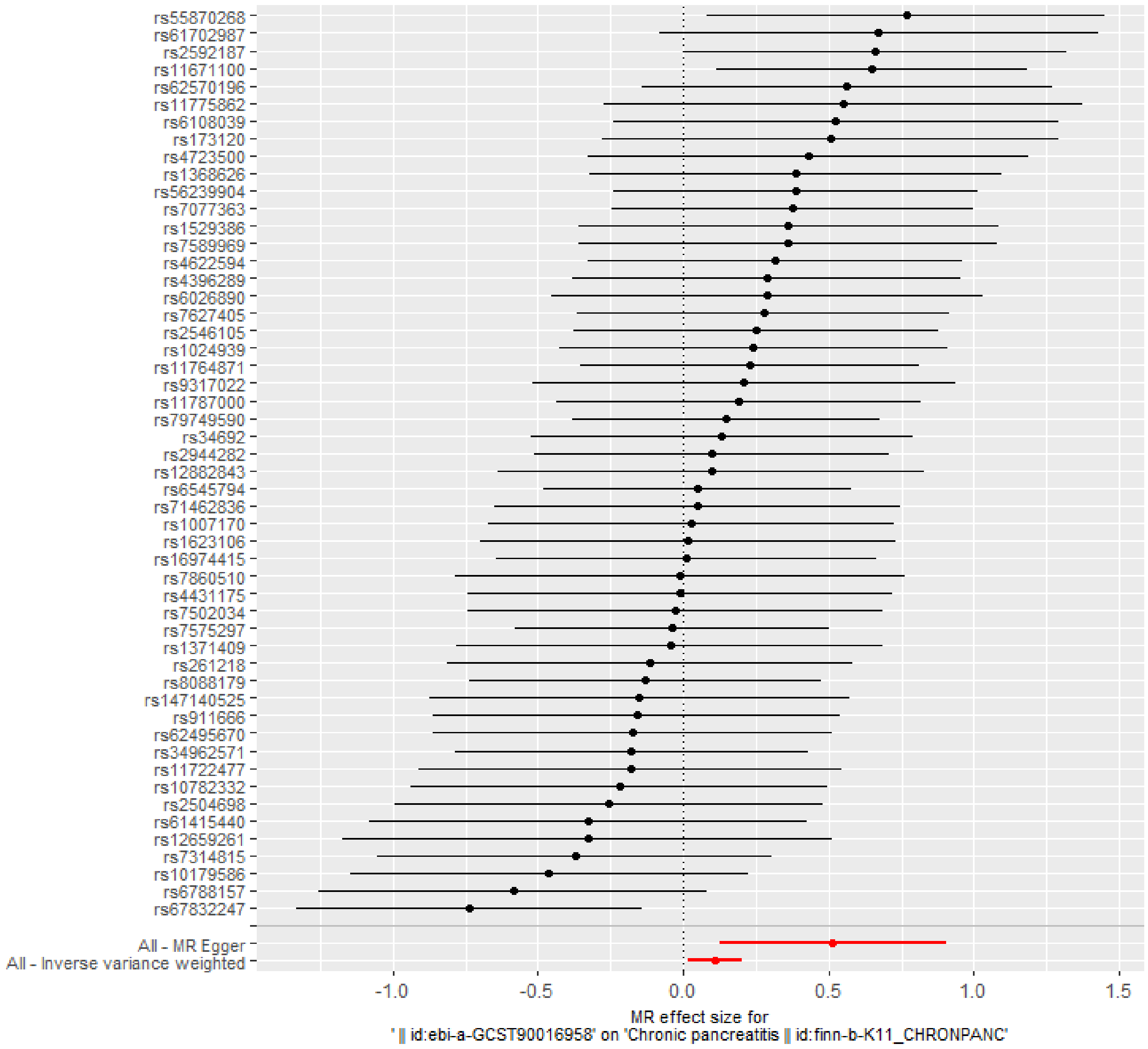
rs55870268
rs61702987
rs2592187
rs11671100
rs62570196
rs11775862
rs6108039
rs173120
rs4723500
rs1368626
rs56239904
rs7077363
rs1529386
rs7589969
rs4622594
rs4396289
rs6026890
rs7627405
rs2546105
rs1024939
rs11764871
rs9317022
rs11787000
rs79749590
rs34692
rs2944282
rs12882843
rs6545794
rs71462836
rs1007170
rs1623106
rs16974415
rs7860510
rs4431175
rs7502034
rs7575297
rs1371409
rs261218
rs8088179
rs147140525
rs911666
rs62495670
rs34962571
rs11722477
rs10782332
rs2504698
rs61415440
rs12659261
rs7314815
rs10179586
rs6788157
rs67832247
All - MR Egger
All - Inverse variance weighted
-1.0
-0.5
0.0
0.5
1.0
1.5
MR effect size for
' || id:ebi-a-GCST90016958' on 'Chronic pancreatitis || id:finn-b-K11_CHRONPANC'

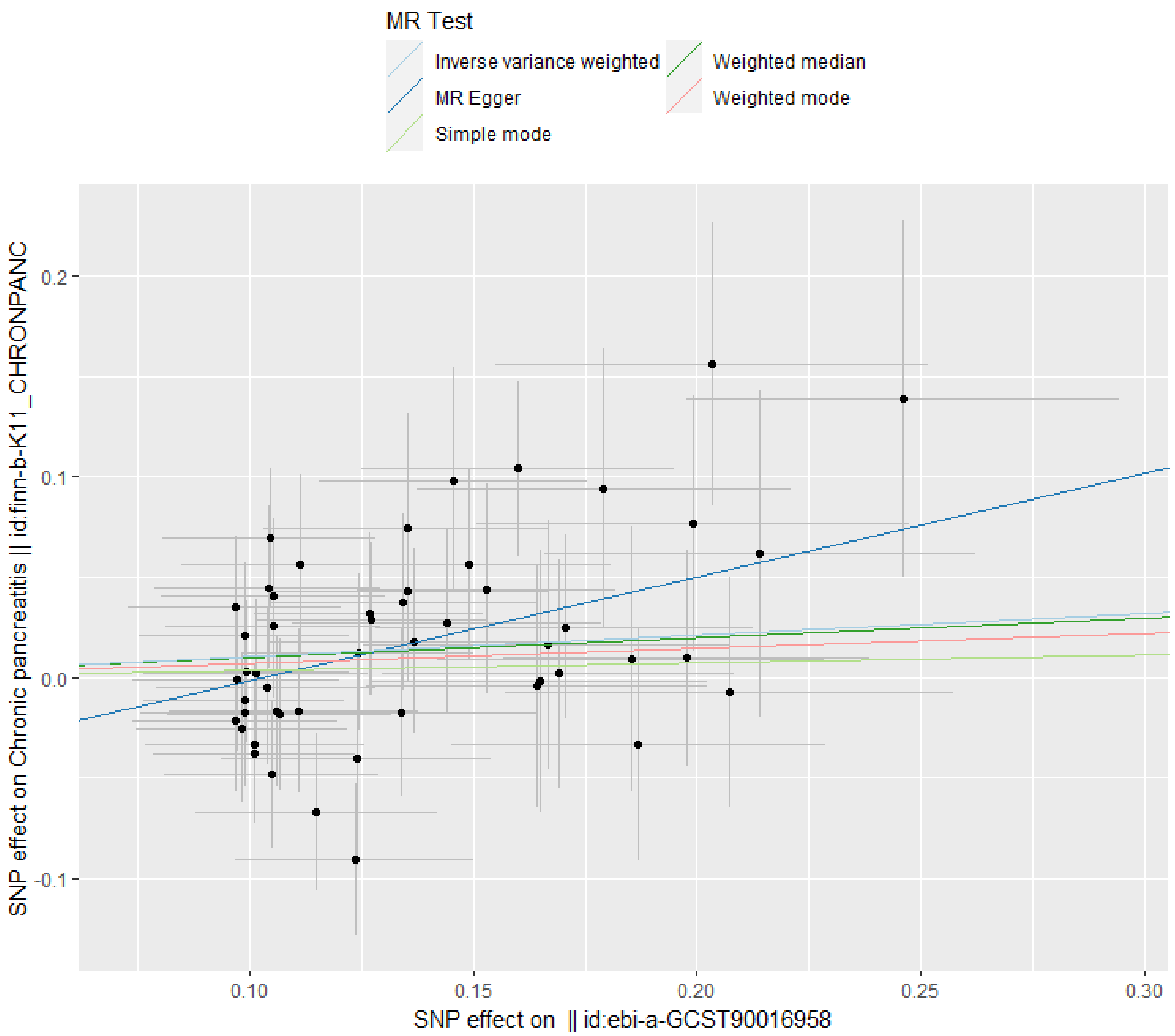

MR Test
Inverse variance weighted
MR Egger
Simple mode
Weighted median
Weighted mode
SNP effect on Chronic pancreatitis || id:finn-b-K11_CHRONPANC
SNP effect on || id:ebi-a-GCST90016958
0.2
0.1
0.0
-0.1
0.10
0.15
0.20
0.25
0.30

Figure 208 Leave-one-out analysis, funnel plot, MR effect size and scatter plot for gut microbiota abundance (genus Marvinbryantia id.2005) on chronic pancreatitis

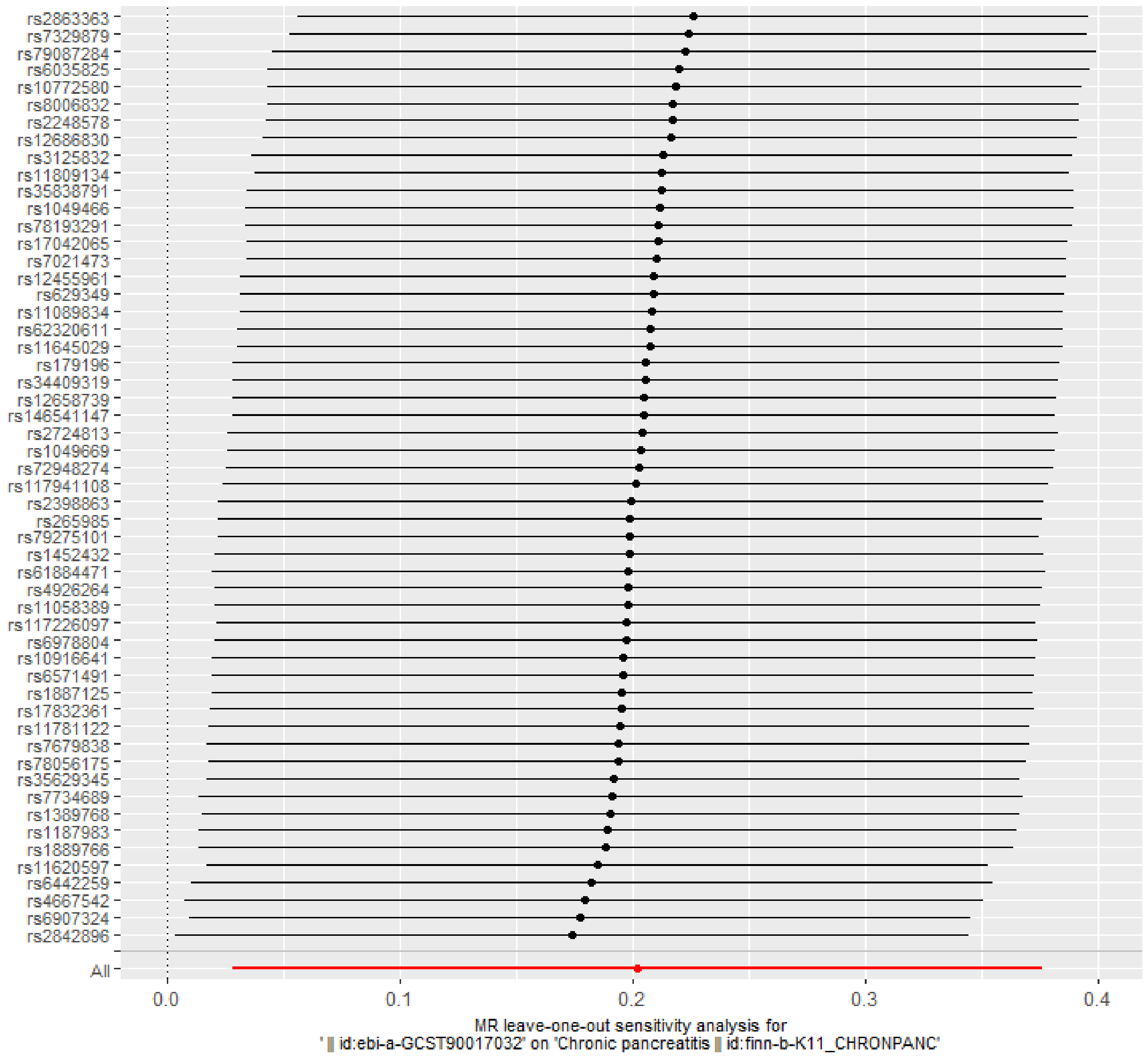

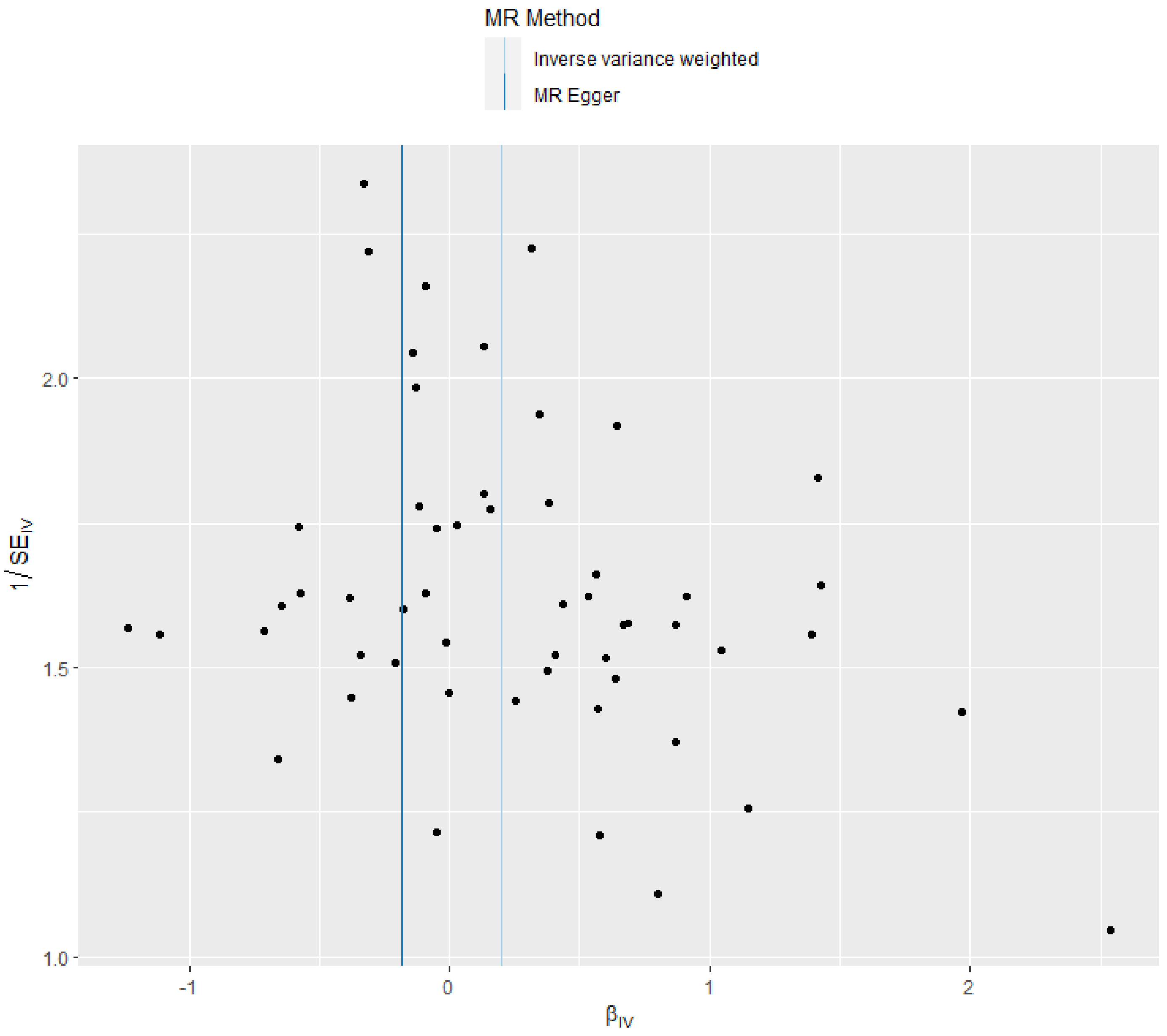

MR Method
Inverse variance weighted
MR Egger
2.0
1.5
1.0
-1
0
1
2
1/SE_IV
β_IV

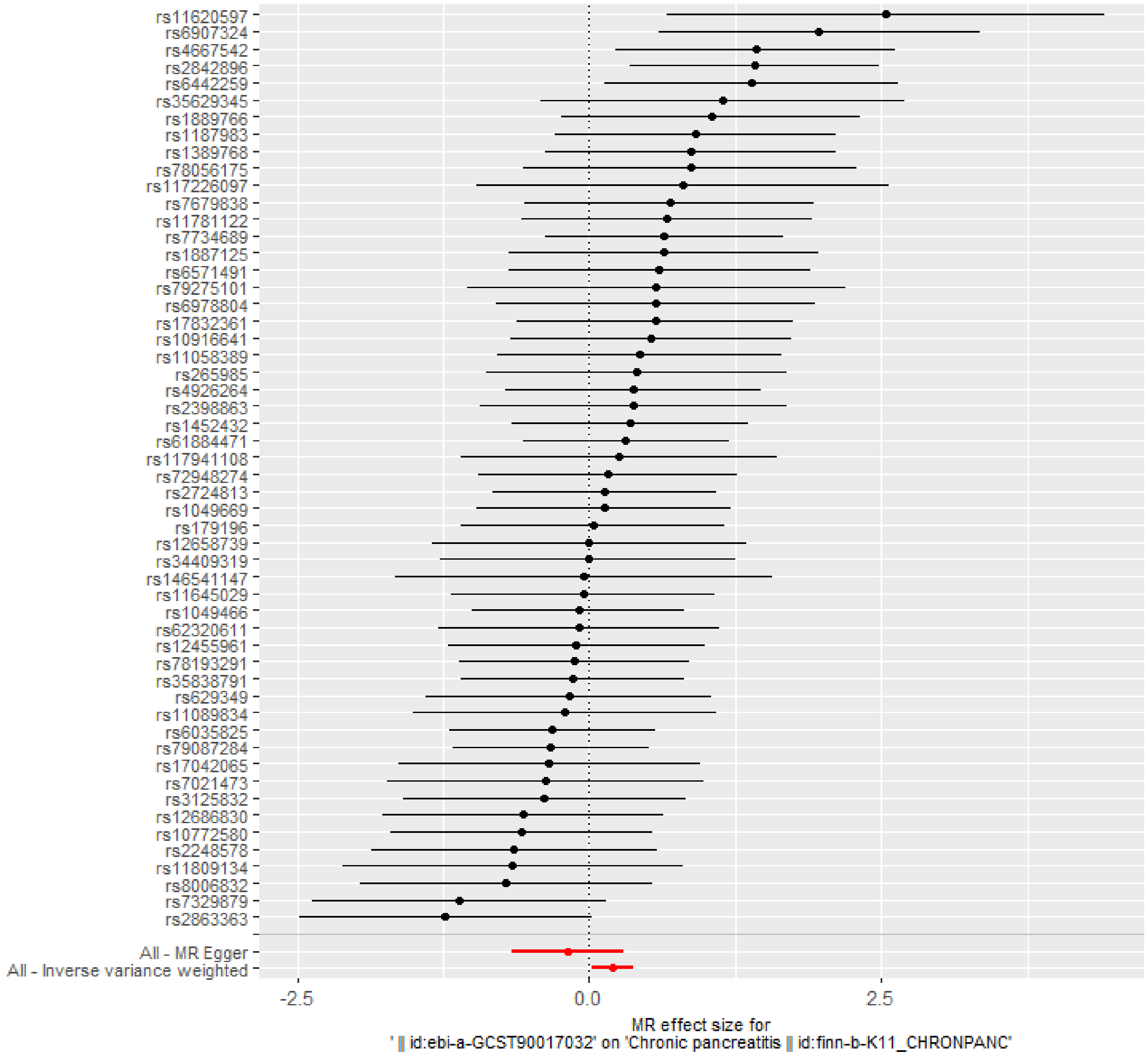
rs11620597
rs6907324
rs4667542
rs2842896
rs6442259
rs35629345
rs1889766
rs1187983
rs1389768
rs78056175
rs117226097
rs7679838
rs11781122
rs7734689
rs1887125
rs6571491
rs79275101
rs6978804
rs17832361
rs10916641
rs11058389
rs265985
rs4926264
rs2398863
rs1452432
rs61884471
rs117941108
rs72948274
rs2724813
rs1049669
rs179196
rs12658739
rs34409319
rs146541147
rs11645029
rs1049466
rs62320611
rs12455961
rs78193291
rs35838791
rs629349
rs11089834
rs6035825
rs79087284
rs17042065
rs7021473
rs3125832
rs12686830
rs10772580
rs2248578
rs11809134
rs8006832
rs7329879
rs2863363
All - MR Egger
All - Inverse variance weighted
-2.5
0.0
2.5
MR effect size for
' || id:ebi-a-GCST90017032' on 'Chronic pancreatitis || id:finn-b-K11_CHRONPANC'

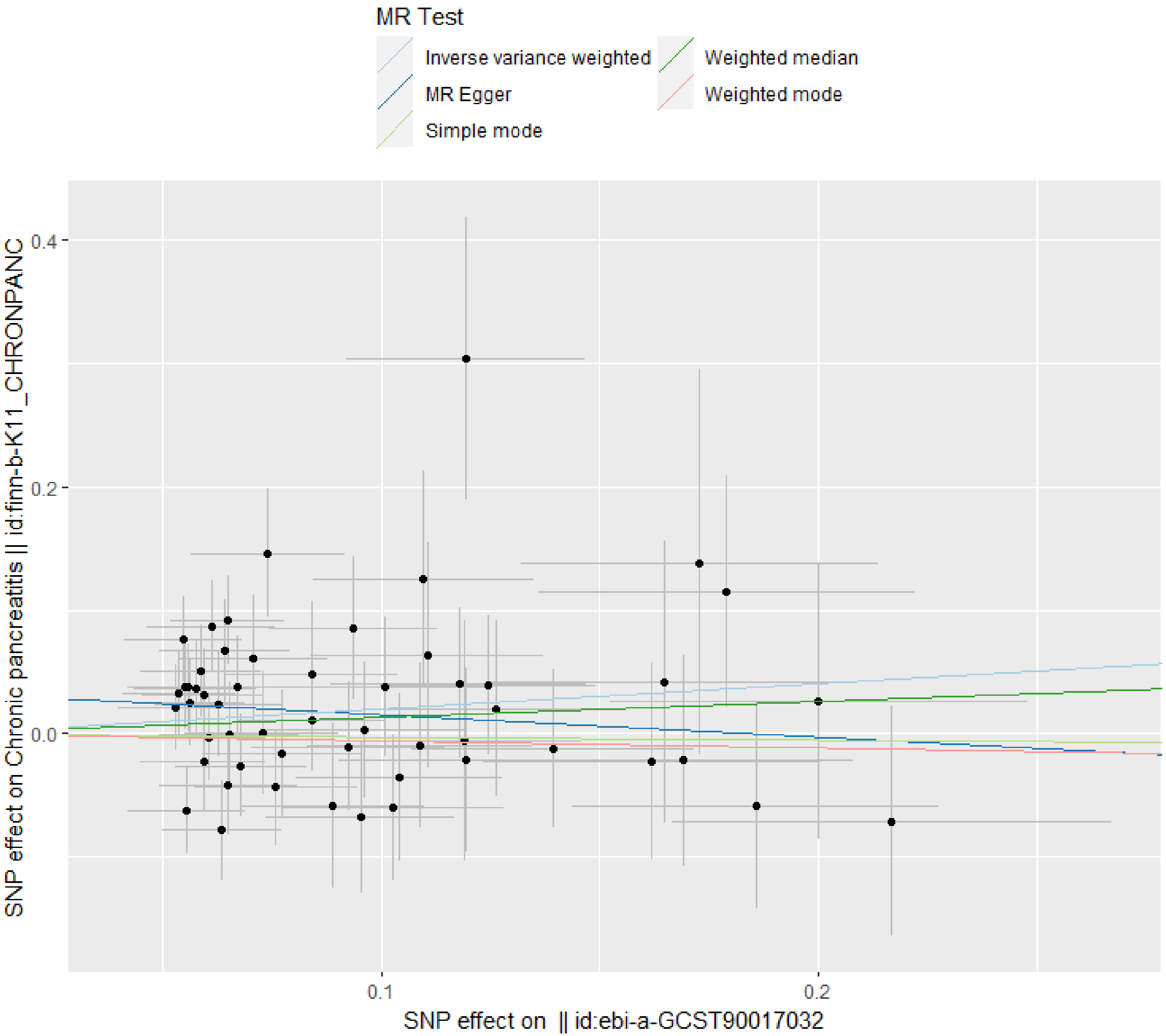

MR Test
Inverse variance weighted
MR Egger
Simple mode
Weighted median
Weighted mode
0.4
0.2
0.0
0.1
0.2
SNP effect on Chronic pancreatitis || id:finn-b-K11_CHRONPANC
SNP effect on || id:ebi-a-GCST90017032

Figure 209 Leave-one-out analysis, funnel plot, MR effect size and scatter plot for gut microbiota abundance (genus Romboutsia id.11347) on chronic pancreatitis

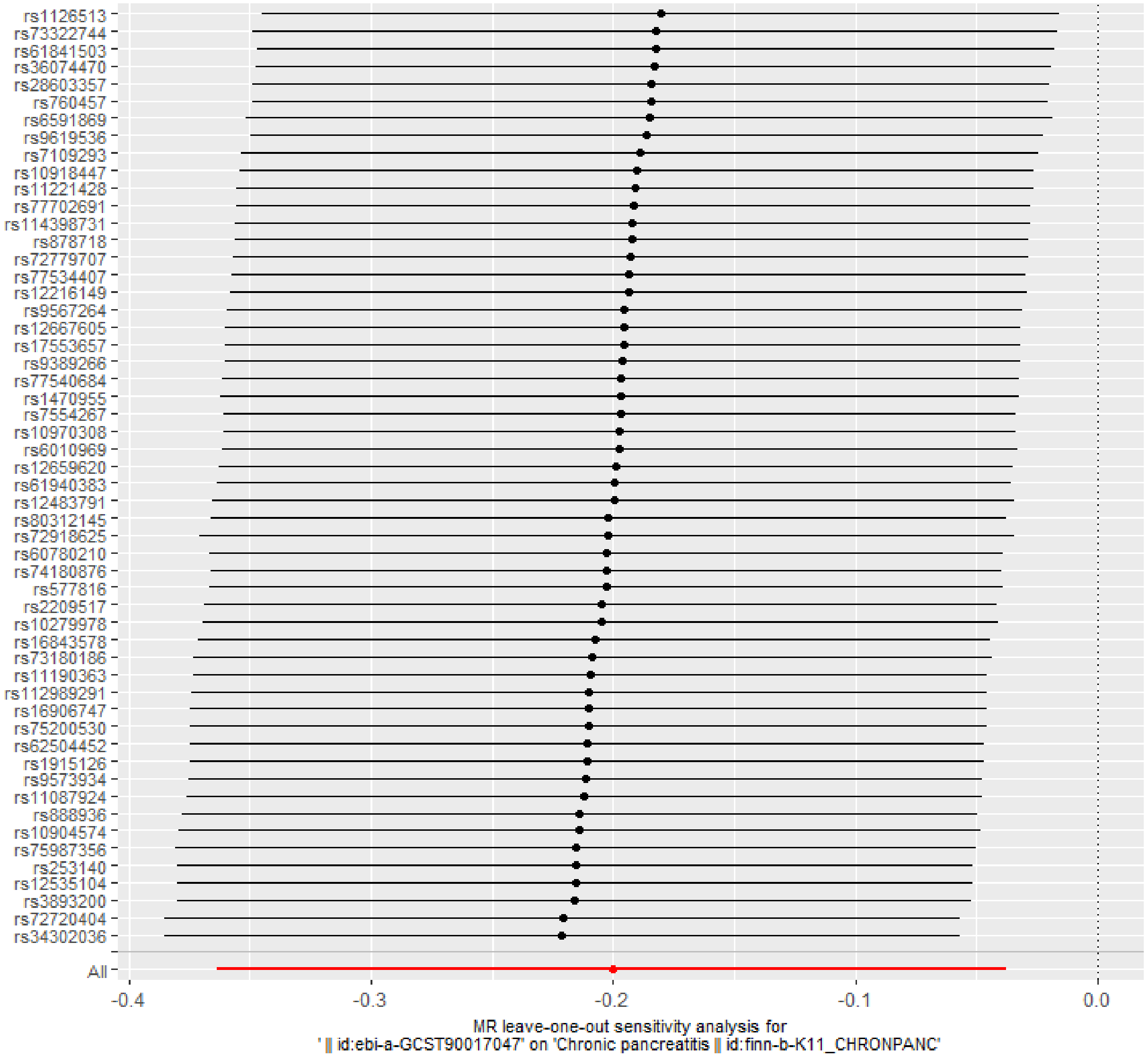

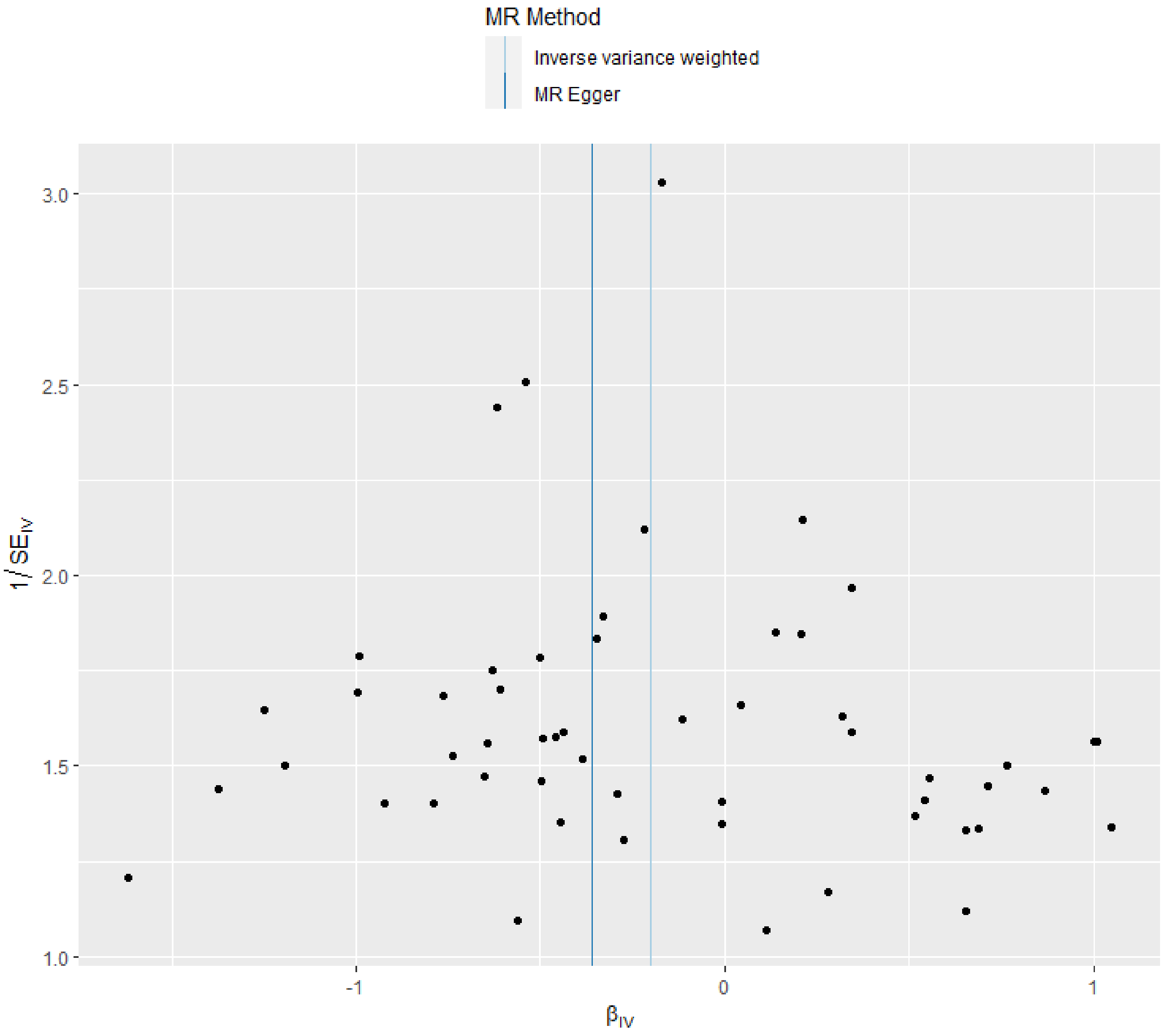

MR Method
Inverse variance weighted
MR Egger
3.0
2.5
2.0
1.5
1.0
-1
0
1
$1/SE_{IV}$
β_{IV}

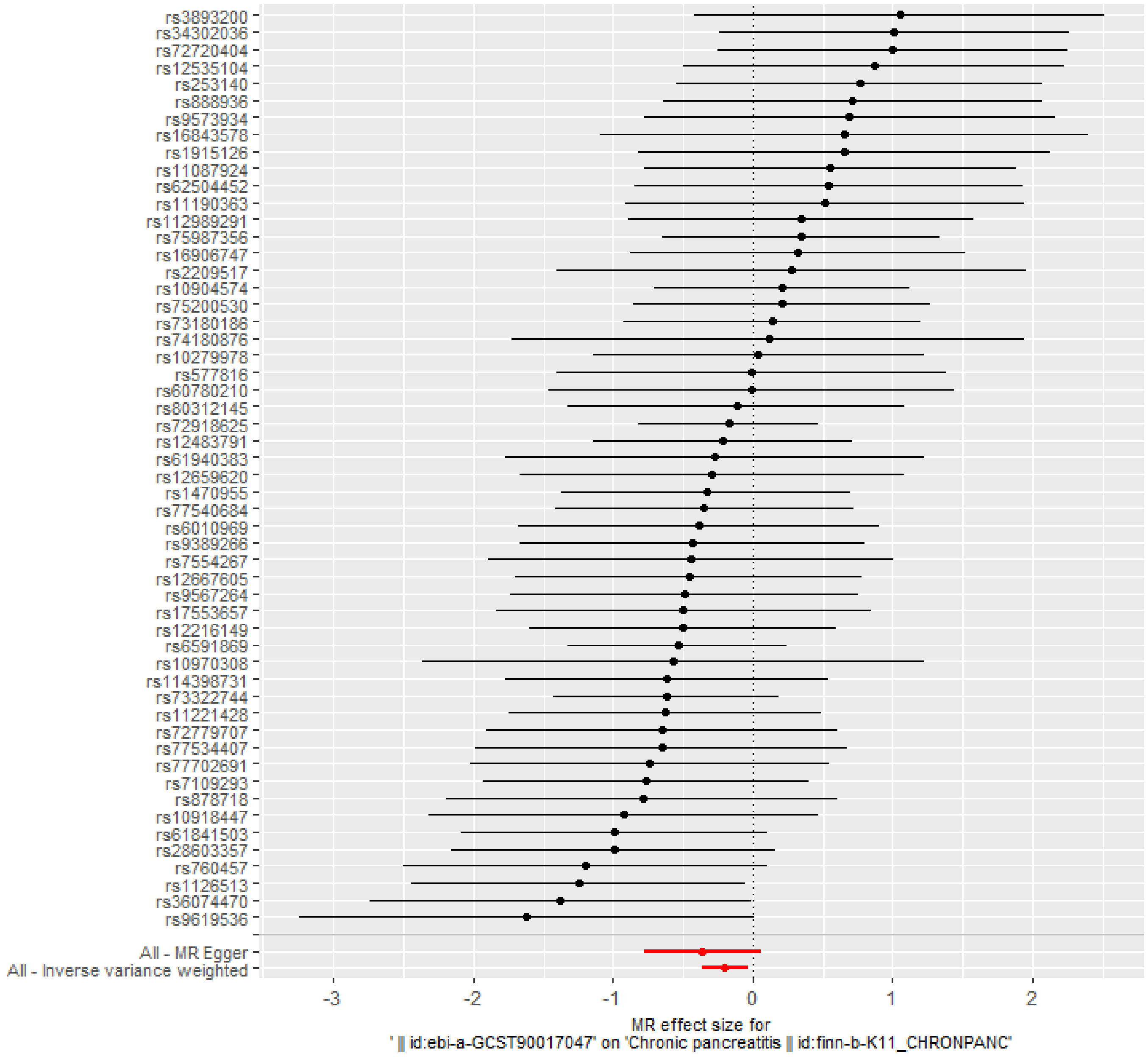
rs3893200
rs34302036
rs72720404
rs12535104
rs253140
rs888936
rs9573934
rs16843578
rs1915126
rs11087924
rs62504452
rs11190363
rs112989291
rs75987356
rs16906747
rs2209517
rs10904574
rs75200530
rs73180186
rs74180876
rs10279978
rs577816
rs60780210
rs80312145
rs72918625
rs12483791
rs61940383
rs12659620
rs1470955
rs77540684
rs6010969
rs9389266
rs7554267
rs12667605
rs9567264
rs17553657
rs12216149
rs6591869
rs10970308
rs114398731
rs73322744
rs11221428
rs72779707
rs77534407
rs77702691
rs7109293
rs878718
rs10918447
rs61841503
rs28603357
rs760457
rs1126513
rs36074470
rs9619536
All - MR Egger
All - Inverse variance weighted
-3
-2
-1
0
1
2
MR effect size for
' || id:ebi-a-GCST90017047' on 'Chronic pancreatitis || id:finn-b-K11_CHRONPANC'

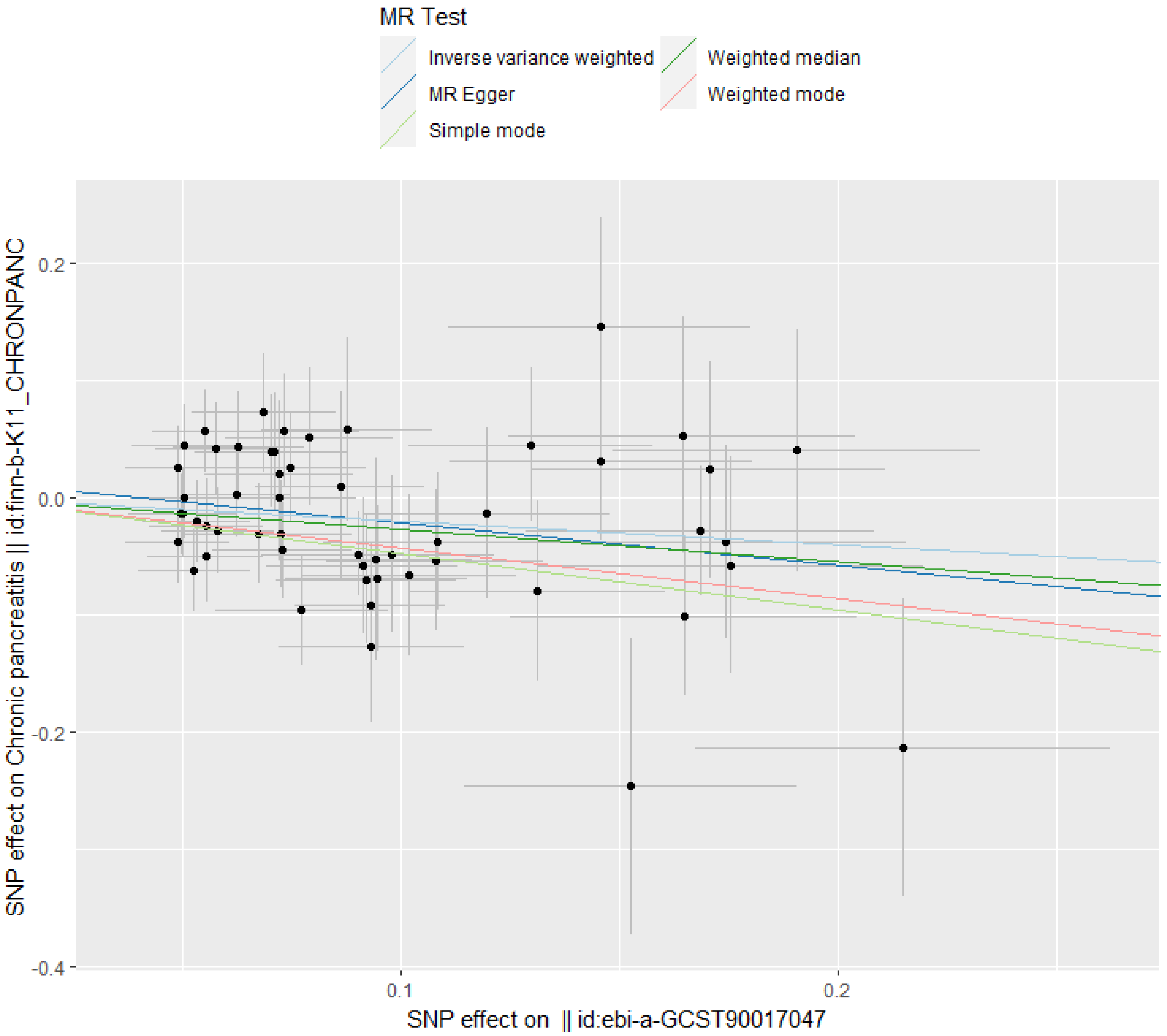

MR Test
Inverse variance weighted
MR Egger
Simple mode
Weighted median
Weighted mode
SNP effect on Chronic pancreatitis || id:finn-b-K11_CHRONPANC
0.2
0.0
-0.2
-0.4
0.1
0.2
SNP effect on || id:ebi-a-GCST90017047

Figure 210 Leave-one-out analysis, funnel plot, MR effect size and scatter plot for gut microbiota abundance (genus Sellimonas id.14369) on chronic pancreatitis

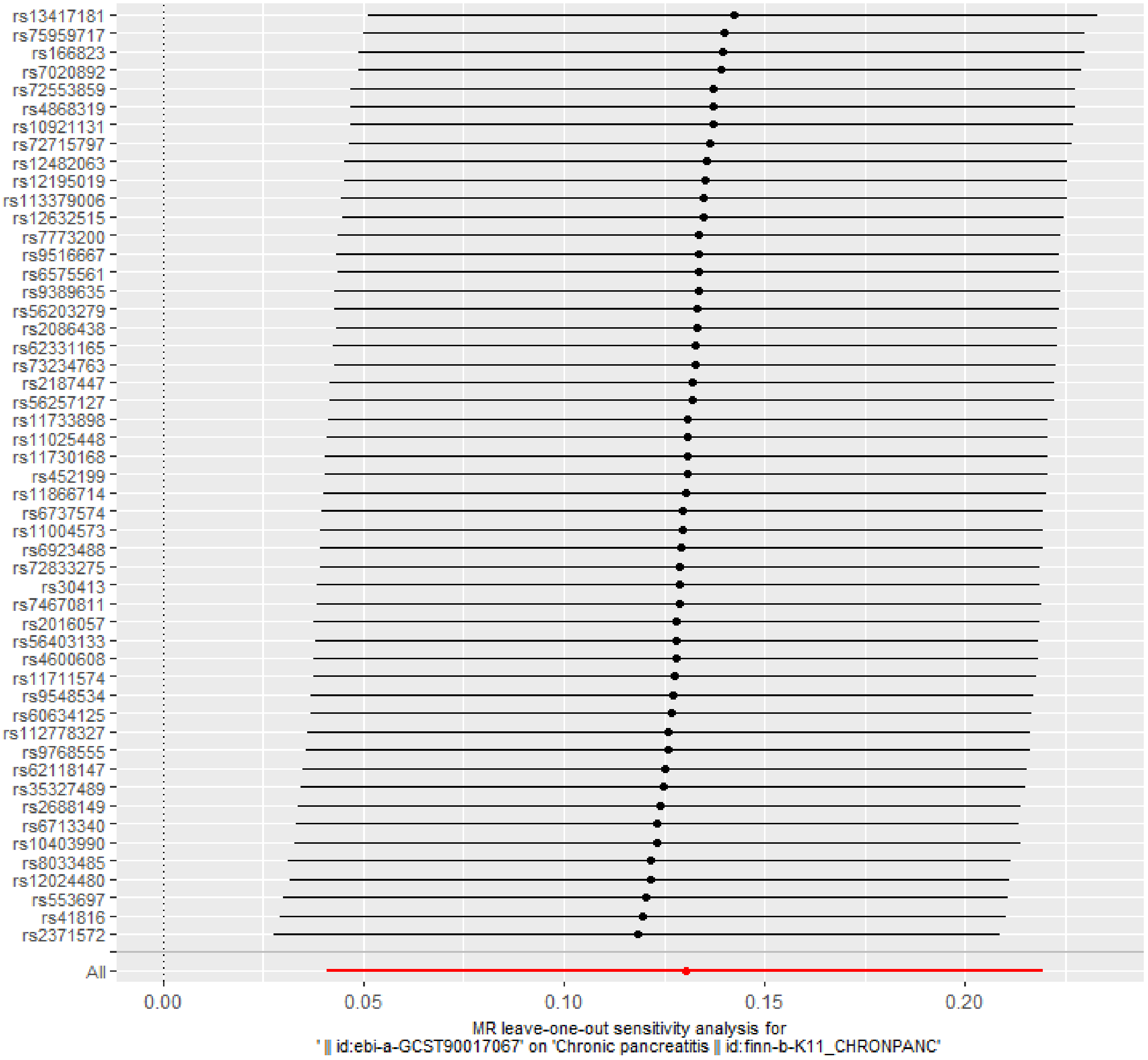

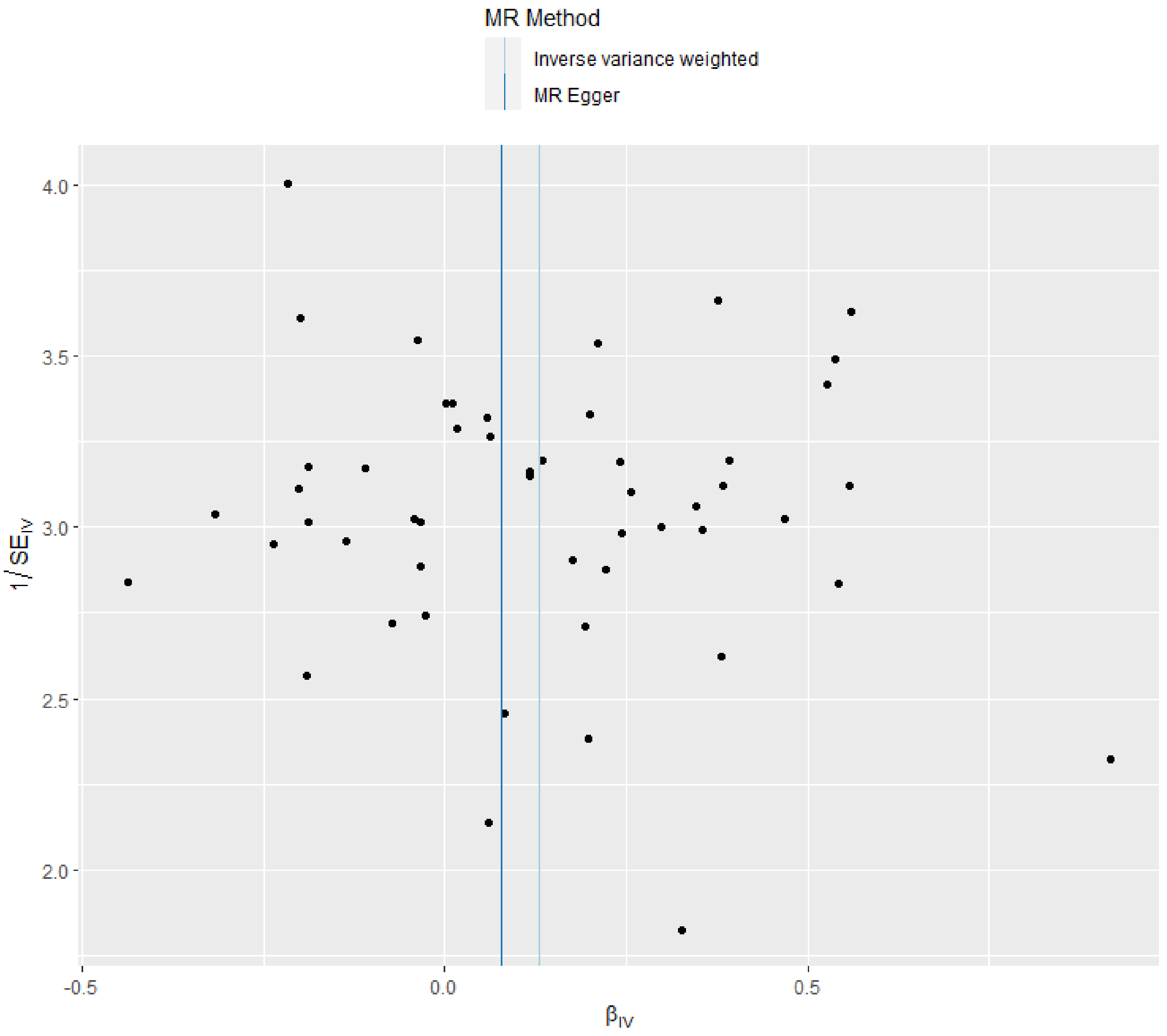
MR Method
Inverse variance weighted
MR Egger
4.0
3.5
3.0
2.5
2.0
1/SE_IV
-0.5
0.0
0.5
β_IV

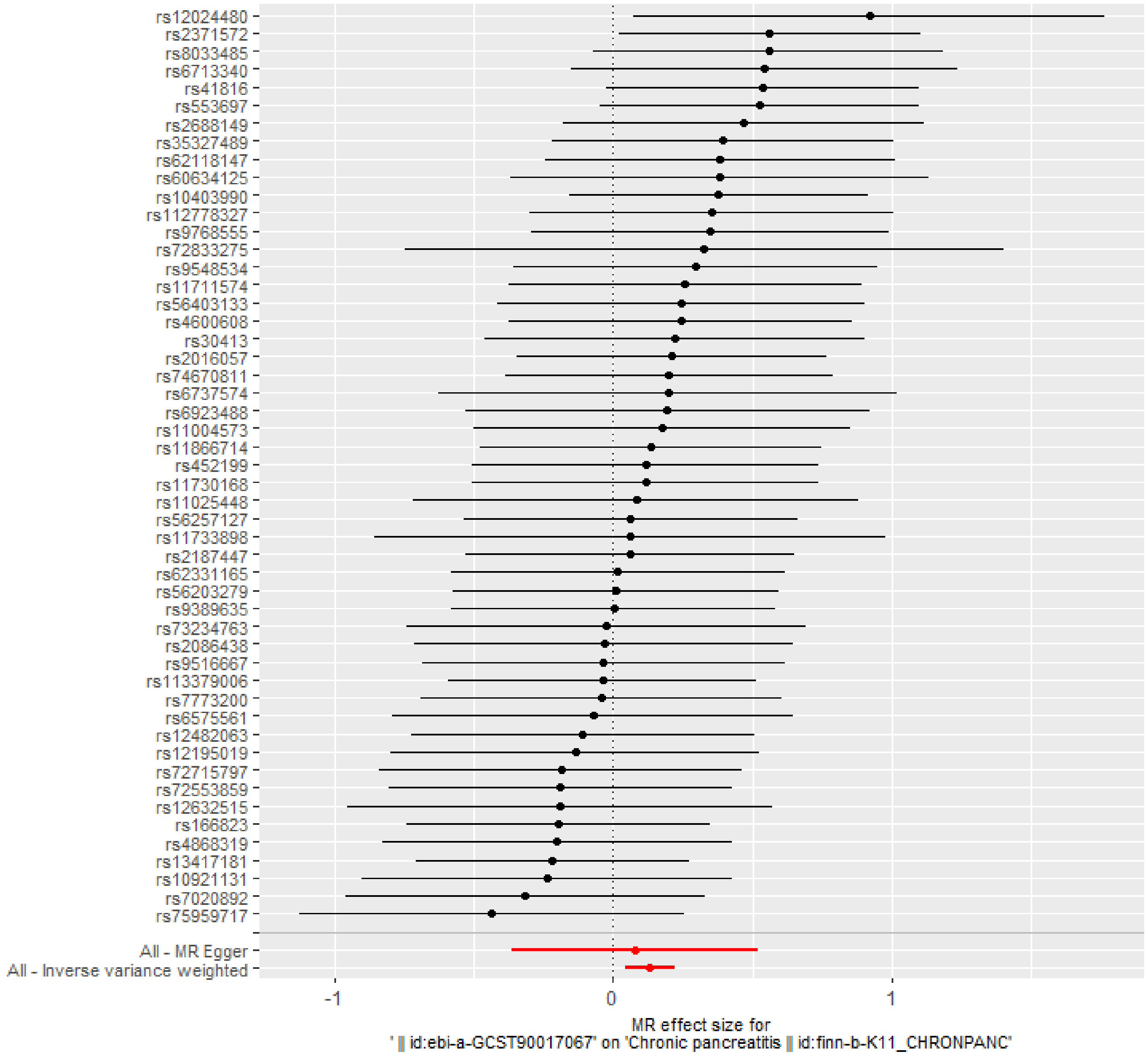

rs12024480
rs2371572
rs8033485
rs6713340
rs41816
rs553697
rs2688149
rs35327489
rs62118147
rs60634125
rs10403990
rs112778327
rs9768555
rs72833275
rs9548534
rs11711574
rs56403133
rs4600608
rs30413
rs2016057
rs74670811
rs6737574
rs6923488
rs11004573
rs11866714
rs452199
rs11730168
rs11025448
rs56257127
rs11733898
rs2187447
rs62331165
rs56203279
rs9389635
rs73234763
rs2086438
rs9516667
rs113379006
rs7773200
rs6575561
rs12482063
rs12195019
rs72715797
rs72553859
rs12632515
rs166823
rs4868319
rs13417181
rs10921131
rs7020892
rs75959717
All - MR Egger
All - Inverse variance weighted
-1
0
1
MR effect size for
' || id:ebi-a-GCST90017067' on 'Chronic pancreatitis || id:finn-b-K11_CHRONPANC'

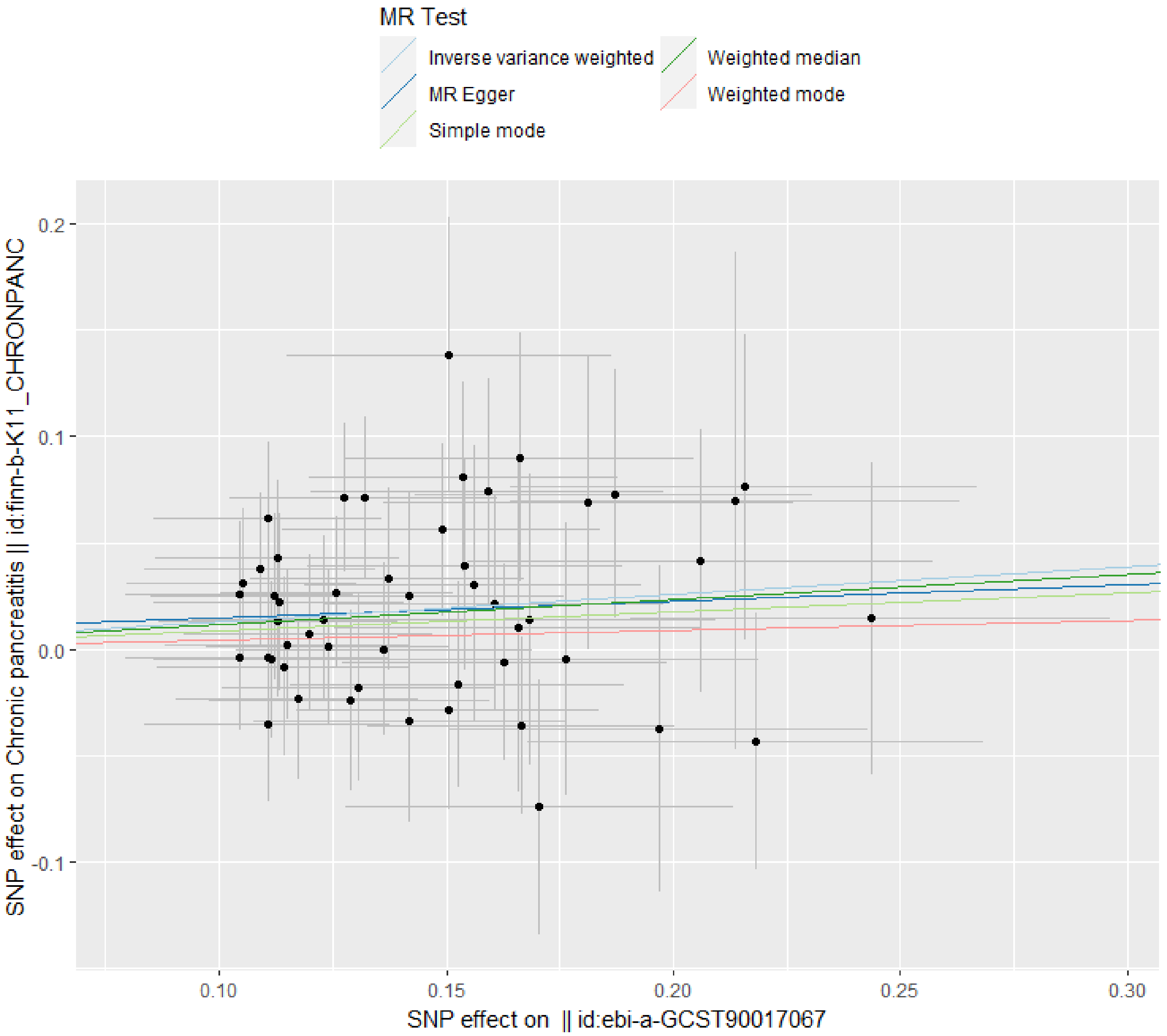

MR Test
Inverse variance weighted
MR Egger
Simple mode
Weighted median
Weighted mode
SNP effect on Chronic pancreatitis || id:finn-b-K11_CHRONPANC
0.2
0.1
0.0
-0.1
0.10
0.15
0.20
0.25
0.30
SNP effect on || id:ebi-a-GCST90017067

Figure 211 Leave-one-out analysis, funnel plot, MR effect size and scatter plot for gut microbiota abundance (genus Slackia id.825) on chronic pancreatitis

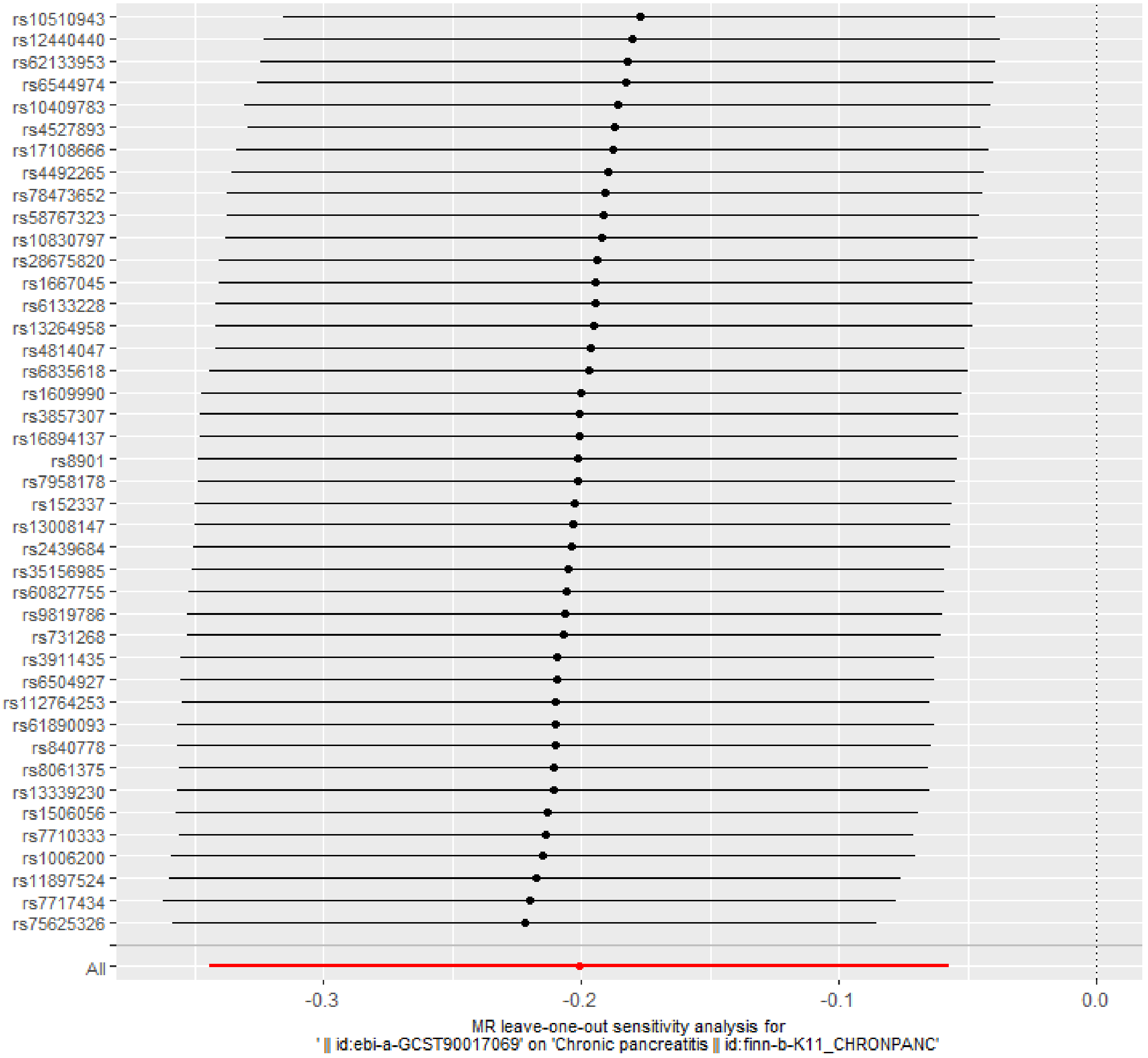

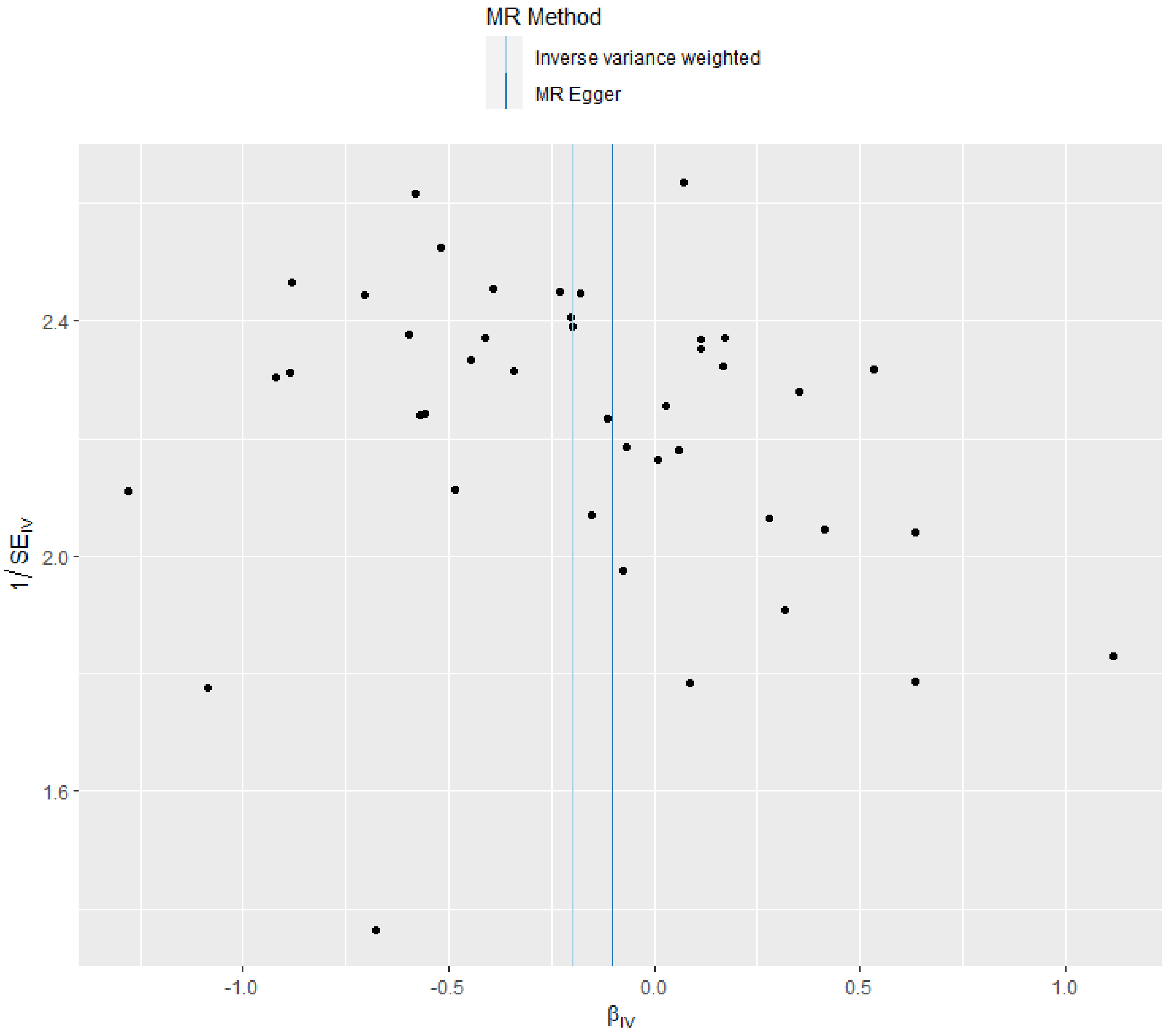
MR Method
Inverse variance weighted
MR Egger
2.4
2.0
1.6
-1.0
-0.5
0.0
0.5
1.0
$1/SE_{IV}$
β_{IV}

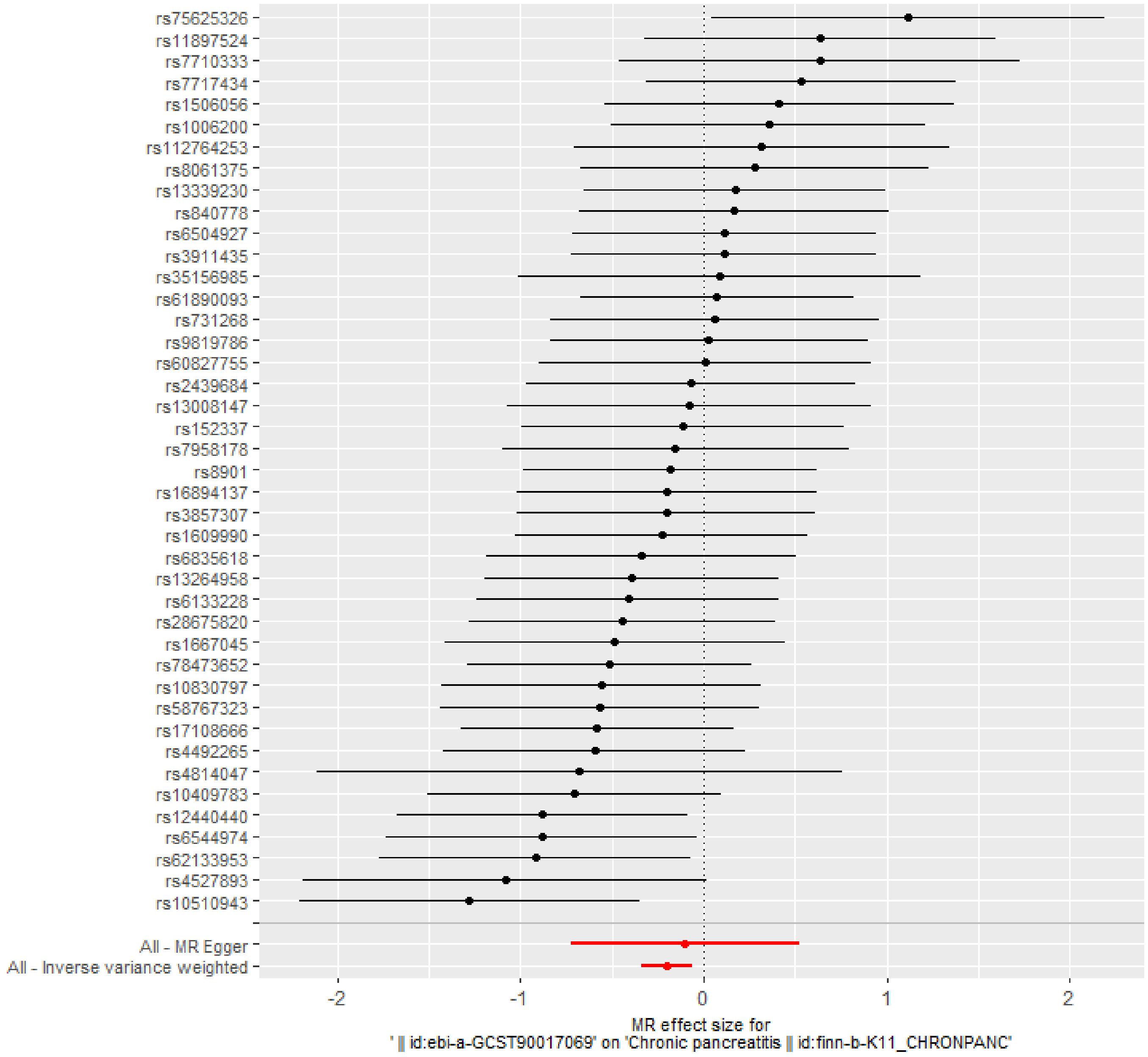
rs75625326
rs11897524
rs7710333
rs7717434
rs1506056
rs1006200
rs112764253
rs8061375
rs13339230
rs840778
rs6504927
rs3911435
rs35156985
rs61890093
rs731268
rs9819786
rs60827755
rs2439684
rs13008147
rs152337
rs7958178
rs8901
rs16894137
rs3857307
rs1609990
rs6835618
rs13264958
rs6133228
rs28675820
rs1667045
rs78473652
rs10830797
rs58767323
rs17108666
rs4492265
rs4814047
rs10409783
rs12440440
rs6544974
rs62133953
rs4527893
rs10510943
All - MR Egger
All - Inverse variance weighted
-2
-1
0
1
2
MR effect size for
' || id:ebi-a-GCST90017069' on 'Chronic pancreatitis || id:finn-b-K11_CHRONPANC'

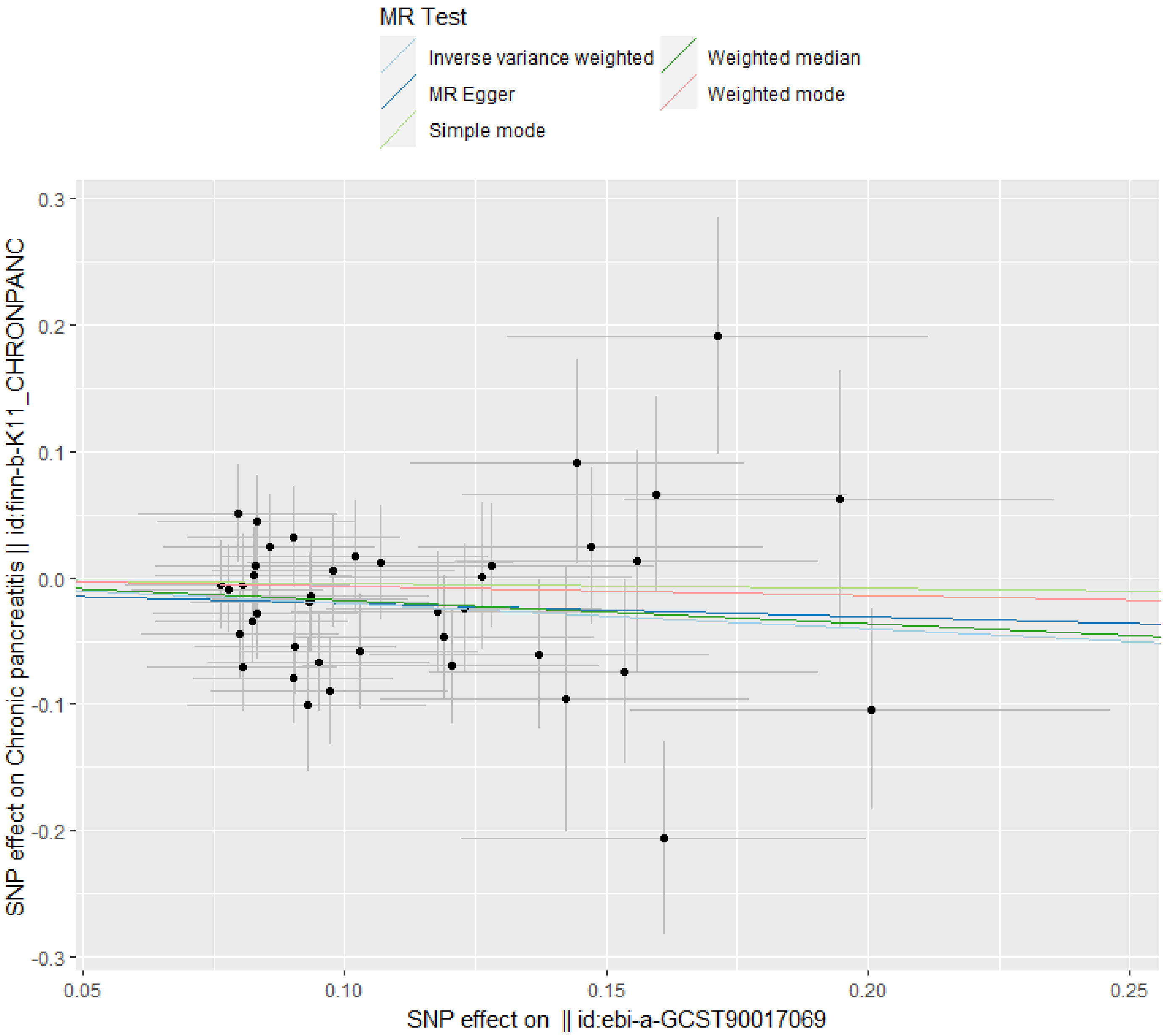
MR Test
Inverse variance weighted
MR Egger
Simple mode
Weighted median
Weighted mode
SNP effect on Chronic pancreatitis || id:finn-b-K11_CHRONPANC
SNP effect on || id:ebi-a-GCST90017069
0.3
0.2
0.1
0.0
-0.1
-0.2
-0.3
0.05
0.10
0.15
0.20
0.25

Figure 212 Leave-one-out analysis, funnel plot, MR effect size and scatter plot for gut microbiota abundance (genus Prevotella9 id.11183) on pancreatic cancer

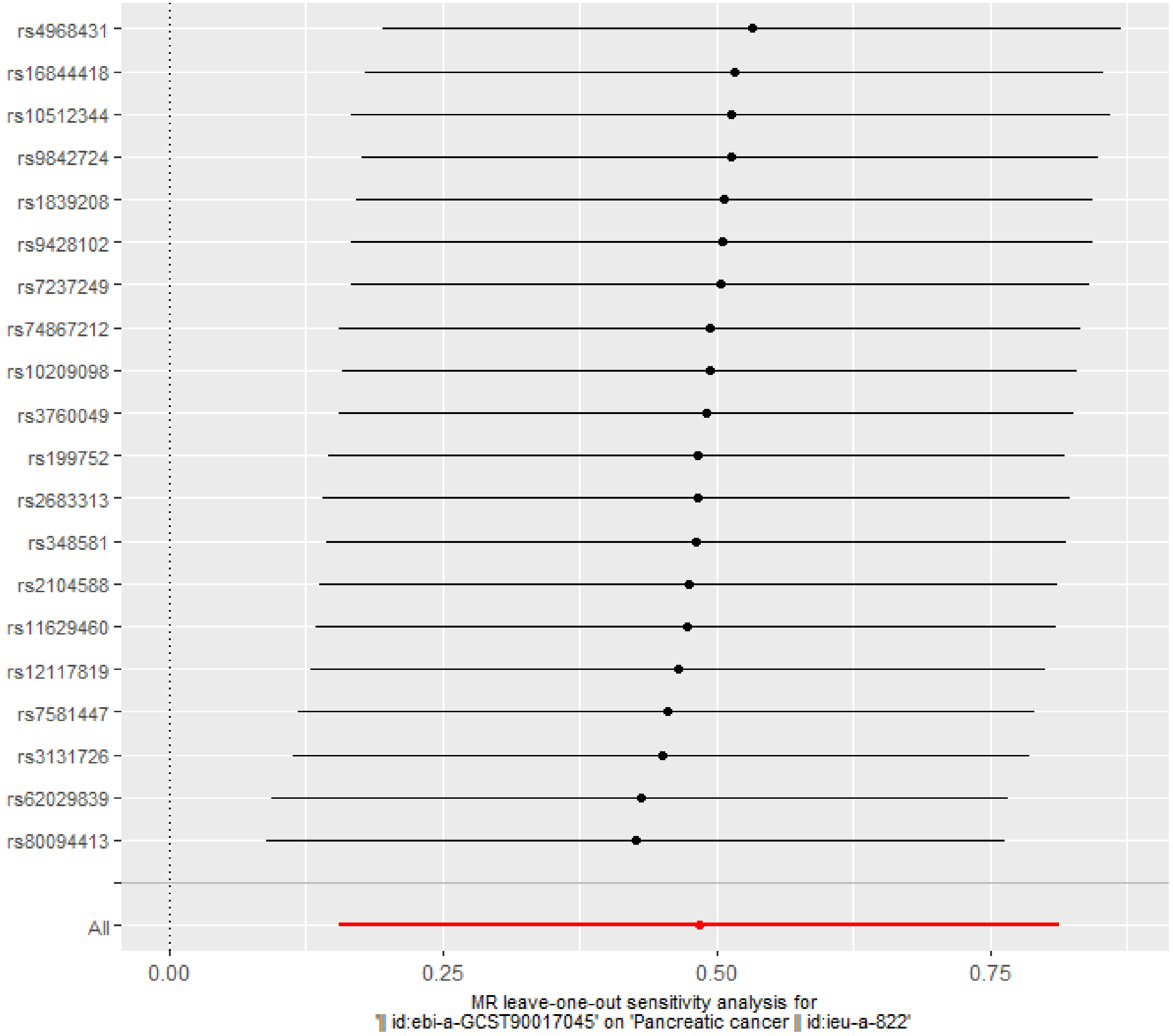

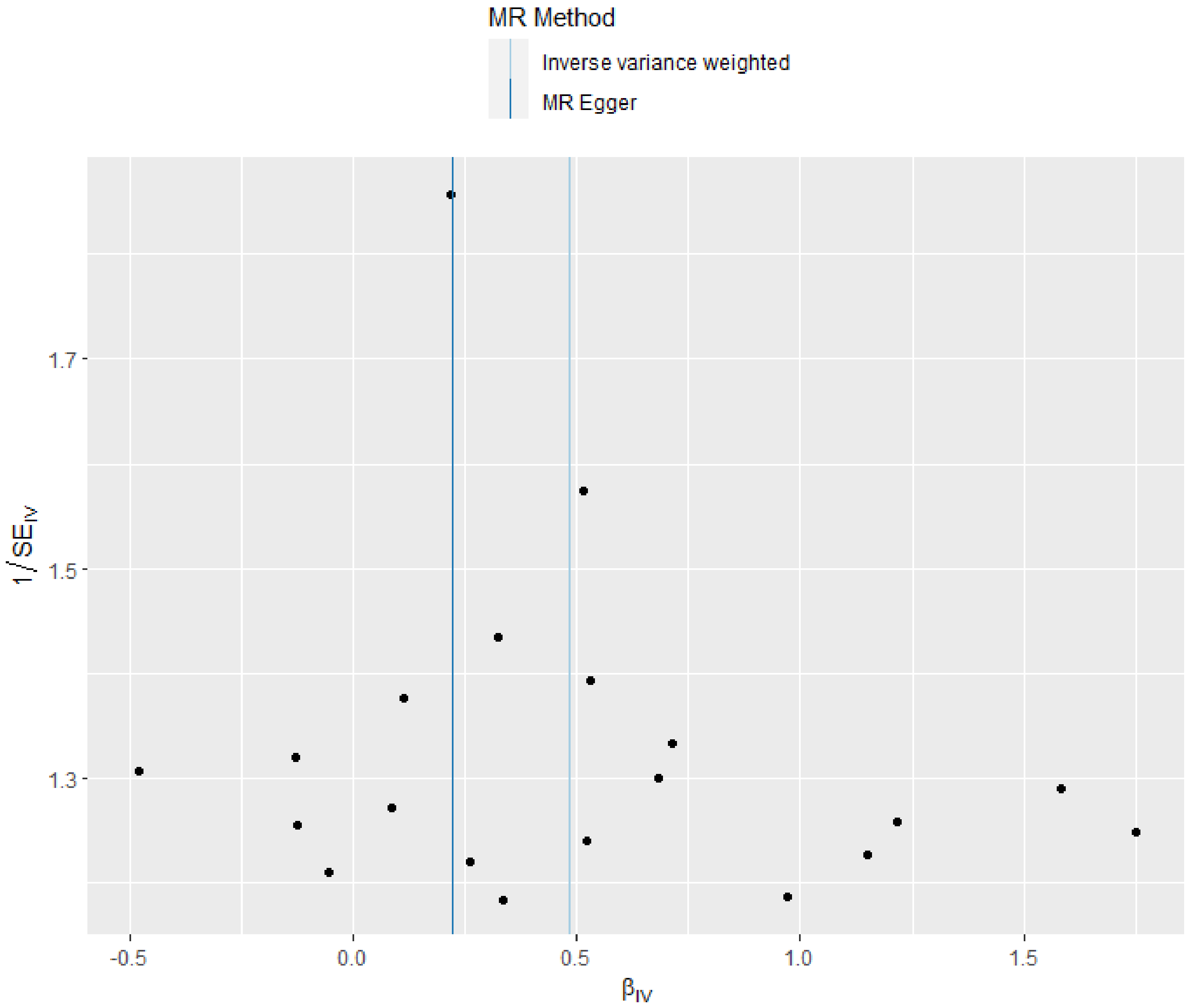

MR Method
Inverse variance weighted
MR Egger
1.7
1.5
1.3
-0.5
0.0
0.5
1.0
1.5
β_{IV}
$1/SE_{IV}$

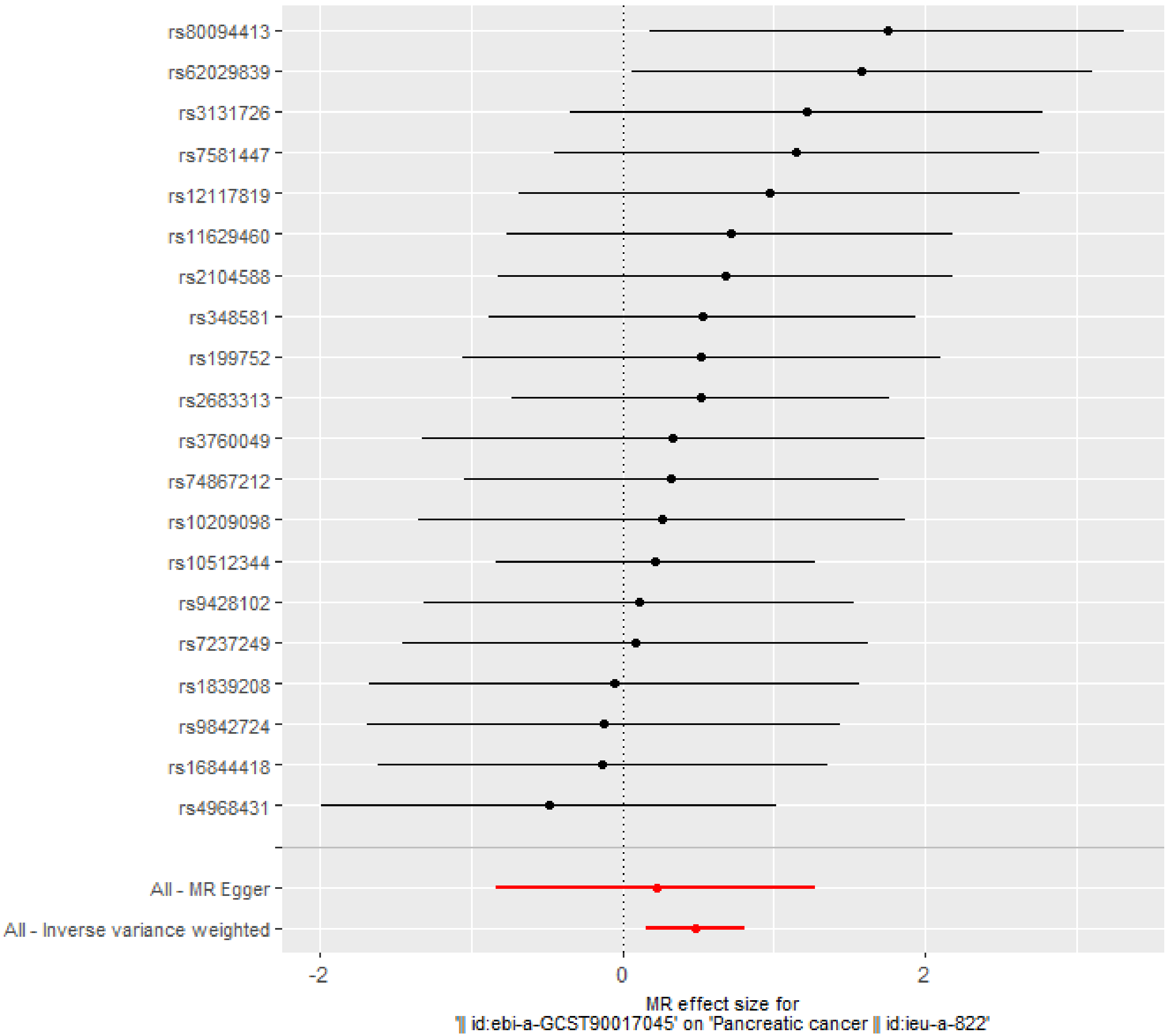
rs80094413
rs62029839
rs3131726
rs7581447
rs12117819
rs11629460
rs2104588
rs348581
rs199752
rs2683313
rs3760049
rs74867212
rs10209098
rs10512344
rs9428102
rs7237249
rs1839208
rs9842724
rs16844418
rs4968431
All - MR Egger
All - Inverse variance weighted
-2
0
2
MR effect size for
'|| id:ebi-a-GCST90017045' on 'Pancreatic cancer || id:ieu-a-822'

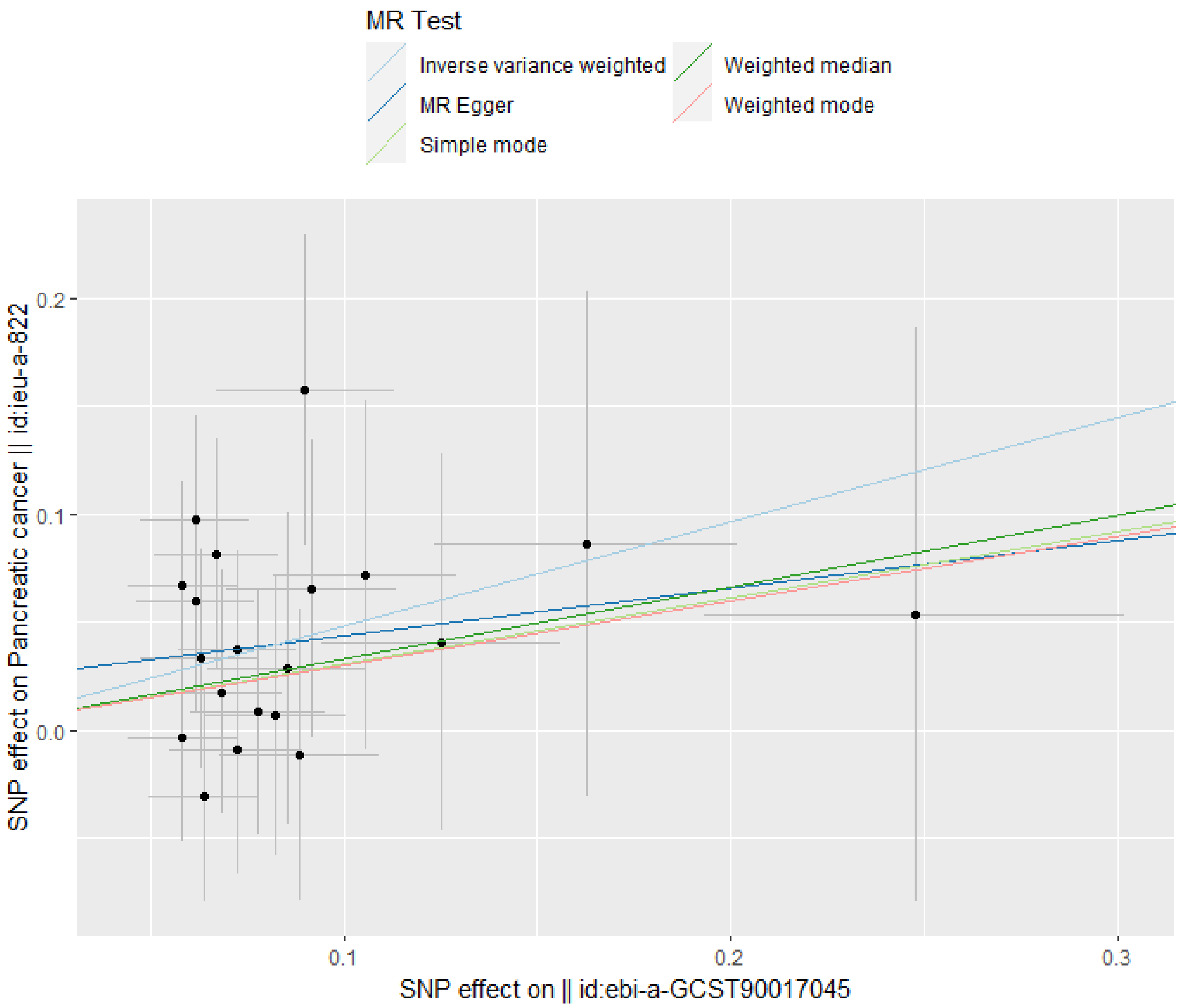
MR Test
Inverse variance weighted
MR Egger
Simple mode
Weighted median
Weighted mode
0.2
0.1
0.0
SNP effect on Pancreatic cancer || id:ieu-a-822
0.1
0.2
0.3
SNP effect on || id:ebi-a-GCST90017045
