# Supplementary material for: Causal relationship between gut microbiota and gastrointestinal diseases: a mendelian randomization study
Source: J Transl Med. 2024 Jan 23;22:92. doi: 10.1186/s12967-024-04894-5 (PMC10804519; doi:10.1186/s12967-024-04894-5)

A. Gastroesophageal reflux disease

1. Gut microbiota abundance (genus Alistipes id.968)

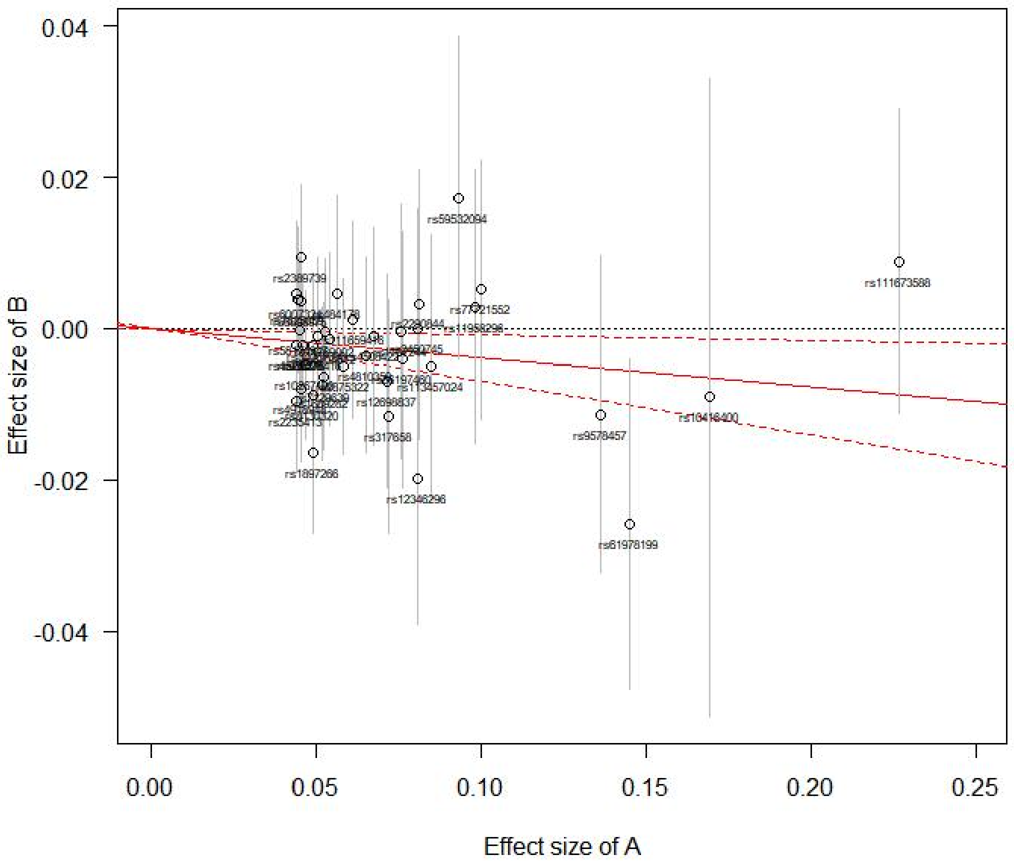

2. Gut microbiota abundance (genus Christensenellaceae R 7group id.11283)

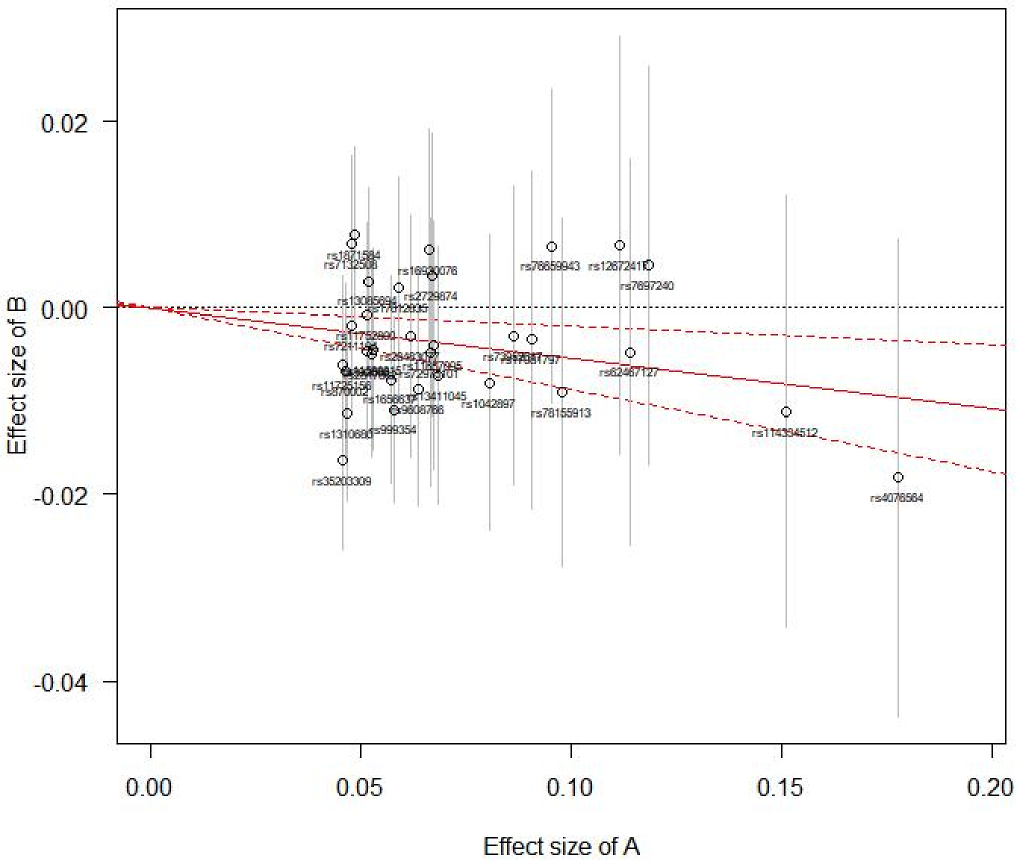

3. Gut microbiota abundance (genus Coprococcus2 id.11302)

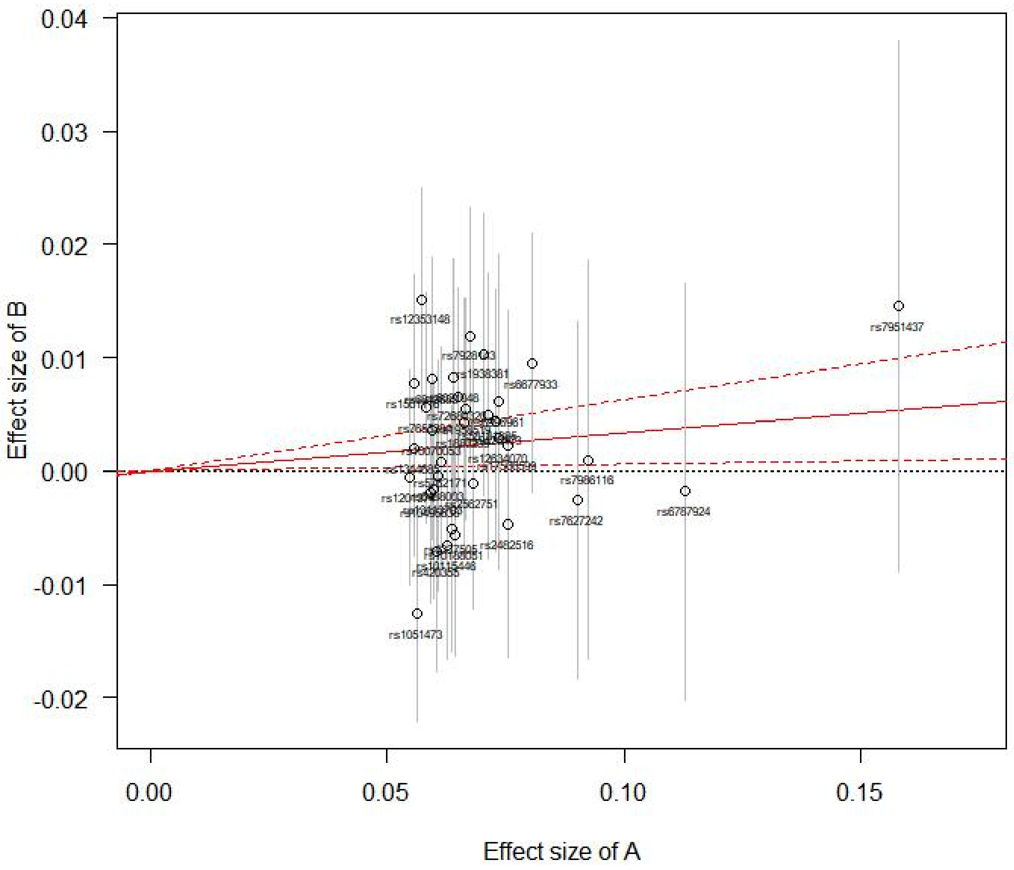

4. Gut microbiota abundance (genus Faecalibacterium id.2057)

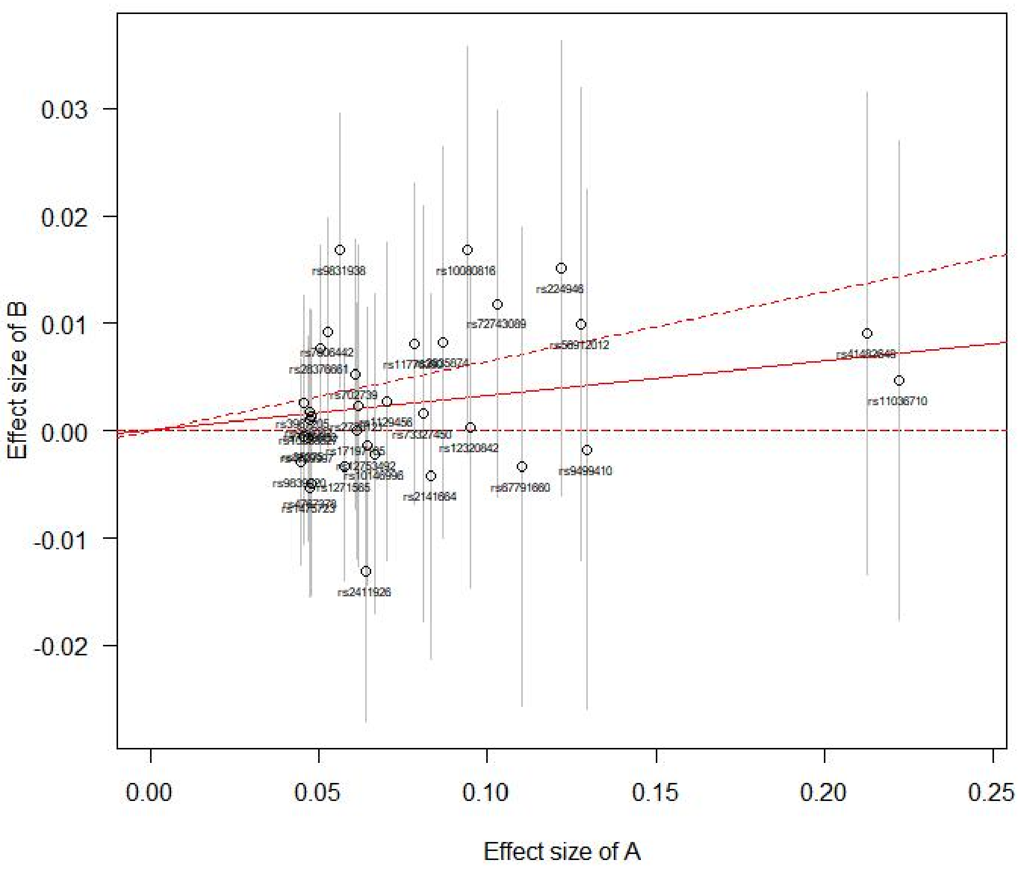

5. Gut microbiota abundance (genus Oscillospira id.2064)

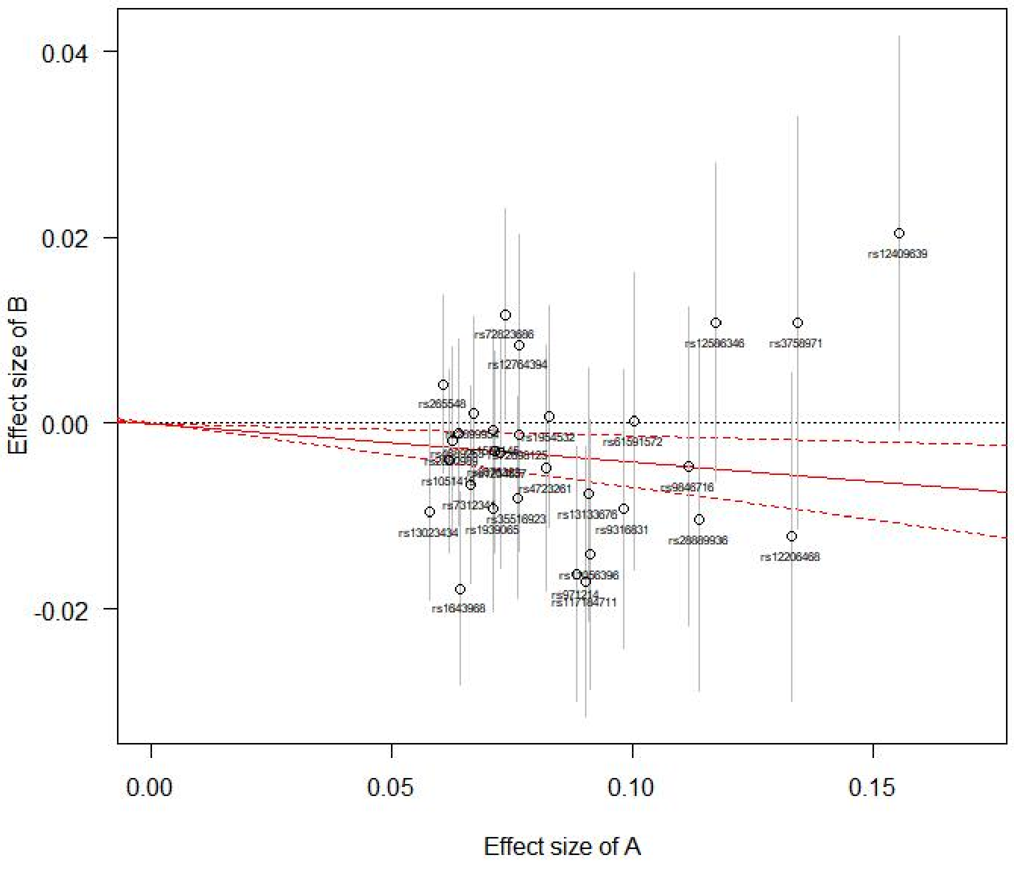

6. Gut microbiota abundance (genus Rikenellaceae RC9 gut group id.11191)

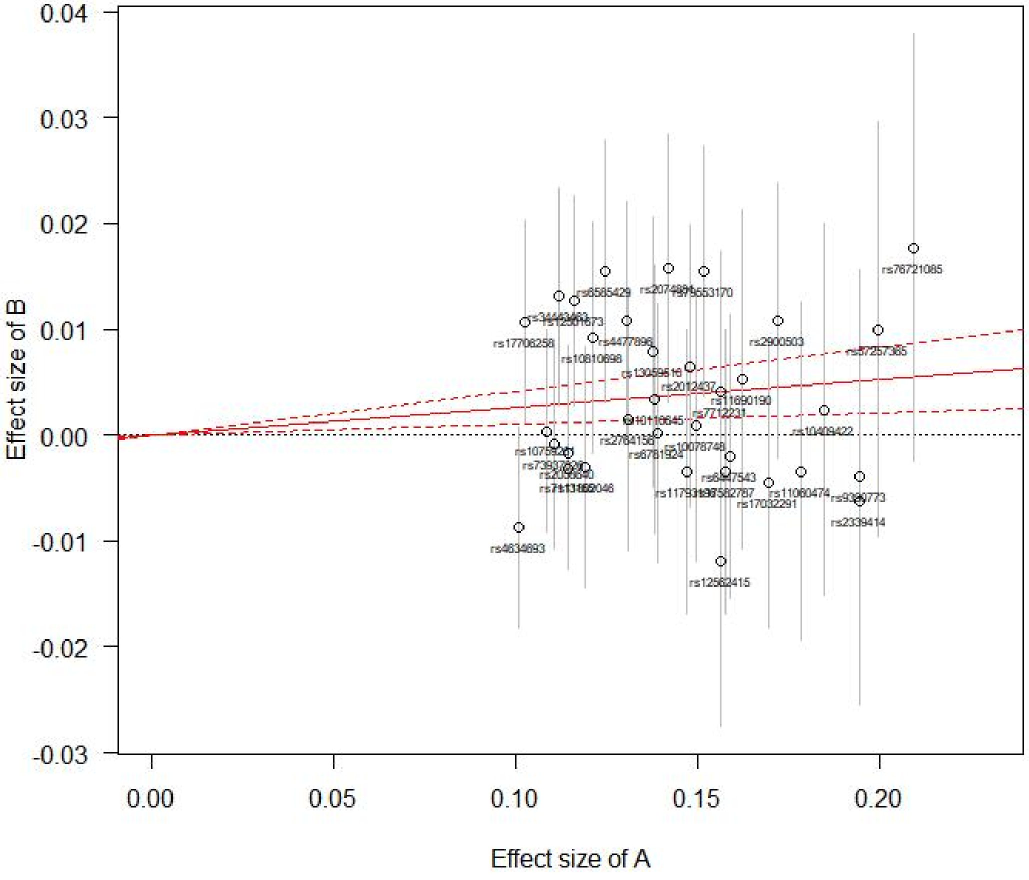

7. Gut microbiota abundance (genus Ruminococcaceae UCG010 id.11367)

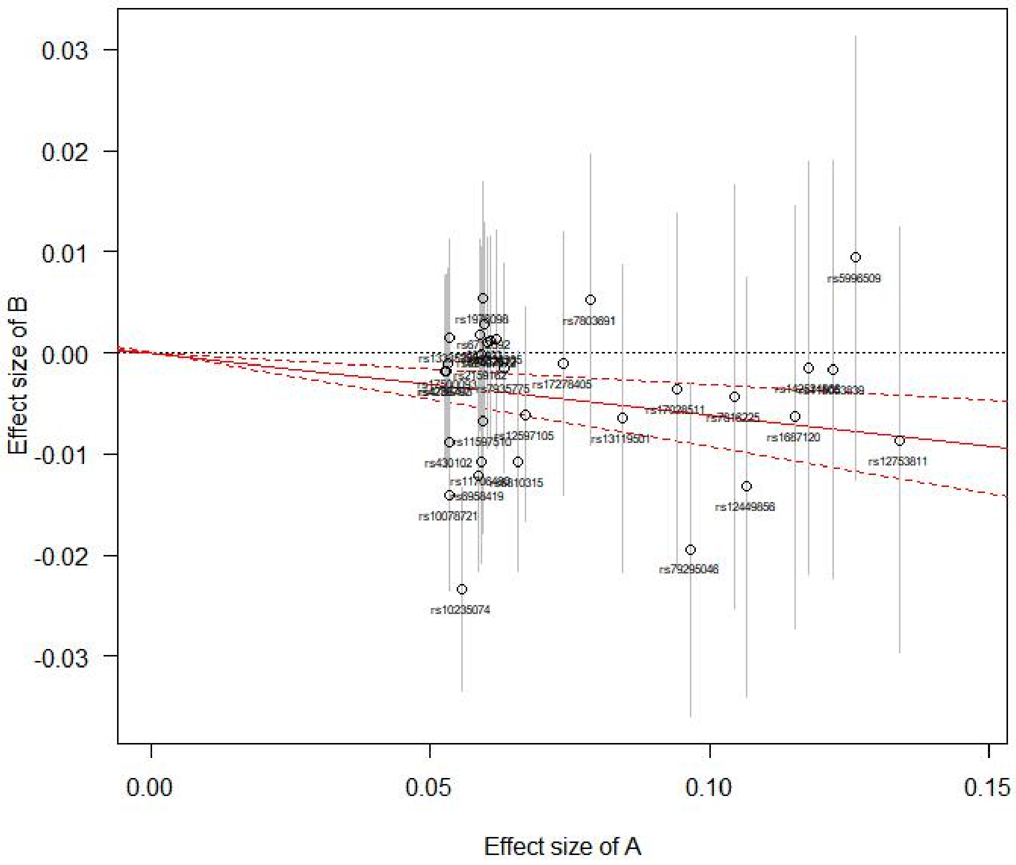

8. Gut microbiota abundance (genus Sellimonas id.14369)

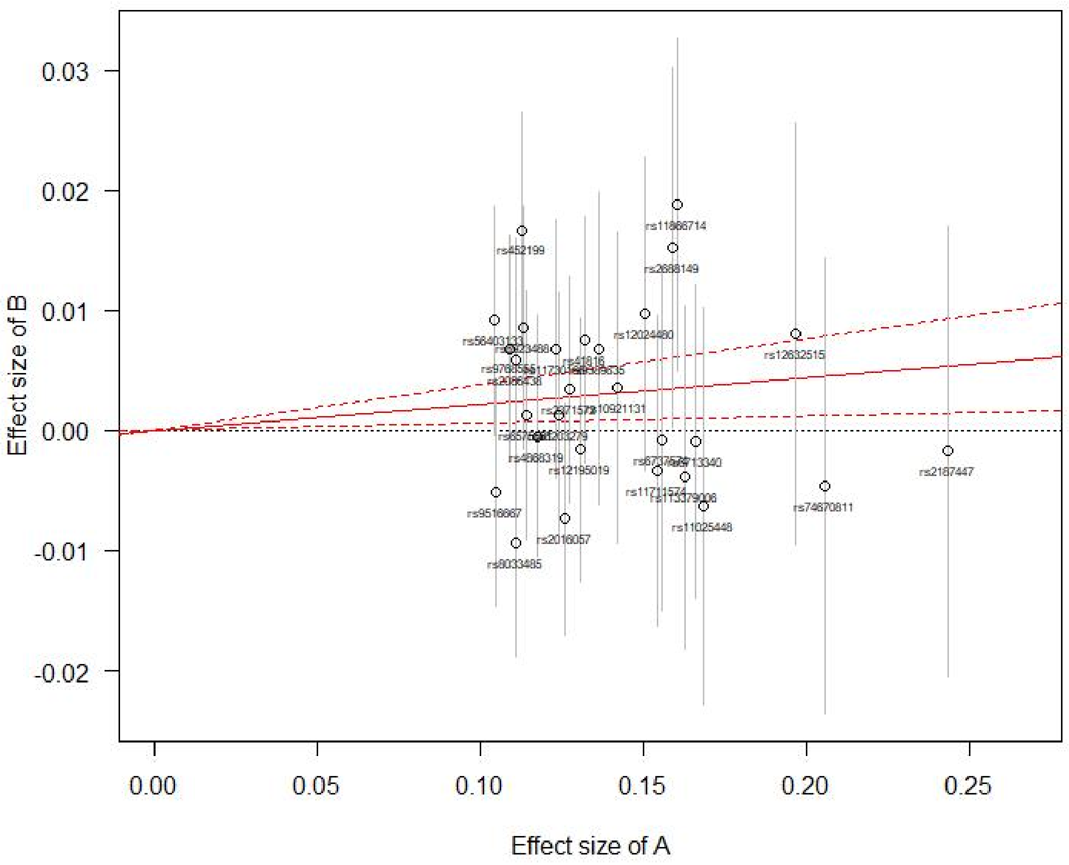

9. Gut microbiota abundance (genus Slackia id.825)

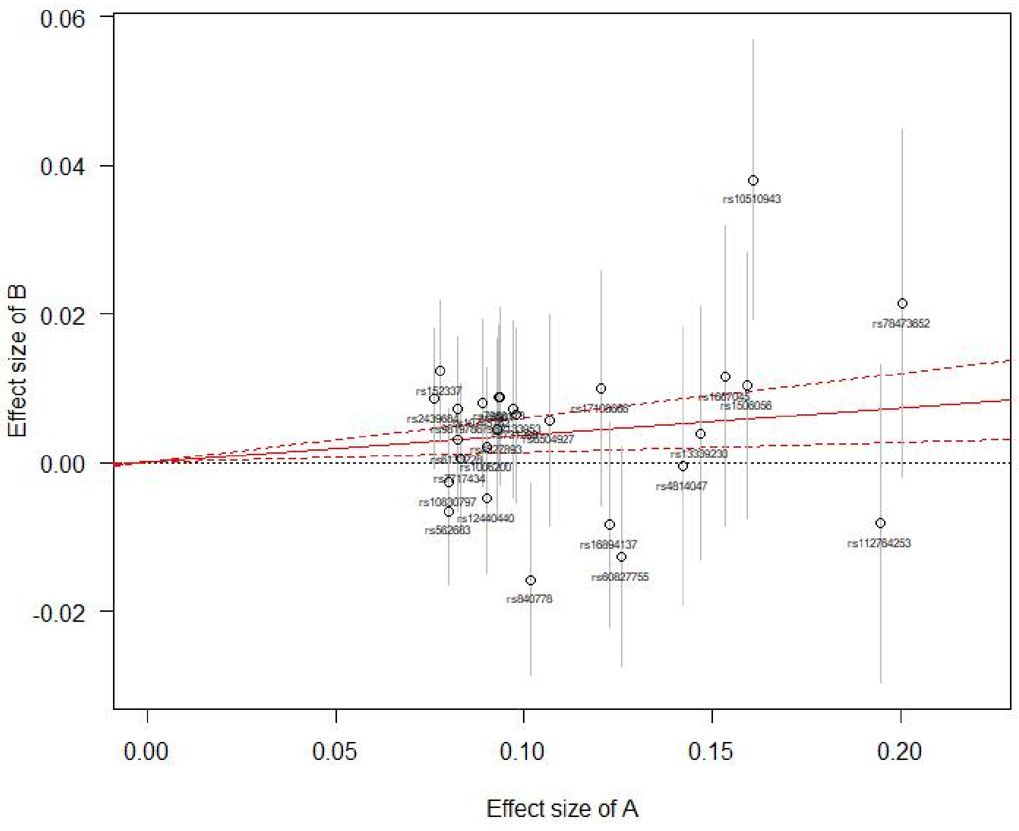

10. Gut microbiota abundance (phylum Firmicutes id.1672)

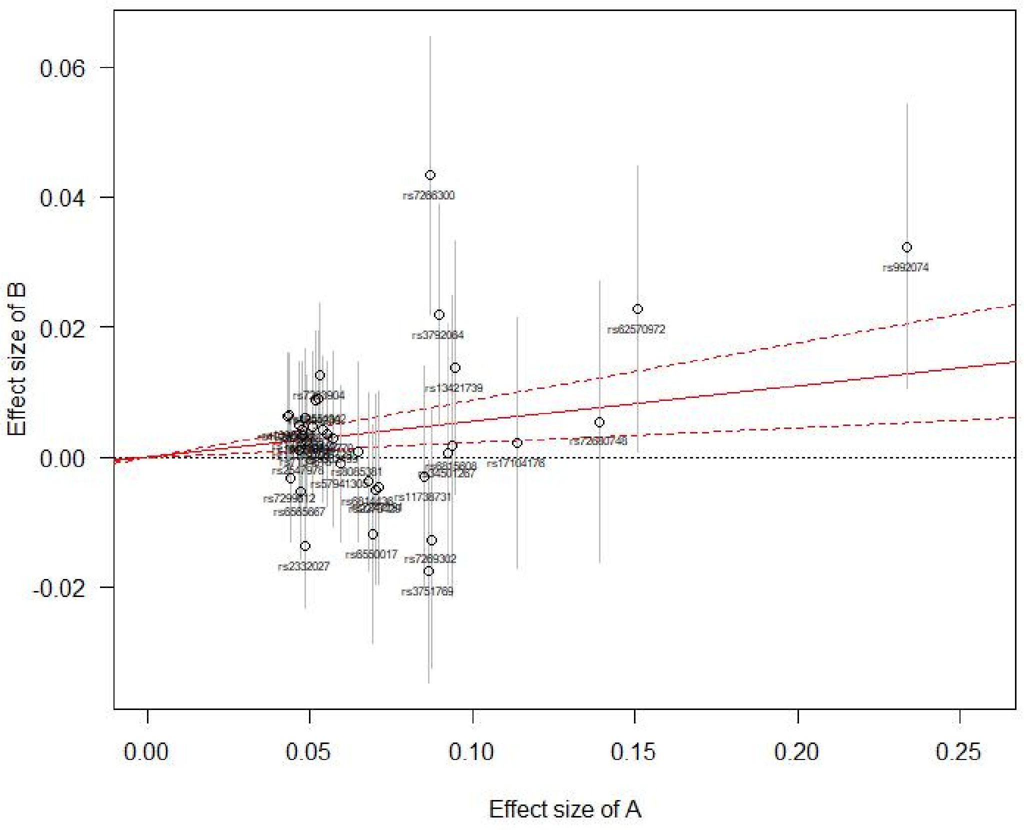

B. Esophageal cancer

1. Gut microbiota abundance (class Actinobacteria id.419)

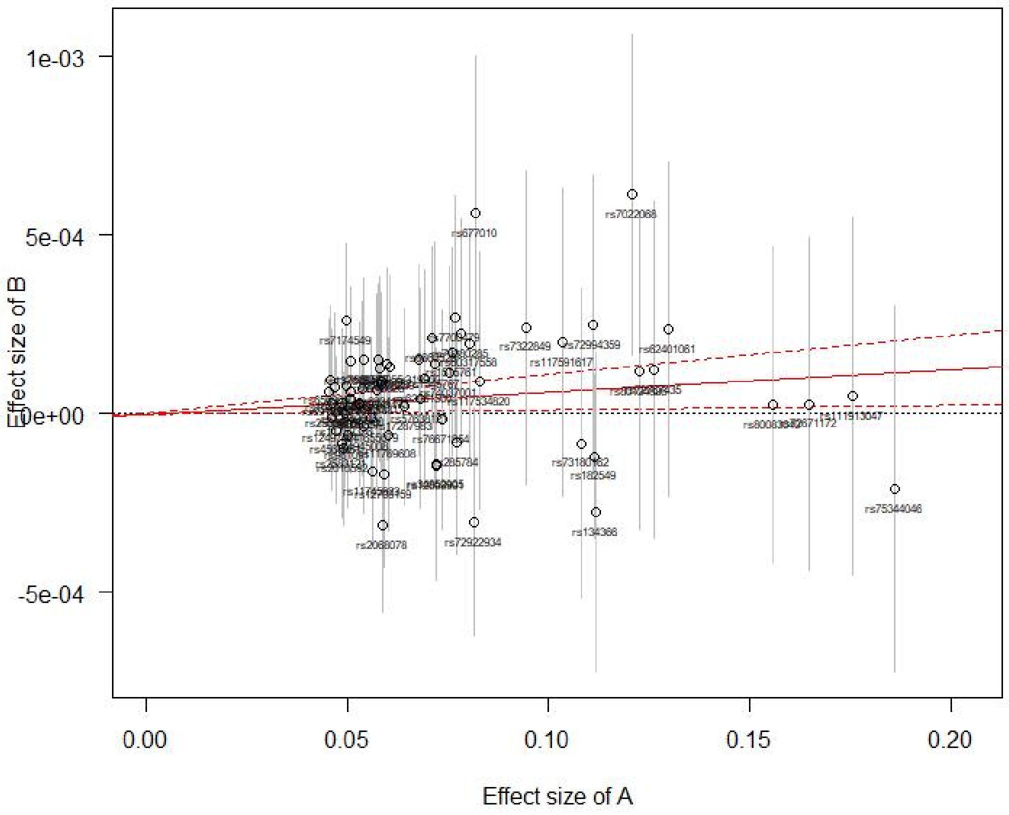

2. Gut microbiota abundance (family Clostridiaceae1 id.1869)

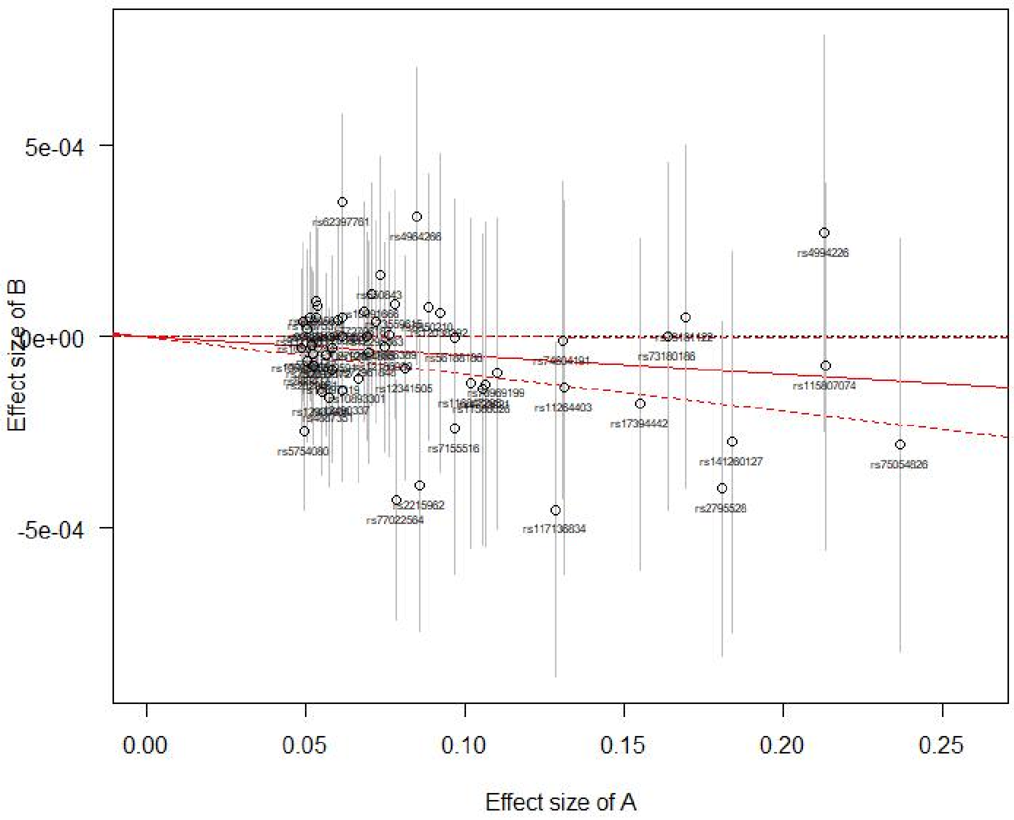

3. Gut microbiota abundance (family Desulfovibrionaceae id.3169)

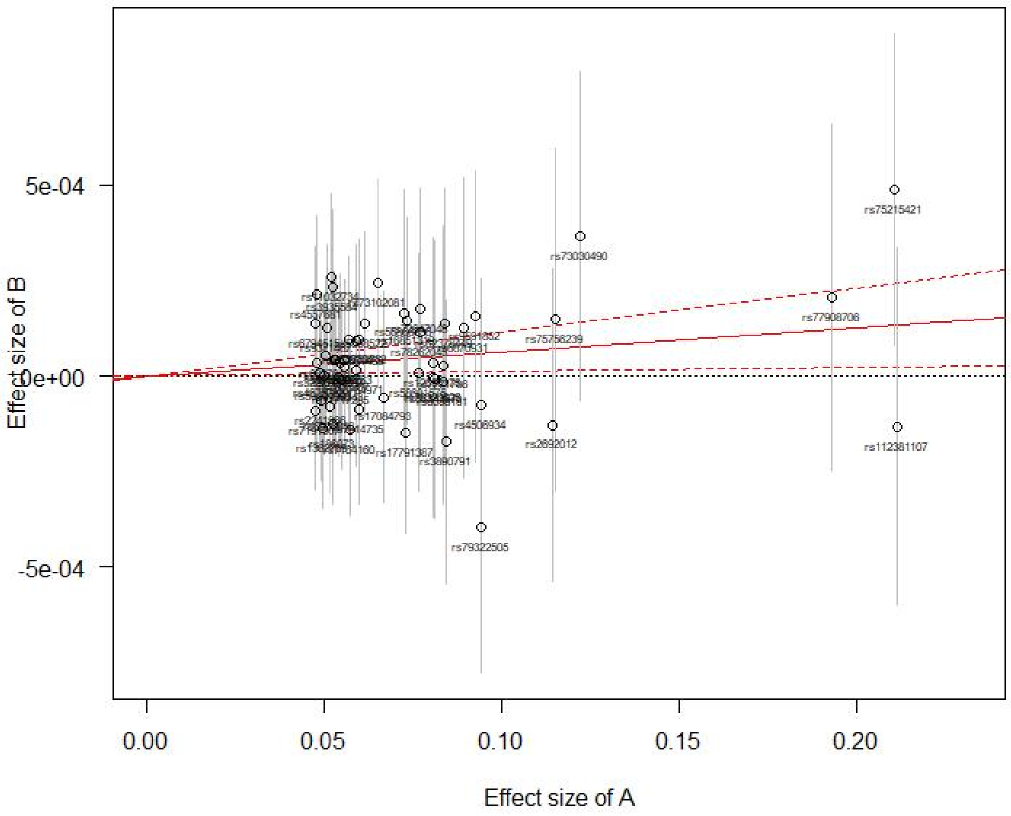

4. Gut microbiota abundance (family Pasteurellaceae id.3689)

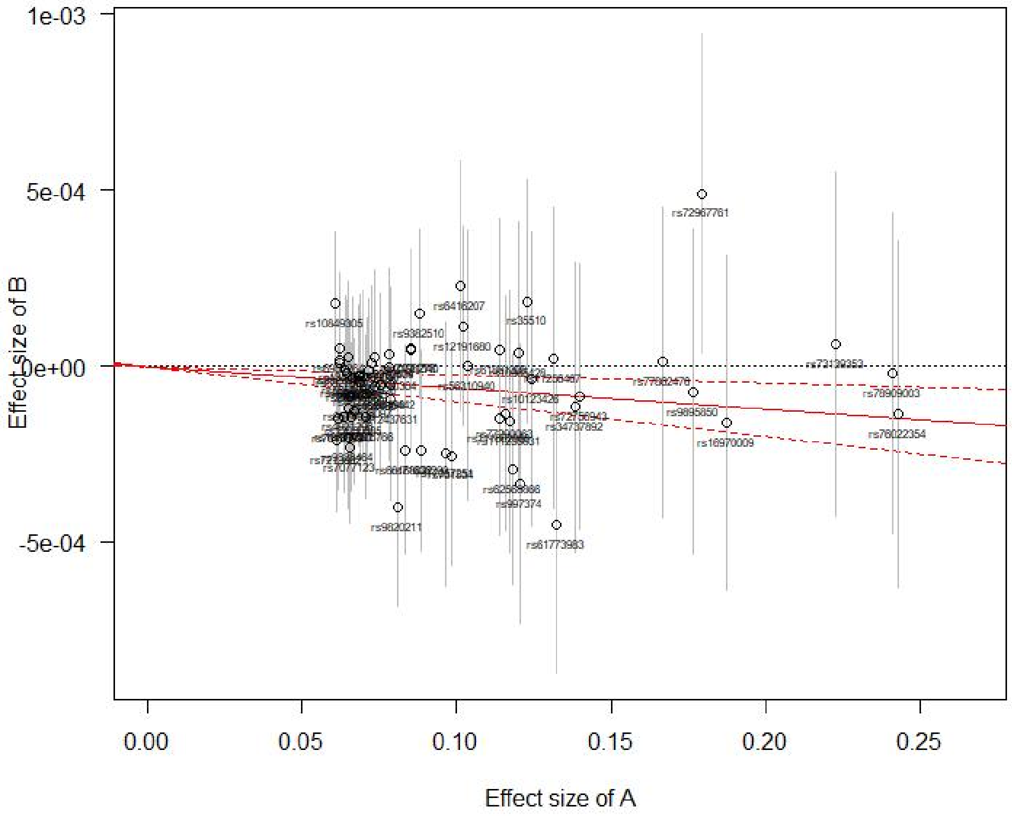

5. Gut microbiota abundance (family Ruminococcaceae id.2050)

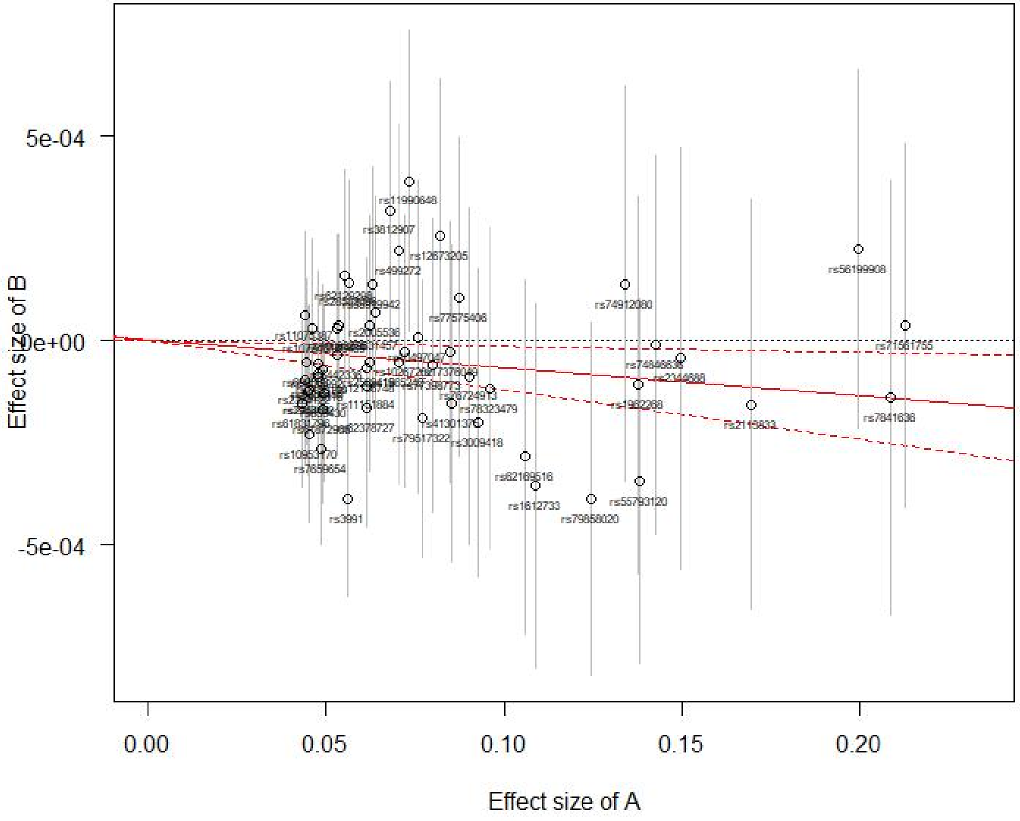

6. Gut microbiota abundance (genus Eisenbergiella id.11304)

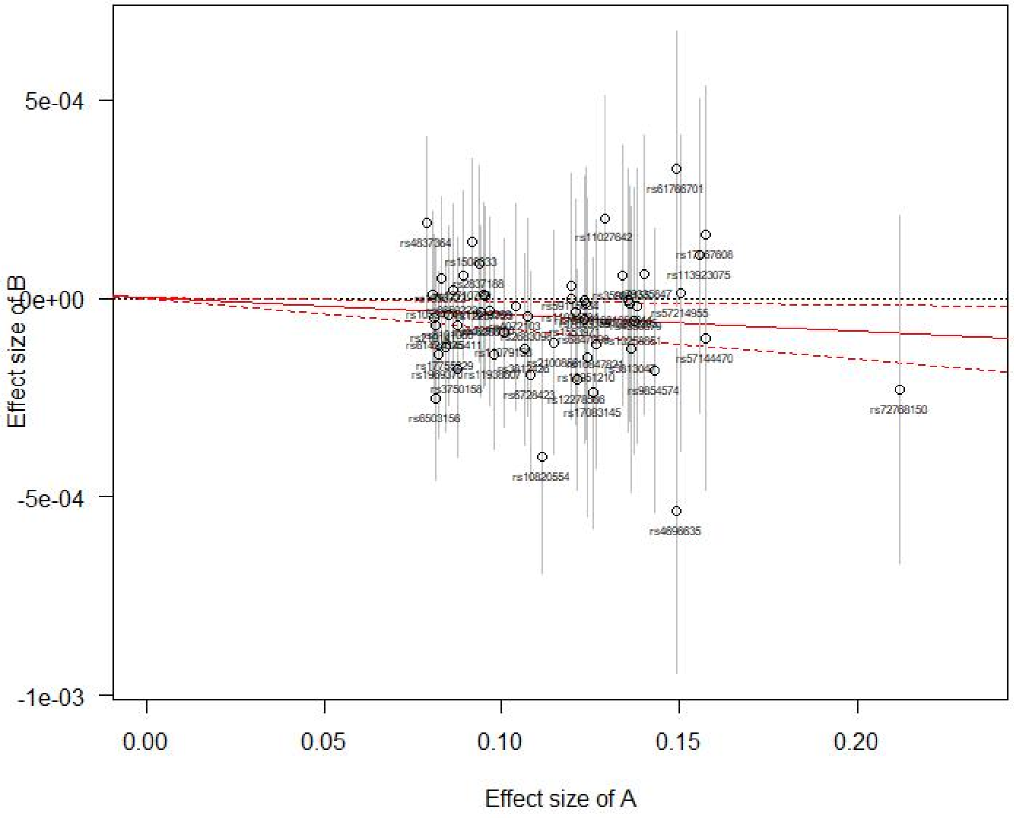

7. Gut microbiota abundance (genus Oxalobacter id.2978)

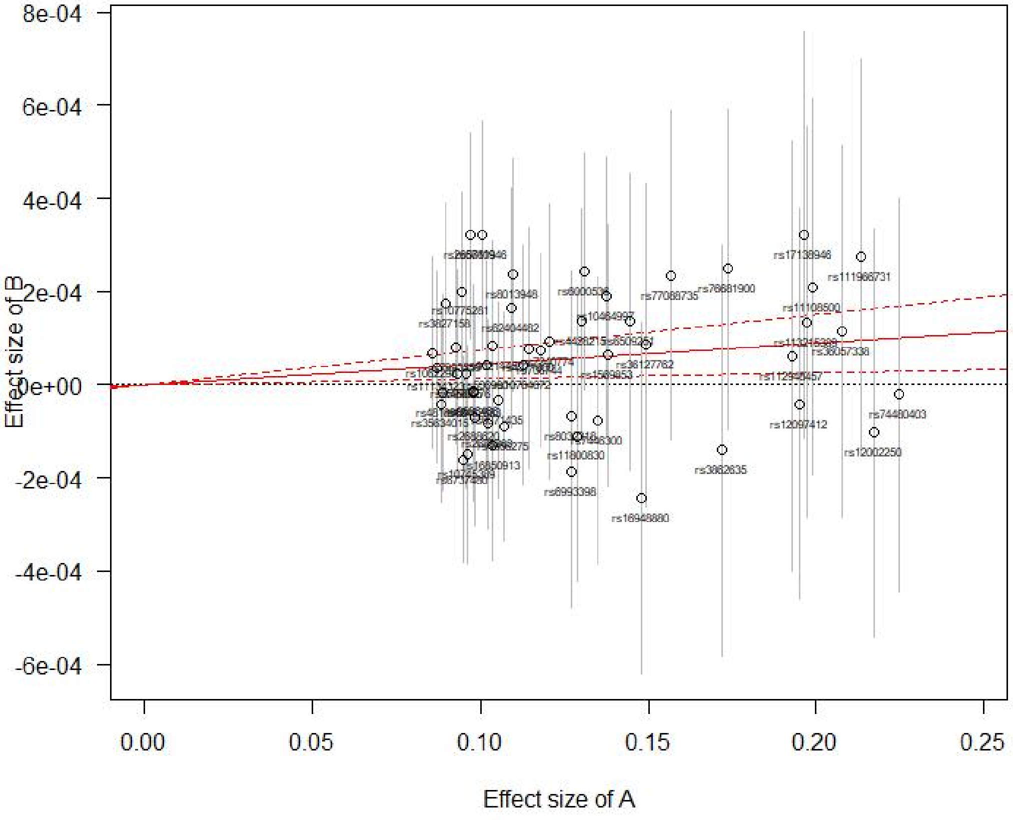

8. Gut microbiota abundance (genus Prevotella9 id.11183)

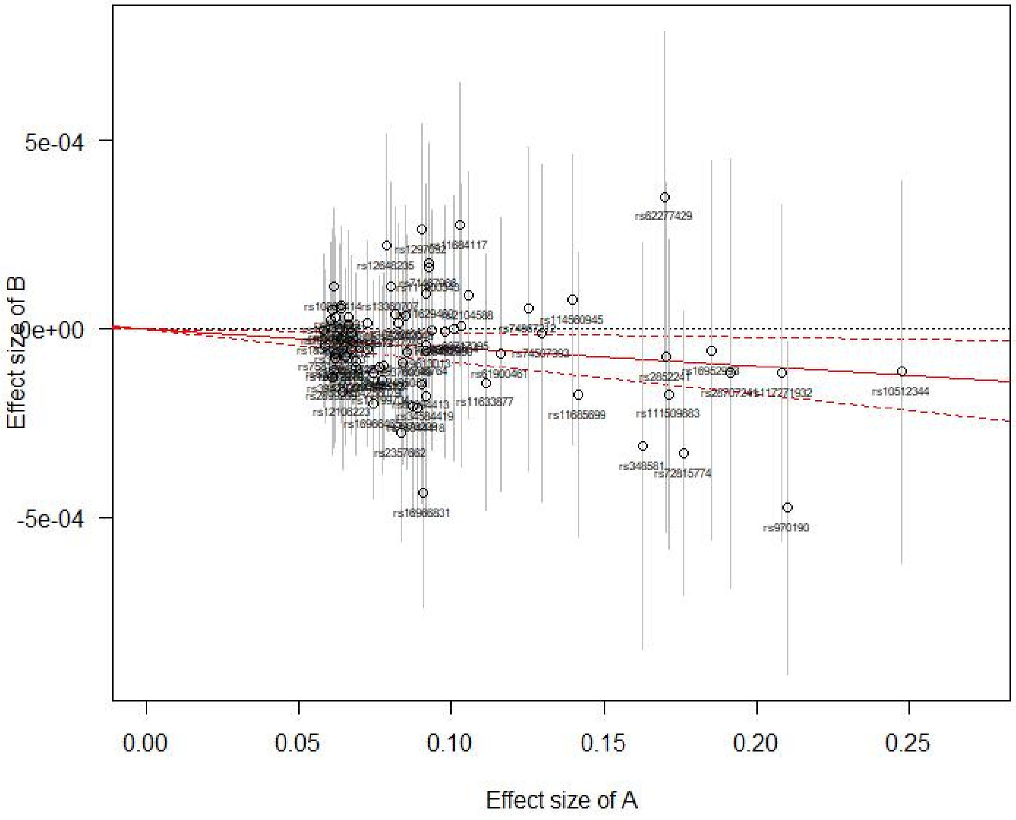

9. Gut microbiota abundance (genus Romboutsia id.11347)

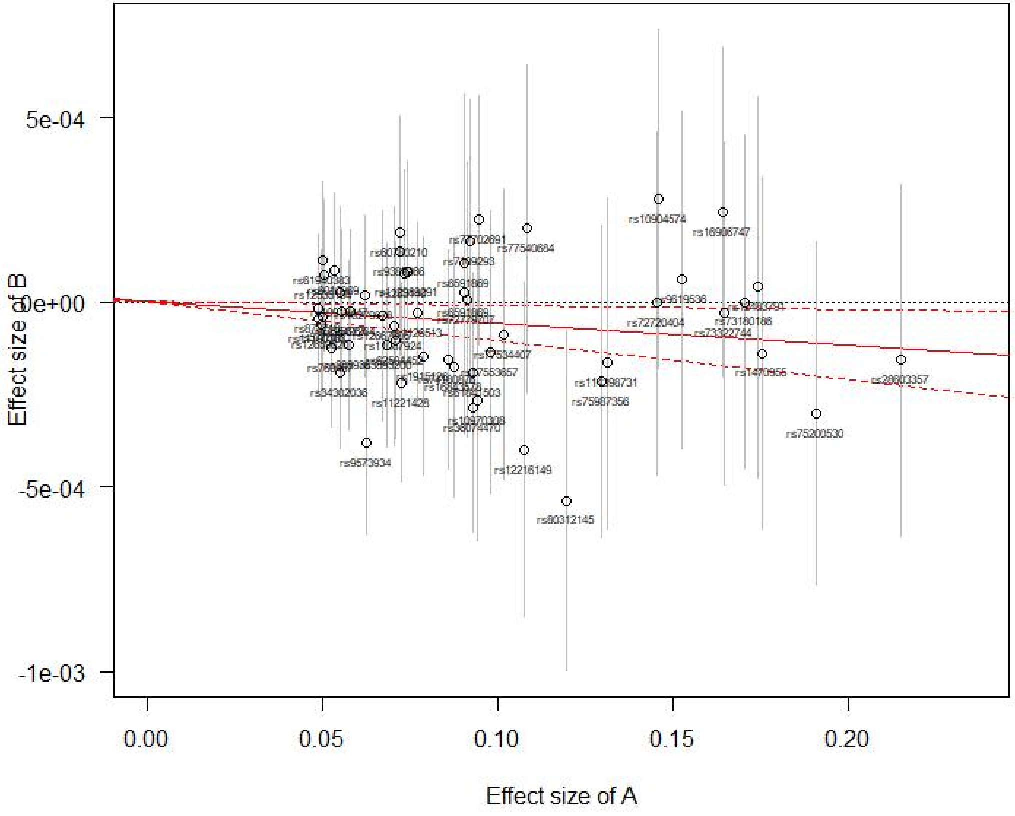

10. Gut microbiota abundance (genus Ruminococcaceae UCG010 id.11367)

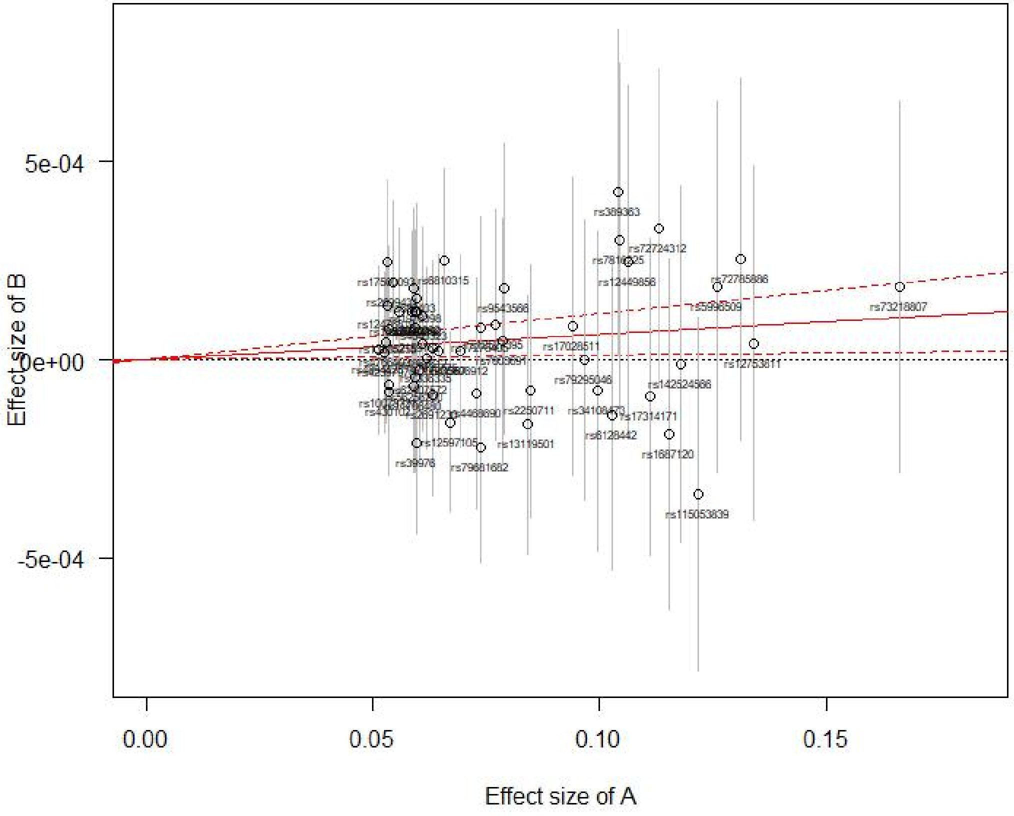

11. Gut microbiota abundance (genus Streptococcus id.1853)

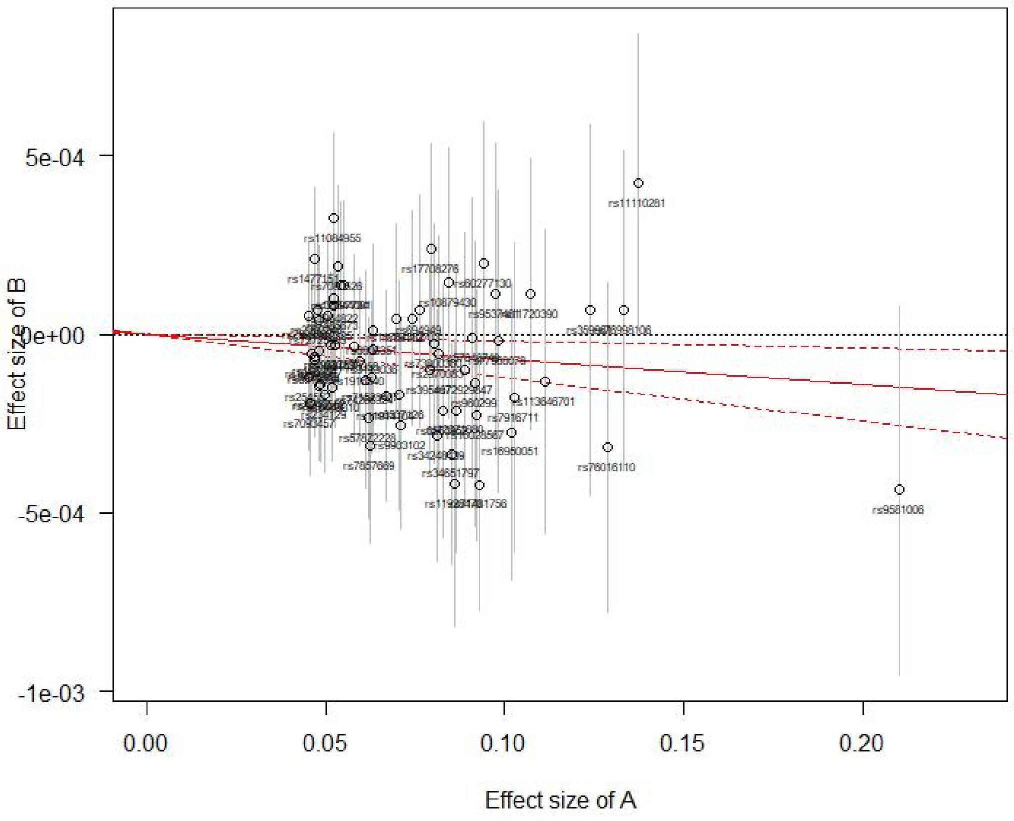

12. Gut microbiota abundance (order Desulfovibrionales id.3156)

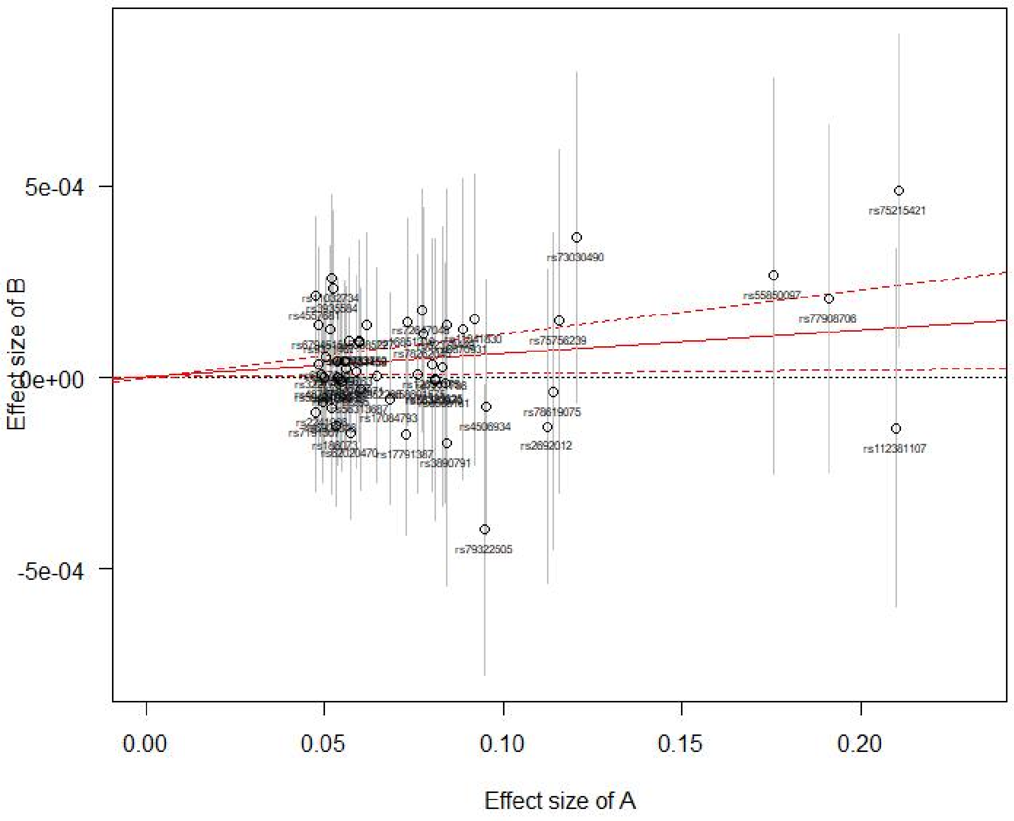

13. Gut microbiota abundance (order Pasteurellales id.3688)

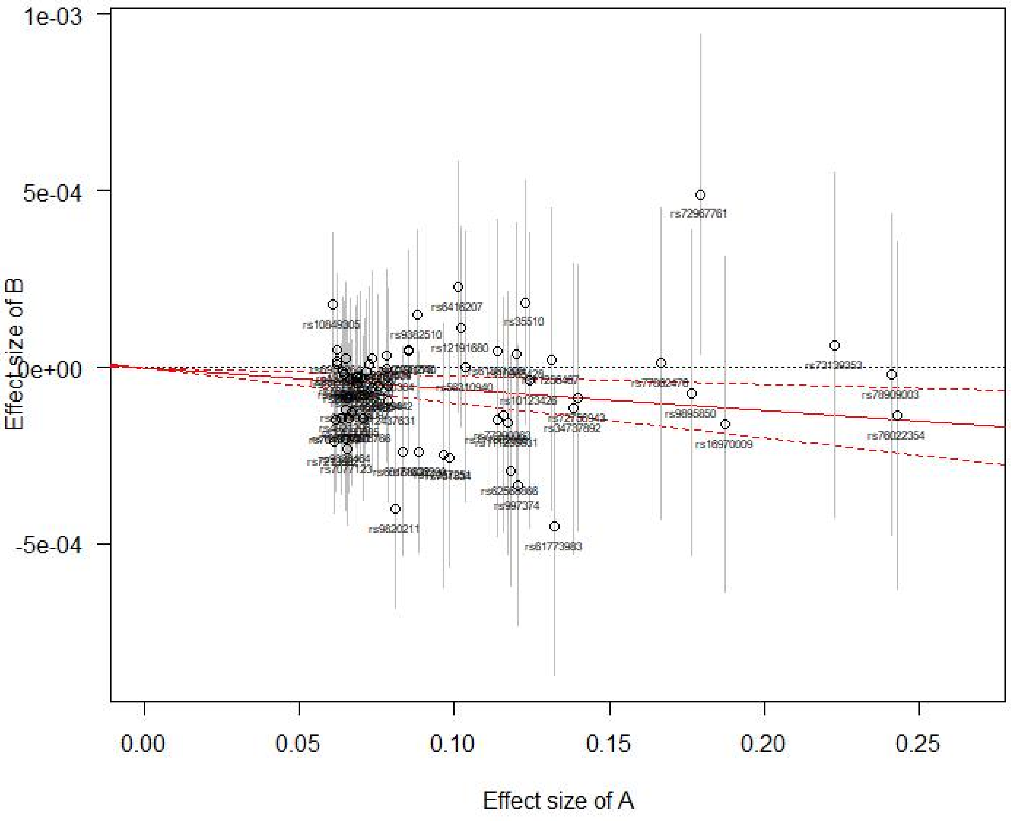

14. Gut microbiota abundance (phylum Actinobacteria id.400)

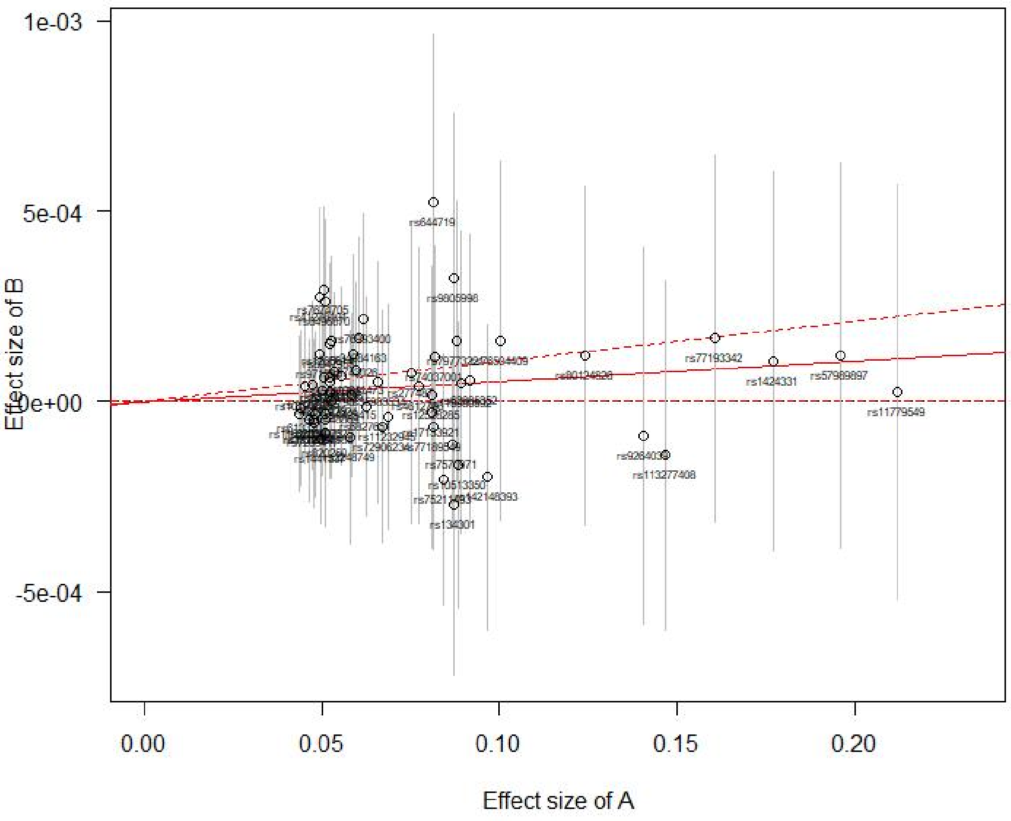

C. Esophagus ulcer

1. Gut microbiota abundance (class Bacteroidia id.912)

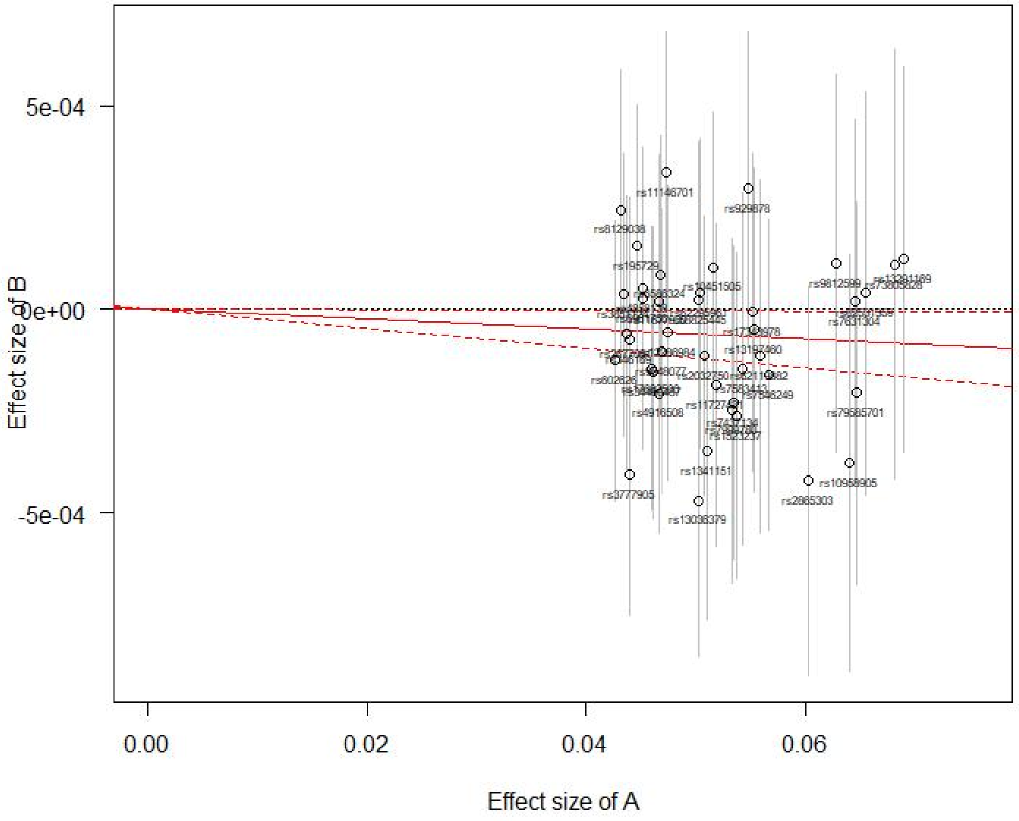

2. Gut microbiota abundance (family Peptostreptococcaceae id.2042)

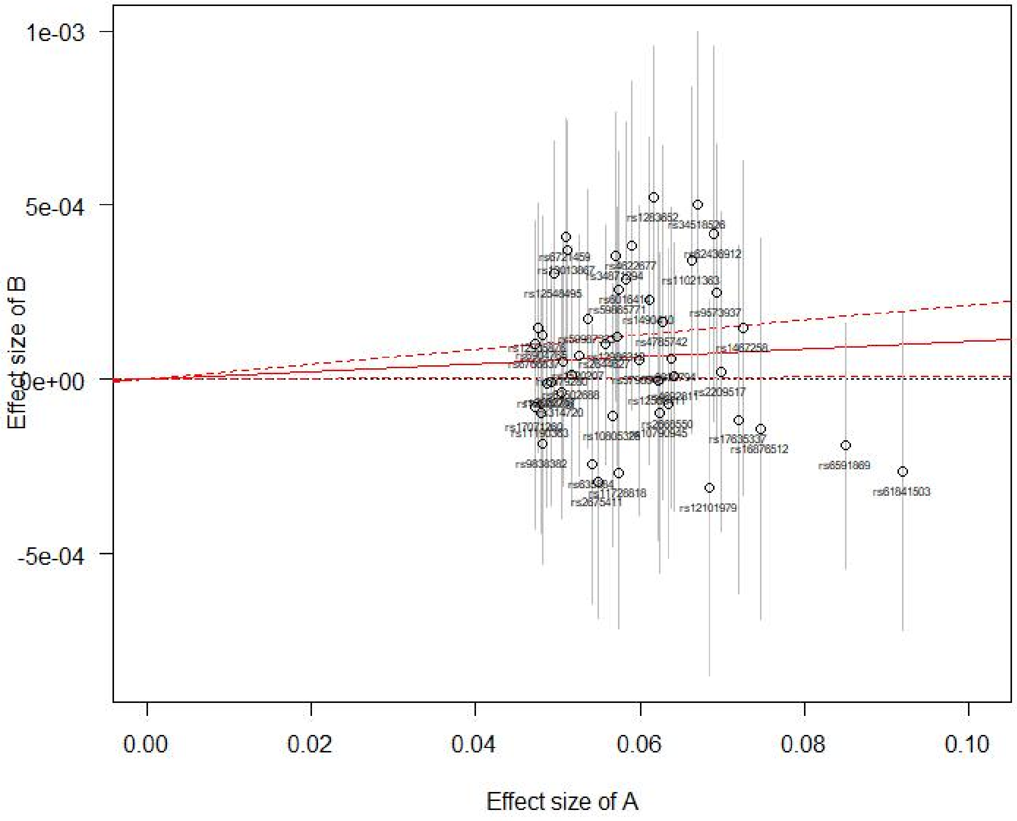

3. Gut microbiota abundance (family Rhodospirillaceae id.2717)

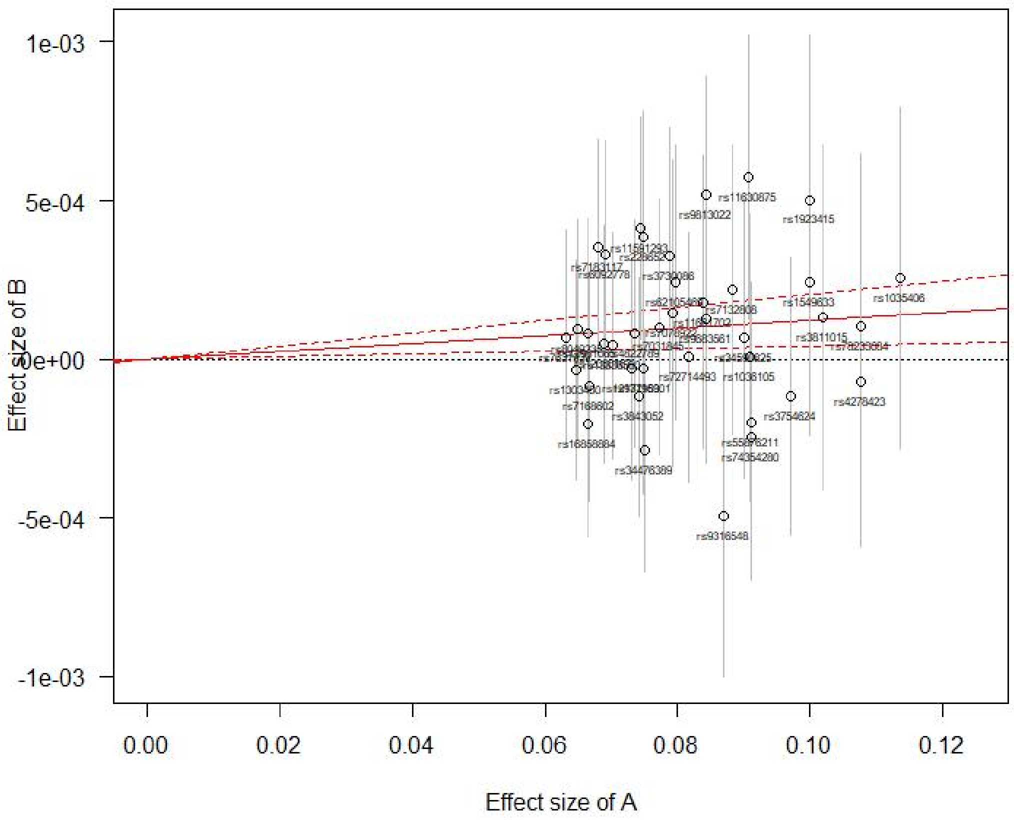

4. Gut microbiota abundance (genus Erysipelatoclostridium id.11381)

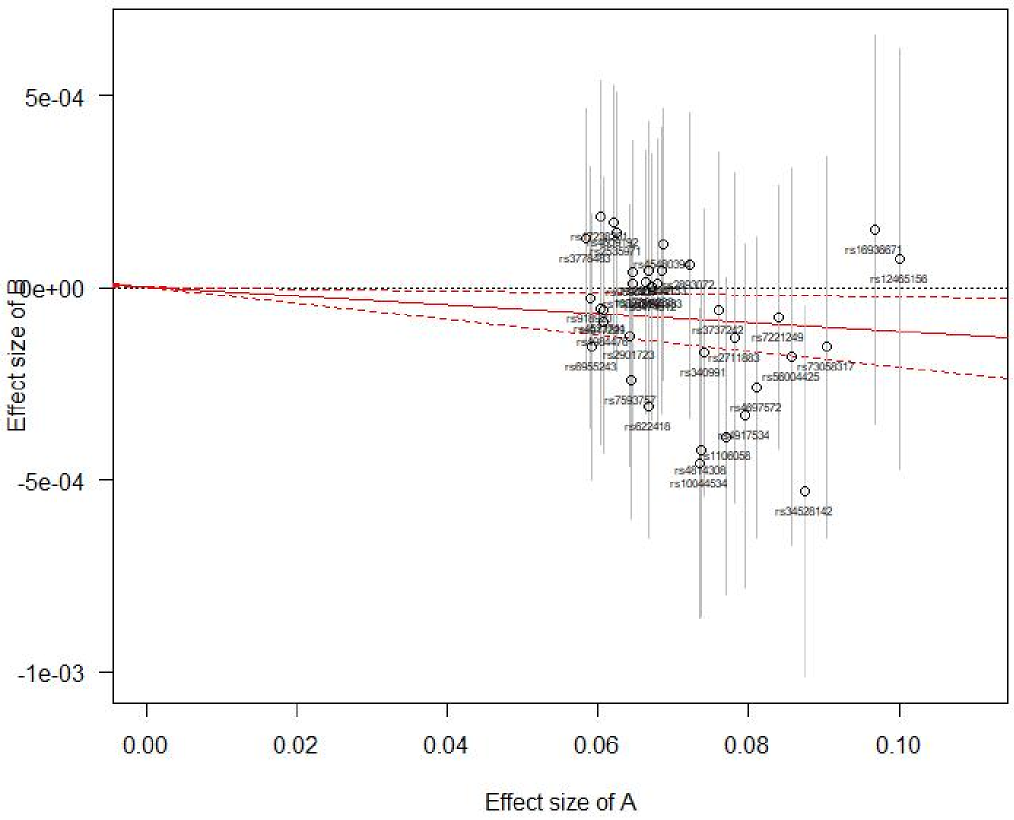

5. Gut microbiota abundance (genus Family XIII AD3011 group id.11293)

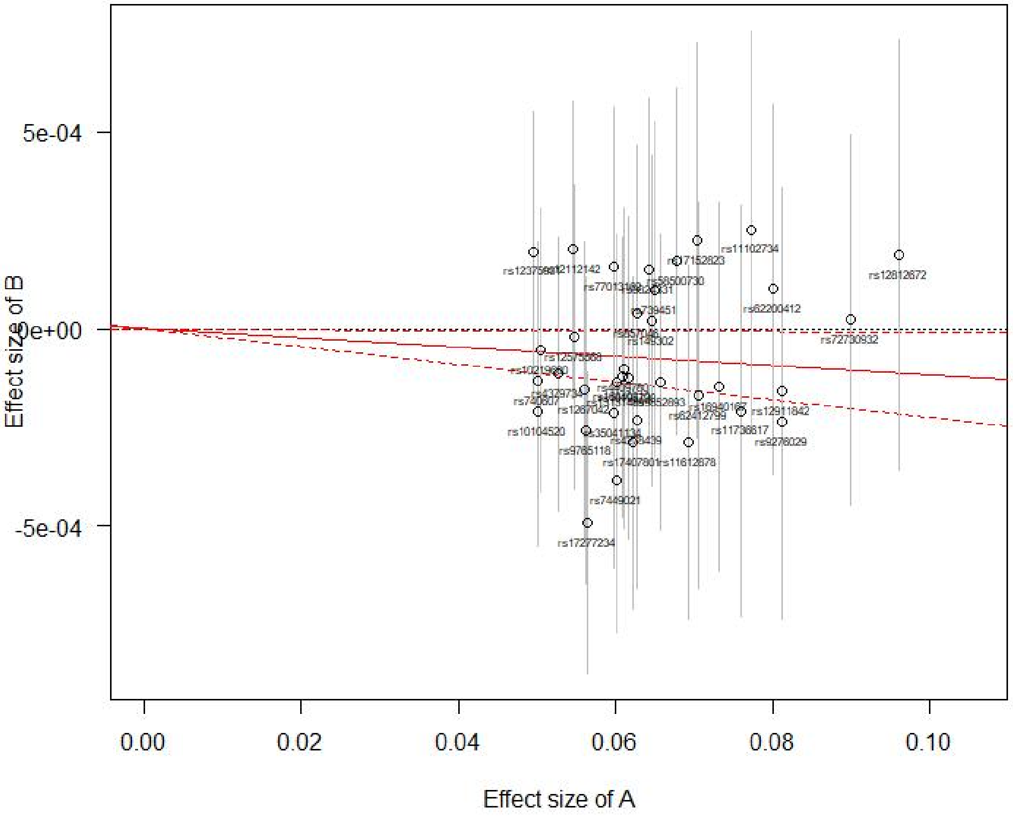

6. Gut microbiota abundance (genus Gordonibacter id.821)

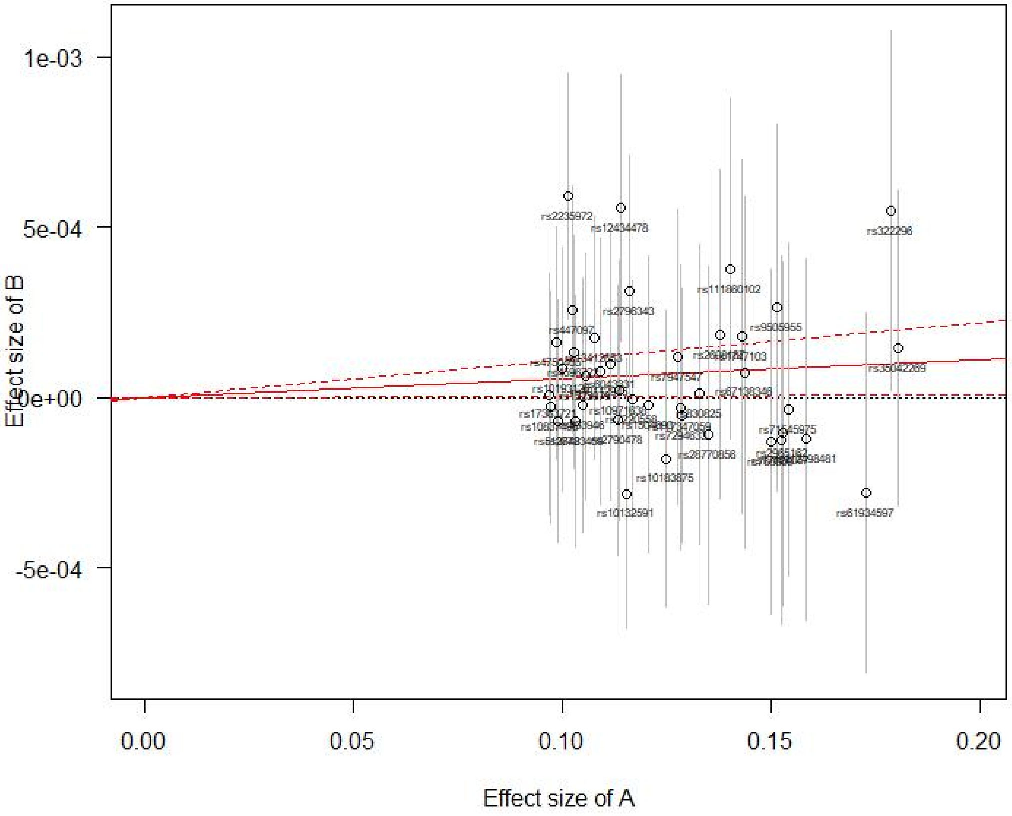

7. Gut microbiota abundance (genus Slackia id.825)

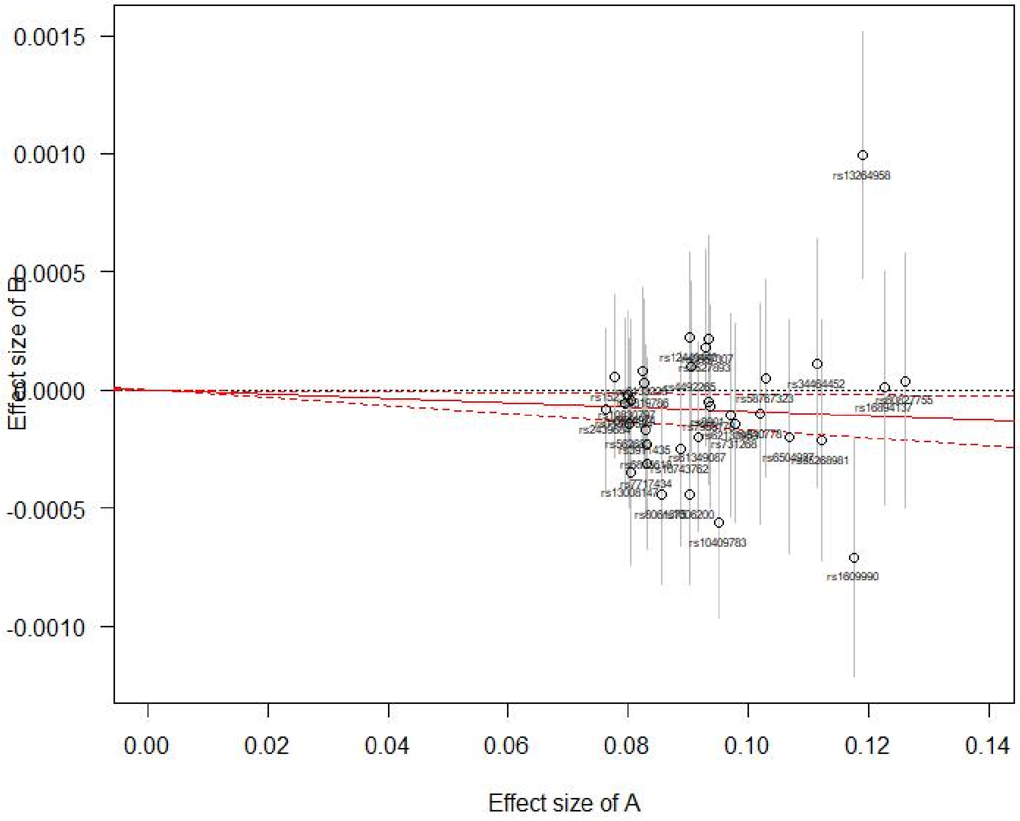

8. Gut microbiota abundance (genus Terrisporobacter id.11348)

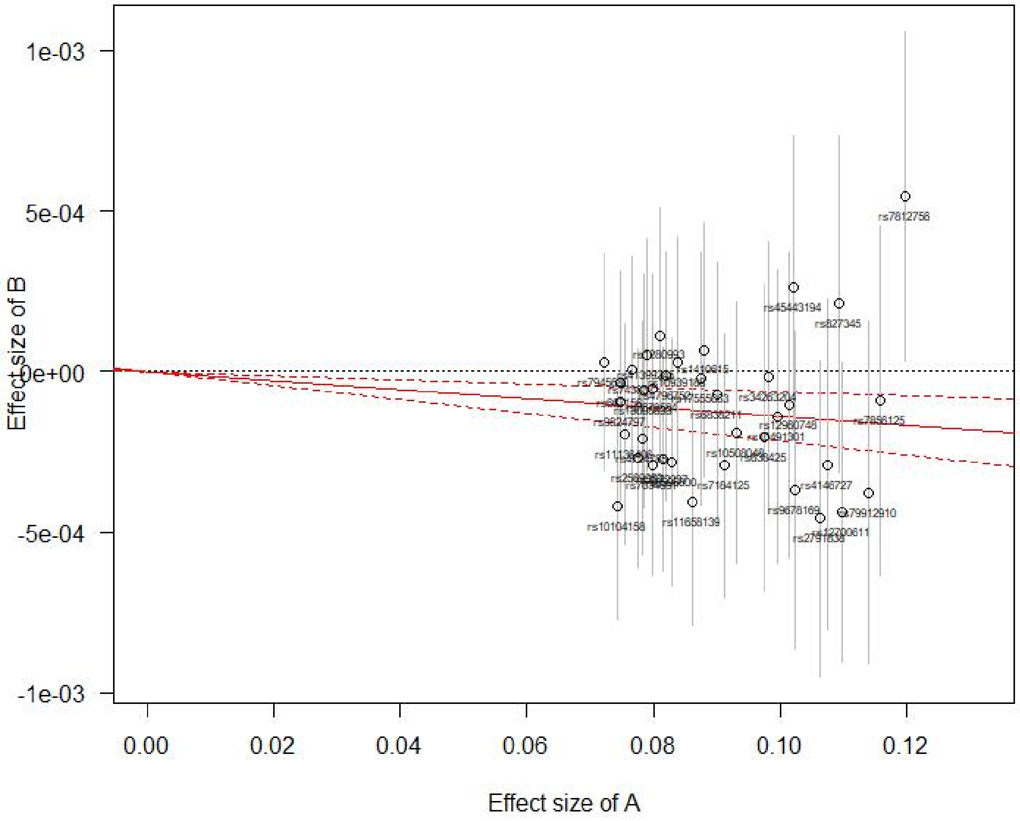

9. Gut microbiota abundance (unknown genus id.2001)

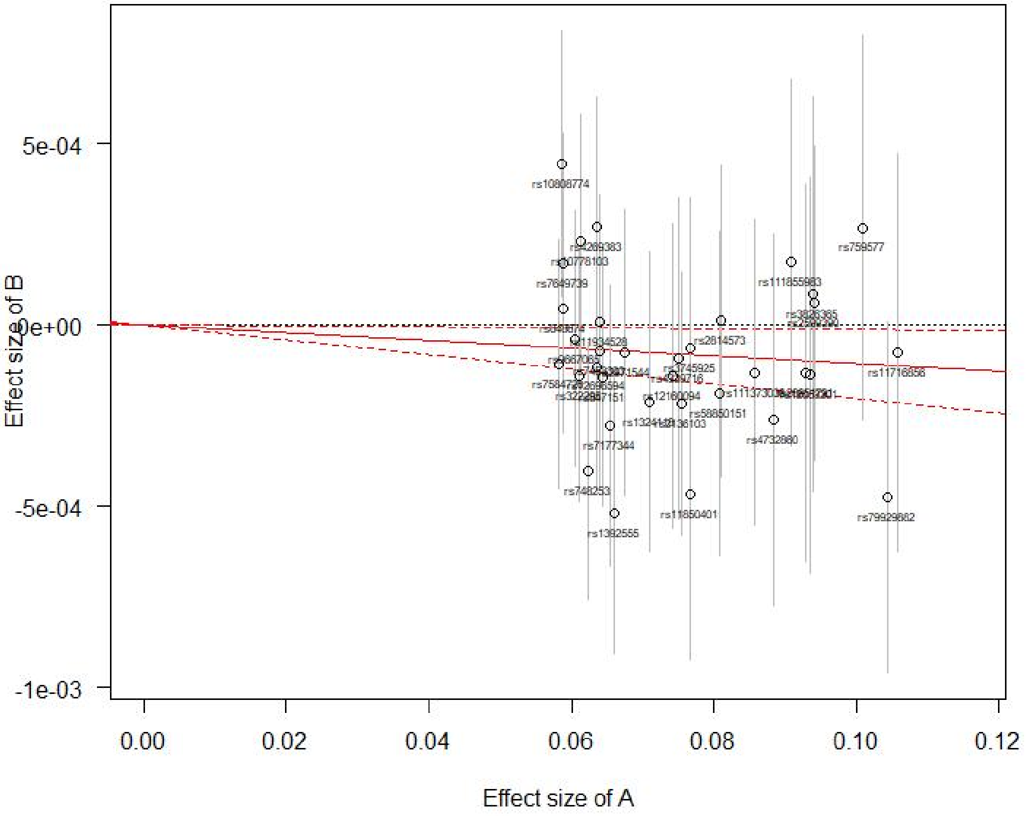

10. Gut microbiota abundance (unknown genus id.2755)

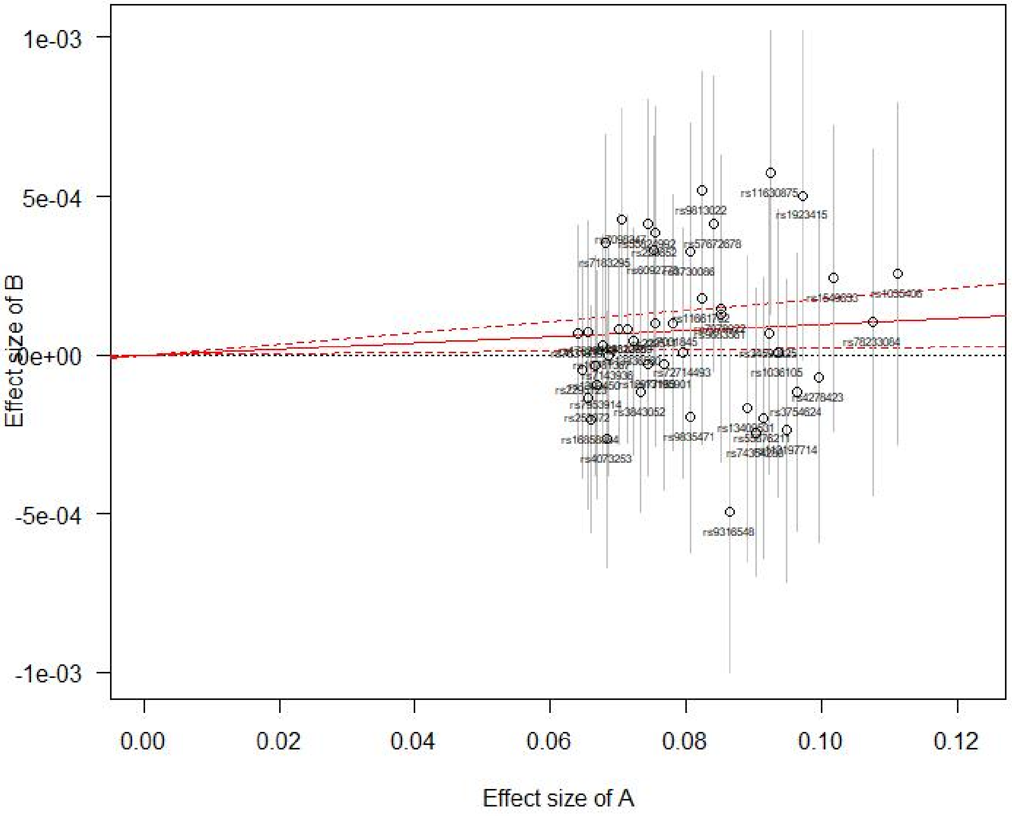

11. Gut microbiota abundance (order Bacillales id.1674)

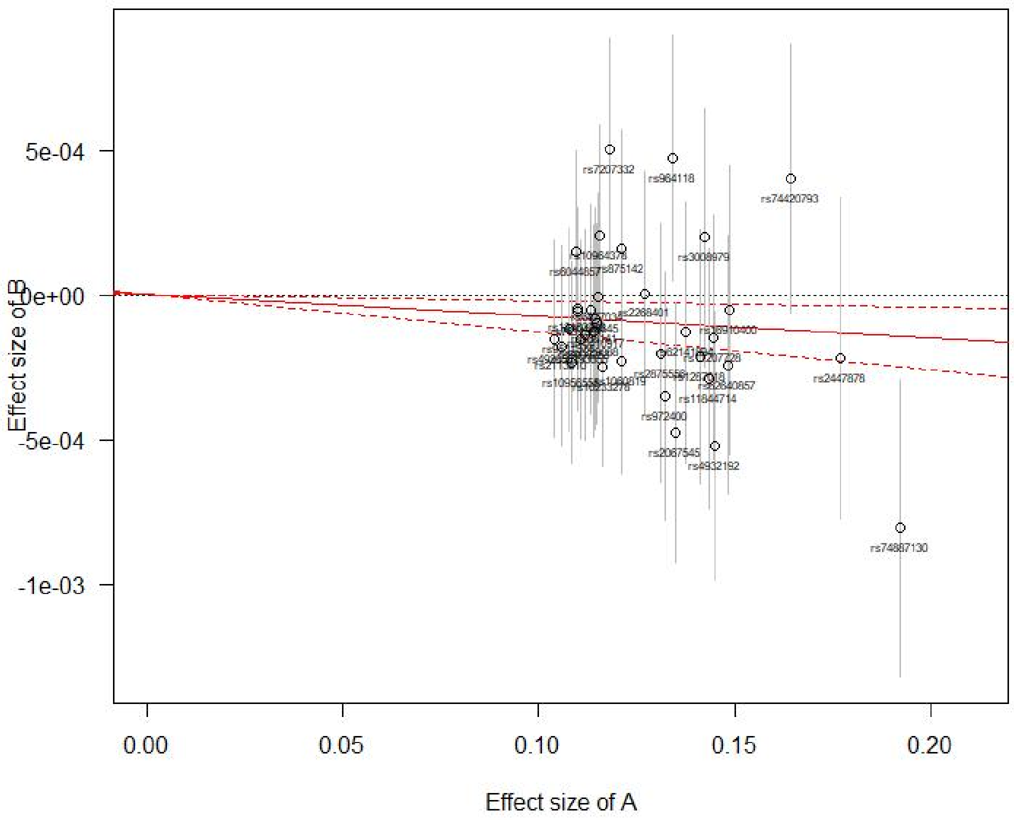

12. Gut microbiota abundance (order Bacteroidales id.913)

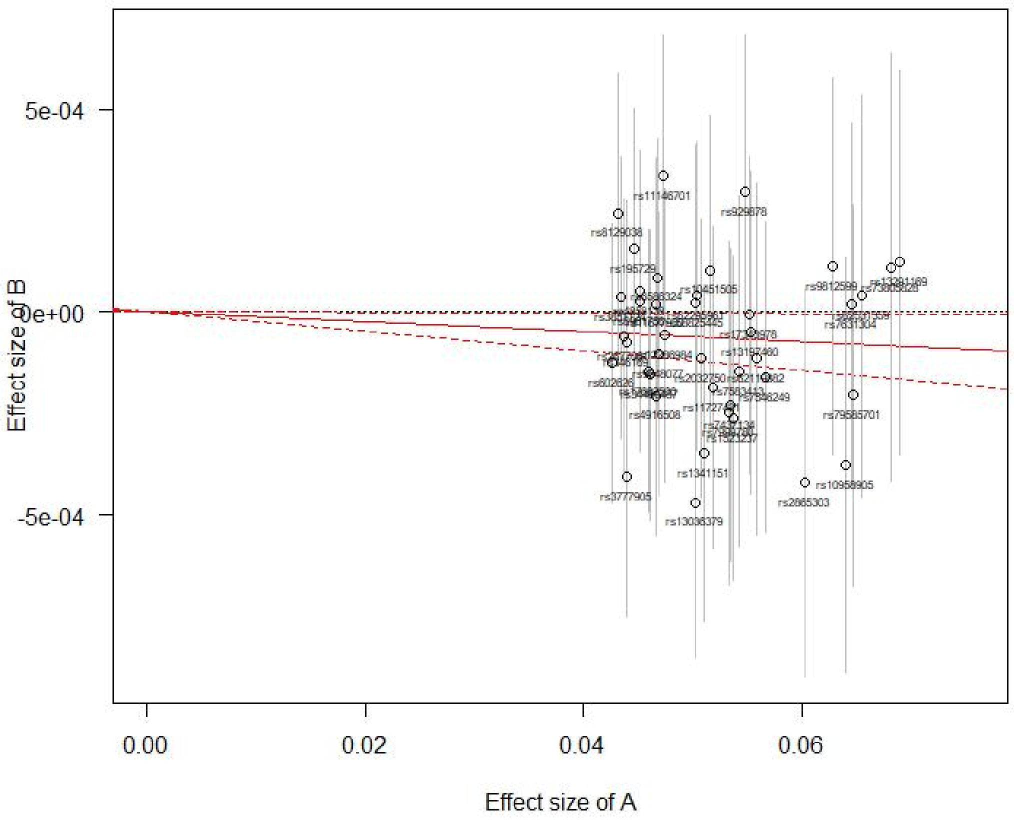

13. Gut microbiota abundance (order Rhodospirillales id.2667)

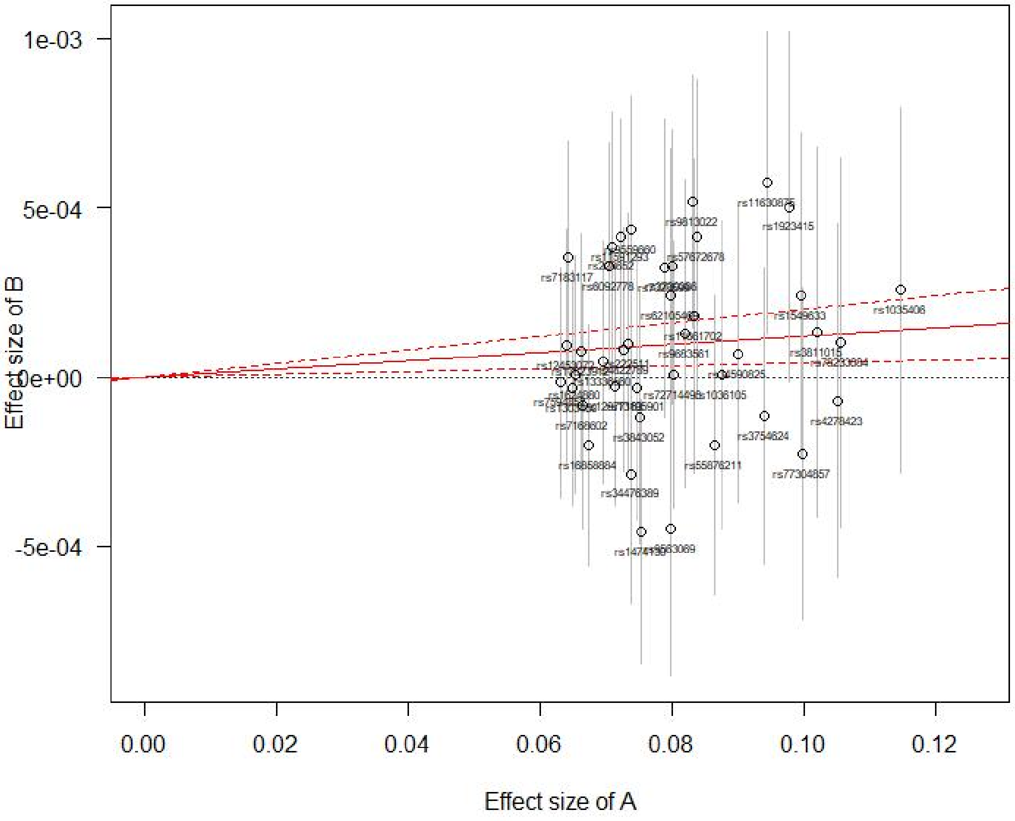

14. Gut microbiota abundance (phylum Cyanobacteria id.1500)

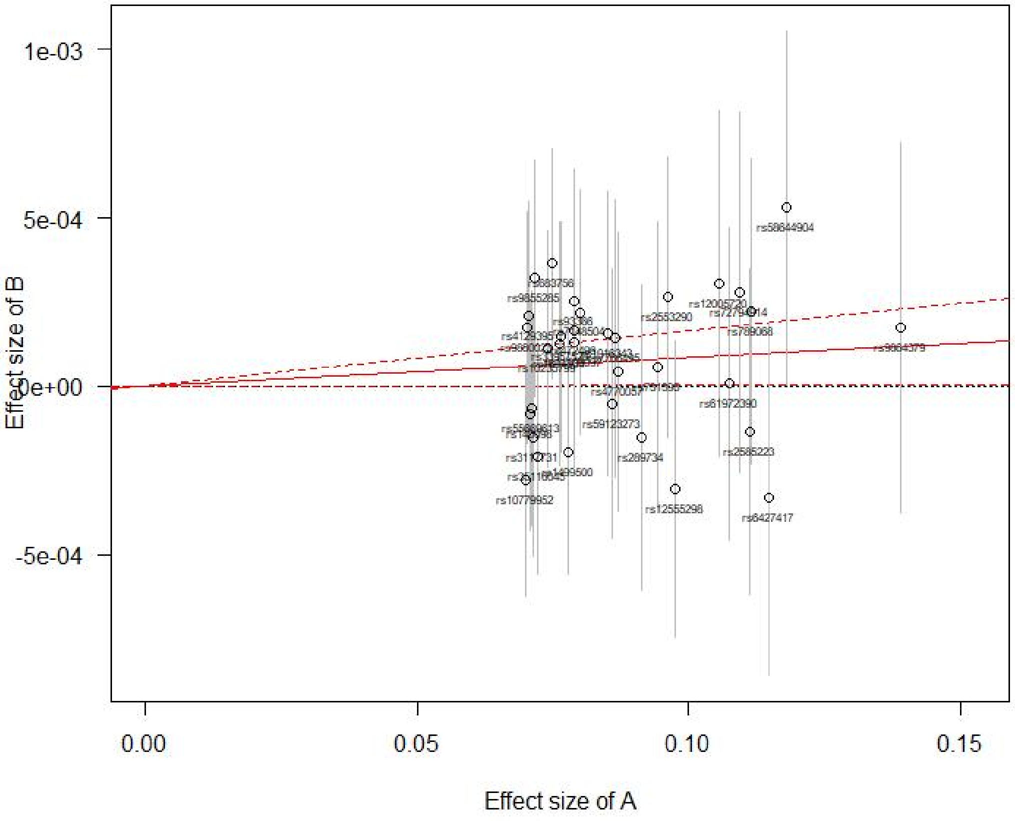

D. Gastric ulcer

1. Gut microbiota abundance (class Bacilli id.1673)

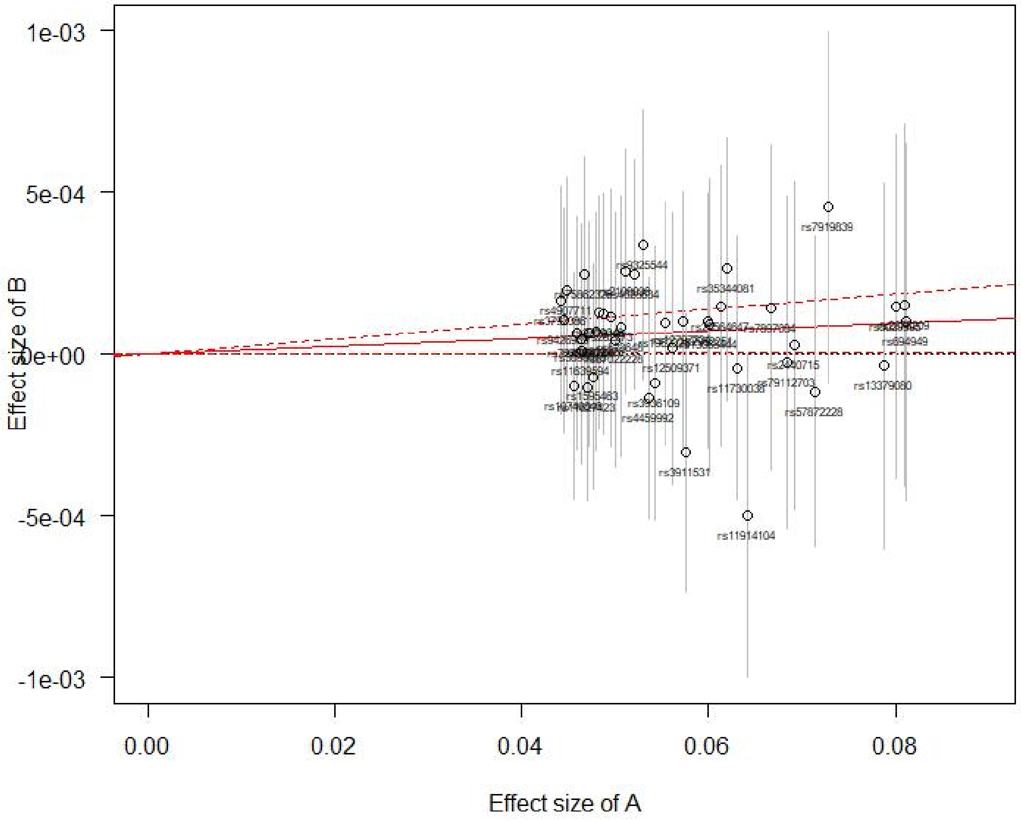

2. Gut microbiota abundance (class Bacteroidia id.912)

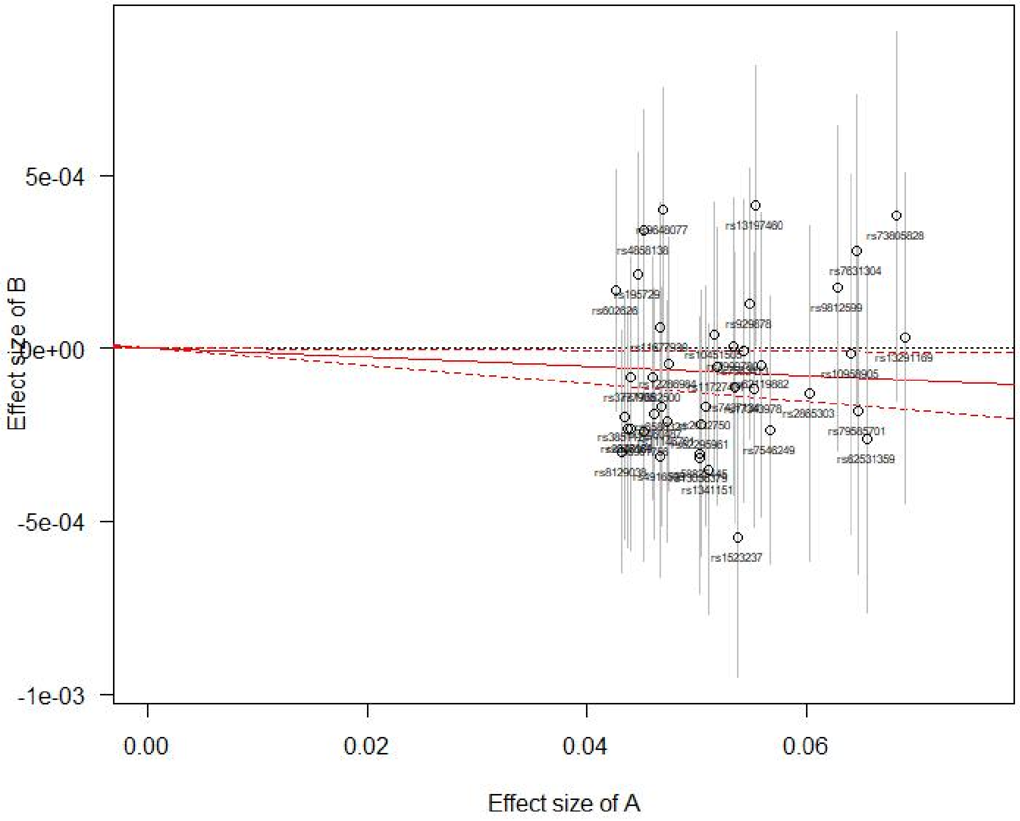

3. Gut microbiota abundance (class Lentisphaeria id.2250)

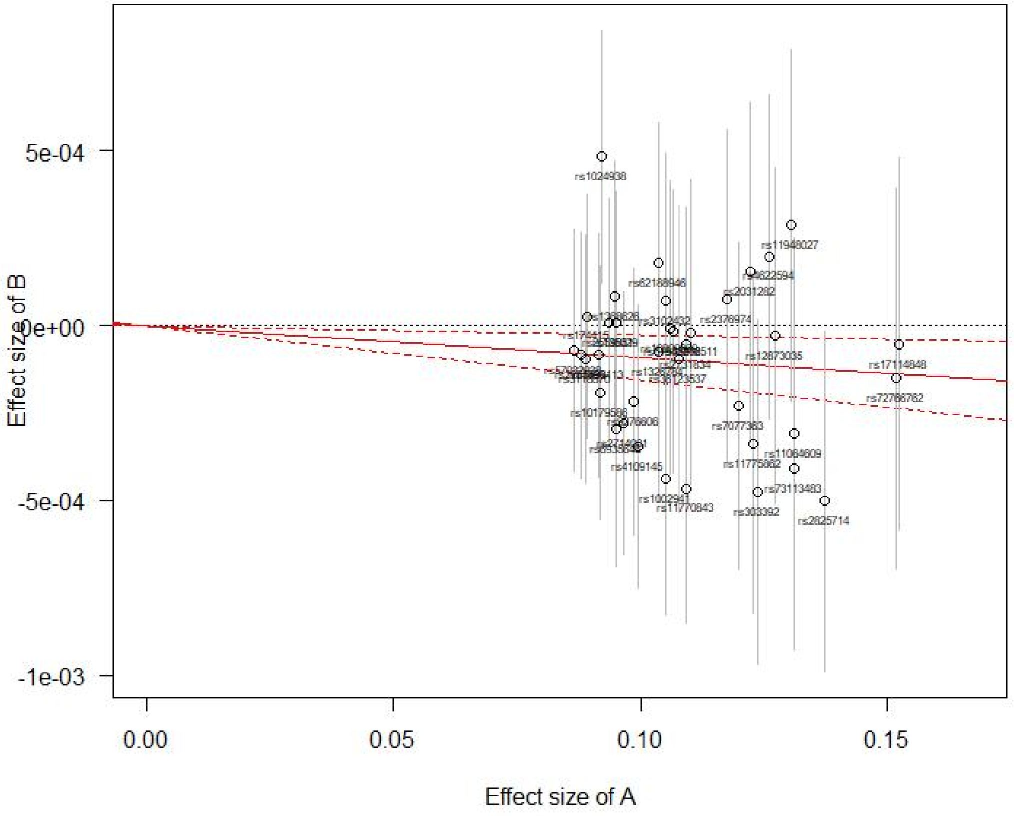

4. Gut microbiota abundance (genus Butyrivibrio id.1993)

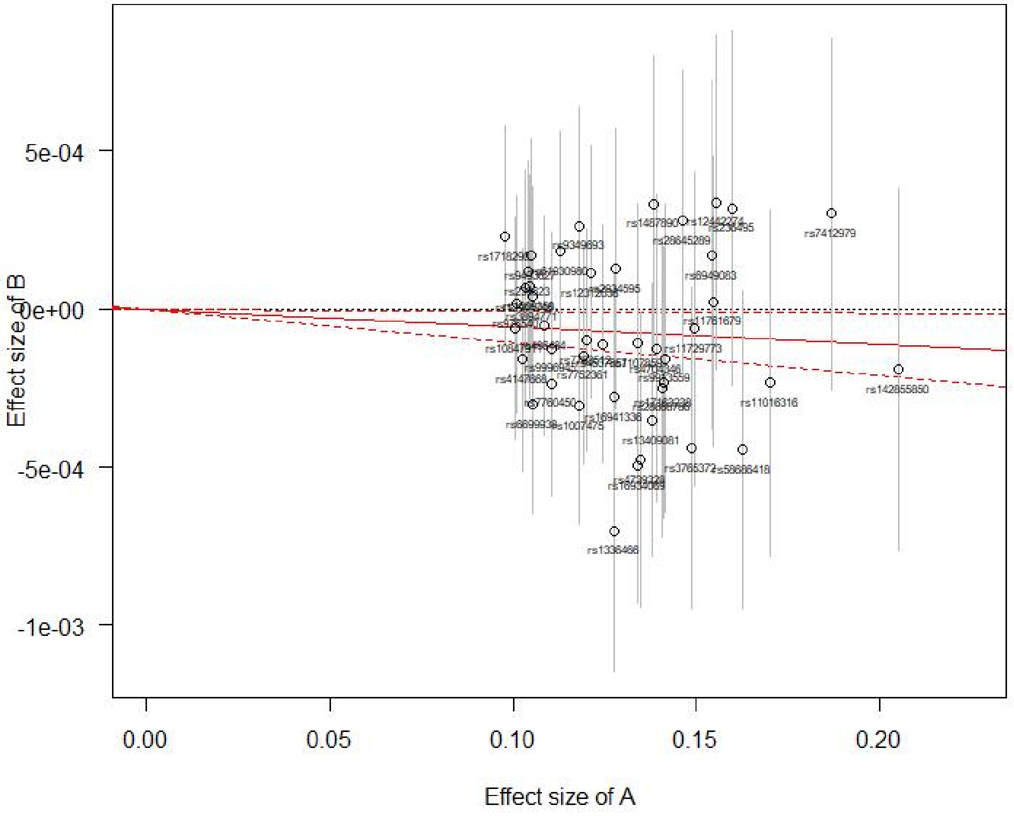

5. Gut microbiota abundance (genus Desulfovibrio id.3173)

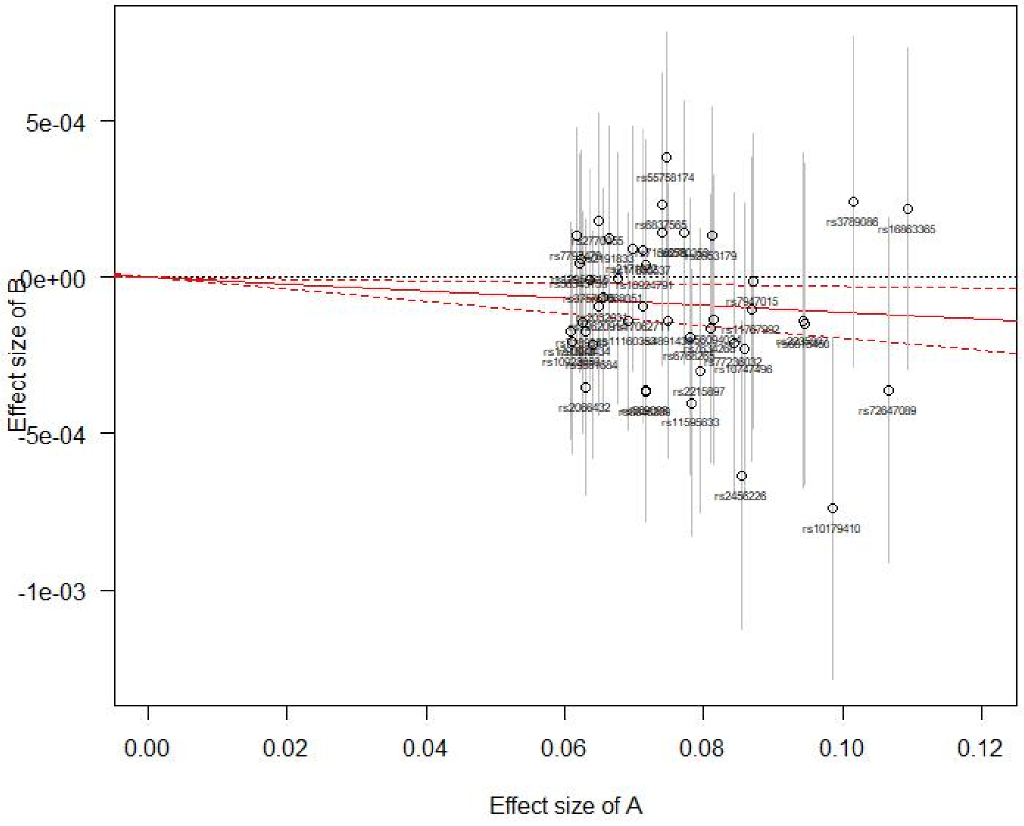

6. Gut microbiota abundance (genus Eubacterium ventriosum group id.11341)

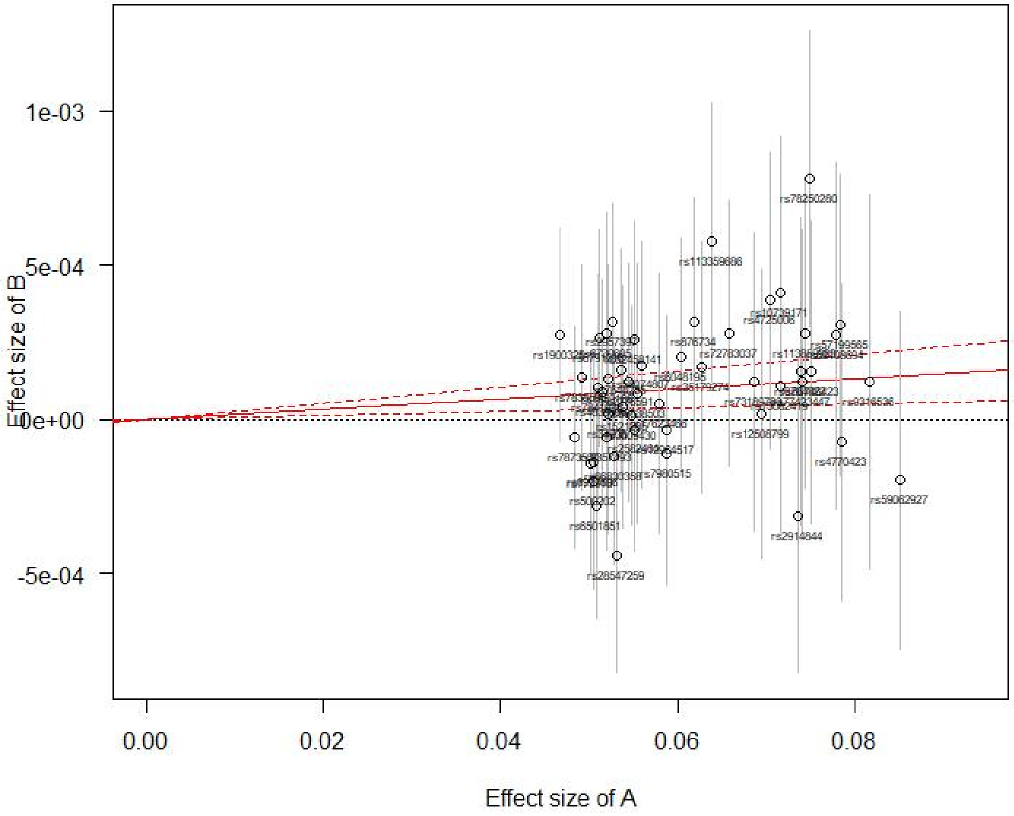

7. Gut microbiota abundance (genus Ruminiclostridium9 id.11357)

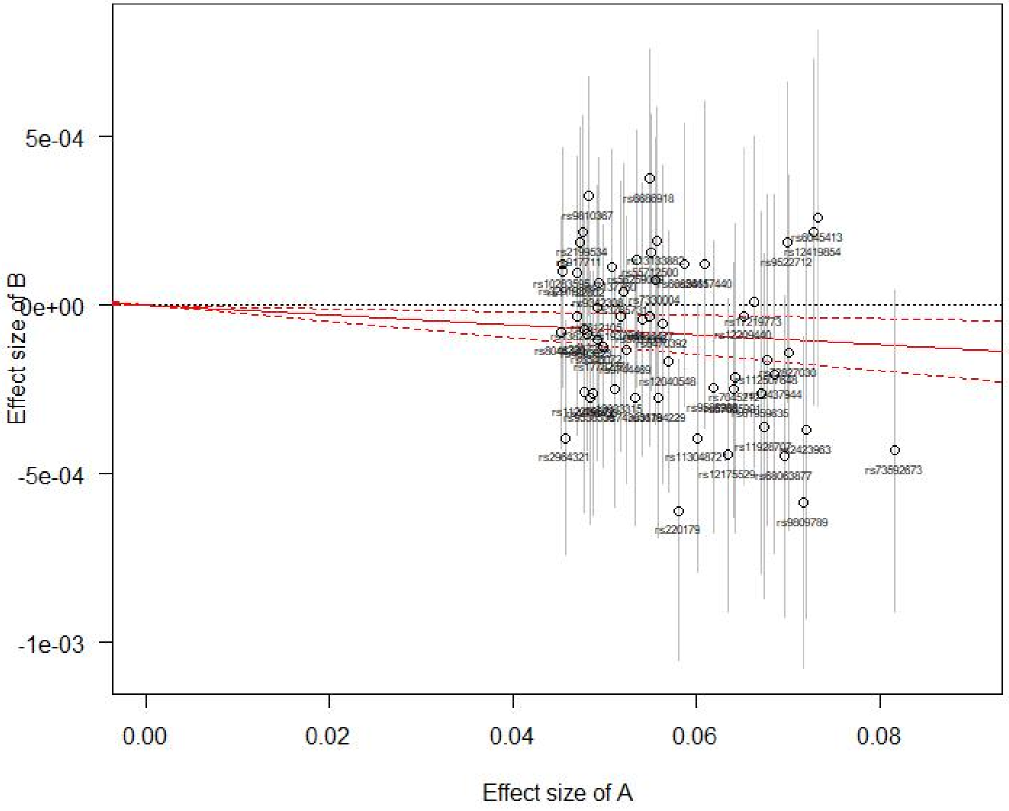

8. Gut microbiota abundance (genus Ruminococcaceae NK4A214 group id.11358)

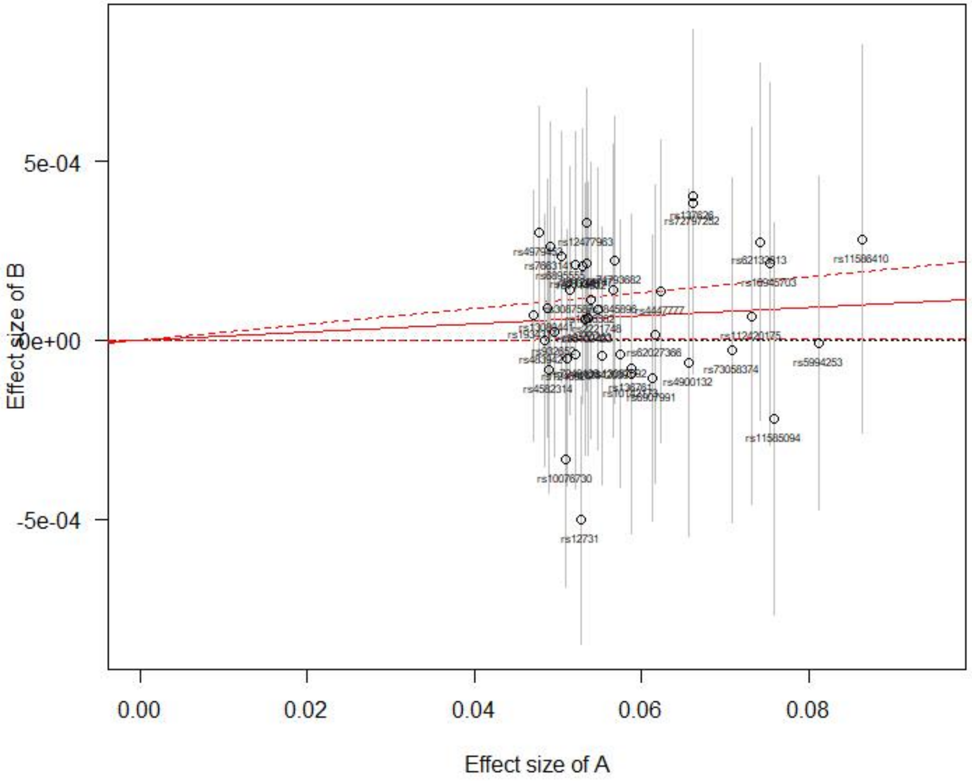

9. Gut microbiota abundance (genus Ruminococcus gauvreauii group id.11342)

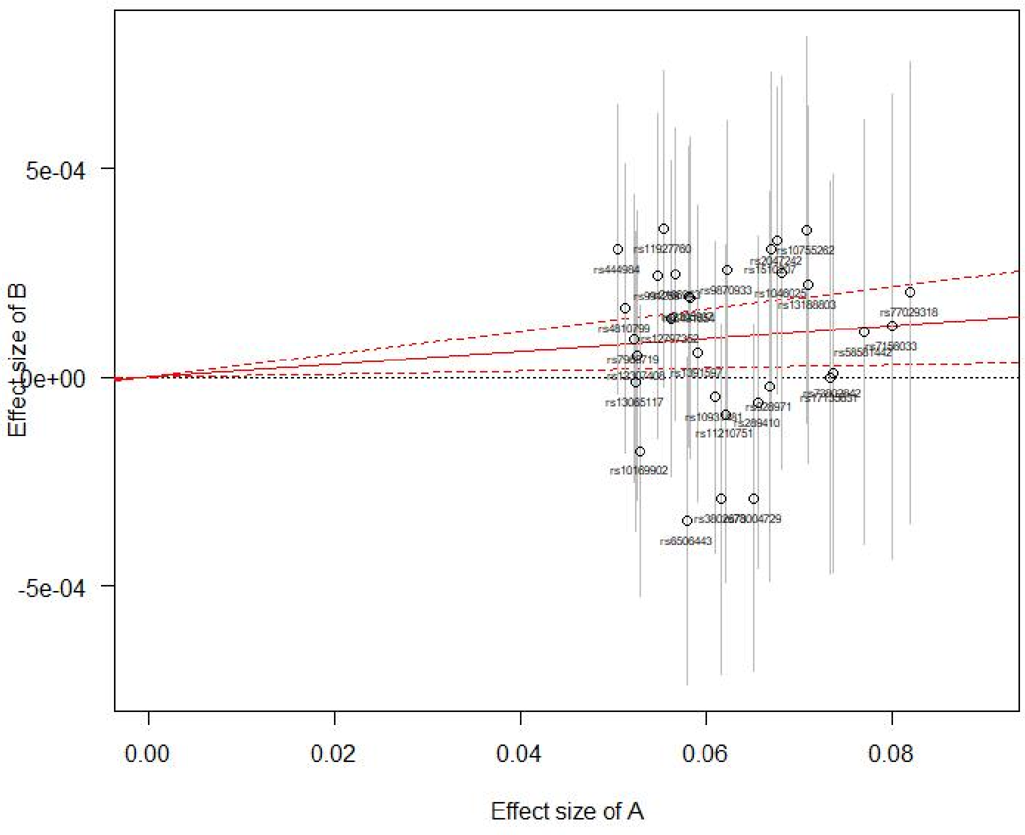

10. Gut microbiota abundance (genus Ruminococcus gnavus group id.14376)

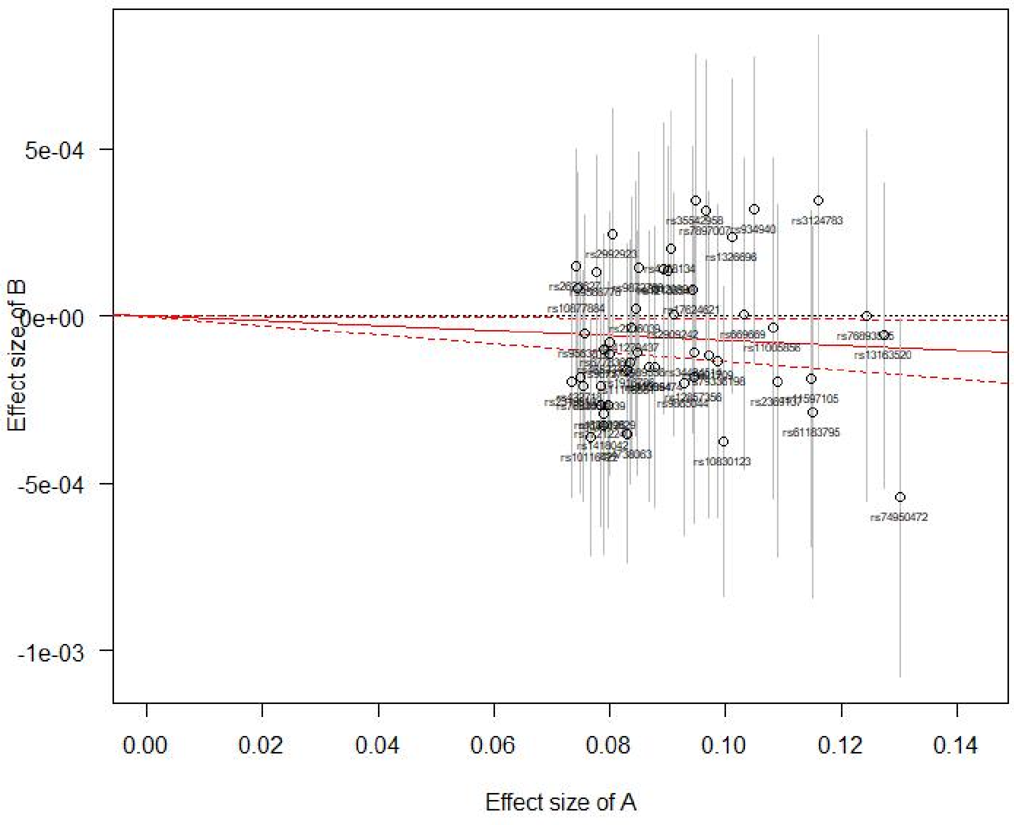

11. Gut microbiota abundance (genus Sutterella id.2896)

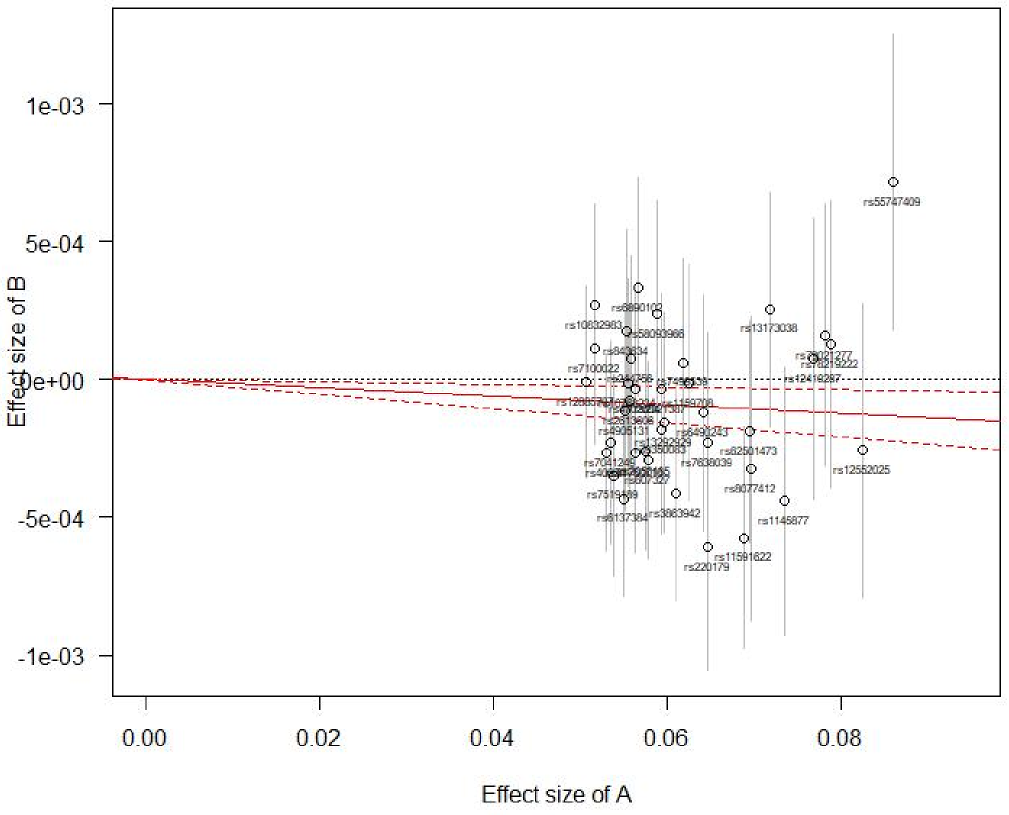

12. Gut microbiota abundance (order Bacteroidales id.913)

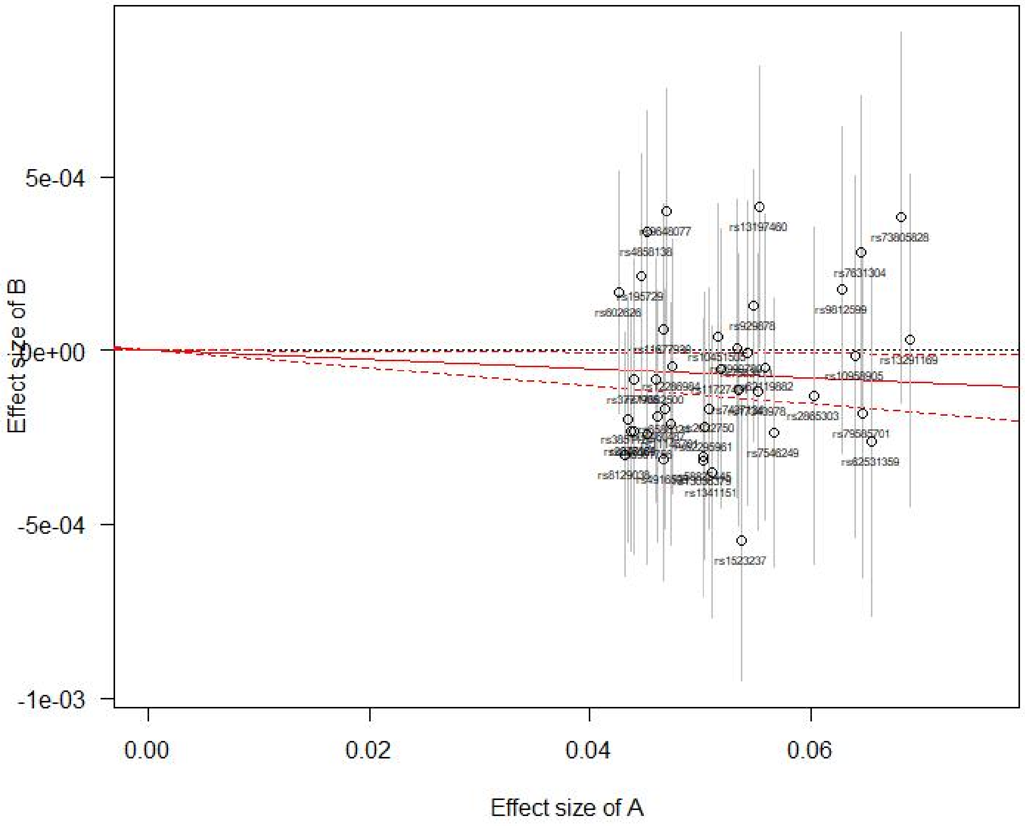

13. Gut microbiota abundance (order Victivallales id.2254)

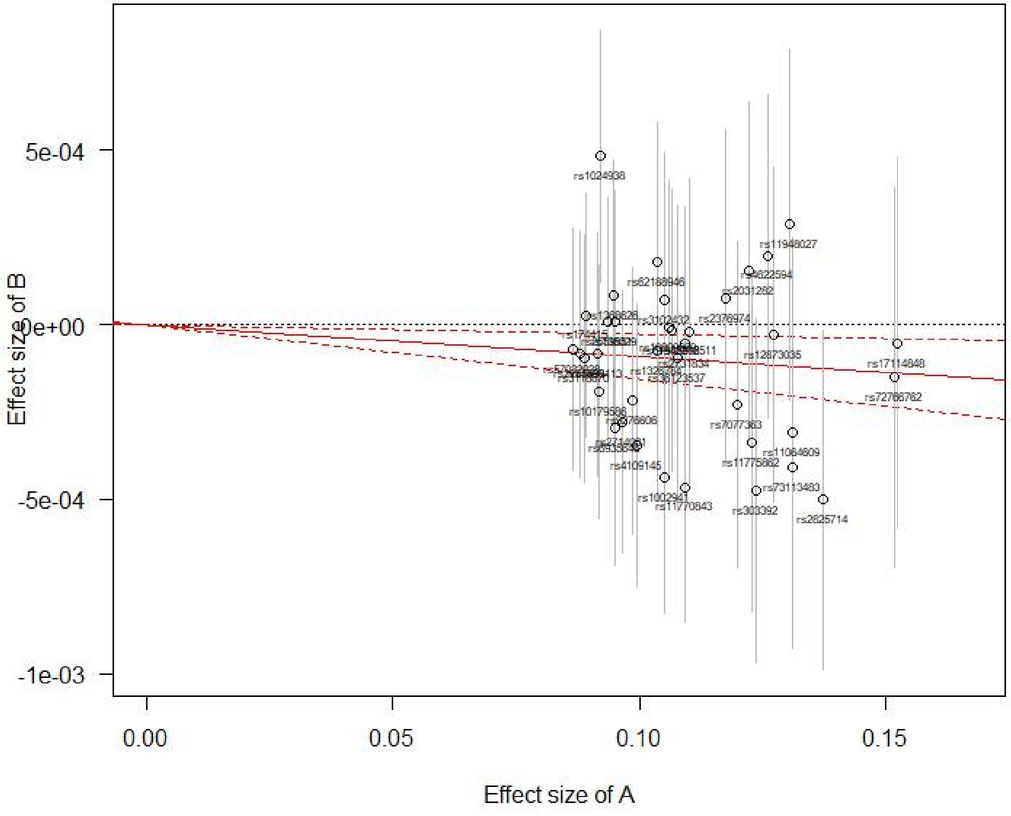

E. Duodenal ulcer

1. Gut microbiota abundance (class Gammaproteobacteria id.3303)

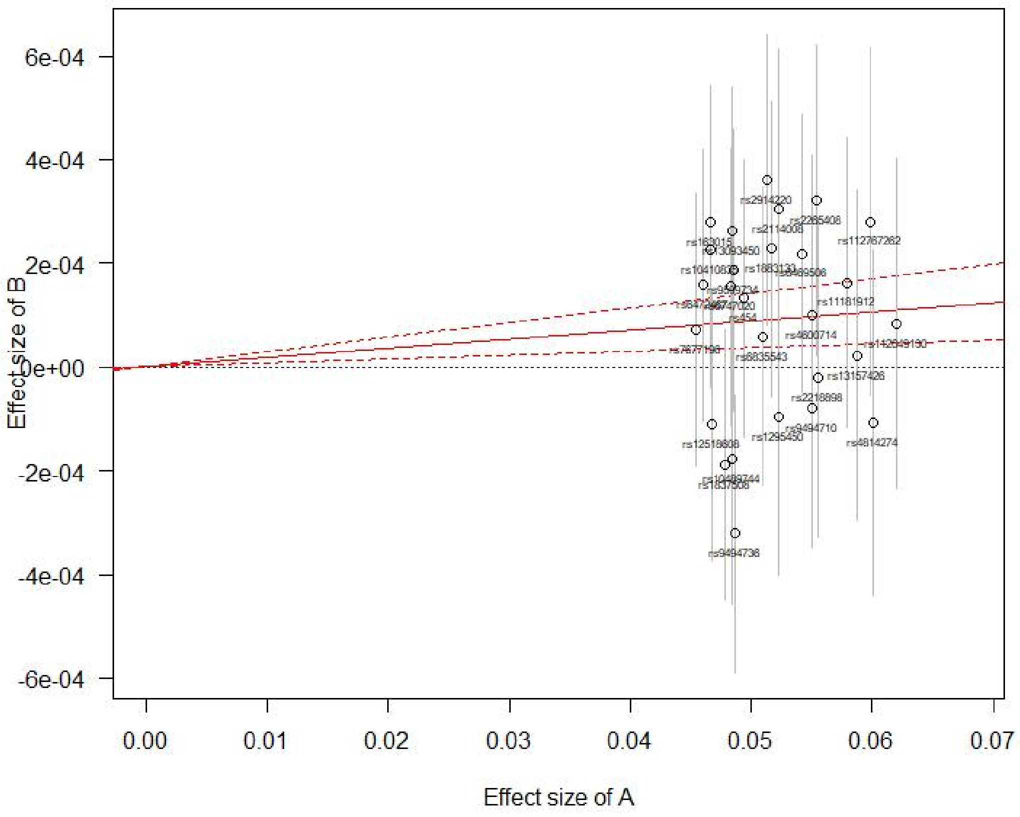

2. Gut microbiota abundance (class Verrucomicrobiae id.4029)

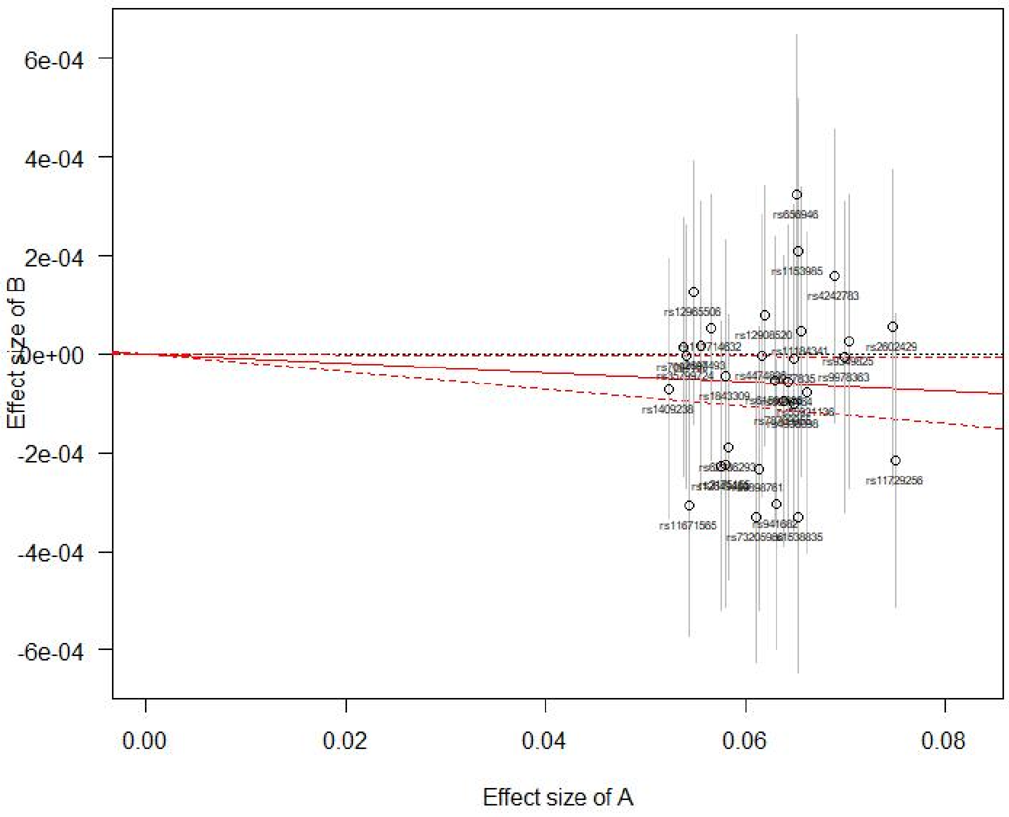

3. Gut microbiota abundance (family Bacteroidaceae id.917)

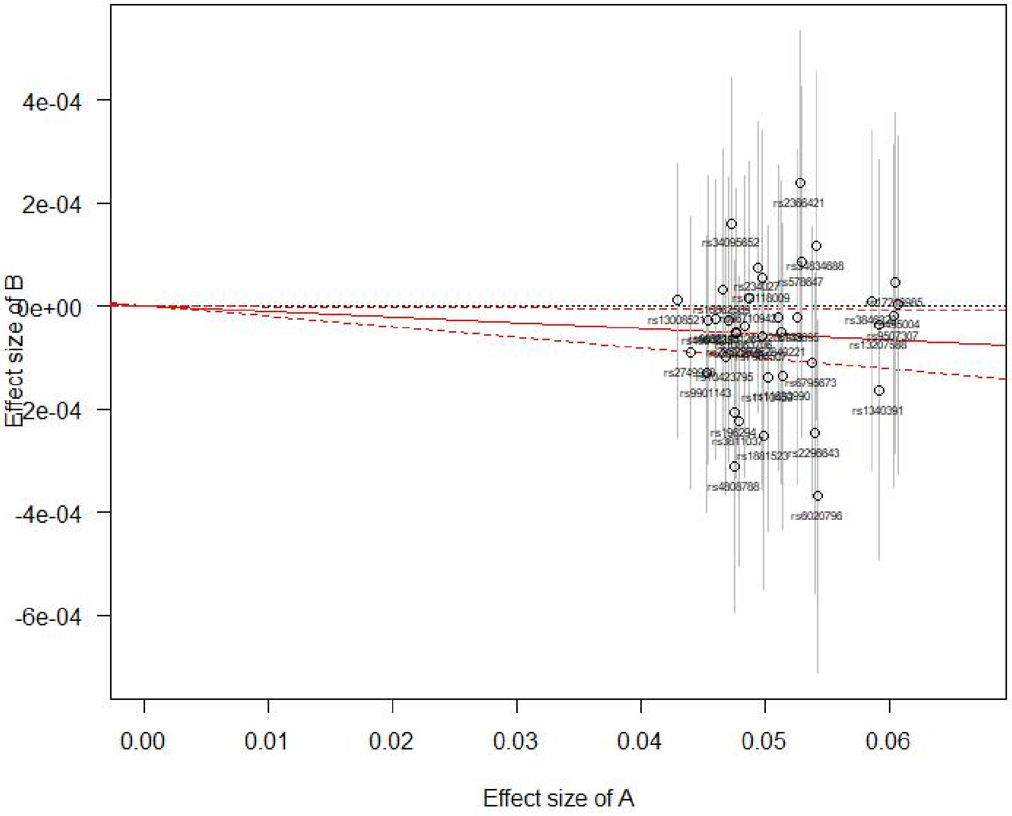

4. Gut microbiota abundance (family Peptostreptococcaceae id.2042)

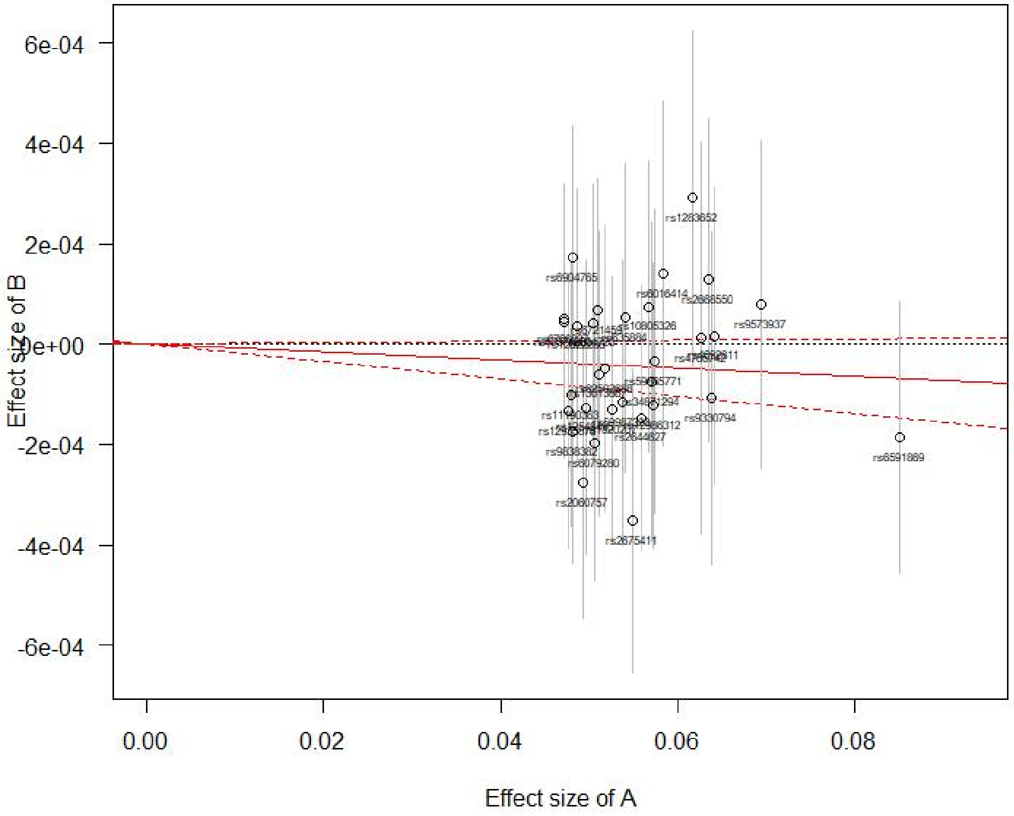

5. Gut microbiota abundance (family Porphyromonadaceae id.943)

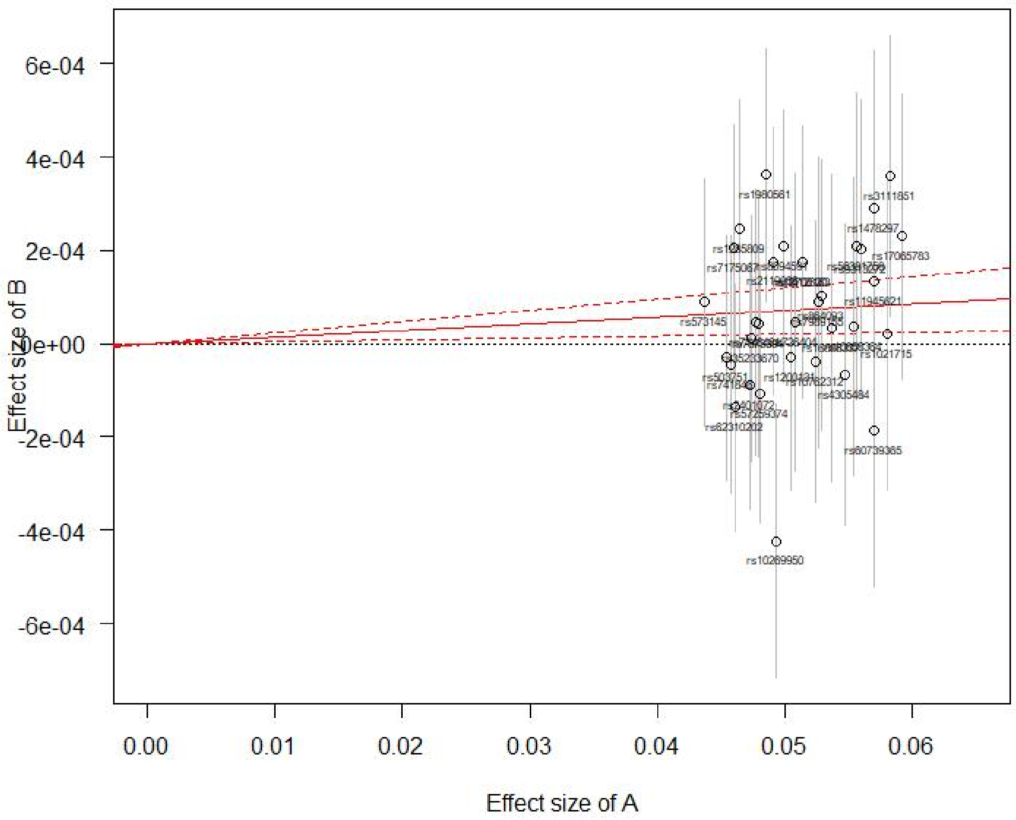

6. Gut microbiota abundance (family Verrucomicrobiaceae id.4036)

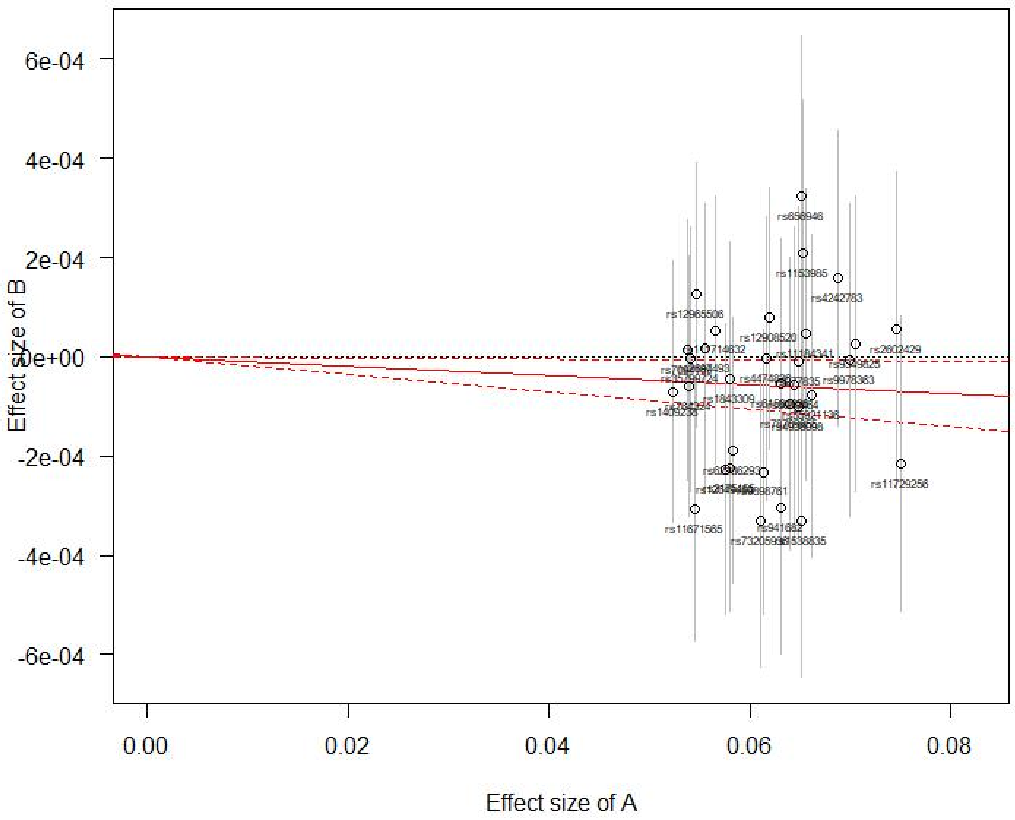

7. Gut microbiota abundance (genus Bacteroides id.918)

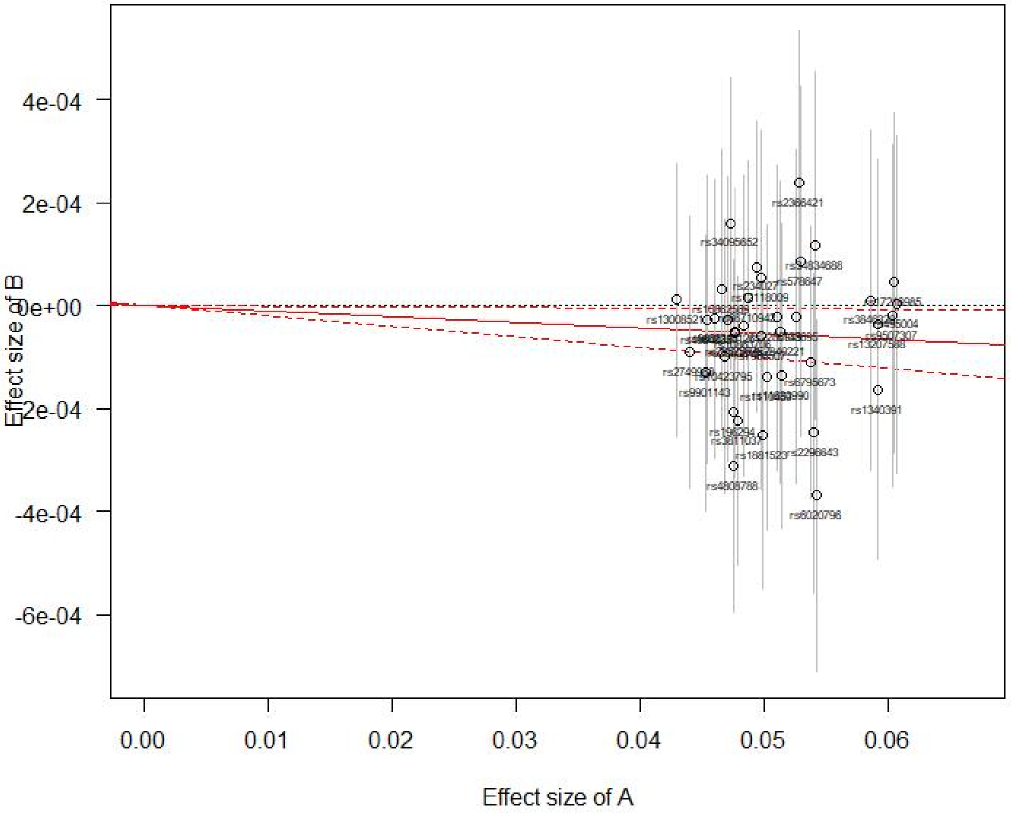

8. Gut microbiota abundance (genus Family XIII UCG001 id.11294)

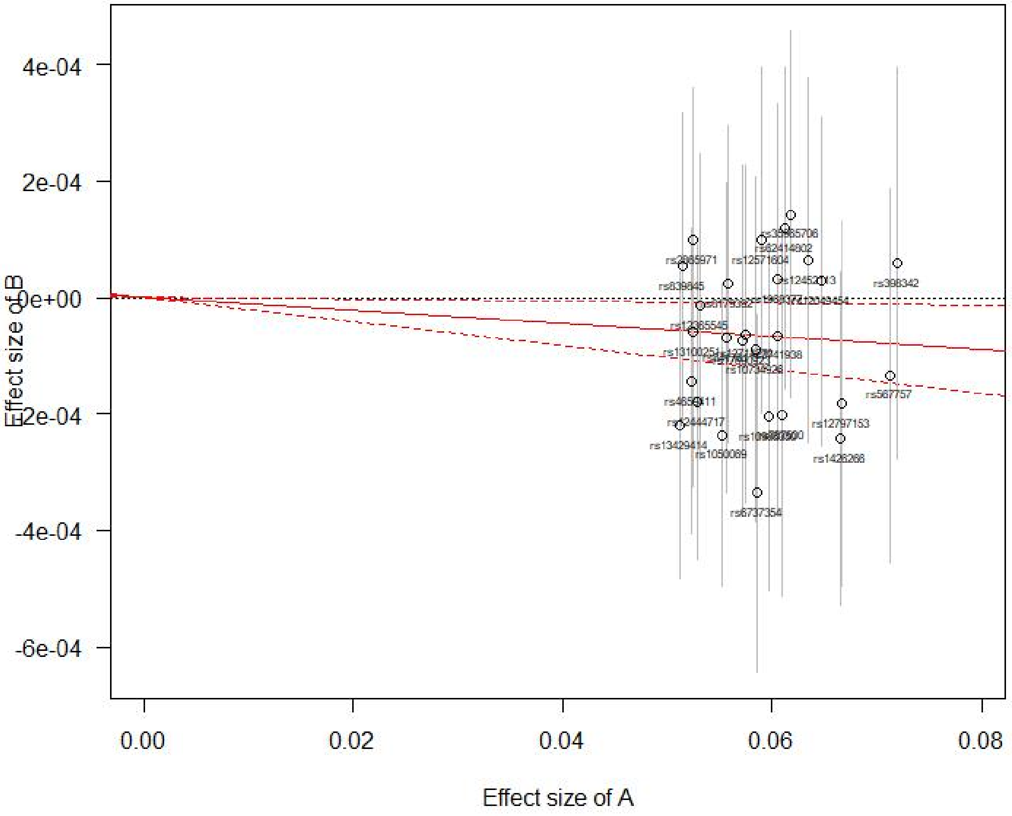

9. Gut microbiota abundance (genus Ruminococcus gauvreauii group id.11342)

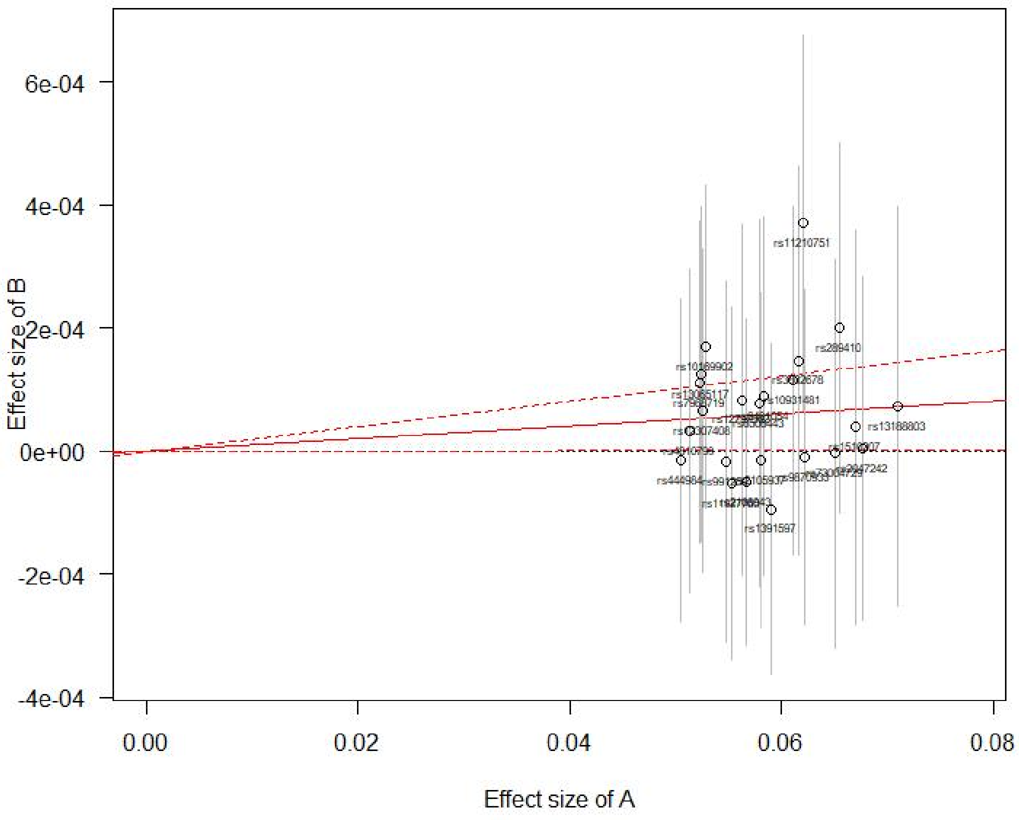

10. Gut microbiota abundance (order Verrucomicrobiales id.4030)

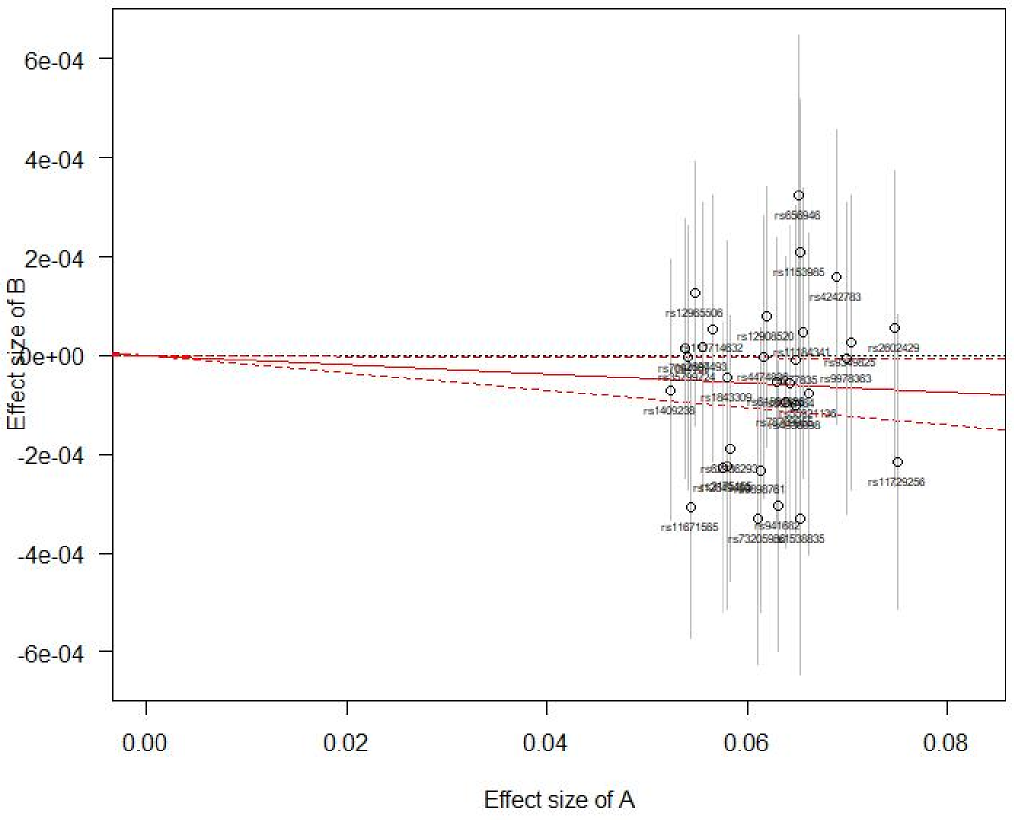

11. Gut microbiota abundance (genus Erysipelotrichaceae UCG003 id.11384)

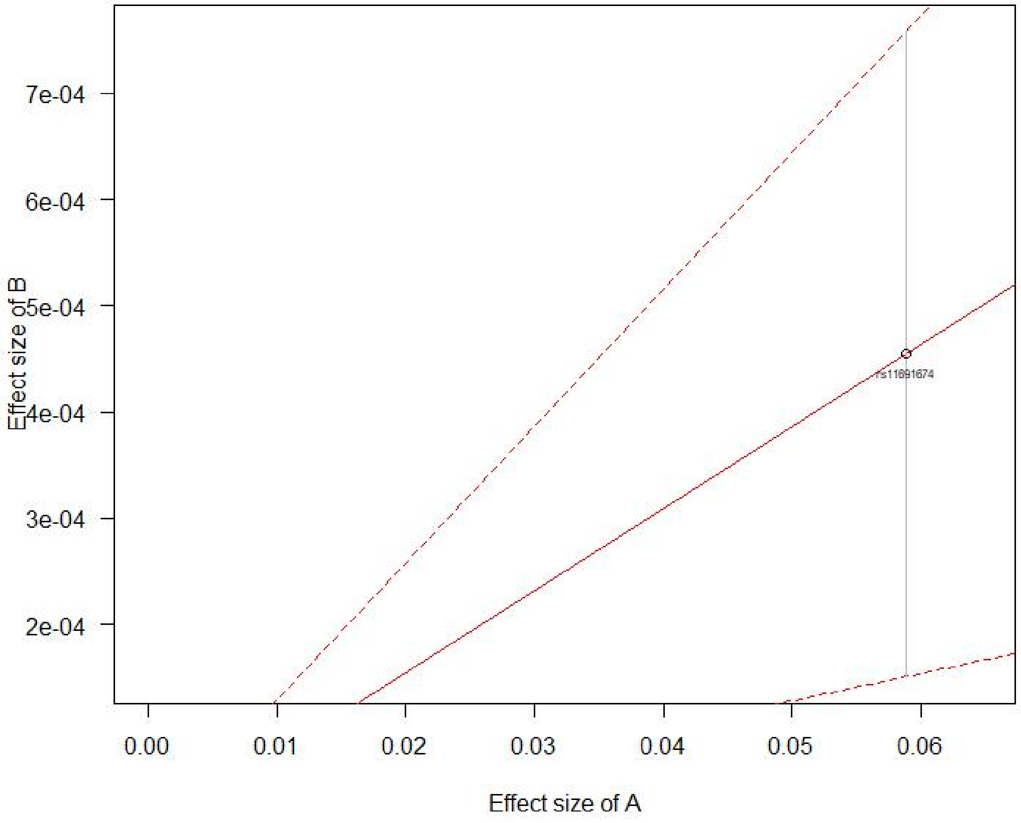

F. Chronic gastritis

1. Gut microbiota abundance (class Verrucomicrobiae id.4029)

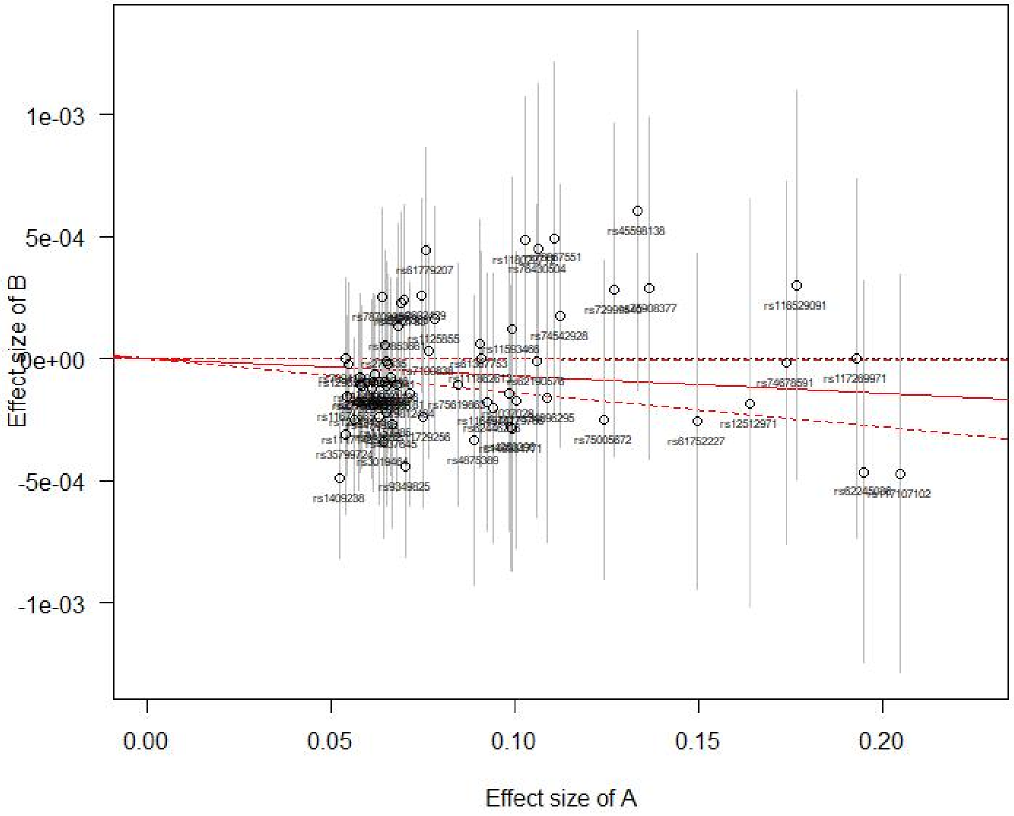

2. Gut microbiota abundance (genus Butyricicoccus id.2055)

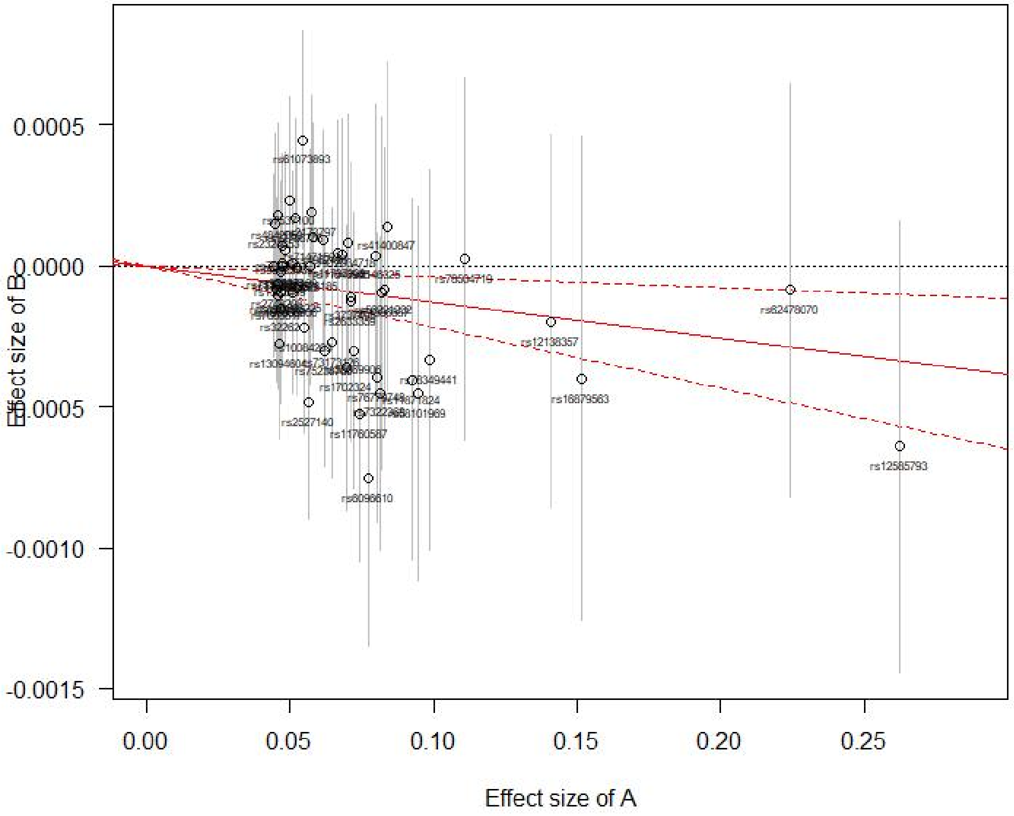

3. Gut microbiota abundance (genus Eubacterium fissicatena group id.14373)

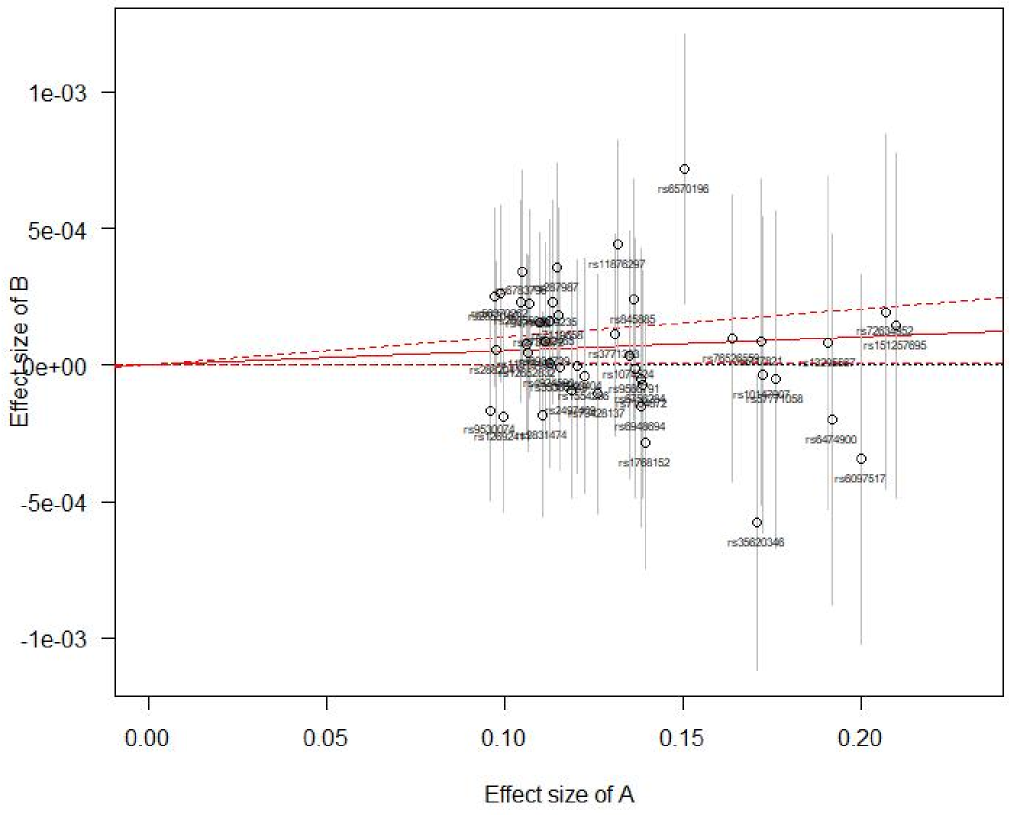

4. Gut microbiota abundance (genus Lachnospiraceae UCG010 id.11330)

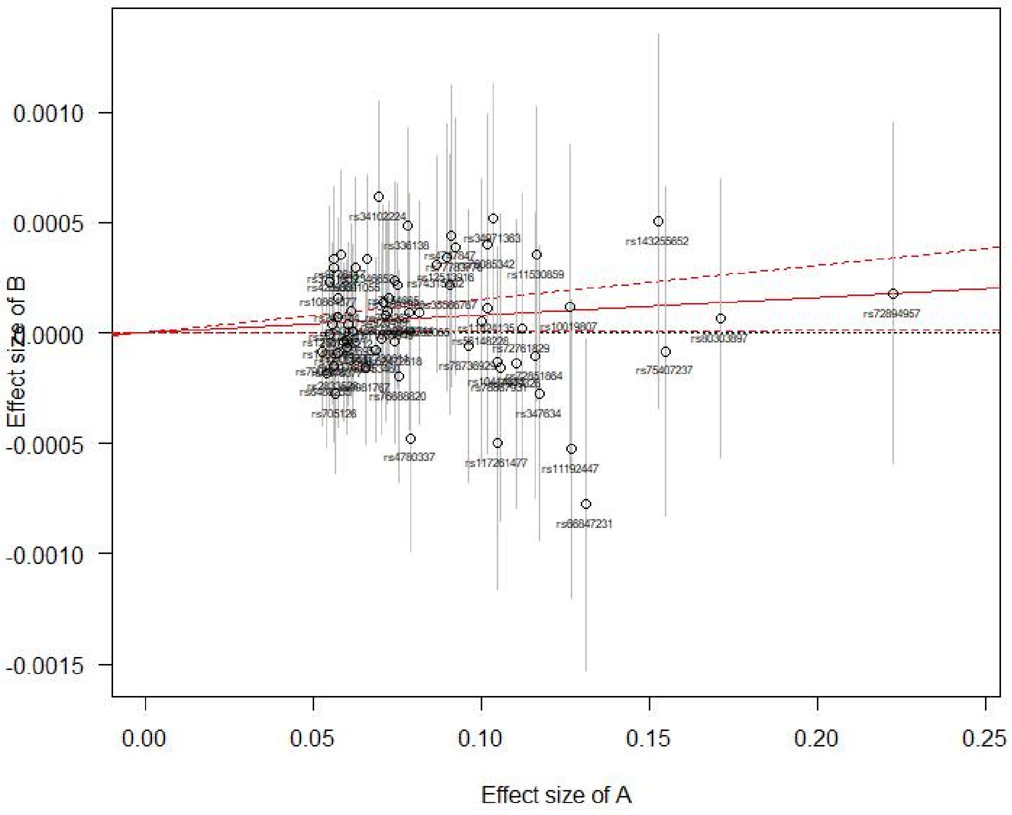

5. Gut microbiota abundance (genus Peptococcus id.2037)

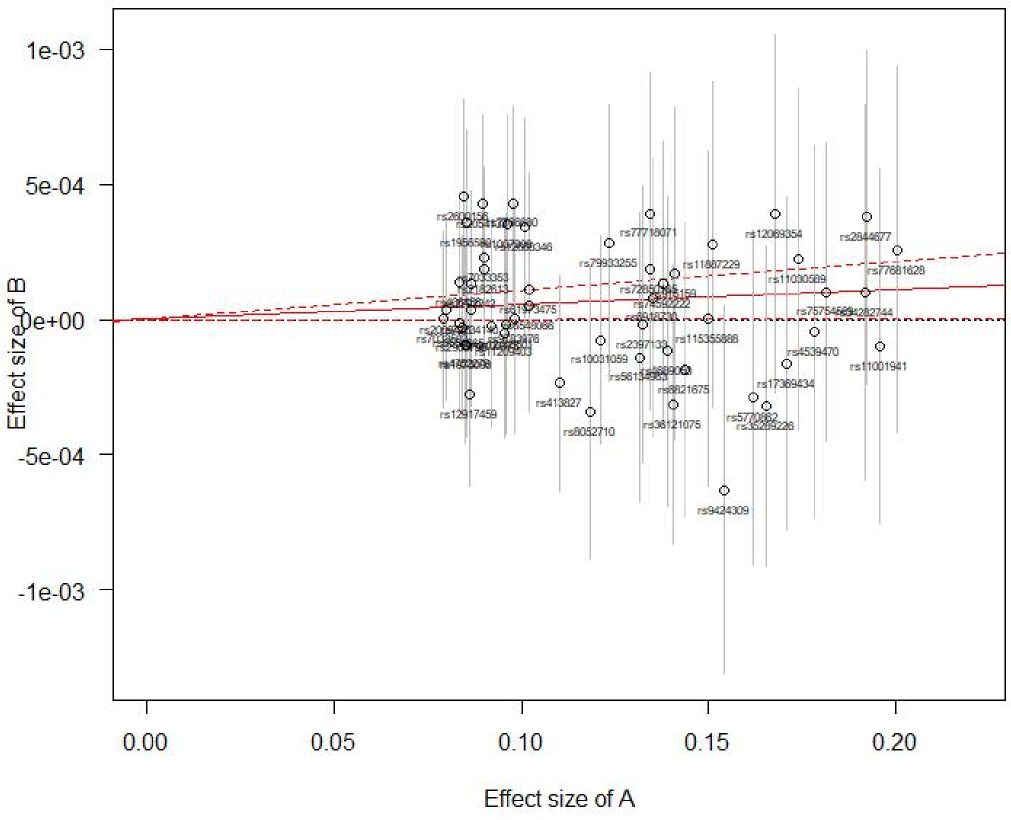

6. Gut microbiota abundance (genus Prevotella7 id.11182)

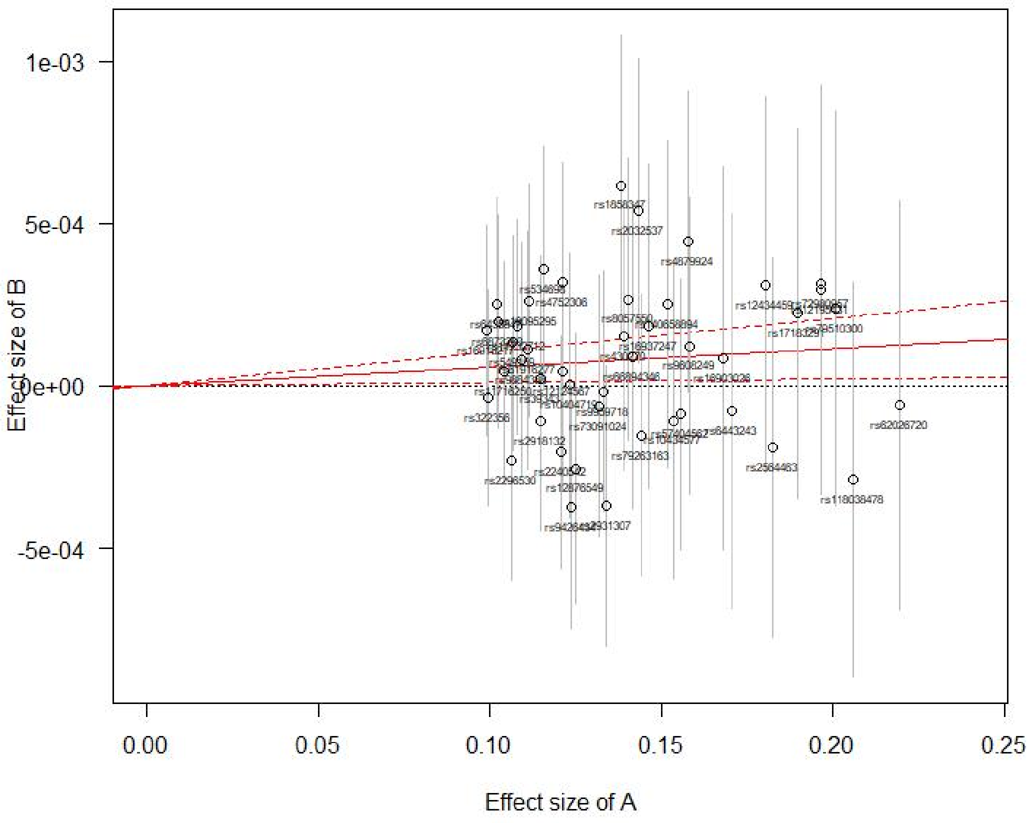

7. Gut microbiota abundance (genus Ruminococcaceae UCG010 id.11367)

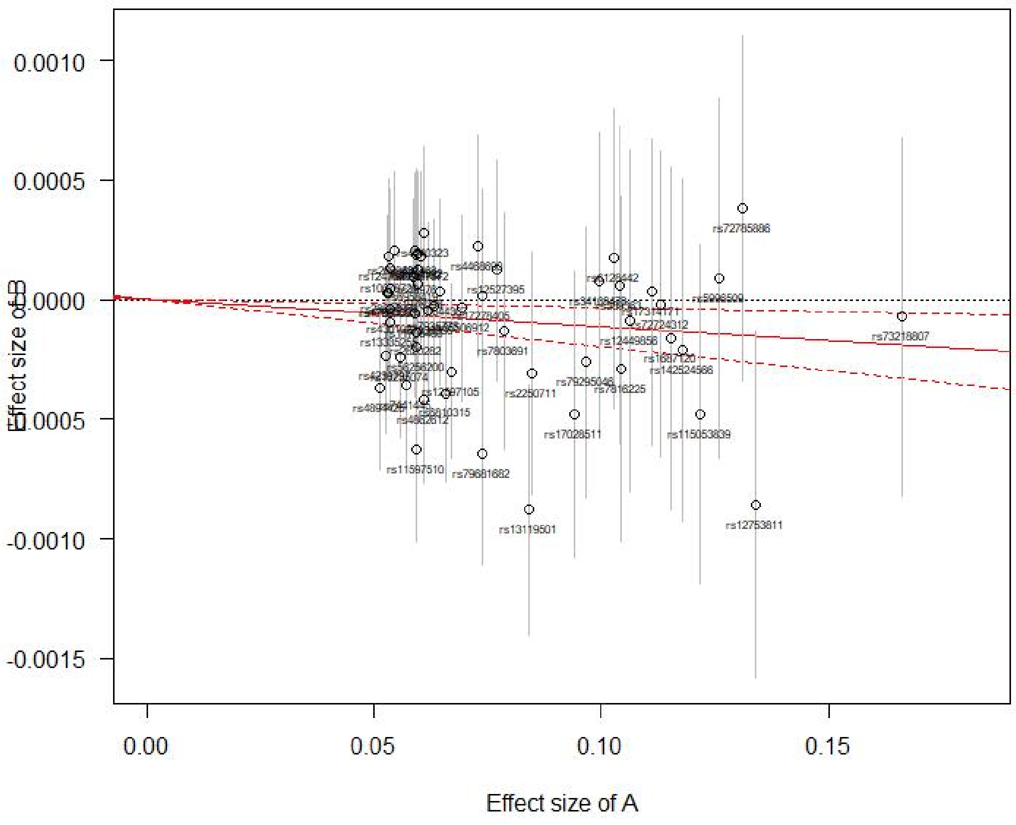

8. Gut microbiota abundance (genus Victivallis id.2256)

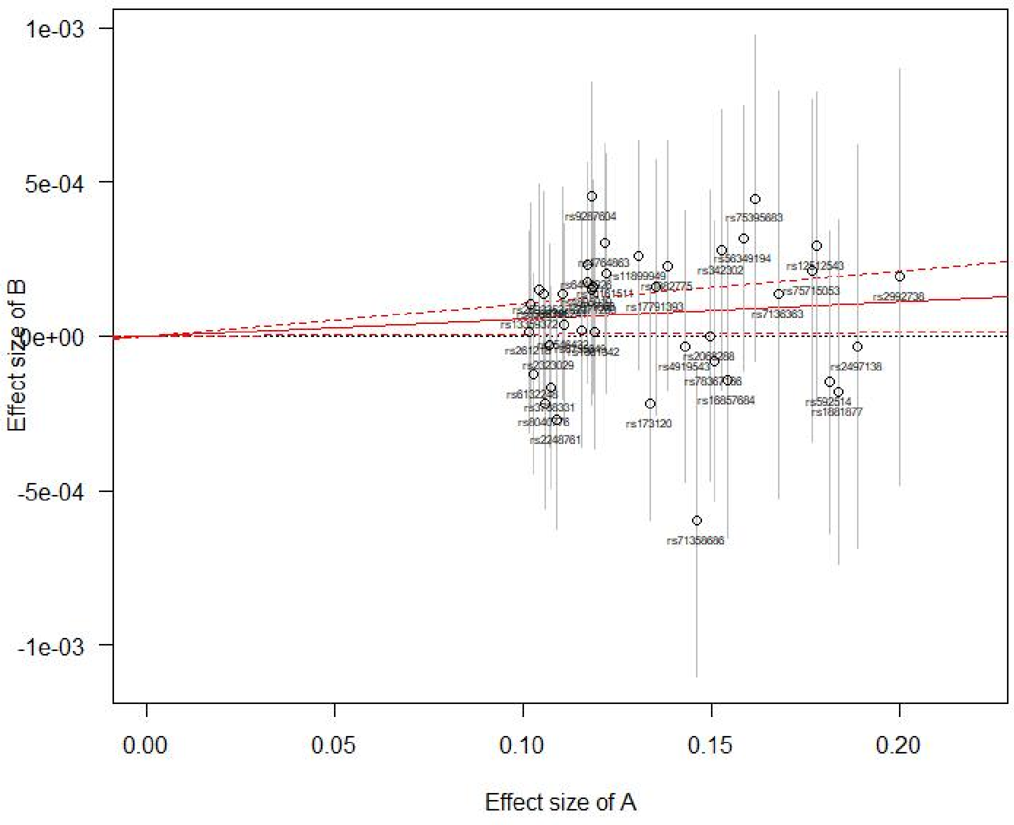

9. Gut microbiota abundance (order Verrucomicrobiales id.4030)

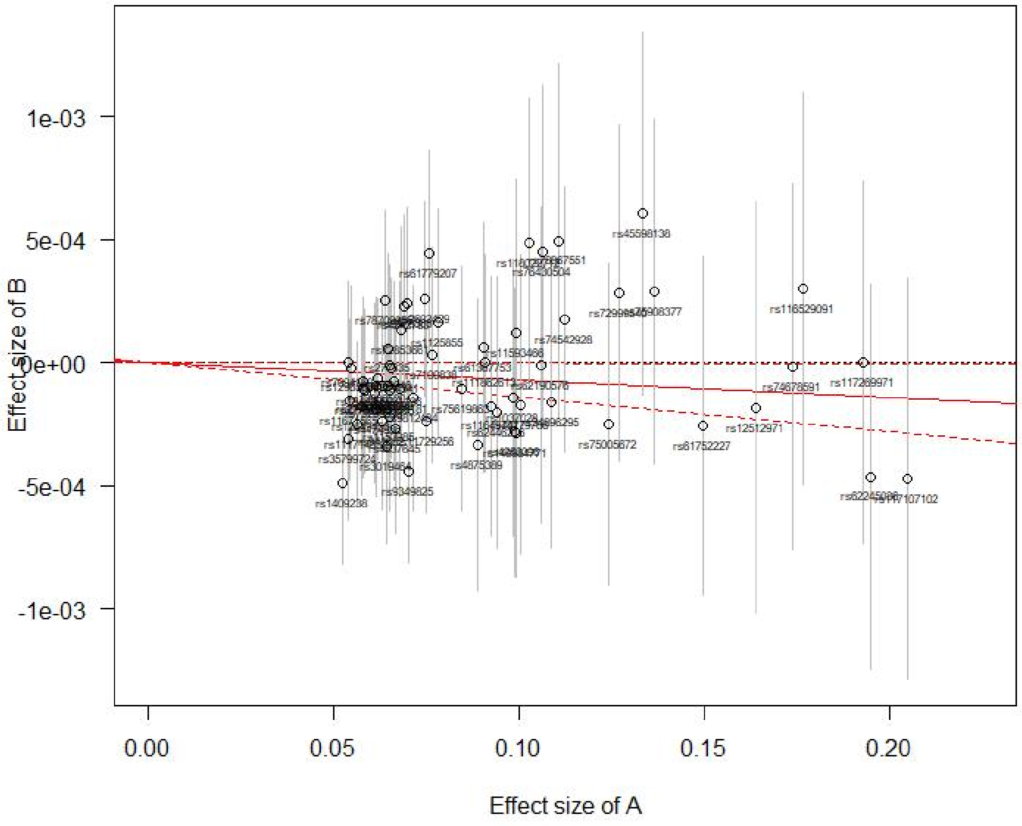

G. Acute gastritis

1. Gut microbiota abundance (class Gammaproteobacteria id.3303)

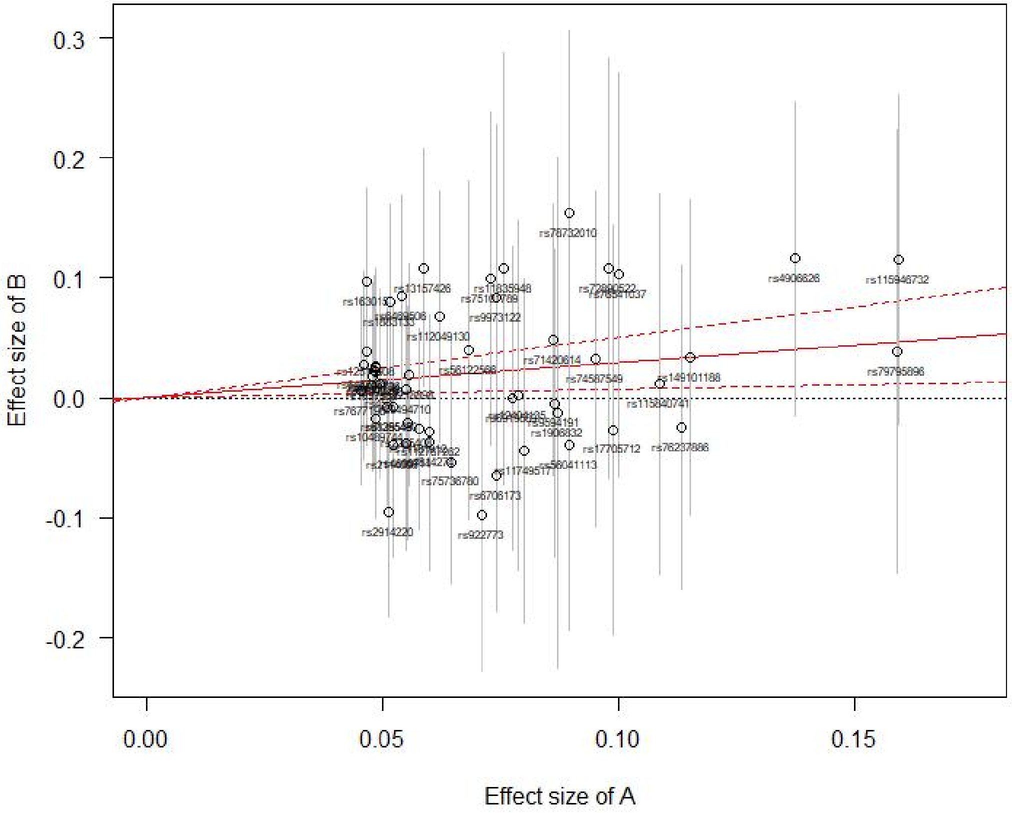

2. Gut microbiota abundance (family Bacteroidaceae id.917)

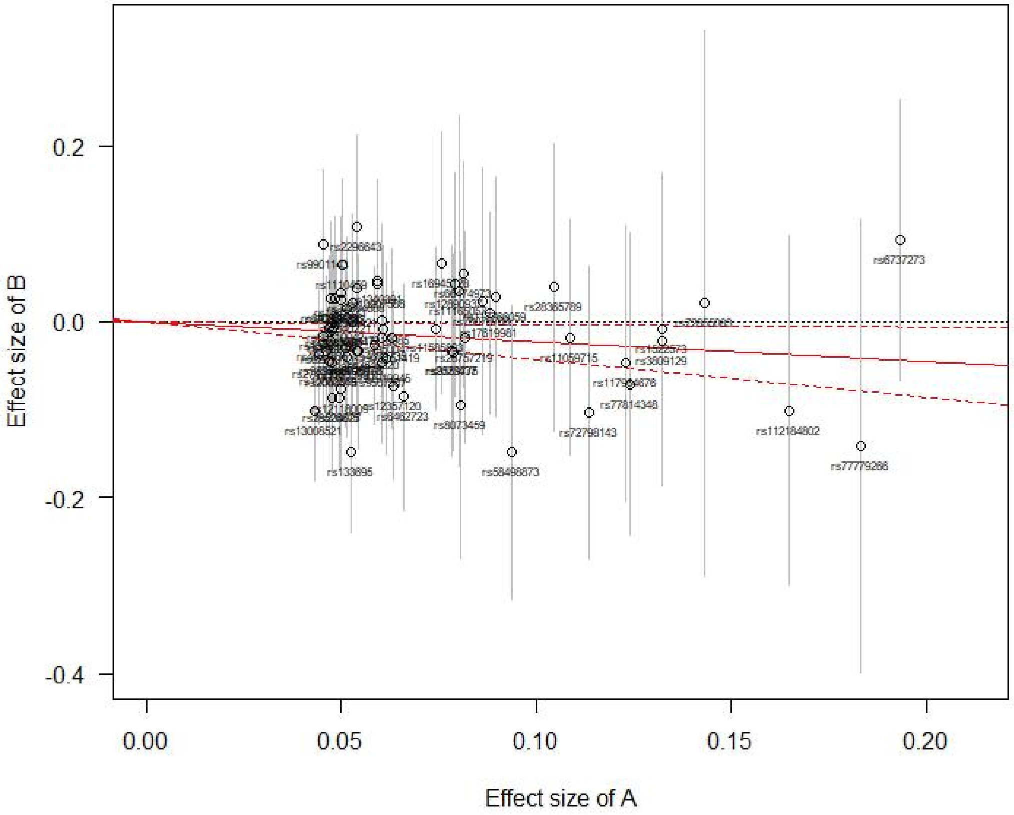

3. Gut microbiota abundance (genus Alloprevotella id.961)

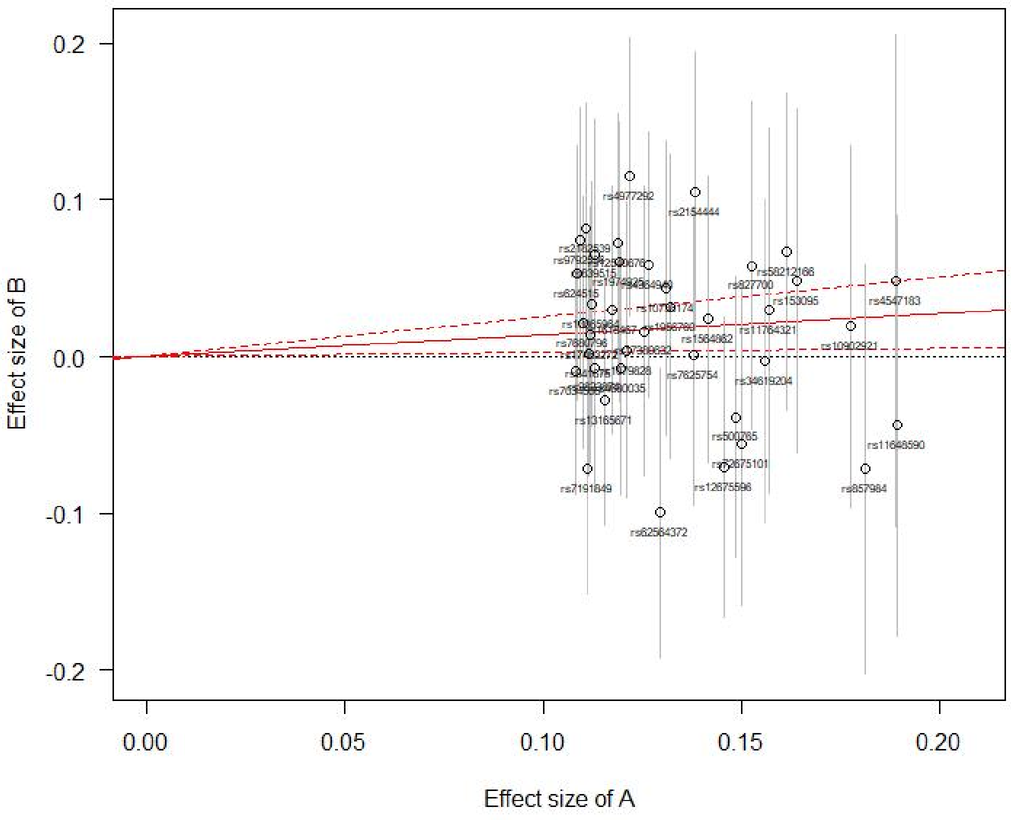

4. Gut microbiota abundance (genus Bacteroides id.918)

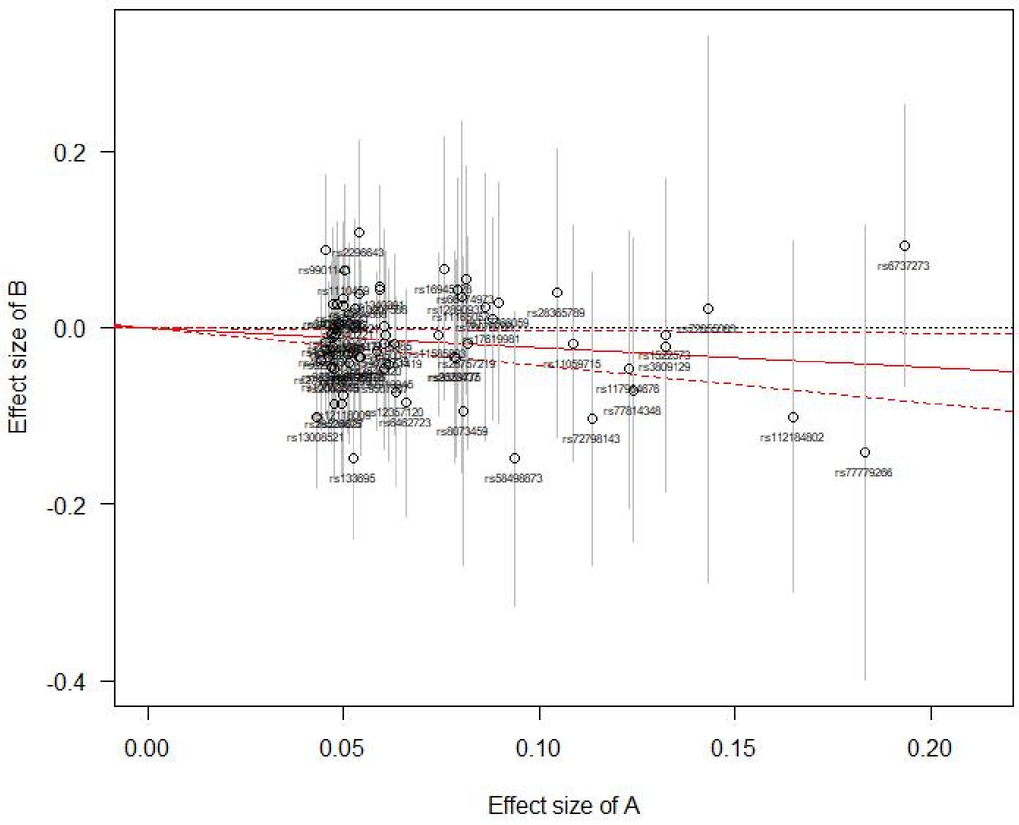

5. Gut microbiota abundance (genus Bilophila id.3170)

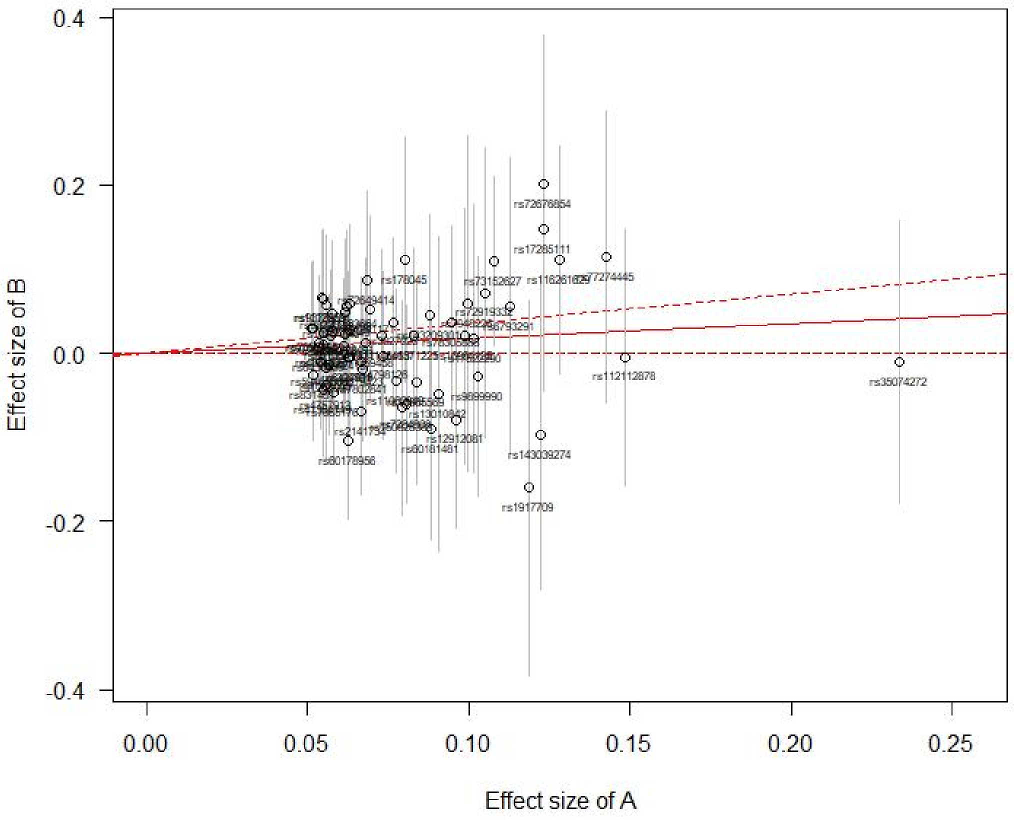

6. Gut microbiota abundance (genus Dialister id.2183)

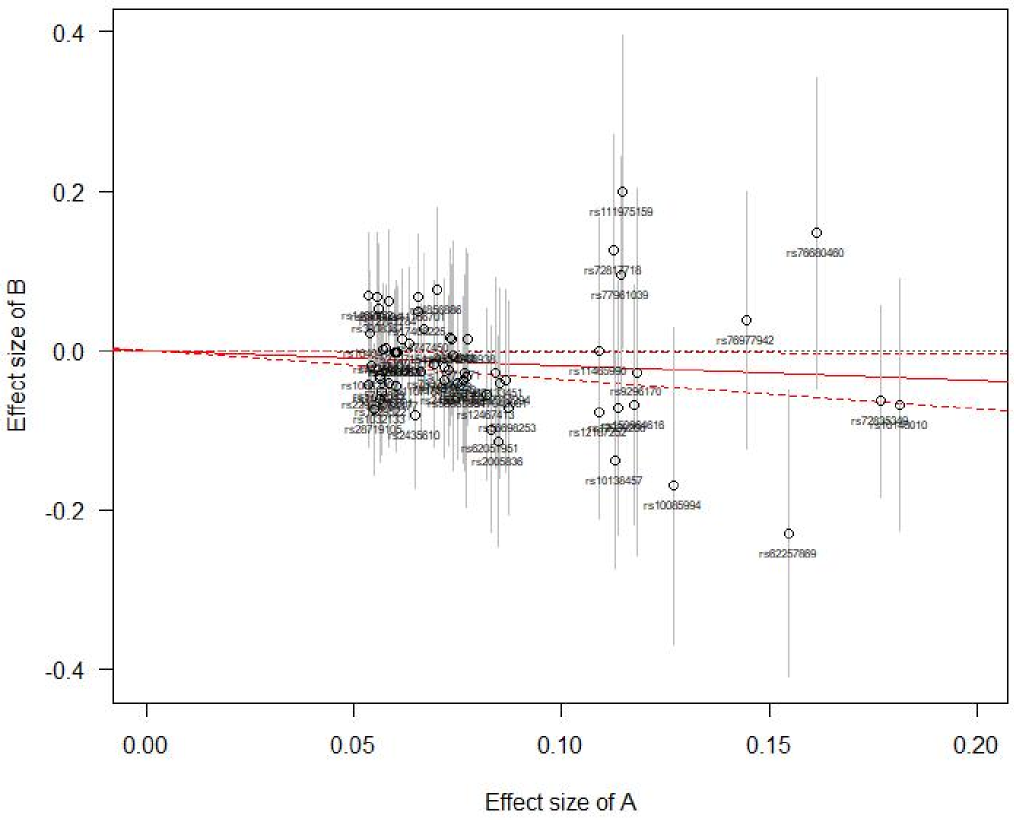

7. Gut microbiota abundance (genus Eubacterium nodatum group id.11297)

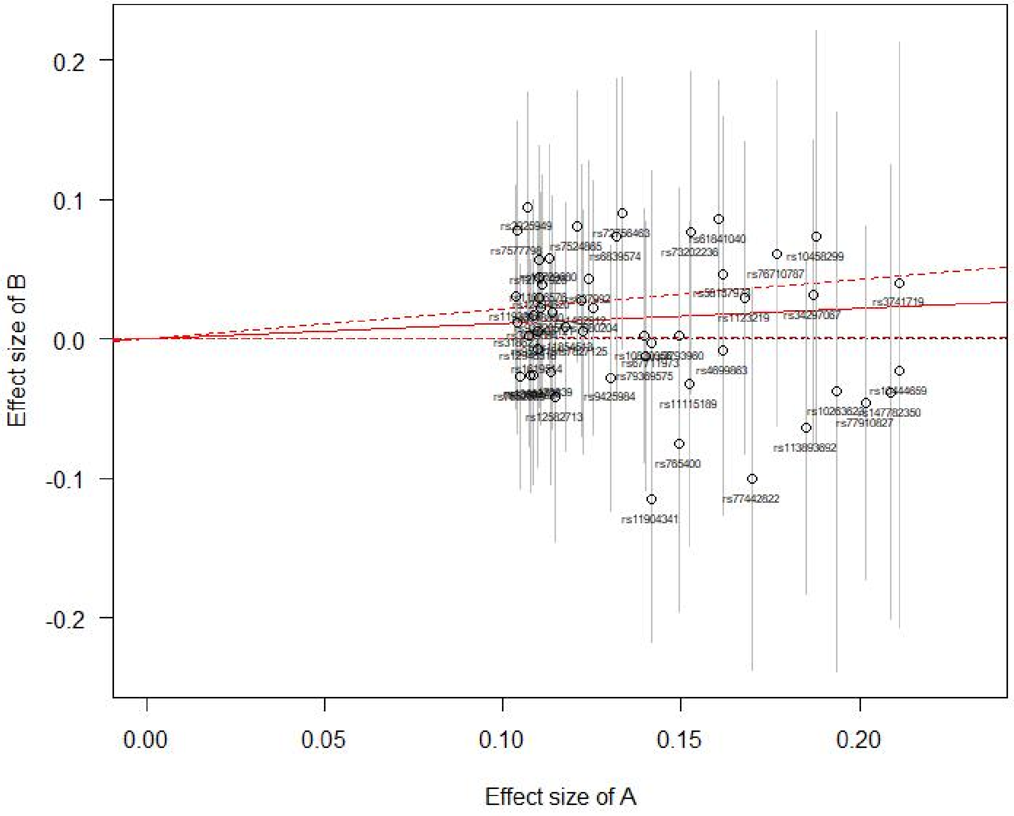

8. Gut microbiota abundance (genus Intestinibacter id.11345)

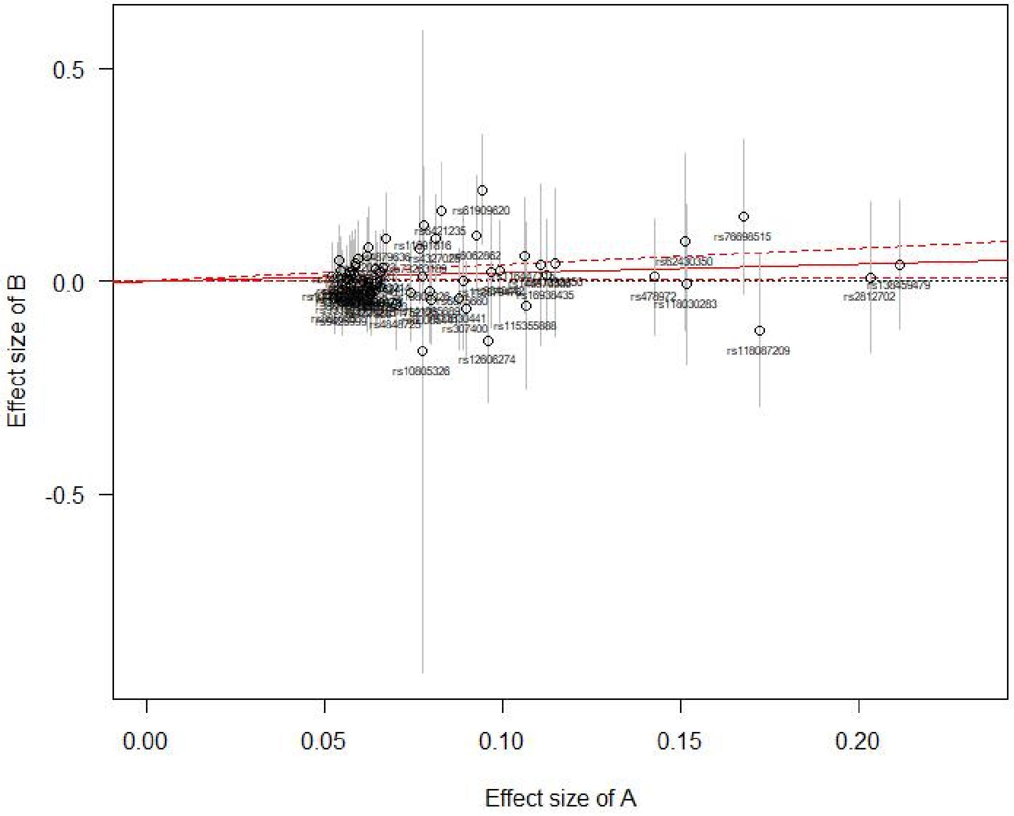

9. Gut microbiota abundance (genus Intestinimonas id.2062)

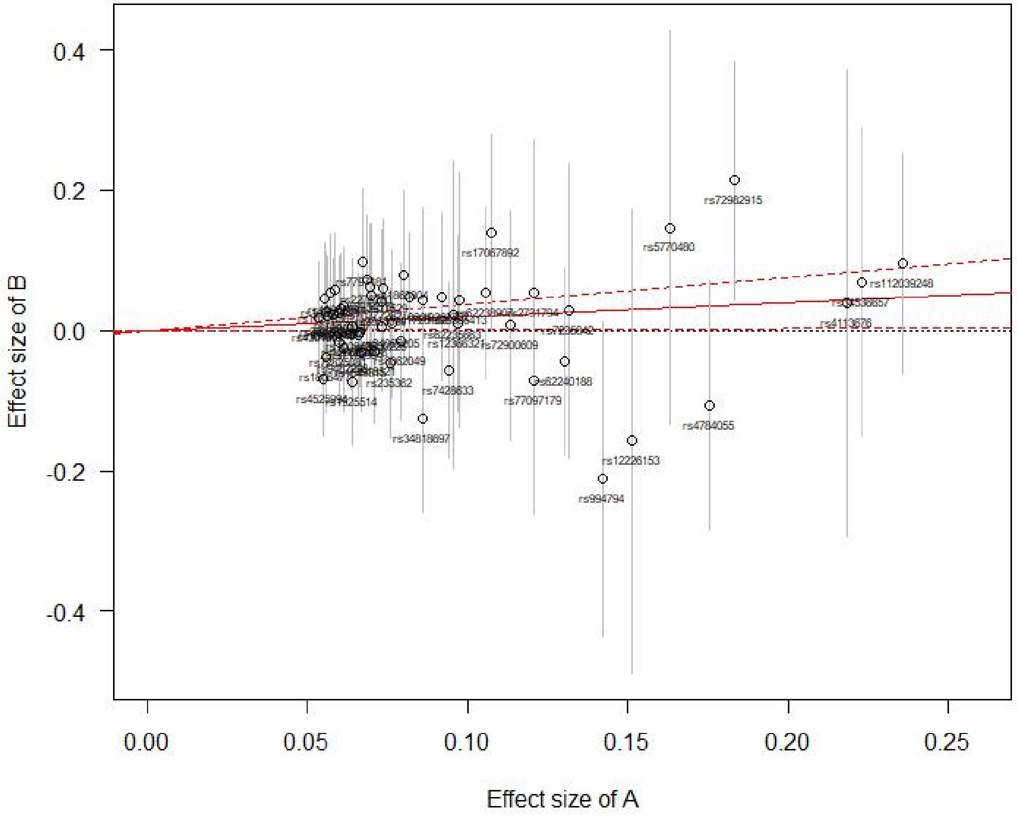

10. Gut microbiota abundance (genus Parabacteroides id.954)

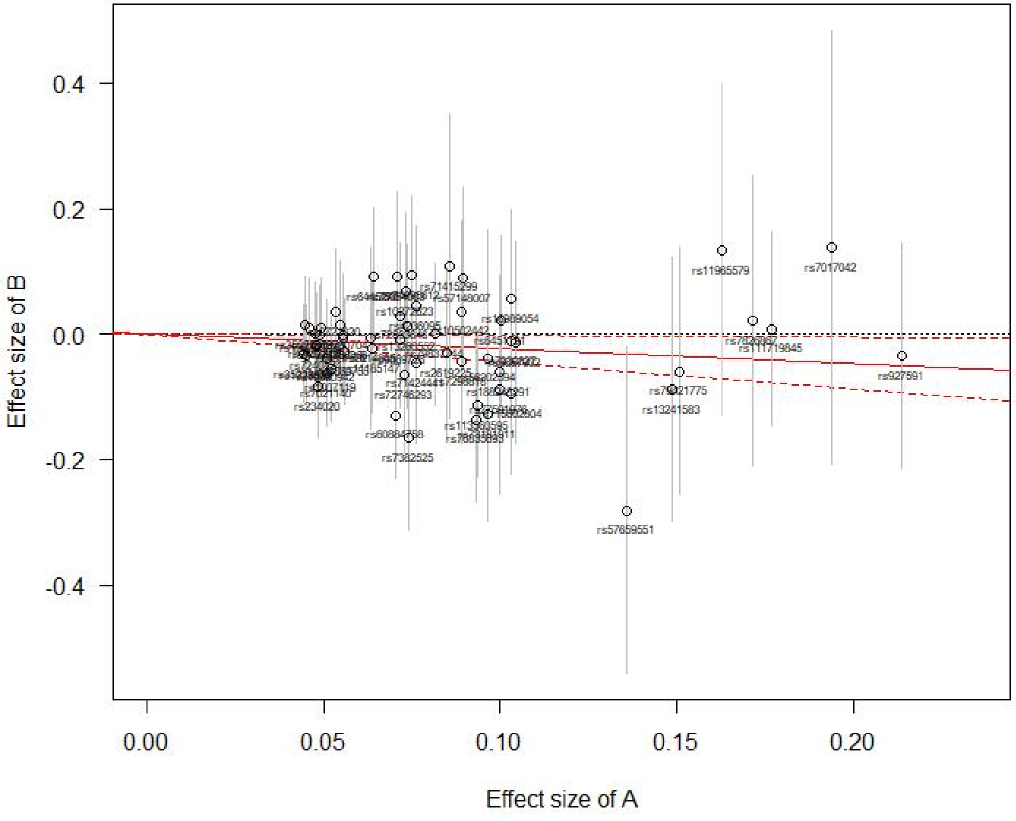

11. Gut microbiota abundance (genus Ruminococcaceae NK4A214 group id.11358)

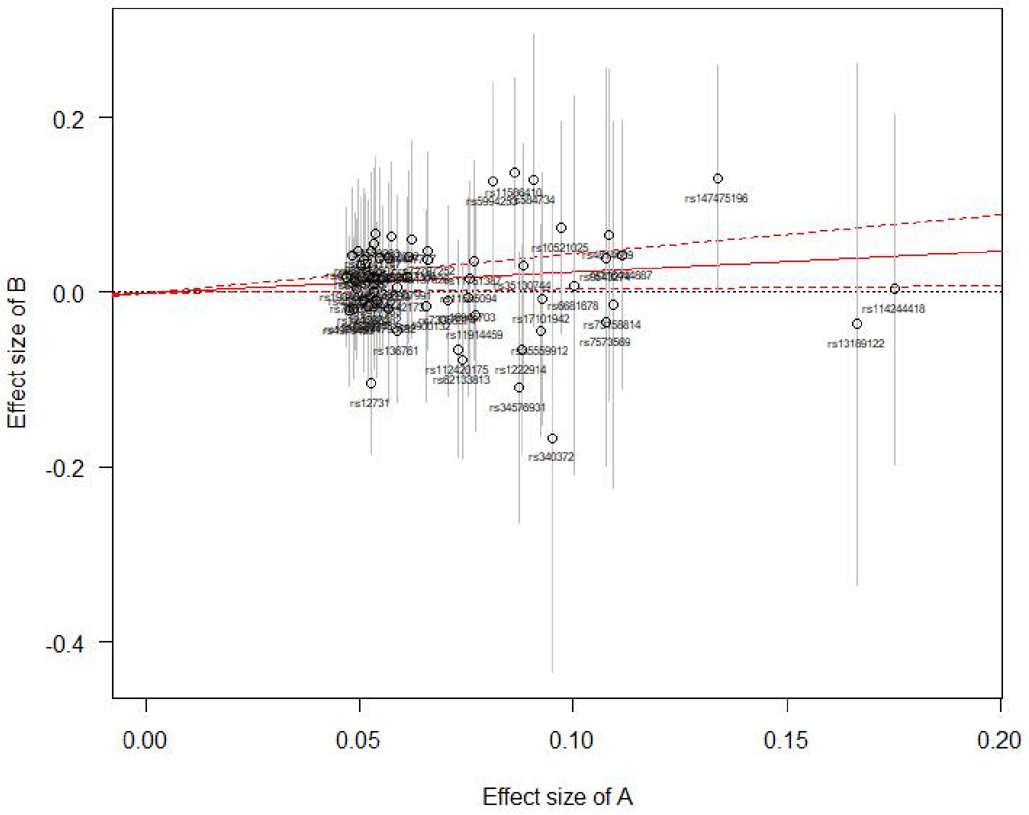

12. Gut microbiota abundance (phylum Verrucomicrobia id.3982)

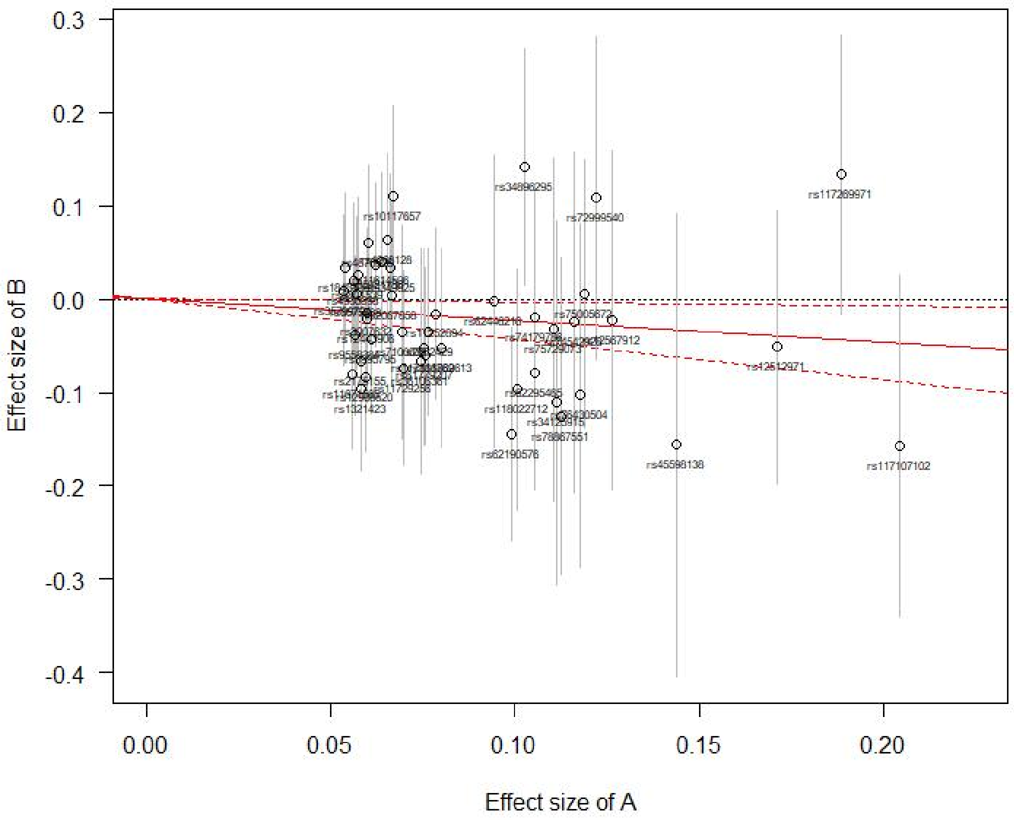

H. Gastric cancer

1. Gut microbiota abundance (class Deltaproteobacteria id.3087)

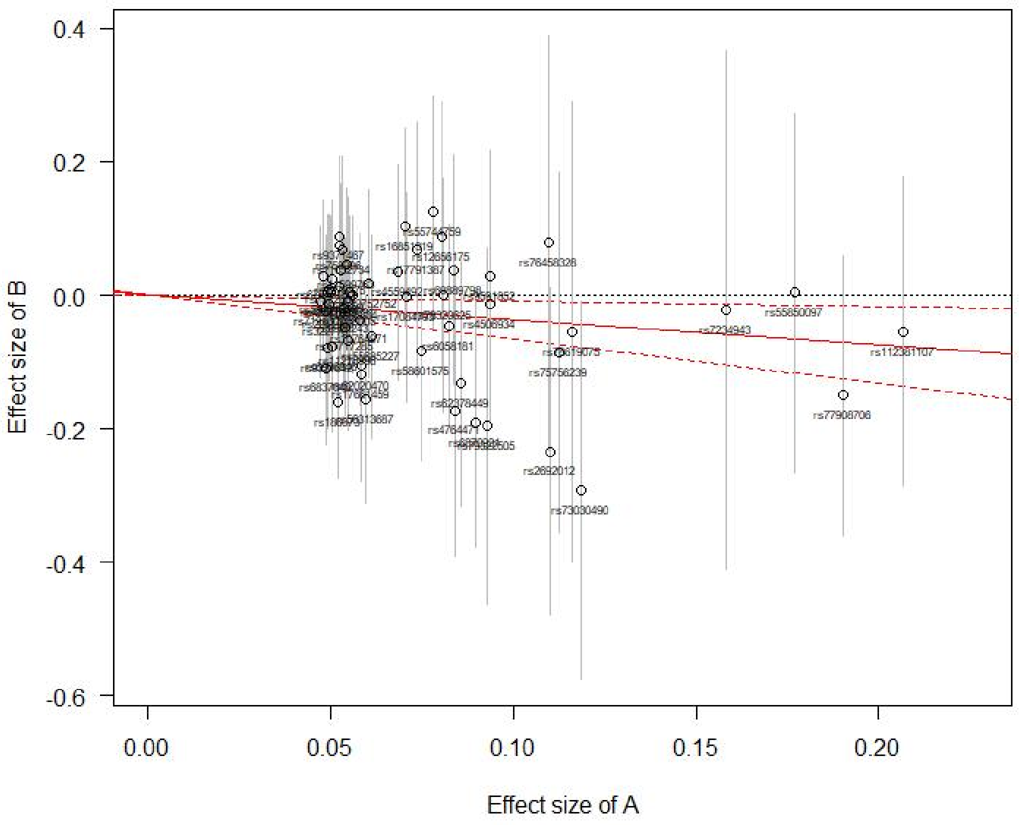

2. Gut microbiota abundance (class Gammaproteobacteria id.3303)

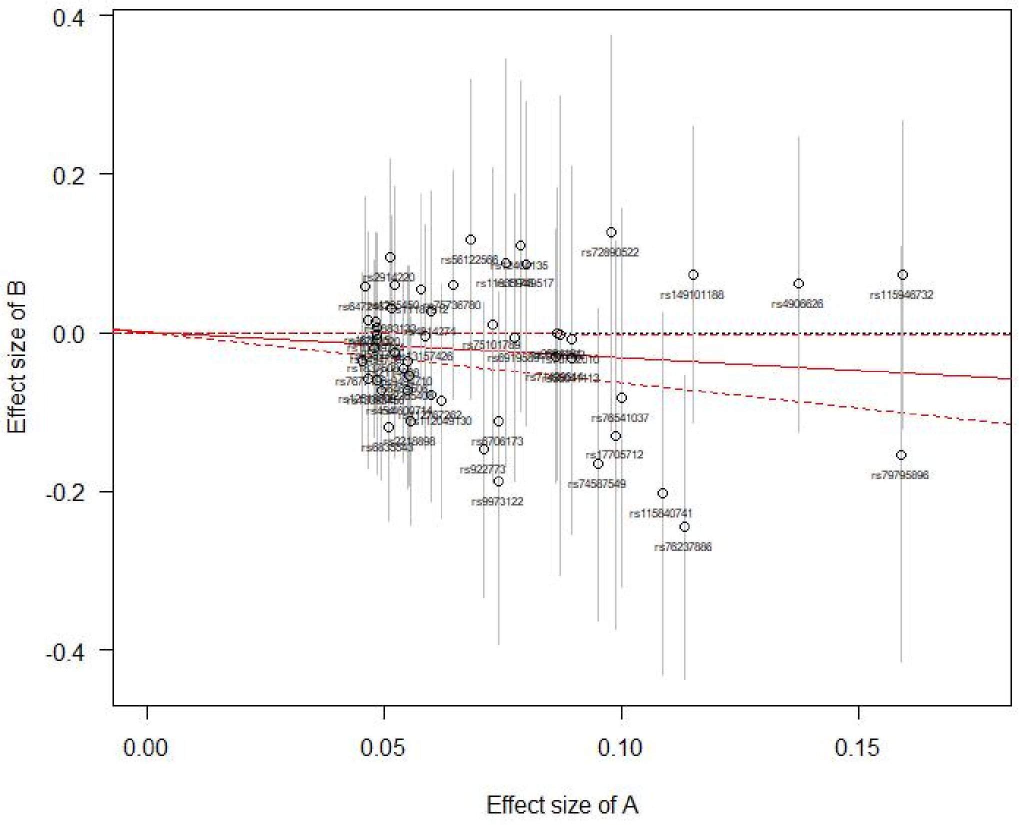

3. Gut microbiota abundance (family Actinomycetaceae id.421)

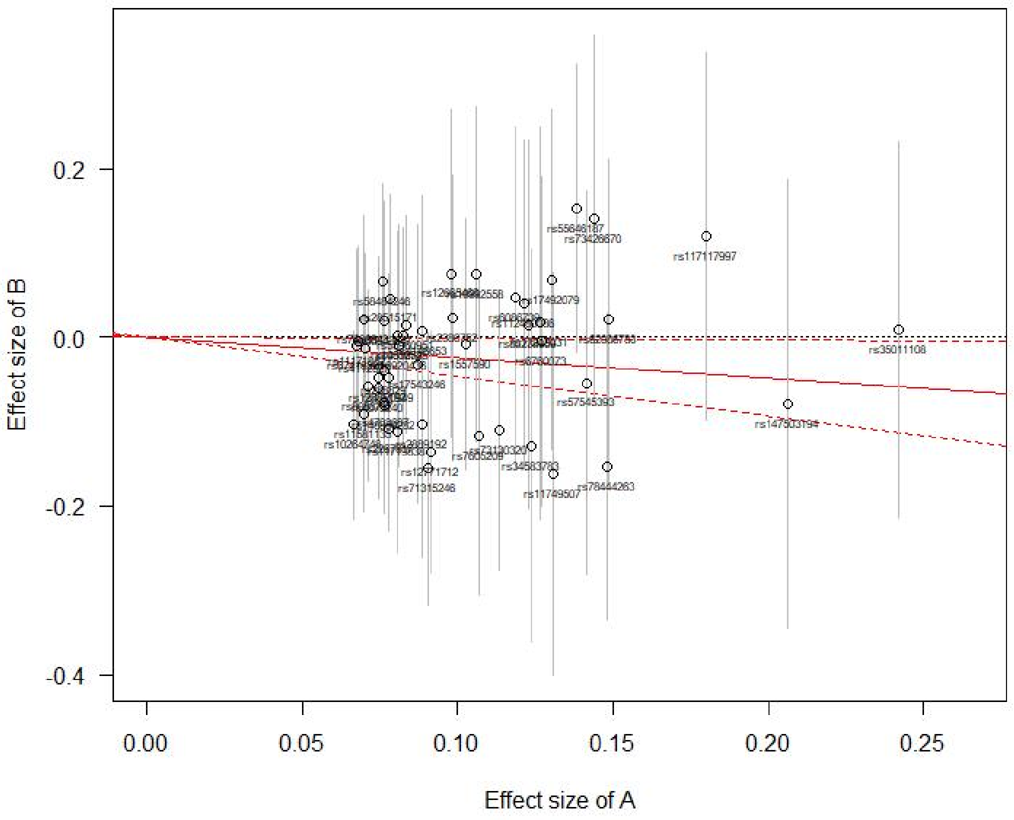

4. Gut microbiota abundance (family Desulfovibrionaceae id.3169)

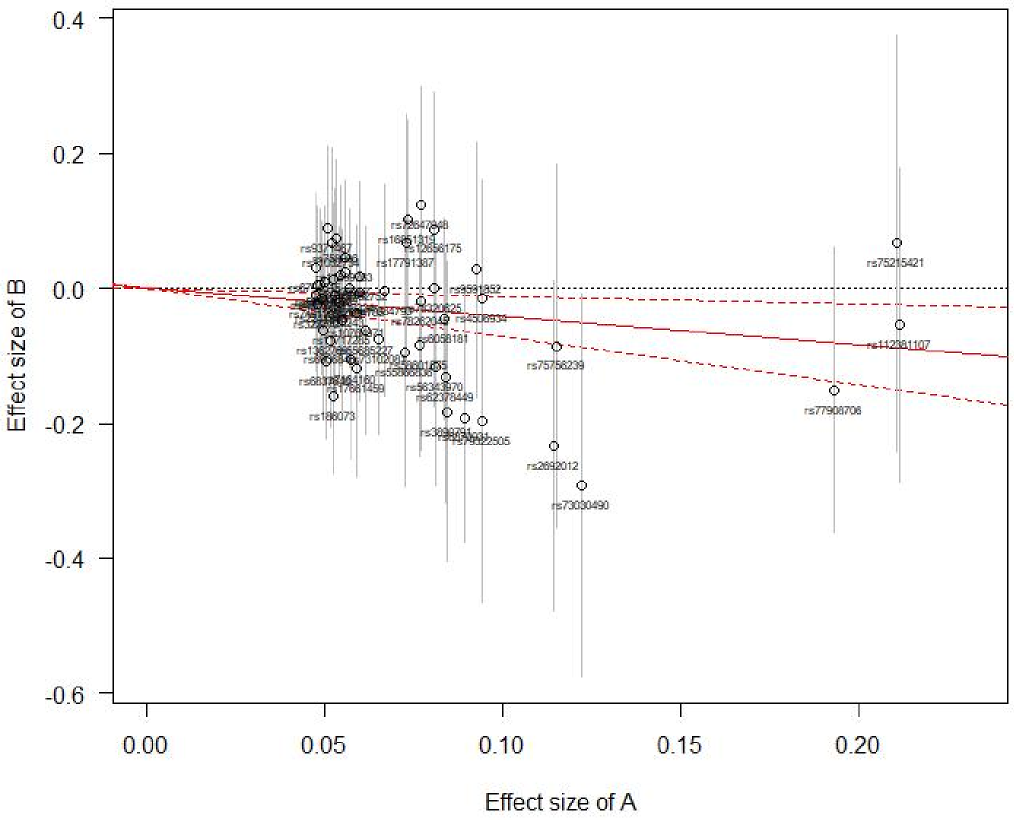

5. Gut microbiota abundance (unknown family id.1000005471)

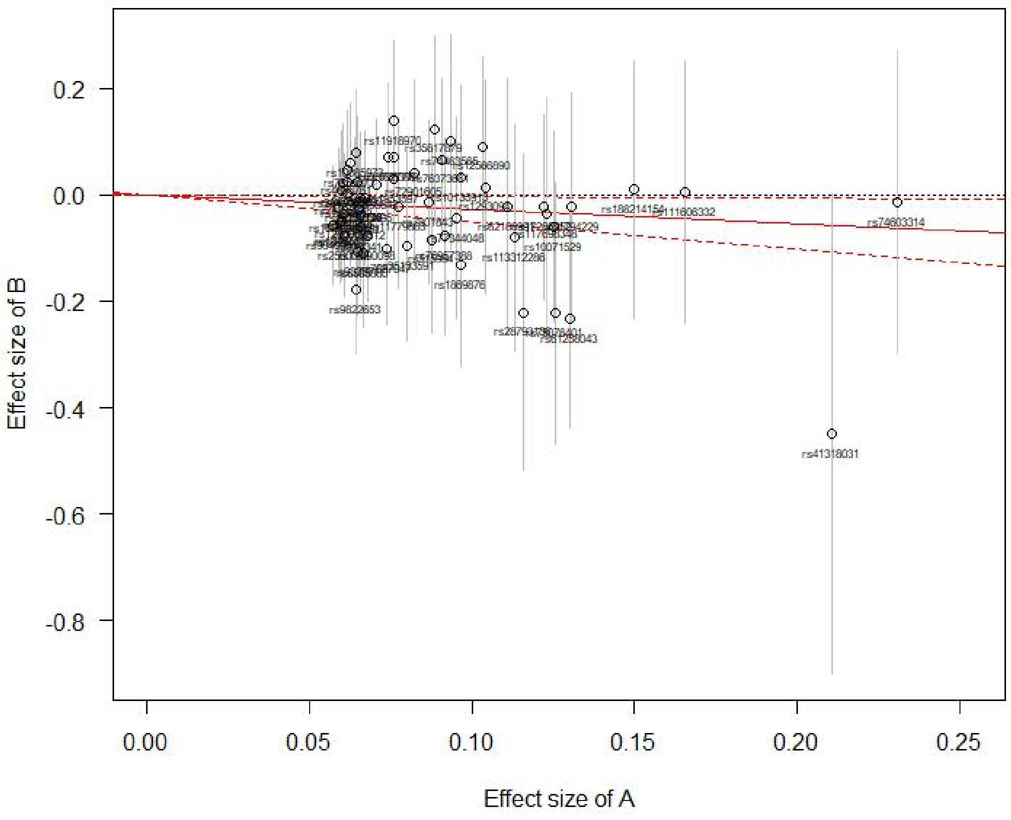

6. Gut microbiota abundance (genus Actinomyces id.423)

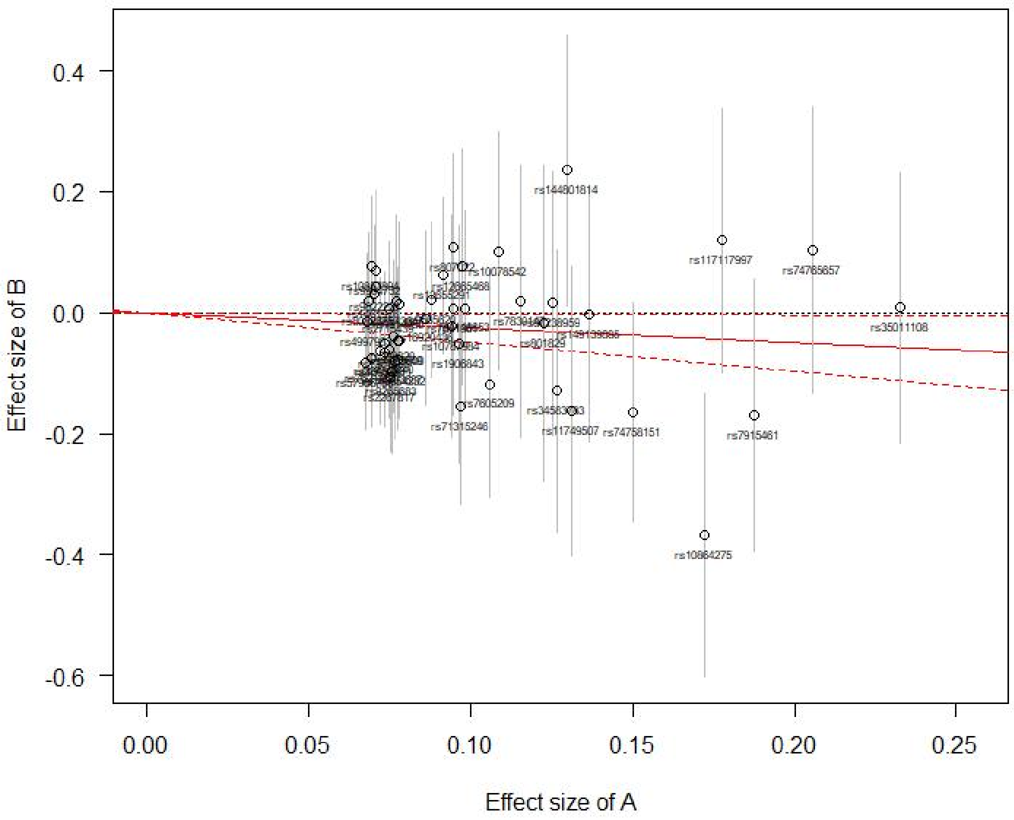

7. Gut microbiota abundance (genus Catenibacterium id.2153)

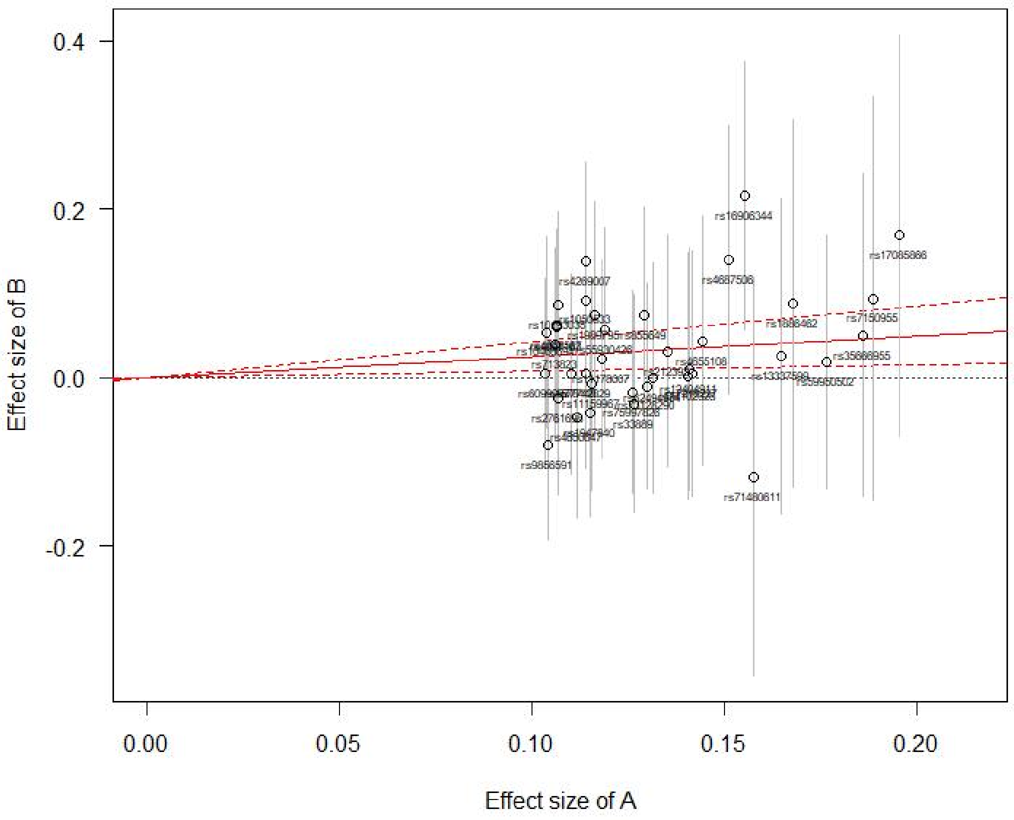

8. Gut microbiota abundance (genus Coprococcus3 id.11303)

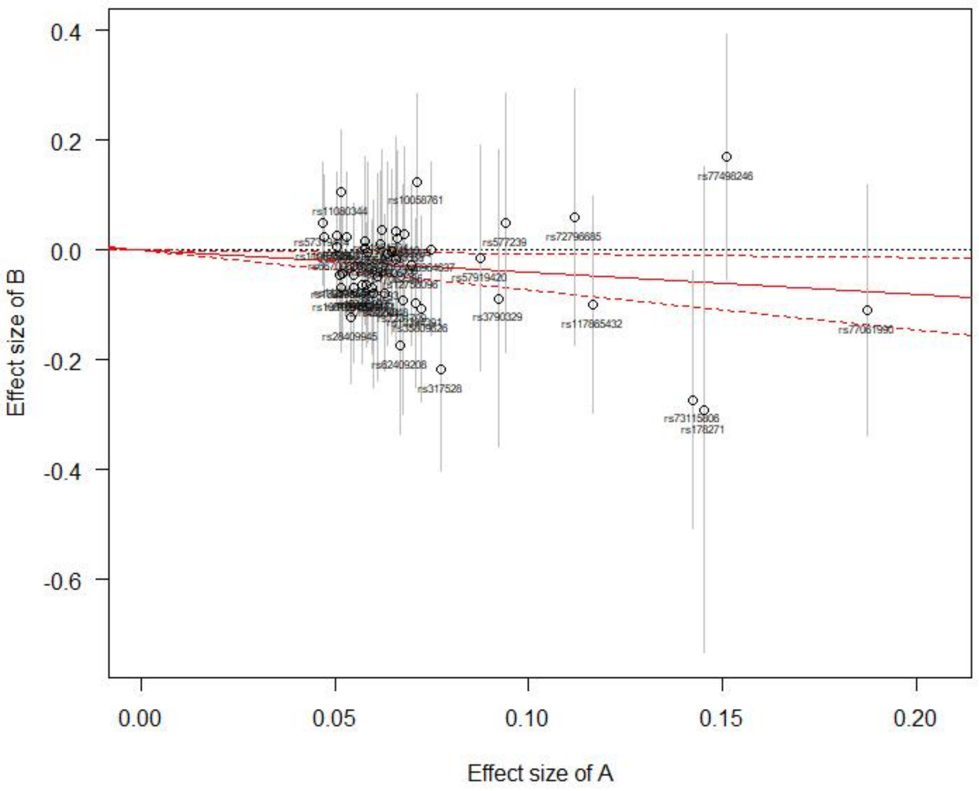

9. Gut microbiota abundance (genus Roseburia id.2012)

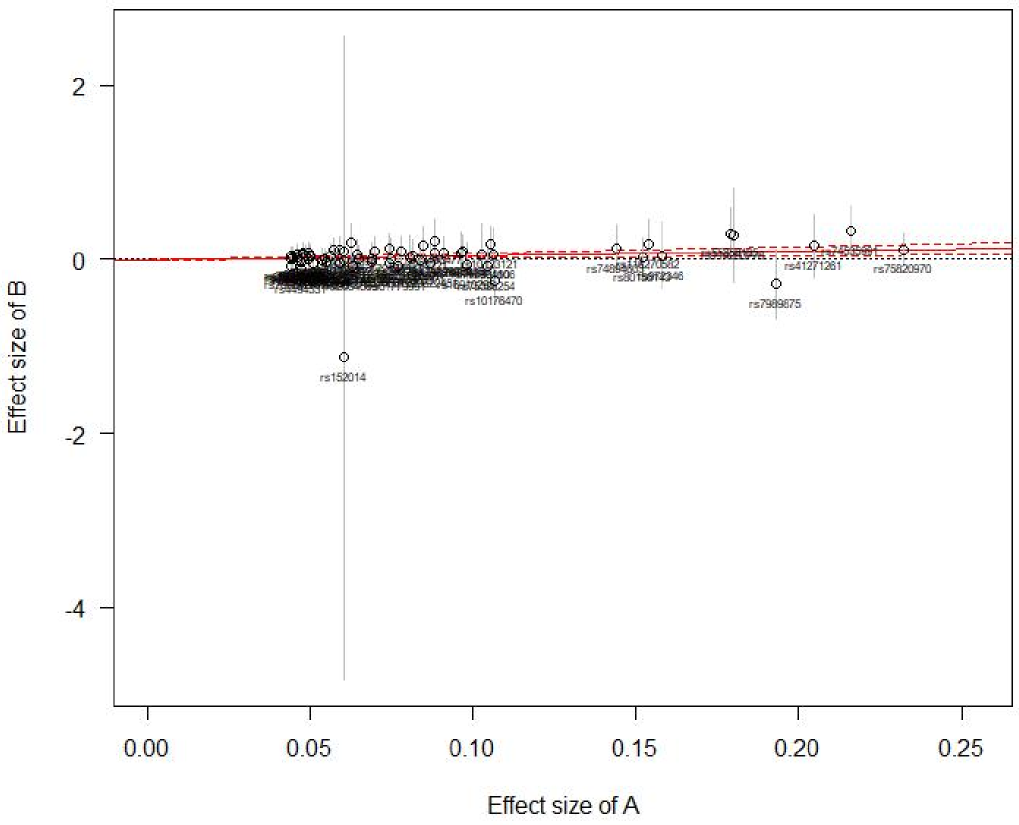

10. Gut microbiota abundance (genus Ruminococcaceae UCG011 id.11368)

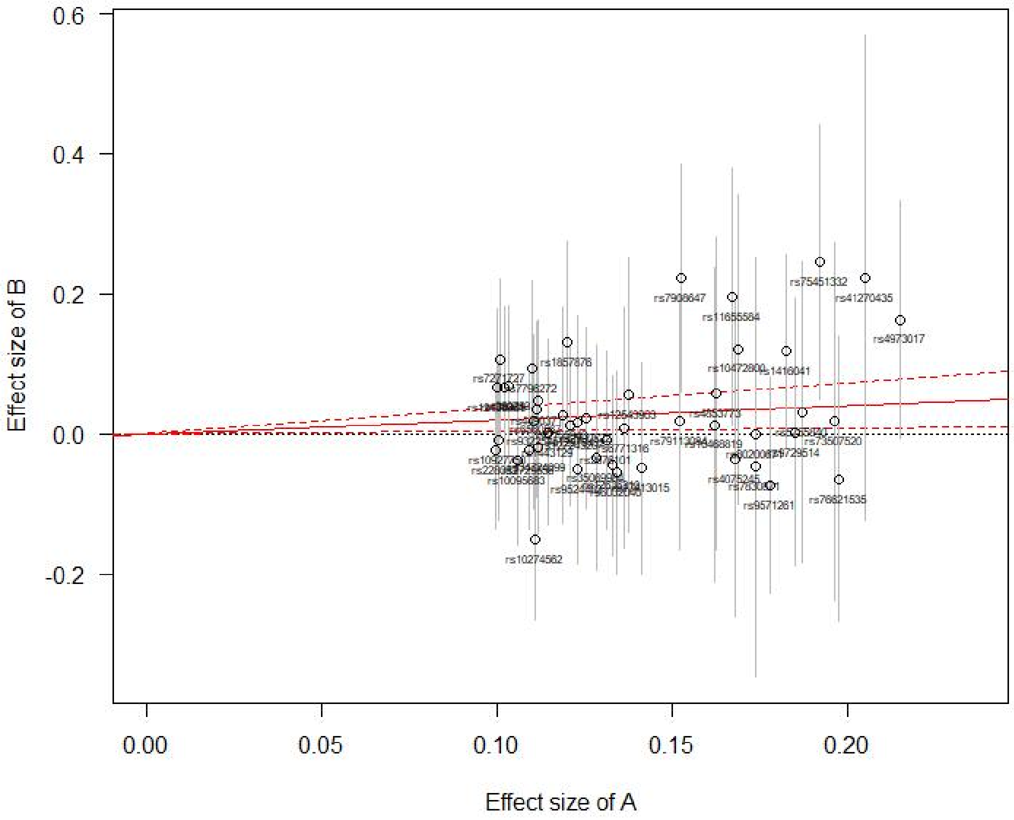

11. Gut microbiota abundance (unknown genus id.1000005472)

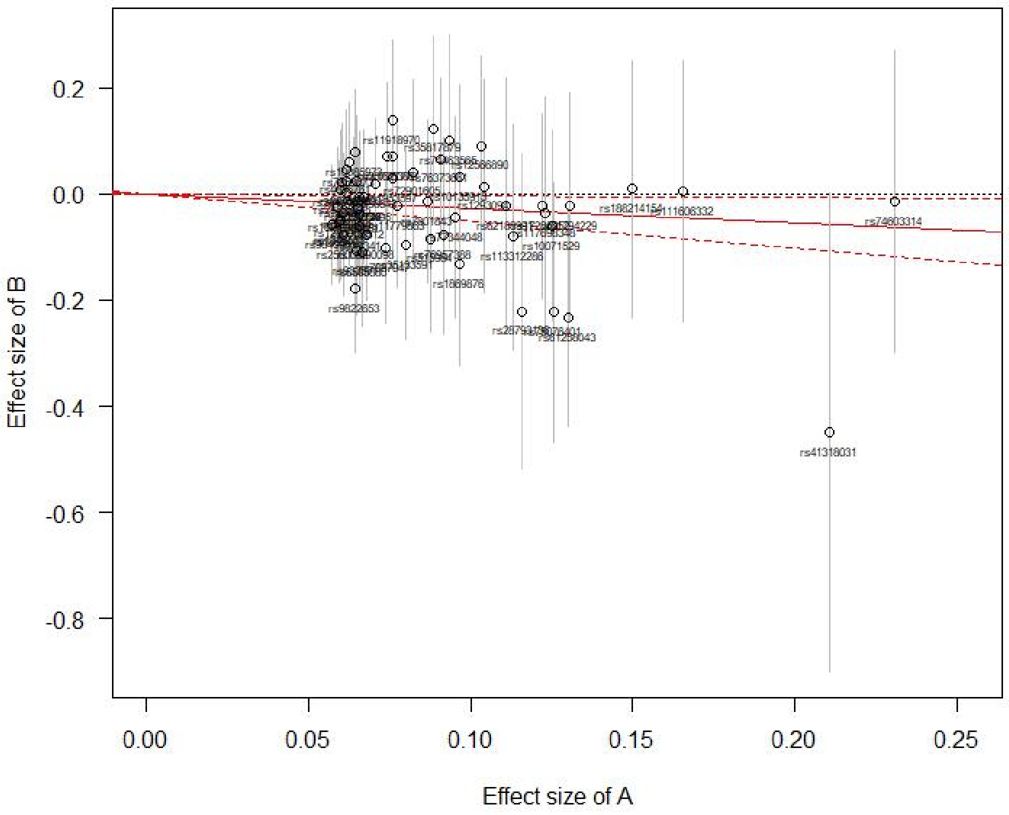

12. Gut microbiota abundance (unknown genus id.2755)

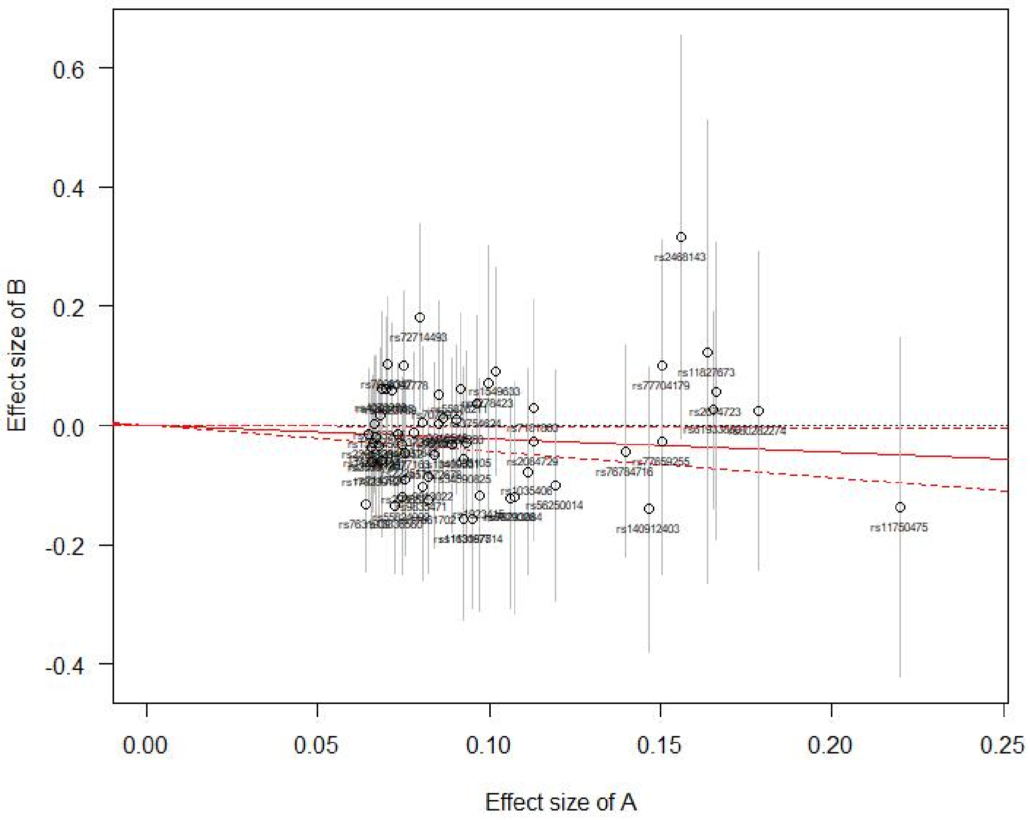

13. Gut microbiota abundance (order Actinomycetales id.420)

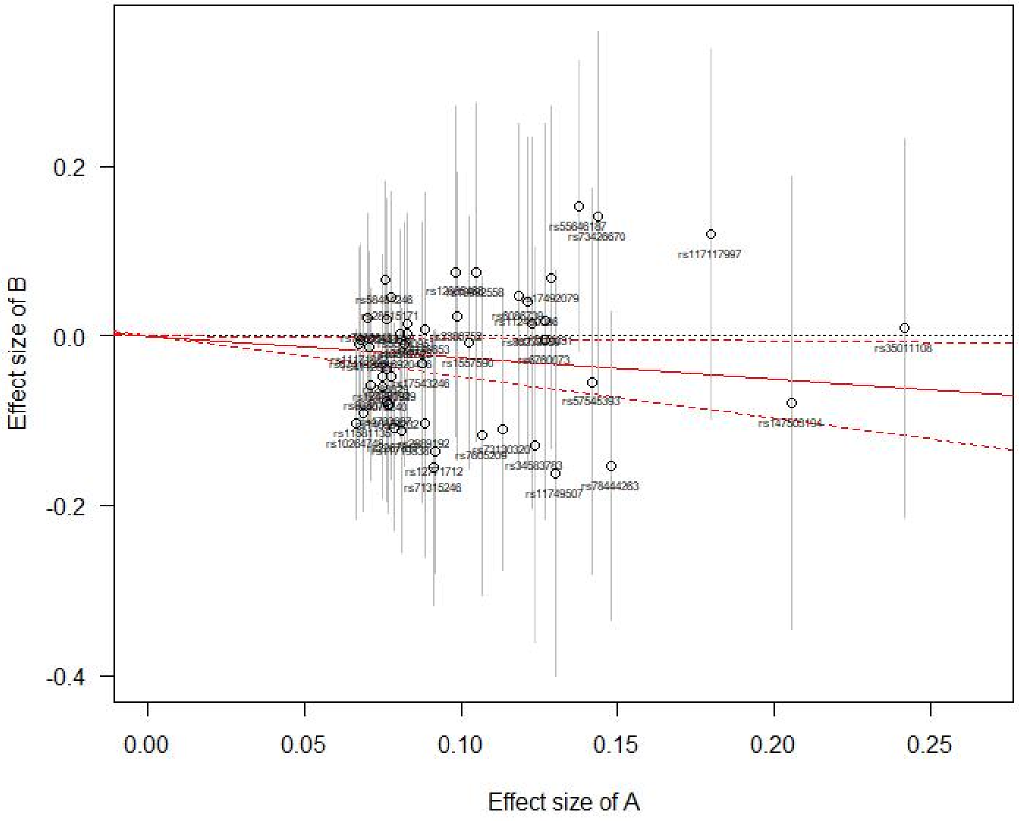

14. Gut microbiota abundance (order Desulfovibrionales id.3156)

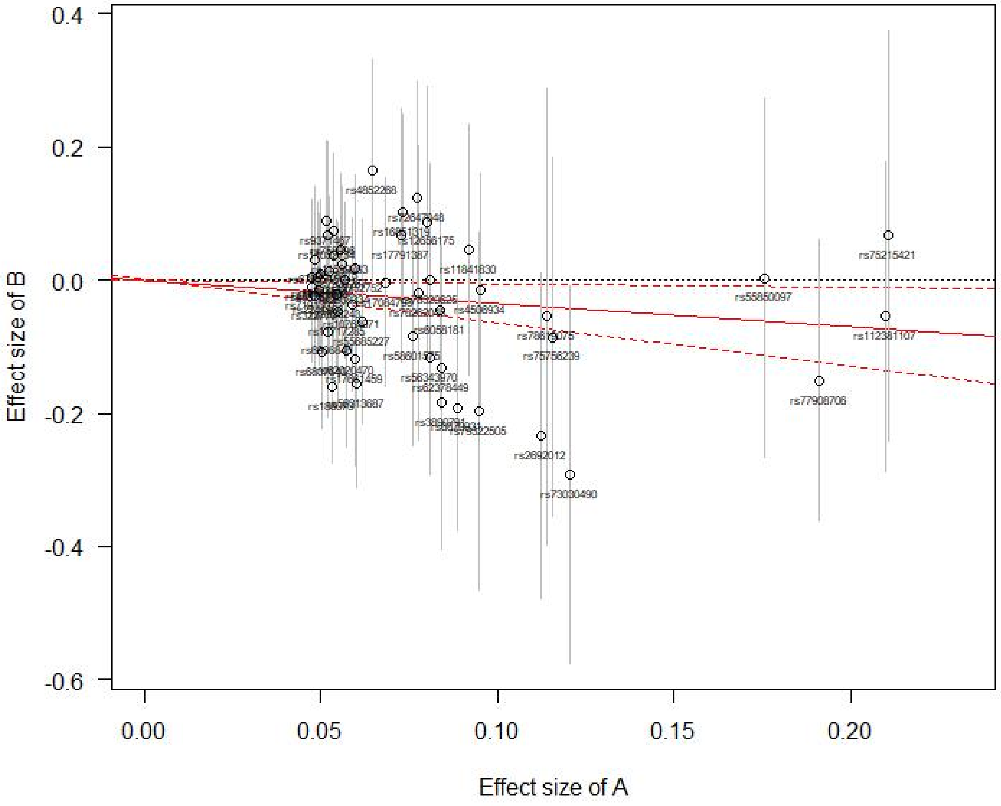

15. Gut microbiota abundance (order Mollicutes RF9 id.11579)

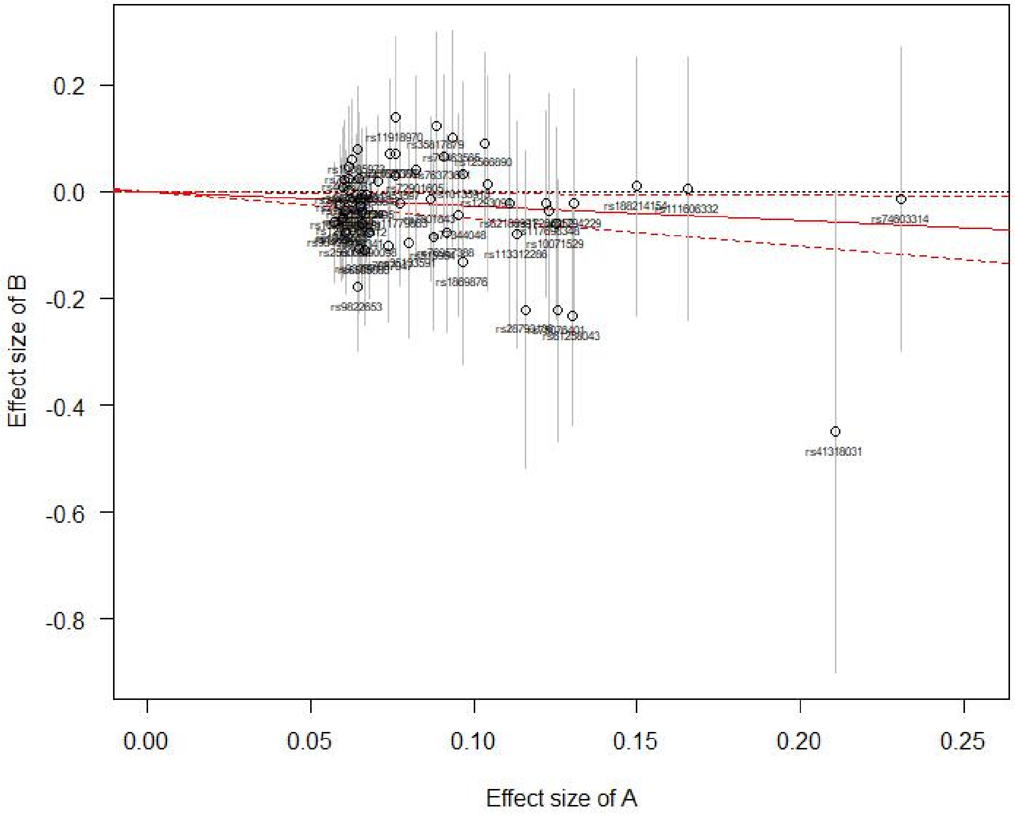

I. Irritable bowel syndrome

1. Gut microbiota abundance (genus Anaerofilum id.2053)

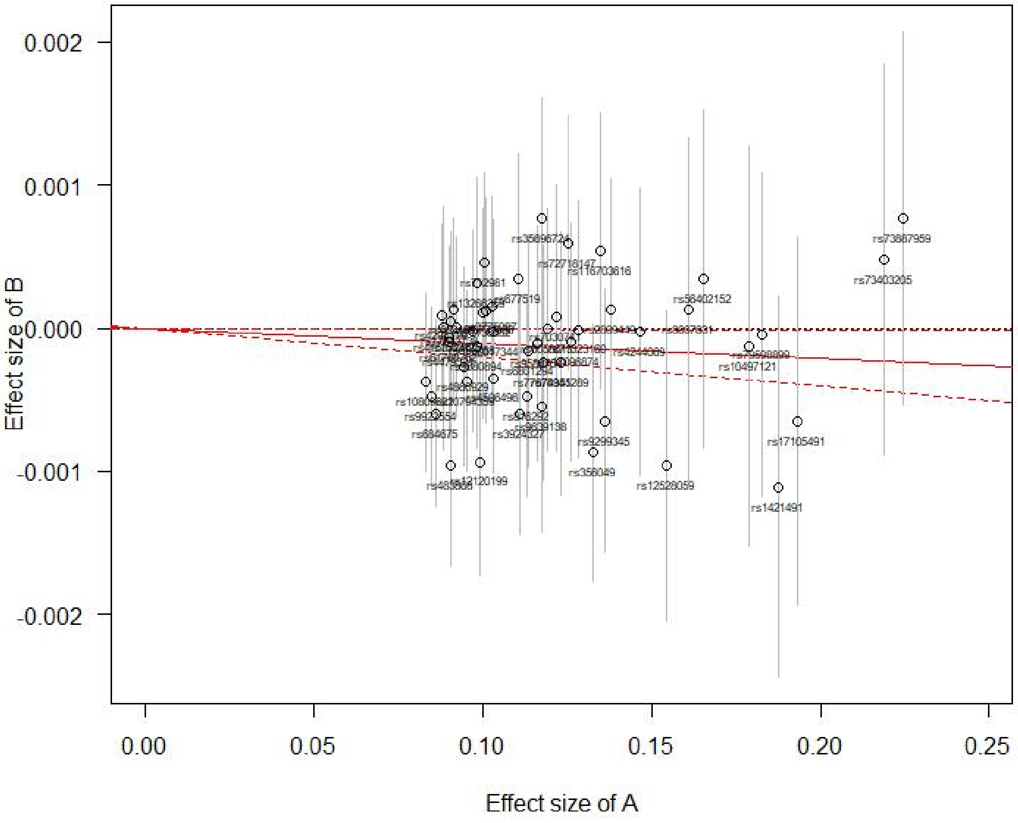

2. Gut microbiota abundance (genus Desulfovibrio id.3173)

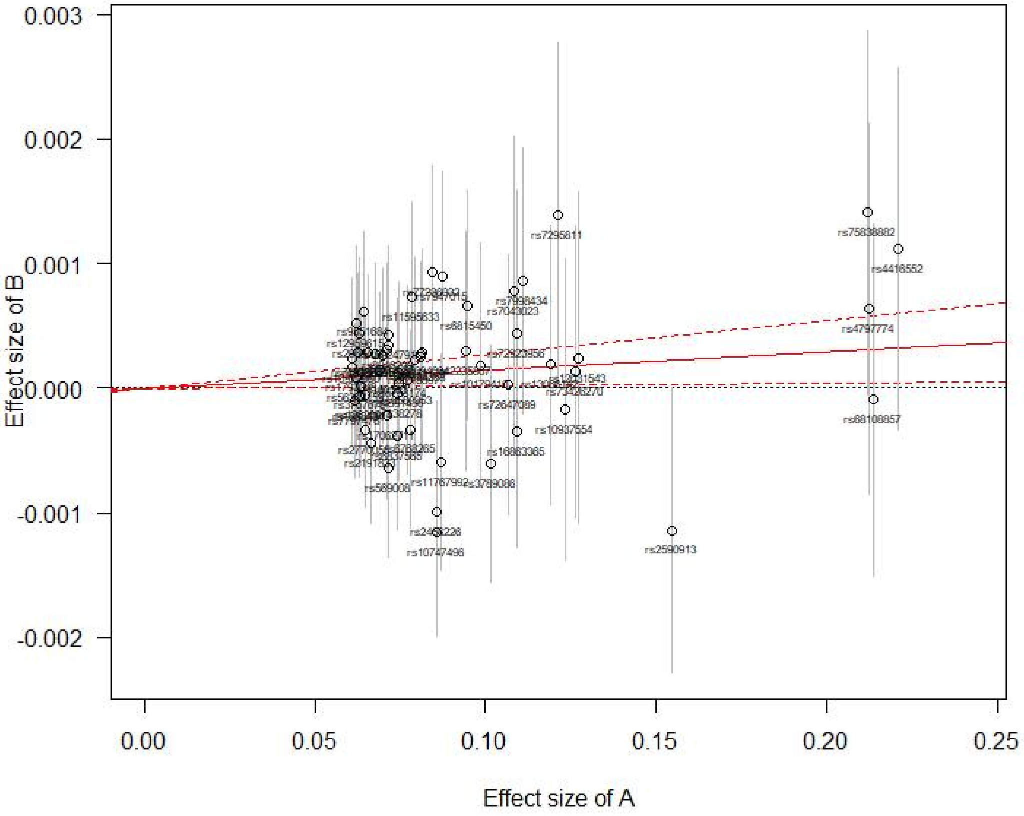

3. Gut microbiota abundance (genus Lachnospiraceae NK4A136 group id.11319)

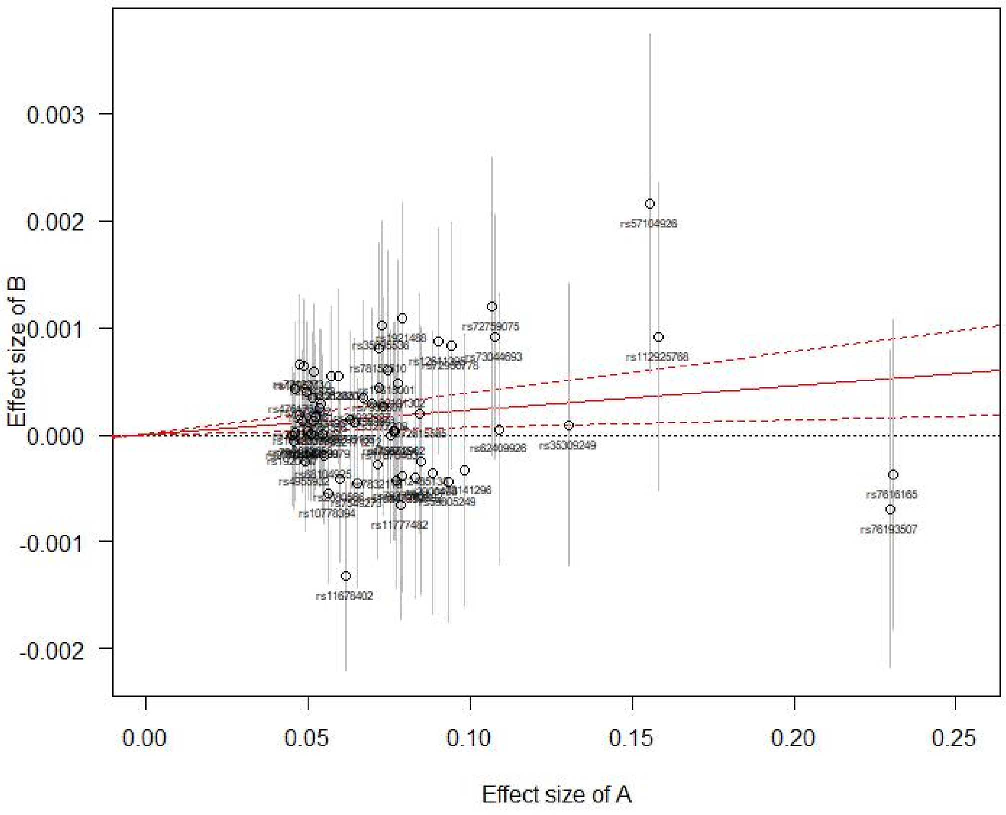

4. Gut microbiota abundance (genus Peptococcus id.2037)

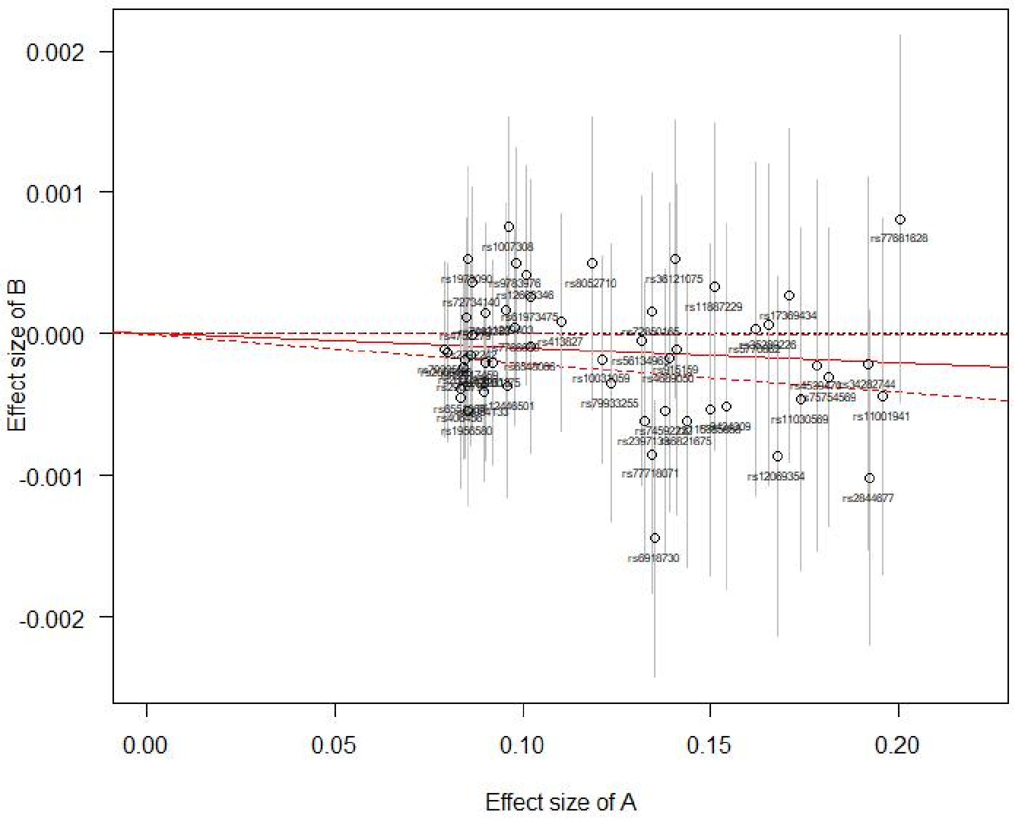

5. Gut microbiota abundance (genus Tyzzerella3 id.11335)

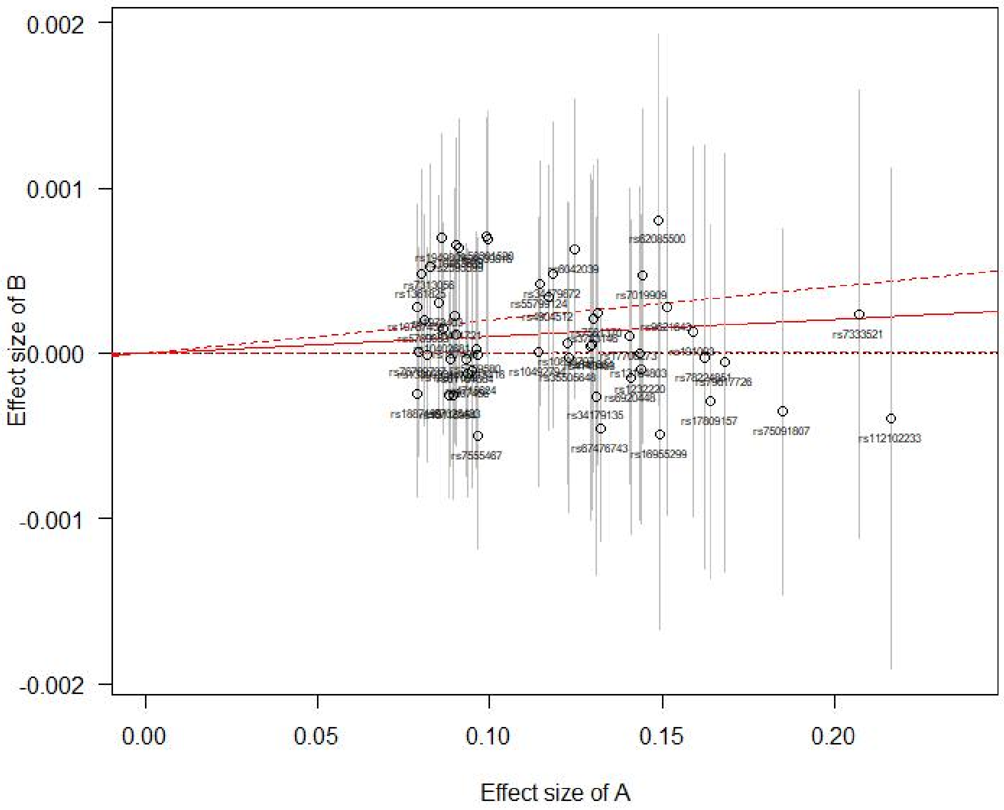

J. Diverticular disease

1. Gut microbiota abundance (class Negativicutes id.2164)

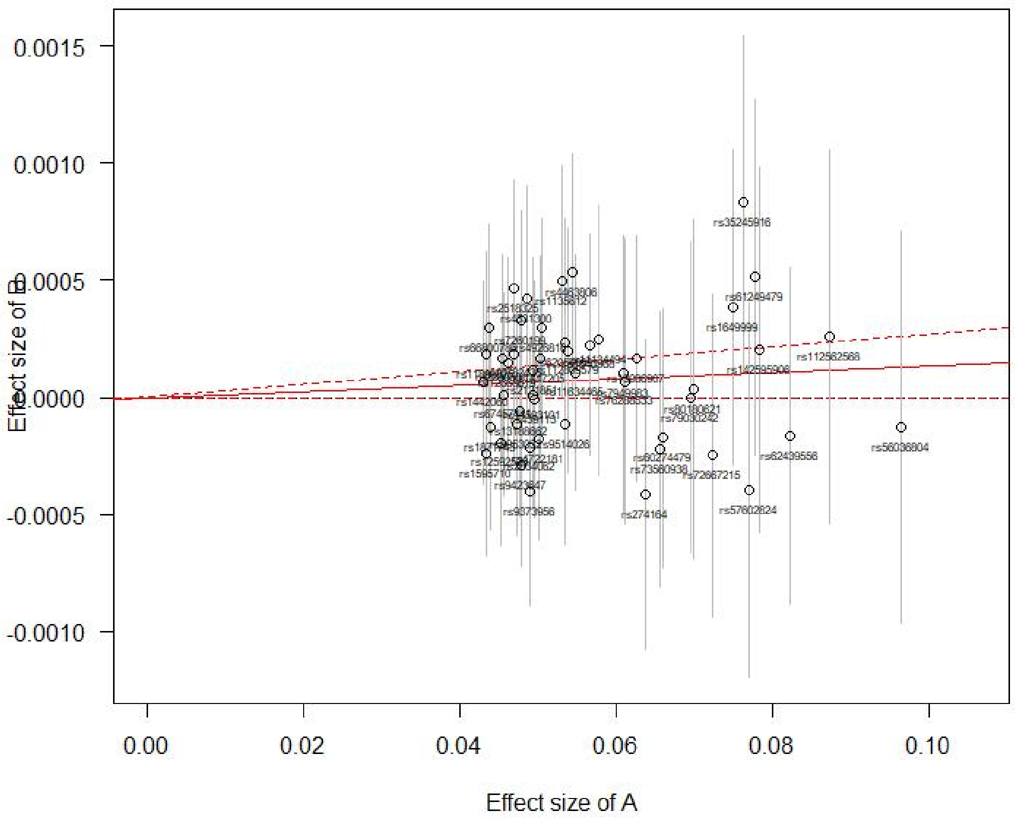

2. Gut microbiota abundance (family Alcaligenaceae id.2875)

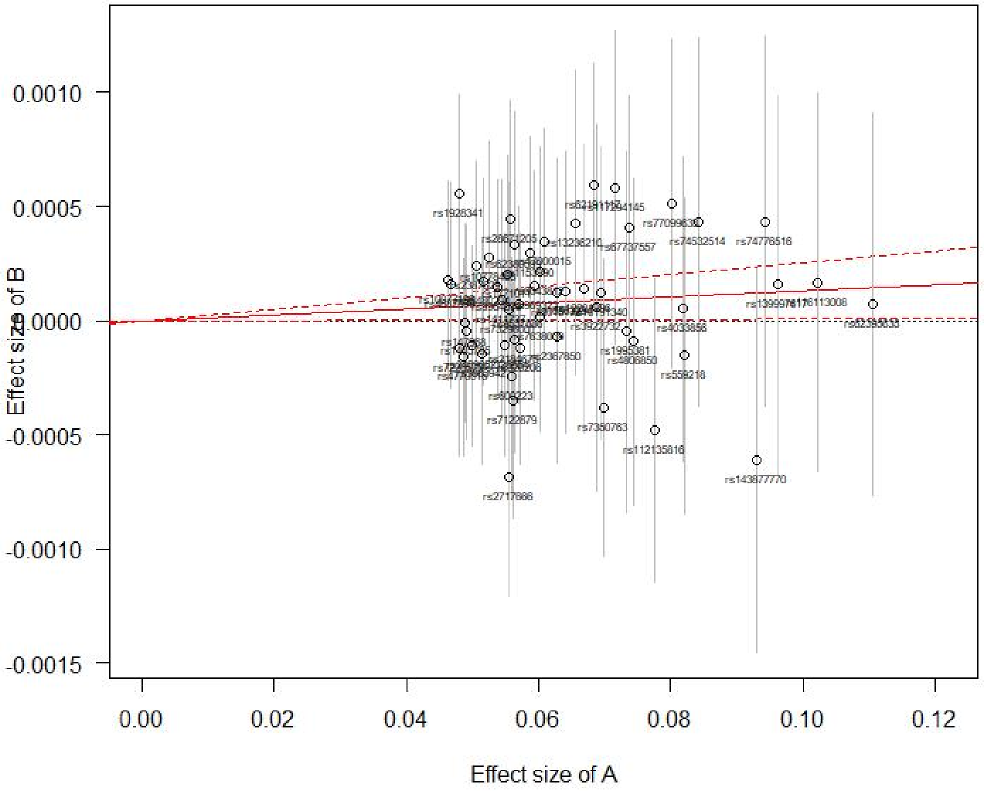

3. Gut microbiota abundance (family Family XIII id.1957)

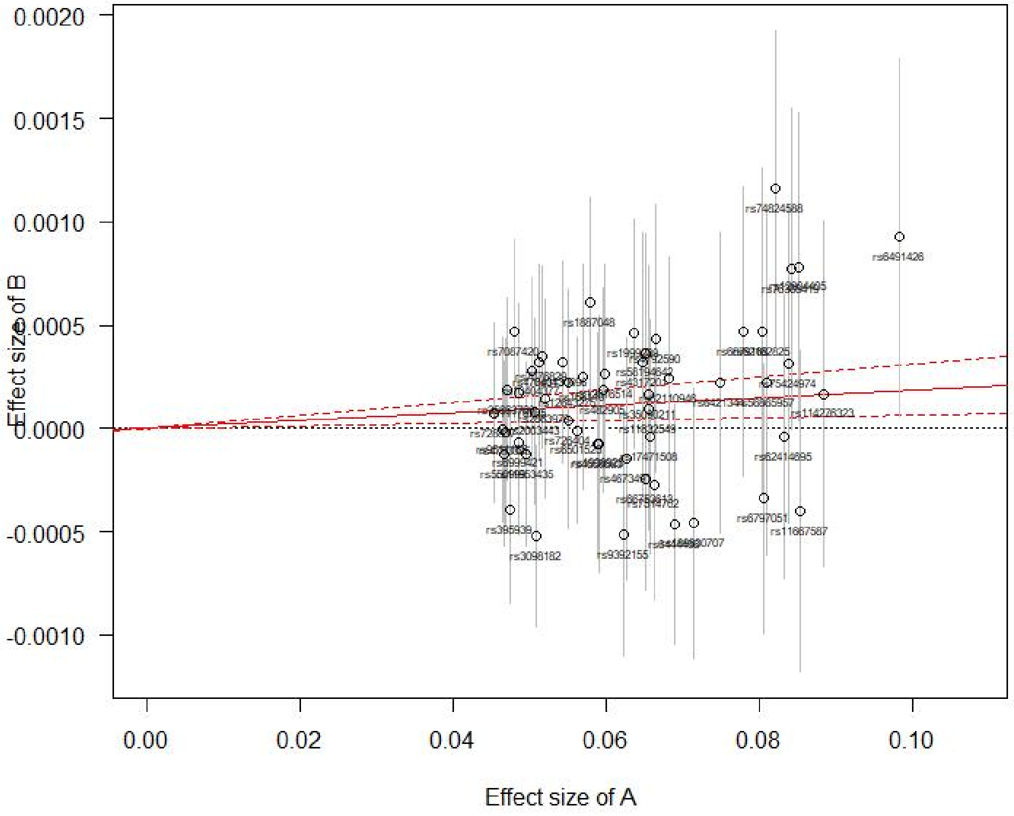

4. Gut microbiota abundance (genus Barnesiella id.944)

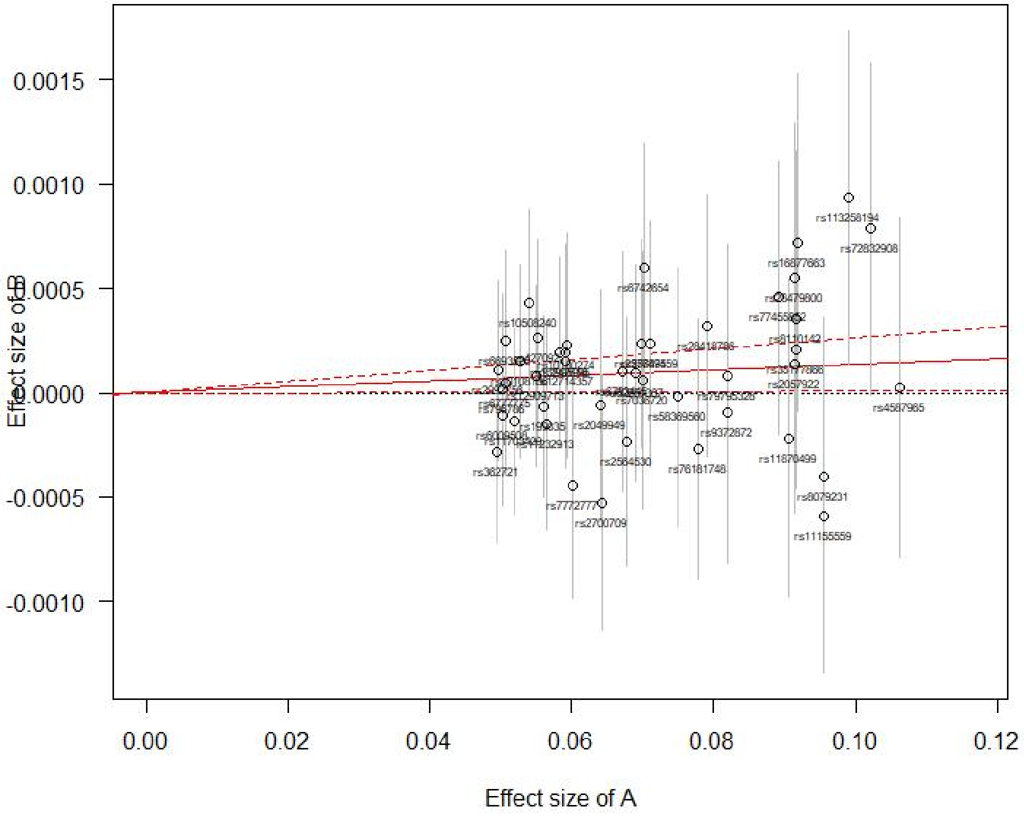

5. Gut microbiota abundance (genus Eubacterium xylanophilum group id.14375)

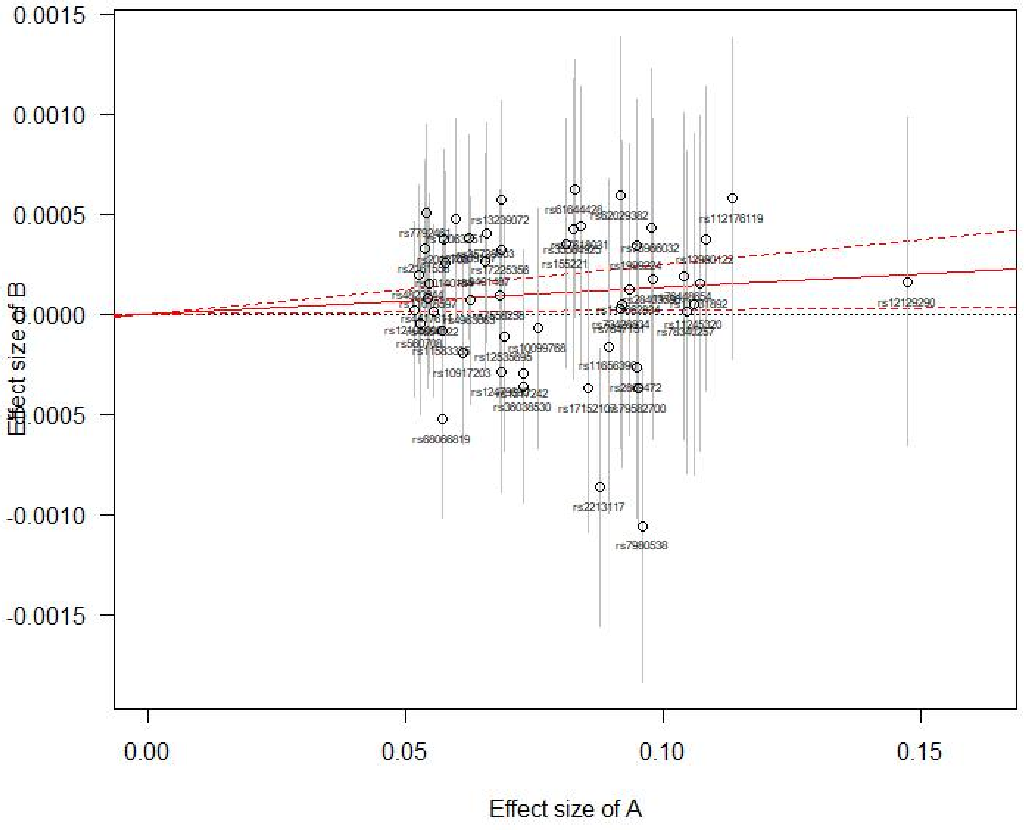

6. Gut microbiota abundance (genus Ruminococcus2 id.11374)

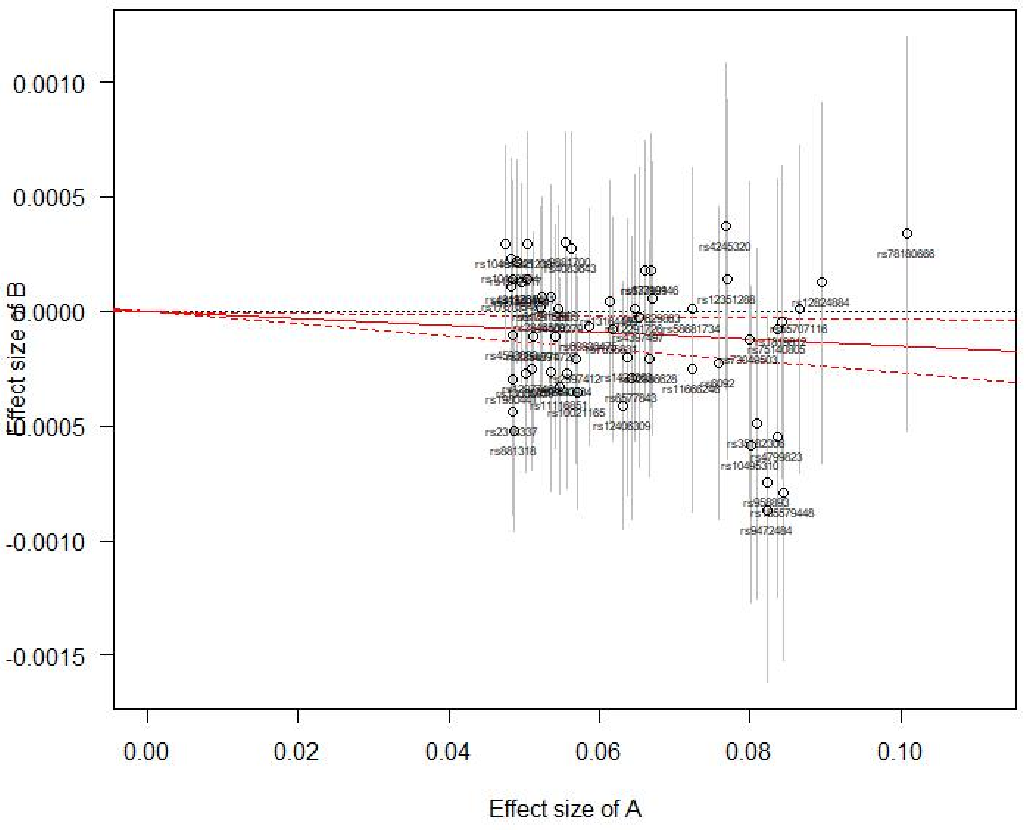

7. Gut microbiota abundance (unknown genus id.1868)

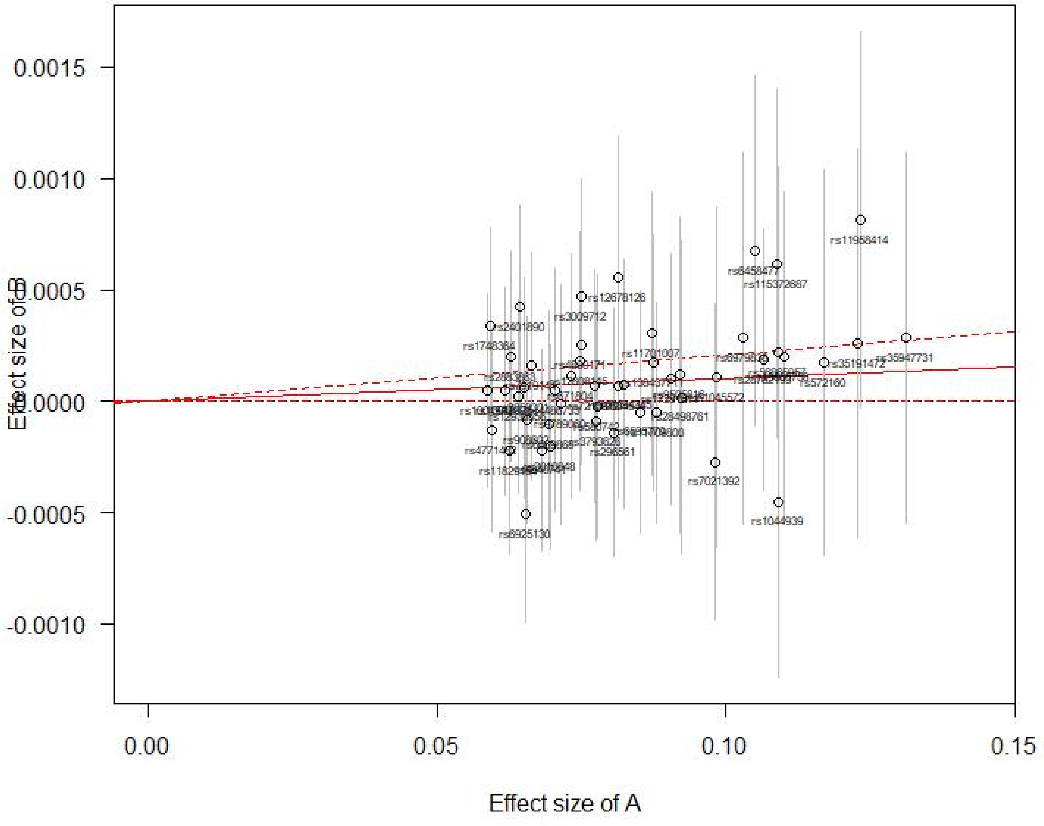

8. Gut microbiota abundance (unknown genus id.959)

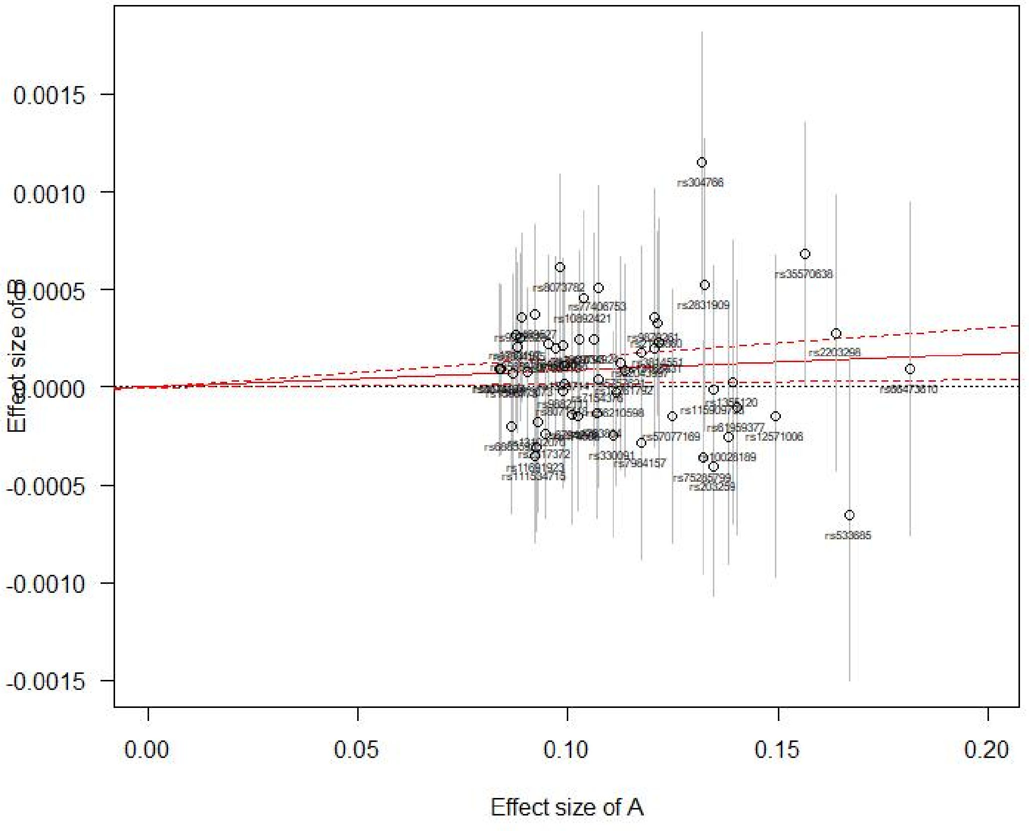

9. Gut microbiota abundance (order Selenomonadales id.2165)

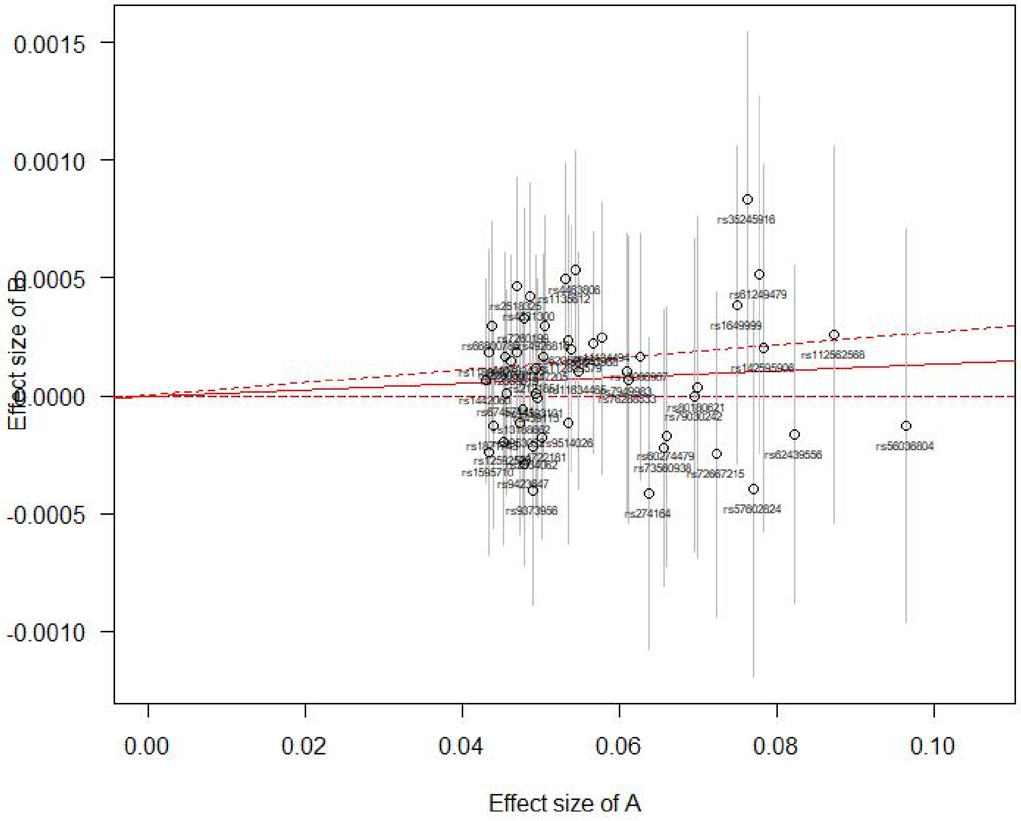

K. Crohn's disease

1. Gut microbiota abundance (class Negativicutes id.2164)

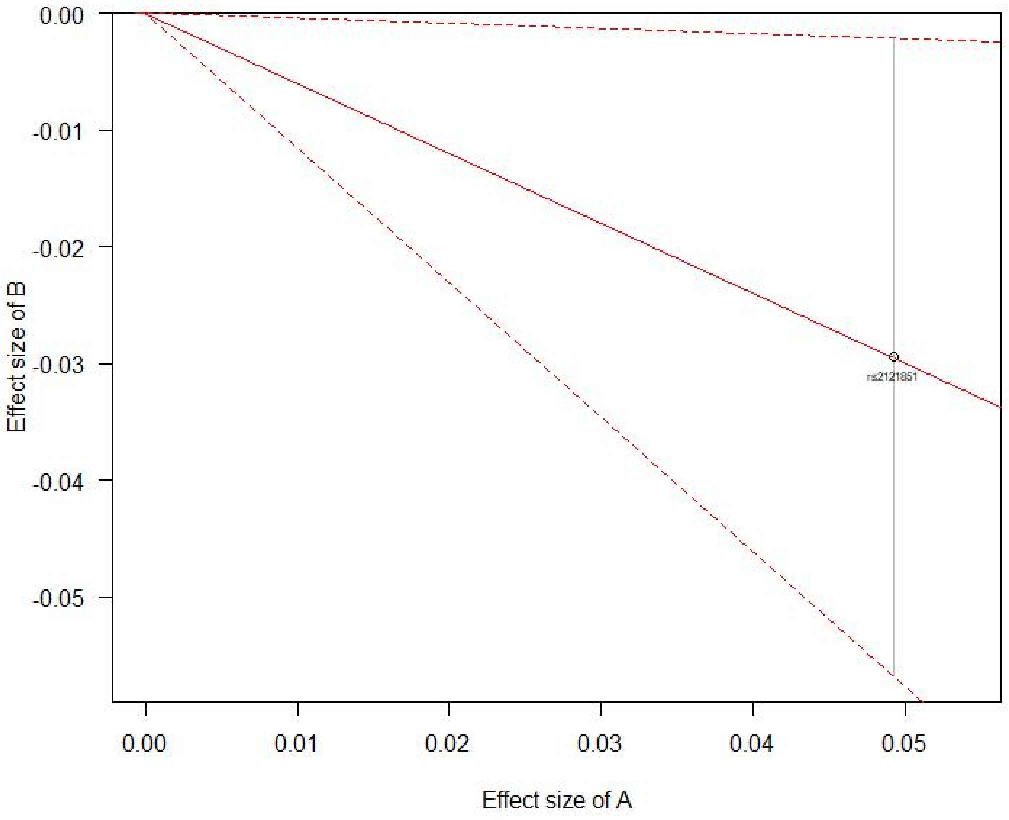

2. Gut microbiota abundance (class Verrucomicrobiae id.4029)

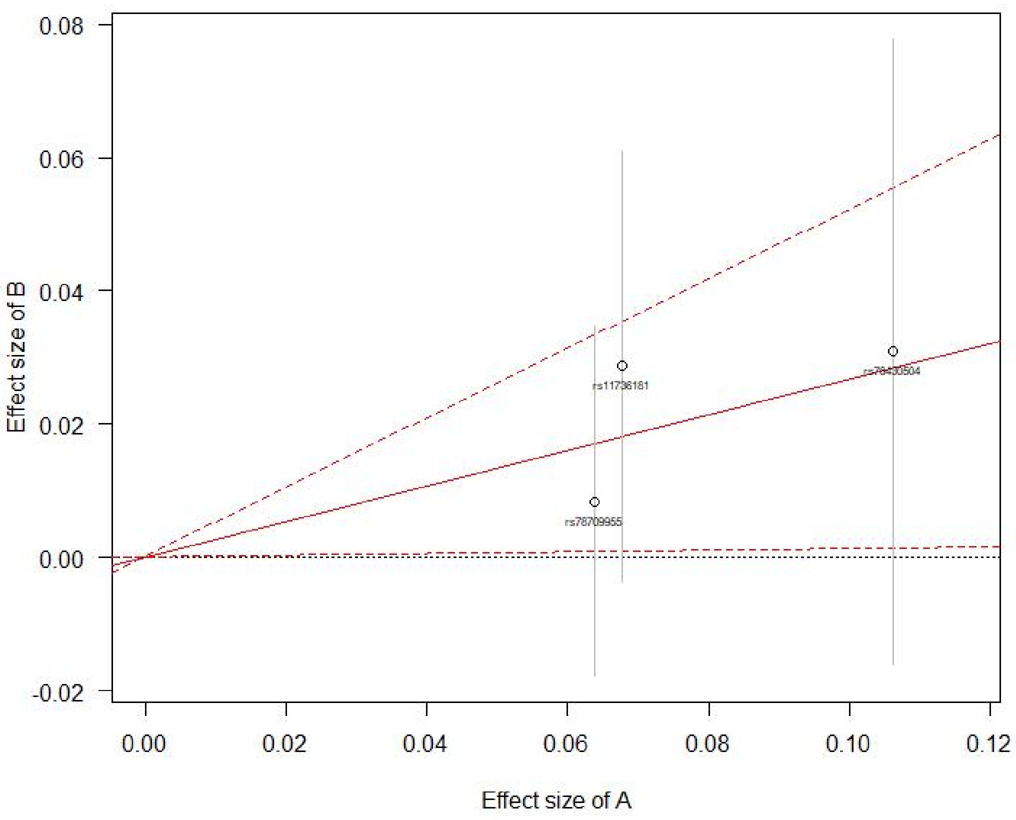

3. Gut microbiota abundance (family Defluviitaleaceae id.1924)

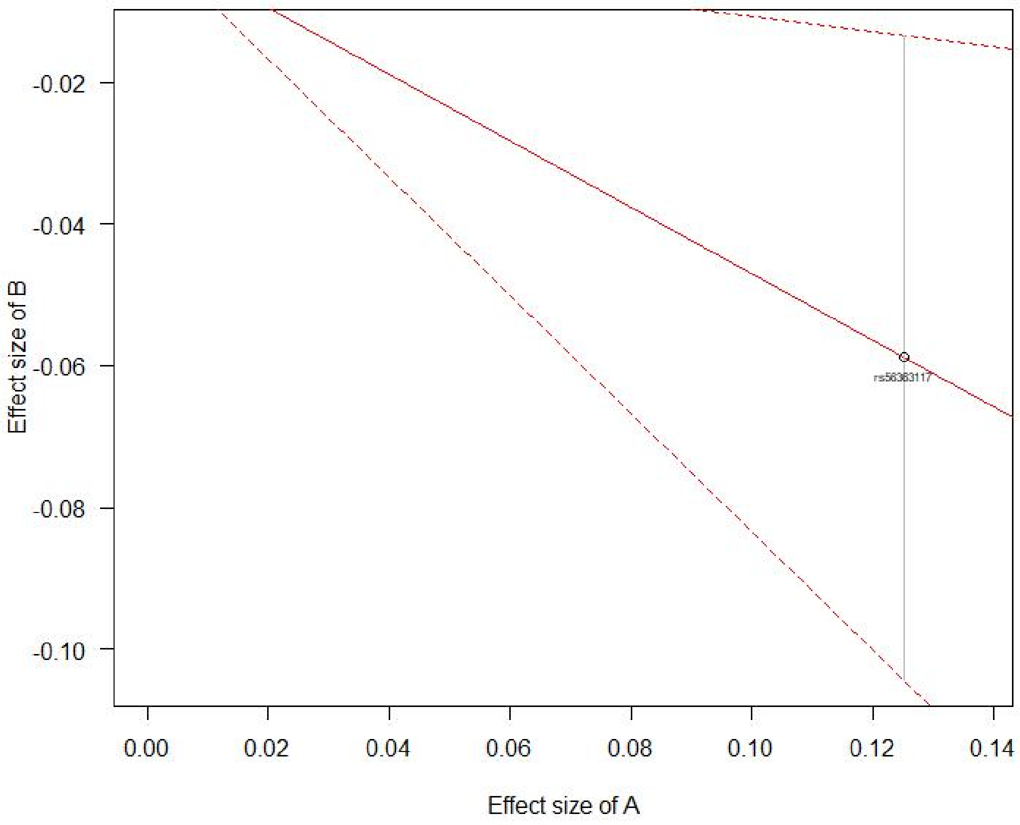

4. Gut microbiota abundance (family Peptostreptococcaceae id.2042)

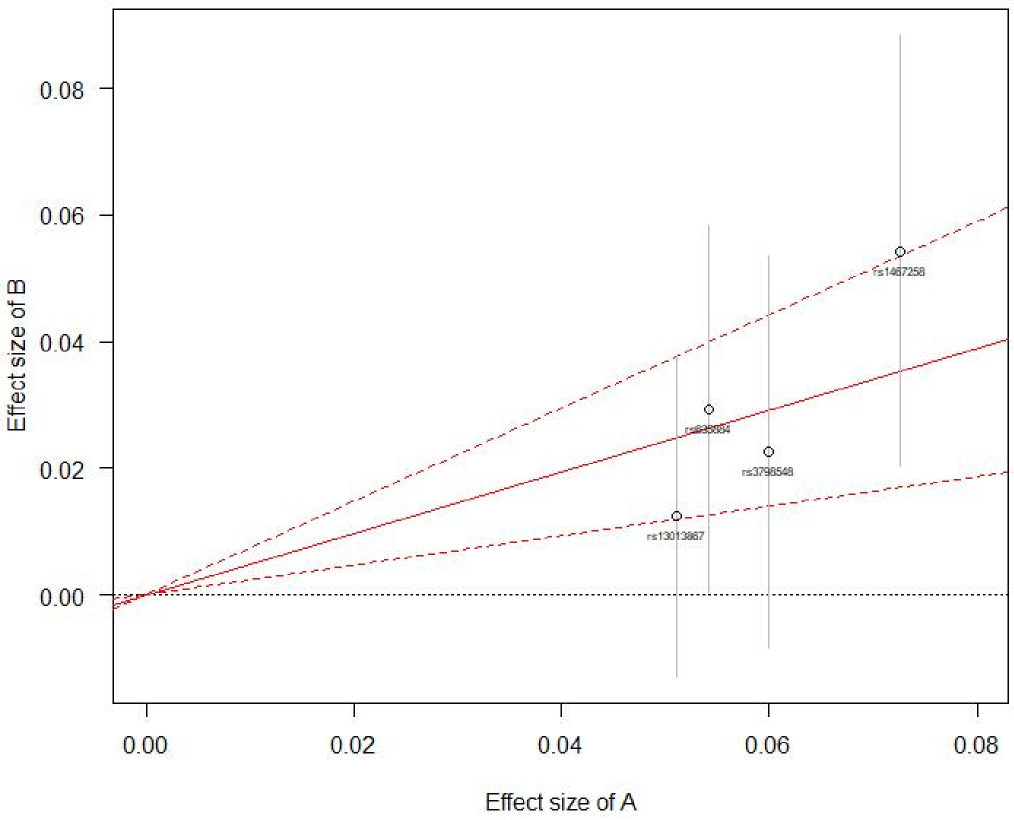

5. Gut microbiota abundance (unknown family id.1000006161)

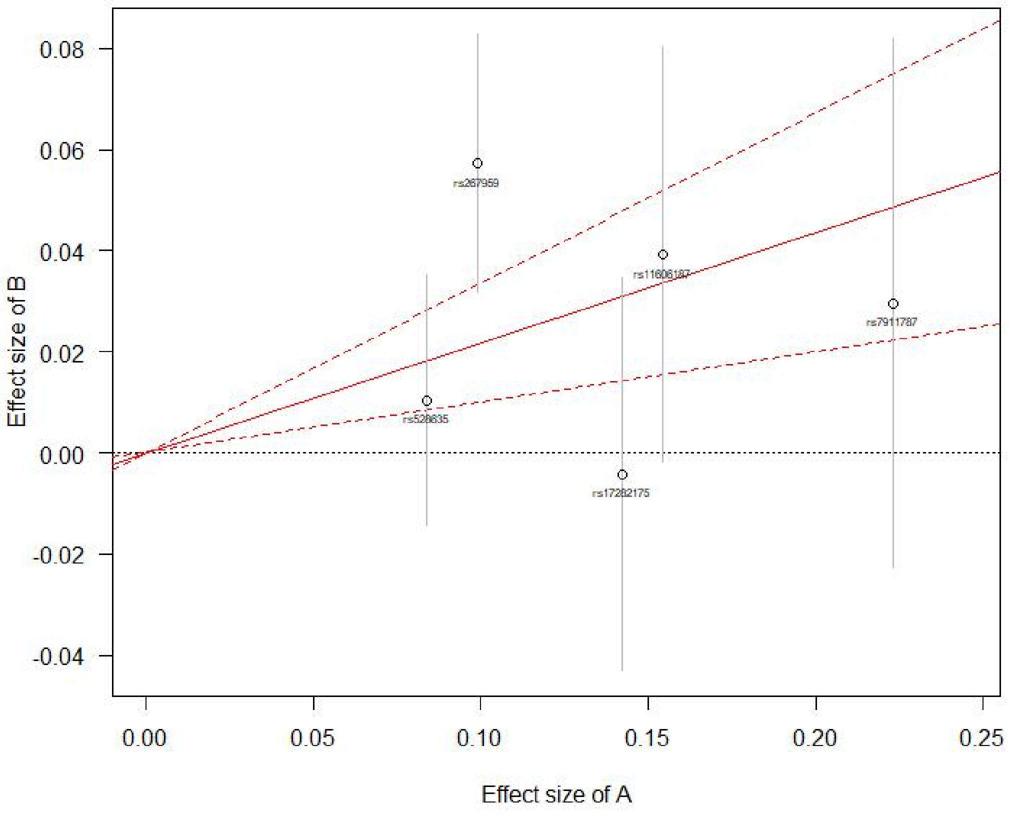

6. Gut microbiota abundance (genus Akkermansia id.4037)

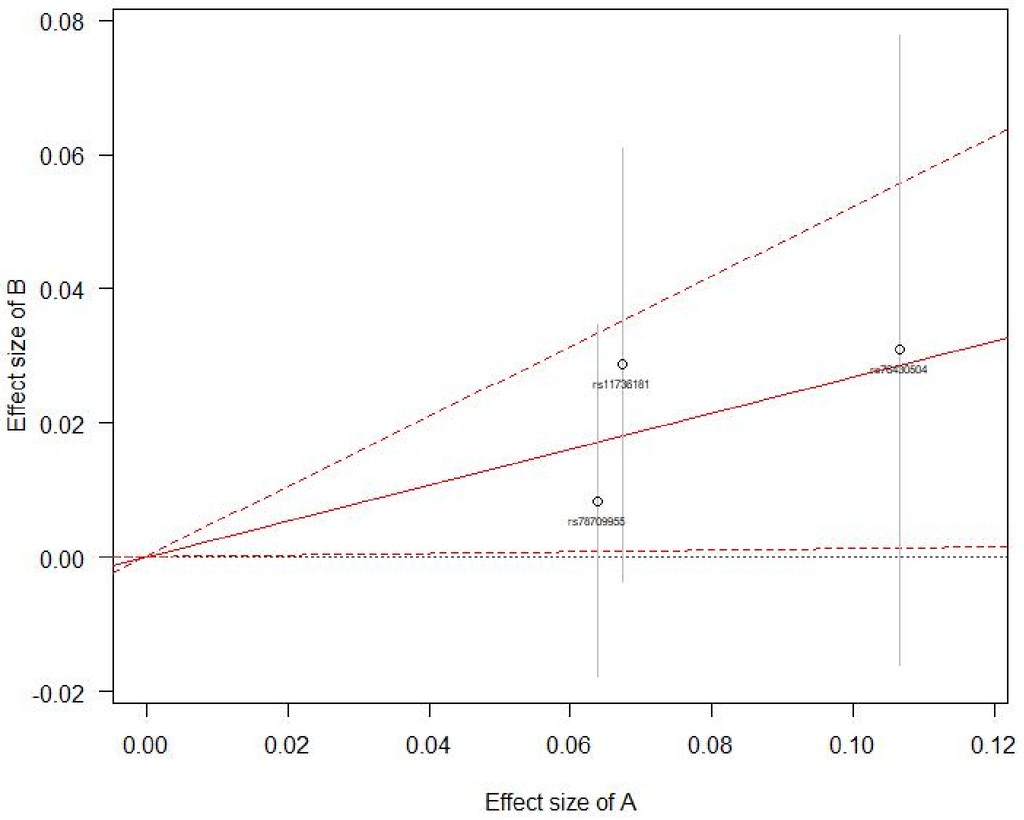

7. Gut microbiota abundance (genus Christensenellaceae R 7group id.11283)

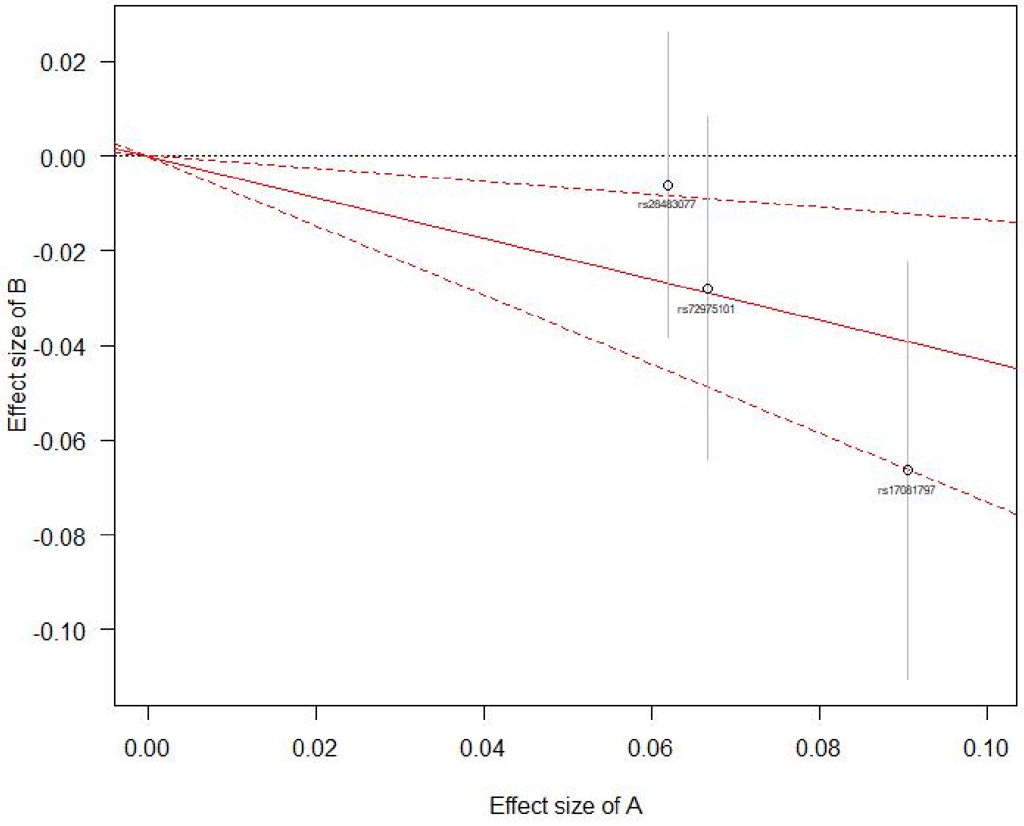

8. Gut microbiota abundance (genus Coprococcus3 id.11303)

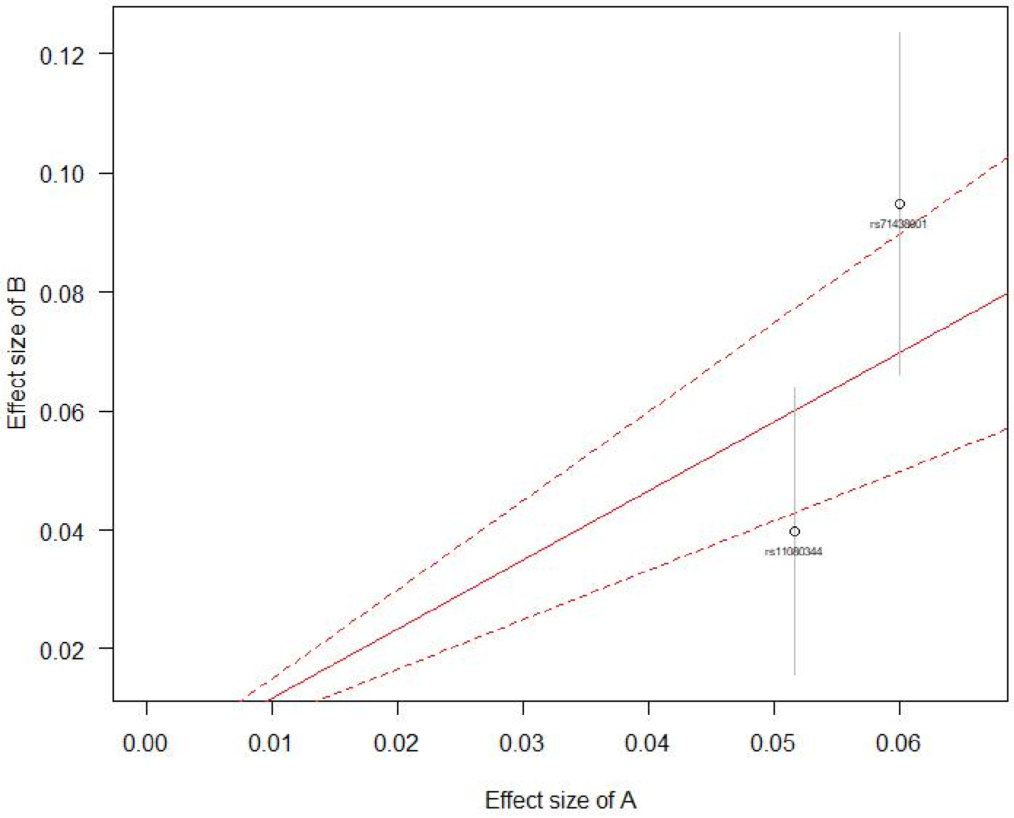

9. Gut microbiota abundance (genus Defluviitaleaceae UCG011 id.11287)

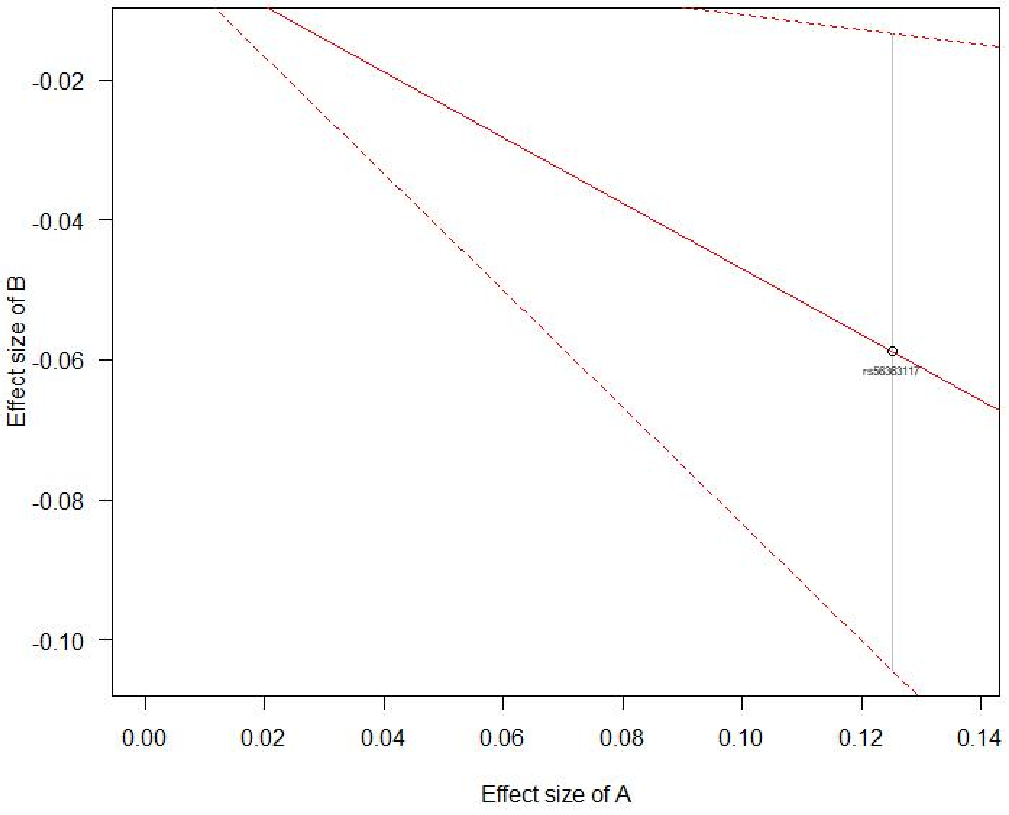

10. Gut microbiota abundance (genus Eubacterium coprostanoligenes group id.11375)

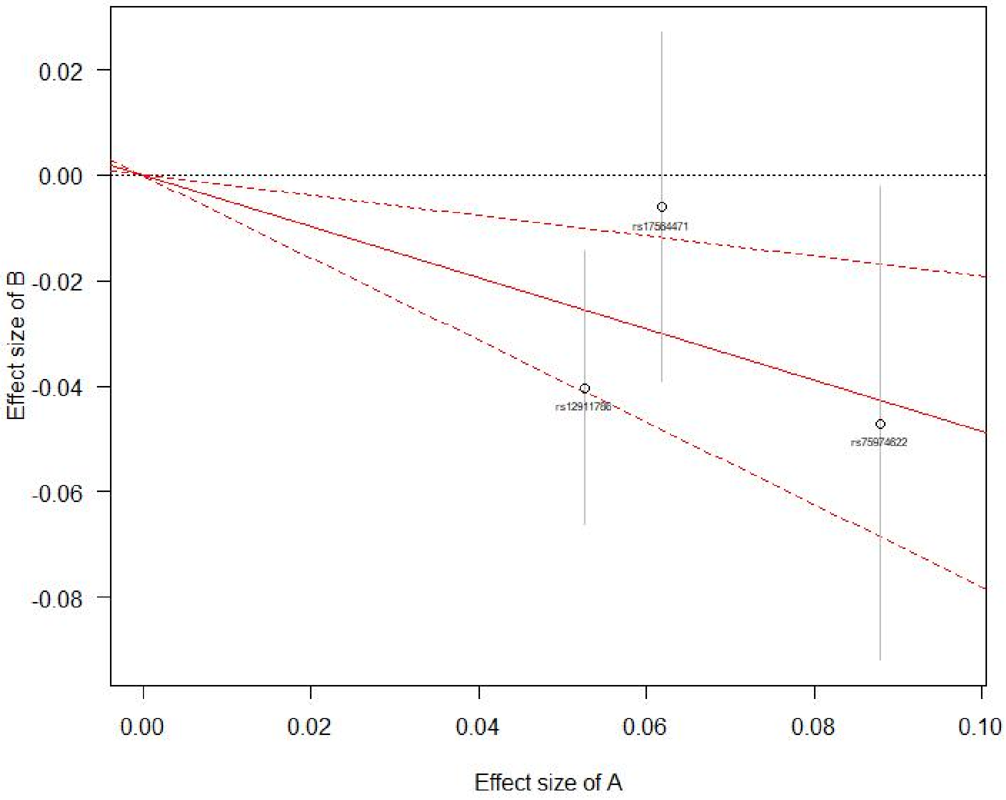

11. Gut microbiota abundance (genus Eubacterium fissicatena group id.14373)

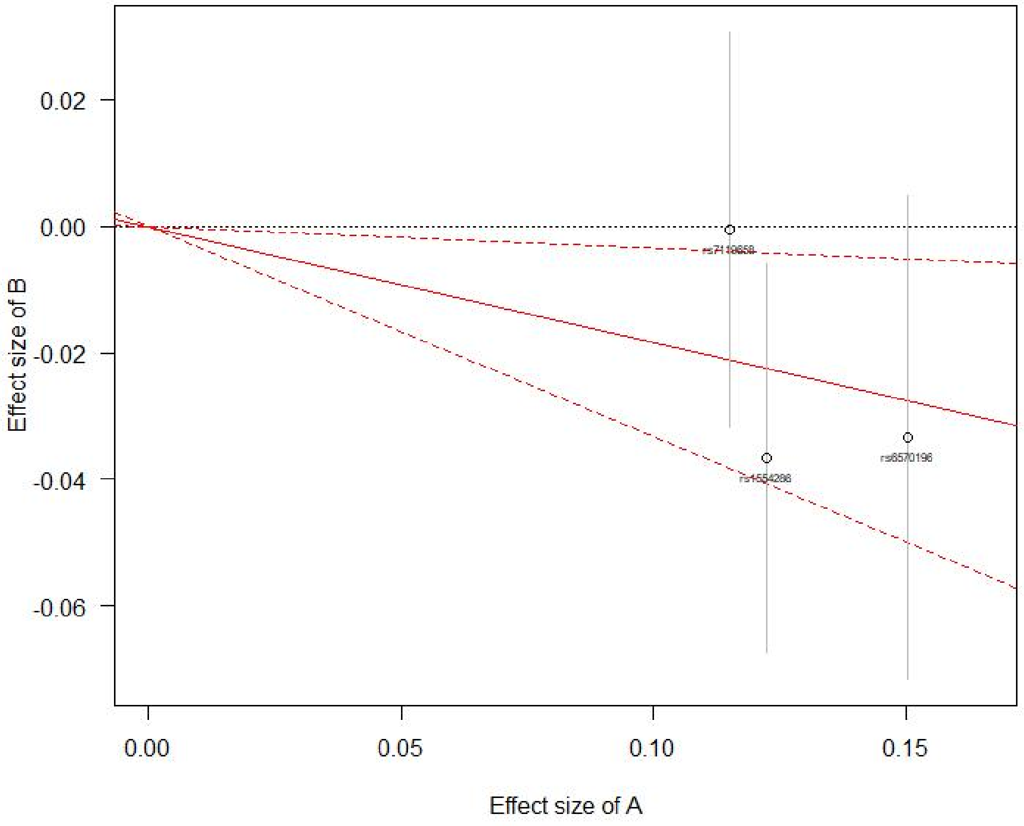

12. Gut microbiota abundance (genus Eubacterium hallii group id.11338)

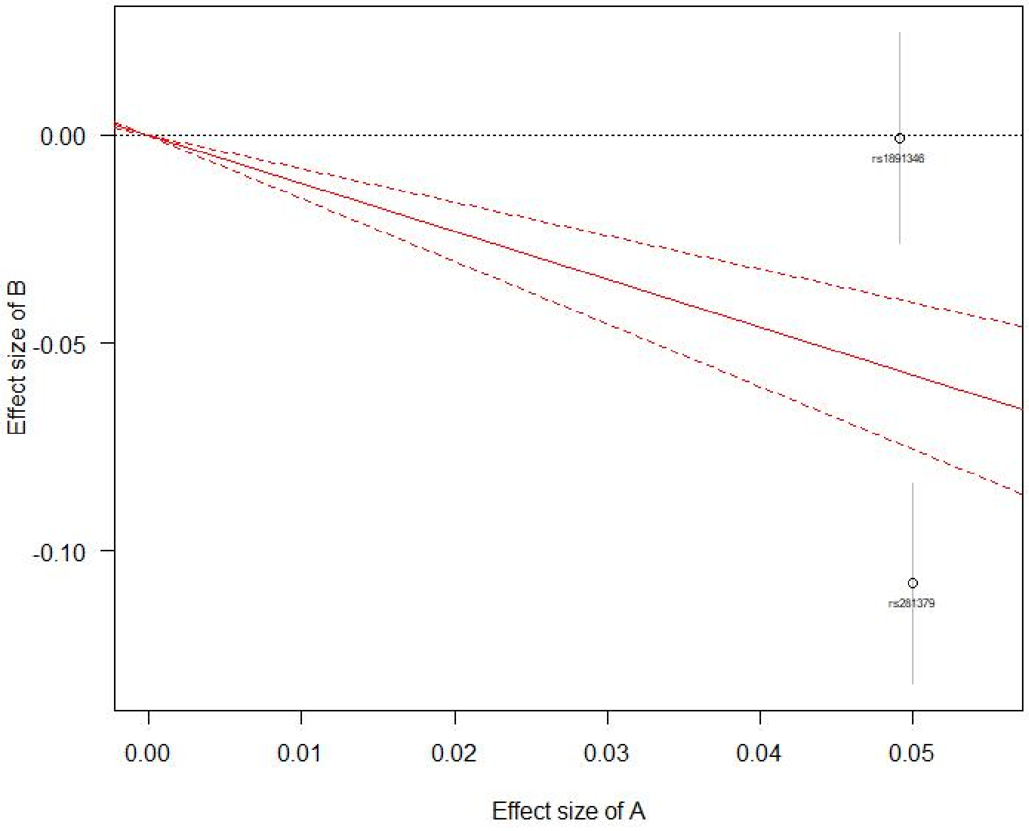

13. Gut microbiota abundance (genus Haemophilus id.3698)

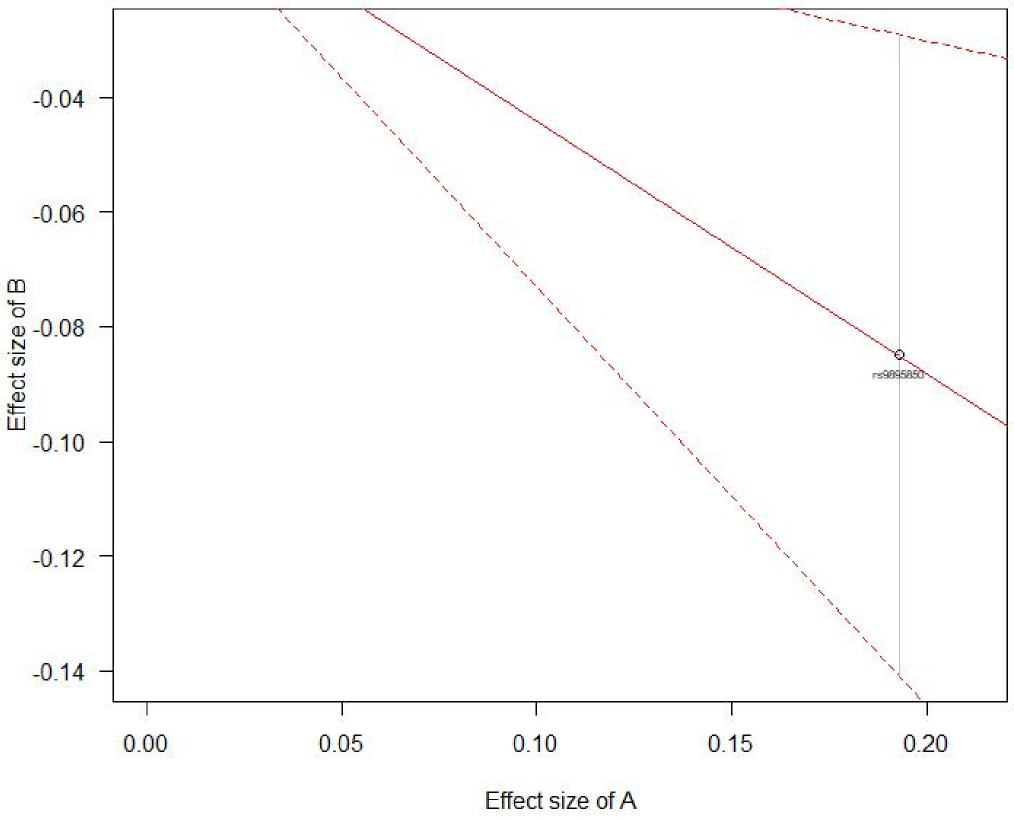

14. Gut microbiota abundance (genus Lachnoclostridium id.11308)

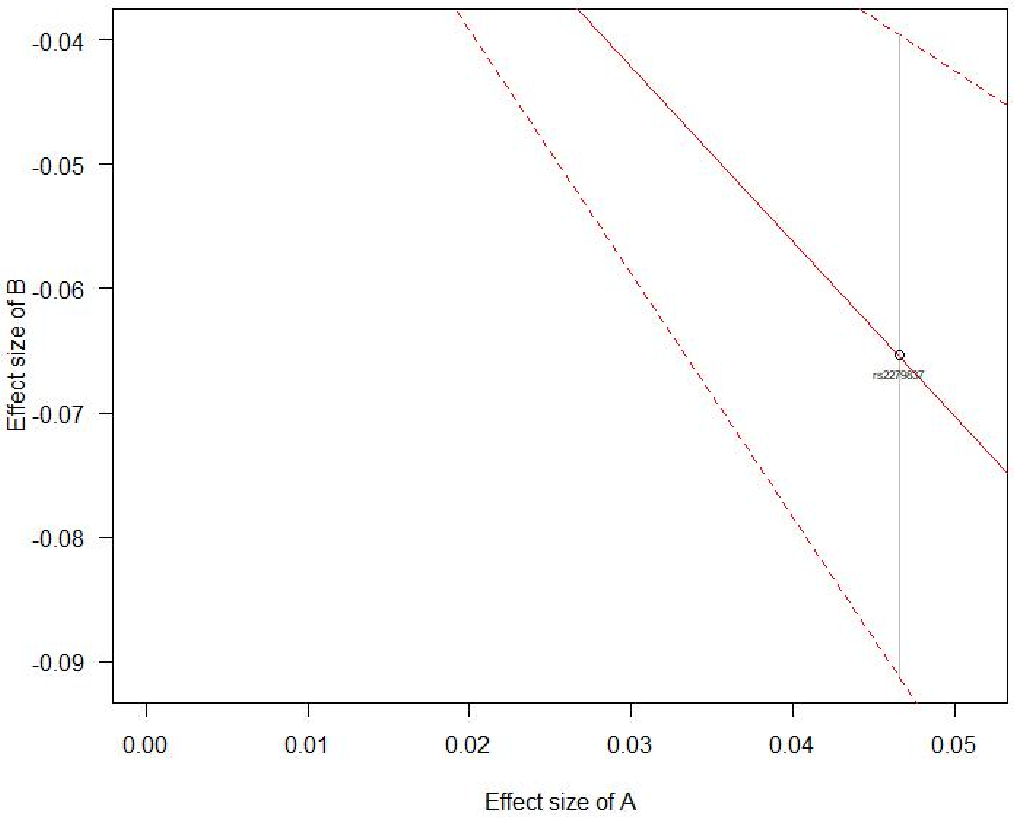

15. Gut microbiota abundance (genus Methanobrevibacter id.123)

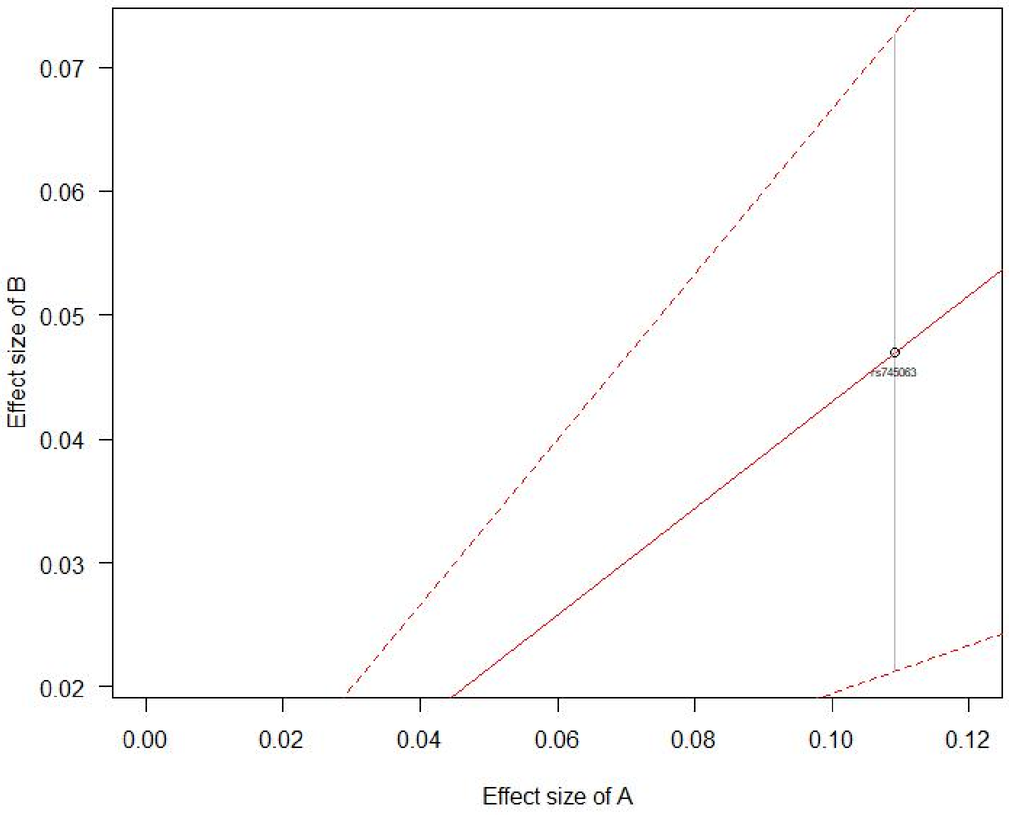

16. Gut microbiota abundance (genus Odoribacter id.952)

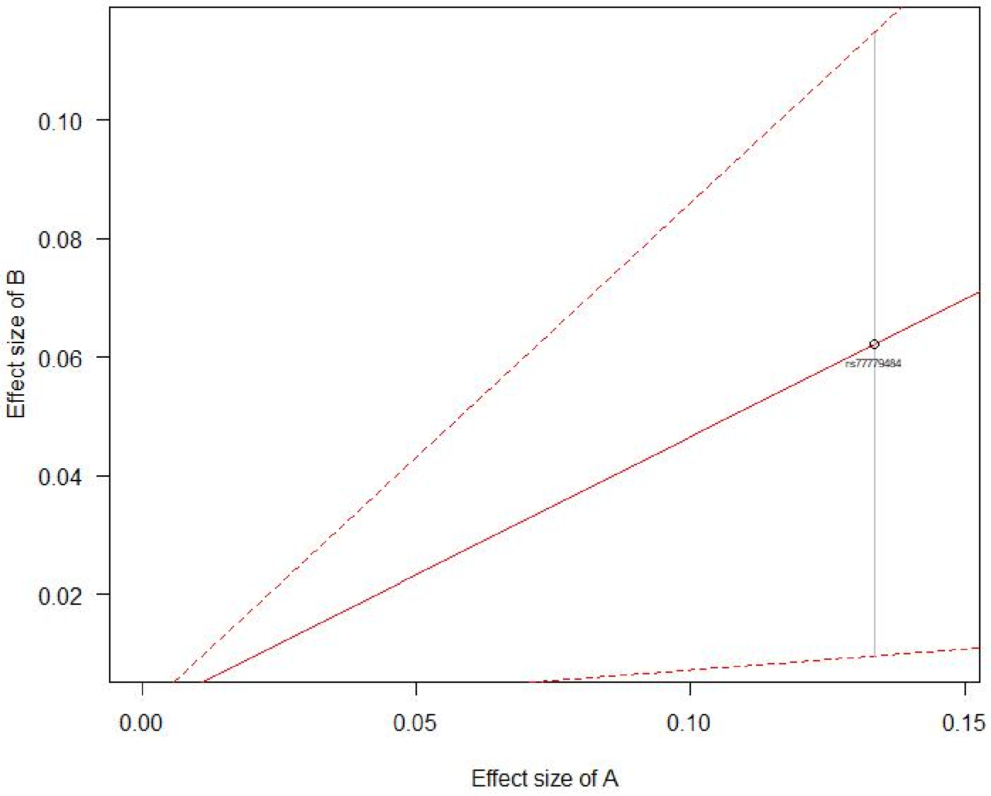

17. Gut microbiota abundance (genus Oscillospira id.2064)

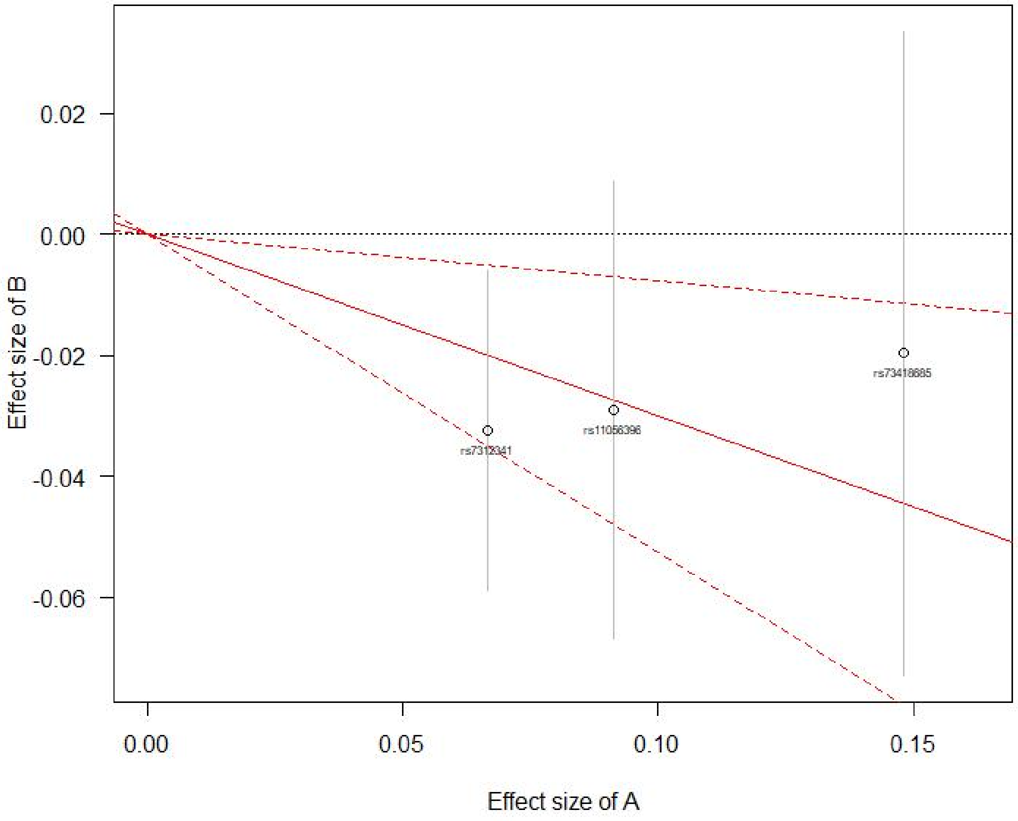

18. Gut microbiota abundance (genus Parabacteroides id.954)

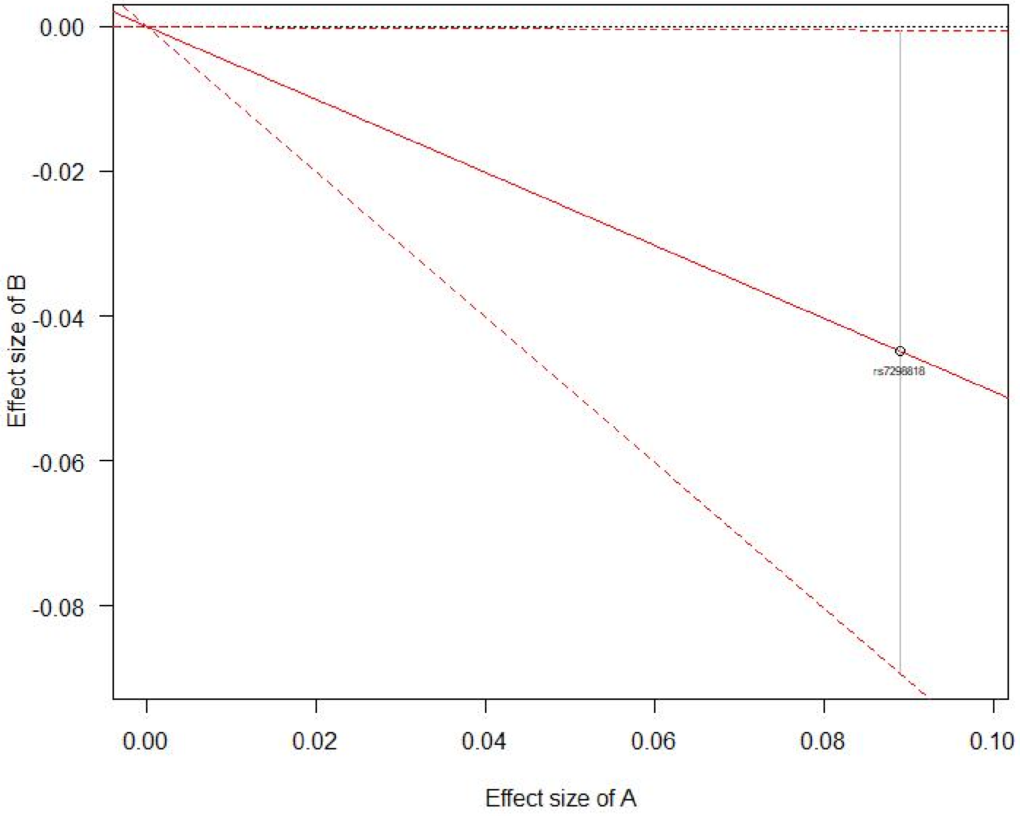

19. Gut microbiota abundance (genus Romboutsia id.11347)

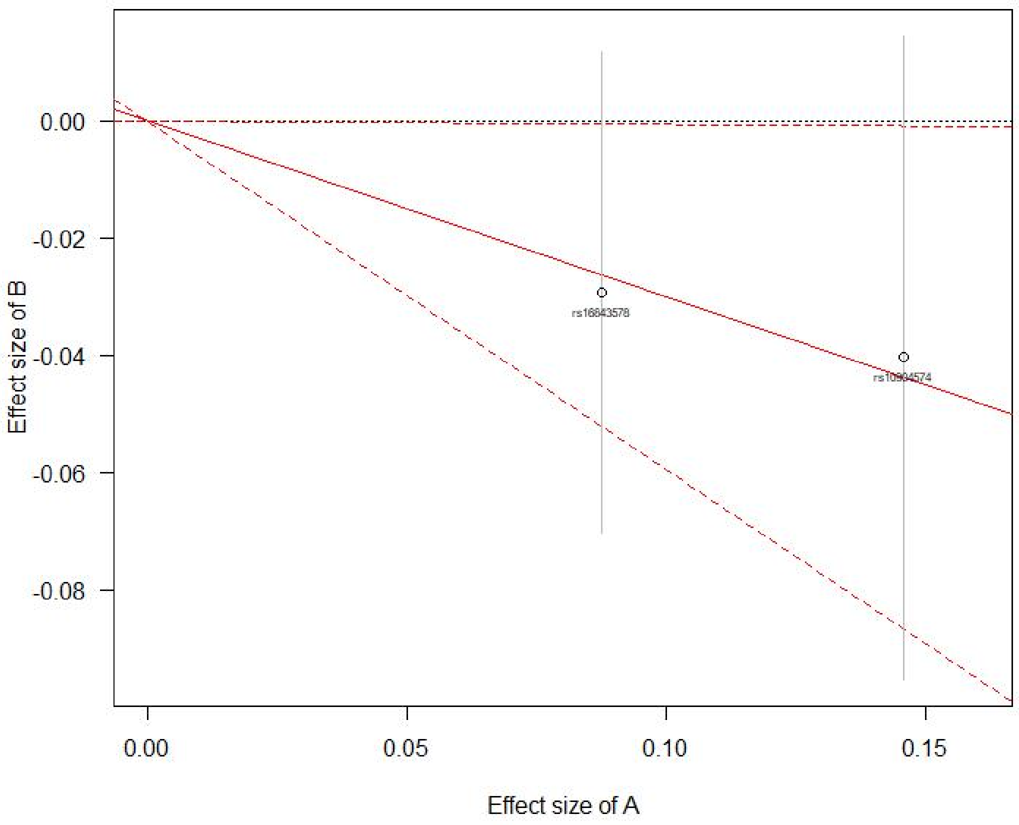

20. Gut microbiota abundance (genus Ruminiclostridium5 id.11355)

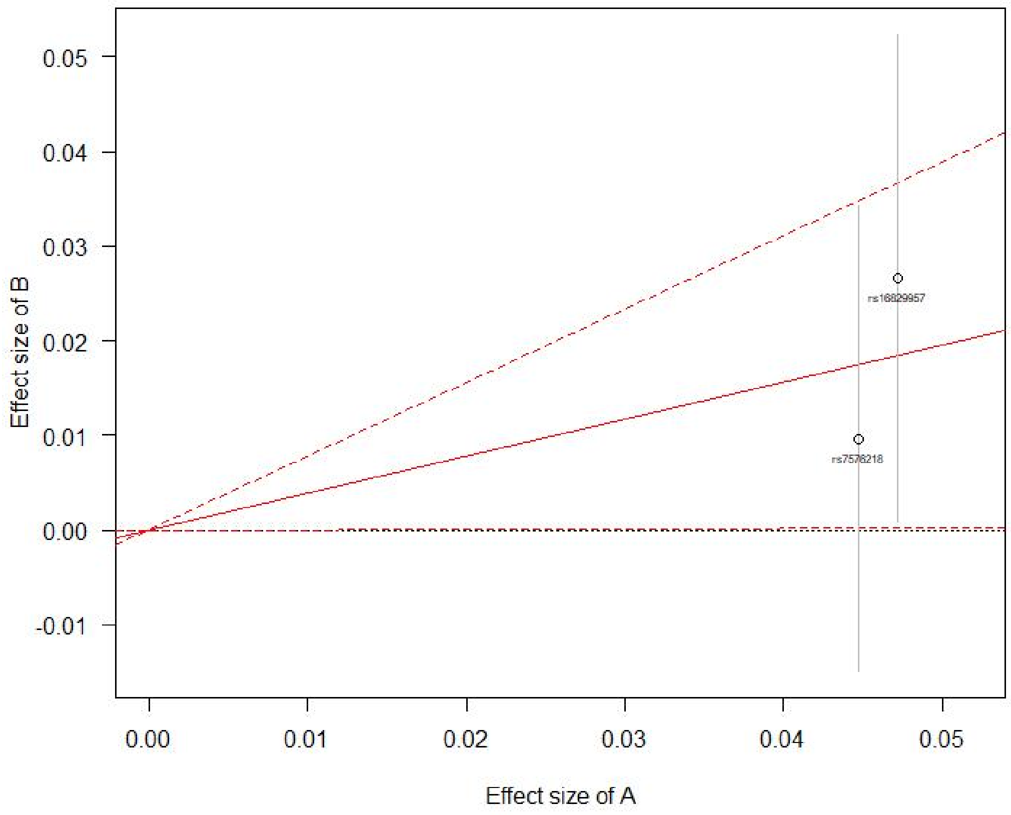

21. Gut microbiota abundance (genus Ruminococcaceae UCG005 id.11363)

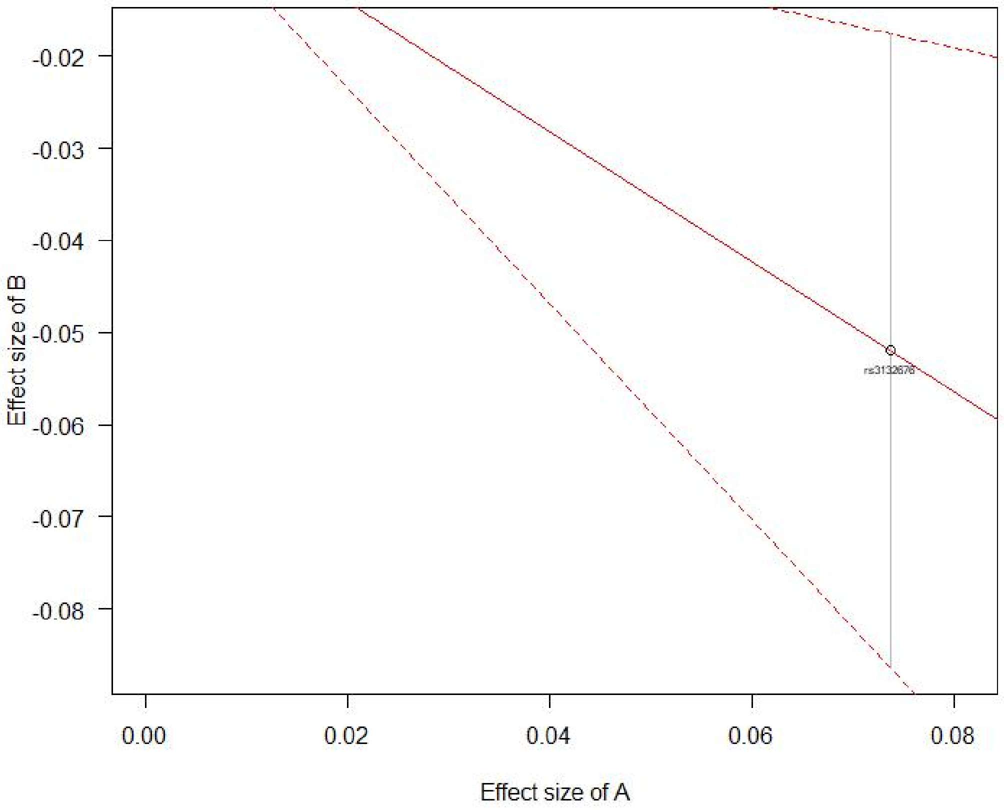

22. Gut microbiota abundance (genus Ruminococcaceae UCG011 id.11368)

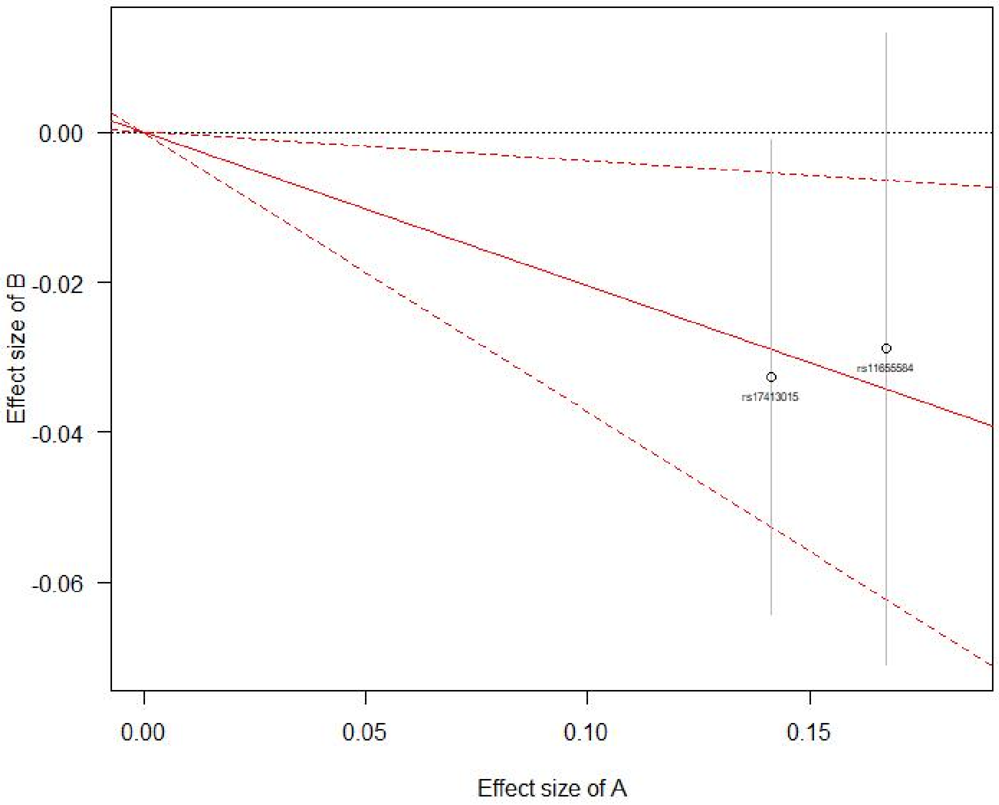

23. Gut microbiota abundance (genus Ruminococcaceae UCG013 id.11370)

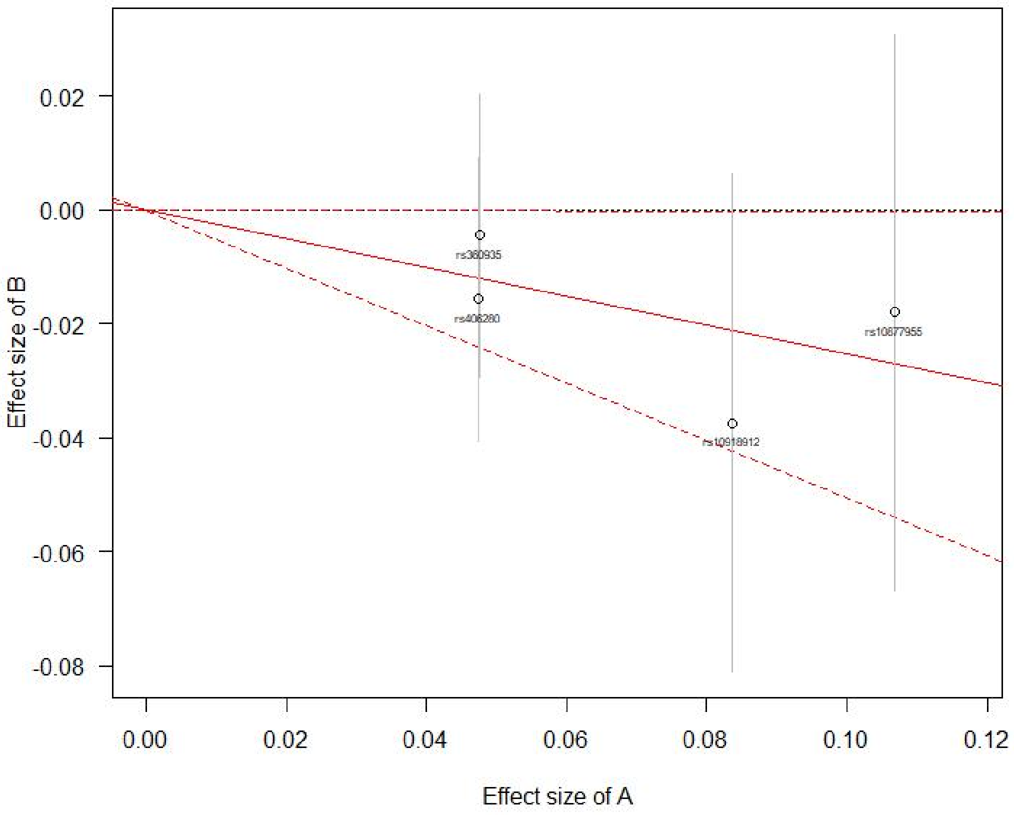

24. Gut microbiota abundance (genus Ruminococcus torques group id.14377)

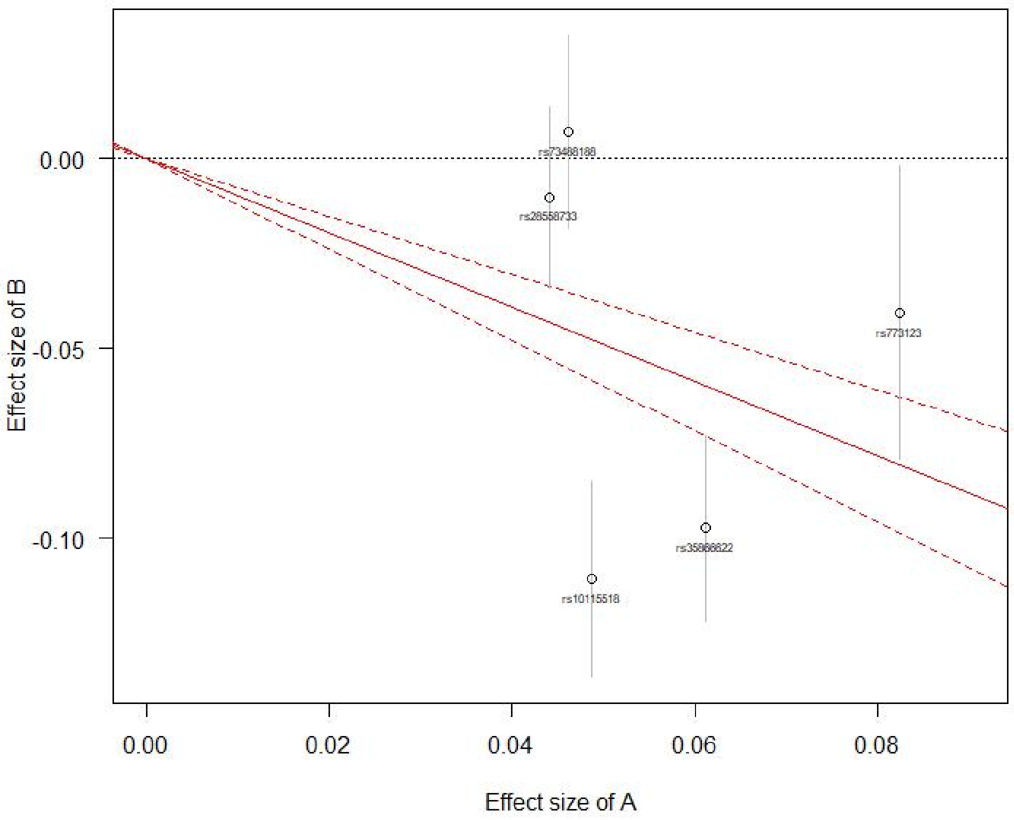

25. Gut microbiota abundance (genus Slackia id.825)

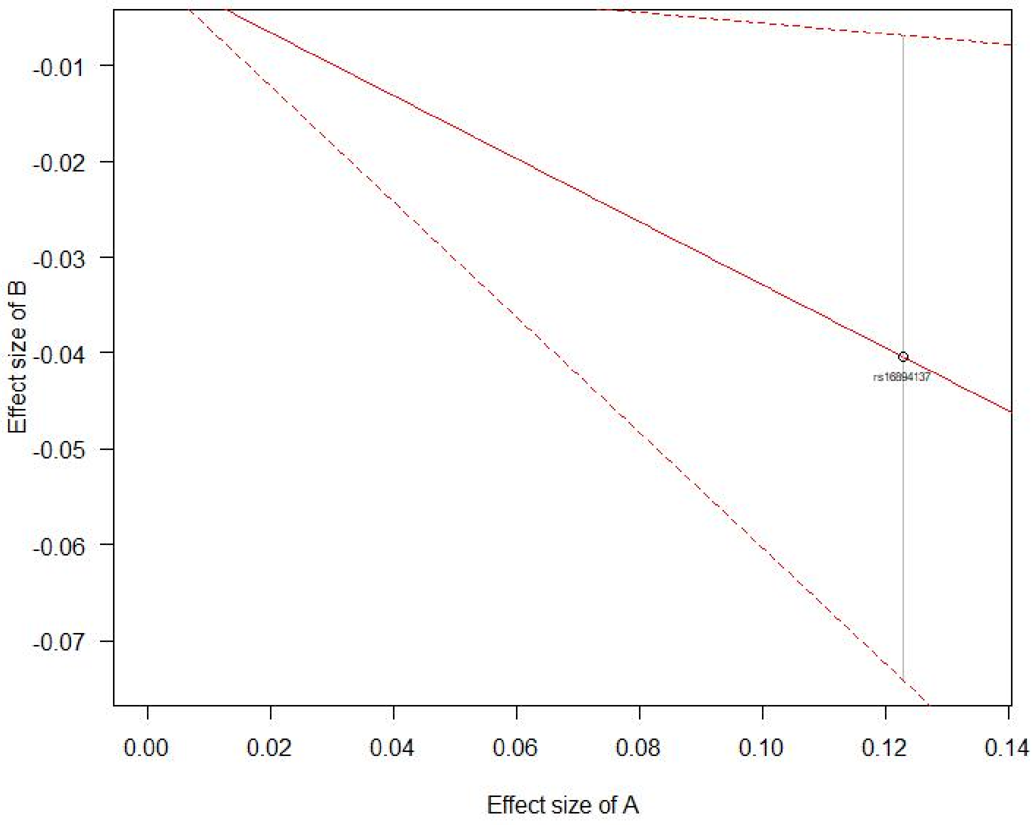

26. Gut microbiota abundance (genus Terrisporobacter id.11348)

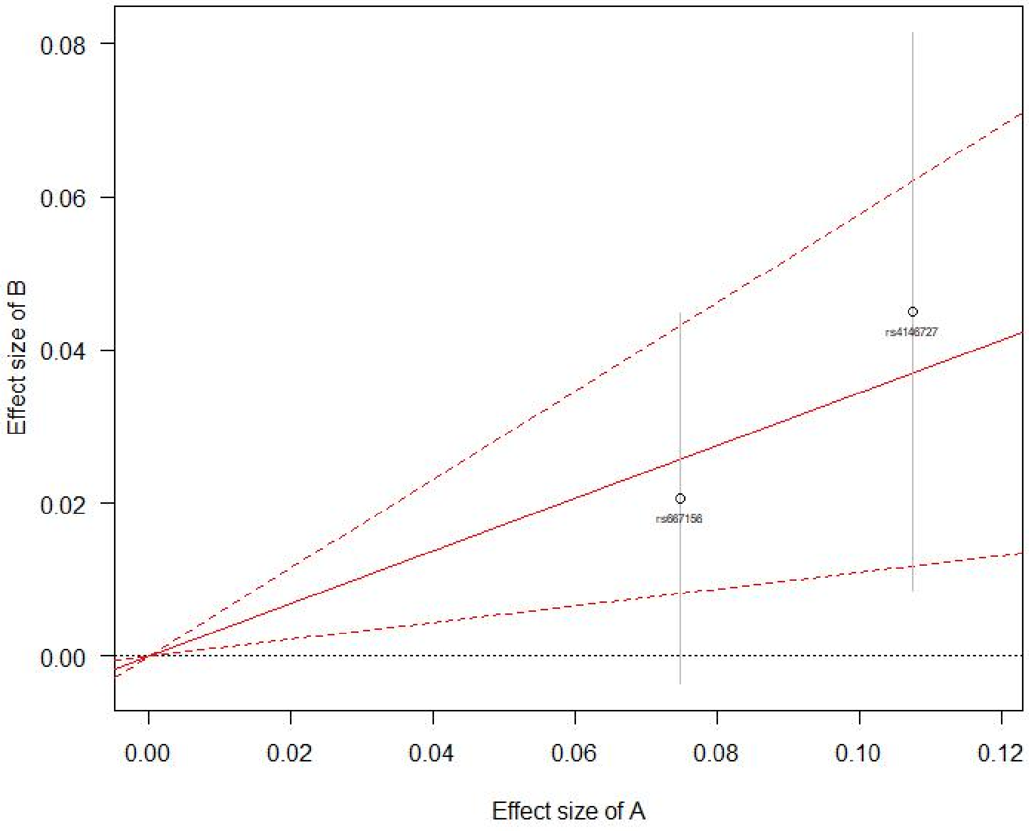

27. Gut microbiota abundance (unknown genus id.1000006162)

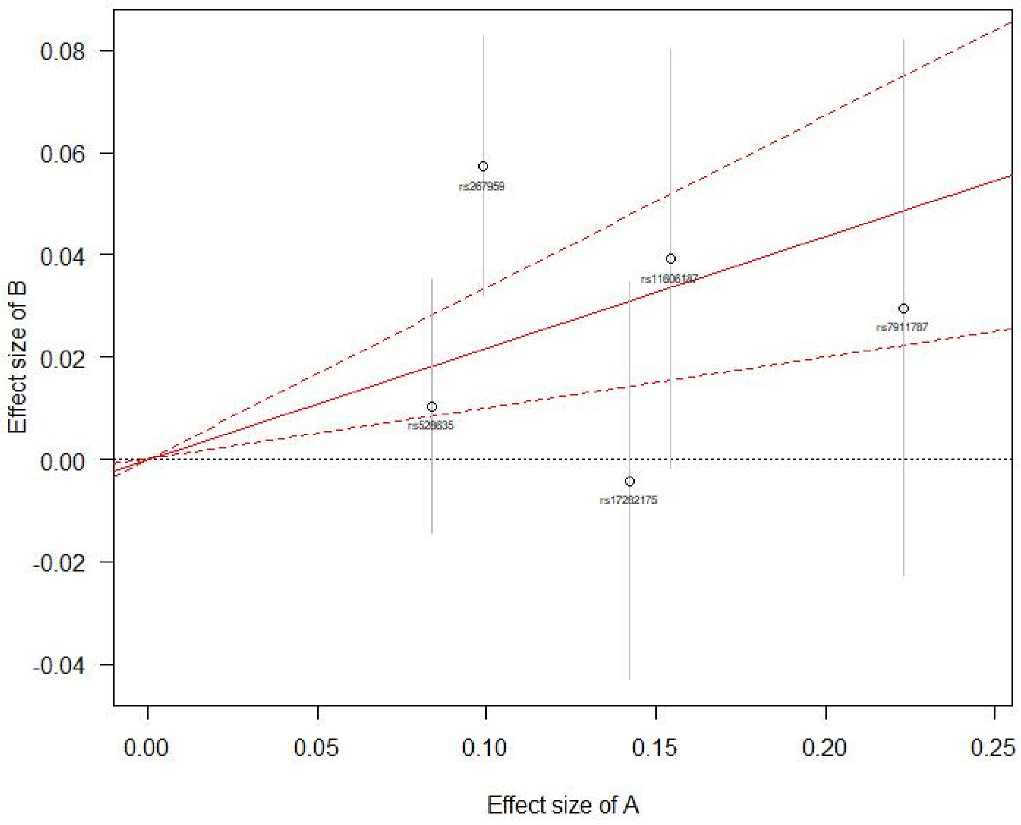

28. Gut microbiota abundance (unknown genus id.2001)

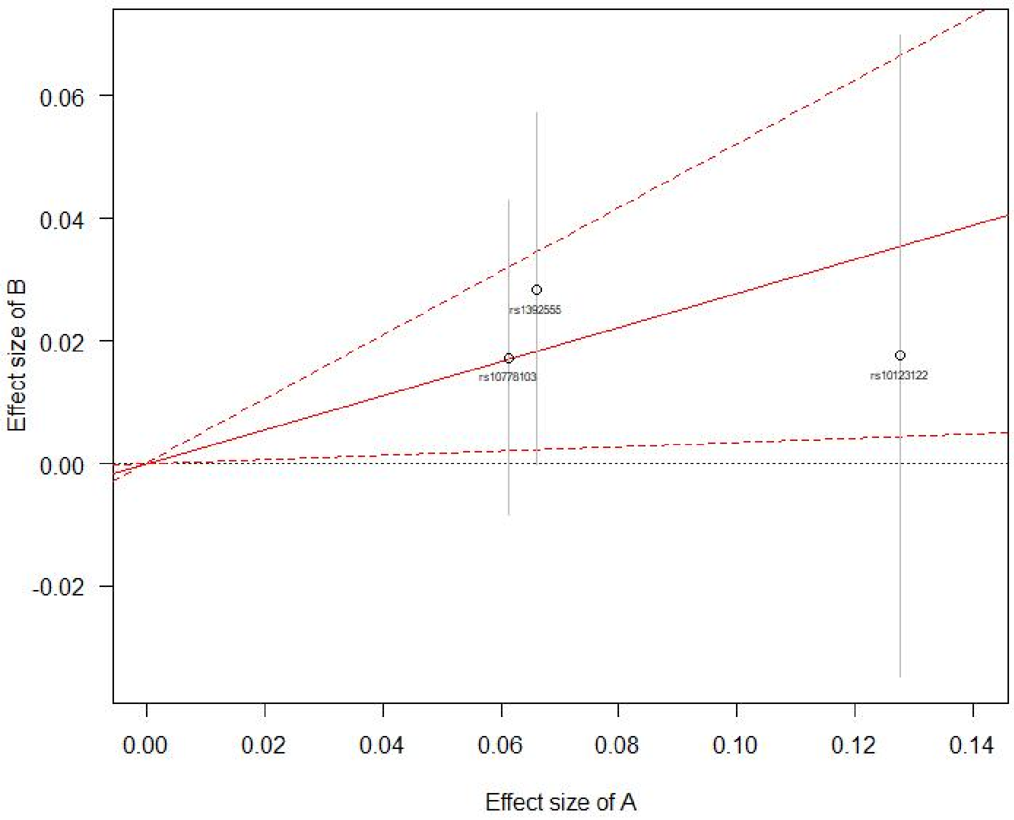

29. Gut microbiota abundance (order NB1n id.3953)

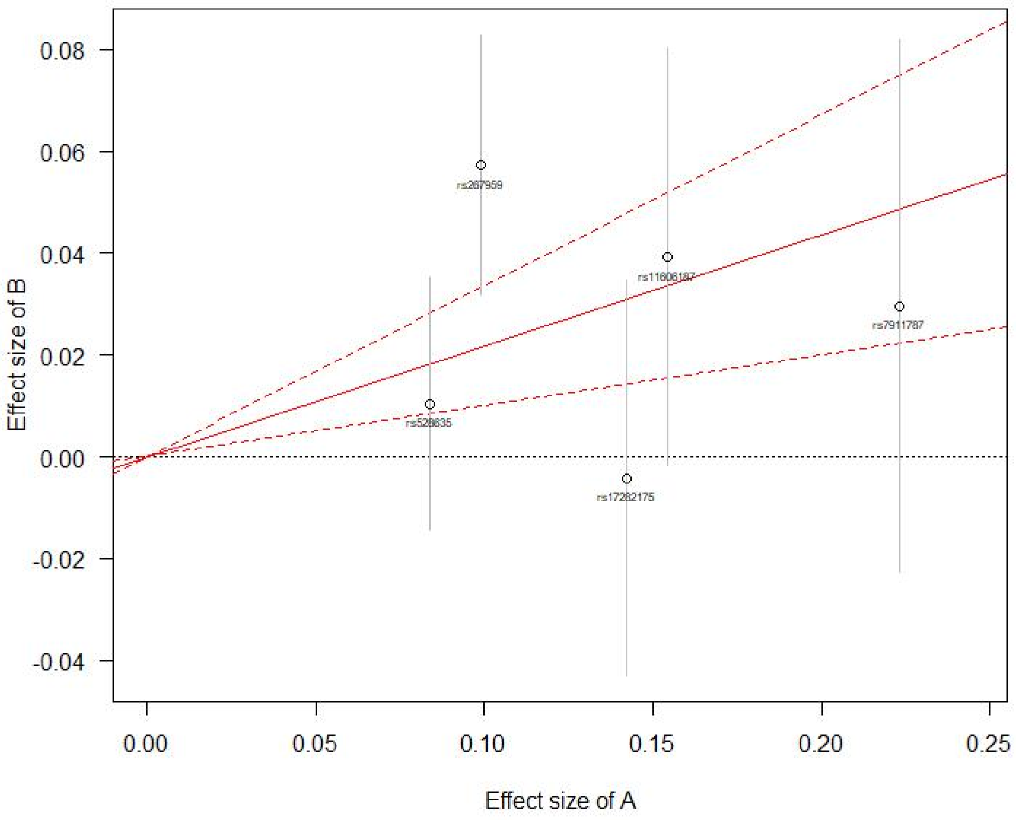

30. Gut microbiota abundance (order Selenomonadales id.2165)

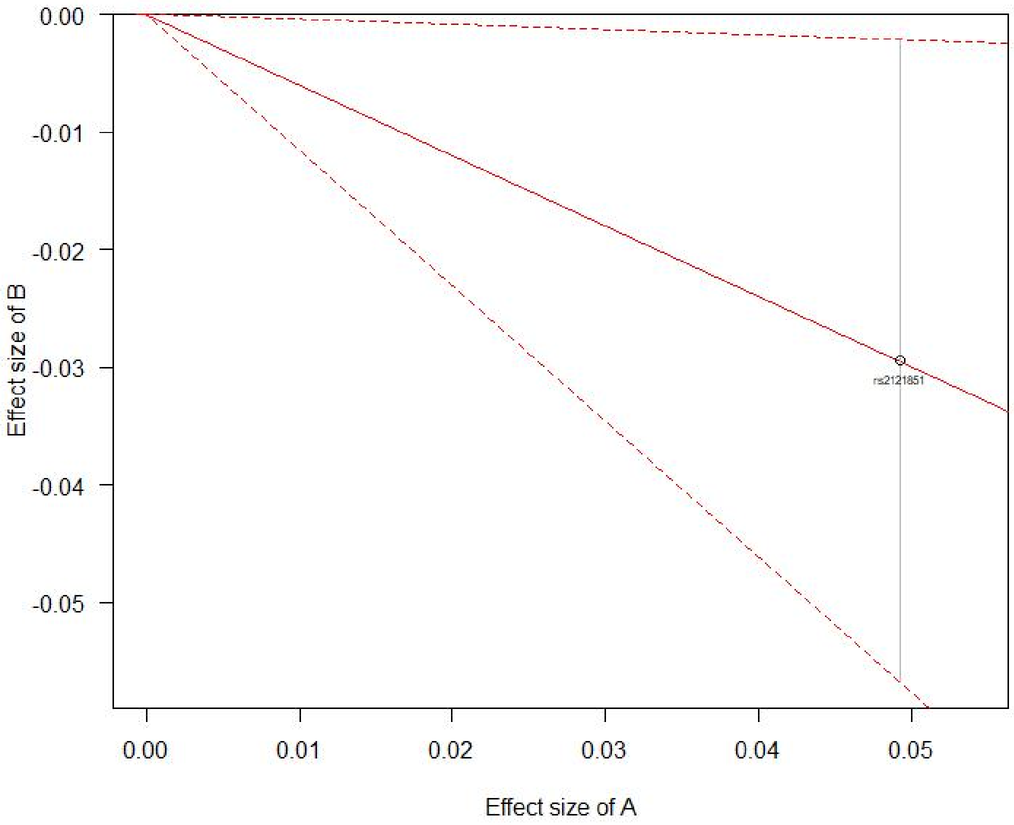

31. Gut microbiota abundance (order Verrucomicrobiales id.4030)

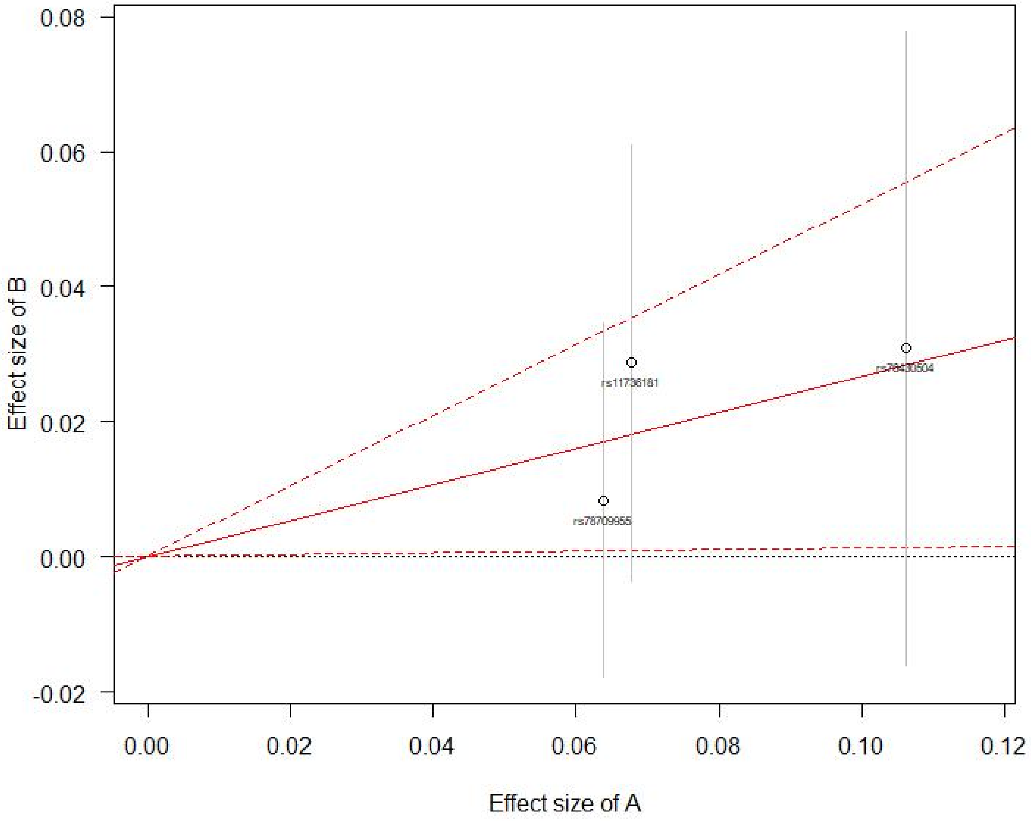

32. Gut microbiota abundance (phylum Verrucomicrobia id.3982)

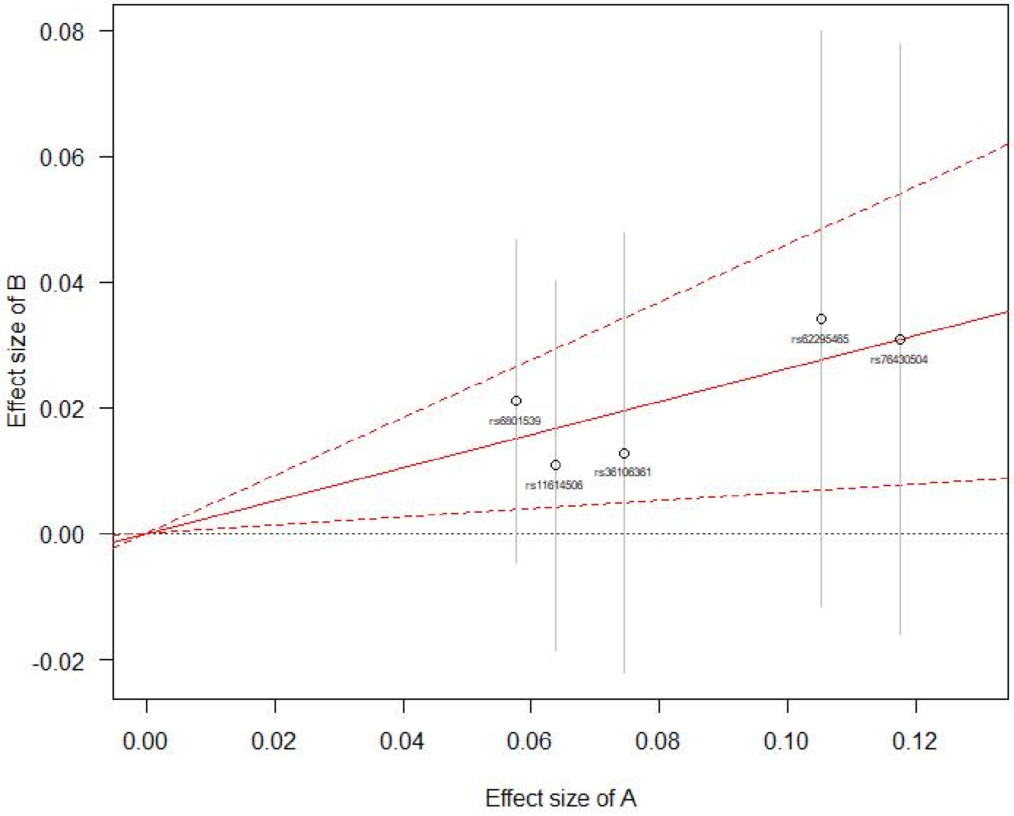

L. Ulcerative colitis

1. Gut microbiota abundance (class Bacilli id.1673)

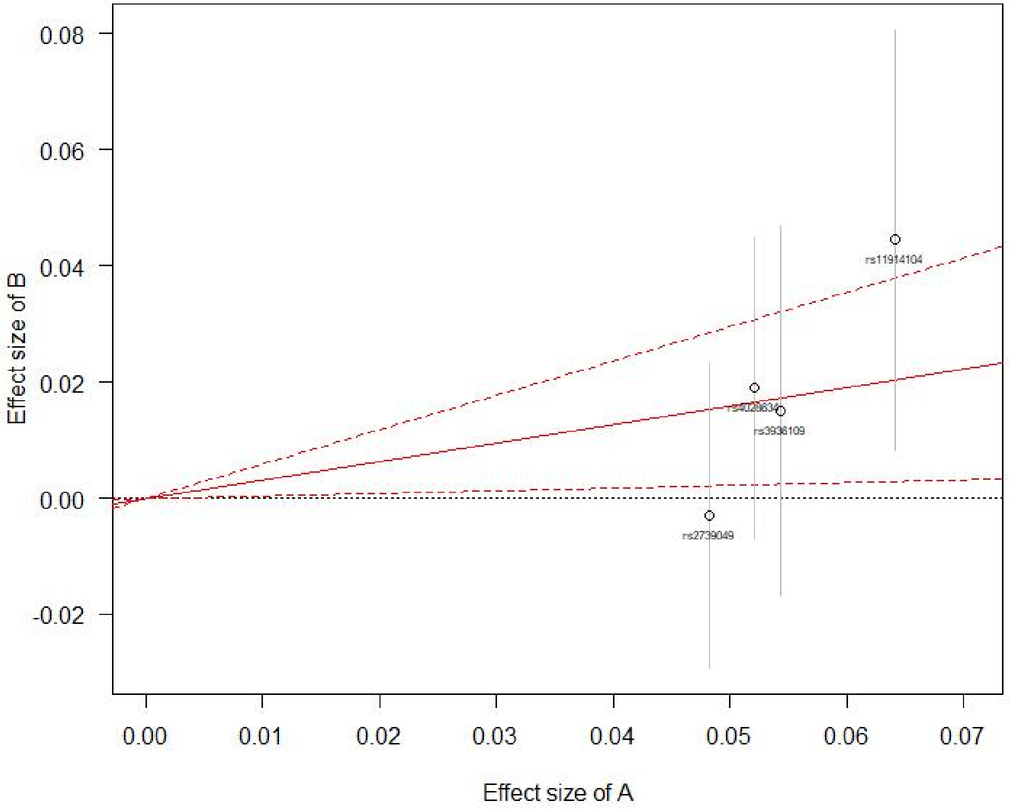

2. Gut microbiota abundance (class Lentisphaeria id.2250)

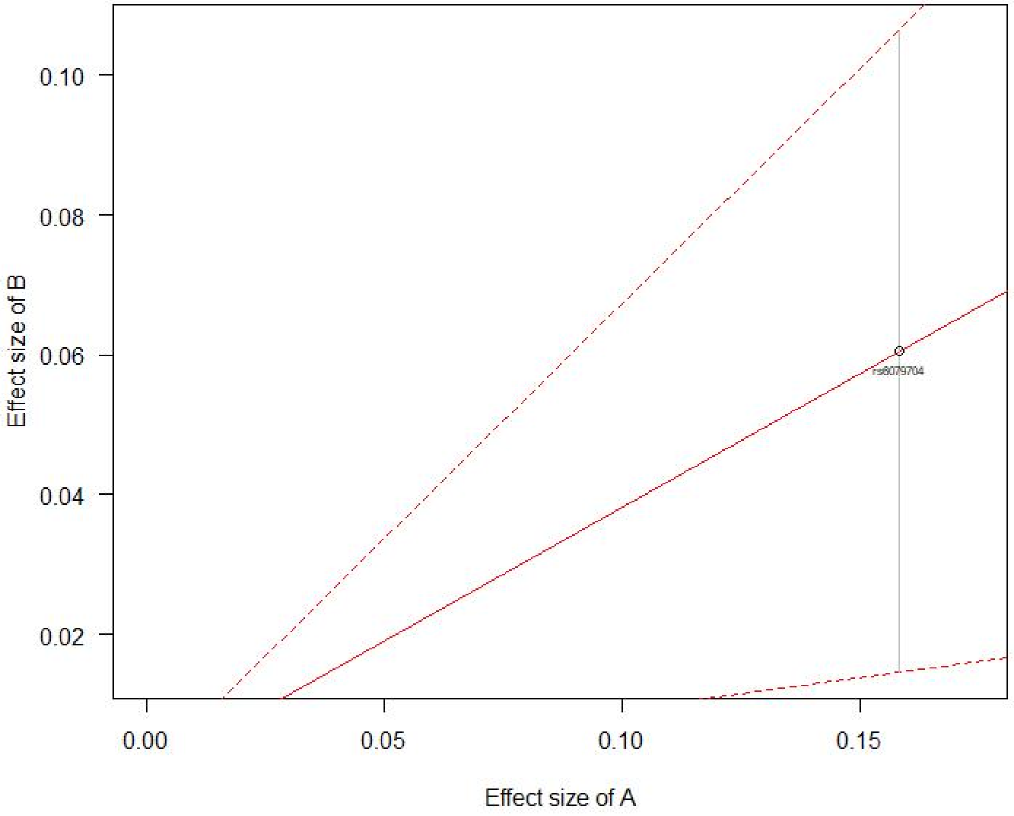

3. Gut microbiota abundance (family Bifidobacteriaceae id.433)

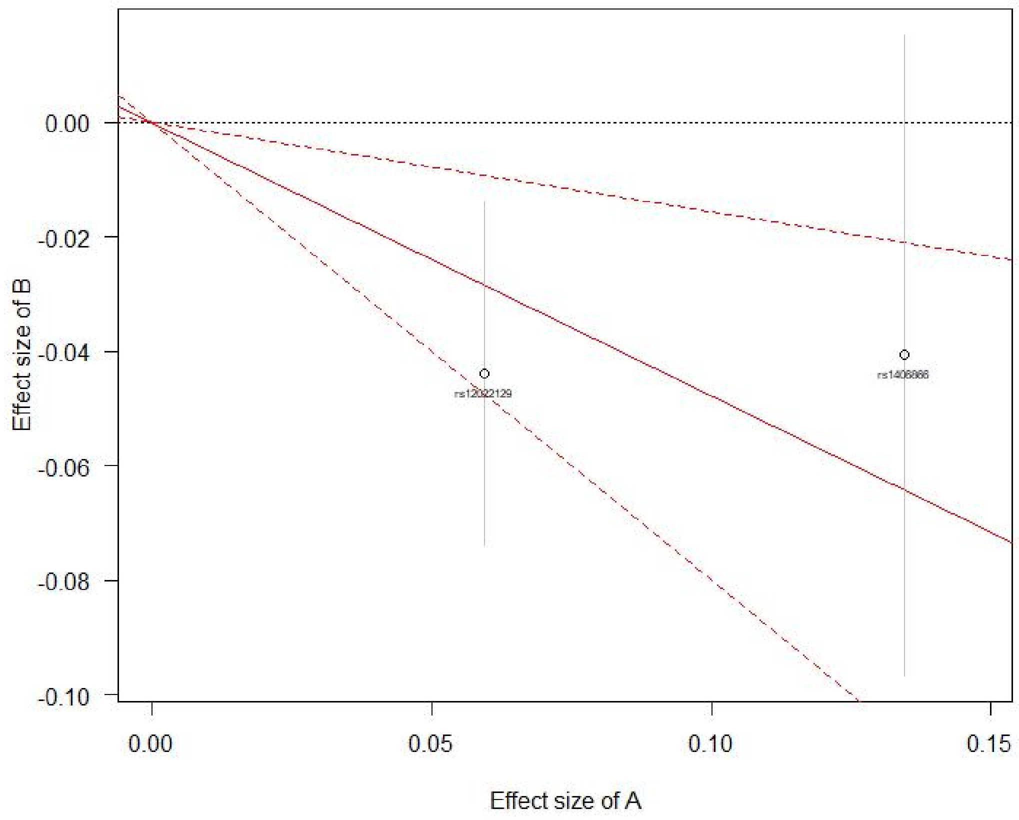

4. Gut microbiota abundance (family Streptococcaceae id.1850)

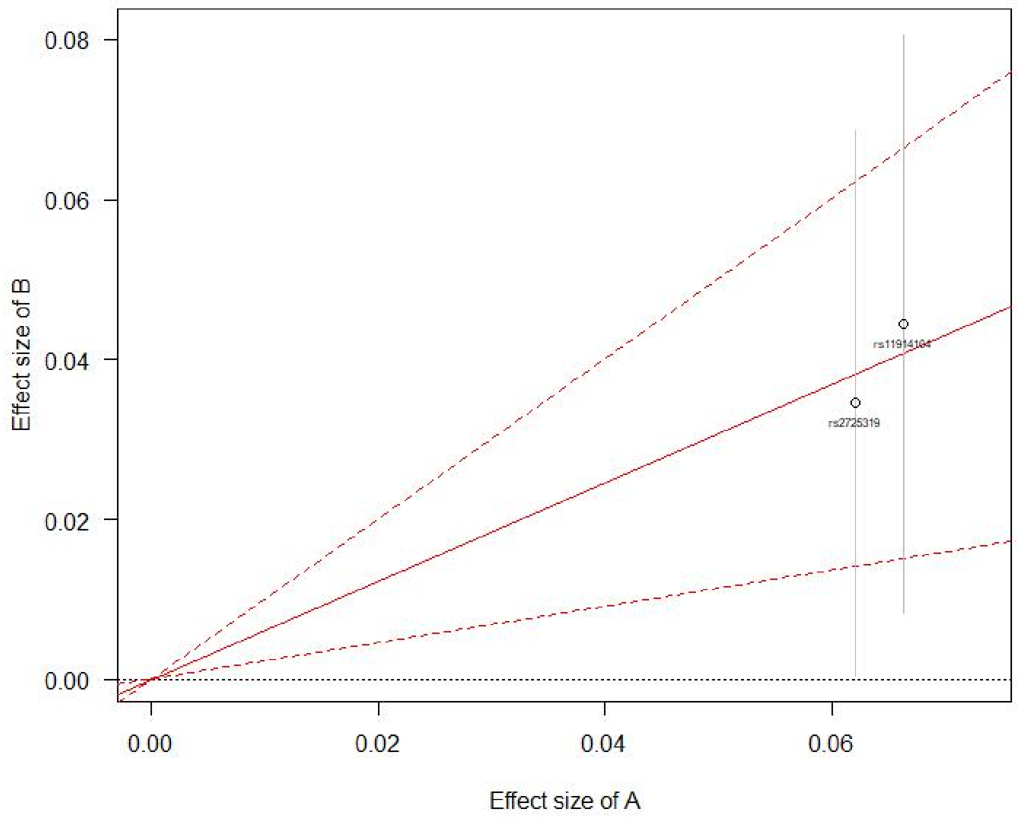

5. Gut microbiota abundance (genus Clostridium innocuum group id.14397)

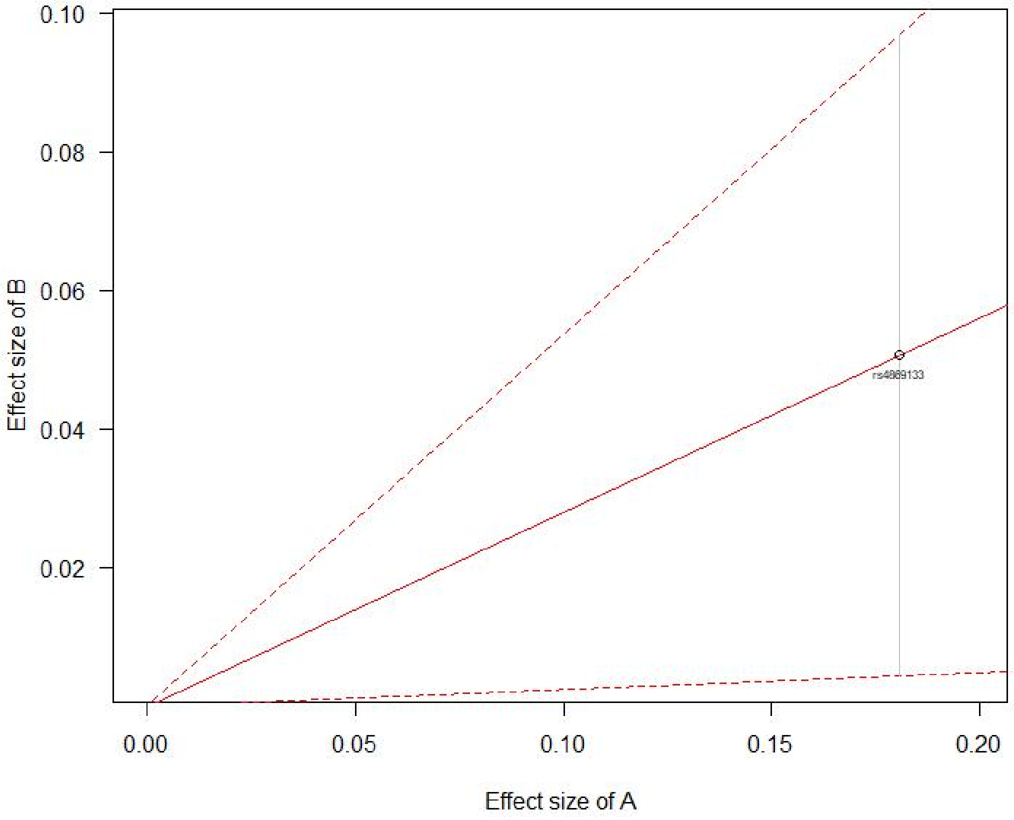

6. Gut microbiota abundance (genus Coprobacter id.949)

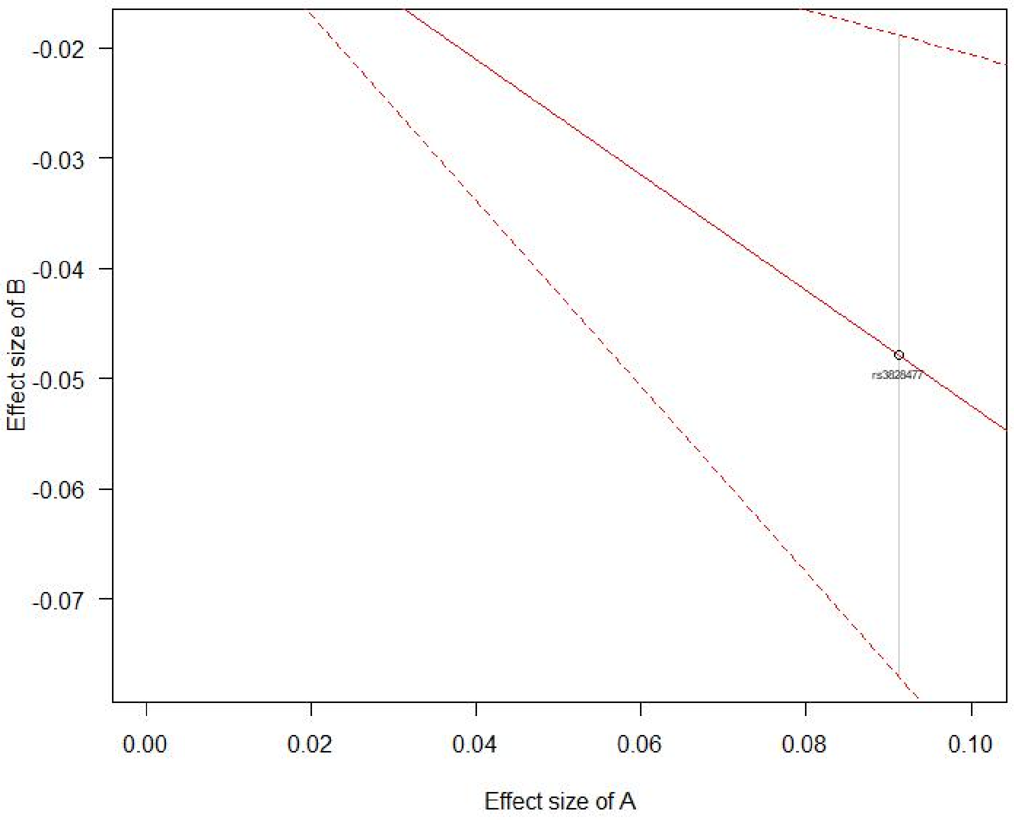

7. Gut microbiota abundance (genus Coprococcus3 id.11303)

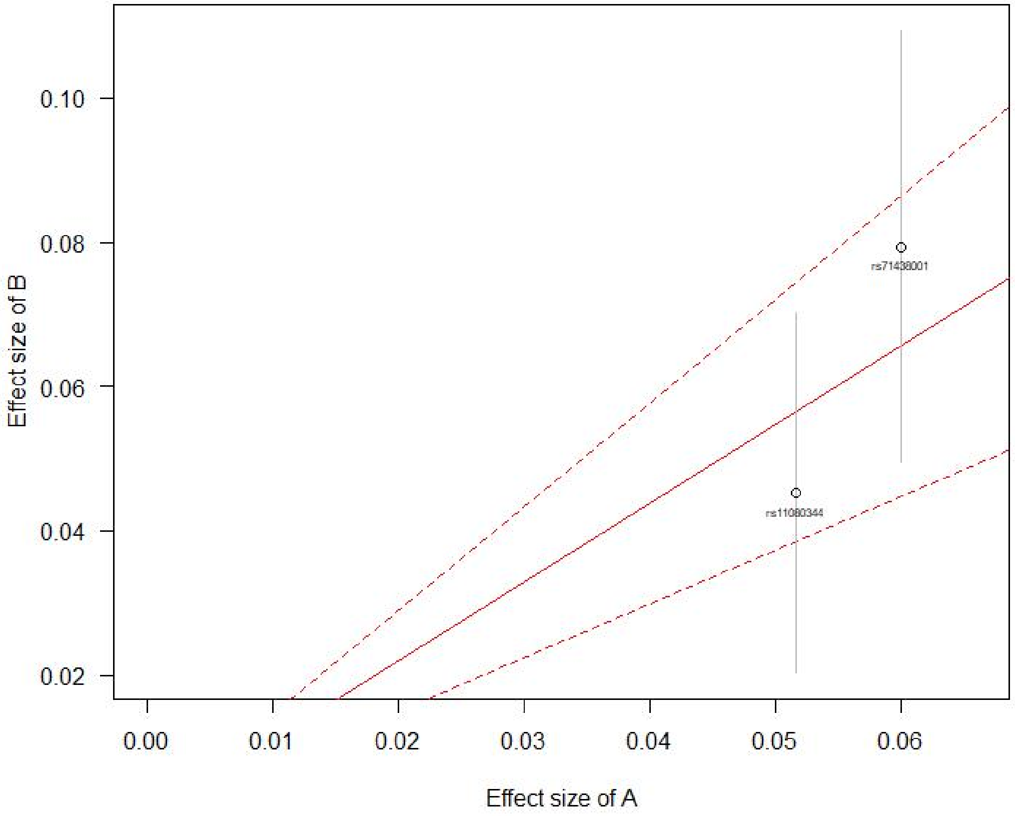

8. Gut microbiota abundance (genus Escherichia Shigella id.3504)

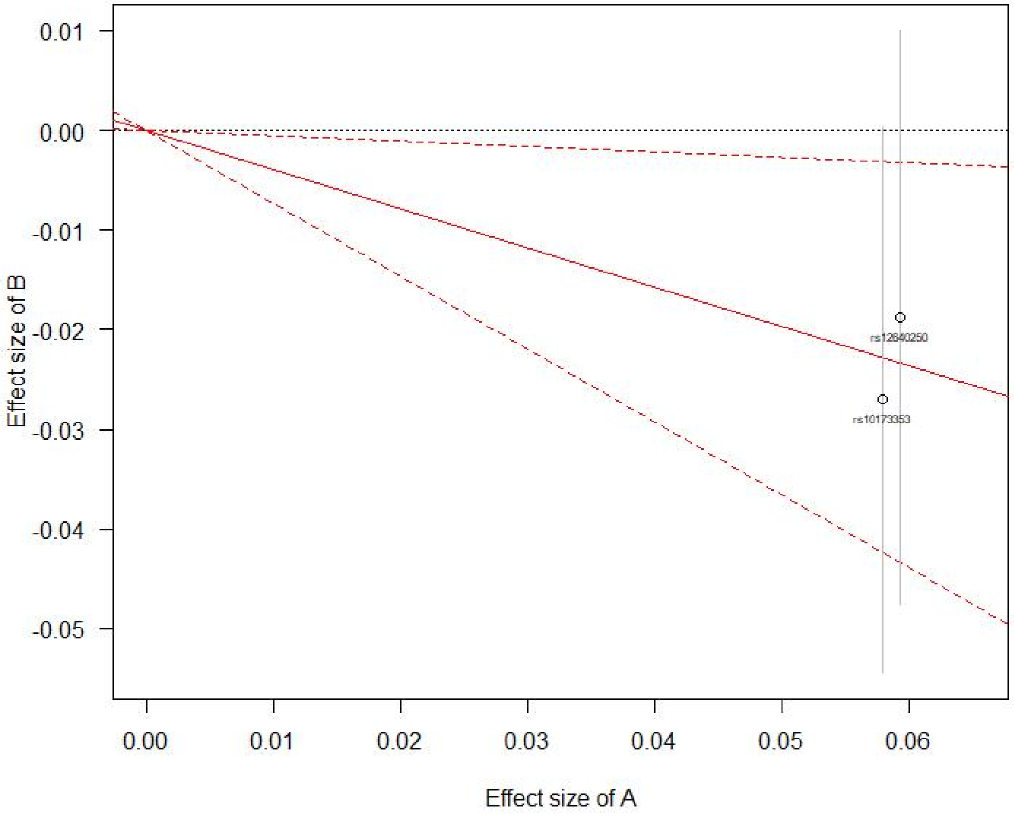

9. Gut microbiota abundance (genus Eubacterium hallii group id.11338)

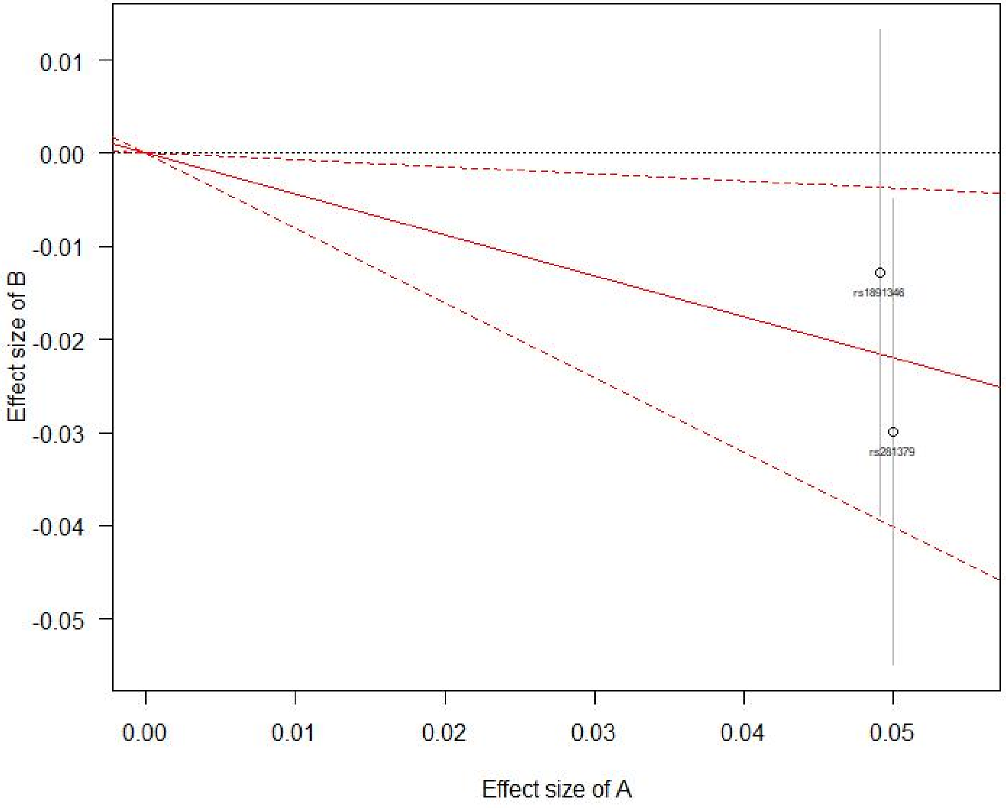

10. Gut microbiota abundance (genus Eubacterium oxidoreducens group id.11339)

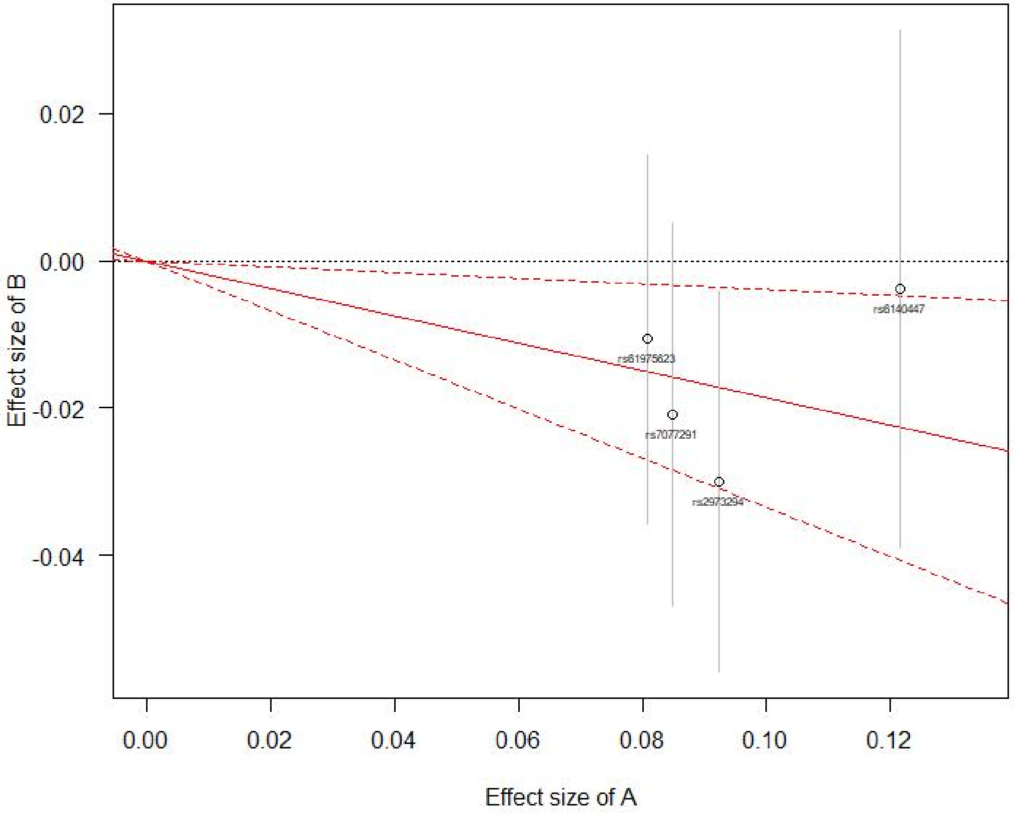

11. Gut microbiota abundance (genus Lachnoclostridium id.11308)

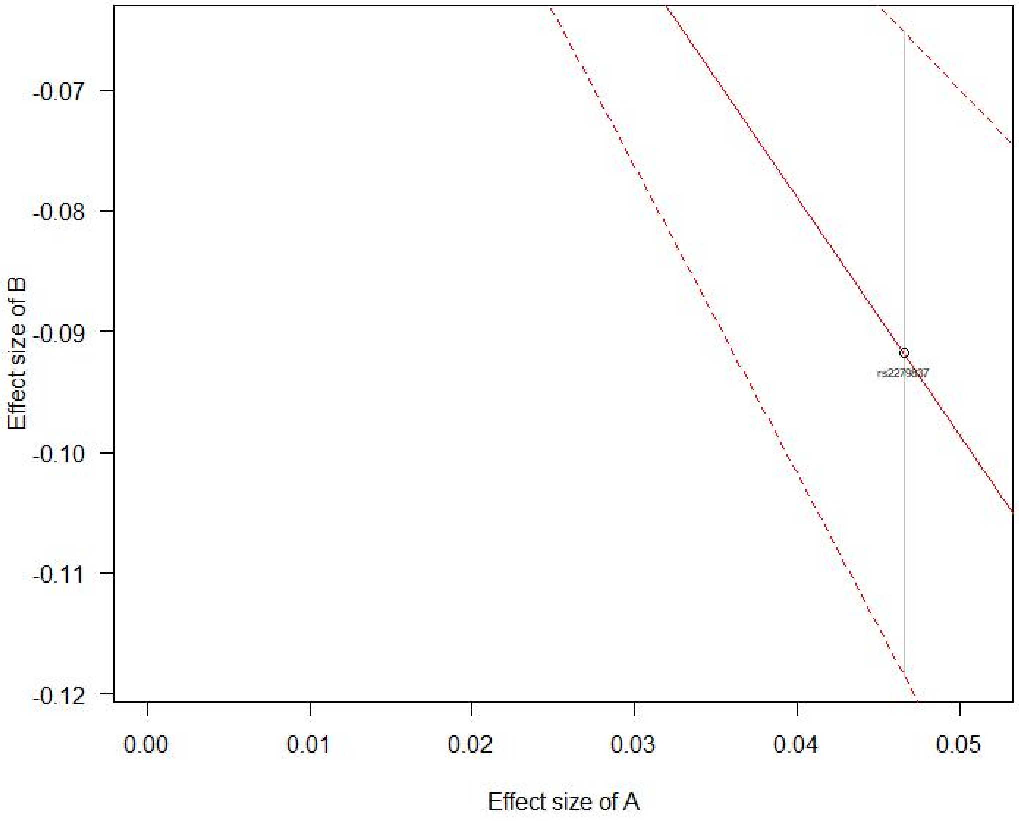

12. Gut microbiota abundance (genus Lachnospiraceae NC2004 group id.11316)

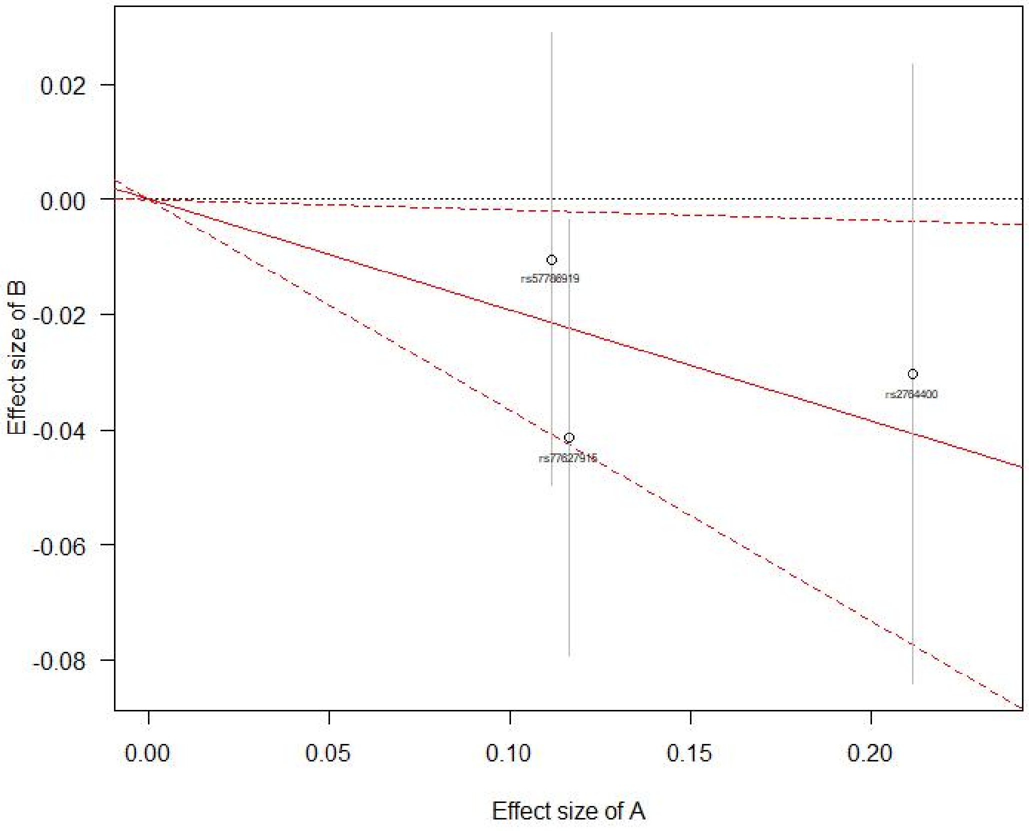

13. Gut microbiota abundance (genus Lachnospira id.2004)

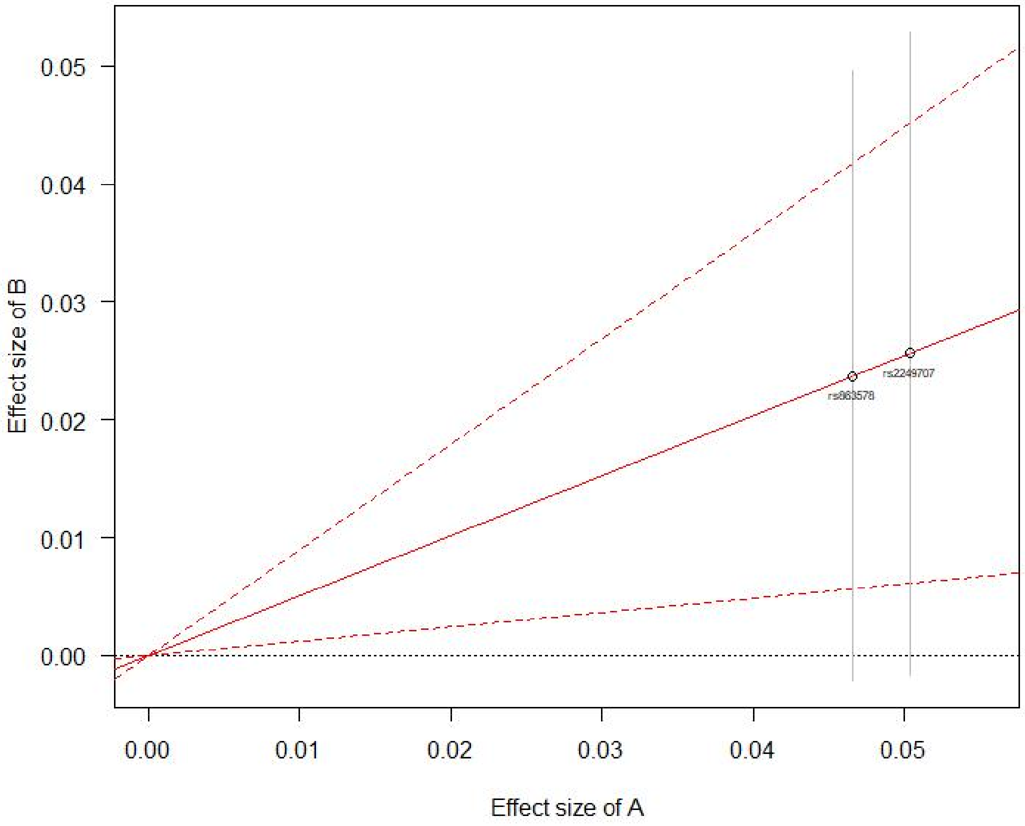

14. Gut microbiota abundance (genus Streptococcus id.1853)

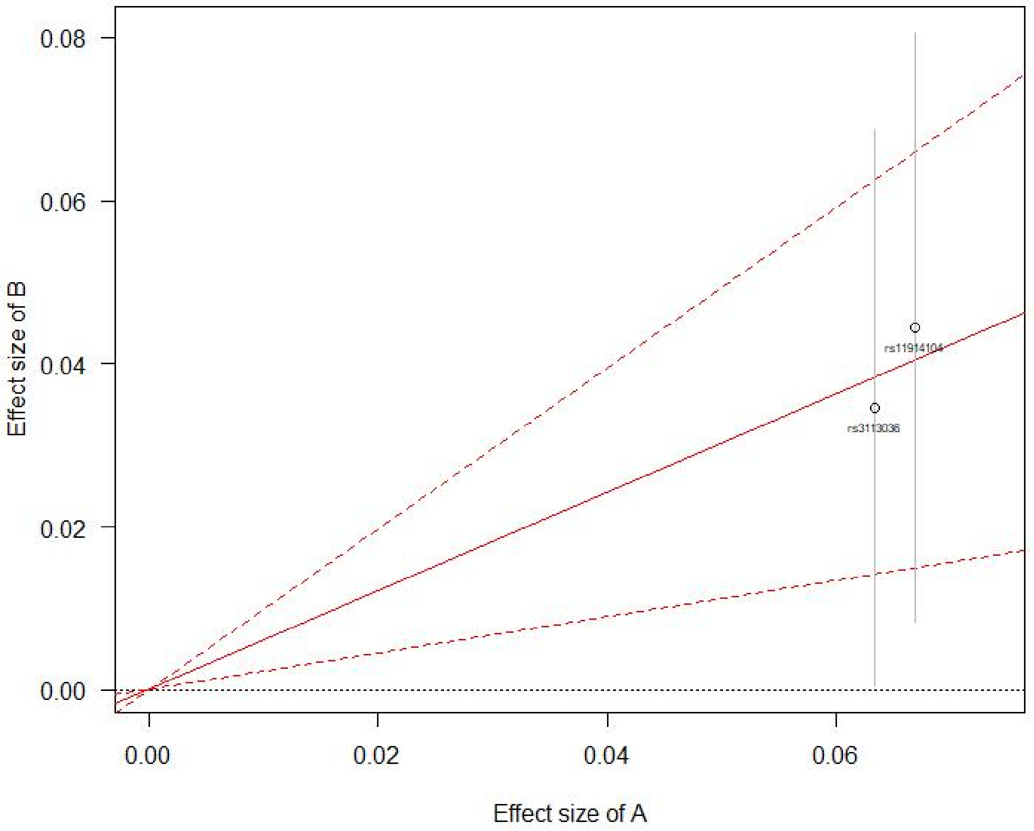

15. Gut microbiota abundance (order Bifidobacteriales id.432)

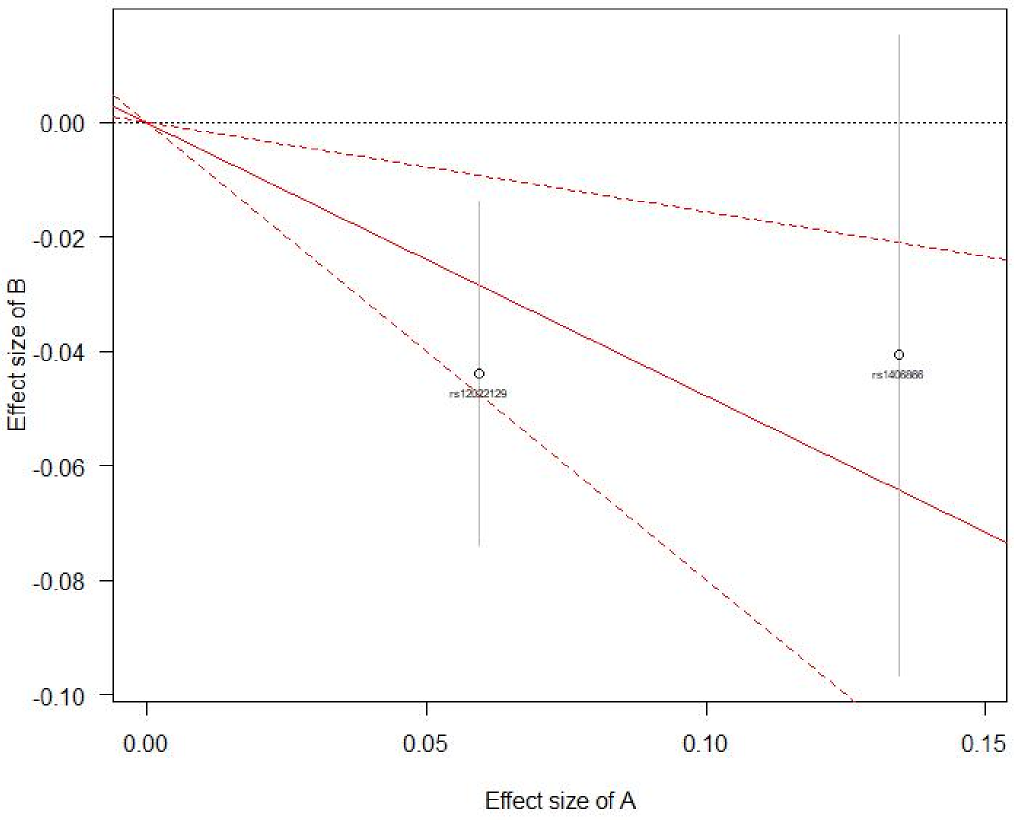

16. Gut microbiota abundance (order Rhodospirillales id.2667)

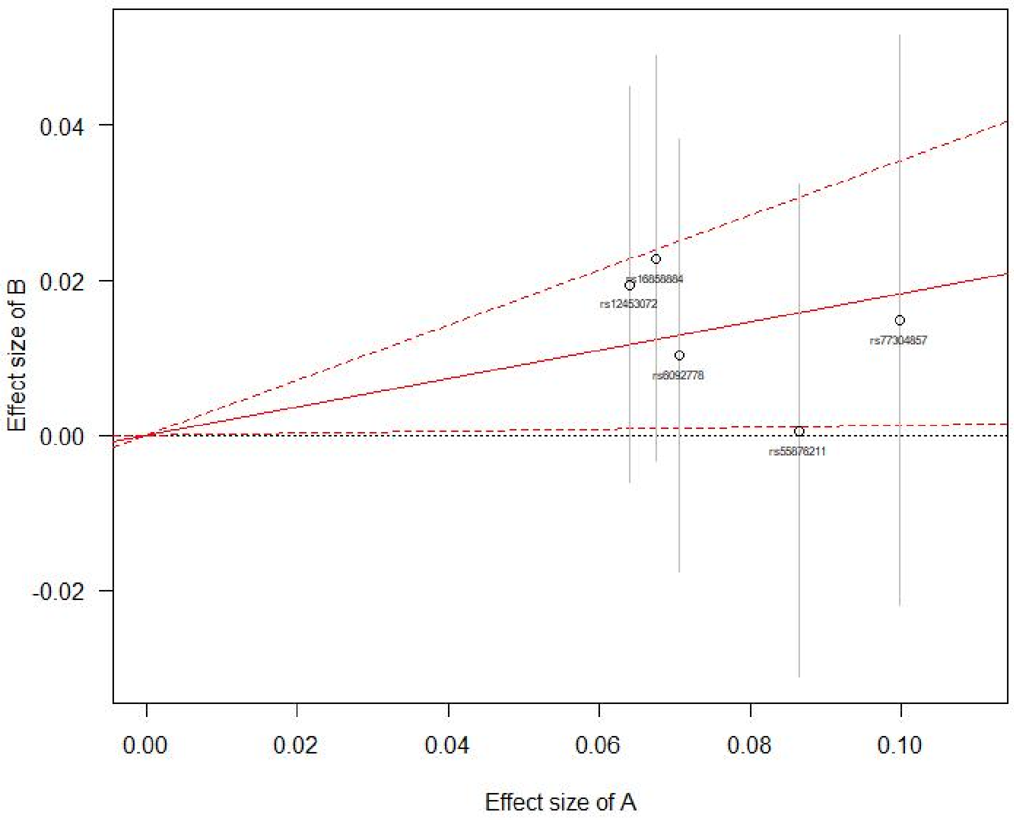

17. Gut microbiota abundance (order Victivallales id.2254)

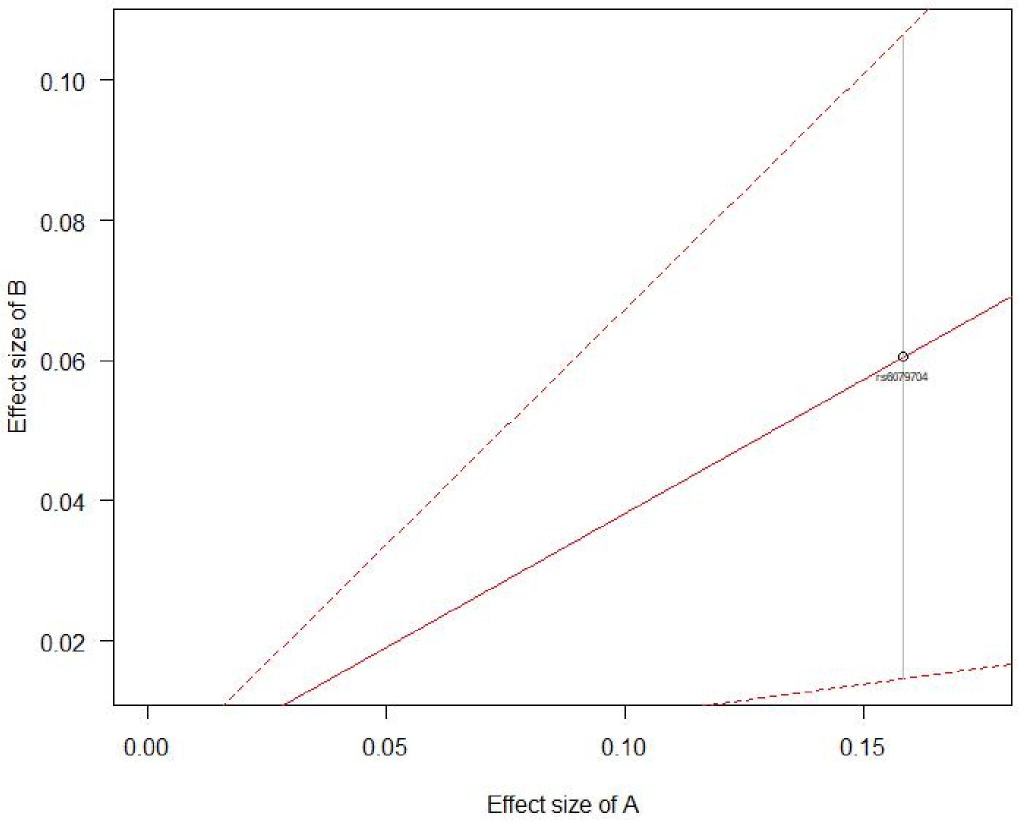

M. Colorectal cancer

1. Gut microbiota abundance (family Enterobacteriaceae id.3469)

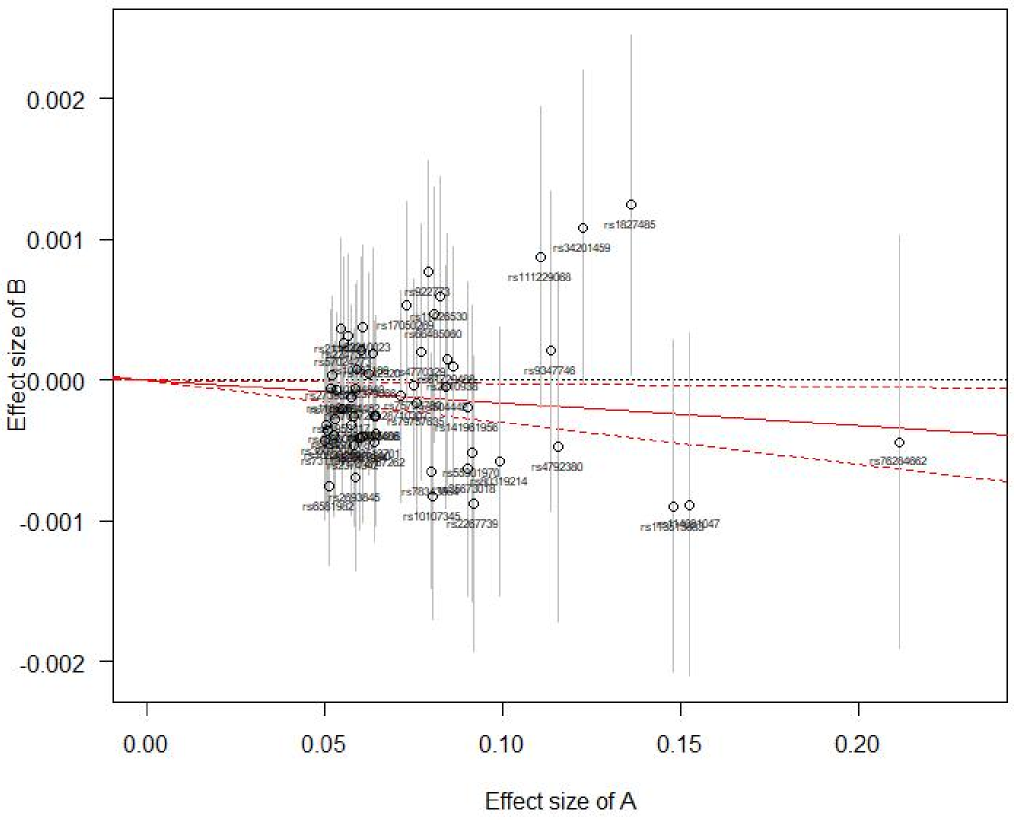

2. Gut microbiota abundance (genus Anaerostipes id.1991)

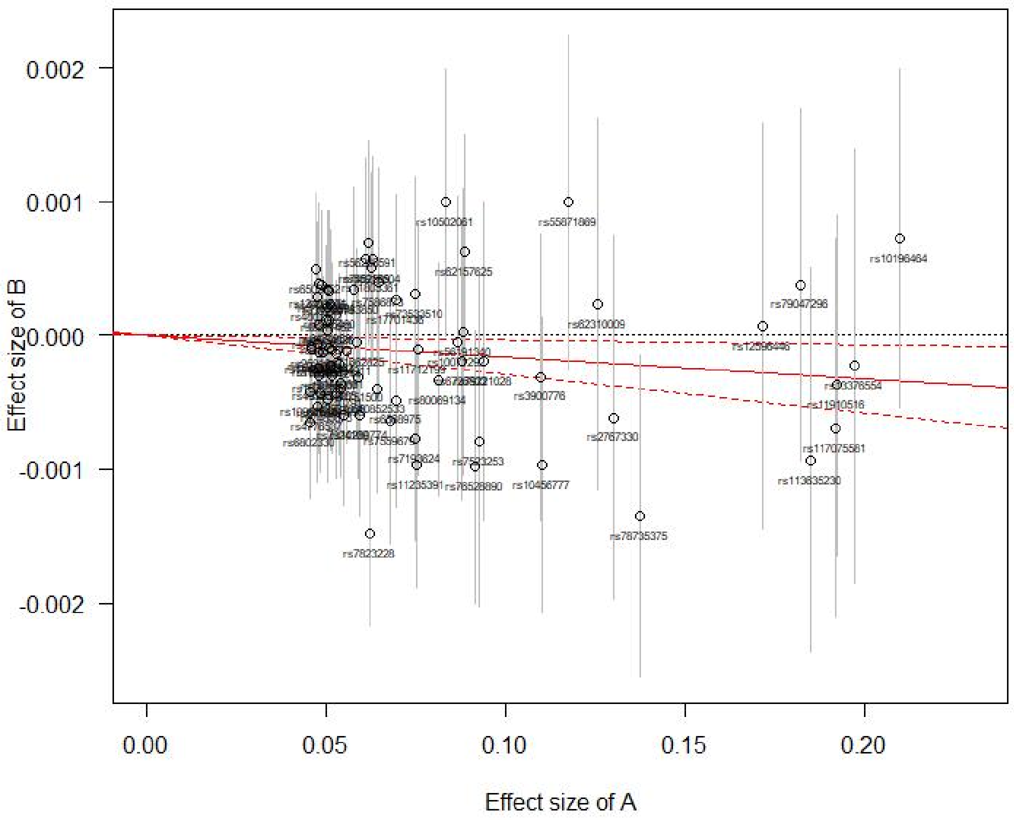

3. Gut microbiota abundance (genus Bilophila id.3170)

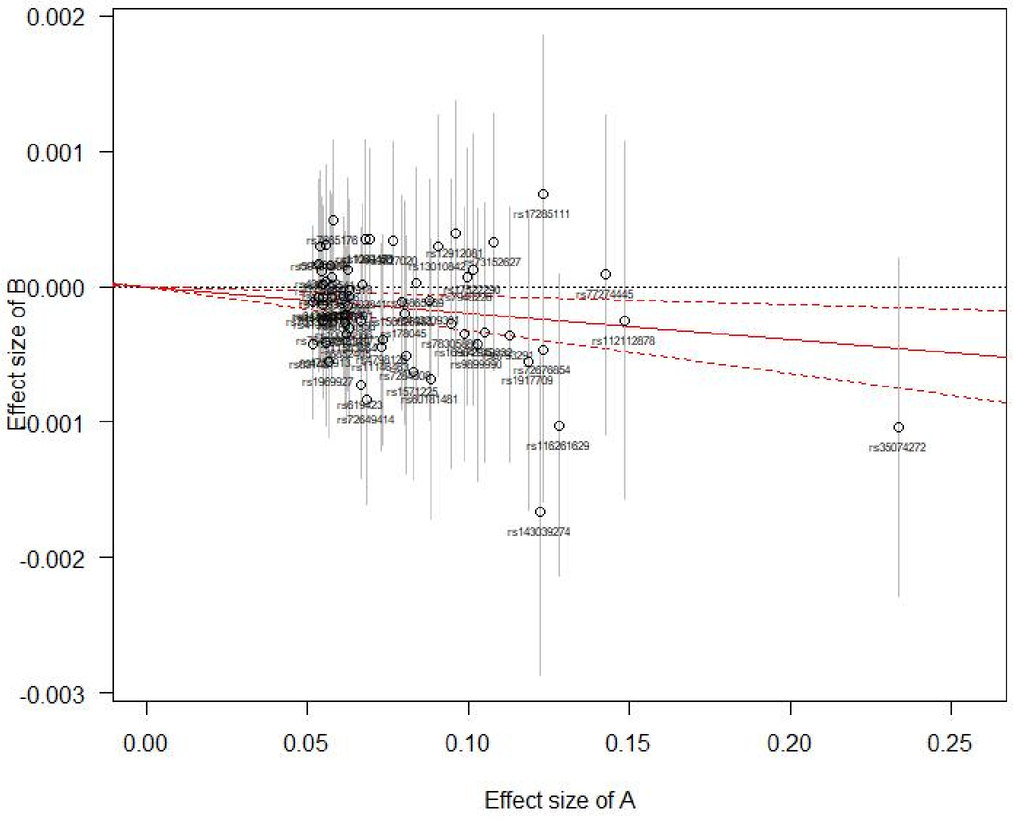

4. Gut microbiota abundance (genus Erysipelatoclostridium id.11381)

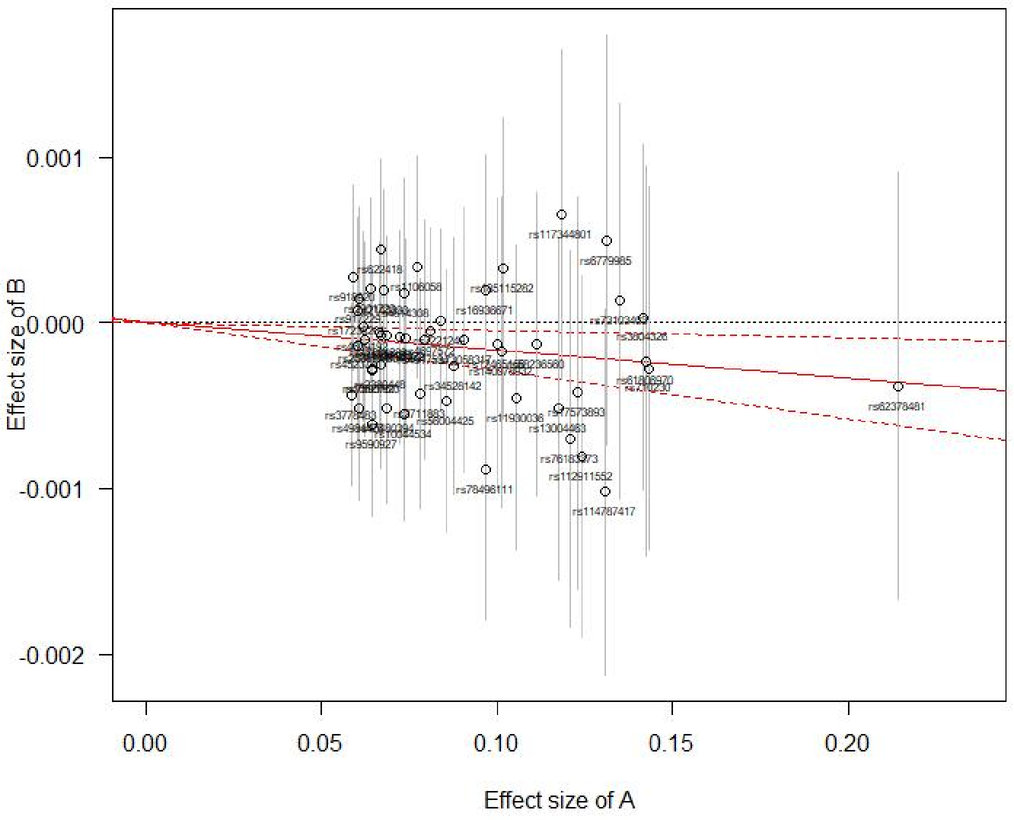

5. Gut microbiota abundance (genus Eubacterium coprostanoligenes group id.11375)

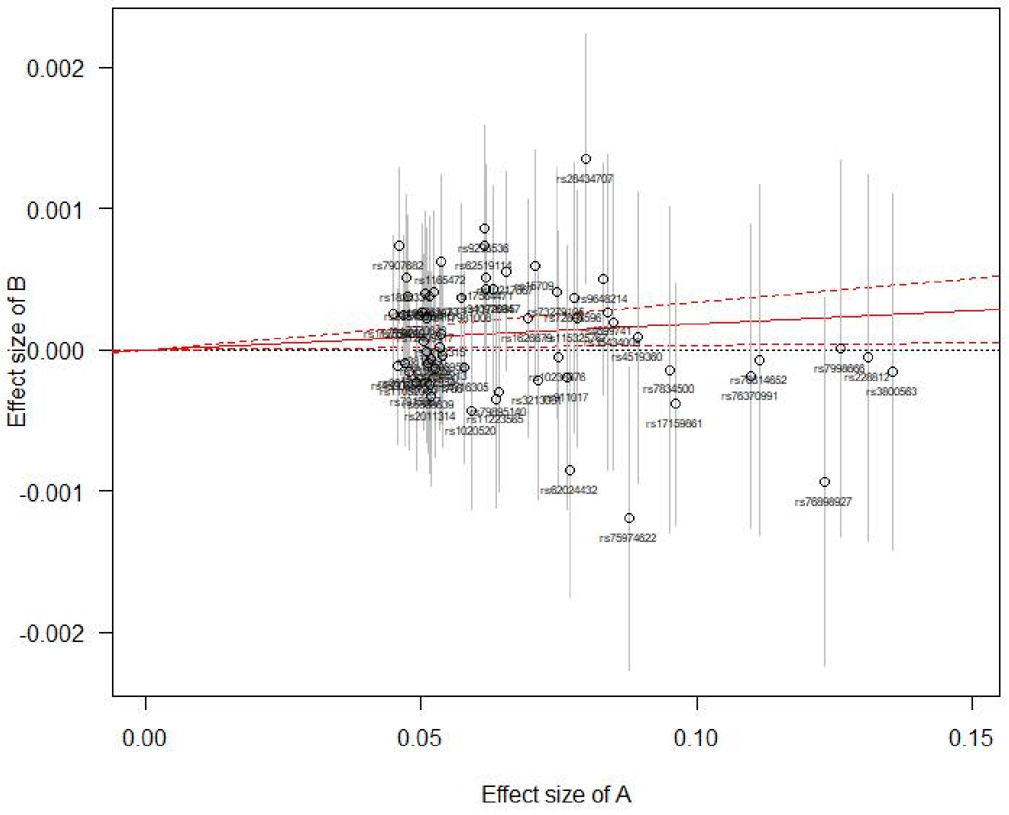

6. Gut microbiota abundance (genus Fusicatenibacter id.11305)

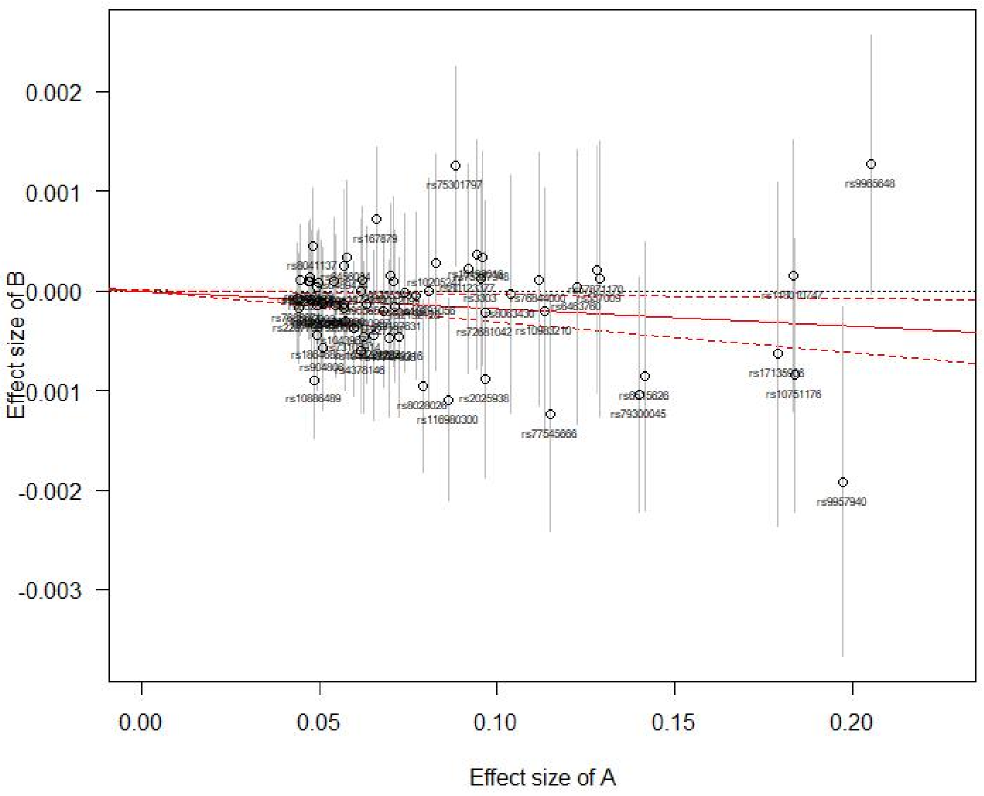

7. Gut microbiota abundance (genus Methanobrevibacter id.123)

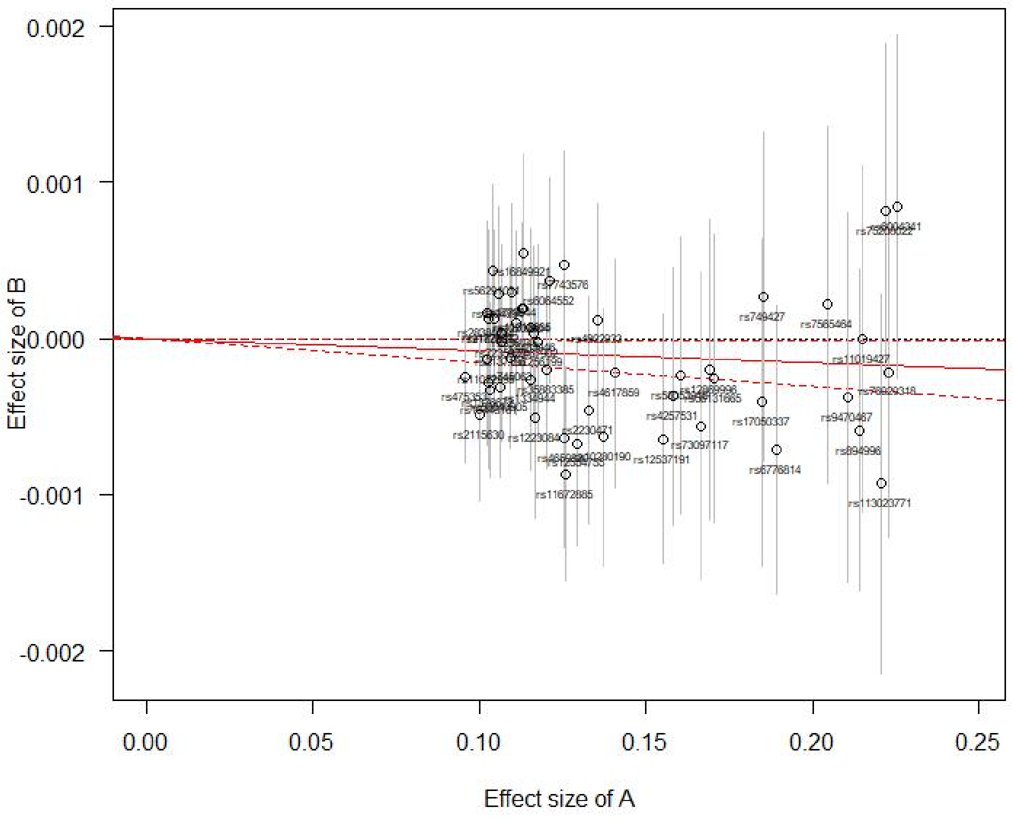

8. Gut microbiota abundance (genus Ruminococcaceae UCG002 id.11360)

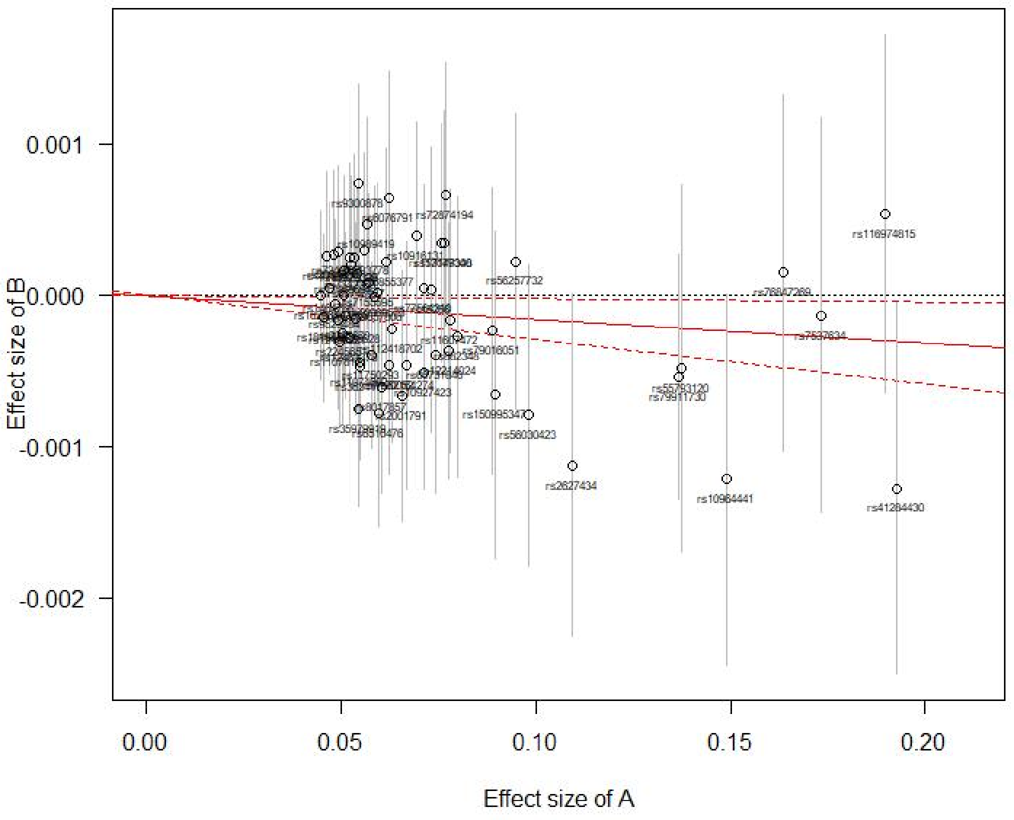

9. Gut microbiota abundance (order Enterobacteriales id.3468)

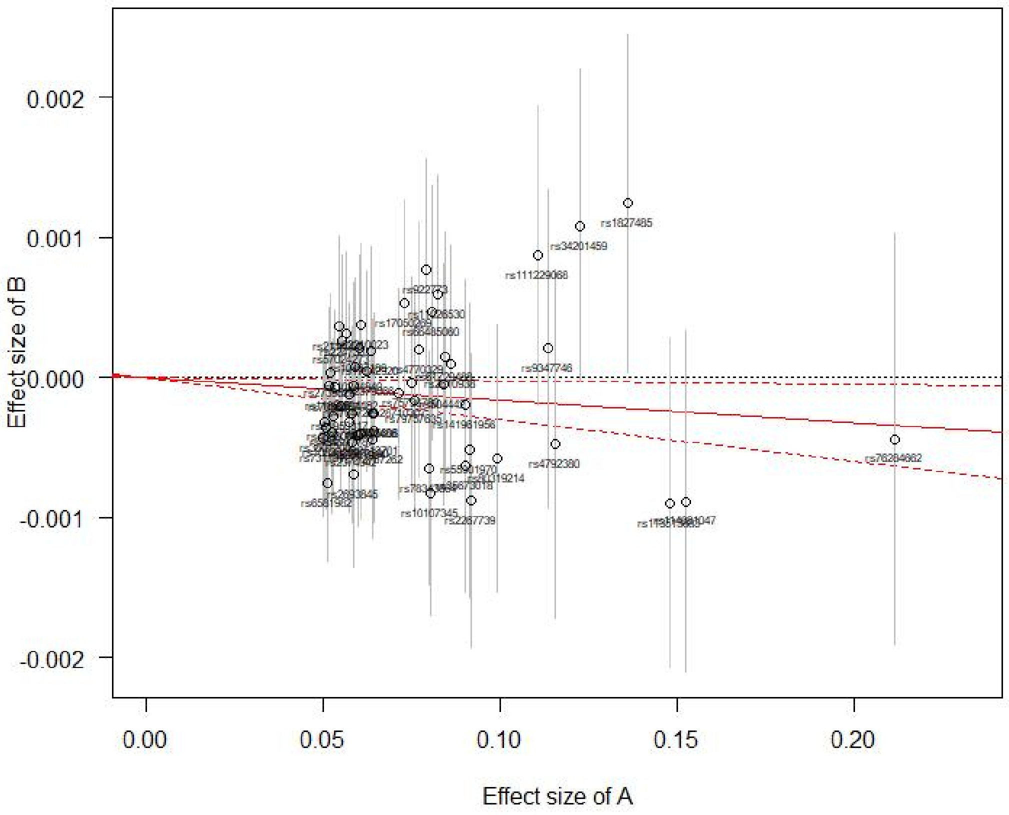

10. Gut microbiota abundance (phylum Proteobacteria id.2375)

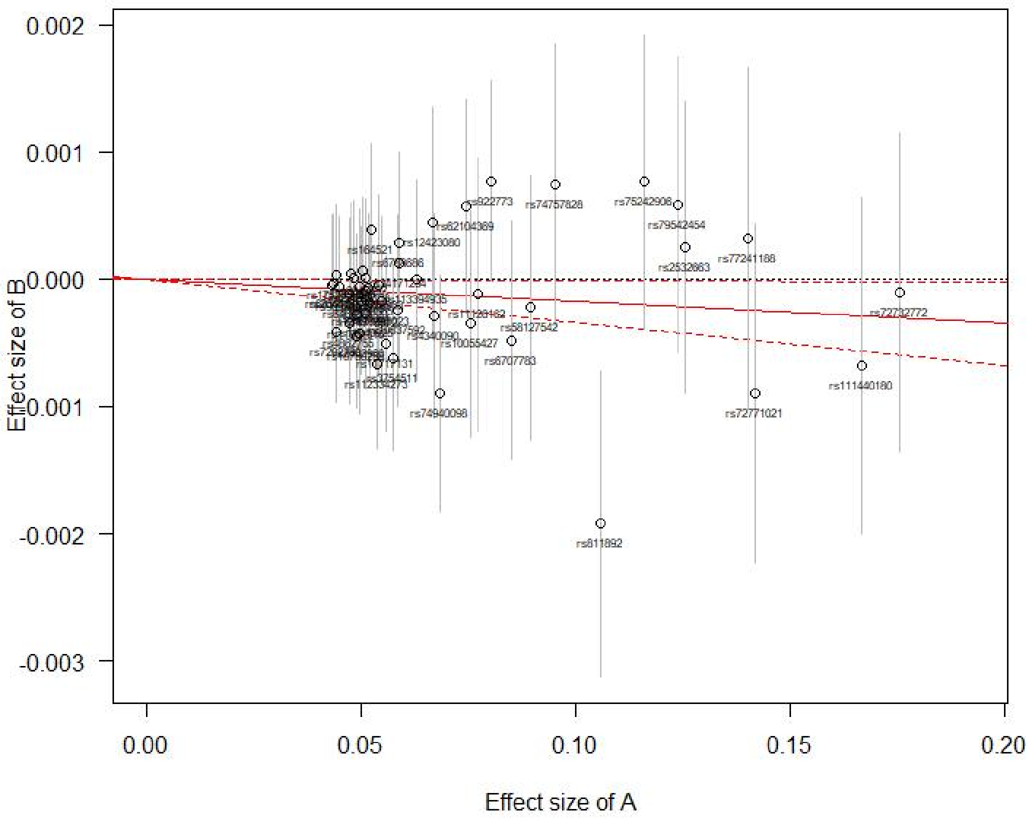

N. Coeliac disease

1. Gut microbiota abundance (class Actinobacteria id.419)

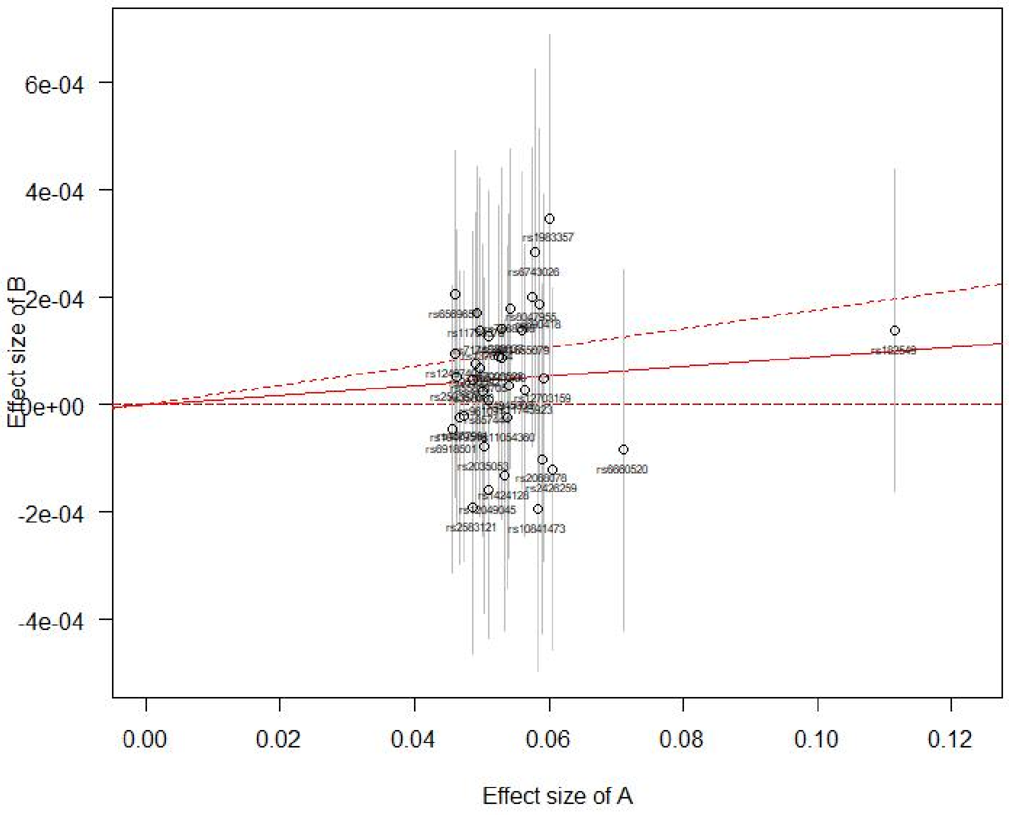

2. Gut microbiota abundance (class Bacilli id.1673)

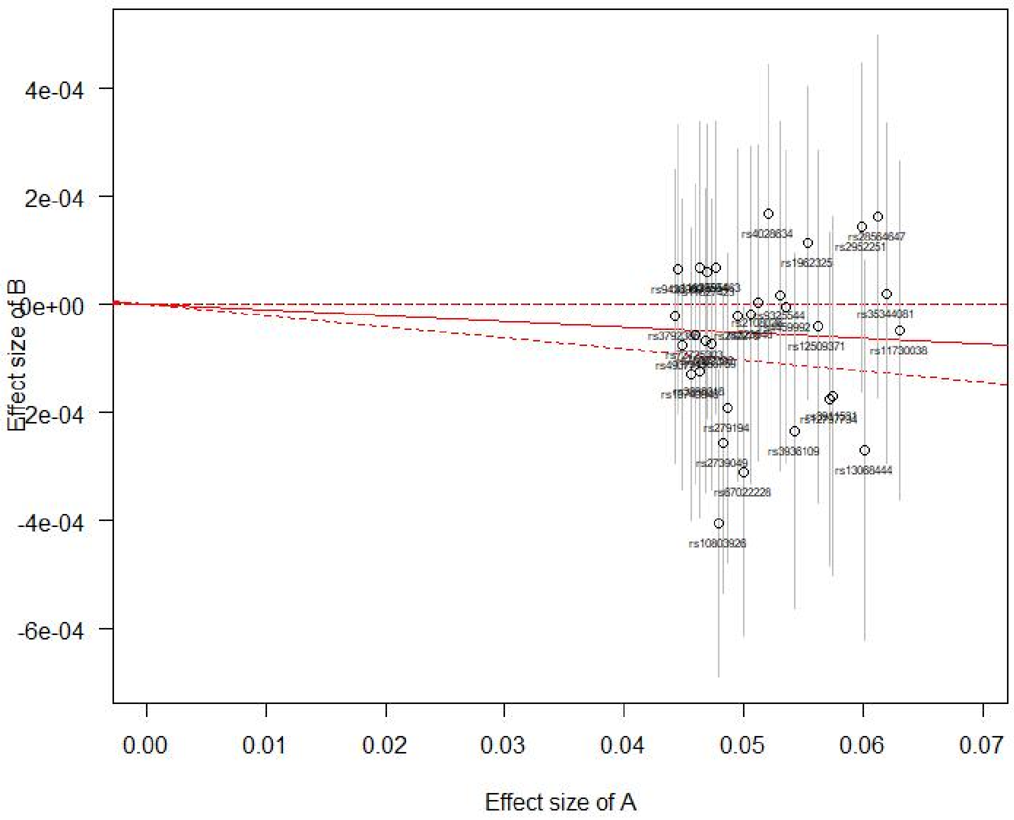

3. Gut microbiota abundance (family Bacteroidaceae id.917)

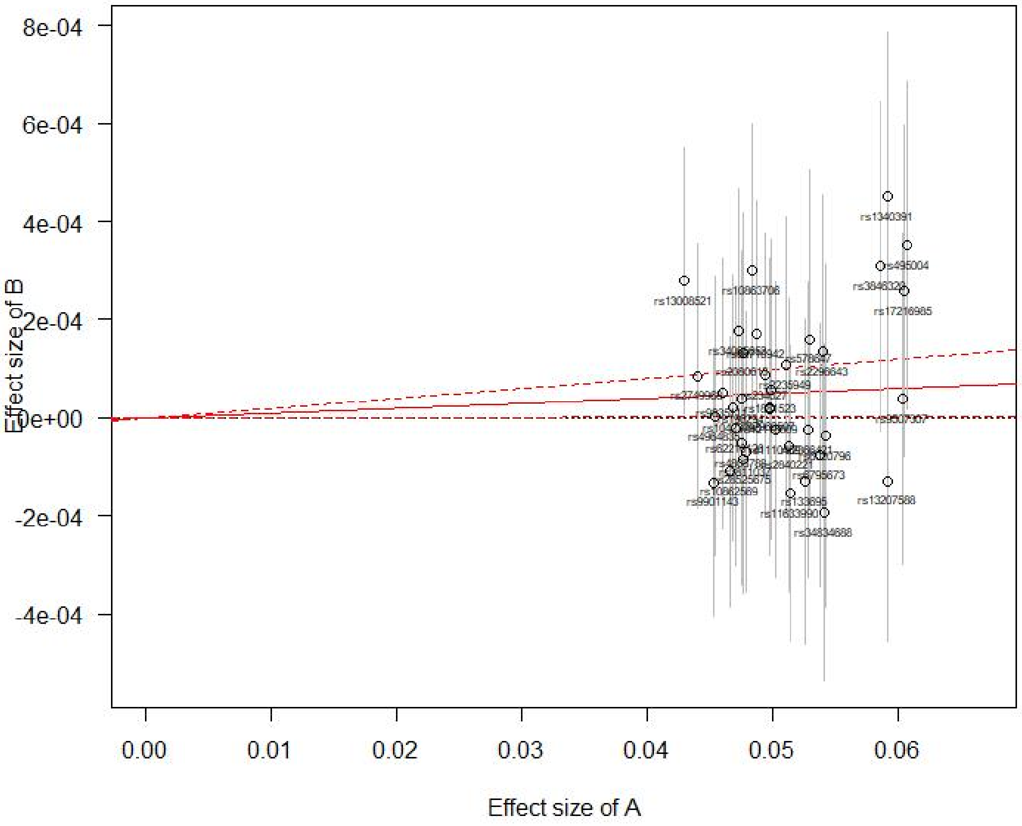

4. Gut microbiota abundance (family Streptococcaceae id.1850)

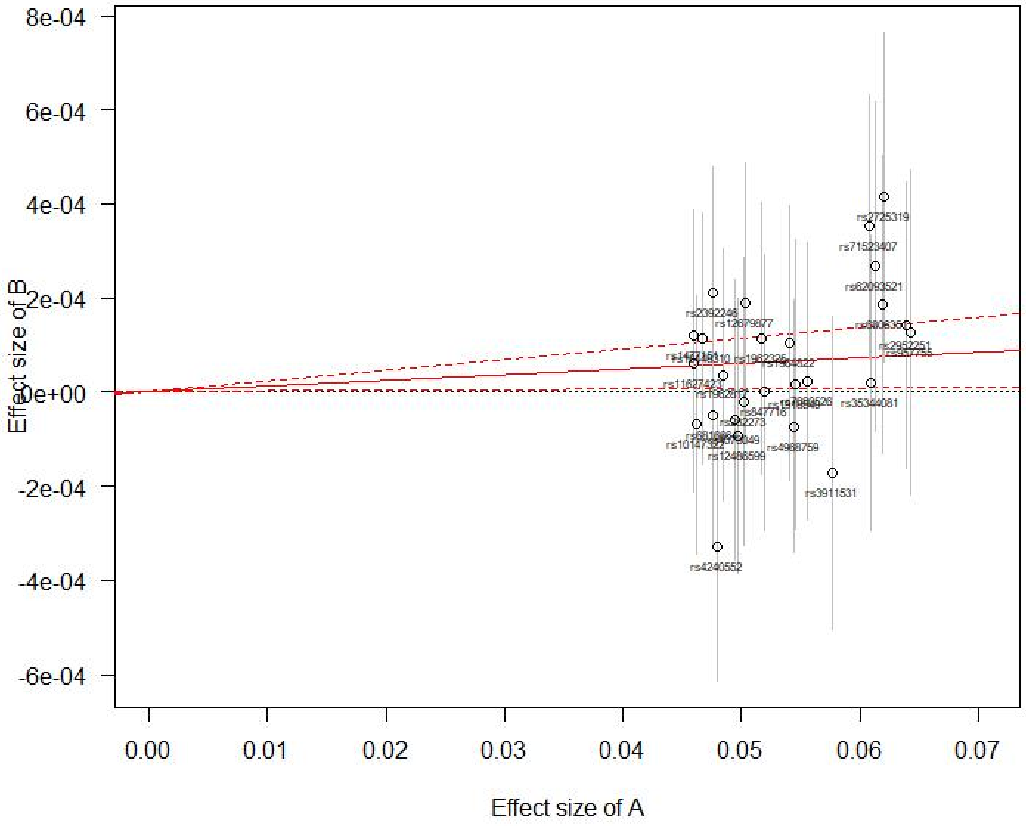

5. Gut microbiota abundance (genus Bacteroides id.918)

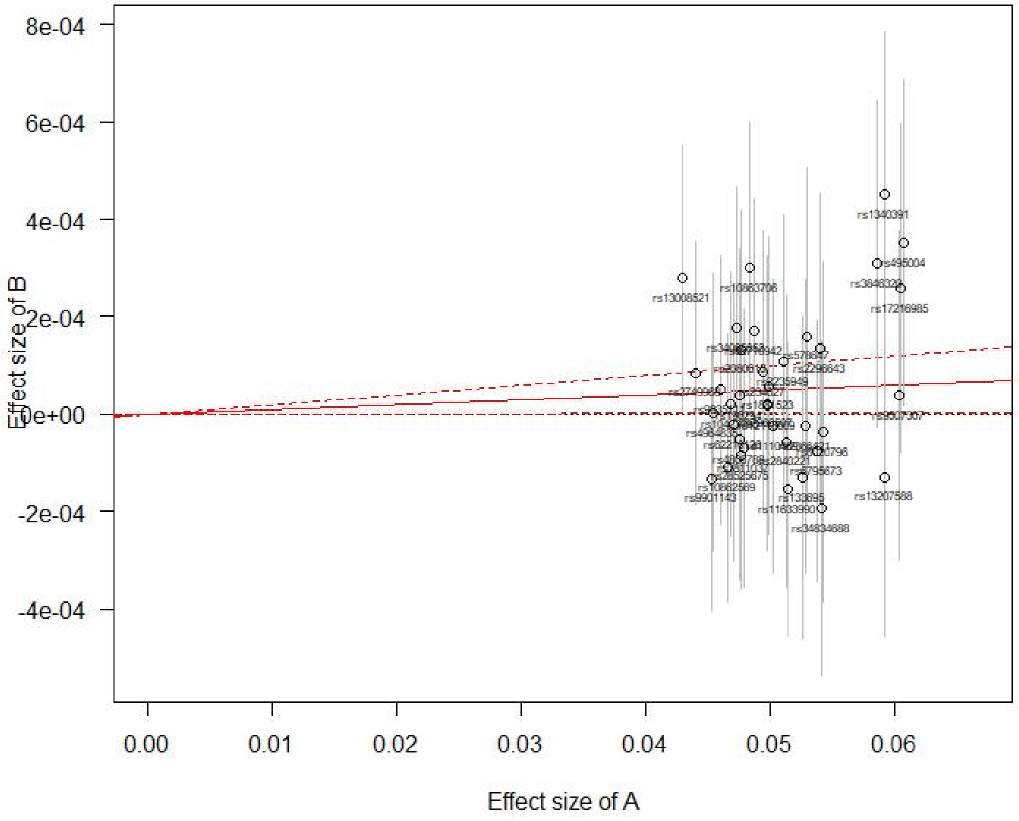

6. Gut microbiota abundance (genus Barnesiella id.944)

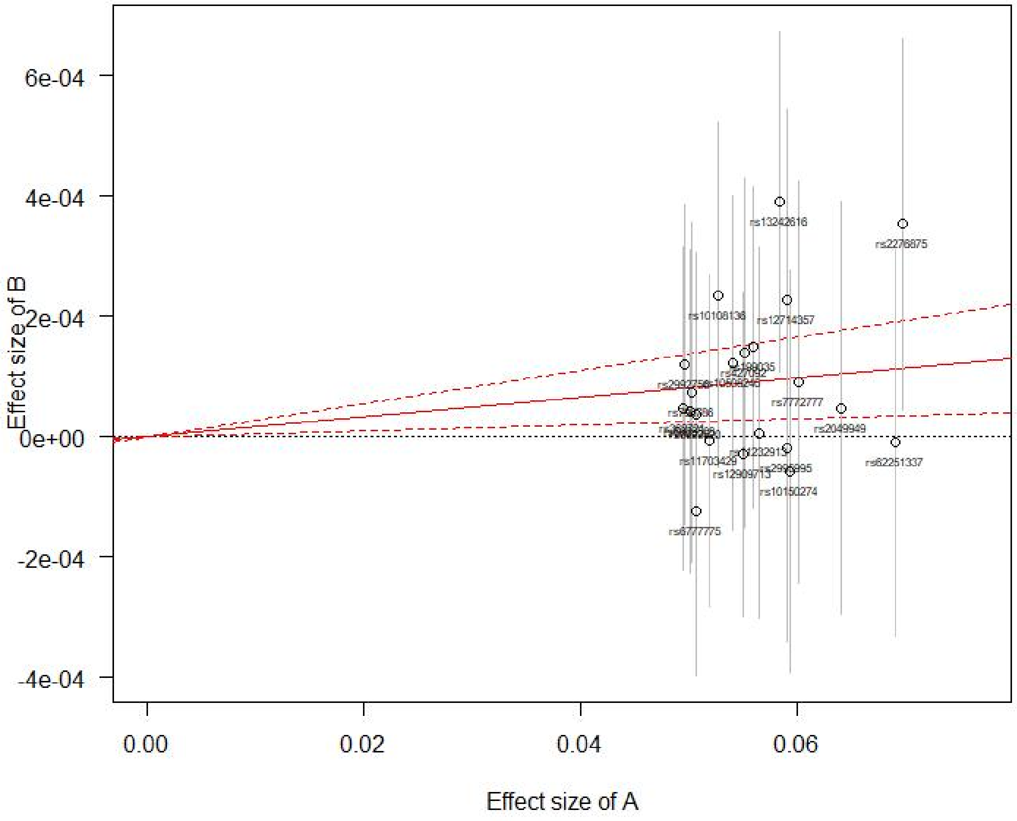

7. Gut microbiota abundance (genus Bifidobacterium id.436)

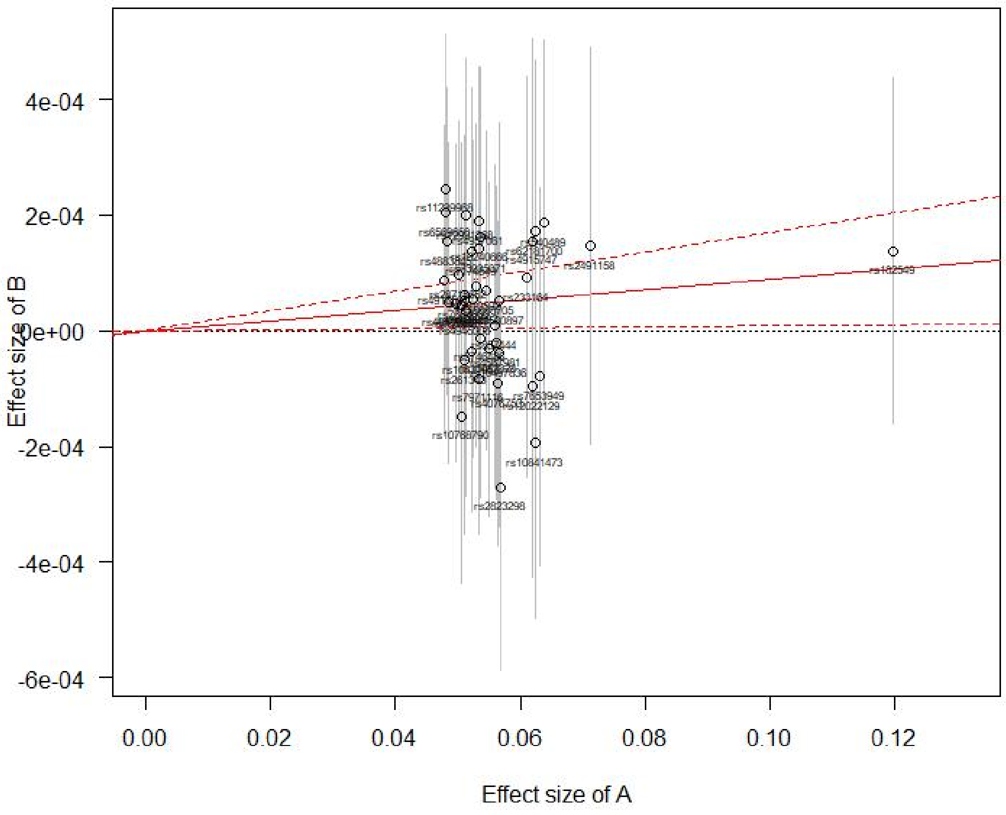

8. Gut microbiota abundance (genus Butyricicoccus id.2055)

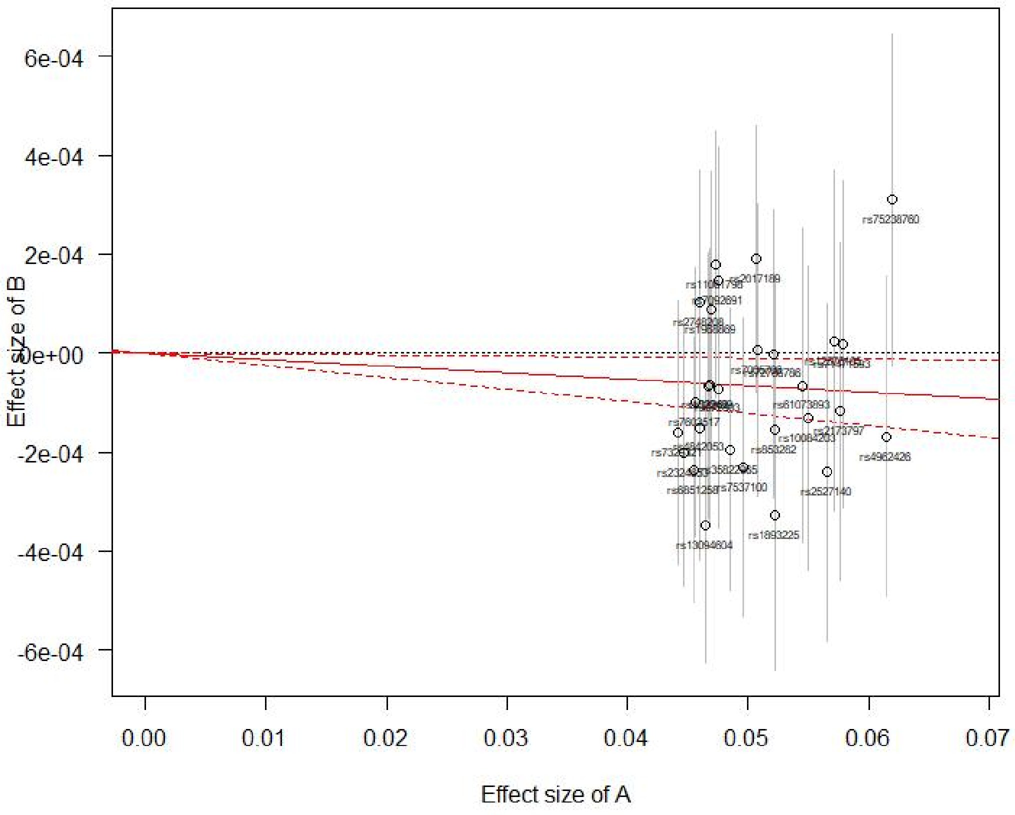

O. Nonalcoholic fatty liver disease

1. Gut microbiota abundance (class Actinobacteria id.419)

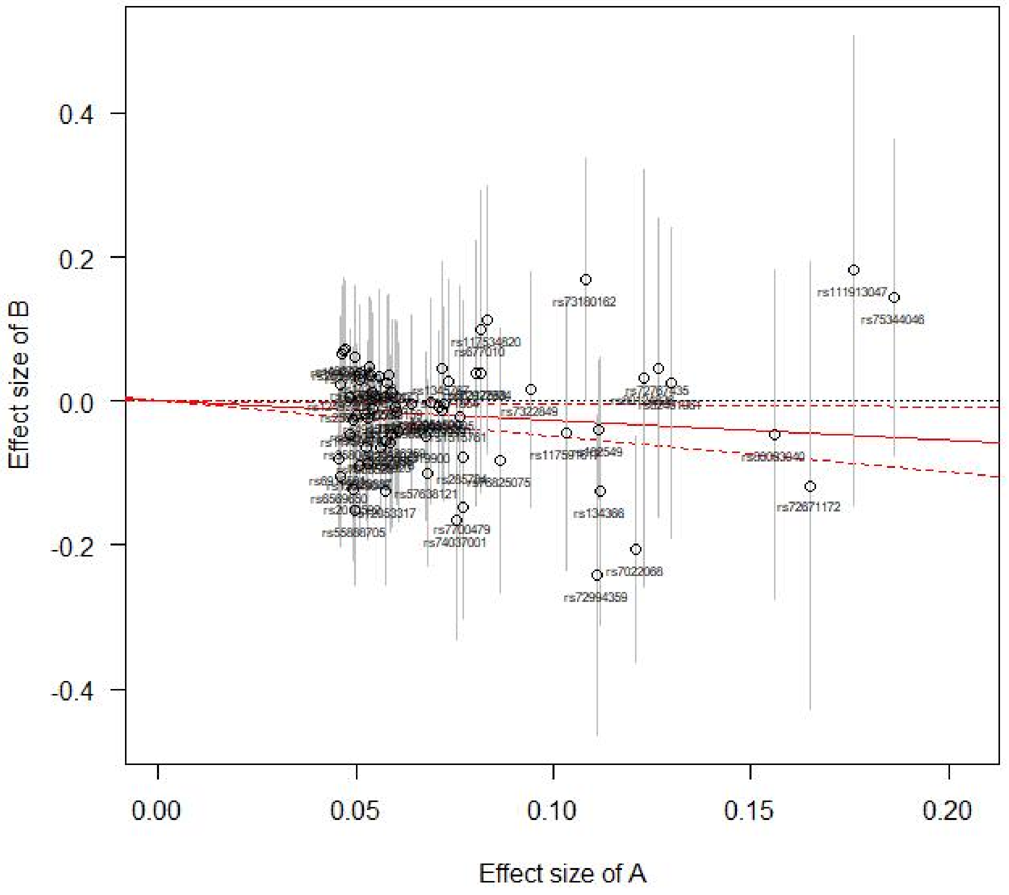

2. Gut microbiota abundance (family Alcaligenaceae id.2875)

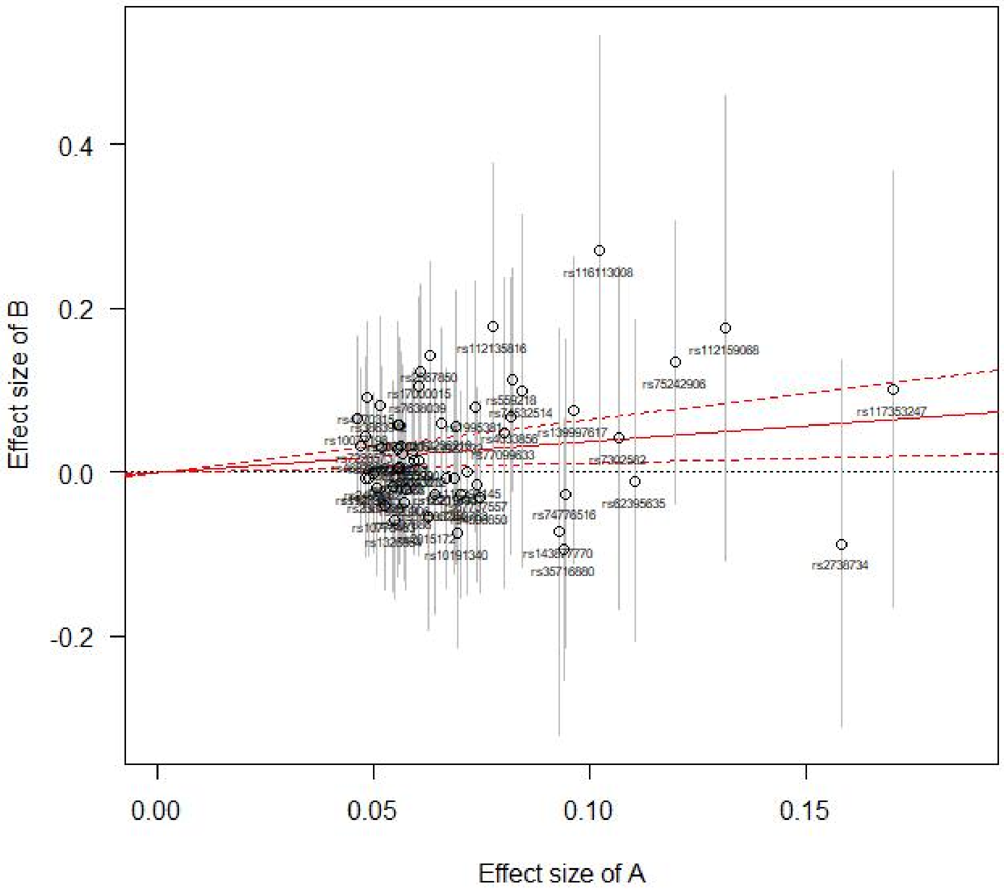

3. Gut microbiota abundance (unknown family id.1000006161)

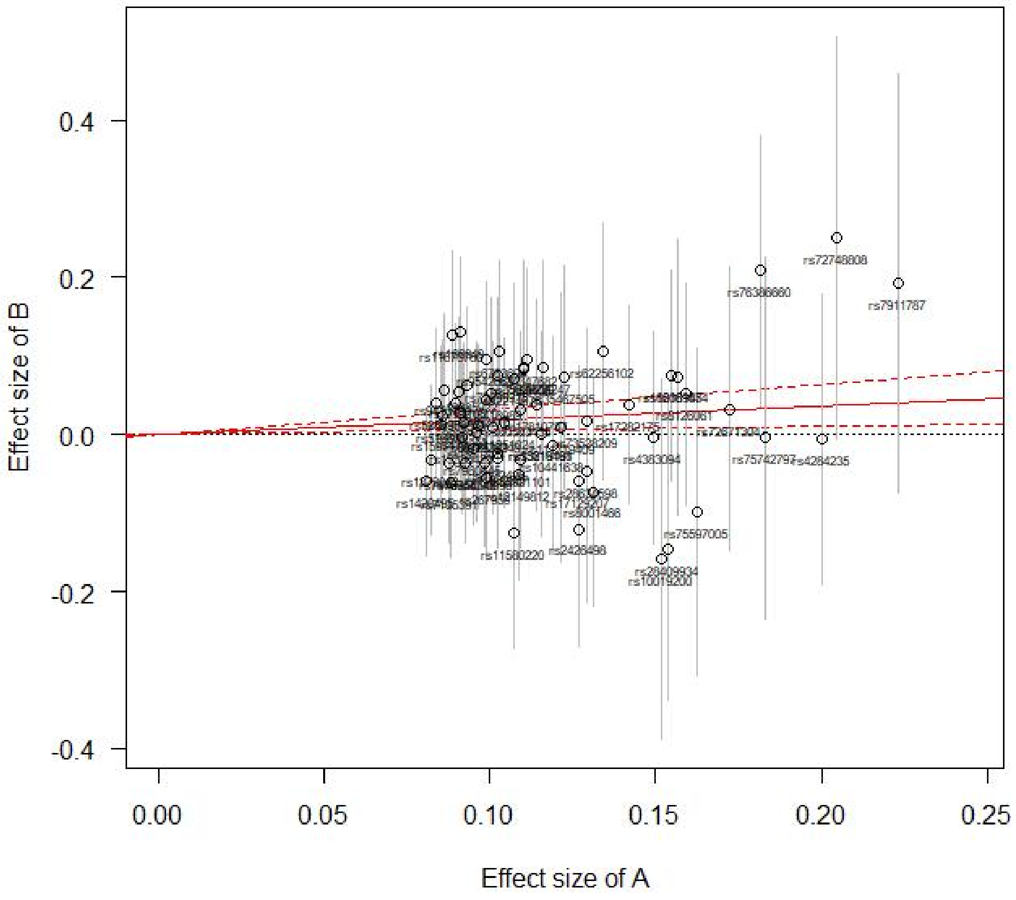

4. Gut microbiota abundance (genus Alloprevotella id.961)

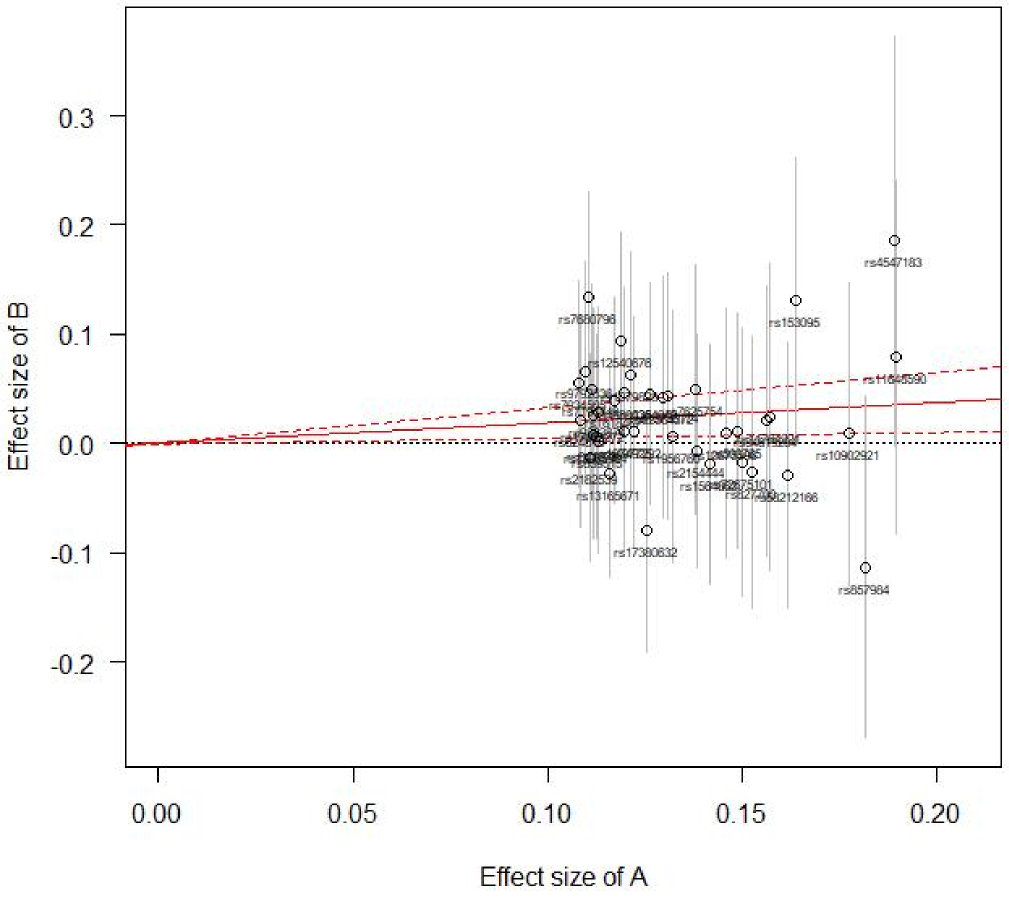

5. Gut microbiota abundance (genus Butyricicoccus id.2055)

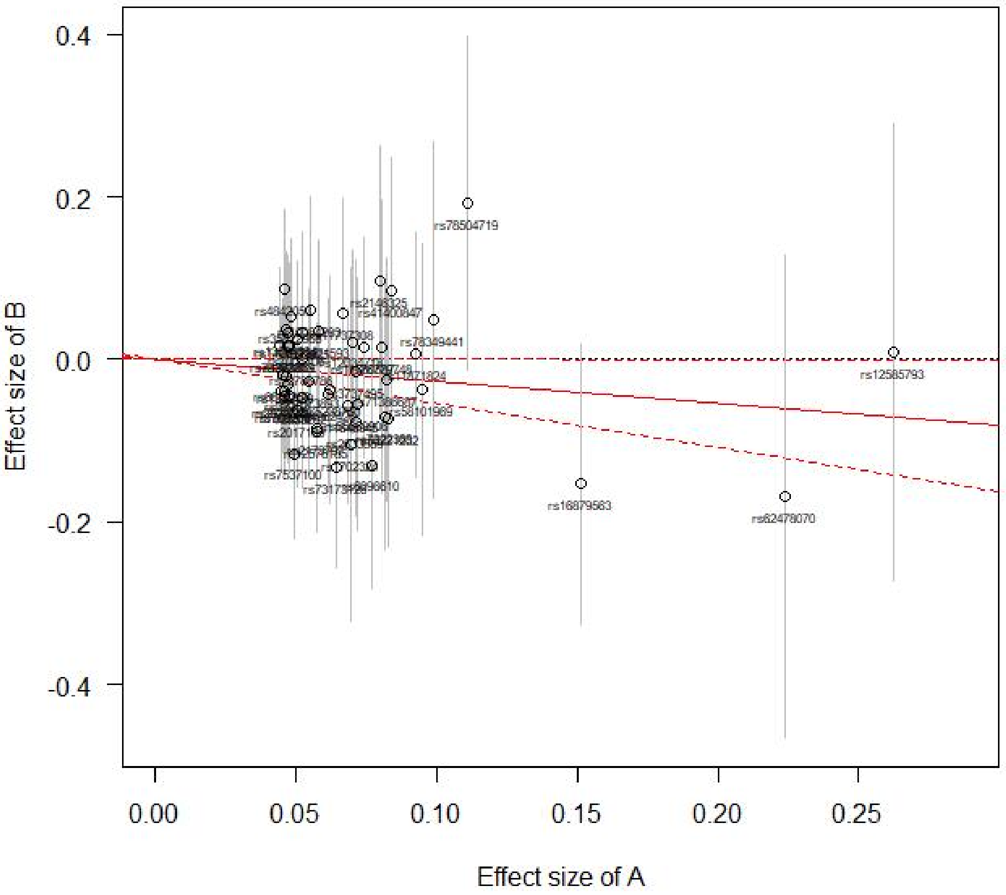

6. Gut microbiota abundance (genus Collinsella id.815)

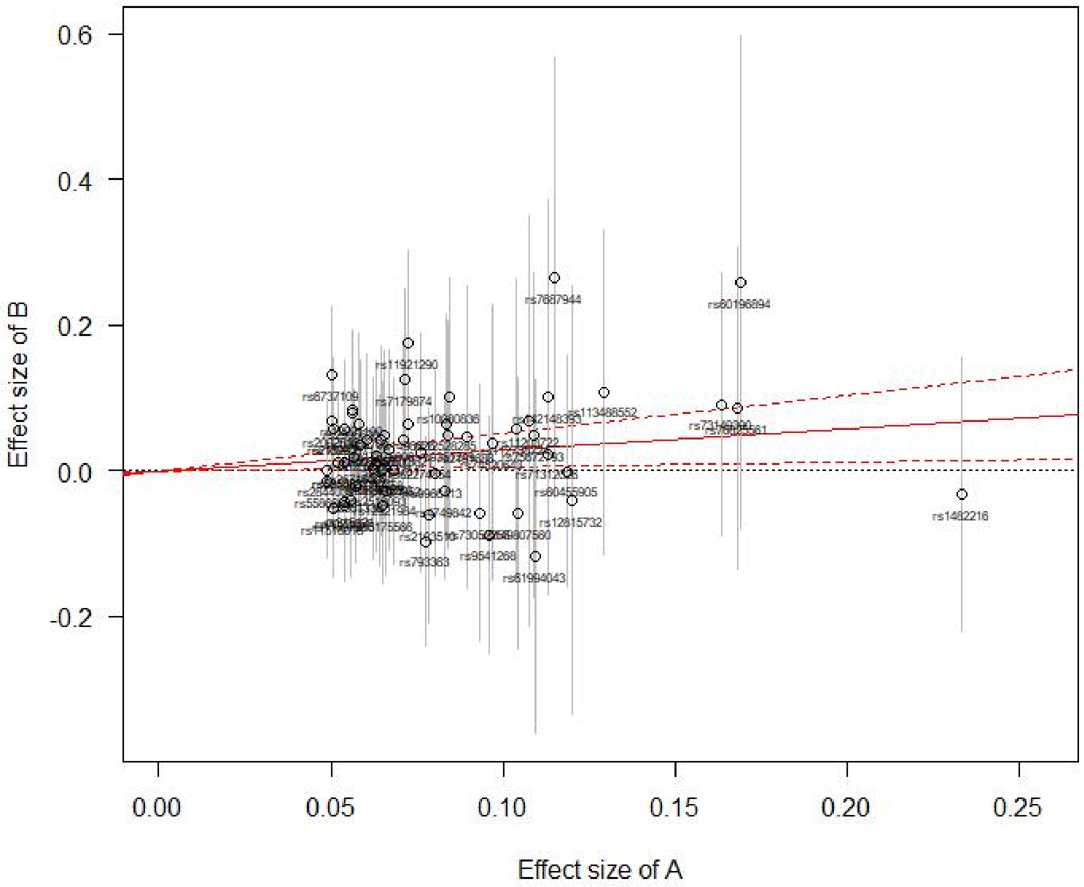

7. Gut microbiota abundance (genus Erysipelatoclostridium id.11381)

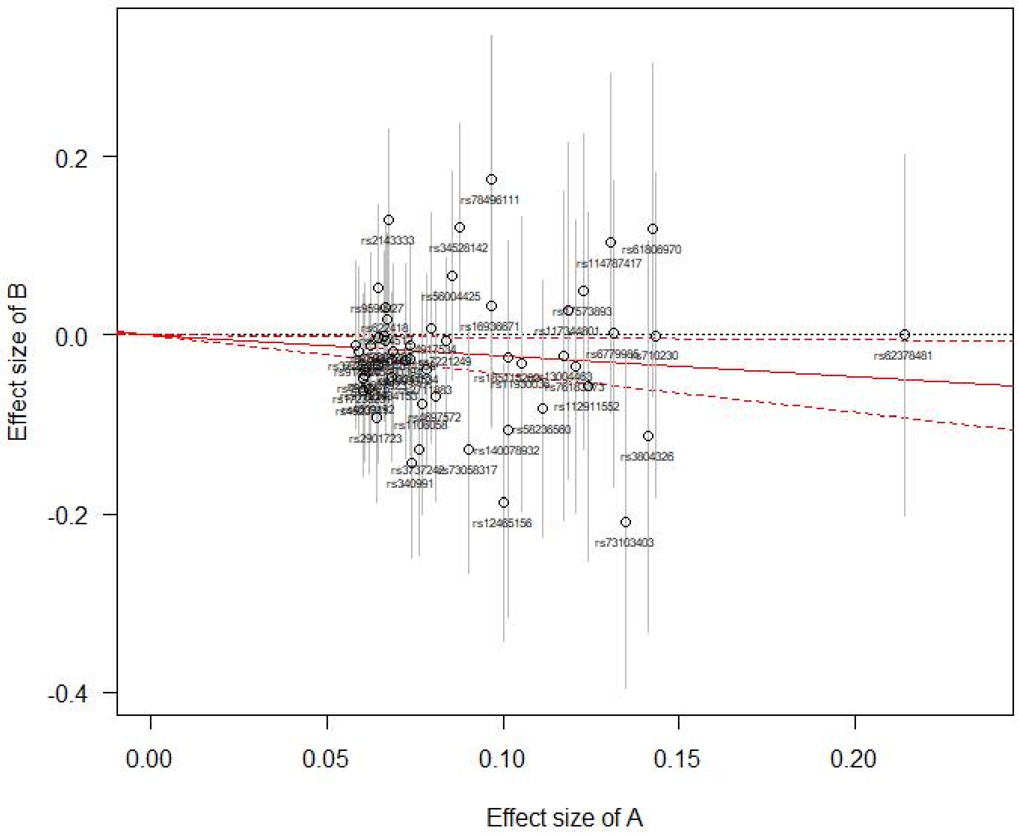

8. Gut microbiota abundance (genus Eubacterium hallii group id.11338)

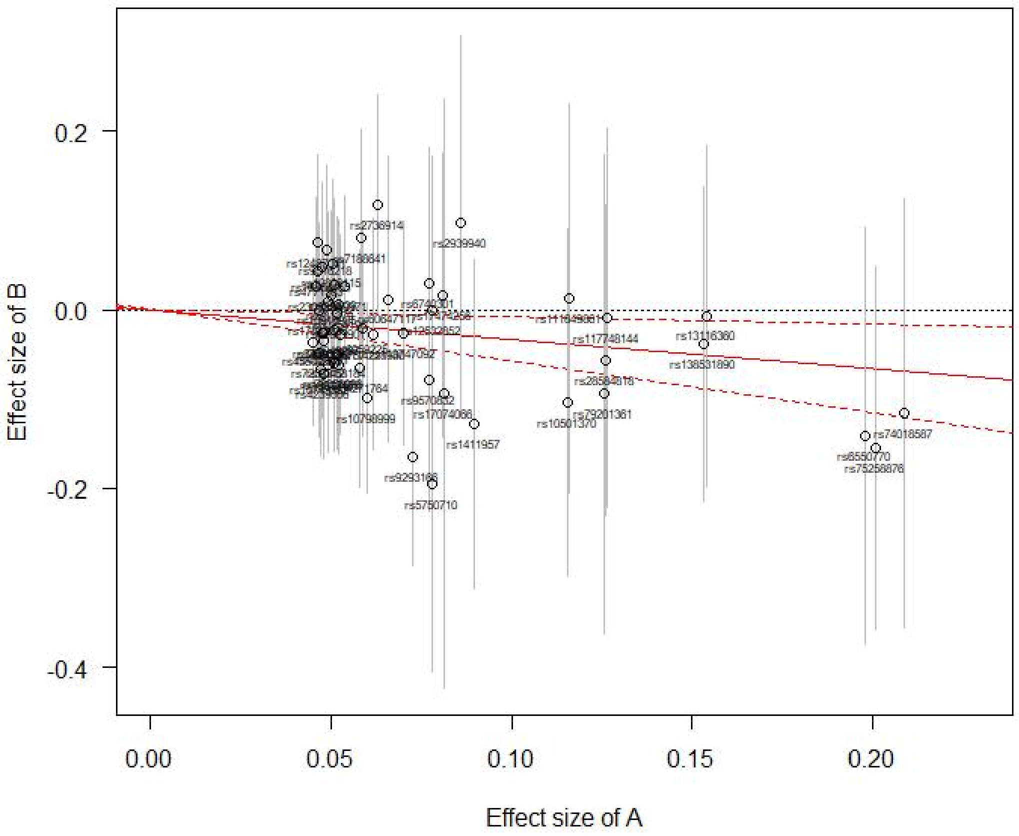

9. Gut microbiota abundance (genus Holdemanella id.11393)

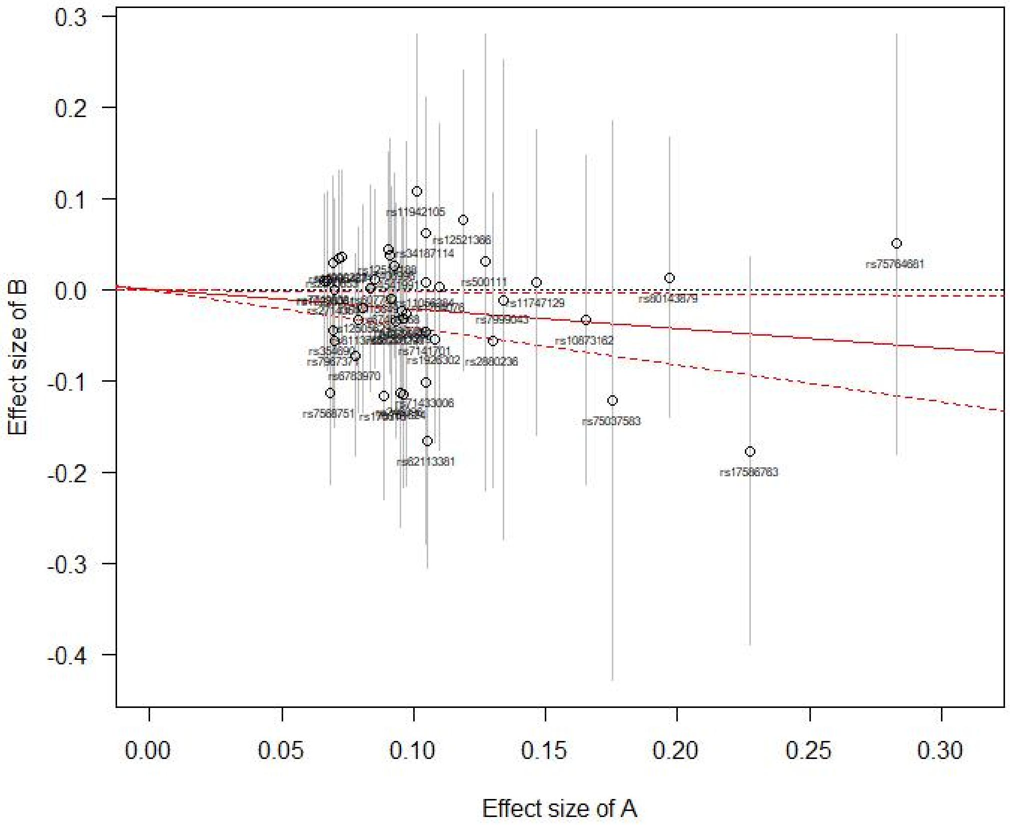

10. Gut microbiota abundance (genus Paraprevotella id.962)

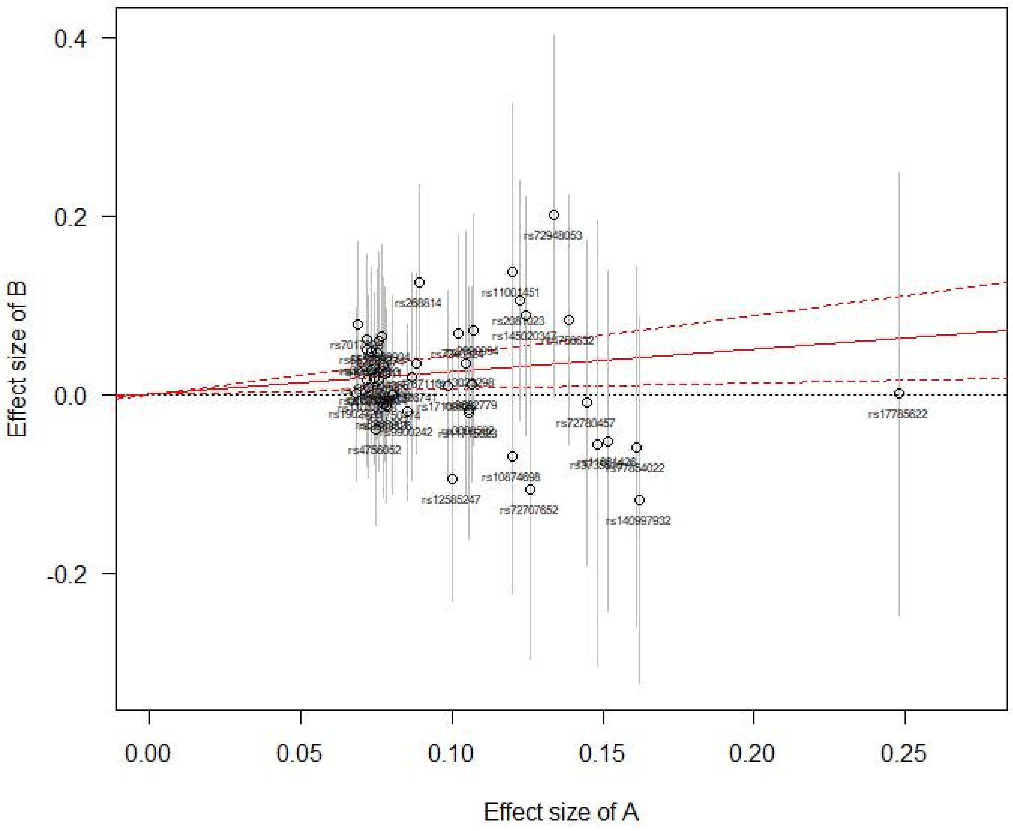

11. Gut microbiota abundance (unknown genus id.1000006162)

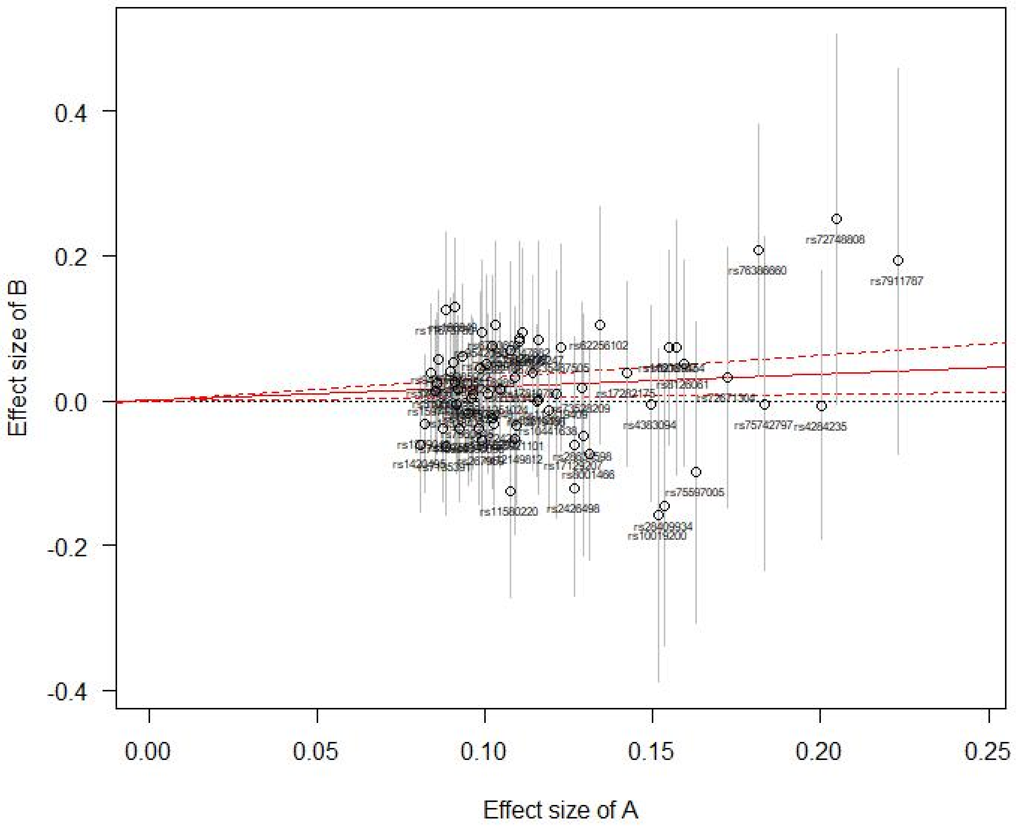

12. Gut microbiota abundance (unknown genus id.959)

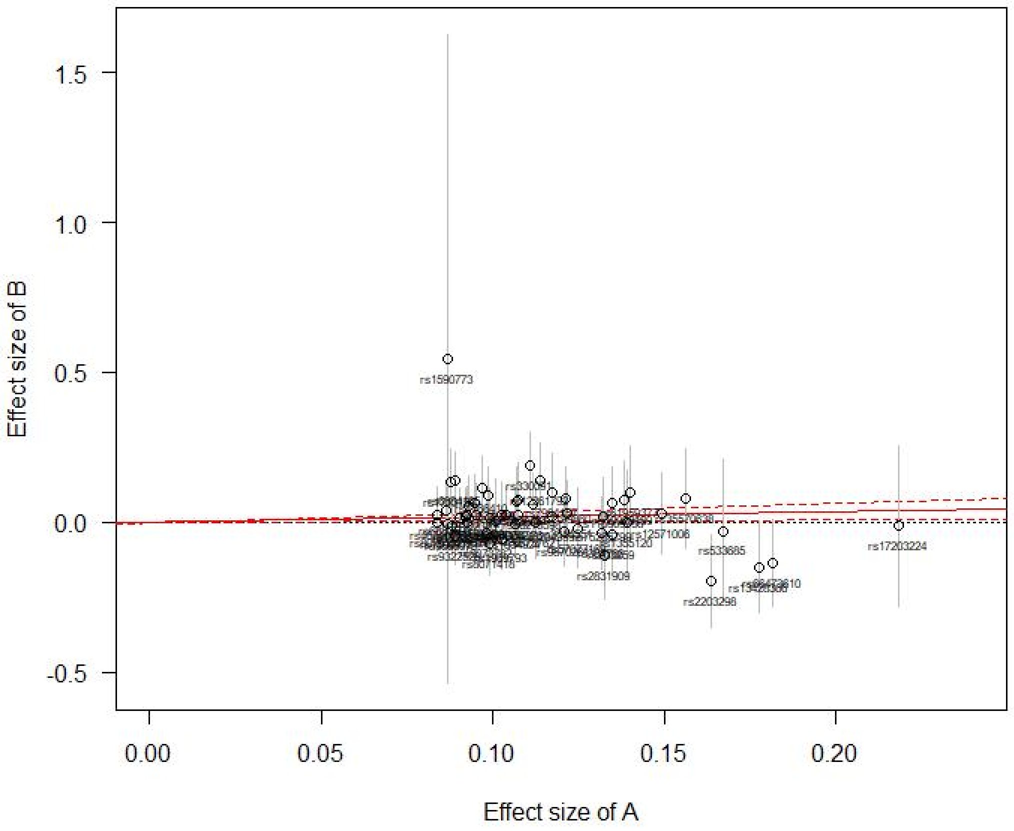

13. Gut microbiota abundance (order NB1n id.3953)

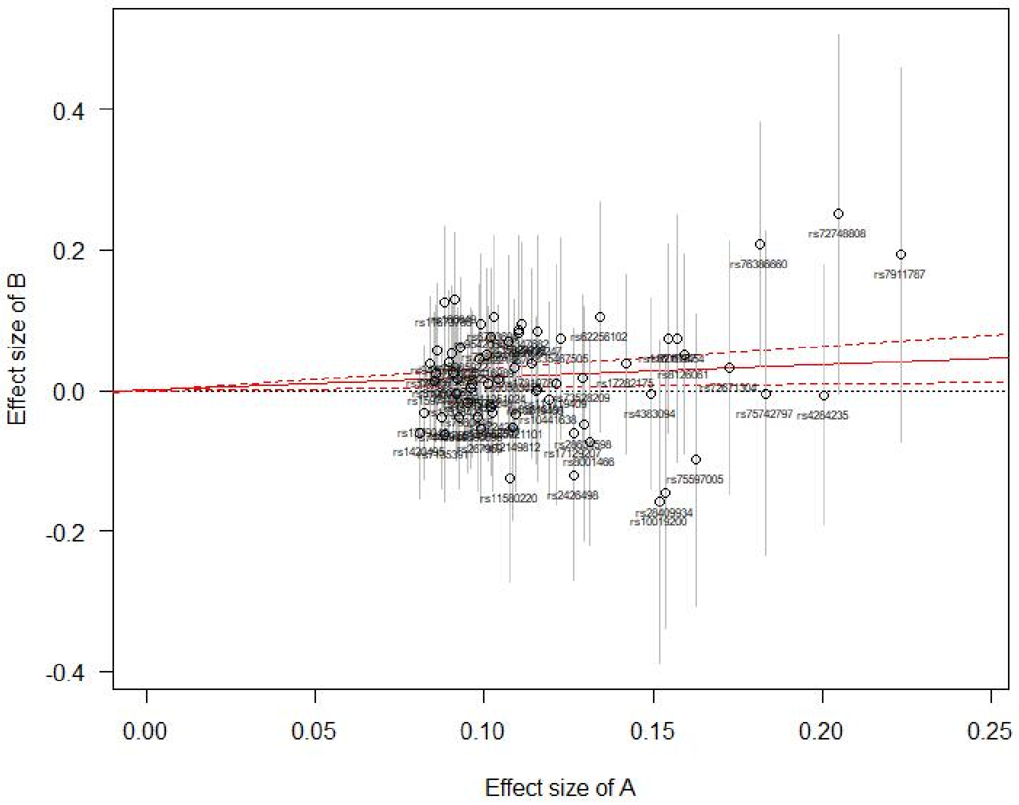

P. Alcoholic liver disease

1. Gut microbiota abundance (class Gammaproteobacteria id.3303)

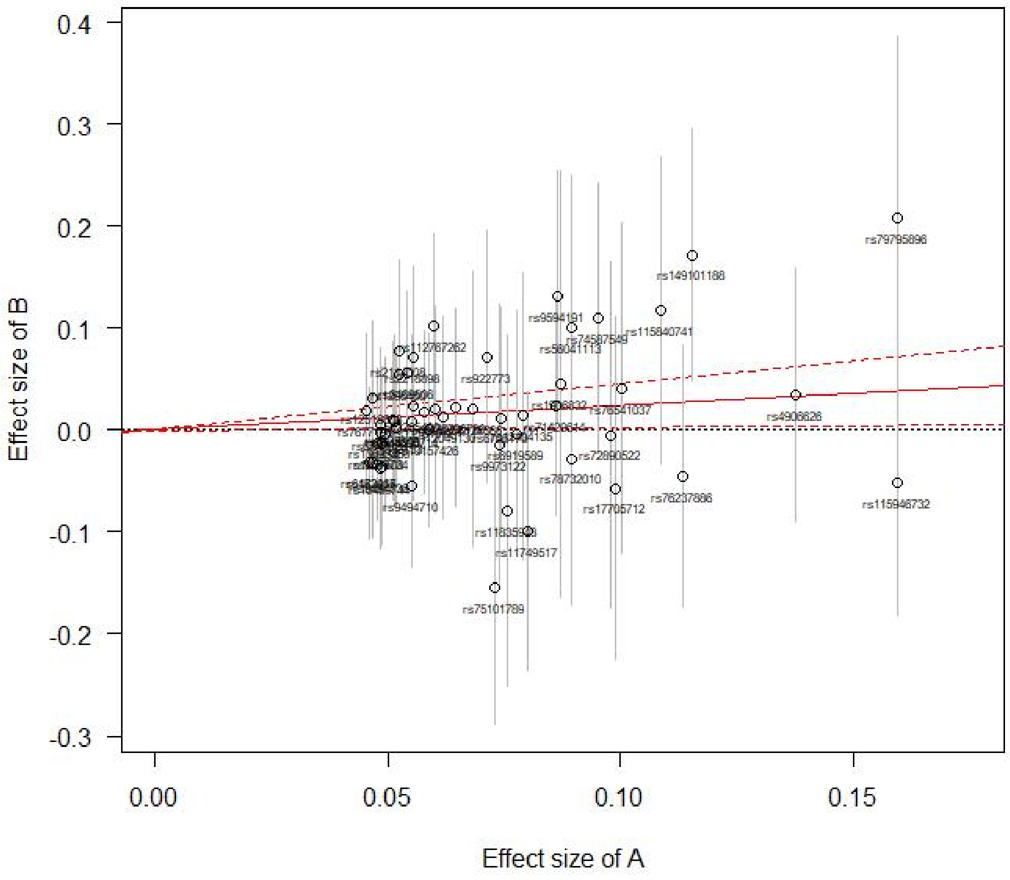

2. Gut microbiota abundance (family Victivallaceae id.2255)

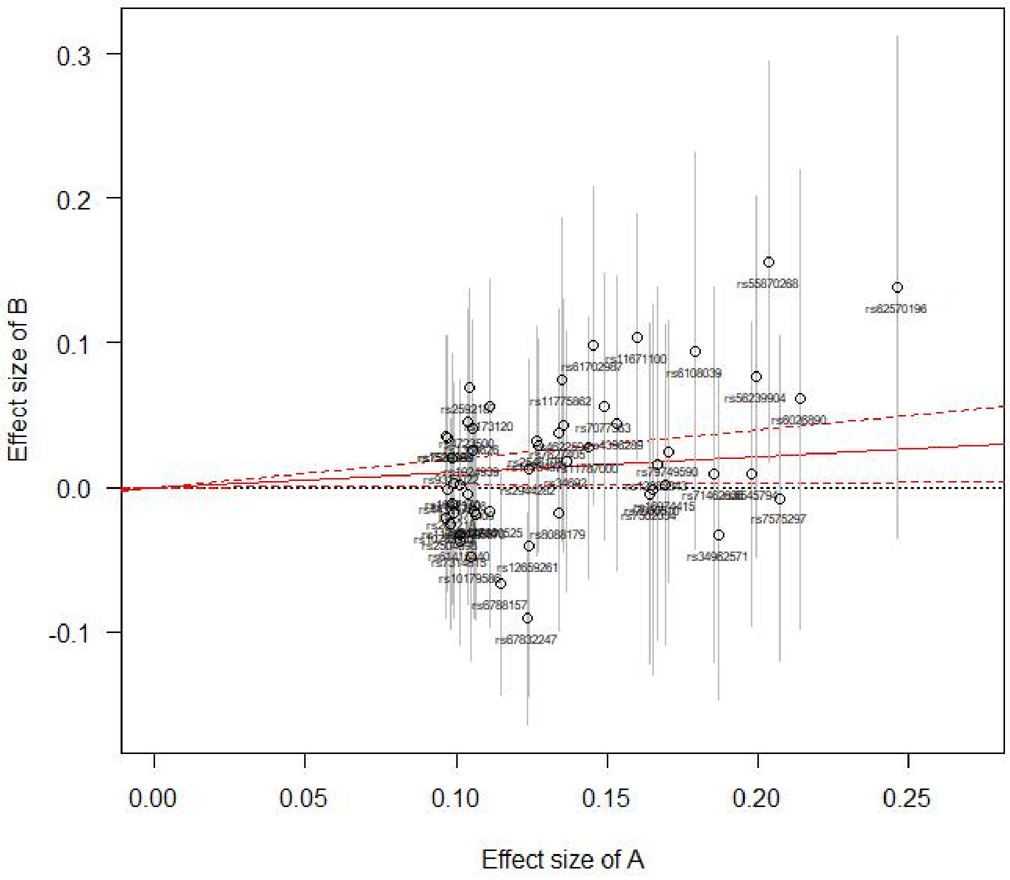

3. Gut microbiota abundance (genus Coprobacter id.949)

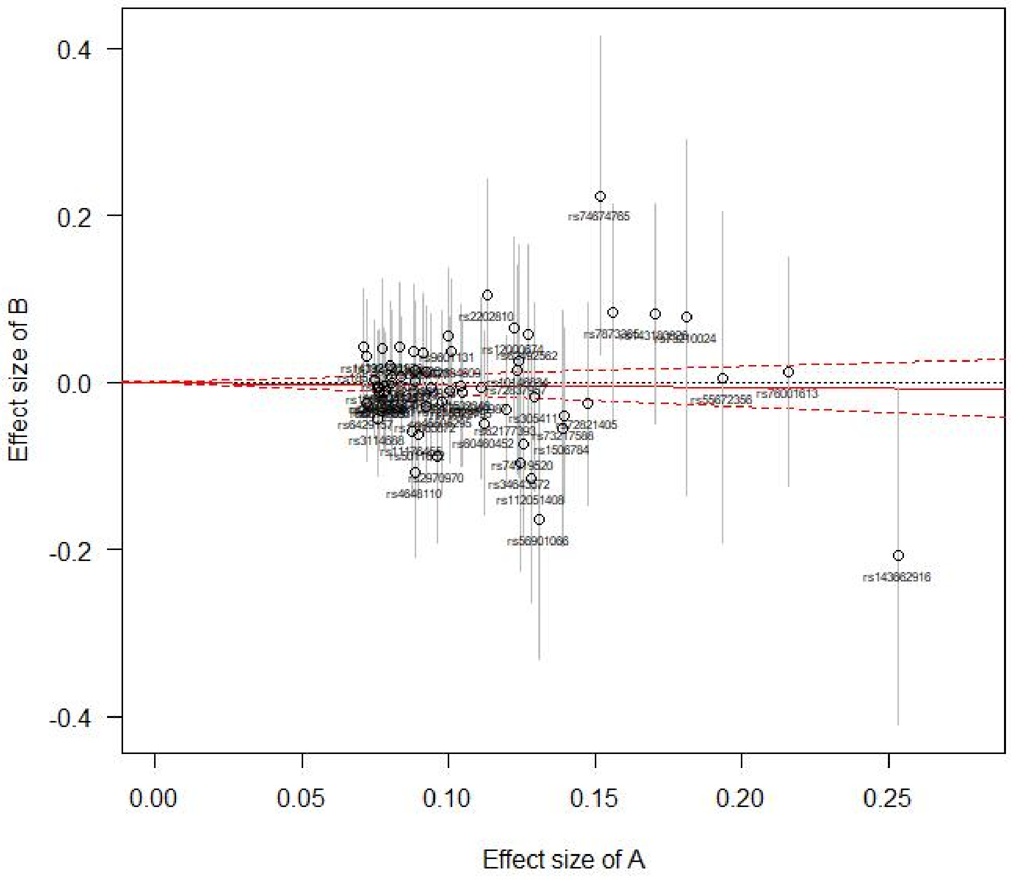

4. Gut microbiota abundance (genus Family XIII UCG001 id.11294)

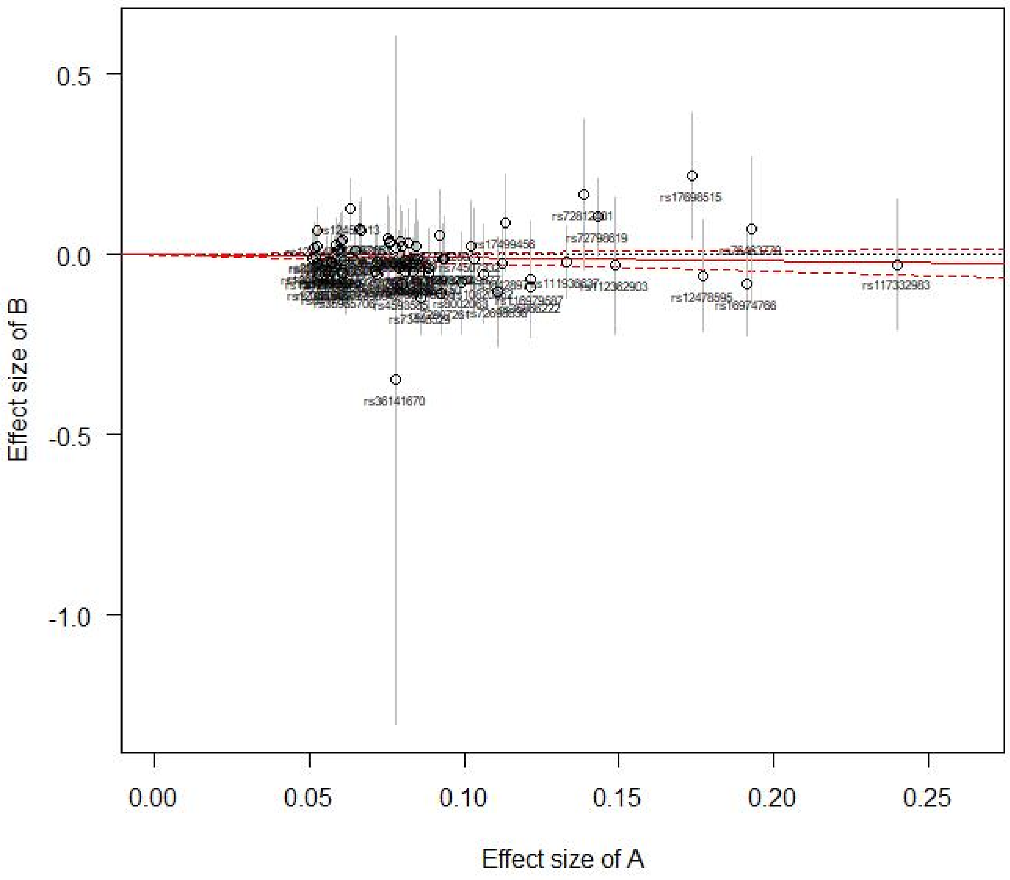

5. Gut microbiota abundance (genus Lactococcus id.1851)

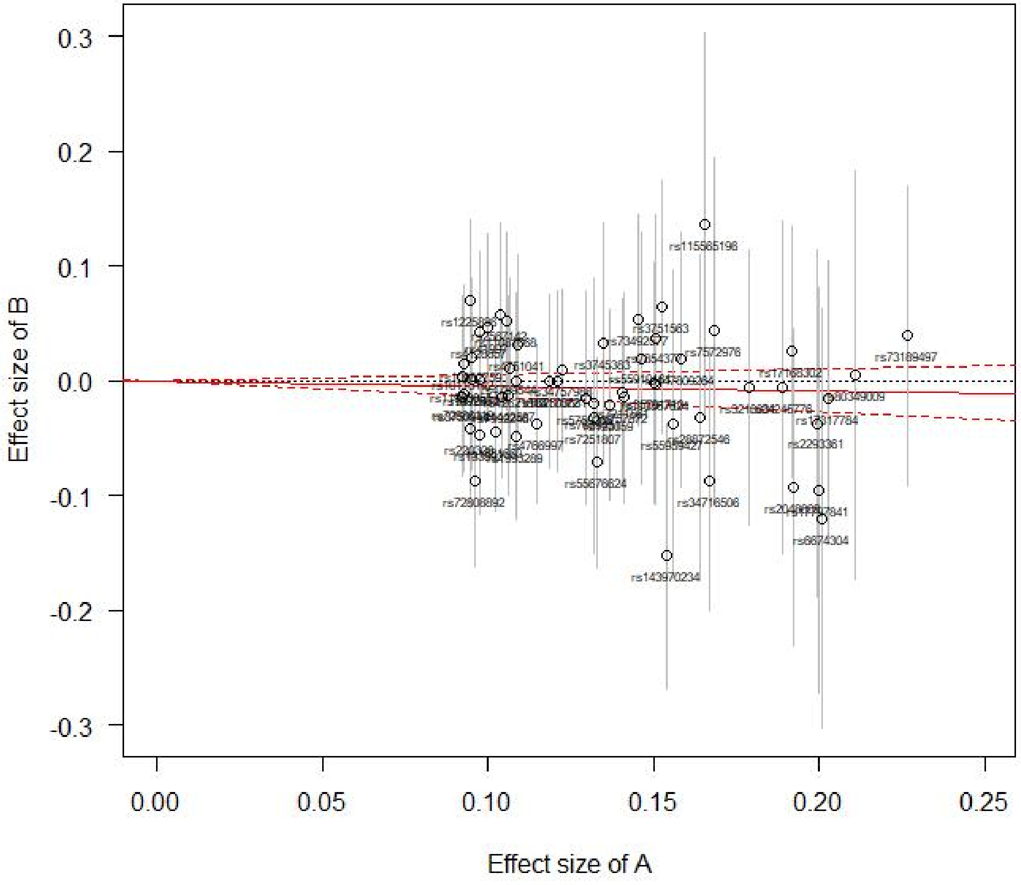

6. Gut microbiota abundance (phylum Actinobacteria id.400)

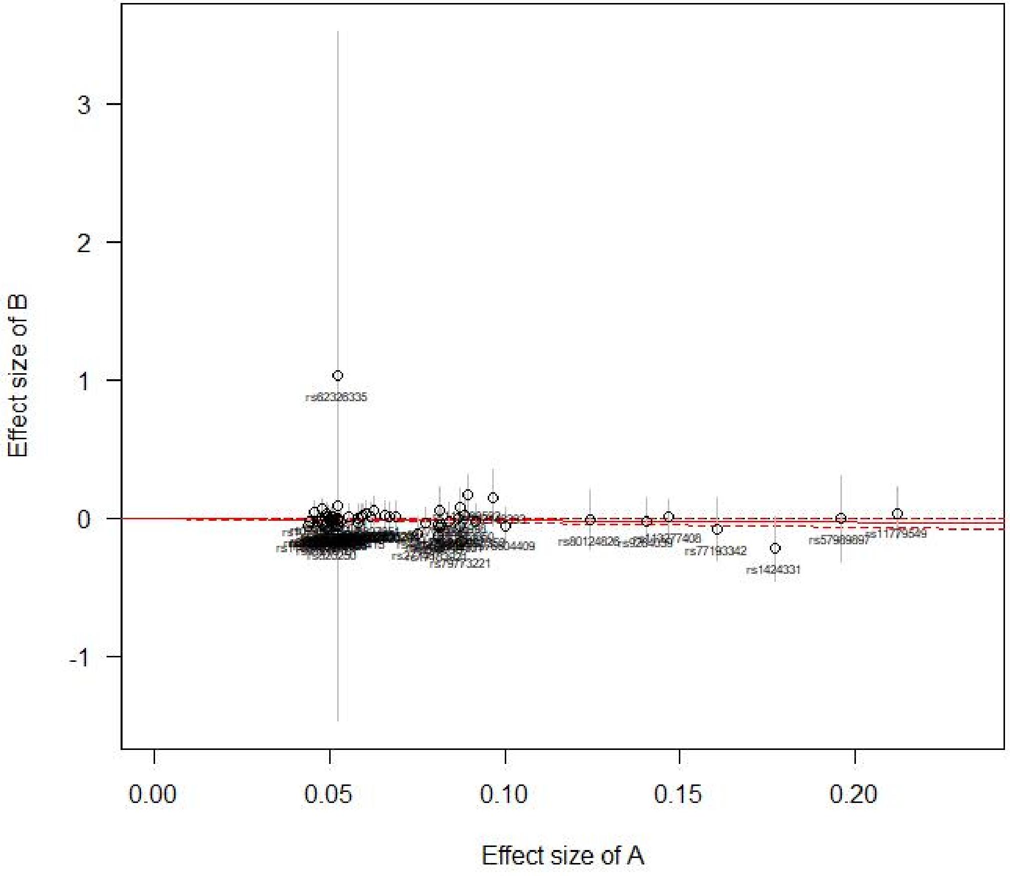

7. Gut microbiota abundance (class Betaproteobacteria id.2867)

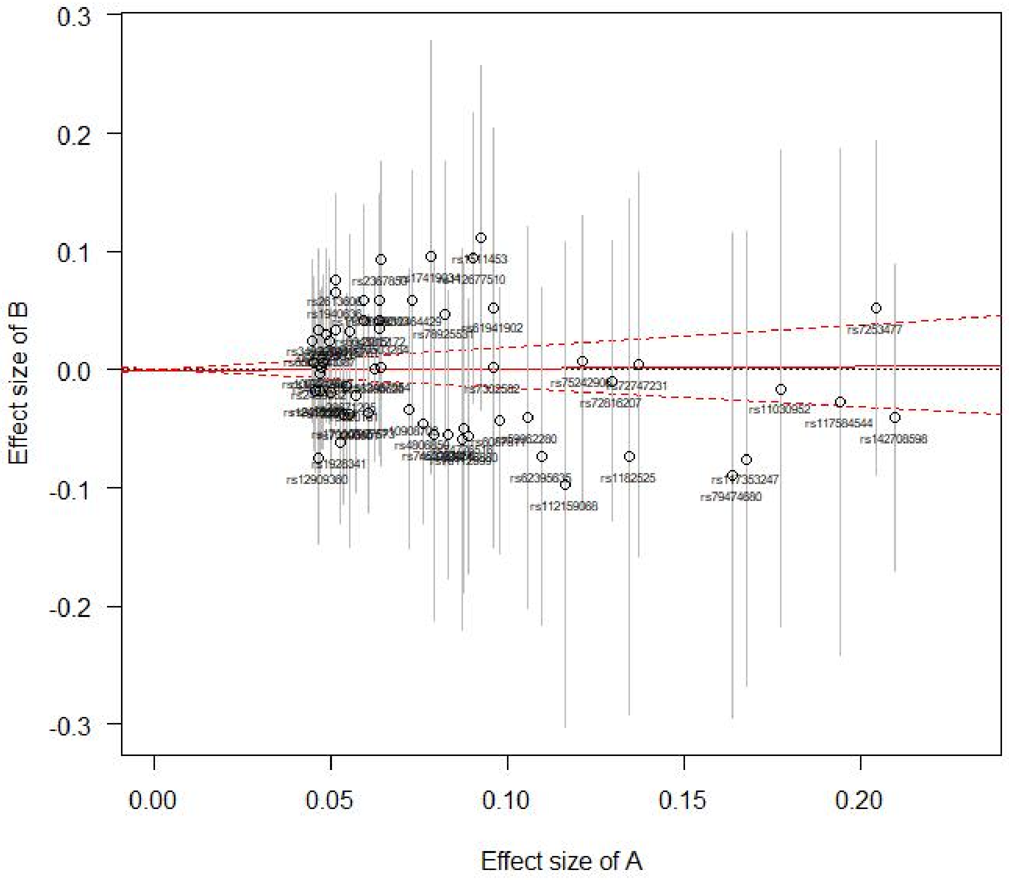

Q. Primary biliary cirrhosis

1. Gut microbiota abundance (family Defluviitaleaceae id.1924)

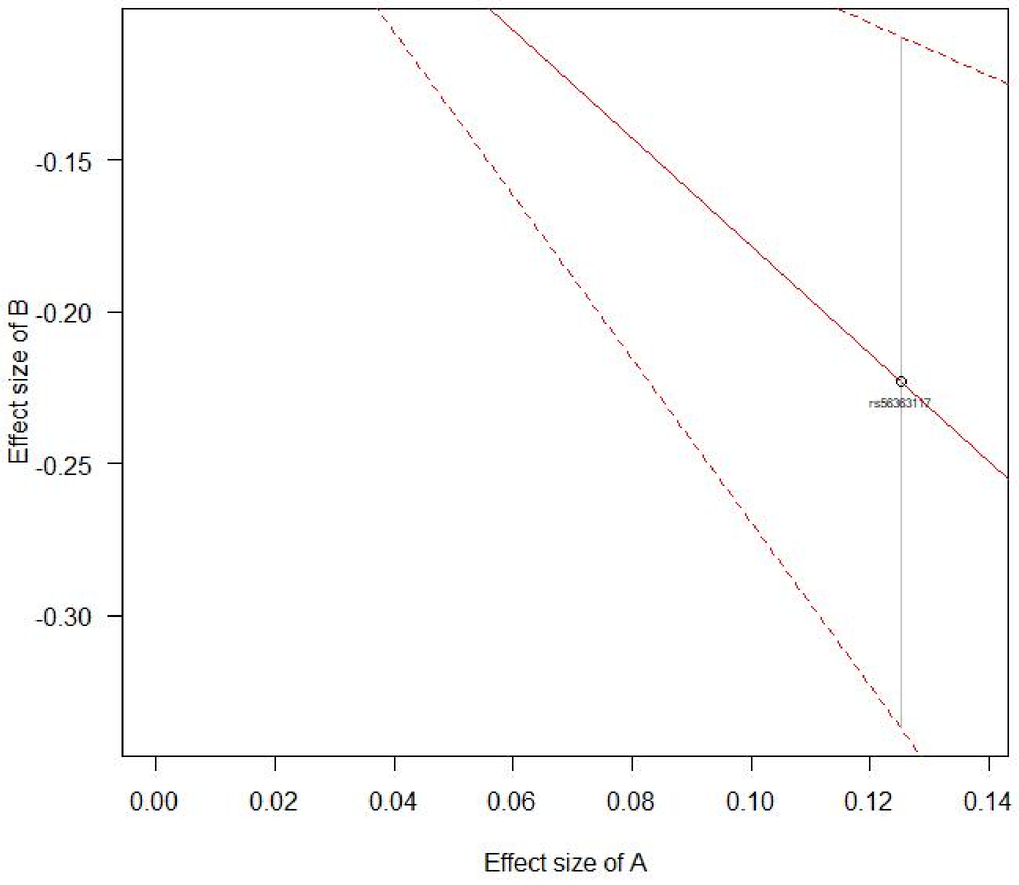

2. Gut microbiota abundance (family Streptococcaceae id.1850)

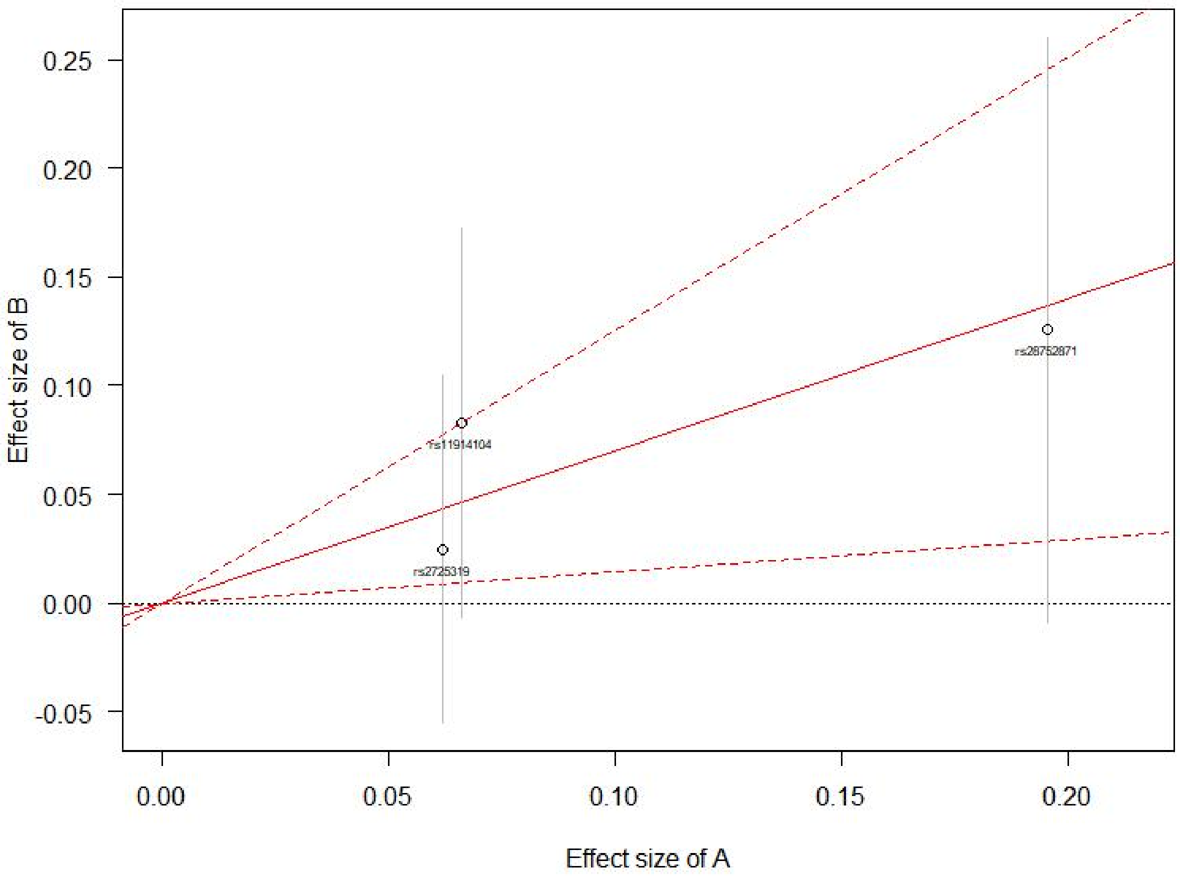

3. Gut microbiota abundance (family Verrucomicrobiaceae id.4036)

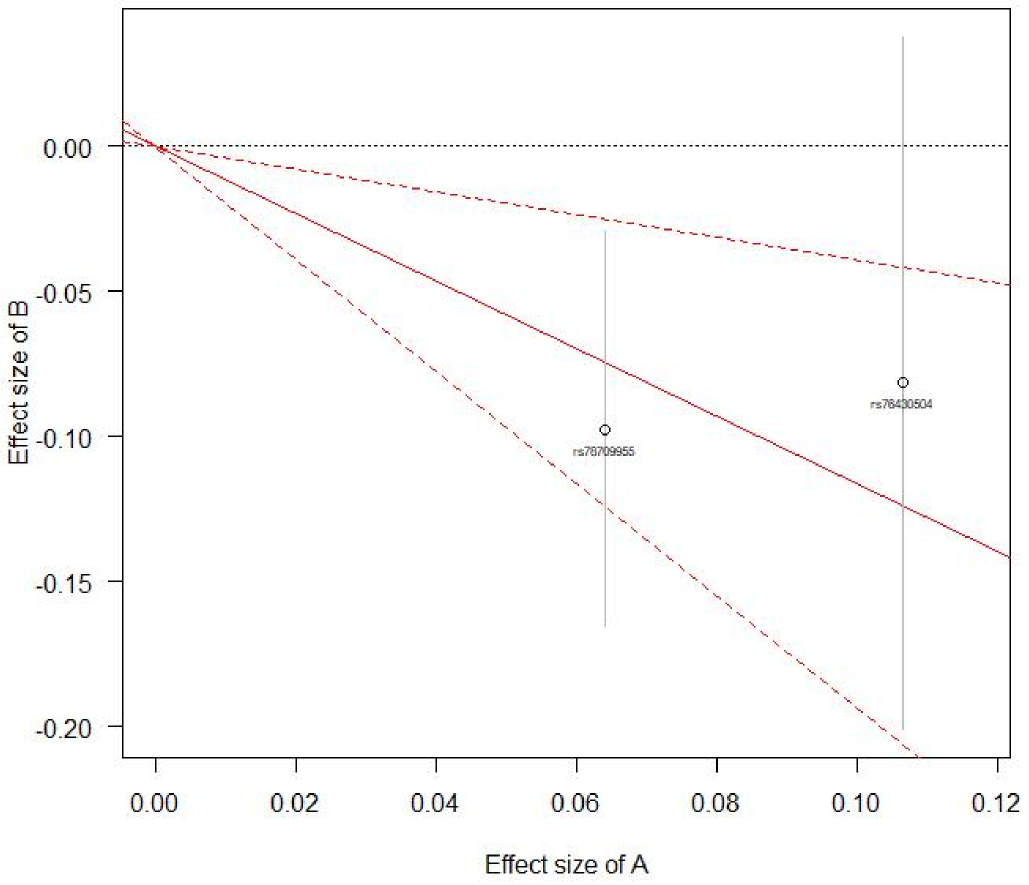

4. Gut microbiota abundance (genus Akkermansia id.4037)

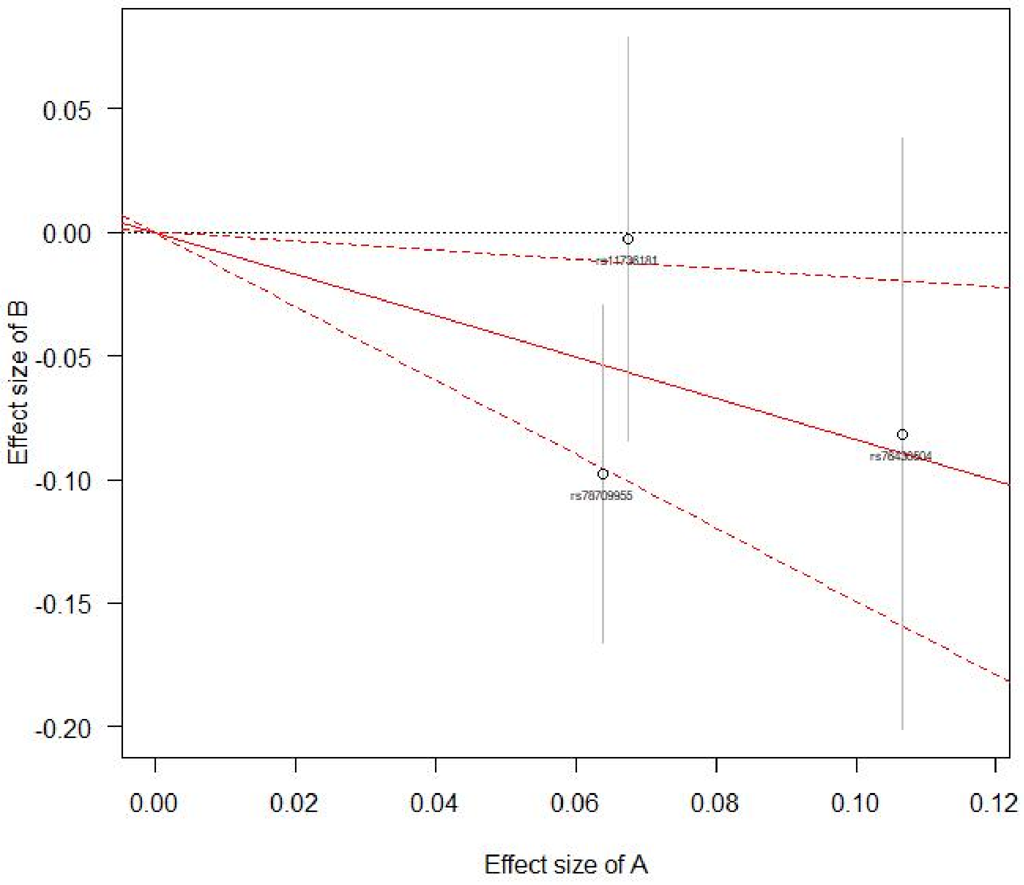

5. Gut microbiota abundance (genus Defluviitaleaceae UCG011 id.11287)

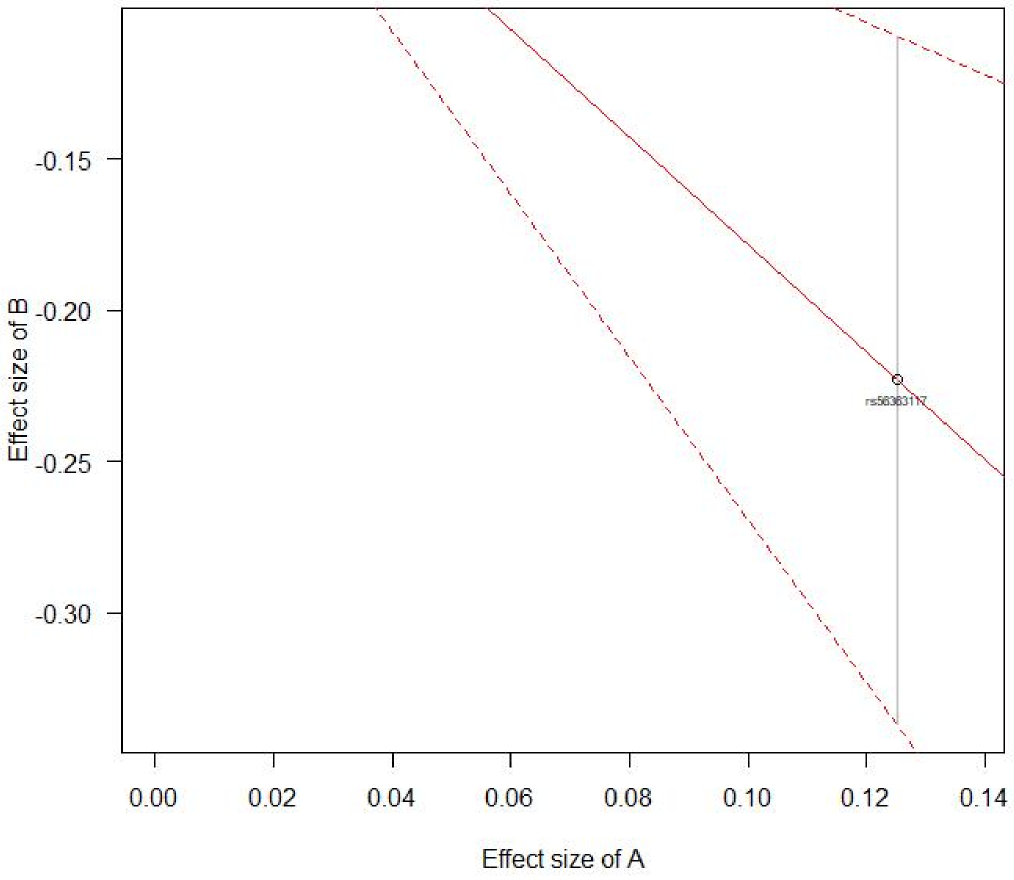

6. Gut microbiota abundance (genus Desulfovibrio id.3173)

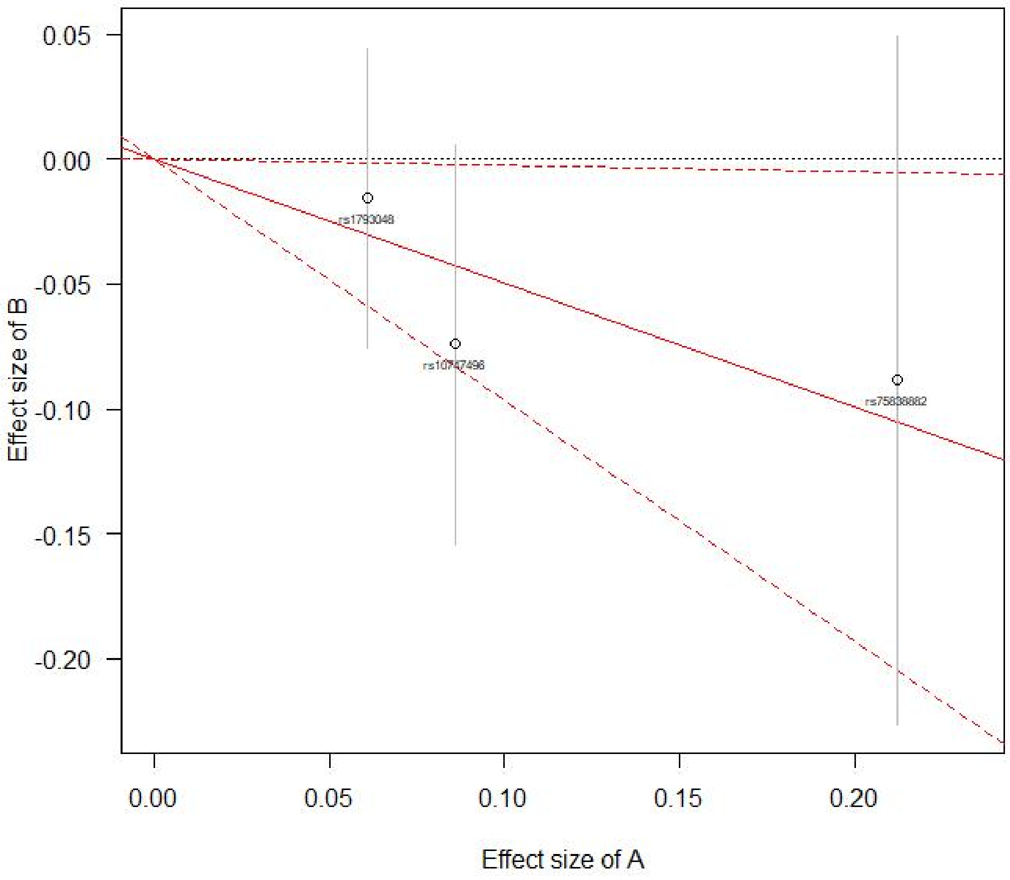

7. Gut microbiota abundance (genus Dialister id.2183)

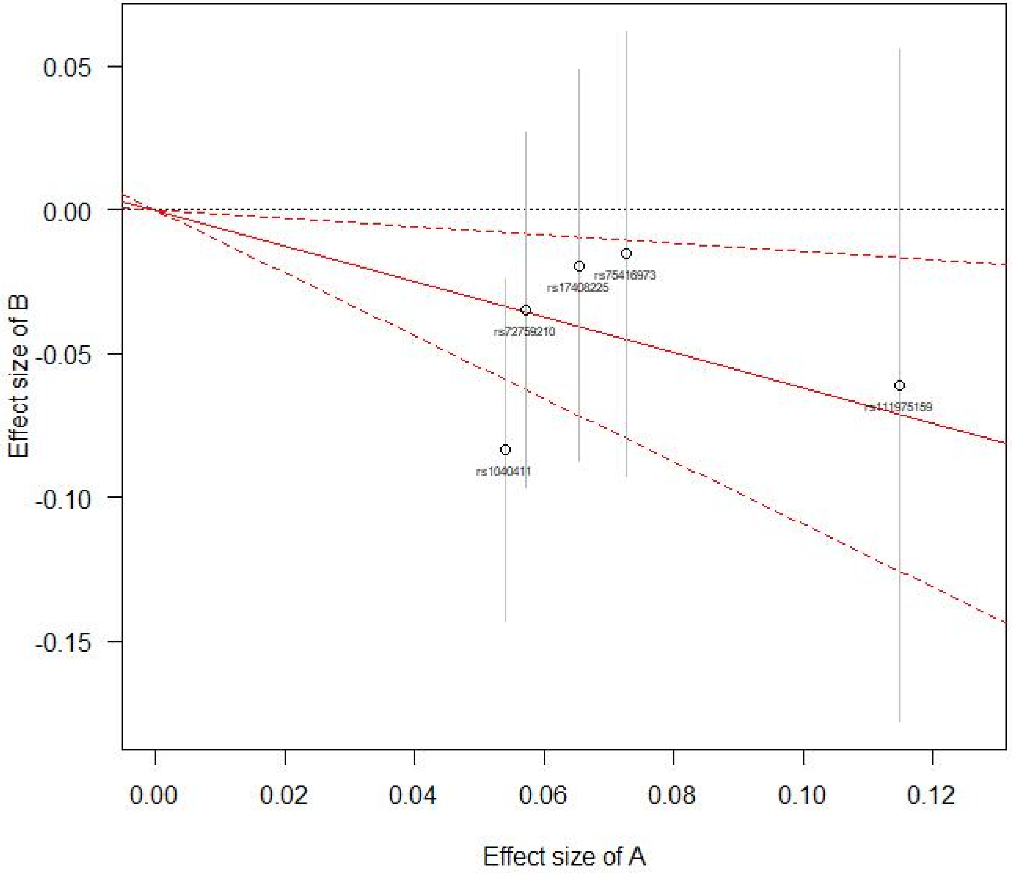

8. Gut microbiota abundance (genus Eubacterium hallii group id.11338)

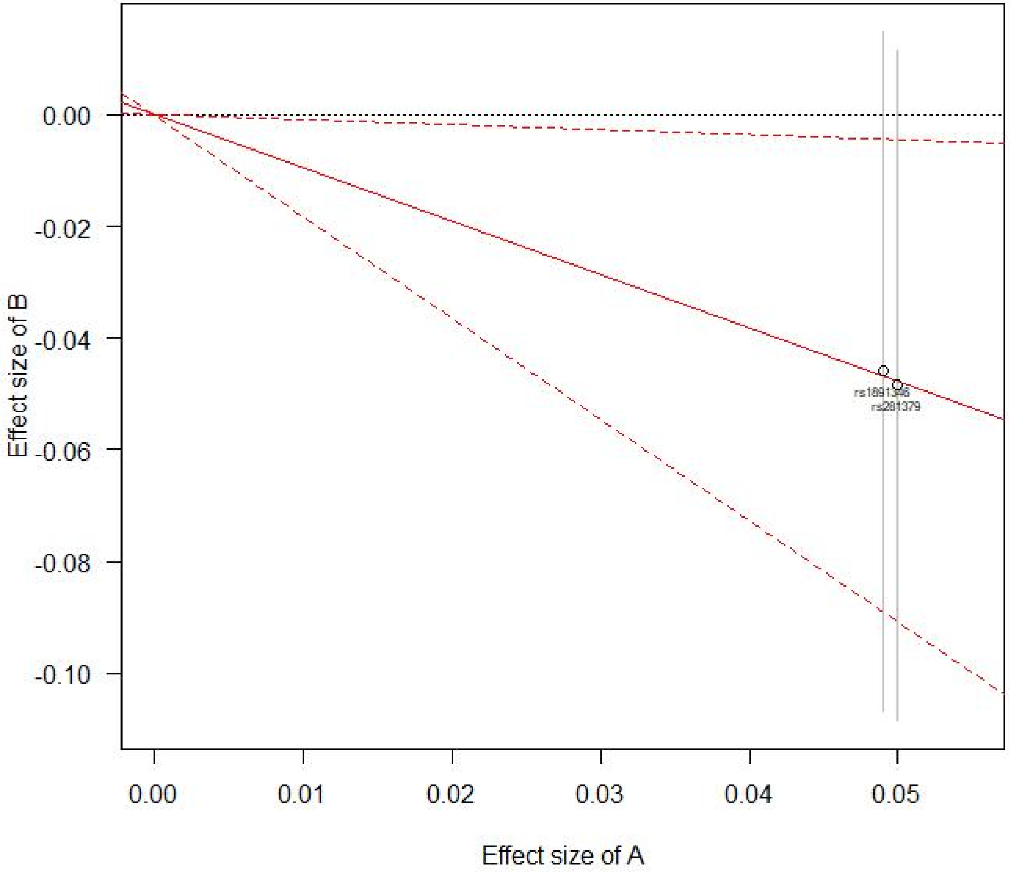

9. Gut microbiota abundance (genus Eubacterium nodatum group id.11297)

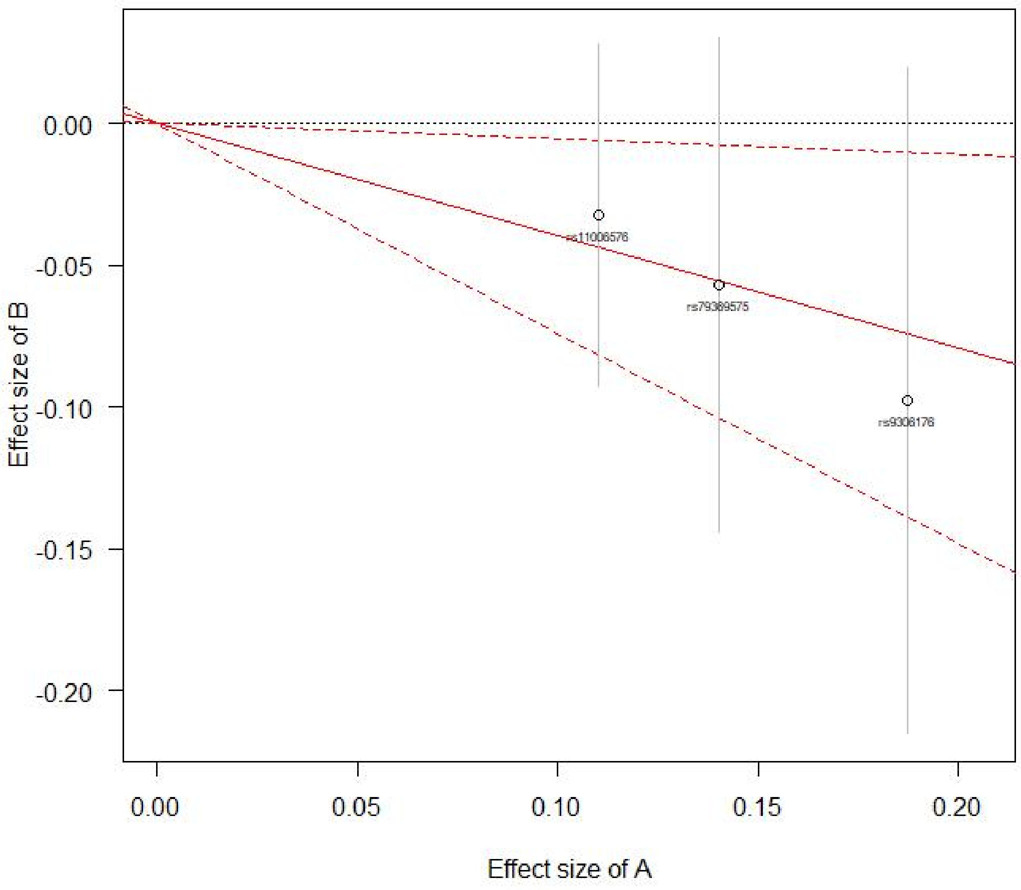

10. Gut microbiota abundance (genus Flavonifractor id.2059)

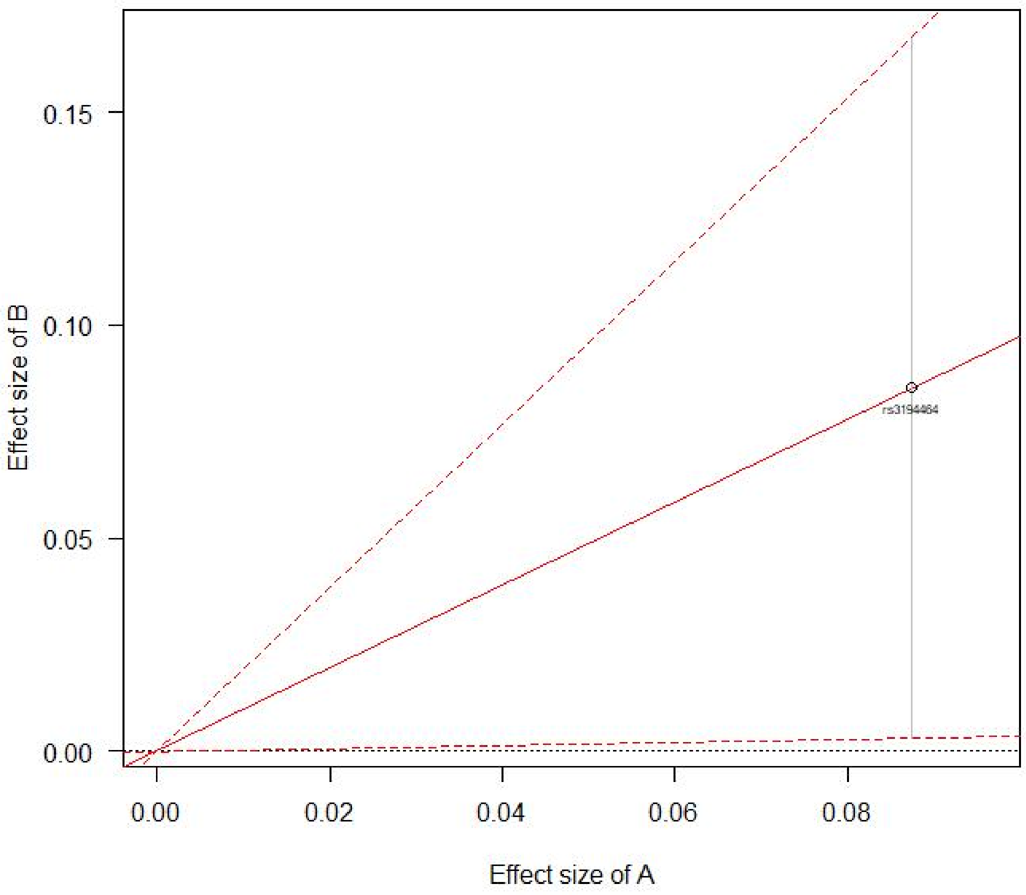

11. Gut microbiota abundance (genus Lachnospiraceae UCG010 id.11330)

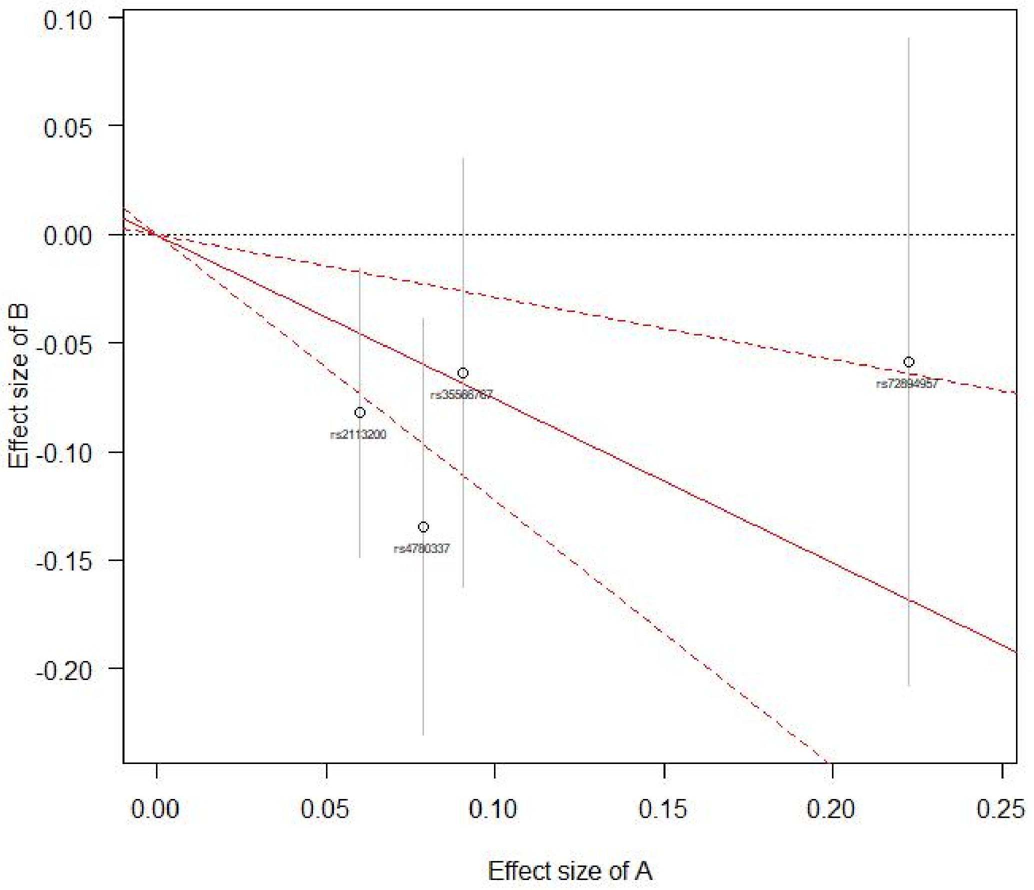

12. Gut microbiota abundance (genus Marvinbryantia id.2005)

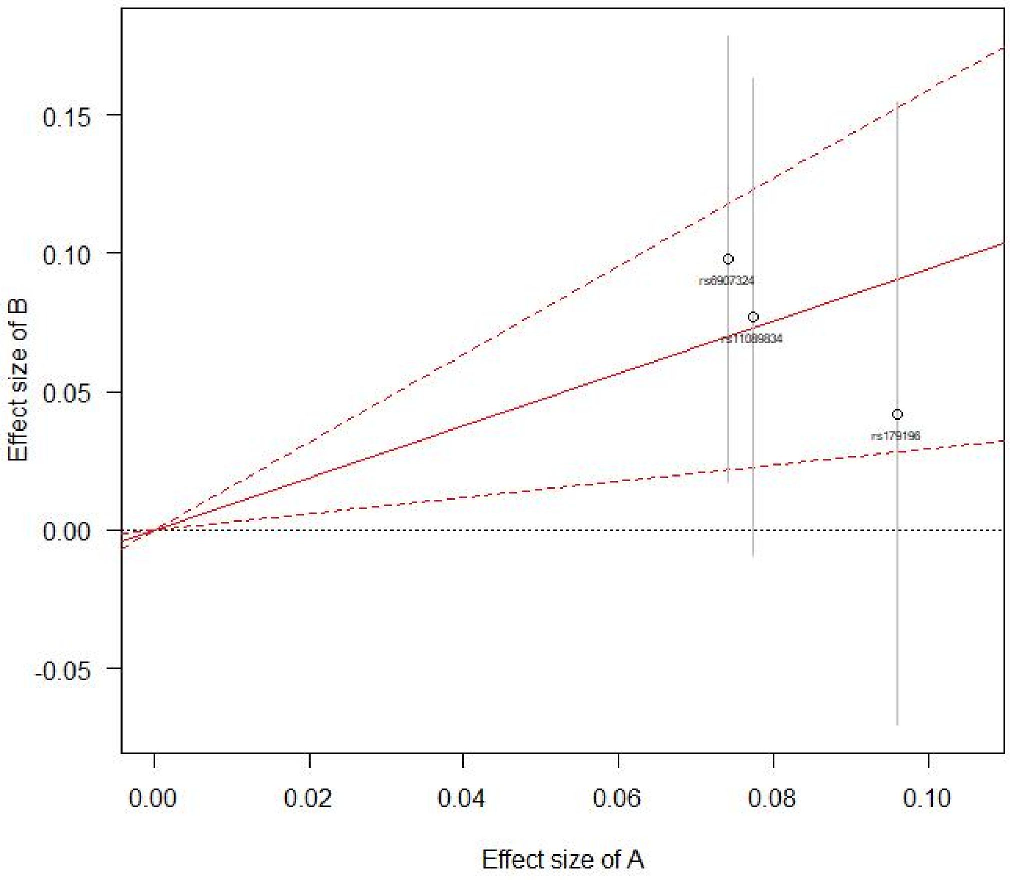

13. Gut microbiota abundance (genus Oscillibacter id.2063)

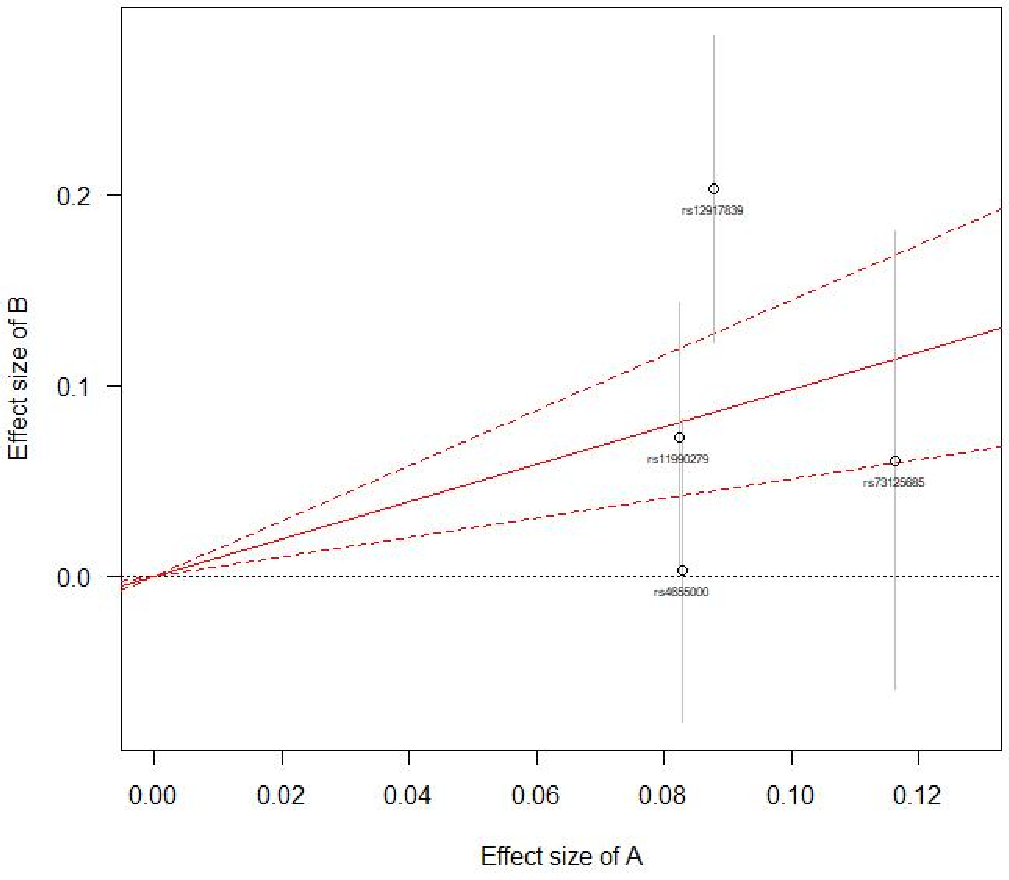

14. Gut microbiota abundance (genus Ruminococcaceae UCG010 id.11367)

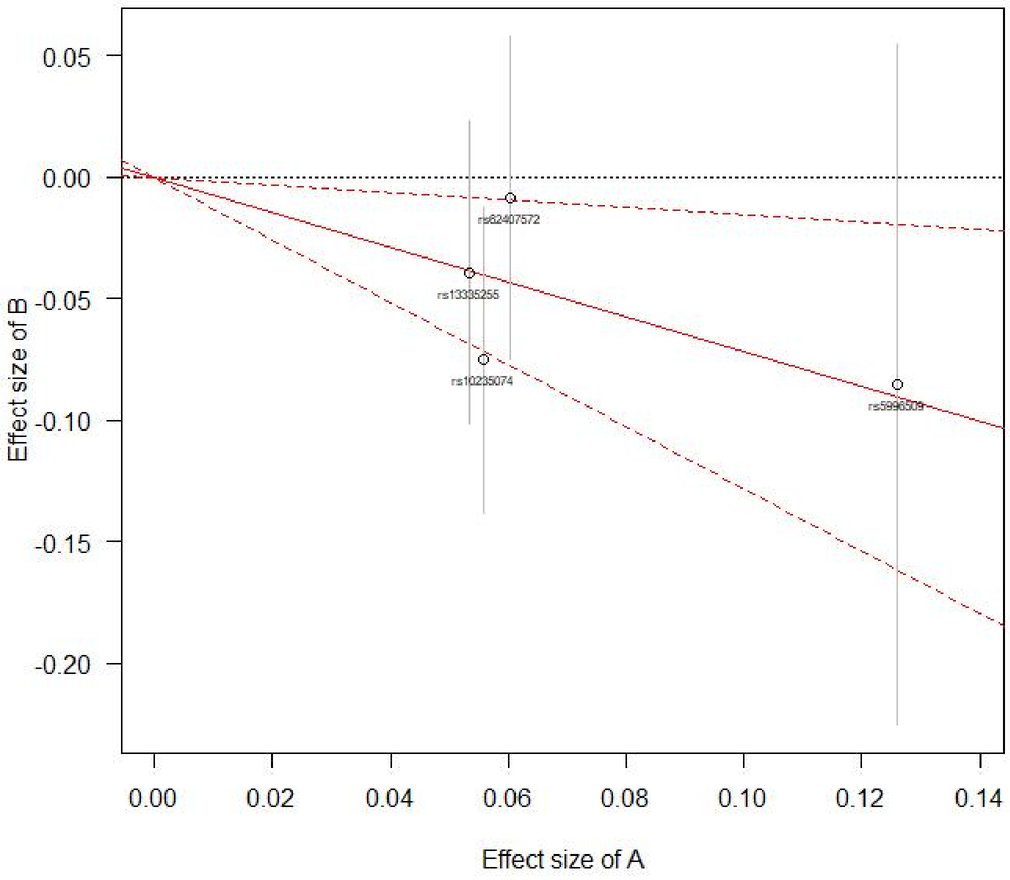

15. Gut microbiota abundance (genus Ruminococcaceae UCG011 id.11368)

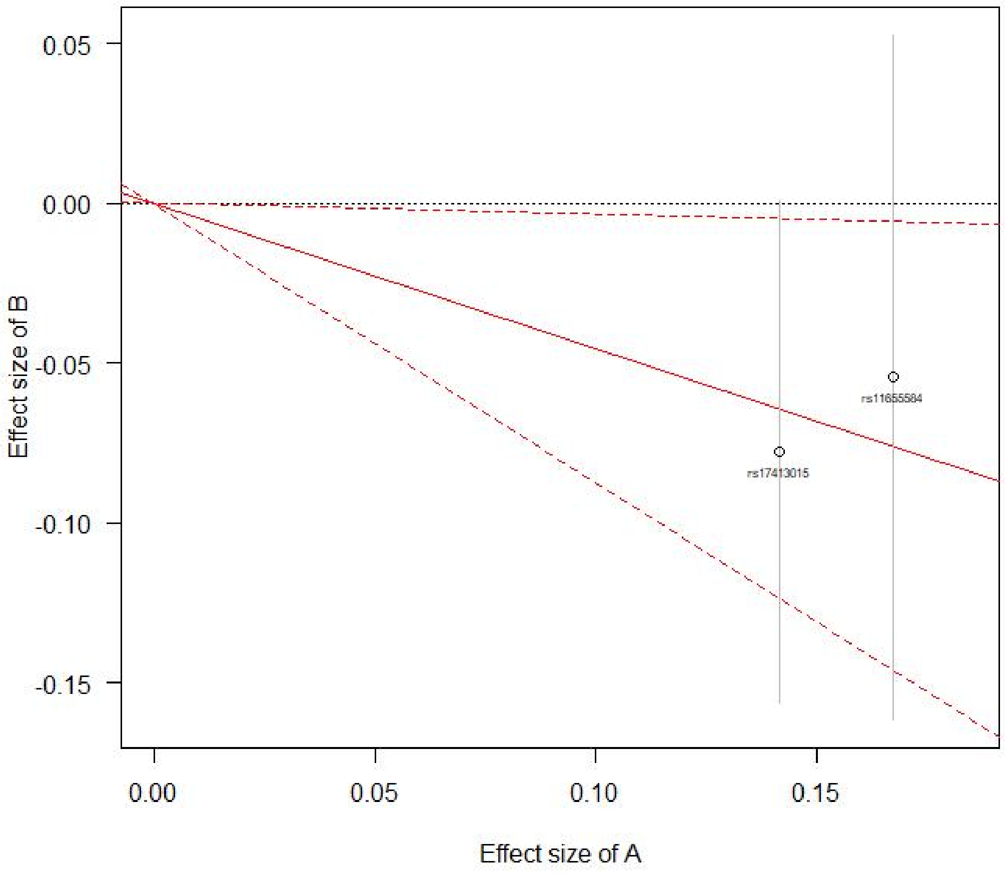

16. Gut microbiota abundance (unknown genus id.826)

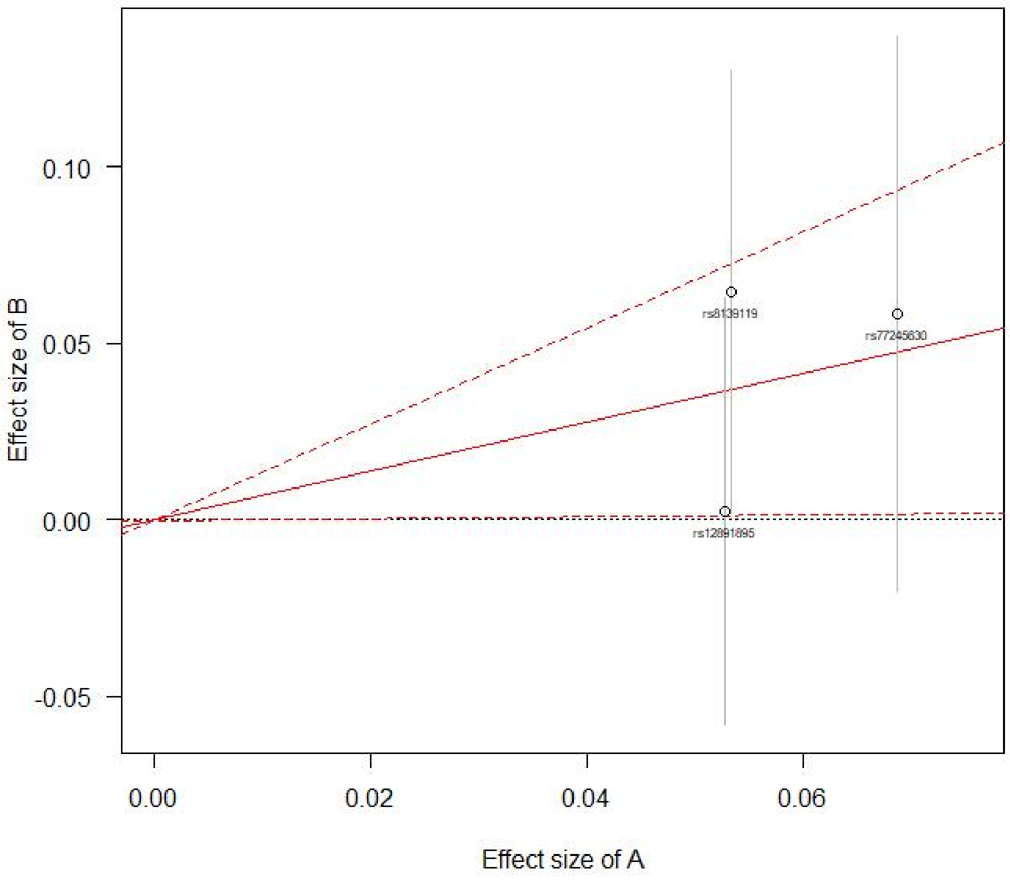

17. Gut microbiota abundance (phylum Actinobacteria id.400)

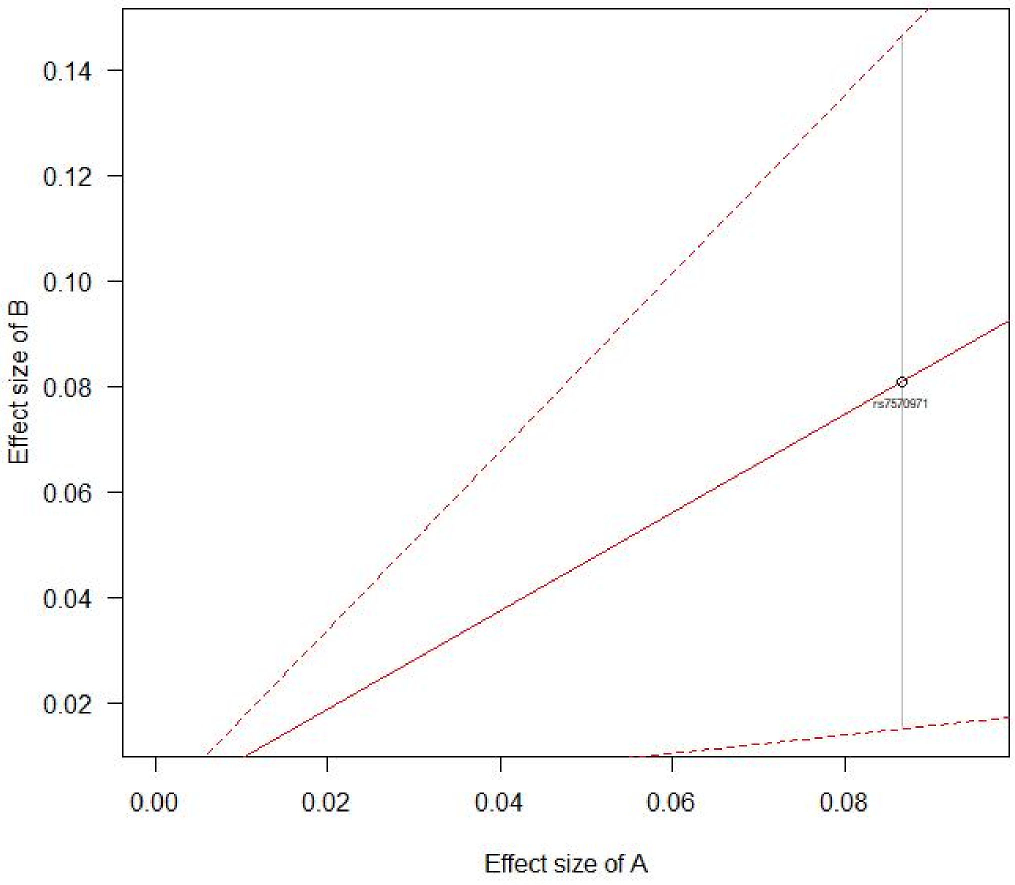

R. Liver cell carcinoma

1. Gut microbiota abundance (family Alcaligenaceae id.2875)

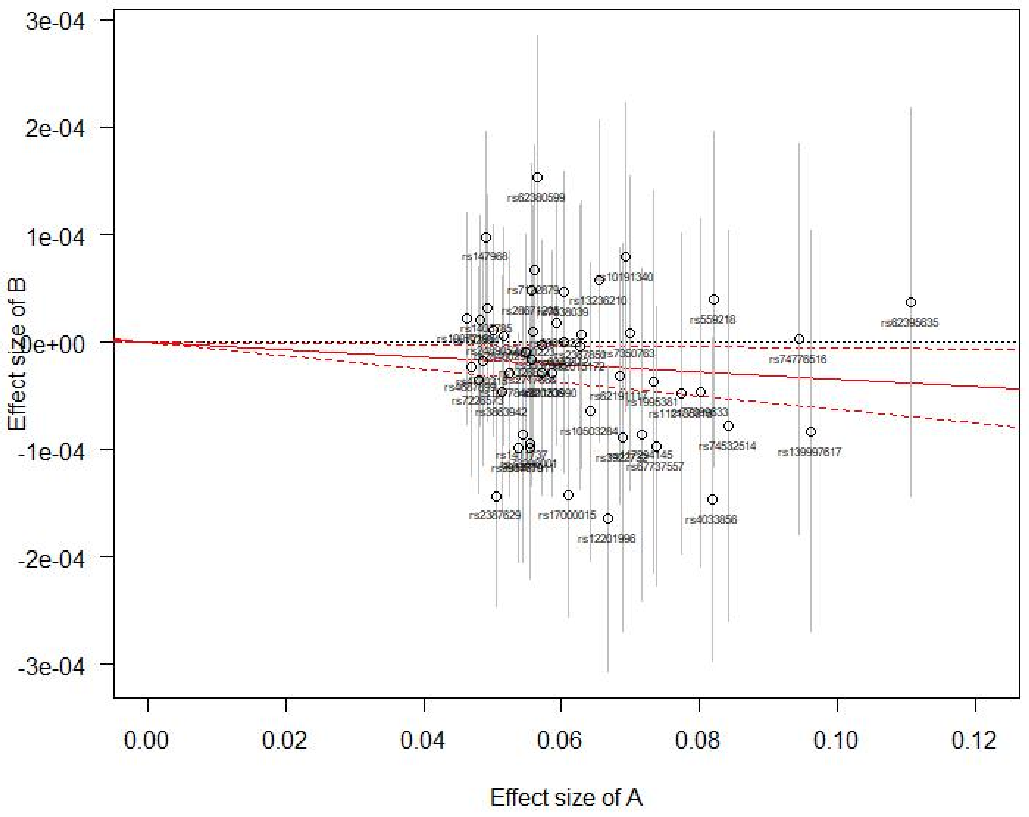

2. Gut microbiota abundance (genus Actinomyces id.423)

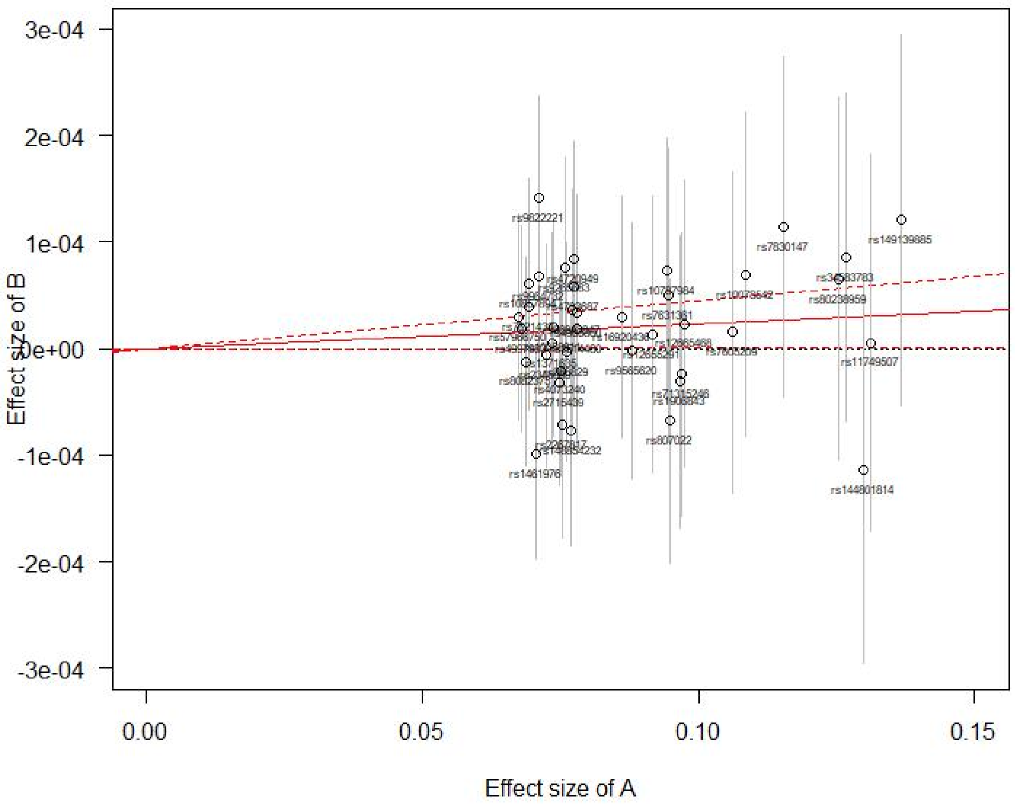

3. Gut microbiota abundance (genus Butyricicoccus id.2055)

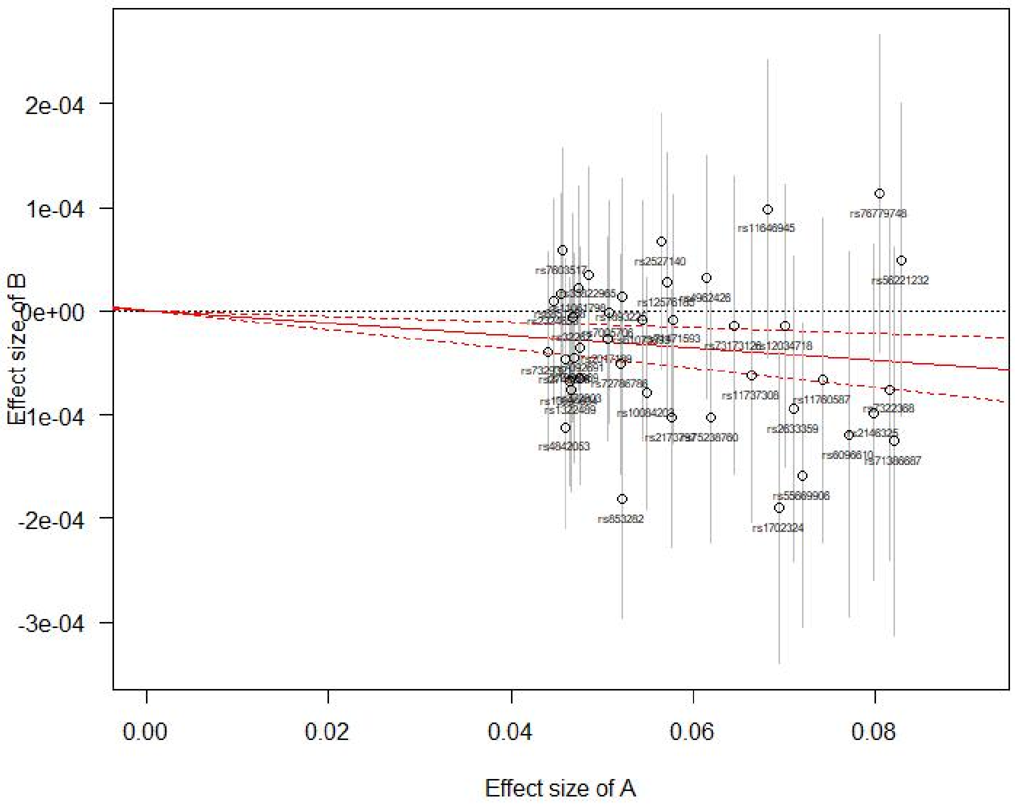

4. Gut microbiota abundance (genus Candidatus Soleaferrea id.11350)

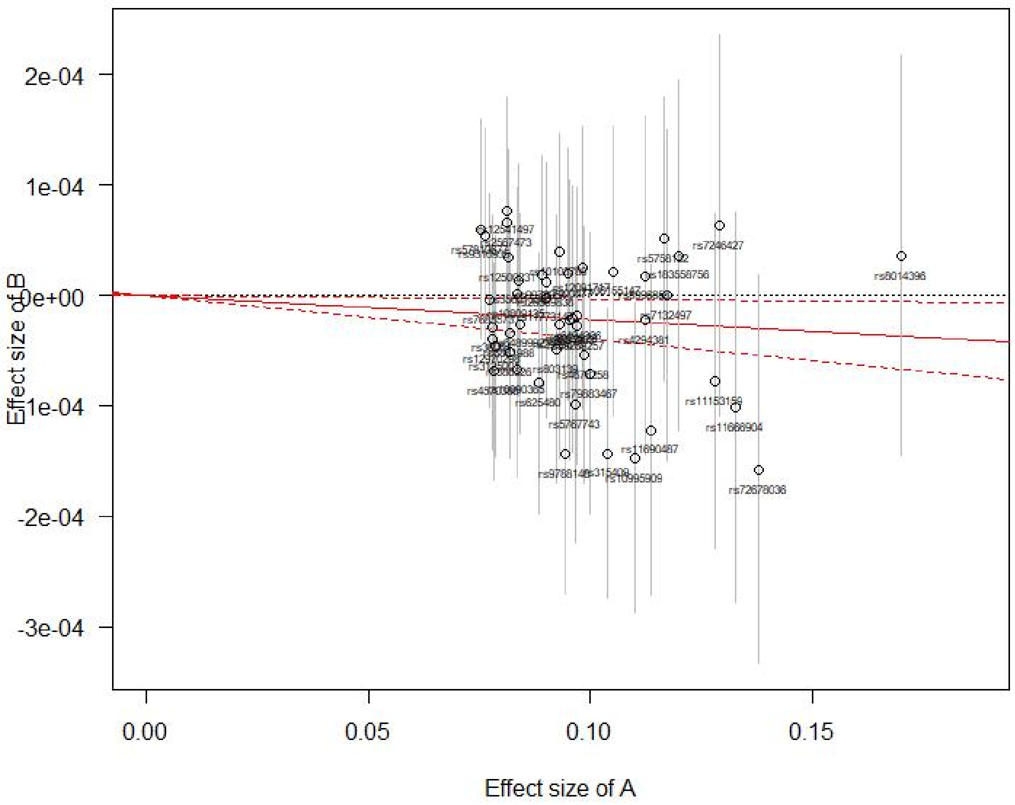

5. Gut microbiota abundance (genus Christensenellaceae R 7group id.11283)

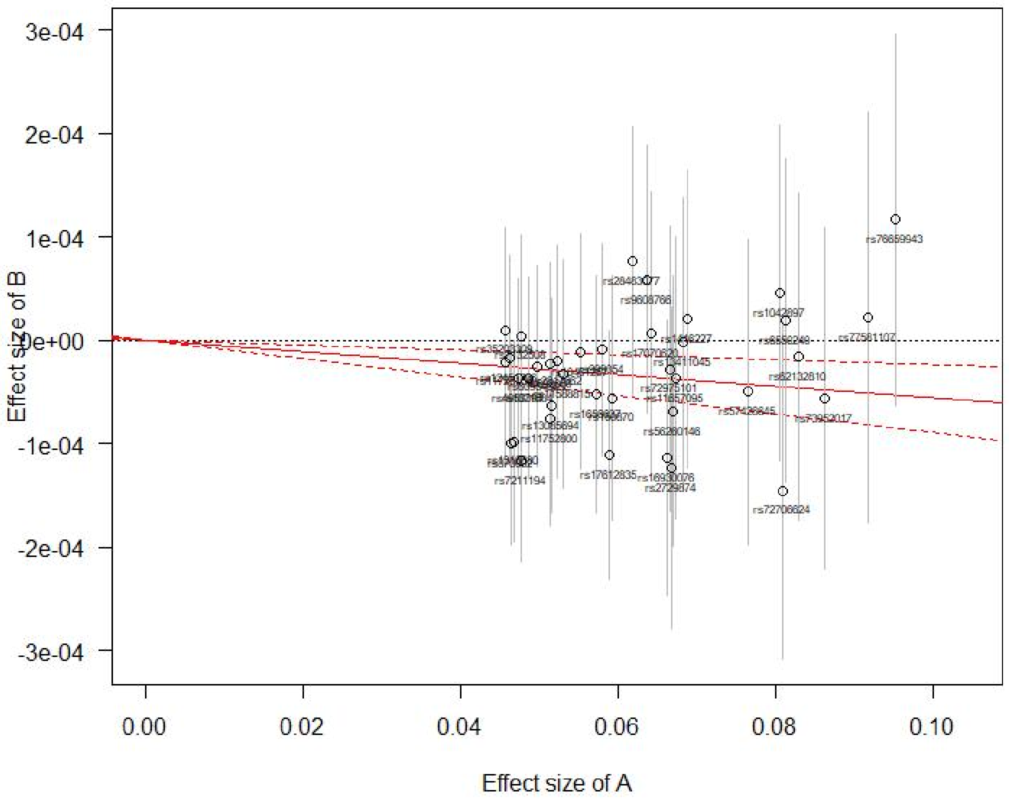

6. Gut microbiota abundance (genus Eubacterium rectale group id.14374)

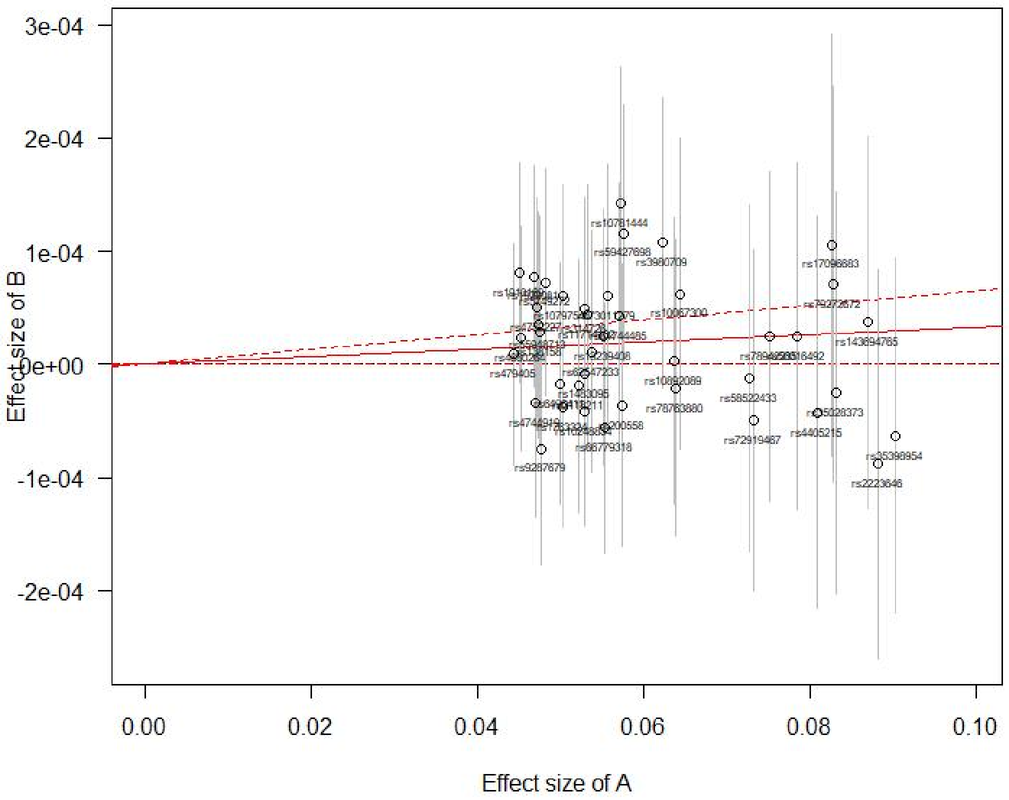

7. Gut microbiota abundance (genus Holdemanella id.11393)

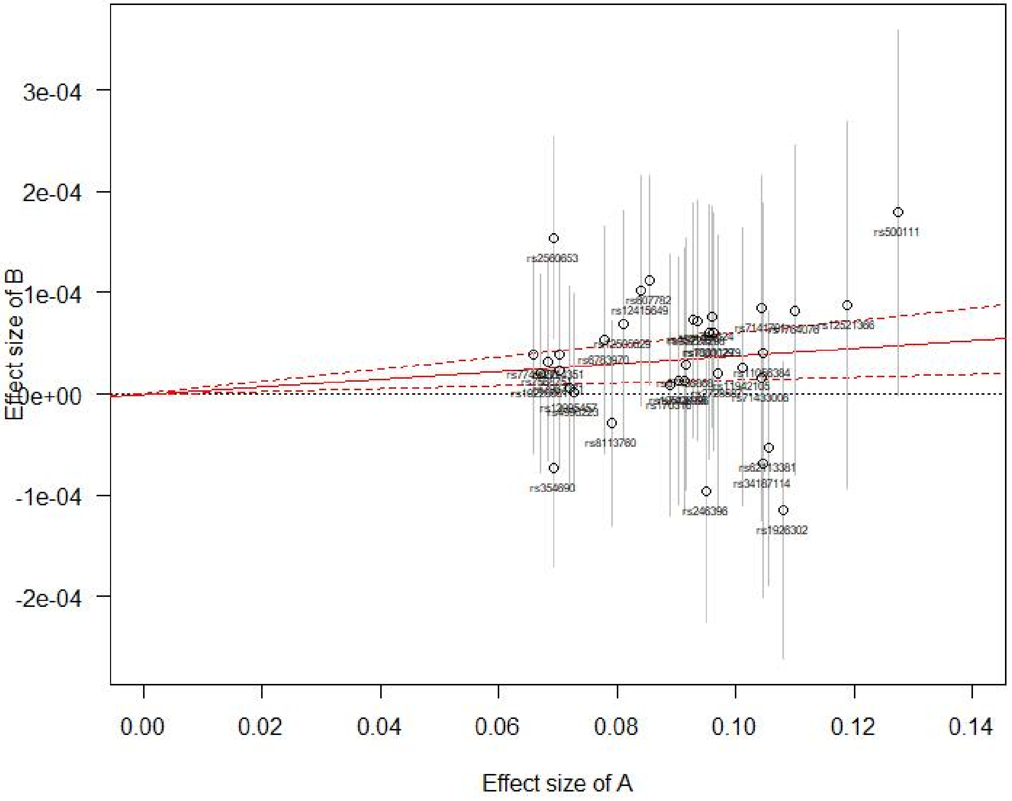

8. Gut microbiota abundance (genus Lachnospiraceae NK4A136 group id.11319)

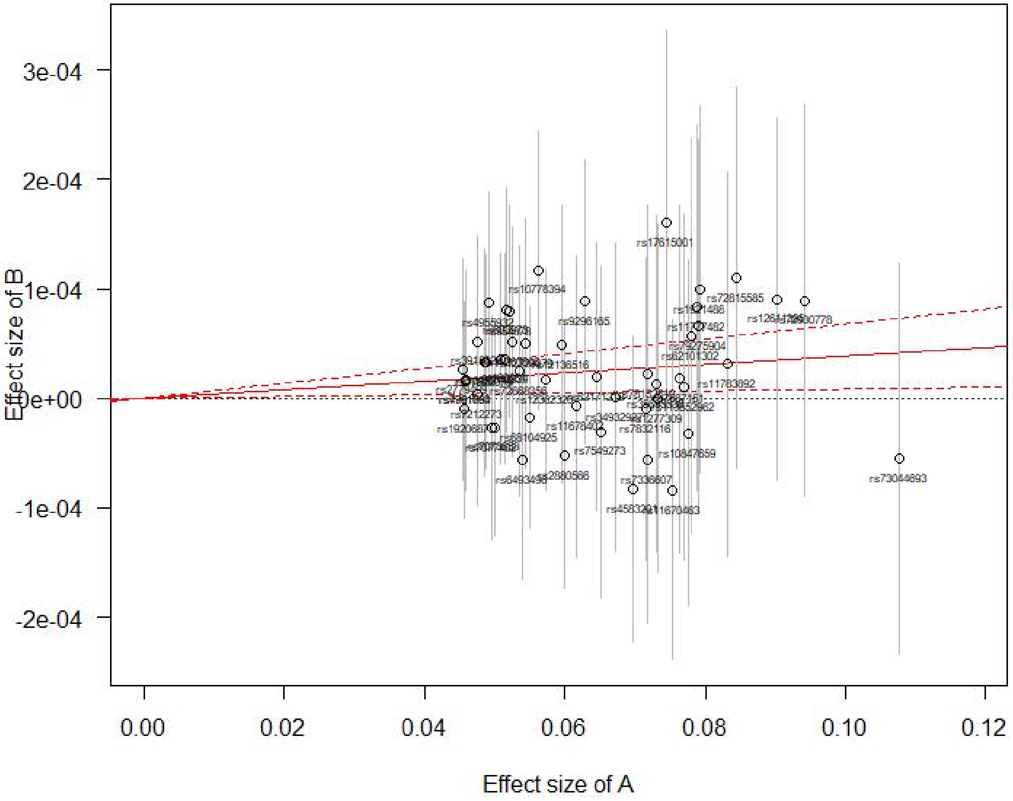

9. Gut microbiota abundance (genus Peptococcus id.2037)

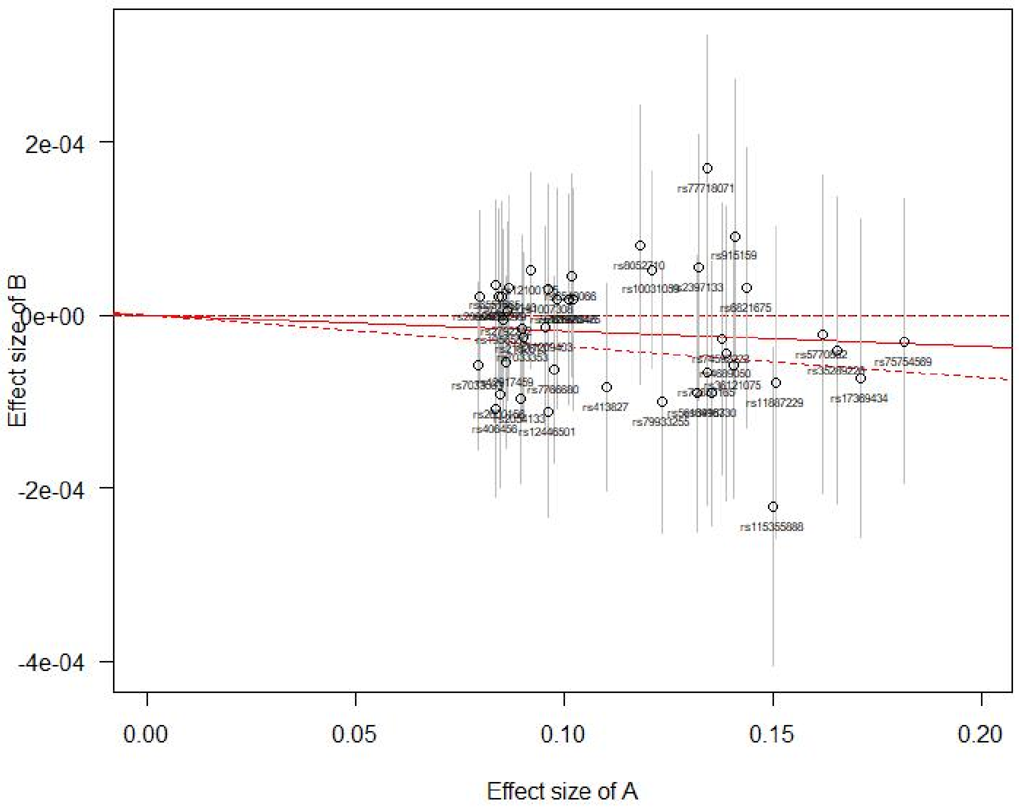

10. Gut microbiota abundance (genus Phascolarctobacterium id.2168)

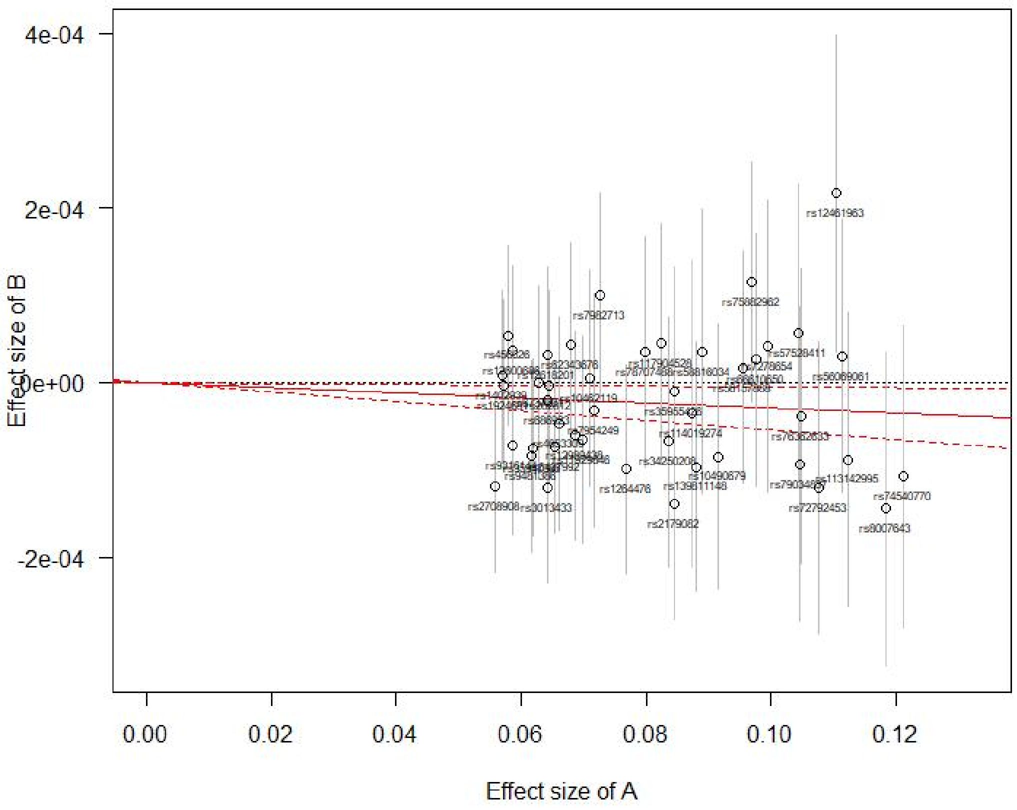

11. Gut microbiota abundance (genus Veillonella id.2198)

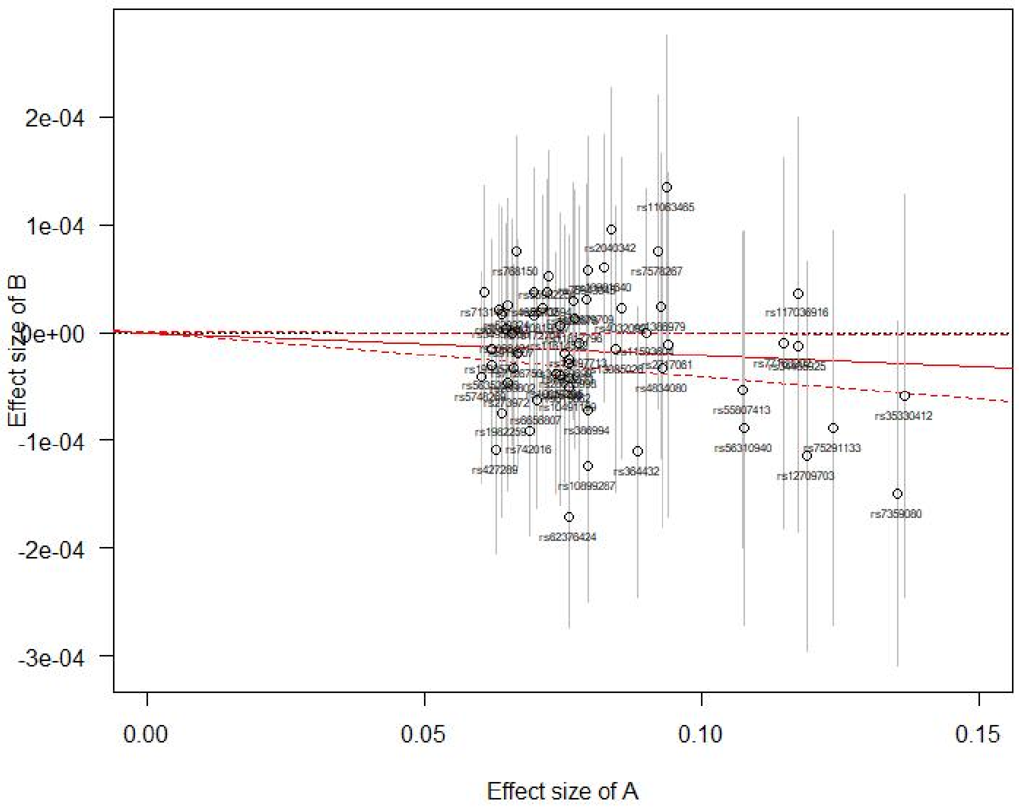

S. Cholelithiasis

1. Gut microbiota abundance (class Alphaproteobacteria id.2379)

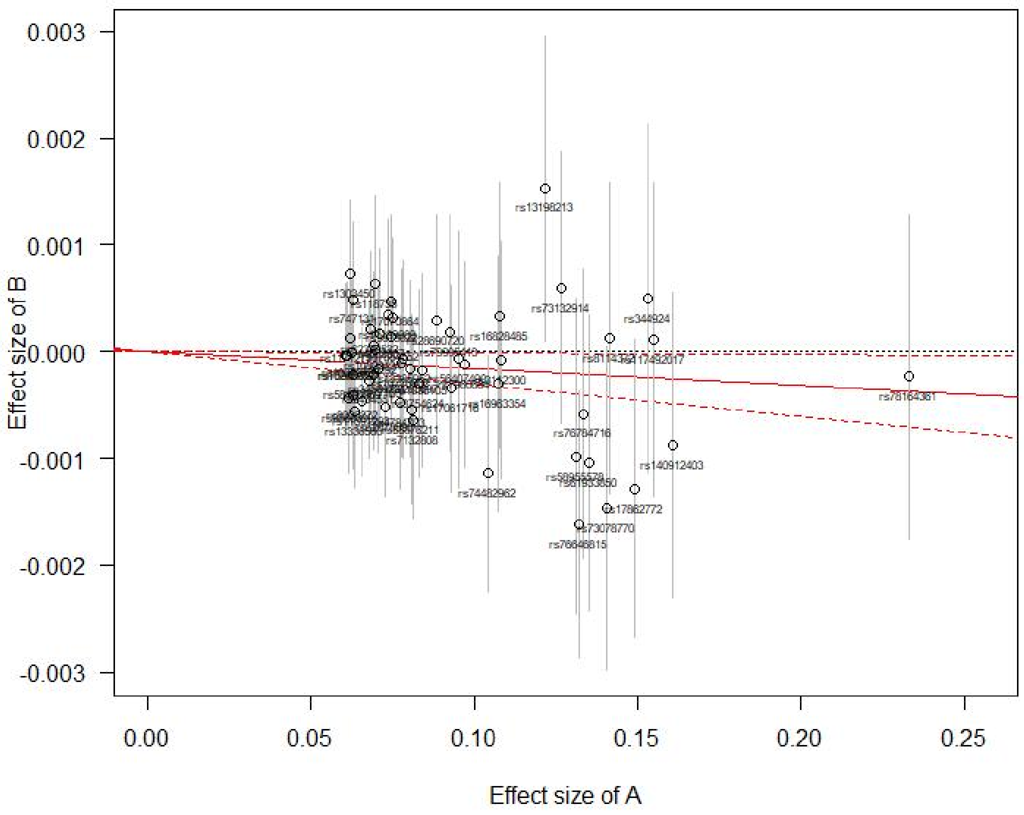

2. Gut microbiota abundance (class Negativicutes id.2164)

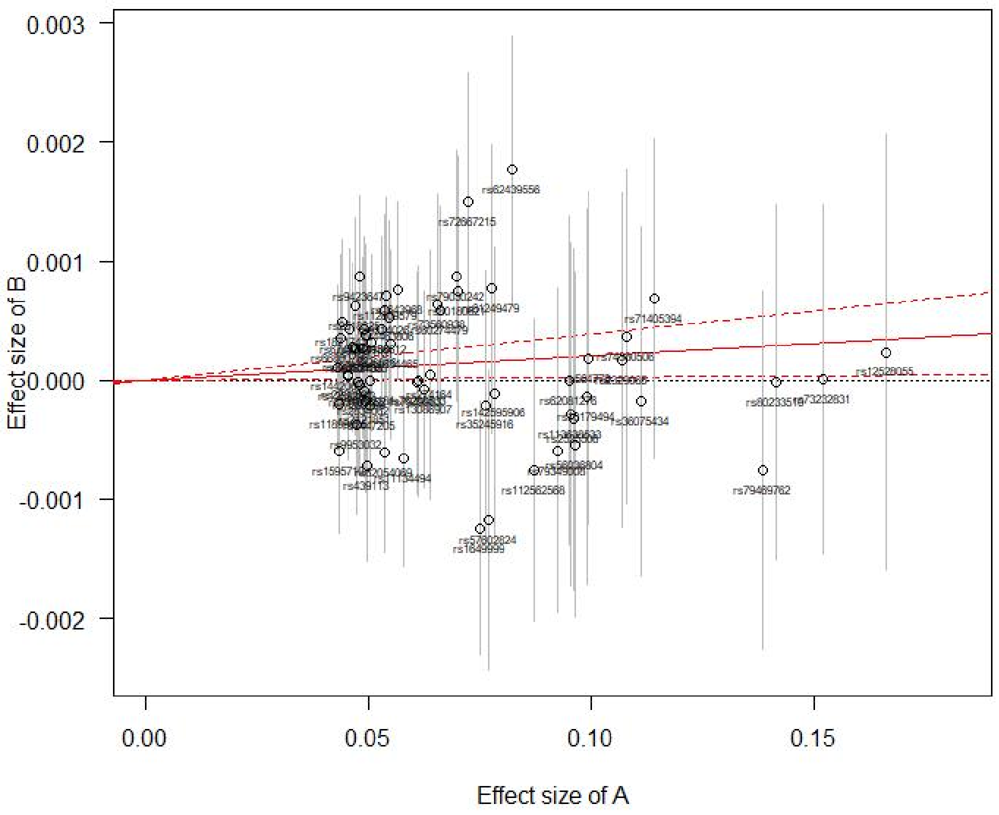

3. Gut microbiota abundance (genus Family XIII AD3011 group id.11293)

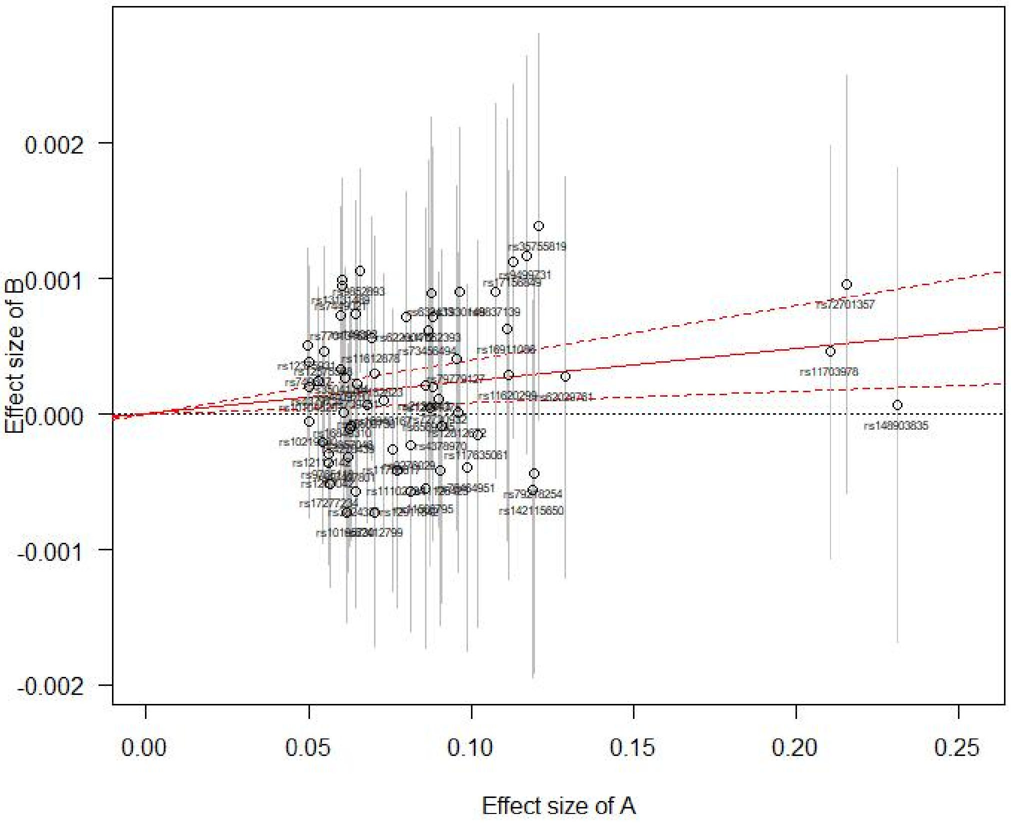

4. Gut microbiota abundance (genus Lachnospiraceae UCG001 id.11321)

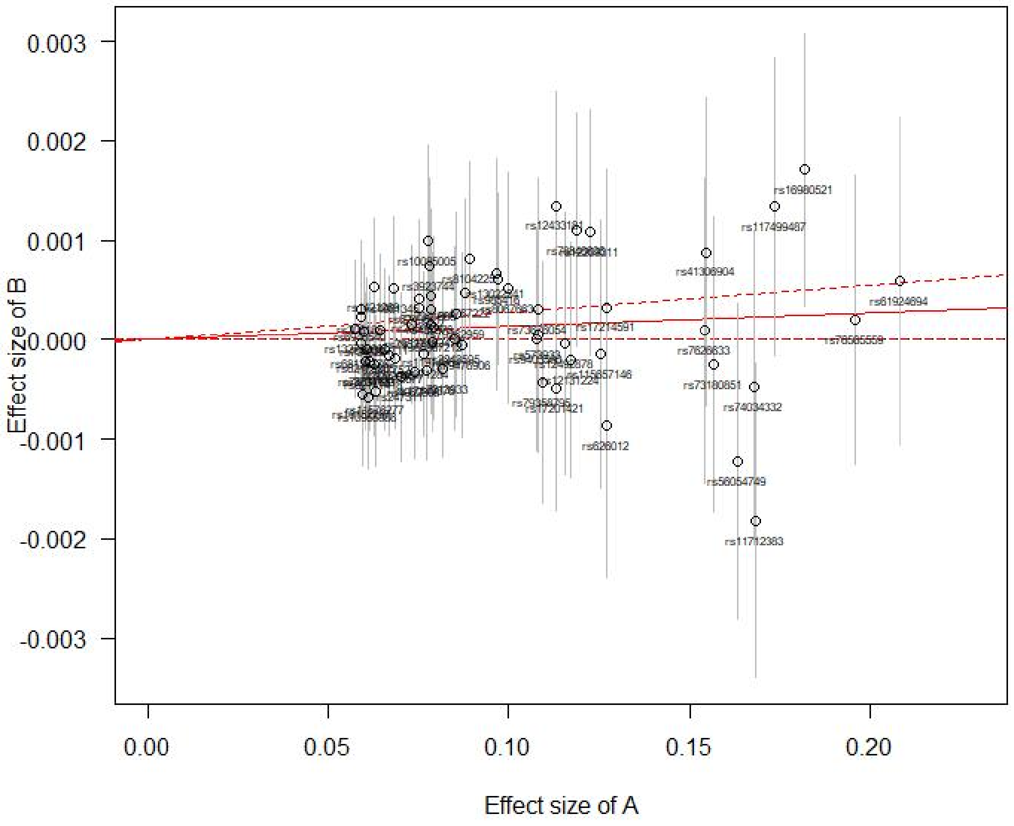

5. Gut microbiota abundance (genus Ruminococcaceae UCG010 id.11367)

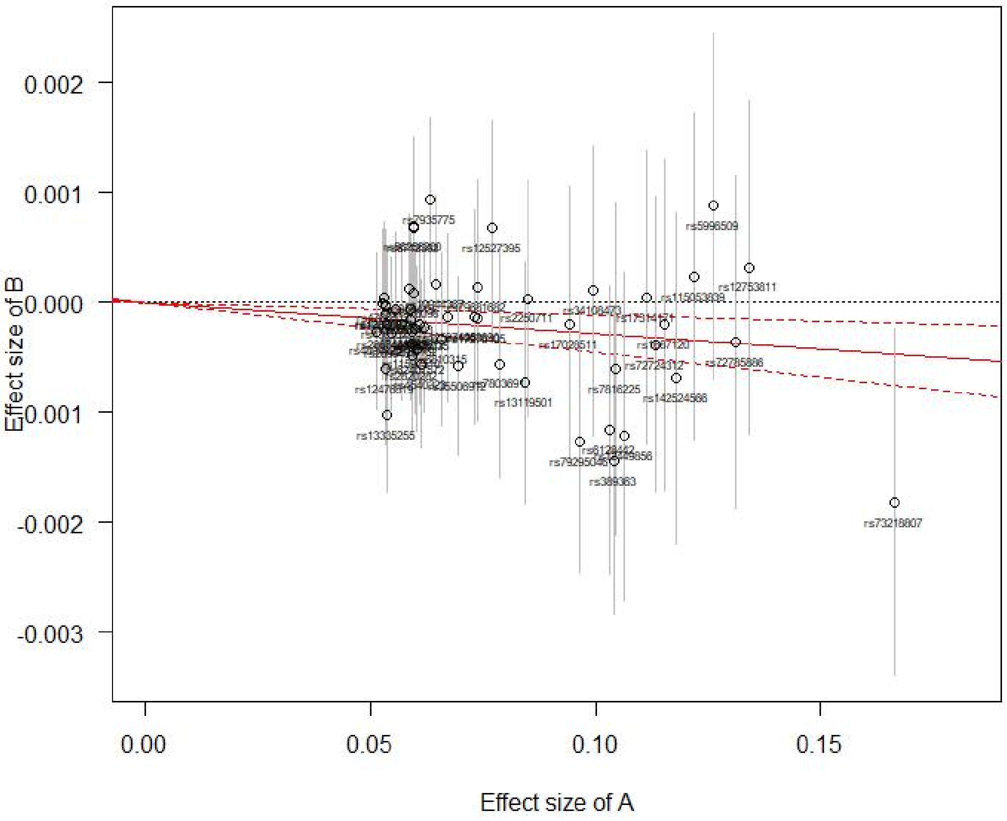

6. Gut microbiota abundance (order Selenomonadales id.2165)

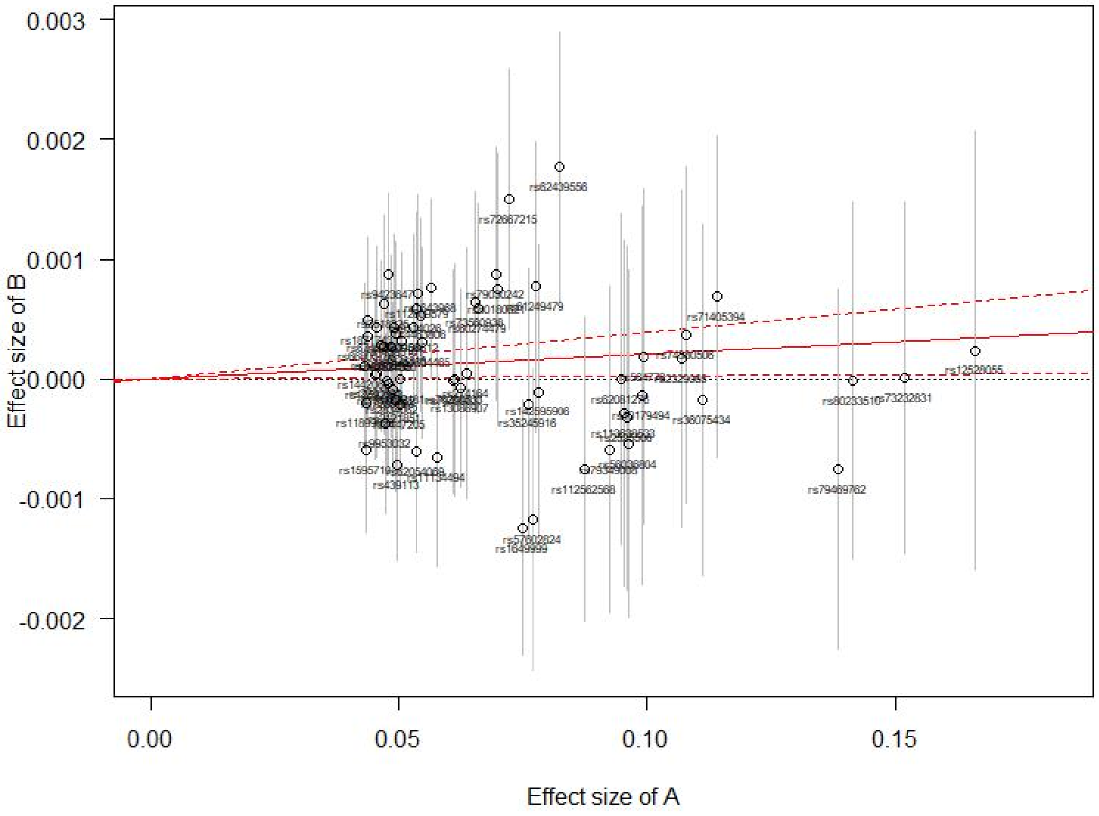

T. Acute pancreatitis

1. Gut microbiota abundance (class Methanobacteria id.119)

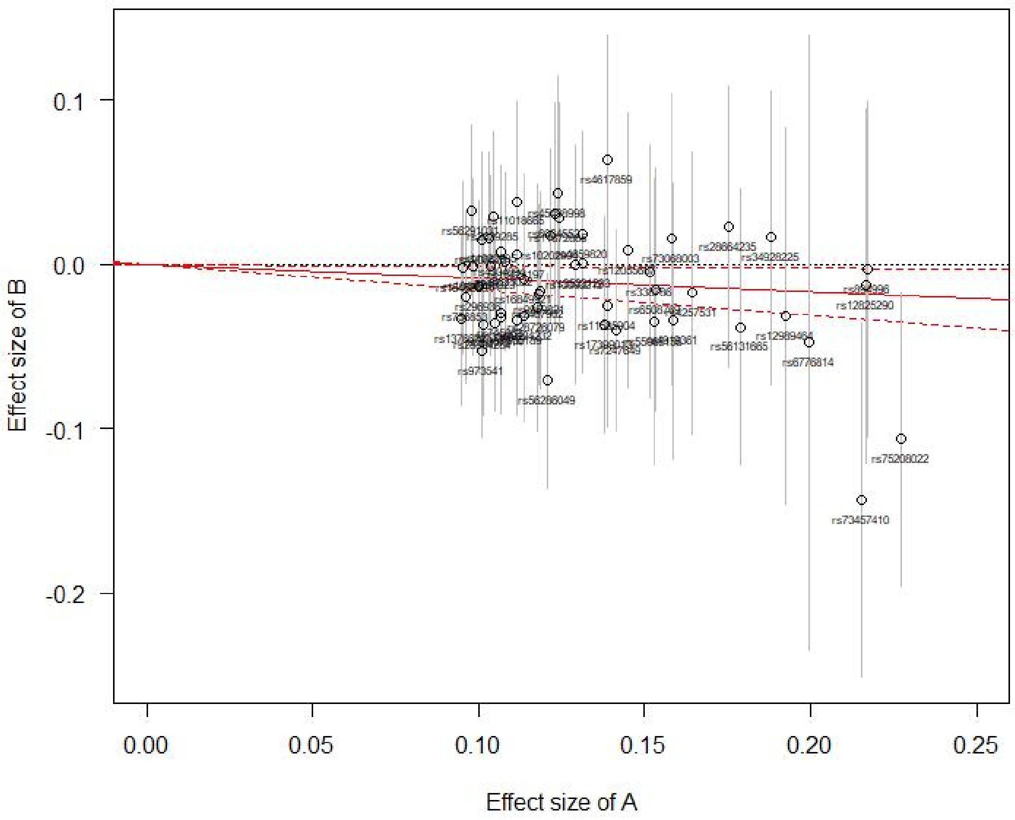

2. Gut microbiota abundance (family Methanobacteriaceae id.121)

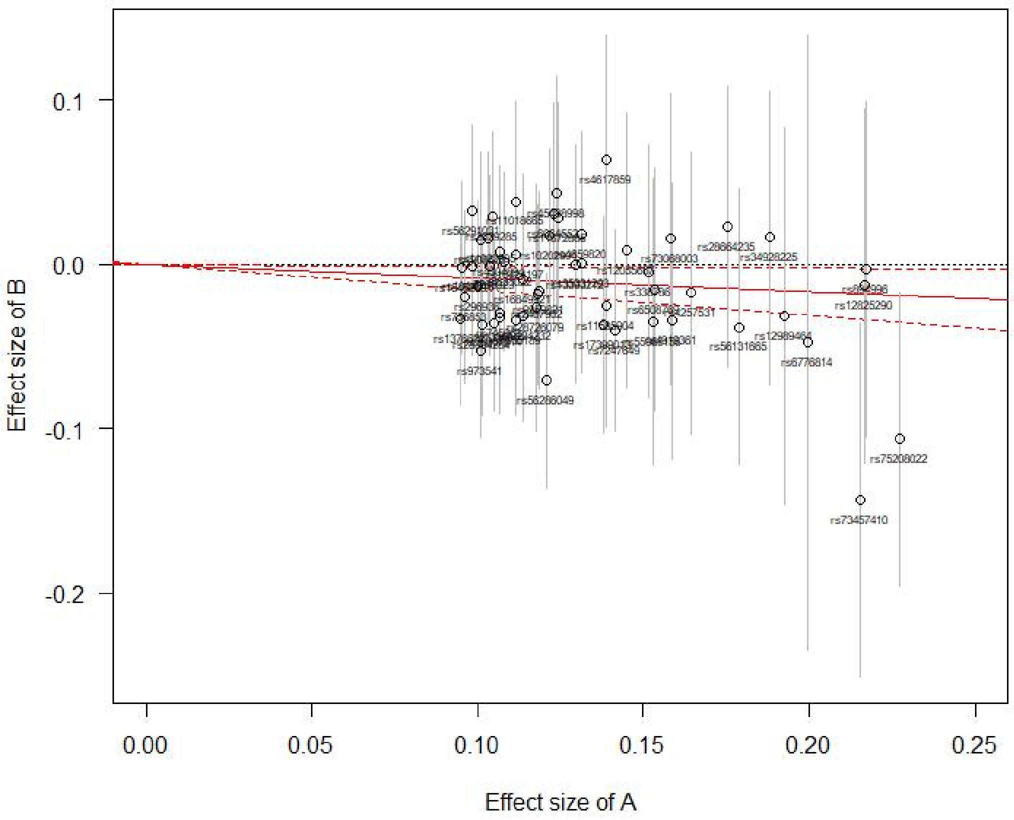

3. Gut microbiota abundance (genus Allisonella id.2174)

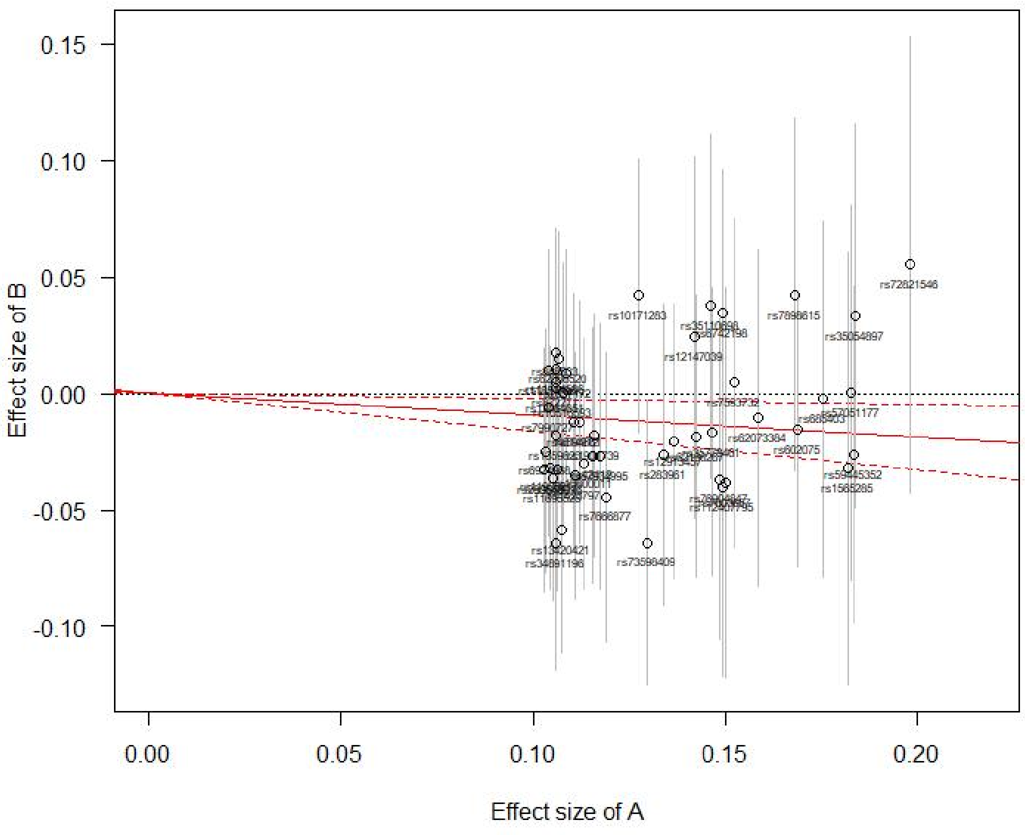

4. Gut microbiota abundance (genus Lachnoclostridium id.11308)

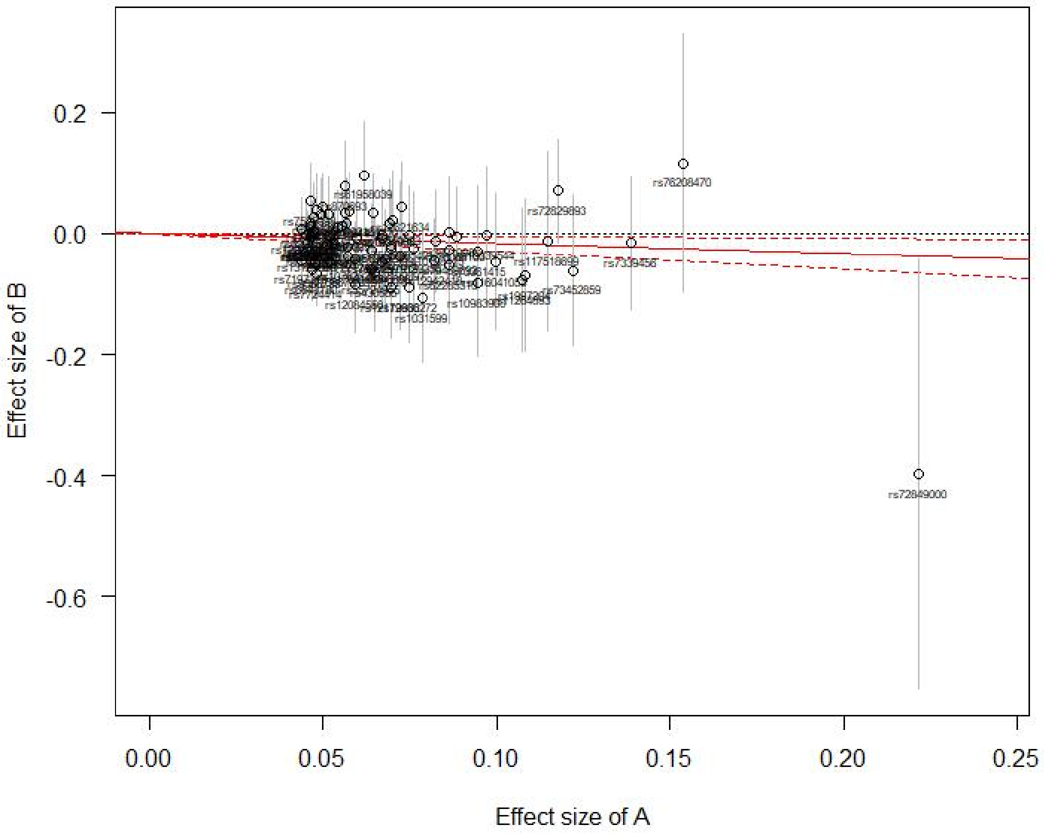

5. Gut microbiota abundance (genus Ruminiclostridium6 id.11356)

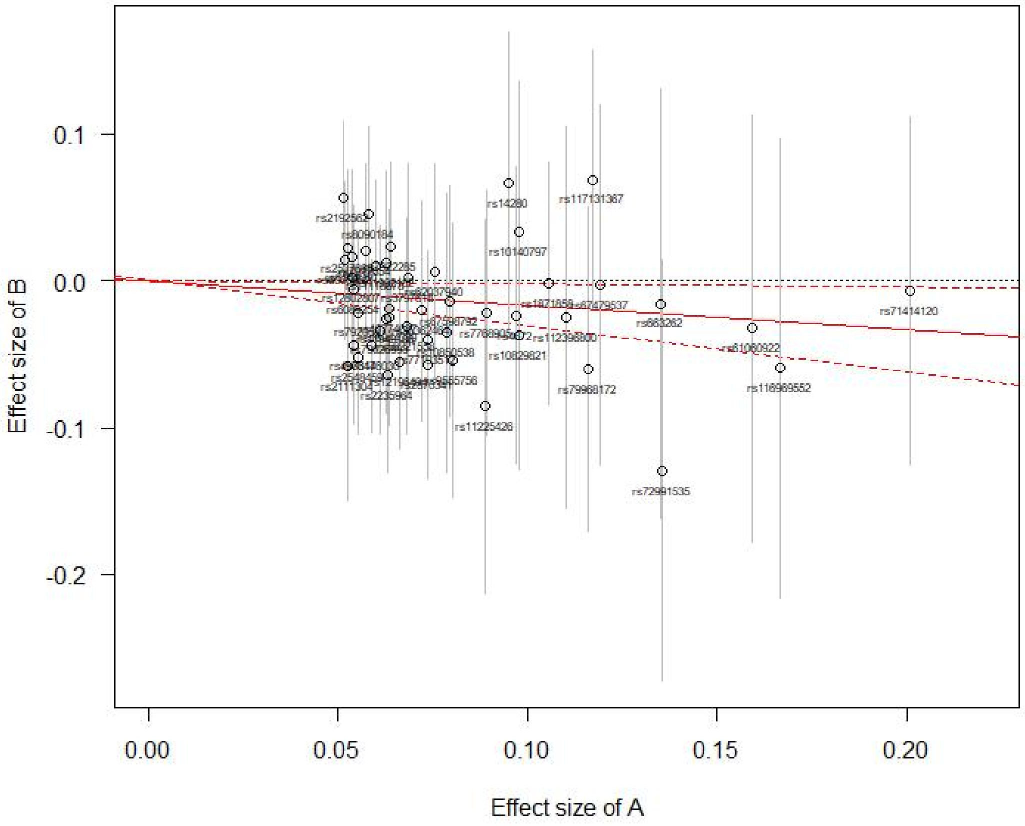

6. Gut microbiota abundance (genus Ruminococcaceae UCG010 id.11367)

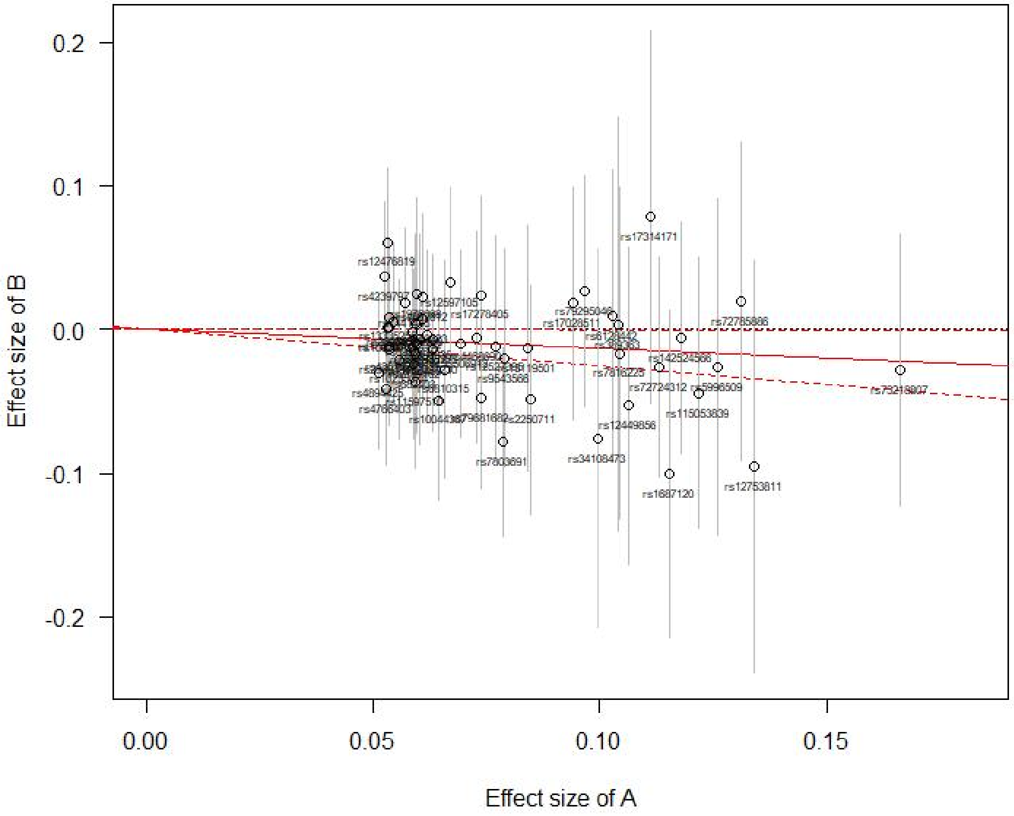

7. Gut microbiota abundance (genus Ruminococcaceae UCG014 id.11371)

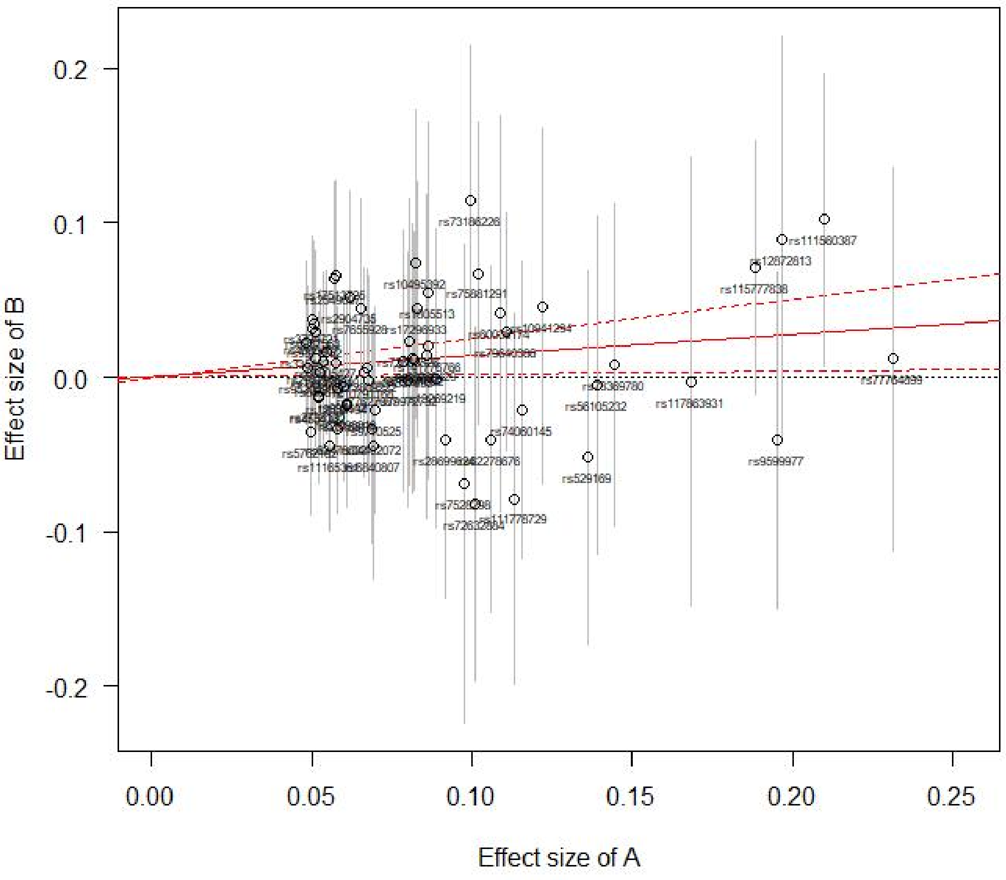

8. Gut microbiota abundance (genus Sellimonas id.14369)

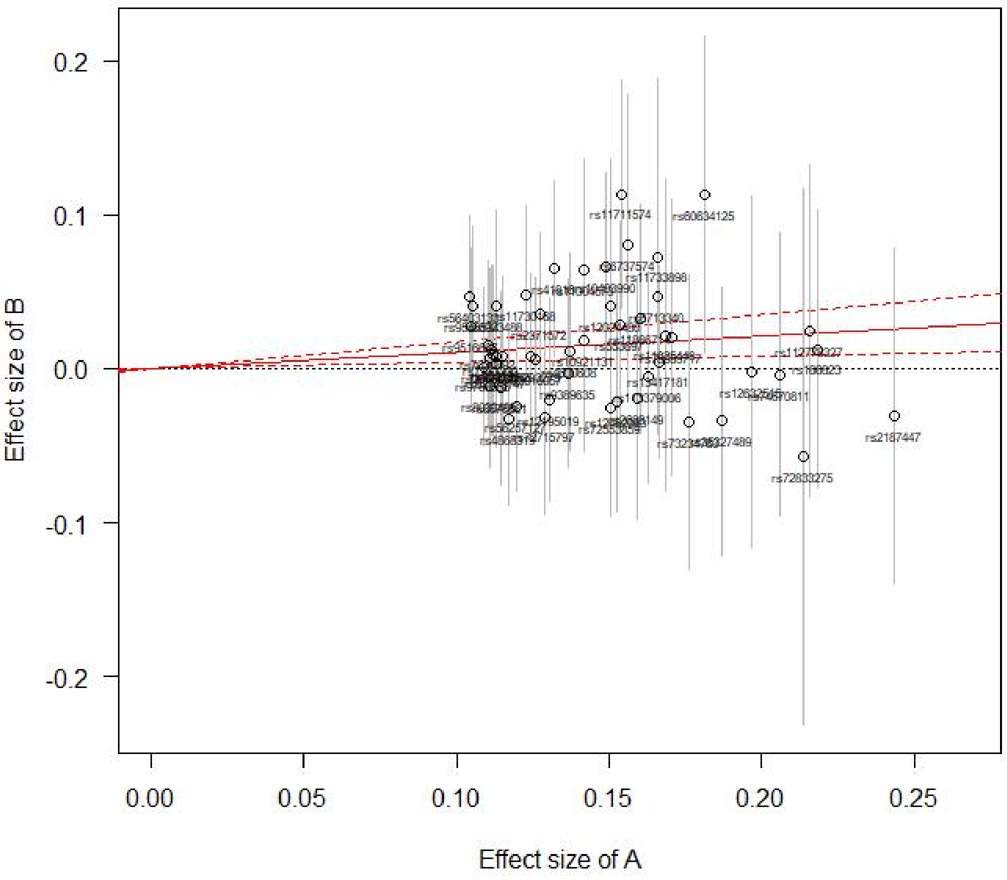

9. Gut microbiota abundance (genus Slackia id.825)

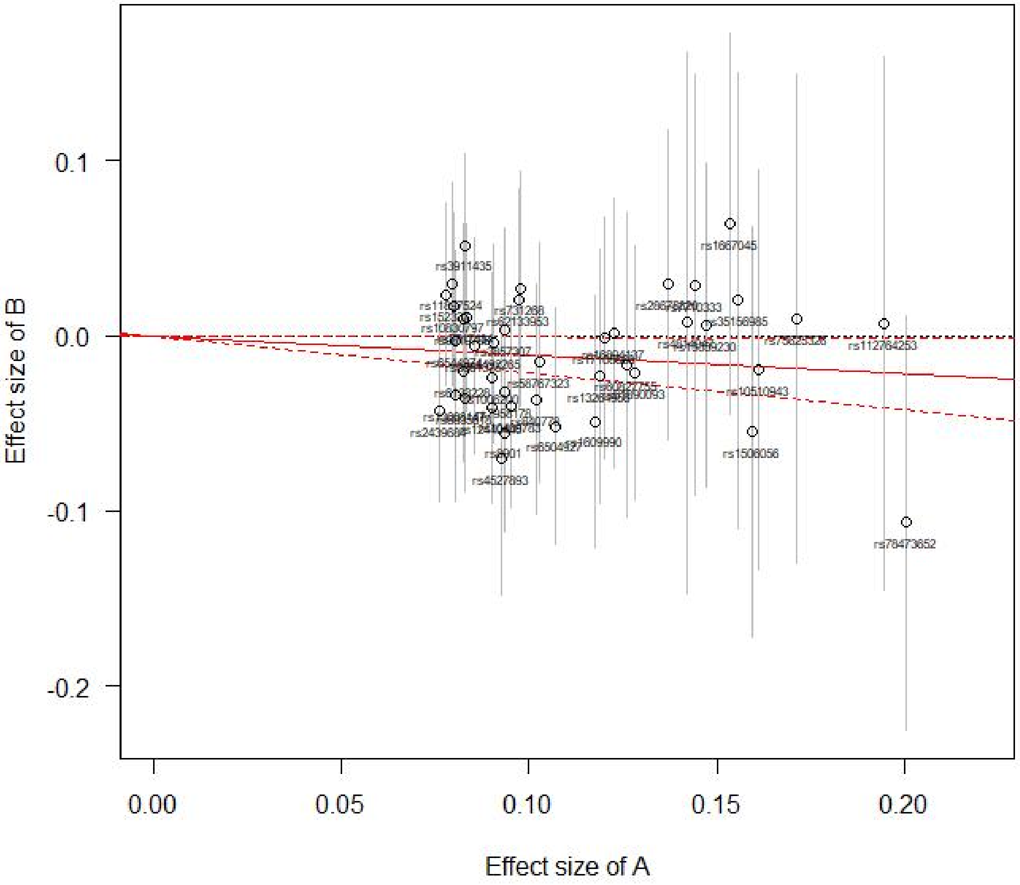

10. Gut microbiota abundance (order Methanobacteriales id.120)

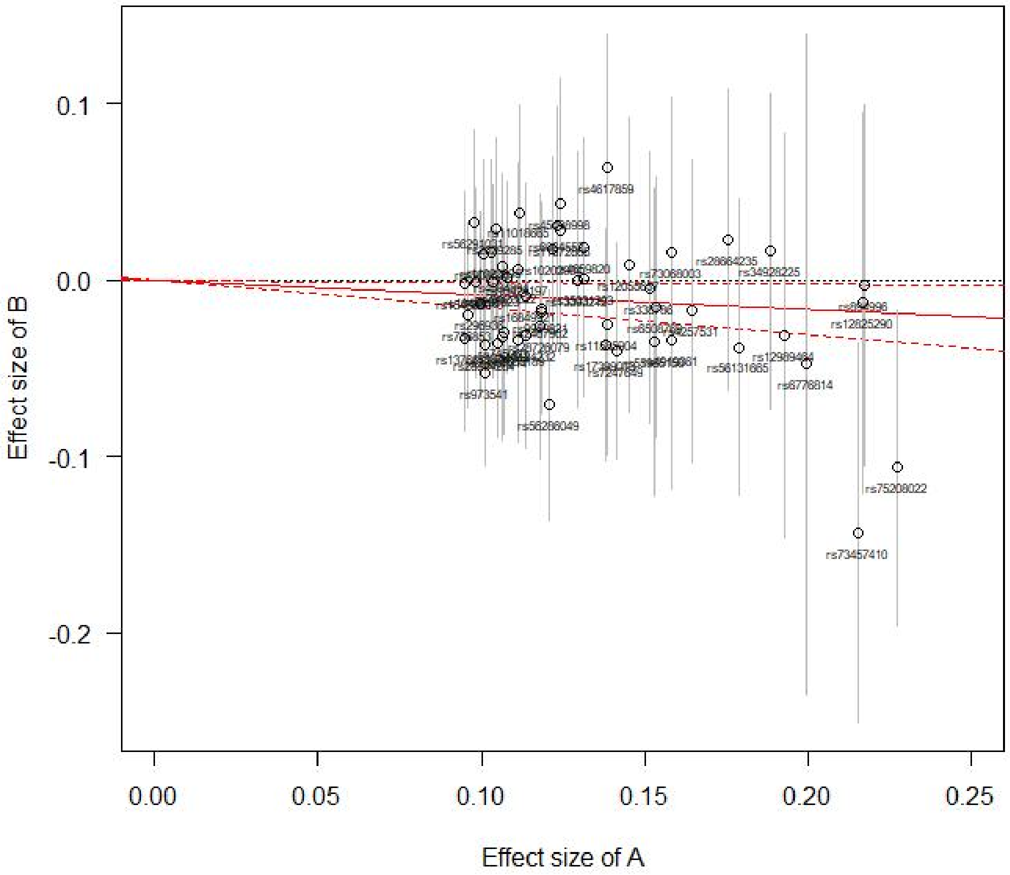

11. Gut microbiota abundance (phylum Verrucomicrobia id.3982)

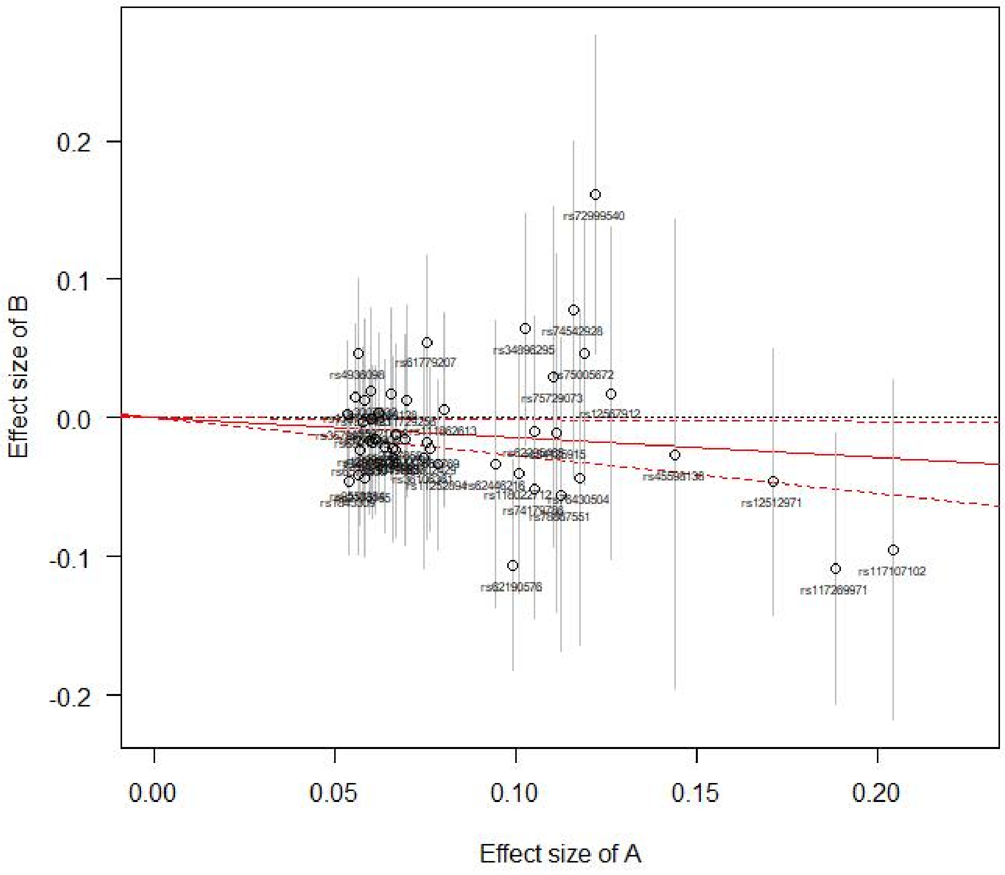

U. Chronic pancreatitis

1. Gut microbiota abundance (family Victivallaceae id.2255)

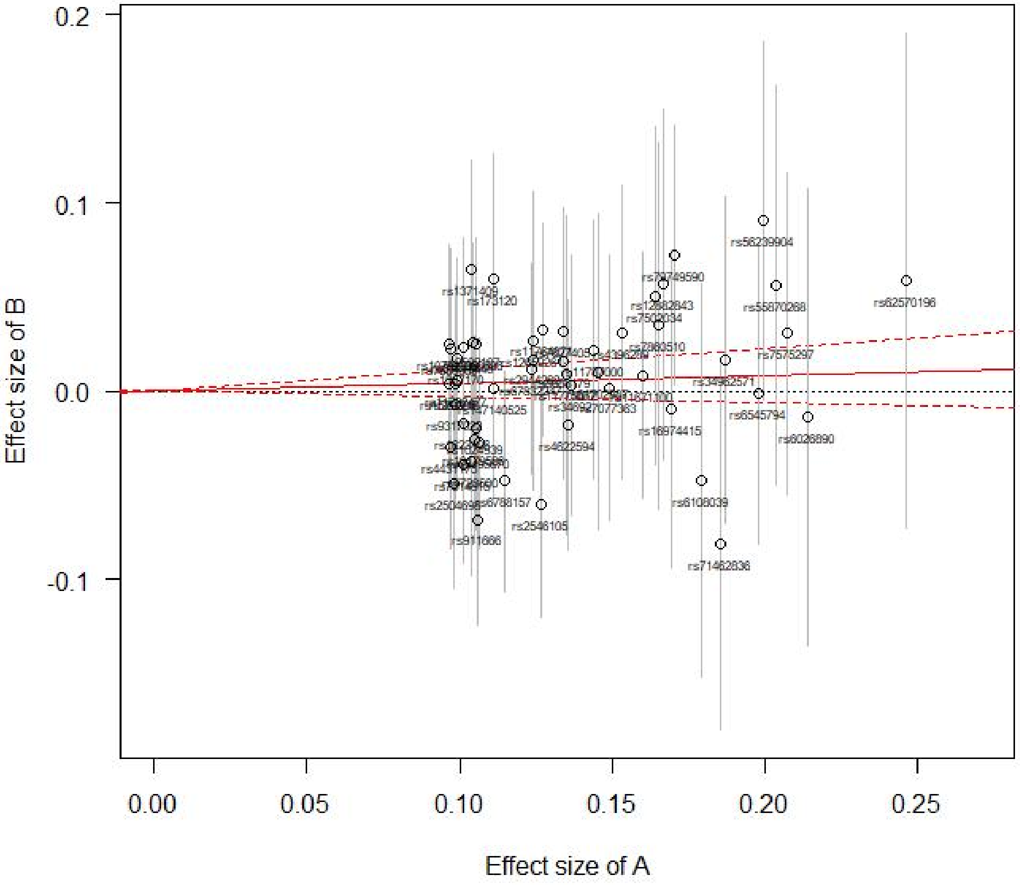

2. Gut microbiota abundance (genus Marvinbryantia id.2005)

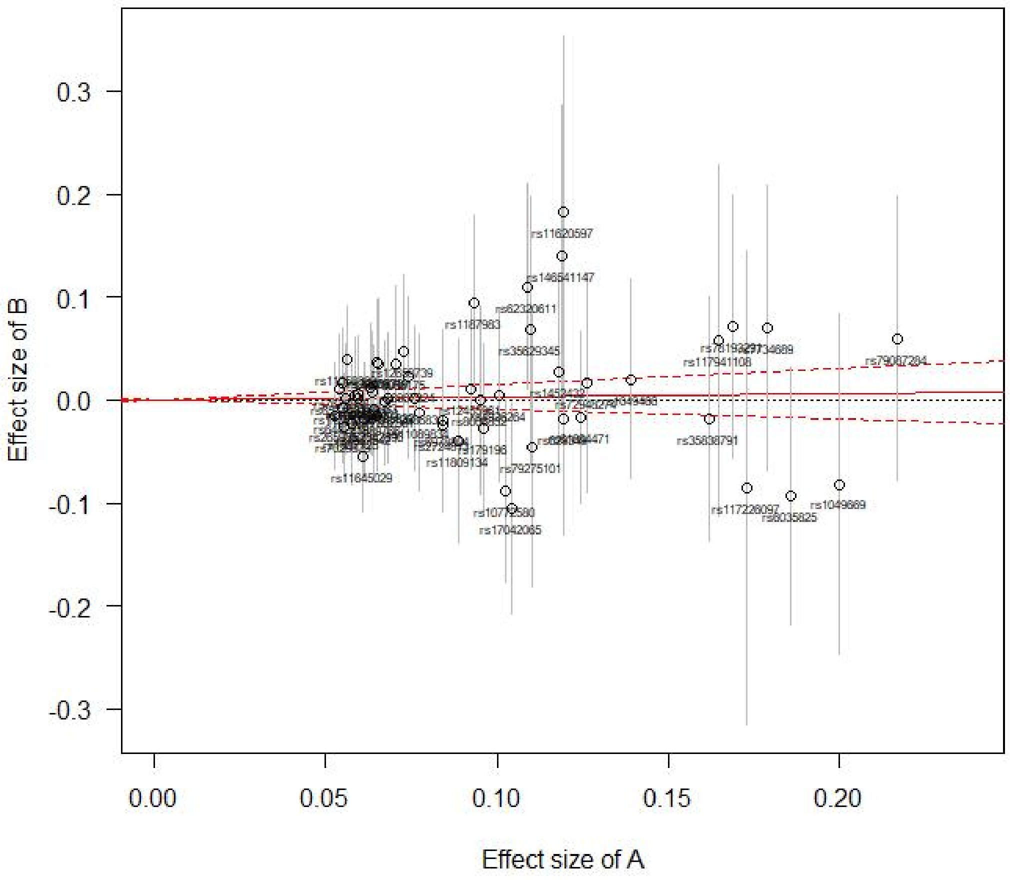

3. Gut microbiota abundance (genus Romboutsia id.11347)

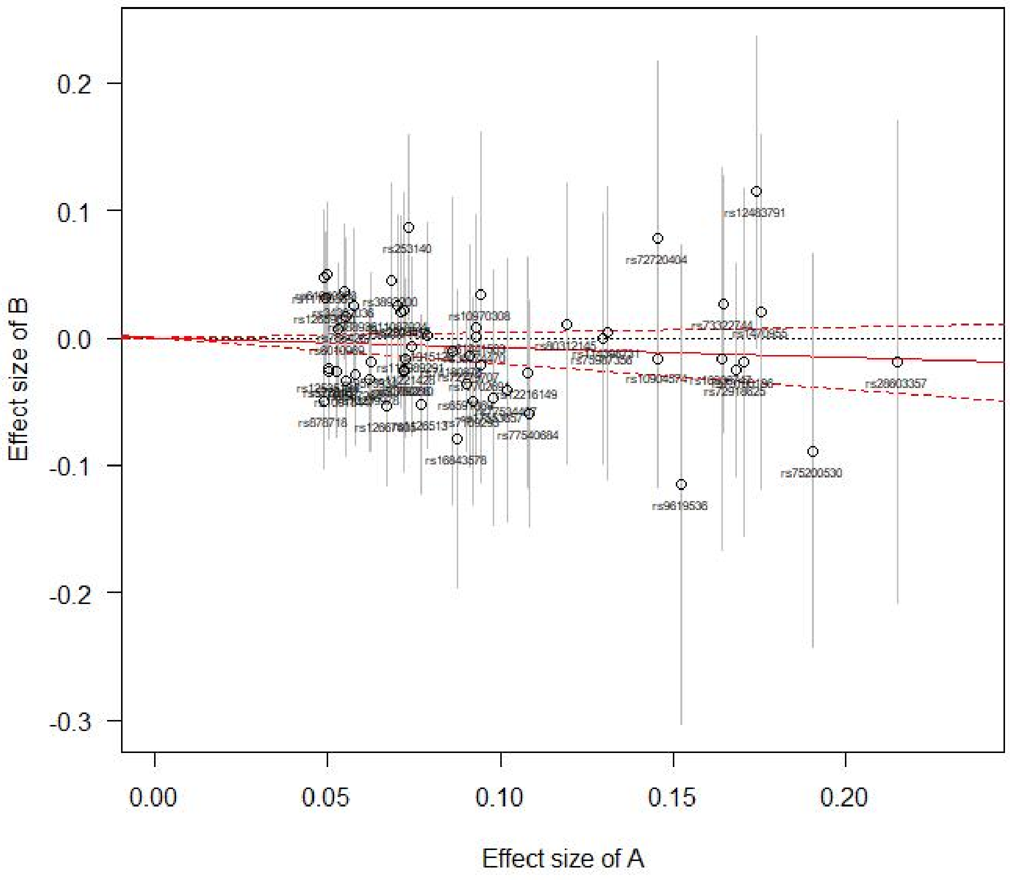

4. Gut microbiota abundance (genus Sellimonas id.14369)

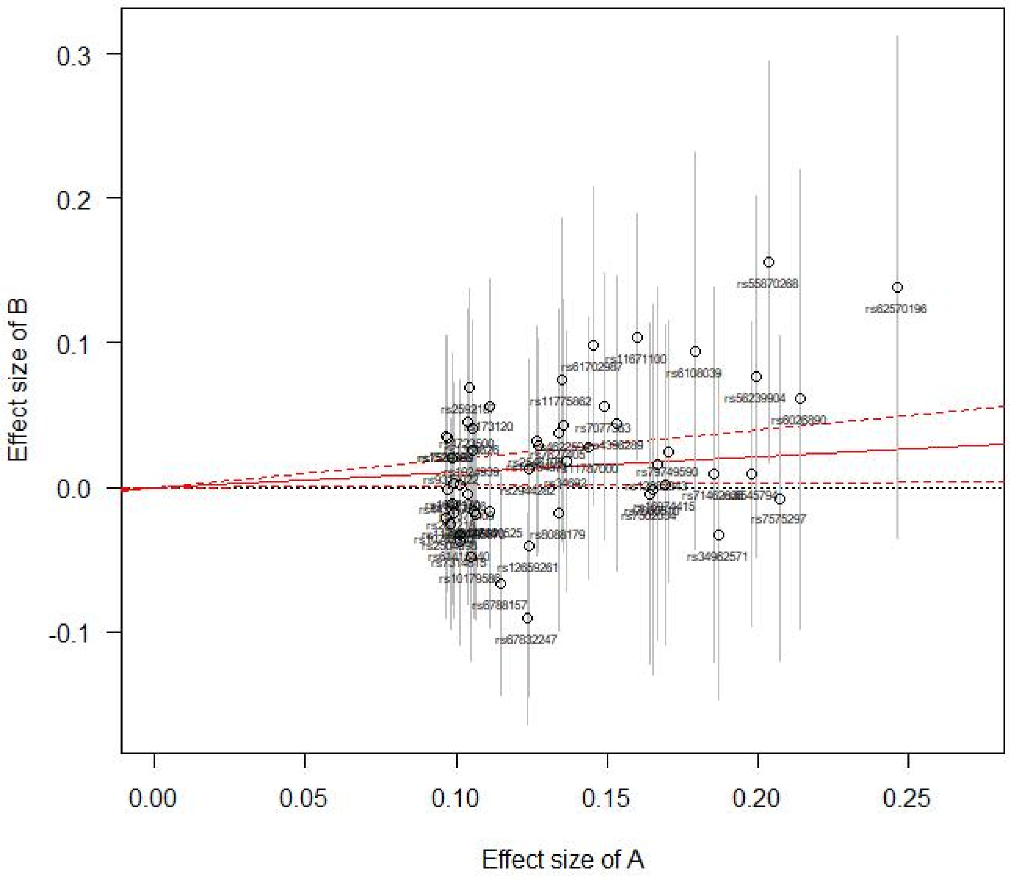

5. Gut microbiota abundance (genus Slackia id.825)

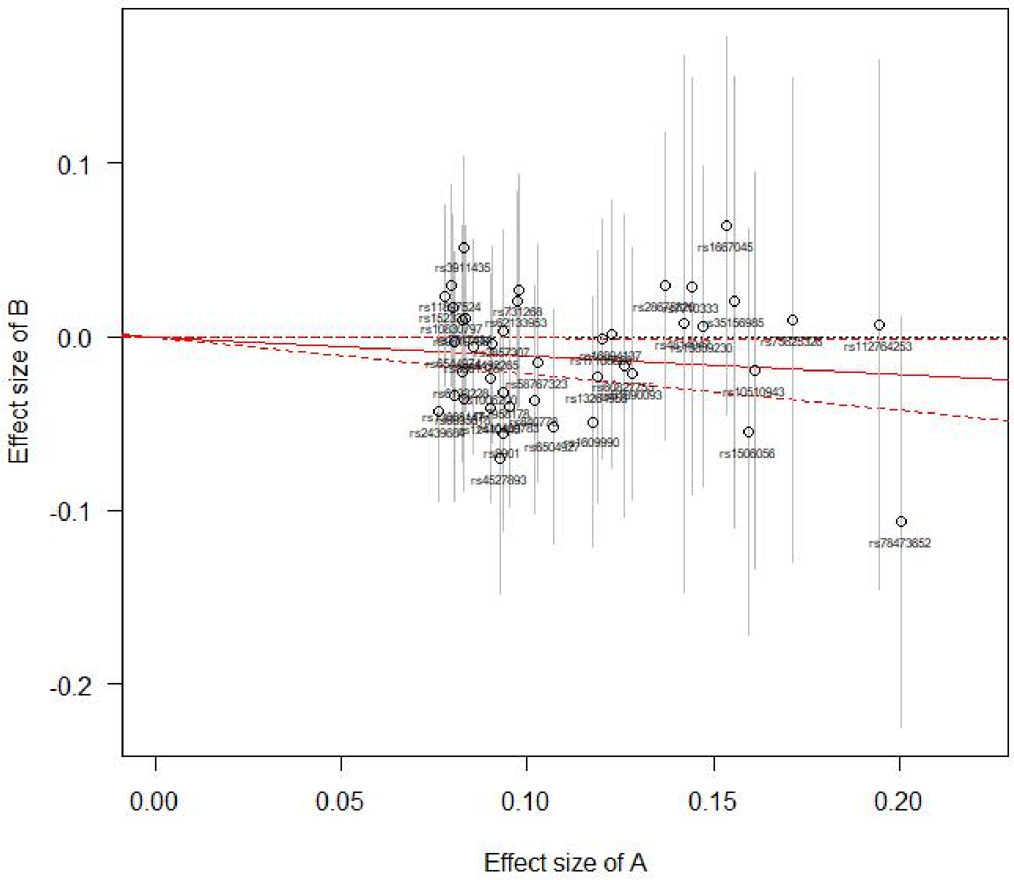

V. Pancreatic cancer

1. Gut microbiota abundance (genus Blautia id.1992)

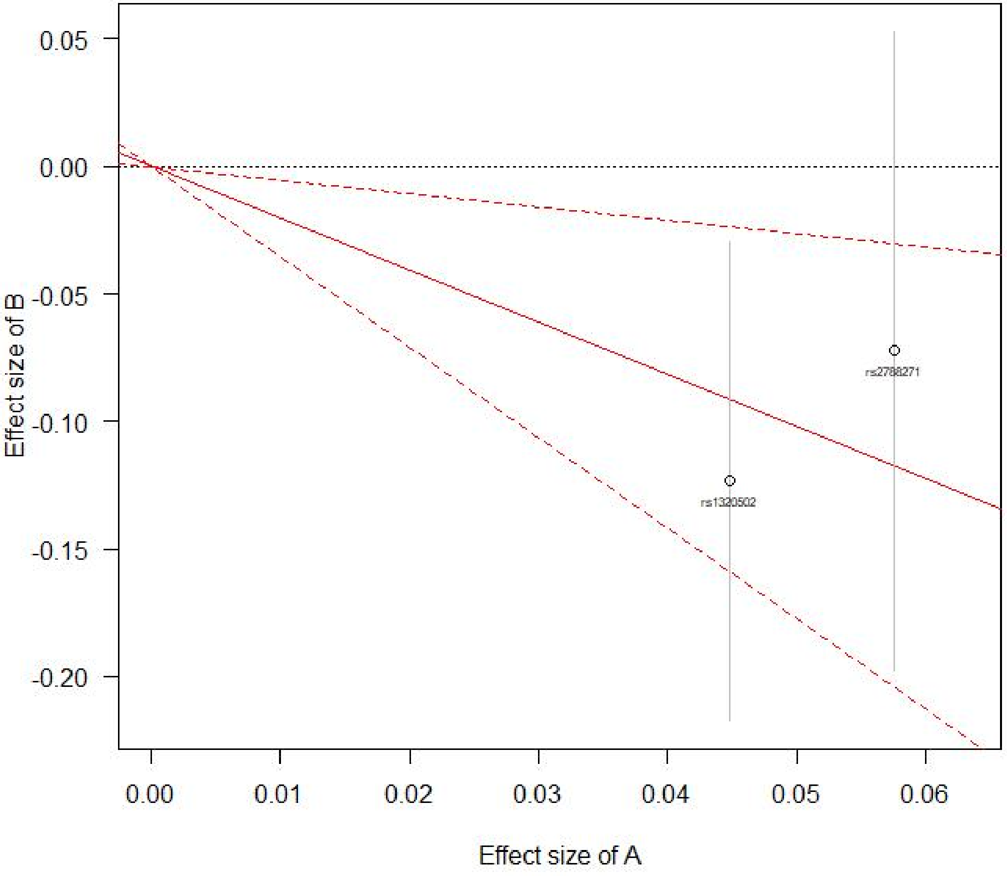

2. Gut microbiota abundance (genus Prevotella9 id.11183)

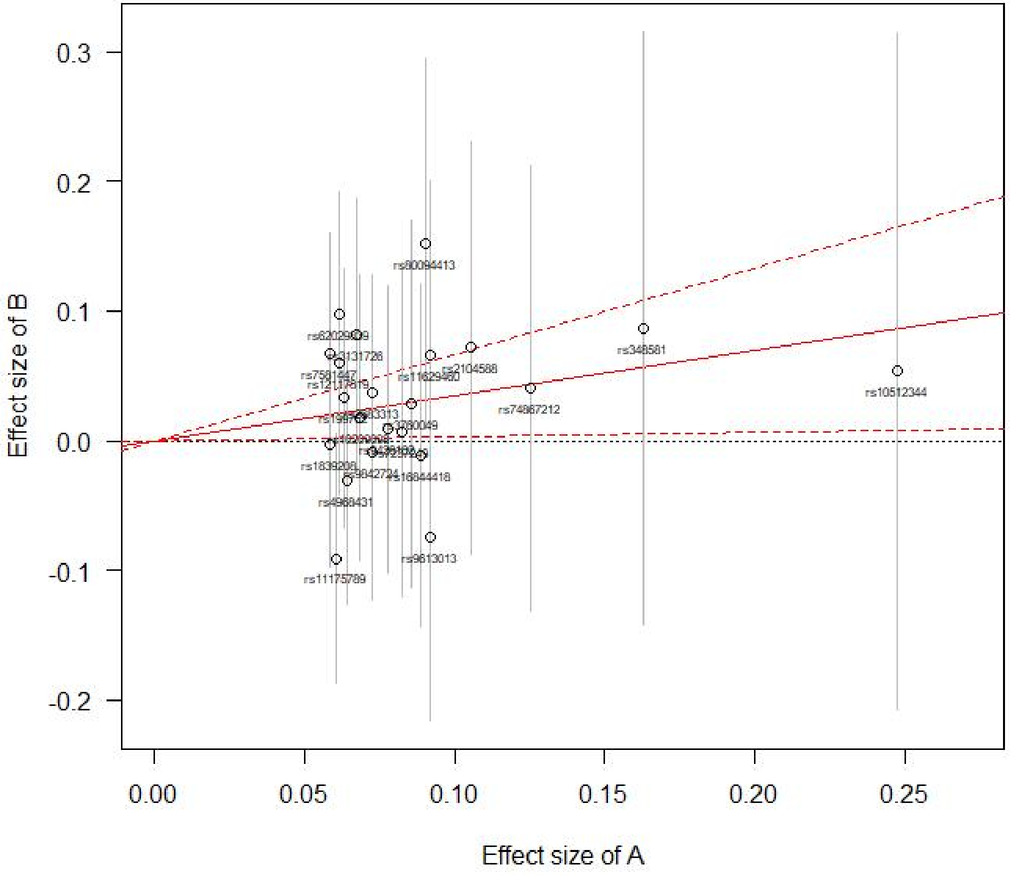

Supplement: Supplementary file 6 — Additional file 6: Genetic risk score GRSgut microbiota for gastrointestinal diseasess. [file 12967_2024_4894_MOESM6_ESM.pdf]
